# Prognostic potential of PRPF3 in hepatocellular carcinoma

**DOI:** 10.18632/aging.102665

**Published:** 2020-01-11

**Authors:** Yinlan Liu, Yuhan Yang, Yan Luo, Juan Wang, Xiangyun Lu, Zongxing Yang, Jin Yang

**Affiliations:** 1 Department of Translational Medicine Center, Affiliated Hospital of Hangzhou Normal University, Hangzhou Normal University, Hangzhou, Zhejiang 310015, P.R. China; 2 Department of Clinical Laboratory, Tongde Hospital of Zhejiang Province, Hangzhou, Zhejiang 310012, P.R. China; 3 State Key Laboratory for Diagnosis and Treatment of Infectious Diseases, National Clinical Research Center for Infectious Diseases, The First Affiliated Hospital, College of Medicine, Zhejiang University, Hangzhou, Zhejiang 310003, P.R. China; 4 The Second Department of Infectious Disease, Xixi Hospital of Hangzhou, The Affiliated Hospital of Zhejiang Chinese Medical University, Hangzhou, Zhejiang 310023, P.R. China

**Keywords:** PRPF3, tumor-infiltrating, spliceosome, prognosis, HCC

## Abstract

pre-mRNA processing factor 3 (PRPF3) is an RNA binding protein in a core component of the exon junction complex. Abnormal *PRPF3* expression is potentially associated with carcinogenesis. However, the biological role of *PRPF3* in hepatocellular carcinoma (HCC) remains to be determined. We analyzed *PRPF3* expression via multiple gene expression databases and identified its genetic alterations and functional networks using cBioPortal. Co-expressed genes with *PRPF3* and its regulators were identified using LinkedOmics. The correlations between *PRPF3* and cancer immune infiltrates were investigated via Tumor Immune Estimation Resource (TIMER). *PRPF3* was found up-regulated with amplification in tumor tissues in multiple HCC cohorts. High *PRPF3* expression was associated with poorer overall survival (OS) and disease-free survival (DFS). Functional network analysis suggested that *PRPF3* regulates spliceosome, DNA replication, and cell cycle signaling via pathways involving several cancer-related kinases and *E2F* family. Notably, *PRPF3* expression was positively correlated with infiltrating levels of CD4+ T and CD8+ T cells, macrophages, neutrophils, and dendritic cells. *PRPF3* expression showed strong correlations with diverse immune marker sets in HCC. These findings suggest that *PRPF3* is correlated with prognosis and immune infiltrating in HCC, laying a foundation for further study of the immune regulatory role of *PRPF3* in HCC.

## INTRODUCTION

Hepatocellular carcinoma (HCC) is the most common form of liver cancers [1], which has an annual incidence of at least 6 per 100,000 individuals and represents the fastest-rising cause of cancer-related death [2]. Due to the high rate of recurrence and metastasis, the five-year survival rate for advanced HCC is poor. However, existing targeted drugs show unsatisfactory efficacy, due to a combination of factors spanning an array of different clinical and biological behaviors, and the development of anti-HCC drug resistance [3]. The molecular mechanisms underlying tumor formation and progression are poorly understood, which further complicates the effective treatment of HCC [4]. In addition, the lack of markers that are specific for tumor type or disease stage represents a critical gap in the current understanding and treatment of HCC.

Pre-mRNA splicing is a fundamental process that plays a considerable role in generating protein diversity. Pre-mRNA splicing is also the key to the pathology of numerous diseases, especially cancers [5]. The connection between cancer biology and splicing regulation is of primary importance to understand the mechanisms leading to disease and also to improve the development of therapeutic approaches [6]. Among the array of splicing factors, pre-mRNA processing factor 3 (*PRPF3*), a component of the U4/U6 di-snRNP, is required for U4/U6•U5 tri-snRNP formation and recruitment to active spliceosomes, which is essential for efficient pre-mRNA splicing [7, 8].

It is also known that one gene pair, *KCNE2-PRPF3* as the signature could robustly predict prognoses of gastric cancer patients treated with 5-FU-based chemotherapy [9]. As a member of the hepatic transcription factor network, Hepatocyte Nuclear Factor 4 Alpha (*HNF4α*) plays a pivotal role in liver development and hepatocellular differentiation. One study indicated that *PRPF3* is an *HNF4α* regulated gene with induced expression in mouse and human HCC [10]. However, the biological function of *PRPF3* in HCC remains to be determined.

Here, we investigated *PRPF3* expression and mutations in data from patients with HCC in The Cancer Genome Atlas (TCGA) and various public databases. Using multi-dimensional analysis, we evaluated genomic alterations and functional networks related to *PRPF3* in HCC and explored its role in tumor immunity. Our results could potentially reveal new targets and strategies for HCC diagnosis and treatment.

## RESULTS

### Elevated expression of PRPF3 in HCC

**Figure 1:**
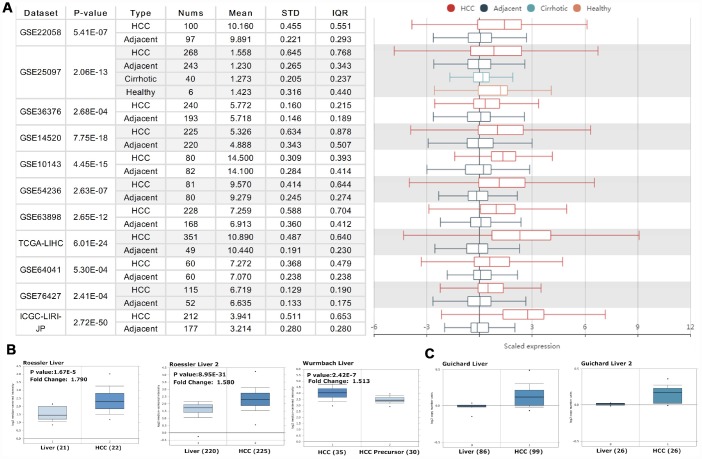
***PRPF3* transcription level in HCC.** (**A**) Chart and plot showing the expression of *PRPF3* in tumor tissues and the adjacent normal tissues, according to t-test in HCCDB. (**B**) Box plot showing *PRPF3* mRNA levels in the Roessler Liver, Roessler Liver 2, and Wurmbach Liver datasets, respectively. (**C**) Box plot showing *PRPF3* copy number in Guichard Liver and Guichard Liver 2 datasets, respectively.

We initially evaluated *PRPF3* transcription levels in multiple HCC studies from TCGA and GEO. Analysis of eleven HCC cohorts in the HCCDB database revealed that mRNA expression of *PRPF3* was significantly higher in HCC tissues than in adjacent normal tissues (Figure 1A). Data in the Oncomine database indicated that *PRPF3* ranked within the top 10% based on mRNA expression (Figure 1B). Levels of *PRPF3* DNA copy number were significantly higher in tumor tissues than in normal tissue (Figure 1C).

**Figure 2:**
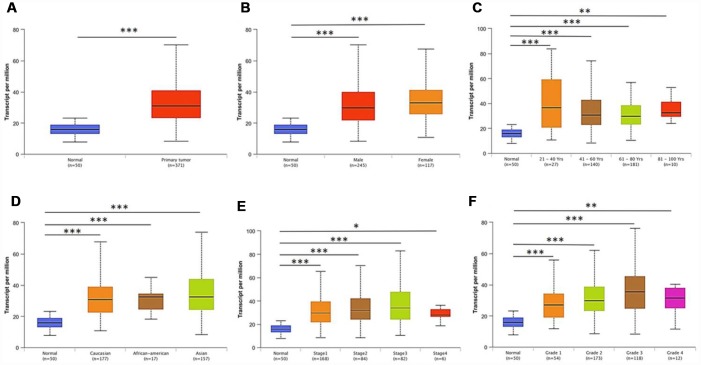
***PRPF3* transcription in subgroups of patients with HCC, stratified based on gender, age and other criteria (UALCAN).** Box-whisker plots showing the expression of PRPF3 in sub groups of LIHC samples. (**A**) Boxplot showing relative expression of PRPF3 in normal and LIHC samples. (**B**) Boxplot showing relative expression of PRPF3 in normal individuals of either gender and male or female LIHC patients, respectively. (**C**) Boxplot showing relative expression of PRPF3 in normal individuals of any age or in LIHC patients aged 21-40, 41-60, 61-80, or 81-100 yr. (**D**) Boxplot showing relative expression of PRPF3 in normal, African American, Caucasian and Asian LIHC patients. (**E**) Boxplot showing relative expression of PRPF3 in normal individuals or in LIHC patients in stages 1, 2, 3 or 4. (**F**) Boxplot showing relative expression of PRPF3 in normal individuals or LIHC patients with grade 1, 2, 3 or 4 tumors. The central mark is the median; the edges of the box are the 25^th^ and 75^th^ percentiles. The t-test was used to estimate the significance of difference in gene expression levels between groups. *, *p* < 0.05; **, *p* < 0.01; ***, *p* < 0.001.

Further sub-group analysis of multiple clinic-pathological features of TCGA-LIHC samples in UALCAN database consistently showed elevated transcription level of *PRPF3*. The expression of *PRPF3* was significantly higher in HCC patients than normal controls in subgroup analysis based on gender, age, ethnicity, disease stages, and tumor grade (Figure 2). Thus, *PRPF3* expression may serve as a potential diagnostic indicator in HCC.

### PRPF3 expression is survival-associated

**Figure 3:**
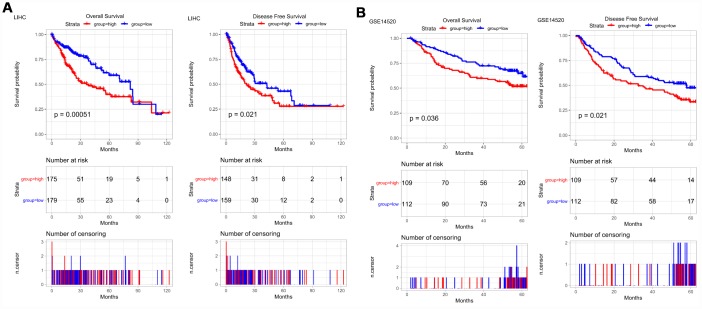
***PRPF3* is associated with survival outcome.** (**A**) Overall survival (OS) and disease-free survival (DFS) in TCGA LIHC cohort. (**B**) OS and DFS of PRPF3 in GSE14520 cohort. The numbers below the figures denote the number of patients at risk in each group.

Then, Kaplan-Meier survival curves were used to assess the association between *PRPF3* expression and the survival outcomes of HCC cohorts with survival information available (Figure 3). The patients were separated into two groups according to the median value of *PRPF3* expression level in each cohort. Generally, the high *PRPF3* expression group had significantly shorter overall survival (OS) (log-rank test, *p* < 0.05) and disease-free survival (DFS) (log-rank test, *p* < 0.05), compared to the low expression group in LIHC cohort (Figure 3A). Similarly, in an independent cohort (GSE14520), the low-risk group had significantly better OS and DFS than the high-risk group (Figure 3B). In addition, high *PRPF3* expression being associated with poor survival was also verified in GSE10141 cohort (Supplementary Figure 1).

### PRPF3 co-expression networks in HCC

**Figure 4:**
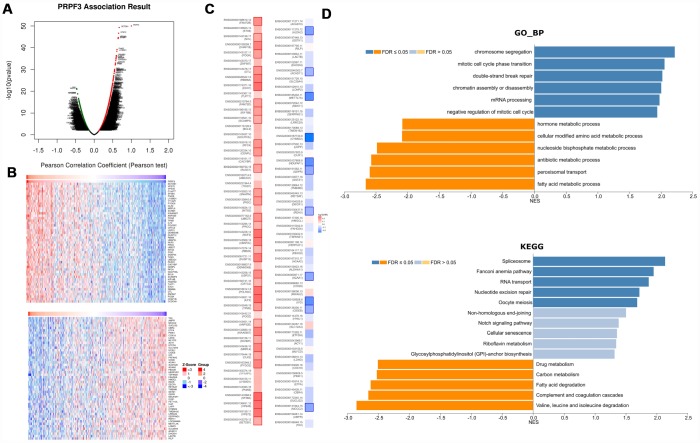
***PRPF3* co-expression genes in HCC (LinkedOmics).** (**A**) The global *PRPF3* highly correlated genes identified by Pearson test in LIHC cohort. (**B**) Heat maps showing top 50 genes positively and negatively correlated with *PRPF3* in LIHC. Red indicates positively correlated genes and blue indicates negatively correlated genes. (**C**) Survival map of the top 50 genes positively and negatively correlated with *PRPF3* in LIHC. (**D**) Significantly enriched GO annotations and KEGG pathways of *PRPF3* in LIHC cohort.

To gain the insight of *PRPF3* biological meaning in HCC, the function module of LinkedOmics was used to examine *PRPF3* co-expression mode in LIHC cohort. As shown in Figure 4A, 3,558 genes (dark red dots) were shown significant positive correlations with *PRPF3*, whereas 1,891 genes (dark green dots) were shown significant negative correlations (false discovery rate, FDR < 0.01). The top 50 significant genes positively and negatively correlated with *PRPF3* were shown in the heat map (Figure 4B). A total description of the co-expressed genes was detailed in Supplementary Table 1.

*PRPF3* expression showed a strong positive association with expression of *SETDB1* (positive rank #1, r = 0.672, *p* = 5.06E-50), *VPS72* (r = 0.658, *p* = 2.19E-47), and *VPS45* (r = 0.647, *p* = 1.95E-45), etc. Notably, the top 50 significantly positive genes showed the high likelihood of being high-risk genes in HCC, in which 34/50 genes were with high hazard ratio (HR) (*p* < 0.05). In contrast, there were 11/50 genes with low HR (*p* < 0.05) in the top 50 negatively significant genes (Figure 4C).

Significant Gene Ontology (GO) term annotation by gene set enrichment analysis (GSEA) showed that *PRPF3* co-expressed genes participate primarily in chromosome segregation, mitotic cell cycle phase transition, double-strand break repair, and mRNA processing, while the activities like fatty acid metabolic process, peroxisomal transport, and multiple metabolic processes were inhibited (Figure 4D and Supplementary Table 2). Kyoto Encyclopedia of Genes and Genomes (KEGG) pathway analysis showed enrichment in the spliceosome, fanconi anemia pathway, RNA transport, and nucleotide excision repair pathways, etc (Figure 4D and Supplementary Table 3). These results suggest that a widespread impact of *PRPF3* on the global transcriptome.

### Regulators of PRPF3 networks in HCC

To further explore the regulators of *PRPF3* in HCC, we analyzed the kinases, miRNAs and transcription factors’ (TF) enrichment of *PRPF3* co-expressed genes. The top 5 most significant kinases related primarily to the cyclin-dependent kinase 1 (*CDK1*), polo like kinase 1 (*PLK1*), Aurora kinase B (*AURKB*), checkpoint kinase 1 (*CHEK1*), and cyclin-dependent kinase 2 (*CDK2*) (Table 1 and Supplementary Table 4). In fact, all of these kinase genes, except *CDK2*, were significantly highly expressed in tumor tissues. In addition, all these kinase genes were significantly associated with the OS of HCC (Supplementary Figure 2).

**Table 1:** The Kinases, miRNAs and transcription factors-target networks of *PRPF3* in HCC.

**Enriched Category**	**Geneset**	**LeadingEdgeNum**	**FDR**
Kinase Target	Kinase_CDK1	85	0.00E+00
	Kinase_PLK1	38	0.00E+00
	Kinase_CHEK1	49	0.00E+00
	Kinase_AURKB	32	0.00E+00
	Kinase_CDK2	118	0.00E+00
miRNA Target	GACTGTT, MIR-212, MIR-132	148	4.32E-01
	AGCGCAG, MIR-191	12	4.56E-01
	CCAGGTT, MIR-490	60	4.58E-01
	GAGCTGG, MIR-337	147	4.67E-01
	ACACTCC, MIR-122A	80	4.70E-01
Transcription Factor	V$E2F4DP2_01	69	0.00E+00
	V$E2F_Q4_01	44	0.00E+00
	KCCGNSWTTT_UNKNOWN	33	8.40E-05
	GCGSCMNTTT_UNKNOWN	30	3.00E-04
	V$ETF_Q6	80	3.20E-04

No significant miRNA was enriched by GSEA for *PRPF3* co-expressed genes (Supplementary Table 5). The enrichment of transcription factors was related mainly to the *E2F* transcription factor family (Supplementary Table 6), including V$E2F_Q6, V$E2F_Q4, V$E2F1_Q6, V$E2F1DP1RB_01, and V$E2F4DP1_01. One recent study, using combinatorial mapping of chromatin occupancy and transcriptome profiling, identified an *E2F*-driven transcriptional program that was associated with the development and progression of HCC [11].

### Genomic alterations of PRPF3 in HCC

**Figure 5:**
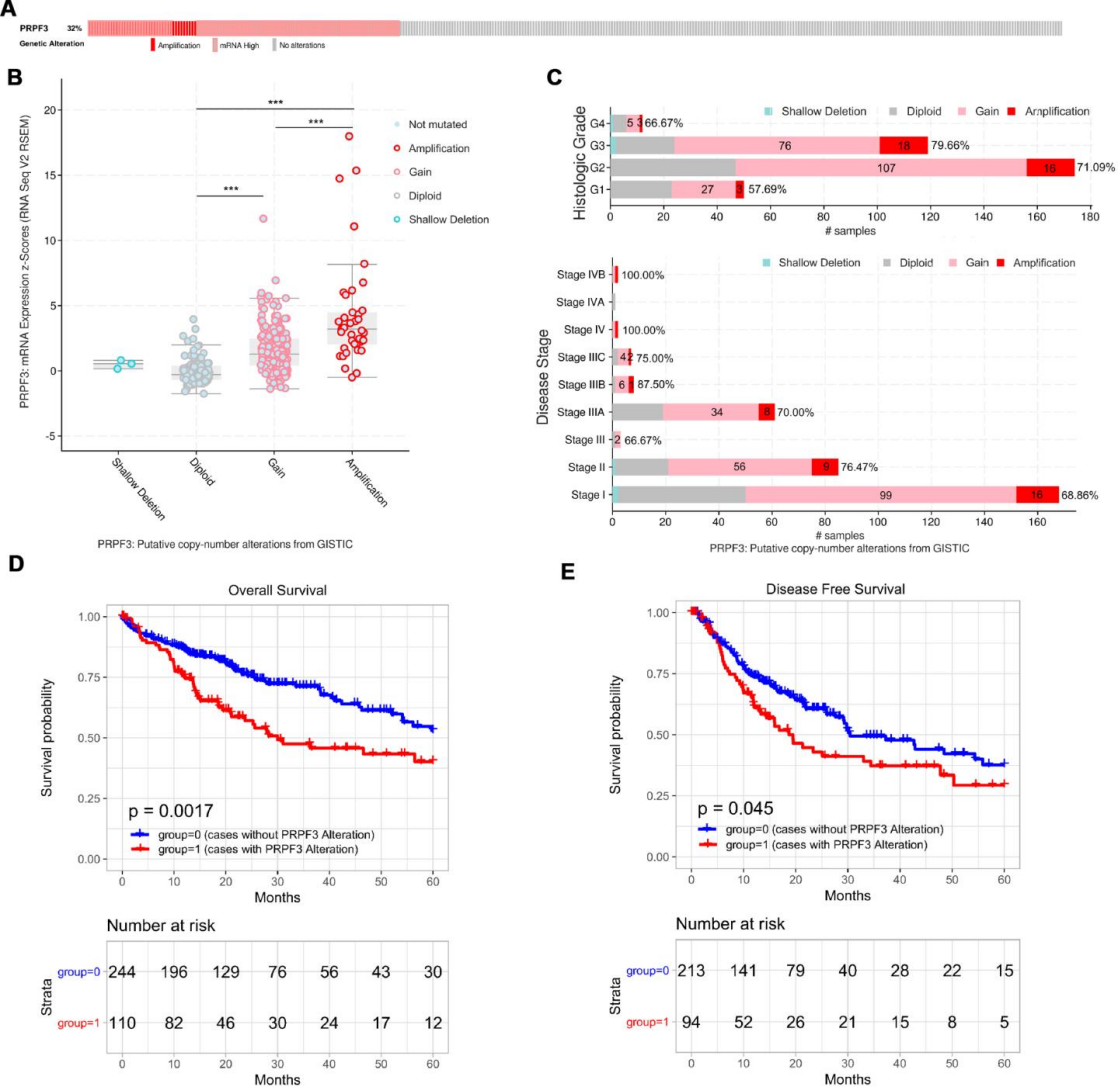
**PRPF3 genomic alterations in HCC (cBioPortal).** (**A**) OncoPrint of *PRPF3* alterations in LIHC cohort. The different types of genetic alterations are highlighted in different colors. (**B**) *PRPF3* expression in different *PRPF3* CNV groups. *PRPF3* amplification (AMP) group has a significantly higher expression level. (**C**) Distribution of *PRPF3* CNV frequency in different stage and grade subgroups. The percentage number on the right of the bar indicates the ratio of patients with PRPF3 gain or AMP in all this subgroup patients. (**D**) To reduce the noise of disease irrelevant deaths, survival time that was greater than five years was truncated to five years. *PRPF3* CNV affects overall survival and disease-free survival. ***, *p* < 0.001.

We then used the cBioPortal tool to determine the types and frequency of *PRPF3* alterations in HCC based on DNA sequencing data from LIHC patients. *PRPF3* was altered in 115 of 370 (32%) LIHC patients (Figure 5A). These alterations include mRNA upregulation in 60 cases (16%), amplification (AMP) in 38 cases (10%), mutation in 1 case (0.3%), and multiple alterations in 19 cases (5%). Thus, AMP is the most common type of *PRPF3* CNV in HCC.

*PRPF3* AMP results in the high expression level of *PRPF3* (Figure 5B). Compared with the diploid group, gain or amplification group has higher *PRPF3* expression levels (*p* < 0.001). Next, the frequency distribution of *PRPF3* CNV patients in different stage and grade groups was presented in Figure 5C, suggesting the high occurrence and an early-event of *PRPF3* CNV alteration in HCC. Moreover, *PRPF3* CNV alteration was significantly associated with the OS and DFS of HCC patients (Figure 5D, 5E). Based on five-years survival, median survival time of samples with *PRPF3* alteration was 29.97 months and 19.47 months for OS, and DFS respectively.

### Gene co-occurrence of PRPF3 alterations in HCC

**Figure 6:**
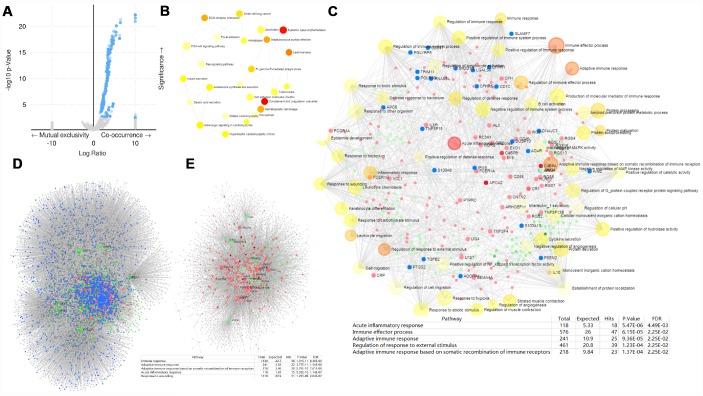
***PRPF3* CNV co-occurrence profiles in HCC.** (**A**) Volcano plot of co-occurrence genes along with *PRPF3* amplification (AMP). (**B**) KEGG pathway analysis of significantly PRPF3 co-occurrence genes. (**C**) GO_BP terms of significantly PRPF3 co-occurrence genes. (**D**) The liver-specific protein-protein interaction (PPI) network of significantly PRPF3 co-occurrence genes. (**E**) Transcription factor-miRNA (TF-miRNA) coregulatory network of significantly *PRPF3* co-occurrence genes.

Gene co-occurrence reflects common genetic risk factors constituting functional relationships, thus we examine the co-occurrence profiles with *PRPF3* AMP in HCC. More than one thousand (1,243) genes were shown having significant co-occurrence with *PRPF3* AMP (Supplementary Table 7). The most frequent alterations were Acidic Nuclear Phosphoprotein 32 Family Member E (*ANP32E*) (34.78%), Aph-1 Homolog A (*APH1A*) (34.78%), and Chromosome 1 Open Reading Frame 54 (*C1orf54*) (34.78%), etc. KEGG pathway analysis of co-occurrence genes showed enrichment in complement and coagulation cascades and systemic lupus erythematosus (Figure 6B). Analysis of significantly enriched GO terms indicated that these genes were primarily involved in acute inflammatory response, immune effector process, and adaptive immune response, etc (Figure 6C and Supplementary Table 8).

Further, the *PRPF3* co-occurrence derived protein-protein interaction (PPI) network was assembled based on liver-specific data collected from the DifferentialNet database [12] (Figure 6D). The top 3 hub genes were Ring Finger Protein 2 (*RNF2*), Myeloid Cell Nuclear Differentiation Antigen (*MNDA*), and Cullin 4A (*CUL4A*). The previous study indicated that loss of *RNF2* inhibited HCC cell growth and promoted apoptosis [13]. While *CUL4A* facilitates hepatocarcinogenesis by promoting cell cycle progression and epithelial-mesenchymal transition [14].

Finally, TF-miRNA coregulatory interactions of the PRPF3 co-occurrence genes was constructed based on the RegNetwork repository (Figure 6E) [15]. The top 3 TFs were Upstream Transcription Factor 1 (*USF1*), POU Class 2 Homeobox 1 (*POU2F1*), and Aryl Hydrocarbon Receptor Nuclear Translocator (*ARNT*). Generally, *USF1* acts as a positive transcription factor, which binds to the basal promoter thus ensuring gene expression in a wide range of tissues including liver [16]. *POU2F1* promotes growth and metastasis of HCC through the FAT Atypical Cadherin 1 (*FAT1*) signaling pathway [17]. Suppression of tumor cell invasion and migration was demonstrated in *ARNT*-silenced HCC cell lines. Silencing of *ARNT* induces anti-tumor effects in hepatoma cell lines under tumor hypoxia [18].

Whether for the liver-specific PPI network or the TF-miRNA coregulatory network, the function annotation implied that *PRPF3* AMP involves in the immune response and inflammatory response.

### PRPF3 is correlated with tumor purity and immune infiltration level in HCC

**Figure 7:**
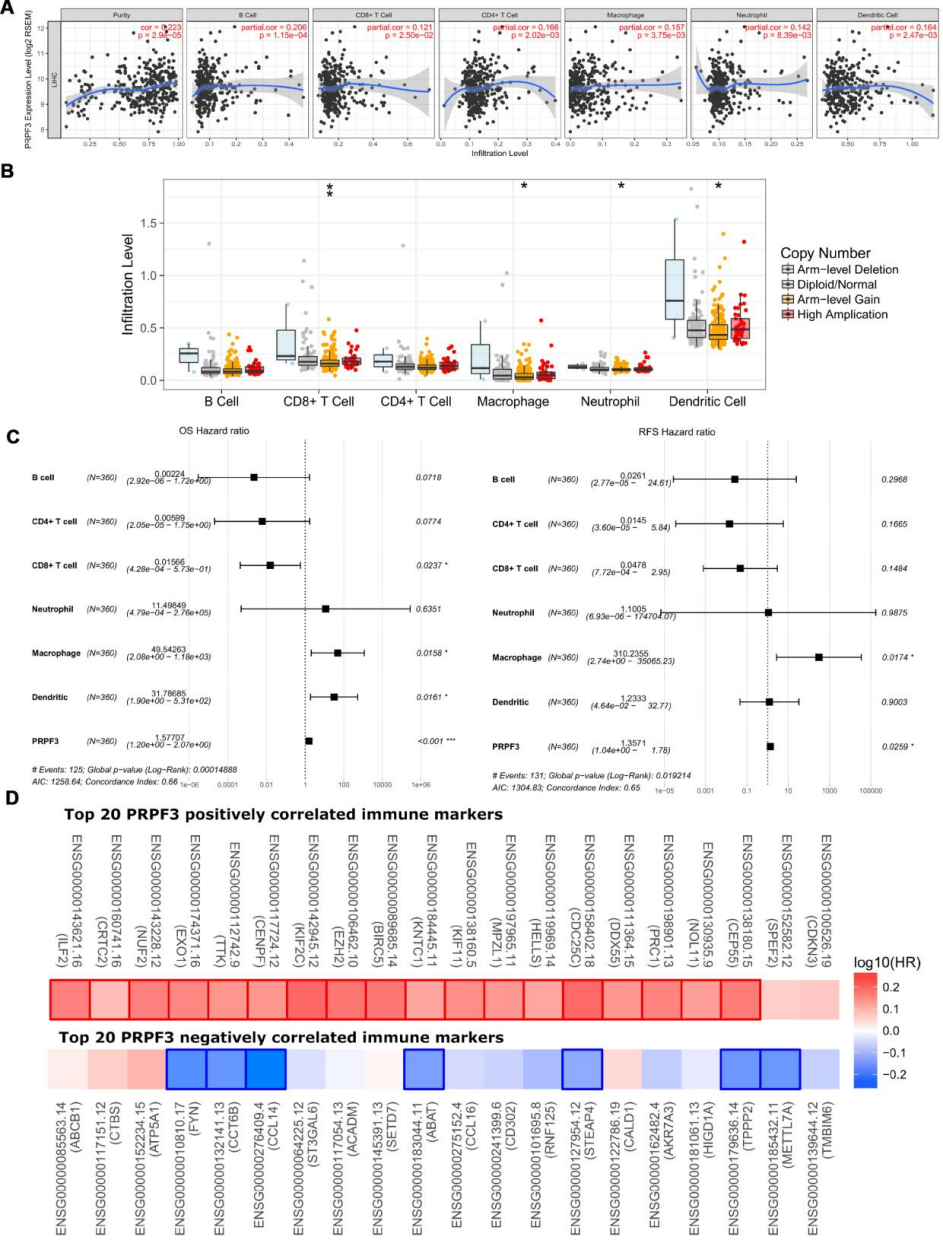
**Correlations of *PRPF3* expression with immune infiltration level in HCC.** (**A**) *PRPF3* expression is significantly related to tumor purity and has significant positive correlations with infiltrating levels of CD8+ T cells, CD4+ T cells, macrophages, neutrophils, and dendritic cells in LIHC. (**B**) *PRPF3* CNV affects the infiltrating levels of CD8+ T cells, macrophages, neutrophils, and dendritic cells in HCC. (**C**) Multivariable hazards models were used to evaluate the impacts of *PRPF3* expression on overall survival and disease-free survival in the presence of infiltrating levels of multiple immune cells. (**D**) Survival maps of top 20 *PRPF3* positively and negatively correlated immune markers in LIHC, respectively. *, *p* < 0.05, **, *p* < 0.01, ***, *p* < 0.001.

Therefore, we investigated whether *PRPF3* expression was correlated with immune infiltration levels in HCC from TIMER database. The results show that *PRPF3* expression has significant correlations with tumor purity (r = 0.223, *p* = 2.90E-05) and significant correlations with the dominant immune cells infiltration levels (Figure 7A). Particularly, *PRPF3* CNV has significant correlations with infiltrating levels of CD8+ T cells, macrophages, neutrophils, and dendritic cells (Figure 7B).

Moreover, multivariable hazards models were used to evaluate the impacts of *PRPF3* expression in the presence of varying immune cells. *PRPF3* had 1.57 times higher risks on OS (*p* < 0.001) and 1.36 times higher risks on DFS (*p* = 0.0259) (Figure 7C).

In addition, *PRPF3* co-occurrence genes with Log Ratio > 10 also showed the significant correlations with tumor purity and varying degree with immune cells (Supplementary Figure 3A). Similar to *PRPF3*, CNV of all these genes have significant correlations with infiltrating levels of CD8+ T cells, macrophages, neutrophils, and dendritic cells (Supplementary Figure 3B).

### PRPF3 expression is associated with immune signatures

Finally, to broaden the understanding of *PRPF3* crosstalk with immune genes, we analyzed the correlations between *PRPF3* expression and various immune signatures, which included immune marker genes of 28 tumor-infiltrating lymphocytes (TILs), immune inhibitory or stimulatory genes (including immune checkpoint gene sets), cytokine-related genes, cancer-testis antigen genes, and major histocompatibility complex (MHC) genes (Table 2 and Supplementary Table 9).

**Table 2:** Correlation analysis between PRPF3 and markers of activated T cells.

**Activated CD8 T cell**	**None**		**Purity**		**Activated CD4 T cell**	**None**		**Purity**	
	**Cor**	**P**	**Cor**	**P**		**Cor**	**P**	**Cor**	**P**
ADRM1	0.0434	4.17E-01	0.0510	3.42E-01	AIM2	0.0519	3.32E-01	0.1657	1.86E-03^*^
AHSA1	0.0464	3.86E-01	0.0498	3.53E-01	BIRC3	0.1174	2.78E-02^*^	0.1768	8.95E-04
C1GALT1C1	-0.0042	9.38E-01	0.0134	8.03E-01	BRIP1	0.1941	2.54E-04^*^	0.1915	3.13E-04^*^
CCT6B	-0.2883	3.82E-08^*^	-0.3117	2.53E-09^*^	CCL20	0.1877	4.06E-04^*^	0.2190	3.59E-05^*^
CD37	-0.0824	1.23E-01	0.0095	8.60E-01	CCL4	-0.1020	5.61E-02	-0.0259	6.29E-01
CD3D	0.0226	6.73E-01	0.1133	3.41E-02^*^	CCL5	-0.1204	2.41E-02^*^	-0.0516	3.35E-01
CD3E	-0.0931	8.14E-02	-0.0112	8.35E-01	CCNB1	0.365	1.67E-12^*^	0.3762	3.31E-13^*^
CD3G	-0.0688	1.99E-01	0.0126	8.14E-01	CCR7	-0.0941	7.82E-02	-0.0267	6.19E-01
CD69	-0.1511	4.56E-03^*^	-0.0892	9.56E-02	DUSP2	-0.0274	6.08E-01	0.043	4.23E-01
CD8A	-0.0993	6.32E-02	-0.0288	5.91E-01	ESCO2	0.3049	5.53E-09^*^	0.3218	7.13E-10^*^
CETN3	0.0194	7.17E-01	0.0206	7.01E-01	ETS1	-0.2332	1.02E-05^*^	-0.1945	2.52E-04^*^
CSE1L	0.2510	1.91E-06^*^	0.2442	3.78E-06^*^	EXO1	0.4795	1.41E-21^*^	0.4902	1.48E-22^*^
GEMIN6	0.0557	2.98E-01	0.0379	4.80E-01	EXOC6	0.2337	9.66E-06^*^	0.2355	8.47E-06^*^
GNLY	-0.0378	4.80E-01	-0.0003	9.95E-01	IARS	0.2431	4.09E-06^*^	0.2496	2.26E-06^*^
GPT2	-0.2661	4.22E-07^*^	-0.2872	4.52E-08^*^	ITK	-0.1523	4.23E-03^*^	-0.0884	9.87E-02
GZMA	-0.1467	5.90E-03^*^	-0.0849	1.13E-01	KIF11	0.4089	1.39E-15^*^	0.4189	2.64E-16^*^
GZMH	-0.1656	1.85E-03^*^	-0.1192	2.57E-02^*^	KNTC1	0.4255	7.19E-17^*^	0.4308	3.02E-17^*^
GZMK	-0.1558	3.44E-03^*^	-0.0950	7.60E-02	NUF2	0.5126	6.60E-25^*^	0.5176	2.25E-25^*^
IL2RB	-0.1291	1.55E-02^*^	-0.0558	2.98E-01	PRC1	0.3915	2.66E-14^*^	0.3994	7.75E-15^*^
LCK	-0.0842	1.15E-01	0.0036	9.47E-01	PSAT1	-0.0417	4.37E-01	-0.0360	5.02E-01
MPZL1	0.3774	2.51E-13^*^	0.4119	9.18E-16^*^	RGS1	0.0037	9.45E-01	0.0929	8.27E-02
NKG7	-0.1198	2.47E-02^*^	-0.0651	2.25E-01	RTKN2	0.3646	1.79E-12^*^	0.3849	8.36E-14^*^
PIK3IP1	-0.1298	1.49E-02^*^	-0.0972	6.93E-02	SAMSN1	-0.1064	4.64E-02^*^	-0.0189	7.25E-01
PTRH2	0.1665	1.74E-03^*^	0.1594	2.78E-03^*^	SELL	-0.0732	1.71E-01	-0.0034	9.50E-01
TIMM13	-0.0588	2.72E-01	-0.0703	1.89E-01	TRAT1	-0.1266	1.77E-02^*^	-0.0603	2.60E-01
ZAP70	-0.0428	4.24E-01	0.0398	4.58E-01					

Cor, R value of Spearman’s correlation; None, correlation without adjustment. Purity, correlation adjusted by purity. ^*^ *p* < 0.05.

After the correlation adjustment by tumor purity, the results revealed the *PRPF3* expression level was significantly correlated with most immune marker sets of various immune cells in LIHC. Table 2 showed the examples of the purity-corrected partial Spearman’s correlation between *PRPF3* and marker genes of activated T cells. In activated CD8 T cells, *PRPF3* is highly correlated with Myelin Protein Zero Like 1 (*MPZL1*). Indeed, AMP of *MPZL1* promotes tumor cell migration through Src-mediated phosphorylation of cortactin in HCC [19]. For activated CD4 T cells, *PRPF3* is significantly correlated with NUF2 Component Of NDC80 Kinetochore Complex (*NUF2*), which was suggested as a valuable prognostic biomarker to predict early recurrence of HCC [20]. Dendritic cell (DC) markers such as TTK Protein Kinase (*TTK*), Kinesin Family Member 2C (*KIF2C*), Centrosomal Protein 55 (*CEP55*), Sperm Flagellar 2 (*SPEF2*), Opa Interacting Protein 5 (*OIP5*), and Tubulin Polymerization Promoting Protein Family Member 2 (*TPPP2*), etc., were also shown significant correlations with PRPF3 expression.

We also found significant correlations between *PRPF3* and marker genes of Treg and myeloid-derived suppressor cell (MDSC), such as Methyltransferase Like 7A (*METTL7A*), Adenosine Deaminase TRNA Specific 2 (*ADAT2*), LDL Receptor Related Protein 1 (*LRP1*), Lysosomal Protein Transmembrane 4 Beta (*LAPTM4B*), Nuclear Factor Erythroid 2-Related Factor 3 (*NFE2L3*), Leucine Rich Repeat Containing 42 (*LRRC42*), CD14, Suppressor Of Cytokine Signaling 2 (*SOCS2*), Hydroxysteroid Dehydrogenase Like 2 (*HSDL2*), and Ankyrin Repeat Domain 10 (*ANKRD10*). Interestingly, LAPTM4B decreases Transforming Growth Factor Beta 1 (*TGF-β1*) production in human Treg cells [21]. A recent study identified the existence of a monocytic subset of MDSCs with the CD14^+^HLA-DR^−^/^low^ phenotype that suppresses the proliferation of T cells [22]. The frequency of CD14^+^HLA-DR^−^/^low^ MDSCs was significantly higher in HCC patients [23].

In immunoinhibitory genes, results showed the expression levels of Cytotoxic T-Lymphocyte Associated Protein 4 (*CTLA4*) and Programmed Cell Death 1 (*PD-1*), and Programmed Cell Death 1 Ligand 2 (*PD-L2*) have positive or negative correlations with *PRPF3* expression, respectively, while TNF Superfamily Member 4 (*TNFSF4*), Inducible T Cell Costimulator Ligand (*ICOSLG*), TNF Superfamily Member 9 (*TNFSF9*), etc., have correlations with *PRPF3* expression in immunostimulator genes. Specifically, we showed chemokine (C-C motif) ligand (CCL)-16, *CCL14*, Interleukin 12A (*IL12A*), *CCL20*, *CCL26*, C-X3-C Motif Chemokine Ligand 1 (*CX3CL1*), *CCL27*, and CD19 Molecule (*CD19*) were significantly correlated with *PRPF3* expression (*p* < 0.0001). Overexpression of the cancer-testis (CT) antigens represents the advanced disease of cancer. High *PRPF3* expression relates to high induction of cancer-testis antigen genes in LIHC, such as the significant positive correlation between *PRPF3* and *NUF2*, *TTK*, *KIF2C*, *CEP55*, *SPEF2*, and *OIP5*, etc.

Generally, the top 5 markers positively correlated with *PRPF3* were Interleukin Enhancer Binding Factor 2 (*ILF2*), CREB Regulated Transcription Coactivator 2 (*CRTC2*), *NUF2*, Exonuclease 1 (*EXO1)*, and *TTK*. And the top 5 markers negatively correlated with *PRPF3* were Transmembrane BAX Inhibitor Motif Containing 6 (*TMBIM6*), *METTL7A*, Tubulin Polymerization Promoting Protein Family Member 2 (*TPPP2*), HIG1 Hypoxia Inducible Domain Family Member 1A (*HIGD1A*), and Aldo-Keto Reductase Family 7 Member A3 (*AKR7A3*). Survival map analysis clearly demonstrated the high risk of *PRPF3* positively correlated marker genes and the low risk of *PRPF3* negatively correlated marker genes (Figure 7D). Therefore, these results further confirm the findings that *PRPF3* is specifically correlated with immune infiltrating cells in HCC, which suggests that *PRPF3* plays a vital role in immune escape in the tumor microenvironment.

## DISCUSSION

Splicing, a key step in gene expression enabling individual genes to encode multiple proteins, is emerging as a major driver of abnormal phenotypic heterogeneity. And it is expected splicing as a potential major source of untapped molecular targets in precision oncology and cancer disparities [24]. *PRPF3*, a core component of the spliceosome complex, is involved in multiple steps of transcription. To gain more detailed insights into the potential functions of *PRPF3* in HCC and its regulatory network, we performed the bioinformatics analysis of public data to guide future research in HCC.

Analysis of transcriptome from more than 3,400 clinical samples comprising six geographic regions and ethnic HCC studies confirmed that *PRPF3* mRNA levels and CNVs are significantly higher in HCC than in normal liver tissue (Figure 1). In addition, high expression of *PRPF3* was significantly related to poor survival and disease-free state in multiple cohorts. Thus, our results suggest that *PRPF3* up-regulation occurs in many cases of HCC and deserves further clinical validation as a potential diagnostic and prognostic marker.

For mining regulators potentially responsible for *PRPF3* dysregulation, we found that *PRPF3* in HCC is associated with a network of kinases including *CDK1*, *PLK1*, *AURKB*, *CHEK1*, and *CDK2*. These kinases regulate genomic stability, mitosis, and the cell cycle, and showed differential expression and survival prognosis in LIHC. In fact, *CDK1* participates in the regulation of mitosis, self-renewal, differentiation, and somatic reprogramming. Various inhibitors of *CDK1*, have been developed, and some have entered phase I and II clinical trials for the treatment of a variety of solid tumors and hematologic malignancies [25]. As a key driver gene, a causal link has recently been established between *PLK1* and hepatocarcinogenesis [26]. In HCC, *PRPF3* may regulate DNA replication, repair, and cell cycle progression via interacted kinases.

Next, the *E2F* family constitute the main transcription factors for *PRPF3* dysregulation. *E2F1* is one of the key links in the cell cycle regulation network. Activated *E2F* oncogenic signaling was always seen in the progression of liver cancer, and studies have shown that dosage-dependent copy number gains in *E2F1* and *E2F3* drive HCC [11]. Our results suggest that *E2F1* is an important regulator of *PRPF3* and that *PRPF3* might act through this factor to regulate the cell cycle and proliferation capacity of HCC. Further studies are needed to test this hypothesis. Our study identified no miRNAs that were significantly associated with *PRPF3*, possibly due to the role of *PRPF3* involving in mRNA splicesome, and keeping away from miRNA cellular machinery.

To probe the signaling events in controlling abnormal *PRPF3* expression, we tested the *PRPF3* co-expression network. Our results suggest that the functional consequence of *PRPF3* mainly include spliceosome, DNA repair, DNA replication, and cell cycle, while it inhibits the metabolic processes, such as fatty acid, lipid, antibiotic, nucleoside bisphosphate, and cellular modified amino acid metabolic process. These findings are consistent with the molecular pathways implicated in HCC carcinogenesis [27].

A recent study found that genomic alteration, such as somatic mutations in the genes encoding components of the spliceosome, occurs frequently in human neoplasms [28]. CNVs can have major genomic implications, such as disrupting genes, altering genetic content, and lead to phenotypic differences. Our study found that the copy number of *PRPF3* was increased in HCC and that the major type of *PRPF3* alteration was AMP, which was associated with shorter survival.

The tumor microenvironment is the non-cancerous cells present in and around a tumor, having a strong influence on the genomic analysis of tumor samples [29]. Since gene dynamics are known to influence belowground genetic diversity and microenvironment processes, co-occurrence analysis was performed. Most co-occurrence genes with *PRPF3* CNV were distributed in 1q21 locus. Further, a gene-level network representing the co-occurrence of genes across HCC genomes was built, which gives the clues of *PRPF3* role in regulating the immune response. Herein, by tumor purity analysis, the network of *PRPF3* alterations is involved in the tumor purity and tumor immunity. Our findings provide a detailed characterization of the association between *PRPF3* and immune marker sets in LIHC patients. Further studies need to be done to elucidate whether *PRPF3* is a crucial factor in mediating T-cell therapy.

In conclusion, this study provides multi-level evidence for the importance of *PRPF3* in hepatocarcinogenesis and its potential as a biomarker in HCC. Our results suggest that *PRPF3* up-regulation in HCC may likely have far-reaching effects in RNA splicing and genomic stability, and at multiple steps of the cell cycle. Further, our results suggest a potential novel immune regulatory role of *PRPF3* in tumor immunity. These findings call for large-scale HCC genomics research and subsequent functional studies.

## MATERIALS AND METHODS

### Databases description

### HCCDB database analysis

HCCDB is a database of HCC expression atlas containing 15 public HCC gene expression datasets containing totally 3917 samples [30], including the data from the Gene Expression Omnibus (GEO), Liver Hepatocellular Carcinoma Project of The Cancer Genome Atlas (TCGA-LIHC) and Liver Cancer - RIKEN, JP Project from International Cancer Genome Consortium (ICGC LIRI-JP). HCCDB provides the visualization for the results from several computational analyses, such as differential expression analysis, tissue-specific and tumor-specific expression analysis.

### Oncomine database analysis

The expression level of the *PRPF3* gene in liver cancers was examined in the Oncomine 4.5 database (https://www.oncomine.org/). Oncomine is a cancer microarray database and web-based data-mining platform. The threshold was determined according to the following values: *p*-value of 0.05, fold change of 1.5, and gene ranking of all.

### UALCAN database analysis

UALCAN (http://ualcan.path.uab.edu) uses TCGA level 3 RNA-seq and clinical data from 31 cancer types [31], allowing analysis of relative expression of genes across tumor and normal samples, as well as in various tumor sub-groups based on individual cancer stages, tumor grade or other clinicopathological features.

### GEPIA database analysis

The Gene Expression Profiling Interactive Analysis (GEPIA) database (http://gepia.cancer-pku.cn/) is an interactive web that includes 9,736 tumors and 8,587 normal samples from TCGA and the GTEx projects [32]. GEPIA was used to generate survival curves, including overall survival (OS) and recurrence-free survival (RFS), based on gene expression with the log-rank test and the Mantel-Cox test in liver cancer.

### c-BioPortal database analysis

The cBio Cancer Genomics Portal (http://cbioportal.org) has multidimensional cancer genomics data sets [33]. Mutation, copy number variation (CNV), and gene co-occurrence of PRPF3 in HCC were analyzed using the c-BioPortal tool. The tab OncoPrint displays an overview of genetic alterations per sample in *PRPF3*.

### LinkedOmics Database Analysis

The LinkedOmics database (http://www.linkedomics. org/login.php) is a web-based platform for analyzing 32 TCGA cancer-associated multi-dimensional datasets [34]. *PRPF3* co-expression was analyzed statistically using Pearson’s correlation coefficient, presenting in volcano plots, heat maps, or scatter plots. Function module of LinkedOmics performs analysis of Gene Ontology biological process (GO_BP), KEGG pathways, kinase-target enrichment, miRNA-target enrichment and transcription factor-target enrichment by the gene set enrichment analysis (GSEA). The rank criterion was FDR < 0.05 and 1000 simulations were performed.

### Networkanalyst database analysis

Network interpreting gene expression was used by NetworkAnalyst 3.0 tool (https://www.network analyst.ca/) [35], which integrates cell-type or tissue-specific protein-protein interaction (PPI) networks, gene regulatory networks, and gene co-expression networks. Function enrichment was based on a similar concept introduced by ClueGO and EnrichmentMap [36].

### TIMER database analysis

TIMER is a comprehensive resource for systematic analysis of immune infiltrates across diverse cancer types from TCGA (https://cistrome.shinyapps.io/timer/), which includes 10,897 samples across 32 cancer types [37]. TIMER applies a deconvolution method [38] to infer the abundance of tumor-infiltrating immune cells (TIICs) from gene expression profiles. We analyzed *PRPF3* expression in LIHC and the correlation of *PRPF3* expression with the abundance of immune infiltrates, including B cells, CD4+ T cells, CD8+ T cells, neutrophils, macrophages, and dendritic cells, as well as the tumor purity.

### Tumor immunology analysis

Tumor purity was estimated using ESTIMATE and a consensus approach, as previously described [29, 39]. ESTIMATE used the single-sample gene-set enrichment analysis (ssGSEA) score to quantify the enrichment levels of immune signatures in tumor.

Further, gene signatures of 28 tumor-infiltrating lymphocytes (TILs) [40], comprising Activated CD8 T cell, Central memory CD8 T cell, Effector memory CD8 T cell, Activated CD4 T cell, Central memory CD4 T cell, Effector memory CD4 T cell, T follicular helper cell, Gamma delta T cell, Type 1 T helper cell, Type 17 T helper cell, Type 2 T helper cell, Regulatory T cell, Activated B cell, Immature B cell, Memory B cell, Natural killer cell (NK), CD56^bright^ NK, CD56^dim^ NK, Myeloid-derived suppressor cell (MDSC), Natural killer T cell (NKT), Activated dendritic cell, Plasmacytoid dendritic cell, Immature dendritic cell, Macrophage, Eosinophil, Mast cell, Monocyte, Neutrophil, Tumor-associated macrophage (TAM), M1 Macrophage, and M2 Macrophage, as well as markers from multiple types of oncoimmunology containing genes associated with immunomodulators and chemokines, were referenced in prior studies [41].

### Statistical analysis

The *t*-test *p* < 0.05 was utilized to determine the statistical significance between groups with different expression level of *PRPF3*. We compared the survival (overall survival (OS) and recurrence-free survival (RFS)) of HCC patients separated by the median expression level of specific genes. Kaplan-Meier curves were used to compare the survival time differences. The log-rank test *p* < 0.05 indicates the significance of survival time differences. The survival analyses were performed by R programming of “survival” and “survminer” package.

We calculated the correlation between *PRPF3* and immune signature score or gene expression levels using the Spearman or partial Spearman method. Tumor purity-corrected partial Spearman’s correlation calculated the correlation between *PRPF3* expression and immune genes while controlling for tumor purity, which was explored using ppcor package [42]. The threshold of *p* < 0.05 indicates the significance of correlation.

## Supplementary Material

Supplementary Figures

Supplementary Table 1

Supplementary Table 2

Supplementary Table 3

Supplementary Table 4

Supplementary Table 5

Supplementary Table 6

Supplementary Table 7

Supplementary Table 8

Supplementary Table 9
