# Supplementary material for: Prognostic potential of PRPF3 in hepatocellular carcinoma
Source: Aging (Albany NY). 2020 Jan 11;12(1):912–30. doi: 10.18632/aging.102665 (PMC6977647; doi:10.18632/aging.102665)
Supplement: Supplementary Figures [file aging-12-102665-s007..pdf]

SUPPLEMENTARY FIGURES

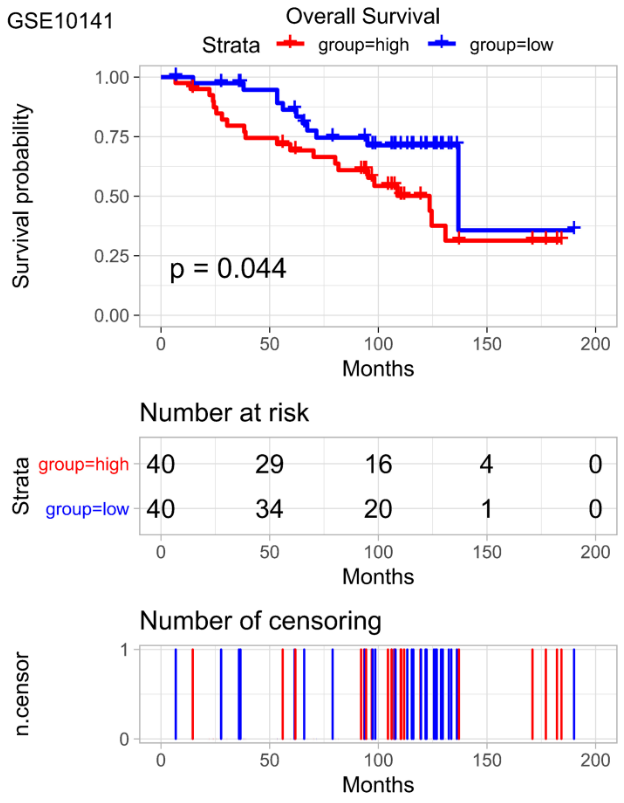

Supplementary Figure 1. High expression of PRPF3 is associated with advanced outcome in GSE10141 cohort.

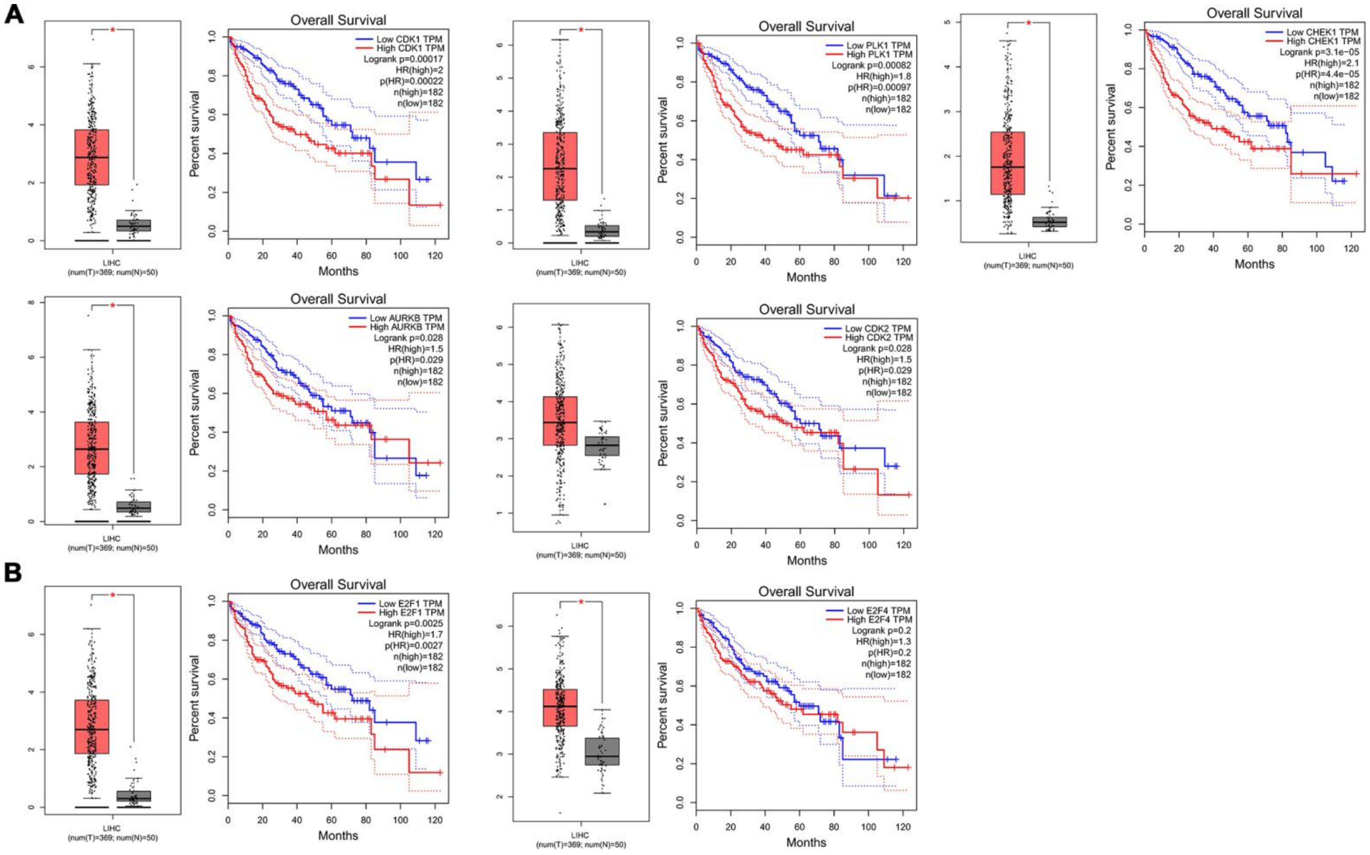

Supplementary Figure 2. Expression and survival outcome of PRPF3-related regulators. (**A**) Top 5 kinase regulators of PRPF3 co-expressed genes. All of these kinase genes, except CDK2, were significantly highly expressed in tumor tissues. All these genes have significant association with OS of HCC. (**B**) E2F regulators of PRPF3 co-expressed genes.

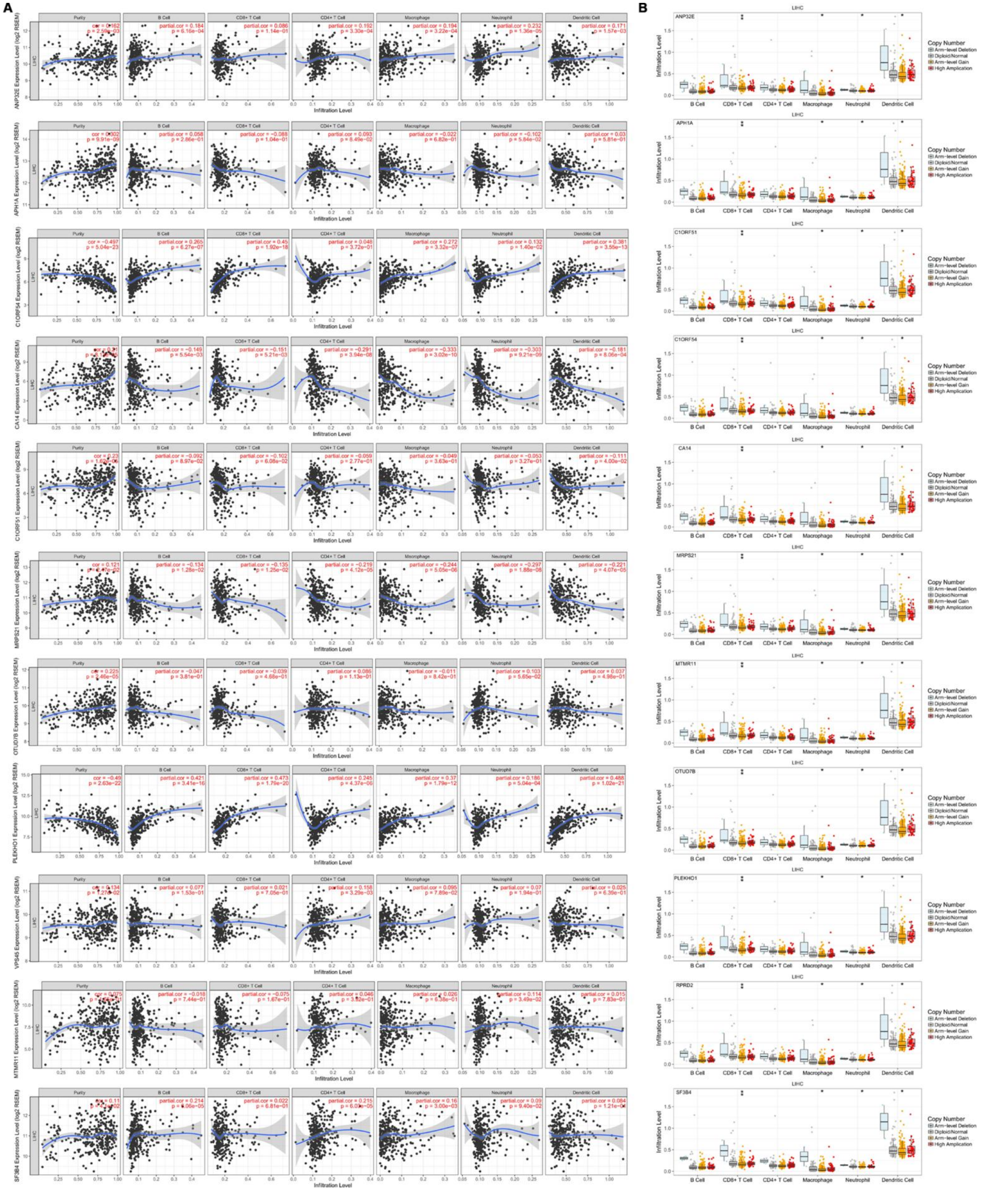

Supplementary Figure 3. Immune infiltration of top PRPF3 co-occurrence genes (Log Ratio > 10). (**A**) Tumor purity and immune infiltration levels. (**B**) CNV affecting the distribution in various immune cells.
