# Supplementary material for: Prognostic potential of PRPF3 in hepatocellular carcinoma
Source: Aging (Albany NY). 2020 Jan 11;12(1):912–30. doi: 10.18632/aging.102665 (PMC6977647; doi:10.18632/aging.102665)
Supplement: Supplementary Table 1 [file aging-12-102665-s010..pdf]

Supplementary Table 1. PRPF3 co-expressed genes.

Query	Correlation coefficient	P-value	FDR (BH)
A1BG	-0.2062	6.30E-05	4.11E-04
A1CF	-0.0253	6.27E-01	7.35E-01
A2BP1	0.1980	1.23E-04	7.30E-04
A2LD1	-0.0994	5.57E-02	1.17E-01
A2ML1	-0.0779	1.34E-01	2.33E-01
A2M	-0.0751	1.49E-01	2.52E-01
A4GALT	0.0087	8.67E-01	9.15E-01
A4GNT	0.2223	1.54E-05	1.23E-04
AAA1	0.1911	2.13E-04	1.16E-03
AAAS	0.1507	3.61E-03	1.26E-02
AACSL	0.0331	5.25E-01	6.48E-01
AACS	0.1716	9.01E-04	3.93E-03
AADACL2	0.0535	3.04E-01	4.33E-01
AADACL3	0.1340	9.79E-03	2.87E-02
AADACL4	0.0328	5.29E-01	6.52E-01
AADAC	-0.1774	5.98E-04	2.76E-03
AADAT	-0.2116	3.97E-05	2.78E-04
AAGAB	0.0314	5.47E-01	6.67E-01
AAK1	0.0727	1.62E-01	2.69E-01
AAMP	0.0017	9.74E-01	9.83E-01
AANAT	0.0377	4.69E-01	5.98E-01
AARS2	0.1865	3.04E-04	1.58E-03
AARSD1	0.1626	1.68E-03	6.65E-03
AARS	-0.0934	7.22E-02	1.44E-01
AASDHPPT	-0.1122	3.07E-02	7.28E-02
AASDH	0.0452	3.85E-01	5.19E-01
AASS	-0.3009	3.35E-09	6.99E-08
AATF	0.3318	5.56E-11	1.73E-09
AATK	0.0666	2.01E-01	3.17E-01
ABAT	-0.2506	1.01E-06	1.15E-05
ABCA10	0.0398	4.45E-01	5.76E-01
ABCA11P	0.2300	7.62E-06	6.61E-05
ABCA12	0.1000	5.43E-02	1.15E-01
ABCA13	-0.0074	8.88E-01	9.28E-01
ABCA17P	0.0949	6.78E-02	1.37E-01
ABCA1	-0.0283	5.87E-01	7.02E-01
ABCA2	0.0195	7.08E-01	8.00E-01
ABCA3	0.0124	8.12E-01	8.76E-01
ABCA4	0.1115	3.18E-02	7.49E-02
ABCA5	0.0448	3.89E-01	5.22E-01
ABCA6	-0.2189	2.10E-05	1.61E-04
ABCA7	-0.0075	8.86E-01	9.27E-01

ABCA8	-0.3029	2.60E-09	5.54E-08
ABCA9	-0.2432	2.13E-06	2.20E-05
ABCB10	0.0886	8.84E-02	1.68E-01
ABCB11	-0.1550	2.75E-03	1.01E-02
ABCB1	-0.2567	5.36E-07	6.51E-06
ABCB4	-0.2676	1.67E-07	2.31E-06
ABCB5	0.1343	9.62E-03	2.83E-02
ABCB6	0.0672	1.96E-01	3.12E-01
ABCB7	-0.0121	8.17E-01	8.80E-01
ABCB8	-0.2210	1.74E-05	1.36E-04
ABCB9	0.0914	7.87E-02	1.54E-01
ABCC10	0.2186	2.16E-05	1.65E-04
ABCC11	-0.1083	3.70E-02	8.47E-02
ABCC12	0.0631	2.25E-01	3.47E-01
ABCC13	0.1093	3.54E-02	8.17E-02
ABCC1	0.1049	4.35E-02	9.65E-02
ABCC2	-0.1057	4.19E-02	9.35E-02
ABCC3	0.0208	6.90E-01	7.86E-01
ABCC4	0.0857	9.92E-02	1.85E-01
ABCC5	0.2116	3.99E-05	2.79E-04
ABCC6P1	-0.0726	1.63E-01	2.70E-01
ABCC6P2	0.0571	2.73E-01	4.00E-01
ABCC6	-0.2056	6.64E-05	4.30E-04
ABCC8	0.0746	1.51E-01	2.56E-01
ABCC9	-0.2356	4.48E-06	4.23E-05
ABCD1	0.2686	1.50E-07	2.10E-06
ABCD2	-0.0691	1.84E-01	2.97E-01
ABCD3	-0.1206	2.01E-02	5.16E-02
ABCD4	-0.1696	1.04E-03	4.44E-03
ABCE1	-0.1060	4.12E-02	9.23E-02
ABCF1	0.2019	8.99E-05	5.58E-04
ABCF2	0.0583	2.62E-01	3.89E-01
ABCF3	0.0223	6.69E-01	7.69E-01
ABCG1	0.0331	5.24E-01	6.48E-01
ABCG2	-0.2375	3.75E-06	3.62E-05
ABCG4	-0.0964	6.37E-02	1.30E-01
ABCG5	-0.0727	1.62E-01	2.69E-01
ABCG8	-0.1036	4.62E-02	1.01E-01
ABHD10	-0.2392	3.17E-06	3.12E-05
ABHD11	0.0659	2.05E-01	3.23E-01
ABHD12B	0.0132	8.00E-01	8.67E-01
ABHD12	0.0082	8.74E-01	9.19E-01
ABHD13	0.0159	7.61E-01	8.39E-01
ABHD14A	-0.0834	1.09E-01	1.99E-01

ABHD14B	-0.3268	1.11E-10	3.22E-09
ABHD15	-0.1029	4.77E-02	1.04E-01
ABHD1	-0.1437	5.57E-03	1.80E-02
ABHD2	-0.3138	6.37E-10	1.57E-08
ABHD3	0.1665	1.29E-03	5.33E-03
ABHD4	-0.0076	8.84E-01	9.25E-01
ABHD5	-0.0544	2.96E-01	4.26E-01
ABHD6	-0.3342	3.93E-11	1.26E-09
ABHD8	-0.0784	1.32E-01	2.29E-01
ABI1	0.0794	1.27E-01	2.23E-01
ABI2	0.1956	1.49E-04	8.60E-04
ABI3BP	-0.1962	1.43E-04	8.31E-04
ABI3	-0.0802	1.23E-01	2.18E-01
ABL1	0.1456	4.95E-03	1.63E-02
ABL2	0.3363	2.91E-11	9.57E-10
ABLIM1	-0.0914	7.87E-02	1.54E-01
ABLIM2	0.0035	9.46E-01	9.67E-01
ABLIM3	-0.2304	7.34E-06	6.42E-05
ABO	-0.0807	1.21E-01	2.15E-01
ABP1	-0.1396	7.08E-03	2.20E-02
ABRA	0.0075	8.85E-01	9.26E-01
ABR	-0.0189	7.17E-01	8.07E-01
ABT1	0.1352	9.13E-03	2.72E-02
ABTB1	0.0452	3.86E-01	5.19E-01
ABTB2	-0.1183	2.27E-02	5.70E-02
ACAA1	-0.3904	5.85E-15	3.97E-13
ACAA2	-0.3872	1.03E-14	6.65E-13
ACACA	0.1262	1.50E-02	4.07E-02
ACACB	-0.1840	3.67E-04	1.85E-03
ACAD10	-0.3484	5.03E-12	1.95E-10
ACAD11	-0.3528	2.59E-12	1.06E-10
ACAD8	-0.2376	3.69E-06	3.57E-05
ACAD9	-0.1064	4.06E-02	9.12E-02
ACADL	-0.1950	1.56E-04	8.93E-04
ACADM	-0.2841	2.55E-08	4.27E-07
ACADSB	-0.2121	3.83E-05	2.70E-04
ACADS	-0.2777	5.40E-08	8.34E-07
ACADVL	-0.2440	1.98E-06	2.08E-05
ACAN	0.1207	2.00E-02	5.14E-02
ACAP1	-0.0480	3.56E-01	4.88E-01
ACAP2	0.0536	3.03E-01	4.33E-01
ACAP3	-0.0042	9.35E-01	9.60E-01
ACAT1	-0.3428	1.15E-11	4.05E-10
ACAT2	-0.0270	6.04E-01	7.16E-01

ACBD3	0.1521	3.31E-03	1.17E-02
ACBD4	-0.2786	4.86E-08	7.60E-07
ACBD5	-0.2379	3.59E-06	3.49E-05
ACBD6	0.4344	1.66E-18	2.75E-16
ACBD7	0.1259	1.53E-02	4.13E-02
ACCN1	-0.1512	3.51E-03	1.23E-02
ACCN2	0.0949	6.79E-02	1.37E-01
ACCN3	0.1547	2.81E-03	1.03E-02
ACCN4	0.2562	5.65E-07	6.79E-06
ACCN5	0.0516	3.22E-01	4.52E-01
ACCSL	-0.0527	3.12E-01	4.41E-01
ACCS	-0.0799	1.24E-01	2.20E-01
ACD	0.2207	1.79E-05	1.40E-04
ACE2	-0.1518	3.39E-03	1.20E-02
ACER1	-0.1478	4.33E-03	1.47E-02
ACER2	-0.1252	1.58E-02	4.24E-02
ACER3	0.0556	2.86E-01	4.14E-01
ACE	-0.0141	7.86E-01	8.58E-01
ACHE	-0.0070	8.94E-01	9.32E-01
ACIN1	0.2849	2.34E-08	3.96E-07
ACLY	0.0684	1.89E-01	3.02E-01
ACMSD	-0.1791	5.29E-04	2.49E-03
ACN9	-0.1049	4.34E-02	9.63E-02
ACO1	-0.2013	9.46E-05	5.81E-04
ACO2	-0.1660	1.34E-03	5.49E-03
ACOT11	0.1627	1.67E-03	6.62E-03
ACOT12	-0.3069	1.55E-09	3.54E-08
ACOT13	-0.2130	3.54E-05	2.53E-04
ACOT1	-0.3059	1.79E-09	4.02E-08
ACOT2	-0.3293	7.84E-11	2.35E-09
ACOT4	-0.1579	2.29E-03	8.61E-03
ACOT6	-0.1952	1.55E-04	8.86E-04
ACOT7	0.1171	2.41E-02	5.97E-02
ACOT8	-0.0271	6.03E-01	7.15E-01
ACOT9	0.0159	7.60E-01	8.39E-01
ACOX1	-0.2756	6.83E-08	1.03E-06
ACOX2	-0.1809	4.62E-04	2.23E-03
ACOX3	-0.1257	1.54E-02	4.15E-02
ACOXL	0.1696	1.04E-03	4.44E-03
ACP1	0.0341	5.13E-01	6.37E-01
ACP2	-0.1062	4.08E-02	9.17E-02
ACP5	-0.0229	6.60E-01	7.61E-01
ACP6	0.3106	9.70E-10	2.30E-08
ACPL2	0.1421	6.10E-03	1.94E-02

ACPP	0.0169	7.46E-01	8.29E-01
ACPT	0.1910	2.15E-04	1.17E-03
ACRBP	0.0543	2.97E-01	4.26E-01
ACRC	0.0939	7.10E-02	1.42E-01
ACRV1	0.3177	3.83E-10	1.00E-08
ACR	-0.1441	5.44E-03	1.77E-02
ACSBG1	-0.1060	4.14E-02	9.26E-02
ACSBG2	0.1282	1.34E-02	3.73E-02
ACSF2	-0.1341	9.74E-03	2.86E-02
ACSF3	-0.2665	1.88E-07	2.57E-06
ACSL1	-0.2719	1.04E-07	1.50E-06
ACSL3	0.0259	6.18E-01	7.28E-01
ACSL4	0.0994	5.58E-02	1.17E-01
ACSL5	-0.1638	1.55E-03	6.22E-03
ACSL6	-0.1444	5.33E-03	1.74E-02
ACSM1	0.0162	7.56E-01	8.36E-01
ACSM2A	-0.2305	7.28E-06	6.38E-05
ACSM2B	-0.1759	6.67E-04	3.04E-03
ACSM3	-0.2518	9.00E-07	1.03E-05
ACSM4	0.0356	4.94E-01	6.21E-01
ACSM5	-0.2545	6.78E-07	8.04E-06
ACSS1	0.0995	5.55E-02	1.17E-01
ACSS2	-0.2130	3.53E-05	2.52E-04
ACSS3	-0.2916	1.05E-08	1.95E-07
ACTA1	0.1983	1.20E-04	7.15E-04
ACTA2	-0.1501	3.76E-03	1.31E-02
ACTBL2	0.0698	1.80E-01	2.92E-01
ACTB	-0.0250	6.31E-01	7.38E-01
ACTC1	-0.1218	1.89E-02	4.91E-02
ACTG1	0.1284	1.33E-02	3.71E-02
ACTG2	-0.0236	6.51E-01	7.54E-01
ACTL6A	0.3711	1.48E-13	7.71E-12
ACTL6B	0.0264	6.13E-01	7.23E-01
ACTL7A	0.0643	2.17E-01	3.37E-01
ACTL7B	0.0888	8.76E-02	1.67E-01
ACTL8	0.0228	6.62E-01	7.63E-01
ACTL9	-0.0996	5.53E-02	1.16E-01
ACTN1	-0.1265	1.48E-02	4.02E-02
ACTN2	0.1291	1.28E-02	3.58E-02
ACTN3	0.0767	1.40E-01	2.41E-01
ACTN4	0.0856	9.98E-02	1.86E-01
ACTR10	0.0659	2.06E-01	3.23E-01
ACTR1A	-0.1946	1.62E-04	9.19E-04
ACTR1B	-0.1297	1.24E-02	3.49E-02

ACTR2	-0.0337	5.17E-01	6.42E-01
ACTR3B	-0.0874	9.29E-02	1.75E-01
ACTR3C	-0.2186	2.16E-05	1.65E-04
ACTR3	0.1038	4.57E-02	1.00E-01
ACTR5	0.2309	7.00E-06	6.18E-05
ACTR6	0.1292	1.27E-02	3.56E-02
ACTR8	0.0274	5.99E-01	7.12E-01
ACTRT1	0.1489	4.05E-03	1.39E-02
ACVR1B	-0.0166	7.50E-01	8.32E-01
ACVR1C	-0.1653	1.40E-03	5.71E-03
ACVR1	0.0582	2.64E-01	3.91E-01
ACVR2A	0.0297	5.69E-01	6.86E-01
ACVR2B	0.2353	4.60E-06	4.33E-05
ACVRL1	-0.0841	1.06E-01	1.94E-01
ACY1	-0.4076	2.80E-16	2.54E-14
ACY3	-0.1958	1.47E-04	8.51E-04
ACYP1	0.3067	1.60E-09	3.63E-08
ACYP2	-0.3425	1.19E-11	4.17E-10
ADAD1	0.0513	3.24E-01	4.54E-01
ADAD2	-0.0036	9.46E-01	9.66E-01
ADAL	-0.1837	3.76E-04	1.88E-03
ADAM10	-0.0027	9.59E-01	9.74E-01
ADAM11	0.1988	1.16E-04	6.92E-04
ADAM12	0.0516	3.21E-01	4.52E-01
ADAM15	0.2400	2.93E-06	2.90E-05
ADAM17	0.0852	1.02E-01	1.88E-01
ADAM18	0.1031	4.71E-02	1.03E-01
ADAM19	0.0122	8.15E-01	8.79E-01
ADAM20	0.0161	7.58E-01	8.37E-01
ADAM21P1	-0.0859	9.85E-02	1.84E-01
ADAM21	-0.0411	4.30E-01	5.62E-01
ADAM22	0.1108	3.29E-02	7.70E-02
ADAM23	0.1262	1.50E-02	4.07E-02
ADAM28	0.0325	5.33E-01	6.55E-01
ADAM29	0.0020	9.70E-01	9.81E-01
ADAM2	0.0471	3.66E-01	4.98E-01
ADAM30	0.0205	6.94E-01	7.89E-01
ADAM32	0.1119	3.12E-02	7.37E-02
ADAM33	-0.0918	7.74E-02	1.52E-01
ADAM3A	0.0900	8.35E-02	1.61E-01
ADAM5P	0.0955	6.62E-02	1.34E-01
ADAM6	-0.0559	2.83E-01	4.11E-01
ADAM7	0.0795	1.26E-01	2.22E-01
ADAM8	0.0977	6.00E-02	1.24E-01

ADAM9	0.0693	1.83E-01	2.95E-01
ADAMDEC1	0.0219	6.75E-01	7.74E-01
ADAMTS10	0.1007	5.26E-02	1.12E-01
ADAMTS12	-0.1195	2.13E-02	5.40E-02
ADAMTS13	-0.0133	7.99E-01	8.67E-01
ADAMTS14	0.1070	3.94E-02	8.91E-02
ADAMTS15	-0.2002	1.03E-04	6.23E-04
ADAMTS16	-0.0070	8.93E-01	9.32E-01
ADAMTS17	-0.0992	5.62E-02	1.18E-01
ADAMTS18	-0.1066	4.02E-02	9.06E-02
ADAMTS19	0.1022	4.92E-02	1.06E-01
ADAMTS1	-0.2421	2.37E-06	2.42E-05
ADAMTS20	0.1291	1.28E-02	3.58E-02
ADAMTS2	-0.1469	4.58E-03	1.53E-02
ADAMTS3	-0.0442	3.96E-01	5.28E-01
ADAMTS4	-0.2059	6.46E-05	4.20E-04
ADAMTS5	0.0444	3.94E-01	5.27E-01
ADAMTS6	0.1248	1.61E-02	4.32E-02
ADAMTS7	-0.0257	6.21E-01	7.30E-01
ADAMTS8	-0.0612	2.40E-01	3.64E-01
ADAMTS9	0.0065	9.00E-01	9.37E-01
ADAMTSL1	-0.1784	5.56E-04	2.60E-03
ADAMTSL2	-0.1242	1.67E-02	4.44E-02
ADAMTSL3	-0.1664	1.30E-03	5.36E-03
ADAMTSL4	-0.0156	7.64E-01	8.41E-01
ADAMTSL5	-0.0618	2.35E-01	3.59E-01
ADAP1	0.1025	4.86E-02	1.05E-01
ADAP2	0.0055	9.15E-01	9.47E-01
ADARB1	-0.1416	6.30E-03	1.99E-02
ADARB2	0.0846	1.04E-01	1.91E-01
ADAR	0.4284	5.39E-18	7.62E-16
ADAT1	-0.1478	4.33E-03	1.47E-02
ADAT2	0.3029	2.59E-09	5.54E-08
ADAT3	-0.0005	9.93E-01	9.96E-01
ADA	0.1338	9.89E-03	2.90E-02
ADCK1	-0.1372	8.12E-03	2.46E-02
ADCK2	-0.0169	7.46E-01	8.28E-01
ADCK4	-0.0698	1.80E-01	2.91E-01
ADCK5	0.0574	2.70E-01	3.98E-01
ADCY10	0.1222	1.85E-02	4.83E-02
ADCY1	-0.0417	4.24E-01	5.56E-01
ADCY2	-0.0507	3.30E-01	4.61E-01
ADCY3	0.0076	8.84E-01	9.26E-01
ADCY4	-0.1778	5.80E-04	2.69E-03

ADCY5	-0.0439	3.99E-01	5.32E-01
ADCY6	0.2271	9.96E-06	8.38E-05
ADCY7	-0.0102	8.44E-01	8.98E-01
ADCY8	-0.0212	6.85E-01	7.81E-01
ADCY9	-0.1347	9.36E-03	2.78E-02
ADCYAP1R1	-0.0829	1.11E-01	2.01E-01
ADCYAP1	-0.2250	1.21E-05	9.91E-05
ADC	-0.0376	4.71E-01	6.00E-01
ADD1	-0.0382	4.63E-01	5.93E-01
ADD2	-0.0181	7.28E-01	8.15E-01
ADD3	0.0829	1.11E-01	2.02E-01
ADH1A	-0.2313	6.78E-06	6.01E-05
ADH1B	-0.2847	2.40E-08	4.05E-07
ADH1C	-0.1398	6.99E-03	2.17E-02
ADH4	-0.2840	2.58E-08	4.31E-07
ADH5	-0.1444	5.33E-03	1.74E-02
ADH6	-0.1801	4.90E-04	2.34E-03
ADH7	-0.0344	5.08E-01	6.33E-01
ADHFE1	-0.2844	2.47E-08	4.15E-07
ADI1	-0.3093	1.15E-09	2.68E-08
ADIG	-0.0954	6.64E-02	1.34E-01
ADIPOQ	-0.0976	6.03E-02	1.25E-01
ADIPOR1	0.1419	6.17E-03	1.96E-02
ADIPOR2	-0.1931	1.82E-04	1.02E-03
ADK	-0.2869	1.85E-08	3.24E-07
ADM2	0.2435	2.08E-06	2.16E-05
ADM	-0.1449	5.16E-03	1.69E-02
ADNP2	0.1546	2.83E-03	1.03E-02
ADNP	0.1469	4.58E-03	1.54E-02
ADORA1	0.1331	1.03E-02	2.99E-02
ADORA2A	0.1103	3.37E-02	7.87E-02
ADORA2B	0.2128	3.60E-05	2.56E-04
ADORA3	0.0067	8.98E-01	9.36E-01
ADO	0.2160	2.71E-05	2.01E-04
ADPGK	0.1498	3.82E-03	1.32E-02
ADPRHL1	0.2344	5.03E-06	4.66E-05
ADPRHL2	0.0595	2.53E-01	3.78E-01
ADPRH	0.0110	8.32E-01	8.90E-01
ADRA1A	-0.2471	1.45E-06	1.57E-05
ADRA1B	-0.2364	4.15E-06	3.95E-05
ADRA1D	0.0028	9.57E-01	9.73E-01
ADRA2A	-0.0922	7.62E-02	1.50E-01
ADRA2B	-0.1268	1.45E-02	3.96E-02
ADRA2C	0.0394	4.49E-01	5.80E-01

ADRB1	-0.1746	7.31E-04	3.29E-03
ADRB2	-0.1824	4.13E-04	2.03E-03
ADRB3	0.0667	2.00E-01	3.17E-01
ADRBK1	0.0294	5.73E-01	6.90E-01
ADRBK2	-0.0657	2.06E-01	3.24E-01
ADRM1	0.0527	3.11E-01	4.41E-01
ADSL	0.2157	2.79E-05	2.06E-04
ADSSL1	-0.1461	4.81E-03	1.60E-02
ADSS	0.2419	2.44E-06	2.48E-05
AEBP1	-0.1266	1.47E-02	4.00E-02
AEBP2	0.1330	1.04E-02	3.01E-02
AEN	-0.1227	1.80E-02	4.72E-02
AES	-0.0720	1.66E-01	2.74E-01
AFAP1L1	-0.0975	6.07E-02	1.25E-01
AFAP1L2	-0.0375	4.72E-01	6.01E-01
AFAP1	0.0059	9.10E-01	9.43E-01
AFARP1	-0.1720	8.82E-04	3.86E-03
AFF1	-0.1197	2.12E-02	5.37E-02
AFF2	0.0815	1.17E-01	2.10E-01
AFF3	0.0426	4.13E-01	5.46E-01
AFF4	-0.2375	3.73E-06	3.61E-05
AFG3L1	0.1401	6.87E-03	2.14E-02
AFG3L2	-0.1742	7.52E-04	3.37E-03
AFMID	-0.0980	5.93E-02	1.23E-01
AFM	-0.1739	7.70E-04	3.44E-03
AFP	0.1871	2.90E-04	1.52E-03
AFTPH	0.0711	1.72E-01	2.81E-01
AG2	-0.2183	2.22E-05	1.69E-04
AGAP11	0.0167	7.48E-01	8.30E-01
AGAP1	0.1295	1.25E-02	3.51E-02
AGAP2	-0.0750	1.49E-01	2.53E-01
AGAP3	-0.0807	1.21E-01	2.15E-01
AGAP4	0.2844	2.47E-08	4.15E-07
AGAP5	0.2358	4.40E-06	4.17E-05
AGAP6	0.2509	9.81E-07	1.11E-05
AGAP7	0.1909	2.16E-04	1.18E-03
AGAP8	0.2304	7.35E-06	6.43E-05
AGA	-0.0592	2.56E-01	3.82E-01
AGBL1	0.0621	2.33E-01	3.56E-01
AGBL2	0.0265	6.10E-01	7.22E-01
AGBL3	0.0957	6.56E-02	1.33E-01
AGBL4	-0.0538	3.01E-01	4.30E-01
AGBL5	0.1615	1.80E-03	7.07E-03
AGER	0.1839	3.71E-04	1.86E-03

AGFG1	0.1065	4.04E-02	9.09E-02
AGFG2	-0.1421	6.12E-03	1.95E-02
AGGF1	0.0205	6.94E-01	7.89E-01
AGK	-0.0419	4.21E-01	5.54E-01
AGL	-0.3117	8.46E-10	2.04E-08
AGMAT	-0.1025	4.85E-02	1.05E-01
AGPAT1	0.0958	6.54E-02	1.33E-01
AGPAT2	-0.0340	5.14E-01	6.38E-01
AGPAT3	-0.0668	1.99E-01	3.16E-01
AGPAT4	0.1274	1.41E-02	3.87E-02
AGPAT5	0.0941	7.02E-02	1.41E-01
AGPAT6	-0.0644	2.16E-01	3.36E-01
AGPAT9	-0.0594	2.53E-01	3.79E-01
AGPHD1	-0.0580	2.65E-01	3.93E-01
AGPS	0.0327	5.30E-01	6.53E-01
AGR2	0.0851	1.02E-01	1.88E-01
AGR3	0.0253	6.28E-01	7.36E-01
AGRN	0.0163	7.54E-01	8.35E-01
AGRP	0.1929	1.85E-04	1.03E-03
AGTPBP1	0.1134	2.90E-02	6.96E-02
AGTR1	-0.1119	3.11E-02	7.36E-02
AGTR2	-0.0581	2.64E-01	3.91E-01
AGTRAP	0.1214	1.93E-02	4.99E-02
AGT	-0.0535	3.04E-01	4.34E-01
AGXT2L1	-0.2571	5.18E-07	6.30E-06
AGXT2L2	-0.2720	1.03E-07	1.49E-06
AGXT2	-0.2167	2.56E-05	1.92E-04
AGXT	-0.1525	3.23E-03	1.15E-02
AHCTF1	0.2429	2.21E-06	2.27E-05
AHCYL1	-0.1590	2.12E-03	8.09E-03
AHCYL2	-0.1666	1.28E-03	5.30E-03
AHCY	-0.1101	3.40E-02	7.92E-02
AHDC1	-0.0002	9.97E-01	9.98E-01
AHI1	0.2125	3.68E-05	2.61E-04
AHNAK2	-0.0021	9.68E-01	9.80E-01
AHNAK	-0.0745	1.52E-01	2.57E-01
AHRR	0.0448	3.89E-01	5.22E-01
AHR	-0.2049	7.05E-05	4.53E-04
AHSA1	0.0451	3.87E-01	5.20E-01
AHSA2	0.2244	1.28E-05	1.04E-04
AHSG	-0.1163	2.51E-02	6.17E-02
AHSP	-0.1384	7.61E-03	2.33E-02
AICDA	0.0131	8.01E-01	8.69E-01
AIDA	0.2151	2.93E-05	2.15E-04

AIF1L	-0.0240	6.45E-01	7.50E-01
AIF1	-0.0513	3.24E-01	4.55E-01
AIFM1	-0.1898	2.36E-04	1.27E-03
AIFM2	0.0581	2.64E-01	3.91E-01
AIFM3	0.1092	3.55E-02	8.19E-02
AIG1	-0.1163	2.51E-02	6.17E-02
AIM1L	0.0425	4.15E-01	5.47E-01
AIM1	-0.1366	8.45E-03	2.55E-02
AIM2	0.0749	1.50E-01	2.53E-01
AIMP1	0.0012	9.81E-01	9.88E-01
AIMP2	-0.1662	1.31E-03	5.40E-03
AIPL1	0.0951	6.72E-02	1.36E-01
AIP	-0.1075	3.85E-02	8.75E-02
AIRE	0.0678	1.93E-01	3.08E-01
AJAP1	-0.0729	1.61E-01	2.68E-01
AK1	-0.0339	5.15E-01	6.39E-01
AK2	-0.1224	1.84E-02	4.79E-02
AK3L1	-0.0199	7.02E-01	7.95E-01
AK3	-0.2357	4.46E-06	4.22E-05
AK5	-0.0202	6.99E-01	7.92E-01
AK7	-0.0052	9.21E-01	9.50E-01
AKAP10	0.0378	4.68E-01	5.98E-01
AKAP11	-0.0826	1.12E-01	2.04E-01
AKAP12	-0.0740	1.55E-01	2.60E-01
AKAP13	-0.1402	6.85E-03	2.14E-02
AKAP14	-0.0293	5.74E-01	6.91E-01
AKAP1	0.0880	9.06E-02	1.72E-01
AKAP2	-0.1182	2.28E-02	5.71E-02
AKAP3	-0.1890	2.51E-04	1.34E-03
AKAP4	0.0263	6.13E-01	7.24E-01
AKAP5	0.0961	6.45E-02	1.31E-01
AKAP6	-0.2030	8.19E-05	5.15E-04
AKAP7	0.0235	6.51E-01	7.55E-01
AKAP8L	0.2142	3.17E-05	2.29E-04
AKAP8	0.1919	2.00E-04	1.10E-03
AKAP9	-0.0867	9.54E-02	1.79E-01
AKD1	0.1286	1.32E-02	3.67E-02
AKIRIN1	-0.0348	5.04E-01	6.29E-01
AKIRIN2	0.0930	7.35E-02	1.46E-01
AKNAD1	0.0731	1.60E-01	2.66E-01
AKNA	-0.0008	9.88E-01	9.93E-01
AKR1A1	-0.1211	1.96E-02	5.06E-02
AKR1B10	0.0661	2.04E-01	3.21E-01
AKR1B15	0.0364	4.85E-01	6.13E-01

AKR1B1	0.0219	6.75E-01	7.74E-01
AKR1C1	-0.1186	2.23E-02	5.62E-02
AKR1C2	-0.0912	7.93E-02	1.55E-01
AKR1C3	0.0766	1.41E-01	2.42E-01
AKR1C4	-0.1788	5.42E-04	2.54E-03
AKR1CL1	-0.0377	4.69E-01	5.99E-01
AKR1D1	-0.1616	1.79E-03	7.03E-03
AKR1E2	0.0598	2.51E-01	3.76E-01
AKR7A2	-0.1862	3.12E-04	1.61E-03
AKR7A3	-0.3033	2.46E-09	5.29E-08
AKR7L	-0.2505	1.03E-06	1.16E-05
AKT1S1	0.0061	9.06E-01	9.41E-01
AKT1	-0.1326	1.06E-02	3.06E-02
AKT2	-0.1852	3.35E-04	1.71E-03
AKT3	-0.0064	9.03E-01	9.39E-01
AKTIP	-0.2862	2.01E-08	3.47E-07
ALAD	-0.1897	2.38E-04	1.28E-03
ALAS1	-0.1857	3.23E-04	1.66E-03
ALAS2	-0.1185	2.24E-02	5.63E-02
ALB	-0.1310	1.15E-02	3.28E-02
ALCAM	-0.0510	3.27E-01	4.58E-01
ALDH16A1	0.0011	9.83E-01	9.89E-01
ALDH18A1	0.0300	5.64E-01	6.82E-01
ALDH1A1	-0.1021	4.93E-02	1.06E-01
ALDH1A2	-0.0331	5.25E-01	6.48E-01
ALDH1A3	-0.1398	7.00E-03	2.17E-02
ALDH1B1	-0.1941	1.68E-04	9.50E-04
ALDH1L1	-0.2207	1.79E-05	1.40E-04
ALDH1L2	-0.1191	2.17E-02	5.50E-02
ALDH2	-0.3496	4.16E-12	1.64E-10
ALDH3A1	-0.0156	7.65E-01	8.41E-01
ALDH3A2	-0.1374	8.05E-03	2.45E-02
ALDH3B1	0.1443	5.35E-03	1.75E-02
ALDH3B2	0.1200	2.08E-02	5.29E-02
ALDH4A1	-0.3890	7.48E-15	4.95E-13
ALDH5A1	-0.2469	1.47E-06	1.59E-05
ALDH6A1	-0.3166	4.43E-10	1.14E-08
ALDH7A1	-0.2456	1.69E-06	1.80E-05
ALDH8A1	-0.1803	4.83E-04	2.31E-03
ALDH9A1	-0.1085	3.67E-02	8.43E-02
ALDOA	0.1032	4.70E-02	1.02E-01
ALDOB	-0.1627	1.67E-03	6.61E-03
ALDOC	0.1104	3.36E-02	7.84E-02
ALG10B	-0.0070	8.94E-01	9.32E-01

ALG10	0.0796	1.26E-01	2.22E-01
ALG11	0.0945	6.90E-02	1.39E-01
ALG12	0.0260	6.18E-01	7.27E-01
ALG13	0.0660	2.05E-01	3.22E-01
ALG14	0.0158	7.62E-01	8.40E-01
ALG1L2	0.0072	8.90E-01	9.30E-01
ALG1L	0.1654	1.39E-03	5.66E-03
ALG1	-0.1699	1.02E-03	4.37E-03
ALG2	0.0504	3.33E-01	4.64E-01
ALG3	0.0612	2.40E-01	3.64E-01
ALG5	-0.1129	2.97E-02	7.09E-02
ALG6	0.2707	1.18E-07	1.69E-06
ALG8	-0.0427	4.12E-01	5.45E-01
ALG9	-0.0333	5.23E-01	6.47E-01
ALKBH1	-0.0052	9.20E-01	9.49E-01
ALKBH2	-0.0072	8.89E-01	9.29E-01
ALKBH3	-0.1542	2.90E-03	1.05E-02
ALKBH4	-0.0617	2.36E-01	3.59E-01
ALKBH5	-0.0974	6.08E-02	1.26E-01
ALKBH6	0.1143	2.77E-02	6.69E-02
ALKBH7	-0.1740	7.65E-04	3.42E-03
ALKBH8	-0.0094	8.57E-01	9.07E-01
ALK	-0.0682	1.90E-01	3.04E-01
ALLC	-0.0086	8.69E-01	9.15E-01
ALMS1P	0.0548	2.92E-01	4.21E-01
ALMS1	0.3297	7.44E-11	2.24E-09
ALOX12B	0.1134	2.89E-02	6.94E-02
ALOX12P2	-0.1129	2.97E-02	7.08E-02
ALOX12	0.0567	2.76E-01	4.04E-01
ALOX15B	0.0290	5.78E-01	6.94E-01
ALOX15	0.0042	9.36E-01	9.60E-01
ALOX5AP	-0.0235	6.52E-01	7.55E-01
ALOX5	0.0702	1.77E-01	2.88E-01
ALOXE3	0.0197	7.05E-01	7.98E-01
ALPI	0.1245	1.64E-02	4.37E-02
ALPK1	-0.1049	4.35E-02	9.63E-02
ALPK2	-0.0704	1.76E-01	2.86E-01
ALPK3	0.0646	2.14E-01	3.34E-01
ALPL	-0.1635	1.57E-03	6.30E-03
ALPPL2	0.0534	3.05E-01	4.34E-01
ALPP	0.1474	4.43E-03	1.49E-02
ALS2CL	0.2040	7.56E-05	4.82E-04
ALS2CR11	0.2007	9.95E-05	6.06E-04
ALS2CR12	0.0129	8.04E-01	8.70E-01

ALS2CR4	0.3126	7.49E-10	1.83E-08
ALS2CR8	-0.1123	3.06E-02	7.26E-02
ALS2	0.1267	1.46E-02	3.98E-02
ALX1	0.1296	1.25E-02	3.50E-02
ALX3	0.1958	1.48E-04	8.52E-04
ALX4	0.0203	6.96E-01	7.90E-01
AMAC1L2	0.1063	4.07E-02	9.15E-02
AMAC1L3	0.0875	9.22E-02	1.74E-01
AMAC1	-0.0167	7.49E-01	8.31E-01
AMACR	-0.3006	3.48E-09	7.25E-08
AMBN	-0.0576	2.68E-01	3.96E-01
AMBP	-0.0682	1.90E-01	3.04E-01
AMBRA1	-0.0625	2.29E-01	3.52E-01
AMD1	0.1023	4.90E-02	1.06E-01
AMDHD1	-0.1752	7.00E-04	3.17E-03
AMDHD2	-0.1442	5.40E-03	1.76E-02
AMELX	0.0240	6.46E-01	7.50E-01
AMELY	0.0451	3.87E-01	5.20E-01
AMFR	-0.4681	1.35E-21	4.07E-19
AMHR2	-0.0029	9.56E-01	9.72E-01
AMH	0.1785	5.53E-04	2.59E-03
AMICA1	0.0221	6.72E-01	7.71E-01
AMIGO1	-0.1041	4.51E-02	9.92E-02
AMIGO2	0.0186	7.21E-01	8.10E-01
AMIGO3	0.3396	1.82E-11	6.21E-10
AMMECR1L	0.1096	3.48E-02	8.06E-02
AMMECR1	0.1541	2.92E-03	1.06E-02
AMN1	0.0774	1.37E-01	2.36E-01
AMN	-0.1303	1.20E-02	3.40E-02
AMOTL1	0.0819	1.15E-01	2.08E-01
AMOTL2	-0.1030	4.74E-02	1.03E-01
AMOT	-0.0744	1.53E-01	2.57E-01
AMPD1	-0.0537	3.03E-01	4.32E-01
AMPD2	-0.0530	3.08E-01	4.38E-01
AMPD3	0.0561	2.82E-01	4.10E-01
AMPH	-0.0127	8.08E-01	8.73E-01
AMTN	0.0736	1.57E-01	2.63E-01
AMT	-0.0731	1.60E-01	2.66E-01
AMY1A	-0.0202	6.99E-01	7.92E-01
AMY2A	-0.0906	8.15E-02	1.58E-01
AMY2B	-0.0241	6.44E-01	7.49E-01
AMZ1	0.0244	6.39E-01	7.45E-01
AMZ2P1	0.2126	3.64E-05	2.59E-04
AMZ2	0.2344	5.03E-06	4.66E-05

ANAPC10	-0.0223	6.69E-01	7.69E-01
ANAPC11	0.1332	1.02E-02	2.97E-02
ANAPC13	-0.0876	9.21E-02	1.74E-01
ANAPC16	-0.0554	2.87E-01	4.16E-01
ANAPC1	0.0768	1.40E-01	2.41E-01
ANAPC2	0.1356	8.94E-03	2.67E-02
ANAPC4	0.3573	1.29E-12	5.64E-11
ANAPC5	0.1366	8.44E-03	2.55E-02
ANAPC7	0.3143	5.96E-10	1.49E-08
ANGEL1	0.1478	4.32E-03	1.47E-02
ANGEL2	0.2601	3.77E-07	4.78E-06
ANGPT1	-0.0889	8.74E-02	1.67E-01
ANGPT2	0.0380	4.66E-01	5.96E-01
ANGPT4	-0.0900	8.34E-02	1.61E-01
ANGPTL1	-0.1716	9.05E-04	3.94E-03
ANGPTL2	-0.1561	2.57E-03	9.47E-03
ANGPTL3	-0.2336	5.45E-06	4.98E-05
ANGPTL4	-0.1438	5.54E-03	1.79E-02
ANGPTL5	-0.0060	9.09E-01	9.43E-01
ANGPTL6	-0.2398	2.98E-06	2.95E-05
ANGPTL7	-0.2099	4.63E-05	3.17E-04
ANG	-0.2372	3.86E-06	3.71E-05
ANK1	0.0154	7.68E-01	8.44E-01
ANK2	0.0693	1.83E-01	2.96E-01
ANK3	0.0018	9.72E-01	9.82E-01
ANKAR	0.1354	9.04E-03	2.70E-02
ANKDD1A	0.2230	1.46E-05	1.17E-04
ANKFN1	-0.0648	2.13E-01	3.32E-01
ANKFY1	-0.1507	3.61E-03	1.26E-02
ANKHD1-EIF4EI	-0.1082	3.72E-02	8.52E-02
ANKHD1	0.1385	7.54E-03	2.31E-02
ANKH	-0.0684	1.89E-01	3.02E-01
ANKIB1	-0.0348	5.03E-01	6.29E-01
ANKK1	0.1624	1.70E-03	6.72E-03
ANKLE1	0.1802	4.87E-04	2.32E-03
ANKLE2	0.2528	8.09E-07	9.39E-06
ANKMY1	0.0791	1.29E-01	2.25E-01
ANKMY2	-0.0234	6.54E-01	7.56E-01
ANKRA2	-0.0758	1.45E-01	2.48E-01
ANKRD10	0.2612	3.33E-07	4.29E-06
ANKRD11	-0.0615	2.37E-01	3.61E-01
ANKRD12	-0.0433	4.06E-01	5.39E-01
ANKRD13A	0.1024	4.88E-02	1.06E-01
ANKRD13B	0.1692	1.07E-03	4.55E-03

ANKRD13C	0.1209	1.98E-02	5.10E-02
ANKRD13D	0.2440	1.98E-06	2.08E-05
ANKRD16	0.2007	9.89E-05	6.03E-04
ANKRD17	-0.0797	1.25E-01	2.21E-01
ANKRD19	0.1164	2.49E-02	6.14E-02
ANKRD1	-0.0398	4.45E-01	5.77E-01
ANKRD20A3	0.0604	2.46E-01	3.70E-01
ANKRD20A4	-0.0371	4.76E-01	6.05E-01
ANKRD20B	-0.0446	3.91E-01	5.24E-01
ANKRD22	0.0797	1.26E-01	2.21E-01
ANKRD23	0.3180	3.65E-10	9.65E-09
ANKRD24	-0.2218	1.62E-05	1.29E-04
ANKRD26P1	0.1157	2.58E-02	6.33E-02
ANKRD26	0.1080	3.77E-02	8.60E-02
ANKRD27	0.1820	4.26E-04	2.08E-03
ANKRD28	-0.0692	1.83E-01	2.96E-01
ANKRD29	-0.0875	9.26E-02	1.75E-01
ANKRD2	0.1035	4.63E-02	1.01E-01
ANKRD30A	0.1295	1.25E-02	3.51E-02
ANKRD30B	-0.0613	2.39E-01	3.63E-01
ANKRD31	0.0594	2.54E-01	3.79E-01
ANKRD32	0.2840	2.59E-08	4.32E-07
ANKRD33	0.0446	3.92E-01	5.25E-01
ANKRD34A	0.0991	5.64E-02	1.18E-01
ANKRD34B	0.1551	2.74E-03	1.00E-02
ANKRD34C	-0.0109	8.35E-01	8.92E-01
ANKRD35	-0.1085	3.67E-02	8.42E-02
ANKRD36BP1	0.0667	2.00E-01	3.16E-01
ANKRD36B	0.2149	2.99E-05	2.19E-04
ANKRD36	0.2896	1.34E-08	2.42E-07
ANKRD37	-0.1776	5.88E-04	2.72E-03
ANKRD39	0.1132	2.93E-02	7.01E-02
ANKRD40	0.1524	3.26E-03	1.16E-02
ANKRD42	-0.0762	1.43E-01	2.45E-01
ANKRD43	0.0466	3.70E-01	5.03E-01
ANKRD44	-0.0465	3.72E-01	5.05E-01
ANKRD45	0.1031	4.72E-02	1.03E-01
ANKRD46	-0.1618	1.76E-03	6.93E-03
ANKRD49	0.2713	1.10E-07	1.58E-06
ANKRD50	-0.1770	6.17E-04	2.84E-03
ANKRD52	0.2694	1.37E-07	1.93E-06
ANKRD53	0.0771	1.38E-01	2.38E-01
ANKRD54	0.1821	4.22E-04	2.07E-03
ANKRD55	-0.1632	1.61E-03	6.41E-03

ANKRD56	-0.1631	1.62E-03	6.47E-03
ANKRD57	-0.1390	7.33E-03	2.26E-02
ANKRD58	0.1857	3.22E-04	1.66E-03
ANKRD5	0.0747	1.51E-01	2.55E-01
ANKRD6	0.1374	8.05E-03	2.45E-02
ANKRD7	0.0574	2.70E-01	3.98E-01
ANKRD9	-0.0788	1.30E-01	2.27E-01
ANKS1A	0.0755	1.47E-01	2.49E-01
ANKS1B	0.1242	1.67E-02	4.43E-02
ANKS3	0.2810	3.68E-08	5.92E-07
ANKS4B	-0.1098	3.45E-02	8.01E-02
ANKS6	0.2736	8.60E-08	1.27E-06
ANKZF1	0.3517	3.06E-12	1.23E-10
ANLN	0.3946	2.85E-15	2.08E-13
ANO10	0.0488	3.48E-01	4.80E-01
ANO1	-0.1776	5.88E-04	2.72E-03
ANO2	0.0071	8.92E-01	9.31E-01
ANO3	-0.1223	1.84E-02	4.81E-02
ANO4	0.0064	9.02E-01	9.38E-01
ANO5	0.0147	7.78E-01	8.52E-01
ANO6	-0.1449	5.17E-03	1.70E-02
ANO7	0.0688	1.86E-01	2.99E-01
ANO8	0.0737	1.57E-01	2.62E-01
ANO9	0.2294	8.08E-06	6.95E-05
ANP32A	0.1087	3.64E-02	8.36E-02
ANP32B	0.2728	9.37E-08	1.37E-06
ANP32C	0.1232	1.76E-02	4.62E-02
ANP32D	0.0528	3.10E-01	4.40E-01
ANP32E	0.5728	9.90E-34	1.31E-30
ANPEP	-0.0059	9.10E-01	9.43E-01
ANTXR1	-0.0998	5.48E-02	1.16E-01
ANTXR2	-0.1579	2.29E-03	8.63E-03
ANTXRL	0.0389	4.55E-01	5.85E-01
ANUBL1	0.0368	4.80E-01	6.09E-01
ANXA10	-0.3093	1.14E-09	2.68E-08
ANXA11	0.0214	6.82E-01	7.79E-01
ANXA13	0.1013	5.11E-02	1.09E-01
ANXA1	-0.1398	7.00E-03	2.17E-02
ANXA2P1	0.1081	3.75E-02	8.56E-02
ANXA2P2	0.0910	8.01E-02	1.56E-01
ANXA2P3	0.1304	1.19E-02	3.37E-02
ANXA2	0.1130	2.95E-02	7.05E-02
ANXA3	-0.0208	6.90E-01	7.86E-01
ANXA4	0.1490	4.01E-03	1.38E-02

ANXA5	0.0533	3.06E-01	4.36E-01
ANXA6	-0.2481	1.31E-06	1.44E-05
ANXA7	-0.1297	1.24E-02	3.48E-02
ANXA8L1	0.1477	4.35E-03	1.47E-02
ANXA8L2	0.0383	4.62E-01	5.92E-01
ANXA8	0.0673	1.96E-01	3.11E-01
ANXA9	0.1426	5.93E-03	1.90E-02
AOAH	-0.0838	1.07E-01	1.96E-01
AOC2	-0.0185	7.22E-01	8.12E-01
AOC3	-0.1966	1.38E-04	8.03E-04
AOX1	-0.2074	5.69E-05	3.79E-04
AOX2P	-0.0085	8.70E-01	9.16E-01
AP1AR	-0.0931	7.33E-02	1.46E-01
AP1B1	-0.0823	1.13E-01	2.05E-01
AP1G1	-0.2329	5.82E-06	5.27E-05
AP1G2	0.2045	7.25E-05	4.65E-04
AP1M1	-0.1284	1.33E-02	3.71E-02
AP1M2	0.1127	3.00E-02	7.14E-02
AP1S1	-0.0645	2.15E-01	3.35E-01
AP1S2	-0.0467	3.70E-01	5.03E-01
AP1S3	0.1158	2.57E-02	6.29E-02
AP2A1	-0.0410	4.31E-01	5.63E-01
AP2A2	0.0253	6.27E-01	7.35E-01
AP2B1	0.0606	2.44E-01	3.68E-01
AP2M1	-0.0238	6.48E-01	7.52E-01
AP2S1	0.0098	8.50E-01	9.03E-01
AP3B1	0.1103	3.37E-02	7.86E-02
AP3B2	0.1548	2.80E-03	1.02E-02
AP3D1	0.0289	5.79E-01	6.96E-01
AP3M1	0.1024	4.88E-02	1.06E-01
AP3M2	0.2211	1.72E-05	1.36E-04
AP3S1	-0.1209	1.98E-02	5.09E-02
AP3S2	-0.3271	1.06E-10	3.09E-09
AP4B1	0.1496	3.87E-03	1.34E-02
AP4E1	0.0284	5.85E-01	7.01E-01
AP4M1	0.1638	1.54E-03	6.20E-03
AP4S1	-0.0792	1.28E-01	2.24E-01
APAF1	0.1667	1.27E-03	5.28E-03
APBA1	-0.1276	1.39E-02	3.83E-02
APBA2	0.0179	7.31E-01	8.18E-01
APBA3	-0.0622	2.32E-01	3.55E-01
APBB1IP	-0.0327	5.30E-01	6.53E-01
APBB1	0.0993	5.61E-02	1.18E-01
APBB2	-0.1562	2.55E-03	9.42E-03

APBB3	0.0653	2.10E-01	3.28E-01
APC2	-0.0819	1.15E-01	2.08E-01
APCDD1L	0.0451	3.86E-01	5.19E-01
APCDD1	0.0397	4.46E-01	5.78E-01
APCS	-0.0926	7.48E-02	1.48E-01
APC	-0.0612	2.39E-01	3.63E-01
APEH	-0.2539	7.23E-07	8.50E-06
APEX1	0.1003	5.36E-02	1.13E-01
APEX2	0.1263	1.49E-02	4.05E-02
APH1A	0.4727	4.77E-22	1.65E-19
APH1B	0.0229	6.60E-01	7.62E-01
API5	-0.1237	1.72E-02	4.54E-02
APIP	0.0383	4.62E-01	5.92E-01
APITD1	0.0875	9.22E-02	1.74E-01
APLF	-0.0216	6.78E-01	7.76E-01
APLNR	-0.2049	6.99E-05	4.50E-04
APLN	0.0761	1.44E-01	2.46E-01
APLP1	0.0061	9.07E-01	9.41E-01
APLP2	-0.1023	4.89E-02	1.06E-01
APOA1BP	0.2587	4.34E-07	5.40E-06
APOA1	-0.1478	4.33E-03	1.47E-02
APOA2	-0.0445	3.93E-01	5.25E-01
APOA4	-0.0724	1.64E-01	2.71E-01
APOA5	-0.1728	8.32E-04	3.67E-03
APOB48R	0.0193	7.11E-01	8.03E-01
APOBEC1	-0.0197	7.05E-01	7.97E-01
APOBEC2	0.1259	1.52E-02	4.11E-02
APOBEC3A	0.0973	6.11E-02	1.26E-01
APOBEC3B	0.1106	3.32E-02	7.76E-02
APOBEC3C	-0.0555	2.86E-01	4.15E-01
APOBEC3D	0.0185	7.22E-01	8.12E-01
APOBEC3F	0.0050	9.24E-01	9.52E-01
APOBEC3G	-0.0257	6.22E-01	7.31E-01
APOBEC3H	-0.0981	5.89E-02	1.22E-01
APOBEC4	0.1237	1.71E-02	4.53E-02
APOB	-0.1449	5.17E-03	1.70E-02
APOC1P1	-0.1943	1.66E-04	9.40E-04
APOC1	-0.1585	2.20E-03	8.32E-03
APOC2	-0.0638	2.20E-01	3.41E-01
APOC3	-0.1373	8.08E-03	2.45E-02
APOC4	-0.1485	4.15E-03	1.42E-02
APOD	-0.0886	8.82E-02	1.68E-01
APOE	-0.1456	4.96E-03	1.64E-02
APOF	-0.2602	3.70E-07	4.71E-06

APOH	-0.0620	2.33E-01	3.56E-01
APOL1	-0.1591	2.12E-03	8.09E-03
APOL2	-0.0261	6.16E-01	7.26E-01
APOL3	-0.0245	6.38E-01	7.44E-01
APOL4	0.0540	3.00E-01	4.29E-01
APOL5	-0.0184	7.23E-01	8.12E-01
APOL6	-0.2817	3.41E-08	5.54E-07
APOLD1	-0.2143	3.15E-05	2.28E-04
APOM	-0.0660	2.05E-01	3.22E-01
APOOL	-0.0413	4.28E-01	5.60E-01
APOO	-0.0110	8.33E-01	8.90E-01
APPBP2	0.1501	3.77E-03	1.31E-02
APPL1	0.0955	6.63E-02	1.34E-01
APPL2	0.1472	4.51E-03	1.52E-02
APP	-0.0489	3.47E-01	4.80E-01
APRT	-0.2081	5.37E-05	3.60E-04
APTX	0.1839	3.69E-04	1.85E-03
AQP10	0.1492	3.97E-03	1.37E-02
AQP11	-0.1030	4.75E-02	1.03E-01
AQP12A	0.0266	6.09E-01	7.21E-01
AQP12B	0.0456	3.82E-01	5.15E-01
AQP1	-0.1553	2.71E-03	9.93E-03
AQP2	-0.0369	4.79E-01	6.08E-01
AQP3	0.0173	7.40E-01	8.24E-01
AQP4	-0.1878	2.76E-04	1.45E-03
AQP5	-0.0096	8.54E-01	9.06E-01
AQP6	-0.1092	3.56E-02	8.20E-02
AQP7P1	-0.1873	2.85E-04	1.49E-03
AQP7P3	-0.1443	5.35E-03	1.75E-02
AQP7	-0.2715	1.09E-07	1.57E-06
AQP8	-0.1305	1.18E-02	3.35E-02
AQP9	-0.2176	2.35E-05	1.78E-04
AQPEP	0.1507	3.62E-03	1.26E-02
AQR	0.0227	6.62E-01	7.63E-01
ARAF	-0.0463	3.73E-01	5.06E-01
ARAP1	0.0786	1.31E-01	2.29E-01
ARAP2	0.0204	6.95E-01	7.89E-01
ARAP3	-0.0472	3.65E-01	4.98E-01
ARCN1	0.0411	4.30E-01	5.62E-01
ARC	-0.0474	3.62E-01	4.95E-01
AREG	-0.0099	8.50E-01	9.02E-01
ARF1	0.1714	9.17E-04	3.99E-03
ARF3	-0.0059	9.10E-01	9.43E-01
ARF4	0.0509	3.28E-01	4.59E-01

ARF5	-0.0204	6.95E-01	7.90E-01
ARF6	-0.0547	2.93E-01	4.23E-01
ARFGAP1	0.2293	8.12E-06	6.98E-05
ARFGAP2	0.0260	6.18E-01	7.27E-01
ARFGAP3	-0.0268	6.07E-01	7.19E-01
ARFGEF1	0.0072	8.90E-01	9.29E-01
ARFGEF2	-0.0628	2.28E-01	3.50E-01
ARFIP1	-0.1698	1.03E-03	4.39E-03
ARFIP2	0.1544	2.86E-03	1.04E-02
ARFRP1	0.0239	6.47E-01	7.51E-01
ARG1	-0.1155	2.61E-02	6.37E-02
ARG2	-0.1200	2.08E-02	5.30E-02
ARGFXP2	0.1881	2.69E-04	1.42E-03
ARGFX	0.0696	1.81E-01	2.93E-01
ARGLU1	0.2995	3.96E-09	8.18E-08
ARHGAP10	-0.1347	9.40E-03	2.78E-02
ARHGAP11A	0.3603	8.17E-13	3.73E-11
ARHGAP11B	0.2728	9.36E-08	1.37E-06
ARHGAP12	-0.0030	9.55E-01	9.72E-01
ARHGAP15	-0.0616	2.36E-01	3.60E-01
ARHGAP17	0.0139	7.90E-01	8.61E-01
ARHGAP18	0.0571	2.72E-01	4.00E-01
ARHGAP19	0.2207	1.80E-05	1.40E-04
ARHGAP1	0.0431	4.08E-01	5.40E-01
ARHGAP20	-0.1276	1.39E-02	3.84E-02
ARHGAP21	-0.0233	6.55E-01	7.57E-01
ARHGAP22	0.0659	2.06E-01	3.23E-01
ARHGAP23	-0.0968	6.25E-02	1.28E-01
ARHGAP24	-0.1334	1.01E-02	2.95E-02
ARHGAP25	-0.0948	6.83E-02	1.38E-01
ARHGAP26	-0.0011	9.84E-01	9.90E-01
ARHGAP27	0.0801	1.24E-01	2.19E-01
ARHGAP28	0.0823	1.13E-01	2.05E-01
ARHGAP29	-0.0733	1.59E-01	2.65E-01
ARHGAP30	-0.0652	2.10E-01	3.28E-01
ARHGAP31	-0.1805	4.78E-04	2.29E-03
ARHGAP32	0.0667	2.00E-01	3.16E-01
ARHGAP33	0.3964	2.06E-15	1.53E-13
ARHGAP36	-0.1630	1.63E-03	6.49E-03
ARHGAP39	0.2443	1.92E-06	2.02E-05
ARHGAP42	-0.1779	5.76E-04	2.68E-03
ARHGAP4	0.1515	3.43E-03	1.21E-02
ARHGAP5	-0.0448	3.90E-01	5.23E-01
ARHGAP6	-0.1470	4.55E-03	1.53E-02

ARHGAP8	0.0671	1.97E-01	3.13E-01
ARHGAP9	-0.0003	9.95E-01	9.97E-01
ARHGDIA	0.1034	4.66E-02	1.02E-01
ARHGDIB	-0.0760	1.44E-01	2.46E-01
ARHGDIG	0.0619	2.34E-01	3.57E-01
ARHGEF10L	0.0234	6.53E-01	7.56E-01
ARHGEF10	-0.1281	1.36E-02	3.76E-02
ARHGEF11	0.3749	7.90E-14	4.25E-12
ARHGEF12	-0.2251	1.20E-05	9.87E-05
ARHGEF15	-0.1901	2.31E-04	1.24E-03
ARHGEF16	0.1404	6.74E-03	2.11E-02
ARHGEF17	0.0056	9.14E-01	9.46E-01
ARHGEF18	0.0480	3.57E-01	4.89E-01
ARHGEF19	0.0927	7.46E-02	1.48E-01
ARHGEF1	0.2617	3.17E-07	4.10E-06
ARHGEF2	0.2857	2.14E-08	3.68E-07
ARHGEF33	0.0856	9.98E-02	1.86E-01
ARHGEF35	0.0244	6.39E-01	7.45E-01
ARHGEF37	-0.0850	1.02E-01	1.88E-01
ARHGEF38	0.1054	4.25E-02	9.46E-02
ARHGEF3	0.0420	4.20E-01	5.52E-01
ARHGEF4	0.0324	5.34E-01	6.56E-01
ARHGEF5	0.0655	2.08E-01	3.26E-01
ARHGEF6	-0.0965	6.35E-02	1.30E-01
ARHGEF7	0.1487	4.11E-03	1.41E-02
ARHGEF9	0.0004	9.93E-01	9.96E-01
ARID1A	0.0506	3.31E-01	4.62E-01
ARID1B	0.1206	2.02E-02	5.17E-02
ARID2	0.1117	3.16E-02	7.44E-02
ARID3A	0.1941	1.69E-04	9.54E-04
ARID3B	0.1959	1.46E-04	8.44E-04
ARID3C	-0.2243	1.29E-05	1.05E-04
ARID4A	-0.0898	8.43E-02	1.62E-01
ARID4B	0.1398	7.01E-03	2.18E-02
ARID5A	-0.1130	2.95E-02	7.06E-02
ARID5B	0.0715	1.70E-01	2.78E-01
ARIH1	-0.1114	3.20E-02	7.52E-02
ARIH2	0.1550	2.76E-03	1.01E-02
ARL10	-0.0972	6.13E-02	1.26E-01
ARL11	-0.0504	3.33E-01	4.64E-01
ARL13A	0.0475	3.61E-01	4.93E-01
ARL13B	0.0240	6.45E-01	7.50E-01
ARL14	0.0142	7.85E-01	8.57E-01
ARL15	-0.1766	6.34E-04	2.91E-03

ARL16	0.2655	2.09E-07	2.82E-06
ARL17A	0.1404	6.74E-03	2.11E-02
ARL17B	0.1520	3.33E-03	1.18E-02
ARL1	0.0013	9.80E-01	9.88E-01
ARL2BP	-0.1528	3.17E-03	1.13E-02
ARL2	0.1524	3.26E-03	1.16E-02
ARL3	0.1778	5.80E-04	2.69E-03
ARL4A	0.1361	8.67E-03	2.60E-02
ARL4C	0.0747	1.51E-01	2.55E-01
ARL4D	0.0008	9.88E-01	9.92E-01
ARL5A	-0.0143	7.84E-01	8.57E-01
ARL5B	-0.0547	2.94E-01	4.23E-01
ARL5C	0.0532	3.07E-01	4.36E-01
ARL6IP1	-0.0687	1.87E-01	3.00E-01
ARL6IP4	-0.0446	3.91E-01	5.24E-01
ARL6IP5	-0.2136	3.34E-05	2.40E-04
ARL6IP6	0.3029	2.60E-09	5.54E-08
ARL6	0.0350	5.02E-01	6.27E-01
ARL8A	0.0070	8.92E-01	9.31E-01
ARL8B	-0.0240	6.45E-01	7.50E-01
ARL9	0.1705	9.74E-04	4.21E-03
ARMC10	-0.1516	3.43E-03	1.21E-02
ARMC1	-0.0874	9.28E-02	1.75E-01
ARMC2	0.2544	6.86E-07	8.10E-06
ARMC3	0.2793	4.49E-08	7.12E-07
ARMC4	0.0379	4.66E-01	5.96E-01
ARMC5	-0.1512	3.51E-03	1.23E-02
ARMC6	-0.0066	9.00E-01	9.37E-01
ARMC7	0.1273	1.41E-02	3.88E-02
ARMC8	0.1175	2.36E-02	5.89E-02
ARMC9	0.0986	5.78E-02	1.20E-01
ARMCX1	0.1385	7.54E-03	2.31E-02
ARMCX2	0.0116	8.23E-01	8.84E-01
ARMCX3	0.0095	8.55E-01	9.06E-01
ARMCX5	0.1142	2.78E-02	6.73E-02
ARMCX6	0.0545	2.95E-01	4.24E-01
ARMS2	-0.0569	2.74E-01	4.03E-01
ARNT2	0.2608	3.48E-07	4.44E-06
ARNTL2	0.1250	1.60E-02	4.28E-02
ARNTL	0.0007	9.89E-01	9.93E-01
ARNT	0.3419	1.29E-11	4.50E-10
ARPC1A	-0.0291	5.76E-01	6.93E-01
ARPC1B	-0.0112	8.29E-01	8.88E-01
ARPC2	0.0635	2.23E-01	3.44E-01

ARPC3	0.0864	9.66E-02	1.81E-01
ARPC4	0.0710	1.72E-01	2.82E-01
ARPC5L	0.1806	4.75E-04	2.27E-03
ARPC5	0.3358	3.14E-11	1.03E-09
ARPM1	-0.0692	1.84E-01	2.96E-01
ARPP19	-0.0063	9.04E-01	9.39E-01
ARPP21	-0.1600	1.99E-03	7.66E-03
ARR3	0.0425	4.14E-01	5.47E-01
ARRB1	0.0567	2.76E-01	4.04E-01
ARRB2	0.1093	3.53E-02	8.17E-02
ARRDC1	0.0327	5.30E-01	6.53E-01
ARRDC2	0.0376	4.71E-01	6.00E-01
ARRDC3	-0.0093	8.59E-01	9.09E-01
ARRDC4	-0.1529	3.15E-03	1.13E-02
ARRDC5	0.0209	6.88E-01	7.84E-01
ARSA	-0.2347	4.91E-06	4.57E-05
ARSB	-0.1108	3.28E-02	7.69E-02
ARSD	-0.1932	1.81E-04	1.01E-03
ARSE	-0.1081	3.74E-02	8.56E-02
ARSF	-0.0427	4.12E-01	5.45E-01
ARSG	-0.0854	1.00E-01	1.86E-01
ARSH	0.0122	8.15E-01	8.78E-01
ARSI	0.1339	9.84E-03	2.89E-02
ARSJ	-0.0321	5.37E-01	6.59E-01
ARSK	-0.0524	3.14E-01	4.44E-01
ART1	-0.1277	1.38E-02	3.82E-02
ART3	0.1275	1.40E-02	3.86E-02
ART4	-0.1042	4.49E-02	9.89E-02
ART5	-0.0053	9.20E-01	9.49E-01
ARTN	0.1607	1.90E-03	7.37E-03
ARV1	0.1439	5.48E-03	1.78E-02
ARVCF	-0.0356	4.95E-01	6.21E-01
ARX	0.0353	4.98E-01	6.24E-01
AR	-0.2067	6.01E-05	3.95E-04
AS3MT	-0.0847	1.03E-01	1.90E-01
ASAH1	-0.0841	1.06E-01	1.94E-01
ASAH2B	-0.0470	3.67E-01	4.99E-01
ASAH2	-0.1221	1.87E-02	4.86E-02
ASAM	-0.0508	3.29E-01	4.60E-01
ASAP1IT1	-0.0117	8.23E-01	8.84E-01
ASAP1	0.1321	1.09E-02	3.12E-02
ASAP2	0.0742	1.54E-01	2.58E-01
ASAP3	-0.1047	4.38E-02	9.69E-02
ASB10	0.0144	7.82E-01	8.55E-01

ASB11	0.1823	4.17E-04	2.05E-03
ASB12	0.1609	1.88E-03	7.31E-03
ASB13	-0.1912	2.12E-04	1.16E-03
ASB14	0.1468	4.62E-03	1.54E-02
ASB15	-0.1267	1.46E-02	3.98E-02
ASB16	0.2875	1.71E-08	3.02E-07
ASB17	0.1312	1.14E-02	3.26E-02
ASB18	0.0226	6.64E-01	7.64E-01
ASB1	0.1177	2.33E-02	5.82E-02
ASB2	-0.0138	7.91E-01	8.61E-01
ASB3	0.1961	1.44E-04	8.35E-04
ASB4	-0.2154	2.86E-05	2.10E-04
ASB5	-0.0921	7.66E-02	1.51E-01
ASB6	0.3365	2.84E-11	9.41E-10
ASB7	-0.1472	4.50E-03	1.51E-02
ASB8	-0.2511	9.65E-07	1.10E-05
ASB9	0.1820	4.26E-04	2.08E-03
ASCC1	-0.1573	2.38E-03	8.90E-03
ASCC2	-0.0032	9.51E-01	9.69E-01
ASCC3	0.0803	1.23E-01	2.18E-01
ASCL1	-0.0650	2.12E-01	3.31E-01
ASCL2	0.1032	4.71E-02	1.03E-01
ASCL3	0.1274	1.40E-02	3.86E-02
ASCL4	0.0733	1.59E-01	2.65E-01
ASF1A	0.2544	6.86E-07	8.10E-06
ASF1B	0.4220	1.87E-17	2.30E-15
ASFMR1	0.1738	7.74E-04	3.45E-03
ASGR1	-0.0859	9.85E-02	1.84E-01
ASGR2	-0.1135	2.88E-02	6.92E-02
ASH1L	0.3165	4.45E-10	1.15E-08
ASH2L	0.0592	2.55E-01	3.81E-01
ASIP	-0.2625	2.89E-07	3.79E-06
ASL	-0.2648	2.26E-07	3.02E-06
ASMTL	-0.1961	1.44E-04	8.35E-04
ASMT	0.1822	4.20E-04	2.06E-03
ASNA1	-0.0697	1.80E-01	2.92E-01
ASNSD1	-0.0397	4.46E-01	5.78E-01
ASNS	0.1287	1.31E-02	3.65E-02
ASPA	-0.2042	7.46E-05	4.76E-04
ASPDH	-0.2685	1.52E-07	2.11E-06
ASPG	-0.1378	7.85E-03	2.40E-02
ASPHD1	0.0240	6.45E-01	7.50E-01
ASPHD2	0.1848	3.47E-04	1.76E-03
ASPH	0.0518	3.19E-01	4.49E-01

ASPM	0.4480	1.02E-19	2.42E-17
ASPN	-0.0755	1.47E-01	2.49E-01
ASPRV1	-0.1206	2.01E-02	5.16E-02
ASPSCR1	0.0131	8.01E-01	8.69E-01
ASRGL1	0.2833	2.82E-08	4.66E-07
ASS1	-0.2610	3.40E-07	4.37E-06
ASTE1	0.1156	2.59E-02	6.35E-02
ASTL	0.0638	2.20E-01	3.40E-01
ASTN1	-0.2137	3.33E-05	2.39E-04
ASTN2	0.0169	7.46E-01	8.28E-01
ASXL1	0.3090	1.20E-09	2.79E-08
ASXL2	-0.0689	1.85E-01	2.98E-01
ASXL3	-0.1767	6.27E-04	2.89E-03
ASZ1	0.0723	1.64E-01	2.72E-01
ATAD1	-0.2072	5.79E-05	3.84E-04
ATAD2B	0.0807	1.21E-01	2.15E-01
ATAD2	0.3048	2.05E-09	4.53E-08
ATAD3A	0.0185	7.22E-01	8.12E-01
ATAD3B	0.1545	2.84E-03	1.03E-02
ATAD3C	-0.0342	5.12E-01	6.36E-01
ATAD5	0.4342	1.72E-18	2.81E-16
ATCAY	-0.0154	7.68E-01	8.43E-01
ATE1	-0.1012	5.15E-02	1.10E-01
ATF1	0.1297	1.24E-02	3.48E-02
ATF2	-0.0170	7.44E-01	8.27E-01
ATF3	-0.1347	9.37E-03	2.78E-02
ATF4	0.1417	6.25E-03	1.98E-02
ATF5	0.0314	5.47E-01	6.67E-01
ATF6B	0.1643	1.49E-03	6.02E-03
ATF6	0.1101	3.39E-02	7.91E-02
ATF7IP2	0.0972	6.15E-02	1.27E-01
ATF7IP	-0.0029	9.56E-01	9.72E-01
ATF7	0.0822	1.14E-01	2.06E-01
ATG10	0.0074	8.86E-01	9.27E-01
ATG12	0.0520	3.18E-01	4.48E-01
ATG16L1	0.0322	5.36E-01	6.58E-01
ATG16L2	0.2464	1.55E-06	1.66E-05
ATG2A	-0.2066	6.08E-05	3.99E-04
ATG2B	-0.0562	2.80E-01	4.09E-01
ATG3	0.0299	5.66E-01	6.84E-01
ATG4A	-0.1913	2.10E-04	1.15E-03
ATG4B	0.2007	9.95E-05	6.06E-04
ATG4C	-0.1008	5.23E-02	1.11E-01
ATG4D	0.0075	8.85E-01	9.26E-01

ATG5	0.1261	1.51E-02	4.08E-02
ATG7	-0.0232	6.57E-01	7.58E-01
ATG9A	0.0493	3.43E-01	4.75E-01
ATG9B	0.1437	5.54E-03	1.79E-02
ATHL1	0.0199	7.03E-01	7.96E-01
ATIC	0.1785	5.51E-04	2.58E-03
ATL1	-0.1112	3.22E-02	7.57E-02
ATL2	0.0451	3.86E-01	5.19E-01
ATL3	-0.1076	3.84E-02	8.73E-02
ATMIN	-0.1933	1.80E-04	1.00E-03
ATM	0.0592	2.56E-01	3.81E-01
ATN1	0.2268	1.03E-05	8.64E-05
ATOH1	0.1317	1.11E-02	3.18E-02
ATOH7	-0.0257	6.21E-01	7.31E-01
ATOH8	-0.1926	1.90E-04	1.06E-03
ATOX1	-0.0945	6.91E-02	1.39E-01
ATP10A	-0.0218	6.76E-01	7.75E-01
ATP10B	0.1443	5.34E-03	1.75E-02
ATP10D	-0.0646	2.14E-01	3.34E-01
ATP11A	0.1394	7.15E-03	2.21E-02
ATP11B	0.0603	2.47E-01	3.71E-01
ATP11C	-0.1139	2.83E-02	6.82E-02
ATP12A	0.1054	4.26E-02	9.47E-02
ATP13A1	0.1010	5.18E-02	1.11E-01
ATP13A2	0.1705	9.77E-04	4.22E-03
ATP13A3	-0.0249	6.32E-01	7.39E-01
ATP13A4	-0.0072	8.90E-01	9.29E-01
ATP13A5	-0.0258	6.20E-01	7.29E-01
ATP1A1	0.1913	2.10E-04	1.15E-03
ATP1A2	-0.1799	4.99E-04	2.37E-03
ATP1A3	-0.0501	3.36E-01	4.67E-01
ATP1A4	0.0619	2.34E-01	3.57E-01
ATP1B1	0.1934	1.79E-04	1.00E-03
ATP1B2	-0.0795	1.27E-01	2.23E-01
ATP1B3	0.1403	6.80E-03	2.12E-02
ATP1B4	-0.0180	7.30E-01	8.17E-01
ATP2A1	0.0360	4.89E-01	6.16E-01
ATP2A2	0.0716	1.69E-01	2.78E-01
ATP2A3	-0.0891	8.67E-02	1.66E-01
ATP2B1	0.0832	1.10E-01	2.00E-01
ATP2B2	-0.0645	2.15E-01	3.35E-01
ATP2B3	0.0083	8.73E-01	9.18E-01
ATP2B4	0.0335	5.20E-01	6.44E-01
ATP2C1	-0.0798	1.25E-01	2.20E-01

ATP2C2	0.2002	1.03E-04	6.24E-04
ATP4A	0.1781	5.69E-04	2.65E-03
ATP4B	0.0609	2.42E-01	3.67E-01
ATP5A1	-0.2871	1.81E-08	3.17E-07
ATP5B	-0.2663	1.92E-07	2.62E-06
ATP5C1	-0.0622	2.32E-01	3.55E-01
ATP5D	-0.1457	4.92E-03	1.63E-02
ATP5EP2	-0.0120	8.17E-01	8.80E-01
ATP5E	0.0237	6.49E-01	7.53E-01
ATP5F1	-0.1644	1.48E-03	5.98E-03
ATP5G1	0.1203	2.04E-02	5.22E-02
ATP5G2	0.1534	3.06E-03	1.10E-02
ATP5G3	-0.1772	6.08E-04	2.80E-03
ATP5H	0.0253	6.27E-01	7.35E-01
ATP5I	-0.0305	5.58E-01	6.77E-01
ATP5J2	-0.0676	1.94E-01	3.09E-01
ATP5J	-0.1085	3.67E-02	8.42E-02
ATP5L2	0.0162	7.56E-01	8.36E-01
ATP5L	-0.0049	9.25E-01	9.52E-01
ATP5O	-0.0526	3.13E-01	4.42E-01
ATP5SL	-0.0154	7.68E-01	8.44E-01
ATP5S	-0.0726	1.63E-01	2.70E-01
ATP6AP1L	-0.0404	4.38E-01	5.70E-01
ATP6AP1	0.1578	2.30E-03	8.67E-03
ATP6AP2	-0.0575	2.69E-01	3.97E-01
ATP6V0A1	-0.0267	6.08E-01	7.20E-01
ATP6V0A2	0.1289	1.29E-02	3.61E-02
ATP6V0A4	0.0764	1.42E-01	2.43E-01
ATP6V0B	-0.0103	8.44E-01	8.98E-01
ATP6V0C	-0.0300	5.65E-01	6.83E-01
ATP6V0D1	-0.2532	7.75E-07	9.03E-06
ATP6V0D2	0.1733	8.02E-04	3.56E-03
ATP6V0E1	-0.1167	2.45E-02	6.06E-02
ATP6V0E2	-0.2369	3.97E-06	3.81E-05
ATP6V1A	-0.1564	2.52E-03	9.33E-03
ATP6V1B1	0.1460	4.83E-03	1.60E-02
ATP6V1B2	-0.1277	1.38E-02	3.82E-02
ATP6V1C1	0.0591	2.56E-01	3.82E-01
ATP6V1C2	0.2730	9.18E-08	1.34E-06
ATP6V1D	-0.0559	2.83E-01	4.11E-01
ATP6V1E1	0.1235	1.74E-02	4.57E-02
ATP6V1E2	0.0533	3.06E-01	4.36E-01
ATP6V1F	-0.0144	7.82E-01	8.55E-01
ATP6V1G1	-0.0241	6.43E-01	7.48E-01

ATP6V1G2	-0.0440	3.98E-01	5.31E-01
ATP6V1G3	0.1134	2.90E-02	6.96E-02
ATP6V1H	-0.0734	1.58E-01	2.64E-01
ATP7A	0.1857	3.23E-04	1.66E-03
ATP7B	-0.1441	5.42E-03	1.77E-02
ATP8A1	-0.0283	5.87E-01	7.02E-01
ATP8A2	-0.0043	9.35E-01	9.59E-01
ATP8B1	-0.1043	4.47E-02	9.84E-02
ATP8B2	0.0871	9.41E-02	1.77E-01
ATP8B3	0.0004	9.94E-01	9.96E-01
ATP8B4	-0.1628	1.65E-03	6.57E-03
ATP8B5P	0.0298	5.67E-01	6.85E-01
ATP9A	0.0441	3.97E-01	5.30E-01
ATP9B	0.1228	1.80E-02	4.72E-02
ATPAF1	-0.1892	2.48E-04	1.32E-03
ATPAF2	-0.1485	4.16E-03	1.42E-02
ATPBD4	-0.0742	1.54E-01	2.59E-01
ATPIF1	-0.1163	2.51E-02	6.17E-02
ATRIP	0.2881	1.60E-08	2.85E-07
ATRNL1	-0.0137	7.92E-01	8.62E-01
ATRN	0.0044	9.32E-01	9.58E-01
ATRX	0.0416	4.24E-01	5.57E-01
ATR	0.0607	2.43E-01	3.68E-01
ATXN10	0.0625	2.30E-01	3.53E-01
ATXN1L	-0.1131	2.94E-02	7.03E-02
ATXN1	-0.0365	4.83E-01	6.12E-01
ATXN2L	0.2819	3.33E-08	5.43E-07
ATXN2	0.1658	1.35E-03	5.53E-03
ATXN3L	0.1053	4.26E-02	9.47E-02
ATXN3	-0.1255	1.56E-02	4.19E-02
ATXN7L1	-0.2311	6.91E-06	6.11E-05
ATXN7L2	0.2536	7.45E-07	8.72E-06
ATXN7L3B	0.0365	4.83E-01	6.12E-01
ATXN7L3	0.2699	1.29E-07	1.83E-06
ATXN7	0.0590	2.57E-01	3.82E-01
AUH	-0.2656	2.08E-07	2.81E-06
AUP1	0.1003	5.36E-02	1.13E-01
AURKAIP1	-0.1095	3.50E-02	8.10E-02
AURKAPS1	0.4231	1.53E-17	1.95E-15
AURKA	0.3496	4.15E-12	1.64E-10
AURKB	0.4100	1.80E-16	1.74E-14
AURKC	-0.0986	5.77E-02	1.20E-01
AUTS2	-0.1138	2.84E-02	6.84E-02
AVEN	-0.1880	2.71E-04	1.43E-03

AVIL	0.0285	5.84E-01	7.00E-01
AVL9	0.0106	8.39E-01	8.94E-01
AVPI1	-0.1587	2.17E-03	8.23E-03
AVPR1A	-0.1340	9.79E-03	2.87E-02
AVPR1B	0.1024	4.88E-02	1.06E-01
AVPR2	-0.1631	1.62E-03	6.47E-03
AVP	0.0612	2.40E-01	3.64E-01
AWAT1	0.0288	5.80E-01	6.96E-01
AWAT2	-0.0705	1.76E-01	2.86E-01
AXIN1	0.1866	3.02E-04	1.57E-03
AXIN2	-0.0159	7.60E-01	8.38E-01
AXL	-0.1115	3.17E-02	7.47E-02
AZGP1	-0.2436	2.05E-06	2.13E-05
AZI1	0.4666	1.85E-21	5.50E-19
AZI2	0.0625	2.30E-01	3.53E-01
AZIN1	-0.0069	8.95E-01	9.33E-01
AZU1	-0.0072	8.89E-01	9.29E-01
B2M	-0.2043	7.36E-05	4.71E-04
B3GALNT1	0.2057	6.55E-05	4.25E-04
B3GALNT2	0.1929	1.85E-04	1.03E-03
B3GALT1	-0.0396	4.47E-01	5.79E-01
B3GALT2	-0.0727	1.62E-01	2.70E-01
B3GALT4	0.0456	3.81E-01	5.15E-01
B3GALT5	0.1014	5.10E-02	1.09E-01
B3GALT6	0.0262	6.15E-01	7.25E-01
B3GALTL	0.0265	6.12E-01	7.23E-01
B3GAT1	0.0957	6.56E-02	1.33E-01
B3GAT2	0.1325	1.06E-02	3.07E-02
B3GAT3	-0.0292	5.75E-01	6.92E-01
B3GNT1	-0.0929	7.40E-02	1.47E-01
B3GNT2	-0.0474	3.62E-01	4.95E-01
B3GNT3	0.1353	9.05E-03	2.70E-02
B3GNT4	0.0791	1.28E-01	2.25E-01
B3GNT5	0.1393	7.19E-03	2.22E-02
B3GNT6	0.0538	3.02E-01	4.31E-01
B3GNT7	0.0961	6.44E-02	1.31E-01
B3GNT8	0.0536	3.03E-01	4.33E-01
B3GNT9	0.0073	8.89E-01	9.29E-01
B3GNTL1	0.2037	7.73E-05	4.90E-04
B4GALNT1	0.0844	1.04E-01	1.92E-01
B4GALNT2	0.2430	2.18E-06	2.25E-05
B4GALNT3	0.0201	6.99E-01	7.92E-01
B4GALNT4	0.1711	9.38E-04	4.07E-03
B4GALT1	0.0029	9.55E-01	9.72E-01

B4GALT2	0.0251	6.30E-01	7.38E-01
B4GALT3	0.4409	4.48E-19	9.20E-17
B4GALT4	0.0635	2.23E-01	3.44E-01
B4GALT5	0.0837	1.07E-01	1.96E-01
B4GALT6	0.0887	8.81E-02	1.68E-01
B4GALT7	0.0135	7.96E-01	8.65E-01
B9D1	0.0011	9.83E-01	9.89E-01
B9D2	0.0172	7.41E-01	8.25E-01
BAALC	-0.1128	2.98E-02	7.10E-02
BAAT	-0.2299	7.70E-06	6.67E-05
BACE1	-0.2391	3.20E-06	3.14E-05
BACE2	0.0559	2.83E-01	4.11E-01
BACH1	0.0389	4.55E-01	5.86E-01
BACH2	-0.0706	1.75E-01	2.85E-01
BAD	0.0166	7.51E-01	8.32E-01
BAG1	-0.0934	7.23E-02	1.44E-01
BAG2	0.1067	4.00E-02	9.02E-02
BAG3	-0.0156	7.65E-01	8.41E-01
BAG4	-0.0372	4.75E-01	6.04E-01
BAG5	0.0528	3.11E-01	4.40E-01
BAGE2	0.0687	1.86E-01	3.00E-01
BAGE	0.0315	5.45E-01	6.65E-01
BAHCC1	0.1568	2.45E-03	9.10E-03
BAHD1	-0.0548	2.92E-01	4.21E-01
BAI1	0.0938	7.12E-02	1.42E-01
BAI2	0.1408	6.62E-03	2.08E-02
BAI3	-0.1103	3.37E-02	7.87E-02
BAIAP2L1	0.0796	1.26E-01	2.22E-01
BAIAP2L2	0.1863	3.09E-04	1.60E-03
BAIAP2	-0.1338	9.89E-03	2.90E-02
BAIAP3	-0.0420	4.20E-01	5.53E-01
BAK1	0.1651	1.41E-03	5.75E-03
BAMBI	0.0523	3.15E-01	4.45E-01
BANF1	0.0984	5.82E-02	1.21E-01
BANF2	0.0965	6.34E-02	1.30E-01
BANK1	0.0675	1.95E-01	3.10E-01
BANP	0.0171	7.43E-01	8.27E-01
BAP1	0.0347	5.05E-01	6.30E-01
BARD1	0.1820	4.27E-04	2.08E-03
BARHL1	0.0148	7.76E-01	8.50E-01
BARHL2	0.1117	3.14E-02	7.42E-02
BARX1	0.0527	3.11E-01	4.41E-01
BARX2	0.1099	3.44E-02	7.99E-02
BASE	0.2465	1.54E-06	1.66E-05

BASP1	-0.1172	2.40E-02	5.97E-02
BAT1	0.2985	4.48E-09	9.08E-08
BAT2L1	0.1684	1.13E-03	4.79E-03
BAT2L2	0.3680	2.44E-13	1.21E-11
BAT2	0.2303	7.40E-06	6.47E-05
BAT3	0.1275	1.40E-02	3.85E-02
BAT4	0.1217	1.91E-02	4.94E-02
BAT5	-0.0572	2.72E-01	3.99E-01
BATF2	0.0173	7.40E-01	8.25E-01
BATF3	-0.0265	6.11E-01	7.22E-01
BATF	-0.0263	6.13E-01	7.24E-01
BAX	0.1270	1.44E-02	3.94E-02
BAZ1A	0.0863	9.70E-02	1.82E-01
BAZ1B	0.0525	3.13E-01	4.42E-01
BAZ2A	0.2402	2.88E-06	2.86E-05
BAZ2B	-0.0967	6.27E-02	1.29E-01
BBC3	0.0953	6.67E-02	1.35E-01
BBOX1	-0.2978	4.92E-09	9.85E-08
BBS10	-0.0007	9.89E-01	9.93E-01
BBS12	0.0201	6.99E-01	7.93E-01
BBS1	-0.1244	1.65E-02	4.39E-02
BBS2	-0.2280	9.19E-06	7.80E-05
BBS4	-0.0392	4.51E-01	5.83E-01
BBS5	0.0751	1.49E-01	2.52E-01
BBS7	0.0717	1.68E-01	2.76E-01
BBS9	-0.0797	1.26E-01	2.22E-01
BBX	-0.0245	6.39E-01	7.45E-01
BCAM	-0.0037	9.44E-01	9.66E-01
BCAN	0.0896	8.49E-02	1.63E-01
BCAP29	-0.1416	6.31E-03	2.00E-02
BCAP31	0.0459	3.78E-01	5.11E-01
BCAR1	-0.1680	1.16E-03	4.87E-03
BCAR3	-0.0265	6.11E-01	7.22E-01
BCAR4	0.0491	3.46E-01	4.78E-01
BCAS1	0.0721	1.66E-01	2.74E-01
BCAS2	0.0523	3.15E-01	4.45E-01
BCAS3	-0.0986	5.77E-02	1.20E-01
BCAS4	0.1060	4.12E-02	9.24E-02
BCAT1	0.0618	2.35E-01	3.58E-01
BCAT2	0.1494	3.92E-03	1.35E-02
BCCIP	-0.0144	7.82E-01	8.55E-01
BCDIN3D	0.0429	4.10E-01	5.42E-01
BCHE	-0.1178	2.33E-02	5.81E-02
BCKDHA	-0.2764	6.25E-08	9.53E-07

BCKDHB	-0.2443	1.92E-06	2.02E-05
BCKDK	-0.3184	3.46E-10	9.21E-09
BCL10	0.1137	2.86E-02	6.87E-02
BCL11A	0.1377	7.92E-03	2.41E-02
BCL11B	-0.0124	8.11E-01	8.76E-01
BCL2A1	0.0245	6.39E-01	7.45E-01
BCL2L10	-0.0294	5.72E-01	6.90E-01
BCL2L11	0.2263	1.08E-05	9.01E-05
BCL2L12	0.2338	5.33E-06	4.89E-05
BCL2L13	-0.1118	3.14E-02	7.41E-02
BCL2L14	0.0984	5.82E-02	1.21E-01
BCL2L15	0.1924	1.93E-04	1.07E-03
BCL2L1	-0.0940	7.06E-02	1.41E-01
BCL2L2	0.0262	6.14E-01	7.24E-01
BCL2	-0.1305	1.19E-02	3.37E-02
BCL3	0.0854	1.00E-01	1.86E-01
BCL6B	-0.1337	9.92E-03	2.90E-02
BCL6	0.0071	8.92E-01	9.31E-01
BCL7A	0.1530	3.13E-03	1.12E-02
BCL7B	-0.1468	4.60E-03	1.54E-02
BCL7C	0.1380	7.77E-03	2.37E-02
BCL8	-0.0234	6.53E-01	7.56E-01
BCL9L	0.1055	4.22E-02	9.40E-02
BCL9	0.4930	4.03E-24	2.06E-21
BCLAF1	0.1759	6.64E-04	3.03E-03
BCMO1	-0.0743	1.53E-01	2.58E-01
BCO2	-0.2462	1.59E-06	1.70E-05
BCORL1	0.1919	2.01E-04	1.11E-03
BCORL2	-0.0017	9.73E-01	9.83E-01
BCOR	0.2114	4.06E-05	2.83E-04
BCR	0.0063	9.04E-01	9.39E-01
BCS1L	0.0357	4.93E-01	6.19E-01
BCYRN1	0.0238	6.48E-01	7.52E-01
BDH1	-0.2928	9.03E-09	1.69E-07
BDH2	-0.2622	2.99E-07	3.90E-06
BDKRB1	0.0151	7.72E-01	8.47E-01
BDKRB2	-0.1224	1.84E-02	4.80E-02
BDNFOS	-0.1902	2.30E-04	1.24E-03
BDNF	-0.0458	3.79E-01	5.12E-01
BDP1	0.0389	4.56E-01	5.86E-01
BEAN	-0.0453	3.84E-01	5.17E-01
BECN1	0.0072	8.90E-01	9.30E-01
BEGAIN	0.0432	4.06E-01	5.39E-01
BEND2	-0.0387	4.57E-01	5.88E-01

BEND3	0.3707	1.59E-13	8.19E-12
BEND4	-0.0528	3.11E-01	4.40E-01
BEND5	-0.0262	6.16E-01	7.25E-01
BEND6	-0.0299	5.66E-01	6.84E-01
BEND7	0.0559	2.83E-01	4.12E-01
BEST1	0.1497	3.85E-03	1.33E-02
BEST2	0.0704	1.76E-01	2.86E-01
BEST3	-0.0882	8.98E-02	1.71E-01
BEST4	0.1683	1.14E-03	4.81E-03
BET1L	-0.0961	6.45E-02	1.31E-01
BET1	-0.1224	1.83E-02	4.78E-02
BET3L	-0.0685	1.88E-01	3.02E-01
BEX1	0.0335	5.20E-01	6.44E-01
BEX2	0.2509	9.85E-07	1.12E-05
BEX4	0.0744	1.52E-01	2.57E-01
BEX5	0.1079	3.78E-02	8.63E-02
BEYLA	0.0795	1.27E-01	2.23E-01
BFAR	-0.0728	1.62E-01	2.69E-01
BFSP1	0.2947	7.17E-09	1.37E-07
BFSP2	0.2554	6.16E-07	7.34E-06
BGLAP	0.2263	1.07E-05	8.96E-05
BGN	-0.1025	4.86E-02	1.05E-01
BHLHA15	-0.0618	2.35E-01	3.58E-01
BHLHB9	0.1027	4.80E-02	1.04E-01
BHLHE22	-0.0579	2.66E-01	3.93E-01
BHLHE40	-0.1420	6.13E-03	1.95E-02
BHLHE41	0.0187	7.19E-01	8.09E-01
BHMT2	-0.1356	8.94E-03	2.67E-02
BHMT	-0.1284	1.33E-02	3.70E-02
BICC1	0.0046	9.30E-01	9.56E-01
BICD1	0.1264	1.49E-02	4.04E-02
BICD2	0.1486	4.11E-03	1.41E-02
BID	0.0423	4.16E-01	5.49E-01
BIK	0.0482	3.55E-01	4.87E-01
BIN1	-0.0691	1.84E-01	2.97E-01
BIN2	-0.0790	1.29E-01	2.26E-01
BIN3	-0.0020	9.70E-01	9.81E-01
BIRC2	0.0830	1.11E-01	2.01E-01
BIRC3	0.1663	1.30E-03	5.37E-03
BIRC5	0.4183	3.81E-17	4.22E-15
BIRC6	-0.0330	5.27E-01	6.49E-01
BIRC7	0.0685	1.88E-01	3.01E-01
BIRC8	0.0929	7.39E-02	1.46E-01
BIVM	0.0531	3.08E-01	4.38E-01

BLCAP	-0.2199	1.93E-05	1.50E-04
BLID	-0.0916	7.79E-02	1.53E-01
BLK	-0.0467	3.70E-01	5.03E-01
BLMH	0.2046	7.19E-05	4.61E-04
BLM	0.4175	4.42E-17	4.84E-15
BLNK	-0.0006	9.90E-01	9.94E-01
BLOC1S1	-0.0226	6.65E-01	7.65E-01
BLOC1S2	-0.1276	1.39E-02	3.84E-02
BLOC1S3	0.1914	2.08E-04	1.14E-03
BLVRA	0.1021	4.94E-02	1.07E-01
BLVRB	-0.1321	1.08E-02	3.12E-02
BLZF1	0.3586	1.06E-12	4.72E-11
BMF	0.1948	1.59E-04	9.07E-04
BMI1	-0.0209	6.89E-01	7.84E-01
BMP10	-0.1950	1.57E-04	8.96E-04
BMP15	-0.0245	6.38E-01	7.45E-01
BMP1	-0.2040	7.57E-05	4.82E-04
BMP2K	-0.1028	4.78E-02	1.04E-01
BMP2	-0.0072	8.90E-01	9.30E-01
BMP3	-0.0330	5.26E-01	6.49E-01
BMP4	-0.0160	7.58E-01	8.38E-01
BMP5	-0.1428	5.87E-03	1.88E-02
BMP6	-0.0673	1.96E-01	3.11E-01
BMP7	0.1283	1.34E-02	3.72E-02
BMP8A	0.1961	1.44E-04	8.34E-04
BMP8B	0.2311	6.89E-06	6.10E-05
BMPER	-0.2045	7.27E-05	4.65E-04
BMPR1A	0.0902	8.28E-02	1.60E-01
BMPR1B	-0.0619	2.34E-01	3.57E-01
BMPR2	-0.0983	5.85E-02	1.22E-01
BMS1P4	0.0884	8.91E-02	1.70E-01
BMS1P5	0.1530	3.14E-03	1.12E-02
BMS1	0.1636	1.56E-03	6.26E-03
BMX	-0.2394	3.12E-06	3.07E-05
BNC1	-0.0618	2.35E-01	3.59E-01
BNC2	-0.1024	4.88E-02	1.06E-01
BNIP1	0.0305	5.58E-01	6.77E-01
BNIP2	-0.0721	1.66E-01	2.74E-01
BNIP3L	-0.0553	2.88E-01	4.17E-01
BNIP3	-0.1773	6.03E-04	2.78E-03
BNIPL	0.1965	1.39E-04	8.07E-04
BOC	-0.1652	1.40E-03	5.71E-03
BOD1L	0.0083	8.73E-01	9.18E-01
BOD1	0.1673	1.22E-03	5.08E-03

BOK	-0.3283	9.02E-11	2.66E-09
BOLA1	0.3555	1.70E-12	7.24E-11
BOLA2	0.1705	9.76E-04	4.22E-03
BOLA3	0.1028	4.78E-02	1.04E-01
BOLL	0.1766	6.31E-04	2.90E-03
BOP1	0.0012	9.81E-01	9.88E-01
BPESC1	0.1299	1.22E-02	3.45E-02
BPGM	-0.0385	4.59E-01	5.90E-01
BPHL	-0.1855	3.28E-04	1.68E-03
BPIL1	0.0740	1.55E-01	2.60E-01
BPIL2	0.1594	2.07E-03	7.93E-03
BPIL3	0.0817	1.16E-01	2.09E-01
BPI	-0.0538	3.01E-01	4.30E-01
BPNT1	0.1015	5.07E-02	1.09E-01
BPTF	0.1792	5.23E-04	2.47E-03
BPY2	-0.0025	9.62E-01	9.76E-01
BRAF	0.0938	7.12E-02	1.42E-01
BRAP	0.1302	1.21E-02	3.42E-02
BRCA1	0.4267	7.54E-18	1.04E-15
BRCA2	0.2056	6.62E-05	4.29E-04
BRCC3	0.1557	2.63E-03	9.68E-03
BRD1	0.1264	1.49E-02	4.04E-02
BRD2	0.0894	8.56E-02	1.64E-01
BRD3	0.1625	1.68E-03	6.66E-03
BRD4	0.0831	1.10E-01	2.01E-01
BRD7P3	0.0004	9.93E-01	9.96E-01
BRD7	-0.0360	4.89E-01	6.17E-01
BRD8	0.2802	4.06E-08	6.47E-07
BRD9	0.2280	9.19E-06	7.80E-05
BRDT	0.1309	1.16E-02	3.31E-02
BREA2	0.1672	1.23E-03	5.11E-03
BRE	-0.0606	2.45E-01	3.69E-01
BRF1	0.0142	7.86E-01	8.58E-01
BRF2	0.2200	1.91E-05	1.48E-04
BRI3BP	0.1301	1.21E-02	3.42E-02
BRI3	-0.0790	1.29E-01	2.26E-01
BRIP1	0.2159	2.75E-05	2.03E-04
BRIX1	0.3690	2.07E-13	1.04E-11
BRMS1L	-0.0384	4.61E-01	5.91E-01
BRMS1	0.1010	5.19E-02	1.11E-01
BRP44L	-0.2166	2.58E-05	1.93E-04
BRP44	0.3521	2.89E-12	1.17E-10
BRPF1	0.2625	2.90E-07	3.80E-06
BRPF3	0.1239	1.70E-02	4.50E-02

BRS3	0.0999	5.46E-02	1.15E-01
BRSK1	0.1675	1.20E-03	5.03E-03
BRSK2	0.0405	4.37E-01	5.68E-01
BRWD1	-0.0402	4.40E-01	5.71E-01
BRWD3	0.0502	3.35E-01	4.67E-01
BSCL2	0.0550	2.91E-01	4.20E-01
BSDC1	-0.2461	1.61E-06	1.72E-05
BSG	0.0137	7.93E-01	8.63E-01
BSND	0.1523	3.28E-03	1.16E-02
BSN	0.0533	3.06E-01	4.36E-01
BSPRY	0.1511	3.53E-03	1.24E-02
BST1	0.0077	8.82E-01	9.25E-01
BST2	-0.1166	2.48E-02	6.10E-02
BSX	-0.0176	7.35E-01	8.21E-01
BTAF1	0.0765	1.42E-01	2.43E-01
BTBD10	0.0906	8.15E-02	1.58E-01
BTBD11	0.0333	5.23E-01	6.47E-01
BTBD12	0.1395	7.11E-03	2.20E-02
BTBD16	-0.1329	1.04E-02	3.01E-02
BTBD17	0.0681	1.91E-01	3.05E-01
BTBD18	0.0656	2.07E-01	3.26E-01
BTBD19	-0.1272	1.42E-02	3.90E-02
BTBD1	-0.1922	1.96E-04	1.08E-03
BTBD2	0.0100	8.48E-01	9.01E-01
BTBD3	0.1344	9.56E-03	2.82E-02
BTBD6	-0.0185	7.22E-01	8.12E-01
BTBD7	0.0819	1.15E-01	2.08E-01
BTBD8	0.1335	1.00E-02	2.93E-02
BTBD9	0.0609	2.42E-01	3.66E-01
BTC	0.0768	1.40E-01	2.41E-01
BTD	-0.3288	8.44E-11	2.51E-09
BTF3L1	0.0309	5.53E-01	6.72E-01
BTF3L4	0.1888	2.55E-04	1.36E-03
BTF3	0.0391	4.52E-01	5.83E-01
BTG1	0.0450	3.87E-01	5.20E-01
BTG2	0.0297	5.68E-01	6.86E-01
BTG3	0.2389	3.25E-06	3.19E-05
BTG4	0.0514	3.23E-01	4.54E-01
BTK	-0.0776	1.36E-01	2.35E-01
BTLA	-0.0248	6.34E-01	7.41E-01
BTN1A1	0.0719	1.67E-01	2.75E-01
BTN2A1	0.1761	6.57E-04	3.00E-03
BTN2A2	0.0864	9.65E-02	1.81E-01
BTN2A3	0.0952	6.71E-02	1.36E-01

BTN3A1	-0.0376	4.70E-01	5.99E-01
BTN3A2	-0.0793	1.27E-01	2.24E-01
BTN3A3	-0.0689	1.85E-01	2.98E-01
BTNL2	0.1012	5.15E-02	1.10E-01
BTNL3	0.0349	5.03E-01	6.28E-01
BTNL8	0.2100	4.56E-05	3.12E-04
BTNL9	-0.2202	1.88E-05	1.46E-04
BTRC	0.0285	5.84E-01	7.00E-01
BUB1B	0.4095	1.98E-16	1.86E-14
BUB1	0.3988	1.36E-15	1.06E-13
BUB3	0.2938	8.00E-09	1.52E-07
BUD13	0.3636	4.84E-13	2.29E-11
BUD31	0.0148	7.76E-01	8.50E-01
BVES	-0.0241	6.43E-01	7.48E-01
BYSL	0.1189	2.20E-02	5.55E-02
BZRAP1	0.1897	2.39E-04	1.28E-03
BZW1	-0.1371	8.20E-03	2.48E-02
BZW2	0.1082	3.73E-02	8.53E-02
C10orf105	-0.0264	6.12E-01	7.23E-01
C10orf107	-0.1101	3.40E-02	7.92E-02
C10orf108	-0.0384	4.61E-01	5.91E-01
C10orf10	-0.0931	7.32E-02	1.45E-01
C10orf110	0.0777	1.35E-01	2.34E-01
C10orf111	0.1276	1.39E-02	3.83E-02
C10orf113	0.0676	1.94E-01	3.10E-01
C10orf114	-0.0140	7.88E-01	8.59E-01
C10orf116	-0.0510	3.27E-01	4.58E-01
C10orf118	-0.0542	2.98E-01	4.27E-01
C10orf119	0.1481	4.26E-03	1.45E-02
C10orf11	-0.1070	3.94E-02	8.91E-02
C10orf120	0.0784	1.32E-01	2.30E-01
C10orf122	-0.0354	4.97E-01	6.23E-01
C10orf125	-0.2419	2.44E-06	2.47E-05
C10orf128	-0.2249	1.23E-05	1.00E-04
C10orf129	0.0275	5.97E-01	7.11E-01
C10orf12	-0.0874	9.28E-02	1.75E-01
C10orf131	-0.0232	6.56E-01	7.58E-01
C10orf137	0.2479	1.34E-06	1.47E-05
C10orf140	0.0872	9.37E-02	1.77E-01
C10orf18	0.1560	2.59E-03	9.54E-03
C10orf25	-0.1338	9.88E-03	2.90E-02
C10orf26	-0.2816	3.43E-08	5.56E-07
C10orf27	-0.0941	7.02E-02	1.41E-01
C10orf28	0.0525	3.13E-01	4.43E-01

C10orf2	0.1262	1.50E-02	4.07E-02
C10orf32	-0.2254	1.17E-05	9.63E-05
C10orf35	0.2130	3.53E-05	2.52E-04
C10orf41	-0.1017	5.04E-02	1.08E-01
C10orf46	-0.0205	6.94E-01	7.89E-01
C10orf47	-0.0607	2.44E-01	3.68E-01
C10orf4	0.1011	5.17E-02	1.10E-01
C10orf50	0.0467	3.69E-01	5.02E-01
C10orf53	-0.0108	8.36E-01	8.92E-01
C10orf54	-0.0663	2.02E-01	3.19E-01
C10orf55	-0.0073	8.88E-01	9.28E-01
C10orf57	-0.2124	3.71E-05	2.63E-04
C10orf58	-0.1357	8.89E-03	2.66E-02
C10orf62	0.1027	4.81E-02	1.04E-01
C10orf67	0.0180	7.29E-01	8.16E-01
C10orf68	0.2829	2.94E-08	4.86E-07
C10orf71	-0.0744	1.53E-01	2.57E-01
C10orf72	-0.1689	1.09E-03	4.64E-03
C10orf75	0.0795	1.27E-01	2.23E-01
C10orf76	-0.1234	1.74E-02	4.58E-02
C10orf78	0.3880	8.91E-15	5.84E-13
C10orf79	0.0938	7.10E-02	1.42E-01
C10orf81	0.2472	1.43E-06	1.55E-05
C10orf82	0.0760	1.44E-01	2.46E-01
C10orf84	-0.0338	5.16E-01	6.41E-01
C10orf88	0.2308	7.11E-06	6.25E-05
C10orf90	-0.0658	2.06E-01	3.24E-01
C10orf91	0.2443	1.92E-06	2.02E-05
C10orf93	-0.0669	1.99E-01	3.15E-01
C10orf95	0.1023	4.91E-02	1.06E-01
C10orf96	0.0635	2.22E-01	3.43E-01
C10orf99	0.0718	1.68E-01	2.76E-01
C11orf10	0.1576	2.34E-03	8.76E-03
C11orf16	0.0760	1.44E-01	2.46E-01
C11orf17	0.1740	7.63E-04	3.41E-03
C11orf1	-0.2278	9.38E-06	7.95E-05
C11orf20	-0.0467	3.70E-01	5.03E-01
C11orf21	-0.0706	1.75E-01	2.85E-01
C11orf24	-0.1130	2.95E-02	7.05E-02
C11orf2	-0.1639	1.54E-03	6.18E-03
C11orf30	0.0631	2.26E-01	3.47E-01
C11orf31	0.1065	4.04E-02	9.09E-02
C11orf34	-0.0767	1.41E-01	2.41E-01
C11orf35	-0.0765	1.41E-01	2.42E-01

C11orf36	0.0344	5.09E-01	6.34E-01
C11orf41	0.1316	1.11E-02	3.19E-02
C11orf42	0.0589	2.58E-01	3.84E-01
C11orf45	0.0642	2.17E-01	3.38E-01
C11orf46	0.0607	2.43E-01	3.68E-01
C11orf48	0.1438	5.51E-03	1.79E-02
C11orf49	0.0597	2.51E-01	3.76E-01
C11orf51	-0.0408	4.34E-01	5.66E-01
C11orf52	-0.0968	6.27E-02	1.29E-01
C11orf53	0.2232	1.43E-05	1.15E-04
C11orf54	-0.2457	1.67E-06	1.78E-05
C11orf57	0.0688	1.86E-01	2.99E-01
C11orf58	-0.0567	2.76E-01	4.04E-01
C11orf59	-0.0059	9.09E-01	9.43E-01
C11orf61	0.3152	5.29E-10	1.33E-08
C11orf63	-0.0632	2.25E-01	3.46E-01
C11orf65	-0.0956	6.58E-02	1.34E-01
C11orf66	-0.2638	2.53E-07	3.36E-06
C11orf67	0.0366	4.83E-01	6.11E-01
C11orf68	0.0047	9.27E-01	9.54E-01
C11orf70	0.0952	6.69E-02	1.35E-01
C11orf71	-0.2494	1.14E-06	1.28E-05
C11orf73	0.1124	3.05E-02	7.24E-02
C11orf74	0.1022	4.92E-02	1.06E-01
C11orf75	0.0851	1.02E-01	1.88E-01
C11orf80	0.1911	2.13E-04	1.16E-03
C11orf82	0.3207	2.55E-10	6.96E-09
C11orf83	0.0387	4.58E-01	5.88E-01
C11orf84	0.3235	1.74E-10	4.89E-09
C11orf85	0.0672	1.97E-01	3.12E-01
C11orf86	-0.0510	3.27E-01	4.57E-01
C11orf87	-0.0978	5.98E-02	1.24E-01
C11orf88	0.0977	6.00E-02	1.24E-01
C11orf90	0.0951	6.72E-02	1.36E-01
C11orf92	0.1898	2.35E-04	1.27E-03
C11orf93	0.3542	2.10E-12	8.76E-11
C11orf94	0.0420	4.19E-01	5.52E-01
C11orf95	-0.0879	9.09E-02	1.72E-01
C11orf9	0.2299	7.68E-06	6.66E-05
C12orf10	-0.0429	4.10E-01	5.43E-01
C12orf11	0.1235	1.74E-02	4.57E-02
C12orf12	0.0500	3.37E-01	4.68E-01
C12orf23	-0.0437	4.01E-01	5.34E-01
C12orf24	-0.0336	5.19E-01	6.43E-01

C12orf26	0.1647	1.45E-03	5.88E-03
C12orf27	0.2856	2.15E-08	3.69E-07
C12orf29	0.0444	3.94E-01	5.27E-01
C12orf32	0.3534	2.34E-12	9.70E-11
C12orf34	0.2240	1.33E-05	1.08E-04
C12orf35	-0.1136	2.87E-02	6.90E-02
C12orf36	0.0071	8.91E-01	9.31E-01
C12orf39	0.0255	6.24E-01	7.33E-01
C12orf40	-0.0583	2.63E-01	3.89E-01
C12orf41	0.2772	5.73E-08	8.82E-07
C12orf42	0.0446	3.91E-01	5.24E-01
C12orf43	0.0517	3.21E-01	4.51E-01
C12orf44	0.0120	8.17E-01	8.80E-01
C12orf45	0.0499	3.38E-01	4.69E-01
C12orf47	0.1840	3.67E-04	1.85E-03
C12orf48	0.3699	1.78E-13	9.14E-12
C12orf49	0.1227	1.81E-02	4.73E-02
C12orf4	0.2791	4.58E-08	7.25E-07
C12orf50	0.0142	7.85E-01	8.57E-01
C12orf51	0.0425	4.14E-01	5.47E-01
C12orf52	-0.0784	1.32E-01	2.29E-01
C12orf53	0.1449	5.16E-03	1.69E-02
C12orf54	0.0558	2.84E-01	4.13E-01
C12orf56	0.1004	5.33E-02	1.13E-01
C12orf57	0.1460	4.83E-03	1.60E-02
C12orf59	0.0182	7.27E-01	8.14E-01
C12orf5	-0.0787	1.30E-01	2.28E-01
C12orf60	0.0520	3.18E-01	4.48E-01
C12orf61	-0.1508	3.59E-03	1.26E-02
C12orf62	-0.0551	2.90E-01	4.19E-01
C12orf63	-0.0078	8.80E-01	9.23E-01
C12orf65	0.2153	2.90E-05	2.13E-04
C12orf66	-0.2097	4.68E-05	3.20E-04
C12orf68	-0.1982	1.22E-04	7.23E-04
C12orf69	-0.1133	2.91E-02	6.98E-02
C12orf70	0.1740	7.62E-04	3.41E-03
C12orf71	0.0183	7.26E-01	8.14E-01
C12orf72	-0.2481	1.31E-06	1.44E-05
C12orf73	0.1265	1.48E-02	4.02E-02
C12orf74	0.0316	5.45E-01	6.65E-01
C12orf75	0.1356	8.92E-03	2.67E-02
C12orf76	0.1461	4.80E-03	1.60E-02
C12orf77	-0.0049	9.25E-01	9.53E-01
C13orf15	-0.1815	4.43E-04	2.15E-03

C13orf16	-0.0126	8.09E-01	8.74E-01
C13orf18	0.0577	2.67E-01	3.95E-01
C13orf1	-0.1843	3.58E-04	1.81E-03
C13orf23	0.2682	1.56E-07	2.17E-06
C13orf26	-0.1337	9.93E-03	2.91E-02
C13orf27	0.0745	1.52E-01	2.56E-01
C13orf29	0.0487	3.50E-01	4.82E-01
C13orf30	0.0823	1.14E-01	2.06E-01
C13orf31	-0.0512	3.26E-01	4.56E-01
C13orf33	-0.0777	1.35E-01	2.34E-01
C13orf34	0.3503	3.76E-12	1.50E-10
C13orf35	0.2841	2.55E-08	4.28E-07
C13orf36	-0.0514	3.23E-01	4.54E-01
C13orf37	0.3221	2.10E-10	5.81E-09
C13orf38	0.0298	5.67E-01	6.84E-01
C13orf39	-0.0524	3.14E-01	4.44E-01
C14orf101	0.1497	3.85E-03	1.33E-02
C14orf102	-0.1229	1.79E-02	4.70E-02
C14orf104	-0.1248	1.62E-02	4.33E-02
C14orf105	0.0385	4.60E-01	5.90E-01
C14orf106	0.1657	1.36E-03	5.57E-03
C14orf109	0.1853	3.33E-04	1.70E-03
C14orf115	0.1029	4.77E-02	1.04E-01
C14orf118	0.0148	7.76E-01	8.50E-01
C14orf119	0.0999	5.44E-02	1.15E-01
C14orf126	-0.1325	1.06E-02	3.07E-02
C14orf128	-0.0274	5.99E-01	7.12E-01
C14orf129	0.0043	9.35E-01	9.59E-01
C14orf132	0.1552	2.72E-03	9.97E-03
C14orf135	0.0415	4.25E-01	5.58E-01
C14orf138	0.1237	1.71E-02	4.53E-02
C14orf139	0.0848	1.03E-01	1.90E-01
C14orf142	0.0608	2.42E-01	3.67E-01
C14orf143	0.0917	7.78E-02	1.52E-01
C14orf145	0.1261	1.51E-02	4.08E-02
C14orf147	-0.0688	1.86E-01	2.99E-01
C14orf148	0.1911	2.14E-04	1.16E-03
C14orf149	-0.0409	4.32E-01	5.65E-01
C14orf153	-0.0348	5.04E-01	6.29E-01
C14orf156	-0.0604	2.45E-01	3.70E-01
C14orf159	-0.3037	2.35E-09	5.08E-08
C14orf162	-0.0388	4.56E-01	5.87E-01
C14orf165	-0.0075	8.85E-01	9.26E-01
C14orf166B	0.1484	4.17E-03	1.42E-02

C14orf166	0.0221	6.71E-01	7.70E-01
C14orf167	-0.2234	1.40E-05	1.13E-04
C14orf169	0.0977	6.01E-02	1.24E-01
C14orf174	0.0791	1.28E-01	2.25E-01
C14orf176	0.0535	3.04E-01	4.34E-01
C14orf178	-0.1393	7.20E-03	2.23E-02
C14orf179	-0.1385	7.54E-03	2.31E-02
C14orf180	-0.0389	4.55E-01	5.85E-01
C14orf181	0.1519	3.37E-03	1.19E-02
C14orf182	-0.0778	1.35E-01	2.34E-01
C14orf183	0.0562	2.80E-01	4.09E-01
C14orf184	0.0059	9.10E-01	9.43E-01
C14orf19	0.1832	3.91E-04	1.95E-03
C14orf1	-0.2301	7.54E-06	6.56E-05
C14orf21	-0.1280	1.36E-02	3.77E-02
C14orf23	0.0748	1.50E-01	2.54E-01
C14orf28	-0.2328	5.85E-06	5.30E-05
C14orf2	-0.0774	1.37E-01	2.36E-01
C14orf33	0.1301	1.21E-02	3.42E-02
C14orf34	0.0557	2.85E-01	4.13E-01
C14orf37	-0.0782	1.33E-01	2.31E-01
C14orf39	0.1366	8.44E-03	2.54E-02
C14orf43	0.0498	3.39E-01	4.70E-01
C14orf45	-0.0641	2.18E-01	3.38E-01
C14orf48	0.0410	4.31E-01	5.64E-01
C14orf49	-0.0541	2.98E-01	4.28E-01
C14orf4	-0.1350	9.21E-03	2.74E-02
C14orf50	0.0022	9.66E-01	9.79E-01
C14orf53	-0.2177	2.35E-05	1.77E-04
C14orf64	0.0127	8.08E-01	8.73E-01
C14orf68	-0.2770	5.80E-08	8.91E-07
C14orf70	0.1501	3.77E-03	1.31E-02
C14orf72	-0.0140	7.88E-01	8.59E-01
C14orf73	0.0050	9.24E-01	9.52E-01
C14orf79	0.0151	7.72E-01	8.46E-01
C14orf80	0.1320	1.09E-02	3.13E-02
C14orf86	0.0749	1.50E-01	2.54E-01
C14orf93	0.1502	3.73E-03	1.30E-02
C15orf17	0.1267	1.46E-02	3.98E-02
C15orf21	0.2025	8.54E-05	5.33E-04
C15orf23	0.2815	3.49E-08	5.64E-07
C15orf24	-0.2093	4.83E-05	3.29E-04
C15orf26	0.0180	7.30E-01	8.17E-01
C15orf27	0.0923	7.57E-02	1.49E-01

C15orf28	0.0939	7.07E-02	1.41E-01
C15orf29	0.1489	4.04E-03	1.39E-02
C15orf2	-0.0850	1.02E-01	1.89E-01
C15orf32	-0.0985	5.80E-02	1.21E-01
C15orf33	-0.1138	2.84E-02	6.84E-02
C15orf34	-0.0556	2.85E-01	4.14E-01
C15orf37	-0.2423	2.34E-06	2.39E-05
C15orf38	0.0624	2.30E-01	3.53E-01
C15orf39	0.2218	1.62E-05	1.28E-04
C15orf40	0.0683	1.89E-01	3.03E-01
C15orf41	0.1189	2.20E-02	5.54E-02
C15orf42	0.4068	3.20E-16	2.89E-14
C15orf43	-0.1889	2.53E-04	1.35E-03
C15orf44	-0.0734	1.58E-01	2.64E-01
C15orf48	0.2063	6.26E-05	4.09E-04
C15orf50	0.0784	1.32E-01	2.30E-01
C15orf51	0.0492	3.44E-01	4.76E-01
C15orf52	0.0507	3.30E-01	4.61E-01
C15orf53	0.0454	3.83E-01	5.17E-01
C15orf54	-0.0353	4.98E-01	6.24E-01
C15orf55	0.0888	8.78E-02	1.67E-01
C15orf56	0.2406	2.77E-06	2.77E-05
C15orf57	-0.0746	1.51E-01	2.55E-01
C15orf58	-0.1007	5.26E-02	1.12E-01
C15orf59	-0.0071	8.91E-01	9.30E-01
C15orf5	-0.0520	3.18E-01	4.48E-01
C15orf60	0.1196	2.12E-02	5.38E-02
C15orf61	-0.0045	9.31E-01	9.57E-01
C15orf62	0.0541	2.98E-01	4.28E-01
C15orf63	0.0256	6.23E-01	7.32E-01
C16orf11	0.1509	3.58E-03	1.25E-02
C16orf13	0.0442	3.96E-01	5.28E-01
C16orf3	0.1192	2.16E-02	5.47E-02
C16orf42	-0.0101	8.47E-01	9.00E-01
C16orf45	-0.1880	2.72E-04	1.43E-03
C16orf46	-0.2325	6.05E-06	5.45E-05
C16orf48	0.0147	7.78E-01	8.52E-01
C16orf52	-0.0358	4.92E-01	6.19E-01
C16orf53	0.1754	6.89E-04	3.13E-03
C16orf54	-0.1019	4.98E-02	1.07E-01
C16orf55	0.0470	3.67E-01	5.00E-01
C16orf57	-0.0814	1.18E-01	2.11E-01
C16orf58	-0.1376	7.93E-03	2.42E-02
C16orf59	0.4001	1.08E-15	8.62E-14

C16orf5	-0.1169	2.43E-02	6.02E-02
C16orf61	-0.0935	7.21E-02	1.44E-01
C16orf62	-0.1739	7.69E-04	3.43E-03
C16orf63	0.0853	1.01E-01	1.87E-01
C16orf68	0.0075	8.85E-01	9.26E-01
C16orf70	-0.2880	1.62E-08	2.87E-07
C16orf71	-0.0100	8.48E-01	9.01E-01
C16orf72	-0.0764	1.42E-01	2.43E-01
C16orf73	0.0916	7.81E-02	1.53E-01
C16orf74	0.0847	1.04E-01	1.91E-01
C16orf75	0.4067	3.29E-16	2.94E-14
C16orf78	-0.0035	9.47E-01	9.67E-01
C16orf79	0.3059	1.78E-09	4.01E-08
C16orf7	-0.1525	3.23E-03	1.15E-02
C16orf80	-0.1860	3.16E-04	1.63E-03
C16orf81	0.1100	3.42E-02	7.96E-02
C16orf82	0.0536	3.03E-01	4.32E-01
C16orf86	-0.2280	9.20E-06	7.80E-05
C16orf87	-0.0835	1.08E-01	1.98E-01
C16orf88	0.2336	5.44E-06	4.97E-05
C16orf89	-0.0640	2.19E-01	3.39E-01
C16orf90	0.0780	1.34E-01	2.32E-01
C16orf91	0.1932	1.81E-04	1.01E-03
C16orf92	0.0792	1.28E-01	2.25E-01
C16orf93	0.1927	1.88E-04	1.05E-03
C17orf100	0.0527	3.12E-01	4.41E-01
C17orf101	-0.0538	3.01E-01	4.30E-01
C17orf102	-0.0774	1.37E-01	2.36E-01
C17orf103	-0.1448	5.19E-03	1.70E-02
C17orf104	0.0781	1.33E-01	2.32E-01
C17orf105	0.1593	2.09E-03	8.01E-03
C17orf106	-0.0841	1.06E-01	1.94E-01
C17orf107	-0.0421	4.19E-01	5.52E-01
C17orf108	-0.0786	1.31E-01	2.28E-01
C17orf28	0.1834	3.85E-04	1.92E-03
C17orf37	0.0992	5.64E-02	1.18E-01
C17orf39	-0.0786	1.31E-01	2.29E-01
C17orf42	0.3573	1.30E-12	5.64E-11
C17orf44	-0.1012	5.14E-02	1.10E-01
C17orf46	0.1063	4.08E-02	9.15E-02
C17orf47	0.0912	7.94E-02	1.55E-01
C17orf48	-0.0516	3.21E-01	4.52E-01
C17orf49	0.0599	2.50E-01	3.75E-01
C17orf50	0.0599	2.50E-01	3.75E-01

C17orf51	-0.0417	4.23E-01	5.55E-01
C17orf53	0.4221	1.84E-17	2.28E-15
C17orf54	0.1231	1.77E-02	4.65E-02
C17orf55	0.1812	4.52E-04	2.19E-03
C17orf56	0.3771	5.57E-14	3.10E-12
C17orf57	0.1304	1.19E-02	3.38E-02
C17orf58	0.0096	8.54E-01	9.06E-01
C17orf59	-0.0079	8.79E-01	9.22E-01
C17orf60	-0.0247	6.36E-01	7.43E-01
C17orf61	-0.1942	1.67E-04	9.45E-04
C17orf62	0.1243	1.66E-02	4.42E-02
C17orf63	0.1846	3.52E-04	1.78E-03
C17orf64	0.0967	6.29E-02	1.29E-01
C17orf65	0.3168	4.30E-10	1.11E-08
C17orf66	0.0273	6.01E-01	7.13E-01
C17orf67	0.1846	3.51E-04	1.78E-03
C17orf68	0.0970	6.19E-02	1.27E-01
C17orf69	0.3498	4.03E-12	1.60E-10
C17orf70	0.0293	5.74E-01	6.91E-01
C17orf71	0.2596	3.98E-07	5.00E-06
C17orf72	-0.0347	5.05E-01	6.30E-01
C17orf73	0.1442	5.39E-03	1.76E-02
C17orf74	0.0083	8.74E-01	9.19E-01
C17orf75	0.2290	8.37E-06	7.17E-05
C17orf76	0.2254	1.17E-05	9.63E-05
C17orf77	0.0454	3.83E-01	5.16E-01
C17orf78	0.1252	1.59E-02	4.25E-02
C17orf79	0.1285	1.33E-02	3.69E-02
C17orf80	0.3511	3.35E-12	1.34E-10
C17orf81	-0.0773	1.37E-01	2.37E-01
C17orf82	0.1299	1.23E-02	3.46E-02
C17orf85	0.0427	4.12E-01	5.45E-01
C17orf86	0.2686	1.50E-07	2.10E-06
C17orf87	-0.0892	8.61E-02	1.65E-01
C17orf88	0.0235	6.52E-01	7.55E-01
C17orf89	0.0008	9.88E-01	9.93E-01
C17orf90	0.1350	9.24E-03	2.75E-02
C17orf91	-0.2137	3.32E-05	2.38E-04
C17orf93	0.2019	9.03E-05	5.59E-04
C17orf95	0.1632	1.61E-03	6.44E-03
C17orf96	-0.0654	2.09E-01	3.27E-01
C17orf97	-0.0477	3.59E-01	4.91E-01
C17orf98	-0.0491	3.45E-01	4.77E-01
C17orf99	0.1992	1.12E-04	6.70E-04

C18orf10	0.1642	1.51E-03	6.08E-03
C18orf16	0.0263	6.14E-01	7.24E-01
C18orf18	-0.1023	4.90E-02	1.06E-01
C18orf19	-0.1821	4.25E-04	2.08E-03
C18orf1	-0.0277	5.95E-01	7.09E-01
C18orf20	-0.1519	3.36E-03	1.19E-02
C18orf21	0.2114	4.07E-05	2.84E-04
C18orf22	-0.1567	2.48E-03	9.18E-03
C18orf25	0.0500	3.37E-01	4.69E-01
C18orf26	0.0187	7.19E-01	8.10E-01
C18orf2	0.0848	1.03E-01	1.89E-01
C18orf32	-0.1724	8.56E-04	3.76E-03
C18orf34	-0.0598	2.50E-01	3.75E-01
C18orf45	0.0431	4.08E-01	5.41E-01
C18orf54	0.2501	1.07E-06	1.20E-05
C18orf55	-0.0356	4.94E-01	6.21E-01
C18orf56	0.1415	6.33E-03	2.00E-02
C18orf62	0.0226	6.64E-01	7.65E-01
C18orf8	-0.0083	8.73E-01	9.18E-01
C19orf10	-0.0074	8.87E-01	9.28E-01
C19orf12	-0.2253	1.18E-05	9.68E-05
C19orf18	0.0708	1.73E-01	2.83E-01
C19orf20	-0.1251	1.59E-02	4.26E-02
C19orf21	0.2259	1.12E-05	9.26E-05
C19orf22	-0.0355	4.96E-01	6.22E-01
C19orf23	0.1105	3.34E-02	7.81E-02
C19orf24	-0.0802	1.23E-01	2.18E-01
C19orf25	0.0293	5.73E-01	6.91E-01
C19orf26	0.0594	2.54E-01	3.79E-01
C19orf28	-0.1857	3.23E-04	1.66E-03
C19orf29	0.0917	7.76E-02	1.52E-01
C19orf2	0.1036	4.61E-02	1.01E-01
C19orf30	0.0004	9.94E-01	9.96E-01
C19orf33	0.1357	8.85E-03	2.65E-02
C19orf34	0.0565	2.78E-01	4.07E-01
C19orf35	-0.0520	3.17E-01	4.48E-01
C19orf36	-0.1365	8.49E-03	2.56E-02
C19orf38	-0.0775	1.36E-01	2.36E-01
C19orf39	-0.1752	7.01E-04	3.17E-03
C19orf40	0.2318	6.45E-06	5.77E-05
C19orf41	0.0880	9.04E-02	1.72E-01
C19orf42	-0.1299	1.23E-02	3.45E-02
C19orf43	-0.0440	3.98E-01	5.31E-01
C19orf44	0.1511	3.54E-03	1.24E-02

C19orf45	0.1236	1.72E-02	4.54E-02
C19orf46	0.0959	6.50E-02	1.32E-01
C19orf47	0.2231	1.44E-05	1.16E-04
C19orf48	0.2301	7.52E-06	6.55E-05
C19orf50	0.0743	1.53E-01	2.58E-01
C19orf51	-0.0206	6.93E-01	7.88E-01
C19orf52	0.0038	9.41E-01	9.64E-01
C19orf53	-0.0387	4.58E-01	5.88E-01
C19orf54	0.0146	7.79E-01	8.52E-01
C19orf55	0.1694	1.05E-03	4.48E-03
C19orf56	-0.0587	2.60E-01	3.86E-01
C19orf57	0.1014	5.10E-02	1.09E-01
C19orf59	0.0376	4.70E-01	6.00E-01
C19orf60	-0.0117	8.22E-01	8.83E-01
C19orf61	0.2349	4.82E-06	4.49E-05
C19orf62	-0.0121	8.16E-01	8.79E-01
C19orf63	-0.0877	9.17E-02	1.74E-01
C19orf66	-0.1315	1.12E-02	3.20E-02
C19orf69	0.1904	2.26E-04	1.23E-03
C19orf6	-0.0715	1.69E-01	2.78E-01
C19orf70	-0.1266	1.47E-02	4.00E-02
C19orf71	-0.0173	7.40E-01	8.25E-01
C19orf73	-0.1114	3.19E-02	7.51E-02
C19orf75	-0.0715	1.69E-01	2.78E-01
C19orf76	0.0417	4.23E-01	5.56E-01
C19orf77	-0.0604	2.46E-01	3.71E-01
C1D	-0.0391	4.53E-01	5.84E-01
C1GALT1C1	0.0182	7.27E-01	8.15E-01
C1GALT1	0.1539	2.96E-03	1.07E-02
C1QA	-0.1059	4.16E-02	9.29E-02
C1QBP	-0.0899	8.39E-02	1.62E-01
C1QB	-0.1004	5.32E-02	1.13E-01
C1QC	-0.0988	5.73E-02	1.20E-01
C1QL1	0.1219	1.88E-02	4.88E-02
C1QL2	-0.0175	7.37E-01	8.22E-01
C1QL3	-0.0045	9.30E-01	9.56E-01
C1QL4	0.1125	3.02E-02	7.19E-02
C1QTNF1	-0.1538	2.97E-03	1.07E-02
C1QTNF2	-0.0007	9.89E-01	9.93E-01
C1QTNF3	-0.0060	9.09E-01	9.43E-01
C1QTNF4	-0.0880	9.05E-02	1.72E-01
C1QTNF6	0.2424	2.32E-06	2.37E-05
C1QTNF7	-0.1944	1.64E-04	9.31E-04
C1QTNF8	0.1022	4.92E-02	1.06E-01

C1QTNF9B	0.0483	3.54E-01	4.86E-01
C1QTNF9	-0.0771	1.38E-01	2.39E-01
C1RL	-0.2104	4.41E-05	3.04E-04
C1R	-0.1960	1.45E-04	8.38E-04
C1S	-0.2103	4.46E-05	3.07E-04
C1orf100	0.0392	4.52E-01	5.83E-01
C1orf101	0.0220	6.73E-01	7.72E-01
C1orf103	-0.0131	8.02E-01	8.69E-01
C1orf104	0.4192	3.23E-17	3.63E-15
C1orf105	-0.0687	1.87E-01	3.00E-01
C1orf106	0.2336	5.42E-06	4.96E-05
C1orf107	0.1421	6.12E-03	1.95E-02
C1orf109	0.0604	2.46E-01	3.70E-01
C1orf110	0.0270	6.05E-01	7.17E-01
C1orf111	-0.0727	1.62E-01	2.69E-01
C1orf112	0.4012	8.83E-16	7.30E-14
C1orf113	0.2329	5.82E-06	5.27E-05
C1orf114	0.0932	7.28E-02	1.45E-01
C1orf115	-0.0301	5.63E-01	6.81E-01
C1orf116	0.1968	1.36E-04	7.95E-04
C1orf122	-0.1924	1.93E-04	1.07E-03
C1orf123	-0.0605	2.45E-01	3.69E-01
C1orf124	0.1781	5.67E-04	2.64E-03
C1orf125	0.2759	6.63E-08	1.01E-06
C1orf126	0.1605	1.93E-03	7.47E-03
C1orf127	0.0394	4.49E-01	5.81E-01
C1orf128	-0.0709	1.73E-01	2.83E-01
C1orf129	0.0567	2.76E-01	4.04E-01
C1orf130	-0.1155	2.60E-02	6.37E-02
C1orf131	0.3159	4.82E-10	1.23E-08
C1orf133	-0.0163	7.54E-01	8.35E-01
C1orf135	0.3358	3.14E-11	1.03E-09
C1orf141	0.0205	6.94E-01	7.89E-01
C1orf144	-0.0748	1.50E-01	2.54E-01
C1orf146	0.0478	3.58E-01	4.90E-01
C1orf14	0.0840	1.06E-01	1.95E-01
C1orf150	-0.0340	5.14E-01	6.38E-01
C1orf151	-0.0770	1.39E-01	2.39E-01
C1orf152	0.1009	5.20E-02	1.11E-01
C1orf156	0.4719	5.73E-22	1.89E-19
C1orf157	0.0638	2.20E-01	3.41E-01
C1orf158	0.1233	1.75E-02	4.59E-02
C1orf159	0.2035	7.89E-05	4.98E-04
C1orf161	-0.1907	2.20E-04	1.20E-03

C1orf162	-0.0977	6.02E-02	1.24E-01
C1orf163	0.0422	4.18E-01	5.51E-01
C1orf168	-0.1196	2.12E-02	5.38E-02
C1orf170	0.0678	1.92E-01	3.07E-01
C1orf172	0.1122	3.08E-02	7.29E-02
C1orf173	-0.0545	2.95E-01	4.25E-01
C1orf174	0.1122	3.07E-02	7.28E-02
C1orf175	0.0443	3.94E-01	5.27E-01
C1orf177	0.0444	3.94E-01	5.27E-01
C1orf180	0.1359	8.77E-03	2.63E-02
C1orf182	0.2096	4.74E-05	3.23E-04
C1orf183	0.0107	8.37E-01	8.93E-01
C1orf185	-0.0214	6.81E-01	7.78E-01
C1orf186	0.1079	3.78E-02	8.63E-02
C1orf187	0.0819	1.15E-01	2.08E-01
C1orf189	0.0628	2.28E-01	3.50E-01
C1orf190	0.0095	8.55E-01	9.06E-01
C1orf192	-0.0062	9.05E-01	9.40E-01
C1orf194	0.0951	6.73E-02	1.36E-01
C1orf198	0.1483	4.19E-03	1.43E-02
C1orf200	0.0059	9.10E-01	9.43E-01
C1orf201	0.1128	2.98E-02	7.10E-02
C1orf203	0.1826	4.08E-04	2.01E-03
C1orf204	0.1956	1.50E-04	8.65E-04
C1orf210	0.0233	6.55E-01	7.57E-01
C1orf212	-0.2197	1.96E-05	1.52E-04
C1orf213	0.2090	4.98E-05	3.37E-04
C1orf216	0.1442	5.39E-03	1.76E-02
C1orf21	0.0087	8.68E-01	9.15E-01
C1orf220	0.2256	1.15E-05	9.50E-05
C1orf223	0.0077	8.82E-01	9.25E-01
C1orf226	0.2039	7.64E-05	4.86E-04
C1orf227	0.0493	3.44E-01	4.76E-01
C1orf228	0.0312	5.49E-01	6.70E-01
C1orf229	0.1328	1.04E-02	3.02E-02
C1orf230	0.1407	6.63E-03	2.08E-02
C1orf25	0.0698	1.80E-01	2.91E-01
C1orf26	0.1875	2.83E-04	1.48E-03
C1orf27	0.2203	1.85E-05	1.44E-04
C1orf31	0.2489	1.20E-06	1.33E-05
C1orf35	0.4391	6.38E-19	1.25E-16
C1orf38	-0.0688	1.86E-01	2.99E-01
C1orf43	0.3413	1.42E-11	4.93E-10
C1orf49	0.0389	4.55E-01	5.86E-01

C1orf50	-0.0811	1.19E-01	2.13E-01
C1orf51	0.1664	1.30E-03	5.36E-03
C1orf52	0.3213	2.34E-10	6.43E-09
C1orf53	-0.0957	6.56E-02	1.33E-01
C1orf54	0.0152	7.71E-01	8.46E-01
C1orf55	0.0863	9.69E-02	1.82E-01
C1orf56	0.2923	9.68E-09	1.80E-07
C1orf57	0.1215	1.92E-02	4.98E-02
C1orf58	0.0319	5.40E-01	6.61E-01
C1orf59	0.1129	2.97E-02	7.09E-02
C1orf61	0.1191	2.18E-02	5.50E-02
C1orf63	0.1488	4.07E-03	1.40E-02
C1orf64	-0.0252	6.28E-01	7.36E-01
C1orf65	0.3237	1.70E-10	4.79E-09
C1orf66	0.1902	2.28E-04	1.24E-03
C1orf68	0.0153	7.69E-01	8.44E-01
C1orf69	-0.0520	3.18E-01	4.48E-01
C1orf70	0.0578	2.67E-01	3.94E-01
C1orf74	0.2582	4.59E-07	5.69E-06
C1orf77	0.6459	3.55E-45	1.42E-41
C1orf83	0.1657	1.36E-03	5.56E-03
C1orf84	0.0844	1.04E-01	1.92E-01
C1orf85	0.2049	7.01E-05	4.51E-04
C1orf86	-0.0450	3.88E-01	5.21E-01
C1orf87	-0.0859	9.87E-02	1.84E-01
C1orf88	0.2119	3.90E-05	2.74E-04
C1orf89	-0.1825	4.12E-04	2.03E-03
C1orf91	0.2550	6.42E-07	7.63E-06
C1orf92	0.0459	3.78E-01	5.11E-01
C1orf93	0.1368	8.34E-03	2.52E-02
C1orf94	0.0548	2.92E-01	4.21E-01
C1orf95	-0.1838	3.72E-04	1.86E-03
C1orf96	0.3365	2.82E-11	9.36E-10
C1orf97	0.0222	6.70E-01	7.70E-01
C1orf9	0.4194	3.09E-17	3.52E-15
C20orf103	0.1116	3.16E-02	7.44E-02
C20orf106	0.2076	5.62E-05	3.75E-04
C20orf107	0.1096	3.48E-02	8.06E-02
C20orf108	-0.2508	9.92E-07	1.12E-05
C20orf111	0.1155	2.62E-02	6.39E-02
C20orf112	0.1205	2.02E-02	5.18E-02
C20orf114	0.1264	1.48E-02	4.04E-02
C20orf117	0.2159	2.73E-05	2.02E-04
C20orf118	0.2991	4.21E-09	8.58E-08

C20orf11	0.1720	8.79E-04	3.85E-03
C20orf123	-0.0410	4.31E-01	5.64E-01
C20orf12	0.1561	2.57E-03	9.49E-03
C20orf132	-0.2198	1.93E-05	1.50E-04
C20orf134	0.1085	3.66E-02	8.41E-02
C20orf135	-0.0307	5.56E-01	6.75E-01
C20orf141	-0.0172	7.42E-01	8.26E-01
C20orf144	0.1078	3.79E-02	8.64E-02
C20orf151	0.0725	1.63E-01	2.71E-01
C20orf152	0.1865	3.04E-04	1.58E-03
C20orf160	-0.2340	5.22E-06	4.82E-05
C20orf165	0.1292	1.28E-02	3.57E-02
C20orf166	0.0124	8.11E-01	8.76E-01
C20orf173	0.0809	1.20E-01	2.14E-01
C20orf177	0.0845	1.04E-01	1.91E-01
C20orf186	0.2340	5.24E-06	4.83E-05
C20orf191	0.0565	2.78E-01	4.07E-01
C20orf194	0.0965	6.34E-02	1.30E-01
C20orf195	0.0879	9.09E-02	1.72E-01
C20orf196	0.0196	7.07E-01	7.99E-01
C20orf197	0.0607	2.44E-01	3.68E-01
C20orf199	0.1001	5.41E-02	1.14E-01
C20orf200	-0.0316	5.44E-01	6.65E-01
C20orf201	0.0534	3.05E-01	4.35E-01
C20orf202	-0.0443	3.95E-01	5.28E-01
C20orf203	0.0545	2.95E-01	4.25E-01
C20orf20	0.2068	6.00E-05	3.94E-04
C20orf24	-0.0116	8.24E-01	8.85E-01
C20orf26	0.0557	2.84E-01	4.13E-01
C20orf27	0.0792	1.28E-01	2.25E-01
C20orf29	-0.1226	1.82E-02	4.75E-02
C20orf30	-0.2311	6.90E-06	6.11E-05
C20orf3	-0.1848	3.47E-04	1.76E-03
C20orf43	0.0684	1.88E-01	3.02E-01
C20orf46	0.0637	2.21E-01	3.42E-01
C20orf4	0.0761	1.44E-01	2.45E-01
C20orf54	0.0202	6.98E-01	7.92E-01
C20orf56	-0.1731	8.11E-04	3.59E-03
C20orf70	0.1911	2.14E-04	1.16E-03
C20orf72	0.2644	2.37E-07	3.15E-06
C20orf7	-0.0732	1.59E-01	2.65E-01
C20orf85	0.0269	6.06E-01	7.18E-01
C20orf94	-0.0024	9.63E-01	9.77E-01
C20orf96	0.1559	2.60E-03	9.57E-03

C21orf119	0.0306	5.57E-01	6.76E-01
C21orf121	-0.1152	2.65E-02	6.47E-02
C21orf122	0.0273	6.00E-01	7.13E-01
C21orf125	0.0898	8.39E-02	1.62E-01
C21orf128	0.0543	2.97E-01	4.26E-01
C21orf129	0.1327	1.05E-02	3.04E-02
C21orf130	0.0875	9.25E-02	1.75E-01
C21orf131	0.0352	4.99E-01	6.25E-01
C21orf15	0.0645	2.16E-01	3.35E-01
C21orf29	0.0205	6.93E-01	7.89E-01
C21orf2	-0.0737	1.56E-01	2.62E-01
C21orf33	-0.2678	1.63E-07	2.26E-06
C21orf34	-0.2599	3.84E-07	4.85E-06
C21orf45	0.2967	5.60E-09	1.11E-07
C21orf49	0.0951	6.74E-02	1.36E-01
C21orf54	-0.0392	4.52E-01	5.83E-01
C21orf56	0.1943	1.66E-04	9.38E-04
C21orf57	0.0049	9.26E-01	9.53E-01
C21orf58	0.3911	5.22E-15	3.60E-13
C21orf59	0.2293	8.17E-06	7.02E-05
C21orf62	0.0556	2.86E-01	4.15E-01
C21orf63	-0.0841	1.06E-01	1.94E-01
C21orf67	-0.1130	2.96E-02	7.06E-02
C21orf70	0.0079	8.79E-01	9.22E-01
C21orf71	0.0033	9.49E-01	9.68E-01
C21orf7	-0.0836	1.08E-01	1.97E-01
C21orf81	0.1692	1.07E-03	4.55E-03
C21orf82	-0.0375	4.71E-01	6.00E-01
C21orf84	-0.0363	4.86E-01	6.14E-01
C21orf88	0.0030	9.54E-01	9.72E-01
C21orf90	-0.0087	8.67E-01	9.14E-01
C21orf91	-0.0114	8.27E-01	8.87E-01
C21orf94	0.0660	2.05E-01	3.22E-01
C21orf96	-0.0424	4.15E-01	5.48E-01
C21orf99	0.1589	2.14E-03	8.15E-03
C22orf13	-0.1699	1.02E-03	4.36E-03
C22orf15	-0.0724	1.64E-01	2.71E-01
C22orf23	0.2247	1.24E-05	1.02E-04
C22orf24	0.0170	7.45E-01	8.28E-01
C22orf25	-0.0541	2.99E-01	4.28E-01
C22orf26	-0.0001	9.98E-01	9.99E-01
C22orf27	0.1311	1.15E-02	3.28E-02
C22orf28	-0.0238	6.48E-01	7.52E-01
C22orf29	0.3339	4.11E-11	1.31E-09

C22orf30	0.2226	1.50E-05	1.20E-04
C22orf31	0.0342	5.12E-01	6.36E-01
C22orf32	-0.0739	1.55E-01	2.61E-01
C22orf33	-0.0861	9.78E-02	1.83E-01
C22orf34	0.0278	5.93E-01	7.07E-01
C22orf36	0.0621	2.33E-01	3.56E-01
C22orf39	0.1539	2.95E-03	1.06E-02
C22orf40	0.1672	1.23E-03	5.11E-03
C22orf41	0.0994	5.58E-02	1.17E-01
C22orf42	-0.0323	5.35E-01	6.57E-01
C22orf43	0.2090	4.97E-05	3.37E-04
C22orf45	0.0083	8.73E-01	9.18E-01
C22orf46	0.1070	3.94E-02	8.90E-02
C22orf9	-0.0096	8.54E-01	9.05E-01
C2CD2L	0.0290	5.77E-01	6.94E-01
C2CD2	0.0720	1.66E-01	2.74E-01
C2CD3	0.1698	1.03E-03	4.40E-03
C2CD4A	0.0021	9.68E-01	9.80E-01
C2CD4B	-0.0786	1.31E-01	2.28E-01
C2CD4C	-0.0501	3.36E-01	4.68E-01
C2CD4D	0.2396	3.05E-06	3.01E-05
C2orf14	0.0491	3.46E-01	4.78E-01
C2orf15	0.1183	2.27E-02	5.69E-02
C2orf16	-0.0473	3.64E-01	4.96E-01
C2orf18	0.0008	9.87E-01	9.92E-01
C2orf24	-0.0963	6.38E-02	1.30E-01
C2orf27A	0.2498	1.10E-06	1.23E-05
C2orf27B	-0.0341	5.12E-01	6.37E-01
C2orf28	0.0888	8.76E-02	1.67E-01
C2orf29	0.2847	2.38E-08	4.03E-07
C2orf34	0.0019	9.70E-01	9.81E-01
C2orf39	0.0293	5.74E-01	6.91E-01
C2orf3	-0.0041	9.38E-01	9.61E-01
C2orf40	-0.1569	2.44E-03	9.09E-03
C2orf42	-0.1186	2.23E-02	5.61E-02
C2orf43	0.1295	1.25E-02	3.51E-02
C2orf44	0.2629	2.80E-07	3.68E-06
C2orf47	-0.2368	3.98E-06	3.82E-05
C2orf48	0.3878	9.26E-15	6.03E-13
C2orf49	0.0474	3.62E-01	4.95E-01
C2orf50	0.1469	4.59E-03	1.54E-02
C2orf51	-0.1331	1.03E-02	2.98E-02
C2orf52	0.1237	1.71E-02	4.52E-02
C2orf53	0.0250	6.32E-01	7.39E-01

C2orf54	0.0674	1.95E-01	3.11E-01
C2orf55	-0.1117	3.15E-02	7.42E-02
C2orf56	-0.1284	1.33E-02	3.71E-02
C2orf57	0.0216	6.78E-01	7.76E-01
C2orf58	-0.0245	6.38E-01	7.45E-01
C2orf60	0.0653	2.10E-01	3.28E-01
C2orf61	0.0810	1.19E-01	2.14E-01
C2orf62	-0.0782	1.33E-01	2.31E-01
C2orf63	0.0489	3.48E-01	4.80E-01
C2orf64	-0.0737	1.57E-01	2.62E-01
C2orf65	0.0425	4.15E-01	5.47E-01
C2orf66	0.0452	3.85E-01	5.19E-01
C2orf67	0.1097	3.47E-02	8.05E-02
C2orf68	0.1831	3.93E-04	1.95E-03
C2orf69	-0.1932	1.80E-04	1.01E-03
C2orf70	0.1483	4.20E-03	1.43E-02
C2orf71	-0.0086	8.70E-01	9.16E-01
C2orf72	-0.0840	1.06E-01	1.95E-01
C2orf73	0.0696	1.81E-01	2.93E-01
C2orf74	-0.0733	1.59E-01	2.65E-01
C2orf76	0.1457	4.92E-03	1.63E-02
C2orf77	0.0450	3.88E-01	5.21E-01
C2orf78	-0.0597	2.52E-01	3.77E-01
C2orf79	-0.0326	5.31E-01	6.53E-01
C2orf7	-0.2291	8.26E-06	7.09E-05
C2orf80	-0.0264	6.12E-01	7.23E-01
C2orf81	0.1152	2.65E-02	6.46E-02
C2orf82	0.0311	5.51E-01	6.71E-01
C2orf83	0.0415	4.25E-01	5.58E-01
C2orf84	-0.0744	1.53E-01	2.57E-01
C2orf85	-0.0493	3.43E-01	4.75E-01
C2orf86	-0.0343	5.10E-01	6.35E-01
C2orf88	-0.0530	3.08E-01	4.38E-01
C2orf89	-0.0760	1.44E-01	2.46E-01
C2	-0.0735	1.57E-01	2.63E-01
C3AR1	-0.0751	1.49E-01	2.52E-01
C3P1	-0.1752	7.00E-04	3.17E-03
C3orf10	-0.0210	6.87E-01	7.83E-01
C3orf14	0.1042	4.49E-02	9.89E-02
C3orf15	0.0026	9.60E-01	9.75E-01
C3orf16	0.0316	5.44E-01	6.65E-01
C3orf17	0.1611	1.85E-03	7.22E-03
C3orf18	0.0324	5.34E-01	6.57E-01
C3orf19	0.1046	4.41E-02	9.74E-02

C3orf1	-0.0626	2.29E-01	3.51E-01
C3orf20	-0.0422	4.17E-01	5.50E-01
C3orf21	0.2046	7.19E-05	4.61E-04
C3orf22	0.0926	7.47E-02	1.48E-01
C3orf23	-0.2125	3.70E-05	2.62E-04
C3orf24	0.0554	2.88E-01	4.16E-01
C3orf26	0.2058	6.54E-05	4.25E-04
C3orf27	0.0598	2.50E-01	3.76E-01
C3orf30	0.0324	5.34E-01	6.56E-01
C3orf31	0.0021	9.68E-01	9.80E-01
C3orf32	0.0369	4.79E-01	6.08E-01
C3orf33	0.1434	5.65E-03	1.82E-02
C3orf34	0.2763	6.29E-08	9.58E-07
C3orf35	0.2069	5.91E-05	3.91E-04
C3orf36	-0.2573	5.06E-07	6.18E-06
C3orf37	0.0865	9.60E-02	1.80E-01
C3orf38	0.1061	4.11E-02	9.21E-02
C3orf39	0.0713	1.70E-01	2.80E-01
C3orf42	0.1033	4.67E-02	1.02E-01
C3orf43	0.1364	8.52E-03	2.56E-02
C3orf45	-0.0098	8.51E-01	9.04E-01
C3orf47	0.1726	8.45E-04	3.72E-03
C3orf48	0.0928	7.42E-02	1.47E-01
C3orf49	-0.0391	4.53E-01	5.84E-01
C3orf50	0.1468	4.60E-03	1.54E-02
C3orf51	-0.0400	4.43E-01	5.74E-01
C3orf52	0.2116	3.99E-05	2.79E-04
C3orf54	0.0335	5.20E-01	6.44E-01
C3orf55	0.1735	7.93E-04	3.53E-03
C3orf57	0.1353	9.08E-03	2.71E-02
C3orf58	0.0100	8.48E-01	9.01E-01
C3orf59	-0.0540	2.99E-01	4.29E-01
C3orf62	0.1754	6.91E-04	3.14E-03
C3orf63	0.1544	2.87E-03	1.04E-02
C3orf64	-0.0653	2.09E-01	3.28E-01
C3orf65	0.1202	2.06E-02	5.25E-02
C3orf66	0.0265	6.10E-01	7.22E-01
C3orf67	0.0399	4.44E-01	5.75E-01
C3orf70	-0.0965	6.35E-02	1.30E-01
C3orf71	0.2277	9.48E-06	8.01E-05
C3orf72	0.1485	4.14E-03	1.41E-02
C3orf74	0.1526	3.22E-03	1.15E-02
C3orf75	-0.0792	1.28E-01	2.25E-01
C3orf77	-0.0349	5.03E-01	6.28E-01

C3orf79	-0.0266	6.10E-01	7.21E-01
C3	-0.1310	1.16E-02	3.29E-02
C4A	-0.1533	3.07E-03	1.10E-02
C4BPA	-0.1355	8.96E-03	2.68E-02
C4BPB	-0.0280	5.91E-01	7.06E-01
C4orf10	0.1282	1.35E-02	3.74E-02
C4orf12	-0.0796	1.26E-01	2.22E-01
C4orf14	0.0539	3.01E-01	4.30E-01
C4orf17	0.1136	2.87E-02	6.91E-02
C4orf19	-0.0481	3.56E-01	4.88E-01
C4orf21	0.2823	3.18E-08	5.21E-07
C4orf22	0.0918	7.74E-02	1.52E-01
C4orf23	0.0624	2.30E-01	3.53E-01
C4orf26	0.1124	3.04E-02	7.23E-02
C4orf27	0.0436	4.02E-01	5.35E-01
C4orf29	-0.1593	2.08E-03	7.98E-03
C4orf31	-0.1365	8.46E-03	2.55E-02
C4orf32	-0.1482	4.23E-03	1.44E-02
C4orf33	-0.1754	6.91E-04	3.13E-03
C4orf34	-0.2407	2.74E-06	2.74E-05
C4orf35	-0.0069	8.95E-01	9.33E-01
C4orf36	-0.0947	6.83E-02	1.38E-01
C4orf37	0.0745	1.52E-01	2.57E-01
C4orf38	-0.1099	3.43E-02	7.98E-02
C4orf39	0.2050	6.97E-05	4.49E-04
C4orf3	-0.3182	3.54E-10	9.39E-09
C4orf40	-0.0172	7.42E-01	8.26E-01
C4orf41	-0.2262	1.09E-05	9.04E-05
C4orf42	0.0836	1.08E-01	1.97E-01
C4orf43	-0.0106	8.38E-01	8.94E-01
C4orf44	0.1555	2.67E-03	9.79E-03
C4orf45	-0.0051	9.22E-01	9.50E-01
C4orf46	0.2898	1.31E-08	2.36E-07
C4orf47	0.1234	1.74E-02	4.59E-02
C4orf48	0.1045	4.42E-02	9.75E-02
C4orf49	-0.1775	5.92E-04	2.74E-03
C4orf50	-0.0286	5.83E-01	6.99E-01
C4orf51	0.0445	3.92E-01	5.25E-01
C4orf52	-0.0069	8.94E-01	9.32E-01
C4orf6	0.1433	5.68E-03	1.83E-02
C4orf7	0.1961	1.44E-04	8.34E-04
C5AR1	-0.1318	1.11E-02	3.17E-02
C5orf13	0.1863	3.10E-04	1.60E-03
C5orf15	-0.0378	4.68E-01	5.98E-01

C5orf20	-0.0111	8.31E-01	8.89E-01
C5orf22	0.1874	2.84E-04	1.49E-03
C5orf23	-0.2255	1.16E-05	9.56E-05
C5orf24	-0.0619	2.34E-01	3.58E-01
C5orf25	0.1256	1.55E-02	4.17E-02
C5orf27	-0.2564	5.56E-07	6.71E-06
C5orf28	0.1403	6.79E-03	2.12E-02
C5orf30	0.1967	1.37E-04	7.97E-04
C5orf32	-0.0700	1.79E-01	2.90E-01
C5orf33	-0.2357	4.45E-06	4.21E-05
C5orf34	0.4374	9.15E-19	1.67E-16
C5orf35	-0.1699	1.02E-03	4.36E-03
C5orf36	-0.1520	3.34E-03	1.18E-02
C5orf38	-0.0230	6.59E-01	7.60E-01
C5orf39	0.1406	6.70E-03	2.10E-02
C5orf40	-0.0263	6.14E-01	7.24E-01
C5orf41	-0.1239	1.70E-02	4.50E-02
C5orf42	0.2107	4.31E-05	2.99E-04
C5orf43	-0.0185	7.23E-01	8.12E-01
C5orf44	0.0337	5.18E-01	6.42E-01
C5orf45	0.0031	9.52E-01	9.70E-01
C5orf46	0.1246	1.63E-02	4.36E-02
C5orf47	-0.0164	7.53E-01	8.34E-01
C5orf48	0.0205	6.94E-01	7.89E-01
C5orf49	0.0730	1.61E-01	2.67E-01
C5orf4	-0.3470	6.13E-12	2.32E-10
C5orf51	0.0599	2.50E-01	3.75E-01
C5orf52	0.0576	2.68E-01	3.96E-01
C5orf53	-0.1233	1.75E-02	4.60E-02
C5orf54	0.4446	2.08E-19	4.45E-17
C5orf55	-0.1421	6.12E-03	1.95E-02
C5orf56	0.0993	5.60E-02	1.17E-01
C5orf58	0.0716	1.69E-01	2.77E-01
C5orf60	0.1579	2.29E-03	8.63E-03
C5orf62	-0.1190	2.18E-02	5.52E-02
C5	-0.0898	8.42E-02	1.62E-01
C6orf103	0.1007	5.26E-02	1.12E-01
C6orf105	0.0235	6.51E-01	7.55E-01
C6orf106	-0.0674	1.95E-01	3.11E-01
C6orf108	-0.0011	9.83E-01	9.89E-01
C6orf10	0.0189	7.17E-01	8.07E-01
C6orf114	-0.0960	6.47E-02	1.32E-01
C6orf115	-0.0791	1.28E-01	2.25E-01
C6orf118	-0.0539	3.01E-01	4.30E-01

C6orf120	0.0539	3.00E-01	4.29E-01
C6orf122	-0.0188	7.19E-01	8.09E-01
C6orf123	-0.1047	4.39E-02	9.71E-02
C6orf124	0.1815	4.42E-04	2.14E-03
C6orf125	0.1705	9.79E-04	4.22E-03
C6orf126	0.1983	1.21E-04	7.15E-04
C6orf127	0.0976	6.03E-02	1.25E-01
C6orf129	0.1610	1.86E-03	7.24E-03
C6orf130	-0.0650	2.11E-01	3.30E-01
C6orf132	0.2092	4.89E-05	3.32E-04
C6orf134	0.3017	3.02E-09	6.37E-08
C6orf136	0.0556	2.85E-01	4.14E-01
C6orf138	-0.2413	2.57E-06	2.59E-05
C6orf141	0.1242	1.67E-02	4.44E-02
C6orf142	-0.1830	3.95E-04	1.96E-03
C6orf145	-0.2696	1.34E-07	1.90E-06
C6orf146	0.0543	2.97E-01	4.26E-01
C6orf147	0.2707	1.19E-07	1.69E-06
C6orf150	0.1000	5.44E-02	1.15E-01
C6orf153	0.1470	4.55E-03	1.53E-02
C6orf154	0.0144	7.82E-01	8.55E-01
C6orf155	-0.0790	1.29E-01	2.26E-01
C6orf15	-0.0432	4.07E-01	5.40E-01
C6orf162	0.2609	3.44E-07	4.41E-06
C6orf163	0.2392	3.16E-06	3.11E-05
C6orf164	0.0680	1.91E-01	3.06E-01
C6orf165	0.0855	1.00E-01	1.86E-01
C6orf167	0.3892	7.24E-15	4.81E-13
C6orf168	0.1423	6.05E-03	1.93E-02
C6orf170	0.1817	4.36E-04	2.12E-03
C6orf174	-0.0371	4.76E-01	6.05E-01
C6orf176	-0.1239	1.69E-02	4.49E-02
C6orf182	0.2364	4.15E-06	3.95E-05
C6orf186	0.0923	7.58E-02	1.49E-01
C6orf191	0.0394	4.50E-01	5.81E-01
C6orf192	0.1069	3.95E-02	8.93E-02
C6orf195	0.2201	1.89E-05	1.47E-04
C6orf1	0.0048	9.26E-01	9.54E-01
C6orf201	0.0327	5.30E-01	6.53E-01
C6orf203	0.0255	6.24E-01	7.33E-01
C6orf204	-0.0088	8.67E-01	9.14E-01
C6orf208	-0.2123	3.75E-05	2.66E-04
C6orf211	-0.0496	3.41E-01	4.72E-01
C6orf217	-0.0210	6.87E-01	7.83E-01

C6orf218	0.0668	1.99E-01	3.15E-01
C6orf221	0.1297	1.24E-02	3.48E-02
C6orf222	0.1260	1.52E-02	4.11E-02
C6orf223	0.2340	5.25E-06	4.84E-05
C6orf225	-0.0122	8.14E-01	8.78E-01
C6orf226	-0.1428	5.86E-03	1.88E-02
C6orf227	0.0268	6.07E-01	7.19E-01
C6orf25	-0.0305	5.58E-01	6.77E-01
C6orf26	0.3372	2.56E-11	8.53E-10
C6orf27	0.0615	2.37E-01	3.61E-01
C6orf35	-0.0754	1.47E-01	2.50E-01
C6orf41	-0.0167	7.49E-01	8.31E-01
C6orf47	0.0677	1.93E-01	3.08E-01
C6orf48	0.1682	1.15E-03	4.83E-03
C6orf52	0.1072	3.91E-02	8.84E-02
C6orf57	0.0815	1.17E-01	2.10E-01
C6orf58	0.0393	4.50E-01	5.82E-01
C6orf59	0.1256	1.55E-02	4.17E-02
C6orf62	0.1567	2.47E-03	9.18E-03
C6orf64	0.2215	1.67E-05	1.32E-04
C6orf70	0.0874	9.27E-02	1.75E-01
C6orf72	-0.0962	6.41E-02	1.31E-01
C6orf81	0.1115	3.19E-02	7.50E-02
C6orf89	0.0425	4.15E-01	5.48E-01
C6orf94	0.1681	1.15E-03	4.86E-03
C6orf97	-0.0600	2.49E-01	3.74E-01
C6	-0.3192	3.10E-10	8.31E-09
C7orf10	-0.1417	6.26E-03	1.98E-02
C7orf11	-0.0242	6.42E-01	7.48E-01
C7orf13	0.1458	4.90E-03	1.62E-02
C7orf16	-0.0735	1.58E-01	2.64E-01
C7orf23	0.1532	3.09E-03	1.11E-02
C7orf25	-0.0024	9.63E-01	9.77E-01
C7orf26	0.1586	2.19E-03	8.30E-03
C7orf27	0.1030	4.73E-02	1.03E-01
C7orf28A	0.2141	3.21E-05	2.32E-04
C7orf28B	0.0941	7.03E-02	1.41E-01
C7orf29	0.1462	4.76E-03	1.58E-02
C7orf30	-0.0621	2.33E-01	3.56E-01
C7orf31	0.1314	1.13E-02	3.23E-02
C7orf33	-0.0200	7.00E-01	7.94E-01
C7orf34	0.1270	1.44E-02	3.93E-02
C7orf36	0.3049	2.01E-09	4.46E-08
C7orf40	0.1979	1.25E-04	7.38E-04

C7orf41	0.0022	9.67E-01	9.79E-01
C7orf42	-0.1158	2.57E-02	6.29E-02
C7orf43	-0.0596	2.52E-01	3.77E-01
C7orf44	0.1820	4.27E-04	2.08E-03
C7orf45	0.0184	7.23E-01	8.12E-01
C7orf46	0.0261	6.17E-01	7.26E-01
C7orf47	0.0988	5.73E-02	1.20E-01
C7orf49	0.1457	4.92E-03	1.63E-02
C7orf4	0.0714	1.70E-01	2.79E-01
C7orf50	0.0713	1.71E-01	2.80E-01
C7orf51	0.1574	2.36E-03	8.84E-03
C7orf52	-0.0465	3.72E-01	5.05E-01
C7orf53	0.0474	3.62E-01	4.95E-01
C7orf54	0.0852	1.01E-01	1.88E-01
C7orf55	-0.1928	1.87E-04	1.04E-03
C7orf57	0.0701	1.78E-01	2.89E-01
C7orf58	-0.3445	8.89E-12	3.24E-10
C7orf59	-0.0236	6.50E-01	7.54E-01
C7orf60	0.0996	5.52E-02	1.16E-01
C7orf61	0.0637	2.21E-01	3.41E-01
C7orf63	-0.1675	1.20E-03	5.02E-03
C7orf64	0.0574	2.70E-01	3.98E-01
C7orf65	0.2324	6.11E-06	5.50E-05
C7orf66	0.1323	1.07E-02	3.09E-02
C7orf68	0.0905	8.19E-02	1.59E-01
C7orf69	0.1627	1.67E-03	6.63E-03
C7orf70	0.2437	2.03E-06	2.12E-05
C7orf71	0.1408	6.60E-03	2.08E-02
C7orf72	0.0359	4.91E-01	6.18E-01
C7	-0.2118	3.92E-05	2.75E-04
C8A	-0.2746	7.68E-08	1.15E-06
C8B	-0.1572	2.39E-03	8.92E-03
C8G	-0.1455	4.98E-03	1.64E-02
C8ORFK29	0.1778	5.82E-04	2.70E-03
C8orf12	0.0243	6.41E-01	7.47E-01
C8orf22	0.0574	2.70E-01	3.98E-01
C8orf30A	-0.0176	7.35E-01	8.21E-01
C8orf31	-0.0196	7.07E-01	7.99E-01
C8orf33	0.1073	3.88E-02	8.80E-02
C8orf34	-0.0306	5.57E-01	6.76E-01
C8orf37	-0.0198	7.04E-01	7.96E-01
C8orf38	0.0998	5.48E-02	1.16E-01
C8orf39	0.1613	1.83E-03	7.14E-03
C8orf40	-0.0881	9.01E-02	1.71E-01

C8orf41	0.0891	8.66E-02	1.66E-01
C8orf42	0.1121	3.08E-02	7.30E-02
C8orf44	0.1752	7.00E-04	3.17E-03
C8orf45	0.1328	1.04E-02	3.03E-02
C8orf46	-0.1345	9.51E-03	2.81E-02
C8orf47	0.1121	3.08E-02	7.30E-02
C8orf48	0.2384	3.43E-06	3.34E-05
C8orf4	-0.1249	1.61E-02	4.30E-02
C8orf51	0.1580	2.27E-03	8.54E-03
C8orf55	-0.0969	6.22E-02	1.28E-01
C8orf56	-0.0035	9.47E-01	9.67E-01
C8orf58	-0.1130	2.96E-02	7.06E-02
C8orf59	0.1438	5.52E-03	1.79E-02
C8orf71	0.1017	5.02E-02	1.08E-01
C8orf73	0.0780	1.34E-01	2.32E-01
C8orf74	0.0318	5.41E-01	6.62E-01
C8orf75	-0.0529	3.09E-01	4.39E-01
C8orf76	0.0704	1.76E-01	2.87E-01
C8orf77	0.2586	4.41E-07	5.47E-06
C8orf79	0.0531	3.08E-01	4.37E-01
C8orf80	-0.1865	3.04E-04	1.58E-03
C8orf83	-0.2034	7.94E-05	5.01E-04
C8orf84	0.0995	5.55E-02	1.17E-01
C8orf85	0.0726	1.63E-01	2.70E-01
C8orf86	-0.0140	7.88E-01	8.59E-01
C9orf100	0.4193	3.12E-17	3.53E-15
C9orf102	-0.1429	5.83E-03	1.87E-02
C9orf103	-0.1379	7.80E-03	2.38E-02
C9orf106	0.0833	1.09E-01	1.99E-01
C9orf109	0.2515	9.20E-07	1.05E-05
C9orf110	0.2152	2.92E-05	2.14E-04
C9orf114	0.1276	1.39E-02	3.84E-02
C9orf116	-0.0787	1.30E-01	2.28E-01
C9orf117	0.2447	1.84E-06	1.95E-05
C9orf119	0.0435	4.03E-01	5.36E-01
C9orf11	0.1039	4.55E-02	9.99E-02
C9orf122	0.1847	3.48E-04	1.77E-03
C9orf123	-0.0779	1.34E-01	2.33E-01
C9orf125	0.1854	3.30E-04	1.69E-03
C9orf128	0.0573	2.71E-01	3.99E-01
C9orf129	-0.0740	1.55E-01	2.60E-01
C9orf130	-0.0735	1.58E-01	2.64E-01
C9orf131	-0.0619	2.34E-01	3.58E-01
C9orf135	0.0549	2.92E-01	4.21E-01

C9orf139	0.0266	6.10E-01	7.21E-01
C9orf140	0.3896	6.82E-15	4.56E-13
C9orf142	0.1569	2.45E-03	9.10E-03
C9orf144B	0.0206	6.93E-01	7.88E-01
C9orf144	0.0297	5.68E-01	6.86E-01
C9orf150	-0.1536	3.01E-03	1.08E-02
C9orf152	0.1052	4.28E-02	9.51E-02
C9orf153	0.0356	4.94E-01	6.21E-01
C9orf156	0.2186	2.16E-05	1.65E-04
C9orf163	0.1821	4.23E-04	2.07E-03
C9orf167	0.0578	2.67E-01	3.94E-01
C9orf169	-0.0306	5.57E-01	6.76E-01
C9orf16	-0.0086	8.70E-01	9.16E-01
C9orf170	0.1418	6.21E-03	1.97E-02
C9orf171	-0.0110	8.33E-01	8.91E-01
C9orf172	0.1641	1.52E-03	6.11E-03
C9orf173	-0.0125	8.10E-01	8.75E-01
C9orf21	0.0590	2.57E-01	3.83E-01
C9orf23	-0.0706	1.75E-01	2.85E-01
C9orf24	-0.0145	7.80E-01	8.54E-01
C9orf25	0.2756	6.86E-08	1.04E-06
C9orf30	0.2992	4.15E-09	8.50E-08
C9orf37	0.1621	1.74E-03	6.84E-03
C9orf3	-0.0308	5.54E-01	6.73E-01
C9orf40	0.2006	1.00E-04	6.09E-04
C9orf41	-0.0697	1.81E-01	2.93E-01
C9orf43	0.0192	7.12E-01	8.03E-01
C9orf44	-0.0591	2.56E-01	3.82E-01
C9orf45	0.3583	1.12E-12	4.97E-11
C9orf46	0.0095	8.55E-01	9.06E-01
C9orf47	0.0476	3.61E-01	4.93E-01
C9orf4	0.0779	1.34E-01	2.32E-01
C9orf50	0.0420	4.20E-01	5.52E-01
C9orf53	0.2194	2.00E-05	1.54E-04
C9orf57	-0.0301	5.64E-01	6.82E-01
C9orf5	-0.0642	2.17E-01	3.37E-01
C9orf64	0.1308	1.17E-02	3.32E-02
C9orf66	0.0464	3.73E-01	5.06E-01
C9orf68	0.0188	7.18E-01	8.09E-01
C9orf69	0.2118	3.90E-05	2.74E-04
C9orf6	0.2158	2.78E-05	2.05E-04
C9orf70	0.0555	2.86E-01	4.15E-01
C9orf71	-0.1998	1.06E-04	6.42E-04
C9orf72	0.0942	7.00E-02	1.40E-01

C9orf78	-0.1677	1.19E-03	4.97E-03
C9orf79	0.0965	6.34E-02	1.30E-01
C9orf7	-0.0334	5.21E-01	6.45E-01
C9orf80	0.1413	6.40E-03	2.02E-02
C9orf82	-0.1084	3.68E-02	8.44E-02
C9orf84	0.0720	1.66E-01	2.74E-01
C9orf85	0.0131	8.01E-01	8.69E-01
C9orf86	0.2954	6.64E-09	1.29E-07
C9orf89	0.1246	1.63E-02	4.35E-02
C9orf91	-0.1285	1.32E-02	3.68E-02
C9orf93	0.0371	4.76E-01	6.05E-01
C9orf95	-0.1231	1.77E-02	4.66E-02
C9orf96	0.2079	5.46E-05	3.65E-04
C9orf98	0.0437	4.01E-01	5.34E-01
C9orf9	0.0343	5.10E-01	6.35E-01
C9	-0.1388	7.43E-03	2.29E-02
CA10	0.0195	7.09E-01	8.01E-01
CA11	0.0434	4.05E-01	5.38E-01
CA12	0.0179	7.31E-01	8.18E-01
CA13	-0.1442	5.38E-03	1.76E-02
CA14	-0.0650	2.12E-01	3.31E-01
CA1	0.0305	5.59E-01	6.77E-01
CA2	-0.1803	4.84E-04	2.31E-03
CA3	-0.0438	4.00E-01	5.33E-01
CA4	-0.1352	9.14E-03	2.72E-02
CA5A	-0.2877	1.67E-08	2.95E-07
CA5BP	0.1798	5.03E-04	2.39E-03
CA5B	-0.0944	6.94E-02	1.39E-01
CA6	0.0396	4.47E-01	5.79E-01
CA7	0.0641	2.18E-01	3.38E-01
CA8	0.0611	2.40E-01	3.64E-01
CA9	0.1827	4.05E-04	2.00E-03
CAB39L	-0.1356	8.91E-03	2.66E-02
CAB39	-0.0404	4.38E-01	5.69E-01
CABC1	-0.0314	5.47E-01	6.67E-01
CABIN1	0.1364	8.50E-03	2.56E-02
CABLES1	-0.0818	1.16E-01	2.08E-01
CABLES2	0.3105	9.84E-10	2.33E-08
CABP1	-0.0374	4.73E-01	6.02E-01
CABP2	-0.0725	1.64E-01	2.71E-01
CABP4	-0.0249	6.33E-01	7.40E-01
CABP5	0.0301	5.64E-01	6.82E-01
CABP7	0.1735	7.89E-04	3.51E-03
CABYR	0.2682	1.56E-07	2.17E-06

CACHD1	-0.0307	5.56E-01	6.75E-01
CACNA1A	0.0075	8.86E-01	9.27E-01
CACNA1B	0.1301	1.22E-02	3.43E-02
CACNA1C	-0.1570	2.43E-03	9.04E-03
CACNA1D	0.0385	4.60E-01	5.90E-01
CACNA1E	0.0540	2.99E-01	4.29E-01
CACNA1F	0.1904	2.25E-04	1.22E-03
CACNA1G	0.1573	2.37E-03	8.87E-03
CACNA1H	0.0152	7.71E-01	8.46E-01
CACNA1I	0.0086	8.68E-01	9.15E-01
CACNA1S	0.0207	6.91E-01	7.86E-01
CACNA2D1	-0.1419	6.17E-03	1.96E-02
CACNA2D2	0.0920	7.69E-02	1.51E-01
CACNA2D3	-0.0369	4.79E-01	6.08E-01
CACNA2D4	0.1069	3.96E-02	8.95E-02
CACNB1	0.1996	1.09E-04	6.55E-04
CACNB2	0.0966	6.30E-02	1.29E-01
CACNB3	0.0672	1.96E-01	3.12E-01
CACNB4	-0.0109	8.35E-01	8.92E-01
CACNG1	0.1150	2.67E-02	6.50E-02
CACNG2	0.0723	1.65E-01	2.73E-01
CACNG3	0.0892	8.62E-02	1.65E-01
CACNG4	0.2285	8.81E-06	7.52E-05
CACNG5	0.0860	9.82E-02	1.83E-01
CACNG6	0.0644	2.16E-01	3.36E-01
CACNG7	0.0414	4.27E-01	5.59E-01
CACNG8	-0.0076	8.83E-01	9.25E-01
CACYBP	0.5053	1.94E-25	1.10E-22
CADM1	-0.1465	4.68E-03	1.56E-02
CADM2	0.0257	6.22E-01	7.31E-01
CADM3	-0.0300	5.64E-01	6.82E-01
CADM4	0.0030	9.55E-01	9.72E-01
CADPS2	-0.0727	1.62E-01	2.69E-01
CADPS	0.1047	4.40E-02	9.71E-02
CAD	0.3216	2.26E-10	6.22E-09
CAGE1	0.1508	3.59E-03	1.26E-02
CALB1	0.0588	2.58E-01	3.84E-01
CALB2	0.0447	3.91E-01	5.24E-01
CALCA	-0.0693	1.83E-01	2.96E-01
CALCB	0.0494	3.42E-01	4.74E-01
CALCOCO1	-0.0277	5.95E-01	7.09E-01
CALCOCO2	-0.0083	8.73E-01	9.18E-01
CALCRL	-0.1998	1.06E-04	6.41E-04
CALCR	0.1637	1.56E-03	6.26E-03

CALD1	-0.3473	5.93E-12	2.26E-10
CALHM1	0.0660	2.04E-01	3.22E-01
CALHM2	-0.1013	5.12E-02	1.10E-01
CALHM3	0.1524	3.25E-03	1.15E-02
CALM1	-0.1477	4.35E-03	1.47E-02
CALM2	0.1067	3.99E-02	9.00E-02
CALM3	0.0770	1.39E-01	2.39E-01
CALML3	-0.0640	2.19E-01	3.39E-01
CALML4	-0.0653	2.09E-01	3.28E-01
CALML5	0.0780	1.34E-01	2.32E-01
CALML6	0.1951	1.56E-04	8.93E-04
CALN1	-0.0354	4.96E-01	6.22E-01
CALR3	0.0717	1.68E-01	2.77E-01
CALR	-0.0304	5.59E-01	6.78E-01
CALU	0.0226	6.64E-01	7.64E-01
CALY	0.0727	1.62E-01	2.69E-01
CAMK1D	-0.0861	9.77E-02	1.83E-01
CAMK1G	-0.0174	7.39E-01	8.23E-01
CAMK1	0.0766	1.41E-01	2.41E-01
CAMK2A	-0.0048	9.27E-01	9.54E-01
CAMK2B	-0.1519	3.36E-03	1.19E-02
CAMK2D	-0.0570	2.73E-01	4.01E-01
CAMK2G	0.0396	4.47E-01	5.78E-01
CAMK2N1	0.0267	6.08E-01	7.20E-01
CAMK2N2	-0.0096	8.54E-01	9.05E-01
CAMK4	-0.1453	5.05E-03	1.66E-02
CAMKK1	-0.0300	5.65E-01	6.83E-01
CAMKK2	0.0171	7.43E-01	8.27E-01
CAMKV	0.2212	1.71E-05	1.34E-04
CAMLG	0.0171	7.43E-01	8.26E-01
CAMP	-0.0488	3.49E-01	4.81E-01
CAMSAP1L1	0.2197	1.95E-05	1.51E-04
CAMSAP1	0.1702	9.97E-04	4.29E-03
CAMTA1	-0.0504	3.33E-01	4.64E-01
CAMTA2	-0.1912	2.12E-04	1.16E-03
CAND1	0.1166	2.47E-02	6.09E-02
CAND2	0.0788	1.30E-01	2.27E-01
CANT1	0.3538	2.23E-12	9.25E-11
CANX	0.0055	9.16E-01	9.47E-01
CAP1	0.0418	4.22E-01	5.55E-01
CAP2	0.0696	1.81E-01	2.93E-01
CAPG	0.1033	4.68E-02	1.02E-01
CAPN10	0.3136	6.52E-10	1.61E-08
CAPN11	-0.1956	1.49E-04	8.60E-04

CAPN12	0.2337	5.38E-06	4.93E-05
CAPN13	0.1056	4.20E-02	9.36E-02
CAPN14	0.1328	1.04E-02	3.03E-02
CAPN1	0.0171	7.42E-01	8.26E-01
CAPN2	0.0262	6.16E-01	7.25E-01
CAPN3	-0.0620	2.33E-01	3.56E-01
CAPN5	-0.0006	9.90E-01	9.94E-01
CAPN6	0.1525	3.24E-03	1.15E-02
CAPN7	-0.0316	5.43E-01	6.64E-01
CAPN8	0.1107	3.31E-02	7.75E-02
CAPN9	0.1771	6.10E-04	2.81E-03
CAPNS1	0.0088	8.66E-01	9.13E-01
CAPNS2	0.0047	9.28E-01	9.54E-01
CAPRIN1	0.2010	9.67E-05	5.92E-04
CAPRIN2	0.1680	1.16E-03	4.87E-03
CAPS2	-0.0156	7.64E-01	8.41E-01
CAPSL	0.1090	3.59E-02	8.26E-02
CAPS	0.2075	5.64E-05	3.76E-04
CAPZA1	0.1139	2.82E-02	6.80E-02
CAPZA2	-0.1308	1.17E-02	3.31E-02
CAPZA3	-0.1122	3.08E-02	7.29E-02
CAPZB	-0.0451	3.86E-01	5.19E-01
CARD10	0.1821	4.23E-04	2.07E-03
CARD11	0.0083	8.73E-01	9.18E-01
CARD14	0.2447	1.85E-06	1.95E-05
CARD16	-0.0404	4.38E-01	5.69E-01
CARD17	0.0802	1.23E-01	2.18E-01
CARD18	0.1595	2.06E-03	7.89E-03
CARD6	-0.0254	6.26E-01	7.35E-01
CARD8	0.0557	2.84E-01	4.13E-01
CARD9	0.1059	4.15E-02	9.28E-02
CARHSP1	0.1198	2.10E-02	5.34E-02
CARKD	-0.0981	5.90E-02	1.22E-01
CARM1	0.0008	9.87E-01	9.92E-01
CARNS1	0.0258	6.20E-01	7.29E-01
CARS2	0.0992	5.63E-02	1.18E-01
CARS	-0.0642	2.17E-01	3.37E-01
CARTPT	0.0026	9.61E-01	9.75E-01
CASC1	0.0886	8.82E-02	1.68E-01
CASC2	0.0925	7.51E-02	1.48E-01
CASC3	0.3322	5.26E-11	1.65E-09
CASC4	-0.1729	8.26E-04	3.64E-03
CASC5	0.1198	2.10E-02	5.35E-02
CASD1	-0.0266	6.10E-01	7.21E-01

CASKIN1	0.2096	4.71E-05	3.21E-04
CASKIN2	0.0419	4.21E-01	5.54E-01
CASK	-0.0092	8.60E-01	9.09E-01
CASP10	0.0028	9.58E-01	9.74E-01
CASP12	-0.0462	3.75E-01	5.08E-01
CASP14	0.0059	9.10E-01	9.43E-01
CASP1	-0.0347	5.05E-01	6.30E-01
CASP2	0.3156	5.06E-10	1.28E-08
CASP3	0.0612	2.40E-01	3.64E-01
CASP4	0.0351	5.00E-01	6.26E-01
CASP5	0.0734	1.58E-01	2.64E-01
CASP6	0.0669	1.98E-01	3.15E-01
CASP7	-0.0091	8.62E-01	9.11E-01
CASP8AP2	0.2697	1.33E-07	1.88E-06
CASP8	0.0742	1.54E-01	2.59E-01
CASP9	0.0242	6.42E-01	7.47E-01
CASQ1	0.1413	6.42E-03	2.03E-02
CASQ2	-0.2246	1.26E-05	1.03E-04
CASR	-0.0223	6.69E-01	7.69E-01
CASS4	-0.2601	3.77E-07	4.78E-06
CAST	-0.1794	5.17E-04	2.45E-03
CASZ1	0.0869	9.47E-02	1.78E-01
CATSPER1	0.1131	2.95E-02	7.04E-02
CATSPER2P1	-0.0967	6.27E-02	1.29E-01
CATSPER2	0.1368	8.34E-03	2.52E-02
CATSPER3	0.1130	2.95E-02	7.05E-02
CATSPER4	0.0577	2.68E-01	3.96E-01
CATSPERB	0.0469	3.68E-01	5.00E-01
CATSPERG	-0.0650	2.12E-01	3.31E-01
CAT	-0.3001	3.68E-09	7.62E-08
CAV1	-0.0610	2.41E-01	3.66E-01
CAV2	-0.1070	3.93E-02	8.89E-02
CAV3	-0.0665	2.01E-01	3.18E-01
CBARA1	-0.1726	8.44E-04	3.71E-03
CBFA2T2	0.4047	4.70E-16	4.07E-14
CBFA2T3	-0.2623	2.97E-07	3.88E-06
CBFB	0.1221	1.87E-02	4.86E-02
CBLB	-0.0238	6.48E-01	7.52E-01
CBLC	0.0419	4.21E-01	5.54E-01
CBLL1	0.0694	1.82E-01	2.94E-01
CBLN1	-0.1655	1.38E-03	5.63E-03
CBLN2	0.0020	9.70E-01	9.81E-01
CBLN3	-0.1316	1.12E-02	3.19E-02
CBLN4	-0.0857	9.94E-02	1.85E-01

CBL	0.0997	5.49E-02	1.16E-01
CBR1	-0.1316	1.12E-02	3.20E-02
CBR3	0.1837	3.75E-04	1.88E-03
CBR4	-0.4453	1.82E-19	3.93E-17
CBS	-0.1808	4.64E-04	2.24E-03
CBWD1	0.0276	5.97E-01	7.10E-01
CBWD2	0.0544	2.96E-01	4.25E-01
CBWD3	0.0454	3.83E-01	5.16E-01
CBWD5	0.1129	2.97E-02	7.08E-02
CBWD6	0.0807	1.21E-01	2.15E-01
CBX1	0.4220	1.89E-17	2.31E-15
CBX2	0.3016	3.08E-09	6.46E-08
CBX3	0.3132	6.89E-10	1.68E-08
CBX4	0.1883	2.64E-04	1.39E-03
CBX5	0.2157	2.79E-05	2.06E-04
CBX6	0.0432	4.06E-01	5.39E-01
CBX7	-0.1146	2.72E-02	6.61E-02
CBX8	0.2183	2.22E-05	1.69E-04
CBY1	0.0843	1.05E-01	1.93E-01
CC2D1A	-0.1110	3.26E-02	7.65E-02
CC2D1B	0.2104	4.40E-05	3.04E-04
CC2D2A	0.0928	7.43E-02	1.47E-01
CC2D2B	-0.1017	5.03E-02	1.08E-01
CCAR1	0.1688	1.10E-03	4.67E-03
CCBE1	-0.2010	9.65E-05	5.92E-04
CCBL1	0.0939	7.08E-02	1.42E-01
CCBL2	-0.1538	2.98E-03	1.07E-02
CCBP2	-0.2365	4.13E-06	3.94E-05
CCDC101	0.0082	8.75E-01	9.19E-01
CCDC102A	0.0450	3.88E-01	5.21E-01
CCDC102B	-0.0737	1.56E-01	2.62E-01
CCDC103	0.1406	6.66E-03	2.09E-02
CCDC104	0.1008	5.25E-02	1.12E-01
CCDC106	-0.1955	1.51E-04	8.68E-04
CCDC107	-0.0230	6.59E-01	7.60E-01
CCDC108	0.1211	1.97E-02	5.07E-02
CCDC109A	0.0988	5.72E-02	1.20E-01
CCDC109B	0.1787	5.45E-04	2.56E-03
CCDC110	0.0486	3.51E-01	4.83E-01
CCDC111	0.0148	7.76E-01	8.50E-01
CCDC112	0.1737	7.81E-04	3.48E-03
CCDC113	-0.0884	8.89E-02	1.69E-01
CCDC114	0.1509	3.57E-03	1.25E-02
CCDC115	-0.0292	5.75E-01	6.92E-01

CCDC116	0.0273	6.00E-01	7.13E-01
CCDC117	0.1362	8.62E-03	2.59E-02
CCDC11	0.0978	5.98E-02	1.24E-01
CCDC120	0.1998	1.07E-04	6.43E-04
CCDC121	0.0468	3.69E-01	5.02E-01
CCDC122	0.1272	1.42E-02	3.90E-02
CCDC123	0.3096	1.11E-09	2.60E-08
CCDC124	-0.0493	3.44E-01	4.76E-01
CCDC125	-0.0620	2.33E-01	3.56E-01
CCDC126	-0.0263	6.14E-01	7.24E-01
CCDC127	0.0945	6.92E-02	1.39E-01
CCDC129	-0.0209	6.88E-01	7.84E-01
CCDC12	0.0652	2.10E-01	3.29E-01
CCDC130	0.1622	1.72E-03	6.80E-03
CCDC132	0.0374	4.73E-01	6.02E-01
CCDC134	0.0602	2.48E-01	3.72E-01
CCDC135	0.0285	5.85E-01	7.00E-01
CCDC136	0.0925	7.51E-02	1.48E-01
CCDC137	0.3437	1.00E-11	3.55E-10
CCDC138	0.1752	7.01E-04	3.17E-03
CCDC13	0.0470	3.66E-01	4.99E-01
CCDC140	0.1033	4.67E-02	1.02E-01
CCDC141	0.0306	5.57E-01	6.76E-01
CCDC142	0.3103	1.01E-09	2.38E-08
CCDC144A	0.0001	9.98E-01	9.99E-01
CCDC144B	-0.0070	8.92E-01	9.31E-01
CCDC144C	0.1325	1.06E-02	3.06E-02
CCDC144NL	0.1081	3.74E-02	8.56E-02
CCDC146	-0.1894	2.44E-04	1.31E-03
CCDC147	-0.1541	2.92E-03	1.06E-02
CCDC148	0.0294	5.73E-01	6.90E-01
CCDC149	0.1101	3.40E-02	7.92E-02
CCDC14	0.3375	2.47E-11	8.23E-10
CCDC150	0.1759	6.65E-04	3.03E-03
CCDC151	0.0129	8.04E-01	8.71E-01
CCDC152	-0.0811	1.19E-01	2.13E-01
CCDC153	0.0619	2.35E-01	3.58E-01
CCDC154	0.3139	6.31E-10	1.56E-08
CCDC155	0.1739	7.69E-04	3.43E-03
CCDC157	0.0637	2.21E-01	3.42E-01
CCDC158	-0.0872	9.35E-02	1.76E-01
CCDC159	-0.0736	1.57E-01	2.63E-01
CCDC15	0.2618	3.14E-07	4.06E-06
CCDC160	-0.0035	9.47E-01	9.67E-01

CCDC163P	0.1327	1.05E-02	3.05E-02
CCDC17	0.0146	7.79E-01	8.52E-01
CCDC18	0.2268	1.03E-05	8.63E-05
CCDC19	0.1766	6.33E-04	2.91E-03
CCDC21	0.2387	3.33E-06	3.25E-05
CCDC22	0.1073	3.88E-02	8.80E-02
CCDC23	0.1203	2.05E-02	5.23E-02
CCDC24	0.0714	1.70E-01	2.79E-01
CCDC25	-0.1968	1.36E-04	7.92E-04
CCDC27	0.0027	9.58E-01	9.74E-01
CCDC28A	-0.0499	3.38E-01	4.69E-01
CCDC28B	0.1085	3.68E-02	8.44E-02
CCDC30	0.0911	7.98E-02	1.55E-01
CCDC33	-0.0433	4.05E-01	5.39E-01
CCDC34	0.2311	6.86E-06	6.08E-05
CCDC36	0.0475	3.61E-01	4.94E-01
CCDC37	0.0656	2.07E-01	3.25E-01
CCDC38	0.0427	4.12E-01	5.45E-01
CCDC39	0.1269	1.45E-02	3.96E-02
CCDC3	-0.1711	9.35E-04	4.06E-03
CCDC40	0.2340	5.24E-06	4.83E-05
CCDC41	0.1847	3.49E-04	1.77E-03
CCDC42B	0.2468	1.50E-06	1.62E-05
CCDC42	-0.0799	1.24E-01	2.20E-01
CCDC43	0.3301	7.05E-11	2.14E-09
CCDC45	0.4822	5.39E-23	2.15E-20
CCDC46	0.1035	4.63E-02	1.01E-01
CCDC47	0.0020	9.69E-01	9.80E-01
CCDC48	-0.1143	2.78E-02	6.71E-02
CCDC50	0.0269	6.05E-01	7.17E-01
CCDC51	0.0177	7.34E-01	8.20E-01
CCDC52	0.1190	2.18E-02	5.52E-02
CCDC53	-0.1736	7.87E-04	3.50E-03
CCDC54	0.0117	8.23E-01	8.84E-01
CCDC55	0.1366	8.42E-03	2.54E-02
CCDC56	0.1112	3.23E-02	7.57E-02
CCDC57	0.1480	4.28E-03	1.45E-02
CCDC58	0.0445	3.93E-01	5.26E-01
CCDC59	0.3019	2.94E-09	6.23E-08
CCDC60	-0.0273	6.00E-01	7.13E-01
CCDC61	0.1483	4.20E-03	1.43E-02
CCDC62	0.1467	4.62E-03	1.54E-02
CCDC63	0.1364	8.52E-03	2.56E-02
CCDC64B	0.1171	2.41E-02	5.99E-02

CCDC64	0.2129	3.55E-05	2.53E-04
CCDC65	0.0813	1.18E-01	2.11E-01
CCDC66	0.3285	8.74E-11	2.59E-09
CCDC67	0.2257	1.13E-05	9.38E-05
CCDC68	-0.0234	6.54E-01	7.56E-01
CCDC69	-0.1599	2.01E-03	7.72E-03
CCDC6	0.1553	2.70E-03	9.88E-03
CCDC70	-0.0083	8.73E-01	9.18E-01
CCDC71	0.0544	2.96E-01	4.25E-01
CCDC72	0.0888	8.77E-02	1.67E-01
CCDC73	0.1934	1.78E-04	9.96E-04
CCDC74A	0.1725	8.47E-04	3.72E-03
CCDC74B	0.2411	2.64E-06	2.64E-05
CCDC75	0.0344	5.09E-01	6.34E-01
CCDC76	0.2069	5.95E-05	3.92E-04
CCDC77	0.2823	3.16E-08	5.18E-07
CCDC78	0.1914	2.09E-04	1.15E-03
CCDC79	0.0427	4.12E-01	5.45E-01
CCDC7	0.1122	3.07E-02	7.27E-02
CCDC80	-0.0897	8.46E-02	1.63E-01
CCDC81	0.1151	2.67E-02	6.50E-02
CCDC82	0.0902	8.28E-02	1.60E-01
CCDC83	0.1277	1.38E-02	3.82E-02
CCDC84	0.2608	3.48E-07	4.44E-06
CCDC85A	-0.1716	9.03E-04	3.94E-03
CCDC85B	-0.0919	7.72E-02	1.52E-01
CCDC85C	-0.0074	8.87E-01	9.28E-01
CCDC86	0.1167	2.46E-02	6.07E-02
CCDC87	0.1037	4.59E-02	1.01E-01
CCDC88A	0.0593	2.54E-01	3.80E-01
CCDC88B	0.0177	7.35E-01	8.20E-01
CCDC88C	0.0726	1.63E-01	2.70E-01
CCDC89	-0.0004	9.94E-01	9.96E-01
CCDC8	-0.0018	9.73E-01	9.83E-01
CCDC90A	-0.0642	2.17E-01	3.37E-01
CCDC90B	-0.0878	9.14E-02	1.73E-01
CCDC91	0.0089	8.65E-01	9.13E-01
CCDC92	-0.0629	2.27E-01	3.48E-01
CCDC93	0.3051	1.96E-09	4.35E-08
CCDC94	-0.0120	8.17E-01	8.80E-01
CCDC96	0.0782	1.32E-01	2.30E-01
CCDC97	0.2803	3.98E-08	6.37E-07
CCDC99	0.3820	2.47E-14	1.48E-12
CCDC9	0.1483	4.20E-03	1.43E-02

CCHCR1	0.3901	6.24E-15	4.20E-13
CCIN	0.0767	1.41E-01	2.41E-01
CCKAR	0.0408	4.33E-01	5.65E-01
CCKBR	0.0659	2.06E-01	3.23E-01
CCK	0.0937	7.14E-02	1.42E-01
CCL11	-0.0425	4.14E-01	5.47E-01
CCL13	-0.0491	3.45E-01	4.77E-01
CCL14-CCL15	-0.0910	8.00E-02	1.56E-01
CCL14	-0.3055	1.88E-09	4.21E-08
CCL15	0.0021	9.68E-01	9.80E-01
CCL16	-0.2623	2.96E-07	3.87E-06
CCL17	0.0123	8.13E-01	8.77E-01
CCL18	-0.1406	6.68E-03	2.10E-02
CCL19	-0.0254	6.25E-01	7.34E-01
CCL1	0.0740	1.55E-01	2.60E-01
CCL20	0.1797	5.06E-04	2.40E-03
CCL21	-0.0631	2.25E-01	3.47E-01
CCL22	-0.0895	8.52E-02	1.64E-01
CCL23	-0.1654	1.39E-03	5.67E-03
CCL24	-0.1054	4.25E-02	9.46E-02
CCL25	0.1241	1.68E-02	4.46E-02
CCL26	0.2117	3.94E-05	2.76E-04
CCL27	0.1591	2.11E-03	8.06E-03
CCL28	0.1085	3.67E-02	8.42E-02
CCL2	-0.1103	3.37E-02	7.86E-02
CCL3L1	0.0609	2.42E-01	3.66E-01
CCL3L3	0.0085	8.70E-01	9.16E-01
CCL3	-0.0938	7.10E-02	1.42E-01
CCL4L2	0.0038	9.42E-01	9.64E-01
CCL4	-0.0818	1.16E-01	2.08E-01
CCL5	-0.0639	2.20E-01	3.40E-01
CCL7	-0.0133	7.99E-01	8.67E-01
CCL8	-0.0341	5.12E-01	6.37E-01
CCM2	-0.2038	7.67E-05	4.87E-04
CCNA1	-0.0128	8.06E-01	8.72E-01
CCNA2	0.3136	6.54E-10	1.61E-08
CCNB1IP1	-0.1834	3.83E-04	1.91E-03
CCNB1	0.3786	4.34E-14	2.48E-12
CCNB2	0.4042	5.14E-16	4.43E-14
CCNB3	0.0158	7.61E-01	8.39E-01
CCNC	0.0523	3.15E-01	4.45E-01
CCND1	-0.2993	4.06E-09	8.34E-08
CCND2	-0.0643	2.17E-01	3.37E-01
CCND3	-0.0196	7.07E-01	7.99E-01

CCNDBP1	-0.1265	1.48E-02	4.02E-02
CCNE1	0.2767	6.03E-08	9.22E-07
CCNE2	0.3979	1.60E-15	1.22E-13
CCNF	0.4468	1.31E-19	3.04E-17
CCNG1	-0.0856	9.98E-02	1.86E-01
CCNG2	-0.0311	5.51E-01	6.71E-01
CCNH	-0.1239	1.70E-02	4.49E-02
CCNI2	0.3830	2.08E-14	1.27E-12
CCNI	-0.0851	1.02E-01	1.88E-01
CCNJL	0.1581	2.25E-03	8.49E-03
CCNJ	0.0522	3.16E-01	4.46E-01
CCNK	0.1742	7.54E-04	3.38E-03
CCNL1	0.1808	4.65E-04	2.24E-03
CCNL2	0.1507	3.61E-03	1.26E-02
CCNO	0.0069	8.94E-01	9.33E-01
CCNT1	-0.0355	4.95E-01	6.21E-01
CCNT2	0.0574	2.70E-01	3.98E-01
CCNYL1	0.1094	3.52E-02	8.14E-02
CCNY	-0.0888	8.77E-02	1.67E-01
CCPG1	-0.2987	4.38E-09	8.90E-08
CCR10	0.0851	1.02E-01	1.88E-01
CCR1	-0.1362	8.63E-03	2.59E-02
CCR2	-0.0837	1.08E-01	1.97E-01
CCR3	0.0801	1.23E-01	2.19E-01
CCR4	-0.1070	3.94E-02	8.90E-02
CCR5	-0.0356	4.94E-01	6.21E-01
CCR6	0.1325	1.06E-02	3.06E-02
CCR7	-0.0457	3.80E-01	5.13E-01
CCR8	-0.0033	9.50E-01	9.69E-01
CCR9	-0.0226	6.65E-01	7.65E-01
CCRL1	0.0214	6.81E-01	7.78E-01
CCRL2	0.0074	8.88E-01	9.28E-01
CCRN4L	-0.0726	1.63E-01	2.70E-01
CCS	-0.1168	2.45E-02	6.05E-02
CCT2	0.2044	7.30E-05	4.67E-04
CCT3	0.4311	3.22E-18	4.86E-16
CCT4	0.1895	2.41E-04	1.29E-03
CCT5	0.1816	4.39E-04	2.13E-03
CCT6A	0.2101	4.51E-05	3.10E-04
CCT6B	-0.2424	2.32E-06	2.38E-05
CCT6P1	0.2359	4.34E-06	4.12E-05
CCT7	0.0660	2.04E-01	3.22E-01
CCT8L2	0.0333	5.23E-01	6.47E-01
CCT8	0.1792	5.22E-04	2.47E-03

CD101	0.0485	3.52E-01	4.84E-01
CD109	0.1217	1.91E-02	4.95E-02
CD14	-0.2458	1.65E-06	1.76E-05
CD151	-0.0187	7.20E-01	8.10E-01
CD160	0.0340	5.14E-01	6.38E-01
CD163L1	-0.0884	8.92E-02	1.70E-01
CD163	-0.1948	1.59E-04	9.07E-04
CD164L2	0.0496	3.41E-01	4.72E-01
CD164	0.0639	2.20E-01	3.40E-01
CD177	0.1043	4.48E-02	9.86E-02
CD180	-0.0813	1.18E-01	2.11E-01
CD19	0.1113	3.22E-02	7.56E-02
CD1A	0.1041	4.52E-02	9.93E-02
CD1B	0.0528	3.10E-01	4.40E-01
CD1C	-0.0657	2.07E-01	3.24E-01
CD1D	-0.1080	3.76E-02	8.58E-02
CD1E	-0.0097	8.52E-01	9.04E-01
CD200R1L	-0.0445	3.93E-01	5.26E-01
CD200R1	-0.1294	1.26E-02	3.53E-02
CD200	-0.0546	2.94E-01	4.23E-01
CD207	0.0625	2.30E-01	3.53E-01
CD209	-0.0988	5.72E-02	1.19E-01
CD226	-0.1410	6.53E-03	2.06E-02
CD22	-0.0017	9.74E-01	9.84E-01
CD244	-0.1064	4.06E-02	9.12E-02
CD247	-0.0573	2.71E-01	3.99E-01
CD248	-0.0213	6.82E-01	7.79E-01
CD24	0.2347	4.90E-06	4.56E-05
CD274	-0.0971	6.18E-02	1.27E-01
CD276	0.0860	9.81E-02	1.83E-01
CD27	0.0211	6.85E-01	7.82E-01
CD28	-0.0797	1.25E-01	2.21E-01
CD2AP	0.2387	3.32E-06	3.25E-05
CD2BP2	0.0459	3.78E-01	5.12E-01
CD2	-0.0224	6.67E-01	7.67E-01
CD300A	-0.0549	2.91E-01	4.20E-01
CD300C	-0.0777	1.35E-01	2.35E-01
CD300E	-0.1398	6.98E-03	2.17E-02
CD300LB	0.0647	2.14E-01	3.33E-01
CD300LD	0.0149	7.75E-01	8.49E-01
CD300LF	-0.0183	7.26E-01	8.14E-01
CD300LG	-0.2505	1.03E-06	1.16E-05
CD302	-0.2770	5.80E-08	8.91E-07
CD320	0.0096	8.54E-01	9.06E-01

CD33	-0.0702	1.77E-01	2.88E-01
CD34	-0.1517	3.41E-03	1.20E-02
CD36	-0.1199	2.09E-02	5.32E-02
CD37	-0.0430	4.09E-01	5.42E-01
CD38	0.0102	8.44E-01	8.98E-01
CD3D	0.0318	5.41E-01	6.62E-01
CD3EAP	0.2887	1.49E-08	2.66E-07
CD3E	-0.0559	2.83E-01	4.11E-01
CD3G	-0.0339	5.15E-01	6.39E-01
CD40LG	-0.1055	4.22E-02	9.41E-02
CD40	-0.1037	4.60E-02	1.01E-01
CD44	-0.1080	3.75E-02	8.58E-02
CD46	0.1119	3.12E-02	7.37E-02
CD47	0.0442	3.96E-01	5.29E-01
CD48	-0.0614	2.38E-01	3.62E-01
CD4	-0.1754	6.91E-04	3.13E-03
CD52	-0.0312	5.49E-01	6.70E-01
CD53	-0.0671	1.98E-01	3.14E-01
CD55	0.0144	7.83E-01	8.55E-01
CD58	0.1783	5.59E-04	2.61E-03
CD59	-0.1569	2.45E-03	9.10E-03
CD5L	-0.1977	1.27E-04	7.46E-04
CD5	-0.0729	1.61E-01	2.68E-01
CD63	-0.0231	6.57E-01	7.59E-01
CD68	-0.1688	1.10E-03	4.67E-03
CD69	-0.1136	2.87E-02	6.91E-02
CD6	-0.0399	4.44E-01	5.76E-01
CD70	0.0205	6.94E-01	7.89E-01
CD72	-0.0515	3.23E-01	4.53E-01
CD74	-0.0635	2.23E-01	3.44E-01
CD79A	0.0110	8.33E-01	8.90E-01
CD79B	-0.0013	9.81E-01	9.88E-01
CD7	0.0784	1.32E-01	2.29E-01
CD80	0.0050	9.23E-01	9.51E-01
CD81	-0.1836	3.79E-04	1.89E-03
CD82	-0.1608	1.89E-03	7.33E-03
CD83	0.0887	8.79E-02	1.68E-01
CD84	-0.1130	2.96E-02	7.07E-02
CD86	-0.0603	2.47E-01	3.71E-01
CD8A	-0.0505	3.32E-01	4.63E-01
CD8B	-0.0175	7.37E-01	8.22E-01
CD93	-0.1732	8.05E-04	3.57E-03
CD96	-0.1010	5.20E-02	1.11E-01
CD97	0.0410	4.31E-01	5.63E-01

CD99L2	-0.0798	1.25E-01	2.21E-01
CD99	-0.0563	2.79E-01	4.08E-01
CD9	-0.0075	8.86E-01	9.27E-01
CDADC1	-0.1953	1.54E-04	8.82E-04
CDAN1	0.2040	7.54E-05	4.80E-04
CDA	-0.0806	1.21E-01	2.16E-01
CDC123	0.1229	1.79E-02	4.69E-02
CDC14A	0.1052	4.28E-02	9.51E-02
CDC14B	-0.2466	1.52E-06	1.64E-05
CDC14C	-0.2143	3.16E-05	2.29E-04
CDC16	0.2307	7.12E-06	6.26E-05
CDC20B	0.0306	5.56E-01	6.75E-01
CDC20	0.4199	2.79E-17	3.25E-15
CDC23	0.1744	7.41E-04	3.33E-03
CDC25A	0.3435	1.02E-11	3.62E-10
CDC25B	0.2054	6.75E-05	4.37E-04
CDC25C	0.4232	1.49E-17	1.92E-15
CDC26	0.1800	4.94E-04	2.35E-03
CDC27	0.2314	6.70E-06	5.97E-05
CDC34	-0.1517	3.40E-03	1.20E-02
CDC37L1	-0.3045	2.11E-09	4.65E-08
CDC37	0.0070	8.93E-01	9.32E-01
CDC40	0.1096	3.48E-02	8.06E-02
CDC42BPA	-0.0606	2.44E-01	3.68E-01
CDC42BPB	-0.0508	3.29E-01	4.60E-01
CDC42BPG	0.1469	4.59E-03	1.54E-02
CDC42EP1	0.1552	2.73E-03	9.98E-03
CDC42EP2	0.0137	7.93E-01	8.63E-01
CDC42EP3	-0.1541	2.91E-03	1.05E-02
CDC42EP4	0.3247	1.48E-10	4.20E-09
CDC42EP5	-0.0878	9.13E-02	1.73E-01
CDC42SE1	0.4175	4.41E-17	4.84E-15
CDC42SE2	0.1193	2.15E-02	5.44E-02
CDC42	0.0081	8.76E-01	9.20E-01
CDC45	0.4390	6.51E-19	1.26E-16
CDC5L	0.0943	6.96E-02	1.40E-01
CDC6	0.4614	5.91E-21	1.71E-18
CDC73	0.1922	1.96E-04	1.08E-03
CDC7	0.3846	1.59E-14	9.88E-13
CDCA2	0.3857	1.32E-14	8.36E-13
CDCA3	0.4085	2.35E-16	2.16E-14
CDCA4	0.4201	2.68E-17	3.14E-15
CDCA5	0.4168	5.03E-17	5.42E-15
CDCA7L	0.2981	4.75E-09	9.56E-08

CDCA7	0.3605	7.88E-13	3.62E-11
CDCA8	0.4229	1.57E-17	1.98E-15
CDCP1	0.1565	2.51E-03	9.28E-03
CDCP2	0.0719	1.67E-01	2.75E-01
CDH10	0.1693	1.06E-03	4.53E-03
CDH11	-0.0809	1.20E-01	2.14E-01
CDH12	0.1204	2.03E-02	5.20E-02
CDH13	-0.2048	7.08E-05	4.55E-04
CDH15	-0.0360	4.89E-01	6.16E-01
CDH16	-0.0073	8.88E-01	9.28E-01
CDH17	0.0917	7.79E-02	1.52E-01
CDH18	0.2617	3.18E-07	4.10E-06
CDH19	-0.0579	2.66E-01	3.93E-01
CDH1	-0.1119	3.11E-02	7.36E-02
CDH20	0.0283	5.86E-01	7.02E-01
CDH22	-0.0621	2.32E-01	3.56E-01
CDH23	-0.0840	1.06E-01	1.95E-01
CDH24	0.1865	3.04E-04	1.58E-03
CDH26	0.0637	2.21E-01	3.41E-01
CDH2	0.0316	5.44E-01	6.65E-01
CDH3	-0.0960	6.47E-02	1.32E-01
CDH4	-0.0572	2.72E-01	4.00E-01
CDH5	-0.2435	2.08E-06	2.16E-05
CDH6	-0.0020	9.69E-01	9.81E-01
CDH7	0.0117	8.22E-01	8.84E-01
CDH8	0.0950	6.76E-02	1.37E-01
CDH9	0.0217	6.78E-01	7.76E-01
CDHR1	-0.0758	1.45E-01	2.47E-01
CDHR2	-0.1082	3.73E-02	8.53E-02
CDHR3	-0.0284	5.85E-01	7.01E-01
CDHR4	0.1393	7.19E-03	2.22E-02
CDHR5	-0.0407	4.35E-01	5.67E-01
CDIPT	-0.1340	9.77E-03	2.87E-02
CDK10	-0.0272	6.02E-01	7.14E-01
CDK11A	0.0543	2.97E-01	4.26E-01
CDK11B	0.0320	5.39E-01	6.61E-01
CDK12	0.0766	1.41E-01	2.41E-01
CDK13	0.0370	4.77E-01	6.06E-01
CDK14	-0.0714	1.70E-01	2.79E-01
CDK15	-0.0907	8.10E-02	1.57E-01
CDK16	0.3114	8.71E-10	2.09E-08
CDK17	0.0007	9.90E-01	9.94E-01
CDK18	-0.0121	8.17E-01	8.80E-01
CDK19	0.3298	7.31E-11	2.21E-09

CDK1	0.4368	1.02E-18	1.84E-16
CDK20	0.1226	1.82E-02	4.75E-02
CDK2AP1	-0.0112	8.30E-01	8.89E-01
CDK2AP2	0.0038	9.42E-01	9.64E-01
CDK2	0.2885	1.52E-08	2.71E-07
CDK3	0.1770	6.16E-04	2.84E-03
CDK4	0.2422	2.36E-06	2.41E-05
CDK5R1	0.2216	1.65E-05	1.31E-04
CDK5R2	0.1555	2.67E-03	9.81E-03
CDK5RAP1	0.1326	1.06E-02	3.06E-02
CDK5RAP2	0.2262	1.09E-05	9.04E-05
CDK5RAP3	0.2849	2.33E-08	3.95E-07
CDK5	-0.1209	1.99E-02	5.11E-02
CDK6	-0.2314	6.72E-06	5.97E-05
CDK7	0.1700	1.01E-03	4.34E-03
CDK8	-0.0117	8.23E-01	8.84E-01
CDK9	0.0419	4.21E-01	5.54E-01
CDKAL1	0.2791	4.60E-08	7.27E-07
CDKL1	0.0696	1.81E-01	2.93E-01
CDKL2	0.0486	3.50E-01	4.83E-01
CDKL3	0.0862	9.75E-02	1.82E-01
CDKL4	0.1397	7.02E-03	2.18E-02
CDKL5	-0.0710	1.72E-01	2.82E-01
CDKN1A	0.0190	7.16E-01	8.06E-01
CDKN1B	0.0563	2.79E-01	4.08E-01
CDKN1C	0.0970	6.19E-02	1.27E-01
CDKN2AIPNL	0.1238	1.70E-02	4.51E-02
CDKN2AIP	-0.1078	3.80E-02	8.66E-02
CDKN2A	0.2788	4.76E-08	7.47E-07
CDKN2BAS	0.2650	2.21E-07	2.97E-06
CDKN2B	0.1369	8.28E-03	2.50E-02
CDKN2C	0.3240	1.64E-10	4.61E-09
CDKN2D	0.2111	4.16E-05	2.89E-04
CDKN3	0.4002	1.05E-15	8.42E-14
CDNF	-0.2151	2.95E-05	2.16E-04
CDO1	-0.2344	5.01E-06	4.65E-05
CDON	0.1281	1.35E-02	3.75E-02
CDR1	0.0602	2.48E-01	3.72E-01
CDR2L	0.0426	4.14E-01	5.46E-01
CDR2	-0.0026	9.60E-01	9.75E-01
CDRT15P	0.0925	7.52E-02	1.48E-01
CDRT15	0.0986	5.79E-02	1.21E-01
CDRT1	0.0742	1.54E-01	2.58E-01
CDRT4	0.1085	3.68E-02	8.43E-02

CDS1	0.1314	1.13E-02	3.23E-02
CDS2	-0.1967	1.37E-04	8.00E-04
CDSN	-0.0124	8.11E-01	8.76E-01
CDT1	0.3991	1.29E-15	1.01E-13
CDV3	0.0622	2.32E-01	3.55E-01
CDX1	-0.0449	3.88E-01	5.21E-01
CDX2	0.2034	7.94E-05	5.01E-04
CDY2B	0.0346	5.07E-01	6.32E-01
CDYL2	-0.1065	4.03E-02	9.06E-02
CDYL	0.1200	2.08E-02	5.29E-02
CEACAM16	0.0172	7.41E-01	8.25E-01
CEACAM18	0.0006	9.91E-01	9.95E-01
CEACAM19	0.1807	4.71E-04	2.26E-03
CEACAM1	-0.1857	3.22E-04	1.66E-03
CEACAM20	0.0242	6.42E-01	7.47E-01
CEACAM21	-0.0541	2.99E-01	4.28E-01
CEACAM22P	-0.0761	1.43E-01	2.45E-01
CEACAM3	0.0365	4.83E-01	6.12E-01
CEACAM4	-0.0348	5.04E-01	6.30E-01
CEACAM5	0.1010	5.19E-02	1.11E-01
CEACAM6	0.0171	7.43E-01	8.27E-01
CEACAM7	0.1196	2.12E-02	5.39E-02
CEACAM8	-0.0647	2.14E-01	3.33E-01
CEBPA	0.1492	3.98E-03	1.37E-02
CEBPB	-0.1155	2.61E-02	6.39E-02
CEBPD	-0.1308	1.17E-02	3.31E-02
CEBPE	0.0082	8.75E-01	9.19E-01
CEBPG	0.0514	3.23E-01	4.54E-01
CEBPZ	0.1621	1.73E-03	6.82E-03
CECR1	-0.1667	1.27E-03	5.27E-03
CECR2	-0.0385	4.60E-01	5.90E-01
CECR4	-0.0201	6.99E-01	7.93E-01
CECR5	-0.1446	5.27E-03	1.72E-02
CECR6	0.0940	7.04E-02	1.41E-01
CECR7	0.1722	8.68E-04	3.80E-03
CELA1	0.0283	5.87E-01	7.02E-01
CELA2A	-0.0086	8.69E-01	9.15E-01
CELA2B	-0.0507	3.30E-01	4.61E-01
CELA3A	0.0079	8.79E-01	9.22E-01
CELA3B	0.0315	5.46E-01	6.66E-01
CELF1	-0.0029	9.56E-01	9.72E-01
CELF2	-0.1142	2.79E-02	6.74E-02
CELF3	0.0642	2.17E-01	3.38E-01
CELF4	0.2431	2.15E-06	2.22E-05

CELF5	0.0692	1.84E-01	2.96E-01
CELF6	0.0450	3.88E-01	5.21E-01
CELP	0.0814	1.18E-01	2.11E-01
CELSR1	0.0994	5.57E-02	1.17E-01
CELSR2	0.0656	2.08E-01	3.26E-01
CELSR3	0.2397	3.03E-06	2.99E-05
CEL	0.2024	8.63E-05	5.37E-04
CEMP1	0.0774	1.37E-01	2.36E-01
CEND1	-0.0406	4.36E-01	5.68E-01
CENPA	0.3900	6.35E-15	4.26E-13
CENPBD1	-0.1112	3.22E-02	7.57E-02
CENPB	0.0582	2.64E-01	3.91E-01
CENPC1	-0.0022	9.67E-01	9.79E-01
CENPE	0.3591	9.82E-13	4.39E-11
CENPF	0.4718	5.79E-22	1.89E-19
CENPH	0.3639	4.67E-13	2.22E-11
CENPI	0.3857	1.33E-14	8.40E-13
CENPJ	0.4503	6.35E-20	1.60E-17
CENPK	0.3768	5.87E-14	3.24E-12
CENPL	0.5046	2.30E-25	1.27E-22
CENPM	0.3787	4.25E-14	2.44E-12
CENPN	-0.0183	7.25E-01	8.14E-01
CENPO	0.2038	7.66E-05	4.87E-04
CENPP	0.2082	5.31E-05	3.57E-04
CENPQ	0.3635	4.96E-13	2.34E-11
CENPT	0.2098	4.65E-05	3.18E-04
CENPV	0.1247	1.62E-02	4.33E-02
CENPW	0.4024	7.12E-16	5.98E-14
CEP110	0.2544	6.85E-07	8.10E-06
CEP120	-0.1021	4.95E-02	1.07E-01
CEP135	0.2123	3.76E-05	2.66E-04
CEP152	0.2951	6.83E-09	1.32E-07
CEP164	0.2826	3.06E-08	5.03E-07
CEP170L	0.1063	4.07E-02	9.15E-02
CEP170	0.1676	1.19E-03	4.98E-03
CEP192	0.1914	2.08E-04	1.14E-03
CEP250	0.3410	1.49E-11	5.16E-10
CEP290	0.2069	5.96E-05	3.92E-04
CEP350	0.2991	4.21E-09	8.58E-08
CEP55	0.4014	8.51E-16	7.06E-14
CEP57	0.1220	1.87E-02	4.87E-02
CEP63	0.0818	1.16E-01	2.09E-01
CEP68	0.0772	1.38E-01	2.37E-01
CEP70	0.0247	6.35E-01	7.42E-01

CEP72	0.2531	7.88E-07	9.15E-06
CEP76	0.0540	3.00E-01	4.29E-01
CEP78	0.3143	5.95E-10	1.49E-08
CEP97	0.1177	2.34E-02	5.83E-02
CEPT1	-0.1795	5.12E-04	2.43E-03
CER1	0.0873	9.31E-02	1.76E-01
CERCAM	0.0022	9.66E-01	9.79E-01
CERKL	0.0032	9.50E-01	9.69E-01
CERK	0.0272	6.01E-01	7.14E-01
CES1	-0.1444	5.31E-03	1.74E-02
CES2	-0.2804	3.96E-08	6.33E-07
CES3	-0.1133	2.92E-02	6.99E-02
CES4	-0.0921	7.65E-02	1.50E-01
CES7	-0.2481	1.31E-06	1.44E-05
CES8	-0.1828	4.03E-04	1.99E-03
CETN1	0.0803	1.23E-01	2.18E-01
CETN2	0.1374	8.04E-03	2.44E-02
CETN3	0.0374	4.73E-01	6.02E-01
CETN4P	-0.0377	4.69E-01	5.99E-01
CETP	-0.1788	5.39E-04	2.53E-03
CFB	-0.1877	2.76E-04	1.45E-03
CFC1B	0.0257	6.21E-01	7.30E-01
CFDP1	-0.1329	1.04E-02	3.01E-02
CFD	-0.0749	1.50E-01	2.54E-01
CFHR1	-0.0597	2.51E-01	3.76E-01
CFHR2	-0.0744	1.53E-01	2.57E-01
CFHR3	-0.2072	5.78E-05	3.83E-04
CFHR4	-0.2472	1.43E-06	1.55E-05
CFHR5	-0.0764	1.42E-01	2.43E-01
CFH	-0.1302	1.21E-02	3.41E-02
CFI	-0.3321	5.33E-11	1.67E-09
CFL1	0.0502	3.35E-01	4.67E-01
CFL2	-0.2535	7.49E-07	8.75E-06
CFLAR	-0.0298	5.67E-01	6.84E-01
CFLP1	0.0214	6.82E-01	7.79E-01
CFP	-0.2075	5.63E-05	3.75E-04
CFTR	-0.0331	5.25E-01	6.48E-01
CG030	0.1897	2.39E-04	1.28E-03
CGA	0.0750	1.50E-01	2.53E-01
CGB1	-0.0170	7.45E-01	8.28E-01
CGB2	0.0120	8.17E-01	8.80E-01
CGB5	0.0315	5.45E-01	6.66E-01
CGB7	0.0156	7.65E-01	8.41E-01
CGB8	0.0063	9.04E-01	9.40E-01

CGB	0.0707	1.74E-01	2.85E-01
CGGBP1	0.0499	3.38E-01	4.69E-01
CGNL1	-0.1324	1.07E-02	3.08E-02
CGN	0.0375	4.72E-01	6.01E-01
CGREF1	-0.0517	3.21E-01	4.51E-01
CGRRF1	0.1203	2.04E-02	5.22E-02
CH25H	-0.1047	4.39E-02	9.70E-02
CHAC1	0.0462	3.75E-01	5.08E-01
CHAC2	0.0674	1.95E-01	3.11E-01
CHADL	-0.0682	1.90E-01	3.04E-01
CHAD	0.0022	9.67E-01	9.80E-01
CHAF1A	0.3938	3.25E-15	2.33E-13
CHAF1B	0.4863	2.05E-23	8.33E-21
CHAT	0.0040	9.38E-01	9.61E-01
CHCHD10	-0.1937	1.75E-04	9.81E-04
CHCHD1	0.0585	2.61E-01	3.87E-01
CHCHD2	0.0377	4.69E-01	5.99E-01
CHCHD3	-0.1272	1.42E-02	3.91E-02
CHCHD4	-0.0652	2.10E-01	3.28E-01
CHCHD5	-0.0901	8.29E-02	1.60E-01
CHCHD6	-0.0036	9.45E-01	9.66E-01
CHCHD7	-0.0654	2.09E-01	3.27E-01
CHCHD8	0.0022	9.66E-01	9.79E-01
CHD1L	0.4024	7.12E-16	5.98E-14
CHD1	0.0703	1.76E-01	2.87E-01
CHD2	-0.0374	4.72E-01	6.01E-01
CHD3	0.0929	7.40E-02	1.47E-01
CHD4	0.2061	6.34E-05	4.14E-04
CHD5	0.0816	1.17E-01	2.10E-01
CHD6	0.0640	2.19E-01	3.39E-01
CHD7	0.0214	6.82E-01	7.79E-01
CHD8	0.1669	1.25E-03	5.21E-03
CHD9	-0.2170	2.50E-05	1.88E-04
CHDH	-0.0994	5.58E-02	1.17E-01
CHEK1	0.4007	9.70E-16	7.92E-14
CHEK2	0.1713	9.23E-04	4.01E-03
CHERP	-0.0003	9.96E-01	9.97E-01
CHFR	0.0380	4.66E-01	5.96E-01
CHGA	0.1205	2.03E-02	5.20E-02
CHGB	-0.0141	7.86E-01	8.58E-01
CHI3L1	-0.0559	2.82E-01	4.11E-01
CHI3L2	0.0487	3.49E-01	4.82E-01
CHIA	0.1115	3.17E-02	7.47E-02
CHIC1	0.0331	5.25E-01	6.48E-01

CHIC2	0.0195	7.09E-01	8.01E-01
CHID1	-0.1682	1.15E-03	4.84E-03
CHIT1	-0.0984	5.84E-02	1.21E-01
CHKA	0.2111	4.17E-05	2.90E-04
CHKB-CPT1B	0.2125	3.68E-05	2.61E-04
CHKB	0.0333	5.23E-01	6.47E-01
CHL1	-0.0852	1.01E-01	1.88E-01
CHML	0.4148	7.39E-17	7.74E-15
CHMP1A	-0.1180	2.31E-02	5.77E-02
CHMP1B	-0.0425	4.14E-01	5.47E-01
CHMP2A	-0.0815	1.17E-01	2.10E-01
CHMP2B	-0.0506	3.31E-01	4.62E-01
CHMP4A	-0.0243	6.41E-01	7.47E-01
CHMP4B	0.0852	1.01E-01	1.88E-01
CHMP4C	0.1647	1.45E-03	5.88E-03
CHMP5	-0.0064	9.02E-01	9.38E-01
CHMP6	0.0212	6.84E-01	7.80E-01
CHMP7	-0.0628	2.28E-01	3.50E-01
CHM	0.0441	3.97E-01	5.30E-01
CHN1	-0.0816	1.17E-01	2.10E-01
CHN2	-0.1857	3.22E-04	1.66E-03
CHODL	0.1218	1.89E-02	4.91E-02
CHORDC1	0.2620	3.07E-07	3.98E-06
CHP2	0.1274	1.41E-02	3.87E-02
CHPF2	-0.0703	1.77E-01	2.88E-01
CHPF	-0.1171	2.41E-02	5.98E-02
CHPT1	-0.2200	1.90E-05	1.48E-04
CHP	-0.3663	3.20E-13	1.56E-11
CHRAC1	0.1401	6.86E-03	2.14E-02
CHRDL1	-0.0108	8.36E-01	8.92E-01
CHRDL2	-0.0315	5.46E-01	6.66E-01
CHRD	-0.0161	7.58E-01	8.37E-01
CHRFAM7A	0.0673	1.96E-01	3.12E-01
CHRM1	0.0861	9.76E-02	1.83E-01
CHRM2	-0.1985	1.19E-04	7.08E-04
CHRM3	0.0787	1.30E-01	2.28E-01
CHRM4	-0.0132	8.00E-01	8.68E-01
CHRM5	0.1391	7.30E-03	2.25E-02
CHRNA10	0.2789	4.70E-08	7.39E-07
CHRNA1	0.0760	1.44E-01	2.46E-01
CHRNA2	-0.0408	4.33E-01	5.65E-01
CHRNA3	-0.0034	9.48E-01	9.68E-01
CHRNA4	-0.0264	6.12E-01	7.23E-01
CHRNA5	0.1177	2.33E-02	5.82E-02

CHRNA6	0.0735	1.57E-01	2.63E-01
CHRNA7	0.0307	5.56E-01	6.75E-01
CHRNA9	0.1168	2.44E-02	6.04E-02
CHRNB1	-0.1057	4.18E-02	9.34E-02
CHRNB2	0.1358	8.84E-03	2.65E-02
CHRNB3	-0.0274	5.99E-01	7.12E-01
CHRNB4	-0.0280	5.90E-01	7.05E-01
CHRND	0.0807	1.21E-01	2.15E-01
CHRNE	-0.1339	9.81E-03	2.88E-02
CHRNG	0.1394	7.17E-03	2.22E-02
CHST10	0.1165	2.48E-02	6.11E-02
CHST11	0.0737	1.57E-01	2.62E-01
CHST12	0.0760	1.44E-01	2.46E-01
CHST13	0.0293	5.74E-01	6.91E-01
CHST14	0.0408	4.33E-01	5.65E-01
CHST15	-0.1597	2.04E-03	7.83E-03
CHST1	0.1501	3.75E-03	1.30E-02
CHST2	-0.0052	9.21E-01	9.50E-01
CHST3	0.0451	3.86E-01	5.19E-01
CHST4	0.0011	9.83E-01	9.89E-01
CHST5	0.0335	5.20E-01	6.44E-01
CHST6	0.0722	1.65E-01	2.73E-01
CHST7	-0.1342	9.68E-03	2.85E-02
CHST8	-0.1345	9.51E-03	2.81E-02
CHST9	-0.0499	3.38E-01	4.69E-01
CHSY1	-0.0860	9.81E-02	1.83E-01
CHSY3	-0.1558	2.62E-03	9.62E-03
CHTF18	0.4174	4.48E-17	4.88E-15
CHTF8	-0.1469	4.58E-03	1.53E-02
CHUK	-0.1258	1.53E-02	4.14E-02
CHURC1	-0.1741	7.55E-04	3.38E-03
CIAO1	-0.1218	1.89E-02	4.92E-02
CIAPIN1	-0.1555	2.66E-03	9.78E-03
CIB1	-0.1589	2.14E-03	8.15E-03
CIB2	0.1622	1.72E-03	6.79E-03
CIB3	0.1086	3.65E-02	8.39E-02
CIB4	0.0124	8.12E-01	8.77E-01
CIC	0.1301	1.21E-02	3.42E-02
CIDEA	-0.0962	6.40E-02	1.31E-01
CIDEB	-0.3986	1.39E-15	1.08E-13
CIDECP	0.1435	5.61E-03	1.81E-02
CIDEC	-0.0496	3.40E-01	4.72E-01
CIITA	0.0350	5.01E-01	6.27E-01
CILP2	0.0805	1.22E-01	2.17E-01

CILP	0.0188	7.18E-01	8.08E-01
CINP	-0.0459	3.78E-01	5.11E-01
CIR1	0.1325	1.06E-02	3.06E-02
CIRBP	-0.0380	4.65E-01	5.95E-01
CIRH1A	0.0091	8.61E-01	9.10E-01
CISD1	-0.1012	5.15E-02	1.10E-01
CISD2	-0.1618	1.77E-03	6.94E-03
CISD3	-0.0737	1.57E-01	2.62E-01
CISH	-0.1016	5.06E-02	1.09E-01
CITED1	0.0030	9.53E-01	9.71E-01
CITED2	-0.0369	4.78E-01	6.07E-01
CITED4	0.1221	1.87E-02	4.86E-02
CIT	0.3438	9.90E-12	3.52E-10
CIZ1	0.3925	4.07E-15	2.87E-13
CKAP2L	0.3887	7.83E-15	5.15E-13
CKAP2	0.2754	7.00E-08	1.05E-06
CKAP4	0.2173	2.42E-05	1.83E-04
CKAP5	0.2740	8.23E-08	1.22E-06
CKB	-0.0426	4.13E-01	5.46E-01
CKLF	0.1512	3.51E-03	1.23E-02
CKMT1A	0.1078	3.79E-02	8.64E-02
CKMT1B	0.1028	4.78E-02	1.04E-01
CKMT2	-0.0215	6.80E-01	7.78E-01
CKM	0.1045	4.42E-02	9.75E-02
CKS1B	0.4726	4.81E-22	1.65E-19
CKS2	0.3514	3.19E-12	1.28E-10
CLASP1	-0.0533	3.06E-01	4.36E-01
CLASP2	0.0858	9.91E-02	1.84E-01
CLCA1	0.0504	3.33E-01	4.64E-01
CLCA2	-0.0831	1.10E-01	2.01E-01
CLCA3P	0.0087	8.68E-01	9.15E-01
CLCA4	0.0586	2.60E-01	3.86E-01
CLCC1	-0.0351	5.00E-01	6.26E-01
CLCF1	0.0955	6.62E-02	1.34E-01
CLCN1	-0.0156	7.64E-01	8.41E-01
CLCN2	0.2292	8.19E-06	7.03E-05
CLCN3	-0.1782	5.64E-04	2.63E-03
CLCN4	-0.0527	3.11E-01	4.41E-01
CLCN5	-0.1193	2.15E-02	5.45E-02
CLCN6	0.0205	6.95E-01	7.89E-01
CLCN7	-0.0130	8.03E-01	8.70E-01
CLCNKA	0.1707	9.63E-04	4.17E-03
CLCNKB	0.1306	1.18E-02	3.35E-02
CLC	-0.0414	4.27E-01	5.59E-01

CLDN10	0.0055	9.16E-01	9.47E-01
CLDN11	0.0754	1.47E-01	2.50E-01
CLDN12	-0.2334	5.53E-06	5.04E-05
CLDN14	-0.0734	1.58E-01	2.64E-01
CLDN15	-0.0296	5.70E-01	6.88E-01
CLDN16	-0.1400	6.92E-03	2.16E-02
CLDN18	0.2229	1.47E-05	1.18E-04
CLDN19	-0.0130	8.03E-01	8.70E-01
CLDN1	0.0661	2.04E-01	3.21E-01
CLDN20	0.1596	2.05E-03	7.86E-03
CLDN22	0.0217	6.77E-01	7.75E-01
CLDN23	-0.0807	1.21E-01	2.16E-01
CLDN25	0.0721	1.66E-01	2.74E-01
CLDN2	-0.1458	4.88E-03	1.62E-02
CLDN3	0.0192	7.12E-01	8.03E-01
CLDN4	0.1581	2.26E-03	8.53E-03
CLDN5	-0.1255	1.56E-02	4.19E-02
CLDN6	0.0495	3.42E-01	4.74E-01
CLDN7	0.0659	2.05E-01	3.23E-01
CLDN8	-0.0734	1.58E-01	2.64E-01
CLDN9	0.0390	4.53E-01	5.84E-01
CLDND1	0.1596	2.05E-03	7.86E-03
CLDND2	-0.0944	6.92E-02	1.39E-01
CLEC10A	-0.0888	8.77E-02	1.67E-01
CLEC11A	-0.0350	5.02E-01	6.27E-01
CLEC12A	-0.0610	2.41E-01	3.66E-01
CLEC12B	0.0785	1.31E-01	2.29E-01
CLEC14A	-0.1960	1.45E-04	8.41E-04
CLEC16A	-0.0203	6.97E-01	7.91E-01
CLEC17A	0.0467	3.70E-01	5.03E-01
CLEC18A	0.0156	7.64E-01	8.41E-01
CLEC18B	0.0260	6.17E-01	7.27E-01
CLEC18C	0.0477	3.60E-01	4.92E-01
CLEC1A	-0.2269	1.02E-05	8.58E-05
CLEC1B	-0.1690	1.08E-03	4.59E-03
CLEC2A	0.0983	5.84E-02	1.21E-01
CLEC2B	-0.0959	6.51E-02	1.32E-01
CLEC2D	0.1668	1.26E-03	5.22E-03
CLEC2L	0.0761	1.44E-01	2.45E-01
CLEC3A	-0.0527	3.12E-01	4.41E-01
CLEC3B	-0.2969	5.51E-09	1.09E-07
CLEC4A	0.0003	9.95E-01	9.97E-01
CLEC4C	0.0459	3.78E-01	5.11E-01
CLEC4D	-0.0059	9.10E-01	9.43E-01

CLEC4E	-0.1150	2.67E-02	6.50E-02
CLEC4F	0.1067	4.00E-02	9.01E-02
CLEC4GP1	-0.0554	2.88E-01	4.16E-01
CLEC4G	-0.1895	2.41E-04	1.29E-03
CLEC4M	-0.1782	5.65E-04	2.64E-03
CLEC5A	0.0688	1.86E-01	2.99E-01
CLEC6A	0.0102	8.44E-01	8.98E-01
CLEC7A	-0.0411	4.30E-01	5.62E-01
CLEC9A	-0.1240	1.68E-02	4.46E-02
CLECL1	0.1154	2.62E-02	6.41E-02
CLGN	0.2423	2.33E-06	2.38E-05
CLIC1	0.2052	6.85E-05	4.43E-04
CLIC2	-0.0705	1.75E-01	2.86E-01
CLIC3	0.0836	1.08E-01	1.97E-01
CLIC4	-0.1246	1.63E-02	4.35E-02
CLIC5	-0.0159	7.61E-01	8.39E-01
CLIC6	-0.0617	2.35E-01	3.59E-01
CLINT1	-0.0873	9.31E-02	1.76E-01
CLIP1	-0.0955	6.61E-02	1.34E-01
CLIP2	-0.0029	9.55E-01	9.72E-01
CLIP3	0.0405	4.36E-01	5.68E-01
CLIP4	-0.0014	9.78E-01	9.86E-01
CLK1	0.0748	1.51E-01	2.55E-01
CLK2P	0.3775	5.18E-14	2.91E-12
CLK2	0.5913	2.33E-36	4.22E-33
CLK3	0.0668	1.99E-01	3.16E-01
CLK4	0.0040	9.38E-01	9.61E-01
CLLU1OS	0.0993	5.60E-02	1.17E-01
CLLU1	0.0311	5.51E-01	6.71E-01
CLMN	-0.1210	1.98E-02	5.09E-02
CLN3	0.1584	2.21E-03	8.35E-03
CLN5	-0.0907	8.10E-02	1.57E-01
CLN6	0.1995	1.09E-04	6.56E-04
CLN8	0.0157	7.63E-01	8.41E-01
CLNK	-0.0341	5.13E-01	6.37E-01
CLNS1A	-0.0644	2.16E-01	3.36E-01
CLOCK	-0.0700	1.79E-01	2.90E-01
CLP1	0.0601	2.48E-01	3.73E-01
CLPB	-0.3387	2.08E-11	7.05E-10
CLPP	-0.1622	1.72E-03	6.78E-03
CLPS	0.0165	7.51E-01	8.32E-01
CLPTM1L	0.0080	8.77E-01	9.21E-01
CLPTM1	-0.1073	3.88E-02	8.80E-02
CLPX	-0.2148	3.02E-05	2.20E-04

CLRN1OS	-0.0653	2.09E-01	3.28E-01
CLRN1	-0.0550	2.90E-01	4.19E-01
CLRN2	-0.0854	1.00E-01	1.86E-01
CLRN3	-0.0522	3.16E-01	4.46E-01
CLSPN	0.3158	4.93E-10	1.25E-08
CLSTN1	0.0994	5.58E-02	1.17E-01
CLSTN2	-0.1231	1.77E-02	4.64E-02
CLSTN3	-0.0319	5.40E-01	6.61E-01
CLTA	0.2105	4.37E-05	3.02E-04
CLTB	-0.0381	4.65E-01	5.95E-01
CLTCL1	-0.0123	8.13E-01	8.77E-01
CLTC	0.0903	8.25E-02	1.60E-01
CLUAP1	0.1385	7.53E-03	2.31E-02
CLUL1	0.1952	1.54E-04	8.85E-04
CLU	-0.1058	4.17E-02	9.32E-02
CLVS1	-0.0967	6.29E-02	1.29E-01
CLVS2	0.0474	3.63E-01	4.95E-01
CLYBL	-0.2434	2.09E-06	2.16E-05
CMA1	-0.1673	1.22E-03	5.08E-03
CMAH	-0.1187	2.22E-02	5.59E-02
CMAS	0.0428	4.11E-01	5.44E-01
CMBL	-0.1424	6.02E-03	1.92E-02
CMC1	-0.0126	8.08E-01	8.74E-01
CMIP	-0.0683	1.89E-01	3.03E-01
CMKLR1	-0.1159	2.56E-02	6.29E-02
CMPK1	-0.1783	5.61E-04	2.62E-03
CMPK2	-0.0148	7.76E-01	8.50E-01
CMTM1	0.1183	2.27E-02	5.69E-02
CMTM2	0.0147	7.78E-01	8.52E-01
CMTM3	0.0435	4.03E-01	5.36E-01
CMTM4	0.1555	2.67E-03	9.79E-03
CMTM5	-0.1438	5.51E-03	1.79E-02
CMTM6	-0.1848	3.45E-04	1.75E-03
CMTM7	0.1347	9.39E-03	2.78E-02
CMTM8	0.0058	9.11E-01	9.44E-01
CMYA5	0.1700	1.01E-03	4.34E-03
CN5H6.4	0.2622	3.00E-07	3.90E-06
CNBD1	0.0428	4.12E-01	5.44E-01
CNBP	-0.1622	1.72E-03	6.78E-03
CNDP1	-0.1030	4.74E-02	1.03E-01
CNDP2	-0.2158	2.75E-05	2.04E-04
CNFN	0.0751	1.49E-01	2.53E-01
CNGA1	-0.0593	2.54E-01	3.80E-01
CNGA2	-0.0567	2.76E-01	4.04E-01

CNGA3	0.1044	4.44E-02	9.80E-02
CNGA4	0.1425	5.96E-03	1.90E-02
CNGB1	0.1258	1.53E-02	4.14E-02
CNGB3	0.1127	3.00E-02	7.14E-02
CNIH2	0.1780	5.71E-04	2.66E-03
CNIH3	-0.0006	9.91E-01	9.94E-01
CNIH4	0.3335	4.35E-11	1.38E-09
CNIH	-0.2107	4.30E-05	2.98E-04
CNKSR1	0.1782	5.62E-04	2.62E-03
CNKSR2	-0.0487	3.50E-01	4.82E-01
CNKSR3	0.1149	2.69E-02	6.53E-02
CNN1	-0.1163	2.51E-02	6.17E-02
CNN2	0.0854	1.00E-01	1.86E-01
CNN3	0.0217	6.77E-01	7.75E-01
CNNM1	-0.0243	6.41E-01	7.47E-01
CNNM2	-0.1853	3.34E-04	1.71E-03
CNNM3	-0.0159	7.61E-01	8.39E-01
CNNM4	0.1983	1.21E-04	7.17E-04
CNOT10	0.3319	5.46E-11	1.70E-09
CNOT1	-0.1051	4.31E-02	9.57E-02
CNOT2	0.2229	1.47E-05	1.18E-04
CNOT3	0.2951	6.87E-09	1.33E-07
CNOT4	-0.0944	6.93E-02	1.39E-01
CNOT6L	-0.0922	7.61E-02	1.50E-01
CNOT6	0.2463	1.57E-06	1.69E-05
CNOT7	0.0344	5.09E-01	6.34E-01
CNOT8	-0.0066	8.99E-01	9.36E-01
CNO	0.1259	1.53E-02	4.13E-02
CNPY1	0.0260	6.18E-01	7.27E-01
CNPY2	0.1230	1.78E-02	4.67E-02
CNPY3	0.2182	2.23E-05	1.70E-04
CNPY4	0.0907	8.10E-02	1.57E-01
CNP	0.2073	5.72E-05	3.80E-04
CNR1	-0.0044	9.32E-01	9.58E-01
CNR2	0.0266	6.10E-01	7.21E-01
CNRIP1	-0.1876	2.79E-04	1.47E-03
CNST	0.0419	4.21E-01	5.54E-01
CNTD1	0.2074	5.72E-05	3.80E-04
CNTD2	0.0485	3.51E-01	4.84E-01
CNTFR	0.0097	8.52E-01	9.04E-01
CNTF	-0.0558	2.83E-01	4.12E-01
CNTLN	0.0795	1.26E-01	2.23E-01
CNTN1	-0.1230	1.78E-02	4.67E-02
CNTN2	0.0439	3.99E-01	5.32E-01

CNTN3	-0.0525	3.13E-01	4.42E-01
CNTN4	-0.1484	4.16E-03	1.42E-02
CNTN5	0.0500	3.37E-01	4.68E-01
CNTN6	-0.0243	6.40E-01	7.46E-01
CNTNAP1	0.1202	2.06E-02	5.25E-02
CNTNAP2	0.0369	4.79E-01	6.08E-01
CNTNAP3	-0.0829	1.11E-01	2.01E-01
CNTNAP4	-0.0034	9.48E-01	9.68E-01
CNTNAP5	0.1405	6.74E-03	2.11E-02
CNTROB	0.1589	2.14E-03	8.15E-03
COASY	0.0536	3.03E-01	4.33E-01
COBLL1	-0.2472	1.43E-06	1.55E-05
COBL	-0.1486	4.12E-03	1.41E-02
COBRA1	0.2559	5.86E-07	7.02E-06
COCH	0.0483	3.53E-01	4.85E-01
COG1	0.3230	1.87E-10	5.25E-09
COG2	0.2635	2.61E-07	3.45E-06
COG3	-0.0125	8.11E-01	8.76E-01
COG4	-0.0276	5.96E-01	7.09E-01
COG5	-0.0874	9.27E-02	1.75E-01
COG6	-0.0534	3.05E-01	4.35E-01
COG7	0.0100	8.48E-01	9.00E-01
COG8	-0.2116	3.97E-05	2.78E-04
COIL	0.3870	1.06E-14	6.86E-13
COL10A1	-0.0466	3.71E-01	5.04E-01
COL11A1	0.0762	1.43E-01	2.45E-01
COL11A2	0.0365	4.84E-01	6.12E-01
COL12A1	-0.0750	1.49E-01	2.53E-01
COL13A1	-0.0302	5.61E-01	6.79E-01
COL14A1	-0.1673	1.22E-03	5.07E-03
COL15A1	-0.1535	3.03E-03	1.09E-02
COL16A1	0.0065	9.01E-01	9.37E-01
COL17A1	-0.0914	7.87E-02	1.54E-01
COL18A1	-0.2544	6.86E-07	8.10E-06
COL19A1	-0.0042	9.36E-01	9.60E-01
COL1A1	-0.0188	7.18E-01	8.09E-01
COL1A2	-0.0352	4.99E-01	6.25E-01
COL20A1	0.1734	7.95E-04	3.53E-03
COL21A1	-0.0893	8.60E-02	1.65E-01
COL22A1	0.1861	3.13E-04	1.62E-03
COL23A1	-0.0607	2.43E-01	3.68E-01
COL24A1	0.1298	1.23E-02	3.46E-02
COL25A1	-0.2011	9.64E-05	5.91E-04
COL27A1	0.0845	1.04E-01	1.91E-01

COL28A1	0.1004	5.33E-02	1.13E-01
COL29A1	-0.0574	2.70E-01	3.98E-01
COL2A1	0.1403	6.79E-03	2.12E-02
COL3A1	-0.0702	1.77E-01	2.88E-01
COL4A1	-0.1003	5.37E-02	1.14E-01
COL4A2	-0.0816	1.17E-01	2.10E-01
COL4A3BP	-0.2560	5.82E-07	6.98E-06
COL4A3	-0.1008	5.23E-02	1.11E-01
COL4A4	-0.1502	3.73E-03	1.30E-02
COL4A5	0.0933	7.27E-02	1.45E-01
COL4A6	0.0822	1.14E-01	2.06E-01
COL5A1	-0.0322	5.36E-01	6.58E-01
COL5A2	-0.0523	3.15E-01	4.45E-01
COL5A3	-0.0669	1.99E-01	3.15E-01
COL6A1	-0.0603	2.47E-01	3.72E-01
COL6A2	-0.0751	1.49E-01	2.52E-01
COL6A3	-0.0576	2.69E-01	3.96E-01
COL6A4P2	0.1201	2.07E-02	5.28E-02
COL6A6	-0.1274	1.41E-02	3.87E-02
COL7A1	0.0515	3.23E-01	4.53E-01
COL8A1	-0.1787	5.42E-04	2.55E-03
COL8A2	0.0028	9.58E-01	9.74E-01
COL9A1	0.0139	7.90E-01	8.61E-01
COL9A2	0.3226	1.98E-10	5.50E-09
COL9A3	0.0558	2.83E-01	4.12E-01
COLEC10	-0.1847	3.48E-04	1.77E-03
COLEC11	-0.1562	2.55E-03	9.42E-03
COLEC12	-0.0313	5.48E-01	6.68E-01
COLQ	-0.0465	3.72E-01	5.05E-01
COMMD10	-0.0325	5.32E-01	6.54E-01
COMMD1	-0.0692	1.84E-01	2.96E-01
COMMD2	0.2384	3.44E-06	3.35E-05
COMMD3	0.0511	3.26E-01	4.57E-01
COMMD4	0.0977	6.01E-02	1.24E-01
COMMD5	0.0692	1.84E-01	2.96E-01
COMMD6	0.0584	2.62E-01	3.88E-01
COMMD7	0.0636	2.22E-01	3.43E-01
COMMD8	0.0888	8.78E-02	1.67E-01
COMMD9	-0.0173	7.39E-01	8.24E-01
COMP	0.0397	4.46E-01	5.77E-01
COMTD1	-0.1092	3.55E-02	8.19E-02
COMT	-0.1314	1.13E-02	3.23E-02
COPA	0.3443	9.16E-12	3.32E-10
COPB1	0.0649	2.12E-01	3.31E-01

COPB2	0.0219	6.74E-01	7.73E-01
COPE	0.0519	3.19E-01	4.49E-01
COPG2	-0.0216	6.78E-01	7.76E-01
COPG	-0.0142	7.85E-01	8.57E-01
COPS2	-0.0540	3.00E-01	4.29E-01
COPS3	0.0897	8.45E-02	1.62E-01
COPS4	-0.0351	5.00E-01	6.26E-01
COPS5	-0.0356	4.95E-01	6.21E-01
COPS6	0.0303	5.61E-01	6.79E-01
COPS7A	-0.0353	4.98E-01	6.24E-01
COPS7B	0.2894	1.37E-08	2.46E-07
COPS8	0.0027	9.58E-01	9.74E-01
COPZ1	0.1540	2.95E-03	1.06E-02
COPZ2	-0.2277	9.44E-06	7.99E-05
COQ10A	-0.1209	1.98E-02	5.10E-02
COQ10B	-0.1872	2.89E-04	1.51E-03
COQ2	-0.1248	1.61E-02	4.31E-02
COQ3	0.0568	2.75E-01	4.03E-01
COQ4	-0.2006	1.00E-04	6.10E-04
COQ5	-0.1032	4.70E-02	1.02E-01
COQ6	-0.3300	7.10E-11	2.15E-09
COQ7	-0.1325	1.06E-02	3.07E-02
COQ9	-0.3915	4.92E-15	3.40E-13
CORIN	-0.0766	1.41E-01	2.42E-01
CORO1A	0.0006	9.91E-01	9.95E-01
CORO1B	-0.0923	7.57E-02	1.49E-01
CORO1C	0.0289	5.80E-01	6.96E-01
CORO2A	0.1988	1.15E-04	6.89E-04
CORO2B	-0.0659	2.05E-01	3.23E-01
CORO6	0.2257	1.13E-05	9.38E-05
CORO7	0.2056	6.61E-05	4.29E-04
CORT	-0.0091	8.61E-01	9.10E-01
COTL1	-0.0515	3.22E-01	4.53E-01
COX10	-0.0232	6.56E-01	7.58E-01
COX11	0.0888	8.76E-02	1.67E-01
COX15	-0.1720	8.81E-04	3.85E-03
COX16	-0.0390	4.54E-01	5.85E-01
COX17	-0.0331	5.25E-01	6.48E-01
COX18	-0.4153	6.74E-17	7.10E-15
COX19	0.1948	1.59E-04	9.06E-04
COX4I1	-0.1955	1.51E-04	8.69E-04
COX4I2	-0.0600	2.49E-01	3.74E-01
COX4NB	-0.1209	1.98E-02	5.09E-02
COX5A	-0.1076	3.84E-02	8.73E-02

COX5B	-0.0876	9.18E-02	1.74E-01
COX6A1	-0.0806	1.21E-01	2.16E-01
COX6A2	-0.0080	8.78E-01	9.21E-01
COX6B1	0.0173	7.39E-01	8.24E-01
COX6B2	0.0873	9.31E-02	1.76E-01
COX6C	-0.0417	4.23E-01	5.56E-01
COX7A1	-0.1489	4.05E-03	1.39E-02
COX7A2L	-0.0349	5.02E-01	6.28E-01
COX7A2	0.0550	2.91E-01	4.20E-01
COX7B2	0.0128	8.06E-01	8.72E-01
COX7B	-0.0816	1.17E-01	2.10E-01
COX7C	-0.0786	1.31E-01	2.28E-01
COX8A	-0.0362	4.87E-01	6.15E-01
COX8C	-0.0052	9.21E-01	9.50E-01
CP110	0.1126	3.02E-02	7.18E-02
CPA1	-0.0549	2.92E-01	4.21E-01
CPA2	0.1130	2.96E-02	7.06E-02
CPA3	-0.1270	1.43E-02	3.93E-02
CPA4	0.0770	1.39E-01	2.39E-01
CPA5	0.0246	6.37E-01	7.43E-01
CPA6	0.0811	1.19E-01	2.13E-01
CPAMD8	-0.0156	7.64E-01	8.41E-01
CPB1	-0.1182	2.27E-02	5.70E-02
CPB2	-0.1608	1.89E-03	7.34E-03
CPD	0.0864	9.66E-02	1.81E-01
CPEB1	-0.0433	4.06E-01	5.39E-01
CPEB2	-0.0724	1.64E-01	2.72E-01
CPEB3	-0.3439	9.75E-12	3.48E-10
CPEB4	-0.1969	1.35E-04	7.88E-04
CPE	-0.0356	4.95E-01	6.21E-01
CPLX1	-0.0732	1.59E-01	2.66E-01
CPLX2	-0.0017	9.74E-01	9.84E-01
CPLX3	0.1118	3.13E-02	7.39E-02
CPLX4	0.0787	1.30E-01	2.28E-01
CPM	-0.0529	3.10E-01	4.39E-01
CPN1	-0.1020	4.96E-02	1.07E-01
CPN2	-0.1829	3.98E-04	1.97E-03
CPNE1	0.1263	1.49E-02	4.05E-02
CPNE2	-0.1078	3.79E-02	8.64E-02
CPNE3	-0.0914	7.87E-02	1.54E-01
CPNE4	0.1194	2.14E-02	5.42E-02
CPNE5	-0.0459	3.78E-01	5.11E-01
CPNE6	0.0827	1.12E-01	2.03E-01
CPNE7	0.1398	7.02E-03	2.18E-02

CPNE8	-0.1448	5.20E-03	1.70E-02
CPNE9	0.0007	9.90E-01	9.94E-01
CPOX	-0.0561	2.81E-01	4.09E-01
CPO	-0.0089	8.64E-01	9.12E-01
CPPED1	-0.1550	2.76E-03	1.01E-02
CPS1	-0.2127	3.62E-05	2.57E-04
CPSF1	0.0773	1.37E-01	2.37E-01
CPSF2	-0.1228	1.79E-02	4.70E-02
CPSF3L	0.0510	3.28E-01	4.58E-01
CPSF3	0.2102	4.50E-05	3.10E-04
CPSF4L	0.2868	1.86E-08	3.24E-07
CPSF4	0.1359	8.75E-03	2.62E-02
CPSF6	0.4054	4.13E-16	3.64E-14
CPSF7	0.1960	1.45E-04	8.37E-04
CPT1A	-0.1726	8.45E-04	3.72E-03
CPT1B	0.2084	5.24E-05	3.52E-04
CPT1C	0.0337	5.18E-01	6.42E-01
CPT2	-0.3195	3.01E-10	8.12E-09
CPVL	-0.0347	5.05E-01	6.30E-01
CPXCR1	-0.0092	8.59E-01	9.09E-01
CPXM1	-0.0102	8.45E-01	8.99E-01
CPXM2	-0.1759	6.68E-04	3.04E-03
CPZ	-0.0700	1.78E-01	2.89E-01
CP	-0.1525	3.23E-03	1.15E-02
CR1L	0.0965	6.34E-02	1.30E-01
CR1	-0.1302	1.21E-02	3.41E-02
CR2	0.1177	2.33E-02	5.82E-02
CRABP1	0.0182	7.27E-01	8.14E-01
CRABP2	0.0108	8.36E-01	8.93E-01
CRADD	-0.3203	2.68E-10	7.27E-09
CRAMP1L	0.2642	2.42E-07	3.21E-06
CRAT	-0.2038	7.71E-05	4.90E-04
CRB1	0.0684	1.89E-01	3.02E-01
CRB2	0.2096	4.74E-05	3.23E-04
CRB3	0.0017	9.74E-01	9.84E-01
CRBN	-0.2190	2.08E-05	1.60E-04
CRCP	0.0194	7.10E-01	8.02E-01
CRCT1	-0.1184	2.25E-02	5.66E-02
CREB1	0.1750	7.11E-04	3.21E-03
CREB3L1	0.0626	2.29E-01	3.51E-01
CREB3L2	-0.0546	2.94E-01	4.23E-01
CREB3L3	-0.0856	9.96E-02	1.85E-01
CREB3L4	0.3602	8.25E-13	3.75E-11
CREB3	0.1593	2.09E-03	8.00E-03

CREB5	0.0178	7.33E-01	8.19E-01
CREBBP	0.0771	1.38E-01	2.38E-01
CREBL2	-0.2384	3.43E-06	3.34E-05
CREBZF	0.3132	6.89E-10	1.68E-08
CREG1	0.0567	2.76E-01	4.05E-01
CREG2	0.1681	1.15E-03	4.86E-03
CRELD1	-0.1254	1.56E-02	4.20E-02
CRELD2	0.1437	5.56E-03	1.80E-02
CREM	-0.1018	5.00E-02	1.08E-01
CRHBP	-0.2098	4.63E-05	3.17E-04
CRHR1	-0.0016	9.75E-01	9.84E-01
CRHR2	-0.0487	3.50E-01	4.82E-01
CRH	-0.0117	8.22E-01	8.84E-01
CRIM1	-0.1151	2.66E-02	6.49E-02
CRIP1	0.0597	2.51E-01	3.76E-01
CRIP2	-0.1265	1.47E-02	4.01E-02
CRIP3	0.1289	1.29E-02	3.61E-02
CRIPAK	0.2062	6.30E-05	4.12E-04
CRIPT	0.1117	3.14E-02	7.42E-02
CRISP1	0.1457	4.94E-03	1.63E-02
CRISP2	0.0801	1.24E-01	2.19E-01
CRISP3	-0.0081	8.76E-01	9.20E-01
CRISPLD1	-0.0947	6.85E-02	1.38E-01
CRISPLD2	-0.1812	4.51E-04	2.18E-03
CRKL	0.1660	1.34E-03	5.48E-03
CRK	-0.2619	3.09E-07	4.00E-06
CRLF1	-0.0695	1.82E-01	2.94E-01
CRLF2	-0.0172	7.41E-01	8.25E-01
CRLF3	0.1691	1.08E-03	4.58E-03
CRLS1	-0.1946	1.62E-04	9.21E-04
CRMP1	0.1533	3.07E-03	1.10E-02
CRNKL1	0.1687	1.11E-03	4.70E-03
CRNN	-0.0513	3.24E-01	4.55E-01
CROCCL1	-0.0180	7.30E-01	8.17E-01
CROCCL2	0.2652	2.18E-07	2.93E-06
CROCC	0.2589	4.28E-07	5.34E-06
CROT	-0.0308	5.55E-01	6.74E-01
CRP	-0.0602	2.48E-01	3.73E-01
CRTAC1	-0.1642	1.51E-03	6.08E-03
CRTAM	-0.0318	5.42E-01	6.63E-01
CRTAP	0.0096	8.55E-01	9.06E-01
CRTC1	0.1432	5.74E-03	1.85E-02
CRTC2	0.5468	2.61E-30	2.60E-27
CRTC3	0.1250	1.60E-02	4.29E-02

CRX	-0.0081	8.76E-01	9.20E-01
CRY1	0.0619	2.34E-01	3.58E-01
CRY2	-0.1487	4.11E-03	1.41E-02
CRYAA	-0.2071	5.84E-05	3.86E-04
CRYAB	-0.0978	5.99E-02	1.24E-01
CRYBA1	0.0285	5.85E-01	7.00E-01
CRYBA2	0.1227	1.80E-02	4.72E-02
CRYBA4	0.0550	2.90E-01	4.19E-01
CRYBB1	-0.0305	5.58E-01	6.76E-01
CRYBB2	0.0507	3.30E-01	4.60E-01
CRYBB3	-0.0242	6.42E-01	7.48E-01
CRYBG3	-0.1288	1.30E-02	3.63E-02
CRYGA	0.1325	1.06E-02	3.06E-02
CRYGC	0.0549	2.92E-01	4.21E-01
CRYGD	0.0609	2.42E-01	3.66E-01
CRYGN	0.0511	3.26E-01	4.57E-01
CRYGS	0.1567	2.46E-03	9.15E-03
CRYL1	-0.2440	1.98E-06	2.08E-05
CRYM	-0.1247	1.62E-02	4.33E-02
CRYZL1	0.1096	3.48E-02	8.07E-02
CRYZ	-0.2417	2.49E-06	2.52E-05
CSAD	-0.0679	1.92E-01	3.06E-01
CSAG1	0.0093	8.58E-01	9.08E-01
CSAG2	0.0583	2.62E-01	3.89E-01
CSAG3	0.0858	9.89E-02	1.84E-01
CSDAP1	0.0553	2.88E-01	4.17E-01
CSDA	0.0255	6.25E-01	7.33E-01
CSDC2	-0.1219	1.89E-02	4.90E-02
CSDE1	-0.0163	7.54E-01	8.35E-01
CSE1L	0.2592	4.12E-07	5.16E-06
CSF1R	-0.1140	2.81E-02	6.79E-02
CSF1	-0.0262	6.15E-01	7.25E-01
CSF2RA	0.0311	5.50E-01	6.70E-01
CSF2RB	-0.0658	2.06E-01	3.24E-01
CSF2	0.0840	1.06E-01	1.95E-01
CSF3R	-0.0038	9.42E-01	9.64E-01
CSF3	-0.1456	4.94E-03	1.63E-02
CSGALNACT1	-0.1328	1.04E-02	3.03E-02
CSGALNACT2	-0.0557	2.85E-01	4.14E-01
CSH1	0.0609	2.42E-01	3.66E-01
CSH2	0.0941	7.04E-02	1.41E-01
CSK	-0.0779	1.34E-01	2.33E-01
CSMD1	0.0385	4.59E-01	5.90E-01
CSMD2	0.0460	3.77E-01	5.10E-01

CSMD3	0.0396	4.48E-01	5.79E-01
CSN1S1	0.0387	4.57E-01	5.88E-01
CSN1S2A	0.0355	4.96E-01	6.22E-01
CSN2	-0.0334	5.21E-01	6.45E-01
CSN3	0.0706	1.75E-01	2.85E-01
CSNK1A1L	0.0360	4.89E-01	6.17E-01
CSNK1A1P	-0.0530	3.09E-01	4.38E-01
CSNK1A1	-0.1626	1.68E-03	6.66E-03
CSNK1D	0.2834	2.80E-08	4.62E-07
CSNK1E	0.2262	1.08E-05	9.01E-05
CSNK1G1	0.1736	7.84E-04	3.49E-03
CSNK1G2	0.0636	2.22E-01	3.42E-01
CSNK1G3	-0.2139	3.26E-05	2.35E-04
CSNK2A1P	0.0355	4.96E-01	6.22E-01
CSNK2A1	0.1551	2.73E-03	9.99E-03
CSNK2A2	-0.2351	4.69E-06	4.40E-05
CSNK2B	0.1117	3.14E-02	7.42E-02
CSPG4PY2	0.0422	4.18E-01	5.50E-01
CSPG4	-0.0084	8.72E-01	9.18E-01
CSPG5	0.2042	7.46E-05	4.76E-04
CSPP1	0.1679	1.17E-03	4.91E-03
CSRNP1	-0.1806	4.74E-04	2.27E-03
CSRNP2	0.1540	2.94E-03	1.06E-02
CSRNP3	0.0715	1.69E-01	2.78E-01
CSRP1	-0.0951	6.74E-02	1.36E-01
CSRP2BP	0.0322	5.37E-01	6.59E-01
CSRP2	-0.0003	9.96E-01	9.97E-01
CSRP3	0.0541	2.99E-01	4.28E-01
CST11	0.0644	2.16E-01	3.36E-01
CST1	-0.0058	9.11E-01	9.44E-01
CST2	0.0701	1.78E-01	2.89E-01
CST3	-0.1313	1.14E-02	3.24E-02
CST4	-0.0038	9.42E-01	9.64E-01
CST5	0.0983	5.87E-02	1.22E-01
CST6	0.0689	1.85E-01	2.98E-01
CST7	-0.0971	6.18E-02	1.27E-01
CST8	0.1175	2.36E-02	5.88E-02
CST9L	0.1658	1.35E-03	5.52E-03
CST9	0.0826	1.12E-01	2.03E-01
CSTA	0.0076	8.84E-01	9.26E-01
CSTB	0.0274	5.99E-01	7.12E-01
CSTF1	0.0419	4.21E-01	5.54E-01
CSTF2T	0.1095	3.50E-02	8.10E-02
CSTF2	0.3425	1.19E-11	4.16E-10

CSTF3	0.2516	9.14E-07	1.05E-05
CSTL1	0.0965	6.34E-02	1.30E-01
CSTT	0.0108	8.36E-01	8.92E-01
CS	0.0870	9.45E-02	1.78E-01
CT45A1	0.0911	7.98E-02	1.55E-01
CT45A2	0.0600	2.49E-01	3.74E-01
CT45A3	0.0575	2.69E-01	3.97E-01
CT45A4	0.0667	2.00E-01	3.16E-01
CT45A5	0.1320	1.09E-02	3.14E-02
CT45A6	-0.0104	8.42E-01	8.97E-01
CT47A10	-0.0427	4.12E-01	5.45E-01
CT47A11	0.0496	3.41E-01	4.72E-01
CT47A1	-0.0234	6.53E-01	7.56E-01
CT47A2	0.0149	7.75E-01	8.50E-01
CT47A6	0.0158	7.61E-01	8.39E-01
CT47A7	0.0589	2.58E-01	3.84E-01
CT47A9	0.0331	5.25E-01	6.48E-01
CT47B1	0.0113	8.29E-01	8.88E-01
CT62	0.1594	2.07E-03	7.94E-03
CTAG1B	0.0687	1.86E-01	3.00E-01
CTAG2	0.0585	2.61E-01	3.87E-01
CTAGE1	-0.1186	2.23E-02	5.61E-02
CTAGE4	0.1922	1.97E-04	1.09E-03
CTAGE5	-0.2027	8.45E-05	5.30E-04
CTAGE6	0.1377	7.89E-03	2.40E-02
CTAGE9	0.1674	1.21E-03	5.05E-03
CTBP1	0.1065	4.04E-02	9.08E-02
CTBP2	-0.0008	9.88E-01	9.93E-01
CTBS	-0.2669	1.79E-07	2.46E-06
CTCFL	0.0512	3.25E-01	4.56E-01
CTCF	-0.0163	7.55E-01	8.35E-01
CTDP1	0.0500	3.37E-01	4.68E-01
CTDSP1	-0.1007	5.26E-02	1.12E-01
CTDSP2	0.1135	2.88E-02	6.93E-02
CTDSPL2	0.1415	6.31E-03	2.00E-02
CTDSPL	0.1937	1.74E-04	9.78E-04
CTF1	-0.0130	8.03E-01	8.70E-01
CTGF	-0.0239	6.46E-01	7.51E-01
CTHRC1	0.1222	1.85E-02	4.83E-02
CTH	-0.1948	1.59E-04	9.07E-04
CTLA4	0.0849	1.03E-01	1.89E-01
CTNNA1	-0.0034	9.48E-01	9.68E-01
CTNNA2	-0.0366	4.82E-01	6.11E-01
CTNNA3	-0.0813	1.18E-01	2.11E-01

CTNNAL1	0.1855	3.29E-04	1.69E-03
CTNNB1	-0.0741	1.54E-01	2.59E-01
CTNNBIP1	0.0318	5.42E-01	6.63E-01
CTNNBL1	-0.0610	2.41E-01	3.66E-01
CTNND1	-0.0949	6.78E-02	1.37E-01
CTNND2	0.2210	1.74E-05	1.37E-04
CTNS	-0.1344	9.53E-03	2.81E-02
CTPS2	0.1608	1.89E-03	7.35E-03
CTPS	0.0159	7.60E-01	8.38E-01
CTR9	-0.0956	6.58E-02	1.34E-01
CTRB1	-0.0232	6.56E-01	7.58E-01
CTRB2	0.0041	9.37E-01	9.61E-01
CTRC	0.0322	5.37E-01	6.59E-01
CTRL	0.1377	7.93E-03	2.41E-02
CTSA	-0.0726	1.63E-01	2.70E-01
CTSB	-0.1793	5.21E-04	2.46E-03
CTSC	0.0317	5.43E-01	6.63E-01
CTSD	-0.1992	1.12E-04	6.72E-04
CTSE	0.2511	9.67E-07	1.10E-05
CTSF	-0.2285	8.79E-06	7.51E-05
CTSG	-0.1762	6.51E-04	2.98E-03
CTSH	0.0058	9.12E-01	9.45E-01
CTSK	0.0468	3.68E-01	5.01E-01
CTSL1	-0.0263	6.13E-01	7.24E-01
CTSL2	0.3439	9.76E-12	3.48E-10
CTSO	-0.2819	3.31E-08	5.39E-07
CTSS	0.0757	1.45E-01	2.48E-01
CTSW	-0.0930	7.37E-02	1.46E-01
CTSZ	-0.1390	7.35E-03	2.27E-02
CTTNBP2NL	0.0057	9.13E-01	9.45E-01
CTTNBP2	-0.0994	5.58E-02	1.17E-01
CTTN	0.0099	8.49E-01	9.02E-01
CTU1	0.1021	4.94E-02	1.07E-01
CTU2	-0.1434	5.67E-03	1.83E-02
CTXN1	0.0362	4.87E-01	6.14E-01
CTXN2	0.1257	1.54E-02	4.15E-02
CTXN3	0.0422	4.18E-01	5.50E-01
CUBN	-0.0165	7.52E-01	8.33E-01
CUEDC1	0.1176	2.35E-02	5.85E-02
CUEDC2	-0.1057	4.19E-02	9.35E-02
CUL1	-0.1059	4.15E-02	9.27E-02
CUL2	0.1603	1.95E-03	7.53E-03
CUL3	0.0271	6.02E-01	7.15E-01
CUL4A	0.0438	4.00E-01	5.33E-01

CUL4B	0.2175	2.39E-05	1.81E-04
CUL5	-0.0587	2.59E-01	3.86E-01
CUL7	0.0739	1.55E-01	2.60E-01
CUL9	0.1562	2.55E-03	9.42E-03
CUTA	0.0939	7.07E-02	1.41E-01
CUTC	-0.0288	5.80E-01	6.97E-01
CUX1	-0.3689	2.09E-13	1.05E-11
CUX2	-0.1153	2.64E-02	6.45E-02
CUZD1	0.2412	2.61E-06	2.62E-05
CWC15	0.1547	2.81E-03	1.02E-02
CWC22	0.2449	1.80E-06	1.90E-05
CWC25	0.2217	1.64E-05	1.30E-04
CWC27	0.2134	3.41E-05	2.45E-04
CWF19L1	0.1273	1.42E-02	3.89E-02
CWF19L2	0.1293	1.27E-02	3.54E-02
CWH43	-0.0860	9.81E-02	1.83E-01
CX3CL1	-0.1846	3.50E-04	1.78E-03
CX3CR1	-0.0898	8.42E-02	1.62E-01
CXADRP2	0.0523	3.15E-01	4.45E-01
CXADRP3	0.1134	2.90E-02	6.95E-02
CXADR	0.0391	4.52E-01	5.83E-01
CXCL10	0.0154	7.67E-01	8.43E-01
CXCL11	-0.0133	7.98E-01	8.66E-01
CXCL12	-0.1736	7.86E-04	3.50E-03
CXCL13	0.0856	9.98E-02	1.86E-01
CXCL14	-0.0656	2.08E-01	3.26E-01
CXCL16	0.0047	9.27E-01	9.54E-01
CXCL17	0.0492	3.45E-01	4.77E-01
CXCL1	0.1046	4.41E-02	9.74E-02
CXCL2	-0.1092	3.55E-02	8.19E-02
CXCL3	0.1293	1.27E-02	3.56E-02
CXCL5	0.1394	7.14E-03	2.21E-02
CXCL6	-0.0215	6.79E-01	7.77E-01
CXCL9	-0.0086	8.69E-01	9.16E-01
CXCR1	-0.0673	1.96E-01	3.11E-01
CXCR2P1	-0.0531	3.08E-01	4.38E-01
CXCR2	-0.0446	3.92E-01	5.25E-01
CXCR3	-0.0151	7.72E-01	8.47E-01
CXCR4	0.0236	6.50E-01	7.54E-01
CXCR5	0.0496	3.41E-01	4.73E-01
CXCR6	-0.0415	4.26E-01	5.58E-01
CXCR7	-0.1385	7.55E-03	2.32E-02
CXXC1	0.2250	1.21E-05	9.92E-05
CXXC4	0.0434	4.05E-01	5.38E-01

CXXC5	0.0470	3.67E-01	5.00E-01
CXorf1	0.0000	1.00E+00	1.00E+00
CXorf21	-0.0068	8.96E-01	9.34E-01
CXorf22	0.1150	2.68E-02	6.52E-02
CXorf23	0.0558	2.84E-01	4.13E-01
CXorf26	0.0933	7.26E-02	1.44E-01
CXorf27	-0.0013	9.80E-01	9.88E-01
CXorf30	0.1927	1.89E-04	1.05E-03
CXorf36	-0.1873	2.86E-04	1.50E-03
CXorf38	-0.1320	1.09E-02	3.14E-02
CXorf40A	0.1349	9.28E-03	2.75E-02
CXorf40B	0.2236	1.38E-05	1.11E-04
CXorf41	0.0030	9.53E-01	9.71E-01
CXorf42	0.1207	2.00E-02	5.14E-02
CXorf48	0.1939	1.71E-04	9.63E-04
CXorf49B	0.0901	8.32E-02	1.61E-01
CXorf50B	-0.0042	9.36E-01	9.60E-01
CXorf51	-0.0470	3.67E-01	5.00E-01
CXorf56	0.0680	1.91E-01	3.05E-01
CXorf57	0.3140	6.19E-10	1.54E-08
CXorf58	-0.0141	7.86E-01	8.58E-01
CXorf59	0.1652	1.40E-03	5.71E-03
CXorf61	0.1852	3.37E-04	1.72E-03
CXorf64	0.0400	4.42E-01	5.74E-01
CXorf65	0.1132	2.93E-02	7.01E-02
CXorf66	-0.1795	5.12E-04	2.43E-03
CYB561D1	0.2273	9.81E-06	8.27E-05
CYB561D2	-0.0333	5.23E-01	6.46E-01
CYB561	0.1742	7.52E-04	3.37E-03
CYB5A	-0.2349	4.80E-06	4.48E-05
CYB5B	-0.1285	1.33E-02	3.69E-02
CYB5D1	-0.1333	1.02E-02	2.96E-02
CYB5D2	-0.3637	4.76E-13	2.25E-11
CYB5R1	0.1026	4.83E-02	1.05E-01
CYB5R2	0.0532	3.07E-01	4.36E-01
CYB5R3	-0.1088	3.63E-02	8.34E-02
CYB5R4	0.0733	1.59E-01	2.64E-01
CYB5RL	0.2035	7.88E-05	4.98E-04
CYBASC3	-0.0850	1.02E-01	1.88E-01
CYBA	0.0677	1.93E-01	3.08E-01
CYBB	-0.0521	3.17E-01	4.47E-01
CYBRD1	-0.1140	2.81E-02	6.79E-02
CYC1	-0.0453	3.84E-01	5.17E-01
CYCSP52	0.0590	2.57E-01	3.83E-01

CYCS	-0.0780	1.34E-01	2.32E-01
CYFIP1	-0.1067	4.00E-02	9.01E-02
CYFIP2	-0.0888	8.75E-02	1.67E-01
CYGB	-0.2103	4.45E-05	3.06E-04
CYHR1	-0.0216	6.79E-01	7.76E-01
CYLC1	-0.0112	8.30E-01	8.89E-01
CYLC2	0.0575	2.69E-01	3.97E-01
CYLD	-0.2007	9.97E-05	6.07E-04
CYMP	0.0501	3.36E-01	4.67E-01
CYP11A1	-0.2318	6.44E-06	5.75E-05
CYP11B1	0.0163	7.55E-01	8.35E-01
CYP11B2	-0.0059	9.09E-01	9.43E-01
CYP17A1	-0.1929	1.86E-04	1.03E-03
CYP19A1	0.1325	1.06E-02	3.07E-02
CYP1A1	-0.1412	6.44E-03	2.03E-02
CYP1A2	-0.2171	2.47E-05	1.86E-04
CYP1B1	-0.0440	3.98E-01	5.31E-01
CYP20A1	0.0351	5.01E-01	6.26E-01
CYP21A2	0.0708	1.74E-01	2.84E-01
CYP24A1	0.0915	7.84E-02	1.53E-01
CYP26A1	-0.1892	2.48E-04	1.32E-03
CYP26B1	0.1877	2.78E-04	1.46E-03
CYP26C1	0.0250	6.32E-01	7.39E-01
CYP27A1	-0.1455	4.99E-03	1.64E-02
CYP27B1	0.1565	2.51E-03	9.29E-03
CYP27C1	0.0143	7.84E-01	8.56E-01
CYP2A13	-0.1057	4.20E-02	9.36E-02
CYP2A6	-0.1968	1.36E-04	7.92E-04
CYP2A7	-0.1858	3.20E-04	1.65E-03
CYP2B6	-0.2096	4.71E-05	3.21E-04
CYP2B7P1	-0.1564	2.52E-03	9.32E-03
CYP2C18	-0.0999	5.46E-02	1.15E-01
CYP2C19	-0.1129	2.97E-02	7.08E-02
CYP2C8	-0.2537	7.34E-07	8.60E-06
CYP2C9	-0.1574	2.36E-03	8.82E-03
CYP2D6	-0.0541	2.99E-01	4.28E-01
CYP2D7P1	-0.0091	8.61E-01	9.10E-01
CYP2E1	-0.1721	8.71E-04	3.81E-03
CYP2F1	0.0974	6.10E-02	1.26E-01
CYP2J2	-0.2692	1.40E-07	1.96E-06
CYP2R1	0.1527	3.19E-03	1.14E-02
CYP2S1	0.0453	3.84E-01	5.17E-01
CYP2U1	-0.2530	7.96E-07	9.25E-06
CYP2W1	0.2510	9.78E-07	1.11E-05

CYP39A1	-0.2939	7.94E-09	1.51E-07
CYP3A43	-0.1312	1.14E-02	3.25E-02
CYP3A4	-0.2318	6.43E-06	5.75E-05
CYP3A5	0.0147	7.78E-01	8.52E-01
CYP3A7	-0.0267	6.08E-01	7.20E-01
CYP46A1	-0.1496	3.87E-03	1.34E-02
CYP4A11	-0.3077	1.41E-09	3.24E-08
CYP4A22	-0.2716	1.08E-07	1.55E-06
CYP4B1	-0.0654	2.09E-01	3.28E-01
CYP4F11	-0.1379	7.83E-03	2.39E-02
CYP4F12	-0.1250	1.60E-02	4.28E-02
CYP4F22	0.0224	6.67E-01	7.67E-01
CYP4F2	-0.2607	3.53E-07	4.50E-06
CYP4F3	-0.2085	5.19E-05	3.50E-04
CYP4F8	-0.0325	5.33E-01	6.55E-01
CYP4V2	-0.3124	7.63E-10	1.86E-08
CYP4X1	-0.1546	2.82E-03	1.03E-02
CYP4Z1	-0.0488	3.49E-01	4.81E-01
CYP4Z2P	0.0990	5.68E-02	1.19E-01
CYP51A1	-0.1819	4.31E-04	2.10E-03
CYP7A1	-0.0927	7.46E-02	1.48E-01
CYP7B1	-0.2221	1.58E-05	1.26E-04
CYP8B1	-0.2650	2.22E-07	2.98E-06
CYR61	-0.1786	5.47E-04	2.57E-03
CYS1	-0.0157	7.63E-01	8.40E-01
CYSLTR1	-0.1905	2.23E-04	1.21E-03
CYSLTR2	-0.0549	2.91E-01	4.20E-01
CYTH1	0.0670	1.98E-01	3.14E-01
CYTH2	0.1344	9.54E-03	2.81E-02
CYTH3	-0.0161	7.58E-01	8.37E-01
CYTH4	-0.0136	7.94E-01	8.63E-01
CYTIP	-0.0556	2.86E-01	4.14E-01
CYTL1	-0.0805	1.22E-01	2.17E-01
CYTSA	-0.0340	5.14E-01	6.38E-01
CYTSB	0.0433	4.06E-01	5.39E-01
CYYR1	-0.2582	4.61E-07	5.70E-06
CYorf15A	-0.0363	4.86E-01	6.14E-01
CYorf15B	-0.0173	7.40E-01	8.25E-01
D2HGDH	-0.1714	9.14E-04	3.98E-03
D4S234E	-0.0905	8.16E-02	1.58E-01
DAAM1	-0.1567	2.47E-03	9.17E-03
DAAM2	-0.1869	2.96E-04	1.54E-03
DAB1	-0.1213	1.94E-02	5.01E-02
DAB2IP	0.1142	2.78E-02	6.72E-02

DAB2	-0.0250	6.32E-01	7.39E-01
DACH1	-0.0627	2.28E-01	3.50E-01
DACH2	-0.0089	8.64E-01	9.12E-01
DACT1	-0.0571	2.73E-01	4.01E-01
DACT2	0.1129	2.97E-02	7.08E-02
DACT3	-0.0508	3.29E-01	4.60E-01
DAD1L	0.1161	2.53E-02	6.20E-02
DAD1	0.1009	5.21E-02	1.11E-01
DAG1	0.0476	3.61E-01	4.93E-01
DAGLA	0.0261	6.16E-01	7.26E-01
DAGLB	-0.0207	6.91E-01	7.87E-01
DAK	-0.2419	2.43E-06	2.47E-05
DALRD3	0.0303	5.61E-01	6.79E-01
DAND5	0.2843	2.52E-08	4.22E-07
DAO	-0.0839	1.07E-01	1.95E-01
DAP3	0.3600	8.57E-13	3.88E-11
DAPK1	0.0407	4.35E-01	5.67E-01
DAPK2	-0.0089	8.64E-01	9.12E-01
DAPK3	-0.0060	9.08E-01	9.42E-01
DAPL1	0.0593	2.55E-01	3.80E-01
DAPP1	-0.0433	4.06E-01	5.39E-01
DAP	-0.1092	3.56E-02	8.20E-02
DARC	-0.2424	2.31E-06	2.37E-05
DARS2	0.3073	1.48E-09	3.39E-08
DARS	0.1067	4.00E-02	9.01E-02
DAXX	0.2432	2.13E-06	2.20E-05
DAZ1	0.0889	8.74E-02	1.67E-01
DAZ2	0.1641	1.52E-03	6.11E-03
DAZ3	0.0863	9.71E-02	1.82E-01
DAZAP1	0.3316	5.65E-11	1.75E-09
DAZAP2	-0.0170	7.45E-01	8.28E-01
DAZL	0.0041	9.37E-01	9.60E-01
DBC1	-0.0251	6.30E-01	7.38E-01
DBF4B	0.4403	4.99E-19	9.94E-17
DBF4	0.2892	1.40E-08	2.51E-07
DBH	-0.0775	1.36E-01	2.36E-01
DBI	-0.0641	2.18E-01	3.38E-01
DBN1	0.0822	1.14E-01	2.06E-01
DBNDD1	-0.1216	1.92E-02	4.97E-02
DBNDD2	0.1904	2.26E-04	1.23E-03
DBNL	-0.0161	7.58E-01	8.38E-01
DBP	0.0909	8.04E-02	1.56E-01
DBR1	0.2026	8.47E-05	5.30E-04
DBT	-0.2912	1.11E-08	2.04E-07

DBX1	0.0322	5.37E-01	6.59E-01
DBX2	0.0194	7.10E-01	8.02E-01
DCAF10	0.0100	8.47E-01	9.00E-01
DCAF11	-0.1756	6.81E-04	3.09E-03
DCAF12L1	0.1115	3.18E-02	7.49E-02
DCAF12L2	0.1016	5.06E-02	1.08E-01
DCAF12	0.1288	1.30E-02	3.62E-02
DCAF13	0.1009	5.20E-02	1.11E-01
DCAF15	0.1197	2.11E-02	5.37E-02
DCAF16	0.2335	5.46E-06	4.99E-05
DCAF17	0.0559	2.83E-01	4.12E-01
DCAF4L1	0.1349	9.28E-03	2.75E-02
DCAF4L2	-0.0197	7.05E-01	7.98E-01
DCAF4	0.0437	4.01E-01	5.34E-01
DCAF5	-0.1028	4.78E-02	1.04E-01
DCAF6	0.1267	1.46E-02	3.99E-02
DCAF7	0.2027	8.41E-05	5.28E-04
DCAF8L1	-0.0214	6.81E-01	7.78E-01
DCAF8L2	0.0545	2.96E-01	4.25E-01
DCAF8	0.0710	1.72E-01	2.82E-01
DCAKD	0.0274	5.98E-01	7.12E-01
DCBLD1	-0.0395	4.48E-01	5.79E-01
DCBLD2	0.0186	7.20E-01	8.10E-01
DCC	-0.0115	8.25E-01	8.85E-01
DCDC1	0.1082	3.73E-02	8.53E-02
DCDC2B	0.1049	4.34E-02	9.62E-02
DCDC2	0.0293	5.73E-01	6.91E-01
DCHS1	-0.1042	4.49E-02	9.88E-02
DCHS2	0.0116	8.24E-01	8.85E-01
DCI	-0.1683	1.14E-03	4.80E-03
DCK	0.1669	1.25E-03	5.19E-03
DCLK1	-0.0836	1.08E-01	1.97E-01
DCLK2	-0.0296	5.70E-01	6.87E-01
DCLK3	0.0563	2.79E-01	4.08E-01
DCLRE1A	0.0785	1.31E-01	2.29E-01
DCLRE1B	0.2405	2.79E-06	2.78E-05
DCLRE1C	0.3388	2.04E-11	6.92E-10
DCN	-0.1472	4.48E-03	1.51E-02
DCP1A	0.1154	2.63E-02	6.42E-02
DCP1B	0.1518	3.38E-03	1.19E-02
DCP2	0.1101	3.41E-02	7.93E-02
DCPS	0.0679	1.92E-01	3.06E-01
DCST1	0.1371	8.20E-03	2.48E-02
DCST2	0.3546	1.98E-12	8.27E-11

DCTD	-0.2738	8.36E-08	1.24E-06
DCTN1	0.1499	3.80E-03	1.32E-02
DCTN2	-0.0088	8.66E-01	9.13E-01
DCTN3	0.0620	2.34E-01	3.57E-01
DCTN4	0.0376	4.70E-01	6.00E-01
DCTN5	0.1979	1.25E-04	7.38E-04
DCTN6	-0.0528	3.10E-01	4.40E-01
DCTPP1	0.1427	5.91E-03	1.89E-02
DCT	-0.0889	8.72E-02	1.67E-01
DCUN1D1	-0.1000	5.42E-02	1.15E-01
DCUN1D2	0.2764	6.23E-08	9.51E-07
DCUN1D3	-0.2209	1.76E-05	1.38E-04
DCUN1D4	0.0003	9.95E-01	9.97E-01
DCUN1D5	0.1782	5.62E-04	2.63E-03
DCXR	-0.2485	1.26E-06	1.39E-05
DCX	0.0856	9.98E-02	1.86E-01
DDA1	0.1151	2.67E-02	6.50E-02
DDAH1	-0.1114	3.19E-02	7.51E-02
DDAH2	0.0525	3.13E-01	4.42E-01
DDB1	-0.0936	7.19E-02	1.43E-01
DDB2	-0.0665	2.01E-01	3.17E-01
DDC	-0.0645	2.15E-01	3.35E-01
DDHD1	0.1942	1.68E-04	9.46E-04
DDHD2	0.0312	5.50E-01	6.70E-01
DDI1	0.0217	6.77E-01	7.75E-01
DDI2	-0.1645	1.47E-03	5.96E-03
DDIT3	0.1201	2.07E-02	5.28E-02
DDIT4L	0.0226	6.65E-01	7.65E-01
DDIT4	-0.0644	2.16E-01	3.36E-01
DDN	0.0880	9.06E-02	1.72E-01
DDOST	0.1210	1.97E-02	5.08E-02
DDO	-0.0896	8.49E-02	1.63E-01
DDR1	0.1802	4.87E-04	2.33E-03
DDR2	-0.0243	6.41E-01	7.47E-01
DDRGK1	-0.1465	4.70E-03	1.57E-02
DDTL	-0.1210	1.98E-02	5.09E-02
DDT	-0.1082	3.72E-02	8.51E-02
DDX10	0.1048	4.36E-02	9.65E-02
DDX11L2	-0.1022	4.91E-02	1.06E-01
DDX11	0.3525	2.68E-12	1.10E-10
DDX12	0.4550	2.36E-20	6.28E-18
DDX17	0.2893	1.38E-08	2.48E-07
DDX18	0.1080	3.76E-02	8.58E-02
DDX19A	-0.2019	9.02E-05	5.59E-04

DDX19B	-0.3106	9.66E-10	2.30E-08
DDX1	0.0755	1.47E-01	2.50E-01
DDX20	0.2488	1.22E-06	1.35E-05
DDX21	-0.0261	6.17E-01	7.26E-01
DDX23	0.2994	4.05E-09	8.33E-08
DDX24	-0.1061	4.11E-02	9.21E-02
DDX25	0.0772	1.38E-01	2.37E-01
DDX26B	0.0347	5.06E-01	6.30E-01
DDX27	0.2702	1.26E-07	1.79E-06
DDX28	-0.1845	3.54E-04	1.79E-03
DDX31	0.3173	4.00E-10	1.04E-08
DDX39	0.2631	2.73E-07	3.59E-06
DDX3X	-0.0834	1.09E-01	1.99E-01
DDX3Y	-0.0240	6.45E-01	7.50E-01
DDX41	0.0066	9.00E-01	9.37E-01
DDX42	0.3004	3.56E-09	7.40E-08
DDX43	0.0082	8.75E-01	9.19E-01
DDX46	0.2321	6.29E-06	5.65E-05
DDX47	0.1490	4.03E-03	1.38E-02
DDX49	0.1235	1.73E-02	4.57E-02
DDX4	0.0617	2.36E-01	3.59E-01
DDX50	0.1838	3.72E-04	1.86E-03
DDX51	0.2125	3.69E-05	2.62E-04
DDX52	0.3010	3.31E-09	6.93E-08
DDX53	-0.0659	2.06E-01	3.23E-01
DDX54	0.0519	3.18E-01	4.48E-01
DDX55	0.3733	1.03E-13	5.46E-12
DDX56	0.0839	1.07E-01	1.95E-01
DDX58	0.0366	4.82E-01	6.10E-01
DDX59	0.2595	4.02E-07	5.05E-06
DDX5	0.0819	1.15E-01	2.08E-01
DDX60L	-0.1387	7.45E-03	2.29E-02
DDX60	-0.1486	4.13E-03	1.41E-02
DDX6	0.0048	9.27E-01	9.54E-01
DEAF1	0.1190	2.19E-02	5.52E-02
1-Dec	-0.0273	6.00E-01	7.13E-01
DECR1	-0.3771	5.57E-14	3.10E-12
DECR2	-0.1442	5.41E-03	1.76E-02
DEDD2	0.1018	5.00E-02	1.08E-01
DEDD	0.4565	1.70E-20	4.70E-18
DEF6	0.0342	5.11E-01	6.36E-01
DEF8	-0.1132	2.92E-02	7.00E-02
DEFA1B	-0.0871	9.40E-02	1.77E-01
DEFA4	0.0554	2.87E-01	4.16E-01

DEFA5	-0.0141	7.86E-01	8.58E-01
DEFA6	0.0423	4.17E-01	5.50E-01
DEFB103B	-0.0121	8.16E-01	8.79E-01
DEFB109P1B	-0.0457	3.80E-01	5.14E-01
DEFB118	0.1337	9.94E-03	2.91E-02
DEFB123	-0.0228	6.62E-01	7.63E-01
DEFB124	0.0913	7.91E-02	1.54E-01
DEFB125	0.0272	6.01E-01	7.14E-01
DEFB126	0.0859	9.85E-02	1.84E-01
DEFB131	0.0766	1.41E-01	2.42E-01
DEFB132	-0.2168	2.54E-05	1.90E-04
DEFB1	0.0232	6.56E-01	7.58E-01
DEFB4A	-0.0297	5.69E-01	6.87E-01
DEGS1	0.1951	1.56E-04	8.93E-04
DEGS2	0.1341	9.73E-03	2.86E-02
DEK	0.3003	3.59E-09	7.45E-08
DEM1	0.2992	4.15E-09	8.50E-08
DENND1A	0.0996	5.53E-02	1.16E-01
DENND1B	0.1647	1.45E-03	5.88E-03
DENND1C	-0.0547	2.94E-01	4.23E-01
DENND2A	-0.0595	2.53E-01	3.78E-01
DENND2C	0.0625	2.30E-01	3.53E-01
DENND2D	0.0335	5.20E-01	6.44E-01
DENND3	0.0463	3.74E-01	5.07E-01
DENND4A	-0.1257	1.54E-02	4.15E-02
DENND4B	0.5442	5.51E-30	4.86E-27
DENND4C	0.0110	8.33E-01	8.91E-01
DENND5A	0.0645	2.15E-01	3.35E-01
DENND5B	-0.1168	2.44E-02	6.04E-02
DENR	0.2014	9.39E-05	5.78E-04
DEPDC1B	0.3307	6.45E-11	1.97E-09
DEPDC1	0.3691	2.05E-13	1.03E-11
DEPDC4	0.1132	2.93E-02	7.02E-02
DEPDC5	-0.0108	8.35E-01	8.92E-01
DEPDC6	-0.1090	3.59E-02	8.26E-02
DEPDC7	-0.2264	1.07E-05	8.94E-05
DERA	-0.2428	2.21E-06	2.28E-05
DERL1	-0.1525	3.23E-03	1.15E-02
DERL2	-0.0901	8.32E-02	1.61E-01
DERL3	-0.0065	9.01E-01	9.37E-01
DES	-0.1186	2.24E-02	5.62E-02
DET1	-0.0780	1.34E-01	2.32E-01
DEXI	-0.2077	5.53E-05	3.69E-04
DFFA	-0.0456	3.81E-01	5.15E-01

DFFB	0.2039	7.65E-05	4.86E-04
DFNA5	0.0231	6.57E-01	7.59E-01
DFNB31	0.2956	6.42E-09	1.25E-07
DFNB59	0.0455	3.82E-01	5.15E-01
DGAT1	-0.0371	4.76E-01	6.05E-01
DGAT2L6	0.0881	9.03E-02	1.71E-01
DGAT2	0.0399	4.43E-01	5.75E-01
DGCR10	0.0089	8.64E-01	9.12E-01
DGCR11	0.1705	9.76E-04	4.22E-03
DGCR14	0.2613	3.30E-07	4.24E-06
DGCR2	0.0514	3.24E-01	4.54E-01
DGCR5	-0.1335	1.00E-02	2.93E-02
DGCR6L	-0.1650	1.43E-03	5.79E-03
DGCR6	-0.1808	4.66E-04	2.24E-03
DGCR8	0.2468	1.49E-06	1.61E-05
DGCR9	0.0655	2.08E-01	3.26E-01
DGKA	-0.0051	9.23E-01	9.51E-01
DGKB	0.0159	7.60E-01	8.38E-01
DGKD	0.1631	1.62E-03	6.46E-03
DGKE	0.0933	7.26E-02	1.44E-01
DGKG	0.0685	1.88E-01	3.01E-01
DGKH	0.0374	4.73E-01	6.02E-01
DGKI	-0.0774	1.37E-01	2.36E-01
DGKK	-0.0565	2.78E-01	4.06E-01
DGKQ	0.0425	4.15E-01	5.47E-01
DGKZ	0.1523	3.28E-03	1.16E-02
DGUOK	0.0818	1.16E-01	2.08E-01
DHCR24	-0.0758	1.45E-01	2.47E-01
DHCR7	-0.1449	5.18E-03	1.70E-02
DHDDS	-0.2026	8.48E-05	5.30E-04
DHDH	0.2472	1.44E-06	1.56E-05
DHDPSL	-0.2121	3.82E-05	2.70E-04
DHFRL1	-0.2003	1.03E-04	6.22E-04
DHFR	0.1050	4.32E-02	9.58E-02
DHH	-0.1867	2.98E-04	1.55E-03
DHODH	-0.3525	2.70E-12	1.10E-10
DHPS	0.0247	6.35E-01	7.42E-01
DHRS11	0.0860	9.80E-02	1.83E-01
DHRS12	-0.3307	6.41E-11	1.97E-09
DHRS13	0.1681	1.15E-03	4.86E-03
DHRS1	-0.2511	9.65E-07	1.10E-05
DHRS2	-0.1377	7.89E-03	2.40E-02
DHRS3	-0.2393	3.13E-06	3.08E-05
DHRS4L1	-0.1474	4.45E-03	1.50E-02

DHRS4L2	-0.1615	1.80E-03	7.06E-03
DHRS4	-0.2045	7.26E-05	4.65E-04
DHRS7B	-0.1221	1.86E-02	4.85E-02
DHRS7C	-0.0028	9.56E-01	9.73E-01
DHRS7	-0.0014	9.78E-01	9.86E-01
DHRS9	-0.1060	4.12E-02	9.23E-02
DHRSX	-0.1328	1.04E-02	3.02E-02
DHTKD1	-0.2456	1.69E-06	1.80E-05
DHX15	0.1860	3.16E-04	1.63E-03
DHX16	0.1926	1.90E-04	1.05E-03
DHX29	-0.0912	7.92E-02	1.55E-01
DHX30	0.1330	1.04E-02	3.00E-02
DHX32	-0.0237	6.49E-01	7.53E-01
DHX33	-0.0659	2.05E-01	3.23E-01
DHX34	0.4349	1.50E-18	2.55E-16
DHX35	0.1393	7.21E-03	2.23E-02
DHX36	-0.0289	5.79E-01	6.96E-01
DHX37	0.1319	1.10E-02	3.16E-02
DHX38	-0.0881	9.00E-02	1.71E-01
DHX40P1	0.0949	6.78E-02	1.37E-01
DHX40	0.0855	1.00E-01	1.86E-01
DHX57	0.2599	3.83E-07	4.84E-06
DHX58	-0.1544	2.86E-03	1.04E-02
DHX8	0.1846	3.52E-04	1.78E-03
DHX9	0.3157	4.98E-10	1.26E-08
DIABLO	-0.0022	9.66E-01	9.79E-01
DIAPH1	-0.1830	3.96E-04	1.97E-03
DIAPH2	0.1469	4.57E-03	1.53E-02
DIAPH3	0.2687	1.48E-07	2.07E-06
DICER1	-0.0238	6.47E-01	7.52E-01
DIDO1	0.2485	1.25E-06	1.38E-05
DIMT1L	0.0323	5.36E-01	6.58E-01
DIO1	-0.1720	8.81E-04	3.86E-03
DIO2	-0.0559	2.83E-01	4.11E-01
DIO3OS	0.0717	1.68E-01	2.77E-01
DIO3	-0.0097	8.52E-01	9.04E-01
DIP2A	0.1144	2.76E-02	6.67E-02
DIP2B	0.0934	7.23E-02	1.44E-01
DIP2C	-0.1091	3.56E-02	8.21E-02
DIRAS1	0.2019	9.00E-05	5.58E-04
DIRAS2	0.1355	8.98E-03	2.68E-02
DIRAS3	-0.1829	4.00E-04	1.98E-03
DIRC1	0.0220	6.72E-01	7.72E-01
DIRC2	0.0137	7.93E-01	8.63E-01

DIRC3	-0.0416	4.24E-01	5.57E-01
DIS3L2	0.0131	8.01E-01	8.69E-01
DIS3L	-0.0516	3.22E-01	4.52E-01
DIS3	-0.0089	8.64E-01	9.12E-01
DISC1	-0.0901	8.30E-02	1.60E-01
DISC2	0.0267	6.08E-01	7.20E-01
DISP1	-0.0307	5.56E-01	6.75E-01
DISP2	-0.0254	6.26E-01	7.34E-01
DIXDC1	-0.1939	1.71E-04	9.63E-04
DKC1	0.3070	1.55E-09	3.54E-08
DKFZP434K028	0.0029	9.55E-01	9.72E-01
DKFZP434L187	0.1651	1.42E-03	5.75E-03
DKFZP586I1420	-0.0215	6.80E-01	7.78E-01
DKFZP686I1521	0.1979	1.25E-04	7.36E-04
DKFZp434J0226	0.1321	1.09E-02	3.13E-02
DKFZp434L192	0.0759	1.45E-01	2.47E-01
DKFZp566F0947	0.0074	8.87E-01	9.28E-01
DKFZp686A1627	0.0808	1.20E-01	2.15E-01
DKFZp686O241	-0.0522	3.16E-01	4.46E-01
DKFZp761E198	-0.1110	3.26E-02	7.65E-02
DKFZp779M065	-0.2294	8.06E-06	6.94E-05
DKK1	0.1101	3.40E-02	7.91E-02
DKK2	-0.1060	4.13E-02	9.24E-02
DKK3	-0.1375	8.01E-03	2.43E-02
DKK4	-0.0142	7.85E-01	8.57E-01
DKKL1	0.1645	1.48E-03	5.97E-03
DLAT	-0.0582	2.64E-01	3.91E-01
DLC1	-0.2408	2.70E-06	2.71E-05
DLD	-0.2543	6.95E-07	8.18E-06
DLEC1	0.0855	1.00E-01	1.86E-01
DLEU1	0.0496	3.41E-01	4.72E-01
DLEU2L	0.2707	1.18E-07	1.69E-06
DLEU2	0.2666	1.86E-07	2.54E-06
DLEU7	0.0748	1.50E-01	2.54E-01
DLG1	0.0350	5.02E-01	6.28E-01
DLG2	-0.2727	9.45E-08	1.38E-06
DLG3	0.1390	7.33E-03	2.26E-02
DLG4	-0.0344	5.09E-01	6.33E-01
DLG5	0.2088	5.04E-05	3.41E-04
DLGAP1	0.0624	2.31E-01	3.53E-01
DLGAP2	-0.0750	1.49E-01	2.53E-01
DLGAP3	0.0112	8.30E-01	8.89E-01
DLGAP4	0.2113	4.09E-05	2.85E-04
DLGAP5	0.3992	1.26E-15	9.90E-14

DLK1	0.0265	6.10E-01	7.22E-01
DLK2	0.1956	1.50E-04	8.65E-04
DLL1	-0.1671	1.24E-03	5.15E-03
DLL3	0.1565	2.50E-03	9.28E-03
DLL4	-0.0515	3.23E-01	4.53E-01
DLST	-0.2447	1.83E-06	1.94E-05
DLX1	0.0888	8.75E-02	1.67E-01
DLX2	0.0756	1.46E-01	2.49E-01
DLX3	0.1403	6.81E-03	2.13E-02
DLX4	0.2337	5.39E-06	4.94E-05
DLX5	0.2033	8.04E-05	5.06E-04
DLX6AS	0.2930	8.88E-09	1.67E-07
DLX6	0.2878	1.65E-08	2.92E-07
DMAP1	0.1175	2.37E-02	5.89E-02
DMBT1	0.2332	5.66E-06	5.14E-05
DMBX1	0.2733	8.86E-08	1.30E-06
DMC1	0.3141	6.12E-10	1.52E-08
DMD	-0.1318	1.10E-02	3.16E-02
DMGDH	-0.2303	7.45E-06	6.50E-05
DMKN	0.1133	2.92E-02	6.99E-02
DMP1	0.1013	5.13E-02	1.10E-01
DMPK	0.1939	1.71E-04	9.65E-04
DMRT1	0.1573	2.37E-03	8.87E-03
DMRT2	0.2298	7.78E-06	6.73E-05
DMRT3	0.0323	5.35E-01	6.57E-01
DMRTA1	-0.0640	2.19E-01	3.39E-01
DMRTA2	0.1784	5.54E-04	2.59E-03
DMRTB1	-0.0254	6.26E-01	7.34E-01
DMRTC1B	0.0755	1.47E-01	2.49E-01
DMRTC1	0.1064	4.06E-02	9.13E-02
DMRTC2	0.1276	1.39E-02	3.83E-02
DMTF1	0.2309	7.01E-06	6.18E-05
DMWD	0.1126	3.02E-02	7.18E-02
DMXL1	-0.0431	4.08E-01	5.41E-01
DMXL2	0.0740	1.55E-01	2.60E-01
DNA2	0.4204	2.57E-17	3.03E-15
DNAH10	0.1806	4.72E-04	2.26E-03
DNAH11	0.0659	2.06E-01	3.23E-01
DNAH12	0.0191	7.14E-01	8.05E-01
DNAH14	0.3578	1.19E-12	5.24E-11
DNAH17	0.2563	5.62E-07	6.76E-06
DNAH1	0.0862	9.74E-02	1.82E-01
DNAH2	-0.0456	3.82E-01	5.15E-01
DNAH3	0.1312	1.14E-02	3.26E-02

DNAH5	-0.0326	5.31E-01	6.53E-01
DNAH6	-0.0805	1.22E-01	2.17E-01
DNAH7	0.0856	9.95E-02	1.85E-01
DNAH8	0.1062	4.10E-02	9.20E-02
DNAH9	-0.1021	4.95E-02	1.07E-01
DNAI1	0.0789	1.29E-01	2.26E-01
DNAI2	0.0476	3.61E-01	4.93E-01
DNAJA1	0.0822	1.14E-01	2.06E-01
DNAJA2	-0.1949	1.58E-04	9.03E-04
DNAJA3	-0.1443	5.35E-03	1.75E-02
DNAJA4	0.0974	6.10E-02	1.26E-01
DNAJB11	0.1664	1.30E-03	5.36E-03
DNAJB12	-0.1060	4.13E-02	9.25E-02
DNAJB13	0.1019	5.00E-02	1.08E-01
DNAJB14	-0.1473	4.45E-03	1.50E-02
DNAJB1	0.0036	9.45E-01	9.66E-01
DNAJB2	-0.0041	9.38E-01	9.61E-01
DNAJB3	0.0633	2.24E-01	3.45E-01
DNAJB4	-0.0059	9.10E-01	9.43E-01
DNAJB5	-0.0695	1.82E-01	2.94E-01
DNAJB6	-0.0741	1.54E-01	2.59E-01
DNAJB7	0.0950	6.75E-02	1.36E-01
DNAJB8	-0.0233	6.55E-01	7.57E-01
DNAJB9	-0.2664	1.91E-07	2.61E-06
DNAJC10	0.1333	1.02E-02	2.97E-02
DNAJC11	-0.0231	6.57E-01	7.59E-01
DNAJC12	-0.0726	1.63E-01	2.70E-01
DNAJC13	-0.0011	9.84E-01	9.90E-01
DNAJC14	0.0808	1.20E-01	2.15E-01
DNAJC15	-0.0149	7.75E-01	8.50E-01
DNAJC16	-0.1893	2.45E-04	1.31E-03
DNAJC17	-0.0536	3.03E-01	4.33E-01
DNAJC18	0.0436	4.02E-01	5.35E-01
DNAJC19	-0.1651	1.42E-03	5.77E-03
DNAJC1	0.0109	8.34E-01	8.91E-01
DNAJC21	0.1483	4.19E-03	1.43E-02
DNAJC22	-0.0797	1.26E-01	2.22E-01
DNAJC24	-0.0492	3.44E-01	4.76E-01
DNAJC25-GNG1	-0.0235	6.52E-01	7.56E-01
DNAJC25	-0.2046	7.20E-05	4.62E-04
DNAJC27	-0.0789	1.29E-01	2.26E-01
DNAJC28	-0.1471	4.53E-03	1.52E-02
DNAJC2	0.1322	1.08E-02	3.11E-02
DNAJC30	-0.3243	1.56E-10	4.44E-09

DNAJC3	-0.0720	1.66E-01	2.74E-01
DNAJC4	-0.0049	9.25E-01	9.53E-01
DNAJC5B	-0.0372	4.75E-01	6.04E-01
DNAJC5G	0.0451	3.87E-01	5.19E-01
DNAJC5	0.1429	5.83E-03	1.87E-02
DNAJC6	0.0670	1.98E-01	3.14E-01
DNAJC7	0.1847	3.48E-04	1.77E-03
DNAJC8	0.1254	1.57E-02	4.21E-02
DNAJC9	0.3230	1.86E-10	5.20E-09
DNAL1	-0.0033	9.50E-01	9.69E-01
DNAL4	0.1946	1.62E-04	9.21E-04
DNALI1	-0.0657	2.07E-01	3.25E-01
DNASE1L1	-0.0054	9.17E-01	9.48E-01
DNASE1L2	0.0669	1.99E-01	3.15E-01
DNASE1L3	-0.3158	4.88E-10	1.24E-08
DNASE1	0.2708	1.17E-07	1.68E-06
DNASE2B	-0.1280	1.36E-02	3.77E-02
DNASE2	-0.0389	4.55E-01	5.86E-01
DND1	-0.0083	8.73E-01	9.18E-01
DNER	-0.0696	1.81E-01	2.93E-01
DNHD1	0.0703	1.77E-01	2.88E-01
DNLZ	0.1146	2.74E-02	6.63E-02
DNM1L	0.2156	2.82E-05	2.08E-04
DNM1P35	0.1771	6.12E-04	2.82E-03
DNM1	0.1493	3.94E-03	1.36E-02
DNM2	-0.0335	5.20E-01	6.44E-01
DNM3	-0.0449	3.88E-01	5.21E-01
DNMBP	-0.1545	2.84E-03	1.03E-02
DNMT1	0.3237	1.69E-10	4.77E-09
DNMT3A	0.2508	9.91E-07	1.12E-05
DNMT3B	0.3471	6.09E-12	2.31E-10
DNMT3L	-0.2018	9.07E-05	5.61E-04
DNPEP	-0.0382	4.64E-01	5.94E-01
DNTTIP1	0.1556	2.65E-03	9.75E-03
DNTTIP2	0.2432	2.14E-06	2.22E-05
DNTT	0.0687	1.87E-01	3.00E-01
DOC2A	0.0856	9.96E-02	1.85E-01
DOC2B	0.0118	8.20E-01	8.82E-01
DOCK10	-0.0264	6.13E-01	7.23E-01
DOCK11	-0.0628	2.28E-01	3.50E-01
DOCK1	-0.0381	4.64E-01	5.94E-01
DOCK2	-0.0804	1.22E-01	2.17E-01
DOCK3	0.2210	1.74E-05	1.36E-04
DOCK4	-0.1698	1.03E-03	4.39E-03

DOCK5	-0.0256	6.24E-01	7.32E-01
DOCK6	-0.0531	3.08E-01	4.37E-01
DOCK7	0.0695	1.82E-01	2.94E-01
DOCK8	-0.0489	3.48E-01	4.80E-01
DOCK9	-0.0817	1.16E-01	2.09E-01
DOHH	0.0305	5.58E-01	6.77E-01
DOK1	0.1119	3.12E-02	7.37E-02
DOK2	-0.0559	2.82E-01	4.11E-01
DOK3	0.0279	5.92E-01	7.06E-01
DOK4	-0.2435	2.08E-06	2.16E-05
DOK5	-0.1448	5.21E-03	1.71E-02
DOK6	0.0229	6.61E-01	7.62E-01
DOK7	-0.0029	9.56E-01	9.73E-01
DOLK	0.1619	1.76E-03	6.90E-03
DOLPP1	0.0578	2.67E-01	3.94E-01
DOM3Z	0.2229	1.46E-05	1.17E-04
DONSON	0.3830	2.07E-14	1.26E-12
DOPEY1	0.1733	8.03E-04	3.56E-03
DOPEY2	0.1388	7.43E-03	2.29E-02
DOT1L	0.2961	6.04E-09	1.18E-07
DPAGT1	0.0630	2.26E-01	3.47E-01
DPCD	-0.0176	7.35E-01	8.21E-01
DPCR1	-0.0624	2.30E-01	3.53E-01
DPEP1	-0.0112	8.30E-01	8.89E-01
DPEP2	-0.1256	1.55E-02	4.17E-02
DPEP3	0.0056	9.15E-01	9.47E-01
DPF1	0.1191	2.17E-02	5.50E-02
DPF2	0.2569	5.30E-07	6.43E-06
DPF3	-0.2850	2.30E-08	3.90E-07
DPH1	-0.0923	7.59E-02	1.50E-01
DPH2	0.1606	1.92E-03	7.43E-03
DPH3B	0.1074	3.86E-02	8.77E-02
DPH3	-0.0057	9.13E-01	9.45E-01
DPH5	0.0500	3.37E-01	4.68E-01
DPM1	0.0518	3.20E-01	4.50E-01
DPM2	0.1761	6.58E-04	3.00E-03
DPM3	0.1295	1.25E-02	3.51E-02
DPP10	0.0260	6.17E-01	7.27E-01
DPP3	-0.0400	4.42E-01	5.74E-01
DPP4	-0.0688	1.86E-01	2.99E-01
DPP6	0.0335	5.20E-01	6.44E-01
DPP7	-0.0360	4.89E-01	6.16E-01
DPP8	-0.0665	2.01E-01	3.18E-01
DPP9	-0.0330	5.26E-01	6.49E-01

DPPA2	0.0768	1.40E-01	2.40E-01
DPPA3	0.0933	7.25E-02	1.44E-01
DPPA4	-0.0257	6.22E-01	7.31E-01
DPPA5	0.0628	2.27E-01	3.49E-01
DPRXP4	0.0981	5.90E-02	1.22E-01
DPRX	-0.0203	6.96E-01	7.91E-01
DPT	-0.1213	1.94E-02	5.01E-02
DPY19L1	-0.0491	3.45E-01	4.77E-01
DPY19L2P1	0.0877	9.16E-02	1.74E-01
DPY19L2P2	0.1682	1.14E-03	4.82E-03
DPY19L2P4	0.0455	3.82E-01	5.15E-01
DPY19L2	0.0241	6.43E-01	7.49E-01
DPY19L3	-0.0043	9.34E-01	9.59E-01
DPY19L4	-0.0286	5.83E-01	6.99E-01
DPY30	0.1295	1.26E-02	3.52E-02
DPYD	-0.0828	1.11E-01	2.03E-01
DPYSL2	-0.2050	6.95E-05	4.48E-04
DPYSL3	-0.0213	6.83E-01	7.80E-01
DPYSL4	0.0773	1.37E-01	2.37E-01
DPYSL5	0.0800	1.24E-01	2.20E-01
DPYS	-0.1954	1.52E-04	8.75E-04
DQX1	0.2816	3.43E-08	5.56E-07
DR1	0.2006	1.00E-04	6.09E-04
DRAM1	0.1007	5.25E-02	1.12E-01
DRAM2	0.0237	6.49E-01	7.53E-01
DRAP1	0.0786	1.31E-01	2.29E-01
DRD1	-0.1035	4.64E-02	1.01E-01
DRD2	0.0249	6.33E-01	7.40E-01
DRD3	-0.0284	5.86E-01	7.01E-01
DRD4	0.0998	5.47E-02	1.15E-01
DRD5	-0.0614	2.38E-01	3.62E-01
DRG1	0.1997	1.08E-04	6.49E-04
DRG2	0.0108	8.35E-01	8.92E-01
DRGX	-0.0232	6.56E-01	7.58E-01
DRP2	0.3292	7.92E-11	2.37E-09
DSC1	-0.0417	4.23E-01	5.55E-01
DSC2	0.0841	1.06E-01	1.94E-01
DSC3	0.0710	1.73E-01	2.82E-01
DSCAML1	0.0213	6.83E-01	7.80E-01
DSCAM	-0.0548	2.93E-01	4.22E-01
DSCC1	0.3055	1.86E-09	4.18E-08
DSCR10	0.1509	3.59E-03	1.25E-02
DSCR3	-0.2226	1.51E-05	1.20E-04
DSCR4	0.0955	6.63E-02	1.34E-01

DSCR6	0.2914	1.07E-08	1.98E-07
DSCR8	0.0603	2.47E-01	3.71E-01
DSCR9	0.2417	2.49E-06	2.52E-05
DSEL	-0.0284	5.86E-01	7.01E-01
DSE	-0.0824	1.13E-01	2.05E-01
DSG1	-0.2744	7.81E-08	1.16E-06
DSG2	0.1886	2.58E-04	1.37E-03
DSG3	-0.0206	6.92E-01	7.87E-01
DSG4	-0.0688	1.86E-01	2.99E-01
DSN1	0.4308	3.39E-18	5.08E-16
DSPP	0.0489	3.47E-01	4.80E-01
DSP	-0.0265	6.11E-01	7.22E-01
DSTN	-0.0636	2.22E-01	3.43E-01
DSTYK	0.2918	1.02E-08	1.90E-07
DST	-0.0634	2.23E-01	3.44E-01
DTD1	0.1015	5.07E-02	1.09E-01
DTHD1	-0.0721	1.66E-01	2.73E-01
DTL	0.4892	1.01E-23	4.39E-21
DTNA	-0.1083	3.70E-02	8.48E-02
DTNBP1	0.2664	1.91E-07	2.61E-06
DTNB	0.1573	2.37E-03	8.87E-03
DTWD1	-0.0405	4.36E-01	5.68E-01
DTWD2	-0.2120	3.83E-05	2.70E-04
DTX1	-0.0934	7.24E-02	1.44E-01
DTX2	0.1077	3.82E-02	8.69E-02
DTX3L	-0.0064	9.02E-01	9.38E-01
DTX3	0.1827	4.04E-04	2.00E-03
DTX4	0.0419	4.22E-01	5.54E-01
DTYMK	0.2781	5.14E-08	7.99E-07
DULLARD	0.1061	4.12E-02	9.23E-02
DUOX1	0.2455	1.70E-06	1.81E-05
DUOX2	0.1346	9.44E-03	2.79E-02
DUOXA1	0.1356	8.94E-03	2.67E-02
DUOXA2	0.1705	9.78E-04	4.22E-03
DUPD1	-0.0208	6.89E-01	7.85E-01
DUS1L	0.0419	4.21E-01	5.54E-01
DUS2L	-0.1066	4.01E-02	9.03E-02
DUS3L	0.0201	7.00E-01	7.93E-01
DUS4L	0.0411	4.30E-01	5.63E-01
DUSP10	-0.0712	1.71E-01	2.81E-01
DUSP11	0.0771	1.38E-01	2.38E-01
DUSP12	0.5441	5.61E-30	4.86E-27
DUSP13	0.0720	1.67E-01	2.75E-01
DUSP14	-0.0441	3.97E-01	5.30E-01

DUSP15	0.1024	4.88E-02	1.06E-01
DUSP16	-0.2132	3.48E-05	2.49E-04
DUSP18	0.1455	4.99E-03	1.64E-02
DUSP19	-0.1440	5.46E-03	1.77E-02
DUSP1	-0.1545	2.85E-03	1.04E-02
DUSP21	0.0467	3.70E-01	5.03E-01
DUSP22	0.0752	1.48E-01	2.52E-01
DUSP23	0.1055	4.22E-02	9.40E-02
DUSP26	0.0893	8.60E-02	1.65E-01
DUSP27	-0.0569	2.75E-01	4.03E-01
DUSP28	0.2382	3.48E-06	3.39E-05
DUSP2	0.0050	9.24E-01	9.52E-01
DUSP3	0.0276	5.96E-01	7.10E-01
DUSP4	0.0131	8.02E-01	8.69E-01
DUSP5P	0.2778	5.30E-08	8.19E-07
DUSP5	-0.0072	8.90E-01	9.30E-01
DUSP6	-0.1637	1.56E-03	6.25E-03
DUSP7	0.0524	3.14E-01	4.44E-01
DUSP8	-0.0489	3.48E-01	4.80E-01
DUSP9	0.3116	8.57E-10	2.06E-08
DUT	0.0557	2.85E-01	4.14E-01
DUXA	0.1002	5.37E-02	1.14E-01
DVL1	-0.0864	9.65E-02	1.81E-01
DVL2	0.2823	3.16E-08	5.18E-07
DVL3	0.2326	5.99E-06	5.41E-05
DVWA	0.1139	2.83E-02	6.82E-02
DYDC1	0.1134	2.90E-02	6.95E-02
DYDC2	-0.0415	4.26E-01	5.58E-01
DYM	0.0574	2.70E-01	3.98E-01
DYNC1H1	0.1035	4.64E-02	1.01E-01
DYNC1I1	-0.0106	8.39E-01	8.94E-01
DYNC1I2	0.0495	3.41E-01	4.73E-01
DYNC1LI1	0.1942	1.67E-04	9.45E-04
DYNC1LI2	-0.0834	1.09E-01	1.98E-01
DYNC2H1	0.0077	8.82E-01	9.25E-01
DYNC2LI1	0.1661	1.32E-03	5.43E-03
DYNLL1	0.1017	5.02E-02	1.08E-01
DYNLL2	-0.0963	6.39E-02	1.31E-01
DYNLRB1	0.1412	6.43E-03	2.03E-02
DYNLRB2	0.0695	1.82E-01	2.94E-01
DYNLT1	0.1755	6.87E-04	3.12E-03
DYNLT3	-0.1167	2.46E-02	6.07E-02
DYRK1A	0.0872	9.35E-02	1.76E-01
DYRK1B	-0.0764	1.42E-01	2.43E-01

DYRK2	0.2277	9.44E-06	7.99E-05
DYRK3	0.0814	1.17E-01	2.11E-01
DYRK4	0.1255	1.56E-02	4.19E-02
DYSFIP1	0.0859	9.85E-02	1.84E-01
DYSF	-0.2941	7.74E-09	1.47E-07
DYX1C1	0.2259	1.12E-05	9.26E-05
DZIP1L	0.2859	2.08E-08	3.58E-07
DZIP1	0.0226	6.65E-01	7.65E-01
DZIP3	-0.0401	4.41E-01	5.73E-01
E2F1	0.3979	1.60E-15	1.22E-13
E2F2	0.3956	2.37E-15	1.75E-13
E2F3	0.2569	5.30E-07	6.44E-06
E2F4	0.1558	2.61E-03	9.62E-03
E2F5	0.0735	1.58E-01	2.64E-01
E2F6	0.3060	1.75E-09	3.96E-08
E2F7	0.3588	1.03E-12	4.60E-11
E2F8	0.3630	5.33E-13	2.50E-11
E4F1	0.1388	7.43E-03	2.29E-02
EAF1	-0.0644	2.16E-01	3.36E-01
EAF2	0.2331	5.68E-06	5.16E-05
EAPP	-0.0589	2.58E-01	3.83E-01
EARS2	-0.2252	1.19E-05	9.79E-05
EBAG9	-0.1661	1.33E-03	5.44E-03
EBF1	-0.0623	2.31E-01	3.54E-01
EBF2	-0.1040	4.53E-02	9.96E-02
EBF3	-0.0802	1.23E-01	2.18E-01
EBF4	0.0245	6.38E-01	7.44E-01
EBI3	-0.0568	2.75E-01	4.03E-01
EBNA1BP2	0.1319	1.10E-02	3.16E-02
EBPL	-0.1273	1.42E-02	3.89E-02
EBP	-0.1165	2.48E-02	6.11E-02
ECD	0.1758	6.73E-04	3.06E-03
ECE1	-0.1174	2.37E-02	5.89E-02
ECE2	-0.0093	8.58E-01	9.09E-01
ECEL1	-0.0106	8.39E-01	8.94E-01
ECH1	-0.1564	2.53E-03	9.35E-03
ECHDC1	-0.1294	1.26E-02	3.54E-02
ECHDC2	-0.1952	1.55E-04	8.86E-04
ECHDC3	-0.0887	8.79E-02	1.68E-01
ECHS1	-0.2631	2.73E-07	3.59E-06
ECM1	-0.0785	1.31E-01	2.29E-01
ECM2	-0.2883	1.57E-08	2.80E-07
ECSCR	-0.1481	4.25E-03	1.44E-02
ECSIT	-0.2284	8.87E-06	7.56E-05

ECT2L	0.1393	7.20E-03	2.23E-02
ECT2	0.3519	2.95E-12	1.19E-10
EDA2R	-0.1715	9.11E-04	3.97E-03
EDARADD	0.1569	2.43E-03	9.06E-03
EDAR	-0.1810	4.59E-04	2.21E-03
EDA	0.0399	4.44E-01	5.75E-01
EDC3	0.2455	1.70E-06	1.81E-05
EDC4	0.1013	5.13E-02	1.10E-01
EDDM3A	-0.0362	4.87E-01	6.15E-01
EDDM3B	0.0445	3.93E-01	5.26E-01
EDEM1	-0.2297	7.84E-06	6.78E-05
EDEM2	0.0352	4.99E-01	6.25E-01
EDEM3	0.1783	5.58E-04	2.61E-03
EDF1	0.0615	2.37E-01	3.61E-01
EDIL3	-0.1042	4.49E-02	9.89E-02
EDN1	-0.0533	3.06E-01	4.36E-01
EDN2	-0.0320	5.39E-01	6.60E-01
EDN3	0.0014	9.78E-01	9.86E-01
EDNRA	-0.0356	4.94E-01	6.21E-01
EDNRB	-0.1551	2.74E-03	1.00E-02
EEA1	-0.0114	8.26E-01	8.86E-01
EED	0.3780	4.75E-14	2.70E-12
EEF1A1P9	0.0397	4.46E-01	5.78E-01
EEF1A1	0.0652	2.10E-01	3.28E-01
EEF1A2	-0.0329	5.28E-01	6.51E-01
EEF1B2	0.1077	3.81E-02	8.68E-02
EEF1DP3	0.0805	1.22E-01	2.16E-01
EEF1D	0.0218	6.76E-01	7.75E-01
EEF1E1	0.1927	1.88E-04	1.05E-03
EEF1G	0.1104	3.35E-02	7.83E-02
EEF2K	-0.0106	8.39E-01	8.94E-01
EEF2	-0.2286	8.67E-06	7.41E-05
EEFSEC	-0.0667	2.00E-01	3.16E-01
EEPD1	0.0186	7.21E-01	8.10E-01
EFCAB10	0.0780	1.34E-01	2.32E-01
EFCAB1	-0.0597	2.52E-01	3.77E-01
EFCAB2	0.1096	3.49E-02	8.08E-02
EFCAB3	0.0919	7.72E-02	1.52E-01
EFCAB4A	0.1101	3.41E-02	7.93E-02
EFCAB4B	0.0077	8.83E-01	9.25E-01
EFCAB5	0.1017	5.04E-02	1.08E-01
EFCAB6	-0.1009	5.20E-02	1.11E-01
EFCAB7	0.2388	3.29E-06	3.23E-05
EFEMP1	-0.0830	1.11E-01	2.01E-01

EFEMP2	-0.0807	1.21E-01	2.16E-01
EFHA1	-0.0518	3.20E-01	4.50E-01
EFHA2	-0.1426	5.95E-03	1.90E-02
EFHB	0.0318	5.41E-01	6.62E-01
EFHC1	0.2525	8.34E-07	9.66E-06
EFHC2	0.1863	3.08E-04	1.60E-03
EFHD1	-0.1259	1.52E-02	4.11E-02
EFHD2	0.0764	1.42E-01	2.43E-01
EFNA1	0.2850	2.30E-08	3.90E-07
EFNA2	-0.0913	7.89E-02	1.54E-01
EFNA3	0.3178	3.78E-10	9.94E-09
EFNA4	0.4098	1.87E-16	1.79E-14
EFNA5	0.0558	2.84E-01	4.12E-01
EFNB1	0.1224	1.83E-02	4.78E-02
EFNB2	-0.1061	4.12E-02	9.23E-02
EFNB3	-0.1473	4.47E-03	1.51E-02
EFR3A	-0.1977	1.27E-04	7.46E-04
EFR3B	0.1670	1.24E-03	5.17E-03
EFS	0.0011	9.82E-01	9.89E-01
EFTUD1	0.0709	1.73E-01	2.83E-01
EFTUD2	0.3000	3.72E-09	7.70E-08
EGFL6	0.0745	1.52E-01	2.56E-01
EGFL7	-0.0420	4.20E-01	5.53E-01
EGFL8	0.0952	6.70E-02	1.36E-01
EGFLAM	-0.1060	4.13E-02	9.24E-02
EGFR	-0.1045	4.42E-02	9.76E-02
EGF	0.1187	2.22E-02	5.59E-02
EGLN1	-0.0403	4.39E-01	5.70E-01
EGLN2	0.0948	6.83E-02	1.38E-01
EGLN3	0.2027	8.41E-05	5.28E-04
EGOT	-0.0289	5.79E-01	6.96E-01
EGR1	-0.1044	4.45E-02	9.81E-02
EGR2	-0.1011	5.18E-02	1.10E-01
EGR3	-0.1514	3.47E-03	1.22E-02
EGR4	-0.0092	8.60E-01	9.10E-01
EHBP1L1	0.1475	4.40E-03	1.49E-02
EHBP1	-0.1610	1.86E-03	7.24E-03
EHD1	-0.0499	3.38E-01	4.69E-01
EHD2	-0.1268	1.45E-02	3.97E-02
EHD3	-0.0842	1.05E-01	1.93E-01
EHD4	-0.0918	7.74E-02	1.52E-01
EHF	0.2001	1.05E-04	6.32E-04
EHHADH	-0.2888	1.48E-08	2.64E-07
EHMT1	0.1060	4.13E-02	9.24E-02

EHMT2	0.3506	3.62E-12	1.45E-10
EI24	-0.1780	5.74E-04	2.67E-03
EID1	-0.1262	1.50E-02	4.08E-02
EID2B	0.2876	1.70E-08	2.99E-07
EID2	0.0860	9.81E-02	1.83E-01
EID3	0.0817	1.16E-01	2.09E-01
EIF1AD	0.2476	1.38E-06	1.50E-05
EIF1AX	-0.0170	7.44E-01	8.27E-01
EIF1AY	-0.0130	8.02E-01	8.70E-01
EIF1B	0.0271	6.03E-01	7.15E-01
EIF1	0.0682	1.90E-01	3.04E-01
EIF2AK1	0.0153	7.69E-01	8.45E-01
EIF2AK2	0.0744	1.53E-01	2.57E-01
EIF2AK3	-0.0169	7.45E-01	8.28E-01
EIF2AK4	-0.2058	6.50E-05	4.23E-04
EIF2A	0.1248	1.62E-02	4.32E-02
EIF2B1	0.2068	5.98E-05	3.93E-04
EIF2B2	-0.1498	3.84E-03	1.33E-02
EIF2B3	-0.0103	8.44E-01	8.98E-01
EIF2B4	0.0666	2.00E-01	3.17E-01
EIF2B5	0.1802	4.86E-04	2.32E-03
EIF2C1	0.0995	5.54E-02	1.17E-01
EIF2C2	0.1583	2.23E-03	8.42E-03
EIF2C3	-0.0137	7.93E-01	8.63E-01
EIF2C4	-0.0821	1.14E-01	2.06E-01
EIF2S1	-0.0572	2.72E-01	4.00E-01
EIF2S2	0.1341	9.69E-03	2.85E-02
EIF2S3	0.1722	8.66E-04	3.80E-03
EIF3A	-0.0494	3.43E-01	4.74E-01
EIF3B	0.1948	1.60E-04	9.10E-04
EIF3CL	-0.0225	6.65E-01	7.65E-01
EIF3C	-0.0038	9.42E-01	9.64E-01
EIF3D	0.2579	4.77E-07	5.87E-06
EIF3E	0.0232	6.56E-01	7.58E-01
EIF3F	0.0577	2.67E-01	3.95E-01
EIF3G	0.0042	9.36E-01	9.60E-01
EIF3H	0.0572	2.72E-01	4.00E-01
EIF3IP1	0.1334	1.01E-02	2.94E-02
EIF3I	-0.0107	8.38E-01	8.93E-01
EIF3J	-0.0370	4.77E-01	6.06E-01
EIF3K	-0.0059	9.10E-01	9.43E-01
EIF3L	0.0497	3.40E-01	4.71E-01
EIF3M	0.1711	9.38E-04	4.07E-03
EIF4A1	-0.0188	7.18E-01	8.09E-01

EIF4A2	0.0520	3.18E-01	4.48E-01
EIF4A3	0.3139	6.28E-10	1.56E-08
EIF4B	-0.0473	3.64E-01	4.96E-01
EIF4E1B	0.0307	5.56E-01	6.75E-01
EIF4E2	0.1234	1.74E-02	4.58E-02
EIF4E3	-0.0242	6.42E-01	7.48E-01
EIF4EBP1	0.0417	4.23E-01	5.56E-01
EIF4EBP2	-0.0719	1.67E-01	2.75E-01
EIF4EBP3	-0.1608	1.89E-03	7.34E-03
EIF4ENIF1	0.2640	2.47E-07	3.28E-06
EIF4E	-0.1046	4.41E-02	9.74E-02
EIF4G1	-0.1120	3.11E-02	7.35E-02
EIF4G2	-0.0241	6.44E-01	7.49E-01
EIF4G3	-0.1258	1.53E-02	4.13E-02
EIF4H	-0.1325	1.06E-02	3.06E-02
EIF5A2	0.0880	9.04E-02	1.72E-01
EIF5AL1	-0.1200	2.08E-02	5.30E-02
EIF5A	-0.1013	5.13E-02	1.10E-01
EIF5B	0.0036	9.46E-01	9.66E-01
EIF5	-0.1420	6.14E-03	1.95E-02
EIF6	0.0331	5.25E-01	6.48E-01
ELAC1	0.0066	9.00E-01	9.37E-01
ELAC2	-0.0770	1.39E-01	2.39E-01
ELANE	-0.0631	2.25E-01	3.47E-01
ELAVL1	0.0504	3.33E-01	4.64E-01
ELAVL2	-0.0954	6.64E-02	1.34E-01
ELAVL3	0.0751	1.49E-01	2.53E-01
ELAVL4	0.1171	2.41E-02	5.98E-02
ELF1	-0.0130	8.02E-01	8.70E-01
ELF2	0.0689	1.86E-01	2.99E-01
ELF3	0.1847	3.48E-04	1.77E-03
ELF4	0.1090	3.59E-02	8.26E-02
ELF5	0.0077	8.82E-01	9.25E-01
ELFN1	-0.2925	9.47E-09	1.77E-07
ELFN2	0.0679	1.92E-01	3.07E-01
ELK1	0.0408	4.33E-01	5.65E-01
ELK3	-0.1381	7.75E-03	2.37E-02
ELK4	0.0797	1.25E-01	2.21E-01
ELL2	-0.0802	1.23E-01	2.18E-01
ELL3	-0.0053	9.19E-01	9.49E-01
ELL	0.0451	3.86E-01	5.19E-01
ELMO1	-0.0822	1.14E-01	2.06E-01
ELMO2	0.0462	3.75E-01	5.08E-01
ELMO3	0.0089	8.64E-01	9.12E-01

ELMOD1	-0.0795	1.26E-01	2.22E-01
ELMOD2	-0.2177	2.34E-05	1.77E-04
ELMOD3	0.0418	4.22E-01	5.55E-01
ELN	-0.1743	7.48E-04	3.35E-03
ELOF1	-0.0803	1.23E-01	2.18E-01
ELOVL1	0.0989	5.71E-02	1.19E-01
ELOVL2	-0.0535	3.04E-01	4.33E-01
ELOVL3	0.1973	1.31E-04	7.69E-04
ELOVL4	0.0152	7.70E-01	8.45E-01
ELOVL5	-0.1682	1.15E-03	4.84E-03
ELOVL6	-0.1678	1.18E-03	4.93E-03
ELOVL7	0.1531	3.10E-03	1.11E-02
ELP2P	0.1607	1.90E-03	7.37E-03
ELP2	-0.0682	1.90E-01	3.04E-01
ELP3	-0.0534	3.05E-01	4.34E-01
ELP4	0.1771	6.12E-04	2.82E-03
ELSPBP1	0.0258	6.21E-01	7.30E-01
ELTD1	-0.2299	7.72E-06	6.69E-05
EMB	-0.0898	8.42E-02	1.62E-01
EMCN	-0.2648	2.27E-07	3.03E-06
EMD	0.1870	2.93E-04	1.53E-03
EME1	0.4485	9.33E-20	2.24E-17
EME2	0.1261	1.51E-02	4.09E-02
EMG1	0.1406	6.69E-03	2.10E-02
EMID1	0.1163	2.51E-02	6.17E-02
EMID2	-0.1824	4.13E-04	2.03E-03
EMILIN1	-0.1006	5.29E-02	1.12E-01
EMILIN2	0.0797	1.25E-01	2.21E-01
EMILIN3	0.1002	5.38E-02	1.14E-01
EML1	-0.1062	4.08E-02	9.17E-02
EML2	0.1420	6.13E-03	1.95E-02
EML3	-0.0856	9.99E-02	1.86E-01
EML4	0.1353	9.08E-03	2.71E-02
EML5	-0.0626	2.29E-01	3.52E-01
EML6	0.0822	1.14E-01	2.06E-01
EMP1	-0.2404	2.81E-06	2.80E-05
EMP2	-0.0245	6.38E-01	7.45E-01
EMP3	-0.0205	6.94E-01	7.89E-01
EMR1	-0.0932	7.28E-02	1.45E-01
EMR2	-0.0654	2.09E-01	3.27E-01
EMR3	0.0204	6.95E-01	7.89E-01
EMR4P	-0.0163	7.54E-01	8.35E-01
EMX1	0.0541	2.99E-01	4.28E-01
EMX2OS	-0.1157	2.58E-02	6.32E-02

EMX2	-0.0984	5.83E-02	1.21E-01
EN1	0.1278	1.37E-02	3.80E-02
EN2	0.1406	6.68E-03	2.10E-02
ENAH	0.2343	5.07E-06	4.69E-05
ENAM	0.1678	1.18E-03	4.95E-03
ENC1	0.0570	2.74E-01	4.02E-01
ENDOD1	-0.1256	1.55E-02	4.17E-02
ENDOG	-0.1705	9.79E-04	4.22E-03
ENDOU	-0.1171	2.41E-02	5.99E-02
ENGASE	0.2641	2.46E-07	3.27E-06
ENG	-0.2902	1.25E-08	2.26E-07
ENHO	-0.0538	3.01E-01	4.30E-01
ENKUR	-0.0807	1.21E-01	2.15E-01
ENO1	0.0623	2.32E-01	3.55E-01
ENO2	0.0976	6.03E-02	1.25E-01
ENO3	-0.2251	1.21E-05	9.88E-05
ENOPH1	0.1703	9.93E-04	4.28E-03
ENOSF1	-0.1378	7.87E-03	2.40E-02
ENOX1	-0.0412	4.29E-01	5.61E-01
ENOX2	0.1972	1.32E-04	7.76E-04
ENPEP	-0.3110	9.15E-10	2.19E-08
ENPP1	-0.0802	1.23E-01	2.18E-01
ENPP2	0.1124	3.04E-02	7.23E-02
ENPP3	-0.0300	5.65E-01	6.83E-01
ENPP4	0.0806	1.21E-01	2.16E-01
ENPP5	0.0720	1.66E-01	2.74E-01
ENPP6	0.0934	7.24E-02	1.44E-01
ENPP7	-0.1521	3.31E-03	1.17E-02
ENSA	0.4480	1.03E-19	2.42E-17
ENTHD1	0.0706	1.75E-01	2.86E-01
ENTPD1	-0.0312	5.49E-01	6.69E-01
ENTPD2	0.2220	1.60E-05	1.27E-04
ENTPD3	0.0715	1.69E-01	2.78E-01
ENTPD4	-0.0028	9.57E-01	9.73E-01
ENTPD5	-0.1916	2.05E-04	1.13E-03
ENTPD6	0.2056	6.63E-05	4.29E-04
ENTPD7	-0.0532	3.07E-01	4.36E-01
ENTPD8	-0.0866	9.57E-02	1.80E-01
ENY2	0.0024	9.64E-01	9.77E-01
EOMES	-0.1047	4.40E-02	9.71E-02
EP300	0.0342	5.12E-01	6.36E-01
EP400NL	0.0611	2.40E-01	3.64E-01
EP400	0.2152	2.91E-05	2.14E-04
EPAS1	-0.2533	7.71E-07	8.99E-06

EPB41L1	0.0794	1.27E-01	2.23E-01
EPB41L2	0.1169	2.44E-02	6.03E-02
EPB41L3	-0.1587	2.17E-03	8.25E-03
EPB41L4A	-0.0139	7.90E-01	8.61E-01
EPB41L4B	-0.0875	9.23E-02	1.75E-01
EPB41L5	-0.0386	4.58E-01	5.89E-01
EPB41	-0.0272	6.01E-01	7.14E-01
EPB42	-0.1950	1.57E-04	8.96E-04
EPB49	-0.0225	6.65E-01	7.65E-01
EPC1	0.0156	7.64E-01	8.41E-01
EPC2	0.0922	7.63E-02	1.50E-01
EPCAM	0.1729	8.24E-04	3.64E-03
EPDR1	-0.0625	2.30E-01	3.52E-01
EPGN	0.1607	1.91E-03	7.39E-03
EPHA10	0.2146	3.06E-05	2.23E-04
EPHA1	-0.0183	7.25E-01	8.14E-01
EPHA2	-0.1879	2.73E-04	1.44E-03
EPHA3	-0.0986	5.79E-02	1.21E-01
EPHA4	-0.1597	2.04E-03	7.82E-03
EPHA5	0.0575	2.69E-01	3.97E-01
EPHA6	0.1349	9.26E-03	2.75E-02
EPHA7	-0.1269	1.45E-02	3.96E-02
EPHA8	0.0921	7.66E-02	1.51E-01
EPHB1	-0.0370	4.77E-01	6.06E-01
EPHB2	-0.0286	5.83E-01	6.99E-01
EPHB3	0.1626	1.68E-03	6.66E-03
EPHB4	-0.0205	6.94E-01	7.89E-01
EPHB6	0.0903	8.24E-02	1.60E-01
EPHX1	-0.0738	1.56E-01	2.61E-01
EPHX2	-0.2023	8.71E-05	5.42E-04
EPHX3	0.0273	6.00E-01	7.13E-01
EPHX4	0.2372	3.84E-06	3.70E-05
EPM2AIP1	-0.1318	1.10E-02	3.16E-02
EPM2A	-0.1571	2.41E-03	8.98E-03
EPN1	-0.1603	1.96E-03	7.55E-03
EPN2	0.1221	1.87E-02	4.86E-02
EPN3	0.1452	5.09E-03	1.67E-02
EPOR	0.1431	5.75E-03	1.85E-02
EPO	0.1147	2.72E-02	6.60E-02
EPPK1	0.2449	1.80E-06	1.91E-05
EPR1	0.3466	6.51E-12	2.44E-10
EPRS	0.2655	2.09E-07	2.82E-06
EPS15L1	0.2458	1.66E-06	1.77E-05
EPS15	-0.0804	1.22E-01	2.17E-01

EPS8L1	0.1160	2.54E-02	6.23E-02
EPS8L2	0.1404	6.77E-03	2.12E-02
EPS8L3	0.2417	2.49E-06	2.52E-05
EPS8	-0.0827	1.12E-01	2.03E-01
EPSTI1	-0.0781	1.33E-01	2.32E-01
EPT1	-0.1038	4.57E-02	1.00E-01
EPX	-0.0012	9.82E-01	9.88E-01
EPYC	0.1171	2.41E-02	5.99E-02
ERAL1	0.1604	1.94E-03	7.52E-03
ERAP1	-0.1315	1.13E-02	3.22E-02
ERAP2	0.0990	5.67E-02	1.19E-01
ERAS	0.0895	8.52E-02	1.64E-01
ERBB2IP	-0.0681	1.91E-01	3.05E-01
ERBB2	-0.1507	3.61E-03	1.26E-02
ERBB3	0.1365	8.45E-03	2.55E-02
ERBB4	0.0130	8.02E-01	8.70E-01
ERC1	-0.0756	1.46E-01	2.49E-01
ERC2	0.0111	8.31E-01	8.89E-01
ERCC1	0.0587	2.60E-01	3.86E-01
ERCC2	0.0056	9.14E-01	9.46E-01
ERCC3	0.2285	8.81E-06	7.52E-05
ERCC4	-0.1074	3.88E-02	8.79E-02
ERCC5	-0.0149	7.75E-01	8.50E-01
ERCC6L	0.4189	3.36E-17	3.77E-15
ERCC6	0.1022	4.91E-02	1.06E-01
ERCC8	0.0378	4.67E-01	5.97E-01
EREG	-0.0113	8.28E-01	8.88E-01
ERF	0.0673	1.96E-01	3.12E-01
ERGIC1	-0.1971	1.33E-04	7.81E-04
ERGIC2	0.0484	3.53E-01	4.84E-01
ERGIC3	-0.0296	5.70E-01	6.88E-01
ERG	-0.2543	6.93E-07	8.17E-06
ERH	0.1882	2.67E-04	1.41E-03
ERI1	0.0818	1.16E-01	2.08E-01
ERI2	-0.0948	6.82E-02	1.37E-01
ERI3	0.1457	4.93E-03	1.63E-02
ERICH1	0.0494	3.43E-01	4.74E-01
ERLEC1	0.0696	1.81E-01	2.93E-01
ERLIN1	-0.1791	5.29E-04	2.49E-03
ERLIN2	-0.1203	2.04E-02	5.22E-02
ERMAP	-0.1763	6.45E-04	2.95E-03
ERMN	0.0475	3.61E-01	4.94E-01
ERMP1	-0.0043	9.34E-01	9.59E-01
ERN1	-0.0709	1.73E-01	2.83E-01

ERN2	0.0256	6.23E-01	7.32E-01
ERO1LB	0.1170	2.42E-02	5.99E-02
ERO1L	0.0149	7.75E-01	8.49E-01
ERP27	0.0799	1.24E-01	2.20E-01
ERP29	-0.1227	1.81E-02	4.73E-02
ERP44	-0.0491	3.45E-01	4.77E-01
ERRFI1	-0.1058	4.16E-02	9.30E-02
ERVFRDE1	-0.1258	1.53E-02	4.14E-02
ESAM	-0.1634	1.59E-03	6.36E-03
ESCO1	0.1313	1.14E-02	3.24E-02
ESCO2	0.3343	3.88E-11	1.25E-09
ESD	-0.1777	5.87E-04	2.72E-03
ESF1	0.0312	5.49E-01	6.69E-01
ESM1	0.1370	8.26E-03	2.50E-02
ESPL1	0.3916	4.78E-15	3.32E-13
ESPNL	0.0299	5.66E-01	6.83E-01
ESPNP	0.0154	7.67E-01	8.43E-01
ESPN	-0.1267	1.46E-02	3.98E-02
ESR1	-0.3346	3.70E-11	1.20E-09
ESR2	0.0690	1.85E-01	2.97E-01
ESRP1	0.1358	8.83E-03	2.64E-02
ESRP2	-0.2815	3.46E-08	5.60E-07
ESRRA	-0.1465	4.69E-03	1.56E-02
ESRRB	-0.0204	6.96E-01	7.90E-01
ESRRG	0.0592	2.56E-01	3.81E-01
ESX1	0.1509	3.57E-03	1.25E-02
ESYT1	-0.0825	1.13E-01	2.04E-01
ESYT2	-0.1280	1.36E-02	3.77E-02
ESYT3	0.2072	5.79E-05	3.84E-04
ETAA1	0.1000	5.44E-02	1.15E-01
ETF1	-0.0254	6.26E-01	7.35E-01
ETFA	-0.4218	1.96E-17	2.39E-15
ETFB	-0.1894	2.43E-04	1.30E-03
ETFDH	-0.4051	4.40E-16	3.84E-14
ETHE1	-0.1994	1.11E-04	6.64E-04
ETNK1	0.1352	9.12E-03	2.72E-02
ETNK2	-0.1253	1.57E-02	4.23E-02
ETS1	-0.1840	3.67E-04	1.85E-03
ETS2	-0.0779	1.34E-01	2.33E-01
ETV1	-0.0511	3.27E-01	4.57E-01
ETV2	0.0535	3.04E-01	4.33E-01
ETV3L	0.1369	8.28E-03	2.50E-02
ETV3	0.0498	3.38E-01	4.70E-01
ETV4	0.2145	3.10E-05	2.25E-04

ETV5	0.1354	9.01E-03	2.69E-02
ETV6	0.0930	7.36E-02	1.46E-01
ETV7	0.1203	2.05E-02	5.23E-02
EVC2	0.2704	1.23E-07	1.75E-06
EVC	0.1552	2.73E-03	9.98E-03
EVI2A	-0.0826	1.12E-01	2.03E-01
EVI2B	-0.0956	6.57E-02	1.34E-01
EVI5L	-0.0081	8.76E-01	9.20E-01
EVI5	-0.0899	8.37E-02	1.61E-01
EVL	-0.0340	5.14E-01	6.38E-01
EVPLL	0.0866	9.58E-02	1.80E-01
EVPL	0.1361	8.67E-03	2.60E-02
EVX1	0.1419	6.17E-03	1.96E-02
EWSR1	0.3975	1.70E-15	1.29E-13
EXD1	0.0806	1.21E-01	2.16E-01
EXD2	0.0008	9.88E-01	9.93E-01
EXD3	0.2122	3.78E-05	2.67E-04
EXO1	0.4900	8.30E-24	3.76E-21
EXOC1	0.0878	9.13E-02	1.73E-01
EXOC2	0.0651	2.11E-01	3.29E-01
EXOC3L2	-0.2125	3.67E-05	2.61E-04
EXOC3L	-0.1100	3.42E-02	7.96E-02
EXOC3	0.1733	8.05E-04	3.57E-03
EXOC4	-0.0875	9.23E-02	1.75E-01
EXOC5	-0.0248	6.34E-01	7.41E-01
EXOC6B	-0.0079	8.80E-01	9.23E-01
EXOC6	0.2256	1.15E-05	9.50E-05
EXOC7	0.1626	1.68E-03	6.65E-03
EXOC8	0.1669	1.25E-03	5.19E-03
EXOG	0.2557	5.97E-07	7.13E-06
EXOSC10	0.1658	1.35E-03	5.53E-03
EXOSC1	0.0520	3.18E-01	4.48E-01
EXOSC2	0.3440	9.54E-12	3.43E-10
EXOSC3	0.3174	3.95E-10	1.03E-08
EXOSC4	-0.0295	5.71E-01	6.89E-01
EXOSC5	0.0846	1.04E-01	1.91E-01
EXOSC6	-0.1716	9.05E-04	3.95E-03
EXOSC7	0.0526	3.12E-01	4.42E-01
EXOSC8	0.2012	9.54E-05	5.86E-04
EXOSC9	0.2163	2.64E-05	1.97E-04
EXPH5	-0.1702	9.95E-04	4.28E-03
EXT1	0.0218	6.76E-01	7.74E-01
EXT2	-0.0688	1.86E-01	2.99E-01
EXTL1	0.0015	9.77E-01	9.85E-01

EXTL2	0.0821	1.14E-01	2.06E-01
EXTL3	0.0678	1.92E-01	3.07E-01
EYA1	0.1484	4.19E-03	1.43E-02
EYA2	0.1071	3.93E-02	8.88E-02
EYA3	0.0110	8.33E-01	8.91E-01
EYA4	0.1013	5.12E-02	1.10E-01
EYS	-0.1207	2.01E-02	5.16E-02
EZH1	0.2647	2.30E-07	3.06E-06
EZH2	0.4526	3.89E-20	1.01E-17
EZR	0.1629	1.65E-03	6.55E-03
F10	-0.1067	4.00E-02	9.01E-02
F11R	0.0972	6.14E-02	1.27E-01
F11	-0.2648	2.27E-07	3.03E-06
F12	-0.1854	3.30E-04	1.69E-03
F13A1	-0.1212	1.95E-02	5.04E-02
F13B	-0.1679	1.17E-03	4.91E-03
F2RL1	0.0154	7.67E-01	8.43E-01
F2RL2	-0.0174	7.39E-01	8.24E-01
F2RL3	-0.0803	1.23E-01	2.18E-01
F2R	-0.1239	1.69E-02	4.49E-02
F2	-0.1084	3.68E-02	8.44E-02
F3	0.0101	8.46E-01	8.99E-01
F5	-0.0637	2.21E-01	3.41E-01
F7	-0.1335	1.00E-02	2.93E-02
F8A1	0.0322	5.37E-01	6.59E-01
F8	-0.1617	1.78E-03	6.99E-03
F9	-0.2262	1.08E-05	9.01E-05
FA2H	0.0562	2.81E-01	4.09E-01
FAAH2	-0.0813	1.18E-01	2.11E-01
FAAH	-0.1174	2.38E-02	5.91E-02
FABP12	0.0127	8.07E-01	8.73E-01
FABP1	-0.1297	1.24E-02	3.48E-02
FABP2	0.0042	9.36E-01	9.60E-01
FABP3	-0.0557	2.85E-01	4.14E-01
FABP4	-0.2232	1.43E-05	1.15E-04
FABP5L3	0.1819	4.29E-04	2.09E-03
FABP5	0.0864	9.67E-02	1.81E-01
FABP6	0.1712	9.32E-04	4.05E-03
FABP7	0.0522	3.16E-01	4.46E-01
FABP9	-0.0563	2.80E-01	4.08E-01
FADD	0.0215	6.80E-01	7.78E-01
FADS1	0.0370	4.77E-01	6.06E-01
FADS2	-0.0377	4.69E-01	5.98E-01
FADS3	0.0013	9.80E-01	9.87E-01

FADS6	0.0794	1.27E-01	2.23E-01
FAF1	0.1679	1.17E-03	4.90E-03
FAF2	0.1679	1.17E-03	4.91E-03
FAHD1	-0.1656	1.37E-03	5.59E-03
FAHD2A	-0.3804	3.22E-14	1.89E-12
FAHD2B	0.0878	9.14E-02	1.73E-01
FAH	-0.2679	1.62E-07	2.24E-06
FAIM2	-0.1137	2.86E-02	6.88E-02
FAIM3	-0.0055	9.15E-01	9.47E-01
FAIM	0.1136	2.87E-02	6.90E-02
FAM100A	-0.0919	7.72E-02	1.51E-01
FAM100B	0.1865	3.05E-04	1.58E-03
FAM101A	0.0528	3.10E-01	4.40E-01
FAM101B	-0.1390	7.33E-03	2.26E-02
FAM102A	0.0927	7.45E-02	1.47E-01
FAM102B	0.1792	5.24E-04	2.47E-03
FAM103A1	0.1423	6.04E-03	1.93E-02
FAM104A	0.3468	6.33E-12	2.38E-10
FAM104B	0.0530	3.08E-01	4.38E-01
FAM105A	0.0251	6.30E-01	7.37E-01
FAM105B	0.0802	1.23E-01	2.18E-01
FAM106A	0.0425	4.14E-01	5.47E-01
FAM106C	0.0510	3.27E-01	4.58E-01
FAM107A	-0.2726	9.58E-08	1.40E-06
FAM107B	-0.2114	4.04E-05	2.82E-04
FAM108A1	0.0498	3.39E-01	4.70E-01
FAM108B1	-0.0438	4.00E-01	5.33E-01
FAM108C1	0.1946	1.62E-04	9.19E-04
FAM109A	0.1732	8.05E-04	3.57E-03
FAM109B	0.0807	1.21E-01	2.15E-01
FAM10A4	0.0037	9.43E-01	9.65E-01
FAM110A	0.2325	6.03E-06	5.44E-05
FAM110B	0.0475	3.61E-01	4.93E-01
FAM110C	-0.0168	7.47E-01	8.30E-01
FAM111A	0.2575	4.95E-07	6.07E-06
FAM111B	0.3690	2.08E-13	1.05E-11
FAM113A	0.0551	2.90E-01	4.19E-01
FAM113B	0.0790	1.29E-01	2.26E-01
FAM114A1	-0.0976	6.05E-02	1.25E-01
FAM114A2	-0.1876	2.81E-04	1.47E-03
FAM115A	-0.1592	2.09E-03	8.01E-03
FAM115C	-0.0155	7.65E-01	8.42E-01
FAM116A	0.1610	1.86E-03	7.24E-03
FAM116B	0.0490	3.47E-01	4.79E-01

FAM117A	-0.1850	3.40E-04	1.73E-03
FAM117B	0.2256	1.15E-05	9.49E-05
FAM118A	0.1942	1.67E-04	9.45E-04
FAM118B	0.0598	2.51E-01	3.76E-01
FAM119A	0.2398	2.99E-06	2.96E-05
FAM119B	-0.0730	1.61E-01	2.67E-01
FAM120AOS	0.2140	3.23E-05	2.33E-04
FAM120A	-0.1216	1.91E-02	4.95E-02
FAM120B	0.0265	6.11E-01	7.22E-01
FAM120C	-0.0064	9.02E-01	9.38E-01
FAM122A	-0.0982	5.88E-02	1.22E-01
FAM122B	0.3343	3.89E-11	1.25E-09
FAM122C	0.1450	5.13E-03	1.69E-02
FAM123A	0.0161	7.57E-01	8.37E-01
FAM123B	0.0823	1.13E-01	2.05E-01
FAM123C	0.0558	2.84E-01	4.12E-01
FAM124A	0.0089	8.64E-01	9.12E-01
FAM124B	-0.1321	1.09E-02	3.12E-02
FAM125A	0.0780	1.34E-01	2.32E-01
FAM125B	0.0974	6.10E-02	1.26E-01
FAM126A	-0.0589	2.58E-01	3.84E-01
FAM126B	0.0523	3.15E-01	4.45E-01
FAM127A	0.1286	1.32E-02	3.67E-02
FAM127B	0.1820	4.26E-04	2.08E-03
FAM127C	0.0562	2.80E-01	4.09E-01
FAM128A	0.1706	9.69E-04	4.19E-03
FAM128B	0.1354	9.04E-03	2.70E-02
FAM129A	-0.1275	1.40E-02	3.86E-02
FAM129B	0.0750	1.49E-01	2.53E-01
FAM129C	0.0329	5.28E-01	6.51E-01
FAM131A	0.2313	6.77E-06	6.01E-05
FAM131B	-0.0459	3.78E-01	5.11E-01
FAM131C	0.0466	3.71E-01	5.04E-01
FAM132A	0.0319	5.41E-01	6.62E-01
FAM133A	0.1394	7.17E-03	2.22E-02
FAM133B	-0.0456	3.81E-01	5.15E-01
FAM134A	-0.0160	7.59E-01	8.38E-01
FAM134B	-0.1565	2.50E-03	9.27E-03
FAM134C	0.0291	5.76E-01	6.93E-01
FAM135A	0.0879	9.08E-02	1.72E-01
FAM135B	-0.0183	7.25E-01	8.13E-01
FAM136A	0.1716	9.03E-04	3.94E-03
FAM136B	0.1300	1.22E-02	3.44E-02
FAM138B	0.0755	1.47E-01	2.50E-01

FAM138D	0.0364	4.84E-01	6.12E-01
FAM138E	0.0487	3.49E-01	4.82E-01
FAM138F	0.0044	9.33E-01	9.58E-01
FAM13AOS	0.1608	1.89E-03	7.33E-03
FAM13A	-0.2406	2.75E-06	2.75E-05
FAM13B	0.0542	2.98E-01	4.27E-01
FAM13C	-0.2908	1.16E-08	2.12E-07
FAM149A	-0.2414	2.56E-06	2.57E-05
FAM149B1	-0.0624	2.31E-01	3.53E-01
FAM150A	0.1182	2.27E-02	5.70E-02
FAM150B	-0.0233	6.55E-01	7.57E-01
FAM151A	-0.1298	1.23E-02	3.47E-02
FAM151B	-0.1388	7.41E-03	2.28E-02
FAM153A	0.1955	1.50E-04	8.65E-04
FAM153B	0.1696	1.04E-03	4.44E-03
FAM153C	0.2021	8.85E-05	5.50E-04
FAM154A	-0.1004	5.33E-02	1.13E-01
FAM154B	0.0480	3.57E-01	4.89E-01
FAM155A	-0.1023	4.89E-02	1.06E-01
FAM155B	0.1663	1.30E-03	5.38E-03
FAM156A	0.3321	5.29E-11	1.66E-09
FAM157A	0.1926	1.90E-04	1.05E-03
FAM157B	0.0141	7.86E-01	8.58E-01
FAM158A	-0.0020	9.70E-01	9.81E-01
FAM159A	0.1986	1.18E-04	7.00E-04
FAM160A1	0.0798	1.25E-01	2.21E-01
FAM160A2	0.1165	2.48E-02	6.11E-02
FAM160B1	-0.1776	5.87E-04	2.72E-03
FAM160B2	0.1004	5.33E-02	1.13E-01
FAM161A	0.1737	7.78E-04	3.47E-03
FAM161B	0.0043	9.35E-01	9.59E-01
FAM162A	-0.1888	2.56E-04	1.36E-03
FAM162B	-0.0175	7.37E-01	8.22E-01
FAM163A	0.1508	3.59E-03	1.26E-02
FAM163B	-0.1708	9.59E-04	4.15E-03
FAM164A	0.1146	2.73E-02	6.62E-02
FAM164C	-0.1786	5.47E-04	2.57E-03
FAM165B	0.0948	6.82E-02	1.37E-01
FAM166A	0.2436	2.05E-06	2.13E-05
FAM166B	0.0358	4.92E-01	6.19E-01
FAM167A	-0.0113	8.29E-01	8.88E-01
FAM167B	-0.2426	2.27E-06	2.33E-05
FAM168A	-0.0430	4.09E-01	5.42E-01
FAM168B	0.1831	3.92E-04	1.95E-03

FAM169A	-0.0551	2.89E-01	4.18E-01
FAM169B	-0.1029	4.77E-02	1.04E-01
FAM170A	0.0369	4.79E-01	6.08E-01
FAM170B	0.0090	8.63E-01	9.11E-01
FAM171A1	-0.0119	8.20E-01	8.82E-01
FAM171A2	0.0989	5.70E-02	1.19E-01
FAM171B	0.0415	4.25E-01	5.58E-01
FAM172A	0.1165	2.49E-02	6.12E-02
FAM173A	-0.1527	3.19E-03	1.14E-02
FAM173B	-0.0960	6.47E-02	1.32E-01
FAM174A	-0.1827	4.06E-04	2.00E-03
FAM174B	-0.1144	2.76E-02	6.67E-02
FAM175A	0.0936	7.17E-02	1.43E-01
FAM175B	-0.0078	8.81E-01	9.24E-01
FAM176A	-0.2669	1.81E-07	2.48E-06
FAM176B	0.0915	7.84E-02	1.53E-01
FAM177A1	-0.1373	8.10E-03	2.46E-02
FAM177B	0.0058	9.11E-01	9.44E-01
FAM178A	0.1515	3.45E-03	1.21E-02
FAM178B	0.0727	1.62E-01	2.69E-01
FAM179A	-0.0388	4.57E-01	5.87E-01
FAM179B	-0.0264	6.12E-01	7.23E-01
FAM180A	-0.2087	5.10E-05	3.44E-04
FAM180B	-0.0158	7.61E-01	8.39E-01
FAM181A	0.0370	4.78E-01	6.06E-01
FAM181B	0.0132	7.99E-01	8.67E-01
FAM182A	0.0811	1.19E-01	2.13E-01
FAM182B	0.1793	5.21E-04	2.47E-03
FAM183A	0.1668	1.26E-03	5.23E-03
FAM183B	0.0433	4.06E-01	5.39E-01
FAM184A	0.0264	6.13E-01	7.23E-01
FAM184B	0.0850	1.02E-01	1.89E-01
FAM185A	-0.2248	1.23E-05	1.01E-04
FAM186A	0.2988	4.35E-09	8.83E-08
FAM186B	-0.2693	1.38E-07	1.95E-06
FAM187B	-0.0169	7.46E-01	8.28E-01
FAM188A	-0.1349	9.27E-03	2.75E-02
FAM188B	0.1899	2.35E-04	1.26E-03
FAM189A1	0.0263	6.13E-01	7.24E-01
FAM189A2	-0.0312	5.49E-01	6.70E-01
FAM189B	0.4295	4.34E-18	6.31E-16
FAM18A	0.1028	4.80E-02	1.04E-01
FAM18B2	-0.0083	8.74E-01	9.18E-01
FAM18B	-0.0984	5.83E-02	1.21E-01

FAM190A	-0.0084	8.72E-01	9.18E-01
FAM190B	-0.1983	1.20E-04	7.14E-04
FAM192A	-0.0273	6.00E-01	7.13E-01
FAM193A	0.2132	3.46E-05	2.48E-04
FAM193B	0.2326	5.99E-06	5.41E-05
FAM194A	0.0129	8.05E-01	8.71E-01
FAM195A	-0.0630	2.26E-01	3.48E-01
FAM195B	0.1192	2.16E-02	5.47E-02
FAM196A	0.0458	3.79E-01	5.12E-01
FAM196B	-0.1097	3.46E-02	8.03E-02
FAM197Y2	-0.0227	6.63E-01	7.64E-01
FAM198A	-0.0860	9.82E-02	1.83E-01
FAM198B	-0.2172	2.45E-05	1.84E-04
FAM199X	0.0880	9.07E-02	1.72E-01
FAM19A1	-0.2444	1.89E-06	1.99E-05
FAM19A2	-0.0393	4.51E-01	5.82E-01
FAM19A3	0.0922	7.62E-02	1.50E-01
FAM19A4	-0.0893	8.59E-02	1.65E-01
FAM19A5	0.0760	1.44E-01	2.46E-01
FAM200A	0.1359	8.76E-03	2.62E-02
FAM200B	0.0999	5.46E-02	1.15E-01
FAM20A	-0.0225	6.66E-01	7.66E-01
FAM20B	0.3565	1.47E-12	6.34E-11
FAM20C	-0.1458	4.89E-03	1.62E-02
FAM21A	0.0268	6.06E-01	7.19E-01
FAM21B	0.1244	1.65E-02	4.39E-02
FAM21C	0.1542	2.89E-03	1.05E-02
FAM22A	-0.0519	3.19E-01	4.49E-01
FAM22D	-0.0751	1.49E-01	2.53E-01
FAM22F	-0.0927	7.45E-02	1.47E-01
FAM22G	0.1808	4.65E-04	2.24E-03
FAM23A	-0.0898	8.40E-02	1.62E-01
FAM24A	-0.0256	6.23E-01	7.32E-01
FAM24B	0.2636	2.59E-07	3.43E-06
FAM25A	0.0124	8.11E-01	8.76E-01
FAM25B	0.0195	7.09E-01	8.01E-01
FAM26D	-0.0015	9.78E-01	9.86E-01
FAM26E	-0.1233	1.75E-02	4.61E-02
FAM26F	0.0205	6.94E-01	7.89E-01
FAM27A	0.1275	1.40E-02	3.85E-02
FAM27B	-0.0267	6.08E-01	7.20E-01
FAM27C	0.1211	1.96E-02	5.06E-02
FAM27L	0.0583	2.63E-01	3.89E-01
FAM32A	-0.0930	7.36E-02	1.46E-01

FAM35A	-0.0776	1.36E-01	2.35E-01
FAM35B2	-0.1284	1.33E-02	3.71E-02
FAM35B	-0.0984	5.83E-02	1.21E-01
FAM36A	0.1348	9.36E-03	2.77E-02
FAM38A	-0.1814	4.46E-04	2.16E-03
FAM38B	0.0443	3.95E-01	5.27E-01
FAM3A	0.0119	8.19E-01	8.81E-01
FAM3B	0.1134	2.89E-02	6.94E-02
FAM3C	-0.1685	1.12E-03	4.75E-03
FAM3D	0.0215	6.79E-01	7.77E-01
FAM40A	0.0427	4.12E-01	5.45E-01
FAM40B	0.1408	6.61E-03	2.08E-02
FAM41C	0.1589	2.14E-03	8.15E-03
FAM43A	-0.1181	2.29E-02	5.72E-02
FAM43B	-0.0742	1.54E-01	2.58E-01
FAM45A	0.0366	4.82E-01	6.10E-01
FAM45B	-0.0398	4.45E-01	5.77E-01
FAM46A	-0.0583	2.63E-01	3.90E-01
FAM46B	0.1007	5.26E-02	1.12E-01
FAM46C	-0.0436	4.02E-01	5.35E-01
FAM46D	0.1300	1.22E-02	3.44E-02
FAM47A	0.0779	1.34E-01	2.33E-01
FAM47B	0.0560	2.82E-01	4.11E-01
FAM47C	-0.0019	9.70E-01	9.81E-01
FAM47E	-0.0340	5.14E-01	6.39E-01
FAM48A	0.2265	1.06E-05	8.83E-05
FAM48B1	-0.0809	1.20E-01	2.14E-01
FAM48B2	-0.0819	1.15E-01	2.08E-01
FAM49A	-0.0414	4.27E-01	5.59E-01
FAM49B	0.1852	3.36E-04	1.72E-03
FAM50A	0.2242	1.30E-05	1.06E-04
FAM50B	-0.0027	9.58E-01	9.74E-01
FAM53A	0.0533	3.06E-01	4.35E-01
FAM53B	0.1135	2.89E-02	6.93E-02
FAM53C	0.0886	8.85E-02	1.69E-01
FAM54A	0.4432	2.81E-19	5.83E-17
FAM54B	-0.1765	6.40E-04	2.93E-03
FAM55A	0.0520	3.18E-01	4.48E-01
FAM55B	-0.0340	5.14E-01	6.38E-01
FAM55C	0.0761	1.43E-01	2.45E-01
FAM55D	0.0324	5.34E-01	6.56E-01
FAM57A	0.1810	4.58E-04	2.21E-03
FAM57B	0.2352	4.67E-06	4.38E-05
FAM58A	0.0750	1.49E-01	2.53E-01

FAM58B	0.0572	2.72E-01	3.99E-01
FAM59A	-0.0478	3.59E-01	4.91E-01
FAM5B	-0.0717	1.68E-01	2.77E-01
FAM5C	-0.0156	7.64E-01	8.41E-01
FAM60A	0.1366	8.44E-03	2.54E-02
FAM63A	0.0449	3.89E-01	5.22E-01
FAM63B	-0.0385	4.59E-01	5.90E-01
FAM64A	0.3359	3.11E-11	1.02E-09
FAM65A	-0.0877	9.16E-02	1.73E-01
FAM65B	-0.1210	1.97E-02	5.07E-02
FAM65C	-0.1034	4.65E-02	1.02E-01
FAM66A	0.0601	2.48E-01	3.73E-01
FAM66C	0.0728	1.61E-01	2.68E-01
FAM66D	0.0086	8.68E-01	9.15E-01
FAM66E	0.0894	8.55E-02	1.64E-01
FAM69A	-0.0938	7.11E-02	1.42E-01
FAM69B	0.0565	2.78E-01	4.07E-01
FAM69C	0.0571	2.73E-01	4.01E-01
FAM70A	0.0367	4.81E-01	6.10E-01
FAM70B	-0.1218	1.89E-02	4.91E-02
FAM71A	0.0485	3.51E-01	4.83E-01
FAM71C	0.0599	2.50E-01	3.75E-01
FAM71D	0.4551	2.31E-20	6.22E-18
FAM71E1	-0.0483	3.54E-01	4.86E-01
FAM71E2	0.1048	4.37E-02	9.67E-02
FAM71F1	-0.0290	5.78E-01	6.95E-01
FAM71F2	-0.0461	3.76E-01	5.09E-01
FAM72A	0.4028	6.58E-16	5.63E-14
FAM72B	0.4779	1.44E-22	5.41E-20
FAM72D	0.4913	6.17E-24	2.93E-21
FAM73A	-0.0685	1.88E-01	3.02E-01
FAM73B	0.1455	4.98E-03	1.64E-02
FAM74A1	-0.0082	8.75E-01	9.19E-01
FAM74A3	0.0069	8.95E-01	9.33E-01
FAM74A4	0.0136	7.94E-01	8.63E-01
FAM75A2	0.0825	1.12E-01	2.04E-01
FAM75A3	0.1003	5.36E-02	1.14E-01
FAM75A5	0.0186	7.21E-01	8.10E-01
FAM75A6	0.0067	8.97E-01	9.35E-01
FAM75C1	0.0634	2.23E-01	3.44E-01
FAM76A	0.0122	8.16E-01	8.79E-01
FAM76B	0.1699	1.02E-03	4.37E-03
FAM78A	-0.0380	4.65E-01	5.95E-01
FAM78B	-0.0538	3.01E-01	4.30E-01

FAM7A2	0.1245	1.64E-02	4.37E-02
FAM7A3	0.1585	2.20E-03	8.33E-03
FAM81A	0.2162	2.66E-05	1.98E-04
FAM81B	0.0352	4.99E-01	6.25E-01
FAM82A1	-0.2421	2.39E-06	2.43E-05
FAM82A2	-0.2423	2.34E-06	2.39E-05
FAM82B	-0.1950	1.57E-04	8.96E-04
FAM83A	0.0006	9.91E-01	9.95E-01
FAM83B	0.0829	1.11E-01	2.02E-01
FAM83C	0.0227	6.63E-01	7.64E-01
FAM83D	0.2457	1.67E-06	1.78E-05
FAM83E	0.0763	1.43E-01	2.44E-01
FAM83F	0.0768	1.40E-01	2.40E-01
FAM83G	0.0522	3.16E-01	4.46E-01
FAM83H	0.0879	9.10E-02	1.72E-01
FAM84A	-0.0173	7.40E-01	8.25E-01
FAM84B	-0.1334	1.01E-02	2.95E-02
FAM86A	0.0390	4.54E-01	5.85E-01
FAM86B1	0.0382	4.64E-01	5.94E-01
FAM86B2	0.0433	4.06E-01	5.39E-01
FAM86C	0.0214	6.81E-01	7.79E-01
FAM86D	0.0579	2.66E-01	3.93E-01
FAM89A	0.0694	1.82E-01	2.95E-01
FAM89B	0.0270	6.05E-01	7.17E-01
FAM8A1	-0.1475	4.42E-03	1.49E-02
FAM90A1	0.1372	8.13E-03	2.47E-02
FAM90A7	0.0567	2.76E-01	4.04E-01
FAM91A1	-0.0251	6.30E-01	7.38E-01
FAM92A1	-0.0177	7.34E-01	8.20E-01
FAM92A3	0.0746	1.51E-01	2.56E-01
FAM92B	-0.0101	8.46E-01	8.99E-01
FAM95B1	0.0635	2.22E-01	3.43E-01
FAM96A	-0.1476	4.39E-03	1.48E-02
FAM96B	-0.1116	3.16E-02	7.45E-02
FAM98A	-0.2272	9.94E-06	8.37E-05
FAM98B	0.0986	5.77E-02	1.20E-01
FAM98C	0.0047	9.29E-01	9.55E-01
FAM99A	-0.0960	6.48E-02	1.32E-01
FAM99B	-0.0766	1.41E-01	2.42E-01
FAM9A	0.0143	7.84E-01	8.56E-01
FAM9B	-0.2056	6.62E-05	4.29E-04
FAM9C	0.0927	7.44E-02	1.47E-01
FANCA	0.2265	1.06E-05	8.82E-05
FANCB	0.3831	2.06E-14	1.26E-12

FANCC	0.0685	1.88E-01	3.01E-01
FANCD2	0.4052	4.31E-16	3.78E-14
FANCE	0.4274	6.63E-18	9.24E-16
FANCF	0.1855	3.29E-04	1.69E-03
FANCG	0.4321	2.60E-18	4.07E-16
FANCI	0.4330	2.19E-18	3.49E-16
FANCL	0.2800	4.12E-08	6.56E-07
FANCM	0.1940	1.70E-04	9.58E-04
FANK1	0.0804	1.22E-01	2.17E-01
FAP	0.0052	9.20E-01	9.49E-01
FAR1	-0.0304	5.59E-01	6.77E-01
FAR2	0.0117	8.22E-01	8.83E-01
FARP1	0.0850	1.02E-01	1.88E-01
FARP2	-0.2782	5.08E-08	7.91E-07
FARS2	-0.1060	4.13E-02	9.24E-02
FARSA	-0.0093	8.58E-01	9.09E-01
FARSB	0.2370	3.93E-06	3.78E-05
FASLG	-0.0460	3.77E-01	5.11E-01
FASN	0.0393	4.51E-01	5.82E-01
FASTKD1	0.0258	6.20E-01	7.29E-01
FASTKD2	-0.0200	7.01E-01	7.94E-01
FASTKD3	0.1896	2.40E-04	1.29E-03
FASTKD5	-0.0420	4.19E-01	5.52E-01
FASTK	-0.0597	2.51E-01	3.76E-01
FAS	-0.2624	2.93E-07	3.82E-06
FAT1	-0.1376	7.94E-03	2.42E-02
FAT2	0.0830	1.11E-01	2.01E-01
FAT3	0.0981	5.89E-02	1.22E-01
FAT4	-0.2955	6.53E-09	1.27E-07
FATE1	0.1018	5.01E-02	1.08E-01
FAU	0.0005	9.92E-01	9.95E-01
FBF1	0.3242	1.58E-10	4.48E-09
FBLIM1	0.0893	8.60E-02	1.65E-01
FBLL1	0.1539	2.96E-03	1.07E-02
FBLN1	0.1604	1.94E-03	7.52E-03
FBLN2	-0.2022	8.81E-05	5.48E-04
FBLN5	-0.2495	1.14E-06	1.27E-05
FBLN7	-0.0580	2.65E-01	3.92E-01
FBL	0.2183	2.22E-05	1.69E-04
FBN1	-0.1390	7.33E-03	2.26E-02
FBN2	0.0706	1.75E-01	2.85E-01
FBN3	0.0332	5.23E-01	6.47E-01
FBP1	-0.2252	1.19E-05	9.80E-05
FBP2	0.1457	4.94E-03	1.63E-02

FBRSL1	0.1684	1.13E-03	4.76E-03
FBRS	0.1645	1.48E-03	5.96E-03
FBXL12	0.0628	2.27E-01	3.49E-01
FBXL13	-0.0172	7.41E-01	8.25E-01
FBXL14	-0.0315	5.45E-01	6.66E-01
FBXL15	-0.1338	9.90E-03	2.90E-02
FBXL16	0.0136	7.93E-01	8.63E-01
FBXL17	-0.1829	3.97E-04	1.97E-03
FBXL18	0.2577	4.85E-07	5.96E-06
FBXL19	0.1543	2.88E-03	1.04E-02
FBXL20	0.1256	1.55E-02	4.17E-02
FBXL21	0.1061	4.12E-02	9.23E-02
FBXL22	0.0122	8.15E-01	8.78E-01
FBXL2	0.1427	5.89E-03	1.88E-02
FBXL3	-0.0907	8.10E-02	1.57E-01
FBXL4	0.0475	3.62E-01	4.94E-01
FBXL5	-0.1926	1.90E-04	1.05E-03
FBXL6	0.0684	1.89E-01	3.02E-01
FBXL7	0.0540	3.00E-01	4.29E-01
FBXL8	-0.1660	1.33E-03	5.47E-03
FBXO10	0.1196	2.12E-02	5.37E-02
FBXO11	0.0697	1.80E-01	2.92E-01
FBXO15	0.0160	7.58E-01	8.38E-01
FBXO16	0.1163	2.51E-02	6.17E-02
FBXO17	-0.0116	8.24E-01	8.85E-01
FBXO18	0.0930	7.35E-02	1.46E-01
FBXO21	0.0567	2.76E-01	4.04E-01
FBXO22OS	0.0227	6.64E-01	7.64E-01
FBXO22	-0.0873	9.31E-02	1.76E-01
FBXO24	0.0279	5.92E-01	7.07E-01
FBXO25	-0.0989	5.70E-02	1.19E-01
FBXO27	-0.0414	4.26E-01	5.59E-01
FBXO28	0.0342	5.12E-01	6.36E-01
FBXO2	-0.1102	3.39E-02	7.90E-02
FBXO30	0.1981	1.23E-04	7.26E-04
FBXO31	-0.2601	3.78E-07	4.78E-06
FBXO32	-0.0918	7.73E-02	1.52E-01
FBXO33	-0.2309	7.00E-06	6.18E-05
FBXO34	0.0555	2.86E-01	4.15E-01
FBXO36	-0.1608	1.88E-03	7.33E-03
FBXO38	-0.0684	1.89E-01	3.02E-01
FBXO39	-0.0773	1.37E-01	2.37E-01
FBXO3	-0.1703	9.90E-04	4.26E-03
FBXO40	-0.0964	6.37E-02	1.30E-01

FBXO41	0.1715	9.14E-04	3.98E-03
FBXO42	-0.0152	7.70E-01	8.45E-01
FBXO43	0.4184	3.72E-17	4.14E-15
FBXO44	0.0016	9.75E-01	9.85E-01
FBXO45	0.2846	2.43E-08	4.09E-07
FBXO46	0.3425	1.20E-11	4.17E-10
FBXO47	0.1619	1.75E-03	6.89E-03
FBXO48	0.1063	4.08E-02	9.15E-02
FBXO4	0.1926	1.89E-04	1.05E-03
FBXO5	0.3624	5.86E-13	2.73E-11
FBXO6	-0.0257	6.22E-01	7.31E-01
FBXO7	-0.1925	1.91E-04	1.06E-03
FBXO8	-0.3854	1.38E-14	8.71E-13
FBXO9	0.1337	9.95E-03	2.91E-02
FBXW10	0.0113	8.28E-01	8.88E-01
FBXW11	-0.0395	4.48E-01	5.80E-01
FBXW12	0.0288	5.80E-01	6.96E-01
FBXW2	0.1593	2.08E-03	7.98E-03
FBXW4	-0.0417	4.23E-01	5.56E-01
FBXW5	-0.0629	2.27E-01	3.48E-01
FBXW7	-0.1159	2.57E-02	6.29E-02
FBXW8	0.0308	5.55E-01	6.74E-01
FBXW9	0.0668	1.99E-01	3.15E-01
FCAMR	-0.1570	2.43E-03	9.04E-03
FCAR	-0.1320	1.09E-02	3.13E-02
FCER1A	-0.0311	5.50E-01	6.70E-01
FCER1G	-0.0165	7.51E-01	8.32E-01
FCER2	0.0252	6.29E-01	7.37E-01
FCF1	-0.0728	1.62E-01	2.69E-01
FCGBP	0.1073	3.88E-02	8.80E-02
FCGR1A	0.0124	8.12E-01	8.76E-01
FCGR1B	0.0319	5.40E-01	6.61E-01
FCGR1C	0.0093	8.59E-01	9.09E-01
FCGR2A	0.0275	5.98E-01	7.11E-01
FCGR2B	-0.0833	1.09E-01	1.99E-01
FCGR2C	-0.0485	3.51E-01	4.83E-01
FCGR3A	-0.0278	5.93E-01	7.08E-01
FCGR3B	-0.0021	9.68E-01	9.80E-01
FCGRT	-0.2991	4.19E-09	8.57E-08
FCHO1	0.1597	2.04E-03	7.83E-03
FCHO2	-0.0934	7.22E-02	1.44E-01
FCHSD1	0.0480	3.56E-01	4.88E-01
FCHSD2	-0.0200	7.00E-01	7.94E-01
FCN1	-0.1311	1.15E-02	3.27E-02

FCN2	-0.1530	3.12E-03	1.12E-02
FCN3	-0.2335	5.46E-06	4.99E-05
FCRL1	0.0560	2.82E-01	4.11E-01
FCRL2	0.0612	2.40E-01	3.64E-01
FCRL3	0.0268	6.07E-01	7.19E-01
FCRL4	0.0598	2.51E-01	3.76E-01
FCRL5	0.0037	9.44E-01	9.65E-01
FCRL6	-0.1459	4.87E-03	1.61E-02
FCRLA	0.0545	2.95E-01	4.24E-01
FCRLB	-0.0658	2.06E-01	3.24E-01
FDFT1	-0.0152	7.70E-01	8.45E-01
FDPSL2A	0.2311	6.89E-06	6.10E-05
FDPS	0.2046	7.21E-05	4.62E-04
FDX1L	-0.0566	2.77E-01	4.05E-01
FDX1	-0.3401	1.69E-11	5.81E-10
FDXACB1	0.0144	7.83E-01	8.55E-01
FDXR	-0.0983	5.84E-02	1.21E-01
FECH	-0.1591	2.12E-03	8.09E-03
FEM1A	-0.1268	1.45E-02	3.96E-02
FEM1B	-0.0725	1.64E-01	2.71E-01
FEM1C	-0.1576	2.33E-03	8.75E-03
FEN1	0.4143	8.07E-17	8.33E-15
FER1L4	0.2196	1.97E-05	1.53E-04
FER1L5	0.1992	1.12E-04	6.69E-04
FER1L6	0.1375	8.00E-03	2.43E-02
FERMT1	0.1240	1.68E-02	4.46E-02
FERMT2	-0.1556	2.65E-03	9.74E-03
FERMT3	-0.0192	7.13E-01	8.04E-01
FER	0.0931	7.34E-02	1.46E-01
FES	0.0991	5.66E-02	1.18E-01
FETUB	-0.1553	2.71E-03	9.91E-03
FEV	0.0808	1.20E-01	2.15E-01
FEZ1	0.0209	6.89E-01	7.85E-01
FEZ2	-0.0305	5.59E-01	6.77E-01
FEZF1	0.0757	1.46E-01	2.48E-01
FEZF2	0.0752	1.48E-01	2.52E-01
FFAR1	0.0897	8.44E-02	1.62E-01
FFAR2	0.0783	1.32E-01	2.30E-01
FFAR3	0.0020	9.69E-01	9.81E-01
FGA	-0.2020	8.95E-05	5.56E-04
FGB	-0.1389	7.36E-03	2.27E-02
FGD1	0.2265	1.05E-05	8.82E-05
FGD2	-0.0645	2.15E-01	3.35E-01
FGD3	0.0642	2.17E-01	3.37E-01

FGD4	-0.1408	6.60E-03	2.08E-02
FGD5	-0.2337	5.38E-06	4.93E-05
FGD6	0.1332	1.02E-02	2.97E-02
FGF10	0.0632	2.24E-01	3.46E-01
FGF11	0.0392	4.52E-01	5.83E-01
FGF12	-0.0582	2.63E-01	3.90E-01
FGF13	0.0416	4.24E-01	5.56E-01
FGF14	-0.1157	2.58E-02	6.32E-02
FGF16	-0.0287	5.81E-01	6.98E-01
FGF17	0.0951	6.73E-02	1.36E-01
FGF18	-0.1287	1.31E-02	3.65E-02
FGF19	-0.0354	4.96E-01	6.22E-01
FGF1	-0.1678	1.18E-03	4.94E-03
FGF20	0.0062	9.06E-01	9.41E-01
FGF21	0.0129	8.04E-01	8.70E-01
FGF22	-0.0124	8.12E-01	8.76E-01
FGF23	-0.0115	8.25E-01	8.85E-01
FGF2	-0.0610	2.42E-01	3.66E-01
FGF3	0.0466	3.71E-01	5.04E-01
FGF4	0.0747	1.51E-01	2.55E-01
FGF5	0.1189	2.20E-02	5.54E-02
FGF7	-0.1893	2.46E-04	1.31E-03
FGF8	0.0162	7.56E-01	8.37E-01
FGF9	0.0483	3.53E-01	4.85E-01
FGFBP1	0.0182	7.27E-01	8.14E-01
FGFBP2	-0.1851	3.38E-04	1.73E-03
FGFBP3	-0.0335	5.20E-01	6.44E-01
FGFR1OP2	0.0664	2.02E-01	3.19E-01
FGFR1OP	0.0598	2.51E-01	3.76E-01
FGFR1	-0.0042	9.36E-01	9.60E-01
FGFR2	0.0638	2.20E-01	3.41E-01
FGFR3	0.2323	6.14E-06	5.52E-05
FGFR4	0.1544	2.86E-03	1.04E-02
FGFRL1	-0.1449	5.18E-03	1.70E-02
FGGY	-0.1532	3.09E-03	1.11E-02
FGG	-0.1506	3.65E-03	1.27E-02
FGL1	-0.1791	5.28E-04	2.49E-03
FGL2	-0.1496	3.87E-03	1.34E-02
FGR	-0.0855	1.00E-01	1.86E-01
FHAD1	0.1715	9.09E-04	3.96E-03
FHDC1	0.2006	9.99E-05	6.08E-04
FHIT	-0.0464	3.73E-01	5.06E-01
FHL1	-0.1684	1.13E-03	4.77E-03
FHL2	-0.0803	1.22E-01	2.18E-01

FHL3	0.1740	7.63E-04	3.41E-03
FHL5	-0.1820	4.27E-04	2.08E-03
FHOD1	-0.0001	9.98E-01	9.99E-01
FHOD3	0.2868	1.86E-08	3.24E-07
FH	-0.0934	7.24E-02	1.44E-01
FIBCD1	0.1585	2.20E-03	8.34E-03
FIBIN	-0.1207	2.01E-02	5.15E-02
FIBP	-0.0165	7.51E-01	8.32E-01
FICD	-0.0055	9.16E-01	9.47E-01
FIG4	0.2945	7.34E-09	1.41E-07
FIGF	-0.0237	6.49E-01	7.53E-01
FIGLA	0.0046	9.29E-01	9.55E-01
FIGNL1	0.2930	8.81E-09	1.66E-07
FIGNL2	0.2155	2.85E-05	2.10E-04
FIGN	0.0637	2.21E-01	3.42E-01
FILIP1L	-0.1197	2.11E-02	5.37E-02
FILIP1	-0.0653	2.09E-01	3.28E-01
FIP1L1	0.2911	1.12E-08	2.05E-07
FIS1	-0.2114	4.05E-05	2.83E-04
FITM1	-0.1230	1.78E-02	4.67E-02
FITM2	-0.2410	2.65E-06	2.66E-05
FIZ1	0.1472	4.51E-03	1.52E-02
FJX1	-0.0446	3.91E-01	5.24E-01
FKBP10	-0.0019	9.71E-01	9.81E-01
FKBP11	0.0417	4.23E-01	5.56E-01
FKBP14	-0.0042	9.36E-01	9.60E-01
FKBP15	0.1648	1.44E-03	5.85E-03
FKBP1AP1	0.1662	1.31E-03	5.41E-03
FKBP1A	0.0840	1.06E-01	1.95E-01
FKBP1B	-0.0578	2.67E-01	3.95E-01
FKBP2	-0.0937	7.14E-02	1.42E-01
FKBP3	0.0055	9.16E-01	9.47E-01
FKBP4	0.0320	5.39E-01	6.61E-01
FKBP5	-0.0911	7.98E-02	1.56E-01
FKBP6	0.0874	9.27E-02	1.75E-01
FKBP7	-0.1346	9.43E-03	2.79E-02
FKBP8	-0.0015	9.78E-01	9.86E-01
FKBP9L	0.0170	7.44E-01	8.27E-01
FKBP9	0.0308	5.55E-01	6.74E-01
FKBPL	0.1346	9.43E-03	2.79E-02
FKRP	-0.0023	9.65E-01	9.79E-01
FKSG29	0.1129	2.97E-02	7.09E-02
FKSG83	0.0337	5.18E-01	6.42E-01
FKTN	0.2225	1.52E-05	1.21E-04

FLAD1	0.3952	2.54E-15	1.86E-13
FLCN	0.0807	1.21E-01	2.16E-01
FLG2	0.0359	4.91E-01	6.18E-01
FLG	0.0048	9.26E-01	9.53E-01
FLI1	-0.1525	3.22E-03	1.15E-02
FLII	-0.0102	8.45E-01	8.99E-01
FLJ10038	-0.0497	3.40E-01	4.71E-01
FLJ10213	0.0646	2.15E-01	3.34E-01
FLJ10357	0.0881	9.02E-02	1.71E-01
FLJ10661	0.0530	3.08E-01	4.38E-01
FLJ11235	-0.1227	1.81E-02	4.74E-02
FLJ12825	0.1534	3.06E-03	1.10E-02
FLJ13197	0.1216	1.91E-02	4.95E-02
FLJ13224	-0.1299	1.23E-02	3.45E-02
FLJ14107	0.0773	1.37E-01	2.37E-01
FLJ16779	0.0675	1.94E-01	3.10E-01
FLJ22536	0.0797	1.25E-01	2.21E-01
FLJ23867	0.1226	1.81E-02	4.74E-02
FLJ25328	0.1486	4.11E-03	1.41E-02
FLJ25363	0.1405	6.71E-03	2.10E-02
FLJ25758	0.1250	1.60E-02	4.28E-02
FLJ26850	0.1234	1.74E-02	4.58E-02
FLJ30679	-0.1078	3.80E-02	8.66E-02
FLJ32063	0.0012	9.81E-01	9.88E-01
FLJ33360	0.1349	9.30E-03	2.76E-02
FLJ33630	-0.1307	1.18E-02	3.33E-02
FLJ34503	0.0644	2.16E-01	3.36E-01
FLJ35024	0.0773	1.37E-01	2.37E-01
FLJ35220	-0.1023	4.90E-02	1.06E-01
FLJ35390	-0.0331	5.25E-01	6.48E-01
FLJ35776	0.0807	1.21E-01	2.15E-01
FLJ36000	0.0859	9.86E-02	1.84E-01
FLJ36031	-0.1586	2.19E-03	8.30E-03
FLJ36777	0.0809	1.20E-01	2.14E-01
FLJ37201	0.1783	5.61E-04	2.62E-03
FLJ37307	-0.0201	7.00E-01	7.93E-01
FLJ37453	-0.0473	3.63E-01	4.96E-01
FLJ37543	0.0288	5.80E-01	6.96E-01
FLJ39582	-0.0806	1.21E-01	2.16E-01
FLJ39609	-0.0901	8.31E-02	1.60E-01
FLJ39653	0.2496	1.12E-06	1.26E-05
FLJ39739	0.1441	5.43E-03	1.77E-02
FLJ40292	0.2297	7.87E-06	6.80E-05
FLJ40330	0.0917	7.76E-02	1.52E-01

FLJ40504	0.0278	5.93E-01	7.08E-01
FLJ40852	-0.0679	1.92E-01	3.06E-01
FLJ41941	0.0817	1.16E-01	2.09E-01
FLJ42289	-0.0598	2.50E-01	3.76E-01
FLJ42393	0.0912	7.94E-02	1.55E-01
FLJ42627	0.2079	5.48E-05	3.66E-04
FLJ42709	-0.1182	2.28E-02	5.71E-02
FLJ42875	0.0610	2.41E-01	3.66E-01
FLJ43390	0.0706	1.75E-01	2.85E-01
FLJ43663	-0.0287	5.82E-01	6.98E-01
FLJ43859	-0.0297	5.68E-01	6.86E-01
FLJ43860	0.1235	1.73E-02	4.57E-02
FLJ43950	0.0282	5.88E-01	7.03E-01
FLJ44054	0.0259	6.20E-01	7.29E-01
FLJ44606	0.1643	1.49E-03	6.02E-03
FLJ44635	-0.0319	5.41E-01	6.62E-01
FLJ45079	0.0109	8.34E-01	8.91E-01
FLJ45244	0.0099	8.49E-01	9.01E-01
FLJ45340	0.0353	4.98E-01	6.24E-01
FLJ45445	0.1759	6.64E-04	3.03E-03
FLJ45983	0.0160	7.59E-01	8.38E-01
FLJ46111	-0.0155	7.66E-01	8.42E-01
FLJ46321	0.1592	2.11E-03	8.04E-03
FLJ46361	-0.0693	1.83E-01	2.95E-01
FLJ90757	0.1582	2.24E-03	8.47E-03
FLNA	0.0624	2.31E-01	3.53E-01
FLNB	0.1348	9.32E-03	2.76E-02
FLNC	0.0718	1.68E-01	2.76E-01
FLOT1	0.0921	7.63E-02	1.50E-01
FLOT2	0.0405	4.36E-01	5.68E-01
FLRT1	0.1824	4.13E-04	2.03E-03
FLRT2	-0.0346	5.07E-01	6.32E-01
FLRT3	-0.1748	7.21E-04	3.25E-03
FLT1	-0.1886	2.59E-04	1.37E-03
FLT3LG	-0.0919	7.70E-02	1.51E-01
FLT3	-0.1589	2.14E-03	8.15E-03
FLT4	-0.2106	4.35E-05	3.01E-04
FLVCR1	0.3201	2.75E-10	7.46E-09
FLVCR2	-0.1628	1.65E-03	6.56E-03
FLYWCH1	0.1059	4.15E-02	9.28E-02
FLYWCH2	0.1829	3.99E-04	1.98E-03
FMN1	0.0882	8.97E-02	1.71E-01
FMN2	0.0900	8.35E-02	1.61E-01
FMNL1	0.0290	5.77E-01	6.94E-01

FMNL2	0.0286	5.83E-01	6.99E-01
FMNL3	0.0063	9.04E-01	9.40E-01
FMO1	0.1417	6.24E-03	1.98E-02
FMO2	-0.2047	7.14E-05	4.58E-04
FMO3	-0.1245	1.64E-02	4.38E-02
FMO4	-0.1933	1.79E-04	1.00E-03
FMO5	-0.0548	2.92E-01	4.21E-01
FMO6P	0.0144	7.82E-01	8.55E-01
FMO9P	0.0122	8.15E-01	8.79E-01
FMOD	-0.0246	6.37E-01	7.44E-01
FMR1NB	0.1435	5.62E-03	1.81E-02
FMR1	0.1181	2.29E-02	5.73E-02
FN1	-0.0740	1.55E-01	2.60E-01
FN3KRP	0.2887	1.49E-08	2.66E-07
FN3K	-0.1181	2.29E-02	5.74E-02
FNBP1L	0.1180	2.30E-02	5.76E-02
FNBP1	0.1692	1.07E-03	4.54E-03
FNBP4	0.2875	1.72E-08	3.04E-07
FNDC1	-0.0280	5.91E-01	7.05E-01
FNDC3A	-0.2017	9.12E-05	5.64E-04
FNDC3B	0.1362	8.64E-03	2.59E-02
FNDC4	-0.1626	1.68E-03	6.65E-03
FNDC5	-0.1466	4.66E-03	1.55E-02
FNDC7	0.1427	5.88E-03	1.88E-02
FNDC8	0.2298	7.78E-06	6.73E-05
FNIP1	-0.1943	1.66E-04	9.40E-04
FNIP2	-0.1246	1.63E-02	4.35E-02
FNTA	0.1462	4.79E-03	1.59E-02
FNTB	0.1097	3.47E-02	8.05E-02
FOLH1B	-0.2983	4.65E-09	9.37E-08
FOLH1	-0.2479	1.33E-06	1.46E-05
FOLR1	0.2007	9.95E-05	6.06E-04
FOLR2	-0.1329	1.04E-02	3.01E-02
FOLR3	0.0762	1.43E-01	2.45E-01
FOLR4	0.1107	3.30E-02	7.72E-02
FOSB	-0.1910	2.15E-04	1.17E-03
FOSL1	-0.1200	2.07E-02	5.29E-02
FOSL2	-0.0939	7.08E-02	1.42E-01
FOS	-0.1466	4.67E-03	1.56E-02
FOXA1	0.0803	1.23E-01	2.18E-01
FOXA2	0.0235	6.51E-01	7.55E-01
FOXA3	0.0899	8.38E-02	1.61E-01
FOXB1	0.0128	8.06E-01	8.72E-01
FOXC1	-0.0744	1.53E-01	2.57E-01

FOXC2	-0.0298	5.67E-01	6.85E-01
FOXD1	0.1559	2.61E-03	9.59E-03
FOXD2	0.1711	9.40E-04	4.08E-03
FOXD3	0.1942	1.68E-04	9.48E-04
FOXD4L1	0.1984	1.19E-04	7.09E-04
FOXD4L2	0.0717	1.68E-01	2.77E-01
FOXD4L3	0.0690	1.85E-01	2.98E-01
FOXD4L5	0.0059	9.10E-01	9.43E-01
FOXD4L6	0.1393	7.20E-03	2.23E-02
FOXD4	0.1434	5.66E-03	1.83E-02
FOXE1	0.0712	1.71E-01	2.81E-01
FOXE3	0.0882	8.99E-02	1.71E-01
FOXF1	-0.1473	4.47E-03	1.51E-02
FOXF2	0.0895	8.51E-02	1.63E-01
FOXG1	0.1082	3.73E-02	8.54E-02
FOXH1	0.0835	1.08E-01	1.98E-01
FOXI1	0.1625	1.69E-03	6.68E-03
FOXI2	-0.0609	2.42E-01	3.66E-01
FOXI3	0.2151	2.94E-05	2.15E-04
FOXJ1	0.2735	8.71E-08	1.28E-06
FOXJ2	0.1038	4.57E-02	1.00E-01
FOXJ3	0.1834	3.83E-04	1.91E-03
FOXK1	0.2950	6.96E-09	1.34E-07
FOXK2	0.3789	4.11E-14	2.37E-12
FOXL1	0.0077	8.83E-01	9.25E-01
FOXL2	0.1113	3.20E-02	7.53E-02
FOXM1	0.3945	2.87E-15	2.08E-13
FOXN1	0.1544	2.86E-03	1.04E-02
FOXN2	0.1033	4.67E-02	1.02E-01
FOXN3	-0.1095	3.49E-02	8.09E-02
FOXN4	0.1060	4.13E-02	9.25E-02
FOXO1	-0.2019	9.00E-05	5.58E-04
FOXO3B	0.1675	1.20E-03	5.01E-03
FOXO3	0.1451	5.11E-03	1.68E-02
FOXO4	-0.0663	2.03E-01	3.20E-01
FOXP1	0.1677	1.19E-03	4.97E-03
FOXP2	-0.1540	2.94E-03	1.06E-02
FOXP3	-0.0799	1.25E-01	2.20E-01
FOXP4	0.0891	8.66E-02	1.66E-01
FOXQ1	0.2714	1.10E-07	1.58E-06
FOXR1	-0.0020	9.69E-01	9.80E-01
FOXR2	0.0942	7.00E-02	1.40E-01
FOXRED1	-0.1096	3.48E-02	8.06E-02
FOXRED2	0.0408	4.33E-01	5.65E-01

FOXS1	0.0413	4.27E-01	5.60E-01
FPGS	0.0589	2.58E-01	3.84E-01
FPGT	0.0148	7.76E-01	8.50E-01
FPR1	-0.0561	2.82E-01	4.10E-01
FPR2	-0.1307	1.17E-02	3.32E-02
FPR3	-0.0830	1.11E-01	2.01E-01
FRAS1	0.1399	6.95E-03	2.16E-02
FRAT1	0.0068	8.96E-01	9.34E-01
FRAT2	0.1092	3.54E-02	8.18E-02
FREM1	0.0830	1.11E-01	2.01E-01
FREM2	-0.0055	9.16E-01	9.47E-01
FRG1B	0.0469	3.67E-01	5.00E-01
FRG1	-0.0632	2.25E-01	3.46E-01
FRG2B	0.0963	6.40E-02	1.31E-01
FRG2C	0.0627	2.28E-01	3.51E-01
FRG2	0.0320	5.38E-01	6.60E-01
FRK	-0.0450	3.88E-01	5.21E-01
FRMD1	0.1238	1.70E-02	4.51E-02
FRMD3	-0.1350	9.25E-03	2.75E-02
FRMD4A	0.1197	2.11E-02	5.36E-02
FRMD4B	-0.0868	9.50E-02	1.79E-01
FRMD5	0.1271	1.43E-02	3.91E-02
FRMD6	-0.0601	2.48E-01	3.73E-01
FRMD7	-0.1127	2.99E-02	7.14E-02
FRMD8	0.2203	1.85E-05	1.44E-04
FRMPD1	-0.0231	6.58E-01	7.59E-01
FRMPD2L1	0.0785	1.31E-01	2.29E-01
FRMPD2	0.1187	2.22E-02	5.60E-02
FRMPD4	-0.0321	5.38E-01	6.60E-01
FRRS1	-0.1240	1.69E-02	4.47E-02
FRS2	0.1421	6.12E-03	1.95E-02
FRS3	0.2320	6.31E-06	5.66E-05
FRYL	0.0288	5.81E-01	6.97E-01
FRY	-0.1245	1.64E-02	4.37E-02
FRZB	-0.0802	1.23E-01	2.18E-01
FSCB	0.0851	1.02E-01	1.88E-01
FSCN1	-0.0017	9.73E-01	9.83E-01
FSCN2	0.1085	3.67E-02	8.42E-02
FSCN3	0.0169	7.46E-01	8.28E-01
FSD1L	0.2670	1.78E-07	2.44E-06
FSD1	-0.0836	1.08E-01	1.98E-01
FSD2	-0.0539	3.00E-01	4.29E-01
FSHR	-0.1401	6.89E-03	2.15E-02
FSIP1	0.0893	8.60E-02	1.65E-01

FSTL1	-0.1274	1.40E-02	3.86E-02
FSTL3	0.0581	2.64E-01	3.91E-01
FSTL4	0.0277	5.94E-01	7.08E-01
FSTL5	0.0430	4.09E-01	5.42E-01
FST	-0.1791	5.29E-04	2.49E-03
FTCD	-0.1176	2.35E-02	5.87E-02
FTH1	0.0246	6.37E-01	7.43E-01
FTHL17	0.1336	9.96E-03	2.91E-02
FTHL3	0.0924	7.56E-02	1.49E-01
FTL	-0.0307	5.56E-01	6.75E-01
FTMT	0.0461	3.76E-01	5.10E-01
FTO	-0.3079	1.37E-09	3.16E-08
FTSJ1	0.2644	2.38E-07	3.16E-06
FTSJ2	0.1662	1.31E-03	5.41E-03
FTSJ3	0.2813	3.54E-08	5.72E-07
FTSJD1	-0.1470	4.56E-03	1.53E-02
FTSJD2	0.0473	3.64E-01	4.96E-01
FUBP1	0.2754	6.99E-08	1.05E-06
FUBP3	0.0400	4.42E-01	5.74E-01
FUCA1	-0.2565	5.48E-07	6.61E-06
FUCA2	0.0473	3.64E-01	4.96E-01
FUK	-0.1568	2.45E-03	9.11E-03
FUNDC1	0.2245	1.26E-05	1.03E-04
FUNDC2P2	0.0621	2.33E-01	3.56E-01
FUNDC2	0.2533	7.68E-07	8.96E-06
FURIN	-0.0725	1.64E-01	2.71E-01
FUS	0.3324	5.11E-11	1.61E-09
FUT10	0.0929	7.39E-02	1.46E-01
FUT11	-0.0247	6.35E-01	7.42E-01
FUT1	0.1887	2.56E-04	1.36E-03
FUT2	0.2820	3.28E-08	5.35E-07
FUT3	0.1530	3.14E-03	1.12E-02
FUT4	0.2491	1.18E-06	1.31E-05
FUT5	-0.1226	1.82E-02	4.75E-02
FUT6	-0.0638	2.20E-01	3.41E-01
FUT7	0.0689	1.86E-01	2.99E-01
FUT8	0.0485	3.52E-01	4.84E-01
FUT9	0.0944	6.93E-02	1.39E-01
FUZ	-0.0385	4.60E-01	5.91E-01
FXC1	0.0634	2.23E-01	3.44E-01
FXN	-0.2031	8.16E-05	5.13E-04
FXR1	0.1158	2.58E-02	6.31E-02
FXR2	-0.0589	2.58E-01	3.83E-01
FXYD1	-0.1440	5.46E-03	1.77E-02

FXYD2	0.0165	7.51E-01	8.32E-01
FXYD3	0.3384	2.16E-11	7.31E-10
FXYD4	0.0988	5.74E-02	1.20E-01
FXYD5	0.0160	7.59E-01	8.38E-01
FXYD6	-0.2163	2.64E-05	1.97E-04
FXYD7	0.0385	4.60E-01	5.91E-01
FYB	-0.0681	1.91E-01	3.05E-01
FYCO1	-0.1255	1.56E-02	4.19E-02
FYN	-0.2979	4.85E-09	9.73E-08
FYTTD1	-0.1385	7.55E-03	2.32E-02
FZD10	0.0031	9.53E-01	9.71E-01
FZD1	0.1445	5.30E-03	1.73E-02
FZD2	0.0635	2.22E-01	3.43E-01
FZD3	0.0412	4.29E-01	5.61E-01
FZD4	-0.1945	1.63E-04	9.26E-04
FZD5	0.1401	6.86E-03	2.14E-02
FZD6	0.0276	5.96E-01	7.10E-01
FZD7	0.1426	5.92E-03	1.90E-02
FZD8	0.0074	8.87E-01	9.28E-01
FZD9	0.1208	2.00E-02	5.13E-02
FZR1	-0.0222	6.71E-01	7.70E-01
G0S2	-0.1149	2.69E-02	6.53E-02
G2E3	0.2165	2.59E-05	1.93E-04
G3BP1	-0.0930	7.36E-02	1.46E-01
G3BP2	-0.0352	4.99E-01	6.25E-01
G6PC2	-0.0237	6.50E-01	7.53E-01
G6PC3	0.1830	3.95E-04	1.96E-03
G6PC	-0.1510	3.54E-03	1.24E-02
G6PD	0.2855	2.17E-08	3.72E-07
GAA	-0.1044	4.45E-02	9.80E-02
GAB1	-0.0785	1.31E-01	2.29E-01
GAB2	-0.0308	5.55E-01	6.74E-01
GAB3	-0.0983	5.85E-02	1.22E-01
GAB4	0.0963	6.39E-02	1.31E-01
GABARAPL1	-0.2800	4.15E-08	6.60E-07
GABARAPL2	-0.2036	7.84E-05	4.96E-04
GABARAPL3	-0.1217	1.90E-02	4.93E-02
GABARAP	-0.0865	9.63E-02	1.81E-01
GABBR1	0.0827	1.12E-01	2.03E-01
GABBR2	0.0262	6.14E-01	7.24E-01
GABPA	0.0843	1.05E-01	1.93E-01
GABPB1	0.1017	5.02E-02	1.08E-01
GABPB2	0.4028	6.68E-16	5.68E-14
GABRA1	-0.0166	7.49E-01	8.31E-01

GABRA2	0.0402	4.40E-01	5.72E-01
GABRA3	0.0872	9.35E-02	1.76E-01
GABRA4	0.0202	6.98E-01	7.92E-01
GABRA5	0.0352	4.99E-01	6.25E-01
GABRA6	0.1095	3.51E-02	8.11E-02
GABRB1	0.0573	2.71E-01	3.99E-01
GABRB2	-0.0659	2.06E-01	3.23E-01
GABRB3	-0.0390	4.54E-01	5.85E-01
GABRD	0.0824	1.13E-01	2.05E-01
GABRE	0.1547	2.82E-03	1.03E-02
GABRG1	0.0423	4.16E-01	5.49E-01
GABRG2	-0.0040	9.39E-01	9.62E-01
GABRG3	0.0576	2.69E-01	3.96E-01
GABRP	-0.0174	7.38E-01	8.23E-01
GABRQ	0.0376	4.70E-01	5.99E-01
GABRR1	0.1751	7.07E-04	3.19E-03
GABRR2	0.0192	7.13E-01	8.04E-01
GABRR3	-0.0228	6.62E-01	7.63E-01
GAD1	0.1734	7.99E-04	3.55E-03
GAD2	0.0717	1.68E-01	2.77E-01
GADD45A	-0.2485	1.26E-06	1.39E-05
GADD45B	-0.1998	1.06E-04	6.41E-04
GADD45GIP1	-0.0370	4.77E-01	6.06E-01
GADD45G	-0.1381	7.72E-03	2.36E-02
GADL1	-0.0638	2.20E-01	3.41E-01
GAGE10	0.0188	7.19E-01	8.09E-01
GAGE12D	0.1406	6.67E-03	2.09E-02
GAGE12F	0.0585	2.61E-01	3.87E-01
GAGE12J	0.1611	1.85E-03	7.23E-03
GAGE13	0.1122	3.08E-02	7.29E-02
GAGE1	0.1069	3.96E-02	8.95E-02
GAGE2A	0.1258	1.54E-02	4.15E-02
GAGE2B	0.1140	2.81E-02	6.79E-02
GAGE2C	0.1015	5.07E-02	1.09E-01
GAGE2D	0.0918	7.73E-02	1.52E-01
GAGE2E	0.0930	7.36E-02	1.46E-01
GAGE4	0.1461	4.80E-03	1.60E-02
GAGE8	0.0834	1.09E-01	1.98E-01
GAK	-0.0505	3.32E-01	4.63E-01
GAL3ST1	0.2193	2.02E-05	1.56E-04
GAL3ST2	0.0993	5.60E-02	1.18E-01
GAL3ST3	0.1295	1.26E-02	3.52E-02
GAL3ST4	0.0304	5.59E-01	6.77E-01
GALC	0.1450	5.14E-03	1.69E-02

GALE	-0.0036	9.45E-01	9.66E-01
GALK1	-0.0474	3.62E-01	4.95E-01
GALK2	-0.0848	1.03E-01	1.89E-01
GALM	-0.1300	1.22E-02	3.43E-02
GALNS	-0.1729	8.23E-04	3.63E-03
GALNT10	-0.0935	7.20E-02	1.44E-01
GALNT11	-0.0741	1.54E-01	2.59E-01
GALNT12	0.0656	2.07E-01	3.25E-01
GALNT13	-0.0552	2.89E-01	4.18E-01
GALNT14	-0.0398	4.45E-01	5.77E-01
GALNT1	-0.0350	5.02E-01	6.28E-01
GALNT2	-0.0482	3.55E-01	4.87E-01
GALNT3	0.0350	5.02E-01	6.27E-01
GALNT4	0.0809	1.20E-01	2.14E-01
GALNT5	0.0281	5.89E-01	7.04E-01
GALNT6	-0.0124	8.12E-01	8.77E-01
GALNT7	-0.0510	3.27E-01	4.58E-01
GALNT8	0.0467	3.69E-01	5.02E-01
GALNT9	0.0210	6.87E-01	7.83E-01
GALNTL1	-0.2029	8.29E-05	5.21E-04
GALNTL2	-0.3499	4.02E-12	1.60E-10
GALNTL4	0.0626	2.29E-01	3.51E-01
GALNTL5	0.0006	9.91E-01	9.94E-01
GALNTL6	-0.0871	9.38E-02	1.77E-01
GALP	-0.0702	1.77E-01	2.88E-01
GALR1	-0.0011	9.84E-01	9.90E-01
GALR2	-0.0020	9.69E-01	9.81E-01
GALR3	-0.1019	4.98E-02	1.07E-01
GALT	-0.0784	1.32E-01	2.29E-01
GAL	0.0209	6.89E-01	7.85E-01
GAMT	-0.1777	5.86E-04	2.71E-03
GANAB	-0.1048	4.37E-02	9.68E-02
GANC	-0.1645	1.48E-03	5.97E-03
GAN	-0.1280	1.36E-02	3.77E-02
GAP43	0.1018	5.02E-02	1.08E-01
GAPDHS	0.0884	8.92E-02	1.70E-01
GAPDH	0.0270	6.05E-01	7.17E-01
GAPT	-0.0031	9.52E-01	9.70E-01
GAPVD1	0.1382	7.70E-03	2.36E-02
GAR1	0.1319	1.10E-02	3.16E-02
GARNL3	-0.0671	1.97E-01	3.13E-01
GARS	-0.0163	7.54E-01	8.35E-01
GART	0.0790	1.29E-01	2.26E-01
GAS1	-0.1075	3.85E-02	8.75E-02

GAS2L1	-0.0102	8.45E-01	8.99E-01
GAS2L2	0.0409	4.32E-01	5.64E-01
GAS2L3	0.1420	6.14E-03	1.95E-02
GAS2	-0.0455	3.82E-01	5.15E-01
GAS5	0.3086	1.26E-09	2.92E-08
GAS6	-0.1256	1.55E-02	4.17E-02
GAS7	0.0279	5.93E-01	7.07E-01
GAS8	-0.1169	2.44E-02	6.03E-02
GAST	0.1291	1.28E-02	3.59E-02
GATA1	-0.1646	1.46E-03	5.91E-03
GATA2	-0.1072	3.90E-02	8.83E-02
GATA3	-0.0602	2.47E-01	3.72E-01
GATA4	0.1542	2.90E-03	1.05E-02
GATA5	0.0340	5.14E-01	6.38E-01
GATA6	-0.0863	9.70E-02	1.82E-01
GATAD1	-0.0775	1.36E-01	2.36E-01
GATAD2A	-0.0567	2.76E-01	4.05E-01
GATAD2B	0.4261	8.54E-18	1.17E-15
GATC	-0.0124	8.12E-01	8.76E-01
GATM	-0.1636	1.57E-03	6.29E-03
GATSL1	-0.0191	7.14E-01	8.05E-01
GATSL2	-0.0777	1.35E-01	2.34E-01
GATSL3	-0.0759	1.45E-01	2.47E-01
GATS	-0.0702	1.77E-01	2.88E-01
GBA2	0.1492	3.99E-03	1.37E-02
GBA3	-0.2487	1.23E-06	1.36E-05
GBAP1	0.2742	8.05E-08	1.20E-06
GBAS	0.0678	1.93E-01	3.08E-01
GBA	0.2610	3.43E-07	4.40E-06
GBE1	-0.1919	2.00E-04	1.10E-03
GBF1	-0.0124	8.13E-01	8.77E-01
GBGT1	0.0214	6.80E-01	7.78E-01
GBP1	-0.0742	1.54E-01	2.59E-01
GBP2	0.0280	5.91E-01	7.05E-01
GBP3	-0.1059	4.14E-02	9.26E-02
GBP4	-0.0565	2.78E-01	4.06E-01
GBP5	-0.0281	5.89E-01	7.04E-01
GBP6	-0.0427	4.12E-01	5.45E-01
GBP7	-0.0855	1.00E-01	1.86E-01
GBX1	0.1046	4.40E-02	9.72E-02
GBX2	-0.0153	7.69E-01	8.44E-01
GCAT	-0.2054	6.76E-05	4.37E-04
GCA	0.0523	3.15E-01	4.45E-01
GCC1	-0.1501	3.77E-03	1.31E-02

GCC2	0.0802	1.23E-01	2.18E-01
GCDH	-0.3472	5.95E-12	2.26E-10
GCET2	0.0748	1.51E-01	2.55E-01
GCFC1	0.3493	4.35E-12	1.71E-10
GCGR	-0.1818	4.32E-04	2.10E-03
GCG	0.1093	3.53E-02	8.16E-02
GCH1	-0.1577	2.32E-03	8.71E-03
GCHFR	-0.1744	7.43E-04	3.34E-03
GCKR	-0.0795	1.26E-01	2.22E-01
GCK	-0.1463	4.76E-03	1.58E-02
GCLC	-0.1681	1.15E-03	4.85E-03
GCLM	0.0646	2.14E-01	3.34E-01
GCM1	0.0873	9.30E-02	1.76E-01
GCM2	0.0152	7.70E-01	8.45E-01
GCN1L1	0.2311	6.91E-06	6.11E-05
GCNT1	0.0718	1.68E-01	2.76E-01
GCNT2	-0.0462	3.75E-01	5.08E-01
GCNT3	0.1628	1.65E-03	6.57E-03
GCNT4	0.0471	3.66E-01	4.98E-01
GCNT7	0.0441	3.97E-01	5.30E-01
GCOM1	0.0272	6.01E-01	7.14E-01
GCSH	-0.2661	1.96E-07	2.67E-06
GC	-0.0993	5.60E-02	1.17E-01
GDAP1L1	0.0837	1.08E-01	1.97E-01
GDAP1	0.1291	1.28E-02	3.58E-02
GDAP2	0.1190	2.18E-02	5.52E-02
GDA	-0.0538	3.01E-01	4.31E-01
GDE1	-0.2052	6.82E-05	4.41E-04
GDEP	-0.0014	9.79E-01	9.86E-01
GDF10	0.0789	1.29E-01	2.26E-01
GDF11	-0.0149	7.75E-01	8.49E-01
GDF15	0.0081	8.76E-01	9.20E-01
GDF1	0.1929	1.85E-04	1.03E-03
GDF2	-0.1690	1.09E-03	4.61E-03
GDF3	-0.0986	5.77E-02	1.20E-01
GDF5	-0.0597	2.51E-01	3.76E-01
GDF6	-0.1107	3.31E-02	7.74E-02
GDF7	-0.2440	1.98E-06	2.08E-05
GDF9	0.0266	6.10E-01	7.21E-01
GDI1	0.2319	6.39E-06	5.72E-05
GDI2	-0.0586	2.60E-01	3.86E-01
GDNF	-0.0003	9.96E-01	9.97E-01
GDPD1	0.1662	1.31E-03	5.41E-03
GDPD2	0.0799	1.25E-01	2.20E-01

GDPD3	0.2438	2.02E-06	2.11E-05
GDPD4	-0.0429	4.10E-01	5.43E-01
GDPD5	0.0572	2.71E-01	3.99E-01
GEFT	-0.0255	6.25E-01	7.33E-01
GEMIN4	0.0951	6.72E-02	1.36E-01
GEMIN5	0.1361	8.67E-03	2.60E-02
GEMIN6	0.0532	3.07E-01	4.36E-01
GEMIN7	0.1661	1.32E-03	5.44E-03
GEMIN8P4	0.1201	2.07E-02	5.28E-02
GEMIN8	0.0630	2.26E-01	3.48E-01
GEM	-0.0946	6.86E-02	1.38E-01
GEN1	0.1585	2.20E-03	8.33E-03
GET4	0.1430	5.80E-03	1.86E-02
GFAP	0.0487	3.50E-01	4.82E-01
GFER	0.0505	3.32E-01	4.63E-01
GFI1B	-0.0408	4.34E-01	5.66E-01
GFI1	0.0135	7.96E-01	8.64E-01
GFM1	-0.1723	8.62E-04	3.78E-03
GFM2	-0.1459	4.87E-03	1.61E-02
GFOD1	-0.2223	1.54E-05	1.23E-04
GFOD2	-0.2633	2.67E-07	3.52E-06
GFPT1	0.0211	6.85E-01	7.82E-01
GFPT2	-0.0162	7.55E-01	8.35E-01
GFRA1	-0.1525	3.23E-03	1.15E-02
GFRA2	-0.2069	5.95E-05	3.92E-04
GFRA3	0.2270	1.01E-05	8.46E-05
GFRA4	-0.0028	9.57E-01	9.73E-01
GFRAL	0.0136	7.94E-01	8.64E-01
GGA1	0.1850	3.40E-04	1.73E-03
GGA2	0.0641	2.18E-01	3.38E-01
GGA3	0.3431	1.09E-11	3.85E-10
GGCT	0.1490	4.02E-03	1.38E-02
GGCX	-0.2238	1.35E-05	1.09E-04
GGH	-0.0335	5.20E-01	6.44E-01
GGNBP1	0.0779	1.34E-01	2.33E-01
GGNBP2	0.3170	4.18E-10	1.09E-08
GGN	0.0437	4.02E-01	5.34E-01
GGPS1	0.2511	9.65E-07	1.10E-05
GGT1	0.0097	8.52E-01	9.04E-01
GGT3P	0.0222	6.70E-01	7.70E-01
GGT5	-0.1825	4.09E-04	2.02E-03
GGT6	0.2252	1.19E-05	9.77E-05
GGT7	-0.0044	9.33E-01	9.58E-01
GGT8P	0.0846	1.04E-01	1.91E-01

GGTA1	-0.1606	1.91E-03	7.42E-03
GGTLC1	0.0374	4.73E-01	6.02E-01
GGTLC2	0.0185	7.22E-01	8.12E-01
GH1	0.0093	8.58E-01	9.08E-01
GH2	0.0111	8.31E-01	8.89E-01
GHDC	-0.0570	2.74E-01	4.02E-01
GHITM	-0.3456	7.63E-12	2.81E-10
GHRHR	-0.0177	7.34E-01	8.20E-01
GHRH	-0.0380	4.65E-01	5.95E-01
GHRLOS	0.2009	9.78E-05	5.99E-04
GHRL	0.1381	7.73E-03	2.36E-02
GHR	-0.2532	7.77E-07	9.04E-06
GHSR	0.0325	5.32E-01	6.54E-01
GIF	0.0479	3.57E-01	4.89E-01
GIGYF1	0.2566	5.47E-07	6.61E-06
GIGYF2	0.1310	1.15E-02	3.28E-02
GIMAP1	-0.1721	8.74E-04	3.83E-03
GIMAP2	-0.1376	7.97E-03	2.43E-02
GIMAP4	-0.1359	8.78E-03	2.63E-02
GIMAP5	-0.0656	2.08E-01	3.26E-01
GIMAP6	-0.2104	4.43E-05	3.05E-04
GIMAP7	-0.1794	5.17E-04	2.45E-03
GIMAP8	-0.2495	1.13E-06	1.26E-05
GIN1	-0.0843	1.05E-01	1.93E-01
GINS1	0.4101	1.77E-16	1.73E-14
GINS2	0.3406	1.57E-11	5.41E-10
GINS3	0.2513	9.47E-07	1.08E-05
GINS4	0.3324	5.07E-11	1.60E-09
GIPC1	-0.0022	9.67E-01	9.79E-01
GIPC2	0.0819	1.15E-01	2.08E-01
GIPC3	-0.1379	7.82E-03	2.39E-02
GIPR	0.1325	1.06E-02	3.07E-02
GIP	0.0835	1.08E-01	1.98E-01
GIT1	0.3534	2.36E-12	9.74E-11
GIT2	0.1514	3.47E-03	1.22E-02
GIYD2	-0.0361	4.88E-01	6.15E-01
GJA10	-0.0172	7.41E-01	8.25E-01
GJA1	-0.1111	3.25E-02	7.62E-02
GJA3	0.0919	7.71E-02	1.51E-01
GJA4	-0.1589	2.15E-03	8.16E-03
GJA5	-0.1475	4.42E-03	1.49E-02
GJA8	-0.0666	2.01E-01	3.17E-01
GJA9	0.1626	1.68E-03	6.65E-03
GJB1	-0.0718	1.67E-01	2.76E-01

GJB2	-0.1392	7.23E-03	2.23E-02
GJB3	0.0732	1.60E-01	2.66E-01
GJB4	-0.0032	9.50E-01	9.69E-01
GJB5	-0.0335	5.20E-01	6.44E-01
GJB6	0.0960	6.48E-02	1.32E-01
GJB7	0.1215	1.92E-02	4.97E-02
GJC1	0.0741	1.54E-01	2.59E-01
GJC2	0.0210	6.87E-01	7.83E-01
GJC3	-0.1820	4.27E-04	2.08E-03
GJD2	0.0054	9.18E-01	9.48E-01
GJD3	-0.1074	3.87E-02	8.78E-02
GJD4	0.1210	1.97E-02	5.08E-02
GK2	0.1998	1.07E-04	6.45E-04
GK3P	-0.2100	4.58E-05	3.14E-04
GK5	0.2308	7.11E-06	6.25E-05
GKAP1	0.2915	1.07E-08	1.97E-07
GKN1	0.0360	4.89E-01	6.16E-01
GKN2	-0.0569	2.75E-01	4.03E-01
GK	-0.2211	1.72E-05	1.35E-04
GLA	0.1436	5.60E-03	1.81E-02
GLB1L2	0.0900	8.34E-02	1.61E-01
GLB1L3	0.0982	5.89E-02	1.22E-01
GLB1L	0.0509	3.28E-01	4.59E-01
GLB1	-0.1207	2.00E-02	5.14E-02
GLCCI1	0.0020	9.70E-01	9.81E-01
GLCE	0.1189	2.20E-02	5.56E-02
GLDC	-0.0127	8.07E-01	8.73E-01
GLDN	0.1462	4.78E-03	1.59E-02
GLE1	0.2324	6.08E-06	5.48E-05
GLG1	-0.0580	2.65E-01	3.93E-01
GLI1	0.0641	2.18E-01	3.38E-01
GLI2	0.0080	8.79E-01	9.22E-01
GLI3	-0.1131	2.94E-02	7.02E-02
GLI4	0.0670	1.98E-01	3.14E-01
GLIPR1L1	0.1420	6.16E-03	1.96E-02
GLIPR1L2	-0.1250	1.60E-02	4.28E-02
GLIPR1	-0.0809	1.20E-01	2.14E-01
GLIPR2	-0.0405	4.37E-01	5.68E-01
GLIS1	-0.0020	9.69E-01	9.81E-01
GLIS2	0.1749	7.17E-04	3.23E-03
GLIS3	0.0368	4.80E-01	6.09E-01
GLMN	0.3110	9.19E-10	2.19E-08
GLO1	0.0989	5.70E-02	1.19E-01
GLOD4	-0.1721	8.76E-04	3.84E-03

GLOD5	-0.2183	2.22E-05	1.69E-04
GLP1R	0.1295	1.26E-02	3.52E-02
GLP2R	-0.0810	1.19E-01	2.13E-01
GLRA1	-0.0427	4.12E-01	5.45E-01
GLRA2	-0.0069	8.94E-01	9.33E-01
GLRA3	-0.0118	8.20E-01	8.82E-01
GLRA4	-0.0183	7.25E-01	8.13E-01
GLRB	0.1015	5.07E-02	1.09E-01
GLRX2	0.0169	7.46E-01	8.29E-01
GLRX3	0.0501	3.36E-01	4.67E-01
GLRX5	-0.1731	8.16E-04	3.61E-03
GLRX	-0.1944	1.64E-04	9.30E-04
GLS2	-0.1843	3.60E-04	1.82E-03
GLS	0.1776	5.89E-04	2.72E-03
GLT1D1	-0.0671	1.98E-01	3.14E-01
GLT25D1	0.1177	2.33E-02	5.82E-02
GLT25D2	0.0449	3.89E-01	5.22E-01
GLT6D1	0.0484	3.53E-01	4.84E-01
GLT8D1	-0.2017	9.13E-05	5.64E-04
GLT8D2	-0.1932	1.82E-04	1.01E-03
GLTPD1	-0.2303	7.44E-06	6.50E-05
GLTPD2	-0.1602	1.97E-03	7.61E-03
GLTP	0.1019	4.98E-02	1.07E-01
GLTSCR1	0.1874	2.84E-04	1.49E-03
GLTSCR2	0.0303	5.61E-01	6.79E-01
GLUD1	-0.2212	1.72E-05	1.35E-04
GLUD2	-0.1489	4.04E-03	1.39E-02
GLUL	-0.0059	9.10E-01	9.43E-01
GLYATL1	-0.3036	2.39E-09	5.18E-08
GLYATL2	0.1815	4.44E-04	2.15E-03
GLYATL3	0.0114	8.26E-01	8.86E-01
GLYAT	-0.3111	9.15E-10	2.19E-08
GLYCTK	-0.1234	1.74E-02	4.58E-02
GLYR1	-0.1237	1.71E-02	4.53E-02
GM2A	-0.0008	9.89E-01	9.93E-01
GMCL1L	0.1682	1.15E-03	4.84E-03
GMCL1	0.0261	6.17E-01	7.27E-01
GMDS	-0.1384	7.58E-03	2.32E-02
GMEB1	0.2611	3.40E-07	4.36E-06
GMEB2	0.3444	8.98E-12	3.27E-10
GMFB	0.1005	5.31E-02	1.13E-01
GMFG	-0.0731	1.60E-01	2.66E-01
GMIP	0.1783	5.61E-04	2.62E-03
GML	0.0476	3.61E-01	4.93E-01

GMNN	0.3162	4.68E-10	1.20E-08
GMPPA	0.0833	1.09E-01	1.99E-01
GMPPB	-0.0969	6.22E-02	1.28E-01
GMPR2	-0.1876	2.79E-04	1.46E-03
GMPR	-0.0971	6.17E-02	1.27E-01
GMPS	0.3528	2.58E-12	1.06E-10
GNA11	-0.0637	2.21E-01	3.41E-01
GNA12	-0.0190	7.15E-01	8.06E-01
GNA13	0.1489	4.05E-03	1.39E-02
GNA14	-0.2836	2.72E-08	4.51E-07
GNA15	-0.0441	3.97E-01	5.30E-01
GNAI1	-0.0516	3.21E-01	4.52E-01
GNAI2	-0.1083	3.70E-02	8.48E-02
GNAI3	0.0904	8.21E-02	1.59E-01
GNAL	0.0292	5.75E-01	6.92E-01
GNAO1	-0.3022	2.85E-09	6.06E-08
GNAQ	0.0229	6.61E-01	7.62E-01
GNASAS	0.1107	3.31E-02	7.74E-02
GNAS	0.1201	2.07E-02	5.27E-02
GNAT1	0.0320	5.39E-01	6.60E-01
GNAT2	-0.1812	4.52E-04	2.19E-03
GNAT3	-0.1344	9.55E-03	2.82E-02
GNAZ	0.2258	1.13E-05	9.35E-05
GNB1L	0.0383	4.62E-01	5.92E-01
GNB1	-0.0363	4.86E-01	6.14E-01
GNB2L1	0.0148	7.77E-01	8.50E-01
GNB2	-0.0776	1.36E-01	2.35E-01
GNB3	0.2156	2.80E-05	2.07E-04
GNB4	-0.0860	9.81E-02	1.83E-01
GNB5	0.0359	4.91E-01	6.18E-01
GNE	-0.2169	2.50E-05	1.88E-04
GNG10	0.0396	4.47E-01	5.78E-01
GNG11	-0.1750	7.13E-04	3.22E-03
GNG12	-0.1258	1.53E-02	4.13E-02
GNG13	0.1591	2.12E-03	8.08E-03
GNG2	-0.0988	5.73E-02	1.20E-01
GNG3	-0.0601	2.48E-01	3.73E-01
GNG4	0.1842	3.61E-04	1.82E-03
GNG5	0.1561	2.57E-03	9.47E-03
GNG7	-0.1581	2.26E-03	8.53E-03
GNG8	0.1262	1.50E-02	4.07E-02
GNGT1	0.0816	1.17E-01	2.10E-01
GNGT2	-0.0553	2.88E-01	4.17E-01
GNL1	0.0675	1.94E-01	3.10E-01

GNL2	0.1869	2.95E-04	1.54E-03
GNL3L	0.1150	2.68E-02	6.51E-02
GNL3	0.1622	1.72E-03	6.78E-03
GNLY	-0.0374	4.72E-01	6.01E-01
GNMT	-0.0650	2.12E-01	3.31E-01
GNPAT	0.1971	1.33E-04	7.78E-04
GNPDA1	0.1658	1.35E-03	5.52E-03
GNPDA2	0.1731	8.15E-04	3.60E-03
GNPNAT1	-0.1776	5.90E-04	2.73E-03
GNPTAB	-0.0178	7.33E-01	8.19E-01
GNPTG	-0.2313	6.75E-06	6.00E-05
GNRH1	0.2700	1.28E-07	1.81E-06
GNRH2	-0.1051	4.31E-02	9.57E-02
GNRHR2	0.1528	3.17E-03	1.13E-02
GNRHR	0.0624	2.31E-01	3.53E-01
GNS	-0.1204	2.03E-02	5.20E-02
GOLGA1	0.0174	7.38E-01	8.23E-01
GOLGA2B	0.3154	5.15E-10	1.30E-08
GOLGA2P3	0.0579	2.66E-01	3.93E-01
GOLGA2	0.0551	2.90E-01	4.19E-01
GOLGA3	0.1647	1.46E-03	5.90E-03
GOLGA4	-0.1077	3.81E-02	8.68E-02
GOLGA5	-0.2032	8.12E-05	5.11E-04
GOLGA6A	-0.1340	9.78E-03	2.87E-02
GOLGA6B	-0.1702	9.97E-04	4.29E-03
GOLGA6C	-0.1669	1.25E-03	5.19E-03
GOLGA6D	-0.0857	9.93E-02	1.85E-01
GOLGA6L10	0.1542	2.90E-03	1.05E-02
GOLGA6L1	0.0849	1.03E-01	1.89E-01
GOLGA6L5	0.0908	8.06E-02	1.57E-01
GOLGA6L6	0.0770	1.39E-01	2.39E-01
GOLGA6L9	0.3144	5.88E-10	1.47E-08
GOLGA7B	0.1634	1.59E-03	6.35E-03
GOLGA7	0.0865	9.60E-02	1.80E-01
GOLGA8A	0.2281	9.13E-06	7.76E-05
GOLGA8B	0.2993	4.10E-09	8.40E-08
GOLGA8C	0.2196	1.98E-05	1.53E-04
GOLGA8DP	0.1615	1.81E-03	7.08E-03
GOLGA8E	0.2069	5.95E-05	3.92E-04
GOLGA8F	0.0639	2.19E-01	3.40E-01
GOLGA8G	0.0534	3.05E-01	4.35E-01
GOLGA9P	0.1030	4.75E-02	1.03E-01
GOLGB1	-0.0093	8.58E-01	9.08E-01
GOLIM4	-0.0478	3.58E-01	4.90E-01

GOLM1	0.0761	1.43E-01	2.45E-01
GOLPH3L	0.4945	2.84E-24	1.49E-21
GOLPH3	-0.0015	9.77E-01	9.86E-01
GOLT1A	0.0251	6.30E-01	7.37E-01
GOLT1B	0.1975	1.29E-04	7.56E-04
GON4L	0.3905	5.80E-15	3.94E-13
GOPC	0.0851	1.02E-01	1.88E-01
GORAB	0.3054	1.90E-09	4.24E-08
GORASP1	-0.0520	3.18E-01	4.48E-01
GORASP2	0.1473	4.46E-03	1.50E-02
GOSR1	0.1506	3.63E-03	1.27E-02
GOSR2	0.2006	1.00E-04	6.10E-04
GOT1L1	0.0504	3.33E-01	4.64E-01
GOT1	-0.2398	2.97E-06	2.94E-05
GOT2	-0.3071	1.52E-09	3.47E-08
GP1BA	0.0047	9.28E-01	9.55E-01
GP2	0.0896	8.48E-02	1.63E-01
GP5	-0.0600	2.49E-01	3.74E-01
GP6	-0.1678	1.18E-03	4.94E-03
GP9	-0.1045	4.43E-02	9.77E-02
GPA33	-0.0227	6.63E-01	7.64E-01
GPAA1	0.0584	2.62E-01	3.89E-01
GPAM	-0.0768	1.40E-01	2.40E-01
GPAT2	-0.0409	4.32E-01	5.65E-01
GPATCH1	0.3586	1.07E-12	4.76E-11
GPATCH2	0.3056	1.84E-09	4.13E-08
GPATCH3	0.0458	3.79E-01	5.12E-01
GPATCH4	0.2976	5.02E-09	1.00E-07
GPATCH8	0.1977	1.27E-04	7.46E-04
GPBAR1	-0.0875	9.25E-02	1.75E-01
GPBP1L1	-0.0669	1.99E-01	3.15E-01
GPBP1	0.2652	2.18E-07	2.93E-06
GPC1	0.0294	5.72E-01	6.89E-01
GPC2	0.1591	2.12E-03	8.08E-03
GPC3	0.2303	7.39E-06	6.46E-05
GPC4	0.0694	1.82E-01	2.95E-01
GPC5	0.1112	3.23E-02	7.58E-02
GPC6	0.0682	1.90E-01	3.04E-01
GPCPD1	-0.0452	3.85E-01	5.19E-01
GPD1L	0.1184	2.26E-02	5.67E-02
GPD1	-0.3384	2.18E-11	7.35E-10
GPD2	0.2912	1.10E-08	2.03E-07
GPER	-0.1325	1.06E-02	3.07E-02
GPHA2	0.1277	1.39E-02	3.83E-02

GPHN	-0.2433	2.12E-06	2.19E-05
GPIHBP1	-0.1588	2.15E-03	8.18E-03
GPI	-0.0756	1.46E-01	2.49E-01
GPKOW	0.1951	1.55E-04	8.88E-04
GPLD1	-0.1662	1.31E-03	5.41E-03
GPM6A	-0.2523	8.49E-07	9.80E-06
GPM6B	0.0169	7.46E-01	8.28E-01
GPN1	0.1906	2.21E-04	1.20E-03
GPN2	0.0142	7.85E-01	8.57E-01
GPN3	0.0834	1.09E-01	1.99E-01
GPNMB	-0.0936	7.16E-02	1.43E-01
GPR101	-0.0042	9.35E-01	9.60E-01
GPR107	0.2296	7.89E-06	6.81E-05
GPR108	-0.0413	4.28E-01	5.60E-01
GPR109A	0.0119	8.20E-01	8.82E-01
GPR109B	0.1018	5.00E-02	1.08E-01
GPR110	-0.0620	2.33E-01	3.57E-01
GPR111	0.1211	1.96E-02	5.06E-02
GPR112	-0.0989	5.70E-02	1.19E-01
GPR113	-0.0107	8.37E-01	8.93E-01
GPR114	0.1265	1.48E-02	4.02E-02
GPR115	0.0807	1.21E-01	2.16E-01
GPR116	-0.2236	1.37E-05	1.11E-04
GPR119	0.0188	7.18E-01	8.09E-01
GPR120	0.1851	3.37E-04	1.72E-03
GPR123	-0.0174	7.38E-01	8.23E-01
GPR124	-0.0836	1.08E-01	1.97E-01
GPR125	-0.1497	3.86E-03	1.34E-02
GPR126	0.0000	1.00E+00	1.00E+00
GPR128	-0.2033	8.04E-05	5.06E-04
GPR12	-0.0979	5.95E-02	1.23E-01
GPR132	-0.0508	3.29E-01	4.60E-01
GPR133	0.0342	5.12E-01	6.36E-01
GPR135	0.0756	1.46E-01	2.48E-01
GPR137B	-0.0091	8.61E-01	9.10E-01
GPR137C	0.2200	1.91E-05	1.48E-04
GPR137	-0.0957	6.55E-02	1.33E-01
GPR141	-0.0603	2.47E-01	3.71E-01
GPR142	-0.0529	3.10E-01	4.39E-01
GPR143	-0.0120	8.18E-01	8.81E-01
GPR144	0.1315	1.12E-02	3.21E-02
GPR146	-0.2988	4.35E-09	8.83E-08
GPR148	-0.0087	8.67E-01	9.15E-01
GPR149	-0.0531	3.08E-01	4.38E-01

GPR150	-0.0978	5.98E-02	1.24E-01
GPR151	0.0300	5.65E-01	6.83E-01
GPR152	-0.0960	6.46E-02	1.32E-01
GPR153	-0.0616	2.37E-01	3.60E-01
GPR155	-0.2241	1.31E-05	1.06E-04
GPR156	0.0560	2.82E-01	4.11E-01
GPR157	-0.0369	4.79E-01	6.07E-01
GPR158	0.0071	8.92E-01	9.31E-01
GPR15	-0.0391	4.53E-01	5.84E-01
GPR160	0.2031	8.14E-05	5.12E-04
GPR161	0.1072	3.91E-02	8.84E-02
GPR162	-0.0186	7.21E-01	8.11E-01
GPR171	-0.0695	1.82E-01	2.94E-01
GPR172A	0.1918	2.02E-04	1.11E-03
GPR172B	0.0852	1.01E-01	1.87E-01
GPR173	-0.0377	4.69E-01	5.99E-01
GPR174	-0.0740	1.55E-01	2.60E-01
GPR176	0.0089	8.65E-01	9.12E-01
GPR179	0.0940	7.04E-02	1.41E-01
GPR17	-0.0821	1.15E-01	2.07E-01
GPR180	-0.0337	5.17E-01	6.42E-01
GPR182	-0.2517	9.11E-07	1.04E-05
GPR183	-0.0677	1.93E-01	3.08E-01
GPR18	-0.0427	4.12E-01	5.45E-01
GPR19	0.3176	3.85E-10	1.01E-08
GPR1	0.0858	9.88E-02	1.84E-01
GPR20	-0.1191	2.18E-02	5.50E-02
GPR21	-0.0114	8.27E-01	8.87E-01
GPR22	0.0281	5.89E-01	7.04E-01
GPR25	0.0424	4.15E-01	5.48E-01
GPR26	-0.0040	9.39E-01	9.62E-01
GPR27	0.1015	5.07E-02	1.09E-01
GPR31	0.0171	7.42E-01	8.26E-01
GPR32	-0.0036	9.45E-01	9.66E-01
GPR34	-0.0655	2.08E-01	3.26E-01
GPR35	0.2295	8.02E-06	6.90E-05
GPR37L1	0.1316	1.12E-02	3.20E-02
GPR37	-0.1422	6.09E-03	1.94E-02
GPR39	-0.0182	7.26E-01	8.14E-01
GPR3	-0.0500	3.37E-01	4.69E-01
GPR44	-0.0212	6.84E-01	7.81E-01
GPR45	0.0054	9.18E-01	9.48E-01
GPR4	-0.1982	1.21E-04	7.18E-04
GPR50	0.0411	4.29E-01	5.62E-01

GPR52	0.0759	1.44E-01	2.46E-01
GPR55	-0.0113	8.29E-01	8.88E-01
GPR56	0.0647	2.14E-01	3.34E-01
GPR61	0.0433	4.06E-01	5.39E-01
GPR62	-0.0383	4.62E-01	5.92E-01
GPR63	0.1572	2.39E-03	8.92E-03
GPR64	0.1182	2.28E-02	5.71E-02
GPR65	-0.0993	5.59E-02	1.17E-01
GPR68	-0.0343	5.10E-01	6.35E-01
GPR6	0.0074	8.87E-01	9.28E-01
GPR75	0.0234	6.53E-01	7.56E-01
GPR77	0.0194	7.10E-01	8.02E-01
GPR78	0.0609	2.42E-01	3.67E-01
GPR81	-0.0029	9.55E-01	9.72E-01
GPR82	-0.0186	7.22E-01	8.11E-01
GPR83	0.1780	5.71E-04	2.66E-03
GPR84	0.1378	7.84E-03	2.39E-02
GPR85	0.0190	7.16E-01	8.06E-01
GPR87	-0.0368	4.80E-01	6.09E-01
GPR88	-0.0100	8.47E-01	9.00E-01
GPR89A	0.3476	5.66E-12	2.16E-10
GPR89B	0.1889	2.53E-04	1.35E-03
GPR89C	0.2882	1.59E-08	2.83E-07
GPR97	0.0790	1.29E-01	2.26E-01
GPR98	-0.0345	5.08E-01	6.32E-01
GPRASP1	0.0880	9.04E-02	1.72E-01
GPRASP2	-0.0405	4.37E-01	5.68E-01
GPRC5A	-0.0656	2.07E-01	3.25E-01
GPRC5B	0.0356	4.94E-01	6.21E-01
GPRC5C	-0.0815	1.17E-01	2.10E-01
GPRC5D	0.3297	7.42E-11	2.24E-09
GPRC6A	0.1359	8.76E-03	2.62E-02
GPRIN1	0.2797	4.29E-08	6.82E-07
GPRIN2	0.1983	1.21E-04	7.16E-04
GPRIN3	-0.0891	8.66E-02	1.66E-01
GPS1	0.0717	1.68E-01	2.77E-01
GPS2	0.0877	9.18E-02	1.74E-01
GPSM1	0.1629	1.65E-03	6.55E-03
GPSM2	0.3970	1.87E-15	1.40E-13
GPSM3	-0.0401	4.41E-01	5.73E-01
GPT2	-0.2508	9.89E-07	1.12E-05
GPT	-0.3166	4.41E-10	1.14E-08
GPX1	-0.1404	6.78E-03	2.12E-02
GPX2	0.0368	4.80E-01	6.09E-01

GPX3	-0.1628	1.65E-03	6.56E-03
GPX4	-0.1369	8.29E-03	2.51E-02
GPX5	0.0115	8.26E-01	8.86E-01
GPX6	0.0501	3.35E-01	4.67E-01
GPX7	0.1263	1.49E-02	4.05E-02
GPX8	-0.0084	8.72E-01	9.17E-01
GRAMD1A	0.2084	5.21E-05	3.51E-04
GRAMD1B	0.0945	6.89E-02	1.39E-01
GRAMD1C	-0.1129	2.97E-02	7.09E-02
GRAMD2	0.0587	2.60E-01	3.86E-01
GRAMD3	-0.1895	2.41E-04	1.29E-03
GRAMD4	-0.0160	7.59E-01	8.38E-01
GRAP2	-0.0574	2.70E-01	3.98E-01
GRAPL	-0.1140	2.82E-02	6.80E-02
GRAP	-0.1588	2.16E-03	8.20E-03
GRASP	-0.1236	1.73E-02	4.56E-02
GRB10	-0.1396	7.08E-03	2.20E-02
GRB14	0.0002	9.98E-01	9.98E-01
GRB2	0.1620	1.74E-03	6.86E-03
GRB7	0.2286	8.73E-06	7.45E-05
GREB1L	0.0172	7.42E-01	8.26E-01
GREB1	-0.1370	8.23E-03	2.49E-02
GREM1	0.0195	7.08E-01	8.00E-01
GREM2	-0.1767	6.31E-04	2.90E-03
GRHL1	0.1115	3.18E-02	7.49E-02
GRHL2	0.0273	6.01E-01	7.13E-01
GRHL3	0.0368	4.80E-01	6.09E-01
GRHPR	-0.3193	3.09E-10	8.31E-09
GRIA1	0.0304	5.59E-01	6.77E-01
GRIA2	0.1971	1.33E-04	7.81E-04
GRIA3	-0.1825	4.10E-04	2.02E-03
GRIA4	0.0641	2.18E-01	3.38E-01
GRID1	-0.0136	7.94E-01	8.63E-01
GRID2IP	0.2069	5.93E-05	3.92E-04
GRID2	0.1054	4.24E-02	9.45E-02
GRIK1	-0.0665	2.01E-01	3.17E-01
GRIK2	0.0580	2.65E-01	3.92E-01
GRIK3	-0.0326	5.31E-01	6.54E-01
GRIK4	0.1227	1.80E-02	4.72E-02
GRIK5	0.2577	4.85E-07	5.96E-06
GRIN1	0.2154	2.85E-05	2.10E-04
GRIN2A	0.1311	1.15E-02	3.26E-02
GRIN2B	0.0015	9.77E-01	9.86E-01
GRIN2C	0.0248	6.34E-01	7.41E-01

GRIN2D	0.0806	1.21E-01	2.16E-01
GRIN3A	-0.0401	4.41E-01	5.73E-01
GRIN3B	0.0282	5.89E-01	7.04E-01
GRINA	-0.0657	2.07E-01	3.25E-01
GRINL1A	-0.0100	8.48E-01	9.00E-01
GRIP1	0.0415	4.26E-01	5.58E-01
GRIP2	0.0060	9.09E-01	9.43E-01
GRIPAP1	0.0668	1.99E-01	3.15E-01
GRK1	0.0992	5.64E-02	1.18E-01
GRK4	-0.0850	1.02E-01	1.89E-01
GRK5	-0.1347	9.38E-03	2.78E-02
GRK6	0.1961	1.44E-04	8.34E-04
GRK7	0.1851	3.39E-04	1.73E-03
GRLF1	-0.1188	2.21E-02	5.58E-02
GRM1	0.1752	7.01E-04	3.17E-03
GRM2	0.0485	3.52E-01	4.84E-01
GRM3	0.0375	4.71E-01	6.00E-01
GRM4	0.2429	2.21E-06	2.28E-05
GRM5	0.1205	2.02E-02	5.19E-02
GRM6	0.0839	1.07E-01	1.95E-01
GRM7	0.0457	3.80E-01	5.14E-01
GRM8	0.0477	3.60E-01	4.92E-01
GRN	0.1528	3.17E-03	1.13E-02
GRPEL1	-0.2545	6.79E-07	8.04E-06
GRPEL2	0.2025	8.58E-05	5.36E-04
GRPR	-0.0689	1.85E-01	2.98E-01
GRP	-0.0376	4.71E-01	6.00E-01
GRRP1	-0.2107	4.31E-05	2.99E-04
GRSF1	-0.1521	3.31E-03	1.17E-02
GRTP1	0.0842	1.06E-01	1.94E-01
GRWD1	0.0032	9.52E-01	9.70E-01
GRXCR2	0.0368	4.80E-01	6.08E-01
GSC	0.0770	1.39E-01	2.39E-01
GSDMA	-0.0165	7.51E-01	8.32E-01
GSDMB	0.2485	1.26E-06	1.39E-05
GSDMC	0.0734	1.58E-01	2.64E-01
GSDMD	-0.0034	9.48E-01	9.68E-01
GSG1L	-0.0274	5.98E-01	7.12E-01
GSG1	-0.0454	3.83E-01	5.17E-01
GSG2	0.4113	1.42E-16	1.41E-14
GSK3A	0.2078	5.49E-05	3.67E-04
GSK3B	-0.0708	1.73E-01	2.83E-01
GSN	-0.1638	1.55E-03	6.22E-03
GSPT1	-0.0488	3.49E-01	4.81E-01

GSPT2	0.1458	4.90E-03	1.62E-02
GSR	0.0661	2.04E-01	3.21E-01
GSS	-0.1208	2.00E-02	5.13E-02
GSTA1	-0.0757	1.45E-01	2.48E-01
GSTA2	-0.0790	1.29E-01	2.25E-01
GSTA3	0.0790	1.29E-01	2.26E-01
GSTA4	0.1660	1.33E-03	5.47E-03
GSTA5	0.0444	3.94E-01	5.27E-01
GSTCD	0.0817	1.16E-01	2.09E-01
GSTK1	-0.3513	3.22E-12	1.29E-10
GSTM1	-0.2067	6.05E-05	3.98E-04
GSTM2P1	-0.1564	2.53E-03	9.35E-03
GSTM2	-0.1779	5.78E-04	2.69E-03
GSTM3	-0.0291	5.76E-01	6.93E-01
GSTM4	-0.1927	1.89E-04	1.05E-03
GSTM5	-0.2427	2.24E-06	2.30E-05
GSTO1	-0.2482	1.30E-06	1.43E-05
GSTO2	0.0687	1.87E-01	3.00E-01
GSTP1	-0.0323	5.36E-01	6.58E-01
GSTT1	-0.1405	6.70E-03	2.10E-02
GSTT2	-0.0656	2.08E-01	3.26E-01
GSTTP1	0.0402	4.40E-01	5.72E-01
GSTTP2	-0.0113	8.28E-01	8.88E-01
GSTZ1	-0.3742	8.92E-14	4.74E-12
GSX1	0.0113	8.29E-01	8.88E-01
GSX2	0.1290	1.29E-02	3.60E-02
GTDC1	0.0368	4.80E-01	6.09E-01
GTF2A1L	-0.1049	4.34E-02	9.62E-02
GTF2A1	-0.0985	5.79E-02	1.21E-01
GTF2A2	0.0889	8.73E-02	1.67E-01
GTF2B	0.0191	7.14E-01	8.05E-01
GTF2E1	0.0447	3.90E-01	5.24E-01
GTF2E2	0.1464	4.72E-03	1.57E-02
GTF2F1	0.0686	1.87E-01	3.00E-01
GTF2F2	0.0527	3.11E-01	4.41E-01
GTF2H1	0.1842	3.61E-04	1.82E-03
GTF2H2B	0.0361	4.88E-01	6.15E-01
GTF2H2C	0.0728	1.62E-01	2.69E-01
GTF2H2	0.0258	6.21E-01	7.30E-01
GTF2H3	-0.0030	9.54E-01	9.71E-01
GTF2H4	0.2149	2.99E-05	2.18E-04
GTF2H5	0.0169	7.46E-01	8.29E-01
GTF2IP1	-0.0176	7.35E-01	8.21E-01
GTF2IRD1	0.2164	2.63E-05	1.96E-04

GTF2IRD2B	-0.1514	3.47E-03	1.22E-02
GTF2IRD2P1	-0.1492	3.98E-03	1.37E-02
GTF2IRD2	-0.1701	1.01E-03	4.32E-03
GTF2I	-0.0978	6.00E-02	1.24E-01
GTF3A	-0.0089	8.64E-01	9.12E-01
GTF3C1	0.0145	7.81E-01	8.55E-01
GTF3C2	0.2150	2.97E-05	2.17E-04
GTF3C3	0.1533	3.07E-03	1.10E-02
GTF3C4	0.0872	9.34E-02	1.76E-01
GTF3C5	0.2385	3.38E-06	3.30E-05
GTF3C6	0.1665	1.29E-03	5.32E-03
GTPBP10	-0.2281	9.10E-06	7.74E-05
GTPBP1	0.1256	1.55E-02	4.17E-02
GTPBP2	0.2697	1.33E-07	1.88E-06
GTPBP3	0.2142	3.17E-05	2.29E-04
GTPBP4	0.1838	3.74E-04	1.87E-03
GTPBP5	-0.0457	3.80E-01	5.14E-01
GTPBP8	-0.0594	2.54E-01	3.79E-01
GTSE1	0.4095	1.96E-16	1.85E-14
GTSF1L	0.0548	2.92E-01	4.21E-01
GTSF1	0.0441	3.97E-01	5.30E-01
GUCA1A	0.0434	4.05E-01	5.38E-01
GUCA1B	-0.0831	1.10E-01	2.00E-01
GUCA1C	0.0399	4.43E-01	5.75E-01
GUCA2A	0.0399	4.44E-01	5.75E-01
GUCA2B	-0.1313	1.14E-02	3.24E-02
GUCY1A2	-0.1650	1.43E-03	5.79E-03
GUCY1A3	-0.0168	7.47E-01	8.30E-01
GUCY1B2	0.2790	4.64E-08	7.30E-07
GUCY1B3	-0.0112	8.30E-01	8.89E-01
GUCY2C	0.0715	1.69E-01	2.78E-01
GUCY2D	0.1674	1.21E-03	5.05E-03
GUCY2E	0.1113	3.21E-02	7.54E-02
GUCY2F	0.0565	2.78E-01	4.06E-01
GUCY2GP	0.0107	8.37E-01	8.93E-01
GUF1	-0.1330	1.03E-02	2.99E-02
GUK1	-0.0130	8.03E-01	8.70E-01
GULP1	0.0716	1.69E-01	2.78E-01
GUSBL1	0.1101	3.40E-02	7.92E-02
GUSBL2	0.2773	5.66E-08	8.72E-07
GUSBP1	0.1979	1.25E-04	7.36E-04
GUSBP3	0.1658	1.35E-03	5.52E-03
GUSB	-0.1244	1.65E-02	4.39E-02
GVIN1	-0.1344	9.57E-03	2.82E-02

GXYLT1	0.0505	3.32E-01	4.63E-01
GXYLT2	-0.0186	7.21E-01	8.11E-01
GYG1	0.0432	4.07E-01	5.40E-01
GYG2	-0.0922	7.61E-02	1.50E-01
GYLTL1B	0.0459	3.78E-01	5.11E-01
GYPA	-0.2149	3.00E-05	2.19E-04
GYPB	-0.1679	1.17E-03	4.90E-03
GYPC	-0.0081	8.77E-01	9.21E-01
GYPE	-0.1436	5.57E-03	1.80E-02
GYS1	0.1219	1.88E-02	4.89E-02
GYS2	-0.2906	1.18E-08	2.15E-07
GZF1	0.0637	2.21E-01	3.42E-01
GZMA	-0.1047	4.38E-02	9.70E-02
GZMB	-0.0714	1.70E-01	2.79E-01
GZMH	-0.1173	2.38E-02	5.92E-02
GZMK	-0.1198	2.10E-02	5.33E-02
GZMM	-0.0903	8.24E-02	1.60E-01
H19	0.0531	3.08E-01	4.37E-01
H1F0	0.1361	8.65E-03	2.60E-02
H1FNT	0.0996	5.54E-02	1.17E-01
H1FOO	-0.0113	8.28E-01	8.87E-01
H1FX	0.2374	3.78E-06	3.65E-05
H2AFB1	0.3181	3.61E-10	9.55E-09
H2AFJ	-0.1141	2.80E-02	6.76E-02
H2AFV	0.1283	1.34E-02	3.72E-02
H2AFX	0.3347	3.69E-11	1.20E-09
H2AFY2	0.0489	3.47E-01	4.79E-01
H2AFY	0.3049	2.03E-09	4.49E-08
H2AFZ	0.2879	1.63E-08	2.89E-07
H2BFM	0.1440	5.45E-03	1.77E-02
H2BFWT	0.0851	1.02E-01	1.88E-01
H2BFXP	0.1036	4.61E-02	1.01E-01
H3F3A	0.2913	1.09E-08	2.01E-07
H3F3B	0.1546	2.83E-03	1.03E-02
H3F3C	0.0405	4.37E-01	5.68E-01
H6PD	-0.1186	2.23E-02	5.61E-02
HAAO	-0.2142	3.18E-05	2.30E-04
HABP2	-0.0391	4.52E-01	5.83E-01
HABP4	-0.0102	8.44E-01	8.98E-01
HACE1	0.2296	7.96E-06	6.86E-05
HACL1	-0.2036	7.84E-05	4.96E-04
HADHA	-0.2696	1.34E-07	1.90E-06
HADHB	-0.3022	2.83E-09	6.03E-08
HADH	-0.2938	8.05E-09	1.53E-07

HAGHL	0.0769	1.39E-01	2.39E-01
HAGH	-0.2363	4.18E-06	3.98E-05
HAL	-0.0159	7.60E-01	8.38E-01
HAMP	-0.0612	2.39E-01	3.64E-01
HAND1	0.0196	7.06E-01	7.99E-01
HAND2	-0.1234	1.74E-02	4.59E-02
HAO1	-0.2419	2.43E-06	2.47E-05
HAO2	-0.1629	1.64E-03	6.53E-03
HAP1	0.1269	1.45E-02	3.96E-02
HAPLN1	0.0006	9.91E-01	9.95E-01
HAPLN2	-0.0090	8.62E-01	9.11E-01
HAPLN3	0.0334	5.21E-01	6.44E-01
HAPLN4	-0.1461	4.81E-03	1.60E-02
HAR1A	-0.0329	5.27E-01	6.50E-01
HAR1B	0.0355	4.96E-01	6.22E-01
HARBI1	-0.0567	2.76E-01	4.05E-01
HARS2	0.0900	8.33E-02	1.61E-01
HARS	-0.0274	5.99E-01	7.12E-01
HAS1	-0.0513	3.24E-01	4.54E-01
HAS2AS	0.0309	5.53E-01	6.73E-01
HAS2	-0.1002	5.38E-02	1.14E-01
HAS3	-0.0031	9.52E-01	9.71E-01
HAT1	0.2523	8.55E-07	9.87E-06
HAUS1	0.3035	2.41E-09	5.20E-08
HAUS2	0.1835	3.81E-04	1.90E-03
HAUS3	0.3577	1.23E-12	5.36E-11
HAUS4	0.0346	5.06E-01	6.31E-01
HAUS5	0.4495	7.45E-20	1.83E-17
HAUS6	0.2647	2.30E-07	3.06E-06
HAUS7	0.2050	6.95E-05	4.48E-04
HAUS8	0.2934	8.42E-09	1.59E-07
HAVCR1	0.0598	2.50E-01	3.75E-01
HAVCR2	-0.0663	2.02E-01	3.19E-01
HAX1	0.3517	3.04E-12	1.23E-10
HBA1	-0.1435	5.63E-03	1.82E-02
HBA2	-0.2165	2.59E-05	1.93E-04
HBBP1	0.0072	8.90E-01	9.29E-01
HBB	-0.1981	1.23E-04	7.27E-04
HBD	-0.1617	1.78E-03	6.99E-03
HBE1	0.0971	6.16E-02	1.27E-01
HBEGF	-0.1000	5.43E-02	1.15E-01
HBG1	-0.1080	3.75E-02	8.58E-02
HBG2	-0.0407	4.34E-01	5.66E-01
HBM	-0.1723	8.63E-04	3.79E-03

HBP1	-0.2260	1.10E-05	9.16E-05
HBQ1	-0.0405	4.37E-01	5.68E-01
HBS1L	0.0611	2.40E-01	3.64E-01
HBXIP	0.0432	4.07E-01	5.39E-01
HBZ	-0.0364	4.85E-01	6.13E-01
HCCS	-0.0405	4.37E-01	5.68E-01
HCFC1R1	0.0123	8.13E-01	8.77E-01
HCFC1	0.3401	1.69E-11	5.81E-10
HCFC2	-0.1334	1.01E-02	2.95E-02
HCG11	-0.1564	2.52E-03	9.34E-03
HCG18	0.2562	5.70E-07	6.84E-06
HCG22	0.0045	9.31E-01	9.57E-01
HCG26	0.0513	3.25E-01	4.55E-01
HCG27	0.2007	9.95E-05	6.06E-04
HCG2P7	0.1524	3.26E-03	1.16E-02
HCG4P6	0.0184	7.24E-01	8.13E-01
HCG4	0.0327	5.29E-01	6.52E-01
HCG9	0.0026	9.60E-01	9.75E-01
HCK	-0.0468	3.69E-01	5.02E-01
HCLS1	-0.1019	4.98E-02	1.07E-01
HCN1	0.0220	6.73E-01	7.72E-01
HCN2	-0.0557	2.84E-01	4.13E-01
HCN3	0.3342	3.96E-11	1.27E-09
HCN4	0.1639	1.54E-03	6.18E-03
HCP5	0.0345	5.08E-01	6.33E-01
HCRTR1	-0.0044	9.33E-01	9.58E-01
HCRTR2	0.0126	8.08E-01	8.74E-01
HCRT	0.1235	1.74E-02	4.57E-02
HCST	-0.0571	2.73E-01	4.01E-01
HDAC10	0.0627	2.28E-01	3.51E-01
HDAC11	0.1830	3.96E-04	1.97E-03
HDAC1	0.2248	1.23E-05	1.01E-04
HDAC2	0.3553	1.77E-12	7.49E-11
HDAC3	-0.0175	7.37E-01	8.22E-01
HDAC4	0.2404	2.82E-06	2.81E-05
HDAC5	0.2197	1.96E-05	1.52E-04
HDAC6	-0.1809	4.61E-04	2.22E-03
HDAC7	0.0970	6.19E-02	1.27E-01
HDAC8	0.0399	4.43E-01	5.75E-01
HDAC9	-0.0265	6.11E-01	7.22E-01
HDC	-0.1464	4.73E-03	1.57E-02
HDDC2	0.2730	9.19E-08	1.34E-06
HDDC3	-0.1627	1.66E-03	6.60E-03
HDGFL1	0.0080	8.79E-01	9.22E-01

HDGFRP2	0.1388	7.42E-03	2.29E-02
HDGFRP3	-0.0894	8.56E-02	1.64E-01
HDGF	0.4019	7.76E-16	6.49E-14
HDHD1A	0.0528	3.11E-01	4.40E-01
HDHD2	-0.1429	5.82E-03	1.87E-02
HDHD3	-0.0935	7.21E-02	1.44E-01
HDLBP	-0.0760	1.44E-01	2.46E-01
HDX	0.0245	6.38E-01	7.44E-01
HEATR1	0.2964	5.84E-09	1.15E-07
HEATR2	0.1668	1.27E-03	5.25E-03
HEATR3	-0.1157	2.58E-02	6.32E-02
HEATR4	-0.0371	4.77E-01	6.05E-01
HEATR5A	-0.0908	8.06E-02	1.57E-01
HEATR5B	0.0080	8.78E-01	9.21E-01
HEATR6	0.2964	5.83E-09	1.15E-07
HEATR7A	0.0200	7.01E-01	7.94E-01
HEATR7B2	0.1436	5.58E-03	1.80E-02
HEBP1	-0.0879	9.10E-02	1.72E-01
HEBP2	0.1399	6.95E-03	2.16E-02
HECA	0.0216	6.78E-01	7.76E-01
HECTD1	-0.1526	3.21E-03	1.14E-02
HECTD2	0.2578	4.81E-07	5.92E-06
HECTD3	-0.0938	7.13E-02	1.42E-01
HECW1	-0.0940	7.05E-02	1.41E-01
HECW2	-0.1762	6.52E-04	2.98E-03
HEG1	-0.0694	1.82E-01	2.94E-01
HELB	0.0698	1.80E-01	2.91E-01
HELLS	0.4006	9.89E-16	8.01E-14
HELQ	-0.0141	7.87E-01	8.58E-01
HELT	0.1921	1.98E-04	1.09E-03
HELZ	0.1664	1.29E-03	5.34E-03
HEMGN	-0.1706	9.71E-04	4.20E-03
HEMK1	-0.0061	9.06E-01	9.41E-01
HEPACAM2	0.1845	3.53E-04	1.79E-03
HEPACAM	-0.2308	7.08E-06	6.23E-05
HEPHL1	0.0694	1.82E-01	2.94E-01
HEPH	-0.1806	4.72E-04	2.26E-03
HEPN1	-0.2184	2.20E-05	1.68E-04
HERC1	-0.0639	2.20E-01	3.40E-01
HERC2P2	0.2365	4.13E-06	3.94E-05
HERC2P4	0.1125	3.03E-02	7.21E-02
HERC2	-0.0273	6.00E-01	7.13E-01
HERC3	-0.1969	1.34E-04	7.86E-04
HERC4	-0.1305	1.19E-02	3.36E-02

HERC5	-0.1102	3.39E-02	7.90E-02
HERC6	-0.1743	7.44E-04	3.34E-03
HERPUD1	-0.3827	2.17E-14	1.32E-12
HERPUD2	-0.1956	1.49E-04	8.60E-04
HES1	0.0520	3.18E-01	4.48E-01
HES2	0.0790	1.29E-01	2.26E-01
HES3	0.0436	4.02E-01	5.35E-01
HES4	0.0839	1.06E-01	1.95E-01
HES5	-0.0688	1.86E-01	2.99E-01
HES6	0.1138	2.84E-02	6.83E-02
HES7	0.0715	1.69E-01	2.78E-01
HESRG	-0.0079	8.80E-01	9.23E-01
HESX1	-0.0337	5.17E-01	6.42E-01
HEXA	-0.2019	9.03E-05	5.59E-04
HEXB	-0.0349	5.03E-01	6.28E-01
HEXDC	0.1391	7.30E-03	2.26E-02
HEXIM1	0.0116	8.24E-01	8.85E-01
HEXIM2	-0.0607	2.43E-01	3.68E-01
HEY1	0.1013	5.11E-02	1.09E-01
HEY2	-0.1109	3.27E-02	7.67E-02
HEYL	-0.0063	9.04E-01	9.39E-01
HFE2	-0.1210	1.97E-02	5.08E-02
HFE	-0.0571	2.73E-01	4.01E-01
HFM1	0.1419	6.19E-03	1.96E-02
HGC6.3	0.1580	2.27E-03	8.55E-03
HGD	-0.2466	1.51E-06	1.63E-05
HGFAC	-0.0801	1.24E-01	2.19E-01
HGF	-0.0826	1.12E-01	2.04E-01
HGSNAT	-0.0029	9.56E-01	9.72E-01
HGS	0.4081	2.56E-16	2.34E-14
HHATL	-0.0486	3.51E-01	4.83E-01
HHAT	0.0274	5.98E-01	7.12E-01
HHEX	0.1895	2.42E-04	1.30E-03
HHIPL1	0.0104	8.42E-01	8.97E-01
HHIPL2	0.1312	1.14E-02	3.25E-02
HHIP	-0.1225	1.83E-02	4.77E-02
HHLA1	0.0184	7.24E-01	8.13E-01
HHLA2	0.1311	1.15E-02	3.26E-02
HHLA3	-0.1296	1.25E-02	3.50E-02
HIAT1	-0.0230	6.58E-01	7.60E-01
HIATL1	0.2839	2.62E-08	4.35E-07
HIATL2	0.2335	5.50E-06	5.02E-05
HIBADH	-0.3222	2.07E-10	5.73E-09
HIBCH	-0.2633	2.66E-07	3.51E-06

HIC1	-0.1722	8.68E-04	3.80E-03
HIC2	0.2597	3.91E-07	4.92E-06
HIF1AN	0.0352	5.00E-01	6.25E-01
HIF1A	0.0348	5.04E-01	6.29E-01
HIF3A	-0.0852	1.01E-01	1.87E-01
HIGD1A	-0.3092	1.17E-09	2.72E-08
HIGD1B	0.0580	2.65E-01	3.92E-01
HIGD1C	0.0344	5.09E-01	6.34E-01
HIGD2A	-0.0519	3.19E-01	4.49E-01
HIGD2B	-0.0503	3.33E-01	4.64E-01
HILS1	0.1575	2.35E-03	8.80E-03
HINFP	0.3694	1.94E-13	9.85E-12
HINT1	-0.0134	7.96E-01	8.65E-01
HINT2	-0.1969	1.35E-04	7.89E-04
HINT3	0.0332	5.24E-01	6.47E-01
HIP1R	0.0674	1.95E-01	3.11E-01
HIP1	-0.0429	4.10E-01	5.43E-01
HIPK1	-0.0227	6.63E-01	7.64E-01
HIPK2	-0.1528	3.16E-03	1.13E-02
HIPK3	-0.0894	8.56E-02	1.64E-01
HIPK4	0.0619	2.34E-01	3.58E-01
HIRA	0.0246	6.37E-01	7.43E-01
HIRIP3	0.0084	8.71E-01	9.17E-01
HIST1H1A	0.0358	4.92E-01	6.19E-01
HIST1H1B	0.1836	3.80E-04	1.90E-03
HIST1H1C	-0.1354	9.02E-03	2.69E-02
HIST1H1D	0.0729	1.61E-01	2.68E-01
HIST1H1E	0.1385	7.55E-03	2.32E-02
HIST1H1T	0.0418	4.22E-01	5.55E-01
HIST1H2AA	0.0721	1.66E-01	2.73E-01
HIST1H2AB	0.0688	1.86E-01	2.99E-01
HIST1H2AC	-0.1841	3.64E-04	1.84E-03
HIST1H2AD	0.0720	1.66E-01	2.74E-01
HIST1H2AE	-0.0882	8.98E-02	1.71E-01
HIST1H2AG	-0.0081	8.77E-01	9.21E-01
HIST1H2AH	0.1919	2.01E-04	1.11E-03
HIST1H2AJ	0.0748	1.51E-01	2.55E-01
HIST1H2AK	0.0613	2.39E-01	3.63E-01
HIST1H2AL	0.1292	1.28E-02	3.57E-02
HIST1H2AM	0.0651	2.11E-01	3.30E-01
HIST1H2BA	0.0460	3.76E-01	5.10E-01
HIST1H2BB	0.1125	3.02E-02	7.19E-02
HIST1H2BC	-0.2104	4.42E-05	3.05E-04
HIST1H2BD	-0.1445	5.30E-03	1.73E-02

HIST1H2BE	-0.1020	4.96E-02	1.07E-01
HIST1H2BF	0.0845	1.04E-01	1.92E-01
HIST1H2BG	0.0710	1.73E-01	2.82E-01
HIST1H2BH	0.0427	4.13E-01	5.45E-01
HIST1H2BI	-0.0306	5.56E-01	6.75E-01
HIST1H2BJ	0.0902	8.27E-02	1.60E-01
HIST1H2BK	-0.1045	4.42E-02	9.75E-02
HIST1H2BL	0.0565	2.78E-01	4.07E-01
HIST1H2BM	0.0468	3.69E-01	5.02E-01
HIST1H2BN	0.0198	7.03E-01	7.96E-01
HIST1H2BO	0.1732	8.10E-04	3.59E-03
HIST1H3A	-0.1010	5.18E-02	1.11E-01
HIST1H3B	0.0245	6.39E-01	7.45E-01
HIST1H3C	0.1656	1.37E-03	5.59E-03
HIST1H3D	0.0086	8.69E-01	9.16E-01
HIST1H3E	-0.1685	1.12E-03	4.74E-03
HIST1H3F	0.0723	1.65E-01	2.73E-01
HIST1H3G	-0.0567	2.76E-01	4.04E-01
HIST1H3H	-0.0318	5.41E-01	6.62E-01
HIST1H3I	0.1265	1.48E-02	4.02E-02
HIST1H3J	0.1831	3.92E-04	1.95E-03
HIST1H4A	0.1360	8.72E-03	2.62E-02
HIST1H4B	0.0941	7.03E-02	1.41E-01
HIST1H4C	0.1282	1.35E-02	3.73E-02
HIST1H4D	0.1831	3.92E-04	1.95E-03
HIST1H4E	0.2025	8.54E-05	5.33E-04
HIST1H4F	0.0032	9.51E-01	9.69E-01
HIST1H4H	-0.0196	7.07E-01	7.99E-01
HIST1H4I	0.0382	4.63E-01	5.93E-01
HIST1H4J	-0.0753	1.48E-01	2.51E-01
HIST1H4K	0.0012	9.81E-01	9.88E-01
HIST1H4L	0.1234	1.74E-02	4.58E-02
HIST2H2AA3	-0.0054	9.18E-01	9.48E-01
HIST2H2AB	0.1455	4.99E-03	1.64E-02
HIST2H2AC	0.1971	1.32E-04	7.77E-04
HIST2H2BA	0.0562	2.81E-01	4.09E-01
HIST2H2BE	0.0682	1.90E-01	3.04E-01
HIST2H2BF	0.1578	2.30E-03	8.66E-03
HIST2H3C	0.2789	4.68E-08	7.35E-07
HIST2H3D	0.1693	1.06E-03	4.51E-03
HIST2H4A	0.2659	2.00E-07	2.71E-06
HIST3H2A	0.1742	7.55E-04	3.38E-03
HIST3H2BB	0.1530	3.13E-03	1.12E-02
HIST3H3	0.0793	1.27E-01	2.24E-01

HIST4H4	0.1670	1.24E-03	5.16E-03
HIVEP1	-0.0684	1.89E-01	3.02E-01
HIVEP2	0.0352	4.99E-01	6.25E-01
HIVEP3	-0.0508	3.29E-01	4.60E-01
HJURP	0.4662	2.02E-21	5.91E-19
HK1	-0.0993	5.59E-02	1.17E-01
HK2	0.0755	1.46E-01	2.49E-01
HK3	-0.0456	3.81E-01	5.14E-01
HKDC1	0.0827	1.12E-01	2.03E-01
HKR1	0.2180	2.27E-05	1.72E-04
HLA-A	-0.0662	2.04E-01	3.21E-01
HLA-B	-0.0845	1.04E-01	1.92E-01
HLA-C	-0.0940	7.06E-02	1.41E-01
HLA-DMA	-0.0142	7.86E-01	8.58E-01
HLA-DMB	-0.0102	8.45E-01	8.99E-01
HLA-DOA	-0.0518	3.20E-01	4.50E-01
HLA-DOB	0.0915	7.83E-02	1.53E-01
HLA-DPA1	-0.0823	1.14E-01	2.05E-01
HLA-DPB1	-0.0509	3.29E-01	4.59E-01
HLA-DPB2	0.0108	8.36E-01	8.93E-01
HLA-DQA1	-0.0533	3.06E-01	4.36E-01
HLA-DQA2	0.0560	2.82E-01	4.11E-01
HLA-DQB1	-0.0602	2.48E-01	3.73E-01
HLA-DQB2	0.0512	3.25E-01	4.56E-01
HLA-DRA	-0.0561	2.81E-01	4.10E-01
HLA-DRB1	-0.0764	1.42E-01	2.43E-01
HLA-DRB5	-0.0612	2.40E-01	3.64E-01
HLA-DRB6	0.0202	6.98E-01	7.91E-01
HLA-E	-0.1849	3.44E-04	1.75E-03
HLA-F	-0.0203	6.97E-01	7.91E-01
HLA-G	-0.0531	3.07E-01	4.37E-01
HLA-H	-0.0164	7.53E-01	8.34E-01
HLA-J	0.0589	2.58E-01	3.84E-01
HLA-L	-0.0327	5.30E-01	6.53E-01
HLCS	-0.0193	7.12E-01	8.03E-01
HLF	-0.1989	1.15E-04	6.87E-04
HLTF	0.0739	1.56E-01	2.61E-01
HLX	-0.1114	3.20E-02	7.52E-02
HM13	0.0920	7.67E-02	1.51E-01
HMBOX1	-0.0768	1.40E-01	2.40E-01
HMBS	0.0057	9.13E-01	9.45E-01
HMCN1	-0.1006	5.28E-02	1.12E-01
HMG20A	-0.0989	5.69E-02	1.19E-01
HMG20B	-0.0376	4.70E-01	6.00E-01

HMGA1	0.3121	7.94E-10	1.93E-08
HMGA2	0.1592	2.10E-03	8.03E-03
HMGB1	0.1671	1.24E-03	5.15E-03
HMGB2	0.3671	2.82E-13	1.39E-11
HMGB3L1	0.0214	6.81E-01	7.78E-01
HMGB3	0.0804	1.22E-01	2.17E-01
HMGB4	-0.1311	1.15E-02	3.27E-02
HMGCLL1	-0.1543	2.88E-03	1.05E-02
HMGCL	-0.3780	4.77E-14	2.71E-12
HMGCR	-0.0575	2.69E-01	3.97E-01
HMGCS1	-0.1440	5.47E-03	1.78E-02
HMGCS2	-0.1352	9.12E-03	2.72E-02
HMGN1	0.2850	2.30E-08	3.90E-07
HMGN2	0.2518	9.01E-07	1.03E-05
HMGN3	0.1137	2.85E-02	6.87E-02
HMGN4	0.2794	4.45E-08	7.06E-07
HMGN5	-0.1066	4.01E-02	9.04E-02
HMGXB3	0.0949	6.78E-02	1.37E-01
HMGXB4	0.2119	3.89E-05	2.73E-04
HMHA1	-0.0261	6.17E-01	7.26E-01
HMHB1	-0.0505	3.32E-01	4.63E-01
HMMR	0.3445	8.87E-12	3.24E-10
HMOX1	-0.1144	2.76E-02	6.67E-02
HMOX2	-0.2227	1.50E-05	1.20E-04
HMP19	0.0094	8.56E-01	9.07E-01
HMSD	0.1152	2.65E-02	6.46E-02
HMX1	-0.0365	4.83E-01	6.11E-01
HMX2	0.2590	4.21E-07	5.26E-06
HMX3	0.2397	3.02E-06	2.98E-05
HN1L	0.1240	1.69E-02	4.47E-02
HN1	0.2762	6.41E-08	9.74E-07
HNF1A	0.0209	6.88E-01	7.84E-01
HNF1B	0.0914	7.86E-02	1.54E-01
HNF4A	-0.0823	1.14E-01	2.06E-01
HNF4G	0.0162	7.56E-01	8.37E-01
HNMT	-0.2163	2.66E-05	1.97E-04
HNRNPA0	0.2600	3.80E-07	4.81E-06
HNRNPA1L2	0.2103	4.44E-05	3.06E-04
HNRNPA1	0.2566	5.47E-07	6.61E-06
HNRNPA2B1	0.3242	1.59E-10	4.50E-09
HNRNPA3P1	0.3747	8.18E-14	4.39E-12
HNRNPA3	0.4683	1.27E-21	3.93E-19
HNRNPAB	0.0770	1.39E-01	2.39E-01
HNRNPCL1	0.1030	4.75E-02	1.03E-01

HNRNPC	0.3177	3.80E-10	9.97E-09
HNRNPD	0.2846	2.41E-08	4.06E-07
HNRNPF	0.0742	1.54E-01	2.59E-01
HNRNPH1	0.3437	1.01E-11	3.56E-10
HNRNPH2	0.0320	5.38E-01	6.60E-01
HNRNPH3	0.2863	1.97E-08	3.41E-07
HNRNPK	0.2526	8.24E-07	9.55E-06
HNRNPL	0.3241	1.61E-10	4.54E-09
HNRNPM	0.3077	1.42E-09	3.25E-08
HNRNPR	0.1664	1.30E-03	5.36E-03
HNRNPUL1	0.2553	6.21E-07	7.39E-06
HNRNPUL2	0.1715	9.13E-04	3.97E-03
HNRNPU	0.4497	7.22E-20	1.80E-17
HNRPDL	0.3077	1.41E-09	3.25E-08
HNRPLL	0.1035	4.63E-02	1.01E-01
HOMER1	-0.0020	9.70E-01	9.81E-01
HOMER2	-0.0989	5.69E-02	1.19E-01
HOMER3	0.1676	1.19E-03	4.99E-03
HOMEZ	0.1080	3.77E-02	8.59E-02
HOOK1	0.0156	7.65E-01	8.41E-01
HOOK2	0.1496	3.88E-03	1.34E-02
HOOK3	0.1134	2.90E-02	6.96E-02
HOPX	-0.0255	6.25E-01	7.33E-01
HORMAD1	0.1488	4.06E-03	1.39E-02
HORMAD2	-0.1648	1.45E-03	5.87E-03
HOTAIR	0.1100	3.42E-02	7.95E-02
HOXA10	0.2343	5.07E-06	4.69E-05
HOXA11AS	0.1442	5.38E-03	1.75E-02
HOXA11	0.2222	1.57E-05	1.25E-04
HOXA13	0.0602	2.48E-01	3.73E-01
HOXA1	0.0054	9.17E-01	9.48E-01
HOXA2	0.0097	8.52E-01	9.04E-01
HOXA3	0.0523	3.15E-01	4.45E-01
HOXA4	-0.0456	3.81E-01	5.15E-01
HOXA5	0.1222	1.85E-02	4.83E-02
HOXA6	0.2166	2.57E-05	1.92E-04
HOXA7	-0.0758	1.45E-01	2.47E-01
HOXA9	0.1462	4.78E-03	1.59E-02
HOXB13	0.2267	1.04E-05	8.68E-05
HOXB1	0.0442	3.96E-01	5.29E-01
HOXB2	-0.0907	8.10E-02	1.57E-01
HOXB3	0.0061	9.06E-01	9.41E-01
HOXB4	-0.1204	2.04E-02	5.22E-02
HOXB5	0.0291	5.77E-01	6.93E-01

HOXB6	0.0314	5.47E-01	6.67E-01
HOXB7	0.1003	5.35E-02	1.13E-01
HOXB8	0.0653	2.09E-01	3.28E-01
HOXB9	0.1300	1.22E-02	3.45E-02
HOXC10	0.1197	2.12E-02	5.37E-02
HOXC11	0.1494	3.91E-03	1.35E-02
HOXC12	0.0509	3.28E-01	4.59E-01
HOXC13	0.0651	2.11E-01	3.30E-01
HOXC4	0.0846	1.04E-01	1.91E-01
HOXC5	0.0619	2.34E-01	3.57E-01
HOXC6	0.0904	8.20E-02	1.59E-01
HOXC8	0.0742	1.54E-01	2.58E-01
HOXC9	0.1363	8.57E-03	2.58E-02
HOXD10	0.0993	5.60E-02	1.18E-01
HOXD11	0.2014	9.39E-05	5.78E-04
HOXD12	0.0602	2.48E-01	3.73E-01
HOXD13	0.1106	3.31E-02	7.75E-02
HOXD1	0.2504	1.03E-06	1.16E-05
HOXD3	0.1839	3.70E-04	1.86E-03
HOXD4	0.2003	1.03E-04	6.21E-04
HOXD8	0.0122	8.15E-01	8.78E-01
HOXD9	0.0548	2.92E-01	4.21E-01
HP1BP3	0.1425	5.97E-03	1.91E-02
HPCAL1	-0.0556	2.85E-01	4.14E-01
HPCAL4	-0.0109	8.35E-01	8.92E-01
HPCA	0.1563	2.54E-03	9.39E-03
HPDL	0.2319	6.39E-06	5.72E-05
HPD	-0.3271	1.06E-10	3.10E-09
HPGDS	0.0293	5.73E-01	6.91E-01
HPGD	-0.1389	7.36E-03	2.27E-02
HPN	-0.1733	8.01E-04	3.55E-03
HPRT1	0.2048	7.06E-05	4.54E-04
HPR	-0.2264	1.06E-05	8.89E-05
HPS1	-0.0043	9.35E-01	9.59E-01
HPS3	0.1645	1.47E-03	5.96E-03
HPS4	0.2008	9.82E-05	6.00E-04
HPS5	-0.1103	3.37E-02	7.87E-02
HPS6	-0.0864	9.66E-02	1.81E-01
HPSE2	0.1717	8.97E-04	3.92E-03
HPSE	0.0594	2.54E-01	3.80E-01
HPVC1	-0.0316	5.44E-01	6.65E-01
HPX	-0.1954	1.53E-04	8.76E-04
HPYR1	0.0715	1.69E-01	2.78E-01
HP	-0.2358	4.42E-06	4.18E-05

HRASLS2	0.0170	7.44E-01	8.27E-01
HRASLS5	0.0750	1.49E-01	2.53E-01
HRASLS	0.0387	4.57E-01	5.88E-01
HRAS	0.1214	1.93E-02	4.99E-02
HRCT1	0.0116	8.24E-01	8.85E-01
HRC	-0.0899	8.37E-02	1.61E-01
HRG	-0.1250	1.60E-02	4.29E-02
HRH1	-0.0359	4.90E-01	6.18E-01
HRH2	-0.0747	1.51E-01	2.55E-01
HRH3	0.0107	8.37E-01	8.93E-01
HRH4	-0.0865	9.62E-02	1.80E-01
HRK	0.0928	7.43E-02	1.47E-01
HRNBP3	0.2535	7.49E-07	8.75E-06
HRNR	0.0281	5.89E-01	7.04E-01
HRSP12	-0.2856	2.15E-08	3.69E-07
HR	0.0363	4.85E-01	6.13E-01
HS1BP3	-0.1070	3.94E-02	8.91E-02
HS2ST1	0.0531	3.07E-01	4.37E-01
HS3ST1	-0.0617	2.35E-01	3.59E-01
HS3ST2	-0.2231	1.44E-05	1.16E-04
HS3ST3A1	-0.1333	1.01E-02	2.96E-02
HS3ST3B1	-0.1743	7.44E-04	3.34E-03
HS3ST4	0.0413	4.28E-01	5.60E-01
HS3ST5	0.0499	3.38E-01	4.69E-01
HS3ST6	0.1777	5.86E-04	2.71E-03
HS6ST1	-0.0526	3.13E-01	4.42E-01
HS6ST2	0.1982	1.21E-04	7.18E-04
HS6ST3	0.0024	9.64E-01	9.78E-01
HSBP1L1	0.0008	9.87E-01	9.92E-01
HSBP1	-0.0380	4.66E-01	5.96E-01
HSCB	-0.1278	1.38E-02	3.81E-02
HSD11B1L	0.0158	7.62E-01	8.40E-01
HSD11B1	-0.2225	1.52E-05	1.21E-04
HSD11B2	0.0842	1.06E-01	1.94E-01
HSD17B10	-0.2009	9.76E-05	5.97E-04
HSD17B11	-0.2073	5.72E-05	3.80E-04
HSD17B12	-0.1472	4.50E-03	1.51E-02
HSD17B13	-0.1878	2.74E-04	1.44E-03
HSD17B14	-0.0128	8.06E-01	8.72E-01
HSD17B1	0.0962	6.41E-02	1.31E-01
HSD17B2	-0.0970	6.20E-02	1.27E-01
HSD17B3	0.0349	5.03E-01	6.28E-01
HSD17B4	-0.3017	3.02E-09	6.37E-08
HSD17B6	-0.2204	1.83E-05	1.43E-04

HSD17B7P2	0.1894	2.43E-04	1.30E-03
HSD17B7	0.1753	6.95E-04	3.15E-03
HSD17B8	-0.2009	9.79E-05	5.99E-04
HSD3B1	0.0396	4.47E-01	5.78E-01
HSD3B2	0.0929	7.40E-02	1.47E-01
HSD3B7	-0.1066	4.02E-02	9.05E-02
HSDL1	-0.0077	8.82E-01	9.25E-01
HSDL2	-0.1731	8.14E-04	3.60E-03
HSF1	0.0484	3.52E-01	4.84E-01
HSF2BP	0.2374	3.76E-06	3.63E-05
HSF2	0.3684	2.27E-13	1.13E-11
HSF4	0.2280	9.16E-06	7.78E-05
HSF5	-0.0221	6.71E-01	7.71E-01
HSFX2	0.1486	4.13E-03	1.41E-02
HSFY2	0.0333	5.22E-01	6.46E-01
HSFYL1	-0.0607	2.43E-01	3.68E-01
HSH2D	0.0262	6.15E-01	7.25E-01
HSN2	0.0539	3.01E-01	4.30E-01
HSP90AA1	0.0557	2.84E-01	4.13E-01
HSP90AB1	0.1458	4.89E-03	1.62E-02
HSP90AB2P	0.1145	2.74E-02	6.64E-02
HSP90AB4P	0.1133	2.91E-02	6.98E-02
HSP90B1	0.0243	6.41E-01	7.47E-01
HSP90B3P	0.0590	2.57E-01	3.83E-01
HSPA12A	0.0726	1.63E-01	2.70E-01
HSPA12B	-0.1828	4.00E-04	1.98E-03
HSPA13	0.1695	1.05E-03	4.48E-03
HSPA14	0.3804	3.21E-14	1.89E-12
HSPA1A	0.1182	2.28E-02	5.71E-02
HSPA1B	0.1356	8.94E-03	2.67E-02
HSPA1L	0.1433	5.68E-03	1.83E-02
HSPA2	0.0435	4.04E-01	5.37E-01
HSPA4L	-0.0322	5.36E-01	6.58E-01
HSPA4	-0.0056	9.14E-01	9.46E-01
HSPA5	0.0909	8.05E-02	1.56E-01
HSPA6	0.1522	3.29E-03	1.17E-02
HSPA7	0.1405	6.72E-03	2.11E-02
HSPA8	-0.0073	8.89E-01	9.29E-01
HSPA9	-0.1892	2.46E-04	1.32E-03
HSPB11	0.3427	1.16E-11	4.09E-10
HSPB1	0.0816	1.17E-01	2.10E-01
HSPB2	-0.0952	6.70E-02	1.36E-01
HSPB3	0.0958	6.54E-02	1.33E-01
HSPB6	-0.0824	1.13E-01	2.05E-01

HSPB7	-0.0363	4.86E-01	6.14E-01
HSPB8	-0.0811	1.19E-01	2.13E-01
HSPB9	0.0616	2.37E-01	3.60E-01
HSPBAP1	0.2277	9.46E-06	8.00E-05
HSPBP1	0.0157	7.63E-01	8.40E-01
HSPC072	0.1852	3.36E-04	1.72E-03
HSPC157	0.0066	9.00E-01	9.37E-01
HSPC159	0.0106	8.39E-01	8.94E-01
HSPD1	0.0321	5.38E-01	6.60E-01
HSPE1	0.0717	1.68E-01	2.77E-01
HSPG2	-0.1231	1.77E-02	4.64E-02
HSPH1	0.1198	2.10E-02	5.35E-02
HTATIP2	-0.0115	8.25E-01	8.86E-01
HTATSF1	0.2638	2.52E-07	3.34E-06
HTA	-0.0630	2.26E-01	3.48E-01
HTN1	0.0590	2.57E-01	3.83E-01
HTN3	0.0873	9.32E-02	1.76E-01
HTR1A	0.0793	1.27E-01	2.23E-01
HTR1B	-0.0309	5.53E-01	6.73E-01
HTR1D	-0.0065	9.01E-01	9.37E-01
HTR1E	0.0287	5.82E-01	6.98E-01
HTR1F	0.0997	5.50E-02	1.16E-01
HTR2A	-0.0722	1.65E-01	2.73E-01
HTR2B	-0.2648	2.26E-07	3.02E-06
HTR2C	0.0788	1.30E-01	2.27E-01
HTR3A	0.1670	1.24E-03	5.17E-03
HTR3B	0.1174	2.37E-02	5.90E-02
HTR3C	-0.0012	9.82E-01	9.89E-01
HTR3D	0.0641	2.18E-01	3.38E-01
HTR3E	0.0586	2.60E-01	3.86E-01
HTR4	0.1465	4.69E-03	1.56E-02
HTR5A	0.0069	8.95E-01	9.33E-01
HTR6	0.0896	8.48E-02	1.63E-01
HTR7P1	-0.0177	7.34E-01	8.20E-01
HTR7	-0.1466	4.67E-03	1.56E-02
HTRA1	-0.2350	4.76E-06	4.46E-05
HTRA2	0.0495	3.42E-01	4.73E-01
HTRA3	0.0499	3.38E-01	4.69E-01
HTRA4	0.0094	8.56E-01	9.07E-01
HTT	-0.0127	8.07E-01	8.73E-01
HULC	-0.1523	3.27E-03	1.16E-02
HUNK	0.2271	9.95E-06	8.38E-05
HUS1B	0.2109	4.25E-05	2.95E-04
HUS1	-0.0009	9.86E-01	9.91E-01

HUWE1	0.1144	2.76E-02	6.67E-02
HVCN1	-0.0649	2.12E-01	3.32E-01
HYAL1	-0.4006	9.86E-16	8.01E-14
HYAL2	-0.1569	2.44E-03	9.09E-03
HYAL3	-0.0043	9.34E-01	9.59E-01
HYAL4	0.1637	1.55E-03	6.23E-03
HYDIN	0.0405	4.37E-01	5.68E-01
HYI	-0.1068	3.97E-02	8.96E-02
HYLS1	0.2367	4.03E-06	3.86E-05
HYMAI	0.1187	2.22E-02	5.59E-02
HYOU1	0.0005	9.93E-01	9.96E-01
IAH1	-0.0513	3.24E-01	4.54E-01
IAPP	-0.0565	2.78E-01	4.06E-01
IARS2	0.0330	5.26E-01	6.49E-01
IARS	0.2319	6.37E-06	5.71E-05
IBSP	0.1121	3.09E-02	7.32E-02
IBTK	0.0549	2.92E-01	4.21E-01
ICA1L	0.1305	1.18E-02	3.35E-02
ICA1	0.0528	3.10E-01	4.40E-01
ICAM1	0.0468	3.69E-01	5.02E-01
ICAM2	-0.0377	4.69E-01	5.99E-01
ICAM3	-0.2559	5.86E-07	7.02E-06
ICAM4	0.0528	3.11E-01	4.40E-01
ICAM5	0.0272	6.02E-01	7.14E-01
ICK	0.0702	1.77E-01	2.88E-01
ICMT	-0.0023	9.65E-01	9.79E-01
ICOSLG	-0.2386	3.36E-06	3.29E-05
ICOS	0.0446	3.91E-01	5.24E-01
ICT1	0.0792	1.28E-01	2.24E-01
ID1	-0.1075	3.84E-02	8.74E-02
ID2B	-0.0642	2.17E-01	3.37E-01
ID2	-0.0760	1.44E-01	2.46E-01
ID3	-0.0702	1.77E-01	2.88E-01
ID4	-0.1008	5.24E-02	1.11E-01
IDE	-0.1593	2.08E-03	7.99E-03
IDH1	-0.1692	1.07E-03	4.55E-03
IDH2	-0.1868	2.98E-04	1.55E-03
IDH3A	-0.1822	4.21E-04	2.07E-03
IDH3B	-0.0687	1.87E-01	3.00E-01
IDH3G	0.0971	6.17E-02	1.27E-01
IDI1	-0.0675	1.95E-01	3.10E-01
IDI2	0.1315	1.12E-02	3.21E-02
IDO1	0.0276	5.96E-01	7.10E-01
IDO2	-0.1539	2.96E-03	1.07E-02

IDS	-0.0240	6.45E-01	7.50E-01
IDUA	0.0667	2.00E-01	3.16E-01
IER2	-0.0459	3.78E-01	5.11E-01
IER3IP1	0.1820	4.25E-04	2.08E-03
IER3	0.1344	9.56E-03	2.82E-02
IER5L	0.0355	4.96E-01	6.22E-01
IER5	0.2880	1.62E-08	2.88E-07
IFFO1	0.0110	8.32E-01	8.90E-01
IFFO2	0.1823	4.17E-04	2.05E-03
IFI16	-0.0156	7.64E-01	8.41E-01
IFI27L1	0.0646	2.14E-01	3.34E-01
IFI27L2	0.1529	3.16E-03	1.13E-02
IFI27	-0.0966	6.31E-02	1.29E-01
IFI30	-0.0115	8.25E-01	8.85E-01
IFI35	0.0561	2.81E-01	4.10E-01
IFI44L	-0.1002	5.38E-02	1.14E-01
IFI44	-0.0868	9.50E-02	1.79E-01
IFI6	0.0110	8.32E-01	8.90E-01
IFIH1	0.0513	3.24E-01	4.55E-01
IFIT1B	-0.1267	1.46E-02	3.98E-02
IFIT1	-0.1835	3.80E-04	1.90E-03
IFIT2	-0.1761	6.57E-04	3.00E-03
IFIT3	-0.1612	1.84E-03	7.19E-03
IFIT5	-0.1469	4.59E-03	1.54E-02
IFITM1	-0.1610	1.87E-03	7.27E-03
IFITM2	-0.1918	2.01E-04	1.11E-03
IFITM3	-0.0635	2.23E-01	3.44E-01
IFITM4P	0.1317	1.11E-02	3.19E-02
IFITM5	0.0223	6.68E-01	7.68E-01
IFLTD1	-0.0042	9.35E-01	9.60E-01
IFNA14	0.0243	6.40E-01	7.46E-01
IFNA1	0.0410	4.31E-01	5.63E-01
IFNA21	0.0645	2.15E-01	3.35E-01
IFNA4	0.0836	1.08E-01	1.97E-01
IFNA5	0.0593	2.55E-01	3.80E-01
IFNA7	0.0579	2.66E-01	3.93E-01
IFNAR1	-0.2061	6.35E-05	4.14E-04
IFNAR2	0.0368	4.80E-01	6.08E-01
IFNB1	0.1074	3.87E-02	8.78E-02
IFNE	0.1275	1.40E-02	3.86E-02
IFNGR1	-0.1257	1.54E-02	4.15E-02
IFNGR2	0.1659	1.34E-03	5.51E-03
IFNG	0.0258	6.21E-01	7.30E-01
IFNK	-0.0318	5.41E-01	6.62E-01

IFNW1	0.0365	4.83E-01	6.11E-01
IFRD1	0.1586	2.19E-03	8.31E-03
IFRD2	0.0079	8.79E-01	9.23E-01
IFT122	0.0124	8.11E-01	8.76E-01
IFT140	0.1491	4.00E-03	1.37E-02
IFT172	0.1877	2.78E-04	1.46E-03
IFT20	0.1319	1.10E-02	3.16E-02
IFT27	0.1054	4.25E-02	9.45E-02
IFT46	-0.1015	5.08E-02	1.09E-01
IFT52	0.2820	3.27E-08	5.33E-07
IFT57	0.1272	1.42E-02	3.90E-02
IFT74	0.1909	2.17E-04	1.18E-03
IFT80	0.2352	4.66E-06	4.38E-05
IFT81	0.2188	2.11E-05	1.62E-04
IFT88	-0.0160	7.58E-01	8.38E-01
IGBP1	0.1062	4.10E-02	9.19E-02
IGDCC3	0.1025	4.84E-02	1.05E-01
IGDCC4	0.1266	1.47E-02	4.00E-02
IGF1R	0.0675	1.94E-01	3.10E-01
IGF1	-0.2314	6.67E-06	5.94E-05
IGF2AS	0.0843	1.05E-01	1.93E-01
IGF2BP1	0.2776	5.42E-08	8.36E-07
IGF2BP2	0.2005	1.01E-04	6.14E-04
IGF2BP3	0.2096	4.74E-05	3.23E-04
IGF2R	0.1084	3.69E-02	8.45E-02
IGF2	0.0238	6.48E-01	7.52E-01
IGFALS	-0.0431	4.08E-01	5.41E-01
IGFBP1	-0.1538	2.98E-03	1.07E-02
IGFBP2	-0.1408	6.62E-03	2.08E-02
IGFBP3	0.0767	1.40E-01	2.41E-01
IGFBP4	-0.2137	3.31E-05	2.38E-04
IGFBP5	-0.1620	1.75E-03	6.88E-03
IGFBP6	-0.0476	3.60E-01	4.92E-01
IGFBP7	-0.1912	2.12E-04	1.16E-03
IGFBPL1	0.1734	7.99E-04	3.55E-03
IGFL1	0.0705	1.75E-01	2.86E-01
IGFL2	0.1221	1.86E-02	4.85E-02
IGFL3	0.0179	7.31E-01	8.18E-01
IGFL4	0.0720	1.67E-01	2.75E-01
IGFN1	-0.0206	6.92E-01	7.87E-01
IGHMBP2	0.1130	2.95E-02	7.05E-02
IGJ	-0.0974	6.10E-02	1.26E-01
IGLL1	0.0452	3.85E-01	5.19E-01
IGLL3	0.1563	2.54E-03	9.38E-03

IGLON5	0.0159	7.60E-01	8.39E-01
IGSF10	-0.1404	6.76E-03	2.11E-02
IGSF11	0.0468	3.69E-01	5.02E-01
IGSF1	0.2206	1.81E-05	1.42E-04
IGSF21	-0.0977	6.01E-02	1.24E-01
IGSF22	0.0958	6.52E-02	1.33E-01
IGSF3	0.1983	1.21E-04	7.16E-04
IGSF5	0.0682	1.90E-01	3.04E-01
IGSF6	-0.0750	1.49E-01	2.53E-01
IGSF8	0.1317	1.11E-02	3.18E-02
IGSF9B	-0.0787	1.30E-01	2.27E-01
IGSF9	0.0038	9.43E-01	9.64E-01
IHH	0.0603	2.47E-01	3.72E-01
IKBIP	0.0629	2.27E-01	3.49E-01
IKBKAP	0.2141	3.21E-05	2.32E-04
IKBKB	0.1295	1.26E-02	3.52E-02
IKBKE	0.2484	1.27E-06	1.40E-05
IKBKG	0.0589	2.58E-01	3.84E-01
IKZF1	-0.1032	4.70E-02	1.02E-01
IKZF2	-0.1272	1.42E-02	3.90E-02
IKZF3	-0.1162	2.52E-02	6.19E-02
IKZF4	0.0670	1.98E-01	3.14E-01
IKZF5	-0.0222	6.70E-01	7.69E-01
IK	0.1895	2.41E-04	1.29E-03
IL10RA	-0.1068	3.98E-02	8.98E-02
IL10RB	0.0536	3.03E-01	4.33E-01
IL10	-0.1130	2.96E-02	7.07E-02
IL11RA	0.1574	2.36E-03	8.82E-03
IL11	0.1984	1.20E-04	7.11E-04
IL12A	0.2826	3.05E-08	5.02E-07
IL12B	-0.0049	9.25E-01	9.52E-01
IL12RB1	-0.0391	4.52E-01	5.84E-01
IL12RB2	-0.0897	8.44E-02	1.62E-01
IL13RA1	-0.1269	1.45E-02	3.96E-02
IL13RA2	0.0047	9.29E-01	9.55E-01
IL13	0.0852	1.01E-01	1.88E-01
IL15RA	0.0415	4.25E-01	5.58E-01
IL15	-0.0217	6.77E-01	7.75E-01
IL16	-0.0788	1.30E-01	2.27E-01
IL17A	-0.0818	1.16E-01	2.09E-01
IL17B	-0.0072	8.91E-01	9.30E-01
IL17C	0.0582	2.64E-01	3.90E-01
IL17D	0.0683	1.90E-01	3.03E-01
IL17F	0.1412	6.43E-03	2.03E-02

IL17RA	-0.0309	5.53E-01	6.73E-01
IL17RB	0.1995	1.09E-04	6.56E-04
IL17RC	-0.1714	9.19E-04	4.00E-03
IL17RD	0.0300	5.64E-01	6.82E-01
IL17REL	0.1255	1.56E-02	4.19E-02
IL17RE	0.1198	2.10E-02	5.34E-02
IL18BP	-0.0477	3.60E-01	4.92E-01
IL18R1	-0.0952	6.71E-02	1.36E-01
IL18RAP	-0.0595	2.53E-01	3.78E-01
IL18	-0.0520	3.18E-01	4.48E-01
IL19	0.0159	7.60E-01	8.38E-01
IL1A	0.0974	6.08E-02	1.26E-01
IL1B	-0.0030	9.55E-01	9.72E-01
IL1F10	0.0537	3.02E-01	4.31E-01
IL1F5	-0.0539	3.01E-01	4.30E-01
IL1F6	0.0096	8.54E-01	9.06E-01
IL1F7	0.1959	1.46E-04	8.45E-04
IL1F8	-0.0135	7.96E-01	8.65E-01
IL1F9	0.0876	9.20E-02	1.74E-01
IL1R1	-0.2139	3.25E-05	2.34E-04
IL1R2	-0.0393	4.50E-01	5.82E-01
IL1RAPL1	0.0348	5.04E-01	6.29E-01
IL1RAPL2	-0.0826	1.12E-01	2.03E-01
IL1RAP	0.0233	6.55E-01	7.57E-01
IL1RL1	-0.2949	7.01E-09	1.35E-07
IL1RL2	-0.0116	8.24E-01	8.85E-01
IL1RN	-0.0916	7.80E-02	1.53E-01
IL20RA	0.2003	1.02E-04	6.21E-04
IL20RB	0.0407	4.34E-01	5.66E-01
IL20	-0.0095	8.55E-01	9.06E-01
IL21R	0.0577	2.67E-01	3.95E-01
IL21	-0.0689	1.85E-01	2.98E-01
IL22RA1	0.1145	2.74E-02	6.63E-02
IL22RA2	0.0569	2.75E-01	4.03E-01
IL22	0.0039	9.41E-01	9.64E-01
IL23A	-0.0041	9.37E-01	9.60E-01
IL23R	-0.0386	4.59E-01	5.89E-01
IL24	-0.0261	6.17E-01	7.26E-01
IL25	-0.0364	4.85E-01	6.13E-01
IL26	0.0218	6.76E-01	7.74E-01
IL27RA	0.1352	9.10E-03	2.71E-02
IL27	-0.0969	6.23E-02	1.28E-01
IL28A	0.0888	8.76E-02	1.67E-01
IL28B	0.0437	4.01E-01	5.34E-01

IL28RA	-0.0252	6.28E-01	7.36E-01
IL29	0.0220	6.73E-01	7.72E-01
IL2RA	-0.0091	8.62E-01	9.11E-01
IL2RB	-0.0896	8.47E-02	1.63E-01
IL2RG	0.0536	3.04E-01	4.33E-01
IL2	-0.0023	9.64E-01	9.78E-01
IL31RA	0.0109	8.35E-01	8.92E-01
IL31	0.0787	1.30E-01	2.28E-01
IL32	-0.0737	1.56E-01	2.62E-01
IL33	-0.1625	1.69E-03	6.70E-03
IL34	-0.0008	9.88E-01	9.93E-01
IL3RA	-0.0734	1.58E-01	2.64E-01
IL3	0.0196	7.07E-01	7.99E-01
IL4I1	0.1470	4.55E-03	1.53E-02
IL4R	0.0623	2.31E-01	3.54E-01
IL4	0.1746	7.31E-04	3.29E-03
IL5RA	-0.1376	7.96E-03	2.42E-02
IL5	0.0458	3.79E-01	5.13E-01
IL6R	-0.0216	6.78E-01	7.76E-01
IL6ST	-0.1461	4.81E-03	1.60E-02
IL6	-0.1487	4.11E-03	1.41E-02
IL7R	-0.1594	2.07E-03	7.94E-03
IL7	-0.0818	1.16E-01	2.08E-01
IL8	0.0886	8.83E-02	1.68E-01
IL9R	0.0679	1.92E-01	3.06E-01
IL9	0.0913	7.90E-02	1.54E-01
ILDR1	0.1917	2.04E-04	1.12E-03
ILDR2	0.1144	2.75E-02	6.66E-02
ILF2	0.5509	7.82E-31	8.65E-28
ILF3	0.3599	8.68E-13	3.92E-11
ILKAP	0.2323	6.16E-06	5.54E-05
ILK	-0.0362	4.87E-01	6.14E-01
ILVBL	-0.2083	5.28E-05	3.55E-04
IMMP1L	0.2302	7.47E-06	6.52E-05
IMMP2L	-0.1924	1.93E-04	1.07E-03
IMMT	-0.1161	2.53E-02	6.21E-02
IMP3	-0.1026	4.83E-02	1.05E-01
IMP4	-0.0761	1.43E-01	2.45E-01
IMP5	0.0083	8.73E-01	9.18E-01
IMPA1	-0.0922	7.60E-02	1.50E-01
IMPA2	0.0959	6.49E-02	1.32E-01
IMPACT	-0.1408	6.61E-03	2.08E-02
IMPAD1	-0.2232	1.43E-05	1.16E-04
IMPDH1	0.1041	4.51E-02	9.91E-02

IMPDH2	0.0648	2.13E-01	3.33E-01
IMPG1	0.1133	2.92E-02	6.99E-02
IMPG2	-0.0488	3.48E-01	4.80E-01
INADL	0.0675	1.95E-01	3.10E-01
INA	-0.0724	1.64E-01	2.71E-01
INCA1	-0.1333	1.02E-02	2.96E-02
INCENP	0.2339	5.30E-06	4.87E-05
INE1	0.0815	1.17E-01	2.11E-01
INE2	0.0379	4.66E-01	5.96E-01
INF2	0.0109	8.35E-01	8.92E-01
ING1	0.0119	8.19E-01	8.81E-01
ING2	-0.0134	7.97E-01	8.65E-01
ING3	-0.0213	6.82E-01	7.79E-01
ING4	0.0363	4.86E-01	6.14E-01
ING5	0.2900	1.28E-08	2.32E-07
INGX	0.0882	8.97E-02	1.71E-01
INHA	0.0963	6.40E-02	1.31E-01
INHBA	-0.2109	4.23E-05	2.94E-04
INHBB	-0.0068	8.96E-01	9.34E-01
INHBC	-0.0955	6.61E-02	1.34E-01
INHBE	-0.0408	4.34E-01	5.65E-01
INMT	-0.1858	3.22E-04	1.66E-03
INO80B	-0.1142	2.78E-02	6.73E-02
INO80C	0.1347	9.37E-03	2.78E-02
INO80D	0.1558	2.62E-03	9.64E-03
INO80E	0.1670	1.24E-03	5.16E-03
INO80	0.0301	5.63E-01	6.81E-01
INPP1	-0.1241	1.68E-02	4.45E-02
INPP4A	0.1831	3.92E-04	1.95E-03
INPP4B	-0.1198	2.10E-02	5.35E-02
INPP5A	0.0209	6.88E-01	7.84E-01
INPP5B	-0.1109	3.27E-02	7.67E-02
INPP5D	-0.1062	4.09E-02	9.18E-02
INPP5E	0.1950	1.57E-04	8.97E-04
INPP5F	0.1169	2.43E-02	6.02E-02
INPP5J	0.2157	2.78E-05	2.05E-04
INPP5K	-0.0446	3.91E-01	5.24E-01
INPPL1	0.0899	8.37E-02	1.61E-01
INS-IGF2	-0.1820	4.27E-04	2.08E-03
INSC	0.0049	9.25E-01	9.52E-01
INSIG1	-0.2069	5.95E-05	3.92E-04
INSIG2	-0.0947	6.84E-02	1.38E-01
INSL3	0.0640	2.19E-01	3.39E-01
INSL4	-0.0487	3.50E-01	4.82E-01

INSL5	-0.0280	5.91E-01	7.05E-01
INSL6	0.1276	1.39E-02	3.84E-02
INSM1	0.0642	2.17E-01	3.38E-01
INSM2	0.0332	5.24E-01	6.47E-01
INSRR	0.1459	4.86E-03	1.61E-02
INSR	-0.2037	7.77E-05	4.93E-04
INS	-0.0663	2.02E-01	3.20E-01
INTS10	-0.0787	1.30E-01	2.27E-01
INTS12	-0.0716	1.69E-01	2.78E-01
INTS1	0.0638	2.20E-01	3.41E-01
INTS2	0.1279	1.37E-02	3.78E-02
INTS3	0.5199	4.47E-27	3.07E-24
INTS4L1	0.0932	7.30E-02	1.45E-01
INTS4L2	0.0605	2.45E-01	3.69E-01
INTS4	0.2969	5.48E-09	1.09E-07
INTS5	-0.1455	4.98E-03	1.64E-02
INTS6	-0.0327	5.30E-01	6.53E-01
INTS7	0.3909	5.41E-15	3.70E-13
INTS8	0.3641	4.49E-13	2.14E-11
INTS9	0.0382	4.63E-01	5.94E-01
INTU	0.2413	2.57E-06	2.59E-05
INVS	0.0788	1.30E-01	2.27E-01
IP6K1	0.2490	1.19E-06	1.32E-05
IP6K2	0.1760	6.62E-04	3.02E-03
IP6K3	0.0071	8.92E-01	9.31E-01
IPCEF1	-0.0713	1.70E-01	2.80E-01
IPMK	-0.0133	7.99E-01	8.67E-01
IPO11	-0.0197	7.05E-01	7.97E-01
IPO13	0.0262	6.14E-01	7.25E-01
IPO4	-0.0402	4.40E-01	5.72E-01
IPO5	0.2003	1.03E-04	6.21E-04
IPO7	0.0653	2.09E-01	3.28E-01
IPO8	0.0419	4.21E-01	5.54E-01
IPO9	0.4264	8.09E-18	1.11E-15
IPPK	0.1069	3.96E-02	8.95E-02
IPP	-0.0466	3.71E-01	5.04E-01
IPW	0.0625	2.30E-01	3.53E-01
IQCA1	-0.0070	8.93E-01	9.32E-01
IQCB1	0.3956	2.41E-15	1.77E-13
IQCC	0.3869	1.07E-14	6.86E-13
IQCD	0.2602	3.72E-07	4.72E-06
IQCE	0.1895	2.41E-04	1.29E-03
IQCF1	0.1886	2.59E-04	1.37E-03
IQCF2	0.0120	8.17E-01	8.80E-01

IQCF3	0.1389	7.38E-03	2.27E-02
IQCF5	0.0320	5.39E-01	6.61E-01
IQCF6	0.0690	1.85E-01	2.98E-01
IQCG	0.0564	2.79E-01	4.07E-01
IQCH	-0.0699	1.79E-01	2.90E-01
IQCJ	0.1018	5.02E-02	1.08E-01
IQCK	0.0535	3.04E-01	4.34E-01
IQGAP1	0.0227	6.63E-01	7.64E-01
IQGAP2	-0.2133	3.44E-05	2.47E-04
IQGAP3	0.4754	2.55E-22	9.25E-20
IQSEC1	-0.0480	3.57E-01	4.89E-01
IQSEC2	-0.0021	9.67E-01	9.80E-01
IQSEC3	-0.1307	1.18E-02	3.33E-02
IQUB	0.0231	6.57E-01	7.59E-01
IRAK1BP1	0.2273	9.79E-06	8.26E-05
IRAK1	0.1978	1.26E-04	7.43E-04
IRAK2	0.0332	5.24E-01	6.47E-01
IRAK3	-0.1178	2.33E-02	5.81E-02
IRAK4	0.0398	4.45E-01	5.77E-01
IREB2	-0.0424	4.15E-01	5.48E-01
IRF1	0.0959	6.50E-02	1.32E-01
IRF2BP1	0.1429	5.82E-03	1.87E-02
IRF2BP2	0.2875	1.73E-08	3.04E-07
IRF2	-0.1496	3.88E-03	1.34E-02
IRF3	0.1968	1.37E-04	7.96E-04
IRF4	-0.0169	7.46E-01	8.28E-01
IRF5	0.1259	1.53E-02	4.13E-02
IRF6	-0.0319	5.40E-01	6.62E-01
IRF7	0.0149	7.74E-01	8.49E-01
IRF8	0.0035	9.46E-01	9.67E-01
IRF9	-0.0427	4.12E-01	5.45E-01
IRGC	0.1095	3.49E-02	8.09E-02
IRGM	-0.0438	4.00E-01	5.33E-01
IRGQ	0.1207	2.01E-02	5.15E-02
IRS1	-0.1272	1.42E-02	3.89E-02
IRS2	-0.0766	1.41E-01	2.42E-01
IRS4	-0.0195	7.08E-01	8.00E-01
IRX1	0.0016	9.75E-01	9.84E-01
IRX2	-0.0318	5.41E-01	6.62E-01
IRX3	0.0414	4.27E-01	5.59E-01
IRX4	-0.0483	3.53E-01	4.85E-01
IRX5	0.0593	2.54E-01	3.80E-01
IRX6	-0.0016	9.75E-01	9.84E-01
ISCA1P1	-0.0321	5.37E-01	6.59E-01

ISCA1	-0.0218	6.75E-01	7.74E-01
ISCA2	-0.0595	2.53E-01	3.78E-01
ISCU	-0.2480	1.32E-06	1.44E-05
ISG15	-0.0634	2.23E-01	3.44E-01
ISG20L2	0.4463	1.46E-19	3.30E-17
ISG20	-0.0131	8.01E-01	8.69E-01
ISL1	0.1468	4.61E-03	1.54E-02
ISL2	0.2121	3.81E-05	2.69E-04
ISLR2	-0.1443	5.37E-03	1.75E-02
ISLR	-0.0558	2.83E-01	4.12E-01
ISM1	-0.1534	3.06E-03	1.10E-02
ISM2	0.1307	1.17E-02	3.33E-02
ISOC1	-0.2014	9.38E-05	5.78E-04
ISOC2	-0.2512	9.54E-07	1.09E-05
ISPD	-0.2404	2.82E-06	2.81E-05
ISX	0.0610	2.41E-01	3.66E-01
ISY1	0.2441	1.96E-06	2.06E-05
ISYNA1	0.0704	1.76E-01	2.87E-01
ITCH	-0.1243	1.66E-02	4.42E-02
ITFG1	-0.3427	1.15E-11	4.06E-10
ITFG2	0.2419	2.43E-06	2.47E-05
ITFG3	-0.0358	4.92E-01	6.19E-01
ITGA10	-0.0595	2.53E-01	3.78E-01
ITGA11	-0.0709	1.73E-01	2.83E-01
ITGA1	-0.2372	3.84E-06	3.70E-05
ITGA2B	0.0367	4.81E-01	6.09E-01
ITGA2	0.0602	2.48E-01	3.73E-01
ITGA3	0.0242	6.42E-01	7.48E-01
ITGA4	-0.0635	2.22E-01	3.43E-01
ITGA5	0.1538	2.97E-03	1.07E-02
ITGA6	0.0157	7.62E-01	8.40E-01
ITGA7	-0.1262	1.50E-02	4.08E-02
ITGA8	-0.1496	3.87E-03	1.34E-02
ITGA9	-0.1841	3.65E-04	1.84E-03
ITGAD	-0.0974	6.09E-02	1.26E-01
ITGAE	0.1272	1.42E-02	3.90E-02
ITGAL	0.0416	4.24E-01	5.57E-01
ITGAM	0.0447	3.91E-01	5.24E-01
ITGAV	0.0462	3.75E-01	5.08E-01
ITGAX	-0.0245	6.39E-01	7.45E-01
ITGB1BP1	0.1903	2.28E-04	1.23E-03
ITGB1BP2	0.0462	3.75E-01	5.09E-01
ITGB1BP3	-0.1261	1.50E-02	4.08E-02
ITGB1	-0.0596	2.52E-01	3.77E-01

ITGB2	-0.0699	1.79E-01	2.91E-01
ITGB3BP	0.3489	4.62E-12	1.80E-10
ITGB3	-0.0751	1.49E-01	2.53E-01
ITGB4	0.0853	1.01E-01	1.87E-01
ITGB5	0.0306	5.56E-01	6.75E-01
ITGB6	0.1048	4.37E-02	9.67E-02
ITGB7	-0.0473	3.63E-01	4.96E-01
ITGB8	0.0771	1.38E-01	2.38E-01
ITGBL1	-0.0824	1.13E-01	2.05E-01
ITIH1	-0.1199	2.09E-02	5.33E-02
ITIH2	-0.0831	1.10E-01	2.00E-01
ITIH3	-0.0799	1.24E-01	2.20E-01
ITIH4	-0.2428	2.23E-06	2.29E-05
ITIH5L	0.0684	1.89E-01	3.02E-01
ITIH5	0.0957	6.56E-02	1.33E-01
ITK	-0.1119	3.12E-02	7.37E-02
ITLN1	0.1058	4.16E-02	9.29E-02
ITLN2	0.0532	3.07E-01	4.37E-01
ITM2A	-0.1161	2.54E-02	6.22E-02
ITM2B	-0.1952	1.55E-04	8.86E-04
ITM2C	0.1428	5.85E-03	1.88E-02
ITPA	0.0333	5.23E-01	6.47E-01
ITPK1	-0.0930	7.37E-02	1.46E-01
ITPKA	0.1896	2.41E-04	1.29E-03
ITPKB	-0.1444	5.32E-03	1.74E-02
ITPKC	0.0123	8.14E-01	8.77E-01
ITPR1	-0.1423	6.02E-03	1.92E-02
ITPR2	-0.1698	1.02E-03	4.38E-03
ITPR3	0.1960	1.45E-04	8.38E-04
ITPRIPL1	0.0480	3.56E-01	4.88E-01
ITPRIPL2	0.0835	1.08E-01	1.98E-01
ITPRIP	-0.3103	1.01E-09	2.39E-08
ITSN1	-0.0109	8.35E-01	8.92E-01
ITSN2	0.0031	9.52E-01	9.70E-01
IVD	-0.3959	2.25E-15	1.67E-13
IVL	-0.0523	3.15E-01	4.45E-01
IVNS1ABP	0.2112	4.14E-05	2.88E-04
IWS1	0.0745	1.52E-01	2.56E-01
IYD	-0.1978	1.25E-04	7.39E-04
IZUMO1	0.2070	5.87E-05	3.88E-04
JAG1	0.0403	4.39E-01	5.70E-01
JAG2	-0.0123	8.14E-01	8.77E-01
JAGN1	0.0044	9.33E-01	9.58E-01
JAK1	-0.1552	2.73E-03	9.98E-03

JAK2	0.0253	6.28E-01	7.36E-01
JAK3	0.0143	7.83E-01	8.56E-01
JAKMIP1	-0.0139	7.89E-01	8.60E-01
JAKMIP2	-0.1527	3.18E-03	1.14E-02
JAKMIP3	0.2105	4.38E-05	3.03E-04
JAM2	-0.1850	3.42E-04	1.74E-03
JAM3	-0.1207	2.01E-02	5.15E-02
JARID2	0.1527	3.19E-03	1.14E-02
JAZF1	-0.1118	3.13E-02	7.40E-02
JDP2	-0.2307	7.12E-06	6.26E-05
JHDM1D	-0.0704	1.76E-01	2.87E-01
JKAMP	-0.0326	5.31E-01	6.54E-01
JMJD1C	-0.0923	7.56E-02	1.49E-01
JMJD4	0.1472	4.50E-03	1.51E-02
JMJD5	-0.2074	5.69E-05	3.78E-04
JMJD6	0.3212	2.39E-10	6.55E-09
JMJD7-PLA2G4	0.0964	6.36E-02	1.30E-01
JMJD8	-0.1803	4.84E-04	2.31E-03
JMY	-0.1135	2.88E-02	6.93E-02
JOSD1	0.2141	3.21E-05	2.32E-04
JOSD2	0.0112	8.30E-01	8.89E-01
JPH1	0.0827	1.12E-01	2.03E-01
JPH2	-0.0604	2.46E-01	3.70E-01
JPH3	-0.0901	8.31E-02	1.60E-01
JPH4	-0.0919	7.72E-02	1.51E-01
JRKL	0.2510	9.75E-07	1.11E-05
JRK	0.3209	2.48E-10	6.78E-09
JSRP1	0.0698	1.80E-01	2.91E-01
JTB	0.3409	1.51E-11	5.21E-10
JUB	-0.0421	4.19E-01	5.52E-01
JUNB	-0.0270	6.04E-01	7.16E-01
JUND	-0.1105	3.33E-02	7.78E-02
JUN	-0.0780	1.34E-01	2.32E-01
JUP	0.0776	1.36E-01	2.35E-01
KAAG1	0.1237	1.71E-02	4.53E-02
KAL1	-0.0591	2.56E-01	3.82E-01
KALRN	-0.1609	1.88E-03	7.31E-03
KANK1	-0.0712	1.71E-01	2.81E-01
KANK2	-0.0256	6.23E-01	7.32E-01
KANK3	-0.2273	9.82E-06	8.28E-05
KANK4	-0.0085	8.71E-01	9.17E-01
KARS	-0.1034	4.65E-02	1.02E-01
KAT2A	0.4035	5.83E-16	5.01E-14
KAT2B	-0.2478	1.35E-06	1.47E-05

KAT5	0.0797	1.25E-01	2.21E-01
KATNA1	0.4438	2.47E-19	5.18E-17
KATNAL1	-0.0290	5.78E-01	6.94E-01
KATNAL2	0.0116	8.24E-01	8.85E-01
KATNB1	-0.0403	4.39E-01	5.71E-01
KAZALD1	-0.1052	4.29E-02	9.53E-02
KAZ	-0.1069	3.97E-02	8.96E-02
KBTBD10	0.0296	5.70E-01	6.88E-01
KBTBD11	-0.1796	5.11E-04	2.42E-03
KBTBD12	0.0928	7.42E-02	1.47E-01
KBTBD13	-0.0160	7.59E-01	8.38E-01
KBTBD2	0.1841	3.64E-04	1.83E-03
KBTBD3	0.0789	1.29E-01	2.27E-01
KBTBD4	0.0185	7.23E-01	8.12E-01
KBTBD5	0.0339	5.15E-01	6.39E-01
KBTBD6	0.0115	8.25E-01	8.85E-01
KBTBD7	-0.0609	2.42E-01	3.67E-01
KBTBD8	-0.1015	5.09E-02	1.09E-01
KC6	0.1226	1.82E-02	4.75E-02
KCMF1	0.1023	4.90E-02	1.06E-01
KCNA10	0.0374	4.73E-01	6.02E-01
KCNA1	0.0026	9.61E-01	9.75E-01
KCNA2	0.0574	2.70E-01	3.98E-01
KCNA3	-0.1501	3.76E-03	1.31E-02
KCNA4	0.1125	3.03E-02	7.21E-02
KCNA5	-0.0840	1.06E-01	1.95E-01
KCNA6	-0.0438	4.00E-01	5.33E-01
KCNA7	0.0766	1.41E-01	2.42E-01
KCNAB1	-0.1291	1.28E-02	3.58E-02
KCNAB2	-0.0838	1.07E-01	1.96E-01
KCNAB3	0.0692	1.83E-01	2.96E-01
KCNB1	-0.2181	2.25E-05	1.71E-04
KCNB2	0.0125	8.11E-01	8.76E-01
KCNC1	0.1802	4.89E-04	2.33E-03
KCNC2	0.0806	1.21E-01	2.16E-01
KCNC3	0.0205	6.94E-01	7.89E-01
KCNC4	0.1085	3.67E-02	8.42E-02
KCND1	0.0598	2.50E-01	3.76E-01
KCND2	-0.1096	3.48E-02	8.07E-02
KCND3	-0.2660	1.99E-07	2.70E-06
KCNE1L	0.2284	8.84E-06	7.54E-05
KCNE1	-0.1304	1.19E-02	3.37E-02
KCNE2	-0.1151	2.67E-02	6.50E-02
KCNE3	0.1052	4.29E-02	9.53E-02

KCNE4	-0.0928	7.41E-02	1.47E-01
KCNF1	0.1128	2.98E-02	7.10E-02
KCNG1	0.1291	1.28E-02	3.58E-02
KCNG2	0.0547	2.94E-01	4.23E-01
KCNG3	0.2192	2.05E-05	1.58E-04
KCNG4	0.0424	4.16E-01	5.49E-01
KCNH1	0.0130	8.03E-01	8.70E-01
KCNH2	0.1436	5.58E-03	1.80E-02
KCNH3	0.2191	2.06E-05	1.59E-04
KCNH4	0.1535	3.03E-03	1.09E-02
KCNH5	0.0743	1.53E-01	2.58E-01
KCNH6	0.0617	2.36E-01	3.59E-01
KCNH7	0.0651	2.11E-01	3.29E-01
KCNH8	0.0719	1.67E-01	2.75E-01
KCNIP1	-0.1062	4.09E-02	9.18E-02
KCNIP2	0.1433	5.70E-03	1.84E-02
KCNIP3	0.1032	4.69E-02	1.02E-01
KCNIP4	0.0121	8.16E-01	8.79E-01
KCNJ10	0.0554	2.87E-01	4.16E-01
KCNJ11	0.2587	4.38E-07	5.44E-06
KCNJ12	-0.0772	1.38E-01	2.38E-01
KCNJ13	0.0182	7.27E-01	8.15E-01
KCNJ14	0.0444	3.93E-01	5.26E-01
KCNJ15	-0.0859	9.85E-02	1.84E-01
KCNJ16	0.0557	2.85E-01	4.13E-01
KCNJ1	0.0672	1.97E-01	3.12E-01
KCNJ2	0.0205	6.94E-01	7.89E-01
KCNJ3	0.0992	5.64E-02	1.18E-01
KCNJ4	-0.0263	6.13E-01	7.24E-01
KCNJ5	-0.0793	1.27E-01	2.23E-01
KCNJ6	-0.0825	1.13E-01	2.05E-01
KCNJ8	-0.2477	1.37E-06	1.49E-05
KCNJ9	0.0669	1.99E-01	3.15E-01
KCNK10	0.1545	2.85E-03	1.04E-02
KCNK12	0.0778	1.35E-01	2.34E-01
KCNK13	0.0272	6.02E-01	7.14E-01
KCNK15	0.0109	8.34E-01	8.91E-01
KCNK16	-0.0603	2.46E-01	3.71E-01
KCNK17	-0.1700	1.01E-03	4.35E-03
KCNK1	0.0019	9.70E-01	9.81E-01
KCNK2	0.1586	2.18E-03	8.26E-03
KCNK3	0.0119	8.20E-01	8.82E-01
KCNK4	-0.0359	4.91E-01	6.18E-01
KCNK5	0.0046	9.30E-01	9.56E-01

KCNK6	-0.0984	5.83E-02	1.21E-01
KCNK7	0.1007	5.27E-02	1.12E-01
KCNK9	0.0859	9.85E-02	1.84E-01
KCNMA1	-0.1871	2.89E-04	1.51E-03
KCNMB1	-0.0370	4.78E-01	6.06E-01
KCNMB2	0.0720	1.67E-01	2.75E-01
KCNMB3	0.2589	4.27E-07	5.33E-06
KCNMB4	-0.0078	8.81E-01	9.24E-01
KCNN1	0.0549	2.92E-01	4.21E-01
KCNN2	-0.1836	3.79E-04	1.89E-03
KCNN3	-0.1133	2.91E-02	6.98E-02
KCNN4	0.0179	7.31E-01	8.18E-01
KCNQ1DN	-0.0635	2.22E-01	3.43E-01
KCNQ1OT1	0.2038	7.70E-05	4.89E-04
KCNQ1	-0.0131	8.02E-01	8.69E-01
KCNQ2	-0.0412	4.28E-01	5.61E-01
KCNQ3	-0.0202	6.98E-01	7.92E-01
KCNQ4	0.1068	3.98E-02	8.98E-02
KCNQ5	-0.0256	6.23E-01	7.32E-01
KCNRG	0.0291	5.76E-01	6.93E-01
KCNS1	0.0856	9.98E-02	1.86E-01
KCNS2	-0.0661	2.04E-01	3.22E-01
KCNS3	0.0376	4.70E-01	6.00E-01
KCNT1	-0.0568	2.75E-01	4.03E-01
KCNT2	0.1190	2.18E-02	5.52E-02
KCNU1	-0.0785	1.31E-01	2.29E-01
KCNV1	0.1683	1.14E-03	4.79E-03
KCNV2	0.2350	4.76E-06	4.46E-05
KCP	0.1489	4.06E-03	1.39E-02
KCTD10	0.0250	6.31E-01	7.39E-01
KCTD11	-0.1104	3.35E-02	7.82E-02
KCTD12	-0.1509	3.57E-03	1.25E-02
KCTD13	0.0561	2.81E-01	4.10E-01
KCTD14	0.0288	5.80E-01	6.97E-01
KCTD15	-0.0653	2.09E-01	3.28E-01
KCTD16	0.0016	9.75E-01	9.84E-01
KCTD17	0.1806	4.72E-04	2.26E-03
KCTD18	-0.0928	7.43E-02	1.47E-01
KCTD19	0.1289	1.30E-02	3.62E-02
KCTD1	0.0500	3.37E-01	4.69E-01
KCTD20	0.0607	2.43E-01	3.68E-01
KCTD21	-0.2251	1.20E-05	9.84E-05
KCTD2	0.0832	1.10E-01	2.00E-01
KCTD3	0.1588	2.16E-03	8.21E-03

KCTD4	0.0048	9.26E-01	9.53E-01
KCTD5	0.0988	5.73E-02	1.20E-01
KCTD6	0.1449	5.16E-03	1.69E-02
KCTD7	0.2521	8.66E-07	9.98E-06
KCTD8	0.0446	3.92E-01	5.25E-01
KCTD9	0.0646	2.15E-01	3.34E-01
KDELC1	0.1907	2.20E-04	1.20E-03
KDELC2	0.0677	1.94E-01	3.09E-01
KDELR1	-0.0140	7.89E-01	8.60E-01
KDELR2	-0.0621	2.33E-01	3.56E-01
KDELR3	0.0220	6.72E-01	7.72E-01
KDM1A	0.1415	6.32E-03	2.00E-02
KDM1B	0.0820	1.15E-01	2.07E-01
KDM2A	0.0638	2.20E-01	3.41E-01
KDM2B	0.0827	1.12E-01	2.03E-01
KDM3A	0.0544	2.96E-01	4.26E-01
KDM3B	0.1522	3.29E-03	1.17E-02
KDM4A	-0.0746	1.51E-01	2.56E-01
KDM4B	-0.0926	7.47E-02	1.48E-01
KDM4C	0.0637	2.21E-01	3.42E-01
KDM4DL	-0.0403	4.39E-01	5.71E-01
KDM4D	0.1167	2.46E-02	6.07E-02
KDM5A	-0.0590	2.57E-01	3.83E-01
KDM5B	0.1623	1.71E-03	6.77E-03
KDM5C	0.1545	2.84E-03	1.03E-02
KDM5D	-0.0335	5.21E-01	6.44E-01
KDM6A	0.1170	2.43E-02	6.00E-02
KDM6B	-0.0595	2.53E-01	3.78E-01
KDR	-0.2579	4.76E-07	5.86E-06
KDSR	-0.2501	1.07E-06	1.20E-05
KEAP1	-0.0452	3.86E-01	5.19E-01
KEL	0.1676	1.20E-03	5.01E-03
KERA	-0.0846	1.04E-01	1.91E-01
KGFLP1	-0.0194	7.09E-01	8.01E-01
KGFLP2	-0.0125	8.11E-01	8.76E-01
KHDC1L	0.1668	1.26E-03	5.23E-03
KHDC1	0.2476	1.38E-06	1.51E-05
KHDRBS1	0.2963	5.88E-09	1.16E-07
KHDRBS2	0.0933	7.28E-02	1.45E-01
KHDRBS3	-0.0641	2.18E-01	3.39E-01
KHK	-0.1315	1.13E-02	3.22E-02
KHNYN	0.0752	1.48E-01	2.52E-01
KHSRP	0.1765	6.39E-04	2.93E-03
KIAA0020	0.0649	2.12E-01	3.32E-01

KIAA0040	0.0482	3.54E-01	4.86E-01
KIAA0087	0.0331	5.25E-01	6.48E-01
KIAA0090	0.0105	8.41E-01	8.95E-01
KIAA0100	0.1868	2.98E-04	1.55E-03
KIAA0101	0.3769	5.76E-14	3.19E-12
KIAA0114	0.0185	7.22E-01	8.11E-01
KIAA0125	0.0651	2.11E-01	3.30E-01
KIAA0141	-0.2339	5.27E-06	4.85E-05
KIAA0146	0.1105	3.33E-02	7.79E-02
KIAA0174	-0.0220	6.73E-01	7.72E-01
KIAA0182	-0.0902	8.28E-02	1.60E-01
KIAA0195	0.2333	5.62E-06	5.11E-05
KIAA0196	0.0680	1.91E-01	3.06E-01
KIAA0226	0.2252	1.19E-05	9.80E-05
KIAA0232	0.0429	4.10E-01	5.43E-01
KIAA0240	-0.0582	2.63E-01	3.90E-01
KIAA0247	-0.0830	1.10E-01	2.01E-01
KIAA0284	0.0036	9.45E-01	9.66E-01
KIAA0317	-0.0486	3.51E-01	4.83E-01
KIAA0319L	-0.0351	5.00E-01	6.26E-01
KIAA0319	0.2070	5.89E-05	3.90E-04
KIAA0355	0.0551	2.90E-01	4.19E-01
KIAA0368	0.1779	5.78E-04	2.69E-03
KIAA0391	-0.0864	9.67E-02	1.81E-01
KIAA0406	0.2041	7.50E-05	4.78E-04
KIAA0408	-0.1680	1.16E-03	4.89E-03
KIAA0415	0.2169	2.52E-05	1.89E-04
KIAA0427	-0.1802	4.88E-04	2.33E-03
KIAA0430	-0.0696	1.81E-01	2.93E-01
KIAA0467	0.1275	1.40E-02	3.85E-02
KIAA0494	-0.1031	4.73E-02	1.03E-01
KIAA0495	0.1023	4.91E-02	1.06E-01
KIAA0513	-0.1474	4.44E-03	1.50E-02
KIAA0528	0.2524	8.46E-07	9.78E-06
KIAA0556	0.1307	1.18E-02	3.34E-02
KIAA0562	-0.0899	8.38E-02	1.62E-01
KIAA0564	-0.2451	1.77E-06	1.88E-05
KIAA0586	0.0384	4.61E-01	5.92E-01
KIAA0649	0.1336	9.99E-03	2.92E-02
KIAA0652	-0.0588	2.59E-01	3.85E-01
KIAA0664P3	0.0379	4.67E-01	5.96E-01
KIAA0664	-0.2672	1.74E-07	2.39E-06
KIAA0748	-0.0514	3.23E-01	4.54E-01
KIAA0753	0.1331	1.03E-02	2.99E-02

KIAA0754	-0.1192	2.16E-02	5.47E-02
KIAA0776	-0.0211	6.86E-01	7.82E-01
KIAA0802	0.1416	6.29E-03	1.99E-02
KIAA0831	-0.0160	7.58E-01	8.38E-01
KIAA0892	0.2453	1.73E-06	1.83E-05
KIAA0895L	0.1264	1.48E-02	4.04E-02
KIAA0895	0.1256	1.55E-02	4.17E-02
KIAA0907	0.5807	7.98E-35	1.14E-31
KIAA0913	-0.0347	5.05E-01	6.30E-01
KIAA0922	-0.2313	6.76E-06	6.00E-05
KIAA0947	0.1436	5.61E-03	1.81E-02
KIAA1009	0.3792	3.91E-14	2.27E-12
KIAA1012	-0.1050	4.33E-02	9.60E-02
KIAA1024	0.1346	9.46E-03	2.80E-02
KIAA1033	-0.0782	1.33E-01	2.31E-01
KIAA1045	-0.0022	9.67E-01	9.80E-01
KIAA1107	0.2101	4.52E-05	3.11E-04
KIAA1109	-0.1235	1.74E-02	4.57E-02
KIAA1143	0.0315	5.45E-01	6.65E-01
KIAA1147	-0.0990	5.67E-02	1.19E-01
KIAA1161	-0.1543	2.88E-03	1.04E-02
KIAA1191	-0.1817	4.35E-04	2.11E-03
KIAA1199	0.0058	9.11E-01	9.44E-01
KIAA1210	0.0684	1.88E-01	3.02E-01
KIAA1211	0.0236	6.50E-01	7.54E-01
KIAA1217	0.0348	5.04E-01	6.29E-01
KIAA1239	0.1344	9.55E-03	2.82E-02
KIAA1244	0.2471	1.45E-06	1.57E-05
KIAA1257	0.1632	1.61E-03	6.43E-03
KIAA1267	0.1987	1.17E-04	6.97E-04
KIAA1274	0.0038	9.42E-01	9.64E-01
KIAA1279	0.0494	3.43E-01	4.75E-01
KIAA1310	0.2013	9.44E-05	5.81E-04
KIAA1324L	0.0077	8.82E-01	9.25E-01
KIAA1324	0.1413	6.43E-03	2.03E-02
KIAA1328	0.2574	5.01E-07	6.13E-06
KIAA1370	-0.1499	3.80E-03	1.32E-02
KIAA1377	0.1884	2.63E-04	1.39E-03
KIAA1383	0.1592	2.10E-03	8.02E-03
KIAA1407	0.2609	3.47E-07	4.44E-06
KIAA1409	-0.1484	4.18E-03	1.43E-02
KIAA1429	0.0844	1.05E-01	1.92E-01
KIAA1430	-0.1868	2.96E-04	1.54E-03
KIAA1432	-0.0384	4.61E-01	5.92E-01

KIAA1462	-0.0277	5.95E-01	7.09E-01
KIAA1467	0.0586	2.61E-01	3.87E-01
KIAA1468	0.0639	2.20E-01	3.40E-01
KIAA1486	0.1248	1.61E-02	4.31E-02
KIAA1522	0.2558	5.89E-07	7.05E-06
KIAA1524	0.3563	1.51E-12	6.46E-11
KIAA1529	0.1952	1.55E-04	8.86E-04
KIAA1530	0.1554	2.69E-03	9.86E-03
KIAA1539	-0.0035	9.46E-01	9.67E-01
KIAA1543	0.0653	2.10E-01	3.28E-01
KIAA1549	0.0148	7.76E-01	8.50E-01
KIAA1586	0.2150	2.95E-05	2.16E-04
KIAA1598	-0.0417	4.23E-01	5.55E-01
KIAA1609	0.1974	1.29E-04	7.61E-04
KIAA1614	0.2790	4.61E-08	7.28E-07
KIAA1632	0.0054	9.18E-01	9.48E-01
KIAA1644	-0.1466	4.65E-03	1.55E-02
KIAA1671	-0.1575	2.35E-03	8.80E-03
KIAA1683	-0.0909	8.05E-02	1.56E-01
KIAA1704	0.0033	9.49E-01	9.68E-01
KIAA1712	-0.0567	2.76E-01	4.04E-01
KIAA1715	-0.1213	1.94E-02	5.02E-02
KIAA1731	0.3923	4.28E-15	2.99E-13
KIAA1737	-0.1945	1.64E-04	9.27E-04
KIAA1751	0.0701	1.78E-01	2.89E-01
KIAA1755	-0.1368	8.35E-03	2.52E-02
KIAA1797	0.2352	4.68E-06	4.39E-05
KIAA1804	0.2685	1.51E-07	2.11E-06
KIAA1826	-0.0226	6.64E-01	7.64E-01
KIAA1841	0.3846	1.60E-14	9.93E-13
KIAA1875	0.1764	6.42E-04	2.94E-03
KIAA1908	0.0524	3.15E-01	4.44E-01
KIAA1919	0.0750	1.49E-01	2.53E-01
KIAA1949	0.1000	5.42E-02	1.15E-01
KIAA1958	0.0435	4.04E-01	5.36E-01
KIAA1967	0.1041	4.51E-02	9.91E-02
KIAA1984	0.1472	4.49E-03	1.51E-02
KIAA2013	-0.2340	5.22E-06	4.82E-05
KIAA2018	-0.1188	2.21E-02	5.57E-02
KIAA2022	0.0753	1.48E-01	2.51E-01
KIAA2026	0.0288	5.80E-01	6.96E-01
KIDINS220	-0.0342	5.12E-01	6.36E-01
KIF11	0.4290	4.87E-18	6.95E-16
KIF12	0.2397	3.01E-06	2.98E-05

KIF13A	-0.0638	2.20E-01	3.41E-01
KIF13B	-0.0967	6.27E-02	1.29E-01
KIF14	0.4342	1.71E-18	2.81E-16
KIF15	0.3832	2.00E-14	1.23E-12
KIF16B	-0.0423	4.16E-01	5.49E-01
KIF17	-0.0726	1.63E-01	2.70E-01
KIF18A	0.4132	9.90E-17	1.01E-14
KIF18B	0.4914	5.95E-24	2.89E-21
KIF19	-0.1514	3.46E-03	1.22E-02
KIF1A	0.0615	2.37E-01	3.61E-01
KIF1B	-0.0911	7.96E-02	1.55E-01
KIF1C	-0.2524	8.40E-07	9.71E-06
KIF20A	0.3665	3.08E-13	1.51E-11
KIF20B	0.3041	2.24E-09	4.88E-08
KIF21A	0.0184	7.23E-01	8.12E-01
KIF21B	0.2354	4.57E-06	4.30E-05
KIF22	0.1849	3.44E-04	1.75E-03
KIF23	0.4136	9.21E-17	9.41E-15
KIF24	0.3456	7.56E-12	2.79E-10
KIF25	0.1195	2.13E-02	5.40E-02
KIF26A	-0.1501	3.76E-03	1.31E-02
KIF26B	0.1073	3.89E-02	8.81E-02
KIF27	0.0537	3.02E-01	4.31E-01
KIF2A	0.1975	1.28E-04	7.56E-04
KIF2B	0.0576	2.68E-01	3.96E-01
KIF2C	0.4493	7.90E-20	1.92E-17
KIF3A	0.0921	7.63E-02	1.50E-01
KIF3B	-0.0995	5.55E-02	1.17E-01
KIF3C	0.1015	5.08E-02	1.09E-01
KIF4A	0.4393	6.21E-19	1.22E-16
KIF4B	0.3193	3.09E-10	8.31E-09
KIF5A	0.0303	5.61E-01	6.79E-01
KIF5B	0.1304	1.19E-02	3.37E-02
KIF5C	-0.0497	3.40E-01	4.72E-01
KIF6	0.0378	4.67E-01	5.97E-01
KIF7	0.0853	1.01E-01	1.87E-01
KIF9	0.0393	4.50E-01	5.82E-01
KIFAP3	0.2593	4.08E-07	5.11E-06
KIFC1	0.4751	2.78E-22	9.89E-20
KIFC2	0.1702	9.95E-04	4.28E-03
KIFC3	0.0140	7.88E-01	8.59E-01
KILLIN	-0.1936	1.75E-04	9.82E-04
KIN	0.2828	2.99E-08	4.93E-07
KIR2DL1	-0.0685	1.88E-01	3.01E-01

KIR2DL3	-0.0194	7.09E-01	8.01E-01
KIR2DL4	0.0009	9.86E-01	9.92E-01
KIR2DS4	-0.0241	6.43E-01	7.48E-01
KIR3DL1	-0.1063	4.06E-02	9.13E-02
KIR3DL2	0.0163	7.54E-01	8.35E-01
KIR3DL3	-0.0703	1.77E-01	2.88E-01
KIR3DP1	-0.0221	6.71E-01	7.71E-01
KIR3DX1	-0.0118	8.21E-01	8.82E-01
KIRREL2	0.1025	4.86E-02	1.05E-01
KIRREL3	0.1705	9.78E-04	4.22E-03
KIRREL	-0.0756	1.46E-01	2.49E-01
KISS1R	0.1577	2.31E-03	8.68E-03
KISS1	0.0675	1.95E-01	3.10E-01
KITLG	0.0786	1.31E-01	2.28E-01
KIT	-0.0467	3.69E-01	5.02E-01
KLB	0.0798	1.25E-01	2.21E-01
KLC1	-0.0108	8.36E-01	8.92E-01
KLC2	0.1715	9.11E-04	3.97E-03
KLC3	0.2556	6.08E-07	7.25E-06
KLC4	-0.1862	3.12E-04	1.61E-03
KLF10	-0.0847	1.03E-01	1.90E-01
KLF11	0.0096	8.54E-01	9.06E-01
KLF12	-0.1660	1.33E-03	5.46E-03
KLF13	0.0674	1.95E-01	3.11E-01
KLF14	-0.0229	6.61E-01	7.62E-01
KLF15	-0.1212	1.95E-02	5.04E-02
KLF16	0.0417	4.23E-01	5.55E-01
KLF17	0.0065	9.01E-01	9.37E-01
KLF1	0.0316	5.45E-01	6.65E-01
KLF2	-0.1110	3.26E-02	7.65E-02
KLF3	0.0124	8.12E-01	8.77E-01
KLF4	0.0461	3.76E-01	5.09E-01
KLF5	0.2183	2.22E-05	1.69E-04
KLF6	-0.1027	4.81E-02	1.04E-01
KLF7	-0.0485	3.51E-01	4.83E-01
KLF8	-0.1035	4.64E-02	1.01E-01
KLF9	-0.1879	2.74E-04	1.44E-03
KLHDC10	-0.1456	4.94E-03	1.63E-02
KLHDC1	-0.0901	8.30E-02	1.60E-01
KLHDC2	-0.2064	6.19E-05	4.05E-04
KLHDC3	0.0949	6.79E-02	1.37E-01
KLHDC4	-0.1606	1.92E-03	7.42E-03
KLHDC5	0.1685	1.12E-03	4.75E-03
KLHDC7A	-0.1490	4.02E-03	1.38E-02

KLHDC7B	0.0526	3.13E-01	4.42E-01
KLHDC8A	0.1788	5.39E-04	2.54E-03
KLHDC8B	0.0706	1.75E-01	2.85E-01
KLHDC9	0.0701	1.78E-01	2.89E-01
KLHL10	-0.0038	9.43E-01	9.64E-01
KLHL11	0.0351	5.00E-01	6.26E-01
KLHL12	0.2139	3.25E-05	2.34E-04
KLHL13	0.0710	1.73E-01	2.82E-01
KLHL14	-0.0075	8.85E-01	9.27E-01
KLHL15	-0.0907	8.09E-02	1.57E-01
KLHL17	0.2854	2.21E-08	3.78E-07
KLHL18	-0.0184	7.24E-01	8.13E-01
KLHL1	0.0334	5.22E-01	6.46E-01
KLHL20	0.1360	8.72E-03	2.62E-02
KLHL21	-0.0451	3.86E-01	5.19E-01
KLHL22	0.1783	5.58E-04	2.61E-03
KLHL23	0.0583	2.62E-01	3.89E-01
KLHL24	-0.1215	1.92E-02	4.98E-02
KLHL25	-0.0780	1.34E-01	2.32E-01
KLHL26	-0.0705	1.75E-01	2.86E-01
KLHL28	-0.0599	2.50E-01	3.75E-01
KLHL29	0.1178	2.33E-02	5.81E-02
KLHL2	-0.2840	2.61E-08	4.34E-07
KLHL30	0.0900	8.33E-02	1.61E-01
KLHL31	0.0113	8.28E-01	8.88E-01
KLHL32	-0.0610	2.41E-01	3.66E-01
KLHL33	-0.0513	3.25E-01	4.55E-01
KLHL34	-0.0676	1.94E-01	3.09E-01
KLHL35	0.0654	2.09E-01	3.28E-01
KLHL36	-0.1412	6.46E-03	2.04E-02
KLHL38	0.1440	5.45E-03	1.77E-02
KLHL3	0.0563	2.79E-01	4.08E-01
KLHL4	-0.0709	1.73E-01	2.83E-01
KLHL5	-0.0553	2.88E-01	4.17E-01
KLHL6	-0.1054	4.24E-02	9.44E-02
KLHL7	0.0722	1.65E-01	2.73E-01
KLHL8	-0.2122	3.78E-05	2.67E-04
KLHL9	0.1183	2.27E-02	5.69E-02
KLK10	-0.1334	1.01E-02	2.95E-02
KLK11	-0.1284	1.33E-02	3.70E-02
KLK12	0.0198	7.03E-01	7.96E-01
KLK13	-0.0224	6.67E-01	7.67E-01
KLK14	0.0654	2.09E-01	3.27E-01
KLK15	0.1070	3.93E-02	8.89E-02

KLK1	0.0985	5.81E-02	1.21E-01
KLK2	-0.0249	6.33E-01	7.40E-01
KLK3	-0.0545	2.95E-01	4.25E-01
KLK4	-0.0721	1.66E-01	2.74E-01
KLK5	0.0466	3.71E-01	5.04E-01
KLK6	0.0926	7.48E-02	1.48E-01
KLK7	0.0642	2.18E-01	3.38E-01
KLK8	0.0575	2.70E-01	3.97E-01
KLK9	0.0225	6.65E-01	7.65E-01
KLKB1	-0.3097	1.09E-09	2.55E-08
KLKP1	-0.0118	8.20E-01	8.82E-01
KLRA1	0.2088	5.04E-05	3.41E-04
KLRAQ1	0.0195	7.08E-01	8.01E-01
KLRB1	-0.1398	6.98E-03	2.17E-02
KLRC1	-0.0277	5.94E-01	7.08E-01
KLRC2	0.0537	3.03E-01	4.32E-01
KLRC3	-0.0169	7.45E-01	8.28E-01
KLRC4	-0.0585	2.61E-01	3.87E-01
KLRD1	-0.1534	3.05E-03	1.10E-02
KLRF1	-0.1303	1.20E-02	3.39E-02
KLRG1	-0.0066	9.00E-01	9.37E-01
KLRG2	0.0398	4.44E-01	5.76E-01
KLRK1	-0.0960	6.46E-02	1.32E-01
KL	-0.2330	5.77E-06	5.23E-05
KMO	-0.2327	5.93E-06	5.36E-05
KNCN	0.1058	4.16E-02	9.29E-02
KNDC1	-0.0115	8.26E-01	8.86E-01
KNG1	-0.1399	6.97E-03	2.17E-02
KNTC1	0.4459	1.61E-19	3.56E-17
KPNA1	-0.2238	1.36E-05	1.10E-04
KPNA2	0.3814	2.71E-14	1.62E-12
KPNA3	-0.0893	8.58E-02	1.65E-01
KPNA4	0.0695	1.81E-01	2.93E-01
KPNA5	0.1906	2.22E-04	1.20E-03
KPNA6	-0.0571	2.73E-01	4.01E-01
KPNA7	0.1153	2.63E-02	6.43E-02
KPNB1	0.2741	8.14E-08	1.21E-06
KPRP	0.1654	1.39E-03	5.66E-03
KPTN	0.1345	9.52E-03	2.81E-02
KRAS	0.1363	8.58E-03	2.58E-02
KRBA1	0.2210	1.74E-05	1.37E-04
KRBA2	0.0410	4.31E-01	5.63E-01
KRCC1	-0.1276	1.39E-02	3.84E-02
KREMEN1	0.1432	5.74E-03	1.85E-02

KREMEN2	0.1334	1.01E-02	2.95E-02
KRI1	0.1902	2.29E-04	1.24E-03
KRIT1	-0.0155	7.67E-01	8.43E-01
KRR1	-0.0019	9.71E-01	9.81E-01
KRT10	0.2026	8.49E-05	5.31E-04
KRT12	0.0075	8.85E-01	9.27E-01
KRT13	-0.0169	7.45E-01	8.28E-01
KRT14	-0.0261	6.16E-01	7.26E-01
KRT15	0.0625	2.30E-01	3.52E-01
KRT16	0.0053	9.19E-01	9.49E-01
KRT17	0.1082	3.73E-02	8.53E-02
KRT18	-0.0552	2.89E-01	4.18E-01
KRT19	0.0623	2.31E-01	3.54E-01
KRT1	-0.1123	3.06E-02	7.26E-02
KRT20	0.0002	9.98E-01	9.98E-01
KRT222	-0.0123	8.13E-01	8.77E-01
KRT23	0.0797	1.26E-01	2.21E-01
KRT24	-0.0250	6.31E-01	7.39E-01
KRT25	0.1118	3.13E-02	7.39E-02
KRT27	-0.1373	8.07E-03	2.45E-02
KRT28	-0.0035	9.46E-01	9.67E-01
KRT2	0.0593	2.55E-01	3.80E-01
KRT31	0.0350	5.02E-01	6.28E-01
KRT32	0.0652	2.10E-01	3.29E-01
KRT33B	-0.0023	9.65E-01	9.79E-01
KRT34	0.0463	3.73E-01	5.06E-01
KRT35	-0.0550	2.91E-01	4.20E-01
KRT36	0.0437	4.02E-01	5.35E-01
KRT37	0.0778	1.35E-01	2.34E-01
KRT38	0.0784	1.32E-01	2.29E-01
KRT39	0.1147	2.72E-02	6.60E-02
KRT3	0.0357	4.93E-01	6.20E-01
KRT40	0.0642	2.18E-01	3.38E-01
KRT4	0.0475	3.61E-01	4.93E-01
KRT5	-0.0937	7.13E-02	1.42E-01
KRT6A	-0.0849	1.03E-01	1.89E-01
KRT6B	-0.0256	6.24E-01	7.32E-01
KRT6C	0.0052	9.20E-01	9.49E-01
KRT71	0.0191	7.14E-01	8.05E-01
KRT72	0.0120	8.18E-01	8.81E-01
KRT73	0.0552	2.89E-01	4.18E-01
KRT74	0.0101	8.47E-01	9.00E-01
KRT75	0.0524	3.14E-01	4.44E-01
KRT76	0.0138	7.91E-01	8.62E-01

KRT77	0.0685	1.88E-01	3.02E-01
KRT78	0.0146	7.80E-01	8.53E-01
KRT79	0.0555	2.86E-01	4.15E-01
KRT7	0.0249	6.32E-01	7.40E-01
KRT80	0.1525	3.23E-03	1.15E-02
KRT81	-0.0001	9.99E-01	1.00E+00
KRT82	-0.0461	3.75E-01	5.09E-01
KRT83	-0.0292	5.75E-01	6.92E-01
KRT84	0.0044	9.33E-01	9.58E-01
KRT85	0.0657	2.07E-01	3.25E-01
KRT86	-0.0053	9.19E-01	9.49E-01
KRT8	-0.0349	5.03E-01	6.28E-01
KRT9	0.1694	1.05E-03	4.48E-03
KRTAP1-1	0.1019	4.99E-02	1.07E-01
KRTAP1-3	0.0055	9.16E-01	9.47E-01
KRTAP1-5	-0.0128	8.05E-01	8.71E-01
KRTAP10-10	0.0337	5.18E-01	6.42E-01
KRTAP10-12	0.0032	9.52E-01	9.70E-01
KRTAP10-1	0.1073	3.89E-02	8.81E-02
KRTAP10-2	0.0558	2.84E-01	4.13E-01
KRTAP10-3	0.0416	4.24E-01	5.57E-01
KRTAP10-4	0.0871	9.40E-02	1.77E-01
KRTAP10-5	-0.0487	3.49E-01	4.81E-01
KRTAP10-6	-0.0928	7.41E-02	1.47E-01
KRTAP10-7	-0.0206	6.93E-01	7.88E-01
KRTAP11-1	-0.0857	9.94E-02	1.85E-01
KRTAP12-1	-0.0093	8.58E-01	9.08E-01
KRTAP12-2	-0.0504	3.33E-01	4.64E-01
KRTAP12-3	0.0497	3.40E-01	4.71E-01
KRTAP13-2	-0.0446	3.91E-01	5.24E-01
KRTAP17-1	0.1078	3.80E-02	8.66E-02
KRTAP19-1	0.1867	3.00E-04	1.56E-03
KRTAP19-3	0.1805	4.77E-04	2.28E-03
KRTAP19-4	0.1862	3.10E-04	1.60E-03
KRTAP19-5	0.1602	1.97E-03	7.60E-03
KRTAP19-8	0.0470	3.67E-01	5.00E-01
KRTAP2-1	0.0454	3.84E-01	5.17E-01
KRTAP2-2	-0.0371	4.76E-01	6.05E-01
KRTAP20-2	0.1696	1.04E-03	4.44E-03
KRTAP20-4	0.2360	4.32E-06	4.10E-05
KRTAP26-1	0.0121	8.16E-01	8.79E-01
KRTAP3-1	0.1563	2.53E-03	9.37E-03
KRTAP3-2	0.1087	3.64E-02	8.36E-02
KRTAP3-3	-0.0115	8.25E-01	8.86E-01

KRTAP4-11	-0.0308	5.55E-01	6.74E-01
KRTAP4-12	0.0015	9.77E-01	9.86E-01
KRTAP4-1	0.1666	1.28E-03	5.31E-03
KRTAP4-2	0.0009	9.85E-01	9.91E-01
KRTAP4-4	0.0275	5.98E-01	7.12E-01
KRTAP4-7	0.0556	2.85E-01	4.14E-01
KRTAP4-8	0.0482	3.54E-01	4.86E-01
KRTAP5-10	0.2625	2.90E-07	3.79E-06
KRTAP5-11	0.0745	1.52E-01	2.56E-01
KRTAP5-1	0.1554	2.69E-03	9.87E-03
KRTAP5-2	-0.0240	6.46E-01	7.50E-01
KRTAP5-3	-0.0469	3.67E-01	5.00E-01
KRTAP5-4	0.0934	7.25E-02	1.44E-01
KRTAP5-5	0.1680	1.16E-03	4.87E-03
KRTAP5-6	0.1272	1.42E-02	3.91E-02
KRTAP5-7	0.1409	6.57E-03	2.07E-02
KRTAP5-8	0.1532	3.09E-03	1.11E-02
KRTAP5-9	0.0781	1.33E-01	2.32E-01
KRTAP6-3	0.2274	9.68E-06	8.18E-05
KRTAP7-1	-0.0325	5.33E-01	6.55E-01
KRTAP8-1	0.0232	6.56E-01	7.58E-01
KRTCAP2	0.2873	1.76E-08	3.09E-07
KRTCAP3	0.1589	2.14E-03	8.13E-03
KRTDAP	0.0914	7.86E-02	1.54E-01
KSR1	-0.0167	7.49E-01	8.31E-01
KSR2	0.0817	1.16E-01	2.09E-01
KTELC1	-0.1503	3.71E-03	1.29E-02
KTI12	0.2669	1.80E-07	2.47E-06
KTN1	-0.0255	6.24E-01	7.33E-01
KYNU	-0.1305	1.18E-02	3.35E-02
KY	0.0033	9.49E-01	9.68E-01
L1CAM	0.0238	6.47E-01	7.52E-01
L1TD1	0.1527	3.20E-03	1.14E-02
L2HGDH	-0.1644	1.49E-03	6.01E-03
L3MBTL2	0.0969	6.23E-02	1.28E-01
L3MBTL3	0.1000	5.42E-02	1.15E-01
L3MBTL4	-0.0648	2.13E-01	3.32E-01
L3MBTL	0.4095	1.94E-16	1.85E-14
LACE1	-0.0512	3.25E-01	4.55E-01
LACTB2	-0.2650	2.22E-07	2.97E-06
LACTB	-0.3523	2.77E-12	1.13E-10
LAD1	0.1179	2.32E-02	5.79E-02
LAG3	0.0783	1.32E-01	2.30E-01
LAGE3	0.2055	6.65E-05	4.31E-04

LAIR1	0.0256	6.24E-01	7.32E-01
LAIR2	0.1322	1.08E-02	3.10E-02
LALBA	0.0550	2.91E-01	4.20E-01
LAMA1	0.0360	4.89E-01	6.17E-01
LAMA2	-0.1615	1.81E-03	7.08E-03
LAMA3	-0.0372	4.75E-01	6.04E-01
LAMA4	-0.0556	2.85E-01	4.14E-01
LAMA5	0.0526	3.12E-01	4.42E-01
LAMB1	0.0452	3.85E-01	5.18E-01
LAMB2L	0.1885	2.61E-04	1.38E-03
LAMB2	-0.2199	1.93E-05	1.50E-04
LAMB3	-0.0019	9.70E-01	9.81E-01
LAMB4	0.0117	8.23E-01	8.84E-01
LAMC1	0.2204	1.85E-05	1.44E-04
LAMC2	0.0194	7.09E-01	8.01E-01
LAMC3	-0.1086	3.65E-02	8.38E-02
LAMP1	-0.2174	2.41E-05	1.82E-04
LAMP2	-0.1168	2.45E-02	6.05E-02
LAMP3	0.1364	8.52E-03	2.56E-02
LANCL1	0.0451	3.86E-01	5.19E-01
LANCL2	-0.0976	6.05E-02	1.25E-01
LANCL3	-0.0185	7.22E-01	8.12E-01
LAP3	-0.2709	1.16E-07	1.67E-06
LAPTM4A	-0.2656	2.07E-07	2.80E-06
LAPTM4B	0.2121	3.83E-05	2.70E-04
LAPTM5	-0.0284	5.86E-01	7.01E-01
LARGE	-0.0862	9.73E-02	1.82E-01
LARP1B	-0.2314	6.66E-06	5.93E-05
LARP1	0.0734	1.58E-01	2.64E-01
LARP4B	0.2943	7.59E-09	1.45E-07
LARP4	-0.2671	1.76E-07	2.42E-06
LARP6	0.0714	1.70E-01	2.79E-01
LARP7	-0.1170	2.43E-02	6.00E-02
LARS2	-0.1949	1.58E-04	9.01E-04
LARS	0.1354	9.01E-03	2.69E-02
LAS1L	0.1839	3.71E-04	1.86E-03
LASP1	0.2052	6.86E-05	4.43E-04
LASS1	0.1543	2.89E-03	1.05E-02
LASS2	0.2249	1.23E-05	1.00E-04
LASS3	0.2084	5.21E-05	3.51E-04
LASS4	-0.2025	8.54E-05	5.33E-04
LASS5	0.3652	3.79E-13	1.82E-11
LASS6	0.0451	3.86E-01	5.19E-01
LAT2	-0.0454	3.83E-01	5.16E-01

LATS1	0.0954	6.65E-02	1.35E-01
LATS2	-0.1512	3.52E-03	1.24E-02
LAT	0.0919	7.71E-02	1.51E-01
LAX1	-0.0561	2.81E-01	4.10E-01
LAYN	0.0114	8.26E-01	8.86E-01
LBH	-0.0541	2.99E-01	4.28E-01
LBP	-0.1459	4.86E-03	1.61E-02
LBR	0.3812	2.80E-14	1.66E-12
LBX1	0.0934	7.22E-02	1.44E-01
LBX2	-0.0940	7.04E-02	1.41E-01
LBXCOR1	0.0132	8.01E-01	8.68E-01
LCA5L	-0.0037	9.44E-01	9.65E-01
LCA5	0.0250	6.31E-01	7.38E-01
LCAT	-0.2691	1.42E-07	1.99E-06
LCE1B	0.0849	1.03E-01	1.89E-01
LCE1C	0.1320	1.09E-02	3.14E-02
LCE1D	-0.0191	7.14E-01	8.05E-01
LCE1E	0.1813	4.49E-04	2.17E-03
LCE1F	0.0560	2.82E-01	4.11E-01
LCE2A	0.0499	3.38E-01	4.69E-01
LCE2B	0.0418	4.22E-01	5.54E-01
LCE2C	0.0308	5.55E-01	6.74E-01
LCE2D	0.0481	3.55E-01	4.88E-01
LCE3A	-0.0065	9.00E-01	9.37E-01
LCE3C	-0.0335	5.20E-01	6.44E-01
LCE3D	-0.0994	5.58E-02	1.17E-01
LCE3E	-0.0824	1.13E-01	2.05E-01
LCE5A	0.0411	4.30E-01	5.62E-01
LCE6A	0.0950	6.75E-02	1.36E-01
LCK	-0.0528	3.11E-01	4.40E-01
LCLAT1	0.1108	3.29E-02	7.71E-02
LCMT1	0.1528	3.18E-03	1.13E-02
LCMT2	0.0377	4.69E-01	5.99E-01
LCN10	0.0046	9.30E-01	9.56E-01
LCN12	-0.0160	7.59E-01	8.38E-01
LCN15	0.0787	1.30E-01	2.27E-01
LCN1	0.1200	2.08E-02	5.30E-02
LCN2	-0.0136	7.95E-01	8.64E-01
LCN6	-0.1175	2.36E-02	5.88E-02
LCN8	-0.0475	3.61E-01	4.94E-01
LCNL1	0.0838	1.07E-01	1.96E-01
LCORL	0.0146	7.79E-01	8.52E-01
LCOR	-0.0379	4.67E-01	5.97E-01
LCP1	-0.0716	1.69E-01	2.78E-01

LCP2	-0.0876	9.22E-02	1.74E-01
LCTL	0.1604	1.94E-03	7.49E-03
LCT	0.0947	6.83E-02	1.38E-01
LDB1	0.0922	7.61E-02	1.50E-01
LDB2	-0.2830	2.90E-08	4.79E-07
LDB3	-0.1023	4.88E-02	1.06E-01
LDHAL6A	0.0661	2.04E-01	3.21E-01
LDHAL6B	0.0949	6.80E-02	1.37E-01
LDHA	-0.1244	1.65E-02	4.40E-02
LDHB	-0.0399	4.44E-01	5.75E-01
LDHC	-0.0231	6.57E-01	7.59E-01
LDHD	-0.4092	2.06E-16	1.92E-14
LDLRAD1	0.0534	3.05E-01	4.34E-01
LDLRAD2	-0.0644	2.16E-01	3.36E-01
LDLRAD3	-0.0891	8.66E-02	1.66E-01
LDLRAP1	-0.0516	3.22E-01	4.52E-01
LDLR	-0.2458	1.65E-06	1.76E-05
LDOC1L	0.0764	1.42E-01	2.43E-01
LDOC1	0.1366	8.44E-03	2.55E-02
LEAP2	-0.1786	5.46E-04	2.56E-03
LECT1	0.0599	2.50E-01	3.75E-01
LECT2	-0.2104	4.41E-05	3.04E-04
LEF1	-0.0262	6.15E-01	7.25E-01
LEFTY1	0.1854	3.32E-04	1.70E-03
LEFTY2	0.1082	3.72E-02	8.51E-02
LEKR1	0.0299	5.66E-01	6.83E-01
LELP1	0.0508	3.29E-01	4.60E-01
LEMD1	0.0393	4.51E-01	5.82E-01
LEMD2	0.2343	5.07E-06	4.69E-05
LEMD3	0.1479	4.30E-03	1.46E-02
LENEP	0.2131	3.48E-05	2.49E-04
LENG1	0.0701	1.78E-01	2.89E-01
LENG8	0.2787	4.82E-08	7.54E-07
LENG9	0.0130	8.02E-01	8.70E-01
LEO1	0.0559	2.83E-01	4.12E-01
LEPRE1	0.1732	8.06E-04	3.57E-03
LEPREL1	-0.0728	1.62E-01	2.69E-01
LEPREL2	-0.0410	4.31E-01	5.63E-01
LEPROTL1	-0.0215	6.80E-01	7.78E-01
LEPROT	-0.1201	2.06E-02	5.27E-02
LEPR	-0.2487	1.24E-06	1.37E-05
LEP	-0.0984	5.82E-02	1.21E-01
LETM1	-0.0436	4.02E-01	5.35E-01
LETM2	0.0923	7.59E-02	1.50E-01

LETMD1	-0.0737	1.56E-01	2.62E-01
LEUTX	0.0418	4.23E-01	5.55E-01
LFNG	-0.0455	3.82E-01	5.15E-01
LGALS12	0.0355	4.95E-01	6.21E-01
LGALS13	0.0951	6.72E-02	1.36E-01
LGALS14	0.2319	6.38E-06	5.71E-05
LGALS1	-0.0084	8.72E-01	9.18E-01
LGALS2	0.0789	1.29E-01	2.26E-01
LGALS3BP	0.0797	1.26E-01	2.22E-01
LGALS3	0.1096	3.49E-02	8.08E-02
LGALS4	0.1203	2.05E-02	5.24E-02
LGALS7B	0.0644	2.16E-01	3.36E-01
LGALS7	0.0591	2.56E-01	3.82E-01
LGALS8	0.0438	4.00E-01	5.33E-01
LGALS9B	0.0124	8.11E-01	8.76E-01
LGALS9C	0.0283	5.88E-01	7.03E-01
LGALS9	0.0309	5.52E-01	6.72E-01
LGI1	-0.0499	3.37E-01	4.69E-01
LGI2	0.0534	3.05E-01	4.34E-01
LGI3	-0.1388	7.42E-03	2.28E-02
LGI4	0.0601	2.48E-01	3.73E-01
LGMN	-0.1661	1.33E-03	5.45E-03
LGR4	-0.0299	5.66E-01	6.83E-01
LGR5	-0.0145	7.80E-01	8.53E-01
LGR6	0.0041	9.38E-01	9.61E-01
LGSN	-0.1898	2.36E-04	1.27E-03
LGTN	0.1760	6.60E-04	3.01E-03
LHB	0.0744	1.53E-01	2.57E-01
LHCGR	-0.1135	2.89E-02	6.94E-02
LHFPL1	-0.0186	7.21E-01	8.10E-01
LHFPL2	0.1190	2.19E-02	5.52E-02
LHFPL3	0.2921	9.89E-09	1.84E-07
LHFPL4	0.2257	1.14E-05	9.40E-05
LHFPL5	0.0917	7.76E-02	1.52E-01
LHFP	-0.1085	3.67E-02	8.43E-02
LHPP	-0.3662	3.22E-13	1.57E-11
LHX1	0.0674	1.95E-01	3.11E-01
LHX2	0.0747	1.51E-01	2.55E-01
LHX3	-0.1201	2.07E-02	5.27E-02
LHX4	0.3382	2.22E-11	7.47E-10
LHX5	0.0476	3.61E-01	4.93E-01
LHX6	-0.1969	1.36E-04	7.91E-04
LHX8	0.1302	1.21E-02	3.41E-02
LHX9	-0.0253	6.27E-01	7.35E-01

LIAS	-0.1904	2.25E-04	1.22E-03
LIFR	-0.0455	3.82E-01	5.15E-01
LIF	0.0380	4.65E-01	5.95E-01
LIG1	0.4223	1.76E-17	2.20E-15
LIG3	0.2691	1.41E-07	1.99E-06
LIG4	-0.0274	5.99E-01	7.12E-01
LILRA1	-0.1286	1.32E-02	3.66E-02
LILRA2	-0.2145	3.09E-05	2.24E-04
LILRA3	-0.0785	1.31E-01	2.29E-01
LILRA4	-0.1097	3.47E-02	8.04E-02
LILRA5	-0.1540	2.94E-03	1.06E-02
LILRA6	-0.0639	2.20E-01	3.40E-01
LILRB1	-0.0896	8.47E-02	1.63E-01
LILRB2	-0.1067	3.99E-02	9.00E-02
LILRB3	-0.0177	7.34E-01	8.20E-01
LILRB4	-0.0321	5.38E-01	6.60E-01
LILRB5	-0.2563	5.63E-07	6.76E-06
LILRP2	0.0222	6.70E-01	7.70E-01
LIM2	0.0325	5.33E-01	6.55E-01
LIMA1	0.0389	4.55E-01	5.86E-01
LIMCH1	-0.0231	6.57E-01	7.59E-01
LIMD1	0.0482	3.55E-01	4.87E-01
LIMD2	0.1148	2.70E-02	6.56E-02
LIME1	-0.1851	3.37E-04	1.72E-03
LIMK1	0.1403	6.81E-03	2.13E-02
LIMK2	0.1777	5.83E-04	2.70E-03
LIMS1	-0.0404	4.38E-01	5.69E-01
LIMS2	-0.1860	3.15E-04	1.63E-03
LIMS3-LOC4408	-0.0065	9.01E-01	9.37E-01
LIMS3	-0.0275	5.98E-01	7.12E-01
LIN28A	0.0046	9.30E-01	9.56E-01
LIN28B	0.0824	1.13E-01	2.05E-01
LIN37	0.1960	1.44E-04	8.37E-04
LIN52	0.0239	6.46E-01	7.51E-01
LIN54	0.0218	6.76E-01	7.74E-01
LIN7A	-0.2355	4.54E-06	4.28E-05
LIN7B	0.0938	7.13E-02	1.42E-01
LIN7C	-0.1935	1.77E-04	9.91E-04
LIN9	0.4600	7.97E-21	2.27E-18
LINGO1	0.0486	3.51E-01	4.83E-01
LINGO2	-0.0586	2.60E-01	3.86E-01
LINGO3	-0.0540	3.00E-01	4.29E-01
LINGO4	0.0085	8.70E-01	9.16E-01
LINS1	0.1409	6.55E-03	2.06E-02

LIPA	-0.2224	1.53E-05	1.22E-04
LIPC	-0.2327	5.92E-06	5.36E-05
LIPE	-0.1146	2.73E-02	6.62E-02
LIPF	0.0503	3.34E-01	4.65E-01
LIPG	-0.1096	3.49E-02	8.08E-02
LIPH	0.0241	6.44E-01	7.49E-01
LIPI	-0.0151	7.72E-01	8.47E-01
LIPJ	-0.0740	1.55E-01	2.60E-01
LIPK	-0.0466	3.71E-01	5.04E-01
LIPM	-0.0220	6.73E-01	7.72E-01
LIPN	-0.0608	2.43E-01	3.67E-01
LIPT1	0.1513	3.49E-03	1.23E-02
LIPT2	0.0454	3.84E-01	5.17E-01
LITAF	-0.0893	8.59E-02	1.65E-01
LIX1L	0.3240	1.62E-10	4.58E-09
LIX1	-0.0074	8.87E-01	9.28E-01
LLGL1	0.0964	6.38E-02	1.30E-01
LLGL2	0.2226	1.50E-05	1.20E-04
LLPH	0.1470	4.55E-03	1.53E-02
LMAN1L	0.0557	2.85E-01	4.14E-01
LMAN1	-0.1384	7.58E-03	2.32E-02
LMAN2L	0.0897	8.44E-02	1.62E-01
LMAN2	-0.0620	2.34E-01	3.57E-01
LMBR1L	0.1378	7.85E-03	2.40E-02
LMBR1	-0.1235	1.73E-02	4.57E-02
LMBRD1	-0.0686	1.87E-01	3.01E-01
LMBRD2	-0.0090	8.64E-01	9.12E-01
LMCD1	0.0392	4.52E-01	5.83E-01
LMF1	-0.2100	4.57E-05	3.13E-04
LMF2	0.0662	2.03E-01	3.21E-01
LMLN	-0.0210	6.87E-01	7.83E-01
LMNA	0.3288	8.41E-11	2.50E-09
LMNB1	0.3810	2.89E-14	1.71E-12
LMNB2	0.3714	1.40E-13	7.35E-12
LMO1	0.0251	6.30E-01	7.38E-01
LMO2	-0.1678	1.18E-03	4.95E-03
LMO3	0.0667	2.00E-01	3.17E-01
LMO4	0.0503	3.34E-01	4.65E-01
LMO7	-0.1500	3.77E-03	1.31E-02
LMOD1	-0.2069	5.95E-05	3.92E-04
LMOD2	-0.1016	5.05E-02	1.08E-01
LMOD3	-0.0745	1.52E-01	2.56E-01
LMTK2	-0.0404	4.37E-01	5.69E-01
LMTK3	0.0990	5.67E-02	1.19E-01

LMX1A	0.1531	3.11E-03	1.11E-02
LMX1B	0.1257	1.54E-02	4.15E-02
LNP1	-0.1165	2.49E-02	6.12E-02
LNPEP	-0.0692	1.83E-01	2.96E-01
LNX1	0.0378	4.68E-01	5.98E-01
LNX2	-0.1102	3.38E-02	7.89E-02
LOC100009676	0.0056	9.15E-01	9.47E-01
LOC100101266	0.1011	5.16E-02	1.10E-01
LOC100101938	0.0308	5.54E-01	6.74E-01
LOC100124692	0.1283	1.34E-02	3.71E-02
LOC100125556	0.2098	4.64E-05	3.17E-04
LOC100126784	-0.1073	3.89E-02	8.82E-02
LOC100127888	0.1628	1.65E-03	6.57E-03
LOC100128023	0.0820	1.15E-01	2.07E-01
LOC100128076	0.0266	6.10E-01	7.21E-01
LOC100128164	0.0738	1.56E-01	2.62E-01
LOC100128191	0.3045	2.12E-09	4.65E-08
LOC100128239	0.0290	5.78E-01	6.95E-01
LOC100128288	0.0629	2.27E-01	3.48E-01
LOC100128292	0.1184	2.25E-02	5.65E-02
LOC100128542	-0.0642	2.18E-01	3.38E-01
LOC100128554	0.0531	3.08E-01	4.37E-01
LOC100128573	0.0773	1.37E-01	2.37E-01
LOC100128640	-0.0867	9.53E-02	1.79E-01
LOC100128675	0.0305	5.58E-01	6.77E-01
LOC100128788	0.0711	1.72E-01	2.81E-01
LOC100128811	-0.0249	6.33E-01	7.40E-01
LOC100128822	0.0747	1.51E-01	2.55E-01
LOC100128842	0.1479	4.30E-03	1.46E-02
LOC100128977	-0.0565	2.78E-01	4.07E-01
LOC100129034	0.0289	5.79E-01	6.95E-01
LOC100129055	0.1714	9.19E-04	4.00E-03
LOC100129066	-0.0021	9.68E-01	9.80E-01
LOC100129387	-0.2163	2.65E-05	1.97E-04
LOC100129534	0.0058	9.11E-01	9.44E-01
LOC100129550	0.1869	2.96E-04	1.54E-03
LOC100129637	0.1777	5.83E-04	2.70E-03
LOC100129716	0.0111	8.31E-01	8.89E-01
LOC100129726	0.0606	2.45E-01	3.69E-01
LOC100129935	0.0752	1.48E-01	2.52E-01
LOC100130015	-0.1650	1.43E-03	5.79E-03
LOC100130093	-0.0964	6.36E-02	1.30E-01
LOC100130148	0.2085	5.19E-05	3.50E-04
LOC100130238	0.0452	3.85E-01	5.18E-01

LOC100130264	0.0004	9.94E-01	9.96E-01
LOC100130274	0.1699	1.02E-03	4.37E-03
LOC100130331	0.0111	8.31E-01	8.89E-01
LOC100130386	-0.0580	2.65E-01	3.92E-01
LOC100130522	0.2097	4.68E-05	3.20E-04
LOC100130557	0.2007	9.90E-05	6.04E-04
LOC100130581	0.0000	1.00E+00	1.00E+00
LOC100130691	0.1604	1.94E-03	7.52E-03
LOC100130776	-0.0712	1.71E-01	2.80E-01
LOC100130872	0.0006	9.90E-01	9.94E-01
LOC100130932	0.2217	1.63E-05	1.29E-04
LOC100130933	0.0405	4.37E-01	5.68E-01
LOC100130987	0.0191	7.13E-01	8.04E-01
LOC100131193	-0.0758	1.45E-01	2.47E-01
LOC100131434	0.2810	3.68E-08	5.92E-07
LOC100131496	-0.1027	4.80E-02	1.04E-01
LOC100131551	0.0539	3.00E-01	4.30E-01
LOC100131691	-0.0552	2.89E-01	4.18E-01
LOC100131726	-0.1781	5.66E-04	2.64E-03
LOC100132111	0.1447	5.23E-03	1.71E-02
LOC100132215	0.0671	1.97E-01	3.14E-01
LOC100132247	0.1539	2.96E-03	1.07E-02
LOC100132287	0.1814	4.45E-04	2.15E-03
LOC100132288	0.0246	6.37E-01	7.43E-01
LOC100132354	0.0502	3.35E-01	4.66E-01
LOC100132707	-0.1618	1.77E-03	6.95E-03
LOC100132724	0.0768	1.40E-01	2.41E-01
LOC100132831	0.0129	8.04E-01	8.70E-01
LOC100132832	-0.0020	9.69E-01	9.81E-01
LOC100133050	-0.0358	4.92E-01	6.19E-01
LOC100133161	0.2000	1.05E-04	6.35E-04
LOC100133308	0.0767	1.40E-01	2.41E-01
LOC100133331	0.0987	5.74E-02	1.20E-01
LOC100133469	0.0347	5.06E-01	6.30E-01
LOC100133545	0.1143	2.77E-02	6.70E-02
LOC100133612	0.0518	3.20E-01	4.50E-01
LOC100133669	0.0703	1.77E-01	2.88E-01
LOC100133893	0.1685	1.13E-03	4.76E-03
LOC100133920	0.0176	7.35E-01	8.21E-01
LOC100133957	0.0027	9.59E-01	9.74E-01
LOC100133985	-0.1336	1.00E-02	2.92E-02
LOC100133991	0.1494	3.93E-03	1.35E-02
LOC100134229	-0.0417	4.24E-01	5.56E-01
LOC100134259	0.0509	3.28E-01	4.59E-01

LOC100134368	0.1673	1.22E-03	5.07E-03
LOC100134713	-0.0267	6.08E-01	7.20E-01
LOC100134868	-0.0632	2.25E-01	3.46E-01
LOC100144603	-0.0103	8.44E-01	8.98E-01
LOC100144604	0.0699	1.79E-01	2.90E-01
LOC100170939	0.1357	8.86E-03	2.65E-02
LOC100188947	0.1163	2.51E-02	6.17E-02
LOC100188949	-0.0545	2.95E-01	4.24E-01
LOC100189589	0.0591	2.56E-01	3.82E-01
LOC100190938	0.0861	9.76E-02	1.83E-01
LOC100190939	-0.0135	7.95E-01	8.64E-01
LOC100190940	-0.0609	2.42E-01	3.67E-01
LOC100190986	0.0954	6.65E-02	1.35E-01
LOC100192378	-0.0061	9.06E-01	9.41E-01
LOC100192379	0.0946	6.89E-02	1.38E-01
LOC100192426	0.0458	3.79E-01	5.12E-01
LOC100216001	0.0152	7.70E-01	8.45E-01
LOC100216545	0.0860	9.82E-02	1.83E-01
LOC100233209	-0.0476	3.60E-01	4.93E-01
LOC100240726	0.0412	4.29E-01	5.61E-01
LOC100240734	0.0010	9.85E-01	9.90E-01
LOC100240735	-0.0071	8.91E-01	9.30E-01
LOC100268168	0.0955	6.63E-02	1.34E-01
LOC100270710	-0.0092	8.60E-01	9.10E-01
LOC100270746	0.0486	3.51E-01	4.83E-01
LOC100270804	0.2621	3.03E-07	3.94E-06
LOC100271722	0.0597	2.51E-01	3.76E-01
LOC100271831	0.0232	6.56E-01	7.58E-01
LOC100271832	0.1155	2.61E-02	6.39E-02
LOC100271836	0.1005	5.31E-02	1.13E-01
LOC100272146	0.2937	8.10E-09	1.53E-07
LOC100272216	-0.0672	1.97E-01	3.13E-01
LOC100272217	0.1167	2.46E-02	6.07E-02
LOC100272228	0.3226	1.98E-10	5.50E-09
LOC100286793	0.3569	1.37E-12	5.95E-11
LOC100286844	0.0550	2.91E-01	4.20E-01
LOC100287227	0.0217	6.77E-01	7.76E-01
LOC100287704	0.0396	4.47E-01	5.79E-01
LOC100287718	0.0844	1.05E-01	1.92E-01
LOC100288778	0.3159	4.84E-10	1.23E-08
LOC100289341	-0.0971	6.17E-02	1.27E-01
LOC100302401	0.0890	8.70E-02	1.66E-01
LOC100302640	0.0575	2.69E-01	3.97E-01
LOC100302650	-0.1399	6.94E-03	2.16E-02

LOC100303728	-0.0317	5.43E-01	6.64E-01
LOC113230	-0.0183	7.25E-01	8.13E-01
LOC115110	0.0290	5.78E-01	6.95E-01
LOC116437	-0.1314	1.13E-02	3.23E-02
LOC121838	0.0213	6.83E-01	7.80E-01
LOC121952	-0.1618	1.77E-03	6.97E-03
LOC126536	0.1759	6.64E-04	3.03E-03
LOC127841	0.3540	2.15E-12	8.93E-11
LOC134466	0.1355	8.98E-03	2.68E-02
LOC143188	-0.0237	6.49E-01	7.53E-01
LOC143666	0.0202	6.98E-01	7.91E-01
LOC144438	0.2089	5.02E-05	3.40E-04
LOC144486	0.2151	2.95E-05	2.16E-04
LOC144571	0.0303	5.61E-01	6.79E-01
LOC144742	-0.0803	1.22E-01	2.18E-01
LOC144776	0.1265	1.48E-02	4.02E-02
LOC145474	0.0289	5.79E-01	6.96E-01
LOC145783	0.1127	3.00E-02	7.15E-02
LOC145820	-0.2459	1.63E-06	1.75E-05
LOC145837	-0.0134	7.96E-01	8.65E-01
LOC145845	0.0767	1.41E-01	2.41E-01
LOC146336	0.1578	2.31E-03	8.67E-03
LOC146481	-0.0113	8.28E-01	8.88E-01
LOC146880	0.4167	5.17E-17	5.51E-15
LOC147727	0.0392	4.51E-01	5.82E-01
LOC147804	0.2730	9.21E-08	1.35E-06
LOC148145	-0.0467	3.70E-01	5.03E-01
LOC148189	-0.0287	5.82E-01	6.98E-01
LOC148413	-0.1069	3.97E-02	8.96E-02
LOC148696	-0.0322	5.36E-01	6.58E-01
LOC148709	0.0824	1.13E-01	2.05E-01
LOC148824	0.1098	3.46E-02	8.02E-02
LOC149134	0.2933	8.50E-09	1.60E-07
LOC149620	0.0850	1.02E-01	1.89E-01
LOC149837	0.1445	5.30E-03	1.73E-02
LOC150185	0.0720	1.66E-01	2.74E-01
LOC150197	0.1618	1.76E-03	6.93E-03
LOC150381	0.1046	4.41E-02	9.73E-02
LOC150527	0.1111	3.24E-02	7.60E-02
LOC150568	0.0887	8.81E-02	1.68E-01
LOC150622	0.0547	2.93E-01	4.22E-01
LOC150776	0.3555	1.70E-12	7.24E-11
LOC150786	-0.0613	2.39E-01	3.63E-01
LOC151009	0.2327	5.93E-06	5.36E-05

LOC151162	0.1196	2.13E-02	5.39E-02
LOC151174	0.0659	2.06E-01	3.23E-01
LOC151300	0.0086	8.69E-01	9.16E-01
LOC151534	-0.1540	2.94E-03	1.06E-02
LOC151658	0.0529	3.09E-01	4.39E-01
LOC152024	0.0803	1.22E-01	2.18E-01
LOC152217	0.0899	8.36E-02	1.61E-01
LOC152225	-0.0227	6.63E-01	7.64E-01
LOC153328	-0.1157	2.58E-02	6.32E-02
LOC153684	0.1070	3.94E-02	8.91E-02
LOC153910	0.0914	7.86E-02	1.54E-01
LOC154449	-0.0653	2.10E-01	3.28E-01
LOC154761	-0.0295	5.71E-01	6.89E-01
LOC154822	0.0276	5.96E-01	7.10E-01
LOC157381	-0.0167	7.48E-01	8.30E-01
LOC157627	0.0151	7.71E-01	8.46E-01
LOC158376	-0.1730	8.19E-04	3.62E-03
LOC158572	0.0172	7.41E-01	8.25E-01
LOC158696	0.1420	6.15E-03	1.95E-02
LOC162632	0.0373	4.73E-01	6.02E-01
LOC168474	0.2033	8.02E-05	5.05E-04
LOC200030	0.2350	4.77E-06	4.46E-05
LOC200726	0.0344	5.09E-01	6.33E-01
LOC201651	-0.2033	7.99E-05	5.04E-04
LOC202181	0.1332	1.02E-02	2.98E-02
LOC202781	-0.0479	3.57E-01	4.89E-01
LOC219347	0.0799	1.24E-01	2.20E-01
LOC220429	-0.0548	2.93E-01	4.22E-01
LOC220594	0.0423	4.17E-01	5.49E-01
LOC220729	0.1588	2.16E-03	8.20E-03
LOC220930	0.0966	6.32E-02	1.29E-01
LOC221122	-0.0261	6.16E-01	7.26E-01
LOC221442	0.1706	9.72E-04	4.20E-03
LOC221710	0.0449	3.89E-01	5.22E-01
LOC222699	0.1347	9.38E-03	2.78E-02
LOC253039	0.3161	4.73E-10	1.21E-08
LOC253724	0.0532	3.07E-01	4.36E-01
LOC254559	0.0900	8.35E-02	1.61E-01
LOC255025	0.1752	7.00E-04	3.17E-03
LOC255167	-0.2523	8.52E-07	9.83E-06
LOC256880	0.3289	8.32E-11	2.48E-09
LOC257358	0.0362	4.87E-01	6.14E-01
LOC25845	0.1608	1.89E-03	7.33E-03
LOC26102	0.0228	6.62E-01	7.63E-01

LOC282997	-0.2408	2.72E-06	2.72E-05
LOC283050	0.2192	2.04E-05	1.57E-04
LOC283070	-0.0916	7.82E-02	1.53E-01
LOC283174	-0.0389	4.55E-01	5.86E-01
LOC283267	0.2208	1.78E-05	1.39E-04
LOC283314	-0.0091	8.62E-01	9.11E-01
LOC283332	0.1236	1.72E-02	4.54E-02
LOC283392	-0.0107	8.37E-01	8.93E-01
LOC283404	0.0622	2.32E-01	3.55E-01
LOC283663	0.0965	6.34E-02	1.30E-01
LOC283731	-0.0808	1.20E-01	2.15E-01
LOC283761	0.0155	7.67E-01	8.43E-01
LOC283856	-0.3178	3.78E-10	9.94E-09
LOC283867	-0.0062	9.06E-01	9.41E-01
LOC283914	0.0337	5.18E-01	6.42E-01
LOC283922	0.0081	8.76E-01	9.20E-01
LOC283999	0.0406	4.35E-01	5.67E-01
LOC284009	0.1797	5.05E-04	2.40E-03
LOC284023	0.2145	3.10E-05	2.25E-04
LOC284100	0.2284	8.85E-06	7.54E-05
LOC284232	0.0031	9.53E-01	9.71E-01
LOC284233	0.1036	4.62E-02	1.01E-01
LOC284276	0.0581	2.64E-01	3.91E-01
LOC284379	0.0383	4.62E-01	5.92E-01
LOC284440	-0.0172	7.42E-01	8.26E-01
LOC284441	-0.0669	1.98E-01	3.15E-01
LOC284551	0.1096	3.48E-02	8.08E-02
LOC284578	0.0696	1.81E-01	2.93E-01
LOC284632	0.0788	1.30E-01	2.27E-01
LOC284661	0.0592	2.55E-01	3.81E-01
LOC284688	0.1197	2.11E-02	5.37E-02
LOC284749	0.2955	6.51E-09	1.26E-07
LOC284788	-0.0420	4.20E-01	5.53E-01
LOC284798	0.1030	4.74E-02	1.03E-01
LOC284837	0.1724	8.53E-04	3.75E-03
LOC284900	0.1985	1.19E-04	7.06E-04
LOC285033	-0.0805	1.22E-01	2.17E-01
LOC285045	0.0205	6.93E-01	7.89E-01
LOC285074	-0.0586	2.60E-01	3.86E-01
LOC285205	0.1167	2.45E-02	6.06E-02
LOC285359	0.3021	2.87E-09	6.10E-08
LOC285370	0.0815	1.17E-01	2.10E-01
LOC285375	0.1504	3.69E-03	1.28E-02
LOC285401	0.0802	1.23E-01	2.18E-01

LOC285419	0.1336	1.00E-02	2.92E-02
LOC285456	-0.1883	2.64E-04	1.39E-03
LOC285501	0.1019	4.98E-02	1.07E-01
LOC285548	0.1293	1.27E-02	3.55E-02
LOC285593	-0.2451	1.77E-06	1.88E-05
LOC285627	0.0154	7.67E-01	8.43E-01
LOC285629	0.2847	2.39E-08	4.03E-07
LOC285692	0.1236	1.73E-02	4.56E-02
LOC285696	-0.1025	4.85E-02	1.05E-01
LOC285733	0.0129	8.05E-01	8.71E-01
LOC285735	0.0572	2.72E-01	4.00E-01
LOC285740	0.0689	1.85E-01	2.98E-01
LOC285768	0.1919	2.01E-04	1.11E-03
LOC285780	-0.1574	2.37E-03	8.86E-03
LOC285796	-0.0223	6.68E-01	7.68E-01
LOC285830	0.2073	5.74E-05	3.81E-04
LOC285847	-0.0965	6.34E-02	1.30E-01
LOC285954	0.0228	6.61E-01	7.63E-01
LOC286002	-0.0061	9.07E-01	9.41E-01
LOC286094	0.0428	4.11E-01	5.44E-01
LOC286135	0.0072	8.90E-01	9.30E-01
LOC286238	-0.0019	9.71E-01	9.81E-01
LOC286359	0.1506	3.65E-03	1.27E-02
LOC286367	0.2000	1.05E-04	6.36E-04
LOC286467	0.0484	3.52E-01	4.84E-01
LOC29034	-0.1219	1.88E-02	4.89E-02
LOC338588	-0.0024	9.63E-01	9.77E-01
LOC338651	0.0418	4.22E-01	5.55E-01
LOC338758	-0.0797	1.26E-01	2.21E-01
LOC338799	0.2161	2.68E-05	1.99E-04
LOC339047	0.3317	5.60E-11	1.74E-09
LOC339240	0.0010	9.85E-01	9.91E-01
LOC339290	-0.0300	5.64E-01	6.82E-01
LOC339524	-0.2063	6.25E-05	4.09E-04
LOC339535	0.1839	3.69E-04	1.85E-03
LOC339674	0.3601	8.45E-13	3.84E-11
LOC339788	0.1100	3.42E-02	7.96E-02
LOC340017	0.0865	9.60E-02	1.80E-01
LOC340074	0.0118	8.21E-01	8.83E-01
LOC340094	0.0016	9.75E-01	9.84E-01
LOC340357	0.0345	5.08E-01	6.32E-01
LOC340508	0.0902	8.27E-02	1.60E-01
LOC341056	0.0778	1.35E-01	2.34E-01
LOC342346	-0.0878	9.11E-02	1.73E-01

LOC344595	-0.0114	8.26E-01	8.86E-01
LOC344967	0.1934	1.78E-04	9.99E-04
LOC347376	0.1116	3.16E-02	7.44E-02
LOC348021	0.0621	2.33E-01	3.56E-01
LOC348840	-0.0526	3.12E-01	4.42E-01
LOC348926	0.0775	1.36E-01	2.36E-01
LOC349114	0.1547	2.81E-03	1.03E-02
LOC349196	0.0844	1.05E-01	1.92E-01
LOC360030	0.0938	7.12E-02	1.42E-01
LOC374443	0.1419	6.18E-03	1.96E-02
LOC374491	0.0605	2.45E-01	3.70E-01
LOC375190	0.1603	1.95E-03	7.55E-03
LOC387646	0.2656	2.08E-07	2.80E-06
LOC387647	0.0268	6.07E-01	7.20E-01
LOC388152	0.2553	6.26E-07	7.44E-06
LOC388242	0.0497	3.40E-01	4.72E-01
LOC388387	0.0028	9.58E-01	9.74E-01
LOC388428	0.0955	6.61E-02	1.34E-01
LOC388588	-0.1028	4.78E-02	1.04E-01
LOC388692	0.1270	1.44E-02	3.94E-02
LOC388789	0.0379	4.67E-01	5.97E-01
LOC388796	0.1563	2.54E-03	9.38E-03
LOC388946	0.0392	4.52E-01	5.83E-01
LOC388955	-0.0543	2.97E-01	4.27E-01
LOC389033	-0.0902	8.28E-02	1.60E-01
LOC389332	0.2201	1.90E-05	1.47E-04
LOC389333	0.0312	5.49E-01	6.69E-01
LOC389458	-0.0099	8.49E-01	9.02E-01
LOC389493	0.1067	3.99E-02	9.00E-02
LOC389634	0.1170	2.42E-02	6.00E-02
LOC389705	0.0357	4.94E-01	6.20E-01
LOC389791	0.2350	4.77E-06	4.46E-05
LOC390595	0.0495	3.41E-01	4.73E-01
LOC390858	-0.0347	5.05E-01	6.30E-01
LOC391322	-0.0320	5.39E-01	6.60E-01
LOC392196	0.0547	2.93E-01	4.22E-01
LOC399744	0.0662	2.03E-01	3.20E-01
LOC399815	0.2581	4.67E-07	5.77E-06
LOC399959	-0.3573	1.30E-12	5.65E-11
LOC400027	-0.0128	8.06E-01	8.72E-01
LOC400043	0.0713	1.71E-01	2.80E-01
LOC400657	0.2116	3.98E-05	2.79E-04
LOC400696	0.1250	1.60E-02	4.28E-02
LOC400752	0.0093	8.58E-01	9.08E-01

LOC400759	-0.0033	9.49E-01	9.68E-01
LOC400794	0.1152	2.65E-02	6.46E-02
LOC400804	0.0597	2.52E-01	3.77E-01
LOC400891	0.0994	5.59E-02	1.17E-01
LOC400927	0.1342	9.66E-03	2.85E-02
LOC400931	0.0761	1.44E-01	2.45E-01
LOC400940	0.0997	5.51E-02	1.16E-01
LOC401010	0.0332	5.23E-01	6.47E-01
LOC401052	0.1652	1.40E-03	5.72E-03
LOC401093	-0.1230	1.78E-02	4.67E-02
LOC401127	-0.1399	6.96E-03	2.17E-02
LOC401387	-0.0712	1.71E-01	2.80E-01
LOC401397	0.0078	8.81E-01	9.24E-01
LOC401431	-0.0842	1.06E-01	1.94E-01
LOC401463	-0.0706	1.75E-01	2.85E-01
LOC401588	0.1662	1.31E-03	5.40E-03
LOC402377	0.1220	1.88E-02	4.88E-02
LOC402644	0.0439	4.00E-01	5.32E-01
LOC407835	0.0578	2.67E-01	3.94E-01
LOC415056	0.0210	6.86E-01	7.83E-01
LOC440040	0.0574	2.70E-01	3.97E-01
LOC440173	0.1466	4.67E-03	1.56E-02
LOC440354	0.0700	1.78E-01	2.89E-01
LOC440356	0.2776	5.43E-08	8.38E-07
LOC440461	0.0339	5.14E-01	6.39E-01
LOC440563	0.1509	3.57E-03	1.25E-02
LOC440896	0.1195	2.13E-02	5.40E-02
LOC440905	0.1352	9.14E-03	2.72E-02
LOC440925	-0.0308	5.55E-01	6.74E-01
LOC440944	0.3458	7.41E-12	2.75E-10
LOC440957	0.0272	6.02E-01	7.14E-01
LOC441046	-0.1485	4.15E-03	1.42E-02
LOC441089	0.1439	5.48E-03	1.78E-02
LOC441177	-0.1112	3.22E-02	7.57E-02
LOC441204	0.0105	8.40E-01	8.94E-01
LOC441208	-0.1481	4.26E-03	1.45E-02
LOC441294	0.1684	1.13E-03	4.77E-03
LOC441454	0.0952	6.70E-02	1.36E-01
LOC441455	0.1148	2.70E-02	6.56E-02
LOC441601	0.1374	8.06E-03	2.45E-02
LOC441666	0.1669	1.26E-03	5.22E-03
LOC441869	0.1499	3.81E-03	1.32E-02
LOC442308	0.0887	8.81E-02	1.68E-01
LOC442421	-0.1395	7.12E-03	2.21E-02

LOC442454	-0.0235	6.52E-01	7.55E-01
LOC442459	0.2148	3.02E-05	2.20E-04
LOC493754	0.2540	7.11E-07	8.37E-06
LOC494141	0.0739	1.56E-01	2.61E-01
LOC541471	0.1927	1.88E-04	1.05E-03
LOC541473	0.0179	7.32E-01	8.18E-01
LOC550112	-0.0153	7.70E-01	8.45E-01
LOC550643	-0.0128	8.06E-01	8.72E-01
LOC554202	0.1612	1.84E-03	7.17E-03
LOC55908	0.0149	7.74E-01	8.49E-01
LOC572558	-0.1320	1.09E-02	3.13E-02
LOC595101	0.0899	8.37E-02	1.61E-01
LOC606724	0.0608	2.42E-01	3.67E-01
LOC613037	0.2079	5.46E-05	3.65E-04
LOC619207	0.0129	8.04E-01	8.71E-01
LOC641298	0.0479	3.57E-01	4.89E-01
LOC641367	0.1267	1.46E-02	3.98E-02
LOC642587	-0.1254	1.56E-02	4.20E-02
LOC642597	-0.2501	1.07E-06	1.20E-05
LOC642826	0.1999	1.06E-04	6.38E-04
LOC642846	0.3979	1.58E-15	1.21E-13
LOC642852	0.3206	2.58E-10	7.03E-09
LOC642929	0.0237	6.50E-01	7.53E-01
LOC643008	0.1082	3.72E-02	8.51E-02
LOC643387	0.2287	8.57E-06	7.33E-05
LOC643486	0.0233	6.55E-01	7.57E-01
LOC643677	0.1406	6.69E-03	2.10E-02
LOC643719	-0.0465	3.72E-01	5.05E-01
LOC643763	0.1215	1.93E-02	4.98E-02
LOC643837	-0.2244	1.28E-05	1.04E-04
LOC643923	-0.0274	5.99E-01	7.12E-01
LOC643955	0.0004	9.94E-01	9.96E-01
LOC644145	-0.0235	6.51E-01	7.55E-01
LOC644165	0.2317	6.53E-06	5.83E-05
LOC644172	-0.1211	1.96E-02	5.06E-02
LOC644538	0.0231	6.57E-01	7.59E-01
LOC644669	0.0619	2.34E-01	3.57E-01
LOC644936	-0.1016	5.06E-02	1.09E-01
LOC645166	0.1931	1.82E-04	1.02E-03
LOC645323	0.0797	1.25E-01	2.21E-01
LOC645332	-0.0204	6.95E-01	7.89E-01
LOC645431	0.0486	3.50E-01	4.83E-01
LOC645676	0.3245	1.53E-10	4.34E-09
LOC645752	-0.0888	8.78E-02	1.67E-01

LOC646214	0.1336	1.00E-02	2.92E-02
LOC646471	0.0077	8.83E-01	9.25E-01
LOC646498	0.0447	3.91E-01	5.24E-01
LOC646627	0.1297	1.24E-02	3.48E-02
LOC646762	0.1602	1.96E-03	7.57E-03
LOC646813	-0.0159	7.61E-01	8.39E-01
LOC646851	0.2973	5.22E-09	1.04E-07
LOC646982	0.0735	1.58E-01	2.64E-01
LOC646999	-0.0722	1.65E-01	2.73E-01
LOC647121	0.0173	7.39E-01	8.24E-01
LOC647288	-0.0964	6.36E-02	1.30E-01
LOC647309	-0.1727	8.39E-04	3.69E-03
LOC647859	-0.0364	4.85E-01	6.13E-01
LOC647946	0.2389	3.26E-06	3.19E-05
LOC647979	0.0244	6.39E-01	7.45E-01
LOC648691	0.1789	5.34E-04	2.52E-03
LOC648740	-0.0008	9.88E-01	9.92E-01
LOC649330	0.1603	1.95E-03	7.53E-03
LOC650368	0.0627	2.28E-01	3.51E-01
LOC650623	-0.0049	9.25E-01	9.52E-01
LOC651250	0.2111	4.16E-05	2.89E-04
LOC652276	0.1242	1.67E-02	4.43E-02
LOC653113	0.0851	1.02E-01	1.88E-01
LOC653501	0.0896	8.47E-02	1.63E-01
LOC653544	-0.1151	2.66E-02	6.49E-02
LOC653566	0.1213	1.95E-02	5.02E-02
LOC653653	-0.0699	1.79E-01	2.91E-01
LOC653786	-0.0867	9.55E-02	1.80E-01
LOC654342	0.1092	3.54E-02	8.18E-02
LOC654433	0.0115	8.25E-01	8.86E-01
LOC678655	-0.0156	7.64E-01	8.41E-01
LOC723809	0.2962	5.98E-09	1.17E-07
LOC723972	0.0828	1.11E-01	2.02E-01
LOC727677	-0.0855	1.00E-01	1.86E-01
LOC727896	0.2007	9.90E-05	6.04E-04
LOC727924	-0.0138	7.92E-01	8.62E-01
LOC728024	0.0380	4.65E-01	5.95E-01
LOC728190	0.1028	4.79E-02	1.04E-01
LOC728264	0.0421	4.19E-01	5.52E-01
LOC728276	-0.0617	2.36E-01	3.59E-01
LOC728323	-0.0153	7.68E-01	8.44E-01
LOC728392	-0.0027	9.59E-01	9.74E-01
LOC728554	0.1276	1.39E-02	3.84E-02
LOC728606	-0.0175	7.36E-01	8.22E-01

LOC728613	0.0646	2.14E-01	3.34E-01
LOC728640	-0.0882	8.98E-02	1.71E-01
LOC728643	0.1927	1.88E-04	1.05E-03
LOC728723	-0.0427	4.13E-01	5.45E-01
LOC728743	0.0026	9.61E-01	9.75E-01
LOC728758	0.1806	4.72E-04	2.26E-03
LOC728819	0.1013	5.11E-02	1.09E-01
LOC728855	0.0991	5.65E-02	1.18E-01
LOC728875	0.0597	2.52E-01	3.77E-01
LOC728989	0.0010	9.84E-01	9.90E-01
LOC729020	-0.0273	6.00E-01	7.13E-01
LOC729082	0.0254	6.26E-01	7.34E-01
LOC729121	0.0573	2.71E-01	3.99E-01
LOC729156	-0.0419	4.21E-01	5.54E-01
LOC729176	0.0264	6.12E-01	7.23E-01
LOC729234	0.1241	1.68E-02	4.46E-02
LOC729375	0.0950	6.77E-02	1.37E-01
LOC729467	0.0434	4.05E-01	5.38E-01
LOC729603	0.0744	1.52E-01	2.57E-01
LOC729609	0.0179	7.31E-01	8.17E-01
LOC729668	0.0594	2.54E-01	3.80E-01
LOC729678	-0.0282	5.88E-01	7.03E-01
LOC729799	0.0091	8.62E-01	9.11E-01
LOC729991-ME	-0.0199	7.03E-01	7.96E-01
LOC729991	0.0907	8.10E-02	1.57E-01
LOC730101	0.2160	2.73E-05	2.02E-04
LOC730668	0.1317	1.11E-02	3.18E-02
LOC731779	0.0942	7.01E-02	1.41E-01
LOC731789	0.0933	7.25E-02	1.44E-01
LOC732275	0.0975	6.06E-02	1.25E-01
LOC80054	0.1990	1.14E-04	6.81E-04
LOC80154	0.2787	4.79E-08	7.51E-07
LOC81691	0.3349	3.55E-11	1.16E-09
LOC84740	0.2263	1.07E-05	8.96E-05
LOC84856	0.0817	1.16E-01	2.09E-01
LOC84931	0.1041	4.52E-02	9.92E-02
LOC84989	0.0863	9.71E-02	1.82E-01
LOC90110	0.0750	1.50E-01	2.53E-01
LOC90246	-0.0176	7.36E-01	8.21E-01
LOC90586	-0.0803	1.22E-01	2.18E-01
LOC90784	0.0728	1.62E-01	2.69E-01
LOC90834	0.1378	7.87E-03	2.40E-02
LOC91149	0.1937	1.74E-04	9.77E-04
LOC91316	0.3440	9.64E-12	3.45E-10

LOC91450	0.0920	7.68E-02	1.51E-01
LOC91948	-0.0492	3.45E-01	4.77E-01
LOC92249	0.0793	1.27E-01	2.24E-01
LOC92659	0.2678	1.64E-07	2.28E-06
LOC92973	0.1357	8.86E-03	2.65E-02
LOC93432	0.1421	6.10E-03	1.94E-02
LOC93622	0.1512	3.50E-03	1.23E-02
LOC96610	-0.0038	9.41E-01	9.64E-01
LOH12CR1	-0.0172	7.41E-01	8.25E-01
LOH12CR2	-0.0567	2.76E-01	4.04E-01
LOH3CR2A	0.0759	1.45E-01	2.47E-01
LONP1	-0.2073	5.75E-05	3.81E-04
LONP2	-0.3553	1.75E-12	7.43E-11
LONRF1	-0.0380	4.65E-01	5.95E-01
LONRF2	0.1536	3.02E-03	1.09E-02
LONRF3	-0.0554	2.88E-01	4.16E-01
LOR	0.0323	5.35E-01	6.57E-01
LOXHD1	-0.0967	6.28E-02	1.29E-01
LOXL1	-0.0490	3.47E-01	4.79E-01
LOXL2	-0.0021	9.68E-01	9.80E-01
LOXL3	0.0204	6.95E-01	7.89E-01
LOXL4	-0.0154	7.68E-01	8.44E-01
LOX	-0.0169	7.45E-01	8.28E-01
LPAL2	0.0555	2.87E-01	4.16E-01
LPAR1	0.0087	8.67E-01	9.14E-01
LPAR2	0.3067	1.61E-09	3.65E-08
LPAR3	-0.0518	3.20E-01	4.50E-01
LPAR4	-0.0559	2.82E-01	4.11E-01
LPAR5	-0.0140	7.88E-01	8.59E-01
LPAR6	-0.0318	5.42E-01	6.63E-01
LPA	-0.0672	1.96E-01	3.12E-01
LPCAT1	0.1483	4.20E-03	1.43E-02
LPCAT2	-0.0281	5.90E-01	7.04E-01
LPCAT3	0.0113	8.28E-01	8.87E-01
LPCAT4	0.1256	1.55E-02	4.17E-02
LPGAT1	0.1407	6.64E-03	2.09E-02
LPHN1	0.0165	7.51E-01	8.32E-01
LPHN2	-0.0415	4.25E-01	5.58E-01
LPHN3	-0.0901	8.29E-02	1.60E-01
LPIN1	-0.1592	2.10E-03	8.02E-03
LPIN2	-0.1369	8.28E-03	2.50E-02
LPIN3	0.1303	1.20E-02	3.39E-02
LPL	-0.1258	1.53E-02	4.14E-02
LPO	0.1705	9.78E-04	4.22E-03

LPPR1	-0.0245	6.38E-01	7.44E-01
LPPR2	-0.0911	7.96E-02	1.55E-01
LPPR3	0.0713	1.71E-01	2.80E-01
LPPR4	0.0052	9.20E-01	9.49E-01
LPPR5	0.0798	1.25E-01	2.21E-01
LPP	-0.0503	3.34E-01	4.64E-01
LPXN	-0.1343	9.59E-03	2.83E-02
LQK1	-0.0206	6.93E-01	7.88E-01
LRAT	-0.0630	2.26E-01	3.48E-01
LRBA	-0.1851	3.38E-04	1.73E-03
LRCH1	-0.1401	6.88E-03	2.14E-02
LRCH2	-0.1543	2.88E-03	1.04E-02
LRCH3	0.1008	5.24E-02	1.11E-01
LRCH4	-0.0258	6.21E-01	7.30E-01
LRDD	0.3161	4.74E-10	1.21E-08
LRFN1	0.1198	2.10E-02	5.35E-02
LRFN2	0.1467	4.62E-03	1.54E-02
LRFN3	-0.0562	2.80E-01	4.09E-01
LRFN4	0.0348	5.05E-01	6.30E-01
LRFN5	0.0826	1.12E-01	2.03E-01
LRG1	-0.1701	1.01E-03	4.32E-03
LRGUK	0.0569	2.74E-01	4.02E-01
LRIG1	-0.1320	1.09E-02	3.14E-02
LRIG2	0.1087	3.63E-02	8.35E-02
LRIG3	0.1888	2.55E-04	1.36E-03
LRIT1	0.0438	4.01E-01	5.33E-01
LRIT2	-0.0419	4.21E-01	5.54E-01
LRIT3	0.0298	5.68E-01	6.85E-01
LRMP	-0.0713	1.70E-01	2.79E-01
LRP10	-0.0647	2.14E-01	3.33E-01
LRP11	0.3338	4.18E-11	1.33E-09
LRP12	0.1106	3.32E-02	7.76E-02
LRP1B	-0.0272	6.01E-01	7.14E-01
LRP1	-0.3033	2.48E-09	5.33E-08
LRP2BP	-0.1047	4.38E-02	9.70E-02
LRP2	0.0001	9.99E-01	9.99E-01
LRP3	0.0311	5.50E-01	6.70E-01
LRP4	-0.0144	7.83E-01	8.55E-01
LRP5L	0.0830	1.10E-01	2.01E-01
LRP5	-0.2125	3.67E-05	2.61E-04
LRP6	-0.0416	4.25E-01	5.57E-01
LRP8	0.2339	5.26E-06	4.84E-05
LRPAP1	-0.1294	1.26E-02	3.54E-02
LRPPRC	0.1209	1.99E-02	5.11E-02

LRRC10B	-0.1127	2.99E-02	7.13E-02
LRRC10	-0.0723	1.65E-01	2.72E-01
LRRC14B	0.1349	9.31E-03	2.76E-02
LRRC14	0.2421	2.37E-06	2.42E-05
LRRC15	-0.0993	5.60E-02	1.18E-01
LRRC16A	0.0451	3.86E-01	5.19E-01
LRRC16B	0.1039	4.55E-02	9.98E-02
LRRC17	-0.0542	2.98E-01	4.28E-01
LRRC18	0.1091	3.56E-02	8.21E-02
LRRC19	-0.0494	3.42E-01	4.74E-01
LRRC1	0.1909	2.18E-04	1.18E-03
LRRC20	-0.0119	8.20E-01	8.82E-01
LRRC23	0.0506	3.32E-01	4.63E-01
LRRC24	-0.0103	8.43E-01	8.97E-01
LRRC25	-0.0727	1.63E-01	2.70E-01
LRRC26	0.1763	6.46E-04	2.96E-03
LRRC27	0.0206	6.92E-01	7.87E-01
LRRC28	-0.0739	1.56E-01	2.61E-01
LRRC29	-0.3622	6.09E-13	2.83E-11
LRRC2	-0.1020	4.96E-02	1.07E-01
LRRC30	-0.0245	6.38E-01	7.44E-01
LRRC31	-0.0120	8.18E-01	8.80E-01
LRRC32	-0.1826	4.08E-04	2.01E-03
LRRC33	-0.1500	3.79E-03	1.32E-02
LRRC34	-0.0016	9.76E-01	9.85E-01
LRRC36	0.1665	1.28E-03	5.31E-03
LRRC37A2	0.0655	2.08E-01	3.26E-01
LRRC37A3	0.1069	3.96E-02	8.95E-02
LRRC37A4	0.0591	2.56E-01	3.82E-01
LRRC37A	0.1498	3.83E-03	1.33E-02
LRRC37B2	0.3391	1.96E-11	6.67E-10
LRRC37B	0.3262	1.21E-10	3.49E-09
LRRC39	0.0575	2.69E-01	3.97E-01
LRRC3B	0.0207	6.91E-01	7.86E-01
LRRC3	-0.2056	6.63E-05	4.29E-04
LRRC40	0.0014	9.79E-01	9.87E-01
LRRC41	0.1227	1.81E-02	4.74E-02
LRRC42	0.2451	1.77E-06	1.88E-05
LRRC43	0.0113	8.29E-01	8.88E-01
LRRC45	0.1387	7.47E-03	2.30E-02
LRRC46	0.2531	7.85E-07	9.13E-06
LRRC47	-0.0591	2.56E-01	3.82E-01
LRRC48	0.1008	5.23E-02	1.11E-01
LRRC49	0.0601	2.48E-01	3.73E-01

LRRC4B	-0.0901	8.30E-02	1.60E-01
LRRC4C	-0.1202	2.05E-02	5.24E-02
LRRC4	-0.0818	1.16E-01	2.08E-01
LRRC50	-0.0345	5.07E-01	6.32E-01
LRRC52	-0.0658	2.06E-01	3.24E-01
LRRC55	-0.0669	1.98E-01	3.15E-01
LRRC56	0.0907	8.10E-02	1.57E-01
LRRC57	0.1342	9.68E-03	2.85E-02
LRRC58	0.0443	3.95E-01	5.27E-01
LRRC59	0.2037	7.79E-05	4.93E-04
LRRC61	-0.0439	4.00E-01	5.33E-01
LRRC66	0.1395	7.14E-03	2.21E-02
LRRC67	-0.0488	3.48E-01	4.80E-01
LRRC69	0.0871	9.40E-02	1.77E-01
LRRC6	-0.0585	2.61E-01	3.87E-01
LRRC70	-0.1719	8.84E-04	3.87E-03
LRRC7	-0.0773	1.37E-01	2.37E-01
LRRC8A	-0.0094	8.57E-01	9.08E-01
LRRC8B	0.1560	2.58E-03	9.51E-03
LRRC8C	-0.0578	2.67E-01	3.94E-01
LRRC8D	-0.0245	6.38E-01	7.44E-01
LRRC8E	0.1663	1.30E-03	5.37E-03
LRRCC1	0.1571	2.41E-03	9.00E-03
LRRFIP1	-0.2216	1.65E-05	1.31E-04
LRRFIP2	0.0180	7.30E-01	8.16E-01
LRRIQ1	0.1438	5.53E-03	1.79E-02
LRRIQ3	-0.0434	4.05E-01	5.38E-01
LRRIQ4	0.1047	4.38E-02	9.70E-02
LRRK1	0.0133	7.98E-01	8.67E-01
LRRK2	-0.1458	4.90E-03	1.62E-02
LRRN1	0.0431	4.08E-01	5.40E-01
LRRN2	0.1296	1.24E-02	3.49E-02
LRRN3	-0.1090	3.59E-02	8.27E-02
LRRN4CL	0.0167	7.48E-01	8.30E-01
LRRN4	0.0949	6.78E-02	1.37E-01
LRRTM1	-0.0273	6.00E-01	7.13E-01
LRRTM2	-0.1469	4.58E-03	1.54E-02
LRRTM3	0.0165	7.51E-01	8.33E-01
LRRTM4	-0.1018	5.01E-02	1.08E-01
LRSAM1	-0.0890	8.70E-02	1.66E-01
LRTM1	0.0304	5.59E-01	6.77E-01
LRTM2	0.0841	1.06E-01	1.94E-01
LRTOMT	-0.1593	2.09E-03	8.00E-03
LRWD1	-0.0994	5.59E-02	1.17E-01

LSAMP	-0.0909	8.03E-02	1.56E-01
LSG1	0.1974	1.30E-04	7.63E-04
LSM10	-0.0472	3.64E-01	4.97E-01
LSM11	0.2501	1.07E-06	1.20E-05
LSM12	0.1887	2.56E-04	1.36E-03
LSM14A	0.2181	2.25E-05	1.71E-04
LSM14B	0.2185	2.18E-05	1.67E-04
LSM1	0.0810	1.19E-01	2.14E-01
LSM2	0.2915	1.06E-08	1.97E-07
LSM3	0.0895	8.50E-02	1.63E-01
LSM4	0.0600	2.49E-01	3.74E-01
LSM5	0.0725	1.63E-01	2.70E-01
LSM6	0.0682	1.90E-01	3.04E-01
LSM7	0.1369	8.30E-03	2.51E-02
LSMD1	-0.0101	8.47E-01	9.00E-01
LSP1	-0.0005	9.92E-01	9.95E-01
LSR	0.0283	5.86E-01	7.02E-01
LSS	-0.0547	2.94E-01	4.23E-01
LST-3TM12	-0.0927	7.44E-02	1.47E-01
LST1	-0.0469	3.67E-01	5.00E-01
LTA4H	0.0472	3.64E-01	4.97E-01
LTA	0.0424	4.16E-01	5.49E-01
LTB4R2	-0.0038	9.42E-01	9.64E-01
LTB4R	0.0440	3.98E-01	5.31E-01
LTBP1	0.0091	8.62E-01	9.11E-01
LTBP2	-0.1416	6.31E-03	1.99E-02
LTBP3	-0.0106	8.39E-01	8.94E-01
LTBP4	-0.1009	5.21E-02	1.11E-01
LTBR	-0.1203	2.04E-02	5.22E-02
LTB	0.0675	1.95E-01	3.10E-01
LTC4S	-0.1150	2.67E-02	6.50E-02
LTF	-0.0136	7.94E-01	8.64E-01
LTK	-0.0043	9.34E-01	9.59E-01
LTV1	0.1967	1.37E-04	7.97E-04
LUC7L2	0.0097	8.52E-01	9.04E-01
LUC7L3	0.3904	5.94E-15	4.01E-13
LUC7L	0.2948	7.14E-09	1.37E-07
LUM	-0.0840	1.06E-01	1.95E-01
LUZP1	-0.1049	4.34E-02	9.63E-02
LUZP2	-0.1487	4.11E-03	1.41E-02
LUZP4	0.0444	3.94E-01	5.27E-01
LUZP6	-0.0519	3.19E-01	4.49E-01
LXN	-0.0357	4.93E-01	6.19E-01
LY6D	-0.0534	3.05E-01	4.35E-01

LY6E	0.0366	4.82E-01	6.10E-01
LY6G5B	0.3852	1.44E-14	8.97E-13
LY6G5C	-0.0703	1.76E-01	2.87E-01
LY6G6C	0.0336	5.19E-01	6.43E-01
LY6G6D	-0.0670	1.98E-01	3.14E-01
LY6G6E	-0.0762	1.43E-01	2.45E-01
LY6G6F	-0.1184	2.26E-02	5.68E-02
LY6H	0.1294	1.26E-02	3.54E-02
LY6K	0.0599	2.50E-01	3.75E-01
LY75	-0.0756	1.46E-01	2.49E-01
LY86	-0.0701	1.78E-01	2.89E-01
LY96	0.0174	7.39E-01	8.24E-01
LY9	-0.0517	3.21E-01	4.51E-01
LYAR	0.1836	3.79E-04	1.90E-03
LYG1	0.1047	4.40E-02	9.71E-02
LYG2	0.1098	3.44E-02	8.00E-02
LYL1	-0.0477	3.59E-01	4.91E-01
LYNX1	0.0437	4.01E-01	5.34E-01
LYN	-0.0951	6.72E-02	1.36E-01
LYPD1	0.0737	1.57E-01	2.62E-01
LYPD2	-0.1278	1.37E-02	3.80E-02
LYPD3	0.1557	2.64E-03	9.69E-03
LYPD4	0.0667	2.00E-01	3.17E-01
LYPD5	-0.0071	8.91E-01	9.30E-01
LYPD6B	0.2005	1.01E-04	6.11E-04
LYPD6	0.2143	3.15E-05	2.28E-04
LYPLA1	-0.1583	2.23E-03	8.43E-03
LYPLA2P1	-0.0527	3.12E-01	4.41E-01
LYPLA2	-0.0432	4.07E-01	5.39E-01
LYPLAL1	-0.0207	6.91E-01	7.86E-01
LYRM1	-0.2780	5.17E-08	8.02E-07
LYRM2	0.2339	5.29E-06	4.86E-05
LYRM4	0.0550	2.91E-01	4.20E-01
LYRM5	-0.1945	1.63E-04	9.23E-04
LYRM7	-0.1887	2.58E-04	1.37E-03
LYSMD1	0.6094	4.39E-39	1.09E-35
LYSMD2	-0.1852	3.37E-04	1.72E-03
LYSMD3	-0.1164	2.50E-02	6.15E-02
LYSMD4	0.1240	1.68E-02	4.46E-02
LYST	0.0769	1.39E-01	2.39E-01
LYVE1	-0.2362	4.24E-06	4.03E-05
LYZL1	0.0200	7.01E-01	7.94E-01
LYZL2	0.0214	6.81E-01	7.78E-01
LYZL4	0.0795	1.26E-01	2.22E-01

LYZL6	-0.0122	8.15E-01	8.79E-01
LYZ	0.0504	3.33E-01	4.64E-01
LZIC	0.0357	4.94E-01	6.20E-01
LZTFL1	-0.0730	1.61E-01	2.67E-01
LZTR1	0.1096	3.49E-02	8.09E-02
LZTS1	-0.0544	2.96E-01	4.25E-01
LZTS2	0.2174	2.40E-05	1.81E-04
M6PR	0.0733	1.59E-01	2.64E-01
MAB21L1	0.0160	7.59E-01	8.38E-01
MAB21L2	0.0095	8.55E-01	9.06E-01
MACC1	0.0612	2.40E-01	3.64E-01
MACF1	-0.0810	1.20E-01	2.14E-01
MACROD1	-0.2990	4.26E-09	8.68E-08
MACROD2	0.1127	3.00E-02	7.14E-02
MAD1L1	-0.0248	6.34E-01	7.41E-01
MAD2L1BP	0.1112	3.23E-02	7.57E-02
MAD2L1	0.3457	7.43E-12	2.76E-10
MAD2L2	0.0210	6.87E-01	7.83E-01
MADCAM1	-0.0304	5.59E-01	6.77E-01
MADD	0.1150	2.67E-02	6.50E-02
MAEA	0.0619	2.35E-01	3.58E-01
MAEL	0.1826	4.07E-04	2.01E-03
MAF1	0.0601	2.49E-01	3.74E-01
MAFA	0.0282	5.88E-01	7.03E-01
MAFB	-0.1261	1.51E-02	4.09E-02
MAFF	-0.0033	9.49E-01	9.68E-01
MAFG	0.2945	7.42E-09	1.42E-07
MAFK	-0.0955	6.63E-02	1.34E-01
MAF	-0.1690	1.08E-03	4.60E-03
MAGEA10	0.1848	3.46E-04	1.76E-03
MAGEA11	0.1252	1.58E-02	4.25E-02
MAGEA12	-0.0218	6.75E-01	7.74E-01
MAGEA1	-0.0051	9.23E-01	9.51E-01
MAGEA2	-0.0038	9.43E-01	9.64E-01
MAGEA3	-0.0068	8.96E-01	9.34E-01
MAGEA4	0.1172	2.40E-02	5.95E-02
MAGEA5	0.2533	7.69E-07	8.97E-06
MAGEA6	0.0000	9.99E-01	1.00E+00
MAGEA8	0.0559	2.83E-01	4.11E-01
MAGEA9B	0.1143	2.78E-02	6.71E-02
MAGEB10	-0.0255	6.25E-01	7.33E-01
MAGEB16	0.0236	6.51E-01	7.55E-01
MAGEB18	0.0752	1.48E-01	2.52E-01
MAGEB1	0.0829	1.11E-01	2.02E-01

MAGEB2	0.0222	6.69E-01	7.69E-01
MAGEB3	0.0892	8.62E-02	1.65E-01
MAGEB4	0.0115	8.26E-01	8.86E-01
MAGEB6	0.0012	9.82E-01	9.89E-01
MAGEC1	0.0815	1.17E-01	2.10E-01
MAGEC2	0.0791	1.29E-01	2.25E-01
MAGEC3	0.2139	3.27E-05	2.35E-04
MAGED1	0.0395	4.48E-01	5.79E-01
MAGED2	0.0891	8.64E-02	1.65E-01
MAGED4B	0.0660	2.05E-01	3.22E-01
MAGED4	0.0835	1.09E-01	1.98E-01
MAGEE1	0.1772	6.08E-04	2.80E-03
MAGEE2	0.1616	1.79E-03	7.01E-03
MAGEF1	0.1032	4.69E-02	1.02E-01
MAGEH1	0.0946	6.88E-02	1.38E-01
MAGEL2	-0.0797	1.25E-01	2.21E-01
MAGI1	-0.1663	1.31E-03	5.39E-03
MAGI2	-0.0561	2.81E-01	4.10E-01
MAGI3	-0.0182	7.27E-01	8.15E-01
MAGIX	-0.0832	1.10E-01	2.00E-01
MAGOHB	0.2589	4.25E-07	5.31E-06
MAGOH	0.2503	1.05E-06	1.18E-05
MAGT1	-0.1171	2.41E-02	5.97E-02
MAG	-0.0160	7.59E-01	8.38E-01
MAK16	0.1219	1.88E-02	4.89E-02
MAK	0.0448	3.89E-01	5.22E-01
MAL2	0.1280	1.36E-02	3.77E-02
MALAT1	0.2351	4.69E-06	4.40E-05
MALL	-0.0911	7.96E-02	1.55E-01
MALT1	0.0772	1.38E-01	2.37E-01
MAL	-0.0285	5.84E-01	7.00E-01
MAMDC2	-0.2058	6.53E-05	4.24E-04
MAMDC4	0.0069	8.95E-01	9.33E-01
MAML1	0.1947	1.60E-04	9.11E-04
MAML2	-0.0409	4.32E-01	5.64E-01
MAML3	-0.1413	6.40E-03	2.02E-02
MAMLD1	0.2193	2.03E-05	1.57E-04
MAMSTR	0.2487	1.23E-06	1.37E-05
MAN1A1	-0.1220	1.87E-02	4.86E-02
MAN1A2	-0.1262	1.50E-02	4.07E-02
MAN1B1	0.1074	3.87E-02	8.78E-02
MAN1C1	-0.2185	2.18E-05	1.66E-04
MAN2A1	-0.1335	1.01E-02	2.94E-02
MAN2A2	0.0347	5.05E-01	6.30E-01

MAN2B1	-0.0854	1.00E-01	1.86E-01
MAN2B2	-0.1274	1.41E-02	3.87E-02
MAN2C1	-0.1777	5.83E-04	2.70E-03
MANBAL	0.0918	7.75E-02	1.52E-01
MANBA	-0.0679	1.92E-01	3.06E-01
MANEAL	-0.0267	6.09E-01	7.20E-01
MANEA	0.0300	5.65E-01	6.82E-01
MANF	0.0602	2.47E-01	3.72E-01
MANSC1	0.1416	6.28E-03	1.99E-02
MAOA	-0.1735	7.92E-04	3.52E-03
MAOB	-0.1620	1.74E-03	6.85E-03
MAP1A	-0.0565	2.78E-01	4.06E-01
MAP1B	-0.1707	9.64E-04	4.17E-03
MAP1D	-0.0725	1.63E-01	2.70E-01
MAP1LC3A	-0.0674	1.95E-01	3.11E-01
MAP1LC3B2	-0.2141	3.22E-05	2.32E-04
MAP1LC3B	-0.2314	6.72E-06	5.97E-05
MAP1LC3C	-0.0380	4.65E-01	5.95E-01
MAP1S	0.2207	1.79E-05	1.40E-04
MAP2K1	-0.1027	4.80E-02	1.04E-01
MAP2K2	0.0076	8.83E-01	9.25E-01
MAP2K3	-0.2390	3.24E-06	3.18E-05
MAP2K4	-0.1167	2.46E-02	6.07E-02
MAP2K5	-0.0995	5.56E-02	1.17E-01
MAP2K6	0.0933	7.26E-02	1.44E-01
MAP2K7	0.0349	5.03E-01	6.28E-01
MAP2	0.0014	9.78E-01	9.86E-01
MAP3K10	0.1043	4.48E-02	9.86E-02
MAP3K11	0.0275	5.98E-01	7.12E-01
MAP3K12	-0.0592	2.55E-01	3.81E-01
MAP3K13	-0.0597	2.52E-01	3.77E-01
MAP3K14	0.1682	1.14E-03	4.82E-03
MAP3K15	0.1213	1.95E-02	5.03E-02
MAP3K1	0.1752	6.98E-04	3.16E-03
MAP3K2	-0.0774	1.37E-01	2.36E-01
MAP3K3	0.1295	1.25E-02	3.51E-02
MAP3K4	0.3160	4.79E-10	1.22E-08
MAP3K5	-0.0952	6.69E-02	1.35E-01
MAP3K6	-0.0261	6.16E-01	7.26E-01
MAP3K7	0.1614	1.82E-03	7.10E-03
MAP3K8	0.0031	9.52E-01	9.71E-01
MAP3K9	0.1785	5.51E-04	2.58E-03
MAP4K1	-0.0187	7.20E-01	8.10E-01
MAP4K2	0.0952	6.71E-02	1.36E-01

MAP4K3	0.0855	9.99E-02	1.86E-01
MAP4K4	0.1105	3.34E-02	7.80E-02
MAP4K5	-0.0089	8.64E-01	9.12E-01
MAP4	-0.0028	9.57E-01	9.73E-01
MAP6D1	0.0208	6.90E-01	7.86E-01
MAP6	-0.0618	2.35E-01	3.58E-01
MAP7D1	0.0758	1.45E-01	2.47E-01
MAP7D2	0.1535	3.04E-03	1.09E-02
MAP7D3	-0.0417	4.24E-01	5.56E-01
MAP7	-0.0215	6.79E-01	7.77E-01
MAP9	-0.0682	1.90E-01	3.04E-01
MAPK10	-0.0065	9.01E-01	9.37E-01
MAPK11	-0.0926	7.48E-02	1.48E-01
MAPK12	0.0442	3.96E-01	5.29E-01
MAPK13	0.2907	1.17E-08	2.14E-07
MAPK14	-0.0013	9.80E-01	9.87E-01
MAPK15	0.1000	5.43E-02	1.15E-01
MAPK1IP1L	0.1699	1.02E-03	4.36E-03
MAPK1	0.0465	3.72E-01	5.05E-01
MAPK3	0.1707	9.60E-04	4.16E-03
MAPK4	-0.0958	6.53E-02	1.33E-01
MAPK6	-0.0398	4.44E-01	5.76E-01
MAPK7	0.2037	7.75E-05	4.92E-04
MAPK8IP1	-0.1019	4.99E-02	1.07E-01
MAPK8IP2	0.2737	8.45E-08	1.25E-06
MAPK8IP3	0.2145	3.10E-05	2.25E-04
MAPK8	-0.0485	3.51E-01	4.84E-01
MAPK9	0.1910	2.14E-04	1.17E-03
MAPKAP1	0.1658	1.35E-03	5.52E-03
MAPKAPK2	0.1506	3.65E-03	1.27E-02
MAPKAPK3	0.0102	8.45E-01	8.99E-01
MAPKAPK5	0.2796	4.31E-08	6.85E-07
MAPKBP1	0.2093	4.86E-05	3.30E-04
MAPKSP1	-0.1518	3.37E-03	1.19E-02
MAPRE1	0.2645	2.35E-07	3.13E-06
MAPRE2	-0.2594	4.03E-07	5.06E-06
MAPRE3	-0.0699	1.79E-01	2.90E-01
MAPT	0.1102	3.38E-02	7.88E-02
10-Mar	0.0869	9.46E-02	1.78E-01
11-Mar	0.0674	1.95E-01	3.11E-01
1-Mar	-0.0021	9.68E-01	9.80E-01
2-Mar	-0.2615	3.25E-07	4.19E-06
3-Mar	0.2139	3.27E-05	2.35E-04
4-Mar	-0.0206	6.93E-01	7.88E-01

5-Mar	-0.2190	2.08E-05	1.60E-04
6-Mar	-0.0156	7.64E-01	8.41E-01
7-Mar	0.1701	1.01E-03	4.33E-03
8-Mar	-0.1034	4.65E-02	1.02E-01
9-Mar	-0.0920	7.67E-02	1.51E-01
MARCKSL1	0.1592	2.11E-03	8.04E-03
MARCKS	0.1738	7.76E-04	3.46E-03
MARCO	-0.1326	1.06E-02	3.06E-02
MARK1	0.0530	3.09E-01	4.38E-01
MARK2	0.1811	4.57E-04	2.21E-03
MARK3	0.0118	8.21E-01	8.83E-01
MARK4	0.1781	5.68E-04	2.65E-03
MARS2	-0.0049	9.25E-01	9.52E-01
MARS	0.1246	1.63E-02	4.36E-02
MARVELD1	-0.0293	5.74E-01	6.91E-01
MARVELD2	0.0053	9.19E-01	9.49E-01
MARVELD3	-0.0027	9.59E-01	9.74E-01
MAS1L	-0.0443	3.95E-01	5.28E-01
MAS1	-0.0092	8.60E-01	9.09E-01
MASP1	-0.1838	3.73E-04	1.87E-03
MASP2	-0.2171	2.47E-05	1.86E-04
MAST1	0.0915	7.84E-02	1.53E-01
MAST2	0.3151	5.37E-10	1.35E-08
MAST3	0.1255	1.56E-02	4.19E-02
MAST4	-0.1312	1.14E-02	3.25E-02
MASTL	0.2365	4.12E-06	3.94E-05
MAT1A	-0.2059	6.45E-05	4.20E-04
MAT2A	0.1090	3.59E-02	8.26E-02
MAT2B	-0.1378	7.84E-03	2.39E-02
MATK	-0.0819	1.15E-01	2.08E-01
MATN1	0.0937	7.15E-02	1.43E-01
MATN2	-0.0637	2.21E-01	3.41E-01
MATN3	0.0082	8.74E-01	9.19E-01
MATN4	0.1198	2.10E-02	5.35E-02
MATR3	0.1962	1.42E-04	8.26E-04
MAVS	0.1456	4.95E-03	1.63E-02
MAX	0.0715	1.69E-01	2.78E-01
MAZ	0.1511	3.53E-03	1.24E-02
MBD1	0.1189	2.19E-02	5.53E-02
MBD2	0.0533	3.06E-01	4.35E-01
MBD3L1	0.0634	2.23E-01	3.44E-01
MBD3L2	-0.0275	5.97E-01	7.10E-01
MBD3L5	-0.0697	1.80E-01	2.92E-01
MBD3	-0.0639	2.20E-01	3.40E-01

MBD4	0.0350	5.02E-01	6.28E-01
MBD5	0.0753	1.48E-01	2.51E-01
MBD6	0.1960	1.45E-04	8.38E-04
MBIP	-0.0336	5.18E-01	6.42E-01
MBL1P	-0.1378	7.88E-03	2.40E-02
MBL2	-0.1205	2.02E-02	5.18E-02
MBLAC1	-0.0577	2.68E-01	3.95E-01
MBLAC2	-0.1331	1.03E-02	2.99E-02
MBNL1	-0.0668	1.99E-01	3.16E-01
MBNL2	-0.2143	3.15E-05	2.28E-04
MBNL3	0.0352	4.99E-01	6.25E-01
MBOAT1	0.1661	1.32E-03	5.43E-03
MBOAT2	0.1189	2.19E-02	5.53E-02
MBOAT4	0.0798	1.25E-01	2.21E-01
MBOAT7	0.1072	3.90E-02	8.83E-02
MBP	-0.0922	7.62E-02	1.50E-01
MBTD1	0.1805	4.76E-04	2.28E-03
MBTPS1	-0.2189	2.11E-05	1.62E-04
MBTPS2	-0.0545	2.95E-01	4.25E-01
MB	-0.0442	3.96E-01	5.28E-01
MC1R	0.0444	3.94E-01	5.27E-01
MC2R	0.0258	6.21E-01	7.30E-01
MC3R	0.0600	2.49E-01	3.74E-01
MC4R	-0.0026	9.60E-01	9.75E-01
MC5R	0.0807	1.21E-01	2.15E-01
MCAM	0.0176	7.35E-01	8.21E-01
MCART1	0.1509	3.57E-03	1.25E-02
MCART2	0.0799	1.24E-01	2.20E-01
MCART3P	0.1115	3.18E-02	7.49E-02
MCART6	-0.0040	9.38E-01	9.61E-01
MCAT	-0.1240	1.69E-02	4.47E-02
MCCC1	-0.1420	6.15E-03	1.95E-02
MCCC2	-0.4461	1.52E-19	3.40E-17
MCCD1	0.1146	2.73E-02	6.62E-02
MCC	-0.1446	5.26E-03	1.72E-02
MCEE	-0.2767	6.07E-08	9.27E-07
MCF2L2	0.0881	9.01E-02	1.71E-01
MCF2L	-0.0575	2.69E-01	3.97E-01
MCF2	0.0366	4.83E-01	6.11E-01
MCFD2	-0.0684	1.89E-01	3.02E-01
MCHR1	-0.0686	1.88E-01	3.01E-01
MCHR2	0.0879	9.09E-02	1.72E-01
MCL1	0.1901	2.31E-04	1.25E-03
MCM10	0.4001	1.07E-15	8.58E-14

MCM2	0.4404	4.96E-19	9.94E-17
MCM3APAS	0.2899	1.29E-08	2.33E-07
MCM3AP	0.1219	1.88E-02	4.89E-02
MCM3	0.4168	5.08E-17	5.45E-15
MCM4	0.3165	4.49E-10	1.15E-08
MCM5	0.4317	2.84E-18	4.35E-16
MCM6	0.4389	6.63E-19	1.26E-16
MCM7	0.3659	3.41E-13	1.65E-11
MCM8	0.3167	4.37E-10	1.13E-08
MCM9	0.2725	9.66E-08	1.40E-06
MCOLN1	-0.1578	2.31E-03	8.68E-03
MCOLN2	0.0217	6.77E-01	7.75E-01
MCOLN3	0.2169	2.51E-05	1.89E-04
MCPH1	0.0360	4.90E-01	6.17E-01
MCRS1	0.2783	5.02E-08	7.81E-07
MCTP1	-0.0361	4.89E-01	6.16E-01
MCTP2	0.0475	3.61E-01	4.93E-01
MCTS1	0.1121	3.08E-02	7.30E-02
MDC1	0.4702	8.42E-22	2.66E-19
MDFIC	-0.1095	3.49E-02	8.09E-02
MDFI	0.0631	2.25E-01	3.47E-01
MDGA1	0.0355	4.96E-01	6.22E-01
MDGA2	0.0049	9.25E-01	9.52E-01
MDH1B	0.1979	1.25E-04	7.36E-04
MDH1	-0.1533	3.08E-03	1.10E-02
MDH2	-0.2672	1.74E-07	2.39E-06
MDK	0.2360	4.32E-06	4.10E-05
MDM1	0.2156	2.80E-05	2.07E-04
MDM2	0.0038	9.41E-01	9.64E-01
MDM4	0.3978	1.63E-15	1.24E-13
MDN1	0.0406	4.35E-01	5.67E-01
MDP1	-0.0554	2.87E-01	4.16E-01
MDS2	0.1508	3.60E-03	1.26E-02
ME1	0.0233	6.55E-01	7.57E-01
ME2	0.1007	5.25E-02	1.12E-01
ME3	0.1041	4.51E-02	9.91E-02
MEA1	0.1402	6.82E-03	2.13E-02
MEAF6	-0.0664	2.02E-01	3.19E-01
MECOM	0.0783	1.32E-01	2.30E-01
MECP2	0.2450	1.79E-06	1.90E-05
MECR	-0.2984	4.57E-09	9.22E-08
MED10	0.2008	9.85E-05	6.02E-04
MED11	-0.1534	3.05E-03	1.10E-02
MED12L	-0.0182	7.27E-01	8.15E-01

MED12	0.2679	1.61E-07	2.24E-06
MED13L	0.0227	6.63E-01	7.64E-01
MED13	0.1662	1.31E-03	5.40E-03
MED14	0.0486	3.50E-01	4.82E-01
MED15	0.3035	2.41E-09	5.20E-08
MED16	-0.0086	8.69E-01	9.16E-01
MED17	0.2267	1.03E-05	8.67E-05
MED18	-0.1902	2.29E-04	1.24E-03
MED19	0.1726	8.41E-04	3.70E-03
MED1	0.0901	8.31E-02	1.60E-01
MED20	0.2178	2.32E-05	1.76E-04
MED21	0.1889	2.53E-04	1.35E-03
MED22	0.3041	2.24E-09	4.88E-08
MED23	0.2346	4.94E-06	4.60E-05
MED24	0.1758	6.69E-04	3.04E-03
MED25	0.0988	5.74E-02	1.20E-01
MED26	0.1584	2.21E-03	8.35E-03
MED27	0.1638	1.55E-03	6.21E-03
MED28	0.1070	3.93E-02	8.89E-02
MED29	-0.0036	9.44E-01	9.66E-01
MED30	0.1592	2.09E-03	8.01E-03
MED31	-0.0449	3.88E-01	5.21E-01
MED4	-0.0250	6.32E-01	7.39E-01
MED6	0.1018	5.02E-02	1.08E-01
MED7	0.1911	2.13E-04	1.16E-03
MED8	0.1967	1.37E-04	8.00E-04
MED9	-0.0492	3.45E-01	4.77E-01
MEF2A	-0.0970	6.21E-02	1.28E-01
MEF2B	0.0660	2.05E-01	3.23E-01
MEF2C	-0.1554	2.68E-03	9.83E-03
MEF2D	0.2909	1.14E-08	2.09E-07
MEFV	-0.0747	1.51E-01	2.55E-01
MEG3	0.0626	2.29E-01	3.52E-01
MEG8	0.0454	3.84E-01	5.17E-01
MEGF10	-0.0515	3.22E-01	4.52E-01
MEGF11	0.0816	1.16E-01	2.09E-01
MEGF6	-0.1022	4.91E-02	1.06E-01
MEGF8	-0.0695	1.82E-01	2.94E-01
MEGF9	-0.0705	1.75E-01	2.86E-01
MEI1	0.0568	2.75E-01	4.03E-01
MEIG1	0.0218	6.76E-01	7.74E-01
MEIS1	0.0990	5.67E-02	1.19E-01
MEIS2	-0.0317	5.43E-01	6.64E-01
MEIS3P1	-0.1890	2.52E-04	1.34E-03

MEIS3	-0.0251	6.30E-01	7.38E-01
MELK	0.4340	1.78E-18	2.88E-16
MEMO1	0.0795	1.26E-01	2.22E-01
MEN1	0.1384	7.61E-03	2.33E-02
MEOX1	-0.0244	6.40E-01	7.46E-01
MEOX2	-0.0708	1.74E-01	2.84E-01
MEP1A	0.1667	1.27E-03	5.28E-03
MEP1B	-0.0873	9.32E-02	1.76E-01
MEPCE	-0.0905	8.16E-02	1.58E-01
MEPE	0.0108	8.36E-01	8.93E-01
MERTK	-0.0668	1.99E-01	3.15E-01
MESDC1	0.1496	3.88E-03	1.34E-02
MESDC2	0.0533	3.06E-01	4.36E-01
MESP1	0.1929	1.86E-04	1.03E-03
MESP2	0.3346	3.74E-11	1.21E-09
MESTIT1	0.0339	5.16E-01	6.40E-01
MEST	0.0805	1.22E-01	2.17E-01
METAP1	0.0774	1.37E-01	2.36E-01
METAP2	0.1124	3.04E-02	7.21E-02
METRNL	-0.0054	9.17E-01	9.48E-01
METRN	-0.1780	5.71E-04	2.66E-03
METT10D	0.0793	1.27E-01	2.24E-01
METT11D1	0.1869	2.95E-04	1.54E-03
METT5D1	-0.1349	9.27E-03	2.75E-02
METTL10	-0.0277	5.94E-01	7.08E-01
METTL11A	-0.0188	7.19E-01	8.09E-01
METTL11B	0.2740	8.22E-08	1.22E-06
METTL12	0.0646	2.15E-01	3.34E-01
METTL13	0.4358	1.25E-18	2.18E-16
METTL14	-0.0828	1.12E-01	2.03E-01
METTL1	0.1899	2.34E-04	1.26E-03
METTL2A	0.2573	5.07E-07	6.19E-06
METTL2B	-0.0691	1.84E-01	2.97E-01
METTL3	0.1929	1.86E-04	1.04E-03
METTL4	0.2291	8.30E-06	7.12E-05
METTL5	0.1743	7.48E-04	3.35E-03
METTL6	0.2270	1.01E-05	8.50E-05
METTL7A	-0.3564	1.48E-12	6.36E-11
METTL7B	-0.1842	3.63E-04	1.83E-03
METTL8	0.0163	7.55E-01	8.35E-01
METTL9	0.2061	6.37E-05	4.16E-04
MET	-0.1275	1.40E-02	3.85E-02
MEX3A	0.3137	6.46E-10	1.59E-08
MEX3B	0.0393	4.50E-01	5.82E-01

MEX3C	0.0853	1.01E-01	1.87E-01
MEX3D	0.1528	3.16E-03	1.13E-02
MFAP1	0.1380	7.78E-03	2.38E-02
MFAP2	0.0808	1.20E-01	2.15E-01
MFAP3L	-0.2303	7.43E-06	6.49E-05
MFAP3	-0.0182	7.27E-01	8.14E-01
MFAP4	-0.1203	2.05E-02	5.24E-02
MFAP5	-0.1653	1.40E-03	5.69E-03
MFF	0.1884	2.62E-04	1.39E-03
MFGE8	-0.0432	4.07E-01	5.39E-01
MFHAS1	-0.0086	8.69E-01	9.15E-01
MFI2	0.2863	1.97E-08	3.41E-07
MFN1	0.0053	9.19E-01	9.49E-01
MFN2	-0.2205	1.83E-05	1.43E-04
MFNG	-0.0318	5.41E-01	6.62E-01
MFRP	-0.0999	5.45E-02	1.15E-01
MFSD10	0.2160	2.71E-05	2.01E-04
MFSD11	-0.0266	6.09E-01	7.21E-01
MFSD1	-0.1759	6.65E-04	3.03E-03
MFSD2A	-0.2308	7.06E-06	6.22E-05
MFSD2B	0.2260	1.10E-05	9.16E-05
MFSD3	-0.2262	1.09E-05	9.06E-05
MFSD4	0.1537	2.99E-03	1.08E-02
MFSD5	0.1153	2.64E-02	6.44E-02
MFSD6L	0.0827	1.12E-01	2.03E-01
MFSD6	0.1431	5.75E-03	1.85E-02
MFSD7	-0.0681	1.91E-01	3.05E-01
MFSD8	-0.1912	2.11E-04	1.15E-03
MFSD9	-0.1692	1.07E-03	4.55E-03
MGAM	0.0734	1.58E-01	2.64E-01
MGAT1	-0.1668	1.27E-03	5.25E-03
MGAT2	-0.2189	2.11E-05	1.62E-04
MGAT3	-0.0244	6.40E-01	7.46E-01
MGAT4A	0.1573	2.37E-03	8.86E-03
MGAT4B	-0.0792	1.28E-01	2.25E-01
MGAT4C	-0.0407	4.35E-01	5.67E-01
MGAT5B	-0.0376	4.70E-01	5.99E-01
MGAT5	-0.0308	5.55E-01	6.74E-01
MGA	0.1374	8.04E-03	2.44E-02
MGC12916	-0.0293	5.74E-01	6.91E-01
MGC12982	0.2664	1.91E-07	2.61E-06
MGC14436	-0.1223	1.84E-02	4.80E-02
MGC15885	0.0314	5.47E-01	6.67E-01
MGC16025	0.0981	5.90E-02	1.22E-01

MGC16121	0.0356	4.94E-01	6.21E-01
MGC16142	0.1007	5.25E-02	1.12E-01
MGC16275	0.1342	9.68E-03	2.85E-02
MGC16384	0.0067	8.97E-01	9.35E-01
MGC16703	-0.0174	7.38E-01	8.23E-01
MGC21881	-0.1233	1.75E-02	4.61E-02
MGC23270	0.0096	8.54E-01	9.06E-01
MGC23284	0.0118	8.21E-01	8.83E-01
MGC26647	-0.0765	1.42E-01	2.43E-01
MGC27382	-0.0793	1.28E-01	2.24E-01
MGC2752	0.0535	3.04E-01	4.34E-01
MGC2889	0.0366	4.83E-01	6.11E-01
MGC29506	-0.0102	8.45E-01	8.99E-01
MGC34034	0.0026	9.60E-01	9.75E-01
MGC3771	0.0034	9.48E-01	9.68E-01
MGC42105	-0.0962	6.42E-02	1.31E-01
MGC4473	0.0795	1.26E-01	2.23E-01
MGC45800	0.0034	9.48E-01	9.68E-01
MGC57346	0.3317	5.63E-11	1.74E-09
MGC70857	-0.1532	3.09E-03	1.11E-02
MGC72080	-0.1571	2.40E-03	8.96E-03
MGC87042	-0.0306	5.57E-01	6.75E-01
MGEA5	-0.1521	3.31E-03	1.17E-02
MGLL	-0.0591	2.56E-01	3.82E-01
MGMT	-0.1108	3.29E-02	7.71E-02
MGP	-0.0732	1.60E-01	2.66E-01
MGRN1	-0.0337	5.17E-01	6.42E-01
MGST1	-0.1914	2.09E-04	1.14E-03
MGST2	-0.2543	6.92E-07	8.16E-06
MGST3	0.0214	6.81E-01	7.78E-01
MIA2	-0.1248	1.61E-02	4.31E-02
MIA3	-0.0912	7.93E-02	1.55E-01
MIAT	0.0721	1.66E-01	2.74E-01
MIA	0.2141	3.22E-05	2.32E-04
MIB1	0.0167	7.49E-01	8.31E-01
MIB2	-0.0516	3.22E-01	4.52E-01
MICAL1	0.1116	3.17E-02	7.47E-02
MICAL2	-0.0971	6.18E-02	1.27E-01
MICAL3	-0.0694	1.82E-01	2.94E-01
MICALCL	-0.1314	1.13E-02	3.23E-02
MICALL1	0.0955	6.61E-02	1.34E-01
MICALL2	0.1601	1.99E-03	7.65E-03
MICA	0.1399	6.95E-03	2.16E-02
MICB	0.1801	4.91E-04	2.34E-03

MID1IP1	0.1371	8.19E-03	2.48E-02
MID1	0.0312	5.49E-01	6.69E-01
MID2	0.0378	4.68E-01	5.98E-01
MIDN	0.0405	4.36E-01	5.68E-01
MIER1	-0.1122	3.07E-02	7.28E-02
MIER2	0.1205	2.02E-02	5.18E-02
MIER3	0.0035	9.47E-01	9.67E-01
MIF4GD	-0.0388	4.57E-01	5.87E-01
MIF	0.0481	3.56E-01	4.88E-01
MIIP	0.1346	9.44E-03	2.79E-02
MIMT1	0.1541	2.93E-03	1.06E-02
MINA	0.0549	2.91E-01	4.20E-01
MINK1	0.0075	8.85E-01	9.27E-01
MINPP1	-0.1744	7.41E-04	3.33E-03
MIOS	0.0136	7.93E-01	8.63E-01
MIOX	0.2334	5.55E-06	5.06E-05
MIPEP	-0.2160	2.71E-05	2.01E-04
MIPOL1	0.2225	1.52E-05	1.21E-04
MIP	-0.2757	6.75E-08	1.02E-06
MIR155HG	0.0705	1.76E-01	2.86E-01
MIR17HG	0.1623	1.71E-03	6.77E-03
MIS12	0.1225	1.82E-02	4.76E-02
MITD1	0.3042	2.22E-09	4.84E-08
MITF	-0.0507	3.30E-01	4.61E-01
MIXL1	0.0936	7.17E-02	1.43E-01
MKI67IP	0.1833	3.88E-04	1.93E-03
MKI67	0.3800	3.42E-14	2.00E-12
MKKS	0.1075	3.85E-02	8.74E-02
MKL1	0.0913	7.91E-02	1.54E-01
MKL2	-0.0662	2.03E-01	3.20E-01
MKLN1	-0.1830	3.96E-04	1.97E-03
MKNK1	0.1186	2.24E-02	5.63E-02
MKNK2	-0.1214	1.93E-02	5.00E-02
MKRN1	-0.0763	1.43E-01	2.44E-01
MKRN2	0.0428	4.11E-01	5.44E-01
MKRN3	-0.0189	7.17E-01	8.07E-01
MKS1	0.2304	7.34E-06	6.43E-05
MKX	-0.1321	1.09E-02	3.13E-02
MLANA	-0.0803	1.23E-01	2.18E-01
MLC1	-0.1593	2.09E-03	8.00E-03
MLEC	-0.0652	2.10E-01	3.29E-01
MLF1IP	0.2924	9.50E-09	1.78E-07
MLF1	0.0175	7.37E-01	8.22E-01
MLF2	-0.1593	2.08E-03	7.98E-03

MLH1	0.0019	9.72E-01	9.82E-01
MLH3	0.1135	2.88E-02	6.93E-02
MLKL	0.0355	4.96E-01	6.22E-01
MLL2	0.1029	4.76E-02	1.04E-01
MLL3	-0.0680	1.91E-01	3.06E-01
MLL4	0.1943	1.67E-04	9.41E-04
MLL5	0.0299	5.65E-01	6.83E-01
MLLT10	0.0839	1.07E-01	1.95E-01
MLLT11	0.2290	8.41E-06	7.19E-05
MLLT1	0.0863	9.71E-02	1.82E-01
MLLT3	-0.0251	6.30E-01	7.38E-01
MLLT4	0.1267	1.46E-02	3.98E-02
MLLT6	0.2866	1.90E-08	3.31E-07
MLL	0.1676	1.19E-03	4.99E-03
MLNR	-0.0422	4.18E-01	5.51E-01
MLN	-0.0096	8.54E-01	9.05E-01
MLPH	-0.0568	2.75E-01	4.04E-01
MLST8	0.0353	4.98E-01	6.24E-01
MLXIPL	-0.0534	3.05E-01	4.34E-01
MLXIP	-0.1366	8.40E-03	2.54E-02
MLX	0.0185	7.23E-01	8.12E-01
MLYCD	-0.4089	2.18E-16	2.02E-14
MMAA	-0.2978	4.93E-09	9.85E-08
MMAB	-0.1615	1.81E-03	7.08E-03
MMACHC	-0.2398	2.99E-06	2.96E-05
MMADHC	-0.1362	8.62E-03	2.59E-02
MMD2	0.1170	2.42E-02	6.00E-02
MMD	0.1892	2.47E-04	1.32E-03
MMEL1	0.0868	9.50E-02	1.79E-01
MME	-0.1427	5.88E-03	1.88E-02
MMGT1	0.2002	1.04E-04	6.27E-04
MMP10	0.1622	1.73E-03	6.80E-03
MMP11	0.0743	1.53E-01	2.58E-01
MMP12	0.0996	5.52E-02	1.16E-01
MMP13	0.1420	6.13E-03	1.95E-02
MMP14	0.0455	3.83E-01	5.16E-01
MMP15	-0.1451	5.10E-03	1.68E-02
MMP16	0.0404	4.38E-01	5.70E-01
MMP17	0.0486	3.51E-01	4.83E-01
MMP19	-0.1250	1.60E-02	4.29E-02
MMP1	0.1807	4.69E-04	2.25E-03
MMP20	0.0237	6.50E-01	7.53E-01
MMP21	0.0255	6.24E-01	7.33E-01
MMP23A	-0.0211	6.86E-01	7.82E-01

MMP23B	-0.1091	3.57E-02	8.23E-02
MMP24	-0.1035	4.63E-02	1.01E-01
MMP25	-0.0054	9.18E-01	9.48E-01
MMP26	0.0403	4.38E-01	5.70E-01
MMP27	0.1664	1.30E-03	5.36E-03
MMP28	-0.0807	1.21E-01	2.15E-01
MMP2	-0.0733	1.59E-01	2.65E-01
MMP3	0.0036	9.44E-01	9.66E-01
MMP7	0.1372	8.15E-03	2.47E-02
MMP8	-0.0193	7.11E-01	8.02E-01
MMP9	0.1009	5.21E-02	1.11E-01
MMRN1	-0.2402	2.88E-06	2.85E-05
MMRN2	-0.2994	4.02E-09	8.28E-08
MMS19	0.0917	7.77E-02	1.52E-01
MN1	-0.0312	5.50E-01	6.70E-01
MNAT1	0.0725	1.63E-01	2.70E-01
MND1	0.3100	1.05E-09	2.46E-08
MNDA	0.0109	8.34E-01	8.91E-01
MNS1	0.1588	2.16E-03	8.22E-03
MNT	0.1008	5.24E-02	1.12E-01
MNX1	0.1461	4.81E-03	1.60E-02
MOAP1	0.0044	9.32E-01	9.58E-01
MOB2	0.0025	9.62E-01	9.76E-01
MOBKL1A	-0.0898	8.43E-02	1.62E-01
MOBKL1B	-0.0839	1.07E-01	1.96E-01
MOBKL2A	0.1543	2.88E-03	1.04E-02
MOBKL2B	0.1428	5.87E-03	1.88E-02
MOBKL2C	-0.0048	9.26E-01	9.53E-01
MOBKL3	0.1843	3.59E-04	1.81E-03
MOBP	0.0589	2.58E-01	3.83E-01
MOCOS	-0.1571	2.41E-03	8.97E-03
MOCS1	-0.2230	1.45E-05	1.17E-04
MOCS2	-0.2943	7.52E-09	1.44E-07
MOCS3	0.0860	9.80E-02	1.83E-01
MOGAT1	-0.2464	1.55E-06	1.67E-05
MOGAT2	-0.2145	3.09E-05	2.24E-04
MOGAT3	0.0550	2.91E-01	4.20E-01
MOGS	0.1338	9.90E-03	2.90E-02
MOG	-0.0515	3.22E-01	4.53E-01
MON1A	-0.0930	7.35E-02	1.46E-01
MON1B	-0.2317	6.51E-06	5.81E-05
MON2	0.0817	1.16E-01	2.09E-01
MORC1	0.0135	7.95E-01	8.64E-01
MORC2	0.3213	2.35E-10	6.44E-09

MORC3	-0.0005	9.93E-01	9.96E-01
MORC4	0.0769	1.39E-01	2.40E-01
MORF4L1	0.1192	2.17E-02	5.48E-02
MORF4L2	0.1470	4.56E-03	1.53E-02
MORF4	0.0348	5.04E-01	6.29E-01
MORN1	0.0134	7.97E-01	8.66E-01
MORN2	0.1331	1.03E-02	2.98E-02
MORN3	0.1471	4.53E-03	1.52E-02
MORN4	-0.0366	4.82E-01	6.10E-01
MORN5	0.1178	2.32E-02	5.80E-02
MOSC1	0.0153	7.69E-01	8.44E-01
MOSC2	-0.2087	5.11E-05	3.44E-04
MOSPD1	0.1763	6.46E-04	2.96E-03
MOSPD2	0.0518	3.19E-01	4.49E-01
MOSPD3	-0.1082	3.72E-02	8.52E-02
MOS	-0.0906	8.13E-02	1.58E-01
MOV10L1	0.0814	1.18E-01	2.11E-01
MOV10	0.0792	1.28E-01	2.25E-01
MOXD1	-0.0176	7.36E-01	8.22E-01
MPDU1	-0.1864	3.06E-04	1.59E-03
MPDZ	-0.2986	4.46E-09	9.05E-08
MPEG1	-0.1605	1.93E-03	7.47E-03
MPG	-0.1439	5.49E-03	1.78E-02
MPHOSPH10	0.0570	2.73E-01	4.01E-01
MPHOSPH6	0.0619	2.34E-01	3.57E-01
MPHOSPH8	0.0125	8.10E-01	8.75E-01
MPHOSPH9	0.0748	1.50E-01	2.54E-01
MPI	-0.2640	2.48E-07	3.29E-06
MPL	-0.0824	1.13E-01	2.05E-01
MPND	-0.2043	7.36E-05	4.71E-04
MPO	-0.1109	3.28E-02	7.69E-02
MPP1	-0.1418	6.21E-03	1.97E-02
MPP2	0.2477	1.36E-06	1.48E-05
MPP3	0.3336	4.30E-11	1.37E-09
MPP4	0.0955	6.63E-02	1.34E-01
MPP5	-0.2125	3.69E-05	2.61E-04
MPP6	0.1837	3.75E-04	1.88E-03
MPP7	0.1147	2.71E-02	6.58E-02
MPPE1	0.0073	8.89E-01	9.29E-01
MPPED1	-0.1513	3.48E-03	1.22E-02
MPPED2	-0.0468	3.69E-01	5.02E-01
MPRIP	-0.0606	2.44E-01	3.68E-01
MPST	-0.0191	7.13E-01	8.04E-01
MPV17L2	-0.1093	3.53E-02	8.16E-02

MPV17L	-0.0386	4.59E-01	5.90E-01
MPV17	0.0724	1.64E-01	2.72E-01
MPZL1	0.3822	2.40E-14	1.44E-12
MPZL2	0.0030	9.55E-01	9.72E-01
MPZL3	-0.0180	7.30E-01	8.17E-01
MPZ	0.0976	6.05E-02	1.25E-01
MR1	-0.0697	1.81E-01	2.93E-01
MRAP2	0.1410	6.52E-03	2.05E-02
MRAP	-0.0956	6.59E-02	1.34E-01
MRAS	-0.0873	9.32E-02	1.76E-01
MRC1	-0.1895	2.41E-04	1.29E-03
MRC2	-0.0191	7.14E-01	8.05E-01
MRE11A	0.2375	3.72E-06	3.60E-05
MREG	0.0910	8.00E-02	1.56E-01
MRFAP1L1	-0.0280	5.90E-01	7.05E-01
MRFAP1	-0.1419	6.17E-03	1.96E-02
MRGPRD	0.0241	6.44E-01	7.49E-01
MRGPRE	-0.0167	7.49E-01	8.31E-01
MRGPRF	-0.1265	1.48E-02	4.02E-02
MRGPRX1	0.0181	7.29E-01	8.16E-01
MRGPRX2	0.0408	4.33E-01	5.65E-01
MRGPRX3	0.0530	3.09E-01	4.38E-01
MRGPRX4	0.1491	3.99E-03	1.37E-02
MRI1	-0.0026	9.60E-01	9.75E-01
MRM1	-0.0540	2.99E-01	4.29E-01
MRO	-0.1637	1.55E-03	6.23E-03
MRP63	-0.0774	1.37E-01	2.36E-01
MRPL10	0.1251	1.59E-02	4.26E-02
MRPL11	0.0861	9.77E-02	1.83E-01
MRPL12	-0.0542	2.98E-01	4.27E-01
MRPL13	0.0011	9.84E-01	9.90E-01
MRPL14	0.0440	3.99E-01	5.31E-01
MRPL15	-0.2138	3.28E-05	2.36E-04
MRPL16	-0.2755	6.89E-08	1.04E-06
MRPL17	0.1421	6.12E-03	1.95E-02
MRPL18	0.2338	5.36E-06	4.91E-05
MRPL19	-0.3048	2.04E-09	4.51E-08
MRPL1	-0.1202	2.06E-02	5.25E-02
MRPL20	-0.0163	7.54E-01	8.35E-01
MRPL21	-0.0041	9.38E-01	9.61E-01
MRPL22	0.0135	7.96E-01	8.65E-01
MRPL23	0.0534	3.05E-01	4.34E-01
MRPL24	0.1964	1.41E-04	8.17E-04
MRPL27	0.0482	3.55E-01	4.87E-01

MRPL28	-0.0620	2.33E-01	3.56E-01
MRPL2	-0.0481	3.56E-01	4.88E-01
MRPL30	-0.0040	9.39E-01	9.62E-01
MRPL32	-0.1844	3.56E-04	1.80E-03
MRPL33	0.1134	2.90E-02	6.95E-02
MRPL34	-0.2920	9.97E-09	1.85E-07
MRPL35	-0.2038	7.68E-05	4.88E-04
MRPL36	-0.0092	8.59E-01	9.09E-01
MRPL37	-0.0674	1.95E-01	3.11E-01
MRPL38	0.0320	5.39E-01	6.61E-01
MRPL39	-0.0833	1.09E-01	1.99E-01
MRPL3	-0.0145	7.81E-01	8.55E-01
MRPL40	-0.1886	2.59E-04	1.37E-03
MRPL41	-0.1602	1.97E-03	7.61E-03
MRPL42P5	-0.0255	6.25E-01	7.33E-01
MRPL42	0.0281	5.90E-01	7.05E-01
MRPL43	-0.0866	9.57E-02	1.80E-01
MRPL44	-0.2365	4.11E-06	3.93E-05
MRPL45	0.1117	3.15E-02	7.43E-02
MRPL46	-0.2964	5.83E-09	1.15E-07
MRPL47	0.1425	5.96E-03	1.90E-02
MRPL48	0.1760	6.63E-04	3.02E-03
MRPL49	-0.0387	4.57E-01	5.88E-01
MRPL4	-0.0769	1.39E-01	2.40E-01
MRPL50	0.2343	5.08E-06	4.70E-05
MRPL51	0.0643	2.17E-01	3.37E-01
MRPL52	0.0536	3.03E-01	4.33E-01
MRPL53	-0.0248	6.34E-01	7.41E-01
MRPL54	-0.1865	3.03E-04	1.58E-03
MRPL55	0.1821	4.23E-04	2.07E-03
MRPL9	0.5881	6.95E-36	1.15E-32
MRPS10	0.0608	2.43E-01	3.67E-01
MRPS11	-0.1210	1.97E-02	5.08E-02
MRPS12	-0.0315	5.46E-01	6.66E-01
MRPS14	0.3016	3.07E-09	6.46E-08
MRPS15	-0.0372	4.75E-01	6.04E-01
MRPS16	-0.0070	8.93E-01	9.32E-01
MRPS17	0.0850	1.02E-01	1.89E-01
MRPS18A	-0.0050	9.23E-01	9.51E-01
MRPS18B	-0.1048	4.38E-02	9.68E-02
MRPS18C	-0.0484	3.53E-01	4.84E-01
MRPS21	0.3329	4.75E-11	1.50E-09
MRPS22	-0.1995	1.09E-04	6.56E-04
MRPS23	0.3396	1.82E-11	6.22E-10

MRPS24	-0.0433	4.06E-01	5.39E-01
MRPS25	-0.1651	1.41E-03	5.75E-03
MRPS26	-0.0170	7.44E-01	8.28E-01
MRPS27	-0.1037	4.59E-02	1.00E-01
MRPS28	-0.1235	1.73E-02	4.57E-02
MRPS2	-0.0720	1.67E-01	2.75E-01
MRPS30	-0.0304	5.59E-01	6.78E-01
MRPS31	-0.0768	1.40E-01	2.40E-01
MRPS33	-0.1808	4.67E-04	2.24E-03
MRPS34	-0.0185	7.23E-01	8.12E-01
MRPS35	-0.0274	5.98E-01	7.12E-01
MRPS36	-0.1212	1.95E-02	5.04E-02
MRPS5	-0.0234	6.53E-01	7.56E-01
MRPS6	0.0140	7.89E-01	8.59E-01
MRPS7	0.0233	6.54E-01	7.57E-01
MRPS9	0.0075	8.85E-01	9.26E-01
MRRF	0.1011	5.17E-02	1.10E-01
MRS2P2	0.0824	1.13E-01	2.05E-01
MRS2	0.1266	1.47E-02	4.00E-02
MRTO4	0.1250	1.60E-02	4.28E-02
MRVI1	-0.1679	1.17E-03	4.91E-03
MS4A10	0.0181	7.28E-01	8.15E-01
MS4A12	0.0578	2.67E-01	3.95E-01
MS4A13	0.1402	6.83E-03	2.13E-02
MS4A14	0.0268	6.06E-01	7.19E-01
MS4A15	0.1573	2.38E-03	8.90E-03
MS4A1	0.0145	7.81E-01	8.54E-01
MS4A2	-0.1388	7.41E-03	2.28E-02
MS4A3	-0.0444	3.94E-01	5.27E-01
MS4A4A	-0.1397	7.05E-03	2.19E-02
MS4A6A	-0.1150	2.68E-02	6.52E-02
MS4A6E	-0.1226	1.82E-02	4.75E-02
MS4A7	-0.1844	3.55E-04	1.80E-03
MS4A8B	0.1859	3.18E-04	1.64E-03
MSC	0.0670	1.98E-01	3.14E-01
MSGN1	0.0290	5.77E-01	6.94E-01
MSH2	0.3996	1.17E-15	9.22E-14
MSH3	-0.2066	6.10E-05	4.00E-04
MSH4	0.0472	3.65E-01	4.97E-01
MSH5	0.3274	1.02E-10	2.97E-09
MSH6	0.3114	8.71E-10	2.09E-08
MSI1	0.1606	1.91E-03	7.42E-03
MSI2	0.1739	7.68E-04	3.43E-03
MSL1	0.2335	5.48E-06	5.00E-05

MSL2	0.1842	3.61E-04	1.82E-03
MSL3L2	0.1561	2.57E-03	9.47E-03
MSL3	0.0278	5.94E-01	7.08E-01
MSLNL	0.0804	1.22E-01	2.17E-01
MSLN	0.0107	8.37E-01	8.93E-01
MSMB	0.0669	1.99E-01	3.15E-01
MSMP	0.0879	9.08E-02	1.72E-01
MSN	-0.0641	2.18E-01	3.38E-01
MSR1	-0.0468	3.68E-01	5.01E-01
MSRA	-0.2757	6.75E-08	1.02E-06
MSRB2	-0.0763	1.42E-01	2.43E-01
MSRB3	-0.0471	3.65E-01	4.98E-01
MST1P2	-0.1081	3.73E-02	8.54E-02
MST1P9	-0.0007	9.89E-01	9.93E-01
MST1R	0.1226	1.82E-02	4.75E-02
MST1	-0.2088	5.07E-05	3.43E-04
MST4	0.2249	1.22E-05	1.00E-04
MSTN	-0.0552	2.89E-01	4.18E-01
MSTO1	0.4360	1.21E-18	2.13E-16
MSTO2P	0.4100	1.79E-16	1.74E-14
MSX1	-0.0164	7.53E-01	8.34E-01
MSX2P1	0.0252	6.29E-01	7.37E-01
MSX2	0.0073	8.89E-01	9.29E-01
MT1A	-0.2221	1.58E-05	1.25E-04
MT1B	-0.0284	5.85E-01	7.01E-01
MT1DP	-0.1508	3.60E-03	1.26E-02
MT1E	-0.2358	4.41E-06	4.18E-05
MT1F	-0.2131	3.51E-05	2.51E-04
MT1G	-0.2333	5.59E-06	5.09E-05
MT1H	-0.1266	1.47E-02	4.00E-02
MT1IP	-0.0652	2.10E-01	3.29E-01
MT1L	-0.0946	6.89E-02	1.39E-01
MT1M	-0.2137	3.32E-05	2.38E-04
MT1X	-0.3100	1.06E-09	2.48E-08
MT2A	-0.2972	5.27E-09	1.05E-07
MT3	-0.0409	4.32E-01	5.64E-01
MT4	-0.0278	5.94E-01	7.08E-01
MTA1	0.1083	3.71E-02	8.49E-02
MTA2	0.1145	2.75E-02	6.65E-02
MTA3	0.3382	2.23E-11	7.49E-10
MTAP	0.2201	1.89E-05	1.47E-04
MTBP	0.3619	6.41E-13	2.96E-11
MTCH1	0.1099	3.43E-02	7.97E-02
MTCH2	-0.2952	6.80E-09	1.31E-07

MTCP1NB	0.0406	4.36E-01	5.68E-01
MTCP1	0.2027	8.45E-05	5.30E-04
MTDH	-0.0391	4.53E-01	5.84E-01
MTERFD1	0.0791	1.28E-01	2.25E-01
MTERFD2	0.0866	9.57E-02	1.80E-01
MTERFD3	0.1957	1.49E-04	8.57E-04
MTERF	0.0042	9.35E-01	9.59E-01
MTF1	0.0700	1.79E-01	2.90E-01
MTF2	0.3016	3.07E-09	6.45E-08
MTFMT	-0.2417	2.47E-06	2.51E-05
MTFR1	-0.1150	2.67E-02	6.50E-02
MTG1	0.0417	4.24E-01	5.56E-01
MTHFD1L	0.2015	9.30E-05	5.73E-04
MTHFD1	-0.3156	5.04E-10	1.27E-08
MTHFD2L	0.0153	7.68E-01	8.44E-01
MTHFD2	0.0012	9.82E-01	9.89E-01
MTHFR	0.0242	6.42E-01	7.47E-01
MTHFSD	-0.0135	7.95E-01	8.64E-01
MTHFS	-0.1990	1.14E-04	6.82E-04
MTIF2	-0.0985	5.82E-02	1.21E-01
MTIF3	-0.0619	2.34E-01	3.57E-01
MTL5	0.2845	2.45E-08	4.12E-07
MTM1	-0.0504	3.33E-01	4.64E-01
MTMR10	-0.1573	2.38E-03	8.88E-03
MTMR11	0.1479	4.32E-03	1.46E-02
MTMR12	0.0285	5.84E-01	6.99E-01
MTMR14	-0.0331	5.25E-01	6.48E-01
MTMR15	-0.0041	9.38E-01	9.61E-01
MTMR1	0.1623	1.71E-03	6.76E-03
MTMR2	0.2166	2.59E-05	1.93E-04
MTMR3	0.1175	2.36E-02	5.87E-02
MTMR4	-0.0155	7.66E-01	8.42E-01
MTMR6	-0.1148	2.70E-02	6.56E-02
MTMR7	0.1509	3.58E-03	1.25E-02
MTMR8	0.1658	1.35E-03	5.52E-03
MTMR9L	-0.0432	4.06E-01	5.39E-01
MTMR9	-0.0141	7.87E-01	8.59E-01
MTNR1A	0.0566	2.77E-01	4.05E-01
MTNR1B	0.1679	1.17E-03	4.91E-03
MTO1	0.0250	6.31E-01	7.38E-01
MTOR	-0.1371	8.16E-03	2.47E-02
MTP18	-0.0190	7.16E-01	8.06E-01
MTPAP	0.1214	1.93E-02	4.99E-02
MTRF1L	0.0657	2.07E-01	3.25E-01

MTRF1	0.0089	8.65E-01	9.13E-01
MTRR	0.0111	8.31E-01	8.89E-01
MTR	0.1040	4.54E-02	9.97E-02
MTSS1L	-0.0641	2.18E-01	3.38E-01
MTSS1	-0.1273	1.41E-02	3.88E-02
MTTP	-0.1570	2.42E-03	9.03E-03
MTUS1	-0.0940	7.06E-02	1.41E-01
MTUS2	-0.0830	1.10E-01	2.01E-01
MTVR2	0.0916	7.81E-02	1.53E-01
MTX1	0.2874	1.74E-08	3.06E-07
MTX2	0.0771	1.38E-01	2.38E-01
MTX3	0.1408	6.60E-03	2.08E-02
MUC12	0.0540	3.00E-01	4.29E-01
MUC13	0.1322	1.08E-02	3.10E-02
MUC15	0.1396	7.10E-03	2.20E-02
MUC16	-0.0199	7.02E-01	7.95E-01
MUC17	0.0366	4.82E-01	6.10E-01
MUC1	0.2100	4.55E-05	3.12E-04
MUC20	0.0000	9.99E-01	1.00E+00
MUC21	-0.0049	9.25E-01	9.52E-01
MUC2	0.0269	6.05E-01	7.17E-01
MUC4	0.0211	6.86E-01	7.82E-01
MUC5B	0.0768	1.40E-01	2.40E-01
MUC6	0.0331	5.25E-01	6.48E-01
MUC7	0.0152	7.71E-01	8.46E-01
MUCL1	-0.1053	4.26E-02	9.47E-02
MUDENG	-0.2307	7.16E-06	6.28E-05
MUL1	-0.2444	1.91E-06	2.01E-05
MUM1L1	0.0416	4.25E-01	5.57E-01
MUM1	0.0864	9.67E-02	1.81E-01
MURC	0.0312	5.49E-01	6.69E-01
MUS81	0.2237	1.37E-05	1.10E-04
MUSK	0.0486	3.50E-01	4.83E-01
MUSTN1	-0.0127	8.08E-01	8.73E-01
MUTED	0.0864	9.64E-02	1.81E-01
MUTYH	0.2366	4.07E-06	3.90E-05
MUT	-0.2094	4.82E-05	3.28E-04
MVD	-0.0513	3.25E-01	4.55E-01
MVK	-0.1847	3.49E-04	1.77E-03
MVP	-0.0609	2.42E-01	3.66E-01
MX1	0.0308	5.54E-01	6.73E-01
MX2	0.0775	1.36E-01	2.36E-01
MXD1	0.1815	4.43E-04	2.15E-03
MXD3	0.2941	7.72E-09	1.47E-07

MXD4	-0.1036	4.61E-02	1.01E-01
MXI1	-0.3249	1.44E-10	4.11E-09
MXRA5	-0.0920	7.67E-02	1.51E-01
MXRA7	0.0915	7.85E-02	1.53E-01
MXRA8	-0.0539	3.01E-01	4.30E-01
MYADML2	0.1817	4.35E-04	2.11E-03
MYADML	0.0608	2.43E-01	3.67E-01
MYADM	0.0560	2.82E-01	4.11E-01
MYBBP1A	0.0975	6.06E-02	1.25E-01
MYBL1	0.2865	1.94E-08	3.37E-07
MYBL2	0.4067	3.27E-16	2.93E-14
MYBPC1	0.0277	5.95E-01	7.09E-01
MYBPC2	0.1056	4.21E-02	9.38E-02
MYBPC3	0.1483	4.19E-03	1.43E-02
MYBPHL	0.1347	9.38E-03	2.78E-02
MYBPH	0.0814	1.18E-01	2.11E-01
MYB	0.2574	5.00E-07	6.12E-06
MYCBP2	-0.1046	4.41E-02	9.74E-02
MYCBPAP	0.3595	9.25E-13	4.17E-11
MYCBP	0.1351	9.19E-03	2.73E-02
MYCL1	-0.1175	2.36E-02	5.87E-02
MYCNOS	0.0719	1.67E-01	2.75E-01
MYCN	0.0864	9.64E-02	1.81E-01
MYCT1	-0.2295	7.96E-06	6.86E-05
MYC	-0.0606	2.44E-01	3.68E-01
MYD88	-0.2605	3.61E-07	4.60E-06
MYEF2	0.1783	5.58E-04	2.61E-03
MYEOV2	0.0669	1.99E-01	3.15E-01
MYEOV	-0.0436	4.03E-01	5.36E-01
MYF6	0.0899	8.37E-02	1.61E-01
MYH10	-0.0723	1.65E-01	2.73E-01
MYH11	-0.1514	3.46E-03	1.22E-02
MYH13	0.0222	6.70E-01	7.70E-01
MYH14	-0.0305	5.58E-01	6.76E-01
MYH15	0.0971	6.18E-02	1.27E-01
MYH16	0.0800	1.24E-01	2.19E-01
MYH1	0.0373	4.74E-01	6.03E-01
MYH2	0.0027	9.59E-01	9.74E-01
MYH3	-0.0329	5.28E-01	6.51E-01
MYH4	0.0648	2.13E-01	3.33E-01
MYH6	0.0755	1.47E-01	2.50E-01
MYH7B	0.0068	8.95E-01	9.33E-01
MYH7	0.0377	4.69E-01	5.99E-01
MYH8	0.0411	4.29E-01	5.62E-01

MYH9	-0.0097	8.52E-01	9.04E-01
MYL10	0.0134	7.97E-01	8.66E-01
MYL12A	-0.1053	4.26E-02	9.47E-02
MYL12B	-0.1046	4.40E-02	9.72E-02
MYL1	-0.0561	2.81E-01	4.09E-01
MYL2	-0.0339	5.15E-01	6.39E-01
MYL3	0.0104	8.42E-01	8.96E-01
MYL4	0.0132	8.00E-01	8.68E-01
MYL5	-0.0995	5.56E-02	1.17E-01
MYL6B	0.0963	6.39E-02	1.30E-01
MYL6	-0.0235	6.52E-01	7.56E-01
MYL7	0.0063	9.03E-01	9.39E-01
MYL9	-0.0935	7.20E-02	1.44E-01
MYLIP	0.0613	2.39E-01	3.63E-01
MYLK2	0.2177	2.33E-05	1.77E-04
MYLK3	0.0592	2.56E-01	3.81E-01
MYLK4	0.0474	3.63E-01	4.95E-01
MYLK	-0.2538	7.27E-07	8.53E-06
MYLPF	-0.0520	3.18E-01	4.48E-01
MYNN	0.1859	3.18E-04	1.64E-03
MYO10	-0.0932	7.29E-02	1.45E-01
MYO15A	0.1106	3.32E-02	7.77E-02
MYO15B	0.0319	5.40E-01	6.61E-01
MYO16	-0.1409	6.55E-03	2.06E-02
MYO18A	-0.0749	1.50E-01	2.54E-01
MYO18B	0.0419	4.20E-01	5.53E-01
MYO19	0.3672	2.77E-13	1.37E-11
MYO1A	0.1765	6.39E-04	2.93E-03
MYO1B	-0.2563	5.63E-07	6.77E-06
MYO1C	-0.0198	7.04E-01	7.97E-01
MYO1D	-0.0425	4.15E-01	5.47E-01
MYO1E	-0.0831	1.10E-01	2.00E-01
MYO1F	-0.0932	7.29E-02	1.45E-01
MYO1G	-0.0205	6.94E-01	7.89E-01
MYO1H	0.1840	3.68E-04	1.85E-03
MYO3A	0.1351	9.18E-03	2.73E-02
MYO3B	-0.0488	3.48E-01	4.80E-01
MYO5A	-0.1134	2.90E-02	6.95E-02
MYO5B	-0.1648	1.44E-03	5.85E-03
MYO5C	0.2262	1.08E-05	9.02E-05
MYO6	0.1478	4.33E-03	1.47E-02
MYO7A	-0.0586	2.60E-01	3.87E-01
MYO7B	-0.0564	2.79E-01	4.07E-01
MYO9A	-0.1140	2.82E-02	6.80E-02

MYO9B	0.1367	8.39E-03	2.53E-02
MYOCD	-0.1640	1.52E-03	6.13E-03
MYOC	-0.0867	9.54E-02	1.79E-01
MYOD1	0.0873	9.30E-02	1.76E-01
MYOF	-0.1075	3.85E-02	8.74E-02
MYOG	0.0950	6.76E-02	1.36E-01
MYOM1	0.0111	8.32E-01	8.90E-01
MYOM2	-0.1957	1.48E-04	8.56E-04
MYOM3	0.0216	6.78E-01	7.76E-01
MYOT	-0.0984	5.82E-02	1.21E-01
MYOZ1	-0.0449	3.88E-01	5.21E-01
MYOZ2	-0.0995	5.55E-02	1.17E-01
MYOZ3	0.0080	8.79E-01	9.22E-01
MYPN	0.1148	2.71E-02	6.57E-02
MYPOP	0.1615	1.81E-03	7.08E-03
MYRIP	-0.2812	3.59E-08	5.79E-07
MYSM1	-0.0171	7.42E-01	8.26E-01
MYST1	0.0028	9.57E-01	9.73E-01
MYST2	0.2762	6.42E-08	9.74E-07
MYST3	0.0550	2.91E-01	4.20E-01
MYST4	-0.0392	4.51E-01	5.82E-01
MYT1L	0.1337	9.91E-03	2.90E-02
MYT1	0.0166	7.50E-01	8.32E-01
MZF1	0.0897	8.44E-02	1.62E-01
N4BP1	-0.1381	7.74E-03	2.37E-02
N4BP2L1	-0.1834	3.86E-04	1.92E-03
N4BP2L2	0.1301	1.21E-02	3.42E-02
N4BP2	-0.0724	1.64E-01	2.72E-01
N4BP3	0.1792	5.23E-04	2.47E-03
N6AMT1	-0.0002	9.97E-01	9.98E-01
N6AMT2	-0.0227	6.63E-01	7.64E-01
NAA10	0.1726	8.41E-04	3.70E-03
NAA11	0.1279	1.37E-02	3.78E-02
NAA15	0.0386	4.59E-01	5.89E-01
NAA16	0.1193	2.16E-02	5.46E-02
NAA20	0.0584	2.62E-01	3.89E-01
NAA25	0.2436	2.05E-06	2.13E-05
NAA30	-0.1650	1.42E-03	5.78E-03
NAA35	0.1578	2.31E-03	8.67E-03
NAA38	0.0944	6.95E-02	1.40E-01
NAA40	0.2787	4.79E-08	7.51E-07
NAA50	0.0978	6.00E-02	1.24E-01
NAAA	-0.1855	3.28E-04	1.68E-03
NAALAD2	0.1432	5.72E-03	1.84E-02

NAALADL1	0.1705	9.74E-04	4.21E-03
NAALADL2	-0.2764	6.28E-08	9.56E-07
NAB1	0.1163	2.50E-02	6.16E-02
NAB2	-0.0115	8.26E-01	8.86E-01
NACA2	0.1700	1.01E-03	4.33E-03
NACAD	-0.1268	1.46E-02	3.98E-02
NACAP1	0.1053	4.28E-02	9.50E-02
NACA	0.1319	1.10E-02	3.14E-02
NACC1	0.0504	3.33E-01	4.64E-01
NACC2	-0.0086	8.69E-01	9.16E-01
NADK	-0.1443	5.36E-03	1.75E-02
NADSYN1	-0.0459	3.78E-01	5.11E-01
NAE1	0.0822	1.14E-01	2.06E-01
NAF1	-0.0771	1.38E-01	2.38E-01
NAGA	-0.1153	2.64E-02	6.45E-02
NAGK	0.0372	4.75E-01	6.04E-01
NAGLU	-0.3345	3.78E-11	1.22E-09
NAGPA	0.0059	9.10E-01	9.43E-01
NAGS	-0.1109	3.27E-02	7.67E-02
NAIF1	0.1617	1.78E-03	6.99E-03
NAIP	0.1039	4.55E-02	9.99E-02
NALCN	0.0050	9.23E-01	9.51E-01
NAMPT	-0.2101	4.53E-05	3.11E-04
NANOG	0.1281	1.35E-02	3.75E-02
NANOS1	0.1499	3.81E-03	1.32E-02
NANOS2	0.0377	4.69E-01	5.99E-01
NANOS3	0.0768	1.40E-01	2.40E-01
NANP	0.0484	3.52E-01	4.84E-01
NANS	0.1014	5.10E-02	1.09E-01
NAP1L1	0.2655	2.11E-07	2.84E-06
NAP1L2	0.0687	1.87E-01	3.00E-01
NAP1L3	0.0202	6.98E-01	7.92E-01
NAP1L4	0.1371	8.17E-03	2.48E-02
NAP1L5	-0.2008	9.87E-05	6.03E-04
NAP1L6	0.1331	1.03E-02	2.99E-02
NAPA	-0.1750	7.11E-04	3.21E-03
NAPB	0.0159	7.61E-01	8.39E-01
NAPEPLD	-0.0814	1.18E-01	2.11E-01
NAPG	0.0486	3.51E-01	4.83E-01
NAPRT1	-0.0741	1.54E-01	2.59E-01
NAPSA	0.0802	1.23E-01	2.18E-01
NAPSB	-0.0280	5.91E-01	7.06E-01
NARFL	0.0169	7.46E-01	8.29E-01
NARF	0.2008	9.84E-05	6.02E-04

NARG2	0.1399	6.96E-03	2.17E-02
NARS2	-0.1201	2.07E-02	5.28E-02
NARS	-0.0163	7.55E-01	8.35E-01
NASP	0.3694	1.95E-13	9.88E-12
NAT10	0.2283	8.95E-06	7.62E-05
NAT14	0.0466	3.71E-01	5.04E-01
NAT15	-0.0014	9.78E-01	9.86E-01
NAT1	-0.1283	1.34E-02	3.72E-02
NAT2	-0.2780	5.19E-08	8.04E-07
NAT6	-0.0727	1.62E-01	2.69E-01
NAT8B	0.0129	8.05E-01	8.71E-01
NAT8L	0.0216	6.78E-01	7.76E-01
NAT8	-0.1457	4.92E-03	1.63E-02
NAT9	0.2869	1.85E-08	3.24E-07
NAV1	-0.0660	2.04E-01	3.22E-01
NAV2	0.0362	4.87E-01	6.15E-01
NAV3	-0.0413	4.28E-01	5.60E-01
NBAS	-0.0551	2.90E-01	4.19E-01
NBEAL1	-0.0643	2.17E-01	3.37E-01
NBEAL2	0.2066	6.10E-05	4.01E-04
NBEA	0.0395	4.48E-01	5.79E-01
NBL1	-0.0741	1.54E-01	2.59E-01
NBLA00301	-0.1511	3.52E-03	1.24E-02
NBN	-0.1428	5.87E-03	1.88E-02
NBPF10	0.0928	7.41E-02	1.47E-01
NBPF14	0.1269	1.45E-02	3.96E-02
NBPF15	0.2093	4.83E-05	3.29E-04
NBPF16	0.1245	1.64E-02	4.38E-02
NBPF1	0.0269	6.05E-01	7.17E-01
NBPF22P	0.0536	3.04E-01	4.33E-01
NBPF3	-0.0111	8.32E-01	8.90E-01
NBPF4	0.1437	5.56E-03	1.80E-02
NBPF6	0.1763	6.45E-04	2.95E-03
NBPF7	0.2064	6.21E-05	4.07E-04
NBPF9	0.0970	6.21E-02	1.27E-01
NBR1	-0.0885	8.88E-02	1.69E-01
NBR2	0.0584	2.62E-01	3.88E-01
NCALD	-0.0203	6.97E-01	7.91E-01
NCAM1	0.0113	8.29E-01	8.88E-01
NCAM2	-0.0941	7.03E-02	1.41E-01
NCAN	-0.0796	1.26E-01	2.22E-01
NCAPD2	0.3383	2.20E-11	7.41E-10
NCAPD3	0.2225	1.52E-05	1.21E-04
NCAPG2	0.3113	8.84E-10	2.12E-08

NCAPG	0.4358	1.26E-18	2.19E-16
NCAPH2	0.3550	1.84E-12	7.72E-11
NCAPH	0.4109	1.51E-16	1.49E-14
NCBP1	0.2216	1.65E-05	1.31E-04
NCBP2	0.2818	3.36E-08	5.47E-07
NCCRP1	0.1469	4.58E-03	1.53E-02
NCDN	0.1047	4.39E-02	9.70E-02
NCEH1	0.1113	3.20E-02	7.53E-02
NCF1B	-0.0156	7.64E-01	8.41E-01
NCF1C	-0.0476	3.61E-01	4.93E-01
NCF1	-0.0013	9.80E-01	9.87E-01
NCF2	0.0689	1.86E-01	2.99E-01
NCF4	-0.0158	7.62E-01	8.40E-01
NCK1	-0.0278	5.93E-01	7.08E-01
NCK2	0.1416	6.28E-03	1.99E-02
NCKAP1L	-0.0704	1.76E-01	2.86E-01
NCKAP1	0.0616	2.37E-01	3.61E-01
NCKAP5L	0.0158	7.61E-01	8.39E-01
NCKAP5	-0.1912	2.12E-04	1.16E-03
NCKIPSD	0.1301	1.21E-02	3.42E-02
NCLN	-0.0148	7.77E-01	8.51E-01
NCL	0.1520	3.35E-03	1.18E-02
NCOA1	-0.0967	6.28E-02	1.29E-01
NCOA2	-0.0961	6.44E-02	1.31E-01
NCOA3	0.0589	2.58E-01	3.84E-01
NCOA4	-0.2170	2.49E-05	1.87E-04
NCOA5	0.4047	4.70E-16	4.07E-14
NCOA6	0.1511	3.53E-03	1.24E-02
NCOA7	0.0492	3.45E-01	4.77E-01
NCOR1	-0.0622	2.32E-01	3.55E-01
NCOR2	0.1828	4.02E-04	1.99E-03
NCR1	-0.1187	2.22E-02	5.59E-02
NCR2	0.0531	3.08E-01	4.37E-01
NCR3	-0.0381	4.64E-01	5.94E-01
NCRNA00028	-0.0213	6.82E-01	7.79E-01
NCRNA00029	0.0512	3.26E-01	4.56E-01
NCRNA00032	0.1617	1.78E-03	6.99E-03
NCRNA00051	0.0457	3.81E-01	5.14E-01
NCRNA00052	-0.0408	4.33E-01	5.65E-01
NCRNA00081	0.0283	5.86E-01	7.02E-01
NCRNA00085	0.1321	1.09E-02	3.13E-02
NCRNA00086	-0.0065	9.01E-01	9.37E-01
NCRNA00087	0.0424	4.15E-01	5.48E-01
NCRNA00092	0.0983	5.86E-02	1.22E-01

NCRNA00093	-0.2110	4.20E-05	2.92E-04
NCRNA00094	0.1420	6.13E-03	1.95E-02
NCRNA00095	0.1968	1.36E-04	7.92E-04
NCRNA00099	-0.0058	9.11E-01	9.44E-01
NCRNA00105	0.1195	2.13E-02	5.40E-02
NCRNA00107	0.2823	3.16E-08	5.18E-07
NCRNA00110	0.1435	5.62E-03	1.81E-02
NCRNA00112	0.0345	5.08E-01	6.32E-01
NCRNA00113	-0.0133	7.98E-01	8.66E-01
NCRNA00114	0.2905	1.21E-08	2.20E-07
NCRNA00115	0.0887	8.79E-02	1.68E-01
NCRNA00116	-0.0499	3.37E-01	4.69E-01
NCRNA00119	0.0172	7.41E-01	8.25E-01
NCRNA00120	-0.0066	8.99E-01	9.36E-01
NCRNA00152	0.1996	1.09E-04	6.53E-04
NCRNA00157	-0.0112	8.30E-01	8.89E-01
NCRNA00158	0.0783	1.32E-01	2.30E-01
NCRNA00159	0.0738	1.56E-01	2.61E-01
NCRNA00160	-0.0605	2.45E-01	3.70E-01
NCRNA00161	-0.0047	9.28E-01	9.54E-01
NCRNA00162	0.1376	7.95E-03	2.42E-02
NCRNA00164	0.1543	2.88E-03	1.05E-02
NCRNA00167	0.1328	1.05E-02	3.03E-02
NCRNA00169	0.0861	9.76E-02	1.83E-01
NCRNA00171	0.1528	3.18E-03	1.13E-02
NCRNA00173	0.1753	6.98E-04	3.16E-03
NCRNA00174	-0.0039	9.40E-01	9.63E-01
NCRNA00175	0.1472	4.49E-03	1.51E-02
NCRNA00176	0.0738	1.56E-01	2.61E-01
NCRNA00181	-0.2985	4.51E-09	9.13E-08
NCRNA00182	0.0044	9.32E-01	9.58E-01
NCRNA00183	0.1965	1.39E-04	8.09E-04
NCRNA00185	-0.0646	2.14E-01	3.34E-01
NCRNA00188	0.1331	1.03E-02	2.99E-02
NCRNA00189	0.0779	1.34E-01	2.33E-01
NCRNA00200	0.1084	3.68E-02	8.44E-02
NCRNA00201	0.3707	1.57E-13	8.11E-12
NCRNA00202	0.2148	3.02E-05	2.20E-04
NCRNA00203	0.1014	5.09E-02	1.09E-01
NCRNA00204B	0.1474	4.43E-03	1.49E-02
NCRNA00207	-0.0604	2.46E-01	3.70E-01
NCRNA00219	0.0498	3.39E-01	4.71E-01
NCRNA00230B	-0.1320	1.09E-02	3.14E-02
NCRNA00235	0.1574	2.36E-03	8.84E-03

NCS1	0.0977	6.00E-02	1.24E-01
NCSTN	0.2976	5.07E-09	1.01E-07
NDC80	0.4249	1.07E-17	1.43E-15
NDE1	0.1844	3.57E-04	1.80E-03
NDEL1	-0.1191	2.18E-02	5.50E-02
NDFIP1	-0.2517	9.04E-07	1.04E-05
NDFIP2	-0.1823	4.16E-04	2.04E-03
NDNL2	0.0431	4.07E-01	5.40E-01
NDN	0.0465	3.72E-01	5.05E-01
NDOR1	0.3248	1.46E-10	4.16E-09
NDP	0.0362	4.86E-01	6.14E-01
NDRG1	0.1820	4.28E-04	2.09E-03
NDRG2	-0.3380	2.28E-11	7.62E-10
NDRG3	0.2633	2.66E-07	3.51E-06
NDRG4	-0.0789	1.29E-01	2.26E-01
NDST1	-0.0540	3.00E-01	4.29E-01
NDST2	0.0536	3.03E-01	4.33E-01
NDST3	-0.1475	4.41E-03	1.49E-02
NDST4	0.0306	5.57E-01	6.76E-01
NDUFA10	-0.1592	2.10E-03	8.03E-03
NDUFA11	-0.0630	2.26E-01	3.48E-01
NDUFA12	0.0259	6.18E-01	7.28E-01
NDUFA13	-0.0485	3.52E-01	4.84E-01
NDUFA1	0.0264	6.12E-01	7.23E-01
NDUFA2	-0.0262	6.15E-01	7.25E-01
NDUFA3	-0.0748	1.51E-01	2.55E-01
NDUFA4L2	0.0907	8.12E-02	1.57E-01
NDUFA4	-0.1258	1.53E-02	4.14E-02
NDUFA5	-0.3105	9.86E-10	2.34E-08
NDUFA6	-0.0388	4.57E-01	5.87E-01
NDUFA7	-0.0736	1.57E-01	2.62E-01
NDUFA8	0.0085	8.70E-01	9.16E-01
NDUFA9	-0.1160	2.55E-02	6.25E-02
NDUFAB1	-0.1231	1.77E-02	4.64E-02
NDUFAF1	-0.3710	1.50E-13	7.83E-12
NDUFAF2	0.1338	9.89E-03	2.90E-02
NDUFAF3	-0.1102	3.39E-02	7.90E-02
NDUFAF4	0.0259	6.19E-01	7.29E-01
NDUFB10	-0.0775	1.36E-01	2.36E-01
NDUFB11	0.0354	4.97E-01	6.23E-01
NDUFB1	-0.1062	4.09E-02	9.17E-02
NDUFB2	-0.1452	5.08E-03	1.67E-02
NDUFB3	0.0901	8.31E-02	1.60E-01
NDUFB4	0.0455	3.82E-01	5.16E-01

NDUFB5	-0.1133	2.91E-02	6.97E-02
NDUFB6	-0.0161	7.58E-01	8.38E-01
NDUFB7	-0.0634	2.23E-01	3.44E-01
NDUFB8	-0.1888	2.54E-04	1.35E-03
NDUFB9	-0.0316	5.44E-01	6.65E-01
NDUFC1	-0.1570	2.42E-03	9.03E-03
NDUFC2	-0.0938	7.11E-02	1.42E-01
NDUFS1	-0.2118	3.91E-05	2.75E-04
NDUFS2	0.0250	6.31E-01	7.39E-01
NDUFS3	-0.0713	1.71E-01	2.80E-01
NDUFS4	-0.0577	2.68E-01	3.95E-01
NDUFS5	-0.0547	2.93E-01	4.23E-01
NDUFS6	0.0761	1.43E-01	2.45E-01
NDUFS7	-0.1855	3.29E-04	1.69E-03
NDUFS8	-0.0878	9.14E-02	1.73E-01
NDUFV1	-0.2230	1.46E-05	1.17E-04
NDUFV2	-0.2300	7.63E-06	6.62E-05
NDUFV3	-0.0079	8.79E-01	9.23E-01
NEAT1	0.1107	3.31E-02	7.75E-02
NEBL	0.0970	6.20E-02	1.27E-01
NEB	0.1038	4.57E-02	1.00E-01
NECAB1	-0.0065	9.01E-01	9.37E-01
NECAB2	-0.1215	1.92E-02	4.98E-02
NECAB3	0.1002	5.38E-02	1.14E-01
NECAP1	-0.0124	8.13E-01	8.77E-01
NECAP2	-0.0550	2.91E-01	4.20E-01
NEDD1	0.2370	3.92E-06	3.77E-05
NEDD4L	0.0297	5.69E-01	6.86E-01
NEDD4	-0.1705	9.79E-04	4.22E-03
NEDD8	0.0814	1.18E-01	2.11E-01
NEDD9	0.0096	8.53E-01	9.05E-01
NEFH	0.1080	3.76E-02	8.58E-02
NEFL	0.0116	8.23E-01	8.85E-01
NEFM	0.0839	1.07E-01	1.95E-01
NEGR1	-0.1373	8.07E-03	2.45E-02
NEIL1	-0.0590	2.57E-01	3.83E-01
NEIL2	-0.1166	2.46E-02	6.08E-02
NEIL3	0.3280	9.37E-11	2.75E-09
NEK10	0.0087	8.67E-01	9.15E-01
NEK11	0.0414	4.27E-01	5.59E-01
NEK1	-0.1736	7.83E-04	3.49E-03
NEK2	0.4712	6.65E-22	2.14E-19
NEK3	0.0572	2.72E-01	3.99E-01
NEK4	0.0974	6.08E-02	1.26E-01

NEK5	0.0570	2.74E-01	4.02E-01
NEK6	-0.1902	2.29E-04	1.24E-03
NEK7	0.1345	9.48E-03	2.80E-02
NEK8	0.1074	3.87E-02	8.78E-02
NEK9	-0.1006	5.27E-02	1.12E-01
NELF	0.0242	6.42E-01	7.48E-01
NELL1	0.1090	3.58E-02	8.25E-02
NELL2	-0.0678	1.92E-01	3.07E-01
NENF	0.1885	2.60E-04	1.38E-03
NEO1	0.0330	5.27E-01	6.49E-01
NES	0.0378	4.68E-01	5.98E-01
NET1	0.0800	1.24E-01	2.19E-01
NETO1	-0.1278	1.37E-02	3.80E-02
NETO2	0.0229	6.60E-01	7.61E-01
NEU1	0.1442	5.40E-03	1.76E-02
NEU2	-0.0027	9.59E-01	9.74E-01
NEU3	-0.0525	3.13E-01	4.42E-01
NEU4	0.0097	8.53E-01	9.05E-01
NEURL1B	0.1038	4.57E-02	1.00E-01
NEURL2	-0.0576	2.68E-01	3.96E-01
NEURL3	0.3100	1.04E-09	2.46E-08
NEURL4	0.1308	1.17E-02	3.31E-02
NEURL	0.0387	4.57E-01	5.88E-01
NEUROD1	0.0736	1.57E-01	2.62E-01
NEUROD2	0.1151	2.66E-02	6.49E-02
NEUROD4	0.1828	4.03E-04	1.99E-03
NEUROD6	0.0696	1.81E-01	2.93E-01
NEUROG1	0.0349	5.03E-01	6.28E-01
NEUROG2	-0.0020	9.70E-01	9.81E-01
NEUROG3	0.0715	1.69E-01	2.78E-01
NEXN	-0.1502	3.74E-03	1.30E-02
NF1P1	0.0217	6.77E-01	7.76E-01
NF1	0.0916	7.82E-02	1.53E-01
NF2	0.1625	1.68E-03	6.67E-03
NFAM1	-0.0920	7.68E-02	1.51E-01
NFASC	-0.1299	1.23E-02	3.45E-02
NFAT5	-0.0555	2.87E-01	4.15E-01
NFATC1	-0.0108	8.35E-01	8.92E-01
NFATC2IP	0.1723	8.62E-04	3.78E-03
NFATC2	-0.0356	4.94E-01	6.21E-01
NFATC3	-0.2247	1.25E-05	1.02E-04
NFATC4	0.1098	3.46E-02	8.03E-02
NFE2L1	-0.0971	6.17E-02	1.27E-01
NFE2L2	-0.0589	2.57E-01	3.83E-01

NFE2L3	0.2507	1.00E-06	1.14E-05
NFE2	-0.0996	5.52E-02	1.16E-01
NFIA	-0.1485	4.15E-03	1.42E-02
NFIB	-0.0182	7.26E-01	8.14E-01
NFIC	-0.1090	3.59E-02	8.26E-02
NFIL3	-0.0839	1.07E-01	1.95E-01
NFIX	-0.1250	1.60E-02	4.28E-02
NFKB1	-0.0903	8.24E-02	1.60E-01
NFKB2	0.2229	1.46E-05	1.18E-04
NFKBIA	-0.0388	4.56E-01	5.87E-01
NFKBIB	-0.0483	3.53E-01	4.85E-01
NFKBID	0.1863	3.07E-04	1.59E-03
NFKBIE	0.1940	1.70E-04	9.60E-04
NFKBIL1	0.1452	5.08E-03	1.67E-02
NFKBIL2	0.3795	3.75E-14	2.18E-12
NFKBIZ	0.1496	3.87E-03	1.34E-02
NFRKB	0.3256	1.32E-10	3.76E-09
NFS1	-0.2403	2.84E-06	2.83E-05
NFU1	0.0744	1.53E-01	2.57E-01
NFX1	0.1010	5.18E-02	1.11E-01
NFXL1	0.1605	1.93E-03	7.48E-03
NFYA	0.2294	8.09E-06	6.95E-05
NFYB	0.0338	5.17E-01	6.41E-01
NFYC	0.2872	1.78E-08	3.12E-07
NGB	0.1319	1.10E-02	3.15E-02
NGDN	0.1469	4.58E-03	1.54E-02
NGEF	0.0102	8.45E-01	8.99E-01
NGFRAP1	0.2058	6.54E-05	4.25E-04
NGFR	-0.1520	3.33E-03	1.18E-02
NGF	-0.0991	5.65E-02	1.18E-01
NGLY1	-0.0588	2.58E-01	3.84E-01
NGRN	0.0156	7.65E-01	8.41E-01
NHEDC1	0.0280	5.91E-01	7.06E-01
NHEDC2	-0.2744	7.81E-08	1.16E-06
NHEG1	0.0489	3.47E-01	4.79E-01
NHEJ1	0.1274	1.40E-02	3.86E-02
NHLH1	0.2183	2.23E-05	1.69E-04
NHLH2	0.0997	5.49E-02	1.16E-01
NHLRC1	0.0278	5.94E-01	7.08E-01
NHLRC2	-0.0853	1.01E-01	1.87E-01
NHLRC3	-0.0722	1.65E-01	2.73E-01
NHLRC4	0.0284	5.86E-01	7.01E-01
NHP2L1	0.1118	3.13E-02	7.40E-02
NHP2	0.0535	3.04E-01	4.33E-01

NHSL1	0.1182	2.28E-02	5.72E-02
NHSL2	-0.2374	3.79E-06	3.65E-05
NHS	-0.0064	9.02E-01	9.38E-01
NICN1	-0.0792	1.28E-01	2.25E-01
NID1	0.0408	4.33E-01	5.65E-01
NID2	-0.0693	1.83E-01	2.95E-01
NIF3L1	0.2227	1.49E-05	1.19E-04
NINJ1	-0.1023	4.89E-02	1.06E-01
NINJ2	-0.0383	4.62E-01	5.93E-01
NINL	0.1241	1.68E-02	4.46E-02
NIN	0.1856	3.27E-04	1.68E-03
NIP7	-0.1189	2.20E-02	5.54E-02
NIPA1	0.1704	9.84E-04	4.24E-03
NIPA2	0.0951	6.72E-02	1.36E-01
NIPAL1	-0.0999	5.46E-02	1.15E-01
NIPAL2	-0.0591	2.56E-01	3.82E-01
NIPAL3	0.0013	9.80E-01	9.87E-01
NIPAL4	0.0290	5.78E-01	6.95E-01
NIPBL	0.1841	3.64E-04	1.84E-03
NIPSNAP1	-0.1910	2.14E-04	1.17E-03
NIPSNAP3A	-0.1743	7.46E-04	3.35E-03
NIPSNAP3B	-0.0008	9.88E-01	9.93E-01
NISCH	0.1625	1.69E-03	6.68E-03
NIT1	0.1069	3.97E-02	8.95E-02
NIT2	-0.2098	4.64E-05	3.17E-04
NKAIN1	0.1526	3.22E-03	1.15E-02
NKAIN2	0.0067	8.98E-01	9.35E-01
NKAIN3	-0.0034	9.48E-01	9.68E-01
NKAIN4	-0.0015	9.77E-01	9.85E-01
NKAPL	-0.2105	4.37E-05	3.02E-04
NKAP	0.2649	2.25E-07	3.01E-06
NKD1	-0.0955	6.62E-02	1.34E-01
NKD2	0.0075	8.86E-01	9.27E-01
NKG7	-0.0821	1.14E-01	2.06E-01
NKIRAS1	-0.1641	1.52E-03	6.10E-03
NKIRAS2	0.2029	8.31E-05	5.22E-04
NKPD1	0.2121	3.83E-05	2.70E-04
NKRF	0.2145	3.09E-05	2.24E-04
NKTR	0.2218	1.63E-05	1.29E-04
NKX1-2	-0.1272	1.42E-02	3.90E-02
NKX2-1	-0.0151	7.73E-01	8.47E-01
NKX2-2	0.0531	3.08E-01	4.37E-01
NKX2-3	-0.0489	3.48E-01	4.80E-01
NKX2-4	-0.0960	6.46E-02	1.32E-01

NKX2-5	0.0537	3.02E-01	4.31E-01
NKX2-6	0.0309	5.53E-01	6.72E-01
NKX2-8	-0.0048	9.27E-01	9.54E-01
NKX3-1	-0.1325	1.06E-02	3.06E-02
NKX3-2	0.1823	4.16E-04	2.04E-03
NKX6-1	0.0702	1.77E-01	2.88E-01
NKX6-2	-0.0930	7.35E-02	1.46E-01
NKX6-3	0.0542	2.98E-01	4.28E-01
NLE1	0.1642	1.50E-03	6.05E-03
NLGN1	-0.0239	6.46E-01	7.51E-01
NLGN2	-0.1044	4.45E-02	9.80E-02
NLGN3	0.0687	1.87E-01	3.00E-01
NLGN4X	-0.0747	1.51E-01	2.55E-01
NLGN4Y	-0.0246	6.37E-01	7.44E-01
NLK	0.1833	3.86E-04	1.93E-03
NLN	-0.1416	6.29E-03	1.99E-02
NLRC3	-0.1048	4.36E-02	9.66E-02
NLRC4	-0.0537	3.02E-01	4.32E-01
NLRC5	0.0454	3.83E-01	5.16E-01
NLRP10	0.0446	3.91E-01	5.24E-01
NLRP11	-0.0626	2.29E-01	3.52E-01
NLRP12	-0.0546	2.94E-01	4.23E-01
NLRP13	0.0316	5.44E-01	6.65E-01
NLRP14	-0.1532	3.10E-03	1.11E-02
NLRP1	-0.0556	2.85E-01	4.14E-01
NLRP2	0.0823	1.13E-01	2.05E-01
NLRP3	-0.1184	2.26E-02	5.67E-02
NLRP4	0.0554	2.87E-01	4.16E-01
NLRP5	0.0744	1.53E-01	2.57E-01
NLRP6	-0.2113	4.09E-05	2.85E-04
NLRP7	0.0596	2.52E-01	3.77E-01
NLRP8	0.0118	8.21E-01	8.83E-01
NLRP9	0.1999	1.06E-04	6.40E-04
NLRX1	-0.0970	6.19E-02	1.27E-01
NMBR	0.0457	3.80E-01	5.14E-01
NMB	0.1786	5.46E-04	2.56E-03
NMD3	-0.1653	1.39E-03	5.68E-03
NME1-NME2	-0.1748	7.20E-04	3.24E-03
NME1	0.1361	8.68E-03	2.60E-02
NME2P1	0.1263	1.49E-02	4.06E-02
NME2	0.0542	2.98E-01	4.28E-01
NME3	0.1043	4.47E-02	9.85E-02
NME4	-0.0265	6.11E-01	7.22E-01
NME5	-0.0533	3.06E-01	4.35E-01

NME6	0.1075	3.85E-02	8.75E-02
NME7	0.4321	2.64E-18	4.10E-16
NMI	0.1089	3.60E-02	8.27E-02
NMNAT1	-0.1867	3.00E-04	1.56E-03
NMNAT2	-0.0121	8.16E-01	8.79E-01
NMNAT3	0.1260	1.51E-02	4.10E-02
NMRAL1	-0.0281	5.89E-01	7.04E-01
NMT1	0.1034	4.66E-02	1.02E-01
NMT2	-0.0487	3.50E-01	4.82E-01
NMUR1	-0.1199	2.08E-02	5.31E-02
NMUR2	0.0098	8.50E-01	9.03E-01
NMU	0.2459	1.63E-06	1.74E-05
NNAT	0.0955	6.60E-02	1.34E-01
NNMT	-0.1498	3.84E-03	1.33E-02
NNT	-0.2934	8.48E-09	1.60E-07
NOB1	-0.0446	3.92E-01	5.25E-01
NOBOX	0.1145	2.74E-02	6.63E-02
NOC2L	-0.0135	7.95E-01	8.64E-01
NOC3L	-0.0138	7.91E-01	8.61E-01
NOC4L	0.1078	3.80E-02	8.66E-02
NOD1	0.0897	8.44E-02	1.62E-01
NOD2	0.1175	2.37E-02	5.89E-02
NODAL	0.1570	2.42E-03	9.02E-03
NOG	-0.0263	6.14E-01	7.24E-01
NOL10	0.1800	4.96E-04	2.36E-03
NOL11	0.3827	2.18E-14	1.32E-12
NOL12	0.3610	7.28E-13	3.35E-11
NOL3	-0.0027	9.58E-01	9.74E-01
NOL4	-0.1097	3.46E-02	8.03E-02
NOL6	0.1239	1.70E-02	4.50E-02
NOL7	0.2403	2.85E-06	2.83E-05
NOL8	0.3027	2.67E-09	5.68E-08
NOL9	0.0550	2.90E-01	4.19E-01
NOLC1	0.1120	3.11E-02	7.35E-02
NOM1	0.0245	6.38E-01	7.45E-01
NOMO1	0.0015	9.77E-01	9.86E-01
NOMO2	0.0550	2.91E-01	4.20E-01
NOMO3	-0.0054	9.17E-01	9.48E-01
NONO	0.3268	1.12E-10	3.23E-09
NOP10	0.0021	9.67E-01	9.80E-01
NOP14	-0.1033	4.69E-02	1.02E-01
NOP16	-0.0484	3.52E-01	4.84E-01
NOP2	0.1595	2.06E-03	7.92E-03
NOP56	0.3400	1.72E-11	5.88E-10

NOP58	0.3763	6.34E-14	3.47E-12
NOS1AP	0.1395	7.13E-03	2.21E-02
NOS1	-0.2127	3.61E-05	2.57E-04
NOS2	-0.0808	1.20E-01	2.15E-01
NOS3	-0.1749	7.14E-04	3.22E-03
NOSIP	0.1133	2.91E-02	6.98E-02
NOSTRIN	-0.2100	4.56E-05	3.12E-04
NOTCH1	0.1822	4.21E-04	2.07E-03
NOTCH2NL	0.2657	2.05E-07	2.77E-06
NOTCH2	0.0286	5.83E-01	6.99E-01
NOTCH3	-0.0955	6.62E-02	1.34E-01
NOTCH4	-0.1621	1.73E-03	6.81E-03
NOTO	0.0818	1.16E-01	2.08E-01
NOTUM	-0.1068	3.99E-02	8.99E-02
NOVA1	-0.0058	9.12E-01	9.45E-01
NOVA2	-0.1066	4.02E-02	9.05E-02
NOV	-0.0545	2.95E-01	4.24E-01
NOX1	0.1031	4.72E-02	1.03E-01
NOX3	0.1450	5.14E-03	1.69E-02
NOX4	-0.0452	3.85E-01	5.18E-01
NOX5	-0.0557	2.84E-01	4.13E-01
NOXA1	0.0110	8.33E-01	8.91E-01
NOXO1	0.0451	3.86E-01	5.19E-01
NPAS1	0.0975	6.06E-02	1.25E-01
NPAS2	0.1368	8.31E-03	2.51E-02
NPAS3	-0.0607	2.44E-01	3.68E-01
NPAS4	0.0540	2.99E-01	4.29E-01
NPAT	0.1300	1.22E-02	3.44E-02
NPBWR1	-0.0840	1.06E-01	1.95E-01
NPB	0.1696	1.04E-03	4.44E-03
NPC1L1	0.0159	7.60E-01	8.38E-01
NPC1	0.1516	3.42E-03	1.21E-02
NPC2	-0.0418	4.23E-01	5.55E-01
NPDC1	-0.1834	3.83E-04	1.91E-03
NPEPL1	0.1631	1.63E-03	6.48E-03
NPEPPS	0.2195	1.99E-05	1.54E-04
NPFFR1	-0.0066	8.99E-01	9.36E-01
NPFFR2	0.0778	1.35E-01	2.33E-01
NPFF	0.2028	8.37E-05	5.26E-04
NPHP1	0.1219	1.88E-02	4.89E-02
NPHP3	0.1886	2.58E-04	1.37E-03
NPHP4	0.0652	2.10E-01	3.29E-01
NPHS1	0.1345	9.51E-03	2.81E-02
NPHS2	0.2743	7.95E-08	1.18E-06

NPIPL3	0.2367	4.03E-06	3.86E-05
NPIP	0.2474	1.40E-06	1.52E-05
NPLOC4	0.2769	5.87E-08	9.01E-07
NPL	0.0632	2.25E-01	3.46E-01
NPM1	0.1305	1.19E-02	3.36E-02
NPM2	0.0064	9.02E-01	9.38E-01
NPM3	0.0647	2.14E-01	3.33E-01
NPNT	-0.0284	5.85E-01	7.01E-01
NPPA	0.0767	1.40E-01	2.41E-01
NPPB	0.0842	1.05E-01	1.94E-01
NPPC	0.1373	8.07E-03	2.45E-02
NPR1	-0.1754	6.89E-04	3.13E-03
NPR2	-0.1224	1.83E-02	4.78E-02
NPR3	-0.2368	4.00E-06	3.83E-05
NPRL2	0.0943	6.98E-02	1.40E-01
NPRL3	-0.0017	9.74E-01	9.84E-01
NPSR1	0.2011	9.64E-05	5.91E-04
NPTN	-0.0139	7.90E-01	8.60E-01
NPTX1	-0.0054	9.17E-01	9.48E-01
NPTX2	0.1049	4.34E-02	9.62E-02
NPTXR	-0.0446	3.91E-01	5.24E-01
NPVF	-0.0392	4.52E-01	5.83E-01
NPW	0.0053	9.18E-01	9.48E-01
NPY1R	-0.1387	7.47E-03	2.30E-02
NPY2R	-0.0560	2.82E-01	4.11E-01
NPY5R	-0.1371	8.20E-03	2.48E-02
NPY6R	-0.1420	6.13E-03	1.95E-02
NPY	-0.0183	7.25E-01	8.14E-01
NQO1	0.1606	1.91E-03	7.41E-03
NQO2	-0.0960	6.48E-02	1.32E-01
NR0B1	0.2225	1.53E-05	1.21E-04
NR0B2	-0.0742	1.53E-01	2.58E-01
NR1D1	0.1142	2.79E-02	6.73E-02
NR1D2	-0.0165	7.52E-01	8.33E-01
NR1H2	-0.0503	3.34E-01	4.65E-01
NR1H3	0.0028	9.58E-01	9.74E-01
NR1H4	-0.0600	2.49E-01	3.74E-01
NR1I2	-0.2075	5.66E-05	3.77E-04
NR1I3	-0.1533	3.08E-03	1.10E-02
NR2C1	0.1611	1.86E-03	7.24E-03
NR2C2AP	0.3331	4.63E-11	1.47E-09
NR2C2	0.0793	1.27E-01	2.24E-01
NR2E1	0.1170	2.42E-02	6.00E-02
NR2E3	0.1127	3.00E-02	7.15E-02

NR2F1	-0.0712	1.71E-01	2.81E-01
NR2F2	-0.0692	1.84E-01	2.96E-01
NR2F6	-0.0464	3.73E-01	5.06E-01
NR3C1	-0.1330	1.03E-02	3.00E-02
NR3C2	-0.2192	2.05E-05	1.58E-04
NR4A1	-0.1543	2.88E-03	1.05E-02
NR4A2	-0.0733	1.59E-01	2.64E-01
NR4A3	-0.1336	1.00E-02	2.92E-02
NR5A1	0.1534	3.06E-03	1.10E-02
NR5A2	0.1528	3.18E-03	1.13E-02
NR6A1	0.1819	4.28E-04	2.09E-03
NRADDP	0.1380	7.76E-03	2.37E-02
NRAP	0.0095	8.56E-01	9.06E-01
NRARP	0.0939	7.09E-02	1.42E-01
NRAS	0.0653	2.09E-01	3.28E-01
NRBF2	-0.2571	5.17E-07	6.30E-06
NRBP1	0.0885	8.86E-02	1.69E-01
NRBP2	-0.0952	6.69E-02	1.35E-01
NRCAM	0.1009	5.22E-02	1.11E-01
NRD1	0.1245	1.64E-02	4.38E-02
NRF1	0.4072	3.02E-16	2.73E-14
NRG1	-0.1515	3.45E-03	1.21E-02
NRG2	0.0183	7.26E-01	8.14E-01
NRG3	0.0664	2.02E-01	3.18E-01
NRG4	0.0645	2.15E-01	3.35E-01
NRGN	0.0358	4.92E-01	6.19E-01
NRIP1	-0.1390	7.34E-03	2.26E-02
NRIP2	-0.0139	7.89E-01	8.60E-01
NRIP3	0.0314	5.46E-01	6.67E-01
NRK	-0.0726	1.63E-01	2.70E-01
NRL	-0.0901	8.29E-02	1.60E-01
NRM	0.4320	2.65E-18	4.10E-16
NRN1L	-0.0905	8.18E-02	1.58E-01
NRN1	-0.1576	2.33E-03	8.74E-03
NRP1	-0.0993	5.60E-02	1.17E-01
NRP2	-0.1871	2.91E-04	1.52E-03
NRSN1	0.1104	3.35E-02	7.83E-02
NRSN2	0.1886	2.58E-04	1.37E-03
NRTN	0.0166	7.50E-01	8.32E-01
NRXN1	-0.0082	8.74E-01	9.19E-01
NRXN2	-0.0129	8.04E-01	8.71E-01
NRXN3	0.0058	9.12E-01	9.45E-01
NSA2	0.0168	7.47E-01	8.30E-01
NSD1	0.1351	9.18E-03	2.73E-02

NSDHL	-0.0113	8.28E-01	8.88E-01
NSFL1C	0.0734	1.58E-01	2.64E-01
NSF	0.1649	1.44E-03	5.84E-03
NSL1	0.2736	8.57E-08	1.27E-06
NSMAF	0.1568	2.45E-03	9.10E-03
NSMCE1	-0.0728	1.61E-01	2.68E-01
NSMCE2	0.1226	1.82E-02	4.75E-02
NSMCE4A	-0.0882	8.98E-02	1.71E-01
NSUN2	0.0606	2.44E-01	3.68E-01
NSUN3	-0.0445	3.93E-01	5.26E-01
NSUN4	0.0816	1.17E-01	2.10E-01
NSUN5P1	0.1585	2.19E-03	8.31E-03
NSUN5P2	0.1846	3.52E-04	1.78E-03
NSUN5	0.2378	3.64E-06	3.53E-05
NSUN6	-0.1570	2.42E-03	9.02E-03
NSUN7	0.2618	3.15E-07	4.08E-06
NT5C1A	-0.0005	9.93E-01	9.96E-01
NT5C1B	0.0472	3.65E-01	4.98E-01
NT5C2	0.0227	6.62E-01	7.63E-01
NT5C3L	0.2081	5.37E-05	3.60E-04
NT5C3	0.2157	2.79E-05	2.06E-04
NT5C	0.1060	4.13E-02	9.25E-02
NT5DC1	-0.1450	5.14E-03	1.69E-02
NT5DC2	0.2629	2.78E-07	3.65E-06
NT5DC3	0.2033	8.02E-05	5.05E-04
NT5E	-0.0512	3.25E-01	4.56E-01
NT5M	0.0692	1.84E-01	2.96E-01
NTAN1	0.0610	2.41E-01	3.66E-01
NTF3	-0.2097	4.69E-05	3.20E-04
NTF4	0.0435	4.03E-01	5.36E-01
NTHL1	-0.0674	1.95E-01	3.11E-01
NTM	0.0115	8.25E-01	8.86E-01
NTN1	-0.0203	6.97E-01	7.91E-01
NTN3	-0.0536	3.03E-01	4.33E-01
NTN4	-0.1509	3.57E-03	1.25E-02
NTN5	0.1009	5.20E-02	1.11E-01
NTNG1	0.0516	3.22E-01	4.52E-01
NTNG2	0.0427	4.12E-01	5.45E-01
NTRK1	0.0394	4.49E-01	5.81E-01
NTRK2	-0.1317	1.11E-02	3.18E-02
NTRK3	-0.0447	3.91E-01	5.24E-01
NTSR1	0.0561	2.81E-01	4.10E-01
NTSR2	-0.0820	1.15E-01	2.07E-01
NTS	0.0229	6.60E-01	7.62E-01

NUAK1	0.1795	5.12E-04	2.43E-03
NUAK2	0.1597	2.03E-03	7.82E-03
NUB1	-0.0785	1.31E-01	2.29E-01
NUBP1	-0.0860	9.81E-02	1.83E-01
NUBP2	-0.0002	9.98E-01	9.98E-01
NUBPL	-0.2278	9.38E-06	7.95E-05
NUCB1	-0.2340	5.22E-06	4.82E-05
NUCB2	0.0925	7.51E-02	1.48E-01
NUCKS1	0.2746	7.70E-08	1.15E-06
NUDCD1	0.1280	1.36E-02	3.77E-02
NUDCD2	-0.0853	1.01E-01	1.87E-01
NUDCD3	-0.0852	1.01E-01	1.87E-01
NUDC	0.0304	5.60E-01	6.78E-01
NUDT10	-0.0927	7.46E-02	1.48E-01
NUDT11	0.0869	9.45E-02	1.78E-01
NUDT12	-0.1534	3.05E-03	1.09E-02
NUDT13	-0.2164	2.62E-05	1.96E-04
NUDT14	0.0138	7.92E-01	8.62E-01
NUDT15	-0.0052	9.21E-01	9.50E-01
NUDT16L1	0.0182	7.27E-01	8.15E-01
NUDT16P1	-0.1629	1.64E-03	6.53E-03
NUDT16	-0.2536	7.44E-07	8.72E-06
NUDT17	0.3202	2.73E-10	7.40E-09
NUDT18	-0.1301	1.22E-02	3.43E-02
NUDT19	-0.0026	9.59E-01	9.75E-01
NUDT1	0.2605	3.60E-07	4.58E-06
NUDT21	-0.0157	7.64E-01	8.41E-01
NUDT22	-0.0798	1.25E-01	2.21E-01
NUDT2	0.1440	5.45E-03	1.77E-02
NUDT3	0.0985	5.80E-02	1.21E-01
NUDT4	-0.0614	2.38E-01	3.62E-01
NUDT5	-0.0089	8.64E-01	9.12E-01
NUDT6	-0.1985	1.18E-04	7.05E-04
NUDT7	-0.2390	3.25E-06	3.19E-05
NUDT8	-0.1988	1.16E-04	6.92E-04
NUDT9P1	0.0059	9.10E-01	9.43E-01
NUDT9	-0.2471	1.45E-06	1.57E-05
NUF2	0.5356	6.34E-29	4.85E-26
NUFIP1	0.0654	2.09E-01	3.27E-01
NUFIP2	0.2365	4.10E-06	3.92E-05
NUMA1	0.0476	3.60E-01	4.93E-01
NUMBL	0.1783	5.61E-04	2.62E-03
NUMB	-0.1505	3.66E-03	1.28E-02
NUP107	0.2998	3.81E-09	7.88E-08

NUP133	0.3030	2.56E-09	5.49E-08
NUP153	0.1098	3.45E-02	8.01E-02
NUP155	0.3030	2.57E-09	5.50E-08
NUP160	0.2004	1.01E-04	6.15E-04
NUP188	0.3265	1.16E-10	3.35E-09
NUP205	0.2254	1.17E-05	9.62E-05
NUP210L	0.0483	3.54E-01	4.86E-01
NUP210	0.3214	2.32E-10	6.39E-09
NUP214	0.3293	7.87E-11	2.36E-09
NUP35	0.1093	3.54E-02	8.17E-02
NUP37	0.2964	5.83E-09	1.15E-07
NUP43	0.3698	1.81E-13	9.26E-12
NUP50	0.1748	7.19E-04	3.24E-03
NUP54	0.1478	4.33E-03	1.47E-02
NUP62CL	0.1558	2.62E-03	9.62E-03
NUP62	0.3480	5.31E-12	2.04E-10
NUP85	0.4561	1.84E-20	5.02E-18
NUP88	0.0380	4.65E-01	5.95E-01
NUP93	0.0994	5.57E-02	1.17E-01
NUP98	0.0691	1.84E-01	2.97E-01
NUPL1	0.1541	2.91E-03	1.05E-02
NUPL2	0.2199	1.93E-05	1.49E-04
NUPR1	0.0182	7.26E-01	8.14E-01
NUS1	0.1012	5.15E-02	1.10E-01
NUSAP1	0.4236	1.38E-17	1.79E-15
NUTF2	-0.0063	9.03E-01	9.39E-01
NVL	0.4810	7.03E-23	2.75E-20
NWD1	0.0740	1.55E-01	2.60E-01
NXF1	0.2318	6.42E-06	5.74E-05
NXF2B	0.0809	1.20E-01	2.14E-01
NXF2	0.0603	2.47E-01	3.72E-01
NXF3	-0.1077	3.82E-02	8.69E-02
NXF4	0.0526	3.13E-01	4.42E-01
NXF5	0.0290	5.78E-01	6.95E-01
NXNL1	-0.0544	2.96E-01	4.26E-01
NXNL2	0.0396	4.46E-01	5.78E-01
NXN	-0.0984	5.83E-02	1.21E-01
NXPH1	-0.0772	1.38E-01	2.38E-01
NXPH2	0.2079	5.45E-05	3.64E-04
NXPH3	-0.0123	8.13E-01	8.77E-01
NXPH4	0.1197	2.11E-02	5.35E-02
NXT1	0.2502	1.05E-06	1.19E-05
NXT2	0.1549	2.78E-03	1.01E-02
NYNRIN	-0.0765	1.41E-01	2.42E-01

NYX	0.1407	6.63E-03	2.08E-02
OAF	-0.2229	1.47E-05	1.18E-04
OAS1	-0.0702	1.77E-01	2.88E-01
OAS2	-0.1370	8.23E-03	2.49E-02
OAS3	0.1034	4.66E-02	1.02E-01
OASL	-0.1404	6.75E-03	2.11E-02
OAT	-0.1446	5.27E-03	1.72E-02
OAZ1	0.0181	7.29E-01	8.16E-01
OAZ2	-0.2936	8.25E-09	1.56E-07
OAZ3	0.2340	5.24E-06	4.83E-05
OBFC1	-0.2981	4.74E-09	9.53E-08
OBFC2A	-0.0155	7.66E-01	8.42E-01
OBFC2B	0.2083	5.26E-05	3.54E-04
OBP2A	0.0621	2.33E-01	3.56E-01
OBP2B	0.0847	1.03E-01	1.90E-01
OBSCN	0.1957	1.49E-04	8.57E-04
OBSL1	-0.0281	5.90E-01	7.05E-01
OC90	-0.0960	6.48E-02	1.32E-01
OCA2	0.1404	6.78E-03	2.12E-02
OCEL1	-0.2790	4.62E-08	7.28E-07
OCIAD1	-0.2041	7.49E-05	4.78E-04
OCIAD2	-0.1170	2.42E-02	6.00E-02
OCLM	0.2403	2.85E-06	2.84E-05
OCLN	0.0697	1.81E-01	2.92E-01
OCM2	-0.0644	2.16E-01	3.36E-01
OCM	0.1563	2.54E-03	9.37E-03
OCRL	0.3440	9.66E-12	3.45E-10
ODAM	-0.0328	5.29E-01	6.51E-01
ODC1	0.2504	1.03E-06	1.16E-05
ODF1	0.0919	7.69E-02	1.51E-01
ODF2L	0.1559	2.61E-03	9.60E-03
ODF2	0.2301	7.54E-06	6.56E-05
ODF3B	-0.1046	4.40E-02	9.72E-02
ODF3L1	-0.0267	6.08E-01	7.20E-01
ODF3L2	-0.1141	2.80E-02	6.76E-02
ODF3	0.0156	7.64E-01	8.41E-01
ODF4	-0.0733	1.59E-01	2.65E-01
ODZ1	-0.0205	6.94E-01	7.89E-01
ODZ2	-0.1638	1.54E-03	6.19E-03
ODZ3	-0.0407	4.34E-01	5.66E-01
ODZ4	-0.0287	5.82E-01	6.98E-01
OFD1	0.3640	4.58E-13	2.18E-11
OGDHL	-0.3115	8.65E-10	2.08E-08
OGDH	-0.2031	8.18E-05	5.14E-04

OGFOD1	-0.2159	2.74E-05	2.02E-04
OGFOD2	0.0558	2.83E-01	4.12E-01
OGFRL1	0.1579	2.28E-03	8.59E-03
OGFR	0.0315	5.45E-01	6.66E-01
OGG1	0.0578	2.67E-01	3.94E-01
OGN	-0.1931	1.82E-04	1.02E-03
OGT	0.2451	1.77E-06	1.88E-05
OIP5	0.3950	2.66E-15	1.94E-13
OIT3	-0.1817	4.37E-04	2.12E-03
OLA1	0.2673	1.73E-07	2.38E-06
OLAH	-0.1654	1.39E-03	5.67E-03
OLFM1	-0.0454	3.84E-01	5.17E-01
OLFM2	-0.1389	7.39E-03	2.28E-02
OLFM3	0.0530	3.09E-01	4.38E-01
OLFM4	0.1083	3.70E-02	8.48E-02
OLFML1	-0.0566	2.77E-01	4.05E-01
OLFML2A	-0.0276	5.96E-01	7.10E-01
OLFML2B	0.0684	1.89E-01	3.02E-01
OLFML3	-0.0335	5.20E-01	6.44E-01
OLIG1	0.0397	4.46E-01	5.77E-01
OLIG2	0.0094	8.57E-01	9.08E-01
OLIG3	0.1809	4.64E-04	2.23E-03
OLR1	-0.0132	8.00E-01	8.68E-01
OMA1	-0.1160	2.55E-02	6.26E-02
OMD	-0.2034	7.96E-05	5.02E-04
OMG	0.0364	4.84E-01	6.12E-01
OMP	-0.0341	5.12E-01	6.37E-01
ONECUT1	0.0621	2.32E-01	3.56E-01
ONECUT2	-0.0846	1.04E-01	1.91E-01
ONECUT3	0.0367	4.81E-01	6.09E-01
OOEP	0.0160	7.59E-01	8.38E-01
OPA1	-0.1655	1.38E-03	5.63E-03
OPA3	0.0179	7.32E-01	8.18E-01
OPALIN	-0.0925	7.51E-02	1.48E-01
OPCML	-0.1276	1.39E-02	3.84E-02
OPHN1	0.0794	1.27E-01	2.23E-01
OPLAH	-0.1722	8.67E-04	3.80E-03
OPN1LW	0.0134	7.97E-01	8.65E-01
OPN1MW	0.0541	2.98E-01	4.28E-01
OPN1SW	0.1752	7.00E-04	3.17E-03
OPN3	0.0405	4.36E-01	5.68E-01
OPN4	-0.1009	5.22E-02	1.11E-01
OPN5	-0.0170	7.45E-01	8.28E-01
OPRD1	0.1068	3.98E-02	8.98E-02

OPRK1	0.0981	5.89E-02	1.22E-01
OPRL1	0.1130	2.95E-02	7.05E-02
OPRM1	0.0499	3.38E-01	4.69E-01
OPTC	0.1064	4.06E-02	9.12E-02
OPTN	-0.0002	9.97E-01	9.98E-01
OR10A2	0.0004	9.94E-01	9.96E-01
OR10A3	0.0571	2.73E-01	4.01E-01
OR10A4	0.0192	7.12E-01	8.03E-01
OR10A5	0.0729	1.61E-01	2.68E-01
OR10A6	0.0543	2.97E-01	4.27E-01
OR10A7	0.1671	1.24E-03	5.14E-03
OR10AD1	0.0354	4.96E-01	6.22E-01
OR10C1	0.1123	3.06E-02	7.25E-02
OR10G2	0.1235	1.73E-02	4.56E-02
OR10G3	0.0852	1.01E-01	1.88E-01
OR10G4	0.0514	3.23E-01	4.54E-01
OR10H1	0.0451	3.86E-01	5.19E-01
OR10H2	0.0687	1.87E-01	3.00E-01
OR10H3	-0.0349	5.03E-01	6.28E-01
OR10H4	0.0814	1.17E-01	2.11E-01
OR10H5	0.0387	4.57E-01	5.88E-01
OR10J3	0.1139	2.83E-02	6.82E-02
OR10J5	-0.0184	7.24E-01	8.13E-01
OR10P1	-0.0474	3.63E-01	4.95E-01
OR10Q1	0.0731	1.60E-01	2.66E-01
OR10V1	-0.0393	4.50E-01	5.82E-01
OR10W1	0.0213	6.82E-01	7.79E-01
OR11A1	0.1684	1.13E-03	4.79E-03
OR11G2	0.0183	7.25E-01	8.13E-01
OR11H12	-0.0312	5.49E-01	6.70E-01
OR11H1	0.0252	6.29E-01	7.37E-01
OR11H4	0.0156	7.64E-01	8.41E-01
OR11H6	0.0280	5.90E-01	7.05E-01
OR11L1	0.0552	2.89E-01	4.18E-01
OR12D2	0.1864	3.06E-04	1.59E-03
OR12D3	0.0851	1.02E-01	1.88E-01
OR13A1	0.1888	2.55E-04	1.35E-03
OR13C2	0.0652	2.10E-01	3.29E-01
OR13C3	0.0074	8.87E-01	9.28E-01
OR13C4	-0.0004	9.94E-01	9.96E-01
OR13C5	-0.0017	9.75E-01	9.84E-01
OR13D1	0.1136	2.86E-02	6.89E-02
OR13F1	-0.0030	9.53E-01	9.71E-01
OR13G1	-0.0943	6.95E-02	1.40E-01

OR13H1	0.0267	6.09E-01	7.20E-01
OR13J1	-0.0605	2.45E-01	3.70E-01
OR14I1	0.0363	4.86E-01	6.14E-01
OR14J1	0.0672	1.97E-01	3.13E-01
OR1B1	0.0723	1.65E-01	2.72E-01
OR1C1	0.0410	4.31E-01	5.64E-01
OR1D4	0.0285	5.84E-01	7.00E-01
OR1E1	-0.0586	2.61E-01	3.87E-01
OR1E2	-0.0432	4.06E-01	5.39E-01
OR1F1	-0.0505	3.32E-01	4.63E-01
OR1F2P	-0.0415	4.25E-01	5.58E-01
OR1G1	-0.0020	9.69E-01	9.80E-01
OR1I1	0.0643	2.16E-01	3.36E-01
OR1J1	0.1173	2.39E-02	5.93E-02
OR1J2	0.1498	3.83E-03	1.33E-02
OR1J4	0.0852	1.01E-01	1.88E-01
OR1K1	-0.0500	3.37E-01	4.69E-01
OR1L3	-0.0420	4.20E-01	5.53E-01
OR1L4	0.1346	9.42E-03	2.79E-02
OR1L6	0.0064	9.02E-01	9.38E-01
OR1L8	-0.1378	7.84E-03	2.39E-02
OR1N1	0.0632	2.24E-01	3.46E-01
OR1N2	0.0919	7.71E-02	1.51E-01
OR1Q1	0.0825	1.13E-01	2.04E-01
OR1S2	0.0844	1.05E-01	1.92E-01
OR2A12	-0.0563	2.79E-01	4.08E-01
OR2A14	0.0168	7.46E-01	8.29E-01
OR2A1	0.0327	5.30E-01	6.53E-01
OR2A25	0.0552	2.89E-01	4.18E-01
OR2A2	0.0565	2.78E-01	4.07E-01
OR2A4	0.0613	2.39E-01	3.63E-01
OR2A5	0.0073	8.88E-01	9.28E-01
OR2A7	0.0666	2.01E-01	3.17E-01
OR2A9P	0.0966	6.32E-02	1.29E-01
OR2AE1	-0.0193	7.11E-01	8.03E-01
OR2AG1	0.0245	6.38E-01	7.44E-01
OR2AG2	-0.0144	7.82E-01	8.55E-01
OR2AK2	0.0066	8.99E-01	9.36E-01
OR2AT4	0.0377	4.70E-01	5.99E-01
OR2B11	0.0405	4.36E-01	5.68E-01
OR2B2	0.1488	4.08E-03	1.40E-02
OR2B3	0.0315	5.46E-01	6.66E-01
OR2B6	0.3069	1.57E-09	3.57E-08
OR2C1	-0.0414	4.27E-01	5.59E-01

OR2C3	0.1618	1.76E-03	6.93E-03
OR2D2	0.1326	1.06E-02	3.06E-02
OR2F1	-0.0460	3.77E-01	5.11E-01
OR2F2	-0.0386	4.59E-01	5.89E-01
OR2G6	0.0696	1.81E-01	2.93E-01
OR2H1	0.1815	4.43E-04	2.15E-03
OR2H2	0.1323	1.08E-02	3.10E-02
OR2J2	-0.0044	9.33E-01	9.58E-01
OR2J3	0.0541	2.99E-01	4.28E-01
OR2K2	0.1327	1.05E-02	3.04E-02
OR2L13	-0.0384	4.61E-01	5.91E-01
OR2L1P	-0.0895	8.53E-02	1.64E-01
OR2L2	0.0984	5.83E-02	1.21E-01
OR2L3	0.0170	7.45E-01	8.28E-01
OR2S2	0.0184	7.24E-01	8.13E-01
OR2T10	0.0278	5.94E-01	7.08E-01
OR2T2	-0.0170	7.44E-01	8.27E-01
OR2T33	-0.0096	8.53E-01	9.05E-01
OR2T34	0.0365	4.83E-01	6.12E-01
OR2T3	0.0729	1.61E-01	2.68E-01
OR2T4	0.0384	4.61E-01	5.92E-01
OR2T5	0.0799	1.24E-01	2.20E-01
OR2T8	-0.0146	7.80E-01	8.53E-01
OR2V2	0.0265	6.11E-01	7.22E-01
OR2W3	-0.1181	2.29E-02	5.73E-02
OR2W5	0.1241	1.68E-02	4.46E-02
OR2Z1	0.1242	1.67E-02	4.43E-02
OR3A1	0.1212	1.95E-02	5.04E-02
OR3A2	0.0603	2.46E-01	3.71E-01
OR3A3	0.0052	9.20E-01	9.49E-01
OR3A4	0.0653	2.10E-01	3.28E-01
OR4A16	0.1799	4.99E-04	2.37E-03
OR4A47	-0.0931	7.34E-02	1.46E-01
OR4C6	0.0242	6.42E-01	7.47E-01
OR4D10	0.0526	3.12E-01	4.42E-01
OR4D11	0.0140	7.88E-01	8.59E-01
OR4D1	-0.0421	4.19E-01	5.52E-01
OR4D6	0.0139	7.90E-01	8.61E-01
OR4D9	0.0093	8.59E-01	9.09E-01
OR4E2	0.0825	1.13E-01	2.04E-01
OR4F17	-0.0304	5.59E-01	6.77E-01
OR4F21	-0.0675	1.94E-01	3.10E-01
OR4F29	0.1232	1.76E-02	4.62E-02
OR4F4	-0.0332	5.24E-01	6.48E-01

OR4F5	-0.0438	4.00E-01	5.33E-01
OR4K14	-0.0355	4.96E-01	6.22E-01
OR4K1	0.0163	7.54E-01	8.35E-01
OR4K2	0.0399	4.43E-01	5.75E-01
OR4M1	0.0008	9.88E-01	9.93E-01
OR4M2	-0.0267	6.09E-01	7.20E-01
OR4N2	-0.0610	2.41E-01	3.66E-01
OR4N3P	-0.0324	5.33E-01	6.56E-01
OR4N4	-0.0403	4.39E-01	5.70E-01
OR4Q3	-0.0721	1.66E-01	2.74E-01
OR51B2	0.0896	8.49E-02	1.63E-01
OR51B4	0.1345	9.47E-03	2.80E-02
OR51B5	0.1238	1.70E-02	4.51E-02
OR51B6	0.0324	5.33E-01	6.56E-01
OR51E1	0.0534	3.05E-01	4.34E-01
OR51E2	-0.0624	2.30E-01	3.53E-01
OR51G2	0.0330	5.26E-01	6.49E-01
OR51I1	0.0752	1.48E-01	2.52E-01
OR51I2	0.0907	8.10E-02	1.57E-01
OR51M1	0.0232	6.56E-01	7.58E-01
OR51Q1	0.1556	2.65E-03	9.72E-03
OR52A4	-0.0064	9.02E-01	9.38E-01
OR52B2	0.0473	3.63E-01	4.96E-01
OR52B4	0.1237	1.71E-02	4.52E-02
OR52B6	0.0448	3.89E-01	5.22E-01
OR52D1	0.1024	4.88E-02	1.06E-01
OR52E2	-0.0483	3.53E-01	4.85E-01
OR52E4	-0.0428	4.11E-01	5.44E-01
OR52E6	0.0287	5.81E-01	6.97E-01
OR52E8	0.1002	5.39E-02	1.14E-01
OR52H1	0.0171	7.42E-01	8.26E-01
OR52I1	0.1016	5.05E-02	1.08E-01
OR52I2	-0.0333	5.23E-01	6.47E-01
OR52K1	-0.1092	3.55E-02	8.19E-02
OR52K2	0.0116	8.24E-01	8.85E-01
OR52L1	0.1019	4.99E-02	1.07E-01
OR52M1	-0.0412	4.28E-01	5.61E-01
OR52N1	-0.0368	4.80E-01	6.09E-01
OR52N2	0.0971	6.16E-02	1.27E-01
OR52N4	0.0636	2.21E-01	3.42E-01
OR52N5	-0.0279	5.92E-01	7.06E-01
OR52R1	-0.0589	2.58E-01	3.84E-01
OR52W1	-0.0240	6.45E-01	7.50E-01
OR56A1	-0.0220	6.73E-01	7.72E-01

OR56A3	0.0530	3.09E-01	4.38E-01
OR56A4	0.0350	5.02E-01	6.28E-01
OR56A5	0.0927	7.46E-02	1.48E-01
OR56B1	-0.0010	9.84E-01	9.90E-01
OR56B4	0.0610	2.41E-01	3.66E-01
OR5A1	0.0871	9.39E-02	1.77E-01
OR5AK2	-0.0809	1.20E-01	2.14E-01
OR5AN1	0.0065	9.01E-01	9.37E-01
OR5AP2	0.0412	4.29E-01	5.62E-01
OR5AU1	0.0237	6.49E-01	7.53E-01
OR5B12	0.1110	3.25E-02	7.63E-02
OR5B21	-0.0081	8.77E-01	9.21E-01
OR5B2	-0.0070	8.93E-01	9.32E-01
OR5C1	0.0333	5.23E-01	6.47E-01
OR5E1P	-0.0622	2.32E-01	3.55E-01
OR5H2	0.0506	3.31E-01	4.62E-01
OR5H6	0.0008	9.88E-01	9.92E-01
OR5K1	0.0405	4.36E-01	5.68E-01
OR5K2	0.0888	8.77E-02	1.67E-01
OR5K3	-0.0755	1.47E-01	2.49E-01
OR5K4	0.0850	1.02E-01	1.88E-01
OR5M10	0.0493	3.43E-01	4.75E-01
OR5M11	0.0574	2.70E-01	3.98E-01
OR5M8	0.0217	6.77E-01	7.75E-01
OR5P3	0.0901	8.31E-02	1.60E-01
OR5V1	0.1061	4.11E-02	9.21E-02
OR6A2	0.0394	4.49E-01	5.80E-01
OR6B1	-0.0092	8.59E-01	9.09E-01
OR6B2	0.0894	8.54E-02	1.64E-01
OR6B3	0.0003	9.95E-01	9.97E-01
OR6C2	0.1124	3.04E-02	7.23E-02
OR6C3	0.1039	4.56E-02	1.00E-01
OR6C68	0.0175	7.38E-01	8.23E-01
OR6C70	0.0691	1.84E-01	2.96E-01
OR6F1	0.0458	3.79E-01	5.12E-01
OR6K3	-0.0057	9.13E-01	9.46E-01
OR6M1	0.0091	8.61E-01	9.10E-01
OR6S1	0.0555	2.86E-01	4.15E-01
OR6T1	0.0853	1.01E-01	1.87E-01
OR6W1P	-0.0078	8.81E-01	9.24E-01
OR7A5	0.1116	3.16E-02	7.45E-02
OR7C1	0.1457	4.92E-03	1.63E-02
OR7D2	-0.0791	1.28E-01	2.25E-01
OR7E156P	0.1458	4.90E-03	1.62E-02

OR7E24	-0.0461	3.76E-01	5.09E-01
OR7E37P	-0.0401	4.41E-01	5.73E-01
OR7E5P	0.1284	1.33E-02	3.71E-02
OR7E91P	0.0686	1.87E-01	3.01E-01
OR8A1	0.1316	1.12E-02	3.19E-02
OR8B12	-0.0159	7.61E-01	8.39E-01
OR8B2	0.0996	5.53E-02	1.16E-01
OR8B3	0.0170	7.44E-01	8.28E-01
OR8B8	0.0909	8.02E-02	1.56E-01
OR8D1	0.0810	1.19E-01	2.14E-01
OR8D2	0.0461	3.76E-01	5.10E-01
OR8G2	0.0946	6.87E-02	1.38E-01
OR8G5	0.0197	7.06E-01	7.98E-01
OR8I2	-0.0563	2.79E-01	4.08E-01
OR8K3	-0.0056	9.14E-01	9.46E-01
OR8S1	0.0273	6.01E-01	7.13E-01
OR9A2	-0.0242	6.42E-01	7.47E-01
OR9A4	0.0531	3.08E-01	4.37E-01
OR9I1	0.1152	2.65E-02	6.47E-02
OR9Q1	0.0208	6.90E-01	7.85E-01
ORAI1	-0.0089	8.64E-01	9.12E-01
ORAI2	0.0118	8.21E-01	8.82E-01
ORAI3	-0.2378	3.65E-06	3.53E-05
ORAOV1	0.1262	1.50E-02	4.07E-02
ORC1L	0.4352	1.41E-18	2.42E-16
ORC2L	0.2673	1.73E-07	2.39E-06
ORC3L	0.3320	5.36E-11	1.67E-09
ORC4L	0.2364	4.14E-06	3.94E-05
ORC5L	-0.0399	4.44E-01	5.76E-01
ORC6L	0.3702	1.72E-13	8.85E-12
ORM1	-0.0807	1.21E-01	2.16E-01
ORM2	-0.1068	3.98E-02	8.99E-02
ORMDL1	0.1092	3.54E-02	8.18E-02
ORMDL2	-0.0187	7.19E-01	8.09E-01
ORMDL3	-0.1787	5.44E-04	2.56E-03
OS9	-0.1810	4.60E-04	2.22E-03
OSBP2	0.3031	2.52E-09	5.42E-08
OSBPL10	0.0485	3.52E-01	4.84E-01
OSBPL11	-0.1826	4.08E-04	2.01E-03
OSBPL1A	-0.1253	1.57E-02	4.22E-02
OSBPL2	0.0360	4.89E-01	6.16E-01
OSBPL3	0.1971	1.33E-04	7.80E-04
OSBPL5	-0.0612	2.40E-01	3.64E-01
OSBPL6	0.0185	7.23E-01	8.12E-01

OSBPL7	0.0383	4.62E-01	5.92E-01
OSBPL8	-0.0037	9.43E-01	9.64E-01
OSBPL9	0.0182	7.26E-01	8.14E-01
OSBP	-0.2083	5.26E-05	3.53E-04
OSCAR	-0.0639	2.19E-01	3.40E-01
OSCP1	0.0365	4.83E-01	6.12E-01
OSGEPL1	0.0483	3.54E-01	4.86E-01
OSGEP	-0.0180	7.30E-01	8.17E-01
OSGIN1	-0.1445	5.28E-03	1.73E-02
OSGIN2	-0.0365	4.83E-01	6.11E-01
OSMR	-0.0481	3.55E-01	4.87E-01
OSM	-0.0698	1.80E-01	2.91E-01
OSR1	-0.0490	3.46E-01	4.78E-01
OSR2	-0.0103	8.44E-01	8.98E-01
OST4	-0.0126	8.08E-01	8.74E-01
OSTBETA	0.0174	7.38E-01	8.23E-01
OSTCL	-0.0043	9.35E-01	9.59E-01
OSTC	-0.0029	9.56E-01	9.73E-01
OSTF1	-0.1182	2.27E-02	5.70E-02
OSTM1	0.0732	1.60E-01	2.66E-01
OSTN	0.0381	4.64E-01	5.94E-01
OSTalpha	-0.1210	1.98E-02	5.09E-02
OTC	-0.2658	2.03E-07	2.75E-06
OTOA	-0.1496	3.87E-03	1.34E-02
OTOF	0.0910	8.00E-02	1.56E-01
OTOL1	0.0610	2.41E-01	3.66E-01
OTOP1	0.0473	3.63E-01	4.96E-01
OTOP2	0.1008	5.25E-02	1.12E-01
OTOP3	0.1123	3.05E-02	7.24E-02
OTOR	0.1049	4.34E-02	9.62E-02
OTOS	-0.0155	7.66E-01	8.42E-01
OTP	0.2272	9.86E-06	8.31E-05
OTUB1	0.0335	5.20E-01	6.44E-01
OTUB2	0.0227	6.63E-01	7.64E-01
OTUD1	0.1404	6.77E-03	2.12E-02
OTUD3	0.3364	2.86E-11	9.44E-10
OTUD4	-0.0914	7.86E-02	1.54E-01
OTUD5	-0.0324	5.34E-01	6.56E-01
OTUD6A	0.0076	8.85E-01	9.26E-01
OTUD6B	0.0564	2.79E-01	4.07E-01
OTUD7A	-0.0738	1.56E-01	2.61E-01
OTUD7B	0.3172	4.09E-10	1.07E-08
OTX1	0.1284	1.33E-02	3.71E-02
OTX2	0.1542	2.91E-03	1.05E-02

OVCA2	-0.0870	9.41E-02	1.77E-01
OVCH1	-0.0046	9.29E-01	9.56E-01
OVCH2	0.1422	6.09E-03	1.94E-02
OVGP1	-0.0345	5.08E-01	6.33E-01
OVOL1	0.1006	5.28E-02	1.12E-01
OVOL2	0.2166	2.57E-05	1.92E-04
OXA1L	-0.1620	1.74E-03	6.85E-03
OXCT1	0.0666	2.00E-01	3.17E-01
OXCT2	0.1165	2.49E-02	6.12E-02
OXER1	0.0533	3.06E-01	4.35E-01
OXGR1	0.0374	4.72E-01	6.01E-01
OXNAD1	-0.2869	1.84E-08	3.23E-07
OXR1	-0.0682	1.90E-01	3.04E-01
OXSM	-0.1902	2.29E-04	1.24E-03
OXSR1	-0.0127	8.08E-01	8.73E-01
OXTR	0.0725	1.63E-01	2.71E-01
OXT	0.0155	7.66E-01	8.42E-01
P2RX1	-0.0720	1.66E-01	2.75E-01
P2RX2	-0.0788	1.30E-01	2.27E-01
P2RX3	-0.1412	6.44E-03	2.03E-02
P2RX4	0.2330	5.74E-06	5.21E-05
P2RX5	0.1505	3.67E-03	1.28E-02
P2RX6	-0.0540	2.99E-01	4.29E-01
P2RX7	-0.0025	9.61E-01	9.76E-01
P2RY10	-0.0480	3.56E-01	4.88E-01
P2RY11	0.1007	5.26E-02	1.12E-01
P2RY12	-0.1718	8.94E-04	3.91E-03
P2RY13	-0.1442	5.39E-03	1.76E-02
P2RY14	-0.1762	6.52E-04	2.98E-03
P2RY1	-0.0673	1.96E-01	3.11E-01
P2RY2	0.0304	5.60E-01	6.78E-01
P2RY4	0.0811	1.19E-01	2.13E-01
P2RY6	0.0481	3.56E-01	4.88E-01
P2RY8	-0.1625	1.68E-03	6.67E-03
P4HA1	-0.1115	3.18E-02	7.49E-02
P4HA2	-0.0518	3.19E-01	4.49E-01
P4HA3	-0.1011	5.18E-02	1.10E-01
P4HB	-0.0621	2.33E-01	3.56E-01
P4HTM	0.0880	9.05E-02	1.72E-01
P704P	0.0808	1.20E-01	2.15E-01
PA2G4P4	0.2470	1.46E-06	1.59E-05
PA2G4	0.2037	7.78E-05	4.93E-04
PAAF1	-0.0242	6.42E-01	7.47E-01
PABPC1L2A	0.0135	7.95E-01	8.64E-01

PABPC1L2B	-0.0580	2.65E-01	3.92E-01
PABPC1L	0.3789	4.12E-14	2.37E-12
PABPC1P2	0.1031	4.72E-02	1.03E-01
PABPC1	0.0710	1.72E-01	2.82E-01
PABPC3	0.0784	1.32E-01	2.30E-01
PABPC4L	-0.0632	2.25E-01	3.46E-01
PABPC4	0.1240	1.69E-02	4.47E-02
PABPC5	-0.0461	3.76E-01	5.10E-01
PABPN1L	-0.0180	7.29E-01	8.16E-01
PABPN1	0.1648	1.44E-03	5.85E-03
PACRGL	0.1070	3.95E-02	8.92E-02
PACRG	-0.2005	1.01E-04	6.15E-04
PACS1	0.0767	1.40E-01	2.41E-01
PACS2	0.0659	2.05E-01	3.23E-01
PACSIN1	0.1403	6.78E-03	2.12E-02
PACSIN2	-0.1303	1.20E-02	3.40E-02
PACSIN3	0.1308	1.17E-02	3.32E-02
PADI1	-0.1059	4.15E-02	9.28E-02
PADI2	0.0164	7.52E-01	8.33E-01
PADI3	0.0194	7.10E-01	8.02E-01
PADI4	-0.1642	1.51E-03	6.08E-03
PADI6	0.0604	2.46E-01	3.70E-01
PAEP	-0.0050	9.24E-01	9.52E-01
PAF1	0.0027	9.58E-01	9.74E-01
PAFAH1B1	-0.0799	1.25E-01	2.20E-01
PAFAH1B2	-0.0604	2.46E-01	3.70E-01
PAFAH1B3	0.2249	1.22E-05	9.97E-05
PAFAH2	-0.3925	4.13E-15	2.90E-13
PAG1	0.0456	3.82E-01	5.15E-01
PAGE1	0.1364	8.51E-03	2.56E-02
PAGE2B	0.0744	1.53E-01	2.57E-01
PAGE2	0.1014	5.09E-02	1.09E-01
PAGE3	0.0663	2.02E-01	3.19E-01
PAGE4	-0.1045	4.44E-02	9.78E-02
PAGE5	-0.0231	6.57E-01	7.59E-01
PAH	-0.1781	5.70E-04	2.65E-03
PAICS	-0.0189	7.17E-01	8.08E-01
PAIP1	-0.0083	8.73E-01	9.18E-01
PAIP2B	-0.1146	2.73E-02	6.61E-02
PAIP2	-0.0372	4.75E-01	6.04E-01
PAK1IP1	0.1615	1.81E-03	7.08E-03
PAK1	0.1940	1.70E-04	9.59E-04
PAK2	0.1154	2.63E-02	6.42E-02
PAK3	-0.0124	8.12E-01	8.76E-01

PAK4	0.2267	1.03E-05	8.65E-05
PAK6	0.2578	4.82E-07	5.92E-06
PAK7	0.1484	4.17E-03	1.42E-02
PALB2	0.1554	2.68E-03	9.83E-03
PALLD	-0.0386	4.59E-01	5.89E-01
PALM2-AKAP2	-0.2738	8.41E-08	1.24E-06
PALM2	-0.2339	5.29E-06	4.86E-05
PALM3	-0.0101	8.46E-01	8.99E-01
PALMD	-0.2106	4.33E-05	3.00E-04
PALM	0.1057	4.19E-02	9.35E-02
PAMR1	-0.1772	6.06E-04	2.79E-03
PAM	-0.1245	1.64E-02	4.37E-02
PAN2	-0.0068	8.97E-01	9.34E-01
PAN3	0.1496	3.89E-03	1.34E-02
PANK1	-0.3305	6.61E-11	2.01E-09
PANK2	0.1237	1.71E-02	4.53E-02
PANK3	-0.0490	3.46E-01	4.78E-01
PANK4	-0.0663	2.02E-01	3.20E-01
PANX1	-0.1406	6.69E-03	2.10E-02
PANX2	0.0034	9.48E-01	9.68E-01
PANX3	0.1170	2.42E-02	6.00E-02
PAOX	-0.2391	3.19E-06	3.13E-05
PAPD4	-0.0749	1.50E-01	2.54E-01
PAPD5	-0.2270	1.00E-05	8.45E-05
PAPD7	0.1373	8.07E-03	2.45E-02
PAPLN	0.1020	4.96E-02	1.07E-01
PAPL	0.1487	4.11E-03	1.41E-02
PAPOLA	0.0461	3.75E-01	5.09E-01
PAPOLB	0.1011	5.17E-02	1.10E-01
PAPOLG	0.0610	2.41E-01	3.66E-01
PAPPA2	-0.1642	1.50E-03	6.05E-03
PAPPA	-0.0891	8.65E-02	1.66E-01
PAPSS1	0.1578	2.30E-03	8.66E-03
PAPSS2	-0.2146	3.08E-05	2.24E-04
PAQR3	-0.0274	5.98E-01	7.12E-01
PAQR4	0.3365	2.86E-11	9.44E-10
PAQR5	0.1681	1.15E-03	4.84E-03
PAQR6	0.2160	2.72E-05	2.02E-04
PAQR7	0.0189	7.17E-01	8.07E-01
PAQR8	0.0833	1.09E-01	1.99E-01
PAQR9	0.0481	3.56E-01	4.88E-01
PAR-SN	0.0830	1.10E-01	2.01E-01
PAR1	0.1178	2.32E-02	5.80E-02
PAR4	-0.0803	1.23E-01	2.18E-01

PAR5	0.0192	7.13E-01	8.04E-01
PARD3B	-0.0732	1.59E-01	2.65E-01
PARD3	0.2051	6.93E-05	4.47E-04
PARD6A	-0.0212	6.84E-01	7.81E-01
PARD6B	0.1387	7.46E-03	2.29E-02
PARD6G	0.2132	3.46E-05	2.48E-04
PARG	0.1158	2.57E-02	6.31E-02
PARK2	-0.1292	1.28E-02	3.57E-02
PARK7	-0.1113	3.21E-02	7.55E-02
PARL	0.0031	9.52E-01	9.70E-01
PARM1	-0.0219	6.74E-01	7.73E-01
PARN	0.0247	6.36E-01	7.43E-01
PARP10	-0.0537	3.02E-01	4.31E-01
PARP11	-0.0924	7.54E-02	1.49E-01
PARP12	0.0852	1.01E-01	1.87E-01
PARP14	0.0173	7.39E-01	8.24E-01
PARP15	0.0263	6.14E-01	7.24E-01
PARP16	-0.0632	2.24E-01	3.46E-01
PARP1	0.2877	1.68E-08	2.96E-07
PARP2	0.2686	1.49E-07	2.08E-06
PARP3	-0.1464	4.71E-03	1.57E-02
PARP4	-0.0391	4.53E-01	5.84E-01
PARP6	-0.0977	6.02E-02	1.24E-01
PARP8	-0.0199	7.02E-01	7.95E-01
PARP9	-0.1041	4.50E-02	9.90E-02
PARS2	0.1552	2.72E-03	9.95E-03
PART1	-0.0039	9.41E-01	9.64E-01
PARVA	-0.0954	6.63E-02	1.34E-01
PARVB	-0.0304	5.60E-01	6.78E-01
PARVG	-0.0358	4.92E-01	6.19E-01
PASD1	0.1108	3.29E-02	7.70E-02
PASK	0.3096	1.11E-09	2.60E-08
PATE1	-0.0358	4.91E-01	6.18E-01
PATE2	0.1455	4.98E-03	1.64E-02
PATE3	-0.0151	7.72E-01	8.47E-01
PATE4	0.0733	1.59E-01	2.65E-01
PATL1	-0.0376	4.70E-01	6.00E-01
PATL2	-0.0230	6.59E-01	7.60E-01
PATZ1	0.1627	1.66E-03	6.60E-03
PAWR	-0.0506	3.31E-01	4.62E-01
PAX1	0.0181	7.28E-01	8.15E-01
PAX2	-0.0579	2.66E-01	3.93E-01
PAX3	0.1353	9.08E-03	2.71E-02
PAX4	0.0008	9.87E-01	9.92E-01

PAX5	-0.0368	4.80E-01	6.09E-01
PAX6	0.1741	7.57E-04	3.39E-03
PAX7	-0.0025	9.62E-01	9.76E-01
PAX8	0.1416	6.29E-03	1.99E-02
PAX9	0.0393	4.51E-01	5.82E-01
PAXIP1	0.1726	8.42E-04	3.71E-03
PBK	0.3783	4.58E-14	2.61E-12
PBLD	-0.2252	1.19E-05	9.80E-05
PBOV1	0.0799	1.24E-01	2.20E-01
PBRM1	0.0593	2.54E-01	3.80E-01
PBX1	-0.0853	1.01E-01	1.87E-01
PBX2	0.1317	1.11E-02	3.18E-02
PBX3	0.1218	1.89E-02	4.91E-02
PBX4	0.0529	3.10E-01	4.39E-01
PBXIP1	0.2102	4.49E-05	3.09E-04
PCA3	-0.0986	5.78E-02	1.21E-01
PCBD1	-0.2421	2.38E-06	2.42E-05
PCBD2	-0.1266	1.47E-02	4.00E-02
PCBP1	-0.0541	2.98E-01	4.28E-01
PCBP2	0.0490	3.47E-01	4.79E-01
PCBP3	-0.0690	1.85E-01	2.98E-01
PCBP4	0.0804	1.22E-01	2.17E-01
PCCA	-0.2356	4.49E-06	4.24E-05
PCCB	-0.2592	4.14E-07	5.19E-06
PCDH10	-0.0888	8.76E-02	1.67E-01
PCDH11X	-0.0101	8.46E-01	8.99E-01
PCDH11Y	-0.0665	2.01E-01	3.18E-01
PCDH12	-0.1832	3.89E-04	1.94E-03
PCDH15	0.0823	1.14E-01	2.06E-01
PCDH17	-0.1040	4.52E-02	9.93E-02
PCDH18	-0.0661	2.04E-01	3.22E-01
PCDH19	0.0357	4.93E-01	6.20E-01
PCDH1	-0.2081	5.35E-05	3.59E-04
PCDH20	-0.0705	1.75E-01	2.86E-01
PCDH7	-0.1286	1.32E-02	3.67E-02
PCDH8	0.0998	5.49E-02	1.16E-01
PCDH9	-0.0389	4.54E-01	5.85E-01
PCDHA10	-0.1420	6.15E-03	1.95E-02
PCDHA11	-0.0756	1.46E-01	2.49E-01
PCDHA12	-0.1096	3.48E-02	8.07E-02
PCDHA13	-0.1216	1.92E-02	4.97E-02
PCDHA1	0.0833	1.09E-01	1.99E-01
PCDHA2	0.0553	2.88E-01	4.17E-01
PCDHA3	0.0599	2.50E-01	3.75E-01

PCDHA4	-0.0326	5.31E-01	6.54E-01
PCDHA5	0.0217	6.76E-01	7.75E-01
PCDHA6	-0.0062	9.06E-01	9.41E-01
PCDHA7	-0.0468	3.68E-01	5.01E-01
PCDHA8	0.0280	5.90E-01	7.05E-01
PCDHA9	0.0187	7.19E-01	8.09E-01
PCDHAC1	-0.1099	3.44E-02	7.98E-02
PCDHAC2	-0.0544	2.96E-01	4.25E-01
PCDHB10	-0.0179	7.31E-01	8.18E-01
PCDHB11	-0.1048	4.37E-02	9.67E-02
PCDHB12	-0.0931	7.32E-02	1.45E-01
PCDHB13	-0.0865	9.63E-02	1.81E-01
PCDHB14	-0.1009	5.22E-02	1.11E-01
PCDHB15	-0.1056	4.20E-02	9.36E-02
PCDHB16	-0.0202	6.98E-01	7.91E-01
PCDHB17	-0.0397	4.46E-01	5.78E-01
PCDHB18	-0.0584	2.62E-01	3.88E-01
PCDHB19P	-0.1033	4.67E-02	1.02E-01
PCDHB1	0.1317	1.11E-02	3.19E-02
PCDHB2	0.0468	3.68E-01	5.01E-01
PCDHB3	0.0315	5.45E-01	6.66E-01
PCDHB4	-0.0928	7.41E-02	1.47E-01
PCDHB5	0.0134	7.97E-01	8.66E-01
PCDHB6	-0.0181	7.28E-01	8.15E-01
PCDHB7	-0.0722	1.65E-01	2.73E-01
PCDHB8	-0.0400	4.42E-01	5.74E-01
PCDHB9	-0.0155	7.67E-01	8.43E-01
PCDHGA10	0.0215	6.80E-01	7.78E-01
PCDHGA11	0.0112	8.30E-01	8.89E-01
PCDHGA12	-0.1493	3.94E-03	1.36E-02
PCDHGA1	-0.0553	2.88E-01	4.17E-01
PCDHGA2	-0.1151	2.67E-02	6.50E-02
PCDHGA3	-0.0873	9.30E-02	1.76E-01
PCDHGA4	-0.0528	3.10E-01	4.40E-01
PCDHGA5	-0.0423	4.17E-01	5.50E-01
PCDHGA6	0.0187	7.19E-01	8.09E-01
PCDHGA7	-0.0244	6.39E-01	7.45E-01
PCDHGA8	0.0667	2.00E-01	3.17E-01
PCDHGA9	-0.0862	9.73E-02	1.82E-01
PCDHGB1	-0.0803	1.23E-01	2.18E-01
PCDHGB2	-0.1133	2.91E-02	6.97E-02
PCDHGB3	-0.0507	3.30E-01	4.61E-01
PCDHGB4	0.0138	7.92E-01	8.62E-01
PCDHGB5	-0.0748	1.50E-01	2.54E-01

PCDHGB6	-0.0063	9.04E-01	9.40E-01
PCDHGB7	-0.1645	1.48E-03	5.97E-03
PCDHGB8P	0.0905	8.18E-02	1.59E-01
PCDHGC3	-0.1645	1.47E-03	5.95E-03
PCDHGC4	0.0484	3.52E-01	4.84E-01
PCDHGC5	0.0131	8.02E-01	8.69E-01
PCDP1	0.0730	1.61E-01	2.68E-01
PCF11	0.1243	1.66E-02	4.42E-02
PCGEM1	0.0239	6.47E-01	7.51E-01
PCGF1	0.1798	5.02E-04	2.39E-03
PCGF2	0.0741	1.54E-01	2.59E-01
PCGF3	0.1615	1.81E-03	7.08E-03
PCGF5	-0.1619	1.76E-03	6.91E-03
PCGF6	-0.0423	4.17E-01	5.50E-01
PCID2	0.2925	9.36E-09	1.75E-07
PCIF1	0.1425	5.95E-03	1.90E-02
PCK1	-0.2627	2.83E-07	3.71E-06
PCK2	-0.2614	3.27E-07	4.22E-06
PCLO	0.1151	2.66E-02	6.48E-02
PCM1	0.0201	7.00E-01	7.93E-01
PCMT1	0.1689	1.09E-03	4.64E-03
PCMTD1	-0.1845	3.54E-04	1.79E-03
PCMTD2	0.0325	5.32E-01	6.55E-01
PCNAP1	-0.0340	5.13E-01	6.38E-01
PCNA	0.3970	1.88E-15	1.40E-13
PCNP	0.1317	1.11E-02	3.19E-02
PCNT	0.1223	1.85E-02	4.82E-02
PCNXL2	0.1904	2.24E-04	1.22E-03
PCNXL3	0.2035	7.89E-05	4.98E-04
PCNX	-0.1517	3.41E-03	1.20E-02
PCOLCE2	0.0626	2.29E-01	3.52E-01
PCOLCE	-0.0908	8.07E-02	1.57E-01
PCOTH	0.1270	1.44E-02	3.94E-02
PCP2	0.2036	7.81E-05	4.94E-04
PCP4L1	-0.0396	4.46E-01	5.78E-01
PCP4	0.0374	4.72E-01	6.01E-01
PCSK1N	0.0722	1.65E-01	2.73E-01
PCSK1	-0.1204	2.03E-02	5.20E-02
PCSK2	-0.0574	2.70E-01	3.98E-01
PCSK4	-0.1112	3.22E-02	7.57E-02
PCSK5	-0.0185	7.23E-01	8.12E-01
PCSK6	-0.2558	5.94E-07	7.11E-06
PCSK7	0.1051	4.30E-02	9.55E-02
PCSK9	0.0838	1.07E-01	1.96E-01

PCTP	-0.2402	2.86E-06	2.84E-05
PCYOX1L	0.1221	1.86E-02	4.85E-02
PCYOX1	-0.2629	2.77E-07	3.64E-06
PCYT1A	-0.1625	1.69E-03	6.68E-03
PCYT1B	0.1123	3.05E-02	7.25E-02
PCYT2	-0.0550	2.91E-01	4.20E-01
PC	-0.2935	8.36E-09	1.58E-07
PDAP1	-0.0032	9.51E-01	9.70E-01
PDCD10	0.1793	5.18E-04	2.45E-03
PDCD11	0.1067	3.99E-02	9.00E-02
PDCD1LG2	-0.1296	1.25E-02	3.50E-02
PDCD1	0.0611	2.41E-01	3.65E-01
PDCD2L	0.1524	3.26E-03	1.16E-02
PDCD2	0.2214	1.68E-05	1.33E-04
PDCD4	-0.0633	2.24E-01	3.45E-01
PDCD5	0.2340	5.25E-06	4.84E-05
PDCD6IP	-0.0990	5.68E-02	1.19E-01
PDCD6	0.1038	4.57E-02	1.00E-01
PDCD7	0.1674	1.21E-03	5.04E-03
PDCL2	0.1480	4.28E-03	1.45E-02
PDCL3	0.1912	2.11E-04	1.15E-03
PDCL	0.2415	2.52E-06	2.54E-05
PDC	0.0620	2.33E-01	3.56E-01
PDDC1	-0.0402	4.40E-01	5.72E-01
PDE10A	-0.1779	5.76E-04	2.68E-03
PDE11A	-0.2176	2.36E-05	1.79E-04
PDE12	-0.0926	7.49E-02	1.48E-01
PDE1A	-0.0418	4.22E-01	5.55E-01
PDE1B	-0.1124	3.05E-02	7.23E-02
PDE1C	-0.1268	1.45E-02	3.96E-02
PDE2A	-0.3298	7.30E-11	2.21E-09
PDE3A	-0.1384	7.60E-03	2.33E-02
PDE3B	-0.1630	1.63E-03	6.49E-03
PDE4A	0.0640	2.19E-01	3.39E-01
PDE4B	-0.1107	3.30E-02	7.72E-02
PDE4C	0.1173	2.39E-02	5.93E-02
PDE4DIP	0.0291	5.76E-01	6.93E-01
PDE4D	-0.1633	1.60E-03	6.39E-03
PDE5A	-0.0409	4.32E-01	5.64E-01
PDE6A	0.0673	1.96E-01	3.11E-01
PDE6B	0.0249	6.32E-01	7.40E-01
PDE6C	0.0017	9.74E-01	9.84E-01
PDE6D	0.1651	1.41E-03	5.75E-03
PDE6G	-0.1254	1.57E-02	4.21E-02

PDE6H	0.0683	1.89E-01	3.03E-01
PDE7A	0.3039	2.30E-09	5.00E-08
PDE7B	-0.2088	5.07E-05	3.42E-04
PDE8A	-0.1442	5.39E-03	1.76E-02
PDE8B	-0.0927	7.46E-02	1.48E-01
PDE9A	0.0978	5.97E-02	1.24E-01
PDF	-0.2520	8.76E-07	1.01E-05
PDGFA	0.0182	7.27E-01	8.14E-01
PDGFB	-0.0371	4.76E-01	6.05E-01
PDGFC	-0.0515	3.22E-01	4.53E-01
PDGFD	-0.1180	2.30E-02	5.76E-02
PDGFRA	-0.0614	2.38E-01	3.62E-01
PDGFRB	-0.1013	5.12E-02	1.10E-01
PDGFRL	0.1464	4.71E-03	1.57E-02
PDHA1	-0.1644	1.49E-03	6.01E-03
PDHA2	0.1195	2.13E-02	5.40E-02
PDHB	-0.1576	2.33E-03	8.74E-03
PDHX	-0.2034	7.95E-05	5.01E-04
PDIA2	0.1441	5.41E-03	1.76E-02
PDIA3P	0.0277	5.95E-01	7.09E-01
PDIA3	0.0103	8.43E-01	8.98E-01
PDIA4	-0.1145	2.74E-02	6.64E-02
PDIA5	-0.1169	2.43E-02	6.02E-02
PDIA6	0.1147	2.72E-02	6.60E-02
PDIK1L	-0.0069	8.95E-01	9.33E-01
PDILT	-0.0127	8.07E-01	8.73E-01
PDK1	-0.0728	1.62E-01	2.68E-01
PDK2	-0.1550	2.76E-03	1.01E-02
PDK3	0.0314	5.46E-01	6.67E-01
PDK4	-0.2965	5.77E-09	1.14E-07
PDLIM1	-0.1399	6.98E-03	2.17E-02
PDLIM2	-0.2390	3.24E-06	3.18E-05
PDLIM3	-0.0175	7.37E-01	8.22E-01
PDLIM4	-0.0386	4.59E-01	5.90E-01
PDLIM5	-0.1866	3.02E-04	1.57E-03
PDLIM7	0.1085	3.68E-02	8.43E-02
PDP1	0.1336	9.97E-03	2.92E-02
PDP2	-0.2070	5.90E-05	3.90E-04
PDPK1	-0.0075	8.85E-01	9.27E-01
PDPN	-0.0393	4.50E-01	5.82E-01
PDPR	-0.0694	1.83E-01	2.95E-01
PDRG1	0.1830	3.96E-04	1.96E-03
PDS5A	0.1270	1.44E-02	3.94E-02
PDS5B	0.0639	2.19E-01	3.40E-01

PDSS1	0.2108	4.25E-05	2.95E-04
PDSS2	-0.1831	3.94E-04	1.96E-03
PDX1	0.2215	1.67E-05	1.32E-04
PDXDC1	-0.1629	1.64E-03	6.55E-03
PDXDC2	0.1103	3.38E-02	7.87E-02
PDXK	0.0083	8.73E-01	9.18E-01
PDXP	-0.1110	3.26E-02	7.65E-02
PDYN	0.0691	1.84E-01	2.97E-01
PDZD11	0.2010	9.65E-05	5.92E-04
PDZD2	-0.0477	3.60E-01	4.92E-01
PDZD3	0.0598	2.50E-01	3.76E-01
PDZD4	-0.1342	9.66E-03	2.85E-02
PDZD7	0.0780	1.34E-01	2.32E-01
PDZD8	-0.0857	9.92E-02	1.85E-01
PDZD9	0.0551	2.90E-01	4.19E-01
PDZK1IP1	-0.0800	1.24E-01	2.19E-01
PDZK1P1	0.1519	3.36E-03	1.19E-02
PDZK1	0.0926	7.48E-02	1.48E-01
PDZRN3	-0.1626	1.68E-03	6.65E-03
PDZRN4	-0.1740	7.64E-04	3.41E-03
PEA15	0.4015	8.40E-16	7.00E-14
PEAR1	-0.1365	8.46E-03	2.55E-02
PEBP1	-0.2813	3.54E-08	5.73E-07
PEBP4	-0.2026	8.51E-05	5.32E-04
PECAM1	-0.1437	5.57E-03	1.80E-02
PECI	-0.2185	2.19E-05	1.67E-04
PECR	-0.2353	4.62E-06	4.35E-05
PEF1	-0.1096	3.48E-02	8.07E-02
PEG10	0.1481	4.25E-03	1.44E-02
PEG3AS	-0.0934	7.25E-02	1.44E-01
PEG3	0.0738	1.56E-01	2.62E-01
PELI1	0.1230	1.77E-02	4.66E-02
PELI2	-0.1080	3.76E-02	8.58E-02
PELI3	0.0142	7.85E-01	8.57E-01
PELO	0.0638	2.20E-01	3.40E-01
PELP1	0.2081	5.38E-05	3.60E-04
PEMT	-0.2770	5.81E-08	8.92E-07
PENK	-0.0387	4.57E-01	5.88E-01
PEPD	-0.2149	2.98E-05	2.18E-04
PER1	-0.1403	6.78E-03	2.12E-02
PER2	0.0124	8.12E-01	8.77E-01
PER3	0.0250	6.31E-01	7.39E-01
PER4	0.0725	1.64E-01	2.71E-01
PERP	0.1124	3.04E-02	7.22E-02

PES1	0.2281	9.10E-06	7.74E-05
PET112L	-0.3675	2.65E-13	1.31E-11
PEX10	-0.1178	2.33E-02	5.81E-02
PEX11A	-0.1585	2.20E-03	8.33E-03
PEX11B	0.3192	3.10E-10	8.31E-09
PEX11G	-0.3442	9.29E-12	3.35E-10
PEX12	-0.1803	4.82E-04	2.31E-03
PEX13	-0.3283	8.98E-11	2.65E-09
PEX14	-0.2201	1.89E-05	1.47E-04
PEX16	-0.0463	3.73E-01	5.06E-01
PEX19	0.0727	1.62E-01	2.69E-01
PEX1	-0.1730	8.17E-04	3.61E-03
PEX26	-0.1430	5.80E-03	1.86E-02
PEX2	-0.0700	1.79E-01	2.90E-01
PEX3	-0.0826	1.12E-01	2.03E-01
PEX5L	0.0784	1.32E-01	2.30E-01
PEX5	-0.1777	5.87E-04	2.72E-03
PEX6	-0.1150	2.68E-02	6.52E-02
PEX7	0.0240	6.46E-01	7.50E-01
PF4V1	-0.0794	1.27E-01	2.23E-01
PF4	-0.0872	9.33E-02	1.76E-01
PFAS	0.1167	2.45E-02	6.06E-02
PFDN1	0.1540	2.94E-03	1.06E-02
PFDN2	0.3475	5.71E-12	2.18E-10
PFDN4	0.2696	1.34E-07	1.90E-06
PFDN5	0.0368	4.80E-01	6.09E-01
PFDN6	0.1404	6.76E-03	2.11E-02
PFKFB1	-0.1841	3.65E-04	1.84E-03
PFKFB2	0.1794	5.18E-04	2.45E-03
PFKFB3	-0.0054	9.18E-01	9.48E-01
PFKFB4	0.1904	2.24E-04	1.22E-03
PFKL	-0.0775	1.36E-01	2.36E-01
PFKM	-0.1562	2.55E-03	9.42E-03
PFKP	0.0408	4.33E-01	5.65E-01
PFN1	0.0088	8.65E-01	9.13E-01
PFN2	0.0340	5.14E-01	6.38E-01
PFN3	0.0215	6.80E-01	7.78E-01
PFN4	0.1128	2.98E-02	7.10E-02
PGA3	0.0197	7.05E-01	7.98E-01
PGA4	0.0333	5.23E-01	6.47E-01
PGA5	0.0508	3.29E-01	4.60E-01
PGAM1	-0.2436	2.05E-06	2.14E-05
PGAM2	0.1301	1.22E-02	3.43E-02
PGAM4	-0.1207	2.01E-02	5.15E-02

PGAM5	-0.0977	6.01E-02	1.24E-01
PGAP1	0.1138	2.84E-02	6.84E-02
PGAP2	0.1323	1.07E-02	3.09E-02
PGAP3	-0.0443	3.94E-01	5.27E-01
PGBD1	0.1897	2.37E-04	1.28E-03
PGBD2	0.2087	5.11E-05	3.44E-04
PGBD3	0.0511	3.26E-01	4.57E-01
PGBD4	-0.0072	8.90E-01	9.30E-01
PGBD5	0.0171	7.43E-01	8.27E-01
PGCP	-0.0505	3.32E-01	4.63E-01
PGC	0.2010	9.69E-05	5.93E-04
PGD	0.0311	5.50E-01	6.70E-01
PGF	0.0398	4.45E-01	5.77E-01
PGGT1B	-0.1490	4.02E-03	1.38E-02
PGK1	0.0115	8.25E-01	8.85E-01
PGK2	0.0658	2.06E-01	3.24E-01
PGLS	-0.1288	1.30E-02	3.63E-02
PGLYRP1	-0.0960	6.48E-02	1.32E-01
PGLYRP2	-0.0286	5.83E-01	6.99E-01
PGLYRP3	0.0995	5.54E-02	1.17E-01
PGLYRP4	0.1780	5.73E-04	2.67E-03
PGM1	-0.2908	1.16E-08	2.12E-07
PGM2L1	0.0266	6.09E-01	7.21E-01
PGM2	-0.0142	7.85E-01	8.57E-01
PGM3	0.1917	2.03E-04	1.12E-03
PGM5P2	-0.0789	1.29E-01	2.26E-01
PGM5	-0.2438	2.02E-06	2.11E-05
PGPEP1L	-0.0240	6.45E-01	7.50E-01
PGPEP1	-0.1166	2.47E-02	6.09E-02
PGP	0.1474	4.45E-03	1.50E-02
PGRMC1	-0.2094	4.80E-05	3.27E-04
PGRMC2	-0.2673	1.73E-07	2.39E-06
PGR	-0.0711	1.72E-01	2.81E-01
PGS1	0.3568	1.39E-12	6.02E-11
PHACTR1	0.1068	3.98E-02	8.98E-02
PHACTR2	-0.0259	6.19E-01	7.28E-01
PHACTR3	-0.1479	4.31E-03	1.46E-02
PHACTR4	-0.0161	7.57E-01	8.37E-01
PHAX	-0.0137	7.92E-01	8.62E-01
PHB2	-0.1258	1.53E-02	4.14E-02
PHB	0.0225	6.65E-01	7.65E-01
PHC1	0.1377	7.93E-03	2.41E-02
PHC2	0.0069	8.95E-01	9.33E-01
PHC3	0.1418	6.22E-03	1.97E-02

PHEX	0.1612	1.84E-03	7.20E-03
PHF10	0.2012	9.53E-05	5.86E-04
PHF11	0.0680	1.91E-01	3.06E-01
PHF12	0.2747	7.58E-08	1.14E-06
PHF13	0.0707	1.74E-01	2.85E-01
PHF14	0.0148	7.77E-01	8.51E-01
PHF15	0.1518	3.38E-03	1.19E-02
PHF16	0.0777	1.35E-01	2.34E-01
PHF17	-0.1438	5.52E-03	1.79E-02
PHF19	0.4170	4.88E-17	5.29E-15
PHF1	0.1494	3.93E-03	1.36E-02
PHF20L1	-0.0354	4.96E-01	6.23E-01
PHF20	0.1460	4.84E-03	1.61E-02
PHF21A	0.1811	4.57E-04	2.21E-03
PHF21B	0.0608	2.42E-01	3.67E-01
PHF23	0.0139	7.89E-01	8.60E-01
PHF2	0.1178	2.33E-02	5.82E-02
PHF3	0.0367	4.81E-01	6.09E-01
PHF5A	0.2333	5.61E-06	5.10E-05
PHF6	0.2902	1.25E-08	2.26E-07
PHF7	-0.1051	4.30E-02	9.55E-02
PHF8	-0.0287	5.81E-01	6.97E-01
PHGDH	-0.0122	8.15E-01	8.78E-01
PHGR1	-0.0593	2.54E-01	3.80E-01
PHIP	0.3284	8.89E-11	2.63E-09
PHKA1	-0.0102	8.45E-01	8.99E-01
PHKA2	0.0802	1.23E-01	2.18E-01
PHKB	-0.2914	1.08E-08	1.99E-07
PHKG1	0.0605	2.45E-01	3.69E-01
PHKG2	-0.0111	8.31E-01	8.89E-01
PHLDA1	-0.2718	1.05E-07	1.52E-06
PHLDA2	0.0985	5.81E-02	1.21E-01
PHLDA3	-0.0866	9.58E-02	1.80E-01
PHLDB1	0.0552	2.89E-01	4.18E-01
PHLDB2	-0.1838	3.71E-04	1.86E-03
PHLDB3	0.0737	1.57E-01	2.62E-01
PHLPP1	-0.1274	1.40E-02	3.86E-02
PHLPP2	-0.0951	6.73E-02	1.36E-01
PHOSPHO1	-0.1168	2.44E-02	6.04E-02
PHOSPHO2	0.2570	5.22E-07	6.35E-06
PHOX2A	0.1856	3.24E-04	1.67E-03
PHOX2B	-0.0142	7.85E-01	8.57E-01
PHPT1	0.2074	5.72E-05	3.80E-04
PHRF1	0.0595	2.53E-01	3.78E-01

PHTF1	-0.0717	1.68E-01	2.77E-01
PHTF2	0.1390	7.32E-03	2.26E-02
PHYHD1	-0.2679	1.62E-07	2.24E-06
PHYHIPL	0.0999	5.46E-02	1.15E-01
PHYHIP	-0.1397	7.05E-03	2.19E-02
PHYH	-0.2042	7.44E-05	4.75E-04
PI15	-0.0102	8.44E-01	8.98E-01
PI16	-0.1268	1.45E-02	3.97E-02
PI3	0.1263	1.49E-02	4.06E-02
PI4K2A	-0.1538	2.97E-03	1.07E-02
PI4K2B	-0.1697	1.04E-03	4.43E-03
PI4KAP1	0.3132	6.87E-10	1.68E-08
PI4KAP2	0.1941	1.68E-04	9.50E-04
PI4KA	0.1199	2.09E-02	5.31E-02
PI4KB	0.6144	7.10E-40	2.02E-36
PIAS1	0.0841	1.06E-01	1.94E-01
PIAS2	-0.0565	2.77E-01	4.06E-01
PIAS3	0.3812	2.81E-14	1.66E-12
PIAS4	0.0467	3.69E-01	5.02E-01
PIBF1	0.1185	2.25E-02	5.65E-02
PICALM	-0.0018	9.73E-01	9.83E-01
PICK1	0.0252	6.28E-01	7.36E-01
PID1	-0.1256	1.55E-02	4.17E-02
PIF1	0.3867	1.11E-14	7.09E-13
PIGA	0.2210	1.74E-05	1.37E-04
PIGB	-0.0009	9.86E-01	9.91E-01
PIGC	0.5152	1.54E-26	1.02E-23
PIGF	0.0580	2.65E-01	3.92E-01
PIGG	0.1201	2.07E-02	5.28E-02
PIGH	-0.0272	6.01E-01	7.14E-01
PIGK	-0.0785	1.31E-01	2.29E-01
PIGL	-0.0858	9.91E-02	1.84E-01
PIGM	0.3496	4.20E-12	1.65E-10
PIGN	0.0147	7.78E-01	8.52E-01
PIGO	-0.0049	9.24E-01	9.52E-01
PIGP	-0.1488	4.06E-03	1.39E-02
PIGQ	-0.1470	4.55E-03	1.53E-02
PIGR	-0.0375	4.72E-01	6.01E-01
PIGS	0.3217	2.22E-10	6.13E-09
PIGT	0.1594	2.07E-03	7.94E-03
PIGU	0.1930	1.84E-04	1.03E-03
PIGV	-0.2665	1.88E-07	2.57E-06
PIGW	0.0585	2.61E-01	3.87E-01
PIGX	0.2544	6.83E-07	8.09E-06

PIGY	-0.1720	8.77E-04	3.84E-03
PIGZ	0.2981	4.72E-09	9.50E-08
PIH1D1	0.1024	4.87E-02	1.05E-01
PIH1D2	0.1131	2.95E-02	7.04E-02
PIK3AP1	-0.0215	6.80E-01	7.77E-01
PIK3C2A	-0.0899	8.37E-02	1.61E-01
PIK3C2B	0.0311	5.50E-01	6.70E-01
PIK3C2G	-0.1784	5.55E-04	2.60E-03
PIK3C3	0.0033	9.49E-01	9.68E-01
PIK3CA	-0.0184	7.23E-01	8.12E-01
PIK3CB	-0.0396	4.47E-01	5.78E-01
PIK3CD	-0.0496	3.41E-01	4.72E-01
PIK3CG	-0.1087	3.63E-02	8.35E-02
PIK3IP1	-0.1053	4.28E-02	9.50E-02
PIK3R1	-0.1612	1.84E-03	7.17E-03
PIK3R2	0.0609	2.42E-01	3.66E-01
PIK3R3	-0.2464	1.55E-06	1.67E-05
PIK3R4	-0.0897	8.44E-02	1.62E-01
PIK3R5	-0.1040	4.52E-02	9.94E-02
PIK3R6	0.0908	8.07E-02	1.57E-01
PIKFYVE	0.0243	6.41E-01	7.47E-01
PILRA	-0.0123	8.14E-01	8.78E-01
PILRB	0.1846	3.52E-04	1.78E-03
PIM1	-0.0164	7.53E-01	8.34E-01
PIM2	0.1148	2.70E-02	6.56E-02
PIM3	-0.0673	1.96E-01	3.11E-01
PIN1L	0.0999	5.46E-02	1.15E-01
PIN1	-0.0265	6.11E-01	7.22E-01
PIN4	0.0649	2.12E-01	3.31E-01
PINK1	-0.4210	2.29E-17	2.74E-15
PINX1	0.0495	3.42E-01	4.74E-01
PION	-0.0935	7.21E-02	1.44E-01
PIP4K2A	0.0687	1.87E-01	3.00E-01
PIP4K2B	0.2517	9.02E-07	1.03E-05
PIP4K2C	0.0493	3.43E-01	4.75E-01
PIP5K1A	0.3889	7.58E-15	5.00E-13
PIP5K1B	-0.0242	6.42E-01	7.47E-01
PIP5K1C	0.0557	2.85E-01	4.14E-01
PIP5K1P1	0.2255	1.15E-05	9.51E-05
PIP5KL1	0.2195	2.00E-05	1.54E-04
PIPOX	-0.1399	6.96E-03	2.17E-02
PIPSL	0.4383	7.52E-19	1.39E-16
PIP	-0.0284	5.85E-01	7.01E-01
PIRT	-0.0021	9.68E-01	9.80E-01

PIR	0.0903	8.23E-02	1.59E-01
PISD	0.0754	1.47E-01	2.50E-01
PITPNA	-0.1871	2.91E-04	1.52E-03
PITPNB	-0.0411	4.30E-01	5.62E-01
PITPNC1	-0.0754	1.47E-01	2.50E-01
PITPNM1	-0.0378	4.68E-01	5.97E-01
PITPNM2	-0.0855	1.00E-01	1.86E-01
PITPNM3	-0.0823	1.14E-01	2.06E-01
PITRM1	-0.0326	5.32E-01	6.54E-01
PITX1	0.1885	2.61E-04	1.38E-03
PITX2	0.0785	1.31E-01	2.29E-01
PITX3	0.0820	1.15E-01	2.07E-01
PIWIL1	0.0483	3.53E-01	4.85E-01
PIWIL2	-0.0799	1.24E-01	2.20E-01
PIWIL3	0.1771	6.11E-04	2.81E-03
PIWIL4	0.2542	7.01E-07	8.24E-06
PJA1	0.1591	2.11E-03	8.07E-03
PJA2	-0.1752	6.98E-04	3.16E-03
PKD1L1	0.0581	2.65E-01	3.92E-01
PKD1L2	0.0713	1.70E-01	2.80E-01
PKD1L3	-0.1261	1.51E-02	4.08E-02
PKD1	0.1679	1.17E-03	4.92E-03
PKD2L1	-0.0131	8.01E-01	8.69E-01
PKD2L2	0.0530	3.09E-01	4.39E-01
PKD2	-0.0555	2.87E-01	4.16E-01
PKDCC	0.1946	1.63E-04	9.23E-04
PKDREJ	0.0032	9.50E-01	9.69E-01
PKHD1L1	0.0016	9.75E-01	9.84E-01
PKHD1	-0.0400	4.42E-01	5.74E-01
PKIA	0.1545	2.84E-03	1.03E-02
PKIB	0.0751	1.49E-01	2.52E-01
PKIG	-0.1826	4.08E-04	2.01E-03
PKLR	-0.0301	5.64E-01	6.82E-01
PKM2	0.2112	4.12E-05	2.87E-04
PKMYT1	0.3666	3.01E-13	1.48E-11
PKN1	0.1509	3.58E-03	1.25E-02
PKN2	0.0635	2.23E-01	3.44E-01
PKN3	0.2445	1.87E-06	1.98E-05
PKNOX1	0.4147	7.51E-17	7.84E-15
PKNOX2	-0.0356	4.94E-01	6.21E-01
PKP1	-0.0468	3.68E-01	5.01E-01
PKP2	-0.1229	1.79E-02	4.69E-02
PKP3	0.0796	1.26E-01	2.22E-01
PKP4	0.0207	6.91E-01	7.86E-01

PL-5283	-0.0683	1.90E-01	3.03E-01
PLA1A	-0.0669	1.99E-01	3.15E-01
PLA2G10	0.1435	5.62E-03	1.81E-02
PLA2G12A	-0.2748	7.45E-08	1.12E-06
PLA2G12B	-0.0576	2.69E-01	3.96E-01
PLA2G15	-0.1614	1.82E-03	7.10E-03
PLA2G16	-0.2155	2.85E-05	2.10E-04
PLA2G1B	0.1001	5.41E-02	1.14E-01
PLA2G2A	-0.0716	1.69E-01	2.78E-01
PLA2G2C	0.0435	4.04E-01	5.37E-01
PLA2G2D	-0.0232	6.56E-01	7.58E-01
PLA2G2E	-0.0010	9.85E-01	9.90E-01
PLA2G2F	-0.0094	8.57E-01	9.08E-01
PLA2G3	0.0514	3.23E-01	4.54E-01
PLA2G4A	-0.0967	6.28E-02	1.29E-01
PLA2G4C	0.0013	9.80E-01	9.87E-01
PLA2G4D	0.2026	8.47E-05	5.30E-04
PLA2G4E	0.1328	1.04E-02	3.02E-02
PLA2G4F	0.1496	3.88E-03	1.34E-02
PLA2G5	-0.1612	1.84E-03	7.17E-03
PLA2G6	0.1651	1.42E-03	5.75E-03
PLA2G7	-0.0130	8.03E-01	8.70E-01
PLA2R1	-0.0486	3.51E-01	4.83E-01
PLAA	0.0203	6.97E-01	7.91E-01
PLAC1	0.2119	3.87E-05	2.73E-04
PLAC2	0.0336	5.19E-01	6.43E-01
PLAC4	0.0307	5.56E-01	6.75E-01
PLAC8L1	0.1327	1.05E-02	3.04E-02
PLAC8	0.0023	9.65E-01	9.78E-01
PLAC9	-0.1343	9.60E-03	2.83E-02
PLAG1	0.0660	2.04E-01	3.22E-01
PLAGL1	0.0719	1.67E-01	2.75E-01
PLAGL2	0.2880	1.62E-08	2.87E-07
PLAT	-0.1875	2.81E-04	1.47E-03
PLAUR	0.0422	4.18E-01	5.50E-01
PLAU	-0.0330	5.27E-01	6.49E-01
PLB1	-0.1168	2.44E-02	6.04E-02
PLBD1	0.1074	3.87E-02	8.79E-02
PLBD2	-0.1984	1.19E-04	7.09E-04
PLCB1	0.1029	4.76E-02	1.04E-01
PLCB2	-0.0632	2.25E-01	3.46E-01
PLCB3	0.2658	2.03E-07	2.75E-06
PLCB4	-0.0472	3.65E-01	4.98E-01
PLCD1	-0.0515	3.23E-01	4.53E-01

PLCD3	0.1064	4.05E-02	9.11E-02
PLCD4	0.2617	3.18E-07	4.10E-06
PLCE1	0.0744	1.53E-01	2.57E-01
PLCG1	0.0025	9.62E-01	9.76E-01
PLCG2	-0.1990	1.14E-04	6.83E-04
PLCH1	0.1141	2.80E-02	6.75E-02
PLCH2	-0.0544	2.96E-01	4.25E-01
PLCL1	-0.0549	2.92E-01	4.21E-01
PLCL2	-0.1179	2.32E-02	5.79E-02
PLCXD1	0.1513	3.50E-03	1.23E-02
PLCXD2	0.0479	3.57E-01	4.89E-01
PLCXD3	-0.0962	6.42E-02	1.31E-01
PLCZ1	0.0703	1.76E-01	2.87E-01
PLD1	0.0080	8.78E-01	9.22E-01
PLD2	0.0033	9.50E-01	9.69E-01
PLD3	-0.0750	1.50E-01	2.53E-01
PLD4	0.0102	8.45E-01	8.99E-01
PLD5	0.1489	4.04E-03	1.39E-02
PLD6	0.1027	4.81E-02	1.04E-01
PLDN	-0.0858	9.90E-02	1.84E-01
PLEC	0.0351	5.01E-01	6.27E-01
PLEK2	0.1947	1.61E-04	9.14E-04
PLEKHA1	-0.0307	5.55E-01	6.75E-01
PLEKHA2	0.0097	8.52E-01	9.04E-01
PLEKHA3	0.0297	5.68E-01	6.86E-01
PLEKHA4	0.0053	9.18E-01	9.48E-01
PLEKHA5	0.0727	1.62E-01	2.69E-01
PLEKHA6	0.1773	6.04E-04	2.79E-03
PLEKHA7	0.0062	9.06E-01	9.41E-01
PLEKHA8	0.0646	2.14E-01	3.34E-01
PLEKHA9	0.1964	1.41E-04	8.17E-04
PLEKHB1	0.1922	1.95E-04	1.08E-03
PLEKHB2	0.0773	1.37E-01	2.37E-01
PLEKHF1	0.0571	2.72E-01	4.00E-01
PLEKHF2	0.1818	4.32E-04	2.10E-03
PLEKHG1	-0.1893	2.46E-04	1.31E-03
PLEKHG2	0.1613	1.83E-03	7.15E-03
PLEKHG3	0.0155	7.66E-01	8.42E-01
PLEKHG4B	0.2085	5.20E-05	3.50E-04
PLEKHG4	0.1299	1.23E-02	3.46E-02
PLEKHG5	-0.0404	4.38E-01	5.70E-01
PLEKHG6	0.2078	5.51E-05	3.68E-04
PLEKHG7	0.0326	5.31E-01	6.54E-01
PLEKHH1	0.1351	9.20E-03	2.74E-02

PLEKHH2	0.1621	1.73E-03	6.81E-03
PLEKHH3	0.1117	3.14E-02	7.42E-02
PLEKHJ1	-0.0583	2.62E-01	3.89E-01
PLEKHM1P	0.2556	6.06E-07	7.24E-06
PLEKHM1	0.0854	1.01E-01	1.87E-01
PLEKHM2	-0.0575	2.69E-01	3.97E-01
PLEKHM3	-0.0644	2.16E-01	3.36E-01
PLEKHN1	0.0541	2.98E-01	4.28E-01
PLEKHO1	0.0446	3.92E-01	5.25E-01
PLEKHO2	-0.0295	5.71E-01	6.89E-01
PLEK	-0.0140	7.89E-01	8.60E-01
PLGLA	-0.2842	2.53E-08	4.25E-07
PLGLB2	-0.1600	1.99E-03	7.65E-03
PLG	-0.1798	5.00E-04	2.38E-03
PLIN1	-0.1885	2.60E-04	1.38E-03
PLIN2	-0.0752	1.49E-01	2.52E-01
PLIN3	0.0557	2.84E-01	4.13E-01
PLIN4	-0.1182	2.28E-02	5.72E-02
PLIN5	-0.0924	7.53E-02	1.49E-01
PLK1S1	0.1569	2.45E-03	9.10E-03
PLK1	0.4214	2.11E-17	2.54E-15
PLK2	-0.1395	7.13E-03	2.21E-02
PLK3	-0.0639	2.20E-01	3.40E-01
PLK4	0.3467	6.43E-12	2.42E-10
PLK5P	0.1847	3.49E-04	1.77E-03
PLLP	0.0129	8.05E-01	8.71E-01
PLN	-0.1697	1.03E-03	4.41E-03
PLOD1	-0.0702	1.77E-01	2.88E-01
PLOD2	0.1022	4.93E-02	1.06E-01
PLOD3	0.0476	3.61E-01	4.93E-01
PLP1	-0.1122	3.07E-02	7.29E-02
PLP2	0.2971	5.38E-09	1.07E-07
PLRG1	-0.0432	4.07E-01	5.40E-01
PLS1	-0.0508	3.29E-01	4.60E-01
PLS3	-0.0764	1.42E-01	2.43E-01
PLSCR1	0.0172	7.42E-01	8.26E-01
PLSCR2	0.2147	3.05E-05	2.22E-04
PLSCR3	-0.0942	6.99E-02	1.40E-01
PLSCR4	-0.1971	1.33E-04	7.80E-04
PLSCR5	0.0257	6.21E-01	7.31E-01
PLTP	-0.0567	2.76E-01	4.05E-01
PLUNC	0.2841	2.56E-08	4.28E-07
PLVAP	-0.2151	2.95E-05	2.16E-04
PLXDC1	0.0910	7.99E-02	1.56E-01

PLXDC2	-0.1291	1.28E-02	3.59E-02
PLXNA1	0.2169	2.50E-05	1.88E-04
PLXNA2	0.0443	3.95E-01	5.27E-01
PLXNA3	0.1573	2.37E-03	8.86E-03
PLXNA4	-0.2364	4.14E-06	3.94E-05
PLXNB1	0.0396	4.47E-01	5.79E-01
PLXNB2	-0.0376	4.71E-01	6.00E-01
PLXNB3	0.1689	1.09E-03	4.62E-03
PLXNC1	-0.0066	8.99E-01	9.36E-01
PLXND1	0.0115	8.25E-01	8.86E-01
PM20D1	-0.0338	5.16E-01	6.41E-01
PM20D2	0.2415	2.53E-06	2.56E-05
PMAIP1	0.1390	7.35E-03	2.27E-02
PMCHL1	0.1065	4.03E-02	9.07E-02
PMCHL2	0.0695	1.82E-01	2.94E-01
PMCH	0.1793	5.20E-04	2.46E-03
PMEPA1	0.0129	8.04E-01	8.70E-01
PMF1	0.3757	6.99E-14	3.80E-12
PMFBP1	0.0176	7.35E-01	8.21E-01
PML	0.0292	5.75E-01	6.92E-01
PMM1	0.0318	5.41E-01	6.62E-01
PMM2	-0.0130	8.02E-01	8.70E-01
PMP22	-0.0659	2.06E-01	3.23E-01
PMP2	-0.0559	2.83E-01	4.12E-01
PMPCA	-0.0620	2.34E-01	3.57E-01
PMPCB	-0.2088	5.06E-05	3.42E-04
PMS1	0.3171	4.15E-10	1.08E-08
PMS2CL	0.1582	2.24E-03	8.45E-03
PMS2L11	0.1719	8.88E-04	3.88E-03
PMS2L1	-0.1329	1.04E-02	3.01E-02
PMS2L2	-0.2000	1.05E-04	6.34E-04
PMS2L3	0.3185	3.43E-10	9.14E-09
PMS2L4	0.1436	5.58E-03	1.80E-02
PMS2L5	-0.1384	7.59E-03	2.33E-02
PMS2	0.0837	1.08E-01	1.97E-01
PMVK	0.1695	1.05E-03	4.47E-03
PNCK	0.1539	2.95E-03	1.06E-02
PNKD	-0.0005	9.92E-01	9.95E-01
PNKP	0.0736	1.57E-01	2.63E-01
PNLDC1	0.1331	1.03E-02	2.99E-02
PNLIPRP1	0.0324	5.34E-01	6.56E-01
PNLIPRP2	0.0605	2.45E-01	3.69E-01
PNLIPRP3	-0.0364	4.85E-01	6.13E-01
PNLIP	-0.0481	3.56E-01	4.88E-01

PNMA1	0.1670	1.24E-03	5.18E-03
PNMA2	-0.0037	9.43E-01	9.65E-01
PNMA3	0.0600	2.49E-01	3.74E-01
PNMA5	0.0476	3.61E-01	4.93E-01
PNMA6A	-0.0395	4.48E-01	5.79E-01
PNMAL1	0.0777	1.35E-01	2.34E-01
PNMAL2	-0.1259	1.52E-02	4.12E-02
PNMT	-0.0207	6.91E-01	7.86E-01
PNN	0.1857	3.23E-04	1.66E-03
PNO1	0.0935	7.22E-02	1.44E-01
PNOC	0.0348	5.03E-01	6.29E-01
PNPLA1	0.0745	1.52E-01	2.56E-01
PNPLA2	-0.1033	4.67E-02	1.02E-01
PNPLA3	-0.1078	3.79E-02	8.64E-02
PNPLA4	-0.3182	3.59E-10	9.50E-09
PNPLA5	-0.1648	1.45E-03	5.86E-03
PNPLA6	-0.0550	2.90E-01	4.19E-01
PNPLA7	-0.2852	2.26E-08	3.85E-07
PNPLA8	-0.2042	7.42E-05	4.74E-04
PNPO	-0.2840	2.59E-08	4.31E-07
PNPT1	0.0639	2.20E-01	3.40E-01
PNP	0.0368	4.80E-01	6.08E-01
PNRC1	0.0036	9.44E-01	9.66E-01
PNRC2	-0.0082	8.75E-01	9.19E-01
POC1A	0.2658	2.04E-07	2.76E-06
POC1B	0.0788	1.30E-01	2.27E-01
POC5	0.2741	8.12E-08	1.20E-06
PODNL1	-0.0363	4.86E-01	6.14E-01
PODN	-0.1338	9.90E-03	2.90E-02
PODXL2	0.1131	2.94E-02	7.04E-02
PODXL	-0.0701	1.78E-01	2.89E-01
POF1B	0.1731	8.14E-04	3.60E-03
POFUT1	-0.0237	6.49E-01	7.53E-01
POFUT2	0.1291	1.28E-02	3.58E-02
POGK	0.4868	1.80E-23	7.47E-21
POGZ	0.5691	3.17E-33	3.94E-30
POLA1	0.4065	3.41E-16	3.04E-14
POLA2	0.3773	5.36E-14	3.00E-12
POLB	0.0666	2.01E-01	3.17E-01
POLD1	0.3911	5.24E-15	3.60E-13
POLD2	0.0402	4.40E-01	5.71E-01
POLD3	0.4099	1.82E-16	1.75E-14
POLD4	-0.2375	3.74E-06	3.61E-05
POLDIP2	-0.1013	5.11E-02	1.09E-01

POLDIP3	0.1526	3.21E-03	1.14E-02
POLE2	0.3605	7.97E-13	3.65E-11
POLE3	0.3061	1.73E-09	3.92E-08
POLE4	0.0301	5.63E-01	6.81E-01
POLE	0.2411	2.62E-06	2.63E-05
POLG2	0.4207	2.40E-17	2.85E-15
POLG	-0.1612	1.84E-03	7.19E-03
POLH	0.1042	4.49E-02	9.89E-02
POLI	0.0390	4.53E-01	5.84E-01
POLK	-0.0249	6.33E-01	7.40E-01
POLL	-0.0111	8.32E-01	8.90E-01
POLM	0.1993	1.11E-04	6.64E-04
POLN	-0.1673	1.22E-03	5.09E-03
POLQ	0.3929	3.83E-15	2.72E-13
POLR1A	0.0231	6.58E-01	7.59E-01
POLR1B	0.0160	7.59E-01	8.38E-01
POLR1C	0.0566	2.77E-01	4.05E-01
POLR1D	0.1255	1.56E-02	4.19E-02
POLR1E	-0.0019	9.71E-01	9.81E-01
POLR2A	0.0082	8.75E-01	9.19E-01
POLR2B	0.0742	1.54E-01	2.58E-01
POLR2C	-0.2621	3.04E-07	3.95E-06
POLR2D	0.2711	1.14E-07	1.63E-06
POLR2E	-0.2033	7.99E-05	5.03E-04
POLR2F	0.0807	1.21E-01	2.15E-01
POLR2G	0.0891	8.66E-02	1.66E-01
POLR2H	0.2089	5.03E-05	3.40E-04
POLR2I	0.1059	4.16E-02	9.29E-02
POLR2J2	0.0159	7.60E-01	8.39E-01
POLR2J3	0.1407	6.63E-03	2.08E-02
POLR2J4	0.2128	3.57E-05	2.55E-04
POLR2J	-0.0158	7.61E-01	8.39E-01
POLR2K	0.1221	1.87E-02	4.86E-02
POLR2L	-0.0795	1.26E-01	2.22E-01
POLR3A	0.0326	5.32E-01	6.54E-01
POLR3B	-0.0230	6.59E-01	7.60E-01
POLR3C	0.5482	1.73E-30	1.82E-27
POLR3D	0.0688	1.86E-01	2.99E-01
POLR3E	-0.0201	7.00E-01	7.93E-01
POLR3F	0.3222	2.07E-10	5.72E-09
POLR3GL	0.1893	2.45E-04	1.31E-03
POLR3G	0.0091	8.61E-01	9.10E-01
POLR3H	-0.0722	1.65E-01	2.73E-01
POLR3K	0.1011	5.17E-02	1.10E-01

POLRMT	-0.0403	4.40E-01	5.71E-01
POM121C	0.1363	8.57E-03	2.58E-02
POM121L10P	0.1860	3.16E-04	1.63E-03
POM121L1P	0.0902	8.26E-02	1.60E-01
POM121L2	0.0833	1.09E-01	1.99E-01
POM121L4P	0.0025	9.62E-01	9.76E-01
POM121L8P	0.0038	9.42E-01	9.64E-01
POM121L9P	-0.0665	2.01E-01	3.17E-01
POM121	0.0934	7.23E-02	1.44E-01
POMC	-0.1181	2.29E-02	5.72E-02
POMGNT1	0.0052	9.20E-01	9.49E-01
POMP	-0.0002	9.97E-01	9.98E-01
POMT1	0.1080	3.77E-02	8.60E-02
POMT2	0.1438	5.52E-03	1.79E-02
POMZP3	0.1018	5.00E-02	1.08E-01
PON1	-0.1870	2.92E-04	1.53E-03
PON2	-0.0509	3.28E-01	4.59E-01
PON3	-0.2437	2.03E-06	2.12E-05
POP1	0.3092	1.16E-09	2.71E-08
POP4	0.1137	2.86E-02	6.87E-02
POP5	0.0705	1.75E-01	2.86E-01
POP7	0.1260	1.51E-02	4.10E-02
POPDC2	-0.0694	1.82E-01	2.94E-01
POPDC3	0.1813	4.49E-04	2.17E-03
PORCN	0.1367	8.39E-03	2.53E-02
POR	-0.2856	2.16E-08	3.70E-07
POSTN	0.0035	9.47E-01	9.67E-01
POT1	-0.0331	5.24E-01	6.48E-01
POTEA	0.1513	3.48E-03	1.22E-02
POTEB	0.0502	3.35E-01	4.66E-01
POTEC	0.0825	1.12E-01	2.04E-01
POTED	0.1377	7.90E-03	2.41E-02
POTEE	0.0175	7.37E-01	8.23E-01
POTEF	0.0862	9.72E-02	1.82E-01
POTEG	0.0479	3.57E-01	4.89E-01
POTEH	0.0337	5.18E-01	6.42E-01
POU1F1	0.0287	5.82E-01	6.98E-01
POU2AF1	0.1060	4.13E-02	9.25E-02
POU2F1	0.1979	1.24E-04	7.36E-04
POU2F2	-0.0061	9.07E-01	9.41E-01
POU2F3	-0.0160	7.58E-01	8.38E-01
POU3F1	0.0581	2.64E-01	3.91E-01
POU3F2	0.0838	1.07E-01	1.96E-01
POU3F3	0.0015	9.77E-01	9.85E-01

POU3F4	0.0362	4.87E-01	6.14E-01
POU4F1	0.0093	8.58E-01	9.09E-01
POU4F2	-0.0267	6.08E-01	7.20E-01
POU4F3	0.0444	3.94E-01	5.27E-01
POU5F1B	0.0547	2.93E-01	4.22E-01
POU5F1	0.1624	1.70E-03	6.72E-03
POU5F2	0.0449	3.89E-01	5.22E-01
POU6F1	-0.1415	6.35E-03	2.01E-02
POU6F2	0.0649	2.12E-01	3.31E-01
PP14571	0.2377	3.68E-06	3.57E-05
PPA1	-0.0654	2.09E-01	3.28E-01
PPA2	-0.2664	1.92E-07	2.61E-06
PPAN-P2RY11	-0.0061	9.06E-01	9.41E-01
PPAN	0.0385	4.60E-01	5.90E-01
PPAP2A	-0.2911	1.11E-08	2.05E-07
PPAP2B	-0.2672	1.75E-07	2.41E-06
PPAP2C	0.2414	2.55E-06	2.57E-05
PPAPDC1A	-0.0309	5.53E-01	6.72E-01
PPAPDC1B	0.1632	1.61E-03	6.42E-03
PPAPDC2	-0.0984	5.84E-02	1.21E-01
PPAPDC3	-0.0519	3.19E-01	4.49E-01
PPARA	-0.1775	5.95E-04	2.75E-03
PPARD	0.1642	1.50E-03	6.05E-03
PPARGC1A	-0.1724	8.56E-04	3.76E-03
PPARGC1B	-0.0910	7.99E-02	1.56E-01
PPARG	-0.0732	1.59E-01	2.66E-01
PPAT	0.1663	1.31E-03	5.39E-03
PPBPL2	0.0793	1.27E-01	2.24E-01
PPBP	-0.1829	3.98E-04	1.97E-03
PPCDC	0.1894	2.43E-04	1.30E-03
PPCS	-0.1559	2.61E-03	9.59E-03
PPDPF	0.0741	1.54E-01	2.59E-01
PPEF1	0.1048	4.37E-02	9.67E-02
PPEF2	0.0563	2.79E-01	4.08E-01
PPFIA1	0.0141	7.86E-01	8.58E-01
PPFIA2	-0.0687	1.87E-01	3.00E-01
PPFIA3	0.0077	8.82E-01	9.25E-01
PPFIA4	0.2835	2.75E-08	4.56E-07
PPFIBP1	-0.0580	2.65E-01	3.93E-01
PPFIBP2	-0.1421	6.13E-03	1.95E-02
PPHLN1	0.2565	5.51E-07	6.65E-06
PPIAL4C	0.0356	4.95E-01	6.21E-01
PPIAL4D	0.0564	2.79E-01	4.07E-01
PPIAL4E	0.0771	1.38E-01	2.38E-01

PPIAL4G	0.0748	1.50E-01	2.54E-01
PPIA	0.0305	5.59E-01	6.77E-01
PPIB	-0.0321	5.37E-01	6.59E-01
PPIC	-0.0254	6.26E-01	7.35E-01
PPID	-0.2215	1.67E-05	1.32E-04
PPIEL	0.1906	2.22E-04	1.21E-03
PPIE	0.2093	4.83E-05	3.29E-04
PPIF	-0.1785	5.53E-04	2.59E-03
PPIG	0.1178	2.33E-02	5.81E-02
PPIH	0.2835	2.75E-08	4.55E-07
PPIL1	0.1927	1.88E-04	1.05E-03
PPIL2	0.1728	8.33E-04	3.67E-03
PPIL3	0.0869	9.46E-02	1.78E-01
PPIL4	0.1204	2.03E-02	5.20E-02
PPIL5	0.2384	3.41E-06	3.33E-05
PPIL6	0.1482	4.24E-03	1.44E-02
PPIP5K1	-0.0276	5.96E-01	7.10E-01
PPIP5K2	-0.0391	4.52E-01	5.83E-01
PPL	-0.0895	8.50E-02	1.63E-01
PPM1A	-0.1494	3.92E-03	1.35E-02
PPM1B	-0.1337	9.91E-03	2.90E-02
PPM1D	0.2291	8.33E-06	7.14E-05
PPM1E	0.0956	6.57E-02	1.33E-01
PPM1F	0.0505	3.32E-01	4.63E-01
PPM1G	0.1730	8.17E-04	3.61E-03
PPM1H	-0.0178	7.32E-01	8.19E-01
PPM1J	-0.1051	4.30E-02	9.55E-02
PPM1K	-0.2234	1.41E-05	1.14E-04
PPM1L	-0.2150	2.98E-05	2.18E-04
PPM1M	0.1802	4.86E-04	2.32E-03
PPM1N	-0.0383	4.63E-01	5.93E-01
PPME1	0.2345	4.98E-06	4.63E-05
PPOX	0.4122	1.19E-16	1.19E-14
PPP1CA	-0.0047	9.28E-01	9.55E-01
PPP1CB	-0.0208	6.90E-01	7.85E-01
PPP1CC	0.2290	8.36E-06	7.16E-05
PPP1R10	0.0158	7.62E-01	8.40E-01
PPP1R11	0.1031	4.72E-02	1.03E-01
PPP1R12A	0.1046	4.40E-02	9.72E-02
PPP1R12B	-0.0335	5.20E-01	6.44E-01
PPP1R12C	-0.0161	7.58E-01	8.37E-01
PPP1R13B	0.0104	8.42E-01	8.97E-01
PPP1R13L	0.1552	2.71E-03	9.94E-03
PPP1R14A	-0.1592	2.09E-03	8.01E-03

PPP1R14B	0.0264	6.12E-01	7.23E-01
PPP1R14C	0.0905	8.18E-02	1.59E-01
PPP1R14D	0.1819	4.29E-04	2.09E-03
PPP1R15A	0.0197	7.05E-01	7.98E-01
PPP1R15B	0.1482	4.22E-03	1.44E-02
PPP1R16A	-0.0445	3.93E-01	5.26E-01
PPP1R16B	-0.1215	1.93E-02	4.98E-02
PPP1R1A	-0.0284	5.85E-01	7.01E-01
PPP1R1B	0.1540	2.94E-03	1.06E-02
PPP1R1C	0.0445	3.92E-01	5.25E-01
PPP1R2P1	-0.0019	9.71E-01	9.81E-01
PPP1R2P3	0.0139	7.89E-01	8.60E-01
PPP1R2P9	0.1123	3.05E-02	7.25E-02
PPP1R2	0.0589	2.58E-01	3.83E-01
PPP1R3A	0.1018	5.01E-02	1.08E-01
PPP1R3B	-0.1567	2.47E-03	9.16E-03
PPP1R3C	0.0505	3.32E-01	4.63E-01
PPP1R3D	0.0598	2.51E-01	3.76E-01
PPP1R3E	0.0504	3.33E-01	4.64E-01
PPP1R3F	-0.0310	5.51E-01	6.71E-01
PPP1R3G	0.0271	6.02E-01	7.15E-01
PPP1R7	-0.0120	8.17E-01	8.80E-01
PPP1R8	0.0542	2.98E-01	4.28E-01
PPP1R9A	0.2696	1.34E-07	1.89E-06
PPP1R9B	0.0386	4.58E-01	5.89E-01
PPP2CA	0.0281	5.89E-01	7.04E-01
PPP2CB	-0.1366	8.45E-03	2.55E-02
PPP2R1A	0.0511	3.26E-01	4.57E-01
PPP2R1B	-0.0562	2.80E-01	4.09E-01
PPP2R2A	-0.0575	2.70E-01	3.97E-01
PPP2R2B	0.0871	9.40E-02	1.77E-01
PPP2R2C	0.1285	1.32E-02	3.68E-02
PPP2R2D	-0.1054	4.25E-02	9.46E-02
PPP2R3A	0.0486	3.51E-01	4.83E-01
PPP2R3B	0.1255	1.56E-02	4.19E-02
PPP2R3C	0.0858	9.88E-02	1.84E-01
PPP2R4	-0.0243	6.41E-01	7.46E-01
PPP2R5A	0.1311	1.15E-02	3.26E-02
PPP2R5B	-0.0755	1.46E-01	2.49E-01
PPP2R5C	-0.0918	7.74E-02	1.52E-01
PPP2R5D	0.2432	2.14E-06	2.22E-05
PPP2R5E	-0.0434	4.05E-01	5.38E-01
PPP3CA	-0.1809	4.64E-04	2.23E-03
PPP3CB	0.0738	1.56E-01	2.61E-01

PPP3CC	-0.1132	2.93E-02	7.01E-02
PPP3R1	-0.0960	6.48E-02	1.32E-01
PPP3R2	0.0936	7.17E-02	1.43E-01
PPP4C	0.0152	7.71E-01	8.46E-01
PPP4R1L	-0.0161	7.57E-01	8.37E-01
PPP4R1	0.0603	2.47E-01	3.71E-01
PPP4R2	-0.0888	8.77E-02	1.67E-01
PPP4R4	-0.1696	1.04E-03	4.45E-03
PPP5C	0.1149	2.69E-02	6.55E-02
PPP6C	0.1231	1.77E-02	4.65E-02
PPPDE1	0.3480	5.29E-12	2.04E-10
PPPDE2	-0.1854	3.31E-04	1.70E-03
PPRC1	0.1312	1.14E-02	3.25E-02
PPT1	0.2381	3.53E-06	3.43E-05
PPT2	0.0289	5.79E-01	6.95E-01
PPTC7	-0.0817	1.16E-01	2.09E-01
PPWD1	0.2360	4.31E-06	4.09E-05
PPY2	0.1692	1.07E-03	4.54E-03
PPYR1	0.0989	5.70E-02	1.19E-01
PPY	0.0499	3.38E-01	4.70E-01
PQBP1	0.2182	2.24E-05	1.70E-04
PQLC1	-0.2807	3.81E-08	6.13E-07
PQLC2	0.0676	1.94E-01	3.09E-01
PQLC3	-0.1140	2.82E-02	6.80E-02
PRAC	0.0477	3.60E-01	4.92E-01
PRAF2	0.0698	1.80E-01	2.91E-01
PRAM1	-0.0272	6.01E-01	7.14E-01
PRAMEF10	-0.1871	2.90E-04	1.51E-03
PRAMEF11	-0.1347	9.41E-03	2.79E-02
PRAMEF12	-0.0179	7.30E-01	8.17E-01
PRAMEF13	0.0077	8.83E-01	9.25E-01
PRAMEF14	0.1030	4.73E-02	1.03E-01
PRAMEF16	0.0087	8.68E-01	9.15E-01
PRAMEF17	0.0417	4.23E-01	5.55E-01
PRAMEF18	0.0062	9.06E-01	9.41E-01
PRAMEF1	-0.0071	8.91E-01	9.30E-01
PRAMEF20	-0.0303	5.61E-01	6.79E-01
PRAMEF22	0.1217	1.90E-02	4.93E-02
PRAMEF2	0.0009	9.86E-01	9.91E-01
PRAMEF4	-0.1610	1.87E-03	7.27E-03
PRAMEF5	-0.1375	7.98E-03	2.43E-02
PRAMEF6	-0.1019	4.98E-02	1.07E-01
PRAMEF8	-0.0280	5.91E-01	7.05E-01
PRAMEF9	-0.1733	8.00E-04	3.55E-03

PRAME	0.2063	6.25E-05	4.09E-04
PRAP1	-0.1032	4.70E-02	1.02E-01
PRB1	-0.1117	3.15E-02	7.42E-02
PRB2	-0.0372	4.75E-01	6.04E-01
PRB3	0.1273	1.42E-02	3.89E-02
PRB4	-0.0521	3.17E-01	4.47E-01
PRC1	0.4210	2.27E-17	2.72E-15
PRCC	0.5277	5.53E-28	4.08E-25
PRCD	0.0364	4.85E-01	6.13E-01
PRCP	-0.2435	2.07E-06	2.16E-05
PRDM10	0.2410	2.66E-06	2.67E-05
PRDM11	-0.0249	6.33E-01	7.40E-01
PRDM12	-0.0619	2.34E-01	3.58E-01
PRDM13	0.1473	4.46E-03	1.50E-02
PRDM14	-0.0098	8.51E-01	9.04E-01
PRDM15	0.2344	5.01E-06	4.65E-05
PRDM16	0.0730	1.61E-01	2.67E-01
PRDM1	-0.0900	8.33E-02	1.61E-01
PRDM2	-0.0117	8.23E-01	8.84E-01
PRDM4	0.2380	3.54E-06	3.44E-05
PRDM5	0.0536	3.03E-01	4.32E-01
PRDM6	-0.0011	9.83E-01	9.89E-01
PRDM7	0.0429	4.10E-01	5.43E-01
PRDM8	-0.0408	4.33E-01	5.65E-01
PRDM9	0.0969	6.21E-02	1.28E-01
PRDX1	0.0796	1.26E-01	2.22E-01
PRDX2	-0.1543	2.89E-03	1.05E-02
PRDX3	-0.2765	6.19E-08	9.44E-07
PRDX4	-0.1148	2.70E-02	6.56E-02
PRDX5	-0.0882	8.98E-02	1.71E-01
PRDX6	0.0817	1.16E-01	2.09E-01
PRDXDD1P	0.2262	1.09E-05	9.04E-05
PREB	-0.0759	1.44E-01	2.46E-01
PRELID1	-0.0006	9.91E-01	9.95E-01
PRELID2	0.2689	1.45E-07	2.04E-06
PRELP	-0.1588	2.16E-03	8.20E-03
PREPL	-0.0177	7.34E-01	8.20E-01
PREP	0.0529	3.09E-01	4.39E-01
PREX1	-0.0534	3.05E-01	4.34E-01
PREX2	-0.0918	7.73E-02	1.52E-01
PRF1	-0.1272	1.42E-02	3.91E-02
PRG1	0.0396	4.47E-01	5.78E-01
PRG2	-0.1971	1.33E-04	7.77E-04
PRG3	-0.0295	5.71E-01	6.89E-01

PRG4	-0.0938	7.12E-02	1.42E-01
PRH1	-0.0059	9.10E-01	9.43E-01
PRH2	0.0004	9.93E-01	9.96E-01
PRHOXNB	0.0261	6.17E-01	7.26E-01
PRIC285	-0.0590	2.57E-01	3.82E-01
PRICKLE1	-0.1279	1.37E-02	3.78E-02
PRICKLE2	-0.1691	1.08E-03	4.59E-03
PRICKLE3	0.0170	7.45E-01	8.28E-01
PRICKLE4	0.0212	6.84E-01	7.81E-01
PRIM1	0.3939	3.21E-15	2.31E-13
PRIM2	0.3658	3.45E-13	1.67E-11
PRIMA1	0.0749	1.50E-01	2.54E-01
PRINS	0.0629	2.27E-01	3.49E-01
PRKAA1	0.0608	2.42E-01	3.67E-01
PRKAA2	0.0450	3.88E-01	5.21E-01
PRKAB1	0.0928	7.43E-02	1.47E-01
PRKAB2	0.1994	1.10E-04	6.59E-04
PRKACA	-0.1287	1.31E-02	3.66E-02
PRKACB	0.0163	7.54E-01	8.35E-01
PRKACG	0.0170	7.44E-01	8.27E-01
PRKAG1	-0.0177	7.35E-01	8.20E-01
PRKAG2	-0.2307	7.13E-06	6.26E-05
PRKAG3	0.1635	1.58E-03	6.32E-03
PRKAR1A	-0.0286	5.83E-01	6.99E-01
PRKAR1B	-0.1504	3.70E-03	1.29E-02
PRKAR2A	-0.0876	9.19E-02	1.74E-01
PRKAR2B	-0.0311	5.50E-01	6.71E-01
PRKCA	0.1937	1.74E-04	9.80E-04
PRKCB	-0.0966	6.30E-02	1.29E-01
PRKCDBP	-0.0207	6.91E-01	7.86E-01
PRKCD	0.1951	1.55E-04	8.89E-04
PRKCE	-0.1224	1.84E-02	4.80E-02
PRKCG	0.0926	7.50E-02	1.48E-01
PRKCH	-0.2301	7.58E-06	6.59E-05
PRKCI	0.2509	9.88E-07	1.12E-05
PRKCQ	-0.0921	7.64E-02	1.50E-01
PRKCSH	-0.0859	9.86E-02	1.84E-01
PRKCZ	-0.1063	4.07E-02	9.13E-02
PRKD1	-0.1496	3.87E-03	1.34E-02
PRKD2	0.1249	1.61E-02	4.30E-02
PRKD3	0.0256	6.23E-01	7.32E-01
PRKDC	0.1646	1.46E-03	5.92E-03
PRKG1	-0.1623	1.71E-03	6.77E-03
PRKG2	0.0142	7.86E-01	8.58E-01

PRKRA	0.2206	1.80E-05	1.41E-04
PRKRIP1	0.1041	4.52E-02	9.92E-02
PRKRIR	0.0420	4.20E-01	5.53E-01
PRKX	0.1237	1.72E-02	4.53E-02
PRKY	0.0014	9.78E-01	9.86E-01
PRLHR	0.0539	3.01E-01	4.30E-01
PRLH	-0.0340	5.13E-01	6.38E-01
PRLR	0.1137	2.85E-02	6.86E-02
PRL	0.1448	5.19E-03	1.70E-02
PRM1	-0.0057	9.12E-01	9.45E-01
PRM2	0.0821	1.14E-01	2.07E-01
PRMT10	-0.0785	1.31E-01	2.29E-01
PRMT1	0.0837	1.08E-01	1.97E-01
PRMT2	0.0784	1.32E-01	2.30E-01
PRMT3	0.2256	1.15E-05	9.47E-05
PRMT5	0.1423	6.04E-03	1.93E-02
PRMT6	-0.0682	1.90E-01	3.04E-01
PRMT7	-0.0443	3.95E-01	5.28E-01
PRMT8	0.0424	4.16E-01	5.48E-01
PRND	0.0400	4.42E-01	5.74E-01
PRNP	-0.0083	8.73E-01	9.18E-01
PRO0611	0.0201	6.99E-01	7.93E-01
PRO0628	-0.0003	9.95E-01	9.97E-01
PRO1768	0.0767	1.40E-01	2.41E-01
PROCA1	0.3046	2.10E-09	4.63E-08
PROCR	-0.0384	4.60E-01	5.91E-01
PROC	-0.1018	5.00E-02	1.08E-01
PRODH2	-0.1208	1.99E-02	5.12E-02
PRODH	-0.1069	3.96E-02	8.94E-02
PROK1	-0.0717	1.68E-01	2.77E-01
PROK2	0.0094	8.57E-01	9.08E-01
PROKR1	0.0743	1.53E-01	2.57E-01
PROKR2	0.0154	7.67E-01	8.43E-01
PROL1	-0.0834	1.09E-01	1.99E-01
PROM1	0.1342	9.67E-03	2.85E-02
PROM2	0.1156	2.59E-02	6.35E-02
PROP1	0.1266	1.47E-02	4.00E-02
PROS1	-0.3190	3.21E-10	8.58E-09
PROSC	-0.0880	9.05E-02	1.72E-01
PROX1	-0.0590	2.57E-01	3.83E-01
PROX2	-0.0146	7.79E-01	8.53E-01
PROZ	-0.2418	2.47E-06	2.50E-05
PRPF18	0.1788	5.41E-04	2.54E-03
PRPF19	0.1681	1.16E-03	4.87E-03

PRPF31	0.0251	6.29E-01	7.37E-01
PRPF38A	0.3869	1.08E-14	6.90E-13
PRPF38B	0.1830	3.96E-04	1.96E-03
PRPF39	0.3141	6.15E-10	1.53E-08
PRPF3	1.0000	1.00E-50	1.00E-46
PRPF40A	0.1807	4.69E-04	2.25E-03
PRPF40B	0.3001	3.72E-09	7.70E-08
PRPF4B	0.2445	1.87E-06	1.98E-05
PRPF4	0.2546	6.69E-07	7.94E-06
PRPF6	0.2811	3.63E-08	5.85E-07
PRPF8	-0.0027	9.59E-01	9.74E-01
PRPH2	0.0464	3.73E-01	5.06E-01
PRPH	0.0406	4.35E-01	5.67E-01
PRPS1L1	0.1692	1.07E-03	4.55E-03
PRPS1	0.1117	3.14E-02	7.41E-02
PRPS2	0.0719	1.67E-01	2.75E-01
PRPSAP1	0.1254	1.57E-02	4.22E-02
PRPSAP2	0.1100	3.42E-02	7.95E-02
PRR11	0.2481	1.31E-06	1.44E-05
PRR12	0.1945	1.63E-04	9.25E-04
PRR13	0.0062	9.06E-01	9.41E-01
PRR14	0.1403	6.80E-03	2.12E-02
PRR15L	0.1101	3.41E-02	7.93E-02
PRR15	0.0245	6.38E-01	7.44E-01
PRR16	-0.0478	3.58E-01	4.91E-01
PRR18	-0.0908	8.07E-02	1.57E-01
PRR19	0.1763	6.48E-04	2.96E-03
PRR22	-0.0234	6.54E-01	7.56E-01
PRR23A	0.0863	9.71E-02	1.82E-01
PRR23B	0.0493	3.43E-01	4.75E-01
PRR23C	-0.0035	9.47E-01	9.67E-01
PRR24	0.1119	3.12E-02	7.37E-02
PRR25	0.0137	7.92E-01	8.62E-01
PRR3	0.2962	5.98E-09	1.17E-07
PRR4	0.1766	6.32E-04	2.90E-03
PRR5-ARHGAP8	0.0837	1.08E-01	1.97E-01
PRR5L	-0.0479	3.58E-01	4.90E-01
PRR5	-0.0748	1.51E-01	2.55E-01
PRR7	0.0119	8.19E-01	8.81E-01
PRRC1	0.0155	7.65E-01	8.42E-01
PRRG1	-0.2734	8.80E-08	1.29E-06
PRRG2	-0.0177	7.35E-01	8.20E-01
PRRG3	0.0718	1.68E-01	2.76E-01
PRRG4	-0.0250	6.32E-01	7.39E-01

PRRT1	-0.0311	5.51E-01	6.71E-01
PRRT2	0.2425	2.29E-06	2.35E-05
PRRT3	-0.0047	9.27E-01	9.54E-01
PRRT4	-0.0057	9.14E-01	9.46E-01
PRRX1	0.0210	6.87E-01	7.83E-01
PRRX2	0.1000	5.43E-02	1.15E-01
PRSS12	0.0239	6.46E-01	7.51E-01
PRSS16	0.1673	1.22E-03	5.08E-03
PRSS1	0.0411	4.30E-01	5.62E-01
PRSS21	0.0976	6.03E-02	1.25E-01
PRSS22	0.1090	3.58E-02	8.24E-02
PRSS23	-0.0323	5.35E-01	6.57E-01
PRSS27	0.0897	8.46E-02	1.63E-01
PRSS30P	0.1602	1.96E-03	7.58E-03
PRSS33	0.1485	4.14E-03	1.41E-02
PRSS35	0.1278	1.38E-02	3.81E-02
PRSS36	-0.1417	6.26E-03	1.98E-02
PRSS37	0.0810	1.19E-01	2.14E-01
PRSS38	0.1926	1.90E-04	1.06E-03
PRSS3	0.1280	1.36E-02	3.77E-02
PRSS41	0.1266	1.47E-02	4.00E-02
PRSS42	0.1166	2.47E-02	6.09E-02
PRSS45	0.1673	1.22E-03	5.08E-03
PRSS48	-0.0298	5.67E-01	6.84E-01
PRSS50	0.1665	1.29E-03	5.32E-03
PRSS53	-0.0657	2.06E-01	3.24E-01
PRSS54	-0.0468	3.69E-01	5.02E-01
PRSS55	0.1156	2.60E-02	6.37E-02
PRSS8	0.0409	4.32E-01	5.64E-01
PRSSL1	-0.0264	6.12E-01	7.23E-01
PRTFDC1	0.3043	2.19E-09	4.78E-08
PRTG	-0.0470	3.66E-01	4.99E-01
PRTN3	0.0499	3.38E-01	4.70E-01
PRUNE2	-0.0170	7.45E-01	8.28E-01
PRUNE	0.3945	2.89E-15	2.09E-13
PRX	0.0918	7.75E-02	1.52E-01
PRY2	-0.0998	5.49E-02	1.16E-01
PSAPL1	0.2210	1.73E-05	1.36E-04
PSAP	-0.1550	2.76E-03	1.01E-02
PSAT1	-0.0459	3.78E-01	5.11E-01
PSCA	0.0776	1.36E-01	2.35E-01
PSD2	-0.1029	4.77E-02	1.04E-01
PSD3	-0.0864	9.64E-02	1.81E-01
PSD4	-0.2160	2.72E-05	2.02E-04

PSD	-0.1733	8.04E-04	3.57E-03
PSEN1	-0.1251	1.59E-02	4.26E-02
PSEN2	0.1013	5.12E-02	1.10E-01
PSENEN	0.0347	5.05E-01	6.30E-01
PSG10	-0.0280	5.91E-01	7.05E-01
PSG11	-0.0388	4.56E-01	5.87E-01
PSG1	0.0634	2.23E-01	3.44E-01
PSG2	-0.0031	9.53E-01	9.71E-01
PSG3	-0.1239	1.69E-02	4.49E-02
PSG4	-0.0226	6.65E-01	7.65E-01
PSG5	0.0354	4.97E-01	6.23E-01
PSG6	-0.0245	6.38E-01	7.44E-01
PSG7	0.0238	6.48E-01	7.52E-01
PSG8	-0.0836	1.08E-01	1.97E-01
PSG9	-0.0173	7.40E-01	8.24E-01
PSIMCT-1	-0.0038	9.42E-01	9.64E-01
PSIP1	0.2520	8.80E-07	1.01E-05
PSKH1	-0.1575	2.35E-03	8.80E-03
PSKH2	0.0800	1.24E-01	2.19E-01
PSMA1	0.1661	1.33E-03	5.44E-03
PSMA2	-0.0978	5.98E-02	1.24E-01
PSMA3	0.0451	3.86E-01	5.19E-01
PSMA4	0.0696	1.81E-01	2.93E-01
PSMA5	0.0378	4.68E-01	5.97E-01
PSMA6	0.1813	4.49E-04	2.17E-03
PSMA7	0.0422	4.18E-01	5.51E-01
PSMA8	0.1342	9.68E-03	2.85E-02
PSMB10	-0.0944	6.93E-02	1.39E-01
PSMB11	-0.0078	8.80E-01	9.23E-01
PSMB1	0.1456	4.95E-03	1.63E-02
PSMB2	0.0930	7.36E-02	1.46E-01
PSMB3	0.1177	2.33E-02	5.82E-02
PSMB4	0.4242	1.24E-17	1.62E-15
PSMB5	-0.0378	4.68E-01	5.98E-01
PSMB6	-0.0841	1.06E-01	1.94E-01
PSMB7	0.0523	3.15E-01	4.45E-01
PSMB8	-0.0478	3.58E-01	4.91E-01
PSMB9	0.0767	1.40E-01	2.41E-01
PSMC1	0.0240	6.45E-01	7.50E-01
PSMC2	-0.1209	1.98E-02	5.10E-02
PSMC3IP	0.4194	3.05E-17	3.52E-15
PSMC3	0.0506	3.31E-01	4.62E-01
PSMC4	0.0778	1.35E-01	2.33E-01
PSMC5	0.1325	1.06E-02	3.06E-02

PSMC6	0.0212	6.83E-01	7.80E-01
PSMD10	0.2094	4.81E-05	3.27E-04
PSMD11	0.1493	3.96E-03	1.36E-02
PSMD12	0.1601	1.97E-03	7.61E-03
PSMD13	0.0450	3.87E-01	5.21E-01
PSMD14	0.1869	2.95E-04	1.54E-03
PSMD1	0.0955	6.62E-02	1.34E-01
PSMD2	0.1003	5.35E-02	1.13E-01
PSMD3	0.1952	1.55E-04	8.86E-04
PSMD4	0.4027	6.71E-16	5.69E-14
PSMD5	0.1507	3.62E-03	1.26E-02
PSMD6	-0.0380	4.66E-01	5.96E-01
PSMD7	-0.1236	1.72E-02	4.54E-02
PSMD8	-0.0056	9.15E-01	9.47E-01
PSMD9	-0.0730	1.60E-01	2.67E-01
PSME1	-0.0299	5.66E-01	6.84E-01
PSME2	0.0049	9.25E-01	9.52E-01
PSME3	0.2413	2.57E-06	2.59E-05
PSME4	0.0479	3.57E-01	4.89E-01
PSMF1	-0.1826	4.09E-04	2.01E-03
PSMG1	0.1401	6.89E-03	2.15E-02
PSMG2	0.0576	2.68E-01	3.96E-01
PSMG3	0.1995	1.09E-04	6.57E-04
PSMG4	0.0514	3.23E-01	4.53E-01
PSORS1C1	0.1476	4.37E-03	1.48E-02
PSORS1C2	0.1361	8.64E-03	2.60E-02
PSORS1C3	0.0800	1.24E-01	2.20E-01
PSPC1	0.1608	1.89E-03	7.33E-03
PSPH	0.2620	3.08E-07	3.99E-06
PSPN	-0.0307	5.56E-01	6.75E-01
PSRC1	0.3553	1.78E-12	7.49E-11
PSTK	-0.0745	1.52E-01	2.57E-01
PSTPIP1	-0.0307	5.55E-01	6.74E-01
PSTPIP2	0.0153	7.69E-01	8.45E-01
PTAFR	-0.0505	3.32E-01	4.63E-01
PTAR1	0.0739	1.56E-01	2.61E-01
PTBP1	0.3347	3.67E-11	1.20E-09
PTBP2	0.3012	3.23E-09	6.77E-08
PTCD1	-0.0689	1.85E-01	2.98E-01
PTCD2	-0.1482	4.22E-03	1.44E-02
PTCD3	-0.0689	1.86E-01	2.99E-01
PTCH1	0.0212	6.84E-01	7.81E-01
PTCH2	-0.0563	2.79E-01	4.08E-01
PTCHD1	-0.0392	4.52E-01	5.83E-01

PTCHD2	0.0708	1.74E-01	2.84E-01
PTCHD3	0.1521	3.32E-03	1.17E-02
PTCRA	-0.0656	2.07E-01	3.25E-01
PTDSS1	-0.0788	1.30E-01	2.27E-01
PTDSS2	0.0543	2.97E-01	4.26E-01
PTENP1	-0.2114	4.04E-05	2.82E-04
PTEN	-0.2470	1.47E-06	1.59E-05
PTER	0.0042	9.36E-01	9.60E-01
PTF1A	0.1766	6.35E-04	2.92E-03
PTGDR	-0.1771	6.09E-04	2.81E-03
PTGDS	-0.0546	2.94E-01	4.23E-01
PTGER1	-0.0509	3.28E-01	4.59E-01
PTGER2	-0.1490	4.03E-03	1.39E-02
PTGER3	-0.1831	3.93E-04	1.95E-03
PTGER4	0.0059	9.10E-01	9.43E-01
PTGES2	0.0955	6.60E-02	1.34E-01
PTGES3	0.2841	2.57E-08	4.29E-07
PTGES	0.1460	4.84E-03	1.61E-02
PTGFRN	0.0588	2.58E-01	3.84E-01
PTGFR	0.0331	5.25E-01	6.48E-01
PTGIR	-0.1147	2.72E-02	6.60E-02
PTGIS	-0.0938	7.12E-02	1.42E-01
PTGR1	-0.1085	3.67E-02	8.43E-02
PTGR2	-0.2401	2.89E-06	2.86E-05
PTGS1	0.0147	7.78E-01	8.52E-01
PTGS2	-0.1203	2.05E-02	5.23E-02
PTH1R	-0.1758	6.70E-04	3.05E-03
PTH2R	-0.1317	1.11E-02	3.18E-02
PTH2	-0.0225	6.66E-01	7.66E-01
PTHLH	0.0719	1.67E-01	2.75E-01
PTH	0.0358	4.92E-01	6.19E-01
PTK2B	-0.1561	2.57E-03	9.49E-03
PTK2	0.0602	2.47E-01	3.72E-01
PTK6	0.0405	4.37E-01	5.68E-01
PTK7	0.1434	5.67E-03	1.83E-02
PTMA	0.2544	6.88E-07	8.12E-06
PTMS	-0.1381	7.72E-03	2.36E-02
PTN	-0.0542	2.97E-01	4.27E-01
PTOV1	0.0022	9.66E-01	9.79E-01
PTP4A1	-0.0579	2.66E-01	3.93E-01
PTP4A2	0.0626	2.29E-01	3.52E-01
PTP4A3	0.1801	4.90E-04	2.34E-03
PTPDC1	0.4141	8.32E-17	8.55E-15
PTPLAD1	-0.1312	1.14E-02	3.25E-02

PTPLAD2	-0.0907	8.10E-02	1.57E-01
PTPLA	0.0583	2.63E-01	3.90E-01
PTPLB	-0.0490	3.47E-01	4.79E-01
PTPMT1	-0.0155	7.66E-01	8.42E-01
PTPN11	-0.1377	7.89E-03	2.40E-02
PTPN12	0.0910	8.01E-02	1.56E-01
PTPN13	-0.0847	1.03E-01	1.90E-01
PTPN14	0.0740	1.55E-01	2.60E-01
PTPN18	-0.0224	6.67E-01	7.67E-01
PTPN1	0.0231	6.58E-01	7.60E-01
PTPN20B	0.1902	2.29E-04	1.24E-03
PTPN21	-0.1399	6.95E-03	2.16E-02
PTPN22	-0.0221	6.71E-01	7.71E-01
PTPN23	0.2227	1.49E-05	1.20E-04
PTPN2	0.1074	3.87E-02	8.78E-02
PTPN3	0.0405	4.36E-01	5.68E-01
PTPN4	-0.1659	1.34E-03	5.50E-03
PTPN5	-0.0510	3.27E-01	4.58E-01
PTPN6	0.1131	2.93E-02	7.02E-02
PTPN7	0.0053	9.19E-01	9.49E-01
PTPN9	-0.0488	3.49E-01	4.81E-01
PTPRA	-0.0271	6.03E-01	7.16E-01
PTPRB	-0.2003	1.02E-04	6.21E-04
PTPRCAP	-0.0212	6.83E-01	7.80E-01
PTPRC	-0.0947	6.86E-02	1.38E-01
PTPRD	-0.0455	3.82E-01	5.15E-01
PTPRE	0.0433	4.06E-01	5.39E-01
PTPRF	0.0719	1.67E-01	2.75E-01
PTPRG	-0.1334	1.01E-02	2.94E-02
PTPRH	0.1245	1.64E-02	4.38E-02
PTPRJ	-0.0206	6.93E-01	7.88E-01
PTPRK	0.1283	1.34E-02	3.71E-02
PTPRM	-0.1021	4.95E-02	1.07E-01
PTPRN2	-0.1704	9.86E-04	4.25E-03
PTPRN	0.0655	2.08E-01	3.27E-01
PTPRO	0.0034	9.48E-01	9.68E-01
PTPRQ	-0.0541	2.99E-01	4.28E-01
PTPRR	-0.0364	4.84E-01	6.12E-01
PTPRS	-0.0211	6.86E-01	7.82E-01
PTPRT	0.1148	2.70E-02	6.56E-02
PTPRU	-0.0798	1.25E-01	2.21E-01
PTPRVP	0.0805	1.22E-01	2.17E-01
PTPRZ1	-0.0911	7.97E-02	1.55E-01
PTRF	-0.1270	1.43E-02	3.93E-02

PTRH1	-0.1204	2.04E-02	5.21E-02
PTRH2	0.1710	9.40E-04	4.08E-03
PTS	-0.1103	3.37E-02	7.86E-02
PTTG1IP	0.0581	2.65E-01	3.92E-01
PTTG1	0.4092	2.07E-16	1.92E-14
PTTG2	0.2630	2.76E-07	3.63E-06
PTTG3P	0.3662	3.25E-13	1.58E-11
PTX3	-0.0353	4.98E-01	6.25E-01
PTX4	0.0178	7.33E-01	8.19E-01
PUF60	0.0806	1.21E-01	2.16E-01
PUM1	0.0223	6.68E-01	7.68E-01
PUM2	0.1456	4.96E-03	1.64E-02
PURA	-0.0349	5.02E-01	6.28E-01
PURB	0.2734	8.79E-08	1.29E-06
PURG	-0.0623	2.31E-01	3.54E-01
PUS10	-0.3070	1.55E-09	3.54E-08
PUS1	0.1390	7.35E-03	2.27E-02
PUS3	-0.1124	3.05E-02	7.23E-02
PUS7L	0.1108	3.28E-02	7.69E-02
PUS7	0.1479	4.30E-03	1.46E-02
PUSL1	0.0371	4.76E-01	6.05E-01
PVALB	-0.1167	2.46E-02	6.08E-02
PVRIG	0.0390	4.54E-01	5.85E-01
PVRL1	0.2579	4.75E-07	5.85E-06
PVRL2	0.0861	9.78E-02	1.83E-01
PVRL3	-0.0612	2.40E-01	3.64E-01
PVRL4	0.1405	6.73E-03	2.11E-02
PVR	-0.0303	5.61E-01	6.79E-01
PVT1	0.1890	2.51E-04	1.34E-03
PWP1	0.1636	1.57E-03	6.29E-03
PWP2	0.2255	1.15E-05	9.52E-05
PWRN1	-0.0870	9.42E-02	1.77E-01
PWRN2	-0.0399	4.44E-01	5.75E-01
PWWP2A	0.1346	9.46E-03	2.80E-02
PWWP2B	0.1159	2.55E-02	6.26E-02
PXDNL	-0.0849	1.03E-01	1.89E-01
PXDN	-0.0546	2.94E-01	4.23E-01
PXK	0.0125	8.11E-01	8.76E-01
PXMP2	-0.2389	3.28E-06	3.21E-05
PXMP4	-0.1132	2.92E-02	7.00E-02
PXN	0.0123	8.13E-01	8.77E-01
PXT1	0.0878	9.13E-02	1.73E-01
PYCARD	0.1558	2.62E-03	9.64E-03
PYCR1	0.1761	6.58E-04	3.00E-03

PYCR2	0.2902	1.25E-08	2.26E-07
PYCRL	-0.0284	5.86E-01	7.01E-01
PYDC1	0.1957	1.49E-04	8.57E-04
PYDC2	0.0584	2.62E-01	3.88E-01
PYGB	0.0321	5.38E-01	6.60E-01
PYGL	-0.1463	4.76E-03	1.58E-02
PYGM	-0.0920	7.68E-02	1.51E-01
PYGO1	-0.1052	4.28E-02	9.51E-02
PYGO2	0.5946	7.73E-37	1.54E-33
PYHIN1	-0.0781	1.33E-01	2.32E-01
PYROXD1	0.0761	1.43E-01	2.45E-01
PYROXD2	-0.0624	2.31E-01	3.53E-01
PYY2	0.2332	5.65E-06	5.14E-05
PYY	0.0688	1.86E-01	2.99E-01
PZP	-0.0415	4.25E-01	5.58E-01
ProSAPiP1	0.0374	4.73E-01	6.02E-01
QARS	0.0183	7.25E-01	8.13E-01
QDPR	-0.3739	9.40E-14	4.98E-12
QKI	0.0703	1.77E-01	2.88E-01
QPCTL	0.0813	1.18E-01	2.11E-01
QPCT	0.0015	9.78E-01	9.86E-01
QPRT	-0.1826	4.07E-04	2.01E-03
QRFPR	-0.1899	2.35E-04	1.27E-03
QRFP	0.0826	1.12E-01	2.03E-01
QRICH1	0.1869	2.94E-04	1.54E-03
QRICH2	0.1886	2.58E-04	1.37E-03
QRSL1	0.2516	9.12E-07	1.04E-05
QSER1	0.3043	2.17E-09	4.76E-08
QSOX1	0.1581	2.25E-03	8.51E-03
QSOX2	0.2684	1.53E-07	2.13E-06
QTRT1	-0.0783	1.32E-01	2.30E-01
QTRTD1	0.1602	1.96E-03	7.57E-03
R3HCC1	0.0165	7.51E-01	8.32E-01
R3HDM1	0.1968	1.36E-04	7.96E-04
R3HDM2	0.0406	4.35E-01	5.67E-01
R3HDML	-0.0619	2.34E-01	3.57E-01
RAB10	0.0527	3.11E-01	4.40E-01
RAB11A	-0.0101	8.46E-01	9.00E-01
RAB11B	-0.1446	5.27E-03	1.72E-02
RAB11FIP1	0.2071	5.85E-05	3.87E-04
RAB11FIP2	-0.0234	6.53E-01	7.56E-01
RAB11FIP3	-0.0305	5.58E-01	6.77E-01
RAB11FIP4	0.2823	3.17E-08	5.19E-07
RAB11FIP5	0.0534	3.05E-01	4.35E-01

RAB12	-0.1037	4.59E-02	1.01E-01
RAB13	0.1476	4.40E-03	1.49E-02
RAB14	0.0225	6.65E-01	7.65E-01
RAB15	0.1680	1.16E-03	4.87E-03
RAB17	-0.1341	9.73E-03	2.86E-02
RAB18	-0.1268	1.45E-02	3.97E-02
RAB19	0.0023	9.66E-01	9.79E-01
RAB1A	-0.0331	5.25E-01	6.48E-01
RAB1B	-0.1617	1.78E-03	6.99E-03
RAB20	-0.0274	5.99E-01	7.12E-01
RAB21	-0.1005	5.31E-02	1.13E-01
RAB22A	0.0257	6.22E-01	7.31E-01
RAB23	0.1039	4.56E-02	1.00E-01
RAB24	0.1394	7.17E-03	2.22E-02
RAB25	0.0326	5.32E-01	6.54E-01
RAB26	-0.2056	6.61E-05	4.29E-04
RAB27A	-0.0092	8.60E-01	9.09E-01
RAB27B	0.0277	5.94E-01	7.08E-01
RAB28	0.2111	4.15E-05	2.89E-04
RAB2A	-0.1308	1.17E-02	3.32E-02
RAB2B	-0.0320	5.39E-01	6.61E-01
RAB30	-0.1361	8.66E-03	2.60E-02
RAB31	-0.0619	2.34E-01	3.57E-01
RAB32	0.1101	3.40E-02	7.93E-02
RAB33A	-0.0132	8.01E-01	8.68E-01
RAB33B	-0.1248	1.62E-02	4.32E-02
RAB34	0.1077	3.81E-02	8.68E-02
RAB35	0.1748	7.23E-04	3.25E-03
RAB36	0.1004	5.33E-02	1.13E-01
RAB37	-0.1321	1.09E-02	3.13E-02
RAB38	0.0529	3.09E-01	4.39E-01
RAB39B	-0.0039	9.41E-01	9.63E-01
RAB39	0.0820	1.15E-01	2.08E-01
RAB3A	0.0128	8.05E-01	8.71E-01
RAB3B	0.1695	1.05E-03	4.47E-03
RAB3C	0.0103	8.44E-01	8.98E-01
RAB3D	0.1054	4.24E-02	9.44E-02
RAB3GAP1	0.0395	4.48E-01	5.79E-01
RAB3GAP2	0.1966	1.38E-04	8.03E-04
RAB3IL1	0.0439	3.99E-01	5.32E-01
RAB3IP	0.1762	6.54E-04	2.99E-03
RAB40AL	0.2850	2.30E-08	3.90E-07
RAB40A	0.2474	1.41E-06	1.53E-05
RAB40B	0.1556	2.65E-03	9.75E-03

RAB40C	0.1332	1.02E-02	2.97E-02
RAB41	0.2044	7.33E-05	4.69E-04
RAB42	0.1736	7.88E-04	3.51E-03
RAB43	-0.1372	8.16E-03	2.47E-02
RAB4A	0.0810	1.19E-01	2.13E-01
RAB4B	-0.0160	7.59E-01	8.38E-01
RAB5A	-0.1575	2.34E-03	8.77E-03
RAB5B	-0.1078	3.79E-02	8.64E-02
RAB5C	0.0563	2.80E-01	4.08E-01
RAB6A	-0.1106	3.32E-02	7.77E-02
RAB6B	0.1038	4.58E-02	1.00E-01
RAB6C	-0.0961	6.44E-02	1.31E-01
RAB7A	-0.1429	5.84E-03	1.87E-02
RAB7L1	0.0983	5.85E-02	1.22E-01
RAB8A	-0.1778	5.81E-04	2.70E-03
RAB8B	0.0134	7.97E-01	8.65E-01
RAB9A	-0.0206	6.93E-01	7.88E-01
RAB9BP1	0.0061	9.08E-01	9.42E-01
RAB9B	0.0759	1.44E-01	2.46E-01
RABAC1	-0.0705	1.75E-01	2.86E-01
RABEP1	-0.1537	3.00E-03	1.08E-02
RABEP2	0.0217	6.77E-01	7.76E-01
RABEPK	-0.1408	6.60E-03	2.08E-02
RABGAP1L	0.1930	1.84E-04	1.03E-03
RABGAP1	0.0921	7.64E-02	1.50E-01
RABGEF1	-0.0142	7.84E-01	8.57E-01
RABGGTA	-0.1039	4.54E-02	9.97E-02
RABGGTB	0.0097	8.53E-01	9.05E-01
RABIF	0.3038	2.33E-09	5.05E-08
RABL2A	0.2119	3.87E-05	2.73E-04
RABL2B	0.1310	1.15E-02	3.28E-02
RABL3	-0.0939	7.09E-02	1.42E-01
RABL5	-0.0120	8.17E-01	8.80E-01
RAC1	0.0618	2.35E-01	3.58E-01
RAC2	-0.0163	7.54E-01	8.35E-01
RAC3	0.0044	9.32E-01	9.58E-01
RACGAP1P	0.3290	8.23E-11	2.46E-09
RACGAP1	0.4384	7.38E-19	1.38E-16
RAD17	-0.0714	1.70E-01	2.79E-01
RAD18	0.1973	1.31E-04	7.67E-04
RAD1	0.3833	1.98E-14	1.22E-12
RAD21L1	-0.0203	6.97E-01	7.91E-01
RAD21	0.1123	3.05E-02	7.25E-02
RAD23A	0.0629	2.27E-01	3.49E-01

RAD23B	0.0528	3.11E-01	4.40E-01
RAD50	-0.0619	2.35E-01	3.58E-01
RAD51AP1	0.3616	6.62E-13	3.05E-11
RAD51AP2	0.0158	7.62E-01	8.39E-01
RAD51C	0.3766	6.04E-14	3.32E-12
RAD51L1	0.0511	3.26E-01	4.57E-01
RAD51L3	0.2389	3.26E-06	3.20E-05
RAD51	0.3777	4.99E-14	2.81E-12
RAD52	0.1773	6.04E-04	2.79E-03
RAD54B	0.3530	2.52E-12	1.04E-10
RAD54L2	-0.1260	1.52E-02	4.11E-02
RAD54L	0.4299	4.06E-18	5.99E-16
RAD9A	0.3201	2.75E-10	7.46E-09
RAD9B	0.0881	9.01E-02	1.71E-01
RADIL	0.0927	7.44E-02	1.47E-01
RAE1	0.3200	2.80E-10	7.57E-09
RAET1E	0.0588	2.58E-01	3.84E-01
RAET1G	0.1050	4.32E-02	9.58E-02
RAET1K	0.2590	4.22E-07	5.28E-06
RAET1L	0.0536	3.03E-01	4.33E-01
RAF1	-0.0258	6.20E-01	7.30E-01
RAG1AP1	0.3343	3.86E-11	1.25E-09
RAG1	-0.0995	5.56E-02	1.17E-01
RAG2	0.0163	7.55E-01	8.35E-01
RAGE	0.1798	5.01E-04	2.38E-03
RAI14	0.1897	2.38E-04	1.28E-03
RAI1	0.1196	2.12E-02	5.39E-02
RAI2	-0.1615	1.81E-03	7.07E-03
RALA	0.0763	1.43E-01	2.44E-01
RALBP1	-0.0803	1.23E-01	2.18E-01
RALB	-0.0835	1.08E-01	1.98E-01
RALGAPA1	-0.0089	8.64E-01	9.12E-01
RALGAPA2	-0.0998	5.48E-02	1.16E-01
RALGAPB	0.1563	2.54E-03	9.39E-03
RALGDS	0.0409	4.32E-01	5.64E-01
RALGPS1	0.0345	5.08E-01	6.33E-01
RALGPS2	0.0488	3.49E-01	4.81E-01
RALYL	-0.0605	2.45E-01	3.69E-01
RALY	0.1811	4.57E-04	2.21E-03
RAMP1	-0.1678	1.18E-03	4.95E-03
RAMP2	-0.1519	3.36E-03	1.19E-02
RAMP3	-0.3340	4.08E-11	1.31E-09
RANBP10	-0.1258	1.53E-02	4.13E-02
RANBP17	0.0160	7.59E-01	8.38E-01

RANBP1	0.2435	2.08E-06	2.16E-05
RANBP2	0.0784	1.32E-01	2.30E-01
RANBP3L	-0.3190	3.21E-10	8.58E-09
RANBP3	0.1036	4.62E-02	1.01E-01
RANBP6	0.0123	8.13E-01	8.77E-01
RANBP9	-0.0137	7.92E-01	8.62E-01
RANGAP1	0.1353	9.06E-03	2.70E-02
RANGRF	0.1093	3.53E-02	8.16E-02
RAN	0.2334	5.52E-06	5.03E-05
RAP1A	-0.1332	1.02E-02	2.97E-02
RAP1B	0.0563	2.80E-01	4.08E-01
RAP1GAP2	0.0267	6.08E-01	7.20E-01
RAP1GAP	0.1060	4.13E-02	9.24E-02
RAP1GDS1	-0.2146	3.08E-05	2.24E-04
RAP2A	0.1764	6.45E-04	2.95E-03
RAP2B	0.0379	4.66E-01	5.96E-01
RAP2C	-0.0239	6.47E-01	7.51E-01
RAPGEF1	0.0560	2.82E-01	4.11E-01
RAPGEF2	-0.2190	2.08E-05	1.60E-04
RAPGEF3	-0.1290	1.29E-02	3.59E-02
RAPGEF4	-0.2065	6.16E-05	4.04E-04
RAPGEF5	-0.0371	4.76E-01	6.05E-01
RAPGEF6	-0.0752	1.48E-01	2.52E-01
RAPGEFL1	0.1808	4.65E-04	2.24E-03
RAPH1	-0.0977	6.01E-02	1.24E-01
RAPSN	0.0701	1.78E-01	2.89E-01
RARA	0.1553	2.71E-03	9.93E-03
RARB	-0.1135	2.88E-02	6.93E-02
RARG	-0.0329	5.28E-01	6.51E-01
RARRES1	-0.0590	2.57E-01	3.83E-01
RARRES2	-0.0901	8.31E-02	1.60E-01
RARRES3	-0.0901	8.32E-02	1.61E-01
RARS2	0.1869	2.94E-04	1.53E-03
RARS	0.0740	1.55E-01	2.60E-01
RASA1	-0.0409	4.32E-01	5.64E-01
RASA2	0.0272	6.02E-01	7.14E-01
RASA3	0.0000	1.00E+00	1.00E+00
RASA4P	0.0163	7.54E-01	8.35E-01
RASA4	-0.0070	8.92E-01	9.31E-01
RASAL1	0.2598	3.88E-07	4.90E-06
RASAL2	0.1264	1.49E-02	4.05E-02
RASAL3	-0.0456	3.81E-01	5.14E-01
RASD1	-0.0285	5.84E-01	7.00E-01
RASD2	0.0151	7.72E-01	8.47E-01

RASEF	0.1226	1.81E-02	4.75E-02
RASGEF1A	0.1546	2.83E-03	1.03E-02
RASGEF1B	-0.1093	3.54E-02	8.17E-02
RASGEF1C	0.1594	2.07E-03	7.94E-03
RASGRF1	-0.0196	7.07E-01	7.99E-01
RASGRF2	-0.1212	1.96E-02	5.05E-02
RASGRP1	-0.0671	1.97E-01	3.13E-01
RASGRP2	-0.0939	7.08E-02	1.42E-01
RASGRP3	-0.0542	2.98E-01	4.27E-01
RASGRP4	-0.1731	8.11E-04	3.59E-03
RASIP1	-0.1463	4.76E-03	1.58E-02
RASL10A	-0.0314	5.47E-01	6.68E-01
RASL10B	0.0030	9.54E-01	9.72E-01
RASL11A	-0.0145	7.80E-01	8.53E-01
RASL11B	-0.0252	6.28E-01	7.37E-01
RASL12	0.0371	4.77E-01	6.05E-01
RASSF10	0.0646	2.14E-01	3.34E-01
RASSF1	0.2119	3.90E-05	2.74E-04
RASSF2	-0.1119	3.12E-02	7.38E-02
RASSF3	0.0669	1.98E-01	3.15E-01
RASSF4	0.1531	3.11E-03	1.11E-02
RASSF5	-0.0225	6.66E-01	7.66E-01
RASSF6	0.0089	8.65E-01	9.13E-01
RASSF7	0.1894	2.44E-04	1.30E-03
RASSF8	0.0355	4.95E-01	6.21E-01
RASSF9	0.0180	7.29E-01	8.16E-01
RAVER1	0.2501	1.07E-06	1.20E-05
RAVER2	0.1824	4.13E-04	2.03E-03
RAX2	0.0564	2.79E-01	4.07E-01
RAX	0.0388	4.56E-01	5.87E-01
RB1CC1	-0.0314	5.47E-01	6.67E-01
RB1	-0.1638	1.54E-03	6.20E-03
RBAK	0.0161	7.57E-01	8.37E-01
RBBP4	0.1093	3.53E-02	8.16E-02
RBBP5	0.1151	2.66E-02	6.49E-02
RBBP6	0.1180	2.30E-02	5.76E-02
RBBP7	0.2321	6.23E-06	5.61E-05
RBBP8	0.2290	8.39E-06	7.18E-05
RBBP9	-0.1897	2.38E-04	1.28E-03
RBCK1	0.1835	3.81E-04	1.90E-03
RBKS	-0.1987	1.16E-04	6.94E-04
RBL1	0.2858	2.09E-08	3.60E-07
RBL2	-0.2387	3.32E-06	3.25E-05
RBM10	0.3553	1.78E-12	7.49E-11

RBM11	0.0972	6.16E-02	1.27E-01
RBM12B	0.2983	4.64E-09	9.37E-08
RBM12	0.2730	9.15E-08	1.34E-06
RBM14	0.4759	2.30E-22	8.50E-20
RBM15B	0.2091	4.93E-05	3.34E-04
RBM15	0.1331	1.03E-02	2.99E-02
RBM16	0.1861	3.13E-04	1.62E-03
RBM17	0.2599	3.83E-07	4.84E-06
RBM18	0.0645	2.15E-01	3.35E-01
RBM19	0.0544	2.96E-01	4.26E-01
RBM20	-0.0671	1.97E-01	3.13E-01
RBM22	0.1962	1.42E-04	8.26E-04
RBM23	-0.0393	4.50E-01	5.82E-01
RBM24	0.0366	4.82E-01	6.11E-01
RBM25	0.1555	2.67E-03	9.79E-03
RBM26	0.1886	2.59E-04	1.37E-03
RBM27	-0.0419	4.21E-01	5.54E-01
RBM28	0.1248	1.61E-02	4.31E-02
RBM33	0.2187	2.14E-05	1.64E-04
RBM34	0.3491	4.49E-12	1.76E-10
RBM38	0.2171	2.46E-05	1.85E-04
RBM39	0.3666	3.04E-13	1.49E-11
RBM3	0.2126	3.65E-05	2.59E-04
RBM41	0.1377	7.92E-03	2.41E-02
RBM42	0.1196	2.12E-02	5.39E-02
RBM43	-0.1320	1.09E-02	3.14E-02
RBM44	-0.0791	1.28E-01	2.25E-01
RBM45	0.0868	9.52E-02	1.79E-01
RBM46	0.0821	1.14E-01	2.07E-01
RBM47	-0.0197	7.06E-01	7.98E-01
RBM4B	0.2994	4.05E-09	8.33E-08
RBM4	0.2068	5.97E-05	3.93E-04
RBM5	0.3261	1.22E-10	3.52E-09
RBM6	0.2901	1.26E-08	2.28E-07
RBM7	-0.0153	7.69E-01	8.44E-01
RBM8A	0.4895	9.53E-24	4.22E-21
RBM9	0.0399	4.43E-01	5.75E-01
RBMS1	-0.0073	8.89E-01	9.29E-01
RBMS2	-0.0116	8.24E-01	8.85E-01
RBMS3	-0.1866	3.01E-04	1.56E-03
RBMX2	0.3006	3.46E-09	7.21E-08
RBMXL1	0.1176	2.35E-02	5.86E-02
RBMXL2	0.0569	2.75E-01	4.03E-01
RBMXL3	0.0367	4.81E-01	6.09E-01

RBMX	0.5425	8.89E-30	7.38E-27
RBMY1A1	-0.0320	5.39E-01	6.61E-01
RBMY1A3P	0.0942	6.98E-02	1.40E-01
RBMY1B	-0.0292	5.75E-01	6.92E-01
RBMY1E	-0.0906	8.14E-02	1.58E-01
RBMY1F	0.0110	8.33E-01	8.91E-01
RBMY1J	-0.0142	7.86E-01	8.58E-01
RBMY2EP	-0.0078	8.80E-01	9.23E-01
RBMY2FP	-0.0343	5.10E-01	6.34E-01
RBMY3AP	0.0497	3.40E-01	4.71E-01
RBP1	0.0074	8.87E-01	9.28E-01
RBP2	0.0326	5.31E-01	6.54E-01
RBP3	0.1584	2.21E-03	8.35E-03
RBP4	-0.2440	1.98E-06	2.08E-05
RBP5	-0.2427	2.25E-06	2.31E-05
RBP7	-0.1560	2.58E-03	9.51E-03
RBPJL	0.0793	1.27E-01	2.24E-01
RBPJ	0.0894	8.53E-02	1.64E-01
RBPMS2	-0.1076	3.84E-02	8.73E-02
RBPMS	-0.0552	2.89E-01	4.18E-01
RBX1	0.1215	1.92E-02	4.97E-02
RC3H1	0.2292	8.20E-06	7.04E-05
RC3H2	-0.1285	1.32E-02	3.68E-02
RCAN1	-0.3382	2.24E-11	7.50E-10
RCAN2	-0.1229	1.79E-02	4.69E-02
RCAN3	0.0557	2.84E-01	4.13E-01
RCBTB1	-0.0310	5.51E-01	6.71E-01
RCBTB2	-0.2018	9.06E-05	5.61E-04
RCC1	0.1218	1.89E-02	4.91E-02
RCC2	0.3108	9.43E-10	2.24E-08
RCCD1	0.2065	6.13E-05	4.02E-04
RCE1	0.2745	7.78E-08	1.16E-06
RCHY1	-0.0631	2.25E-01	3.47E-01
RCL1	-0.2050	6.98E-05	4.49E-04
RCN1	-0.0768	1.40E-01	2.41E-01
RCN2	0.1965	1.39E-04	8.08E-04
RCN3	0.0283	5.86E-01	7.02E-01
RCOR1	0.0227	6.63E-01	7.64E-01
RCOR2	0.2232	1.43E-05	1.16E-04
RCOR3	0.2977	4.95E-09	9.90E-08
RCSD1	-0.0922	7.61E-02	1.50E-01
RCVRN	-0.0498	3.39E-01	4.70E-01
RD3	0.0116	8.24E-01	8.85E-01
RDBP	0.2744	7.79E-08	1.16E-06

RDH10	-0.0365	4.83E-01	6.11E-01
RDH11	-0.3582	1.13E-12	4.98E-11
RDH12	0.0429	4.10E-01	5.43E-01
RDH13	-0.1298	1.23E-02	3.47E-02
RDH14	-0.1861	3.13E-04	1.62E-03
RDH16	-0.2130	3.54E-05	2.53E-04
RDH5	-0.3779	4.87E-14	2.76E-12
RDH8	0.0811	1.19E-01	2.13E-01
RDM1	0.3288	8.44E-11	2.51E-09
RDX	-0.0754	1.47E-01	2.50E-01
REC8	0.1164	2.50E-02	6.15E-02
RECK	0.0256	6.23E-01	7.32E-01
RECQL4	0.3699	1.79E-13	9.19E-12
RECQL5	0.2762	6.41E-08	9.74E-07
RECQL	0.1121	3.08E-02	7.30E-02
REEP1	0.0006	9.91E-01	9.95E-01
REEP2	0.0709	1.73E-01	2.83E-01
REEP3	-0.1708	9.59E-04	4.16E-03
REEP4	0.1314	1.13E-02	3.22E-02
REEP5	-0.3426	1.17E-11	4.12E-10
REEP6	-0.1818	4.32E-04	2.10E-03
REG1A	0.0100	8.48E-01	9.01E-01
REG1B	-0.0407	4.35E-01	5.66E-01
REG1P	-0.0469	3.68E-01	5.01E-01
REG3A	0.0081	8.77E-01	9.21E-01
REG3G	0.0427	4.12E-01	5.45E-01
REG4	0.1490	4.02E-03	1.38E-02
RELA	0.0547	2.93E-01	4.23E-01
RELB	0.2130	3.51E-05	2.51E-04
RELL1	0.1134	2.89E-02	6.94E-02
RELL2	0.0819	1.15E-01	2.08E-01
RELN	0.0111	8.32E-01	8.90E-01
RELT	0.1625	1.69E-03	6.68E-03
REL	-0.0311	5.51E-01	6.71E-01
REM1	-0.1072	3.90E-02	8.83E-02
REM2	0.2305	7.27E-06	6.38E-05
RENBP	0.0596	2.52E-01	3.78E-01
REN	-0.0823	1.13E-01	2.05E-01
REP15	0.1472	4.50E-03	1.51E-02
REPIN1	-0.0024	9.63E-01	9.77E-01
REPS1	0.0860	9.80E-02	1.83E-01
REPS2	-0.1590	2.13E-03	8.10E-03
RER1	-0.1675	1.20E-03	5.02E-03
RERE	0.1315	1.12E-02	3.21E-02

RERGL	-0.1298	1.24E-02	3.48E-02
RERG	-0.0940	7.05E-02	1.41E-01
RESP18	0.0004	9.93E-01	9.96E-01
REST	-0.0802	1.23E-01	2.18E-01
RETNLB	0.0677	1.93E-01	3.08E-01
RETN	0.0498	3.39E-01	4.70E-01
RETSAT	-0.3755	7.20E-14	3.91E-12
RET	-0.1494	3.92E-03	1.35E-02
REV1	0.1851	3.37E-04	1.72E-03
REV3L	-0.0262	6.14E-01	7.24E-01
REXO1L1	0.0055	9.15E-01	9.47E-01
REXO1	0.0922	7.60E-02	1.50E-01
REXO2	-0.1047	4.39E-02	9.70E-02
REXO4	0.1802	4.87E-04	2.32E-03
RFC1	0.1195	2.13E-02	5.40E-02
RFC2	0.2405	2.78E-06	2.78E-05
RFC3	0.2779	5.26E-08	8.14E-07
RFC4	0.4967	1.64E-24	8.81E-22
RFC5	0.3227	1.93E-10	5.39E-09
RFESD	0.0567	2.76E-01	4.04E-01
RFFL	-0.0420	4.19E-01	5.52E-01
RFK	-0.0180	7.30E-01	8.17E-01
RFNG	-0.0685	1.88E-01	3.01E-01
RFPL1S	0.0680	1.91E-01	3.05E-01
RFPL1	0.1036	4.61E-02	1.01E-01
RFPL2	0.0371	4.76E-01	6.05E-01
RFPL3S	0.2603	3.68E-07	4.67E-06
RFPL3	0.0582	2.63E-01	3.90E-01
RFPL4A	-0.0280	5.91E-01	7.06E-01
RFPL4B	0.0861	9.79E-02	1.83E-01
RFT1	0.0940	7.05E-02	1.41E-01
RFTN1	-0.0236	6.50E-01	7.54E-01
RFTN2	-0.0263	6.14E-01	7.24E-01
RFWD2	0.3861	1.24E-14	7.89E-13
RFWD3	0.2719	1.03E-07	1.49E-06
RFX1	0.0908	8.06E-02	1.57E-01
RFX2	0.0711	1.72E-01	2.81E-01
RFX3	0.1441	5.44E-03	1.77E-02
RFX4	0.1217	1.91E-02	4.94E-02
RFX5	0.2523	8.49E-07	9.80E-06
RFX6	0.0758	1.45E-01	2.48E-01
RFX7	0.0462	3.75E-01	5.08E-01
RFX8	0.0674	1.95E-01	3.11E-01
RFXANK	0.2244	1.28E-05	1.04E-04

RFXAP	0.2949	6.98E-09	1.34E-07
RG9MTD1	0.0545	2.95E-01	4.25E-01
RG9MTD2	-0.0157	7.63E-01	8.41E-01
RG9MTD3	0.3709	1.54E-13	7.97E-12
RGAG1	-0.0811	1.19E-01	2.13E-01
RGAG4	-0.2711	1.13E-07	1.62E-06
RGL1	-0.0645	2.15E-01	3.35E-01
RGL2	0.1537	2.99E-03	1.08E-02
RGL3	0.1222	1.86E-02	4.84E-02
RGL4	0.0613	2.39E-01	3.63E-01
RGMA	-0.0907	8.11E-02	1.57E-01
RGMB	-0.1003	5.36E-02	1.13E-01
RGNEF	-0.0485	3.51E-01	4.83E-01
RGN	-0.1949	1.58E-04	9.03E-04
RGP1	-0.0277	5.95E-01	7.09E-01
RGPD1	0.0055	9.15E-01	9.47E-01
RGPD3	0.0789	1.29E-01	2.26E-01
RGPD4	0.1454	5.03E-03	1.66E-02
RGPD5	0.1345	9.51E-03	2.81E-02
RGPD6	0.1935	1.77E-04	9.95E-04
RGPD8	0.0426	4.13E-01	5.46E-01
RGR	0.0941	7.01E-02	1.41E-01
RGS10	0.0572	2.72E-01	3.99E-01
RGS11	-0.1089	3.60E-02	8.27E-02
RGS12	0.0556	2.85E-01	4.14E-01
RGS13	0.0288	5.80E-01	6.96E-01
RGS14	0.0107	8.38E-01	8.93E-01
RGS16	0.0215	6.80E-01	7.78E-01
RGS17	0.1591	2.12E-03	8.08E-03
RGS18	-0.1140	2.82E-02	6.80E-02
RGS19	0.0917	7.79E-02	1.52E-01
RGS1	0.0432	4.07E-01	5.39E-01
RGS20	0.1827	4.04E-04	2.00E-03
RGS21	-0.0010	9.85E-01	9.90E-01
RGS22	-0.1017	5.02E-02	1.08E-01
RGS2	0.0444	3.93E-01	5.26E-01
RGS3	-0.0026	9.60E-01	9.75E-01
RGS4	-0.0504	3.33E-01	4.64E-01
RGS5	-0.0896	8.47E-02	1.63E-01
RGS6	-0.1797	5.05E-04	2.40E-03
RGS7BP	-0.1143	2.77E-02	6.71E-02
RGS7	0.0622	2.32E-01	3.55E-01
RGS8	0.0157	7.63E-01	8.41E-01
RGS9BP	0.0782	1.33E-01	2.31E-01

RGS9	-0.0657	2.07E-01	3.25E-01
RGSL1	-0.0918	7.75E-02	1.52E-01
RHAG	-0.0810	1.19E-01	2.14E-01
RHBDD1	-0.1985	1.19E-04	7.08E-04
RHBDD2	-0.1792	5.24E-04	2.47E-03
RHBDD3	0.0530	3.09E-01	4.38E-01
RHBDF1	0.0178	7.32E-01	8.19E-01
RHBDF2	0.0988	5.73E-02	1.20E-01
RHBDL1	0.0832	1.10E-01	2.00E-01
RHBDL2	0.1497	3.84E-03	1.33E-02
RHBDL3	0.0757	1.45E-01	2.48E-01
RHBG	0.0703	1.76E-01	2.87E-01
RHCE	-0.0708	1.73E-01	2.83E-01
RHCG	-0.2011	9.61E-05	5.90E-04
RHD	-0.0642	2.17E-01	3.37E-01
RHEBL1	0.3643	4.40E-13	2.10E-11
RHEB	0.0750	1.49E-01	2.53E-01
RHOA	0.0589	2.58E-01	3.84E-01
RHOBTB1	-0.0137	7.93E-01	8.63E-01
RHOBTB2	0.0556	2.86E-01	4.14E-01
RHOBTB3	-0.0326	5.32E-01	6.54E-01
RHOB	-0.2783	5.00E-08	7.81E-07
RHOC	-0.1155	2.61E-02	6.37E-02
RHOD	-0.1161	2.53E-02	6.21E-02
RHOF	0.1693	1.06E-03	4.51E-03
RHOG	0.0674	1.95E-01	3.11E-01
RHOH	-0.0283	5.87E-01	7.02E-01
RHOJ	-0.1810	4.61E-04	2.22E-03
RHOQ	-0.0512	3.26E-01	4.56E-01
RHOT1	0.2023	8.69E-05	5.41E-04
RHOT2	0.1680	1.16E-03	4.87E-03
RHOU	0.0055	9.16E-01	9.47E-01
RHOV	0.1431	5.76E-03	1.85E-02
RHOXF1	0.0798	1.25E-01	2.21E-01
RHOXF2B	0.1036	4.62E-02	1.01E-01
RHO	0.1346	9.42E-03	2.79E-02
RHPN1	0.1888	2.55E-04	1.36E-03
RHPN2	-0.0055	9.16E-01	9.47E-01
RIBC1	0.1746	7.29E-04	3.28E-03
RIBC2	0.4084	2.42E-16	2.22E-14
RIC3	0.0232	6.56E-01	7.58E-01
RIC8A	0.0813	1.18E-01	2.11E-01
RIC8B	0.1350	9.21E-03	2.74E-02
RICH2	0.0985	5.79E-02	1.21E-01

RICTOR	0.1351	9.20E-03	2.74E-02
RIF1	0.0927	7.45E-02	1.47E-01
RILPL1	0.0610	2.41E-01	3.66E-01
RILPL2	-0.1585	2.20E-03	8.34E-03
RILP	-0.3522	2.83E-12	1.15E-10
RIMBP2	-0.0506	3.31E-01	4.62E-01
RIMBP3C	0.2167	2.56E-05	1.92E-04
RIMBP3	0.1499	3.80E-03	1.32E-02
RIMKLA	0.0340	5.14E-01	6.39E-01
RIMKLB	-0.0711	1.72E-01	2.81E-01
RIMS1	0.0005	9.92E-01	9.95E-01
RIMS2	0.0362	4.87E-01	6.15E-01
RIMS3	0.3133	6.80E-10	1.67E-08
RIMS4	0.0314	5.47E-01	6.67E-01
RIN1	0.0016	9.76E-01	9.85E-01
RIN2	-0.0637	2.21E-01	3.42E-01
RIN3	-0.0568	2.75E-01	4.03E-01
RING1	0.0729	1.61E-01	2.68E-01
RINL	0.0828	1.11E-01	2.02E-01
RINT1	-0.0315	5.45E-01	6.66E-01
RIOK1	0.2363	4.20E-06	3.99E-05
RIOK2	0.0489	3.47E-01	4.79E-01
RIOK3	-0.1020	4.96E-02	1.07E-01
RIPK1	-0.0446	3.91E-01	5.24E-01
RIPK2	0.0239	6.46E-01	7.50E-01
RIPK3	-0.0398	4.45E-01	5.77E-01
RIPK4	-0.0827	1.12E-01	2.03E-01
RIPPLY1	-0.1057	4.18E-02	9.33E-02
RIPPLY2	0.1983	1.20E-04	7.15E-04
RIT1	0.4157	6.25E-17	6.62E-15
RIT2	0.0297	5.68E-01	6.85E-01
RLBP1	0.0961	6.44E-02	1.31E-01
RLF	0.0845	1.04E-01	1.91E-01
RLIM	0.0981	5.90E-02	1.22E-01
RLN1	-0.0258	6.21E-01	7.30E-01
RLN2	0.1503	3.70E-03	1.29E-02
RLN3	0.2273	9.79E-06	8.26E-05
RLTPR	0.0170	7.45E-01	8.28E-01
RMI1	0.3306	6.50E-11	1.99E-09
RMND1	0.0284	5.86E-01	7.01E-01
RMND5A	-0.0971	6.17E-02	1.27E-01
RMND5B	-0.0003	9.96E-01	9.97E-01
RMRP	0.0160	7.58E-01	8.38E-01
RMST	0.1696	1.04E-03	4.43E-03

RNASE10	0.0459	3.78E-01	5.11E-01
RNASE11	0.0563	2.79E-01	4.08E-01
RNASE12	-0.0085	8.71E-01	9.17E-01
RNASE13	0.0352	4.99E-01	6.25E-01
RNASE1	-0.0847	1.03E-01	1.90E-01
RNASE2	0.1664	1.30E-03	5.37E-03
RNASE3	-0.0022	9.67E-01	9.79E-01
RNASE4	-0.2307	7.17E-06	6.29E-05
RNASE6	-0.0555	2.86E-01	4.15E-01
RNASE7	-0.0177	7.35E-01	8.20E-01
RNASE8	0.0438	4.00E-01	5.33E-01
RNASE9	-0.0288	5.81E-01	6.97E-01
RNASEH1	0.0468	3.69E-01	5.02E-01
RNASEH2A	0.3569	1.39E-12	6.01E-11
RNASEH2B	0.2195	1.99E-05	1.54E-04
RNASEH2C	0.0519	3.19E-01	4.49E-01
RNASEK	-0.0875	9.25E-02	1.75E-01
RNASEL	-0.0088	8.66E-01	9.14E-01
RNASEN	0.3812	2.80E-14	1.66E-12
RNASET2	0.1585	2.20E-03	8.34E-03
RND1	-0.0880	9.04E-02	1.72E-01
RND2	0.0149	7.74E-01	8.49E-01
RND3	-0.1778	5.82E-04	2.70E-03
RNF103	-0.1067	3.99E-02	9.00E-02
RNF10	-0.1216	1.91E-02	4.95E-02
RNF111	0.0073	8.88E-01	9.29E-01
RNF112	-0.0085	8.70E-01	9.16E-01
RNF113A	0.2191	2.07E-05	1.59E-04
RNF113B	0.0052	9.21E-01	9.50E-01
RNF114	0.1199	2.09E-02	5.33E-02
RNF115	0.2527	8.17E-07	9.47E-06
RNF11	-0.1905	2.23E-04	1.21E-03
RNF121	0.0203	6.96E-01	7.90E-01
RNF122	0.0719	1.67E-01	2.75E-01
RNF123	-0.2714	1.09E-07	1.57E-06
RNF125	-0.3074	1.46E-09	3.35E-08
RNF126P1	0.1093	3.53E-02	8.16E-02
RNF126	-0.0210	6.86E-01	7.83E-01
RNF128	-0.1590	2.13E-03	8.13E-03
RNF130	-0.1658	1.35E-03	5.52E-03
RNF133	0.0734	1.58E-01	2.64E-01
RNF135	-0.0694	1.83E-01	2.95E-01
RNF138P1	0.0246	6.37E-01	7.43E-01
RNF138	0.1450	5.14E-03	1.69E-02

RNF139	-0.0613	2.39E-01	3.63E-01
RNF13	-0.1173	2.38E-02	5.92E-02
RNF141	-0.1034	4.65E-02	1.02E-01
RNF144A	0.3122	7.87E-10	1.91E-08
RNF144B	-0.2351	4.70E-06	4.41E-05
RNF145	0.0554	2.87E-01	4.16E-01
RNF146	0.1422	6.09E-03	1.94E-02
RNF148	0.0988	5.74E-02	1.20E-01
RNF149	0.0631	2.25E-01	3.47E-01
RNF14	-0.2347	4.88E-06	4.55E-05
RNF150	-0.1418	6.22E-03	1.97E-02
RNF151	0.2693	1.39E-07	1.96E-06
RNF152	-0.2968	5.55E-09	1.10E-07
RNF157	0.0865	9.61E-02	1.80E-01
RNF160	0.0111	8.31E-01	8.89E-01
RNF165	-0.1076	3.83E-02	8.71E-02
RNF166	0.0384	4.61E-01	5.92E-01
RNF167	-0.1334	1.01E-02	2.95E-02
RNF168	-0.0110	8.33E-01	8.90E-01
RNF169	0.0729	1.61E-01	2.68E-01
RNF170	-0.0845	1.04E-01	1.92E-01
RNF175	-0.0343	5.11E-01	6.35E-01
RNF17	0.1599	2.01E-03	7.72E-03
RNF180	-0.1151	2.66E-02	6.48E-02
RNF181	0.0309	5.53E-01	6.73E-01
RNF182	-0.1192	2.17E-02	5.49E-02
RNF183	0.0899	8.39E-02	1.62E-01
RNF185	-0.1170	2.42E-02	5.99E-02
RNF186	0.1269	1.44E-02	3.95E-02
RNF187	0.0418	4.22E-01	5.55E-01
RNF19A	0.0211	6.86E-01	7.82E-01
RNF19B	0.0743	1.53E-01	2.58E-01
RNF207	0.1332	1.02E-02	2.97E-02
RNF208	-0.0161	7.58E-01	8.37E-01
RNF20	0.2730	9.19E-08	1.34E-06
RNF212	-0.0384	4.61E-01	5.92E-01
RNF213	0.1674	1.21E-03	5.07E-03
RNF214	0.2124	3.70E-05	2.62E-04
RNF215	0.0105	8.41E-01	8.96E-01
RNF216L	0.0423	4.17E-01	5.49E-01
RNF216	0.1916	2.06E-04	1.13E-03
RNF217	-0.0838	1.07E-01	1.96E-01
RNF219	0.1950	1.57E-04	8.95E-04
RNF220	0.3034	2.43E-09	5.24E-08

RNF222	0.1062	4.09E-02	9.17E-02
RNF24	0.2571	5.17E-07	6.29E-06
RNF25	0.1126	3.01E-02	7.17E-02
RNF26	0.1483	4.20E-03	1.43E-02
RNF2	0.1976	1.28E-04	7.51E-04
RNF31	0.1754	6.89E-04	3.13E-03
RNF32	0.0610	2.41E-01	3.66E-01
RNF34	0.2601	3.75E-07	4.76E-06
RNF38	0.1367	8.39E-03	2.53E-02
RNF39	0.1372	8.15E-03	2.47E-02
RNF40	0.0521	3.17E-01	4.47E-01
RNF41	0.0240	6.45E-01	7.50E-01
RNF43	0.0414	4.27E-01	5.59E-01
RNF44	0.2075	5.63E-05	3.76E-04
RNF4	0.0891	8.66E-02	1.66E-01
RNF5P1	-0.0451	3.86E-01	5.19E-01
RNF5	0.0047	9.29E-01	9.55E-01
RNF6	-0.0527	3.12E-01	4.41E-01
RNF7	-0.0491	3.46E-01	4.78E-01
RNF8	0.3162	4.65E-10	1.19E-08
RNFT1	0.0798	1.25E-01	2.21E-01
RNFT2	0.2609	3.47E-07	4.44E-06
RNGTT	0.1728	8.34E-04	3.67E-03
RNH1	-0.1240	1.68E-02	4.46E-02
RNLS	-0.2699	1.29E-07	1.83E-06
RNMTL1	-0.1259	1.53E-02	4.13E-02
RNMT	0.1114	3.19E-02	7.50E-02
RNPC3	0.1686	1.12E-03	4.73E-03
RNPEPL1	-0.1012	5.15E-02	1.10E-01
RNPEP	-0.0167	7.48E-01	8.30E-01
RNPS1	0.3179	3.70E-10	9.77E-09
RNU11	0.0905	8.17E-02	1.58E-01
RNU4ATAC	-0.0271	6.03E-01	7.15E-01
RNU6ATAC	0.1026	4.83E-02	1.05E-01
ROBLD3	0.1075	3.85E-02	8.74E-02
ROBO1	0.1096	3.49E-02	8.08E-02
ROBO2	0.0126	8.09E-01	8.74E-01
ROBO3	0.0562	2.80E-01	4.09E-01
ROBO4	-0.2708	1.17E-07	1.67E-06
ROCK1	-0.0529	3.10E-01	4.39E-01
ROCK2	-0.0480	3.56E-01	4.88E-01
ROD1	0.3227	1.93E-10	5.39E-09
ROGDI	-0.0547	2.93E-01	4.22E-01
ROM1	-0.1381	7.73E-03	2.36E-02

ROMO1	0.0863	9.68E-02	1.81E-01
ROPN1B	-0.0238	6.48E-01	7.52E-01
ROPN1L	0.0591	2.57E-01	3.82E-01
ROPN1	0.0391	4.53E-01	5.84E-01
ROR1	0.0282	5.89E-01	7.03E-01
ROR2	-0.0338	5.17E-01	6.41E-01
RORA	-0.2188	2.13E-05	1.63E-04
RORB	-0.0606	2.45E-01	3.69E-01
RORC	0.0175	7.36E-01	8.22E-01
ROS1	-0.0570	2.74E-01	4.02E-01
RP1-177G6.2	0.0480	3.57E-01	4.89E-01
RP1L1	-0.0816	1.16E-01	2.09E-01
RP1	-0.0489	3.47E-01	4.80E-01
RP2	-0.0624	2.30E-01	3.53E-01
RP9P	0.2148	3.01E-05	2.19E-04
RP9	0.0927	7.47E-02	1.48E-01
RPA1	0.2119	3.88E-05	2.73E-04
RPA2	0.1498	3.82E-03	1.32E-02
RPA3	0.1326	1.06E-02	3.05E-02
RPA4	0.1701	1.00E-03	4.30E-03
RPAIN	0.0273	6.00E-01	7.13E-01
RPAP1	0.1368	8.33E-03	2.52E-02
RPAP2	0.1074	3.87E-02	8.78E-02
RPAP3	0.2137	3.32E-05	2.38E-04
RPE65	0.0615	2.38E-01	3.61E-01
RPE	0.0257	6.21E-01	7.30E-01
RPF1	0.1244	1.65E-02	4.39E-02
RPF2	0.1845	3.52E-04	1.78E-03
RPGRIP1L	0.1056	4.20E-02	9.37E-02
RPGRIP1	0.1049	4.35E-02	9.63E-02
RPGR	0.0288	5.81E-01	6.97E-01
RPH3AL	-0.2649	2.24E-07	3.00E-06
RPH3A	-0.0408	4.33E-01	5.65E-01
RPIA	0.3229	1.90E-10	5.31E-09
RPL10A	0.0917	7.79E-02	1.52E-01
RPL10L	0.0525	3.14E-01	4.43E-01
RPL10	0.1468	4.59E-03	1.54E-02
RPL11	0.0358	4.92E-01	6.19E-01
RPL12	0.0639	2.19E-01	3.40E-01
RPL13AP17	-0.0606	2.44E-01	3.69E-01
RPL13AP20	0.0524	3.15E-01	4.44E-01
RPL13AP3	0.0356	4.95E-01	6.21E-01
RPL13AP6	0.1283	1.34E-02	3.72E-02
RPL13A	0.0398	4.45E-01	5.77E-01

RPL13P5	0.1731	8.11E-04	3.59E-03
RPL13	-0.0606	2.44E-01	3.68E-01
RPL14	0.0844	1.04E-01	1.92E-01
RPL15	0.0025	9.62E-01	9.76E-01
RPL17	0.1463	4.76E-03	1.58E-02
RPL18A	0.0636	2.22E-01	3.43E-01
RPL18	0.0910	7.99E-02	1.56E-01
RPL19P12	0.1256	1.55E-02	4.18E-02
RPL19	0.1190	2.19E-02	5.52E-02
RPL21P44	0.0022	9.66E-01	9.79E-01
RPL21	0.0990	5.68E-02	1.19E-01
RPL22L1	0.0432	4.07E-01	5.40E-01
RPL22	0.0496	3.41E-01	4.72E-01
RPL23AP32	0.1075	3.85E-02	8.75E-02
RPL23AP53	0.1070	3.94E-02	8.91E-02
RPL23AP64	0.0701	1.78E-01	2.89E-01
RPL23AP7	0.0008	9.88E-01	9.93E-01
RPL23AP82	0.0114	8.27E-01	8.87E-01
RPL23A	0.1915	2.07E-04	1.14E-03
RPL23P8	0.1227	1.80E-02	4.72E-02
RPL23	0.1814	4.46E-04	2.16E-03
RPL24	0.0767	1.40E-01	2.41E-01
RPL26L1	-0.0895	8.50E-02	1.63E-01
RPL26	0.0134	7.97E-01	8.65E-01
RPL27A	0.0913	7.89E-02	1.54E-01
RPL27	0.2128	3.59E-05	2.56E-04
RPL28	0.0342	5.12E-01	6.36E-01
RPL29P2	0.0119	8.19E-01	8.81E-01
RPL29	0.0420	4.19E-01	5.52E-01
RPL30	0.0956	6.59E-02	1.34E-01
RPL31P11	0.1344	9.52E-03	2.81E-02
RPL31	0.1021	4.93E-02	1.06E-01
RPL32P3	0.2481	1.31E-06	1.44E-05
RPL32	0.1022	4.92E-02	1.06E-01
RPL34	-0.0020	9.69E-01	9.81E-01
RPL35A	0.2008	9.83E-05	6.01E-04
RPL35	0.1224	1.84E-02	4.80E-02
RPL36AL	-0.1183	2.27E-02	5.70E-02
RPL36A	0.2165	2.60E-05	1.94E-04
RPL36	0.0408	4.33E-01	5.65E-01
RPL37A	0.1135	2.89E-02	6.93E-02
RPL37	0.1754	6.92E-04	3.14E-03
RPL38	0.2173	2.42E-05	1.82E-04
RPL39L	0.1794	5.16E-04	2.44E-03

RPL39	0.1575	2.34E-03	8.78E-03
RPL3L	0.0210	6.87E-01	7.83E-01
RPL3	-0.0368	4.80E-01	6.09E-01
RPL41	0.0809	1.20E-01	2.14E-01
RPL4	0.0345	5.07E-01	6.32E-01
RPL5	0.1307	1.17E-02	3.33E-02
RPL6	0.0891	8.66E-02	1.66E-01
RPL7A	0.1232	1.76E-02	4.63E-02
RPL7L1	-0.0060	9.08E-01	9.42E-01
RPL7	0.0370	4.78E-01	6.06E-01
RPL8	0.0868	9.50E-02	1.79E-01
RPL9	-0.0133	7.98E-01	8.67E-01
RPLP0P2	0.0909	8.03E-02	1.56E-01
RPLP0	0.0998	5.48E-02	1.16E-01
RPLP1	0.1066	4.02E-02	9.04E-02
RPLP2	0.1371	8.18E-03	2.48E-02
RPN1	-0.0350	5.02E-01	6.28E-01
RPN2	0.1368	8.32E-03	2.51E-02
RPP14	-0.2160	2.71E-05	2.01E-04
RPP21	0.3018	3.00E-09	6.34E-08
RPP25	0.1344	9.54E-03	2.81E-02
RPP30	0.2152	2.90E-05	2.13E-04
RPP38	0.1850	3.41E-04	1.74E-03
RPP40	0.0517	3.21E-01	4.51E-01
RPPH1	-0.0204	6.96E-01	7.90E-01
RPRD1A	0.2112	4.13E-05	2.88E-04
RPRD1B	-0.1086	3.66E-02	8.40E-02
RPRD2	0.4335	1.96E-18	3.15E-16
RPRML	-0.0552	2.89E-01	4.18E-01
RPRM	0.0910	8.00E-02	1.56E-01
RPS10P7	0.1119	3.12E-02	7.37E-02
RPS10	0.1384	7.57E-03	2.32E-02
RPS11	0.0799	1.24E-01	2.20E-01
RPS12	0.1756	6.83E-04	3.10E-03
RPS13	0.1106	3.33E-02	7.78E-02
RPS14	0.0603	2.46E-01	3.71E-01
RPS15AP10	0.1118	3.13E-02	7.40E-02
RPS15A	0.1176	2.35E-02	5.85E-02
RPS15	0.0514	3.24E-01	4.54E-01
RPS16	0.1270	1.43E-02	3.93E-02
RPS17	0.0967	6.28E-02	1.29E-01
RPS18	0.1647	1.45E-03	5.89E-03
RPS19BP1	0.0756	1.46E-01	2.49E-01
RPS19	0.0935	7.20E-02	1.43E-01

RPS20	0.0214	6.82E-01	7.79E-01
RPS21	0.1970	1.34E-04	7.82E-04
RPS23	0.0819	1.15E-01	2.08E-01
RPS24	0.0987	5.76E-02	1.20E-01
RPS25	0.1093	3.53E-02	8.16E-02
RPS26P11	-0.0231	6.57E-01	7.59E-01
RPS26	0.0414	4.26E-01	5.59E-01
RPS27A	0.1290	1.29E-02	3.60E-02
RPS27L	-0.1778	5.81E-04	2.70E-03
RPS27	0.1204	2.04E-02	5.21E-02
RPS28	-0.0402	4.40E-01	5.72E-01
RPS29	0.0716	1.69E-01	2.77E-01
RPS2P32	0.1479	4.29E-03	1.46E-02
RPS2	0.0614	2.38E-01	3.62E-01
RPS3A	0.0763	1.43E-01	2.44E-01
RPS3	0.1612	1.84E-03	7.19E-03
RPS4X	0.1277	1.38E-02	3.82E-02
RPS4Y1	-0.0362	4.86E-01	6.14E-01
RPS4Y2	0.0136	7.93E-01	8.63E-01
RPS5	0.1165	2.48E-02	6.11E-02
RPS6KA1	-0.0404	4.38E-01	5.69E-01
RPS6KA2	-0.1884	2.63E-04	1.39E-03
RPS6KA3	0.0136	7.94E-01	8.63E-01
RPS6KA4	0.0514	3.23E-01	4.54E-01
RPS6KA5	0.1213	1.95E-02	5.03E-02
RPS6KA6	0.0664	2.02E-01	3.18E-01
RPS6KB1	0.2148	3.01E-05	2.19E-04
RPS6KB2	-0.0419	4.21E-01	5.54E-01
RPS6KC1	0.3267	1.12E-10	3.26E-09
RPS6KL1	-0.1139	2.83E-02	6.82E-02
RPS6	0.1420	6.15E-03	1.95E-02
RPS7	0.1309	1.16E-02	3.30E-02
RPS8	0.1361	8.65E-03	2.60E-02
RPS9	0.0597	2.51E-01	3.76E-01
RPSAP52	0.0981	5.92E-02	1.23E-01
RPSAP58	0.1373	8.08E-03	2.45E-02
RPSAP9	0.1059	4.15E-02	9.28E-02
RPSA	0.1384	7.60E-03	2.33E-02
RPTN	0.0940	7.06E-02	1.41E-01
RPTOR	0.1288	1.30E-02	3.63E-02
RPUSD1	0.1101	3.40E-02	7.92E-02
RPUSD2	-0.0451	3.87E-01	5.20E-01
RPUSD3	-0.0238	6.47E-01	7.51E-01
RPUSD4	-0.0687	1.86E-01	3.00E-01

RQCD1	0.1666	1.28E-03	5.30E-03
RRAD	0.0112	8.30E-01	8.89E-01
RRAGA	-0.0758	1.45E-01	2.47E-01
RRAGB	-0.0195	7.08E-01	8.00E-01
RRAGC	0.0173	7.40E-01	8.25E-01
RRAGD	0.0701	1.78E-01	2.89E-01
RRAS2	0.0043	9.34E-01	9.59E-01
RRAS	-0.0227	6.64E-01	7.64E-01
RRBP1	-0.0592	2.55E-01	3.81E-01
RREB1	-0.0141	7.86E-01	8.58E-01
RRH	-0.0455	3.82E-01	5.15E-01
RRM1	0.3754	7.34E-14	3.97E-12
RRM2B	-0.1701	1.01E-03	4.32E-03
RRM2	0.3489	4.63E-12	1.80E-10
RRN3P1	0.0638	2.20E-01	3.40E-01
RRN3P2	-0.0135	7.96E-01	8.64E-01
RRN3P3	0.0973	6.11E-02	1.26E-01
RRN3	-0.1089	3.61E-02	8.29E-02
RRP12	0.0888	8.76E-02	1.67E-01
RRP15	0.2604	3.64E-07	4.63E-06
RRP1B	0.1499	3.80E-03	1.32E-02
RRP1	0.2014	9.34E-05	5.75E-04
RRP7A	-0.0138	7.91E-01	8.62E-01
RRP7B	0.1999	1.06E-04	6.38E-04
RRP8	-0.0193	7.10E-01	8.02E-01
RRP9	0.1657	1.36E-03	5.56E-03
RRS1	0.0039	9.41E-01	9.64E-01
RS1	0.2264	1.06E-05	8.87E-05
RSAD1	-0.1038	4.56E-02	1.00E-01
RSAD2	-0.0339	5.15E-01	6.39E-01
RSBN1L	-0.0515	3.23E-01	4.53E-01
RSBN1	-0.0128	8.06E-01	8.72E-01
RSC1A1	-0.1762	6.51E-04	2.98E-03
RSF1	-0.0112	8.30E-01	8.89E-01
RSL1D1	0.1383	7.62E-03	2.33E-02
RSL24D1	0.0619	2.34E-01	3.57E-01
RSPH10B2	0.0286	5.83E-01	6.99E-01
RSPH1	0.2507	1.01E-06	1.14E-05
RSPH3	0.1057	4.18E-02	9.34E-02
RSPH4A	0.0049	9.25E-01	9.52E-01
RSPH6A	0.0871	9.38E-02	1.77E-01
RSPH9	0.0850	1.02E-01	1.89E-01
RSPO1	-0.0745	1.52E-01	2.57E-01
RSPO2	-0.1234	1.74E-02	4.58E-02

RSPO3	-0.1781	5.70E-04	2.65E-03
RSPO4	0.0942	7.00E-02	1.40E-01
RSPRY1	-0.1462	4.79E-03	1.59E-02
RSRC1	0.0466	3.71E-01	5.03E-01
RSRC2	0.1216	1.91E-02	4.95E-02
RSU1	-0.0400	4.43E-01	5.75E-01
RTBDN	0.0043	9.34E-01	9.59E-01
RTCD1	0.1133	2.91E-02	6.98E-02
RTDR1	0.2248	1.24E-05	1.01E-04
RTEL1	0.2085	5.20E-05	3.50E-04
RTF1	0.0049	9.26E-01	9.53E-01
RTKN2	0.3697	1.84E-13	9.35E-12
RTKN	0.1175	2.36E-02	5.88E-02
RTL1	-0.0267	6.09E-01	7.20E-01
RTN1	-0.0298	5.67E-01	6.85E-01
RTN2	0.0728	1.62E-01	2.69E-01
RTN3	0.1056	4.20E-02	9.36E-02
RTN4IP1	0.0446	3.92E-01	5.25E-01
RTN4RL1	0.0379	4.67E-01	5.96E-01
RTN4RL2	-0.0061	9.06E-01	9.41E-01
RTN4R	0.0826	1.12E-01	2.04E-01
RTN4	-0.2714	1.09E-07	1.57E-06
RTP1	-0.0092	8.59E-01	9.09E-01
RTP2	-0.0813	1.18E-01	2.11E-01
RTP3	-0.2687	1.48E-07	2.07E-06
RTP4	-0.0085	8.71E-01	9.17E-01
RTTN	0.1826	4.07E-04	2.01E-03
RUFY1	0.0648	2.13E-01	3.32E-01
RUFY2	0.1260	1.52E-02	4.10E-02
RUFY3	-0.0598	2.51E-01	3.76E-01
RUFY4	0.1331	1.03E-02	2.99E-02
RUNDC1	0.3146	5.75E-10	1.44E-08
RUNDC2A	0.0261	6.16E-01	7.26E-01
RUNDC2C	0.0592	2.56E-01	3.81E-01
RUNDC3A	0.2042	7.41E-05	4.74E-04
RUNDC3B	-0.2792	4.55E-08	7.20E-07
RUNX1T1	-0.2226	1.50E-05	1.20E-04
RUNX1	0.1439	5.50E-03	1.79E-02
RUNX2	-0.0311	5.50E-01	6.70E-01
RUNX3	-0.0617	2.36E-01	3.60E-01
RUSC1	0.5076	1.10E-25	6.43E-23
RUSC2	0.0946	6.86E-02	1.38E-01
RUVBL1	0.1631	1.62E-03	6.47E-03
RUVBL2	0.0855	1.00E-01	1.86E-01

RWDD1	0.1169	2.44E-02	6.03E-02
RWDD2A	0.2309	7.02E-06	6.19E-05
RWDD2B	-0.2329	5.78E-06	5.24E-05
RWDD3	0.1004	5.34E-02	1.13E-01
RWDD4A	-0.2422	2.37E-06	2.42E-05
RXFP1	-0.0137	7.92E-01	8.62E-01
RXFP2	-0.0002	9.97E-01	9.98E-01
RXFP3	0.0668	1.99E-01	3.16E-01
RXFP4	0.2428	2.23E-06	2.29E-05
RXRA	0.0004	9.94E-01	9.96E-01
RXRB	0.0556	2.85E-01	4.14E-01
RXRG	-0.0368	4.80E-01	6.09E-01
RYBP	-0.0305	5.58E-01	6.77E-01
RYK	0.1486	4.11E-03	1.41E-02
RYR1	-0.0048	9.27E-01	9.54E-01
RYR2	0.0340	5.14E-01	6.38E-01
RYR3	0.1227	1.80E-02	4.72E-02
S100A10	0.1893	2.45E-04	1.31E-03
S100A11	0.1139	2.83E-02	6.81E-02
S100A12	-0.1049	4.35E-02	9.63E-02
S100A13	0.1970	1.34E-04	7.84E-04
S100A14	0.1972	1.32E-04	7.76E-04
S100A16	0.1758	6.70E-04	3.05E-03
S100A1	0.1564	2.53E-03	9.35E-03
S100A2	0.1807	4.69E-04	2.25E-03
S100A3	0.0762	1.43E-01	2.45E-01
S100A4	0.0264	6.12E-01	7.23E-01
S100A5	0.0872	9.34E-02	1.76E-01
S100A6	0.0921	7.63E-02	1.50E-01
S100A7A	-0.0059	9.10E-01	9.43E-01
S100A7	0.0203	6.97E-01	7.91E-01
S100A8	0.0302	5.62E-01	6.80E-01
S100A9	0.1169	2.43E-02	6.02E-02
S100B	0.0796	1.26E-01	2.22E-01
S100G	0.0269	6.05E-01	7.17E-01
S100PBP	0.3766	6.07E-14	3.33E-12
S100P	0.2049	7.03E-05	4.52E-04
S100Z	-0.0160	7.59E-01	8.38E-01
S1PR1	-0.2144	3.13E-05	2.27E-04
S1PR2	0.1468	4.60E-03	1.54E-02
S1PR3	-0.0361	4.88E-01	6.15E-01
S1PR4	-0.1028	4.79E-02	1.04E-01
S1PR5	0.0071	8.92E-01	9.31E-01
SAA1	-0.1785	5.52E-04	2.59E-03

SAA2	-0.1924	1.93E-04	1.07E-03
SAA3P	0.0309	5.54E-01	6.73E-01
SAA4	-0.1483	4.20E-03	1.43E-02
SAAL1	0.1621	1.74E-03	6.84E-03
SAC3D1	0.2952	6.73E-09	1.30E-07
SACM1L	-0.0914	7.87E-02	1.54E-01
SACS	0.1555	2.67E-03	9.80E-03
SAE1	0.1707	9.63E-04	4.17E-03
SAFB2	0.1668	1.26E-03	5.24E-03
SAFB	0.1564	2.53E-03	9.35E-03
SAGE1	0.1417	6.26E-03	1.98E-02
SAG	0.0440	3.98E-01	5.31E-01
SALL1	-0.1553	2.70E-03	9.90E-03
SALL2	0.2804	3.95E-08	6.33E-07
SALL3	0.0656	2.08E-01	3.26E-01
SALL4	0.3043	2.18E-09	4.77E-08
SAMD10	0.1161	2.53E-02	6.21E-02
SAMD11	-0.0694	1.82E-01	2.94E-01
SAMD12	0.0750	1.50E-01	2.53E-01
SAMD13	0.1302	1.21E-02	3.41E-02
SAMD14	0.0422	4.18E-01	5.51E-01
SAMD1	0.1591	2.11E-03	8.05E-03
SAMD3	-0.0833	1.09E-01	1.99E-01
SAMD4A	-0.1641	1.51E-03	6.09E-03
SAMD4B	0.1197	2.11E-02	5.35E-02
SAMD5	-0.0507	3.30E-01	4.61E-01
SAMD7	-0.0102	8.45E-01	8.99E-01
SAMD8	-0.0852	1.01E-01	1.87E-01
SAMD9L	-0.0291	5.77E-01	6.94E-01
SAMD9	-0.0709	1.73E-01	2.82E-01
SAMHD1	-0.1465	4.68E-03	1.56E-02
SAMM50	-0.1056	4.20E-02	9.36E-02
SAMSN1	-0.0623	2.31E-01	3.54E-01
SAP130	0.2724	9.76E-08	1.42E-06
SAP18	-0.0251	6.30E-01	7.37E-01
SAP30BP	0.2616	3.19E-07	4.12E-06
SAP30L	0.0243	6.41E-01	7.47E-01
SAP30	0.1608	1.89E-03	7.33E-03
SAPS1	0.1130	2.95E-02	7.04E-02
SAPS2	-0.0462	3.75E-01	5.08E-01
SAPS3	0.0187	7.20E-01	8.10E-01
SAR1A	-0.0747	1.51E-01	2.55E-01
SAR1B	-0.1959	1.46E-04	8.43E-04
SARDH	-0.2440	1.98E-06	2.08E-05

SARM1	0.1188	2.21E-02	5.56E-02
SARNP	0.1245	1.65E-02	4.38E-02
SARS2	-0.0791	1.28E-01	2.25E-01
SARS	-0.0730	1.61E-01	2.68E-01
SART1	0.1530	3.12E-03	1.12E-02
SART3	0.2294	8.11E-06	6.97E-05
SASH1	-0.1104	3.36E-02	7.84E-02
SASH3	-0.0578	2.67E-01	3.94E-01
SASS6	0.3482	5.19E-12	2.00E-10
SAT1	-0.3053	1.92E-09	4.27E-08
SAT2	-0.1484	4.17E-03	1.42E-02
SATB1	-0.1742	7.50E-04	3.36E-03
SATB2	-0.1149	2.69E-02	6.53E-02
SATL1	0.0022	9.67E-01	9.79E-01
SAV1	-0.1808	4.66E-04	2.24E-03
SBDSP1	-0.1162	2.52E-02	6.20E-02
SBDS	-0.3014	3.16E-09	6.61E-08
SBF1P1	0.0924	7.54E-02	1.49E-01
SBF1	0.1341	9.71E-03	2.86E-02
SBF2	-0.1120	3.10E-02	7.33E-02
SBK1	0.2352	4.67E-06	4.38E-05
SBK2	-0.0517	3.20E-01	4.51E-01
SBNO1	-0.0376	4.70E-01	6.00E-01
SBNO2	0.0442	3.96E-01	5.28E-01
SBSN	0.0070	8.93E-01	9.31E-01
SC4MOL	-0.2903	1.23E-08	2.23E-07
SC5DL	-0.2575	4.95E-07	6.07E-06
SC65	0.2317	6.48E-06	5.78E-05
SCAF1	0.2149	2.99E-05	2.19E-04
SCAI	0.2532	7.80E-07	9.07E-06
SCAMP1	-0.1117	3.15E-02	7.43E-02
SCAMP2	0.0050	9.23E-01	9.51E-01
SCAMP3	0.4927	4.40E-24	2.19E-21
SCAMP4	0.1150	2.67E-02	6.51E-02
SCAMP5	0.1528	3.17E-03	1.13E-02
SCAND1	-0.0786	1.31E-01	2.28E-01
SCAND2	0.1276	1.39E-02	3.83E-02
SCAND3	0.1080	3.76E-02	8.59E-02
SCAPER	0.0334	5.22E-01	6.45E-01
SCAP	-0.0937	7.14E-02	1.42E-01
SCARA3	0.0393	4.51E-01	5.82E-01
SCARA5	-0.1048	4.36E-02	9.65E-02
SCARB1	-0.0504	3.33E-01	4.64E-01
SCARB2	-0.1524	3.26E-03	1.16E-02

SCARF1	-0.2062	6.28E-05	4.10E-04
SCARF2	-0.1430	5.78E-03	1.86E-02
SCARNA10	0.0041	9.38E-01	9.61E-01
SCARNA11	0.0861	9.76E-02	1.83E-01
SCARNA12	0.2584	4.49E-07	5.56E-06
SCARNA14	0.0841	1.06E-01	1.94E-01
SCARNA15	0.0540	3.00E-01	4.29E-01
SCARNA16	0.1986	1.18E-04	7.02E-04
SCARNA17	-0.1081	3.74E-02	8.56E-02
SCARNA18	0.0663	2.03E-01	3.20E-01
SCARNA1	0.0437	4.01E-01	5.34E-01
SCARNA21	0.0203	6.97E-01	7.91E-01
SCARNA22	0.0796	1.26E-01	2.22E-01
SCARNA2	-0.0707	1.74E-01	2.85E-01
SCARNA3	-0.0064	9.03E-01	9.38E-01
SCARNA4	0.0223	6.69E-01	7.69E-01
SCARNA5	-0.0704	1.76E-01	2.86E-01
SCARNA6	0.0464	3.73E-01	5.06E-01
SCARNA7	-0.0531	3.08E-01	4.38E-01
SCARNA8	0.0634	2.23E-01	3.44E-01
SCARNA9L	0.0097	8.53E-01	9.04E-01
SCARNA9	-0.0711	1.72E-01	2.81E-01
SCCPDH	-0.0091	8.61E-01	9.10E-01
SCD5	-0.0457	3.80E-01	5.14E-01
SCD	-0.1217	1.90E-02	4.93E-02
SCEL	-0.0154	7.67E-01	8.43E-01
SCFD1	-0.0432	4.06E-01	5.39E-01
SCFD2	-0.0549	2.92E-01	4.21E-01
SCG2	0.0060	9.09E-01	9.43E-01
SCG3	0.0054	9.18E-01	9.48E-01
SCG5	0.0640	2.18E-01	3.39E-01
SCGB1A1	0.0702	1.77E-01	2.88E-01
SCGB1C1	-0.0815	1.17E-01	2.10E-01
SCGB1D2	0.1046	4.42E-02	9.75E-02
SCGB2A1	0.2191	2.06E-05	1.59E-04
SCGB2A2	-0.0263	6.14E-01	7.24E-01
SCGB3A1	-0.1481	4.25E-03	1.44E-02
SCGB3A2	-0.0292	5.75E-01	6.92E-01
SCGBL	-0.1354	9.03E-03	2.69E-02
SCGN	0.0516	3.22E-01	4.52E-01
SCHIP1	-0.1045	4.44E-02	9.78E-02
SCIN	0.0751	1.49E-01	2.52E-01
SCLT1	0.1429	5.82E-03	1.87E-02
SCLY	0.0145	7.81E-01	8.54E-01

SCMH1	0.3085	1.27E-09	2.94E-08
SCML1	0.0931	7.34E-02	1.46E-01
SCML2	0.2865	1.94E-08	3.37E-07
SCML4	-0.1245	1.64E-02	4.38E-02
SCN10A	-0.0884	8.89E-02	1.69E-01
SCN11A	-0.1039	4.55E-02	9.99E-02
SCN1A	0.0106	8.39E-01	8.94E-01
SCN1B	-0.0984	5.82E-02	1.21E-01
SCN2A	-0.0009	9.86E-01	9.91E-01
SCN2B	-0.1255	1.56E-02	4.19E-02
SCN3A	-0.0535	3.04E-01	4.33E-01
SCN3B	0.0140	7.88E-01	8.59E-01
SCN4A	-0.1744	7.39E-04	3.32E-03
SCN4B	-0.2173	2.42E-05	1.83E-04
SCN5A	0.0437	4.02E-01	5.35E-01
SCN7A	-0.1341	9.73E-03	2.86E-02
SCN8A	0.0349	5.02E-01	6.28E-01
SCN9A	0.0116	8.23E-01	8.84E-01
SCNM1	0.5857	1.55E-35	2.37E-32
SCNN1A	-0.0528	3.10E-01	4.40E-01
SCNN1B	0.0833	1.09E-01	1.99E-01
SCNN1D	0.0887	8.79E-02	1.68E-01
SCNN1G	0.0317	5.42E-01	6.63E-01
SCO1	-0.2167	2.55E-05	1.91E-04
SCO2	-0.0332	5.24E-01	6.48E-01
SCOC	-0.1781	5.68E-04	2.65E-03
SCP2	-0.2608	3.48E-07	4.44E-06
SCPEP1	0.0356	4.94E-01	6.21E-01
SCRG1	-0.0938	7.10E-02	1.42E-01
SCRIB	0.1215	1.93E-02	4.98E-02
SCRN1	0.0698	1.80E-01	2.92E-01
SCRN2	-0.1818	4.33E-04	2.11E-03
SCRN3	-0.0693	1.83E-01	2.96E-01
SCRT1	0.0998	5.47E-02	1.15E-01
SCRT2	0.0117	8.22E-01	8.83E-01
SCTR	0.0893	8.60E-02	1.65E-01
SCT	0.0931	7.32E-02	1.45E-01
SCUBE1	-0.0916	7.82E-02	1.53E-01
SCUBE2	-0.0705	1.76E-01	2.86E-01
SCUBE3	0.0424	4.16E-01	5.48E-01
SCXB	0.2790	4.62E-08	7.28E-07
SCYL1	-0.1874	2.83E-04	1.48E-03
SCYL2	-0.1025	4.86E-02	1.05E-01
SCYL3	0.3185	3.44E-10	9.17E-09

SDAD1	-0.0218	6.76E-01	7.75E-01
SDC1	-0.2163	2.66E-05	1.97E-04
SDC2	-0.0865	9.61E-02	1.80E-01
SDC3	0.0123	8.14E-01	8.77E-01
SDC4P	0.0138	7.91E-01	8.61E-01
SDC4	-0.0484	3.52E-01	4.84E-01
SDCBP2	-0.1471	4.53E-03	1.52E-02
SDCBP	-0.2209	1.76E-05	1.38E-04
SDCCAG1	-0.0005	9.93E-01	9.96E-01
SDCCAG3	-0.0025	9.61E-01	9.76E-01
SDCCAG8	0.2069	5.92E-05	3.91E-04
SDF2L1	0.0832	1.10E-01	2.00E-01
SDF2	0.1566	2.49E-03	9.25E-03
SDF4	-0.1744	7.43E-04	3.33E-03
SDHAF1	0.0585	2.61E-01	3.87E-01
SDHAF2	0.0643	2.17E-01	3.37E-01
SDHAP1	0.3048	2.04E-09	4.52E-08
SDHAP2	0.2706	1.19E-07	1.70E-06
SDHAP3	0.0304	5.59E-01	6.77E-01
SDHA	-0.3443	9.15E-12	3.32E-10
SDHB	-0.3298	7.29E-11	2.21E-09
SDHC	0.1414	6.39E-03	2.02E-02
SDHD	-0.3306	6.51E-11	1.99E-09
SDK1	0.1078	3.80E-02	8.65E-02
SDK2	0.0158	7.62E-01	8.40E-01
SDPR	-0.1884	2.63E-04	1.39E-03
SDR16C5	-0.0392	4.52E-01	5.83E-01
SDR39U1	-0.1053	4.26E-02	9.47E-02
SDR42E1	0.0159	7.61E-01	8.39E-01
SDR9C7	0.1221	1.87E-02	4.85E-02
SDSL	-0.1914	2.08E-04	1.14E-03
SDS	-0.1821	4.25E-04	2.08E-03
SEBOX	0.0221	6.72E-01	7.71E-01
SEC11A	-0.0177	7.34E-01	8.20E-01
SEC11C	0.0556	2.85E-01	4.14E-01
SEC13	-0.0231	6.57E-01	7.59E-01
SEC14L1	-0.1166	2.47E-02	6.09E-02
SEC14L2	-0.2590	4.25E-07	5.31E-06
SEC14L3	-0.1646	1.46E-03	5.91E-03
SEC14L4	0.0623	2.31E-01	3.54E-01
SEC14L5	-0.0270	6.04E-01	7.17E-01
SEC16A	0.0831	1.10E-01	2.01E-01
SEC16B	0.0032	9.51E-01	9.70E-01
SEC1	0.0644	2.16E-01	3.36E-01

SEC22A	0.2268	1.03E-05	8.62E-05
SEC22B	-0.0734	1.58E-01	2.64E-01
SEC22C	-0.0878	9.13E-02	1.73E-01
SEC23A	-0.2204	1.84E-05	1.43E-04
SEC23B	0.1083	3.71E-02	8.49E-02
SEC23IP	-0.0343	5.10E-01	6.35E-01
SEC24A	-0.1718	8.95E-04	3.91E-03
SEC24B	-0.1413	6.41E-03	2.02E-02
SEC24C	0.0619	2.35E-01	3.58E-01
SEC24D	-0.1438	5.52E-03	1.79E-02
SEC31A	-0.0804	1.22E-01	2.17E-01
SEC31B	-0.0641	2.18E-01	3.38E-01
SEC61A1	0.0413	4.27E-01	5.60E-01
SEC61A2	0.1461	4.82E-03	1.60E-02
SEC61B	0.0657	2.06E-01	3.24E-01
SEC61G	0.1324	1.07E-02	3.08E-02
SEC62	-0.2486	1.25E-06	1.38E-05
SEC63	0.0887	8.80E-02	1.68E-01
SECISBP2L	-0.1400	6.90E-03	2.15E-02
SECISBP2	0.0191	7.13E-01	8.04E-01
SECTM1	0.1240	1.69E-02	4.47E-02
SEH1L	0.1575	2.35E-03	8.79E-03
SEL1L2	-0.0397	4.46E-01	5.77E-01
SEL1L3	0.1191	2.17E-02	5.49E-02
SEL1L	-0.2295	7.97E-06	6.87E-05
SELENBP1	-0.0346	5.07E-01	6.32E-01
SELE	-0.2719	1.04E-07	1.50E-06
SELK	0.0097	8.52E-01	9.04E-01
SELL	-0.0546	2.94E-01	4.23E-01
SELM	0.1574	2.36E-03	8.82E-03
SELO	-0.2428	2.22E-06	2.29E-05
SELPLG	-0.0875	9.25E-02	1.75E-01
SELP	-0.2216	1.65E-05	1.31E-04
SELS	0.0160	7.58E-01	8.38E-01
SELT	-0.0751	1.49E-01	2.53E-01
SELV	0.0785	1.31E-01	2.29E-01
SEMA3A	0.0044	9.32E-01	9.58E-01
SEMA3B	-0.0107	8.37E-01	8.93E-01
SEMA3C	-0.0289	5.79E-01	6.96E-01
SEMA3D	-0.0281	5.90E-01	7.04E-01
SEMA3E	0.0952	6.69E-02	1.35E-01
SEMA3F	-0.0406	4.36E-01	5.67E-01
SEMA3G	-0.1667	1.27E-03	5.28E-03
SEMA4A	0.1117	3.14E-02	7.42E-02

SEMA4B	0.0201	7.00E-01	7.93E-01
SEMA4C	0.1235	1.73E-02	4.57E-02
SEMA4D	0.0963	6.38E-02	1.30E-01
SEMA4F	0.1806	4.72E-04	2.26E-03
SEMA4G	0.0020	9.69E-01	9.81E-01
SEMA5A	-0.0069	8.95E-01	9.33E-01
SEMA5B	0.1253	1.57E-02	4.22E-02
SEMA6A	0.0705	1.75E-01	2.86E-01
SEMA6B	-0.0668	1.99E-01	3.16E-01
SEMA6C	0.3440	9.63E-12	3.45E-10
SEMA6D	-0.0348	5.03E-01	6.29E-01
SEMA7A	-0.0491	3.45E-01	4.77E-01
SEMG1	0.1012	5.13E-02	1.10E-01
SEMG2	-0.0028	9.57E-01	9.73E-01
SENP1	0.2505	1.03E-06	1.16E-05
SENP2	0.0323	5.35E-01	6.58E-01
SENP3	0.1442	5.39E-03	1.76E-02
SENP5	0.1754	6.91E-04	3.13E-03
SENP6	0.1168	2.45E-02	6.05E-02
SENP7	0.0707	1.74E-01	2.84E-01
SENP8	-0.1819	4.31E-04	2.10E-03
15-Sep	-0.0106	8.38E-01	8.94E-01
SEPHS1	0.0998	5.47E-02	1.15E-01
SEPHS2	-0.1890	2.52E-04	1.34E-03
SEPN1	0.0818	1.16E-01	2.09E-01
SEPP1	-0.1664	1.30E-03	5.36E-03
SEPSECS	-0.2101	4.54E-05	3.11E-04
10-Sep	-0.0433	4.06E-01	5.39E-01
11-Sep	-0.1658	1.35E-03	5.52E-03
12-Sep	0.2070	5.88E-05	3.89E-04
14-Sep	0.1814	4.45E-04	2.16E-03
1-Sep	-0.0166	7.50E-01	8.31E-01
2-Sep	0.1242	1.67E-02	4.44E-02
3-Sep	0.2781	5.14E-08	7.99E-07
4-Sep	-0.1630	1.63E-03	6.50E-03
5-Sep	0.1729	8.27E-04	3.65E-03
6-Sep	0.0823	1.13E-01	2.05E-01
SEPT7L	0.1011	5.17E-02	1.10E-01
SEPT7P2	0.2051	6.92E-05	4.47E-04
7-Sep	0.0195	7.09E-01	8.01E-01
8-Sep	0.1719	8.87E-04	3.88E-03
9-Sep	0.2015	9.30E-05	5.73E-04
SEPW1	0.0275	5.98E-01	7.12E-01
SEPX1	-0.1793	5.22E-04	2.47E-03

SERAC1	0.2145	3.10E-05	2.25E-04
SERBP1	0.0625	2.29E-01	3.52E-01
SERF1A	0.1993	1.12E-04	6.68E-04
SERF2	-0.0735	1.58E-01	2.64E-01
SERGEF	0.1113	3.21E-02	7.55E-02
SERHL2	0.0447	3.91E-01	5.24E-01
SERHL	0.1307	1.18E-02	3.33E-02
SERINC1	-0.1524	3.25E-03	1.15E-02
SERINC2	-0.1788	5.41E-04	2.54E-03
SERINC3	-0.1995	1.10E-04	6.58E-04
SERINC4	0.1828	4.03E-04	1.99E-03
SERINC5	-0.0969	6.23E-02	1.28E-01
SERP1	-0.0439	3.99E-01	5.32E-01
SERP2	0.0544	2.96E-01	4.25E-01
SERPINA10	-0.1447	5.22E-03	1.71E-02
SERPINA11	-0.1222	1.86E-02	4.84E-02
SERPINA12	0.1575	2.35E-03	8.81E-03
SERPINA13	0.1076	3.84E-02	8.73E-02
SERPINA1	-0.1387	7.47E-03	2.30E-02
SERPINA3	-0.0703	1.77E-01	2.87E-01
SERPINA4	-0.1210	1.98E-02	5.09E-02
SERPINA5	-0.0084	8.71E-01	9.17E-01
SERPINA6	-0.1114	3.20E-02	7.52E-02
SERPINA7	-0.0763	1.42E-01	2.44E-01
SERPINA9	0.0353	4.98E-01	6.24E-01
SERPINB10	0.0802	1.23E-01	2.18E-01
SERPINB11	0.0014	9.79E-01	9.86E-01
SERPINB12	-0.0303	5.61E-01	6.79E-01
SERPINB13	-0.0622	2.32E-01	3.55E-01
SERPINB1	0.0584	2.62E-01	3.88E-01
SERPINB2	0.0613	2.39E-01	3.63E-01
SERPINB3	-0.0544	2.96E-01	4.25E-01
SERPINB4	-0.0606	2.44E-01	3.69E-01
SERPINB5	-0.0028	9.57E-01	9.73E-01
SERPINB6	0.0469	3.68E-01	5.01E-01
SERPINB7	0.0056	9.15E-01	9.47E-01
SERPINB8	-0.1090	3.58E-02	8.24E-02
SERPINB9	-0.0525	3.13E-01	4.42E-01
SERPINC1	-0.1147	2.71E-02	6.58E-02
SERPIND1	-0.0875	9.25E-02	1.75E-01
SERPINE1	0.0056	9.15E-01	9.47E-01
SERPINE2	0.0491	3.45E-01	4.77E-01
SERPINE3	0.0414	4.27E-01	5.59E-01
SERPINF1	-0.1658	1.35E-03	5.54E-03

SERPINF2	-0.0697	1.80E-01	2.92E-01
SERPING1	-0.3603	8.17E-13	3.73E-11
SERPINH1	0.1308	1.17E-02	3.32E-02
SERPINI1	0.1076	3.82E-02	8.70E-02
SERPINI2	0.0529	3.10E-01	4.39E-01
SERTAD1	-0.1582	2.24E-03	8.45E-03
SERTAD2	0.1249	1.61E-02	4.30E-02
SERTAD3	0.0541	2.98E-01	4.28E-01
SERTAD4	-0.0412	4.29E-01	5.61E-01
SESN1	-0.0620	2.34E-01	3.57E-01
SESN2	-0.1906	2.22E-04	1.20E-03
SESN3	-0.1884	2.64E-04	1.39E-03
SESTD1	0.1387	7.44E-03	2.29E-02
SETBP1	-0.0815	1.17E-01	2.10E-01
SETD1A	0.1477	4.35E-03	1.47E-02
SETD1B	0.1017	5.04E-02	1.08E-01
SETD2	0.0349	5.03E-01	6.28E-01
SETD3	-0.2068	5.96E-05	3.92E-04
SETD4	0.2384	3.44E-06	3.35E-05
SETD5	0.1740	7.62E-04	3.41E-03
SETD6	-0.0665	2.01E-01	3.18E-01
SETD7	-0.3014	3.13E-09	6.57E-08
SETD8	0.2279	9.27E-06	7.85E-05
SETDB1	0.6717	5.07E-50	5.05E-46
SETDB2	0.0763	1.42E-01	2.44E-01
SETMAR	0.0621	2.33E-01	3.56E-01
SETX	0.0594	2.54E-01	3.79E-01
SET	0.2840	2.59E-08	4.32E-07
SEZ6L2	0.0443	3.95E-01	5.28E-01
SEZ6L	0.0293	5.74E-01	6.91E-01
SEZ6	0.2050	6.97E-05	4.49E-04
SF1	0.2265	1.05E-05	8.82E-05
SF3A1	0.1357	8.89E-03	2.66E-02
SF3A2	0.3548	1.91E-12	7.99E-11
SF3A3	0.2227	1.50E-05	1.20E-04
SF3B14	0.1359	8.77E-03	2.63E-02
SF3B1	0.1569	2.44E-03	9.08E-03
SF3B2	0.1733	8.01E-04	3.55E-03
SF3B3	0.0728	1.62E-01	2.69E-01
SF3B4	0.6445	6.25E-45	2.07E-41
SF3B5	0.1404	6.75E-03	2.11E-02
SF4	0.2081	5.38E-05	3.60E-04
SFI1	0.3823	2.33E-14	1.41E-12
SFMBT1	0.0387	4.57E-01	5.88E-01

SFMBT2	-0.1369	8.27E-03	2.50E-02
SFN	0.1563	2.54E-03	9.38E-03
SFPQ	0.4384	7.44E-19	1.38E-16
SFRP1	-0.1568	2.46E-03	9.13E-03
SFRP2	-0.0596	2.52E-01	3.78E-01
SFRP4	-0.1164	2.50E-02	6.15E-02
SFRP5	0.1543	2.88E-03	1.04E-02
SFRS11	0.2354	4.56E-06	4.30E-05
SFRS12IP1	0.0880	9.06E-02	1.72E-01
SFRS12	0.1475	4.42E-03	1.49E-02
SFRS13A	0.1707	9.66E-04	4.18E-03
SFRS13B	0.2596	3.97E-07	4.99E-06
SFRS14	0.2723	9.97E-08	1.45E-06
SFRS15	0.1674	1.21E-03	5.06E-03
SFRS16	0.3179	3.71E-10	9.78E-09
SFRS17A	0.2806	3.85E-08	6.19E-07
SFRS18	0.2781	5.15E-08	8.00E-07
SFRS1	0.3557	1.67E-12	7.13E-11
SFRS2B	-0.1634	1.59E-03	6.37E-03
SFRS2IP	0.0149	7.75E-01	8.50E-01
SFRS2	0.4109	1.52E-16	1.49E-14
SFRS3	0.3991	1.30E-15	1.01E-13
SFRS4	0.0413	4.28E-01	5.61E-01
SFRS5	-0.1080	3.76E-02	8.58E-02
SFRS6	0.2010	9.67E-05	5.92E-04
SFRS7	0.3745	8.53E-14	4.57E-12
SFRS8	0.3017	3.04E-09	6.40E-08
SFRS9	0.1015	5.08E-02	1.09E-01
SFT2D1	0.2637	2.55E-07	3.37E-06
SFT2D2	0.1747	7.24E-04	3.26E-03
SFT2D3	0.1358	8.83E-03	2.64E-02
SFTA1P	-0.1040	4.52E-02	9.93E-02
SFTA2	-0.0133	7.98E-01	8.67E-01
SFTA3	0.0527	3.11E-01	4.40E-01
SFTPA1	-0.0342	5.12E-01	6.36E-01
SFTPA2	0.0541	2.98E-01	4.28E-01
SFTPB	-0.0151	7.71E-01	8.46E-01
SFTPC	-0.0477	3.59E-01	4.91E-01
SFTPD	-0.1105	3.34E-02	7.80E-02
SFXN1	-0.3093	1.15E-09	2.68E-08
SFXN2	-0.1702	9.97E-04	4.29E-03
SFXN3	0.0048	9.27E-01	9.54E-01
SFXN4	-0.0751	1.49E-01	2.52E-01
SFXN5	-0.1350	9.23E-03	2.74E-02

SGCA	-0.0663	2.03E-01	3.20E-01
SGCB	0.0354	4.96E-01	6.22E-01
SGCD	-0.1401	6.89E-03	2.15E-02
SGCE	0.1322	1.08E-02	3.10E-02
SGCG	0.0785	1.31E-01	2.29E-01
SGCZ	0.1260	1.52E-02	4.10E-02
SGEF	-0.1765	6.40E-04	2.93E-03
SGIP1	0.0487	3.49E-01	4.81E-01
SGK196	0.0710	1.72E-01	2.82E-01
SGK1	-0.0705	1.75E-01	2.86E-01
SGK223	-0.0611	2.40E-01	3.64E-01
SGK269	-0.0802	1.23E-01	2.18E-01
SGK2	-0.1028	4.79E-02	1.04E-01
SGK3	-0.1577	2.31E-03	8.69E-03
SGK494	0.4195	3.04E-17	3.52E-15
SGMS1	-0.1384	7.58E-03	2.33E-02
SGMS2	-0.1536	3.01E-03	1.08E-02
SGOL1	0.3621	6.16E-13	2.85E-11
SGOL2	0.4307	3.44E-18	5.12E-16
SGPL1	-0.1625	1.69E-03	6.69E-03
SGPP1	-0.1509	3.58E-03	1.25E-02
SGPP2	0.0710	1.72E-01	2.82E-01
SGSH	0.1442	5.39E-03	1.76E-02
SGSM1	0.2022	8.78E-05	5.46E-04
SGSM2	0.0501	3.36E-01	4.68E-01
SGSM3	0.1018	5.01E-02	1.08E-01
SGTA	-0.0295	5.71E-01	6.88E-01
SGTB	0.1015	5.08E-02	1.09E-01
SH2B1	0.0363	4.85E-01	6.13E-01
SH2B2	0.0637	2.21E-01	3.41E-01
SH2B3	-0.0850	1.02E-01	1.89E-01
SH2D1A	-0.0771	1.38E-01	2.38E-01
SH2D1B	-0.0870	9.41E-02	1.77E-01
SH2D2A	0.0718	1.68E-01	2.76E-01
SH2D3A	0.0565	2.78E-01	4.07E-01
SH2D3C	-0.1233	1.75E-02	4.61E-02
SH2D4A	0.0179	7.32E-01	8.18E-01
SH2D4B	0.0092	8.60E-01	9.10E-01
SH2D5	0.1086	3.66E-02	8.40E-02
SH2D6	0.1065	4.03E-02	9.07E-02
SH2D7	0.1465	4.68E-03	1.56E-02
SH3BGRL2	-0.1628	1.66E-03	6.59E-03
SH3BGRL3	0.0321	5.38E-01	6.60E-01
SH3BGRL	-0.0758	1.45E-01	2.47E-01

SH3BGR	-0.1450	5.13E-03	1.69E-02
SH3BP1	0.2101	4.52E-05	3.10E-04
SH3BP2	-0.0877	9.18E-02	1.74E-01
SH3BP4	-0.0396	4.46E-01	5.78E-01
SH3BP5L	0.2632	2.69E-07	3.54E-06
SH3BP5	-0.2868	1.85E-08	3.24E-07
SH3D19	-0.3154	5.19E-10	1.30E-08
SH3D20	-0.0152	7.71E-01	8.46E-01
SH3GL1	0.0105	8.40E-01	8.95E-01
SH3GL2	0.0182	7.27E-01	8.15E-01
SH3GL3	-0.0665	2.01E-01	3.18E-01
SH3GLB1	-0.0508	3.29E-01	4.60E-01
SH3GLB2	0.2101	4.52E-05	3.10E-04
SH3KBP1	-0.1634	1.59E-03	6.37E-03
SH3PXD2A	0.0785	1.31E-01	2.29E-01
SH3PXD2B	0.1146	2.73E-02	6.61E-02
SH3RF1	-0.0100	8.47E-01	9.00E-01
SH3RF2	0.0974	6.09E-02	1.26E-01
SH3RF3	-0.0756	1.46E-01	2.49E-01
SH3TC1	0.0142	7.85E-01	8.57E-01
SH3TC2	-0.0003	9.96E-01	9.97E-01
SH3YL1	0.0364	4.84E-01	6.12E-01
SHANK1	-0.0650	2.12E-01	3.31E-01
SHANK2	-0.0036	9.44E-01	9.66E-01
SHANK3	-0.1755	6.85E-04	3.11E-03
SHARPIN	0.0540	3.00E-01	4.29E-01
SHBG	-0.1201	2.06E-02	5.27E-02
SHB	-0.1331	1.03E-02	2.99E-02
SHC1	0.3054	1.89E-09	4.23E-08
SHC2	-0.2525	8.36E-07	9.68E-06
SHC3	-0.0722	1.65E-01	2.73E-01
SHC4	-0.1019	4.98E-02	1.07E-01
SHCBP1	0.3471	6.05E-12	2.30E-10
SHD	-0.0973	6.12E-02	1.26E-01
SHE	-0.0207	6.91E-01	7.86E-01
SHFM1	0.0265	6.11E-01	7.22E-01
SHF	-0.1738	7.76E-04	3.46E-03
SHH	-0.2309	7.01E-06	6.18E-05
SHISA2	-0.0014	9.79E-01	9.86E-01
SHISA3	-0.1602	1.97E-03	7.60E-03
SHISA4	0.0034	9.47E-01	9.67E-01
SHISA5	-0.1192	2.16E-02	5.47E-02
SHISA6	-0.0419	4.21E-01	5.54E-01
SHISA7	0.0554	2.87E-01	4.16E-01

SHISA9	0.0552	2.89E-01	4.18E-01
SHKBP1	0.1515	3.44E-03	1.21E-02
SHMT1	-0.2302	7.51E-06	6.54E-05
SHMT2	-0.2095	4.78E-05	3.26E-04
SHOC2	-0.0791	1.28E-01	2.25E-01
SHOX2	0.2674	1.71E-07	2.36E-06
SHOX	-0.0224	6.68E-01	7.68E-01
SHPK	-0.3279	9.57E-11	2.81E-09
SHPRH	0.1036	4.61E-02	1.01E-01
SHQ1	0.0313	5.47E-01	6.68E-01
SHROOM1	-0.0669	1.99E-01	3.15E-01
SHROOM2	-0.0760	1.44E-01	2.46E-01
SHROOM3	-0.0216	6.78E-01	7.76E-01
SHROOM4	-0.2250	1.22E-05	9.97E-05
SIAE	-0.3060	1.76E-09	3.96E-08
SIAH1	-0.0710	1.73E-01	2.82E-01
SIAH2	-0.0669	1.99E-01	3.15E-01
SIAH3	0.0571	2.72E-01	4.00E-01
SIDT1	-0.1338	9.88E-03	2.90E-02
SIDT2	-0.2091	4.94E-05	3.35E-04
SIGIRR	-0.0690	1.85E-01	2.98E-01
SIGLEC10	0.0228	6.62E-01	7.63E-01
SIGLEC11	-0.1206	2.02E-02	5.17E-02
SIGLEC12	0.0172	7.41E-01	8.25E-01
SIGLEC14	-0.0734	1.59E-01	2.64E-01
SIGLEC15	-0.0938	7.12E-02	1.42E-01
SIGLEC16	-0.0792	1.28E-01	2.25E-01
SIGLEC1	-0.1304	1.19E-02	3.37E-02
SIGLEC5	-0.0782	1.33E-01	2.31E-01
SIGLEC6	-0.0569	2.74E-01	4.02E-01
SIGLEC7	-0.1067	4.00E-02	9.01E-02
SIGLEC8	-0.1006	5.28E-02	1.12E-01
SIGLEC9	-0.0531	3.08E-01	4.37E-01
SIGLECP3	-0.0615	2.37E-01	3.61E-01
SIGMAR1	-0.0744	1.53E-01	2.57E-01
SIK1	-0.1101	3.41E-02	7.93E-02
SIK2	-0.0764	1.42E-01	2.43E-01
SIK3	0.0363	4.86E-01	6.14E-01
SIKE1	-0.0106	8.39E-01	8.94E-01
SIL1	-0.1928	1.87E-04	1.04E-03
SILV	-0.1263	1.50E-02	4.06E-02
SIM1	-0.0121	8.17E-01	8.80E-01
SIM2	0.1224	1.84E-02	4.80E-02
SIN3A	0.1247	1.62E-02	4.33E-02

SIN3B	0.1525	3.23E-03	1.15E-02
SIP1	0.2728	9.38E-08	1.37E-06
SIPA1L1	-0.1770	6.17E-04	2.84E-03
SIPA1L2	0.0662	2.03E-01	3.20E-01
SIPA1L3	0.1826	4.07E-04	2.01E-03
SIPA1	-0.0197	7.05E-01	7.98E-01
SIRPA	-0.0508	3.29E-01	4.60E-01
SIRPB1	-0.0725	1.64E-01	2.71E-01
SIRPB2	-0.1352	9.13E-03	2.72E-02
SIRPD	-0.0037	9.43E-01	9.65E-01
SIRPG	0.0385	4.60E-01	5.90E-01
SIRT1	0.0361	4.88E-01	6.15E-01
SIRT2	-0.1425	5.97E-03	1.91E-02
SIRT3	-0.1443	5.34E-03	1.75E-02
SIRT4	-0.0040	9.38E-01	9.62E-01
SIRT5	-0.1562	2.55E-03	9.40E-03
SIRT6	0.0940	7.05E-02	1.41E-01
SIRT7	0.3004	3.56E-09	7.40E-08
SIT1	0.0175	7.36E-01	8.22E-01
SIVA1	0.0561	2.81E-01	4.10E-01
SIX1	0.0880	9.05E-02	1.72E-01
SIX2	0.1896	2.40E-04	1.29E-03
SIX3	0.1711	9.38E-04	4.07E-03
SIX4	0.1297	1.24E-02	3.49E-02
SIX5	0.2305	7.25E-06	6.36E-05
SIX6	0.0604	2.46E-01	3.71E-01
SI	0.0490	3.47E-01	4.79E-01
SKA1	0.3894	7.01E-15	4.67E-13
SKA2	0.2314	6.71E-06	5.97E-05
SKA3	0.4465	1.40E-19	3.22E-17
SKAP1	-0.1085	3.67E-02	8.43E-02
SKAP2	0.0546	2.94E-01	4.23E-01
SKIL	0.0806	1.21E-01	2.16E-01
SKINTL	0.0701	1.78E-01	2.89E-01
SKIV2L2	0.1238	1.71E-02	4.51E-02
SKIV2L	0.1247	1.63E-02	4.34E-02
SKI	-0.0304	5.59E-01	6.78E-01
SKP1	-0.0141	7.87E-01	8.58E-01
SKP2	0.1703	9.94E-04	4.28E-03
SLA2	-0.0566	2.77E-01	4.05E-01
SLAIN1	-0.0949	6.78E-02	1.37E-01
SLAIN2	-0.1058	4.16E-02	9.30E-02
SLAMF1	-0.0789	1.29E-01	2.26E-01
SLAMF6	-0.1131	2.93E-02	7.02E-02

SLAMF7	-0.0144	7.82E-01	8.55E-01
SLAMF8	0.0091	8.62E-01	9.11E-01
SLAMF9	-0.0303	5.60E-01	6.78E-01
SLA	-0.1135	2.89E-02	6.93E-02
SLBP	0.3309	6.28E-11	1.93E-09
SLC10A1	-0.2367	4.03E-06	3.86E-05
SLC10A2	0.0076	8.84E-01	9.26E-01
SLC10A3	0.2482	1.30E-06	1.43E-05
SLC10A4	0.1754	6.92E-04	3.14E-03
SLC10A5	-0.0774	1.37E-01	2.36E-01
SLC10A6	-0.2007	9.93E-05	6.06E-04
SLC10A7	-0.0867	9.56E-02	1.80E-01
SLC11A1	-0.0252	6.28E-01	7.36E-01
SLC11A2	-0.0054	9.18E-01	9.48E-01
SLC12A1	-0.2309	7.02E-06	6.19E-05
SLC12A2	-0.0014	9.79E-01	9.86E-01
SLC12A3	0.0337	5.17E-01	6.42E-01
SLC12A4	-0.4007	9.66E-16	7.92E-14
SLC12A5	-0.0453	3.84E-01	5.17E-01
SLC12A6	-0.0699	1.79E-01	2.90E-01
SLC12A7	0.1084	3.69E-02	8.46E-02
SLC12A8	0.0291	5.76E-01	6.93E-01
SLC12A9	0.0708	1.74E-01	2.84E-01
SLC13A1	0.0393	4.51E-01	5.82E-01
SLC13A2	0.1583	2.22E-03	8.40E-03
SLC13A3	-0.1917	2.03E-04	1.12E-03
SLC13A4	0.2014	9.34E-05	5.75E-04
SLC13A5	-0.1954	1.52E-04	8.70E-04
SLC14A1	-0.1347	9.41E-03	2.78E-02
SLC14A2	-0.1554	2.69E-03	9.85E-03
SLC15A1	0.1193	2.16E-02	5.47E-02
SLC15A2	0.1475	4.41E-03	1.49E-02
SLC15A3	-0.0969	6.23E-02	1.28E-01
SLC15A4	0.0036	9.44E-01	9.66E-01
SLC16A10	-0.0155	7.66E-01	8.42E-01
SLC16A11	-0.1603	1.96E-03	7.55E-03
SLC16A12	-0.2019	8.99E-05	5.58E-04
SLC16A13	-0.1920	1.99E-04	1.10E-03
SLC16A14	0.0063	9.04E-01	9.39E-01
SLC16A1	-0.0883	8.93E-02	1.70E-01
SLC16A2	-0.2904	1.22E-08	2.21E-07
SLC16A3	0.1672	1.23E-03	5.12E-03
SLC16A4	-0.2229	1.47E-05	1.18E-04
SLC16A5	-0.1092	3.55E-02	8.19E-02

SLC16A6	0.0122	8.15E-01	8.79E-01
SLC16A7	-0.1754	6.89E-04	3.13E-03
SLC16A8	0.1166	2.47E-02	6.08E-02
SLC16A9	0.0884	8.91E-02	1.70E-01
SLC17A1	-0.1810	4.59E-04	2.21E-03
SLC17A2	-0.1913	2.10E-04	1.15E-03
SLC17A3	-0.1380	7.76E-03	2.37E-02
SLC17A4	-0.0963	6.39E-02	1.31E-01
SLC17A5	0.1521	3.31E-03	1.17E-02
SLC17A6	0.0681	1.91E-01	3.05E-01
SLC17A7	-0.1024	4.87E-02	1.05E-01
SLC17A8	0.0029	9.56E-01	9.72E-01
SLC17A9	0.0752	1.48E-01	2.52E-01
SLC18A1	-0.0151	7.71E-01	8.46E-01
SLC18A2	-0.1304	1.20E-02	3.38E-02
SLC18A3	-0.0051	9.22E-01	9.50E-01
SLC19A1	0.0310	5.51E-01	6.71E-01
SLC19A2	0.0834	1.09E-01	1.99E-01
SLC19A3	-0.0910	8.01E-02	1.56E-01
SLC1A1	-0.1635	1.58E-03	6.33E-03
SLC1A2	-0.2566	5.44E-07	6.59E-06
SLC1A3	-0.0193	7.11E-01	8.03E-01
SLC1A4	0.0873	9.30E-02	1.76E-01
SLC1A5	0.2072	5.77E-05	3.83E-04
SLC1A6	0.0454	3.84E-01	5.17E-01
SLC1A7	0.0967	6.27E-02	1.29E-01
SLC20A1	0.1089	3.60E-02	8.28E-02
SLC20A2	-0.1603	1.95E-03	7.54E-03
SLC22A10	-0.0865	9.60E-02	1.80E-01
SLC22A11	-0.0432	4.07E-01	5.39E-01
SLC22A12	-0.0923	7.58E-02	1.49E-01
SLC22A13	-0.0072	8.90E-01	9.30E-01
SLC22A14	0.0152	7.70E-01	8.46E-01
SLC22A15	0.1380	7.77E-03	2.37E-02
SLC22A16	0.0538	3.01E-01	4.30E-01
SLC22A17	-0.0245	6.39E-01	7.45E-01
SLC22A18AS	-0.1060	4.13E-02	9.25E-02
SLC22A18	-0.1544	2.86E-03	1.04E-02
SLC22A1	-0.1972	1.32E-04	7.76E-04
SLC22A20	0.2082	5.30E-05	3.56E-04
SLC22A23	0.1184	2.25E-02	5.65E-02
SLC22A24	0.1325	1.07E-02	3.07E-02
SLC22A25	-0.1591	2.12E-03	8.08E-03
SLC22A2	-0.0289	5.79E-01	6.96E-01

SLC22A3	-0.1395	7.13E-03	2.21E-02
SLC22A4	-0.0806	1.21E-01	2.16E-01
SLC22A5	0.1194	2.14E-02	5.42E-02
SLC22A6	0.0077	8.83E-01	9.25E-01
SLC22A7	-0.1509	3.58E-03	1.25E-02
SLC22A8	0.0614	2.38E-01	3.62E-01
SLC22A9	0.0085	8.70E-01	9.16E-01
SLC23A1	0.0360	4.90E-01	6.17E-01
SLC23A2	-0.1586	2.18E-03	8.28E-03
SLC23A3	-0.0675	1.94E-01	3.10E-01
SLC24A1	-0.1903	2.27E-04	1.23E-03
SLC24A2	0.0849	1.03E-01	1.89E-01
SLC24A3	-0.0922	7.63E-02	1.50E-01
SLC24A4	-0.1312	1.14E-02	3.25E-02
SLC24A5	0.1633	1.60E-03	6.38E-03
SLC24A6	-0.0245	6.38E-01	7.44E-01
SLC25A10	-0.1388	7.41E-03	2.28E-02
SLC25A11	-0.2300	7.61E-06	6.61E-05
SLC25A12	0.0340	5.14E-01	6.38E-01
SLC25A13	-0.3035	2.40E-09	5.19E-08
SLC25A14	0.2757	6.75E-08	1.02E-06
SLC25A15	-0.2022	8.81E-05	5.48E-04
SLC25A16	-0.0330	5.26E-01	6.49E-01
SLC25A17	0.0178	7.32E-01	8.19E-01
SLC25A18	-0.1007	5.27E-02	1.12E-01
SLC25A19	0.2724	9.86E-08	1.43E-06
SLC25A1	-0.0861	9.77E-02	1.83E-01
SLC25A20	-0.2401	2.89E-06	2.86E-05
SLC25A21	0.0307	5.56E-01	6.75E-01
SLC25A22	-0.0309	5.54E-01	6.73E-01
SLC25A23	-0.2240	1.32E-05	1.07E-04
SLC25A24	0.0557	2.85E-01	4.13E-01
SLC25A25	-0.1008	5.23E-02	1.11E-01
SLC25A26	-0.1426	5.94E-03	1.90E-02
SLC25A27	-0.0193	7.10E-01	8.02E-01
SLC25A28	-0.0430	4.08E-01	5.41E-01
SLC25A29	0.0775	1.36E-01	2.36E-01
SLC25A2	0.0054	9.18E-01	9.48E-01
SLC25A30	-0.0809	1.20E-01	2.14E-01
SLC25A31	0.0455	3.82E-01	5.16E-01
SLC25A32	-0.0625	2.30E-01	3.53E-01
SLC25A33	-0.0161	7.57E-01	8.37E-01
SLC25A34	-0.1488	4.06E-03	1.39E-02
SLC25A35	0.1934	1.79E-04	1.00E-03

SLC25A36	0.0323	5.36E-01	6.58E-01
SLC25A37	0.0321	5.38E-01	6.60E-01
SLC25A38	-0.1839	3.69E-04	1.85E-03
SLC25A39	0.1359	8.78E-03	2.63E-02
SLC25A3	-0.1747	7.28E-04	3.28E-03
SLC25A40	0.0053	9.19E-01	9.49E-01
SLC25A41	-0.0044	9.32E-01	9.58E-01
SLC25A42	-0.2778	5.31E-08	8.21E-07
SLC25A43	0.0744	1.53E-01	2.57E-01
SLC25A44	0.1269	1.44E-02	3.95E-02
SLC25A45	0.1200	2.08E-02	5.30E-02
SLC25A46	-0.2381	3.53E-06	3.43E-05
SLC25A4	-0.3545	2.00E-12	8.34E-11
SLC25A5	-0.0120	8.17E-01	8.80E-01
SLC25A6	0.0891	8.65E-02	1.66E-01
SLC26A10	0.1228	1.80E-02	4.72E-02
SLC26A11	0.2978	4.91E-09	9.84E-08
SLC26A1	-0.2598	3.89E-07	4.90E-06
SLC26A2	0.1578	2.30E-03	8.66E-03
SLC26A3	-0.0687	1.87E-01	3.00E-01
SLC26A4	-0.0137	7.92E-01	8.62E-01
SLC26A5	-0.0349	5.03E-01	6.28E-01
SLC26A6	0.2355	4.53E-06	4.28E-05
SLC26A7	0.0234	6.54E-01	7.56E-01
SLC26A8	-0.0987	5.76E-02	1.20E-01
SLC26A9	0.1789	5.36E-04	2.52E-03
SLC27A1	-0.0729	1.61E-01	2.68E-01
SLC27A2	-0.2522	8.61E-07	9.92E-06
SLC27A3	0.1738	7.75E-04	3.46E-03
SLC27A4	-0.2011	9.63E-05	5.91E-04
SLC27A5	-0.3483	5.11E-12	1.98E-10
SLC27A6	0.0709	1.73E-01	2.83E-01
SLC28A1	-0.2416	2.50E-06	2.53E-05
SLC28A2	0.0087	8.67E-01	9.14E-01
SLC28A3	0.0181	7.28E-01	8.15E-01
SLC29A1	0.1440	5.44E-03	1.77E-02
SLC29A2	0.1127	3.00E-02	7.15E-02
SLC29A3	-0.0350	5.02E-01	6.27E-01
SLC29A4	0.0517	3.21E-01	4.51E-01
SLC2A10	-0.1926	1.90E-04	1.05E-03
SLC2A11	0.0041	9.38E-01	9.61E-01
SLC2A12	-0.0797	1.25E-01	2.21E-01
SLC2A13	0.0360	4.90E-01	6.17E-01
SLC2A14	-0.0594	2.54E-01	3.79E-01

SLC2A1	0.1241	1.68E-02	4.45E-02
SLC2A2	-0.1640	1.53E-03	6.13E-03
SLC2A3	-0.1280	1.36E-02	3.76E-02
SLC2A4RG	-0.0095	8.55E-01	9.06E-01
SLC2A4	-0.1649	1.44E-03	5.83E-03
SLC2A5	0.0011	9.84E-01	9.90E-01
SLC2A6	0.1796	5.10E-04	2.42E-03
SLC2A7	0.0863	9.70E-02	1.82E-01
SLC2A8	0.0658	2.06E-01	3.24E-01
SLC2A9	-0.1469	4.58E-03	1.53E-02
SLC30A10	-0.0340	5.14E-01	6.39E-01
SLC30A1	-0.1501	3.77E-03	1.31E-02
SLC30A2	-0.0294	5.73E-01	6.90E-01
SLC30A3	0.1280	1.36E-02	3.76E-02
SLC30A4	-0.1795	5.12E-04	2.43E-03
SLC30A5	0.0585	2.61E-01	3.87E-01
SLC30A6	0.0066	9.00E-01	9.37E-01
SLC30A7	0.0293	5.74E-01	6.91E-01
SLC30A8	0.1171	2.41E-02	5.97E-02
SLC30A9	-0.0727	1.62E-01	2.69E-01
SLC31A1	-0.2368	4.01E-06	3.84E-05
SLC31A2	-0.1657	1.36E-03	5.56E-03
SLC32A1	-0.0564	2.79E-01	4.07E-01
SLC33A1	-0.0316	5.44E-01	6.65E-01
SLC34A1	-0.0907	8.09E-02	1.57E-01
SLC34A2	0.0641	2.18E-01	3.38E-01
SLC34A3	0.1719	8.87E-04	3.88E-03
SLC35A1	0.1156	2.59E-02	6.35E-02
SLC35A2	0.0235	6.52E-01	7.56E-01
SLC35A3	-0.1230	1.78E-02	4.66E-02
SLC35A4	0.0559	2.83E-01	4.12E-01
SLC35A5	-0.1567	2.47E-03	9.18E-03
SLC35B1	0.0708	1.74E-01	2.84E-01
SLC35B2	0.0572	2.72E-01	4.00E-01
SLC35B3	0.0184	7.24E-01	8.13E-01
SLC35B4	-0.0539	3.00E-01	4.30E-01
SLC35C1	-0.1798	5.01E-04	2.38E-03
SLC35C2	-0.0945	6.91E-02	1.39E-01
SLC35D1	-0.2349	4.78E-06	4.46E-05
SLC35D2	0.0837	1.08E-01	1.97E-01
SLC35D3	0.0430	4.09E-01	5.42E-01
SLC35E1	-0.0357	4.92E-01	6.19E-01
SLC35E2	0.0127	8.08E-01	8.74E-01
SLC35E3	0.0684	1.88E-01	3.02E-01

SLC35E4	0.2296	7.93E-06	6.85E-05
SLC35F1	0.0032	9.52E-01	9.70E-01
SLC35F2	0.1365	8.48E-03	2.55E-02
SLC35F3	0.0966	6.30E-02	1.29E-01
SLC35F4	-0.0054	9.17E-01	9.48E-01
SLC35F5	-0.0805	1.22E-01	2.16E-01
SLC36A1	0.2000	1.05E-04	6.34E-04
SLC36A2	0.1742	7.52E-04	3.37E-03
SLC36A3	0.0563	2.80E-01	4.08E-01
SLC36A4	0.1608	1.89E-03	7.35E-03
SLC37A1	0.1917	2.04E-04	1.12E-03
SLC37A2	-0.0359	4.91E-01	6.18E-01
SLC37A3	0.0616	2.36E-01	3.60E-01
SLC37A4	-0.2404	2.83E-06	2.82E-05
SLC38A10	0.1079	3.78E-02	8.62E-02
SLC38A11	-0.1003	5.35E-02	1.13E-01
SLC38A1	0.0852	1.01E-01	1.87E-01
SLC38A2	-0.2767	6.01E-08	9.19E-07
SLC38A3	-0.1123	3.05E-02	7.24E-02
SLC38A4	-0.1301	1.21E-02	3.42E-02
SLC38A5	-0.0137	7.93E-01	8.63E-01
SLC38A6	0.1257	1.54E-02	4.15E-02
SLC38A7	-0.1175	2.37E-02	5.89E-02
SLC38A8	-0.1208	2.00E-02	5.13E-02
SLC38A9	0.0784	1.32E-01	2.29E-01
SLC39A10	0.1712	9.29E-04	4.04E-03
SLC39A11	-0.1929	1.86E-04	1.04E-03
SLC39A12	-0.0950	6.75E-02	1.36E-01
SLC39A13	0.1140	2.81E-02	6.78E-02
SLC39A14	-0.2188	2.12E-05	1.63E-04
SLC39A1	0.4096	1.94E-16	1.84E-14
SLC39A2	-0.1283	1.34E-02	3.71E-02
SLC39A3	0.0059	9.09E-01	9.43E-01
SLC39A4	0.1152	2.65E-02	6.47E-02
SLC39A5	-0.0954	6.63E-02	1.34E-01
SLC39A6	0.0867	9.56E-02	1.80E-01
SLC39A7	-0.0098	8.51E-01	9.03E-01
SLC39A8	-0.2301	7.55E-06	6.57E-05
SLC39A9	-0.2184	2.19E-05	1.67E-04
SLC3A1	-0.0834	1.09E-01	1.98E-01
SLC3A2	-0.1228	1.80E-02	4.71E-02
SLC40A1	-0.1734	7.99E-04	3.55E-03
SLC41A1	0.2477	1.36E-06	1.48E-05
SLC41A2	-0.2415	2.52E-06	2.55E-05

SLC41A3	0.1568	2.46E-03	9.15E-03
SLC43A1	-0.1340	9.76E-03	2.87E-02
SLC43A2	0.0198	7.04E-01	7.97E-01
SLC43A3	0.0361	4.88E-01	6.15E-01
SLC44A1	0.1734	7.97E-04	3.54E-03
SLC44A2	0.0055	9.15E-01	9.47E-01
SLC44A3	0.1711	9.35E-04	4.06E-03
SLC44A4	0.1203	2.04E-02	5.22E-02
SLC44A5	0.0361	4.88E-01	6.16E-01
SLC45A1	-0.1409	6.55E-03	2.06E-02
SLC45A2	-0.1977	1.27E-04	7.46E-04
SLC45A3	-0.0102	8.45E-01	8.99E-01
SLC45A4	0.1818	4.31E-04	2.10E-03
SLC46A1	0.0334	5.22E-01	6.46E-01
SLC46A2	0.0975	6.07E-02	1.25E-01
SLC46A3	-0.2105	4.38E-05	3.03E-04
SLC47A1	-0.1383	7.65E-03	2.34E-02
SLC47A2	0.0276	5.97E-01	7.10E-01
SLC48A1	-0.0031	9.52E-01	9.70E-01
SLC4A10	-0.1060	4.12E-02	9.24E-02
SLC4A11	0.0884	8.92E-02	1.70E-01
SLC4A1AP	0.1469	4.58E-03	1.53E-02
SLC4A1	-0.1474	4.45E-03	1.50E-02
SLC4A2	-0.0210	6.86E-01	7.83E-01
SLC4A3	0.1217	1.91E-02	4.94E-02
SLC4A4	-0.2588	4.30E-07	5.36E-06
SLC4A5	0.1489	4.04E-03	1.39E-02
SLC4A7	0.0307	5.56E-01	6.75E-01
SLC4A8	0.0319	5.41E-01	6.62E-01
SLC4A9	0.0693	1.83E-01	2.95E-01
SLC5A10	-0.0122	8.15E-01	8.78E-01
SLC5A11	0.0735	1.58E-01	2.64E-01
SLC5A12	-0.0744	1.52E-01	2.57E-01
SLC5A1	0.0801	1.24E-01	2.19E-01
SLC5A2	-0.0425	4.14E-01	5.47E-01
SLC5A3	0.0080	8.78E-01	9.21E-01
SLC5A4	-0.2081	5.37E-05	3.60E-04
SLC5A5	0.0907	8.11E-02	1.57E-01
SLC5A6	-0.0825	1.12E-01	2.04E-01
SLC5A7	0.0453	3.84E-01	5.17E-01
SLC5A8	0.0327	5.30E-01	6.53E-01
SLC5A9	0.0177	7.34E-01	8.20E-01
SLC6A10P	0.1222	1.86E-02	4.84E-02
SLC6A11	0.1008	5.23E-02	1.11E-01

SLC6A12	-0.2956	6.41E-09	1.25E-07
SLC6A13	-0.2636	2.59E-07	3.43E-06
SLC6A14	0.2298	7.74E-06	6.71E-05
SLC6A15	0.1639	1.54E-03	6.17E-03
SLC6A16	-0.0946	6.86E-02	1.38E-01
SLC6A17	-0.0384	4.61E-01	5.92E-01
SLC6A18	0.1095	3.50E-02	8.10E-02
SLC6A19	0.1564	2.52E-03	9.32E-03
SLC6A1	-0.1230	1.78E-02	4.67E-02
SLC6A20	-0.0392	4.51E-01	5.82E-01
SLC6A2	-0.0420	4.20E-01	5.53E-01
SLC6A3	0.0202	6.99E-01	7.92E-01
SLC6A4	0.0497	3.40E-01	4.72E-01
SLC6A5	0.0414	4.26E-01	5.59E-01
SLC6A6	0.0220	6.73E-01	7.72E-01
SLC6A7	-0.0721	1.66E-01	2.74E-01
SLC6A8	0.1077	3.82E-02	8.69E-02
SLC6A9	-0.0030	9.55E-01	9.72E-01
SLC7A10	0.2016	9.23E-05	5.69E-04
SLC7A11	0.0650	2.12E-01	3.31E-01
SLC7A13	0.0112	8.30E-01	8.89E-01
SLC7A14	-0.0273	6.00E-01	7.13E-01
SLC7A1	0.1969	1.35E-04	7.91E-04
SLC7A2	-0.1240	1.69E-02	4.47E-02
SLC7A3	0.0170	7.45E-01	8.28E-01
SLC7A4	-0.0734	1.58E-01	2.64E-01
SLC7A5P1	0.0553	2.88E-01	4.17E-01
SLC7A5P2	0.1862	3.11E-04	1.61E-03
SLC7A5	0.0871	9.39E-02	1.77E-01
SLC7A6OS	-0.0209	6.88E-01	7.84E-01
SLC7A6	0.0572	2.71E-01	3.99E-01
SLC7A7	0.0457	3.80E-01	5.14E-01
SLC7A8	-0.0894	8.57E-02	1.64E-01
SLC7A9	-0.0114	8.27E-01	8.87E-01
SLC8A1	-0.1596	2.04E-03	7.85E-03
SLC8A2	0.1034	4.65E-02	1.02E-01
SLC8A3	-0.2462	1.58E-06	1.70E-05
SLC9A10	0.1790	5.31E-04	2.50E-03
SLC9A11	0.0094	8.56E-01	9.07E-01
SLC9A1	0.0038	9.42E-01	9.64E-01
SLC9A2	0.0800	1.24E-01	2.19E-01
SLC9A3R1	0.0622	2.32E-01	3.55E-01
SLC9A3R2	-0.1691	1.07E-03	4.56E-03
SLC9A3	0.1021	4.94E-02	1.07E-01

SLC9A4	0.1153	2.64E-02	6.44E-02
SLC9A5	0.0440	3.98E-01	5.31E-01
SLC9A6	0.1497	3.85E-03	1.33E-02
SLC9A7	0.0388	4.56E-01	5.87E-01
SLC9A8	0.0808	1.20E-01	2.15E-01
SLC9A9	-0.0375	4.71E-01	6.00E-01
SLCO1A2	-0.0988	5.73E-02	1.20E-01
SLCO1B1	-0.2465	1.54E-06	1.66E-05
SLCO1B3	-0.1178	2.32E-02	5.80E-02
SLCO1C1	-0.1416	6.30E-03	1.99E-02
SLCO2A1	-0.3087	1.24E-09	2.87E-08
SLCO2B1	-0.2514	9.31E-07	1.07E-05
SLCO3A1	0.0716	1.69E-01	2.78E-01
SLCO4A1	-0.0182	7.27E-01	8.15E-01
SLCO4C1	0.0715	1.69E-01	2.78E-01
SLCO5A1	0.0753	1.48E-01	2.51E-01
SLCO6A1	0.1011	5.18E-02	1.11E-01
SLED1	-0.0386	4.58E-01	5.89E-01
SLFN11	-0.0206	6.93E-01	7.88E-01
SLFN12L	-0.1037	4.58E-02	1.00E-01
SLFN12	-0.0436	4.02E-01	5.35E-01
SLFN13	0.1628	1.65E-03	6.57E-03
SLFN14	0.0181	7.28E-01	8.15E-01
SLFN5	-0.1790	5.32E-04	2.51E-03
SLFNL1	0.1074	3.86E-02	8.77E-02
SLIT1	0.0464	3.73E-01	5.06E-01
SLIT2	-0.1155	2.61E-02	6.39E-02
SLIT3	-0.1777	5.84E-04	2.71E-03
SLITRK1	0.2073	5.73E-05	3.81E-04
SLITRK2	0.0104	8.42E-01	8.96E-01
SLITRK3	-0.0953	6.68E-02	1.35E-01
SLITRK4	-0.0562	2.81E-01	4.09E-01
SLITRK5	0.1047	4.39E-02	9.70E-02
SLITRK6	-0.0761	1.43E-01	2.45E-01
SLK	-0.0962	6.41E-02	1.31E-01
SLMAP	0.0279	5.93E-01	7.07E-01
SLMO1	0.1682	1.15E-03	4.83E-03
SLMO2	0.1909	2.16E-04	1.18E-03
SLN	0.0012	9.81E-01	9.88E-01
SLPI	0.0578	2.67E-01	3.95E-01
SLTM	0.0858	9.89E-02	1.84E-01
SLU7	0.0587	2.59E-01	3.85E-01
SLURP1	-0.0234	6.53E-01	7.56E-01
SMAD1	-0.2482	1.29E-06	1.42E-05

SMAD2	0.0762	1.43E-01	2.45E-01
SMAD3	0.1738	7.75E-04	3.46E-03
SMAD4	0.0347	5.05E-01	6.30E-01
SMAD5OS	0.0462	3.75E-01	5.09E-01
SMAD5	0.1535	3.03E-03	1.09E-02
SMAD6	-0.2035	7.89E-05	4.98E-04
SMAD7	-0.0182	7.27E-01	8.14E-01
SMAD9	-0.1464	4.73E-03	1.57E-02
SMAGP	0.0978	5.98E-02	1.24E-01
SMAP1	0.2573	5.05E-07	6.17E-06
SMAP2	-0.1028	4.79E-02	1.04E-01
SMARCA1	0.0703	1.77E-01	2.88E-01
SMARCA2	-0.1942	1.68E-04	9.48E-04
SMARCA4	0.2240	1.33E-05	1.07E-04
SMARCA5	0.0246	6.37E-01	7.43E-01
SMARCAD1	-0.0290	5.77E-01	6.94E-01
SMARCAL1	0.1476	4.39E-03	1.48E-02
SMARCB1	0.1661	1.32E-03	5.44E-03
SMARCC1	0.2013	9.44E-05	5.80E-04
SMARCC2	0.2355	4.52E-06	4.26E-05
SMARCD1	0.2493	1.16E-06	1.30E-05
SMARCD2	0.1039	4.54E-02	9.97E-02
SMARCD3	0.0603	2.47E-01	3.72E-01
SMARCE1	0.3307	6.42E-11	1.97E-09
SMC1A	0.1198	2.10E-02	5.34E-02
SMC1B	0.3125	7.54E-10	1.84E-08
SMC2	0.2925	9.41E-09	1.76E-07
SMC3	0.1652	1.40E-03	5.71E-03
SMC4	0.3314	5.88E-11	1.82E-09
SMC5	0.2566	5.45E-07	6.59E-06
SMC6	0.1340	9.76E-03	2.87E-02
SMCHD1	0.1990	1.14E-04	6.82E-04
SMCP	0.0992	5.63E-02	1.18E-01
SMCR5	0.1020	4.96E-02	1.07E-01
SMCR7L	0.0557	2.85E-01	4.14E-01
SMCR7	0.0156	7.65E-01	8.41E-01
SMCR8	0.1181	2.29E-02	5.74E-02
SMEK1	0.0861	9.76E-02	1.83E-01
SMEK2	0.0240	6.45E-01	7.50E-01
SMEK3P	0.0593	2.55E-01	3.80E-01
SMG1	0.0541	2.98E-01	4.28E-01
SMG5	0.3906	5.67E-15	3.87E-13
SMG6	-0.1624	1.70E-03	6.71E-03
SMG7	0.4131	1.01E-16	1.03E-14

SMN1	0.1365	8.47E-03	2.55E-02
SMN2	0.1650	1.43E-03	5.79E-03
SMNDC1	0.0662	2.03E-01	3.20E-01
SMOC1	-0.1917	2.03E-04	1.12E-03
SMOC2	-0.1585	2.20E-03	8.34E-03
SMOX	0.0534	3.05E-01	4.34E-01
SMO	0.0222	6.70E-01	7.70E-01
SMPD1	-0.2913	1.09E-08	2.01E-07
SMPD2	0.2623	2.97E-07	3.88E-06
SMPD3	-0.0972	6.16E-02	1.27E-01
SMPD4	0.3654	3.69E-13	1.78E-11
SMPDL3A	-0.0763	1.42E-01	2.44E-01
SMPDL3B	0.1670	1.25E-03	5.18E-03
SMPX	-0.1059	4.15E-02	9.28E-02
SMR3A	-0.0630	2.26E-01	3.47E-01
SMR3B	-0.0579	2.66E-01	3.93E-01
SMS	0.1127	3.00E-02	7.14E-02
SMTNL1	-0.0282	5.88E-01	7.03E-01
SMTNL2	-0.0286	5.83E-01	6.99E-01
SMTN	0.1136	2.87E-02	6.91E-02
SMU1	0.1943	1.66E-04	9.41E-04
SMUG1	0.1789	5.37E-04	2.53E-03
SMURF1	0.0053	9.18E-01	9.48E-01
SMURF2	0.2687	1.48E-07	2.07E-06
SMYD1	-0.0384	4.61E-01	5.92E-01
SMYD2	0.0315	5.45E-01	6.66E-01
SMYD3	0.3060	1.76E-09	3.96E-08
SMYD4	0.0606	2.44E-01	3.69E-01
SMYD5	0.2088	5.06E-05	3.42E-04
SNAI1	-0.1108	3.28E-02	7.70E-02
SNAI2	-0.1273	1.41E-02	3.88E-02
SNAI3	-0.0086	8.69E-01	9.16E-01
SNAP23	-0.1707	9.64E-04	4.17E-03
SNAP25	0.1336	9.98E-03	2.92E-02
SNAP29	0.0266	6.09E-01	7.21E-01
SNAP47	0.1731	8.15E-04	3.60E-03
SNAP91	0.0620	2.34E-01	3.57E-01
SNAPC1	0.0948	6.81E-02	1.37E-01
SNAPC2	-0.0386	4.58E-01	5.89E-01
SNAPC3	-0.0658	2.06E-01	3.24E-01
SNAPC4	0.2112	4.11E-05	2.87E-04
SNAPC5	-0.0108	8.36E-01	8.93E-01
SNAPIN	0.5138	2.19E-26	1.41E-23
SNAR-B2	0.0127	8.07E-01	8.73E-01

SNAR-C4	0.0433	4.06E-01	5.39E-01
SNAR-G1	0.0378	4.68E-01	5.98E-01
SNCAIP	-0.0892	8.64E-02	1.65E-01
SNCA	-0.1283	1.34E-02	3.72E-02
SNCB	0.0143	7.83E-01	8.56E-01
SNCG	0.0939	7.10E-02	1.42E-01
SND1	-0.1237	1.71E-02	4.53E-02
SNED1	-0.1296	1.25E-02	3.50E-02
SNF8	0.1619	1.75E-03	6.89E-03
SNHG10	0.2597	3.92E-07	4.93E-06
SNHG11	0.1017	5.04E-02	1.08E-01
SNHG12	0.2952	6.77E-09	1.31E-07
SNHG1	0.3750	7.84E-14	4.23E-12
SNHG3-RCC1	0.0652	2.10E-01	3.29E-01
SNHG3	0.2340	5.21E-06	4.82E-05
SNHG4	0.2470	1.47E-06	1.59E-05
SNHG5	0.0653	2.10E-01	3.28E-01
SNHG6	0.1419	6.19E-03	1.96E-02
SNHG7	0.2326	6.00E-06	5.41E-05
SNHG8	-0.0509	3.28E-01	4.59E-01
SNHG9	0.0347	5.05E-01	6.30E-01
SNIP1	0.0434	4.04E-01	5.37E-01
SNN	-0.0105	8.40E-01	8.95E-01
SNORA10	0.1444	5.32E-03	1.74E-02
SNORA11B	0.1010	5.19E-02	1.11E-01
SNORA11	0.0795	1.26E-01	2.22E-01
SNORA12	0.1363	8.55E-03	2.57E-02
SNORA13	0.0220	6.73E-01	7.72E-01
SNORA14A	-0.0067	8.97E-01	9.34E-01
SNORA14B	-0.0073	8.88E-01	9.28E-01
SNORA15	0.0304	5.59E-01	6.77E-01
SNORA16A	-0.0306	5.56E-01	6.75E-01
SNORA16B	0.0459	3.78E-01	5.11E-01
SNORA18	-0.0277	5.95E-01	7.09E-01
SNORA1	0.0445	3.93E-01	5.26E-01
SNORA20	0.0224	6.67E-01	7.67E-01
SNORA21	0.1179	2.31E-02	5.78E-02
SNORA22	-0.0094	8.57E-01	9.08E-01
SNORA23	0.0383	4.62E-01	5.92E-01
SNORA24	0.0412	4.29E-01	5.61E-01
SNORA25	0.0970	6.21E-02	1.27E-01
SNORA26	0.0770	1.39E-01	2.39E-01
SNORA27	0.0944	6.94E-02	1.39E-01
SNORA28	0.0456	3.81E-01	5.14E-01

SNORA29	0.1036	4.62E-02	1.01E-01
SNORA2A	0.0375	4.72E-01	6.01E-01
SNORA2B	-0.0311	5.51E-01	6.71E-01
SNORA31	0.0848	1.03E-01	1.90E-01
SNORA32	0.0975	6.06E-02	1.25E-01
SNORA34	0.0745	1.52E-01	2.56E-01
SNORA36A	-0.0260	6.18E-01	7.27E-01
SNORA37	0.0283	5.87E-01	7.02E-01
SNORA38B	-0.0392	4.52E-01	5.83E-01
SNORA38	0.0729	1.61E-01	2.68E-01
SNORA39	0.1727	8.37E-04	3.69E-03
SNORA3	0.0283	5.87E-01	7.02E-01
SNORA40	0.0457	3.80E-01	5.14E-01
SNORA41	0.1172	2.40E-02	5.95E-02
SNORA42	0.0621	2.33E-01	3.56E-01
SNORA44	0.0424	4.16E-01	5.49E-01
SNORA45	0.0657	2.07E-01	3.25E-01
SNORA46	0.0712	1.71E-01	2.80E-01
SNORA47	-0.0255	6.25E-01	7.33E-01
SNORA48	0.0268	6.07E-01	7.19E-01
SNORA49	0.0312	5.49E-01	6.70E-01
SNORA4	0.0060	9.08E-01	9.42E-01
SNORA50	0.0644	2.16E-01	3.36E-01
SNORA51	-0.0064	9.02E-01	9.38E-01
SNORA52	0.0738	1.56E-01	2.62E-01
SNORA53	0.0143	7.83E-01	8.56E-01
SNORA54	0.0398	4.45E-01	5.77E-01
SNORA55	-0.0041	9.37E-01	9.61E-01
SNORA56	0.0661	2.04E-01	3.22E-01
SNORA57	0.1631	1.62E-03	6.47E-03
SNORA58	0.0074	8.87E-01	9.28E-01
SNORA59B	0.0651	2.11E-01	3.30E-01
SNORA5A	-0.0050	9.23E-01	9.52E-01
SNORA5B	0.0822	1.14E-01	2.06E-01
SNORA5C	0.0108	8.36E-01	8.93E-01
SNORA61	0.0623	2.32E-01	3.55E-01
SNORA62	0.0823	1.13E-01	2.05E-01
SNORA63	0.1093	3.53E-02	8.15E-02
SNORA64	0.0017	9.74E-01	9.83E-01
SNORA65	0.0508	3.29E-01	4.60E-01
SNORA66	0.0136	7.94E-01	8.63E-01
SNORA67	0.1956	1.50E-04	8.63E-04
SNORA68	0.0384	4.61E-01	5.91E-01
SNORA6	0.0949	6.77E-02	1.37E-01

SNORA70	0.0545	2.95E-01	4.24E-01
SNORA71A	0.0185	7.23E-01	8.12E-01
SNORA71B	0.0200	7.02E-01	7.95E-01
SNORA71C	-0.0004	9.94E-01	9.96E-01
SNORA71D	0.0260	6.17E-01	7.27E-01
SNORA72	0.0857	9.92E-02	1.85E-01
SNORA74A	0.0328	5.29E-01	6.52E-01
SNORA74B	0.0554	2.87E-01	4.16E-01
SNORA75	-0.0003	9.95E-01	9.97E-01
SNORA76	0.1855	3.29E-04	1.69E-03
SNORA77	-0.0499	3.38E-01	4.69E-01
SNORA79	0.0408	4.33E-01	5.65E-01
SNORA7B	0.0378	4.68E-01	5.98E-01
SNORA80	-0.0506	3.31E-01	4.62E-01
SNORA81	0.0926	7.48E-02	1.48E-01
SNORA84	-0.0170	7.45E-01	8.28E-01
SNORA8	0.2188	2.12E-05	1.63E-04
SNORA9	-0.0113	8.29E-01	8.88E-01
SNORD10	0.1146	2.72E-02	6.61E-02
SNORD115-13	-0.0473	3.64E-01	4.96E-01
SNORD115-26	-0.1172	2.40E-02	5.96E-02
SNORD115-7	-0.0695	1.82E-01	2.94E-01
SNORD116-20	0.0034	9.48E-01	9.68E-01
SNORD116-28	0.0949	6.80E-02	1.37E-01
SNORD116-4	-0.0187	7.20E-01	8.10E-01
SNORD15A	0.0739	1.55E-01	2.61E-01
SNORD15B	0.0281	5.90E-01	7.04E-01
SNORD17	0.0570	2.73E-01	4.01E-01
SNORD1C	0.1870	2.94E-04	1.53E-03
SNORD22	0.0545	2.95E-01	4.24E-01
SNORD89	0.0873	9.31E-02	1.76E-01
SNORD94	0.0186	7.22E-01	8.11E-01
SNORD97	-0.0233	6.55E-01	7.57E-01
SNPH	-0.0343	5.10E-01	6.34E-01
SNRK	-0.2263	1.08E-05	9.00E-05
SNRNP200	0.2061	6.36E-05	4.15E-04
SNRNP25	0.0253	6.27E-01	7.35E-01
SNRNP27	0.0463	3.74E-01	5.07E-01
SNRNP35	0.0458	3.79E-01	5.12E-01
SNRNP40	0.2121	3.82E-05	2.70E-04
SNRNP48	0.2005	1.01E-04	6.13E-04
SNRNP70	0.3117	8.45E-10	2.04E-08
SNRPA1	0.2924	9.52E-09	1.78E-07
SNRPA	0.2891	1.42E-08	2.54E-07

SNRPB2	0.2060	6.42E-05	4.18E-04
SNRPB	0.3092	1.17E-09	2.72E-08
SNRPC	0.2195	1.99E-05	1.54E-04
SNRPD1	0.3392	1.92E-11	6.53E-10
SNRPD2	0.2163	2.65E-05	1.97E-04
SNRPD3	0.1704	9.83E-04	4.24E-03
SNRPE	0.3957	2.36E-15	1.75E-13
SNRPF	0.1783	5.59E-04	2.61E-03
SNRPG	0.2961	6.10E-09	1.19E-07
SNRPN	0.0318	5.42E-01	6.63E-01
SNTA1	-0.0126	8.09E-01	8.74E-01
SNTB1	-0.1025	4.86E-02	1.05E-01
SNTB2	-0.0489	3.47E-01	4.80E-01
SNTG1	-0.1148	2.71E-02	6.57E-02
SNTG2	-0.0027	9.58E-01	9.74E-01
SNTN	0.1071	3.92E-02	8.88E-02
SNUPN	0.1440	5.45E-03	1.77E-02
SNURF	0.0413	4.27E-01	5.60E-01
SNW1	0.1449	5.17E-03	1.70E-02
SNX10	0.0016	9.76E-01	9.85E-01
SNX11	0.1300	1.22E-02	3.44E-02
SNX12	-0.0484	3.53E-01	4.85E-01
SNX13	-0.0563	2.80E-01	4.08E-01
SNX14	0.0736	1.57E-01	2.63E-01
SNX15	0.1475	4.41E-03	1.49E-02
SNX16	0.0159	7.60E-01	8.38E-01
SNX17	-0.1194	2.15E-02	5.44E-02
SNX18	-0.0175	7.37E-01	8.22E-01
SNX19	-0.0485	3.52E-01	4.84E-01
SNX1	-0.1323	1.07E-02	3.09E-02
SNX20	-0.0774	1.37E-01	2.36E-01
SNX21	-0.0253	6.27E-01	7.36E-01
SNX22	0.0978	6.00E-02	1.24E-01
SNX24	-0.0020	9.69E-01	9.81E-01
SNX25	-0.1331	1.02E-02	2.98E-02
SNX27	0.4251	1.04E-17	1.40E-15
SNX29	0.0948	6.83E-02	1.38E-01
SNX2	-0.0737	1.56E-01	2.62E-01
SNX30	-0.0021	9.69E-01	9.80E-01
SNX31	-0.1185	2.24E-02	5.64E-02
SNX32	0.0193	7.11E-01	8.03E-01
SNX33	-0.1066	4.01E-02	9.04E-02
SNX3	0.0283	5.86E-01	7.02E-01
SNX4	-0.1441	5.41E-03	1.76E-02

SNX5	0.0390	4.53E-01	5.84E-01
SNX6	-0.0378	4.68E-01	5.98E-01
SNX7	0.0685	1.88E-01	3.01E-01
SNX8	0.1595	2.06E-03	7.90E-03
SNX9	-0.0233	6.55E-01	7.57E-01
SOAT1	0.2769	5.89E-08	9.03E-07
SOAT2	0.0497	3.40E-01	4.71E-01
SOBP	0.0019	9.70E-01	9.81E-01
SOCS1	-0.0024	9.64E-01	9.78E-01
SOCS2	-0.2062	6.28E-05	4.10E-04
SOCS3	-0.1337	9.93E-03	2.91E-02
SOCS4	0.0956	6.60E-02	1.34E-01
SOCS5	0.1996	1.08E-04	6.51E-04
SOCS6	-0.0336	5.18E-01	6.42E-01
SOCS7	0.2699	1.30E-07	1.84E-06
SOD1	-0.1160	2.55E-02	6.25E-02
SOD2	-0.0782	1.33E-01	2.31E-01
SOD3	-0.0058	9.11E-01	9.44E-01
SOHLH1	0.0896	8.47E-02	1.63E-01
SOHLH2	0.0247	6.35E-01	7.42E-01
SOLH	0.2113	4.09E-05	2.85E-04
SON	0.0373	4.74E-01	6.02E-01
SORBS1	-0.2399	2.96E-06	2.93E-05
SORBS2	-0.2129	3.55E-05	2.53E-04
SORBS3	-0.1492	3.98E-03	1.37E-02
SORCS1	0.1875	2.81E-04	1.48E-03
SORCS2	0.0020	9.69E-01	9.80E-01
SORCS3	0.0343	5.10E-01	6.35E-01
SORD	-0.2090	4.98E-05	3.38E-04
SORL1	-0.2225	1.52E-05	1.21E-04
SORT1	-0.0014	9.78E-01	9.86E-01
SOS1	-0.0106	8.38E-01	8.94E-01
SOS2	-0.0375	4.71E-01	6.00E-01
SOSTDC1	0.0973	6.13E-02	1.26E-01
SOST	-0.0193	7.11E-01	8.03E-01
SOX10	-0.0375	4.72E-01	6.01E-01
SOX11	0.1330	1.03E-02	3.00E-02
SOX12	0.0783	1.32E-01	2.30E-01
SOX13	0.2673	1.73E-07	2.39E-06
SOX14	-0.0058	9.12E-01	9.44E-01
SOX15	0.0011	9.83E-01	9.90E-01
SOX17	-0.1435	5.63E-03	1.82E-02
SOX18	-0.1042	4.49E-02	9.89E-02
SOX1	-0.0306	5.57E-01	6.76E-01

SOX21	-0.0087	8.68E-01	9.15E-01
SOX2OT	-0.1515	3.44E-03	1.21E-02
SOX2	-0.0269	6.06E-01	7.18E-01
SOX30	0.0135	7.95E-01	8.64E-01
SOX3	0.0346	5.06E-01	6.31E-01
SOX4	0.1602	1.97E-03	7.60E-03
SOX5	-0.1201	2.06E-02	5.27E-02
SOX6	-0.1417	6.26E-03	1.98E-02
SOX7	-0.2111	4.17E-05	2.90E-04
SOX8	-0.0435	4.04E-01	5.37E-01
SOX9	0.1916	2.05E-04	1.13E-03
SP100	-0.2125	3.69E-05	2.62E-04
SP110	0.0988	5.73E-02	1.20E-01
SP140L	0.0799	1.24E-01	2.20E-01
SP140	0.0982	5.89E-02	1.22E-01
SP1	0.1667	1.27E-03	5.27E-03
SP2	0.1472	4.50E-03	1.51E-02
SP3	0.1788	5.41E-04	2.54E-03
SP4	0.1721	8.75E-04	3.83E-03
SP5	0.0736	1.57E-01	2.63E-01
SP6	0.0330	5.26E-01	6.49E-01
SP7	0.0803	1.23E-01	2.18E-01
SP8	0.2492	1.17E-06	1.30E-05
SP9	0.1733	8.03E-04	3.56E-03
SPA17	0.2166	2.58E-05	1.93E-04
SPACA1	-0.0666	2.01E-01	3.17E-01
SPACA3	0.0260	6.18E-01	7.27E-01
SPACA4	0.1008	5.23E-02	1.11E-01
SPACA5	0.0912	7.94E-02	1.55E-01
SPAG11A	0.0453	3.84E-01	5.17E-01
SPAG11B	0.0494	3.42E-01	4.74E-01
SPAG16	0.0005	9.92E-01	9.95E-01
SPAG17	0.2050	6.98E-05	4.49E-04
SPAG1	0.1124	3.04E-02	7.23E-02
SPAG4	-0.0357	4.92E-01	6.19E-01
SPAG5	0.3779	4.89E-14	2.76E-12
SPAG6	0.2535	7.52E-07	8.78E-06
SPAG7	-0.1664	1.29E-03	5.35E-03
SPAG8	0.1503	3.71E-03	1.29E-02
SPAG9	0.0638	2.21E-01	3.41E-01
SPAM1	0.0018	9.72E-01	9.82E-01
SPANXA2	0.1192	2.16E-02	5.47E-02
SPANXB2	0.1549	2.78E-03	1.02E-02
SPANXC	0.1207	2.01E-02	5.15E-02

SPANXE	0.1993	1.12E-04	6.68E-04
SPANXN1	0.1309	1.16E-02	3.31E-02
SPANXN2	-0.0262	6.15E-01	7.25E-01
SPANXN3	0.0802	1.23E-01	2.18E-01
SPANXN4	-0.0077	8.82E-01	9.25E-01
SPANXN5	0.0449	3.89E-01	5.22E-01
SPARCL1	-0.2084	5.24E-05	3.53E-04
SPARC	-0.0991	5.65E-02	1.18E-01
SPAST	0.0993	5.61E-02	1.18E-01
SPATA12	0.0522	3.16E-01	4.46E-01
SPATA13	-0.0101	8.46E-01	8.99E-01
SPATA16	-0.0107	8.38E-01	8.93E-01
SPATA17	0.2433	2.11E-06	2.19E-05
SPATA18	-0.2141	3.22E-05	2.32E-04
SPATA19	0.0449	3.89E-01	5.22E-01
SPATA1	-0.0227	6.63E-01	7.64E-01
SPATA20	-0.0882	8.97E-02	1.71E-01
SPATA21	0.0354	4.97E-01	6.23E-01
SPATA22	-0.1162	2.52E-02	6.19E-02
SPATA24	0.0381	4.64E-01	5.95E-01
SPATA2L	-0.0982	5.87E-02	1.22E-01
SPATA2	0.2022	8.79E-05	5.47E-04
SPATA3	0.0836	1.08E-01	1.97E-01
SPATA4	0.0236	6.51E-01	7.55E-01
SPATA5L1	0.1777	5.85E-04	2.71E-03
SPATA5	0.0284	5.86E-01	7.01E-01
SPATA6	0.0771	1.38E-01	2.38E-01
SPATA7	0.0405	4.37E-01	5.69E-01
SPATA8	0.1009	5.21E-02	1.11E-01
SPATA9	-0.0309	5.53E-01	6.72E-01
SPATC1	0.0818	1.16E-01	2.09E-01
SPATS1	0.0940	7.06E-02	1.41E-01
SPATS2L	0.1995	1.10E-04	6.58E-04
SPATS2	0.3482	5.14E-12	1.99E-10
SPC24	0.2800	4.12E-08	6.56E-07
SPC25	0.4247	1.11E-17	1.47E-15
SPCS1	-0.1499	3.81E-03	1.32E-02
SPCS2	0.1151	2.66E-02	6.49E-02
SPCS3	-0.2892	1.40E-08	2.51E-07
SPDEF	0.0086	8.68E-01	9.15E-01
SPDYA	0.2049	7.01E-05	4.51E-04
SPDYC	-0.2162	2.66E-05	1.98E-04
SPDYE1	0.2071	5.82E-05	3.85E-04
SPDYE2	0.1054	4.24E-02	9.44E-02

SPDYE3	-0.0446	3.91E-01	5.24E-01
SPDYE4	0.0436	4.02E-01	5.35E-01
SPDYE5	0.2017	9.11E-05	5.64E-04
SPDYE6	0.2106	4.32E-05	2.99E-04
SPDYE7P	0.1290	1.29E-02	3.60E-02
SPDYE8P	-0.0189	7.17E-01	8.08E-01
SPEF1	0.0485	3.52E-01	4.84E-01
SPEF2	0.3841	1.72E-14	1.06E-12
SPEG	-0.0142	7.86E-01	8.58E-01
SPEM1	0.0849	1.03E-01	1.89E-01
SPEN	0.0563	2.80E-01	4.08E-01
SPERT	0.1820	4.27E-04	2.08E-03
SPESP1	-0.0503	3.34E-01	4.65E-01
SPG11	-0.1206	2.01E-02	5.16E-02
SPG20	-0.1226	1.81E-02	4.75E-02
SPG21	-0.0256	6.23E-01	7.32E-01
SPG7	-0.3021	2.88E-09	6.11E-08
SPHAR	0.0949	6.80E-02	1.37E-01
SPHK1	0.1936	1.75E-04	9.84E-04
SPHK2	0.0037	9.44E-01	9.65E-01
SPHKAP	0.0251	6.30E-01	7.38E-01
SPI1	-0.0396	4.47E-01	5.79E-01
SPIB	0.2324	6.11E-06	5.50E-05
SPIC	-0.1387	7.46E-03	2.29E-02
SPIN1	0.1616	1.79E-03	7.03E-03
SPIN2A	0.0529	3.10E-01	4.39E-01
SPIN2B	0.1030	4.74E-02	1.03E-01
SPIN3	0.1888	2.55E-04	1.36E-03
SPIN4	0.2477	1.36E-06	1.49E-05
SPINK13	0.1179	2.32E-02	5.79E-02
SPINK14	-0.0362	4.87E-01	6.14E-01
SPINK1	0.0478	3.58E-01	4.91E-01
SPINK2	0.0760	1.44E-01	2.46E-01
SPINK4	0.1380	7.77E-03	2.37E-02
SPINK5	0.0204	6.95E-01	7.89E-01
SPINK6	-0.0145	7.80E-01	8.53E-01
SPINK7	-0.0575	2.69E-01	3.97E-01
SPINK8	-0.0311	5.51E-01	6.71E-01
SPINK9	0.0792	1.28E-01	2.25E-01
SPINLW1	0.0521	3.16E-01	4.46E-01
SPINT1	0.1900	2.32E-04	1.25E-03
SPINT2	0.1277	1.38E-02	3.82E-02
SPINT3	-0.0505	3.32E-01	4.63E-01
SPIRE1	-0.0082	8.75E-01	9.20E-01

SPIRE2	0.0902	8.27E-02	1.60E-01
SPNS1	0.1293	1.27E-02	3.55E-02
SPNS2	-0.3030	2.57E-09	5.50E-08
SPNS3	0.0166	7.49E-01	8.31E-01
SPN	-0.0927	7.44E-02	1.47E-01
SPO11	0.0310	5.52E-01	6.72E-01
SPOCD1	0.1836	3.79E-04	1.89E-03
SPOCK1	-0.0233	6.54E-01	7.57E-01
SPOCK2	-0.0778	1.35E-01	2.34E-01
SPOCK3	0.0764	1.42E-01	2.43E-01
SPON1	-0.1207	2.00E-02	5.15E-02
SPON2	0.0185	7.23E-01	8.12E-01
SPOPL	0.0838	1.07E-01	1.96E-01
SPOP	0.1925	1.92E-04	1.06E-03
SPP1	0.1206	2.02E-02	5.17E-02
SPP2	-0.1955	1.51E-04	8.67E-04
SPPL2A	-0.2118	3.92E-05	2.75E-04
SPPL2B	0.1036	4.61E-02	1.01E-01
SPPL3	0.0781	1.33E-01	2.32E-01
SPRED1	0.0353	4.98E-01	6.24E-01
SPRED2	-0.0142	7.85E-01	8.57E-01
SPRED3	-0.0220	6.73E-01	7.72E-01
SPRN	0.0848	1.03E-01	1.90E-01
SPRR1A	-0.0735	1.58E-01	2.64E-01
SPRR1B	-0.0759	1.45E-01	2.47E-01
SPRR2A	-0.1221	1.86E-02	4.85E-02
SPRR2B	-0.0848	1.03E-01	1.90E-01
SPRR2C	-0.0773	1.37E-01	2.37E-01
SPRR2D	-0.0511	3.26E-01	4.56E-01
SPRR2E	-0.0607	2.43E-01	3.68E-01
SPRR2F	-0.0079	8.80E-01	9.23E-01
SPRR2G	-0.0527	3.11E-01	4.41E-01
SPRR3	-0.0649	2.12E-01	3.31E-01
SPRR4	-0.0017	9.74E-01	9.83E-01
SPRY1	-0.2365	4.12E-06	3.94E-05
SPRY2	-0.1022	4.93E-02	1.06E-01
SPRY3	-0.0056	9.14E-01	9.46E-01
SPRY4	-0.0228	6.61E-01	7.63E-01
SPRYD3	-0.0638	2.20E-01	3.41E-01
SPRYD4	-0.3185	3.41E-10	9.11E-09
SPRYD5	-0.0427	4.12E-01	5.45E-01
SPR	-0.2466	1.52E-06	1.64E-05
SPSB1	-0.1522	3.29E-03	1.17E-02
SPSB2	0.2004	1.02E-04	6.17E-04

SPSB3	0.0525	3.13E-01	4.43E-01
SPSB4	-0.1075	3.86E-02	8.76E-02
SPTA1	0.0356	4.95E-01	6.21E-01
SPTAN1	0.1944	1.65E-04	9.33E-04
SPTBN1	-0.1016	5.05E-02	1.08E-01
SPTBN2	-0.0946	6.89E-02	1.38E-01
SPTBN4	-0.1313	1.14E-02	3.24E-02
SPTBN5	0.1038	4.58E-02	1.00E-01
SPTB	0.1095	3.50E-02	8.09E-02
SPTLC1	0.2075	5.64E-05	3.76E-04
SPTLC2	-0.1395	7.12E-03	2.21E-02
SPTLC3	-0.0364	4.85E-01	6.13E-01
SPTY2D1	0.0692	1.83E-01	2.96E-01
SPZ1	0.1656	1.37E-03	5.60E-03
SQLE	0.1100	3.41E-02	7.93E-02
SQRDL	0.0274	5.98E-01	7.12E-01
SQSTM1	0.1168	2.44E-02	6.04E-02
SR140	0.3171	4.11E-10	1.07E-08
SRA1	-0.0308	5.54E-01	6.74E-01
SRBD1	-0.1381	7.71E-03	2.36E-02
SRCAP	0.1317	1.11E-02	3.18E-02
SRCIN1	0.0630	2.26E-01	3.48E-01
SRCRB4D	0.0251	6.30E-01	7.37E-01
SRC	0.1978	1.25E-04	7.40E-04
SRD5A1	-0.2573	5.05E-07	6.17E-06
SRD5A2	-0.2839	2.63E-08	4.37E-07
SRD5A3	-0.0281	5.89E-01	7.04E-01
SREBF1	0.0215	6.80E-01	7.78E-01
SREBF2	0.1040	4.53E-02	9.95E-02
SRFBP1	0.0475	3.62E-01	4.94E-01
SRF	0.1981	1.22E-04	7.24E-04
SRGAP1	0.0847	1.03E-01	1.91E-01
SRGAP2	0.2021	8.87E-05	5.51E-04
SRGAP3	-0.1074	3.88E-02	8.79E-02
SRGN	-0.1487	4.09E-03	1.40E-02
SRI	0.0696	1.81E-01	2.93E-01
SRL	-0.2720	1.02E-07	1.48E-06
SRMS	0.0982	5.89E-02	1.22E-01
SRM	0.0022	9.67E-01	9.79E-01
SRP14	-0.0026	9.61E-01	9.75E-01
SRP19	0.0989	5.70E-02	1.19E-01
SRP54	-0.0393	4.50E-01	5.81E-01
SRP68	0.2497	1.11E-06	1.24E-05
SRP72	0.1072	3.91E-02	8.85E-02

SRP9	0.2022	8.76E-05	5.45E-04
SRPK1	0.1436	5.58E-03	1.80E-02
SRPK2	-0.0234	6.53E-01	7.56E-01
SRPK3	0.2309	7.01E-06	6.18E-05
SRPRB	-0.0354	4.96E-01	6.22E-01
SRPR	-0.1256	1.55E-02	4.17E-02
SRPX2	-0.0368	4.80E-01	6.08E-01
SRPX	-0.1571	2.40E-03	8.96E-03
SRRD	0.1397	7.06E-03	2.19E-02
SRRM1	0.0310	5.51E-01	6.71E-01
SRRM2	0.1258	1.53E-02	4.14E-02
SRRM3	0.0353	4.97E-01	6.24E-01
SRRM4	0.1077	3.81E-02	8.68E-02
SRRM5	0.1408	6.61E-03	2.08E-02
SRRT	0.4194	3.09E-17	3.52E-15
SRR	-0.2611	3.37E-07	4.33E-06
SRXN1	0.1299	1.23E-02	3.46E-02
SRY	-0.0375	4.71E-01	6.00E-01
SS18L1	-0.0869	9.48E-02	1.78E-01
SS18L2	0.0940	7.05E-02	1.41E-01
SS18	0.0099	8.49E-01	9.02E-01
SSBP1	0.0182	7.26E-01	8.14E-01
SSBP2	0.0687	1.87E-01	3.00E-01
SSBP3	-0.0780	1.34E-01	2.32E-01
SSBP4	0.0720	1.66E-01	2.74E-01
SSB	0.2353	4.62E-06	4.35E-05
SSC5D	-0.0784	1.31E-01	2.29E-01
SSFA2	-0.0890	8.70E-02	1.66E-01
SSH1	-0.0240	6.45E-01	7.50E-01
SSH2	0.2186	2.17E-05	1.65E-04
SSH3	0.0813	1.18E-01	2.11E-01
SSNA1	0.1694	1.06E-03	4.50E-03
SSPN	0.0211	6.85E-01	7.82E-01
SSPO	-0.0498	3.39E-01	4.70E-01
SSR1	0.1624	1.70E-03	6.72E-03
SSR2	0.4511	5.31E-20	1.36E-17
SSR3	0.0371	4.76E-01	6.05E-01
SSR4	0.1514	3.47E-03	1.22E-02
SSRP1	0.2877	1.67E-08	2.96E-07
SSSCA1	0.0221	6.72E-01	7.71E-01
SSTR1	-0.0183	7.26E-01	8.14E-01
SSTR2	0.0556	2.86E-01	4.15E-01
SSTR3	0.1048	4.36E-02	9.65E-02
SSTR4	-0.0249	6.33E-01	7.40E-01

SSTR5	0.1353	9.06E-03	2.70E-02
SST	0.0363	4.86E-01	6.14E-01
SSU72	-0.0672	1.97E-01	3.12E-01
SSX1	0.0435	4.03E-01	5.36E-01
SSX2IP	0.1170	2.42E-02	6.00E-02
SSX2	0.0516	3.21E-01	4.52E-01
SSX3	0.0632	2.24E-01	3.46E-01
SSX4	0.1082	3.73E-02	8.53E-02
SSX5	0.0495	3.41E-01	4.73E-01
SSX6	0.1059	4.15E-02	9.27E-02
SSX7	0.1218	1.89E-02	4.91E-02
SSX8	0.0768	1.40E-01	2.41E-01
ST13	-0.0454	3.83E-01	5.17E-01
ST14	0.0379	4.67E-01	5.97E-01
ST18	0.0648	2.13E-01	3.32E-01
ST20	0.0418	4.22E-01	5.55E-01
ST3GAL1	-0.2726	9.61E-08	1.40E-06
ST3GAL2	-0.2041	7.53E-05	4.80E-04
ST3GAL3	-0.1422	6.09E-03	1.94E-02
ST3GAL4	-0.0520	3.18E-01	4.48E-01
ST3GAL5	-0.1098	3.45E-02	8.01E-02
ST3GAL6	-0.2627	2.84E-07	3.73E-06
ST5	0.1225	1.82E-02	4.76E-02
ST6GAL1	-0.0983	5.85E-02	1.22E-01
ST6GAL2	-0.1018	5.00E-02	1.08E-01
ST6GALNAC1	-0.0819	1.15E-01	2.08E-01
ST6GALNAC2	0.1172	2.40E-02	5.95E-02
ST6GALNAC3	-0.2250	1.21E-05	9.92E-05
ST6GALNAC4	0.1426	5.93E-03	1.90E-02
ST6GALNAC5	0.1308	1.17E-02	3.32E-02
ST6GALNAC6	-0.2203	1.85E-05	1.45E-04
ST7L	0.1186	2.23E-02	5.61E-02
ST7OT1	0.0047	9.28E-01	9.55E-01
ST7OT2	0.0110	8.33E-01	8.91E-01
ST7OT3	0.0347	5.05E-01	6.30E-01
ST7OT4	0.0000	1.00E+00	1.00E+00
ST7	-0.2528	8.06E-07	9.36E-06
ST8SIA1	-0.0136	7.94E-01	8.63E-01
ST8SIA2	-0.0779	1.34E-01	2.33E-01
ST8SIA3	-0.0293	5.74E-01	6.91E-01
ST8SIA4	-0.0455	3.82E-01	5.16E-01
ST8SIA5	-0.0442	3.96E-01	5.28E-01
ST8SIA6	-0.2244	1.28E-05	1.04E-04
STAB1	-0.0853	1.01E-01	1.87E-01

STAB2	-0.1405	6.71E-03	2.10E-02
STAC2	0.0881	9.00E-02	1.71E-01
STAC3	-0.0284	5.86E-01	7.01E-01
STAC	0.0373	4.74E-01	6.03E-01
STAG1	0.0382	4.63E-01	5.93E-01
STAG2	0.1515	3.43E-03	1.21E-02
STAG3L1	0.0149	7.75E-01	8.50E-01
STAG3L2	-0.0321	5.37E-01	6.59E-01
STAG3L3	-0.0199	7.03E-01	7.96E-01
STAG3L4	0.1227	1.80E-02	4.72E-02
STAG3	-0.1183	2.26E-02	5.69E-02
STAM2	0.0110	8.33E-01	8.91E-01
STAMBPL1	0.0599	2.50E-01	3.75E-01
STAMBP	-0.0030	9.53E-01	9.71E-01
STAM	0.1253	1.57E-02	4.22E-02
STAP1	0.0271	6.03E-01	7.16E-01
STAP2	-0.1155	2.60E-02	6.37E-02
STARD10	-0.2271	9.98E-06	8.40E-05
STARD13	-0.1308	1.17E-02	3.31E-02
STARD3NL	0.1325	1.06E-02	3.06E-02
STARD3	0.1170	2.42E-02	6.00E-02
STARD4	-0.0900	8.34E-02	1.61E-01
STARD5	-0.2968	5.57E-09	1.10E-07
STARD6	0.1156	2.60E-02	6.36E-02
STARD7	0.0354	4.96E-01	6.22E-01
STARD8	-0.0332	5.24E-01	6.48E-01
STAR	0.1430	5.81E-03	1.87E-02
STAT1	0.0727	1.62E-01	2.69E-01
STAT2	-0.1052	4.29E-02	9.53E-02
STAT3	-0.0791	1.28E-01	2.25E-01
STAT4	0.0430	4.09E-01	5.42E-01
STAT5A	-0.0666	2.00E-01	3.17E-01
STAT5B	-0.0428	4.11E-01	5.44E-01
STAT6	-0.0406	4.36E-01	5.68E-01
STAU1	-0.0756	1.46E-01	2.49E-01
STAU2	-0.0076	8.84E-01	9.26E-01
STBD1	-0.2571	5.18E-07	6.30E-06
STC1	-0.0761	1.43E-01	2.45E-01
STC2	0.0329	5.27E-01	6.50E-01
STEAP1	-0.1690	1.09E-03	4.61E-03
STEAP2	-0.0966	6.30E-02	1.29E-01
STEAP3	-0.2338	5.33E-06	4.89E-05
STEAP4	-0.3102	1.02E-09	2.40E-08
STH	0.0379	4.67E-01	5.96E-01

STIL	0.4277	6.27E-18	8.80E-16
STIM1	-0.0513	3.24E-01	4.55E-01
STIM2	0.1172	2.39E-02	5.95E-02
STIP1	0.3287	8.50E-11	2.52E-09
STK10	-0.1030	4.73E-02	1.03E-01
STK11IP	0.2199	1.92E-05	1.49E-04
STK11	0.0023	9.64E-01	9.78E-01
STK16	-0.2704	1.22E-07	1.74E-06
STK17A	0.1038	4.58E-02	1.00E-01
STK17B	-0.0055	9.16E-01	9.47E-01
STK19	0.2224	1.53E-05	1.22E-04
STK24	0.2662	1.95E-07	2.65E-06
STK25	0.1684	1.13E-03	4.77E-03
STK31	0.1236	1.73E-02	4.56E-02
STK32A	-0.0144	7.82E-01	8.55E-01
STK32B	0.0151	7.73E-01	8.47E-01
STK32C	0.0820	1.15E-01	2.07E-01
STK33	0.1263	1.50E-02	4.06E-02
STK35	0.0869	9.47E-02	1.78E-01
STK36	0.1934	1.78E-04	9.97E-04
STK38L	0.0025	9.62E-01	9.76E-01
STK38	0.1731	8.14E-04	3.60E-03
STK39	0.1674	1.21E-03	5.05E-03
STK3	0.0600	2.49E-01	3.74E-01
STK40	-0.1273	1.42E-02	3.89E-02
STK4	0.1339	9.80E-03	2.88E-02
STL	0.0788	1.30E-01	2.27E-01
STMN1	0.3654	3.70E-13	1.78E-11
STMN2	-0.0075	8.86E-01	9.27E-01
STMN3	-0.0535	3.04E-01	4.33E-01
STMN4	0.0234	6.54E-01	7.56E-01
STOML1	-0.1349	9.26E-03	2.75E-02
STOML2	0.0585	2.61E-01	3.87E-01
STOML3	0.0158	7.61E-01	8.39E-01
STOM	-0.1335	1.00E-02	2.93E-02
STON1-GTF2A1	-0.0782	1.33E-01	2.31E-01
STON1	-0.0576	2.69E-01	3.96E-01
STON2	-0.0927	7.46E-02	1.48E-01
STOX1	0.1633	1.60E-03	6.41E-03
STOX2	-0.0063	9.04E-01	9.40E-01
STRA13	0.1214	1.94E-02	5.00E-02
STRA6	0.0860	9.81E-02	1.83E-01
STRA8	0.1111	3.25E-02	7.62E-02
STRADA	0.3632	5.19E-13	2.44E-11

STRADB	-0.2309	7.01E-06	6.18E-05
STRAP	0.0298	5.68E-01	6.85E-01
STRBP	0.2226	1.51E-05	1.21E-04
STRC	0.1499	3.79E-03	1.32E-02
STRN3	-0.1326	1.06E-02	3.05E-02
STRN4	0.1806	4.72E-04	2.26E-03
STRN	-0.0075	8.85E-01	9.26E-01
STS	-0.2512	9.54E-07	1.09E-05
STT3A	0.2191	2.07E-05	1.59E-04
STT3B	-0.0737	1.57E-01	2.62E-01
STUB1	-0.0739	1.56E-01	2.61E-01
STX10	0.0911	7.97E-02	1.55E-01
STX11	-0.1007	5.26E-02	1.12E-01
STX12	-0.1436	5.58E-03	1.80E-02
STX16	0.2486	1.25E-06	1.38E-05
STX17	0.0142	7.86E-01	8.58E-01
STX18	0.0948	6.82E-02	1.37E-01
STX19	-0.0065	9.01E-01	9.37E-01
STX1A	0.2259	1.12E-05	9.28E-05
STX1B	-0.0793	1.27E-01	2.23E-01
STX2	0.1824	4.12E-04	2.03E-03
STX3	0.0541	2.99E-01	4.28E-01
STX4	0.1469	4.58E-03	1.54E-02
STX5	0.0030	9.54E-01	9.72E-01
STX6	0.4799	9.18E-23	3.52E-20
STX7	0.0973	6.13E-02	1.26E-01
STX8	0.0109	8.35E-01	8.92E-01
STXBP1	-0.0184	7.24E-01	8.13E-01
STXBP2	0.0878	9.11E-02	1.73E-01
STXBP3	0.0158	7.61E-01	8.39E-01
STXBP4	0.1879	2.73E-04	1.44E-03
STXBP5L	0.0012	9.81E-01	9.88E-01
STXBP5	0.1610	1.86E-03	7.25E-03
STXBP6	0.1841	3.65E-04	1.84E-03
STYK1	0.0055	9.16E-01	9.47E-01
STYXL1	-0.0988	5.73E-02	1.20E-01
STYX	-0.1644	1.49E-03	6.00E-03
SUB1	0.2535	7.49E-07	8.75E-06
SUCLA2	-0.1244	1.65E-02	4.40E-02
SUCLG1	-0.1921	1.97E-04	1.09E-03
SUCLG2	-0.4456	1.70E-19	3.72E-17
SUCNR1	-0.1477	4.36E-03	1.47E-02
SUDS3	0.1084	3.69E-02	8.46E-02
SUFU	-0.1148	2.70E-02	6.56E-02

SUGT1L1	0.0500	3.37E-01	4.69E-01
SUGT1P1	0.0496	3.41E-01	4.72E-01
SUGT1	-0.0480	3.56E-01	4.88E-01
SULF1	-0.0395	4.49E-01	5.80E-01
SULF2	-0.0901	8.32E-02	1.61E-01
SULT1A1	-0.1612	1.85E-03	7.20E-03
SULT1A2	-0.1790	5.34E-04	2.51E-03
SULT1A3	0.1263	1.49E-02	4.05E-02
SULT1B1	-0.2991	4.21E-09	8.58E-08
SULT1C2	0.1410	6.53E-03	2.06E-02
SULT1C3	0.0342	5.12E-01	6.36E-01
SULT1C4	0.0252	6.29E-01	7.37E-01
SULT1E1	-0.1129	2.97E-02	7.09E-02
SULT2A1	-0.1177	2.34E-02	5.84E-02
SULT2B1	-0.0041	9.37E-01	9.61E-01
SULT4A1	-0.0721	1.66E-01	2.74E-01
SULT6B1	-0.0077	8.82E-01	9.25E-01
SUMF1	-0.2374	3.77E-06	3.64E-05
SUMF2	-0.1137	2.86E-02	6.88E-02
SUMO1P1	0.0958	6.52E-02	1.33E-01
SUMO1P3	0.0975	6.05E-02	1.25E-01
SUMO1	0.0148	7.76E-01	8.50E-01
SUMO2	0.3461	7.03E-12	2.62E-10
SUMO3	-0.0182	7.26E-01	8.14E-01
SUMO4	-0.0493	3.43E-01	4.75E-01
SUN1	0.1374	8.03E-03	2.44E-02
SUN2	-0.1607	1.90E-03	7.37E-03
SUN3	0.1766	6.32E-04	2.90E-03
SUOX	-0.3050	2.00E-09	4.46E-08
SUPT16H	0.2610	3.43E-07	4.40E-06
SUPT3H	0.2438	2.02E-06	2.11E-05
SUPT4H1	0.2656	2.08E-07	2.80E-06
SUPT5H	0.2693	1.39E-07	1.96E-06
SUPT6H	0.1353	9.08E-03	2.71E-02
SUPT7L	0.2156	2.81E-05	2.07E-04
SUPV3L1	0.0272	6.01E-01	7.14E-01
SURF1	-0.1323	1.08E-02	3.10E-02
SURF2	0.1175	2.36E-02	5.87E-02
SURF4	-0.0412	4.29E-01	5.61E-01
SURF6	0.2298	7.78E-06	6.73E-05
SUSD1	0.1250	1.60E-02	4.28E-02
SUSD2	-0.1250	1.60E-02	4.29E-02
SUSD3	-0.0484	3.53E-01	4.85E-01
SUSD4	0.2853	2.24E-08	3.82E-07

SUSD5	-0.0239	6.46E-01	7.51E-01
SUV39H1	0.2037	7.75E-05	4.92E-04
SUV39H2	0.3999	1.11E-15	8.85E-14
SUV420H1	-0.0366	4.83E-01	6.11E-01
SUV420H2	0.2459	1.64E-06	1.75E-05
SUZ12P	0.4063	3.51E-16	3.11E-14
SUZ12	0.2912	1.11E-08	2.03E-07
SV2A	0.1063	4.06E-02	9.13E-02
SV2B	0.0464	3.73E-01	5.06E-01
SV2C	0.0449	3.89E-01	5.22E-01
SVEP1	-0.1206	2.02E-02	5.17E-02
SVIL	0.0381	4.64E-01	5.94E-01
SVIP	0.1478	4.32E-03	1.46E-02
SVOPL	0.1776	5.88E-04	2.72E-03
SVOP	0.0852	1.01E-01	1.87E-01
SWAP70	0.0446	3.91E-01	5.24E-01
SYAP1	-0.1161	2.54E-02	6.22E-02
SYBU	-0.2080	5.40E-05	3.61E-04
SYCE1L	0.1425	5.96E-03	1.91E-02
SYCE1	0.0273	6.00E-01	7.13E-01
SYCE2	0.0239	6.46E-01	7.51E-01
SYCN	0.0090	8.63E-01	9.12E-01
SYCP1	0.0805	1.22E-01	2.16E-01
SYCP2L	-0.0133	7.99E-01	8.67E-01
SYCP2	0.1535	3.03E-03	1.09E-02
SYCP3	0.0536	3.03E-01	4.33E-01
SYDE1	0.0164	7.53E-01	8.34E-01
SYDE2	0.0153	7.69E-01	8.45E-01
SYF2	0.0201	7.00E-01	7.93E-01
SYK	0.0320	5.38E-01	6.60E-01
SYMPK	0.0697	1.80E-01	2.92E-01
SYN1	0.0342	5.11E-01	6.36E-01
SYN2	-0.0601	2.48E-01	3.73E-01
SYN3	0.0361	4.88E-01	6.16E-01
SYNCRIP	0.2084	5.22E-05	3.52E-04
SYNC	0.1054	4.24E-02	9.44E-02
SYNE1	-0.0939	7.08E-02	1.42E-01
SYNE2	-0.0889	8.74E-02	1.67E-01
SYNGAP1	0.0053	9.20E-01	9.49E-01
SYNGR1	0.0765	1.41E-01	2.42E-01
SYNGR2	0.1723	8.61E-04	3.78E-03
SYNGR3	0.0627	2.29E-01	3.51E-01
SYNGR4	0.1716	9.03E-04	3.94E-03
SYNJ1	0.0509	3.28E-01	4.59E-01

SYNJ2BP	-0.2420	2.42E-06	2.46E-05
SYNJ2	0.1866	3.01E-04	1.56E-03
SYNM	0.1051	4.30E-02	9.55E-02
SYNPO2L	-0.0310	5.52E-01	6.72E-01
SYNPO2	-0.2667	1.85E-07	2.53E-06
SYNPO	-0.1546	2.84E-03	1.03E-02
SYNPR	-0.0193	7.11E-01	8.02E-01
SYNRG	0.1169	2.43E-02	6.02E-02
SYPL1	-0.1183	2.27E-02	5.70E-02
SYPL2	-0.1931	1.82E-04	1.02E-03
SYP	0.0709	1.73E-01	2.83E-01
SYS1-DBNDD2	0.0001	9.99E-01	1.00E+00
SYS1	-0.1360	8.71E-03	2.61E-02
SYT10	-0.0851	1.02E-01	1.88E-01
SYT11	-0.0690	1.85E-01	2.98E-01
SYT12	0.0478	3.59E-01	4.91E-01
SYT13	0.0738	1.56E-01	2.61E-01
SYT14L	0.0053	9.19E-01	9.49E-01
SYT14	0.0263	6.13E-01	7.24E-01
SYT15	-0.0553	2.88E-01	4.17E-01
SYT16	0.1228	1.80E-02	4.71E-02
SYT17	-0.0376	4.70E-01	6.00E-01
SYT1	0.0272	6.01E-01	7.14E-01
SYT2	0.0994	5.59E-02	1.17E-01
SYT3	0.0015	9.77E-01	9.86E-01
SYT4	0.0030	9.55E-01	9.72E-01
SYT5	0.1807	4.69E-04	2.25E-03
SYT6	0.0423	4.17E-01	5.49E-01
SYT7	-0.0919	7.72E-02	1.52E-01
SYT8	0.1980	1.24E-04	7.32E-04
SYT9	-0.1839	3.70E-04	1.86E-03
SYTL1	0.0437	4.01E-01	5.34E-01
SYTL2	-0.1163	2.51E-02	6.17E-02
SYTL3	-0.0413	4.28E-01	5.60E-01
SYTL4	-0.0431	4.08E-01	5.40E-01
SYTL5	-0.2622	3.02E-07	3.92E-06
SYVN1	-0.1241	1.68E-02	4.46E-02
TAAR1	0.0009	9.86E-01	9.92E-01
TAAR5	0.0004	9.94E-01	9.96E-01
TAAR6	0.0898	8.41E-02	1.62E-01
TAAR8	0.0683	1.89E-01	3.03E-01
TAAR9	0.0982	5.89E-02	1.22E-01
TAB1	0.1435	5.63E-03	1.82E-02
TAB2	0.0213	6.82E-01	7.79E-01

TAB3	-0.0508	3.29E-01	4.60E-01
TAC1	-0.0133	7.98E-01	8.66E-01
TAC3	0.1612	1.84E-03	7.19E-03
TAC4	-0.0129	8.04E-01	8.70E-01
TACC1	-0.0331	5.25E-01	6.48E-01
TACC2	-0.0508	3.29E-01	4.60E-01
TACC3	0.3926	4.01E-15	2.83E-13
TACO1	-0.1213	1.94E-02	5.01E-02
TACR1	-0.1843	3.59E-04	1.81E-03
TACR2	0.0487	3.50E-01	4.82E-01
TACR3	0.0648	2.13E-01	3.33E-01
TACSTD2	0.0114	8.27E-01	8.87E-01
TADA1	0.1876	2.79E-04	1.47E-03
TADA2A	0.2124	3.73E-05	2.64E-04
TADA2B	-0.1133	2.91E-02	6.98E-02
TADA3	-0.1341	9.72E-03	2.86E-02
TAF10	-0.0881	9.02E-02	1.71E-01
TAF11	0.2376	3.69E-06	3.57E-05
TAF12	0.0946	6.86E-02	1.38E-01
TAF13	-0.1102	3.39E-02	7.90E-02
TAF15	0.3078	1.39E-09	3.21E-08
TAF1A	0.2796	4.34E-08	6.89E-07
TAF1B	0.1561	2.58E-03	9.50E-03
TAF1C	0.0453	3.85E-01	5.18E-01
TAF1D	0.2154	2.86E-05	2.10E-04
TAF1L	0.0041	9.37E-01	9.61E-01
TAF1	0.1092	3.55E-02	8.19E-02
TAF2	0.0129	8.04E-01	8.70E-01
TAF3	0.1961	1.44E-04	8.34E-04
TAF4B	0.0163	7.54E-01	8.35E-01
TAF4	0.2660	1.98E-07	2.69E-06
TAF5L	0.1994	1.10E-04	6.59E-04
TAF5	0.2058	6.51E-05	4.23E-04
TAF6L	-0.0276	5.96E-01	7.10E-01
TAF6	0.2224	1.53E-05	1.22E-04
TAF7L	-0.0692	1.84E-01	2.96E-01
TAF7	0.1504	3.69E-03	1.29E-02
TAF8	-0.0270	6.04E-01	7.16E-01
TAF9B	0.1767	6.29E-04	2.89E-03
TAF9	0.0811	1.19E-01	2.13E-01
TAGAP	-0.0742	1.54E-01	2.59E-01
TAGLN2	0.1803	4.83E-04	2.31E-03
TAGLN3	0.1214	1.94E-02	5.00E-02
TAGLN	-0.1518	3.38E-03	1.19E-02

TAL1	-0.2328	5.84E-06	5.28E-05
TAL2	0.1514	3.46E-03	1.22E-02
TALDO1	0.0382	4.63E-01	5.94E-01
TANC1	-0.0157	7.63E-01	8.40E-01
TANC2	0.0762	1.43E-01	2.45E-01
TANK	-0.0673	1.96E-01	3.11E-01
TAOK1	0.0006	9.91E-01	9.95E-01
TAOK2	-0.0053	9.19E-01	9.49E-01
TAOK3	-0.0252	6.28E-01	7.36E-01
TAP1	0.1151	2.66E-02	6.49E-02
TAP2	0.0195	7.08E-01	8.00E-01
TAPBPL	-0.1768	6.25E-04	2.88E-03
TAPBP	-0.0406	4.36E-01	5.68E-01
TAPT1	-0.2068	6.01E-05	3.95E-04
TARBP1	0.2866	1.92E-08	3.33E-07
TARBP2	-0.0234	6.54E-01	7.56E-01
TARDBP	0.2845	2.44E-08	4.10E-07
TARM1	-0.0245	6.39E-01	7.45E-01
TARP	-0.1428	5.86E-03	1.88E-02
TARS2	0.1598	2.02E-03	7.75E-03
TARSL2	-0.0565	2.78E-01	4.07E-01
TARS	0.1188	2.21E-02	5.57E-02
TAS1R1	-0.0674	1.95E-01	3.11E-01
TAS1R2	0.0870	9.44E-02	1.78E-01
TAS1R3	0.0518	3.20E-01	4.50E-01
TAS2R10	0.0862	9.75E-02	1.82E-01
TAS2R13	0.0595	2.53E-01	3.78E-01
TAS2R14	0.0917	7.76E-02	1.52E-01
TAS2R19	0.1073	3.89E-02	8.81E-02
TAS2R1	0.1420	6.14E-03	1.95E-02
TAS2R20	0.2017	9.14E-05	5.65E-04
TAS2R30	0.0287	5.82E-01	6.98E-01
TAS2R31	0.1158	2.57E-02	6.31E-02
TAS2R38	0.1563	2.53E-03	9.35E-03
TAS2R3	0.0688	1.86E-01	2.99E-01
TAS2R40	-0.0599	2.50E-01	3.75E-01
TAS2R42	0.0084	8.72E-01	9.18E-01
TAS2R43	0.1427	5.90E-03	1.89E-02
TAS2R46	0.1030	4.75E-02	1.03E-01
TAS2R4	0.1859	3.18E-04	1.64E-03
TAS2R50	-0.0276	5.97E-01	7.10E-01
TAS2R5	0.1267	1.46E-02	3.98E-02
TAS2R60	-0.0063	9.04E-01	9.39E-01
TAS2R8	-0.0235	6.52E-01	7.55E-01

TAS2R9	0.1014	5.11E-02	1.09E-01
TASP1	0.1039	4.56E-02	1.00E-01
TATDN1	0.0979	5.95E-02	1.23E-01
TATDN2	0.1274	1.41E-02	3.87E-02
TATDN3	0.0151	7.72E-01	8.47E-01
TAT	-0.3183	3.52E-10	9.35E-09
TAX1BP1	-0.0867	9.55E-02	1.79E-01
TAX1BP3	0.0589	2.58E-01	3.84E-01
TAZ	0.2132	3.48E-05	2.49E-04
TBC1D10A	-0.0516	3.21E-01	4.52E-01
TBC1D10B	0.1916	2.05E-04	1.13E-03
TBC1D10C	-0.0263	6.14E-01	7.24E-01
TBC1D12	0.0819	1.15E-01	2.08E-01
TBC1D13	0.2333	5.60E-06	5.10E-05
TBC1D14	0.1368	8.31E-03	2.51E-02
TBC1D15	-0.0902	8.28E-02	1.60E-01
TBC1D16	0.0282	5.89E-01	7.04E-01
TBC1D17	0.0078	8.81E-01	9.24E-01
TBC1D19	-0.0111	8.31E-01	8.89E-01
TBC1D1	0.0581	2.64E-01	3.91E-01
TBC1D20	-0.0708	1.74E-01	2.84E-01
TBC1D21	-0.0232	6.56E-01	7.58E-01
TBC1D22A	0.0016	9.76E-01	9.85E-01
TBC1D22B	0.3072	1.51E-09	3.46E-08
TBC1D23	-0.1385	7.55E-03	2.32E-02
TBC1D24	-0.0594	2.54E-01	3.79E-01
TBC1D25	0.0513	3.24E-01	4.55E-01
TBC1D26	0.0979	5.97E-02	1.24E-01
TBC1D28	0.0106	8.39E-01	8.94E-01
TBC1D29	0.0061	9.07E-01	9.41E-01
TBC1D2B	-0.1307	1.17E-02	3.33E-02
TBC1D2	-0.0713	1.71E-01	2.80E-01
TBC1D3B	0.3116	8.50E-10	2.05E-08
TBC1D3C	0.2116	3.99E-05	2.79E-04
TBC1D3G	0.0235	6.52E-01	7.55E-01
TBC1D3H	0.2118	3.91E-05	2.74E-04
TBC1D3P2	0.0983	5.85E-02	1.22E-01
TBC1D3	0.3280	9.33E-11	2.74E-09
TBC1D4	-0.1205	2.03E-02	5.19E-02
TBC1D5	-0.0662	2.04E-01	3.21E-01
TBC1D7	0.0113	8.29E-01	8.88E-01
TBC1D8B	-0.0627	2.29E-01	3.51E-01
TBC1D8	0.0871	9.38E-02	1.77E-01
TBC1D9B	-0.1340	9.76E-03	2.87E-02

TBC1D9	-0.1093	3.54E-02	8.17E-02
TBCA	0.0006	9.91E-01	9.94E-01
TBCB	0.1039	4.56E-02	1.00E-01
TBCCD1	0.0574	2.70E-01	3.98E-01
TBCC	0.2302	7.47E-06	6.52E-05
TBCD	0.0887	8.80E-02	1.68E-01
TBCEL	-0.0095	8.55E-01	9.06E-01
TBCE	0.2153	2.90E-05	2.13E-04
TBCK	-0.1993	1.11E-04	6.68E-04
TBK1	0.0166	7.49E-01	8.31E-01
TBKBP1	0.0366	4.82E-01	6.11E-01
TBL1XR1	0.1757	6.77E-04	3.08E-03
TBL1X	-0.1669	1.26E-03	5.22E-03
TBL1Y	-0.0411	4.30E-01	5.62E-01
TBL2	-0.0371	4.77E-01	6.05E-01
TBL3	0.0003	9.95E-01	9.97E-01
TBPL1	0.2024	8.61E-05	5.37E-04
TBPL2	0.0363	4.86E-01	6.14E-01
TBP	0.3935	3.46E-15	2.47E-13
TBR1	0.1681	1.15E-03	4.86E-03
TBRG1	-0.0790	1.29E-01	2.25E-01
TBRG4	-0.0376	4.70E-01	6.00E-01
TBX10	-0.0247	6.36E-01	7.43E-01
TBX15	-0.0832	1.09E-01	2.00E-01
TBX18	0.1095	3.50E-02	8.10E-02
TBX19	0.4003	1.04E-15	8.40E-14
TBX1	0.0739	1.55E-01	2.60E-01
TBX20	-0.1695	1.05E-03	4.47E-03
TBX21	-0.0924	7.53E-02	1.49E-01
TBX22	-0.0135	7.96E-01	8.65E-01
TBX2	-0.0402	4.40E-01	5.72E-01
TBX3	-0.1342	9.68E-03	2.85E-02
TBX4	0.1431	5.76E-03	1.85E-02
TBX5	0.0391	4.53E-01	5.84E-01
TBX6	0.0524	3.14E-01	4.44E-01
TBXA2R	-0.1631	1.62E-03	6.47E-03
TBXAS1	-0.0918	7.73E-02	1.52E-01
TC2N	0.0586	2.61E-01	3.87E-01
TCAM1P	0.0814	1.18E-01	2.11E-01
TCAP	0.0182	7.26E-01	8.14E-01
TCEA1	-0.0203	6.97E-01	7.91E-01
TCEA2	-0.1211	1.96E-02	5.06E-02
TCEA3	0.0151	7.72E-01	8.47E-01
TCEAL1	0.0649	2.12E-01	3.32E-01

TCEAL2	0.0188	7.19E-01	8.09E-01
TCEAL3	0.0715	1.69E-01	2.78E-01
TCEAL4	0.0360	4.89E-01	6.16E-01
TCEAL5	0.1150	2.68E-02	6.52E-02
TCEAL6	0.0707	1.74E-01	2.85E-01
TCEAL7	0.0024	9.63E-01	9.77E-01
TCEAL8	0.1817	4.35E-04	2.11E-03
TCEANC	0.0446	3.92E-01	5.25E-01
TCEB1	0.0780	1.34E-01	2.32E-01
TCEB2	0.0263	6.14E-01	7.24E-01
TCEB3B	0.0685	1.88E-01	3.01E-01
TCEB3C	0.0085	8.71E-01	9.17E-01
TCEB3	-0.0757	1.46E-01	2.48E-01
TCERG1L	-0.0239	6.47E-01	7.51E-01
TCERG1	0.3326	4.91E-11	1.55E-09
TCF12	-0.0213	6.82E-01	7.79E-01
TCF15	-0.0592	2.55E-01	3.81E-01
TCF19	0.4345	1.62E-18	2.71E-16
TCF20	0.1767	6.29E-04	2.89E-03
TCF21	-0.1452	5.06E-03	1.67E-02
TCF23	-0.1127	3.01E-02	7.15E-02
TCF25	-0.2854	2.21E-08	3.78E-07
TCF3	0.2743	7.89E-08	1.18E-06
TCF4	-0.1917	2.03E-04	1.12E-03
TCF7L1	0.1213	1.94E-02	5.01E-02
TCF7L2	0.0440	3.98E-01	5.31E-01
TCF7	0.0815	1.17E-01	2.11E-01
TCFL5	0.1204	2.04E-02	5.22E-02
TCHHL1	0.0141	7.87E-01	8.59E-01
TCHH	-0.1451	5.10E-03	1.68E-02
TCHP	0.2424	2.32E-06	2.37E-05
TCIRG1	0.1868	2.98E-04	1.55E-03
TCL1A	0.0194	7.09E-01	8.01E-01
TCL1B	0.2336	5.46E-06	4.99E-05
TCL6	0.1611	1.86E-03	7.24E-03
TCN1	-0.0362	4.87E-01	6.15E-01
TCN2	-0.0138	7.91E-01	8.61E-01
TCOF1	0.3136	6.56E-10	1.61E-08
TCP10L2	0.1085	3.66E-02	8.41E-02
TCP10L	-0.0746	1.51E-01	2.55E-01
TCP10	0.1163	2.51E-02	6.17E-02
TCP11L1	-0.0271	6.02E-01	7.15E-01
TCP11L2	-0.1013	5.12E-02	1.10E-01
TCP11	-0.1292	1.27E-02	3.56E-02

TCP1	0.2567	5.37E-07	6.52E-06
TCTA	-0.1051	4.31E-02	9.57E-02
TCTE1	0.0228	6.61E-01	7.63E-01
TCTE3	0.2817	3.40E-08	5.54E-07
TCTEX1D1	-0.0327	5.30E-01	6.53E-01
TCTEX1D2	0.2862	2.01E-08	3.47E-07
TCTEX1D4	-0.0992	5.63E-02	1.18E-01
TCTN1	0.0201	6.99E-01	7.92E-01
TCTN2	0.2959	6.20E-09	1.21E-07
TCTN3	-0.2788	4.77E-08	7.48E-07
TDGF1	-0.0851	1.02E-01	1.88E-01
TDGF3	-0.0990	5.68E-02	1.19E-01
TDG	0.2463	1.57E-06	1.69E-05
TDH	0.1007	5.26E-02	1.12E-01
TDO2	-0.1524	3.25E-03	1.15E-02
TDP1	0.1765	6.40E-04	2.93E-03
TDP2	-0.0136	7.94E-01	8.63E-01
TDRD10	0.1712	9.32E-04	4.05E-03
TDRD12	0.0477	3.59E-01	4.92E-01
TDRD1	0.0972	6.14E-02	1.26E-01
TDRD3	-0.0771	1.38E-01	2.38E-01
TDRD5	0.2660	1.99E-07	2.70E-06
TDRD6	-0.0691	1.84E-01	2.96E-01
TDRD7	0.1793	5.22E-04	2.47E-03
TDRD9	0.0165	7.52E-01	8.33E-01
TDRG1	0.0487	3.50E-01	4.82E-01
TDRKH	0.4367	1.05E-18	1.88E-16
TEAD1	-0.0306	5.57E-01	6.76E-01
TEAD2	0.1605	1.93E-03	7.46E-03
TEAD3	0.2805	3.89E-08	6.25E-07
TEAD4	0.0345	5.07E-01	6.32E-01
TECPR1	-0.1191	2.17E-02	5.50E-02
TECPR2	-0.1893	2.45E-04	1.31E-03
TECRL	-0.0028	9.57E-01	9.73E-01
TECR	-0.0447	3.90E-01	5.24E-01
TECTA	-0.0502	3.35E-01	4.67E-01
TECTB	-0.0235	6.52E-01	7.55E-01
TEC	-0.1193	2.16E-02	5.46E-02
TEDDM1	0.0827	1.12E-01	2.03E-01
TEF	0.0052	9.21E-01	9.50E-01
TEKT1	-0.1222	1.85E-02	4.83E-02
TEKT2	0.1571	2.41E-03	8.98E-03
TEKT3	0.0022	9.67E-01	9.80E-01
TEKT4	-0.0033	9.49E-01	9.68E-01

TEKT5	-0.0109	8.35E-01	8.92E-01
TEK	-0.2623	2.98E-07	3.89E-06
TELO2	0.1780	5.71E-04	2.66E-03
TENC1	-0.1704	9.85E-04	4.24E-03
TEP1	-0.0053	9.19E-01	9.49E-01
TEPP	-0.0310	5.51E-01	6.71E-01
TERC	0.1004	5.33E-02	1.13E-01
TERF1	-0.0538	3.02E-01	4.31E-01
TERF2IP	-0.2438	2.02E-06	2.11E-05
TERF2	-0.1762	6.50E-04	2.97E-03
TERT	0.1788	5.39E-04	2.54E-03
TESC	0.0859	9.84E-02	1.84E-01
TESK1	0.1575	2.34E-03	8.77E-03
TESK2	-0.0379	4.67E-01	5.97E-01
TES	0.0351	5.01E-01	6.26E-01
TET1	0.2353	4.62E-06	4.35E-05
TET2	-0.0410	4.31E-01	5.63E-01
TET3	0.1900	2.33E-04	1.26E-03
TEX101	-0.0218	6.75E-01	7.74E-01
TEX10	0.2829	2.97E-08	4.89E-07
TEX11	0.0479	3.57E-01	4.89E-01
TEX12	0.0827	1.12E-01	2.03E-01
TEX13A	-0.0066	8.99E-01	9.36E-01
TEX13B	0.0262	6.15E-01	7.25E-01
TEX14	0.1848	3.45E-04	1.76E-03
TEX15	0.0696	1.81E-01	2.93E-01
TEX19	0.2321	6.26E-06	5.62E-05
TEX261	0.0283	5.87E-01	7.02E-01
TEX264	-0.2157	2.80E-05	2.06E-04
TEX2	0.0743	1.53E-01	2.58E-01
TEX9	0.0924	7.55E-02	1.49E-01
TFAMP1	0.1861	3.12E-04	1.62E-03
TFAM	0.0377	4.69E-01	5.99E-01
TFAP2A	0.2025	8.60E-05	5.36E-04
TFAP2B	0.1459	4.85E-03	1.61E-02
TFAP2C	0.1115	3.18E-02	7.49E-02
TFAP2D	0.0213	6.82E-01	7.79E-01
TFAP2E	0.2573	5.04E-07	6.16E-06
TFAP4	0.3305	6.60E-11	2.01E-09
TFB1M	-0.0081	8.76E-01	9.20E-01
TFB2M	-0.0050	9.23E-01	9.51E-01
TFCP2L1	0.1171	2.41E-02	5.97E-02
TFCP2	0.1104	3.36E-02	7.84E-02
TFDP1	0.2955	6.53E-09	1.27E-07

TFDP2	0.1390	7.32E-03	2.26E-02
TFDP3	-0.0765	1.41E-01	2.42E-01
TFE3	0.1846	3.51E-04	1.78E-03
TFEB	0.0235	6.52E-01	7.55E-01
TFEC	-0.0571	2.72E-01	4.00E-01
TFF1	0.0360	4.89E-01	6.16E-01
TFF2	0.1215	1.93E-02	4.98E-02
TFF3	0.0171	7.42E-01	8.26E-01
TFG	-0.0001	9.99E-01	1.00E+00
TFIP11	0.1767	6.27E-04	2.88E-03
TFPI2	-0.0367	4.81E-01	6.10E-01
TFPI	-0.1260	1.51E-02	4.10E-02
TFPT	0.1327	1.05E-02	3.04E-02
TFR2	-0.1889	2.53E-04	1.35E-03
TFRC	0.1268	1.46E-02	3.98E-02
TF	-0.0585	2.61E-01	3.88E-01
TGDS	0.0453	3.84E-01	5.17E-01
TGFA	0.1255	1.56E-02	4.19E-02
TGFB1I1	-0.0660	2.05E-01	3.22E-01
TGFB1	-0.0332	5.24E-01	6.47E-01
TGFB2	0.0182	7.27E-01	8.15E-01
TGFB3	-0.1005	5.30E-02	1.12E-01
TGFBI	-0.0740	1.55E-01	2.60E-01
TGFBR1	0.0444	3.94E-01	5.27E-01
TGFBR2	-0.2862	2.00E-08	3.46E-07
TGFBR3	-0.1362	8.64E-03	2.59E-02
TGFBRAP1	-0.0295	5.72E-01	6.89E-01
TGIF1	0.0679	1.92E-01	3.07E-01
TGIF2LX	0.0362	4.87E-01	6.14E-01
TGIF2LY	0.0459	3.78E-01	5.11E-01
TGIF2	0.1081	3.73E-02	8.54E-02
TGM1	0.2059	6.45E-05	4.20E-04
TGM2	-0.1592	2.11E-03	8.05E-03
TGM3	0.0282	5.88E-01	7.03E-01
TGM4	0.0395	4.48E-01	5.79E-01
TGM5	-0.0739	1.55E-01	2.60E-01
TGM6	-0.0322	5.36E-01	6.58E-01
TGM7	0.0453	3.84E-01	5.17E-01
TGOLN2	-0.1393	7.20E-03	2.23E-02
TGS1	0.1345	9.48E-03	2.80E-02
TG	-0.0436	4.02E-01	5.35E-01
TH1L	0.1422	6.08E-03	1.94E-02
THADA	0.0403	4.39E-01	5.71E-01
THAP10	0.0782	1.33E-01	2.31E-01

THAP11	0.0315	5.46E-01	6.66E-01
THAP1	0.0890	8.69E-02	1.66E-01
THAP2	0.0853	1.01E-01	1.87E-01
THAP3	0.0293	5.74E-01	6.91E-01
THAP4	-0.0060	9.08E-01	9.43E-01
THAP5	-0.1132	2.93E-02	7.01E-02
THAP6	-0.0162	7.56E-01	8.36E-01
THAP7	0.0665	2.01E-01	3.17E-01
THAP8	0.1810	4.59E-04	2.21E-03
THAP9	0.0195	7.09E-01	8.01E-01
THBD	-0.2201	1.88E-05	1.46E-04
THBS1	-0.1210	1.97E-02	5.07E-02
THBS2	-0.1028	4.80E-02	1.04E-01
THBS3	0.1243	1.66E-02	4.42E-02
THBS4	-0.0589	2.58E-01	3.84E-01
THEG	0.0343	5.10E-01	6.35E-01
THEM4	0.4592	9.53E-21	2.67E-18
THEM5	0.1820	4.26E-04	2.08E-03
THEMIS	-0.1057	4.19E-02	9.35E-02
THG1L	-0.1075	3.85E-02	8.74E-02
THNSL1	-0.2424	2.31E-06	2.37E-05
THNSL2	-0.0781	1.33E-01	2.32E-01
THOC1	0.3463	6.86E-12	2.56E-10
THOC2	0.2888	1.47E-08	2.64E-07
THOC3	0.1826	4.06E-04	2.01E-03
THOC4	0.3742	8.87E-14	4.74E-12
THOC5	0.2520	8.78E-07	1.01E-05
THOC6	0.1679	1.17E-03	4.91E-03
THOC7	0.2300	7.59E-06	6.60E-05
THOP1	-0.0412	4.29E-01	5.61E-01
THPO	-0.0501	3.36E-01	4.67E-01
THRAP3	0.1107	3.31E-02	7.74E-02
THRA	-0.0621	2.33E-01	3.56E-01
THRB	-0.1076	3.84E-02	8.73E-02
THRSP	-0.2742	8.01E-08	1.19E-06
THSD1P1	0.0031	9.53E-01	9.71E-01
THSD1	-0.2153	2.89E-05	2.12E-04
THSD4	-0.0433	4.06E-01	5.39E-01
THSD7A	-0.1091	3.56E-02	8.21E-02
THSD7B	-0.0562	2.81E-01	4.09E-01
THTPA	-0.0964	6.37E-02	1.30E-01
THUMPD1	0.0428	4.11E-01	5.44E-01
THUMPD2	0.3045	2.13E-09	4.67E-08
THUMPD3	0.0167	7.49E-01	8.31E-01

THY1	-0.0267	6.08E-01	7.20E-01
THYN1	-0.0084	8.72E-01	9.18E-01
TH	0.0296	5.70E-01	6.88E-01
TIA1	0.2432	2.13E-06	2.21E-05
TIAF1	-0.0231	6.57E-01	7.59E-01
TIAL1	0.0908	8.07E-02	1.57E-01
TIAM1	-0.0760	1.44E-01	2.46E-01
TIAM2	0.1912	2.12E-04	1.16E-03
TICAM1	0.0603	2.47E-01	3.71E-01
TICAM2	0.0889	8.74E-02	1.67E-01
TIE1	-0.2197	1.96E-05	1.52E-04
TIFAB	0.0551	2.90E-01	4.19E-01
TIFA	-0.0021	9.68E-01	9.80E-01
TIGD1	0.5110	4.61E-26	2.87E-23
TIGD2	-0.2036	7.83E-05	4.96E-04
TIGD3	0.1771	6.10E-04	2.81E-03
TIGD4	0.0912	7.94E-02	1.55E-01
TIGD5	0.1727	8.35E-04	3.68E-03
TIGD6	0.0394	4.50E-01	5.81E-01
TIGD7	0.3423	1.24E-11	4.31E-10
TIGIT	0.0052	9.20E-01	9.49E-01
TIMD4	-0.0794	1.27E-01	2.23E-01
TIMELESS	0.3368	2.71E-11	8.99E-10
TIMM10	-0.0194	7.10E-01	8.02E-01
TIMM13	-0.0857	9.92E-02	1.85E-01
TIMM16	0.0710	1.72E-01	2.82E-01
TIMM17A	0.0243	6.41E-01	7.46E-01
TIMM17B	0.1693	1.06E-03	4.51E-03
TIMM22	-0.0211	6.85E-01	7.82E-01
TIMM44	-0.0797	1.25E-01	2.21E-01
TIMM50	0.1146	2.73E-02	6.61E-02
TIMM8A	-0.0352	4.99E-01	6.25E-01
TIMM8B	-0.0144	7.82E-01	8.55E-01
TIMM9	0.1699	1.02E-03	4.37E-03
TIMP1	-0.0408	4.34E-01	5.65E-01
TIMP2	0.0022	9.66E-01	9.79E-01
TIMP3	-0.3041	2.25E-09	4.88E-08
TIMP4	-0.0728	1.62E-01	2.69E-01
TINAGL1	-0.1452	5.06E-03	1.67E-02
TINAG	0.0698	1.80E-01	2.92E-01
TINF2	-0.0231	6.58E-01	7.59E-01
TIPARP	-0.1044	4.44E-02	9.79E-02
TIPIN	0.3717	1.34E-13	7.05E-12
TIPRL	0.4544	2.68E-20	7.02E-18

TIRAP	0.0831	1.10E-01	2.00E-01
TJAP1	0.4010	9.12E-16	7.51E-14
TJP1	-0.0189	7.16E-01	8.07E-01
TJP2	0.1277	1.38E-02	3.82E-02
TJP3	0.0185	7.23E-01	8.12E-01
TK1	0.2945	7.41E-09	1.42E-07
TK2	-0.4722	5.28E-22	1.78E-19
TKTL1	0.1510	3.55E-03	1.25E-02
TKTL2	0.1684	1.13E-03	4.78E-03
TKT	0.0783	1.32E-01	2.30E-01
TLCD1	0.1952	1.54E-04	8.84E-04
TLE1	0.1748	7.21E-04	3.25E-03
TLE2	-0.2180	2.27E-05	1.72E-04
TLE3	0.0751	1.49E-01	2.52E-01
TLE4	-0.0873	9.31E-02	1.76E-01
TLE6	-0.0245	6.38E-01	7.44E-01
TLK1	0.0774	1.37E-01	2.36E-01
TLK2	0.3629	5.42E-13	2.54E-11
TLL1	-0.1525	3.23E-03	1.15E-02
TLL2	0.1291	1.28E-02	3.58E-02
TLN1	0.0555	2.87E-01	4.15E-01
TLN2	-0.1043	4.48E-02	9.86E-02
TLR10	0.1641	1.52E-03	6.10E-03
TLR1	0.0352	4.99E-01	6.25E-01
TLR2	-0.0235	6.51E-01	7.55E-01
TLR3	-0.2023	8.68E-05	5.41E-04
TLR4	-0.1740	7.61E-04	3.41E-03
TLR5	0.2562	5.67E-07	6.81E-06
TLR6	0.0091	8.61E-01	9.10E-01
TLR7	-0.0284	5.85E-01	7.01E-01
TLR8	-0.0806	1.21E-01	2.16E-01
TLR9	0.1364	8.54E-03	2.57E-02
TLX1NB	0.0671	1.97E-01	3.13E-01
TLX1	0.0003	9.95E-01	9.97E-01
TLX2	0.1547	2.81E-03	1.03E-02
TLX3	-0.0053	9.18E-01	9.48E-01
TM2D1	0.0747	1.51E-01	2.55E-01
TM2D2	-0.0014	9.79E-01	9.87E-01
TM2D3	-0.1837	3.76E-04	1.88E-03
TM4SF18	-0.1290	1.29E-02	3.60E-02
TM4SF19	0.0630	2.26E-01	3.48E-01
TM4SF1	0.0912	7.93E-02	1.55E-01
TM4SF20	0.1003	5.37E-02	1.14E-01
TM4SF4	-0.0941	7.03E-02	1.41E-01

TM4SF5	-0.0264	6.12E-01	7.23E-01
TM6SF1	-0.0723	1.65E-01	2.73E-01
TM6SF2	-0.2577	4.87E-07	5.98E-06
TM7SF2	-0.1722	8.66E-04	3.80E-03
TM7SF3	-0.0129	8.04E-01	8.71E-01
TM7SF4	-0.1486	4.13E-03	1.41E-02
TM9SF1	-0.1102	3.39E-02	7.90E-02
TM9SF2	-0.0510	3.28E-01	4.58E-01
TM9SF3	-0.1483	4.20E-03	1.43E-02
TM9SF4	-0.0270	6.04E-01	7.17E-01
TMBIM1	-0.0098	8.51E-01	9.03E-01
TMBIM4	-0.0608	2.43E-01	3.67E-01
TMBIM6	-0.3742	8.91E-14	4.74E-12
TMC1	0.0200	7.01E-01	7.94E-01
TMC2	0.1991	1.13E-04	6.74E-04
TMC3	0.1207	2.00E-02	5.14E-02
TMC4	0.0683	1.90E-01	3.03E-01
TMC5	0.0732	1.59E-01	2.65E-01
TMC6	0.0944	6.95E-02	1.40E-01
TMC7	0.1066	4.02E-02	9.05E-02
TMC8	-0.0540	2.99E-01	4.29E-01
TMCC1	-0.0820	1.15E-01	2.07E-01
TMCC2	-0.0326	5.31E-01	6.53E-01
TMCC3	0.0180	7.30E-01	8.17E-01
TMCO1	0.3072	1.50E-09	3.43E-08
TMCO2	0.1520	3.34E-03	1.18E-02
TMCO3	0.1797	5.04E-04	2.39E-03
TMCO4	-0.0318	5.41E-01	6.62E-01
TMCO5A	0.0597	2.51E-01	3.76E-01
TMCO6	0.0472	3.64E-01	4.97E-01
TMCO7	-0.2129	3.56E-05	2.53E-04
TMED10P1	-0.0015	9.77E-01	9.86E-01
TMED10	-0.2094	4.80E-05	3.27E-04
TMED1	-0.2128	3.58E-05	2.55E-04
TMED2	0.0000	1.00E+00	1.00E+00
TMED3	0.1609	1.88E-03	7.31E-03
TMED4	-0.1257	1.54E-02	4.15E-02
TMED5	-0.0530	3.09E-01	4.38E-01
TMED6	-0.0510	3.27E-01	4.57E-01
TMED7-TICAM2	-0.1122	3.07E-02	7.28E-02
TMED7	-0.1316	1.12E-02	3.20E-02
TMED8	0.1576	2.33E-03	8.75E-03
TMED9	0.0356	4.94E-01	6.21E-01
TMEFF1	0.1387	7.48E-03	2.30E-02

TMEFF2	-0.0168	7.47E-01	8.29E-01
TMEM100	-0.2482	1.29E-06	1.42E-05
TMEM101	0.1827	4.03E-04	1.99E-03
TMEM102	-0.0517	3.21E-01	4.51E-01
TMEM104	0.1174	2.37E-02	5.90E-02
TMEM105	0.0815	1.17E-01	2.10E-01
TMEM106A	-0.0101	8.46E-01	8.99E-01
TMEM106B	-0.0496	3.40E-01	4.72E-01
TMEM106C	0.3360	3.04E-11	9.99E-10
TMEM107	0.1650	1.43E-03	5.79E-03
TMEM108	-0.0083	8.73E-01	9.18E-01
TMEM109	0.0201	7.00E-01	7.93E-01
TMEM110	-0.2754	6.98E-08	1.05E-06
TMEM111	-0.1587	2.16E-03	8.22E-03
TMEM114	-0.0435	4.03E-01	5.36E-01
TMEM115	0.1175	2.36E-02	5.88E-02
TMEM116	0.1459	4.85E-03	1.61E-02
TMEM117	-0.0752	1.48E-01	2.52E-01
TMEM119	-0.0472	3.65E-01	4.97E-01
TMEM11	0.0775	1.36E-01	2.36E-01
TMEM120A	-0.2139	3.28E-05	2.35E-04
TMEM120B	0.2749	7.39E-08	1.11E-06
TMEM121	-0.0909	8.05E-02	1.57E-01
TMEM123	-0.0011	9.84E-01	9.90E-01
TMEM125	0.0892	8.62E-02	1.65E-01
TMEM126A	0.0489	3.48E-01	4.80E-01
TMEM126B	0.1002	5.39E-02	1.14E-01
TMEM127	-0.2281	9.12E-06	7.76E-05
TMEM128	0.0525	3.13E-01	4.42E-01
TMEM129	-0.2760	6.56E-08	9.96E-07
TMEM130	0.1792	5.24E-04	2.47E-03
TMEM131	0.1091	3.57E-02	8.23E-02
TMEM132A	0.0930	7.34E-02	1.46E-01
TMEM132B	-0.0571	2.73E-01	4.01E-01
TMEM132C	-0.1583	2.22E-03	8.40E-03
TMEM132D	-0.0244	6.40E-01	7.46E-01
TMEM132E	-0.1064	4.05E-02	9.11E-02
TMEM133	-0.0922	7.60E-02	1.50E-01
TMEM134	-0.2988	4.34E-09	8.83E-08
TMEM135	-0.1434	5.66E-03	1.83E-02
TMEM136	0.2248	1.24E-05	1.01E-04
TMEM138	0.0061	9.06E-01	9.41E-01
TMEM139	0.1041	4.50E-02	9.91E-02
TMEM140	-0.3121	8.01E-10	1.94E-08

TMEM141	-0.0223	6.69E-01	7.69E-01
TMEM143	-0.2008	9.86E-05	6.02E-04
TMEM144	-0.0188	7.17E-01	8.08E-01
TMEM145	0.0786	1.31E-01	2.28E-01
TMEM146	0.0266	6.10E-01	7.21E-01
TMEM147	0.1413	6.42E-03	2.03E-02
TMEM149	-0.0336	5.19E-01	6.43E-01
TMEM14A	0.1307	1.17E-02	3.33E-02
TMEM14B	0.2439	1.99E-06	2.09E-05
TMEM14C	0.1053	4.26E-02	9.47E-02
TMEM14E	0.0225	6.66E-01	7.66E-01
TMEM150A	-0.2490	1.19E-06	1.32E-05
TMEM150B	-0.0001	9.98E-01	9.99E-01
TMEM150C	-0.1650	1.43E-03	5.79E-03
TMEM151A	0.0335	5.20E-01	6.44E-01
TMEM151B	0.2315	6.63E-06	5.91E-05
TMEM154	-0.1435	5.61E-03	1.81E-02
TMEM155	0.1197	2.11E-02	5.35E-02
TMEM156	-0.0134	7.97E-01	8.65E-01
TMEM158	-0.0454	3.84E-01	5.17E-01
TMEM159	0.0669	1.98E-01	3.15E-01
TMEM160	0.0044	9.32E-01	9.58E-01
TMEM161A	-0.0874	9.29E-02	1.75E-01
TMEM161B	-0.0334	5.21E-01	6.45E-01
TMEM163	-0.0136	7.94E-01	8.64E-01
TMEM164	0.1714	9.17E-04	3.99E-03
TMEM165	0.1332	1.02E-02	2.97E-02
TMEM167A	-0.0453	3.84E-01	5.17E-01
TMEM167B	-0.0942	7.00E-02	1.40E-01
TMEM168	0.1381	7.72E-03	2.36E-02
TMEM169	0.0512	3.26E-01	4.56E-01
TMEM170A	-0.1778	5.79E-04	2.69E-03
TMEM170B	0.0254	6.26E-01	7.34E-01
TMEM171	0.1601	1.98E-03	7.63E-03
TMEM173	-0.1298	1.23E-02	3.46E-02
TMEM174	-0.0370	4.77E-01	6.06E-01
TMEM175	-0.1526	3.22E-03	1.15E-02
TMEM176A	-0.1757	6.77E-04	3.08E-03
TMEM176B	-0.2139	3.27E-05	2.35E-04
TMEM177	-0.0445	3.93E-01	5.26E-01
TMEM178	-0.0457	3.80E-01	5.14E-01
TMEM179B	-0.1468	4.61E-03	1.54E-02
TMEM179	0.1179	2.31E-02	5.78E-02
TMEM17	-0.0046	9.29E-01	9.56E-01

TMEM180	0.1122	3.07E-02	7.28E-02
TMEM181	0.1832	3.91E-04	1.95E-03
TMEM182	0.0479	3.57E-01	4.89E-01
TMEM183A	0.2611	3.39E-07	4.36E-06
TMEM184A	-0.0191	7.13E-01	8.04E-01
TMEM184B	0.1269	1.45E-02	3.96E-02
TMEM184C	-0.1764	6.41E-04	2.94E-03
TMEM185A	0.1839	3.70E-04	1.86E-03
TMEM185B	0.0923	7.58E-02	1.49E-01
TMEM186	0.0758	1.45E-01	2.47E-01
TMEM187	-0.0359	4.91E-01	6.18E-01
TMEM188	-0.1348	9.34E-03	2.77E-02
TMEM189-UBE2	-0.0276	5.96E-01	7.10E-01
TMEM189	0.1459	4.88E-03	1.62E-02
TMEM18	0.0903	8.24E-02	1.60E-01
TMEM190	0.1535	3.04E-03	1.09E-02
TMEM191A	0.0525	3.13E-01	4.43E-01
TMEM192	-0.3628	5.49E-13	2.56E-11
TMEM194A	0.2945	7.37E-09	1.41E-07
TMEM194B	0.1993	1.11E-04	6.66E-04
TMEM195	-0.1453	5.04E-03	1.66E-02
TMEM196	-0.0220	6.72E-01	7.72E-01
TMEM198	0.0095	8.55E-01	9.06E-01
TMEM199	0.1263	1.50E-02	4.06E-02
TMEM19	-0.1515	3.45E-03	1.22E-02
TMEM200A	-0.0344	5.09E-01	6.33E-01
TMEM200B	0.0369	4.78E-01	6.07E-01
TMEM200C	0.1029	4.75E-02	1.03E-01
TMEM201	0.3577	1.22E-12	5.33E-11
TMEM202	-0.0374	4.73E-01	6.02E-01
TMEM203	0.0712	1.71E-01	2.80E-01
TMEM204	-0.2026	8.46E-05	5.30E-04
TMEM205	-0.2339	5.26E-06	4.84E-05
TMEM206	0.2231	1.44E-05	1.16E-04
TMEM207	0.1484	4.17E-03	1.42E-02
TMEM208	-0.2211	1.73E-05	1.36E-04
TMEM209	0.0583	2.63E-01	3.89E-01
TMEM20	-0.0601	2.48E-01	3.73E-01
TMEM211	0.0299	5.66E-01	6.83E-01
TMEM212	0.1390	7.33E-03	2.26E-02
TMEM213	0.1092	3.55E-02	8.19E-02
TMEM214	0.0186	7.21E-01	8.10E-01
TMEM215	-0.0293	5.73E-01	6.91E-01
TMEM216	0.2256	1.14E-05	9.45E-05

TMEM217	-0.1118	3.13E-02	7.40E-02
TMEM218	0.2436	2.05E-06	2.14E-05
TMEM219	-0.1541	2.92E-03	1.06E-02
TMEM220	-0.2163	2.64E-05	1.97E-04
TMEM222	-0.1140	2.82E-02	6.80E-02
TMEM223	0.0005	9.93E-01	9.96E-01
TMEM229A	-0.0267	6.08E-01	7.20E-01
TMEM229B	-0.0035	9.47E-01	9.67E-01
TMEM22	0.0928	7.41E-02	1.47E-01
TMEM231	-0.0719	1.67E-01	2.75E-01
TMEM232	-0.1506	3.64E-03	1.27E-02
TMEM233	-0.2214	1.68E-05	1.33E-04
TMEM25	-0.1528	3.18E-03	1.13E-02
TMEM26	-0.1145	2.74E-02	6.64E-02
TMEM27	-0.1243	1.66E-02	4.42E-02
TMEM2	-0.0632	2.25E-01	3.46E-01
TMEM30A	-0.0411	4.30E-01	5.62E-01
TMEM30B	-0.1779	5.78E-04	2.68E-03
TMEM30C	0.0795	1.26E-01	2.22E-01
TMEM31	0.0199	7.02E-01	7.95E-01
TMEM33	-0.1337	9.92E-03	2.90E-02
TMEM35	0.0468	3.69E-01	5.02E-01
TMEM37	-0.1428	5.88E-03	1.88E-02
TMEM38A	-0.0922	7.60E-02	1.50E-01
TMEM38B	0.0248	6.35E-01	7.42E-01
TMEM39A	0.2504	1.03E-06	1.16E-05
TMEM39B	0.1023	4.90E-02	1.06E-01
TMEM40	0.0847	1.03E-01	1.90E-01
TMEM41A	0.0120	8.18E-01	8.81E-01
TMEM41B	0.1139	2.82E-02	6.80E-02
TMEM42	0.0055	9.17E-01	9.47E-01
TMEM43	0.0301	5.63E-01	6.81E-01
TMEM44	0.1476	4.40E-03	1.49E-02
TMEM45A	-0.0220	6.73E-01	7.72E-01
TMEM45B	0.0599	2.50E-01	3.75E-01
TMEM47	-0.2051	6.89E-05	4.45E-04
TMEM48	0.1174	2.37E-02	5.89E-02
TMEM49	0.2153	2.88E-05	2.12E-04
TMEM50A	-0.0143	7.84E-01	8.56E-01
TMEM50B	0.0232	6.57E-01	7.58E-01
TMEM51	0.1336	1.00E-02	2.92E-02
TMEM52	0.0479	3.58E-01	4.90E-01
TMEM53	-0.3149	5.51E-10	1.38E-08
TMEM54	-0.0022	9.66E-01	9.79E-01

TMEM55A	0.1115	3.18E-02	7.49E-02
TMEM55B	0.0193	7.12E-01	8.03E-01
TMEM56	-0.1954	1.52E-04	8.70E-04
TMEM57	-0.1576	2.33E-03	8.75E-03
TMEM59L	-0.0251	6.30E-01	7.38E-01
TMEM59	-0.1667	1.27E-03	5.27E-03
TMEM5	0.0820	1.15E-01	2.07E-01
TMEM60	0.0851	1.02E-01	1.88E-01
TMEM61	0.2580	4.72E-07	5.82E-06
TMEM62	0.1125	3.02E-02	7.19E-02
TMEM63A	-0.1334	1.01E-02	2.95E-02
TMEM63B	-0.0613	2.39E-01	3.63E-01
TMEM63C	0.0046	9.29E-01	9.56E-01
TMEM64	-0.1170	2.42E-02	6.00E-02
TMEM65	0.2021	8.88E-05	5.51E-04
TMEM66	-0.2384	3.42E-06	3.34E-05
TMEM67	0.1859	3.19E-04	1.64E-03
TMEM68	0.0611	2.41E-01	3.65E-01
TMEM69	0.1369	8.30E-03	2.51E-02
TMEM70	-0.1433	5.69E-03	1.83E-02
TMEM71	-0.1122	3.08E-02	7.29E-02
TMEM72	0.1822	4.19E-04	2.06E-03
TMEM74	0.0163	7.54E-01	8.34E-01
TMEM79	0.4314	3.02E-18	4.59E-16
TMEM80	0.1026	4.84E-02	1.05E-01
TMEM81	0.2557	5.97E-07	7.13E-06
TMEM82	-0.2180	2.28E-05	1.73E-04
TMEM84	0.0930	7.36E-02	1.46E-01
TMEM85	-0.0679	1.92E-01	3.07E-01
TMEM86A	-0.1166	2.47E-02	6.09E-02
TMEM86B	-0.0108	8.35E-01	8.92E-01
TMEM87A	0.1025	4.85E-02	1.05E-01
TMEM87B	0.1237	1.71E-02	4.53E-02
TMEM88B	0.0612	2.40E-01	3.64E-01
TMEM88	-0.2348	4.86E-06	4.53E-05
TMEM89	0.0353	4.98E-01	6.25E-01
TMEM8A	-0.1425	5.97E-03	1.91E-02
TMEM8B	0.1187	2.22E-02	5.59E-02
TMEM8C	0.0862	9.73E-02	1.82E-01
TMEM90A	-0.0293	5.74E-01	6.91E-01
TMEM90B	-0.0638	2.21E-01	3.41E-01
TMEM91	0.2016	9.23E-05	5.69E-04
TMEM92	-0.0169	7.45E-01	8.28E-01
TMEM93	-0.0698	1.79E-01	2.91E-01

TMEM95	0.0183	7.25E-01	8.13E-01
TMEM97	0.1524	3.25E-03	1.15E-02
TMEM98	0.2343	5.08E-06	4.70E-05
TMEM99	0.1028	4.79E-02	1.04E-01
TMEM9B	-0.1591	2.12E-03	8.08E-03
TMEM9	0.2805	3.91E-08	6.28E-07
TMF1	0.0227	6.63E-01	7.64E-01
TMIE	0.2608	3.48E-07	4.44E-06
TMIGD1	0.1274	1.41E-02	3.87E-02
TMIGD2	-0.0927	7.44E-02	1.47E-01
TMLHE	-0.0891	8.66E-02	1.66E-01
TMOD1	-0.1312	1.14E-02	3.26E-02
TMOD2	-0.1173	2.38E-02	5.92E-02
TMOD3	-0.0349	5.02E-01	6.28E-01
TMOD4	0.1944	1.65E-04	9.33E-04
TMPO	0.3007	3.41E-09	7.11E-08
TMPPE	0.0575	2.69E-01	3.97E-01
TMPRSS11A	-0.0558	2.83E-01	4.12E-01
TMPRSS11BNL	-0.0300	5.65E-01	6.83E-01
TMPRSS11B	-0.0304	5.60E-01	6.78E-01
TMPRSS11D	-0.0325	5.33E-01	6.55E-01
TMPRSS11F	-0.0607	2.44E-01	3.68E-01
TMPRSS12	0.1623	1.71E-03	6.77E-03
TMPRSS13	0.0898	8.42E-02	1.62E-01
TMPRSS15	0.1229	1.79E-02	4.70E-02
TMPRSS2	-0.0820	1.15E-01	2.07E-01
TMPRSS3	0.2564	5.59E-07	6.72E-06
TMPRSS4	0.0779	1.34E-01	2.33E-01
TMPRSS5	0.0169	7.46E-01	8.28E-01
TMPRSS6	-0.1085	3.67E-02	8.42E-02
TMPRSS7	0.1825	4.11E-04	2.02E-03
TMPRSS9	-0.0088	8.67E-01	9.14E-01
TMSB10	0.1032	4.70E-02	1.03E-01
TMSB15A	0.1916	2.05E-04	1.12E-03
TMSB15B	0.0861	9.79E-02	1.83E-01
TMSB4Y	-0.0381	4.65E-01	5.95E-01
TMSL3	0.0133	7.98E-01	8.67E-01
TMTC1	-0.0890	8.71E-02	1.66E-01
TMTC2	0.0604	2.45E-01	3.70E-01
TMTC3	0.0920	7.67E-02	1.51E-01
TMTC4	0.1290	1.29E-02	3.60E-02
TMUB1	-0.0872	9.34E-02	1.76E-01
TMUB2	0.2344	5.01E-06	4.65E-05
TMX1	-0.0512	3.25E-01	4.56E-01

TMX2	0.0147	7.78E-01	8.52E-01
TMX3	0.1120	3.10E-02	7.33E-02
TMX4	0.0514	3.24E-01	4.54E-01
TNC	-0.0661	2.04E-01	3.21E-01
TNFAIP1	-0.0520	3.18E-01	4.48E-01
TNFAIP2	0.1141	2.80E-02	6.76E-02
TNFAIP3	0.0748	1.51E-01	2.55E-01
TNFAIP6	0.0750	1.49E-01	2.53E-01
TNFAIP8L1	-0.2069	5.95E-05	3.92E-04
TNFAIP8L2	-0.0041	9.37E-01	9.61E-01
TNFAIP8L3	0.0337	5.18E-01	6.42E-01
TNFAIP8	0.0429	4.10E-01	5.43E-01
TNFRSF10A	-0.0811	1.19E-01	2.13E-01
TNFRSF10B	-0.0630	2.26E-01	3.48E-01
TNFRSF10C	-0.0019	9.71E-01	9.81E-01
TNFRSF10D	-0.0844	1.05E-01	1.92E-01
TNFRSF11A	0.1320	1.10E-02	3.14E-02
TNFRSF11B	0.0921	7.63E-02	1.50E-01
TNFRSF12A	-0.0413	4.28E-01	5.60E-01
TNFRSF13B	0.0596	2.52E-01	3.77E-01
TNFRSF13C	0.1648	1.45E-03	5.86E-03
TNFRSF14	-0.0514	3.23E-01	4.54E-01
TNFRSF17	-0.0020	9.70E-01	9.81E-01
TNFRSF18	0.1194	2.14E-02	5.43E-02
TNFRSF19	-0.0058	9.12E-01	9.45E-01
TNFRSF1A	-0.1299	1.23E-02	3.45E-02
TNFRSF1B	-0.1116	3.17E-02	7.47E-02
TNFRSF21	0.0232	6.56E-01	7.58E-01
TNFRSF25	0.1523	3.27E-03	1.16E-02
TNFRSF4	0.1423	6.04E-03	1.93E-02
TNFRSF6B	0.0813	1.18E-01	2.12E-01
TNFRSF8	-0.0138	7.91E-01	8.61E-01
TNFRSF9	0.0140	7.88E-01	8.59E-01
TNFSF10	0.0090	8.63E-01	9.11E-01
TNFSF11	-0.0542	2.98E-01	4.27E-01
TNFSF12-TNFS	-0.0373	4.73E-01	6.02E-01
TNFSF12	0.0085	8.70E-01	9.16E-01
TNFSF13B	-0.0603	2.46E-01	3.71E-01
TNFSF13	-0.0836	1.08E-01	1.97E-01
TNFSF14	0.0962	6.42E-02	1.31E-01
TNFSF15	0.0390	4.54E-01	5.84E-01
TNFSF18	-0.0419	4.21E-01	5.54E-01
TNFSF4	0.2513	9.42E-07	1.08E-05
TNFSF8	-0.1471	4.51E-03	1.52E-02

TNFSF9	0.1627	1.66E-03	6.61E-03
TNF	0.0141	7.86E-01	8.58E-01
TNIK	0.0822	1.14E-01	2.06E-01
TNIP1	-0.1200	2.08E-02	5.30E-02
TNIP2	-0.0255	6.25E-01	7.34E-01
TNIP3	-0.0320	5.39E-01	6.61E-01
TNK1	0.1510	3.55E-03	1.24E-02
TNK2	0.0320	5.39E-01	6.61E-01
TNKS1BP1	-0.1422	6.08E-03	1.94E-02
TNKS2	-0.0581	2.64E-01	3.91E-01
TNKS	0.0906	8.13E-02	1.58E-01
TNMD	-0.1758	6.71E-04	3.05E-03
TNNC1	0.1569	2.44E-03	9.08E-03
TNNC2	-0.0116	8.24E-01	8.85E-01
TNNI1	0.0766	1.41E-01	2.41E-01
TNNI2	0.0786	1.31E-01	2.28E-01
TNNI3K	0.0291	5.76E-01	6.93E-01
TNNI3	0.1948	1.59E-04	9.06E-04
TNNT1	0.1937	1.75E-04	9.80E-04
TNNT2	0.3170	4.21E-10	1.09E-08
TNNT3	-0.0239	6.46E-01	7.51E-01
TNN	-0.0351	5.01E-01	6.27E-01
TNP1	0.0186	7.21E-01	8.11E-01
TNPO1	0.0755	1.47E-01	2.50E-01
TNPO2	0.0035	9.47E-01	9.67E-01
TNPO3	-0.0703	1.77E-01	2.88E-01
TNRC18	0.2297	7.81E-06	6.75E-05
TNRC6A	-0.0363	4.86E-01	6.14E-01
TNRC6B	0.0193	7.11E-01	8.03E-01
TNRC6C	-0.0090	8.63E-01	9.12E-01
TNR	0.0303	5.60E-01	6.79E-01
TNS1	-0.0602	2.47E-01	3.72E-01
TNS3	-0.0689	1.85E-01	2.98E-01
TNS4	-0.0220	6.73E-01	7.72E-01
TNXB	-0.1579	2.29E-03	8.62E-03
TOB1	-0.0193	7.10E-01	8.02E-01
TOB2	0.0779	1.34E-01	2.33E-01
TOE1	0.1804	4.81E-04	2.30E-03
TOLLIP	-0.2934	8.46E-09	1.59E-07
TOM1L1	-0.0140	7.88E-01	8.59E-01
TOM1L2	0.0109	8.34E-01	8.91E-01
TOM1	-0.0818	1.16E-01	2.09E-01
TOMM20L	0.0677	1.93E-01	3.08E-01
TOMM20	0.2547	6.64E-07	7.88E-06

TOMM22	0.0997	5.51E-02	1.16E-01
TOMM34	0.0802	1.23E-01	2.18E-01
TOMM40L	0.2751	7.24E-08	1.09E-06
TOMM40	0.1504	3.68E-03	1.28E-02
TOMM5	0.2489	1.21E-06	1.34E-05
TOMM6	0.1653	1.40E-03	5.69E-03
TOMM70A	-0.1590	2.13E-03	8.12E-03
TOMM7	0.0504	3.33E-01	4.64E-01
TOP1MT	0.0286	5.83E-01	6.99E-01
TOP1P1	0.0533	3.06E-01	4.36E-01
TOP1P2	0.0810	1.19E-01	2.14E-01
TOP1	-0.0198	7.04E-01	7.97E-01
TOP2A	0.4327	2.33E-18	3.69E-16
TOP2B	0.1436	5.59E-03	1.81E-02
TOP3A	0.1016	5.05E-02	1.08E-01
TOP3B	0.3086	1.26E-09	2.92E-08
TOPBP1	0.3325	4.99E-11	1.57E-09
TOPORS	-0.0014	9.79E-01	9.86E-01
TOR1AIP1	0.0975	6.06E-02	1.25E-01
TOR1AIP2	0.0109	8.34E-01	8.92E-01
TOR1A	0.0135	7.96E-01	8.65E-01
TOR1B	-0.0039	9.40E-01	9.63E-01
TOR2A	0.0898	8.41E-02	1.62E-01
TOR3A	0.3257	1.28E-10	3.68E-09
TOX2	0.0106	8.39E-01	8.94E-01
TOX3	0.0296	5.69E-01	6.87E-01
TOX4	0.1235	1.73E-02	4.56E-02
TOX	-0.0040	9.39E-01	9.62E-01
TP53AIP1	0.1365	8.47E-03	2.55E-02
TP53BP1	0.1398	7.02E-03	2.18E-02
TP53BP2	0.2274	9.72E-06	8.20E-05
TP53I11	0.0087	8.68E-01	9.15E-01
TP53I13	0.1939	1.71E-04	9.63E-04
TP53I3	-0.0422	4.18E-01	5.51E-01
TP53INP1	-0.0265	6.10E-01	7.22E-01
TP53INP2	-0.2039	7.63E-05	4.85E-04
TP53RK	0.0711	1.72E-01	2.82E-01
TP53TG1	-0.1821	4.23E-04	2.07E-03
TP53TG3B	0.1233	1.75E-02	4.60E-02
TP53TG5	0.0316	5.44E-01	6.65E-01
TP53	0.1688	1.10E-03	4.65E-03
TP63	-0.0606	2.44E-01	3.68E-01
TP73	0.1954	1.52E-04	8.70E-04
TPBG	0.0959	6.51E-02	1.32E-01

TPCN1	0.0107	8.37E-01	8.93E-01
TPCN2	-0.0262	6.15E-01	7.25E-01
TPD52L1	-0.1041	4.52E-02	9.92E-02
TPD52L2	0.2401	2.90E-06	2.87E-05
TPD52L3	0.0203	6.97E-01	7.91E-01
TPD52	0.0594	2.54E-01	3.79E-01
TPH1	0.1270	1.44E-02	3.94E-02
TPH2	-0.0553	2.88E-01	4.17E-01
TPI1P2	-0.1715	9.09E-04	3.96E-03
TPI1P3	0.0227	6.63E-01	7.64E-01
TPI1	-0.0381	4.64E-01	5.94E-01
TPK1	-0.2238	1.35E-05	1.09E-04
TPM1	-0.0546	2.94E-01	4.23E-01
TPM2	0.0294	5.72E-01	6.89E-01
TPM3	0.5664	7.28E-33	8.53E-30
TPM4	0.1328	1.04E-02	3.02E-02
TPMT	-0.2157	2.79E-05	2.06E-04
TPO	-0.0484	3.53E-01	4.85E-01
TPP1	-0.1934	1.78E-04	9.98E-04
TPP2	0.1105	3.33E-02	7.78E-02
TPPP2	-0.3311	6.11E-11	1.88E-09
TPPP3	-0.0035	9.47E-01	9.67E-01
TPPP	-0.0327	5.30E-01	6.53E-01
TPRA1	0.0564	2.79E-01	4.07E-01
TPRG1L	-0.2579	4.73E-07	5.83E-06
TPRG1	-0.2583	4.57E-07	5.66E-06
TPRKB	0.2957	6.40E-09	1.25E-07
TPRN	0.1913	2.11E-04	1.15E-03
TPRX1	-0.0303	5.61E-01	6.79E-01
TPRXL	0.0995	5.54E-02	1.17E-01
TPR	0.3595	9.30E-13	4.18E-11
TPSAB1	-0.1611	1.85E-03	7.21E-03
TPSB2	-0.1425	5.98E-03	1.91E-02
TPSD1	-0.0362	4.87E-01	6.15E-01
TPSG1	-0.0373	4.74E-01	6.03E-01
TPST1	-0.1202	2.06E-02	5.25E-02
TPST2	-0.0324	5.34E-01	6.56E-01
TPT1	-0.1469	4.57E-03	1.53E-02
TPTE2P1	0.0912	7.93E-02	1.55E-01
TPTE2P3	0.1610	1.87E-03	7.27E-03
TPTE2	0.0127	8.07E-01	8.73E-01
TPTE	0.1330	1.04E-02	3.01E-02
TPX2	0.4222	1.80E-17	2.25E-15
TRA2A	0.1853	3.33E-04	1.70E-03

TRA2B	0.2802	4.05E-08	6.47E-07
TRABD	0.1273	1.42E-02	3.89E-02
TRADD	-0.0860	9.83E-02	1.83E-01
TRAF1	0.1216	1.91E-02	4.95E-02
TRAF2	0.4404	4.94E-19	9.94E-17
TRAF3IP1	0.0051	9.21E-01	9.50E-01
TRAF3IP2	0.0076	8.83E-01	9.25E-01
TRAF3IP3	-0.0532	3.07E-01	4.36E-01
TRAF3	0.0502	3.35E-01	4.66E-01
TRAF4	0.1281	1.36E-02	3.76E-02
TRAF5	0.3266	1.14E-10	3.30E-09
TRAF6	-0.0184	7.24E-01	8.13E-01
TRAF7	0.0219	6.75E-01	7.73E-01
TRAFD1	0.2110	4.18E-05	2.91E-04
TRAIP	0.3929	3.81E-15	2.71E-13
TRAK1	0.0794	1.27E-01	2.23E-01
TRAK2	0.0737	1.57E-01	2.62E-01
TRAM1L1	0.2769	5.92E-08	9.07E-07
TRAM1	-0.0963	6.40E-02	1.31E-01
TRAM2	-0.0714	1.70E-01	2.79E-01
TRANK1	-0.2032	8.08E-05	5.09E-04
TRAP1	-0.0762	1.43E-01	2.45E-01
TRAPPC10	-0.0435	4.04E-01	5.37E-01
TRAPPC1	-0.0217	6.78E-01	7.76E-01
TRAPPC2L	-0.1423	6.04E-03	1.93E-02
TRAPPC2P1	0.1375	8.02E-03	2.44E-02
TRAPPC2	0.1060	4.13E-02	9.25E-02
TRAPPC3	-0.0056	9.15E-01	9.47E-01
TRAPPC4	0.1898	2.35E-04	1.27E-03
TRAPPC5	-0.0985	5.80E-02	1.21E-01
TRAPPC6A	-0.0226	6.65E-01	7.65E-01
TRAPPC6B	-0.1120	3.10E-02	7.33E-02
TRAPPC9	-0.0481	3.55E-01	4.88E-01
TRAT1	-0.0785	1.31E-01	2.29E-01
TRDMT1	0.1535	3.04E-03	1.09E-02
TRDN	0.0226	6.65E-01	7.65E-01
TREH	-0.1985	1.19E-04	7.08E-04
TREM1	0.1582	2.24E-03	8.46E-03
TREM2	0.0851	1.02E-01	1.88E-01
TREML1	0.1031	4.72E-02	1.03E-01
TREML2P1	0.0461	3.76E-01	5.10E-01
TREML2	0.0251	6.30E-01	7.38E-01
TREML3	0.1158	2.57E-02	6.29E-02
TREML4	0.0456	3.82E-01	5.15E-01

TRERF1	-0.0396	4.46E-01	5.78E-01
TREX1	-0.0180	7.30E-01	8.17E-01
TREX2	0.0986	5.79E-02	1.21E-01
TRHDE	-0.0804	1.22E-01	2.17E-01
TRHR	-0.0255	6.24E-01	7.33E-01
TRH	0.0781	1.33E-01	2.32E-01
TRIAP1	-0.0800	1.24E-01	2.19E-01
TRIB1	-0.0089	8.64E-01	9.12E-01
TRIB2	-0.0237	6.49E-01	7.53E-01
TRIB3	0.1071	3.93E-02	8.89E-02
TRIL	-0.1203	2.04E-02	5.22E-02
TRIM10	0.0239	6.46E-01	7.51E-01
TRIM11	0.4289	4.88E-18	6.95E-16
TRIM13	0.0728	1.62E-01	2.69E-01
TRIM14	-0.0485	3.52E-01	4.84E-01
TRIM15	0.0530	3.08E-01	4.38E-01
TRIM16L	0.0890	8.69E-02	1.66E-01
TRIM16	0.1483	4.20E-03	1.43E-02
TRIM17	0.2951	6.90E-09	1.33E-07
TRIM21	0.0327	5.31E-01	6.53E-01
TRIM22	-0.1585	2.20E-03	8.34E-03
TRIM23	-0.0851	1.02E-01	1.88E-01
TRIM24	0.0905	8.17E-02	1.58E-01
TRIM25	0.2504	1.03E-06	1.16E-05
TRIM26	0.0030	9.55E-01	9.72E-01
TRIM27	0.1074	3.87E-02	8.78E-02
TRIM28	0.2368	4.01E-06	3.85E-05
TRIM29	0.0852	1.02E-01	1.88E-01
TRIM2	-0.1749	7.15E-04	3.22E-03
TRIM31	0.1878	2.75E-04	1.44E-03
TRIM32	0.2491	1.18E-06	1.32E-05
TRIM33	0.0687	1.87E-01	3.00E-01
TRIM34	-0.0377	4.69E-01	5.98E-01
TRIM35	-0.1455	4.97E-03	1.64E-02
TRIM36	-0.0203	6.97E-01	7.91E-01
TRIM37	0.2436	2.06E-06	2.15E-05
TRIM38	-0.1126	3.02E-02	7.18E-02
TRIM39	0.0804	1.22E-01	2.17E-01
TRIM3	0.1017	5.03E-02	1.08E-01
TRIM40	0.0422	4.17E-01	5.50E-01
TRIM41	-0.1472	4.50E-03	1.51E-02
TRIM42	0.0586	2.61E-01	3.87E-01
TRIM43	-0.0609	2.42E-01	3.66E-01
TRIM44	-0.1340	9.77E-03	2.87E-02

TRIM45	0.4296	4.31E-18	6.31E-16
TRIM46	0.1864	3.06E-04	1.59E-03
TRIM47	0.2403	2.85E-06	2.83E-05
TRIM48	-0.0503	3.34E-01	4.65E-01
TRIM49L	-0.0675	1.94E-01	3.10E-01
TRIM49	0.0111	8.31E-01	8.89E-01
TRIM4	-0.1460	4.83E-03	1.60E-02
TRIM50	0.1816	4.38E-04	2.13E-03
TRIM52	0.1787	5.45E-04	2.56E-03
TRIM53	0.0397	4.45E-01	5.77E-01
TRIM54	0.1751	7.03E-04	3.18E-03
TRIM55	-0.1874	2.84E-04	1.49E-03
TRIM56	-0.0958	6.54E-02	1.33E-01
TRIM58	-0.1263	1.49E-02	4.05E-02
TRIM59	0.3580	1.16E-12	5.09E-11
TRIM5	-0.1709	9.47E-04	4.11E-03
TRIM6-TRIM34	-0.0166	7.50E-01	8.32E-01
TRIM60	0.0530	3.09E-01	4.38E-01
TRIM61	-0.0090	8.63E-01	9.11E-01
TRIM62	0.0152	7.71E-01	8.46E-01
TRIM63	-0.1337	9.93E-03	2.91E-02
TRIM65	0.2938	8.04E-09	1.52E-07
TRIM66	0.0377	4.69E-01	5.99E-01
TRIM67	0.3162	4.63E-10	1.19E-08
TRIM68	0.1351	9.18E-03	2.73E-02
TRIM69	-0.1122	3.08E-02	7.29E-02
TRIM6	0.0591	2.56E-01	3.82E-01
TRIM71	0.1170	2.42E-02	6.00E-02
TRIM72	0.1880	2.71E-04	1.43E-03
TRIM74	0.0494	3.42E-01	4.74E-01
TRIM77	-0.0053	9.19E-01	9.49E-01
TRIM78P	0.0651	2.11E-01	3.29E-01
TRIM7	0.1005	5.30E-02	1.12E-01
TRIM8	-0.0898	8.42E-02	1.62E-01
TRIM9	-0.0303	5.61E-01	6.79E-01
TRIML1	-0.1153	2.63E-02	6.43E-02
TRIML2	-0.0246	6.37E-01	7.44E-01
TRIOBP	0.0664	2.02E-01	3.19E-01
TRIO	0.0426	4.13E-01	5.46E-01
TRIP10	0.1323	1.07E-02	3.09E-02
TRIP11	-0.1011	5.17E-02	1.10E-01
TRIP12	0.0265	6.11E-01	7.22E-01
TRIP13	0.3791	3.95E-14	2.29E-12
TRIP4	0.2543	6.95E-07	8.18E-06

TRIP6	-0.0521	3.17E-01	4.47E-01
TRIT1	0.1180	2.31E-02	5.77E-02
TRMT112	0.1345	9.48E-03	2.80E-02
TRMT11	0.0043	9.34E-01	9.59E-01
TRMT12	0.0814	1.18E-01	2.11E-01
TRMT1	0.1875	2.82E-04	1.48E-03
TRMT2A	0.2103	4.45E-05	3.06E-04
TRMT2B	0.1870	2.92E-04	1.53E-03
TRMT5	0.0389	4.55E-01	5.86E-01
TRMT61A	0.0191	7.14E-01	8.05E-01
TRMT61B	0.0855	1.00E-01	1.86E-01
TRMT6	0.2301	7.57E-06	6.58E-05
TRMU	0.1336	9.99E-03	2.92E-02
TRNAU1AP	0.2446	1.86E-06	1.97E-05
TRNP1	0.0970	6.19E-02	1.27E-01
TRNT1	-0.0080	8.77E-01	9.21E-01
TROAP	0.4390	6.59E-19	1.26E-16
TROVE2	0.3454	7.77E-12	2.85E-10
TRO	-0.0462	3.75E-01	5.08E-01
TRPA1	0.1182	2.27E-02	5.70E-02
TRPC1	0.1575	2.34E-03	8.78E-03
TRPC2	0.0667	2.00E-01	3.16E-01
TRPC3	0.1326	1.06E-02	3.06E-02
TRPC4AP	0.0589	2.58E-01	3.84E-01
TRPC4	-0.1481	4.24E-03	1.44E-02
TRPC5	-0.2043	7.38E-05	4.71E-04
TRPC6	-0.0363	4.86E-01	6.14E-01
TRPC7	0.0042	9.36E-01	9.60E-01
TRPM1	-0.0665	2.01E-01	3.17E-01
TRPM2	0.0905	8.16E-02	1.58E-01
TRPM3	-0.0404	4.38E-01	5.69E-01
TRPM4	-0.1050	4.32E-02	9.59E-02
TRPM5	0.1106	3.32E-02	7.76E-02
TRPM6	-0.0670	1.98E-01	3.14E-01
TRPM7	-0.0919	7.69E-02	1.51E-01
TRPM8	-0.0343	5.11E-01	6.35E-01
TRPS1	-0.0560	2.82E-01	4.10E-01
TRPT1	0.0445	3.93E-01	5.26E-01
TRPV1	-0.0130	8.03E-01	8.70E-01
TRPV2	-0.0332	5.24E-01	6.48E-01
TRPV3	-0.0597	2.51E-01	3.76E-01
TRPV4	-0.0425	4.14E-01	5.47E-01
TRPV5	0.0981	5.90E-02	1.22E-01
TRPV6	0.1182	2.27E-02	5.70E-02

TRRAP	0.0451	3.86E-01	5.19E-01
TRUB1	-0.1282	1.35E-02	3.74E-02
TRUB2	-0.0802	1.23E-01	2.18E-01
TRY6	0.0400	4.43E-01	5.75E-01
TRYX3	0.1314	1.13E-02	3.23E-02
TSC1	0.3594	9.35E-13	4.20E-11
TSC22D1	-0.1557	2.63E-03	9.68E-03
TSC22D2	-0.1010	5.19E-02	1.11E-01
TSC22D3	-0.1009	5.22E-02	1.11E-01
TSC22D4	0.0298	5.68E-01	6.85E-01
TSC2	0.0757	1.46E-01	2.48E-01
TSEN15	0.3410	1.49E-11	5.14E-10
TSEN2	0.1228	1.79E-02	4.70E-02
TSEN34	0.1701	1.00E-03	4.32E-03
TSEN54	0.3466	6.49E-12	2.43E-10
TSFM	-0.1169	2.44E-02	6.03E-02
TSG101	0.1013	5.13E-02	1.10E-01
TSG1	-0.1049	4.34E-02	9.62E-02
TSGA10IP	0.0383	4.62E-01	5.92E-01
TSGA10	0.0194	7.10E-01	8.02E-01
TSGA13	-0.0184	7.24E-01	8.12E-01
TSGA14	0.0955	6.61E-02	1.34E-01
TSHB	-0.0319	5.40E-01	6.62E-01
TSHR	0.0532	3.06E-01	4.36E-01
TSHZ1	-0.0014	9.79E-01	9.86E-01
TSHZ2	-0.0285	5.84E-01	7.00E-01
TSHZ3	-0.1402	6.86E-03	2.14E-02
TSIX	0.0672	1.97E-01	3.12E-01
TSKS	0.0821	1.14E-01	2.06E-01
TSKU	-0.1822	4.21E-04	2.06E-03
TSLP	-0.2295	8.01E-06	6.90E-05
TSNARE1	0.1561	2.56E-03	9.46E-03
TSNAX-DISC1	-0.0319	5.40E-01	6.62E-01
TSNAXIP1	-0.0732	1.59E-01	2.65E-01
TSNAX	0.2108	4.26E-05	2.95E-04
TSN	0.2645	2.34E-07	3.11E-06
TSPAN10	0.0263	6.14E-01	7.24E-01
TSPAN11	-0.0606	2.44E-01	3.69E-01
TSPAN12	-0.1183	2.27E-02	5.70E-02
TSPAN13	-0.0108	8.36E-01	8.92E-01
TSPAN14	-0.0324	5.33E-01	6.56E-01
TSPAN15	0.1257	1.54E-02	4.15E-02
TSPAN16	0.0518	3.19E-01	4.49E-01
TSPAN17	0.0160	7.58E-01	8.38E-01

TSPAN18	-0.1547	2.81E-03	1.02E-02
TSPAN19	0.1211	1.97E-02	5.07E-02
TSPAN1	-0.1171	2.41E-02	5.98E-02
TSPAN2	-0.1044	4.44E-02	9.79E-02
TSPAN31	-0.3807	3.03E-14	1.79E-12
TSPAN32	0.0084	8.73E-01	9.18E-01
TSPAN33	-0.0818	1.16E-01	2.08E-01
TSPAN3	0.0775	1.36E-01	2.36E-01
TSPAN4	-0.1336	9.99E-03	2.92E-02
TSPAN5	-0.0391	4.53E-01	5.84E-01
TSPAN6	-0.0713	1.70E-01	2.80E-01
TSPAN7	0.0118	8.21E-01	8.82E-01
TSPAN8	0.0309	5.53E-01	6.72E-01
TSPAN9	-0.2621	3.05E-07	3.96E-06
TSPO2	0.0277	5.95E-01	7.09E-01
TSPO	-0.0209	6.88E-01	7.84E-01
TSPY1	-0.0314	5.47E-01	6.67E-01
TSPY2	-0.0022	9.67E-01	9.80E-01
TSPY3	-0.0107	8.37E-01	8.93E-01
TSPY4	-0.0575	2.70E-01	3.97E-01
TSPYL1	-0.1820	4.25E-04	2.08E-03
TSPYL2	0.1422	6.08E-03	1.94E-02
TSPYL3	0.0453	3.84E-01	5.18E-01
TSPYL4	0.0388	4.57E-01	5.87E-01
TSPYL5	-0.1460	4.85E-03	1.61E-02
TSPYL6	0.0776	1.36E-01	2.35E-01
TSR1	0.0149	7.75E-01	8.49E-01
TSR2	-0.0122	8.15E-01	8.78E-01
TSSC1	0.1548	2.79E-03	1.02E-02
TSSC4	0.1298	1.24E-02	3.47E-02
TSSK1B	-0.1080	3.76E-02	8.58E-02
TSSK3	0.1010	5.18E-02	1.11E-01
TSSK4	0.0625	2.30E-01	3.53E-01
TSSK6	0.0195	7.08E-01	8.01E-01
TSTA3	-0.0582	2.63E-01	3.90E-01
TSTD1	0.0211	6.85E-01	7.82E-01
TSTD2	-0.0304	5.59E-01	6.77E-01
TST	-0.3052	1.93E-09	4.31E-08
TTBK1	-0.0291	5.76E-01	6.93E-01
TTBK2	-0.0828	1.11E-01	2.02E-01
TTC12	0.0608	2.43E-01	3.67E-01
TTC13	0.4365	1.08E-18	1.92E-16
TTC14	0.1865	3.05E-04	1.58E-03
TTC15	-0.0473	3.63E-01	4.96E-01

TTC16	0.0144	7.83E-01	8.55E-01
TTC17	-0.0558	2.84E-01	4.13E-01
TTC18	-0.0047	9.29E-01	9.55E-01
TTC19	-0.1599	2.00E-03	7.70E-03
TTC1	0.0384	4.60E-01	5.91E-01
TTC21A	0.1239	1.70E-02	4.50E-02
TTC21B	0.1978	1.26E-04	7.42E-04
TTC22	0.1075	3.85E-02	8.74E-02
TTC23L	0.1251	1.59E-02	4.26E-02
TTC23	0.0169	7.46E-01	8.28E-01
TTC24	0.0237	6.49E-01	7.53E-01
TTC25	0.0369	4.79E-01	6.08E-01
TTC26	0.1350	9.23E-03	2.74E-02
TTC27	0.2492	1.17E-06	1.30E-05
TTC28	-0.0320	5.39E-01	6.60E-01
TTC29	0.0693	1.83E-01	2.95E-01
TTC30A	0.0521	3.17E-01	4.47E-01
TTC30B	0.1002	5.39E-02	1.14E-01
TTC31	0.0475	3.62E-01	4.94E-01
TTC32	0.1027	4.82E-02	1.04E-01
TTC33	0.0453	3.85E-01	5.18E-01
TTC35	0.0512	3.25E-01	4.56E-01
TTC36	-0.3377	2.41E-11	8.03E-10
TTC37	-0.0575	2.69E-01	3.97E-01
TTC38	-0.3020	2.92E-09	6.18E-08
TTC39A	0.1545	2.85E-03	1.04E-02
TTC39B	-0.0596	2.52E-01	3.78E-01
TTC39C	-0.0774	1.37E-01	2.36E-01
TTC3	0.0088	8.66E-01	9.13E-01
TTC4	0.1685	1.12E-03	4.76E-03
TTC5	0.0294	5.72E-01	6.89E-01
TTC7A	0.1255	1.56E-02	4.19E-02
TTC7B	-0.1837	3.75E-04	1.88E-03
TTC8	-0.0828	1.11E-01	2.02E-01
TTC9B	0.0307	5.55E-01	6.74E-01
TTC9C	0.1609	1.88E-03	7.32E-03
TTC9	-0.1508	3.60E-03	1.26E-02
TTF1	0.3463	6.88E-12	2.57E-10
TTF2	0.2581	4.64E-07	5.73E-06
TTK	0.4683	1.28E-21	3.93E-19
TTLL10	-0.0429	4.10E-01	5.43E-01
TTLL11	0.0483	3.53E-01	4.85E-01
TTLL12	0.0076	8.84E-01	9.25E-01
TTLL13	-0.0348	5.04E-01	6.29E-01

TTLL1	0.1503	3.71E-03	1.29E-02
TTLL2	0.0535	3.04E-01	4.33E-01
TTLL3	0.2483	1.29E-06	1.42E-05
TTLL4	0.3270	1.07E-10	3.12E-09
TTLL5	0.1052	4.29E-02	9.54E-02
TTLL6	0.1243	1.66E-02	4.42E-02
TTLL7	0.1301	1.21E-02	3.42E-02
TTLL8	0.0271	6.02E-01	7.15E-01
TTLL9	0.1578	2.31E-03	8.68E-03
TTL	-0.0387	4.57E-01	5.88E-01
TTN	0.1708	9.56E-04	4.14E-03
TTPAL	-0.1539	2.95E-03	1.07E-02
TTPA	-0.2174	2.41E-05	1.82E-04
TTR	-0.1467	4.64E-03	1.55E-02
TTTY10	-0.0768	1.40E-01	2.40E-01
TTTY14	-0.0519	3.19E-01	4.49E-01
TTTY15	0.0229	6.60E-01	7.61E-01
TTTY1B	0.0637	2.21E-01	3.42E-01
TTTY20	0.0303	5.61E-01	6.79E-01
TTTY2	0.0805	1.22E-01	2.17E-01
TTTY4C	-0.0587	2.59E-01	3.85E-01
TTTY5	0.0681	1.90E-01	3.04E-01
TTTY6B	0.0908	8.06E-02	1.57E-01
TTTY6	-0.0119	8.19E-01	8.81E-01
TTTY8	0.0734	1.58E-01	2.64E-01
TTTY9B	-0.0555	2.86E-01	4.15E-01
TTYH1	0.1121	3.08E-02	7.30E-02
TTYH2	0.0608	2.43E-01	3.67E-01
TTYH3	0.0737	1.56E-01	2.62E-01
TUBA1A	0.0650	2.11E-01	3.30E-01
TUBA1B	0.3581	1.15E-12	5.06E-11
TUBA1C	0.3165	4.46E-10	1.15E-08
TUBA3C	0.1261	1.51E-02	4.09E-02
TUBA3D	0.1370	8.23E-03	2.49E-02
TUBA3E	0.1576	2.33E-03	8.74E-03
TUBA4A	0.1430	5.79E-03	1.86E-02
TUBA4B	0.0326	5.31E-01	6.54E-01
TUBA8	0.0474	3.62E-01	4.94E-01
TUBAL3	0.1720	8.76E-04	3.84E-03
TUBB1	0.0398	4.45E-01	5.77E-01
TUBB2A	0.0335	5.20E-01	6.44E-01
TUBB2B	-0.0099	8.49E-01	9.02E-01
TUBB2C	0.2068	5.96E-05	3.92E-04
TUBB3	0.0965	6.35E-02	1.30E-01

TUBB4Q	0.1923	1.94E-04	1.07E-03
TUBB4	0.0697	1.80E-01	2.92E-01
TUBB6	-0.0961	6.45E-02	1.32E-01
TUBB8	0.2170	2.48E-05	1.87E-04
TUBBP5	0.2214	1.69E-05	1.33E-04
TUBB	0.2916	1.05E-08	1.95E-07
TUBD1	0.4237	1.35E-17	1.76E-15
TUBE1	-0.1314	1.13E-02	3.23E-02
TUBG1	0.3110	9.20E-10	2.19E-08
TUBG2	0.2035	7.87E-05	4.98E-04
TUBGCP2	0.0780	1.34E-01	2.32E-01
TUBGCP3	0.2319	6.35E-06	5.70E-05
TUBGCP4	-0.0068	8.96E-01	9.34E-01
TUBGCP5	0.0194	7.09E-01	8.01E-01
TUBGCP6	0.1324	1.07E-02	3.08E-02
TUB	0.1156	2.59E-02	6.35E-02
TUFM	-0.2119	3.90E-05	2.74E-04
TUFT1	0.4903	7.90E-24	3.66E-21
TUG1	0.3346	3.74E-11	1.21E-09
TULP1	0.2064	6.17E-05	4.04E-04
TULP2	0.0599	2.50E-01	3.75E-01
TULP3	0.2315	6.63E-06	5.91E-05
TULP4	0.1658	1.35E-03	5.54E-03
TUSC1	0.0273	6.00E-01	7.13E-01
TUSC2	0.0010	9.84E-01	9.90E-01
TUSC3	-0.0037	9.43E-01	9.64E-01
TUSC5	-0.1457	4.93E-03	1.63E-02
TUT1	-0.0490	3.47E-01	4.79E-01
TWF1	-0.0172	7.41E-01	8.25E-01
TWF2	0.0945	6.91E-02	1.39E-01
TWIST1	0.0220	6.72E-01	7.72E-01
TWIST2	-0.1578	2.30E-03	8.66E-03
TWISTNB	0.0194	7.10E-01	8.02E-01
TWSG1	-0.0597	2.51E-01	3.76E-01
TXK	-0.1404	6.78E-03	2.12E-02
TXLNA	0.0388	4.56E-01	5.86E-01
TXLNB	0.1041	4.52E-02	9.93E-02
TXLNG	0.0430	4.09E-01	5.42E-01
TXN2	-0.1590	2.12E-03	8.09E-03
TXNDC11	-0.1193	2.15E-02	5.46E-02
TXNDC12	0.0654	2.09E-01	3.27E-01
TXNDC15	-0.2351	4.71E-06	4.41E-05
TXNDC16	0.0535	3.04E-01	4.33E-01
TXNDC17	-0.1065	4.04E-02	9.09E-02

TXNDC2	0.0504	3.33E-01	4.64E-01
TXNDC3	0.0187	7.20E-01	8.10E-01
TXNDC5	-0.0316	5.43E-01	6.64E-01
TXNDC6	0.1001	5.40E-02	1.14E-01
TXNDC9	0.2246	1.26E-05	1.02E-04
TXNIP	-0.0608	2.42E-01	3.67E-01
TXNL1	-0.0753	1.48E-01	2.51E-01
TXNL4A	0.0923	7.59E-02	1.50E-01
TXNL4B	-0.1685	1.12E-03	4.74E-03
TXNRD1	0.0846	1.04E-01	1.91E-01
TXNRD2	-0.1125	3.03E-02	7.20E-02
TXNRD3IT1	-0.2744	7.81E-08	1.16E-06
TXN	0.1842	3.62E-04	1.83E-03
TYK2	0.0307	5.55E-01	6.75E-01
TYMP	-0.0175	7.36E-01	8.22E-01
TYMS	0.3499	3.99E-12	1.59E-10
TYRO3	0.2566	5.45E-07	6.59E-06
TYROBP	-0.0507	3.30E-01	4.61E-01
TYRP1	-0.0854	1.00E-01	1.86E-01
TYR	-0.0626	2.29E-01	3.51E-01
TYSND1	0.1258	1.53E-02	4.14E-02
TYW1B	-0.0750	1.49E-01	2.53E-01
TYW1	-0.1205	2.02E-02	5.19E-02
TYW3	0.0478	3.59E-01	4.91E-01
T	0.0550	2.91E-01	4.20E-01
U2AF1L4	0.2025	8.60E-05	5.36E-04
U2AF1	0.3120	8.09E-10	1.96E-08
U2AF2	0.3017	3.03E-09	6.39E-08
UACA	-0.0061	9.07E-01	9.41E-01
UAP1L1	0.0970	6.20E-02	1.27E-01
UAP1	0.2007	9.92E-05	6.05E-04
UBA1	-0.0320	5.39E-01	6.61E-01
UBA2	0.3045	2.12E-09	4.66E-08
UBA3	0.0406	4.36E-01	5.67E-01
UBA52	0.0666	2.01E-01	3.17E-01
UBA5	-0.1253	1.57E-02	4.22E-02
UBA6	-0.0038	9.42E-01	9.64E-01
UBA7	-0.1099	3.43E-02	7.97E-02
UBAC1	0.0914	7.87E-02	1.54E-01
UBAC2	0.1224	1.83E-02	4.78E-02
UBAP1	0.0788	1.30E-01	2.27E-01
UBAP2L	0.5365	4.85E-29	3.86E-26
UBAP2	0.2791	4.59E-08	7.25E-07
UBASH3A	-0.0398	4.45E-01	5.77E-01

UBASH3B	0.0402	4.40E-01	5.71E-01
UBB	-0.1727	8.37E-04	3.69E-03
UBC	-0.0437	4.01E-01	5.34E-01
UBD	0.2010	9.68E-05	5.93E-04
UBE2A	0.2286	8.69E-06	7.43E-05
UBE2B	-0.0241	6.43E-01	7.48E-01
UBE2CBP	0.2479	1.34E-06	1.46E-05
UBE2C	0.4119	1.26E-16	1.25E-14
UBE2D1	0.1623	1.71E-03	6.77E-03
UBE2D2	0.1799	4.98E-04	2.37E-03
UBE2D3	-0.2385	3.39E-06	3.31E-05
UBE2D4	-0.1830	3.95E-04	1.96E-03
UBE2DNL	-0.0102	8.44E-01	8.98E-01
UBE2E1	0.3136	6.53E-10	1.61E-08
UBE2E2	-0.1855	3.28E-04	1.68E-03
UBE2E3	0.1387	7.48E-03	2.30E-02
UBE2F	-0.1090	3.58E-02	8.24E-02
UBE2G1	-0.1351	9.18E-03	2.73E-02
UBE2G2	-0.0282	5.88E-01	7.03E-01
UBE2H	-0.2346	4.92E-06	4.58E-05
UBE2I	0.3320	5.39E-11	1.68E-09
UBE2J1	0.1395	7.11E-03	2.20E-02
UBE2J2	0.0913	7.90E-02	1.54E-01
UBE2K	0.0789	1.29E-01	2.26E-01
UBE2L3	0.1598	2.02E-03	7.78E-03
UBE2L6	-0.1467	4.64E-03	1.55E-02
UBE2MP1	0.1493	3.95E-03	1.36E-02
UBE2M	0.1641	1.52E-03	6.11E-03
UBE2NL	0.0940	7.05E-02	1.41E-01
UBE2N	0.1675	1.20E-03	5.02E-03
UBE2O	0.3059	1.78E-09	4.01E-08
UBE2Q1	0.5095	6.72E-26	4.06E-23
UBE2Q2P1	0.0223	6.69E-01	7.68E-01
UBE2Q2	0.0678	1.92E-01	3.07E-01
UBE2QL1	-0.2268	1.03E-05	8.62E-05
UBE2R2	0.0969	6.22E-02	1.28E-01
UBE2S	0.3048	2.04E-09	4.51E-08
UBE2T	0.5240	1.48E-27	1.06E-24
UBE2U	0.1302	1.21E-02	3.41E-02
UBE2V1	0.1123	3.05E-02	7.25E-02
UBE2V2	-0.0062	9.05E-01	9.40E-01
UBE2W	-0.1281	1.36E-02	3.76E-02
UBE2Z	0.3058	1.80E-09	4.05E-08
UBE3A	-0.1344	9.52E-03	2.81E-02

UBE3B	0.0201	6.99E-01	7.92E-01
UBE3C	-0.1480	4.28E-03	1.45E-02
UBE4A	0.0386	4.59E-01	5.89E-01
UBE4B	0.0465	3.72E-01	5.05E-01
UBFD1	-0.0901	8.31E-02	1.60E-01
UBIAD1	-0.0980	5.94E-02	1.23E-01
UBL3	-0.1656	1.37E-03	5.60E-03
UBL4A	0.1984	1.20E-04	7.13E-04
UBL4B	0.1242	1.67E-02	4.43E-02
UBL5	0.0275	5.98E-01	7.12E-01
UBL7	0.0440	3.99E-01	5.31E-01
UBLCP1	0.0497	3.40E-01	4.71E-01
UBN1	0.2040	7.57E-05	4.82E-04
UBN2	0.0804	1.22E-01	2.17E-01
UBOX5	0.0590	2.57E-01	3.82E-01
UBP1	-0.0435	4.04E-01	5.37E-01
UBQLN1	0.0668	1.99E-01	3.16E-01
UBQLN2	0.0718	1.67E-01	2.76E-01
UBQLN3	0.0465	3.72E-01	5.05E-01
UBQLN4	0.3971	1.82E-15	1.38E-13
UBQLNL	0.1221	1.87E-02	4.86E-02
UBR1	-0.1045	4.42E-02	9.75E-02
UBR2	-0.0418	4.22E-01	5.55E-01
UBR3	-0.1058	4.17E-02	9.31E-02
UBR4	-0.0215	6.80E-01	7.78E-01
UBR5	0.0801	1.23E-01	2.19E-01
UBR7	0.1124	3.04E-02	7.23E-02
UBTD1	-0.1458	4.90E-03	1.62E-02
UBTD2	0.0792	1.28E-01	2.24E-01
UBTFL1	0.1099	3.44E-02	7.98E-02
UBTF	0.2318	6.42E-06	5.74E-05
UBXN10	-0.1952	1.55E-04	8.86E-04
UBXN11	0.1734	7.94E-04	3.53E-03
UBXN1	0.0647	2.14E-01	3.33E-01
UBXN2A	0.1828	4.01E-04	1.99E-03
UBXN2B	-0.0992	5.62E-02	1.18E-01
UBXN4	0.0077	8.83E-01	9.25E-01
UBXN6	-0.2910	1.13E-08	2.08E-07
UBXN7	0.0767	1.40E-01	2.41E-01
UBXN8	-0.1881	2.70E-04	1.42E-03
UCA1	0.0202	6.98E-01	7.92E-01
UCHL1	0.0665	2.01E-01	3.18E-01
UCHL3	-0.1055	4.23E-02	9.41E-02
UCHL5	0.2538	7.26E-07	8.52E-06

UCK1	0.0286	5.83E-01	6.99E-01
UCK2	0.3920	4.50E-15	3.14E-13
UCKL1AS	0.2740	8.22E-08	1.22E-06
UCKL1	0.3289	8.32E-11	2.48E-09
UCMA	-0.0362	4.87E-01	6.14E-01
UCN2	0.1143	2.77E-02	6.70E-02
UCN3	0.0939	7.09E-02	1.42E-01
UCN	-0.0082	8.75E-01	9.19E-01
UCP1	0.0748	1.51E-01	2.55E-01
UCP2	-0.0282	5.88E-01	7.03E-01
UCP3	0.2977	4.98E-09	9.93E-08
UEVLD	-0.0056	9.14E-01	9.46E-01
UFC1	0.3770	5.63E-14	3.12E-12
UFD1L	0.1346	9.43E-03	2.79E-02
UFM1	-0.0670	1.98E-01	3.14E-01
UFSP1	-0.1327	1.05E-02	3.04E-02
UFSP2	-0.2735	8.71E-08	1.28E-06
UGCG	0.0125	8.11E-01	8.76E-01
UGDH	0.0874	9.28E-02	1.75E-01
UGGT1	0.0439	3.99E-01	5.32E-01
UGGT2	0.0859	9.87E-02	1.84E-01
UGP2	-0.2783	5.01E-08	7.81E-07
UGT1A10	0.1582	2.24E-03	8.46E-03
UGT1A1	-0.1971	1.33E-04	7.79E-04
UGT1A3	-0.2139	3.26E-05	2.35E-04
UGT1A4	-0.2163	2.65E-05	1.97E-04
UGT1A5	-0.1662	1.31E-03	5.40E-03
UGT1A6	-0.1059	4.15E-02	9.27E-02
UGT1A7	0.1336	9.99E-03	2.92E-02
UGT1A8	0.0330	5.27E-01	6.49E-01
UGT1A9	-0.1314	1.13E-02	3.22E-02
UGT2A1	0.0667	2.00E-01	3.17E-01
UGT2A3	-0.1199	2.09E-02	5.31E-02
UGT2B10	-0.2937	8.16E-09	1.54E-07
UGT2B11	-0.0632	2.24E-01	3.46E-01
UGT2B15	-0.1953	1.53E-04	8.80E-04
UGT2B28	-0.2054	6.76E-05	4.37E-04
UGT2B4	-0.0564	2.79E-01	4.07E-01
UGT2B7	-0.2261	1.09E-05	9.07E-05
UGT3A1	-0.0107	8.37E-01	8.93E-01
UGT3A2	0.0277	5.95E-01	7.09E-01
UGT8	0.0936	7.19E-02	1.43E-01
UHMK1	-0.0075	8.85E-01	9.26E-01
UHRF1BP1L	-0.0726	1.63E-01	2.70E-01

UHRF1BP1	0.1528	3.18E-03	1.13E-02
UHRF1	0.4246	1.13E-17	1.49E-15
UHRF2	0.2508	9.96E-07	1.13E-05
UIMC1	0.1900	2.32E-04	1.25E-03
ULBP1	0.1704	9.84E-04	4.24E-03
ULBP2	0.0838	1.07E-01	1.96E-01
ULBP3	-0.0127	8.08E-01	8.73E-01
ULK1	0.1470	4.56E-03	1.53E-02
ULK2	0.0546	2.94E-01	4.23E-01
ULK3	0.0593	2.54E-01	3.80E-01
ULK4	-0.0185	7.23E-01	8.12E-01
UMODL1	0.1719	8.88E-04	3.88E-03
UMOD	0.0915	7.85E-02	1.54E-01
UMPS	0.1493	3.94E-03	1.36E-02
UNC119B	0.1820	4.25E-04	2.08E-03
UNC119	0.1596	2.04E-03	7.85E-03
UNC13A	0.0854	1.01E-01	1.86E-01
UNC13B	0.0167	7.48E-01	8.30E-01
UNC13C	0.1002	5.39E-02	1.14E-01
UNC13D	0.0430	4.09E-01	5.41E-01
UNC45A	0.0132	7.99E-01	8.67E-01
UNC45B	-0.0948	6.81E-02	1.37E-01
UNC50	0.0830	1.10E-01	2.01E-01
UNC5A	-0.0049	9.25E-01	9.52E-01
UNC5B	0.0783	1.32E-01	2.30E-01
UNC5CL	0.0332	5.24E-01	6.48E-01
UNC5C	-0.1230	1.78E-02	4.67E-02
UNC5D	-0.0115	8.25E-01	8.85E-01
UNC80	0.0289	5.79E-01	6.95E-01
UNC93A	0.0692	1.84E-01	2.96E-01
UNC93B1	0.0571	2.73E-01	4.01E-01
UNG	0.1997	1.08E-04	6.48E-04
UNKL	0.2755	6.95E-08	1.05E-06
UNK	0.3878	9.15E-15	5.98E-13
UOX	0.0737	1.57E-01	2.62E-01
UPB1	-0.1376	7.97E-03	2.43E-02
UPF0639	0.0254	6.25E-01	7.34E-01
UPF1	-0.0289	5.79E-01	6.96E-01
UPF2	0.0862	9.74E-02	1.82E-01
UPF3A	0.2826	3.07E-08	5.05E-07
UPF3B	0.3871	1.04E-14	6.75E-13
UPK1A	0.1947	1.61E-04	9.13E-04
UPK1B	0.0264	6.12E-01	7.23E-01
UPK2	0.0172	7.42E-01	8.26E-01

UPK3A	0.1429	5.82E-03	1.87E-02
UPK3BL	0.1185	2.24E-02	5.63E-02
UPK3B	-0.0608	2.43E-01	3.68E-01
UPP1	0.0836	1.08E-01	1.97E-01
UPP2	-0.2721	1.02E-07	1.48E-06
UPRT	-0.0309	5.53E-01	6.72E-01
UQCC	-0.1139	2.82E-02	6.80E-02
UQCR10	-0.0808	1.20E-01	2.15E-01
UQCR11	-0.1567	2.48E-03	9.19E-03
UQCRB	-0.0611	2.41E-01	3.65E-01
UQCRC1	-0.2585	4.46E-07	5.54E-06
UQCRC2	-0.2773	5.62E-08	8.66E-07
UQCRFS1	-0.1463	4.74E-03	1.58E-02
UQCRHL	0.2025	8.54E-05	5.33E-04
UQCRH	0.1567	2.47E-03	9.17E-03
UQCRQ	-0.1725	8.49E-04	3.73E-03
URB1	0.0381	4.64E-01	5.94E-01
URB2	0.2545	6.75E-07	8.00E-06
URGCP	-0.0809	1.20E-01	2.14E-01
URM1	0.1636	1.56E-03	6.26E-03
UROC1	-0.1419	6.17E-03	1.96E-02
UROD	-0.1080	3.76E-02	8.58E-02
UROS	-0.1514	3.46E-03	1.22E-02
USE1	-0.0726	1.63E-01	2.70E-01
USF1	0.4251	1.04E-17	1.40E-15
USF2	-0.0278	5.94E-01	7.08E-01
USH1C	0.1212	1.95E-02	5.03E-02
USH1G	0.2180	2.28E-05	1.73E-04
USH2A	-0.0553	2.88E-01	4.17E-01
USHBP1	-0.1729	8.26E-04	3.65E-03
USMG5	-0.0790	1.29E-01	2.25E-01
USO1	-0.1180	2.30E-02	5.76E-02
USP10	-0.1162	2.52E-02	6.19E-02
USP11	0.0817	1.16E-01	2.09E-01
USP12	-0.0805	1.22E-01	2.17E-01
USP13	0.1149	2.69E-02	6.55E-02
USP14	0.1473	4.45E-03	1.50E-02
USP15	-0.0415	4.26E-01	5.58E-01
USP16	0.0769	1.39E-01	2.39E-01
USP17L2	0.0157	7.63E-01	8.40E-01
USP17	0.0454	3.83E-01	5.17E-01
USP18	-0.0607	2.43E-01	3.68E-01
USP19	0.0336	5.19E-01	6.43E-01
USP1	0.3582	1.13E-12	5.00E-11

USP20	0.1747	7.28E-04	3.28E-03
USP21	0.5454	3.84E-30	3.64E-27
USP22	0.0942	6.99E-02	1.40E-01
USP24	0.1238	1.70E-02	4.51E-02
USP25	-0.0892	8.64E-02	1.65E-01
USP26	0.0877	9.16E-02	1.73E-01
USP27X	0.0633	2.24E-01	3.45E-01
USP28	0.1527	3.19E-03	1.14E-02
USP29	-0.0259	6.19E-01	7.28E-01
USP2	-0.1335	1.00E-02	2.93E-02
USP30	-0.0954	6.65E-02	1.35E-01
USP31	0.1317	1.11E-02	3.18E-02
USP32	0.0528	3.10E-01	4.40E-01
USP33	0.0924	7.54E-02	1.49E-01
USP34	0.0794	1.27E-01	2.23E-01
USP35	-0.1812	4.53E-04	2.19E-03
USP36	0.3308	6.35E-11	1.95E-09
USP37	0.3478	5.50E-12	2.11E-10
USP38	-0.2493	1.16E-06	1.29E-05
USP39	0.2109	4.25E-05	2.95E-04
USP3	0.0650	2.11E-01	3.30E-01
USP40	0.0100	8.48E-01	9.01E-01
USP42	0.1407	6.64E-03	2.09E-02
USP43	0.1217	1.91E-02	4.94E-02
USP44	0.0851	1.02E-01	1.88E-01
USP45	0.1379	7.83E-03	2.39E-02
USP46	0.1207	2.00E-02	5.14E-02
USP47	-0.2027	8.44E-05	5.29E-04
USP48	0.0989	5.70E-02	1.19E-01
USP49	0.0597	2.51E-01	3.76E-01
USP4	-0.0212	6.84E-01	7.80E-01
USP50	0.0233	6.54E-01	7.57E-01
USP51	0.0500	3.37E-01	4.68E-01
USP53	-0.1593	2.09E-03	8.01E-03
USP54	0.0369	4.79E-01	6.07E-01
USP5	0.0464	3.73E-01	5.06E-01
USP6NL	0.0306	5.57E-01	6.76E-01
USP6	0.1617	1.78E-03	6.98E-03
USP7	-0.0104	8.41E-01	8.96E-01
USP8	-0.1061	4.12E-02	9.23E-02
USP9X	-0.0662	2.04E-01	3.21E-01
USP9Y	-0.0067	8.98E-01	9.36E-01
USPL1	0.1601	1.98E-03	7.62E-03
UST	-0.1021	4.93E-02	1.06E-01

UTF1	0.0621	2.33E-01	3.56E-01
UTP11L	0.1817	4.35E-04	2.12E-03
UTP14A	0.3268	1.11E-10	3.22E-09
UTP14C	-0.0858	9.90E-02	1.84E-01
UTP15	-0.0032	9.51E-01	9.69E-01
UTP18	0.3443	9.14E-12	3.32E-10
UTP20	0.1306	1.18E-02	3.35E-02
UTP23	0.1424	6.02E-03	1.92E-02
UTP3	-0.0334	5.21E-01	6.45E-01
UTP6	0.4438	2.47E-19	5.18E-17
UTRN	0.1003	5.37E-02	1.14E-01
UTS2D	-0.0374	4.73E-01	6.02E-01
UTS2R	0.1164	2.50E-02	6.14E-02
UTS2	0.0957	6.55E-02	1.33E-01
UTY	-0.0355	4.95E-01	6.22E-01
UVRAG	-0.0115	8.25E-01	8.86E-01
UXS1	0.1661	1.32E-03	5.44E-03
UXT	0.0287	5.82E-01	6.98E-01
VAC14	-0.1579	2.29E-03	8.61E-03
VAMP1	0.1045	4.43E-02	9.77E-02
VAMP2	-0.0396	4.47E-01	5.78E-01
VAMP3	-0.1055	4.23E-02	9.42E-02
VAMP4	0.2995	3.99E-09	8.24E-08
VAMP5	-0.1793	5.21E-04	2.46E-03
VAMP7	-0.0711	1.72E-01	2.82E-01
VAMP8	-0.0289	5.80E-01	6.96E-01
VANGL1	0.1452	5.09E-03	1.67E-02
VANGL2	0.1027	4.81E-02	1.04E-01
VAPA	0.0220	6.73E-01	7.72E-01
VAPB	-0.0906	8.13E-02	1.58E-01
VARS2	-0.0330	5.26E-01	6.49E-01
VARS	0.0129	8.04E-01	8.70E-01
VASH1	0.0440	3.98E-01	5.31E-01
VASH2	0.1332	1.02E-02	2.97E-02
VASN	-0.0565	2.78E-01	4.07E-01
VASP	0.1503	3.71E-03	1.29E-02
VAT1L	0.0804	1.22E-01	2.17E-01
VAT1	0.0861	9.79E-02	1.83E-01
VAV1	-0.0619	2.34E-01	3.57E-01
VAV2	0.1041	4.51E-02	9.91E-02
VAV3	-0.0492	3.44E-01	4.76E-01
VAX1	0.1747	7.26E-04	3.27E-03
VAX2	0.1214	1.93E-02	4.99E-02
VBP1	0.2852	2.25E-08	3.85E-07

VCAM1	-0.0357	4.93E-01	6.20E-01
VCAN	-0.0243	6.41E-01	7.47E-01
VCL	0.0491	3.45E-01	4.77E-01
VCPIP1	-0.0994	5.58E-02	1.17E-01
VCP	-0.0322	5.36E-01	6.58E-01
VCX2	0.1072	3.91E-02	8.85E-02
VCX3A	0.0568	2.75E-01	4.04E-01
VCX3B	0.1015	5.07E-02	1.09E-01
VCX	0.0671	1.97E-01	3.14E-01
VCY	0.0317	5.42E-01	6.63E-01
VDAC1	0.0330	5.26E-01	6.49E-01
VDAC2	0.0424	4.15E-01	5.48E-01
VDAC3	0.0586	2.60E-01	3.87E-01
VDR	0.1163	2.50E-02	6.16E-02
VEGFA	0.1783	5.60E-04	2.62E-03
VEGFB	0.0805	1.22E-01	2.17E-01
VEGFC	-0.1546	2.82E-03	1.03E-02
VENTXP1	0.0850	1.02E-01	1.89E-01
VENTXP7	0.1095	3.50E-02	8.10E-02
VENTX	-0.0091	8.61E-01	9.10E-01
VEPH1	0.0619	2.34E-01	3.58E-01
VEZF1	0.3259	1.25E-10	3.59E-09
VEZT	0.1249	1.61E-02	4.31E-02
VGF	0.0378	4.68E-01	5.98E-01
VGLL1	0.0572	2.72E-01	4.00E-01
VGLL2	0.1261	1.51E-02	4.09E-02
VGLL3	-0.1695	1.05E-03	4.48E-03
VGLL4	0.0351	5.00E-01	6.26E-01
VHLL	0.1303	1.20E-02	3.39E-02
VHL	0.0584	2.62E-01	3.88E-01
VIL1	0.1585	2.20E-03	8.33E-03
VILL	0.0253	6.27E-01	7.35E-01
VIM	-0.0968	6.26E-02	1.28E-01
VIPAR	-0.0030	9.55E-01	9.72E-01
VIPR1	-0.2888	1.47E-08	2.64E-07
VIPR2	0.0209	6.88E-01	7.84E-01
VIP	0.0545	2.95E-01	4.24E-01
VIT	0.0563	2.79E-01	4.08E-01
VKORC1L1	-0.0549	2.91E-01	4.20E-01
VKORC1	-0.2008	9.87E-05	6.03E-04
VLDLR	-0.0290	5.78E-01	6.95E-01
VMA21	0.3256	1.31E-10	3.74E-09
VMAC	0.0937	7.14E-02	1.42E-01
VMO1	-0.1024	4.88E-02	1.06E-01

VN1R1	0.2284	8.90E-06	7.58E-05
VN1R2	-0.0470	3.66E-01	4.99E-01
VN1R5	0.1905	2.23E-04	1.21E-03
VNN1	-0.0576	2.69E-01	3.96E-01
VNN2	0.0939	7.09E-02	1.42E-01
VNN3	-0.0363	4.86E-01	6.14E-01
VOPP1	0.0351	5.00E-01	6.26E-01
VPRBP	0.0573	2.71E-01	3.99E-01
VPREB1	0.0413	4.27E-01	5.60E-01
VPREB3	-0.0282	5.88E-01	7.03E-01
VPS11	0.1397	7.05E-03	2.19E-02
VPS13A	0.0602	2.48E-01	3.72E-01
VPS13B	-0.0251	6.31E-01	7.38E-01
VPS13C	-0.1382	7.66E-03	2.35E-02
VPS13D	-0.2105	4.39E-05	3.03E-04
VPS16	0.0974	6.09E-02	1.26E-01
VPS18	-0.1602	1.97E-03	7.61E-03
VPS24	0.1271	1.43E-02	3.92E-02
VPS25	0.1499	3.80E-03	1.32E-02
VPS26A	0.1855	3.28E-04	1.68E-03
VPS26B	-0.1043	4.46E-02	9.82E-02
VPS28	-0.0221	6.72E-01	7.71E-01
VPS29	0.0967	6.28E-02	1.29E-01
VPS33A	0.0723	1.65E-01	2.72E-01
VPS33B	0.1350	9.25E-03	2.75E-02
VPS35	0.0175	7.36E-01	8.22E-01
VPS36	-0.2106	4.34E-05	3.00E-04
VPS37A	0.0159	7.60E-01	8.38E-01
VPS37B	0.1340	9.79E-03	2.87E-02
VPS37C	0.2475	1.39E-06	1.51E-05
VPS37D	-0.1411	6.48E-03	2.04E-02
VPS39	0.0658	2.06E-01	3.24E-01
VPS41	-0.1438	5.51E-03	1.79E-02
VPS45	0.6473	1.96E-45	9.76E-42
VPS4A	-0.1191	2.18E-02	5.51E-02
VPS4B	-0.1490	4.03E-03	1.38E-02
VPS52	0.0814	1.17E-01	2.11E-01
VPS53	-0.0847	1.03E-01	1.90E-01
VPS54	0.1357	8.84E-03	2.65E-02
VPS72	0.6580	2.20E-47	1.46E-43
VPS8	0.1355	8.99E-03	2.68E-02
VRK1	0.2816	3.43E-08	5.56E-07
VRK2	0.1635	1.58E-03	6.33E-03
VRK3	-0.1058	4.17E-02	9.32E-02

VSIG10L	-0.0449	3.89E-01	5.22E-01
VSIG10	0.1455	4.98E-03	1.64E-02
VSIG1	0.0232	6.57E-01	7.58E-01
VSIG2	-0.1799	4.99E-04	2.38E-03
VSIG4	-0.1151	2.66E-02	6.49E-02
VSIG8	0.1427	5.89E-03	1.89E-02
VSNL1	-0.0201	6.99E-01	7.93E-01
VSTM1	0.0321	5.37E-01	6.59E-01
VSTM2A	-0.0387	4.57E-01	5.88E-01
VSTM2B	0.0157	7.63E-01	8.40E-01
VSTM2L	-0.1533	3.07E-03	1.10E-02
VSX1	0.0665	2.02E-01	3.18E-01
VSX2	0.1854	3.30E-04	1.69E-03
VTA1	0.1932	1.81E-04	1.01E-03
VTCN1	0.0499	3.38E-01	4.69E-01
VTI1A	-0.0562	2.80E-01	4.09E-01
VTI1B	-0.2419	2.44E-06	2.48E-05
VTN	-0.0899	8.37E-02	1.61E-01
VWA1	-0.0741	1.54E-01	2.59E-01
VWA2	-0.0049	9.25E-01	9.53E-01
VWA3A	0.0749	1.50E-01	2.54E-01
VWA3B	0.0025	9.61E-01	9.76E-01
VWA5A	-0.0217	6.77E-01	7.76E-01
VWA5B1	0.0208	6.90E-01	7.85E-01
VWA5B2	0.1695	1.05E-03	4.46E-03
VWC2L	0.0746	1.51E-01	2.55E-01
VWC2	-0.0407	4.34E-01	5.66E-01
VWCE	0.0509	3.28E-01	4.59E-01
VWDE	0.1111	3.24E-02	7.60E-02
VWF	-0.2790	4.61E-08	7.28E-07
WAC	0.2466	1.52E-06	1.64E-05
WAPAL	-0.0339	5.16E-01	6.40E-01
WARS2	0.0099	8.49E-01	9.01E-01
WARS	-0.0389	4.55E-01	5.86E-01
WASF1	0.3443	9.24E-12	3.33E-10
WASF2	0.1850	3.42E-04	1.74E-03
WASF3	-0.1094	3.52E-02	8.14E-02
WASH2P	0.2346	4.95E-06	4.61E-05
WASH3P	0.2866	1.90E-08	3.31E-07
WASH5P	0.1688	1.10E-03	4.65E-03
WASH7P	0.3488	4.72E-12	1.83E-10
WASL	-0.1970	1.34E-04	7.86E-04
WAS	-0.0247	6.35E-01	7.42E-01
WBP11P1	0.0656	2.08E-01	3.26E-01

WBP11	0.1441	5.41E-03	1.76E-02
WBP1	-0.0471	3.66E-01	4.99E-01
WBP2NL	0.1459	4.86E-03	1.61E-02
WBP2	-0.0184	7.25E-01	8.13E-01
WBP4	-0.0092	8.60E-01	9.10E-01
WBP5	0.2061	6.34E-05	4.14E-04
WBSCR16	-0.2000	1.05E-04	6.34E-04
WBSCR17	-0.1349	9.30E-03	2.76E-02
WBSCR22	-0.0028	9.58E-01	9.74E-01
WBSCR26	-0.0239	6.46E-01	7.50E-01
WBSCR27	0.1002	5.38E-02	1.14E-01
WBSCR28	0.1925	1.91E-04	1.06E-03
WDFY1	-0.0084	8.71E-01	9.17E-01
WDFY2	0.0604	2.46E-01	3.71E-01
WDFY3	-0.0521	3.17E-01	4.47E-01
WDFY4	-0.0445	3.93E-01	5.26E-01
WDHD1	0.3853	1.40E-14	8.79E-13
WDR11	-0.0965	6.34E-02	1.30E-01
WDR12	0.1812	4.54E-04	2.19E-03
WDR13	-0.0356	4.95E-01	6.21E-01
WDR16	0.1959	1.46E-04	8.44E-04
WDR17	-0.0448	3.89E-01	5.22E-01
WDR18	-0.0313	5.47E-01	6.68E-01
WDR19	0.1329	1.04E-02	3.02E-02
WDR1	-0.0315	5.46E-01	6.66E-01
WDR20	-0.0011	9.83E-01	9.89E-01
WDR24	-0.0710	1.72E-01	2.82E-01
WDR25	-0.0903	8.25E-02	1.60E-01
WDR26	0.1710	9.43E-04	4.09E-03
WDR27	0.3450	8.22E-12	3.01E-10
WDR31	0.0414	4.26E-01	5.59E-01
WDR33	0.3455	7.73E-12	2.84E-10
WDR34	0.2803	4.00E-08	6.39E-07
WDR35	0.0226	6.64E-01	7.64E-01
WDR36	-0.1243	1.66E-02	4.41E-02
WDR37	0.0617	2.35E-01	3.59E-01
WDR38	0.0921	7.64E-02	1.50E-01
WDR3	-0.0003	9.95E-01	9.97E-01
WDR41	0.0380	4.66E-01	5.96E-01
WDR43	0.0346	5.06E-01	6.31E-01
WDR44	-0.0592	2.55E-01	3.81E-01
WDR45L	0.1501	3.75E-03	1.31E-02
WDR45	-0.1073	3.89E-02	8.81E-02
WDR46	0.1470	4.56E-03	1.53E-02

WDR47	0.0989	5.69E-02	1.19E-01
WDR48	0.0092	8.59E-01	9.09E-01
WDR49	0.1408	6.62E-03	2.08E-02
WDR4	0.2091	4.95E-05	3.35E-04
WDR52	0.2825	3.09E-08	5.08E-07
WDR53	0.3470	6.18E-12	2.33E-10
WDR54	0.1064	4.05E-02	9.10E-02
WDR55	-0.0695	1.81E-01	2.93E-01
WDR59	-0.1464	4.72E-03	1.57E-02
WDR5B	0.2090	4.96E-05	3.36E-04
WDR5	0.2732	8.98E-08	1.32E-06
WDR60	-0.0013	9.81E-01	9.88E-01
WDR61	-0.0851	1.02E-01	1.88E-01
WDR62	0.4292	4.61E-18	6.66E-16
WDR63	-0.0531	3.07E-01	4.37E-01
WDR64	-0.0034	9.48E-01	9.68E-01
WDR65	-0.1471	4.53E-03	1.52E-02
WDR66	0.0130	8.03E-01	8.70E-01
WDR67	0.2598	3.90E-07	4.91E-06
WDR69	0.0949	6.78E-02	1.37E-01
WDR6	0.1290	1.29E-02	3.60E-02
WDR70	0.2566	5.42E-07	6.57E-06
WDR72	0.0027	9.59E-01	9.74E-01
WDR73	0.1422	6.09E-03	1.94E-02
WDR74	0.0707	1.74E-01	2.84E-01
WDR75	0.3257	1.29E-10	3.70E-09
WDR76	0.4124	1.14E-16	1.15E-14
WDR77	0.1335	1.01E-02	2.94E-02
WDR78	-0.0739	1.55E-01	2.60E-01
WDR7	-0.2148	3.01E-05	2.19E-04
WDR81	-0.1405	6.71E-03	2.10E-02
WDR82	0.0234	6.53E-01	7.56E-01
WDR83	0.0166	7.51E-01	8.32E-01
WDR85	0.3712	1.45E-13	7.57E-12
WDR86	-0.1217	1.91E-02	4.94E-02
WDR87	0.1540	2.93E-03	1.06E-02
WDR88	0.2413	2.58E-06	2.59E-05
WDR89	0.0455	3.82E-01	5.15E-01
WDR8	0.1232	1.76E-02	4.63E-02
WDR90	0.3207	2.56E-10	6.99E-09
WDR91	0.1482	4.22E-03	1.44E-02
WDR92	0.0140	7.88E-01	8.59E-01
WDR93	0.1351	9.15E-03	2.72E-02
WDSUB1	0.2676	1.68E-07	2.32E-06

WDTC1	-0.1438	5.51E-03	1.79E-02
WDYHV1	0.2058	6.53E-05	4.24E-04
WEE1	0.1446	5.25E-03	1.72E-02
WEE2	-0.0329	5.27E-01	6.50E-01
WFDC10A	0.0916	7.81E-02	1.53E-01
WFDC10B	0.0946	6.87E-02	1.38E-01
WFDC12	0.0504	3.33E-01	4.64E-01
WFDC13	0.1099	3.43E-02	7.98E-02
WFDC1	-0.1253	1.57E-02	4.22E-02
WFDC2	0.0471	3.65E-01	4.98E-01
WFDC3	0.1089	3.61E-02	8.30E-02
WFDC5	0.0107	8.38E-01	8.93E-01
WFDC6	0.1405	6.72E-03	2.10E-02
WFDC8	0.0941	7.03E-02	1.41E-01
WFIKKN1	0.0631	2.25E-01	3.47E-01
WFIKKN2	-0.0413	4.28E-01	5.60E-01
WFS1	0.0721	1.66E-01	2.74E-01
WHAMML1	0.0123	8.14E-01	8.78E-01
WHAMML2	0.0350	5.01E-01	6.27E-01
WHAMM	-0.0305	5.58E-01	6.77E-01
WHSC1L1	0.1652	1.40E-03	5.71E-03
WHSC1	0.3759	6.71E-14	3.66E-12
WHSC2	0.1921	1.97E-04	1.09E-03
WIBG	0.0672	1.97E-01	3.12E-01
WIF1	0.1250	1.60E-02	4.28E-02
WIPF1	-0.0585	2.61E-01	3.87E-01
WIPF2	0.1462	4.78E-03	1.59E-02
WIPF3	-0.0018	9.72E-01	9.82E-01
WIPI1	-0.0286	5.83E-01	6.99E-01
WIPI2	-0.0741	1.54E-01	2.59E-01
WISP1	-0.1542	2.90E-03	1.05E-02
WISP2	-0.1749	7.17E-04	3.23E-03
WISP3	0.1016	5.05E-02	1.08E-01
WIT1	0.1300	1.22E-02	3.44E-02
WIZ	-0.0344	5.09E-01	6.34E-01
WLS	-0.2036	7.81E-05	4.94E-04
WNK1	0.0439	3.99E-01	5.32E-01
WNK2	0.2065	6.13E-05	4.02E-04
WNK3	-0.0186	7.22E-01	8.11E-01
WNK4	0.1112	3.22E-02	7.57E-02
WNT10A	0.0840	1.06E-01	1.95E-01
WNT10B	0.0631	2.26E-01	3.47E-01
WNT11	0.0205	6.94E-01	7.89E-01
WNT16	0.0306	5.57E-01	6.76E-01

WNT1	-0.1074	3.87E-02	8.78E-02
WNT2B	0.0101	8.46E-01	8.99E-01
WNT2	-0.1178	2.33E-02	5.81E-02
WNT3A	0.0977	6.01E-02	1.24E-01
WNT3	0.1088	3.62E-02	8.32E-02
WNT4	0.0694	1.82E-01	2.94E-01
WNT5A	-0.0628	2.27E-01	3.49E-01
WNT5B	-0.0648	2.13E-01	3.33E-01
WNT6	0.0222	6.69E-01	7.69E-01
WNT7A	-0.0909	8.05E-02	1.56E-01
WNT7B	0.1043	4.48E-02	9.86E-02
WNT8A	-0.0376	4.70E-01	6.00E-01
WNT8B	-0.0066	8.99E-01	9.36E-01
WNT9A	-0.0124	8.12E-01	8.76E-01
WNT9B	-0.2017	9.13E-05	5.64E-04
WRAP53	0.1803	4.83E-04	2.31E-03
WRB	0.0547	2.93E-01	4.23E-01
WRNIP1	0.0823	1.14E-01	2.06E-01
WRN	0.0809	1.20E-01	2.14E-01
WSB1	0.1897	2.38E-04	1.28E-03
WSB2	0.1026	4.83E-02	1.05E-01
WSCD1	0.1449	5.17E-03	1.70E-02
WSCD2	-0.2478	1.35E-06	1.47E-05
WT1	0.1337	9.93E-03	2.91E-02
WTAP	0.2653	2.14E-07	2.88E-06
WTIP	-0.0358	4.92E-01	6.19E-01
WWC1	-0.0524	3.14E-01	4.44E-01
WWC2	-0.1969	1.35E-04	7.88E-04
WWC3	0.0609	2.42E-01	3.66E-01
WWOX	-0.2539	7.24E-07	8.50E-06
WWP1	-0.1167	2.45E-02	6.06E-02
WWP2	-0.1208	1.99E-02	5.13E-02
WWTR1	0.0565	2.77E-01	4.06E-01
XAB2	0.0859	9.86E-02	1.84E-01
XAF1	-0.1454	5.02E-03	1.65E-02
XAGE1D	0.0221	6.72E-01	7.71E-01
XAGE2	0.1915	2.07E-04	1.14E-03
XAGE3	0.0110	8.33E-01	8.90E-01
XAGE5	0.0631	2.26E-01	3.47E-01
XBP1	-0.0358	4.92E-01	6.19E-01
XCL1	0.0823	1.13E-01	2.05E-01
XCL2	0.0234	6.53E-01	7.56E-01
XCR1	-0.1021	4.94E-02	1.07E-01
XDH	-0.1808	4.67E-04	2.24E-03

XG	-0.1221	1.86E-02	4.85E-02
XIAP	0.0340	5.14E-01	6.38E-01
XIRP1	-0.0146	7.80E-01	8.53E-01
XIRP2	0.0766	1.41E-01	2.42E-01
XIST	0.0185	7.23E-01	8.12E-01
XKR3	0.0263	6.13E-01	7.24E-01
XKR4	0.0534	3.05E-01	4.35E-01
XKR5	0.1599	2.00E-03	7.71E-03
XKR6	-0.0102	8.45E-01	8.99E-01
XKR7	-0.0165	7.51E-01	8.32E-01
XKR8	-0.0639	2.19E-01	3.40E-01
XKR9	-0.1132	2.92E-02	7.00E-02
XKRX	0.0912	7.95E-02	1.55E-01
XKRY2	-0.0050	9.23E-01	9.52E-01
XK	-0.0441	3.97E-01	5.30E-01
XPA	0.1433	5.68E-03	1.83E-02
XPC	-0.1181	2.29E-02	5.73E-02
XPNPEP1	0.0585	2.61E-01	3.87E-01
XPNPEP2	-0.0327	5.30E-01	6.53E-01
XPNPEP3	0.0617	2.36E-01	3.59E-01
XPO1	0.3411	1.47E-11	5.11E-10
XPO4	0.1304	1.19E-02	3.37E-02
XPO5	0.2417	2.48E-06	2.51E-05
XPO6	0.1414	6.39E-03	2.02E-02
XPO7	0.1178	2.33E-02	5.81E-02
XPOT	0.0637	2.21E-01	3.42E-01
XPR1	0.2752	7.13E-08	1.07E-06
XRCC1	0.2555	6.09E-07	7.26E-06
XRCC2	0.3492	4.42E-12	1.73E-10
XRCC3	0.3997	1.17E-15	9.21E-14
XRCC4	0.0633	2.24E-01	3.45E-01
XRCC5	0.2050	6.94E-05	4.48E-04
XRCC6BP1	0.1182	2.28E-02	5.72E-02
XRCC6	0.2169	2.50E-05	1.88E-04
XRN1	-0.0363	4.86E-01	6.14E-01
XRN2	-0.0096	8.54E-01	9.06E-01
XRRA1	0.2201	1.89E-05	1.47E-04
XYLB	-0.1151	2.66E-02	6.49E-02
XYLT1	-0.0859	9.86E-02	1.84E-01
XYLT2	0.1392	7.25E-03	2.24E-02
YAF2	-0.1186	2.24E-02	5.63E-02
YAP1	-0.0389	4.55E-01	5.86E-01
YARS2	0.0087	8.67E-01	9.15E-01
YARS	0.0085	8.71E-01	9.17E-01

YBX1	0.0431	4.08E-01	5.41E-01
YBX2	0.0655	2.08E-01	3.27E-01
YDJC	0.1170	2.42E-02	6.00E-02
YEATS2	0.2850	2.30E-08	3.90E-07
YEATS4	0.1628	1.65E-03	6.57E-03
YES1	-0.0562	2.80E-01	4.09E-01
YIF1A	-0.1396	7.09E-03	2.20E-02
YIF1B	0.0375	4.72E-01	6.01E-01
YIPF1	-0.0811	1.19E-01	2.13E-01
YIPF2	-0.1312	1.14E-02	3.25E-02
YIPF3	-0.0789	1.29E-01	2.26E-01
YIPF4	-0.1455	4.98E-03	1.64E-02
YIPF5	0.0456	3.81E-01	5.15E-01
YIPF6	-0.0458	3.79E-01	5.13E-01
YIPF7	-0.0434	4.05E-01	5.38E-01
YJEFN3	0.3041	2.24E-09	4.88E-08
YKT6	0.1313	1.13E-02	3.23E-02
YLPM1	0.0035	9.47E-01	9.67E-01
YME1L1	0.0048	9.27E-01	9.54E-01
YOD1	0.3337	4.25E-11	1.35E-09
YPEL1	-0.0641	2.18E-01	3.38E-01
YPEL2	0.0104	8.42E-01	8.97E-01
YPEL3	0.0111	8.32E-01	8.90E-01
YPEL4	0.0322	5.37E-01	6.59E-01
YPEL5	-0.1098	3.45E-02	8.01E-02
YRDC	-0.0345	5.07E-01	6.32E-01
YSK4	-0.0083	8.73E-01	9.18E-01
YTHDC1	0.1271	1.43E-02	3.92E-02
YTHDC2	-0.0802	1.23E-01	2.18E-01
YTHDF1	0.2054	6.73E-05	4.36E-04
YTHDF2	0.0371	4.76E-01	6.05E-01
YTHDF3	-0.1480	4.29E-03	1.46E-02
YWHAB	0.0622	2.32E-01	3.55E-01
YWHAE	-0.0725	1.63E-01	2.71E-01
YWHAG	-0.1281	1.35E-02	3.75E-02
YWHAH	0.1587	2.17E-03	8.22E-03
YWHAQ	0.1579	2.29E-03	8.61E-03
YWHAZ	0.1640	1.53E-03	6.15E-03
YY1AP1	0.5965	4.02E-37	8.91E-34
YY1	-0.0642	2.17E-01	3.37E-01
YY2	-0.0359	4.91E-01	6.18E-01
ZACN	0.1430	5.80E-03	1.86E-02
ZADH2	-0.1753	6.94E-04	3.15E-03
ZAK	-0.0802	1.23E-01	2.18E-01

ZAN	0.1005	5.32E-02	1.13E-01
ZAP70	-0.0104	8.41E-01	8.96E-01
ZAR1L	0.1080	3.75E-02	8.58E-02
ZAR1	0.0860	9.80E-02	1.83E-01
ZBBX	0.0772	1.38E-01	2.37E-01
ZBED1	-0.1277	1.39E-02	3.83E-02
ZBED2	0.1059	4.16E-02	9.29E-02
ZBED3	-0.0862	9.72E-02	1.82E-01
ZBED4	0.2519	8.91E-07	1.02E-05
ZBED5	0.2896	1.33E-08	2.41E-07
ZBP1	0.0217	6.77E-01	7.75E-01
ZBTB10	-0.0775	1.36E-01	2.36E-01
ZBTB11	0.1310	1.15E-02	3.28E-02
ZBTB12	0.3088	1.23E-09	2.85E-08
ZBTB16	-0.2708	1.18E-07	1.68E-06
ZBTB17	0.1056	4.21E-02	9.37E-02
ZBTB1	0.0031	9.52E-01	9.70E-01
ZBTB20	-0.0873	9.32E-02	1.76E-01
ZBTB22	0.0317	5.42E-01	6.63E-01
ZBTB24	0.2812	3.61E-08	5.82E-07
ZBTB25	0.0389	4.55E-01	5.86E-01
ZBTB26	0.1542	2.91E-03	1.05E-02
ZBTB2	0.2178	2.32E-05	1.76E-04
ZBTB32	0.0630	2.26E-01	3.47E-01
ZBTB33	-0.0596	2.52E-01	3.77E-01
ZBTB34	0.3443	9.23E-12	3.33E-10
ZBTB37	0.0205	6.94E-01	7.89E-01
ZBTB38	-0.1438	5.52E-03	1.79E-02
ZBTB39	0.0908	8.09E-02	1.57E-01
ZBTB3	0.1145	2.75E-02	6.65E-02
ZBTB40	0.2610	3.42E-07	4.39E-06
ZBTB41	0.2981	4.76E-09	9.56E-08
ZBTB42	-0.0127	8.07E-01	8.73E-01
ZBTB43	0.0207	6.92E-01	7.87E-01
ZBTB44	0.0481	3.55E-01	4.87E-01
ZBTB45	0.2313	6.78E-06	6.01E-05
ZBTB46	-0.0328	5.29E-01	6.52E-01
ZBTB47	-0.0973	6.12E-02	1.26E-01
ZBTB48	0.0272	6.01E-01	7.14E-01
ZBTB49	0.1724	8.55E-04	3.76E-03
ZBTB4	-0.0044	9.33E-01	9.58E-01
ZBTB5	0.2785	4.89E-08	7.63E-07
ZBTB6	0.2016	9.20E-05	5.68E-04
ZBTB7A	-0.1278	1.38E-02	3.81E-02

ZBTB7B	0.3054	1.89E-09	4.23E-08
ZBTB7C	-0.2862	2.00E-08	3.46E-07
ZBTB8A	0.1006	5.29E-02	1.12E-01
ZBTB8B	0.1386	7.49E-03	2.30E-02
ZBTB8OS	0.0092	8.60E-01	9.10E-01
ZBTB9	0.3159	4.85E-10	1.23E-08
ZC3H10	0.2330	5.75E-06	5.21E-05
ZC3H11A	0.3456	7.55E-12	2.79E-10
ZC3H12A	0.0281	5.90E-01	7.05E-01
ZC3H12B	0.1446	5.27E-03	1.72E-02
ZC3H12C	-0.0359	4.90E-01	6.18E-01
ZC3H12D	0.0127	8.08E-01	8.74E-01
ZC3H13	-0.1248	1.62E-02	4.32E-02
ZC3H14	-0.0056	9.14E-01	9.46E-01
ZC3H15	0.0249	6.32E-01	7.39E-01
ZC3H18	0.0003	9.95E-01	9.97E-01
ZC3H3	0.1520	3.34E-03	1.18E-02
ZC3H4	0.2216	1.66E-05	1.31E-04
ZC3H6	-0.0118	8.20E-01	8.82E-01
ZC3H7A	0.0196	7.07E-01	7.99E-01
ZC3H7B	-0.0498	3.39E-01	4.70E-01
ZC3H8	0.0662	2.03E-01	3.21E-01
ZC3HAV1L	0.0974	6.08E-02	1.26E-01
ZC3HAV1	0.0540	2.99E-01	4.29E-01
ZC3HC1	-0.0752	1.48E-01	2.52E-01
ZC4H2	0.1399	6.96E-03	2.17E-02
ZCCHC10	0.1092	3.54E-02	8.18E-02
ZCCHC11	0.1438	5.51E-03	1.79E-02
ZCCHC12	0.0724	1.64E-01	2.72E-01
ZCCHC13	0.0438	4.00E-01	5.33E-01
ZCCHC14	-0.1012	5.15E-02	1.10E-01
ZCCHC16	-0.1813	4.50E-04	2.18E-03
ZCCHC17	0.2374	3.78E-06	3.65E-05
ZCCHC18	-0.0217	6.77E-01	7.76E-01
ZCCHC24	-0.2573	5.08E-07	6.20E-06
ZCCHC2	0.0124	8.11E-01	8.76E-01
ZCCHC3	0.2074	5.72E-05	3.80E-04
ZCCHC4	0.0674	1.95E-01	3.11E-01
ZCCHC5	0.0083	8.74E-01	9.18E-01
ZCCHC6	-0.1429	5.83E-03	1.87E-02
ZCCHC7	0.2247	1.25E-05	1.02E-04
ZCCHC8	0.1717	8.99E-04	3.92E-03
ZCCHC9	0.1346	9.43E-03	2.79E-02
ZCRB1	0.0905	8.16E-02	1.58E-01

ZCWPW1	-0.0561	2.81E-01	4.09E-01
ZCWPW2	0.0606	2.44E-01	3.69E-01
ZDBF2	0.1294	1.26E-02	3.54E-02
ZDHHC11	0.2280	9.18E-06	7.79E-05
ZDHHC12	0.1256	1.55E-02	4.17E-02
ZDHHC13	0.2499	1.09E-06	1.22E-05
ZDHHC14	0.1278	1.38E-02	3.81E-02
ZDHHC15	-0.0365	4.84E-01	6.12E-01
ZDHHC16	0.0177	7.34E-01	8.20E-01
ZDHHC17	0.1151	2.66E-02	6.49E-02
ZDHHC18	0.1740	7.61E-04	3.41E-03
ZDHHC19	-0.0870	9.41E-02	1.77E-01
ZDHHC1	0.0174	7.38E-01	8.23E-01
ZDHHC20	-0.0077	8.83E-01	9.25E-01
ZDHHC21	0.1235	1.73E-02	4.57E-02
ZDHHC22	0.0015	9.78E-01	9.86E-01
ZDHHC23	0.1479	4.31E-03	1.46E-02
ZDHHC24	-0.0894	8.57E-02	1.64E-01
ZDHHC2	0.0371	4.76E-01	6.05E-01
ZDHHC3	-0.0743	1.53E-01	2.58E-01
ZDHHC4	-0.0858	9.88E-02	1.84E-01
ZDHHC5	-0.0106	8.39E-01	8.94E-01
ZDHHC6	0.0774	1.37E-01	2.36E-01
ZDHHC7	0.0020	9.69E-01	9.81E-01
ZDHHC8P1	0.2252	1.19E-05	9.79E-05
ZDHHC8	-0.2019	9.03E-05	5.59E-04
ZDHHC9	0.0489	3.47E-01	4.80E-01
ZEB1	-0.0696	1.81E-01	2.93E-01
ZEB2	-0.1576	2.33E-03	8.73E-03
ZER1	0.1662	1.32E-03	5.42E-03
ZFAND1	0.0079	8.79E-01	9.22E-01
ZFAND2A	0.0828	1.11E-01	2.02E-01
ZFAND2B	0.0085	8.71E-01	9.17E-01
ZFAND3	0.0229	6.61E-01	7.62E-01
ZFAND5	-0.0787	1.30E-01	2.28E-01
ZFAND6	-0.0544	2.96E-01	4.25E-01
ZFATAS	0.0299	5.66E-01	6.84E-01
ZFAT	0.0924	7.54E-02	1.49E-01
ZFC3H1	0.1559	2.59E-03	9.56E-03
ZFHX3	0.0225	6.66E-01	7.66E-01
ZFHX4	-0.0990	5.68E-02	1.19E-01
ZFP106	-0.0316	5.44E-01	6.65E-01
ZFP112	0.1699	1.02E-03	4.37E-03
ZFP14	0.1588	2.15E-03	8.18E-03

ZFP161	-0.0111	8.32E-01	8.90E-01
ZFP1	-0.2064	6.19E-05	4.06E-04
ZFP28	0.0856	9.99E-02	1.86E-01
ZFP2	0.0610	2.41E-01	3.66E-01
ZFP30	0.0984	5.82E-02	1.21E-01
ZFP36L1	-0.0464	3.73E-01	5.06E-01
ZFP36L2	0.0590	2.57E-01	3.83E-01
ZFP36	-0.1656	1.37E-03	5.58E-03
ZFP37	0.1684	1.13E-03	4.77E-03
ZFP3	0.0294	5.72E-01	6.89E-01
ZFP41	0.0667	2.00E-01	3.16E-01
ZFP42	0.0888	8.78E-02	1.67E-01
ZFP57	-0.0109	8.34E-01	8.91E-01
ZFP62	0.2503	1.05E-06	1.18E-05
ZFP64	0.1925	1.91E-04	1.06E-03
ZFP82	0.1237	1.71E-02	4.53E-02
ZFP90	-0.0661	2.04E-01	3.22E-01
ZFP91-CNTF	-0.0886	8.83E-02	1.68E-01
ZFP91	-0.0532	3.07E-01	4.37E-01
ZFP92	0.1186	2.23E-02	5.61E-02
ZFPL1	0.0957	6.57E-02	1.33E-01
ZFPM1	-0.0616	2.37E-01	3.60E-01
ZFPM2	-0.1899	2.34E-04	1.26E-03
ZFR2	0.0003	9.96E-01	9.97E-01
ZFR	0.0836	1.08E-01	1.97E-01
ZFX	0.0958	6.52E-02	1.33E-01
ZFYVE16	0.0989	5.70E-02	1.19E-01
ZFYVE19	0.0070	8.93E-01	9.32E-01
ZFYVE1	-0.2506	1.02E-06	1.15E-05
ZFYVE20	0.0728	1.61E-01	2.68E-01
ZFYVE21	-0.2600	3.82E-07	4.83E-06
ZFYVE26	0.0717	1.68E-01	2.77E-01
ZFYVE27	-0.0515	3.23E-01	4.53E-01
ZFYVE28	-0.1968	1.36E-04	7.91E-04
ZFYVE9	-0.0776	1.35E-01	2.35E-01
ZFY	-0.0438	4.00E-01	5.33E-01
ZG16B	0.1078	3.80E-02	8.66E-02
ZG16	-0.1436	5.57E-03	1.80E-02
ZGLP1	0.1642	1.51E-03	6.07E-03
ZGPAT	-0.1190	2.19E-02	5.53E-02
ZHX1	-0.0866	9.60E-02	1.80E-01
ZHX2	-0.0098	8.51E-01	9.03E-01
ZHX3	-0.2367	4.05E-06	3.88E-05
ZIC1	-0.0503	3.34E-01	4.65E-01

ZIC2	0.2460	1.62E-06	1.73E-05
ZIC3	0.0549	2.92E-01	4.21E-01
ZIC4	0.0195	7.08E-01	8.00E-01
ZIC5	0.2838	2.65E-08	4.41E-07
ZIK1	0.0715	1.69E-01	2.78E-01
ZIM2	0.1838	3.72E-04	1.86E-03
ZIM3	0.0882	8.97E-02	1.71E-01
ZKSCAN1	-0.0890	8.70E-02	1.66E-01
ZKSCAN2	-0.1390	7.33E-03	2.26E-02
ZKSCAN3	0.2852	2.26E-08	3.85E-07
ZKSCAN4	0.1481	4.25E-03	1.45E-02
ZKSCAN5	0.0148	7.76E-01	8.50E-01
ZMAT1	-0.0164	7.54E-01	8.34E-01
ZMAT2	-0.0310	5.52E-01	6.72E-01
ZMAT3	-0.2003	1.03E-04	6.22E-04
ZMAT4	0.0748	1.51E-01	2.55E-01
ZMAT5	0.0916	7.82E-02	1.53E-01
ZMIZ1	0.1223	1.85E-02	4.81E-02
ZMIZ2	0.2732	8.98E-08	1.32E-06
ZMPSTE24	-0.1126	3.02E-02	7.18E-02
ZMYM1	0.2617	3.15E-07	4.08E-06
ZMYM2	0.0943	6.97E-02	1.40E-01
ZMYM3	0.1731	8.13E-04	3.60E-03
ZMYM4	0.1432	5.74E-03	1.85E-02
ZMYM5	0.0703	1.77E-01	2.88E-01
ZMYM6	0.0531	3.08E-01	4.38E-01
ZMYND10	0.2197	1.96E-05	1.52E-04
ZMYND11	-0.0436	4.02E-01	5.35E-01
ZMYND12	-0.2244	1.28E-05	1.04E-04
ZMYND15	-0.0336	5.18E-01	6.42E-01
ZMYND17	0.0582	2.64E-01	3.91E-01
ZMYND19	0.3162	4.63E-10	1.19E-08
ZMYND8	0.0391	4.52E-01	5.83E-01
ZNF100	0.1658	1.36E-03	5.54E-03
ZNF101	0.2321	6.28E-06	5.64E-05
ZNF107	0.2120	3.85E-05	2.71E-04
ZNF10	0.0731	1.60E-01	2.66E-01
ZNF114	-0.0004	9.94E-01	9.96E-01
ZNF117	0.1735	7.91E-04	3.52E-03
ZNF121	-0.0190	7.16E-01	8.07E-01
ZNF124	0.2110	4.21E-05	2.92E-04
ZNF12	0.1834	3.84E-04	1.91E-03
ZNF131	0.2373	3.81E-06	3.67E-05
ZNF132	-0.0327	5.30E-01	6.52E-01

ZNF133	0.3377	2.40E-11	8.01E-10
ZNF134	0.0610	2.41E-01	3.66E-01
ZNF135	0.1184	2.26E-02	5.67E-02
ZNF136	0.0035	9.47E-01	9.67E-01
ZNF137	0.1875	2.81E-04	1.47E-03
ZNF138	0.0357	4.93E-01	6.20E-01
ZNF140	0.0825	1.13E-01	2.04E-01
ZNF141	0.1171	2.41E-02	5.99E-02
ZNF142	0.3207	2.56E-10	6.99E-09
ZNF143	0.1837	3.77E-04	1.88E-03
ZNF146	0.1158	2.57E-02	6.30E-02
ZNF148	0.0263	6.14E-01	7.24E-01
ZNF14	0.1683	1.14E-03	4.81E-03
ZNF154	-0.0892	8.62E-02	1.65E-01
ZNF155	0.1430	5.78E-03	1.86E-02
ZNF157	0.1636	1.57E-03	6.28E-03
ZNF160	0.0779	1.34E-01	2.33E-01
ZNF165	0.2119	3.87E-05	2.73E-04
ZNF167	0.1044	4.45E-02	9.80E-02
ZNF169	0.3559	1.62E-12	6.91E-11
ZNF16	0.1819	4.29E-04	2.09E-03
ZNF174	0.2187	2.13E-05	1.63E-04
ZNF175	-0.0120	8.18E-01	8.81E-01
ZNF177	0.1708	9.58E-04	4.15E-03
ZNF17	0.0694	1.83E-01	2.95E-01
ZNF180	0.1099	3.44E-02	8.00E-02
ZNF181	0.0328	5.29E-01	6.52E-01
ZNF182	0.1707	9.60E-04	4.16E-03
ZNF184	0.1555	2.68E-03	9.82E-03
ZNF185	0.1841	3.65E-04	1.84E-03
ZNF187	0.1937	1.74E-04	9.79E-04
ZNF189	0.2054	6.75E-05	4.37E-04
ZNF18	-0.1312	1.14E-02	3.25E-02
ZNF192	0.0009	9.87E-01	9.92E-01
ZNF193	0.2849	2.34E-08	3.97E-07
ZNF195	0.2258	1.12E-05	9.31E-05
ZNF197	0.0465	3.72E-01	5.05E-01
ZNF19	0.0372	4.75E-01	6.04E-01
ZNF200	0.1369	8.27E-03	2.50E-02
ZNF202	0.3725	1.17E-13	6.18E-12
ZNF204P	0.1997	1.08E-04	6.48E-04
ZNF205	0.1951	1.56E-04	8.91E-04
ZNF207	0.4347	1.57E-18	2.66E-16
ZNF208	0.0447	3.90E-01	5.24E-01

ZNF20	0.0975	6.05E-02	1.25E-01
ZNF211	0.2089	5.03E-05	3.40E-04
ZNF212	0.0326	5.31E-01	6.54E-01
ZNF213	0.3648	4.02E-13	1.93E-11
ZNF214	-0.0380	4.65E-01	5.95E-01
ZNF215	0.1285	1.33E-02	3.69E-02
ZNF217	0.0706	1.75E-01	2.85E-01
ZNF219	-0.0496	3.41E-01	4.73E-01
ZNF221	0.0972	6.15E-02	1.27E-01
ZNF222	0.3039	2.28E-09	4.96E-08
ZNF223	0.1621	1.73E-03	6.82E-03
ZNF224	0.1431	5.76E-03	1.85E-02
ZNF225	0.1262	1.50E-02	4.07E-02
ZNF226	0.2115	4.00E-05	2.80E-04
ZNF227	0.1427	5.90E-03	1.89E-02
ZNF229	0.1490	4.01E-03	1.38E-02
ZNF22	-0.0390	4.54E-01	5.85E-01
ZNF230	0.2406	2.77E-06	2.77E-05
ZNF232	0.2139	3.25E-05	2.34E-04
ZNF233	0.1888	2.56E-04	1.36E-03
ZNF234	0.2590	4.24E-07	5.30E-06
ZNF235	0.2210	1.74E-05	1.37E-04
ZNF236	0.0940	7.05E-02	1.41E-01
ZNF238	0.0870	9.41E-02	1.77E-01
ZNF239	0.2411	2.64E-06	2.65E-05
ZNF23	-0.0642	2.17E-01	3.37E-01
ZNF248	0.3140	6.24E-10	1.55E-08
ZNF24	-0.0056	9.14E-01	9.46E-01
ZNF250	0.1362	8.60E-03	2.59E-02
ZNF251	0.2595	4.02E-07	5.05E-06
ZNF252	-0.0879	9.10E-02	1.72E-01
ZNF253	0.0757	1.46E-01	2.48E-01
ZNF254	-0.0049	9.24E-01	9.52E-01
ZNF256	0.2027	8.43E-05	5.28E-04
ZNF257	0.1477	4.35E-03	1.47E-02
ZNF259	0.0844	1.05E-01	1.92E-01
ZNF25	-0.0016	9.76E-01	9.85E-01
ZNF260	0.2004	1.01E-04	6.15E-04
ZNF263	0.2743	7.95E-08	1.18E-06
ZNF264	0.0856	9.98E-02	1.86E-01
ZNF266	0.1737	7.77E-04	3.46E-03
ZNF267	0.1146	2.73E-02	6.62E-02
ZNF268	-0.0645	2.15E-01	3.35E-01
ZNF26	0.3476	5.65E-12	2.16E-10

ZNF271	0.0329	5.27E-01	6.50E-01
ZNF273	0.2047	7.15E-05	4.59E-04
ZNF274	0.0979	5.96E-02	1.24E-01
ZNF275	0.0210	6.87E-01	7.83E-01
ZNF276	-0.0321	5.38E-01	6.59E-01
ZNF277	-0.2962	6.00E-09	1.18E-07
ZNF280A	0.1902	2.29E-04	1.24E-03
ZNF280B	0.1141	2.80E-02	6.77E-02
ZNF280C	0.2787	4.82E-08	7.54E-07
ZNF280D	-0.0472	3.65E-01	4.97E-01
ZNF281	0.0541	2.99E-01	4.28E-01
ZNF282	0.1064	4.05E-02	9.11E-02
ZNF283	0.2564	5.57E-07	6.71E-06
ZNF284	0.2177	2.33E-05	1.77E-04
ZNF285	0.2171	2.46E-05	1.85E-04
ZNF286A	0.2097	4.69E-05	3.20E-04
ZNF286B	0.2024	8.63E-05	5.37E-04
ZNF287	0.0857	9.95E-02	1.85E-01
ZNF28	0.1656	1.37E-03	5.59E-03
ZNF292	0.1800	4.96E-04	2.36E-03
ZNF295	-0.0906	8.12E-02	1.58E-01
ZNF296	0.3529	2.54E-12	1.05E-10
ZNF2	0.2047	7.11E-05	4.57E-04
ZNF300	0.2588	4.33E-07	5.39E-06
ZNF302	0.2346	4.95E-06	4.61E-05
ZNF304	0.1247	1.62E-02	4.33E-02
ZNF30	0.2226	1.50E-05	1.20E-04
ZNF311	0.0856	9.99E-02	1.86E-01
ZNF317	-0.0358	4.92E-01	6.19E-01
ZNF318	0.2016	9.22E-05	5.69E-04
ZNF319	0.0123	8.14E-01	8.77E-01
ZNF320	0.1499	3.81E-03	1.32E-02
ZNF321	0.1796	5.08E-04	2.41E-03
ZNF322A	0.1951	1.56E-04	8.93E-04
ZNF322B	0.1997	1.08E-04	6.48E-04
ZNF323	0.0852	1.01E-01	1.88E-01
ZNF324B	0.2105	4.38E-05	3.03E-04
ZNF324	0.0724	1.64E-01	2.72E-01
ZNF326	0.0345	5.08E-01	6.33E-01
ZNF329	0.0931	7.33E-02	1.46E-01
ZNF32	0.1810	4.58E-04	2.21E-03
ZNF330	-0.3335	4.37E-11	1.39E-09
ZNF331	0.0552	2.89E-01	4.18E-01
ZNF333	0.1437	5.54E-03	1.79E-02

ZNF334	-0.0615	2.37E-01	3.61E-01
ZNF335	0.3852	1.43E-14	8.97E-13
ZNF337	0.3116	8.50E-10	2.05E-08
ZNF33A	0.1456	4.96E-03	1.64E-02
ZNF33B	0.1301	1.21E-02	3.42E-02
ZNF341	0.2026	8.50E-05	5.32E-04
ZNF343	0.1927	1.89E-04	1.05E-03
ZNF345	0.2042	7.43E-05	4.74E-04
ZNF346	0.2580	4.69E-07	5.79E-06
ZNF347	0.0928	7.44E-02	1.47E-01
ZNF34	0.0619	2.35E-01	3.58E-01
ZNF350	0.1920	1.99E-04	1.10E-03
ZNF354A	0.1892	2.47E-04	1.32E-03
ZNF354B	0.1649	1.43E-03	5.82E-03
ZNF354C	0.0527	3.12E-01	4.41E-01
ZNF358	-0.0959	6.49E-02	1.32E-01
ZNF35	0.1670	1.25E-03	5.18E-03
ZNF362	0.0964	6.37E-02	1.30E-01
ZNF365	0.0382	4.63E-01	5.93E-01
ZNF366	-0.1749	7.17E-04	3.23E-03
ZNF367	0.2702	1.26E-07	1.79E-06
ZNF37A	0.0825	1.13E-01	2.04E-01
ZNF37B	0.3676	2.59E-13	1.29E-11
ZNF382	0.0989	5.71E-02	1.19E-01
ZNF383	0.1966	1.38E-04	8.04E-04
ZNF384	0.3226	1.96E-10	5.46E-09
ZNF385A	0.0095	8.56E-01	9.06E-01
ZNF385B	-0.1744	7.42E-04	3.33E-03
ZNF385D	-0.0685	1.88E-01	3.02E-01
ZNF389	0.1494	3.92E-03	1.35E-02
ZNF391	0.1861	3.13E-04	1.62E-03
ZNF394	-0.0015	9.77E-01	9.85E-01
ZNF395	0.0327	5.30E-01	6.52E-01
ZNF396	0.0822	1.14E-01	2.06E-01
ZNF397OS	0.1613	1.83E-03	7.15E-03
ZNF397	0.0010	9.85E-01	9.91E-01
ZNF398	0.0377	4.69E-01	5.98E-01
ZNF3	-0.0539	3.01E-01	4.30E-01
ZNF404	0.2587	4.36E-07	5.43E-06
ZNF407	0.0646	2.14E-01	3.34E-01
ZNF408	0.1957	1.48E-04	8.55E-04
ZNF410	0.1369	8.30E-03	2.51E-02
ZNF414	0.0921	7.66E-02	1.51E-01
ZNF415	0.1077	3.81E-02	8.67E-02

ZNF416	0.2315	6.63E-06	5.91E-05
ZNF417	0.1009	5.22E-02	1.11E-01
ZNF418	0.1125	3.02E-02	7.19E-02
ZNF419	0.2302	7.49E-06	6.53E-05
ZNF41	-0.0165	7.52E-01	8.33E-01
ZNF420	0.0875	9.24E-02	1.75E-01
ZNF423	-0.1792	5.22E-04	2.47E-03
ZNF425	-0.1339	9.81E-03	2.88E-02
ZNF426	0.0123	8.13E-01	8.77E-01
ZNF428	-0.0546	2.94E-01	4.23E-01
ZNF429	-0.0381	4.65E-01	5.95E-01
ZNF430	0.0723	1.65E-01	2.72E-01
ZNF431	0.1656	1.37E-03	5.59E-03
ZNF432	0.1848	3.46E-04	1.76E-03
ZNF433	0.1000	5.43E-02	1.15E-01
ZNF434	0.0992	5.62E-02	1.18E-01
ZNF436	0.0107	8.37E-01	8.93E-01
ZNF438	0.1279	1.37E-02	3.78E-02
ZNF439	0.0863	9.68E-02	1.81E-01
ZNF43	0.1103	3.37E-02	7.87E-02
ZNF440	0.1083	3.70E-02	8.48E-02
ZNF441	-0.0548	2.93E-01	4.22E-01
ZNF442	0.0363	4.86E-01	6.14E-01
ZNF443	0.1290	1.29E-02	3.60E-02
ZNF444	-0.0139	7.89E-01	8.60E-01
ZNF445	0.1629	1.65E-03	6.56E-03
ZNF446	0.2687	1.48E-07	2.07E-06
ZNF449	0.2099	4.59E-05	3.15E-04
ZNF44	0.1589	2.15E-03	8.17E-03
ZNF451	0.0746	1.52E-01	2.56E-01
ZNF454	0.0951	6.72E-02	1.36E-01
ZNF45	0.1861	3.12E-04	1.62E-03
ZNF460	-0.0330	5.26E-01	6.49E-01
ZNF461	0.2922	9.77E-09	1.82E-07
ZNF462	-0.1411	6.47E-03	2.04E-02
ZNF467	-0.0950	6.75E-02	1.36E-01
ZNF468	0.1174	2.38E-02	5.91E-02
ZNF469	0.0025	9.62E-01	9.76E-01
ZNF470	0.0958	6.53E-02	1.33E-01
ZNF471	0.0783	1.32E-01	2.30E-01
ZNF473	0.3200	2.81E-10	7.57E-09
ZNF474	-0.0041	9.37E-01	9.61E-01
ZNF479	0.0593	2.54E-01	3.80E-01
ZNF480	0.1094	3.52E-02	8.15E-02

ZNF483	0.0803	1.23E-01	2.18E-01
ZNF484	0.1759	6.65E-04	3.03E-03
ZNF485	0.2047	7.16E-05	4.60E-04
ZNF486	0.1097	3.46E-02	8.03E-02
ZNF487	-0.0916	7.81E-02	1.53E-01
ZNF488	0.2021	8.87E-05	5.51E-04
ZNF48	0.2094	4.79E-05	3.27E-04
ZNF490	0.0519	3.19E-01	4.49E-01
ZNF491	0.2074	5.68E-05	3.78E-04
ZNF492	0.1382	7.70E-03	2.36E-02
ZNF493	0.1121	3.09E-02	7.32E-02
ZNF496	0.1411	6.49E-03	2.05E-02
ZNF497	0.0997	5.50E-02	1.16E-01
ZNF498	0.0745	1.52E-01	2.57E-01
ZNF500	0.1510	3.55E-03	1.24E-02
ZNF501	0.0841	1.06E-01	1.94E-01
ZNF502	0.0770	1.39E-01	2.39E-01
ZNF503	-0.0521	3.17E-01	4.47E-01
ZNF506	0.1666	1.28E-03	5.31E-03
ZNF507	0.2362	4.25E-06	4.04E-05
ZNF510	0.0660	2.05E-01	3.22E-01
ZNF511	-0.0391	4.53E-01	5.84E-01
ZNF512B	0.1313	1.14E-02	3.24E-02
ZNF512	0.2853	2.21E-08	3.79E-07
ZNF513	0.0037	9.43E-01	9.65E-01
ZNF514	0.2549	6.50E-07	7.73E-06
ZNF516	0.0484	3.53E-01	4.84E-01
ZNF517	0.1834	3.85E-04	1.92E-03
ZNF518A	0.1376	7.95E-03	2.42E-02
ZNF518B	0.0192	7.12E-01	8.03E-01
ZNF519	0.3142	6.08E-10	1.52E-08
ZNF521	-0.2417	2.49E-06	2.52E-05
ZNF524	-0.0229	6.60E-01	7.61E-01
ZNF525	0.1815	4.43E-04	2.15E-03
ZNF526	0.2431	2.16E-06	2.23E-05
ZNF527	0.2069	5.93E-05	3.91E-04
ZNF528	0.1201	2.07E-02	5.27E-02
ZNF529	0.2878	1.66E-08	2.94E-07
ZNF530	0.3457	7.46E-12	2.76E-10
ZNF532	0.1539	2.96E-03	1.07E-02
ZNF534	0.0979	5.97E-02	1.24E-01
ZNF536	0.1036	4.61E-02	1.01E-01
ZNF540	0.0747	1.51E-01	2.55E-01
ZNF541	-0.1163	2.51E-02	6.17E-02

ZNF542	-0.0005	9.93E-01	9.96E-01
ZNF543	0.1440	5.47E-03	1.78E-02
ZNF544	0.2237	1.37E-05	1.10E-04
ZNF546	0.0911	7.96E-02	1.55E-01
ZNF547	0.2016	9.23E-05	5.69E-04
ZNF548	0.2013	9.43E-05	5.80E-04
ZNF549	0.0419	4.21E-01	5.54E-01
ZNF550	0.2378	3.64E-06	3.53E-05
ZNF551	0.1389	7.37E-03	2.27E-02
ZNF552	0.2579	4.73E-07	5.83E-06
ZNF554	0.1571	2.41E-03	8.97E-03
ZNF555	0.1282	1.34E-02	3.73E-02
ZNF556	0.0923	7.58E-02	1.49E-01
ZNF557	0.0699	1.79E-01	2.91E-01
ZNF558	0.0686	1.88E-01	3.01E-01
ZNF559	0.1402	6.83E-03	2.13E-02
ZNF560	0.1806	4.73E-04	2.27E-03
ZNF561	-0.0327	5.30E-01	6.53E-01
ZNF562	0.0293	5.73E-01	6.91E-01
ZNF563	-0.0508	3.29E-01	4.60E-01
ZNF564	0.0183	7.25E-01	8.13E-01
ZNF565	0.2538	7.28E-07	8.53E-06
ZNF566	0.1018	5.02E-02	1.08E-01
ZNF567	0.2489	1.20E-06	1.33E-05
ZNF568	0.0972	6.15E-02	1.27E-01
ZNF569	0.2081	5.35E-05	3.59E-04
ZNF570	0.0850	1.02E-01	1.89E-01
ZNF571	0.1437	5.55E-03	1.80E-02
ZNF572	0.1767	6.27E-04	2.89E-03
ZNF573	0.0889	8.74E-02	1.67E-01
ZNF574	0.1580	2.28E-03	8.59E-03
ZNF575	-0.0736	1.57E-01	2.63E-01
ZNF576	0.0160	7.59E-01	8.38E-01
ZNF577	0.1646	1.47E-03	5.92E-03
ZNF578	0.1266	1.46E-02	3.99E-02
ZNF579	0.1055	4.22E-02	9.40E-02
ZNF57	0.1419	6.20E-03	1.97E-02
ZNF580	0.1055	4.23E-02	9.42E-02
ZNF581	0.1268	1.46E-02	3.98E-02
ZNF582	0.0251	6.30E-01	7.37E-01
ZNF583	0.1110	3.25E-02	7.64E-02
ZNF584	-0.1077	3.80E-02	8.67E-02
ZNF585A	0.2354	4.56E-06	4.30E-05
ZNF585B	0.1328	1.04E-02	3.03E-02

ZNF586	0.1438	5.52E-03	1.79E-02
ZNF587	0.1397	7.03E-03	2.18E-02
ZNF589	0.2864	1.96E-08	3.40E-07
ZNF592	0.0352	4.99E-01	6.25E-01
ZNF593	-0.0833	1.09E-01	1.99E-01
ZNF594	0.2276	9.54E-06	8.06E-05
ZNF595	0.0775	1.36E-01	2.36E-01
ZNF596	0.0850	1.02E-01	1.88E-01
ZNF597	-0.0025	9.61E-01	9.76E-01
ZNF598	0.1151	2.66E-02	6.49E-02
ZNF599	0.1541	2.93E-03	1.06E-02
ZNF600	0.1637	1.56E-03	6.24E-03
ZNF605	0.2062	6.32E-05	4.12E-04
ZNF606	0.1717	8.99E-04	3.92E-03
ZNF607	0.2179	2.29E-05	1.74E-04
ZNF608	0.0806	1.21E-01	2.16E-01
ZNF609	-0.0167	7.49E-01	8.31E-01
ZNF610	0.0927	7.46E-02	1.47E-01
ZNF611	0.1491	4.00E-03	1.38E-02
ZNF613	0.2074	5.67E-05	3.78E-04
ZNF614	0.1601	1.98E-03	7.65E-03
ZNF615	0.1540	2.95E-03	1.06E-02
ZNF616	-0.0442	3.96E-01	5.29E-01
ZNF618	0.2430	2.17E-06	2.24E-05
ZNF619	0.0148	7.77E-01	8.51E-01
ZNF620	0.2230	1.45E-05	1.17E-04
ZNF621	0.1095	3.51E-02	8.11E-02
ZNF622	0.0802	1.23E-01	2.18E-01
ZNF623	0.1659	1.34E-03	5.51E-03
ZNF624	0.1408	6.61E-03	2.08E-02
ZNF625	0.0835	1.08E-01	1.98E-01
ZNF626	0.0358	4.92E-01	6.19E-01
ZNF627	0.0615	2.37E-01	3.61E-01
ZNF628	0.1266	1.47E-02	4.00E-02
ZNF629	0.0756	1.46E-01	2.49E-01
ZNF630	0.1328	1.05E-02	3.03E-02
ZNF638	0.2167	2.54E-05	1.91E-04
ZNF639	0.1876	2.79E-04	1.47E-03
ZNF641	-0.0293	5.74E-01	6.91E-01
ZNF642	0.1388	7.40E-03	2.28E-02
ZNF643	0.1190	2.19E-02	5.53E-02
ZNF644	0.1499	3.81E-03	1.32E-02
ZNF645	0.0762	1.43E-01	2.44E-01
ZNF646	0.2221	1.58E-05	1.26E-04

ZNF648	-0.0772	1.38E-01	2.38E-01
ZNF649	0.0888	8.77E-02	1.67E-01
ZNF652	0.1476	4.37E-03	1.48E-02
ZNF653	-0.0191	7.13E-01	8.04E-01
ZNF654	-0.0147	7.78E-01	8.52E-01
ZNF655	-0.0983	5.86E-02	1.22E-01
ZNF658	0.1885	2.61E-04	1.38E-03
ZNF660	0.0569	2.75E-01	4.03E-01
ZNF662	0.0214	6.81E-01	7.78E-01
ZNF664	0.0616	2.37E-01	3.60E-01
ZNF665	0.1339	9.84E-03	2.89E-02
ZNF667	0.0965	6.35E-02	1.30E-01
ZNF668	0.1792	5.24E-04	2.47E-03
ZNF669	0.2159	2.74E-05	2.03E-04
ZNF670	0.1233	1.75E-02	4.61E-02
ZNF671	0.0071	8.91E-01	9.31E-01
ZNF672	0.2241	1.32E-05	1.07E-04
ZNF673	0.1442	5.38E-03	1.75E-02
ZNF674	0.0692	1.83E-01	2.96E-01
ZNF675	0.1739	7.70E-04	3.44E-03
ZNF676	0.0298	5.67E-01	6.84E-01
ZNF677	0.0202	6.98E-01	7.91E-01
ZNF678	0.0946	6.89E-02	1.38E-01
ZNF679	0.0055	9.16E-01	9.47E-01
ZNF680	-0.1097	3.47E-02	8.05E-02
ZNF681	0.2074	5.67E-05	3.78E-04
ZNF682	0.2071	5.82E-05	3.86E-04
ZNF683	-0.0559	2.83E-01	4.12E-01
ZNF684	-0.0671	1.97E-01	3.13E-01
ZNF687	0.4874	1.57E-23	6.65E-21
ZNF688	-0.1215	1.92E-02	4.98E-02
ZNF689	-0.0074	8.87E-01	9.28E-01
ZNF691	0.1939	1.71E-04	9.63E-04
ZNF692	0.4229	1.57E-17	1.98E-15
ZNF695	0.2869	1.85E-08	3.23E-07
ZNF696	0.1605	1.93E-03	7.48E-03
ZNF697	0.0108	8.35E-01	8.92E-01
ZNF699	-0.0476	3.61E-01	4.93E-01
ZNF69	-0.0069	8.95E-01	9.33E-01
ZNF700	0.2803	4.01E-08	6.41E-07
ZNF701	0.1533	3.07E-03	1.10E-02
ZNF702P	0.1183	2.26E-02	5.69E-02
ZNF703	-0.0419	4.21E-01	5.54E-01
ZNF704	-0.0442	3.96E-01	5.29E-01

ZNF705A	0.0280	5.91E-01	7.05E-01
ZNF705D	-0.0480	3.56E-01	4.88E-01
ZNF706	0.0355	4.96E-01	6.22E-01
ZNF707	0.1843	3.59E-04	1.81E-03
ZNF708	0.1613	1.83E-03	7.14E-03
ZNF709	0.1011	5.16E-02	1.10E-01
ZNF70	0.1387	7.46E-03	2.30E-02
ZNF710	0.0094	8.57E-01	9.08E-01
ZNF711	0.1941	1.69E-04	9.53E-04
ZNF713	0.2060	6.39E-05	4.16E-04
ZNF714	0.2038	7.70E-05	4.89E-04
ZNF716	0.0345	5.07E-01	6.32E-01
ZNF717	-0.0491	3.46E-01	4.78E-01
ZNF718	0.1906	2.22E-04	1.20E-03
ZNF71	0.2414	2.54E-06	2.57E-05
ZNF720	-0.1074	3.87E-02	8.78E-02
ZNF721	0.0336	5.19E-01	6.43E-01
ZNF727	0.0901	8.29E-02	1.60E-01
ZNF732	0.0967	6.27E-02	1.29E-01
ZNF735	-0.0250	6.32E-01	7.39E-01
ZNF737	0.1134	2.89E-02	6.94E-02
ZNF738	0.2907	1.17E-08	2.14E-07
ZNF740	0.1446	5.27E-03	1.72E-02
ZNF746	0.0762	1.43E-01	2.45E-01
ZNF747	-0.0840	1.06E-01	1.95E-01
ZNF749	0.1364	8.51E-03	2.56E-02
ZNF74	0.2671	1.77E-07	2.43E-06
ZNF750	-0.0383	4.62E-01	5.92E-01
ZNF75A	0.2351	4.69E-06	4.40E-05
ZNF75D	0.1071	3.92E-02	8.88E-02
ZNF761	0.1411	6.47E-03	2.04E-02
ZNF763	0.0034	9.48E-01	9.68E-01
ZNF764	0.2724	9.82E-08	1.43E-06
ZNF765	0.2448	1.82E-06	1.93E-05
ZNF766	0.2421	2.37E-06	2.42E-05
ZNF767	0.2451	1.77E-06	1.88E-05
ZNF768	0.0696	1.81E-01	2.93E-01
ZNF76	0.2144	3.12E-05	2.26E-04
ZNF770	-0.1581	2.26E-03	8.53E-03
ZNF771	0.0310	5.51E-01	6.71E-01
ZNF772	0.1354	9.03E-03	2.69E-02
ZNF773	0.1290	1.29E-02	3.60E-02
ZNF774	0.1046	4.41E-02	9.74E-02
ZNF775	0.1807	4.70E-04	2.26E-03

ZNF776	-0.0209	6.88E-01	7.84E-01
ZNF777	-0.0166	7.50E-01	8.32E-01
ZNF778	-0.1839	3.69E-04	1.85E-03
ZNF77	0.1465	4.70E-03	1.57E-02
ZNF780A	0.0243	6.41E-01	7.47E-01
ZNF780B	0.0747	1.51E-01	2.55E-01
ZNF781	0.1598	2.02E-03	7.75E-03
ZNF782	0.3497	4.12E-12	1.63E-10
ZNF784	-0.0120	8.18E-01	8.81E-01
ZNF785	0.1230	1.77E-02	4.66E-02
ZNF786	0.0303	5.61E-01	6.79E-01
ZNF787	0.1191	2.18E-02	5.50E-02
ZNF788	0.0355	4.96E-01	6.22E-01
ZNF789	0.3682	2.35E-13	1.17E-11
ZNF790	-0.0909	8.03E-02	1.56E-01
ZNF791	-0.0331	5.25E-01	6.48E-01
ZNF792	0.2585	4.47E-07	5.55E-06
ZNF793	0.1955	1.51E-04	8.68E-04
ZNF799	-0.0123	8.13E-01	8.77E-01
ZNF79	0.1260	1.52E-02	4.11E-02
ZNF7	0.2304	7.33E-06	6.42E-05
ZNF800	-0.1165	2.48E-02	6.11E-02
ZNF804A	0.0173	7.40E-01	8.25E-01
ZNF804B	-0.0746	1.52E-01	2.56E-01
ZNF805	0.0700	1.79E-01	2.90E-01
ZNF808	0.1359	8.77E-03	2.63E-02
ZNF80	0.0326	5.31E-01	6.54E-01
ZNF813	0.0576	2.68E-01	3.96E-01
ZNF814	0.0960	6.48E-02	1.32E-01
ZNF815	0.2414	2.56E-06	2.57E-05
ZNF816A	0.1487	4.10E-03	1.40E-02
ZNF81	0.0486	3.50E-01	4.82E-01
ZNF821	0.0766	1.41E-01	2.42E-01
ZNF823	0.0132	7.99E-01	8.67E-01
ZNF826	0.1383	7.65E-03	2.34E-02
ZNF827	-0.0609	2.42E-01	3.66E-01
ZNF828	0.0916	7.80E-02	1.53E-01
ZNF829	0.1577	2.32E-03	8.70E-03
ZNF830	0.1737	7.77E-04	3.46E-03
ZNF831	-0.0775	1.36E-01	2.36E-01
ZNF833	0.0794	1.27E-01	2.23E-01
ZNF835	-0.0066	8.99E-01	9.36E-01
ZNF836	0.1693	1.06E-03	4.53E-03
ZNF837	-0.2082	5.30E-05	3.56E-04

ZNF839	0.1164	2.49E-02	6.13E-02
ZNF83	0.1841	3.66E-04	1.84E-03
ZNF841	0.2958	6.26E-09	1.22E-07
ZNF843	-0.0618	2.35E-01	3.58E-01
ZNF844	0.0490	3.47E-01	4.79E-01
ZNF845	0.1023	4.90E-02	1.06E-01
ZNF846	-0.0591	2.56E-01	3.82E-01
ZNF84	0.3277	9.78E-11	2.87E-09
ZNF853	-0.0240	6.45E-01	7.50E-01
ZNF85	0.1857	3.23E-04	1.66E-03
ZNF860	0.1283	1.34E-02	3.72E-02
ZNF862	0.0702	1.77E-01	2.88E-01
ZNF876P	0.0941	7.03E-02	1.41E-01
ZNF878	0.1819	4.30E-04	2.09E-03
ZNF879	0.1826	4.07E-04	2.01E-03
ZNF880	0.0880	9.05E-02	1.72E-01
ZNF883	0.1790	5.33E-04	2.51E-03
ZNF8	0.2781	5.12E-08	7.96E-07
ZNF90	0.2212	1.70E-05	1.34E-04
ZNF91	0.0345	5.08E-01	6.33E-01
ZNF92	0.1251	1.59E-02	4.27E-02
ZNF93	0.2723	9.91E-08	1.44E-06
ZNF98	0.1146	2.73E-02	6.62E-02
ZNF99	0.0064	9.02E-01	9.38E-01
ZNFX1	-0.0465	3.72E-01	5.05E-01
ZNHIT1	-0.2689	1.45E-07	2.04E-06
ZNHIT2	-0.0989	5.71E-02	1.19E-01
ZNHIT3	0.3047	2.08E-09	4.59E-08
ZNHIT6	0.1290	1.29E-02	3.60E-02
ZNRD1	0.1705	9.77E-04	4.22E-03
ZNRF1	-0.1195	2.13E-02	5.41E-02
ZNRF2	-0.1318	1.11E-02	3.17E-02
ZNRF3	0.0309	5.53E-01	6.73E-01
ZNRF4	-0.0654	2.09E-01	3.28E-01
ZP1	0.1397	7.06E-03	2.19E-02
ZP2	-0.1190	2.19E-02	5.52E-02
ZP3	0.2359	4.34E-06	4.12E-05
ZPBP2	0.0023	9.65E-01	9.78E-01
ZPBP	-0.0218	6.76E-01	7.75E-01
ZPLD1	0.1267	1.46E-02	3.98E-02
ZRANB1	-0.1690	1.08E-03	4.60E-03
ZRANB2	0.1897	2.38E-04	1.28E-03
ZRANB3	0.0160	7.58E-01	8.38E-01
ZRSR2	0.1850	3.40E-04	1.73E-03

ZSCAN10	0.1091	3.58E-02	8.24E-02
ZSCAN12P1	0.0953	6.68E-02	1.35E-01
ZSCAN12	0.1611	1.86E-03	7.24E-03
ZSCAN16	0.2822	3.20E-08	5.23E-07
ZSCAN18	0.1274	1.41E-02	3.87E-02
ZSCAN1	0.0370	4.77E-01	6.06E-01
ZSCAN20	0.1464	4.72E-03	1.57E-02
ZSCAN21	-0.0448	3.89E-01	5.22E-01
ZSCAN22	0.2338	5.31E-06	4.88E-05
ZSCAN23	0.0144	7.82E-01	8.55E-01
ZSCAN29	0.1166	2.47E-02	6.10E-02
ZSCAN2	0.1235	1.73E-02	4.57E-02
ZSCAN4	0.0159	7.61E-01	8.39E-01
ZSCAN5A	0.0218	6.75E-01	7.74E-01
ZSCAN5B	0.1215	1.92E-02	4.98E-02
ZSWIM1	0.2436	2.06E-06	2.15E-05
ZSWIM2	-0.0125	8.11E-01	8.76E-01
ZSWIM3	0.2116	3.98E-05	2.79E-04
ZSWIM4	0.1476	4.39E-03	1.49E-02
ZSWIM5	0.1508	3.59E-03	1.26E-02
ZSWIM6	-0.0052	9.20E-01	9.49E-01
ZSWIM7	-0.1049	4.35E-02	9.64E-02
ZUFSP	0.2907	1.16E-08	2.13E-07
ZW10	0.1861	3.13E-04	1.62E-03
ZWILCH	0.2554	6.16E-07	7.34E-06
ZWINT	0.4144	7.95E-17	8.25E-15
ZXDA	0.0444	3.93E-01	5.26E-01
ZXDB	0.0074	8.87E-01	9.28E-01
ZXDC	0.2564	5.56E-07	6.70E-06
ZYG11A	0.1527	3.18E-03	1.14E-02
ZYG11B	-0.1685	1.12E-03	4.76E-03
ZYX	0.0368	4.80E-01	6.08E-01
ZZEF1	-0.0034	9.48E-01	9.68E-01
ZZZ3	0.0713	1.71E-01	2.80E-01
psiTPTE22	-0.1972	1.32E-04	7.75E-04
tAKR	-0.2467	1.50E-06	1.62E-05
