# Supplementary material for: Prognostic potential of PRPF3 in hepatocellular carcinoma
Source: Aging (Albany NY). 2020 Jan 11;12(1):912–30. doi: 10.18632/aging.102665 (PMC6977647; doi:10.18632/aging.102665)
Supplement: Supplementary Table 2 [file aging-12-102665-s003..pdf]

Supplementary Table 2. GO term annotation of PRPF3 co-expressed genes.

geneSet	description	ES	NES	pValue	FDR	link	size	leadingEdgeNum	leadingEdgeId	userId
GO:0006260	DNA replication	0.7862	2.2709	0.00E+00	0.00E+00	http://amigo.geneontology.org/amigo/term/GO:0006260	233	93	5984;9156;51514;8208;990;146956;4171;80174;8318;4175;983;79915;4174;672;3978;11232;1763;641;63922;4172;2237;9837;10714;5422;1111;55388;81620;9134;5111;5557;10036;10721;5424;80119;11169;8317;29980;5888;23649;6240;54962;9401;4678;4176;5558;79733;26271;5427;144455;10535;1663;10919;6241;57695;9984;7270;51659;8	ATAD5;ATRIP;BLM;BRCA1;CCNA2;CCNE1;CCNE2;CDC45;CDC6;CDC7;CDK1;CDK2;CDT1;CHAF1A;CHAF1B;CHEK1;CHTF18;CLSPN;DBF4;DBF4B;DDX11;DHX9;DNA2;DONSON;DSCC1;DTL;E2F7;E2F8;EHMT2;EME1;ESCO2;EXO1;FAM111A;FBXO5;FEN1;GINS1;GINS2;GINS3;GINS4;GMNN;HMGA1;ING5;KIN;LIG1;LIG3;MCM10;MCM2;MCM3;MCM4;MCM5;MC

GO:0007059	chromosome segregation	0.7706	2.2171	0.00E+00	0.00E+00	http://amigo.geneontology.org/amigo/term/GO:0007059	263	93	23126;83540;146909;3833;1063;4751;7272;55355;990;3192;11004;146956;221150;24137;29127;6421;55154;64151;7756;9928;7153;79980;672;10403;57405;51203;55143;5347;9055;991;332;641;113130;11130;2237;9493;81930;23397;9212;701;9232;2177;387103;55165;7517;9787;81620;699;9134;11339;10460;9700;220134;4288;9319;891;106	ANAPC4;AURKB;BIRC5;BLM;BRCA1;BUB1;BUB1B;BUB3;CCNB1;CCNE1;CCNE2;CDC20;CDC6;CDCA5;CDCA8;CDT1;CENPE;CENPF;CENPQ;CENPW;CEP55;DDX11;DLGAP5;DMC1;DSCC1;DSN1;ECT2;EME1;ESCO2;ESPL1;FANCD2;FBXO5;FEN1;H2AFY;HJURP;HNRNPU;KIF14;KIF18A;KIF18B;KIF23;KIF2C;KIF4A;KIF4B;KIFC1;KPNB1;MAD2L1;MKI67;MSH5;MS

GO:0044839	cell cycle G2/M phase transition	0.7327	2.0988	0.00E+00	0.00E+00	http://amigo.geneontology.org/amigo/term/GO:0044839	192	77	51514;1063;4751;990;55835;23354;2029;80174;983;9928;79915;9833;672;995;22974;5347;641;9212;51512;9133;1111;2305;8317;29980;9814;891;5889;9401;9088;8481;26271;79441;6790;10733;3161;11113;993;11190;57504;11073;7840;63967;84131;890;2956;7283;55920;9555;115106;10657;22995;93323;203068;1017;80279;1453;22897;979	AKAP8L;ALMS1;ATAD5;AURKA;AURKB;BLM;BRCA1;CCNA2;CCNB1;CCNB2;CDC25A;CDC25B;CDC25C;CDC6;CDC7;CDK1;CDK2;CDK4;CDK5RAP2;CDK5RAP3;CENPF;CENPJ;CEP135;CEP152;CEP164;CEP250;CEP290;CEP72;CEP78;CHEK1;CIT;CKAP5;CLSPN;CSNK1D;DBF4B;DONSON;DTL;ENSA;FBXO5;FOXM1;GTSE1;H2AFY;HAUS1;HAUS3;HAUS5;HAUS6

GO:0007051	spindle organization	0.7485	2.0954	0.00E+00	0.00E+00	http://amigo.geneontology.org/amigo/term/GO:0007051	152	59	83540;3833;4751;7272;3192;23354;259266;24137;29127;55154;7756;284403;3832;6491;10403;57405;22974;5347;9055;991;9493;9212;4605;29899;10460;9700;891;10615;3925;8481;26271;7175;1062;79441;84722;6790;23636;8480;285643;7283;115106;440270;93323;203068;1453;3837;9793;25886;54801;22919;55722;114327;4204;5901;104	ASPM;AURKA;AURKB;CCNB1;CDC20;CENPE;CEP72;CKAP5;CSNK1D;EFHC1;ESPL1;FBXO5;GOLGA8A;GOLGA8B;GPSM2;HAUS1;HAUS3;HAUS5;HAUS6;HAUS8;HNRNPU;KIF11;KIF23;KIF4A;KIF4B;KIFC1;KPNB1;LZTS2;MAPRE1;MECP2;MSTO1;MYBL2;NDC80;NEK2;NUF2;NUP62;OFD1;PLK1;POC1A;PRC1;PSRC1;RACGAP1;RAE1;RAN;SPAG5;SPC25;STIL;ST

GO:0000075	cell cycle checkpoint	0.7359	2.0951	0.00E+00	0.00E+00	http://amigo.geneontology.org/amigo/term/GO:0000075	196	77	5546;65123;51514;1063;9656;7272;990;261726;146956;9735;8318;983;7756;7153;672;10403;995;5347;1763;991;641;11130;79968;9212;51512;701;8682;1111;7517;4436;81620;699;1869;4194;5111;25896;5810;29980;9319;891;54962;25988;79733;7175;144455;6790;9984;4085;8914;3014;11073;25904;5883;63967;835;2956;84444;7027;4849;918	ATRIP;AURKA;AURKB;BLM;BRCA1;BRIP1;BUB1;BUB1B;BUB3;CASP2;CCNB1;CDC20;CDC25C;CDC45;CDC6;CDK1;CDK2;CDK5RAP2;CDK5RAP3;CDT1;CENPF;CHEK1;CLSPN;CNOT10;CNOT2;CNOT3;CNOT6;DCLRE1B;DNA2;DONSON;DOT1L;DTL;E2F1;E2F7;E2F8;EME1;GTSE1;H2AFX;HINFP;HUS1B;INTS3;INTS7;KNTC1;MAD2L1;MDC1;MDM4;MSH2;MSH6;MU

GO:0050000	chromosome localization	0.8010	2.0809	0.00E+00	0.00E+00	http://amigo.geneontology.org/amigo/term/GO:0050000	68	19	83540;3833;1063;3192;11004;9928;10403;55143;113130;81930;55165;9787;81620;891;10615;55166;1062;84722;23636	CCNB1;CDCA5;CDCA8;CDT1;CENPE;CENPF;CENPQ;CEP55;DLGAP5;HNRNPU;KIF14;KIF18A;KIF2C;KIFC1;NDC80;NUF2;NUP62;PSRC1;SPAG

GO:0044772	mitotic cell cycle phase transition	0.7012	2.0559	0.00E+00	0.00E+00	http://amigo.geneontology.org/amigo/term/GO:0044772	453	161	51514;128239;1163;1063;4751;7272;990;2146;55835;23354;2029;9735;4171;80174;8318;4175;983;7756;9928;79915;9833;4174;672;10403;995;22974;5347;991;641;113130;4172;11065;9212;51512;701;5422;9133;1033;55388;7517;9787;81620;699;1869;9134;4194;5111;54443;2305;5557;10460;9700;8317;29980;9814;9319;891;23649;5889;9401;2	ALMS1;ANAPC4;ANAPC7;ANLN;ATAD5;AURKA;AURKB;BLM;BRCA1;BUB1;BUB1B;BUB3;CASP2;CCNA2;CCNB1;CCNB2;CCNE1;CCNE2;CDC16;CDC20;CDC25A;CDC25B;CDC25C;CDC27;CDC45;CDC6;CDC7;CDCA5;CDK1;CDK2;CDK4;CDK5RAP2;CDK5RAP3;CDKN2A;CDKN2C;CDKN2D;CDKN3;CDT1;CENPE;CENPF;CENPJ;CEP135;CEP152;CEP164;CEP

GO:0032200	telomere organization	0.7302	2.0303	0.00E+00	0.00E+00	http://amigo.geneontology.org/amigo/term/GO:0032200	136	69	5984;9156;4751;3192;7203;1763;641;2237;9887;9212;10714;5422;7517;9134;5111;5557;5424;23381;80119;5888;23649;5889;9401;5558;64421;3181;5985;3183;4216;1736;84444;3020;142;3184;10728;8550;5983;898;6950;23137;3178;7515;5982;64858;55226;65057;2547;6117;135458;908;51750;675;7520;10576;6714;26272;10575;22948;55135;	ACD;AURKB;BLM;BRCA2;CCNE1;CCNE2;CCT2;CCT3;CCT4;CCT5;CCT6A;CCT8;DCLRE1B;DCLRE1C;DKC1;DNA2;DOT1L;EXO1;EXOSC10;FBXO4;FEN1;GNL3;H3F3A;HIST4H4;HNRNPA1;HNRNPA2B1;HNRNPC;HNRNPD;HNRNPU;HUS1B;MAP3K4;MAPK3;MAPKAPK5;NAT10;NEK2;PARP1;PCNA;PIF1;POLA1;POLA2;POLD1;POLD3;PRIM1;PRIM2;PRKDC;PTGES

GO:0006302	double-strand break repair	0.7137	2.0217	0.00E+00	0.00E+00	http://amigo.geneontology.org/amigo/term/GO:0006302	180	79	9156;9656;146956;8318;6421;8438;672;1763;641;113130;2237;5422;1111;7517;4436;2305;10721;8317;2187;9319;5888;5889;9401;10635;25788;1663;7516;51659;64421;8914;3014;157570;84296;80010;84515;9025;11144;7913;63979;142;9400;64710;254394;55159;3980;10038;55100;23137;7515;64858;79913;5932;80198;2547;83990;401720;	ACTR5;APTX;BARD1;BLM;BRCA1;BRCA2;BRIP1;CCDC155;CDC45;CDC7;CDCA5;CHEK1;CHEK2;DCLRE1B;DCLRE1C;DDX11;DEK;DMC1;DNA2;EME1;ESCO2;EXO1;FANCB;FEN1;FIGNL1;FIGNL2;FOXM1;GINS2;GINS4;H2AFX;HIST4H4;HUS1B;LIG3;MCM8;MCM9;MDC1;MSH2;MUS81;NIPBL;NUCKS1;PARP1;PARP2;PAXIP1;POLA1;POLM;POLQ;PRPF19;PSM

GO:0006338	chromatin remodeling	0.7179	2.0166	0.00E+00	0.00E+00	http://amigo.geneontology.org/amigo/term/GO:0006338	141	54	6944;81611;91687;55355;57459;2648;9557;387103;1111;3070;79723;11339;1058;2491;80336;79019;64105;86;4678;3148;64946;55166;7175;3066;23421;6605;3183;9025;11335;3159;22955;54107;3020;1029;6827;4602;5757;6602;1616;9759;11176;6601;5931;3065;6597;10014;6871;80152;401541;1108;79172;6599;1810;23347	ACTL6A;ANP32E;BAZ2A;CBX3;CDKN2A;CENPA;CENPH;CENPI;CENPK;CENPL;CENPM;CENPO;CENPP;CENPQ;CENPT;CENPW;CHD1L;CHD4;CHEK1;DAXX;DR1;GATAD2B;H3F3A;HDAC1;HDAC2;HDAC4;HDAC5;HELLS;HJURP;HMGA1;HMGB2;HNRNPC;ITGB3BP;KAT2A;MYB;NASP;OIP5;PABPC1L;POLE3;PTMA;RBBP7;RNF8;SCMH1;SMARCA4;SMARCC1;S

GO:0048285	organelle fission	0.6870	2.0139	0.00E+00	0.00E+00	http://amigo.geneontology.org/amigo/term/GO:0048285	404	141	23126;83540;146909;3833;1063;4751;7272;990;3192;11004;146956;259266;899;9735;24137;29127;11022;55154;64151;7756;9928;7153;79980;3832;10403;51203;995;55143;22974;5347;9055;991;286151;641;113130;11130;9493;81930;11065;23397;9212;701;9232;4605;2177;9133;55165;1111;7517;9787;81620;699;9134;54443;10460;9700;58	AKAP8;AKAP8L;ANAPC4;ANLN;ASPM;AURKA;AURKB;BLM;BRCA2;BRIP1;BUB1;BUB1B;BUB3;C11orf80;CCNA2;CCNB1;CCNB2;CCNE1;CCNE2;CCNF;CCNI2;CDC16;CDC20;CDC25B;CDC25C;CDC27;CDC6;CDCA5;CDCA8;CDK5RAP2;CDT1;CENPE;CENPF;CEP192;CEP55;CHEK1;CKS2;CNTD1;CYP26B1;DDHD1;DLGAP5;DMC1;DNM1L;DSCC1;DSN1;DYN

GO:0033044	regulation of chromosome organization	0.6823	2.0085	0.00E+00	0.00E+00	http://amigo.geneontology.org/amigo/term/GO:0033044	305	115	9869;90780;1063;4751;7272;990;3192;4171;8318;6421;7756;7153;7203;672;10403;5347;991;26147;113130;2237;9212;701;9232;2648;1111;7517;9787;81620;699;10460;9700;23381;80119;63925;4288;9319;891;8726;11011;26271;7175;1062;29945;1663;1789;4085;4000;3181;1786;3183;4216;3159;1736;51043;9555;29028;22823;9184;55795;302	ACD;AKAP8;AKAP8L;ANAPC4;ASF1A;ATAD2;AURKB;BCOR;BRCA1;BUB1;BUB1B;BUB3;CCNB1;CCT2;CCT3;CCT4;CCT5;CCT6A;CCT8;CDC20;CDC23;CDC26;CDC27;CDC45;CDC6;CDCA5;CDK5RAP2;CDT1;CENPE;CENPF;CHEK1;DDX11;DKC1;DLGAP5;DNMT1;DNMT3B;DYNC1LI1;EED;ESPL1;FBXO4;FBXO5;FEN1;H1FX;H2AFY;H3F3A;HDAC5;HIST1H1B;HM

GO:0006333	chromatin assembly or disassembly	0.7103	2.0033	0.00E+00	0.00E+00	http://amigo.geneontology.org/amigo/term/GO:0006333	138	52	91687;8208;55355;4171;55723;387103;3070;79723;11339;10036;1058;2491;79019;64105;4678;3148;64946;55166;11011;7175;23421;3014;6605;474382;3159;54107;64754;9555;3020;6418;126961;1029;10541;4673;11198;25842;8520;6602;1616;8971;6601;5931;9733;6597;55201;80152;401541;146059;29855;79172;6599;23347	ANP32B;ASF1A;ASF1B;CDAN1;CDKN2A;CENPA;CENPH;CENPI;CENPK;CENPL;CENPM;CENPO;CENPP;CENPQ;CENPT;CENPW;CHAF1A;CHAF1B;DAXX;H1FX;H2AFB1;H2AFX;H2AFY;H3F3A;HAT1;HELLS;HIST2H3C;HJURP;HMGA1;HMGB2;ITGB3BP;MAP1S;MCM2;NAP1L1;NASP;OIP5;POLE3;RBBP7;SART3;SET;SMARCA4;SMARCC1;SMARCC2;SMARCD1;SM

GO:0008380	RNA splicing	0.6939	1.9951	0.00E+00	0.00E+00	http://amigo.geneontology.org/amigo/term/GO:0008380	363	162	9129;10262;1196;27316;5546;9939;220988;3192;6421;51593;6635;51747;65109;84950;9584;84811;55094;8241;8175;283989;9984;55339;3187;1478;116461;6632;5725;2521;22794;26528;4841;10181;3181;3191;23210;10921;3183;1660;55015;9775;6625;6628;4670;11338;58155;51634;11325;25766;9343;9416;83759;10657;6637;55692;6627;5	ACIN1;AKAP8L;BUD13;CASC3;CCAR1;CELF4;CLK2;CPSF3;CPSF7;CRNKL1;CSTF2;CSTF3;CWC22;CWC25;CWC27;DAZAP1;DBR1;DDX17;DDX20;DDX23;DDX42;DDX46;DHX15;DHX16;DHX8;DHX9;ECD;EFTUD2;EIF4A3;FIP1L1;FUS;GEMIN7;GPATCH1;GPKOW;HMX2;HNRNPA0;HNRNPA1;HNRNPA1L2;HNRNPA2B1;HNRNPA3;HNRNPC;HNRNPD;HNRNPH1;

GO:0036297	interstrand cross-link repair	0.8107	1.9893	0.00E+00	0.00E+00	http://amigo.geneontology.org/amigo/term/GO:0036297	39	23	29089;146956;55215;2189;2178;2177;7517;2187;5888;10635;64421;84515;9025;2956;84126;55120;64710;254394;55159;64858;2175;80198;6117	ATRIP;DCLRE1B;DCLRE1C;EME1;FANCA;FANCB;FANCD2;FANCE;FANCG;FANCI;FANCL;MCM8;MCM9;MSH6;MUS81;NUCKS1;RAD51;RAD51AP1;RFWD3;RNF8;RPA
GO:0022616	DNA strand elongation	0.8880	1.9851	0.00E+00	0.00E+00	http://amigo.geneontology.org/amigo/term/GO:0022616	23	16	5984;3978;1763;2237;9837;5422;5111;10535;51659;84296;5983;64710;3980;64785;5426;64858	DCLRE1B;DNA2;FEN1;GINS1;GINS2;GINS3;GINS4;LIG1;LIG3;NUCKS1;PCNA;POLA1;POLE;RFC3;RFC4;RNASEH2A

GO:0006397	mRNA processing	0.6852	1.9765	0.00E+00	0.00E+00	http://amigo.geneontology.org/amigo/term/GO:0006397	423	180	9129;10262;27316;5546;9939;220988;3192;6421;103;51593;11052;6635;51747;65109;84950;80336;891;9584;84811;55094;8241;8175;283989;9984;9877;55339;3187;1478;116461;6632;5725;2521;22794;26528;7884;4841;10181;3181;3191;23210;25888;474382;10921;3183;1660;55015;9775;6625;6628;4670;11338;58155;51634;11325;25766;9	ACIN1;ADAR;AKAP8L;BARD1;BUD13;CASC3;CCAR1;CCNB1;CDC73;CDK7;CELF4;CPSF3;CPSF4L;CPSF6;CPSF7;CRNKL1;CSTF2;CSTF3;CWC22;CWC25;CWC27;DAZAP1;DBR1;DDX17;DDX20;DDX23;DDX42;DDX46;DHX15;DHX16;DHX8;DHX9;ECD;EFTUD2;EIF4A3;ERCC3;FIP1L1;FUS;GEMIN7;GPATCH1;GPKOW;GTF2H1;GTF2H4;H2AFB1;HMX2;HNRNPA0;HN

GO:0006403	RNA localization	0.6784	1.9515	0.00E+00	0.00E+00	http://amigo.geneontology.org/amigo/term/GO:0006403	208	90	9939;220988;79902;3192;6421;7203;9887;11052;23381;65109;51602;348995;7175;7248;23636;9984;9877;55339;23404;7514;22794;7884;8021;23511;3181;23225;8480;10921;51010;1660;9775;1736;9326;55746;9631;11338;51634;57122;79023;10657;55795;81608;57187;65110;22916;24148;10642;9793;5433;55110;6950;3178;9804;8563;411	AHCTF1;AKAP8L;CASC3;CCT2;CCT3;CCT4;CCT5;CCT6A;CCT8;CKAP5;CPSF3;CPSF6;DHX9;DKC1;EIF4A3;EXOSC2;EXOSC3;FBL;FIP1L1;GLE1;HHEX;HNRNPA1;HNRNPA1L2;HNRNPA2B1;HNRNPA3;HNRNPU;IGF2BP1;IGF2BP2;IGF2BP3;KHDRBS1;KHSRP;LTV1;MAGOH;MAGOHB;MRPL18;NCBP1;NCBP2;NOP58;NUP107;NUP133;NUP155;NUP160;NUP188;NUP2

GO:0045930	negative regulation of mitotic cell cycle	0.6758	1.9407	0.00E+00	0.00E+00	http://amigo.geneontology.org/amigo/term/GO:0045930	237	63	5546;65123;1063;9656;7272;990;2146;146956;9735;983;7756;7153;672;10403;995;5347;991;641;11130;9212;51512;701;9232;1111;7517;4436;81620;699;1869;4194;5111;29980;9319;891;54962;79733;26271;27085;7175;144455;6790;4085;11073;25904;5883;63967;835;2956;7027;4849;9184;55795;1017;5933;80279;9400;55159;10744;90806;55	ANGEL2;AURKA;AURKB;BLM;BRCA1;BTG3;BUB1;BUB1B;BUB3;CASP2;CCNB1;CDC20;CDC25C;CDC6;CDK1;CDK2;CDK5RAP3;CDT1;CENPF;CHEK1;CLSPN;CNOT10;CNOT3;CNOT6;DCLRE1B;DONSON;E2F1;E2F7;E2F8;EME1;EZH2;FBXO5;GTSE1;INTS3;KNTC1;MAD2L1;MDC1;MDM4;MSH2;MSH6;MTBP;NDC80;PCID2;PCNA;PLK1;PRCC;PTTG1;PTTG2;RA

GO:0031023	microtubule organizing center organization	0.7022	1.9382	0.00E+00	0.00E+00	http://amigo.geneontology.org/amigo/term/GO:0031023	121	50	4751;55835;23354;899;983;284403;3832;6491;672;5347;2648;1111;7517;8481;79441;6790;7516;163786;23636;10733;7514;11190;115106;22995;93323;1017;9793;25886;54801;26973;9696;55722;4591;4957;55755;56890;84552;9662;10806;675;55559;163688;55125;124093;91147;51199;253012;54820;55142;25914	AURKA;BRCA1;BRCA2;CALML6;CCDC78;CCNF;CDK1;CDK2;CDK5RAP2;CENPJ;CEP135;CEP152;CEP192;CEP250;CEP72;CHEK1;CHORDC1;CKAP5;CROCC;HAUS1;HAUS2;HAUS3;HAUS5;HAUS6;HAUS7;HAUS8;HEPACAM2;KAT2A;KIF11;MDM1;NDE1;NEK2;NIN;NUP62;ODF2;OFD1;PARD6G;PLK1;PLK4;POC1A;RTTN;SASS6;SDCCAG8;STIL;TMEM67;TRI

GO:0034502	protein localization to chromosome	0.7447	1.9359	0.00E+00	0.00E+00	http://amigo.geneontology.org/amigo/term/GO:0034502	68	38	7272;2146;983;7203;10403;5347;113130;9212;701;4436;81620;1058;55166;27085;157570;84515;55920;1736;9555;9184;254394;6950;221496;65057;6117;908;675;7520;10576;10575;3550;9183;25836;3009;22948;55135;10694;7015	ACD;AURKB;BRCA2;BUB1B;BUB3;CCT2;CCT3;CCT4;CCT5;CCT6A;CCT8;CDCA5;CDK1;CDT1;CENPA;CENPQ;DKC1;ESCO2;EZH2;H2AFY;HIST1H1B;IK;LEMD2;MCM8;MCM9;MSH2;MTBP;NDC80;NIPBL;PLK1;RCC2;RPA1;TCP1;TERT;TTK;WRA

GO:0000910	cytokinesis	0.6866	1.9275	0.00E+00	0.00E+00	http://amigo.geneontology.org/amigo/term/GO:0000910	150	47	990;24137;29127;9928;51203;5347;9055;9493;9212;55165;81620;54443;9700;1058;80179;10112;3925;79733;144455;28964;1894;6790;23636;11113;10899;285643;129531;9585;84440;55964;26586;114327;25909;3619;84445;124404;675;994;10801;56155;346288;55660;403;11021;5413;23176;375	AHCTF1;ANLN;ARF1;ARL3;AURKA;AURKB;BRCA2;CDC25B;CDC6;CDT1;CENPA;CEP55;CIT;CKAP2;E2F7;E2F8;ECT2;EFHC1;ESPL1;GIT1;INCENP;JTB;KIF14;KIF20A;KIF20B;KIF23;KIF4A;KIF4B;LZTS2;MITD1;MYO19;NUP62;NUSAP1;PLK1;PRC1;PRPF40A;RAB11FIP4;RAB35;RACGAP1;SEPT12;SEPT14;SEPT3;SEPT5;

GO:0006310	DNA recombination	0.6765	1.9266	0.00E+00	0.00E+00	http://amigo.geneontology.org/amigo/term/GO:0006310	233	91	9156;146956;8318;6421;79915;7153;8438;672;3978;29893;641;2237;1111;7517;4436;10721;80119;8317;2187;3838;9319;5888;5889;86;9401;3148;10635;25788;7516;9984;51659;64421;3014;84296;80010;4439;4841;4798;51010;84515;9025;11144;2956;84057;63979;142;22944;10445;9400;64710;6929;254394;55159;3980;7247;23137;7515;513	ACTL6A;ACTR5;ATAD5;BLM;BRCA1;BRCA2;BRIP1;C11orf80;CCDC155;CDC45;CDC7;CHEK1;CNTD1;DCLRE1C;DMC1;EME1;ERCC3;EXO1;EXOSC3;FANCB;FANCM;FEN1;FIGNL1;FIGNL2;GINS2;GINS4;H1FX;H2AFX;HIST1H1B;HMGB1;HMGB2;HUS1B;IL4;INO80E;KIN;KPNA2;LIG1;LIG3;MCM8;MCM9;MCRS1;MND1;MSH2;MSH5;MSH6;MUS81;NFRKB;NIPBL;NO

GO:0045787	positive regulation of cell cycle	0.6548	1.9151	0.00E+00	0.00E+00	http://amigo.geneontology.org/amigo/term/GO:0045787	353	102	51514;1163;990;2146;55835;3192;899;80174;8318;29127;6421;983;9928;79915;672;10403;51203;995;113130;2237;9493;11065;9212;51512;9133;1111;7517;9787;81620;1869;9134;4194;29899;5111;9700;8317;645121;891;10615;5889;9401;79733;26271;27085;7175;144455;29945;84722;1663;1894;1164;6790;23636;10733;9984;4085;11113;99	ANAPC4;ARID3A;ATAD5;AURKA;AURKB;BCL2L11;BRCA1;BRCA2;CASP2;CCNA2;CCNB1;CCNB2;CCNE1;CCNE2;CCNF;CCNI2;CDC25A;CDC25B;CDC25C;CDC45;CDC6;CDC7;CDC73;CDCA5;CDK1;CDK2;CDK4;CDK5R1;CDT1;CENPJ;CHEK1;CIT;CKS1B;CKS2;CNOT10;CNOT2;CNOT3;CNOT6;CUL4B;DBF4B;DDX11;DLGAP5;DTL;DYNC1LI1;E2F1;E2F7;E2F8;

GO:0031123	RNA 3'-end processing	0.6963	1.9091	0.00E+00	0.00E+00	http://amigo.geneontology.org/amigo/term/GO:0031123	111	47	9939;11052;25896;65109;80336;891;55656;9984;9877;55339;23404;1478;22794;7884;25888;10921;51010;9775;1736;11338;81608;6626;57187;642843;22916;56254;5433;6829;55110;8563;1479;4116;134353;6741;80145;4686;2091;5393;51692;199746;11340;6227;79869;79577;84321;580;114034	BARD1;CASC3;CCNB1;CDC73;CPSF3;CPSF4L;CPSF6;CPSF7;CSTF2;CSTF3;DKC1;EIF4A3;EXOSC2;EXOSC3;EXOSC8;EXOSC9;FBL;FIP1L1;INTS7;INTS8;LSM11;MAGOH;MAGOHB;NCBP1;NCBP2;PABPC1L;POLR2D;RBM8A;RNF20;RNPS1;RPS21;SLBP;SNRPA;SSB;SUPT5H;THOC1;THOC2;THOC3;THOC5;THOC7;TOE1;U2AF1L4;U2AF2;UPF

GO:0006720	isoprenoid metabolic process	-0.5443	-2.0236	0.00E+00	0.00E+00	http://amigo.geneontology.org/amigo/term/GO:0006720	116	41	112724;54600;3158;345;224;220;1543;3157;128;338;348;7276;335;1559;1571;1109;64577;57107;4598;57665;1592;54658;79947;5264;10901;54659;6382;1544;1576;55825;9249;5950;83875;6715;127;4035;285440;8228;51109;54884;5959	ADH4;ADH5;AKR1C4;ALDH1A3;ALDH3A2;ALDH8A1;APOA1;APOB;APOC3;APOE;BCO2;CYP1A1;CYP1A2;CYP26A1;CYP2C9;CYP2E1;CYP3A4;CYP4V2;DHDDS;DHRS3;DHRS4;HMGCS1;HMGCS2;LRP1;MVK;PDSS2;PECR;PHYH;PNPLA4;RBP4;RDH11;RDH13;RDH14;RDH5;RETSAT;SDC1;SRD5A1;TTR;UGT1A1;UGT1

GO:0019748	secondary metabolic process	-0.6039	-2.0591	0.00E+00	0.00E+00	http://amigo.geneontology.org/amigo/term/GO:0019748	56	26	1553;1652;4644;1645;5066;596;1497;1543;9839;1109;1549;8574;1491;1548;51151;23474;2327;1544;9693;5223;5446;56898;434;22977;30;84869	ACAA1;AKR1C1;AKR1C4;AKR7A2;AKR7A3;ASIP;BCL2;BDH2;CBR4;CTH;CTNS;CYP1A1;CYP1A2;CYP2A13;CYP2A6;CYP2A7;DDT;ETHE1;FMO2;MYO5A;PAM;PGAM1;PON3;RAPGEF2;SLC4

GO:0009259	ribonucleotide metabolic process	-0.4837	-2.0594	0.00E+00	0.00E+00	http://amigo.geneontology.org/amigo/term/GO:0009259	441	142	3157;5151;65220;23305;133;4708;120892;56975;122622;2645;7386;51144;1346;6999;5164;79717;5213;523;4717;55034;5162;122970;4705;6817;229;5144;51703;5160;2977;4976;79071;60481;1355;10891;27089;4881;348158;5465;196883;10846;51727;10105;6799;168537;477;339123;32;5207;4598;374291;670;64802;51292;1969;4714;54995;	AADAT;AASS;ABHD14B;ACACB;ACAT1;ACOT1;ACOT12;ACOT13;ACOT2;ACOT4;ACOT6;ACSF3;ACSL1;ACSL5;ACSL6;ACSM2A;ACSM2B;ACSM3;ACSM5;ACSS2;ADCY4;ADCYAP1;ADK;ADM;ADSSL1;AK3;ALDOB;APRT;ATP1A2;ATP6V1A;BAAT;BPHL;CBFA2T3;CHCHD10;CLPX;CMPK1;COQ9;COX15;COX4I1;COX7A1;DHODH;DHTKD1;DLD;DLG2;DLST;DNAJ

GO:0002526	acute inflammatory response	-0.5354	-2.0640	0.00E+00	0.00E+00	http://amigo.geneontology.org/amigo/term/GO:0002526	151	62	1191;712;5005;2147;5294;11315;462;1051;197;114548;5743;4153;11331;1378;3075;718;728;722;4889;5265;735;196527;733;3929;3572;6291;3569;720;1910;966;732;1361;2157;6288;1370;5733;975;3133;2161;629;7036;6289;9332;715;54658;5199;716;730;116;3250;3240;3700;5547;10877;445;731;3818;1776;5627;729;3426;710	ADCYAP1;AHSG;ANO6;ASS1;C1QA;C1R;C1S;C3;C4A;C4BPA;C5AR1;C6;C7;C8A;C8B;C8G;C9;CD163;CD59;CD81;CEBPB;CFB;CFH;CFHR4;CFI;CFP;CLU;CPB2;CPN2;CR1;DNASE1L3;EDNRB;F12;F2;F8;HLA-E;HP;HPR;IL6;IL6ST;ITIH4;KLKB1;LBP;MBL2;NLRP3;NPY5R;ORM2;PARK7;PHB2;PIK3CG;PRCP;PROS1;PTGER3;PTGS2;SAA1;SAA2;SAA4;SER

GO:0006766	vitamin metabolic process	-0.5425	-2.0690	0.00E+00	0.00E+00	http://amigo.geneontology.org/amigo/term/GO:0006766	124	49	1727;6615;57026;60496;160428;10327;66002;6591;6515;81693;8942;873;2350;27249;57834;1543;56922;9962;326625;5641;2729;5826;1592;9104;10588;79001;316;6472;23498;7274;10840;27010;6470;1583;1576;8564;1528;5095;25974;171425;9446;5096;8529;55163;5091;166785;4522;686;64087	AASDHPPT;ABCD4;AKR1A1;ALDH1L1;ALDH1L2;AMN;AOX1;BTD;CBR1;CLYBL;CYB5A;CYB5R3;CYP11A1;CYP1A1;CYP26A1;CYP3A4;CYP4F11;CYP4F12;CYP4F2;FOLR2;GCLC;GSTO1;HAAO;KMO;KYNU;LGMN;MCCC1;MCCC2;MMAA;MMAB;MMACHC;MMADHC;MTHFD1;MTHFS;PC;PCCA;PCCB;PDXP;PNPO;RGN;SHMT1;SHMT2;SLC23A2;SLC2A3;SNAI1;S

GO:0042445	hormone metabolic process	-0.5373	-2.0807	0.00E+00	0.00E+00	http://amigo.geneontology.org/amigo/term/GO:0042445	208	75	866;10269;6783;151056;1889;1645;5122;1428;1359;112724;1312;54600;64377;27122;5781;10202;1522;220;1543;4311;653;1717;133;7276;51144;335;59272;3416;6817;6718;8879;1215;1733;10891;3249;1511;1109;64577;57665;1592;1586;54658;389434;7923;10901;51170;1393;54659;8630;3290;7364;6469;1583;1576;9249;5446;5950;83875;26	ACAA1;ACE2;ADH4;ADM;AKR1C1;AKR1C4;AKR1D1;ALDH1A3;ALDH8A1;APOA1;BCO2;BMP5;CHST8;CMA1;COMT;CPA3;CRHBP;CRYM;CTSG;CTSZ;CYP11A1;CYP17A1;CYP1A1;CYP26A1;CYP3A4;DHCR7;DHRS2;DHRS3;DHRS4;DIO1;DKK3;ECE1;ENPEP;ESR1;FDX1;GHR;HPN;HSD11B1;HSD17B11;HSD17B12;HSD17B4;HSD17B6;HSD17B8;IDE;IYD;MME;PCS

GO:0006575	cellular modified amino acid metabolic process	-0.5152	-2.0848	0.00E+00	0.00E+00	http://amigo.geneontology.org/amigo/term/GO:0006575	181	71	5625;223;191;11315;5033;60496;10549;6647;10841;160428;2937;1428;635;2350;1497;23743;2879;51268;2876;2952;9027;57515;2643;5322;2628;2729;3417;1733;26873;1374;3249;2593;2946;347735;2687;4257;2948;33;389434;10588;23474;10955;1384;2944;6472;11145;55748;10840;6470;29958;3029;25974;2949;1757;501;7915;2346;4258;44	AASDHPPT;ACADL;ACADM;AHCY;ALDH1L1;ALDH1L2;ALDH4A1;ALDH5A1;ALDH7A1;ALDH9A1;ASS1;BBOX1;BHMT;BHMT2;CNDP2;CPT1A;CRAT;CRYM;CTNS;DIO1;DMGDH;ETHE1;FOLH1;FOLR2;FTCD;GAMT;GATM;GCH1;GCLC;GGT5;GOT2;GPX1;GPX4;GSS;GSTK1;GSTM1;GSTM2;GSTM4;GSTM5;GSTT1;GSTZ1;HAGH;HPN;IDH1;IYD;MGST1;MGST2;MMACH

GO:0008202	steroid metabolic process	-0.5073	-2.0936	0.00E+00	0.00E+00	http://amigo.geneontology.org/amigo/term/GO:0008202	291	101	3158;27122;10202;1543;653;3157;1066;1717;338;133;1593;348;51144;335;10858;2538;8647;1559;341;8542;6817;6718;7555;8879;5641;2246;79071;1571;7108;10891;116519;1109;1071;6799;10613;8309;1595;32;4598;5444;1592;3490;1586;33;7366;1548;84681;54658;7923;2053;10901;54490;8578;3638;51170;8856;5007;1555;64788;1544;6095;8	ABCB11;ACAA1;ACAA2;ACACB;ACADL;ACADVL;ACOX2;ADM;AKR1C4;AKR1D1;AMACR;ANGPTL3;APOA1;APOA5;APOB;APOC1;APOE;APOF;APOL1;BAAT;BMP5;CAT;CES1;CETP;CNBP;CYP11A1;CYP17A1;CYP1A1;CYP1A2;CYP26A1;CYP27A1;CYP2A6;CYP2B6;CYP2C8;CYP2C9;CYP2E1;CYP39A1;CYP3A4;CYP46A1;CYP4V2;CYP51A1;CYP7B1;CYP8B1;

GO:0006638	neutral lipid metabolic process	-0.5767	-2.1020	0.00E+00	0.00E+00	http://amigo.geneontology.org/amigo/term/GO:0006638	111	44	4023;6622;2168;718;9663;345;5781;2876;338;348;335;2538;341;23175;150379;2822;1571;1374;116519;13;1071;9104;3638;64788;1373;80168;11145;2710;2167;6400;3990;283;128486;3949;116255;5106;5105;2180;7069;847;11057;8228;57406;10998	AADAC;ABHD2;ABHD6;ACSL1;ANG;APOA1;APOA5;APOB;APOC1;APOC3;APOE;C3;CAT;CETP;CPS1;CPT1A;CYP2E1;FABP1;FABP4;FITM2;G6PC;GK;GPLD1;GPX1;INSIG1;LDLR;LIPC;LMF1;LPIN1;LPIN2;LPL;MOGAT1;MOGAT2;PCK1;PCK2;PLA2G16;PNPLA4;PNPLA5;PTPN11;RGN;SEL1L;

GO:0006090	pyruvate metabolic process	-0.5702	-2.1333	0.00E+00	0.00E+00	http://amigo.geneontology.org/amigo/term/GO:0006090	108	36	2645;189;5164;5213;5162;229;5160;10891;5465;339123;10993;5207;957;4967;8050;3643;57546;2027;2203;51422;3479;3029;5223;55526;1738;63826;5106;863;5105;5236;5091;5166;134526;55753;2819;2954	ACOT12;AGXT;ALDOB;CBFA2T3;DHTKD1;DLD;ENO3;ENTPD5;FBP1;GCK;GPD1;GSTZ1;HAGH;IGF1;INSR;JMJD8;OGDH;OGDHL;PC;PCK1;PCK2;PDHA1;PDHB;PDHX;PDK2;PDK4;PDP2;PFKFB1;PFKM;PGAM1;PGM1;PPARA;PPARGC1A;PRKAG

GO:0042180	cellular ketone metabolic process	-0.5298	-2.2103	0.00E+00	0.00E+00	http://amigo.geneontology.org/amigo/term/GO:0042180	176	71	1645;5743;11160;93058;5581;3667;6622;635;2168;8942;1312;10229;207;8013;552;2475;345;27122;57834;301;653;23038;133;6999;169355;5164;23408;341;60481;10891;1374;116519;5465;1109;130013;10613;57107;32;208;634;8574;80219;1586;79602;9104;33;51117;10901;3638;57546;1555;51166;51422;8564;3029;37;2531;6715;8529;6716;12	AADAT;ACACB;ACADL;ACADVL;ACMSD;ADH4;ADIPOR2;ADM;AKR1C1;AKR1C4;AKR7A2;AKT1;AKT2;ANXA1;APOA5;APOC1;APOC3;AVPR1A;BHMT;BMP5;CBR4;CEACAM1;COMT;COQ10A;COQ10B;COQ4;COQ6;COQ7;COQ9;CPT1A;CYP17A1;CYP2B6;CYP4F11;CYP4F2;DHRS4;DKK3;ELOVL5;ERLIN1;ERLIN2;FABP1;GOT2;GPD1;GSTZ1;HAGH;IDO2;INSIG1;IR

GO:0042537	benzene-containing compound metabolic process	-0.8131	-2.2607	0.00E+00	0.00E+00	http://amigo.geneontology.org/amigo/term/GO:0042537	22	14	6999;169355;1571;2946;2948;54658;2944;51166;23498;8564;5446;6716;2806;30	AADAT;ACAA1;CYP2E1;GOT2;GSTM1;GSTM2;GSTM4;HAAO;IDO2;KMO;PON3;SRD5A2;TDO2;UGT1A

GO:0030258	lipid modification	-0.5375	-2.2739	0.00E+00	0.00E+00	http://amigo.geneontology.org/amigo/term/GO:0030258	270	99	27349;3628;8310;3667;4233;8817;55862;2168;207;8013;9021;2475;224;5781;1543;26063;348;335;2064;3084;55361;1891;341;5295;51179;2246;55300;10891;1374;116519;5465;5288;55343;8309;145567;32;208;2252;2109;83667;79602;33;55268;23167;2053;26061;1384;6483;5264;8835;114757;54928;51422;1576;9365;55825;54363;37;8503;572	ACAA1;ACAA2;ACACB;ACAD10;ACAD11;ACADL;ACADM;ACADS;ACADVL;ACAT1;ACOX1;ACOX2;ACOX3;ADIPOR2;AKT1;AKT2;ALDH3A2;AMACR;APOA1;APOA5;APOC1;APOE;AUH;BDH2;CPT1A;CPT2;CRAT;CYGB;CYP1A1;CYP2C8;CYP3A4;CYP4A22;CYP4V2;DECR1;DECR2;ECH1;ECHDC1;ECHDC2;ECHS1;EFR3A;EHHADH;EPHX2;ERBB2;ESR1;ETFA;ETFB

GO:0006091	generation of precursor metabolites and energy	-0.5466	-2.2915	0.00E+00	0.00E+00	http://amigo.geneontology.org/amigo/term/GO:0006091	418	162	441531;93058;10327;4706;3939;8803;55022;4697;3667;83931;51341;6622;3795;9852;6834;10229;207;8013;552;3158;2475;126;23410;128;4708;3572;2645;5836;7386;1346;2538;4190;54511;57019;5213;10975;79660;4717;5162;2235;4705;3416;5018;4129;229;5160;81889;50;3417;728294;1355;10891;27089;51218;664;5465;10105;1109;130;3391	ACADVL;ACAT1;ACO1;ACO2;ACOX1;ACSS2;ACSS3;ADH1A;ADH1B;ADH1C;ADH4;ADH5;ADH6;AGL;AKR1A1;AKR1C4;AKR7A2;AKR7A3;AKT1;AKT2;ALDH1B1;ALDH2;ALDH4A1;ALDH5A1;ALDOB;AOX1;AQP7;ASIP;AVPR1A;BDH1;BDH2;BNIP3;CAT;CBFA2T3;CHCHD10;CIAPIN1;COQ10A;COQ10B;COQ7;COQ9;COX15;COX4I1;COX7A1;CYB5A;CYP1A2;D2HGDH;DH

GO:0006081	cellular aldehyde metabolic process	-0.6508	-2.2933	0.00E+00	0.00E+00	http://amigo.geneontology.org/amigo/term/GO:0006081	69	37	223;57026;11315;1645;9563;58510;10327;132158;25796;3795;224;27122;220;653;128;189;229;3417;2098;64577;8574;3418;64080;64902;3029;51071;5223;501;83875;55163;127;22977;2806;9380;23729;51109;8659	ADH4;ADH5;AGXT;AGXT2;AKR1A1;AKR1C1;AKR7A2;AKR7A3;ALDH1A3;ALDH3A2;ALDH4A1;ALDH7A1;ALDH8A1;ALDH9A1;ALDOB;BCO2;BMP5;DERA;DKK3;ESD;GLYCTK;GOT2;GRHPR;H6PD;HAGH;IDH1;IDH2;KHK;PARK7;PDXP;PGAM1;PGLS;PNPO;PRODH

GO:0006790	sulfur compound metabolic process	-0.5723	-2.3002	0.00E+00	0.00E+00	http://amigo.geneontology.org/amigo/term/GO:0006790	335	143	8460;2799;2720;2937;10327;6319;9391;8803;150274;6622;635;8942;1312;55790;80221;56548;1497;64377;3158;23743;2879;27430;1468;51268;2876;2952;56922;6487;3157;23305;9027;51144;1634;9348;6999;189;5164;57019;337876;5162;122970;5549;25828;51363;6817;51703;5160;79071;2729;60481;3417;26873;2588;9953;348158;2098;25	AADAT;AASS;ABHD14B;ACACB;ACAT1;ACOT1;ACOT12;ACOT13;ACOT2;ACOT4;ACOT6;ACSF2;ACSF3;ACSL1;ACSL5;ACSL6;ACSM2A;ACSM2B;ACSM3;ACSM5;ACSS2;ADI1;AGXT;AKR1A1;ALDH5A1;BAAT;BHMT;BHMT2;BTD;CDO1;CHST15;CHST7;CHST8;CHSY3;CIAO1;CIAPIN1;CNDP2;COMT;CPS1;CSGALNACT1;CTH;CTNS;DCN;DLD;DLST;ELOVL5;ELOVL

GO:0006520	cellular amino acid metabolic process	-0.5671	-2.3377	0.00E+00	0.00E+00	http://amigo.geneontology.org/amigo/term/GO:0006520	301	113	1497;23743;51733;55556;374569;51268;131669;56922;4942;122622;3067;2747;6999;189;169355;54511;25828;2628;23569;7965;2729;4846;144193;2593;5053;130013;10993;2687;27165;670;64802;22928;51557;113675;1491;23395;1807;389434;10588;10955;3028;443;23464;4143;6472;56954;51091;51166;36;1373;4842;23498;5184;3176;649	AADAT;AASS;ABAT;ACAD8;ACADSB;ACAT1;ACMSD;ACY1;ADHFE1;ADI1;ADSSL1;AGXT;AGXT2;AIMP2;ALDH4A1;ALDH5A1;ALDH6A1;ALDH7A1;AMDHD1;ASL;ASPA;ASPG;ASS1;AUH;BAAT;BCKDHA;BCKDHB;BCKDK;BHMT2;BPHL;CDO1;CPS1;CTH;CTNS;DLST;DPYS;EARS2;ENOSF1;FAH;FOLH1;FOLH1B;GAMT;GATM;GCAT;GCDH;GCLC;GCSH;GGT5;GLS2;

GO:0033865	nucleoside bisphosphate metabolic process	-0.6236	-2.4816	0.00E+00	0.00E+00	http://amigo.geneontology.org/amigo/term/GO:0033865	128	59	80221;3158;51268;3157;23305;51144;6999;5164;79717;5162;122970;6817;51703;5160;79071;60481;348158;6799;32;4598;54995;8802;641372;4967;8050;57546;51166;55856;55902;9060;54928;570;123876;283927;1743;6296;1738;54988;10861;197322;2180;84693;5166;27284;10157;3295;641371;134526;10249;84836;10965;53354;38;2639;	AADAT;AASS;ABHD14B;ACACB;ACAT1;ACOT1;ACOT12;ACOT13;ACOT2;ACOT4;ACOT6;ACSF2;ACSF3;ACSL1;ACSL5;ACSL6;ACSM2A;ACSM2B;ACSM3;ACSM5;ACSS2;BAAT;DLD;DLST;ELOVL5;ELOVL6;GCDH;GLYAT;GSTZ1;HMGCL;HMGCS1;HMGCS2;HSD17B12;HSD17B4;IMPAD1;MCCC2;MCEE;MLYCD;MVK;NUDT7;OGDH;OXSM;PANK1;PAPSS2;PDHA1

GO:0016053	organic acid biosynthetic process	-0.5564	-2.5189	0.00E+00	0.00E+00	http://amigo.geneontology.org/amigo/term/GO:0016053	383	153	5743;11160;5825;441531;10327;6319;56624;4706;27349;3939;8803;114876;4023;51341;635;8942;873;552;23743;2879;345;51733;374569;3248;301;220;1543;84696;23038;26063;4942;1593;2645;51144;1634;2747;10858;7390;189;8647;5213;1559;2643;341;5322;6718;229;2628;55301;79071;60481;1571;10891;116519;348158;5465;2593;5053;11	AASS;ABAT;ABCB11;ABCD3;ABHD1;ABHD2;ACACB;ACADL;ACADVL;ACMSD;ACOT12;ACOX2;ACSF3;ACSM2A;ACSM2B;ACSM3;ACSM5;ACSS2;ADI1;ADIPOR2;AGXT;AGXT2;AKR1A1;AKR1C4;AKR1D1;ALDH1A3;ALDH8A1;ALDOB;AMACR;ANXA1;APOA5;APOC1;APOC3;ASAH2;ASL;ASPA;ASPG;ASS1;AVPR1A;BAAT;BHMT;BHMT2;CBFA2T3;CBR1;CBR4;CDO

GO:0016042	lipid catabolic process	-0.6168	-2.5373	0.00E+00	0.00E+00	http://amigo.geneontology.org/amigo/term/GO:0016042	302	139	6783;23491;3991;4668;51390;151056;2166;6822;9784;5825;56624;5581;27349;66002;8310;4023;3667;55862;2168;207;9663;2475;345;224;374569;22933;7879;84696;26063;1066;338;348;335;125981;10858;339221;5660;1891;341;23175;5322;23659;6718;8879;51179;5140;150379;2822;3417;1374;116519;13;5465;8309;32;208;5346;1592;2109;8	AADAC;ABCD3;ABHD1;ABHD2;ABHD6;ACAA1;ACAA2;ACACB;ACAD10;ACAD11;ACADL;ACADM;ACADS;ACADVL;ACAT1;ACBD5;ACER1;ACOX1;ACOX2;ACOX3;AIG1;AKR1D1;AKT1;AKT2;ALDH3A2;AMACR;ANGPTL3;APOA1;APOA5;APOB;APOC1;APOC3;APOE;ASAH2;ASPG;AUH;BCO2;BDH2;CES1;CES3;CPS1;CPT1A;CPT2;CRAT;CYP1A2;CYP26A1;CYP3

GO:0007031	peroxisome organization	-0.7015	-2.5385	0.00E+00	0.00E+00	http://amigo.geneontology.org/amigo/term/GO:0007031	79	45	51268;55670;65084;26063;189;1891;122970;8800;3416;51179;3417;7314;5189;5830;5193;8309;2053;26061;1384;5264;10901;51024;11145;5195;570;55825;7323;283927;196743;54363;11001;6342;51;1962;847;23600;3295;5194;10965;92960;373156;83752;3155;30;23417	ACAA1;ACOT2;ACOT4;ACOX1;ACOX2;AGXT;AMACR;BAAT;CAT;CRAT;DECR2;DHRS4;ECH1;EHHADH;EPHX2;FIS1;GSTK1;HACL1;HAO1;HAO2;HMGCL;HSD17B4;IDE;IDH1;LONP2;MLYCD;NUDT7;PAOX;PECR;PEX1;PEX11A;PEX11G;PEX12;PEX13;PEX14;PEX26;PEX5;PHYH;PIPOX;PLA2G16;SCP2;SLC27A2;TMEM1

GO:0016999	antibiotic metabolic process	-0.6627	-2.5725	0.00E+00	0.00E+00	http://amigo.geneontology.org/amigo/term/GO:0016999	141	62	126;2876;1543;131669;3039;51005;128;4190;7001;5162;6817;4129;2878;5160;81889;50;3417;3042;144193;2098;1109;6799;130;10013;3419;8574;3418;8802;219;54658;3043;10588;48;4967;55902;10020;1544;124;3240;1743;55526;7915;5106;5105;4191;6482;10935;6716;127;125;23530;2534;27284;847;55753;6390;6392;6389;217;65018;84869;8	ACO1;ACO2;ACSS2;ADH1A;ADH1B;ADH1C;ADH4;ADH5;ADH6;AKR1C4;AKR7A2;ALDH1B1;ALDH2;ALDH5A1;AMDHD1;AMDHD2;CAT;CBR4;CYP1A1;CYP1A2;DHTKD1;DLST;ESD;FAHD1;FYN;GNE;GPX1;GPX3;HBA1;HBB;HBM;HDAC6;HP;IDH1;IDH2;IDH3A;MAOB;MDH1;MDH2;MTHFS;NNT;OGDH;OGDHL;PCK1;PCK2;PDHA1;PDHB;PINK1;PRDX2;PRDX3;SDHA;S

GO:0042737	drug catabolic process	-0.6651	-2.5757	0.00E+00	0.00E+00	http://amigo.geneontology.org/amigo/term/GO:0042737	130	60	51268;2876;3039;51005;7001;1559;3416;6817;4129;2878;1571;3042;4128;5053;6799;10993;23475;161823;1549;113675;2329;219;1807;1548;3043;10588;23464;8856;1555;51166;4842;1544;1576;3240;196743;54363;5092;1743;3081;113612;1558;5106;5105;2653;1486;2184;1573;10935;5447;622;23530;27284;847;10157;6898;3242;38;217;5860	AADAT;AASS;ACAT1;ADAL;ALDH1B1;ALDH2;AMDHD2;BDH1;CAT;CTBS;CYP1A2;CYP2A6;CYP2A7;CYP2B6;CYP2C8;CYP2C9;CYP2E1;CYP2J2;CYP2U1;CYP3A4;DLST;DPYS;FAH;FMO4;GCAT;GCSH;GPX1;GPX3;GSTZ1;HAO1;HBA1;HBB;HBM;HGD;HP;HPD;IDE;MAOA;MAOB;MTHFS;NNT;NOS1;NR1I2;PAH;PAOX;PCBD1;PCK1;PCK2;PIPOX;POR;PRDX2;PR

GO:0043574	peroxisomal transport	-0.7497	-2.5953	0.00E+00	0.00E+00	http://amigo.geneontology.org/amigo/term/GO:0043574	68	40	51268;55670;26063;189;1891;122970;3416;51179;3417;7314;5189;5830;5193;8309;2053;26061;1384;5264;10901;5195;570;55825;7323;283927;196743;54363;11001;6342;51;1962;847;23600;3295;5194;10965;373156;83752;3155;30;23417	ACAA1;ACOT2;ACOT4;ACOX1;ACOX2;AGXT;AMACR;BAAT;CAT;CRAT;DECR2;DHRS4;ECH1;EHHADH;EPHX2;GSTK1;HACL1;HAO1;HAO2;HMGCL;HSD17B4;IDE;IDH1;LONP2;MLYCD;NUDT7;PAOX;PECR;PEX1;PEX12;PEX13;PEX14;PEX26;PEX5;PHYH;PIPOX;SCP2;SLC27A

GO:0006631	fatty acid metabolic process	-0.6449	-2.6585	0.00E+00	0.00E+00	http://amigo.geneontology.org/amigo/term/GO:0006631	339	168	948;5743;11160;5825;6319;56624;4706;27349;5066;66002;8310;4023;3667;6622;55862;2168;7132;718;873;207;8013;552;80221;2879;9663;2475;345;224;57834;3248;301;2876;1543;84696;23038;26063;23305;1066;51144;5164;1891;1559;122970;10434;341;23175;23659;8879;51179;51703;55301;79071;60481;1571;10891;1374;116519;84266;2	ABCD3;ABHD1;ABHD2;ACAA1;ACAA2;ACACB;ACAD10;ACAD11;ACADL;ACADM;ACADS;ACADSB;ACADVL;ACAT1;ACBD5;ACOT1;ACOT12;ACOT2;ACOT4;ACOT6;ACOX1;ACOX2;ACOX3;ACSF2;ACSF3;ACSL1;ACSL5;ACSL6;ACSM2A;ACSM2B;ACSM3;ACSM5;ACSS2;ADIPOR2;AKR1C4;AKT1;AKT2;ALDH3A2;ALDH5A1;ALKBH7;AMACR;ANGPTL3;ANXA1;APOA
