# Supplementary material for: Prognostic potential of PRPF3 in hepatocellular carcinoma
Source: Aging (Albany NY). 2020 Jan 11;12(1):912–30. doi: 10.18632/aging.102665 (PMC6977647; doi:10.18632/aging.102665)
Supplement: Supplementary Table 3 [file aging-12-102665-s004..pdf]

Supplementary Table 3. KEGG annotation of PRPF3 co-expressed genes.

geneSet	description	ES	NES	pValue	FDR	link	size	leadingEdgeNum	leadingEdgeId	userId
hsa03040	Spliceosome	0.7712	2.1347	0.00E+00	0.00E+00	http://www.kegg.jp/kegg-bin/show_pathway?hsa03040+9129+10262+27316+9939+220988+3192+6635+84950+8175+9984+6632+10915+3183+9775+7307+6625+6628+4670+11338+11325+25766+9343+9416+6637+	115	69	9129;10262;27316;9939;220988;3192;6635;84950;8175;9984;6632;10915;3183;9775;7307;6625;6628;4670;11338;11325;25766;9343;9416;6637;6627;57819;6626;57187;22985;10465;22916;24148;6434;84991;55110;3178;9128;3190;4116;57461;84844;9879;10946;4686;6631;10084;6633;9410;10713;144983;10450;23020;6629;199746;55696;51645;8449;1665;29896;1659;55119;84321;55660;8559;6636;10992;6	ACIN1;CRNKL1;DDX23;DDX42;DDX46;DHX15;DHX16;DHX8;EFTUD2;EIF4A3;HNRNPA1;HNRNPA1L2;HNRNPA3;HNRNPC;HNRNPK;HNRNPM;HNRNPU;ISY1;LSM2;MAGOH;MAGOHB;NCBP1;NCBP2;PHF5A;

hsa03030	DNA replication	0.8588	2.0923	0.00E+00	0.00E+00	http://www.kegg.jp/kegg-bin/show_pathway?hsa03030+5984+4171+4175+4174+3978+1763+4172+2237+10714+5422+5111+5557+5424+23649+4176+5558+5427+10535+5985+4173+54107+5983+5	36	26	5984;4171;4175;4174;3978;1763;4172;2237;10714;5422;5111;5557;5424;23649;4176;5558;5427;10535;5985;4173;54107;5983;5426;5982;79621;6117	DNA2;FEN1;LIG1;MCM2;MCM3;MCM4;MCM5;MCM6;MCM7;PCNA;POLA1;POLA2;POLD1;POLD3;POLE;POLE2;POLE3;PRIM1;PRIM2;RFC2;RFC3;RFC4;RFC5;RNASEH2A;RNASEH2B;RPA1

hsa04110	Cell cycle	0.7296	2.0171	0.00E+00	0.00E+00	http://www.kegg.jp/kegg-bin/show_pathway?hsa04110+7272+990+4171+8318+4175+983+4174+995+5347+991+4172+701+9232+9133+1111+699+1869+9134+5111+1870+9700+8317+891+9088+4176+29945+3066+	118	47	7272;990;4171;8318;4175;983;4174;995;5347;991;4172;701;9232;9133;1111;699;1869;9134;5111;1870;9700;8317;891;9088;4176;29945;3066;4085;993;1031;4173;51434;890;27127;7027;9184;10926;1017;5933;1029;898;10744;1871;1019;996;8881;3065	ANAPC4;ANAPC7;BUB1;BUB1B;BUB3;CCNA2;CCNB1;CCNB2;CCNE1;CCNE2;CDC16;CDC20;CDC25A;CDC25C;CDC27;CDC45;CDC6;CDC7;CDK1;CDK2;CDK4;CDKN2A;CDKN2C;CHEK1;DBF4;E2F1;E2F2;

hsa03460	Fanconi anemia pathway	0.7839	1.9485	0.00E+00	0.00E+00	http://www.kegg.jp/kegg-bin/show_pathway?hsa03460+29089+146956+55215+2189+2178+672+641+2177+2187+5888+5889+7398+80010+8940+84126+55120+2175+80198+83990+6117+6	44	24	29089;146956;55215;2189;2178;672;641;2177;2187;5888;5889;7398;80010;8940;84126;55120;2175;80198;83990;6117;675;57697;2188;51455	ATRIP;BLM;BRCA1;BRCA2;BRIP1;EME1;FANCA;FANCB;FANCD2;FANCE;FANCF;FANCG;FANCI;FANCL;FANCM;MUS81;RAD51;RAD51C;REV1;RMI1;RPA1;TOP3B;UBE2T;USP1

hsa01230	Biosynthesis of amino acids	-0.5903	-2.0781	0.00E+00	0.00E+00	http://www.kegg.jp/kegg-bin/show_pathway?hsa01230+5213+229+50+3417+5053+875+10993+3419+3418+113675+1491+48+4143+6472+1373+2027+6470+2805+5223+84706+445+435+5009+5091	69	28	5213;229;50;3417;5053;875;10993;3419;3418;113675;1491;48;4143;6472;1373;2027;6470;2805;5223;84706;445;435;5009;5091;2806;2875;6898;95	ACO1;ACO2;ACY1;ALDOB;ASL;ASS1;CBS;CPS1;CTH;ENO3;GOT1;GOT2;GPT;GPT2;IDH1;IDH2;IDH3A;MAT1A;OTC;PAH;PC;PFKM;PGAM1;SDS;SDSL;SHMT1;SHMT2;TAT

hsa05012	Parkinson disease	-0.5683	-2.1450	0.00E+00	0.00E+00	http://www.kegg.jp/kegg-bin/show_pathway?hsa05012+4707+9377+2771+7318+11315+4711+4704+4706+4697+6622+5566+6571+7326+2861+4708+120892+7386+9246+1346+10975+4717+4705+27089+7314+1010	115	39	4707;9377;2771;7318;11315;4711;4704;4706;4697;6622;5566;6571;7326;2861;4708;120892;7386;9246;1346;10975;4717;4705;27089;7314;10105;374291;4714;1327;4719;4723;4729;7384;7385;4698;6390;6392;6389;291;65018	COX4I1;COX5A;COX7A1;GNAI2;GPR37;LRRK2;NDUFA10;NDUFA4;NDUFA5;NDUFA9;NDUFAB1;NDUFB1;NDUFB2;NDUFB5;NDUFB8;NDUFC1;NDUFS1;NDUFS7;NDUFV1;NDUFV2;PARK7;PINK1;PPIF;PRK

hsa00010	Glycolysis / Gluconeogenesis	-0.6367	-2.1522	0.00E+00	0.00E+00	http://www.kegg.jp/kegg-bin/show_pathway?hsa00010+441531+10327+3939+130589+224+220+126+128+2645+2538+5213+5162+229+5160+9562+130+219+55902+2027+2203+124+5223+501+1738+5106+5105+12	66	30	441531;10327;3939;130589;224;220;126;128;2645;2538;5213;5162;229;5160;9562;130;219;55902;2027;2203;124;5223;501;1738;5106;5105;127;125;5236;217	ACSS2;ADH1A;ADH1B;ADH1C;ADH4;ADH5;ADH6;AKR1A1;ALDH1A3;ALDH1B1;ALDH2;ALDH3A2;ALDH7A1;ALDOB;DLD;ENO3;FBP1;G6PC;GALM;GCK;LDHA;MINPP1;PCK1;PCK2;PDHA1;PDHB;PFKM;PGAM

hsa00120	Primary bile acid biosynthesis	-0.8369	-2.1878	0.00E+00	0.00E+00	http://www.kegg.jp/kegg-bin/show_pathway?hsa00120+1593+10858+6718+1109+8309+9420+570+6342+1582+51302+23600+32	17	13	1593;10858;6718;1109;8309;9420;570;6342;1582;51302;23600;3295;10998	ACOX2;AKR1C4;AKR1D1;AMACR;BAAT;CYP27A1;CYP39A1;CYP46A1;CYP7B1;CYP8B1;HSD17B4;SCP2;SLC27A5

hsa00620	Pyruvate metabolism	-0.7278	-2.2046	0.00E+00	0.00E+00	http://www.kegg.jp/kegg-bin/show_pathway?hsa00620+4190+5162+5160+32+219+55902+3029+501+1738+5106+5105+4191+5091+134526+9380+98+38+	38	19	4190;5162;5160;32;219;55902;3029;501;1738;5106;5105;4191;5091;134526;9380;98;38;217;197257	ACACB;ACAT1;ACOT12;ACSS2;ACYP2;ALDH1B1;ALDH2;ALDH7A1;DLD;GRHPR;HAGH;LDHD;MDH1;MDH2;PC;PCK1;PCK2;PDHA1;PDHB

hsa00140	Steroid hormone biosynthesis	-0.6704	-2.2158	0.00E+00	0.00E+00	http://www.kegg.jp/kegg-bin/show_pathway?hsa00140+6783+1645+79799+1312+54600+1543+51144+220074+6718+54579+1571+1109+1586+7366+54658+7923+54490+54659+54657+1544+8630+9420+3290+7364	57	30	6783;1645;79799;1312;54600;1543;51144;220074;6718;54579;1571;1109;1586;7366;54658;7923;54490;54659;54657;1544;8630;9420;3290;7364;1583;1576;412;6715;6716;7365	AKR1C1;AKR1C4;AKR1D1;COMT;CYP11A1;CYP17A1;CYP1A1;CYP1A2;CYP2E1;CYP3A4;CYP7B1;HSD11B1;HSD17B12;HSD17B6;HSD17B8;LRTOMT;SRD5A1;SRD5A2;STS;SULT1E1;UGT1A1;UGT1A3;UGT1A4;UGT1

hsa00220	Arginine biosynthesis	-0.8058	-2.2280	0.00E+00	0.00E+00	http://www.kegg.jp/kegg-bin/show_pathway?hsa00220+4846+27165+1373+4842+2746+2805+84706+445+435+5009+28	20	13	4846;27165;1373;4842;2746;2805;84706;445;435;5009;2806;2875;95	ACY1;ASL;ASS1;CPS1;GLS2;GLUD1;GOT1;GOT2;GPT;GPT2;NOS1;NOS3;OTC

hsa00260	Glycine, serine and threonine metabolism	-0.7477	-2.2547	0.00E+00	0.00E+00	http://www.kegg.jp/kegg-bin/show_pathway?hsa00260+212+441531+132158+635+51268+189+4129+2628+4128+2593+875+10993+211+113675+1491+8639+23464+6472+64902+6470+29958+5223+1757+501+1738	39	28	212;441531;132158;635;51268;189;4129;2628;4128;2593;875;10993;211;113675;1491;8639;23464;6472;64902;6470;29958;5223;1757;501;1738;63826;2653;9380	AGXT;AGXT2;ALAS1;ALAS2;ALDH7A1;AOC3;BHMT;CBS;CTH;DLD;DMGDH;GAMT;GATM;GCAT;GCSH;GLYCTK;GRHPR;MAOA;MAOB;PGAM1;PGAM4;PIPOX;SARDH;SDS;SDSL;SHMT1;SHMT2;SRR

hsa00410	beta-Alanine metabolism	-0.7669	-2.2799	0.00E+00	0.00E+00	http://www.kegg.jp/kegg-bin/show_pathway?hsa00410+219+1807+8639+55748+501+18+1892+26275+3030+34+1962+4329+217	31	14	219;1807;8639;55748;501;18;1892;26275;3030;34;1962;4329;217;23417	ABAT;ACADM;ALDH1B1;ALDH2;ALDH6A1;ALDH7A1;AOC3;CNDP2;DPYS;ECHS1;EHHADH;HADHA;HIBCH;MLYCD

hsa00650	Butanoate metabolism	-0.7993	-2.3206	0.00E+00	0.00E+00	http://www.kegg.jp/kegg-bin/show_pathway?hsa00650+3158+3157+54511+79944+348158+123876+7915+18+6296+54988+56898+1892+3030+35+1962+6	27	19	3158;3157;54511;79944;348158;123876;7915;18;6296;54988;56898;1892;3030;35;1962;622;3033;38;3155	ABAT;ACADS;ACAT1;ACSM2A;ACSM2B;ACSM3;ACSM5;ALDH5A1;BDH1;BDH2;ECHS1;EHHADH;HADH;HADHA;HMGCL;HMGCLL1;HMGCS1;HMGC

hsa00350	Tyrosine metabolism	-0.7705	-2.3252	0.00E+00	0.00E+00	http://www.kegg.jp/kegg-bin/show_pathway?hsa00350+1312+220+126+128+220074+4129+81889+4128+130+8639+316+124+2805+3081+2184+127+125+2806+68	36	21	1312;220;126;128;220074;4129;81889;4128;130;8639;316;124;2805;3081;2184;127;125;2806;6898;3242;2954	ADH1A;ADH1B;ADH1C;ADH4;ADH5;ADH6;ALDH1A3;AOC3;AOX1;COMT;FAH;FAHD1;GOT1;GOT2;GSTZ1;HGD;HPD;LRTOMT;MAOA;MAOB;TAT

hsa00980	Metabolism of xenobiotics by cytochrome P450	-0.6753	-2.3533	0.00E+00	0.00E+00	http://www.kegg.jp/kegg-bin/show_pathway?hsa00980+6822+1645+79799+54600+873+220+126+2952+1543+128+1559+54579+1571+2946+130+8574+4257+2948+7366+1548+54658+54490+2944+1555+54659+546	70	39	6822;1645;79799;54600;873;220;126;2952;1543;128;1559;54579;1571;2946;130;8574;4257;2948;7366;1548;54658;54490;2944;1555;54659;54657;1544;3290;7364;124;1576;2949;9446;4258;127;125;7365;22977;373156	ADH1A;ADH1B;ADH1C;ADH4;ADH5;ADH6;AKR1C1;AKR7A2;AKR7A3;ALDH1A3;CBR1;CYP1A1;CYP1A2;CYP2A6;CYP2B6;CYP2C9;CYP2E1;CYP3A4;GSTK1;GSTM1;GSTM2;GSTM4;GSTM5;GSTO1;GSTT1;HSD11B

hsa00630	Glyoxylate and dicarboxylate metabolism	-0.8082	-2.3656	0.00E+00	0.00E+00	http://www.kegg.jp/kegg-bin/show_pathway?hsa00630+189+4190+51179+50+48+6472+55902+6470+5095+54363+1738+5096+2653+4191+84693	28	18	189;4190;51179;50;48;6472;55902;6470;5095;54363;1738;5096;2653;4191;84693;847;9380;38	ACAT1;ACO1;ACO2;ACSS2;AGXT;CAT;DLD;GCSH;GRHPR;HAO1;HAO2;MCEE;MDH1;MDH2;PCCA;PCCB;SHMT1;SHMT2

hsa03320	PPAR signaling pathway	-0.6871	-2.4064	0.00E+00	0.00E+00	http://www.kegg.jp/kegg-bin/show_pathway?hsa03320+729359+948+6319+8310+4023+2168+3158+345+51129+23305+1593+335+51703+1374+116519+5465+8309+5346+33+10999+2710+2167+10580+11001+6342	74	38	729359;948;6319;8310;4023;2168;3158;345;51129;23305;1593;335;51703;1374;116519;5465;8309;5346;33;10999;2710;2167;10580;11001;6342;5106;5105;1582;364;284541;2180;51;34;1962;1579;1376;10998;30	ACAA1; ACADL; ACADM; ACOX1; ACOX2; ACOX3; ACSL1;ACSL5;ACSL6;ANGPTL4;APOA1;APOA5;APOC3;AQP7;CD36;CPT1A;CPT2;CYP27A1;CYP4A11;CYP4A22;CYP8B1;EHHADH;FABP1;FABP4;GK;HMGCS2;

hsa00380	Tryptophan metabolism	-0.7876	-2.4184	0.00E+00	0.00E+00	http://www.kegg.jp/kegg-bin/show_pathway?hsa00380+8942+224+1543+6999+169355+4129+4128+130013+11185+219+4967+316+51166+23498+1544+8564+501+1892+3030+1962+3033+847+557	37	26	8942;224;1543;6999;169355;4129;4128;130013;11185;219;4967;316;51166;23498;1544;8564;501;1892;3030;1962;3033;847;55753;38;2639;217	AADAT;ACAT1;ACMSD;ALDH1B1;ALDH2;ALDH3A2;ALDH7A1;AOX1;CAT;CYP1A1;CYP1A2;ECHS1;EHHADH;GCDH;HAAO;HADH;HADHA;IDO2;INMT;KMO;KYNU;MAOA;MAOB;OGDH;OGDHL;TDO

hsa00020	Citrate cycle (TCA cycle)	-0.8227	-2.4549	0.00E+00	0.00E+00	http://www.kegg.jp/kegg-bin/show_pathway?hsa00020+4190+5162+5160+50+3417+3419+3418+8802+48+4967+1743+1738+5106+5105+4191+5091+55753+6390+	30	21	4190;5162;5160;50;3417;3419;3418;8802;48;4967;1743;1738;5106;5105;4191;5091;55753;6390;6392;6389;8801	ACO1;ACO2;DLD;DLST;IDH1;IDH2;IDH3A;MDH1;MDH2;OGDH;OGDHL;PC;PCK1;PCK2;PDHA1;PDHB;SDHA;SDHB;SDHD;SUCLG1;SUCLG2

hsa01212	Fatty acid metabolism	-0.7575	-2.5006	0.00E+00	0.00E+00	http://www.kegg.jp/kegg-bin/show_pathway?hsa01212+23305+51144+51703+79071+60481+1374+54995+33+36+55825+37+1892+3030+2180+51+35+34+1962+3033+51102+3032+1376+38+10449+	44	25	23305;51144;51703;79071;60481;1374;54995;33;36;55825;37;1892;3030;2180;51;35;34;1962;3033;51102;3032;1376;38;10449;30	ACAA1;ACAA2;ACADL;ACADM;ACADS;ACADSB;ACADVL;ACAT1;ACOX1;ACSL1;ACSL5;ACSL6;CPT1A;CPT2;ECHS1;EHHADH;ELOVL5;ELOVL6;HADH;HADHA;HADHB;HSD17B12;MECR;OXS

hsa00982	Drug metabolism	-0.7208	-2.5060	0.00E+00	0.00E+00	http://www.kegg.jp/kegg-bin/show_pathway?hsa00982+220+126+2952+128+1559+4129+54579+1571+4128+2946+130+4257+2948+2329+7366+1548+54658+2327+54490+2944+316+1555+54659+54657+1544+7364	66	36	220;126;2952;128;1559;4129;54579;1571;4128;2946;130;4257;2948;2329;7366;1548;54658;2327;54490;2944;316;1555;54659;54657;1544;7364;124;1576;2949;9446;1558;4258;127;125;7365;373156	ADH1A;ADH1B;ADH1C;ADH4;ADH5;ADH6;ALDH1A3;AOX1;CYP1A2;CYP2A6;CYP2B6;CYP2C8;CYP2C9;CYP2E1;CYP3A4;FMO2;FMO4;GSTK1;GSTM1;GSTM2;GSTM4;GSTM5;GSTO1;GSTT1;MAOA;MAOB

hsa00830	Retinol metabolism	-0.7648	-2.5200	0.00E+00	0.00E+00	http://www.kegg.jp/kegg-bin/show_pathway?hsa00830+126+1543+128+728635+1559+317749+54579+130+1592+7366+1548+54658+10901+54490+316+1555+8608+54659+54657+1544+8630+7364+124+1576+9249	63	35	126;1543;128;728635;1559;317749;54579;130;1592;7366;1548;54658;10901;54490;316;1555;8608;54659;54657;1544;8630;7364;124;1576;9249;1558;284541;127;125;7365;1579;8228;51109;54884;5959	ADH1A;ADH1B;ADH1C;ADH4;ADH5;ADH6;AOX1;CYP1A1;CYP1A2;CYP26A1;CYP2A6;CYP2B6;CYP2C8;CYP2C9;CYP3A4;CYP4A11;CYP4A22;DHRS3;DHRS4;DHRS4L1;DHRS4L2;HSD17B6;PNPLA4;RDH11;RDH16;

hsa01200	Carbon metabolism	-0.6904	-2.5225	0.00E+00	0.00E+00	http://www.kegg.jp/kegg-bin/show_pathway?hsa01200+128+2645+2747+189+4190+5213+5162+229+51179+5160+50+3417+2098+10993+3419+3418+113675+8802+9104+48+4967+6472+1373+55902+2746+2027+2	110	56	128;2645;2747;189;4190;5213;5162;229;51179;5160;50;3417;2098;10993;3419;3418;113675;8802;9104;48;4967;6472;1373;55902;2746;2027;2203;6470;5095;2805;54363;5223;1743;84706;1738;5096;1892;26275;2653;4191;3030;84693;35;34;1962;5091;847;2806;55753;4329;2875;6390;6392;38;6389;8801	ACADM;ACADS;ACAT1;ACO1;ACO2;ACSS2;ADH5;AGXT;ALDH6A1;ALDOB;CAT;CPS1;DLD;DLST;ECHS1;EHHADH;ENO3;ESD;FBP1;GCK;GCSH;GLUD1;GLUD2;GOT1;GOT2;GPT;GPT2;HADHA;HAO1;HAO

hsa00640	Propanoate metabolism	-0.8687	-2.5403	0.00E+00	0.00E+00	http://www.kegg.jp/kegg-bin/show_pathway?hsa00640+32+8802+55902+5095+594+18+1738+5096+1892+26275+3030+593+84693+34+1962+1629+79611+4329+3	31	21	32;8802;55902;5095;594;18;1738;5096;1892;26275;3030;593;84693;34;1962;1629;79611;4329;38;23417;8801	ABAT;ACACB;ACADM;ACAT1;ACSS2;ACSS3;ALDH6A1;BCKDHA;BCKDHB;DBT;DLD;ECHS1;EHHADH;HADHA;HIBCH;MCEE;MLYCD;PCCA;PCCB;SUCLG1;S

hsa04146	Peroxisome	-0.7215	-2.6067	0.00E+00	0.00E+00	http://www.kegg.jp/kegg-bin/show_pathway?hsa04146+51268+55670+26063+23305+728635+189+83594+54511+1891+8800+51179+51703+3417+5826+5189+5830+5193+7498+8309+4598+3418+2053+26061+1384	82	47	51268;55670;26063;23305;728635;189;83594;54511;1891;8800;51179;51703;3417;5826;5189;5830;5193;7498;8309;4598;3418;2053;26061;1384;5264;10901;5195;570;55825;5827;283927;196743;54363;11001;6342;2180;51;1962;847;23600;3295;5194;92960;373156;3155;30;23417	ABCD4;ACAA1;ACOX1;ACOX2;ACSL1;ACSL5;ACSL6;AGXT;AMACR;BAAT;CAT;CRAT;DECR2;DHRS4;DHRS4L1;ECH1;EHHADH;EPHX2;GSTK1;HACL1;HAO1;HAO2;HMGCL;HMGCLL1;HSD17B4;IDH1;IDH2;

hsa00071	Fatty acid degradation	-0.8221	-2.6228	0.00E+00	0.00E+00	http://www.kegg.jp/kegg-bin/show_pathway?hsa00071+224+126+128+23305+51703+1374+130+219+33+36+124+37+501+1892+3030+284541+2180+51+35+127+34+125+1962+3033+3032+1579+1376+38+2639+217	42	32	224;126;128;23305;51703;1374;130;219;33;36;124;37;501;1892;3030;284541;2180;51;35;127;34;125;1962;3033;3032;1579;1376;38;2639;217;10449;30	ACAA1;ACAA2;ACADL;ACADM;ACADS;ACADSB;ACADVL;ACAT1;ACOX1;ACSL1;ACSL5;ACSL6;ADH1A;ADH1B;ADH1C;ADH4;ADH5;ADH6;ALDH1B1;ALDH2;ALDH3A2;ALDH7A1;CPT1A;CPT2;CYP4A11;CYP4A2

hsa04610	Complement and coagulation cascades	-0.7374	-2.6597	0.00E+00	0.00E+00	http://www.kegg.jp/kegg-bin/show_pathway?hsa04610+714+713+5624+1191+712+2159+2147+462+11326+4153+2162+624+2149+7035+1378+3075+718+728+2155+722+5265+735+2244+3827+733+2266+720+966+	78	52	714;713;5624;1191;712;2159;2147;462;11326;4153;2162;624;2149;7035;1378;3075;718;728;2155;722;5265;735;2244;3827;733;2266;720;966;732;1361;2157;2165;5340;5648;2161;5327;629;715;2243;716;730;10747;7056;2158;2160;731;7450;3818;5627;729;3426;710	BDKRB2;C1QA;C1QB;C1QC;C1R;C1S;C3;C4A;C4BPA;C5AR1;C6;C7;C8A;C8B;C8G;C9;CD59;CFB;CFH;CFI;CLU;CPB2;CR1;F10;F11;F12;F13A1;F13B;F2;F2R;F7;F8;F9;FGA;FGB;FGG;KLKB1;KNG1;MASP1;MAS

hsa00280	Valine, leucine and isoleucine degradation	-0.8633	-2.8511	0.00E+00	0.00E+00	http://www.kegg.jp/kegg-bin/show_pathway?hsa00280+219+3028+316+36+64902+5095+27034+594+501+18+1738+5096+1892+26275+549+197322+3030+593+84693+35+34+1962+1629+3033+3032+4329+11112+3	47	34	219;3028;316;36;64902;5095;27034;594;501;18;1738;5096;1892;26275;549;197322;3030;593;84693;35;34;1962;1629;3033;3032;4329;11112;38;217;3155;10449;30;3712;64087	ABAT;ACAA1;ACAA2;ACAD8;ACADM;ACADS;ACADSB;ACAT1;ACSF3;AGXT2;ALDH1B1;ALDH2;ALDH6A1;ALDH7A1;AOX1;AUH;BCKDHA;BCKDHB;DBT;DLD;ECHS1;EHHADH;HADH;HADHA;HADHB;HIBADH;

hsa03013	RNA transport	0.6616	1.8694	0.00E+00	9.44E-05	http://www.kegg.jp/kegg-bin/show_pathway?hsa03013+9939+79902+10460+65109+80336+348995+7175+23636+9984+7514+22794+7329+8021+23511+23225+8480+10921+9775+10940+55746+9631+79897+57122	157	70	9939;79902;10460;65109;80336;348995;7175;23636;9984;7514;22794;7329;8021;23511;23225;8480;10921;9775;10940;55746;9631;79897;57122;79023;57187;22985;65110;22916;3837;55110;8664;8563;4116;29107;11218;57510;5901;10482;80145;23165;4686;11097;10556;1967;23279;8662;5411;10557;84321;8893;10762;1968;79228;79760;6607;81929;55916;8086;53371;285190;10073;10419;6606;100101	AAAS;ACIN1;CASC3;DDX20;EIF2B1;EIF2B5;EIF2S2;EIF2S3;EIF3B;EIF3D;EIF4A3;GEMIN5;GEMIN7;KPNB1;MAGOH;MAGOHB;NCBP1;NCBP2;NUP107;NUP133;NUP155;NUP160;NUP188;NUP205;NUP210;NUP214

hsa03410	Base excision repair	0.7685	1.8462	0.00E+00	2.36E-04	http://www.kegg.jp/kegg-bin/show_pathway?hsa03410+3978+2237+10714+5111+5424+5427+55247+54107+142+3980+10038+7515+699	33	15	3978;2237;10714;5111;5424;5427;55247;54107;142;3980;10038;7515;6996;5426;4595	FEN1;LIG1;LIG3;MUTYH;NEIL3;PARP1;PARP2;PCNA;POLD1;POLD3;POLE;POLE2;POLE3;TDG;XRCC1

hsa03015	mRNA surveillance pathway	0.6753	1.8259	0.00E+00	4.72E-04	http://www.kegg.jp/kegg-bin/show_pathway?hsa03015+9939+9887+11052+23381+65109+80336+55339+1478+22794+26528+10921+9775+81608+22985+65110+22916+55110+1479+4116+29107+5528+104	87	25	9939;9887;11052;23381;65109;80336;55339;1478;22794;26528;10921;9775;81608;22985;65110;22916;55110;1479;4116;29107;5528;10482;5501;4686;51692	ACIN1;CASC3;CPSF3;CPSF6;CSTF2;CSTF3;DAZAP1;EIF4A3;FIP1L1;MAGOH;MAGOHB;NCBP1;NCBP2;NXF1;NXT1;PABPC1L;PPP1CC;PPP2R5D;RBM8A;RNPS1;SMG5;SMG7;UPF3A;UPF3B;WDR33

hsa03440	Homologous recombination	0.7649	1.8269	0.00E+00	5.39E-04	http://www.kegg.jp/kegg-bin/show_pathway?hsa03440+146956+8438+672+641+10714+7517+5424+5888+5889+25788+7516+11073+8940+5932+80198+83990+6117+675+51720+5	34	22	146956;8438;672;641;10714;7517;5424;5888;5889;25788;7516;11073;8940;5932;80198;83990;6117;675;51720;580;5893;29935	BARD1;BLM;BRCA1;BRCA2;BRIP1;EME1;MUS81;POLD1;POLD3;RAD51;RAD51C;RAD52;RAD54B;RAD54L;RBBP8;RPA1;RPA4;TOP3B;TOPBP1;UIMC1;XRCC2;XRCC3

hsa03430	Mismatch repair	0.7844	1.7900	0.00E+00	8.92E-04	http://www.kegg.jp/kegg-bin/show_pathway?hsa03430+5984+9156+3978+10714+4436+5111+5424+5985+2956	23	11	5984;9156;3978;10714;4436;5111;5424;5985;2956;5983;5982	EXO1;LIG1;MSH2;MSH6;PCNA;POLD1;POLD3;RFC2;RFC3;RFC4;RFC5
hsa03420	Nucleotide excision repair	0.6963	1.7182	2.47E-03	5.62E-03	http://www.kegg.jp/kegg-bin/show_pathway?hsa03420+5984+3978+10714+5111+5424+5427+5985+54107+5983+5426+5982+2071+8450	45	15	5984;3978;10714;5111;5424;5427;5985;54107;5983;5426;5982;2071;8450;2968;6117	CUL4B;ERCC3;GTF2H4;LIG1;PCNA;POLD1;POLD3;POLE;POLE2;POLE3;RFC2;RFC3;RFC4;RFC5;RPA1

hsa04114	Oocyte meiosis	0.6051	1.6829	0.00E+00	8.67E-03	http://www.kegg.jp/kegg-bin/show_pathway?hsa04114+983+995+5347+991+286151+9232+9133+699+9134+9700+891+9088+26271+29945+6790+4085+51434+27127+1017+898+10744+5528+996+8881+5501+112+	118	32	983;995;5347;991;286151;9232;9133;699;9134;9700;891;9088;26271;29945;6790;4085;51434;27127;1017;898;10744;5528;996;8881;5501;112;729597;285955;245711;442590;3710;163688	ADCY6;ANAPC4;ANAPC7;AURKA;BUB1;CALML6;CCNB1;CCNB2;CCNE1;CCNE2;CDC16;CDC20;CDC25C;CDC27;CDK1;CDK2;ESPL1;FBXO43;FBXO5;ITPR3;MAD2L1;PKMYT1;PLK1;PPP1CC;PPP2R5D;PTT

hsa03008	Ribosome biogenesis in eukaryotes	0.6284	1.6340	1.14E-03	1.77E-02	http://www.kegg.jp/kegg-bin/show_pathway?hsa03008+4931+55813+51602+51096+7514+10528+10813+84128+6949+10940+1736+55127+29107+83732+5901+10482+55226+5822+2091+10556+55341+298	69	24	4931;55813;51602;51096;7514;10528;10813;84128;6949;10940;1736;55127;29107;83732;5901;10482;55226;5822;2091;10556;55341;29889;10557;23560	DKC1;FBL;GNL2;GTPBP4;HEATR1;LSG1;NAT10;NOP56;NOP58;NVL;NXF1;NXT1;POP1;PWP2;RAN;RIOK1;RPP30;RPP38;TCOF1;UTP14A;UTP18;UTP6;WDR75;XPO1

hsa00240	Pyrimidine metabolism	0.5906	1.6000	0.00E+00	2.61E-02	http://www.kegg.jp/kegg-bin/show_pathway?hsa00240+10623+29922+10714+5422+5557+7371+5424+23649+6240+5558+5427+124583+7298+6241+54963+10621+790+54107+708	96	21	10623;29922;10714;5422;5557;7371;5424;23649;6240;5558;5427;124583;7298;6241;54963;10621;790;54107;7083;1841;5433	CAD;CANT1;DTYMK;NME7;POLA1;POLA2;POLD1;POLD3;POLE2;POLE3;POLR2D;POLR3C;POLR3F;PRIM1;PRIM2;RRM1;RRM2;TK1;TYMS;UCK2;UCKL1

hsa04914	Progesterone-mediated oocyte maturation	0.5858	1.6015	0.00E+00	2.73E-02	http://www.kegg.jp/kegg-bin/show_pathway?hsa04914+983+995+5347+9133+699+891+9088+29945+6790+4085+993+51434+890+5603+1017+996+8881+112+729597+285955+994+245711+442590+5601+3835+246	94	28	983;995;5347;9133;699;891;9088;29945;6790;4085;993;51434;890;5603;1017;996;8881;112;729597;285955;994;245711;442590;5601;3835;246184;8697;5595	ADCY6;ANAPC4;ANAPC7;AURKA;BUB1;CCNA2;CCNB1;CCNB2;CDC16;CDC23;CDC25A;CDC25B;CDC25C;CDC26;CDC27;CDK1;CDK2;KIF22;MAD2L1;MAPK13;MAPK3;MAPK9;PKMYT1;PLK1;SPDYA;S

hsa03022	Basal transcription factors	0.6338	1.5840	5.97E-03	3.14E-02	http://www.kegg.jp/kegg-bin/show_pathway?hsa03022+6908+8148+6874+6882+2071+6878+9569+2968+6877+9519+27097+83860+2965+51616+	44	17	6908;8148;6874;6882;2071;6878;9569;2968;6877;9519;27097;83860;2965;51616;1022;6879;2961	CDK7;ERCC3;GTF2E2;GTF2H1;GTF2H4;GTF2IRD1;TAF11;TAF15;TAF3;TAF4;TAF5;TAF5L;TAF6;TAF7;TAF9B;TBP;TBPL1

hsa05206	MicroRNAs in cancer	0.5457	1.5378	0.00E+00	5.68E-02	http://www.kegg.jp/kegg-bin/show_pathway?hsa05206+2146+672+995+113130+9493+1869+9134+4194+1870+3925+1789+993+7329+1786+1944+6464+1029+10642+898+1871+3190+1788+9759+2261+10298+1001	149	46	2146;672;995;113130;9493;1869;9134;4194;1870;3925;1789;993;7329;1786;1944;6464;1029;10642;898;1871;3190;1788;9759;2261;10298;10018;3065;11186;994;5598;6541;90427;5578;4170;4851;7422;2744;4082;7157;27086;1399;578;7430;2885;6659;8091	BAK1;BCL2L11;BMF;BRCA1;CCNE1;CCNE2;CDC25A;CDC25B;CDC25C;CDCA5;CDKN2A;CRKL;DNMT1;DNMT3A;DNMT3B;E2F1;E2F2;E2F3;EFNA3;EZH2;EZR;FGFR3;FOXP1;GLS;GRB2;HDAC1;HDAC4;H

hsa03450	Non-homologous end-joining	0.7514	1.5019	3.52E-02	8.63E-02	http://www.kegg.jp/kegg-bin/show_pathway?hsa03450+2237+64421+2547+7520+274	12	6	2237;64421;2547;7520;27434;5591	DCLRE1C;FEN1;POLM;PRKDC;XRCC5;XRCC6

hsa04120	Ubiquitin mediated proteolysis	0.5321	1.4944	2.12E-03	8.99E-02	http://www.kegg.jp/kegg-bin/show_pathway?hsa04120+55585+672+991+11065+10401+29945+7329+51434+7324+63893+65264+27338+10054+55120+64750+22954+4591+996+8881+7319+8450+26272+246184+73	133	36	55585;672;991;11065;10401;29945;7329;51434;7324;63893;65264;27338;10054;55120;64750;22954;4591;996;8881;7319;8450;26272;246184;7322;4214;8697;23759;10055;6502;27339;7334;330;9040;7321;8453;7332	ANAPC4;ANAPC7;BIRC3;BRCA1;CDC16;CDC20;CDC23;CDC26;CDC27;CUL2;CUL4B;FANCL;FBXO4;MAP3K1;PIAS3;PPIL2;PRPF19;SAE1;SKP2;SMURF2;TRIM32;TRIM37;UBA2;UBE2A;UBE2C;UBE2D1;U

hsa05166	Human T-cell leukemia virus 1 infection	0.4993	1.4372	0.00E+00	1.66E-01	http://www.kegg.jp/kegg-bin/show_pathway?hsa05166+200186+991+10714+701+9232+4605+9133+2648+1111+1869+5111+1870+6908+5424+5427+29945+4085+7514+1031+51434+54107+9184+1856+1029+6929+	252	37	200186;991;10714;701;9232;4605;9133;2648;1111;1869;5111;1870;6908;5424;5427;29945;4085;7514;1031;51434;54107;9184;1856;1029;6929;10744;4602;1871;5902;1019;5426;5901;1857;996;8881;112;4791	ADCY6;ANAPC4;ANAPC7;BUB1B;BUB3;CCNB2;CDC16;CDC20;CDC27;CDK4;CDKN2A;CDKN2C;CHEK1;CRTC2;DVL2;DVL3;E2F1;E2F2;E2F3;KAT2A;MAD2L1;MYB;MYBL2;NFKB2;PCNA;POLD1;POLD3;POL

hsa04330	Notch signaling pathway	0.5457	1.3839	4.81E-02	2.73E-01	http://www.kegg.jp/kegg-bin/show_pathway?hsa04330+51107+2648+3066+23385+1856+1857+3065+9794+9612+19640	48	12	51107;2648;3066;23385;1856;1857;3065;9794;9612;196403;4851;9253	APH1A;DTX3;DVL2;DVL3;HDAC1;HDAC2;KAT2A;MAML1;NCOR2;NCSTN;NOTCH1;NUMBL

hsa04218	Cellular senescence	0.4798	1.3560	2.70E-02	3.37E-01	http://www.kegg.jp/kegg-bin/show_pathway?hsa04218+286826+983+4605+9133+1111+1869+9134+1870+2305+5810+891+7248+993+5883+890+5603+1017+5933+1029+8	155	22	286826;983;4605;9133;1111;1869;9134;1870;2305;5810;891;7248;993;5883;890;5603;1017;5933;1029;898;1871;1019	CCNA2;CCNB1;CCNB2;CCNE1;CCNE2;CDC25A;CDK1;CDK2;CDK4;CDKN2A;CHEK1;E2F1;E2F2;E2F3;FOXM1;LIN9;MAPK13;MYBL2;RAD1;RAD9A;RBL1;TSC1

hsa04115	p53 signaling pathway	0.5051	1.3396	5.52E-02	3.54E-01	http://www.kegg.jp/kegg-bin/show_pathway?hsa04115+983+51512+9133+1111+9134+4194+891+6241+92344+1017+1029+898+	69	14	983;51512;9133;1111;9134;4194;891;6241;92344;1017;1029;898;1019;8493	CCNB1;CCNB2;CCNE1;CCNE2;CDK1;CDK2;CDK4;CDKN2A;CHEK1;GORAB;GTSE1;MDM4;PPM1D;RRM2

hsa05203	Viral carcinogenesis	0.4633	1.3341	2.06E-02	3.56E-01	http://www.kegg.jp/kegg-bin/show_pathway?hsa05203+7186+983+991+2648+1111+9134+6908+148327+3066+7188+890+1017+5933+1029+898+8370+3190+5902+1019+9759+3065+4791+10014+2968+1108+8367+	197	58	7186;983;991;2648;1111;9134;6908;148327;3066;7188;890;1017;5933;1029;898;8370;3190;5902;1019;9759;3065;4791;10014;2968;1108;8367;9519;6722;6714;3661;2965;8360;79885;1385;245972;8348;5595;6502;7157;121504;578;1388;7534;2885;10488;7533;10971;440689;128312;9261;2961;22938;286436;468;5366;1030;3845;8359	ATF4;ATF6B;ATP6V0D2;BAK1;CCNA2;CCNE1;CCNE2;CDC20;CDK1;CDK2;CDK4;CDKN2A;CDKN2B;CHD4;CHEK1;CREB1;CREB3;CREB3L4;GRB2;GTF2E2;GTF2H1;GTF2H4;H2BFM;HDAC1;HDAC11;HDAC2;

hsa00740	Riboflavin metabolism	0.7270	1.3433	1.31E-01	3.58E-01	http://www.kegg.jp/kegg-bin/show_pathway?hsa00740+803	8	1	80308	FLAD1
hsa00563	Glycosylphosphatidylinositol (GPI)-anchor biosynthesis	0.5876	1.3167	1.06E-01	3.81E-01	http://www.kegg.jp/kegg-bin/show_pathway?hsa00563+5279+93183+94005+80235+54965+5277+128869+8818	25	9	5279;93183;94005;80235;54965;5277;128869;8818;51604	DPM2;PIGA;PIGC;PIGM;PIGS;PIGT;PIGU;PIGX;PIGZ
