# Supplementary material for: Prognostic potential of PRPF3 in hepatocellular carcinoma
Source: Aging (Albany NY). 2020 Jan 11;12(1):912–30. doi: 10.18632/aging.102665 (PMC6977647; doi:10.18632/aging.102665)
Supplement: Supplementary Table 4 [file aging-12-102665-s006..pdf]

Supplementary Table 4. Kinases enrichment of PRPF3 co-expressed genes.

geneSet	description	ES	NES	pValue	FDR	link	size	leadingEdgeNum	leadingEdgeId	userId
Kinase_CDK1	cyclin dependent kinase 1	0.7313	2.1071	0.00E+00	0.00E+00	https://www.ncbi.nlm.nih.gov/gene/?term=CDK1	257	85	5298;9898;5984;51514;2146;11004;64151;7153;2189;3832;672;29128;51203;995;3978;22974;9055;991;54821;286151;332;641;113130;2237;701;55165;1111;9787;699;1869;4194;2305;9700;1058;8317;3930;4001;4288;3607;891;55872;10615;84823;4176;3925;7175;7248;7398;29945;8833;1894;6241;9877;993;7514;3054;7329;7884;4000;1786;5985;5883;6949;3159;9585;11338;7112;7083;29920;10849;10389;5983;2	ANAPC4;BCL2L11;BIRC5;BLM;BRCA1;BUB1;BUB1B;CCNB1;CD3EAP;CDC16;CDC20;CDC25A;CDC25C;CDC27;CDC7;CDCA5;CENPA;CEP55;CHEK1;CKAP2;DLGAP5;DNMT1;DTL;E2F1;ECT2;ERCC6L;ESPL1;E

Kinase_PLK1	polo like kinase 1	0.7706	2.0963	0.00E+00	0.00E+00	https://www.ncbi.nlm.nih.gov/gene/?term=PLK1	91	38	990;3192;11004;29127;7153;6491;672;995;9055;54821;286151;332;51512;701;55165;2305;9700;891;5888;9088;55166;26271;29945;993;63967;51434;23512;1856;121441;5901;996;8881;55200;675;994;3796;7161;55125	ANAPC4;ANAPC7;BIRC5;BRCA1;BRCA2;BUB1B;CCNB1;CDC16;CDC25A;CDC25B;CDC25C;CDC27;CDC6;CENPQ;CEP192;CEP55;CLSPN;DVL2;ERCC6L;ESPL1;FBXO43;FBXO5;FOXM1;GTSE1;HNRNPU;KIF2A;

Kinase_CHEK1	checkpoint kinase 1	0.7257	2.0144	0.00E+00	0.00E+00	https://www.ncbi.nlm.nih.gov/gene/?term=CHEK1	130	49	9129;10432;3833;9656;29127;983;4174;2178;995;3978;641;4172;2237;9212;2177;1111;4194;3838;4288;5888;84811;11011;4085;993;4000;3181;63967;4670;1876;7027;3020;9181;2058;1871;125950;8473;5528;10155;51400;54883;3915;2547;27332;11186;994;9782;7161;51720;64062	ARHGEF2;AURKB;BLM;BUD13;CDC25A;CDC25B;CDC25C;CDK1;CHEK1;CLSPN;CWC25;E2F3;E2F6;EPRS;FANCD2;FANCE;FEN1;H3F3A;HNRNPA2B1;HNRNPM;KIFC1;KPNA2;LAMC1;LIG1;LMNA;MAD2L1;

Kinase_AURKB	aurora kinase B	0.7379	2.0069	0.00E+00	0.00E+00	https://www.ncbi.nlm.nih.gov/gene/?term=AURKB	87	32	27316;10432;11004;24137;29127;79980;10403;51203;55143;5347;332;113130;9493;9212;1058;157313;4288;79801;11056;7913;3020;126961;26586;11198;51535;3151;3619;10014;23468;5935;11186;56288	AURKB;BIRC5;CBX5;CDCA2;CDCA5;CDCA8;CENPA;CKAP2;DDX52;DEK;DSN1;H3F3A;HDAC5;HIST2H3C;HMGN2;INCENP;KIF23;KIF2C;KIF4A;MKI67;NDC80;NUSAP1;PARD3;PLK1;PPHLN1;RACGAP1;RAS

Kinase_CDK2	cyclin dependent kinase 2	0.6879	1.9825	0.00E+00	0.00E+00	https://www.ncbi.nlm.nih.gov/gene/?term=CDK2	278	118	9129;9869;200186;23557;51514;1063;9656;990;2146;4171;672;29128;995;3978;22974;991;641;4172;23397;4605;1111;9787;81620;1869;1870;2305;8317;4288;3607;84823;4176;3925;3609;7175;29945;8833;23421;6241;57695;9877;3187;3054;11073;1786;5883;4173;51434;9775;890;6949;3159;6625;5127;7283;63893;55746;57122;7083;29920;84295;84289;1017;84126;10389;5933;3184;898;64710;6929;6829;3980;	AARS2;ANAPC4;ANAPC7;ANKLE2;ARHGAP19;ATRIP;BARD1;BAZ2A;BLM;BRCA1;BRCA2;C9orf40;CAMSAP1;CCNA2;CCNE1;CDC16;CDC20;CDC23;CDC25C;CDC26;CDC27;CDC6;CDC7;CDK16;CDK2;CDK7;

Kinase_ATR	ATR serine/threonine kinase	0.7255	1.8887	0.00E+00	0.00E+00	https://www.ncbi.nlm.nih.gov/gene/?term=ATR	66	36	9656;4171;55215;672;641;63922;4172;2177;1111;7517;1869;2130;5810;51659;64421;3014;5883;63967;10926;84126;22897;1029;1616;6601;5932;2175;4848;11186;51720;55775;1385;11200;7157;1633;6667;5591	ATRIP;BLM;BRCA1;CDKN2A;CEP164;CHEK1;CHEK2;CHTF18;CLSPN;CNOT2;CREB1;DAXX;DBF4;DCK;DCLRE1C;E2F1;EWSR1;FANCA;FANCD2;FANCI;GINS2;H2AFX;MCM2;MCM3;MDC1;PRKDC;RAD1;RA

Kinase_CHEK2	checkpoint kinase 2	0.8068	1.8495	0.00E+00	1.36E-04	https://www.ncbi.nlm.nih.gov/gene/?term=CHEK2	27	13	7272;672;995;641;1869;4194;2305;993;26574;1871;7515;10197;10155	AATF;BLM;BRCA1;CDC25A;CDC25C;E2F1;E2F3;FOXM1;MDM4;PSME3;TRIM28;TTK

Kinase_ATM	ATM serine/threonine kinase	0.6596	1.8157	0.00E+00	3.58E-04	https://www.ncbi.nlm.nih.gov/gene/?term=ATM	123	58	9156;9656;2146;55215;672;51203;641;4172;2177;1111;699;1869;4194;2130;64421;3014;11073;2521;26574;5883;3159;10926;22897;56254;55159;9696;1616;5528;10155;6601;5932;8445;6597;57621;2547;6117;11186;1108;56852;51720;8570;55775;1385;5496;11200;7157;1633;6667;9126;5591;8091;8030;57646;9815;6118;25;51444;7507	AATF;ABL1;BLM;BRCA1;BUB1;CCDC6;CEP164;CHD4;CHEK1;CHEK2;CREB1;CROCC;DAXX;DBF4;DCK;DCLRE1C;DYRK2;E2F1;EWSR1;EXO1;EZH2;FANCD2;FANCI;FUS;GIT2;H2AFX;HMGA1;HMGA2;KH

Kinase_PRKDC	protein kinase, DNA-activated, catalytic polypeptide	0.7037	1.7689	0.00E+00	2.22E-03	https://www.ncbi.nlm.nih.gov/gene/?term=PRKDC	46	24	3192;7391;64421;3014;2521;835;7515;10155;4791;3207;3651;2547;5971;7520;6722;5451;3661;55775;11200;7157;5591;6118;25;7507	ABL1;CASP2;CHEK2;DCLRE1C;FUS;H2AFX;HNRNPU;HOXA11;IRF3;NFKB2;PDX1;POU2F1;PRKDC;RELB;RPA2;SRF;TDP1;TP53;TRIM28;USF1;XPA;XRCC1;

Kinase_AURKA	aurora kinase A	0.7007	1.7645	0.00E+00	2.29E-03	https://www.ncbi.nlm.nih.gov/gene/?term=AURKA	46	19	55835;4171;672;10403;51203;22974;5347;9787;10460;1058;6790;51053;10445;121441;10018;11186;994;56288;7161	AURKA;BCL2L11;BRCA1;CDC25B;CENPA;CENPJ;DLGAP5;GMNN;MCM2;MCRS1;NDC80;NEDD1;NUSAP1;PARD3;PLK1;RASSF1;TACC3;TP7
Kinase_TTK	TTK protein kinase	0.8839	1.7573	0.00E+00	2.69E-03	https://www.ncbi.nlm.nih.gov/gene/?term=T	12	5	7272;55143;9055;641;701	BLM;BUB1B;CDCA8;PRC1;TTK

Kinase_CSNK2A1	casein kinase 2 alpha 1	0.5727	1.6470	0.00E+00	2.48E-02	https://www.ncbi.nlm.nih.gov/gene/?term=CSNK2A1	255	76	9129;9656;4171;983;64151;7153;672;995;3978;10951;332;23397;4605;5810;5888;3066;7298;405;3054;7884;5883;10921;3183;51053;835;3020;6749;4603;3150;23435;10728;6418;1856;126961;10541;8370;4602;3178;7515;1616;5082;6741;9141;1857;996;2071;6804;3065;5393;114798;994;8367;7020;23;6722;5058;5601;51720;3087;26088;10163;8356;8360;9612;4851;9894;51447;11319;1385;7088;653604;7157;11	ABCF1;ANP32B;ARNT;BIRC5;BRCA1;CASP2;CBX1;CDC25B;CDC25C;CDC27;CDK1;CREB1;DAXX;DVL2;DVL3;ECD;ERCC3;EXOSC9;FAF1;GGA1;GMNN;H3F3A;HCFC1;HDAC1;HDAC2;HHEX;HIST1H3C;HIST1

Kinase_PRKCI	protein kinase C iota	0.7396	1.6489	6.71E-03	2.63E-02	https://www.ncbi.nlm.nih.gov/gene/?term=PRKCI	21	9	4001;84823;1894;4000;56288;1999;2011;1022;7430	CDK7;ECT2;ELF3;EZR;LMNA;LMNB1;LMNB2;MARK2;PA
Kinase_PRKD3	protein kinase D3	0.8476	1.5750	3.12E-03	7.69E-02	https://www.ncbi.nlm.nih.gov/gene/?term=PRKD3	8	5	4001;84823;28964;4000;10014	GIT1;HDAC5;LMNA;LMNB1;LMNB2
Kinase_PLK4	polo like kinase 4	0.9601	1.5685	1.63E-03	7.96E-02	https://www.ncbi.nlm.nih.gov/gene/?term=P	5	3	55835;6491;10733	CENPJ;PLK4;STIL
Kinase_WEE1	WEE1 G2 checkpoint kinase	0.9457	1.5622	1.60E-03	8.23E-02	https://www.ncbi.nlm.nih.gov/gene/?term=W	5	4	983;9133;891;1017	CCNB1;CCNB2;CDK1;CDK2
Kinase_CAMK2B	calcium/calmodulin dependent protein kinase II beta	0.8059	1.5555	1.04E-02	8.56E-02	https://www.ncbi.nlm.nih.gov/gene/?term=CAMK2B	11	3	995;286151;3925	CDC25C;FBXO43;STMN1

Kinase_NEK1	NIMA related kinase 1	0.9680	1.5426	0.00E+00	9.58E-02	https://www.ncbi.nlm.nih.gov/gene/?term=N	4	2	8438;10403	NDC80;RAD54L
Kinase_STK39	serine/threonine kinase 39	-0.8680	-1.8124	0.00E+00	1.13E-01	https://www.ncbi.nlm.nih.gov/gene/?term=S	8	3	6557;8671;6560	SLC12A1;SLC12A4;SLC4A4
Kinase_PKN2	protein kinase N2	0.8498	1.5216	2.65E-02	1.24E-01	https://www.ncbi.nlm.nih.gov/gene/?term=P	7	4	4001;84823;4000;994	CDC25B;LMNA;LMNB1;LMNB2
Kinase_CAMK4	calcium/calmodulin dependent protein kinase IV	0.6947	1.5123	2.37E-02	1.33E-01	https://www.ncbi.nlm.nih.gov/gene/?term=CAMK4	18	8	3925;148327;3191;10014;1385;1388;10488;468	ATF4;ATF6B;CREB1;CREB3;CREB3L4;HDAC5;HNRNPL;
Kinase_NEK2	NIMA related kinase 2	0.8344	1.5034	2.61E-02	1.43E-01	https://www.ncbi.nlm.nih.gov/gene/?term=N	8	5	4751;10403;11190;5501;2175	CEP250;FANCA;NDC80;NEK2;PPP1CC

Kinase_AKT1	AKT serine/threonine kinase 1	0.5276	1.4999	1.04E-03	1.43E-01	https://www.ncbi.nlm.nih.gov/gene/?term=AKT1	175	50	90780;1196;55355;2146;672;8682;1111;4194;83879;3609;7248;11073;4000;1786;1017;22985;3178;80153;2175;10018;5971;2931;10576;3654;3661;4794;4234;4084;9891;5208;7015;8570;4214;1385;4088;6667;79109;7534;4771;6188;5914;1457;23647;4150;2138;9097;51230;2309;7465;3757	ACIN1;ARFIP2;BCL2L11;BRCA1;CCT2;CDCA7;CDK2;CHEK1;CLK2;CREB1;CSNK2A1;DNMT1;EDC3;EYA1;EZH2;FANCA;FOXO3;GSK3A;HJURP;HNRNPA1;ILF3;IRAK1;IRF3;KCNH2;KHSRP;LMNA;MAP3K1;MA

Kinase_PLK3	polo like kinase 3	0.6939	1.4955	2.61E-02	1.44E-01	https://www.ncbi.nlm.nih.gov/gene/?term=P	20	5	7153;995;891;993;7443	CCNB1;CDC25A;CDC25C;TOP2A;VRK1
Kinase_CAMK2D	calcium/calmodulin dependent protein kinase II delta	0.7052	1.4831	3.54E-02	1.50E-01	https://www.ncbi.nlm.nih.gov/gene/?term=CAMK2D	16	7	995;286151;3767;9759;2891;7273;6885	CDC25C;FBXO43;GRIA2;HDAC4;KCNJ11;MAP3K7;TTN
Kinase_BRAF	B-Raf proto-oncogene, serine/threonine kinase	0.7055	1.4859	3.40E-02	1.50E-01	https://www.ncbi.nlm.nih.gov/gene/?term=B	16	4	7272;5603;5601;5595	MAPK13;MAPK3;MAPK9;TTK
Kinase_BTK	Bruton tyrosine kinase	-0.7062	-1.5612	2.74E-02	5.90E-01	https://www.ncbi.nlm.nih.gov/gene/?term=B	9	6	695;2969;10320;3702;5336;660	BMX;BTK;GTF2I;IKZF1;ITK;PLCG2
Kinase_PAK6	p21 (RAC1) activated kinase 6	-0.4529	-1.0728	3.56E-01	7.04E-01	https://www.ncbi.nlm.nih.gov/gene/?term=P	12	3	367;5606;4638	AR;MAP2K3;MYLK

Kinase_DAPK3	death associated protein kinase 3	-0.4296	-1.0647	3.72E-01	7.06E-01	https://www.ncbi.nlm.nih.gov/gene/?term=DAPK3	17	6	10398;4636;103910;10627;2908;94274	MYL12A;MYL12B;MYL5;MYL9;NR3C1;PPP1R14A
Kinase_EIF2AK1	eukaryotic translation initiation factor 2 alpha kinase 1	-0.6845	-1.0938	4.30E-01	7.07E-01	https://www.ncbi.nlm.nih.gov/gene/?term=EIF2AK1	3	3	4792;4793;1965	EIF2S1;NFKBIA;NFKBIB
Kinase_EIF2AK3	eukaryotic translation initiation factor 2 alpha kinase 3	-0.6845	-1.0938	4.30E-01	7.07E-01	https://www.ncbi.nlm.nih.gov/gene/?term=EIF2AK3	3	3	4792;4793;1965	EIF2S1;NFKBIA;NFKBIB
Kinase_MAP3K9	mitogen-activated protein kinase kinase kinase 9	-0.6782	-1.1637	3.56E-01	7.11E-01	https://www.ncbi.nlm.nih.gov/gene/?term=MAP3K9	4	2	6416;5606	MAP2K3;MAP2K4

Kinase_TGFBR1	transforming growth factor beta receptor 1	-0.5075	-1.0766	3.78E-01	7.11E-01	https://www.ncbi.nlm.nih.gov/gene/?term=TGFBR1	8	2	7049;2022	ENG;TGFBR3
Kinase_PIM1	Pim-1 proto-oncogene, serine/threonine kinase	-0.4016	-1.0840	3.52E-01	7.12E-01	https://www.ncbi.nlm.nih.gov/gene/?term=PIM1	19	8	8660;50943;4217;8350;3667;4824;367;9429	ABCG2;AR;FOXP3;HIST1H3A;IRS1;IRS2;MAP3K5;NKX3-1
Kinase_PHKG1	phosphorylase kinase catalytic subunit gamma 1	-0.7283	-1.1698	3.49E-01	7.16E-01	https://www.ncbi.nlm.nih.gov/gene/?term=PHKG1	3	2	5837;5836	PYGL;PYGM
Kinase_PHKG2	phosphorylase kinase catalytic subunit gamma 2	-0.7283	-1.1698	3.49E-01	7.16E-01	https://www.ncbi.nlm.nih.gov/gene/?term=PHKG2	3	2	5837;5836	PYGL;PYGM

Kinase_MYLK	myosin light chain kinase	-0.4935	-1.1043	3.42E-01	7.17E-01	https://www.ncbi.nlm.nih.gov/gene/?term=M	10	4	10398;4636;103910;10627	MYL12A;MYL12B;MYL5;MYL9
Kinase_MYLK3	myosin light chain kinase 3	-0.4935	-1.1043	3.42E-01	7.17E-01	https://www.ncbi.nlm.nih.gov/gene/?term=MYLK3	10	4	10398;4636;103910;10627	MYL12A;MYL12B;MYL5;MYL9
Kinase_MYLK4	myosin light chain kinase family member 4	-0.4935	-1.1043	3.42E-01	7.17E-01	https://www.ncbi.nlm.nih.gov/gene/?term=MYLK4	10	4	10398;4636;103910;10627	MYL12A;MYL12B;MYL5;MYL9
Kinase_MAP3K6	mitogen-activated protein kinase kinase kinase 6	-0.6369	-1.0406	4.91E-01	7.26E-01	https://www.ncbi.nlm.nih.gov/gene/?term=MAP3K6	3	2	9064;5599	MAP3K6;MAPK8
Kinase_TNK2	tyrosine kinase non receptor 2	-0.8633	-1.6082	2.58E-02	7.26E-01	https://www.ncbi.nlm.nih.gov/gene/?term=T	5	2	367;51741	AR;WWOX

Kinase_PAK2	p21 (RAC1) activated kinase 2	-0.4284	-1.1398	3.04E-01	7.28E-01	https://www.ncbi.nlm.nih.gov/gene/?term=P	21	3	5606;4638;800	CALD1;MAP2K3;MYLK
Kinase_PTK2B	protein tyrosine kinase 2 beta	-0.5816	-1.1470	3.29E-01	7.30E-01	https://www.ncbi.nlm.nih.gov/gene/?term=PTK2B	6	3	6622;2185;4846	NOS3;PTK2B;SNCA
Kinase_JAK1	Janus kinase 1	-0.5460	-1.0443	4.46E-01	7.33E-01	https://www.ncbi.nlm.nih.gov/gene/?term=J	6	5	6777;6776;6774;4217;6773	MAP3K5;STAT2;STAT3;STAT5A;STAT5B
Kinase_PTK6	protein tyrosine kinase 6	-0.5535	-1.1741	2.82E-01	7.49E-01	https://www.ncbi.nlm.nih.gov/gene/?term=PTK6	9	6	6777;394;1499;55620;207;9564	AKT1;ARHGAP5;BCAR1;CTNNB1;STAP2;STAT5B
Kinase_TYK2	tyrosine kinase 2	-0.5773	-1.1093	3.56E-01	7.61E-01	https://www.ncbi.nlm.nih.gov/gene/?term=T	6	3	6774;6773;3454	IFNAR1;STAT2;STAT3

Kinase_ADRBK1	G protein-coupled receptor kinase 2	-0.3959	-1.0077	4.51E-01	7.61E-01	https://www.ncbi.nlm.nih.gov/gene/?term=ADRBK1	18	5	6622;10411;6915;154;1129	ADRB2;CHRM2;RAPGEF3;SNCA;TBXA2R
Kinase_TEC	tec protein tyrosine kinase	-0.7579	-1.4495	9.90E-02	7.64E-01	https://www.ncbi.nlm.nih.gov/gene/?term=T	5	4	695;3702;5336;660	BMX;BTK;ITK;PLCG2
Kinase_GRK4	G protein-coupled receptor kinase 4	-0.6397	-1.0138	5.42E-01	7.65E-01	https://www.ncbi.nlm.nih.gov/gene/?term=G	3	2	2868;624	BDKRB2;GRK4
Kinase_YES1	YES proto-oncogene 1, Src family tyrosine kinase	-0.5229	-1.1155	3.27E-01	7.65E-01	https://www.ncbi.nlm.nih.gov/gene/?term=Y	9	2	1808;2534	DPYSL2;FYN
