# Supplementary material for: Prognostic potential of PRPF3 in hepatocellular carcinoma
Source: Aging (Albany NY). 2020 Jan 11;12(1):912–30. doi: 10.18632/aging.102665 (PMC6977647; doi:10.18632/aging.102665)
Supplement: Supplementary Table 5 [file aging-12-102665-s002..pdf]

Supplementary Table 5. miRNA enrichment of PRPF3 co-expressed genes.

geneSet	ES	NES	pValue	FDR	link	size	leadingEdgeNum	leadingEdgeI	userId
GACTGTT,MIR-212,MIR-132	0.5070	1.4324	8.40E-03	4.32E-01	http://www.broadinstitute.org/gsea/msigdb/cards/GACTGTT,MIR-212,MIR-132	148	44	9191;3192;672;10951;8682;93643;9826;1852;8148;4670;58155;3015;8880;57649;5814;6874;90957;134353;4204;8899;112476;4800;126526;85464;10042;5411;66037;5496;26520;143684;376267;79109;10336;6659;5718;8091;3776;196441;3021;84146;3670;2309;5991;51496	ARHGEF11;BOLL;BRCA1;C19orf47;CBX1;CTDSPL2;DEDD;DHX57;DUSP9;FAM76B;FOXO3;FUBP1;H2AFZ;H3F3B;HMGA2;HMGXB4;HNRNPM;HN

AGCGCAG,MIR-191	0.6699	1.3554	1.06E-01	4.56E-01	http://www.broadinstitute.org/gsea/msigdb/cards/AGCGCAG,MIR-191	12	5	6625;55833;8473;149041;3146	HMGB1;OGT;RC3H1;SNRNP70;UBAP2

CCAGGTT,MIR-490	0.5352	1.3970	3.79E-02	4.58E-01	http://www.broadinstitute.org/gsea/msigdb/cards/CCAGGTT,MIR-490	60	23	115703;55692;10521;23119;6602;8473;25909;10197;57532;51400;6877;22976;23598;4440;8091;54620;55687;6664;4869;89792;9462;31;5613	ACACA;AHCTF1;ARHGAP33;DDX17;FBXL19;GAL3ST3;HIC2;HMGA2;LUC7L;MSI1;NPM1;NUFIP2;OGT;PATZ1;PAXIP1;PPME1;PRKX;PSME3;

GAGCTGG,MIR-337	0.4590	1.2849	4.60E-02	4.67E-01	http://www.broadinstitute.org/gsea/msigdb/cards/GAGCTGG,MIR-337	147	23	23126;8703;55154;79915;81609;28964;10919;10963;5326;11273;7005;10445;115426;388228;79089;79012;57655;22838;1108;23;149428;2870;114785	ABCF1;ATAD5;ATXN2L;B4GALT3;BNIPL;CAMKV;CHD4;EHMT2;GIT1;GRAMD1A;GRK6;MBD6;MCRS1;MSTO1;PLAGL2;POGZ;RNF44;SBK1;SNX

ACACTCC,MIR-122A	0.4931	1.2878	1.09E-01	4.70E-01	http://www.broadinstitute.org/gsea/msigdb/cards/ACACTCC,MIR-122A	65	20	3192;28964;199699;90324;8428;7862;6602;4204;145282;3915;9520;2976;93380;26000;114815;132789;8030;10479;587;2581	BCAT2;BRPF1;CCDC6;CCDC97;DAND5;GALC;GIT1;GNPDA2;GTF3C2;HNRNPU;LAMC1;MECP2;MIPOL1;MMGT1;NPEPPS;SLC9A6;SMARCD1;

GTGGTGA,MIR-197	0.5138	1.3578	4.64E-02	4.73E-01	http://www.broadinstitute.org/gsea/msigdb/cards/GTGGTGA,MIR-197	67	21	672;57459;1663;159090;11056;3184;23615;10695;55544;2902;9364;10322;2891;51701;405753;146223;139728;605;1759;79102;30819	BCL7A;BRCA1;CMTM4;CNPY3;DDX11;DDX52;DNM1;DUOXA2;FAM122B;GATAD2B;GRIA2;GRIN1;HNRNPD;KCNIP2;NLK;PNCK;PYY2;RA

GGCAGCT,MIR-22	0.4529	1.2895	2.96E-02	4.78E-01	http://www.broadinstitute.org/gsea/msigdb/cards/GGCAGCT,MIR-22	215	57	607;57645;84950;11011;57695;8936;140459;7716;55920;64708;5326;23360;5814;29789;90957;60682;5977;7301;9807;80153;4204;9759;10655;4800;8625;3915;5935;4582;1677;1045;4185;10620;55227;51571;7266;55167;4084;51195;115019;22874;66037;164633;552900;27086;27347;6667;448834;64319;799;60509;56650;3021;54207;2	ADAM11;AGBL5;ARFIP2;ARID3B;ASB6;BCL9;BOLA2;BOLL;CABP7;CALCR;CDX2;CLDND1;COPS7B;DFFB;DHX57;DMRT2;DNAJC7;DPF2;EDC3;FAM

AACTGAC,MIR-223	0.4955	1.3438	5.84E-02	4.81E-01	http://www.broadinstitute.org/gsea/msigdb/cards/AACTGAC,MIR-223	89	25	24137;284403;5316;27173;3925;58155;5326;1942;5814;80196;6602;7343;7319;401720;6198;54522;5955;481;3087;538;6670;5911;53944;80232;27347	ANKRD16;ATP1B1;ATP7A;CSNK1G1;EFNA1;FIGNL2;HHEX;KIF4A;PKNOX1;PLAGL2;PTBP2;PURB;RAP2A;RCN2;RNF34;RPS6KB1;SLC39A1;S

GGCAGTG,MIR-324-3P	0.5358	1.4338	1.10E-02	4.86E-01	http://www.broadinstitute.org/gsea/msigdb/cards/GGCAGTG,MIR-324-3P	93	18	1196;6421;10401;28964;11113;10963;9783;10642;57649;29789;996;9988;10014;10625;23131;26118;114815;10163	CDC27;CIT;CLK2;DMTF1;GIT1;GPATCH8;HDAC5;IGF2BP1;IVNS1ABP;OLA1;PHF12;PIAS3;RIMS3;SFPQ;SORCS1;STIP1;WA

TCTAGAG,MIR-517	0.5453	1.3619	6.42E-02	4.86E-01	http://www.broadinstitute.org/gsea/msigdb/cards/TCTAGAG,MIR-517	45	15	388695;5316;3609;55082;10481;57332;5935;29081;57181;64319;9441;3670;6616;121536;3219	AEBP2;ARGLU1;CBX8;FBRS;HOXB13;HOXB9;ILF3;ISL1;LYSMD1;MED26;METTL5;PKNOX1;RBM3;SLC39A10

CAGGTCC,MIR-492	0.4899	1.2688	1.22E-01	4.91E-01	http://www.broadinstitute.org/gsea/msigdb/cards/CAGGTCC,MIR-492	59	10	607;55215;63893;9184;5326;6434;5814;81610;9684;51400	BCL9;BUB3;FAM83D;FANCI;LRRC14;PLAGL2;PPME1;PURB;TRA2B;UBE2O
TCCGTCC,MIR-184	0.6557	1.2896	1.71E-01	4.94E-01	http://www.broadinstitute.org/gsea/msigdb/cards/TCCGTCC,MIR-184	11	3	11273;55833;7536	ATXN2L;SF1;UBAP2
ACAACCT,MIR-453	0.5164	1.2748	1.16E-01	4.94E-01	http://www.broadinstitute.org/gsea/msigdb/cards/ACAACCT,MIR-453	39	7	81609;1663;200035;58487;4097;10695;6198	CNPY3;CREBZF;DDX11;MAFG;NUDT17;RPS6KB1;SNX2

GGATCCG,MIR-127	0.6589	1.2708	1.93E-01	4.97E-01	http://www.broadinstitute.org/gsea/msigdb/cards/GGATCCG,MIR-127	10	3	90957;1677;10616	DFFB;DHX57;RBCK1
CCACACA,MIR-147	0.5410	1.3980	4.62E-02	4.98E-01	http://www.broadinstitute.org/gsea/msigdb/cards/CCACACA,MIR-147	57	15	55204;57645;6626;90957;1788;4204;85366;54880;10217;476;23446;5110;26027;6745;827	ACOT11;ATP1A1;BCOR;CAPN6;CTDSPL;DHX57;DNMT3A;GOLPH3L;MECP2;MYLK2;PCMT1;POGK;SLC44A1;SNRPA

TCTGGAC,MIR-198	0.5467	1.4601	1.35E-02	4.99E-01	http://www.broadinstitute.org/gsea/msigdb/cards/TCTGGAC,MIR-198	82	10	23126;51107;3192;25788;89884;26528;3159;3020;84295;57649	APH1A;DAZAP1;H3F3A;HMGA1;HNRNPU;LHX4;PHF12;PHF6;POGZ;RAD

GTGACTT,MIR-224	0.4693	1.3134	3.54E-02	5.03E-01	http://www.broadinstitute.org/gsea/msigdb/cards/GTGACTT,MIR-224	150	29	3192;7756;9493;3925;57695;11113;23163;55432;10915;22794;1944;79893;389677;80742;4097;6434;55964;54676;9589;8899;11176;55740;126526;130574;1810;91461;11321;26118;51571	BAZ2A;C19orf47;CASC3;CIT;DR1;EFNA3;ENAH;FAM49B;GGA3;GGNBP2;GPN1;GTPBP2;HNRNPU;KIF23;LYPD6;MAFG;PKDCC;PRPF4B;PR

GTAAACC,MIR-299-5P	0.5215	1.3084	1.00E-01	5.05E-01	http://www.broadinstitute.org/gsea/msigdb/cards/GTAAACC,MIR-299-5P	47	20	4849;4097;57649;5814;4204;9759;90843;23598;6659;3021;23369;5525;9474;6091;4144;2290;7543;118424;4618;1431	ATG5;CNOT3;CS;FOXG1;H3F3B;HDAC4;MAFG;MAT2A;MECP2;MYF6;PATZ1;PHF12;PPP2R5A;PUM2;PURB;ROBO1;SOX4;TCEAL

GAGACTG,MIR-452	0.4911	1.3327	5.09E-02	5.09E-01	http://www.broadinstitute.org/gsea/msigdb/cards/GAGACTG,MIR-452	91	25	10432;81609;10951;347240;4952;23163;6874;90957;8473;256356;9520;10057;10492;55288;79869;3706;6942;11021;5496;552900;6667;2049;196441;84146;2309	ABCC5;BOLA2;CBX1;CPSF7;DHX57;EPHB3;FOXO3;GGA3;GK5;ITPKA;KIF24;NPEPPS;OCRL;OGT;PPM1G;RAB35;RBM14;RHOT1;SNX27;SP1;SYN

AGCTCCT,MIR-28	0.5041	1.3718	4.55E-02	5.10E-01	http://www.broadinstitute.org/gsea/msigdb/cards/AGCTCCT,MIR-28	83	31	9869;56882;4209;6749;84450;3190;9684;85464;148266;4076;6722;124801;54820;55167;4088;53944;164633;81929;8242;827;9643;147179;340554;2020;6010;9266;4215;63946;8193;23098;54802	CABP7;CAPN6;CAPRIN1;CDC42SE1;CSNK1G1;CYTH2;DMRTC2;DPF1;EN2;HNRNPK;KDM5C;LRRC14;LSM12;MAP3K3;MEF2D;MORF4L2;

ACCAATC,MIR-509	0.5175	1.2899	1.20E-01	5.10E-01	http://www.broadinstitute.org/gsea/msigdb/cards/ACCAATC,MIR-509	44	5	10262;9191;7716;5814;11176	BAZ2A;DEDD;PURB;SF3B4;VEZ

ATCATGA,MIR-433	0.4764	1.3020	5.75E-02	5.13E-01	http://www.broadinstitute.org/gsea/msigdb/cards/ATCATGA,MIR-433	106	28	55835;8703;79573;126626;23381;5883;5326;163589;9589;1871;57472;7072;10298;3915;9662;375133;23531;55167;55119;7322;2186;165055;11329;7273;55749;9859;10236;2885	B4GALT3;BPTF;CCAR1;CCDC138;CENPJ;CEP135;CEP170;CNOT6;E2F3;GABPB2;GRB2;HNRNPR;LAMC1;MMD;MSL2;PAK4;PI4KAP2;PLAGL2;

CACGTTT,MIR-302A	0.5902	1.3629	9.24E-02	5.16E-01	http://www.broadinstitute.org/gsea/msigdb/cards/CACGTTT,MIR-302A	27	11	10642;51377;8899;6877;130074;8453;5015;23592;131034;10933;2139	CPNE4;CUL2;EYA2;FAM168B;IGF2BP1;LEMD3;MORF4L1;OTX2;PRPF4B;TAF5;UCHL5

GAGCCAG,MIR-149	0.5344	1.4968	2.10E-03	5.18E-01	http://www.broadinstitute.org/gsea/msigdb/cards/GAGCCAG,MIR-149	135	34	200186;7203;10951;51747;63925;28964;84923;7023;835;55633;55833;10642;5814;3178;8396;64858;83723;7536;57621;10322;83850;90161;6714;6045;91461;124801;22874;8570;6667;7534;2049;4771;65108;8030	CASP2;CBX1;CCDC6;CCT3;CRTC2;DCLRE1B;EPHB3;ESYT3;FAM104A;FAM57B;GIT1;HNRNPA1;HS6ST2;IGF2BP1;KHSRP;LSM12;LUC7L3;MA

GCACCTT,MIR-18A,MIR-18B	0.4709	1.2914	6.46E-02	5.21E-01	http://www.broadinstitute.org/gsea/msigdb/cards/GCACCTT,MIR-18A,MIR-18B	112	21	1196;26147;10401;3298;23097;203547;65264;4209;235435;11273;5814;195828;64599;10950;55288;10644;9655;90355;10217;55897;26118	ATXN2L;BTG3;C5orf30;CDK19;CLK2;CTDSPL;GIGYF1;HSF2;IGF2BP2;MEF2D;MESP1;PHF19;PIAS3;PURB;RHOT1;SOCS5;TARDBP;UBE2Z;V

GGGCATT,MIR-365	-0.2406	-0.8884	7.81E-01	8.81E-01	http://www.broadinstitute.org/gsea/msigdb/cards/GGGCATT,MIR-365	104	24	9901;1837;54762;23314;3920;84937;64399;8539;64755;133;2101;23678;8997;81876;5729;2113;55327;58476;649;11069;4306;54861;4638;220441	ADM;API5;BMP1;C16orf58;DTNA;ESRRA;ETS1;GRAMD1C;HHIP;KALRN;LAMP2;LIN7C;MYLK;NR3C2;PTGDR;RAB1B;RAPGEF4;RNF152;SATB

GTGCCAA,MIR-96	-0.2235	-0.9011	8.33E-01	9.25E-01	http://www.broadinstitute.org/gsea/msigdb/cards/GTGCCAA,MIR-96	284	52	3667;9788;8139;55089;26018;83478;8013;7326;2475;55553;1983;6487;84272;23242;59277;7092;57631;775;23136;84333;30011;6505;55752;9223;6907;3709;9194;10613;64651;25987;5733;284612;80315;2308;11069;114757;23365;9695;5606;55108;7095;18;2823;7982;6506;284439;25924;9079;9467;220441;2534;7009	ABAT;ARHGAP24;ARHGEF12;BSDC1;CACNA1C;COBL;CPEB4;CSRNP1;CYGB;EDEM1;EIF5;EPB41L3;ERLIN1;FOXO1;FYN;GAN;GPM6A;IRS1;I

GTAAGAT,MIR-200A	-0.2787	-0.9130	6.23E-01	9.78E-01	http://www.broadinstitute.org/gsea/msigdb/cards/GTAAGAT,MIR-200A	45	12	8139;7337;4774;3164;10777;84333;23767;687;10135;6672;23516;10395	ARPP21;DLC1;FLRT3;GAN;KLF9;NAMPT;NFIA;NR4A1;PCGF5;SLC39A14;SP100;UBE
TCGATGG,MIR-213	-0.3129	-0.5831	9.21E-01	9.82E-01	http://www.broadinstitute.org/gsea/msigdb/cards/TCGATGG,MIR-213	5	2	23193;23314	GANAB;SATB2

GTGTCAA,MIR-514	-0.3759	-1.2815	5.97E-02	1.00E+00	http://www.broadinstitute.org/gsea/msigdb/cards/GTGTCAA,MIR-514	58	13	166336;23321;81558;8322;367;730;6886;5095;27125;5728;4430;10867;4947	AFF4;AR;C7;FAM117A;FZD4;MYO1B;OAZ2;PCCA;PRICKLE2;PTEN;TAL1;TRIM2;TSP
TAGGTCA,MIR-192,MIR-215	-0.3439	-1.0834	2.82E-01	1.00E+00	http://www.broadinstitute.org/gsea/msigdb/cards/TAGGTCA,MIR-192,MIR-215	42	9	5862;8204;9839;158763;5925;10904;9208;113251;1629	ARHGAP36;BLCAP;DBT;LARP4;LRRFIP1;NRIP1;RAB2A;RB1;ZEB

CGGTGTG,MIR-220	-0.6301	-1.1586	3.37E-01	1.00E+00	http://www.broadinstitute.org/gsea/msigdb/cards/CGGTGTG,MIR-220	5	3	7068;57731;2876	GPX1;SPTBN4;THRB
CTACTAG,MIR-325	-0.4359	-1.0778	3.42E-01	1.00E+00	http://www.broadinstitute.org/gsea/msigdb/cards/CTACTAG,MIR-325	16	5	91624;1452;570;407738;1398	BAAT;CRK;CSNK1A1;FAM19A1;NE

GTATTAT,MIR-369-3P	-0.2392	-0.9800	5.00E-01	1.00E+00	http://www.broadinstitute.org/gsea/msigdb/cards/GTATTAT,MIR-369-3P	193	44	22982;56137;56135;5142;1051;11342;10138;56136;6529;25932;830;1756;56139;6487;395;253943;23568;7424;9839;51363;25777;7555;5144;6815;9552;23767;23090;114885;7325;23414;26994;23300;96459;2308;2675;27115;10150;7323;23621;54363;1073;2534;4035;267	AMFR;ARHGAP6;ARL2BP;ATMIN;BACE1;CAPZA2;CEBPB;CFL2;CHST15;CLIC4;CNBP;DIP2C;DMD;FLRT3;FNIP1;FOXO1;FYN;GFRA2;HAO1;LR

GCTCTTG,MIR-335	-0.2640	-0.9386	5.94E-01	1.00E+00	http://www.broadinstitute.org/gsea/msigdb/cards/GCTCTTG,MIR-335	77	19	10269;51232;1264;11059;8460;1176;2162;84656;8013;7326;9728;6444;5463;29801;83641;123879;7328;55108;9467	AP3S1;BSDC1;CNN1;CRIM1;DCUN1D3;F13A1;FAM107B;GLYR1;NR4A3;POU6F1;SECISBP2L;SGCD;SH3BP5;TPST1;UBE2G1;UBE2H;WW

CTTTGCA,MIR-527	-0.2327	-0.9451	6.92E-01	1.00E+00	http://www.broadinstitute.org/gsea/msigdb/cards/CTTTGCA,MIR-527	222	45	23443;8539;83452;151;23114;223082;5906;9021;84248;5587;1960;6299;4208;2185;9839;118429;23034;122830;55752;11278;113201;83716;3419;3778;23414;6595;8322;10294;80315;22862;4967;202018;27115;10150;11102;80205;4306;26959;164;23169;5728;1647;388;80004;800	ADRA2B;ANTXR2;AP1G1;API5;CALD1;CASC4;CHD9;CPEB4;CRISPLD2;DNAJA2;EGR3;ESRP2;FNDC3A;FYTTD1;FZD4;GADD45A;HBP1;IDH3A

GCTTGAA,MIR-498	-0.2532	-0.9207	7.14E-01	1.00E+00	http://www.broadinstitute.org/gsea/msigdb/cards/GCTTGAA,MIR-498	103	27	1837;3751;5870;5142;2697;5066;11252;830;9021;1497;84248;144402;54921;3660;10979;9839;5295;11278;51727;5530;64756;26994;10972;26959;54861;114299;54941	ATPAF1;CAPZA2;CHTF8;CMPK1;CPNE8;CTNS;DTNA;FERMT2;FYTTD1;GJA1;HBP1;IRF2;KCND2;KLF12;PACSIN2;PALM2;PAM;PDE4B;PIK3R1

ACTTTAT,MIR-142-5P	-0.2229	-0.9509	7.27E-01	1.00E+00	http://www.broadinstitute.org/gsea/msigdb/cards/ACTTTAT,MIR-142-5P	270	40	2353;9690;338645;1960;3084;2926;80176;54511;337876;2643;79589;7538;5775;60592;1182;4289;23414;53339;80315;22862;548645;196;83641;80205;6386;3479;122525;164;27125;10580;9510;407738;2034;8671;79038;1398;7048;55664;22849;9709	ADAMTS1;AFF4;AHR;AP1G1;BTBD1;C14orf28;CDC37L1;CHD9;CHSY3;CLCN3;CPEB3;CPEB4;CRK;DNAJC25;EGR3;EPAS1;FAM107B;FAM19A

AACGGTT,MIR-451	-0.3290	-0.7396	7.94E-01	1.00E+00	http://www.broadinstitute.org/gsea/msigdb/cards/AACGGTT,MIR-451	10	1	64400	AKTIP
TTCCGTT,MIR-191	-0.2308	-0.6655	9.72E-01	1.00E+00	http://www.broadinstitute.org/gsea/msigdb/cards/TTCCGTT,MIR-191	29	2	164;219654	AP1G1;ZCCHC24
