# Supplementary material for: Prognostic potential of PRPF3 in hepatocellular carcinoma
Source: Aging (Albany NY). 2020 Jan 11;12(1):912–30. doi: 10.18632/aging.102665 (PMC6977647; doi:10.18632/aging.102665)
Supplement: Supplementary Table 6 [file aging-12-102665-s005..pdf]

Supplementary Table 6. Transcription factor enrichment of PRPF3 co-expressed genes.

geneSet	ES	NES	pValue	FDR	link	size	leadingEdgeNum	leadingEdgeId	userId
V$E2F_Q6	0.74	2.16	0.00E+00	0.00E+00	http://www.broadinstitute.org/gsea/msigdb/cards/V$E2F_Q6	211	82	57592;51107;990;2146;4171;8318;4175;983;84320;79915;284403;4172;10714;5422;2177;4436;81620;1869;5111;5424;25896;51747;8726;5888;23649;4678;9088;4176;3925;79733;26271;83879;5427;9824;3609;144455;6241;163786;23636;57695;993;6632;124222;57657;11073;7023;1786;23234;56957;84515;4173;51053;63967;29028;22823;7112;83463;10521;10849;3015;5933;3184;6434;6646;3837;195828;9994;1871;7301;3178;11100;147929;3151;64785;125950;8899;5902;57532;	ACBD6;APH1A;ARHGAP11A;ATAD2;ATAD5;CASP8AP2;CD3EAP;CDC25A;CDC45;CDC6;CDCA7;CDK1;CDT1;CLSPN;DDX17;DNAJC9;DNMT1;E2F1;E2F3;E2F7;E2F8;EED;EZH2;FANCD2;FBXO5;GINS3;GMNN;H2AFZ;HCN3;HMGN2;HNRNPA1;HNRNPD;HNRNPUL1;ILF3;INTS7;KPNB1;LUC7L3;MCM2;MCM3;MCM4;MCM6;MCM7;MCM8;MSH2;MTF2;MXD3;NASP;NUFIP2;NUP62

V$E2F_Q4	0.74	2.14	0.00E+00	0.00E+00	http://www.broadinstitute.org/gsea/msigdb/cards/V$E2F_Q4	212	81	57592;51107;990;2146;4171;8318;4175;983;84320;79915;284403;4172;10714;5422;2177;4436;81620;1869;5111;5424;25896;51747;8726;5888;23649;4678;9088;4176;3925;79733;26271;83879;5427;9824;3609;144455;6241;163786;23636;57695;993;6632;124222;57657;11073;7023;1786;23234;56957;84515;4173;51053;63967;29028;22823;7112;83463;10521;10849;3015;5933;3184;6434;6646;3837;195828;9994;1871;7301;3178;11100;147929;3151;64785;125950;5902;57532;8484	ACBD6;APH1A;ARHGAP11A;ATAD2;ATAD5;CASP8AP2;CD3EAP;CDC25A;CDC45;CDC6;CDCA7;CDK1;CDT1;CLSPN;DDX17;DNAJC9;DNMT1;E2F1;E2F3;E2F7;E2F8;EED;EZH2;FANCD2;FBXO5;GINS3;GMNN;H2AFZ;HCN3;HMGN2;HNRNPA1;HNRNPD;HNRNPUL1;ILF3;INTS7;KPNB1;LUC7L3;MCM2;MCM3;MCM4;MCM6;MCM7;MCM8;MSH2;MTF2;MXD3;NASP;NUFIP2;NUP62

V$E2F1_Q6	0.73	2.12	0.00E+00	0.00E+00	http://www.broadinstitute.org/gsea/msigdb/cards/V$E2F1_Q6	213	85	57592;51107;990;2146;4171;4175;983;84320;79915;2189;284403;4172;10714;5422;2177;4436;81620;1869;5111;5424;51747;84950;8726;4678;9088;4176;3925;79733;26271;83879;5427;9824;3609;144455;6241;163786;23636;57695;993;6632;124222;57657;11073;7023;4439;3181;1786;23234;56957;84515;4173;51053;63967;11335;3159;29028;22823;7112;83463;10849;3015;5933;3184;6434;6646;3837;195828;9994;1871;7301;11100;147929;64785;125950;8899;5902;57532;84844;	ACBD6;APH1A;ARHGAP11A;ATAD2;ATAD5;CASP8AP2;CBX3;CD3EAP;CDC25A;CDC6;CDCA7;CDK1;CDT1;CLSPN;CTNND2;DNAJC9;DNMT1;E2F1;E2F3;E2F7;E2F8;EED;EZH2;FANCD2;FANCG;FBXO5;GINS3;GMNN;H2AFZ;HCN3;HMGA1;HMGXB4;HNRNPA2B1;HNRNPD;HNRNPUL1;ILF3;KLF5;KPNB1;LUC7L3;MCM2;MCM3;MCM4;MCM6;MCM7;MCM8;MSH2;MSH5;MTF2;MX

V$E2F1DP1RB_01	0.73	2.12	0.00E+00	0.00E+00	http://www.broadinstitute.org/gsea/msigdb/cards/V$E2F1DP1RB_01	210	75	57592;51107;990;2146;4171;4175;983;84320;79915;284403;3978;4172;10714;5422;4436;81620;1869;5111;5424;25896;51747;8726;5888;23649;4678;9088;4176;3925;79733;26271;83879;5427;9824;3609;144455;6241;163786;23636;57695;993;6632;124222;57657;11073;7023;1786;23234;56957;84515;51053;63967;29028;22823;7112;83463;10849;3015;3184;6646;9016;3837;195828;9994;7301;3178;11100;147929;3151;64785;125950;57532;5931;51605;8493;5501	ACBD6;APH1A;ARHGAP11A;ATAD2;ATAD5;CASP8AP2;CD3EAP;CDC25A;CDC6;CDCA7;CDK1;CDT1;CLSPN;DNAJC9;DNMT1;E2F1;E2F7;E2F8;EED;EZH2;FBXO5;GINS3;GMNN;H2AFZ;HCN3;HMGN2;HNRNPA1;HNRNPD;HNRNPUL1;ILF3;INTS7;KPNB1;LIG1;LUC7L3;MCM2;MCM3;MCM6;MCM7;MCM8;MSH2;MTF2;MXD3;NASP;NUFIP2;NUP62;OTUD7B;PAQR4;PCNA;PKM

V$E2F4DP1_01	0.73	2.11	0.00E+00	0.00E+00	http://www.broadinstitute.org/gsea/msigdb/cards/V$E2F4DP1_01	220	80	57592;51107;990;2146;4171;4175;983;84320;79915;2189;284403;3978;4172;10714;5422;2177;4436;1869;5111;5424;51747;8726;4678;9088;4176;3925;79733;26271;83879;5427;9824;3609;144455;6241;163786;23636;57695;993;6632;124222;57657;11073;7023;4439;3181;1786;23234;56957;84515;4173;51053;63967;11335;3159;29028;22823;7112;83463;10849;3015;5933;3184;6434;6646;9016;195828;9994;1871;7301;11100;147929;64785;125950;8899;5902;57532;84844;5931;	ACBD6;APH1A;ARHGAP11A;ATAD2;ATAD5;CASP8AP2;CBX3;CD3EAP;CDC25A;CDC6;CDCA7;CDK1;CLSPN;DNAJC9;DNMT1;E2F1;E2F3;E2F7;E2F8;EED;EZH2;FANCD2;FANCG;FBXO5;GINS3;GMNN;H2AFZ;HCN3;HMGA1;HNRNPA2B1;HNRNPD;HNRNPUL1;ILF3;LIG1;LUC7L3;MCM2;MCM3;MCM4;MCM6;MCM7;MCM8;MSH2;MSH5;MTF2;MXD3;NASP;NUFIP2;NUP62;OTU

V$E2F1DP1_01	0.73	2.11	0.00E+00	0.00E+00	http://www.broadinstitute.org/gsea/msigdb/cards/V$E2F1DP1_01	218	80	57592;51107;990;2146;4171;4175;983;84320;79915;2189;284403;4172;10714;5422;2177;4436;1869;5111;5424;51747;8726;4678;9088;4176;3925;79733;26271;83879;5427;9824;3609;144455;6241;163786;23636;57695;993;6632;124222;57657;11073;7023;4439;3181;1786;23234;56957;84515;4173;51053;63967;11335;3159;29028;22823;7112;83463;10849;3015;5933;3184;6434;6646;195828;9994;65056;7862;1871;7301;11100;5757;147929;64785;125950;8899;5902;57532;84844;	ACBD6;APH1A;ARHGAP11A;ATAD2;ATAD5;BRPF1;CASP8AP2;CBX3;CD3EAP;CDC25A;CDC6;CDCA7;CDK1;CLSPN;DNAJC9;DNMT1;E2F1;E2F3;E2F7;E2F8;EED;EZH2;FANCD2;FANCG;FBXO5;GINS3;GMNN;GPBP1;H2AFZ;HCN3;HMGA1;HNRNPA2B1;HNRNPD;HNRNPUL1;ILF3;LUC7L3;MCM2;MCM3;MCM4;MCM6;MCM7;MCM8;MSH2;MSH5;MTF2;MXD3;NASP;NUFIP2;NUP

V$E2F1DP2_01	0.73	2.11	0.00E+00	0.00E+00	http://www.broadinstitute.org/gsea/msigdb/cards/V$E2F1DP2_01	218	80	57592;51107;990;2146;4171;4175;983;84320;79915;2189;284403;4172;10714;5422;2177;4436;1869;5111;5424;51747;8726;4678;9088;4176;3925;79733;26271;83879;5427;9824;3609;144455;6241;163786;23636;57695;993;6632;124222;57657;11073;7023;4439;3181;1786;23234;56957;84515;4173;51053;63967;11335;3159;29028;22823;7112;83463;10849;3015;5933;3184;6434;6646;195828;9994;65056;7862;1871;7301;11100;5757;147929;64785;125950;8899;5902;57532;84844;	ACBD6;APH1A;ARHGAP11A;ATAD2;ATAD5;BRPF1;CASP8AP2;CBX3;CD3EAP;CDC25A;CDC6;CDCA7;CDK1;CLSPN;DNAJC9;DNMT1;E2F1;E2F3;E2F7;E2F8;EED;EZH2;FANCD2;FANCG;FBXO5;GINS3;GMNN;GPBP1;H2AFZ;HCN3;HMGA1;HNRNPA2B1;HNRNPD;HNRNPUL1;ILF3;LUC7L3;MCM2;MCM3;MCM4;MCM6;MCM7;MCM8;MSH2;MSH5;MTF2;MXD3;NASP;NUFIP2;NUP

V$E2F4DP2_01	0.73	2.11	0.00E+00	0.00E+00	http://www.broadinstitute.org/gsea/msigdb/cards/V$E2F4DP2_01	218	80	57592;51107;990;2146;4171;4175;983;84320;79915;2189;284403;4172;10714;5422;2177;4436;1869;5111;5424;51747;8726;4678;9088;4176;3925;79733;26271;83879;5427;9824;3609;144455;6241;163786;23636;57695;993;6632;124222;57657;11073;7023;4439;3181;1786;23234;56957;84515;4173;51053;63967;11335;3159;29028;22823;7112;83463;10849;3015;5933;3184;6434;6646;195828;9994;65056;7862;1871;7301;11100;5757;147929;64785;125950;8899;5902;57532;84844;	ACBD6;APH1A;ARHGAP11A;ATAD2;ATAD5;BRPF1;CASP8AP2;CBX3;CD3EAP;CDC25A;CDC6;CDCA7;CDK1;CLSPN;DNAJC9;DNMT1;E2F1;E2F3;E2F7;E2F8;EED;EZH2;FANCD2;FANCG;FBXO5;GINS3;GMNN;GPBP1;H2AFZ;HCN3;HMGA1;HNRNPA2B1;HNRNPD;HNRNPUL1;ILF3;LUC7L3;MCM2;MCM3;MCM4;MCM6;MCM7;MCM8;MSH2;MSH5;MTF2;MXD3;NASP;NUFIP2;NUP

V$E2F_02	0.73	2.10	0.00E+00	0.00E+00	http://www.broadinstitute.org/gsea/msigdb/cards/V$E2F_02	218	80	57592;51107;990;2146;4171;4175;983;84320;79915;2189;284403;4172;10714;5422;2177;4436;1869;5111;5424;51747;8726;4678;9088;4176;3925;79733;26271;83879;5427;9824;3609;144455;6241;163786;23636;57695;993;6632;124222;57657;11073;7023;4439;3181;1786;23234;56957;84515;4173;51053;63967;11335;3159;29028;22823;7112;83463;10849;3015;5933;3184;6434;6646;195828;9994;65056;7862;1871;7301;11100;5757;147929;64785;125950;8899;5902;57532;84844;	ACBD6;APH1A;ARHGAP11A;ATAD2;ATAD5;BRPF1;CASP8AP2;CBX3;CD3EAP;CDC25A;CDC6;CDCA7;CDK1;CLSPN;DNAJC9;DNMT1;E2F1;E2F3;E2F7;E2F8;EED;EZH2;FANCD2;FANCG;FBXO5;GINS3;GMNN;GPBP1;H2AFZ;HCN3;HMGA1;HNRNPA2B1;HNRNPD;HNRNPUL1;ILF3;LUC7L3;MCM2;MCM3;MCM4;MCM6;MCM7;MCM8;MSH2;MSH5;MTF2;MXD3;NASP;NUFIP2;NUP

V$E2F1_Q3	0.72	2.08	0.00E+00	0.00E+00	http://www.broadinstitute.org/gsea/msigdb/cards/V$E2F1_Q3	225	78	57592;51107;990;2146;4171;4175;983;84320;79915;284403;4172;10714;5422;4436;1869;5111;5424;51747;84950;56992;8726;54962;4678;9088;4176;3925;79733;26271;83879;5427;9824;3609;144455;7398;6241;163786;23636;57695;10733;993;6632;124222;57657;11073;7023;1786;23234;56957;84515;51053;63967;29028;22823;7112;4849;83463;10849;3015;5933;3184;6646;57649;195828;9994;1871;6950;7301;11100;147929;3151;64785;125950;91107;57532;29074;147912;51605	ACBD6;APH1A;ARHGAP11A;ATAD2;ATAD5;CASP8AP2;CD3EAP;CDC25A;CDC6;CDCA7;CDK1;CLSPN;CNOT3;DNAJC9;DNMT1;E2F1;E2F3;E2F7;E2F8;EED;EZH2;FBXO5;GINS3;GMNN;H2AFZ;HCN3;HMGN2;HNRNPD;HNRNPUL1;ILF3;KIF15;LUC7L3;MCM2;MCM3;MCM6;MCM7;MCM8;MRPL18;MSH2;MTF2;MXD3;NASP;NUFIP2;NUP62;OTUD7B;PAQR4;PCNA;PHF12;PKMYT

SGCGSSAAA_V$E2F1DP2_01	0.72	2.06	0.00E+00	0.00E+00	http://www.broadinstitute.org/gsea/msigdb/cards/SGCGSSAAA_V$E2F1DP2_01	155	60	57592;990;4171;4175;84320;79915;2189;284403;4172;10714;5422;4436;1869;5111;5424;51747;8726;4678;9088;4176;79733;26271;83879;5427;3609;6241;23636;57695;993;6632;124222;57657;11073;7023;4439;1786;23234;4173;51053;63967;3159;29028;22823;83463;3015;3184;6434;195828;9994;1871;7301;11100;5757;147929;64785;125950;8899;5902;84844;8493	ACBD6;ATAD2;ATAD5;CASP8AP2;CDC25A;CDC6;CDCA7;CLSPN;DNAJC9;DNMT1;E2F1;E2F3;E2F8;EED;FANCG;FBXO5;GINS3;GMNN;H2AFZ;HCN3;HMGA1;HNRNPD;HNRNPUL1;ILF3;LUC7L3;MCM2;MCM3;MCM4;MCM6;MCM7;MSH2;MSH5;MTF2;MXD3;NASP;NUP62;PAQR4;PCNA;PHF5A;PKMYT1;POLA1;POLD1;POLD3;POLE2;PPM1D;PRPF4B;PTMA;RANBP1;RAVER1;RR

V$E2F1_Q6_01	0.69	2.00	0.00E+00	0.00E+00	http://www.broadinstitute.org/gsea/msigdb/cards/V$E2F1_Q6_01	218	79	2146;4171;8318;4175;84320;79915;2189;4172;10714;5422;4436;1869;5111;9700;25896;84950;8726;5888;23649;4678;9088;4176;3925;79733;26271;83879;5427;9824;3609;163786;57695;993;124222;57657;11073;7023;1786;23234;56957;84515;4173;51053;63967;835;29028;22823;7112;4849;83463;10521;10849;5933;3184;195828;9994;8847;1871;7301;3178;11100;5757;3151;125950;5902;57532;84844;51605;8493;4686;1501;688;23468;27037;10492;1108;6839;7374;5451;79869	ACBD6;ARHGAP11A;ATAD2;ATAD5;CASP2;CASP8AP2;CBX5;CD3EAP;CDC25A;CDC45;CDCA7;CHD4;CLSPN;CNOT3;CPSF7;CTNND2;DDX17;DLEU2;DNAJC9;DNMT1;E2F1;E2F3;E2F8;EED;ESPL1;EZH2;FANCG;FBXO5;GMNN;HCN3;HMGN2;HNRNPA1;HNRNPD;HNRNPUL1;ILF3;INTS7;KLF5;MCM2;MCM3;MCM4;MCM6;MCM7;MCM8;MSH2;MTF2;MXD3;NASP;NCBP1;NUFIP2

V$E2F_Q3	0.69	2.00	0.00E+00	0.00E+00	http://www.broadinstitute.org/gsea/msigdb/cards/V$E2F_Q3	204	72	990;2146;4171;8318;4175;983;3978;4172;5422;1869;5111;25896;84950;56992;5888;23649;54962;4678;9088;4176;3925;79733;26271;83879;5427;9824;144455;7398;163786;10733;993;124222;11073;1786;56957;84515;4173;51053;29028;4849;83463;10521;3015;3184;9016;57649;3837;195828;9994;1871;6950;3178;147929;127002;3151;5902;91107;51400;29074;5931;147912;51605;5501;1501;688;58495;23468;1211;27037;27043;3927;124925	ARHGAP11A;ATAD2;ATXN7L2;CASP8AP2;CBX5;CDC25A;CDC45;CDC6;CDCA7;CDK1;CLTA;CNOT3;CTNND2;DDX17;DNMT1;E2F1;E2F3;E2F7;E2F8;EZH2;FBXO5;GMNN;H2AFZ;HMGN2;HNRNPA1;HNRNPD;INTS7;KIF15;KLF5;KPNB1;LASP1;LIG1;MCM2;MCM3;MCM4;MCM6;MCM7;MCM8;MRPL18;MXD3;NASP;OTUD7B;OVOL2;PAQR4;PCNA;PELP1;PHF12;PKMYT1;PLK4;

V$E2F_Q4_01	0.69	1.99	0.00E+00	0.00E+00	http://www.broadinstitute.org/gsea/msigdb/cards/V$E2F_Q4_01	215	69	81611;57592;990;2146;9735;4171;8318;4175;84320;9833;2189;284403;4172;5422;1869;5111;5557;25896;84950;5888;23649;54962;4678;9088;4176;3925;79733;26271;83879;5427;9824;144455;163786;10733;993;124222;11073;7884;1786;4173;51053;835;3159;29028;22823;83463;10592;10521;3015;3184;3837;195828;9994;1871;3178;5757;147929;3151;64785;5902;1616;8493;283248;1501;688;58495;23468;2118;27037	ACBD6;ANP32E;ARHGAP11A;ATAD2;CASP2;CASP8AP2;CBX5;CDC25A;CDC45;CDC6;CDCA7;CTNND2;DAXX;DDX17;DNMT1;E2F1;E2F3;E2F7;E2F8;ETV4;EZH2;FANCG;FBXO5;GINS3;GMNN;H2AFZ;HMGA1;HMGN2;HNRNPA1;HNRNPD;INTS7;KLF5;KNTC1;KPNB1;MCM2;MCM3;MCM4;MCM6;MCM7;MELK;MTF2;MXD3;NASP;OVOL2;PAQR4;PCNA;PKMYT1;PLK4;POLA1;

V$E2F_Q6_01	0.68	1.98	0.00E+00	0.00E+00	http://www.broadinstitute.org/gsea/msigdb/cards/V$E2F_Q6_01	219	72	81611;57592;990;2146;4171;8318;4175;84320;9833;2189;3978;4172;5422;1869;5111;5557;5424;25896;84950;5888;54962;4678;9088;4176;3925;79733;26271;83879;5427;9824;144455;6241;163786;10733;993;124222;11073;1786;4173;51053;835;29028;22823;83463;10592;10521;3015;5933;22985;3184;55120;3837;195828;9994;8847;1871;3178;5757;147929;3151;5902;1616;57510;8493;283248;1501;688;58495;23468;2118;54880;27037	ACBD6;ACIN1;ANP32E;ARHGAP11A;ATAD2;BCOR;CASP2;CASP8AP2;CBX5;CDC25A;CDC45;CDC6;CDCA7;CTNND2;DAXX;DDX17;DLEU2;DNMT1;E2F1;E2F3;E2F7;E2F8;ETV4;EZH2;FANCG;FANCL;FBXO5;GMNN;H2AFZ;HMGN2;HNRNPA1;HNRNPD;INTS7;KLF5;KPNB1;LIG1;MCM2;MCM3;MCM4;MCM6;MCM7;MELK;MTF2;MXD3;NASP;OVOL2;PAQR4;PCNA;PKMYT1;PL

V$E2F_03	0.68	1.96	0.00E+00	0.00E+00	http://www.broadinstitute.org/gsea/msigdb/cards/V$E2F_03	224	68	81611;57592;990;2146;9735;4171;8318;4175;9833;2189;3978;4172;5422;126626;1869;5557;5424;25896;84950;5888;23649;54962;4678;9088;4176;3925;79733;26271;83879;144455;163786;10733;993;124222;11073;2521;7884;1786;23234;4173;51053;11144;29028;22823;83463;10592;10521;3015;5933;3184;195828;9994;10949;1871;6950;3178;5757;147929;3151;5902;1616;112476;29074;8493;283248;688;58495;23468	ANP32E;ATAD2;CASP8AP2;CBX5;CDC25A;CDC45;CDC6;CDCA7;DAXX;DDX17;DMC1;DNAJC9;DNMT1;E2F1;E2F3;E2F7;E2F8;EZH2;FANCG;FBXO5;FUS;GABPB2;GMNN;H2AFZ;HMGN2;HNRNPA0;HNRNPA1;HNRNPD;INTS7;KLF5;KNTC1;LIG1;MCM2;MCM3;MCM4;MCM6;MCM7;MELK;MRPL18;MTF2;MXD3;NASP;OVOL2;PAQR4;PKMYT1;PLK4;POLA1;POLA2;POLD1;PPM1

V$E2F_Q3_01	0.67	1.93	0.00E+00	0.00E+00	http://www.broadinstitute.org/gsea/msigdb/cards/V$E2F_Q3_01	215	69	57592;990;2146;4171;8318;4175;983;84320;9833;2189;284403;4172;5422;1869;5111;25896;51747;5888;23649;54962;4678;9088;4176;3925;79733;26271;83879;5427;9824;144455;7398;163786;993;124222;11073;1786;56957;84515;4173;51053;835;3159;29028;9631;22823;83463;10592;10521;3015;3184;3837;195828;9994;1871;3178;5757;147929;3151;64785;5902;1616;91107;51605;1501;688;58495;23468;27037;27043	ACBD6;ARHGAP11A;ATAD2;CASP2;CASP8AP2;CBX5;CDC25A;CDC45;CDC6;CDCA7;CDK1;CTNND2;DAXX;DDX17;DNMT1;E2F1;E2F3;E2F7;E2F8;EZH2;FANCG;FBXO5;GINS3;GMNN;H2AFZ;HMGA1;HMGN2;HNRNPA1;HNRNPD;INTS7;KLF5;KPNB1;LUC7L3;MCM2;MCM3;MCM4;MCM6;MCM7;MCM8;MELK;MTF2;MXD3;NASP;NUP155;OTUD7B;OVOL2;PAQR4;PCNA;PELP1;

V$E2F1_Q4_01	0.66	1.92	0.00E+00	0.00E+00	http://www.broadinstitute.org/gsea/msigdb/cards/V$E2F1_Q4_01	210	64	57592;990;2146;4171;8318;4175;983;84320;9833;2189;284403;4172;5422;1869;5111;25896;51747;5888;23649;4678;9088;4176;3925;79733;26271;83879;5427;9824;7398;163786;993;124222;11073;1786;56957;84515;4173;51053;835;3159;29028;9631;22823;83463;10592;10521;3015;3184;3837;195828;9994;1871;3178;5757;147929;3151;64785;5902;1616;51605;1501;688;58495;23468	ACBD6;ARHGAP11A;ATAD2;CASP2;CASP8AP2;CBX5;CDC25A;CDC45;CDC6;CDCA7;CDK1;CTNND2;DAXX;DDX17;DNMT1;E2F1;E2F3;E2F8;EZH2;FANCG;FBXO5;GINS3;GMNN;H2AFZ;HMGA1;HMGN2;HNRNPA1;HNRNPD;INTS7;KLF5;KPNB1;LUC7L3;MCM2;MCM3;MCM4;MCM6;MCM7;MCM8;MELK;MTF2;MXD3;NASP;NUP155;OTUD7B;OVOL2;PAQR4;PCNA;PKMYT1;POL

KTGGYRSGAA_UNKNOWN	0.72	1.89	0.00E+00	0.00E+00	http://www.broadinstitute.org/gsea/msigdb/cards/KTGGYRSGAA_UNKNOWN	68	23	5984;2146;4175;29128;3978;4172;5557;5424;84950;4176;64946;83879;11073;5902;1616;112476;2664;9130;283248;22839;27037;23616;10492	CDCA7;CENPH;DAXX;DLGAP4;EZH2;FAM50A;GDI1;LIG1;MCM3;MCM6;MCM7;POLD1;PRIM1;PRPF38A;PRRT2;RANBP1;RCOR2;RFC4;SH3BP1;SYNCRIP;TOPBP1;TRMT2A;UHRF1

V$MAZ_Q6	0.66	1.88	0.00E+00	0.00E+00	http://www.broadinstitute.org/gsea/msigdb/cards/V$MAZ_Q6	181	52	9869;23126;7170;200186;607;57592;10228;1163;2029;23248;10712;9887;5498;126626;4194;8394;10401;79733;89884;4000;56957;6464;55182;200185;4802;9181;1942;9213;5331;127002;5993;3151;51322;4192;3065;283248;2224;4076;5451;6045;79869;476;84265;80128;2965;79009;7702;51317;3746;22874;5080;2275	ARHGEF2;ATP1A1;ATXN7L2;BCL9;CAPRIN1;CKS1B;CPSF7;CRTC2;DDX50;E2F8;EFNA1;ENSA;FAM189B;FDPS;FHL3;GABPB2;GTF2H1;HDAC1;HMGN2;KCNC1;KRTCAP2;LHX4;LMNA;MDK;MDM4;NFYC;OTUD7B;PAX6;PHF21A;PIAS3;PIP5K1A;PLCB3;PLEKHA6;POGZ;POLR3GL;POU2F1;PPOX;RCOR2;RFX5;RNF2;RNF220;RPRD2;SETDB1;SHC1;SMG7;STX6;TPM3;TRIM46;WA

KCCGNSWTTT_UNKNOWN	0.67	1.81	0.00E+00	8.35E-05	http://www.broadinstitute.org/gsea/msigdb/cards/KCCGNSWTTT_UNKNOWN	95	44	2029;126626;9584;7818;3609;55339;3187;7514;4841;3181;890;11335;8148;58155;4849;10521;6434;1871;5757;3190;8473;9988;7536;10643;10492;5936;5451;7552;10270;55227;1665;25836;51317;6667;1399;9126;26575;5015;5863;3720;10735;84922;23394;2309	ADNP;AKAP8;CBX3;CCNA2;CNOT3;CRKL;DAP3;DDX17;DHX15;DMTF1;E2F3;ENSA;FIZ1;FOXO3;GABPB2;HNRNPA2B1;HNRNPH1;HNRNPK;IGF2BP3;ILF3;JARID2;LRRC1;NIPBL;NONO;OGT;OTX2;PHF21A;POU2F1;PTBP2;PTMA;RBM39;RBM4;RGL2;RGS17;SF1;SMC3;SP1;STAG2;SYNCRIP;TAF15;TRA2B;WDR33

GCGSCMNTTT_UNKNOWN	0.67	1.77	0.00E+00	2.79E-04	http://www.broadinstitute.org/gsea/msigdb/cards/GCGSCMNTTT_UNKNOWN	65	33	27316;5422;126626;79723;51747;4176;10051;56957;58486;23435;57649;8847;65056;11198;1434;56980;57560;54540;2719;9662;28511;5451;5307;29896;7266;7702;9774;5471;9179;55269;6294;10735;84919	AP4M1;BCLAF1;CEP135;CSE1L;DLEU2;DNAJC7;FAM193B;GABPB2;GPBP1;GPC3;IFT80;LUC7L3;MCM7;NKIRAS2;OTUD7B;PHF12;PITX1;POLA1;POU2F1;PPAT;PPP1R15B;PRDM10;PSPC1;RBMX;SAFB;SMC4;STAG2;SUPT16H;SUV39H2;TARDBP;TRA2A;

V$E2F1_Q4	0.61	1.75	0.00E+00	3.81E-04	http://www.broadinstitute.org/gsea/msigdb/cards/V$E2F1_Q4	222	63	220988;990;2146;9735;24137;8318;9833;55705;63922;1869;5111;5557;25896;5888;23649;4678;9088;3925;79733;26271;5427;144455;163786;11113;25912;55000;7884;3181;56957;4173;63967;11335;11325;10521;4802;3184;3837;1871;3178;3151;54487;5902;1616;112476;10155;79089;4800;5501;30827;3207;1501;23468;2118;27037;51163;51248;7374;79596;8535;2079;10163;55646;55119	C1orf43;CBX3;CBX4;CBX5;CDC45;CDC6;CHTF18;CIT;CLSPN;CTNND2;CXXC1;DAXX;DBR1;DDX17;DDX42;DGCR8;E2F1;E2F3;E2F7;E2F8;ERH;ETV4;EZH2;FBXO5;HMGN2;HNRNPA1;HNRNPA2B1;HNRNPA3;HNRNPD;HOXA11;INTS7;IPO9;KIF4A;KNTC1;KPNB1;LYAR;MCM4;MELK;NASP;NFYA;NFYC;OTUD7B;PCNA;PDZD11;PKMYT1;POLA2;POLE2;PPP1CC;PRIM1;PRPF38

V$ETF_Q6	0.64	1.74	0.00E+00	4.38E-04	http://www.broadinstitute.org/gsea/msigdb/cards/V$ETF_Q6	105	30	200186;27005;23623;57592;23248;79723;8317;7818;25912;57147;1944;55870;23139;55182;360023;3020;4802;9213;9580;51377;127002;3151;254428;51322;7804;1810;5451;6045;27022;79577	ASH1L;ATXN7L2;C1orf43;CDC7;CDC73;CRTC2;DAP3;DR1;EFNA3;FOXD3;H3F3A;HMGN2;LRP8;MAST2;NFYC;POU2F1;RNF2;RNF220;RPRD2;RUSC1;SCYL3;SLC41A1;SOX13;SUV39H2;UCHL5;USP21;WAC;XPR
V$E2F_01	0.65	1.71	0.00E+00	1.12E-03	http://www.broadinstitute.org/gsea/msigdb/cards/V$E2F_01	64	26	4171;4172;1869;5111;8726;4678;144455;3014;11073;10051;1786;5985;4173;835;58155;3184;1871;5902;84445;10042;27037;1108;85235;8570;8348;23546	CASP2;CHD4;DNMT1;E2F1;E2F3;E2F7;EED;H2AFX;HIST1H2AH;HIST1H2BO;HMGXB4;HNRNPD;KHSRP;LZTS2;MCM2;MCM3;MCM4;NASP;PCNA;PTBP2;RANBP1;RFC5;SMC4;SYNGR4;TOPBP1

V$SRF_01	-0.50	-1.62	1.24E-02	3.66E-01	http://www.broadinstitute.org/gsea/msigdb/cards/V$SRF	49	16	2843;8061;3214;7057;87;2353;1960;4629;10335;64102;60592;2354;85458;309;800;291	ACTN1;ANXA6;CALD1;DIXDC1;EGR3;FOS;FOSB;FOSL1;GPR20;HOXB4;MRVI1;MYH11;SCOC;SLC25A4;THBS1;TNMD

GATAAGR_V$GATA_C	-0.33	-1.36	0.00E+00	6.28E-01	http://www.broadinstitute.org/gsea/msigdb/cards/GATAAGR_V$GATA_C	278	67	162466;23554;6660;3214;27254;340419;3667;3795;1756;124637;762;10912;3708;653808;3157;6943;6521;4774;947;2313;10602;6299;4208;54893;7148;3488;2628;2623;4005;5350;3090;10891;6304;151126;23767;64388;9843;123228;5648;5207;8828;7036;2252;2329;8322;27302;2038;55268;26468;5553;4485;4908;80168;862;151556;4775;116;80177;92162;255488;23051;407738;2078;5009;3931;63891;1523	ADCYAP1;BMP10;CA4;CD34;CDC42EP3;CSDC2;CUX1;CYB5D1;DMD;ECHDC2;EPB42;ERG;FAM19A1;FGF7;FLI1;FLRT3;FMO4;FZD4;GADD45G;GATA1;GATM;GPR155;GREM2;HEPH;HIC1;HMGCS1;HOXB4;IGFBP5;IRS1;ITPR1;KHK;LCAT;LHX6;LMO2;MASP1;MEF2C;MOGAT2;MST1;MTMR10;MYCT1;NFATC3;NFIA;NRP2;NTF3;OTC;PFKFB1;PHOSPHO1;PLN;PPARGC1A;PRG2;

V$CEBPB_01	-0.31	-1.31	0.00E+00	6.33E-01	http://www.broadinstitute.org/gsea/msigdb/cards/V$CEBPB_01	245	66	7798;23019;89857;9341;54206;6441;3702;5153;3586;5915;5739;10549;51232;2259;55294;162466;2045;9788;54542;718;213;54832;9517;2876;124997;53829;65220;1634;5494;59272;93986;10602;1843;10777;5428;5295;6718;3488;55640;51703;5098;6907;10390;154;8828;344148;9104;55268;84570;716;6095;23460;2167;2158;6557;164;5606;660;58488;407738;1073;1740;388;4035;1827;10449	ABCA6;ACAA2;ACE2;ACSL5;ADRB2;AKR1D1;ALB;AP1G1;ARPP21;BMX;C1S;C3;CDC42EP3;CEPT1;CFL2;CNOT1;COL25A1;CRIM1;DCN;DLG2;DUSP1;ECHDC2;EPHA7;ERRFI1;F9;FABP4;FAM19A1;FBXW7;FGF14;FLVCR2;FOXP2;GPX1;IGFBP5;IL10;ITK;KLHL6;LRP1;LUZP1;MAP2K3;MTSS1;NADK;NCKAP5;NRP2;P2RY13;PCDHGC3;PCTP;PDE1B;PHOSPHO1;PIK3R1;POLG;PP

V$HOX13_01	-0.41	-1.32	8.00E-02	7.00E-01	http://www.broadinstitute.org/gsea/msigdb/cards/V$HOX13_0	44	9	8334;10584;64093;4908;3479;80762;595;5138;800	CALD1;CCND1;COLEC10;HIST1H2AC;IGF1;NDFIP1;NTF3;PDE2A;SMOC1

TTAYRTAA_V$E4BP4_01	-0.27	-1.10	1.67E-01	7.16E-01	http://www.broadinstitute.org/gsea/msigdb/cards/TTAYRTAA_V$E4BP4_01	236	65	23017;51232;2259;55294;384;2872;8633;10129;10260;22807;51341;144165;7532;90627;213;1756;9099;54847;257194;9728;124997;3572;23705;9348;801;253943;2538;1306;8848;5592;55118;266629;79071;26273;64388;64841;55107;10468;9628;154;6376;687;51557;127687;114905;10294;26468;57222;1540;11345;1808;10218;64398;4842;10150;7328;50808;5606;8503;2823;4638;64400;54414;2028;9709	ADRB2;AK3;AKTIP;ALB;ANGPTL7;ANO1;ARG2;C1QTNF7;C1orf122;CADM1;CALM1;COL15A1;CRIM1;CRTAC1;CX3CL1;CYLD;DENND4A;DMD;DNAJA2;DPYSL2;ELOVL6;ENPEP;ERGIC1;FAIM2;FBXO3;FBXW7;FGF14;FRY;FST;G6PC;GABARAPL2;GNPNAT1;GPM6A;GREM2;HERPUD1;IKZF2;IL6ST;KLF9;LGSN;LHX6;MAP2K3;MBNL2;MKNK2;MPP5;MYLK;NDST3;NEGR1;NOS1;PIK

V$GATA_Q6	-0.32	-1.28	4.00E-02	7.21E-01	http://www.broadinstitute.org/gsea/msigdb/cards/V$GATA_Q6	185	36	133;6521;1286;947;2313;93986;10602;7148;57617;3488;2628;2623;11278;4005;3090;10891;80344;64388;5648;5207;81558;8828;9104;27302;2038;55268;26468;80168;862;151556;80177;407738;2078;10867;3931;267	ADM;AMFR;BMP10;CD34;CDC42EP3;COL4A4;DCAF11;ECHDC2;EPB42;ERG;FAM117A;FAM19A1;FLI1;FOXP2;GATA1;GATM;GPR155;GREM2;HIC1;IGFBP5;KLF12;LCAT;LHX6;LMO2;MASP1;MOGAT2;MYCT1;NRP2;PFKFB1;PPARGC1A;RGN;RUNX1T1;SLC4A1;TNXB;TSPA

V$HNF3B_01	-0.27	-1.10	5.00E-02	7.31E-01	http://www.broadinstitute.org/gsea/msigdb/cards/V$HNF3B_01	196	40	1756;10458;301;57616;91179;3707;7276;8516;2674;93986;10602;1843;23002;9839;117581;11278;6907;389136;4734;9068;10891;29951;167465;284273;64102;1124;23414;127733;80315;3624;6095;862;1021;25925;5950;2823;2160;1573;2028;800	ANGPTL1;ANXA1;BAIAP2;CALD1;CDC42EP3;CDK6;CHN2;CPEB4;CYP2J2;DAAM1;DMD;DUSP1;ENPEP;F11;FOXP2;GFRA1;GPM6A;INHBA;ITGA8;ITPKB;KLF12;NEDD4;PDZRN4;PPARGC1A;RBP4;RORA;RUNX1T1;SCARF2;TBL1X;TNMD;TSHZ3;TTR;TWIST2;UBXN10;VGLL3;ZADH2;ZEB2;ZFPM2;ZNF3

V$FREAC4_01	-0.30	-1.14	1.64E-01	7.34E-01	http://www.broadinstitute.org/gsea/msigdb/cards/V$FREAC4_01	139	37	3339;10129;22807;23239;80380;79873;375449;4163;23705;2674;93986;3164;1843;11278;9528;4734;29951;151126;153;284273;64102;5745;9843;127733;25890;23474;3624;10150;862;3479;7328;1317;2012;407738;2078;254827;800	ABI3BP;ADRB1;CADM1;CALD1;DUSP1;EMP1;ERG;ETHE1;FAM19A1;FOXP2;FRY;GFRA1;HEPH;HSPG2;IGF1;IKZF2;INHBA;KLF12;MAST4;MBNL2;MCC;NAALADL2;NEDD4;NR4A1;NUDT18;PDCD1LG2;PDZRN4;PHLPP1;PTH1R;RUNX1T1;SLC31A1;TMEM59;TNMD;UBE2H;UBXN10;ZADH

YATGNWAAT_V$OCT_C	-0.26	-1.11	0.00E+00	7.40E-01	http://www.broadinstitute.org/gsea/msigdb/cards/YATGNWAAT_V$OCT_C	331	59	653;377677;4163;133;1440;2101;23705;1634;56670;4774;6299;6422;5868;9839;28514;4094;166336;4734;4131;29951;60592;5207;8334;81558;208;143686;6925;8322;26468;2828;11162;266812;84570;58476;25837;8347;4842;10150;123879;23365;6038;220296;222255;4883;283;93129;10580;5728;2078;1740;57149;388;79777;9079;595;85450;202151;1827;1523	ACBD4;ADM;AKT2;ANG;ARHGEF12;ATXN7L1;BMP5;CA13;CADM1;CCND1;COL25A1;CSF3;CUX1;DCN;DCUN1D3;DLG2;DLL1;ERG;ESRRA;FAM117A;FZD4;GPR4;HEPACAM;HIST1H2AC;HIST1H2BC;ITPRIP;LDB2;LHX6;LYRM1;MAF;MAP1B;MBNL2;MCC;NAP1L5;NEDD4;NFIA;NOS1;NPR3;NUDT6;ORAI3;PDZRN4;PFKFB1;PRICKLE2;PTEN;RAB26;RAB5A;RANBP3L;RCAN1;RHO

V$STAT5B_01	-0.32	-1.37	0.00E+00	7.44E-01	http://www.broadinstitute.org/gsea/msigdb/cards/V$STAT5B_01	226	67	6347;25820;999;3782;286410;6647;5816;6919;10282;582;7122;320;207;8013;7106;2887;9124;653;5348;9230;2353;3660;55361;114897;11346;4547;8406;9839;3908;158763;57188;51266;9732;23086;5662;10468;23090;170392;54918;1124;84675;25927;687;1592;10783;55246;2243;9507;4143;8835;5097;730;6382;10747;29100;2710;2158;6886;5606;10599;3764;55273;2078;10087;6609;5627;710	ADAMTS4;ADAMTSL3;AKT1;APBA1;ARHGAP36;ARIH1;ATP1C;BBS1;BET1;BMP5;C1QTNF1;C7;CCDC25;CCL2;CDH1;CHN2;CLDN5;CLEC1B;CMTM6;CNRIP1;COL4A3BP;CYP26A1;DOCK4;ERG;EXPH5;F9;FGA;FOS;FST;FXYD1;GK;GRB10;IRF2;KCNJ8;KCNN3;KLF9;LAMA2;MAP2K3;MASP2;MAT1A;MTTP;NEK6;NR4A3;OIT3;PCDH1;PDLIM1;PI4K2A;PROS1;PSD;PVALB;RAB11B;

V$SRF_C	-0.28	-1.12	4.35E-02	7.44E-01	http://www.broadinstitute.org/gsea/msigdb/cards/V$SRF_C	201	53	2843;8061;3214;7057;70;80303;3679;116535;87;90627;1756;10474;27314;10912;124997;53834;2353;1960;4629;6876;93986;3164;9839;25777;8997;1848;6517;9552;28514;10335;5350;60592;1295;57381;143425;10611;84675;2354;85458;8639;443;83604;4041;123879;309;1073;4638;4430;1398;57149;6716;800;291	ACTC1;ACTN1;ANXA6;AOC3;ASPA;CALD1;CFL2;COL8A1;CRK;DCUN1D3;DIXDC1;DLL1;DMD;DUSP6;EFHD1;EGR3;FGFRL1;FOS;FOSB;FOSL1;FOXP2;GADD45G;GPR20;HOXB4;ITGA7;KALRN;LRP5;LYRM1;MRGPRF;MRVI1;MYH11;MYLK;MYO1B;NR4A1;PDLIM5;PLN;RAB30;RHOJ;SCOC;SLC25A4;SLC2A4;SPAG7;SRD5A2;STARD13;SUN2;SYT9;TADA3;TAGLN;THBS1;TME

V$SRF_Q4	-0.28	-1.09	1.36E-01	7.44E-01	http://www.broadinstitute.org/gsea/msigdb/cards/V$SRF_Q4	210	50	2843;3214;7057;70;27044;80303;116535;87;7111;1756;127435;10474;27314;10912;53834;6450;2101;2353;1960;4629;6876;93986;3164;1848;6907;28514;90525;64102;60592;92949;10468;57381;6376;10611;2354;85458;148534;443;83604;123879;4883;309;1073;4638;4430;1740;57149;6716;800;291	ACTC1;ACTN1;ADAMTSL1;ANXA6;ASPA;CALD1;CFL2;CX3CL1;DCUN1D3;DIXDC1;DLG2;DLL1;DMD;DUSP6;EFHD1;EGR3;ESRRA;FGFRL1;FOS;FOSB;FOXP2;FST;GADD45G;GPR20;HOXB4;LYRM1;MRGPRF;MYH11;MYLK;MYO1B;NPR3;NR4A1;PDLIM5;PODN;RAB30;RHOJ;SCOC;SH3BGR;SHF;SLC25A4;SND1;SRD5A2;TADA3;TAGLN;TBL1X;THBS1;TMEM4

YGACNNYACAR_UNKNOWN	-0.31	-1.11	1.75E-01	7.47E-01	http://www.broadinstitute.org/gsea/msigdb/cards/YGACNNYACAR_UNKNO	86	15	203190;5024;1960;8848;6907;60481;29951;4967;4091;58476;1373;7328;407738;2034;364	AQP7;CPS1;EGR3;ELOVL5;EPAS1;FAM19A1;LGI3;OGDH;P2RX3;PDZRN4;SMAD6;TBL1X;TP53INP2;TSC22D1;UBE2H

V$CDC5_01	-0.28	-1.14	5.26E-02	7.50E-01	http://www.broadinstitute.org/gsea/msigdb/cards/V$CDC5_01	230	40	3707;6943;23705;2674;128434;93986;10602;6299;775;4547;23678;5295;51179;5098;11278;10891;29951;143686;23414;10783;6595;80315;266812;10999;1456;6382;6095;2710;6469;8825;407738;8503;6540;1740;2534;6303;6578;4601;800;1523	CACNA1C;CADM1;CALD1;CDC42EP3;CPEB4;CSNK1G3;CUX1;DLG2;FAM19A1;FOXP2;FYN;GFRA1;GK;HAO2;ITPKB;KLF12;LIN7A;MTTP;MXI1;NAP1L5;NEK6;PCDHGC3;PDZRN4;PIK3R1;PIK3R3;PPARGC1A;RORA;SALL1;SAT1;SDC1;SESN3;SGK3;SHH;SLC27A4;SLC6A13;SLCO2A1;SMARCA2;TCF2
V$IRF2_01	-0.29	-1.08	2.73E-01	7.52E-01	http://www.broadinstitute.org/gsea/msigdb/cards/V$IRF	116	15	443;567;4306;91614;1021;4883;10580;5239;54386;55650;54407;9079;220965;1776;2099	ASPA;B2M;CDK6;DEPDC7;DNASE1L3;ESR1;FAM13C;LDB2;NPR3;NR3C2;PGM5;PIGV;SLC38A2;SORBS1;TERF2IP

V$HMEF2_Q6	-0.29	-1.10	2.03E-01	7.57E-01	http://www.broadinstitute.org/gsea/msigdb/cards/V$HMEF2_Q6	130	37	4067;51725;5607;3037;7798;23676;1112;4649;162466;26051;4784;58189;3679;2045;90627;1756;9124;2353;10602;3164;8848;10777;166336;4734;151126;2791;23414;22899;10294;127733;55031;10150;1901;845;2027;27125;6578	AFF4;ARHGEF15;ARPP21;CASQ2;CDC42EP3;DMD;DNAJA2;ENO3;EPHA7;FBXO40;FOS;FOXN3;GNG11;HAS2;ITGA7;LUZP1;LYN;MAP2K5;MBNL2;MYO9A;NEDD4;NFIX;NR4A1;PDLIM1;PHOSPHO1;PPP1R16B;PRICKLE2;S1PR1;SLCO2A1;SMPX;STARD13;TSC22D1;UBXN10;USP47;WF

RAAGNYNNCTTY_UNKNOWN	-0.29	-1.12	1.02E-01	7.61E-01	http://www.broadinstitute.org/gsea/msigdb/cards/RAAGNYNNCTTY_UNKNOWN	133	26	144402;395;4837;93986;30011;4005;10891;51218;6304;10468;1729;54873;51666;6095;9420;862;3479;23051;3672;27125;129303;10516;1740;8291;220441;4601	AFF4;ARHGAP6;ASB4;CPNE8;CYP7B1;DIAPH1;DLG2;DYSF;FBLN5;FOXP2;FST;GLRX5;IGF1;ITGA1;LMO2;MXI1;NNMT;PALMD;PPARGC1A;RNF152;RORA;RUNX1T1;SATB1;SH3KBP1;TMEM150A;

V$STAT5A_01	-0.27	-1.12	1.05E-01	7.65E-01	http://www.broadinstitute.org/gsea/msigdb/cards/V$STAT5A_01	231	48	8013;7106;2887;9124;9230;1440;2353;4774;3660;114897;11346;4547;8406;3908;158763;57188;5662;23090;170392;54918;1124;687;2321;1592;91703;55246;84570;2243;9507;8835;730;81575;6382;10747;29100;2710;2158;6886;5606;10599;3764;55273;10087;6609;79689;5627;710;26231	ACY3;ADAMTS4;ADAMTSL3;APOLD1;ARHGAP36;C1QTNF1;C7;CCDC25;CHN2;CMTM6;COL25A1;COL4A3BP;CSF3;CYP26A1;F9;FGA;FLT1;FOS;GK;GRB10;IRF2;KCNJ8;KLF9;LAMA2;LRRC29;MAP2K3;MASP2;MTTP;NFIA;NR4A3;OIT3;PDLIM1;PROS1;PSD;RAB11B;SDC1;SERPING1;SLCO1B1;SMPD1;SOCS2;SRPX;STEAP4;SYNPO;TAL1;TMEM100;TMEM208;

V$LMO2COM_02	-0.28	-1.14	1.00E-01	7.87E-01	http://www.broadinstitute.org/gsea/msigdb/cards/V$LMO2COM_02	231	45	90627;2908;124637;339122;10912;55534;133;3363;6521;5229;947;55361;93986;3164;6299;57617;3488;2623;2822;4005;286016;10891;5830;92949;831;5648;8828;7036;8322;54675;2038;55268;26468;80315;80168;862;92162;1003;6547;3931;23788;2534;57447;124936;267	ADAMTSL1;ADM;AMFR;CAST;CD34;CDH5;CPEB4;CRLS1;CYB5D1;CYB5D2;ECHDC2;EPB42;FOXP2;FYN;FZD4;GADD45G;GATA1;GPLD1;HTR7;IGFBP5;LCAT;LHX6;LMO2;MAML3;MASP1;MOGAT2;MTCH2;NDRG2;NR3C1;NR4A1;NRP2;PEX5;PGGT1B;PI4K2A;PPARGC1A;RAB43;RUNX1T1;SALL1;SLC4A1;SLC8A3;STARD13;TFR

V$HNF3ALPHA_Q6	-0.26	-1.00	5.20E-01	7.87E-01	http://www.broadinstitute.org/gsea/msigdb/cards/V$HNF3ALPHA_Q6	194	43	1756;83891;5793;8013;10458;57616;653808;3707;4163;9075;4774;1356;93986;10602;4208;732;23678;117581;9185;5144;1848;11278;4094;389136;9068;284273;23584;127687;84913;6595;54873;4041;2167;91614;64116;407738;2823;23710;80004;622;2775;666;800	ANGPTL1;ATOH8;BAIAP2;BDH1;BOK;C1orf122;C8B;CALD1;CDC42EP3;CLDN2;CP;DEPDC7;DMD;DUSP6;ESRP2;FABP4;FAM19A1;FOXP2;GABARAPL1;GNAO1;GPM6A;ITPKB;KLF12;LRP5;MAF;MCC;MEF2C;NFIA;NR4A3;PALMD;PDE4D;PTPRG;REPS2;SGK3;SLC39A8;SMARCA2;SNX25;TSHZ3;TWIST2;VGLL3;VSI

V$STAT4_01	-0.26	-1.02	3.12E-01	7.94E-01	http://www.broadinstitute.org/gsea/msigdb/cards/V$STAT4_01	241	57	4023;87;1359;131450;9099;255877;22877;9689;84248;2876;85456;653;53829;89970;8516;91624;3084;7092;2313;3164;4208;7957;56929;9839;81876;4855;5144;5412;9732;4734;9068;85476;10891;6304;151126;55080;5288;57381;2113;50846;2321;55268;80315;29801;5097;6386;3290;51267;5583;3479;7323;64236;10243;80762;2078;5243;54407	ABCB1;ACTN1;ANGPTL1;BCL6B;BMP5;BZW1;CD200R1;CLEC1A;CPA3;CPEB4;DHH;DOCK4;ECHDC2;EPM2A;ERG;ETS1;FEM1C;FLI1;FLT1;FYTTD1;GFM1;GPHN;GPX1;HSD11B1;IGF1;ITGA8;LPL;MEF2C;MLXIP;NDFIP1;NEDD4;NEXN;NOTCH4;NR4A1;NRG1;P2RY13;PCDH1;PDE4D;PDLIM2;PIK3C2G;PPARGC1A;PRKCH;RAB1B;RHOJ;RSPRY1;SATB1;SDCBP;SLC38A2;TA

RYTAAWNNNTGAY_UNKNOWN	-0.31	-1.06	2.99E-01	7.95E-01	http://www.broadinstitute.org/gsea/msigdb/cards/RYTAAWNNNTGAY_UNKN	61	20	81603;55959;116085;2638;1958;57555;5915;55294;2981;762;93986;5745;687;23414;344148;54658;660;54363;5105;6716	BMX;CA4;EGR1;FBXW7;FOXP2;GC;GUCA2B;HAO1;KLF9;NCKAP5;NLGN2;PCK1;PTH1R;RARB;SLC22A12;SRD5A2;SULF2;TRIM8;UGT1A1;ZFPM2

V$MEF2_02	-0.26	-1.07	1.74E-01	7.96E-01	http://www.broadinstitute.org/gsea/msigdb/cards/V$MEF2_02	207	41	81792;6660;3214;70;161247;1272;58189;3679;2045;3667;1756;9099;467;3248;3164;358;4208;8848;10777;90952;93649;6517;10891;151126;26037;1295;477;115294;27303;6595;9172;80315;1901;845;6547;148;1740;2099;57447;291;1523	ACTC1;ADAMTS12;ADRA1A;AQP1;ARPP21;ATF3;ATP1A2;CASQ2;CNTN1;COL8A1;CPEB4;CUX1;DLG2;DMD;EPHA7;ESAM;ESR1;FITM1;HOXB4;HPGD;IRS1;ITGA7;MEF2C;MYOCD;MYOM2;NDRG2;NR4A1;PCMTD1;PPARGC1A;RBMS3;S1PR1;SIPA1L1;SLC25A4;SLC2A4;SLC8A3;SMARCA2;SOX5;TSC22D1;USP2;WF
V$MEF2_04	-0.36	-1.00	4.17E-01	8.00E-01	http://www.broadinstitute.org/gsea/msigdb/cards/V$MEF	23	4	7223;10858;10891;1523	CUX1;CYP46A1;PPARGC1A;TRPC4

V$OCT1_07	-0.28	-1.07	2.11E-01	8.03E-01	http://www.broadinstitute.org/gsea/msigdb/cards/V$OCT1_07	147	27	1440;23705;4774;93986;6299;166336;4734;29951;284273;64388;60592;9843;85458;8322;80315;11162;25837;4842;123879;862;25940;220296;57149;9079;595;10165;1827	CADM1;CCND1;CPEB4;CSF3;DCUN1D3;DIXDC1;FAM98A;FOXP2;FZD4;GREM2;HEPACAM;HEPH;LDB2;LYRM1;NEDD4;NFIA;NOS1;NUDT6;PDZRN4;PRICKLE2;RAB26;RCAN1;RUNX1T1;SALL1;SCOC;SLC25A1
