# Supplementary material for: Prognostic potential of PRPF3 in hepatocellular carcinoma
Source: Aging (Albany NY). 2020 Jan 11;12(1):912–30. doi: 10.18632/aging.102665 (PMC6977647; doi:10.18632/aging.102665)
Supplement: Supplementary Table 7 [file aging-12-102665-s009..pdf]

Supplementary Table 7. PRPF3 AMP co-occurrence genes.

Gene	Cytoband	Samples with alteration in altered group	Samples with alteration in unaltered group	Log Ratio	p-Value	q-Value	Tendency
ANP32E	1q21.2	40 (34.78%)	0 (0.00%)	>10	6.26E-23	1.68E-19	Co-occurrence
APH1A	1q21.2	40 (34.78%)	0 (0.00%)	>10	6.26E-23	1.68E-19	Co-occurrence
C1ORF54	1q21.2	40 (34.78%)	0 (0.00%)	>10	6.26E-23	1.68E-19	Co-occurrence
CA14	1q21.2	40 (34.78%)	0 (0.00%)	>10	6.26E-23	1.68E-19	Co-occurrence
CIART	1q21.2	40 (34.78%)	0 (0.00%)	>10	6.26E-23	1.68E-19	Co-occurrence
MRPS21	1q21.2	40 (34.78%)	0 (0.00%)	>10	6.26E-23	1.68E-19	Co-occurrence
RN7SL480P	1q21.2	40 (34.78%)	0 (0.00%)	>10	6.26E-23	1.68E-19	Co-occurrence
OTUD7B	1q21.2	39 (33.91%)	0 (0.00%)	>10	2.65E-22	4.96E-19	Co-occurrence
PLEKHO1	1q21.2	39 (33.91%)	0 (0.00%)	>10	2.65E-22	4.96E-19	Co-occurrence
VPS45	1q21.2	39 (33.91%)	0 (0.00%)	>10	2.65E-22	4.96E-19	Co-occurrence
MIR-4257/4257		41 (35.65%)	1 (0.41%)	6.45	4.77E-22	8.13E-19	Co-occurrence
MTMR11	1q21.2	38 (33.04%)	0 (0.00%)	>10	1.11E-21	1.60E-18	Co-occurrence
SF3B4	1q21.2	38 (33.04%)	0 (0.00%)	>10	1.11E-21	1.60E-18	Co-occurrence
ECM1	1q21.2	40 (34.78%)	1 (0.41%)	6.41	1.98E-21	2.19E-18	Co-occurrence
FALEC	1q21.2	40 (34.78%)	1 (0.41%)	6.41	1.98E-21	2.19E-18	Co-occurrence
RPRD2	1q21.2	40 (34.78%)	1 (0.41%)	6.41	1.98E-21	2.19E-18	Co-occurrence
TARS2	1q21.2	40 (34.78%)	1 (0.41%)	6.41	1.98E-21	2.19E-18	Co-occurrence
ADAMTSL4	1q21.2	41 (35.65%)	2 (0.82%)	5.45	7.93E-21	7.08E-18	Co-occurrence
ADAMTSL4-AS1	1q21.2	41 (35.65%)	2 (0.82%)	5.45	7.93E-21	7.08E-18	Co-occurrence
RN7SL473P	1q21.2	41 (35.65%)	2 (0.82%)	5.45	7.93E-21	7.08E-18	Co-occurrence
RN7SL600P	1q21.2	41 (35.65%)	2 (0.82%)	5.45	7.93E-21	7.08E-18	Co-occurrence
ENSA	1q21.3	41 (35.65%)	3 (1.22%)	4.86	8.98E-20	7.32E-17	Co-occurrence
MCL1	1q21.2	41 (35.65%)	3 (1.22%)	4.86	8.98E-20	7.32E-17	Co-occurrence
CDC42SE1	1q21.3	39 (33.91%)	2 (0.82%)	5.38	1.28E-19	8.91E-17	Co-occurrence
GABPB2	1q21.3	39 (33.91%)	2 (0.82%)	5.38	1.28E-19	8.91E-17	Co-occurrence

MLLT11	1q21.3	39 (33.91%)	2 (0.82%)	5.38	1.28E-19	8.91E-17	Co-occurrence
SEMA6C	1q21.3	39 (33.91%)	2 (0.82%)	5.38	1.28E-19	8.91E-17	Co-occurrence
BNIPL	1q21.3	38 (33.04%)	2 (0.82%)	5.34	5.07E-19	3.17E-16	Co-occurrence
C1ORF56	1q21.3	38 (33.04%)	2 (0.82%)	5.34	5.07E-19	3.17E-16	Co-occurrence
TNFAIP8L2	1q21.3	38 (33.04%)	2 (0.82%)	5.34	5.07E-19	3.17E-16	Co-occurrence
SV2A	1q21.2	36 (31.30%)	1 (0.41%)	6.26	5.30E-19	3.21E-16	Co-occurrence
AQP10	1q21.3	41 (35.65%)	4 (1.63%)	4.45	7.79E-19	3.66E-16	Co-occurrence
ATP8B2	1q21.3	41 (35.65%)	4 (1.63%)	4.45	7.79E-19	3.66E-16	Co-occurrence
HAX1	1q21.3	41 (35.65%)	4 (1.63%)	4.45	7.79E-19	3.66E-16	Co-occurrence
NUP210L	1q21.3	41 (35.65%)	4 (1.63%)	4.45	7.79E-19	3.66E-16	Co-occurrence
CTSK	1q21.3	39 (33.91%)	3 (1.22%)	4.79	1.38E-18	3.66E-16	Co-occurrence
PIP5K1A	1q21.3	39 (33.91%)	3 (1.22%)	4.79	1.38E-18	3.66E-16	Co-occurrence
ACP6	1q21.2	35 (30.43%)	1 (0.41%)	6.22	2.09E-18	3.66E-16	Co-occurrence
ANKRD20A12P	-	35 (30.43%)	1 (0.41%)	6.22	2.09E-18	3.66E-16	Co-occurrence
ANKRD34A	1q21.1	35 (30.43%)	1 (0.41%)	6.22	2.09E-18	3.66E-16	Co-occurrence
ANKRD35	1q21.1	35 (30.43%)	1 (0.41%)	6.22	2.09E-18	3.66E-16	Co-occurrence
BCL9	1q21.2	35 (30.43%)	1 (0.41%)	6.22	2.09E-18	3.66E-16	Co-occurrence
BOLA1	1q21.2	35 (30.43%)	1 (0.41%)	6.22	2.09E-18	3.66E-16	Co-occurrence
CD160	1q21.1	35 (30.43%)	1 (0.41%)	6.22	2.09E-18	3.66E-16	Co-occurrence
CHD1L	1q21.1	35 (30.43%)	1 (0.41%)	6.22	2.09E-18	3.66E-16	Co-occurrence
FAM72B	1p11.2	35 (30.43%)	1 (0.41%)	6.22	2.09E-18	3.66E-16	Co-occurrence
FAM72C	1q21.1	35 (30.43%)	1 (0.41%)	6.22	2.09E-18	3.66E-16	Co-occurrence
FAM72D	1q21.1	35 (30.43%)	1 (0.41%)	6.22	2.09E-18	3.66E-16	Co-occurrence
FCGR1A	1q21.2	35 (30.43%)	1 (0.41%)	6.22	2.09E-18	3.66E-16	Co-occurrence
FCGR1B	1p11.2	35 (30.43%)	1 (0.41%)	6.22	2.09E-18	3.66E-16	Co-occurrence
FCGR1CP	1q21.1	35 (30.43%)	1 (0.41%)	6.22	2.09E-18	3.66E-16	Co-occurrence
FMO5	1q21.1	35 (30.43%)	1 (0.41%)	6.22	2.09E-18	3.66E-16	Co-occurrence
GJA5	1q21.2	35 (30.43%)	1 (0.41%)	6.22	2.09E-18	3.66E-16	Co-occurrence
GJA8	1q21.2	35 (30.43%)	1 (0.41%)	6.22	2.09E-18	3.66E-16	Co-occurrence

GNRHR2	1q21.1	35 (30.43%)	1 (0.41%)	6.22	2.09E-18	3.66E-16	Co-occurrence
GPR89A	1q21.1	35 (30.43%)	1 (0.41%)	6.22	2.09E-18	3.66E-16	Co-occurrence
GPR89B	1q21.2	35 (30.43%)	1 (0.41%)	6.22	2.09E-18	3.66E-16	Co-occurrence
HIST2H2AA3	1q21.2	35 (30.43%)	1 (0.41%)	6.22	2.09E-18	3.66E-16	Co-occurrence
HIST2H2AA4	1q21.2	35 (30.43%)	1 (0.41%)	6.22	2.09E-18	3.66E-16	Co-occurrence
HIST2H2AB	1q21.2	35 (30.43%)	1 (0.41%)	6.22	2.09E-18	3.66E-16	Co-occurrence
HIST2H2AC	1q21.2	35 (30.43%)	1 (0.41%)	6.22	2.09E-18	3.66E-16	Co-occurrence
HIST2H2BA	1p11.2	35 (30.43%)	1 (0.41%)	6.22	2.09E-18	3.66E-16	Co-occurrence
HIST2H2BE	1q21.2	35 (30.43%)	1 (0.41%)	6.22	2.09E-18	3.66E-16	Co-occurrence
HIST2H2BF	1q21.2	35 (30.43%)	1 (0.41%)	6.22	2.09E-18	3.66E-16	Co-occurrence
HIST2H3A	1q21.2	35 (30.43%)	1 (0.41%)	6.22	2.09E-18	3.66E-16	Co-occurrence
HIST2H3C	1q21.2	35 (30.43%)	1 (0.41%)	6.22	2.09E-18	3.66E-16	Co-occurrence
HIST2H3D	1q21.2	35 (30.43%)	1 (0.41%)	6.22	2.09E-18	3.66E-16	Co-occurrence
HIST2H4A	1q21.2	35 (30.43%)	1 (0.41%)	6.22	2.09E-18	3.66E-16	Co-occurrence
HIST2H4B	1q21.2	35 (30.43%)	1 (0.41%)	6.22	2.09E-18	3.66E-16	Co-occurrence
HJV	1q21.1	35 (30.43%)	1 (0.41%)	6.22	2.09E-18	3.66E-16	Co-occurrence
HYDIN2	1q21.1	35 (30.43%)	1 (0.41%)	6.22	2.09E-18	3.66E-16	Co-occurrence
ITGA10	1q21.1	35 (30.43%)	1 (0.41%)	6.22	2.09E-18	3.66E-16	Co-occurrence
LINC00623	1p11.2	35 (30.43%)	1 (0.41%)	6.22	2.09E-18	3.66E-16	Co-occurrence
LINC00624	1q21.1-q21.2	35 (30.43%)	1 (0.41%)	6.22	2.09E-18	3.66E-16	Co-occurrence
LIX1L	1q21.1	35 (30.43%)	1 (0.41%)	6.22	2.09E-18	3.66E-16	Co-occurrence
NBPF10	1q21.1	35 (30.43%)	1 (0.41%)	6.22	2.09E-18	3.66E-16	Co-occurrence
NBPF11	1q21.2	35 (30.43%)	1 (0.41%)	6.22	2.09E-18	3.66E-16	Co-occurrence
NBPF12	1q21.1	35 (30.43%)	1 (0.41%)	6.22	2.09E-18	3.66E-16	Co-occurrence
NBPF14	1q21.2	35 (30.43%)	1 (0.41%)	6.22	2.09E-18	3.66E-16	Co-occurrence
NBPF15	1q21.1	35 (30.43%)	1 (0.41%)	6.22	2.09E-18	3.66E-16	Co-occurrence
NBPF20	1q21.1	35 (30.43%)	1 (0.41%)	6.22	2.09E-18	3.66E-16	Co-occurrence
NBPF8	1p11.2	35 (30.43%)	1 (0.41%)	6.22	2.09E-18	3.66E-16	Co-occurrence
NBPF9	1q21.2	35 (30.43%)	1 (0.41%)	6.22	2.09E-18	3.66E-16	Co-occurrence

NOTCH2	1p12	35 (30.43%)	1 (0.41%)	6.22	2.09E-18	3.66E-16	Co-occurrence
NOTCH2NLA	1q21.1	35 (30.43%)	1 (0.41%)	6.22	2.09E-18	3.66E-16	Co-occurrence
NUDT17	1q21.1	35 (30.43%)	1 (0.41%)	6.22	2.09E-18	3.66E-16	Co-occurrence
PDE4DIP	1q21.2	35 (30.43%)	1 (0.41%)	6.22	2.09E-18	3.66E-16	Co-occurrence
PDZK1	1q21.1	35 (30.43%)	1 (0.41%)	6.22	2.09E-18	3.66E-16	Co-occurrence
PDZK1P1	1q21.2	35 (30.43%)	1 (0.41%)	6.22	2.09E-18	3.66E-16	Co-occurrence
PEX11B	1q21.1	35 (30.43%)	1 (0.41%)	6.22	2.09E-18	3.66E-16	Co-occurrence
PIAS3	1q21.1	35 (30.43%)	1 (0.41%)	6.22	2.09E-18	3.66E-16	Co-occurrence
POLR3C	1q21.1	35 (30.43%)	1 (0.41%)	6.22	2.09E-18	3.66E-16	Co-occurrence
POLR3GL	1q21.1	35 (30.43%)	1 (0.41%)	6.22	2.09E-18	3.66E-16	Co-occurrence
PPIAL4A	1p11.2	35 (30.43%)	1 (0.41%)	6.22	2.09E-18	3.66E-16	Co-occurrence
PPIAL4C	1q21.2	35 (30.43%)	1 (0.41%)	6.22	2.09E-18	3.66E-16	Co-occurrence
PPIAL4D	1q21.1	35 (30.43%)	1 (0.41%)	6.22	2.09E-18	3.66E-16	Co-occurrence
PPIAL4G	1q21.2	35 (30.43%)	1 (0.41%)	6.22	2.09E-18	3.66E-16	Co-occurrence
PRKAB2	1q21.1	35 (30.43%)	1 (0.41%)	6.22	2.09E-18	3.66E-16	Co-occurrence
RBM8A	1q21.1	35 (30.43%)	1 (0.41%)	6.22	2.09E-18	3.66E-16	Co-occurrence
RN7SKP88	1q21.2	35 (30.43%)	1 (0.41%)	6.22	2.09E-18	3.66E-16	Co-occurrence
RN7SL261P	1q21.2	35 (30.43%)	1 (0.41%)	6.22	2.09E-18	3.66E-16	Co-occurrence
RNA5SP57	1q21.2	35 (30.43%)	1 (0.41%)	6.22	2.09E-18	3.66E-16	Co-occurrence
RNA5SP59	1q21.1	35 (30.43%)	1 (0.41%)	6.22	2.09E-18	3.66E-16	Co-occurrence
RNF115	1q21.1	35 (30.43%)	1 (0.41%)	6.22	2.09E-18	3.66E-16	Co-occurrence
SEC22B	1p12	35 (30.43%)	1 (0.41%)	6.22	2.09E-18	3.66E-16	Co-occurrence
SRGAP2B	1q21.1	35 (30.43%)	1 (0.41%)	6.22	2.09E-18	3.66E-16	Co-occurrence
TXNIP	1q21.1	35 (30.43%)	1 (0.41%)	6.22	2.09E-18	3.66E-16	Co-occurrence
C1ORF189	1q21.3	40 (34.78%)	4 (1.63%)	4.41	2.99E-18	4.46E-16	Co-occurrence
C1ORF43	1q21.3	40 (34.78%)	4 (1.63%)	4.41	2.99E-18	4.46E-16	Co-occurrence
CREB3L4	1q21.3	40 (34.78%)	4 (1.63%)	4.41	2.99E-18	4.46E-16	Co-occurrence
CRTC2	1q21.3	40 (34.78%)	4 (1.63%)	4.41	2.99E-18	4.46E-16	Co-occurrence
DENND4B	1q21.3	40 (34.78%)	4 (1.63%)	4.41	2.99E-18	4.46E-16	Co-occurrence

GATAD2B	1q21.3	40 (34.78%)	4 (1.63%)	4.41	2.99E-18	4.46E-16	Co-occurrence
GOLPH3L	1q21.3	40 (34.78%)	4 (1.63%)	4.41	2.99E-18	4.46E-16	Co-occurrence
HORMAD1	1q21.3	40 (34.78%)	4 (1.63%)	4.41	2.99E-18	4.46E-16	Co-occurrence
IL6R	1q21.3	40 (34.78%)	4 (1.63%)	4.41	2.99E-18	4.46E-16	Co-occurrence
JTB	1q21.3	40 (34.78%)	4 (1.63%)	4.41	2.99E-18	4.46E-16	Co-occurrence
MIR-5698/5698		40 (34.78%)	4 (1.63%)	4.41	2.99E-18	4.46E-16	Co-occurrence
RAB13	1q21.3	40 (34.78%)	4 (1.63%)	4.41	2.99E-18	4.46E-16	Co-occurrence
RN7SL431P	1q21.3	40 (34.78%)	4 (1.63%)	4.41	2.99E-18	4.46E-16	Co-occurrence
RPS27	1q21.3	40 (34.78%)	4 (1.63%)	4.41	2.99E-18	4.46E-16	Co-occurrence
SHE	1q21.3	40 (34.78%)	4 (1.63%)	4.41	2.99E-18	4.46E-16	Co-occurrence
SLC39A1	1q21.3	40 (34.78%)	4 (1.63%)	4.41	2.99E-18	4.46E-16	Co-occurrence
TDRD10	1q21.3	40 (34.78%)	4 (1.63%)	4.41	2.99E-18	4.46E-16	Co-occurrence
TPM3	1q21.3	40 (34.78%)	4 (1.63%)	4.41	2.99E-18	4.46E-16	Co-occurrence
UBAP2L	1q21.3	40 (34.78%)	4 (1.63%)	4.41	2.99E-18	4.46E-16	Co-occurrence
ANXA9	1q21.3	38 (33.04%)	3 (1.22%)	4.75	5.31E-18	5.16E-16	Co-occurrence
C1ORF68	1q21.3	38 (33.04%)	3 (1.22%)	4.75	5.31E-18	5.16E-16	Co-occurrence
C2CD4D	1q21.3	38 (33.04%)	3 (1.22%)	4.75	5.31E-18	5.16E-16	Co-occurrence
CERS2	1q21.3	38 (33.04%)	3 (1.22%)	4.75	5.31E-18	5.16E-16	Co-occurrence
CRNN	1q21.3	38 (33.04%)	3 (1.22%)	4.75	5.31E-18	5.16E-16	Co-occurrence
FLG	1q21.3	38 (33.04%)	3 (1.22%)	4.75	5.31E-18	5.16E-16	Co-occurrence
FLG2	1q21.3	38 (33.04%)	3 (1.22%)	4.75	5.31E-18	5.16E-16	Co-occurrence
HRNR	1q21.3	38 (33.04%)	3 (1.22%)	4.75	5.31E-18	5.16E-16	Co-occurrence
IVL	1q21.3	38 (33.04%)	3 (1.22%)	4.75	5.31E-18	5.16E-16	Co-occurrence
KPRP	1q21.3	38 (33.04%)	3 (1.22%)	4.75	5.31E-18	5.16E-16	Co-occurrence
LCE1A	1q21.3	38 (33.04%)	3 (1.22%)	4.75	5.31E-18	5.16E-16	Co-occurrence
LCE1B	1q21.3	38 (33.04%)	3 (1.22%)	4.75	5.31E-18	5.16E-16	Co-occurrence
LCE1C	1q21.3	38 (33.04%)	3 (1.22%)	4.75	5.31E-18	5.16E-16	Co-occurrence
LCE1D	1q21.3	38 (33.04%)	3 (1.22%)	4.75	5.31E-18	5.16E-16	Co-occurrence
LCE1E	1q21.3	38 (33.04%)	3 (1.22%)	4.75	5.31E-18	5.16E-16	Co-occurrence

LCE1F	1q21.3	38 (33.04%)	3 (1.22%)	4.75	5.31E-18	5.16E-16	Co-occurrence
LCE2A	1q21.3	38 (33.04%)	3 (1.22%)	4.75	5.31E-18	5.16E-16	Co-occurrence
LCE2B	1q21.3	38 (33.04%)	3 (1.22%)	4.75	5.31E-18	5.16E-16	Co-occurrence
LCE2C	1q21.3	38 (33.04%)	3 (1.22%)	4.75	5.31E-18	5.16E-16	Co-occurrence
LCE2D	1q21.3	38 (33.04%)	3 (1.22%)	4.75	5.31E-18	5.16E-16	Co-occurrence
LCE4A	1q21.3	38 (33.04%)	3 (1.22%)	4.75	5.31E-18	5.16E-16	Co-occurrence
LCE6A	1q21.3	38 (33.04%)	3 (1.22%)	4.75	5.31E-18	5.16E-16	Co-occurrence
LELP1	1q21.3	38 (33.04%)	3 (1.22%)	4.75	5.31E-18	5.16E-16	Co-occurrence
LINC00302	1q21.3	38 (33.04%)	3 (1.22%)	4.75	5.31E-18	5.16E-16	Co-occurrence
LINGO4	1q21.3	38 (33.04%)	3 (1.22%)	4.75	5.31E-18	5.16E-16	Co-occurrence
LOR	1q21.3	38 (33.04%)	3 (1.22%)	4.75	5.31E-18	5.16E-16	Co-occurrence
LYSMD1	1q21.3	38 (33.04%)	3 (1.22%)	4.75	5.31E-18	5.16E-16	Co-occurrence
MINDY1	1q21.3	38 (33.04%)	3 (1.22%)	4.75	5.31E-18	5.16E-16	Co-occurrence
NBPF18P	1q21.3	38 (33.04%)	3 (1.22%)	4.75	5.31E-18	5.16E-16	Co-occurrence
PGLYRP3	1q21.3	38 (33.04%)	3 (1.22%)	4.75	5.31E-18	5.16E-16	Co-occurrence
PGLYRP4	1q21.3	38 (33.04%)	3 (1.22%)	4.75	5.31E-18	5.16E-16	Co-occurrence
PRR9	1q21.3	38 (33.04%)	3 (1.22%)	4.75	5.31E-18	5.16E-16	Co-occurrence
PRUNE1	1q21.3	38 (33.04%)	3 (1.22%)	4.75	5.31E-18	5.16E-16	Co-occurrence
RN7SL44P	1q21.3	38 (33.04%)	3 (1.22%)	4.75	5.31E-18	5.16E-16	Co-occurrence
RORC	1q21.3	38 (33.04%)	3 (1.22%)	4.75	5.31E-18	5.16E-16	Co-occurrence
RPTN	1q21.3	38 (33.04%)	3 (1.22%)	4.75	5.31E-18	5.16E-16	Co-occurrence
S100A10	1q21.3	38 (33.04%)	3 (1.22%)	4.75	5.31E-18	5.16E-16	Co-occurrence
S100A11	1q21.3	38 (33.04%)	3 (1.22%)	4.75	5.31E-18	5.16E-16	Co-occurrence
S100A12	1q21.3	38 (33.04%)	3 (1.22%)	4.75	5.31E-18	5.16E-16	Co-occurrence
S100A2	1q21.3	38 (33.04%)	3 (1.22%)	4.75	5.31E-18	5.16E-16	Co-occurrence
S100A3	1q21.3	38 (33.04%)	3 (1.22%)	4.75	5.31E-18	5.16E-16	Co-occurrence
S100A4	1q21.3	38 (33.04%)	3 (1.22%)	4.75	5.31E-18	5.16E-16	Co-occurrence
S100A5	1q21.3	38 (33.04%)	3 (1.22%)	4.75	5.31E-18	5.16E-16	Co-occurrence
S100A6	1q21.3	38 (33.04%)	3 (1.22%)	4.75	5.31E-18	5.16E-16	Co-occurrence

S100A7	1q21.3	38 (33.04%)	3 (1.22%)	4.75	5.31E-18	5.16E-16	Co-occurrence
S100A7A	1q21.3	38 (33.04%)	3 (1.22%)	4.75	5.31E-18	5.16E-16	Co-occurrence
S100A7L2	1q21.3	38 (33.04%)	3 (1.22%)	4.75	5.31E-18	5.16E-16	Co-occurrence
S100A8	1q21.3	38 (33.04%)	3 (1.22%)	4.75	5.31E-18	5.16E-16	Co-occurrence
S100A9	1q21.3	38 (33.04%)	3 (1.22%)	4.75	5.31E-18	5.16E-16	Co-occurrence
SCNM1	1q21.3	38 (33.04%)	3 (1.22%)	4.75	5.31E-18	5.16E-16	Co-occurrence
SMCP	1q21.3	38 (33.04%)	3 (1.22%)	4.75	5.31E-18	5.16E-16	Co-occurrence
SPRR1A	1q21.3	38 (33.04%)	3 (1.22%)	4.75	5.31E-18	5.16E-16	Co-occurrence
SPRR1B	1q21.3	38 (33.04%)	3 (1.22%)	4.75	5.31E-18	5.16E-16	Co-occurrence
SPRR2A	1q21.3	38 (33.04%)	3 (1.22%)	4.75	5.31E-18	5.16E-16	Co-occurrence
SPRR2B	1q21.3	38 (33.04%)	3 (1.22%)	4.75	5.31E-18	5.16E-16	Co-occurrence
SPRR2D	1q21.3	38 (33.04%)	3 (1.22%)	4.75	5.31E-18	5.16E-16	Co-occurrence
SPRR2E	1q21.3	38 (33.04%)	3 (1.22%)	4.75	5.31E-18	5.16E-16	Co-occurrence
SPRR2F	1q21.3	38 (33.04%)	3 (1.22%)	4.75	5.31E-18	5.16E-16	Co-occurrence
SPRR2G	1q21.3	38 (33.04%)	3 (1.22%)	4.75	5.31E-18	5.16E-16	Co-occurrence
SPRR3	1q21.3	38 (33.04%)	3 (1.22%)	4.75	5.31E-18	5.16E-16	Co-occurrence
SPRR4	1q21.3	38 (33.04%)	3 (1.22%)	4.75	5.31E-18	5.16E-16	Co-occurrence
TCHH	1q21.3	38 (33.04%)	3 (1.22%)	4.75	5.31E-18	5.16E-16	Co-occurrence
TCHHL1	1q21.3	38 (33.04%)	3 (1.22%)	4.75	5.31E-18	5.16E-16	Co-occurrence
THEM4	1q21.3	38 (33.04%)	3 (1.22%)	4.75	5.31E-18	5.16E-16	Co-occurrence
THEM5	1q21.3	38 (33.04%)	3 (1.22%)	4.75	5.31E-18	5.16E-16	Co-occurrence
TMOD4	1q21.3	38 (33.04%)	3 (1.22%)	4.75	5.31E-18	5.16E-16	Co-occurrence
VPS72	1q21.3	38 (33.04%)	3 (1.22%)	4.75	5.31E-18	5.16E-16	Co-occurrence
CTSS	1q21.3	41 (35.65%)	5 (2.04%)	4.13	5.53E-18	5.34E-16	Co-occurrence
ADAR	1q21.3	39 (33.91%)	4 (1.63%)	4.38	1.14E-17	1.06E-15	Co-occurrence
ARNT	1q21.3	39 (33.91%)	4 (1.63%)	4.38	1.14E-17	1.06E-15	Co-occurrence
CHRNB2	1q21.3	39 (33.91%)	4 (1.63%)	4.38	1.14E-17	1.06E-15	Co-occurrence
PSMD4	1q21.3	39 (33.91%)	4 (1.63%)	4.38	1.14E-17	1.06E-15	Co-occurrence
RN7SL444P	1q21.3	39 (33.91%)	4 (1.63%)	4.38	1.14E-17	1.06E-15	Co-occurrence

SETDB1	1q21.3	39 (33.91%)	4 (1.63%)	4.38	1.14E-17	1.06E-15	Co-occurrence
UBE2Q1	1q21.3	39 (33.91%)	4 (1.63%)	4.38	1.14E-17	1.06E-15	Co-occurrence
ZNF687	1q21.3	39 (33.91%)	4 (1.63%)	4.38	1.14E-17	1.06E-15	Co-occurrence
CRCT1	1q21.3	37 (32.17%)	3 (1.22%)	4.72	2.02E-17	1.81E-15	Co-occurrence
LCE3A	1q21.3	37 (32.17%)	3 (1.22%)	4.72	2.02E-17	1.81E-15	Co-occurrence
LCE3B	1q21.3	37 (32.17%)	3 (1.22%)	4.72	2.02E-17	1.81E-15	Co-occurrence
LCE3C	1q21.3	37 (32.17%)	3 (1.22%)	4.72	2.02E-17	1.81E-15	Co-occurrence
LCE3D	1q21.3	37 (32.17%)	3 (1.22%)	4.72	2.02E-17	1.81E-15	Co-occurrence
LCE3E	1q21.3	37 (32.17%)	3 (1.22%)	4.72	2.02E-17	1.81E-15	Co-occurrence
LCE5A	1q21.3	37 (32.17%)	3 (1.22%)	4.72	2.02E-17	1.81E-15	Co-occurrence
CELF3	1q21.3	38 (33.04%)	4 (1.63%)	4.34	4.26E-17	3.57E-15	Co-occurrence
INTS3	1q21.3	38 (33.04%)	4 (1.63%)	4.34	4.26E-17	3.57E-15	Co-occurrence
MRPL9	1q21.3	38 (33.04%)	4 (1.63%)	4.34	4.26E-17	3.57E-15	Co-occurrence
OAZ3	1q21.3	38 (33.04%)	4 (1.63%)	4.34	4.26E-17	3.57E-15	Co-occurrence
POGZ	1q21.3	38 (33.04%)	4 (1.63%)	4.34	4.26E-17	3.57E-15	Co-occurrence
RIIAD1	1q21.3	38 (33.04%)	4 (1.63%)	4.34	4.26E-17	3.57E-15	Co-occurrence
RN7SL372P	1q21.3	38 (33.04%)	4 (1.63%)	4.34	4.26E-17	3.57E-15	Co-occurrence
RNY4P25	1q21.3	38 (33.04%)	4 (1.63%)	4.34	4.26E-17	3.57E-15	Co-occurrence
S100A1	1q21.3	38 (33.04%)	4 (1.63%)	4.34	4.26E-17	3.57E-15	Co-occurrence
S100A13	1q21.3	38 (33.04%)	4 (1.63%)	4.34	4.26E-17	3.57E-15	Co-occurrence
S100A14	1q21.3	38 (33.04%)	4 (1.63%)	4.34	4.26E-17	3.57E-15	Co-occurrence
S100A16	1q21.3	38 (33.04%)	4 (1.63%)	4.34	4.26E-17	3.57E-15	Co-occurrence
SLC27A3	1q21.3	38 (33.04%)	4 (1.63%)	4.34	4.26E-17	3.57E-15	Co-occurrence
SNX27	1q21.3	38 (33.04%)	4 (1.63%)	4.34	4.26E-17	3.57E-15	Co-occurrence
TDRKH	1q21.3	38 (33.04%)	4 (1.63%)	4.34	4.26E-17	3.57E-15	Co-occurrence
EFNA1	1q22	42 (36.52%)	7 (2.86%)	3.68	4.86E-17	4.05E-15	Co-occurrence
PI4KB	1q21.3	39 (33.91%)	5 (2.04%)	4.05	7.67E-17	6.34E-15	Co-occurrence
SEMA4A	1q22	39 (33.91%)	5 (2.04%)	4.05	7.67E-17	6.34E-15	Co-occurrence
PSMB4	1q21.3	37 (32.17%)	4 (1.63%)	4.3	1.58E-16	1.29E-14	Co-occurrence

SELENBP1	1q21.3	37 (32.17%)	4 (1.63%)	4.3	1.58E-16	1.29E-14	Co-occurrence
DPM3	1q22	42 (36.52%)	8 (3.27%)	3.48	2.34E-16	1.84E-14	Co-occurrence
EFNA3	1q21.3	42 (36.52%)	8 (3.27%)	3.48	2.34E-16	1.84E-14	Co-occurrence
GBA	1q22	42 (36.52%)	8 (3.27%)	3.48	2.34E-16	1.84E-14	Co-occurrence
GBAP1	1q22	42 (36.52%)	8 (3.27%)	3.48	2.34E-16	1.84E-14	Co-occurrence
KRTCAP2	1q22	42 (36.52%)	8 (3.27%)	3.48	2.34E-16	1.84E-14	Co-occurrence
MTX1	1q22	42 (36.52%)	8 (3.27%)	3.48	2.34E-16	1.84E-14	Co-occurrence
MUC1	1q22	42 (36.52%)	8 (3.27%)	3.48	2.34E-16	1.84E-14	Co-occurrence
SLC50A1	1q22	42 (36.52%)	8 (3.27%)	3.48	2.34E-16	1.84E-14	Co-occurrence
THBS3	1q22	42 (36.52%)	8 (3.27%)	3.48	2.34E-16	1.84E-14	Co-occurrence
TRIM46	1q22	42 (36.52%)	8 (3.27%)	3.48	2.34E-16	1.84E-14	Co-occurrence
BCAN	1q23.1	38 (33.04%)	5 (2.04%)	4.02	2.80E-16	2.03E-14	Co-occurrence
BGLAP	1q22	38 (33.04%)	5 (2.04%)	4.02	2.80E-16	2.03E-14	Co-occurrence
CGN	1q21.3	38 (33.04%)	5 (2.04%)	4.02	2.80E-16	2.03E-14	Co-occurrence
CHTOP	1q21.3	38 (33.04%)	5 (2.04%)	4.02	2.80E-16	2.03E-14	Co-occurrence
CRABP2	1q23.1	38 (33.04%)	5 (2.04%)	4.02	2.80E-16	2.03E-14	Co-occurrence
FCRL3	1q23.1	38 (33.04%)	5 (2.04%)	4.02	2.80E-16	2.03E-14	Co-occurrence
FCRL4	1q23.1	38 (33.04%)	5 (2.04%)	4.02	2.80E-16	2.03E-14	Co-occurrence
FCRL5	1q23.1	38 (33.04%)	5 (2.04%)	4.02	2.80E-16	2.03E-14	Co-occurrence
INSRR	1q23.1	38 (33.04%)	5 (2.04%)	4.02	2.80E-16	2.03E-14	Co-occurrence
MIR-554/554		38 (33.04%)	5 (2.04%)	4.02	2.80E-16	2.03E-14	Co-occurrence
NES	1q23.1	38 (33.04%)	5 (2.04%)	4.02	2.80E-16	2.03E-14	Co-occurrence
NTRK1	1q23.1	38 (33.04%)	5 (2.04%)	4.02	2.80E-16	2.03E-14	Co-occurrence
PAQR6	1q22	38 (33.04%)	5 (2.04%)	4.02	2.80E-16	2.03E-14	Co-occurrence
PMF1	1q22	38 (33.04%)	5 (2.04%)	4.02	2.80E-16	2.03E-14	Co-occurrence
RFX5	1q21.3	38 (33.04%)	5 (2.04%)	4.02	2.80E-16	2.03E-14	Co-occurrence
SH2D2A	1q23.1	38 (33.04%)	5 (2.04%)	4.02	2.80E-16	2.03E-14	Co-occurrence
SLC25A44	1q22	38 (33.04%)	5 (2.04%)	4.02	2.80E-16	2.03E-14	Co-occurrence
SNAPIN	1q21.3	38 (33.04%)	5 (2.04%)	4.02	2.80E-16	2.03E-14	Co-occurrence

SNORA44	1p35.3	38 (33.04%)	5 (2.04%)	4.02	2.80E-16	2.03E-14	Co-occurrence
TUFT1	1q21.3	38 (33.04%)	5 (2.04%)	4.02	2.80E-16	2.03E-14	Co-occurrence
DAP3	1q22	39 (33.91%)	6 (2.45%)	3.79	4.40E-16	3.17E-14	Co-occurrence
KCNN3	1q21.3	39 (33.91%)	6 (2.45%)	3.79	4.40E-16	3.17E-14	Co-occurrence
ARHGEF2	1q22	40 (34.78%)	7 (2.86%)	3.61	6.29E-16	4.40E-14	Co-occurrence
LAMTOR2	1q22	40 (34.78%)	7 (2.86%)	3.61	6.29E-16	4.40E-14	Co-occurrence
LMNA	1q22	40 (34.78%)	7 (2.86%)	3.61	6.29E-16	4.40E-14	Co-occurrence
MEX3A	1q22	40 (34.78%)	7 (2.86%)	3.61	6.29E-16	4.40E-14	Co-occurrence
RAB25	1q22	40 (34.78%)	7 (2.86%)	3.61	6.29E-16	4.40E-14	Co-occurrence
SSR2	1q22	40 (34.78%)	7 (2.86%)	3.61	6.29E-16	4.40E-14	Co-occurrence
UBQLN4	1q22	40 (34.78%)	7 (2.86%)	3.61	6.29E-16	4.40E-14	Co-occurrence
CKS1B	1q21.3	41 (35.65%)	8 (3.27%)	3.45	8.29E-16	5.46E-14	Co-occurrence
DCST1	1q21.3	41 (35.65%)	8 (3.27%)	3.45	8.29E-16	5.46E-14	Co-occurrence
DCST2	1q21.3	41 (35.65%)	8 (3.27%)	3.45	8.29E-16	5.46E-14	Co-occurrence
EFNA4	1q21.3	41 (35.65%)	8 (3.27%)	3.45	8.29E-16	5.46E-14	Co-occurrence
FAM189B	1q22	41 (35.65%)	8 (3.27%)	3.45	8.29E-16	5.46E-14	Co-occurrence
FDPS	1q22	41 (35.65%)	8 (3.27%)	3.45	8.29E-16	5.46E-14	Co-occurrence
FLAD1	1q21.3	41 (35.65%)	8 (3.27%)	3.45	8.29E-16	5.46E-14	Co-occurrence
HCN3	1q22	41 (35.65%)	8 (3.27%)	3.45	8.29E-16	5.46E-14	Co-occurrence
LENEP	1q21.3	41 (35.65%)	8 (3.27%)	3.45	8.29E-16	5.46E-14	Co-occurrence
MIR-4258/4258		41 (35.65%)	8 (3.27%)	3.45	8.29E-16	5.46E-14	Co-occurrence
PBXIP1	1q21.3	41 (35.65%)	8 (3.27%)	3.45	8.29E-16	5.46E-14	Co-occurrence
PKLR	1q22	41 (35.65%)	8 (3.27%)	3.45	8.29E-16	5.46E-14	Co-occurrence
PYGO2	1q21.3	41 (35.65%)	8 (3.27%)	3.45	8.29E-16	5.46E-14	Co-occurrence
RUSC1	1q22	41 (35.65%)	8 (3.27%)	3.45	8.29E-16	5.46E-14	Co-occurrence
SCAMP3	1q22	41 (35.65%)	8 (3.27%)	3.45	8.29E-16	5.46E-14	Co-occurrence
SHC1	1q21.3	41 (35.65%)	8 (3.27%)	3.45	8.29E-16	5.46E-14	Co-occurrence
ZBTB7B	1q21.3	41 (35.65%)	8 (3.27%)	3.45	8.29E-16	5.46E-14	Co-occurrence
CD5L	1q23.1	37 (32.17%)	5 (2.04%)	3.98	1.01E-15	6.59E-14	Co-occurrence

FCRL1	1q23.1	37 (32.17%)	5 (2.04%)	3.98	1.01E-15	6.59E-14	Co-occurrence
FCRL2	1q23.1	37 (32.17%)	5 (2.04%)	3.98	1.01E-15	6.59E-14	Co-occurrence
ARHGEF11	1q23.1	38 (33.04%)	6 (2.45%)	3.75	1.57E-15	9.32E-14	Co-occurrence
C1ORF61	1q22	38 (33.04%)	6 (2.45%)	3.75	1.57E-15	9.32E-14	Co-occurrence
CCT3	1q22	38 (33.04%)	6 (2.45%)	3.75	1.57E-15	9.32E-14	Co-occurrence
ETV3	1q23.1	38 (33.04%)	6 (2.45%)	3.75	1.57E-15	9.32E-14	Co-occurrence
ETV3L	1q23.1	38 (33.04%)	6 (2.45%)	3.75	1.57E-15	9.32E-14	Co-occurrence
GLMP	1q22	38 (33.04%)	6 (2.45%)	3.75	1.57E-15	9.32E-14	Co-occurrence
GPATCH4	1q22-q23.1	38 (33.04%)	6 (2.45%)	3.75	1.57E-15	9.32E-14	Co-occurrence
HAPLN2	1q23.1	38 (33.04%)	6 (2.45%)	3.75	1.57E-15	9.32E-14	Co-occurrence
HDGF	1q23.1	38 (33.04%)	6 (2.45%)	3.75	1.57E-15	9.32E-14	Co-occurrence
ILF2	1q21.3	38 (33.04%)	6 (2.45%)	3.75	1.57E-15	9.32E-14	Co-occurrence
IQGAP3	1q22	38 (33.04%)	6 (2.45%)	3.75	1.57E-15	9.32E-14	Co-occurrence
ISG20L2	1q23.1	38 (33.04%)	6 (2.45%)	3.75	1.57E-15	9.32E-14	Co-occurrence
LRRC71	1q23.1	38 (33.04%)	6 (2.45%)	3.75	1.57E-15	9.32E-14	Co-occurrence
MEF2D	1q22	38 (33.04%)	6 (2.45%)	3.75	1.57E-15	9.32E-14	Co-occurrence
MIR-765/765		38 (33.04%)	6 (2.45%)	3.75	1.57E-15	9.32E-14	Co-occurrence
MRPL24	1q23.1	38 (33.04%)	6 (2.45%)	3.75	1.57E-15	9.32E-14	Co-occurrence
NAXE	1q22	38 (33.04%)	6 (2.45%)	3.75	1.57E-15	9.32E-14	Co-occurrence
NPR1	1q21.3	38 (33.04%)	6 (2.45%)	3.75	1.57E-15	9.32E-14	Co-occurrence
PEAR1	1q23.1	38 (33.04%)	6 (2.45%)	3.75	1.57E-15	9.32E-14	Co-occurrence
PRCC	1q23.1	38 (33.04%)	6 (2.45%)	3.75	1.57E-15	9.32E-14	Co-occurrence
RHBG	1q22	38 (33.04%)	6 (2.45%)	3.75	1.57E-15	9.32E-14	Co-occurrence
RN7SL612P	1q23.1	38 (33.04%)	6 (2.45%)	3.75	1.57E-15	9.32E-14	Co-occurrence
RRNAD1	1q23.1	38 (33.04%)	6 (2.45%)	3.75	1.57E-15	9.32E-14	Co-occurrence
SMG5	1q22	38 (33.04%)	6 (2.45%)	3.75	1.57E-15	9.32E-14	Co-occurrence
TMEM79	1q22	38 (33.04%)	6 (2.45%)	3.75	1.57E-15	9.32E-14	Co-occurrence
TSACC	1q22	38 (33.04%)	6 (2.45%)	3.75	1.57E-15	9.32E-14	Co-occurrence
TTC24	1q22	38 (33.04%)	6 (2.45%)	3.75	1.57E-15	9.32E-14	Co-occurrence

VHLL	1q22	38 (33.04%)	6 (2.45%)	3.75	1.57E-15	9.32E-14	Co-occurrence
GON4L	1q22	39 (33.91%)	7 (2.86%)	3.57	2.21E-15	1.29E-13	Co-occurrence
MSTO1	1q22	39 (33.91%)	7 (2.86%)	3.57	2.21E-15	1.29E-13	Co-occurrence
RXFP4	1q22	39 (33.91%)	7 (2.86%)	3.57	2.21E-15	1.29E-13	Co-occurrence
SYT11	1q22	39 (33.91%)	7 (2.86%)	3.57	2.21E-15	1.29E-13	Co-occurrence
YY1AP1	1q22	39 (33.91%)	7 (2.86%)	3.57	2.21E-15	1.29E-13	Co-occurrence
ASH1L	1q22	40 (34.78%)	8 (3.27%)	3.41	2.89E-15	1.67E-13	Co-occurrence
CLK2	1q22	40 (34.78%)	8 (3.27%)	3.41	2.89E-15	1.67E-13	Co-occurrence
MIR-555/555		40 (34.78%)	8 (3.27%)	3.41	2.89E-15	1.67E-13	Co-occurrence
PMVK	1q21.3	40 (34.78%)	8 (3.27%)	3.41	2.89E-15	1.67E-13	Co-occurrence
ADAM15	1q21.3	41 (35.65%)	9 (3.67%)	3.28	3.54E-15	2.04E-13	Co-occurrence
KHDC4	1q22	39 (33.91%)	8 (3.27%)	3.38	9.95E-15	5.67E-13	Co-occurrence
RIT1	1q22	39 (33.91%)	8 (3.27%)	3.38	9.95E-15	5.67E-13	Co-occurrence
SCARNA4	1q22	39 (33.91%)	8 (3.27%)	3.38	9.95E-15	5.67E-13	Co-occurrence
CD1D	1q23.1	35 (30.43%)	5 (2.04%)	3.9	1.27E-14	7.21E-13	Co-occurrence
CFAP126	1q23.3	37 (32.17%)	7 (2.86%)	3.49	2.64E-14	1.49E-12	Co-occurrence
MPZ	1q23.3	37 (32.17%)	7 (2.86%)	3.49	2.64E-14	1.49E-12	Co-occurrence
SDHC	1q23.3	37 (32.17%)	7 (2.86%)	3.49	2.64E-14	1.49E-12	Co-occurrence
CD1A	1q23.1	34 (29.57%)	5 (2.04%)	3.86	4.41E-14	2.35E-12	Co-occurrence
CD1B	1q23.1	34 (29.57%)	5 (2.04%)	3.86	4.41E-14	2.35E-12	Co-occurrence
CD1C	1q23.1	34 (29.57%)	5 (2.04%)	3.86	4.41E-14	2.35E-12	Co-occurrence
CD1E	1q23.1	34 (29.57%)	5 (2.04%)	3.86	4.41E-14	2.35E-12	Co-occurrence
KIRREL1	1q23.1	34 (29.57%)	5 (2.04%)	3.86	4.41E-14	2.35E-12	Co-occurrence
OR10K1	1q23.1	34 (29.57%)	5 (2.04%)	3.86	4.41E-14	2.35E-12	Co-occurrence
OR10K2	1q23.1	34 (29.57%)	5 (2.04%)	3.86	4.41E-14	2.35E-12	Co-occurrence
OR10R2	1q23.1	34 (29.57%)	5 (2.04%)	3.86	4.41E-14	2.35E-12	Co-occurrence
OR10T2	1q23.1	34 (29.57%)	5 (2.04%)	3.86	4.41E-14	2.35E-12	Co-occurrence
OR10X1	1q23.1	34 (29.57%)	5 (2.04%)	3.86	4.41E-14	2.35E-12	Co-occurrence
OR10Z1	1q23.1	34 (29.57%)	5 (2.04%)	3.86	4.41E-14	2.35E-12	Co-occurrence

OR6K2	1q23.1	34 (29.57%)	5 (2.04%)	3.86	4.41E-14	2.35E-12	Co-occurrence
OR6K3	1q23.1	34 (29.57%)	5 (2.04%)	3.86	4.41E-14	2.35E-12	Co-occurrence
OR6K6	1q23.1	34 (29.57%)	5 (2.04%)	3.86	4.41E-14	2.35E-12	Co-occurrence
OR6N1	1q23.1	34 (29.57%)	5 (2.04%)	3.86	4.41E-14	2.35E-12	Co-occurrence
OR6N2	1q23.1	34 (29.57%)	5 (2.04%)	3.86	4.41E-14	2.35E-12	Co-occurrence
OR6P1	1q23.1	34 (29.57%)	5 (2.04%)	3.86	4.41E-14	2.35E-12	Co-occurrence
OR6Y1	1q23.1	34 (29.57%)	5 (2.04%)	3.86	4.41E-14	2.35E-12	Co-occurrence
SPTA1	1q23.1	34 (29.57%)	5 (2.04%)	3.86	4.41E-14	2.35E-12	Co-occurrence
ARHGAP30	1q23.3	35 (30.43%)	6 (2.45%)	3.64	6.56E-14	3.23E-12	Co-occurrence
ATP1A2	1q23.2	35 (30.43%)	6 (2.45%)	3.64	6.56E-14	3.23E-12	Co-occurrence
ATP1A4	1q23.2	35 (30.43%)	6 (2.45%)	3.64	6.56E-14	3.23E-12	Co-occurrence
CASQ1	1q23.2	35 (30.43%)	6 (2.45%)	3.64	6.56E-14	3.23E-12	Co-occurrence
CD244	1q23.3	35 (30.43%)	6 (2.45%)	3.64	6.56E-14	3.23E-12	Co-occurrence
CD48	1q23.3	35 (30.43%)	6 (2.45%)	3.64	6.56E-14	3.23E-12	Co-occurrence
CD84	1q23.3	35 (30.43%)	6 (2.45%)	3.64	6.56E-14	3.23E-12	Co-occurrence
COPA	1q23.2	35 (30.43%)	6 (2.45%)	3.64	6.56E-14	3.23E-12	Co-occurrence
DCAF8	1q23.2	35 (30.43%)	6 (2.45%)	3.64	6.56E-14	3.23E-12	Co-occurrence
F11R	1q23.3	35 (30.43%)	6 (2.45%)	3.64	6.56E-14	3.23E-12	Co-occurrence
IGSF8	1q23.2	35 (30.43%)	6 (2.45%)	3.64	6.56E-14	3.23E-12	Co-occurrence
ITLN1	1q23.3	35 (30.43%)	6 (2.45%)	3.64	6.56E-14	3.23E-12	Co-occurrence
ITLN2	1q23.3	35 (30.43%)	6 (2.45%)	3.64	6.56E-14	3.23E-12	Co-occurrence
KCNJ10	1q23.2	35 (30.43%)	6 (2.45%)	3.64	6.56E-14	3.23E-12	Co-occurrence
KCNJ9	1q23.2	35 (30.43%)	6 (2.45%)	3.64	6.56E-14	3.23E-12	Co-occurrence
KLHDC9	1q23.3	35 (30.43%)	6 (2.45%)	3.64	6.56E-14	3.23E-12	Co-occurrence
LY9	1q23.3	35 (30.43%)	6 (2.45%)	3.64	6.56E-14	3.23E-12	Co-occurrence
NCSTN	1q23.2	35 (30.43%)	6 (2.45%)	3.64	6.56E-14	3.23E-12	Co-occurrence
NECTIN4	1q23.3	35 (30.43%)	6 (2.45%)	3.64	6.56E-14	3.23E-12	Co-occurrence
NHLH1	1q23.2	35 (30.43%)	6 (2.45%)	3.64	6.56E-14	3.23E-12	Co-occurrence
PEA15	1q23.2	35 (30.43%)	6 (2.45%)	3.64	6.56E-14	3.23E-12	Co-occurrence

PEX19	1q23.2	35 (30.43%)	6 (2.45%)	3.64	6.56E-14	3.23E-12	Co-occurrence
PIGM	1q23.2	35 (30.43%)	6 (2.45%)	3.64	6.56E-14	3.23E-12	Co-occurrence
SLAMF1	1q23.3	35 (30.43%)	6 (2.45%)	3.64	6.56E-14	3.23E-12	Co-occurrence
SLAMF6	1q23.2-q23.3	35 (30.43%)	6 (2.45%)	3.64	6.56E-14	3.23E-12	Co-occurrence
SLAMF7	1q23.3	35 (30.43%)	6 (2.45%)	3.64	6.56E-14	3.23E-12	Co-occurrence
TSTD1	1q23.3	35 (30.43%)	6 (2.45%)	3.64	6.56E-14	3.23E-12	Co-occurrence
USF1	1q23.3	35 (30.43%)	6 (2.45%)	3.64	6.56E-14	3.23E-12	Co-occurrence
VANGL2	1q23.2	35 (30.43%)	6 (2.45%)	3.64	6.56E-14	3.23E-12	Co-occurrence
B4GALT3	1q23.3	36 (31.30%)	7 (2.86%)	3.45	8.93E-14	4.31E-12	Co-occurrence
DEDD	1q23.3	36 (31.30%)	7 (2.86%)	3.45	8.93E-14	4.31E-12	Co-occurrence
NIT1	1q23.3	36 (31.30%)	7 (2.86%)	3.45	8.93E-14	4.31E-12	Co-occurrence
PFDN2	1q23.3	36 (31.30%)	7 (2.86%)	3.45	8.93E-14	4.31E-12	Co-occurrence
PPOX	1q23.3	36 (31.30%)	7 (2.86%)	3.45	8.93E-14	4.31E-12	Co-occurrence
SNORA78	16p13.3	36 (31.30%)	7 (2.86%)	3.45	8.93E-14	4.31E-12	Co-occurrence
UFC1	1q23.3	36 (31.30%)	7 (2.86%)	3.45	8.93E-14	4.31E-12	Co-occurrence
USP21	1q23.3	36 (31.30%)	7 (2.86%)	3.45	8.93E-14	4.31E-12	Co-occurrence
APOA2	1q23.3	37 (32.17%)	8 (3.27%)	3.3	1.13E-13	5.36E-12	Co-occurrence
FCER1G	1q23.3	37 (32.17%)	8 (3.27%)	3.3	1.13E-13	5.36E-12	Co-occurrence
NDUFS2	1q23.3	37 (32.17%)	8 (3.27%)	3.3	1.13E-13	5.36E-12	Co-occurrence
NR1I3	1q23.3	37 (32.17%)	8 (3.27%)	3.3	1.13E-13	5.36E-12	Co-occurrence
PCP4L1	1q23.3	37 (32.17%)	8 (3.27%)	3.3	1.13E-13	5.36E-12	Co-occurrence
TOMM40L	1q23.3	37 (32.17%)	8 (3.27%)	3.3	1.13E-13	5.36E-12	Co-occurrence
PBX1	1q23.3	33 (28.70%)	5 (2.04%)	3.81	1.51E-13	7.16E-12	Co-occurrence
ACKR1	1q23.2	34 (29.57%)	6 (2.45%)	3.59	2.22E-13	9.84E-12	Co-occurrence
AIM2	1q23.1-q23.2	34 (29.57%)	6 (2.45%)	3.59	2.22E-13	9.84E-12	Co-occurrence
ALDH9A1	1q24.1	34 (29.57%)	6 (2.45%)	3.59	2.22E-13	9.84E-12	Co-occurrence
APCS	1q23.2	34 (29.57%)	6 (2.45%)	3.59	2.22E-13	9.84E-12	Co-occurrence
CADM3	1q23.2	34 (29.57%)	6 (2.45%)	3.59	2.22E-13	9.84E-12	Co-occurrence
CFAP45	1q23.2	34 (29.57%)	6 (2.45%)	3.59	2.22E-13	9.84E-12	Co-occurrence

CREG1	1q24.2	34 (29.57%)	6 (2.45%)	3.59	2.22E-13	9.84E-12	Co-occurrence
CRP	1q23.2	34 (29.57%)	6 (2.45%)	3.59	2.22E-13	9.84E-12	Co-occurrence
DUSP23	1q23.2	34 (29.57%)	6 (2.45%)	3.59	2.22E-13	9.84E-12	Co-occurrence
FCER1A	1q23.2	34 (29.57%)	6 (2.45%)	3.59	2.22E-13	9.84E-12	Co-occurrence
FCRL6	1q23.2	34 (29.57%)	6 (2.45%)	3.59	2.22E-13	9.84E-12	Co-occurrence
IFI16	1q23.1	34 (29.57%)	6 (2.45%)	3.59	2.22E-13	9.84E-12	Co-occurrence
IGSF9	1q23.2	34 (29.57%)	6 (2.45%)	3.59	2.22E-13	9.84E-12	Co-occurrence
MNDA	1q23.1	34 (29.57%)	6 (2.45%)	3.59	2.22E-13	9.84E-12	Co-occurrence
OR10J1	1q23.2	34 (29.57%)	6 (2.45%)	3.59	2.22E-13	9.84E-12	Co-occurrence
OR10J3	1q23.2	34 (29.57%)	6 (2.45%)	3.59	2.22E-13	9.84E-12	Co-occurrence
OR10J4	1q23.2	34 (29.57%)	6 (2.45%)	3.59	2.22E-13	9.84E-12	Co-occurrence
OR10J5	1q23.2	34 (29.57%)	6 (2.45%)	3.59	2.22E-13	9.84E-12	Co-occurrence
PYHIN1	1q23.1	34 (29.57%)	6 (2.45%)	3.59	2.22E-13	9.84E-12	Co-occurrence
RCSD1	1q24.2	34 (29.57%)	6 (2.45%)	3.59	2.22E-13	9.84E-12	Co-occurrence
RNA5SP60	1q23.2	34 (29.57%)	6 (2.45%)	3.59	2.22E-13	9.84E-12	Co-occurrence
SLAMF8	1q23.2	34 (29.57%)	6 (2.45%)	3.59	2.22E-13	9.84E-12	Co-occurrence
SLAMF9	1q23.2	34 (29.57%)	6 (2.45%)	3.59	2.22E-13	9.84E-12	Co-occurrence
SNHG28	1q23.2	34 (29.57%)	6 (2.45%)	3.59	2.22E-13	9.84E-12	Co-occurrence
SNORD64	15q11.2	34 (29.57%)	6 (2.45%)	3.59	2.22E-13	9.84E-12	Co-occurrence
TAGLN2	1q23.2	34 (29.57%)	6 (2.45%)	3.59	2.22E-13	9.84E-12	Co-occurrence
VSIG8	1q23.2	34 (29.57%)	6 (2.45%)	3.59	2.22E-13	9.84E-12	Co-occurrence
FCGR2A	1q23.3	35 (30.43%)	7 (2.86%)	3.41	2.98E-13	1.30E-11	Co-occurrence
FCGR2B	1q23.3	35 (30.43%)	7 (2.86%)	3.41	2.98E-13	1.30E-11	Co-occurrence
FCGR2C	1q23.3	35 (30.43%)	7 (2.86%)	3.41	2.98E-13	1.30E-11	Co-occurrence
FCGR3A	1q23.3	35 (30.43%)	7 (2.86%)	3.41	2.98E-13	1.30E-11	Co-occurrence
FCGR3B	1q23.3	35 (30.43%)	7 (2.86%)	3.41	2.98E-13	1.30E-11	Co-occurrence
HSPA6	1q23.3	35 (30.43%)	7 (2.86%)	3.41	2.98E-13	1.30E-11	Co-occurrence
MIR-4654/4654		35 (30.43%)	7 (2.86%)	3.41	2.98E-13	1.30E-11	Co-occurrence
RPL31P11	1q23.3	35 (30.43%)	7 (2.86%)	3.41	2.98E-13	1.30E-11	Co-occurrence

ADAMTS4	1q23.3	36 (31.30%)	8 (3.27%)	3.26	3.72E-13	1.62E-11	Co-occurrence
NOS1AP	1q23.3	37 (32.17%)	9 (3.67%)	3.13	4.38E-13	1.90E-11	Co-occurrence
CD247	1q24.2	33 (28.70%)	6 (2.45%)	3.55	7.40E-13	3.10E-11	Co-occurrence
DUSP27	1q24.1	33 (28.70%)	6 (2.45%)	3.55	7.40E-13	3.10E-11	Co-occurrence
FAM78B	1q24.1	33 (28.70%)	6 (2.45%)	3.55	7.40E-13	3.10E-11	Co-occurrence
FMO9P	1q24.1	33 (28.70%)	6 (2.45%)	3.55	7.40E-13	3.10E-11	Co-occurrence
GPA33	1q24.1	33 (28.70%)	6 (2.45%)	3.55	7.40E-13	3.10E-11	Co-occurrence
ILDR2	1q24.1	33 (28.70%)	6 (2.45%)	3.55	7.40E-13	3.10E-11	Co-occurrence
LRRC52	1q24.1	33 (28.70%)	6 (2.45%)	3.55	7.40E-13	3.10E-11	Co-occurrence
MAEL	1q24.1	33 (28.70%)	6 (2.45%)	3.55	7.40E-13	3.10E-11	Co-occurrence
MGST3	1q24.1	33 (28.70%)	6 (2.45%)	3.55	7.40E-13	3.10E-11	Co-occurrence
MIR-921/921		33 (28.70%)	6 (2.45%)	3.55	7.40E-13	3.10E-11	Co-occurrence
POGK	1q24.1	33 (28.70%)	6 (2.45%)	3.55	7.40E-13	3.10E-11	Co-occurrence
RNA5SP64	1q24.1	33 (28.70%)	6 (2.45%)	3.55	7.40E-13	3.10E-11	Co-occurrence
RNA5SP65	1q24.1	33 (28.70%)	6 (2.45%)	3.55	7.40E-13	3.10E-11	Co-occurrence
RXRG	1q23.3	33 (28.70%)	6 (2.45%)	3.55	7.40E-13	3.10E-11	Co-occurrence
TADA1	1q24.1	33 (28.70%)	6 (2.45%)	3.55	7.40E-13	3.10E-11	Co-occurrence
ADCY10	1q24.2	34 (29.57%)	7 (2.86%)	3.37	9.81E-13	4.03E-11	Co-occurrence
ANKRD36BP1	1q24.2	34 (29.57%)	7 (2.86%)	3.37	9.81E-13	4.03E-11	Co-occurrence
DCAF6	1q24.2	34 (29.57%)	7 (2.86%)	3.37	9.81E-13	4.03E-11	Co-occurrence
GPR161	1q24.2	34 (29.57%)	7 (2.86%)	3.37	9.81E-13	4.03E-11	Co-occurrence
MPC2	1q24.2	34 (29.57%)	7 (2.86%)	3.37	9.81E-13	4.03E-11	Co-occurrence
MPZL1	1q24.2	34 (29.57%)	7 (2.86%)	3.37	9.81E-13	4.03E-11	Co-occurrence
SFT2D2	1q24.2	34 (29.57%)	7 (2.86%)	3.37	9.81E-13	4.03E-11	Co-occurrence
TBX19	1q24.2	34 (29.57%)	7 (2.86%)	3.37	9.81E-13	4.03E-11	Co-occurrence
TIPRL	1q24.2	34 (29.57%)	7 (2.86%)	3.37	9.81E-13	4.03E-11	Co-occurrence
CCDC190	1q23.3	35 (30.43%)	8 (3.27%)	3.22	1.21E-12	4.93E-11	Co-occurrence
HSD17B7	1q23.3	35 (30.43%)	8 (3.27%)	3.22	1.21E-12	4.93E-11	Co-occurrence
RN7SL861P	1q23.3	35 (30.43%)	8 (3.27%)	3.22	1.21E-12	4.93E-11	Co-occurrence

RNA5SP61	1q23.3	35 (30.43%)	8 (3.27%)	3.22	1.21E-12	4.93E-11	Co-occurrence
LMX1A	1q23.3	33 (28.70%)	7 (2.86%)	3.33	3.18E-12	1.28E-10	Co-occurrence
MIR-557/557		33 (28.70%)	7 (2.86%)	3.33	3.18E-12	1.28E-10	Co-occurrence
POU2F1	1q24.2	33 (28.70%)	7 (2.86%)	3.33	3.18E-12	1.28E-10	Co-occurrence
TMCO1	1q24.1	33 (28.70%)	7 (2.86%)	3.33	3.18E-12	1.28E-10	Co-occurrence
UCK2	1q24.1	33 (28.70%)	7 (2.86%)	3.33	3.18E-12	1.28E-10	Co-occurrence
ATF6	1q23.3	34 (29.57%)	8 (3.27%)	3.18	3.88E-12	1.53E-10	Co-occurrence
DUSP12	1q23.3	34 (29.57%)	8 (3.27%)	3.18	3.88E-12	1.53E-10	Co-occurrence
FCRLA	1q23.3	34 (29.57%)	8 (3.27%)	3.18	3.88E-12	1.53E-10	Co-occurrence
FCRLB	1q23.3	34 (29.57%)	8 (3.27%)	3.18	3.88E-12	1.53E-10	Co-occurrence
NUF2	1q23.3	34 (29.57%)	8 (3.27%)	3.18	3.88E-12	1.53E-10	Co-occurrence
OLFML2B	1q23.3	34 (29.57%)	8 (3.27%)	3.18	3.88E-12	1.53E-10	Co-occurrence
RGS4	1q23.3	34 (29.57%)	8 (3.27%)	3.18	3.88E-12	1.53E-10	Co-occurrence
RGS5	1q23.3	34 (29.57%)	8 (3.27%)	3.18	3.88E-12	1.53E-10	Co-occurrence
RN7SL466P	1q23.3	34 (29.57%)	8 (3.27%)	3.18	3.88E-12	1.53E-10	Co-occurrence
RNA5SP62	1q23.3	34 (29.57%)	8 (3.27%)	3.18	3.88E-12	1.53E-10	Co-occurrence
RNA5SP63	1q23.3	34 (29.57%)	8 (3.27%)	3.18	3.88E-12	1.53E-10	Co-occurrence
C1ORF226	1q23.3	35 (30.43%)	9 (3.67%)	3.05	4.47E-12	1.74E-10	Co-occurrence
DDR2	1q23.3	35 (30.43%)	9 (3.67%)	3.05	4.47E-12	1.74E-10	Co-occurrence
SH2D1B	1q23.3	35 (30.43%)	9 (3.67%)	3.05	4.47E-12	1.74E-10	Co-occurrence
SPATA46	1q23.3	35 (30.43%)	9 (3.67%)	3.05	4.47E-12	1.74E-10	Co-occurrence
UAP1	1q23.3	35 (30.43%)	9 (3.67%)	3.05	4.47E-12	1.74E-10	Co-occurrence
UHMK1	1q23.3	35 (30.43%)	9 (3.67%)	3.05	4.47E-12	1.74E-10	Co-occurrence
C1ORF105	1q24.3	30 (26.09%)	5 (2.04%)	3.68	5.65E-12	2.15E-10	Co-occurrence
DNM3	1q24.3	30 (26.09%)	5 (2.04%)	3.68	5.65E-12	2.15E-10	Co-occurrence
DNM3OS	1q24.3	30 (26.09%)	5 (2.04%)	3.68	5.65E-12	2.15E-10	Co-occurrence
FASLG	1q24.3	30 (26.09%)	5 (2.04%)	3.68	5.65E-12	2.15E-10	Co-occurrence
PIGC	1q24.3	30 (26.09%)	5 (2.04%)	3.68	5.65E-12	2.15E-10	Co-occurrence
PRDX6	1q25.1	30 (26.09%)	5 (2.04%)	3.68	5.65E-12	2.15E-10	Co-occurrence

SLC9C2	1q25.1	30 (26.09%)	5 (2.04%)	3.68	5.65E-12	2.15E-10	Co-occurrence
SUCO	1q24.3	30 (26.09%)	5 (2.04%)	3.68	5.65E-12	2.15E-10	Co-occurrence
TNFSF18	1q25.1	30 (26.09%)	5 (2.04%)	3.68	5.65E-12	2.15E-10	Co-occurrence
TNFSF4	1q25.1	30 (26.09%)	5 (2.04%)	3.68	5.65E-12	2.15E-10	Co-occurrence
C1ORF112	1q24.2	31 (26.96%)	6 (2.45%)	3.46	7.91E-12	2.98E-10	Co-occurrence
F5	1q24.2	31 (26.96%)	6 (2.45%)	3.46	7.91E-12	2.98E-10	Co-occurrence
SELE	1q24.2	31 (26.96%)	6 (2.45%)	3.46	7.91E-12	2.98E-10	Co-occurrence
SELL	1q24.2	31 (26.96%)	6 (2.45%)	3.46	7.91E-12	2.98E-10	Co-occurrence
SELP	1q24.2	31 (26.96%)	6 (2.45%)	3.46	7.91E-12	2.98E-10	Co-occurrence
DPT	1q24.2	32 (27.83%)	7 (2.86%)	3.28	1.02E-11	3.82E-10	Co-occurrence
XCL1	1q24.2	32 (27.83%)	7 (2.86%)	3.28	1.02E-11	3.82E-10	Co-occurrence
XCL2	1q24.2	32 (27.83%)	7 (2.86%)	3.28	1.02E-11	3.82E-10	Co-occurrence
ANKRD45	1q25.1	29 (25.22%)	5 (2.04%)	3.63	1.84E-11	6.87E-10	Co-occurrence
RN7SKP160	1q25.1	29 (25.22%)	5 (2.04%)	3.63	1.84E-11	6.87E-10	Co-occurrence
BLZF1	1q24.2	30 (26.09%)	6 (2.45%)	3.41	2.53E-11	9.02E-10	Co-occurrence
CCDC181	1q24.2	30 (26.09%)	6 (2.45%)	3.41	2.53E-11	9.02E-10	Co-occurrence
COP1	1q25.1-q25.2	30 (26.09%)	6 (2.45%)	3.41	2.53E-11	9.02E-10	Co-occurrence
EEF1AKNMT	1q24.3	30 (26.09%)	6 (2.45%)	3.41	2.53E-11	9.02E-10	Co-occurrence
FMO3	1q24.3	30 (26.09%)	6 (2.45%)	3.41	2.53E-11	9.02E-10	Co-occurrence
KIFAP3	1q24.2	30 (26.09%)	6 (2.45%)	3.41	2.53E-11	9.02E-10	Co-occurrence
METTL11B	1q24.2	30 (26.09%)	6 (2.45%)	3.41	2.53E-11	9.02E-10	Co-occurrence
METTL18	1q24.2	30 (26.09%)	6 (2.45%)	3.41	2.53E-11	9.02E-10	Co-occurrence
MROH9	1q24.3	30 (26.09%)	6 (2.45%)	3.41	2.53E-11	9.02E-10	Co-occurrence
MYOC	1q24.3	30 (26.09%)	6 (2.45%)	3.41	2.53E-11	9.02E-10	Co-occurrence
PRRC2C	1q24.3	30 (26.09%)	6 (2.45%)	3.41	2.53E-11	9.02E-10	Co-occurrence
PRRX1	1q24.2	30 (26.09%)	6 (2.45%)	3.41	2.53E-11	9.02E-10	Co-occurrence
RC3H1	1q25.1	30 (26.09%)	6 (2.45%)	3.41	2.53E-11	9.02E-10	Co-occurrence
RN7SL269P	1q24.2	30 (26.09%)	6 (2.45%)	3.41	2.53E-11	9.02E-10	Co-occurrence
RN7SL333P	1q24.2	30 (26.09%)	6 (2.45%)	3.41	2.53E-11	9.02E-10	Co-occurrence

RN7SL425P	1q24.3	30 (26.09%)	6 (2.45%)	3.41	2.53E-11	9.02E-10	Co-occurrence
RNA5SP67	1q25.1	30 (26.09%)	6 (2.45%)	3.41	2.53E-11	9.02E-10	Co-occurrence
RNA5SP68	1q25.1	30 (26.09%)	6 (2.45%)	3.41	2.53E-11	9.02E-10	Co-occurrence
SCARNA20	17q23.2	30 (26.09%)	6 (2.45%)	3.41	2.53E-11	9.02E-10	Co-occurrence
SCARNA3	1q25.1	30 (26.09%)	6 (2.45%)	3.41	2.53E-11	9.02E-10	Co-occurrence
SCYL3	1q24.2	30 (26.09%)	6 (2.45%)	3.41	2.53E-11	9.02E-10	Co-occurrence
SERPINC1	1q25.1	30 (26.09%)	6 (2.45%)	3.41	2.53E-11	9.02E-10	Co-occurrence
SLC19A2	1q24.2	30 (26.09%)	6 (2.45%)	3.41	2.53E-11	9.02E-10	Co-occurrence
VAMP4	1q24.3	30 (26.09%)	6 (2.45%)	3.41	2.53E-11	9.02E-10	Co-occurrence
LINC00626	1q24.2	31 (26.96%)	7 (2.86%)	3.24	3.22E-11	1.14E-09	Co-occurrence
LINC00970	1q24.2	31 (26.96%)	7 (2.86%)	3.24	3.22E-11	1.14E-09	Co-occurrence
CENPL	1q25.1	29 (25.22%)	6 (2.45%)	3.36	8.01E-11	2.77E-09	Co-occurrence
DARS2	1q25.1	29 (25.22%)	6 (2.45%)	3.36	8.01E-11	2.77E-09	Co-occurrence
FMO1	1q24.3	29 (25.22%)	6 (2.45%)	3.36	8.01E-11	2.77E-09	Co-occurrence
FMO2	1q24.3	29 (25.22%)	6 (2.45%)	3.36	8.01E-11	2.77E-09	Co-occurrence
FMO4	1q24.3	29 (25.22%)	6 (2.45%)	3.36	8.01E-11	2.77E-09	Co-occurrence
FMO6P	1q24.3	29 (25.22%)	6 (2.45%)	3.36	8.01E-11	2.77E-09	Co-occurrence
GAS5	1q25.1	29 (25.22%)	6 (2.45%)	3.36	8.01E-11	2.77E-09	Co-occurrence
KLHL20	1q25.1	29 (25.22%)	6 (2.45%)	3.36	8.01E-11	2.77E-09	Co-occurrence
PAPPA2	1q25.2	29 (25.22%)	6 (2.45%)	3.36	8.01E-11	2.77E-09	Co-occurrence
RABGAP1L	1q25.1	29 (25.22%)	6 (2.45%)	3.36	8.01E-11	2.77E-09	Co-occurrence
SNORD78	1q25.1	29 (25.22%)	6 (2.45%)	3.36	8.01E-11	2.77E-09	Co-occurrence
TNR	1q25.1	29 (25.22%)	6 (2.45%)	3.36	8.01E-11	2.77E-09	Co-occurrence
ZBTB37	1q25.1	29 (25.22%)	6 (2.45%)	3.36	8.01E-11	2.77E-09	Co-occurrence
ATP1B1	1q24.2	30 (26.09%)	7 (2.86%)	3.19	1.00E-10	3.44E-09	Co-occurrence
GORAB	1q24.2	30 (26.09%)	7 (2.86%)	3.19	1.00E-10	3.44E-09	Co-occurrence
NME7	1q24.2	30 (26.09%)	7 (2.86%)	3.19	1.00E-10	3.44E-09	Co-occurrence
RNA5SP66	1q24.2	30 (26.09%)	7 (2.86%)	3.19	1.00E-10	3.44E-09	Co-occurrence
ASTN1	1q25.2	28 (24.35%)	6 (2.45%)	3.31	2.49E-10	8.51E-09	Co-occurrence

BRINP2	1q25.2	28 (24.35%)	6 (2.45%)	3.31	2.49E-10	8.51E-09	Co-occurrence
GPR52	1q25.1	28 (24.35%)	6 (2.45%)	3.31	2.49E-10	8.51E-09	Co-occurrence
SEC16B	1q25.2	28 (24.35%)	6 (2.45%)	3.31	2.49E-10	8.51E-09	Co-occurrence
ABL2	1q25.2	29 (25.22%)	7 (2.86%)	3.14	3.07E-10	1.00E-08	Co-occurrence
ACBD6	1q25.2-q25.3	29 (25.22%)	7 (2.86%)	3.14	3.07E-10	1.00E-08	Co-occurrence
ANGPTL1	1q25.2	29 (25.22%)	7 (2.86%)	3.14	3.07E-10	1.00E-08	Co-occurrence
AXDND1	1q25.2	29 (25.22%)	7 (2.86%)	3.14	3.07E-10	1.00E-08	Co-occurrence
CACYBP	1q25.1	29 (25.22%)	7 (2.86%)	3.14	3.07E-10	1.00E-08	Co-occurrence
CEP350	1q25.2	29 (25.22%)	7 (2.86%)	3.14	3.07E-10	1.00E-08	Co-occurrence
FAM163A	1q25.2	29 (25.22%)	7 (2.86%)	3.14	3.07E-10	1.00E-08	Co-occurrence
IER5	1q25.3	29 (25.22%)	7 (2.86%)	3.14	3.07E-10	1.00E-08	Co-occurrence
KIAA0040	1q25.1	29 (25.22%)	7 (2.86%)	3.14	3.07E-10	1.00E-08	Co-occurrence
LHX4	1q25.2	29 (25.22%)	7 (2.86%)	3.14	3.07E-10	1.00E-08	Co-occurrence
MR1	1q25.3	29 (25.22%)	7 (2.86%)	3.14	3.07E-10	1.00E-08	Co-occurrence
MRPS14	1q25.1	29 (25.22%)	7 (2.86%)	3.14	3.07E-10	1.00E-08	Co-occurrence
NPHS2	1q25.2	29 (25.22%)	7 (2.86%)	3.14	3.07E-10	1.00E-08	Co-occurrence
QSOX1	1q25.2	29 (25.22%)	7 (2.86%)	3.14	3.07E-10	1.00E-08	Co-occurrence
RALGPS2	1q25.2	29 (25.22%)	7 (2.86%)	3.14	3.07E-10	1.00E-08	Co-occurrence
RN7SL230P	1q25.2	29 (25.22%)	7 (2.86%)	3.14	3.07E-10	1.00E-08	Co-occurrence
SNORA67	17p13.1	29 (25.22%)	7 (2.86%)	3.14	3.07E-10	1.00E-08	Co-occurrence
SOAT1	1q25.2	29 (25.22%)	7 (2.86%)	3.14	3.07E-10	1.00E-08	Co-occurrence
TDRD5	1q25.2	29 (25.22%)	7 (2.86%)	3.14	3.07E-10	1.00E-08	Co-occurrence
TOR1AIP1	1q25.2	29 (25.22%)	7 (2.86%)	3.14	3.07E-10	1.00E-08	Co-occurrence
TOR1AIP2	1q25.2	29 (25.22%)	7 (2.86%)	3.14	3.07E-10	1.00E-08	Co-occurrence
TOR3A	1q25.2	29 (25.22%)	7 (2.86%)	3.14	3.07E-10	1.00E-08	Co-occurrence
XPR1	1q25.3	29 (25.22%)	7 (2.86%)	3.14	3.07E-10	1.00E-08	Co-occurrence
C1ORF220	1q25.2	28 (24.35%)	7 (2.86%)	3.09	9.27E-10	2.99E-08	Co-occurrence
CLEC20A	1q25.2	28 (24.35%)	7 (2.86%)	3.09	9.27E-10	2.99E-08	Co-occurrence
GLUL	1q25.3	28 (24.35%)	7 (2.86%)	3.09	9.27E-10	2.99E-08	Co-occurrence

LINC00272	1q25.3	28 (24.35%)	7 (2.86%)	3.09	9.27E-10	2.99E-08	Co-occurrence
MIR-4424/4424		28 (24.35%)	7 (2.86%)	3.09	9.27E-10	2.99E-08	Co-occurrence
RASAL2	1q25.2	28 (24.35%)	7 (2.86%)	3.09	9.27E-10	2.99E-08	Co-occurrence
RNA5SP69	1q25.2	28 (24.35%)	7 (2.86%)	3.09	9.27E-10	2.99E-08	Co-occurrence
TEDDM1	1q25.3	28 (24.35%)	7 (2.86%)	3.09	9.27E-10	2.99E-08	Co-occurrence
TEX35	1q25.2	28 (24.35%)	7 (2.86%)	3.09	9.27E-10	2.99E-08	Co-occurrence
FAM20B	1q25.2	29 (25.22%)	8 (3.27%)	2.95	1.06E-09	3.37E-08	Co-occurrence
KIAA1614	1q25.3	29 (25.22%)	8 (3.27%)	2.95	1.06E-09	3.37E-08	Co-occurrence
RN7SKP229	1q25.3	29 (25.22%)	8 (3.27%)	2.95	1.06E-09	3.37E-08	Co-occurrence
RNA5SP70	1q25.3	29 (25.22%)	8 (3.27%)	2.95	1.06E-09	3.37E-08	Co-occurrence
STX6	1q25.3	29 (25.22%)	8 (3.27%)	2.95	1.06E-09	3.37E-08	Co-occurrence
TNN	1q25.1	29 (25.22%)	8 (3.27%)	2.95	1.06E-09	3.37E-08	Co-occurrence
DHX9	1q25.3	27 (23.48%)	7 (2.86%)	3.04	2.76E-09	8.61E-08	Co-occurrence
LAMC1	1q25.3	27 (23.48%)	7 (2.86%)	3.04	2.76E-09	8.61E-08	Co-occurrence
LAMC2	1q25.3	27 (23.48%)	7 (2.86%)	3.04	2.76E-09	8.61E-08	Co-occurrence
NMNAT2	1q25.3	27 (23.48%)	7 (2.86%)	3.04	2.76E-09	8.61E-08	Co-occurrence
NPL	1q25.3	27 (23.48%)	7 (2.86%)	3.04	2.76E-09	8.61E-08	Co-occurrence
RGSL1	1q25.3	27 (23.48%)	7 (2.86%)	3.04	2.76E-09	8.61E-08	Co-occurrence
RN7SL654P	1q25.3	27 (23.48%)	7 (2.86%)	3.04	2.76E-09	8.61E-08	Co-occurrence
RNA5SP71	1q25.3	27 (23.48%)	7 (2.86%)	3.04	2.76E-09	8.61E-08	Co-occurrence
RNA5SP72	1q25.3	27 (23.48%)	7 (2.86%)	3.04	2.76E-09	8.61E-08	Co-occurrence
RNASEL	1q25.3	27 (23.48%)	7 (2.86%)	3.04	2.76E-09	8.61E-08	Co-occurrence
RNF2	1q25.3	27 (23.48%)	7 (2.86%)	3.04	2.76E-09	8.61E-08	Co-occurrence
SHCBP1L	1q25.3	27 (23.48%)	7 (2.86%)	3.04	2.76E-09	8.61E-08	Co-occurrence
TRMT1L	1q25.3	27 (23.48%)	7 (2.86%)	3.04	2.76E-09	8.61E-08	Co-occurrence
SWT1	1q25.3	28 (24.35%)	8 (3.27%)	2.9	3.10E-09	9.65E-08	Co-occurrence
CACNA1E	1q25.3	29 (25.22%)	9 (3.67%)	2.78	3.32E-09	1.03E-07	Co-occurrence
APOBEC4	1q25.3	27 (23.48%)	8 (3.27%)	2.85	8.94E-09	2.73E-07	Co-occurrence
ARPC5	1q25.3	27 (23.48%)	8 (3.27%)	2.85	8.94E-09	2.73E-07	Co-occurrence

COLGALT2	1q25.3	27 (23.48%)	8 (3.27%)	2.85	8.94E-09	2.73E-07	Co-occurrence
NCF2	1q25.3	27 (23.48%)	8 (3.27%)	2.85	8.94E-09	2.73E-07	Co-occurrence
NIBAN1	1q25.3	27 (23.48%)	8 (3.27%)	2.85	8.94E-09	2.73E-07	Co-occurrence
RGL1	1q25.3	27 (23.48%)	8 (3.27%)	2.85	8.94E-09	2.73E-07	Co-occurrence
RGS16	1q25.3	27 (23.48%)	8 (3.27%)	2.85	8.94E-09	2.73E-07	Co-occurrence
RGS8	1q25.3	27 (23.48%)	8 (3.27%)	2.85	8.94E-09	2.73E-07	Co-occurrence
SMG7	1q25.3	27 (23.48%)	8 (3.27%)	2.85	8.94E-09	2.73E-07	Co-occurrence
TSEN15	1q25.3	27 (23.48%)	8 (3.27%)	2.85	8.94E-09	2.73E-07	Co-occurrence
ZNF648	1q25.3	27 (23.48%)	8 (3.27%)	2.85	8.94E-09	2.73E-07	Co-occurrence
OCLM	1q31.1	25 (21.74%)	7 (2.86%)	2.93	2.34E-08	7.07E-07	Co-occurrence
ODR4	1q31.1	25 (21.74%)	7 (2.86%)	2.93	2.34E-08	7.07E-07	Co-occurrence
PDC	1q31.1	25 (21.74%)	7 (2.86%)	2.93	2.34E-08	7.07E-07	Co-occurrence
PLA2G4A	1q31.1	25 (21.74%)	7 (2.86%)	2.93	2.34E-08	7.07E-07	Co-occurrence
PTGS2	1q31.1	25 (21.74%)	7 (2.86%)	2.93	2.34E-08	7.07E-07	Co-occurrence
TPR	1q31.1	25 (21.74%)	7 (2.86%)	2.93	2.34E-08	7.07E-07	Co-occurrence
IVNS1ABP	1q25.3	26 (22.61%)	8 (3.27%)	2.79	2.54E-08	7.68E-07	Co-occurrence
C1ORF21	1q25.3	27 (23.48%)	9 (3.67%)	2.68	2.65E-08	7.98E-07	Co-occurrence
EDEM3	1q25.3	27 (23.48%)	9 (3.67%)	2.68	2.65E-08	7.98E-07	Co-occurrence
KCNT2	1q31.3	29 (25.22%)	12 (4.90%)	2.36	6.58E-08	1.98E-06	Co-occurrence
F13B	1q31.3	28 (24.35%)	11 (4.49%)	2.44	6.99E-08	2.10E-06	Co-occurrence
BRINP3	1q31.1	25 (21.74%)	8 (3.27%)	2.74	7.10E-08	2.12E-06	Co-occurrence
PRG4	1q31.1	25 (21.74%)	8 (3.27%)	2.74	7.10E-08	2.12E-06	Co-occurrence
RN7SKP156	1q31.1	25 (21.74%)	8 (3.27%)	2.74	7.10E-08	2.12E-06	Co-occurrence
RNA5SP73	1q31.1	25 (21.74%)	8 (3.27%)	2.74	7.10E-08	2.12E-06	Co-occurrence
HMCN1	1q25.3-q31.1	26 (22.61%)	9 (3.67%)	2.62	7.30E-08	2.17E-06	Co-occurrence
CFH	1q31.3	28 (24.35%)	12 (4.90%)	2.31	1.72E-07	5.09E-06	Co-occurrence
CFHR1	1q31.3	28 (24.35%)	12 (4.90%)	2.31	1.72E-07	5.09E-06	Co-occurrence
CFHR2	1q31.3	28 (24.35%)	12 (4.90%)	2.31	1.72E-07	5.09E-06	Co-occurrence
CFHR3	1q31.3	28 (24.35%)	12 (4.90%)	2.31	1.72E-07	5.09E-06	Co-occurrence

CFHR4	1q31.3	28 (24.35%)	12 (4.90%)	2.31	1.72E-07	5.09E-06	Co-occurrence
CFHR5	1q31.3	28 (24.35%)	12 (4.90%)	2.31	1.72E-07	5.09E-06	Co-occurrence
ASPM	1q31.3	27 (23.48%)	11 (4.49%)	2.39	1.85E-07	5.42E-06	Co-occurrence
C1ORF53	1q31.3	27 (23.48%)	11 (4.49%)	2.39	1.85E-07	5.42E-06	Co-occurrence
LHX9	1q31.3	27 (23.48%)	11 (4.49%)	2.39	1.85E-07	5.42E-06	Co-occurrence
NEK7	1q31.3	27 (23.48%)	11 (4.49%)	2.39	1.85E-07	5.42E-06	Co-occurrence
CTSE	1q32.1	26 (22.61%)	10 (4.08%)	2.47	1.94E-07	5.68E-06	Co-occurrence
ATP6V1G3	1q31.3	26 (22.61%)	11 (4.49%)	2.33	4.81E-07	1.37E-05	Co-occurrence
AVPR1B	1q32.1	26 (22.61%)	11 (4.49%)	2.33	4.81E-07	1.37E-05	Co-occurrence
C1ORF116	1q32.1	26 (22.61%)	11 (4.49%)	2.33	4.81E-07	1.37E-05	Co-occurrence
CRB1	1q31.3	26 (22.61%)	11 (4.49%)	2.33	4.81E-07	1.37E-05	Co-occurrence
DENND1B	1q31.3	26 (22.61%)	11 (4.49%)	2.33	4.81E-07	1.37E-05	Co-occurrence
FCAMR	1q32.1	26 (22.61%)	11 (4.49%)	2.33	4.81E-07	1.37E-05	Co-occurrence
IL10	1q32.1	26 (22.61%)	11 (4.49%)	2.33	4.81E-07	1.37E-05	Co-occurrence
IL19	1q32.1	26 (22.61%)	11 (4.49%)	2.33	4.81E-07	1.37E-05	Co-occurrence
IL20	1q32.1	26 (22.61%)	11 (4.49%)	2.33	4.81E-07	1.37E-05	Co-occurrence
LAD1	1q32.1	26 (22.61%)	11 (4.49%)	2.33	4.81E-07	1.37E-05	Co-occurrence
MAPKAPK2	1q32.1	26 (22.61%)	11 (4.49%)	2.33	4.81E-07	1.37E-05	Co-occurrence
MIR181A1HG	1q32.1	26 (22.61%)	11 (4.49%)	2.33	4.81E-07	1.37E-05	Co-occurrence
PHLDA3	1q32.1	26 (22.61%)	11 (4.49%)	2.33	4.81E-07	1.37E-05	Co-occurrence
PTPRC	1q31.3-q32.1	26 (22.61%)	11 (4.49%)	2.33	4.81E-07	1.37E-05	Co-occurrence
RHEX	1q32.1	26 (22.61%)	11 (4.49%)	2.33	4.81E-07	1.37E-05	Co-occurrence
RPS10P7	1q32.1	26 (22.61%)	11 (4.49%)	2.33	4.81E-07	1.37E-05	Co-occurrence
SNORD60	16p13.3	26 (22.61%)	11 (4.49%)	2.33	4.81E-07	1.37E-05	Co-occurrence
TNNI1	1q32.1	26 (22.61%)	11 (4.49%)	2.33	4.81E-07	1.37E-05	Co-occurrence
TNNT2	1q32.1	26 (22.61%)	11 (4.49%)	2.33	4.81E-07	1.37E-05	Co-occurrence
ZBTB41	1q31.3	26 (22.61%)	11 (4.49%)	2.33	4.81E-07	1.37E-05	Co-occurrence
C1ORF147	1q32.1	26 (22.61%)	12 (4.90%)	2.21	1.12E-06	3.07E-05	Co-occurrence
C4BPA	1q32.2	26 (22.61%)	12 (4.90%)	2.21	1.12E-06	3.07E-05	Co-occurrence

C4BPB	1q32.1	26 (22.61%)	12 (4.90%)	2.21	1.12E-06	3.07E-05	Co-occurrence
CSRP1	1q32.1	26 (22.61%)	12 (4.90%)	2.21	1.12E-06	3.07E-05	Co-occurrence
DYRK3	1q32.1	26 (22.61%)	12 (4.90%)	2.21	1.12E-06	3.07E-05	Co-occurrence
EIF2D	1q32.1	26 (22.61%)	12 (4.90%)	2.21	1.12E-06	3.07E-05	Co-occurrence
ELF3	1q32.1	26 (22.61%)	12 (4.90%)	2.21	1.12E-06	3.07E-05	Co-occurrence
FCMR	1q32.1	26 (22.61%)	12 (4.90%)	2.21	1.12E-06	3.07E-05	Co-occurrence
IKBKE	1q32.1	26 (22.61%)	12 (4.90%)	2.21	1.12E-06	3.07E-05	Co-occurrence
IL24	1q32.1	26 (22.61%)	12 (4.90%)	2.21	1.12E-06	3.07E-05	Co-occurrence
NAV1	1q32.1	26 (22.61%)	12 (4.90%)	2.21	1.12E-06	3.07E-05	Co-occurrence
PFKFB2	1q32.1	26 (22.61%)	12 (4.90%)	2.21	1.12E-06	3.07E-05	Co-occurrence
PIGR	1q32.1	26 (22.61%)	12 (4.90%)	2.21	1.12E-06	3.07E-05	Co-occurrence
RASSF5	1q32.1	26 (22.61%)	12 (4.90%)	2.21	1.12E-06	3.07E-05	Co-occurrence
RNPEP	1q32.1	26 (22.61%)	12 (4.90%)	2.21	1.12E-06	3.07E-05	Co-occurrence
SRGAP2	1q32.1	26 (22.61%)	12 (4.90%)	2.21	1.12E-06	3.07E-05	Co-occurrence
YOD1	1q32.1	26 (22.61%)	12 (4.90%)	2.21	1.12E-06	3.07E-05	Co-occurrence
ASCL5	1q32.1	25 (21.74%)	11 (4.49%)	2.28	1.23E-06	3.07E-05	Co-occurrence
CACNA1S	1q32.1	25 (21.74%)	11 (4.49%)	2.28	1.23E-06	3.07E-05	Co-occurrence
CD34	1q32.2	25 (21.74%)	11 (4.49%)	2.28	1.23E-06	3.07E-05	Co-occurrence
CD46	1q32.2	25 (21.74%)	11 (4.49%)	2.28	1.23E-06	3.07E-05	Co-occurrence
CD55	1q32.2	25 (21.74%)	11 (4.49%)	2.28	1.23E-06	3.07E-05	Co-occurrence
CDK18	1q32.1	25 (21.74%)	11 (4.49%)	2.28	1.23E-06	3.07E-05	Co-occurrence
CNTN2	1q32.1	25 (21.74%)	11 (4.49%)	2.28	1.23E-06	3.07E-05	Co-occurrence
CR1	1q32.2	25 (21.74%)	11 (4.49%)	2.28	1.23E-06	3.07E-05	Co-occurrence
CR1L	1q32.2	25 (21.74%)	11 (4.49%)	2.28	1.23E-06	3.07E-05	Co-occurrence
CR2	1q32.2	25 (21.74%)	11 (4.49%)	2.28	1.23E-06	3.07E-05	Co-occurrence
DSTYK	1q32.1	25 (21.74%)	11 (4.49%)	2.28	1.23E-06	3.07E-05	Co-occurrence
KIF21B	1q32.1	25 (21.74%)	11 (4.49%)	2.28	1.23E-06	3.07E-05	Co-occurrence
KLHDC8A	1q32.1	25 (21.74%)	11 (4.49%)	2.28	1.23E-06	3.07E-05	Co-occurrence
LEMD1	1q32.1	25 (21.74%)	11 (4.49%)	2.28	1.23E-06	3.07E-05	Co-occurrence

LINC00303	1q32.1	25 (21.74%)	11 (4.49%)	2.28	1.23E-06	3.07E-05	Co-occurrence
LYPD8	1q44	25 (21.74%)	11 (4.49%)	2.28	1.23E-06	3.07E-05	Co-occurrence
MIR29B2CHG	1q32.2	25 (21.74%)	11 (4.49%)	2.28	1.23E-06	3.07E-05	Co-occurrence
NUAK2	1q32.1	25 (21.74%)	11 (4.49%)	2.28	1.23E-06	3.07E-05	Co-occurrence
NUCKS1	1q32.1	25 (21.74%)	11 (4.49%)	2.28	1.23E-06	3.07E-05	Co-occurrence
OR11L1	1q44	25 (21.74%)	11 (4.49%)	2.28	1.23E-06	3.07E-05	Co-occurrence
OR13G1	1q44	25 (21.74%)	11 (4.49%)	2.28	1.23E-06	3.07E-05	Co-occurrence
OR14A16	1q44	25 (21.74%)	11 (4.49%)	2.28	1.23E-06	3.07E-05	Co-occurrence
OR14A2	1q44	25 (21.74%)	11 (4.49%)	2.28	1.23E-06	3.07E-05	Co-occurrence
OR14C36	1q44	25 (21.74%)	11 (4.49%)	2.28	1.23E-06	3.07E-05	Co-occurrence
OR14I1	1q44	25 (21.74%)	11 (4.49%)	2.28	1.23E-06	3.07E-05	Co-occurrence
OR14K1	1q44	25 (21.74%)	11 (4.49%)	2.28	1.23E-06	3.07E-05	Co-occurrence
OR1C1	1q44	25 (21.74%)	11 (4.49%)	2.28	1.23E-06	3.07E-05	Co-occurrence
OR2AJ1	1q44	25 (21.74%)	11 (4.49%)	2.28	1.23E-06	3.07E-05	Co-occurrence
OR2AK2	1q44	25 (21.74%)	11 (4.49%)	2.28	1.23E-06	3.07E-05	Co-occurrence
OR2G6	1q44	25 (21.74%)	11 (4.49%)	2.28	1.23E-06	3.07E-05	Co-occurrence
OR2L13	1q44	25 (21.74%)	11 (4.49%)	2.28	1.23E-06	3.07E-05	Co-occurrence
OR2L2	1q44	25 (21.74%)	11 (4.49%)	2.28	1.23E-06	3.07E-05	Co-occurrence
OR2L3	1q44	25 (21.74%)	11 (4.49%)	2.28	1.23E-06	3.07E-05	Co-occurrence
OR2L5	1q44	25 (21.74%)	11 (4.49%)	2.28	1.23E-06	3.07E-05	Co-occurrence
OR2L8	1q44	25 (21.74%)	11 (4.49%)	2.28	1.23E-06	3.07E-05	Co-occurrence
OR2M2	1q44	25 (21.74%)	11 (4.49%)	2.28	1.23E-06	3.07E-05	Co-occurrence
OR2M3	1q44	25 (21.74%)	11 (4.49%)	2.28	1.23E-06	3.07E-05	Co-occurrence
OR2M4	1q44	25 (21.74%)	11 (4.49%)	2.28	1.23E-06	3.07E-05	Co-occurrence
OR2M5	1q44	25 (21.74%)	11 (4.49%)	2.28	1.23E-06	3.07E-05	Co-occurrence
OR2M7	1q44	25 (21.74%)	11 (4.49%)	2.28	1.23E-06	3.07E-05	Co-occurrence
OR2T1	1q44	25 (21.74%)	11 (4.49%)	2.28	1.23E-06	3.07E-05	Co-occurrence
OR2T10	1q44	25 (21.74%)	11 (4.49%)	2.28	1.23E-06	3.07E-05	Co-occurrence
OR2T11	1q44	25 (21.74%)	11 (4.49%)	2.28	1.23E-06	3.07E-05	Co-occurrence

OR2T12	1q44	25 (21.74%)	11 (4.49%)	2.28	1.23E-06	3.07E-05	Co-occurrence
OR2T2	1q44	25 (21.74%)	11 (4.49%)	2.28	1.23E-06	3.07E-05	Co-occurrence
OR2T27	1q44	25 (21.74%)	11 (4.49%)	2.28	1.23E-06	3.07E-05	Co-occurrence
OR2T29	1q44	25 (21.74%)	11 (4.49%)	2.28	1.23E-06	3.07E-05	Co-occurrence
OR2T3	1q44	25 (21.74%)	11 (4.49%)	2.28	1.23E-06	3.07E-05	Co-occurrence
OR2T33	1q44	25 (21.74%)	11 (4.49%)	2.28	1.23E-06	3.07E-05	Co-occurrence
OR2T34	1q44	25 (21.74%)	11 (4.49%)	2.28	1.23E-06	3.07E-05	Co-occurrence
OR2T35	1q44	25 (21.74%)	11 (4.49%)	2.28	1.23E-06	3.07E-05	Co-occurrence
OR2T4	1q44	25 (21.74%)	11 (4.49%)	2.28	1.23E-06	3.07E-05	Co-occurrence
OR2T5	1q44	25 (21.74%)	11 (4.49%)	2.28	1.23E-06	3.07E-05	Co-occurrence
OR2T6	1q44	25 (21.74%)	11 (4.49%)	2.28	1.23E-06	3.07E-05	Co-occurrence
OR2T7	1q44	25 (21.74%)	11 (4.49%)	2.28	1.23E-06	3.07E-05	Co-occurrence
OR2T8	1q44	25 (21.74%)	11 (4.49%)	2.28	1.23E-06	3.07E-05	Co-occurrence
OR2W3	1q44	25 (21.74%)	11 (4.49%)	2.28	1.23E-06	3.07E-05	Co-occurrence
OR6F1	1q44	25 (21.74%)	11 (4.49%)	2.28	1.23E-06	3.07E-05	Co-occurrence
PGBD2	1q44	25 (21.74%)	11 (4.49%)	2.28	1.23E-06	3.07E-05	Co-occurrence
PKP1	1q32.1	25 (21.74%)	11 (4.49%)	2.28	1.23E-06	3.07E-05	Co-occurrence
PM20D1	1q32.1	25 (21.74%)	11 (4.49%)	2.28	1.23E-06	3.07E-05	Co-occurrence
RAB29	1q32.1	25 (21.74%)	11 (4.49%)	2.28	1.23E-06	3.07E-05	Co-occurrence
RBBP5	1q32.1	25 (21.74%)	11 (4.49%)	2.28	1.23E-06	3.07E-05	Co-occurrence
SH3BP5L	1q44	25 (21.74%)	11 (4.49%)	2.28	1.23E-06	3.07E-05	Co-occurrence
SLC41A1	1q32.1	25 (21.74%)	11 (4.49%)	2.28	1.23E-06	3.07E-05	Co-occurrence
SLC45A3	1q32.1	25 (21.74%)	11 (4.49%)	2.28	1.23E-06	3.07E-05	Co-occurrence
SNORA72	8q22.2	25 (21.74%)	11 (4.49%)	2.28	1.23E-06	3.07E-05	Co-occurrence
TMCC2	1q32.1	25 (21.74%)	11 (4.49%)	2.28	1.23E-06	3.07E-05	Co-occurrence
TMEM81	1q32.1	25 (21.74%)	11 (4.49%)	2.28	1.23E-06	3.07E-05	Co-occurrence
TRIM58	1q44	25 (21.74%)	11 (4.49%)	2.28	1.23E-06	3.07E-05	Co-occurrence
UBE2T	1q32.1	25 (21.74%)	11 (4.49%)	2.28	1.23E-06	3.07E-05	Co-occurrence
ZNF672	1q44	25 (21.74%)	11 (4.49%)	2.28	1.23E-06	3.07E-05	Co-occurrence

ZNF692	1q44	25 (21.74%)	11 (4.49%)	2.28	1.23E-06	3.07E-05	Co-occurrence
FAM72A	1q32.1	24 (20.87%)	10 (4.08%)	2.35	1.32E-06	3.28E-05	Co-occurrence
SLC26A9	1q32.1	24 (20.87%)	10 (4.08%)	2.35	1.32E-06	3.28E-05	Co-occurrence
SLC30A10	1q41	22 (19.13%)	8 (3.27%)	2.55	1.40E-06	3.48E-05	Co-occurrence
ATP2B4	1q32.1	25 (21.74%)	12 (4.90%)	2.15	2.78E-06	6.68E-05	Co-occurrence
BTG2	1q32.1	25 (21.74%)	12 (4.90%)	2.15	2.78E-06	6.68E-05	Co-occurrence
CAMSAP2	1q32.1	25 (21.74%)	12 (4.90%)	2.15	2.78E-06	6.68E-05	Co-occurrence
CHIT1	1q32.1	25 (21.74%)	12 (4.90%)	2.15	2.78E-06	6.68E-05	Co-occurrence
DDX59	1q32.1	25 (21.74%)	12 (4.90%)	2.15	2.78E-06	6.68E-05	Co-occurrence
ELK4	1q32.1	25 (21.74%)	12 (4.90%)	2.15	2.78E-06	6.68E-05	Co-occurrence
FMOD	1q32.1	25 (21.74%)	12 (4.90%)	2.15	2.78E-06	6.68E-05	Co-occurrence
GPR25	1q32.1	25 (21.74%)	12 (4.90%)	2.15	2.78E-06	6.68E-05	Co-occurrence
IGFN1	1q32.1	25 (21.74%)	12 (4.90%)	2.15	2.78E-06	6.68E-05	Co-occurrence
INAVA	1q32.1	25 (21.74%)	12 (4.90%)	2.15	2.78E-06	6.68E-05	Co-occurrence
IPO9	1q32.1	25 (21.74%)	12 (4.90%)	2.15	2.78E-06	6.68E-05	Co-occurrence
KIF14	1q32.1	25 (21.74%)	12 (4.90%)	2.15	2.78E-06	6.68E-05	Co-occurrence
LGR6	1q32.1	25 (21.74%)	12 (4.90%)	2.15	2.78E-06	6.68E-05	Co-occurrence
LINC00862	1q32.1	25 (21.74%)	12 (4.90%)	2.15	2.78E-06	6.68E-05	Co-occurrence
LMOD1	1q32.1	25 (21.74%)	12 (4.90%)	2.15	2.78E-06	6.68E-05	Co-occurrence
MFSD4A	1q32.1	25 (21.74%)	12 (4.90%)	2.15	2.78E-06	6.68E-05	Co-occurrence
MIR-1231/1231		25 (21.74%)	12 (4.90%)	2.15	2.78E-06	6.68E-05	Co-occurrence
MIR-5191/5191		25 (21.74%)	12 (4.90%)	2.15	2.78E-06	6.68E-05	Co-occurrence
NFASC	1q32.1	25 (21.74%)	12 (4.90%)	2.15	2.78E-06	6.68E-05	Co-occurrence
OPTC	1q32.1	25 (21.74%)	12 (4.90%)	2.15	2.78E-06	6.68E-05	Co-occurrence
PPP1R12B	1q32.1	25 (21.74%)	12 (4.90%)	2.15	2.78E-06	6.68E-05	Co-occurrence
PRELP	1q32.1	25 (21.74%)	12 (4.90%)	2.15	2.78E-06	6.68E-05	Co-occurrence
RGS18	1q31.2	25 (21.74%)	12 (4.90%)	2.15	2.78E-06	6.68E-05	Co-occurrence
SHISA4	1q32.1	25 (21.74%)	12 (4.90%)	2.15	2.78E-06	6.68E-05	Co-occurrence
TIMM17A	1q32.1	25 (21.74%)	12 (4.90%)	2.15	2.78E-06	6.68E-05	Co-occurrence

TMEM9	1q32.1	25 (21.74%)	12 (4.90%)	2.15	2.78E-06	6.68E-05	Co-occurrence
ZNF281	1q32.1	25 (21.74%)	12 (4.90%)	2.15	2.78E-06	6.68E-05	Co-occurrence
C1ORF74	1q32.2	24 (20.87%)	11 (4.49%)	2.22	3.07E-06	7.20E-05	Co-occurrence
CAMK1G	1q32.2	24 (20.87%)	11 (4.49%)	2.22	3.07E-06	7.20E-05	Co-occurrence
G0S2	1q32.2	24 (20.87%)	11 (4.49%)	2.22	3.07E-06	7.20E-05	Co-occurrence
GCSAML	1q44	24 (20.87%)	11 (4.49%)	2.22	3.07E-06	7.20E-05	Co-occurrence
IRF6	1q32.2	24 (20.87%)	11 (4.49%)	2.22	3.07E-06	7.20E-05	Co-occurrence
KIF26B	1q44	24 (20.87%)	11 (4.49%)	2.22	3.07E-06	7.20E-05	Co-occurrence
LAMB3	1q32.2	24 (20.87%)	11 (4.49%)	2.22	3.07E-06	7.20E-05	Co-occurrence
MDM4	1q32.1	24 (20.87%)	11 (4.49%)	2.22	3.07E-06	7.20E-05	Co-occurrence
MIR-4260/4260		24 (20.87%)	11 (4.49%)	2.22	3.07E-06	7.20E-05	Co-occurrence
MIR205HG	1q32.2	24 (20.87%)	11 (4.49%)	2.22	3.07E-06	7.20E-05	Co-occurrence
OR2C3	1q44	24 (20.87%)	11 (4.49%)	2.22	3.07E-06	7.20E-05	Co-occurrence
OR2G2	1q44	24 (20.87%)	11 (4.49%)	2.22	3.07E-06	7.20E-05	Co-occurrence
OR2G3	1q44	24 (20.87%)	11 (4.49%)	2.22	3.07E-06	7.20E-05	Co-occurrence
OR2W5	1q44	24 (20.87%)	11 (4.49%)	2.22	3.07E-06	7.20E-05	Co-occurrence
RNA5SP74	1q32.1	24 (20.87%)	11 (4.49%)	2.22	3.07E-06	7.20E-05	Co-occurrence
SNRPE	1q32.1	24 (20.87%)	11 (4.49%)	2.22	3.07E-06	7.20E-05	Co-occurrence
TRAF3IP3	1q32.2	24 (20.87%)	11 (4.49%)	2.22	3.07E-06	7.20E-05	Co-occurrence
UTP25	1q32.2	24 (20.87%)	11 (4.49%)	2.22	3.07E-06	7.20E-05	Co-occurrence
ZBED6	1q32.1	24 (20.87%)	11 (4.49%)	2.22	3.07E-06	7.20E-05	Co-occurrence
ZC3H11A	1q32.1	24 (20.87%)	11 (4.49%)	2.22	3.07E-06	7.20E-05	Co-occurrence
EDARADD	1q42.3-q43	23 (20.00%)	10 (4.08%)	2.29	3.33E-06	7.77E-05	Co-occurrence
ERO1B	1q42.3	23 (20.00%)	10 (4.08%)	2.29	3.33E-06	7.77E-05	Co-occurrence
SMYD2	1q32.3	23 (20.00%)	10 (4.08%)	2.29	3.33E-06	7.77E-05	Co-occurrence
BTNL10	1q42.13	22 (19.13%)	9 (3.67%)	2.38	3.53E-06	7.95E-05	Co-occurrence
EPRS	1q41	22 (19.13%)	9 (3.67%)	2.38	3.53E-06	7.95E-05	Co-occurrence
HIST3H2A	1q42.13	22 (19.13%)	9 (3.67%)	2.38	3.53E-06	7.95E-05	Co-occurrence
HIST3H2BB	1q42.13	22 (19.13%)	9 (3.67%)	2.38	3.53E-06	7.95E-05	Co-occurrence

HIST3H3	1q42.13	22 (19.13%)	9 (3.67%)	2.38	3.53E-06	7.95E-05	Co-occurrence
OBSCN	1q42.13	22 (19.13%)	9 (3.67%)	2.38	3.53E-06	7.95E-05	Co-occurrence
PTPN14	1q32.3-q41	22 (19.13%)	9 (3.67%)	2.38	3.53E-06	7.95E-05	Co-occurrence
RNA5S1	1q42.13	22 (19.13%)	9 (3.67%)	2.38	3.53E-06	7.95E-05	Co-occurrence
RNA5S10	1q42.13	22 (19.13%)	9 (3.67%)	2.38	3.53E-06	7.95E-05	Co-occurrence
RNA5S11	1q42.13	22 (19.13%)	9 (3.67%)	2.38	3.53E-06	7.95E-05	Co-occurrence
RNA5S12	1q42.13	22 (19.13%)	9 (3.67%)	2.38	3.53E-06	7.95E-05	Co-occurrence
RNA5S13	1q42.13	22 (19.13%)	9 (3.67%)	2.38	3.53E-06	7.95E-05	Co-occurrence
RNA5S14	1q42.13	22 (19.13%)	9 (3.67%)	2.38	3.53E-06	7.95E-05	Co-occurrence
RNA5S15	1q42.13	22 (19.13%)	9 (3.67%)	2.38	3.53E-06	7.95E-05	Co-occurrence
RNA5S16	1q42.13	22 (19.13%)	9 (3.67%)	2.38	3.53E-06	7.95E-05	Co-occurrence
RNA5S17	1q42.13	22 (19.13%)	9 (3.67%)	2.38	3.53E-06	7.95E-05	Co-occurrence
RNA5S2	1q42.13	22 (19.13%)	9 (3.67%)	2.38	3.53E-06	7.95E-05	Co-occurrence
RNA5S3	1q42.13	22 (19.13%)	9 (3.67%)	2.38	3.53E-06	7.95E-05	Co-occurrence
RNA5S4	1q42.13	22 (19.13%)	9 (3.67%)	2.38	3.53E-06	7.95E-05	Co-occurrence
RNA5S5	1q42.13	22 (19.13%)	9 (3.67%)	2.38	3.53E-06	7.95E-05	Co-occurrence
RNA5S6	1q42.13	22 (19.13%)	9 (3.67%)	2.38	3.53E-06	7.95E-05	Co-occurrence
RNA5S7	1q42.13	22 (19.13%)	9 (3.67%)	2.38	3.53E-06	7.95E-05	Co-occurrence
RNA5S8	1q42.13	22 (19.13%)	9 (3.67%)	2.38	3.53E-06	7.95E-05	Co-occurrence
RNA5S9	1q42.13	22 (19.13%)	9 (3.67%)	2.38	3.53E-06	7.95E-05	Co-occurrence
RNA5SP18	1q42.13	22 (19.13%)	9 (3.67%)	2.38	3.53E-06	7.95E-05	Co-occurrence
RNA5SP19	1q42.13	22 (19.13%)	9 (3.67%)	2.38	3.53E-06	7.95E-05	Co-occurrence
RNF187	1q42.13	22 (19.13%)	9 (3.67%)	2.38	3.53E-06	7.95E-05	Co-occurrence
TRIM11	1q42.13	22 (19.13%)	9 (3.67%)	2.38	3.53E-06	7.95E-05	Co-occurrence
TRIM17	1q42.13	22 (19.13%)	9 (3.67%)	2.38	3.53E-06	7.95E-05	Co-occurrence
BPNT1	1q41	21 (18.26%)	8 (3.27%)	2.48	3.64E-06	8.08E-05	Co-occurrence
C1ORF115	1q41	21 (18.26%)	8 (3.27%)	2.48	3.64E-06	8.08E-05	Co-occurrence
HDAC1P2	1q41	21 (18.26%)	8 (3.27%)	2.48	3.64E-06	8.08E-05	Co-occurrence
HLX	1q41	21 (18.26%)	8 (3.27%)	2.48	3.64E-06	8.08E-05	Co-occurrence

IARS2	1q41	21 (18.26%)	8 (3.27%)	2.48	3.64E-06	8.08E-05	Co-occurrence
1-Mar	1q41	21 (18.26%)	8 (3.27%)	2.48	3.64E-06	8.08E-05	Co-occurrence
2-Mar	1q41	21 (18.26%)	8 (3.27%)	2.48	3.64E-06	8.08E-05	Co-occurrence
MARK1	1q41	21 (18.26%)	8 (3.27%)	2.48	3.64E-06	8.08E-05	Co-occurrence
RAB3GAP2	1q41	21 (18.26%)	8 (3.27%)	2.48	3.64E-06	8.08E-05	Co-occurrence
RN7SL464P	1q41	21 (18.26%)	8 (3.27%)	2.48	3.64E-06	8.08E-05	Co-occurrence
RNA5SP76	1q41	21 (18.26%)	8 (3.27%)	2.48	3.64E-06	8.08E-05	Co-occurrence
RNU6ATAC35P	1q41	21 (18.26%)	8 (3.27%)	2.48	3.64E-06	8.08E-05	Co-occurrence
SNORA36B	1q41	21 (18.26%)	8 (3.27%)	2.48	3.64E-06	8.08E-05	Co-occurrence
NR5A2	1q32.1	26 (22.61%)	14 (5.71%)	1.98	5.21E-06	1.15E-04	Co-occurrence
ADIPOR1	1q32.1	25 (21.74%)	13 (5.31%)	2.03	5.97E-06	1.29E-04	Co-occurrence
ADORA1	1q32.1	25 (21.74%)	13 (5.31%)	2.03	5.97E-06	1.29E-04	Co-occurrence
CHI3L1	1q32.1	25 (21.74%)	13 (5.31%)	2.03	5.97E-06	1.29E-04	Co-occurrence
CYB5R1	1q32.1	25 (21.74%)	13 (5.31%)	2.03	5.97E-06	1.29E-04	Co-occurrence
KDM5B	1q32.1	25 (21.74%)	13 (5.31%)	2.03	5.97E-06	1.29E-04	Co-occurrence
KLHL12	1q32.1	25 (21.74%)	13 (5.31%)	2.03	5.97E-06	1.29E-04	Co-occurrence
MYBPH	1q32.1	25 (21.74%)	13 (5.31%)	2.03	5.97E-06	1.29E-04	Co-occurrence
MYOG	1q32.1	25 (21.74%)	13 (5.31%)	2.03	5.97E-06	1.29E-04	Co-occurrence
PCAT6	1q32.1	25 (21.74%)	13 (5.31%)	2.03	5.97E-06	1.29E-04	Co-occurrence
PLXNA2	1q32.2	25 (21.74%)	13 (5.31%)	2.03	5.97E-06	1.29E-04	Co-occurrence
PPFIA4	1q32.1	25 (21.74%)	13 (5.31%)	2.03	5.97E-06	1.29E-04	Co-occurrence
RABIF	1q32.1	25 (21.74%)	13 (5.31%)	2.03	5.97E-06	1.29E-04	Co-occurrence
RGS1	1q31.2	25 (21.74%)	13 (5.31%)	2.03	5.97E-06	1.29E-04	Co-occurrence
RGS13	1q31.2	25 (21.74%)	13 (5.31%)	2.03	5.97E-06	1.29E-04	Co-occurrence
RGS2	1q31.2	25 (21.74%)	13 (5.31%)	2.03	5.97E-06	1.29E-04	Co-occurrence
RGS21	1q31.2	25 (21.74%)	13 (5.31%)	2.03	5.97E-06	1.29E-04	Co-occurrence
RN7SKP126	1q31.2	25 (21.74%)	13 (5.31%)	2.03	5.97E-06	1.29E-04	Co-occurrence
SYT2	1q32.1	25 (21.74%)	13 (5.31%)	2.03	5.97E-06	1.29E-04	Co-occurrence
TMEM183A	1q32.1	25 (21.74%)	13 (5.31%)	2.03	5.97E-06	1.29E-04	Co-occurrence

ETNK2	1q32.1	24 (20.87%)	12 (4.90%)	2.09	6.76E-06	1.45E-04	Co-occurrence
GOLT1A	1q32.1	24 (20.87%)	12 (4.90%)	2.09	6.76E-06	1.45E-04	Co-occurrence
LAX1	1q32.1	24 (20.87%)	12 (4.90%)	2.09	6.76E-06	1.45E-04	Co-occurrence
LINC00184	1q42.3	24 (20.87%)	12 (4.90%)	2.09	6.76E-06	1.45E-04	Co-occurrence
LRRN2	1q32.1	24 (20.87%)	12 (4.90%)	2.09	6.76E-06	1.45E-04	Co-occurrence
PIK3C2B	1q32.1	24 (20.87%)	12 (4.90%)	2.09	6.76E-06	1.45E-04	Co-occurrence
PLEKHA6	1q32.1	24 (20.87%)	12 (4.90%)	2.09	6.76E-06	1.45E-04	Co-occurrence
PPP1R15B	1q32.1	24 (20.87%)	12 (4.90%)	2.09	6.76E-06	1.45E-04	Co-occurrence
REN	1q32.1	24 (20.87%)	12 (4.90%)	2.09	6.76E-06	1.45E-04	Co-occurrence
RNA5SP75	1q32.1	24 (20.87%)	12 (4.90%)	2.09	6.76E-06	1.45E-04	Co-occurrence
SOX13	1q32.1	24 (20.87%)	12 (4.90%)	2.09	6.76E-06	1.45E-04	Co-occurrence
ATP4B	13q34	12 (10.43%)	1 (0.41%)	4.68	7.09E-06	1.50E-04	Co-occurrence
GAS6	13q34	12 (10.43%)	1 (0.41%)	4.68	7.09E-06	1.50E-04	Co-occurrence
GRK1	13q34	12 (10.43%)	1 (0.41%)	4.68	7.09E-06	1.50E-04	Co-occurrence
GRTP1	13q34	12 (10.43%)	1 (0.41%)	4.68	7.09E-06	1.50E-04	Co-occurrence
LINC00452	13q34	12 (10.43%)	1 (0.41%)	4.68	7.09E-06	1.50E-04	Co-occurrence
LINC00454	13q34	12 (10.43%)	1 (0.41%)	4.68	7.09E-06	1.50E-04	Co-occurrence
LINC00565	13q34	12 (10.43%)	1 (0.41%)	4.68	7.09E-06	1.50E-04	Co-occurrence
RASA3	13q34	12 (10.43%)	1 (0.41%)	4.68	7.09E-06	1.50E-04	Co-occurrence
TFDP1	13q34	12 (10.43%)	1 (0.41%)	4.68	7.09E-06	1.50E-04	Co-occurrence
TMEM255B	13q34	12 (10.43%)	1 (0.41%)	4.68	7.09E-06	1.50E-04	Co-occurrence
CATSPERE	1q44	23 (20.00%)	11 (4.49%)	2.16	7.54E-06	1.55E-04	Co-occurrence
CHML	1q43	23 (20.00%)	11 (4.49%)	2.16	7.54E-06	1.55E-04	Co-occurrence
COX20	1q44	23 (20.00%)	11 (4.49%)	2.16	7.54E-06	1.55E-04	Co-occurrence
DESI2	1q44	23 (20.00%)	11 (4.49%)	2.16	7.54E-06	1.55E-04	Co-occurrence
EFCAB2	1q44	23 (20.00%)	11 (4.49%)	2.16	7.54E-06	1.55E-04	Co-occurrence
FH	1q43	23 (20.00%)	11 (4.49%)	2.16	7.54E-06	1.55E-04	Co-occurrence
HHAT	1q32.2	23 (20.00%)	11 (4.49%)	2.16	7.54E-06	1.55E-04	Co-occurrence
HNRNPU	1q44	23 (20.00%)	11 (4.49%)	2.16	7.54E-06	1.55E-04	Co-occurrence

IRF2BP2	1q42.3	23 (20.00%)	11 (4.49%)	2.16	7.54E-06	1.55E-04	Co-occurrence
KMO	1q43	23 (20.00%)	11 (4.49%)	2.16	7.54E-06	1.55E-04	Co-occurrence
LYST	1q42.3	23 (20.00%)	11 (4.49%)	2.16	7.54E-06	1.55E-04	Co-occurrence
MIR-3916/3916		23 (20.00%)	11 (4.49%)	2.16	7.54E-06	1.55E-04	Co-occurrence
NLRP3	1q44	23 (20.00%)	11 (4.49%)	2.16	7.54E-06	1.55E-04	Co-occurrence
OPN3	1q43	23 (20.00%)	11 (4.49%)	2.16	7.54E-06	1.55E-04	Co-occurrence
OR2B11	1q44	23 (20.00%)	11 (4.49%)	2.16	7.54E-06	1.55E-04	Co-occurrence
PROX1	1q32.3	23 (20.00%)	11 (4.49%)	2.16	7.54E-06	1.55E-04	Co-occurrence
RN7SKP55	1q44	23 (20.00%)	11 (4.49%)	2.16	7.54E-06	1.55E-04	Co-occurrence
RNA5SP82	1q44	23 (20.00%)	11 (4.49%)	2.16	7.54E-06	1.55E-04	Co-occurrence
SERTAD4	1q32.2	23 (20.00%)	11 (4.49%)	2.16	7.54E-06	1.55E-04	Co-occurrence
SLC35F3	1q42.2	23 (20.00%)	11 (4.49%)	2.16	7.54E-06	1.55E-04	Co-occurrence
SNORD118	17p13.1	23 (20.00%)	11 (4.49%)	2.16	7.54E-06	1.55E-04	Co-occurrence
SYT14	1q32.2	23 (20.00%)	11 (4.49%)	2.16	7.54E-06	1.55E-04	Co-occurrence
TBCE	1q42.3	23 (20.00%)	11 (4.49%)	2.16	7.54E-06	1.55E-04	Co-occurrence
ZNF124	1q44	23 (20.00%)	11 (4.49%)	2.16	7.54E-06	1.55E-04	Co-occurrence
ZNF496	1q44	23 (20.00%)	11 (4.49%)	2.16	7.54E-06	1.55E-04	Co-occurrence
ACTN2	1q43	22 (19.13%)	10 (4.08%)	2.23	8.27E-06	1.65E-04	Co-occurrence
ANGEL2	1q32.3	22 (19.13%)	10 (4.08%)	2.23	8.27E-06	1.65E-04	Co-occurrence
ATF3	1q32.3	22 (19.13%)	10 (4.08%)	2.23	8.27E-06	1.65E-04	Co-occurrence
BATF3	1q32.3	22 (19.13%)	10 (4.08%)	2.23	8.27E-06	1.65E-04	Co-occurrence
FAM71A	1q32.3	22 (19.13%)	10 (4.08%)	2.23	8.27E-06	1.65E-04	Co-occurrence
FLVCR1	1q32.3	22 (19.13%)	10 (4.08%)	2.23	8.27E-06	1.65E-04	Co-occurrence
FMN2	1q43	22 (19.13%)	10 (4.08%)	2.23	8.27E-06	1.65E-04	Co-occurrence
GPR137B	1q42.3	22 (19.13%)	10 (4.08%)	2.23	8.27E-06	1.65E-04	Co-occurrence
GREM2	1q43	22 (19.13%)	10 (4.08%)	2.23	8.27E-06	1.65E-04	Co-occurrence
HEATR1	1q43	22 (19.13%)	10 (4.08%)	2.23	8.27E-06	1.65E-04	Co-occurrence
LGALS8	1q43	22 (19.13%)	10 (4.08%)	2.23	8.27E-06	1.65E-04	Co-occurrence
MIR-3123/3123		22 (19.13%)	10 (4.08%)	2.23	8.27E-06	1.65E-04	Co-occurrence

MIR-4428/4428		22 (19.13%)	10 (4.08%)	2.23	8.27E-06	1.65E-04	Co-occurrence
MT1HL1	1q43	22 (19.13%)	10 (4.08%)	2.23	8.27E-06	1.65E-04	Co-occurrence
MTR	1q43	22 (19.13%)	10 (4.08%)	2.23	8.27E-06	1.65E-04	Co-occurrence
MTRNR2L11	1q43	22 (19.13%)	10 (4.08%)	2.23	8.27E-06	1.65E-04	Co-occurrence
NENF	1q32.3	22 (19.13%)	10 (4.08%)	2.23	8.27E-06	1.65E-04	Co-occurrence
NID1	1q42.3	22 (19.13%)	10 (4.08%)	2.23	8.27E-06	1.65E-04	Co-occurrence
NSL1	1q32.3	22 (19.13%)	10 (4.08%)	2.23	8.27E-06	1.65E-04	Co-occurrence
PACC1	1q32.3	22 (19.13%)	10 (4.08%)	2.23	8.27E-06	1.65E-04	Co-occurrence
PPP2R5A	1q32.3	22 (19.13%)	10 (4.08%)	2.23	8.27E-06	1.65E-04	Co-occurrence
RAB4A	1q42.13	22 (19.13%)	10 (4.08%)	2.23	8.27E-06	1.65E-04	Co-occurrence
RHOU	1q42.13	22 (19.13%)	10 (4.08%)	2.23	8.27E-06	1.65E-04	Co-occurrence
RN7SKP195	1q43	22 (19.13%)	10 (4.08%)	2.23	8.27E-06	1.65E-04	Co-occurrence
RPS6KC1	1q32.3	22 (19.13%)	10 (4.08%)	2.23	8.27E-06	1.65E-04	Co-occurrence
SNORA16B	1q32.3	22 (19.13%)	10 (4.08%)	2.23	8.27E-06	1.65E-04	Co-occurrence
SPATA45	1q32.3	22 (19.13%)	10 (4.08%)	2.23	8.27E-06	1.65E-04	Co-occurrence
TATDN3	1q32.3	22 (19.13%)	10 (4.08%)	2.23	8.27E-06	1.65E-04	Co-occurrence
TMEM78	1q42.13	22 (19.13%)	10 (4.08%)	2.23	8.27E-06	1.65E-04	Co-occurrence
ZP4	1q43	22 (19.13%)	10 (4.08%)	2.23	8.27E-06	1.65E-04	Co-occurrence
CCDC185	1q41	21 (18.26%)	9 (3.67%)	2.31	8.88E-06	1.76E-04	Co-occurrence
CDC42BPA	1q42.13	21 (18.26%)	9 (3.67%)	2.31	8.88E-06	1.76E-04	Co-occurrence
CENPF	1q41	21 (18.26%)	9 (3.67%)	2.31	8.88E-06	1.76E-04	Co-occurrence
KCNK2	1q41	21 (18.26%)	9 (3.67%)	2.31	8.88E-06	1.76E-04	Co-occurrence
TP53BP2	1q41	21 (18.26%)	9 (3.67%)	2.31	8.88E-06	1.76E-04	Co-occurrence
CNIH3	1q42.12	20 (17.39%)	8 (3.27%)	2.41	9.31E-06	1.84E-04	Co-occurrence
ESRRG	1q41	20 (17.39%)	8 (3.27%)	2.41	9.31E-06	1.84E-04	Co-occurrence
WDR26	1q42.11-q42.12	20 (17.39%)	8 (3.27%)	2.41	9.31E-06	1.84E-04	Co-occurrence
ARL8A	1q32.1	25 (21.74%)	14 (5.71%)	1.93	1.22E-05	2.40E-04	Co-occurrence
GPR37L1	1q32.1	25 (21.74%)	14 (5.71%)	1.93	1.22E-05	2.40E-04	Co-occurrence
PTPN7	1q32.1	25 (21.74%)	14 (5.71%)	1.93	1.22E-05	2.40E-04	Co-occurrence

PTPRVP	1q32.1	25 (21.74%)	14 (5.71%)	1.93	1.22E-05	2.40E-04	Co-occurrence
HSD11B1	1q32.2	24 (20.87%)	13 (5.31%)	1.98	1.41E-05	2.77E-04	Co-occurrence
KISS1	1q32.1	24 (20.87%)	13 (5.31%)	1.98	1.41E-05	2.77E-04	Co-occurrence
AHCTF1	1q44	23 (20.00%)	12 (4.90%)	2.03	1.61E-05	3.13E-04	Co-occurrence
B3GALNT2	1q42.3	23 (20.00%)	12 (4.90%)	2.03	1.61E-05	3.13E-04	Co-occurrence
C1ORF229	1q44	23 (20.00%)	12 (4.90%)	2.03	1.61E-05	3.13E-04	Co-occurrence
CNST	1q44	23 (20.00%)	12 (4.90%)	2.03	1.61E-05	3.13E-04	Co-occurrence
SCCPDH	1q44	23 (20.00%)	12 (4.90%)	2.03	1.61E-05	3.13E-04	Co-occurrence
SMYD3	1q44	23 (20.00%)	12 (4.90%)	2.03	1.61E-05	3.13E-04	Co-occurrence
TFB2M	1q44	23 (20.00%)	12 (4.90%)	2.03	1.61E-05	3.13E-04	Co-occurrence
WDR64	1q43	23 (20.00%)	12 (4.90%)	2.03	1.61E-05	3.13E-04	Co-occurrence
ZNF669	1q44	23 (20.00%)	12 (4.90%)	2.03	1.61E-05	3.13E-04	Co-occurrence
ZNF670	1q44	23 (20.00%)	12 (4.90%)	2.03	1.61E-05	3.13E-04	Co-occurrence
ZNF695	1q44	23 (20.00%)	12 (4.90%)	2.03	1.61E-05	3.13E-04	Co-occurrence
ADSS	1q44	22 (19.13%)	11 (4.49%)	2.09	1.81E-05	3.43E-04	Co-occurrence
AKT3	1q43-q44	22 (19.13%)	11 (4.49%)	2.09	1.81E-05	3.43E-04	Co-occurrence
ARID4B	1q42.3	22 (19.13%)	11 (4.49%)	2.09	1.81E-05	3.43E-04	Co-occurrence
C1ORF100	1q44	22 (19.13%)	11 (4.49%)	2.09	1.81E-05	3.43E-04	Co-occurrence
CEP170	1q43	22 (19.13%)	11 (4.49%)	2.09	1.81E-05	3.43E-04	Co-occurrence
CHRM3	1q43	22 (19.13%)	11 (4.49%)	2.09	1.81E-05	3.43E-04	Co-occurrence
ENAH	1q42.12	22 (19.13%)	11 (4.49%)	2.09	1.81E-05	3.43E-04	Co-occurrence
EXO1	1q43	22 (19.13%)	11 (4.49%)	2.09	1.81E-05	3.43E-04	Co-occurrence
GGPS1	1q42.3	22 (19.13%)	11 (4.49%)	2.09	1.81E-05	3.43E-04	Co-occurrence
GNG4	1q42.3	22 (19.13%)	11 (4.49%)	2.09	1.81E-05	3.43E-04	Co-occurrence
INTS7	1q32.3	22 (19.13%)	11 (4.49%)	2.09	1.81E-05	3.43E-04	Co-occurrence
MAP1LC3C	1q43	22 (19.13%)	11 (4.49%)	2.09	1.81E-05	3.43E-04	Co-occurrence
PLD5	1q43	22 (19.13%)	11 (4.49%)	2.09	1.81E-05	3.43E-04	Co-occurrence
RBM34	1q42.3	22 (19.13%)	11 (4.49%)	2.09	1.81E-05	3.43E-04	Co-occurrence
RGS7	1q43|1q23.1	22 (19.13%)	11 (4.49%)	2.09	1.81E-05	3.43E-04	Co-occurrence

RN7SKP12	1q43	22 (19.13%)	11 (4.49%)	2.09	1.81E-05	3.43E-04	Co-occurrence
RN7SL148P	1q44	22 (19.13%)	11 (4.49%)	2.09	1.81E-05	3.43E-04	Co-occurrence
RNA5SP81		22 (19.13%)	11 (4.49%)	2.09	1.81E-05	3.43E-04	Co-occurrence
RNY4P16	1q42.3	22 (19.13%)	11 (4.49%)	2.09	1.81E-05	3.43E-04	Co-occurrence
RYR2	1q43	22 (19.13%)	11 (4.49%)	2.09	1.81E-05	3.43E-04	Co-occurrence
SDCCAG8	1q43-q44	22 (19.13%)	11 (4.49%)	2.09	1.81E-05	3.43E-04	Co-occurrence
SNORA14B	1q42.3	22 (19.13%)	11 (4.49%)	2.09	1.81E-05	3.43E-04	Co-occurrence
TOMM20	1q42.3	22 (19.13%)	11 (4.49%)	2.09	1.81E-05	3.43E-04	Co-occurrence
VASH2	1q32.3	22 (19.13%)	11 (4.49%)	2.09	1.81E-05	3.43E-04	Co-occurrence
ZBTB18	1q44	22 (19.13%)	11 (4.49%)	2.09	1.81E-05	3.43E-04	Co-occurrence
ACTA1	1q42.13	21 (18.26%)	10 (4.08%)	2.16	2.01E-05	3.75E-04	Co-occurrence
CAPN2	1q41	21 (18.26%)	10 (4.08%)	2.16	2.01E-05	3.75E-04	Co-occurrence
CAPN8	1q41	21 (18.26%)	10 (4.08%)	2.16	2.01E-05	3.75E-04	Co-occurrence
CCSAP	1q42.13	21 (18.26%)	10 (4.08%)	2.16	2.01E-05	3.75E-04	Co-occurrence
DTL	1q32.3	21 (18.26%)	10 (4.08%)	2.16	2.01E-05	3.75E-04	Co-occurrence
DUSP10	1q41	21 (18.26%)	10 (4.08%)	2.16	2.01E-05	3.75E-04	Co-occurrence
MIR-3122/3122		21 (18.26%)	10 (4.08%)	2.16	2.01E-05	3.75E-04	Co-occurrence
RN7SKP276	1q42.13	21 (18.26%)	10 (4.08%)	2.16	2.01E-05	3.75E-04	Co-occurrence
RN7SKP98	1q32.3	21 (18.26%)	10 (4.08%)	2.16	2.01E-05	3.75E-04	Co-occurrence
RNA5SP78	1q42.13	21 (18.26%)	10 (4.08%)	2.16	2.01E-05	3.75E-04	Co-occurrence
SPHAR	1q42.13	21 (18.26%)	10 (4.08%)	2.16	2.01E-05	3.75E-04	Co-occurrence
SUSD4	1q41	21 (18.26%)	10 (4.08%)	2.16	2.01E-05	3.75E-04	Co-occurrence
TAF5L	1q42.13	21 (18.26%)	10 (4.08%)	2.16	2.01E-05	3.75E-04	Co-occurrence
TLR5	1q41	21 (18.26%)	10 (4.08%)	2.16	2.01E-05	3.75E-04	Co-occurrence
ACBD3	1q42.12	20 (17.39%)	9 (3.67%)	2.24	2.19E-05	3.85E-04	Co-occurrence
AIDA	1q41	20 (17.39%)	9 (3.67%)	2.24	2.19E-05	3.85E-04	Co-occurrence
ARF1	1q42.13	20 (17.39%)	9 (3.67%)	2.24	2.19E-05	3.85E-04	Co-occurrence
BROX	1q41	20 (17.39%)	9 (3.67%)	2.24	2.19E-05	3.85E-04	Co-occurrence
C1ORF143	1q41	20 (17.39%)	9 (3.67%)	2.24	2.19E-05	3.85E-04	Co-occurrence

C1ORF35	1q42.13	20 (17.39%)	9 (3.67%)	2.24	2.19E-05	3.85E-04	Co-occurrence
CNIH4	1q42.11	20 (17.39%)	9 (3.67%)	2.24	2.19E-05	3.85E-04	Co-occurrence
DISP1	1q41	20 (17.39%)	9 (3.67%)	2.24	2.19E-05	3.85E-04	Co-occurrence
DNAH14	1q42.12	20 (17.39%)	9 (3.67%)	2.24	2.19E-05	3.85E-04	Co-occurrence
FAM177B	1q41	20 (17.39%)	9 (3.67%)	2.24	2.19E-05	3.85E-04	Co-occurrence
GJC2	1q42.13	20 (17.39%)	9 (3.67%)	2.24	2.19E-05	3.85E-04	Co-occurrence
GPATCH2	1q41	20 (17.39%)	9 (3.67%)	2.24	2.19E-05	3.85E-04	Co-occurrence
GUK1	1q42.13	20 (17.39%)	9 (3.67%)	2.24	2.19E-05	3.85E-04	Co-occurrence
H3F3A	1q42.12	20 (17.39%)	9 (3.67%)	2.24	2.19E-05	3.85E-04	Co-occurrence
HHIPL2	1q41	20 (17.39%)	9 (3.67%)	2.24	2.19E-05	3.85E-04	Co-occurrence
IBA57	1q42.13	20 (17.39%)	9 (3.67%)	2.24	2.19E-05	3.85E-04	Co-occurrence
IBA57-DT	1q42.13	20 (17.39%)	9 (3.67%)	2.24	2.19E-05	3.85E-04	Co-occurrence
JMJD4	1q42.13	20 (17.39%)	9 (3.67%)	2.24	2.19E-05	3.85E-04	Co-occurrence
LBR	1q42.12	20 (17.39%)	9 (3.67%)	2.24	2.19E-05	3.85E-04	Co-occurrence
LEFTY1	1q42.12	20 (17.39%)	9 (3.67%)	2.24	2.19E-05	3.85E-04	Co-occurrence
LEFTY2	1q42.12	20 (17.39%)	9 (3.67%)	2.24	2.19E-05	3.85E-04	Co-occurrence
LIN9	1q42.12	20 (17.39%)	9 (3.67%)	2.24	2.19E-05	3.85E-04	Co-occurrence
LINC00210	1q41	20 (17.39%)	9 (3.67%)	2.24	2.19E-05	3.85E-04	Co-occurrence
LYPLAL1	1q41	20 (17.39%)	9 (3.67%)	2.24	2.19E-05	3.85E-04	Co-occurrence
MIA3	1q41	20 (17.39%)	9 (3.67%)	2.24	2.19E-05	3.85E-04	Co-occurrence
MIXL1	1q42.12	20 (17.39%)	9 (3.67%)	2.24	2.19E-05	3.85E-04	Co-occurrence
MRPL55	1q42.13	20 (17.39%)	9 (3.67%)	2.24	2.19E-05	3.85E-04	Co-occurrence
OBSCN-AS1	1q42.13	20 (17.39%)	9 (3.67%)	2.24	2.19E-05	3.85E-04	Co-occurrence
PARP1	1q42.12	20 (17.39%)	9 (3.67%)	2.24	2.19E-05	3.85E-04	Co-occurrence
PRSS38	1q42.13	20 (17.39%)	9 (3.67%)	2.24	2.19E-05	3.85E-04	Co-occurrence
PSEN2	1q42.13	20 (17.39%)	9 (3.67%)	2.24	2.19E-05	3.85E-04	Co-occurrence
PYCR2	1q42.12	20 (17.39%)	9 (3.67%)	2.24	2.19E-05	3.85E-04	Co-occurrence
RRP15	1q41	20 (17.39%)	9 (3.67%)	2.24	2.19E-05	3.85E-04	Co-occurrence
SDE2	1q42.12	20 (17.39%)	9 (3.67%)	2.24	2.19E-05	3.85E-04	Co-occurrence

SNAP47	1q42.13	20 (17.39%)	9 (3.67%)	2.24	2.19E-05	3.85E-04	Co-occurrence
SPATA17	1q41	20 (17.39%)	9 (3.67%)	2.24	2.19E-05	3.85E-04	Co-occurrence
TAF1A	1q41	20 (17.39%)	9 (3.67%)	2.24	2.19E-05	3.85E-04	Co-occurrence
TGFB2	1q41	20 (17.39%)	9 (3.67%)	2.24	2.19E-05	3.85E-04	Co-occurrence
WNT3A	1q42.13	20 (17.39%)	9 (3.67%)	2.24	2.19E-05	3.85E-04	Co-occurrence
WNT9A	1q42.13	20 (17.39%)	9 (3.67%)	2.24	2.19E-05	3.85E-04	Co-occurrence
ADPRHL1	13q34	11 (9.57%)	1 (0.41%)	4.55	2.20E-05	3.85E-04	Co-occurrence
ATP11A	13q34	11 (9.57%)	1 (0.41%)	4.55	2.20E-05	3.85E-04	Co-occurrence
ATP11AUN	13q34	11 (9.57%)	1 (0.41%)	4.55	2.20E-05	3.85E-04	Co-occurrence
CDC16	13q34	11 (9.57%)	1 (0.41%)	4.55	2.20E-05	3.85E-04	Co-occurrence
CHAMP1	13q34	11 (9.57%)	1 (0.41%)	4.55	2.20E-05	3.85E-04	Co-occurrence
CUL4A	13q34	11 (9.57%)	1 (0.41%)	4.55	2.20E-05	3.85E-04	Co-occurrence
DCUN1D2	13q34	11 (9.57%)	1 (0.41%)	4.55	2.20E-05	3.85E-04	Co-occurrence
F10	13q34	11 (9.57%)	1 (0.41%)	4.55	2.20E-05	3.85E-04	Co-occurrence
F7	13q34	11 (9.57%)	1 (0.41%)	4.55	2.20E-05	3.85E-04	Co-occurrence
LAMP1	13q34	11 (9.57%)	1 (0.41%)	4.55	2.20E-05	3.85E-04	Co-occurrence
LINC00354	13q34	11 (9.57%)	1 (0.41%)	4.55	2.20E-05	3.85E-04	Co-occurrence
LINC00404	13q34	11 (9.57%)	1 (0.41%)	4.55	2.20E-05	3.85E-04	Co-occurrence
MCF2L	13q34	11 (9.57%)	1 (0.41%)	4.55	2.20E-05	3.85E-04	Co-occurrence
MIR-4502/4502		11 (9.57%)	1 (0.41%)	4.55	2.20E-05	3.85E-04	Co-occurrence
PCID2	13q34	11 (9.57%)	1 (0.41%)	4.55	2.20E-05	3.85E-04	Co-occurrence
PROZ	13q34	11 (9.57%)	1 (0.41%)	4.55	2.20E-05	3.85E-04	Co-occurrence
SNORD44	1q25.1	11 (9.57%)	1 (0.41%)	4.55	2.20E-05	3.85E-04	Co-occurrence
SOX1	13q34	11 (9.57%)	1 (0.41%)	4.55	2.20E-05	3.85E-04	Co-occurrence
SOX1-OT	13q34	11 (9.57%)	1 (0.41%)	4.55	2.20E-05	3.85E-04	Co-occurrence
SPACA7	13q34	11 (9.57%)	1 (0.41%)	4.55	2.20E-05	3.85E-04	Co-occurrence
TMCO3	13q34	11 (9.57%)	1 (0.41%)	4.55	2.20E-05	3.85E-04	Co-occurrence
TUBGCP3	13q34	11 (9.57%)	1 (0.41%)	4.55	2.20E-05	3.85E-04	Co-occurrence
UPF3A	13q34	11 (9.57%)	1 (0.41%)	4.55	2.20E-05	3.85E-04	Co-occurrence

B3GALT2	1q31.2	23 (20.00%)	13 (5.31%)	1.91	3.27E-05	5.70E-04	Co-occurrence
CDC73	1q31.2	23 (20.00%)	13 (5.31%)	1.91	3.27E-05	5.70E-04	Co-occurrence
KCNH1	1q32.2	23 (20.00%)	13 (5.31%)	1.91	3.27E-05	5.70E-04	Co-occurrence
MIR-1278/1278		23 (20.00%)	13 (5.31%)	1.91	3.27E-05	5.70E-04	Co-occurrence
RO60	1q31.2	23 (20.00%)	13 (5.31%)	1.91	3.27E-05	5.70E-04	Co-occurrence
UCHL5	1q31.2	23 (20.00%)	13 (5.31%)	1.91	3.27E-05	5.70E-04	Co-occurrence
CARS2	13q34	12 (10.43%)	2 (0.82%)	3.68	3.61E-05	6.27E-04	Co-occurrence
COL4A2	13q34	12 (10.43%)	2 (0.82%)	3.68	3.61E-05	6.27E-04	Co-occurrence
NAXD	13q34	12 (10.43%)	2 (0.82%)	3.68	3.61E-05	6.27E-04	Co-occurrence
RAB20	13q34	12 (10.43%)	2 (0.82%)	3.68	3.61E-05	6.27E-04	Co-occurrence
MAP10	1q42.2	22 (19.13%)	12 (4.90%)	1.97	3.76E-05	6.51E-04	Co-occurrence
PCNX2	1q42.2	22 (19.13%)	12 (4.90%)	1.97	3.76E-05	6.51E-04	Co-occurrence
RN7SL668P	1q42.3	22 (19.13%)	12 (4.90%)	1.97	3.76E-05	6.51E-04	Co-occurrence
TARBP1	1q42.2	22 (19.13%)	12 (4.90%)	1.97	3.76E-05	6.51E-04	Co-occurrence
ABCB10	1q42.13	21 (18.26%)	11 (4.49%)	2.02	4.27E-05	7.35E-04	Co-occurrence
COA6	1q42.2	21 (18.26%)	11 (4.49%)	2.02	4.27E-05	7.35E-04	Co-occurrence
DISC1	1q42.2	21 (18.26%)	11 (4.49%)	2.02	4.27E-05	7.35E-04	Co-occurrence
LPGAT1	1q32.3	21 (18.26%)	11 (4.49%)	2.02	4.27E-05	7.35E-04	Co-occurrence
NUP133	1q42.13	21 (18.26%)	11 (4.49%)	2.02	4.27E-05	7.35E-04	Co-occurrence
RN7SL344P	1q32.3	21 (18.26%)	11 (4.49%)	2.02	4.27E-05	7.35E-04	Co-occurrence
TSNAX	1q42.2	21 (18.26%)	11 (4.49%)	2.02	4.27E-05	7.35E-04	Co-occurrence
USH2A	1q41	21 (18.26%)	11 (4.49%)	2.02	4.27E-05	7.35E-04	Co-occurrence
COQ8A	1q42.13	20 (17.39%)	10 (4.08%)	2.09	4.79E-05	8.14E-04	Co-occurrence
EPHX1	1q42.12	20 (17.39%)	10 (4.08%)	2.09	4.79E-05	8.14E-04	Co-occurrence
GALNT2	1q42.13	20 (17.39%)	10 (4.08%)	2.09	4.79E-05	8.14E-04	Co-occurrence
ITPKB	1q42.12	20 (17.39%)	10 (4.08%)	2.09	4.79E-05	8.14E-04	Co-occurrence
NVL	1q42.11	20 (17.39%)	10 (4.08%)	2.09	4.79E-05	8.14E-04	Co-occurrence
PGBD5	1q42.13	20 (17.39%)	10 (4.08%)	2.09	4.79E-05	8.14E-04	Co-occurrence
RN7SKP165	1q42.12	20 (17.39%)	10 (4.08%)	2.09	4.79E-05	8.14E-04	Co-occurrence

RNA5SP77	1q42.13	20 (17.39%)	10 (4.08%)	2.09	4.79E-05	8.14E-04	Co-occurrence
SRP9	1q42.12	20 (17.39%)	10 (4.08%)	2.09	4.79E-05	8.14E-04	Co-occurrence
STUM	1q42.12	20 (17.39%)	10 (4.08%)	2.09	4.79E-05	8.14E-04	Co-occurrence
TMEM63A	1q42.12	20 (17.39%)	10 (4.08%)	2.09	4.79E-05	8.14E-04	Co-occurrence
URB2	1q42.13	20 (17.39%)	10 (4.08%)	2.09	4.79E-05	8.14E-04	Co-occurrence
ZNF678	1q42.13	20 (17.39%)	10 (4.08%)	2.09	4.79E-05	8.14E-04	Co-occurrence
FBXO28	1q42.11	19 (16.52%)	9 (3.67%)	2.17	5.28E-05	8.96E-04	Co-occurrence
RN7SKP49	1q42.11	19 (16.52%)	9 (3.67%)	2.17	5.28E-05	8.96E-04	Co-occurrence
GLRX2	1q31.2	22 (19.13%)	13 (5.31%)	1.85	7.39E-05	1.25E-03	Co-occurrence
LINC00467	1q32.3	22 (19.13%)	13 (5.31%)	1.85	7.39E-05	1.25E-03	Co-occurrence
RCOR3	1q32.2-q32.3	22 (19.13%)	13 (5.31%)	1.85	7.39E-05	1.25E-03	Co-occurrence
TRAF5	1q32.3	22 (19.13%)	13 (5.31%)	1.85	7.39E-05	1.25E-03	Co-occurrence
KCNK1	1q42.2	21 (18.26%)	12 (4.90%)	1.9	8.57E-05	1.45E-03	Co-occurrence
MIR-4427/4427		21 (18.26%)	12 (4.90%)	1.9	8.57E-05	1.45E-03	Co-occurrence
NEK2	1q32.3	21 (18.26%)	12 (4.90%)	1.9	8.57E-05	1.45E-03	Co-occurrence
ARV1	1q42.2	20 (17.39%)	11 (4.49%)	1.95	9.84E-05	1.63E-03	Co-occurrence
C1ORF131	1q42.2	20 (17.39%)	11 (4.49%)	1.95	9.84E-05	1.63E-03	Co-occurrence
COG2	1q42.2	20 (17.39%)	11 (4.49%)	1.95	9.84E-05	1.63E-03	Co-occurrence
EGLN1	1q42.2	20 (17.39%)	11 (4.49%)	1.95	9.84E-05	1.63E-03	Co-occurrence
EXOC8	1q42.2	20 (17.39%)	11 (4.49%)	1.95	9.84E-05	1.63E-03	Co-occurrence
FAM89A	1q42.2	20 (17.39%)	11 (4.49%)	1.95	9.84E-05	1.63E-03	Co-occurrence
GNPAT	1q42.2	20 (17.39%)	11 (4.49%)	1.95	9.84E-05	1.63E-03	Co-occurrence
KCTD3	1q41	20 (17.39%)	11 (4.49%)	1.95	9.84E-05	1.63E-03	Co-occurrence
LINC00582	1q42.2	20 (17.39%)	11 (4.49%)	1.95	9.84E-05	1.63E-03	Co-occurrence
MIR-1182/1182		20 (17.39%)	11 (4.49%)	1.95	9.84E-05	1.63E-03	Co-occurrence
RN7SL299P	1q42.2	20 (17.39%)	11 (4.49%)	1.95	9.84E-05	1.63E-03	Co-occurrence
RN7SL837P	1q42.2	20 (17.39%)	11 (4.49%)	1.95	9.84E-05	1.63E-03	Co-occurrence
RNA5SP80	1q42.2	20 (17.39%)	11 (4.49%)	1.95	9.84E-05	1.63E-03	Co-occurrence
SIPA1L2	1q42.2	20 (17.39%)	11 (4.49%)	1.95	9.84E-05	1.63E-03	Co-occurrence

SPRTN	1q42.2	20 (17.39%)	11 (4.49%)	1.95	9.84E-05	1.63E-03	Co-occurrence
TRIM67	1q42.2	20 (17.39%)	11 (4.49%)	1.95	9.84E-05	1.63E-03	Co-occurrence
TTC13	1q42.2	20 (17.39%)	11 (4.49%)	1.95	9.84E-05	1.63E-03	Co-occurrence
ANKRD10	13q34	11 (9.57%)	2 (0.82%)	3.55	1.04E-04	1.71E-03	Co-occurrence
ARHGEF7	13q34	11 (9.57%)	2 (0.82%)	3.55	1.04E-04	1.71E-03	Co-occurrence
ING1	13q34	11 (9.57%)	2 (0.82%)	3.55	1.04E-04	1.71E-03	Co-occurrence
LINC00346	13q34	11 (9.57%)	2 (0.82%)	3.55	1.04E-04	1.71E-03	Co-occurrence
LINC00567	13q34	11 (9.57%)	2 (0.82%)	3.55	1.04E-04	1.71E-03	Co-occurrence
TEX29	13q34	11 (9.57%)	2 (0.82%)	3.55	1.04E-04	1.71E-03	Co-occurrence
DEGS1	1q42.11	19 (16.52%)	10 (4.08%)	2.02	1.12E-04	1.84E-03	Co-occurrence
MAP3K21	1q42.2	21 (18.26%)	13 (5.31%)	1.78	1.64E-04	2.70E-03	Co-occurrence
AGT	1q42.2	20 (17.39%)	12 (4.90%)	1.83	1.91E-04	3.14E-03	Co-occurrence
C1ORF198	1q42.2	20 (17.39%)	12 (4.90%)	1.83	1.91E-04	3.14E-03	Co-occurrence
CAPN9	1q42.2	20 (17.39%)	12 (4.90%)	1.83	1.91E-04	3.14E-03	Co-occurrence
NTPCR	1q42.2	20 (17.39%)	12 (4.90%)	1.83	1.91E-04	3.14E-03	Co-occurrence
RNA5SP79	1q42.2	20 (17.39%)	12 (4.90%)	1.83	1.91E-04	3.14E-03	Co-occurrence
DNAJC3	13q32.1	9 (7.83%)	1 (0.41%)	4.26	2.02E-04	3.32E-03	Co-occurrence
SLC30A1	1q32.3	22 (19.13%)	15 (6.12%)	1.64	2.50E-04	4.09E-03	Co-occurrence
COL4A1	13q34	11 (9.57%)	3 (1.22%)	2.97	3.52E-04	5.74E-03	Co-occurrence
IRS2	13q34	11 (9.57%)	3 (1.22%)	2.97	3.52E-04	5.74E-03	Co-occurrence
LINC00676	13q34	11 (9.57%)	3 (1.22%)	2.97	3.52E-04	5.74E-03	Co-occurrence
MYO16	13q33.3	11 (9.57%)	3 (1.22%)	2.97	3.52E-04	5.74E-03	Co-occurrence
RN7SKP10	13q34	11 (9.57%)	3 (1.22%)	2.97	3.52E-04	5.74E-03	Co-occurrence
RN7SL783P	13q34	11 (9.57%)	3 (1.22%)	2.97	3.52E-04	5.74E-03	Co-occurrence
RD3	1q32.3	22 (19.13%)	16 (6.53%)	1.55	4.32E-04	7.04E-03	Co-occurrence
ABHD13	13q33.3	9 (7.83%)	2 (0.82%)	3.26	8.11E-04	0.0129	Co-occurrence
ARGLU1	13q33.3	9 (7.83%)	2 (0.82%)	3.26	8.11E-04	0.0129	Co-occurrence
CLDN10	13q32.1	9 (7.83%)	2 (0.82%)	3.26	8.11E-04	0.0129	Co-occurrence
DCT	13q32.1	9 (7.83%)	2 (0.82%)	3.26	8.11E-04	0.0129	Co-occurrence

DZIP1	13q32.1	9 (7.83%)	2 (0.82%)	3.26	8.11E-04	0.0129	Co-occurrence
EFNB2	13q33.3	9 (7.83%)	2 (0.82%)	3.26	8.11E-04	0.0129	Co-occurrence
FAM155A	13q33.3	9 (7.83%)	2 (0.82%)	3.26	8.11E-04	0.0129	Co-occurrence
GPR180	13q32.1	9 (7.83%)	2 (0.82%)	3.26	8.11E-04	0.0129	Co-occurrence
LIG4	13q33.3	9 (7.83%)	2 (0.82%)	3.26	8.11E-04	0.0129	Co-occurrence
LINC00391	13q32.1	9 (7.83%)	2 (0.82%)	3.26	8.11E-04	0.0129	Co-occurrence
LINC00443	13q33.3	9 (7.83%)	2 (0.82%)	3.26	8.11E-04	0.0129	Co-occurrence
LINC00460	13q33.2	9 (7.83%)	2 (0.82%)	3.26	8.11E-04	0.0129	Co-occurrence
LINC00551	13q33.3	9 (7.83%)	2 (0.82%)	3.26	8.11E-04	0.0129	Co-occurrence
MIR-1267/1267		9 (7.83%)	2 (0.82%)	3.26	8.11E-04	0.0129	Co-occurrence
NALCN	13q32.3-q33.1	9 (7.83%)	2 (0.82%)	3.26	8.11E-04	0.0129	Co-occurrence
RN7SL164P	13q32.1	9 (7.83%)	2 (0.82%)	3.26	8.11E-04	0.0129	Co-occurrence
RN7SL585P	13q32.1	9 (7.83%)	2 (0.82%)	3.26	8.11E-04	0.0129	Co-occurrence
RNA5SP35	13q31.3	9 (7.83%)	2 (0.82%)	3.26	8.11E-04	0.0129	Co-occurrence
RNA5SP36	13q32.1	9 (7.83%)	2 (0.82%)	3.26	8.11E-04	0.0129	Co-occurrence
RNA5SP39	13q33.3	9 (7.83%)	2 (0.82%)	3.26	8.11E-04	0.0129	Co-occurrence
SNORD22	11q12.3	9 (7.83%)	2 (0.82%)	3.26	8.11E-04	0.0129	Co-occurrence
SNORD31	11q12.3	9 (7.83%)	2 (0.82%)	3.26	8.11E-04	0.0129	Co-occurrence
SOX21	13q32.1	9 (7.83%)	2 (0.82%)	3.26	8.11E-04	0.0129	Co-occurrence
TGDS	13q32.1	9 (7.83%)	2 (0.82%)	3.26	8.11E-04	0.0129	Co-occurrence
TNFSF13B	13q33.3	9 (7.83%)	2 (0.82%)	3.26	8.11E-04	0.0129	Co-occurrence
UGGT2	13q32.1	9 (7.83%)	2 (0.82%)	3.26	8.11E-04	0.0129	Co-occurrence
GPC5	13q31.3	10 (8.70%)	3 (1.22%)	2.83	9.24E-04	0.0147	Co-occurrence
LIMD1	3p21.31	6 (5.22%)	0 (0.00%)	>10	9.71E-04	0.0154	Co-occurrence
LINC00351	13q31.1	7 (6.09%)	1 (0.41%)	3.9	1.75E-03	0.0277	Co-occurrence
LINC00433	13q31.2	7 (6.09%)	1 (0.41%)	3.9	1.75E-03	0.0277	Co-occurrence
LINC00560	13q31.2	7 (6.09%)	1 (0.41%)	3.9	1.75E-03	0.0277	Co-occurrence
MIR-4500/4500		7 (6.09%)	1 (0.41%)	3.9	1.75E-03	0.0277	Co-occurrence
MIR4500HG	13q31.2	7 (6.09%)	1 (0.41%)	3.9	1.75E-03	0.0277	Co-occurrence

SLITRK5	13q31.2	7 (6.09%)	1 (0.41%)	3.9	1.75E-03	0.0277	Co-occurrence
SLITRK6	13q31.1	7 (6.09%)	1 (0.41%)	3.9	1.75E-03	0.0277	Co-occurrence
PRKCA	17q24.2	13 (11.30%)	7 (2.86%)	1.98	1.84E-03	0.0291	Co-occurrence
APOH	17q24.2	12 (10.43%)	6 (2.45%)	2.09	2.05E-03	0.0323	Co-occurrence
HELZ	17q24.2	12 (10.43%)	6 (2.45%)	2.09	2.05E-03	0.0323	Co-occurrence
RN7SL735P	17q24.2	12 (10.43%)	6 (2.45%)	2.09	2.05E-03	0.0323	Co-occurrence
RNA5SP444	17q24.2	12 (10.43%)	6 (2.45%)	2.09	2.05E-03	0.0323	Co-occurrence
ABCC4	13q32.1	8 (6.96%)	2 (0.82%)	3.09	2.19E-03	0.0339	Co-occurrence
BIVM	13q33.1	8 (6.96%)	2 (0.82%)	3.09	2.19E-03	0.0339	Co-occurrence
CCDC168	13q33.1	8 (6.96%)	2 (0.82%)	3.09	2.19E-03	0.0339	Co-occurrence
DAOA	13q33.2|13q34	8 (6.96%)	2 (0.82%)	3.09	2.19E-03	0.0339	Co-occurrence
ERCC5	13q33.1	8 (6.96%)	2 (0.82%)	3.09	2.19E-03	0.0339	Co-occurrence
LINC00283	-	8 (6.96%)	2 (0.82%)	3.09	2.19E-03	0.0339	Co-occurrence
LINC00343	13q33.2	8 (6.96%)	2 (0.82%)	3.09	2.19E-03	0.0339	Co-occurrence
LINC00555	13q33.1	8 (6.96%)	2 (0.82%)	3.09	2.19E-03	0.0339	Co-occurrence
LINC00557	13q32.1	8 (6.96%)	2 (0.82%)	3.09	2.19E-03	0.0339	Co-occurrence
METTL21C	13q33.1	8 (6.96%)	2 (0.82%)	3.09	2.19E-03	0.0339	Co-occurrence
METTL21EP	13q33.1	8 (6.96%)	2 (0.82%)	3.09	2.19E-03	0.0339	Co-occurrence
MIR-4705/4705		8 (6.96%)	2 (0.82%)	3.09	2.19E-03	0.0339	Co-occurrence
POGLUT2	13q33.1	8 (6.96%)	2 (0.82%)	3.09	2.19E-03	0.0339	Co-occurrence
RNA5SP38	13q33.2	8 (6.96%)	2 (0.82%)	3.09	2.19E-03	0.0339	Co-occurrence
RNY1P2	13q33.1	8 (6.96%)	2 (0.82%)	3.09	2.19E-03	0.0339	Co-occurrence
RNY3P8	13q32.1	8 (6.96%)	2 (0.82%)	3.09	2.19E-03	0.0339	Co-occurrence
RNY4P27	13q32.1	8 (6.96%)	2 (0.82%)	3.09	2.19E-03	0.0339	Co-occurrence
RNY5P8	13q33.1	8 (6.96%)	2 (0.82%)	3.09	2.19E-03	0.0339	Co-occurrence
SLC10A2	13q33.1	8 (6.96%)	2 (0.82%)	3.09	2.19E-03	0.0339	Co-occurrence
TEX30	13q33.1	8 (6.96%)	2 (0.82%)	3.09	2.19E-03	0.0339	Co-occurrence
TPP2	13q33.1	8 (6.96%)	2 (0.82%)	3.09	2.19E-03	0.0339	Co-occurrence
CLYBL	13q32.3	10 (8.70%)	4 (1.63%)	2.41	2.35E-03	0.0359	Co-occurrence

GGACT	13q32.3	10 (8.70%)	4 (1.63%)	2.41	2.35E-03	0.0359	Co-occurrence
MIR-4306/4306		10 (8.70%)	4 (1.63%)	2.41	2.35E-03	0.0359	Co-occurrence
FGF14	13q33.1	9 (7.83%)	3 (1.22%)	2.68	2.37E-03	0.0359	Co-occurrence
GPC6	13q31.3-q32.1	9 (7.83%)	3 (1.22%)	2.68	2.37E-03	0.0359	Co-occurrence
HS6ST3	13q32.1	9 (7.83%)	3 (1.22%)	2.68	2.37E-03	0.0359	Co-occurrence
IPO5	13q32.2	9 (7.83%)	3 (1.22%)	2.68	2.37E-03	0.0359	Co-occurrence
ITGBL1	13q33.1	9 (7.83%)	3 (1.22%)	2.68	2.37E-03	0.0359	Co-occurrence
LINC00359	13q32.1	9 (7.83%)	3 (1.22%)	2.68	2.37E-03	0.0359	Co-occurrence
LINC00379	13q31.3	9 (7.83%)	3 (1.22%)	2.68	2.37E-03	0.0359	Co-occurrence
LINC00410	13q31.3	9 (7.83%)	3 (1.22%)	2.68	2.37E-03	0.0359	Co-occurrence
LINC00411	13q32.3	9 (7.83%)	3 (1.22%)	2.68	2.37E-03	0.0359	Co-occurrence
MBNL2	13q32.1	9 (7.83%)	3 (1.22%)	2.68	2.37E-03	0.0359	Co-occurrence
MIR-3170/3170		9 (7.83%)	3 (1.22%)	2.68	2.37E-03	0.0359	Co-occurrence
MIR17HG	13q31.3	9 (7.83%)	3 (1.22%)	2.68	2.37E-03	0.0359	Co-occurrence
OXGR1	13q32.1	9 (7.83%)	3 (1.22%)	2.68	2.37E-03	0.0359	Co-occurrence
RAP2A	13q32.1	9 (7.83%)	3 (1.22%)	2.68	2.37E-03	0.0359	Co-occurrence
RN7SKP7	13q32.1	9 (7.83%)	3 (1.22%)	2.68	2.37E-03	0.0359	Co-occurrence
RN7SKP8	13q32.2	9 (7.83%)	3 (1.22%)	2.68	2.37E-03	0.0359	Co-occurrence
RNA5SP37	13q32.1	9 (7.83%)	3 (1.22%)	2.68	2.37E-03	0.0359	Co-occurrence
RNF113B	13q32.2	9 (7.83%)	3 (1.22%)	2.68	2.37E-03	0.0359	Co-occurrence
BCAS3	17q23.2	14 (12.17%)	9 (3.67%)	1.73	3.02E-03	0.0458	Co-occurrence
HMGCS2	1p12	5 (4.35%)	0 (0.00%)	>10	3.13E-03	0.0473	Co-occurrence
LARS2	3p21.31	5 (4.35%)	0 (0.00%)	>10	3.13E-03	0.0473	Co-occurrence
NF1	17q11.2	5 (4.35%)	0 (0.00%)	>10	3.13E-03	0.0473	Co-occurrence
RAB11FIP4	17q11.2	5 (4.35%)	0 (0.00%)	>10	3.13E-03	0.0473	Co-occurrence
REG4	1p12	5 (4.35%)	0 (0.00%)	>10	3.13E-03	0.0473	Co-occurrence
RN7SL45P	17q11.2	5 (4.35%)	0 (0.00%)	>10	3.13E-03	0.0473	Co-occurrence
RN7SL79P	17q11.2	5 (4.35%)	0 (0.00%)	>10	3.13E-03	0.0473	Co-occurrence
RNU6ATAC7P	17q11.2	5 (4.35%)	0 (0.00%)	>10	3.13E-03	0.0473	Co-occurrence

CACNG5	17q24.2	11 (9.57%)	6 (2.45%)	1.97	4.60E-03	0.0689	Co-occurrence
CEP112	17q24.1	11 (9.57%)	6 (2.45%)	1.97	4.60E-03	0.0689	Co-occurrence
GDPD1	17q22	11 (9.57%)	6 (2.45%)	1.97	4.60E-03	0.0689	Co-occurrence
MIR-635/635		11 (9.57%)	6 (2.45%)	1.97	4.60E-03	0.0689	Co-occurrence
PCCA	13q32.3	11 (9.57%)	6 (2.45%)	1.97	4.60E-03	0.0689	Co-occurrence
PRKAR1A	17q24.2	11 (9.57%)	6 (2.45%)	1.97	4.60E-03	0.0689	Co-occurrence
PSMD12	17q24.2	11 (9.57%)	6 (2.45%)	1.97	4.60E-03	0.0689	Co-occurrence
RNA5SP446	17q24.2	11 (9.57%)	6 (2.45%)	1.97	4.60E-03	0.0689	Co-occurrence
RNA5SP447	17q24.2	11 (9.57%)	6 (2.45%)	1.97	4.60E-03	0.0689	Co-occurrence
WIPI1	17q24.2	11 (9.57%)	6 (2.45%)	1.97	4.60E-03	0.0689	Co-occurrence
LINC00333	13q31.1	6 (5.22%)	1 (0.41%)	3.68	5.00E-03	0.0747	Co-occurrence
PHGDH	1p12	6 (5.22%)	1 (0.41%)	3.68	5.00E-03	0.0747	Co-occurrence
LINC00554	13q32.3	10 (8.70%)	5 (2.04%)	2.09	5.14E-03	0.0766	Co-occurrence
TMTC4	13q32.3	10 (8.70%)	5 (2.04%)	2.09	5.14E-03	0.0766	Co-occurrence
ZIC2	13q32.3	10 (8.70%)	5 (2.04%)	2.09	5.14E-03	0.0766	Co-occurrence
ZIC5	13q32.3	10 (8.70%)	5 (2.04%)	2.09	5.14E-03	0.0766	Co-occurrence
GPR18	13q32.3	9 (7.83%)	4 (1.63%)	2.26	5.61E-03	0.0829	Co-occurrence
GPR183	13q32.3	9 (7.83%)	4 (1.63%)	2.26	5.61E-03	0.0829	Co-occurrence
LINC00449	13q32.3	9 (7.83%)	4 (1.63%)	2.26	5.61E-03	0.0829	Co-occurrence
MIR-623/623		9 (7.83%)	4 (1.63%)	2.26	5.61E-03	0.0829	Co-occurrence
RN7SKP9	13q32.3	9 (7.83%)	4 (1.63%)	2.26	5.61E-03	0.0829	Co-occurrence
RNY3P6	13q32.3	9 (7.83%)	4 (1.63%)	2.26	5.61E-03	0.0829	Co-occurrence
STK24	13q32.2	9 (7.83%)	4 (1.63%)	2.26	5.61E-03	0.0829	Co-occurrence
TM9SF2	13q32.3	9 (7.83%)	4 (1.63%)	2.26	5.61E-03	0.0829	Co-occurrence
UBAC2	13q32.3	9 (7.83%)	4 (1.63%)	2.26	5.61E-03	0.0829	Co-occurrence
LINC00353	13q31.3	7 (6.09%)	2 (0.82%)	2.9	5.78E-03	0.0851	Co-occurrence
LINC00440	13q31.2	7 (6.09%)	2 (0.82%)	2.9	5.78E-03	0.0851	Co-occurrence
LINC00559	13q31.3	7 (6.09%)	2 (0.82%)	2.9	5.78E-03	0.0851	Co-occurrence
MIR-622/622		7 (6.09%)	2 (0.82%)	2.9	5.78E-03	0.0851	Co-occurrence

RNA5SP34	13q31.3	7 (6.09%)	2 (0.82%)	2.9	5.78E-03	0.0851	Co-occurrence
RNU4ATAC3P	13q31.3	8 (6.96%)	3 (1.22%)	2.51	5.88E-03	0.0866	Co-occurrence
ABCA10	17q24.3	11 (9.57%)	7 (2.86%)	1.74	8.61E-03	0.126	Co-occurrence
ARSG	17q24.2	11 (9.57%)	7 (2.86%)	1.74	8.61E-03	0.126	Co-occurrence
PITPNC1	17q24.2	11 (9.57%)	7 (2.86%)	1.74	8.61E-03	0.126	Co-occurrence
PRR11	17q22	11 (9.57%)	7 (2.86%)	1.74	8.61E-03	0.126	Co-occurrence
SKA2	17q22	11 (9.57%)	7 (2.86%)	1.74	8.61E-03	0.126	Co-occurrence
SMG8	17q22	11 (9.57%)	7 (2.86%)	1.74	8.61E-03	0.126	Co-occurrence
YPEL2	17q22	11 (9.57%)	7 (2.86%)	1.74	8.61E-03	0.126	Co-occurrence
BPTF	17q24.2	10 (8.70%)	6 (2.45%)	1.83	9.99E-03	0.142	Co-occurrence
C17ORF58	17q24.2	10 (8.70%)	6 (2.45%)	1.83	9.99E-03	0.142	Co-occurrence
CACNG1	17q24.2	10 (8.70%)	6 (2.45%)	1.83	9.99E-03	0.142	Co-occurrence
CACNG4	17q24.2	10 (8.70%)	6 (2.45%)	1.83	9.99E-03	0.142	Co-occurrence
FAM20A	17q24.2	10 (8.70%)	6 (2.45%)	1.83	9.99E-03	0.142	Co-occurrence
KPNA2	17q24.2	10 (8.70%)	6 (2.45%)	1.83	9.99E-03	0.142	Co-occurrence
MIR-634/634		10 (8.70%)	6 (2.45%)	1.83	9.99E-03	0.142	Co-occurrence
NOL11	17q24.2	10 (8.70%)	6 (2.45%)	1.83	9.99E-03	0.142	Co-occurrence
RN7SL622P	17q24.2	10 (8.70%)	6 (2.45%)	1.83	9.99E-03	0.142	Co-occurrence
RN7SL756P	17q24.2	10 (8.70%)	6 (2.45%)	1.83	9.99E-03	0.142	Co-occurrence
RNA5SP445	17q24.2	10 (8.70%)	6 (2.45%)	1.83	9.99E-03	0.142	Co-occurrence
SNORA38B	17q24.2	10 (8.70%)	6 (2.45%)	1.83	9.99E-03	0.142	Co-occurrence
ARHGAP29	1p22.1	4 (3.48%)	0 (0.00%)	>10	0.01	0.142	Co-occurrence
BEST3	12q15	4 (3.48%)	0 (0.00%)	>10	0.01	0.142	Co-occurrence
CCT2	12q15	4 (3.48%)	0 (0.00%)	>10	0.01	0.142	Co-occurrence
CPSF6	12q15	4 (3.48%)	0 (0.00%)	>10	0.01	0.142	Co-occurrence
EVI2A	17q11.2	4 (3.48%)	0 (0.00%)	>10	0.01	0.142	Co-occurrence
EVI2B	17q11.2	4 (3.48%)	0 (0.00%)	>10	0.01	0.142	Co-occurrence
FAM187B	19q13.12	4 (3.48%)	0 (0.00%)	>10	0.01	0.142	Co-occurrence
FRS2	12q15	4 (3.48%)	0 (0.00%)	>10	0.01	0.142	Co-occurrence

GRAMD1A	19q13.11	4 (3.48%)	0 (0.00%)	>10	0.01	0.142	Co-occurrence
HPN	19q13.11	4 (3.48%)	0 (0.00%)	>10	0.01	0.142	Co-occurrence
IFNG	12q15	4 (3.48%)	0 (0.00%)	>10	0.01	0.142	Co-occurrence
LINC00904	19q13.11	4 (3.48%)	0 (0.00%)	>10	0.01	0.142	Co-occurrence
LRRC10	12q15	4 (3.48%)	0 (0.00%)	>10	0.01	0.142	Co-occurrence
LSR	19q13.12	4 (3.48%)	0 (0.00%)	>10	0.01	0.142	Co-occurrence
LYZ	12q15	4 (3.48%)	0 (0.00%)	>10	0.01	0.142	Co-occurrence
MEGF9	9q33.2	4 (3.48%)	0 (0.00%)	>10	0.01	0.142	Co-occurrence
MYRFL	12q15	4 (3.48%)	0 (0.00%)	>10	0.01	0.142	Co-occurrence
OMG	17q11.2	4 (3.48%)	0 (0.00%)	>10	0.01	0.142	Co-occurrence
RAB3IP	12q15	4 (3.48%)	0 (0.00%)	>10	0.01	0.142	Co-occurrence
RN7SL804P	12q15	4 (3.48%)	0 (0.00%)	>10	0.01	0.142	Co-occurrence
SCN1B	19q13.11	4 (3.48%)	0 (0.00%)	>10	0.01	0.142	Co-occurrence
SFI1	22q12.2	4 (3.48%)	0 (0.00%)	>10	0.01	0.142	Co-occurrence
SNORA70G	12q15	4 (3.48%)	0 (0.00%)	>10	0.01	0.142	Co-occurrence
USF2	19q13.12	4 (3.48%)	0 (0.00%)	>10	0.01	0.142	Co-occurrence
YEATS4	12q15	4 (3.48%)	0 (0.00%)	>10	0.01	0.142	Co-occurrence
ZNF30	19q13.11	4 (3.48%)	0 (0.00%)	>10	0.01	0.142	Co-occurrence
ZNF302	19q13.11	4 (3.48%)	0 (0.00%)	>10	0.01	0.142	Co-occurrence
ZNF599	19q13.11	4 (3.48%)	0 (0.00%)	>10	0.01	0.142	Co-occurrence
ZNF697	1p12	4 (3.48%)	0 (0.00%)	>10	0.01	0.142	Co-occurrence
ZNF792	19q13.11	4 (3.48%)	0 (0.00%)	>10	0.01	0.142	Co-occurrence
ACTG1	17q25.3	13 (11.30%)	10 (4.08%)	1.47	0.0104	0.147	Co-occurrence
CA4	17q23.1	13 (11.30%)	10 (4.08%)	1.47	0.0104	0.147	Co-occurrence
FAAP100	17q25.3	13 (11.30%)	10 (4.08%)	1.47	0.0104	0.147	Co-occurrence
FSCN2	17q25.3	13 (11.30%)	10 (4.08%)	1.47	0.0104	0.147	Co-occurrence
FARP1	13q32.2	9 (7.83%)	5 (2.04%)	1.94	0.0115	0.162	Co-occurrence
RN7SL60P	13q32.2	9 (7.83%)	5 (2.04%)	1.94	0.0115	0.162	Co-occurrence
APPBP2	17q23.2	12 (10.43%)	9 (3.67%)	1.51	0.0125	0.175	Co-occurrence

C17ORF64	17q23.2	12 (10.43%)	9 (3.67%)	1.51	0.0125	0.175	Co-occurrence
PPM1D	17q23.2	12 (10.43%)	9 (3.67%)	1.51	0.0125	0.175	Co-occurrence
RN7SL606P	17q23.2	12 (10.43%)	9 (3.67%)	1.51	0.0125	0.175	Co-occurrence
RPL12P38	17q23.2	12 (10.43%)	9 (3.67%)	1.51	0.0125	0.175	Co-occurrence
ALB	4q13.3	5 (4.35%)	1 (0.41%)	3.41	0.0139	0.195	Co-occurrence
HERC1	15q22.31	5 (4.35%)	1 (0.41%)	3.41	0.0139	0.195	Co-occurrence
MAFF	22q13.1	5 (4.35%)	1 (0.41%)	3.41	0.0139	0.195	Co-occurrence
MIR-422A/422A		5 (4.35%)	1 (0.41%)	3.41	0.0139	0.195	Co-occurrence
PICK1	22q13.1	5 (4.35%)	1 (0.41%)	3.41	0.0139	0.195	Co-occurrence
PLA2G6	22q13.1	5 (4.35%)	1 (0.41%)	3.41	0.0139	0.195	Co-occurrence
RN7SL704P	22q13.1	5 (4.35%)	1 (0.41%)	3.41	0.0139	0.195	Co-occurrence
SCYL2	12q23.1	5 (4.35%)	1 (0.41%)	3.41	0.0139	0.195	Co-occurrence
SLITRK1	13q31.1	5 (4.35%)	1 (0.41%)	3.41	0.0139	0.195	Co-occurrence
TMEM184B	22q13.1	5 (4.35%)	1 (0.41%)	3.41	0.0139	0.195	Co-occurrence
DAB1	1p32.2	6 (5.22%)	2 (0.82%)	2.68	0.0148	0.206	Co-occurrence
CLTC	17q23.1	11 (9.57%)	8 (3.27%)	1.55	0.0149	0.207	Co-occurrence
DHX40	17q23.1	11 (9.57%)	8 (3.27%)	1.55	0.0149	0.207	Co-occurrence
NPLOC4	17q25.3	13 (11.30%)	11 (4.49%)	1.33	0.0165	0.23	Co-occurrence
ABCA5	17q24.3	10 (8.70%)	7 (2.86%)	1.61	0.0177	0.239	Co-occurrence
ABCA6	17q24.2-q24.3	10 (8.70%)	7 (2.86%)	1.61	0.0177	0.239	Co-occurrence
AMZ2	17q24.2	10 (8.70%)	7 (2.86%)	1.61	0.0177	0.239	Co-occurrence
CD79B	17q23.3	10 (8.70%)	7 (2.86%)	1.61	0.0177	0.239	Co-occurrence
CSH1	17q23.3	10 (8.70%)	7 (2.86%)	1.61	0.0177	0.239	Co-occurrence
CSH2	17q23.3	10 (8.70%)	7 (2.86%)	1.61	0.0177	0.239	Co-occurrence
CSHL1	17q23.3	10 (8.70%)	7 (2.86%)	1.61	0.0177	0.239	Co-occurrence
DDX42	17q23.3	10 (8.70%)	7 (2.86%)	1.61	0.0177	0.239	Co-occurrence
EFCAB3	17q23.2	10 (8.70%)	7 (2.86%)	1.61	0.0177	0.239	Co-occurrence
ERN1	17q23.3	10 (8.70%)	7 (2.86%)	1.61	0.0177	0.239	Co-occurrence
FTSJ3	17q23.3	10 (8.70%)	7 (2.86%)	1.61	0.0177	0.239	Co-occurrence

GH1	17q23.3	10 (8.70%)	7 (2.86%)	1.61	0.0177	0.239	Co-occurrence
GH2	17q23.3	10 (8.70%)	7 (2.86%)	1.61	0.0177	0.239	Co-occurrence
ICAM2	17q23.3	10 (8.70%)	7 (2.86%)	1.61	0.0177	0.239	Co-occurrence
KCNJ16	17q24.3	10 (8.70%)	7 (2.86%)	1.61	0.0177	0.239	Co-occurrence
KCNJ2	17q24.3	10 (8.70%)	7 (2.86%)	1.61	0.0177	0.239	Co-occurrence
LIMD2	17q23.3	10 (8.70%)	7 (2.86%)	1.61	0.0177	0.239	Co-occurrence
LINC00511	17q24.3	10 (8.70%)	7 (2.86%)	1.61	0.0177	0.239	Co-occurrence
LINC00674	17q24.2	10 (8.70%)	7 (2.86%)	1.61	0.0177	0.239	Co-occurrence
LRRC37A16P	17q24.2	10 (8.70%)	7 (2.86%)	1.61	0.0177	0.239	Co-occurrence
MAP2K6	17q24.3	10 (8.70%)	7 (2.86%)	1.61	0.0177	0.239	Co-occurrence
MAP3K3	17q23.3	10 (8.70%)	7 (2.86%)	1.61	0.0177	0.239	Co-occurrence
METTL2A	17q23.2	10 (8.70%)	7 (2.86%)	1.61	0.0177	0.239	Co-occurrence
MILR1	17q23.3	10 (8.70%)	7 (2.86%)	1.61	0.0177	0.239	Co-occurrence
MIR-4729/4729		10 (8.70%)	7 (2.86%)	1.61	0.0177	0.239	Co-occurrence
PPM1E	17q22	10 (8.70%)	7 (2.86%)	1.61	0.0177	0.239	Co-occurrence
PRR29	17q23.3	10 (8.70%)	7 (2.86%)	1.61	0.0177	0.239	Co-occurrence
PSMC5	17q23.3	10 (8.70%)	7 (2.86%)	1.61	0.0177	0.239	Co-occurrence
RGS9	17q24.1	10 (8.70%)	7 (2.86%)	1.61	0.0177	0.239	Co-occurrence
RN7SKP180	17q24.3	10 (8.70%)	7 (2.86%)	1.61	0.0177	0.239	Co-occurrence
RN7SL716P	17q22	10 (8.70%)	7 (2.86%)	1.61	0.0177	0.239	Co-occurrence
SCN4A	17q23.3	10 (8.70%)	7 (2.86%)	1.61	0.0177	0.239	Co-occurrence
SLC16A6	17q24.2	10 (8.70%)	7 (2.86%)	1.61	0.0177	0.239	Co-occurrence
SMARCD2	17q23.3	10 (8.70%)	7 (2.86%)	1.61	0.0177	0.239	Co-occurrence
SNORD104	17q23.3	10 (8.70%)	7 (2.86%)	1.61	0.0177	0.239	Co-occurrence
SOX9	17q24.3	10 (8.70%)	7 (2.86%)	1.61	0.0177	0.239	Co-occurrence
STRADA	17q23.3	10 (8.70%)	7 (2.86%)	1.61	0.0177	0.239	Co-occurrence
TBC1D3P2	17q23.2	10 (8.70%)	7 (2.86%)	1.61	0.0177	0.239	Co-occurrence
TCAM1P	17q23.3	10 (8.70%)	7 (2.86%)	1.61	0.0177	0.239	Co-occurrence
TEX2	17q23.3	10 (8.70%)	7 (2.86%)	1.61	0.0177	0.239	Co-occurrence

TLK2	17q23.2	10 (8.70%)	7 (2.86%)	1.61	0.0177	0.239	Co-occurrence
TRIM37	17q22	10 (8.70%)	7 (2.86%)	1.61	0.0177	0.239	Co-occurrence
EIF4A3	17q25.3	12 (10.43%)	10 (4.08%)	1.35	0.02	0.269	Co-occurrence
USP32	17q23.1-q23.2	12 (10.43%)	10 (4.08%)	1.35	0.02	0.269	Co-occurrence
AXIN2	17q24.1	9 (7.83%)	6 (2.45%)	1.68	0.0209	0.281	Co-occurrence
DOCK9	13q32.3	9 (7.83%)	6 (2.45%)	1.68	0.0209	0.281	Co-occurrence
SLC15A1	13q32.2-q32.3	9 (7.83%)	6 (2.45%)	1.68	0.0209	0.281	Co-occurrence
SUPT4H1	17q22	9 (7.83%)	6 (2.45%)	1.68	0.0209	0.281	Co-occurrence
CPT1A	11q13.3	1 (0.87%)	14 (5.71%)	-2.72	0.0229	0.307	Mutual exclusivity
MRPL21	11q13.3	1 (0.87%)	14 (5.71%)	-2.72	0.0229	0.307	Mutual exclusivity
ABCA9	17q24.2	11 (9.57%)	9 (3.67%)	1.38	0.0241	0.323	Co-occurrence
ADGRV1	5q14.3	7 (6.09%)	4 (1.63%)	1.9	0.0286	0.359	Co-occurrence
C17ORF80	17q25.1	10 (8.70%)	8 (3.27%)	1.41	0.0291	0.359	Co-occurrence
CDC42EP4	17q25.1	10 (8.70%)	8 (3.27%)	1.41	0.0291	0.359	Co-occurrence
CEP95	17q23.3	10 (8.70%)	8 (3.27%)	1.41	0.0291	0.359	Co-occurrence
COG1	17q25.1	10 (8.70%)	8 (3.27%)	1.41	0.0291	0.359	Co-occurrence
CPSF4L	17q25.1	10 (8.70%)	8 (3.27%)	1.41	0.0291	0.359	Co-occurrence
DDX5	17q23.3	10 (8.70%)	8 (3.27%)	1.41	0.0291	0.359	Co-occurrence
FAM104A	17q25.1	10 (8.70%)	8 (3.27%)	1.41	0.0291	0.359	Co-occurrence
10-Mar	17q23.2	10 (8.70%)	8 (3.27%)	1.41	0.0291	0.359	Co-occurrence
MED13	17q23.2	10 (8.70%)	8 (3.27%)	1.41	0.0291	0.359	Co-occurrence
MIR-5047/5047		10 (8.70%)	8 (3.27%)	1.41	0.0291	0.359	Co-occurrence
MRC2	17q23.2	10 (8.70%)	8 (3.27%)	1.41	0.0291	0.359	Co-occurrence
POLG2	17q23.3	10 (8.70%)	8 (3.27%)	1.41	0.0291	0.359	Co-occurrence
PTRH2	17q23.1	10 (8.70%)	8 (3.27%)	1.41	0.0291	0.359	Co-occurrence
RN7SL800P	17q23.2	10 (8.70%)	8 (3.27%)	1.41	0.0291	0.359	Co-occurrence
SDK2	17q25.1	10 (8.70%)	8 (3.27%)	1.41	0.0291	0.359	Co-occurrence
SLC39A11	17q24.3-q25.1	10 (8.70%)	8 (3.27%)	1.41	0.0291	0.359	Co-occurrence
SMURF2	17q23.3-q24.1	10 (8.70%)	8 (3.27%)	1.41	0.0291	0.359	Co-occurrence

SSTR2	17q25.1	10 (8.70%)	8 (3.27%)	1.41	0.0291	0.359	Co-occurrence
ACY3	11q13.2	0 (0.00%)	9 (3.67%)	<-10	0.0299	0.359	Mutual exclusivity
ALDH3B1	11q13.2	0 (0.00%)	9 (3.67%)	<-10	0.0299	0.359	Mutual exclusivity
ALDH3B2	11q13.2	0 (0.00%)	9 (3.67%)	<-10	0.0299	0.359	Mutual exclusivity
C11ORF72	11q13.2	0 (0.00%)	9 (3.67%)	<-10	0.0299	0.359	Mutual exclusivity
DOC2GP	11q13.2	0 (0.00%)	9 (3.67%)	<-10	0.0299	0.359	Mutual exclusivity
FAM86C2P	11q13.2	0 (0.00%)	9 (3.67%)	<-10	0.0299	0.359	Mutual exclusivity
GSTP1	11q13.2	0 (0.00%)	9 (3.67%)	<-10	0.0299	0.359	Mutual exclusivity
NDUFV1	11q13.2	0 (0.00%)	9 (3.67%)	<-10	0.0299	0.359	Mutual exclusivity
NUDT8	11q13.2	0 (0.00%)	9 (3.67%)	<-10	0.0299	0.359	Mutual exclusivity
POLD4	11q13.2	0 (0.00%)	9 (3.67%)	<-10	0.0299	0.359	Mutual exclusivity
SSH3	11q13.2	0 (0.00%)	9 (3.67%)	<-10	0.0299	0.359	Mutual exclusivity
TBX10	11q13.2	0 (0.00%)	9 (3.67%)	<-10	0.0299	0.359	Mutual exclusivity
UNC93B1	11q13.2	0 (0.00%)	9 (3.67%)	<-10	0.0299	0.359	Mutual exclusivity
ARHGDIA	17q25.3	12 (10.43%)	11 (4.49%)	1.22	0.0304	0.359	Co-occurrence
ARL16	17q25.3	12 (10.43%)	11 (4.49%)	1.22	0.0304	0.359	Co-occurrence
CARD14	17q25.3	12 (10.43%)	11 (4.49%)	1.22	0.0304	0.359	Co-occurrence
CCDC137	17q25.3	12 (10.43%)	11 (4.49%)	1.22	0.0304	0.359	Co-occurrence
GCGR	17q25.3	12 (10.43%)	11 (4.49%)	1.22	0.0304	0.359	Co-occurrence
HEATR6	17q23.1	12 (10.43%)	11 (4.49%)	1.22	0.0304	0.359	Co-occurrence
HGS	17q25.3	12 (10.43%)	11 (4.49%)	1.22	0.0304	0.359	Co-occurrence
MCRIP1	17q25.3	12 (10.43%)	11 (4.49%)	1.22	0.0304	0.359	Co-occurrence
MIR-4737/4737		12 (10.43%)	11 (4.49%)	1.22	0.0304	0.359	Co-occurrence
MRPL12	17q25.3	12 (10.43%)	11 (4.49%)	1.22	0.0304	0.359	Co-occurrence
OXLD1	17q25.3	12 (10.43%)	11 (4.49%)	1.22	0.0304	0.359	Co-occurrence
P4HB	17q25.3	12 (10.43%)	11 (4.49%)	1.22	0.0304	0.359	Co-occurrence
PDE6G	17q25.3	12 (10.43%)	11 (4.49%)	1.22	0.0304	0.359	Co-occurrence
PPP1R27	17q25.3	12 (10.43%)	11 (4.49%)	1.22	0.0304	0.359	Co-occurrence
RNFT1	17q23.1	12 (10.43%)	11 (4.49%)	1.22	0.0304	0.359	Co-occurrence

RPS6KB1	17q23.1	12 (10.43%)	11 (4.49%)	1.22	0.0304	0.359	Co-occurrence
SLC25A10	17q25.3	12 (10.43%)	11 (4.49%)	1.22	0.0304	0.359	Co-occurrence
TBC1D16	17q25.3	12 (10.43%)	11 (4.49%)	1.22	0.0304	0.359	Co-occurrence
TERT	5p15.33	12 (10.43%)	11 (4.49%)	1.22	0.0304	0.359	Co-occurrence
ABCA4	1p22.1	3 (2.61%)	0 (0.00%)	>10	0.032	0.359	Co-occurrence
ACR	22q13.33	3 (2.61%)	0 (0.00%)	>10	0.032	0.359	Co-occurrence
ADGRL3	4q13.1	3 (2.61%)	0 (0.00%)	>10	0.032	0.359	Co-occurrence
ADH4	4q23	3 (2.61%)	0 (0.00%)	>10	0.032	0.359	Co-occurrence
ADM2	22q13.33	3 (2.61%)	0 (0.00%)	>10	0.032	0.359	Co-occurrence
AFM	4q13.3	3 (2.61%)	0 (0.00%)	>10	0.032	0.359	Co-occurrence
ALAD	9q32	3 (2.61%)	0 (0.00%)	>10	0.032	0.359	Co-occurrence
ALDOC	17q11.2	3 (2.61%)	0 (0.00%)	>10	0.032	0.359	Co-occurrence
ALG12	22q13.33	3 (2.61%)	0 (0.00%)	>10	0.032	0.359	Co-occurrence
ALG14	1p21.3	3 (2.61%)	0 (0.00%)	>10	0.032	0.359	Co-occurrence
ANKS1B	12q23.1	3 (2.61%)	0 (0.00%)	>10	0.032	0.359	Co-occurrence
ANO4	12q23.1	3 (2.61%)	0 (0.00%)	>10	0.032	0.359	Co-occurrence
APOBEC3A	22q13.1	3 (2.61%)	0 (0.00%)	>10	0.032	0.359	Co-occurrence
APOBEC3B	22q13.1	3 (2.61%)	0 (0.00%)	>10	0.032	0.359	Co-occurrence
APOBEC3C	22q13.1	3 (2.61%)	0 (0.00%)	>10	0.032	0.359	Co-occurrence
APOBEC3D	22q13.1	3 (2.61%)	0 (0.00%)	>10	0.032	0.359	Co-occurrence
APOBEC3F	22q13.1	3 (2.61%)	0 (0.00%)	>10	0.032	0.359	Co-occurrence
APOBEC3G	22q13.1	3 (2.61%)	0 (0.00%)	>10	0.032	0.359	Co-occurrence
APOBEC3H	22q13.1	3 (2.61%)	0 (0.00%)	>10	0.032	0.359	Co-occurrence
ARHGAP33	19q13.12	3 (2.61%)	0 (0.00%)	>10	0.032	0.359	Co-occurrence
ARSA	22q13.33	3 (2.61%)	0 (0.00%)	>10	0.032	0.359	Co-occurrence
ASS1	9q34.11	3 (2.61%)	0 (0.00%)	>10	0.032	0.359	Co-occurrence
ATF1	12q13.12	3 (2.61%)	0 (0.00%)	>10	0.032	0.359	Co-occurrence
ATP4A	19q13.12	3 (2.61%)	0 (0.00%)	>10	0.032	0.359	Co-occurrence
ATP8B5P	9p13.3	3 (2.61%)	0 (0.00%)	>10	0.032	0.359	Co-occurrence

BRD1	22q13.33	3 (2.61%)	0 (0.00%)	>10	0.032	0.359	Co-occurrence
BSPRY	9q32	3 (2.61%)	0 (0.00%)	>10	0.032	0.359	Co-occurrence
C12ORF29	12q21.32	3 (2.61%)	0 (0.00%)	>10	0.032	0.359	Co-occurrence
C12ORF49	12q24.22	3 (2.61%)	0 (0.00%)	>10	0.032	0.359	Co-occurrence
C12ORF50	12q21.32	3 (2.61%)	0 (0.00%)	>10	0.032	0.359	Co-occurrence
C22ORF34	22q13.33	3 (2.61%)	0 (0.00%)	>10	0.032	0.359	Co-occurrence
C9ORF43	9q32	3 (2.61%)	0 (0.00%)	>10	0.032	0.359	Co-occurrence
CAND1	12q14.3-q15	3 (2.61%)	0 (0.00%)	>10	0.032	0.359	Co-occurrence
CBX6	22q13.1	3 (2.61%)	0 (0.00%)	>10	0.032	0.359	Co-occurrence
CBX7	22q13.1	3 (2.61%)	0 (0.00%)	>10	0.032	0.359	Co-occurrence
CCT8L2	22q11.1	3 (2.61%)	0 (0.00%)	>10	0.032	0.359	Co-occurrence
CD22	19q13.12	3 (2.61%)	0 (0.00%)	>10	0.032	0.359	Co-occurrence
CDHR4	3p21.31	3 (2.61%)	0 (0.00%)	>10	0.032	0.359	Co-occurrence
CECR7	22q11.1	3 (2.61%)	0 (0.00%)	>10	0.032	0.359	Co-occurrence
CEP290	12q21.32	3 (2.61%)	0 (0.00%)	>10	0.032	0.359	Co-occurrence
CHKB	22q13.33	3 (2.61%)	0 (0.00%)	>10	0.032	0.359	Co-occurrence
CNOT2	12q15	3 (2.61%)	0 (0.00%)	>10	0.032	0.359	Co-occurrence
COX6B1	19q13.12	3 (2.61%)	0 (0.00%)	>10	0.032	0.359	Co-occurrence
CPT1B	22q13.33	3 (2.61%)	0 (0.00%)	>10	0.032	0.359	Co-occurrence
CRELD2	22q13.33	3 (2.61%)	0 (0.00%)	>10	0.032	0.359	Co-occurrence
CRYBA1	17q11.2	3 (2.61%)	0 (0.00%)	>10	0.032	0.359	Co-occurrence
DCHS2	4q31.3	3 (2.61%)	0 (0.00%)	>10	0.032	0.359	Co-occurrence
DENND6B	22q13.33	3 (2.61%)	0 (0.00%)	>10	0.032	0.359	Co-occurrence
DIAPH3	13q21.2	3 (2.61%)	0 (0.00%)	>10	0.032	0.359	Co-occurrence
DMKN	19q13.12	3 (2.61%)	0 (0.00%)	>10	0.032	0.359	Co-occurrence
DNAH10	12q24.31	3 (2.61%)	0 (0.00%)	>10	0.032	0.359	Co-occurrence
DNAL4	22q13.1	3 (2.61%)	0 (0.00%)	>10	0.032	0.359	Co-occurrence
DRG1	22q12.2	3 (2.61%)	0 (0.00%)	>10	0.032	0.359	Co-occurrence
DYRK2	12q15	3 (2.61%)	0 (0.00%)	>10	0.032	0.359	Co-occurrence

EIF4ENIF1	22q12.2	3 (2.61%)	0 (0.00%)	>10	0.032	0.359	Co-occurrence
ENO1	1p36.23	3 (2.61%)	0 (0.00%)	>10	0.032	0.359	Co-occurrence
EPS15	1p32.3	3 (2.61%)	0 (0.00%)	>10	0.032	0.359	Co-occurrence
ETV2	19q13.12	3 (2.61%)	0 (0.00%)	>10	0.032	0.359	Co-occurrence
FAM71C	12q23.1	3 (2.61%)	0 (0.00%)	>10	0.032	0.359	Co-occurrence
FBXW8	12q24.22	3 (2.61%)	0 (0.00%)	>10	0.032	0.359	Co-occurrence
FFAR1	19q13.12	3 (2.61%)	0 (0.00%)	>10	0.032	0.359	Co-occurrence
FFAR2	19q13.12	3 (2.61%)	0 (0.00%)	>10	0.032	0.359	Co-occurrence
FFAR3	19q13.12	3 (2.61%)	0 (0.00%)	>10	0.032	0.359	Co-occurrence
FOXN1	17q11.2	3 (2.61%)	0 (0.00%)	>10	0.032	0.359	Co-occurrence
GAB4	22q11.1	3 (2.61%)	0 (0.00%)	>10	0.032	0.359	Co-occurrence
GAPDHS	19q13.12	3 (2.61%)	0 (0.00%)	>10	0.032	0.359	Co-occurrence
GAREM1	18q12.1	3 (2.61%)	0 (0.00%)	>10	0.032	0.359	Co-occurrence
GOSR1	17q11.2	3 (2.61%)	0 (0.00%)	>10	0.032	0.359	Co-occurrence
GPI	19q13.11	3 (2.61%)	0 (0.00%)	>10	0.032	0.359	Co-occurrence
GPR42	19q13.12	3 (2.61%)	0 (0.00%)	>10	0.032	0.359	Co-occurrence
HAMP	19q13.12	3 (2.61%)	0 (0.00%)	>10	0.032	0.359	Co-occurrence
HAUS5	19q13.12	3 (2.61%)	0 (0.00%)	>10	0.032	0.359	Co-occurrence
HDAC10	22q13.33	3 (2.61%)	0 (0.00%)	>10	0.032	0.359	Co-occurrence
HDHD3	9q32	3 (2.61%)	0 (0.00%)	>10	0.032	0.359	Co-occurrence
HMCN2	9q34.11	3 (2.61%)	0 (0.00%)	>10	0.032	0.359	Co-occurrence
HRK	12q24.22	3 (2.61%)	0 (0.00%)	>10	0.032	0.359	Co-occurrence
HSD3B1	1p12	3 (2.61%)	0 (0.00%)	>10	0.032	0.359	Co-occurrence
HSFY1P1	22q11.1	3 (2.61%)	0 (0.00%)	>10	0.032	0.359	Co-occurrence
HSPB6	19q13.12	3 (2.61%)	0 (0.00%)	>10	0.032	0.359	Co-occurrence
IGFLR1	19q13.12	3 (2.61%)	0 (0.00%)	>10	0.032	0.359	Co-occurrence
IL17REL	22q13.33	3 (2.61%)	0 (0.00%)	>10	0.032	0.359	Co-occurrence
INKA1	3p21.31	3 (2.61%)	0 (0.00%)	>10	0.032	0.359	Co-occurrence
KCNJ3	2q24.1	3 (2.61%)	0 (0.00%)	>10	0.032	0.359	Co-occurrence

KCNMB3P1	22q11.1	3 (2.61%)	0 (0.00%)	>10	0.032	0.359	Co-occurrence
KCNMB4	12q15	3 (2.61%)	0 (0.00%)	>10	0.032	0.359	Co-occurrence
KCTD17	22q12.3	3 (2.61%)	0 (0.00%)	>10	0.032	0.359	Co-occurrence
KIAA0100	17q11.2	3 (2.61%)	0 (0.00%)	>10	0.032	0.359	Co-occurrence
KIAA0355	19q13.11	3 (2.61%)	0 (0.00%)	>10	0.032	0.359	Co-occurrence
KITLG	12q21.32	3 (2.61%)	0 (0.00%)	>10	0.032	0.359	Co-occurrence
KLHDC7B	22q13.33	3 (2.61%)	0 (0.00%)	>10	0.032	0.359	Co-occurrence
KMT2B	19q13.12	3 (2.61%)	0 (0.00%)	>10	0.032	0.359	Co-occurrence
KRT18P55	17q11.2	3 (2.61%)	0 (0.00%)	>10	0.032	0.359	Co-occurrence
KRTDAP	19q13.12	3 (2.61%)	0 (0.00%)	>10	0.032	0.359	Co-occurrence
LIN37	19q13.12	3 (2.61%)	0 (0.00%)	>10	0.032	0.359	Co-occurrence
LINC00173	12q24.22	3 (2.61%)	0 (0.00%)	>10	0.032	0.359	Co-occurrence
LINC00358	13q21.31	3 (2.61%)	0 (0.00%)	>10	0.032	0.359	Co-occurrence
LINC00448	13q21.31	3 (2.61%)	0 (0.00%)	>10	0.032	0.359	Co-occurrence
LINC00458	13q14.3	3 (2.61%)	0 (0.00%)	>10	0.032	0.359	Co-occurrence
LINC00459	13q21.31	3 (2.61%)	0 (0.00%)	>10	0.032	0.359	Co-occurrence
LINC00558	13q14.3	3 (2.61%)	0 (0.00%)	>10	0.032	0.359	Co-occurrence
LINC00622	1p12	3 (2.61%)	0 (0.00%)	>10	0.032	0.359	Co-occurrence
LINC00877	3p13	3 (2.61%)	0 (0.00%)	>10	0.032	0.359	Co-occurrence
LMF2	22q13.33	3 (2.61%)	0 (0.00%)	>10	0.032	0.359	Co-occurrence
LRIG3	12q14.1	3 (2.61%)	0 (0.00%)	>10	0.032	0.359	Co-occurrence
LSM14A	19q13.11	3 (2.61%)	0 (0.00%)	>10	0.032	0.359	Co-occurrence
MAG	19q13.12	3 (2.61%)	0 (0.00%)	>10	0.032	0.359	Co-occurrence
MAP1LC3B2	12q24.22	3 (2.61%)	0 (0.00%)	>10	0.032	0.359	Co-occurrence
MAPK11	22q13.33	3 (2.61%)	0 (0.00%)	>10	0.032	0.359	Co-occurrence
MAPK12	22q13.33	3 (2.61%)	0 (0.00%)	>10	0.032	0.359	Co-occurrence
MAPK8IP2	22q13.33	3 (2.61%)	0 (0.00%)	>10	0.032	0.359	Co-occurrence
MED13L	12q24.21	3 (2.61%)	0 (0.00%)	>10	0.032	0.359	Co-occurrence
MIOX	22q13.33	3 (2.61%)	0 (0.00%)	>10	0.032	0.359	Co-occurrence

MIR-1251/1251		3 (2.61%)	0 (0.00%)	>10	0.032	0.359	Co-occurrence
MIR-4303/4303		3 (2.61%)	0 (0.00%)	>10	0.032	0.359	Co-occurrence
MIR-4421/4421		3 (2.61%)	0 (0.00%)	>10	0.032	0.359	Co-occurrence
MIR-4495/4495		3 (2.61%)	0 (0.00%)	>10	0.032	0.359	Co-occurrence
MIR-4523/4523		3 (2.61%)	0 (0.00%)	>10	0.032	0.359	Co-occurrence
MKRN9P	12q21.32	3 (2.61%)	0 (0.00%)	>10	0.032	0.359	Co-occurrence
MLC1	22q13.33	3 (2.61%)	0 (0.00%)	>10	0.032	0.359	Co-occurrence
MOV10L1	22q13.33	3 (2.61%)	0 (0.00%)	>10	0.032	0.359	Co-occurrence
NCAPH2	22q13.33	3 (2.61%)	0 (0.00%)	>10	0.032	0.359	Co-occurrence
NEDD1	12q23.1	3 (2.61%)	0 (0.00%)	>10	0.032	0.359	Co-occurrence
NEK8	17q11.2	3 (2.61%)	0 (0.00%)	>10	0.032	0.359	Co-occurrence
NOS2	17q11.2	3 (2.61%)	0 (0.00%)	>10	0.032	0.359	Co-occurrence
NPTXR	22q13.1	3 (2.61%)	0 (0.00%)	>10	0.032	0.359	Co-occurrence
NR4A2	2q24.1	3 (2.61%)	0 (0.00%)	>10	0.032	0.359	Co-occurrence
NUFIP2	17q11.2	3 (2.61%)	0 (0.00%)	>10	0.032	0.359	Co-occurrence
ODF3B	22q13.33	3 (2.61%)	0 (0.00%)	>10	0.032	0.359	Co-occurrence
OR11H1	22q11.1	3 (2.61%)	0 (0.00%)	>10	0.032	0.359	Co-occurrence
OR6C1	12q13.2	3 (2.61%)	0 (0.00%)	>10	0.032	0.359	Co-occurrence
OR6C3	12q13.2	3 (2.61%)	0 (0.00%)	>10	0.032	0.359	Co-occurrence
OR6C65	12q13.2	3 (2.61%)	0 (0.00%)	>10	0.032	0.359	Co-occurrence
OR6C75	12q13.2	3 (2.61%)	0 (0.00%)	>10	0.032	0.359	Co-occurrence
PANX2	22q13.33	3 (2.61%)	0 (0.00%)	>10	0.032	0.359	Co-occurrence
PATZ1	22q12.2	3 (2.61%)	0 (0.00%)	>10	0.032	0.359	Co-occurrence
PCDH17	13q21.1	3 (2.61%)	0 (0.00%)	>10	0.032	0.359	Co-occurrence
PCNAP1	4q23	3 (2.61%)	0 (0.00%)	>10	0.032	0.359	Co-occurrence
PDGFB	22q13.1	3 (2.61%)	0 (0.00%)	>10	0.032	0.359	Co-occurrence
PIGS	17q11.2	3 (2.61%)	0 (0.00%)	>10	0.032	0.359	Co-occurrence
PIK3IP1	22q12.2	3 (2.61%)	0 (0.00%)	>10	0.032	0.359	Co-occurrence
PIM3	22q13.33	3 (2.61%)	0 (0.00%)	>10	0.032	0.359	Co-occurrence

PIWIL2	8p21.3	3 (2.61%)	0 (0.00%)	>10	0.032	0.359	Co-occurrence
PLXNB2	22q13.33	3 (2.61%)	0 (0.00%)	>10	0.032	0.359	Co-occurrence
POLE3	9q32	3 (2.61%)	0 (0.00%)	>10	0.032	0.359	Co-occurrence
POTEH	22q11.1	3 (2.61%)	0 (0.00%)	>10	0.032	0.359	Co-occurrence
PPP3CC	8p21.3	3 (2.61%)	0 (0.00%)	>10	0.032	0.359	Co-occurrence
PPP6R2	22q13.33	3 (2.61%)	0 (0.00%)	>10	0.032	0.359	Co-occurrence
PROCA1	17q11.2	3 (2.61%)	0 (0.00%)	>10	0.032	0.359	Co-occurrence
PRODH2	19q13.12	3 (2.61%)	0 (0.00%)	>10	0.032	0.359	Co-occurrence
PROSER3	19q13.12	3 (2.61%)	0 (0.00%)	>10	0.032	0.359	Co-occurrence
PRR20A	13q21.1	3 (2.61%)	0 (0.00%)	>10	0.032	0.359	Co-occurrence
PRR20B	13q21.1	3 (2.61%)	0 (0.00%)	>10	0.032	0.359	Co-occurrence
PRR20C	13q21.1	3 (2.61%)	0 (0.00%)	>10	0.032	0.359	Co-occurrence
PRR20D	13q21.1	3 (2.61%)	0 (0.00%)	>10	0.032	0.359	Co-occurrence
PRR20E	13q21.1	3 (2.61%)	0 (0.00%)	>10	0.032	0.359	Co-occurrence
PRRC2B	9q34.13	3 (2.61%)	0 (0.00%)	>10	0.032	0.359	Co-occurrence
PSENEN	19q13.12	3 (2.61%)	0 (0.00%)	>10	0.032	0.359	Co-occurrence
RAB34	17q11.2	3 (2.61%)	0 (0.00%)	>10	0.032	0.359	Co-occurrence
RABL2B	22q13.33	3 (2.61%)	0 (0.00%)	>10	0.032	0.359	Co-occurrence
RBFOX2	22q12.3	3 (2.61%)	0 (0.00%)	>10	0.032	0.359	Co-occurrence
RBM42	19q13.12	3 (2.61%)	0 (0.00%)	>10	0.032	0.359	Co-occurrence
RMST	12q23.1|12q21	3 (2.61%)	0 (0.00%)	>10	0.032	0.359	Co-occurrence
RN7SKP214	22q12.3	3 (2.61%)	0 (0.00%)	>10	0.032	0.359	Co-occurrence
RN7SKP252	22q13.33	3 (2.61%)	0 (0.00%)	>10	0.032	0.359	Co-occurrence
RN7SKP263	12q22	3 (2.61%)	0 (0.00%)	>10	0.032	0.359	Co-occurrence
RN7SKP50	11p15.4	3 (2.61%)	0 (0.00%)	>10	0.032	0.359	Co-occurrence
RN7SL375P	13q21.2	3 (2.61%)	0 (0.00%)	>10	0.032	0.359	Co-occurrence
RN7SL440P	1p22.1	3 (2.61%)	0 (0.00%)	>10	0.032	0.359	Co-occurrence
RN7SL491P	19q13.12	3 (2.61%)	0 (0.00%)	>10	0.032	0.359	Co-occurrence
RN7SL500P	22q13.33	3 (2.61%)	0 (0.00%)	>10	0.032	0.359	Co-occurrence

RN7SL665P	9q34.11	3 (2.61%)	0 (0.00%)	>10	0.032	0.359	Co-occurrence
RNA5SP107	2q24.1	3 (2.61%)	0 (0.00%)	>10	0.032	0.359	Co-occurrence
RNA5SP364	12q21.32	3 (2.61%)	0 (0.00%)	>10	0.032	0.359	Co-occurrence
RNA5SP496	22q12.2	3 (2.61%)	0 (0.00%)	>10	0.032	0.359	Co-occurrence
RNFT2	12q24.22	3 (2.61%)	0 (0.00%)	>10	0.032	0.359	Co-occurrence
RPL23A	17q11.2	3 (2.61%)	0 (0.00%)	>10	0.032	0.359	Co-occurrence
RPL3	22q13.1	3 (2.61%)	0 (0.00%)	>10	0.032	0.359	Co-occurrence
RSKR	17q11.2	3 (2.61%)	0 (0.00%)	>10	0.032	0.359	Co-occurrence
RUSC2	9p13.3	3 (2.61%)	0 (0.00%)	>10	0.032	0.359	Co-occurrence
SBF1	22q13.33	3 (2.61%)	0 (0.00%)	>10	0.032	0.359	Co-occurrence
SBSN	19q13.12	3 (2.61%)	0 (0.00%)	>10	0.032	0.359	Co-occurrence
SCGB1B2P	19q13.11	3 (2.61%)	0 (0.00%)	>10	0.032	0.359	Co-occurrence
SCGB2B2	19q13.11	3 (2.61%)	0 (0.00%)	>10	0.032	0.359	Co-occurrence
SCO2	22q13.33	3 (2.61%)	0 (0.00%)	>10	0.032	0.359	Co-occurrence
SDF2	17q11.2	3 (2.61%)	0 (0.00%)	>10	0.032	0.359	Co-occurrence
SH3KBP1	Xp22.12	3 (2.61%)	0 (0.00%)	>10	0.032	0.359	Co-occurrence
SHANK3	22q13.33	3 (2.61%)	0 (0.00%)	>10	0.032	0.359	Co-occurrence
SLC39A14	8p21.3	3 (2.61%)	0 (0.00%)	>10	0.032	0.359	Co-occurrence
SNORD42A	17q11.2	3 (2.61%)	0 (0.00%)	>10	0.032	0.359	Co-occurrence
SNORD42B	17q11.2	3 (2.61%)	0 (0.00%)	>10	0.032	0.359	Co-occurrence
SNORD4A	17q11.2	3 (2.61%)	0 (0.00%)	>10	0.032	0.359	Co-occurrence
SNORD4B	17q11.2	3 (2.61%)	0 (0.00%)	>10	0.032	0.359	Co-occurrence
SNORD83A	22q13.1	3 (2.61%)	0 (0.00%)	>10	0.032	0.359	Co-occurrence
SNORD83B	22q13.1	3 (2.61%)	0 (0.00%)	>10	0.032	0.359	Co-occurrence
SPAG5	17q11.2	3 (2.61%)	0 (0.00%)	>10	0.032	0.359	Co-occurrence
SUPT6H	17q11.2	3 (2.61%)	0 (0.00%)	>10	0.032	0.359	Co-occurrence
SWAP70	11p15.4	3 (2.61%)	0 (0.00%)	>10	0.032	0.359	Co-occurrence
SYCE3	22q13.33	3 (2.61%)	0 (0.00%)	>10	0.032	0.359	Co-occurrence
SYNGR1	22q13.1	3 (2.61%)	0 (0.00%)	>10	0.032	0.359	Co-occurrence

TAOK1	17q11.2	3 (2.61%)	0 (0.00%)	>10	0.032	0.359	Co-occurrence
TLCD1	17q11.2	3 (2.61%)	0 (0.00%)	>10	0.032	0.359	Co-occurrence
TMEM147	19q13.12	3 (2.61%)	0 (0.00%)	>10	0.032	0.359	Co-occurrence
TMTC3	12q21.32	3 (2.61%)	0 (0.00%)	>10	0.032	0.359	Co-occurrence
TPTEP1	22q11.1	3 (2.61%)	0 (0.00%)	>10	0.032	0.359	Co-occurrence
TRABD	22q13.33	3 (2.61%)	0 (0.00%)	>10	0.032	0.359	Co-occurrence
TRAF4	17q11.2	3 (2.61%)	0 (0.00%)	>10	0.032	0.359	Co-occurrence
TTLL8	22q13.33	3 (2.61%)	0 (0.00%)	>10	0.032	0.359	Co-occurrence
TUBGCP6	22q13.33	3 (2.61%)	0 (0.00%)	>10	0.032	0.359	Co-occurrence
TYMP	22q13.33	3 (2.61%)	0 (0.00%)	>10	0.032	0.359	Co-occurrence
U2AF1L4	19q13.12	3 (2.61%)	0 (0.00%)	>10	0.032	0.359	Co-occurrence
UBA2	19q13.11	3 (2.61%)	0 (0.00%)	>10	0.032	0.359	Co-occurrence
UBA7	3p21.31	3 (2.61%)	0 (0.00%)	>10	0.032	0.359	Co-occurrence
UGCG	9q31.3	3 (2.61%)	0 (0.00%)	>10	0.032	0.359	Co-occurrence
UNC119	17q11.2	3 (2.61%)	0 (0.00%)	>10	0.032	0.359	Co-occurrence
UNC13B	9p13.3	3 (2.61%)	0 (0.00%)	>10	0.032	0.359	Co-occurrence
UPK1A	19q13.12	3 (2.61%)	0 (0.00%)	>10	0.032	0.359	Co-occurrence
WEE1	11p15.4	3 (2.61%)	0 (0.00%)	>10	0.032	0.359	Co-occurrence
WTIP	19q13.11	3 (2.61%)	0 (0.00%)	>10	0.032	0.359	Co-occurrence
XKR3	22q11.1	3 (2.61%)	0 (0.00%)	>10	0.032	0.359	Co-occurrence
ZBED4	22q13.33	3 (2.61%)	0 (0.00%)	>10	0.032	0.359	Co-occurrence
ZBTB32	19q13.12	3 (2.61%)	0 (0.00%)	>10	0.032	0.359	Co-occurrence
ZNF143	11p15.4	3 (2.61%)	0 (0.00%)	>10	0.032	0.359	Co-occurrence
EYS	6q12	1 (0.87%)	13 (5.31%)	-2.61	0.0321	0.36	Mutual exclusivity
KCNJ12	17p11.2	6 (5.22%)	3 (1.22%)	2.09	0.0327	0.366	Co-occurrence
MYEOV	11q13.3	4 (3.48%)	23 (9.39%)	-1.43	0.033	0.37	Mutual exclusivity
ACE	17q23.3	9 (7.83%)	7 (2.86%)	1.45	0.035	0.388	Co-occurrence
C17ORF47	17q22	9 (7.83%)	7 (2.86%)	1.45	0.035	0.388	Co-occurrence
CCDC47	17q23.3	9 (7.83%)	7 (2.86%)	1.45	0.035	0.388	Co-occurrence

CYB561	17q23.3	9 (7.83%)	7 (2.86%)	1.45	0.035	0.388	Co-occurrence
DCAF7	17q23.3	9 (7.83%)	7 (2.86%)	1.45	0.035	0.388	Co-occurrence
GNA13	17q24.1	9 (7.83%)	7 (2.86%)	1.45	0.035	0.388	Co-occurrence
HSF5	17q22	9 (7.83%)	7 (2.86%)	1.45	0.035	0.388	Co-occurrence
KCNH6	17q23.3	9 (7.83%)	7 (2.86%)	1.45	0.035	0.388	Co-occurrence
MTMR4	17q22	9 (7.83%)	7 (2.86%)	1.45	0.035	0.388	Co-occurrence
RAD51C	17q22	9 (7.83%)	7 (2.86%)	1.45	0.035	0.388	Co-occurrence
RNF43	17q22	9 (7.83%)	7 (2.86%)	1.45	0.035	0.388	Co-occurrence
SEPTIN4	17q22	9 (7.83%)	7 (2.86%)	1.45	0.035	0.388	Co-occurrence
TACO1	17q23.3	9 (7.83%)	7 (2.86%)	1.45	0.035	0.388	Co-occurrence
TEX14	17q22	9 (7.83%)	7 (2.86%)	1.45	0.035	0.388	Co-occurrence
ARFGAP1	20q13.33	5 (4.35%)	2 (0.82%)	2.41	0.0363	0.388	Co-occurrence
BHLHE23	20q13.33	5 (4.35%)	2 (0.82%)	2.41	0.0363	0.388	Co-occurrence
BIRC7	20q13.33	5 (4.35%)	2 (0.82%)	2.41	0.0363	0.388	Co-occurrence
COL9A3	20q13.33	5 (4.35%)	2 (0.82%)	2.41	0.0363	0.388	Co-occurrence
CUL2	10p11.21	5 (4.35%)	2 (0.82%)	2.41	0.0363	0.388	Co-occurrence
DAPK2	15q22.31	5 (4.35%)	2 (0.82%)	2.41	0.0363	0.388	Co-occurrence
DIDO1	20q13.33	5 (4.35%)	2 (0.82%)	2.41	0.0363	0.388	Co-occurrence
FAM27E5	17p11.2	5 (4.35%)	2 (0.82%)	2.41	0.0363	0.388	Co-occurrence
GID8	20q13.33	5 (4.35%)	2 (0.82%)	2.41	0.0363	0.388	Co-occurrence
HAR1A	20q13.33	5 (4.35%)	2 (0.82%)	2.41	0.0363	0.388	Co-occurrence
HAR1B	20q13.33	5 (4.35%)	2 (0.82%)	2.41	0.0363	0.388	Co-occurrence
HSD17B6	12q13.3	5 (4.35%)	2 (0.82%)	2.41	0.0363	0.388	Co-occurrence
LINC00029	20q13.33	5 (4.35%)	2 (0.82%)	2.41	0.0363	0.388	Co-occurrence
LINC00659	20q13.33	5 (4.35%)	2 (0.82%)	2.41	0.0363	0.388	Co-occurrence
LINC00686	-	5 (4.35%)	2 (0.82%)	2.41	0.0363	0.388	Co-occurrence
LINC02693	17p11.2	5 (4.35%)	2 (0.82%)	2.41	0.0363	0.388	Co-occurrence
MDM2	12q15	5 (4.35%)	2 (0.82%)	2.41	0.0363	0.388	Co-occurrence
MIR-3196/3196		5 (4.35%)	2 (0.82%)	2.41	0.0363	0.388	Co-occurrence

MIR-3611/3611		5 (4.35%)	2 (0.82%)	2.41	0.0363	0.388	Co-occurrence
MIR-4326/4326		5 (4.35%)	2 (0.82%)	2.41	0.0363	0.388	Co-occurrence
MIR1-1HG	20q13.33	5 (4.35%)	2 (0.82%)	2.41	0.0363	0.388	Co-occurrence
MRGBP	20q13.33	5 (4.35%)	2 (0.82%)	2.41	0.0363	0.388	Co-occurrence
MTND1P15	17p11.2	5 (4.35%)	2 (0.82%)	2.41	0.0363	0.388	Co-occurrence
MTRNR2L1	17p11.2	5 (4.35%)	2 (0.82%)	2.41	0.0363	0.388	Co-occurrence
MYO1D	17q11.2	5 (4.35%)	2 (0.82%)	2.41	0.0363	0.388	Co-occurrence
NKAIN4	20q13.33	5 (4.35%)	2 (0.82%)	2.41	0.0363	0.388	Co-occurrence
NTSR1	20q13.33	5 (4.35%)	2 (0.82%)	2.41	0.0363	0.388	Co-occurrence
NUP107	12q15	5 (4.35%)	2 (0.82%)	2.41	0.0363	0.388	Co-occurrence
OGFR	20q13.33	5 (4.35%)	2 (0.82%)	2.41	0.0363	0.388	Co-occurrence
PARD3	10p11.22-p11.21	5 (4.35%)	2 (0.82%)	2.41	0.0363	0.388	Co-occurrence
SLC17A8	12q23.1	5 (4.35%)	2 (0.82%)	2.41	0.0363	0.388	Co-occurrence
SLC17A9	20q13.33	5 (4.35%)	2 (0.82%)	2.41	0.0363	0.388	Co-occurrence
SLC35E3	12q15	5 (4.35%)	2 (0.82%)	2.41	0.0363	0.388	Co-occurrence
SLCO4A1	20q13.33	5 (4.35%)	2 (0.82%)	2.41	0.0363	0.388	Co-occurrence
TCFL5	20q13.33	5 (4.35%)	2 (0.82%)	2.41	0.0363	0.388	Co-occurrence
TMEM98	17q11.2	5 (4.35%)	2 (0.82%)	2.41	0.0363	0.388	Co-occurrence
UBBP4	17p11.2	5 (4.35%)	2 (0.82%)	2.41	0.0363	0.388	Co-occurrence
YTHDF1	20q13.33	5 (4.35%)	2 (0.82%)	2.41	0.0363	0.388	Co-occurrence
ZBTB39	12q13.3	5 (4.35%)	2 (0.82%)	2.41	0.0363	0.388	Co-occurrence
AATK	17q25.3	11 (9.57%)	10 (4.08%)	1.23	0.0369	0.388	Co-occurrence
BAIAP2	17q25.3	11 (9.57%)	10 (4.08%)	1.23	0.0369	0.388	Co-occurrence
CBX2	17q25.3	11 (9.57%)	10 (4.08%)	1.23	0.0369	0.388	Co-occurrence
CEP131	17q25.3	11 (9.57%)	10 (4.08%)	1.23	0.0369	0.388	Co-occurrence
CHMP6	17q25.3	11 (9.57%)	10 (4.08%)	1.23	0.0369	0.388	Co-occurrence
ENPP7	17q25.3	11 (9.57%)	10 (4.08%)	1.23	0.0369	0.388	Co-occurrence
GAA	17q25.3	11 (9.57%)	10 (4.08%)	1.23	0.0369	0.388	Co-occurrence
LINC00482	17q25.3	11 (9.57%)	10 (4.08%)	1.23	0.0369	0.388	Co-occurrence

MIR-4739/4739		11 (9.57%)	10 (4.08%)	1.23	0.0369	0.388	Co-occurrence
MIR-657/657		11 (9.57%)	10 (4.08%)	1.23	0.0369	0.388	Co-occurrence
NDUFAF8	17q25.3	11 (9.57%)	10 (4.08%)	1.23	0.0369	0.388	Co-occurrence
RPTOR	17q25.3	11 (9.57%)	10 (4.08%)	1.23	0.0369	0.388	Co-occurrence
SLC26A11	17q25.3	11 (9.57%)	10 (4.08%)	1.23	0.0369	0.388	Co-occurrence
SLC38A10	17q25.3	11 (9.57%)	10 (4.08%)	1.23	0.0369	0.388	Co-occurrence
ST6GALNAC1	17q25.1	11 (9.57%)	10 (4.08%)	1.23	0.0369	0.388	Co-occurrence
TEPSIN	17q25.3	11 (9.57%)	10 (4.08%)	1.23	0.0369	0.388	Co-occurrence
TMEM105	17q25.3	11 (9.57%)	10 (4.08%)	1.23	0.0369	0.388	Co-occurrence
ADAP1	7p22.3	4 (3.48%)	1 (0.41%)	3.09	0.0377	0.388	Co-occurrence
AGAP5	10q22.2	4 (3.48%)	1 (0.41%)	3.09	0.0377	0.388	Co-occurrence
AK4	1p31.3	4 (3.48%)	1 (0.41%)	3.09	0.0377	0.388	Co-occurrence
ANKRD54	22q13.1	4 (3.48%)	1 (0.41%)	3.09	0.0377	0.388	Co-occurrence
AOC2	17q21.31	4 (3.48%)	1 (0.41%)	3.09	0.0377	0.388	Co-occurrence
BAIAP2L2	22q13.1	4 (3.48%)	1 (0.41%)	3.09	0.0377	0.388	Co-occurrence
BECN1	17q21.31	4 (3.48%)	1 (0.41%)	3.09	0.0377	0.388	Co-occurrence
BMS1P4	10q22.2	4 (3.48%)	1 (0.41%)	3.09	0.0377	0.388	Co-occurrence
C7ORF50	7p22.3	4 (3.48%)	1 (0.41%)	3.09	0.0377	0.388	Co-occurrence
CEP19	3q29	4 (3.48%)	1 (0.41%)	3.09	0.0377	0.388	Co-occurrence
CLIP2	7q11.23	4 (3.48%)	1 (0.41%)	3.09	0.0377	0.388	Co-occurrence
CNTD1	17q21.2-q21.31	4 (3.48%)	1 (0.41%)	3.09	0.0377	0.388	Co-occurrence
CNTNAP1	17q21.2	4 (3.48%)	1 (0.41%)	3.09	0.0377	0.388	Co-occurrence
COA3	17q21.2	4 (3.48%)	1 (0.41%)	3.09	0.0377	0.388	Co-occurrence
COL28A1	7p21.3	4 (3.48%)	1 (0.41%)	3.09	0.0377	0.388	Co-occurrence
COPRS	17q11.2	4 (3.48%)	1 (0.41%)	3.09	0.0377	0.388	Co-occurrence
COX19	7p22.3	4 (3.48%)	1 (0.41%)	3.09	0.0377	0.388	Co-occurrence
CYP2W1	7p22.3	4 (3.48%)	1 (0.41%)	3.09	0.0377	0.388	Co-occurrence
DLG1	3q29	4 (3.48%)	1 (0.41%)	3.09	0.0377	0.388	Co-occurrence
EMC9	14q12	4 (3.48%)	1 (0.41%)	3.09	0.0377	0.388	Co-occurrence

EZH1	17q21.2	4 (3.48%)	1 (0.41%)	3.09	0.0377	0.388	Co-occurrence
FITM1	14q12	4 (3.48%)	1 (0.41%)	3.09	0.0377	0.388	Co-occurrence
FXYD1	19q13.12	4 (3.48%)	1 (0.41%)	3.09	0.0377	0.388	Co-occurrence
FXYD3	19q13.12	4 (3.48%)	1 (0.41%)	3.09	0.0377	0.388	Co-occurrence
FXYD5	19q13.12	4 (3.48%)	1 (0.41%)	3.09	0.0377	0.388	Co-occurrence
FXYD7	19q13.12	4 (3.48%)	1 (0.41%)	3.09	0.0377	0.388	Co-occurrence
GALR3	22q13.1	4 (3.48%)	1 (0.41%)	3.09	0.0377	0.388	Co-occurrence
GAS2L3	12q23.1	4 (3.48%)	1 (0.41%)	3.09	0.0377	0.388	Co-occurrence
GCAT	22q13.1	4 (3.48%)	1 (0.41%)	3.09	0.0377	0.388	Co-occurrence
GET4	7p22.3	4 (3.48%)	1 (0.41%)	3.09	0.0377	0.388	Co-occurrence
GLUD1P3	10q22.2	4 (3.48%)	1 (0.41%)	3.09	0.0377	0.388	Co-occurrence
GPER1	7p22.3	4 (3.48%)	1 (0.41%)	3.09	0.0377	0.388	Co-occurrence
GPR146	7p22.3	4 (3.48%)	1 (0.41%)	3.09	0.0377	0.388	Co-occurrence
H1F0	22q13.1	4 (3.48%)	1 (0.41%)	3.09	0.0377	0.388	Co-occurrence
IRF9	14q12	4 (3.48%)	1 (0.41%)	3.09	0.0377	0.388	Co-occurrence
LGI4	19q13.12|19q13.11	4 (3.48%)	1 (0.41%)	3.09	0.0377	0.388	Co-occurrence
LINC00564	13q31.1	4 (3.48%)	1 (0.41%)	3.09	0.0377	0.388	Co-occurrence
LRP1	12q13.3	4 (3.48%)	1 (0.41%)	3.09	0.0377	0.388	Co-occurrence
LRRC37B	17q11.2	4 (3.48%)	1 (0.41%)	3.09	0.0377	0.388	Co-occurrence
MELTF	3q29	4 (3.48%)	1 (0.41%)	3.09	0.0377	0.388	Co-occurrence
MIOS	7p21.3	4 (3.48%)	1 (0.41%)	3.09	0.0377	0.388	Co-occurrence
MIR-658/658		4 (3.48%)	1 (0.41%)	3.09	0.0377	0.388	Co-occurrence
MYOZ1	10q22.2	4 (3.48%)	1 (0.41%)	3.09	0.0377	0.388	Co-occurrence
NAB2	12q13.3	4 (3.48%)	1 (0.41%)	3.09	0.0377	0.388	Co-occurrence
NCBP2	3q29	4 (3.48%)	1 (0.41%)	3.09	0.0377	0.388	Co-occurrence
NDFIP2	13q31.1	4 (3.48%)	1 (0.41%)	3.09	0.0377	0.388	Co-occurrence
NEMP1	12q13.3	4 (3.48%)	1 (0.41%)	3.09	0.0377	0.388	Co-occurrence
NR1H4	12q23.1	4 (3.48%)	1 (0.41%)	3.09	0.0377	0.388	Co-occurrence
NRROS	3q29	4 (3.48%)	1 (0.41%)	3.09	0.0377	0.388	Co-occurrence

PAK2	3q29	4 (3.48%)	1 (0.41%)	3.09	0.0377	0.388	Co-occurrence
PIGX	3q29	4 (3.48%)	1 (0.41%)	3.09	0.0377	0.388	Co-occurrence
PIGZ	3q29	4 (3.48%)	1 (0.41%)	3.09	0.0377	0.388	Co-occurrence
PSME1	14q12	4 (3.48%)	1 (0.41%)	3.09	0.0377	0.388	Co-occurrence
PSME2	14q12	4 (3.48%)	1 (0.41%)	3.09	0.0377	0.388	Co-occurrence
PSME3	17q21.31	4 (3.48%)	1 (0.41%)	3.09	0.0377	0.388	Co-occurrence
PTMAP5	13q31.1	4 (3.48%)	1 (0.41%)	3.09	0.0377	0.388	Co-occurrence
RAMP2	17q21.2	4 (3.48%)	1 (0.41%)	3.09	0.0377	0.388	Co-occurrence
RAP1B	12q15	4 (3.48%)	1 (0.41%)	3.09	0.0377	0.388	Co-occurrence
RBM26	13q31.1	4 (3.48%)	1 (0.41%)	3.09	0.0377	0.388	Co-occurrence
REC8	14q12	4 (3.48%)	1 (0.41%)	3.09	0.0377	0.388	Co-occurrence
RNA5SP320	10q22.2	4 (3.48%)	1 (0.41%)	3.09	0.0377	0.388	Co-occurrence
RNA5SP33	13q31.1	4 (3.48%)	1 (0.41%)	3.09	0.0377	0.388	Co-occurrence
RNA5SP383	14q12	4 (3.48%)	1 (0.41%)	3.09	0.0377	0.388	Co-occurrence
RNF31	14q12	4 (3.48%)	1 (0.41%)	3.09	0.0377	0.388	Co-occurrence
RPA3	7p21.3	4 (3.48%)	1 (0.41%)	3.09	0.0377	0.388	Co-occurrence
SEC24C	10q22.2	4 (3.48%)	1 (0.41%)	3.09	0.0377	0.388	Co-occurrence
SENP5	3q29	4 (3.48%)	1 (0.41%)	3.09	0.0377	0.388	Co-occurrence
SH3GL1P1	17q11.2	4 (3.48%)	1 (0.41%)	3.09	0.0377	0.388	Co-occurrence
SLC16A8	22q13.1	4 (3.48%)	1 (0.41%)	3.09	0.0377	0.388	Co-occurrence
SPRY2	13q31.1	4 (3.48%)	1 (0.41%)	3.09	0.0377	0.388	Co-occurrence
STAT6	12q13.3	4 (3.48%)	1 (0.41%)	3.09	0.0377	0.388	Co-occurrence
SUN1	7p22.3	4 (3.48%)	1 (0.41%)	3.09	0.0377	0.388	Co-occurrence
SYNPO2L	10q22.2	4 (3.48%)	1 (0.41%)	3.09	0.0377	0.388	Co-occurrence
USP54	10q22.2	4 (3.48%)	1 (0.41%)	3.09	0.0377	0.388	Co-occurrence
UTP6	17q11.2	4 (3.48%)	1 (0.41%)	3.09	0.0377	0.388	Co-occurrence
VPS25	17q21.2	4 (3.48%)	1 (0.41%)	3.09	0.0377	0.388	Co-occurrence
WNK4	17q21.2	4 (3.48%)	1 (0.41%)	3.09	0.0377	0.388	Co-occurrence
ZFAND2A	7p22.3	4 (3.48%)	1 (0.41%)	3.09	0.0377	0.388	Co-occurrence

ZNF181	19q13.11	4 (3.48%)	1 (0.41%)	3.09	0.0377	0.388	Co-occurrence
DYNLL2	17q22	8 (6.96%)	6 (2.45%)	1.51	0.0421	0.432	Co-occurrence
MIR-4736/4736		8 (6.96%)	6 (2.45%)	1.51	0.0421	0.432	Co-occurrence
MPO	17q22	8 (6.96%)	6 (2.45%)	1.51	0.0421	0.432	Co-occurrence
TSPOAP1	17q22	8 (6.96%)	6 (2.45%)	1.51	0.0421	0.432	Co-occurrence
ALYREF	17q25.3	12 (10.43%)	12 (4.90%)	1.09	0.0442	0.451	Co-occurrence
ANAPC11	17q25.3	12 (10.43%)	12 (4.90%)	1.09	0.0442	0.451	Co-occurrence
MAFG	17q25.3	12 (10.43%)	12 (4.90%)	1.09	0.0442	0.451	Co-occurrence
MYADML2	17q25.3	12 (10.43%)	12 (4.90%)	1.09	0.0442	0.451	Co-occurrence
NPB	17q25.3	12 (10.43%)	12 (4.90%)	1.09	0.0442	0.451	Co-occurrence
PCYT2	17q25.3	12 (10.43%)	12 (4.90%)	1.09	0.0442	0.451	Co-occurrence
PYCR1	17q25.3	12 (10.43%)	12 (4.90%)	1.09	0.0442	0.451	Co-occurrence
SIRT7	17q25.3	12 (10.43%)	12 (4.90%)	1.09	0.0442	0.451	Co-occurrence
AIP	11q13.2	0 (0.00%)	8 (3.27%)	<-10	0.0443	0.451	Mutual exclusivity
CABP2	11q13.2	0 (0.00%)	8 (3.27%)	<-10	0.0443	0.451	Mutual exclusivity
CABP4	11q13.2	0 (0.00%)	8 (3.27%)	<-10	0.0443	0.451	Mutual exclusivity
CDK2AP2	11q13.2	0 (0.00%)	8 (3.27%)	<-10	0.0443	0.451	Mutual exclusivity
GPR152	11q13.2	0 (0.00%)	8 (3.27%)	<-10	0.0443	0.451	Mutual exclusivity
PITPNM1	11q13.2	0 (0.00%)	8 (3.27%)	<-10	0.0443	0.451	Mutual exclusivity
TMEM134	11q13.2	0 (0.00%)	8 (3.27%)	<-10	0.0443	0.451	Mutual exclusivity
ABCA8	17q24.2	10 (8.70%)	9 (3.67%)	1.24	0.0448	0.456	Co-occurrence
VMP1	17q23.1	10 (8.70%)	9 (3.67%)	1.24	0.0448	0.456	Co-occurrence
MSI2	17q22	7 (6.09%)	5 (2.04%)	1.58	0.0505	0.513	Co-occurrence
RN7SL449P	17q22	7 (6.09%)	5 (2.04%)	1.58	0.0505	0.513	Co-occurrence
MRGPRD	11q13.3	2 (1.74%)	15 (6.12%)	-1.82	0.052	0.528	Mutual exclusivity
CBX4	17q25.3	11 (9.57%)	11 (4.49%)	1.09	0.0538	0.542	Co-occurrence
CBX8	17q25.3	11 (9.57%)	11 (4.49%)	1.09	0.0538	0.542	Co-occurrence
CCDC40	17q25.3	11 (9.57%)	11 (4.49%)	1.09	0.0538	0.542	Co-occurrence
ENDOV	17q25.3	11 (9.57%)	11 (4.49%)	1.09	0.0538	0.542	Co-occurrence

MIR-4635/4635		11 (9.57%)	11 (4.49%)	1.09	0.0538	0.542	Co-occurrence
MIR-4730/4730		11 (9.57%)	11 (4.49%)	1.09	0.0538	0.542	Co-occurrence
NKD2	5p15.33	11 (9.57%)	11 (4.49%)	1.09	0.0538	0.542	Co-occurrence
NPTX1	17q25.3	11 (9.57%)	11 (4.49%)	1.09	0.0538	0.542	Co-occurrence
RBFOX3	17q25.3	11 (9.57%)	11 (4.49%)	1.09	0.0538	0.542	Co-occurrence
RNF213	17q25.3	11 (9.57%)	11 (4.49%)	1.09	0.0538	0.542	Co-occurrence
SGSH	17q25.3	11 (9.57%)	11 (4.49%)	1.09	0.0538	0.542	Co-occurrence
SLC12A7	5p15.33	11 (9.57%)	11 (4.49%)	1.09	0.0538	0.542	Co-occurrence
SLC6A18	5p15.33	11 (9.57%)	11 (4.49%)	1.09	0.0538	0.542	Co-occurrence
SLC6A19	5p15.33	11 (9.57%)	11 (4.49%)	1.09	0.0538	0.542	Co-occurrence
TSPAN10	17q25.3	11 (9.57%)	11 (4.49%)	1.09	0.0538	0.542	Co-occurrence
TUBD1	17q23.1	11 (9.57%)	11 (4.49%)	1.09	0.0538	0.542	Co-occurrence
AMZ2P1	17q24.1	9 (7.83%)	8 (3.27%)	1.26	0.0545	0.545	Co-occurrence
BRIP1	17q23.2	9 (7.83%)	8 (3.27%)	1.26	0.0545	0.545	Co-occurrence
INTS2	17q23.2	9 (7.83%)	8 (3.27%)	1.26	0.0545	0.545	Co-occurrence
LRRC37A3	17q24.1	9 (7.83%)	8 (3.27%)	1.26	0.0545	0.545	Co-occurrence
MIR-633/633		9 (7.83%)	8 (3.27%)	1.26	0.0545	0.545	Co-occurrence
NACA2	17q23.2	9 (7.83%)	8 (3.27%)	1.26	0.0545	0.545	Co-occurrence
PLEKHM1P1	17q24.1	9 (7.83%)	8 (3.27%)	1.26	0.0545	0.545	Co-occurrence
RN7SL404P	17q24.1	9 (7.83%)	8 (3.27%)	1.26	0.0545	0.545	Co-occurrence
SLC16A6P1	17q24.1	9 (7.83%)	8 (3.27%)	1.26	0.0545	0.545	Co-occurrence
TANC2	17q23.2-q23.3	9 (7.83%)	8 (3.27%)	1.26	0.0545	0.545	Co-occurrence
TBX4	17q23.2	9 (7.83%)	8 (3.27%)	1.26	0.0545	0.545	Co-occurrence
SLC7A10	19q13.11	6 (5.22%)	4 (1.63%)	1.68	0.0605	0.593	Co-occurrence
DUS1L	17q25.3	12 (10.43%)	13 (5.31%)	0.98	0.062	0.593	Co-occurrence
FASN	17q25.3	12 (10.43%)	13 (5.31%)	0.98	0.062	0.593	Co-occurrence
C11ORF24	11q13.2	1 (0.87%)	11 (4.49%)	-2.37	0.0623	0.593	Mutual exclusivity
LRP5	11q13.2	1 (0.87%)	11 (4.49%)	-2.37	0.0623	0.593	Mutual exclusivity
PPP6R3	11q13.2	1 (0.87%)	11 (4.49%)	-2.37	0.0623	0.593	Mutual exclusivity

AFMID	17q25.3	10 (8.70%)	10 (4.08%)	1.09	0.0655	0.593	Co-occurrence
BIRC5	17q25.3	10 (8.70%)	10 (4.08%)	1.09	0.0655	0.593	Co-occurrence
C17ORF99	17q25.3	10 (8.70%)	10 (4.08%)	1.09	0.0655	0.593	Co-occurrence
C1QTNF1	17q25.3	10 (8.70%)	10 (4.08%)	1.09	0.0655	0.593	Co-occurrence
CANT1	17q25.3	10 (8.70%)	10 (4.08%)	1.09	0.0655	0.593	Co-occurrence
CYTH1	17q25.3	10 (8.70%)	10 (4.08%)	1.09	0.0655	0.593	Co-occurrence
DNAH17	17q25.3	10 (8.70%)	10 (4.08%)	1.09	0.0655	0.593	Co-occurrence
ENGASE	17q25.3	10 (8.70%)	10 (4.08%)	1.09	0.0655	0.593	Co-occurrence
JMJD6	17q25.1	10 (8.70%)	10 (4.08%)	1.09	0.0655	0.593	Co-occurrence
LGALS3BP	17q25.3	10 (8.70%)	10 (4.08%)	1.09	0.0655	0.593	Co-occurrence
LINC01973	17q25.3	10 (8.70%)	10 (4.08%)	1.09	0.0655	0.593	Co-occurrence
METTL23	17q25.1	10 (8.70%)	10 (4.08%)	1.09	0.0655	0.593	Co-occurrence
MFSD11	17q25.1-q25.2	10 (8.70%)	10 (4.08%)	1.09	0.0655	0.593	Co-occurrence
MIR-4316/4316		10 (8.70%)	10 (4.08%)	1.09	0.0655	0.593	Co-occurrence
MIR-636/636		10 (8.70%)	10 (4.08%)	1.09	0.0655	0.593	Co-occurrence
MXRA7	17q25.1	10 (8.70%)	10 (4.08%)	1.09	0.0655	0.593	Co-occurrence
PGS1	17q25.3	10 (8.70%)	10 (4.08%)	1.09	0.0655	0.593	Co-occurrence
PRPSAP1	17q25.1	10 (8.70%)	10 (4.08%)	1.09	0.0655	0.593	Co-occurrence
RN7SL236P	17q25.3	10 (8.70%)	10 (4.08%)	1.09	0.0655	0.593	Co-occurrence
RN7SL454P	17q25.3	10 (8.70%)	10 (4.08%)	1.09	0.0655	0.593	Co-occurrence
SEPTIN9	17q25.3	10 (8.70%)	10 (4.08%)	1.09	0.0655	0.593	Co-occurrence
SOCS3	17q25.3	10 (8.70%)	10 (4.08%)	1.09	0.0655	0.593	Co-occurrence
SPHK1	17q25.1	10 (8.70%)	10 (4.08%)	1.09	0.0655	0.593	Co-occurrence
SRSF2	17q25.1	10 (8.70%)	10 (4.08%)	1.09	0.0655	0.593	Co-occurrence
SYNGR2	17q25.3	10 (8.70%)	10 (4.08%)	1.09	0.0655	0.593	Co-occurrence
TIMP2	17q25.3	10 (8.70%)	10 (4.08%)	1.09	0.0655	0.593	Co-occurrence
TK1	17q25.3	10 (8.70%)	10 (4.08%)	1.09	0.0655	0.593	Co-occurrence
TMC6	17q25.3	10 (8.70%)	10 (4.08%)	1.09	0.0655	0.593	Co-occurrence
TMC8	17q25.3	10 (8.70%)	10 (4.08%)	1.09	0.0655	0.593	Co-occurrence

TMEM235	17q25.3	10 (8.70%)	10 (4.08%)	1.09	0.0655	0.593	Co-occurrence
TNRC6C	17q25.3	10 (8.70%)	10 (4.08%)	1.09	0.0655	0.593	Co-occurrence
UBE2O	17q25.1	10 (8.70%)	10 (4.08%)	1.09	0.0655	0.593	Co-occurrence
USP36	17q25.3	10 (8.70%)	10 (4.08%)	1.09	0.0655	0.593	Co-occurrence
EPX	17q22	8 (6.96%)	7 (2.86%)	1.28	0.0663	0.593	Co-occurrence
LPO	17q22	8 (6.96%)	7 (2.86%)	1.28	0.0663	0.593	Co-occurrence
MKS1	17q22	8 (6.96%)	7 (2.86%)	1.28	0.0663	0.593	Co-occurrence
OR4D1	17q22	8 (6.96%)	7 (2.86%)	1.28	0.0663	0.593	Co-occurrence
OR4D2	17q22	8 (6.96%)	7 (2.86%)	1.28	0.0663	0.593	Co-occurrence
SLC35B3	6p24.3	8 (6.96%)	7 (2.86%)	1.28	0.0663	0.593	Co-occurrence
IGHMBP2	11q13.3	2 (1.74%)	14 (5.71%)	-1.72	0.0695	0.593	Mutual exclusivity
MIR-3164/3164		3 (2.61%)	17 (6.94%)	-1.41	0.0715	0.593	Mutual exclusivity
TPCN2	11q13.3	3 (2.61%)	17 (6.94%)	-1.41	0.0715	0.593	Mutual exclusivity
ADRM1	20q13.33	5 (4.35%)	3 (1.22%)	1.83	0.0722	0.593	Co-occurrence
AFF4	5q31.1	5 (4.35%)	3 (1.22%)	1.83	0.0722	0.593	Co-occurrence
CABLES2	20q13.33	5 (4.35%)	3 (1.22%)	1.83	0.0722	0.593	Co-occurrence
CDH4	20q13.33	5 (4.35%)	3 (1.22%)	1.83	0.0722	0.593	Co-occurrence
COL20A1	20q13.33	5 (4.35%)	3 (1.22%)	1.83	0.0722	0.593	Co-occurrence
CPM	12q15	5 (4.35%)	3 (1.22%)	1.83	0.0722	0.593	Co-occurrence
DPY19L2P4	7q21.13	5 (4.35%)	3 (1.22%)	1.83	0.0722	0.593	Co-occurrence
GADD45A	1p31.3	5 (4.35%)	3 (1.22%)	1.83	0.0722	0.593	Co-occurrence
GATA5	20q13.33	5 (4.35%)	3 (1.22%)	1.83	0.0722	0.593	Co-occurrence
GNG12	1p31.3	5 (4.35%)	3 (1.22%)	1.83	0.0722	0.593	Co-occurrence
HRH3	20q13.33	5 (4.35%)	3 (1.22%)	1.83	0.0722	0.593	Co-occurrence
LAMA5	20q13.33	5 (4.35%)	3 (1.22%)	1.83	0.0722	0.593	Co-occurrence
LEAP2	5q31.1	5 (4.35%)	3 (1.22%)	1.83	0.0722	0.593	Co-occurrence
LRP3	19q13.11	5 (4.35%)	3 (1.22%)	1.83	0.0722	0.593	Co-occurrence
LSM14B	20q13.33	5 (4.35%)	3 (1.22%)	1.83	0.0722	0.593	Co-occurrence
MIR-1257/1257		5 (4.35%)	3 (1.22%)	1.83	0.0722	0.593	Co-occurrence

MTG2	20q13.33	5 (4.35%)	3 (1.22%)	1.83	0.0722	0.593	Co-occurrence
NME1	17q21.33	5 (4.35%)	3 (1.22%)	1.83	0.0722	0.593	Co-occurrence
NME2	17q21.33	5 (4.35%)	3 (1.22%)	1.83	0.0722	0.593	Co-occurrence
OSBPL2	20q13.33	5 (4.35%)	3 (1.22%)	1.83	0.0722	0.593	Co-occurrence
PSMA7	20q13.33	5 (4.35%)	3 (1.22%)	1.83	0.0722	0.593	Co-occurrence
RBBP8NL	20q13.33	5 (4.35%)	3 (1.22%)	1.83	0.0722	0.593	Co-occurrence
RPS21	20q13.33	5 (4.35%)	3 (1.22%)	1.83	0.0722	0.593	Co-occurrence
SS18L1	20q13.33	5 (4.35%)	3 (1.22%)	1.83	0.0722	0.593	Co-occurrence
STEAP1	7q21.13	5 (4.35%)	3 (1.22%)	1.83	0.0722	0.593	Co-occurrence
STEAP2	7q21.13	5 (4.35%)	3 (1.22%)	1.83	0.0722	0.593	Co-occurrence
TAC3	12q13.3	5 (4.35%)	3 (1.22%)	1.83	0.0722	0.593	Co-occurrence
TAF4	20q13.33	5 (4.35%)	3 (1.22%)	1.83	0.0722	0.593	Co-occurrence
TEX47	7q21.13	5 (4.35%)	3 (1.22%)	1.83	0.0722	0.593	Co-occurrence
UQCRQ	5q31.1	5 (4.35%)	3 (1.22%)	1.83	0.0722	0.593	Co-occurrence
ZNF804B	7q21.13	5 (4.35%)	3 (1.22%)	1.83	0.0722	0.593	Co-occurrence
CENPX	17q25.3	11 (9.57%)	12 (4.90%)	0.97	0.0753	0.593	Co-occurrence
DCXR	17q25.3	11 (9.57%)	12 (4.90%)	0.97	0.0753	0.593	Co-occurrence
LRRC45	17q25.3	11 (9.57%)	12 (4.90%)	0.97	0.0753	0.593	Co-occurrence
NOTUM	17q25.3	11 (9.57%)	12 (4.90%)	0.97	0.0753	0.593	Co-occurrence
RAC3	17q25.3	11 (9.57%)	12 (4.90%)	0.97	0.0753	0.593	Co-occurrence
C17ORF82	17q23.2	9 (7.83%)	9 (3.67%)	1.09	0.0798	0.593	Co-occurrence
RN7SL448P	17q23.2	9 (7.83%)	9 (3.67%)	1.09	0.0798	0.593	Co-occurrence
TBX2	17q23.2	9 (7.83%)	9 (3.67%)	1.09	0.0798	0.593	Co-occurrence
TNFSF10	3q26.31	9 (7.83%)	9 (3.67%)	1.09	0.0798	0.593	Co-occurrence
CEBPA	19q13.11	7 (6.09%)	6 (2.45%)	1.31	0.0808	0.593	Co-occurrence
MRPS23	17q22	7 (6.09%)	6 (2.45%)	1.31	0.0808	0.593	Co-occurrence
RN7SKP94	17q22	7 (6.09%)	6 (2.45%)	1.31	0.0808	0.593	Co-occurrence
SRSF1	17q22	7 (6.09%)	6 (2.45%)	1.31	0.0808	0.593	Co-occurrence
CCDC57	17q25.3	12 (10.43%)	14 (5.71%)	0.87	0.0839	0.593	Co-occurrence

ADNP	20q13.13	4 (3.48%)	2 (0.82%)	2.09	0.0852	0.593	Co-occurrence
AGAP2	12q14.1	4 (3.48%)	2 (0.82%)	2.09	0.0852	0.593	Co-occurrence
ALDH1A2	15q21.3	4 (3.48%)	2 (0.82%)	2.09	0.0852	0.593	Co-occurrence
AP1M1	19p13.11	4 (3.48%)	2 (0.82%)	2.09	0.0852	0.593	Co-occurrence
APOC1	19q13.32	4 (3.48%)	2 (0.82%)	2.09	0.0852	0.593	Co-occurrence
APOE	19q13.32	4 (3.48%)	2 (0.82%)	2.09	0.0852	0.593	Co-occurrence
ASIC2	17q11.2-q12	4 (3.48%)	2 (0.82%)	2.09	0.0852	0.593	Co-occurrence
ATP9A	20q13.2	4 (3.48%)	2 (0.82%)	2.09	0.0852	0.593	Co-occurrence
BUD23	7q11.23	4 (3.48%)	2 (0.82%)	2.09	0.0852	0.593	Co-occurrence
C17ORF75	17q11.2	4 (3.48%)	2 (0.82%)	2.09	0.0852	0.593	Co-occurrence
C22ORF23	22q13.1	4 (3.48%)	2 (0.82%)	2.09	0.0852	0.593	Co-occurrence
CARMIL3	14q11.2	4 (3.48%)	2 (0.82%)	2.09	0.0852	0.593	Co-occurrence
CCNY	10p11.21	4 (3.48%)	2 (0.82%)	2.09	0.0852	0.593	Co-occurrence
CDC27	17q21.32	4 (3.48%)	2 (0.82%)	2.09	0.0852	0.593	Co-occurrence
CDK2	12q13.2	4 (3.48%)	2 (0.82%)	2.09	0.0852	0.593	Co-occurrence
CDK4	12q14.1	4 (3.48%)	2 (0.82%)	2.09	0.0852	0.593	Co-occurrence
CDK5R1	17q11.2	4 (3.48%)	2 (0.82%)	2.09	0.0852	0.593	Co-occurrence
CIB3	19p13.11	4 (3.48%)	2 (0.82%)	2.09	0.0852	0.593	Co-occurrence
CNP	17q21.2	4 (3.48%)	2 (0.82%)	2.09	0.0852	0.593	Co-occurrence
CPD	17q11.2	4 (3.48%)	2 (0.82%)	2.09	0.0852	0.593	Co-occurrence
CPNE6	14q11.2	4 (3.48%)	2 (0.82%)	2.09	0.0852	0.593	Co-occurrence
CREM	10p11.21	4 (3.48%)	2 (0.82%)	2.09	0.0852	0.593	Co-occurrence
CYP27B1	12q14.1	4 (3.48%)	2 (0.82%)	2.09	0.0852	0.593	Co-occurrence
DDX17	22q13.1	4 (3.48%)	2 (0.82%)	2.09	0.0852	0.593	Co-occurrence
DGKA	12q13.2	4 (3.48%)	2 (0.82%)	2.09	0.0852	0.593	Co-occurrence
DHRS13	17q11.2	4 (3.48%)	2 (0.82%)	2.09	0.0852	0.593	Co-occurrence
DMC1	22q13.1	4 (3.48%)	2 (0.82%)	2.09	0.0852	0.593	Co-occurrence
DNAJC7	17q21.2	4 (3.48%)	2 (0.82%)	2.09	0.0852	0.593	Co-occurrence
DPM1	20q13.13	4 (3.48%)	2 (0.82%)	2.09	0.0852	0.593	Co-occurrence

ERBB3	12q13.2	4 (3.48%)	2 (0.82%)	2.09	0.0852	0.593	Co-occurrence
ESYT1	12q13.2	4 (3.48%)	2 (0.82%)	2.09	0.0852	0.593	Co-occurrence
FAM227A	22q13.1	4 (3.48%)	2 (0.82%)	2.09	0.0852	0.593	Co-occurrence
FAM32A	19p13.11	4 (3.48%)	2 (0.82%)	2.09	0.0852	0.593	Co-occurrence
FMNL1	17q21.31	4 (3.48%)	2 (0.82%)	2.09	0.0852	0.593	Co-occurrence
GLI1	12q13.3	4 (3.48%)	2 (0.82%)	2.09	0.0852	0.593	Co-occurrence
GPR182	12q13.3	4 (3.48%)	2 (0.82%)	2.09	0.0852	0.593	Co-occurrence
HBCBP	12q13.3	4 (3.48%)	2 (0.82%)	2.09	0.0852	0.593	Co-occurrence
HSH2D	19p13.11	4 (3.48%)	2 (0.82%)	2.09	0.0852	0.593	Co-occurrence
INHBC	12q13.3	4 (3.48%)	2 (0.82%)	2.09	0.0852	0.593	Co-occurrence
INHBE	12q13.3	4 (3.48%)	2 (0.82%)	2.09	0.0852	0.593	Co-occurrence
ITGB3	17q21.32	4 (3.48%)	2 (0.82%)	2.09	0.0852	0.593	Co-occurrence
KCNG1	20q13.13	4 (3.48%)	2 (0.82%)	2.09	0.0852	0.593	Co-occurrence
LGALS1	22q13.1	4 (3.48%)	2 (0.82%)	2.09	0.0852	0.593	Co-occurrence
LINC00838	10p11.22	4 (3.48%)	2 (0.82%)	2.09	0.0852	0.593	Co-occurrence
LYZL2	10p11.23	4 (3.48%)	2 (0.82%)	2.09	0.0852	0.593	Co-occurrence
MAP3K14	17q21.31	4 (3.48%)	2 (0.82%)	2.09	0.0852	0.593	Co-occurrence
9-Mar	12q14.1	4 (3.48%)	2 (0.82%)	2.09	0.0852	0.593	Co-occurrence
MIR-2117/2117		4 (3.48%)	2 (0.82%)	2.09	0.0852	0.593	Co-occurrence
MIR-4534/4534		4 (3.48%)	2 (0.82%)	2.09	0.0852	0.593	Co-occurrence
MIR-632/632		4 (3.48%)	2 (0.82%)	2.09	0.0852	0.593	Co-occurrence
MOCS3	20q13.13	4 (3.48%)	2 (0.82%)	2.09	0.0852	0.593	Co-occurrence
MYL4	17q21.32	4 (3.48%)	2 (0.82%)	2.09	0.0852	0.593	Co-occurrence
MYO1A	12q13.3	4 (3.48%)	2 (0.82%)	2.09	0.0852	0.593	Co-occurrence
NDUFA4L2	12q13.3	4 (3.48%)	2 (0.82%)	2.09	0.0852	0.593	Co-occurrence
NFATC2	20q13.2	4 (3.48%)	2 (0.82%)	2.09	0.0852	0.593	Co-occurrence
NOL12	22q13.1	4 (3.48%)	2 (0.82%)	2.09	0.0852	0.593	Co-occurrence
NRL	14q11.2-q12	4 (3.48%)	2 (0.82%)	2.09	0.0852	0.593	Co-occurrence
NRP1	10p11.22	4 (3.48%)	2 (0.82%)	2.09	0.0852	0.593	Co-occurrence

NXPH4	12q13.3	4 (3.48%)	2 (0.82%)	2.09	0.0852	0.593	Co-occurrence
OS9	12q13.3-q14.1	4 (3.48%)	2 (0.82%)	2.09	0.0852	0.593	Co-occurrence
PA2G4	12q13.2	4 (3.48%)	2 (0.82%)	2.09	0.0852	0.593	Co-occurrence
PCK2	14q11.2-q12	4 (3.48%)	2 (0.82%)	2.09	0.0852	0.593	Co-occurrence
PHF12	17q11.2	4 (3.48%)	2 (0.82%)	2.09	0.0852	0.593	Co-occurrence
PMEL	12q13.2	4 (3.48%)	2 (0.82%)	2.09	0.0852	0.593	Co-occurrence
POLR2F	22q13.1	4 (3.48%)	2 (0.82%)	2.09	0.0852	0.593	Co-occurrence
PSMD11	17q11.2	4 (3.48%)	2 (0.82%)	2.09	0.0852	0.593	Co-occurrence
PYM1	12q13.2	4 (3.48%)	2 (0.82%)	2.09	0.0852	0.593	Co-occurrence
RAB8A	19p13.11	4 (3.48%)	2 (0.82%)	2.09	0.0852	0.593	Co-occurrence
RBM6	3p21.31	4 (3.48%)	2 (0.82%)	2.09	0.0852	0.593	Co-occurrence
RERE	1p36.23	4 (3.48%)	2 (0.82%)	2.09	0.0852	0.593	Co-occurrence
RHOT1	17q11.2	4 (3.48%)	2 (0.82%)	2.09	0.0852	0.593	Co-occurrence
RN7SL398P	10p11.22	4 (3.48%)	2 (0.82%)	2.09	0.0852	0.593	Co-occurrence
RNA5SP437	17q11.2	4 (3.48%)	2 (0.82%)	2.09	0.0852	0.593	Co-occurrence
RPL41	12q13.2	4 (3.48%)	2 (0.82%)	2.09	0.0852	0.593	Co-occurrence
SALL4	20q13.2	4 (3.48%)	2 (0.82%)	2.09	0.0852	0.593	Co-occurrence
SHMT2	12q13.3	4 (3.48%)	2 (0.82%)	2.09	0.0852	0.593	Co-occurrence
SOX10	22q13.1	4 (3.48%)	2 (0.82%)	2.09	0.0852	0.593	Co-occurrence
SPACA3	17q11.2	4 (3.48%)	2 (0.82%)	2.09	0.0852	0.593	Co-occurrence
SPATA32	17q21.31	4 (3.48%)	2 (0.82%)	2.09	0.0852	0.593	Co-occurrence
STAC3	12q13.3	4 (3.48%)	2 (0.82%)	2.09	0.0852	0.593	Co-occurrence
STX1A	7q11.23	4 (3.48%)	2 (0.82%)	2.09	0.0852	0.593	Co-occurrence
SUZ12	17q11.2	4 (3.48%)	2 (0.82%)	2.09	0.0852	0.593	Co-occurrence
TCTEX1D2	3q29	4 (3.48%)	2 (0.82%)	2.09	0.0852	0.593	Co-occurrence
TM4SF19	3q29	4 (3.48%)	2 (0.82%)	2.09	0.0852	0.593	Co-occurrence
TOMM40	19q13.32	4 (3.48%)	2 (0.82%)	2.09	0.0852	0.593	Co-occurrence
TPM4	19p13.12-p13.11	4 (3.48%)	2 (0.82%)	2.09	0.0852	0.593	Co-occurrence
TRIOBP	22q13.1	4 (3.48%)	2 (0.82%)	2.09	0.0852	0.593	Co-occurrence

TSPAN31	12q14.1	4 (3.48%)	2 (0.82%)	2.09	0.0852	0.593	Co-occurrence
TTC25	17q21.2	4 (3.48%)	2 (0.82%)	2.09	0.0852	0.593	Co-occurrence
UBXN7	3q29	4 (3.48%)	2 (0.82%)	2.09	0.0852	0.593	Co-occurrence
ZC3H10	12q13.2	4 (3.48%)	2 (0.82%)	2.09	0.0852	0.593	Co-occurrence
ZNF207	17q11.2	4 (3.48%)	2 (0.82%)	2.09	0.0852	0.593	Co-occurrence
GAL	11q13.2	1 (0.87%)	10 (4.08%)	-2.23	0.086	0.593	Mutual exclusivity
TESMIN	11q13.3	1 (0.87%)	10 (4.08%)	-2.23	0.086	0.593	Mutual exclusivity
GALK1	17q25.1	10 (8.70%)	11 (4.49%)	0.95	0.0915	0.593	Co-occurrence
H3F3B	17q25.1	10 (8.70%)	11 (4.49%)	0.95	0.0915	0.593	Co-occurrence
ITGB4	17q25.1	10 (8.70%)	11 (4.49%)	0.95	0.0915	0.593	Co-occurrence
LINC00868	17q25.2	10 (8.70%)	11 (4.49%)	0.95	0.0915	0.593	Co-occurrence
RECQL5	17q25.1	10 (8.70%)	11 (4.49%)	0.95	0.0915	0.593	Co-occurrence
SAP30BP	17q25.1	10 (8.70%)	11 (4.49%)	0.95	0.0915	0.593	Co-occurrence
SEC14L1	17q25.2-q25.3	10 (8.70%)	11 (4.49%)	0.95	0.0915	0.593	Co-occurrence
SNHG20	17q25.2	10 (8.70%)	11 (4.49%)	0.95	0.0915	0.593	Co-occurrence
TRIM47	17q25.1	10 (8.70%)	11 (4.49%)	0.95	0.0915	0.593	Co-occurrence
TRIM65	17q25.1	10 (8.70%)	11 (4.49%)	0.95	0.0915	0.593	Co-occurrence
UNC13D	17q25.1	10 (8.70%)	11 (4.49%)	0.95	0.0915	0.593	Co-occurrence
UNK	17q25.1	10 (8.70%)	11 (4.49%)	0.95	0.0915	0.593	Co-occurrence
WBP2	17q25.1	10 (8.70%)	11 (4.49%)	0.95	0.0915	0.593	Co-occurrence
CD99L2	Xq28	0 (0.00%)	6 (2.45%)	<-10	0.0974	0.593	Mutual exclusivity
GRM8	7q31.33	0 (0.00%)	6 (2.45%)	<-10	0.0974	0.593	Mutual exclusivity
HMGB3	Xq28	0 (0.00%)	6 (2.45%)	<-10	0.0974	0.593	Mutual exclusivity
INTS4	11q14.1	0 (0.00%)	6 (2.45%)	<-10	0.0974	0.593	Mutual exclusivity
LRGUK	7q33	0 (0.00%)	6 (2.45%)	<-10	0.0974	0.593	Mutual exclusivity
MTM1	Xq28	0 (0.00%)	6 (2.45%)	<-10	0.0974	0.593	Mutual exclusivity
PLB1	2p23.2	0 (0.00%)	6 (2.45%)	<-10	0.0974	0.593	Mutual exclusivity
SCGB1A1	11q12.3	0 (0.00%)	6 (2.45%)	<-10	0.0974	0.593	Mutual exclusivity
TCOF1	5q32-q33.1	0 (0.00%)	6 (2.45%)	<-10	0.0974	0.593	Mutual exclusivity

TNPO3	7q32.1	0 (0.00%)	6 (2.45%)	<-10	0.0974	0.593	Mutual exclusivity
XRRA1	11q13.4	0 (0.00%)	6 (2.45%)	<-10	0.0974	0.593	Mutual exclusivity
BLOC1S5	6p24.3	8 (6.96%)	8 (3.27%)	1.09	0.0975	0.593	Co-occurrence
BMP6	6p24.3	8 (6.96%)	8 (3.27%)	1.09	0.0975	0.593	Co-occurrence
CAGE1	6p24.3	8 (6.96%)	8 (3.27%)	1.09	0.0975	0.593	Co-occurrence
CAP2	6p22.3	8 (6.96%)	8 (3.27%)	1.09	0.0975	0.593	Co-occurrence
DSP	6p24.3	8 (6.96%)	8 (3.27%)	1.09	0.0975	0.593	Co-occurrence
EDN1	6p24.1	8 (6.96%)	8 (3.27%)	1.09	0.0975	0.593	Co-occurrence
EEF1E1	6p24.3	8 (6.96%)	8 (3.27%)	1.09	0.0975	0.593	Co-occurrence
F13A1	6p25.1	8 (6.96%)	8 (3.27%)	1.09	0.0975	0.593	Co-occurrence
FARS2	6p25.1	8 (6.96%)	8 (3.27%)	1.09	0.0975	0.593	Co-occurrence
HULC	6p24.3	8 (6.96%)	8 (3.27%)	1.09	0.0975	0.593	Co-occurrence
LY86	6p25.1	8 (6.96%)	8 (3.27%)	1.09	0.0975	0.593	Co-occurrence
MIR-5683/5683		8 (6.96%)	8 (3.27%)	1.09	0.0975	0.593	Co-occurrence
NRN1	6p25.1	8 (6.96%)	8 (3.27%)	1.09	0.0975	0.593	Co-occurrence
RIOK1	6p24.3	8 (6.96%)	8 (3.27%)	1.09	0.0975	0.593	Co-occurrence
RN7SKP293	6p24.1	8 (6.96%)	8 (3.27%)	1.09	0.0975	0.593	Co-occurrence
RN7SL221P	6p25.1	8 (6.96%)	8 (3.27%)	1.09	0.0975	0.593	Co-occurrence
RREB1	6p24.3	8 (6.96%)	8 (3.27%)	1.09	0.0975	0.593	Co-occurrence
SNRNP48	6p24.3	8 (6.96%)	8 (3.27%)	1.09	0.0975	0.593	Co-occurrence
SSR1	6p24.3	8 (6.96%)	8 (3.27%)	1.09	0.0975	0.593	Co-occurrence
TXNDC5	6p24.3	8 (6.96%)	8 (3.27%)	1.09	0.0975	0.593	Co-occurrence
AATF	17q12	3 (2.61%)	1 (0.41%)	2.68	0.0979	0.593	Co-occurrence
ABHD11	7q11.23	3 (2.61%)	1 (0.41%)	2.68	0.0979	0.593	Co-occurrence
ABHD11-AS1	7q11.23	3 (2.61%)	1 (0.41%)	2.68	0.0979	0.593	Co-occurrence
ACOD1	13q22.3	3 (2.61%)	1 (0.41%)	2.68	0.0979	0.593	Co-occurrence
ACTR6	12q23.1	3 (2.61%)	1 (0.41%)	2.68	0.0979	0.593	Co-occurrence
ADCY4	14q12	3 (2.61%)	1 (0.41%)	2.68	0.0979	0.593	Co-occurrence
ANKRD18DP	3q29	3 (2.61%)	1 (0.41%)	2.68	0.0979	0.593	Co-occurrence

AOC3	17q21.31	3 (2.61%)	1 (0.41%)	2.68	0.0979	0.593	Co-occurrence
AOC4P	17q21.31	3 (2.61%)	1 (0.41%)	2.68	0.0979	0.593	Co-occurrence
AP2B1	17q12	3 (2.61%)	1 (0.41%)	2.68	0.0979	0.593	Co-occurrence
APOF	12q13.3	3 (2.61%)	1 (0.41%)	2.68	0.0979	0.593	Co-occurrence
ARHGEF25	12q13.3	3 (2.61%)	1 (0.41%)	2.68	0.0979	0.593	Co-occurrence
ATP5F1B	12q13.3	3 (2.61%)	1 (0.41%)	2.68	0.0979	0.593	Co-occurrence
B4GALNT1	12q13.3	3 (2.61%)	1 (0.41%)	2.68	0.0979	0.593	Co-occurrence
BAZ2A	12q13.3	3 (2.61%)	1 (0.41%)	2.68	0.0979	0.593	Co-occurrence
BDH1	3q29	3 (2.61%)	1 (0.41%)	2.68	0.0979	0.593	Co-occurrence
BLM	15q26.1	3 (2.61%)	1 (0.41%)	2.68	0.0979	0.593	Co-occurrence
BRAT1	7p22.3	3 (2.61%)	1 (0.41%)	2.68	0.0979	0.593	Co-occurrence
C17ORF50	17q12	3 (2.61%)	1 (0.41%)	2.68	0.0979	0.593	Co-occurrence
C1QTNF6	22q12.3	3 (2.61%)	1 (0.41%)	2.68	0.0979	0.593	Co-occurrence
C22ORF24	22q12.3	3 (2.61%)	1 (0.41%)	2.68	0.0979	0.593	Co-occurrence
C3ORF18	3p21.31	3 (2.61%)	1 (0.41%)	2.68	0.0979	0.593	Co-occurrence
CACHD1	1p31.3	3 (2.61%)	1 (0.41%)	2.68	0.0979	0.593	Co-occurrence
CALML4	15q23	3 (2.61%)	1 (0.41%)	2.68	0.0979	0.593	Co-occurrence
CAMK2G	10q22.2	3 (2.61%)	1 (0.41%)	2.68	0.0979	0.593	Co-occurrence
CARD10	22q13.1	3 (2.61%)	1 (0.41%)	2.68	0.0979	0.593	Co-occurrence
CASK	Xp11.4	3 (2.61%)	1 (0.41%)	2.68	0.0979	0.593	Co-occurrence
CBLN3	14q12	3 (2.61%)	1 (0.41%)	2.68	0.0979	0.593	Co-occurrence
CBY1	22q13.1	3 (2.61%)	1 (0.41%)	2.68	0.0979	0.593	Co-occurrence
CCL14	17q12	3 (2.61%)	1 (0.41%)	2.68	0.0979	0.593	Co-occurrence
CCL15	17q12	3 (2.61%)	1 (0.41%)	2.68	0.0979	0.593	Co-occurrence
CCL16	17q12	3 (2.61%)	1 (0.41%)	2.68	0.0979	0.593	Co-occurrence
CCL18	17q12	3 (2.61%)	1 (0.41%)	2.68	0.0979	0.593	Co-occurrence
CCL23	17q12	3 (2.61%)	1 (0.41%)	2.68	0.0979	0.593	Co-occurrence
CCL3	17q12	3 (2.61%)	1 (0.41%)	2.68	0.0979	0.593	Co-occurrence
CCL3L1	17q21.1	3 (2.61%)	1 (0.41%)	2.68	0.0979	0.593	Co-occurrence

CCL3L3	17q12	3 (2.61%)	1 (0.41%)	2.68	0.0979	0.593	Co-occurrence
CCL4	17q12	3 (2.61%)	1 (0.41%)	2.68	0.0979	0.593	Co-occurrence
CCL4L1	17q12	3 (2.61%)	1 (0.41%)	2.68	0.0979	0.593	Co-occurrence
CCL4L2	17q12	3 (2.61%)	1 (0.41%)	2.68	0.0979	0.593	Co-occurrence
CCL5	17q12	3 (2.61%)	1 (0.41%)	2.68	0.0979	0.593	Co-occurrence
CCR10	17q21.2	3 (2.61%)	1 (0.41%)	2.68	0.0979	0.593	Co-occurrence
CDC42EP1	22q13.1	3 (2.61%)	1 (0.41%)	2.68	0.0979	0.593	Co-occurrence
CHCHD1	10q22.2	3 (2.61%)	1 (0.41%)	2.68	0.0979	0.593	Co-occurrence
CHMP4A	14q12	3 (2.61%)	1 (0.41%)	2.68	0.0979	0.593	Co-occurrence
CHST12	7p22.3	3 (2.61%)	1 (0.41%)	2.68	0.0979	0.593	Co-occurrence
CHST8	19q13.11	3 (2.61%)	1 (0.41%)	2.68	0.0979	0.593	Co-occurrence
CIB2	15q25.1	3 (2.61%)	1 (0.41%)	2.68	0.0979	0.593	Co-occurrence
CIDEB	14q12	3 (2.61%)	1 (0.41%)	2.68	0.0979	0.593	Co-occurrence
CISH	3p21.2	3 (2.61%)	1 (0.41%)	2.68	0.0979	0.593	Co-occurrence
CLASRP	19q13.32	3 (2.61%)	1 (0.41%)	2.68	0.0979	0.593	Co-occurrence
CLEC19A	16p12.3	3 (2.61%)	1 (0.41%)	2.68	0.0979	0.593	Co-occurrence
CLN5	13q22.3	3 (2.61%)	1 (0.41%)	2.68	0.0979	0.593	Co-occurrence
COASY	17q21.2	3 (2.61%)	1 (0.41%)	2.68	0.0979	0.593	Co-occurrence
CRADD	12q22	3 (2.61%)	1 (0.41%)	2.68	0.0979	0.593	Co-occurrence
CRTC3	15q26.1	3 (2.61%)	1 (0.41%)	2.68	0.0979	0.593	Co-occurrence
CSNK1G1	15q22.31	3 (2.61%)	1 (0.41%)	2.68	0.0979	0.593	Co-occurrence
CYTH4	22q13.1	3 (2.61%)	1 (0.41%)	2.68	0.0979	0.593	Co-occurrence
DCTN2	12q13.3	3 (2.61%)	1 (0.41%)	2.68	0.0979	0.593	Co-occurrence
DDX27	20q13.13	3 (2.61%)	1 (0.41%)	2.68	0.0979	0.593	Co-occurrence
DDX52	17q12	3 (2.61%)	1 (0.41%)	2.68	0.0979	0.593	Co-occurrence
DEPDC4	12q23.1	3 (2.61%)	1 (0.41%)	2.68	0.0979	0.593	Co-occurrence
DHRS1	14q12	3 (2.61%)	1 (0.41%)	2.68	0.0979	0.593	Co-occurrence
DHRS11	17q12	3 (2.61%)	1 (0.41%)	2.68	0.0979	0.593	Co-occurrence
DNAAF5	7p22.3	3 (2.61%)	1 (0.41%)	2.68	0.0979	0.593	Co-occurrence

DOCK3	3p21.2	3 (2.61%)	1 (0.41%)	2.68	0.0979	0.593	Co-occurrence
DPP8	15q22.31	3 (2.61%)	1 (0.41%)	2.68	0.0979	0.593	Co-occurrence
DTX3	12q13.3	3 (2.61%)	1 (0.41%)	2.68	0.0979	0.593	Co-occurrence
EDNRB	13q22.3	3 (2.61%)	1 (0.41%)	2.68	0.0979	0.593	Co-occurrence
EFCAB5	17q11.2	3 (2.61%)	1 (0.41%)	2.68	0.0979	0.593	Co-occurrence
EIF3B	7p22.3	3 (2.61%)	1 (0.41%)	2.68	0.0979	0.593	Co-occurrence
EIF4H	7q11.23	3 (2.61%)	1 (0.41%)	2.68	0.0979	0.593	Co-occurrence
ELFN1	7p22.3	3 (2.61%)	1 (0.41%)	2.68	0.0979	0.593	Co-occurrence
ELFN2	22q13.1	3 (2.61%)	1 (0.41%)	2.68	0.0979	0.593	Co-occurrence
FAM157A	3q29	3 (2.61%)	1 (0.41%)	2.68	0.0979	0.593	Co-occurrence
FAM20C	7p22.3	3 (2.61%)	1 (0.41%)	2.68	0.0979	0.593	Co-occurrence
FBXL22	15q22.31	3 (2.61%)	1 (0.41%)	2.68	0.0979	0.593	Co-occurrence
FBXL3	13q22.3	3 (2.61%)	1 (0.41%)	2.68	0.0979	0.593	Co-occurrence
FBXW2	9q33.2	3 (2.61%)	1 (0.41%)	2.68	0.0979	0.593	Co-occurrence
FUT11	10q22.2	3 (2.61%)	1 (0.41%)	2.68	0.0979	0.593	Co-occurrence
FYTTD1	3q29	3 (2.61%)	1 (0.41%)	2.68	0.0979	0.593	Co-occurrence
GABARAPL3	15q26.1	3 (2.61%)	1 (0.41%)	2.68	0.0979	0.593	Co-occurrence
GAS2L2	17q12	3 (2.61%)	1 (0.41%)	2.68	0.0979	0.593	Co-occurrence
GGNBP2	17q12	3 (2.61%)	1 (0.41%)	2.68	0.0979	0.593	Co-occurrence
GLS2	12q13.3	3 (2.61%)	1 (0.41%)	2.68	0.0979	0.593	Co-occurrence
GMPR2	14q12	3 (2.61%)	1 (0.41%)	2.68	0.0979	0.593	Co-occurrence
GOLGA2P5	12q23.1	3 (2.61%)	1 (0.41%)	2.68	0.0979	0.593	Co-occurrence
GPD2	2q24.1	3 (2.61%)	1 (0.41%)	2.68	0.0979	0.593	Co-occurrence
GRIFIN	7p22.3	3 (2.61%)	1 (0.41%)	2.68	0.0979	0.593	Co-occurrence
GTPBP1	22q13.1	3 (2.61%)	1 (0.41%)	2.68	0.0979	0.593	Co-occurrence
HEATR9	17q12	3 (2.61%)	1 (0.41%)	2.68	0.0979	0.593	Co-occurrence
HEMK1	3p21.31	3 (2.61%)	1 (0.41%)	2.68	0.0979	0.593	Co-occurrence
HMGB1P24	17q12	3 (2.61%)	1 (0.41%)	2.68	0.0979	0.593	Co-occurrence
HSD17B1	17q21.2	3 (2.61%)	1 (0.41%)	2.68	0.0979	0.593	Co-occurrence

ICA1	7p21.3	3 (2.61%)	1 (0.41%)	2.68	0.0979	0.593	Co-occurrence
IDH2	15q26.1	3 (2.61%)	1 (0.41%)	2.68	0.0979	0.593	Co-occurrence
IDH3A	15q25.1	3 (2.61%)	1 (0.41%)	2.68	0.0979	0.593	Co-occurrence
IGDCC3	15q22.31	3 (2.61%)	1 (0.41%)	2.68	0.0979	0.593	Co-occurrence
IGDCC4	15q22.31	3 (2.61%)	1 (0.41%)	2.68	0.0979	0.593	Co-occurrence
IL17RA	22q11.1	3 (2.61%)	1 (0.41%)	2.68	0.0979	0.593	Co-occurrence
IL22	12q15	3 (2.61%)	1 (0.41%)	2.68	0.0979	0.593	Co-occurrence
IL23A	12q13.3	3 (2.61%)	1 (0.41%)	2.68	0.0979	0.593	Co-occurrence
IL26	12q15	3 (2.61%)	1 (0.41%)	2.68	0.0979	0.593	Co-occurrence
IL2RB	22q12.3	3 (2.61%)	1 (0.41%)	2.68	0.0979	0.593	Co-occurrence
INTS1	7p22.3	3 (2.61%)	1 (0.41%)	2.68	0.0979	0.593	Co-occurrence
IPO4	14q12	3 (2.61%)	1 (0.41%)	2.68	0.0979	0.593	Co-occurrence
IQCG	3q29	3 (2.61%)	1 (0.41%)	2.68	0.0979	0.593	Co-occurrence
IQGAP1	15q26.1	3 (2.61%)	1 (0.41%)	2.68	0.0979	0.593	Co-occurrence
JAK1	1p31.3	3 (2.61%)	1 (0.41%)	2.68	0.0979	0.593	Co-occurrence
JOSD1	22q13.1	3 (2.61%)	1 (0.41%)	2.68	0.0979	0.593	Co-occurrence
KCTD12	13q22.3	3 (2.61%)	1 (0.41%)	2.68	0.0979	0.593	Co-occurrence
KCTD15	19q13.11	3 (2.61%)	1 (0.41%)	2.68	0.0979	0.593	Co-occurrence
KHNYN	14q12	3 (2.61%)	1 (0.41%)	2.68	0.0979	0.593	Co-occurrence
KIF5A	12q13.3	3 (2.61%)	1 (0.41%)	2.68	0.0979	0.593	Co-occurrence
LACTB	15q22.2	3 (2.61%)	1 (0.41%)	2.68	0.0979	0.593	Co-occurrence
LAMC3	9q34.12	3 (2.61%)	1 (0.41%)	2.68	0.0979	0.593	Co-occurrence
LARGE1	22q12.3	3 (2.61%)	1 (0.41%)	2.68	0.0979	0.593	Co-occurrence
LAT2	7q11.23	3 (2.61%)	1 (0.41%)	2.68	0.0979	0.593	Co-occurrence
LFNG	7p22.3	3 (2.61%)	1 (0.41%)	2.68	0.0979	0.593	Co-occurrence
LGALS2	22q13.1	3 (2.61%)	1 (0.41%)	2.68	0.0979	0.593	Co-occurrence
LGALS7B	19q13.2	3 (2.61%)	1 (0.41%)	2.68	0.0979	0.593	Co-occurrence
LHX1	17q12	3 (2.61%)	1 (0.41%)	2.68	0.0979	0.593	Co-occurrence
LINC00331	13q31.1	3 (2.61%)	1 (0.41%)	2.68	0.0979	0.593	Co-occurrence

LINC00446	13q22.3	3 (2.61%)	1 (0.41%)	2.68	0.0979	0.593	Co-occurrence
LINC00671	17q21.31	3 (2.61%)	1 (0.41%)	2.68	0.0979	0.593	Co-occurrence
LITAF	16p13.13	3 (2.61%)	1 (0.41%)	2.68	0.0979	0.593	Co-occurrence
LMLN	3q29	3 (2.61%)	1 (0.41%)	2.68	0.0979	0.593	Co-occurrence
LRCH3	3q29	3 (2.61%)	1 (0.41%)	2.68	0.0979	0.593	Co-occurrence
LRP8	1p32.3	3 (2.61%)	1 (0.41%)	2.68	0.0979	0.593	Co-occurrence
LTB4R	14q12	3 (2.61%)	1 (0.41%)	2.68	0.0979	0.593	Co-occurrence
LTB4R2	14q12	3 (2.61%)	1 (0.41%)	2.68	0.0979	0.593	Co-occurrence
LYZL6	17q12	3 (2.61%)	1 (0.41%)	2.68	0.0979	0.593	Co-occurrence
MAFK	7p22.3	3 (2.61%)	1 (0.41%)	2.68	0.0979	0.593	Co-occurrence
MAP2K5	15q23	3 (2.61%)	1 (0.41%)	2.68	0.0979	0.593	Co-occurrence
MAPKAPK3	3p21.2	3 (2.61%)	1 (0.41%)	2.68	0.0979	0.593	Co-occurrence
MDM1	12q15	3 (2.61%)	1 (0.41%)	2.68	0.0979	0.593	Co-occurrence
MDP1	14q12	3 (2.61%)	1 (0.41%)	2.68	0.0979	0.593	Co-occurrence
MESD	15q25.1	3 (2.61%)	1 (0.41%)	2.68	0.0979	0.593	Co-occurrence
MFNG	22q13.1	3 (2.61%)	1 (0.41%)	2.68	0.0979	0.593	Co-occurrence
MICALL2	7p22.3	3 (2.61%)	1 (0.41%)	2.68	0.0979	0.593	Co-occurrence
MID1	Xp22.2	3 (2.61%)	1 (0.41%)	2.68	0.0979	0.593	Co-occurrence
MIP	12q13.3	3 (2.61%)	1 (0.41%)	2.68	0.0979	0.593	Co-occurrence
MIR-2909/2909		3 (2.61%)	1 (0.41%)	2.68	0.0979	0.593	Co-occurrence
MIR-3665/3665		3 (2.61%)	1 (0.41%)	2.68	0.0979	0.593	Co-occurrence
MIR-3671/3671		3 (2.61%)	1 (0.41%)	2.68	0.0979	0.593	Co-occurrence
MIR-4284/4284		3 (2.61%)	1 (0.41%)	2.68	0.0979	0.593	Co-occurrence
MIR-4514/4514		3 (2.61%)	1 (0.41%)	2.68	0.0979	0.593	Co-occurrence
MIR-4648/4648		3 (2.61%)	1 (0.41%)	2.68	0.0979	0.593	Co-occurrence
MIR-922/922		3 (2.61%)	1 (0.41%)	2.68	0.0979	0.593	Co-occurrence
MLX	17q21.2	3 (2.61%)	1 (0.41%)	2.68	0.0979	0.593	Co-occurrence
MMP28	17q12	3 (2.61%)	1 (0.41%)	2.68	0.0979	0.593	Co-occurrence
MRM1	17q12	3 (2.61%)	1 (0.41%)	2.68	0.0979	0.593	Co-occurrence

MYCBP2	13q22.3	3 (2.61%)	1 (0.41%)	2.68	0.0979	0.593	Co-occurrence
MYO19	17q12	3 (2.61%)	1 (0.41%)	2.68	0.0979	0.593	Co-occurrence
NAGLU	17q21.2	3 (2.61%)	1 (0.41%)	2.68	0.0979	0.593	Co-occurrence
NAV2	11p15.1	3 (2.61%)	1 (0.41%)	2.68	0.0979	0.593	Co-occurrence
NDST2	10q22.2	3 (2.61%)	1 (0.41%)	2.68	0.0979	0.593	Co-occurrence
NEDD8	14q12	3 (2.61%)	1 (0.41%)	2.68	0.0979	0.593	Co-occurrence
NFATC4	14q12	3 (2.61%)	1 (0.41%)	2.68	0.0979	0.593	Co-occurrence
NGRN	15q26.1	3 (2.61%)	1 (0.41%)	2.68	0.0979	0.593	Co-occurrence
NOP9	14q12	3 (2.61%)	1 (0.41%)	2.68	0.0979	0.593	Co-occurrence
NTNG1	1p13.3	3 (2.61%)	1 (0.41%)	2.68	0.0979	0.593	Co-occurrence
NXPH1	7p21.3	3 (2.61%)	1 (0.41%)	2.68	0.0979	0.593	Co-occurrence
NYNRIN	14q12	3 (2.61%)	1 (0.41%)	2.68	0.0979	0.593	Co-occurrence
PADI6	1p36.13	3 (2.61%)	1 (0.41%)	2.68	0.0979	0.593	Co-occurrence
PCDH9	13q21.32	3 (2.61%)	1 (0.41%)	2.68	0.0979	0.593	Co-occurrence
PDE4B	1p31.3	3 (2.61%)	1 (0.41%)	2.68	0.0979	0.593	Co-occurrence
PDGFA	7p22.3	3 (2.61%)	1 (0.41%)	2.68	0.0979	0.593	Co-occurrence
PEX12	17q12	3 (2.61%)	1 (0.41%)	2.68	0.0979	0.593	Co-occurrence
PHF7	3p21.1	3 (2.61%)	1 (0.41%)	2.68	0.0979	0.593	Co-occurrence
PIAS1	15q23	3 (2.61%)	1 (0.41%)	2.68	0.0979	0.593	Co-occurrence
PIGW	17q12	3 (2.61%)	1 (0.41%)	2.68	0.0979	0.593	Co-occurrence
PIP4K2C	12q13.3	3 (2.61%)	1 (0.41%)	2.68	0.0979	0.593	Co-occurrence
PLEKHH3	17q21.2	3 (2.61%)	1 (0.41%)	2.68	0.0979	0.593	Co-occurrence
PLRG1	4q31.3	3 (2.61%)	1 (0.41%)	2.68	0.0979	0.593	Co-occurrence
POU4F1	13q31.1	3 (2.61%)	1 (0.41%)	2.68	0.0979	0.593	Co-occurrence
PPIB	15q22.31	3 (2.61%)	1 (0.41%)	2.68	0.0979	0.593	Co-occurrence
PRKAR1B	7p22.3	3 (2.61%)	1 (0.41%)	2.68	0.0979	0.593	Co-occurrence
PSMC3IP	17q21.2	3 (2.61%)	1 (0.41%)	2.68	0.0979	0.593	Co-occurrence
PSMG3	7p22.3	3 (2.61%)	1 (0.41%)	2.68	0.0979	0.593	Co-occurrence
RABGGTA	14q12	3 (2.61%)	1 (0.41%)	2.68	0.0979	0.593	Co-occurrence

RAC2	22q13.1	3 (2.61%)	1 (0.41%)	2.68	0.0979	0.593	Co-occurrence
RASL10B	17q12	3 (2.61%)	1 (0.41%)	2.68	0.0979	0.593	Co-occurrence
RAVER2	1p31.3	3 (2.61%)	1 (0.41%)	2.68	0.0979	0.593	Co-occurrence
RBMS2	12q13.3	3 (2.61%)	1 (0.41%)	2.68	0.0979	0.593	Co-occurrence
RCC2	1p36.13	3 (2.61%)	1 (0.41%)	2.68	0.0979	0.593	Co-occurrence
RDM1	17q12	3 (2.61%)	1 (0.41%)	2.68	0.0979	0.593	Co-occurrence
RETREG3	17q21.2	3 (2.61%)	1 (0.41%)	2.68	0.0979	0.593	Co-occurrence
RFC2	7q11.23	3 (2.61%)	1 (0.41%)	2.68	0.0979	0.593	Co-occurrence
RHOA	3p21.31	3 (2.61%)	1 (0.41%)	2.68	0.0979	0.593	Co-occurrence
RIPK3	14q12	3 (2.61%)	1 (0.41%)	2.68	0.0979	0.593	Co-occurrence
RN7SKP250	12q24.11	3 (2.61%)	1 (0.41%)	2.68	0.0979	0.593	Co-occurrence
RN7SL150P	19q13.11	3 (2.61%)	1 (0.41%)	2.68	0.0979	0.593	Co-occurrence
RN7SL176P	12q23.1	3 (2.61%)	1 (0.41%)	2.68	0.0979	0.593	Co-occurrence
RN7SL265P	7q11.23	3 (2.61%)	1 (0.41%)	2.68	0.0979	0.593	Co-occurrence
RN7SL301P	17q12	3 (2.61%)	1 (0.41%)	2.68	0.0979	0.593	Co-occurrence
RN7SL736P	15q26.1	3 (2.61%)	1 (0.41%)	2.68	0.0979	0.593	Co-occurrence
RN7SL810P	13q22.3	3 (2.61%)	1 (0.41%)	2.68	0.0979	0.593	Co-occurrence
RNA5SP110	2q24.3	3 (2.61%)	1 (0.41%)	2.68	0.0979	0.593	Co-occurrence
RNA5SP111	2q24.3	3 (2.61%)	1 (0.41%)	2.68	0.0979	0.593	Co-occurrence
RNA5SP439	17q12	3 (2.61%)	1 (0.41%)	2.68	0.0979	0.593	Co-occurrence
RNA5SP497	22q12.3	3 (2.61%)	1 (0.41%)	2.68	0.0979	0.593	Co-occurrence
RNF219	13q31.1	3 (2.61%)	1 (0.41%)	2.68	0.0979	0.593	Co-occurrence
RNY3P3	13q22.3	3 (2.61%)	1 (0.41%)	2.68	0.0979	0.593	Co-occurrence
RNY3P7	13q22.3	3 (2.61%)	1 (0.41%)	2.68	0.0979	0.593	Co-occurrence
RNY4P13	17q11.2	3 (2.61%)	1 (0.41%)	2.68	0.0979	0.593	Co-occurrence
RPL35A	3q29	3 (2.61%)	1 (0.41%)	2.68	0.0979	0.593	Co-occurrence
RPS27L	15q22.2	3 (2.61%)	1 (0.41%)	2.68	0.0979	0.593	Co-occurrence
RUBCN	3q29	3 (2.61%)	1 (0.41%)	2.68	0.0979	0.593	Co-occurrence
SCEL	13q22.3	3 (2.61%)	1 (0.41%)	2.68	0.0979	0.593	Co-occurrence

SCN3A	2q24.3	3 (2.61%)	1 (0.41%)	2.68	0.0979	0.593	Co-occurrence
SEMA3G	3p21.1	3 (2.61%)	1 (0.41%)	2.68	0.0979	0.593	Co-occurrence
SKOR1	15q23	3 (2.61%)	1 (0.41%)	2.68	0.0979	0.593	Co-occurrence
SLAIN1	13q22.3	3 (2.61%)	1 (0.41%)	2.68	0.0979	0.593	Co-occurrence
SLC16A7	12q14.1	3 (2.61%)	1 (0.41%)	2.68	0.0979	0.593	Co-occurrence
SLC22A14	3p22.2	3 (2.61%)	1 (0.41%)	2.68	0.0979	0.593	Co-occurrence
SLC26A10	12q13.3	3 (2.61%)	1 (0.41%)	2.68	0.0979	0.593	Co-occurrence
SLFN11	17q12	3 (2.61%)	1 (0.41%)	2.68	0.0979	0.593	Co-occurrence
SLFN12	17q12	3 (2.61%)	1 (0.41%)	2.68	0.0979	0.593	Co-occurrence
SLFN12L	17q12	3 (2.61%)	1 (0.41%)	2.68	0.0979	0.593	Co-occurrence
SLFN13	17q12	3 (2.61%)	1 (0.41%)	2.68	0.0979	0.593	Co-occurrence
SLFN14	17q12	3 (2.61%)	1 (0.41%)	2.68	0.0979	0.593	Co-occurrence
SLFN5	17q12	3 (2.61%)	1 (0.41%)	2.68	0.0979	0.593	Co-occurrence
SNN	16p13.13	3 (2.61%)	1 (0.41%)	2.68	0.0979	0.593	Co-occurrence
SNORD59A	12q13.3	3 (2.61%)	1 (0.41%)	2.68	0.0979	0.593	Co-occurrence
SNORD7	17q12	3 (2.61%)	1 (0.41%)	2.68	0.0979	0.593	Co-occurrence
SNX22	15q22.31	3 (2.61%)	1 (0.41%)	2.68	0.0979	0.593	Co-occurrence
SNX8	7p22.3	3 (2.61%)	1 (0.41%)	2.68	0.0979	0.593	Co-occurrence
SPRYD4	12q13.3	3 (2.61%)	1 (0.41%)	2.68	0.0979	0.593	Co-occurrence
SSTR3	22q13.1	3 (2.61%)	1 (0.41%)	2.68	0.0979	0.593	Co-occurrence
STAB2	12q23.3	3 (2.61%)	1 (0.41%)	2.68	0.0979	0.593	Co-occurrence
STAT2	12q13.3	3 (2.61%)	1 (0.41%)	2.68	0.0979	0.593	Co-occurrence
STAT3	17q21.2	3 (2.61%)	1 (0.41%)	2.68	0.0979	0.593	Co-occurrence
STAT5A	17q21.2	3 (2.61%)	1 (0.41%)	2.68	0.0979	0.593	Co-occurrence
STAT5B	17q21.2	3 (2.61%)	1 (0.41%)	2.68	0.0979	0.593	Co-occurrence
SUN2	22q13.1	3 (2.61%)	1 (0.41%)	2.68	0.0979	0.593	Co-occurrence
SYN3	22q12.3	3 (2.61%)	1 (0.41%)	2.68	0.0979	0.593	Co-occurrence
SYNRG	17q12	3 (2.61%)	1 (0.41%)	2.68	0.0979	0.593	Co-occurrence
TAF15	17q12	3 (2.61%)	1 (0.41%)	2.68	0.0979	0.593	Co-occurrence

TBC1D3B	17q12	3 (2.61%)	1 (0.41%)	2.68	0.0979	0.593	Co-occurrence
TBC1D3C	17q12	3 (2.61%)	1 (0.41%)	2.68	0.0979	0.593	Co-occurrence
TBC1D3G	17q12	3 (2.61%)	1 (0.41%)	2.68	0.0979	0.593	Co-occurrence
TBC1D3H	17q12	3 (2.61%)	1 (0.41%)	2.68	0.0979	0.593	Co-occurrence
TGM1	14q12	3 (2.61%)	1 (0.41%)	2.68	0.0979	0.593	Co-occurrence
TIMELESS	12q13.3	3 (2.61%)	1 (0.41%)	2.68	0.0979	0.593	Co-occurrence
TIMP3	22q12.3	3 (2.61%)	1 (0.41%)	2.68	0.0979	0.593	Co-occurrence
TINF2	14q12	3 (2.61%)	1 (0.41%)	2.68	0.0979	0.593	Co-occurrence
TLNRD1	15q25.1	3 (2.61%)	1 (0.41%)	2.68	0.0979	0.593	Co-occurrence
TM9SF1	14q12	3 (2.61%)	1 (0.41%)	2.68	0.0979	0.593	Co-occurrence
TMEM184A	7p22.3	3 (2.61%)	1 (0.41%)	2.68	0.0979	0.593	Co-occurrence
TMPRSS11E	4q13.2	3 (2.61%)	1 (0.41%)	2.68	0.0979	0.593	Co-occurrence
TMPRSS6	22q12.3	3 (2.61%)	1 (0.41%)	2.68	0.0979	0.593	Co-occurrence
TOMM22	22q13.1	3 (2.61%)	1 (0.41%)	2.68	0.0979	0.593	Co-occurrence
TPM1	15q22.2	3 (2.61%)	1 (0.41%)	2.68	0.0979	0.593	Co-occurrence
TSSK4	14q12	3 (2.61%)	1 (0.41%)	2.68	0.0979	0.593	Co-occurrence
TTLL13P	15q26.1	3 (2.61%)	1 (0.41%)	2.68	0.0979	0.593	Co-occurrence
TUBG1	17q21.2	3 (2.61%)	1 (0.41%)	2.68	0.0979	0.593	Co-occurrence
TUBG2	17q21.2	3 (2.61%)	1 (0.41%)	2.68	0.0979	0.593	Co-occurrence
TXNDC11	16p13.13	3 (2.61%)	1 (0.41%)	2.68	0.0979	0.593	Co-occurrence
UGT2A3	4q13.2	3 (2.61%)	1 (0.41%)	2.68	0.0979	0.593	Co-occurrence
UGT2B10	4q13.2	3 (2.61%)	1 (0.41%)	2.68	0.0979	0.593	Co-occurrence
UGT2B11	4q13.2	3 (2.61%)	1 (0.41%)	2.68	0.0979	0.593	Co-occurrence
UGT2B15	4q13.2	3 (2.61%)	1 (0.41%)	2.68	0.0979	0.593	Co-occurrence
UGT2B17	4q13.2	3 (2.61%)	1 (0.41%)	2.68	0.0979	0.593	Co-occurrence
UGT2B28	4q13.2	3 (2.61%)	1 (0.41%)	2.68	0.0979	0.593	Co-occurrence
UGT2B4	4q13.3	3 (2.61%)	1 (0.41%)	2.68	0.0979	0.593	Co-occurrence
UGT2B7	4q13.2	3 (2.61%)	1 (0.41%)	2.68	0.0979	0.593	Co-occurrence
UHRF1BP1L	12q23.1	3 (2.61%)	1 (0.41%)	2.68	0.0979	0.593	Co-occurrence

UNCX	7p22.3	3 (2.61%)	1 (0.41%)	2.68	0.0979	0.593	Co-occurrence
USP3	15q22.31	3 (2.61%)	1 (0.41%)	2.68	0.0979	0.593	Co-occurrence
USP4	3p21.31	3 (2.61%)	1 (0.41%)	2.68	0.0979	0.593	Co-occurrence
VCL	10q22.2	3 (2.61%)	1 (0.41%)	2.68	0.0979	0.593	Co-occurrence
WARS2	1p12	3 (2.61%)	1 (0.41%)	2.68	0.0979	0.593	Co-occurrence
XIRP2	2q24.3	3 (2.61%)	1 (0.41%)	2.68	0.0979	0.593	Co-occurrence
YTHDC1	4q13.2	3 (2.61%)	1 (0.41%)	2.68	0.0979	0.593	Co-occurrence
YWHAH	22q12.3	3 (2.61%)	1 (0.41%)	2.68	0.0979	0.593	Co-occurrence
ZNF507	19q13.11	3 (2.61%)	1 (0.41%)	2.68	0.0979	0.593	Co-occurrence
ZNF774	15q26.1	3 (2.61%)	1 (0.41%)	2.68	0.0979	0.593	Co-occurrence
ZNHIT3	17q12	3 (2.61%)	1 (0.41%)	2.68	0.0979	0.593	Co-occurrence
ZSWIM8	10q22.2	3 (2.61%)	1 (0.41%)	2.68	0.0979	0.593	Co-occurrence
AKAP1	17q22	6 (5.22%)	5 (2.04%)	1.35	0.0989	0.593	Co-occurrence
C17ORF67	17q22	6 (5.22%)	5 (2.04%)	1.35	0.0989	0.593	Co-occurrence
COIL	17q22	6 (5.22%)	5 (2.04%)	1.35	0.0989	0.593	Co-occurrence
COX11	17q22	6 (5.22%)	5 (2.04%)	1.35	0.0989	0.593	Co-occurrence
DGKE	17q22	6 (5.22%)	5 (2.04%)	1.35	0.0989	0.593	Co-occurrence
KIF2B	17q22	6 (5.22%)	5 (2.04%)	1.35	0.0989	0.593	Co-occurrence
MMD	17q22	6 (5.22%)	5 (2.04%)	1.35	0.0989	0.593	Co-occurrence
NOG	17q22	6 (5.22%)	5 (2.04%)	1.35	0.0989	0.593	Co-occurrence
RN7SKP14	17q22	6 (5.22%)	5 (2.04%)	1.35	0.0989	0.593	Co-occurrence
SCPEP1	17q22	6 (5.22%)	5 (2.04%)	1.35	0.0989	0.593	Co-occurrence
STXBP4	17q22	6 (5.22%)	5 (2.04%)	1.35	0.0989	0.593	Co-occurrence
TOM1L1	17q22	6 (5.22%)	5 (2.04%)	1.35	0.0989	0.593	Co-occurrence
TRIM25	17q22	6 (5.22%)	5 (2.04%)	1.35	0.0989	0.593	Co-occurrence
A2M	12p13.31	2 (1.74%)	0 (0.00%)	>10	0.101	0.593	Co-occurrence
A4GALT	22q13.2	2 (1.74%)	0 (0.00%)	>10	0.101	0.593	Co-occurrence
ABCD3	1p21.3	2 (1.74%)	0 (0.00%)	>10	0.101	0.593	Co-occurrence
ABHD15	17q11.2	2 (1.74%)	0 (0.00%)	>10	0.101	0.593	Co-occurrence

ABO	9q34.2	2 (1.74%)	0 (0.00%)	>10	0.101	0.593	Co-occurrence
ACAA1	3p22.2	2 (1.74%)	0 (0.00%)	>10	0.101	0.593	Co-occurrence
ACOT7	1p36.31	2 (1.74%)	0 (0.00%)	>10	0.101	0.593	Co-occurrence
ACSS3	12q21.31	2 (1.74%)	0 (0.00%)	>10	0.101	0.593	Co-occurrence
ADAMTS13	9q34.2	2 (1.74%)	0 (0.00%)	>10	0.101	0.593	Co-occurrence
ADSL	22q13.1	2 (1.74%)	0 (0.00%)	>10	0.101	0.593	Co-occurrence
AIF1L	9q34.12-q34.13	2 (1.74%)	0 (0.00%)	>10	0.101	0.593	Co-occurrence
AK5	1p31.1	2 (1.74%)	0 (0.00%)	>10	0.101	0.593	Co-occurrence
AK8	9q34.13	2 (1.74%)	0 (0.00%)	>10	0.101	0.593	Co-occurrence
ALAS2	Xp11.21	2 (1.74%)	0 (0.00%)	>10	0.101	0.593	Co-occurrence
ALG6	1p31.3	2 (1.74%)	0 (0.00%)	>10	0.101	0.593	Co-occurrence
ALKBH2	12q24.11	2 (1.74%)	0 (0.00%)	>10	0.101	0.593	Co-occurrence
ALOX5AP	13q12.3	2 (1.74%)	0 (0.00%)	>10	0.101	0.593	Co-occurrence
ALS2CL	3p21.31	2 (1.74%)	0 (0.00%)	>10	0.101	0.593	Co-occurrence
ALX1	12q21.31	2 (1.74%)	0 (0.00%)	>10	0.101	0.593	Co-occurrence
AMELX	Xp22.2	2 (1.74%)	0 (0.00%)	>10	0.101	0.593	Co-occurrence
ANGPTL3	1p31.3	2 (1.74%)	0 (0.00%)	>10	0.101	0.593	Co-occurrence
ANKRD13B	17q11.2	2 (1.74%)	0 (0.00%)	>10	0.101	0.593	Co-occurrence
AP5B1	11q13.1	2 (1.74%)	0 (0.00%)	>10	0.101	0.593	Co-occurrence
APAF1	12q23.1	2 (1.74%)	0 (0.00%)	>10	0.101	0.593	Co-occurrence
APEX2	Xp11.21	2 (1.74%)	0 (0.00%)	>10	0.101	0.593	Co-occurrence
APOL1	22q12.3	2 (1.74%)	0 (0.00%)	>10	0.101	0.593	Co-occurrence
APOL2	22q12.3	2 (1.74%)	0 (0.00%)	>10	0.101	0.593	Co-occurrence
APOL3	22q12.3	2 (1.74%)	0 (0.00%)	>10	0.101	0.593	Co-occurrence
APOL4	22q12.3	2 (1.74%)	0 (0.00%)	>10	0.101	0.593	Co-occurrence
APOL5	22q12.3	2 (1.74%)	0 (0.00%)	>10	0.101	0.593	Co-occurrence
APOL6	22q12.3	2 (1.74%)	0 (0.00%)	>10	0.101	0.593	Co-occurrence
APPL2	12q23.3	2 (1.74%)	0 (0.00%)	>10	0.101	0.593	Co-occurrence
ARFGAP3	22q13.2	2 (1.74%)	0 (0.00%)	>10	0.101	0.593	Co-occurrence

ARHGAP8	22q13.31	2 (1.74%)	0 (0.00%)	>10	0.101	0.593	Co-occurrence
ARL1	12q23.2	2 (1.74%)	0 (0.00%)	>10	0.101	0.593	Co-occurrence
ARL2	11q13.1	2 (1.74%)	0 (0.00%)	>10	0.101	0.593	Co-occurrence
ARMH1	1p34.1	2 (1.74%)	0 (0.00%)	>10	0.101	0.593	Co-occurrence
ARPP21	3p22.3	2 (1.74%)	0 (0.00%)	>10	0.101	0.593	Co-occurrence
ASB6	9q34.11	2 (1.74%)	0 (0.00%)	>10	0.101	0.593	Co-occurrence
ASCL1	12q23.2	2 (1.74%)	0 (0.00%)	>10	0.101	0.593	Co-occurrence
ATAD5	17q11.2	2 (1.74%)	0 (0.00%)	>10	0.101	0.593	Co-occurrence
ATF4	22q13.1	2 (1.74%)	0 (0.00%)	>10	0.101	0.593	Co-occurrence
ATG2A	11q13.1	2 (1.74%)	0 (0.00%)	>10	0.101	0.593	Co-occurrence
ATG4C	1p31.3	2 (1.74%)	0 (0.00%)	>10	0.101	0.593	Co-occurrence
ATP2B1	12q21.33	2 (1.74%)	0 (0.00%)	>10	0.101	0.593	Co-occurrence
ATP2B1-AS1	12q21.33	2 (1.74%)	0 (0.00%)	>10	0.101	0.593	Co-occurrence
ATP5F1EP2	13q12.2	2 (1.74%)	0 (0.00%)	>10	0.101	0.593	Co-occurrence
ATP5MGL	22q13.2	2 (1.74%)	0 (0.00%)	>10	0.101	0.593	Co-occurrence
ATP6V0A2	12q24.31	2 (1.74%)	0 (0.00%)	>10	0.101	0.593	Co-occurrence
ATP6V1A	3q13.31	2 (1.74%)	0 (0.00%)	>10	0.101	0.593	Co-occurrence
ATRIP	3p21.31	2 (1.74%)	0 (0.00%)	>10	0.101	0.593	Co-occurrence
ATXN10	22q13.31	2 (1.74%)	0 (0.00%)	>10	0.101	0.593	Co-occurrence
ATXN7L3B	12q21.1	2 (1.74%)	0 (0.00%)	>10	0.101	0.593	Co-occurrence
AVPR1A	12q14.2	2 (1.74%)	0 (0.00%)	>10	0.101	0.593	Co-occurrence
AZU1	19p13.3	2 (1.74%)	0 (0.00%)	>10	0.101	0.593	Co-occurrence
B3GLCT	13q12.3	2 (1.74%)	0 (0.00%)	>10	0.101	0.593	Co-occurrence
BARHL1	9q34.13	2 (1.74%)	0 (0.00%)	>10	0.101	0.593	Co-occurrence
BATF2	11q13.1	2 (1.74%)	0 (0.00%)	>10	0.101	0.593	Co-occurrence
BBS10	12q21.2	2 (1.74%)	0 (0.00%)	>10	0.101	0.593	Co-occurrence
BCLAF3	Xp22.12	2 (1.74%)	0 (0.00%)	>10	0.101	0.593	Co-occurrence
BEGAIN	14q32.2	2 (1.74%)	0 (0.00%)	>10	0.101	0.593	Co-occurrence
BIK	22q13.2	2 (1.74%)	0 (0.00%)	>10	0.101	0.593	Co-occurrence

BIN2	12q13.13	2 (1.74%)	0 (0.00%)	>10	0.101	0.593	Co-occurrence
BLOC1S1	12q13.2	2 (1.74%)	0 (0.00%)	>10	0.101	0.593	Co-occurrence
BMP8A	1p34.3	2 (1.74%)	0 (0.00%)	>10	0.101	0.593	Co-occurrence
BOC	3q13.2	2 (1.74%)	0 (0.00%)	>10	0.101	0.593	Co-occurrence
BTG1	12q21.33	2 (1.74%)	0 (0.00%)	>10	0.101	0.593	Co-occurrence
C12ORF56	12q14.2	2 (1.74%)	0 (0.00%)	>10	0.101	0.593	Co-occurrence
C12ORF66	12q14.2	2 (1.74%)	0 (0.00%)	>10	0.101	0.593	Co-occurrence
C12ORF74	12q22	2 (1.74%)	0 (0.00%)	>10	0.101	0.593	Co-occurrence
C12ORF75	12q23.3	2 (1.74%)	0 (0.00%)	>10	0.101	0.593	Co-occurrence
C1ORF185	1p32.3	2 (1.74%)	0 (0.00%)	>10	0.101	0.593	Co-occurrence
C3ORF35	3p22.2	2 (1.74%)	0 (0.00%)	>10	0.101	0.593	Co-occurrence
C9ORF50	9q34.11	2 (1.74%)	0 (0.00%)	>10	0.101	0.593	Co-occurrence
CA6	1p36.23	2 (1.74%)	0 (0.00%)	>10	0.101	0.593	Co-occurrence
CACFD1	9q34.2	2 (1.74%)	0 (0.00%)	>10	0.101	0.593	Co-occurrence
CACNA1I	22q13.1	2 (1.74%)	0 (0.00%)	>10	0.101	0.593	Co-occurrence
CACNG2	22q12.3	2 (1.74%)	0 (0.00%)	>10	0.101	0.593	Co-occurrence
CAMKV	3p21.31	2 (1.74%)	0 (0.00%)	>10	0.101	0.593	Co-occurrence
CAPN1	11q13.1	2 (1.74%)	0 (0.00%)	>10	0.101	0.593	Co-occurrence
CAPS2	12q21.1-q21.2	2 (1.74%)	0 (0.00%)	>10	0.101	0.593	Co-occurrence
CBLL2	Xp22.11	2 (1.74%)	0 (0.00%)	>10	0.101	0.593	Co-occurrence
CCDC158	4q21.1	2 (1.74%)	0 (0.00%)	>10	0.101	0.593	Co-occurrence
CCDC38	12q23.1	2 (1.74%)	0 (0.00%)	>10	0.101	0.593	Co-occurrence
CCDC51	3p21.31	2 (1.74%)	0 (0.00%)	>10	0.101	0.593	Co-occurrence
CCDC59	12q21.31	2 (1.74%)	0 (0.00%)	>10	0.101	0.593	Co-occurrence
CCDC62	12q24.31	2 (1.74%)	0 (0.00%)	>10	0.101	0.593	Co-occurrence
CCDC85B	11q13.1	2 (1.74%)	0 (0.00%)	>10	0.101	0.593	Co-occurrence
CCDC92	12q24.31	2 (1.74%)	0 (0.00%)	>10	0.101	0.593	Co-occurrence
CCER1	12q21.33	2 (1.74%)	0 (0.00%)	>10	0.101	0.593	Co-occurrence
CCIN	9p13.3	2 (1.74%)	0 (0.00%)	>10	0.101	0.593	Co-occurrence

CCN2	6q23.2	2 (1.74%)	0 (0.00%)	>10	0.101	0.593	Co-occurrence
CCNA2	4q27	2 (1.74%)	0 (0.00%)	>10	0.101	0.593	Co-occurrence
CD63	12q13.2	2 (1.74%)	0 (0.00%)	>10	0.101	0.593	Co-occurrence
CD72	9p13.3	2 (1.74%)	0 (0.00%)	>10	0.101	0.593	Co-occurrence
CDC26	9q32	2 (1.74%)	0 (0.00%)	>10	0.101	0.593	Co-occurrence
CDC42BPG	11q13.1	2 (1.74%)	0 (0.00%)	>10	0.101	0.593	Co-occurrence
CDC42EP2	11q13.1	2 (1.74%)	0 (0.00%)	>10	0.101	0.593	Co-occurrence
CDCA5	11q13.1	2 (1.74%)	0 (0.00%)	>10	0.101	0.593	Co-occurrence
CDK17	12q23.1	2 (1.74%)	0 (0.00%)	>10	0.101	0.593	Co-occurrence
CDK5RAP2	9q33.2	2 (1.74%)	0 (0.00%)	>10	0.101	0.593	Co-occurrence
CDKN2C	1p32.3	2 (1.74%)	0 (0.00%)	>10	0.101	0.593	Co-occurrence
CDX2	13q12.2	2 (1.74%)	0 (0.00%)	>10	0.101	0.593	Co-occurrence
CEL	9q34.13	2 (1.74%)	0 (0.00%)	>10	0.101	0.593	Co-occurrence
CELA1	12q13.13	2 (1.74%)	0 (0.00%)	>10	0.101	0.593	Co-occurrence
CELP	9q34.13	2 (1.74%)	0 (0.00%)	>10	0.101	0.593	Co-occurrence
CELSR1	22q13.31	2 (1.74%)	0 (0.00%)	>10	0.101	0.593	Co-occurrence
CEP83	12q22	2 (1.74%)	0 (0.00%)	>10	0.101	0.593	Co-occurrence
CFAP299	4q21.21	2 (1.74%)	0 (0.00%)	>10	0.101	0.593	Co-occurrence
CFAP44	3q13.2	2 (1.74%)	0 (0.00%)	>10	0.101	0.593	Co-occurrence
CFAP54	12q23.1	2 (1.74%)	0 (0.00%)	>10	0.101	0.593	Co-occurrence
CFAP77	9q34.13	2 (1.74%)	0 (0.00%)	>10	0.101	0.593	Co-occurrence
CFL1	11q13.1	2 (1.74%)	0 (0.00%)	>10	0.101	0.593	Co-occurrence
CHPT1	12q23.2	2 (1.74%)	0 (0.00%)	>10	0.101	0.593	Co-occurrence
CLCA3P	1p22.3	2 (1.74%)	0 (0.00%)	>10	0.101	0.593	Co-occurrence
CLCN4	Xp22.2	2 (1.74%)	0 (0.00%)	>10	0.101	0.593	Co-occurrence
CLLU1	12q22	2 (1.74%)	0 (0.00%)	>10	0.101	0.593	Co-occurrence
CLLU1OS	12q22	2 (1.74%)	0 (0.00%)	>10	0.101	0.593	Co-occurrence
CLTA	9p13.3	2 (1.74%)	0 (0.00%)	>10	0.101	0.593	Co-occurrence
COL7A1	3p21.31	2 (1.74%)	0 (0.00%)	>10	0.101	0.593	Co-occurrence

COL9A2	1p34.2	2 (1.74%)	0 (0.00%)	>10	0.101	0.593	Co-occurrence
CORO1C	12q24.11	2 (1.74%)	0 (0.00%)	>10	0.101	0.593	Co-occurrence
CORO6	17q11.2	2 (1.74%)	0 (0.00%)	>10	0.101	0.593	Co-occurrence
CREB3	9p13.3	2 (1.74%)	0 (0.00%)	>10	0.101	0.593	Co-occurrence
CRLF3	17q11.2	2 (1.74%)	0 (0.00%)	>10	0.101	0.593	Co-occurrence
CRY1	12q23.3	2 (1.74%)	0 (0.00%)	>10	0.101	0.593	Co-occurrence
CSF2RB	22q12.3	2 (1.74%)	0 (0.00%)	>10	0.101	0.593	Co-occurrence
CSN1S1	4q13.3	2 (1.74%)	0 (0.00%)	>10	0.101	0.593	Co-occurrence
CSRNP2	12q13.12	2 (1.74%)	0 (0.00%)	>10	0.101	0.593	Co-occurrence
CSRP2	12q21.2	2 (1.74%)	0 (0.00%)	>10	0.101	0.593	Co-occurrence
CTAGE1	18q11.2	2 (1.74%)	0 (0.00%)	>10	0.101	0.593	Co-occurrence
CTDSPL	3p22.2	2 (1.74%)	0 (0.00%)	>10	0.101	0.593	Co-occurrence
CTSW	11q13.1	2 (1.74%)	0 (0.00%)	>10	0.101	0.593	Co-occurrence
CXCL1	4q13.3	2 (1.74%)	0 (0.00%)	>10	0.101	0.593	Co-occurrence
CXCL6	4q13.3	2 (1.74%)	0 (0.00%)	>10	0.101	0.593	Co-occurrence
CXCL8	4q13.3	2 (1.74%)	0 (0.00%)	>10	0.101	0.593	Co-occurrence
CYB5R3	22q13.2	2 (1.74%)	0 (0.00%)	>10	0.101	0.593	Co-occurrence
CYP2D6	22q13.2	2 (1.74%)	0 (0.00%)	>10	0.101	0.593	Co-occurrence
CYP2D7	22q13.2	2 (1.74%)	0 (0.00%)	>10	0.101	0.593	Co-occurrence
DAB2IP	9q33.2	2 (1.74%)	0 (0.00%)	>10	0.101	0.593	Co-occurrence
DAO	12q24.11	2 (1.74%)	0 (0.00%)	>10	0.101	0.593	Co-occurrence
DAZAP2	12q13.13	2 (1.74%)	0 (0.00%)	>10	0.101	0.593	Co-occurrence
DCN	12q21.33	2 (1.74%)	0 (0.00%)	>10	0.101	0.593	Co-occurrence
DDX31	9q34.13	2 (1.74%)	0 (0.00%)	>10	0.101	0.593	Co-occurrence
DDX53	Xp22.11	2 (1.74%)	0 (0.00%)	>10	0.101	0.593	Co-occurrence
DDX55	12q24.31	2 (1.74%)	0 (0.00%)	>10	0.101	0.593	Co-occurrence
DDX6	11q23.3	2 (1.74%)	0 (0.00%)	>10	0.101	0.593	Co-occurrence
DENR	12q24.31	2 (1.74%)	0 (0.00%)	>10	0.101	0.593	Co-occurrence
DEPDC1	1p31.3	2 (1.74%)	0 (0.00%)	>10	0.101	0.593	Co-occurrence

DIP2B	12q13.12	2 (1.74%)	0 (0.00%)	>10	0.101	0.593	Co-occurrence
DIRAS3	1p31.3	2 (1.74%)	0 (0.00%)	>10	0.101	0.593	Co-occurrence
DLEC1	3p22.2	2 (1.74%)	0 (0.00%)	>10	0.101	0.593	Co-occurrence
DLEU2L	1p31.3	2 (1.74%)	0 (0.00%)	>10	0.101	0.593	Co-occurrence
DMRTA2	1p32.3	2 (1.74%)	0 (0.00%)	>10	0.101	0.593	Co-occurrence
DNAH10OS	12q24.31	2 (1.74%)	0 (0.00%)	>10	0.101	0.593	Co-occurrence
DNAJB4	1p31.1	2 (1.74%)	0 (0.00%)	>10	0.101	0.593	Co-occurrence
DNAJC11	1p36.31	2 (1.74%)	0 (0.00%)	>10	0.101	0.593	Co-occurrence
DOCK7	1p31.3	2 (1.74%)	0 (0.00%)	>10	0.101	0.593	Co-occurrence
DOT1L	19p13.3	2 (1.74%)	0 (0.00%)	>10	0.101	0.593	Co-occurrence
DPF2	11q13.1	2 (1.74%)	0 (0.00%)	>10	0.101	0.593	Co-occurrence
DPY19L2	12q14.2	2 (1.74%)	0 (0.00%)	>10	0.101	0.593	Co-occurrence
DRAM1	12q23.2	2 (1.74%)	0 (0.00%)	>10	0.101	0.593	Co-occurrence
DUSP6	12q21.33	2 (1.74%)	0 (0.00%)	>10	0.101	0.593	Co-occurrence
E2F7	12q21.2	2 (1.74%)	0 (0.00%)	>10	0.101	0.593	Co-occurrence
EEA1	12q22	2 (1.74%)	0 (0.00%)	>10	0.101	0.593	Co-occurrence
EEF1AKMT1	13q12.11	2 (1.74%)	0 (0.00%)	>10	0.101	0.593	Co-occurrence
EFCAB6	22q13.2-q13.31	2 (1.74%)	0 (0.00%)	>10	0.101	0.593	Co-occurrence
EFCAB7	1p31.3	2 (1.74%)	0 (0.00%)	>10	0.101	0.593	Co-occurrence
EFEMP2	11q13.1	2 (1.74%)	0 (0.00%)	>10	0.101	0.593	Co-occurrence
EHBP1L1	11q13.1	2 (1.74%)	0 (0.00%)	>10	0.101	0.593	Co-occurrence
EHD1	11q13.1	2 (1.74%)	0 (0.00%)	>10	0.101	0.593	Co-occurrence
EIF1AX	Xp22.12	2 (1.74%)	0 (0.00%)	>10	0.101	0.593	Co-occurrence
EIF2B1	12q24.31	2 (1.74%)	0 (0.00%)	>10	0.101	0.593	Co-occurrence
EIF3D	22q12.3	2 (1.74%)	0 (0.00%)	>10	0.101	0.593	Co-occurrence
ELK3	12q23.1	2 (1.74%)	0 (0.00%)	>10	0.101	0.593	Co-occurrence
ENTHD1	22q13.1	2 (1.74%)	0 (0.00%)	>10	0.101	0.593	Co-occurrence
EP300	22q13.2	2 (1.74%)	0 (0.00%)	>10	0.101	0.593	Co-occurrence
EPYC	12q21.33	2 (1.74%)	0 (0.00%)	>10	0.101	0.593	Co-occurrence

ERBIN	5q12.3	2 (1.74%)	0 (0.00%)	>10	0.101	0.593	Co-occurrence
ERCC3	2q14.3	2 (1.74%)	0 (0.00%)	>10	0.101	0.593	Co-occurrence
EWSR1	22q12.2	2 (1.74%)	0 (0.00%)	>10	0.101	0.593	Co-occurrence
EXOSC9	4q27	2 (1.74%)	0 (0.00%)	>10	0.101	0.593	Co-occurrence
FAF1	1p32.3	2 (1.74%)	0 (0.00%)	>10	0.101	0.593	Co-occurrence
FAM118A	22q13.31	2 (1.74%)	0 (0.00%)	>10	0.101	0.593	Co-occurrence
FAM166B	9p13.3	2 (1.74%)	0 (0.00%)	>10	0.101	0.593	Co-occurrence
FAM221B	9p13.3	2 (1.74%)	0 (0.00%)	>10	0.101	0.593	Co-occurrence
FAM78A	9q34.13	2 (1.74%)	0 (0.00%)	>10	0.101	0.593	Co-occurrence
FAM83F	22q13.1	2 (1.74%)	0 (0.00%)	>10	0.101	0.593	Co-occurrence
FAM89B	11q13.1	2 (1.74%)	0 (0.00%)	>10	0.101	0.593	Co-occurrence
FANCB	Xp22.2	2 (1.74%)	0 (0.00%)	>10	0.101	0.593	Co-occurrence
FAU	11q13.1	2 (1.74%)	0 (0.00%)	>10	0.101	0.593	Co-occurrence
FBLN1	22q13.31	2 (1.74%)	0 (0.00%)	>10	0.101	0.593	Co-occurrence
FBXO21	12q24.22	2 (1.74%)	0 (0.00%)	>10	0.101	0.593	Co-occurrence
FGD1	Xp11.22	2 (1.74%)	0 (0.00%)	>10	0.101	0.593	Co-occurrence
FGD6	12q22	2 (1.74%)	0 (0.00%)	>10	0.101	0.593	Co-occurrence
FIBP	11q13.1	2 (1.74%)	0 (0.00%)	>10	0.101	0.593	Co-occurrence
FLT1	13q12.3	2 (1.74%)	0 (0.00%)	>10	0.101	0.593	Co-occurrence
FLT3	13q12.2	2 (1.74%)	0 (0.00%)	>10	0.101	0.593	Co-occurrence
FOSL1	11q13.1	2 (1.74%)	0 (0.00%)	>10	0.101	0.593	Co-occurrence
FOXD3	1p31.3	2 (1.74%)	0 (0.00%)	>10	0.101	0.593	Co-occurrence
FRMD8	11q13.1	2 (1.74%)	0 (0.00%)	>10	0.101	0.593	Co-occurrence
FUBP1	1p31.1	2 (1.74%)	0 (0.00%)	>10	0.101	0.593	Co-occurrence
GALNT13	2q23.3-q24.1	2 (1.74%)	0 (0.00%)	>10	0.101	0.593	Co-occurrence
GALNT4	12q21.33	2 (1.74%)	0 (0.00%)	>10	0.101	0.593	Co-occurrence
GAS2L1	22q12.2	2 (1.74%)	0 (0.00%)	>10	0.101	0.593	Co-occurrence
GBA2	9p13.3	2 (1.74%)	0 (0.00%)	>10	0.101	0.593	Co-occurrence
GBGT1	9q34.2	2 (1.74%)	0 (0.00%)	>10	0.101	0.593	Co-occurrence

GDAP2	1p12	2 (1.74%)	0 (0.00%)	>10	0.101	0.593	Co-occurrence
GDF11	12q13.2	2 (1.74%)	0 (0.00%)	>10	0.101	0.593	Co-occurrence
GEMIN8	Xp22.2	2 (1.74%)	0 (0.00%)	>10	0.101	0.593	Co-occurrence
GFI1B	9q34.13	2 (1.74%)	0 (0.00%)	>10	0.101	0.593	Co-occurrence
GIPC2	1p31.1	2 (1.74%)	0 (0.00%)	>10	0.101	0.593	Co-occurrence
GIT1	17q11.2	2 (1.74%)	0 (0.00%)	>10	0.101	0.593	Co-occurrence
GLIPR1	12q21.2	2 (1.74%)	0 (0.00%)	>10	0.101	0.593	Co-occurrence
GLIPR1L1	12q21.2	2 (1.74%)	0 (0.00%)	>10	0.101	0.593	Co-occurrence
GLIPR1L2	12q21.2	2 (1.74%)	0 (0.00%)	>10	0.101	0.593	Co-occurrence
GLIPR2	9p13.3	2 (1.74%)	0 (0.00%)	>10	0.101	0.593	Co-occurrence
GLRA2	Xp22.2	2 (1.74%)	0 (0.00%)	>10	0.101	0.593	Co-occurrence
GNE	9p13.3	2 (1.74%)	0 (0.00%)	>10	0.101	0.593	Co-occurrence
GNL3L	Xp11.22	2 (1.74%)	0 (0.00%)	>10	0.101	0.593	Co-occurrence
GNPTAB	12q23.2	2 (1.74%)	0 (0.00%)	>10	0.101	0.593	Co-occurrence
GNS	12q14.3	2 (1.74%)	0 (0.00%)	>10	0.101	0.593	Co-occurrence
GOLGA4	3p22.2	2 (1.74%)	0 (0.00%)	>10	0.101	0.593	Co-occurrence
GPHA2	11q13.1	2 (1.74%)	0 (0.00%)	>10	0.101	0.593	Co-occurrence
GPM6B	Xp22.2	2 (1.74%)	0 (0.00%)	>10	0.101	0.593	Co-occurrence
GPR107	9q34.11	2 (1.74%)	0 (0.00%)	>10	0.101	0.593	Co-occurrence
GPR153	1p36.31	2 (1.74%)	0 (0.00%)	>10	0.101	0.593	Co-occurrence
GRAP2	22q13.1	2 (1.74%)	0 (0.00%)	>10	0.101	0.593	Co-occurrence
GRIP1	12q14.3	2 (1.74%)	0 (0.00%)	>10	0.101	0.593	Co-occurrence
GSX1	13q12.2	2 (1.74%)	0 (0.00%)	>10	0.101	0.593	Co-occurrence
GTF2H3	12q24.31	2 (1.74%)	0 (0.00%)	>10	0.101	0.593	Co-occurrence
GTF3A	13q12.2	2 (1.74%)	0 (0.00%)	>10	0.101	0.593	Co-occurrence
GTF3C4	9q34.13	2 (1.74%)	0 (0.00%)	>10	0.101	0.593	Co-occurrence
GTF3C5	9q34.13	2 (1.74%)	0 (0.00%)	>10	0.101	0.593	Co-occurrence
GTSE1	22q13.31	2 (1.74%)	0 (0.00%)	>10	0.101	0.593	Co-occurrence
HAO2	1p12	2 (1.74%)	0 (0.00%)	>10	0.101	0.593	Co-occurrence

HCAR1	12q24.31	2 (1.74%)	0 (0.00%)	>10	0.101	0.593	Co-occurrence
HCAR2	12q24.31	2 (1.74%)	0 (0.00%)	>10	0.101	0.593	Co-occurrence
HCAR3	12q24.31	2 (1.74%)	0 (0.00%)	>10	0.101	0.593	Co-occurrence
HCCS	Xp22.2	2 (1.74%)	0 (0.00%)	>10	0.101	0.593	Co-occurrence
HELB	12q14.3|12q	2 (1.74%)	0 (0.00%)	>10	0.101	0.593	Co-occurrence
HES2	1p36.31	2 (1.74%)	0 (0.00%)	>10	0.101	0.593	Co-occurrence
HIGD1C	12q13.12	2 (1.74%)	0 (0.00%)	>10	0.101	0.593	Co-occurrence
HINT2	9p13.3	2 (1.74%)	0 (0.00%)	>10	0.101	0.593	Co-occurrence
HINT3	6q22.32	2 (1.74%)	0 (0.00%)	>10	0.101	0.593	Co-occurrence
HIP1R	12q24.31	2 (1.74%)	0 (0.00%)	>10	0.101	0.593	Co-occurrence
HMGA2	12q14.3	2 (1.74%)	0 (0.00%)	>10	0.101	0.593	Co-occurrence
HMGB1	13q12.3	2 (1.74%)	0 (0.00%)	>10	0.101	0.593	Co-occurrence
HMGN2P46	15q21.1	2 (1.74%)	0 (0.00%)	>10	0.101	0.593	Co-occurrence
HMGXB4	22q12.3	2 (1.74%)	0 (0.00%)	>10	0.101	0.593	Co-occurrence
HMOX1	22q12.3	2 (1.74%)	0 (0.00%)	>10	0.101	0.593	Co-occurrence
HNRNPA1P30	13q12.11	2 (1.74%)	0 (0.00%)	>10	0.101	0.593	Co-occurrence
HRCT1	9p13.3	2 (1.74%)	0 (0.00%)	>10	0.101	0.593	Co-occurrence
HS2ST1	1p22.3	2 (1.74%)	0 (0.00%)	>10	0.101	0.593	Co-occurrence
HSPH1	13q12.3	2 (1.74%)	0 (0.00%)	>10	0.101	0.593	Co-occurrence
IFT20	17q11.2	2 (1.74%)	0 (0.00%)	>10	0.101	0.593	Co-occurrence
IFT27	22q12.3	2 (1.74%)	0 (0.00%)	>10	0.101	0.593	Co-occurrence
IFT88	13q12.11	2 (1.74%)	0 (0.00%)	>10	0.101	0.593	Co-occurrence
IGF1	12q23.2	2 (1.74%)	0 (0.00%)	>10	0.101	0.593	Co-occurrence
IGSF22	11p15.1	2 (1.74%)	0 (0.00%)	>10	0.101	0.593	Co-occurrence
IKBIP	12q23.1	2 (1.74%)	0 (0.00%)	>10	0.101	0.593	Co-occurrence
IL17D	13q12.11	2 (1.74%)	0 (0.00%)	>10	0.101	0.593	Co-occurrence
INPP5J	22q12.2	2 (1.74%)	0 (0.00%)	>10	0.101	0.593	Co-occurrence
IPO7	11p15.4	2 (1.74%)	0 (0.00%)	>10	0.101	0.593	Co-occurrence
IRAK3	12q14.3	2 (1.74%)	0 (0.00%)	>10	0.101	0.593	Co-occurrence

ISX	22q12.3	2 (1.74%)	0 (0.00%)	>10	0.101	0.593	Co-occurrence
ITGA7	12q13.2	2 (1.74%)	0 (0.00%)	>10	0.101	0.593	Co-occurrence
ITGB3BP	1p31.3	2 (1.74%)	0 (0.00%)	>10	0.101	0.593	Co-occurrence
ITIH6	Xp11.22	2 (1.74%)	0 (0.00%)	>10	0.101	0.593	Co-occurrence
IWS1	2q14.3	2 (1.74%)	0 (0.00%)	>10	0.101	0.593	Co-occurrence
KANK4	1p31.3	2 (1.74%)	0 (0.00%)	>10	0.101	0.593	Co-occurrence
KAT5	11q13.1	2 (1.74%)	0 (0.00%)	>10	0.101	0.593	Co-occurrence
KATNAL1	13q12.3	2 (1.74%)	0 (0.00%)	>10	0.101	0.593	Co-occurrence
KCNC2	12q21.1	2 (1.74%)	0 (0.00%)	>10	0.101	0.593	Co-occurrence
KCNK7	11q13.1	2 (1.74%)	0 (0.00%)	>10	0.101	0.593	Co-occurrence
KERA	12q21.33	2 (1.74%)	0 (0.00%)	>10	0.101	0.593	Co-occurrence
KIAA0930	22q13.31	2 (1.74%)	0 (0.00%)	>10	0.101	0.593	Co-occurrence
KIF2C	1p34.1	2 (1.74%)	0 (0.00%)	>10	0.101	0.593	Co-occurrence
KLHL18	3p21.31	2 (1.74%)	0 (0.00%)	>10	0.101	0.593	Co-occurrence
KNTC1	12q24.31	2 (1.74%)	0 (0.00%)	>10	0.101	0.593	Co-occurrence
KRR1	12q21.2	2 (1.74%)	0 (0.00%)	>10	0.101	0.593	Co-occurrence
KRT19P2	12q22	2 (1.74%)	0 (0.00%)	>10	0.101	0.593	Co-occurrence
KSR1	17q11.2	2 (1.74%)	0 (0.00%)	>10	0.101	0.593	Co-occurrence
L1TD1	1p31.3	2 (1.74%)	0 (0.00%)	>10	0.101	0.593	Co-occurrence
LATS2	13q12.11	2 (1.74%)	0 (0.00%)	>10	0.101	0.593	Co-occurrence
LDHAL6CP	12q14.2	2 (1.74%)	0 (0.00%)	>10	0.101	0.593	Co-occurrence
LDLRAD1	1p32.3	2 (1.74%)	0 (0.00%)	>10	0.101	0.593	Co-occurrence
LEMD3	12q14.3	2 (1.74%)	0 (0.00%)	>10	0.101	0.593	Co-occurrence
LETMD1	12q13.12	2 (1.74%)	0 (0.00%)	>10	0.101	0.593	Co-occurrence
LGALS9	17q11.2	2 (1.74%)	0 (0.00%)	>10	0.101	0.593	Co-occurrence
LGR5	12q21.1	2 (1.74%)	0 (0.00%)	>10	0.101	0.593	Co-occurrence
LHFPL1	Xq23	2 (1.74%)	0 (0.00%)	>10	0.101	0.593	Co-occurrence
LIMK2	22q12.2	2 (1.74%)	0 (0.00%)	>10	0.101	0.593	Co-occurrence
LIN7A	12q21.31	2 (1.74%)	0 (0.00%)	>10	0.101	0.593	Co-occurrence

LINC00207	22q13.31	2 (1.74%)	0 (0.00%)	>10	0.101	0.593	Co-occurrence
LINC00229	22q13.31	2 (1.74%)	0 (0.00%)	>10	0.101	0.593	Co-occurrence
LINC00297	13q12.3	2 (1.74%)	0 (0.00%)	>10	0.101	0.593	Co-occurrence
LINC00365	13q12.3	2 (1.74%)	0 (0.00%)	>10	0.101	0.593	Co-occurrence
LINC00378	13q21.2	2 (1.74%)	0 (0.00%)	>10	0.101	0.593	Co-occurrence
LINC00398	13q12.3	2 (1.74%)	0 (0.00%)	>10	0.101	0.593	Co-occurrence
LINC00426	13q12.3	2 (1.74%)	0 (0.00%)	>10	0.101	0.593	Co-occurrence
LINC00427	-	2 (1.74%)	0 (0.00%)	>10	0.101	0.593	Co-occurrence
LINC00434	13q21.2	2 (1.74%)	0 (0.00%)	>10	0.101	0.593	Co-occurrence
LINC00485	12q23.2	2 (1.74%)	0 (0.00%)	>10	0.101	0.593	Co-occurrence
LINC00543	13q12.2	2 (1.74%)	0 (0.00%)	>10	0.101	0.593	Co-occurrence
LINC00544	13q12.3	2 (1.74%)	0 (0.00%)	>10	0.101	0.593	Co-occurrence
LINC00545	13q12.3	2 (1.74%)	0 (0.00%)	>10	0.101	0.593	Co-occurrence
LINC00572	13q12.3	2 (1.74%)	0 (0.00%)	>10	0.101	0.593	Co-occurrence
LINC00615	12q21.33	2 (1.74%)	0 (0.00%)	>10	0.101	0.593	Co-occurrence
LINC00963	9q34.11	2 (1.74%)	0 (0.00%)	>10	0.101	0.593	Co-occurrence
LINC01465	12q14.1	2 (1.74%)	0 (0.00%)	>10	0.101	0.593	Co-occurrence
LINC01619	12q21.33	2 (1.74%)	0 (0.00%)	>10	0.101	0.593	Co-occurrence
LLPH	12q14.3	2 (1.74%)	0 (0.00%)	>10	0.101	0.593	Co-occurrence
LMO4	1p22.3	2 (1.74%)	0 (0.00%)	>10	0.101	0.593	Co-occurrence
LNX2	13q12.2	2 (1.74%)	0 (0.00%)	>10	0.101	0.593	Co-occurrence
LPAR3	1p22.3	2 (1.74%)	0 (0.00%)	>10	0.101	0.593	Co-occurrence
LRRC2	3p21.31	2 (1.74%)	0 (0.00%)	>10	0.101	0.593	Co-occurrence
LRRC37BP1	17q11.2	2 (1.74%)	0 (0.00%)	>10	0.101	0.593	Co-occurrence
LRRIQ1	12q21.31	2 (1.74%)	0 (0.00%)	>10	0.101	0.593	Co-occurrence
LTBP3	11q13.1	2 (1.74%)	0 (0.00%)	>10	0.101	0.593	Co-occurrence
LUM	12q21.33	2 (1.74%)	0 (0.00%)	>10	0.101	0.593	Co-occurrence
LUZP2	11p14.3	2 (1.74%)	0 (0.00%)	>10	0.101	0.593	Co-occurrence
LYRM9	17q11.2	2 (1.74%)	0 (0.00%)	>10	0.101	0.593	Co-occurrence

MAGED2	Xp11.21	2 (1.74%)	0 (0.00%)	>10	0.101	0.593	Co-occurrence
MAGEH1	Xp11.21	2 (1.74%)	0 (0.00%)	>10	0.101	0.593	Co-occurrence
MAJIN	11q13.1	2 (1.74%)	0 (0.00%)	>10	0.101	0.593	Co-occurrence
MALAT1	11q13.1	2 (1.74%)	0 (0.00%)	>10	0.101	0.593	Co-occurrence
MAN1A2	1p12	2 (1.74%)	0 (0.00%)	>10	0.101	0.593	Co-occurrence
MAP3K11	11q13.1	2 (1.74%)	0 (0.00%)	>10	0.101	0.593	Co-occurrence
MAP3K2	2q14.3	2 (1.74%)	0 (0.00%)	>10	0.101	0.593	Co-occurrence
MAP7D2	Xp22.12	2 (1.74%)	0 (0.00%)	>10	0.101	0.593	Co-occurrence
MAST2	1p34.1	2 (1.74%)	0 (0.00%)	>10	0.101	0.593	Co-occurrence
MB	22q12.3	2 (1.74%)	0 (0.00%)	>10	0.101	0.593	Co-occurrence
MCAT	22q13.2	2 (1.74%)	0 (0.00%)	>10	0.101	0.593	Co-occurrence
MCM5	22q12.3	2 (1.74%)	0 (0.00%)	>10	0.101	0.593	Co-occurrence
MCOLN2	1p22.3	2 (1.74%)	0 (0.00%)	>10	0.101	0.593	Co-occurrence
MCOLN3	1p22.3	2 (1.74%)	0 (0.00%)	>10	0.101	0.593	Co-occurrence
MED22	9q34.2	2 (1.74%)	0 (0.00%)	>10	0.101	0.593	Co-occurrence
MED27	9q34.13	2 (1.74%)	0 (0.00%)	>10	0.101	0.593	Co-occurrence
MEDAG	13q12.3	2 (1.74%)	0 (0.00%)	>10	0.101	0.593	Co-occurrence
MEP1B	18q12.1	2 (1.74%)	0 (0.00%)	>10	0.101	0.593	Co-occurrence
METAP2	12q22	2 (1.74%)	0 (0.00%)	>10	0.101	0.593	Co-occurrence
METTL25	12q21.31	2 (1.74%)	0 (0.00%)	>10	0.101	0.593	Co-occurrence
METTL7A	12q13.12	2 (1.74%)	0 (0.00%)	>10	0.101	0.593	Co-occurrence
METTL7B	12q13.2	2 (1.74%)	0 (0.00%)	>10	0.101	0.593	Co-occurrence
MGAT3	22q13.1	2 (1.74%)	0 (0.00%)	>10	0.101	0.593	Co-occurrence
MGAT4C	12q21.31-q21.32	2 (1.74%)	0 (0.00%)	>10	0.101	0.593	Co-occurrence
MICU1	10q22.1	2 (1.74%)	0 (0.00%)	>10	0.101	0.593	Co-occurrence
MIEF1	22q13.1	2 (1.74%)	0 (0.00%)	>10	0.101	0.593	Co-occurrence
MIR-1227/1227		2 (1.74%)	0 (0.00%)	>10	0.101	0.593	Co-occurrence
MIR-1262/1262		2 (1.74%)	0 (0.00%)	>10	0.101	0.593	Co-occurrence
MIR-1281/1281		2 (1.74%)	0 (0.00%)	>10	0.101	0.593	Co-occurrence

MIR-23C/23C		2 (1.74%)	0 (0.00%)	>10	0.101	0.593	Co-occurrence
MIR-3169/3169		2 (1.74%)	0 (0.00%)	>10	0.101	0.593	Co-occurrence
MIR-3612/3612		2 (1.74%)	0 (0.00%)	>10	0.101	0.593	Co-occurrence
MIR-3909/3909		2 (1.74%)	0 (0.00%)	>10	0.101	0.593	Co-occurrence
MIR-4252/4252		2 (1.74%)	0 (0.00%)	>10	0.101	0.593	Co-occurrence
MIR-4489/4489		2 (1.74%)	0 (0.00%)	>10	0.101	0.593	Co-occurrence
MIR-4522/4522		2 (1.74%)	0 (0.00%)	>10	0.101	0.593	Co-occurrence
MIR-4535/4535		2 (1.74%)	0 (0.00%)	>10	0.101	0.593	Co-occurrence
MIR-492/492		2 (1.74%)	0 (0.00%)	>10	0.101	0.593	Co-occurrence
MIR-548AW/548AW		2 (1.74%)	0 (0.00%)	>10	0.101	0.593	Co-occurrence
MIR-5700/5700		2 (1.74%)	0 (0.00%)	>10	0.101	0.593	Co-occurrence
MIR-617/617		2 (1.74%)	0 (0.00%)	>10	0.101	0.593	Co-occurrence
MIR-618/618		2 (1.74%)	0 (0.00%)	>10	0.101	0.593	Co-occurrence
MIR-620/620		2 (1.74%)	0 (0.00%)	>10	0.101	0.593	Co-occurrence
MIR-711/711		2 (1.74%)	0 (0.00%)	>10	0.101	0.593	Co-occurrence
MISP	19p13.3	2 (1.74%)	0 (0.00%)	>10	0.101	0.593	Co-occurrence
MON1A	3p21.31	2 (1.74%)	0 (0.00%)	>10	0.101	0.593	Co-occurrence
MON2	12q14.1	2 (1.74%)	0 (0.00%)	>10	0.101	0.593	Co-occurrence
MORN3	12q24.31	2 (1.74%)	0 (0.00%)	>10	0.101	0.593	Co-occurrence
MOSPD2	Xp22.2	2 (1.74%)	0 (0.00%)	>10	0.101	0.593	Co-occurrence
MPPED1	22q13.2	2 (1.74%)	0 (0.00%)	>10	0.101	0.593	Co-occurrence
MPST	22q12.3	2 (1.74%)	0 (0.00%)	>10	0.101	0.593	Co-occurrence
MRGPRX1	11p15.1|11	2 (1.74%)	0 (0.00%)	>10	0.101	0.593	Co-occurrence
MRGPRX2	11p15.1	2 (1.74%)	0 (0.00%)	>10	0.101	0.593	Co-occurrence
MRPL42	12q22	2 (1.74%)	0 (0.00%)	>10	0.101	0.593	Co-occurrence
MRPL49	11q13.1	2 (1.74%)	0 (0.00%)	>10	0.101	0.593	Co-occurrence
MSMP	9p13.3	2 (1.74%)	0 (0.00%)	>10	0.101	0.593	Co-occurrence
MSRB3	12q14.3	2 (1.74%)	0 (0.00%)	>10	0.101	0.593	Co-occurrence
MST1R	3p21.31	2 (1.74%)	0 (0.00%)	>10	0.101	0.593	Co-occurrence

MTERF2	12q23.3	2 (1.74%)	0 (0.00%)	>10	0.101	0.593	Co-occurrence
MTIF3	13q12.2	2 (1.74%)	0 (0.00%)	>10	0.101	0.593	Co-occurrence
MTUS2	13q12.3	2 (1.74%)	0 (0.00%)	>10	0.101	0.593	Co-occurrence
MUS81	11q13.1	2 (1.74%)	0 (0.00%)	>10	0.101	0.593	Co-occurrence
MYBPC1	12q23.2	2 (1.74%)	0 (0.00%)	>10	0.101	0.593	Co-occurrence
MYD88	3p22.2	2 (1.74%)	0 (0.00%)	>10	0.101	0.593	Co-occurrence
MYF5	12q21.31	2 (1.74%)	0 (0.00%)	>10	0.101	0.593	Co-occurrence
MYF6	12q21.31	2 (1.74%)	0 (0.00%)	>10	0.101	0.593	Co-occurrence
MYH9	22q12.3	2 (1.74%)	0 (0.00%)	>10	0.101	0.593	Co-occurrence
MYMK	9q34.2	2 (1.74%)	0 (0.00%)	>10	0.101	0.593	Co-occurrence
NAA50	3q13.31	2 (1.74%)	0 (0.00%)	>10	0.101	0.593	Co-occurrence
NAALADL1	11q13.1	2 (1.74%)	0 (0.00%)	>10	0.101	0.593	Co-occurrence
NAP1L1	12q21.2	2 (1.74%)	0 (0.00%)	>10	0.101	0.593	Co-occurrence
NAV3	12q21.2	2 (1.74%)	0 (0.00%)	>10	0.101	0.593	Co-occurrence
NBEAL2	3p21.31	2 (1.74%)	0 (0.00%)	>10	0.101	0.593	Co-occurrence
NCF4	22q12.3	2 (1.74%)	0 (0.00%)	>10	0.101	0.593	Co-occurrence
NCS1	9q34.11	2 (1.74%)	0 (0.00%)	>10	0.101	0.593	Co-occurrence
NDUFA12	12q22	2 (1.74%)	0 (0.00%)	>10	0.101	0.593	Co-occurrence
NEAT1	11q13.1	2 (1.74%)	0 (0.00%)	>10	0.101	0.593	Co-occurrence
NEXN	1p31.1	2 (1.74%)	0 (0.00%)	>10	0.101	0.593	Co-occurrence
NF2	22q12.2	2 (1.74%)	0 (0.00%)	>10	0.101	0.593	Co-occurrence
NIPSNAP1	22q12.2	2 (1.74%)	0 (0.00%)	>10	0.101	0.593	Co-occurrence
NLK	17q11.2	2 (1.74%)	0 (0.00%)	>10	0.101	0.593	Co-occurrence
NPHS1	19q13.12	2 (1.74%)	0 (0.00%)	>10	0.101	0.593	Co-occurrence
NPR2	9p13.3	2 (1.74%)	0 (0.00%)	>10	0.101	0.593	Co-occurrence
NR2C1	12q22	2 (1.74%)	0 (0.00%)	>10	0.101	0.593	Co-occurrence
NSUN4	1p33	2 (1.74%)	0 (0.00%)	>10	0.101	0.593	Co-occurrence
NTMT1	9q34.11	2 (1.74%)	0 (0.00%)	>10	0.101	0.593	Co-occurrence
NTN4	12q22	2 (1.74%)	0 (0.00%)	>10	0.101	0.593	Co-occurrence

NTNG2	9q34.13	2 (1.74%)	0 (0.00%)	>10	0.101	0.593	Co-occurrence
NTS	12q21.31	2 (1.74%)	0 (0.00%)	>10	0.101	0.593	Co-occurrence
NUDT4	12q22	2 (1.74%)	0 (0.00%)	>10	0.101	0.593	Co-occurrence
NUP214	9q34.13	2 (1.74%)	0 (0.00%)	>10	0.101	0.593	Co-occurrence
NUP37	12q23.2	2 (1.74%)	0 (0.00%)	>10	0.101	0.593	Co-occurrence
NUP50	22q13.31	2 (1.74%)	0 (0.00%)	>10	0.101	0.593	Co-occurrence
OBP2B	9q34.2	2 (1.74%)	0 (0.00%)	>10	0.101	0.593	Co-occurrence
OFD1	Xp22.2	2 (1.74%)	0 (0.00%)	>10	0.101	0.593	Co-occurrence
OIT3	10q22.1	2 (1.74%)	0 (0.00%)	>10	0.101	0.593	Co-occurrence
OR10P1	12q13.2	2 (1.74%)	0 (0.00%)	>10	0.101	0.593	Co-occurrence
OR13J1	9p13.3	2 (1.74%)	0 (0.00%)	>10	0.101	0.593	Co-occurrence
OR2AP1	12q13.2	2 (1.74%)	0 (0.00%)	>10	0.101	0.593	Co-occurrence
OR2S2	9p13.3	2 (1.74%)	0 (0.00%)	>10	0.101	0.593	Co-occurrence
OR6C2	12q13.2	2 (1.74%)	0 (0.00%)	>10	0.101	0.593	Co-occurrence
OR6C4	12q13.2	2 (1.74%)	0 (0.00%)	>10	0.101	0.593	Co-occurrence
OR6C6	12q13.2	2 (1.74%)	0 (0.00%)	>10	0.101	0.593	Co-occurrence
OR6C68	12q13.2	2 (1.74%)	0 (0.00%)	>10	0.101	0.593	Co-occurrence
OR6C70	12q13.2	2 (1.74%)	0 (0.00%)	>10	0.101	0.593	Co-occurrence
OR6C76	12q13.2	2 (1.74%)	0 (0.00%)	>10	0.101	0.593	Co-occurrence
ORAI1	12q24.31	2 (1.74%)	0 (0.00%)	>10	0.101	0.593	Co-occurrence
OSBPL10	3p23	2 (1.74%)	0 (0.00%)	>10	0.101	0.593	Co-occurrence
OSBPL8	12q21.2	2 (1.74%)	0 (0.00%)	>10	0.101	0.593	Co-occurrence
OSBPL9	1p32.3	2 (1.74%)	0 (0.00%)	>10	0.101	0.593	Co-occurrence
OTOGL	12q21.31	2 (1.74%)	0 (0.00%)	>10	0.101	0.593	Co-occurrence
OVOL1	11q13.1	2 (1.74%)	0 (0.00%)	>10	0.101	0.593	Co-occurrence
OXSR1	3p22.2	2 (1.74%)	0 (0.00%)	>10	0.101	0.593	Co-occurrence
PABPC4	1p34.3	2 (1.74%)	0 (0.00%)	>10	0.101	0.593	Co-occurrence
PACSIN2	22q13.2	2 (1.74%)	0 (0.00%)	>10	0.101	0.593	Co-occurrence
PALM2	9q31.3	2 (1.74%)	0 (0.00%)	>10	0.101	0.593	Co-occurrence

PAN3	13q12.2	2 (1.74%)	0 (0.00%)	>10	0.101	0.593	Co-occurrence
PARPBP	12q23.2	2 (1.74%)	0 (0.00%)	>10	0.101	0.593	Co-occurrence
PARVB	22q13.31	2 (1.74%)	0 (0.00%)	>10	0.101	0.593	Co-occurrence
PARVG	22q13.31	2 (1.74%)	0 (0.00%)	>10	0.101	0.593	Co-occurrence
PATJ	1p31.3	2 (1.74%)	0 (0.00%)	>10	0.101	0.593	Co-occurrence
PAWR	12q21.2	2 (1.74%)	0 (0.00%)	>10	0.101	0.593	Co-occurrence
PCDH20	13q21.2	2 (1.74%)	0 (0.00%)	>10	0.101	0.593	Co-occurrence
PCNX3	11q13.1	2 (1.74%)	0 (0.00%)	>10	0.101	0.593	Co-occurrence
PDCD2L	19q13.11	2 (1.74%)	0 (0.00%)	>10	0.101	0.593	Co-occurrence
PDX1	13q12.2	2 (1.74%)	0 (0.00%)	>10	0.101	0.593	Co-occurrence
PEBP1	12q24.23	2 (1.74%)	0 (0.00%)	>10	0.101	0.593	Co-occurrence
PF4V1	4q13.3	2 (1.74%)	0 (0.00%)	>10	0.101	0.593	Co-occurrence
PFKFB1	Xp11.21	2 (1.74%)	0 (0.00%)	>10	0.101	0.593	Co-occurrence
PFKFB4	3p21.31	2 (1.74%)	0 (0.00%)	>10	0.101	0.593	Co-occurrence
PGAM1P5	12q22	2 (1.74%)	0 (0.00%)	>10	0.101	0.593	Co-occurrence
PGM1	1p31.3	2 (1.74%)	0 (0.00%)	>10	0.101	0.593	Co-occurrence
PHEX	Xp22.11	2 (1.74%)	0 (0.00%)	>10	0.101	0.593	Co-occurrence
PHF21B	22q13.31	2 (1.74%)	0 (0.00%)	>10	0.101	0.593	Co-occurrence
PHLDA1	12q21.2	2 (1.74%)	0 (0.00%)	>10	0.101	0.593	Co-occurrence
PIGK	1p31.1	2 (1.74%)	0 (0.00%)	>10	0.101	0.593	Co-occurrence
PIK3R1	5q13.1	2 (1.74%)	0 (0.00%)	>10	0.101	0.593	Co-occurrence
PIK3R3	1p34.1	2 (1.74%)	0 (0.00%)	>10	0.101	0.593	Co-occurrence
PISD	22q12.2	2 (1.74%)	0 (0.00%)	>10	0.101	0.593	Co-occurrence
PLA2G12B	10q22.1	2 (1.74%)	0 (0.00%)	>10	0.101	0.593	Co-occurrence
PLA2G3	22q12.2	2 (1.74%)	0 (0.00%)	>10	0.101	0.593	Co-occurrence
PLCD1	3p22.2	2 (1.74%)	0 (0.00%)	>10	0.101	0.593	Co-occurrence
PLEKHA8P1	12q12	2 (1.74%)	0 (0.00%)	>10	0.101	0.593	Co-occurrence
PLEKHG7	12q22	2 (1.74%)	0 (0.00%)	>10	0.101	0.593	Co-occurrence
PLEKHJ1	19p13.3	2 (1.74%)	0 (0.00%)	>10	0.101	0.593	Co-occurrence

PLPP7	9q34.13	2 (1.74%)	0 (0.00%)	>10	0.101	0.593	Co-occurrence
PLPPR3	19p13.3	2 (1.74%)	0 (0.00%)	>10	0.101	0.593	Co-occurrence
PLXNB1	3p21.31	2 (1.74%)	0 (0.00%)	>10	0.101	0.593	Co-occurrence
PLXNC1	12q22	2 (1.74%)	0 (0.00%)	>10	0.101	0.593	Co-occurrence
PMCH	12q23.2	2 (1.74%)	0 (0.00%)	>10	0.101	0.593	Co-occurrence
POC1B	12q21.33	2 (1.74%)	0 (0.00%)	>10	0.101	0.593	Co-occurrence
POLA2	11q13.1	2 (1.74%)	0 (0.00%)	>10	0.101	0.593	Co-occurrence
POLDIP2	17q11.2	2 (1.74%)	0 (0.00%)	>10	0.101	0.593	Co-occurrence
POLDIP3	22q13.2	2 (1.74%)	0 (0.00%)	>10	0.101	0.593	Co-occurrence
POLR1D	13q12.2	2 (1.74%)	0 (0.00%)	>10	0.101	0.593	Co-occurrence
POLR3B	12q23.3	2 (1.74%)	0 (0.00%)	>10	0.101	0.593	Co-occurrence
POMGNT1	1p34.1	2 (1.74%)	0 (0.00%)	>10	0.101	0.593	Co-occurrence
POMP	13q12.3	2 (1.74%)	0 (0.00%)	>10	0.101	0.593	Co-occurrence
POMT1	9q34.13	2 (1.74%)	0 (0.00%)	>10	0.101	0.593	Co-occurrence
POU6F1	12q13.13	2 (1.74%)	0 (0.00%)	>10	0.101	0.593	Co-occurrence
PPFIA2	12q21.31	2 (1.74%)	0 (0.00%)	>10	0.101	0.593	Co-occurrence
PPM1H	12q14.1-q14.2	2 (1.74%)	0 (0.00%)	>10	0.101	0.593	Co-occurrence
PPP1R12A	12q21.2-q21.31	2 (1.74%)	0 (0.00%)	>10	0.101	0.593	Co-occurrence
PPP2R5B	11q13.1	2 (1.74%)	0 (0.00%)	>10	0.101	0.593	Co-occurrence
PPP6C	9q33.3	2 (1.74%)	0 (0.00%)	>10	0.101	0.593	Co-occurrence
PPY2P	17q11.2	2 (1.74%)	0 (0.00%)	>10	0.101	0.593	Co-occurrence
PRMT6	1p13.3	2 (1.74%)	0 (0.00%)	>10	0.101	0.593	Co-occurrence
PROC	2q14.3	2 (1.74%)	0 (0.00%)	>10	0.101	0.593	Co-occurrence
PRPF4	9q32	2 (1.74%)	0 (0.00%)	>10	0.101	0.593	Co-occurrence
PRR5	22q13.31	2 (1.74%)	0 (0.00%)	>10	0.101	0.593	Co-occurrence
PRRX2	9q34.11	2 (1.74%)	0 (0.00%)	>10	0.101	0.593	Co-occurrence
PRSS42P	3p21.31	2 (1.74%)	0 (0.00%)	>10	0.101	0.593	Co-occurrence
PRSS44P	3p21.31	2 (1.74%)	0 (0.00%)	>10	0.101	0.593	Co-occurrence
PRSS45P	3p21.31	2 (1.74%)	0 (0.00%)	>10	0.101	0.593	Co-occurrence

PRSS46P	3p21.31	2 (1.74%)	0 (0.00%)	>10	0.101	0.593	Co-occurrence
PRSS50	3p21.31	2 (1.74%)	0 (0.00%)	>10	0.101	0.593	Co-occurrence
PSMD5	9q33.2	2 (1.74%)	0 (0.00%)	>10	0.101	0.593	Co-occurrence
PTBP1	19p13.3	2 (1.74%)	0 (0.00%)	>10	0.101	0.593	Co-occurrence
PTPN23	3p21.31	2 (1.74%)	0 (0.00%)	>10	0.101	0.593	Co-occurrence
PTPN5	11p15.1	2 (1.74%)	0 (0.00%)	>10	0.101	0.593	Co-occurrence
PTPRB	12q15	2 (1.74%)	0 (0.00%)	>10	0.101	0.593	Co-occurrence
PTPRQ	12q21.31	2 (1.74%)	0 (0.00%)	>10	0.101	0.593	Co-occurrence
PTPRR	12q15	2 (1.74%)	0 (0.00%)	>10	0.101	0.593	Co-occurrence
PVALB	22q12.3	2 (1.74%)	0 (0.00%)	>10	0.101	0.593	Co-occurrence
PYY2	17q11.2	2 (1.74%)	0 (0.00%)	>10	0.101	0.593	Co-occurrence
RABEPK	9q33.3	2 (1.74%)	0 (0.00%)	>10	0.101	0.593	Co-occurrence
RALGDS	9q34.13-q34.2	2 (1.74%)	0 (0.00%)	>10	0.101	0.593	Co-occurrence
RAPGEF1	9q34.13	2 (1.74%)	0 (0.00%)	>10	0.101	0.593	Co-occurrence
RASD2	22q12.3	2 (1.74%)	0 (0.00%)	>10	0.101	0.593	Co-occurrence
RASGEF1B	4q21.21	2 (1.74%)	0 (0.00%)	>10	0.101	0.593	Co-occurrence
RASL11A	13q12.2	2 (1.74%)	0 (0.00%)	>10	0.101	0.593	Co-occurrence
RASSF3	12q14.2	2 (1.74%)	0 (0.00%)	>10	0.101	0.593	Co-occurrence
RASSF6	4q13.3	2 (1.74%)	0 (0.00%)	>10	0.101	0.593	Co-occurrence
RASSF9	12q21.31	2 (1.74%)	0 (0.00%)	>10	0.101	0.593	Co-occurrence
RBX1	22q13.2	2 (1.74%)	0 (0.00%)	>10	0.101	0.593	Co-occurrence
RDH5	12q13.2	2 (1.74%)	0 (0.00%)	>10	0.101	0.593	Co-occurrence
RECK	9p13.3	2 (1.74%)	0 (0.00%)	>10	0.101	0.593	Co-occurrence
RELA	11q13.1	2 (1.74%)	0 (0.00%)	>10	0.101	0.593	Co-occurrence
REXO4	9q34.2	2 (1.74%)	0 (0.00%)	>10	0.101	0.593	Co-occurrence
RFC5	12q24.23	2 (1.74%)	0 (0.00%)	>10	0.101	0.593	Co-occurrence
RFT1	3p21.1	2 (1.74%)	0 (0.00%)	>10	0.101	0.593	Co-occurrence
RFX4	12q23.3	2 (1.74%)	0 (0.00%)	>10	0.101	0.593	Co-occurrence
RGP1	9p13.3	2 (1.74%)	0 (0.00%)	>10	0.101	0.593	Co-occurrence

RIBC2	22q13.31	2 (1.74%)	0 (0.00%)	>10	0.101	0.593	Co-occurrence
RIC8B	12q23.3	2 (1.74%)	0 (0.00%)	>10	0.101	0.593	Co-occurrence
RLF	1p34.2	2 (1.74%)	0 (0.00%)	>10	0.101	0.593	Co-occurrence
RN7SKP11	12q23.1	2 (1.74%)	0 (0.00%)	>10	0.101	0.593	Co-occurrence
RN7SKP166	12q14.3	2 (1.74%)	0 (0.00%)	>10	0.101	0.593	Co-occurrence
RN7SKP172	12q21.2	2 (1.74%)	0 (0.00%)	>10	0.101	0.593	Co-occurrence
RN7SKP183	Xp22.12	2 (1.74%)	0 (0.00%)	>10	0.101	0.593	Co-occurrence
RN7SKP210	22q13.1	2 (1.74%)	0 (0.00%)	>10	0.101	0.593	Co-occurrence
RN7SKP261	12q21.31	2 (1.74%)	0 (0.00%)	>10	0.101	0.593	Co-occurrence
RN7SKP6	13q21.1	2 (1.74%)	0 (0.00%)	>10	0.101	0.593	Co-occurrence
RN7SKP80	22q13.2	2 (1.74%)	0 (0.00%)	>10	0.101	0.593	Co-occurrence
RN7SL130P	1p31.3	2 (1.74%)	0 (0.00%)	>10	0.101	0.593	Co-occurrence
RN7SL145P	3p21.31	2 (1.74%)	0 (0.00%)	>10	0.101	0.593	Co-occurrence
RN7SL154P	19q13.11	2 (1.74%)	0 (0.00%)	>10	0.101	0.593	Co-occurrence
RN7SL159P	9q34.11	2 (1.74%)	0 (0.00%)	>10	0.101	0.593	Co-occurrence
RN7SL180P	1p31.3	2 (1.74%)	0 (0.00%)	>10	0.101	0.593	Co-occurrence
RN7SL217P	3p21.31	2 (1.74%)	0 (0.00%)	>10	0.101	0.593	Co-occurrence
RN7SL272P	13q12.2	2 (1.74%)	0 (0.00%)	>10	0.101	0.593	Co-occurrence
RN7SL309P	11q13.1	2 (1.74%)	0 (0.00%)	>10	0.101	0.593	Co-occurrence
RN7SL316P	17q11.2	2 (1.74%)	0 (0.00%)	>10	0.101	0.593	Co-occurrence
RN7SL328P	9q34.13	2 (1.74%)	0 (0.00%)	>10	0.101	0.593	Co-occurrence
RN7SL330P	12q22	2 (1.74%)	0 (0.00%)	>10	0.101	0.593	Co-occurrence
RN7SL356P	14q24.3	2 (1.74%)	0 (0.00%)	>10	0.101	0.593	Co-occurrence
RN7SL370P	1p31.1	2 (1.74%)	0 (0.00%)	>10	0.101	0.593	Co-occurrence
RN7SL451P	1p36.23	2 (1.74%)	0 (0.00%)	>10	0.101	0.593	Co-occurrence
RN7SL483P	12q22	2 (1.74%)	0 (0.00%)	>10	0.101	0.593	Co-occurrence
RN7SL488P	1p31.3	2 (1.74%)	0 (0.00%)	>10	0.101	0.593	Co-occurrence
RN7SL519P	12q13.12	2 (1.74%)	0 (0.00%)	>10	0.101	0.593	Co-occurrence
RN7SL618P	13q14.3	2 (1.74%)	0 (0.00%)	>10	0.101	0.593	Co-occurrence

RN7SL630P	12q22	2 (1.74%)	0 (0.00%)	>10	0.101	0.593	Co-occurrence
RN7SL734P	12q21.2	2 (1.74%)	0 (0.00%)	>10	0.101	0.593	Co-occurrence
RN7SL737P	12q22	2 (1.74%)	0 (0.00%)	>10	0.101	0.593	Co-occurrence
RN7SL767P	3q13.2	2 (1.74%)	0 (0.00%)	>10	0.101	0.593	Co-occurrence
RN7SL793P	12q23.2	2 (1.74%)	0 (0.00%)	>10	0.101	0.593	Co-occurrence
RN7SL88P	12q23.1	2 (1.74%)	0 (0.00%)	>10	0.101	0.593	Co-occurrence
RNA5SP127	3p23	2 (1.74%)	0 (0.00%)	>10	0.101	0.593	Co-occurrence
RNA5SP129	3p22.2	2 (1.74%)	0 (0.00%)	>10	0.101	0.593	Co-occurrence
RNA5SP20	1p31.1	2 (1.74%)	0 (0.00%)	>10	0.101	0.593	Co-occurrence
RNA5SP22	1p31.1	2 (1.74%)	0 (0.00%)	>10	0.101	0.593	Co-occurrence
RNA5SP30	13q21.1	2 (1.74%)	0 (0.00%)	>10	0.101	0.593	Co-occurrence
RNA5SP31	13q21.2	2 (1.74%)	0 (0.00%)	>10	0.101	0.593	Co-occurrence
RNA5SP361	12q12	2 (1.74%)	0 (0.00%)	>10	0.101	0.593	Co-occurrence
RNA5SP362	12q14.3	2 (1.74%)	0 (0.00%)	>10	0.101	0.593	Co-occurrence
RNA5SP363	12q21.2	2 (1.74%)	0 (0.00%)	>10	0.101	0.593	Co-occurrence
RNA5SP365	12q21.33	2 (1.74%)	0 (0.00%)	>10	0.101	0.593	Co-occurrence
RNA5SP366	12q23.1	2 (1.74%)	0 (0.00%)	>10	0.101	0.593	Co-occurrence
RNA5SP367	12q23.2	2 (1.74%)	0 (0.00%)	>10	0.101	0.593	Co-occurrence
RNA5SP368	12q23.2	2 (1.74%)	0 (0.00%)	>10	0.101	0.593	Co-occurrence
RNA5SP369	12q23.2	2 (1.74%)	0 (0.00%)	>10	0.101	0.593	Co-occurrence
RNA5SP372	12q24.11	2 (1.74%)	0 (0.00%)	>10	0.101	0.593	Co-occurrence
RNA5SP376	12q24.33	2 (1.74%)	0 (0.00%)	>10	0.101	0.593	Co-occurrence
RNA5SP453	18q12.1	2 (1.74%)	0 (0.00%)	>10	0.101	0.593	Co-occurrence
RNA5SP52	1p22.3	2 (1.74%)	0 (0.00%)	>10	0.101	0.593	Co-occurrence
RNA5SP56	1p12	2 (1.74%)	0 (0.00%)	>10	0.101	0.593	Co-occurrence
RNASEH2C	11q13.1	2 (1.74%)	0 (0.00%)	>10	0.101	0.593	Co-occurrence
RNF125	18q12.1	2 (1.74%)	0 (0.00%)	>10	0.101	0.593	Co-occurrence
RNF138	18q12.1	2 (1.74%)	0 (0.00%)	>10	0.101	0.593	Co-occurrence
RNF183	9q32	2 (1.74%)	0 (0.00%)	>10	0.101	0.593	Co-occurrence

RNF185	22q12.2	2 (1.74%)	0 (0.00%)	>10	0.101	0.593	Co-occurrence
RNF220	1p34.1	2 (1.74%)	0 (0.00%)	>10	0.101	0.593	Co-occurrence
RNF38	9p13.2	2 (1.74%)	0 (0.00%)	>10	0.101	0.593	Co-occurrence
RNU12	22q13.2	2 (1.74%)	0 (0.00%)	>10	0.101	0.593	Co-occurrence
RNU6ATAC33P	11p15.4	2 (1.74%)	0 (0.00%)	>10	0.101	0.593	Co-occurrence
RNU6ATAC42P	12q14.3	2 (1.74%)	0 (0.00%)	>10	0.101	0.593	Co-occurrence
RNY1P1	13q12.2	2 (1.74%)	0 (0.00%)	>10	0.101	0.593	Co-occurrence
RNY3P5	13q21.2	2 (1.74%)	0 (0.00%)	>10	0.101	0.593	Co-occurrence
RNY4P28	13q21.2	2 (1.74%)	0 (0.00%)	>10	0.101	0.593	Co-occurrence
RNY4P31	13q21.2	2 (1.74%)	0 (0.00%)	>10	0.101	0.593	Co-occurrence
RNY4P7	2q14.3	2 (1.74%)	0 (0.00%)	>10	0.101	0.593	Co-occurrence
RPE65	1p31.3	2 (1.74%)	0 (0.00%)	>10	0.101	0.593	Co-occurrence
RPL21	13q12.2	2 (1.74%)	0 (0.00%)	>10	0.101	0.593	Co-occurrence
RPL7A	9q34.2	2 (1.74%)	0 (0.00%)	>10	0.101	0.593	Co-occurrence
RPS11P6	12q14.2	2 (1.74%)	0 (0.00%)	>10	0.101	0.593	Co-occurrence
RPS19BP1	22q13.1	2 (1.74%)	0 (0.00%)	>10	0.101	0.593	Co-occurrence
RPSAP52	12q14.3	2 (1.74%)	0 (0.00%)	>10	0.101	0.593	Co-occurrence
RRP7A	22q13.2	2 (1.74%)	0 (0.00%)	>10	0.101	0.593	Co-occurrence
RRP7BP	22q13.2	2 (1.74%)	0 (0.00%)	>10	0.101	0.593	Co-occurrence
RSRC2	12q24.31	2 (1.74%)	0 (0.00%)	>10	0.101	0.593	Co-occurrence
RTL6	22q13.31	2 (1.74%)	0 (0.00%)	>10	0.101	0.593	Co-occurrence
RXYLT1	12q14.2	2 (1.74%)	0 (0.00%)	>10	0.101	0.593	Co-occurrence
SAC3D1	11q13.1	2 (1.74%)	0 (0.00%)	>10	0.101	0.593	Co-occurrence
SACM1L	3p21.31	2 (1.74%)	0 (0.00%)	>10	0.101	0.593	Co-occurrence
SAMM50	22q13.31	2 (1.74%)	0 (0.00%)	>10	0.101	0.593	Co-occurrence
SARM1	17q11.2	2 (1.74%)	0 (0.00%)	>10	0.101	0.593	Co-occurrence
SCUBE1	22q13.2	2 (1.74%)	0 (0.00%)	>10	0.101	0.593	Co-occurrence
SCYL1	11q13.1	2 (1.74%)	0 (0.00%)	>10	0.101	0.593	Co-occurrence
SEBOX	17q11.2	2 (1.74%)	0 (0.00%)	>10	0.101	0.593	Co-occurrence

SERHL	22q13.2	2 (1.74%)	0 (0.00%)	>10	0.101	0.593	Co-occurrence
SERHL2	22q13.2	2 (1.74%)	0 (0.00%)	>10	0.101	0.593	Co-occurrence
SETD2	3p21.31	2 (1.74%)	0 (0.00%)	>10	0.101	0.593	Co-occurrence
SETX	9q34.13	2 (1.74%)	0 (0.00%)	>10	0.101	0.593	Co-occurrence
SFT2D3	2q14.3	2 (1.74%)	0 (0.00%)	>10	0.101	0.593	Co-occurrence
SGSM3	22q13.1	2 (1.74%)	0 (0.00%)	>10	0.101	0.593	Co-occurrence
SHISA5	3p21.31	2 (1.74%)	0 (0.00%)	>10	0.101	0.593	Co-occurrence
SHISAL1	22q13.31	2 (1.74%)	0 (0.00%)	>10	0.101	0.593	Co-occurrence
SHROOM2	Xp22.2	2 (1.74%)	0 (0.00%)	>10	0.101	0.593	Co-occurrence
SIDT1	3q13.2	2 (1.74%)	0 (0.00%)	>10	0.101	0.593	Co-occurrence
SIPA1	11q13.1	2 (1.74%)	0 (0.00%)	>10	0.101	0.593	Co-occurrence
SIT1	9p13.3	2 (1.74%)	0 (0.00%)	>10	0.101	0.593	Co-occurrence
SLC11A2	12q13.12	2 (1.74%)	0 (0.00%)	>10	0.101	0.593	Co-occurrence
SLC13A2	17q11.2	2 (1.74%)	0 (0.00%)	>10	0.101	0.593	Co-occurrence
SLC22A20P	11q13.1	2 (1.74%)	0 (0.00%)	>10	0.101	0.593	Co-occurrence
SLC25A3	12q23.1	2 (1.74%)	0 (0.00%)	>10	0.101	0.593	Co-occurrence
SLC25A45	11q13.1	2 (1.74%)	0 (0.00%)	>10	0.101	0.593	Co-occurrence
SLC26A6	3p21.31	2 (1.74%)	0 (0.00%)	>10	0.101	0.593	Co-occurrence
SLC2A5	1p36.23	2 (1.74%)	0 (0.00%)	>10	0.101	0.593	Co-occurrence
SLC2A6	9q34.2	2 (1.74%)	0 (0.00%)	>10	0.101	0.593	Co-occurrence
SLC2A7	1p36.23	2 (1.74%)	0 (0.00%)	>10	0.101	0.593	Co-occurrence
SLC30A4	15q21.1|15q21.1	2 (1.74%)	0 (0.00%)	>10	0.101	0.593	Co-occurrence
SLC31A1	9q32	2 (1.74%)	0 (0.00%)	>10	0.101	0.593	Co-occurrence
SLC46A1	17q11.2	2 (1.74%)	0 (0.00%)	>10	0.101	0.593	Co-occurrence
SLC46A3	13q12.3	2 (1.74%)	0 (0.00%)	>10	0.101	0.593	Co-occurrence
SLC5A8	12q23.1-q23.2	2 (1.74%)	0 (0.00%)	>10	0.101	0.593	Co-occurrence
SLC6A15	12q21.31	2 (1.74%)	0 (0.00%)	>10	0.101	0.593	Co-occurrence
SLC7A1	13q12.3	2 (1.74%)	0 (0.00%)	>10	0.101	0.593	Co-occurrence
SLC9A7P1	12q23.1	2 (1.74%)	0 (0.00%)	>10	0.101	0.593	Co-occurrence

SMAGP	12q13.13	2 (1.74%)	0 (0.00%)	>10	0.101	0.593	Co-occurrence
SMAP2	1p34.2	2 (1.74%)	0 (0.00%)	>10	0.101	0.593	Co-occurrence
SMC1B	22q13.31	2 (1.74%)	0 (0.00%)	>10	0.101	0.593	Co-occurrence
SMDT1	22q13.2	2 (1.74%)	0 (0.00%)	>10	0.101	0.593	Co-occurrence
SMTN	22q12.2	2 (1.74%)	0 (0.00%)	>10	0.101	0.593	Co-occurrence
SMURF2P1	17q11.2	2 (1.74%)	0 (0.00%)	>10	0.101	0.593	Co-occurrence
SNORA23	11p15.4	2 (1.74%)	0 (0.00%)	>10	0.101	0.593	Co-occurrence
SNORA40	11q21	2 (1.74%)	0 (0.00%)	>10	0.101	0.593	Co-occurrence
SNORA53	12q23.1	2 (1.74%)	0 (0.00%)	>10	0.101	0.593	Co-occurrence
SNORA55	1p34.3	2 (1.74%)	0 (0.00%)	>10	0.101	0.593	Co-occurrence
SNORA9	7p13	2 (1.74%)	0 (0.00%)	>10	0.101	0.593	Co-occurrence
SNORD102	13q12.2	2 (1.74%)	0 (0.00%)	>10	0.101	0.593	Co-occurrence
SNORD24	9q34.2	2 (1.74%)	0 (0.00%)	>10	0.101	0.593	Co-occurrence
SNORD36A	9q34.2	2 (1.74%)	0 (0.00%)	>10	0.101	0.593	Co-occurrence
SNORD36B	9q34.2	2 (1.74%)	0 (0.00%)	>10	0.101	0.593	Co-occurrence
SNORD36C	9q34.2	2 (1.74%)	0 (0.00%)	>10	0.101	0.593	Co-occurrence
SNORD62A	9q34.13	2 (1.74%)	0 (0.00%)	>10	0.101	0.593	Co-occurrence
SNORD62B	9q34.13	2 (1.74%)	0 (0.00%)	>10	0.101	0.593	Co-occurrence
SNORD77	1q25.1	2 (1.74%)	0 (0.00%)	>10	0.101	0.593	Co-occurrence
SNRPF	12q23.1	2 (1.74%)	0 (0.00%)	>10	0.101	0.593	Co-occurrence
SNX15	11q13.1	2 (1.74%)	0 (0.00%)	>10	0.101	0.593	Co-occurrence
SNX32	11q13.1	2 (1.74%)	0 (0.00%)	>10	0.101	0.593	Co-occurrence
SOCS2	12q22	2 (1.74%)	0 (0.00%)	>10	0.101	0.593	Co-occurrence
SPAAR	9p13.3	2 (1.74%)	0 (0.00%)	>10	0.101	0.593	Co-occurrence
SPACA9	9q34.13	2 (1.74%)	0 (0.00%)	>10	0.101	0.593	Co-occurrence
SPAG17	1p12	2 (1.74%)	0 (0.00%)	>10	0.101	0.593	Co-occurrence
SPAG8	9p13.3	2 (1.74%)	0 (0.00%)	>10	0.101	0.593	Co-occurrence
SPATA13	13q12.12	2 (1.74%)	0 (0.00%)	>10	0.101	0.593	Co-occurrence
SPDYC	11q13.1	2 (1.74%)	0 (0.00%)	>10	0.101	0.593	Co-occurrence

SPIC	12q23.2	2 (1.74%)	0 (0.00%)	>10	0.101	0.593	Co-occurrence
SPICE1	3q13.2	2 (1.74%)	0 (0.00%)	>10	0.101	0.593	Co-occurrence
SREK1	5q12.3	2 (1.74%)	0 (0.00%)	>10	0.101	0.593	Co-occurrence
SSH1	12q24.11	2 (1.74%)	0 (0.00%)	>10	0.101	0.593	Co-occurrence
ST6GALNAC5	1p31.1	2 (1.74%)	0 (0.00%)	>10	0.101	0.593	Co-occurrence
STAC	3p22.3-p22.2	2 (1.74%)	0 (0.00%)	>10	0.101	0.593	Co-occurrence
STKLD1	9q34.2	2 (1.74%)	0 (0.00%)	>10	0.101	0.593	Co-occurrence
STT3B	3p23	2 (1.74%)	0 (0.00%)	>10	0.101	0.593	Co-occurrence
SULT1E1	4q13.3	2 (1.74%)	0 (0.00%)	>10	0.101	0.593	Co-occurrence
SULT4A1	22q13.31	2 (1.74%)	0 (0.00%)	>10	0.101	0.593	Co-occurrence
SUMF1	3p26.1	2 (1.74%)	0 (0.00%)	>10	0.101	0.593	Co-occurrence
SURF1	9q34.2	2 (1.74%)	0 (0.00%)	>10	0.101	0.593	Co-occurrence
SURF2	9q34.2	2 (1.74%)	0 (0.00%)	>10	0.101	0.593	Co-occurrence
SURF4	9q34.2	2 (1.74%)	0 (0.00%)	>10	0.101	0.593	Co-occurrence
SURF6	9q34.2	2 (1.74%)	0 (0.00%)	>10	0.101	0.593	Co-occurrence
SUZ12P1	17q11.2	2 (1.74%)	0 (0.00%)	>10	0.101	0.593	Co-occurrence
SYCP3	12q23.2	2 (1.74%)	0 (0.00%)	>10	0.101	0.593	Co-occurrence
SYT1	12q21.2	2 (1.74%)	0 (0.00%)	>10	0.101	0.593	Co-occurrence
SYVN1	11q13.1	2 (1.74%)	0 (0.00%)	>10	0.101	0.593	Co-occurrence
TAB1	22q13.1	2 (1.74%)	0 (0.00%)	>10	0.101	0.593	Co-occurrence
TAFA2	12q14.1	2 (1.74%)	0 (0.00%)	>10	0.101	0.593	Co-occurrence
TAFA5	22q13.32	2 (1.74%)	0 (0.00%)	>10	0.101	0.593	Co-occurrence
TAOK3	12q24.23	2 (1.74%)	0 (0.00%)	>10	0.101	0.593	Co-occurrence
TBC1D29P	17q11.2	2 (1.74%)	0 (0.00%)	>10	0.101	0.593	Co-occurrence
TBC1D30	12q14.3	2 (1.74%)	0 (0.00%)	>10	0.101	0.593	Co-occurrence
TBC1D3P5	17q11.2	2 (1.74%)	0 (0.00%)	>10	0.101	0.593	Co-occurrence
TBK1	12q14.2	2 (1.74%)	0 (0.00%)	>10	0.101	0.593	Co-occurrence
TCP11L2	12q23.3	2 (1.74%)	0 (0.00%)	>10	0.101	0.593	Co-occurrence
TCTN2	12q24.31	2 (1.74%)	0 (0.00%)	>10	0.101	0.593	Co-occurrence

TDGF1	3p21.31	2 (1.74%)	0 (0.00%)	>10	0.101	0.593	Co-occurrence
TDRD3	13q21.2	2 (1.74%)	0 (0.00%)	>10	0.101	0.593	Co-occurrence
TEFM	17q11.2	2 (1.74%)	0 (0.00%)	>10	0.101	0.593	Co-occurrence
TENT5C	1p12	2 (1.74%)	0 (0.00%)	>10	0.101	0.593	Co-occurrence
TESC	12q24.22	2 (1.74%)	0 (0.00%)	>10	0.101	0.593	Co-occurrence
TESK1	9p13.3	2 (1.74%)	0 (0.00%)	>10	0.101	0.593	Co-occurrence
TEX26	13q12.3	2 (1.74%)	0 (0.00%)	>10	0.101	0.593	Co-occurrence
TEX33	22q12.3	2 (1.74%)	0 (0.00%)	>10	0.101	0.593	Co-occurrence
TEX41	2q22.3	2 (1.74%)	0 (0.00%)	>10	0.101	0.593	Co-occurrence
TFCP2	12q13.12-q13.13	2 (1.74%)	0 (0.00%)	>10	0.101	0.593	Co-occurrence
THAP2	12q21.1	2 (1.74%)	0 (0.00%)	>10	0.101	0.593	Co-occurrence
TIGD3	11q13.1	2 (1.74%)	0 (0.00%)	>10	0.101	0.593	Co-occurrence
TLCD4	1p21.3	2 (1.74%)	0 (0.00%)	>10	0.101	0.593	Co-occurrence
TM7SF2	11q13.1	2 (1.74%)	0 (0.00%)	>10	0.101	0.593	Co-occurrence
TMA7	3p21.31	2 (1.74%)	0 (0.00%)	>10	0.101	0.593	Co-occurrence
TMBIM4	12q14.3	2 (1.74%)	0 (0.00%)	>10	0.101	0.593	Co-occurrence
TMCC3	12q22	2 (1.74%)	0 (0.00%)	>10	0.101	0.593	Co-occurrence
TMCO2	1p34.2	2 (1.74%)	0 (0.00%)	>10	0.101	0.593	Co-occurrence
TMED2	12q24.31	2 (1.74%)	0 (0.00%)	>10	0.101	0.593	Co-occurrence
TMEM132C	12q24.32-q24.33	2 (1.74%)	0 (0.00%)	>10	0.101	0.593	Co-occurrence
TMEM19	12q21.1	2 (1.74%)	0 (0.00%)	>10	0.101	0.593	Co-occurrence
TMEM199	17q11.2	2 (1.74%)	0 (0.00%)	>10	0.101	0.593	Co-occurrence
TMEM263	12q23.3	2 (1.74%)	0 (0.00%)	>10	0.101	0.593	Co-occurrence
TMEM53	1p34.1	2 (1.74%)	0 (0.00%)	>10	0.101	0.593	Co-occurrence
TMEM59	1p32.3	2 (1.74%)	0 (0.00%)	>10	0.101	0.593	Co-occurrence
TMEM86A	11p15.1	2 (1.74%)	0 (0.00%)	>10	0.101	0.593	Co-occurrence
TMEM89	3p21.31	2 (1.74%)	0 (0.00%)	>10	0.101	0.593	Co-occurrence
TMEM8B	9p13.3	2 (1.74%)	0 (0.00%)	>10	0.101	0.593	Co-occurrence
TMEM97	17q11.2	2 (1.74%)	0 (0.00%)	>10	0.101	0.593	Co-occurrence

TMIE	3p21.31	2 (1.74%)	0 (0.00%)	>10	0.101	0.593	Co-occurrence
TMPO	12q23.1	2 (1.74%)	0 (0.00%)	>10	0.101	0.593	Co-occurrence
TMPRSS12	12q13.12	2 (1.74%)	0 (0.00%)	>10	0.101	0.593	Co-occurrence
TNFAIP1	17q11.2	2 (1.74%)	0 (0.00%)	>10	0.101	0.593	Co-occurrence
TNRC6B	22q13.1	2 (1.74%)	0 (0.00%)	>10	0.101	0.593	Co-occurrence
TOM1	22q12.3	2 (1.74%)	0 (0.00%)	>10	0.101	0.593	Co-occurrence
TP53I11	11p11.2	2 (1.74%)	0 (0.00%)	>10	0.101	0.593	Co-occurrence
TP53I13	17q11.2	2 (1.74%)	0 (0.00%)	>10	0.101	0.593	Co-occurrence
TPH2	12q21.1	2 (1.74%)	0 (0.00%)	>10	0.101	0.593	Co-occurrence
TRAIP	3p21.31	2 (1.74%)	0 (0.00%)	>10	0.101	0.593	Co-occurrence
TRAPPC8	18q12.1	2 (1.74%)	0 (0.00%)	>10	0.101	0.593	Co-occurrence
TREX1	3p21.31	2 (1.74%)	0 (0.00%)	>10	0.101	0.593	Co-occurrence
TRIM3	11p15.4	2 (1.74%)	0 (0.00%)	>10	0.101	0.593	Co-occurrence
TRMU	22q13.31	2 (1.74%)	0 (0.00%)	>10	0.101	0.593	Co-occurrence
TRO	Xp11.21	2 (1.74%)	0 (0.00%)	>10	0.101	0.593	Co-occurrence
TSC1	9q34.13	2 (1.74%)	0 (0.00%)	>10	0.101	0.593	Co-occurrence
TSPAN1	1p34.1	2 (1.74%)	0 (0.00%)	>10	0.101	0.593	Co-occurrence
TSPAN18	11p11.2	2 (1.74%)	0 (0.00%)	>10	0.101	0.593	Co-occurrence
TSPAN19	12q21.31	2 (1.74%)	0 (0.00%)	>10	0.101	0.593	Co-occurrence
TSPAN8	12q21.1	2 (1.74%)	0 (0.00%)	>10	0.101	0.593	Co-occurrence
TSPO	22q13.2	2 (1.74%)	0 (0.00%)	>10	0.101	0.593	Co-occurrence
TSR2	Xp11.22	2 (1.74%)	0 (0.00%)	>10	0.101	0.593	Co-occurrence
TST	22q12.3	2 (1.74%)	0 (0.00%)	>10	0.101	0.593	Co-occurrence
TTF1	9q34.13	2 (1.74%)	0 (0.00%)	>10	0.101	0.593	Co-occurrence
TTLL1	22q13.2	2 (1.74%)	0 (0.00%)	>10	0.101	0.593	Co-occurrence
TTLL11	9q33.2	2 (1.74%)	0 (0.00%)	>10	0.101	0.593	Co-occurrence
TTLL12	22q13.2	2 (1.74%)	0 (0.00%)	>10	0.101	0.593	Co-occurrence
TTR	18q12.1	2 (1.74%)	0 (0.00%)	>10	0.101	0.593	Co-occurrence
TUT4	1p32.3	2 (1.74%)	0 (0.00%)	>10	0.101	0.593	Co-occurrence

TXNDC12	1p32.3	2 (1.74%)	0 (0.00%)	>10	0.101	0.593	Co-occurrence
UBC	12q24.31	2 (1.74%)	0 (0.00%)	>10	0.101	0.593	Co-occurrence
UBE2N	12q22	2 (1.74%)	0 (0.00%)	>10	0.101	0.593	Co-occurrence
UBL3	13q12.3	2 (1.74%)	0 (0.00%)	>10	0.101	0.593	Co-occurrence
UCK1	9q34.13	2 (1.74%)	0 (0.00%)	>10	0.101	0.593	Co-occurrence
UCN2	3p21.31	2 (1.74%)	0 (0.00%)	>10	0.101	0.593	Co-occurrence
UNG	12q24.11	2 (1.74%)	0 (0.00%)	>10	0.101	0.593	Co-occurrence
UPK3A	22q13.31	2 (1.74%)	0 (0.00%)	>10	0.101	0.593	Co-occurrence
UQCRC1	3p21.31	2 (1.74%)	0 (0.00%)	>10	0.101	0.593	Co-occurrence
URAD	13q12.2	2 (1.74%)	0 (0.00%)	>10	0.101	0.593	Co-occurrence
USF3	3q13.2	2 (1.74%)	0 (0.00%)	>10	0.101	0.593	Co-occurrence
USP1	1p31.3	2 (1.74%)	0 (0.00%)	>10	0.101	0.593	Co-occurrence
USP15	12q14.1	2 (1.74%)	0 (0.00%)	>10	0.101	0.593	Co-occurrence
USP30	12q24.11	2 (1.74%)	0 (0.00%)	>10	0.101	0.593	Co-occurrence
USP44	12q22	2 (1.74%)	0 (0.00%)	>10	0.101	0.593	Co-occurrence
USP51	Xp11.21	2 (1.74%)	0 (0.00%)	>10	0.101	0.593	Co-occurrence
USPL1	13q12.3	2 (1.74%)	0 (0.00%)	>10	0.101	0.593	Co-occurrence
UTP20	12q23.2	2 (1.74%)	0 (0.00%)	>10	0.101	0.593	Co-occurrence
VEZT	12q22	2 (1.74%)	0 (0.00%)	>10	0.101	0.593	Co-occurrence
VILL	3p22.2	2 (1.74%)	0 (0.00%)	>10	0.101	0.593	Co-occurrence
VPS37B	12q24.31	2 (1.74%)	0 (0.00%)	>10	0.101	0.593	Co-occurrence
VPS51	11q13.1	2 (1.74%)	0 (0.00%)	>10	0.101	0.593	Co-occurrence
VSIG10	12q24.23	2 (1.74%)	0 (0.00%)	>10	0.101	0.593	Co-occurrence
VTN	17q11.2	2 (1.74%)	0 (0.00%)	>10	0.101	0.593	Co-occurrence
WASHC3	12q23.2	2 (1.74%)	0 (0.00%)	>10	0.101	0.593	Co-occurrence
WASHC4	12q23.3	2 (1.74%)	0 (0.00%)	>10	0.101	0.593	Co-occurrence
WDR25	14q32.2	2 (1.74%)	0 (0.00%)	>10	0.101	0.593	Co-occurrence
WDR3	1p12	2 (1.74%)	0 (0.00%)	>10	0.101	0.593	Co-occurrence
WDR31	9q32	2 (1.74%)	0 (0.00%)	>10	0.101	0.593	Co-occurrence

WDR33	2q14.3	2 (1.74%)	0 (0.00%)	>10	0.101	0.593	Co-occurrence
WDR63	1p22.3	2 (1.74%)	0 (0.00%)	>10	0.101	0.593	Co-occurrence
WIF1	12q14.3	2 (1.74%)	0 (0.00%)	>10	0.101	0.593	Co-occurrence
WLS	1p31.3	2 (1.74%)	0 (0.00%)	>10	0.101	0.593	Co-occurrence
WNT7B	22q13.31	2 (1.74%)	0 (0.00%)	>10	0.101	0.593	Co-occurrence
WSB1	17q11.1	2 (1.74%)	0 (0.00%)	>10	0.101	0.593	Co-occurrence
WSB2	12q24.23	2 (1.74%)	0 (0.00%)	>10	0.101	0.593	Co-occurrence
WWC3	Xp22.2	2 (1.74%)	0 (0.00%)	>10	0.101	0.593	Co-occurrence
XPNPEP3	22q13.2	2 (1.74%)	0 (0.00%)	>10	0.101	0.593	Co-occurrence
XPO4	13q12.11	2 (1.74%)	0 (0.00%)	>10	0.101	0.593	Co-occurrence
XPOT	12q14.2	2 (1.74%)	0 (0.00%)	>10	0.101	0.593	Co-occurrence
ZBTB20	3q13.31	2 (1.74%)	0 (0.00%)	>10	0.101	0.593	Co-occurrence
ZCCHC8	12q24.31	2 (1.74%)	0 (0.00%)	>10	0.101	0.593	Co-occurrence
ZDHHC13	11p15.1	2 (1.74%)	0 (0.00%)	>10	0.101	0.593	Co-occurrence
ZDHHC17	12q21.2	2 (1.74%)	0 (0.00%)	>10	0.101	0.593	Co-occurrence
ZFC3H1	12q21.1	2 (1.74%)	0 (0.00%)	>10	0.101	0.593	Co-occurrence
ZFPL1	11q13.1	2 (1.74%)	0 (0.00%)	>10	0.101	0.593	Co-occurrence
ZFX	Xp22.11	2 (1.74%)	0 (0.00%)	>10	0.101	0.593	Co-occurrence
ZMPSTE24	1p34.2	2 (1.74%)	0 (0.00%)	>10	0.101	0.593	Co-occurrence
ZNF569	19q13.12	2 (1.74%)	0 (0.00%)	>10	0.101	0.593	Co-occurrence
ZNF570	19q13.12	2 (1.74%)	0 (0.00%)	>10	0.101	0.593	Co-occurrence
ZNHIT2	11q13.1	2 (1.74%)	0 (0.00%)	>10	0.101	0.593	Co-occurrence
ZNRD2	11q13.1	2 (1.74%)	0 (0.00%)	>10	0.101	0.593	Co-occurrence
ZZZ3	1p31.1	2 (1.74%)	0 (0.00%)	>10	0.101	0.593	Co-occurrence
ASPSCR1	17q25.3	11 (9.57%)	13 (5.31%)	0.85	0.102	0.593	Co-occurrence
GPS1	17q25.3	11 (9.57%)	13 (5.31%)	0.85	0.102	0.593	Co-occurrence
RFNG	17q25.3	11 (9.57%)	13 (5.31%)	0.85	0.102	0.593	Co-occurrence
ENPP2	8q24.12	16 (13.91%)	49 (20.00%)	-0.52	0.104	0.605	Mutual exclusivity
CCND1	11q13.3	5 (4.35%)	21 (8.57%)	-0.98	0.107	0.627	Mutual exclusivity

LTO1	11q13.3	5 (4.35%)	21 (8.57%)	-0.98	0.107	0.627	Mutual exclusivity
DERL1	8q24.13	17 (14.78%)	51 (20.82%)	-0.49	0.11	0.643	Mutual exclusivity
AANAT	17q25.1	9 (7.83%)	10 (4.08%)	0.94	0.111	0.647	Co-occurrence
CYGB	17q25.1	9 (7.83%)	10 (4.08%)	0.94	0.111	0.647	Co-occurrence
FOXJ1	17q25.1	9 (7.83%)	10 (4.08%)	0.94	0.111	0.647	Co-occurrence
PRCD	17q25.1	9 (7.83%)	10 (4.08%)	0.94	0.111	0.647	Co-occurrence
QRICH2	17q25.1	9 (7.83%)	10 (4.08%)	0.94	0.111	0.647	Co-occurrence
RHBDF2	17q25.1	9 (7.83%)	10 (4.08%)	0.94	0.111	0.647	Co-occurrence
RNF157	17q25.1	9 (7.83%)	10 (4.08%)	0.94	0.111	0.647	Co-occurrence
SNHG16	17q25.1	9 (7.83%)	10 (4.08%)	0.94	0.111	0.647	Co-occurrence
SNORD1B	17q25.1	9 (7.83%)	10 (4.08%)	0.94	0.111	0.647	Co-occurrence
ST6GALNAC2	17q25.1	9 (7.83%)	10 (4.08%)	0.94	0.111	0.647	Co-occurrence
UBALD2	17q25.1	9 (7.83%)	10 (4.08%)	0.94	0.111	0.647	Co-occurrence
MIR-2053/2053		15 (13.04%)	46 (18.78%)	-0.53	0.114	0.66	Mutual exclusivity
ANKRD13D	11q13.2	1 (0.87%)	9 (3.67%)	-2.08	0.118	0.674	Mutual exclusivity
GRK2	11q13.2	1 (0.87%)	9 (3.67%)	-2.08	0.118	0.674	Mutual exclusivity
NDUFS8	11q13.2	1 (0.87%)	9 (3.67%)	-2.08	0.118	0.674	Mutual exclusivity
PHF3	6q12	1 (0.87%)	9 (3.67%)	-2.08	0.118	0.674	Mutual exclusivity
PPP1R3A	7q31.1	1 (0.87%)	9 (3.67%)	-2.08	0.118	0.674	Mutual exclusivity
TCIRG1	11q13.2	1 (0.87%)	9 (3.67%)	-2.08	0.118	0.674	Mutual exclusivity
FGF4	11q13.3	3 (2.61%)	15 (6.12%)	-1.23	0.119	0.674	Mutual exclusivity
MRGPRF	11q13.3	3 (2.61%)	15 (6.12%)	-1.23	0.119	0.674	Mutual exclusivity
ADTRP	6p24.1	7 (6.09%)	7 (2.86%)	1.09	0.12	0.674	Co-occurrence
C6ORF52	6p24.2	7 (6.09%)	7 (2.86%)	1.09	0.12	0.674	Co-occurrence
CUEDC1	17q22	7 (6.09%)	7 (2.86%)	1.09	0.12	0.674	Co-occurrence
GCM2	6p24.2	7 (6.09%)	7 (2.86%)	1.09	0.12	0.674	Co-occurrence
GCNT2P1	6p24.2	7 (6.09%)	7 (2.86%)	1.09	0.12	0.674	Co-occurrence
LINC00518	6p24.3	7 (6.09%)	7 (2.86%)	1.09	0.12	0.674	Co-occurrence
MAK	6p24.2	7 (6.09%)	7 (2.86%)	1.09	0.12	0.674	Co-occurrence

NEDD9	6p24.2	7 (6.09%)	7 (2.86%)	1.09	0.12	0.674	Co-occurrence
PAK1IP1	6p24.2	7 (6.09%)	7 (2.86%)	1.09	0.12	0.674	Co-occurrence
RNA5SP203	6p24.2	7 (6.09%)	7 (2.86%)	1.09	0.12	0.674	Co-occurrence
RNU6ATAC21P	6p24.3	7 (6.09%)	7 (2.86%)	1.09	0.12	0.674	Co-occurrence
SMIM13	6p24.2	7 (6.09%)	7 (2.86%)	1.09	0.12	0.674	Co-occurrence
SYCP2L	6p24.2	7 (6.09%)	7 (2.86%)	1.09	0.12	0.674	Co-occurrence
TFAP2A	6p24.3	7 (6.09%)	7 (2.86%)	1.09	0.12	0.674	Co-occurrence
TMEM14B	6p24.2	7 (6.09%)	7 (2.86%)	1.09	0.12	0.674	Co-occurrence
TMEM14C	6p24.2	7 (6.09%)	7 (2.86%)	1.09	0.12	0.674	Co-occurrence
TMEM170B	6p24.2	7 (6.09%)	7 (2.86%)	1.09	0.12	0.674	Co-occurrence
VEZF1	17q22	7 (6.09%)	7 (2.86%)	1.09	0.12	0.674	Co-occurrence
AOX2P	2q33.1	5 (4.35%)	4 (1.63%)	1.41	0.122	0.674	Co-occurrence
CFAP69	7q21.13	5 (4.35%)	4 (1.63%)	1.41	0.122	0.674	Co-occurrence
CHRNA4	20q13.33	5 (4.35%)	4 (1.63%)	1.41	0.122	0.674	Co-occurrence
COPZ2	17q21.32	5 (4.35%)	4 (1.63%)	1.41	0.122	0.674	Co-occurrence
GDF9	5q31.1	5 (4.35%)	4 (1.63%)	1.41	0.122	0.674	Co-occurrence
KCNQ2	20q13.33	5 (4.35%)	4 (1.63%)	1.41	0.122	0.674	Co-occurrence
PTPN1	20q13.13	5 (4.35%)	4 (1.63%)	1.41	0.122	0.674	Co-occurrence
RNA5SP192	5q31.1	5 (4.35%)	4 (1.63%)	1.41	0.122	0.674	Co-occurrence
SHROOM1	5q31.1	5 (4.35%)	4 (1.63%)	1.41	0.122	0.674	Co-occurrence
MGAT5B	17q25.2	10 (8.70%)	12 (4.90%)	0.83	0.123	0.674	Co-occurrence
CALB1	8q21.3	12 (10.43%)	38 (15.51%)	-0.57	0.127	0.674	Mutual exclusivity
ATAD2	8q24.13	17 (14.78%)	50 (20.41%)	-0.47	0.128	0.674	Mutual exclusivity
C8ORF76	8q24.13	17 (14.78%)	50 (20.41%)	-0.47	0.128	0.674	Mutual exclusivity
EXT1	8q24.11	17 (14.78%)	50 (20.41%)	-0.47	0.128	0.674	Mutual exclusivity
RNY4P5	8q24.13	17 (14.78%)	50 (20.41%)	-0.47	0.128	0.674	Mutual exclusivity
TBC1D31	8q24.13	17 (14.78%)	50 (20.41%)	-0.47	0.128	0.674	Mutual exclusivity
ZHX1	8q24.13	17 (14.78%)	50 (20.41%)	-0.47	0.128	0.674	Mutual exclusivity
RNF19A	8q22.2	15 (13.04%)	45 (18.37%)	-0.49	0.132	0.674	Mutual exclusivity

TMEM74	8q23.1	15 (13.04%)	45 (18.37%)	-0.49	0.132	0.674	Mutual exclusivity
TRHR	8q23.1	15 (13.04%)	45 (18.37%)	-0.49	0.132	0.674	Mutual exclusivity
CSNK1D	17q25.3	11 (9.57%)	14 (5.71%)	0.74	0.133	0.674	Co-occurrence
SLC16A3	17q25.3	11 (9.57%)	14 (5.71%)	0.74	0.133	0.674	Co-occurrence
LYRM4	6p25.1	8 (6.96%)	9 (3.67%)	0.92	0.136	0.674	Co-occurrence
PPP1R3G	6p25.1	8 (6.96%)	9 (3.67%)	0.92	0.136	0.674	Co-occurrence
PRPF4B	6p25.2	8 (6.96%)	9 (3.67%)	0.92	0.136	0.674	Co-occurrence
RN7SL554P	6p25.1	8 (6.96%)	9 (3.67%)	0.92	0.136	0.674	Co-occurrence
RNA5SP202	6p25.1	8 (6.96%)	9 (3.67%)	0.92	0.136	0.674	Co-occurrence
RPP40	6p25.1	8 (6.96%)	9 (3.67%)	0.92	0.136	0.674	Co-occurrence
COL14A1	8q24.12	16 (13.91%)	47 (19.18%)	-0.46	0.14	0.674	Mutual exclusivity
CSMD3	8q23.3	16 (13.91%)	47 (19.18%)	-0.46	0.14	0.674	Mutual exclusivity
DEPTOR	8q24.12	16 (13.91%)	47 (19.18%)	-0.46	0.14	0.674	Mutual exclusivity
DSCC1	8q24.12	16 (13.91%)	47 (19.18%)	-0.46	0.14	0.674	Mutual exclusivity
LINC00536	8q23.3	16 (13.91%)	47 (19.18%)	-0.46	0.14	0.674	Mutual exclusivity
MRPL13	8q24.12	16 (13.91%)	47 (19.18%)	-0.46	0.14	0.674	Mutual exclusivity
MTBP	8q24.12	16 (13.91%)	47 (19.18%)	-0.46	0.14	0.674	Mutual exclusivity
RN7SKP153	8q24.12	16 (13.91%)	47 (19.18%)	-0.46	0.14	0.674	Mutual exclusivity
RN7SL396P	8q24.12	16 (13.91%)	47 (19.18%)	-0.46	0.14	0.674	Mutual exclusivity
RNA5SP276	8q23.3	16 (13.91%)	47 (19.18%)	-0.46	0.14	0.674	Mutual exclusivity
RNA5SP277	8q24.12	16 (13.91%)	47 (19.18%)	-0.46	0.14	0.674	Mutual exclusivity
TAF2	8q24.12	16 (13.91%)	47 (19.18%)	-0.46	0.14	0.674	Mutual exclusivity
TRPS1	8q23.3	16 (13.91%)	47 (19.18%)	-0.46	0.14	0.674	Mutual exclusivity
AAMDC	11q14.1	0 (0.00%)	5 (2.04%)	<-10	0.144	0.674	Mutual exclusivity
ABCG5	2p21	0 (0.00%)	5 (2.04%)	<-10	0.144	0.674	Mutual exclusivity
ABCG8	2p21	0 (0.00%)	5 (2.04%)	<-10	0.144	0.674	Mutual exclusivity
AHCYL2	7q32.1	0 (0.00%)	5 (2.04%)	<-10	0.144	0.674	Mutual exclusivity
ARF5	7q32.1	0 (0.00%)	5 (2.04%)	<-10	0.144	0.674	Mutual exclusivity
ARL10	5q35.2	0 (0.00%)	5 (2.04%)	<-10	0.144	0.674	Mutual exclusivity

ARSI	5q32	0 (0.00%)	5 (2.04%)	<-10	0.144	0.674	Mutual exclusivity
ASRGL1	11q12.3	0 (0.00%)	5 (2.04%)	<-10	0.144	0.674	Mutual exclusivity
ATP6V1F	7q32.1	0 (0.00%)	5 (2.04%)	<-10	0.144	0.674	Mutual exclusivity
C5ORF60	5q35.3	0 (0.00%)	5 (2.04%)	<-10	0.144	0.674	Mutual exclusivity
C9	5p13.1	0 (0.00%)	5 (2.04%)	<-10	0.144	0.674	Mutual exclusivity
CALU	7q32.1	0 (0.00%)	5 (2.04%)	<-10	0.144	0.674	Mutual exclusivity
CBY3	5q35.3	0 (0.00%)	5 (2.04%)	<-10	0.144	0.674	Mutual exclusivity
CCDC136	7q32.1	0 (0.00%)	5 (2.04%)	<-10	0.144	0.674	Mutual exclusivity
CDHR2	5q35.2	0 (0.00%)	5 (2.04%)	<-10	0.144	0.674	Mutual exclusivity
CDKN2AIPNL	5q31.1	0 (0.00%)	5 (2.04%)	<-10	0.144	0.674	Mutual exclusivity
CLTB	5q35.2	0 (0.00%)	5 (2.04%)	<-10	0.144	0.674	Mutual exclusivity
CNOT4	7q33	0 (0.00%)	5 (2.04%)	<-10	0.144	0.674	Mutual exclusivity
DAB2	5p13.1	0 (0.00%)	5 (2.04%)	<-10	0.144	0.674	Mutual exclusivity
DYNC2LI1	2p21	0 (0.00%)	5 (2.04%)	<-10	0.144	0.674	Mutual exclusivity
FAF2	5q35.2	0 (0.00%)	5 (2.04%)	<-10	0.144	0.674	Mutual exclusivity
FAM153A	5q35.3	0 (0.00%)	5 (2.04%)	<-10	0.144	0.674	Mutual exclusivity
FAM153B	5q35.2	0 (0.00%)	5 (2.04%)	<-10	0.144	0.674	Mutual exclusivity
FAM153CP	5q35.3	0 (0.00%)	5 (2.04%)	<-10	0.144	0.674	Mutual exclusivity
FAM180A	7q33	0 (0.00%)	5 (2.04%)	<-10	0.144	0.674	Mutual exclusivity
FAM71F1	7q32.1	0 (0.00%)	5 (2.04%)	<-10	0.144	0.674	Mutual exclusivity
FAM71F2	7q32.1	0 (0.00%)	5 (2.04%)	<-10	0.144	0.674	Mutual exclusivity
FLNC	7q32.1	0 (0.00%)	5 (2.04%)	<-10	0.144	0.674	Mutual exclusivity
FSCN3	7q32.1	0 (0.00%)	5 (2.04%)	<-10	0.144	0.674	Mutual exclusivity
FSD1	19p13.3	0 (0.00%)	5 (2.04%)	<-10	0.144	0.674	Mutual exclusivity
FYB1	5p13.1	0 (0.00%)	5 (2.04%)	<-10	0.144	0.674	Mutual exclusivity
GCC1	7q32.1	0 (0.00%)	5 (2.04%)	<-10	0.144	0.674	Mutual exclusivity
GPRIN1	5q35.2	0 (0.00%)	5 (2.04%)	<-10	0.144	0.674	Mutual exclusivity
HIGD2A	5q35.2	0 (0.00%)	5 (2.04%)	<-10	0.144	0.674	Mutual exclusivity
HILPDA	7q32.1	0 (0.00%)	5 (2.04%)	<-10	0.144	0.674	Mutual exclusivity

HNRNPAB	5q35.3	0 (0.00%)	5 (2.04%)	<-10	0.144	0.674	Mutual exclusivity
IMPDH1	7q32.1	0 (0.00%)	5 (2.04%)	<-10	0.144	0.674	Mutual exclusivity
IRF5	7q32.1	0 (0.00%)	5 (2.04%)	<-10	0.144	0.674	Mutual exclusivity
KCP	7q32.1	0 (0.00%)	5 (2.04%)	<-10	0.144	0.674	Mutual exclusivity
KCTD14	11q14.1	0 (0.00%)	5 (2.04%)	<-10	0.144	0.674	Mutual exclusivity
KIAA1191	5q35.2	0 (0.00%)	5 (2.04%)	<-10	0.144	0.674	Mutual exclusivity
LEP	7q32.1	0 (0.00%)	5 (2.04%)	<-10	0.144	0.674	Mutual exclusivity
LINC00603	5p13.1	0 (0.00%)	5 (2.04%)	<-10	0.144	0.674	Mutual exclusivity
LRRC4	7q32.1	0 (0.00%)	5 (2.04%)	<-10	0.144	0.674	Mutual exclusivity
LUZP6	7q33	0 (0.00%)	5 (2.04%)	<-10	0.144	0.674	Mutual exclusivity
METTL2B	7q32.1	0 (0.00%)	5 (2.04%)	<-10	0.144	0.674	Mutual exclusivity
MIR-592/592		0 (0.00%)	5 (2.04%)	<-10	0.144	0.674	Mutual exclusivity
MTMR1	Xq28	0 (0.00%)	5 (2.04%)	<-10	0.144	0.674	Mutual exclusivity
MTPN	7q33	0 (0.00%)	5 (2.04%)	<-10	0.144	0.674	Mutual exclusivity
MYO7A	11q13.5	0 (0.00%)	5 (2.04%)	<-10	0.144	0.674	Mutual exclusivity
N4BP3	5q35.3	0 (0.00%)	5 (2.04%)	<-10	0.144	0.674	Mutual exclusivity
NEU3	11q13.4	0 (0.00%)	5 (2.04%)	<-10	0.144	0.674	Mutual exclusivity
NHP2	5q35.3	0 (0.00%)	5 (2.04%)	<-10	0.144	0.674	Mutual exclusivity
NOP16	5q35.2	0 (0.00%)	5 (2.04%)	<-10	0.144	0.674	Mutual exclusivity
OPN1SW	7q32.1	0 (0.00%)	5 (2.04%)	<-10	0.144	0.674	Mutual exclusivity
PAX4	7q32.1	0 (0.00%)	5 (2.04%)	<-10	0.144	0.674	Mutual exclusivity
PHYKPL	5q35.3	0 (0.00%)	5 (2.04%)	<-10	0.144	0.674	Mutual exclusivity
PLEKHH2	2p21	0 (0.00%)	5 (2.04%)	<-10	0.144	0.674	Mutual exclusivity
PPIC	5q23.2	0 (0.00%)	5 (2.04%)	<-10	0.144	0.674	Mutual exclusivity
PRDM6	5q23.2	0 (0.00%)	5 (2.04%)	<-10	0.144	0.674	Mutual exclusivity
PROP1	5q35.3	0 (0.00%)	5 (2.04%)	<-10	0.144	0.674	Mutual exclusivity
PRRT4	7q32.1	0 (0.00%)	5 (2.04%)	<-10	0.144	0.674	Mutual exclusivity
RBM28	7q32.1	0 (0.00%)	5 (2.04%)	<-10	0.144	0.674	Mutual exclusivity
RMND5B	5q35.3	0 (0.00%)	5 (2.04%)	<-10	0.144	0.674	Mutual exclusivity

RN7SKP66	2p21	0 (0.00%)	5 (2.04%)	<-10	0.144	0.674	Mutual exclusivity
RN7SL551P	18q22.3	0 (0.00%)	5 (2.04%)	<-10	0.144	0.674	Mutual exclusivity
RN7SL684P	5q35.2	0 (0.00%)	5 (2.04%)	<-10	0.144	0.674	Mutual exclusivity
RN7SL711P	5q23.2	0 (0.00%)	5 (2.04%)	<-10	0.144	0.674	Mutual exclusivity
RN7SL81P	7q32.1	0 (0.00%)	5 (2.04%)	<-10	0.144	0.674	Mutual exclusivity
RNA5SP242	7q32.1	0 (0.00%)	5 (2.04%)	<-10	0.144	0.674	Mutual exclusivity
RNA5SP243	7q32.1	0 (0.00%)	5 (2.04%)	<-10	0.144	0.674	Mutual exclusivity
RNA5SP513	Xq25	0 (0.00%)	5 (2.04%)	<-10	0.144	0.674	Mutual exclusivity
RNF169	11q13.4	0 (0.00%)	5 (2.04%)	<-10	0.144	0.674	Mutual exclusivity
RNF44	5q35.2	0 (0.00%)	5 (2.04%)	<-10	0.144	0.674	Mutual exclusivity
SIMC1	5q35.2	0 (0.00%)	5 (2.04%)	<-10	0.144	0.674	Mutual exclusivity
SLIT3	5q34-q35.1	0 (0.00%)	5 (2.04%)	<-10	0.144	0.674	Mutual exclusivity
SMARCA1	Xq25-q26.1	0 (0.00%)	5 (2.04%)	<-10	0.144	0.674	Mutual exclusivity
SND1	7q32.1	0 (0.00%)	5 (2.04%)	<-10	0.144	0.674	Mutual exclusivity
SPCS2	11q13.4	0 (0.00%)	5 (2.04%)	<-10	0.144	0.674	Mutual exclusivity
SYNPO	5q33.1	0 (0.00%)	5 (2.04%)	<-10	0.144	0.674	Mutual exclusivity
TENM2	5q34	0 (0.00%)	5 (2.04%)	<-10	0.144	0.674	Mutual exclusivity
THOC3	5q35.2	0 (0.00%)	5 (2.04%)	<-10	0.144	0.674	Mutual exclusivity
UVRAG	11q13.5	0 (0.00%)	5 (2.04%)	<-10	0.144	0.674	Mutual exclusivity
ZNF354A	5q35.3	0 (0.00%)	5 (2.04%)	<-10	0.144	0.674	Mutual exclusivity
ZNF800	7q31.33	0 (0.00%)	5 (2.04%)	<-10	0.144	0.674	Mutual exclusivity
ANKFN1	17q22	6 (5.22%)	6 (2.45%)	1.09	0.147	0.674	Co-occurrence
C17ORF112	17q22	6 (5.22%)	6 (2.45%)	1.09	0.147	0.674	Co-occurrence
CEBPG	19q13.11	6 (5.22%)	6 (2.45%)	1.09	0.147	0.674	Co-occurrence
HLF	17q22	6 (5.22%)	6 (2.45%)	1.09	0.147	0.674	Co-occurrence
PCTP	17q22	6 (5.22%)	6 (2.45%)	1.09	0.147	0.674	Co-occurrence
RNF126P1	17q22	6 (5.22%)	6 (2.45%)	1.09	0.147	0.674	Co-occurrence
TMEM100	17q22	6 (5.22%)	6 (2.45%)	1.09	0.147	0.674	Co-occurrence
ANXA13	8q24.13	17 (14.78%)	49 (20.00%)	-0.44	0.147	0.674	Mutual exclusivity

CASC8	8q24.21	17 (14.78%)	49 (20.00%)	-0.44	0.147	0.674	Mutual exclusivity
FAM83A	8q24.13	17 (14.78%)	49 (20.00%)	-0.44	0.147	0.674	Mutual exclusivity
FAM91A1	8q24.13	17 (14.78%)	49 (20.00%)	-0.44	0.147	0.674	Mutual exclusivity
FBXO32	8q24.13	17 (14.78%)	49 (20.00%)	-0.44	0.147	0.674	Mutual exclusivity
MIR-4662B/4662B		17 (14.78%)	49 (20.00%)	-0.44	0.147	0.674	Mutual exclusivity
MIR-4663/4663		17 (14.78%)	49 (20.00%)	-0.44	0.147	0.674	Mutual exclusivity
PCAT1	8q24.21	17 (14.78%)	49 (20.00%)	-0.44	0.147	0.674	Mutual exclusivity
PCAT2	8q24.21	17 (14.78%)	49 (20.00%)	-0.44	0.147	0.674	Mutual exclusivity
WDYHV1	8q24.13	17 (14.78%)	49 (20.00%)	-0.44	0.147	0.674	Mutual exclusivity
ZHX2	8q24.13	17 (14.78%)	49 (20.00%)	-0.44	0.147	0.674	Mutual exclusivity
BTBD17	17q25.1	9 (7.83%)	11 (4.49%)	0.8	0.149	0.674	Co-occurrence
C17ORF77	17q25.1	9 (7.83%)	11 (4.49%)	0.8	0.149	0.674	Co-occurrence
CD300A	17q25.1	9 (7.83%)	11 (4.49%)	0.8	0.149	0.674	Co-occurrence
CD300C	17q25.1	9 (7.83%)	11 (4.49%)	0.8	0.149	0.674	Co-occurrence
CD300E	17q25.1	9 (7.83%)	11 (4.49%)	0.8	0.149	0.674	Co-occurrence
CD300LB	17q25.1	9 (7.83%)	11 (4.49%)	0.8	0.149	0.674	Co-occurrence
CD300LD	17q25.1	9 (7.83%)	11 (4.49%)	0.8	0.149	0.674	Co-occurrence
CD300LF	17q25.1	9 (7.83%)	11 (4.49%)	0.8	0.149	0.674	Co-occurrence
CDK3	17q25.1	9 (7.83%)	11 (4.49%)	0.8	0.149	0.674	Co-occurrence
DNAI2	17q25.1	9 (7.83%)	11 (4.49%)	0.8	0.149	0.674	Co-occurrence
EVPL	17q25.1	9 (7.83%)	11 (4.49%)	0.8	0.149	0.674	Co-occurrence
EXOC7	17q25.1	9 (7.83%)	11 (4.49%)	0.8	0.149	0.674	Co-occurrence
FADS6	17q25.1	9 (7.83%)	11 (4.49%)	0.8	0.149	0.674	Co-occurrence
FBF1	17q25.1	9 (7.83%)	11 (4.49%)	0.8	0.149	0.674	Co-occurrence
FDXR	17q25.1	9 (7.83%)	11 (4.49%)	0.8	0.149	0.674	Co-occurrence
GALR2	17q25.1	9 (7.83%)	11 (4.49%)	0.8	0.149	0.674	Co-occurrence
GPR142	17q25.1	9 (7.83%)	11 (4.49%)	0.8	0.149	0.674	Co-occurrence
GPRC5C	17q25.1	9 (7.83%)	11 (4.49%)	0.8	0.149	0.674	Co-occurrence
GRIN2C	17q25.1	9 (7.83%)	11 (4.49%)	0.8	0.149	0.674	Co-occurrence

KIF19	17q25.1	9 (7.83%)	11 (4.49%)	0.8	0.149	0.674	Co-occurrence
LINC00469	17q25.1	9 (7.83%)	11 (4.49%)	0.8	0.149	0.674	Co-occurrence
LLGL2	17q25.1	9 (7.83%)	11 (4.49%)	0.8	0.149	0.674	Co-occurrence
MIR-3615/3615		9 (7.83%)	11 (4.49%)	0.8	0.149	0.674	Co-occurrence
MRPL38	17q25.1	9 (7.83%)	11 (4.49%)	0.8	0.149	0.674	Co-occurrence
MYO15B	17q25.1	9 (7.83%)	11 (4.49%)	0.8	0.149	0.674	Co-occurrence
NAT9	17q25.1	9 (7.83%)	11 (4.49%)	0.8	0.149	0.674	Co-occurrence
RAB37	17q25.1	9 (7.83%)	11 (4.49%)	0.8	0.149	0.674	Co-occurrence
RNA5SP448	17q25.1	9 (7.83%)	11 (4.49%)	0.8	0.149	0.674	Co-occurrence
RPL38	17q25.1	9 (7.83%)	11 (4.49%)	0.8	0.149	0.674	Co-occurrence
SLC9A3R1	17q25.1	9 (7.83%)	11 (4.49%)	0.8	0.149	0.674	Co-occurrence
SMIM5	17q25.1	9 (7.83%)	11 (4.49%)	0.8	0.149	0.674	Co-occurrence
SMIM6	17q25.1	9 (7.83%)	11 (4.49%)	0.8	0.149	0.674	Co-occurrence
SRP68	17q25.1	9 (7.83%)	11 (4.49%)	0.8	0.149	0.674	Co-occurrence
TEN1	17q25.1	9 (7.83%)	11 (4.49%)	0.8	0.149	0.674	Co-occurrence
TMEM104	17q25.1	9 (7.83%)	11 (4.49%)	0.8	0.149	0.674	Co-occurrence
TSEN54	17q25.1	9 (7.83%)	11 (4.49%)	0.8	0.149	0.674	Co-occurrence
TTYH2	17q25.1	9 (7.83%)	11 (4.49%)	0.8	0.149	0.674	Co-occurrence
ZACN	17q25.1	9 (7.83%)	11 (4.49%)	0.8	0.149	0.674	Co-occurrence
DECR1	8q21.3	12 (10.43%)	37 (15.10%)	-0.53	0.149	0.674	Mutual exclusivity
LINC00534	8q21.3	12 (10.43%)	37 (15.10%)	-0.53	0.149	0.674	Mutual exclusivity
NBN	8q21.3	12 (10.43%)	37 (15.10%)	-0.53	0.149	0.674	Mutual exclusivity
OSGIN2	8q21.3	12 (10.43%)	37 (15.10%)	-0.53	0.149	0.674	Mutual exclusivity
RN7SKP231	8q21.3	12 (10.43%)	37 (15.10%)	-0.53	0.149	0.674	Mutual exclusivity
ACBD4	17q21.31	4 (3.48%)	3 (1.22%)	1.51	0.15	0.674	Co-occurrence
AVIL	12q14.1	4 (3.48%)	3 (1.22%)	1.51	0.15	0.674	Co-occurrence
BCAS4	20q13.13	4 (3.48%)	3 (1.22%)	1.51	0.15	0.674	Co-occurrence
CHRNA6	8p11.21	4 (3.48%)	3 (1.22%)	1.51	0.15	0.674	Co-occurrence
CTDSP2	12q14.1	4 (3.48%)	3 (1.22%)	1.51	0.15	0.674	Co-occurrence

CYP4F22	19p13.12	4 (3.48%)	3 (1.22%)	1.51	0.15	0.674	Co-occurrence
DHX8	17q21.31	4 (3.48%)	3 (1.22%)	1.51	0.15	0.674	Co-occurrence
DNAJC30	7q11.23	4 (3.48%)	3 (1.22%)	1.51	0.15	0.674	Co-occurrence
EEF1AKMT3	12q14.1	4 (3.48%)	3 (1.22%)	1.51	0.15	0.674	Co-occurrence
EIF3L	22q13.1	4 (3.48%)	3 (1.22%)	1.51	0.15	0.674	Co-occurrence
ETV4	17q21.31	4 (3.48%)	3 (1.22%)	1.51	0.15	0.674	Co-occurrence
FKBP7	2q31.2	4 (3.48%)	3 (1.22%)	1.51	0.15	0.674	Co-occurrence
FLOT2	17q11.2	4 (3.48%)	3 (1.22%)	1.51	0.15	0.674	Co-occurrence
GLIS3	9p24.2	4 (3.48%)	3 (1.22%)	1.51	0.15	0.674	Co-occurrence
HEXIM1	17q21.31	4 (3.48%)	3 (1.22%)	1.51	0.15	0.674	Co-occurrence
HEXIM2	17q21.31	4 (3.48%)	3 (1.22%)	1.51	0.15	0.674	Co-occurrence
HOOK3	8p11.21	4 (3.48%)	3 (1.22%)	1.51	0.15	0.674	Co-occurrence
IKZF4	12q13.2	4 (3.48%)	3 (1.22%)	1.51	0.15	0.674	Co-occurrence
ITGB1	10p11.22	4 (3.48%)	3 (1.22%)	1.51	0.15	0.674	Co-occurrence
LBP	20q11.23	4 (3.48%)	3 (1.22%)	1.51	0.15	0.674	Co-occurrence
LINC00885	3q29	4 (3.48%)	3 (1.22%)	1.51	0.15	0.674	Co-occurrence
METTL1	12q14.1	4 (3.48%)	3 (1.22%)	1.51	0.15	0.674	Co-occurrence
MICALL1	22q13.1	4 (3.48%)	3 (1.22%)	1.51	0.15	0.674	Co-occurrence
MIR-4469/4469		4 (3.48%)	3 (1.22%)	1.51	0.15	0.674	Co-occurrence
MIR-645/645		4 (3.48%)	3 (1.22%)	1.51	0.15	0.674	Co-occurrence
NECTIN2	19q13.32	4 (3.48%)	3 (1.22%)	1.51	0.15	0.674	Co-occurrence
PCYT1A	3q29	4 (3.48%)	3 (1.22%)	1.51	0.15	0.674	Co-occurrence
PDE11A	2q31.2	4 (3.48%)	3 (1.22%)	1.51	0.15	0.674	Co-occurrence
PEPD	19q13.11	4 (3.48%)	3 (1.22%)	1.51	0.15	0.674	Co-occurrence
PGLYRP2	19p13.12	4 (3.48%)	3 (1.22%)	1.51	0.15	0.674	Co-occurrence
PJVK	2q31.2	4 (3.48%)	3 (1.22%)	1.51	0.15	0.674	Co-occurrence
PLCD3	17q21.31	4 (3.48%)	3 (1.22%)	1.51	0.15	0.674	Co-occurrence
PLEKHA3	2q31.2	4 (3.48%)	3 (1.22%)	1.51	0.15	0.674	Co-occurrence
PRKRA	2q31.2	4 (3.48%)	3 (1.22%)	1.51	0.15	0.674	Co-occurrence

R3HDM2	12q13.3	4 (3.48%)	3 (1.22%)	1.51	0.15	0.674	Co-occurrence
RAB5B	12q13.2	4 (3.48%)	3 (1.22%)	1.51	0.15	0.674	Co-occurrence
RHBDL3	17q11.2	4 (3.48%)	3 (1.22%)	1.51	0.15	0.674	Co-occurrence
RIPOR3	20q13.13	4 (3.48%)	3 (1.22%)	1.51	0.15	0.674	Co-occurrence
RN7SL392P	1p31.3	4 (3.48%)	3 (1.22%)	1.51	0.15	0.674	Co-occurrence
RN7SL806P	8p11.21	4 (3.48%)	3 (1.22%)	1.51	0.15	0.674	Co-occurrence
RN7SL847P	10p11.22	4 (3.48%)	3 (1.22%)	1.51	0.15	0.674	Co-occurrence
RNF170	8p11.21	4 (3.48%)	3 (1.22%)	1.51	0.15	0.674	Co-occurrence
RPS26	12q13.2	4 (3.48%)	3 (1.22%)	1.51	0.15	0.674	Co-occurrence
SDR9C7	12q13.3	4 (3.48%)	3 (1.22%)	1.51	0.15	0.674	Co-occurrence
SEZ6	17q11.2	4 (3.48%)	3 (1.22%)	1.51	0.15	0.674	Co-occurrence
SLC51A	3q29	4 (3.48%)	3 (1.22%)	1.51	0.15	0.674	Co-occurrence
SUOX	12q13.2	4 (3.48%)	3 (1.22%)	1.51	0.15	0.674	Co-occurrence
TFRC	3q29	4 (3.48%)	3 (1.22%)	1.51	0.15	0.674	Co-occurrence
THAP1	8p11.21	4 (3.48%)	3 (1.22%)	1.51	0.15	0.674	Co-occurrence
TSFM	12q14.1	4 (3.48%)	3 (1.22%)	1.51	0.15	0.674	Co-occurrence
TSHZ2	20q13.2	4 (3.48%)	3 (1.22%)	1.51	0.15	0.674	Co-occurrence
TTN	2q31.2	4 (3.48%)	3 (1.22%)	1.51	0.15	0.674	Co-occurrence
ZDHHC19	3q29	4 (3.48%)	3 (1.22%)	1.51	0.15	0.674	Co-occurrence
ZNF217	20q13.2	4 (3.48%)	3 (1.22%)	1.51	0.15	0.674	Co-occurrence
EBAG9	8q23.2	15 (13.04%)	44 (17.96%)	-0.46	0.153	0.674	Mutual exclusivity
EIF3E	8q23.1	15 (13.04%)	44 (17.96%)	-0.46	0.153	0.674	Mutual exclusivity
EMC2	8q23.1	15 (13.04%)	44 (17.96%)	-0.46	0.153	0.674	Mutual exclusivity
ENY2	8q23.1	15 (13.04%)	44 (17.96%)	-0.46	0.153	0.674	Mutual exclusivity
KCNV1	8q23.2	15 (13.04%)	44 (17.96%)	-0.46	0.153	0.674	Mutual exclusivity
MIR-4471/4471		15 (13.04%)	44 (17.96%)	-0.46	0.153	0.674	Mutual exclusivity
NUDCD1	8q23.1	15 (13.04%)	44 (17.96%)	-0.46	0.153	0.674	Mutual exclusivity
PKHD1L1	8q23.1-q23.2	15 (13.04%)	44 (17.96%)	-0.46	0.153	0.674	Mutual exclusivity
RNA5SP275	8q23.1	15 (13.04%)	44 (17.96%)	-0.46	0.153	0.674	Mutual exclusivity

RSPO2	8q23.1	15 (13.04%)	44 (17.96%)	-0.46	0.153	0.674	Mutual exclusivity
SYBU	8q23.2	15 (13.04%)	44 (17.96%)	-0.46	0.153	0.674	Mutual exclusivity
NIPAL2	8q22.2	13 (11.30%)	39 (15.92%)	-0.49	0.159	0.674	Mutual exclusivity
BMT2	7q31.1	1 (0.87%)	8 (3.27%)	-1.91	0.16	0.674	Mutual exclusivity
CARNS1	11q13.2	1 (0.87%)	8 (3.27%)	-1.91	0.16	0.674	Mutual exclusivity
CLCF1	11q13.2	1 (0.87%)	8 (3.27%)	-1.91	0.16	0.674	Mutual exclusivity
CORO1B	11q13.2	1 (0.87%)	8 (3.27%)	-1.91	0.16	0.674	Mutual exclusivity
DOCK4	7q31.1	1 (0.87%)	8 (3.27%)	-1.91	0.16	0.674	Mutual exclusivity
IFRD1	7q31.1	1 (0.87%)	8 (3.27%)	-1.91	0.16	0.674	Mutual exclusivity
KDM2A	11q13.2	1 (0.87%)	8 (3.27%)	-1.91	0.16	0.674	Mutual exclusivity
LAMB1	7q31.1	1 (0.87%)	8 (3.27%)	-1.91	0.16	0.674	Mutual exclusivity
LRRN3	7q31.1	1 (0.87%)	8 (3.27%)	-1.91	0.16	0.674	Mutual exclusivity
LSMEM1	7q31.1	1 (0.87%)	8 (3.27%)	-1.91	0.16	0.674	Mutual exclusivity
PTPRCAP	11q13.2	1 (0.87%)	8 (3.27%)	-1.91	0.16	0.674	Mutual exclusivity
RN7SKP187	7q31.1	1 (0.87%)	8 (3.27%)	-1.91	0.16	0.674	Mutual exclusivity
RN7SKP239	11q13.2	1 (0.87%)	8 (3.27%)	-1.91	0.16	0.674	Mutual exclusivity
RNA5SP237	7q31.1	1 (0.87%)	8 (3.27%)	-1.91	0.16	0.674	Mutual exclusivity
RPS6KB2	11q13.2	1 (0.87%)	8 (3.27%)	-1.91	0.16	0.674	Mutual exclusivity
TBC1D10C	11q13.2	1 (0.87%)	8 (3.27%)	-1.91	0.16	0.674	Mutual exclusivity
TMEM168	7q31.1	1 (0.87%)	8 (3.27%)	-1.91	0.16	0.674	Mutual exclusivity
TSRM	7q31.1	1 (0.87%)	8 (3.27%)	-1.91	0.16	0.674	Mutual exclusivity
ZNF277	7q31.1	1 (0.87%)	8 (3.27%)	-1.91	0.16	0.674	Mutual exclusivity
E2F3	6p22.3	7 (6.09%)	8 (3.27%)	0.9	0.166	0.674	Co-occurrence
ELOVL2	6p24.2	7 (6.09%)	8 (3.27%)	0.9	0.166	0.674	Co-occurrence
FNDC3B	3q26.31	7 (6.09%)	8 (3.27%)	0.9	0.166	0.674	Co-occurrence
GCNT2	6p24.3-p24.2	7 (6.09%)	8 (3.27%)	0.9	0.166	0.674	Co-occurrence
GHSR	3q26.31	7 (6.09%)	8 (3.27%)	0.9	0.166	0.674	Co-occurrence
GMPR	6p22.3	7 (6.09%)	8 (3.27%)	0.9	0.166	0.674	Co-occurrence
HIVEP1	6p24.1	7 (6.09%)	8 (3.27%)	0.9	0.166	0.674	Co-occurrence

MBOAT1	6p22.3	7 (6.09%)	8 (3.27%)	0.9	0.166	0.674	Co-occurrence
MIR-5689/5689		7 (6.09%)	8 (3.27%)	0.9	0.166	0.674	Co-occurrence
MYLIP	6p22.3	7 (6.09%)	8 (3.27%)	0.9	0.166	0.674	Co-occurrence
OFCC1	6p24.3	7 (6.09%)	8 (3.27%)	0.9	0.166	0.674	Co-occurrence
RN7SL128P	6p22.3	7 (6.09%)	8 (3.27%)	0.9	0.166	0.674	Co-occurrence
SDC2	8q22.1	14 (12.17%)	41 (16.73%)	-0.46	0.168	0.674	Mutual exclusivity
AARD	8q24.11	17 (14.78%)	48 (19.59%)	-0.41	0.169	0.674	Mutual exclusivity
CCAT1	8q24.21	17 (14.78%)	48 (19.59%)	-0.41	0.169	0.674	Mutual exclusivity
COLEC10	8q24.12	17 (14.78%)	48 (19.59%)	-0.41	0.169	0.674	Mutual exclusivity
EIF3H	8q23.3-q24.11	17 (14.78%)	48 (19.59%)	-0.41	0.169	0.674	Mutual exclusivity
FER1L6	8q24.13	17 (14.78%)	48 (19.59%)	-0.41	0.169	0.674	Mutual exclusivity
KLHL38	8q24.13	17 (14.78%)	48 (19.59%)	-0.41	0.169	0.674	Mutual exclusivity
LINC00964	8q24.13	17 (14.78%)	48 (19.59%)	-0.41	0.169	0.674	Mutual exclusivity
LRATD2	8q24.21	17 (14.78%)	48 (19.59%)	-0.41	0.169	0.674	Mutual exclusivity
MED30	8q24.11	17 (14.78%)	48 (19.59%)	-0.41	0.169	0.674	Mutual exclusivity
MIR-3610/3610		17 (14.78%)	48 (19.59%)	-0.41	0.169	0.674	Mutual exclusivity
MTSS1	8q24.13	17 (14.78%)	48 (19.59%)	-0.41	0.169	0.674	Mutual exclusivity
NSMCE2	8q24.13	17 (14.78%)	48 (19.59%)	-0.41	0.169	0.674	Mutual exclusivity
RAD21	8q24.11	17 (14.78%)	48 (19.59%)	-0.41	0.169	0.674	Mutual exclusivity
RN7SKP155	8q24.13	17 (14.78%)	48 (19.59%)	-0.41	0.169	0.674	Mutual exclusivity
RN7SL228P	8q24.11	17 (14.78%)	48 (19.59%)	-0.41	0.169	0.674	Mutual exclusivity
RN7SL826P	8q24.11	17 (14.78%)	48 (19.59%)	-0.41	0.169	0.674	Mutual exclusivity
SAMD12	8q24.11-q24.12	17 (14.78%)	48 (19.59%)	-0.41	0.169	0.674	Mutual exclusivity
SLC30A8	8q24.11	17 (14.78%)	48 (19.59%)	-0.41	0.169	0.674	Mutual exclusivity
SNTB1	8q24.12	17 (14.78%)	48 (19.59%)	-0.41	0.169	0.674	Mutual exclusivity
TRIB1	8q24.13	17 (14.78%)	48 (19.59%)	-0.41	0.169	0.674	Mutual exclusivity
UTP23	8q24.11	17 (14.78%)	48 (19.59%)	-0.41	0.169	0.674	Mutual exclusivity
RIPK2	8q21.3	12 (10.43%)	36 (14.69%)	-0.49	0.174	0.674	Mutual exclusivity
RNA5SP273	8q21.3	12 (10.43%)	36 (14.69%)	-0.49	0.174	0.674	Mutual exclusivity

BPHL	6p25.2	8 (6.96%)	10 (4.08%)	0.77	0.181	0.674	Co-occurrence
C6ORF201	6p25.2	8 (6.96%)	10 (4.08%)	0.77	0.181	0.674	Co-occurrence
CDKAL1	6p22.3	8 (6.96%)	10 (4.08%)	0.77	0.181	0.674	Co-occurrence
CDYL	6p25.1	8 (6.96%)	10 (4.08%)	0.77	0.181	0.674	Co-occurrence
ECI2	6p25.2	8 (6.96%)	10 (4.08%)	0.77	0.181	0.674	Co-occurrence
FAM217A	6p25.2	8 (6.96%)	10 (4.08%)	0.77	0.181	0.674	Co-occurrence
FAM50B	6p25.2	8 (6.96%)	10 (4.08%)	0.77	0.181	0.674	Co-occurrence
PXDC1	6p25.2	8 (6.96%)	10 (4.08%)	0.77	0.181	0.674	Co-occurrence
RNA5SP201	6p25.2	8 (6.96%)	10 (4.08%)	0.77	0.181	0.674	Co-occurrence
TUBB2A	6p25.2	8 (6.96%)	10 (4.08%)	0.77	0.181	0.674	Co-occurrence
TUBB2B	6p25.2	8 (6.96%)	10 (4.08%)	0.77	0.181	0.674	Co-occurrence
TRIM55	8q13.1	8 (6.96%)	26 (10.61%)	-0.61	0.182	0.674	Mutual exclusivity
ABCC3	17q21.33	5 (4.35%)	5 (2.04%)	1.09	0.183	0.674	Co-occurrence
MIR-3128/3128		5 (4.35%)	5 (2.04%)	1.09	0.183	0.674	Co-occurrence
NFE2L2	2q31.2	5 (4.35%)	5 (2.04%)	1.09	0.183	0.674	Co-occurrence
NLGN1	3q26.31	5 (4.35%)	5 (2.04%)	1.09	0.183	0.674	Co-occurrence
C8ORF87	8q22.1	13 (11.30%)	38 (15.51%)	-0.46	0.183	0.674	Mutual exclusivity
LINC00535	8q22.1	13 (11.30%)	38 (15.51%)	-0.46	0.183	0.674	Mutual exclusivity
RNA5SP274	8q22.1	13 (11.30%)	38 (15.51%)	-0.46	0.183	0.674	Mutual exclusivity
RUNX1T1	8q21.3	13 (11.30%)	38 (15.51%)	-0.46	0.183	0.674	Mutual exclusivity
TRIQK	8q22.1	13 (11.30%)	38 (15.51%)	-0.46	0.183	0.674	Mutual exclusivity
KHDRBS3	8q24.23	16 (13.91%)	45 (18.37%)	-0.4	0.185	0.674	Mutual exclusivity
AARSD1	17q21.31	3 (2.61%)	2 (0.82%)	1.68	0.188	0.674	Co-occurrence
ACACA	17q12	3 (2.61%)	2 (0.82%)	1.68	0.188	0.674	Co-occurrence
ACLY	17q21.2	3 (2.61%)	2 (0.82%)	1.68	0.188	0.674	Co-occurrence
ADAM11	17q21.31	3 (2.61%)	2 (0.82%)	1.68	0.188	0.674	Co-occurrence
ADAMTSL3	15q25.2	3 (2.61%)	2 (0.82%)	1.68	0.188	0.674	Co-occurrence
AFP	4q13.3	3 (2.61%)	2 (0.82%)	1.68	0.188	0.674	Co-occurrence
AKT2	19q13.2	3 (2.61%)	2 (0.82%)	1.68	0.188	0.674	Co-occurrence

ALAS1	3p21.2	3 (2.61%)	2 (0.82%)	1.68	0.188	0.674	Co-occurrence
ANKRD60	20q13.32	3 (2.61%)	2 (0.82%)	1.68	0.188	0.674	Co-occurrence
APCDD1L	20q13.32	3 (2.61%)	2 (0.82%)	1.68	0.188	0.674	Co-occurrence
APOC1P1	19q13.32	3 (2.61%)	2 (0.82%)	1.68	0.188	0.674	Co-occurrence
APOC2	19q13.32	3 (2.61%)	2 (0.82%)	1.68	0.188	0.674	Co-occurrence
APOC4	19q13.32	3 (2.61%)	2 (0.82%)	1.68	0.188	0.674	Co-occurrence
ARHGAP23	17q12	3 (2.61%)	2 (0.82%)	1.68	0.188	0.674	Co-occurrence
ARHGAP9	12q13.3	3 (2.61%)	2 (0.82%)	1.68	0.188	0.674	Co-occurrence
ARHGEF10L	1p36.13	3 (2.61%)	2 (0.82%)	1.68	0.188	0.674	Co-occurrence
ARL4D	17q21.31	3 (2.61%)	2 (0.82%)	1.68	0.188	0.674	Co-occurrence
ATP6V0A1	17q21.2	3 (2.61%)	2 (0.82%)	1.68	0.188	0.674	Co-occurrence
ATXN7L3	17q21.31	3 (2.61%)	2 (0.82%)	1.68	0.188	0.674	Co-occurrence
BAZ1B	7q11.23	3 (2.61%)	2 (0.82%)	1.68	0.188	0.674	Co-occurrence
BBS4	15q24.1	3 (2.61%)	2 (0.82%)	1.68	0.188	0.674	Co-occurrence
BMP7	20q13.31	3 (2.61%)	2 (0.82%)	1.68	0.188	0.674	Co-occurrence
C10ORF55	10q22.2	3 (2.61%)	2 (0.82%)	1.68	0.188	0.674	Co-occurrence
C17ORF102	17q12	3 (2.61%)	2 (0.82%)	1.68	0.188	0.674	Co-occurrence
C17ORF78	17q12	3 (2.61%)	2 (0.82%)	1.68	0.188	0.674	Co-occurrence
C20ORF85	20q13.32	3 (2.61%)	2 (0.82%)	1.68	0.188	0.674	Co-occurrence
CACNA2D2	3p21.31	3 (2.61%)	2 (0.82%)	1.68	0.188	0.674	Co-occurrence
CARD9	9q34.3	3 (2.61%)	2 (0.82%)	1.68	0.188	0.674	Co-occurrence
CAVIN1	17q21.2	3 (2.61%)	2 (0.82%)	1.68	0.188	0.674	Co-occurrence
CBFA2T2	20q11.21-q11.22	3 (2.61%)	2 (0.82%)	1.68	0.188	0.674	Co-occurrence
CCDC200	17q21.31	3 (2.61%)	2 (0.82%)	1.68	0.188	0.674	Co-occurrence
CCDC43	17q21.31	3 (2.61%)	2 (0.82%)	1.68	0.188	0.674	Co-occurrence
CCDC7	10p11.22	3 (2.61%)	2 (0.82%)	1.68	0.188	0.674	Co-occurrence
CCL1	17q12	3 (2.61%)	2 (0.82%)	1.68	0.188	0.674	Co-occurrence
CCL11	17q12	3 (2.61%)	2 (0.82%)	1.68	0.188	0.674	Co-occurrence
CCL13	17q12	3 (2.61%)	2 (0.82%)	1.68	0.188	0.674	Co-occurrence

CCL2	17q12	3 (2.61%)	2 (0.82%)	1.68	0.188	0.674	Co-occurrence
CCL7	17q12	3 (2.61%)	2 (0.82%)	1.68	0.188	0.674	Co-occurrence
CCL8	17q12	3 (2.61%)	2 (0.82%)	1.68	0.188	0.674	Co-occurrence
CCR7	17q21.2	3 (2.61%)	2 (0.82%)	1.68	0.188	0.674	Co-occurrence
CFAP161	15q25.1	3 (2.61%)	2 (0.82%)	1.68	0.188	0.674	Co-occurrence
CIAO2A	15q22.31	3 (2.61%)	2 (0.82%)	1.68	0.188	0.674	Co-occurrence
CIB1	15q26.1	3 (2.61%)	2 (0.82%)	1.68	0.188	0.674	Co-occurrence
CISD3	17q12	3 (2.61%)	2 (0.82%)	1.68	0.188	0.674	Co-occurrence
CLDN3	7q11.23	3 (2.61%)	2 (0.82%)	1.68	0.188	0.674	Co-occurrence
CLDN4	7q11.23	3 (2.61%)	2 (0.82%)	1.68	0.188	0.674	Co-occurrence
CLPTM1	19q13.32	3 (2.61%)	2 (0.82%)	1.68	0.188	0.674	Co-occurrence
CNPY2	12q13.3	3 (2.61%)	2 (0.82%)	1.68	0.188	0.674	Co-occurrence
CNTD2	19q13.2	3 (2.61%)	2 (0.82%)	1.68	0.188	0.674	Co-occurrence
COBLL1	2q24.3	3 (2.61%)	2 (0.82%)	1.68	0.188	0.674	Co-occurrence
COQ7	16p12.3	3 (2.61%)	2 (0.82%)	1.68	0.188	0.674	Co-occurrence
CSNK1E	22q13.1	3 (2.61%)	2 (0.82%)	1.68	0.188	0.674	Co-occurrence
CSPG4P5	15q25.2	3 (2.61%)	2 (0.82%)	1.68	0.188	0.674	Co-occurrence
CYP2B6	19q13.2	3 (2.61%)	2 (0.82%)	1.68	0.188	0.674	Co-occurrence
CYP4F11	19p13.12	3 (2.61%)	2 (0.82%)	1.68	0.188	0.674	Co-occurrence
CYP4F12	19p13.12	3 (2.61%)	2 (0.82%)	1.68	0.188	0.674	Co-occurrence
CYP4F2	19p13.12	3 (2.61%)	2 (0.82%)	1.68	0.188	0.674	Co-occurrence
CYP4F23P	19p13.12	3 (2.61%)	2 (0.82%)	1.68	0.188	0.674	Co-occurrence
CYP4F24P	19p13.12	3 (2.61%)	2 (0.82%)	1.68	0.188	0.674	Co-occurrence
CYP4F3	19p13.12	3 (2.61%)	2 (0.82%)	1.68	0.188	0.674	Co-occurrence
CYP4F8	19p13.12	3 (2.61%)	2 (0.82%)	1.68	0.188	0.674	Co-occurrence
DBF4B	17q21.31|17q21	3 (2.61%)	2 (0.82%)	1.68	0.188	0.674	Co-occurrence
DCAF11	14q12	3 (2.61%)	2 (0.82%)	1.68	0.188	0.674	Co-occurrence
DCAKD	17q21.31	3 (2.61%)	2 (0.82%)	1.68	0.188	0.674	Co-occurrence
DDIT3	12q13.3	3 (2.61%)	2 (0.82%)	1.68	0.188	0.674	Co-occurrence

DHRS4L1	14q11.2	3 (2.61%)	2 (0.82%)	1.68	0.188	0.674	Co-occurrence
DKFZP434A062	9q34.3	3 (2.61%)	2 (0.82%)	1.68	0.188	0.674	Co-occurrence
DNLZ	9q34.3	3 (2.61%)	2 (0.82%)	1.68	0.188	0.674	Co-occurrence
DUSP14	17q12	3 (2.61%)	2 (0.82%)	1.68	0.188	0.674	Co-occurrence
EFCAB13	17q21.32	3 (2.61%)	2 (0.82%)	1.68	0.188	0.674	Co-occurrence
EFL1P1	15q25.2	3 (2.61%)	2 (0.82%)	1.68	0.188	0.674	Co-occurrence
EGLN2	19q13.2	3 (2.61%)	2 (0.82%)	1.68	0.188	0.674	Co-occurrence
EMP2	16p13.13	3 (2.61%)	2 (0.82%)	1.68	0.188	0.674	Co-occurrence
EPC1	10p11.22	3 (2.61%)	2 (0.82%)	1.68	0.188	0.674	Co-occurrence
EPOP	17q12	3 (2.61%)	2 (0.82%)	1.68	0.188	0.674	Co-occurrence
FAM171A2	17q21.31	3 (2.61%)	2 (0.82%)	1.68	0.188	0.674	Co-occurrence
FAM209A	20q13.31	3 (2.61%)	2 (0.82%)	1.68	0.188	0.674	Co-occurrence
FAM209B	20q13.31	3 (2.61%)	2 (0.82%)	1.68	0.188	0.674	Co-occurrence
FAM83G	17p11.2	3 (2.61%)	2 (0.82%)	1.68	0.188	0.674	Co-occurrence
FBXO45	3q29	3 (2.61%)	2 (0.82%)	1.68	0.188	0.674	Co-occurrence
FCGBP	19q13.2	3 (2.61%)	2 (0.82%)	1.68	0.188	0.674	Co-occurrence
FGB	4q31.3	3 (2.61%)	2 (0.82%)	1.68	0.188	0.674	Co-occurrence
FZD2	17q21.31	3 (2.61%)	2 (0.82%)	1.68	0.188	0.674	Co-occurrence
FZD8	10p11.21	3 (2.61%)	2 (0.82%)	1.68	0.188	0.674	Co-occurrence
FZD9	7q11.23	3 (2.61%)	2 (0.82%)	1.68	0.188	0.674	Co-occurrence
G6PC	17q21.31	3 (2.61%)	2 (0.82%)	1.68	0.188	0.674	Co-occurrence
G6PC2	2q31.1	3 (2.61%)	2 (0.82%)	1.68	0.188	0.674	Co-occurrence
GCNT7	20q13.31	3 (2.61%)	2 (0.82%)	1.68	0.188	0.674	Co-occurrence
GDPGP1	15q26.1	3 (2.61%)	2 (0.82%)	1.68	0.188	0.674	Co-occurrence
GJC1	17q21.31	3 (2.61%)	2 (0.82%)	1.68	0.188	0.674	Co-occurrence
GJD4	10p11.21	3 (2.61%)	2 (0.82%)	1.68	0.188	0.674	Co-occurrence
GLCCI1	7p21.3	3 (2.61%)	2 (0.82%)	1.68	0.188	0.674	Co-occurrence
GNAS	20q13.32	3 (2.61%)	2 (0.82%)	1.68	0.188	0.674	Co-occurrence
GOLGA6B	15q24.1	3 (2.61%)	2 (0.82%)	1.68	0.188	0.674	Co-occurrence

GOLGA6L4	15q25.2	3 (2.61%)	2 (0.82%)	1.68	0.188	0.674	Co-occurrence
GOLGA6L5P	15q25.2	3 (2.61%)	2 (0.82%)	1.68	0.188	0.674	Co-occurrence
GPATCH1	19q13.11	3 (2.61%)	2 (0.82%)	1.68	0.188	0.674	Co-occurrence
GPATCH8	17q21.31	3 (2.61%)	2 (0.82%)	1.68	0.188	0.674	Co-occurrence
GPSM1	9q34.3	3 (2.61%)	2 (0.82%)	1.68	0.188	0.674	Co-occurrence
GRAP	17p11.2	3 (2.61%)	2 (0.82%)	1.68	0.188	0.674	Co-occurrence
GRAPL	17p11.2	3 (2.61%)	2 (0.82%)	1.68	0.188	0.674	Co-occurrence
GRB14	2q24.3	3 (2.61%)	2 (0.82%)	1.68	0.188	0.674	Co-occurrence
GRN	17q21.31	3 (2.61%)	2 (0.82%)	1.68	0.188	0.674	Co-occurrence
HIGD2B	15q24.1	3 (2.61%)	2 (0.82%)	1.68	0.188	0.674	Co-occurrence
HNF1B	17q12	3 (2.61%)	2 (0.82%)	1.68	0.188	0.674	Co-occurrence
IGIP	5q31.3	3 (2.61%)	2 (0.82%)	1.68	0.188	0.674	Co-occurrence
IL16	15q25.1	3 (2.61%)	2 (0.82%)	1.68	0.188	0.674	Co-occurrence
ITGA2B	17q21.31	3 (2.61%)	2 (0.82%)	1.68	0.188	0.674	Co-occurrence
ITPRIPL2	16p12.3	3 (2.61%)	2 (0.82%)	1.68	0.188	0.674	Co-occurrence
KCNJ4	22q13.1	3 (2.61%)	2 (0.82%)	1.68	0.188	0.674	Co-occurrence
KDELR3	22q13.1	3 (2.61%)	2 (0.82%)	1.68	0.188	0.674	Co-occurrence
KLF2	19p13.11	3 (2.61%)	2 (0.82%)	1.68	0.188	0.674	Co-occurrence
LHX3	9q34.3	3 (2.61%)	2 (0.82%)	1.68	0.188	0.674	Co-occurrence
LINC00661	19p13.12	3 (2.61%)	2 (0.82%)	1.68	0.188	0.674	Co-occurrence
LINC00905	19p13.12	3 (2.61%)	2 (0.82%)	1.68	0.188	0.674	Co-occurrence
LINC00910	17q21.31	3 (2.61%)	2 (0.82%)	1.68	0.188	0.674	Co-occurrence
LINC00933	15q25.2	3 (2.61%)	2 (0.82%)	1.68	0.188	0.674	Co-occurrence
MAIP1	2q33.1	3 (2.61%)	2 (0.82%)	1.68	0.188	0.674	Co-occurrence
MAP3K10	19q13.2	3 (2.61%)	2 (0.82%)	1.68	0.188	0.674	Co-occurrence
MARS	12q13.3	3 (2.61%)	2 (0.82%)	1.68	0.188	0.674	Co-occurrence
MBD6	12q13.3	3 (2.61%)	2 (0.82%)	1.68	0.188	0.674	Co-occurrence
MEIOC	17q21.31	3 (2.61%)	2 (0.82%)	1.68	0.188	0.674	Co-occurrence
MEOX1	17q21.31	3 (2.61%)	2 (0.82%)	1.68	0.188	0.674	Co-occurrence

MIR-298/298		3 (2.61%)	2 (0.82%)	1.68	0.188	0.674	Co-occurrence
MIR-4325/4325		3 (2.61%)	2 (0.82%)	1.68	0.188	0.674	Co-occurrence
MIR-4532/4532		3 (2.61%)	2 (0.82%)	1.68	0.188	0.674	Co-occurrence
MIR-4683/4683		3 (2.61%)	2 (0.82%)	1.68	0.188	0.674	Co-occurrence
MIR-4734/4734		3 (2.61%)	2 (0.82%)	1.68	0.188	0.674	Co-occurrence
MLLT6	17q12	3 (2.61%)	2 (0.82%)	1.68	0.188	0.674	Co-occurrence
MTRNR2L3	20q13.31	3 (2.61%)	2 (0.82%)	1.68	0.188	0.674	Co-occurrence
NACA	12q13.3	3 (2.61%)	2 (0.82%)	1.68	0.188	0.674	Co-occurrence
NBR1	17q21.31	3 (2.61%)	2 (0.82%)	1.68	0.188	0.674	Co-occurrence
NBR2	17q21.31	3 (2.61%)	2 (0.82%)	1.68	0.188	0.674	Co-occurrence
NELFCD	20q13.32	3 (2.61%)	2 (0.82%)	1.68	0.188	0.674	Co-occurrence
NMB	15q25.2	3 (2.61%)	2 (0.82%)	1.68	0.188	0.674	Co-occurrence
NPEPL1	20q13.32	3 (2.61%)	2 (0.82%)	1.68	0.188	0.674	Co-occurrence
NRG2	5q31.2	3 (2.61%)	2 (0.82%)	1.68	0.188	0.674	Co-occurrence
NSRP1	17q11.2	3 (2.61%)	2 (0.82%)	1.68	0.188	0.674	Co-occurrence
NUBP1	16p13.13	3 (2.61%)	2 (0.82%)	1.68	0.188	0.674	Co-occurrence
OR10H1	19p13.12	3 (2.61%)	2 (0.82%)	1.68	0.188	0.674	Co-occurrence
OR10H2	19p13.12	3 (2.61%)	2 (0.82%)	1.68	0.188	0.674	Co-occurrence
OR10H3	19p13.12	3 (2.61%)	2 (0.82%)	1.68	0.188	0.674	Co-occurrence
OR10H4	19p13.12	3 (2.61%)	2 (0.82%)	1.68	0.188	0.674	Co-occurrence
OR10H5	19p13.12	3 (2.61%)	2 (0.82%)	1.68	0.188	0.674	Co-occurrence
PAN2	12q13.3	3 (2.61%)	2 (0.82%)	1.68	0.188	0.674	Co-occurrence
PCGF2	17q12	3 (2.61%)	2 (0.82%)	1.68	0.188	0.674	Co-occurrence
PLAU	10q22.2	3 (2.61%)	2 (0.82%)	1.68	0.188	0.674	Co-occurrence
PLCL1	2q33.1	3 (2.61%)	2 (0.82%)	1.68	0.188	0.674	Co-occurrence
PMEPA1	20q13.31	3 (2.61%)	2 (0.82%)	1.68	0.188	0.674	Co-occurrence
PPM1M	3p21.2	3 (2.61%)	2 (0.82%)	1.68	0.188	0.674	Co-occurrence
PPP4R1L	20q13.32	3 (2.61%)	2 (0.82%)	1.68	0.188	0.674	Co-occurrence
PRIM1	12q13.3	3 (2.61%)	2 (0.82%)	1.68	0.188	0.674	Co-occurrence

PROSER2	10p14	3 (2.61%)	2 (0.82%)	1.68	0.188	0.674	Co-occurrence
PSMB3	17q12	3 (2.61%)	2 (0.82%)	1.68	0.188	0.674	Co-occurrence
PSMC4	19q13.2	3 (2.61%)	2 (0.82%)	1.68	0.188	0.674	Co-occurrence
PTGES3	12q13.3|12	3 (2.61%)	2 (0.82%)	1.68	0.188	0.674	Co-occurrence
PTGES3L	17q21.31	3 (2.61%)	2 (0.82%)	1.68	0.188	0.674	Co-occurrence
PURA	5q31.3	3 (2.61%)	2 (0.82%)	1.68	0.188	0.674	Co-occurrence
QSOX2	9q34.3	3 (2.61%)	2 (0.82%)	1.68	0.188	0.674	Co-occurrence
RAB22A	20q13.32	3 (2.61%)	2 (0.82%)	1.68	0.188	0.674	Co-occurrence
RAB4B	19q13.2	3 (2.61%)	2 (0.82%)	1.68	0.188	0.674	Co-occurrence
RAE1	20q13.31	3 (2.61%)	2 (0.82%)	1.68	0.188	0.674	Co-occurrence
RBM5	3p21.31	3 (2.61%)	2 (0.82%)	1.68	0.188	0.674	Co-occurrence
RELB	19q13.32	3 (2.61%)	2 (0.82%)	1.68	0.188	0.674	Co-occurrence
RGS3	9q32	3 (2.61%)	2 (0.82%)	1.68	0.188	0.674	Co-occurrence
RN7SL170P	20q13.31	3 (2.61%)	2 (0.82%)	1.68	0.188	0.674	Co-occurrence
RN7SL258P	17q21.31	3 (2.61%)	2 (0.82%)	1.68	0.188	0.674	Co-occurrence
RN7SL331P	15q25.2	3 (2.61%)	2 (0.82%)	1.68	0.188	0.674	Co-occurrence
RN7SL340P	19q12	3 (2.61%)	2 (0.82%)	1.68	0.188	0.674	Co-occurrence
RN7SL346P	15q26.1	3 (2.61%)	2 (0.82%)	1.68	0.188	0.674	Co-occurrence
RN7SL417P	15q25.2	3 (2.61%)	2 (0.82%)	1.68	0.188	0.674	Co-occurrence
RN7SL434P	3q29	3 (2.61%)	2 (0.82%)	1.68	0.188	0.674	Co-occurrence
RN7SL507P	17q21.31	3 (2.61%)	2 (0.82%)	1.68	0.188	0.674	Co-occurrence
RN7SL738P	3q29	3 (2.61%)	2 (0.82%)	1.68	0.188	0.674	Co-occurrence
RN7SL809P	12q13.3	3 (2.61%)	2 (0.82%)	1.68	0.188	0.674	Co-occurrence
RN7SL819P	17q21.31	3 (2.61%)	2 (0.82%)	1.68	0.188	0.674	Co-occurrence
RN7SL853P	15q24.1	3 (2.61%)	2 (0.82%)	1.68	0.188	0.674	Co-occurrence
RNA5SP438	17q12	3 (2.61%)	2 (0.82%)	1.68	0.188	0.674	Co-occurrence
RNA5SP440	17q12	3 (2.61%)	2 (0.82%)	1.68	0.188	0.674	Co-occurrence
RNA5SP443	17q21.31	3 (2.61%)	2 (0.82%)	1.68	0.188	0.674	Co-occurrence
RNF168	3q29	3 (2.61%)	2 (0.82%)	1.68	0.188	0.674	Co-occurrence

RNY4P2	17q21.31	3 (2.61%)	2 (0.82%)	1.68	0.188	0.674	Co-occurrence
RPL27	17q21.31	3 (2.61%)	2 (0.82%)	1.68	0.188	0.674	Co-occurrence
RTF2	20q13.31	3 (2.61%)	2 (0.82%)	1.68	0.188	0.674	Co-occurrence
RUNDC1	17q21.31	3 (2.61%)	2 (0.82%)	1.68	0.188	0.674	Co-occurrence
RUNDC3A	17q21.31	3 (2.61%)	2 (0.82%)	1.68	0.188	0.674	Co-occurrence
SCAND2P	15q25.2	3 (2.61%)	2 (0.82%)	1.68	0.188	0.674	Co-occurrence
SEC11A	15q25.2-q25.3	3 (2.61%)	2 (0.82%)	1.68	0.188	0.674	Co-occurrence
SEMA4B	15q26.1	3 (2.61%)	2 (0.82%)	1.68	0.188	0.674	Co-occurrence
SERINC3	20q13.12	3 (2.61%)	2 (0.82%)	1.68	0.188	0.674	Co-occurrence
SH2D7	15q25.1	3 (2.61%)	2 (0.82%)	1.68	0.188	0.674	Co-occurrence
SLC25A39	17q21.31	3 (2.61%)	2 (0.82%)	1.68	0.188	0.674	Co-occurrence
SLC38A11	2q24.3	3 (2.61%)	2 (0.82%)	1.68	0.188	0.674	Co-occurrence
SLC38A9	5q11.2	3 (2.61%)	2 (0.82%)	1.68	0.188	0.674	Co-occurrence
SLC45A1	1p36.23	3 (2.61%)	2 (0.82%)	1.68	0.188	0.674	Co-occurrence
SLC4A1	17q21.31	3 (2.61%)	2 (0.82%)	1.68	0.188	0.674	Co-occurrence
SLC51B	15q22.31	3 (2.61%)	2 (0.82%)	1.68	0.188	0.674	Co-occurrence
SLC5A10	17p11.2	3 (2.61%)	2 (0.82%)	1.68	0.188	0.674	Co-occurrence
SLC6A4	17q11.2	3 (2.61%)	2 (0.82%)	1.68	0.188	0.674	Co-occurrence
SMCO1	3q29	3 (2.61%)	2 (0.82%)	1.68	0.188	0.674	Co-occurrence
SNAPC4	9q34.3	3 (2.61%)	2 (0.82%)	1.68	0.188	0.674	Co-occurrence
SNORA70F	2q24.3	3 (2.61%)	2 (0.82%)	1.68	0.188	0.674	Co-occurrence
SNORD12	20q13.13	3 (2.61%)	2 (0.82%)	1.68	0.188	0.674	Co-occurrence
SNORD12B	20q13.13	3 (2.61%)	2 (0.82%)	1.68	0.188	0.674	Co-occurrence
SNORD12C	20q13.13	3 (2.61%)	2 (0.82%)	1.68	0.188	0.674	Co-occurrence
SNORD3A	17p11.2	3 (2.61%)	2 (0.82%)	1.68	0.188	0.674	Co-occurrence
SNORD3C	17p11.2	3 (2.61%)	2 (0.82%)	1.68	0.188	0.674	Co-occurrence
SNORD3D	17p11.2	3 (2.61%)	2 (0.82%)	1.68	0.188	0.674	Co-occurrence
SNX1	15q22.31	3 (2.61%)	2 (0.82%)	1.68	0.188	0.674	Co-occurrence
SPC25	2q24.3	3 (2.61%)	2 (0.82%)	1.68	0.188	0.674	Co-occurrence

SPO11	20q13.31	3 (2.61%)	2 (0.82%)	1.68	0.188	0.674	Co-occurrence
SRCIN1	17q12	3 (2.61%)	2 (0.82%)	1.68	0.188	0.674	Co-occurrence
STX16	20q13.32	3 (2.61%)	2 (0.82%)	1.68	0.188	0.674	Co-occurrence
SYT17	16p12.3	3 (2.61%)	2 (0.82%)	1.68	0.188	0.674	Co-occurrence
TADA2A	17q12	3 (2.61%)	2 (0.82%)	1.68	0.188	0.674	Co-occurrence
TBC1D3	17q12	3 (2.61%)	2 (0.82%)	1.68	0.188	0.674	Co-occurrence
TBC1D3F	17q12	3 (2.61%)	2 (0.82%)	1.68	0.188	0.674	Co-occurrence
TEKT5	16p13.13	3 (2.61%)	2 (0.82%)	1.68	0.188	0.674	Co-occurrence
TFAP2C	20q13.31	3 (2.61%)	2 (0.82%)	1.68	0.188	0.674	Co-occurrence
TLN2	15q22.2	3 (2.61%)	2 (0.82%)	1.68	0.188	0.674	Co-occurrence
TLR9	3p21.2	3 (2.61%)	2 (0.82%)	1.68	0.188	0.674	Co-occurrence
TMC7	16p12.3	3 (2.61%)	2 (0.82%)	1.68	0.188	0.674	Co-occurrence
TMEM106A	17q21.31	3 (2.61%)	2 (0.82%)	1.68	0.188	0.674	Co-occurrence
TMEM132E	17q12	3 (2.61%)	2 (0.82%)	1.68	0.188	0.674	Co-occurrence
TMIGD1	17q11.2	3 (2.61%)	2 (0.82%)	1.68	0.188	0.674	Co-occurrence
TMUB2	17q21.31	3 (2.61%)	2 (0.82%)	1.68	0.188	0.674	Co-occurrence
TTC9B	19q13.2	3 (2.61%)	2 (0.82%)	1.68	0.188	0.674	Co-occurrence
TTPAL	20q13.12	3 (2.61%)	2 (0.82%)	1.68	0.188	0.674	Co-occurrence
TVP23A	16p13.13	3 (2.61%)	2 (0.82%)	1.68	0.188	0.674	Co-occurrence
TWF2	3p21.2	3 (2.61%)	2 (0.82%)	1.68	0.188	0.674	Co-occurrence
UBE2Q2P1	15q25.2	3 (2.61%)	2 (0.82%)	1.68	0.188	0.674	Co-occurrence
UBTF	17q21.31	3 (2.61%)	2 (0.82%)	1.68	0.188	0.674	Co-occurrence
UCA1	19p13.12	3 (2.61%)	2 (0.82%)	1.68	0.188	0.674	Co-occurrence
UPP2	2q24.1	3 (2.61%)	2 (0.82%)	1.68	0.188	0.674	Co-occurrence
UQCRFS1	19q12	3 (2.61%)	2 (0.82%)	1.68	0.188	0.674	Co-occurrence
VAPB	20q13.32	3 (2.61%)	2 (0.82%)	1.68	0.188	0.674	Co-occurrence
WDR53	3q29	3 (2.61%)	2 (0.82%)	1.68	0.188	0.674	Co-occurrence
WDR73	15q25.2	3 (2.61%)	2 (0.82%)	1.68	0.188	0.674	Co-occurrence
WDR82	3p21.2	3 (2.61%)	2 (0.82%)	1.68	0.188	0.674	Co-occurrence

ZBP1	20q13.31	3 (2.61%)	2 (0.82%)	1.68	0.188	0.674	Co-occurrence
ZEB1	10p11.22	3 (2.61%)	2 (0.82%)	1.68	0.188	0.674	Co-occurrence
ZFAS1	20q13.13	3 (2.61%)	2 (0.82%)	1.68	0.188	0.674	Co-occurrence
ZFP64	20q13.2	3 (2.61%)	2 (0.82%)	1.68	0.188	0.674	Co-occurrence
ZNF546	19q13.2	3 (2.61%)	2 (0.82%)	1.68	0.188	0.674	Co-occurrence
ZNF780A	19q13.2	3 (2.61%)	2 (0.82%)	1.68	0.188	0.674	Co-occurrence
ZNF780B	19q13.2	3 (2.61%)	2 (0.82%)	1.68	0.188	0.674	Co-occurrence
ZNF831	20q13.32	3 (2.61%)	2 (0.82%)	1.68	0.188	0.674	Co-occurrence
ZNFX1	20q13.13	3 (2.61%)	2 (0.82%)	1.68	0.188	0.674	Co-occurrence
ZSCAN2	15q25.2	3 (2.61%)	2 (0.82%)	1.68	0.188	0.674	Co-occurrence
STAU2	8q21.11	11 (9.57%)	33 (13.47%)	-0.49	0.19	0.674	Mutual exclusivity
FGF3	11q13.3	3 (2.61%)	13 (5.31%)	-1.02	0.191	0.674	Mutual exclusivity
ACOX1	17q25.1	9 (7.83%)	12 (4.90%)	0.68	0.192	0.674	Co-occurrence
B3GNTL1	17q25.3	9 (7.83%)	12 (4.90%)	0.68	0.192	0.674	Co-occurrence
FN3K	17q25.3	9 (7.83%)	12 (4.90%)	0.68	0.192	0.674	Co-occurrence
FN3KRP	17q25.3	9 (7.83%)	12 (4.90%)	0.68	0.192	0.674	Co-occurrence
METRNL	17q25.3	9 (7.83%)	12 (4.90%)	0.68	0.192	0.674	Co-occurrence
TBCD	17q25.3	9 (7.83%)	12 (4.90%)	0.68	0.192	0.674	Co-occurrence
ZNF750	17q25.3	9 (7.83%)	12 (4.90%)	0.68	0.192	0.674	Co-occurrence
CCN3	8q24.12	17 (14.78%)	47 (19.18%)	-0.38	0.193	0.674	Mutual exclusivity
HAS2	8q24.13	17 (14.78%)	47 (19.18%)	-0.38	0.193	0.674	Mutual exclusivity
MAL2	8q24.12	17 (14.78%)	47 (19.18%)	-0.38	0.193	0.674	Mutual exclusivity
MIR-1208/1208		17 (14.78%)	47 (19.18%)	-0.38	0.193	0.674	Mutual exclusivity
MYC	8q24.21	17 (14.78%)	47 (19.18%)	-0.38	0.193	0.674	Mutual exclusivity
NDUFB9	8q24.13	17 (14.78%)	47 (19.18%)	-0.38	0.193	0.674	Mutual exclusivity
PVT1	8q24.21	17 (14.78%)	47 (19.18%)	-0.38	0.193	0.674	Mutual exclusivity
RN7SKP226	8q24.21	17 (14.78%)	47 (19.18%)	-0.38	0.193	0.674	Mutual exclusivity
RN7SL329P	8q24.13	17 (14.78%)	47 (19.18%)	-0.38	0.193	0.674	Mutual exclusivity
RNF139	8q24.13	17 (14.78%)	47 (19.18%)	-0.38	0.193	0.674	Mutual exclusivity

SNORA32	11q21	17 (14.78%)	47 (19.18%)	-0.38	0.193	0.674	Mutual exclusivity
SQLE	8q24.13	17 (14.78%)	47 (19.18%)	-0.38	0.193	0.674	Mutual exclusivity
TATDN1	8q24.13	17 (14.78%)	47 (19.18%)	-0.38	0.193	0.674	Mutual exclusivity
TMEM65	8q24.13	17 (14.78%)	47 (19.18%)	-0.38	0.193	0.674	Mutual exclusivity
TNFRSF11B	8q24.12	17 (14.78%)	47 (19.18%)	-0.38	0.193	0.674	Mutual exclusivity
TRMT12	8q24.13	17 (14.78%)	47 (19.18%)	-0.38	0.193	0.674	Mutual exclusivity
WASHC5	8q24.13	17 (14.78%)	47 (19.18%)	-0.38	0.193	0.674	Mutual exclusivity
ZNF572	8q24.13	17 (14.78%)	47 (19.18%)	-0.38	0.193	0.674	Mutual exclusivity
CPQ	8q22.1	14 (12.17%)	40 (16.33%)	-0.42	0.193	0.674	Mutual exclusivity
MATN2	8q22.1-q22.2	14 (12.17%)	40 (16.33%)	-0.42	0.193	0.674	Mutual exclusivity
MTERF3	8q22.1	14 (12.17%)	40 (16.33%)	-0.42	0.193	0.674	Mutual exclusivity
PTDSS1	8q22.1	14 (12.17%)	40 (16.33%)	-0.42	0.193	0.674	Mutual exclusivity
RPL30	8q22.2	14 (12.17%)	40 (16.33%)	-0.42	0.193	0.674	Mutual exclusivity
UQCRB	8q22.1	14 (12.17%)	40 (16.33%)	-0.42	0.193	0.674	Mutual exclusivity
FGF19	11q13.3	5 (4.35%)	18 (7.35%)	-0.76	0.199	0.674	Mutual exclusivity
CD7	17q25.3	10 (8.70%)	14 (5.71%)	0.61	0.201	0.674	Co-occurrence
SECTM1	17q25.3	10 (8.70%)	14 (5.71%)	0.61	0.201	0.674	Co-occurrence
TEX19	17q25.3	10 (8.70%)	14 (5.71%)	0.61	0.201	0.674	Co-occurrence
ANKRD46	8q22.3	15 (13.04%)	42 (17.14%)	-0.39	0.202	0.674	Mutual exclusivity
COX6C	8q22.2	15 (13.04%)	42 (17.14%)	-0.39	0.202	0.674	Mutual exclusivity
FBXO43	8q22.2	15 (13.04%)	42 (17.14%)	-0.39	0.202	0.674	Mutual exclusivity
NDUFAF6	8q22.1	15 (13.04%)	42 (17.14%)	-0.39	0.202	0.674	Mutual exclusivity
POLR2K	8q22.2	15 (13.04%)	42 (17.14%)	-0.39	0.202	0.674	Mutual exclusivity
RGS22	8q22.2	15 (13.04%)	42 (17.14%)	-0.39	0.202	0.674	Mutual exclusivity
SNX31	8q22.3	15 (13.04%)	42 (17.14%)	-0.39	0.202	0.674	Mutual exclusivity
SPAG1	8q22.2	15 (13.04%)	42 (17.14%)	-0.39	0.202	0.674	Mutual exclusivity
ABRA	8q23.1	16 (13.91%)	44 (17.96%)	-0.37	0.211	0.674	Mutual exclusivity
ANGPT1	8q23.1	16 (13.91%)	44 (17.96%)	-0.37	0.211	0.674	Mutual exclusivity
OXR1	8q23.1	16 (13.91%)	44 (17.96%)	-0.37	0.211	0.674	Mutual exclusivity

CNBD1	8q21.3	13 (11.30%)	37 (15.10%)	-0.42	0.211	0.674	Mutual exclusivity
DCAF4L2	8q21.3	13 (11.30%)	37 (15.10%)	-0.42	0.211	0.674	Mutual exclusivity
LRRC69	8q21.3	13 (11.30%)	37 (15.10%)	-0.42	0.211	0.674	Mutual exclusivity
NECAB1	8q21.3	13 (11.30%)	37 (15.10%)	-0.42	0.211	0.674	Mutual exclusivity
OTUD6B	8q21.3	13 (11.30%)	37 (15.10%)	-0.42	0.211	0.674	Mutual exclusivity
PIP4P2	8q21.3	13 (11.30%)	37 (15.10%)	-0.42	0.211	0.674	Mutual exclusivity
SLC26A7	8q21.3	13 (11.30%)	37 (15.10%)	-0.42	0.211	0.674	Mutual exclusivity
TMEM64	8q21.3	13 (11.30%)	37 (15.10%)	-0.42	0.211	0.674	Mutual exclusivity
AIFM1	Xq26.1	0 (0.00%)	4 (1.63%)	<-10	0.213	0.674	Mutual exclusivity
AKR1B10	7q33	0 (0.00%)	4 (1.63%)	<-10	0.213	0.674	Mutual exclusivity
AKR1B15	7q33	0 (0.00%)	4 (1.63%)	<-10	0.213	0.674	Mutual exclusivity
ALG8	11q14.1	0 (0.00%)	4 (1.63%)	<-10	0.213	0.674	Mutual exclusivity
ALK	2p23.2-p23.1	0 (0.00%)	4 (1.63%)	<-10	0.213	0.674	Mutual exclusivity
APLN	Xq26.1	0 (0.00%)	4 (1.63%)	<-10	0.213	0.674	Mutual exclusivity
AQP11	11q14.1	0 (0.00%)	4 (1.63%)	<-10	0.213	0.674	Mutual exclusivity
ARHGAP36	Xq26.1	0 (0.00%)	4 (1.63%)	<-10	0.213	0.674	Mutual exclusivity
ARRB1	11q13.4	0 (0.00%)	4 (1.63%)	<-10	0.213	0.674	Mutual exclusivity
ATP13A1	19p13.11	0 (0.00%)	4 (1.63%)	<-10	0.213	0.674	Mutual exclusivity
ATP6V0E1	5q35.1	0 (0.00%)	4 (1.63%)	<-10	0.213	0.674	Mutual exclusivity
BASP1	5p15.1	0 (0.00%)	4 (1.63%)	<-10	0.213	0.674	Mutual exclusivity
BCORL1	Xq26.1	0 (0.00%)	4 (1.63%)	<-10	0.213	0.674	Mutual exclusivity
BEST1	11q12.3	0 (0.00%)	4 (1.63%)	<-10	0.213	0.674	Mutual exclusivity
BIRC6	2p22.3	0 (0.00%)	4 (1.63%)	<-10	0.213	0.674	Mutual exclusivity
BNIP1	5q35.1	0 (0.00%)	4 (1.63%)	<-10	0.213	0.674	Mutual exclusivity
BORCS8	19p13.11	0 (0.00%)	4 (1.63%)	<-10	0.213	0.674	Mutual exclusivity
BPGM	7q33	0 (0.00%)	4 (1.63%)	<-10	0.213	0.674	Mutual exclusivity
BTNL8	5q35.3	0 (0.00%)	4 (1.63%)	<-10	0.213	0.674	Mutual exclusivity
C5ORF52	5q33.3	0 (0.00%)	4 (1.63%)	<-10	0.213	0.674	Mutual exclusivity
C5ORF58	5q35.1	0 (0.00%)	4 (1.63%)	<-10	0.213	0.674	Mutual exclusivity

CAMK2A	5q32	0 (0.00%)	4 (1.63%)	<-10	0.213	0.674	Mutual exclusivity
CAPN13	2p23.1	0 (0.00%)	4 (1.63%)	<-10	0.213	0.674	Mutual exclusivity
CAPN14	2p23.1	0 (0.00%)	4 (1.63%)	<-10	0.213	0.674	Mutual exclusivity
CCNG1	5q34	0 (0.00%)	4 (1.63%)	<-10	0.213	0.674	Mutual exclusivity
CDX1	5q32	0 (0.00%)	4 (1.63%)	<-10	0.213	0.674	Mutual exclusivity
CHAF1A	19p13.3	0 (0.00%)	4 (1.63%)	<-10	0.213	0.674	Mutual exclusivity
CILP2	19p13.11	0 (0.00%)	4 (1.63%)	<-10	0.213	0.674	Mutual exclusivity
CLIP4	2p23.2	0 (0.00%)	4 (1.63%)	<-10	0.213	0.674	Mutual exclusivity
CLK4	5q35.3	0 (0.00%)	4 (1.63%)	<-10	0.213	0.674	Mutual exclusivity
CNGA2	Xq28	0 (0.00%)	4 (1.63%)	<-10	0.213	0.674	Mutual exclusivity
CNOT6	5q35.3	0 (0.00%)	4 (1.63%)	<-10	0.213	0.674	Mutual exclusivity
COL23A1	5q35.3	0 (0.00%)	4 (1.63%)	<-10	0.213	0.674	Mutual exclusivity
COL26A1	7q22.1	0 (0.00%)	4 (1.63%)	<-10	0.213	0.674	Mutual exclusivity
CPLX2	5q35.2	0 (0.00%)	4 (1.63%)	<-10	0.213	0.674	Mutual exclusivity
CREBRF	5q35.1	0 (0.00%)	4 (1.63%)	<-10	0.213	0.674	Mutual exclusivity
CYB561A3	11q12.2	0 (0.00%)	4 (1.63%)	<-10	0.213	0.674	Mutual exclusivity
CYB5A	18q22.3	0 (0.00%)	4 (1.63%)	<-10	0.213	0.674	Mutual exclusivity
CYREN	7q33	0 (0.00%)	4 (1.63%)	<-10	0.213	0.674	Mutual exclusivity
DAGLA	11q12.2	0 (0.00%)	4 (1.63%)	<-10	0.213	0.674	Mutual exclusivity
DCTN4	5q33.1	0 (0.00%)	4 (1.63%)	<-10	0.213	0.674	Mutual exclusivity
DGAT2	11q13.5	0 (0.00%)	4 (1.63%)	<-10	0.213	0.674	Mutual exclusivity
DNAJC2	7q22.1	0 (0.00%)	4 (1.63%)	<-10	0.213	0.674	Mutual exclusivity
DOCK2	5q35.1	0 (0.00%)	4 (1.63%)	<-10	0.213	0.674	Mutual exclusivity
DPY30	2p22.3	0 (0.00%)	4 (1.63%)	<-10	0.213	0.674	Mutual exclusivity
DRD1	5q35.2	0 (0.00%)	4 (1.63%)	<-10	0.213	0.674	Mutual exclusivity
EFCAB9	5q35.1	0 (0.00%)	4 (1.63%)	<-10	0.213	0.674	Mutual exclusivity
EHBP1	2p15	0 (0.00%)	4 (1.63%)	<-10	0.213	0.674	Mutual exclusivity
EHD3	2p23.1	0 (0.00%)	4 (1.63%)	<-10	0.213	0.674	Mutual exclusivity
ELF4	Xq26.1	0 (0.00%)	4 (1.63%)	<-10	0.213	0.674	Mutual exclusivity

ENOX2	Xq26.1	0 (0.00%)	4 (1.63%)	<-10	0.213	0.674	Mutual exclusivity
ERICH1	8p23.3	0 (0.00%)	4 (1.63%)	<-10	0.213	0.674	Mutual exclusivity
FADS1	11q12.2	0 (0.00%)	4 (1.63%)	<-10	0.213	0.674	Mutual exclusivity
FADS2	11q12.2	0 (0.00%)	4 (1.63%)	<-10	0.213	0.674	Mutual exclusivity
FADS3	11q12.2	0 (0.00%)	4 (1.63%)	<-10	0.213	0.674	Mutual exclusivity
FAM45BP	Xq26.1	0 (0.00%)	4 (1.63%)	<-10	0.213	0.674	Mutual exclusivity
FAM98A	2p22.3	0 (0.00%)	4 (1.63%)	<-10	0.213	0.674	Mutual exclusivity
FATE1	Xq28	0 (0.00%)	4 (1.63%)	<-10	0.213	0.674	Mutual exclusivity
FBLL1	5q34	0 (0.00%)	4 (1.63%)	<-10	0.213	0.674	Mutual exclusivity
FBXO25	8p23.3	0 (0.00%)	4 (1.63%)	<-10	0.213	0.674	Mutual exclusivity
FBXW11	5q35.1	0 (0.00%)	4 (1.63%)	<-10	0.213	0.674	Mutual exclusivity
FEN1	11q12.2	0 (0.00%)	4 (1.63%)	<-10	0.213	0.674	Mutual exclusivity
FGF18	5q35.1	0 (0.00%)	4 (1.63%)	<-10	0.213	0.674	Mutual exclusivity
FLT4	5q35.3	0 (0.00%)	4 (1.63%)	<-10	0.213	0.674	Mutual exclusivity
FOXI1	5q35.1	0 (0.00%)	4 (1.63%)	<-10	0.213	0.674	Mutual exclusivity
FRMD7	Xq26.2	0 (0.00%)	4 (1.63%)	<-10	0.213	0.674	Mutual exclusivity
FTH1	11q12.3	0 (0.00%)	4 (1.63%)	<-10	0.213	0.674	Mutual exclusivity
GABRA3	Xq28	0 (0.00%)	4 (1.63%)	<-10	0.213	0.674	Mutual exclusivity
GABRP	5q35.1	0 (0.00%)	4 (1.63%)	<-10	0.213	0.674	Mutual exclusivity
GALNT14	2p23.1	0 (0.00%)	4 (1.63%)	<-10	0.213	0.674	Mutual exclusivity
GATAD2A	19p13.11	0 (0.00%)	4 (1.63%)	<-10	0.213	0.674	Mutual exclusivity
GFPT2	5q35.3	0 (0.00%)	4 (1.63%)	<-10	0.213	0.674	Mutual exclusivity
GHR	5p13.1-p12	0 (0.00%)	4 (1.63%)	<-10	0.213	0.674	Mutual exclusivity
GMIP	19p13.11	0 (0.00%)	4 (1.63%)	<-10	0.213	0.674	Mutual exclusivity
GPR119	Xq26.1	0 (0.00%)	4 (1.63%)	<-10	0.213	0.674	Mutual exclusivity
GRAMD2B	5q23.2	0 (0.00%)	4 (1.63%)	<-10	0.213	0.674	Mutual exclusivity
HDGFL2	19p13.3	0 (0.00%)	4 (1.63%)	<-10	0.213	0.674	Mutual exclusivity
HMMR	5q34	0 (0.00%)	4 (1.63%)	<-10	0.213	0.674	Mutual exclusivity
HRH2	5q35.2	0 (0.00%)	4 (1.63%)	<-10	0.213	0.674	Mutual exclusivity

HS3ST4	16p12.1	0 (0.00%)	4 (1.63%)	<-10	0.213	0.674	Mutual exclusivity
IGSF1	Xq26.1	0 (0.00%)	4 (1.63%)	<-10	0.213	0.674	Mutual exclusivity
IL4R	16p12.1	0 (0.00%)	4 (1.63%)	<-10	0.213	0.674	Mutual exclusivity
INCENP	11q12.3	0 (0.00%)	4 (1.63%)	<-10	0.213	0.674	Mutual exclusivity
INSYN2B	5q35.1	0 (0.00%)	4 (1.63%)	<-10	0.213	0.674	Mutual exclusivity
IZUMO2	19q13.33	0 (0.00%)	4 (1.63%)	<-10	0.213	0.674	Mutual exclusivity
KCNIP1	5q35.1	0 (0.00%)	4 (1.63%)	<-10	0.213	0.674	Mutual exclusivity
KCNMB1	5q35.1	0 (0.00%)	4 (1.63%)	<-10	0.213	0.674	Mutual exclusivity
KCNQ5	6q13	0 (0.00%)	4 (1.63%)	<-10	0.213	0.674	Mutual exclusivity
LBH	2p23.1	0 (0.00%)	4 (1.63%)	<-10	0.213	0.674	Mutual exclusivity
LCLAT1	2p23.1	0 (0.00%)	4 (1.63%)	<-10	0.213	0.674	Mutual exclusivity
LCP2	5q35.1	0 (0.00%)	4 (1.63%)	<-10	0.213	0.674	Mutual exclusivity
LINC00486	2p22.3	0 (0.00%)	4 (1.63%)	<-10	0.213	0.674	Mutual exclusivity
LINC00604	-	0 (0.00%)	4 (1.63%)	<-10	0.213	0.674	Mutual exclusivity
LINC00847	5q35.3	0 (0.00%)	4 (1.63%)	<-10	0.213	0.674	Mutual exclusivity
LPAR2	19p13.11	0 (0.00%)	4 (1.63%)	<-10	0.213	0.674	Mutual exclusivity
LRG1	19p13.3	0 (0.00%)	4 (1.63%)	<-10	0.213	0.674	Mutual exclusivity
LRPPRC	2p21	0 (0.00%)	4 (1.63%)	<-10	0.213	0.674	Mutual exclusivity
LSM11	5q33.3	0 (0.00%)	4 (1.63%)	<-10	0.213	0.674	Mutual exclusivity
LTBP1	2p22.3	0 (0.00%)	4 (1.63%)	<-10	0.213	0.674	Mutual exclusivity
MAMLD1	Xq28	0 (0.00%)	4 (1.63%)	<-10	0.213	0.674	Mutual exclusivity
MAPK9	5q35.3	0 (0.00%)	4 (1.63%)	<-10	0.213	0.674	Mutual exclusivity
MAT2B	5q34	0 (0.00%)	4 (1.63%)	<-10	0.213	0.674	Mutual exclusivity
MAU2	19p13.11	0 (0.00%)	4 (1.63%)	<-10	0.213	0.674	Mutual exclusivity
MEF2B	19p13.11	0 (0.00%)	4 (1.63%)	<-10	0.213	0.674	Mutual exclusivity
MEMO1	2p22.3	0 (0.00%)	4 (1.63%)	<-10	0.213	0.674	Mutual exclusivity
MGAT1	5q35.3	0 (0.00%)	4 (1.63%)	<-10	0.213	0.674	Mutual exclusivity
MIPOL1	14q13.3-q21.1	0 (0.00%)	4 (1.63%)	<-10	0.213	0.674	Mutual exclusivity
MIR-326/326		0 (0.00%)	4 (1.63%)	<-10	0.213	0.674	Mutual exclusivity

MIR-378E/378E		0 (0.00%)	4 (1.63%)	<-10	0.213	0.674	Mutual exclusivity
MIR-4454/4454		0 (0.00%)	4 (1.63%)	<-10	0.213	0.674	Mutual exclusivity
MIR-4634/4634		0 (0.00%)	4 (1.63%)	<-10	0.213	0.674	Mutual exclusivity
MIR-4765/4765		0 (0.00%)	4 (1.63%)	<-10	0.213	0.674	Mutual exclusivity
MIR-548AL/548AL		0 (0.00%)	4 (1.63%)	<-10	0.213	0.674	Mutual exclusivity
MIR-548W/548W		0 (0.00%)	4 (1.63%)	<-10	0.213	0.674	Mutual exclusivity
MIR-558/558		0 (0.00%)	4 (1.63%)	<-10	0.213	0.674	Mutual exclusivity
MIR-611/611		0 (0.00%)	4 (1.63%)	<-10	0.213	0.674	Mutual exclusivity
MIR-640/640		0 (0.00%)	4 (1.63%)	<-10	0.213	0.674	Mutual exclusivity
MOGAT2	11q13.5	0 (0.00%)	4 (1.63%)	<-10	0.213	0.674	Mutual exclusivity
MPND	19p13.3	0 (0.00%)	4 (1.63%)	<-10	0.213	0.674	Mutual exclusivity
MRPL22	5q33.2	0 (0.00%)	4 (1.63%)	<-10	0.213	0.674	Mutual exclusivity
MSX2	5q35.2	0 (0.00%)	4 (1.63%)	<-10	0.213	0.674	Mutual exclusivity
MYADML	2p22.3	0 (0.00%)	4 (1.63%)	<-10	0.213	0.674	Mutual exclusivity
MYDGF	19p13.3	0 (0.00%)	4 (1.63%)	<-10	0.213	0.674	Mutual exclusivity
MYH14	19q13.33	0 (0.00%)	4 (1.63%)	<-10	0.213	0.674	Mutual exclusivity
MYL10	7q22.1	0 (0.00%)	4 (1.63%)	<-10	0.213	0.674	Mutual exclusivity
MYOM2	8p23.3	0 (0.00%)	4 (1.63%)	<-10	0.213	0.674	Mutual exclusivity
MYOZ3	5q33.1	0 (0.00%)	4 (1.63%)	<-10	0.213	0.674	Mutual exclusivity
MYRF	11q12.2	0 (0.00%)	4 (1.63%)	<-10	0.213	0.674	Mutual exclusivity
NDUFA13	19p13.11	0 (0.00%)	4 (1.63%)	<-10	0.213	0.674	Mutual exclusivity
NDUFC2	11q14.1	0 (0.00%)	4 (1.63%)	<-10	0.213	0.674	Mutual exclusivity
NLRC4	2p22.3	0 (0.00%)	4 (1.63%)	<-10	0.213	0.674	Mutual exclusivity
NPM1	5q35.1	0 (0.00%)	4 (1.63%)	<-10	0.213	0.674	Mutual exclusivity
NR2C2AP	19p13.11	0 (0.00%)	4 (1.63%)	<-10	0.213	0.674	Mutual exclusivity
NRF1	7q32.2	0 (0.00%)	4 (1.63%)	<-10	0.213	0.674	Mutual exclusivity
NUDCD2	5q34	0 (0.00%)	4 (1.63%)	<-10	0.213	0.674	Mutual exclusivity
NUP205	7q33	0 (0.00%)	4 (1.63%)	<-10	0.213	0.674	Mutual exclusivity
OR13H1	Xq26.2	0 (0.00%)	4 (1.63%)	<-10	0.213	0.674	Mutual exclusivity

OR2AT4	11q13.4	0 (0.00%)	4 (1.63%)	<-10	0.213	0.674	Mutual exclusivity
OR2Y1	5q35.3	0 (0.00%)	4 (1.63%)	<-10	0.213	0.674	Mutual exclusivity
OR4F21	8p23.3	0 (0.00%)	4 (1.63%)	<-10	0.213	0.674	Mutual exclusivity
P4HA3	11q13.4	0 (0.00%)	4 (1.63%)	<-10	0.213	0.674	Mutual exclusivity
PANK3	5q34	0 (0.00%)	4 (1.63%)	<-10	0.213	0.674	Mutual exclusivity
PASD1	Xq28	0 (0.00%)	4 (1.63%)	<-10	0.213	0.674	Mutual exclusivity
PBX4	19p13.11	0 (0.00%)	4 (1.63%)	<-10	0.213	0.674	Mutual exclusivity
PCARE	2p23.2	0 (0.00%)	4 (1.63%)	<-10	0.213	0.674	Mutual exclusivity
PDE7B	6q23.3	0 (0.00%)	4 (1.63%)	<-10	0.213	0.674	Mutual exclusivity
PGM2L1	11q13.4	0 (0.00%)	4 (1.63%)	<-10	0.213	0.674	Mutual exclusivity
PLIN4	19p13.3	0 (0.00%)	4 (1.63%)	<-10	0.213	0.674	Mutual exclusivity
PLIN5	19p13.3	0 (0.00%)	4 (1.63%)	<-10	0.213	0.674	Mutual exclusivity
PMPCB	7q22.1	0 (0.00%)	4 (1.63%)	<-10	0.213	0.674	Mutual exclusivity
PPM1B	2p21	0 (0.00%)	4 (1.63%)	<-10	0.213	0.674	Mutual exclusivity
PREPL	2p21	0 (0.00%)	4 (1.63%)	<-10	0.213	0.674	Mutual exclusivity
PRKCE	2p21	0 (0.00%)	4 (1.63%)	<-10	0.213	0.674	Mutual exclusivity
PRRG3	Xq28	0 (0.00%)	4 (1.63%)	<-10	0.213	0.674	Mutual exclusivity
PSMC2	7q22.1	0 (0.00%)	4 (1.63%)	<-10	0.213	0.674	Mutual exclusivity
RAB33A	Xq26.1	0 (0.00%)	4 (1.63%)	<-10	0.213	0.674	Mutual exclusivity
RAB3IL1	11q12.2-q12.3	0 (0.00%)	4 (1.63%)	<-10	0.213	0.674	Mutual exclusivity
RANBP17	5q35.1	0 (0.00%)	4 (1.63%)	<-10	0.213	0.674	Mutual exclusivity
RAP2C	Xq26.2	0 (0.00%)	4 (1.63%)	<-10	0.213	0.674	Mutual exclusivity
RARS	5q34	0 (0.00%)	4 (1.63%)	<-10	0.213	0.674	Mutual exclusivity
RASGRP3	2p22.3	0 (0.00%)	4 (1.63%)	<-10	0.213	0.674	Mutual exclusivity
RBM22	5q33.1	0 (0.00%)	4 (1.63%)	<-10	0.213	0.674	Mutual exclusivity
RBMX2	Xq26.1	0 (0.00%)	4 (1.63%)	<-10	0.213	0.674	Mutual exclusivity
RELN	7q22.1	0 (0.00%)	4 (1.63%)	<-10	0.213	0.674	Mutual exclusivity
RFXANK	19p13.11	0 (0.00%)	4 (1.63%)	<-10	0.213	0.674	Mutual exclusivity
RN7SKP148	5q35.2	0 (0.00%)	4 (1.63%)	<-10	0.213	0.674	Mutual exclusivity

RN7SKP70	5q35.3	0 (0.00%)	4 (1.63%)	<-10	0.213	0.674	Mutual exclusivity
RN7SL121P	19p13.3	0 (0.00%)	4 (1.63%)	<-10	0.213	0.674	Mutual exclusivity
RN7SL191P	Xq26.1	0 (0.00%)	4 (1.63%)	<-10	0.213	0.674	Mutual exclusivity
RN7SL239P	11q13.4	0 (0.00%)	4 (1.63%)	<-10	0.213	0.674	Mutual exclusivity
RN7SL306P	7q32.1	0 (0.00%)	4 (1.63%)	<-10	0.213	0.674	Mutual exclusivity
RN7SL339P	5q35.1	0 (0.00%)	4 (1.63%)	<-10	0.213	0.674	Mutual exclusivity
RN7SL391P	12p13.31	0 (0.00%)	4 (1.63%)	<-10	0.213	0.674	Mutual exclusivity
RN7SL516P	2p23.2	0 (0.00%)	4 (1.63%)	<-10	0.213	0.674	Mutual exclusivity
RN7SL623P	5q35.1	0 (0.00%)	4 (1.63%)	<-10	0.213	0.674	Mutual exclusivity
RN7SL646P	5q35.3	0 (0.00%)	4 (1.63%)	<-10	0.213	0.674	Mutual exclusivity
RN7SL689P	5q23.2	0 (0.00%)	4 (1.63%)	<-10	0.213	0.674	Mutual exclusivity
RN7SL786P	11q13.5	0 (0.00%)	4 (1.63%)	<-10	0.213	0.674	Mutual exclusivity
RN7SL860P	19p12	0 (0.00%)	4 (1.63%)	<-10	0.213	0.674	Mutual exclusivity
RNA5SP180	5p15.1	0 (0.00%)	4 (1.63%)	<-10	0.213	0.674	Mutual exclusivity
RNA5SP244	7q32.2	0 (0.00%)	4 (1.63%)	<-10	0.213	0.674	Mutual exclusivity
RNA5SP405	16p12.1	0 (0.00%)	4 (1.63%)	<-10	0.213	0.674	Mutual exclusivity
RNA5SP514	Xq26.2	0 (0.00%)	4 (1.63%)	<-10	0.213	0.674	Mutual exclusivity
RNA5SP89	2p23.2	0 (0.00%)	4 (1.63%)	<-10	0.213	0.674	Mutual exclusivity
RNA5SP90	2p23.1	0 (0.00%)	4 (1.63%)	<-10	0.213	0.674	Mutual exclusivity
RNA5SP91	2p22.3	0 (0.00%)	4 (1.63%)	<-10	0.213	0.674	Mutual exclusivity
RNA5SP92	2p22.3	0 (0.00%)	4 (1.63%)	<-10	0.213	0.674	Mutual exclusivity
RPL23AP53	8p23.3	0 (0.00%)	4 (1.63%)	<-10	0.213	0.674	Mutual exclusivity
RPLP0P2	11q12.2	0 (0.00%)	4 (1.63%)	<-10	0.213	0.674	Mutual exclusivity
RSF1	11q14.1	0 (0.00%)	4 (1.63%)	<-10	0.213	0.674	Mutual exclusivity
SASH3	Xq26.1	0 (0.00%)	4 (1.63%)	<-10	0.213	0.674	Mutual exclusivity
SCGB3A1	5q35.3	0 (0.00%)	4 (1.63%)	<-10	0.213	0.674	Mutual exclusivity
SEMA6B	19p13.3	0 (0.00%)	4 (1.63%)	<-10	0.213	0.674	Mutual exclusivity
SERPINA1	14q32.13	0 (0.00%)	4 (1.63%)	<-10	0.213	0.674	Mutual exclusivity
SFXN1	5q35.2	0 (0.00%)	4 (1.63%)	<-10	0.213	0.674	Mutual exclusivity

SH3GL1	19p13.3	0 (0.00%)	4 (1.63%)	<-10	0.213	0.674	Mutual exclusivity
SH3PXD2B	5q35.1	0 (0.00%)	4 (1.63%)	<-10	0.213	0.674	Mutual exclusivity
SHD	19p13.3	0 (0.00%)	4 (1.63%)	<-10	0.213	0.674	Mutual exclusivity
SIX2	2p21	0 (0.00%)	4 (1.63%)	<-10	0.213	0.674	Mutual exclusivity
SIX3	2p21	0 (0.00%)	4 (1.63%)	<-10	0.213	0.674	Mutual exclusivity
SLC13A4	7q33	0 (0.00%)	4 (1.63%)	<-10	0.213	0.674	Mutual exclusivity
SLC25A14	Xq26.1	0 (0.00%)	4 (1.63%)	<-10	0.213	0.674	Mutual exclusivity
SLC26A5	7q22.1	0 (0.00%)	4 (1.63%)	<-10	0.213	0.674	Mutual exclusivity
SLC30A6	2p22.3	0 (0.00%)	4 (1.63%)	<-10	0.213	0.674	Mutual exclusivity
SLC3A1	2p21	0 (0.00%)	4 (1.63%)	<-10	0.213	0.674	Mutual exclusivity
SLC6A7	5q32	0 (0.00%)	4 (1.63%)	<-10	0.213	0.674	Mutual exclusivity
SLCO2B1	11q13.4	0 (0.00%)	4 (1.63%)	<-10	0.213	0.674	Mutual exclusivity
SMIM23	5q35.1	0 (0.00%)	4 (1.63%)	<-10	0.213	0.674	Mutual exclusivity
SMKR1	7q32.1	0 (0.00%)	4 (1.63%)	<-10	0.213	0.674	Mutual exclusivity
SNORA64	16p13.3	0 (0.00%)	4 (1.63%)	<-10	0.213	0.674	Mutual exclusivity
SNORA74B	5q35.1	0 (0.00%)	4 (1.63%)	<-10	0.213	0.674	Mutual exclusivity
SNX2	5q23.2	0 (0.00%)	4 (1.63%)	<-10	0.213	0.674	Mutual exclusivity
SNX24	5q23.2	0 (0.00%)	4 (1.63%)	<-10	0.213	0.674	Mutual exclusivity
SPAST	2p22.3	0 (0.00%)	4 (1.63%)	<-10	0.213	0.674	Mutual exclusivity
SPDL1	5q35.1	0 (0.00%)	4 (1.63%)	<-10	0.213	0.674	Mutual exclusivity
SRD5A2	2p23.1	0 (0.00%)	4 (1.63%)	<-10	0.213	0.674	Mutual exclusivity
STAP2	19p13.3	0 (0.00%)	4 (1.63%)	<-10	0.213	0.674	Mutual exclusivity
STK10	5q35.1	0 (0.00%)	4 (1.63%)	<-10	0.213	0.674	Mutual exclusivity
STK26	Xq26.2	0 (0.00%)	4 (1.63%)	<-10	0.213	0.674	Mutual exclusivity
STMP1	7q33	0 (0.00%)	4 (1.63%)	<-10	0.213	0.674	Mutual exclusivity
STRA8	7q33	0 (0.00%)	4 (1.63%)	<-10	0.213	0.674	Mutual exclusivity
STRIP2	7q32.1	0 (0.00%)	4 (1.63%)	<-10	0.213	0.674	Mutual exclusivity
TDRP	8p23.3	0 (0.00%)	4 (1.63%)	<-10	0.213	0.674	Mutual exclusivity
THG1L	5q33.3	0 (0.00%)	4 (1.63%)	<-10	0.213	0.674	Mutual exclusivity

THRSP	11q14.1	0 (0.00%)	4 (1.63%)	<-10	0.213	0.674	Mutual exclusivity
TKFC	11q12.2	0 (0.00%)	4 (1.63%)	<-10	0.213	0.674	Mutual exclusivity
TLX3	5q35.1	0 (0.00%)	4 (1.63%)	<-10	0.213	0.674	Mutual exclusivity
TMEM138	11q12.2	0 (0.00%)	4 (1.63%)	<-10	0.213	0.674	Mutual exclusivity
TMEM140	7q33	0 (0.00%)	4 (1.63%)	<-10	0.213	0.674	Mutual exclusivity
TMEM161A	19p13.11	0 (0.00%)	4 (1.63%)	<-10	0.213	0.674	Mutual exclusivity
TMEM216	11q12.2	0 (0.00%)	4 (1.63%)	<-10	0.213	0.674	Mutual exclusivity
TMEM258	11q12.2	0 (0.00%)	4 (1.63%)	<-10	0.213	0.674	Mutual exclusivity
TMIGD2	19p13.3	0 (0.00%)	4 (1.63%)	<-10	0.213	0.674	Mutual exclusivity
TNFAIP8L1	19p13.3	0 (0.00%)	4 (1.63%)	<-10	0.213	0.674	Mutual exclusivity
TPBGL	11q13.4	0 (0.00%)	4 (1.63%)	<-10	0.213	0.674	Mutual exclusivity
TSSK6	19p13.11	0 (0.00%)	4 (1.63%)	<-10	0.213	0.674	Mutual exclusivity
TTC27	2p22.3	0 (0.00%)	4 (1.63%)	<-10	0.213	0.674	Mutual exclusivity
UBE2B	5q31.1	0 (0.00%)	4 (1.63%)	<-10	0.213	0.674	Mutual exclusivity
UBTD2	5q35.1	0 (0.00%)	4 (1.63%)	<-10	0.213	0.674	Mutual exclusivity
UBXN6	19p13.3	0 (0.00%)	4 (1.63%)	<-10	0.213	0.674	Mutual exclusivity
USP26	Xq26.2	0 (0.00%)	4 (1.63%)	<-10	0.213	0.674	Mutual exclusivity
USP39	2p11.2	0 (0.00%)	4 (1.63%)	<-10	0.213	0.674	Mutual exclusivity
UTP14A	Xq26.1	0 (0.00%)	4 (1.63%)	<-10	0.213	0.674	Mutual exclusivity
VMA21	Xq28	0 (0.00%)	4 (1.63%)	<-10	0.213	0.674	Mutual exclusivity
WDR1	4p16.1	0 (0.00%)	4 (1.63%)	<-10	0.213	0.674	Mutual exclusivity
WDR91	7q33	0 (0.00%)	4 (1.63%)	<-10	0.213	0.674	Mutual exclusivity
WWC1	5q34	0 (0.00%)	4 (1.63%)	<-10	0.213	0.674	Mutual exclusivity
XDH	2p23.1	0 (0.00%)	4 (1.63%)	<-10	0.213	0.674	Mutual exclusivity
YIPF4	2p22.3	0 (0.00%)	4 (1.63%)	<-10	0.213	0.674	Mutual exclusivity
YJEFN3	19p13.11	0 (0.00%)	4 (1.63%)	<-10	0.213	0.674	Mutual exclusivity
YPEL5	2p23.1	0 (0.00%)	4 (1.63%)	<-10	0.213	0.674	Mutual exclusivity
ZDHHC9	Xq26.1	0 (0.00%)	4 (1.63%)	<-10	0.213	0.674	Mutual exclusivity
ZFP62	5q35.3	0 (0.00%)	4 (1.63%)	<-10	0.213	0.674	Mutual exclusivity

ZKSCAN2	16p12.1	0 (0.00%)	4 (1.63%)	<-10	0.213	0.674	Mutual exclusivity
ZNF101	19p13.11	0 (0.00%)	4 (1.63%)	<-10	0.213	0.674	Mutual exclusivity
ZNF208	19p12	0 (0.00%)	4 (1.63%)	<-10	0.213	0.674	Mutual exclusivity
ZNF257	19p12	0 (0.00%)	4 (1.63%)	<-10	0.213	0.674	Mutual exclusivity
ZNF280C	Xq26.1	0 (0.00%)	4 (1.63%)	<-10	0.213	0.674	Mutual exclusivity
ZNF492	19p12	0 (0.00%)	4 (1.63%)	<-10	0.213	0.674	Mutual exclusivity
ZNF596	8p23.3	0 (0.00%)	4 (1.63%)	<-10	0.213	0.674	Mutual exclusivity
ZNF608	5q23.2	0 (0.00%)	4 (1.63%)	<-10	0.213	0.674	Mutual exclusivity
ZNF676	19p12	0 (0.00%)	4 (1.63%)	<-10	0.213	0.674	Mutual exclusivity
ZNF729	19p12	0 (0.00%)	4 (1.63%)	<-10	0.213	0.674	Mutual exclusivity
ZNF98	19p12	0 (0.00%)	4 (1.63%)	<-10	0.213	0.674	Mutual exclusivity
ZNF99	19p12	0 (0.00%)	4 (1.63%)	<-10	0.213	0.674	Mutual exclusivity
CRH	8q13.1	8 (6.96%)	25 (10.20%)	-0.55	0.214	0.674	Mutual exclusivity
LINC00967	8q13.1	8 (6.96%)	25 (10.20%)	-0.55	0.214	0.674	Mutual exclusivity
ATPSCKMT	5p15.2	1 (0.87%)	7 (2.86%)	-1.72	0.216	0.674	Mutual exclusivity
CBLL1	7q22.3	1 (0.87%)	7 (2.86%)	-1.72	0.216	0.674	Mutual exclusivity
CCT5	5p15.2	1 (0.87%)	7 (2.86%)	-1.72	0.216	0.674	Mutual exclusivity
CMBL	5p15.2	1 (0.87%)	7 (2.86%)	-1.72	0.216	0.674	Mutual exclusivity
DLD	7q31.1	1 (0.87%)	7 (2.86%)	-1.72	0.216	0.674	Mutual exclusivity
EXOC4	7q33	1 (0.87%)	7 (2.86%)	-1.72	0.216	0.674	Mutual exclusivity
FAM3C	7q31.31	1 (0.87%)	7 (2.86%)	-1.72	0.216	0.674	Mutual exclusivity
FGFR4	5q35.2	1 (0.87%)	7 (2.86%)	-1.72	0.216	0.674	Mutual exclusivity
GPR85	7q31.1	1 (0.87%)	7 (2.86%)	-1.72	0.216	0.674	Mutual exclusivity
IL12RB1	19p13.11	1 (0.87%)	7 (2.86%)	-1.72	0.216	0.674	Mutual exclusivity
LAMB4	7q31.1	1 (0.87%)	7 (2.86%)	-1.72	0.216	0.674	Mutual exclusivity
LRFN4	11q13.2	1 (0.87%)	7 (2.86%)	-1.72	0.216	0.674	Mutual exclusivity
6-Mar	5p15.2	1 (0.87%)	7 (2.86%)	-1.72	0.216	0.674	Mutual exclusivity
MAST3	19p13.11	1 (0.87%)	7 (2.86%)	-1.72	0.216	0.674	Mutual exclusivity
NSD1	5q35.3	1 (0.87%)	7 (2.86%)	-1.72	0.216	0.674	Mutual exclusivity

PIK3R2	19p13.11	1 (0.87%)	7 (2.86%)	-1.72	0.216	0.674	Mutual exclusivity
SLC26A3	7q22.3-q31.1	1 (0.87%)	7 (2.86%)	-1.72	0.216	0.674	Mutual exclusivity
SLC26A4	7q22.3	1 (0.87%)	7 (2.86%)	-1.72	0.216	0.674	Mutual exclusivity
WNT16	7q31.31	1 (0.87%)	7 (2.86%)	-1.72	0.216	0.674	Mutual exclusivity
ZMAT4	8p11.21	1 (0.87%)	7 (2.86%)	-1.72	0.216	0.674	Mutual exclusivity
CCDC26	8q24.21	17 (14.78%)	46 (18.78%)	-0.34	0.219	0.674	Mutual exclusivity
CCN4	8q24.22	17 (14.78%)	46 (18.78%)	-0.34	0.219	0.674	Mutual exclusivity
LINC00861	8q24.13	17 (14.78%)	46 (18.78%)	-0.34	0.219	0.674	Mutual exclusivity
LINC00977	8q24.21	17 (14.78%)	46 (18.78%)	-0.34	0.219	0.674	Mutual exclusivity
MIR-1205/1205		17 (14.78%)	46 (18.78%)	-0.34	0.219	0.674	Mutual exclusivity
MIR-3686/3686		17 (14.78%)	46 (18.78%)	-0.34	0.219	0.674	Mutual exclusivity
NDRG1	8q24.22	17 (14.78%)	46 (18.78%)	-0.34	0.219	0.674	Mutual exclusivity
POU5F1B	8q24.21	17 (14.78%)	46 (18.78%)	-0.34	0.219	0.674	Mutual exclusivity
RN7SKP206	8q24.21	17 (14.78%)	46 (18.78%)	-0.34	0.219	0.674	Mutual exclusivity
SLA	8q24.22	17 (14.78%)	46 (18.78%)	-0.34	0.219	0.674	Mutual exclusivity
ST3GAL1	8q24.22	17 (14.78%)	46 (18.78%)	-0.34	0.219	0.674	Mutual exclusivity
TG	8q24.22	17 (14.78%)	46 (18.78%)	-0.34	0.219	0.674	Mutual exclusivity
TMEM75	8q24.21	17 (14.78%)	46 (18.78%)	-0.34	0.219	0.674	Mutual exclusivity
AHRR	5p15.33	7 (6.09%)	9 (3.67%)	0.73	0.22	0.674	Co-occurrence
ATXN1	6p22.3	7 (6.09%)	9 (3.67%)	0.73	0.22	0.674	Co-occurrence
BRD9	5p15.33	7 (6.09%)	9 (3.67%)	0.73	0.22	0.674	Co-occurrence
CCDC127	5p15.33	7 (6.09%)	9 (3.67%)	0.73	0.22	0.674	Co-occurrence
CEP72	5p15.33	7 (6.09%)	9 (3.67%)	0.73	0.22	0.674	Co-occurrence
EXOC3	5p15.33	7 (6.09%)	9 (3.67%)	0.73	0.22	0.674	Co-occurrence
EXOC3-AS1	5p15.33	7 (6.09%)	9 (3.67%)	0.73	0.22	0.674	Co-occurrence
HDGFL1	6p22.3	7 (6.09%)	9 (3.67%)	0.73	0.22	0.674	Co-occurrence
ID4	6p22.3	7 (6.09%)	9 (3.67%)	0.73	0.22	0.674	Co-occurrence
JARID2	6p22.3	7 (6.09%)	9 (3.67%)	0.73	0.22	0.674	Co-occurrence
LRRC14B	5p15.33	7 (6.09%)	9 (3.67%)	0.73	0.22	0.674	Co-occurrence

MIR-4456/4456		7 (6.09%)	9 (3.67%)	0.73	0.22	0.674	Co-occurrence
PDCD6	5p15.33	7 (6.09%)	9 (3.67%)	0.73	0.22	0.674	Co-occurrence
PLEKHG4B	5p15.33	7 (6.09%)	9 (3.67%)	0.73	0.22	0.674	Co-occurrence
PRL	6p22.3	7 (6.09%)	9 (3.67%)	0.73	0.22	0.674	Co-occurrence
RBM24	6p22.3	7 (6.09%)	9 (3.67%)	0.73	0.22	0.674	Co-occurrence
RN7SKP240	6p22.3	7 (6.09%)	9 (3.67%)	0.73	0.22	0.674	Co-occurrence
RN7SL140P	2p24.1	7 (6.09%)	9 (3.67%)	0.73	0.22	0.674	Co-occurrence
RNA5SP205	6p22.3	7 (6.09%)	9 (3.67%)	0.73	0.22	0.674	Co-occurrence
SDHA	5p15.33	7 (6.09%)	9 (3.67%)	0.73	0.22	0.674	Co-occurrence
SLC9A3	5p15.33	7 (6.09%)	9 (3.67%)	0.73	0.22	0.674	Co-occurrence
SOX4	6p22.3	7 (6.09%)	9 (3.67%)	0.73	0.22	0.674	Co-occurrence
STMND1	6p22.3	7 (6.09%)	9 (3.67%)	0.73	0.22	0.674	Co-occurrence
TPPP	5p15.33	7 (6.09%)	9 (3.67%)	0.73	0.22	0.674	Co-occurrence
TRIP13	5p15.33	7 (6.09%)	9 (3.67%)	0.73	0.22	0.674	Co-occurrence
WFIKKN2	17q21.33	7 (6.09%)	9 (3.67%)	0.73	0.22	0.674	Co-occurrence
ZDHHC11	5p15.33	7 (6.09%)	9 (3.67%)	0.73	0.22	0.674	Co-occurrence
ZDHHC11B	5p15.33	7 (6.09%)	9 (3.67%)	0.73	0.22	0.674	Co-occurrence
ERICH5	8q22.2	14 (12.17%)	39 (15.92%)	-0.39	0.221	0.674	Mutual exclusivity
ESRP1	8q22.1	14 (12.17%)	39 (15.92%)	-0.39	0.221	0.674	Mutual exclusivity
GDF6	8q22.1	14 (12.17%)	39 (15.92%)	-0.39	0.221	0.674	Mutual exclusivity
KCNS2	8q22.2	14 (12.17%)	39 (15.92%)	-0.39	0.221	0.674	Mutual exclusivity
MTDH	8q22.1	14 (12.17%)	39 (15.92%)	-0.39	0.221	0.674	Mutual exclusivity
POP1	8q22.2	14 (12.17%)	39 (15.92%)	-0.39	0.221	0.674	Mutual exclusivity
RIDA	8q22.2	14 (12.17%)	39 (15.92%)	-0.39	0.221	0.674	Mutual exclusivity
TSPYL5	8q22.1	14 (12.17%)	39 (15.92%)	-0.39	0.221	0.674	Mutual exclusivity
DNAJC5B	8q13.1	9 (7.83%)	27 (11.02%)	-0.49	0.228	0.674	Mutual exclusivity
ABCB1	7q21.12	4 (3.48%)	4 (1.63%)	1.09	0.229	0.674	Co-occurrence
ABHD16B	20q13.33	4 (3.48%)	4 (1.63%)	1.09	0.229	0.674	Co-occurrence
ARFRP1	20q13.33	4 (3.48%)	4 (1.63%)	1.09	0.229	0.674	Co-occurrence

BCAM	19q13.32	4 (3.48%)	4 (1.63%)	1.09	0.229	0.674	Co-occurrence
C20ORF204	20q13.33	4 (3.48%)	4 (1.63%)	1.09	0.229	0.674	Co-occurrence
CACNA1G	17q21.33	4 (3.48%)	4 (1.63%)	1.09	0.229	0.674	Co-occurrence
CALML3	10p15.1	4 (3.48%)	4 (1.63%)	1.09	0.229	0.674	Co-occurrence
CBX1	17q21.32	4 (3.48%)	4 (1.63%)	1.09	0.229	0.674	Co-occurrence
CCNE1	19q12	4 (3.48%)	4 (1.63%)	1.09	0.229	0.674	Co-occurrence
CDK5RAP3	17q21.32	4 (3.48%)	4 (1.63%)	1.09	0.229	0.674	Co-occurrence
CHRNB3	8p11.21	4 (3.48%)	4 (1.63%)	1.09	0.229	0.674	Co-occurrence
CYP3A43	7q22.1	4 (3.48%)	4 (1.63%)	1.09	0.229	0.674	Co-occurrence
DNAJC5	20q13.33	4 (3.48%)	4 (1.63%)	1.09	0.229	0.674	Co-occurrence
EEF1A2	20q13.33	4 (3.48%)	4 (1.63%)	1.09	0.229	0.674	Co-occurrence
FNDC11	20q13.33	4 (3.48%)	4 (1.63%)	1.09	0.229	0.674	Co-occurrence
GMEB2	20q13.33	4 (3.48%)	4 (1.63%)	1.09	0.229	0.674	Co-occurrence
GTPBP10	7q21.13	4 (3.48%)	4 (1.63%)	1.09	0.229	0.674	Co-occurrence
HELZ2	20q13.33	4 (3.48%)	4 (1.63%)	1.09	0.229	0.674	Co-occurrence
HNRNPA3	2q31.2	4 (3.48%)	4 (1.63%)	1.09	0.229	0.674	Co-occurrence
LIME1	20q13.33	4 (3.48%)	4 (1.63%)	1.09	0.229	0.674	Co-occurrence
LKAAEAR1	20q13.33	4 (3.48%)	4 (1.63%)	1.09	0.229	0.674	Co-occurrence
LRRC15	3q29	4 (3.48%)	4 (1.63%)	1.09	0.229	0.674	Co-occurrence
MBTD1	17q21.33	4 (3.48%)	4 (1.63%)	1.09	0.229	0.674	Co-occurrence
MIR-647/647		4 (3.48%)	4 (1.63%)	1.09	0.229	0.674	Co-occurrence
MYT1	20q13.33	4 (3.48%)	4 (1.63%)	1.09	0.229	0.674	Co-occurrence
NFE2L1	17q21.32	4 (3.48%)	4 (1.63%)	1.09	0.229	0.674	Co-occurrence
NPBWR2	20q13.33	4 (3.48%)	4 (1.63%)	1.09	0.229	0.674	Co-occurrence
OPRL1	20q13.33	4 (3.48%)	4 (1.63%)	1.09	0.229	0.674	Co-occurrence
PCMTD2	20q13.33	4 (3.48%)	4 (1.63%)	1.09	0.229	0.674	Co-occurrence
PIPOX	17q11.2	4 (3.48%)	4 (1.63%)	1.09	0.229	0.674	Co-occurrence
PPDPF	20q13.33	4 (3.48%)	4 (1.63%)	1.09	0.229	0.674	Co-occurrence
PRPF6	20q13.33	4 (3.48%)	4 (1.63%)	1.09	0.229	0.674	Co-occurrence

PTK6	20q13.33	4 (3.48%)	4 (1.63%)	1.09	0.229	0.674	Co-occurrence
RBM45	2q31.2	4 (3.48%)	4 (1.63%)	1.09	0.229	0.674	Co-occurrence
RDH16	12q13.3	4 (3.48%)	4 (1.63%)	1.09	0.229	0.674	Co-occurrence
RGS19	20q13.33	4 (3.48%)	4 (1.63%)	1.09	0.229	0.674	Co-occurrence
RN7SL672P	20q13.13	4 (3.48%)	4 (1.63%)	1.09	0.229	0.674	Co-occurrence
RTEL1	20q13.33	4 (3.48%)	4 (1.63%)	1.09	0.229	0.674	Co-occurrence
SAMD10	20q13.33	4 (3.48%)	4 (1.63%)	1.09	0.229	0.674	Co-occurrence
SLC20A2	8p11.21	4 (3.48%)	4 (1.63%)	1.09	0.229	0.674	Co-occurrence
SLC2A4RG	20q13.33	4 (3.48%)	4 (1.63%)	1.09	0.229	0.674	Co-occurrence
SMIM19	8p11.21	4 (3.48%)	4 (1.63%)	1.09	0.229	0.674	Co-occurrence
SOX18	20q13.33	4 (3.48%)	4 (1.63%)	1.09	0.229	0.674	Co-occurrence
SPATA2	20q13.13	4 (3.48%)	4 (1.63%)	1.09	0.229	0.674	Co-occurrence
SRMS	20q13.33	4 (3.48%)	4 (1.63%)	1.09	0.229	0.674	Co-occurrence
STMN3	20q13.33	4 (3.48%)	4 (1.63%)	1.09	0.229	0.674	Co-occurrence
TCEA2	20q13.33	4 (3.48%)	4 (1.63%)	1.09	0.229	0.674	Co-occurrence
TMEM44	3q29	4 (3.48%)	4 (1.63%)	1.09	0.229	0.674	Co-occurrence
TNFRSF6B	20q13.33	4 (3.48%)	4 (1.63%)	1.09	0.229	0.674	Co-occurrence
TPD52L2	20q13.33	4 (3.48%)	4 (1.63%)	1.09	0.229	0.674	Co-occurrence
UCKL1	20q13.33	4 (3.48%)	4 (1.63%)	1.09	0.229	0.674	Co-occurrence
ZBTB46	20q13.33	4 (3.48%)	4 (1.63%)	1.09	0.229	0.674	Co-occurrence
ZGPAT	20q13.33	4 (3.48%)	4 (1.63%)	1.09	0.229	0.674	Co-occurrence
ZNF512B	20q13.33	4 (3.48%)	4 (1.63%)	1.09	0.229	0.674	Co-occurrence
ATP6V1C1	8q22.3	15 (13.04%)	41 (16.73%)	-0.36	0.23	0.674	Mutual exclusivity
AZIN1	8q22.3	15 (13.04%)	41 (16.73%)	-0.36	0.23	0.674	Mutual exclusivity
BAALC	8q22.3	15 (13.04%)	41 (16.73%)	-0.36	0.23	0.674	Mutual exclusivity
BAALC-AS2	8q22.3	15 (13.04%)	41 (16.73%)	-0.36	0.23	0.674	Mutual exclusivity
CTHRC1	8q22.3	15 (13.04%)	41 (16.73%)	-0.36	0.23	0.674	Mutual exclusivity
DCAF13	8q22.3	15 (13.04%)	41 (16.73%)	-0.36	0.23	0.674	Mutual exclusivity
FZD6	8q22.3	15 (13.04%)	41 (16.73%)	-0.36	0.23	0.674	Mutual exclusivity

MIR-1273A/1273A		15 (13.04%)	41 (16.73%)	-0.36	0.23	0.674	Mutual exclusivity
SLC25A32	8q22.3	15 (13.04%)	41 (16.73%)	-0.36	0.23	0.674	Mutual exclusivity
CASKIN2	17q25.1	8 (6.96%)	11 (4.49%)	0.63	0.231	0.674	Co-occurrence
DUSP22	6p25.3	8 (6.96%)	11 (4.49%)	0.63	0.231	0.674	Co-occurrence
EXOC2	6p25.3	8 (6.96%)	11 (4.49%)	0.63	0.231	0.674	Co-occurrence
FOXC1	6p25.3	8 (6.96%)	11 (4.49%)	0.63	0.231	0.674	Co-occurrence
FOXF2	6p25.3	8 (6.96%)	11 (4.49%)	0.63	0.231	0.674	Co-occurrence
FOXQ1	6p25.3	8 (6.96%)	11 (4.49%)	0.63	0.231	0.674	Co-occurrence
HUS1B	6p25.3	8 (6.96%)	11 (4.49%)	0.63	0.231	0.674	Co-occurrence
IRF4	6p25.3	8 (6.96%)	11 (4.49%)	0.63	0.231	0.674	Co-occurrence
LINC01600	6p25.2	8 (6.96%)	11 (4.49%)	0.63	0.231	0.674	Co-occurrence
MYLK4	6p25.2	8 (6.96%)	11 (4.49%)	0.63	0.231	0.674	Co-occurrence
OTOP2	17q25.1	8 (6.96%)	11 (4.49%)	0.63	0.231	0.674	Co-occurrence
OTOP3	17q25.1	8 (6.96%)	11 (4.49%)	0.63	0.231	0.674	Co-occurrence
PSMG4	6p25.2	8 (6.96%)	11 (4.49%)	0.63	0.231	0.674	Co-occurrence
RIPK1	6p25.2	8 (6.96%)	11 (4.49%)	0.63	0.231	0.674	Co-occurrence
RN7SL352P	6p25.3	8 (6.96%)	11 (4.49%)	0.63	0.231	0.674	Co-occurrence
SERPINB1	6p25.2	8 (6.96%)	11 (4.49%)	0.63	0.231	0.674	Co-occurrence
TMEM94	17q25.1	8 (6.96%)	11 (4.49%)	0.63	0.231	0.674	Co-occurrence
USH1G	17q25.1	8 (6.96%)	11 (4.49%)	0.63	0.231	0.674	Co-occurrence
WRNIP1	6p25.2	8 (6.96%)	11 (4.49%)	0.63	0.231	0.674	Co-occurrence
FAM110B	8q12.1	7 (6.09%)	22 (8.98%)	-0.56	0.235	0.674	Mutual exclusivity
TNFRSF21	6p12.3	3 (2.61%)	12 (4.90%)	-0.91	0.238	0.674	Mutual exclusivity
DPYS	8q22.3	16 (13.91%)	43 (17.55%)	-0.34	0.239	0.674	Mutual exclusivity
LRP12	8q22.3	16 (13.91%)	43 (17.55%)	-0.34	0.239	0.674	Mutual exclusivity
ZFPM2	8q23.1	16 (13.91%)	43 (17.55%)	-0.34	0.239	0.674	Mutual exclusivity
MIR-4525/4525		9 (7.83%)	13 (5.31%)	0.56	0.24	0.674	Co-occurrence
RAB40B	17q25.3	9 (7.83%)	13 (5.31%)	0.56	0.24	0.674	Co-occurrence
WDR45B	17q25.3	9 (7.83%)	13 (5.31%)	0.56	0.24	0.674	Co-occurrence

AAGAB	15q23	2 (1.74%)	1 (0.41%)	2.09	0.24	0.674	Co-occurrence
ABHD17C	15q25.1	2 (1.74%)	1 (0.41%)	2.09	0.24	0.674	Co-occurrence
ABHD2	15q26.1	2 (1.74%)	1 (0.41%)	2.09	0.24	0.674	Co-occurrence
ACSBG1	15q25.1	2 (1.74%)	1 (0.41%)	2.09	0.24	0.674	Co-occurrence
ACTB	7p22.1	2 (1.74%)	1 (0.41%)	2.09	0.24	0.674	Co-occurrence
ACTL8	1p36.13	2 (1.74%)	1 (0.41%)	2.09	0.24	0.674	Co-occurrence
ACVR1	2q24.1	2 (1.74%)	1 (0.41%)	2.09	0.24	0.674	Co-occurrence
ACVR1C	2q24.1	2 (1.74%)	1 (0.41%)	2.09	0.24	0.674	Co-occurrence
ADA2	22q11.1	2 (1.74%)	1 (0.41%)	2.09	0.24	0.674	Co-occurrence
ADAMTS7	15q25.1	2 (1.74%)	1 (0.41%)	2.09	0.24	0.674	Co-occurrence
ADAMTSL2	9q34.2	2 (1.74%)	1 (0.41%)	2.09	0.24	0.674	Co-occurrence
ADAP2	17q11.2	2 (1.74%)	1 (0.41%)	2.09	0.24	0.674	Co-occurrence
ADK	10q22.2|10q11-q24	2 (1.74%)	1 (0.41%)	2.09	0.24	0.674	Co-occurrence
ADM	11p15.4	2 (1.74%)	1 (0.41%)	2.09	0.24	0.674	Co-occurrence
AEN	15q26.1	2 (1.74%)	1 (0.41%)	2.09	0.24	0.674	Co-occurrence
AGBL1	15q25.3	2 (1.74%)	1 (0.41%)	2.09	0.24	0.674	Co-occurrence
AGPAT2	9q34.3	2 (1.74%)	1 (0.41%)	2.09	0.24	0.674	Co-occurrence
AIMP2	7p22.1	2 (1.74%)	1 (0.41%)	2.09	0.24	0.674	Co-occurrence
AK1	9q34.11	2 (1.74%)	1 (0.41%)	2.09	0.24	0.674	Co-occurrence
AKAP13	15q25.3	2 (1.74%)	1 (0.41%)	2.09	0.24	0.674	Co-occurrence
AKAP7	6q23.2	2 (1.74%)	1 (0.41%)	2.09	0.24	0.674	Co-occurrence
AKNAD1	1p13.3	2 (1.74%)	1 (0.41%)	2.09	0.24	0.674	Co-occurrence
ALDH1L2	12q23.3	2 (1.74%)	1 (0.41%)	2.09	0.24	0.674	Co-occurrence
AMBP	9q32	2 (1.74%)	1 (0.41%)	2.09	0.24	0.674	Co-occurrence
AMDHD1	12q23.1	2 (1.74%)	1 (0.41%)	2.09	0.24	0.674	Co-occurrence
AMH	19p13.3	2 (1.74%)	1 (0.41%)	2.09	0.24	0.674	Co-occurrence
AMPD3	11p15.4	2 (1.74%)	1 (0.41%)	2.09	0.24	0.674	Co-occurrence
AMT	3p21.31	2 (1.74%)	1 (0.41%)	2.09	0.24	0.674	Co-occurrence
AMZ1	7p22.3	2 (1.74%)	1 (0.41%)	2.09	0.24	0.674	Co-occurrence

ANKRD27	19q13.11	2 (1.74%)	1 (0.41%)	2.09	0.24	0.674	Co-occurrence
ANKRD34C	15q25.1	2 (1.74%)	1 (0.41%)	2.09	0.24	0.674	Co-occurrence
ANKRD61	7p22.1	2 (1.74%)	1 (0.41%)	2.09	0.24	0.674	Co-occurrence
ANP32A	15q23	2 (1.74%)	1 (0.41%)	2.09	0.24	0.674	Co-occurrence
ANPEP	15q26.1	2 (1.74%)	1 (0.41%)	2.09	0.24	0.674	Co-occurrence
ANXA2	15q22.2	2 (1.74%)	1 (0.41%)	2.09	0.24	0.674	Co-occurrence
AP1B1P1	22q12.3	2 (1.74%)	1 (0.41%)	2.09	0.24	0.674	Co-occurrence
AP3M1	10q22.2	2 (1.74%)	1 (0.41%)	2.09	0.24	0.674	Co-occurrence
AP3S2	15q26.1	2 (1.74%)	1 (0.41%)	2.09	0.24	0.674	Co-occurrence
AP5Z1	7p22.1	2 (1.74%)	1 (0.41%)	2.09	0.24	0.674	Co-occurrence
APH1B	15q22.2	2 (1.74%)	1 (0.41%)	2.09	0.24	0.674	Co-occurrence
AQP9	15q21.3	2 (1.74%)	1 (0.41%)	2.09	0.24	0.674	Co-occurrence
ARG1	6q23.2	2 (1.74%)	1 (0.41%)	2.09	0.24	0.674	Co-occurrence
ARHGAP5	14q12	2 (1.74%)	1 (0.41%)	2.09	0.24	0.674	Co-occurrence
ARHGAP6	Xp22.2	2 (1.74%)	1 (0.41%)	2.09	0.24	0.674	Co-occurrence
ARHGEF39	9p13.3	2 (1.74%)	1 (0.41%)	2.09	0.24	0.674	Co-occurrence
ARID3B	15q24.1	2 (1.74%)	1 (0.41%)	2.09	0.24	0.674	Co-occurrence
ARNT2	15q25.1	2 (1.74%)	1 (0.41%)	2.09	0.24	0.674	Co-occurrence
ARPIN	15q26.1	2 (1.74%)	1 (0.41%)	2.09	0.24	0.674	Co-occurrence
ARRDC1	9q34.3	2 (1.74%)	1 (0.41%)	2.09	0.24	0.674	Co-occurrence
ARRDC1-AS1	9q34.3	2 (1.74%)	1 (0.41%)	2.09	0.24	0.674	Co-occurrence
ATP13A2	1p36.13	2 (1.74%)	1 (0.41%)	2.09	0.24	0.674	Co-occurrence
ATP23	12q14.1	2 (1.74%)	1 (0.41%)	2.09	0.24	0.674	Co-occurrence
ATXN8OS	13q21.33	2 (1.74%)	1 (0.41%)	2.09	0.24	0.674	Co-occurrence
BAP1	3p21.1	2 (1.74%)	1 (0.41%)	2.09	0.24	0.674	Co-occurrence
BCAR4	16p13.13	2 (1.74%)	1 (0.41%)	2.09	0.24	0.674	Co-occurrence
BCL2A1	15q25.1	2 (1.74%)	1 (0.41%)	2.09	0.24	0.674	Co-occurrence
BCL2L2	14q11.2	2 (1.74%)	1 (0.41%)	2.09	0.24	0.674	Co-occurrence
BNIP2	15q22.2	2 (1.74%)	1 (0.41%)	2.09	0.24	0.674	Co-occurrence

BORA	13q21.33	2 (1.74%)	1 (0.41%)	2.09	0.24	0.674	Co-occurrence
BPIFC	22q12.3	2 (1.74%)	1 (0.41%)	2.09	0.24	0.674	Co-occurrence
C12ORF42	12q23.2-q23.3	2 (1.74%)	1 (0.41%)	2.09	0.24	0.674	Co-occurrence
C12ORF45	12q23.3	2 (1.74%)	1 (0.41%)	2.09	0.24	0.674	Co-occurrence
C12ORF71	12p11.23	2 (1.74%)	1 (0.41%)	2.09	0.24	0.674	Co-occurrence
C12ORF73	12q23.3	2 (1.74%)	1 (0.41%)	2.09	0.24	0.674	Co-occurrence
C15ORF39	15q24.2	2 (1.74%)	1 (0.41%)	2.09	0.24	0.674	Co-occurrence
C15ORF61	15q23	2 (1.74%)	1 (0.41%)	2.09	0.24	0.674	Co-occurrence
C15ORF65	15q21.3	2 (1.74%)	1 (0.41%)	2.09	0.24	0.674	Co-occurrence
C1GALT1	7p22.1-p21.3	2 (1.74%)	1 (0.41%)	2.09	0.24	0.674	Co-occurrence
C1ORF141	1p31.3	2 (1.74%)	1 (0.41%)	2.09	0.24	0.674	Co-occurrence
C22ORF42	22q12.3	2 (1.74%)	1 (0.41%)	2.09	0.24	0.674	Co-occurrence
C2CD4A	15q22.2	2 (1.74%)	1 (0.41%)	2.09	0.24	0.674	Co-occurrence
C2CD4B	15q22.2	2 (1.74%)	1 (0.41%)	2.09	0.24	0.674	Co-occurrence
C3ORF62	3p21.31	2 (1.74%)	1 (0.41%)	2.09	0.24	0.674	Co-occurrence
C7ORF26	7p22.1	2 (1.74%)	1 (0.41%)	2.09	0.24	0.674	Co-occurrence
C9ORF116	9q34.3	2 (1.74%)	1 (0.41%)	2.09	0.24	0.674	Co-occurrence
C9ORF163	9q34.3	2 (1.74%)	1 (0.41%)	2.09	0.24	0.674	Co-occurrence
C9ORF78	9q34.11	2 (1.74%)	1 (0.41%)	2.09	0.24	0.674	Co-occurrence
CA12	15q22.2	2 (1.74%)	1 (0.41%)	2.09	0.24	0.674	Co-occurrence
CA9	9p13.3	2 (1.74%)	1 (0.41%)	2.09	0.24	0.674	Co-occurrence
CACNA1B	9q34.3	2 (1.74%)	1 (0.41%)	2.09	0.24	0.674	Co-occurrence
CAMSAP1	9q34.3	2 (1.74%)	1 (0.41%)	2.09	0.24	0.674	Co-occurrence
CAP1	1p34.2	2 (1.74%)	1 (0.41%)	2.09	0.24	0.674	Co-occurrence
CAPN12	19q13.2	2 (1.74%)	1 (0.41%)	2.09	0.24	0.674	Co-occurrence
CARD11	7p22.2	2 (1.74%)	1 (0.41%)	2.09	0.24	0.674	Co-occurrence
CASC3	17q21.1	2 (1.74%)	1 (0.41%)	2.09	0.24	0.674	Co-occurrence
CCDC107	9p13.3	2 (1.74%)	1 (0.41%)	2.09	0.24	0.674	Co-occurrence
CCDC33	15q24.1	2 (1.74%)	1 (0.41%)	2.09	0.24	0.674	Co-occurrence

CCNB2	15q22.2	2 (1.74%)	1 (0.41%)	2.09	0.24	0.674	Co-occurrence
CCP110	16p12.3	2 (1.74%)	1 (0.41%)	2.09	0.24	0.674	Co-occurrence
CCPG1	15q21.3	2 (1.74%)	1 (0.41%)	2.09	0.24	0.674	Co-occurrence
CCT6B	17q12	2 (1.74%)	1 (0.41%)	2.09	0.24	0.674	Co-occurrence
CCZ1	7p22.1	2 (1.74%)	1 (0.41%)	2.09	0.24	0.674	Co-occurrence
CCZ1B	7p22.1	2 (1.74%)	1 (0.41%)	2.09	0.24	0.674	Co-occurrence
CD276	15q24.1	2 (1.74%)	1 (0.41%)	2.09	0.24	0.674	Co-occurrence
CDC6	17q21.2	2 (1.74%)	1 (0.41%)	2.09	0.24	0.674	Co-occurrence
CDH23	10q22.1	2 (1.74%)	1 (0.41%)	2.09	0.24	0.674	Co-occurrence
CDPF1	22q13.31	2 (1.74%)	1 (0.41%)	2.09	0.24	0.674	Co-occurrence
CEACAM1	19q13.2	2 (1.74%)	1 (0.41%)	2.09	0.24	0.674	Co-occurrence
CEACAM8	19q13.2	2 (1.74%)	1 (0.41%)	2.09	0.24	0.674	Co-occurrence
CELSR2	1p13.3	2 (1.74%)	1 (0.41%)	2.09	0.24	0.674	Co-occurrence
CEMIP	15q25.1	2 (1.74%)	1 (0.41%)	2.09	0.24	0.674	Co-occurrence
CEND1	11p15.5	2 (1.74%)	1 (0.41%)	2.09	0.24	0.674	Co-occurrence
CEP89	19q13.11	2 (1.74%)	1 (0.41%)	2.09	0.24	0.674	Co-occurrence
CFAP100	3q21.3	2 (1.74%)	1 (0.41%)	2.09	0.24	0.674	Co-occurrence
CFAP57	1p34.2	2 (1.74%)	1 (0.41%)	2.09	0.24	0.674	Co-occurrence
CHRNA3	15q25.1	2 (1.74%)	1 (0.41%)	2.09	0.24	0.674	Co-occurrence
CHRNA5	15q25.1	2 (1.74%)	1 (0.41%)	2.09	0.24	0.674	Co-occurrence
CHRNB4	15q25.1	2 (1.74%)	1 (0.41%)	2.09	0.24	0.674	Co-occurrence
CHST11	12q23.3	2 (1.74%)	1 (0.41%)	2.09	0.24	0.674	Co-occurrence
CIC	19q13.2	2 (1.74%)	1 (0.41%)	2.09	0.24	0.674	Co-occurrence
CKAP4	12q23.3	2 (1.74%)	1 (0.41%)	2.09	0.24	0.674	Co-occurrence
CLIP1	12q24.31	2 (1.74%)	1 (0.41%)	2.09	0.24	0.674	Co-occurrence
CLK3	15q24.1	2 (1.74%)	1 (0.41%)	2.09	0.24	0.674	Co-occurrence
CLN6	15q23	2 (1.74%)	1 (0.41%)	2.09	0.24	0.674	Co-occurrence
CMA1	14q12	2 (1.74%)	1 (0.41%)	2.09	0.24	0.674	Co-occurrence
CMTM5	14q11.2	2 (1.74%)	1 (0.41%)	2.09	0.24	0.674	Co-occurrence

COL5A1	9q34.3	2 (1.74%)	1 (0.41%)	2.09	0.24	0.674	Co-occurrence
COMMD6	13q22.2	2 (1.74%)	1 (0.41%)	2.09	0.24	0.674	Co-occurrence
CORO2B	15q23	2 (1.74%)	1 (0.41%)	2.09	0.24	0.674	Co-occurrence
COX5A	15q24.2	2 (1.74%)	1 (0.41%)	2.09	0.24	0.674	Co-occurrence
CPLX3	15q24.1	2 (1.74%)	1 (0.41%)	2.09	0.24	0.674	Co-occurrence
CPPED1	16p13.12	2 (1.74%)	1 (0.41%)	2.09	0.24	0.674	Co-occurrence
CPT2	1p32.3	2 (1.74%)	1 (0.41%)	2.09	0.24	0.674	Co-occurrence
CRABP1	15q25.1	2 (1.74%)	1 (0.41%)	2.09	0.24	0.674	Co-occurrence
CROCC	1p36.13	2 (1.74%)	1 (0.41%)	2.09	0.24	0.674	Co-occurrence
CROCCP2	1p36.13	2 (1.74%)	1 (0.41%)	2.09	0.24	0.674	Co-occurrence
CROCCP3	1p36.13	2 (1.74%)	1 (0.41%)	2.09	0.24	0.674	Co-occurrence
CSF3	17q21.1	2 (1.74%)	1 (0.41%)	2.09	0.24	0.674	Co-occurrence
CSK	15q24.1	2 (1.74%)	1 (0.41%)	2.09	0.24	0.674	Co-occurrence
CSPG4	15q24.2	2 (1.74%)	1 (0.41%)	2.09	0.24	0.674	Co-occurrence
CSPG4P12	15q25.3	2 (1.74%)	1 (0.41%)	2.09	0.24	0.674	Co-occurrence
CSRNP3	2q24.3	2 (1.74%)	1 (0.41%)	2.09	0.24	0.674	Co-occurrence
CTAGE9	6q23.2	2 (1.74%)	1 (0.41%)	2.09	0.24	0.674	Co-occurrence
CXCL17	19q13.2	2 (1.74%)	1 (0.41%)	2.09	0.24	0.674	Co-occurrence
CYB561D2	3p21.31	2 (1.74%)	1 (0.41%)	2.09	0.24	0.674	Co-occurrence
CYP11A1	15q24.1	2 (1.74%)	1 (0.41%)	2.09	0.24	0.674	Co-occurrence
CYP1A1	15q24.1	2 (1.74%)	1 (0.41%)	2.09	0.24	0.674	Co-occurrence
CYP1A2	15q24.1	2 (1.74%)	1 (0.41%)	2.09	0.24	0.674	Co-occurrence
CYTH3	7p22.1	2 (1.74%)	1 (0.41%)	2.09	0.24	0.674	Co-occurrence
CYTIP	2q24.1	2 (1.74%)	1 (0.41%)	2.09	0.24	0.674	Co-occurrence
CZIB	1p32.3	2 (1.74%)	1 (0.41%)	2.09	0.24	0.674	Co-occurrence
DACH1	13q21.33	2 (1.74%)	1 (0.41%)	2.09	0.24	0.674	Co-occurrence
DAGLB	7p22.1	2 (1.74%)	1 (0.41%)	2.09	0.24	0.674	Co-occurrence
DBH	9q34.2	2 (1.74%)	1 (0.41%)	2.09	0.24	0.674	Co-occurrence
DDC	7p12.2-p12.1	2 (1.74%)	1 (0.41%)	2.09	0.24	0.674	Co-occurrence

DENND4A	15q22.31	2 (1.74%)	1 (0.41%)	2.09	0.24	0.674	Co-occurrence
DET1	15q26.1	2 (1.74%)	1 (0.41%)	2.09	0.24	0.674	Co-occurrence
DHRS4	14q11.2	2 (1.74%)	1 (0.41%)	2.09	0.24	0.674	Co-occurrence
DIPK1B	9q34.3	2 (1.74%)	1 (0.41%)	2.09	0.24	0.674	Co-occurrence
DIS3	13q21.33	2 (1.74%)	1 (0.41%)	2.09	0.24	0.674	Co-occurrence
DIS3L	15q22.31	2 (1.74%)	1 (0.41%)	2.09	0.24	0.674	Co-occurrence
DMRTB1	1p32.3	2 (1.74%)	1 (0.41%)	2.09	0.24	0.674	Co-occurrence
DNAAF4	15q21.3	2 (1.74%)	1 (0.41%)	2.09	0.24	0.674	Co-occurrence
DNAH1	3p21.1	2 (1.74%)	1 (0.41%)	2.09	0.24	0.674	Co-occurrence
DNAJA4	15q25.1	2 (1.74%)	1 (0.41%)	2.09	0.24	0.674	Co-occurrence
DNAJC14	12q13.2	2 (1.74%)	1 (0.41%)	2.09	0.24	0.674	Co-occurrence
DNM1P34	15q24.2	2 (1.74%)	1 (0.41%)	2.09	0.24	0.674	Co-occurrence
DNM1P35	15q24.2	2 (1.74%)	1 (0.41%)	2.09	0.24	0.674	Co-occurrence
DPH7	9q34.3	2 (1.74%)	1 (0.41%)	2.09	0.24	0.674	Co-occurrence
DPM2	9q34.11	2 (1.74%)	1 (0.41%)	2.09	0.24	0.674	Co-occurrence
DPP4	2q24.2	2 (1.74%)	1 (0.41%)	2.09	0.24	0.674	Co-occurrence
DPY19L3	19q13.11	2 (1.74%)	1 (0.41%)	2.09	0.24	0.674	Co-occurrence
DUSP15	20q11.21	2 (1.74%)	1 (0.41%)	2.09	0.24	0.674	Co-occurrence
DUSP7	3p21.2	2 (1.74%)	1 (0.41%)	2.09	0.24	0.674	Co-occurrence
ECH1	19q13.2	2 (1.74%)	1 (0.41%)	2.09	0.24	0.674	Co-occurrence
EDC3	15q24.1	2 (1.74%)	1 (0.41%)	2.09	0.24	0.674	Co-occurrence
EFS	14q11.2	2 (1.74%)	1 (0.41%)	2.09	0.24	0.674	Co-occurrence
EGFL7	9q34.3	2 (1.74%)	1 (0.41%)	2.09	0.24	0.674	Co-occurrence
EHMT1	9q34.3	2 (1.74%)	1 (0.41%)	2.09	0.24	0.674	Co-occurrence
EID3	12q23.3	2 (1.74%)	1 (0.41%)	2.09	0.24	0.674	Co-occurrence
EIF1	17q21.2	2 (1.74%)	1 (0.41%)	2.09	0.24	0.674	Co-occurrence
EIF2AK1	7p22.1	2 (1.74%)	1 (0.41%)	2.09	0.24	0.674	Co-occurrence
ELN	7q11.23	2 (1.74%)	1 (0.41%)	2.09	0.24	0.674	Co-occurrence
ENPP1	6q23.2	2 (1.74%)	1 (0.41%)	2.09	0.24	0.674	Co-occurrence

ENPP3	6q23.2	2 (1.74%)	1 (0.41%)	2.09	0.24	0.674	Co-occurrence
ENTPD8	9q34.3	2 (1.74%)	1 (0.41%)	2.09	0.24	0.674	Co-occurrence
ERMN	2q24.1	2 (1.74%)	1 (0.41%)	2.09	0.24	0.674	Co-occurrence
ESPN	1p36.31	2 (1.74%)	1 (0.41%)	2.09	0.24	0.674	Co-occurrence
ESPNP	1p36.13	2 (1.74%)	1 (0.41%)	2.09	0.24	0.674	Co-occurrence
ETFA	15q24.2-q24.3	2 (1.74%)	1 (0.41%)	2.09	0.24	0.674	Co-occurrence
EWSAT1	15q23	2 (1.74%)	1 (0.41%)	2.09	0.24	0.674	Co-occurrence
EXD3	9q34.3	2 (1.74%)	1 (0.41%)	2.09	0.24	0.674	Co-occurrence
FAAP24	19q13.11	2 (1.74%)	1 (0.41%)	2.09	0.24	0.674	Co-occurrence
FAM102A	9q34.11	2 (1.74%)	1 (0.41%)	2.09	0.24	0.674	Co-occurrence
FAM102B	1p13.3	2 (1.74%)	1 (0.41%)	2.09	0.24	0.674	Co-occurrence
FAM157B	9q34.3	2 (1.74%)	1 (0.41%)	2.09	0.24	0.674	Co-occurrence
FAM163B	9q34.2	2 (1.74%)	1 (0.41%)	2.09	0.24	0.674	Co-occurrence
FAM219B	15q24.1-q24.2	2 (1.74%)	1 (0.41%)	2.09	0.24	0.674	Co-occurrence
FAM220A	7p22.1	2 (1.74%)	1 (0.41%)	2.09	0.24	0.674	Co-occurrence
FAM222A	12q24.11	2 (1.74%)	1 (0.41%)	2.09	0.24	0.674	Co-occurrence
FAM47E	4q21.1	2 (1.74%)	1 (0.41%)	2.09	0.24	0.674	Co-occurrence
FAM81A	15q22.2	2 (1.74%)	1 (0.41%)	2.09	0.24	0.674	Co-occurrence
FANCI	15q26.1	2 (1.74%)	1 (0.41%)	2.09	0.24	0.674	Co-occurrence
FAP	2q24.2	2 (1.74%)	1 (0.41%)	2.09	0.24	0.674	Co-occurrence
FBXL18	7p22.1	2 (1.74%)	1 (0.41%)	2.09	0.24	0.674	Co-occurrence
FBXO22	15q24.2	2 (1.74%)	1 (0.41%)	2.09	0.24	0.674	Co-occurrence
FBXO7	22q12.3	2 (1.74%)	1 (0.41%)	2.09	0.24	0.674	Co-occurrence
FCN1	9q34.3	2 (1.74%)	1 (0.41%)	2.09	0.24	0.674	Co-occurrence
FCN2	9q34.3	2 (1.74%)	1 (0.41%)	2.09	0.24	0.674	Co-occurrence
FEM1B	15q23	2 (1.74%)	1 (0.41%)	2.09	0.24	0.674	Co-occurrence
FES	15q26.1	2 (1.74%)	1 (0.41%)	2.09	0.24	0.674	Co-occurrence
FGFR1OP2	12p11.23	2 (1.74%)	1 (0.41%)	2.09	0.24	0.674	Co-occurrence
FGGY	1p32.1	2 (1.74%)	1 (0.41%)	2.09	0.24	0.674	Co-occurrence

FHAD1	1p36.21	2 (1.74%)	1 (0.41%)	2.09	0.24	0.674	Co-occurrence
FHIT	3p14.2	2 (1.74%)	1 (0.41%)	2.09	0.24	0.674	Co-occurrence
FIBCD1	9q34.12	2 (1.74%)	1 (0.41%)	2.09	0.24	0.674	Co-occurrence
FIGN	2q24.3	2 (1.74%)	1 (0.41%)	2.09	0.24	0.674	Co-occurrence
FIGNL1	7p12.2	2 (1.74%)	1 (0.41%)	2.09	0.24	0.674	Co-occurrence
FKBP10	17q21.2	2 (1.74%)	1 (0.41%)	2.09	0.24	0.674	Co-occurrence
FKSG48		2 (1.74%)	1 (0.41%)	2.09	0.24	0.674	Co-occurrence
FNBP1	9q34.11	2 (1.74%)	1 (0.41%)	2.09	0.24	0.674	Co-occurrence
FNDC7	1p13.3	2 (1.74%)	1 (0.41%)	2.09	0.24	0.674	Co-occurrence
FNDC8	17q12	2 (1.74%)	1 (0.41%)	2.09	0.24	0.674	Co-occurrence
FOXB1	15q22.2	2 (1.74%)	1 (0.41%)	2.09	0.24	0.674	Co-occurrence
FOXK1	7p22.1	2 (1.74%)	1 (0.41%)	2.09	0.24	0.674	Co-occurrence
FOXRED2	22q12.3	2 (1.74%)	1 (0.41%)	2.09	0.24	0.674	Co-occurrence
FOXS1	20q11.21	2 (1.74%)	1 (0.41%)	2.09	0.24	0.674	Co-occurrence
FSCN1	7p22.1	2 (1.74%)	1 (0.41%)	2.09	0.24	0.674	Co-occurrence
FUBP3	9q34.11-q34.12	2 (1.74%)	1 (0.41%)	2.09	0.24	0.674	Co-occurrence
FURIN	15q26.1	2 (1.74%)	1 (0.41%)	2.09	0.24	0.674	Co-occurrence
GALNT3	2q24.3	2 (1.74%)	1 (0.41%)	2.09	0.24	0.674	Co-occurrence
GALNT5	2q24.1	2 (1.74%)	1 (0.41%)	2.09	0.24	0.674	Co-occurrence
GARNL3	9q33.3	2 (1.74%)	1 (0.41%)	2.09	0.24	0.674	Co-occurrence
GAST	17q21.2	2 (1.74%)	1 (0.41%)	2.09	0.24	0.674	Co-occurrence
GATA6	18q11.2	2 (1.74%)	1 (0.41%)	2.09	0.24	0.674	Co-occurrence
GATD1	11p15.5	2 (1.74%)	1 (0.41%)	2.09	0.24	0.674	Co-occurrence
GATM	15q21.1	2 (1.74%)	1 (0.41%)	2.09	0.24	0.674	Co-occurrence
GCA	2q24.2	2 (1.74%)	1 (0.41%)	2.09	0.24	0.674	Co-occurrence
GCG	2q24.2	2 (1.74%)	1 (0.41%)	2.09	0.24	0.674	Co-occurrence
GDE1	16p12.3	2 (1.74%)	1 (0.41%)	2.09	0.24	0.674	Co-occurrence
GEMIN7	19q13.32	2 (1.74%)	1 (0.41%)	2.09	0.24	0.674	Co-occurrence
GHDC	17q21.2	2 (1.74%)	1 (0.41%)	2.09	0.24	0.674	Co-occurrence

GJD3	17q21.2	2 (1.74%)	1 (0.41%)	2.09	0.24	0.674	Co-occurrence
GLCE	15q23	2 (1.74%)	1 (0.41%)	2.09	0.24	0.674	Co-occurrence
GLT6D1	9q34.3	2 (1.74%)	1 (0.41%)	2.09	0.24	0.674	Co-occurrence
GLT8D2	12q23.3	2 (1.74%)	1 (0.41%)	2.09	0.24	0.674	Co-occurrence
GLYCTK	3p21.2	2 (1.74%)	1 (0.41%)	2.09	0.24	0.674	Co-occurrence
GNA12	7p22.3-p22.2	2 (1.74%)	1 (0.41%)	2.09	0.24	0.674	Co-occurrence
GOLGA6A	15q24.1	2 (1.74%)	1 (0.41%)	2.09	0.24	0.674	Co-occurrence
GOLGA6C	15q24.2	2 (1.74%)	1 (0.41%)	2.09	0.24	0.674	Co-occurrence
GOLGA6D	15q24.2	2 (1.74%)	1 (0.41%)	2.09	0.24	0.674	Co-occurrence
GPR139	16p12.3	2 (1.74%)	1 (0.41%)	2.09	0.24	0.674	Co-occurrence
GPR17	2q14.3	2 (1.74%)	1 (0.41%)	2.09	0.24	0.674	Co-occurrence
GPRC5B	16p12.3	2 (1.74%)	1 (0.41%)	2.09	0.24	0.674	Co-occurrence
GPX1	3p21.31	2 (1.74%)	1 (0.41%)	2.09	0.24	0.674	Co-occurrence
GRAMD4	22q13.31	2 (1.74%)	1 (0.41%)	2.09	0.24	0.674	Co-occurrence
GRB10	7p12.1	2 (1.74%)	1 (0.41%)	2.09	0.24	0.674	Co-occurrence
GRID2IP	7p22.1	2 (1.74%)	1 (0.41%)	2.09	0.24	0.674	Co-occurrence
GSDMA	17q21.1	2 (1.74%)	1 (0.41%)	2.09	0.24	0.674	Co-occurrence
GSDMB	17q21.1	2 (1.74%)	1 (0.41%)	2.09	0.24	0.674	Co-occurrence
GSPT1	16p13.13	2 (1.74%)	1 (0.41%)	2.09	0.24	0.674	Co-occurrence
GTF2H1	11p15.1	2 (1.74%)	1 (0.41%)	2.09	0.24	0.674	Co-occurrence
GTF2H2	5q13.2	2 (1.74%)	1 (0.41%)	2.09	0.24	0.674	Co-occurrence
GTF2H2B	5q13.2	2 (1.74%)	1 (0.41%)	2.09	0.24	0.674	Co-occurrence
GTF2H2C	5q13.2	2 (1.74%)	1 (0.41%)	2.09	0.24	0.674	Co-occurrence
GUCY2GP	10q25.2	2 (1.74%)	1 (0.41%)	2.09	0.24	0.674	Co-occurrence
GUSBP3	5q13.2	2 (1.74%)	1 (0.41%)	2.09	0.24	0.674	Co-occurrence
HABP2	10q25.3	2 (1.74%)	1 (0.41%)	2.09	0.24	0.674	Co-occurrence
HACD3	15q22.31	2 (1.74%)	1 (0.41%)	2.09	0.24	0.674	Co-occurrence
HAL	12q23.1	2 (1.74%)	1 (0.41%)	2.09	0.24	0.674	Co-occurrence
HAP1	17q21.2	2 (1.74%)	1 (0.41%)	2.09	0.24	0.674	Co-occurrence

HCFC2	12q23.3	2 (1.74%)	1 (0.41%)	2.09	0.24	0.674	Co-occurrence
HCN4	15q24.1	2 (1.74%)	1 (0.41%)	2.09	0.24	0.674	Co-occurrence
HCRT	17q21.2	2 (1.74%)	1 (0.41%)	2.09	0.24	0.674	Co-occurrence
HDDC3	15q26.1	2 (1.74%)	1 (0.41%)	2.09	0.24	0.674	Co-occurrence
HDHD5	22q11.1	2 (1.74%)	1 (0.41%)	2.09	0.24	0.674	Co-occurrence
HENMT1	1p13.3	2 (1.74%)	1 (0.41%)	2.09	0.24	0.674	Co-occurrence
HMG20A	15q24.3	2 (1.74%)	1 (0.41%)	2.09	0.24	0.674	Co-occurrence
HOMEZ	14q11.2	2 (1.74%)	1 (0.41%)	2.09	0.24	0.674	Co-occurrence
HPCA	1p35.1	2 (1.74%)	1 (0.41%)	2.09	0.24	0.674	Co-occurrence
HPS5	11p15.1	2 (1.74%)	1 (0.41%)	2.09	0.24	0.674	Co-occurrence
HPX	11p15.4	2 (1.74%)	1 (0.41%)	2.09	0.24	0.674	Co-occurrence
HSP90B1	12q23.3	2 (1.74%)	1 (0.41%)	2.09	0.24	0.674	Co-occurrence
HYAL1	3p21.31	2 (1.74%)	1 (0.41%)	2.09	0.24	0.674	Co-occurrence
HYAL3	3p21.31	2 (1.74%)	1 (0.41%)	2.09	0.24	0.674	Co-occurrence
HYKK	15q25.1	2 (1.74%)	1 (0.41%)	2.09	0.24	0.674	Co-occurrence
ICE2	15q22.2	2 (1.74%)	1 (0.41%)	2.09	0.24	0.674	Co-occurrence
IFIH1	2q24.2	2 (1.74%)	1 (0.41%)	2.09	0.24	0.674	Co-occurrence
IFNL1	19q13.2	2 (1.74%)	1 (0.41%)	2.09	0.24	0.674	Co-occurrence
IFNL2	19q13.2	2 (1.74%)	1 (0.41%)	2.09	0.24	0.674	Co-occurrence
IFNL3	19q13.2	2 (1.74%)	1 (0.41%)	2.09	0.24	0.674	Co-occurrence
IFNL4	19q13.2	2 (1.74%)	1 (0.41%)	2.09	0.24	0.674	Co-occurrence
IFRD2	3p21.31	2 (1.74%)	1 (0.41%)	2.09	0.24	0.674	Co-occurrence
IKZF3	17q12-q21.1	2 (1.74%)	1 (0.41%)	2.09	0.24	0.674	Co-occurrence
IL23R	1p31.3	2 (1.74%)	1 (0.41%)	2.09	0.24	0.674	Co-occurrence
IL25	14q11.2	2 (1.74%)	1 (0.41%)	2.09	0.24	0.674	Co-occurrence
IMP3	15q24.2	2 (1.74%)	1 (0.41%)	2.09	0.24	0.674	Co-occurrence
INSL5	1p31.3	2 (1.74%)	1 (0.41%)	2.09	0.24	0.674	Co-occurrence
INSYN1	15q24.1	2 (1.74%)	1 (0.41%)	2.09	0.24	0.674	Co-occurrence
INTS13	12p11.23	2 (1.74%)	1 (0.41%)	2.09	0.24	0.674	Co-occurrence

INTS14	15q22.31	2 (1.74%)	1 (0.41%)	2.09	0.24	0.674	Co-occurrence
IP6K1	3p21.31	2 (1.74%)	1 (0.41%)	2.09	0.24	0.674	Co-occurrence
IQCE	7p22.3	2 (1.74%)	1 (0.41%)	2.09	0.24	0.674	Co-occurrence
IQCH	15q23	2 (1.74%)	1 (0.41%)	2.09	0.24	0.674	Co-occurrence
IQCK	16p12.3	2 (1.74%)	1 (0.41%)	2.09	0.24	0.674	Co-occurrence
IREB2	15q25.1	2 (1.74%)	1 (0.41%)	2.09	0.24	0.674	Co-occurrence
ISG20	15q26.1	2 (1.74%)	1 (0.41%)	2.09	0.24	0.674	Co-occurrence
ISL2	15q24.3	2 (1.74%)	1 (0.41%)	2.09	0.24	0.674	Co-occurrence
ISLR	15q24.1	2 (1.74%)	1 (0.41%)	2.09	0.24	0.674	Co-occurrence
ISLR2	15q24.1	2 (1.74%)	1 (0.41%)	2.09	0.24	0.674	Co-occurrence
ITGA11	15q23	2 (1.74%)	1 (0.41%)	2.09	0.24	0.674	Co-occurrence
ITGA9	3p22.2	2 (1.74%)	1 (0.41%)	2.09	0.24	0.674	Co-occurrence
JSRP1	19p13.3	2 (1.74%)	1 (0.41%)	2.09	0.24	0.674	Co-occurrence
JUP	17q21.2	2 (1.74%)	1 (0.41%)	2.09	0.24	0.674	Co-occurrence
KCNH7	2q24.2	2 (1.74%)	1 (0.41%)	2.09	0.24	0.674	Co-occurrence
KCNMA1	10q22.3	2 (1.74%)	1 (0.41%)	2.09	0.24	0.674	Co-occurrence
KCNT1	9q34.3	2 (1.74%)	1 (0.41%)	2.09	0.24	0.674	Co-occurrence
KDELR2	7p22.1	2 (1.74%)	1 (0.41%)	2.09	0.24	0.674	Co-occurrence
KDM4A	1p34.2-p34.1	2 (1.74%)	1 (0.41%)	2.09	0.24	0.674	Co-occurrence
KIAA1324	1p13.3	2 (1.74%)	1 (0.41%)	2.09	0.24	0.674	Co-occurrence
KIF23	15q23	2 (1.74%)	1 (0.41%)	2.09	0.24	0.674	Co-occurrence
KIF7	15q26.1	2 (1.74%)	1 (0.41%)	2.09	0.24	0.674	Co-occurrence
KIF9	3p21.31	2 (1.74%)	1 (0.41%)	2.09	0.24	0.674	Co-occurrence
KLF12	13q22.1	2 (1.74%)	1 (0.41%)	2.09	0.24	0.674	Co-occurrence
KLF5	13q22.1	2 (1.74%)	1 (0.41%)	2.09	0.24	0.674	Co-occurrence
KLHL1	13q21.33	2 (1.74%)	1 (0.41%)	2.09	0.24	0.674	Co-occurrence
KLHL21	1p36.31	2 (1.74%)	1 (0.41%)	2.09	0.24	0.674	Co-occurrence
KLHL25	15q25.3	2 (1.74%)	1 (0.41%)	2.09	0.24	0.674	Co-occurrence
KNOP1	16p12.3	2 (1.74%)	1 (0.41%)	2.09	0.24	0.674	Co-occurrence

KRT13	17q21.2	2 (1.74%)	1 (0.41%)	2.09	0.24	0.674	Co-occurrence
KRT14	17q21.2	2 (1.74%)	1 (0.41%)	2.09	0.24	0.674	Co-occurrence
KRT15	17q21.2	2 (1.74%)	1 (0.41%)	2.09	0.24	0.674	Co-occurrence
KRT16	17q21.2	2 (1.74%)	1 (0.41%)	2.09	0.24	0.674	Co-occurrence
KRT17	17q21.2	2 (1.74%)	1 (0.41%)	2.09	0.24	0.674	Co-occurrence
KRT19	17q21.2	2 (1.74%)	1 (0.41%)	2.09	0.24	0.674	Co-occurrence
KRT31	17q21.2	2 (1.74%)	1 (0.41%)	2.09	0.24	0.674	Co-occurrence
KRT32	17q21.2	2 (1.74%)	1 (0.41%)	2.09	0.24	0.674	Co-occurrence
KRT33A	17q21.2	2 (1.74%)	1 (0.41%)	2.09	0.24	0.674	Co-occurrence
KRT33B	17q21.2	2 (1.74%)	1 (0.41%)	2.09	0.24	0.674	Co-occurrence
KRT34	17q21.2	2 (1.74%)	1 (0.41%)	2.09	0.24	0.674	Co-occurrence
KRT35	17q21.2	2 (1.74%)	1 (0.41%)	2.09	0.24	0.674	Co-occurrence
KRT36	17q21.2	2 (1.74%)	1 (0.41%)	2.09	0.24	0.674	Co-occurrence
KRT37	17q21.2	2 (1.74%)	1 (0.41%)	2.09	0.24	0.674	Co-occurrence
KRT38	17q21.2	2 (1.74%)	1 (0.41%)	2.09	0.24	0.674	Co-occurrence
KRT39	17q21.2	2 (1.74%)	1 (0.41%)	2.09	0.24	0.674	Co-occurrence
KRT40	17q21.2	2 (1.74%)	1 (0.41%)	2.09	0.24	0.674	Co-occurrence
KRT42P	17q21.2	2 (1.74%)	1 (0.41%)	2.09	0.24	0.674	Co-occurrence
KRT9	17q21.2	2 (1.74%)	1 (0.41%)	2.09	0.24	0.674	Co-occurrence
LCN1	9q34.3	2 (1.74%)	1 (0.41%)	2.09	0.24	0.674	Co-occurrence
LCN10	9q34.3	2 (1.74%)	1 (0.41%)	2.09	0.24	0.674	Co-occurrence
LCN15	9q34.3	2 (1.74%)	1 (0.41%)	2.09	0.24	0.674	Co-occurrence
LCN6	9q34.3	2 (1.74%)	1 (0.41%)	2.09	0.24	0.674	Co-occurrence
LCN8	9q34.3	2 (1.74%)	1 (0.41%)	2.09	0.24	0.674	Co-occurrence
LCN9	9q34.3	2 (1.74%)	1 (0.41%)	2.09	0.24	0.674	Co-occurrence
LCTL	15q22.31	2 (1.74%)	1 (0.41%)	2.09	0.24	0.674	Co-occurrence
LDHAL6B	15q22.2	2 (1.74%)	1 (0.41%)	2.09	0.24	0.674	Co-occurrence
LGALS4	19q13.2	2 (1.74%)	1 (0.41%)	2.09	0.24	0.674	Co-occurrence
LGALS7	19q13.2	2 (1.74%)	1 (0.41%)	2.09	0.24	0.674	Co-occurrence

LIG3	17q12	2 (1.74%)	1 (0.41%)	2.09	0.24	0.674	Co-occurrence
LIMK1	7q11.23	2 (1.74%)	1 (0.41%)	2.09	0.24	0.674	Co-occurrence
LIMS2	2q14.3	2 (1.74%)	1 (0.41%)	2.09	0.24	0.674	Co-occurrence
LINC00052	15q25.3	2 (1.74%)	1 (0.41%)	2.09	0.24	0.674	Co-occurrence
LINC00347	13q22.1	2 (1.74%)	1 (0.41%)	2.09	0.24	0.674	Co-occurrence
LINC00348	13q21.33	2 (1.74%)	1 (0.41%)	2.09	0.24	0.674	Co-occurrence
LINC00355	13q21.31	2 (1.74%)	1 (0.41%)	2.09	0.24	0.674	Co-occurrence
LINC00364	13q21.32	2 (1.74%)	1 (0.41%)	2.09	0.24	0.674	Co-occurrence
LINC00381	13q22.1	2 (1.74%)	1 (0.41%)	2.09	0.24	0.674	Co-occurrence
LINC00392	13q22.1	2 (1.74%)	1 (0.41%)	2.09	0.24	0.674	Co-occurrence
LINC00393	13q22.1	2 (1.74%)	1 (0.41%)	2.09	0.24	0.674	Co-occurrence
LINC00395	13q21.31	2 (1.74%)	1 (0.41%)	2.09	0.24	0.674	Co-occurrence
LINC00402	-	2 (1.74%)	1 (0.41%)	2.09	0.24	0.674	Co-occurrence
LINC00466	1p31.3	2 (1.74%)	1 (0.41%)	2.09	0.24	0.674	Co-occurrence
LINC00477	12p12.1	2 (1.74%)	1 (0.41%)	2.09	0.24	0.674	Co-occurrence
LINC00561	13q22.2	2 (1.74%)	1 (0.41%)	2.09	0.24	0.674	Co-occurrence
LINC00596	14q11.2	2 (1.74%)	1 (0.41%)	2.09	0.24	0.674	Co-occurrence
LINC00662	19q11	2 (1.74%)	1 (0.41%)	2.09	0.24	0.674	Co-occurrence
LINC00694		2 (1.74%)	1 (0.41%)	2.09	0.24	0.674	Co-occurrence
LINC00696	3p21.2	2 (1.74%)	1 (0.41%)	2.09	0.24	0.674	Co-occurrence
LINC00856	10q22.3	2 (1.74%)	1 (0.41%)	2.09	0.24	0.674	Co-occurrence
LINC00898	22q13.31	2 (1.74%)	1 (0.41%)	2.09	0.24	0.674	Co-occurrence
LINC00899	22q13.31	2 (1.74%)	1 (0.41%)	2.09	0.24	0.674	Co-occurrence
LINC00927	15q25.1	2 (1.74%)	1 (0.41%)	2.09	0.24	0.674	Co-occurrence
LINC00928	15q26.1	2 (1.74%)	1 (0.41%)	2.09	0.24	0.674	Co-occurrence
LINC00974	17q21.2	2 (1.74%)	1 (0.41%)	2.09	0.24	0.674	Co-occurrence
LINGO1	15q24.3	2 (1.74%)	1 (0.41%)	2.09	0.24	0.674	Co-occurrence
LMAN1L	15q24.1	2 (1.74%)	1 (0.41%)	2.09	0.24	0.674	Co-occurrence
LMO7	13q22.2	2 (1.74%)	1 (0.41%)	2.09	0.24	0.674	Co-occurrence

LMO7DN	13q22.2	2 (1.74%)	1 (0.41%)	2.09	0.24	0.674	Co-occurrence
LOXL1	15q24.1	2 (1.74%)	1 (0.41%)	2.09	0.24	0.674	Co-occurrence
LRFN1	19q13.2	2 (1.74%)	1 (0.41%)	2.09	0.24	0.674	Co-occurrence
LRRC3C	17q21.1	2 (1.74%)	1 (0.41%)	2.09	0.24	0.674	Co-occurrence
LSMEM2	3p21.31	2 (1.74%)	1 (0.41%)	2.09	0.24	0.674	Co-occurrence
LTA4H	12q23.1	2 (1.74%)	1 (0.41%)	2.09	0.24	0.674	Co-occurrence
LZTFL1	3p21.31	2 (1.74%)	1 (0.41%)	2.09	0.24	0.674	Co-occurrence
MACF1	1p34.3	2 (1.74%)	1 (0.41%)	2.09	0.24	0.674	Co-occurrence
MAD1L1	7p22.3	2 (1.74%)	1 (0.41%)	2.09	0.24	0.674	Co-occurrence
MAGOH	1p32.3	2 (1.74%)	1 (0.41%)	2.09	0.24	0.674	Co-occurrence
MAN2A2	15q26.1	2 (1.74%)	1 (0.41%)	2.09	0.24	0.674	Co-occurrence
MAP2K1	15q22.31	2 (1.74%)	1 (0.41%)	2.09	0.24	0.674	Co-occurrence
MARVELD2	5q13.2	2 (1.74%)	1 (0.41%)	2.09	0.24	0.674	Co-occurrence
MCU	10q22.1	2 (1.74%)	1 (0.41%)	2.09	0.24	0.674	Co-occurrence
MDGA2	14q21.3	2 (1.74%)	1 (0.41%)	2.09	0.24	0.674	Co-occurrence
MED21	12p11.23	2 (1.74%)	1 (0.41%)	2.09	0.24	0.674	Co-occurrence
MED23	6q23.2	2 (1.74%)	1 (0.41%)	2.09	0.24	0.674	Co-occurrence
MEGF11	15q22.31	2 (1.74%)	1 (0.41%)	2.09	0.24	0.674	Co-occurrence
METTL27	7q11.23	2 (1.74%)	1 (0.41%)	2.09	0.24	0.674	Co-occurrence
MFAP2	1p36.13	2 (1.74%)	1 (0.41%)	2.09	0.24	0.674	Co-occurrence
MIER1	1p31.3	2 (1.74%)	1 (0.41%)	2.09	0.24	0.674	Co-occurrence
MIGA1	1p31.1	2 (1.74%)	1 (0.41%)	2.09	0.24	0.674	Co-occurrence
MINAR1	15q25.1	2 (1.74%)	1 (0.41%)	2.09	0.24	0.674	Co-occurrence
MINDY2	15q21.3-q22.1	2 (1.74%)	1 (0.41%)	2.09	0.24	0.674	Co-occurrence
MIR-1179/1179		2 (1.74%)	1 (0.41%)	2.09	0.24	0.674	Co-occurrence
MIR-1276/1276		2 (1.74%)	1 (0.41%)	2.09	0.24	0.674	Co-occurrence
MIR-184/184		2 (1.74%)	1 (0.41%)	2.09	0.24	0.674	Co-occurrence
MIR-3159/3159		2 (1.74%)	1 (0.41%)	2.09	0.24	0.674	Co-occurrence
MIR-3174/3174		2 (1.74%)	1 (0.41%)	2.09	0.24	0.674	Co-occurrence

MIR-3652/3652		2 (1.74%)	1 (0.41%)	2.09	0.24	0.674	Co-occurrence
MIR-3683/3683		2 (1.74%)	1 (0.41%)	2.09	0.24	0.674	Co-occurrence
MIR-3685/3685		2 (1.74%)	1 (0.41%)	2.09	0.24	0.674	Co-occurrence
MIR-3689C/3689C		2 (1.74%)	1 (0.41%)	2.09	0.24	0.674	Co-occurrence
MIR-3689E/3689E		2 (1.74%)	1 (0.41%)	2.09	0.24	0.674	Co-occurrence
MIR-3689F/3689F		2 (1.74%)	1 (0.41%)	2.09	0.24	0.674	Co-occurrence
MIR-3713/3713		2 (1.74%)	1 (0.41%)	2.09	0.24	0.674	Co-occurrence
MIR-3972/3972		2 (1.74%)	1 (0.41%)	2.09	0.24	0.674	Co-occurrence
MIR-4271/4271		2 (1.74%)	1 (0.41%)	2.09	0.24	0.674	Co-occurrence
MIR-4311/4311		2 (1.74%)	1 (0.41%)	2.09	0.24	0.674	Co-occurrence
MIR-4312/4312		2 (1.74%)	1 (0.41%)	2.09	0.24	0.674	Co-occurrence
MIR-4313/4313		2 (1.74%)	1 (0.41%)	2.09	0.24	0.674	Co-occurrence
MIR-4321/4321		2 (1.74%)	1 (0.41%)	2.09	0.24	0.674	Co-occurrence
MIR-4511/4511		2 (1.74%)	1 (0.41%)	2.09	0.24	0.674	Co-occurrence
MIR-4512/4512		2 (1.74%)	1 (0.41%)	2.09	0.24	0.674	Co-occurrence
MIR-4513/4513		2 (1.74%)	1 (0.41%)	2.09	0.24	0.674	Co-occurrence
MIR-4656/4656		2 (1.74%)	1 (0.41%)	2.09	0.24	0.674	Co-occurrence
MIR-4672/4672		2 (1.74%)	1 (0.41%)	2.09	0.24	0.674	Co-occurrence
MIR-4673/4673		2 (1.74%)	1 (0.41%)	2.09	0.24	0.674	Co-occurrence
MIR-4674/4674		2 (1.74%)	1 (0.41%)	2.09	0.24	0.674	Co-occurrence
MIR-4718/4718		2 (1.74%)	1 (0.41%)	2.09	0.24	0.674	Co-occurrence
MIR-4770/4770		2 (1.74%)	1 (0.41%)	2.09	0.24	0.674	Co-occurrence
MIR-4794/4794		2 (1.74%)	1 (0.41%)	2.09	0.24	0.674	Co-occurrence
MIR-602/602		2 (1.74%)	1 (0.41%)	2.09	0.24	0.674	Co-occurrence
MIR9-3HG	15q26.1	2 (1.74%)	1 (0.41%)	2.09	0.24	0.674	Co-occurrence
MMD2	10p	2 (1.74%)	1 (0.41%)	2.09	0.24	0.674	Co-occurrence
MMP19	12q13.2	2 (1.74%)	1 (0.41%)	2.09	0.24	0.674	Co-occurrence
MNS1	15q21.3	2 (1.74%)	1 (0.41%)	2.09	0.24	0.674	Co-occurrence
MORF4L1	15q25.1	2 (1.74%)	1 (0.41%)	2.09	0.24	0.674	Co-occurrence

MPI	15q24.1	2 (1.74%)	1 (0.41%)	2.09	0.24	0.674	Co-occurrence
MRGPRX3	11p15.1	2 (1.74%)	1 (0.41%)	2.09	0.24	0.674	Co-occurrence
MRGPRX4	11p15.1	2 (1.74%)	1 (0.41%)	2.09	0.24	0.674	Co-occurrence
MRM2	7p22.3	2 (1.74%)	1 (0.41%)	2.09	0.24	0.674	Co-occurrence
MRPL41	9q34.3	2 (1.74%)	1 (0.41%)	2.09	0.24	0.674	Co-occurrence
MRPL46	15q25.3	2 (1.74%)	1 (0.41%)	2.09	0.24	0.674	Co-occurrence
MRPS11	15q25.3	2 (1.74%)	1 (0.41%)	2.09	0.24	0.674	Co-occurrence
MRPS2	9q34.3	2 (1.74%)	1 (0.41%)	2.09	0.24	0.674	Co-occurrence
MSL1	17q21.1	2 (1.74%)	1 (0.41%)	2.09	0.24	0.674	Co-occurrence
MST1L	1p36.13	2 (1.74%)	1 (0.41%)	2.09	0.24	0.674	Co-occurrence
MTHFS	15q25.1	2 (1.74%)	1 (0.41%)	2.09	0.24	0.674	Co-occurrence
MYBL2	20q13.12	2 (1.74%)	1 (0.41%)	2.09	0.24	0.674	Co-occurrence
MYBPHL	1p13.3	2 (1.74%)	1 (0.41%)	2.09	0.24	0.674	Co-occurrence
MYH6	14q11.2	2 (1.74%)	1 (0.41%)	2.09	0.24	0.674	Co-occurrence
MYH7	14q11.2	2 (1.74%)	1 (0.41%)	2.09	0.24	0.674	Co-occurrence
MYLK2	20q11.21	2 (1.74%)	1 (0.41%)	2.09	0.24	0.674	Co-occurrence
MYO1E	15q22.2	2 (1.74%)	1 (0.41%)	2.09	0.24	0.674	Co-occurrence
MYO7B	2q14.3	2 (1.74%)	1 (0.41%)	2.09	0.24	0.674	Co-occurrence
MZT1	13q21.33	2 (1.74%)	1 (0.41%)	2.09	0.24	0.674	Co-occurrence
NAA80	3p21.31	2 (1.74%)	1 (0.41%)	2.09	0.24	0.674	Co-occurrence
NACC2	9q34.3	2 (1.74%)	1 (0.41%)	2.09	0.24	0.674	Co-occurrence
NAIP	5q13.2	2 (1.74%)	1 (0.41%)	2.09	0.24	0.674	Co-occurrence
NBPF1	1p36.13	2 (1.74%)	1 (0.41%)	2.09	0.24	0.674	Co-occurrence
NBPF4	1p13.3	2 (1.74%)	1 (0.41%)	2.09	0.24	0.674	Co-occurrence
NBPF5P	1p13.3	2 (1.74%)	1 (0.41%)	2.09	0.24	0.674	Co-occurrence
NBPF6	1p13.3	2 (1.74%)	1 (0.41%)	2.09	0.24	0.674	Co-occurrence
NEDD4	15q21.3	2 (1.74%)	1 (0.41%)	2.09	0.24	0.674	Co-occurrence
NELFB	9q34.3	2 (1.74%)	1 (0.41%)	2.09	0.24	0.674	Co-occurrence
NFAM1	22q13.2	2 (1.74%)	1 (0.41%)	2.09	0.24	0.674	Co-occurrence

NFIA	1p31.3	2 (1.74%)	1 (0.41%)	2.09	0.24	0.674	Co-occurrence
NFYB	12q23.3	2 (1.74%)	1 (0.41%)	2.09	0.24	0.674	Co-occurrence
NICN1	3p21.31	2 (1.74%)	1 (0.41%)	2.09	0.24	0.674	Co-occurrence
NISCH	3p21.1	2 (1.74%)	1 (0.41%)	2.09	0.24	0.674	Co-occurrence
NKIRAS2	17q21.2	2 (1.74%)	1 (0.41%)	2.09	0.24	0.674	Co-occurrence
NKPD1	19q13.32	2 (1.74%)	1 (0.41%)	2.09	0.24	0.674	Co-occurrence
NLE1	17q12	2 (1.74%)	1 (0.41%)	2.09	0.24	0.674	Co-occurrence
NOL9	1p36.31	2 (1.74%)	1 (0.41%)	2.09	0.24	0.674	Co-occurrence
NOP53	19q13.33	2 (1.74%)	1 (0.41%)	2.09	0.24	0.674	Co-occurrence
NOS1	12q24.22	2 (1.74%)	1 (0.41%)	2.09	0.24	0.674	Co-occurrence
NOTCH1	9q34.3	2 (1.74%)	1 (0.41%)	2.09	0.24	0.674	Co-occurrence
NOX5	15q23	2 (1.74%)	1 (0.41%)	2.09	0.24	0.674	Co-occurrence
NOXA1	9q34.3	2 (1.74%)	1 (0.41%)	2.09	0.24	0.674	Co-occurrence
NPPB	1p36.22	2 (1.74%)	1 (0.41%)	2.09	0.24	0.674	Co-occurrence
NPRL2	3p21.31	2 (1.74%)	1 (0.41%)	2.09	0.24	0.674	Co-occurrence
NPTN	15q24.1	2 (1.74%)	1 (0.41%)	2.09	0.24	0.674	Co-occurrence
NR1D1	17q21.1	2 (1.74%)	1 (0.41%)	2.09	0.24	0.674	Co-occurrence
NRARP	9q34.3	2 (1.74%)	1 (0.41%)	2.09	0.24	0.674	Co-occurrence
NRG4	15q24.2	2 (1.74%)	1 (0.41%)	2.09	0.24	0.674	Co-occurrence
NSMF	9q34.3	2 (1.74%)	1 (0.41%)	2.09	0.24	0.674	Co-occurrence
NTRK3	15q25.3	2 (1.74%)	1 (0.41%)	2.09	0.24	0.674	Co-occurrence
NUDT1	7p22.3	2 (1.74%)	1 (0.41%)	2.09	0.24	0.674	Co-occurrence
NUDT13	10q22.2	2 (1.74%)	1 (0.41%)	2.09	0.24	0.674	Co-occurrence
NUDT19	19q13.11	2 (1.74%)	1 (0.41%)	2.09	0.24	0.674	Co-occurrence
NYX	Xp11.4	2 (1.74%)	1 (0.41%)	2.09	0.24	0.674	Co-occurrence
OAZ1	19p13.3	2 (1.74%)	1 (0.41%)	2.09	0.24	0.674	Co-occurrence
OBP2A	9q34.3	2 (1.74%)	1 (0.41%)	2.09	0.24	0.674	Co-occurrence
OCLN	5q13.2	2 (1.74%)	1 (0.41%)	2.09	0.24	0.674	Co-occurrence
OCM	7p22.1	2 (1.74%)	1 (0.41%)	2.09	0.24	0.674	Co-occurrence

ODF3L1	15q24.2	2 (1.74%)	1 (0.41%)	2.09	0.24	0.674	Co-occurrence
OLFM3	1p21.1	2 (1.74%)	1 (0.41%)	2.09	0.24	0.674	Co-occurrence
OR2A4	6q23.2	2 (1.74%)	1 (0.41%)	2.09	0.24	0.674	Co-occurrence
ORMDL2	12q13.2	2 (1.74%)	1 (0.41%)	2.09	0.24	0.674	Co-occurrence
ORMDL3	17q21.1	2 (1.74%)	1 (0.41%)	2.09	0.24	0.674	Co-occurrence
P3H4	17q21.2	2 (1.74%)	1 (0.41%)	2.09	0.24	0.674	Co-occurrence
P4HA1	10q22.1	2 (1.74%)	1 (0.41%)	2.09	0.24	0.674	Co-occurrence
PABPN1	14q11.2	2 (1.74%)	1 (0.41%)	2.09	0.24	0.674	Co-occurrence
PADI1	1p36.13	2 (1.74%)	1 (0.41%)	2.09	0.24	0.674	Co-occurrence
PADI2	1p36.13	2 (1.74%)	1 (0.41%)	2.09	0.24	0.674	Co-occurrence
PADI3	1p36.13	2 (1.74%)	1 (0.41%)	2.09	0.24	0.674	Co-occurrence
PADI4	1p36.13	2 (1.74%)	1 (0.41%)	2.09	0.24	0.674	Co-occurrence
PAEP	9q34.3	2 (1.74%)	1 (0.41%)	2.09	0.24	0.674	Co-occurrence
PAFAH1B3	19q13.2	2 (1.74%)	1 (0.41%)	2.09	0.24	0.674	Co-occurrence
PAH	12q23.2	2 (1.74%)	1 (0.41%)	2.09	0.24	0.674	Co-occurrence
PAPOLB	7p22.1	2 (1.74%)	1 (0.41%)	2.09	0.24	0.674	Co-occurrence
PAQR5	15q23	2 (1.74%)	1 (0.41%)	2.09	0.24	0.674	Co-occurrence
PARP16	15q22.31	2 (1.74%)	1 (0.41%)	2.09	0.24	0.674	Co-occurrence
PCDH15	10q21.1	2 (1.74%)	1 (0.41%)	2.09	0.24	0.674	Co-occurrence
PCLAF	15q22.31	2 (1.74%)	1 (0.41%)	2.09	0.24	0.674	Co-occurrence
PDCD5	19q13.11	2 (1.74%)	1 (0.41%)	2.09	0.24	0.674	Co-occurrence
PDE8A	15q25.3	2 (1.74%)	1 (0.41%)	2.09	0.24	0.674	Co-occurrence
PEAK1	15q24.3	2 (1.74%)	1 (0.41%)	2.09	0.24	0.674	Co-occurrence
PEAK3	19p13.3	2 (1.74%)	1 (0.41%)	2.09	0.24	0.674	Co-occurrence
PEX11A	15q26.1	2 (1.74%)	1 (0.41%)	2.09	0.24	0.674	Co-occurrence
PEX14	1p36.22	2 (1.74%)	1 (0.41%)	2.09	0.24	0.674	Co-occurrence
PHF13	1p36.31	2 (1.74%)	1 (0.41%)	2.09	0.24	0.674	Co-occurrence
PIBF1	13q21.33-q22.1	2 (1.74%)	1 (0.41%)	2.09	0.24	0.674	Co-occurrence
PIEZO2	18p11.22-p11.21	2 (1.74%)	1 (0.41%)	2.09	0.24	0.674	Co-occurrence

PIP5KL1	9q34.11	2 (1.74%)	1 (0.41%)	2.09	0.24	0.674	Co-occurrence
PKDREJ	22q13.31	2 (1.74%)	1 (0.41%)	2.09	0.24	0.674	Co-occurrence
PLEKHG5	1p36.31	2 (1.74%)	1 (0.41%)	2.09	0.24	0.674	Co-occurrence
PLIN1	15q26.1	2 (1.74%)	1 (0.41%)	2.09	0.24	0.674	Co-occurrence
PLPP1	5q11.2	2 (1.74%)	1 (0.41%)	2.09	0.24	0.674	Co-occurrence
PLPPR4	1p21.3-p21.2	2 (1.74%)	1 (0.41%)	2.09	0.24	0.674	Co-occurrence
PLPPR5	1p21.3	2 (1.74%)	1 (0.41%)	2.09	0.24	0.674	Co-occurrence
PML	15q24.1	2 (1.74%)	1 (0.41%)	2.09	0.24	0.674	Co-occurrence
PMS2	7p22.1	2 (1.74%)	1 (0.41%)	2.09	0.24	0.674	Co-occurrence
PMS2CL	7p22.1	2 (1.74%)	1 (0.41%)	2.09	0.24	0.674	Co-occurrence
PNPLA7	9q34.3	2 (1.74%)	1 (0.41%)	2.09	0.24	0.674	Co-occurrence
POC1A	3p21.2	2 (1.74%)	1 (0.41%)	2.09	0.24	0.674	Co-occurrence
PODN	1p32.3	2 (1.74%)	1 (0.41%)	2.09	0.24	0.674	Co-occurrence
POLG	15q26.1	2 (1.74%)	1 (0.41%)	2.09	0.24	0.674	Co-occurrence
POLR3D	8p21.3	2 (1.74%)	1 (0.41%)	2.09	0.24	0.674	Co-occurrence
POU6F2	7p14.1	2 (1.74%)	1 (0.41%)	2.09	0.24	0.674	Co-occurrence
PPARA	22q13.31	2 (1.74%)	1 (0.41%)	2.09	0.24	0.674	Co-occurrence
PPCDC	15q24.2	2 (1.74%)	1 (0.41%)	2.09	0.24	0.674	Co-occurrence
PPP1R26	9q34.3	2 (1.74%)	1 (0.41%)	2.09	0.24	0.674	Co-occurrence
PPP1R37	19q13.32	2 (1.74%)	1 (0.41%)	2.09	0.24	0.674	Co-occurrence
PPP1R3E	14q11.2	2 (1.74%)	1 (0.41%)	2.09	0.24	0.674	Co-occurrence
PPP3CB	10q22.2	2 (1.74%)	1 (0.41%)	2.09	0.24	0.674	Co-occurrence
PPT1	1p34.2	2 (1.74%)	1 (0.41%)	2.09	0.24	0.674	Co-occurrence
PRC1	15q26.1	2 (1.74%)	1 (0.41%)	2.09	0.24	0.674	Co-occurrence
PRDM10	11q24.3	2 (1.74%)	1 (0.41%)	2.09	0.24	0.674	Co-occurrence
PREX1	20q13.13	2 (1.74%)	1 (0.41%)	2.09	0.24	0.674	Co-occurrence
PRKCD	3p21.1	2 (1.74%)	1 (0.41%)	2.09	0.24	0.674	Co-occurrence
PRPF38B	1p13.3	2 (1.74%)	1 (0.41%)	2.09	0.24	0.674	Co-occurrence
PRR19	19q13.2	2 (1.74%)	1 (0.41%)	2.09	0.24	0.674	Co-occurrence

PRR34	22q13.31	2 (1.74%)	1 (0.41%)	2.09	0.24	0.674	Co-occurrence
PRTG	15q21.3	2 (1.74%)	1 (0.41%)	2.09	0.24	0.674	Co-occurrence
PSMA4	15q25.1	2 (1.74%)	1 (0.41%)	2.09	0.24	0.674	Co-occurrence
PSMD14	2q24.2	2 (1.74%)	1 (0.41%)	2.09	0.24	0.674	Co-occurrence
PSMD3	17q21.1	2 (1.74%)	1 (0.41%)	2.09	0.24	0.674	Co-occurrence
PSRC1	1p13.3	2 (1.74%)	1 (0.41%)	2.09	0.24	0.674	Co-occurrence
PSTPIP1	15q24.3	2 (1.74%)	1 (0.41%)	2.09	0.24	0.674	Co-occurrence
PTGES	9q34.11	2 (1.74%)	1 (0.41%)	2.09	0.24	0.674	Co-occurrence
PTPN9	15q24.2	2 (1.74%)	1 (0.41%)	2.09	0.24	0.674	Co-occurrence
PYGO1	15q21.3	2 (1.74%)	1 (0.41%)	2.09	0.24	0.674	Co-occurrence
QRFP	9q34.12	2 (1.74%)	1 (0.41%)	2.09	0.24	0.674	Co-occurrence
RAB11A	15q22.31	2 (1.74%)	1 (0.41%)	2.09	0.24	0.674	Co-occurrence
RAB21	12q21.1	2 (1.74%)	1 (0.41%)	2.09	0.24	0.674	Co-occurrence
RAB8B	15q22.2	2 (1.74%)	1 (0.41%)	2.09	0.24	0.674	Co-occurrence
RAC1	7p22.1	2 (1.74%)	1 (0.41%)	2.09	0.24	0.674	Co-occurrence
RAD51D	17q12	2 (1.74%)	1 (0.41%)	2.09	0.24	0.674	Co-occurrence
RADIL	7p22.1	2 (1.74%)	1 (0.41%)	2.09	0.24	0.674	Co-occurrence
RALGPS1	9q33.3	2 (1.74%)	1 (0.41%)	2.09	0.24	0.674	Co-occurrence
RAPGEFL1	17q21.1	2 (1.74%)	1 (0.41%)	2.09	0.24	0.674	Co-occurrence
RARA	17q21.2	2 (1.74%)	1 (0.41%)	2.09	0.24	0.674	Co-occurrence
RASSF1	3p21.31	2 (1.74%)	1 (0.41%)	2.09	0.24	0.674	Co-occurrence
RBAK	7p22.1	2 (1.74%)	1 (0.41%)	2.09	0.24	0.674	Co-occurrence
RCCD1	15q26.1	2 (1.74%)	1 (0.41%)	2.09	0.24	0.674	Co-occurrence
RCN2	15q24.3	2 (1.74%)	1 (0.41%)	2.09	0.24	0.674	Co-occurrence
REC114	15q24.1	2 (1.74%)	1 (0.41%)	2.09	0.24	0.674	Co-occurrence
RFFL	17q12	2 (1.74%)	1 (0.41%)	2.09	0.24	0.674	Co-occurrence
RFPL2	22q12.3	2 (1.74%)	1 (0.41%)	2.09	0.24	0.674	Co-occurrence
RFPL3	22q12.3	2 (1.74%)	1 (0.41%)	2.09	0.24	0.674	Co-occurrence
RFPL3S	22q12.3	2 (1.74%)	1 (0.41%)	2.09	0.24	0.674	Co-occurrence

RFX7	15q21.3	2 (1.74%)	1 (0.41%)	2.09	0.24	0.674	Co-occurrence
RGS9BP	19q13.11	2 (1.74%)	1 (0.41%)	2.09	0.24	0.674	Co-occurrence
RHCG	15q26.1	2 (1.74%)	1 (0.41%)	2.09	0.24	0.674	Co-occurrence
RHPN2	19q13.11	2 (1.74%)	1 (0.41%)	2.09	0.24	0.674	Co-occurrence
RLBP1	15q26.1	2 (1.74%)	1 (0.41%)	2.09	0.24	0.674	Co-occurrence
RMRP	9p13.3	2 (1.74%)	1 (0.41%)	2.09	0.24	0.674	Co-occurrence
RN7SKP130	7p22.2	2 (1.74%)	1 (0.41%)	2.09	0.24	0.674	Co-occurrence
RN7SKP152	2q24.3	2 (1.74%)	1 (0.41%)	2.09	0.24	0.674	Co-occurrence
RN7SKP205	14q11.2	2 (1.74%)	1 (0.41%)	2.09	0.24	0.674	Co-occurrence
RN7SKP217	15q24.3	2 (1.74%)	1 (0.41%)	2.09	0.24	0.674	Co-occurrence
RN7SKP22	19q13.11	2 (1.74%)	1 (0.41%)	2.09	0.24	0.674	Co-occurrence
RN7SKP245	6q23.2	2 (1.74%)	1 (0.41%)	2.09	0.24	0.674	Co-occurrence
RN7SKP281	2q24.1	2 (1.74%)	1 (0.41%)	2.09	0.24	0.674	Co-occurrence
RN7SKP65	12q14.1	2 (1.74%)	1 (0.41%)	2.09	0.24	0.674	Co-occurrence
RN7SKP95	15q21.3	2 (1.74%)	1 (0.41%)	2.09	0.24	0.674	Co-occurrence
RN7SL138P	17q11.2	2 (1.74%)	1 (0.41%)	2.09	0.24	0.674	Co-occurrence
RN7SL214P	15q24.3	2 (1.74%)	1 (0.41%)	2.09	0.24	0.674	Co-occurrence
RN7SL22P	9p13.3	2 (1.74%)	1 (0.41%)	2.09	0.24	0.674	Co-occurrence
RN7SL251P	2p11.2	2 (1.74%)	1 (0.41%)	2.09	0.24	0.674	Co-occurrence
RN7SL278P	15q24.3	2 (1.74%)	1 (0.41%)	2.09	0.24	0.674	Co-occurrence
RN7SL305P	22q12.3	2 (1.74%)	1 (0.41%)	2.09	0.24	0.674	Co-occurrence
RN7SL319P	15q24.2	2 (1.74%)	1 (0.41%)	2.09	0.24	0.674	Co-occurrence
RN7SL327P	15q24.2	2 (1.74%)	1 (0.41%)	2.09	0.24	0.674	Co-occurrence
RN7SL363P	15q26.1	2 (1.74%)	1 (0.41%)	2.09	0.24	0.674	Co-occurrence
RN7SL38P	12p12.1	2 (1.74%)	1 (0.41%)	2.09	0.24	0.674	Co-occurrence
RN7SL399P	17q21.2	2 (1.74%)	1 (0.41%)	2.09	0.24	0.674	Co-occurrence
RN7SL423P	2q24.2	2 (1.74%)	1 (0.41%)	2.09	0.24	0.674	Co-occurrence
RN7SL428P	15q25.3	2 (1.74%)	1 (0.41%)	2.09	0.24	0.674	Co-occurrence
RN7SL429P	15q24.1	2 (1.74%)	1 (0.41%)	2.09	0.24	0.674	Co-occurrence

RN7SL438P	15q23	2 (1.74%)	1 (0.41%)	2.09	0.24	0.674	Co-occurrence
RN7SL455P	2p21	2 (1.74%)	1 (0.41%)	2.09	0.24	0.674	Co-occurrence
RN7SL489P	15q24.2	2 (1.74%)	1 (0.41%)	2.09	0.24	0.674	Co-occurrence
RN7SL510P	15q24.2	2 (1.74%)	1 (0.41%)	2.09	0.24	0.674	Co-occurrence
RN7SL556P	7p22.1	2 (1.74%)	1 (0.41%)	2.09	0.24	0.674	Co-occurrence
RN7SL568P	15q21.3	2 (1.74%)	1 (0.41%)	2.09	0.24	0.674	Co-occurrence
RN7SL571P	13q22.2	2 (1.74%)	1 (0.41%)	2.09	0.24	0.674	Co-occurrence
RN7SL614P	1p36.22	2 (1.74%)	1 (0.41%)	2.09	0.24	0.674	Co-occurrence
RN7SL616P	5q13.2	2 (1.74%)	1 (0.41%)	2.09	0.24	0.674	Co-occurrence
RN7SL761P	13q21.33	2 (1.74%)	1 (0.41%)	2.09	0.24	0.674	Co-occurrence
RN7SL789P	19q13.11	2 (1.74%)	1 (0.41%)	2.09	0.24	0.674	Co-occurrence
RN7SL851P	7p22.1	2 (1.74%)	1 (0.41%)	2.09	0.24	0.674	Co-occurrence
RN7SL854P	1p31.3	2 (1.74%)	1 (0.41%)	2.09	0.24	0.674	Co-occurrence
RNA5SP109	2q24.2	2 (1.74%)	1 (0.41%)	2.09	0.24	0.674	Co-occurrence
RNA5SP130	3p21.31	2 (1.74%)	1 (0.41%)	2.09	0.24	0.674	Co-occurrence
RNA5SP131	3p21.31	2 (1.74%)	1 (0.41%)	2.09	0.24	0.674	Co-occurrence
RNA5SP21	1p31.1	2 (1.74%)	1 (0.41%)	2.09	0.24	0.674	Co-occurrence
RNA5SP32		2 (1.74%)	1 (0.41%)	2.09	0.24	0.674	Co-occurrence
RNA5SP334	11p15.1	2 (1.74%)	1 (0.41%)	2.09	0.24	0.674	Co-occurrence
RNA5SP370	12q23.3	2 (1.74%)	1 (0.41%)	2.09	0.24	0.674	Co-occurrence
RNA5SP396	15q22.2	2 (1.74%)	1 (0.41%)	2.09	0.24	0.674	Co-occurrence
RNA5SP397	15q22.2	2 (1.74%)	1 (0.41%)	2.09	0.24	0.674	Co-occurrence
RNA5SP398		2 (1.74%)	1 (0.41%)	2.09	0.24	0.674	Co-occurrence
RNA5SP400	15q25.3	2 (1.74%)	1 (0.41%)	2.09	0.24	0.674	Co-occurrence
RNA5SP424	16q12.1	2 (1.74%)	1 (0.41%)	2.09	0.24	0.674	Co-occurrence
RNA5SP425	16q12.1	2 (1.74%)	1 (0.41%)	2.09	0.24	0.674	Co-occurrence
RNA5SP441	17q21.2	2 (1.74%)	1 (0.41%)	2.09	0.24	0.674	Co-occurrence
RNA5SP442	17q21.2	2 (1.74%)	1 (0.41%)	2.09	0.24	0.674	Co-occurrence
RNA5SP471	19q12	2 (1.74%)	1 (0.41%)	2.09	0.24	0.674	Co-occurrence

RNA5SP472	19q13.11	2 (1.74%)	1 (0.41%)	2.09	0.24	0.674	Co-occurrence
RNA5SP49	1p31.3	2 (1.74%)	1 (0.41%)	2.09	0.24	0.674	Co-occurrence
RNF11	1p32.3	2 (1.74%)	1 (0.41%)	2.09	0.24	0.674	Co-occurrence
RNF111	15q22.1-q22.2	2 (1.74%)	1 (0.41%)	2.09	0.24	0.674	Co-occurrence
RNF135	17q11.2	2 (1.74%)	1 (0.41%)	2.09	0.24	0.674	Co-occurrence
RNF19B	1p35.1	2 (1.74%)	1 (0.41%)	2.09	0.24	0.674	Co-occurrence
RNF212B	14q11.2	2 (1.74%)	1 (0.41%)	2.09	0.24	0.674	Co-occurrence
RNF216	7p22.1	2 (1.74%)	1 (0.41%)	2.09	0.24	0.674	Co-occurrence
RNF216P1	7p22.1	2 (1.74%)	1 (0.41%)	2.09	0.24	0.674	Co-occurrence
RNU1-1	1p36.13	2 (1.74%)	1 (0.41%)	2.09	0.24	0.674	Co-occurrence
RNU4ATAC4P	1p31.3	2 (1.74%)	1 (0.41%)	2.09	0.24	0.674	Co-occurrence
RNU6ATAC20P	18q11.2	2 (1.74%)	1 (0.41%)	2.09	0.24	0.674	Co-occurrence
RNY1P5	13q22.1	2 (1.74%)	1 (0.41%)	2.09	0.24	0.674	Co-occurrence
RNY1P8	13q22.1	2 (1.74%)	1 (0.41%)	2.09	0.24	0.674	Co-occurrence
RNY3P10	13q21.33	2 (1.74%)	1 (0.41%)	2.09	0.24	0.674	Co-occurrence
RNY4P29	13q21.1	2 (1.74%)	1 (0.41%)	2.09	0.24	0.674	Co-occurrence
RNY4P8	17q21.2	2 (1.74%)	1 (0.41%)	2.09	0.24	0.674	Co-occurrence
ROR1	1p31.3	2 (1.74%)	1 (0.41%)	2.09	0.24	0.674	Co-occurrence
RPL4	15q22.31	2 (1.74%)	1 (0.41%)	2.09	0.24	0.674	Co-occurrence
RPLP1	15q23	2 (1.74%)	1 (0.41%)	2.09	0.24	0.674	Co-occurrence
RPP25	15q24.2	2 (1.74%)	1 (0.41%)	2.09	0.24	0.674	Co-occurrence
RPS6KA3	Xp22.12	2 (1.74%)	1 (0.41%)	2.09	0.24	0.674	Co-occurrence
RSL1D1	16p13.13	2 (1.74%)	1 (0.41%)	2.09	0.24	0.674	Co-occurrence
RSPH10B	7p22.1	2 (1.74%)	1 (0.41%)	2.09	0.24	0.674	Co-occurrence
RSPH10B2	7p22.1	2 (1.74%)	1 (0.41%)	2.09	0.24	0.674	Co-occurrence
RTCB	22q12.3	2 (1.74%)	1 (0.41%)	2.09	0.24	0.674	Co-occurrence
S1PR1	1p21.2	2 (1.74%)	1 (0.41%)	2.09	0.24	0.674	Co-occurrence
SAA1	11p15.1	2 (1.74%)	1 (0.41%)	2.09	0.24	0.674	Co-occurrence
SAA3P	11p15.1	2 (1.74%)	1 (0.41%)	2.09	0.24	0.674	Co-occurrence

SAAL1	11p15.1	2 (1.74%)	1 (0.41%)	2.09	0.24	0.674	Co-occurrence
SARDH	9q34.2	2 (1.74%)	1 (0.41%)	2.09	0.24	0.674	Co-occurrence
SARNP	12q13.2	2 (1.74%)	1 (0.41%)	2.09	0.24	0.674	Co-occurrence
SARS	1p13.3	2 (1.74%)	1 (0.41%)	2.09	0.24	0.674	Co-occurrence
SBF2	11p15.4	2 (1.74%)	1 (0.41%)	2.09	0.24	0.674	Co-occurrence
SCAMP2	15q24.1	2 (1.74%)	1 (0.41%)	2.09	0.24	0.674	Co-occurrence
SCAMP5	15q24.2	2 (1.74%)	1 (0.41%)	2.09	0.24	0.674	Co-occurrence
SCAPER	15q24.3	2 (1.74%)	1 (0.41%)	2.09	0.24	0.674	Co-occurrence
SCARB1	12q24.31	2 (1.74%)	1 (0.41%)	2.09	0.24	0.674	Co-occurrence
SCARNA14	15q22.31	2 (1.74%)	1 (0.41%)	2.09	0.24	0.674	Co-occurrence
SCN1A	2q24.3	2 (1.74%)	1 (0.41%)	2.09	0.24	0.674	Co-occurrence
SCN2A	2q24.3	2 (1.74%)	1 (0.41%)	2.09	0.24	0.674	Co-occurrence
SCN7A	2q24.3	2 (1.74%)	1 (0.41%)	2.09	0.24	0.674	Co-occurrence
SCN9A	2q24.3	2 (1.74%)	1 (0.41%)	2.09	0.24	0.674	Co-occurrence
SDHB	1p36.13	2 (1.74%)	1 (0.41%)	2.09	0.24	0.674	Co-occurrence
SDK1	7p22.2	2 (1.74%)	1 (0.41%)	2.09	0.24	0.674	Co-occurrence
SDR39U1	14q12	2 (1.74%)	1 (0.41%)	2.09	0.24	0.674	Co-occurrence
SEC16A	9q34.3	2 (1.74%)	1 (0.41%)	2.09	0.24	0.674	Co-occurrence
SEC22C	3p22.1	2 (1.74%)	1 (0.41%)	2.09	0.24	0.674	Co-occurrence
SELENOW	19q13.33	2 (1.74%)	1 (0.41%)	2.09	0.24	0.674	Co-occurrence
SEMA7A	15q24.1	2 (1.74%)	1 (0.41%)	2.09	0.24	0.674	Co-occurrence
SERF1A	5q13.2	2 (1.74%)	1 (0.41%)	2.09	0.24	0.674	Co-occurrence
SERF1B	5q13.2	2 (1.74%)	1 (0.41%)	2.09	0.24	0.674	Co-occurrence
SERGEF	11p15.1	2 (1.74%)	1 (0.41%)	2.09	0.24	0.674	Co-occurrence
SF3A2	19p13.3	2 (1.74%)	1 (0.41%)	2.09	0.24	0.674	Co-occurrence
SFMBT1	3p21.1	2 (1.74%)	1 (0.41%)	2.09	0.24	0.674	Co-occurrence
SGIP1	1p31.3	2 (1.74%)	1 (0.41%)	2.09	0.24	0.674	Co-occurrence
SH2D6	2p11.2	2 (1.74%)	1 (0.41%)	2.09	0.24	0.674	Co-occurrence
SHISA9	16p13.12	2 (1.74%)	1 (0.41%)	2.09	0.24	0.674	Co-occurrence

SLC1A7	1p32.3	2 (1.74%)	1 (0.41%)	2.09	0.24	0.674	Co-occurrence
SLC22A13	3p22.2	2 (1.74%)	1 (0.41%)	2.09	0.24	0.674	Co-occurrence
SLC22A17	14q11.2	2 (1.74%)	1 (0.41%)	2.09	0.24	0.674	Co-occurrence
SLC24A1	15q22.31	2 (1.74%)	1 (0.41%)	2.09	0.24	0.674	Co-occurrence
SLC25A24	1p13.3	2 (1.74%)	1 (0.41%)	2.09	0.24	0.674	Co-occurrence
SLC25A24P1	1p13.3	2 (1.74%)	1 (0.41%)	2.09	0.24	0.674	Co-occurrence
SLC25A3P1	1p32.3	2 (1.74%)	1 (0.41%)	2.09	0.24	0.674	Co-occurrence
SLC29A4	7p22.1	2 (1.74%)	1 (0.41%)	2.09	0.24	0.674	Co-occurrence
SLC35D1	1p31.3	2 (1.74%)	1 (0.41%)	2.09	0.24	0.674	Co-occurrence
SLC35G3	17q12	2 (1.74%)	1 (0.41%)	2.09	0.24	0.674	Co-occurrence
SLC41A2	12q23.3	2 (1.74%)	1 (0.41%)	2.09	0.24	0.674	Co-occurrence
SLC4A10	2q24.2	2 (1.74%)	1 (0.41%)	2.09	0.24	0.674	Co-occurrence
SLC5A1	22q12.3	2 (1.74%)	1 (0.41%)	2.09	0.24	0.674	Co-occurrence
SLC5A4	22q12.3	2 (1.74%)	1 (0.41%)	2.09	0.24	0.674	Co-occurrence
SLC6A20	3p21.31	2 (1.74%)	1 (0.41%)	2.09	0.24	0.674	Co-occurrence
SLC7A9	19q13.11	2 (1.74%)	1 (0.41%)	2.09	0.24	0.674	Co-occurrence
SLTM	15q22.1	2 (1.74%)	1 (0.41%)	2.09	0.24	0.674	Co-occurrence
SMAD3	15q22.33	2 (1.74%)	1 (0.41%)	2.09	0.24	0.674	Co-occurrence
SMAD6	15q22.31	2 (1.74%)	1 (0.41%)	2.09	0.24	0.674	Co-occurrence
SMAD7	18q21.1	2 (1.74%)	1 (0.41%)	2.09	0.24	0.674	Co-occurrence
SMN1	5q13.2	2 (1.74%)	1 (0.41%)	2.09	0.24	0.674	Co-occurrence
SMN2	5q13.2	2 (1.74%)	1 (0.41%)	2.09	0.24	0.674	Co-occurrence
SNAPC5	15q22.31	2 (1.74%)	1 (0.41%)	2.09	0.24	0.674	Co-occurrence
SNHG11	20q11.23	2 (1.74%)	1 (0.41%)	2.09	0.24	0.674	Co-occurrence
SNHG7	9q34.3	2 (1.74%)	1 (0.41%)	2.09	0.24	0.674	Co-occurrence
SNORA31	13q14.13	2 (1.74%)	1 (0.41%)	2.09	0.24	0.674	Co-occurrence
SNORA60	20q11.23	2 (1.74%)	1 (0.41%)	2.09	0.24	0.674	Co-occurrence
SNORD16	15q22.31	2 (1.74%)	1 (0.41%)	2.09	0.24	0.674	Co-occurrence
SNORD18A	15q22.31	2 (1.74%)	1 (0.41%)	2.09	0.24	0.674	Co-occurrence

SNORD18B	15q22.31	2 (1.74%)	1 (0.41%)	2.09	0.24	0.674	Co-occurrence
SNORD18C	15q22.31	2 (1.74%)	1 (0.41%)	2.09	0.24	0.674	Co-occurrence
SNORD37	19p13.3	2 (1.74%)	1 (0.41%)	2.09	0.24	0.674	Co-occurrence
SNTA1	20q11.21	2 (1.74%)	1 (0.41%)	2.09	0.24	0.674	Co-occurrence
SNUPN	15q24.2	2 (1.74%)	1 (0.41%)	2.09	0.24	0.674	Co-occurrence
SNX29	16p13.13-p13.12	2 (1.74%)	1 (0.41%)	2.09	0.24	0.674	Co-occurrence
SNX33	15q24.2	2 (1.74%)	1 (0.41%)	2.09	0.24	0.674	Co-occurrence
SOHLH1	9q34.3	2 (1.74%)	1 (0.41%)	2.09	0.24	0.674	Co-occurrence
SORT1	1p13.3|1p21.3-p13.1	2 (1.74%)	1 (0.41%)	2.09	0.24	0.674	Co-occurrence
SPATA5L1	15q21.1	2 (1.74%)	1 (0.41%)	2.09	0.24	0.674	Co-occurrence
SPESP1	15q23	2 (1.74%)	1 (0.41%)	2.09	0.24	0.674	Co-occurrence
SPTY2D1	11p15.1	2 (1.74%)	1 (0.41%)	2.09	0.24	0.674	Co-occurrence
SRGAP1	12q14.2	2 (1.74%)	1 (0.41%)	2.09	0.24	0.674	Co-occurrence
SS18L2	3p22.1	2 (1.74%)	1 (0.41%)	2.09	0.24	0.674	Co-occurrence
SSH2	17q11.2	2 (1.74%)	1 (0.41%)	2.09	0.24	0.674	Co-occurrence
ST20	15q25.1	2 (1.74%)	1 (0.41%)	2.09	0.24	0.674	Co-occurrence
ST20-AS1	15q25.1	2 (1.74%)	1 (0.41%)	2.09	0.24	0.674	Co-occurrence
ST6GALNAC4	9q34.11	2 (1.74%)	1 (0.41%)	2.09	0.24	0.674	Co-occurrence
ST6GALNAC6	9q34.11	2 (1.74%)	1 (0.41%)	2.09	0.24	0.674	Co-occurrence
STAU1	20q13.13	2 (1.74%)	1 (0.41%)	2.09	0.24	0.674	Co-occurrence
STIMATE	3p21.1	2 (1.74%)	1 (0.41%)	2.09	0.24	0.674	Co-occurrence
STK38L	12p11.23	2 (1.74%)	1 (0.41%)	2.09	0.24	0.674	Co-occurrence
STOML1	15q24.1	2 (1.74%)	1 (0.41%)	2.09	0.24	0.674	Co-occurrence
STPG3	9q34.3	2 (1.74%)	1 (0.41%)	2.09	0.24	0.674	Co-occurrence
STRA6	15q24.1	2 (1.74%)	1 (0.41%)	2.09	0.24	0.674	Co-occurrence
STXBP3	1p13.3	2 (1.74%)	1 (0.41%)	2.09	0.24	0.674	Co-occurrence
SUSD1	9q31.3-q32	2 (1.74%)	1 (0.41%)	2.09	0.24	0.674	Co-occurrence
SV2B	15q26.1	2 (1.74%)	1 (0.41%)	2.09	0.24	0.674	Co-occurrence
SV2C	5q13.3	2 (1.74%)	1 (0.41%)	2.09	0.24	0.674	Co-occurrence

SVOP	12q24.11	2 (1.74%)	1 (0.41%)	2.09	0.24	0.674	Co-occurrence
TALDO1	11p15.5	2 (1.74%)	1 (0.41%)	2.09	0.24	0.674	Co-occurrence
TANC1	2q24.2	2 (1.74%)	1 (0.41%)	2.09	0.24	0.674	Co-occurrence
TANK	2q24.2	2 (1.74%)	1 (0.41%)	2.09	0.24	0.674	Co-occurrence
TAS1R1	1p36.31	2 (1.74%)	1 (0.41%)	2.09	0.24	0.674	Co-occurrence
TBC1D15	12q21.1	2 (1.74%)	1 (0.41%)	2.09	0.24	0.674	Co-occurrence
TBC1D21	15q24.1	2 (1.74%)	1 (0.41%)	2.09	0.24	0.674	Co-occurrence
TBC1D4	13q22.2	2 (1.74%)	1 (0.41%)	2.09	0.24	0.674	Co-occurrence
TBR1	2q24.2	2 (1.74%)	1 (0.41%)	2.09	0.24	0.674	Co-occurrence
TBX15	1p12	2 (1.74%)	1 (0.41%)	2.09	0.24	0.674	Co-occurrence
TCF20	22q13.2|22q13.3	2 (1.74%)	1 (0.41%)	2.09	0.24	0.674	Co-occurrence
TCTA	3p21.31	2 (1.74%)	1 (0.41%)	2.09	0.24	0.674	Co-occurrence
TCTEX1D1	1p31.3	2 (1.74%)	1 (0.41%)	2.09	0.24	0.674	Co-occurrence
TDG	12q23.3	2 (1.74%)	1 (0.41%)	2.09	0.24	0.674	Co-occurrence
TDRD12	19q13.11	2 (1.74%)	1 (0.41%)	2.09	0.24	0.674	Co-occurrence
TEX9	15q21.3	2 (1.74%)	1 (0.41%)	2.09	0.24	0.674	Co-occurrence
THAP3	1p36.31	2 (1.74%)	1 (0.41%)	2.09	0.24	0.674	Co-occurrence
THEG5	19q12	2 (1.74%)	1 (0.41%)	2.09	0.24	0.674	Co-occurrence
TICAM1	19p13.3	2 (1.74%)	1 (0.41%)	2.09	0.24	0.674	Co-occurrence
TICRR	15q26.1	2 (1.74%)	1 (0.41%)	2.09	0.24	0.674	Co-occurrence
TIPIN	15q22.31	2 (1.74%)	1 (0.41%)	2.09	0.24	0.674	Co-occurrence
TLL2	10q24.1	2 (1.74%)	1 (0.41%)	2.09	0.24	0.674	Co-occurrence
TLN1	9p13.3	2 (1.74%)	1 (0.41%)	2.09	0.24	0.674	Co-occurrence
TM2D1	1p31.3	2 (1.74%)	1 (0.41%)	2.09	0.24	0.674	Co-occurrence
TM7SF3	12p11.23	2 (1.74%)	1 (0.41%)	2.09	0.24	0.674	Co-occurrence
TM9SF3	10q24.1	2 (1.74%)	1 (0.41%)	2.09	0.24	0.674	Co-occurrence
TMC3	15q25.1	2 (1.74%)	1 (0.41%)	2.09	0.24	0.674	Co-occurrence
TMC5	16p12.3	2 (1.74%)	1 (0.41%)	2.09	0.24	0.674	Co-occurrence
TMED3	15q25.1	2 (1.74%)	1 (0.41%)	2.09	0.24	0.674	Co-occurrence

TMEM115	3p21.31	2 (1.74%)	1 (0.41%)	2.09	0.24	0.674	Co-occurrence
TMEM120B	12q24.31	2 (1.74%)	1 (0.41%)	2.09	0.24	0.674	Co-occurrence
TMEM121B	22q11.1	2 (1.74%)	1 (0.41%)	2.09	0.24	0.674	Co-occurrence
TMEM141	9q34.3	2 (1.74%)	1 (0.41%)	2.09	0.24	0.674	Co-occurrence
TMEM145	19q13.2	2 (1.74%)	1 (0.41%)	2.09	0.24	0.674	Co-occurrence
TMEM198B	12q13.2	2 (1.74%)	1 (0.41%)	2.09	0.24	0.674	Co-occurrence
TMEM201	1p36.22	2 (1.74%)	1 (0.41%)	2.09	0.24	0.674	Co-occurrence
TMEM250	9q34.3	2 (1.74%)	1 (0.41%)	2.09	0.24	0.674	Co-occurrence
TMEM270	7q11.23	2 (1.74%)	1 (0.41%)	2.09	0.24	0.674	Co-occurrence
TMEM54	1p35.1	2 (1.74%)	1 (0.41%)	2.09	0.24	0.674	Co-occurrence
TMTC2	12q21.31	2 (1.74%)	1 (0.41%)	2.09	0.24	0.674	Co-occurrence
TNFRSF17	16p13.13	2 (1.74%)	1 (0.41%)	2.09	0.24	0.674	Co-occurrence
TNFRSF25	1p36.31	2 (1.74%)	1 (0.41%)	2.09	0.24	0.674	Co-occurrence
TNNC1	3p21.1	2 (1.74%)	1 (0.41%)	2.09	0.24	0.674	Co-occurrence
TNRC18	7p22.1	2 (1.74%)	1 (0.41%)	2.09	0.24	0.674	Co-occurrence
TOP2A	17q21.2	2 (1.74%)	1 (0.41%)	2.09	0.24	0.674	Co-occurrence
TOR1A	9q34.11	2 (1.74%)	1 (0.41%)	2.09	0.24	0.674	Co-occurrence
TOR1B	9q34.11	2 (1.74%)	1 (0.41%)	2.09	0.24	0.674	Co-occurrence
TOR4A	9q34.3	2 (1.74%)	1 (0.41%)	2.09	0.24	0.674	Co-occurrence
TP53INP2	20q11.22	2 (1.74%)	1 (0.41%)	2.09	0.24	0.674	Co-occurrence
TPH1	11p15.1	2 (1.74%)	1 (0.41%)	2.09	0.24	0.674	Co-occurrence
TPM2	9p13.3	2 (1.74%)	1 (0.41%)	2.09	0.24	0.674	Co-occurrence
TPX2	20q11.21	2 (1.74%)	1 (0.41%)	2.09	0.24	0.674	Co-occurrence
TRABD2B	1p33	2 (1.74%)	1 (0.41%)	2.09	0.24	0.674	Co-occurrence
TRHDE	12q21.1	2 (1.74%)	1 (0.41%)	2.09	0.24	0.674	Co-occurrence
TRIP4	15q22.31	2 (1.74%)	1 (0.41%)	2.09	0.24	0.674	Co-occurrence
TSHZ3	19q12	2 (1.74%)	1 (0.41%)	2.09	0.24	0.674	Co-occurrence
TSPAN3	15q24.3	2 (1.74%)	1 (0.41%)	2.09	0.24	0.674	Co-occurrence
TTC21B	2q24.3	2 (1.74%)	1 (0.41%)	2.09	0.24	0.674	Co-occurrence

TTC38	22q13.31	2 (1.74%)	1 (0.41%)	2.09	0.24	0.674	Co-occurrence
TTC39A	1p32.3	2 (1.74%)	1 (0.41%)	2.09	0.24	0.674	Co-occurrence
TTYH3	7p22.3	2 (1.74%)	1 (0.41%)	2.09	0.24	0.674	Co-occurrence
TUBBP5	9q34.3	2 (1.74%)	1 (0.41%)	2.09	0.24	0.674	Co-occurrence
TUSC2	3p21.31	2 (1.74%)	1 (0.41%)	2.09	0.24	0.674	Co-occurrence
TXN2	22q12.3	2 (1.74%)	1 (0.41%)	2.09	0.24	0.674	Co-occurrence
TXNRD1	12q23.3	2 (1.74%)	1 (0.41%)	2.09	0.24	0.674	Co-occurrence
UBAC1	9q34.3	2 (1.74%)	1 (0.41%)	2.09	0.24	0.674	Co-occurrence
UBE2Q2	15q24.2	2 (1.74%)	1 (0.41%)	2.09	0.24	0.674	Co-occurrence
UBE2U	1p31.3	2 (1.74%)	1 (0.41%)	2.09	0.24	0.674	Co-occurrence
UBL7	15q24.1	2 (1.74%)	1 (0.41%)	2.09	0.24	0.674	Co-occurrence
UCHL3	13q22.2	2 (1.74%)	1 (0.41%)	2.09	0.24	0.674	Co-occurrence
UEVLD	11p15.1	2 (1.74%)	1 (0.41%)	2.09	0.24	0.674	Co-occurrence
UGT2A1	4q13.3	2 (1.74%)	1 (0.41%)	2.09	0.24	0.674	Co-occurrence
UGT2A2	4q13.3	2 (1.74%)	1 (0.41%)	2.09	0.24	0.674	Co-occurrence
ULK3	15q24.1	2 (1.74%)	1 (0.41%)	2.09	0.24	0.674	Co-occurrence
UNC45A	15q26.1	2 (1.74%)	1 (0.41%)	2.09	0.24	0.674	Co-occurrence
UNC45B	17q12	2 (1.74%)	1 (0.41%)	2.09	0.24	0.674	Co-occurrence
UNC5D	8p12	2 (1.74%)	1 (0.41%)	2.09	0.24	0.674	Co-occurrence
USP20	9q34.11	2 (1.74%)	1 (0.41%)	2.09	0.24	0.674	Co-occurrence
USP33	1p31.1	2 (1.74%)	1 (0.41%)	2.09	0.24	0.674	Co-occurrence
USP42	7p22.1	2 (1.74%)	1 (0.41%)	2.09	0.24	0.674	Co-occurrence
VPS33B	15q26.1	2 (1.74%)	1 (0.41%)	2.09	0.24	0.674	Co-occurrence
VPS35L	16p12.3	2 (1.74%)	1 (0.41%)	2.09	0.24	0.674	Co-occurrence
WBP11P1	18q12.1	2 (1.74%)	1 (0.41%)	2.09	0.24	0.674	Co-occurrence
WDR61	15q25.1	2 (1.74%)	1 (0.41%)	2.09	0.24	0.674	Co-occurrence
WDR72	15q21.3	2 (1.74%)	1 (0.41%)	2.09	0.24	0.674	Co-occurrence
WDR78	1p31.3	2 (1.74%)	1 (0.41%)	2.09	0.24	0.674	Co-occurrence
WDSUB1	2q24.2	2 (1.74%)	1 (0.41%)	2.09	0.24	0.674	Co-occurrence

WIPF2	17q21.2	2 (1.74%)	1 (0.41%)	2.09	0.24	0.674	Co-occurrence
WIPI2	7p22.1	2 (1.74%)	1 (0.41%)	2.09	0.24	0.674	Co-occurrence
WNK3	Xp11.22	2 (1.74%)	1 (0.41%)	2.09	0.24	0.674	Co-occurrence
ZBTB48	1p36.31	2 (1.74%)	1 (0.41%)	2.09	0.24	0.674	Co-occurrence
ZC3H7A	16p13.13	2 (1.74%)	1 (0.41%)	2.09	0.24	0.674	Co-occurrence
ZDHHC4	7p22.1	2 (1.74%)	1 (0.41%)	2.09	0.24	0.674	Co-occurrence
ZFAND6	15q25.1	2 (1.74%)	1 (0.41%)	2.09	0.24	0.674	Co-occurrence
ZMYND10	3p21.31	2 (1.74%)	1 (0.41%)	2.09	0.24	0.674	Co-occurrence
ZMYND19	9q34.3	2 (1.74%)	1 (0.41%)	2.09	0.24	0.674	Co-occurrence
ZNF12	7p22.1	2 (1.74%)	1 (0.41%)	2.09	0.24	0.674	Co-occurrence
ZNF254	19p12	2 (1.74%)	1 (0.41%)	2.09	0.24	0.674	Co-occurrence
ZNF296	19q13.32	2 (1.74%)	1 (0.41%)	2.09	0.24	0.674	Co-occurrence
ZNF385C	17q21.2	2 (1.74%)	1 (0.41%)	2.09	0.24	0.674	Co-occurrence
ZNF710	15q26.1	2 (1.74%)	1 (0.41%)	2.09	0.24	0.674	Co-occurrence
ZNF726	19p12	2 (1.74%)	1 (0.41%)	2.09	0.24	0.674	Co-occurrence
ZNF733P	7q11.21	2 (1.74%)	1 (0.41%)	2.09	0.24	0.674	Co-occurrence
ZNF815P	7p22.1	2 (1.74%)	1 (0.41%)	2.09	0.24	0.674	Co-occurrence
ZNF830	17q12	2 (1.74%)	1 (0.41%)	2.09	0.24	0.674	Co-occurrence
ZNF853	7p22.1	2 (1.74%)	1 (0.41%)	2.09	0.24	0.674	Co-occurrence
ZNF890P	7p22.1	2 (1.74%)	1 (0.41%)	2.09	0.24	0.674	Co-occurrence
ZPBP2	17q21.1	2 (1.74%)	1 (0.41%)	2.09	0.24	0.674	Co-occurrence
ZWILCH	15q22.31	2 (1.74%)	1 (0.41%)	2.09	0.24	0.674	Co-occurrence
CNGB3	8q21.3	13 (11.30%)	36 (14.69%)	-0.38	0.241	0.674	Mutual exclusivity
CPNE3	8q21.3	13 (11.30%)	36 (14.69%)	-0.38	0.241	0.674	Mutual exclusivity
MMP16	8q21.3	13 (11.30%)	36 (14.69%)	-0.38	0.241	0.674	Mutual exclusivity
PAG1	8q21.13	13 (11.30%)	36 (14.69%)	-0.38	0.241	0.674	Mutual exclusivity
RMDN1	8q21.3	13 (11.30%)	36 (14.69%)	-0.38	0.241	0.674	Mutual exclusivity
SLC7A13	8q21.3	13 (11.30%)	36 (14.69%)	-0.38	0.241	0.674	Mutual exclusivity
WWP1	8q21.3	13 (11.30%)	36 (14.69%)	-0.38	0.241	0.674	Mutual exclusivity

EFR3A	8q24.22	17 (14.78%)	45 (18.37%)	-0.31	0.247	0.674	Mutual exclusivity
FAM49B	8q24.21	17 (14.78%)	45 (18.37%)	-0.31	0.247	0.674	Mutual exclusivity
GSDMC	8q24.21	17 (14.78%)	45 (18.37%)	-0.31	0.247	0.674	Mutual exclusivity
HHLA1	8q24.22	17 (14.78%)	45 (18.37%)	-0.31	0.247	0.674	Mutual exclusivity
HPYR1	8q24.22	17 (14.78%)	45 (18.37%)	-0.31	0.247	0.674	Mutual exclusivity
KCNQ3	8q24.22	17 (14.78%)	45 (18.37%)	-0.31	0.247	0.674	Mutual exclusivity
LRRC6	8q24.22	17 (14.78%)	45 (18.37%)	-0.31	0.247	0.674	Mutual exclusivity
MIR-5194/5194		17 (14.78%)	45 (18.37%)	-0.31	0.247	0.674	Mutual exclusivity
OC90	8q24.22	17 (14.78%)	45 (18.37%)	-0.31	0.247	0.674	Mutual exclusivity
PHF20L1	8q24.22	17 (14.78%)	45 (18.37%)	-0.31	0.247	0.674	Mutual exclusivity
TMEM71	8q24.22	17 (14.78%)	45 (18.37%)	-0.31	0.247	0.674	Mutual exclusivity
ADHFE1	8q13.1	8 (6.96%)	24 (9.80%)	-0.49	0.251	0.674	Mutual exclusivity
ARMC1	8q13.1	8 (6.96%)	24 (9.80%)	-0.49	0.251	0.674	Mutual exclusivity
C8ORF44	8q13.1	8 (6.96%)	24 (9.80%)	-0.49	0.251	0.674	Mutual exclusivity
MTFR1	8q13.1	8 (6.96%)	24 (9.80%)	-0.49	0.251	0.674	Mutual exclusivity
PDE7A	8q13.1	8 (6.96%)	24 (9.80%)	-0.49	0.251	0.674	Mutual exclusivity
RRS1	8q13.1	8 (6.96%)	24 (9.80%)	-0.49	0.251	0.674	Mutual exclusivity
SGK3	8q13.1	8 (6.96%)	24 (9.80%)	-0.49	0.251	0.674	Mutual exclusivity
VCPIP1	8q13.1	8 (6.96%)	24 (9.80%)	-0.49	0.251	0.674	Mutual exclusivity
VXN	8q13.1	8 (6.96%)	24 (9.80%)	-0.49	0.251	0.674	Mutual exclusivity
CDH17	8q22.1	14 (12.17%)	38 (15.51%)	-0.35	0.251	0.674	Mutual exclusivity
FAM92A	8q22.1	14 (12.17%)	38 (15.51%)	-0.35	0.251	0.674	Mutual exclusivity
FSBP	8q22.1	14 (12.17%)	38 (15.51%)	-0.35	0.251	0.674	Mutual exclusivity
GEM	8q22.1	14 (12.17%)	38 (15.51%)	-0.35	0.251	0.674	Mutual exclusivity
PDP1	8q22.1	14 (12.17%)	38 (15.51%)	-0.35	0.251	0.674	Mutual exclusivity
RAD54B	8q22.1	14 (12.17%)	38 (15.51%)	-0.35	0.251	0.674	Mutual exclusivity
RBM12B	8q22.1	14 (12.17%)	38 (15.51%)	-0.35	0.251	0.674	Mutual exclusivity
TMEM67	8q22.1	14 (12.17%)	38 (15.51%)	-0.35	0.251	0.674	Mutual exclusivity
VIRMA	8q22.1	14 (12.17%)	38 (15.51%)	-0.35	0.251	0.674	Mutual exclusivity

B4GALT5	20q13.13	5 (4.35%)	6 (2.45%)	0.83	0.252	0.674	Co-occurrence
SLC9A8	20q13.13	5 (4.35%)	6 (2.45%)	0.83	0.252	0.674	Co-occurrence
CRISPLD1	8q21.13	11 (9.57%)	31 (12.65%)	-0.4	0.253	0.674	Mutual exclusivity
ELOC	8q21.11	11 (9.57%)	31 (12.65%)	-0.4	0.253	0.674	Mutual exclusivity
GDAP1	8q21.11	11 (9.57%)	31 (12.65%)	-0.4	0.253	0.674	Mutual exclusivity
JPH1	8q21.11	11 (9.57%)	31 (12.65%)	-0.4	0.253	0.674	Mutual exclusivity
LY96	8q21.11	11 (9.57%)	31 (12.65%)	-0.4	0.253	0.674	Mutual exclusivity
MSC	8q13.3	11 (9.57%)	31 (12.65%)	-0.4	0.253	0.674	Mutual exclusivity
NCOA2	8q13.3	11 (9.57%)	31 (12.65%)	-0.4	0.253	0.674	Mutual exclusivity
PI15	8q21.13	11 (9.57%)	31 (12.65%)	-0.4	0.253	0.674	Mutual exclusivity
PRDM14	8q13.3	11 (9.57%)	31 (12.65%)	-0.4	0.253	0.674	Mutual exclusivity
TMEM70	8q21.11	11 (9.57%)	31 (12.65%)	-0.4	0.253	0.674	Mutual exclusivity
GRHL2	8q22.3	15 (13.04%)	40 (16.33%)	-0.32	0.26	0.674	Mutual exclusivity
KLF10	8q22.3	15 (13.04%)	40 (16.33%)	-0.32	0.26	0.674	Mutual exclusivity
LAPTM4B	8q22.1	15 (13.04%)	40 (16.33%)	-0.32	0.26	0.674	Mutual exclusivity
MIR-5680/5680		15 (13.04%)	40 (16.33%)	-0.32	0.26	0.674	Mutual exclusivity
MIR-599/599		15 (13.04%)	40 (16.33%)	-0.32	0.26	0.674	Mutual exclusivity
NACA4P	8q22.3	15 (13.04%)	40 (16.33%)	-0.32	0.26	0.674	Mutual exclusivity
NCALD	8q22.3	15 (13.04%)	40 (16.33%)	-0.32	0.26	0.674	Mutual exclusivity
ODF1	8q22.3	15 (13.04%)	40 (16.33%)	-0.32	0.26	0.674	Mutual exclusivity
PABPC1	8q22.3	15 (13.04%)	40 (16.33%)	-0.32	0.26	0.674	Mutual exclusivity
RN7SKP249	8q22.3	15 (13.04%)	40 (16.33%)	-0.32	0.26	0.674	Mutual exclusivity
RN7SL350P	8q22.2	15 (13.04%)	40 (16.33%)	-0.32	0.26	0.674	Mutual exclusivity
RN7SL563P	8q22.3	15 (13.04%)	40 (16.33%)	-0.32	0.26	0.674	Mutual exclusivity
RN7SL685P	8q22.3	15 (13.04%)	40 (16.33%)	-0.32	0.26	0.674	Mutual exclusivity
RNU6ATAC41P	8q22.3	15 (13.04%)	40 (16.33%)	-0.32	0.26	0.674	Mutual exclusivity
RNU6ATAC8P	8q22.3	15 (13.04%)	40 (16.33%)	-0.32	0.26	0.674	Mutual exclusivity
RRM2B	8q22.3	15 (13.04%)	40 (16.33%)	-0.32	0.26	0.674	Mutual exclusivity
TP53INP1	8q22.1	15 (13.04%)	40 (16.33%)	-0.32	0.26	0.674	Mutual exclusivity

UBR5	8q22.3	15 (13.04%)	40 (16.33%)	-0.32	0.26	0.674	Mutual exclusivity
YWHAZ	8q22.3	15 (13.04%)	40 (16.33%)	-0.32	0.26	0.674	Mutual exclusivity
ZNF706	8q22.3	15 (13.04%)	40 (16.33%)	-0.32	0.26	0.674	Mutual exclusivity
CAV2	7q31.2	2 (1.74%)	9 (3.67%)	-1.08	0.261	0.674	Mutual exclusivity
CHKA	11q13.2	2 (1.74%)	9 (3.67%)	-1.08	0.261	0.674	Mutual exclusivity
FOXP2	7q31.1	2 (1.74%)	9 (3.67%)	-1.08	0.261	0.674	Mutual exclusivity
MDFIC	7q31.1-q31.2	2 (1.74%)	9 (3.67%)	-1.08	0.261	0.674	Mutual exclusivity
MIR-3666/3666		2 (1.74%)	9 (3.67%)	-1.08	0.261	0.674	Mutual exclusivity
PTP4A1	6q12	2 (1.74%)	9 (3.67%)	-1.08	0.261	0.674	Mutual exclusivity
RNA5SP238	7q31.1	2 (1.74%)	9 (3.67%)	-1.08	0.261	0.674	Mutual exclusivity
TES	7q31.2	2 (1.74%)	9 (3.67%)	-1.08	0.261	0.674	Mutual exclusivity
TFEC	7q31.2	2 (1.74%)	9 (3.67%)	-1.08	0.261	0.674	Mutual exclusivity
DEK	6p22.3	6 (5.22%)	8 (3.27%)	0.68	0.268	0.674	Co-occurrence
DTNBP1	6p22.3	6 (5.22%)	8 (3.27%)	0.68	0.268	0.674	Co-occurrence
FAM8A1	6p22.3	6 (5.22%)	8 (3.27%)	0.68	0.268	0.674	Co-occurrence
KIF13A	6p22.3	6 (5.22%)	8 (3.27%)	0.68	0.268	0.674	Co-occurrence
NCEH1	3q26.31	6 (5.22%)	8 (3.27%)	0.68	0.268	0.674	Co-occurrence
NHLRC1	6p22.3	6 (5.22%)	8 (3.27%)	0.68	0.268	0.674	Co-occurrence
RN7SL332P	6p23	6 (5.22%)	8 (3.27%)	0.68	0.268	0.674	Co-occurrence
RNA5SP204	6p22.3	6 (5.22%)	8 (3.27%)	0.68	0.268	0.674	Co-occurrence
TPMT	6p22.3	6 (5.22%)	8 (3.27%)	0.68	0.268	0.674	Co-occurrence
DCSTAMP	8q22.3	16 (13.91%)	42 (17.14%)	-0.3	0.269	0.674	Mutual exclusivity
RIMS2	8q22.3	16 (13.91%)	42 (17.14%)	-0.3	0.269	0.674	Mutual exclusivity
CA1	8q21.2	13 (11.30%)	35 (14.29%)	-0.34	0.274	0.674	Mutual exclusivity
CA13	8q21.2	13 (11.30%)	35 (14.29%)	-0.34	0.274	0.674	Mutual exclusivity
CA2	8q21.2	13 (11.30%)	35 (14.29%)	-0.34	0.274	0.674	Mutual exclusivity
CA3	8q21.2	13 (11.30%)	35 (14.29%)	-0.34	0.274	0.674	Mutual exclusivity
E2F5	8q21.2	13 (11.30%)	35 (14.29%)	-0.34	0.274	0.674	Mutual exclusivity
LRRCC1	8q21.2	13 (11.30%)	35 (14.29%)	-0.34	0.274	0.674	Mutual exclusivity

RBIS	8q21.2	13 (11.30%)	35 (14.29%)	-0.34	0.274	0.674	Mutual exclusivity
RNA5SP272	8q21.3	13 (11.30%)	35 (14.29%)	-0.34	0.274	0.674	Mutual exclusivity
CASC15	6p22.3	7 (6.09%)	10 (4.08%)	0.58	0.278	0.674	Co-occurrence
ASAP1	8q24.21-q24.22	18 (15.65%)	46 (18.78%)	-0.26	0.285	0.674	Mutual exclusivity
ARMC7	17q25.1	8 (6.96%)	12 (4.90%)	0.51	0.286	0.674	Co-occurrence
ATP5PD	17q25.1	8 (6.96%)	12 (4.90%)	0.51	0.286	0.674	Co-occurrence
CDR2L	17q25.1	8 (6.96%)	12 (4.90%)	0.51	0.286	0.674	Co-occurrence
GGA3	17q25.1	8 (6.96%)	12 (4.90%)	0.51	0.286	0.674	Co-occurrence
GRB2	17q25.1	8 (6.96%)	12 (4.90%)	0.51	0.286	0.674	Co-occurrence
HID1	17q25.1	8 (6.96%)	12 (4.90%)	0.51	0.286	0.674	Co-occurrence
JPT1	17q25.1	8 (6.96%)	12 (4.90%)	0.51	0.286	0.674	Co-occurrence
KCTD2	17q25.1	8 (6.96%)	12 (4.90%)	0.51	0.286	0.674	Co-occurrence
MIF4GD	17q25.1	8 (6.96%)	12 (4.90%)	0.51	0.286	0.674	Co-occurrence
MRPL58	17q25.1	8 (6.96%)	12 (4.90%)	0.51	0.286	0.674	Co-occurrence
MRPS7	17q25.1	8 (6.96%)	12 (4.90%)	0.51	0.286	0.674	Co-occurrence
NQO2	6p25.2	8 (6.96%)	12 (4.90%)	0.51	0.286	0.674	Co-occurrence
NT5C	17q25.1	8 (6.96%)	12 (4.90%)	0.51	0.286	0.674	Co-occurrence
NUP85	17q25.1	8 (6.96%)	12 (4.90%)	0.51	0.286	0.674	Co-occurrence
RN7SL573P	17q25.1	8 (6.96%)	12 (4.90%)	0.51	0.286	0.674	Co-occurrence
SERPINB6	6p25.2	8 (6.96%)	12 (4.90%)	0.51	0.286	0.674	Co-occurrence
SERPINB9	6p25.2	8 (6.96%)	12 (4.90%)	0.51	0.286	0.674	Co-occurrence
SLC16A5	17q25.1	8 (6.96%)	12 (4.90%)	0.51	0.286	0.674	Co-occurrence
SLC22A23	6p25.2	8 (6.96%)	12 (4.90%)	0.51	0.286	0.674	Co-occurrence
SLC25A19	17q25.1	8 (6.96%)	12 (4.90%)	0.51	0.286	0.674	Co-occurrence
SUMO2	17q25.1	8 (6.96%)	12 (4.90%)	0.51	0.286	0.674	Co-occurrence
AASS	7q31.32	1 (0.87%)	6 (2.45%)	-1.49	0.287	0.674	Mutual exclusivity
ADAMTS2	5q35.3	1 (0.87%)	6 (2.45%)	-1.49	0.287	0.674	Mutual exclusivity
AHNAK	11q12.3	1 (0.87%)	6 (2.45%)	-1.49	0.287	0.674	Mutual exclusivity
B4GALT7	5q35.3	1 (0.87%)	6 (2.45%)	-1.49	0.287	0.674	Mutual exclusivity

BCAP29	7q22.3	1 (0.87%)	6 (2.45%)	-1.49	0.287	0.674	Mutual exclusivity
C11ORF80	11q13.2	1 (0.87%)	6 (2.45%)	-1.49	0.287	0.674	Mutual exclusivity
C5ORF38	5p15.33	1 (0.87%)	6 (2.45%)	-1.49	0.287	0.674	Mutual exclusivity
C7ORF66	7q31.1	1 (0.87%)	6 (2.45%)	-1.49	0.287	0.674	Mutual exclusivity
CD74	5q33.1	1 (0.87%)	6 (2.45%)	-1.49	0.287	0.674	Mutual exclusivity
CDH12	5p14.3	1 (0.87%)	6 (2.45%)	-1.49	0.287	0.674	Mutual exclusivity
COMP	19p13.11	1 (0.87%)	6 (2.45%)	-1.49	0.287	0.674	Mutual exclusivity
CRLF1	19p13.11	1 (0.87%)	6 (2.45%)	-1.49	0.287	0.674	Mutual exclusivity
CRTC1	19p13.11	1 (0.87%)	6 (2.45%)	-1.49	0.287	0.674	Mutual exclusivity
DNAJB9	7q31.1|14q24.2-q24.3	1 (0.87%)	6 (2.45%)	-1.49	0.287	0.674	Mutual exclusivity
DUS4L	7q22.3	1 (0.87%)	6 (2.45%)	-1.49	0.287	0.674	Mutual exclusivity
DYNC1I1	7q21.3	1 (0.87%)	6 (2.45%)	-1.49	0.287	0.674	Mutual exclusivity
FEZF1	7q31.32	1 (0.87%)	6 (2.45%)	-1.49	0.287	0.674	Mutual exclusivity
FKBP1C	6q12	1 (0.87%)	6 (2.45%)	-1.49	0.287	0.674	Mutual exclusivity
FOSL2	2p23.2	1 (0.87%)	6 (2.45%)	-1.49	0.287	0.674	Mutual exclusivity
GDPD4	11q13.5	1 (0.87%)	6 (2.45%)	-1.49	0.287	0.674	Mutual exclusivity
GPR22	7q22.3	1 (0.87%)	6 (2.45%)	-1.49	0.287	0.674	Mutual exclusivity
IFI30	19p13.11	1 (0.87%)	6 (2.45%)	-1.49	0.287	0.674	Mutual exclusivity
IRX2	5p15.33	1 (0.87%)	6 (2.45%)	-1.49	0.287	0.674	Mutual exclusivity
MKLN1	7q32.3	1 (0.87%)	6 (2.45%)	-1.49	0.287	0.674	Mutual exclusivity
MPV17L2	19p13.11	1 (0.87%)	6 (2.45%)	-1.49	0.287	0.674	Mutual exclusivity
MXD3	5q35.3	1 (0.87%)	6 (2.45%)	-1.49	0.287	0.674	Mutual exclusivity
NDST1	5q33.1	1 (0.87%)	6 (2.45%)	-1.49	0.287	0.674	Mutual exclusivity
PDE4C	19p13.11	1 (0.87%)	6 (2.45%)	-1.49	0.287	0.674	Mutual exclusivity
PFN3	5q35.3	1 (0.87%)	6 (2.45%)	-1.49	0.287	0.674	Mutual exclusivity
PNPLA8	7q31.1	1 (0.87%)	6 (2.45%)	-1.49	0.287	0.674	Mutual exclusivity
PRELID1	5q35.3	1 (0.87%)	6 (2.45%)	-1.49	0.287	0.674	Mutual exclusivity
PTPRZ1	7q31.32	1 (0.87%)	6 (2.45%)	-1.49	0.287	0.674	Mutual exclusivity
RAB24	5q35.3	1 (0.87%)	6 (2.45%)	-1.49	0.287	0.674	Mutual exclusivity

RAB3A	19p13.11	1 (0.87%)	6 (2.45%)	-1.49	0.287	0.674	Mutual exclusivity
RBM33	7q36.3	1 (0.87%)	6 (2.45%)	-1.49	0.287	0.674	Mutual exclusivity
RCE1	11q13.2	1 (0.87%)	6 (2.45%)	-1.49	0.287	0.674	Mutual exclusivity
REX1BD	19p13.11	1 (0.87%)	6 (2.45%)	-1.49	0.287	0.674	Mutual exclusivity
RN7SKP277	7q31.32	1 (0.87%)	6 (2.45%)	-1.49	0.287	0.674	Mutual exclusivity
RN7SL12P	11q13.2	1 (0.87%)	6 (2.45%)	-1.49	0.287	0.674	Mutual exclusivity
RNF133	7q31.32	1 (0.87%)	6 (2.45%)	-1.49	0.287	0.674	Mutual exclusivity
RNF148	7q31.32	1 (0.87%)	6 (2.45%)	-1.49	0.287	0.674	Mutual exclusivity
RPS14	5q33.1	1 (0.87%)	6 (2.45%)	-1.49	0.287	0.674	Mutual exclusivity
SLC34A1	5q35.3	1 (0.87%)	6 (2.45%)	-1.49	0.287	0.674	Mutual exclusivity
SLC35B4	7q33	1 (0.87%)	6 (2.45%)	-1.49	0.287	0.674	Mutual exclusivity
SPTBN2	11q13.2	1 (0.87%)	6 (2.45%)	-1.49	0.287	0.674	Mutual exclusivity
THAP5	7q31.1	1 (0.87%)	6 (2.45%)	-1.49	0.287	0.674	Mutual exclusivity
TMED9	5q35.3	1 (0.87%)	6 (2.45%)	-1.49	0.287	0.674	Mutual exclusivity
UBA52	19p13.11	1 (0.87%)	6 (2.45%)	-1.49	0.287	0.674	Mutual exclusivity
UBE2H	7q32.2	1 (0.87%)	6 (2.45%)	-1.49	0.287	0.674	Mutual exclusivity
UIMC1	5q35.2	1 (0.87%)	6 (2.45%)	-1.49	0.287	0.674	Mutual exclusivity
UPF1	19p13.11	1 (0.87%)	6 (2.45%)	-1.49	0.287	0.674	Mutual exclusivity
ZNF346	5q35.2	1 (0.87%)	6 (2.45%)	-1.49	0.287	0.674	Mutual exclusivity
CASC9	8q21.13	11 (9.57%)	30 (12.24%)	-0.36	0.289	0.674	Mutual exclusivity
EYA1	8q13.3	11 (9.57%)	30 (12.24%)	-0.36	0.289	0.674	Mutual exclusivity
HNF4G	8q21.13	11 (9.57%)	30 (12.24%)	-0.36	0.289	0.674	Mutual exclusivity
KCNB2	8q21.11	11 (9.57%)	30 (12.24%)	-0.36	0.289	0.674	Mutual exclusivity
RDH10	8q21.11	11 (9.57%)	30 (12.24%)	-0.36	0.289	0.674	Mutual exclusivity
RNA5SP271	8q21.11	11 (9.57%)	30 (12.24%)	-0.36	0.289	0.674	Mutual exclusivity
RPL7	8q21.11	11 (9.57%)	30 (12.24%)	-0.36	0.289	0.674	Mutual exclusivity
SBSPON	8q21.11	11 (9.57%)	30 (12.24%)	-0.36	0.289	0.674	Mutual exclusivity
TERF1	8q21.11	11 (9.57%)	30 (12.24%)	-0.36	0.289	0.674	Mutual exclusivity
TRPA1	8q21.11	11 (9.57%)	30 (12.24%)	-0.36	0.289	0.674	Mutual exclusivity

BHLHE22	8q12.3	8 (6.96%)	23 (9.39%)	-0.43	0.291	0.674	Mutual exclusivity
CYP7B1	8q12.3	8 (6.96%)	23 (9.39%)	-0.43	0.291	0.674	Mutual exclusivity
GGH	8q12.3	8 (6.96%)	23 (9.39%)	-0.43	0.291	0.674	Mutual exclusivity
MIR124-2HG	8q12.3	8 (6.96%)	23 (9.39%)	-0.43	0.291	0.674	Mutual exclusivity
RN7SL135P	8q12.3	8 (6.96%)	23 (9.39%)	-0.43	0.291	0.674	Mutual exclusivity
TTPA	8q12.3	8 (6.96%)	23 (9.39%)	-0.43	0.291	0.674	Mutual exclusivity
YTHDF3	8q12.3	8 (6.96%)	23 (9.39%)	-0.43	0.291	0.674	Mutual exclusivity
ACAP2	3q29	3 (2.61%)	3 (1.22%)	1.09	0.291	0.674	Co-occurrence
ADAM22	7q21.12	3 (2.61%)	3 (1.22%)	1.09	0.291	0.674	Co-occurrence
AKAP8	19p13.12	3 (2.61%)	3 (1.22%)	1.09	0.291	0.674	Co-occurrence
AKAP8L	19p13.12	3 (2.61%)	3 (1.22%)	1.09	0.291	0.674	Co-occurrence
AOX1	2q33.1	3 (2.61%)	3 (1.22%)	1.09	0.291	0.674	Co-occurrence
APOD	3q29	3 (2.61%)	3 (1.22%)	1.09	0.291	0.674	Co-occurrence
ASB16	17q21.31	3 (2.61%)	3 (1.22%)	1.09	0.291	0.674	Co-occurrence
ATP5F1E	20q13.32	3 (2.61%)	3 (1.22%)	1.09	0.291	0.674	Co-occurrence
AURKA	20q13.2	3 (2.61%)	3 (1.22%)	1.09	0.291	0.674	Co-occurrence
AZGP1	7q22.1	3 (2.61%)	3 (1.22%)	1.09	0.291	0.674	Co-occurrence
BCAS1	20q13.2	3 (2.61%)	3 (1.22%)	1.09	0.291	0.674	Co-occurrence
BCL7B	7q11.23	3 (2.61%)	3 (1.22%)	1.09	0.291	0.674	Co-occurrence
BLMH	17q11.2	3 (2.61%)	3 (1.22%)	1.09	0.291	0.674	Co-occurrence
BPI	20q11.23	3 (2.61%)	3 (1.22%)	1.09	0.291	0.674	Co-occurrence
BRD4	19p13.12	3 (2.61%)	3 (1.22%)	1.09	0.291	0.674	Co-occurrence
C17ORF53	17q21.31	3 (2.61%)	3 (1.22%)	1.09	0.291	0.674	Co-occurrence
C19ORF44	19p13.11	3 (2.61%)	3 (1.22%)	1.09	0.291	0.674	Co-occurrence
C5ORF56	5q31.1	3 (2.61%)	3 (1.22%)	1.09	0.291	0.674	Co-occurrence
CAMK2B	7p13	3 (2.61%)	3 (1.22%)	1.09	0.291	0.674	Co-occurrence
CASS4	20q13.31	3 (2.61%)	3 (1.22%)	1.09	0.291	0.674	Co-occurrence
CCDC141	2q31.2	3 (2.61%)	3 (1.22%)	1.09	0.291	0.674	Co-occurrence
CCDC144NL	17p11.2	3 (2.61%)	3 (1.22%)	1.09	0.291	0.674	Co-occurrence

CCNI2	5q31.1	3 (2.61%)	3 (1.22%)	1.09	0.291	0.674	Co-occurrence
CD300LG	17q21.31	3 (2.61%)	3 (1.22%)	1.09	0.291	0.674	Co-occurrence
CEBPB	20q13.13	3 (2.61%)	3 (1.22%)	1.09	0.291	0.674	Co-occurrence
CFAP97D1	17q21.31	3 (2.61%)	3 (1.22%)	1.09	0.291	0.674	Co-occurrence
COL1A1	17q21.33	3 (2.61%)	3 (1.22%)	1.09	0.291	0.674	Co-occurrence
CSTF1	20q13.2-q13.31	3 (2.61%)	3 (1.22%)	1.09	0.291	0.674	Co-occurrence
CTCFL	20q13.31	3 (2.61%)	3 (1.22%)	1.09	0.291	0.674	Co-occurrence
CTSZ	20q13.32	3 (2.61%)	3 (1.22%)	1.09	0.291	0.674	Co-occurrence
CYP24A1	20q13.2	3 (2.61%)	3 (1.22%)	1.09	0.291	0.674	Co-occurrence
CYP2A6	19q13.2	3 (2.61%)	3 (1.22%)	1.09	0.291	0.674	Co-occurrence
CYP2A7	19q13.2	3 (2.61%)	3 (1.22%)	1.09	0.291	0.674	Co-occurrence
CYP2B7P	19q13.2	3 (2.61%)	3 (1.22%)	1.09	0.291	0.674	Co-occurrence
CYP2G1P	19q13.2	3 (2.61%)	3 (1.22%)	1.09	0.291	0.674	Co-occurrence
CYSTM1	5q31.3	3 (2.61%)	3 (1.22%)	1.09	0.291	0.674	Co-occurrence
DHRS7B	17p11.2	3 (2.61%)	3 (1.22%)	1.09	0.291	0.674	Co-occurrence
DKK4	8p11.21	3 (2.61%)	3 (1.22%)	1.09	0.291	0.674	Co-occurrence
DNAI1	9p13.3	3 (2.61%)	3 (1.22%)	1.09	0.291	0.674	Co-occurrence
DNAJC6	1p31.3	3 (2.61%)	3 (1.22%)	1.09	0.291	0.674	Co-occurrence
DOK5	20q13.2	3 (2.61%)	3 (1.22%)	1.09	0.291	0.674	Co-occurrence
DUSP3	17q21.31	3 (2.61%)	3 (1.22%)	1.09	0.291	0.674	Co-occurrence
EPN2	17p11.2	3 (2.61%)	3 (1.22%)	1.09	0.291	0.674	Co-occurrence
ERAL1	17q11.2	3 (2.61%)	3 (1.22%)	1.09	0.291	0.674	Co-occurrence
EVX2	2q31.1	3 (2.61%)	3 (1.22%)	1.09	0.291	0.674	Co-occurrence
FAM210B	20q13.2	3 (2.61%)	3 (1.22%)	1.09	0.291	0.674	Co-occurrence
FAM222B	17q11.2	3 (2.61%)	3 (1.22%)	1.09	0.291	0.674	Co-occurrence
FAM43A	3q29	3 (2.61%)	3 (1.22%)	1.09	0.291	0.674	Co-occurrence
G6PC3	17q21.31	3 (2.61%)	3 (1.22%)	1.09	0.291	0.674	Co-occurrence
GALNTL5	7q36.1	3 (2.61%)	3 (1.22%)	1.09	0.291	0.674	Co-occurrence
GFER	16p13.3	3 (2.61%)	3 (1.22%)	1.09	0.291	0.674	Co-occurrence

GGA1	22q13.1	3 (2.61%)	3 (1.22%)	1.09	0.291	0.674	Co-occurrence
GJC3	7q22.1	3 (2.61%)	3 (1.22%)	1.09	0.291	0.674	Co-occurrence
HDAC5	17q21.31	3 (2.61%)	3 (1.22%)	1.09	0.291	0.674	Co-occurrence
HIGD1B	17q21.31	3 (2.61%)	3 (1.22%)	1.09	0.291	0.674	Co-occurrence
HILS1	17q21.33	3 (2.61%)	3 (1.22%)	1.09	0.291	0.674	Co-occurrence
HOXD1	2q31.1	3 (2.61%)	3 (1.22%)	1.09	0.291	0.674	Co-occurrence
HOXD10	2q31.1	3 (2.61%)	3 (1.22%)	1.09	0.291	0.674	Co-occurrence
HOXD11	2q31.1	3 (2.61%)	3 (1.22%)	1.09	0.291	0.674	Co-occurrence
HOXD12	2q31.1	3 (2.61%)	3 (1.22%)	1.09	0.291	0.674	Co-occurrence
HOXD13	2q31.1	3 (2.61%)	3 (1.22%)	1.09	0.291	0.674	Co-occurrence
HOXD3	2q31.1	3 (2.61%)	3 (1.22%)	1.09	0.291	0.674	Co-occurrence
HOXD4	2q31.1	3 (2.61%)	3 (1.22%)	1.09	0.291	0.674	Co-occurrence
HOXD8	2q31.1	3 (2.61%)	3 (1.22%)	1.09	0.291	0.674	Co-occurrence
HOXD9	2q31.1	3 (2.61%)	3 (1.22%)	1.09	0.291	0.674	Co-occurrence
IFI35	17q21.31	3 (2.61%)	3 (1.22%)	1.09	0.291	0.674	Co-occurrence
IL13	5q31.1	3 (2.61%)	3 (1.22%)	1.09	0.291	0.674	Co-occurrence
IL4	5q31.1	3 (2.61%)	3 (1.22%)	1.09	0.291	0.674	Co-occurrence
IL5	5q31.1	3 (2.61%)	3 (1.22%)	1.09	0.291	0.674	Co-occurrence
INHCAP	3q22.1	3 (2.61%)	3 (1.22%)	1.09	0.291	0.674	Co-occurrence
IRF1	5q31.1	3 (2.61%)	3 (1.22%)	1.09	0.291	0.674	Co-occurrence
ITGA3	17q21.33	3 (2.61%)	3 (1.22%)	1.09	0.291	0.674	Co-occurrence
JADE2	5q31.1	3 (2.61%)	3 (1.22%)	1.09	0.291	0.674	Co-occurrence
KCNB1	20q13.13	3 (2.61%)	3 (1.22%)	1.09	0.291	0.674	Co-occurrence
KIF3A	5q31.1	3 (2.61%)	3 (1.22%)	1.09	0.291	0.674	Co-occurrence
LEPR	1p31.3	3 (2.61%)	3 (1.22%)	1.09	0.291	0.674	Co-occurrence
LEPROT	1p31.3	3 (2.61%)	3 (1.22%)	1.09	0.291	0.674	Co-occurrence
LINC00924	15q26.2	3 (2.61%)	3 (1.22%)	1.09	0.291	0.674	Co-occurrence
LINC01620	20q13.12	3 (2.61%)	3 (1.22%)	1.09	0.291	0.674	Co-occurrence
LNPK	2q31.1	3 (2.61%)	3 (1.22%)	1.09	0.291	0.674	Co-occurrence

LSG1	3q29	3 (2.61%)	3 (1.22%)	1.09	0.291	0.674	Co-occurrence
LSM12	17q21.31	3 (2.61%)	3 (1.22%)	1.09	0.291	0.674	Co-occurrence
MAP2K3	17p11.2	3 (2.61%)	3 (1.22%)	1.09	0.291	0.674	Co-occurrence
MIR-1469/1469		3 (2.61%)	3 (1.22%)	1.09	0.291	0.674	Co-occurrence
MIR-1470/1470		3 (2.61%)	3 (1.22%)	1.09	0.291	0.674	Co-occurrence
MIR-3137/3137		3 (2.61%)	3 (1.22%)	1.09	0.291	0.674	Co-occurrence
MIR570HG	3q29	3 (2.61%)	3 (1.22%)	1.09	0.291	0.674	Co-occurrence
MLXIPL	7q11.23	3 (2.61%)	3 (1.22%)	1.09	0.291	0.674	Co-occurrence
MPP3	17q21.31	3 (2.61%)	3 (1.22%)	1.09	0.291	0.674	Co-occurrence
MTX2	2q31.1	3 (2.61%)	3 (1.22%)	1.09	0.291	0.674	Co-occurrence
MUC20	3q29	3 (2.61%)	3 (1.22%)	1.09	0.291	0.674	Co-occurrence
MUC4	3q29	3 (2.61%)	3 (1.22%)	1.09	0.291	0.674	Co-occurrence
MYCL	1p34.2	3 (2.61%)	3 (1.22%)	1.09	0.291	0.674	Co-occurrence
MYL6	12q13.2	3 (2.61%)	3 (1.22%)	1.09	0.291	0.674	Co-occurrence
MYL6B	12q13.2	3 (2.61%)	3 (1.22%)	1.09	0.291	0.674	Co-occurrence
MYO18A	17q11.2	3 (2.61%)	3 (1.22%)	1.09	0.291	0.674	Co-occurrence
NAGS	17q21.31	3 (2.61%)	3 (1.22%)	1.09	0.291	0.674	Co-occurrence
NATD1	17p11.2	3 (2.61%)	3 (1.22%)	1.09	0.291	0.674	Co-occurrence
NDUFB10	16p13.3	3 (2.61%)	3 (1.22%)	1.09	0.291	0.674	Co-occurrence
NMT1	17q21.31	3 (2.61%)	3 (1.22%)	1.09	0.291	0.674	Co-occurrence
NOXO1	16p13.3	3 (2.61%)	3 (1.22%)	1.09	0.291	0.674	Co-occurrence
NPW	16p13.3	3 (2.61%)	3 (1.22%)	1.09	0.291	0.674	Co-occurrence
NR2F2	15q26.2	3 (2.61%)	3 (1.22%)	1.09	0.291	0.674	Co-occurrence
NTHL1	16p13.3	3 (2.61%)	3 (1.22%)	1.09	0.291	0.674	Co-occurrence
PARD6B	20q13.13	3 (2.61%)	3 (1.22%)	1.09	0.291	0.674	Co-occurrence
PCK1	20q13.31	3 (2.61%)	3 (1.22%)	1.09	0.291	0.674	Co-occurrence
PDK2	17q21.33	3 (2.61%)	3 (1.22%)	1.09	0.291	0.674	Co-occurrence
PDXP	22q13.1	3 (2.61%)	3 (1.22%)	1.09	0.291	0.674	Co-occurrence
PFDN4	20q13.2	3 (2.61%)	3 (1.22%)	1.09	0.291	0.674	Co-occurrence

PFKP	10p15.2	3 (2.61%)	3 (1.22%)	1.09	0.291	0.674	Co-occurrence
PKD1	16p13.3	3 (2.61%)	3 (1.22%)	1.09	0.291	0.674	Co-occurrence
POLB	8p11.21	3 (2.61%)	3 (1.22%)	1.09	0.291	0.674	Co-occurrence
PPP1R2	3q29	3 (2.61%)	3 (1.22%)	1.09	0.291	0.674	Co-occurrence
PPP1R9B	17q21.33	3 (2.61%)	3 (1.22%)	1.09	0.291	0.674	Co-occurrence
PPY	17q21.31	3 (2.61%)	3 (1.22%)	1.09	0.291	0.674	Co-occurrence
PRELID3B	20q13.32	3 (2.61%)	3 (1.22%)	1.09	0.291	0.674	Co-occurrence
PRKAG2	7q36.1	3 (2.61%)	3 (1.22%)	1.09	0.291	0.674	Co-occurrence
PYY	17q21.31	3 (2.61%)	3 (1.22%)	1.09	0.291	0.674	Co-occurrence
RASAL3	19p13.12	3 (2.61%)	3 (1.22%)	1.09	0.291	0.674	Co-occurrence
RASL12	15q22.31	3 (2.61%)	3 (1.22%)	1.09	0.291	0.674	Co-occurrence
RBM38	20q13.31	3 (2.61%)	3 (1.22%)	1.09	0.291	0.674	Co-occurrence
RN7SKP181	15q26.2	3 (2.61%)	3 (1.22%)	1.09	0.291	0.674	Co-occurrence
RN7SKP184	20q13.2	3 (2.61%)	3 (1.22%)	1.09	0.291	0.674	Co-occurrence
RN7SKP254	15q26.2	3 (2.61%)	3 (1.22%)	1.09	0.291	0.674	Co-occurrence
RN7SL219P	16p13.3	3 (2.61%)	3 (1.22%)	1.09	0.291	0.674	Co-occurrence
RN7SL36P	3q29	3 (2.61%)	3 (1.22%)	1.09	0.291	0.674	Co-occurrence
RN7SL385P	22q13.1	3 (2.61%)	3 (1.22%)	1.09	0.291	0.674	Co-occurrence
RN7SL405P	17q21.31	3 (2.61%)	3 (1.22%)	1.09	0.291	0.674	Co-occurrence
RN7SL426P	17p11.2	3 (2.61%)	3 (1.22%)	1.09	0.291	0.674	Co-occurrence
RN7SL677P	15q26.2	3 (2.61%)	3 (1.22%)	1.09	0.291	0.674	Co-occurrence
RND2	17q21.31	3 (2.61%)	3 (1.22%)	1.09	0.291	0.674	Co-occurrence
RNF151	16p13.3	3 (2.61%)	3 (1.22%)	1.09	0.291	0.674	Co-occurrence
RNU4ATAC7P	20q13.2	3 (2.61%)	3 (1.22%)	1.09	0.291	0.674	Co-occurrence
RNU6ATAC14P	2q31.1	3 (2.61%)	3 (1.22%)	1.09	0.291	0.674	Co-occurrence
RNU6ATAC24P	3q29	3 (2.61%)	3 (1.22%)	1.09	0.291	0.674	Co-occurrence
RPS2	16p13.3	3 (2.61%)	3 (1.22%)	1.09	0.291	0.674	Co-occurrence
RUNDC3B	7q21.12	3 (2.61%)	3 (1.22%)	1.09	0.291	0.674	Co-occurrence
RXRA	9q34.2	3 (2.61%)	3 (1.22%)	1.09	0.291	0.674	Co-occurrence

SAMD14	17q21.33	3 (2.61%)	3 (1.22%)	1.09	0.291	0.674	Co-occurrence
SDHAP1	3q29	3 (2.61%)	3 (1.22%)	1.09	0.291	0.674	Co-occurrence
SGCA	17q21.33	3 (2.61%)	3 (1.22%)	1.09	0.291	0.674	Co-occurrence
SH3BP1	22q13.1	3 (2.61%)	3 (1.22%)	1.09	0.291	0.674	Co-occurrence
SHROOM3	4q21.1	3 (2.61%)	3 (1.22%)	1.09	0.291	0.674	Co-occurrence
SLC22A5	5q31.1	3 (2.61%)	3 (1.22%)	1.09	0.291	0.674	Co-occurrence
SLC9A3R2	16p13.3	3 (2.61%)	3 (1.22%)	1.09	0.291	0.674	Co-occurrence
SMARCC2	12q13.2	3 (2.61%)	3 (1.22%)	1.09	0.291	0.674	Co-occurrence
SNHG9	16p13.3	3 (2.61%)	3 (1.22%)	1.09	0.291	0.674	Co-occurrence
SNORA10	16p13.3	3 (2.61%)	3 (1.22%)	1.09	0.291	0.674	Co-occurrence
SOST	17q21.31	3 (2.61%)	3 (1.22%)	1.09	0.291	0.674	Co-occurrence
SPATA8	15q26.2	3 (2.61%)	3 (1.22%)	1.09	0.291	0.674	Co-occurrence
SRI	7q21.12	3 (2.61%)	3 (1.22%)	1.09	0.291	0.674	Co-occurrence
SRPRB	3q22.1	3 (2.61%)	3 (1.22%)	1.09	0.291	0.674	Co-occurrence
STEAP4	7q21.12	3 (2.61%)	3 (1.22%)	1.09	0.291	0.674	Co-occurrence
SYNGR3	16p13.3	3 (2.61%)	3 (1.22%)	1.09	0.291	0.674	Co-occurrence
TBL2	7q11.23	3 (2.61%)	3 (1.22%)	1.09	0.291	0.674	Co-occurrence
TBL3	16p13.3	3 (2.61%)	3 (1.22%)	1.09	0.291	0.674	Co-occurrence
TF	3q22.1	3 (2.61%)	3 (1.22%)	1.09	0.291	0.674	Co-occurrence
TIAF1	17q11.2	3 (2.61%)	3 (1.22%)	1.09	0.291	0.674	Co-occurrence
TMEM101	17q21.31	3 (2.61%)	3 (1.22%)	1.09	0.291	0.674	Co-occurrence
TMEM11	17p11.2	3 (2.61%)	3 (1.22%)	1.09	0.291	0.674	Co-occurrence
TMEM92	17q21.33	3 (2.61%)	3 (1.22%)	1.09	0.291	0.674	Co-occurrence
TNK2	3q29	3 (2.61%)	3 (1.22%)	1.09	0.291	0.674	Co-occurrence
TRIM4	7q22.1	3 (2.61%)	3 (1.22%)	1.09	0.291	0.674	Co-occurrence
TRIT1	1p34.2	3 (2.61%)	3 (1.22%)	1.09	0.291	0.674	Co-occurrence
TSC2	16p13.3	3 (2.61%)	3 (1.22%)	1.09	0.291	0.674	Co-occurrence
TTC30A	2q31.2	3 (2.61%)	3 (1.22%)	1.09	0.291	0.674	Co-occurrence
TTC30B	2q31.2	3 (2.61%)	3 (1.22%)	1.09	0.291	0.674	Co-occurrence

TUBB1	20q13.32	3 (2.61%)	3 (1.22%)	1.09	0.291	0.674	Co-occurrence
UBE2K	4p14	3 (2.61%)	3 (1.22%)	1.09	0.291	0.674	Co-occurrence
UBE2V1	20q13.13	3 (2.61%)	3 (1.22%)	1.09	0.291	0.674	Co-occurrence
ULK1	12q24.33	3 (2.61%)	3 (1.22%)	1.09	0.291	0.674	Co-occurrence
URI1	19q12	3 (2.61%)	3 (1.22%)	1.09	0.291	0.674	Co-occurrence
VAT1	17q21.31	3 (2.61%)	3 (1.22%)	1.09	0.291	0.674	Co-occurrence
VDAC3	8p11.21	3 (2.61%)	3 (1.22%)	1.09	0.291	0.674	Co-occurrence
VPS37D	7q11.23	3 (2.61%)	3 (1.22%)	1.09	0.291	0.674	Co-occurrence
WDR70	5p13.2	3 (2.61%)	3 (1.22%)	1.09	0.291	0.674	Co-occurrence
WDR88	19q13.11	3 (2.61%)	3 (1.22%)	1.09	0.291	0.674	Co-occurrence
WIZ	19p13.12	3 (2.61%)	3 (1.22%)	1.09	0.291	0.674	Co-occurrence
XYLT2	17q21.33	3 (2.61%)	3 (1.22%)	1.09	0.291	0.674	Co-occurrence
ZNF3	7q22.1	3 (2.61%)	3 (1.22%)	1.09	0.291	0.674	Co-occurrence
ZNF598	16p13.3	3 (2.61%)	3 (1.22%)	1.09	0.291	0.674	Co-occurrence
ZSCAN21	7q22.1	3 (2.61%)	3 (1.22%)	1.09	0.291	0.674	Co-occurrence
CYBC1	17q25.3	9 (7.83%)	14 (5.71%)	0.45	0.291	0.674	Co-occurrence
FOXK2	17q25.3	9 (7.83%)	14 (5.71%)	0.45	0.291	0.674	Co-occurrence
HEXD	17q25.3	9 (7.83%)	14 (5.71%)	0.45	0.291	0.674	Co-occurrence
NARF	17q25.3	9 (7.83%)	14 (5.71%)	0.45	0.291	0.674	Co-occurrence
OGFOD3	17q25.3	9 (7.83%)	14 (5.71%)	0.45	0.291	0.674	Co-occurrence
UTS2R	17q25.3	9 (7.83%)	14 (5.71%)	0.45	0.291	0.674	Co-occurrence
C8ORF37	8q22.1	15 (13.04%)	39 (15.92%)	-0.29	0.293	0.674	Mutual exclusivity
CCNE2	8q22.1	15 (13.04%)	39 (15.92%)	-0.29	0.293	0.674	Mutual exclusivity
INTS8	8q22.1	15 (13.04%)	39 (15.92%)	-0.29	0.293	0.674	Mutual exclusivity
OSR2	8q22.2	15 (13.04%)	39 (15.92%)	-0.29	0.293	0.674	Mutual exclusivity
PLEKHF2	8q22.1	15 (13.04%)	39 (15.92%)	-0.29	0.293	0.674	Mutual exclusivity
RN7SKP85	8q22.2	15 (13.04%)	39 (15.92%)	-0.29	0.293	0.674	Mutual exclusivity
STK3	8q22.2	15 (13.04%)	39 (15.92%)	-0.29	0.293	0.674	Mutual exclusivity
ADGRF1	6p12.3|6	3 (2.61%)	11 (4.49%)	-0.78	0.294	0.674	Mutual exclusivity

BYSL	6p21.1	3 (2.61%)	11 (4.49%)	-0.78	0.294	0.674	Mutual exclusivity
C6ORF132	6p21.1	3 (2.61%)	11 (4.49%)	-0.78	0.294	0.674	Mutual exclusivity
CCND3	6p21.1	3 (2.61%)	11 (4.49%)	-0.78	0.294	0.674	Mutual exclusivity
CD2AP	6p12.3	3 (2.61%)	11 (4.49%)	-0.78	0.294	0.674	Mutual exclusivity
GUCA1A	6p21.1	3 (2.61%)	11 (4.49%)	-0.78	0.294	0.674	Mutual exclusivity
UBE2W	8q21.11	12 (10.43%)	32 (13.06%)	-0.32	0.3	0.674	Mutual exclusivity
VPS13B	8q22.2	16 (13.91%)	41 (16.73%)	-0.27	0.302	0.674	Mutual exclusivity
ALKAL1	8q11.23	6 (5.22%)	18 (7.35%)	-0.49	0.305	0.674	Mutual exclusivity
LYPLA1	8q11.23	6 (5.22%)	18 (7.35%)	-0.49	0.305	0.674	Mutual exclusivity
MRPL15	8q11.23	6 (5.22%)	18 (7.35%)	-0.49	0.305	0.674	Mutual exclusivity
NPBWR1	8q11.23	6 (5.22%)	18 (7.35%)	-0.49	0.305	0.674	Mutual exclusivity
OPRK1	8q11.23	6 (5.22%)	18 (7.35%)	-0.49	0.305	0.674	Mutual exclusivity
RB1CC1	8q11.23	6 (5.22%)	18 (7.35%)	-0.49	0.305	0.674	Mutual exclusivity
RGS20	8q11.23	6 (5.22%)	18 (7.35%)	-0.49	0.305	0.674	Mutual exclusivity
RNU6ATAC32P	8q11.23	6 (5.22%)	18 (7.35%)	-0.49	0.305	0.674	Mutual exclusivity
TCEA1	8q11.23	6 (5.22%)	18 (7.35%)	-0.49	0.305	0.674	Mutual exclusivity
FABP5	8q21.13	13 (11.30%)	34 (13.88%)	-0.3	0.31	0.674	Mutual exclusivity
MIR-5708/5708		13 (11.30%)	34 (13.88%)	-0.3	0.31	0.674	Mutual exclusivity
MRPS28	8q21.13	13 (11.30%)	34 (13.88%)	-0.3	0.31	0.674	Mutual exclusivity
PMP2	8q21.13	13 (11.30%)	34 (13.88%)	-0.3	0.31	0.674	Mutual exclusivity
RN7SL308P	8q21.13	13 (11.30%)	34 (13.88%)	-0.3	0.31	0.674	Mutual exclusivity
RN7SL41P	8q21.13	13 (11.30%)	34 (13.88%)	-0.3	0.31	0.674	Mutual exclusivity
TPD52	8q21.13	13 (11.30%)	34 (13.88%)	-0.3	0.31	0.674	Mutual exclusivity
ZBTB10	8q21.13	13 (11.30%)	34 (13.88%)	-0.3	0.31	0.674	Mutual exclusivity
AGO2	8q24.3	17 (14.78%)	43 (17.55%)	-0.25	0.31	0.674	Mutual exclusivity
CASC7	8q24.3	17 (14.78%)	43 (17.55%)	-0.25	0.31	0.674	Mutual exclusivity
CHRAC1	8q24.3	17 (14.78%)	43 (17.55%)	-0.25	0.31	0.674	Mutual exclusivity
DENND3	8q24.3	17 (14.78%)	43 (17.55%)	-0.25	0.31	0.674	Mutual exclusivity
EEF1D	8q24.3	17 (14.78%)	43 (17.55%)	-0.25	0.31	0.674	Mutual exclusivity

GSDMD	8q24.3	17 (14.78%)	43 (17.55%)	-0.25	0.31	0.674	Mutual exclusivity
MROH6	8q24.3	17 (14.78%)	43 (17.55%)	-0.25	0.31	0.674	Mutual exclusivity
NAPRT	8q24.3	17 (14.78%)	43 (17.55%)	-0.25	0.31	0.674	Mutual exclusivity
PARP10	8q24.3	17 (14.78%)	43 (17.55%)	-0.25	0.31	0.674	Mutual exclusivity
PTK2	8q24.3	17 (14.78%)	43 (17.55%)	-0.25	0.31	0.674	Mutual exclusivity
PYCR3	8q24.3	17 (14.78%)	43 (17.55%)	-0.25	0.31	0.674	Mutual exclusivity
RNA5SP278	8q24.3	17 (14.78%)	43 (17.55%)	-0.25	0.31	0.674	Mutual exclusivity
SLC45A4	8q24.3	17 (14.78%)	43 (17.55%)	-0.25	0.31	0.674	Mutual exclusivity
TIGD5	8q24.3	17 (14.78%)	43 (17.55%)	-0.25	0.31	0.674	Mutual exclusivity
TSTA3	8q24.3	17 (14.78%)	43 (17.55%)	-0.25	0.31	0.674	Mutual exclusivity
ZC3H3	8q24.3	17 (14.78%)	43 (17.55%)	-0.25	0.31	0.674	Mutual exclusivity
ZNF623	8q24.3	17 (14.78%)	43 (17.55%)	-0.25	0.31	0.674	Mutual exclusivity
A1BG	19q13.43	0 (0.00%)	3 (1.22%)	<-10	0.314	0.674	Mutual exclusivity
ACER3	11q13.5	0 (0.00%)	3 (1.22%)	<-10	0.314	0.674	Mutual exclusivity
ACOX2	3p14.3	0 (0.00%)	3 (1.22%)	<-10	0.314	0.674	Mutual exclusivity
ACP4	19q13.33	0 (0.00%)	3 (1.22%)	<-10	0.314	0.674	Mutual exclusivity
ACSL4	Xq23	0 (0.00%)	3 (1.22%)	<-10	0.314	0.674	Mutual exclusivity
ADAM19	5q33.3	0 (0.00%)	3 (1.22%)	<-10	0.314	0.674	Mutual exclusivity
ADCK2	7q34	0 (0.00%)	3 (1.22%)	<-10	0.314	0.674	Mutual exclusivity
ADGRA3	4p15.2	0 (0.00%)	3 (1.22%)	<-10	0.314	0.674	Mutual exclusivity
ADGRB3	6q12-q13	0 (0.00%)	3 (1.22%)	<-10	0.314	0.674	Mutual exclusivity
ADGRG1	16q21	0 (0.00%)	3 (1.22%)	<-10	0.314	0.674	Mutual exclusivity
ADM5	19q13.33	0 (0.00%)	3 (1.22%)	<-10	0.314	0.674	Mutual exclusivity
ADRA1B	5q33.3	0 (0.00%)	3 (1.22%)	<-10	0.314	0.674	Mutual exclusivity
AFF2	Xq28	0 (0.00%)	3 (1.22%)	<-10	0.314	0.674	Mutual exclusivity
AGK	7q34	0 (0.00%)	3 (1.22%)	<-10	0.314	0.674	Mutual exclusivity
AGR2	7p21.1	0 (0.00%)	3 (1.22%)	<-10	0.314	0.674	Mutual exclusivity
AGR3	7p21.1	0 (0.00%)	3 (1.22%)	<-10	0.314	0.674	Mutual exclusivity
AKT1S1	19q13.33	0 (0.00%)	3 (1.22%)	<-10	0.314	0.674	Mutual exclusivity

ALDH7A1	5q23.2	0 (0.00%)	3 (1.22%)	<-10	0.314	0.674	Mutual exclusivity
ALKBH4	7q22.1	0 (0.00%)	3 (1.22%)	<-10	0.314	0.674	Mutual exclusivity
ANKRD55	5q11.2	0 (0.00%)	3 (1.22%)	<-10	0.314	0.674	Mutual exclusivity
ANXA2R	5p12	0 (0.00%)	3 (1.22%)	<-10	0.314	0.674	Mutual exclusivity
ANXA6	5q33.1	0 (0.00%)	3 (1.22%)	<-10	0.314	0.674	Mutual exclusivity
AP1S1	7q22.1	0 (0.00%)	3 (1.22%)	<-10	0.314	0.674	Mutual exclusivity
APBB2	4p14-p13	0 (0.00%)	3 (1.22%)	<-10	0.314	0.674	Mutual exclusivity
APOBR	16p12.1	0 (0.00%)	3 (1.22%)	<-10	0.314	0.674	Mutual exclusivity
AQP8	16p12.1	0 (0.00%)	3 (1.22%)	<-10	0.314	0.674	Mutual exclusivity
ARAP1	11q13.4	0 (0.00%)	3 (1.22%)	<-10	0.314	0.674	Mutual exclusivity
ARHGAP17	16p12.1	0 (0.00%)	3 (1.22%)	<-10	0.314	0.674	Mutual exclusivity
ARHGEF10	8p23.3	0 (0.00%)	3 (1.22%)	<-10	0.314	0.674	Mutual exclusivity
ARHGEF33	2p22.1	0 (0.00%)	3 (1.22%)	<-10	0.314	0.674	Mutual exclusivity
ARHGEF35	7q35	0 (0.00%)	3 (1.22%)	<-10	0.314	0.674	Mutual exclusivity
ARHGEF5	7q35	0 (0.00%)	3 (1.22%)	<-10	0.314	0.674	Mutual exclusivity
ARMC10	7q22.1	0 (0.00%)	3 (1.22%)	<-10	0.314	0.674	Mutual exclusivity
ASPDH	19q13.33	0 (0.00%)	3 (1.22%)	<-10	0.314	0.674	Mutual exclusivity
ATF5	19q13.33	0 (0.00%)	3 (1.22%)	<-10	0.314	0.674	Mutual exclusivity
ATOH8	2p11.2	0 (0.00%)	3 (1.22%)	<-10	0.314	0.674	Mutual exclusivity
ATP10D	4p12	0 (0.00%)	3 (1.22%)	<-10	0.314	0.674	Mutual exclusivity
ATP2A1	16p11.2	0 (0.00%)	3 (1.22%)	<-10	0.314	0.674	Mutual exclusivity
ATP8B1	18q21.31	0 (0.00%)	3 (1.22%)	<-10	0.314	0.674	Mutual exclusivity
ATXN2L	16p11.2	0 (0.00%)	3 (1.22%)	<-10	0.314	0.674	Mutual exclusivity
AURKC	19q13.43	0 (0.00%)	3 (1.22%)	<-10	0.314	0.674	Mutual exclusivity
B3GNT6	11q13.5	0 (0.00%)	3 (1.22%)	<-10	0.314	0.674	Mutual exclusivity
BCL2L12	19q13.33	0 (0.00%)	3 (1.22%)	<-10	0.314	0.674	Mutual exclusivity
BEND4	4p13	0 (0.00%)	3 (1.22%)	<-10	0.314	0.674	Mutual exclusivity
BIRC8	19q13.42	0 (0.00%)	3 (1.22%)	<-10	0.314	0.674	Mutual exclusivity
BMP4	14q22.2	0 (0.00%)	3 (1.22%)	<-10	0.314	0.674	Mutual exclusivity

BRAF	7q34	0 (0.00%)	3 (1.22%)	<-10	0.314	0.674	Mutual exclusivity
BRSK1	19q13.42	0 (0.00%)	3 (1.22%)	<-10	0.314	0.674	Mutual exclusivity
BTNL3	5q35.3	0 (0.00%)	3 (1.22%)	<-10	0.314	0.674	Mutual exclusivity
BTNL9	5q35.3	0 (0.00%)	3 (1.22%)	<-10	0.314	0.674	Mutual exclusivity
C16ORF82	16p12.1	0 (0.00%)	3 (1.22%)	<-10	0.314	0.674	Mutual exclusivity
C18ORF63	18q22.3	0 (0.00%)	3 (1.22%)	<-10	0.314	0.674	Mutual exclusivity
C19ORF18	19q13.43	0 (0.00%)	3 (1.22%)	<-10	0.314	0.674	Mutual exclusivity
C19ORF48	19q13.33	0 (0.00%)	3 (1.22%)	<-10	0.314	0.674	Mutual exclusivity
C19ORF81	19q13.33	0 (0.00%)	3 (1.22%)	<-10	0.314	0.674	Mutual exclusivity
C2CD6	2q33.1	0 (0.00%)	3 (1.22%)	<-10	0.314	0.674	Mutual exclusivity
C2ORF68	2p11.2	0 (0.00%)	3 (1.22%)	<-10	0.314	0.674	Mutual exclusivity
C5ORF34	5p12	0 (0.00%)	3 (1.22%)	<-10	0.314	0.674	Mutual exclusivity
C5ORF63	5q23.2	0 (0.00%)	3 (1.22%)	<-10	0.314	0.674	Mutual exclusivity
CACNG3	16p12.1	0 (0.00%)	3 (1.22%)	<-10	0.314	0.674	Mutual exclusivity
CACNG6	19q13.42	0 (0.00%)	3 (1.22%)	<-10	0.314	0.674	Mutual exclusivity
CACNG7	19q13.42	0 (0.00%)	3 (1.22%)	<-10	0.314	0.674	Mutual exclusivity
CACNG8	19q13.42	0 (0.00%)	3 (1.22%)	<-10	0.314	0.674	Mutual exclusivity
CAPN5	11q13.5	0 (0.00%)	3 (1.22%)	<-10	0.314	0.674	Mutual exclusivity
CASP2	7q34	0 (0.00%)	3 (1.22%)	<-10	0.314	0.674	Mutual exclusivity
CATSPER3	5q31.1	0 (0.00%)	3 (1.22%)	<-10	0.314	0.674	Mutual exclusivity
CCDC106	19q13.42	0 (0.00%)	3 (1.22%)	<-10	0.314	0.674	Mutual exclusivity
CCDC69	5q33.1	0 (0.00%)	3 (1.22%)	<-10	0.314	0.674	Mutual exclusivity
CCL28	5p12	0 (0.00%)	3 (1.22%)	<-10	0.314	0.674	Mutual exclusivity
CD19	16p11.2	0 (0.00%)	3 (1.22%)	<-10	0.314	0.674	Mutual exclusivity
CD38	4p15.32	0 (0.00%)	3 (1.22%)	<-10	0.314	0.674	Mutual exclusivity
CD5	11q12.2	0 (0.00%)	3 (1.22%)	<-10	0.314	0.674	Mutual exclusivity
CD6	11q12.2	0 (0.00%)	3 (1.22%)	<-10	0.314	0.674	Mutual exclusivity
CD9	12p13.31	0 (0.00%)	3 (1.22%)	<-10	0.314	0.674	Mutual exclusivity
CDC42EP3	2p22.2	0 (0.00%)	3 (1.22%)	<-10	0.314	0.674	Mutual exclusivity

CDC42EP5	19q13.42	0 (0.00%)	3 (1.22%)	<-10	0.314	0.674	Mutual exclusivity
CDH18	5p14.3	0 (0.00%)	3 (1.22%)	<-10	0.314	0.674	Mutual exclusivity
CDKL3	5q31.1	0 (0.00%)	3 (1.22%)	<-10	0.314	0.674	Mutual exclusivity
CDKL4	2p22.1	0 (0.00%)	3 (1.22%)	<-10	0.314	0.674	Mutual exclusivity
CDKN3	14q22.2	0 (0.00%)	3 (1.22%)	<-10	0.314	0.674	Mutual exclusivity
CDR2	16p12.2	0 (0.00%)	3 (1.22%)	<-10	0.314	0.674	Mutual exclusivity
CEBPZ	2p22.2	0 (0.00%)	3 (1.22%)	<-10	0.314	0.674	Mutual exclusivity
CGRRF1	14q22.2	0 (0.00%)	3 (1.22%)	<-10	0.314	0.674	Mutual exclusivity
CHMP2A	19q13.43	0 (0.00%)	3 (1.22%)	<-10	0.314	0.674	Mutual exclusivity
CHRDL2	11q13.4	0 (0.00%)	3 (1.22%)	<-10	0.314	0.674	Mutual exclusivity
CLCN1	7q34	0 (0.00%)	3 (1.22%)	<-10	0.314	0.674	Mutual exclusivity
CLDN15	7q22.1	0 (0.00%)	3 (1.22%)	<-10	0.314	0.674	Mutual exclusivity
CLEC11A	19q13.33	0 (0.00%)	3 (1.22%)	<-10	0.314	0.674	Mutual exclusivity
CLEC5A	7q34	0 (0.00%)	3 (1.22%)	<-10	0.314	0.674	Mutual exclusivity
CLN3	16p12.1	0 (0.00%)	3 (1.22%)	<-10	0.314	0.674	Mutual exclusivity
CLN8	8p23.3	0 (0.00%)	3 (1.22%)	<-10	0.314	0.674	Mutual exclusivity
CLRN2	4p15.32	0 (0.00%)	3 (1.22%)	<-10	0.314	0.674	Mutual exclusivity
CLSTN3	12p13.31	0 (0.00%)	3 (1.22%)	<-10	0.314	0.674	Mutual exclusivity
CNGA1	4p12	0 (0.00%)	3 (1.22%)	<-10	0.314	0.674	Mutual exclusivity
CNIH1	14q22.2	0 (0.00%)	3 (1.22%)	<-10	0.314	0.674	Mutual exclusivity
COMMD8	4p12	0 (0.00%)	3 (1.22%)	<-10	0.314	0.674	Mutual exclusivity
CORIN	4p12	0 (0.00%)	3 (1.22%)	<-10	0.314	0.674	Mutual exclusivity
COX6B2	19q13.42	0 (0.00%)	3 (1.22%)	<-10	0.314	0.674	Mutual exclusivity
CPSF7	11q12.2	0 (0.00%)	3 (1.22%)	<-10	0.314	0.674	Mutual exclusivity
CPT1C	19q13.33	0 (0.00%)	3 (1.22%)	<-10	0.314	0.674	Mutual exclusivity
CRIM1	2p22.2	0 (0.00%)	3 (1.22%)	<-10	0.314	0.674	Mutual exclusivity
CTAGE4	7q35	0 (0.00%)	3 (1.22%)	<-10	0.314	0.674	Mutual exclusivity
CTAGE8	7q35	0 (0.00%)	3 (1.22%)	<-10	0.314	0.674	Mutual exclusivity
CUL4B	Xq24	0 (0.00%)	3 (1.22%)	<-10	0.314	0.674	Mutual exclusivity

CUX1	7q22.1	0 (0.00%)	3 (1.22%)	<-10	0.314	0.674	Mutual exclusivity
CWH43	4p11	0 (0.00%)	3 (1.22%)	<-10	0.314	0.674	Mutual exclusivity
CXORF40B	Xq28	0 (0.00%)	3 (1.22%)	<-10	0.314	0.674	Mutual exclusivity
CXORF51A	Xq27.3	0 (0.00%)	3 (1.22%)	<-10	0.314	0.674	Mutual exclusivity
CXORF51B	Xq27.3	0 (0.00%)	3 (1.22%)	<-10	0.314	0.674	Mutual exclusivity
CYFIP2	5q33.3	0 (0.00%)	3 (1.22%)	<-10	0.314	0.674	Mutual exclusivity
CYP1B1	2p22.2	0 (0.00%)	3 (1.22%)	<-10	0.314	0.674	Mutual exclusivity
DCAF16	4p15.31	0 (0.00%)	3 (1.22%)	<-10	0.314	0.674	Mutual exclusivity
DCAF4L1	4p13	0 (0.00%)	3 (1.22%)	<-10	0.314	0.674	Mutual exclusivity
DDB1	11q12.2	0 (0.00%)	3 (1.22%)	<-10	0.314	0.674	Mutual exclusivity
DELE1	5q31.3	0 (0.00%)	3 (1.22%)	<-10	0.314	0.674	Mutual exclusivity
DENND2A	7q34	0 (0.00%)	3 (1.22%)	<-10	0.314	0.674	Mutual exclusivity
DHX15	4p15.2	0 (0.00%)	3 (1.22%)	<-10	0.314	0.674	Mutual exclusivity
DHX57	2p22.1	0 (0.00%)	3 (1.22%)	<-10	0.314	0.674	Mutual exclusivity
DKFZP434E111	11q14.1	0 (0.00%)	3 (1.22%)	<-10	0.314	0.674	Mutual exclusivity
DLGAP2	8p23.3	0 (0.00%)	3 (1.22%)	<-10	0.314	0.674	Mutual exclusivity
DNAAF3	19q13.42	0 (0.00%)	3 (1.22%)	<-10	0.314	0.674	Mutual exclusivity
DOCK11	Xq24	0 (0.00%)	3 (1.22%)	<-10	0.314	0.674	Mutual exclusivity
DPRX	19q13.42	0 (0.00%)	3 (1.22%)	<-10	0.314	0.674	Mutual exclusivity
DPY19L2P2	7q22.1	0 (0.00%)	3 (1.22%)	<-10	0.314	0.674	Mutual exclusivity
DUXA	19q13.43	0 (0.00%)	3 (1.22%)	<-10	0.314	0.674	Mutual exclusivity
EBF1	5q33.3	0 (0.00%)	3 (1.22%)	<-10	0.314	0.674	Mutual exclusivity
EBI3	19p13.3	0 (0.00%)	3 (1.22%)	<-10	0.314	0.674	Mutual exclusivity
EEF1G	11q12.3	0 (0.00%)	3 (1.22%)	<-10	0.314	0.674	Mutual exclusivity
EEF2K	16p12.2	0 (0.00%)	3 (1.22%)	<-10	0.314	0.674	Mutual exclusivity
EIF2AK2	2p22.2	0 (0.00%)	3 (1.22%)	<-10	0.314	0.674	Mutual exclusivity
EIF3C	16p11.2	0 (0.00%)	3 (1.22%)	<-10	0.314	0.674	Mutual exclusivity
EIF3CL	16p12.1	0 (0.00%)	3 (1.22%)	<-10	0.314	0.674	Mutual exclusivity
EMC10	19q13.33	0 (0.00%)	3 (1.22%)	<-10	0.314	0.674	Mutual exclusivity

EMSY	11q13.5	0 (0.00%)	3 (1.22%)	<-10	0.314	0.674	Mutual exclusivity
EPHA1	7q34-q35	0 (0.00%)	3 (1.22%)	<-10	0.314	0.674	Mutual exclusivity
EPHB6	7q34	0 (0.00%)	3 (1.22%)	<-10	0.314	0.674	Mutual exclusivity
EPN1	19q13.42	0 (0.00%)	3 (1.22%)	<-10	0.314	0.674	Mutual exclusivity
EPO	7q22.1	0 (0.00%)	3 (1.22%)	<-10	0.314	0.674	Mutual exclusivity
EPS8L1	19q13.42	0 (0.00%)	3 (1.22%)	<-10	0.314	0.674	Mutual exclusivity
FAM114A2	5q33.2	0 (0.00%)	3 (1.22%)	<-10	0.314	0.674	Mutual exclusivity
FAM117B	2q33.2	0 (0.00%)	3 (1.22%)	<-10	0.314	0.674	Mutual exclusivity
FAM126A	7p15.3	0 (0.00%)	3 (1.22%)	<-10	0.314	0.674	Mutual exclusivity
FAM131B	7q34	0 (0.00%)	3 (1.22%)	<-10	0.314	0.674	Mutual exclusivity
FAM177A1	14q13.2	0 (0.00%)	3 (1.22%)	<-10	0.314	0.674	Mutual exclusivity
FAM184B	4p15.32-p15.31	0 (0.00%)	3 (1.22%)	<-10	0.314	0.674	Mutual exclusivity
FAM185A	7q22.1	0 (0.00%)	3 (1.22%)	<-10	0.314	0.674	Mutual exclusivity
FAM193A	4p16.3	0 (0.00%)	3 (1.22%)	<-10	0.314	0.674	Mutual exclusivity
FAM71B	5q33.3	0 (0.00%)	3 (1.22%)	<-10	0.314	0.674	Mutual exclusivity
FAM71E1	19q13.33	0 (0.00%)	3 (1.22%)	<-10	0.314	0.674	Mutual exclusivity
FAM71E2	19q13.42	0 (0.00%)	3 (1.22%)	<-10	0.314	0.674	Mutual exclusivity
FAM90A27P	19q13.42	0 (0.00%)	3 (1.22%)	<-10	0.314	0.674	Mutual exclusivity
FAM90A28P	19q13.42	0 (0.00%)	3 (1.22%)	<-10	0.314	0.674	Mutual exclusivity
FBLN7	2q13-q14.1	0 (0.00%)	3 (1.22%)	<-10	0.314	0.674	Mutual exclusivity
FBXL13	7q22.1	0 (0.00%)	3 (1.22%)	<-10	0.314	0.674	Mutual exclusivity
FBXL21P	5q31.1	0 (0.00%)	3 (1.22%)	<-10	0.314	0.674	Mutual exclusivity
FBXO15	18q22.3	0 (0.00%)	3 (1.22%)	<-10	0.314	0.674	Mutual exclusivity
FCAR	19q13.42	0 (0.00%)	3 (1.22%)	<-10	0.314	0.674	Mutual exclusivity
FEZ2	2p22.2	0 (0.00%)	3 (1.22%)	<-10	0.314	0.674	Mutual exclusivity
FGF1	5q31.3	0 (0.00%)	3 (1.22%)	<-10	0.314	0.674	Mutual exclusivity
FGFBP1	4p15.32	0 (0.00%)	3 (1.22%)	<-10	0.314	0.674	Mutual exclusivity
FIS1	7q22.1	0 (0.00%)	3 (1.22%)	<-10	0.314	0.674	Mutual exclusivity
FIZ1	19q13.42	0 (0.00%)	3 (1.22%)	<-10	0.314	0.674	Mutual exclusivity

FMR1	Xq27.3	0 (0.00%)	3 (1.22%)	<-10	0.314	0.674	Mutual exclusivity
FMR1NB	Xq27.3-q28	0 (0.00%)	3 (1.22%)	<-10	0.314	0.674	Mutual exclusivity
FNDC9	5q33.3	0 (0.00%)	3 (1.22%)	<-10	0.314	0.674	Mutual exclusivity
FOXA1	14q21.1	0 (0.00%)	3 (1.22%)	<-10	0.314	0.674	Mutual exclusivity
FRMD6	14q22.1	0 (0.00%)	3 (1.22%)	<-10	0.314	0.674	Mutual exclusivity
FRYL	4p11	0 (0.00%)	3 (1.22%)	<-10	0.314	0.674	Mutual exclusivity
FTH1P10	5p15.1	0 (0.00%)	3 (1.22%)	<-10	0.314	0.674	Mutual exclusivity
FTMT	5q23.1	0 (0.00%)	3 (1.22%)	<-10	0.314	0.674	Mutual exclusivity
GAB2	11q14.1	0 (0.00%)	3 (1.22%)	<-10	0.314	0.674	Mutual exclusivity
GABRA4	4p12	0 (0.00%)	3 (1.22%)	<-10	0.314	0.674	Mutual exclusivity
GABRB1	4p12	0 (0.00%)	3 (1.22%)	<-10	0.314	0.674	Mutual exclusivity
GABRE	Xq28	0 (0.00%)	3 (1.22%)	<-10	0.314	0.674	Mutual exclusivity
GALM	2p22.1	0 (0.00%)	3 (1.22%)	<-10	0.314	0.674	Mutual exclusivity
GALNT10	5q33.2	0 (0.00%)	3 (1.22%)	<-10	0.314	0.674	Mutual exclusivity
GALP	19q13.43	0 (0.00%)	3 (1.22%)	<-10	0.314	0.674	Mutual exclusivity
GBA3	4p15.2	0 (0.00%)	3 (1.22%)	<-10	0.314	0.674	Mutual exclusivity
GEMIN6	2p22.1	0 (0.00%)	3 (1.22%)	<-10	0.314	0.674	Mutual exclusivity
GGCX	2p11.2	0 (0.00%)	3 (1.22%)	<-10	0.314	0.674	Mutual exclusivity
GLRX	5q15	0 (0.00%)	3 (1.22%)	<-10	0.314	0.674	Mutual exclusivity
GM2A	5q33.1	0 (0.00%)	3 (1.22%)	<-10	0.314	0.674	Mutual exclusivity
GMFB	14q22.2	0 (0.00%)	3 (1.22%)	<-10	0.314	0.674	Mutual exclusivity
GNPDA1	5q31.3	0 (0.00%)	3 (1.22%)	<-10	0.314	0.674	Mutual exclusivity
GP6	19q13.42	0 (0.00%)	3 (1.22%)	<-10	0.314	0.674	Mutual exclusivity
GPATCH11	2p22.2	0 (0.00%)	3 (1.22%)	<-10	0.314	0.674	Mutual exclusivity
GPC3	Xq26.2	0 (0.00%)	3 (1.22%)	<-10	0.314	0.674	Mutual exclusivity
GPC4	Xq26.2	0 (0.00%)	3 (1.22%)	<-10	0.314	0.674	Mutual exclusivity
GPR32	19q13.33	0 (0.00%)	3 (1.22%)	<-10	0.314	0.674	Mutual exclusivity
GPX3	5q33.1	0 (0.00%)	3 (1.22%)	<-10	0.314	0.674	Mutual exclusivity
GRIA1	5q33.2	0 (0.00%)	3 (1.22%)	<-10	0.314	0.674	Mutual exclusivity

GSG1L	16p12.1	0 (0.00%)	3 (1.22%)	<-10	0.314	0.674	Mutual exclusivity
GSTK1	7q34	0 (0.00%)	3 (1.22%)	<-10	0.314	0.674	Mutual exclusivity
GUSBP1	5p14.3	0 (0.00%)	3 (1.22%)	<-10	0.314	0.674	Mutual exclusivity
GXYLT2	3p13	0 (0.00%)	3 (1.22%)	<-10	0.314	0.674	Mutual exclusivity
H1FOO	3q22.1	0 (0.00%)	3 (1.22%)	<-10	0.314	0.674	Mutual exclusivity
HAND1	5q33.2	0 (0.00%)	3 (1.22%)	<-10	0.314	0.674	Mutual exclusivity
HAVCR1	5q33.3	0 (0.00%)	3 (1.22%)	<-10	0.314	0.674	Mutual exclusivity
HAVCR2	5q33.3	0 (0.00%)	3 (1.22%)	<-10	0.314	0.674	Mutual exclusivity
HEATR5B	2p22.2	0 (0.00%)	3 (1.22%)	<-10	0.314	0.674	Mutual exclusivity
HMGCS1	5p12	0 (0.00%)	3 (1.22%)	<-10	0.314	0.674	Mutual exclusivity
HNRNPLL	2p22.1	0 (0.00%)	3 (1.22%)	<-10	0.314	0.674	Mutual exclusivity
HS3ST2	16p12.2	0 (0.00%)	3 (1.22%)	<-10	0.314	0.674	Mutual exclusivity
HSFX1	Xq28	0 (0.00%)	3 (1.22%)	<-10	0.314	0.674	Mutual exclusivity
HSFX2	Xq28	0 (0.00%)	3 (1.22%)	<-10	0.314	0.674	Mutual exclusivity
HSPBP1	19q13.42	0 (0.00%)	3 (1.22%)	<-10	0.314	0.674	Mutual exclusivity
IFNLR1	1p36.11	0 (0.00%)	3 (1.22%)	<-10	0.314	0.674	Mutual exclusivity
IFT122	3q21.3-q22.1	0 (0.00%)	3 (1.22%)	<-10	0.314	0.674	Mutual exclusivity
IFT22	7q22.1	0 (0.00%)	3 (1.22%)	<-10	0.314	0.674	Mutual exclusivity
IGSF6	16p12.2	0 (0.00%)	3 (1.22%)	<-10	0.314	0.674	Mutual exclusivity
IL11	19q13.42	0 (0.00%)	3 (1.22%)	<-10	0.314	0.674	Mutual exclusivity
IL12B	5q33.3	0 (0.00%)	3 (1.22%)	<-10	0.314	0.674	Mutual exclusivity
IL13RA1	Xq24	0 (0.00%)	3 (1.22%)	<-10	0.314	0.674	Mutual exclusivity
IL21R	16p12.1	0 (0.00%)	3 (1.22%)	<-10	0.314	0.674	Mutual exclusivity
IL27	16p12.1-p11.2	0 (0.00%)	3 (1.22%)	<-10	0.314	0.674	Mutual exclusivity
IL4I1	19q13.33	0 (0.00%)	3 (1.22%)	<-10	0.314	0.674	Mutual exclusivity
IL6	7p15.3	0 (0.00%)	3 (1.22%)	<-10	0.314	0.674	Mutual exclusivity
IL6ST	5q11.2	0 (0.00%)	3 (1.22%)	<-10	0.314	0.674	Mutual exclusivity
IL9	5q31.1	0 (0.00%)	3 (1.22%)	<-10	0.314	0.674	Mutual exclusivity
IRF3	19q13.33	0 (0.00%)	3 (1.22%)	<-10	0.314	0.674	Mutual exclusivity

IRGM	5q33.1	0 (0.00%)	3 (1.22%)	<-10	0.314	0.674	Mutual exclusivity
ISOC2	19q13.42	0 (0.00%)	3 (1.22%)	<-10	0.314	0.674	Mutual exclusivity
ITK	5q33.3	0 (0.00%)	3 (1.22%)	<-10	0.314	0.674	Mutual exclusivity
JDP2	14q24.3	0 (0.00%)	3 (1.22%)	<-10	0.314	0.674	Mutual exclusivity
JOSD2	19q13.33	0 (0.00%)	3 (1.22%)	<-10	0.314	0.674	Mutual exclusivity
KBTBD11	8p23.3	0 (0.00%)	3 (1.22%)	<-10	0.314	0.674	Mutual exclusivity
KCNC3	19q13.33	0 (0.00%)	3 (1.22%)	<-10	0.314	0.674	Mutual exclusivity
KCNIP4	4p15.31-p15.2	0 (0.00%)	3 (1.22%)	<-10	0.314	0.674	Mutual exclusivity
KCTD6	3p14.3	0 (0.00%)	3 (1.22%)	<-10	0.314	0.674	Mutual exclusivity
KDM8	16p12.1	0 (0.00%)	3 (1.22%)	<-10	0.314	0.674	Mutual exclusivity
KEL	7q34	0 (0.00%)	3 (1.22%)	<-10	0.314	0.674	Mutual exclusivity
KHDC1	6q13	0 (0.00%)	3 (1.22%)	<-10	0.314	0.674	Mutual exclusivity
KHDC1L	6q13	0 (0.00%)	3 (1.22%)	<-10	0.314	0.674	Mutual exclusivity
KIAA0556	16p12.1	0 (0.00%)	3 (1.22%)	<-10	0.314	0.674	Mutual exclusivity
KIAA0825	5q15	0 (0.00%)	3 (1.22%)	<-10	0.314	0.674	Mutual exclusivity
KIAA1147	7q34	0 (0.00%)	3 (1.22%)	<-10	0.314	0.674	Mutual exclusivity
KIAA1210	Xq24	0 (0.00%)	3 (1.22%)	<-10	0.314	0.674	Mutual exclusivity
KIF4B	5q33.2	0 (0.00%)	3 (1.22%)	<-10	0.314	0.674	Mutual exclusivity
KIR2DL1	19q13.42	0 (0.00%)	3 (1.22%)	<-10	0.314	0.674	Mutual exclusivity
KIR2DL3	19q13.42	0 (0.00%)	3 (1.22%)	<-10	0.314	0.674	Mutual exclusivity
KIR2DL4	19q13.42	0 (0.00%)	3 (1.22%)	<-10	0.314	0.674	Mutual exclusivity
KIR2DS4	19q13.42	0 (0.00%)	3 (1.22%)	<-10	0.314	0.674	Mutual exclusivity
KIR3DL1	19q13.42	0 (0.00%)	3 (1.22%)	<-10	0.314	0.674	Mutual exclusivity
KIR3DL2	19q13.42	0 (0.00%)	3 (1.22%)	<-10	0.314	0.674	Mutual exclusivity
KIR3DL3	19q13.42	0 (0.00%)	3 (1.22%)	<-10	0.314	0.674	Mutual exclusivity
KIR3DX1	19q13.42	0 (0.00%)	3 (1.22%)	<-10	0.314	0.674	Mutual exclusivity
KLHL35	11q13.4	0 (0.00%)	3 (1.22%)	<-10	0.314	0.674	Mutual exclusivity
KLK1	19q13.33	0 (0.00%)	3 (1.22%)	<-10	0.314	0.674	Mutual exclusivity
KLK10	19q13.41	0 (0.00%)	3 (1.22%)	<-10	0.314	0.674	Mutual exclusivity

KLK11	19q13.41	0 (0.00%)	3 (1.22%)	<-10	0.314	0.674	Mutual exclusivity
KLK12	19q13.41	0 (0.00%)	3 (1.22%)	<-10	0.314	0.674	Mutual exclusivity
KLK15	19q13.33	0 (0.00%)	3 (1.22%)	<-10	0.314	0.674	Mutual exclusivity
KLK2	19q13.33	0 (0.00%)	3 (1.22%)	<-10	0.314	0.674	Mutual exclusivity
KLK3	19q13.33	0 (0.00%)	3 (1.22%)	<-10	0.314	0.674	Mutual exclusivity
KLK4	19q13.41	0 (0.00%)	3 (1.22%)	<-10	0.314	0.674	Mutual exclusivity
KLK5	19q13.41	0 (0.00%)	3 (1.22%)	<-10	0.314	0.674	Mutual exclusivity
KLK6	19q13.41	0 (0.00%)	3 (1.22%)	<-10	0.314	0.674	Mutual exclusivity
KLK7	19q13.41	0 (0.00%)	3 (1.22%)	<-10	0.314	0.674	Mutual exclusivity
KLK8	19q13.41	0 (0.00%)	3 (1.22%)	<-10	0.314	0.674	Mutual exclusivity
KLK9	19q13.41	0 (0.00%)	3 (1.22%)	<-10	0.314	0.674	Mutual exclusivity
KLKP1	19q13.33	0 (0.00%)	3 (1.22%)	<-10	0.314	0.674	Mutual exclusivity
KMT5C	19q13.42	0 (0.00%)	3 (1.22%)	<-10	0.314	0.674	Mutual exclusivity
LAIR1	19q13.42	0 (0.00%)	3 (1.22%)	<-10	0.314	0.674	Mutual exclusivity
LAIR2	19q13.42	0 (0.00%)	3 (1.22%)	<-10	0.314	0.674	Mutual exclusivity
LAP3	4p15.32	0 (0.00%)	3 (1.22%)	<-10	0.314	0.674	Mutual exclusivity
LAT	16p11.2	0 (0.00%)	3 (1.22%)	<-10	0.314	0.674	Mutual exclusivity
LCMT1	16p12.1	0 (0.00%)	3 (1.22%)	<-10	0.314	0.674	Mutual exclusivity
LCORL	4p15.31	0 (0.00%)	3 (1.22%)	<-10	0.314	0.674	Mutual exclusivity
LDB3	10q23.2	0 (0.00%)	3 (1.22%)	<-10	0.314	0.674	Mutual exclusivity
LECT2	5q31.1	0 (0.00%)	3 (1.22%)	<-10	0.314	0.674	Mutual exclusivity
LENG8	19q13.42	0 (0.00%)	3 (1.22%)	<-10	0.314	0.674	Mutual exclusivity
LENG9	19q13.42	0 (0.00%)	3 (1.22%)	<-10	0.314	0.674	Mutual exclusivity
LILRA1	19q13.42	0 (0.00%)	3 (1.22%)	<-10	0.314	0.674	Mutual exclusivity
LILRA2	19q13.42	0 (0.00%)	3 (1.22%)	<-10	0.314	0.674	Mutual exclusivity
LILRB1	19q13.42	0 (0.00%)	3 (1.22%)	<-10	0.314	0.674	Mutual exclusivity
LILRB4	19q13.42	0 (0.00%)	3 (1.22%)	<-10	0.314	0.674	Mutual exclusivity
LINC00211	2p22.2	0 (0.00%)	3 (1.22%)	<-10	0.314	0.674	Mutual exclusivity
LINC00301	11q12.2	0 (0.00%)	3 (1.22%)	<-10	0.314	0.674	Mutual exclusivity

LINC00619	10q11.21	0 (0.00%)	3 (1.22%)	<-10	0.314	0.674	Mutual exclusivity
LINC00839	10q11.21	0 (0.00%)	3 (1.22%)	<-10	0.314	0.674	Mutual exclusivity
LINC00840	10q11.21	0 (0.00%)	3 (1.22%)	<-10	0.314	0.674	Mutual exclusivity
LINC00841	10q11.21	0 (0.00%)	3 (1.22%)	<-10	0.314	0.674	Mutual exclusivity
LINC00894	Xq28	0 (0.00%)	3 (1.22%)	<-10	0.314	0.674	Mutual exclusivity
LINC01554	5q15	0 (0.00%)	3 (1.22%)	<-10	0.314	0.674	Mutual exclusivity
LIPG	18q21.1	0 (0.00%)	3 (1.22%)	<-10	0.314	0.674	Mutual exclusivity
LLCFC1	7q34	0 (0.00%)	3 (1.22%)	<-10	0.314	0.674	Mutual exclusivity
LMNB1	5q23.2	0 (0.00%)	3 (1.22%)	<-10	0.314	0.674	Mutual exclusivity
LOX	5q23.1	0 (0.00%)	3 (1.22%)	<-10	0.314	0.674	Mutual exclusivity
LRRC10B	11q12.2	0 (0.00%)	3 (1.22%)	<-10	0.314	0.674	Mutual exclusivity
LRRC17	7q22.1	0 (0.00%)	3 (1.22%)	<-10	0.314	0.674	Mutual exclusivity
LRRC4B	19q13.33	0 (0.00%)	3 (1.22%)	<-10	0.314	0.674	Mutual exclusivity
LRRC75A	17p11.2	0 (0.00%)	3 (1.22%)	<-10	0.314	0.674	Mutual exclusivity
LRWD1	7q22.1	0 (0.00%)	3 (1.22%)	<-10	0.314	0.674	Mutual exclusivity
MAGEA10	Xq28	0 (0.00%)	3 (1.22%)	<-10	0.314	0.674	Mutual exclusivity
MAGEA11	Xq28	0 (0.00%)	3 (1.22%)	<-10	0.314	0.674	Mutual exclusivity
MAGEA4	Xq28	0 (0.00%)	3 (1.22%)	<-10	0.314	0.674	Mutual exclusivity
MAGEA8	Xq28	0 (0.00%)	3 (1.22%)	<-10	0.314	0.674	Mutual exclusivity
MAGEA9	Xq28	0 (0.00%)	3 (1.22%)	<-10	0.314	0.674	Mutual exclusivity
MAGEA9B	Xq28	0 (0.00%)	3 (1.22%)	<-10	0.314	0.674	Mutual exclusivity
3-Mar	5q23.2	0 (0.00%)	3 (1.22%)	<-10	0.314	0.674	Mutual exclusivity
MCTS1	Xq24	0 (0.00%)	3 (1.22%)	<-10	0.314	0.674	Mutual exclusivity
MED28	4p15.32	0 (0.00%)	3 (1.22%)	<-10	0.314	0.674	Mutual exclusivity
MED7	5q33.3	0 (0.00%)	3 (1.22%)	<-10	0.314	0.674	Mutual exclusivity
MEGF10	5q23.2	0 (0.00%)	3 (1.22%)	<-10	0.314	0.674	Mutual exclusivity
METTL9	16p12.2	0 (0.00%)	3 (1.22%)	<-10	0.314	0.674	Mutual exclusivity
MGAM	7q34	0 (0.00%)	3 (1.22%)	<-10	0.314	0.674	Mutual exclusivity
MIMT1	19q13.43	0 (0.00%)	3 (1.22%)	<-10	0.314	0.674	Mutual exclusivity

MIR-1294/1294		0 (0.00%)	3 (1.22%)	<-10	0.314	0.674	Mutual exclusivity
MIR-1303/1303		0 (0.00%)	3 (1.22%)	<-10	0.314	0.674	Mutual exclusivity
MIR-1323/1323		0 (0.00%)	3 (1.22%)	<-10	0.314	0.674	Mutual exclusivity
MIR-3138/3138		0 (0.00%)	3 (1.22%)	<-10	0.314	0.674	Mutual exclusivity
MIR-3141/3141		0 (0.00%)	3 (1.22%)	<-10	0.314	0.674	Mutual exclusivity
MIR-3654/3654		0 (0.00%)	3 (1.22%)	<-10	0.314	0.674	Mutual exclusivity
MIR-4285/4285		0 (0.00%)	3 (1.22%)	<-10	0.314	0.674	Mutual exclusivity
MIR-4467/4467		0 (0.00%)	3 (1.22%)	<-10	0.314	0.674	Mutual exclusivity
MIR-4488/4488		0 (0.00%)	3 (1.22%)	<-10	0.314	0.674	Mutual exclusivity
MIR-4503/4503		0 (0.00%)	3 (1.22%)	<-10	0.314	0.674	Mutual exclusivity
MIR-4517/4517		0 (0.00%)	3 (1.22%)	<-10	0.314	0.674	Mutual exclusivity
MIR-4696/4696		0 (0.00%)	3 (1.22%)	<-10	0.314	0.674	Mutual exclusivity
MIR-4721/4721		0 (0.00%)	3 (1.22%)	<-10	0.314	0.674	Mutual exclusivity
MIR-4751/4751		0 (0.00%)	3 (1.22%)	<-10	0.314	0.674	Mutual exclusivity
MIR-4754/4754		0 (0.00%)	3 (1.22%)	<-10	0.314	0.674	Mutual exclusivity
MIR-507/507		0 (0.00%)	3 (1.22%)	<-10	0.314	0.674	Mutual exclusivity
MIR-513B/513B		0 (0.00%)	3 (1.22%)	<-10	0.314	0.674	Mutual exclusivity
MIR-513C/513C		0 (0.00%)	3 (1.22%)	<-10	0.314	0.674	Mutual exclusivity
MIR-518B/518B		0 (0.00%)	3 (1.22%)	<-10	0.314	0.674	Mutual exclusivity
MIR-520H/520H		0 (0.00%)	3 (1.22%)	<-10	0.314	0.674	Mutual exclusivity
MIR-527/527		0 (0.00%)	3 (1.22%)	<-10	0.314	0.674	Mutual exclusivity
MIR-573/573		0 (0.00%)	3 (1.22%)	<-10	0.314	0.674	Mutual exclusivity
MIR-596/596		0 (0.00%)	3 (1.22%)	<-10	0.314	0.674	Mutual exclusivity
MIR-890/890		0 (0.00%)	3 (1.22%)	<-10	0.314	0.674	Mutual exclusivity
MIR-891B/891B		0 (0.00%)	3 (1.22%)	<-10	0.314	0.674	Mutual exclusivity
MIR-892A/892A		0 (0.00%)	3 (1.22%)	<-10	0.314	0.674	Mutual exclusivity
MIR-892B/892B		0 (0.00%)	3 (1.22%)	<-10	0.314	0.674	Mutual exclusivity
MIR-935/935		0 (0.00%)	3 (1.22%)	<-10	0.314	0.674	Mutual exclusivity
MKRN1	7q34	0 (0.00%)	3 (1.22%)	<-10	0.314	0.674	Mutual exclusivity

MMP27	11q22.2	0 (0.00%)	3 (1.22%)	<-10	0.314	0.674	Mutual exclusivity
MOGAT3	7q22.1	0 (0.00%)	3 (1.22%)	<-10	0.314	0.674	Mutual exclusivity
MORN2	2p22.1	0 (0.00%)	3 (1.22%)	<-10	0.314	0.674	Mutual exclusivity
MOSMO	16p12.2	0 (0.00%)	3 (1.22%)	<-10	0.314	0.674	Mutual exclusivity
MPC1	6q27	0 (0.00%)	3 (1.22%)	<-10	0.314	0.674	Mutual exclusivity
MPP4	2q33.1	0 (0.00%)	3 (1.22%)	<-10	0.314	0.674	Mutual exclusivity
MRPS33	7q34	0 (0.00%)	3 (1.22%)	<-10	0.314	0.674	Mutual exclusivity
MS4A1	11q12.2	0 (0.00%)	3 (1.22%)	<-10	0.314	0.674	Mutual exclusivity
MS4A10	11q12.2	0 (0.00%)	3 (1.22%)	<-10	0.314	0.674	Mutual exclusivity
MS4A12	11q12.2	0 (0.00%)	3 (1.22%)	<-10	0.314	0.674	Mutual exclusivity
MS4A13	11q12.2	0 (0.00%)	3 (1.22%)	<-10	0.314	0.674	Mutual exclusivity
MS4A15	11q12.2	0 (0.00%)	3 (1.22%)	<-10	0.314	0.674	Mutual exclusivity
MS4A18	11q12.2	0 (0.00%)	3 (1.22%)	<-10	0.314	0.674	Mutual exclusivity
MS4A8	11q12.2	0 (0.00%)	3 (1.22%)	<-10	0.314	0.674	Mutual exclusivity
MTRNR2L6	7q34	0 (0.00%)	3 (1.22%)	<-10	0.314	0.674	Mutual exclusivity
MUC12	7q22.1	0 (0.00%)	3 (1.22%)	<-10	0.314	0.674	Mutual exclusivity
MUC17	7q22.1	0 (0.00%)	3 (1.22%)	<-10	0.314	0.674	Mutual exclusivity
MUC3A	7q22.1	0 (0.00%)	3 (1.22%)	<-10	0.314	0.674	Mutual exclusivity
MYADM	19q13.42	0 (0.00%)	3 (1.22%)	<-10	0.314	0.674	Mutual exclusivity
MYBPC2	19q13.33	0 (0.00%)	3 (1.22%)	<-10	0.314	0.674	Mutual exclusivity
MYO10	5p15.1	0 (0.00%)	3 (1.22%)	<-10	0.314	0.674	Mutual exclusivity
MZF1	19q13.43	0 (0.00%)	3 (1.22%)	<-10	0.314	0.674	Mutual exclusivity
NAPEPLD	7q22.1	0 (0.00%)	3 (1.22%)	<-10	0.314	0.674	Mutual exclusivity
NAPSA	19q13.33	0 (0.00%)	3 (1.22%)	<-10	0.314	0.674	Mutual exclusivity
NAPSB	19q13.33	0 (0.00%)	3 (1.22%)	<-10	0.314	0.674	Mutual exclusivity
NARS2	11q14.1	0 (0.00%)	3 (1.22%)	<-10	0.314	0.674	Mutual exclusivity
NAT14	19q13.42	0 (0.00%)	3 (1.22%)	<-10	0.314	0.674	Mutual exclusivity
NAT16	7q22.1	0 (0.00%)	3 (1.22%)	<-10	0.314	0.674	Mutual exclusivity
NCAPG	4p15.31	0 (0.00%)	3 (1.22%)	<-10	0.314	0.674	Mutual exclusivity

NCR1	19q13.42	0 (0.00%)	3 (1.22%)	<-10	0.314	0.674	Mutual exclusivity
NDUFA3	19q13.42	0 (0.00%)	3 (1.22%)	<-10	0.314	0.674	Mutual exclusivity
NDUFAF7	2p22.2	0 (0.00%)	3 (1.22%)	<-10	0.314	0.674	Mutual exclusivity
NDUFB2	7q34	0 (0.00%)	3 (1.22%)	<-10	0.314	0.674	Mutual exclusivity
NFATC2IP	16p11.2	0 (0.00%)	3 (1.22%)	<-10	0.314	0.674	Mutual exclusivity
NFXL1	4p12	0 (0.00%)	3 (1.22%)	<-10	0.314	0.674	Mutual exclusivity
NIM1K	5p12	0 (0.00%)	3 (1.22%)	<-10	0.314	0.674	Mutual exclusivity
NIPAL1	4p12	0 (0.00%)	3 (1.22%)	<-10	0.314	0.674	Mutual exclusivity
NIPAL4	5q33.3	0 (0.00%)	3 (1.22%)	<-10	0.314	0.674	Mutual exclusivity
NLRP11	19q13.42-q13.43	0 (0.00%)	3 (1.22%)	<-10	0.314	0.674	Mutual exclusivity
NLRP12	19q13.42	0 (0.00%)	3 (1.22%)	<-10	0.314	0.674	Mutual exclusivity
NLRP13	19q13.43	0 (0.00%)	3 (1.22%)	<-10	0.314	0.674	Mutual exclusivity
NLRP2	19q13.42	0 (0.00%)	3 (1.22%)	<-10	0.314	0.674	Mutual exclusivity
NLRP4	19q13.43	0 (0.00%)	3 (1.22%)	<-10	0.314	0.674	Mutual exclusivity
NLRP5	19q13.43	0 (0.00%)	3 (1.22%)	<-10	0.314	0.674	Mutual exclusivity
NLRP7	19q13.42	0 (0.00%)	3 (1.22%)	<-10	0.314	0.674	Mutual exclusivity
NLRP8	19q13.43	0 (0.00%)	3 (1.22%)	<-10	0.314	0.674	Mutual exclusivity
NLRP9	19q13.42	0 (0.00%)	3 (1.22%)	<-10	0.314	0.674	Mutual exclusivity
NMUR2	5q33.1	0 (0.00%)	3 (1.22%)	<-10	0.314	0.674	Mutual exclusivity
NNT	5p12	0 (0.00%)	3 (1.22%)	<-10	0.314	0.674	Mutual exclusivity
NOBOX	7q35	0 (0.00%)	3 (1.22%)	<-10	0.314	0.674	Mutual exclusivity
NOL4L	20q11.21	0 (0.00%)	3 (1.22%)	<-10	0.314	0.674	Mutual exclusivity
NOSIP	19q13.33	0 (0.00%)	3 (1.22%)	<-10	0.314	0.674	Mutual exclusivity
NPIPB11	16p11.2	0 (0.00%)	3 (1.22%)	<-10	0.314	0.674	Mutual exclusivity
NPIPB3	16p12.2	0 (0.00%)	3 (1.22%)	<-10	0.314	0.674	Mutual exclusivity
NPIPB4	16p12.2	0 (0.00%)	3 (1.22%)	<-10	0.314	0.674	Mutual exclusivity
NPIPB5	16p12.2	0 (0.00%)	3 (1.22%)	<-10	0.314	0.674	Mutual exclusivity
NPIPB6	16p12.1	0 (0.00%)	3 (1.22%)	<-10	0.314	0.674	Mutual exclusivity
NPIPB7	16p12.1	0 (0.00%)	3 (1.22%)	<-10	0.314	0.674	Mutual exclusivity

NPIPB8	16p11.2	0 (0.00%)	3 (1.22%)	<-10	0.314	0.674	Mutual exclusivity
NPIPB9	16p11.2	0 (0.00%)	3 (1.22%)	<-10	0.314	0.674	Mutual exclusivity
NR1H2	19q13.33	0 (0.00%)	3 (1.22%)	<-10	0.314	0.674	Mutual exclusivity
NRDE2	14q32.11	0 (0.00%)	3 (1.22%)	<-10	0.314	0.674	Mutual exclusivity
NSMCE1	16p12.1	0 (0.00%)	3 (1.22%)	<-10	0.314	0.674	Mutual exclusivity
NUP62	19q13.33	0 (0.00%)	3 (1.22%)	<-10	0.314	0.674	Mutual exclusivity
OCIAD1	4p11	0 (0.00%)	3 (1.22%)	<-10	0.314	0.674	Mutual exclusivity
OCIAD2	4p11	0 (0.00%)	3 (1.22%)	<-10	0.314	0.674	Mutual exclusivity
OPN4	10q23.2	0 (0.00%)	3 (1.22%)	<-10	0.314	0.674	Mutual exclusivity
OR2A1	7q35	0 (0.00%)	3 (1.22%)	<-10	0.314	0.674	Mutual exclusivity
OR2A14	7q35	0 (0.00%)	3 (1.22%)	<-10	0.314	0.674	Mutual exclusivity
OR2A20P	7q35	0 (0.00%)	3 (1.22%)	<-10	0.314	0.674	Mutual exclusivity
OR2A42	7q35	0 (0.00%)	3 (1.22%)	<-10	0.314	0.674	Mutual exclusivity
OR2A7	7q35	0 (0.00%)	3 (1.22%)	<-10	0.314	0.674	Mutual exclusivity
OR2A9P	7q35	0 (0.00%)	3 (1.22%)	<-10	0.314	0.674	Mutual exclusivity
OR2V1	5q35.3	0 (0.00%)	3 (1.22%)	<-10	0.314	0.674	Mutual exclusivity
OR2V2	5q35.3	0 (0.00%)	3 (1.22%)	<-10	0.314	0.674	Mutual exclusivity
OR4F3	5q35.3	0 (0.00%)	3 (1.22%)	<-10	0.314	0.674	Mutual exclusivity
OR6V1	7q34	0 (0.00%)	3 (1.22%)	<-10	0.314	0.674	Mutual exclusivity
OR6W1P	7q34	0 (0.00%)	3 (1.22%)	<-10	0.314	0.674	Mutual exclusivity
OR9A1P	7q34	0 (0.00%)	3 (1.22%)	<-10	0.314	0.674	Mutual exclusivity
OR9A2	7q34	0 (0.00%)	3 (1.22%)	<-10	0.314	0.674	Mutual exclusivity
OR9A3P	7q34	0 (0.00%)	3 (1.22%)	<-10	0.314	0.674	Mutual exclusivity
OR9A4	7q34	0 (0.00%)	3 (1.22%)	<-10	0.314	0.674	Mutual exclusivity
ORAI2	7q22.1	0 (0.00%)	3 (1.22%)	<-10	0.314	0.674	Mutual exclusivity
ORC5	7q22.1-q22.2	0 (0.00%)	3 (1.22%)	<-10	0.314	0.674	Mutual exclusivity
OSCAR	19q13.42	0 (0.00%)	3 (1.22%)	<-10	0.314	0.674	Mutual exclusivity
OTOA	16p12.2|16p12.2	0 (0.00%)	3 (1.22%)	<-10	0.314	0.674	Mutual exclusivity
PAIP1	5p12	0 (0.00%)	3 (1.22%)	<-10	0.314	0.674	Mutual exclusivity

PAX9	14q13.3	0 (0.00%)	3 (1.22%)	<-10	0.314	0.674	Mutual exclusivity
PCBD2	5q31.1	0 (0.00%)	3 (1.22%)	<-10	0.314	0.674	Mutual exclusivity
PCDH1	5q31.3	0 (0.00%)	3 (1.22%)	<-10	0.314	0.674	Mutual exclusivity
PCDH12	5q31.3	0 (0.00%)	3 (1.22%)	<-10	0.314	0.674	Mutual exclusivity
PDE2A	11q13.4	0 (0.00%)	3 (1.22%)	<-10	0.314	0.674	Mutual exclusivity
PDGFRB	5q32	0 (0.00%)	3 (1.22%)	<-10	0.314	0.674	Mutual exclusivity
PDHB	3p14.3	0 (0.00%)	3 (1.22%)	<-10	0.314	0.674	Mutual exclusivity
PDZD9	16p12.2	0 (0.00%)	3 (1.22%)	<-10	0.314	0.674	Mutual exclusivity
PEG3	19q13.43	0 (0.00%)	3 (1.22%)	<-10	0.314	0.674	Mutual exclusivity
PGA3	11q12.2	0 (0.00%)	3 (1.22%)	<-10	0.314	0.674	Mutual exclusivity
PGA4	11q12.2	0 (0.00%)	3 (1.22%)	<-10	0.314	0.674	Mutual exclusivity
PGA5	11q12.2	0 (0.00%)	3 (1.22%)	<-10	0.314	0.674	Mutual exclusivity
PGM2	4p14	0 (0.00%)	3 (1.22%)	<-10	0.314	0.674	Mutual exclusivity
PGRMC1	Xq24	0 (0.00%)	3 (1.22%)	<-10	0.314	0.674	Mutual exclusivity
PHAX	5q23.2	0 (0.00%)	3 (1.22%)	<-10	0.314	0.674	Mutual exclusivity
PIP	7q34	0 (0.00%)	3 (1.22%)	<-10	0.314	0.674	Mutual exclusivity
PITX1	5q31.1	0 (0.00%)	3 (1.22%)	<-10	0.314	0.674	Mutual exclusivity
PLOD3	7q22.1	0 (0.00%)	3 (1.22%)	<-10	0.314	0.674	Mutual exclusivity
PLXND1	3q22.1	0 (0.00%)	3 (1.22%)	<-10	0.314	0.674	Mutual exclusivity
PMCHL1	5p14.3	0 (0.00%)	3 (1.22%)	<-10	0.314	0.674	Mutual exclusivity
POLD1	19q13.33	0 (0.00%)	3 (1.22%)	<-10	0.314	0.674	Mutual exclusivity
POLR2J	7q22.1	0 (0.00%)	3 (1.22%)	<-10	0.314	0.674	Mutual exclusivity
POLR2J2	7q22.1	0 (0.00%)	3 (1.22%)	<-10	0.314	0.674	Mutual exclusivity
POLR2J3	7q22.1	0 (0.00%)	3 (1.22%)	<-10	0.314	0.674	Mutual exclusivity
POLR3E	16p12.2	0 (0.00%)	3 (1.22%)	<-10	0.314	0.674	Mutual exclusivity
PPARGC1A	4p15.2	0 (0.00%)	3 (1.22%)	<-10	0.314	0.674	Mutual exclusivity
PPME1	11q13.4	0 (0.00%)	3 (1.22%)	<-10	0.314	0.674	Mutual exclusivity
PPP1R12C	19q13.42	0 (0.00%)	3 (1.22%)	<-10	0.314	0.674	Mutual exclusivity
PPP1R32	11q12.2	0 (0.00%)	3 (1.22%)	<-10	0.314	0.674	Mutual exclusivity

PPP2R3C	14q13.2	0 (0.00%)	3 (1.22%)	<-10	0.314	0.674	Mutual exclusivity
PPP6R1	19q13.42	0 (0.00%)	3 (1.22%)	<-10	0.314	0.674	Mutual exclusivity
PRKCB	16p12.2-p12.1	0 (0.00%)	3 (1.22%)	<-10	0.314	0.674	Mutual exclusivity
PRKCG	19q13.42	0 (0.00%)	3 (1.22%)	<-10	0.314	0.674	Mutual exclusivity
PRKD3	2p22.2	0 (0.00%)	3 (1.22%)	<-10	0.314	0.674	Mutual exclusivity
PRKRIP1	7q22.1	0 (0.00%)	3 (1.22%)	<-10	0.314	0.674	Mutual exclusivity
PRMT1	19q13.33	0 (0.00%)	3 (1.22%)	<-10	0.314	0.674	Mutual exclusivity
PRPF19	11q12.2	0 (0.00%)	3 (1.22%)	<-10	0.314	0.674	Mutual exclusivity
PRPF31	19q13.42	0 (0.00%)	3 (1.22%)	<-10	0.314	0.674	Mutual exclusivity
PRR12	19q13.33	0 (0.00%)	3 (1.22%)	<-10	0.314	0.674	Mutual exclusivity
PRR18	6q27	0 (0.00%)	3 (1.22%)	<-10	0.314	0.674	Mutual exclusivity
PRRG2	19q13.33	0 (0.00%)	3 (1.22%)	<-10	0.314	0.674	Mutual exclusivity
PRSS1	7q34	0 (0.00%)	3 (1.22%)	<-10	0.314	0.674	Mutual exclusivity
PRSS37	7q34	0 (0.00%)	3 (1.22%)	<-10	0.314	0.674	Mutual exclusivity
PRSS3P2	7q34	0 (0.00%)	3 (1.22%)	<-10	0.314	0.674	Mutual exclusivity
PRSS3P3	7q34	0 (0.00%)	3 (1.22%)	<-10	0.314	0.674	Mutual exclusivity
PRSS58	7q34	0 (0.00%)	3 (1.22%)	<-10	0.314	0.674	Mutual exclusivity
PTPRH	19q13.42	0 (0.00%)	3 (1.22%)	<-10	0.314	0.674	Mutual exclusivity
PWWP2A	5q33.3	0 (0.00%)	3 (1.22%)	<-10	0.314	0.674	Mutual exclusivity
QDPR	4p15.32	0 (0.00%)	3 (1.22%)	<-10	0.314	0.674	Mutual exclusivity
QPCT	2p22.2	0 (0.00%)	3 (1.22%)	<-10	0.314	0.674	Mutual exclusivity
RAB19	7q34	0 (0.00%)	3 (1.22%)	<-10	0.314	0.674	Mutual exclusivity
RAB7A	3q21.3	0 (0.00%)	3 (1.22%)	<-10	0.314	0.674	Mutual exclusivity
RABEP2	16p11.2	0 (0.00%)	3 (1.22%)	<-10	0.314	0.674	Mutual exclusivity
RACK1	5q35.3	0 (0.00%)	3 (1.22%)	<-10	0.314	0.674	Mutual exclusivity
RAD51B	14q24.1	0 (0.00%)	3 (1.22%)	<-10	0.314	0.674	Mutual exclusivity
RAPGEF5	7p15.3	0 (0.00%)	3 (1.22%)	<-10	0.314	0.674	Mutual exclusivity
RASA4	7q22.1	0 (0.00%)	3 (1.22%)	<-10	0.314	0.674	Mutual exclusivity
RASA4B	7q22.1	0 (0.00%)	3 (1.22%)	<-10	0.314	0.674	Mutual exclusivity

RBBP6	16p12.1	0 (0.00%)	3 (1.22%)	<-10	0.314	0.674	Mutual exclusivity
RBP5	12p13.31	0 (0.00%)	3 (1.22%)	<-10	0.314	0.674	Mutual exclusivity
RDH13	19q13.42	0 (0.00%)	3 (1.22%)	<-10	0.314	0.674	Mutual exclusivity
RELL1	4p14	0 (0.00%)	3 (1.22%)	<-10	0.314	0.674	Mutual exclusivity
RFPL4A	19q13.42	0 (0.00%)	3 (1.22%)	<-10	0.314	0.674	Mutual exclusivity
RFPL4AL1	19q13.42	0 (0.00%)	3 (1.22%)	<-10	0.314	0.674	Mutual exclusivity
RGPD8	2q14.1	0 (0.00%)	3 (1.22%)	<-10	0.314	0.674	Mutual exclusivity
RHO	3q22.1	0 (0.00%)	3 (1.22%)	<-10	0.314	0.674	Mutual exclusivity
RHOBTB3	5q15	0 (0.00%)	3 (1.22%)	<-10	0.314	0.674	Mutual exclusivity
RIMS1	6q13	0 (0.00%)	3 (1.22%)	<-10	0.314	0.674	Mutual exclusivity
RMDN2	2p22.2	0 (0.00%)	3 (1.22%)	<-10	0.314	0.674	Mutual exclusivity
RN7SKP109	19q13.42	0 (0.00%)	3 (1.22%)	<-10	0.314	0.674	Mutual exclusivity
RN7SKP117	5q23.2	0 (0.00%)	3 (1.22%)	<-10	0.314	0.674	Mutual exclusivity
RN7SKP133	5p15.1	0 (0.00%)	3 (1.22%)	<-10	0.314	0.674	Mutual exclusivity
RN7SKP149	Xq27.3	0 (0.00%)	3 (1.22%)	<-10	0.314	0.674	Mutual exclusivity
RN7SKP174	7q35	0 (0.00%)	3 (1.22%)	<-10	0.314	0.674	Mutual exclusivity
RN7SKP189	Xq27.3	0 (0.00%)	3 (1.22%)	<-10	0.314	0.674	Mutual exclusivity
RN7SKP198	7q22.1	0 (0.00%)	3 (1.22%)	<-10	0.314	0.674	Mutual exclusivity
RN7SKP257	14q13.3	0 (0.00%)	3 (1.22%)	<-10	0.314	0.674	Mutual exclusivity
RN7SKP267	Xq28	0 (0.00%)	3 (1.22%)	<-10	0.314	0.674	Mutual exclusivity
RN7SKP54	7q22.1	0 (0.00%)	3 (1.22%)	<-10	0.314	0.674	Mutual exclusivity
RN7SKP81	Xq27.3	0 (0.00%)	3 (1.22%)	<-10	0.314	0.674	Mutual exclusivity
RN7SKP86	7q22.1	0 (0.00%)	3 (1.22%)	<-10	0.314	0.674	Mutual exclusivity
RN7SL126P	2p11.2	0 (0.00%)	3 (1.22%)	<-10	0.314	0.674	Mutual exclusivity
RN7SL16P	4p15.2	0 (0.00%)	3 (1.22%)	<-10	0.314	0.674	Mutual exclusivity
RN7SL177P	5q33.2	0 (0.00%)	3 (1.22%)	<-10	0.314	0.674	Mutual exclusivity
RN7SL18P	2p15	0 (0.00%)	3 (1.22%)	<-10	0.314	0.674	Mutual exclusivity
RN7SL23P	11q12.2	0 (0.00%)	3 (1.22%)	<-10	0.314	0.674	Mutual exclusivity
RN7SL317P	19q13.42	0 (0.00%)	3 (1.22%)	<-10	0.314	0.674	Mutual exclusivity

RN7SL380P	12p13.31	0 (0.00%)	3 (1.22%)	<-10	0.314	0.674	Mutual exclusivity
RN7SL414P	2p21	0 (0.00%)	3 (1.22%)	<-10	0.314	0.674	Mutual exclusivity
RN7SL481P	7q34	0 (0.00%)	3 (1.22%)	<-10	0.314	0.674	Mutual exclusivity
RN7SL525P	19q13.43	0 (0.00%)	3 (1.22%)	<-10	0.314	0.674	Mutual exclusivity
RN7SL526P	19q13.43	0 (0.00%)	3 (1.22%)	<-10	0.314	0.674	Mutual exclusivity
RN7SL535P	7q34	0 (0.00%)	3 (1.22%)	<-10	0.314	0.674	Mutual exclusivity
RN7SL557P	16p12.1	0 (0.00%)	3 (1.22%)	<-10	0.314	0.674	Mutual exclusivity
RN7SL58P	5p14.3	0 (0.00%)	3 (1.22%)	<-10	0.314	0.674	Mutual exclusivity
RN7SL602P	2p22.3	0 (0.00%)	3 (1.22%)	<-10	0.314	0.674	Mutual exclusivity
RN7SL655P	5q33.2	0 (0.00%)	3 (1.22%)	<-10	0.314	0.674	Mutual exclusivity
RN7SL693P	19q13.43	0 (0.00%)	3 (1.22%)	<-10	0.314	0.674	Mutual exclusivity
RN7SL69P	12p13.31	0 (0.00%)	3 (1.22%)	<-10	0.314	0.674	Mutual exclusivity
RN7SL752P	3q22.1	0 (0.00%)	3 (1.22%)	<-10	0.314	0.674	Mutual exclusivity
RN7SL771P	7q34	0 (0.00%)	3 (1.22%)	<-10	0.314	0.674	Mutual exclusivity
RN7SL96P	2p22.1	0 (0.00%)	3 (1.22%)	<-10	0.314	0.674	Mutual exclusivity
RNA5SP155	4p16.1	0 (0.00%)	3 (1.22%)	<-10	0.314	0.674	Mutual exclusivity
RNA5SP179	5p15.1	0 (0.00%)	3 (1.22%)	<-10	0.314	0.674	Mutual exclusivity
RNA5SP208	6q12	0 (0.00%)	3 (1.22%)	<-10	0.314	0.674	Mutual exclusivity
RNA5SP227	7p15.3	0 (0.00%)	3 (1.22%)	<-10	0.314	0.674	Mutual exclusivity
RNA5SP247	7q34	0 (0.00%)	3 (1.22%)	<-10	0.314	0.674	Mutual exclusivity
RNA5SP248	7q34	0 (0.00%)	3 (1.22%)	<-10	0.314	0.674	Mutual exclusivity
RNA5SP343	11q13.4	0 (0.00%)	3 (1.22%)	<-10	0.314	0.674	Mutual exclusivity
RNA5SP473	19q13.43	0 (0.00%)	3 (1.22%)	<-10	0.314	0.674	Mutual exclusivity
RNA5SP516	Xq27.2	0 (0.00%)	3 (1.22%)	<-10	0.314	0.674	Mutual exclusivity
RNA5SP517	Xq27.3	0 (0.00%)	3 (1.22%)	<-10	0.314	0.674	Mutual exclusivity
RNF14	5q31.3	0 (0.00%)	3 (1.22%)	<-10	0.314	0.674	Mutual exclusivity
RNF145	5q33.3	0 (0.00%)	3 (1.22%)	<-10	0.314	0.674	Mutual exclusivity
RNF181	2p11.2	0 (0.00%)	3 (1.22%)	<-10	0.314	0.674	Mutual exclusivity
RNU4ATAC2P	5q33.3	0 (0.00%)	3 (1.22%)	<-10	0.314	0.674	Mutual exclusivity

RNU6ATAC40P	7q35	0 (0.00%)	3 (1.22%)	<-10	0.314	0.674	Mutual exclusivity
RPL23AP79	19q13.43	0 (0.00%)	3 (1.22%)	<-10	0.314	0.674	Mutual exclusivity
RPL28	19q13.42	0 (0.00%)	3 (1.22%)	<-10	0.314	0.674	Mutual exclusivity
RPS3	11q13.4	0 (0.00%)	3 (1.22%)	<-10	0.314	0.674	Mutual exclusivity
RPS5	19q13.43	0 (0.00%)	3 (1.22%)	<-10	0.314	0.674	Mutual exclusivity
RPS6KA2	6q27	0 (0.00%)	3 (1.22%)	<-10	0.314	0.674	Mutual exclusivity
RRAS	19q13.33	0 (0.00%)	3 (1.22%)	<-10	0.314	0.674	Mutual exclusivity
RRN3P1	16p12.2	0 (0.00%)	3 (1.22%)	<-10	0.314	0.674	Mutual exclusivity
RRN3P2	16p11.2	0 (0.00%)	3 (1.22%)	<-10	0.314	0.674	Mutual exclusivity
RRN3P3	16p12.2	0 (0.00%)	3 (1.22%)	<-10	0.314	0.674	Mutual exclusivity
SAP30L	5q33.2	0 (0.00%)	3 (1.22%)	<-10	0.314	0.674	Mutual exclusivity
SBK1	16p12.1	0 (0.00%)	3 (1.22%)	<-10	0.314	0.674	Mutual exclusivity
SBK2	19q13.42	0 (0.00%)	3 (1.22%)	<-10	0.314	0.674	Mutual exclusivity
SBK3	19q13.42	0 (0.00%)	3 (1.22%)	<-10	0.314	0.674	Mutual exclusivity
SCAF1	19q13.33	0 (0.00%)	3 (1.22%)	<-10	0.314	0.674	Mutual exclusivity
SCGB1D1	11q12.3	0 (0.00%)	3 (1.22%)	<-10	0.314	0.674	Mutual exclusivity
SCGB1D2	11q12.3	0 (0.00%)	3 (1.22%)	<-10	0.314	0.674	Mutual exclusivity
SCGB1D4	11q12.3	0 (0.00%)	3 (1.22%)	<-10	0.314	0.674	Mutual exclusivity
SCGB2A1	11q12.3	0 (0.00%)	3 (1.22%)	<-10	0.314	0.674	Mutual exclusivity
SCGB2A2	11q12.3	0 (0.00%)	3 (1.22%)	<-10	0.314	0.674	Mutual exclusivity
SDHAF2	11q12.2	0 (0.00%)	3 (1.22%)	<-10	0.314	0.674	Mutual exclusivity
SDR42E2	16p12.2	0 (0.00%)	3 (1.22%)	<-10	0.314	0.674	Mutual exclusivity
SERPINA10	14q32.13	0 (0.00%)	3 (1.22%)	<-10	0.314	0.674	Mutual exclusivity
SERPINA6	14q32.13	0 (0.00%)	3 (1.22%)	<-10	0.314	0.674	Mutual exclusivity
SERPINE1	7q22.1	0 (0.00%)	3 (1.22%)	<-10	0.314	0.674	Mutual exclusivity
SFT2D1	6q27	0 (0.00%)	3 (1.22%)	<-10	0.314	0.674	Mutual exclusivity
SFTPB	2p11.2	0 (0.00%)	3 (1.22%)	<-10	0.314	0.674	Mutual exclusivity
SH2B1	16p11.2	0 (0.00%)	3 (1.22%)	<-10	0.314	0.674	Mutual exclusivity
SH2B2	7q22.1	0 (0.00%)	3 (1.22%)	<-10	0.314	0.674	Mutual exclusivity

SHANK1	19q13.33	0 (0.00%)	3 (1.22%)	<-10	0.314	0.674	Mutual exclusivity
SHISA7	19q13.42	0 (0.00%)	3 (1.22%)	<-10	0.314	0.674	Mutual exclusivity
SIGLEC11	19q13.33	0 (0.00%)	3 (1.22%)	<-10	0.314	0.674	Mutual exclusivity
SIGLEC15	18q12.3	0 (0.00%)	3 (1.22%)	<-10	0.314	0.674	Mutual exclusivity
SIGLEC16	19q13.33	0 (0.00%)	3 (1.22%)	<-10	0.314	0.674	Mutual exclusivity
SLAIN2	4p11	0 (0.00%)	3 (1.22%)	<-10	0.314	0.674	Mutual exclusivity
SLC10A4	4p11	0 (0.00%)	3 (1.22%)	<-10	0.314	0.674	Mutual exclusivity
SLC15A3	11q12.2	0 (0.00%)	3 (1.22%)	<-10	0.314	0.674	Mutual exclusivity
SLC25A21	14q13.3	0 (0.00%)	3 (1.22%)	<-10	0.314	0.674	Mutual exclusivity
SLC25A48	5q31.1	0 (0.00%)	3 (1.22%)	<-10	0.314	0.674	Mutual exclusivity
SLC27A5	19q13.43	0 (0.00%)	3 (1.22%)	<-10	0.314	0.674	Mutual exclusivity
SLC2A9	4p16.1	0 (0.00%)	3 (1.22%)	<-10	0.314	0.674	Mutual exclusivity
SLC30A9	4p13	0 (0.00%)	3 (1.22%)	<-10	0.314	0.674	Mutual exclusivity
SLC37A3	7q34	0 (0.00%)	3 (1.22%)	<-10	0.314	0.674	Mutual exclusivity
SLC5A11	16p12.1	0 (0.00%)	3 (1.22%)	<-10	0.314	0.674	Mutual exclusivity
SLITRK2	Xq27.3	0 (0.00%)	3 (1.22%)	<-10	0.314	0.674	Mutual exclusivity
SLITRK4	Xq27.3	0 (0.00%)	3 (1.22%)	<-10	0.314	0.674	Mutual exclusivity
SMAD5	5q31.1	0 (0.00%)	3 (1.22%)	<-10	0.314	0.674	Mutual exclusivity
SMG1P1	16p12.2	0 (0.00%)	3 (1.22%)	<-10	0.314	0.674	Mutual exclusivity
SMIM17	19q13.43	0 (0.00%)	3 (1.22%)	<-10	0.314	0.674	Mutual exclusivity
SMIM3	5q33.1	0 (0.00%)	3 (1.22%)	<-10	0.314	0.674	Mutual exclusivity
SNCAIP	5q23.2	0 (0.00%)	3 (1.22%)	<-10	0.314	0.674	Mutual exclusivity
SNORA24	4q26	0 (0.00%)	3 (1.22%)	<-10	0.314	0.674	Mutual exclusivity
SNORA33	6q23.2	0 (0.00%)	3 (1.22%)	<-10	0.314	0.674	Mutual exclusivity
SNORA68	19p13.11	0 (0.00%)	3 (1.22%)	<-10	0.314	0.674	Mutual exclusivity
SNORD15A	11q13.4	0 (0.00%)	3 (1.22%)	<-10	0.314	0.674	Mutual exclusivity
SNORD15B	11q13.4	0 (0.00%)	3 (1.22%)	<-10	0.314	0.674	Mutual exclusivity
SNORD88A	19q13.33	0 (0.00%)	3 (1.22%)	<-10	0.314	0.674	Mutual exclusivity
SNORD88B	19q13.33	0 (0.00%)	3 (1.22%)	<-10	0.314	0.674	Mutual exclusivity

SNORD88C	19q13.33	0 (0.00%)	3 (1.22%)	<-10	0.314	0.674	Mutual exclusivity
SNORD93	7p15.3	0 (0.00%)	3 (1.22%)	<-10	0.314	0.674	Mutual exclusivity
SNORD95	5q35.3	0 (0.00%)	3 (1.22%)	<-10	0.314	0.674	Mutual exclusivity
SNORD96A	5q35.3	0 (0.00%)	3 (1.22%)	<-10	0.314	0.674	Mutual exclusivity
SORCS2	4p16.1	0 (0.00%)	3 (1.22%)	<-10	0.314	0.674	Mutual exclusivity
SOS1	2p22.1	0 (0.00%)	3 (1.22%)	<-10	0.314	0.674	Mutual exclusivity
SOX30	5q33.3	0 (0.00%)	3 (1.22%)	<-10	0.314	0.674	Mutual exclusivity
SPANXN1	Xq27.3	0 (0.00%)	3 (1.22%)	<-10	0.314	0.674	Mutual exclusivity
SPANXN2	Xq27.3	0 (0.00%)	3 (1.22%)	<-10	0.314	0.674	Mutual exclusivity
SPANXN4	Xq27.3	0 (0.00%)	3 (1.22%)	<-10	0.314	0.674	Mutual exclusivity
SPATA9	5q15	0 (0.00%)	3 (1.22%)	<-10	0.314	0.674	Mutual exclusivity
SPDYE2	7q22.1	0 (0.00%)	3 (1.22%)	<-10	0.314	0.674	Mutual exclusivity
SPDYE2B	7q22.1	0 (0.00%)	3 (1.22%)	<-10	0.314	0.674	Mutual exclusivity
SPIB	19q13.33	0 (0.00%)	3 (1.22%)	<-10	0.314	0.674	Mutual exclusivity
SPNS1	16p11.2	0 (0.00%)	3 (1.22%)	<-10	0.314	0.674	Mutual exclusivity
SRFBP1	5q23.1	0 (0.00%)	3 (1.22%)	<-10	0.314	0.674	Mutual exclusivity
SRP54	14q13.2	0 (0.00%)	3 (1.22%)	<-10	0.314	0.674	Mutual exclusivity
SRSF7	2p22.1	0 (0.00%)	3 (1.22%)	<-10	0.314	0.674	Mutual exclusivity
SSBP1	7q34	0 (0.00%)	3 (1.22%)	<-10	0.314	0.674	Mutual exclusivity
SSC5D	19q13.42	0 (0.00%)	3 (1.22%)	<-10	0.314	0.674	Mutual exclusivity
STARD10	11q13.4	0 (0.00%)	3 (1.22%)	<-10	0.314	0.674	Mutual exclusivity
STEAP1B	7p15.3	0 (0.00%)	3 (1.22%)	<-10	0.314	0.674	Mutual exclusivity
STK35	20p13	0 (0.00%)	3 (1.22%)	<-10	0.314	0.674	Mutual exclusivity
STRADB	2q33.1	0 (0.00%)	3 (1.22%)	<-10	0.314	0.674	Mutual exclusivity
STRN	2p22.2	0 (0.00%)	3 (1.22%)	<-10	0.314	0.674	Mutual exclusivity
STRN3	14q12	0 (0.00%)	3 (1.22%)	<-10	0.314	0.674	Mutual exclusivity
SULT1A1	16p11.2	0 (0.00%)	3 (1.22%)	<-10	0.314	0.674	Mutual exclusivity
SULT1A2	16p11.2	0 (0.00%)	3 (1.22%)	<-10	0.314	0.674	Mutual exclusivity
SULT6B1	2p22.2	0 (0.00%)	3 (1.22%)	<-10	0.314	0.674	Mutual exclusivity

SUSD2	22q11.23	0 (0.00%)	3 (1.22%)	<-10	0.314	0.674	Mutual exclusivity
SYT3	19q13.33	0 (0.00%)	3 (1.22%)	<-10	0.314	0.674	Mutual exclusivity
SYT5	19q13.42|11p	0 (0.00%)	3 (1.22%)	<-10	0.314	0.674	Mutual exclusivity
SYT7	11q12.2	0 (0.00%)	3 (1.22%)	<-10	0.314	0.674	Mutual exclusivity
TARM1	19q13.42	0 (0.00%)	3 (1.22%)	<-10	0.314	0.674	Mutual exclusivity
TAS2R3	7q34	0 (0.00%)	3 (1.22%)	<-10	0.314	0.674	Mutual exclusivity
TAS2R38	7q34	0 (0.00%)	3 (1.22%)	<-10	0.314	0.674	Mutual exclusivity
TAS2R39	7q34	0 (0.00%)	3 (1.22%)	<-10	0.314	0.674	Mutual exclusivity
TAS2R4	7q34	0 (0.00%)	3 (1.22%)	<-10	0.314	0.674	Mutual exclusivity
TAS2R40	7q34	0 (0.00%)	3 (1.22%)	<-10	0.314	0.674	Mutual exclusivity
TAS2R41	7q35	0 (0.00%)	3 (1.22%)	<-10	0.314	0.674	Mutual exclusivity
TAS2R5	7q34	0 (0.00%)	3 (1.22%)	<-10	0.314	0.674	Mutual exclusivity
TAS2R60	7q35	0 (0.00%)	3 (1.22%)	<-10	0.314	0.674	Mutual exclusivity
TBC1D17	19q13.33	0 (0.00%)	3 (1.22%)	<-10	0.314	0.674	Mutual exclusivity
TBXT	6q27	0 (0.00%)	3 (1.22%)	<-10	0.314	0.674	Mutual exclusivity
TEC	19q13.2	0 (0.00%)	3 (1.22%)	<-10	0.314	0.674	Mutual exclusivity
TENM1	Xq25	0 (0.00%)	3 (1.22%)	<-10	0.314	0.674	Mutual exclusivity
TENM4	11q14.1	0 (0.00%)	3 (1.22%)	<-10	0.314	0.674	Mutual exclusivity
TEX43	5q23.2	0 (0.00%)	3 (1.22%)	<-10	0.314	0.674	Mutual exclusivity
TFDP3	Xq26.2	0 (0.00%)	3 (1.22%)	<-10	0.314	0.674	Mutual exclusivity
TFPT	19q13.42	0 (0.00%)	3 (1.22%)	<-10	0.314	0.674	Mutual exclusivity
TGFBI	5q31.1	0 (0.00%)	3 (1.22%)	<-10	0.314	0.674	Mutual exclusivity
THAP12	11q13.5	0 (0.00%)	3 (1.22%)	<-10	0.314	0.674	Mutual exclusivity
TIE1	1p34.2	0 (0.00%)	3 (1.22%)	<-10	0.314	0.674	Mutual exclusivity
TIMM21	18q22.3	0 (0.00%)	3 (1.22%)	<-10	0.314	0.674	Mutual exclusivity
TM4SF20	2q36.3	0 (0.00%)	3 (1.22%)	<-10	0.314	0.674	Mutual exclusivity
TMCC1	3q22.1	0 (0.00%)	3 (1.22%)	<-10	0.314	0.674	Mutual exclusivity
TMEM109	11q12.2	0 (0.00%)	3 (1.22%)	<-10	0.314	0.674	Mutual exclusivity
TMEM132A	11q12.2	0 (0.00%)	3 (1.22%)	<-10	0.314	0.674	Mutual exclusivity

TMEM139	7q34	0 (0.00%)	3 (1.22%)	<-10	0.314	0.674	Mutual exclusivity
TMEM150A	2p11.2	0 (0.00%)	3 (1.22%)	<-10	0.314	0.674	Mutual exclusivity
TMEM150B	19q13.42	0 (0.00%)	3 (1.22%)	<-10	0.314	0.674	Mutual exclusivity
TMEM17	2p15	0 (0.00%)	3 (1.22%)	<-10	0.314	0.674	Mutual exclusivity
TMEM178B	7q34	0 (0.00%)	3 (1.22%)	<-10	0.314	0.674	Mutual exclusivity
TMEM185A	Xq28	0 (0.00%)	3 (1.22%)	<-10	0.314	0.674	Mutual exclusivity
TMEM190	19q13.42	0 (0.00%)	3 (1.22%)	<-10	0.314	0.674	Mutual exclusivity
TMEM237	2q33.1	0 (0.00%)	3 (1.22%)	<-10	0.314	0.674	Mutual exclusivity
TMEM238	19q13.42	0 (0.00%)	3 (1.22%)	<-10	0.314	0.674	Mutual exclusivity
TMEM257	Xq27.3	0 (0.00%)	3 (1.22%)	<-10	0.314	0.674	Mutual exclusivity
TMEM267	5p12	0 (0.00%)	3 (1.22%)	<-10	0.314	0.674	Mutual exclusivity
TMEM86B	19q13.42	0 (0.00%)	3 (1.22%)	<-10	0.314	0.674	Mutual exclusivity
TMEM87B	2q13	0 (0.00%)	3 (1.22%)	<-10	0.314	0.674	Mutual exclusivity
TNIP1	5q33.1	0 (0.00%)	3 (1.22%)	<-10	0.314	0.674	Mutual exclusivity
TNNI3	19q13.42	0 (0.00%)	3 (1.22%)	<-10	0.314	0.674	Mutual exclusivity
TNNT1	19q13.42	0 (0.00%)	3 (1.22%)	<-10	0.314	0.674	Mutual exclusivity
TNRC6A	16p12.1	0 (0.00%)	3 (1.22%)	<-10	0.314	0.674	Mutual exclusivity
TOMM7	7p15.3	0 (0.00%)	3 (1.22%)	<-10	0.314	0.674	Mutual exclusivity
TPK1	7q35	0 (0.00%)	3 (1.22%)	<-10	0.314	0.674	Mutual exclusivity
TPM3P9	19q13.42	0 (0.00%)	3 (1.22%)	<-10	0.314	0.674	Mutual exclusivity
TRAK2	2q33.1	0 (0.00%)	3 (1.22%)	<-10	0.314	0.674	Mutual exclusivity
TRAPPC2	Xp22.2	0 (0.00%)	3 (1.22%)	<-10	0.314	0.674	Mutual exclusivity
TRBC2	7q34	0 (0.00%)	3 (1.22%)	<-10	0.314	0.674	Mutual exclusivity
TRBV19	7q34	0 (0.00%)	3 (1.22%)	<-10	0.314	0.674	Mutual exclusivity
TRBV2	7q34	0 (0.00%)	3 (1.22%)	<-10	0.314	0.674	Mutual exclusivity
TRBV27	7q34	0 (0.00%)	3 (1.22%)	<-10	0.314	0.674	Mutual exclusivity
TRBV28	7q34	0 (0.00%)	3 (1.22%)	<-10	0.314	0.674	Mutual exclusivity
TRBV30	7q34	0 (0.00%)	3 (1.22%)	<-10	0.314	0.674	Mutual exclusivity
TRBV9	7q34	0 (0.00%)	3 (1.22%)	<-10	0.314	0.674	Mutual exclusivity

TRIM28	19q13.43	0 (0.00%)	3 (1.22%)	<-10	0.314	0.674	Mutual exclusivity
TRIM41	5q35.3	0 (0.00%)	3 (1.22%)	<-10	0.314	0.674	Mutual exclusivity
TRIM52	5q35.3	0 (0.00%)	3 (1.22%)	<-10	0.314	0.674	Mutual exclusivity
TRIM56	7q22.1	0 (0.00%)	3 (1.22%)	<-10	0.314	0.674	Mutual exclusivity
TRIM7	5q35.3	0 (0.00%)	3 (1.22%)	<-10	0.314	0.674	Mutual exclusivity
TRPV5	7q34	0 (0.00%)	3 (1.22%)	<-10	0.314	0.674	Mutual exclusivity
TRPV6	7q34	0 (0.00%)	3 (1.22%)	<-10	0.314	0.674	Mutual exclusivity
TSPAN13	7p21.1	0 (0.00%)	3 (1.22%)	<-10	0.314	0.674	Mutual exclusivity
TTC1	5q33.3	0 (0.00%)	3 (1.22%)	<-10	0.314	0.674	Mutual exclusivity
TTC6	14q21.1	0 (0.00%)	3 (1.22%)	<-10	0.314	0.674	Mutual exclusivity
TTYH1	19q13.42	0 (0.00%)	3 (1.22%)	<-10	0.314	0.674	Mutual exclusivity
TUFM	16p11.2	0 (0.00%)	3 (1.22%)	<-10	0.314	0.674	Mutual exclusivity
TXK	4p12	0 (0.00%)	3 (1.22%)	<-10	0.314	0.674	Mutual exclusivity
U2AF2	19q13.42	0 (0.00%)	3 (1.22%)	<-10	0.314	0.674	Mutual exclusivity
UBE2M	19q13.43	0 (0.00%)	3 (1.22%)	<-10	0.314	0.674	Mutual exclusivity
UBE2NL	Xq27.3	0 (0.00%)	3 (1.22%)	<-10	0.314	0.674	Mutual exclusivity
UBE2S	19q13.42	0 (0.00%)	3 (1.22%)	<-10	0.314	0.674	Mutual exclusivity
UBLCP1	5q33.3	0 (0.00%)	3 (1.22%)	<-10	0.314	0.674	Mutual exclusivity
UPK3BL1	7q22.1	0 (0.00%)	3 (1.22%)	<-10	0.314	0.674	Mutual exclusivity
UQCRC2	16p12.2	0 (0.00%)	3 (1.22%)	<-10	0.314	0.674	Mutual exclusivity
USP29	19q13.43	0 (0.00%)	3 (1.22%)	<-10	0.314	0.674	Mutual exclusivity
USP35	11q14.1	0 (0.00%)	3 (1.22%)	<-10	0.314	0.674	Mutual exclusivity
VAMP5	2p11.2	0 (0.00%)	3 (1.22%)	<-10	0.314	0.674	Mutual exclusivity
VAMP8	2p11.2	0 (0.00%)	3 (1.22%)	<-10	0.314	0.674	Mutual exclusivity
VGF	7q22.1	0 (0.00%)	3 (1.22%)	<-10	0.314	0.674	Mutual exclusivity
VIT	2p22.2	0 (0.00%)	3 (1.22%)	<-10	0.314	0.674	Mutual exclusivity
VN1R1	19q13.43	0 (0.00%)	3 (1.22%)	<-10	0.314	0.674	Mutual exclusivity
VPS37C	11q12.2	0 (0.00%)	3 (1.22%)	<-10	0.314	0.674	Mutual exclusivity
VRK3	19q13.33	0 (0.00%)	3 (1.22%)	<-10	0.314	0.674	Mutual exclusivity

VSTM1	19q13.42	0 (0.00%)	3 (1.22%)	<-10	0.314	0.674	Mutual exclusivity
VWA3A	16p12.2	0 (0.00%)	3 (1.22%)	<-10	0.314	0.674	Mutual exclusivity
VWCE	11q12.2	0 (0.00%)	3 (1.22%)	<-10	0.314	0.674	Mutual exclusivity
VWF	12p13.31	0 (0.00%)	3 (1.22%)	<-10	0.314	0.674	Mutual exclusivity
WAPL	10q23.2	0 (0.00%)	3 (1.22%)	<-10	0.314	0.674	Mutual exclusivity
WEE2	7q34	0 (0.00%)	3 (1.22%)	<-10	0.314	0.674	Mutual exclusivity
YJU2	19p13.3	0 (0.00%)	3 (1.22%)	<-10	0.314	0.674	Mutual exclusivity
ZAR1	4p11	0 (0.00%)	3 (1.22%)	<-10	0.314	0.674	Mutual exclusivity
ZBTB45	19q13.43	0 (0.00%)	3 (1.22%)	<-10	0.314	0.674	Mutual exclusivity
ZC3H6	2q14.1	0 (0.00%)	3 (1.22%)	<-10	0.314	0.674	Mutual exclusivity
ZDHHC14	6q25.3	0 (0.00%)	3 (1.22%)	<-10	0.314	0.674	Mutual exclusivity
ZFP28	19q13.43	0 (0.00%)	3 (1.22%)	<-10	0.314	0.674	Mutual exclusivity
ZIK1	19q13.43	0 (0.00%)	3 (1.22%)	<-10	0.314	0.674	Mutual exclusivity
ZIM2	19q13.43	0 (0.00%)	3 (1.22%)	<-10	0.314	0.674	Mutual exclusivity
ZIM3	19q13.43	0 (0.00%)	3 (1.22%)	<-10	0.314	0.674	Mutual exclusivity
ZNF131	5p12	0 (0.00%)	3 (1.22%)	<-10	0.314	0.674	Mutual exclusivity
ZNF132	19q13.43	0 (0.00%)	3 (1.22%)	<-10	0.314	0.674	Mutual exclusivity
ZNF134	19q13.43	0 (0.00%)	3 (1.22%)	<-10	0.314	0.674	Mutual exclusivity
ZNF135	19q13.43	0 (0.00%)	3 (1.22%)	<-10	0.314	0.674	Mutual exclusivity
ZNF154	19q13.43	0 (0.00%)	3 (1.22%)	<-10	0.314	0.674	Mutual exclusivity
ZNF17	19q13.43	0 (0.00%)	3 (1.22%)	<-10	0.314	0.674	Mutual exclusivity
ZNF211	19q13.43	0 (0.00%)	3 (1.22%)	<-10	0.314	0.674	Mutual exclusivity
ZNF239	10q11.21	0 (0.00%)	3 (1.22%)	<-10	0.314	0.674	Mutual exclusivity
ZNF256	19q13.43	0 (0.00%)	3 (1.22%)	<-10	0.314	0.674	Mutual exclusivity
ZNF264	19q13.43	0 (0.00%)	3 (1.22%)	<-10	0.314	0.674	Mutual exclusivity
ZNF274	19q13.43	0 (0.00%)	3 (1.22%)	<-10	0.314	0.674	Mutual exclusivity
ZNF300	5q33.1	0 (0.00%)	3 (1.22%)	<-10	0.314	0.674	Mutual exclusivity
ZNF300P1	5q33.1	0 (0.00%)	3 (1.22%)	<-10	0.314	0.674	Mutual exclusivity
ZNF304	19q13.43	0 (0.00%)	3 (1.22%)	<-10	0.314	0.674	Mutual exclusivity

ZNF32	10q11.21	0 (0.00%)	3 (1.22%)	<-10	0.314	0.674	Mutual exclusivity
ZNF324	19q13.43	0 (0.00%)	3 (1.22%)	<-10	0.314	0.674	Mutual exclusivity
ZNF324B	19q13.43	0 (0.00%)	3 (1.22%)	<-10	0.314	0.674	Mutual exclusivity
ZNF329	19q13.43	0 (0.00%)	3 (1.22%)	<-10	0.314	0.674	Mutual exclusivity
ZNF331	19q13.42	0 (0.00%)	3 (1.22%)	<-10	0.314	0.674	Mutual exclusivity
ZNF37BP	10q11.21	0 (0.00%)	3 (1.22%)	<-10	0.314	0.674	Mutual exclusivity
ZNF416	19q13.43	0 (0.00%)	3 (1.22%)	<-10	0.314	0.674	Mutual exclusivity
ZNF417	19q13.43	0 (0.00%)	3 (1.22%)	<-10	0.314	0.674	Mutual exclusivity
ZNF418	19q13.43	0 (0.00%)	3 (1.22%)	<-10	0.314	0.674	Mutual exclusivity
ZNF419	19q13.43	0 (0.00%)	3 (1.22%)	<-10	0.314	0.674	Mutual exclusivity
ZNF444	19q13.43	0 (0.00%)	3 (1.22%)	<-10	0.314	0.674	Mutual exclusivity
ZNF446	19q13.43	0 (0.00%)	3 (1.22%)	<-10	0.314	0.674	Mutual exclusivity
ZNF460	19q13.43	0 (0.00%)	3 (1.22%)	<-10	0.314	0.674	Mutual exclusivity
ZNF470	19q13.43	0 (0.00%)	3 (1.22%)	<-10	0.314	0.674	Mutual exclusivity
ZNF471	19q13.43	0 (0.00%)	3 (1.22%)	<-10	0.314	0.674	Mutual exclusivity
ZNF473	19q13.33	0 (0.00%)	3 (1.22%)	<-10	0.314	0.674	Mutual exclusivity
ZNF474	5q23.2	0 (0.00%)	3 (1.22%)	<-10	0.314	0.674	Mutual exclusivity
ZNF485	10q11.21	0 (0.00%)	3 (1.22%)	<-10	0.314	0.674	Mutual exclusivity
ZNF497	19q13.43	0 (0.00%)	3 (1.22%)	<-10	0.314	0.674	Mutual exclusivity
ZNF524	19q13.42	0 (0.00%)	3 (1.22%)	<-10	0.314	0.674	Mutual exclusivity
ZNF525	19q13.42	0 (0.00%)	3 (1.22%)	<-10	0.314	0.674	Mutual exclusivity
ZNF528	19q13.41	0 (0.00%)	3 (1.22%)	<-10	0.314	0.674	Mutual exclusivity
ZNF530	19q13.43	0 (0.00%)	3 (1.22%)	<-10	0.314	0.674	Mutual exclusivity
ZNF534	19q13.41	0 (0.00%)	3 (1.22%)	<-10	0.314	0.674	Mutual exclusivity
ZNF542P	19q13.43	0 (0.00%)	3 (1.22%)	<-10	0.314	0.674	Mutual exclusivity
ZNF543	19q13.43	0 (0.00%)	3 (1.22%)	<-10	0.314	0.674	Mutual exclusivity
ZNF544	19q13.43	0 (0.00%)	3 (1.22%)	<-10	0.314	0.674	Mutual exclusivity
ZNF547	19q13.43	0 (0.00%)	3 (1.22%)	<-10	0.314	0.674	Mutual exclusivity
ZNF548	19q13.43	0 (0.00%)	3 (1.22%)	<-10	0.314	0.674	Mutual exclusivity

ZNF549	19q13.43	0 (0.00%)	3 (1.22%)	<-10	0.314	0.674	Mutual exclusivity
ZNF550	19q13.43	0 (0.00%)	3 (1.22%)	<-10	0.314	0.674	Mutual exclusivity
ZNF551	19q13.43	0 (0.00%)	3 (1.22%)	<-10	0.314	0.674	Mutual exclusivity
ZNF552	19q13.43	0 (0.00%)	3 (1.22%)	<-10	0.314	0.674	Mutual exclusivity
ZNF578	19q13.41	0 (0.00%)	3 (1.22%)	<-10	0.314	0.674	Mutual exclusivity
ZNF579	19q13.42	0 (0.00%)	3 (1.22%)	<-10	0.314	0.674	Mutual exclusivity
ZNF580	19q13.42	0 (0.00%)	3 (1.22%)	<-10	0.314	0.674	Mutual exclusivity
ZNF581	19q13.42	0 (0.00%)	3 (1.22%)	<-10	0.314	0.674	Mutual exclusivity
ZNF582	19q13.43	0 (0.00%)	3 (1.22%)	<-10	0.314	0.674	Mutual exclusivity
ZNF583	19q13.43	0 (0.00%)	3 (1.22%)	<-10	0.314	0.674	Mutual exclusivity
ZNF584	19q13.43	0 (0.00%)	3 (1.22%)	<-10	0.314	0.674	Mutual exclusivity
ZNF586	19q13.43	0 (0.00%)	3 (1.22%)	<-10	0.314	0.674	Mutual exclusivity
ZNF587	19q13.43	0 (0.00%)	3 (1.22%)	<-10	0.314	0.674	Mutual exclusivity
ZNF587B	19q13.43	0 (0.00%)	3 (1.22%)	<-10	0.314	0.674	Mutual exclusivity
ZNF606	19q13.43	0 (0.00%)	3 (1.22%)	<-10	0.314	0.674	Mutual exclusivity
ZNF610	19q13.41	0 (0.00%)	3 (1.22%)	<-10	0.314	0.674	Mutual exclusivity
ZNF628	19q13.42	0 (0.00%)	3 (1.22%)	<-10	0.314	0.674	Mutual exclusivity
ZNF667	19q13.43	0 (0.00%)	3 (1.22%)	<-10	0.314	0.674	Mutual exclusivity
ZNF671	19q13.43	0 (0.00%)	3 (1.22%)	<-10	0.314	0.674	Mutual exclusivity
ZNF71	19q13.43	0 (0.00%)	3 (1.22%)	<-10	0.314	0.674	Mutual exclusivity
ZNF724	19p12	0 (0.00%)	3 (1.22%)	<-10	0.314	0.674	Mutual exclusivity
ZNF728	19p12	0 (0.00%)	3 (1.22%)	<-10	0.314	0.674	Mutual exclusivity
ZNF730	19p12	0 (0.00%)	3 (1.22%)	<-10	0.314	0.674	Mutual exclusivity
ZNF749	19q13.43	0 (0.00%)	3 (1.22%)	<-10	0.314	0.674	Mutual exclusivity
ZNF761	19q13.42	0 (0.00%)	3 (1.22%)	<-10	0.314	0.674	Mutual exclusivity
ZNF765	19q13.42	0 (0.00%)	3 (1.22%)	<-10	0.314	0.674	Mutual exclusivity
ZNF772	19q13.43	0 (0.00%)	3 (1.22%)	<-10	0.314	0.674	Mutual exclusivity
ZNF773	19q13.43	0 (0.00%)	3 (1.22%)	<-10	0.314	0.674	Mutual exclusivity
ZNF776	19q13.43	0 (0.00%)	3 (1.22%)	<-10	0.314	0.674	Mutual exclusivity

ZNF784	19q13.42	0 (0.00%)	3 (1.22%)	<-10	0.314	0.674	Mutual exclusivity
ZNF787	19q13.43	0 (0.00%)	3 (1.22%)	<-10	0.314	0.674	Mutual exclusivity
ZNF8	19q13.43	0 (0.00%)	3 (1.22%)	<-10	0.314	0.674	Mutual exclusivity
ZNF805	19q13.43	0 (0.00%)	3 (1.22%)	<-10	0.314	0.674	Mutual exclusivity
ZNF808	19q13.41	0 (0.00%)	3 (1.22%)	<-10	0.314	0.674	Mutual exclusivity
ZNF813	19q13.42	0 (0.00%)	3 (1.22%)	<-10	0.314	0.674	Mutual exclusivity
ZNF814	19q13.43	0 (0.00%)	3 (1.22%)	<-10	0.314	0.674	Mutual exclusivity
ZNF835	19q13.43	0 (0.00%)	3 (1.22%)	<-10	0.314	0.674	Mutual exclusivity
ZNF837	19q13.43	0 (0.00%)	3 (1.22%)	<-10	0.314	0.674	Mutual exclusivity
ZNF845	19q13.42	0 (0.00%)	3 (1.22%)	<-10	0.314	0.674	Mutual exclusivity
ZNF865	19q13.42	0 (0.00%)	3 (1.22%)	<-10	0.314	0.674	Mutual exclusivity
ZNF880	19q13.41	0 (0.00%)	3 (1.22%)	<-10	0.314	0.674	Mutual exclusivity
ZNF91	19p12	0 (0.00%)	3 (1.22%)	<-10	0.314	0.674	Mutual exclusivity
ZNHIT1	7q22.1	0 (0.00%)	3 (1.22%)	<-10	0.314	0.674	Mutual exclusivity
ZP1	11q12.2	0 (0.00%)	3 (1.22%)	<-10	0.314	0.674	Mutual exclusivity
ZSCAN1	19q13.43	0 (0.00%)	3 (1.22%)	<-10	0.314	0.674	Mutual exclusivity
ZSCAN18	19q13.43	0 (0.00%)	3 (1.22%)	<-10	0.314	0.674	Mutual exclusivity
ZSCAN22	19q13.43	0 (0.00%)	3 (1.22%)	<-10	0.314	0.674	Mutual exclusivity
ZSCAN4	19q13.43	0 (0.00%)	3 (1.22%)	<-10	0.314	0.674	Mutual exclusivity
ZSCAN5A	19q13.43	0 (0.00%)	3 (1.22%)	<-10	0.314	0.674	Mutual exclusivity
ZSCAN5B	19q13.43	0 (0.00%)	3 (1.22%)	<-10	0.314	0.674	Mutual exclusivity
ZSCAN5C	19q13.43	0 (0.00%)	3 (1.22%)	<-10	0.314	0.674	Mutual exclusivity
ZSCAN5DP	19q13.43	0 (0.00%)	3 (1.22%)	<-10	0.314	0.674	Mutual exclusivity
ZYX	7q34	0 (0.00%)	3 (1.22%)	<-10	0.314	0.674	Mutual exclusivity
ANKRD40	17q21.33	4 (3.48%)	5 (2.04%)	0.77	0.314	0.674	Co-occurrence
CA10	17q21.33-q22	4 (3.48%)	5 (2.04%)	0.77	0.314	0.674	Co-occurrence
EPN3	17q21.33	4 (3.48%)	5 (2.04%)	0.77	0.314	0.674	Co-occurrence
KPNA7	7q22.1	4 (3.48%)	5 (2.04%)	0.77	0.314	0.674	Co-occurrence
LINC00578	3q26.32	4 (3.48%)	5 (2.04%)	0.77	0.314	0.674	Co-occurrence

MIR-3609/3609		4 (3.48%)	5 (2.04%)	0.77	0.314	0.674	Co-occurrence
MYCBPAP	17q21.33	4 (3.48%)	5 (2.04%)	0.77	0.314	0.674	Co-occurrence
OSBPL6	2q31.2	4 (3.48%)	5 (2.04%)	0.77	0.314	0.674	Co-occurrence
PNPO	17q21.32	4 (3.48%)	5 (2.04%)	0.77	0.314	0.674	Co-occurrence
PRR15L	17q21.32	4 (3.48%)	5 (2.04%)	0.77	0.314	0.674	Co-occurrence
PTGIS	20q13.13	4 (3.48%)	5 (2.04%)	0.77	0.314	0.674	Co-occurrence
SMURF1	7q22.1	4 (3.48%)	5 (2.04%)	0.77	0.314	0.674	Co-occurrence
SPATA20	17q21.33	4 (3.48%)	5 (2.04%)	0.77	0.314	0.674	Co-occurrence
TMEM130	7q22.1	4 (3.48%)	5 (2.04%)	0.77	0.314	0.674	Co-occurrence
TPRG1	3q28	4 (3.48%)	5 (2.04%)	0.77	0.314	0.674	Co-occurrence
TRRAP	7q22.1	4 (3.48%)	5 (2.04%)	0.77	0.314	0.674	Co-occurrence
ADCY8	8q24.22	18 (15.65%)	45 (18.37%)	-0.23	0.318	0.674	Mutual exclusivity
ZFAT	8q24.22	18 (15.65%)	45 (18.37%)	-0.23	0.318	0.674	Mutual exclusivity
BICRAL	6p21.1	4 (3.48%)	13 (5.31%)	-0.61	0.319	0.674	Mutual exclusivity
C6ORF226	6p21.1	4 (3.48%)	13 (5.31%)	-0.61	0.319	0.674	Mutual exclusivity
CNPY3	6p21.1	4 (3.48%)	13 (5.31%)	-0.61	0.319	0.674	Mutual exclusivity
CUL9	6p21.1	4 (3.48%)	13 (5.31%)	-0.61	0.319	0.674	Mutual exclusivity
PTCRA	6p21.1	4 (3.48%)	13 (5.31%)	-0.61	0.319	0.674	Mutual exclusivity
RPL7L1	6p21.1	4 (3.48%)	13 (5.31%)	-0.61	0.319	0.674	Mutual exclusivity
ATP6V0D2	8q21.3	14 (12.17%)	36 (14.69%)	-0.27	0.319	0.674	Mutual exclusivity
RALYL	8q21.2	14 (12.17%)	36 (14.69%)	-0.27	0.319	0.674	Mutual exclusivity
A2MP1	12p13.31	1 (0.87%)	0 (0.00%)	>10	0.319	0.674	Co-occurrence
AACS	12q24.31	1 (0.87%)	0 (0.00%)	>10	0.319	0.674	Co-occurrence
AAR2	20q11.23	1 (0.87%)	0 (0.00%)	>10	0.319	0.674	Co-occurrence
ABCC9	12p12.1	1 (0.87%)	0 (0.00%)	>10	0.319	0.674	Co-occurrence
ABCD2	12q12	1 (0.87%)	0 (0.00%)	>10	0.319	0.674	Co-occurrence
ABHD10	3q13.2	1 (0.87%)	0 (0.00%)	>10	0.319	0.674	Co-occurrence
ABHD17A	19p13.3	1 (0.87%)	0 (0.00%)	>10	0.319	0.674	Co-occurrence
ABR	17p13.3	1 (0.87%)	0 (0.00%)	>10	0.319	0.674	Co-occurrence

ACACB	12q24.11	1 (0.87%)	0 (0.00%)	>10	0.319	0.674	Co-occurrence
ACAD10	12q24.12	1 (0.87%)	0 (0.00%)	>10	0.319	0.674	Co-occurrence
ACADM	1p31.1	1 (0.87%)	0 (0.00%)	>10	0.319	0.674	Co-occurrence
ACADS	12q24.31	1 (0.87%)	0 (0.00%)	>10	0.319	0.674	Co-occurrence
ACAP3	1p36.33	1 (0.87%)	0 (0.00%)	>10	0.319	0.674	Co-occurrence
ACCS	11p11.2	1 (0.87%)	0 (0.00%)	>10	0.319	0.674	Co-occurrence
ACCSL	11p11.2	1 (0.87%)	0 (0.00%)	>10	0.319	0.674	Co-occurrence
ACE2	Xp22.2	1 (0.87%)	0 (0.00%)	>10	0.319	0.674	Co-occurrence
ACO1	9p21.1	1 (0.87%)	0 (0.00%)	>10	0.319	0.674	Co-occurrence
ACOT9	Xp22.11	1 (0.87%)	0 (0.00%)	>10	0.319	0.674	Co-occurrence
ACP2	11p11.2|11p12-p11	1 (0.87%)	0 (0.00%)	>10	0.319	0.674	Co-occurrence
ACRV1	11q24.2	1 (0.87%)	0 (0.00%)	>10	0.319	0.674	Co-occurrence
ACSBG2	19p13.3	1 (0.87%)	0 (0.00%)	>10	0.319	0.674	Co-occurrence
ACSM6	10q23.33	1 (0.87%)	0 (0.00%)	>10	0.319	0.674	Co-occurrence
ACTBP12	1p22.1	1 (0.87%)	0 (0.00%)	>10	0.319	0.674	Co-occurrence
ACTC1	15q14	1 (0.87%)	0 (0.00%)	>10	0.319	0.674	Co-occurrence
ACTRT2	1p36.32	1 (0.87%)	0 (0.00%)	>10	0.319	0.674	Co-occurrence
ACVR1B	12q13.13	1 (0.87%)	0 (0.00%)	>10	0.319	0.674	Co-occurrence
ACVR2B	3p22.2	1 (0.87%)	0 (0.00%)	>10	0.319	0.674	Co-occurrence
ADAM1A	12q24.12-q24.13	1 (0.87%)	0 (0.00%)	>10	0.319	0.674	Co-occurrence
ADAM20P1	14q24.2	1 (0.87%)	0 (0.00%)	>10	0.319	0.674	Co-occurrence
ADAM21	14q24.2	1 (0.87%)	0 (0.00%)	>10	0.319	0.674	Co-occurrence
ADAMTS14	10q22.1	1 (0.87%)	0 (0.00%)	>10	0.319	0.674	Co-occurrence
ADAMTS18	16q23.1	1 (0.87%)	0 (0.00%)	>10	0.319	0.674	Co-occurrence
ADAMTS20	12q12	1 (0.87%)	0 (0.00%)	>10	0.319	0.674	Co-occurrence
ADAMTS3	4q13.3	1 (0.87%)	0 (0.00%)	>10	0.319	0.674	Co-occurrence
ADAT3	19p13.3	1 (0.87%)	0 (0.00%)	>10	0.319	0.674	Co-occurrence
ADD3	10q25.1-q25.2	1 (0.87%)	0 (0.00%)	>10	0.319	0.674	Co-occurrence
ADGRD1	12q24.33	1 (0.87%)	0 (0.00%)	>10	0.319	0.674	Co-occurrence

ADGRD2	9q33.3	1 (0.87%)	0 (0.00%)	>10	0.319	0.674	Co-occurrence
ADGRG2	Xp22.13	1 (0.87%)	0 (0.00%)	>10	0.319	0.674	Co-occurrence
ADGRL2	1p31.1	1 (0.87%)	0 (0.00%)	>10	0.319	0.674	Co-occurrence
ADGRL4	1p31.1	1 (0.87%)	0 (0.00%)	>10	0.319	0.674	Co-occurrence
ADH1B	4q23	1 (0.87%)	0 (0.00%)	>10	0.319	0.674	Co-occurrence
ADPRHL2	1p34.3	1 (0.87%)	0 (0.00%)	>10	0.319	0.674	Co-occurrence
ADSSL1	14q32.33	1 (0.87%)	0 (0.00%)	>10	0.319	0.674	Co-occurrence
AGAP6	10q11.23	1 (0.87%)	0 (0.00%)	>10	0.319	0.674	Co-occurrence
AGAP7P	10q11.22	1 (0.87%)	0 (0.00%)	>10	0.319	0.674	Co-occurrence
AGBL2	11p11.2	1 (0.87%)	0 (0.00%)	>10	0.319	0.674	Co-occurrence
AGBL4	1p33	1 (0.87%)	0 (0.00%)	>10	0.319	0.674	Co-occurrence
AGO1	1p34.3	1 (0.87%)	0 (0.00%)	>10	0.319	0.674	Co-occurrence
AGO3	1p34.3	1 (0.87%)	0 (0.00%)	>10	0.319	0.674	Co-occurrence
AGO4	1p34.3	1 (0.87%)	0 (0.00%)	>10	0.319	0.674	Co-occurrence
AGRN	1p36.33	1 (0.87%)	0 (0.00%)	>10	0.319	0.674	Co-occurrence
AGRP	16q22.1	1 (0.87%)	0 (0.00%)	>10	0.319	0.674	Co-occurrence
AHDC1	1p36.11-p35.3	1 (0.87%)	0 (0.00%)	>10	0.319	0.674	Co-occurrence
AHNAK2	14q32.33	1 (0.87%)	0 (0.00%)	>10	0.319	0.674	Co-occurrence
AIFM2	10q22.1	1 (0.87%)	0 (0.00%)	>10	0.319	0.674	Co-occurrence
AJAP1	1p36.32	1 (0.87%)	0 (0.00%)	>10	0.319	0.674	Co-occurrence
AKAP17A	Xp22.33 and Yp11.2	1 (0.87%)	0 (0.00%)	>10	0.319	0.674	Co-occurrence
AKAP2	9q31.3	1 (0.87%)	0 (0.00%)	>10	0.319	0.674	Co-occurrence
AKAP5	14q23.3	1 (0.87%)	0 (0.00%)	>10	0.319	0.674	Co-occurrence
AKIP1	11p15.4	1 (0.87%)	0 (0.00%)	>10	0.319	0.674	Co-occurrence
AKR1A1	1p34.1	1 (0.87%)	0 (0.00%)	>10	0.319	0.674	Co-occurrence
AKT1	14q32.33	1 (0.87%)	0 (0.00%)	>10	0.319	0.674	Co-occurrence
ALDH1B1	9p13.1	1 (0.87%)	0 (0.00%)	>10	0.319	0.674	Co-occurrence
ALDH2	12q24.12	1 (0.87%)	0 (0.00%)	>10	0.319	0.674	Co-occurrence
ALDH6A1	14q24.3	1 (0.87%)	0 (0.00%)	>10	0.319	0.674	Co-occurrence

ALG10	12p11.1	1 (0.87%)	0 (0.00%)	>10	0.319	0.674	Co-occurrence
ALG10B	12q12	1 (0.87%)	0 (0.00%)	>10	0.319	0.674	Co-occurrence
ALG11	13q14.3	1 (0.87%)	0 (0.00%)	>10	0.319	0.674	Co-occurrence
ALKBH3	11p11.2	1 (0.87%)	0 (0.00%)	>10	0.319	0.674	Co-occurrence
ALKBH6	19q13.12	1 (0.87%)	0 (0.00%)	>10	0.319	0.674	Co-occurrence
ALX4	11p11.2	1 (0.87%)	0 (0.00%)	>10	0.319	0.674	Co-occurrence
AMBN	4q13.3	1 (0.87%)	0 (0.00%)	>10	0.319	0.674	Co-occurrence
AMBRA1	11p11.2	1 (0.87%)	0 (0.00%)	>10	0.319	0.674	Co-occurrence
AMER2	13q12.13	1 (0.87%)	0 (0.00%)	>10	0.319	0.674	Co-occurrence
AMER3	2q21.1	1 (0.87%)	0 (0.00%)	>10	0.319	0.674	Co-occurrence
AMMECR1L	2q14.3	1 (0.87%)	0 (0.00%)	>10	0.319	0.674	Co-occurrence
AMN1	12p11.21	1 (0.87%)	0 (0.00%)	>10	0.319	0.674	Co-occurrence
AMOT	Xq23	1 (0.87%)	0 (0.00%)	>10	0.319	0.674	Co-occurrence
AMTN	4q13.3	1 (0.87%)	0 (0.00%)	>10	0.319	0.674	Co-occurrence
ANAPC16	10q22.1	1 (0.87%)	0 (0.00%)	>10	0.319	0.674	Co-occurrence
ANAPC5	12q24.31	1 (0.87%)	0 (0.00%)	>10	0.319	0.674	Co-occurrence
ANAPC7	12q24.11	1 (0.87%)	0 (0.00%)	>10	0.319	0.674	Co-occurrence
ANK2	4q25-q26	1 (0.87%)	0 (0.00%)	>10	0.319	0.674	Co-occurrence
ANKRD13C	1p31.1	1 (0.87%)	0 (0.00%)	>10	0.319	0.674	Co-occurrence
ANKRD17	4q13.3	1 (0.87%)	0 (0.00%)	>10	0.319	0.674	Co-occurrence
ANKRD18A	9p13.1	1 (0.87%)	0 (0.00%)	>10	0.319	0.674	Co-occurrence
ANKRD20A1	9q21.11	1 (0.87%)	0 (0.00%)	>10	0.319	0.674	Co-occurrence
ANKRD20A19P	13q12.12	1 (0.87%)	0 (0.00%)	>10	0.319	0.674	Co-occurrence
ANKRD20A2	9p11.2	1 (0.87%)	0 (0.00%)	>10	0.319	0.674	Co-occurrence
ANKRD20A3	9q21.11	1 (0.87%)	0 (0.00%)	>10	0.319	0.674	Co-occurrence
ANKRD20A4	9q13	1 (0.87%)	0 (0.00%)	>10	0.319	0.674	Co-occurrence
ANKRD29	18q11.2	1 (0.87%)	0 (0.00%)	>10	0.319	0.674	Co-occurrence
ANKRD50	4q28.1	1 (0.87%)	0 (0.00%)	>10	0.319	0.674	Co-occurrence
ANKRD65	1p36.33	1 (0.87%)	0 (0.00%)	>10	0.319	0.674	Co-occurrence

ANKRD9	14q32.31	1 (0.87%)	0 (0.00%)	>10	0.319	0.674	Co-occurrence
ANKS6	9q22.33	1 (0.87%)	0 (0.00%)	>10	0.319	0.674	Co-occurrence
ANO3	11p14.3-p14.2	1 (0.87%)	0 (0.00%)	>10	0.319	0.674	Co-occurrence
ANOS1	Xp22.31	1 (0.87%)	0 (0.00%)	>10	0.319	0.674	Co-occurrence
ANTXR2	4q21.21	1 (0.87%)	0 (0.00%)	>10	0.319	0.674	Co-occurrence
ANXA5	4q27	1 (0.87%)	0 (0.00%)	>10	0.319	0.674	Co-occurrence
AP1B1	22q12.2	1 (0.87%)	0 (0.00%)	>10	0.319	0.674	Co-occurrence
AP1S2	Xp22.2	1 (0.87%)	0 (0.00%)	>10	0.319	0.674	Co-occurrence
AP3D1	19p13.3	1 (0.87%)	0 (0.00%)	>10	0.319	0.674	Co-occurrence
AP4B1	1p13.2	1 (0.87%)	0 (0.00%)	>10	0.319	0.674	Co-occurrence
APBA3	19p13.3	1 (0.87%)	0 (0.00%)	>10	0.319	0.674	Co-occurrence
API5	11p12	1 (0.87%)	0 (0.00%)	>10	0.319	0.674	Co-occurrence
APLP1	19q13.12	1 (0.87%)	0 (0.00%)	>10	0.319	0.674	Co-occurrence
AQP4	18q11.2	1 (0.87%)	0 (0.00%)	>10	0.319	0.674	Co-occurrence
AQP7	9p13.3	1 (0.87%)	0 (0.00%)	>10	0.319	0.674	Co-occurrence
ARAF	Xp11.3	1 (0.87%)	0 (0.00%)	>10	0.319	0.674	Co-occurrence
AREG	4q13.3	1 (0.87%)	0 (0.00%)	>10	0.319	0.674	Co-occurrence
ARFGAP2	11p11.2	1 (0.87%)	0 (0.00%)	>10	0.319	0.674	Co-occurrence
ARFIP2	11p15.4	1 (0.87%)	0 (0.00%)	>10	0.319	0.674	Co-occurrence
ARHGAP1	11p11.2	1 (0.87%)	0 (0.00%)	>10	0.319	0.674	Co-occurrence
ARHGEF16	1p36.32	1 (0.87%)	0 (0.00%)	>10	0.319	0.674	Co-occurrence
ARHGEF4	2q21.1	1 (0.87%)	0 (0.00%)	>10	0.319	0.674	Co-occurrence
ARID3A	19p13.3	1 (0.87%)	0 (0.00%)	>10	0.319	0.674	Co-occurrence
ARL13B	3q11.1-q11.2	1 (0.87%)	0 (0.00%)	>10	0.319	0.674	Co-occurrence
ARL5A	2q23.3	1 (0.87%)	0 (0.00%)	>10	0.319	0.674	Co-occurrence
ARL6IP4	12q24.31	1 (0.87%)	0 (0.00%)	>10	0.319	0.674	Co-occurrence
ARL6IP6	2q23.3	1 (0.87%)	0 (0.00%)	>10	0.319	0.674	Co-occurrence
ARL8B	3p26.1	1 (0.87%)	0 (0.00%)	>10	0.319	0.674	Co-occurrence
ARNTL	11p15.3	1 (0.87%)	0 (0.00%)	>10	0.319	0.674	Co-occurrence

ARPC3	12q24.11	1 (0.87%)	0 (0.00%)	>10	0.319	0.674	Co-occurrence
ARPC5L	9q33.3	1 (0.87%)	0 (0.00%)	>10	0.319	0.674	Co-occurrence
ARPP19	15q21.2	1 (0.87%)	0 (0.00%)	>10	0.319	0.674	Co-occurrence
ARSD	Xp22.33	1 (0.87%)	0 (0.00%)	>10	0.319	0.674	Co-occurrence
ARSE	Xp22.33	1 (0.87%)	0 (0.00%)	>10	0.319	0.674	Co-occurrence
ARSF	Xp22.33	1 (0.87%)	0 (0.00%)	>10	0.319	0.674	Co-occurrence
ARSH	Xp22.33	1 (0.87%)	0 (0.00%)	>10	0.319	0.674	Co-occurrence
ARSJ	4q26	1 (0.87%)	0 (0.00%)	>10	0.319	0.674	Co-occurrence
ART3	4q21.1|4p15.1-p14	1 (0.87%)	0 (0.00%)	>10	0.319	0.674	Co-occurrence
ARTN	1p34.1	1 (0.87%)	0 (0.00%)	>10	0.319	0.674	Co-occurrence
ARX	Xp21.3	1 (0.87%)	0 (0.00%)	>10	0.319	0.674	Co-occurrence
ASAH2	10q11.23	1 (0.87%)	0 (0.00%)	>10	0.319	0.674	Co-occurrence
ASB11	Xp22.2	1 (0.87%)	0 (0.00%)	>10	0.319	0.674	Co-occurrence
ASB17	1p31.1	1 (0.87%)	0 (0.00%)	>10	0.319	0.674	Co-occurrence
ASB9	Xp22.2	1 (0.87%)	0 (0.00%)	>10	0.319	0.674	Co-occurrence
ASCC1	10q22.1	1 (0.87%)	0 (0.00%)	>10	0.319	0.674	Co-occurrence
ASCC3	6q16.3	1 (0.87%)	0 (0.00%)	>10	0.319	0.674	Co-occurrence
ASCL3	11p15.4	1 (0.87%)	0 (0.00%)	>10	0.319	0.674	Co-occurrence
ASCL4	12q23.3	1 (0.87%)	0 (0.00%)	>10	0.319	0.674	Co-occurrence
ASMT	Xp22.33 and Yp11.2	1 (0.87%)	0 (0.00%)	>10	0.319	0.674	Co-occurrence
ASMTL	Xp22.33 and Yp11.2	1 (0.87%)	0 (0.00%)	>10	0.319	0.674	Co-occurrence
ASTN2	9q33.1	1 (0.87%)	0 (0.00%)	>10	0.319	0.674	Co-occurrence
ATAD3A	1p36.33	1 (0.87%)	0 (0.00%)	>10	0.319	0.674	Co-occurrence
ATAD3B	1p36.33	1 (0.87%)	0 (0.00%)	>10	0.319	0.674	Co-occurrence
ATAD3C	1p36.33	1 (0.87%)	0 (0.00%)	>10	0.319	0.674	Co-occurrence
ATCAY	19p13.3	1 (0.87%)	0 (0.00%)	>10	0.319	0.674	Co-occurrence
ATG101	12q13.13	1 (0.87%)	0 (0.00%)	>10	0.319	0.674	Co-occurrence
ATG13	11p11.2	1 (0.87%)	0 (0.00%)	>10	0.319	0.674	Co-occurrence
ATG3	3q13.2	1 (0.87%)	0 (0.00%)	>10	0.319	0.674	Co-occurrence

ATL3	11q13.1	1 (0.87%)	0 (0.00%)	>10	0.319	0.674	Co-occurrence
ATOH1	4q22.2	1 (0.87%)	0 (0.00%)	>10	0.319	0.674	Co-occurrence
ATP12A	13q12.12|13q12.1-q12.3	1 (0.87%)	0 (0.00%)	>10	0.319	0.674	Co-occurrence
ATP2A2	12q24.11	1 (0.87%)	0 (0.00%)	>10	0.319	0.674	Co-occurrence
ATP6AP2	Xp11.4	1 (0.87%)	0 (0.00%)	>10	0.319	0.674	Co-occurrence
ATP6V0B	1p34.1	1 (0.87%)	0 (0.00%)	>10	0.319	0.674	Co-occurrence
ATP6V0D1	16q22.1	1 (0.87%)	0 (0.00%)	>10	0.319	0.674	Co-occurrence
ATP6V1E1	22q11.21	1 (0.87%)	0 (0.00%)	>10	0.319	0.674	Co-occurrence
ATP6V1G1	9q32	1 (0.87%)	0 (0.00%)	>10	0.319	0.674	Co-occurrence
ATP7A	Xq21.1	1 (0.87%)	0 (0.00%)	>10	0.319	0.674	Co-occurrence
ATP7B	13q14.3	1 (0.87%)	0 (0.00%)	>10	0.319	0.674	Co-occurrence
ATP8A2	13q12.13	1 (0.87%)	0 (0.00%)	>10	0.319	0.674	Co-occurrence
ATP8B3	19p13.3	1 (0.87%)	0 (0.00%)	>10	0.319	0.674	Co-occurrence
ATRX	Xq21.1	1 (0.87%)	0 (0.00%)	>10	0.319	0.674	Co-occurrence
ATXN2	12q24.12	1 (0.87%)	0 (0.00%)	>10	0.319	0.674	Co-occurrence
AURKAIP1	1p36.33	1 (0.87%)	0 (0.00%)	>10	0.319	0.674	Co-occurrence
AZI2	3p24.1	1 (0.87%)	0 (0.00%)	>10	0.319	0.674	Co-occurrence
B3GALT6	1p36.33	1 (0.87%)	0 (0.00%)	>10	0.319	0.674	Co-occurrence
B3GNT4	12q24.31	1 (0.87%)	0 (0.00%)	>10	0.319	0.674	Co-occurrence
B3GNT9	16q22.1	1 (0.87%)	0 (0.00%)	>10	0.319	0.674	Co-occurrence
B4GALT2	1p34.1	1 (0.87%)	0 (0.00%)	>10	0.319	0.674	Co-occurrence
B4GALT6	18q12.1	1 (0.87%)	0 (0.00%)	>10	0.319	0.674	Co-occurrence
BAG5	14q32.33	1 (0.87%)	0 (0.00%)	>10	0.319	0.674	Co-occurrence
BANK1	4q24	1 (0.87%)	0 (0.00%)	>10	0.319	0.674	Co-occurrence
BARHL2	1p22.2	1 (0.87%)	0 (0.00%)	>10	0.319	0.674	Co-occurrence
BBOF1	14q24.3	1 (0.87%)	0 (0.00%)	>10	0.319	0.674	Co-occurrence
BBOX1	11p14.2	1 (0.87%)	0 (0.00%)	>10	0.319	0.674	Co-occurrence
BBS7	4q27	1 (0.87%)	0 (0.00%)	>10	0.319	0.674	Co-occurrence
BCAR3	1p22.1	1 (0.87%)	0 (0.00%)	>10	0.319	0.674	Co-occurrence

BCL10	1p22.3	1 (0.87%)	0 (0.00%)	>10	0.319	0.674	Co-occurrence
BCL2L10	15q21.2	1 (0.87%)	0 (0.00%)	>10	0.319	0.674	Co-occurrence
BCL2L15	1p13.2	1 (0.87%)	0 (0.00%)	>10	0.319	0.674	Co-occurrence
BDNF	11p14.1	1 (0.87%)	0 (0.00%)	>10	0.319	0.674	Co-occurrence
BEND2	Xp22.13	1 (0.87%)	0 (0.00%)	>10	0.319	0.674	Co-occurrence
BEND3	6q21	1 (0.87%)	0 (0.00%)	>10	0.319	0.674	Co-occurrence
BEND5	1p33	1 (0.87%)	0 (0.00%)	>10	0.319	0.674	Co-occurrence
BEST4	1p34.1	1 (0.87%)	0 (0.00%)	>10	0.319	0.674	Co-occurrence
BHLHA9	17p13.3	1 (0.87%)	0 (0.00%)	>10	0.319	0.674	Co-occurrence
BHLHE40	3p26.1	1 (0.87%)	0 (0.00%)	>10	0.319	0.674	Co-occurrence
BICD1	12p11.21	1 (0.87%)	0 (0.00%)	>10	0.319	0.674	Co-occurrence
BICDL1	12q24.23	1 (0.87%)	0 (0.00%)	>10	0.319	0.674	Co-occurrence
BIN1	2q14.3	1 (0.87%)	0 (0.00%)	>10	0.319	0.674	Co-occurrence
BLOC1S6	15q21.1	1 (0.87%)	0 (0.00%)	>10	0.319	0.674	Co-occurrence
BMP1	8p21.3	1 (0.87%)	0 (0.00%)	>10	0.319	0.674	Co-occurrence
BMP3	4q21.21	1 (0.87%)	0 (0.00%)	>10	0.319	0.674	Co-occurrence
BMX	Xp22.2	1 (0.87%)	0 (0.00%)	>10	0.319	0.674	Co-occurrence
BRAP	12q24.12	1 (0.87%)	0 (0.00%)	>10	0.319	0.674	Co-occurrence
BRDT	1p22.1	1 (0.87%)	0 (0.00%)	>10	0.319	0.674	Co-occurrence
BRI3BP	12q24.31	1 (0.87%)	0 (0.00%)	>10	0.319	0.674	Co-occurrence
BRINP1	9q33.1	1 (0.87%)	0 (0.00%)	>10	0.319	0.674	Co-occurrence
BRSK2	11p15.5	1 (0.87%)	0 (0.00%)	>10	0.319	0.674	Co-occurrence
BSDC1	1p35.1	1 (0.87%)	0 (0.00%)	>10	0.319	0.674	Co-occurrence
BSG	19p13.3	1 (0.87%)	0 (0.00%)	>10	0.319	0.674	Co-occurrence
BSND	1p32.3	1 (0.87%)	0 (0.00%)	>10	0.319	0.674	Co-occurrence
BTBD10	11p15.3	1 (0.87%)	0 (0.00%)	>10	0.319	0.674	Co-occurrence
BTBD11	12q23.3	1 (0.87%)	0 (0.00%)	>10	0.319	0.674	Co-occurrence
BTBD19	1p34.1	1 (0.87%)	0 (0.00%)	>10	0.319	0.674	Co-occurrence
BTBD2	19p13.3	1 (0.87%)	0 (0.00%)	>10	0.319	0.674	Co-occurrence

BTBD7	14q32.12	1 (0.87%)	0 (0.00%)	>10	0.319	0.674	Co-occurrence
BTBD8	1p22.1	1 (0.87%)	0 (0.00%)	>10	0.319	0.674	Co-occurrence
BTC	4q13.3	1 (0.87%)	0 (0.00%)	>10	0.319	0.674	Co-occurrence
BTF3L4	1p32.3	1 (0.87%)	0 (0.00%)	>10	0.319	0.674	Co-occurrence
BTLA	3q13.2	1 (0.87%)	0 (0.00%)	>10	0.319	0.674	Co-occurrence
BUB1B	15q15.1	1 (0.87%)	0 (0.00%)	>10	0.319	0.674	Co-occurrence
C10ORF105	10q22.1	1 (0.87%)	0 (0.00%)	>10	0.319	0.674	Co-occurrence
C10ORF91	10q26.3	1 (0.87%)	0 (0.00%)	>10	0.319	0.674	Co-occurrence
C11ORF16	11p15.4	1 (0.87%)	0 (0.00%)	>10	0.319	0.674	Co-occurrence
C11ORF40	11p15.4	1 (0.87%)	0 (0.00%)	>10	0.319	0.674	Co-occurrence
C11ORF49	11p11.2	1 (0.87%)	0 (0.00%)	>10	0.319	0.674	Co-occurrence
C11ORF58	11p15.2	1 (0.87%)	0 (0.00%)	>10	0.319	0.674	Co-occurrence
C11ORF94	11p11.2	1 (0.87%)	0 (0.00%)	>10	0.319	0.674	Co-occurrence
C11ORF96	11p11.2	1 (0.87%)	0 (0.00%)	>10	0.319	0.674	Co-occurrence
C12ORF43	12q24.31	1 (0.87%)	0 (0.00%)	>10	0.319	0.674	Co-occurrence
C13ORF42	13q14.3	1 (0.87%)	0 (0.00%)	>10	0.319	0.674	Co-occurrence
C15ORF48	15q21.1	1 (0.87%)	0 (0.00%)	>10	0.319	0.674	Co-occurrence
C16ORF70	16q22.1	1 (0.87%)	0 (0.00%)	>10	0.319	0.674	Co-occurrence
C16ORF95	16q24.2	1 (0.87%)	0 (0.00%)	>10	0.319	0.674	Co-occurrence
C17ORF97	17p13.3	1 (0.87%)	0 (0.00%)	>10	0.319	0.674	Co-occurrence
C19ORF33	19q13.2	1 (0.87%)	0 (0.00%)	>10	0.319	0.674	Co-occurrence
C19ORF71	19p13.3	1 (0.87%)	0 (0.00%)	>10	0.319	0.674	Co-occurrence
C1ORF109	1p34.3	1 (0.87%)	0 (0.00%)	>10	0.319	0.674	Co-occurrence
C1ORF122	1p34.3	1 (0.87%)	0 (0.00%)	>10	0.319	0.674	Co-occurrence
C1ORF146	1p22.1	1 (0.87%)	0 (0.00%)	>10	0.319	0.674	Co-occurrence
C1ORF159	1p36.33	1 (0.87%)	0 (0.00%)	>10	0.319	0.674	Co-occurrence
C1ORF174	1p36.32	1 (0.87%)	0 (0.00%)	>10	0.319	0.674	Co-occurrence
C1ORF216	1p34.3	1 (0.87%)	0 (0.00%)	>10	0.319	0.674	Co-occurrence
C1ORF52	1p22.3	1 (0.87%)	0 (0.00%)	>10	0.319	0.674	Co-occurrence

C1ORF87	1p32.1	1 (0.87%)	0 (0.00%)	>10	0.319	0.674	Co-occurrence
C1QTNF12	1p36.33	1 (0.87%)	0 (0.00%)	>10	0.319	0.674	Co-occurrence
C1QTNF4	11p11.2	1 (0.87%)	0 (0.00%)	>10	0.319	0.674	Co-occurrence
C1QTNF9	13q12.12	1 (0.87%)	0 (0.00%)	>10	0.319	0.674	Co-occurrence
C1QTNF9B	13q12.12	1 (0.87%)	0 (0.00%)	>10	0.319	0.674	Co-occurrence
C22ORF31	22q12.1	1 (0.87%)	0 (0.00%)	>10	0.319	0.674	Co-occurrence
C22ORF46	22q13.2	1 (0.87%)	0 (0.00%)	>10	0.319	0.674	Co-occurrence
C2CD5	12p12.1	1 (0.87%)	0 (0.00%)	>10	0.319	0.674	Co-occurrence
C3ORF52	3q13.2	1 (0.87%)	0 (0.00%)	>10	0.319	0.674	Co-occurrence
C5	9q33.2	1 (0.87%)	0 (0.00%)	>10	0.319	0.674	Co-occurrence
C9ORF131	9p13.3	1 (0.87%)	0 (0.00%)	>10	0.319	0.674	Co-occurrence
C9ORF147	9q32	1 (0.87%)	0 (0.00%)	>10	0.319	0.674	Co-occurrence
C9ORF152	9q31.3	1 (0.87%)	0 (0.00%)	>10	0.319	0.674	Co-occurrence
CA5B	Xp22.2	1 (0.87%)	0 (0.00%)	>10	0.319	0.674	Co-occurrence
CA5BP1	Xp22.2	1 (0.87%)	0 (0.00%)	>10	0.319	0.674	Co-occurrence
CABLES1	18q11.2	1 (0.87%)	0 (0.00%)	>10	0.319	0.674	Co-occurrence
CABP7	22q12.2	1 (0.87%)	0 (0.00%)	>10	0.319	0.674	Co-occurrence
CABS1	4q13.3	1 (0.87%)	0 (0.00%)	>10	0.319	0.674	Co-occurrence
CABYR	18q11.2	1 (0.87%)	0 (0.00%)	>10	0.319	0.674	Co-occurrence
CACNB4	2q23.3	1 (0.87%)	0 (0.00%)	>10	0.319	0.674	Co-occurrence
CACTIN	19p13.3	1 (0.87%)	0 (0.00%)	>10	0.319	0.674	Co-occurrence
CALCA	11p15.2	1 (0.87%)	0 (0.00%)	>10	0.319	0.674	Co-occurrence
CALCB	11p15.2	1 (0.87%)	0 (0.00%)	>10	0.319	0.674	Co-occurrence
CALML6	1p36.33	1 (0.87%)	0 (0.00%)	>10	0.319	0.674	Co-occurrence
CAMK2D	4q26	1 (0.87%)	0 (0.00%)	>10	0.319	0.674	Co-occurrence
CAMKK2	12q24.31	1 (0.87%)	0 (0.00%)	>10	0.319	0.674	Co-occurrence
CAMP	3p21.31	1 (0.87%)	0 (0.00%)	>10	0.319	0.674	Co-occurrence
CAMTA1	1p36.31-p36.23	1 (0.87%)	0 (0.00%)	>10	0.319	0.674	Co-occurrence
CAPNS1	19q13.12	1 (0.87%)	0 (0.00%)	>10	0.319	0.674	Co-occurrence

CAPRIN2	12p11.21	1 (0.87%)	0 (0.00%)	>10	0.319	0.674	Co-occurrence
CASC4	15q15.3	1 (0.87%)	0 (0.00%)	>10	0.319	0.674	Co-occurrence
CASP7	10q25.3	1 (0.87%)	0 (0.00%)	>10	0.319	0.674	Co-occurrence
CASTOR1	22q12.2	1 (0.87%)	0 (0.00%)	>10	0.319	0.674	Co-occurrence
CATSPERG	19q13.2	1 (0.87%)	0 (0.00%)	>10	0.319	0.674	Co-occurrence
CBFB	16q22.1	1 (0.87%)	0 (0.00%)	>10	0.319	0.674	Co-occurrence
CBWD3	9q21.11	1 (0.87%)	0 (0.00%)	>10	0.319	0.674	Co-occurrence
CBWD5	9q21.11	1 (0.87%)	0 (0.00%)	>10	0.319	0.674	Co-occurrence
CBWD6	9p11.2	1 (0.87%)	0 (0.00%)	>10	0.319	0.674	Co-occurrence
CBWD7	9p12	1 (0.87%)	0 (0.00%)	>10	0.319	0.674	Co-occurrence
CC2D1B	1p32.3	1 (0.87%)	0 (0.00%)	>10	0.319	0.674	Co-occurrence
CCDC115	2q21.1	1 (0.87%)	0 (0.00%)	>10	0.319	0.674	Co-occurrence
CCDC117	22q12.1	1 (0.87%)	0 (0.00%)	>10	0.319	0.674	Co-occurrence
CCDC12	3p21.31	1 (0.87%)	0 (0.00%)	>10	0.319	0.674	Co-occurrence
CCDC134	22q13.2	1 (0.87%)	0 (0.00%)	>10	0.319	0.674	Co-occurrence
CCDC157	22q12.2	1 (0.87%)	0 (0.00%)	>10	0.319	0.674	Co-occurrence
CCDC163	1p34.1	1 (0.87%)	0 (0.00%)	>10	0.319	0.674	Co-occurrence
CCDC17	1p34.1	1 (0.87%)	0 (0.00%)	>10	0.319	0.674	Co-occurrence
CCDC178	18q12.1	1 (0.87%)	0 (0.00%)	>10	0.319	0.674	Co-occurrence
CCDC179	11p14.3	1 (0.87%)	0 (0.00%)	>10	0.319	0.674	Co-occurrence
CCDC18	1p22.1	1 (0.87%)	0 (0.00%)	>10	0.319	0.674	Co-occurrence
CCDC191	3q13.31	1 (0.87%)	0 (0.00%)	>10	0.319	0.674	Co-occurrence
CCDC24	1p34.1	1 (0.87%)	0 (0.00%)	>10	0.319	0.674	Co-occurrence
CCDC27	1p36.32	1 (0.87%)	0 (0.00%)	>10	0.319	0.674	Co-occurrence
CCDC28B	1p35.2	1 (0.87%)	0 (0.00%)	>10	0.319	0.674	Co-occurrence
CCDC34	11p14.1	1 (0.87%)	0 (0.00%)	>10	0.319	0.674	Co-occurrence
CCDC60	12q24.23	1 (0.87%)	0 (0.00%)	>10	0.319	0.674	Co-occurrence
CCDC63	12q24.11	1 (0.87%)	0 (0.00%)	>10	0.319	0.674	Co-occurrence
CCDC70	13q14.3	1 (0.87%)	0 (0.00%)	>10	0.319	0.674	Co-occurrence

CCDC74B	2q21.1	1 (0.87%)	0 (0.00%)	>10	0.319	0.674	Co-occurrence
CCDC80	3q13.2	1 (0.87%)	0 (0.00%)	>10	0.319	0.674	Co-occurrence
CCK	3p22.1	1 (0.87%)	0 (0.00%)	>10	0.319	0.674	Co-occurrence
CCL21	9p13.3	1 (0.87%)	0 (0.00%)	>10	0.319	0.674	Co-occurrence
CCN1	1p22.3	1 (0.87%)	0 (0.00%)	>10	0.319	0.674	Co-occurrence
CCNA1	13q13.3	1 (0.87%)	0 (0.00%)	>10	0.319	0.674	Co-occurrence
CCNC	6q16.2	1 (0.87%)	0 (0.00%)	>10	0.319	0.674	Co-occurrence
CCNI	4q21.1	1 (0.87%)	0 (0.00%)	>10	0.319	0.674	Co-occurrence
CCNL2	1p36.33	1 (0.87%)	0 (0.00%)	>10	0.319	0.674	Co-occurrence
CCR1	3p21.31	1 (0.87%)	0 (0.00%)	>10	0.319	0.674	Co-occurrence
CCR2	3p21.31	1 (0.87%)	0 (0.00%)	>10	0.319	0.674	Co-occurrence
CCR3	3p21.31	1 (0.87%)	0 (0.00%)	>10	0.319	0.674	Co-occurrence
CCR4	3p22.3	1 (0.87%)	0 (0.00%)	>10	0.319	0.674	Co-occurrence
CCR5	3p21.31	1 (0.87%)	0 (0.00%)	>10	0.319	0.674	Co-occurrence
CCR8	3p22.1	1 (0.87%)	0 (0.00%)	>10	0.319	0.674	Co-occurrence
CCRL2	3p21.31	1 (0.87%)	0 (0.00%)	>10	0.319	0.674	Co-occurrence
CD101	1p13.1	1 (0.87%)	0 (0.00%)	>10	0.319	0.674	Co-occurrence
CD164L2	1p36.11	1 (0.87%)	0 (0.00%)	>10	0.319	0.674	Co-occurrence
CD180	5q12.3	1 (0.87%)	0 (0.00%)	>10	0.319	0.674	Co-occurrence
CD200	3q13.2	1 (0.87%)	0 (0.00%)	>10	0.319	0.674	Co-occurrence
CD200R1	3q13.2	1 (0.87%)	0 (0.00%)	>10	0.319	0.674	Co-occurrence
CD200R1L	3q13.2	1 (0.87%)	0 (0.00%)	>10	0.319	0.674	Co-occurrence
CD320	19p13.2	1 (0.87%)	0 (0.00%)	>10	0.319	0.674	Co-occurrence
CD44	11p13	1 (0.87%)	0 (0.00%)	>10	0.319	0.674	Co-occurrence
CD69	12p13.31	1 (0.87%)	0 (0.00%)	>10	0.319	0.674	Co-occurrence
CD82	11p11.2	1 (0.87%)	0 (0.00%)	>10	0.319	0.674	Co-occurrence
CD86	3q13.33	1 (0.87%)	0 (0.00%)	>10	0.319	0.674	Co-occurrence
CD96	3q13.13-q13.2	1 (0.87%)	0 (0.00%)	>10	0.319	0.674	Co-occurrence
CD99	Xp22.33 and Yp11.2	1 (0.87%)	0 (0.00%)	>10	0.319	0.674	Co-occurrence

CD99P1	Xp22.33 and Yp11.2	1 (0.87%)	0 (0.00%)	>10	0.319	0.674	Co-occurrence
CDC25A	3p21.31	1 (0.87%)	0 (0.00%)	>10	0.319	0.674	Co-occurrence
CDC34	19p13.3	1 (0.87%)	0 (0.00%)	>10	0.319	0.674	Co-occurrence
CDCA4	14q32.33	1 (0.87%)	0 (0.00%)	>10	0.319	0.674	Co-occurrence
CDCA8	1p34.3	1 (0.87%)	0 (0.00%)	>10	0.319	0.674	Co-occurrence
CDCP1	3p21.31	1 (0.87%)	0 (0.00%)	>10	0.319	0.674	Co-occurrence
CDCP2	1p32.3	1 (0.87%)	0 (0.00%)	>10	0.319	0.674	Co-occurrence
CDH2	18q12.1	1 (0.87%)	0 (0.00%)	>10	0.319	0.674	Co-occurrence
CDK11A	1p36.33	1 (0.87%)	0 (0.00%)	>10	0.319	0.674	Co-occurrence
CDK11B	1p36.33	1 (0.87%)	0 (0.00%)	>10	0.319	0.674	Co-occurrence
CDK16	Xp11.3	1 (0.87%)	0 (0.00%)	>10	0.319	0.674	Co-occurrence
CDK8	13q12.13	1 (0.87%)	0 (0.00%)	>10	0.319	0.674	Co-occurrence
CDKL2	4q21.1	1 (0.87%)	0 (0.00%)	>10	0.319	0.674	Co-occurrence
CDON	11q24.2	1 (0.87%)	0 (0.00%)	>10	0.319	0.674	Co-occurrence
CECR2	22q11.1-q11.21	1 (0.87%)	0 (0.00%)	>10	0.319	0.674	Co-occurrence
CELF1	11p11.2	1 (0.87%)	0 (0.00%)	>10	0.319	0.674	Co-occurrence
CELSR3	3p21.31	1 (0.87%)	0 (0.00%)	>10	0.319	0.674	Co-occurrence
CENPC	4q13.2	1 (0.87%)	0 (0.00%)	>10	0.319	0.674	Co-occurrence
CENPJ	13q12.12-q12.13	1 (0.87%)	0 (0.00%)	>10	0.319	0.674	Co-occurrence
CENPK	5q12.3	1 (0.87%)	0 (0.00%)	>10	0.319	0.674	Co-occurrence
CENPM	22q13.2	1 (0.87%)	0 (0.00%)	>10	0.319	0.674	Co-occurrence
CEP104	1p36.32	1 (0.87%)	0 (0.00%)	>10	0.319	0.674	Co-occurrence
CEP170B	14q32.33	1 (0.87%)	0 (0.00%)	>10	0.319	0.674	Co-occurrence
CEP44	4q34.1	1 (0.87%)	0 (0.00%)	>10	0.319	0.674	Co-occurrence
CEP78	9q21.2	1 (0.87%)	0 (0.00%)	>10	0.319	0.674	Co-occurrence
CFAP53	18q21.1	1 (0.87%)	0 (0.00%)	>10	0.319	0.674	Co-occurrence
CFAP73	12q24.13	1 (0.87%)	0 (0.00%)	>10	0.319	0.674	Co-occurrence
CFAP74	1p36.33	1 (0.87%)	0 (0.00%)	>10	0.319	0.674	Co-occurrence
CFC1	2q21.1	1 (0.87%)	0 (0.00%)	>10	0.319	0.674	Co-occurrence

CFC1B	2q21.1	1 (0.87%)	0 (0.00%)	>10	0.319	0.674	Co-occurrence
CFD	19p13.3	1 (0.87%)	0 (0.00%)	>10	0.319	0.674	Co-occurrence
CFP	Xp11.23	1 (0.87%)	0 (0.00%)	>10	0.319	0.674	Co-occurrence
CHAC1	15q15.1	1 (0.87%)	0 (0.00%)	>10	0.319	0.674	Co-occurrence
CHD5	1p36.31	1 (0.87%)	0 (0.00%)	>10	0.319	0.674	Co-occurrence
CHD6	20q12	1 (0.87%)	0 (0.00%)	>10	0.319	0.674	Co-occurrence
CHEK1	11q24.2	1 (0.87%)	0 (0.00%)	>10	0.319	0.674	Co-occurrence
CHEK2	22q12.1	1 (0.87%)	0 (0.00%)	>10	0.319	0.674	Co-occurrence
CHEK2P2	15q11.1	1 (0.87%)	0 (0.00%)	>10	0.319	0.674	Co-occurrence
CHGA	14q32.12	1 (0.87%)	0 (0.00%)	>10	0.319	0.674	Co-occurrence
CHGB	20p12.3	1 (0.87%)	0 (0.00%)	>10	0.319	0.674	Co-occurrence
CHL1	3p26.3	1 (0.87%)	0 (0.00%)	>10	0.319	0.674	Co-occurrence
CHM	Xq21.2	1 (0.87%)	0 (0.00%)	>10	0.319	0.674	Co-occurrence
CHRM4	11p11.2	1 (0.87%)	0 (0.00%)	>10	0.319	0.674	Co-occurrence
CHRNA2	8p21.2	1 (0.87%)	0 (0.00%)	>10	0.319	0.674	Co-occurrence
CHST1	11p11.2	1 (0.87%)	0 (0.00%)	>10	0.319	0.674	Co-occurrence
CHST3	10q22.1	1 (0.87%)	0 (0.00%)	>10	0.319	0.674	Co-occurrence
CHST9	18q11.2	1 (0.87%)	0 (0.00%)	>10	0.319	0.674	Co-occurrence
CIT	12q24.23	1 (0.87%)	0 (0.00%)	>10	0.319	0.674	Co-occurrence
CKAP2	13q14.3	1 (0.87%)	0 (0.00%)	>10	0.319	0.674	Co-occurrence
CKAP5	11p11.2	1 (0.87%)	0 (0.00%)	>10	0.319	0.674	Co-occurrence
CKB	14q32.33	1 (0.87%)	0 (0.00%)	>10	0.319	0.674	Co-occurrence
CLASP2	3p22.3	1 (0.87%)	0 (0.00%)	>10	0.319	0.674	Co-occurrence
CLBA1	14q32.33	1 (0.87%)	0 (0.00%)	>10	0.319	0.674	Co-occurrence
CLCA1	1p22.3	1 (0.87%)	0 (0.00%)	>10	0.319	0.674	Co-occurrence
CLCA2	1p22.3	1 (0.87%)	0 (0.00%)	>10	0.319	0.674	Co-occurrence
CLCA4	1p22.3	1 (0.87%)	0 (0.00%)	>10	0.319	0.674	Co-occurrence
CLDN14	21q22.13	1 (0.87%)	0 (0.00%)	>10	0.319	0.674	Co-occurrence
CLDN2	Xq22.3	1 (0.87%)	0 (0.00%)	>10	0.319	0.674	Co-occurrence

CLEC12A	12p13.31	1 (0.87%)	0 (0.00%)	>10	0.319	0.674	Co-occurrence
CLEC12B	12p13.2	1 (0.87%)	0 (0.00%)	>10	0.319	0.674	Co-occurrence
CLEC1A	12p13.2	1 (0.87%)	0 (0.00%)	>10	0.319	0.674	Co-occurrence
CLEC1B	12p13.31-p13.2	1 (0.87%)	0 (0.00%)	>10	0.319	0.674	Co-occurrence
CLEC2A	12p13.31	1 (0.87%)	0 (0.00%)	>10	0.319	0.674	Co-occurrence
CLEC2B	12p13.31	1 (0.87%)	0 (0.00%)	>10	0.319	0.674	Co-occurrence
CLEC2D	12p13.31	1 (0.87%)	0 (0.00%)	>10	0.319	0.674	Co-occurrence
CLEC3B	3p21.31	1 (0.87%)	0 (0.00%)	>10	0.319	0.674	Co-occurrence
CLEC7A	12p13.2	1 (0.87%)	0 (0.00%)	>10	0.319	0.674	Co-occurrence
CLEC9A	12p13.2	1 (0.87%)	0 (0.00%)	>10	0.319	0.674	Co-occurrence
CLECL1	12p13.31	1 (0.87%)	0 (0.00%)	>10	0.319	0.674	Co-occurrence
CLIP3	19q13.12	1 (0.87%)	0 (0.00%)	>10	0.319	0.674	Co-occurrence
CLSPN	1p34.3	1 (0.87%)	0 (0.00%)	>10	0.319	0.674	Co-occurrence
CLTRN	Xp22.2	1 (0.87%)	0 (0.00%)	>10	0.319	0.674	Co-occurrence
CMAS	12p12.1	1 (0.87%)	0 (0.00%)	>10	0.319	0.674	Co-occurrence
CMC1	3p24.1	1 (0.87%)	0 (0.00%)	>10	0.319	0.674	Co-occurrence
CMKLR1	12q23.3	1 (0.87%)	0 (0.00%)	>10	0.319	0.674	Co-occurrence
CMTM6	3p22.3	1 (0.87%)	0 (0.00%)	>10	0.319	0.674	Co-occurrence
CMTM7	3p22.3	1 (0.87%)	0 (0.00%)	>10	0.319	0.674	Co-occurrence
CNBD2	20q11.23	1 (0.87%)	0 (0.00%)	>10	0.319	0.674	Co-occurrence
CNKSR2	Xp22.12	1 (0.87%)	0 (0.00%)	>10	0.319	0.674	Co-occurrence
CNMD	13q14.3	1 (0.87%)	0 (0.00%)	>10	0.319	0.674	Co-occurrence
CNN3	1p21.3	1 (0.87%)	0 (0.00%)	>10	0.319	0.674	Co-occurrence
CNOT10	3p22.3	1 (0.87%)	0 (0.00%)	>10	0.319	0.674	Co-occurrence
CNTN4	3p26.3-p26.2	1 (0.87%)	0 (0.00%)	>10	0.319	0.674	Co-occurrence
CNTN6	3p26.3	1 (0.87%)	0 (0.00%)	>10	0.319	0.674	Co-occurrence
CNTNAP3	9p12	1 (0.87%)	0 (0.00%)	>10	0.319	0.674	Co-occurrence
CNTNAP3B	9p11.2	1 (0.87%)	0 (0.00%)	>10	0.319	0.674	Co-occurrence
CNTRL	9q33.2	1 (0.87%)	0 (0.00%)	>10	0.319	0.674	Co-occurrence

COA7	1p32.3	1 (0.87%)	0 (0.00%)	>10	0.319	0.674	Co-occurrence
COL24A1	1p22.3	1 (0.87%)	0 (0.00%)	>10	0.319	0.674	Co-occurrence
COL8A2	1p34.3	1 (0.87%)	0 (0.00%)	>10	0.319	0.674	Co-occurrence
COPB1	11p15.2	1 (0.87%)	0 (0.00%)	>10	0.319	0.674	Co-occurrence
COQ3	6q16.2	1 (0.87%)	0 (0.00%)	>10	0.319	0.674	Co-occurrence
COQ5	12q24.31	1 (0.87%)	0 (0.00%)	>10	0.319	0.674	Co-occurrence
COX16	14q24.2	1 (0.87%)	0 (0.00%)	>10	0.319	0.674	Co-occurrence
COX18	4q13.3	1 (0.87%)	0 (0.00%)	>10	0.319	0.674	Co-occurrence
COX6A1	12q24.31|12q24.2	1 (0.87%)	0 (0.00%)	>10	0.319	0.674	Co-occurrence
COX7A1	19q13.12	1 (0.87%)	0 (0.00%)	>10	0.319	0.674	Co-occurrence
COX7B	Xq21.1	1 (0.87%)	0 (0.00%)	>10	0.319	0.674	Co-occurrence
COX8C	14q32.12	1 (0.87%)	0 (0.00%)	>10	0.319	0.674	Co-occurrence
CPNE1	20q11.22	1 (0.87%)	0 (0.00%)	>10	0.319	0.674	Co-occurrence
CPNE8	12q12	1 (0.87%)	0 (0.00%)	>10	0.319	0.674	Co-occurrence
CPTP	1p36.33	1 (0.87%)	0 (0.00%)	>10	0.319	0.674	Co-occurrence
CRAT	9q34.11	1 (0.87%)	0 (0.00%)	>10	0.319	0.674	Co-occurrence
CRB2	9q33.3	1 (0.87%)	0 (0.00%)	>10	0.319	0.674	Co-occurrence
CRBN	3p26.2	1 (0.87%)	0 (0.00%)	>10	0.319	0.674	Co-occurrence
CREB3L1	11p11.2	1 (0.87%)	0 (0.00%)	>10	0.319	0.674	Co-occurrence
CRLF2	Xp22.33 and Yp11.2	1 (0.87%)	0 (0.00%)	>10	0.319	0.674	Co-occurrence
CRLS1	20p12.3	1 (0.87%)	0 (0.00%)	>10	0.319	0.674	Co-occurrence
CRTAP	3p22.3	1 (0.87%)	0 (0.00%)	>10	0.319	0.674	Co-occurrence
CRY2	11p11.2	1 (0.87%)	0 (0.00%)	>10	0.319	0.674	Co-occurrence
CRYL1	13q12.11	1 (0.87%)	0 (0.00%)	>10	0.319	0.674	Co-occurrence
CRYZ	1p31.1	1 (0.87%)	0 (0.00%)	>10	0.319	0.674	Co-occurrence
CSF2RA	Xp22.33 and Yp11.2	1 (0.87%)	0 (0.00%)	>10	0.319	0.674	Co-occurrence
CSF3R	1p34.3	1 (0.87%)	0 (0.00%)	>10	0.319	0.674	Co-occurrence
CSN1S2AP	4q13.3	1 (0.87%)	0 (0.00%)	>10	0.319	0.674	Co-occurrence
CSN2	4q13.3	1 (0.87%)	0 (0.00%)	>10	0.319	0.674	Co-occurrence

CSN3	4q13.3	1 (0.87%)	0 (0.00%)	>10	0.319	0.674	Co-occurrence
CSNK1G2	19p13.3	1 (0.87%)	0 (0.00%)	>10	0.319	0.674	Co-occurrence
CSNK2A3	11p15.4	1 (0.87%)	0 (0.00%)	>10	0.319	0.674	Co-occurrence
CSPG5	3p21.31	1 (0.87%)	0 (0.00%)	>10	0.319	0.674	Co-occurrence
CSRNP1	3p22.2	1 (0.87%)	0 (0.00%)	>10	0.319	0.674	Co-occurrence
CSRP3	11p15.1	1 (0.87%)	0 (0.00%)	>10	0.319	0.674	Co-occurrence
CTBS	1p22.3	1 (0.87%)	0 (0.00%)	>10	0.319	0.674	Co-occurrence
CTCF	16q22.1	1 (0.87%)	0 (0.00%)	>10	0.319	0.674	Co-occurrence
CTDSPL2	15q15.3-q21.1	1 (0.87%)	0 (0.00%)	>10	0.319	0.674	Co-occurrence
CTH	1p31.1	1 (0.87%)	0 (0.00%)	>10	0.319	0.674	Co-occurrence
CTNNB1	3p22.1	1 (0.87%)	0 (0.00%)	>10	0.319	0.674	Co-occurrence
CTPS2	Xp22.2	1 (0.87%)	0 (0.00%)	>10	0.319	0.674	Co-occurrence
CTR9	11p15.4	1 (0.87%)	0 (0.00%)	>10	0.319	0.674	Co-occurrence
CUX2	12q24.11-q24.12	1 (0.87%)	0 (0.00%)	>10	0.319	0.674	Co-occurrence
CX3CR1	3p22.2	1 (0.87%)	0 (0.00%)	>10	0.319	0.674	Co-occurrence
CXCL10	4q21.1	1 (0.87%)	0 (0.00%)	>10	0.319	0.674	Co-occurrence
CXCL11	4q21.1	1 (0.87%)	0 (0.00%)	>10	0.319	0.674	Co-occurrence
CXCL2	4q13.3	1 (0.87%)	0 (0.00%)	>10	0.319	0.674	Co-occurrence
CXCL3	4q13.3	1 (0.87%)	0 (0.00%)	>10	0.319	0.674	Co-occurrence
CXCL5	4q13.3	1 (0.87%)	0 (0.00%)	>10	0.319	0.674	Co-occurrence
CXCL9	4q21.1	1 (0.87%)	0 (0.00%)	>10	0.319	0.674	Co-occurrence
CXORF38	Xp11.4	1 (0.87%)	0 (0.00%)	>10	0.319	0.674	Co-occurrence
CXORF58	Xp22.11	1 (0.87%)	0 (0.00%)	>10	0.319	0.674	Co-occurrence
CXXC1	18q21.1	1 (0.87%)	0 (0.00%)	>10	0.319	0.674	Co-occurrence
CXXC1P1	Xp11.23	1 (0.87%)	0 (0.00%)	>10	0.319	0.674	Co-occurrence
CYB5R2	11p15.4	1 (0.87%)	0 (0.00%)	>10	0.319	0.674	Co-occurrence
CYFIP1	15q11.2	1 (0.87%)	0 (0.00%)	>10	0.319	0.674	Co-occurrence
CYP2J2	1p32.1	1 (0.87%)	0 (0.00%)	>10	0.319	0.674	Co-occurrence
CYP2R1	11p15.2	1 (0.87%)	0 (0.00%)	>10	0.319	0.674	Co-occurrence

CYP4F30P	2q21.1	1 (0.87%)	0 (0.00%)	>10	0.319	0.674	Co-occurrence
CYP4F62P	2q21.1	1 (0.87%)	0 (0.00%)	>10	0.319	0.674	Co-occurrence
DACH2	Xq21.2	1 (0.87%)	0 (0.00%)	>10	0.319	0.674	Co-occurrence
DAPK3	19p13.3	1 (0.87%)	0 (0.00%)	>10	0.319	0.674	Co-occurrence
DBIL5P	17p13.3	1 (0.87%)	0 (0.00%)	>10	0.319	0.674	Co-occurrence
DBX1	11p15.1	1 (0.87%)	0 (0.00%)	>10	0.319	0.674	Co-occurrence
DBX2	12q12	1 (0.87%)	0 (0.00%)	>10	0.319	0.674	Co-occurrence
DCAF1	3p21.2	1 (0.87%)	0 (0.00%)	>10	0.319	0.674	Co-occurrence
DCAF4	14q24.2	1 (0.87%)	0 (0.00%)	>10	0.319	0.674	Co-occurrence
DCD	12q13.2	1 (0.87%)	0 (0.00%)	>10	0.319	0.674	Co-occurrence
DCDC2B	1p35.2	1 (0.87%)	0 (0.00%)	>10	0.319	0.674	Co-occurrence
DCHS1	11p15.4	1 (0.87%)	0 (0.00%)	>10	0.319	0.674	Co-occurrence
DCK	4q13.3	1 (0.87%)	0 (0.00%)	>10	0.319	0.674	Co-occurrence
DCLK3	3p22.2	1 (0.87%)	0 (0.00%)	>10	0.319	0.674	Co-occurrence
DCLRE1B	1p13.2	1 (0.87%)	0 (0.00%)	>10	0.319	0.674	Co-occurrence
DCPS	11q24.2	1 (0.87%)	0 (0.00%)	>10	0.319	0.674	Co-occurrence
DDB2	11p11.2	1 (0.87%)	0 (0.00%)	>10	0.319	0.674	Co-occurrence
DDX11L1	1p36.33	1 (0.87%)	0 (0.00%)	>10	0.319	0.674	Co-occurrence
DDX25	11q24.2	1 (0.87%)	0 (0.00%)	>10	0.319	0.674	Co-occurrence
DDX54	12q24.13	1 (0.87%)	0 (0.00%)	>10	0.319	0.674	Co-occurrence
DDX58	9p21.1	1 (0.87%)	0 (0.00%)	>10	0.319	0.674	Co-occurrence
1-Dec	9q33.1	1 (0.87%)	0 (0.00%)	>10	0.319	0.674	Co-occurrence
DENND1A	9q33.3	1 (0.87%)	0 (0.00%)	>10	0.319	0.674	Co-occurrence
DENND5A	11p15.4	1 (0.87%)	0 (0.00%)	>10	0.319	0.674	Co-occurrence
DENND5B	12p11.21	1 (0.87%)	0 (0.00%)	>10	0.319	0.674	Co-occurrence
DFFB	1p36.32	1 (0.87%)	0 (0.00%)	>10	0.319	0.674	Co-occurrence
DGKZ	11p11.2	1 (0.87%)	0 (0.00%)	>10	0.319	0.674	Co-occurrence
DGUOK	2p13.1	1 (0.87%)	0 (0.00%)	>10	0.319	0.674	Co-occurrence
DHCR24	1p32.3	1 (0.87%)	0 (0.00%)	>10	0.319	0.674	Co-occurrence

DHRS12	13q14.3	1 (0.87%)	0 (0.00%)	>10	0.319	0.674	Co-occurrence
DHRSX	Xp22.33 and Yp11.2	1 (0.87%)	0 (0.00%)	>10	0.319	0.674	Co-occurrence
DHX30	3p21.31	1 (0.87%)	0 (0.00%)	>10	0.319	0.674	Co-occurrence
DHX37	12q24.31	1 (0.87%)	0 (0.00%)	>10	0.319	0.674	Co-occurrence
DIABLO	12q24.31	1 (0.87%)	0 (0.00%)	>10	0.319	0.674	Co-occurrence
DIO1	1p32.3	1 (0.87%)	0 (0.00%)	>10	0.319	0.674	Co-occurrence
DIPK1A	1p22.1	1 (0.87%)	0 (0.00%)	>10	0.319	0.674	Co-occurrence
DKFZP547L112	15q11.2	1 (0.87%)	0 (0.00%)	>10	0.319	0.674	Co-occurrence
DLEU1	13q14.2-q14.3	1 (0.87%)	0 (0.00%)	>10	0.319	0.674	Co-occurrence
DLEU2	13q14.2	1 (0.87%)	0 (0.00%)	>10	0.319	0.674	Co-occurrence
DLEU7	13q14.3	1 (0.87%)	0 (0.00%)	>10	0.319	0.674	Co-occurrence
DLGAP3	1p34.3	1 (0.87%)	0 (0.00%)	>10	0.319	0.674	Co-occurrence
DLK1	14q32.2	1 (0.87%)	0 (0.00%)	>10	0.319	0.674	Co-occurrence
DMAP1	1p34.1	1 (0.87%)	0 (0.00%)	>10	0.319	0.674	Co-occurrence
DMBX1	1p33	1 (0.87%)	0 (0.00%)	>10	0.319	0.674	Co-occurrence
DMTN	8p21.3	1 (0.87%)	0 (0.00%)	>10	0.319	0.674	Co-occurrence
DNAJB5	9p13.3	1 (0.87%)	0 (0.00%)	>10	0.319	0.674	Co-occurrence
DNAJB7	22q13.2	1 (0.87%)	0 (0.00%)	>10	0.319	0.674	Co-occurrence
DNALI1	1p34.3	1 (0.87%)	0 (0.00%)	>10	0.319	0.674	Co-occurrence
DNASE2B	1p31.1-p22.3	1 (0.87%)	0 (0.00%)	>10	0.319	0.674	Co-occurrence
DNHD1	11p15.4	1 (0.87%)	0 (0.00%)	>10	0.319	0.674	Co-occurrence
DNM1L	12p11.21	1 (0.87%)	0 (0.00%)	>10	0.319	0.674	Co-occurrence
DNTTIP2	1p22.1	1 (0.87%)	0 (0.00%)	>10	0.319	0.674	Co-occurrence
DOC2B	17p13.3	1 (0.87%)	0 (0.00%)	>10	0.319	0.674	Co-occurrence
DOHH	19p13.3	1 (0.87%)	0 (0.00%)	>10	0.319	0.674	Co-occurrence
DOK2	8p21.3	1 (0.87%)	0 (0.00%)	>10	0.319	0.674	Co-occurrence
DOLK	9q34.11	1 (0.87%)	0 (0.00%)	>10	0.319	0.674	Co-occurrence
DOLPP1	9q34.11	1 (0.87%)	0 (0.00%)	>10	0.319	0.674	Co-occurrence
DPF1	19q13.2	1 (0.87%)	0 (0.00%)	>10	0.319	0.674	Co-occurrence

DPH2	1p34.1	1 (0.87%)	0 (0.00%)	>10	0.319	0.674	Co-occurrence
DPYSL4	10q26.3	1 (0.87%)	0 (0.00%)	>10	0.319	0.674	Co-occurrence
DR1	1p22.1	1 (0.87%)	0 (0.00%)	>10	0.319	0.674	Co-occurrence
DRD3	3q13.31	1 (0.87%)	0 (0.00%)	>10	0.319	0.674	Co-occurrence
DSC1	18q12.1	1 (0.87%)	0 (0.00%)	>10	0.319	0.674	Co-occurrence
DSC2	18q12.1	1 (0.87%)	0 (0.00%)	>10	0.319	0.674	Co-occurrence
DSC3	18q12.1	1 (0.87%)	0 (0.00%)	>10	0.319	0.674	Co-occurrence
DSG1	18q12.1	1 (0.87%)	0 (0.00%)	>10	0.319	0.674	Co-occurrence
DSG2	18q12.1	1 (0.87%)	0 (0.00%)	>10	0.319	0.674	Co-occurrence
DSG3	18q12.1	1 (0.87%)	0 (0.00%)	>10	0.319	0.674	Co-occurrence
DSG4	18q12.1	1 (0.87%)	0 (0.00%)	>10	0.319	0.674	Co-occurrence
DTX1	12q24.13	1 (0.87%)	0 (0.00%)	>10	0.319	0.674	Co-occurrence
DUOX1	15q21.1	1 (0.87%)	0 (0.00%)	>10	0.319	0.674	Co-occurrence
DUOX2	15q21.1	1 (0.87%)	0 (0.00%)	>10	0.319	0.674	Co-occurrence
DUOXA1	15q21.1	1 (0.87%)	0 (0.00%)	>10	0.319	0.674	Co-occurrence
DUOXA2	15q21.1	1 (0.87%)	0 (0.00%)	>10	0.319	0.674	Co-occurrence
DUSP18	22q12.2	1 (0.87%)	0 (0.00%)	>10	0.319	0.674	Co-occurrence
DUSP21	Xp11.3	1 (0.87%)	0 (0.00%)	>10	0.319	0.674	Co-occurrence
DUSP8	11p15.5	1 (0.87%)	0 (0.00%)	>10	0.319	0.674	Co-occurrence
DVL1	1p36.33	1 (0.87%)	0 (0.00%)	>10	0.319	0.674	Co-occurrence
DYDC1	10q23.1	1 (0.87%)	0 (0.00%)	>10	0.319	0.674	Co-occurrence
DYDC2	10q23.1	1 (0.87%)	0 (0.00%)	>10	0.319	0.674	Co-occurrence
DYNC1LI1	3p22.3	1 (0.87%)	0 (0.00%)	>10	0.319	0.674	Co-occurrence
DYNLL1	12q24.31	1 (0.87%)	0 (0.00%)	>10	0.319	0.674	Co-occurrence
E2F4	16q22.1	1 (0.87%)	0 (0.00%)	>10	0.319	0.674	Co-occurrence
E2F8	11p15.1	1 (0.87%)	0 (0.00%)	>10	0.319	0.674	Co-occurrence
EBP	Xp11.23	1 (0.87%)	0 (0.00%)	>10	0.319	0.674	Co-occurrence
ECHDC2	1p32.3	1 (0.87%)	0 (0.00%)	>10	0.319	0.674	Co-occurrence
EDEM1	3p26.1	1 (0.87%)	0 (0.00%)	>10	0.319	0.674	Co-occurrence

EEF2	19p13.3	1 (0.87%)	0 (0.00%)	>10	0.319	0.674	Co-occurrence
EFHB	3p24.3	1 (0.87%)	0 (0.00%)	>10	0.319	0.674	Co-occurrence
EFHC2	Xp11.3	1 (0.87%)	0 (0.00%)	>10	0.319	0.674	Co-occurrence
EGFL6	Xp22.2	1 (0.87%)	0 (0.00%)	>10	0.319	0.674	Co-occurrence
EGOT	3p26.1	1 (0.87%)	0 (0.00%)	>10	0.319	0.674	Co-occurrence
EHF	11p13	1 (0.87%)	0 (0.00%)	>10	0.319	0.674	Co-occurrence
EIF1B	3p22.1	1 (0.87%)	0 (0.00%)	>10	0.319	0.674	Co-occurrence
EIF2B3	1p34.1	1 (0.87%)	0 (0.00%)	>10	0.319	0.674	Co-occurrence
EIF2S3	Xp22.11	1 (0.87%)	0 (0.00%)	>10	0.319	0.674	Co-occurrence
EIF3F	11p15.4	1 (0.87%)	0 (0.00%)	>10	0.319	0.674	Co-occurrence
EIF3I	1p35.2	1 (0.87%)	0 (0.00%)	>10	0.319	0.674	Co-occurrence
EIF4E3	3p13	1 (0.87%)	0 (0.00%)	>10	0.319	0.674	Co-occurrence
EIF4EBP2	10q22.1	1 (0.87%)	0 (0.00%)	>10	0.319	0.674	Co-occurrence
EIF4G2	11p15.4	1 (0.87%)	0 (0.00%)	>10	0.319	0.674	Co-occurrence
EIF5	14q32.32	1 (0.87%)	0 (0.00%)	>10	0.319	0.674	Co-occurrence
EIF6	20q11.22	1 (0.87%)	0 (0.00%)	>10	0.319	0.674	Co-occurrence
ELANE	19p13.3	1 (0.87%)	0 (0.00%)	>10	0.319	0.674	Co-occurrence
ELAVL2	9p21.3	1 (0.87%)	0 (0.00%)	>10	0.319	0.674	Co-occurrence
ELAVL4	1p33-p32.3	1 (0.87%)	0 (0.00%)	>10	0.319	0.674	Co-occurrence
ELK1	Xp11.23	1 (0.87%)	0 (0.00%)	>10	0.319	0.674	Co-occurrence
ELMO3	16q22.1	1 (0.87%)	0 (0.00%)	>10	0.319	0.674	Co-occurrence
ELMSAN1	14q24.3	1 (0.87%)	0 (0.00%)	>10	0.319	0.674	Co-occurrence
ELP6	3p21.31	1 (0.87%)	0 (0.00%)	>10	0.319	0.674	Co-occurrence
EMID1	22q12.2	1 (0.87%)	0 (0.00%)	>10	0.319	0.674	Co-occurrence
EMILIN3	20q12	1 (0.87%)	0 (0.00%)	>10	0.319	0.674	Co-occurrence
EML5	14q31.3	1 (0.87%)	0 (0.00%)	>10	0.319	0.674	Co-occurrence
EMX2	10q26.11	1 (0.87%)	0 (0.00%)	>10	0.319	0.674	Co-occurrence
ENAM	4q13.3	1 (0.87%)	0 (0.00%)	>10	0.319	0.674	Co-occurrence
ENDOG	9q34.11	1 (0.87%)	0 (0.00%)	>10	0.319	0.674	Co-occurrence

ENO4	10q25.3	1 (0.87%)	0 (0.00%)	>10	0.319	0.674	Co-occurrence
ENOPH1	4q21.22	1 (0.87%)	0 (0.00%)	>10	0.319	0.674	Co-occurrence
ENPEP	4q25	1 (0.87%)	0 (0.00%)	>10	0.319	0.674	Co-occurrence
ENTPD3	3p22.1	1 (0.87%)	0 (0.00%)	>10	0.319	0.674	Co-occurrence
ENTPD5	14q24.3	1 (0.87%)	0 (0.00%)	>10	0.319	0.674	Co-occurrence
EOMES	3p24.1	1 (0.87%)	0 (0.00%)	>10	0.319	0.674	Co-occurrence
EPB41L1	20q11.23	1 (0.87%)	0 (0.00%)	>10	0.319	0.674	Co-occurrence
EPB41L5	2q14.2	1 (0.87%)	0 (0.00%)	>10	0.319	0.674	Co-occurrence
EPGN	4q13.3	1 (0.87%)	0 (0.00%)	>10	0.319	0.674	Co-occurrence
EPHA10	1p34.3	1 (0.87%)	0 (0.00%)	>10	0.319	0.674	Co-occurrence
EPHA3	3p11.1	1 (0.87%)	0 (0.00%)	>10	0.319	0.674	Co-occurrence
EPHA5	4q13.1-q13.2	1 (0.87%)	0 (0.00%)	>10	0.319	0.674	Co-occurrence
EPHX2	8p21.2-p21.1	1 (0.87%)	0 (0.00%)	>10	0.319	0.674	Co-occurrence
EPHX4	1p22.1	1 (0.87%)	0 (0.00%)	>10	0.319	0.674	Co-occurrence
EPM2AIP1	3p22.2	1 (0.87%)	0 (0.00%)	>10	0.319	0.674	Co-occurrence
EREG	4q13.3	1 (0.87%)	0 (0.00%)	>10	0.319	0.674	Co-occurrence
ERGIC2	12p11.22	1 (0.87%)	0 (0.00%)	>10	0.319	0.674	Co-occurrence
ERI1	8p23.1	1 (0.87%)	0 (0.00%)	>10	0.319	0.674	Co-occurrence
ERI3	1p34.1	1 (0.87%)	0 (0.00%)	>10	0.319	0.674	Co-occurrence
ERICH3	1p31.1	1 (0.87%)	0 (0.00%)	>10	0.319	0.674	Co-occurrence
ERP29	12q24.13	1 (0.87%)	0 (0.00%)	>10	0.319	0.674	Co-occurrence
ERRFI1	1p36.23	1 (0.87%)	0 (0.00%)	>10	0.319	0.674	Co-occurrence
ESR2	14q23.2-q23.3	1 (0.87%)	0 (0.00%)	>10	0.319	0.674	Co-occurrence
ESRRAP2	13q12.11	1 (0.87%)	0 (0.00%)	>10	0.319	0.674	Co-occurrence
ETFBKMT	12p11.21	1 (0.87%)	0 (0.00%)	>10	0.319	0.674	Co-occurrence
ETNK1	12p12.1	1 (0.87%)	0 (0.00%)	>10	0.319	0.674	Co-occurrence
EVA1B	1p34.3	1 (0.87%)	0 (0.00%)	>10	0.319	0.674	Co-occurrence
EVI5	1p22.1	1 (0.87%)	0 (0.00%)	>10	0.319	0.674	Co-occurrence
EXO5	1p34.2	1 (0.87%)	0 (0.00%)	>10	0.319	0.674	Co-occurrence

EXOC3L1	16q22.1	1 (0.87%)	0 (0.00%)	>10	0.319	0.674	Co-occurrence
EXOG	3p22.2	1 (0.87%)	0 (0.00%)	>10	0.319	0.674	Co-occurrence
EXOSC7	3p21.31	1 (0.87%)	0 (0.00%)	>10	0.319	0.674	Co-occurrence
EXT2	11p11.2	1 (0.87%)	0 (0.00%)	>10	0.319	0.674	Co-occurrence
F2	11p11.2	1 (0.87%)	0 (0.00%)	>10	0.319	0.674	Co-occurrence
F3	1p21.3	1 (0.87%)	0 (0.00%)	>10	0.319	0.674	Co-occurrence
FAAH	1p33	1 (0.87%)	0 (0.00%)	>10	0.319	0.674	Co-occurrence
FAAH2	Xp11.21	1 (0.87%)	0 (0.00%)	>10	0.319	0.674	Co-occurrence
FAAP20	1p36.33	1 (0.87%)	0 (0.00%)	>10	0.319	0.674	Co-occurrence
FAM104B	Xp11.21	1 (0.87%)	0 (0.00%)	>10	0.319	0.674	Co-occurrence
FAM118B	11q24.2	1 (0.87%)	0 (0.00%)	>10	0.319	0.674	Co-occurrence
FAM120C	Xp11.22	1 (0.87%)	0 (0.00%)	>10	0.319	0.674	Co-occurrence
FAM124A	13q14.3	1 (0.87%)	0 (0.00%)	>10	0.319	0.674	Co-occurrence
FAM138A	1p36.33	1 (0.87%)	0 (0.00%)	>10	0.319	0.674	Co-occurrence
FAM13C	10q21.1	1 (0.87%)	0 (0.00%)	>10	0.319	0.674	Co-occurrence
FAM160B2	8p21.3	1 (0.87%)	0 (0.00%)	>10	0.319	0.674	Co-occurrence
FAM167B	1p35.2	1 (0.87%)	0 (0.00%)	>10	0.319	0.674	Co-occurrence
FAM180B	11p11.2	1 (0.87%)	0 (0.00%)	>10	0.319	0.674	Co-occurrence
FAM182A	20p11.1	1 (0.87%)	0 (0.00%)	>10	0.319	0.674	Co-occurrence
FAM201A	9p13.1	1 (0.87%)	0 (0.00%)	>10	0.319	0.674	Co-occurrence
FAM205A	9p13.3	1 (0.87%)	0 (0.00%)	>10	0.319	0.674	Co-occurrence
FAM205BP	9p13.3	1 (0.87%)	0 (0.00%)	>10	0.319	0.674	Co-occurrence
FAM205C	9p13.3	1 (0.87%)	0 (0.00%)	>10	0.319	0.674	Co-occurrence
FAM214A	15q21.2-q21.3	1 (0.87%)	0 (0.00%)	>10	0.319	0.674	Co-occurrence
FAM214B	9p13.3	1 (0.87%)	0 (0.00%)	>10	0.319	0.674	Co-occurrence
FAM229A	1p35.1	1 (0.87%)	0 (0.00%)	>10	0.319	0.674	Co-occurrence
FAM25D	10q11.23	1 (0.87%)	0 (0.00%)	>10	0.319	0.674	Co-occurrence
FAM27B	9q21.11	1 (0.87%)	0 (0.00%)	>10	0.319	0.674	Co-occurrence
FAM27C	9q13	1 (0.87%)	0 (0.00%)	>10	0.319	0.674	Co-occurrence

FAM27D1	9p11.2	1 (0.87%)	0 (0.00%)	>10	0.319	0.674	Co-occurrence
FAM27E2	9p11.2	1 (0.87%)	0 (0.00%)	>10	0.319	0.674	Co-occurrence
FAM27E3	9q21.11	1 (0.87%)	0 (0.00%)	>10	0.319	0.674	Co-occurrence
FAM41C	1p36.33	1 (0.87%)	0 (0.00%)	>10	0.319	0.674	Co-occurrence
FAM74A1	9p12	1 (0.87%)	0 (0.00%)	>10	0.319	0.674	Co-occurrence
FAM74A3	9q21.11	1 (0.87%)	0 (0.00%)	>10	0.319	0.674	Co-occurrence
FAM74A4	9q12	1 (0.87%)	0 (0.00%)	>10	0.319	0.674	Co-occurrence
FAM74A6	9q12	1 (0.87%)	0 (0.00%)	>10	0.319	0.674	Co-occurrence
FAM83C	20q11.22	1 (0.87%)	0 (0.00%)	>10	0.319	0.674	Co-occurrence
FAM87B	1p36.33	1 (0.87%)	0 (0.00%)	>10	0.319	0.674	Co-occurrence
FAM95B1	9p11.2	1 (0.87%)	0 (0.00%)	>10	0.319	0.674	Co-occurrence
FAM98C	19q13.2	1 (0.87%)	0 (0.00%)	>10	0.319	0.674	Co-occurrence
FAM9A	Xp22.31	1 (0.87%)	0 (0.00%)	>10	0.319	0.674	Co-occurrence
FAM9B	Xp22.31	1 (0.87%)	0 (0.00%)	>10	0.319	0.674	Co-occurrence
FANCF	11p14.3	1 (0.87%)	0 (0.00%)	>10	0.319	0.674	Co-occurrence
FANCG	9p13.3	1 (0.87%)	0 (0.00%)	>10	0.319	0.674	Co-occurrence
FAR1	11p15.3	1 (0.87%)	0 (0.00%)	>10	0.319	0.674	Co-occurrence
FAR2	12p11.22	1 (0.87%)	0 (0.00%)	>10	0.319	0.674	Co-occurrence
FAXC	6q16.2	1 (0.87%)	0 (0.00%)	>10	0.319	0.674	Co-occurrence
FBXL2	3p22.3	1 (0.87%)	0 (0.00%)	>10	0.319	0.674	Co-occurrence
FBXL8	16q22.1	1 (0.87%)	0 (0.00%)	>10	0.319	0.674	Co-occurrence
FBXO31	16q24.2	1 (0.87%)	0 (0.00%)	>10	0.319	0.674	Co-occurrence
FBXO8	4q34.1	1 (0.87%)	0 (0.00%)	>10	0.319	0.674	Co-occurrence
FBXW12	3p21.31	1 (0.87%)	0 (0.00%)	>10	0.319	0.674	Co-occurrence
FDCSP	4q13.3|4q13	1 (0.87%)	0 (0.00%)	>10	0.319	0.674	Co-occurrence
FENDRR	16q24.1	1 (0.87%)	0 (0.00%)	>10	0.319	0.674	Co-occurrence
FERMT1	20p12.3	1 (0.87%)	0 (0.00%)	>10	0.319	0.674	Co-occurrence
FEZ1	11q24.2	1 (0.87%)	0 (0.00%)	>10	0.319	0.674	Co-occurrence
FGD4	12p11.21	1 (0.87%)	0 (0.00%)	>10	0.319	0.674	Co-occurrence

FGF16	Xq21.1	1 (0.87%)	0 (0.00%)	>10	0.319	0.674	Co-occurrence
FGF17	8p21.3	1 (0.87%)	0 (0.00%)	>10	0.319	0.674	Co-occurrence
FGF22	19p13.3	1 (0.87%)	0 (0.00%)	>10	0.319	0.674	Co-occurrence
FGF5	4q21.21	1 (0.87%)	0 (0.00%)	>10	0.319	0.674	Co-occurrence
FGF9	13q12.11	1 (0.87%)	0 (0.00%)	>10	0.319	0.674	Co-occurrence
FGR	1p35.3	1 (0.87%)	0 (0.00%)	>10	0.319	0.674	Co-occurrence
FHL3	1p34.3	1 (0.87%)	0 (0.00%)	>10	0.319	0.674	Co-occurrence
FHOD1	16q22.1	1 (0.87%)	0 (0.00%)	>10	0.319	0.674	Co-occurrence
FIBIN	11p14.2	1 (0.87%)	0 (0.00%)	>10	0.319	0.674	Co-occurrence
FICD	12q23.3	1 (0.87%)	0 (0.00%)	>10	0.319	0.674	Co-occurrence
FMNL2	2q23.3	1 (0.87%)	0 (0.00%)	>10	0.319	0.674	Co-occurrence
FNBP1L	1p22.1	1 (0.87%)	0 (0.00%)	>10	0.319	0.674	Co-occurrence
FNBP4	11p11.2	1 (0.87%)	0 (0.00%)	>10	0.319	0.674	Co-occurrence
FNDC10	1p36.33	1 (0.87%)	0 (0.00%)	>10	0.319	0.674	Co-occurrence
FOLH1	11p11.12	1 (0.87%)	0 (0.00%)	>10	0.319	0.674	Co-occurrence
FOXC2	16q24.1	1 (0.87%)	0 (0.00%)	>10	0.319	0.674	Co-occurrence
FOXD4L3	9q21.11	1 (0.87%)	0 (0.00%)	>10	0.319	0.674	Co-occurrence
FOXD4L4	9q21.11	1 (0.87%)	0 (0.00%)	>10	0.319	0.674	Co-occurrence
FOXD4L5	9q21.11	1 (0.87%)	0 (0.00%)	>10	0.319	0.674	Co-occurrence
FOXD4L6	9p11.2	1 (0.87%)	0 (0.00%)	>10	0.319	0.674	Co-occurrence
FOXF1	16q24.1	1 (0.87%)	0 (0.00%)	>10	0.319	0.674	Co-occurrence
FOXL1	16q24.1	1 (0.87%)	0 (0.00%)	>10	0.319	0.674	Co-occurrence
FOXN4	12q24.11	1 (0.87%)	0 (0.00%)	>10	0.319	0.674	Co-occurrence
FOXO1	13q14.11	1 (0.87%)	0 (0.00%)	>10	0.319	0.674	Co-occurrence
FOXR2	Xp11.21	1 (0.87%)	0 (0.00%)	>10	0.319	0.674	Co-occurrence
FOXRED1	11q24.2	1 (0.87%)	0 (0.00%)	>10	0.319	0.674	Co-occurrence
FPGT	1p31.1	1 (0.87%)	0 (0.00%)	>10	0.319	0.674	Co-occurrence
FREM2	13q13.3	1 (0.87%)	0 (0.00%)	>10	0.319	0.674	Co-occurrence
FRMPD4	Xp22.2	1 (0.87%)	0 (0.00%)	>10	0.319	0.674	Co-occurrence

FSTL3	19p13.3	1 (0.87%)	0 (0.00%)	>10	0.319	0.674	Co-occurrence
FTLP10	4q13.2	1 (0.87%)	0 (0.00%)	>10	0.319	0.674	Co-occurrence
FTSJ1	Xp11.23	1 (0.87%)	0 (0.00%)	>10	0.319	0.674	Co-occurrence
FUNDC1	Xp11.3	1 (0.87%)	0 (0.00%)	>10	0.319	0.674	Co-occurrence
FZD10	12q24.33	1 (0.87%)	0 (0.00%)	>10	0.319	0.674	Co-occurrence
FZR1	19p13.3	1 (0.87%)	0 (0.00%)	>10	0.319	0.674	Co-occurrence
G3BP2	4q21.1	1 (0.87%)	0 (0.00%)	>10	0.319	0.674	Co-occurrence
GABPB1	15q21.2	1 (0.87%)	0 (0.00%)	>10	0.319	0.674	Co-occurrence
GABRD	1p36.33	1 (0.87%)	0 (0.00%)	>10	0.319	0.674	Co-occurrence
GAGE1	Xp11.23	1 (0.87%)	0 (0.00%)	>10	0.319	0.674	Co-occurrence
GALNT18	11p15.4	1 (0.87%)	0 (0.00%)	>10	0.319	0.674	Co-occurrence
GALNT6	12q13.13	1 (0.87%)	0 (0.00%)	>10	0.319	0.674	Co-occurrence
GAP43	3q13.31	1 (0.87%)	0 (0.00%)	>10	0.319	0.674	Co-occurrence
GAPVD1	9q33.3	1 (0.87%)	0 (0.00%)	>10	0.319	0.674	Co-occurrence
GAS2	11p14.3	1 (0.87%)	0 (0.00%)	>10	0.319	0.674	Co-occurrence
GATC	12q24.31	1 (0.87%)	0 (0.00%)	>10	0.319	0.674	Co-occurrence
GBP1	1p22.2	1 (0.87%)	0 (0.00%)	>10	0.319	0.674	Co-occurrence
GBP1P1	1p22.2	1 (0.87%)	0 (0.00%)	>10	0.319	0.674	Co-occurrence
GBP2	1p22.2	1 (0.87%)	0 (0.00%)	>10	0.319	0.674	Co-occurrence
GBP3	1p22.2	1 (0.87%)	0 (0.00%)	>10	0.319	0.674	Co-occurrence
GBP4	1p22.2	1 (0.87%)	0 (0.00%)	>10	0.319	0.674	Co-occurrence
GBP5	1p22.2	1 (0.87%)	0 (0.00%)	>10	0.319	0.674	Co-occurrence
GBP6	1p22.2	1 (0.87%)	0 (0.00%)	>10	0.319	0.674	Co-occurrence
GBP7	1p22.2	1 (0.87%)	0 (0.00%)	>10	0.319	0.674	Co-occurrence
GC	4q13.3	1 (0.87%)	0 (0.00%)	>10	0.319	0.674	Co-occurrence
GCLM	1p22.1	1 (0.87%)	0 (0.00%)	>10	0.319	0.674	Co-occurrence
GCN1	12q24.23	1 (0.87%)	0 (0.00%)	>10	0.319	0.674	Co-occurrence
GCSAM	3q13.2	1 (0.87%)	0 (0.00%)	>10	0.319	0.674	Co-occurrence
GEMIN4	17p13.3	1 (0.87%)	0 (0.00%)	>10	0.319	0.674	Co-occurrence

GFI1	1p22.1	1 (0.87%)	0 (0.00%)	>10	0.319	0.674	Co-occurrence
GFRA2	8p21.3	1 (0.87%)	0 (0.00%)	>10	0.319	0.674	Co-occurrence
GGN	19q13.2	1 (0.87%)	0 (0.00%)	>10	0.319	0.674	Co-occurrence
GGTA1P	9q33.2	1 (0.87%)	0 (0.00%)	>10	0.319	0.674	Co-occurrence
GINS2	16q24.1	1 (0.87%)	0 (0.00%)	>10	0.319	0.674	Co-occurrence
GIPC3	19p13.3	1 (0.87%)	0 (0.00%)	>10	0.319	0.674	Co-occurrence
GJA3	13q12.11	1 (0.87%)	0 (0.00%)	>10	0.319	0.674	Co-occurrence
GJA4	1p34.3	1 (0.87%)	0 (0.00%)	>10	0.319	0.674	Co-occurrence
GJB2	13q12.11	1 (0.87%)	0 (0.00%)	>10	0.319	0.674	Co-occurrence
GJB3	1p34.3	1 (0.87%)	0 (0.00%)	>10	0.319	0.674	Co-occurrence
GJB4	1p34.3	1 (0.87%)	0 (0.00%)	>10	0.319	0.674	Co-occurrence
GJB5	1p34.3	1 (0.87%)	0 (0.00%)	>10	0.319	0.674	Co-occurrence
GJB6	13q12.11	1 (0.87%)	0 (0.00%)	>10	0.319	0.674	Co-occurrence
GK2	4q21.21	1 (0.87%)	0 (0.00%)	>10	0.319	0.674	Co-occurrence
GLB1	3p22.3	1 (0.87%)	0 (0.00%)	>10	0.319	0.674	Co-occurrence
GLIS1	1p32.3	1 (0.87%)	0 (0.00%)	>10	0.319	0.674	Co-occurrence
GLMN	1p22.1	1 (0.87%)	0 (0.00%)	>10	0.319	0.674	Co-occurrence
GLOD4	17p13.3	1 (0.87%)	0 (0.00%)	>10	0.319	0.674	Co-occurrence
GLT1D1	12q24.33	1 (0.87%)	0 (0.00%)	>10	0.319	0.674	Co-occurrence
GLYCAM1	12q13.2	1 (0.87%)	0 (0.00%)	>10	0.319	0.674	Co-occurrence
GNB1	1p36.33	1 (0.87%)	0 (0.00%)	>10	0.319	0.674	Co-occurrence
GNB5	15q21.2	1 (0.87%)	0 (0.00%)	>10	0.319	0.674	Co-occurrence
GNG5	1p22.3	1 (0.87%)	0 (0.00%)	>10	0.319	0.674	Co-occurrence
GNL2	1p34.3	1 (0.87%)	0 (0.00%)	>10	0.319	0.674	Co-occurrence
GNRHR	4q13.2	1 (0.87%)	0 (0.00%)	>10	0.319	0.674	Co-occurrence
GOLGA1	9q33.3	1 (0.87%)	0 (0.00%)	>10	0.319	0.674	Co-occurrence
GOLGA5	14q32.12	1 (0.87%)	0 (0.00%)	>10	0.319	0.674	Co-occurrence
GOLGA6L1	15q11.2	1 (0.87%)	0 (0.00%)	>10	0.319	0.674	Co-occurrence
GOLGA6L2	15q11.2	1 (0.87%)	0 (0.00%)	>10	0.319	0.674	Co-occurrence

GOLGA6L6	15q11.2	1 (0.87%)	0 (0.00%)	>10	0.319	0.674	Co-occurrence
GOLGA8DP	15q11.2	1 (0.87%)	0 (0.00%)	>10	0.319	0.674	Co-occurrence
GOLGA8EP	15q11.2	1 (0.87%)	0 (0.00%)	>10	0.319	0.674	Co-occurrence
GOLGA8IP	15q11.2	1 (0.87%)	0 (0.00%)	>10	0.319	0.674	Co-occurrence
GOLGA8S	15q11.2	1 (0.87%)	0 (0.00%)	>10	0.319	0.674	Co-occurrence
GON7	14q32.12	1 (0.87%)	0 (0.00%)	>10	0.319	0.674	Co-occurrence
GORASP1	3p22.2	1 (0.87%)	0 (0.00%)	>10	0.319	0.674	Co-occurrence
GPBP1L1	1p34.1	1 (0.87%)	0 (0.00%)	>10	0.319	0.674	Co-occurrence
GPCPD1	20p12.3	1 (0.87%)	0 (0.00%)	>10	0.319	0.674	Co-occurrence
GPD1L	3p22.3	1 (0.87%)	0 (0.00%)	>10	0.319	0.674	Co-occurrence
GPHB5	14q23.2	1 (0.87%)	0 (0.00%)	>10	0.319	0.674	Co-occurrence
GPN3	12q24.11	1 (0.87%)	0 (0.00%)	>10	0.319	0.674	Co-occurrence
GPR12	13q12.13	1 (0.87%)	0 (0.00%)	>10	0.319	0.674	Co-occurrence
GPR132	14q32.33	1 (0.87%)	0 (0.00%)	>10	0.319	0.674	Co-occurrence
GPR143	Xp22.2	1 (0.87%)	0 (0.00%)	>10	0.319	0.674	Co-occurrence
GPR148	2q21.1	1 (0.87%)	0 (0.00%)	>10	0.319	0.674	Co-occurrence
GPR157	1p36.22	1 (0.87%)	0 (0.00%)	>10	0.319	0.674	Co-occurrence
GPR21	9q33.2	1 (0.87%)	0 (0.00%)	>10	0.319	0.674	Co-occurrence
GPR27	3p13	1 (0.87%)	0 (0.00%)	>10	0.319	0.674	Co-occurrence
GPR3	1p36.11	1 (0.87%)	0 (0.00%)	>10	0.319	0.674	Co-occurrence
GPX7	1p32.3	1 (0.87%)	0 (0.00%)	>10	0.319	0.674	Co-occurrence
GRAMD1C	3q13.31	1 (0.87%)	0 (0.00%)	>10	0.319	0.674	Co-occurrence
GRASP	12q13.13	1 (0.87%)	0 (0.00%)	>10	0.319	0.674	Co-occurrence
GRIK3	1p34.3	1 (0.87%)	0 (0.00%)	>10	0.319	0.674	Co-occurrence
GRM2	3p21.2	1 (0.87%)	0 (0.00%)	>10	0.319	0.674	Co-occurrence
GRM7	3p26.1	1 (0.87%)	0 (0.00%)	>10	0.319	0.674	Co-occurrence
GRPR	Xp22.2	1 (0.87%)	0 (0.00%)	>10	0.319	0.674	Co-occurrence
GRSF1	4q13.3	1 (0.87%)	0 (0.00%)	>10	0.319	0.674	Co-occurrence
GSE1	16q24.1	1 (0.87%)	0 (0.00%)	>10	0.319	0.674	Co-occurrence

GSN	9q33.2	1 (0.87%)	0 (0.00%)	>10	0.319	0.674	Co-occurrence
GTDC1	2q22.3	1 (0.87%)	0 (0.00%)	>10	0.319	0.674	Co-occurrence
GTF2B	1p22.2	1 (0.87%)	0 (0.00%)	>10	0.319	0.674	Co-occurrence
GTPBP6	Xp22.33 and Yp11.31	1 (0.87%)	0 (0.00%)	>10	0.319	0.674	Co-occurrence
GTPBP8	3q13.2	1 (0.87%)	0 (0.00%)	>10	0.319	0.674	Co-occurrence
GTSF1	12q13.13	1 (0.87%)	0 (0.00%)	>10	0.319	0.674	Co-occurrence
GUCY1B2	13q14.3	1 (0.87%)	0 (0.00%)	>10	0.319	0.674	Co-occurrence
GVINP1	11p15.4	1 (0.87%)	0 (0.00%)	>10	0.319	0.674	Co-occurrence
GXYLT1	12q12	1 (0.87%)	0 (0.00%)	>10	0.319	0.674	Co-occurrence
GYG2	Xp22.33	1 (0.87%)	0 (0.00%)	>10	0.319	0.674	Co-occurrence
GYPC	2q14.3	1 (0.87%)	0 (0.00%)	>10	0.319	0.674	Co-occurrence
GZMM	19p13.3	1 (0.87%)	0 (0.00%)	>10	0.319	0.674	Co-occurrence
H2AFY2	10q22.1	1 (0.87%)	0 (0.00%)	>10	0.319	0.674	Co-occurrence
H3F3C	12p11.21	1 (0.87%)	0 (0.00%)	>10	0.319	0.674	Co-occurrence
HAO1	20p12.3	1 (0.87%)	0 (0.00%)	>10	0.319	0.674	Co-occurrence
HARBI1	11p11.2	1 (0.87%)	0 (0.00%)	>10	0.319	0.674	Co-occurrence
HBB	11p15.4	1 (0.87%)	0 (0.00%)	>10	0.319	0.674	Co-occurrence
HBBP1	11p15.4	1 (0.87%)	0 (0.00%)	>10	0.319	0.674	Co-occurrence
HBD	-	1 (0.87%)	0 (0.00%)	>10	0.319	0.674	Co-occurrence
HBE1	11p15.4	1 (0.87%)	0 (0.00%)	>10	0.319	0.674	Co-occurrence
HBG1	11p15.4	1 (0.87%)	0 (0.00%)	>10	0.319	0.674	Co-occurrence
HBG2	11p15.4	1 (0.87%)	0 (0.00%)	>10	0.319	0.674	Co-occurrence
HCN2	19p13.3	1 (0.87%)	0 (0.00%)	>10	0.319	0.674	Co-occurrence
HCST	19q13.12	1 (0.87%)	0 (0.00%)	>10	0.319	0.674	Co-occurrence
HDAC1	1p35.2-p35.1	1 (0.87%)	0 (0.00%)	>10	0.319	0.674	Co-occurrence
HDAC2	6q21	1 (0.87%)	0 (0.00%)	>10	0.319	0.674	Co-occurrence
HDC	15q21.2	1 (0.87%)	0 (0.00%)	>10	0.319	0.674	Co-occurrence
HECTD3	1p34.1	1 (0.87%)	0 (0.00%)	>10	0.319	0.674	Co-occurrence
HERC2P2	15q11.2	1 (0.87%)	0 (0.00%)	>10	0.319	0.674	Co-occurrence

HERC2P3	15q11.1-q11.2	1 (0.87%)	0 (0.00%)	>10	0.319	0.674	Co-occurrence
HES3	1p36.31	1 (0.87%)	0 (0.00%)	>10	0.319	0.674	Co-occurrence
HES4	1p36.33	1 (0.87%)	0 (0.00%)	>10	0.319	0.674	Co-occurrence
HES5	1p36.32	1 (0.87%)	0 (0.00%)	>10	0.319	0.674	Co-occurrence
HHLA3	1p31.1	1 (0.87%)	0 (0.00%)	>10	0.319	0.674	Co-occurrence
HIF1AN	10q24.31	1 (0.87%)	0 (0.00%)	>10	0.319	0.674	Co-occurrence
HIPK1	1p13.2	1 (0.87%)	0 (0.00%)	>10	0.319	0.674	Co-occurrence
HMG20B	19p13.3	1 (0.87%)	0 (0.00%)	>10	0.319	0.674	Co-occurrence
HMGB1P5	3p24.3	1 (0.87%)	0 (0.00%)	>10	0.319	0.674	Co-occurrence
HNF1A	12q24.31	1 (0.87%)	0 (0.00%)	>10	0.319	0.674	Co-occurrence
HNRNPA1L2	13q14.3	1 (0.87%)	0 (0.00%)	>10	0.319	0.674	Co-occurrence
HNRNPD	4q21.22	1 (0.87%)	0 (0.00%)	>10	0.319	0.674	Co-occurrence
HNRNPDL	4q21.22	1 (0.87%)	0 (0.00%)	>10	0.319	0.674	Co-occurrence
HNRNPKP3	11p12	1 (0.87%)	0 (0.00%)	>10	0.319	0.674	Co-occurrence
HOOK1	1p32.1	1 (0.87%)	0 (0.00%)	>10	0.319	0.674	Co-occurrence
HORMAD2	22q12.2	1 (0.87%)	0 (0.00%)	>10	0.319	0.674	Co-occurrence
HPDL	1p34.1	1 (0.87%)	0 (0.00%)	>10	0.319	0.674	Co-occurrence
HPS4	22q12.1	1 (0.87%)	0 (0.00%)	>10	0.319	0.674	Co-occurrence
HR	8p21.3	1 (0.87%)	0 (0.00%)	>10	0.319	0.674	Co-occurrence
HRH4	18q11.2	1 (0.87%)	0 (0.00%)	>10	0.319	0.674	Co-occurrence
HSD11B2	16q22.1	1 (0.87%)	0 (0.00%)	>10	0.319	0.674	Co-occurrence
HSD17B12	11p11.2	1 (0.87%)	0 (0.00%)	>10	0.319	0.674	Co-occurrence
HSDL2	9q32	1 (0.87%)	0 (0.00%)	>10	0.319	0.674	Co-occurrence
HSF4	16q22.1	1 (0.87%)	0 (0.00%)	>10	0.319	0.674	Co-occurrence
HSPA2	14q23.3	1 (0.87%)	0 (0.00%)	>10	0.319	0.674	Co-occurrence
HSPA5	9q33.3	1 (0.87%)	0 (0.00%)	>10	0.319	0.674	Co-occurrence
HSPB11	1p32.3	1 (0.87%)	0 (0.00%)	>10	0.319	0.674	Co-occurrence
HSPB8	12q24.23	1 (0.87%)	0 (0.00%)	>10	0.319	0.674	Co-occurrence
HSPD1P6	3p22.2	1 (0.87%)	0 (0.00%)	>10	0.319	0.674	Co-occurrence

HTATIP2	11p15.1	1 (0.87%)	0 (0.00%)	>10	0.319	0.674	Co-occurrence
HTN1	4q13.3	1 (0.87%)	0 (0.00%)	>10	0.319	0.674	Co-occurrence
HTN3	4q13.3	1 (0.87%)	0 (0.00%)	>10	0.319	0.674	Co-occurrence
HTR7	10q23.31	1 (0.87%)	0 (0.00%)	>10	0.319	0.674	Co-occurrence
HYLS1	11q24.2	1 (0.87%)	0 (0.00%)	>10	0.319	0.674	Co-occurrence
IAPP	12p12.1	1 (0.87%)	0 (0.00%)	>10	0.319	0.674	Co-occurrence
ICMT	1p36.31	1 (0.87%)	0 (0.00%)	>10	0.319	0.674	Co-occurrence
IDNK	9q21.32	1 (0.87%)	0 (0.00%)	>10	0.319	0.674	Co-occurrence
IER5L	9q34.11	1 (0.87%)	0 (0.00%)	>10	0.319	0.674	Co-occurrence
IFI44	1p31.1	1 (0.87%)	0 (0.00%)	>10	0.319	0.674	Co-occurrence
IFI44L	1p31.1	1 (0.87%)	0 (0.00%)	>10	0.319	0.674	Co-occurrence
IGFBPL1	9p13.1	1 (0.87%)	0 (0.00%)	>10	0.319	0.674	Co-occurrence
IGSF11	3q13.32	1 (0.87%)	0 (0.00%)	>10	0.319	0.674	Co-occurrence
IL3RA	Xp22.33 and Yp11.2	1 (0.87%)	0 (0.00%)	>10	0.319	0.674	Co-occurrence
IL5RA	3p26.2	1 (0.87%)	0 (0.00%)	>10	0.319	0.674	Co-occurrence
ILK	11p15.4	1 (0.87%)	0 (0.00%)	>10	0.319	0.674	Co-occurrence
IMP4	2q21.1	1 (0.87%)	0 (0.00%)	>10	0.319	0.674	Co-occurrence
IMPACT	18q11.2	1 (0.87%)	0 (0.00%)	>10	0.319	0.674	Co-occurrence
INE1	Xp11.3	1 (0.87%)	0 (0.00%)	>10	0.319	0.674	Co-occurrence
INF2	14q32.33	1 (0.87%)	0 (0.00%)	>10	0.319	0.674	Co-occurrence
INIP	9q32	1 (0.87%)	0 (0.00%)	>10	0.319	0.674	Co-occurrence
INPP5A	10q26.3	1 (0.87%)	0 (0.00%)	>10	0.319	0.674	Co-occurrence
INPP5B	1p34.3	1 (0.87%)	0 (0.00%)	>10	0.319	0.674	Co-occurrence
INSC	11p15.2	1 (0.87%)	0 (0.00%)	>10	0.319	0.674	Co-occurrence
INTS11	1p36.33	1 (0.87%)	0 (0.00%)	>10	0.319	0.674	Co-occurrence
INTS6	13q14.3	1 (0.87%)	0 (0.00%)	>10	0.319	0.674	Co-occurrence
IP6K2	3p21.31	1 (0.87%)	0 (0.00%)	>10	0.319	0.674	Co-occurrence
IPO13	1p34.1	1 (0.87%)	0 (0.00%)	>10	0.319	0.674	Co-occurrence
IPO8	12p11.21	1 (0.87%)	0 (0.00%)	>10	0.319	0.674	Co-occurrence

IPP	1p34.1	1 (0.87%)	0 (0.00%)	>10	0.319	0.674	Co-occurrence
IQCC	1p35.2	1 (0.87%)	0 (0.00%)	>10	0.319	0.674	Co-occurrence
IQCD	12q24.13	1 (0.87%)	0 (0.00%)	>10	0.319	0.674	Co-occurrence
IQCF2	3p21.2	1 (0.87%)	0 (0.00%)	>10	0.319	0.674	Co-occurrence
IQCF3	3p21.2	1 (0.87%)	0 (0.00%)	>10	0.319	0.674	Co-occurrence
IQCF6	3p21.2	1 (0.87%)	0 (0.00%)	>10	0.319	0.674	Co-occurrence
IRAK4	12q12	1 (0.87%)	0 (0.00%)	>10	0.319	0.674	Co-occurrence
IRF2BPL	14q24.3	1 (0.87%)	0 (0.00%)	>10	0.319	0.674	Co-occurrence
ISCU	12q23.3	1 (0.87%)	0 (0.00%)	>10	0.319	0.674	Co-occurrence
ISG15	1p36.33	1 (0.87%)	0 (0.00%)	>10	0.319	0.674	Co-occurrence
ITPK1	14q32.12	1 (0.87%)	0 (0.00%)	>10	0.319	0.674	Co-occurrence
IZUMO4	19p13.3	1 (0.87%)	0 (0.00%)	>10	0.319	0.674	Co-occurrence
JAKMIP3	10q26.3	1 (0.87%)	0 (0.00%)	>10	0.319	0.674	Co-occurrence
JCHAIN	4q13.3	1 (0.87%)	0 (0.00%)	>10	0.319	0.674	Co-occurrence
JPH2	20q13.12	1 (0.87%)	0 (0.00%)	>10	0.319	0.674	Co-occurrence
JUN	1p32.1	1 (0.87%)	0 (0.00%)	>10	0.319	0.674	Co-occurrence
KBTBD4	11p11.2	1 (0.87%)	0 (0.00%)	>10	0.319	0.674	Co-occurrence
KCMF1	2p11.2	1 (0.87%)	0 (0.00%)	>10	0.319	0.674	Co-occurrence
KCNAB2	1p36.31	1 (0.87%)	0 (0.00%)	>10	0.319	0.674	Co-occurrence
KCNJ8	12p12.1	1 (0.87%)	0 (0.00%)	>10	0.319	0.674	Co-occurrence
KCNK18	10q25.3	1 (0.87%)	0 (0.00%)	>10	0.319	0.674	Co-occurrence
KCNK6	19q13.2	1 (0.87%)	0 (0.00%)	>10	0.319	0.674	Co-occurrence
KCNQ4	1p34.2	1 (0.87%)	0 (0.00%)	>10	0.319	0.674	Co-occurrence
KCNRG	13q14.2	1 (0.87%)	0 (0.00%)	>10	0.319	0.674	Co-occurrence
KCTD1	18q11.2	1 (0.87%)	0 (0.00%)	>10	0.319	0.674	Co-occurrence
KCTD19	16q22.1	1 (0.87%)	0 (0.00%)	>10	0.319	0.674	Co-occurrence
KDM2B	12q24.31	1 (0.87%)	0 (0.00%)	>10	0.319	0.674	Co-occurrence
KDM6A	Xp11.3	1 (0.87%)	0 (0.00%)	>10	0.319	0.674	Co-occurrence
KIAA0319L	1p34.3	1 (0.87%)	0 (0.00%)	>10	0.319	0.674	Co-occurrence

KIAA0895L	16q22.1	1 (0.87%)	0 (0.00%)	>10	0.319	0.674	Co-occurrence
KIAA1107	1p22.1	1 (0.87%)	0 (0.00%)	>10	0.319	0.674	Co-occurrence
KIAA1143	3p21.31	1 (0.87%)	0 (0.00%)	>10	0.319	0.674	Co-occurrence
KIAA1958	9q32	1 (0.87%)	0 (0.00%)	>10	0.319	0.674	Co-occurrence
KIF15	3p21.31	1 (0.87%)	0 (0.00%)	>10	0.319	0.674	Co-occurrence
KIF21A	12q12	1 (0.87%)	0 (0.00%)	>10	0.319	0.674	Co-occurrence
KIRREL2	19q13.12	1 (0.87%)	0 (0.00%)	>10	0.319	0.674	Co-occurrence
KISS1R	19p13.3	1 (0.87%)	0 (0.00%)	>10	0.319	0.674	Co-occurrence
KLF16	19p13.3	1 (0.87%)	0 (0.00%)	>10	0.319	0.674	Co-occurrence
KLF17	1p34.1	1 (0.87%)	0 (0.00%)	>10	0.319	0.674	Co-occurrence
KLHL14	18q12.1	1 (0.87%)	0 (0.00%)	>10	0.319	0.674	Co-occurrence
KLHL15	Xp22.11	1 (0.87%)	0 (0.00%)	>10	0.319	0.674	Co-occurrence
KLHL17	1p36.33	1 (0.87%)	0 (0.00%)	>10	0.319	0.674	Co-occurrence
KLHL34	Xp22.12	1 (0.87%)	0 (0.00%)	>10	0.319	0.674	Co-occurrence
KLRB1	12p13.31	1 (0.87%)	0 (0.00%)	>10	0.319	0.674	Co-occurrence
KLRF1	12p13.31	1 (0.87%)	0 (0.00%)	>10	0.319	0.674	Co-occurrence
KLRF2	12p13.31	1 (0.87%)	0 (0.00%)	>10	0.319	0.674	Co-occurrence
KLRG1	12p13.31	1 (0.87%)	0 (0.00%)	>10	0.319	0.674	Co-occurrence
KMT5A	12q24.31	1 (0.87%)	0 (0.00%)	>10	0.319	0.674	Co-occurrence
KNCN	1p33	1 (0.87%)	0 (0.00%)	>10	0.319	0.674	Co-occurrence
KREMEN1	22q12.1	1 (0.87%)	0 (0.00%)	>10	0.319	0.674	Co-occurrence
KRT80	12q13.13	1 (0.87%)	0 (0.00%)	>10	0.319	0.674	Co-occurrence
KTI12	1p32.3	1 (0.87%)	0 (0.00%)	>10	0.319	0.674	Co-occurrence
KYAT1	9q34.11	1 (0.87%)	0 (0.00%)	>10	0.319	0.674	Co-occurrence
KYAT3	1p22.2	1 (0.87%)	0 (0.00%)	>10	0.319	0.674	Co-occurrence
LACRT	12q13.2	1 (0.87%)	0 (0.00%)	>10	0.319	0.674	Co-occurrence
LARGE2	11p11.2	1 (0.87%)	0 (0.00%)	>10	0.319	0.674	Co-occurrence
LCK	1p35.2	1 (0.87%)	0 (0.00%)	>10	0.319	0.674	Co-occurrence
LDHB	12p12.1	1 (0.87%)	0 (0.00%)	>10	0.319	0.674	Co-occurrence

LEXM	1p32.3	1 (0.87%)	0 (0.00%)	>10	0.319	0.674	Co-occurrence
LGI3	8p21.3	1 (0.87%)	0 (0.00%)	>10	0.319	0.674	Co-occurrence
LGR4	11p14.1	1 (0.87%)	0 (0.00%)	>10	0.319	0.674	Co-occurrence
LHX2	9q33.3	1 (0.87%)	0 (0.00%)	>10	0.319	0.674	Co-occurrence
LHX5	12q24.13	1 (0.87%)	0 (0.00%)	>10	0.319	0.674	Co-occurrence
LHX6	9q33.2	1 (0.87%)	0 (0.00%)	>10	0.319	0.674	Co-occurrence
LIN52	14q24.3	1 (0.87%)	0 (0.00%)	>10	0.319	0.674	Co-occurrence
LIN54	4q21.22	1 (0.87%)	0 (0.00%)	>10	0.319	0.674	Co-occurrence
LIN7C	11p14.1	1 (0.87%)	0 (0.00%)	>10	0.319	0.674	Co-occurrence
LINC00102	Xp22.33 and Yp11.2	1 (0.87%)	0 (0.00%)	>10	0.319	0.674	Co-occurrence
LINC00106	Xp22.33 and Yp11.2	1 (0.87%)	0 (0.00%)	>10	0.319	0.674	Co-occurrence
LINC00115	1p36.33	1 (0.87%)	0 (0.00%)	>10	0.319	0.674	Co-occurrence
LINC00313	21q22.3	1 (0.87%)	0 (0.00%)	>10	0.319	0.674	Co-occurrence
LINC00326	6q23.2	1 (0.87%)	0 (0.00%)	>10	0.319	0.674	Co-occurrence
LINC00327	13q12.12	1 (0.87%)	0 (0.00%)	>10	0.319	0.674	Co-occurrence
LINC00337	1p36.31	1 (0.87%)	0 (0.00%)	>10	0.319	0.674	Co-occurrence
LINC00345	13q14.3	1 (0.87%)	0 (0.00%)	>10	0.319	0.674	Co-occurrence
LINC00350	13q12.11	1 (0.87%)	0 (0.00%)	>10	0.319	0.674	Co-occurrence
LINC00352	13q12.12	1 (0.87%)	0 (0.00%)	>10	0.319	0.674	Co-occurrence
LINC00362	13q12.12	1 (0.87%)	0 (0.00%)	>10	0.319	0.674	Co-occurrence
LINC00366	13q13.3	1 (0.87%)	0 (0.00%)	>10	0.319	0.674	Co-occurrence
LINC00415	13q12.13	1 (0.87%)	0 (0.00%)	>10	0.319	0.674	Co-occurrence
LINC00421	13q12.11	1 (0.87%)	0 (0.00%)	>10	0.319	0.674	Co-occurrence
LINC00424	13q12.11	1 (0.87%)	0 (0.00%)	>10	0.319	0.674	Co-occurrence
LINC00474	9q33.1	1 (0.87%)	0 (0.00%)	>10	0.319	0.674	Co-occurrence
LINC00505	1p33	1 (0.87%)	0 (0.00%)	>10	0.319	0.674	Co-occurrence
LINC00507	12q24.32	1 (0.87%)	0 (0.00%)	>10	0.319	0.674	Co-occurrence
LINC00508	12q24.32	1 (0.87%)	0 (0.00%)	>10	0.319	0.674	Co-occurrence
LINC00523	14q32.2	1 (0.87%)	0 (0.00%)	>10	0.319	0.674	Co-occurrence

LINC00556	13q12.11	1 (0.87%)	0 (0.00%)	>10	0.319	0.674	Co-occurrence
LINC00566	13q12.12	1 (0.87%)	0 (0.00%)	>10	0.319	0.674	Co-occurrence
LINC00575	4q21.22	1 (0.87%)	0 (0.00%)	>10	0.319	0.674	Co-occurrence
LINC00612	12p13.31	1 (0.87%)	0 (0.00%)	>10	0.319	0.674	Co-occurrence
LINC00621	13q12.12	1 (0.87%)	0 (0.00%)	>10	0.319	0.674	Co-occurrence
LINC00634	22q13.2	1 (0.87%)	0 (0.00%)	>10	0.319	0.674	Co-occurrence
LINC00638	14q32.33	1 (0.87%)	0 (0.00%)	>10	0.319	0.674	Co-occurrence
LINC00654	20p12.3	1 (0.87%)	0 (0.00%)	>10	0.319	0.674	Co-occurrence
LINC00658	20p12.3	1 (0.87%)	0 (0.00%)	>10	0.319	0.674	Co-occurrence
LINC00665	19q13.12	1 (0.87%)	0 (0.00%)	>10	0.319	0.674	Co-occurrence
LINC00678	11p14.1	1 (0.87%)	0 (0.00%)	>10	0.319	0.674	Co-occurrence
LINC00685	Xp22.33 and Yp11.31	1 (0.87%)	0 (0.00%)	>10	0.319	0.674	Co-occurrence
LINC00691	3p24.2	1 (0.87%)	0 (0.00%)	>10	0.319	0.674	Co-occurrence
LINC00692	3p24.2	1 (0.87%)	0 (0.00%)	>10	0.319	0.674	Co-occurrence
LINC00693	3p24.1	1 (0.87%)	0 (0.00%)	>10	0.319	0.674	Co-occurrence
LINC00843	10q11.23	1 (0.87%)	0 (0.00%)	>10	0.319	0.674	Co-occurrence
LINC00870	3p13	1 (0.87%)	0 (0.00%)	>10	0.319	0.674	Co-occurrence
LINC00901	3q13.31	1 (0.87%)	0 (0.00%)	>10	0.319	0.674	Co-occurrence
LINC00903	3q13.31	1 (0.87%)	0 (0.00%)	>10	0.319	0.674	Co-occurrence
LINC00917	16q24.1	1 (0.87%)	0 (0.00%)	>10	0.319	0.674	Co-occurrence
LINC00939	12q24.32	1 (0.87%)	0 (0.00%)	>10	0.319	0.674	Co-occurrence
LINC00941	12p11.21	1 (0.87%)	0 (0.00%)	>10	0.319	0.674	Co-occurrence
LINC00943	12q24.32	1 (0.87%)	0 (0.00%)	>10	0.319	0.674	Co-occurrence
LINC00944	12q24.32	1 (0.87%)	0 (0.00%)	>10	0.319	0.674	Co-occurrence
LINC00987	12p13.31	1 (0.87%)	0 (0.00%)	>10	0.319	0.674	Co-occurrence
LINC00989	4q21.21	1 (0.87%)	0 (0.00%)	>10	0.319	0.674	Co-occurrence
LINC01193	15q11.2	1 (0.87%)	0 (0.00%)	>10	0.319	0.674	Co-occurrence
LINC01546	Xp22.33	1 (0.87%)	0 (0.00%)	>10	0.319	0.674	Co-occurrence
LINC01555	1p22.3	1 (0.87%)	0 (0.00%)	>10	0.319	0.674	Co-occurrence

LINC01560	Xp11.3	1 (0.87%)	0 (0.00%)	>10	0.319	0.674	Co-occurrence
LINC02603	9q22.32	1 (0.87%)	0 (0.00%)	>10	0.319	0.674	Co-occurrence
LMO1	11p15.4	1 (0.87%)	0 (0.00%)	>10	0.319	0.674	Co-occurrence
LMX1B	9q33.3	1 (0.87%)	0 (0.00%)	>10	0.319	0.674	Co-occurrence
LOC105378503		1 (0.87%)	0 (0.00%)	>10	0.319	0.674	Co-occurrence
LPAR1	9q31.3	1 (0.87%)	0 (0.00%)	>10	0.319	0.674	Co-occurrence
LPIN3	20q12	1 (0.87%)	0 (0.00%)	>10	0.319	0.674	Co-occurrence
LRFN3	19q13.12	1 (0.87%)	0 (0.00%)	>10	0.319	0.674	Co-occurrence
LRP4	11p11.2	1 (0.87%)	0 (0.00%)	>10	0.319	0.674	Co-occurrence
LRRC2-AS1	3p21.31	1 (0.87%)	0 (0.00%)	>10	0.319	0.674	Co-occurrence
LRRC20	10q22.1	1 (0.87%)	0 (0.00%)	>10	0.319	0.674	Co-occurrence
LRRC27	10q26.3	1 (0.87%)	0 (0.00%)	>10	0.319	0.674	Co-occurrence
LRRC29	16q22.1	1 (0.87%)	0 (0.00%)	>10	0.319	0.674	Co-occurrence
LRRC37A7P	18q12.1	1 (0.87%)	0 (0.00%)	>10	0.319	0.674	Co-occurrence
LRRC40	1p31.1	1 (0.87%)	0 (0.00%)	>10	0.319	0.674	Co-occurrence
LRRC41	1p34.1-p33	1 (0.87%)	0 (0.00%)	>10	0.319	0.674	Co-occurrence
LRRC42	1p32.3	1 (0.87%)	0 (0.00%)	>10	0.319	0.674	Co-occurrence
LRRC47	1p36.32	1 (0.87%)	0 (0.00%)	>10	0.319	0.674	Co-occurrence
LRRC53	1p31.3	1 (0.87%)	0 (0.00%)	>10	0.319	0.674	Co-occurrence
LRRC7	1p31.1	1 (0.87%)	0 (0.00%)	>10	0.319	0.674	Co-occurrence
LRRC8A	9q34.11	1 (0.87%)	0 (0.00%)	>10	0.319	0.674	Co-occurrence
LRRC8B	1p22.2	1 (0.87%)	0 (0.00%)	>10	0.319	0.674	Co-occurrence
LRRFIP2	3p22.2	1 (0.87%)	0 (0.00%)	>10	0.319	0.674	Co-occurrence
LRRIQ3	1p31.1	1 (0.87%)	0 (0.00%)	>10	0.319	0.674	Co-occurrence
LRRN1	3p26.2	1 (0.87%)	0 (0.00%)	>10	0.319	0.674	Co-occurrence
LRRN4	20p12.3	1 (0.87%)	0 (0.00%)	>10	0.319	0.674	Co-occurrence
LSAMP	3q13.31	1 (0.87%)	0 (0.00%)	>10	0.319	0.674	Co-occurrence
LSM10	1p34.3	1 (0.87%)	0 (0.00%)	>10	0.319	0.674	Co-occurrence
LTF	3p21.31	1 (0.87%)	0 (0.00%)	>10	0.319	0.674	Co-occurrence

LURAP1	1p34.1	1 (0.87%)	0 (0.00%)	>10	0.319	0.674	Co-occurrence
LYVE1	11p15.4	1 (0.87%)	0 (0.00%)	>10	0.319	0.674	Co-occurrence
LYZL4	3p22.1	1 (0.87%)	0 (0.00%)	>10	0.319	0.674	Co-occurrence
M6PR	12p13.31	1 (0.87%)	0 (0.00%)	>10	0.319	0.674	Co-occurrence
MAATS1	3q13.33	1 (0.87%)	0 (0.00%)	>10	0.319	0.674	Co-occurrence
MACROD2	20p12.1	1 (0.87%)	0 (0.00%)	>10	0.319	0.674	Co-occurrence
MADCAM1	19p13.3	1 (0.87%)	0 (0.00%)	>10	0.319	0.674	Co-occurrence
MADD	11p11.2	1 (0.87%)	0 (0.00%)	>10	0.319	0.674	Co-occurrence
MAFB	20q12	1 (0.87%)	0 (0.00%)	>10	0.319	0.674	Co-occurrence
MAGEB17	Xp22.2	1 (0.87%)	0 (0.00%)	>10	0.319	0.674	Co-occurrence
MAGEB18	Xp21.3	1 (0.87%)	0 (0.00%)	>10	0.319	0.674	Co-occurrence
MAGEB5	Xp21.3	1 (0.87%)	0 (0.00%)	>10	0.319	0.674	Co-occurrence
MAGEB6	Xp21.3	1 (0.87%)	0 (0.00%)	>10	0.319	0.674	Co-occurrence
MAGI3	1p13.2	1 (0.87%)	0 (0.00%)	>10	0.319	0.674	Co-occurrence
MAGT1	Xq21.1	1 (0.87%)	0 (0.00%)	>10	0.319	0.674	Co-occurrence
MANEAL	1p34.3	1 (0.87%)	0 (0.00%)	>10	0.319	0.674	Co-occurrence
MANF	3p21.2	1 (0.87%)	0 (0.00%)	>10	0.319	0.674	Co-occurrence
MAP1LC3B	16q24.2	1 (0.87%)	0 (0.00%)	>10	0.319	0.674	Co-occurrence
MAP3K15	Xp22.12	1 (0.87%)	0 (0.00%)	>10	0.319	0.674	Co-occurrence
MAP4	3p21.31	1 (0.87%)	0 (0.00%)	>10	0.319	0.674	Co-occurrence
MAP4K2	11q13.1	1 (0.87%)	0 (0.00%)	>10	0.319	0.674	Co-occurrence
MAP7D1	1p34.3	1 (0.87%)	0 (0.00%)	>10	0.319	0.674	Co-occurrence
MAPK6	15q21.2	1 (0.87%)	0 (0.00%)	>10	0.319	0.674	Co-occurrence
MAPK8IP1	11p11.2	1 (0.87%)	0 (0.00%)	>10	0.319	0.674	Co-occurrence
MAPKAP1	9q33.3	1 (0.87%)	0 (0.00%)	>10	0.319	0.674	Co-occurrence
MAPKAPK5	12q24.12-q24.13	1 (0.87%)	0 (0.00%)	>10	0.319	0.674	Co-occurrence
MARCKS	6q21	1 (0.87%)	0 (0.00%)	>10	0.319	0.674	Co-occurrence
MARCKSL1	1p35.1	1 (0.87%)	0 (0.00%)	>10	0.319	0.674	Co-occurrence
MARK3	14q32.32-q32.33	1 (0.87%)	0 (0.00%)	>10	0.319	0.674	Co-occurrence

MAST4	5q12.3	1 (0.87%)	0 (0.00%)	>10	0.319	0.674	Co-occurrence
MAT1A	10q22.3	1 (0.87%)	0 (0.00%)	>10	0.319	0.674	Co-occurrence
MATK	19p13.3	1 (0.87%)	0 (0.00%)	>10	0.319	0.674	Co-occurrence
MBD1	18q21.1	1 (0.87%)	0 (0.00%)	>10	0.319	0.674	Co-occurrence
MBL2	10q21.1	1 (0.87%)	0 (0.00%)	>10	0.319	0.674	Co-occurrence
MBTPS2	Xp22.12	1 (0.87%)	0 (0.00%)	>10	0.319	0.674	Co-occurrence
MCHR2	6q16.2	1 (0.87%)	0 (0.00%)	>10	0.319	0.674	Co-occurrence
MCM8	20p12.3	1 (0.87%)	0 (0.00%)	>10	0.319	0.674	Co-occurrence
MDK	11p11.2	1 (0.87%)	0 (0.00%)	>10	0.319	0.674	Co-occurrence
MEAF6	1p34.3	1 (0.87%)	0 (0.00%)	>10	0.319	0.674	Co-occurrence
MED14	Xp11.4	1 (0.87%)	0 (0.00%)	>10	0.319	0.674	Co-occurrence
MED16	19p13.3	1 (0.87%)	0 (0.00%)	>10	0.319	0.674	Co-occurrence
MEG3	14q32.2	1 (0.87%)	0 (0.00%)	>10	0.319	0.674	Co-occurrence
MEGF6	1p36.32	1 (0.87%)	0 (0.00%)	>10	0.319	0.674	Co-occurrence
MEI1	22q13.2	1 (0.87%)	0 (0.00%)	>10	0.319	0.674	Co-occurrence
MEN1	11q13.1	1 (0.87%)	0 (0.00%)	>10	0.319	0.674	Co-occurrence
MFSD12	19p13.3	1 (0.87%)	0 (0.00%)	>10	0.319	0.674	Co-occurrence
MFSD14B	9q22.32	1 (0.87%)	0 (0.00%)	>10	0.319	0.674	Co-occurrence
MIB2	1p36.33	1 (0.87%)	0 (0.00%)	>10	0.319	0.674	Co-occurrence
MICAL2	11p15.3	1 (0.87%)	0 (0.00%)	>10	0.319	0.674	Co-occurrence
MICALCL	11p15.3	1 (0.87%)	0 (0.00%)	>10	0.319	0.674	Co-occurrence
MICU2	13q12.11	1 (0.87%)	0 (0.00%)	>10	0.319	0.674	Co-occurrence
MID1IP1	Xp11.4	1 (0.87%)	0 (0.00%)	>10	0.319	0.674	Co-occurrence
MIGA2	9q34.11	1 (0.87%)	0 (0.00%)	>10	0.319	0.674	Co-occurrence
MIPEP	13q12.12	1 (0.87%)	0 (0.00%)	>10	0.319	0.674	Co-occurrence
MIPEPP3	13q12.11	1 (0.87%)	0 (0.00%)	>10	0.319	0.674	Co-occurrence
MIR-1273F/1273F		1 (0.87%)	0 (0.00%)	>10	0.319	0.674	Co-occurrence
MIR-1299/1299		1 (0.87%)	0 (0.00%)	>10	0.319	0.674	Co-occurrence
MIR-2113/2113		1 (0.87%)	0 (0.00%)	>10	0.319	0.674	Co-occurrence

MIR-2276/2276		1 (0.87%)	0 (0.00%)	>10	0.319	0.674	Co-occurrence
MIR-2278/2278		1 (0.87%)	0 (0.00%)	>10	0.319	0.674	Co-occurrence
MIR-2392/2392		1 (0.87%)	0 (0.00%)	>10	0.319	0.674	Co-occurrence
MIR-297/297		1 (0.87%)	0 (0.00%)	>10	0.319	0.674	Co-occurrence
MIR-302E/302E		1 (0.87%)	0 (0.00%)	>10	0.319	0.674	Co-occurrence
MIR-3161/3161		1 (0.87%)	0 (0.00%)	>10	0.319	0.674	Co-occurrence
MIR-3183/3183		1 (0.87%)	0 (0.00%)	>10	0.319	0.674	Co-occurrence
MIR-3201/3201		1 (0.87%)	0 (0.00%)	>10	0.319	0.674	Co-occurrence
MIR-325/325		1 (0.87%)	0 (0.00%)	>10	0.319	0.674	Co-occurrence
MIR-3657/3657		1 (0.87%)	0 (0.00%)	>10	0.319	0.674	Co-occurrence
MIR-3659/3659		1 (0.87%)	0 (0.00%)	>10	0.319	0.674	Co-occurrence
MIR-378G/378G		1 (0.87%)	0 (0.00%)	>10	0.319	0.674	Co-occurrence
MIR-378I/378I		1 (0.87%)	0 (0.00%)	>10	0.319	0.674	Co-occurrence
MIR-3908/3908		1 (0.87%)	0 (0.00%)	>10	0.319	0.674	Co-occurrence
MIR-3924/3924		1 (0.87%)	0 (0.00%)	>10	0.319	0.674	Co-occurrence
MIR-4255/4255		1 (0.87%)	0 (0.00%)	>10	0.319	0.674	Co-occurrence
MIR-429/429		1 (0.87%)	0 (0.00%)	>10	0.319	0.674	Co-occurrence
MIR-4299/4299		1 (0.87%)	0 (0.00%)	>10	0.319	0.674	Co-occurrence
MIR-4304/4304		1 (0.87%)	0 (0.00%)	>10	0.319	0.674	Co-occurrence
MIR-4309/4309		1 (0.87%)	0 (0.00%)	>10	0.319	0.674	Co-occurrence
MIR-4329/4329		1 (0.87%)	0 (0.00%)	>10	0.319	0.674	Co-occurrence
MIR-4417/4417		1 (0.87%)	0 (0.00%)	>10	0.319	0.674	Co-occurrence
MIR-4419B/4419B		1 (0.87%)	0 (0.00%)	>10	0.319	0.674	Co-occurrence
MIR-4422/4422		1 (0.87%)	0 (0.00%)	>10	0.319	0.674	Co-occurrence
MIR-4443/4443		1 (0.87%)	0 (0.00%)	>10	0.319	0.674	Co-occurrence
MIR-4447/4447		1 (0.87%)	0 (0.00%)	>10	0.319	0.674	Co-occurrence
MIR-4477A/4477A		1 (0.87%)	0 (0.00%)	>10	0.319	0.674	Co-occurrence
MIR-4478/4478		1 (0.87%)	0 (0.00%)	>10	0.319	0.674	Co-occurrence
MIR-4486/4486		1 (0.87%)	0 (0.00%)	>10	0.319	0.674	Co-occurrence

MIR-4487/4487		1 (0.87%)	0 (0.00%)	>10	0.319	0.674	Co-occurrence
MIR-4498/4498		1 (0.87%)	0 (0.00%)	>10	0.319	0.674	Co-occurrence
MIR-4499/4499		1 (0.87%)	0 (0.00%)	>10	0.319	0.674	Co-occurrence
MIR-4660/4660		1 (0.87%)	0 (0.00%)	>10	0.319	0.674	Co-occurrence
MIR-4688/4688		1 (0.87%)	0 (0.00%)	>10	0.319	0.674	Co-occurrence
MIR-4689/4689		1 (0.87%)	0 (0.00%)	>10	0.319	0.674	Co-occurrence
MIR-4710/4710		1 (0.87%)	0 (0.00%)	>10	0.319	0.674	Co-occurrence
MIR-4741/4741		1 (0.87%)	0 (0.00%)	>10	0.319	0.674	Co-occurrence
MIR-4791/4791		1 (0.87%)	0 (0.00%)	>10	0.319	0.674	Co-occurrence
MIR-4792/4792		1 (0.87%)	0 (0.00%)	>10	0.319	0.674	Co-occurrence
MIR-5095/5095		1 (0.87%)	0 (0.00%)	>10	0.319	0.674	Co-occurrence
MIR-5188/5188		1 (0.87%)	0 (0.00%)	>10	0.319	0.674	Co-occurrence
MIR-548AN/548AN		1 (0.87%)	0 (0.00%)	>10	0.319	0.674	Co-occurrence
MIR-548M/548M		1 (0.87%)	0 (0.00%)	>10	0.319	0.674	Co-occurrence
MIR-551A/551A		1 (0.87%)	0 (0.00%)	>10	0.319	0.674	Co-occurrence
MIR-564/564		1 (0.87%)	0 (0.00%)	>10	0.319	0.674	Co-occurrence
MIR-567/567		1 (0.87%)	0 (0.00%)	>10	0.319	0.674	Co-occurrence
MIR-568/568		1 (0.87%)	0 (0.00%)	>10	0.319	0.674	Co-occurrence
MIR-5691/5691		1 (0.87%)	0 (0.00%)	>10	0.319	0.674	Co-occurrence
MIR-5693/5693		1 (0.87%)	0 (0.00%)	>10	0.319	0.674	Co-occurrence
MIR-575/575		1 (0.87%)	0 (0.00%)	>10	0.319	0.674	Co-occurrence
MIR-577/577		1 (0.87%)	0 (0.00%)	>10	0.319	0.674	Co-occurrence
MIR-601/601		1 (0.87%)	0 (0.00%)	>10	0.319	0.674	Co-occurrence
MIR-637/637		1 (0.87%)	0 (0.00%)	>10	0.319	0.674	Co-occurrence
MIR-670/670		1 (0.87%)	0 (0.00%)	>10	0.319	0.674	Co-occurrence
MIR-759/759		1 (0.87%)	0 (0.00%)	>10	0.319	0.674	Co-occurrence
MIR-760/760		1 (0.87%)	0 (0.00%)	>10	0.319	0.674	Co-occurrence
MIR-761/761		1 (0.87%)	0 (0.00%)	>10	0.319	0.674	Co-occurrence
MIR-920/920		1 (0.87%)	0 (0.00%)	>10	0.319	0.674	Co-occurrence

MIR181A2HG	9q33.3	1 (0.87%)	0 (0.00%)	>10	0.319	0.674	Co-occurrence
MIR600HG	9q33.3	1 (0.87%)	0 (0.00%)	>10	0.319	0.674	Co-occurrence
MIR99AHG	21q21.1	1 (0.87%)	0 (0.00%)	>10	0.319	0.674	Co-occurrence
MKNK1	1p33	1 (0.87%)	0 (0.00%)	>10	0.319	0.674	Co-occurrence
MKNK2	19p13.3	1 (0.87%)	0 (0.00%)	>10	0.319	0.674	Co-occurrence
MLEC	12q24.31	1 (0.87%)	0 (0.00%)	>10	0.319	0.674	Co-occurrence
MLH1	3p22.2	1 (0.87%)	0 (0.00%)	>10	0.319	0.674	Co-occurrence
MLLT1	19p13.3	1 (0.87%)	0 (0.00%)	>10	0.319	0.674	Co-occurrence
MMACHC	1p34.1	1 (0.87%)	0 (0.00%)	>10	0.319	0.674	Co-occurrence
MMEL1	1p36.32	1 (0.87%)	0 (0.00%)	>10	0.319	0.674	Co-occurrence
MMP23B	1p36.33	1 (0.87%)	0 (0.00%)	>10	0.319	0.674	Co-occurrence
MMP24	20q11.22	1 (0.87%)	0 (0.00%)	>10	0.319	0.674	Co-occurrence
MMP26	11p15.4	1 (0.87%)	0 (0.00%)	>10	0.319	0.674	Co-occurrence
MOAP1	14q32.12	1 (0.87%)	0 (0.00%)	>10	0.319	0.674	Co-occurrence
MOB1B	4q13.3	1 (0.87%)	0 (0.00%)	>10	0.319	0.674	Co-occurrence
MOB2	11p15.5	1 (0.87%)	0 (0.00%)	>10	0.319	0.674	Co-occurrence
MOB3A	19p13.3	1 (0.87%)	0 (0.00%)	>10	0.319	0.674	Co-occurrence
MOB3C	1p33	1 (0.87%)	0 (0.00%)	>10	0.319	0.674	Co-occurrence
MOBP	3p22.1	1 (0.87%)	0 (0.00%)	>10	0.319	0.674	Co-occurrence
MORC2	22q12.2	1 (0.87%)	0 (0.00%)	>10	0.319	0.674	Co-occurrence
MORC4	Xq22.3	1 (0.87%)	0 (0.00%)	>10	0.319	0.674	Co-occurrence
MORN1	1p36.32	1 (0.87%)	0 (0.00%)	>10	0.319	0.674	Co-occurrence
MORN5	9q33.2	1 (0.87%)	0 (0.00%)	>10	0.319	0.674	Co-occurrence
MOXD1	6q23.2	1 (0.87%)	0 (0.00%)	>10	0.319	0.674	Co-occurrence
MPHOSPH8	13q12.11	1 (0.87%)	0 (0.00%)	>10	0.319	0.674	Co-occurrence
MPHOSPH9	12q24.31	1 (0.87%)	0 (0.00%)	>10	0.319	0.674	Co-occurrence
MRM3	17p13.3	1 (0.87%)	0 (0.00%)	>10	0.319	0.674	Co-occurrence
MRPL17	11p15.4	1 (0.87%)	0 (0.00%)	>10	0.319	0.674	Co-occurrence
MRPL20	1p36.33	1 (0.87%)	0 (0.00%)	>10	0.319	0.674	Co-occurrence

MRPL54	19p13.3	1 (0.87%)	0 (0.00%)	>10	0.319	0.674	Co-occurrence
MRPL57	13q12.11	1 (0.87%)	0 (0.00%)	>10	0.319	0.674	Co-occurrence
MRPS15	1p34.3	1 (0.87%)	0 (0.00%)	>10	0.319	0.674	Co-occurrence
MRPS31	13q14.11	1 (0.87%)	0 (0.00%)	>10	0.319	0.674	Co-occurrence
MRRF	9q33.2	1 (0.87%)	0 (0.00%)	>10	0.319	0.674	Co-occurrence
MRVI1	11p15.4	1 (0.87%)	0 (0.00%)	>10	0.319	0.674	Co-occurrence
MSH4	1p31.1	1 (0.87%)	0 (0.00%)	>10	0.319	0.674	Co-occurrence
MSI1	12q24.31	1 (0.87%)	0 (0.00%)	>10	0.319	0.674	Co-occurrence
MSL3	Xp22.2	1 (0.87%)	0 (0.00%)	>10	0.319	0.674	Co-occurrence
MSMB	10q11.22	1 (0.87%)	0 (0.00%)	>10	0.319	0.674	Co-occurrence
MT4	16q13	1 (0.87%)	0 (0.00%)	>10	0.319	0.674	Co-occurrence
MTCH2	11p11.2	1 (0.87%)	0 (0.00%)	>10	0.319	0.674	Co-occurrence
MTF1	1p34.3	1 (0.87%)	0 (0.00%)	>10	0.319	0.674	Co-occurrence
MTF2	1p22.1	1 (0.87%)	0 (0.00%)	>10	0.319	0.674	Co-occurrence
MTFP1	22q12.2	1 (0.87%)	0 (0.00%)	>10	0.319	0.674	Co-occurrence
MTHFD1	14q23.3	1 (0.87%)	0 (0.00%)	>10	0.319	0.674	Co-occurrence
MTHFD2L	4q13.3	1 (0.87%)	0 (0.00%)	>10	0.319	0.674	Co-occurrence
MTHFSD	16q24.1	1 (0.87%)	0 (0.00%)	>10	0.319	0.674	Co-occurrence
MTMR6	13q12.13	1 (0.87%)	0 (0.00%)	>10	0.319	0.674	Co-occurrence
MTMR9LP	1p35.2	1 (0.87%)	0 (0.00%)	>10	0.319	0.674	Co-occurrence
MTRNR2L10	Xp11.21	1 (0.87%)	0 (0.00%)	>10	0.319	0.674	Co-occurrence
MTRNR2L13	4q26	1 (0.87%)	0 (0.00%)	>10	0.319	0.674	Co-occurrence
MTRNR2L5	10q21.1	1 (0.87%)	0 (0.00%)	>10	0.319	0.674	Co-occurrence
MTRNR2L8	11p15.4	1 (0.87%)	0 (0.00%)	>10	0.319	0.674	Co-occurrence
MUC15	11p14.2	1 (0.87%)	0 (0.00%)	>10	0.319	0.674	Co-occurrence
MUC7	4q13.3	1 (0.87%)	0 (0.00%)	>10	0.319	0.674	Co-occurrence
MUCL1	12q13.2	1 (0.87%)	0 (0.00%)	>10	0.319	0.674	Co-occurrence
MUSK	9q31.3	1 (0.87%)	0 (0.00%)	>10	0.319	0.674	Co-occurrence
MUTYH	1p34.1	1 (0.87%)	0 (0.00%)	>10	0.319	0.674	Co-occurrence

MXI1	10q25.2	1 (0.87%)	0 (0.00%)	>10	0.319	0.674	Co-occurrence
MXRA5	Xp22.33	1 (0.87%)	0 (0.00%)	>10	0.319	0.674	Co-occurrence
MXRA8	1p36.33	1 (0.87%)	0 (0.00%)	>10	0.319	0.674	Co-occurrence
MYBPC3	11p11.2	1 (0.87%)	0 (0.00%)	>10	0.319	0.674	Co-occurrence
MYL2	12q24.11	1 (0.87%)	0 (0.00%)	>10	0.319	0.674	Co-occurrence
MYL3	3p21.31	1 (0.87%)	0 (0.00%)	>10	0.319	0.674	Co-occurrence
MYO18B	22q12.1	1 (0.87%)	0 (0.00%)	>10	0.319	0.674	Co-occurrence
MYO5A	15q21.2	1 (0.87%)	0 (0.00%)	>10	0.319	0.674	Co-occurrence
MYO5B	18q21.1	1 (0.87%)	0 (0.00%)	>10	0.319	0.674	Co-occurrence
MYO5C	15q21.2	1 (0.87%)	0 (0.00%)	>10	0.319	0.674	Co-occurrence
MYRIP	3p22.1	1 (0.87%)	0 (0.00%)	>10	0.319	0.674	Co-occurrence
MZT2B	2q21.1	1 (0.87%)	0 (0.00%)	>10	0.319	0.674	Co-occurrence
NAA11	4q21.21	1 (0.87%)	0 (0.00%)	>10	0.319	0.674	Co-occurrence
NAA25	12q24.13	1 (0.87%)	0 (0.00%)	>10	0.319	0.674	Co-occurrence
NAAA	4q21.1	1 (0.87%)	0 (0.00%)	>10	0.319	0.674	Co-occurrence
NADK	1p36.33	1 (0.87%)	0 (0.00%)	>10	0.319	0.674	Co-occurrence
NAGA	22q13.2	1 (0.87%)	0 (0.00%)	>10	0.319	0.674	Co-occurrence
NASP	1p34.1	1 (0.87%)	0 (0.00%)	>10	0.319	0.674	Co-occurrence
NBEAP1	15q11.2	1 (0.87%)	0 (0.00%)	>10	0.319	0.674	Co-occurrence
NCBP2L	Xq22.3	1 (0.87%)	0 (0.00%)	>10	0.319	0.674	Co-occurrence
NCDN	1p34.3	1 (0.87%)	0 (0.00%)	>10	0.319	0.674	Co-occurrence
NCKAP1L	12q13.13-q13.2	1 (0.87%)	0 (0.00%)	>10	0.319	0.674	Co-occurrence
NCKIPSD	3p21.31	1 (0.87%)	0 (0.00%)	>10	0.319	0.674	Co-occurrence
NCOA4	10q11.22	1 (0.87%)	0 (0.00%)	>10	0.319	0.674	Co-occurrence
NCOR1P1	20p11.1	1 (0.87%)	0 (0.00%)	>10	0.319	0.674	Co-occurrence
NCOR2	12q24.31	1 (0.87%)	0 (0.00%)	>10	0.319	0.674	Co-occurrence
NDC1	1p32.3	1 (0.87%)	0 (0.00%)	>10	0.319	0.674	Co-occurrence
NDST4	4q26	1 (0.87%)	0 (0.00%)	>10	0.319	0.674	Co-occurrence
NDUFA6	22q13.2	1 (0.87%)	0 (0.00%)	>10	0.319	0.674	Co-occurrence

NDUFA7	19p13.2	1 (0.87%)	0 (0.00%)	>10	0.319	0.674	Co-occurrence
NDUFA8	9q33.2	1 (0.87%)	0 (0.00%)	>10	0.319	0.674	Co-occurrence
NDUFB11	Xp11.3	1 (0.87%)	0 (0.00%)	>10	0.319	0.674	Co-occurrence
NDUFS3	11p11.2	1 (0.87%)	0 (0.00%)	>10	0.319	0.674	Co-occurrence
NEB	2q23.3	1 (0.87%)	0 (0.00%)	>10	0.319	0.674	Co-occurrence
NEFH	22q12.2	1 (0.87%)	0 (0.00%)	>10	0.319	0.674	Co-occurrence
NEGR1	1p31.1	1 (0.87%)	0 (0.00%)	>10	0.319	0.674	Co-occurrence
NEK10	3p24.1	1 (0.87%)	0 (0.00%)	>10	0.319	0.674	Co-occurrence
NEK6	9q33.3	1 (0.87%)	0 (0.00%)	>10	0.319	0.674	Co-occurrence
NELL1	11p15.1	1 (0.87%)	0 (0.00%)	>10	0.319	0.674	Co-occurrence
NELL2	12q12	1 (0.87%)	0 (0.00%)	>10	0.319	0.674	Co-occurrence
NEPRO	3q13.2	1 (0.87%)	0 (0.00%)	>10	0.319	0.674	Co-occurrence
NFKBID	19q13.12	1 (0.87%)	0 (0.00%)	>10	0.319	0.674	Co-occurrence
NFS1	20q11.22	1 (0.87%)	0 (0.00%)	>10	0.319	0.674	Co-occurrence
NFYC	1p34.2	1 (0.87%)	0 (0.00%)	>10	0.319	0.674	Co-occurrence
NHLRC3	13q13.3	1 (0.87%)	0 (0.00%)	>10	0.319	0.674	Co-occurrence
NHS	Xp22.2-p22.13	1 (0.87%)	0 (0.00%)	>10	0.319	0.674	Co-occurrence
NIPA1	15q11.2	1 (0.87%)	0 (0.00%)	>10	0.319	0.674	Co-occurrence
NIPA2	15q11.2	1 (0.87%)	0 (0.00%)	>10	0.319	0.674	Co-occurrence
NKD1	16q12.1	1 (0.87%)	0 (0.00%)	>10	0.319	0.674	Co-occurrence
NKIRAS1	3p24.2	1 (0.87%)	0 (0.00%)	>10	0.319	0.674	Co-occurrence
NLRP10	11p15.4	1 (0.87%)	0 (0.00%)	>10	0.319	0.674	Co-occurrence
NLRP14	11p15.4	1 (0.87%)	0 (0.00%)	>10	0.319	0.674	Co-occurrence
NME6	3p21.31	1 (0.87%)	0 (0.00%)	>10	0.319	0.674	Co-occurrence
NMRK2	19p13.3	1 (0.87%)	0 (0.00%)	>10	0.319	0.674	Co-occurrence
NOC2L	1p36.33	1 (0.87%)	0 (0.00%)	>10	0.319	0.674	Co-occurrence
NOD2	16q12.1	1 (0.87%)	0 (0.00%)	>10	0.319	0.674	Co-occurrence
NODAL	10q22.1	1 (0.87%)	0 (0.00%)	>10	0.319	0.674	Co-occurrence
NOL3	16q22.1	1 (0.87%)	0 (0.00%)	>10	0.319	0.674	Co-occurrence

NOL6	9p13.3	1 (0.87%)	0 (0.00%)	>10	0.319	0.674	Co-occurrence
NORAD	20q11.23	1 (0.87%)	0 (0.00%)	>10	0.319	0.674	Co-occurrence
NPC1	18q11.2	1 (0.87%)	0 (0.00%)	>10	0.319	0.674	Co-occurrence
NPFFR1	10q22.1	1 (0.87%)	0 (0.00%)	>10	0.319	0.674	Co-occurrence
NPFFR2	4q13.3	1 (0.87%)	0 (0.00%)	>10	0.319	0.674	Co-occurrence
NPHP4	1p36.31	1 (0.87%)	0 (0.00%)	>10	0.319	0.674	Co-occurrence
NPM2	8p21.3	1 (0.87%)	0 (0.00%)	>10	0.319	0.674	Co-occurrence
NR1D2	3p24.2	1 (0.87%)	0 (0.00%)	>10	0.319	0.674	Co-occurrence
NR1H3	11p11.2	1 (0.87%)	0 (0.00%)	>10	0.319	0.674	Co-occurrence
NR4A1	12q13.13	1 (0.87%)	0 (0.00%)	>10	0.319	0.674	Co-occurrence
NR5A1	9q33.3	1 (0.87%)	0 (0.00%)	>10	0.319	0.674	Co-occurrence
NR6A1	9q33.3	1 (0.87%)	0 (0.00%)	>10	0.319	0.674	Co-occurrence
NRDC	1p32.3	1 (0.87%)	0 (0.00%)	>10	0.319	0.674	Co-occurrence
NRIP3	11p15.4	1 (0.87%)	0 (0.00%)	>10	0.319	0.674	Co-occurrence
NRON	9q33.3|9	1 (0.87%)	0 (0.00%)	>10	0.319	0.674	Co-occurrence
NT5C3AP1	4q26	1 (0.87%)	0 (0.00%)	>10	0.319	0.674	Co-occurrence
NUDT18	8p21.3	1 (0.87%)	0 (0.00%)	>10	0.319	0.674	Co-occurrence
NUP160	11p11.2	1 (0.87%)	0 (0.00%)	>10	0.319	0.674	Co-occurrence
NUP188	9q34.11	1 (0.87%)	0 (0.00%)	>10	0.319	0.674	Co-occurrence
NUP54	4q21.1	1 (0.87%)	0 (0.00%)	>10	0.319	0.674	Co-occurrence
NUP58	13q12.13	1 (0.87%)	0 (0.00%)	>10	0.319	0.674	Co-occurrence
NUTM2F	9q22.32	1 (0.87%)	0 (0.00%)	>10	0.319	0.674	Co-occurrence
NXN	17p13.3	1 (0.87%)	0 (0.00%)	>10	0.319	0.674	Co-occurrence
OAS1	12q24.13	1 (0.87%)	0 (0.00%)	>10	0.319	0.674	Co-occurrence
OAS2	12q24.13	1 (0.87%)	0 (0.00%)	>10	0.319	0.674	Co-occurrence
OAS3	12q24.13	1 (0.87%)	0 (0.00%)	>10	0.319	0.674	Co-occurrence
OASL	12q24.31	1 (0.87%)	0 (0.00%)	>10	0.319	0.674	Co-occurrence
ODAM	4q13.3	1 (0.87%)	0 (0.00%)	>10	0.319	0.674	Co-occurrence
ODAPH	4q21.1	1 (0.87%)	0 (0.00%)	>10	0.319	0.674	Co-occurrence

ODF2L	1p22.3	1 (0.87%)	0 (0.00%)	>10	0.319	0.674	Co-occurrence
ODF3L2	19p13.3	1 (0.87%)	0 (0.00%)	>10	0.319	0.674	Co-occurrence
OGFOD2	12q24.31	1 (0.87%)	0 (0.00%)	>10	0.319	0.674	Co-occurrence
OLFM4	13q14.3	1 (0.87%)	0 (0.00%)	>10	0.319	0.674	Co-occurrence
OLFML1	11p15.4	1 (0.87%)	0 (0.00%)	>10	0.319	0.674	Co-occurrence
OLFML2A	9q33.3	1 (0.87%)	0 (0.00%)	>10	0.319	0.674	Co-occurrence
OLFML3	1p13.2	1 (0.87%)	0 (0.00%)	>10	0.319	0.674	Co-occurrence
OLR1	12p13.2	1 (0.87%)	0 (0.00%)	>10	0.319	0.674	Co-occurrence
OMA1	1p32.2-p32.1	1 (0.87%)	0 (0.00%)	>10	0.319	0.674	Co-occurrence
ONECUT1	15q21.3	1 (0.87%)	0 (0.00%)	>10	0.319	0.674	Co-occurrence
ONECUT3	19p13.3	1 (0.87%)	0 (0.00%)	>10	0.319	0.674	Co-occurrence
OPRPN	4q13.3	1 (0.87%)	0 (0.00%)	>10	0.319	0.674	Co-occurrence
OR10A2	11p15.4	1 (0.87%)	0 (0.00%)	>10	0.319	0.674	Co-occurrence
OR10A3	11p15.4	1 (0.87%)	0 (0.00%)	>10	0.319	0.674	Co-occurrence
OR10A4	11p15.4	1 (0.87%)	0 (0.00%)	>10	0.319	0.674	Co-occurrence
OR10A5	11p15.4	1 (0.87%)	0 (0.00%)	>10	0.319	0.674	Co-occurrence
OR10A6	11p15.4	1 (0.87%)	0 (0.00%)	>10	0.319	0.674	Co-occurrence
OR10A7	12q13.2	1 (0.87%)	0 (0.00%)	>10	0.319	0.674	Co-occurrence
OR10AB1P	11p15.4	1 (0.87%)	0 (0.00%)	>10	0.319	0.674	Co-occurrence
OR1B1	9q33.2	1 (0.87%)	0 (0.00%)	>10	0.319	0.674	Co-occurrence
OR1J1	9q33.2	1 (0.87%)	0 (0.00%)	>10	0.319	0.674	Co-occurrence
OR1J2	9q33.2	1 (0.87%)	0 (0.00%)	>10	0.319	0.674	Co-occurrence
OR1J4	9q33.2	1 (0.87%)	0 (0.00%)	>10	0.319	0.674	Co-occurrence
OR1K1	9q33.2	1 (0.87%)	0 (0.00%)	>10	0.319	0.674	Co-occurrence
OR1L1	9q33.2	1 (0.87%)	0 (0.00%)	>10	0.319	0.674	Co-occurrence
OR1L3	9q33.2	1 (0.87%)	0 (0.00%)	>10	0.319	0.674	Co-occurrence
OR1L4	9q33.2	1 (0.87%)	0 (0.00%)	>10	0.319	0.674	Co-occurrence
OR1L6	9q33.2	1 (0.87%)	0 (0.00%)	>10	0.319	0.674	Co-occurrence
OR1L8	9q33.2	1 (0.87%)	0 (0.00%)	>10	0.319	0.674	Co-occurrence

OR1N1	9q33.2	1 (0.87%)	0 (0.00%)	>10	0.319	0.674	Co-occurrence
OR1N2	9q33.2	1 (0.87%)	0 (0.00%)	>10	0.319	0.674	Co-occurrence
OR1Q1	9q33.2	1 (0.87%)	0 (0.00%)	>10	0.319	0.674	Co-occurrence
OR2AG1	11p15.4	1 (0.87%)	0 (0.00%)	>10	0.319	0.674	Co-occurrence
OR2AG2	11p15.4	1 (0.87%)	0 (0.00%)	>10	0.319	0.674	Co-occurrence
OR2D2	11p15.4	1 (0.87%)	0 (0.00%)	>10	0.319	0.674	Co-occurrence
OR2D3	11p15.4	1 (0.87%)	0 (0.00%)	>10	0.319	0.674	Co-occurrence
OR4A47	11p11.2	1 (0.87%)	0 (0.00%)	>10	0.319	0.674	Co-occurrence
OR4B1	11p11.2	1 (0.87%)	0 (0.00%)	>10	0.319	0.674	Co-occurrence
OR4C12	11p11.12	1 (0.87%)	0 (0.00%)	>10	0.319	0.674	Co-occurrence
OR4C13	11p11.12	1 (0.87%)	0 (0.00%)	>10	0.319	0.674	Co-occurrence
OR4C3	11p11.2	1 (0.87%)	0 (0.00%)	>10	0.319	0.674	Co-occurrence
OR4C5	11p11.2	1 (0.87%)	0 (0.00%)	>10	0.319	0.674	Co-occurrence
OR4D10	11q12.1	1 (0.87%)	0 (0.00%)	>10	0.319	0.674	Co-occurrence
OR4D11	11q12.1	1 (0.87%)	0 (0.00%)	>10	0.319	0.674	Co-occurrence
OR4D6	11q12.1	1 (0.87%)	0 (0.00%)	>10	0.319	0.674	Co-occurrence
OR4D9	11q12.1	1 (0.87%)	0 (0.00%)	>10	0.319	0.674	Co-occurrence
OR4F16	1p36.33	1 (0.87%)	0 (0.00%)	>10	0.319	0.674	Co-occurrence
OR4F29	1p36.33	1 (0.87%)	0 (0.00%)	>10	0.319	0.674	Co-occurrence
OR4F5	1p36.33	1 (0.87%)	0 (0.00%)	>10	0.319	0.674	Co-occurrence
OR4M2	15q11.2	1 (0.87%)	0 (0.00%)	>10	0.319	0.674	Co-occurrence
OR4N4	15q11.2	1 (0.87%)	0 (0.00%)	>10	0.319	0.674	Co-occurrence
OR4S1	11p11.2	1 (0.87%)	0 (0.00%)	>10	0.319	0.674	Co-occurrence
OR4X1	11p11.2	1 (0.87%)	0 (0.00%)	>10	0.319	0.674	Co-occurrence
OR4X2	11p11.2	1 (0.87%)	0 (0.00%)	>10	0.319	0.674	Co-occurrence
OR51A2	11p15.4	1 (0.87%)	0 (0.00%)	>10	0.319	0.674	Co-occurrence
OR51A4	11p15.4	1 (0.87%)	0 (0.00%)	>10	0.319	0.674	Co-occurrence
OR51A7	11p15.4	1 (0.87%)	0 (0.00%)	>10	0.319	0.674	Co-occurrence
OR51B2	11p15.4	1 (0.87%)	0 (0.00%)	>10	0.319	0.674	Co-occurrence

OR51B4	11p15.4	1 (0.87%)	0 (0.00%)	>10	0.319	0.674	Co-occurrence
OR51B5	11p15.4	1 (0.87%)	0 (0.00%)	>10	0.319	0.674	Co-occurrence
OR51B6	11p15.4	1 (0.87%)	0 (0.00%)	>10	0.319	0.674	Co-occurrence
OR51C1P	11p15.4	1 (0.87%)	0 (0.00%)	>10	0.319	0.674	Co-occurrence
OR51D1	11p15.4	1 (0.87%)	0 (0.00%)	>10	0.319	0.674	Co-occurrence
OR51E1	11p15.4	1 (0.87%)	0 (0.00%)	>10	0.319	0.674	Co-occurrence
OR51E2	11p15.4	1 (0.87%)	0 (0.00%)	>10	0.319	0.674	Co-occurrence
OR51F1	11p15.4	1 (0.87%)	0 (0.00%)	>10	0.319	0.674	Co-occurrence
OR51F2	11p15.4	1 (0.87%)	0 (0.00%)	>10	0.319	0.674	Co-occurrence
OR51G1	11p15.4	1 (0.87%)	0 (0.00%)	>10	0.319	0.674	Co-occurrence
OR51G2	11p15.4	1 (0.87%)	0 (0.00%)	>10	0.319	0.674	Co-occurrence
OR51H1	11p15.4	1 (0.87%)	0 (0.00%)	>10	0.319	0.674	Co-occurrence
OR51I1	11p15.4	1 (0.87%)	0 (0.00%)	>10	0.319	0.674	Co-occurrence
OR51I2	11p15.4	1 (0.87%)	0 (0.00%)	>10	0.319	0.674	Co-occurrence
OR51J1	11p15.4	1 (0.87%)	0 (0.00%)	>10	0.319	0.674	Co-occurrence
OR51L1	11p15.4	1 (0.87%)	0 (0.00%)	>10	0.319	0.674	Co-occurrence
OR51M1	11p15.4	1 (0.87%)	0 (0.00%)	>10	0.319	0.674	Co-occurrence
OR51Q1	11p15.4	1 (0.87%)	0 (0.00%)	>10	0.319	0.674	Co-occurrence
OR51S1	11p15.4	1 (0.87%)	0 (0.00%)	>10	0.319	0.674	Co-occurrence
OR51T1	11p15.4	1 (0.87%)	0 (0.00%)	>10	0.319	0.674	Co-occurrence
OR51V1	11p15.4	1 (0.87%)	0 (0.00%)	>10	0.319	0.674	Co-occurrence
OR52A1	11p15.4	1 (0.87%)	0 (0.00%)	>10	0.319	0.674	Co-occurrence
OR52A4P	11p15.4	1 (0.87%)	0 (0.00%)	>10	0.319	0.674	Co-occurrence
OR52A5	11p15.4	1 (0.87%)	0 (0.00%)	>10	0.319	0.674	Co-occurrence
OR52B2	11p15.4	1 (0.87%)	0 (0.00%)	>10	0.319	0.674	Co-occurrence
OR52B6	11p15.4	1 (0.87%)	0 (0.00%)	>10	0.319	0.674	Co-occurrence
OR52D1	11p15.4	1 (0.87%)	0 (0.00%)	>10	0.319	0.674	Co-occurrence
OR52E2	11p15.4	1 (0.87%)	0 (0.00%)	>10	0.319	0.674	Co-occurrence
OR52E4	11p15.4	1 (0.87%)	0 (0.00%)	>10	0.319	0.674	Co-occurrence

OR52E6	11p15.4	1 (0.87%)	0 (0.00%)	>10	0.319	0.674	Co-occurrence
OR52E8	11p15.4	1 (0.87%)	0 (0.00%)	>10	0.319	0.674	Co-occurrence
OR52H1	11p15.4	1 (0.87%)	0 (0.00%)	>10	0.319	0.674	Co-occurrence
OR52I1	11p15.4	1 (0.87%)	0 (0.00%)	>10	0.319	0.674	Co-occurrence
OR52I2	11p15.4	1 (0.87%)	0 (0.00%)	>10	0.319	0.674	Co-occurrence
OR52J3	11p15.4	1 (0.87%)	0 (0.00%)	>10	0.319	0.674	Co-occurrence
OR52K1	11p15.4	1 (0.87%)	0 (0.00%)	>10	0.319	0.674	Co-occurrence
OR52L1	11p15.4	1 (0.87%)	0 (0.00%)	>10	0.319	0.674	Co-occurrence
OR52M1	11p15.4	1 (0.87%)	0 (0.00%)	>10	0.319	0.674	Co-occurrence
OR52N1	11p15.4	1 (0.87%)	0 (0.00%)	>10	0.319	0.674	Co-occurrence
OR52N2	11p15.4	1 (0.87%)	0 (0.00%)	>10	0.319	0.674	Co-occurrence
OR52N4	11p15.4	1 (0.87%)	0 (0.00%)	>10	0.319	0.674	Co-occurrence
OR52N5	11p15.4	1 (0.87%)	0 (0.00%)	>10	0.319	0.674	Co-occurrence
OR52R1	11p15.4	1 (0.87%)	0 (0.00%)	>10	0.319	0.674	Co-occurrence
OR52W1	11p15.4	1 (0.87%)	0 (0.00%)	>10	0.319	0.674	Co-occurrence
OR56A1	11p15.4	1 (0.87%)	0 (0.00%)	>10	0.319	0.674	Co-occurrence
OR56A3	11p15.4	1 (0.87%)	0 (0.00%)	>10	0.319	0.674	Co-occurrence
OR56A4	11p15.4	1 (0.87%)	0 (0.00%)	>10	0.319	0.674	Co-occurrence
OR56B1	11p15.4	1 (0.87%)	0 (0.00%)	>10	0.319	0.674	Co-occurrence
OR56B4	11p15.4	1 (0.87%)	0 (0.00%)	>10	0.319	0.674	Co-occurrence
OR5A1	11q12.1	1 (0.87%)	0 (0.00%)	>10	0.319	0.674	Co-occurrence
OR5A2	11q12.1	1 (0.87%)	0 (0.00%)	>10	0.319	0.674	Co-occurrence
OR5AN1	11q12.1	1 (0.87%)	0 (0.00%)	>10	0.319	0.674	Co-occurrence
OR5C1	9q33.2	1 (0.87%)	0 (0.00%)	>10	0.319	0.674	Co-occurrence
OR5P2	11p15.4	1 (0.87%)	0 (0.00%)	>10	0.319	0.674	Co-occurrence
OR5P3	11p15.4	1 (0.87%)	0 (0.00%)	>10	0.319	0.674	Co-occurrence
OR6A2	11p15.4	1 (0.87%)	0 (0.00%)	>10	0.319	0.674	Co-occurrence
OR6C74	12q13.2	1 (0.87%)	0 (0.00%)	>10	0.319	0.674	Co-occurrence
OR7E14P	11p15.1	1 (0.87%)	0 (0.00%)	>10	0.319	0.674	Co-occurrence

OR7E47P	12q13.13	1 (0.87%)	0 (0.00%)	>10	0.319	0.674	Co-occurrence
OR7E94P	4q21.21	1 (0.87%)	0 (0.00%)	>10	0.319	0.674	Co-occurrence
OR9K2	12q13.2	1 (0.87%)	0 (0.00%)	>10	0.319	0.674	Co-occurrence
ORC1	1p32.3	1 (0.87%)	0 (0.00%)	>10	0.319	0.674	Co-occurrence
OSBP2	22q12.2	1 (0.87%)	0 (0.00%)	>10	0.319	0.674	Co-occurrence
OSBPL1A	18q11.2	1 (0.87%)	0 (0.00%)	>10	0.319	0.674	Co-occurrence
OSCP1	1p34.3	1 (0.87%)	0 (0.00%)	>10	0.319	0.674	Co-occurrence
OSER1	20q13.12	1 (0.87%)	0 (0.00%)	>10	0.319	0.674	Co-occurrence
OSM	22q12.2	1 (0.87%)	0 (0.00%)	>10	0.319	0.674	Co-occurrence
OTC	Xp11.4	1 (0.87%)	0 (0.00%)	>10	0.319	0.674	Co-occurrence
OVCH1	12p11.22	1 (0.87%)	0 (0.00%)	>10	0.319	0.674	Co-occurrence
OVCH2	11p15.4	1 (0.87%)	0 (0.00%)	>10	0.319	0.674	Co-occurrence
OVOL3	19q13.12	1 (0.87%)	0 (0.00%)	>10	0.319	0.674	Co-occurrence
P2RX4	12q24.31	1 (0.87%)	0 (0.00%)	>10	0.319	0.674	Co-occurrence
P2RX7	12q24.31	1 (0.87%)	0 (0.00%)	>10	0.319	0.674	Co-occurrence
P2RY8	Xp22.33 and Yp11.2	1 (0.87%)	0 (0.00%)	>10	0.319	0.674	Co-occurrence
PABPC3	13q12.13	1 (0.87%)	0 (0.00%)	>10	0.319	0.674	Co-occurrence
PABPC4L	4q28.3	1 (0.87%)	0 (0.00%)	>10	0.319	0.674	Co-occurrence
PACSIN3	11p11.2	1 (0.87%)	0 (0.00%)	>10	0.319	0.674	Co-occurrence
PAGE1	Xp11.23	1 (0.87%)	0 (0.00%)	>10	0.319	0.674	Co-occurrence
PAGE2	Xp11.21	1 (0.87%)	0 (0.00%)	>10	0.319	0.674	Co-occurrence
PAGE2B	Xp11.21	1 (0.87%)	0 (0.00%)	>10	0.319	0.674	Co-occurrence
PAGE3	Xp11.21	1 (0.87%)	0 (0.00%)	>10	0.319	0.674	Co-occurrence
PAGE5	Xp11.21	1 (0.87%)	0 (0.00%)	>10	0.319	0.674	Co-occurrence
PALD1	10q22.1	1 (0.87%)	0 (0.00%)	>10	0.319	0.674	Co-occurrence
PALLD	4q32.3	1 (0.87%)	0 (0.00%)	>10	0.319	0.674	Co-occurrence
PALM	19p13.3	1 (0.87%)	0 (0.00%)	>10	0.319	0.674	Co-occurrence
PANK4	1p36.32	1 (0.87%)	0 (0.00%)	>10	0.319	0.674	Co-occurrence
PAPPA	9q33.1	1 (0.87%)	0 (0.00%)	>10	0.319	0.674	Co-occurrence

PARG	10q11.23	1 (0.87%)	0 (0.00%)	>10	0.319	0.674	Co-occurrence
PARK7	1p36.23	1 (0.87%)	0 (0.00%)	>10	0.319	0.674	Co-occurrence
PARM1	4q13.3	1 (0.87%)	0 (0.00%)	>10	0.319	0.674	Co-occurrence
PARP4	13q12.12	1 (0.87%)	0 (0.00%)	>10	0.319	0.674	Co-occurrence
PARS2	1p32.3	1 (0.87%)	0 (0.00%)	>10	0.319	0.674	Co-occurrence
PARVA	11p15.3	1 (0.87%)	0 (0.00%)	>10	0.319	0.674	Co-occurrence
PATE1	11q24.2	1 (0.87%)	0 (0.00%)	>10	0.319	0.674	Co-occurrence
PATE2	11q24.2	1 (0.87%)	0 (0.00%)	>10	0.319	0.674	Co-occurrence
PATE3	11q24.2	1 (0.87%)	0 (0.00%)	>10	0.319	0.674	Co-occurrence
PATE4	11q24.2	1 (0.87%)	0 (0.00%)	>10	0.319	0.674	Co-occurrence
PAX2	10q24.31	1 (0.87%)	0 (0.00%)	>10	0.319	0.674	Co-occurrence
PBX3	9q33.3	1 (0.87%)	0 (0.00%)	>10	0.319	0.674	Co-occurrence
PCAT4	4q21.21	1 (0.87%)	0 (0.00%)	>10	0.319	0.674	Co-occurrence
PCAT7	9q22.32	1 (0.87%)	0 (0.00%)	>10	0.319	0.674	Co-occurrence
PCBD1	10q22.1	1 (0.87%)	0 (0.00%)	>10	0.319	0.674	Co-occurrence
PCDH10	4q28.3	1 (0.87%)	0 (0.00%)	>10	0.319	0.674	Co-occurrence
PCDH11X	Xq21.31	1 (0.87%)	0 (0.00%)	>10	0.319	0.674	Co-occurrence
PCDH8	13q14.3	1 (0.87%)	0 (0.00%)	>10	0.319	0.674	Co-occurrence
PCSK9	1p32.3	1 (0.87%)	0 (0.00%)	>10	0.319	0.674	Co-occurrence
PCYT1B	Xp22.11	1 (0.87%)	0 (0.00%)	>10	0.319	0.674	Co-occurrence
PDCD6IP	3p22.3	1 (0.87%)	0 (0.00%)	>10	0.319	0.674	Co-occurrence
PDCL	9q33.2	1 (0.87%)	0 (0.00%)	>10	0.319	0.674	Co-occurrence
PDE1B	12q13.2	1 (0.87%)	0 (0.00%)	>10	0.319	0.674	Co-occurrence
PDE3A	12p12.2	1 (0.87%)	0 (0.00%)	>10	0.319	0.674	Co-occurrence
PDE3B	11p15.2	1 (0.87%)	0 (0.00%)	>10	0.319	0.674	Co-occurrence
PDHA1	Xp22.12	1 (0.87%)	0 (0.00%)	>10	0.319	0.674	Co-occurrence
PDK3	Xp22.11	1 (0.87%)	0 (0.00%)	>10	0.319	0.674	Co-occurrence
PDLIM2	8p21.3	1 (0.87%)	0 (0.00%)	>10	0.319	0.674	Co-occurrence
PDLIM5	4q22.3	1 (0.87%)	0 (0.00%)	>10	0.319	0.674	Co-occurrence

PDZD8	10q25.3-q26.11	1 (0.87%)	0 (0.00%)	>10	0.319	0.674	Co-occurrence
PDZRN4	12q12	1 (0.87%)	0 (0.00%)	>10	0.319	0.674	Co-occurrence
PER3	1p36.23	1 (0.87%)	0 (0.00%)	>10	0.319	0.674	Co-occurrence
PERM1	1p36.33	1 (0.87%)	0 (0.00%)	>10	0.319	0.674	Co-occurrence
PES1	22q12.2	1 (0.87%)	0 (0.00%)	>10	0.319	0.674	Co-occurrence
PEX10	1p36.32	1 (0.87%)	0 (0.00%)	>10	0.319	0.674	Co-occurrence
PEX16	11p11.2	1 (0.87%)	0 (0.00%)	>10	0.319	0.674	Co-occurrence
PF4	4q13.3	1 (0.87%)	0 (0.00%)	>10	0.319	0.674	Co-occurrence
PGAM4	Xq21.1	1 (0.87%)	0 (0.00%)	>10	0.319	0.674	Co-occurrence
PGK1	Xq21.1	1 (0.87%)	0 (0.00%)	>10	0.319	0.674	Co-occurrence
PGM5	9q21.11	1 (0.87%)	0 (0.00%)	>10	0.319	0.674	Co-occurrence
PGM5P2	9p11.2	1 (0.87%)	0 (0.00%)	>10	0.319	0.674	Co-occurrence
PHC1	12p13.31	1 (0.87%)	0 (0.00%)	>10	0.319	0.674	Co-occurrence
PHETA1	12q24.12	1 (0.87%)	0 (0.00%)	>10	0.319	0.674	Co-occurrence
PHETA2	22q13.2	1 (0.87%)	0 (0.00%)	>10	0.319	0.674	Co-occurrence
PHF19	9q33.2	1 (0.87%)	0 (0.00%)	>10	0.319	0.674	Co-occurrence
PHF20	20q11.22-q11.23	1 (0.87%)	0 (0.00%)	>10	0.319	0.674	Co-occurrence
PHF21A	11p11.2	1 (0.87%)	0 (0.00%)	>10	0.319	0.674	Co-occurrence
PHF24	9p13.3	1 (0.87%)	0 (0.00%)	>10	0.319	0.674	Co-occurrence
PHF8	Xp11.22	1 (0.87%)	0 (0.00%)	>10	0.319	0.674	Co-occurrence
PHKA2	Xp22.13	1 (0.87%)	0 (0.00%)	>10	0.319	0.674	Co-occurrence
PHLDB2	3q13.2	1 (0.87%)	0 (0.00%)	>10	0.319	0.674	Co-occurrence
PHTF1	1p13.2	1 (0.87%)	0 (0.00%)	>10	0.319	0.674	Co-occurrence
PHYHD1	9q34.11	1 (0.87%)	0 (0.00%)	>10	0.319	0.674	Co-occurrence
PHYHIPL	10q21.1	1 (0.87%)	0 (0.00%)	>10	0.319	0.674	Co-occurrence
PIGA	Xp22.2	1 (0.87%)	0 (0.00%)	>10	0.319	0.674	Co-occurrence
PIGO	9p13.3	1 (0.87%)	0 (0.00%)	>10	0.319	0.674	Co-occurrence
PIN1P1	1p31.1	1 (0.87%)	0 (0.00%)	>10	0.319	0.674	Co-occurrence
PIP5K1C	19p13.3	1 (0.87%)	0 (0.00%)	>10	0.319	0.674	Co-occurrence

PIR	Xp22.2	1 (0.87%)	0 (0.00%)	>10	0.319	0.674	Co-occurrence
PITPNM2	12q24.31	1 (0.87%)	0 (0.00%)	>10	0.319	0.674	Co-occurrence
PITX2	4q25	1 (0.87%)	0 (0.00%)	>10	0.319	0.674	Co-occurrence
PIWIL1	12q24.33	1 (0.87%)	0 (0.00%)	>10	0.319	0.674	Co-occurrence
PKN2	1p22.2	1 (0.87%)	0 (0.00%)	>10	0.319	0.674	Co-occurrence
PKNOX2	11q24.2	1 (0.87%)	0 (0.00%)	>10	0.319	0.674	Co-occurrence
PKP2	12p11.21	1 (0.87%)	0 (0.00%)	>10	0.319	0.674	Co-occurrence
PLA2G1B	12q24.31	1 (0.87%)	0 (0.00%)	>10	0.319	0.674	Co-occurrence
PLCB1	20p12.3	1 (0.87%)	0 (0.00%)	>10	0.319	0.674	Co-occurrence
PLCH2	1p36.32	1 (0.87%)	0 (0.00%)	>10	0.319	0.674	Co-occurrence
PLCXD1	Xp22.33 and Yp11.32-p11.3	1 (0.87%)	0 (0.00%)	>10	0.319	0.674	Co-occurrence
PLCXD2	3q13.2	1 (0.87%)	0 (0.00%)	>10	0.319	0.674	Co-occurrence
PLD4	14q32.33	1 (0.87%)	0 (0.00%)	>10	0.319	0.674	Co-occurrence
PLEKHA5	12p12.3	1 (0.87%)	0 (0.00%)	>10	0.319	0.674	Co-occurrence
PLEKHA7	11p15.2-p15.1	1 (0.87%)	0 (0.00%)	>10	0.319	0.674	Co-occurrence
PLEKHG3	14q23.3	1 (0.87%)	0 (0.00%)	>10	0.319	0.674	Co-occurrence
PLEKHG4	16q22.1	1 (0.87%)	0 (0.00%)	>10	0.319	0.674	Co-occurrence
PLEKHN1	1p36.33	1 (0.87%)	0 (0.00%)	>10	0.319	0.674	Co-occurrence
PLEKHS1	10q25.3	1 (0.87%)	0 (0.00%)	>10	0.319	0.674	Co-occurrence
PLK3	1p34.1	1 (0.87%)	0 (0.00%)	>10	0.319	0.674	Co-occurrence
PNISR	6q16.2	1 (0.87%)	0 (0.00%)	>10	0.319	0.674	Co-occurrence
PNPLA3	22q13.31	1 (0.87%)	0 (0.00%)	>10	0.319	0.674	Co-occurrence
PNPLA5	22q13.31	1 (0.87%)	0 (0.00%)	>10	0.319	0.674	Co-occurrence
POLA1	Xp22.11-p21.3	1 (0.87%)	0 (0.00%)	>10	0.319	0.674	Co-occurrence
POLR2D	2q14.3	1 (0.87%)	0 (0.00%)	>10	0.319	0.674	Co-occurrence
POLR2I	19q13.12	1 (0.87%)	0 (0.00%)	>10	0.319	0.674	Co-occurrence
POLRMT	19p13.3	1 (0.87%)	0 (0.00%)	>10	0.319	0.674	Co-occurrence
POP5	12q24.31	1 (0.87%)	0 (0.00%)	>10	0.319	0.674	Co-occurrence
PORCN	Xp11.23	1 (0.87%)	0 (0.00%)	>10	0.319	0.674	Co-occurrence

POTEB	15q11.2	1 (0.87%)	0 (0.00%)	>10	0.319	0.674	Co-occurrence
POTEB2	15q11.2	1 (0.87%)	0 (0.00%)	>10	0.319	0.674	Co-occurrence
POTEF	2q21.1	1 (0.87%)	0 (0.00%)	>10	0.319	0.674	Co-occurrence
POTEI	2q21.1	1 (0.87%)	0 (0.00%)	>10	0.319	0.674	Co-occurrence
POTEJ	2q21.1	1 (0.87%)	0 (0.00%)	>10	0.319	0.674	Co-occurrence
POU3F1	1p34.3	1 (0.87%)	0 (0.00%)	>10	0.319	0.674	Co-occurrence
PP2D1	3p24.3	1 (0.87%)	0 (0.00%)	>10	0.319	0.674	Co-occurrence
PPA1	10q22.1	1 (0.87%)	0 (0.00%)	>10	0.319	0.674	Co-occurrence
PPBP	4q13.3	1 (0.87%)	0 (0.00%)	>10	0.319	0.674	Co-occurrence
PPBPP2	4q13.3	1 (0.87%)	0 (0.00%)	>10	0.319	0.674	Co-occurrence
PPEF1	Xp22.13	1 (0.87%)	0 (0.00%)	>10	0.319	0.674	Co-occurrence
PPEF2	4q21.1	1 (0.87%)	0 (0.00%)	>10	0.319	0.674	Co-occurrence
PPFIBP2	11p15.4	1 (0.87%)	0 (0.00%)	>10	0.319	0.674	Co-occurrence
PPHLN1	12q12	1 (0.87%)	0 (0.00%)	>10	0.319	0.674	Co-occurrence
PPIF	10q22.3	1 (0.87%)	0 (0.00%)	>10	0.319	0.674	Co-occurrence
PPP1R14A	19q13.2	1 (0.87%)	0 (0.00%)	>10	0.319	0.674	Co-occurrence
PPP1R1A	12q13.2	1 (0.87%)	0 (0.00%)	>10	0.319	0.674	Co-occurrence
PPP1R36	14q23.3	1 (0.87%)	0 (0.00%)	>10	0.319	0.674	Co-occurrence
PPP1R3B	8p23.1	1 (0.87%)	0 (0.00%)	>10	0.319	0.674	Co-occurrence
PPP2R3B	Xp22.33 and Yp11.31	1 (0.87%)	0 (0.00%)	>10	0.319	0.674	Co-occurrence
PPP2R5E	14q23.2	1 (0.87%)	0 (0.00%)	>10	0.319	0.674	Co-occurrence
PPWD1	5q12.3	1 (0.87%)	0 (0.00%)	>10	0.319	0.674	Co-occurrence
PRDM11	11p11.2	1 (0.87%)	0 (0.00%)	>10	0.319	0.674	Co-occurrence
PRDM13	6q16.2	1 (0.87%)	0 (0.00%)	>10	0.319	0.674	Co-occurrence
PRDM16	1p36.32	1 (0.87%)	0 (0.00%)	>10	0.319	0.674	Co-occurrence
PRDM16-DT	1p36.32	1 (0.87%)	0 (0.00%)	>10	0.319	0.674	Co-occurrence
PRDM4	12q23.3	1 (0.87%)	0 (0.00%)	>10	0.319	0.674	Co-occurrence
PRDM8	4q21.21	1 (0.87%)	0 (0.00%)	>10	0.319	0.674	Co-occurrence
PRDX1	1p34.1	1 (0.87%)	0 (0.00%)	>10	0.319	0.674	Co-occurrence

PRDX4	Xp22.11	1 (0.87%)	0 (0.00%)	>10	0.319	0.674	Co-occurrence
PRF1	10q22.1	1 (0.87%)	0 (0.00%)	>10	0.319	0.674	Co-occurrence
PRICKLE1	12q12	1 (0.87%)	0 (0.00%)	>10	0.319	0.674	Co-occurrence
PRKAB1	12q24.23	1 (0.87%)	0 (0.00%)	>10	0.319	0.674	Co-occurrence
PRKACB	1p31.1	1 (0.87%)	0 (0.00%)	>10	0.319	0.674	Co-occurrence
PRKCZ	1p36.33	1 (0.87%)	0 (0.00%)	>10	0.319	0.674	Co-occurrence
PRKX	Xp22.33	1 (0.87%)	0 (0.00%)	>10	0.319	0.674	Co-occurrence
PROK2	3p13	1 (0.87%)	0 (0.00%)	>10	0.319	0.674	Co-occurrence
PROS1	3q11.1	1 (0.87%)	0 (0.00%)	>10	0.319	0.674	Co-occurrence
PROSER1	13q13.3	1 (0.87%)	0 (0.00%)	>10	0.319	0.674	Co-occurrence
PRPF38A	1p32.3	1 (0.87%)	0 (0.00%)	>10	0.319	0.674	Co-occurrence
PRPF40A	2q23.3	1 (0.87%)	0 (0.00%)	>10	0.319	0.674	Co-occurrence
PRPS2	Xp22.2	1 (0.87%)	0 (0.00%)	>10	0.319	0.674	Co-occurrence
PRR27	4q13.3	1 (0.87%)	0 (0.00%)	>10	0.319	0.674	Co-occurrence
PRSS57	19p13.3	1 (0.87%)	0 (0.00%)	>10	0.319	0.674	Co-occurrence
PRTN3	19p13.3	1 (0.87%)	0 (0.00%)	>10	0.319	0.674	Co-occurrence
PRXL2B	1p36.32	1 (0.87%)	0 (0.00%)	>10	0.319	0.674	Co-occurrence
PSAP	10q22.1	1 (0.87%)	0 (0.00%)	>10	0.319	0.674	Co-occurrence
PSMA1	11p15.2	1 (0.87%)	0 (0.00%)	>10	0.319	0.674	Co-occurrence
PSMA8	18q11.2	1 (0.87%)	0 (0.00%)	>10	0.319	0.674	Co-occurrence
PSMB7	9q33.3	1 (0.87%)	0 (0.00%)	>10	0.319	0.674	Co-occurrence
PSMC3	11p11.2	1 (0.87%)	0 (0.00%)	>10	0.319	0.674	Co-occurrence
PSMD8	19q13.2	1 (0.87%)	0 (0.00%)	>10	0.319	0.674	Co-occurrence
PSPC1	13q12.11	1 (0.87%)	0 (0.00%)	>10	0.319	0.674	Co-occurrence
PTBP2	1p21.3	1 (0.87%)	0 (0.00%)	>10	0.319	0.674	Co-occurrence
PTBP3	9q32	1 (0.87%)	0 (0.00%)	>10	0.319	0.674	Co-occurrence
PTCH2	1p34.1	1 (0.87%)	0 (0.00%)	>10	0.319	0.674	Co-occurrence
PTCHD1	Xp22.11	1 (0.87%)	0 (0.00%)	>10	0.319	0.674	Co-occurrence
PTGER3	1p31.1	1 (0.87%)	0 (0.00%)	>10	0.319	0.674	Co-occurrence

PTGFR	1p31.1	1 (0.87%)	0 (0.00%)	>10	0.319	0.674	Co-occurrence
PTGFRN	1p13.1	1 (0.87%)	0 (0.00%)	>10	0.319	0.674	Co-occurrence
PTGR2	14q24.3	1 (0.87%)	0 (0.00%)	>10	0.319	0.674	Co-occurrence
PTGS1	9q33.2	1 (0.87%)	0 (0.00%)	>10	0.319	0.674	Co-occurrence
PTH	11p15.3	1 (0.87%)	0 (0.00%)	>10	0.319	0.674	Co-occurrence
PTH1R	3p21.31	1 (0.87%)	0 (0.00%)	>10	0.319	0.674	Co-occurrence
PTPA	9q34.11	1 (0.87%)	0 (0.00%)	>10	0.319	0.674	Co-occurrence
PTPDC1	9q22.32	1 (0.87%)	0 (0.00%)	>10	0.319	0.674	Co-occurrence
PTPMT1	11p11.2	1 (0.87%)	0 (0.00%)	>10	0.319	0.674	Co-occurrence
PTPN18	2q21.1	1 (0.87%)	0 (0.00%)	>10	0.319	0.674	Co-occurrence
PTPN21	14q31.3	1 (0.87%)	0 (0.00%)	>10	0.319	0.674	Co-occurrence
PTPN22	1p13.2	1 (0.87%)	0 (0.00%)	>10	0.319	0.674	Co-occurrence
PTPRG	3p14.2	1 (0.87%)	0 (0.00%)	>10	0.319	0.674	Co-occurrence
PTPRJ	11p11.2	1 (0.87%)	0 (0.00%)	>10	0.319	0.674	Co-occurrence
PUS3	11q24.2	1 (0.87%)	0 (0.00%)	>10	0.319	0.674	Co-occurrence
PUS7L	12q12	1 (0.87%)	0 (0.00%)	>10	0.319	0.674	Co-occurrence
PUSL1	1p36.33	1 (0.87%)	0 (0.00%)	>10	0.319	0.674	Co-occurrence
PWP1	12q23.3	1 (0.87%)	0 (0.00%)	>10	0.319	0.674	Co-occurrence
PWWP2B	10q26.3	1 (0.87%)	0 (0.00%)	>10	0.319	0.674	Co-occurrence
PXN	12q24.23	1 (0.87%)	0 (0.00%)	>10	0.319	0.674	Co-occurrence
PYROXD1	12p12.1	1 (0.87%)	0 (0.00%)	>10	0.319	0.674	Co-occurrence
PZP	12p13.31	1 (0.87%)	0 (0.00%)	>10	0.319	0.674	Co-occurrence
QTRT2	3q13.31	1 (0.87%)	0 (0.00%)	>10	0.319	0.674	Co-occurrence
R3HDM4	19p13.3	1 (0.87%)	0 (0.00%)	>10	0.319	0.674	Co-occurrence
RAB14	9q33.2	1 (0.87%)	0 (0.00%)	>10	0.319	0.674	Co-occurrence
RAB35	12q24.23	1 (0.87%)	0 (0.00%)	>10	0.319	0.674	Co-occurrence
RAB3B	1p32.3	1 (0.87%)	0 (0.00%)	>10	0.319	0.674	Co-occurrence
RAB5A	3p24.3	1 (0.87%)	0 (0.00%)	>10	0.319	0.674	Co-occurrence
RAB6C	2q21.1	1 (0.87%)	0 (0.00%)	>10	0.319	0.674	Co-occurrence

RAB9A	Xp22.2	1 (0.87%)	0 (0.00%)	>10	0.319	0.674	Co-occurrence
RABGAP1	9q33.2-q33.3	1 (0.87%)	0 (0.00%)	>10	0.319	0.674	Co-occurrence
RABGGTB	1p31.1	1 (0.87%)	0 (0.00%)	>10	0.319	0.674	Co-occurrence
RAD54L	1p34.1	1 (0.87%)	0 (0.00%)	>10	0.319	0.674	Co-occurrence
RAD54L2	3p21.2	1 (0.87%)	0 (0.00%)	>10	0.319	0.674	Co-occurrence
RAI2	Xp22.13	1 (0.87%)	0 (0.00%)	>10	0.319	0.674	Co-occurrence
RAN	12q24.33	1 (0.87%)	0 (0.00%)	>10	0.319	0.674	Co-occurrence
RAPSN	11p11.2	1 (0.87%)	0 (0.00%)	>10	0.319	0.674	Co-occurrence
RASAL1	12q24.13	1 (0.87%)	0 (0.00%)	>10	0.319	0.674	Co-occurrence
RASGRP4	19q13.2	1 (0.87%)	0 (0.00%)	>10	0.319	0.674	Co-occurrence
RASL10A	22q12.2	1 (0.87%)	0 (0.00%)	>10	0.319	0.674	Co-occurrence
RAX2	19p13.3	1 (0.87%)	0 (0.00%)	>10	0.319	0.674	Co-occurrence
RBBP8	18q11.2	1 (0.87%)	0 (0.00%)	>10	0.319	0.674	Co-occurrence
RBM10	Xp11.3	1 (0.87%)	0 (0.00%)	>10	0.319	0.674	Co-occurrence
RBM12	20q11.22	1 (0.87%)	0 (0.00%)	>10	0.319	0.674	Co-occurrence
RBM15B	3p21.2	1 (0.87%)	0 (0.00%)	>10	0.319	0.674	Co-occurrence
RBM18	9q33.2	1 (0.87%)	0 (0.00%)	>10	0.319	0.674	Co-occurrence
RBM19	12q24.13-q24.21	1 (0.87%)	0 (0.00%)	>10	0.319	0.674	Co-occurrence
RBM25	14q24.2	1 (0.87%)	0 (0.00%)	>10	0.319	0.674	Co-occurrence
RBM3	Xp11.23	1 (0.87%)	0 (0.00%)	>10	0.319	0.674	Co-occurrence
RBM39	20q11.22	1 (0.87%)	0 (0.00%)	>10	0.319	0.674	Co-occurrence
RBM46	4q32.1	1 (0.87%)	0 (0.00%)	>10	0.319	0.674	Co-occurrence
RBMS3	3p24.1	1 (0.87%)	0 (0.00%)	>10	0.319	0.674	Co-occurrence
RBMXL1	1p22.2	1 (0.87%)	0 (0.00%)	>10	0.319	0.674	Co-occurrence
RBMXL2	11p15.4	1 (0.87%)	0 (0.00%)	>10	0.319	0.674	Co-occurrence
RC3H2	9q33.2	1 (0.87%)	0 (0.00%)	>10	0.319	0.674	Co-occurrence
RCHY1	4q21.1	1 (0.87%)	0 (0.00%)	>10	0.319	0.674	Co-occurrence
RCOR1	14q32.31-q32.32	1 (0.87%)	0 (0.00%)	>10	0.319	0.674	Co-occurrence
RECQL	12p12.1	1 (0.87%)	0 (0.00%)	>10	0.319	0.674	Co-occurrence

REEP3	10q21.3	1 (0.87%)	0 (0.00%)	>10	0.319	0.674	Co-occurrence
REEP4	8p21.3	1 (0.87%)	0 (0.00%)	>10	0.319	0.674	Co-occurrence
RER1	1p36.32	1 (0.87%)	0 (0.00%)	>10	0.319	0.674	Co-occurrence
RESF1	12p11.21	1 (0.87%)	0 (0.00%)	>10	0.319	0.674	Co-occurrence
REXO1	19p13.3	1 (0.87%)	0 (0.00%)	>10	0.319	0.674	Co-occurrence
RFLNA	12q24.31	1 (0.87%)	0 (0.00%)	>10	0.319	0.674	Co-occurrence
RFLNB	17p13.3	1 (0.87%)	0 (0.00%)	>10	0.319	0.674	Co-occurrence
RFPL1	22q12.2	1 (0.87%)	0 (0.00%)	>10	0.319	0.674	Co-occurrence
RFPL1S	22q12.2	1 (0.87%)	0 (0.00%)	>10	0.319	0.674	Co-occurrence
RFX2	19p13.3	1 (0.87%)	0 (0.00%)	>10	0.319	0.674	Co-occurrence
RGN	Xp11.3	1 (0.87%)	0 (0.00%)	>10	0.319	0.674	Co-occurrence
RHBDD3	22q12.2	1 (0.87%)	0 (0.00%)	>10	0.319	0.674	Co-occurrence
RHOJ	14q23.2	1 (0.87%)	0 (0.00%)	>10	0.319	0.674	Co-occurrence
RIC3	11p15.4	1 (0.87%)	0 (0.00%)	>10	0.319	0.674	Co-occurrence
RIF1	2q23.3	1 (0.87%)	0 (0.00%)	>10	0.319	0.674	Co-occurrence
RILPL1	12q24.31	1 (0.87%)	0 (0.00%)	>10	0.319	0.674	Co-occurrence
RILPL2	12q24.31	1 (0.87%)	0 (0.00%)	>10	0.319	0.674	Co-occurrence
RIMBP2	12q24.33	1 (0.87%)	0 (0.00%)	>10	0.319	0.674	Co-occurrence
RIMS3	1p34.2	1 (0.87%)	0 (0.00%)	>10	0.319	0.674	Co-occurrence
RIN2	20p11.23	1 (0.87%)	0 (0.00%)	>10	0.319	0.674	Co-occurrence
RIOK3	18q11.2	1 (0.87%)	0 (0.00%)	>10	0.319	0.674	Co-occurrence
RIPOR1	16q22.1	1 (0.87%)	0 (0.00%)	>10	0.319	0.674	Co-occurrence
RIPPLY1	Xq22.3	1 (0.87%)	0 (0.00%)	>10	0.319	0.674	Co-occurrence
RITA1	12q24.13	1 (0.87%)	0 (0.00%)	>10	0.319	0.674	Co-occurrence
RMC1	18q11.2	1 (0.87%)	0 (0.00%)	>10	0.319	0.674	Co-occurrence
RN7SKP123	1p22.1	1 (0.87%)	0 (0.00%)	>10	0.319	0.674	Co-occurrence
RN7SKP125	9q33.1	1 (0.87%)	0 (0.00%)	>10	0.319	0.674	Co-occurrence
RN7SKP128	9q33.1	1 (0.87%)	0 (0.00%)	>10	0.319	0.674	Co-occurrence
RN7SKP144	3p26.3	1 (0.87%)	0 (0.00%)	>10	0.319	0.674	Co-occurrence

RN7SKP151	11p15.3	1 (0.87%)	0 (0.00%)	>10	0.319	0.674	Co-occurrence
RN7SKP161	12p13.2	1 (0.87%)	0 (0.00%)	>10	0.319	0.674	Co-occurrence
RN7SKP169	22q12.1	1 (0.87%)	0 (0.00%)	>10	0.319	0.674	Co-occurrence
RN7SKP19	1p31.1	1 (0.87%)	0 (0.00%)	>10	0.319	0.674	Co-occurrence
RN7SKP196	10q21.1	1 (0.87%)	0 (0.00%)	>10	0.319	0.674	Co-occurrence
RN7SKP197	12q24.23	1 (0.87%)	0 (0.00%)	>10	0.319	0.674	Co-occurrence
RN7SKP20	Xp22.2	1 (0.87%)	0 (0.00%)	>10	0.319	0.674	Co-occurrence
RN7SKP216	12q24.21	1 (0.87%)	0 (0.00%)	>10	0.319	0.674	Co-occurrence
RN7SKP227	3p22.2	1 (0.87%)	0 (0.00%)	>10	0.319	0.674	Co-occurrence
RN7SKP247	1p31.1	1 (0.87%)	0 (0.00%)	>10	0.319	0.674	Co-occurrence
RN7SKP270	1p21.3	1 (0.87%)	0 (0.00%)	>10	0.319	0.674	Co-occurrence
RN7SKP271	20q11.22	1 (0.87%)	0 (0.00%)	>10	0.319	0.674	Co-occurrence
RN7SKP287	11p11.2	1 (0.87%)	0 (0.00%)	>10	0.319	0.674	Co-occurrence
RN7SKP291	1p32.3	1 (0.87%)	0 (0.00%)	>10	0.319	0.674	Co-occurrence
RN7SKP44	18q12.1	1 (0.87%)	0 (0.00%)	>10	0.319	0.674	Co-occurrence
RN7SKP71	12q24.13	1 (0.87%)	0 (0.00%)	>10	0.319	0.674	Co-occurrence
RN7SKP90	11p15.2	1 (0.87%)	0 (0.00%)	>10	0.319	0.674	Co-occurrence
RN7SL106P	15q11.2	1 (0.87%)	0 (0.00%)	>10	0.319	0.674	Co-occurrence
RN7SL120P	3p26.3	1 (0.87%)	0 (0.00%)	>10	0.319	0.674	Co-occurrence
RN7SL122P	1p35.1	1 (0.87%)	0 (0.00%)	>10	0.319	0.674	Co-occurrence
RN7SL131P	1p34.3	1 (0.87%)	0 (0.00%)	>10	0.319	0.674	Co-occurrence
RN7SL162P	22q12.1	1 (0.87%)	0 (0.00%)	>10	0.319	0.674	Co-occurrence
RN7SL166P	13q12.11	1 (0.87%)	0 (0.00%)	>10	0.319	0.674	Co-occurrence
RN7SL181P	9q33.2	1 (0.87%)	0 (0.00%)	>10	0.319	0.674	Co-occurrence
RN7SL184P	4q26	1 (0.87%)	0 (0.00%)	>10	0.319	0.674	Co-occurrence
RN7SL187P	9q33.2	1 (0.87%)	0 (0.00%)	>10	0.319	0.674	Co-occurrence
RN7SL188P	11p15.2	1 (0.87%)	0 (0.00%)	>10	0.319	0.674	Co-occurrence
RN7SL202P	19p13.3	1 (0.87%)	0 (0.00%)	>10	0.319	0.674	Co-occurrence
RN7SL216P	3p24.2	1 (0.87%)	0 (0.00%)	>10	0.319	0.674	Co-occurrence

RN7SL218P	4q13.3	1 (0.87%)	0 (0.00%)	>10	0.319	0.674	Co-occurrence
RN7SL240P	11p14.1	1 (0.87%)	0 (0.00%)	>10	0.319	0.674	Co-occurrence
RN7SL242P	1p31.1	1 (0.87%)	0 (0.00%)	>10	0.319	0.674	Co-occurrence
RN7SL266P	Xq23	1 (0.87%)	0 (0.00%)	>10	0.319	0.674	Co-occurrence
RN7SL271P	3p13	1 (0.87%)	0 (0.00%)	>10	0.319	0.674	Co-occurrence
RN7SL290P	1p32.3	1 (0.87%)	0 (0.00%)	>10	0.319	0.674	Co-occurrence
RN7SL291P	Xp11.3	1 (0.87%)	0 (0.00%)	>10	0.319	0.674	Co-occurrence
RN7SL296P	3p22.3	1 (0.87%)	0 (0.00%)	>10	0.319	0.674	Co-occurrence
RN7SL302P	9q33.3	1 (0.87%)	0 (0.00%)	>10	0.319	0.674	Co-occurrence
RN7SL30P	9q33.3	1 (0.87%)	0 (0.00%)	>10	0.319	0.674	Co-occurrence
RN7SL310P	18q21.1	1 (0.87%)	0 (0.00%)	>10	0.319	0.674	Co-occurrence
RN7SL320P	13q14.3	1 (0.87%)	0 (0.00%)	>10	0.319	0.674	Co-occurrence
RN7SL321P	3p21.31	1 (0.87%)	0 (0.00%)	>10	0.319	0.674	Co-occurrence
RN7SL326P	1p34.2	1 (0.87%)	0 (0.00%)	>10	0.319	0.674	Co-occurrence
RN7SL338P	9p13.3	1 (0.87%)	0 (0.00%)	>10	0.319	0.674	Co-occurrence
RN7SL343P	9p11.2	1 (0.87%)	0 (0.00%)	>10	0.319	0.674	Co-occurrence
RN7SL351P	11q24.2	1 (0.87%)	0 (0.00%)	>10	0.319	0.674	Co-occurrence
RN7SL379P	Xq21.33	1 (0.87%)	0 (0.00%)	>10	0.319	0.674	Co-occurrence
RN7SL400P	15q11.2	1 (0.87%)	0 (0.00%)	>10	0.319	0.674	Co-occurrence
RN7SL402P	19q13.12	1 (0.87%)	0 (0.00%)	>10	0.319	0.674	Co-occurrence
RN7SL411P	3p22.1	1 (0.87%)	0 (0.00%)	>10	0.319	0.674	Co-occurrence
RN7SL413P	13q14.3	1 (0.87%)	0 (0.00%)	>10	0.319	0.674	Co-occurrence
RN7SL422P	9q21.11	1 (0.87%)	0 (0.00%)	>10	0.319	0.674	Co-occurrence
RN7SL430P	9q32	1 (0.87%)	0 (0.00%)	>10	0.319	0.674	Co-occurrence
RN7SL435P	11q12.1	1 (0.87%)	0 (0.00%)	>10	0.319	0.674	Co-occurrence
RN7SL460P	Xq21.1	1 (0.87%)	0 (0.00%)	>10	0.319	0.674	Co-occurrence
RN7SL462P	9q12	1 (0.87%)	0 (0.00%)	>10	0.319	0.674	Co-occurrence
RN7SL475P	1p32.1	1 (0.87%)	0 (0.00%)	>10	0.319	0.674	Co-occurrence
RN7SL479P	1p34.1	1 (0.87%)	0 (0.00%)	>10	0.319	0.674	Co-occurrence

RN7SL48P	Xp22.13	1 (0.87%)	0 (0.00%)	>10	0.319	0.674	Co-occurrence
RN7SL495P	15q11.2	1 (0.87%)	0 (0.00%)	>10	0.319	0.674	Co-occurrence
RN7SL498P	20p12.3	1 (0.87%)	0 (0.00%)	>10	0.319	0.674	Co-occurrence
RN7SL503P	1p34.3	1 (0.87%)	0 (0.00%)	>10	0.319	0.674	Co-occurrence
RN7SL504P	3p21.2	1 (0.87%)	0 (0.00%)	>10	0.319	0.674	Co-occurrence
RN7SL508P	12q24.23	1 (0.87%)	0 (0.00%)	>10	0.319	0.674	Co-occurrence
RN7SL536P	15q11.2	1 (0.87%)	0 (0.00%)	>10	0.319	0.674	Co-occurrence
RN7SL538P	1p31.1	1 (0.87%)	0 (0.00%)	>10	0.319	0.674	Co-occurrence
RN7SL544P	9q13	1 (0.87%)	0 (0.00%)	>10	0.319	0.674	Co-occurrence
RN7SL545P	15q11.2	1 (0.87%)	0 (0.00%)	>10	0.319	0.674	Co-occurrence
RN7SL546P	14q32.31	1 (0.87%)	0 (0.00%)	>10	0.319	0.674	Co-occurrence
RN7SL547P	20p12.3	1 (0.87%)	0 (0.00%)	>10	0.319	0.674	Co-occurrence
RN7SL553P	3p26.1	1 (0.87%)	0 (0.00%)	>10	0.319	0.674	Co-occurrence
RN7SL565P	9p11.2	1 (0.87%)	0 (0.00%)	>10	0.319	0.674	Co-occurrence
RN7SL574P	1p36.32	1 (0.87%)	0 (0.00%)	>10	0.319	0.674	Co-occurrence
RN7SL578P	Xp22.33	1 (0.87%)	0 (0.00%)	>10	0.319	0.674	Co-occurrence
RN7SL57P	9q32	1 (0.87%)	0 (0.00%)	>10	0.319	0.674	Co-occurrence
RN7SL582P	3q13.31	1 (0.87%)	0 (0.00%)	>10	0.319	0.674	Co-occurrence
RN7SL583P	1p22.2	1 (0.87%)	0 (0.00%)	>10	0.319	0.674	Co-occurrence
RN7SL584P	15q11.1	1 (0.87%)	0 (0.00%)	>10	0.319	0.674	Co-occurrence
RN7SL586P	14q24.2	1 (0.87%)	0 (0.00%)	>10	0.319	0.674	Co-occurrence
RN7SL591P	10q21.2	1 (0.87%)	0 (0.00%)	>10	0.319	0.674	Co-occurrence
RN7SL615P	20q12	1 (0.87%)	0 (0.00%)	>10	0.319	0.674	Co-occurrence
RN7SL62P	1p32.3	1 (0.87%)	0 (0.00%)	>10	0.319	0.674	Co-occurrence
RN7SL633P	22q12.2	1 (0.87%)	0 (0.00%)	>10	0.319	0.674	Co-occurrence
RN7SL640P	9p12	1 (0.87%)	0 (0.00%)	>10	0.319	0.674	Co-occurrence
RN7SL652P	11p11.2	1 (0.87%)	0 (0.00%)	>10	0.319	0.674	Co-occurrence
RN7SL653P	1p22.1	1 (0.87%)	0 (0.00%)	>10	0.319	0.674	Co-occurrence
RN7SL657P	1p36.33	1 (0.87%)	0 (0.00%)	>10	0.319	0.674	Co-occurrence

RN7SL658P	Xp22.2	1 (0.87%)	0 (0.00%)	>10	0.319	0.674	Co-occurrence
RN7SL663P	19q13.2	1 (0.87%)	0 (0.00%)	>10	0.319	0.674	Co-occurrence
RN7SL664P	3p21.31	1 (0.87%)	0 (0.00%)	>10	0.319	0.674	Co-occurrence
RN7SL692P	1p22.1	1 (0.87%)	0 (0.00%)	>10	0.319	0.674	Co-occurrence
RN7SL713P	1p32.1	1 (0.87%)	0 (0.00%)	>10	0.319	0.674	Co-occurrence
RN7SL720P	9p22.2	1 (0.87%)	0 (0.00%)	>10	0.319	0.674	Co-occurrence
RN7SL722P	9q13	1 (0.87%)	0 (0.00%)	>10	0.319	0.674	Co-occurrence
RN7SL741P	13q12.13	1 (0.87%)	0 (0.00%)	>10	0.319	0.674	Co-occurrence
RN7SL745P	18q11.2	1 (0.87%)	0 (0.00%)	>10	0.319	0.674	Co-occurrence
RN7SL759P	15q11.2	1 (0.87%)	0 (0.00%)	>10	0.319	0.674	Co-occurrence
RN7SL763P	9p12	1 (0.87%)	0 (0.00%)	>10	0.319	0.674	Co-occurrence
RN7SL766P	13q12.11	1 (0.87%)	0 (0.00%)	>10	0.319	0.674	Co-occurrence
RN7SL769P	12q24.11	1 (0.87%)	0 (0.00%)	>10	0.319	0.674	Co-occurrence
RN7SL785P	Xp11.3	1 (0.87%)	0 (0.00%)	>10	0.319	0.674	Co-occurrence
RN7SL787P	9q21.11	1 (0.87%)	0 (0.00%)	>10	0.319	0.674	Co-occurrence
RN7SL788P	1p32.3	1 (0.87%)	0 (0.00%)	>10	0.319	0.674	Co-occurrence
RN7SL808P	4q26	1 (0.87%)	0 (0.00%)	>10	0.319	0.674	Co-occurrence
RN7SL815P	3q13.31	1 (0.87%)	0 (0.00%)	>10	0.319	0.674	Co-occurrence
RN7SL824P	1p22.1	1 (0.87%)	0 (0.00%)	>10	0.319	0.674	Co-occurrence
RN7SL831P	1p21.3	1 (0.87%)	0 (0.00%)	>10	0.319	0.674	Co-occurrence
RN7SL843P	22q11.1	1 (0.87%)	0 (0.00%)	>10	0.319	0.674	Co-occurrence
RN7SL865P	12q24.21	1 (0.87%)	0 (0.00%)	>10	0.319	0.674	Co-occurrence
RN7SL870P	3p21.31	1 (0.87%)	0 (0.00%)	>10	0.319	0.674	Co-occurrence
RN7SL91P	Xp21.3	1 (0.87%)	0 (0.00%)	>10	0.319	0.674	Co-occurrence
RN7SL97P	18q11.2	1 (0.87%)	0 (0.00%)	>10	0.319	0.674	Co-occurrence
RNA5SP125	3p24.2	1 (0.87%)	0 (0.00%)	>10	0.319	0.674	Co-occurrence
RNA5SP126	3p24.2	1 (0.87%)	0 (0.00%)	>10	0.319	0.674	Co-occurrence
RNA5SP128	3p22.3	1 (0.87%)	0 (0.00%)	>10	0.319	0.674	Co-occurrence
RNA5SP132	3p21.2	1 (0.87%)	0 (0.00%)	>10	0.319	0.674	Co-occurrence

RNA5SP163	4q13.3	1 (0.87%)	0 (0.00%)	>10	0.319	0.674	Co-occurrence
RNA5SP164	4q22.2	1 (0.87%)	0 (0.00%)	>10	0.319	0.674	Co-occurrence
RNA5SP173	4q34.3	1 (0.87%)	0 (0.00%)	>10	0.319	0.674	Co-occurrence
RNA5SP218	6q23.2	1 (0.87%)	0 (0.00%)	>10	0.319	0.674	Co-occurrence
RNA5SP23	1p31.1	1 (0.87%)	0 (0.00%)	>10	0.319	0.674	Co-occurrence
RNA5SP25	13q12.11	1 (0.87%)	0 (0.00%)	>10	0.319	0.674	Co-occurrence
RNA5SP28	13q14.3	1 (0.87%)	0 (0.00%)	>10	0.319	0.674	Co-occurrence
RNA5SP281	9p21.1	1 (0.87%)	0 (0.00%)	>10	0.319	0.674	Co-occurrence
RNA5SP283	9q13	1 (0.87%)	0 (0.00%)	>10	0.319	0.674	Co-occurrence
RNA5SP284	9q13	1 (0.87%)	0 (0.00%)	>10	0.319	0.674	Co-occurrence
RNA5SP29	13q14.3	1 (0.87%)	0 (0.00%)	>10	0.319	0.674	Co-occurrence
RNA5SP295	9q32	1 (0.87%)	0 (0.00%)	>10	0.319	0.674	Co-occurrence
RNA5SP317	10q11.23	1 (0.87%)	0 (0.00%)	>10	0.319	0.674	Co-occurrence
RNA5SP329	11p15.4	1 (0.87%)	0 (0.00%)	>10	0.319	0.674	Co-occurrence
RNA5SP330	11p15.4	1 (0.87%)	0 (0.00%)	>10	0.319	0.674	Co-occurrence
RNA5SP331	11p15.2	1 (0.87%)	0 (0.00%)	>10	0.319	0.674	Co-occurrence
RNA5SP332	11p15.2	1 (0.87%)	0 (0.00%)	>10	0.319	0.674	Co-occurrence
RNA5SP335	11p15.1	1 (0.87%)	0 (0.00%)	>10	0.319	0.674	Co-occurrence
RNA5SP336	11p15.1	1 (0.87%)	0 (0.00%)	>10	0.319	0.674	Co-occurrence
RNA5SP337	11p15.1	1 (0.87%)	0 (0.00%)	>10	0.319	0.674	Co-occurrence
RNA5SP338	11p14.3	1 (0.87%)	0 (0.00%)	>10	0.319	0.674	Co-occurrence
RNA5SP339	11p14.1	1 (0.87%)	0 (0.00%)	>10	0.319	0.674	Co-occurrence
RNA5SP340	11p11.2	1 (0.87%)	0 (0.00%)	>10	0.319	0.674	Co-occurrence
RNA5SP356	12p11.22	1 (0.87%)	0 (0.00%)	>10	0.319	0.674	Co-occurrence
RNA5SP358	12q12	1 (0.87%)	0 (0.00%)	>10	0.319	0.674	Co-occurrence
RNA5SP359	12q12	1 (0.87%)	0 (0.00%)	>10	0.319	0.674	Co-occurrence
RNA5SP360	12q12	1 (0.87%)	0 (0.00%)	>10	0.319	0.674	Co-occurrence
RNA5SP371	12q23.3	1 (0.87%)	0 (0.00%)	>10	0.319	0.674	Co-occurrence
RNA5SP373	12q24.12	1 (0.87%)	0 (0.00%)	>10	0.319	0.674	Co-occurrence

RNA5SP374	12q24.23	1 (0.87%)	0 (0.00%)	>10	0.319	0.674	Co-occurrence
RNA5SP375	12q24.31	1 (0.87%)	0 (0.00%)	>10	0.319	0.674	Co-occurrence
RNA5SP43	1p34.3	1 (0.87%)	0 (0.00%)	>10	0.319	0.674	Co-occurrence
RNA5SP452	18q11.2	1 (0.87%)	0 (0.00%)	>10	0.319	0.674	Co-occurrence
RNA5SP457	18q21.1	1 (0.87%)	0 (0.00%)	>10	0.319	0.674	Co-occurrence
RNA5SP458	18q21.1	1 (0.87%)	0 (0.00%)	>10	0.319	0.674	Co-occurrence
RNA5SP47	1p34.1	1 (0.87%)	0 (0.00%)	>10	0.319	0.674	Co-occurrence
RNA5SP48	1p32.3	1 (0.87%)	0 (0.00%)	>10	0.319	0.674	Co-occurrence
RNA5SP483	20q11.22	1 (0.87%)	0 (0.00%)	>10	0.319	0.674	Co-occurrence
RNA5SP484	20q12	1 (0.87%)	0 (0.00%)	>10	0.319	0.674	Co-occurrence
RNA5SP498	Xp22.33 and Yp11.2	1 (0.87%)	0 (0.00%)	>10	0.319	0.674	Co-occurrence
RNA5SP499	Xp22.31	1 (0.87%)	0 (0.00%)	>10	0.319	0.674	Co-occurrence
RNA5SP50	1p31.1	1 (0.87%)	0 (0.00%)	>10	0.319	0.674	Co-occurrence
RNA5SP503	Xp11.23	1 (0.87%)	0 (0.00%)	>10	0.319	0.674	Co-occurrence
RNA5SP505	Xp11.22	1 (0.87%)	0 (0.00%)	>10	0.319	0.674	Co-occurrence
RNA5SP508	Xq13.3	1 (0.87%)	0 (0.00%)	>10	0.319	0.674	Co-occurrence
RNA5SP509	Xq21.1	1 (0.87%)	0 (0.00%)	>10	0.319	0.674	Co-occurrence
RNA5SP51	1p22.3	1 (0.87%)	0 (0.00%)	>10	0.319	0.674	Co-occurrence
RNA5SP510	Xq21.33	1 (0.87%)	0 (0.00%)	>10	0.319	0.674	Co-occurrence
RNA5SP53	1p22.1	1 (0.87%)	0 (0.00%)	>10	0.319	0.674	Co-occurrence
RNA5SP55	1p13.1	1 (0.87%)	0 (0.00%)	>10	0.319	0.674	Co-occurrence
RNA5SP97	2p13.1	1 (0.87%)	0 (0.00%)	>10	0.319	0.674	Co-occurrence
RNASEH2B	13q14.3	1 (0.87%)	0 (0.00%)	>10	0.319	0.674	Co-occurrence
RNF10	12q24.31	1 (0.87%)	0 (0.00%)	>10	0.319	0.674	Co-occurrence
RNF126	19p13.3	1 (0.87%)	0 (0.00%)	>10	0.319	0.674	Co-occurrence
RNF128	Xq22.3	1 (0.87%)	0 (0.00%)	>10	0.319	0.674	Co-occurrence
RNF141	11p15.4|11p15	1 (0.87%)	0 (0.00%)	>10	0.319	0.674	Co-occurrence
RNF17	13q12.12	1 (0.87%)	0 (0.00%)	>10	0.319	0.674	Co-occurrence
RNF175	4q31.3	1 (0.87%)	0 (0.00%)	>10	0.319	0.674	Co-occurrence

RNF207	1p36.31	1 (0.87%)	0 (0.00%)	>10	0.319	0.674	Co-occurrence
RNF215	22q12.2	1 (0.87%)	0 (0.00%)	>10	0.319	0.674	Co-occurrence
RNF223	1p36.33	1 (0.87%)	0 (0.00%)	>10	0.319	0.674	Co-occurrence
RNF34	12q24.31	1 (0.87%)	0 (0.00%)	>10	0.319	0.674	Co-occurrence
RNF6	13q12.13	1 (0.87%)	0 (0.00%)	>10	0.319	0.674	Co-occurrence
RNU4ATAC11P	9p13.3	1 (0.87%)	0 (0.00%)	>10	0.319	0.674	Co-occurrence
RNU4ATAC8P	1p31.1	1 (0.87%)	0 (0.00%)	>10	0.319	0.674	Co-occurrence
RNU4ATAC9P	4q13.3	1 (0.87%)	0 (0.00%)	>10	0.319	0.674	Co-occurrence
RNU6ATAC22P	22q13.2	1 (0.87%)	0 (0.00%)	>10	0.319	0.674	Co-occurrence
RNU6ATAC29P	3p21.2	1 (0.87%)	0 (0.00%)	>10	0.319	0.674	Co-occurrence
RNU6ATAC4P	3p22.2	1 (0.87%)	0 (0.00%)	>10	0.319	0.674	Co-occurrence
RNU6ATAC5P	4q13.3	1 (0.87%)	0 (0.00%)	>10	0.319	0.674	Co-occurrence
RNY1P3	13q12.13	1 (0.87%)	0 (0.00%)	>10	0.319	0.674	Co-occurrence
RNY1P6	13q14.3	1 (0.87%)	0 (0.00%)	>10	0.319	0.674	Co-occurrence
RNY1P7	13q12.12	1 (0.87%)	0 (0.00%)	>10	0.319	0.674	Co-occurrence
RNY3P4	13q12.12	1 (0.87%)	0 (0.00%)	>10	0.319	0.674	Co-occurrence
RNY4P18	9q31.3	1 (0.87%)	0 (0.00%)	>10	0.319	0.674	Co-occurrence
RNY4P22	3p24.3	1 (0.87%)	0 (0.00%)	>10	0.319	0.674	Co-occurrence
RNY4P24	13q14.3	1 (0.87%)	0 (0.00%)	>10	0.319	0.674	Co-occurrence
RNY5P1	1p34.3	1 (0.87%)	0 (0.00%)	>10	0.319	0.674	Co-occurrence
ROMO1	20q11.22	1 (0.87%)	0 (0.00%)	>10	0.319	0.674	Co-occurrence
RPAP2	1p22.1	1 (0.87%)	0 (0.00%)	>10	0.319	0.674	Co-occurrence
RPF1	1p22.3	1 (0.87%)	0 (0.00%)	>10	0.319	0.674	Co-occurrence
RPH3A	12q24.13	1 (0.87%)	0 (0.00%)	>10	0.319	0.674	Co-occurrence
RPH3AL	17p13.3	1 (0.87%)	0 (0.00%)	>10	0.319	0.674	Co-occurrence
RPL14	3p22.1	1 (0.87%)	0 (0.00%)	>10	0.319	0.674	Co-occurrence
RPL15	3p24.2	1 (0.87%)	0 (0.00%)	>10	0.319	0.674	Co-occurrence
RPL22	1p36.31	1 (0.87%)	0 (0.00%)	>10	0.319	0.674	Co-occurrence
RPL22P19	12q24.31	1 (0.87%)	0 (0.00%)	>10	0.319	0.674	Co-occurrence

RPL27A	11p15.4	1 (0.87%)	0 (0.00%)	>10	0.319	0.674	Co-occurrence
RPL35	9q33.3	1 (0.87%)	0 (0.00%)	>10	0.319	0.674	Co-occurrence
RPL5	1p22.1	1 (0.87%)	0 (0.00%)	>10	0.319	0.674	Co-occurrence
RPLP0	12q24.23	1 (0.87%)	0 (0.00%)	>10	0.319	0.674	Co-occurrence
RPRM	2q23.3	1 (0.87%)	0 (0.00%)	>10	0.319	0.674	Co-occurrence
RPS12	6q23.2	1 (0.87%)	0 (0.00%)	>10	0.319	0.674	Co-occurrence
RPS13	11p15.1	1 (0.87%)	0 (0.00%)	>10	0.319	0.674	Co-occurrence
RPS27P25	12q24.31	1 (0.87%)	0 (0.00%)	>10	0.319	0.674	Co-occurrence
RPS4XP16	13q14.3	1 (0.87%)	0 (0.00%)	>10	0.319	0.674	Co-occurrence
RPS8	1p34.1	1 (0.87%)	0 (0.00%)	>10	0.319	0.674	Co-occurrence
RPSA	3p22.1	1 (0.87%)	0 (0.00%)	>10	0.319	0.674	Co-occurrence
RPUSD4	11q24.2	1 (0.87%)	0 (0.00%)	>10	0.319	0.674	Co-occurrence
RRAGB	Xp11.21	1 (0.87%)	0 (0.00%)	>10	0.319	0.674	Co-occurrence
RRAS2	11p15.2	1 (0.87%)	0 (0.00%)	>10	0.319	0.674	Co-occurrence
RRP8	11p15.4	1 (0.87%)	0 (0.00%)	>10	0.319	0.674	Co-occurrence
RS1	Xp22.13	1 (0.87%)	0 (0.00%)	>10	0.319	0.674	Co-occurrence
RSBN1	1p13.2	1 (0.87%)	0 (0.00%)	>10	0.319	0.674	Co-occurrence
RSPO1	1p34.3	1 (0.87%)	0 (0.00%)	>10	0.319	0.674	Co-occurrence
RTKN2	10q21.2	1 (0.87%)	0 (0.00%)	>10	0.319	0.674	Co-occurrence
RTL4	Xq23	1 (0.87%)	0 (0.00%)	>10	0.319	0.674	Co-occurrence
RTP3	3p21.31	1 (0.87%)	0 (0.00%)	>10	0.319	0.674	Co-occurrence
RUFY3	4q13.3	1 (0.87%)	0 (0.00%)	>10	0.319	0.674	Co-occurrence
RWDD3	1p21.3	1 (0.87%)	0 (0.00%)	>10	0.319	0.674	Co-occurrence
S100G	Xp22.2	1 (0.87%)	0 (0.00%)	>10	0.319	0.674	Co-occurrence
SACS	13q12.12	1 (0.87%)	0 (0.00%)	>10	0.319	0.674	Co-occurrence
SALL1	16q12.1	1 (0.87%)	0 (0.00%)	>10	0.319	0.674	Co-occurrence
SAMD11	1p36.33	1 (0.87%)	0 (0.00%)	>10	0.319	0.674	Co-occurrence
SAMD13	1p31.1	1 (0.87%)	0 (0.00%)	>10	0.319	0.674	Co-occurrence
SAP130	2q14.3	1 (0.87%)	0 (0.00%)	>10	0.319	0.674	Co-occurrence

SAP18	13q12.11	1 (0.87%)	0 (0.00%)	>10	0.319	0.674	Co-occurrence
SAR1A	10q22.1	1 (0.87%)	0 (0.00%)	>10	0.319	0.674	Co-occurrence
SART3	12q23.3	1 (0.87%)	0 (0.00%)	>10	0.319	0.674	Co-occurrence
SAT1	Xp22.11	1 (0.87%)	0 (0.00%)	>10	0.319	0.674	Co-occurrence
SATB1	3p24.3	1 (0.87%)	0 (0.00%)	>10	0.319	0.674	Co-occurrence
SBNO1	12q24.31	1 (0.87%)	0 (0.00%)	>10	0.319	0.674	Co-occurrence
SCAI	9q33.3	1 (0.87%)	0 (0.00%)	>10	0.319	0.674	Co-occurrence
SCAMP4	19p13.3	1 (0.87%)	0 (0.00%)	>10	0.319	0.674	Co-occurrence
SCAND1	20q11.23	1 (0.87%)	0 (0.00%)	>10	0.319	0.674	Co-occurrence
SCAP	3p21.31	1 (0.87%)	0 (0.00%)	>10	0.319	0.674	Co-occurrence
SCARNA16	17q25.2	1 (0.87%)	0 (0.00%)	>10	0.319	0.674	Co-occurrence
SCARNA23	Xp22.11	1 (0.87%)	0 (0.00%)	>10	0.319	0.674	Co-occurrence
SCD5	4q21.22	1 (0.87%)	0 (0.00%)	>10	0.319	0.674	Co-occurrence
SCML1	Xp22.13	1 (0.87%)	0 (0.00%)	>10	0.319	0.674	Co-occurrence
SCN10A	3p22.2	1 (0.87%)	0 (0.00%)	>10	0.319	0.674	Co-occurrence
SCN11A	3p22.2	1 (0.87%)	0 (0.00%)	>10	0.319	0.674	Co-occurrence
SCN5A	3p22.2	1 (0.87%)	0 (0.00%)	>10	0.319	0.674	Co-occurrence
SCN8A	12q13.13	1 (0.87%)	0 (0.00%)	>10	0.319	0.674	Co-occurrence
SCNN1D	1p36.33	1 (0.87%)	0 (0.00%)	>10	0.319	0.674	Co-occurrence
SCUBE2	11p15.4	1 (0.87%)	0 (0.00%)	>10	0.319	0.674	Co-occurrence
SDAD1	4q21.1	1 (0.87%)	0 (0.00%)	>10	0.319	0.674	Co-occurrence
SDF4	1p36.33	1 (0.87%)	0 (0.00%)	>10	0.319	0.674	Co-occurrence
SDHAF1	19q13.12	1 (0.87%)	0 (0.00%)	>10	0.319	0.674	Co-occurrence
SEC14L2	22q12.2	1 (0.87%)	0 (0.00%)	>10	0.319	0.674	Co-occurrence
SEC14L3	22q12.2	1 (0.87%)	0 (0.00%)	>10	0.319	0.674	Co-occurrence
SEC14L4	22q12.2	1 (0.87%)	0 (0.00%)	>10	0.319	0.674	Co-occurrence
SEC31A	4q21.22	1 (0.87%)	0 (0.00%)	>10	0.319	0.674	Co-occurrence
SELPLG	12q24.11	1 (0.87%)	0 (0.00%)	>10	0.319	0.674	Co-occurrence
SEPTIN11	4q21.1	1 (0.87%)	0 (0.00%)	>10	0.319	0.674	Co-occurrence

SEPTIN3	22q13.2	1 (0.87%)	0 (0.00%)	>10	0.319	0.674	Co-occurrence
SERPINE3	13q14.3	1 (0.87%)	0 (0.00%)	>10	0.319	0.674	Co-occurrence
SETMAR	3p26.1	1 (0.87%)	0 (0.00%)	>10	0.319	0.674	Co-occurrence
SEZ6L	22q12.1	1 (0.87%)	0 (0.00%)	>10	0.319	0.674	Co-occurrence
SF3A1	22q12.2	1 (0.87%)	0 (0.00%)	>10	0.319	0.674	Co-occurrence
SF3A3	1p34.3	1 (0.87%)	0 (0.00%)	>10	0.319	0.674	Co-occurrence
SFRP2	4q31.3	1 (0.87%)	0 (0.00%)	>10	0.319	0.674	Co-occurrence
SFTPC	8p21.3	1 (0.87%)	0 (0.00%)	>10	0.319	0.674	Co-occurrence
SGCG	13q12.12	1 (0.87%)	0 (0.00%)	>10	0.319	0.674	Co-occurrence
SGK1	6q23.2	1 (0.87%)	0 (0.00%)	>10	0.319	0.674	Co-occurrence
SGMS1	10q11.23	1 (0.87%)	0 (0.00%)	>10	0.319	0.674	Co-occurrence
SGO1	3p24.3	1 (0.87%)	0 (0.00%)	>10	0.319	0.674	Co-occurrence
SGPL1	10q22.1	1 (0.87%)	0 (0.00%)	>10	0.319	0.674	Co-occurrence
SGPP1	14q23.2	1 (0.87%)	0 (0.00%)	>10	0.319	0.674	Co-occurrence
SGTB	5q12.3	1 (0.87%)	0 (0.00%)	>10	0.319	0.674	Co-occurrence
SH3D21	1p34.3	1 (0.87%)	0 (0.00%)	>10	0.319	0.674	Co-occurrence
SH3GLB1	1p22.3	1 (0.87%)	0 (0.00%)	>10	0.319	0.674	Co-occurrence
SH3GLB2	9q34.11	1 (0.87%)	0 (0.00%)	>10	0.319	0.674	Co-occurrence
SHF	15q21.1	1 (0.87%)	0 (0.00%)	>10	0.319	0.674	Co-occurrence
SHISA2	13q12.13	1 (0.87%)	0 (0.00%)	>10	0.319	0.674	Co-occurrence
SHISA8	22q13.2	1 (0.87%)	0 (0.00%)	>10	0.319	0.674	Co-occurrence
SHISAL2A	1p32.3	1 (0.87%)	0 (0.00%)	>10	0.319	0.674	Co-occurrence
SHLD1	20p12.3	1 (0.87%)	0 (0.00%)	>10	0.319	0.674	Co-occurrence
SHOC1	9q31.3	1 (0.87%)	0 (0.00%)	>10	0.319	0.674	Co-occurrence
SHOX	Xp22.33 and Yp11.2	1 (0.87%)	0 (0.00%)	>10	0.319	0.674	Co-occurrence
SHTN1	10q25.3	1 (0.87%)	0 (0.00%)	>10	0.319	0.674	Co-occurrence
SIM1	6q16.3	1 (0.87%)	0 (0.00%)	>10	0.319	0.674	Co-occurrence
SIM2	21q22.13	1 (0.87%)	0 (0.00%)	>10	0.319	0.674	Co-occurrence
SIRT4	12q24.23-q24.31	1 (0.87%)	0 (0.00%)	>10	0.319	0.674	Co-occurrence

SIVA1	14q32.33	1 (0.87%)	0 (0.00%)	>10	0.319	0.674	Co-occurrence
SKA3	13q12.11	1 (0.87%)	0 (0.00%)	>10	0.319	0.674	Co-occurrence
SKI	1p36.33-p36.32	1 (0.87%)	0 (0.00%)	>10	0.319	0.674	Co-occurrence
SLC15A4	12q24.33	1 (0.87%)	0 (0.00%)	>10	0.319	0.674	Co-occurrence
SLC17A6	11p14.3	1 (0.87%)	0 (0.00%)	>10	0.319	0.674	Co-occurrence
SLC18A2	10q25.3	1 (0.87%)	0 (0.00%)	>10	0.319	0.674	Co-occurrence
SLC18B1	6q23.2	1 (0.87%)	0 (0.00%)	>10	0.319	0.674	Co-occurrence
SLC25A18	22q11.21	1 (0.87%)	0 (0.00%)	>10	0.319	0.674	Co-occurrence
SLC25A38	3p22.1	1 (0.87%)	0 (0.00%)	>10	0.319	0.674	Co-occurrence
SLC25A52	18q12.1	1 (0.87%)	0 (0.00%)	>10	0.319	0.674	Co-occurrence
SLC25A6	Xp22.33 and Yp11.2	1 (0.87%)	0 (0.00%)	>10	0.319	0.674	Co-occurrence
SLC35A5	3q13.2	1 (0.87%)	0 (0.00%)	>10	0.319	0.674	Co-occurrence
SLC35C1	11p11.2	1 (0.87%)	0 (0.00%)	>10	0.319	0.674	Co-occurrence
SLC35E2A	1p36.33	1 (0.87%)	0 (0.00%)	>10	0.319	0.674	Co-occurrence
SLC35E2B	1p36.33	1 (0.87%)	0 (0.00%)	>10	0.319	0.674	Co-occurrence
SLC35E4	22q12.2	1 (0.87%)	0 (0.00%)	>10	0.319	0.674	Co-occurrence
SLC38A2	12q13.11	1 (0.87%)	0 (0.00%)	>10	0.319	0.674	Co-occurrence
SLC38A5	Xp11.23	1 (0.87%)	0 (0.00%)	>10	0.319	0.674	Co-occurrence
SLC39A13	11p11.2	1 (0.87%)	0 (0.00%)	>10	0.319	0.674	Co-occurrence
SLC44A3	1p21.3	1 (0.87%)	0 (0.00%)	>10	0.319	0.674	Co-occurrence
SLC46A2	9q32	1 (0.87%)	0 (0.00%)	>10	0.319	0.674	Co-occurrence
SLC4A4	4q13.3	1 (0.87%)	0 (0.00%)	>10	0.319	0.674	Co-occurrence
SLC4A7	3p24.1	1 (0.87%)	0 (0.00%)	>10	0.319	0.674	Co-occurrence
SLC4A8	12q13.13	1 (0.87%)	0 (0.00%)	>10	0.319	0.674	Co-occurrence
SLC5A12	11p14.2	1 (0.87%)	0 (0.00%)	>10	0.319	0.674	Co-occurrence
SLC5A9	1p33	1 (0.87%)	0 (0.00%)	>10	0.319	0.674	Co-occurrence
SLC6A5	11p15.1	1 (0.87%)	0 (0.00%)	>10	0.319	0.674	Co-occurrence
SLC9A5	16q22.1	1 (0.87%)	0 (0.00%)	>10	0.319	0.674	Co-occurrence
SLC9C1	3q13.2	1 (0.87%)	0 (0.00%)	>10	0.319	0.674	Co-occurrence

SLCO1A2	12p12.1	1 (0.87%)	0 (0.00%)	>10	0.319	0.674	Co-occurrence
SLCO1B1	12p12.1	1 (0.87%)	0 (0.00%)	>10	0.319	0.674	Co-occurrence
SLCO1B3	12p12.2	1 (0.87%)	0 (0.00%)	>10	0.319	0.674	Co-occurrence
SLCO1B7	12p12.2	1 (0.87%)	0 (0.00%)	>10	0.319	0.674	Co-occurrence
SLCO1C1	12p12.2	1 (0.87%)	0 (0.00%)	>10	0.319	0.674	Co-occurrence
SMARCC1	3p21.31	1 (0.87%)	0 (0.00%)	>10	0.319	0.674	Co-occurrence
SMIM1	1p36.32	1 (0.87%)	0 (0.00%)	>10	0.319	0.674	Co-occurrence
SMIM12	1p34.3	1 (0.87%)	0 (0.00%)	>10	0.319	0.674	Co-occurrence
SMNDC1	10q25.2	1 (0.87%)	0 (0.00%)	>10	0.319	0.674	Co-occurrence
SMPD4	2q21.1	1 (0.87%)	0 (0.00%)	>10	0.319	0.674	Co-occurrence
SMPX	Xp22.12	1 (0.87%)	0 (0.00%)	>10	0.319	0.674	Co-occurrence
SMR3A	4q13.3	1 (0.87%)	0 (0.00%)	>10	0.319	0.674	Co-occurrence
SMR3B	4q13.3	1 (0.87%)	0 (0.00%)	>10	0.319	0.674	Co-occurrence
SMS	Xp22.11	1 (0.87%)	0 (0.00%)	>10	0.319	0.674	Co-occurrence
SNIP1	1p34.3	1 (0.87%)	0 (0.00%)	>10	0.319	0.674	Co-occurrence
SNORA11C	Xp11.3	1 (0.87%)	0 (0.00%)	>10	0.319	0.674	Co-occurrence
SNORA26	4q12	1 (0.87%)	0 (0.00%)	>10	0.319	0.674	Co-occurrence
SNORA28	14q32.32	1 (0.87%)	0 (0.00%)	>10	0.319	0.674	Co-occurrence
SNORA3A	11p15.4	1 (0.87%)	0 (0.00%)	>10	0.319	0.674	Co-occurrence
SNORA3B	11p15.4	1 (0.87%)	0 (0.00%)	>10	0.319	0.674	Co-occurrence
SNORA50C	17q23.3	1 (0.87%)	0 (0.00%)	>10	0.319	0.674	Co-occurrence
SNORA51	20p13	1 (0.87%)	0 (0.00%)	>10	0.319	0.674	Co-occurrence
SNORA58	3q22.1	1 (0.87%)	0 (0.00%)	>10	0.319	0.674	Co-occurrence
SNORA6	3p22.1	1 (0.87%)	0 (0.00%)	>10	0.319	0.674	Co-occurrence
SNORA62	3p22.1	1 (0.87%)	0 (0.00%)	>10	0.319	0.674	Co-occurrence
SNORA63	3q27.3	1 (0.87%)	0 (0.00%)	>10	0.319	0.674	Co-occurrence
SNORA66	1p22.1	1 (0.87%)	0 (0.00%)	>10	0.319	0.674	Co-occurrence
SNORA70C	9q33.1	1 (0.87%)	0 (0.00%)	>10	0.319	0.674	Co-occurrence
SNORA77	1q32.1	1 (0.87%)	0 (0.00%)	>10	0.319	0.674	Co-occurrence

SNORD100	6q23.2	1 (0.87%)	0 (0.00%)	>10	0.319	0.674	Co-occurrence
SNORD101	6q23.2	1 (0.87%)	0 (0.00%)	>10	0.319	0.674	Co-occurrence
SNORD112	14q32.2	1 (0.87%)	0 (0.00%)	>10	0.319	0.674	Co-occurrence
SNORD125	22q12.2	1 (0.87%)	0 (0.00%)	>10	0.319	0.674	Co-occurrence
SNORD14A	11p15.1	1 (0.87%)	0 (0.00%)	>10	0.319	0.674	Co-occurrence
SNORD14B	11p15.1	1 (0.87%)	0 (0.00%)	>10	0.319	0.674	Co-occurrence
SNORD21	1p22.1	1 (0.87%)	0 (0.00%)	>10	0.319	0.674	Co-occurrence
SNORD38A	1p34.1	1 (0.87%)	0 (0.00%)	>10	0.319	0.674	Co-occurrence
SNORD38B	1p34.1	1 (0.87%)	0 (0.00%)	>10	0.319	0.674	Co-occurrence
SNORD45A	1p31.1	1 (0.87%)	0 (0.00%)	>10	0.319	0.674	Co-occurrence
SNORD45B	1p31.1	1 (0.87%)	0 (0.00%)	>10	0.319	0.674	Co-occurrence
SNORD45C	1p31.1	1 (0.87%)	0 (0.00%)	>10	0.319	0.674	Co-occurrence
SNORD46	1p34.1	1 (0.87%)	0 (0.00%)	>10	0.319	0.674	Co-occurrence
SNORD55	1p34.1	1 (0.87%)	0 (0.00%)	>10	0.319	0.674	Co-occurrence
SNORD56	20p13	1 (0.87%)	0 (0.00%)	>10	0.319	0.674	Co-occurrence
SNORD65	17p11.2	1 (0.87%)	0 (0.00%)	>10	0.319	0.674	Co-occurrence
SNORD67	11p11.2	1 (0.87%)	0 (0.00%)	>10	0.319	0.674	Co-occurrence
SNORD81	1q25.1	1 (0.87%)	0 (0.00%)	>10	0.319	0.674	Co-occurrence
SNORD90	9q33.2	1 (0.87%)	0 (0.00%)	>10	0.319	0.674	Co-occurrence
SNORD97	11p15.4	1 (0.87%)	0 (0.00%)	>10	0.319	0.674	Co-occurrence
SNRNP35	12q24.31	1 (0.87%)	0 (0.00%)	>10	0.319	0.674	Co-occurrence
SNRNP40	1p35.2	1 (0.87%)	0 (0.00%)	>10	0.319	0.674	Co-occurrence
SNU13	22q13.2	1 (0.87%)	0 (0.00%)	>10	0.319	0.674	Co-occurrence
SNX18P3	9p13.1	1 (0.87%)	0 (0.00%)	>10	0.319	0.674	Co-occurrence
SNX20	16q12.1	1 (0.87%)	0 (0.00%)	>10	0.319	0.674	Co-occurrence
SNX30	9q32	1 (0.87%)	0 (0.00%)	>10	0.319	0.674	Co-occurrence
SORBS3	8p21.3	1 (0.87%)	0 (0.00%)	>10	0.319	0.674	Co-occurrence
SORD	15q21.1	1 (0.87%)	0 (0.00%)	>10	0.319	0.674	Co-occurrence
SOX5	12p12.1	1 (0.87%)	0 (0.00%)	>10	0.319	0.674	Co-occurrence

SOX6	11p15.2	1 (0.87%)	0 (0.00%)	>10	0.319	0.674	Co-occurrence
SPAG4	20q11.22	1 (0.87%)	0 (0.00%)	>10	0.319	0.674	Co-occurrence
SPATA1	1p22.3	1 (0.87%)	0 (0.00%)	>10	0.319	0.674	Co-occurrence
SPATA31A1	9p12	1 (0.87%)	0 (0.00%)	>10	0.319	0.674	Co-occurrence
SPATA31A3	9q21.11	1 (0.87%)	0 (0.00%)	>10	0.319	0.674	Co-occurrence
SPATA31A5	9q12	1 (0.87%)	0 (0.00%)	>10	0.319	0.674	Co-occurrence
SPATA31A6	9p11.2	1 (0.87%)	0 (0.00%)	>10	0.319	0.674	Co-occurrence
SPATA31A7	9q12	1 (0.87%)	0 (0.00%)	>10	0.319	0.674	Co-occurrence
SPATA6	1p33	1 (0.87%)	0 (0.00%)	>10	0.319	0.674	Co-occurrence
SPI1	11p11.2	1 (0.87%)	0 (0.00%)	>10	0.319	0.674	Co-occurrence
SPINK8	3p21.31	1 (0.87%)	0 (0.00%)	>10	0.319	0.674	Co-occurrence
SPINT2	19q13.2	1 (0.87%)	0 (0.00%)	>10	0.319	0.674	Co-occurrence
SPON1	11p15.2	1 (0.87%)	0 (0.00%)	>10	0.319	0.674	Co-occurrence
SPOUT1	9q34.11	1 (0.87%)	0 (0.00%)	>10	0.319	0.674	Co-occurrence
SPPL3	12q24.31	1 (0.87%)	0 (0.00%)	>10	0.319	0.674	Co-occurrence
SPRED3	19q13.2	1 (0.87%)	0 (0.00%)	>10	0.319	0.674	Co-occurrence
SPRY1	4q28.1	1 (0.87%)	0 (0.00%)	>10	0.319	0.674	Co-occurrence
SPRYD7	13q14.2	1 (0.87%)	0 (0.00%)	>10	0.319	0.674	Co-occurrence
SREBF2	22q13.2	1 (0.87%)	0 (0.00%)	>10	0.319	0.674	Co-occurrence
SRPRA	11q24.2	1 (0.87%)	0 (0.00%)	>10	0.319	0.674	Co-occurrence
SRRD	22q12.1	1 (0.87%)	0 (0.00%)	>10	0.319	0.674	Co-occurrence
SRRM4	12q24.23	1 (0.87%)	0 (0.00%)	>10	0.319	0.674	Co-occurrence
SRSF11	1p31.1	1 (0.87%)	0 (0.00%)	>10	0.319	0.674	Co-occurrence
SRSF9	12q24.31	1 (0.87%)	0 (0.00%)	>10	0.319	0.674	Co-occurrence
SS18	18q11.2	1 (0.87%)	0 (0.00%)	>10	0.319	0.674	Co-occurrence
SSBP3	1p32.3	1 (0.87%)	0 (0.00%)	>10	0.319	0.674	Co-occurrence
SSU72	1p36.33	1 (0.87%)	0 (0.00%)	>10	0.319	0.674	Co-occurrence
SSX1	Xp11.23	1 (0.87%)	0 (0.00%)	>10	0.319	0.674	Co-occurrence
SSX2IP	1p22.3	1 (0.87%)	0 (0.00%)	>10	0.319	0.674	Co-occurrence

SSX3	Xp11.23	1 (0.87%)	0 (0.00%)	>10	0.319	0.674	Co-occurrence
SSX4	Xp11.23	1 (0.87%)	0 (0.00%)	>10	0.319	0.674	Co-occurrence
SSX4B	Xp11.23	1 (0.87%)	0 (0.00%)	>10	0.319	0.674	Co-occurrence
SSX5	Xp11.23	1 (0.87%)	0 (0.00%)	>10	0.319	0.674	Co-occurrence
SSX9P	Xp11.23	1 (0.87%)	0 (0.00%)	>10	0.319	0.674	Co-occurrence
ST13	22q13.2	1 (0.87%)	0 (0.00%)	>10	0.319	0.674	Co-occurrence
ST3GAL4	11q24.2	1 (0.87%)	0 (0.00%)	>10	0.319	0.674	Co-occurrence
ST5	11p15.4	1 (0.87%)	0 (0.00%)	>10	0.319	0.674	Co-occurrence
ST6GALNAC3	1p31.1	1 (0.87%)	0 (0.00%)	>10	0.319	0.674	Co-occurrence
ST8SIA1	12p12.1	1 (0.87%)	0 (0.00%)	>10	0.319	0.674	Co-occurrence
STAM2	2q23.3	1 (0.87%)	0 (0.00%)	>10	0.319	0.674	Co-occurrence
STAP1	4q13.2	1 (0.87%)	0 (0.00%)	>10	0.319	0.674	Co-occurrence
STATH	4q13.3	1 (0.87%)	0 (0.00%)	>10	0.319	0.674	Co-occurrence
STK32C	10q26.3	1 (0.87%)	0 (0.00%)	>10	0.319	0.674	Co-occurrence
STK33	11p15.4	1 (0.87%)	0 (0.00%)	>10	0.319	0.674	Co-occurrence
STK40	1p34.3	1 (0.87%)	0 (0.00%)	>10	0.319	0.674	Co-occurrence
STMN1P1	12p11.21	1 (0.87%)	0 (0.00%)	>10	0.319	0.674	Co-occurrence
STOM	9q33.2	1 (0.87%)	0 (0.00%)	>10	0.319	0.674	Co-occurrence
STOML2	9p13.3	1 (0.87%)	0 (0.00%)	>10	0.319	0.674	Co-occurrence
STOML3	13q13.3	1 (0.87%)	0 (0.00%)	>10	0.319	0.674	Co-occurrence
STRBP	9q33.3	1 (0.87%)	0 (0.00%)	>10	0.319	0.674	Co-occurrence
STT3A	11q24.2	1 (0.87%)	0 (0.00%)	>10	0.319	0.674	Co-occurrence
STX19	3q11.2	1 (0.87%)	0 (0.00%)	>10	0.319	0.674	Co-occurrence
STX2	12q24.33	1 (0.87%)	0 (0.00%)	>10	0.319	0.674	Co-occurrence
STX7	6q23.2	1 (0.87%)	0 (0.00%)	>10	0.319	0.674	Co-occurrence
SUDS3	12q24.23	1 (0.87%)	0 (0.00%)	>10	0.319	0.674	Co-occurrence
SUGT1	13q14.3	1 (0.87%)	0 (0.00%)	>10	0.319	0.674	Co-occurrence
SUSD5	3p22.3	1 (0.87%)	0 (0.00%)	>10	0.319	0.674	Co-occurrence
SVIP	11p14.3	1 (0.87%)	0 (0.00%)	>10	0.319	0.674	Co-occurrence

SYAP1	Xp22.2	1 (0.87%)	0 (0.00%)	>10	0.319	0.674	Co-occurrence
SYDE2	1p22.3	1 (0.87%)	0 (0.00%)	>10	0.319	0.674	Co-occurrence
SYN1	Xp11.3-p11.23	1 (0.87%)	0 (0.00%)	>10	0.319	0.674	Co-occurrence
SYNE2	14q23.2	1 (0.87%)	0 (0.00%)	>10	0.319	0.674	Co-occurrence
SYNE4	19q13.12	1 (0.87%)	0 (0.00%)	>10	0.319	0.674	Co-occurrence
SYNJ2BP	14q24.2	1 (0.87%)	0 (0.00%)	>10	0.319	0.674	Co-occurrence
SYT10	12p11.1	1 (0.87%)	0 (0.00%)	>10	0.319	0.674	Co-occurrence
SYT13	11p11.2	1 (0.87%)	0 (0.00%)	>10	0.319	0.674	Co-occurrence
SYT6	1p13.2	1 (0.87%)	0 (0.00%)	>10	0.319	0.674	Co-occurrence
TAAR1	6q23.2	1 (0.87%)	0 (0.00%)	>10	0.319	0.674	Co-occurrence
TAAR2	6q23.2	1 (0.87%)	0 (0.00%)	>10	0.319	0.674	Co-occurrence
TAAR3P	6q23.2	1 (0.87%)	0 (0.00%)	>10	0.319	0.674	Co-occurrence
TAAR5	6q23.2	1 (0.87%)	0 (0.00%)	>10	0.319	0.674	Co-occurrence
TAAR6	6q23.2	1 (0.87%)	0 (0.00%)	>10	0.319	0.674	Co-occurrence
TAAR8	6q23.2	1 (0.87%)	0 (0.00%)	>10	0.319	0.674	Co-occurrence
TAAR9	6q23.2	1 (0.87%)	0 (0.00%)	>10	0.319	0.674	Co-occurrence
TACSTD2	1p32.1	1 (0.87%)	0 (0.00%)	>10	0.319	0.674	Co-occurrence
TAF10	11p15.4	1 (0.87%)	0 (0.00%)	>10	0.319	0.674	Co-occurrence
TAF4B	18q11.2	1 (0.87%)	0 (0.00%)	>10	0.319	0.674	Co-occurrence
TAF9B	Xq21.1	1 (0.87%)	0 (0.00%)	>10	0.319	0.674	Co-occurrence
TAGLN3	3q13.2	1 (0.87%)	0 (0.00%)	>10	0.319	0.674	Co-occurrence
TAS1R3	1p36.33	1 (0.87%)	0 (0.00%)	>10	0.319	0.674	Co-occurrence
TBATA	10q22.1	1 (0.87%)	0 (0.00%)	>10	0.319	0.674	Co-occurrence
TBC1D10A	22q12.2	1 (0.87%)	0 (0.00%)	>10	0.319	0.674	Co-occurrence
TBC1D25	Xp11.23	1 (0.87%)	0 (0.00%)	>10	0.319	0.674	Co-occurrence
TBCB	19q13.12	1 (0.87%)	0 (0.00%)	>10	0.319	0.674	Co-occurrence
TBL1X	Xp22.31-p22.2	1 (0.87%)	0 (0.00%)	>10	0.319	0.674	Co-occurrence
TBX3	12q24.21	1 (0.87%)	0 (0.00%)	>10	0.319	0.674	Co-occurrence
TBX5	12q24.21	1 (0.87%)	0 (0.00%)	>10	0.319	0.674	Co-occurrence

TBXA2R	19p13.3	1 (0.87%)	0 (0.00%)	>10	0.319	0.674	Co-occurrence
TCEANC	Xp22.2	1 (0.87%)	0 (0.00%)	>10	0.319	0.674	Co-occurrence
TCF7L1	2p11.2	1 (0.87%)	0 (0.00%)	>10	0.319	0.674	Co-occurrence
TCN2	22q12.2	1 (0.87%)	0 (0.00%)	>10	0.319	0.674	Co-occurrence
TCTEX1D4	1p34.1	1 (0.87%)	0 (0.00%)	>10	0.319	0.674	Co-occurrence
TECPR2	14q32.31	1 (0.87%)	0 (0.00%)	>10	0.319	0.674	Co-occurrence
TECRL	4q13.1	1 (0.87%)	0 (0.00%)	>10	0.319	0.674	Co-occurrence
TEKT2	1p34.3	1 (0.87%)	0 (0.00%)	>10	0.319	0.674	Co-occurrence
TERB2	15q21.1	1 (0.87%)	0 (0.00%)	>10	0.319	0.674	Co-occurrence
TESK2	1p34.1	1 (0.87%)	0 (0.00%)	>10	0.319	0.674	Co-occurrence
TESPA1	12q13.2	1 (0.87%)	0 (0.00%)	>10	0.319	0.674	Co-occurrence
TEX264	3p21.2	1 (0.87%)	0 (0.00%)	>10	0.319	0.674	Co-occurrence
TFAM	10q21.1	1 (0.87%)	0 (0.00%)	>10	0.319	0.674	Co-occurrence
TFIP11	22q12.1	1 (0.87%)	0 (0.00%)	>10	0.319	0.674	Co-occurrence
TGFBR2	3p24.1	1 (0.87%)	0 (0.00%)	>10	0.319	0.674	Co-occurrence
TGM4	3p21.31	1 (0.87%)	0 (0.00%)	>10	0.319	0.674	Co-occurrence
TGOLN2	2p11.2	1 (0.87%)	0 (0.00%)	>10	0.319	0.674	Co-occurrence
THAP6	4q21.1	1 (0.87%)	0 (0.00%)	>10	0.319	0.674	Co-occurrence
THAP8	19q13.12	1 (0.87%)	0 (0.00%)	>10	0.319	0.674	Co-occurrence
THAP9	4q21.22	1 (0.87%)	0 (0.00%)	>10	0.319	0.674	Co-occurrence
THOC5	22q12.2	1 (0.87%)	0 (0.00%)	>10	0.319	0.674	Co-occurrence
THRAP3	1p34.3	1 (0.87%)	0 (0.00%)	>10	0.319	0.674	Co-occurrence
THRB	3p24.2	1 (0.87%)	0 (0.00%)	>10	0.319	0.674	Co-occurrence
THSD1	13q14.3	1 (0.87%)	0 (0.00%)	>10	0.319	0.674	Co-occurrence
THSD7B	2q22.1	1 (0.87%)	0 (0.00%)	>10	0.319	0.674	Co-occurrence
TIGIT	3q13.31	1 (0.87%)	0 (0.00%)	>10	0.319	0.674	Co-occurrence
TIMM10B	11p15.4	1 (0.87%)	0 (0.00%)	>10	0.319	0.674	Co-occurrence
TIMM22	17p13.3	1 (0.87%)	0 (0.00%)	>10	0.319	0.674	Co-occurrence
TIMM23	10q11.22	1 (0.87%)	0 (0.00%)	>10	0.319	0.674	Co-occurrence

TIMM23B	10q11.23	1 (0.87%)	0 (0.00%)	>10	0.319	0.674	Co-occurrence
TIMP1	Xp11.3	1 (0.87%)	0 (0.00%)	>10	0.319	0.674	Co-occurrence
TIRAP	11q24.2	1 (0.87%)	0 (0.00%)	>10	0.319	0.674	Co-occurrence
TJP3	19p13.3	1 (0.87%)	0 (0.00%)	>10	0.319	0.674	Co-occurrence
TLCD3A	17p13.3	1 (0.87%)	0 (0.00%)	>10	0.319	0.674	Co-occurrence
TLR2	4q31.3	1 (0.87%)	0 (0.00%)	>10	0.319	0.674	Co-occurrence
TLR4	9q33.1	1 (0.87%)	0 (0.00%)	>10	0.319	0.674	Co-occurrence
TMED5	1p22.1	1 (0.87%)	0 (0.00%)	>10	0.319	0.674	Co-occurrence
TMEM116	12q24.12-q24.13	1 (0.87%)	0 (0.00%)	>10	0.319	0.674	Co-occurrence
TMEM117	12q12	1 (0.87%)	0 (0.00%)	>10	0.319	0.674	Co-occurrence
TMEM119	12q23.3	1 (0.87%)	0 (0.00%)	>10	0.319	0.674	Co-occurrence
TMEM132B	12q24.31-q24.32	1 (0.87%)	0 (0.00%)	>10	0.319	0.674	Co-occurrence
TMEM132D	12q24.33	1 (0.87%)	0 (0.00%)	>10	0.319	0.674	Co-occurrence
TMEM150C	4q21.22	1 (0.87%)	0 (0.00%)	>10	0.319	0.674	Co-occurrence
TMEM155	4q27	1 (0.87%)	0 (0.00%)	>10	0.319	0.674	Co-occurrence
TMEM158	3p21.31	1 (0.87%)	0 (0.00%)	>10	0.319	0.674	Co-occurrence
TMEM208	16q22.1	1 (0.87%)	0 (0.00%)	>10	0.319	0.674	Co-occurrence
TMEM233	12q24.23	1 (0.87%)	0 (0.00%)	>10	0.319	0.674	Co-occurrence
TMEM234	1p35.2	1 (0.87%)	0 (0.00%)	>10	0.319	0.674	Co-occurrence
TMEM240	1p36.33	1 (0.87%)	0 (0.00%)	>10	0.319	0.674	Co-occurrence
TMEM241	18q11.2	1 (0.87%)	0 (0.00%)	>10	0.319	0.674	Co-occurrence
TMEM251	14q32.12	1 (0.87%)	0 (0.00%)	>10	0.319	0.674	Co-occurrence
TMEM268	9q32	1 (0.87%)	0 (0.00%)	>10	0.319	0.674	Co-occurrence
TMEM35B	1p34.3	1 (0.87%)	0 (0.00%)	>10	0.319	0.674	Co-occurrence
TMEM41B	11p15.4	1 (0.87%)	0 (0.00%)	>10	0.319	0.674	Co-occurrence
TMEM42	3p21.31	1 (0.87%)	0 (0.00%)	>10	0.319	0.674	Co-occurrence
TMEM52	1p36.33	1 (0.87%)	0 (0.00%)	>10	0.319	0.674	Co-occurrence
TMEM52B	12p13.2	1 (0.87%)	0 (0.00%)	>10	0.319	0.674	Co-occurrence
TMEM69	1p34.1	1 (0.87%)	0 (0.00%)	>10	0.319	0.674	Co-occurrence

TMEM88B	1p36.33	1 (0.87%)	0 (0.00%)	>10	0.319	0.674	Co-occurrence
TMEM9B	11p15.4	1 (0.87%)	0 (0.00%)	>10	0.319	0.674	Co-occurrence
TMPPE	3p22.3	1 (0.87%)	0 (0.00%)	>10	0.319	0.674	Co-occurrence
TMPRSS11A	4q13.2	1 (0.87%)	0 (0.00%)	>10	0.319	0.674	Co-occurrence
TMPRSS11B	4q13.2	1 (0.87%)	0 (0.00%)	>10	0.319	0.674	Co-occurrence
TMPRSS11BNL	4q13.2	1 (0.87%)	0 (0.00%)	>10	0.319	0.674	Co-occurrence
TMPRSS11D	4q13.2	1 (0.87%)	0 (0.00%)	>10	0.319	0.674	Co-occurrence
TMPRSS11F	4q13.2	1 (0.87%)	0 (0.00%)	>10	0.319	0.674	Co-occurrence
TMPRSS11GP	4q13.2	1 (0.87%)	0 (0.00%)	>10	0.319	0.674	Co-occurrence
TMPRSS7	3q13.2	1 (0.87%)	0 (0.00%)	>10	0.319	0.674	Co-occurrence
TMSB10	2p11.2	1 (0.87%)	0 (0.00%)	>10	0.319	0.674	Co-occurrence
TMTC1	12p11.22	1 (0.87%)	0 (0.00%)	>10	0.319	0.674	Co-occurrence
TMX4	20p12.3	1 (0.87%)	0 (0.00%)	>10	0.319	0.674	Co-occurrence
TNFRSF10C	8p21.3	1 (0.87%)	0 (0.00%)	>10	0.319	0.674	Co-occurrence
TNFRSF13C	22q13.2	1 (0.87%)	0 (0.00%)	>10	0.319	0.674	Co-occurrence
TNFRSF14	1p36.32	1 (0.87%)	0 (0.00%)	>10	0.319	0.674	Co-occurrence
TNFRSF18	1p36.33	1 (0.87%)	0 (0.00%)	>10	0.319	0.674	Co-occurrence
TNFRSF19	13q12.12	1 (0.87%)	0 (0.00%)	>10	0.319	0.674	Co-occurrence
TNFRSF4	1p36.33	1 (0.87%)	0 (0.00%)	>10	0.319	0.674	Co-occurrence
TNFRSF9	1p36.23	1 (0.87%)	0 (0.00%)	>10	0.319	0.674	Co-occurrence
TNFSF8	9q32-q33.1	1 (0.87%)	0 (0.00%)	>10	0.319	0.674	Co-occurrence
TNNI3K	1p31.1	1 (0.87%)	0 (0.00%)	>10	0.319	0.674	Co-occurrence
TOE1	1p34.1	1 (0.87%)	0 (0.00%)	>10	0.319	0.674	Co-occurrence
TOLLIP	11p15.5	1 (0.87%)	0 (0.00%)	>10	0.319	0.674	Co-occurrence
TOM1L2	17p11.2	1 (0.87%)	0 (0.00%)	>10	0.319	0.674	Co-occurrence
TP73	1p36.32	1 (0.87%)	0 (0.00%)	>10	0.319	0.674	Co-occurrence
TPCN1	12q24.13	1 (0.87%)	0 (0.00%)	>10	0.319	0.674	Co-occurrence
TPGS1	19p13.3	1 (0.87%)	0 (0.00%)	>10	0.319	0.674	Co-occurrence
TPP1	11p15.4	1 (0.87%)	0 (0.00%)	>10	0.319	0.674	Co-occurrence

TPRG1L	1p36.32	1 (0.87%)	0 (0.00%)	>10	0.319	0.674	Co-occurrence
TPTE2	13q12.11	1 (0.87%)	0 (0.00%)	>10	0.319	0.674	Co-occurrence
TPTE2P1	13q12.13	1 (0.87%)	0 (0.00%)	>10	0.319	0.674	Co-occurrence
TPTE2P6	13q12.12	1 (0.87%)	0 (0.00%)	>10	0.319	0.674	Co-occurrence
TRABD2A	2p11.2	1 (0.87%)	0 (0.00%)	>10	0.319	0.674	Co-occurrence
TRADD	16q22.1	1 (0.87%)	0 (0.00%)	>10	0.319	0.674	Co-occurrence
TRAFD1	12q24.13	1 (0.87%)	0 (0.00%)	>10	0.319	0.674	Co-occurrence
TRAK1	3p22.1	1 (0.87%)	0 (0.00%)	>10	0.319	0.674	Co-occurrence
TRAM1L1	4q26	1 (0.87%)	0 (0.00%)	>10	0.319	0.674	Co-occurrence
TRANK1	3p22.2	1 (0.87%)	0 (0.00%)	>10	0.319	0.674	Co-occurrence
TRAPPC13	5q12.3	1 (0.87%)	0 (0.00%)	>10	0.319	0.674	Co-occurrence
TRAPPC3	1p34.3	1 (0.87%)	0 (0.00%)	>10	0.319	0.674	Co-occurrence
TRARG1	17p13.3	1 (0.87%)	0 (0.00%)	>10	0.319	0.674	Co-occurrence
TRIAP1	12q24.31	1 (0.87%)	0 (0.00%)	>10	0.319	0.674	Co-occurrence
TRIM13	13q14.2	1 (0.87%)	0 (0.00%)	>10	0.319	0.674	Co-occurrence
TRIM22	11p15.4	1 (0.87%)	0 (0.00%)	>10	0.319	0.674	Co-occurrence
TRIM23	5q12.3	1 (0.87%)	0 (0.00%)	>10	0.319	0.674	Co-occurrence
TRIM29	11q23.3	1 (0.87%)	0 (0.00%)	>10	0.319	0.674	Co-occurrence
TRIM32	9q33.1	1 (0.87%)	0 (0.00%)	>10	0.319	0.674	Co-occurrence
TRIM34	11p15.4	1 (0.87%)	0 (0.00%)	>10	0.319	0.674	Co-occurrence
TRIM45	1p13.1	1 (0.87%)	0 (0.00%)	>10	0.319	0.674	Co-occurrence
TRIM49B	11p11.12	1 (0.87%)	0 (0.00%)	>10	0.319	0.674	Co-occurrence
TRIM5	11p15.4	1 (0.87%)	0 (0.00%)	>10	0.319	0.674	Co-occurrence
TRIM6	11p15.4	1 (0.87%)	0 (0.00%)	>10	0.319	0.674	Co-occurrence
TRIM64C	11p11.12	1 (0.87%)	0 (0.00%)	>10	0.319	0.674	Co-occurrence
TRIM66	11p15.4	1 (0.87%)	0 (0.00%)	>10	0.319	0.674	Co-occurrence
TRIM68	11p15.4	1 (0.87%)	0 (0.00%)	>10	0.319	0.674	Co-occurrence
TRIM71	3p22.3	1 (0.87%)	0 (0.00%)	>10	0.319	0.674	Co-occurrence
TRMT6	20p12.3	1 (0.87%)	0 (0.00%)	>10	0.319	0.674	Co-occurrence

TRMT61A	14q32.33	1 (0.87%)	0 (0.00%)	>10	0.319	0.674	Co-occurrence
TRNT1	3p26.2	1 (0.87%)	0 (0.00%)	>10	0.319	0.674	Co-occurrence
TSC22D3	Xq22.3	1 (0.87%)	0 (0.00%)	>10	0.319	0.674	Co-occurrence
TSPAN11	12p11.21	1 (0.87%)	0 (0.00%)	>10	0.319	0.674	Co-occurrence
TSSK3	1p35.1	1 (0.87%)	0 (0.00%)	>10	0.319	0.674	Co-occurrence
TSTD3	6q16.2	1 (0.87%)	0 (0.00%)	>10	0.319	0.674	Co-occurrence
TTC17	11p12-p11.2	1 (0.87%)	0 (0.00%)	>10	0.319	0.674	Co-occurrence
TTC21A	3p22.2	1 (0.87%)	0 (0.00%)	>10	0.319	0.674	Co-occurrence
TTC22	1p32.3	1 (0.87%)	0 (0.00%)	>10	0.319	0.674	Co-occurrence
TTC28	22q12.1	1 (0.87%)	0 (0.00%)	>10	0.319	0.674	Co-occurrence
TTC34	1p36.32	1 (0.87%)	0 (0.00%)	>10	0.319	0.674	Co-occurrence
TTC39C	18q11.2	1 (0.87%)	0 (0.00%)	>10	0.319	0.674	Co-occurrence
TTC4	1p32.3	1 (0.87%)	0 (0.00%)	>10	0.319	0.674	Co-occurrence
TTF2	1p13.1	1 (0.87%)	0 (0.00%)	>10	0.319	0.674	Co-occurrence
TTLL10	1p36.33	1 (0.87%)	0 (0.00%)	>10	0.319	0.674	Co-occurrence
TTLL7	1p31.1	1 (0.87%)	0 (0.00%)	>10	0.319	0.674	Co-occurrence
TTLL9	20q11.21	1 (0.87%)	0 (0.00%)	>10	0.319	0.674	Co-occurrence
TUB	11p15.4	1 (0.87%)	0 (0.00%)	>10	0.319	0.674	Co-occurrence
TUBA3E	2q21.1	1 (0.87%)	0 (0.00%)	>10	0.319	0.674	Co-occurrence
TUBGCP5	15q11.2	1 (0.87%)	0 (0.00%)	>10	0.319	0.674	Co-occurrence
TUG1	22q12.2	1 (0.87%)	0 (0.00%)	>10	0.319	0.674	Co-occurrence
TUSC7	3q13.31	1 (0.87%)	0 (0.00%)	>10	0.319	0.674	Co-occurrence
TUT7	9q21.33	1 (0.87%)	0 (0.00%)	>10	0.319	0.674	Co-occurrence
TWF1	12q12	1 (0.87%)	0 (0.00%)	>10	0.319	0.674	Co-occurrence
TXN	9q31.3	1 (0.87%)	0 (0.00%)	>10	0.319	0.674	Co-occurrence
TXNDC8	9q31.3	1 (0.87%)	0 (0.00%)	>10	0.319	0.674	Co-occurrence
TYROBP	19q13.12	1 (0.87%)	0 (0.00%)	>10	0.319	0.674	Co-occurrence
TYSND1	10q22.1	1 (0.87%)	0 (0.00%)	>10	0.319	0.674	Co-occurrence
TYW3	1p31.1	1 (0.87%)	0 (0.00%)	>10	0.319	0.674	Co-occurrence

UBA1	Xp11.3	1 (0.87%)	0 (0.00%)	>10	0.319	0.674	Co-occurrence
UBA6	4q13.2	1 (0.87%)	0 (0.00%)	>10	0.319	0.674	Co-occurrence
UBE2D1	10q21.1	1 (0.87%)	0 (0.00%)	>10	0.319	0.674	Co-occurrence
UBE2E1	3p24.2	1 (0.87%)	0 (0.00%)	>10	0.319	0.674	Co-occurrence
UBE2E2	3p24.3	1 (0.87%)	0 (0.00%)	>10	0.319	0.674	Co-occurrence
UBE2J2	1p36.33	1 (0.87%)	0 (0.00%)	>10	0.319	0.674	Co-occurrence
UBP1	3p22.3	1 (0.87%)	0 (0.00%)	>10	0.319	0.674	Co-occurrence
UBQLN2	Xp11.21	1 (0.87%)	0 (0.00%)	>10	0.319	0.674	Co-occurrence
UBQLN3	11p15.4	1 (0.87%)	0 (0.00%)	>10	0.319	0.674	Co-occurrence
UBQLNL	11p15.4	1 (0.87%)	0 (0.00%)	>10	0.319	0.674	Co-occurrence
UBR7	14q32.12	1 (0.87%)	0 (0.00%)	>10	0.319	0.674	Co-occurrence
UFM1	13q13.3	1 (0.87%)	0 (0.00%)	>10	0.319	0.674	Co-occurrence
UGGT1	2q14.3	1 (0.87%)	0 (0.00%)	>10	0.319	0.674	Co-occurrence
UGT8	4q26	1 (0.87%)	0 (0.00%)	>10	0.319	0.674	Co-occurrence
UNC119B	12q24.31	1 (0.87%)	0 (0.00%)	>10	0.319	0.674	Co-occurrence
UNC79	14q32.12	1 (0.87%)	0 (0.00%)	>10	0.319	0.674	Co-occurrence
UOX	1p31.1	1 (0.87%)	0 (0.00%)	>10	0.319	0.674	Co-occurrence
UQCR10	22q12.2	1 (0.87%)	0 (0.00%)	>10	0.319	0.674	Co-occurrence
UQCRH	1p33	1 (0.87%)	0 (0.00%)	>10	0.319	0.674	Co-occurrence
UROD	1p34.1	1 (0.87%)	0 (0.00%)	>10	0.319	0.674	Co-occurrence
USO1	4q21.1	1 (0.87%)	0 (0.00%)	>10	0.319	0.674	Co-occurrence
USP11	Xp11.3	1 (0.87%)	0 (0.00%)	>10	0.319	0.674	Co-occurrence
USP12	13q12.13	1 (0.87%)	0 (0.00%)	>10	0.319	0.674	Co-occurrence
USP2	11q23.3	1 (0.87%)	0 (0.00%)	>10	0.319	0.674	Co-occurrence
USP24	1p32.3	1 (0.87%)	0 (0.00%)	>10	0.319	0.674	Co-occurrence
USP45	6q16.2	1 (0.87%)	0 (0.00%)	>10	0.319	0.674	Co-occurrence
UTP11	1p34.3	1 (0.87%)	0 (0.00%)	>10	0.319	0.674	Co-occurrence
UTP14C	13q14.3	1 (0.87%)	0 (0.00%)	>10	0.319	0.674	Co-occurrence
UTP3	4q13.3	1 (0.87%)	0 (0.00%)	>10	0.319	0.674	Co-occurrence

UTS2	1p36.23	1 (0.87%)	0 (0.00%)	>10	0.319	0.674	Co-occurrence
UXT	Xp11.23	1 (0.87%)	0 (0.00%)	>10	0.319	0.674	Co-occurrence
VAMP3	1p36.23	1 (0.87%)	0 (0.00%)	>10	0.319	0.674	Co-occurrence
VAX1	10q25.3	1 (0.87%)	0 (0.00%)	>10	0.319	0.674	Co-occurrence
VCP	9p13.3	1 (0.87%)	0 (0.00%)	>10	0.319	0.674	Co-occurrence
VCX3B	Xp22.31	1 (0.87%)	0 (0.00%)	>10	0.319	0.674	Co-occurrence
VEGFD	Xp22.2	1 (0.87%)	0 (0.00%)	>10	0.319	0.674	Co-occurrence
VNN1	6q23.2	1 (0.87%)	0 (0.00%)	>10	0.319	0.674	Co-occurrence
VNN2	6q23.2	1 (0.87%)	0 (0.00%)	>10	0.319	0.674	Co-occurrence
VNN3	6q23.2	1 (0.87%)	0 (0.00%)	>10	0.319	0.674	Co-occurrence
VPS33A	12q24.31	1 (0.87%)	0 (0.00%)	>10	0.319	0.674	Co-occurrence
VPS36	13q14.3	1 (0.87%)	0 (0.00%)	>10	0.319	0.674	Co-occurrence
VPS53	17p13.3	1 (0.87%)	0 (0.00%)	>10	0.319	0.674	Co-occurrence
VSIR	10q22.1	1 (0.87%)	0 (0.00%)	>10	0.319	0.674	Co-occurrence
VTCN1	1p13.1-p12	1 (0.87%)	0 (0.00%)	>10	0.319	0.674	Co-occurrence
VWA1	1p36.33	1 (0.87%)	0 (0.00%)	>10	0.319	0.674	Co-occurrence
WASF2	1p36.11	1 (0.87%)	0 (0.00%)	>10	0.319	0.674	Co-occurrence
WASF3	13q12.13	1 (0.87%)	0 (0.00%)	>10	0.319	0.674	Co-occurrence
WBP2NL	22q13.2	1 (0.87%)	0 (0.00%)	>10	0.319	0.674	Co-occurrence
WDFY2	13q14.3	1 (0.87%)	0 (0.00%)	>10	0.319	0.674	Co-occurrence
WDR13	Xp11.23	1 (0.87%)	0 (0.00%)	>10	0.319	0.674	Co-occurrence
WDR38	9q33.3	1 (0.87%)	0 (0.00%)	>10	0.319	0.674	Co-occurrence
WDR48	3p22.2	1 (0.87%)	0 (0.00%)	>10	0.319	0.674	Co-occurrence
WDR62	19q13.12	1 (0.87%)	0 (0.00%)	>10	0.319	0.674	Co-occurrence
WDR87	19q13.13	1 (0.87%)	0 (0.00%)	>10	0.319	0.674	Co-occurrence
WDR89	14q23.2	1 (0.87%)	0 (0.00%)	>10	0.319	0.674	Co-occurrence
WHAMMP3	15q11.2	1 (0.87%)	0 (0.00%)	>10	0.319	0.674	Co-occurrence
WRAP73	1p36.32	1 (0.87%)	0 (0.00%)	>10	0.319	0.674	Co-occurrence
WSCD1	17p13.2	1 (0.87%)	0 (0.00%)	>10	0.319	0.674	Co-occurrence

WSCD2	12q23.3	1 (0.87%)	0 (0.00%)	>10	0.319	0.674	Co-occurrence
XBP1	22q12.1|22q12	1 (0.87%)	0 (0.00%)	>10	0.319	0.674	Co-occurrence
XCR1	3p21.31	1 (0.87%)	0 (0.00%)	>10	0.319	0.674	Co-occurrence
XG	Xp22.33	1 (0.87%)	0 (0.00%)	>10	0.319	0.674	Co-occurrence
XIRP1	3p22.2	1 (0.87%)	0 (0.00%)	>10	0.319	0.674	Co-occurrence
XPNPEP1	10q25.1	1 (0.87%)	0 (0.00%)	>10	0.319	0.674	Co-occurrence
XPO7	8p21.3	1 (0.87%)	0 (0.00%)	>10	0.319	0.674	Co-occurrence
XYLB	3p22.2	1 (0.87%)	0 (0.00%)	>10	0.319	0.674	Co-occurrence
YAF2	12q12	1 (0.87%)	0 (0.00%)	>10	0.319	0.674	Co-occurrence
YARS2	12p11.21	1 (0.87%)	0 (0.00%)	>10	0.319	0.674	Co-occurrence
YIF1B	19q13.2	1 (0.87%)	0 (0.00%)	>10	0.319	0.674	Co-occurrence
YIPF1	1p32.3	1 (0.87%)	0 (0.00%)	>10	0.319	0.674	Co-occurrence
YRDC	1p34.3	1 (0.87%)	0 (0.00%)	>10	0.319	0.674	Co-occurrence
YWHAE	17p13.3	1 (0.87%)	0 (0.00%)	>10	0.319	0.674	Co-occurrence
YY2	Xp22.12	1 (0.87%)	0 (0.00%)	>10	0.319	0.674	Co-occurrence
ZBED1	Xp22.33 and Yp11.2	1 (0.87%)	0 (0.00%)	>10	0.319	0.674	Co-occurrence
ZBED2	3q13.13	1 (0.87%)	0 (0.00%)	>10	0.319	0.674	Co-occurrence
ZBED5	11p15.4	1 (0.87%)	0 (0.00%)	>10	0.319	0.674	Co-occurrence
ZBTB1	14q23.3	1 (0.87%)	0 (0.00%)	>10	0.319	0.674	Co-occurrence
ZBTB25	14q23.3	1 (0.87%)	0 (0.00%)	>10	0.319	0.674	Co-occurrence
ZBTB26	9q33.2	1 (0.87%)	0 (0.00%)	>10	0.319	0.674	Co-occurrence
ZBTB34	9q33.3	1 (0.87%)	0 (0.00%)	>10	0.319	0.674	Co-occurrence
ZBTB42	14q32.33	1 (0.87%)	0 (0.00%)	>10	0.319	0.674	Co-occurrence
ZBTB43	9q33.3	1 (0.87%)	0 (0.00%)	>10	0.319	0.674	Co-occurrence
ZBTB6	9q33.2	1 (0.87%)	0 (0.00%)	>10	0.319	0.674	Co-occurrence
ZBTB8B	1p35.1	1 (0.87%)	0 (0.00%)	>10	0.319	0.674	Co-occurrence
ZC3H12A	1p34.3	1 (0.87%)	0 (0.00%)	>10	0.319	0.674	Co-occurrence
ZC3H14	14q31.3	1 (0.87%)	0 (0.00%)	>10	0.319	0.674	Co-occurrence
ZCCHC14	16q24.2	1 (0.87%)	0 (0.00%)	>10	0.319	0.674	Co-occurrence

ZCCHC17	1p35.2	1 (0.87%)	0 (0.00%)	>10	0.319	0.674	Co-occurrence
ZCCHC24	10q22.3	1 (0.87%)	0 (0.00%)	>10	0.319	0.674	Co-occurrence
ZCRB1	12q12	1 (0.87%)	0 (0.00%)	>10	0.319	0.674	Co-occurrence
ZCWPW2	3p24.1	1 (0.87%)	0 (0.00%)	>10	0.319	0.674	Co-occurrence
ZDHHC20	13q12.11	1 (0.87%)	0 (0.00%)	>10	0.319	0.674	Co-occurrence
ZDHHC23	3q13.31	1 (0.87%)	0 (0.00%)	>10	0.319	0.674	Co-occurrence
ZDHHC3	3p21.31	1 (0.87%)	0 (0.00%)	>10	0.319	0.674	Co-occurrence
ZFP14	19q13.12	1 (0.87%)	0 (0.00%)	>10	0.319	0.674	Co-occurrence
ZFP30	19q13.12	1 (0.87%)	0 (0.00%)	>10	0.319	0.674	Co-occurrence
ZFP69	1p34.2	1 (0.87%)	0 (0.00%)	>10	0.319	0.674	Co-occurrence
ZFP69B	1p34.2	1 (0.87%)	0 (0.00%)	>10	0.319	0.674	Co-occurrence
ZFP82	19q13.12	1 (0.87%)	0 (0.00%)	>10	0.319	0.674	Co-occurrence
ZFR2	19p13.3	1 (0.87%)	0 (0.00%)	>10	0.319	0.674	Co-occurrence
ZFYVE1	14q24.2	1 (0.87%)	0 (0.00%)	>10	0.319	0.674	Co-occurrence
ZFYVE9	1p32.3	1 (0.87%)	0 (0.00%)	>10	0.319	0.674	Co-occurrence
ZKSCAN7	3p21.31	1 (0.87%)	0 (0.00%)	>10	0.319	0.674	Co-occurrence
ZMAT5	22q12.2	1 (0.87%)	0 (0.00%)	>10	0.319	0.674	Co-occurrence
ZMYM1	1p34.3	1 (0.87%)	0 (0.00%)	>10	0.319	0.674	Co-occurrence
ZMYM2	13q12.11	1 (0.87%)	0 (0.00%)	>10	0.319	0.674	Co-occurrence
ZMYM4	1p34.3	1 (0.87%)	0 (0.00%)	>10	0.319	0.674	Co-occurrence
ZMYM5	13q12.11	1 (0.87%)	0 (0.00%)	>10	0.319	0.674	Co-occurrence
ZMYM6	1p34.3	1 (0.87%)	0 (0.00%)	>10	0.319	0.674	Co-occurrence
ZNF146	19q13.12	1 (0.87%)	0 (0.00%)	>10	0.319	0.674	Co-occurrence
ZNF157	Xp11.3	1 (0.87%)	0 (0.00%)	>10	0.319	0.674	Co-occurrence
ZNF169	9q22.32	1 (0.87%)	0 (0.00%)	>10	0.319	0.674	Co-occurrence
ZNF197	3p21.31	1 (0.87%)	0 (0.00%)	>10	0.319	0.674	Co-occurrence
ZNF214	11p15.4	1 (0.87%)	0 (0.00%)	>10	0.319	0.674	Co-occurrence
ZNF215	11p15.4	1 (0.87%)	0 (0.00%)	>10	0.319	0.674	Co-occurrence
ZNF260	19q13.12	1 (0.87%)	0 (0.00%)	>10	0.319	0.674	Co-occurrence

ZNF345	19q13.12	1 (0.87%)	0 (0.00%)	>10	0.319	0.674	Co-occurrence
ZNF35	3p21.31	1 (0.87%)	0 (0.00%)	>10	0.319	0.674	Co-occurrence
ZNF382	19q13.12	1 (0.87%)	0 (0.00%)	>10	0.319	0.674	Co-occurrence
ZNF383	19q13.12	1 (0.87%)	0 (0.00%)	>10	0.319	0.674	Co-occurrence
ZNF385D	3p24.3	1 (0.87%)	0 (0.00%)	>10	0.319	0.674	Co-occurrence
ZNF408	11p11.2	1 (0.87%)	0 (0.00%)	>10	0.319	0.674	Co-occurrence
ZNF41	Xp11.3	1 (0.87%)	0 (0.00%)	>10	0.319	0.674	Co-occurrence
ZNF420	19q13.12	1 (0.87%)	0 (0.00%)	>10	0.319	0.674	Co-occurrence
ZNF461	19q13.12	1 (0.87%)	0 (0.00%)	>10	0.319	0.674	Co-occurrence
ZNF501	3p21.31	1 (0.87%)	0 (0.00%)	>10	0.319	0.674	Co-occurrence
ZNF502	3p21.31	1 (0.87%)	0 (0.00%)	>10	0.319	0.674	Co-occurrence
ZNF521	18q11.2	1 (0.87%)	0 (0.00%)	>10	0.319	0.674	Co-occurrence
ZNF527	19q13.12	1 (0.87%)	0 (0.00%)	>10	0.319	0.674	Co-occurrence
ZNF529	19q13.12	1 (0.87%)	0 (0.00%)	>10	0.319	0.674	Co-occurrence
ZNF532	18q21.32	1 (0.87%)	0 (0.00%)	>10	0.319	0.674	Co-occurrence
ZNF540	19q13.12	1 (0.87%)	0 (0.00%)	>10	0.319	0.674	Co-occurrence
ZNF565	19q13.12	1 (0.87%)	0 (0.00%)	>10	0.319	0.674	Co-occurrence
ZNF566	19q13.12	1 (0.87%)	0 (0.00%)	>10	0.319	0.674	Co-occurrence
ZNF567	19q13.12	1 (0.87%)	0 (0.00%)	>10	0.319	0.674	Co-occurrence
ZNF568	19q13.12	1 (0.87%)	0 (0.00%)	>10	0.319	0.674	Co-occurrence
ZNF571	19q13.12	1 (0.87%)	0 (0.00%)	>10	0.319	0.674	Co-occurrence
ZNF573	19q13.12	1 (0.87%)	0 (0.00%)	>10	0.319	0.674	Co-occurrence
ZNF585A	19q13.12	1 (0.87%)	0 (0.00%)	>10	0.319	0.674	Co-occurrence
ZNF585B	19q13.12	1 (0.87%)	0 (0.00%)	>10	0.319	0.674	Co-occurrence
ZNF589	3p21.31	1 (0.87%)	0 (0.00%)	>10	0.319	0.674	Co-occurrence
ZNF607	19q13.12	1 (0.87%)	0 (0.00%)	>10	0.319	0.674	Co-occurrence
ZNF619	3p22.1	1 (0.87%)	0 (0.00%)	>10	0.319	0.674	Co-occurrence
ZNF620	3p22.1	1 (0.87%)	0 (0.00%)	>10	0.319	0.674	Co-occurrence
ZNF621	3p22.1	1 (0.87%)	0 (0.00%)	>10	0.319	0.674	Co-occurrence

ZNF644	1p22.2	1 (0.87%)	0 (0.00%)	>10	0.319	0.674	Co-occurrence
ZNF658	9q21.11	1 (0.87%)	0 (0.00%)	>10	0.319	0.674	Co-occurrence
ZNF660	3p21.31	1 (0.87%)	0 (0.00%)	>10	0.319	0.674	Co-occurrence
ZNF664	12q24.31	1 (0.87%)	0 (0.00%)	>10	0.319	0.674	Co-occurrence
ZNF684	1p34.2	1 (0.87%)	0 (0.00%)	>10	0.319	0.674	Co-occurrence
ZNF781	19q13.12	1 (0.87%)	0 (0.00%)	>10	0.319	0.674	Co-occurrence
ZNF790	19q13.12	1 (0.87%)	0 (0.00%)	>10	0.319	0.674	Co-occurrence
ZNF793	19q13.12	1 (0.87%)	0 (0.00%)	>10	0.319	0.674	Co-occurrence
ZNF80	3q13.31	1 (0.87%)	0 (0.00%)	>10	0.319	0.674	Co-occurrence
ZNF829	19q13.12	1 (0.87%)	0 (0.00%)	>10	0.319	0.674	Co-occurrence
ZNF850	19q13.12	1 (0.87%)	0 (0.00%)	>10	0.319	0.674	Co-occurrence
ZNF852	3p21.31	1 (0.87%)	0 (0.00%)	>10	0.319	0.674	Co-occurrence
ZNF860	3p23-p22.3	1 (0.87%)	0 (0.00%)	>10	0.319	0.674	Co-occurrence
ZNF875	19q13.12	1 (0.87%)	0 (0.00%)	>10	0.319	0.674	Co-occurrence
ZNHIT6	1p22.3	1 (0.87%)	0 (0.00%)	>10	0.319	0.674	Co-occurrence
ZNRF3	22q12.1	1 (0.87%)	0 (0.00%)	>10	0.319	0.674	Co-occurrence
ZRANB2	1p31.1	1 (0.87%)	0 (0.00%)	>10	0.319	0.674	Co-occurrence
ZRSR2	Xp22.2	1 (0.87%)	0 (0.00%)	>10	0.319	0.674	Co-occurrence
ZSWIM5	1p34.1	1 (0.87%)	0 (0.00%)	>10	0.319	0.674	Co-occurrence
ZXDA	Xp11.21	1 (0.87%)	0 (0.00%)	>10	0.319	0.674	Co-occurrence
ZYG11A	1p32.3	1 (0.87%)	0 (0.00%)	>10	0.319	0.674	Co-occurrence
ZYG11B	1p32.3	1 (0.87%)	0 (0.00%)	>10	0.319	0.674	Co-occurrence
CHD7	8q12.2	7 (6.09%)	20 (8.16%)	-0.42	0.321	0.677	Mutual exclusivity
IMPAD1	8q12.1	7 (6.09%)	20 (8.16%)	-0.42	0.321	0.677	Mutual exclusivity
LINC00588	8q12.1	7 (6.09%)	20 (8.16%)	-0.42	0.321	0.677	Mutual exclusivity
LINC00968	8q12.1	7 (6.09%)	20 (8.16%)	-0.42	0.321	0.677	Mutual exclusivity
PENK	8q12.1	7 (6.09%)	20 (8.16%)	-0.42	0.321	0.677	Mutual exclusivity
RAB2A	8q12.1-q12.2	7 (6.09%)	20 (8.16%)	-0.42	0.321	0.677	Mutual exclusivity
RNA5SP266	8q12.1	7 (6.09%)	20 (8.16%)	-0.42	0.321	0.677	Mutual exclusivity

SDR16C5	8q12.1	7 (6.09%)	20 (8.16%)	-0.42	0.321	0.677	Mutual exclusivity
SDR16C6P	8q12.1	7 (6.09%)	20 (8.16%)	-0.42	0.321	0.677	Mutual exclusivity
COL21A1	6p12.1|6p12.3-p11.2	5 (4.35%)	7 (2.86%)	0.61	0.327	0.686	Co-occurrence
ECT2	3q26.31	5 (4.35%)	7 (2.86%)	0.61	0.327	0.686	Co-occurrence
ITPR3	6p21.31	5 (4.35%)	7 (2.86%)	0.61	0.327	0.686	Co-occurrence
LEMD2	6p21.31	5 (4.35%)	7 (2.86%)	0.61	0.327	0.686	Co-occurrence
MEGF8	19q13.2	5 (4.35%)	7 (2.86%)	0.61	0.327	0.686	Co-occurrence
PIM1	6p21.2	5 (4.35%)	7 (2.86%)	0.61	0.327	0.686	Co-occurrence
PP13439	3q26.31	5 (4.35%)	7 (2.86%)	0.61	0.327	0.686	Co-occurrence
RHOB	2p24.1	5 (4.35%)	7 (2.86%)	0.61	0.327	0.686	Co-occurrence
RN7SL141P	3q26.31	5 (4.35%)	7 (2.86%)	0.61	0.327	0.686	Co-occurrence
RN7SL636P	20q13.13	5 (4.35%)	7 (2.86%)	0.61	0.327	0.686	Co-occurrence
SPATA16	3q26.31	5 (4.35%)	7 (2.86%)	0.61	0.327	0.686	Co-occurrence
TMEM212	3q26.31	5 (4.35%)	7 (2.86%)	0.61	0.327	0.686	Co-occurrence
TMEM217	6p21.2	5 (4.35%)	7 (2.86%)	0.61	0.327	0.686	Co-occurrence
TNIK	3q26.2-q26.31	5 (4.35%)	7 (2.86%)	0.61	0.327	0.686	Co-occurrence
TOB1	17q21.33	5 (4.35%)	7 (2.86%)	0.61	0.327	0.686	Co-occurrence
UQCC2	6p21.31	5 (4.35%)	7 (2.86%)	0.61	0.327	0.686	Co-occurrence
LACTB2	8q13.3	11 (9.57%)	29 (11.84%)	-0.31	0.328	0.686	Mutual exclusivity
RN7SL19P	8q13.3	11 (9.57%)	29 (11.84%)	-0.31	0.328	0.686	Mutual exclusivity
XKR9	8q13.3	11 (9.57%)	29 (11.84%)	-0.31	0.328	0.686	Mutual exclusivity
DPY19L4	8q22.1	15 (13.04%)	38 (15.51%)	-0.25	0.328	0.686	Mutual exclusivity
ANKRD7	7q31.31	2 (1.74%)	8 (3.27%)	-0.91	0.33	0.686	Mutual exclusivity
ASZ1	7q31.2	2 (1.74%)	8 (3.27%)	-0.91	0.33	0.686	Mutual exclusivity
CFTR	7q31.2	2 (1.74%)	8 (3.27%)	-0.91	0.33	0.686	Mutual exclusivity
CTTNBP2	7q31.31	2 (1.74%)	8 (3.27%)	-0.91	0.33	0.686	Mutual exclusivity
IMMP2L	7q31.1	2 (1.74%)	8 (3.27%)	-0.91	0.33	0.686	Mutual exclusivity
KCND2	7q31.31	2 (1.74%)	8 (3.27%)	-0.91	0.33	0.686	Mutual exclusivity
NAA38	17p13.1	2 (1.74%)	8 (3.27%)	-0.91	0.33	0.686	Mutual exclusivity

PPP1CA	11q13.2	2 (1.74%)	8 (3.27%)	-0.91	0.33	0.686	Mutual exclusivity
RAD9A	11q13.2	2 (1.74%)	8 (3.27%)	-0.91	0.33	0.686	Mutual exclusivity
SHANK2	11q13.3-q13.4	2 (1.74%)	8 (3.27%)	-0.91	0.33	0.686	Mutual exclusivity
WNT2	7q31.2	2 (1.74%)	8 (3.27%)	-0.91	0.33	0.686	Mutual exclusivity
CD83	6p23	6 (5.22%)	9 (3.67%)	0.51	0.335	0.686	Co-occurrence
GNL1	6p21.33	6 (5.22%)	9 (3.67%)	0.51	0.335	0.686	Co-occurrence
HCG17	6p22.1	6 (5.22%)	9 (3.67%)	0.51	0.335	0.686	Co-occurrence
HCG18	6p22.1	6 (5.22%)	9 (3.67%)	0.51	0.335	0.686	Co-occurrence
HCG9	6p22.1	6 (5.22%)	9 (3.67%)	0.51	0.335	0.686	Co-occurrence
KDM1B	6p22.3	6 (5.22%)	9 (3.67%)	0.51	0.335	0.686	Co-occurrence
NRM	6p21.33	6 (5.22%)	9 (3.67%)	0.51	0.335	0.686	Co-occurrence
NUP153	6p22.3	6 (5.22%)	9 (3.67%)	0.51	0.335	0.686	Co-occurrence
PHACTR1	6p24.1	6 (5.22%)	9 (3.67%)	0.51	0.335	0.686	Co-occurrence
PPP1R11	6p22.1	6 (5.22%)	9 (3.67%)	0.51	0.335	0.686	Co-occurrence
PPP1R18	6p21.33	6 (5.22%)	9 (3.67%)	0.51	0.335	0.686	Co-occurrence
PRR3	6p21.33	6 (5.22%)	9 (3.67%)	0.51	0.335	0.686	Co-occurrence
RNF182	6p23	6 (5.22%)	9 (3.67%)	0.51	0.335	0.686	Co-occurrence
RPP21	6p22.1	6 (5.22%)	9 (3.67%)	0.51	0.335	0.686	Co-occurrence
SDC1	2p24.1	6 (5.22%)	9 (3.67%)	0.51	0.335	0.686	Co-occurrence
TBC1D7	6p24.1	6 (5.22%)	9 (3.67%)	0.51	0.335	0.686	Co-occurrence
TRIM10	6p22.1	6 (5.22%)	9 (3.67%)	0.51	0.335	0.686	Co-occurrence
TRIM15	6p22.1	6 (5.22%)	9 (3.67%)	0.51	0.335	0.686	Co-occurrence
TRIM26	6p22.1	6 (5.22%)	9 (3.67%)	0.51	0.335	0.686	Co-occurrence
TRIM39	6p22.1	6 (5.22%)	9 (3.67%)	0.51	0.335	0.686	Co-occurrence
TRIM40	6p22.1	6 (5.22%)	9 (3.67%)	0.51	0.335	0.686	Co-occurrence
ZNRD1	6p22.1	6 (5.22%)	9 (3.67%)	0.51	0.335	0.686	Co-occurrence
CYP7A1	8q12.1	8 (6.96%)	22 (8.98%)	-0.37	0.335	0.686	Mutual exclusivity
RN7SKP135	8q12.3	8 (6.96%)	22 (8.98%)	-0.37	0.335	0.686	Mutual exclusivity
RNA5SP267	8q12.1	8 (6.96%)	22 (8.98%)	-0.37	0.335	0.686	Mutual exclusivity

UBXN2B	8q12.1	8 (6.96%)	22 (8.98%)	-0.37	0.335	0.686	Mutual exclusivity
RN7SKP204	6p23	7 (6.09%)	11 (4.49%)	0.44	0.34	0.686	Co-occurrence
GMDS	6p25.3	8 (6.96%)	13 (5.31%)	0.39	0.344	0.686	Co-occurrence
ADCK5	8q24.3	17 (14.78%)	42 (17.14%)	-0.21	0.344	0.686	Mutual exclusivity
ARC	8q24.3	17 (14.78%)	42 (17.14%)	-0.21	0.344	0.686	Mutual exclusivity
ARHGAP39	8q24.3	17 (14.78%)	42 (17.14%)	-0.21	0.344	0.686	Mutual exclusivity
BOP1	8q24.3	17 (14.78%)	42 (17.14%)	-0.21	0.344	0.686	Mutual exclusivity
C8ORF31	8q24.3	17 (14.78%)	42 (17.14%)	-0.21	0.344	0.686	Mutual exclusivity
C8ORF33	8q24.3	17 (14.78%)	42 (17.14%)	-0.21	0.344	0.686	Mutual exclusivity
C8ORF82	8q24.3	17 (14.78%)	42 (17.14%)	-0.21	0.344	0.686	Mutual exclusivity
CCDC166	8q24.3	17 (14.78%)	42 (17.14%)	-0.21	0.344	0.686	Mutual exclusivity
COMMD5	8q24.3	17 (14.78%)	42 (17.14%)	-0.21	0.344	0.686	Mutual exclusivity
CPSF1	8q24.3	17 (14.78%)	42 (17.14%)	-0.21	0.344	0.686	Mutual exclusivity
CYHR1	8q24.3	17 (14.78%)	42 (17.14%)	-0.21	0.344	0.686	Mutual exclusivity
CYP11B1	8q24.3	17 (14.78%)	42 (17.14%)	-0.21	0.344	0.686	Mutual exclusivity
CYP11B2	8q24.3	17 (14.78%)	42 (17.14%)	-0.21	0.344	0.686	Mutual exclusivity
DGAT1	8q24.3	17 (14.78%)	42 (17.14%)	-0.21	0.344	0.686	Mutual exclusivity
EPPK1	8q24.3	17 (14.78%)	42 (17.14%)	-0.21	0.344	0.686	Mutual exclusivity
FAM83H	8q24.3	17 (14.78%)	42 (17.14%)	-0.21	0.344	0.686	Mutual exclusivity
FBXL6	8q24.3	17 (14.78%)	42 (17.14%)	-0.21	0.344	0.686	Mutual exclusivity
FOXH1	8q24.3	17 (14.78%)	42 (17.14%)	-0.21	0.344	0.686	Mutual exclusivity
GLI4	8q24.3	17 (14.78%)	42 (17.14%)	-0.21	0.344	0.686	Mutual exclusivity
GML	8q24.3	17 (14.78%)	42 (17.14%)	-0.21	0.344	0.686	Mutual exclusivity
GPIHBP1	8q24.3	17 (14.78%)	42 (17.14%)	-0.21	0.344	0.686	Mutual exclusivity
GPR20	8q24.3	17 (14.78%)	42 (17.14%)	-0.21	0.344	0.686	Mutual exclusivity
GPT	8q24.3	17 (14.78%)	42 (17.14%)	-0.21	0.344	0.686	Mutual exclusivity
GRINA	8q24.3	17 (14.78%)	42 (17.14%)	-0.21	0.344	0.686	Mutual exclusivity
HSF1	8q24.3	17 (14.78%)	42 (17.14%)	-0.21	0.344	0.686	Mutual exclusivity
JRK	8q24.3	17 (14.78%)	42 (17.14%)	-0.21	0.344	0.686	Mutual exclusivity

KIFC2	8q24.3	17 (14.78%)	42 (17.14%)	-0.21	0.344	0.686	Mutual exclusivity
LINC00051	8q24.3	17 (14.78%)	42 (17.14%)	-0.21	0.344	0.686	Mutual exclusivity
LRRC14	8q24.3	17 (14.78%)	42 (17.14%)	-0.21	0.344	0.686	Mutual exclusivity
LRRC24	8q24.3	17 (14.78%)	42 (17.14%)	-0.21	0.344	0.686	Mutual exclusivity
LY6E	8q24.3	17 (14.78%)	42 (17.14%)	-0.21	0.344	0.686	Mutual exclusivity
LY6H	8q24.3	17 (14.78%)	42 (17.14%)	-0.21	0.344	0.686	Mutual exclusivity
MAFA	8q24.3	17 (14.78%)	42 (17.14%)	-0.21	0.344	0.686	Mutual exclusivity
MAPK15	-	17 (14.78%)	42 (17.14%)	-0.21	0.344	0.686	Mutual exclusivity
MFSD3	8q24.3	17 (14.78%)	42 (17.14%)	-0.21	0.344	0.686	Mutual exclusivity
MIR-661/661		17 (14.78%)	42 (17.14%)	-0.21	0.344	0.686	Mutual exclusivity
MIR-939/939		17 (14.78%)	42 (17.14%)	-0.21	0.344	0.686	Mutual exclusivity
MROH5	8q24.3	17 (14.78%)	42 (17.14%)	-0.21	0.344	0.686	Mutual exclusivity
NRBP2	8q24.3	17 (14.78%)	42 (17.14%)	-0.21	0.344	0.686	Mutual exclusivity
PLEC	8q24.3	17 (14.78%)	42 (17.14%)	-0.21	0.344	0.686	Mutual exclusivity
PPP1R16A	8q24.3	17 (14.78%)	42 (17.14%)	-0.21	0.344	0.686	Mutual exclusivity
PTP4A3	8q24.3	17 (14.78%)	42 (17.14%)	-0.21	0.344	0.686	Mutual exclusivity
PUF60	8q24.3	17 (14.78%)	42 (17.14%)	-0.21	0.344	0.686	Mutual exclusivity
RECQL4	8q24.3	17 (14.78%)	42 (17.14%)	-0.21	0.344	0.686	Mutual exclusivity
RHPN1	8q24.3	17 (14.78%)	42 (17.14%)	-0.21	0.344	0.686	Mutual exclusivity
RN7SL395P	8q24.3	17 (14.78%)	42 (17.14%)	-0.21	0.344	0.686	Mutual exclusivity
RPL8	8q24.3	17 (14.78%)	42 (17.14%)	-0.21	0.344	0.686	Mutual exclusivity
SCRIB	8q24.3	17 (14.78%)	42 (17.14%)	-0.21	0.344	0.686	Mutual exclusivity
SCRT1	8q24.3	17 (14.78%)	42 (17.14%)	-0.21	0.344	0.686	Mutual exclusivity
SCX	8q24.3	17 (14.78%)	42 (17.14%)	-0.21	0.344	0.686	Mutual exclusivity
SLC39A4	8q24.3	17 (14.78%)	42 (17.14%)	-0.21	0.344	0.686	Mutual exclusivity
SLC52A2	8q24.3	17 (14.78%)	42 (17.14%)	-0.21	0.344	0.686	Mutual exclusivity
TMEM249	8q24.3	17 (14.78%)	42 (17.14%)	-0.21	0.344	0.686	Mutual exclusivity
TONSL	8q24.3	17 (14.78%)	42 (17.14%)	-0.21	0.344	0.686	Mutual exclusivity
TOP1MT	8q24.3	17 (14.78%)	42 (17.14%)	-0.21	0.344	0.686	Mutual exclusivity

TSNARE1	8q24.3	17 (14.78%)	42 (17.14%)	-0.21	0.344	0.686	Mutual exclusivity
VPS28	8q24.3	17 (14.78%)	42 (17.14%)	-0.21	0.344	0.686	Mutual exclusivity
ZFP41	8q24.3	17 (14.78%)	42 (17.14%)	-0.21	0.344	0.686	Mutual exclusivity
ZNF16	8q24.3	17 (14.78%)	42 (17.14%)	-0.21	0.344	0.686	Mutual exclusivity
ZNF250	8q24.3	17 (14.78%)	42 (17.14%)	-0.21	0.344	0.686	Mutual exclusivity
ZNF251	8q24.3	17 (14.78%)	42 (17.14%)	-0.21	0.344	0.686	Mutual exclusivity
ZNF252P	8q24.3	17 (14.78%)	42 (17.14%)	-0.21	0.344	0.686	Mutual exclusivity
ZNF34	8q24.3	17 (14.78%)	42 (17.14%)	-0.21	0.344	0.686	Mutual exclusivity
ZNF517	8q24.3	17 (14.78%)	42 (17.14%)	-0.21	0.344	0.686	Mutual exclusivity
ZNF696	8q24.3	17 (14.78%)	42 (17.14%)	-0.21	0.344	0.686	Mutual exclusivity
ZNF7	8q24.3	17 (14.78%)	42 (17.14%)	-0.21	0.344	0.686	Mutual exclusivity
ZNF707	8q24.3	17 (14.78%)	42 (17.14%)	-0.21	0.344	0.686	Mutual exclusivity
FNTA	8p11.21	9 (7.83%)	15 (6.12%)	0.35	0.346	0.686	Co-occurrence
HGSNAT	8p11.21-p11.1	9 (7.83%)	15 (6.12%)	0.35	0.346	0.686	Co-occurrence
POMK	8p11.21	9 (7.83%)	15 (6.12%)	0.35	0.346	0.686	Co-occurrence
POTEA	8p11.1	9 (7.83%)	15 (6.12%)	0.35	0.346	0.686	Co-occurrence
CHMP4C	8q21.13	13 (11.30%)	33 (13.47%)	-0.25	0.348	0.686	Mutual exclusivity
FABP12	8q21.13	13 (11.30%)	33 (13.47%)	-0.25	0.348	0.686	Mutual exclusivity
FABP4	8q21.13	13 (11.30%)	33 (13.47%)	-0.25	0.348	0.686	Mutual exclusivity
FABP9	8q21.13	13 (11.30%)	33 (13.47%)	-0.25	0.348	0.686	Mutual exclusivity
HEY1	8q21.13	13 (11.30%)	33 (13.47%)	-0.25	0.348	0.686	Mutual exclusivity
IMPA1	8q21.13	13 (11.30%)	33 (13.47%)	-0.25	0.348	0.686	Mutual exclusivity
IMPA1P1	8q21.13	13 (11.30%)	33 (13.47%)	-0.25	0.348	0.686	Mutual exclusivity
RN7SL675P	8q13.3	13 (11.30%)	33 (13.47%)	-0.25	0.348	0.686	Mutual exclusivity
RNA5SP270	8q13.3	13 (11.30%)	33 (13.47%)	-0.25	0.348	0.686	Mutual exclusivity
SLC10A5	8q21.13	13 (11.30%)	33 (13.47%)	-0.25	0.348	0.686	Mutual exclusivity
SLCO5A1	8q13.3	13 (11.30%)	33 (13.47%)	-0.25	0.348	0.686	Mutual exclusivity
SNX16	8q21.13	13 (11.30%)	33 (13.47%)	-0.25	0.348	0.686	Mutual exclusivity
STMN2	8q21.13	13 (11.30%)	33 (13.47%)	-0.25	0.348	0.686	Mutual exclusivity

ZFAND1	8q21.13	13 (11.30%)	33 (13.47%)	-0.25	0.348	0.686	Mutual exclusivity
ARFGEF1	8q13.2	9 (7.83%)	24 (9.80%)	-0.32	0.348	0.686	Mutual exclusivity
COPS5	8q13.1	9 (7.83%)	24 (9.80%)	-0.32	0.348	0.686	Mutual exclusivity
CSPP1	8q13.1-q13.2	9 (7.83%)	24 (9.80%)	-0.32	0.348	0.686	Mutual exclusivity
MCMDC2	8q13.1	9 (7.83%)	24 (9.80%)	-0.32	0.348	0.686	Mutual exclusivity
MYBL1	8q13.1	9 (7.83%)	24 (9.80%)	-0.32	0.348	0.686	Mutual exclusivity
PPP1R42	8q13.1	9 (7.83%)	24 (9.80%)	-0.32	0.348	0.686	Mutual exclusivity
RNA5SP268	8q13.1	9 (7.83%)	24 (9.80%)	-0.32	0.348	0.686	Mutual exclusivity
SNHG6	8q13.1|8q13	9 (7.83%)	24 (9.80%)	-0.32	0.348	0.686	Mutual exclusivity
SNORD87	8q13.1	9 (7.83%)	24 (9.80%)	-0.32	0.348	0.686	Mutual exclusivity
TCF24	8q13.1	9 (7.83%)	24 (9.80%)	-0.32	0.348	0.686	Mutual exclusivity
GTPBP2	6p21.1	6 (5.22%)	17 (6.94%)	-0.41	0.356	0.686	Mutual exclusivity
MAD2L1BP	6p21.1	6 (5.22%)	17 (6.94%)	-0.41	0.356	0.686	Mutual exclusivity
PSKH2	8q21.3	14 (12.17%)	35 (14.29%)	-0.23	0.357	0.686	Mutual exclusivity
ADGRF2	6p12.3	3 (2.61%)	10 (4.08%)	-0.65	0.358	0.686	Mutual exclusivity
ADGRF4	6p12.3	3 (2.61%)	10 (4.08%)	-0.65	0.358	0.686	Mutual exclusivity
ADGRF5	6p12.3	3 (2.61%)	10 (4.08%)	-0.65	0.358	0.686	Mutual exclusivity
CLIC5	6p21.1	3 (2.61%)	10 (4.08%)	-0.65	0.358	0.686	Mutual exclusivity
ENPP4	6p21.1	3 (2.61%)	10 (4.08%)	-0.65	0.358	0.686	Mutual exclusivity
GSTA5	6p12.2	3 (2.61%)	10 (4.08%)	-0.65	0.358	0.686	Mutual exclusivity
GUCA1B	6p21.1	3 (2.61%)	10 (4.08%)	-0.65	0.358	0.686	Mutual exclusivity
KMT5B	11q13.2	3 (2.61%)	10 (4.08%)	-0.65	0.358	0.686	Mutual exclusivity
MRPS10	6p21.1	3 (2.61%)	10 (4.08%)	-0.65	0.358	0.686	Mutual exclusivity
PLA2G7	6p12.3	3 (2.61%)	10 (4.08%)	-0.65	0.358	0.686	Mutual exclusivity
RCAN2	6p12.3	3 (2.61%)	10 (4.08%)	-0.65	0.358	0.686	Mutual exclusivity
RN7SKP116	6p12.3	3 (2.61%)	10 (4.08%)	-0.65	0.358	0.686	Mutual exclusivity
TAF8	6p21.1	3 (2.61%)	10 (4.08%)	-0.65	0.358	0.686	Mutual exclusivity
TRERF1	6p21.1	3 (2.61%)	10 (4.08%)	-0.65	0.358	0.686	Mutual exclusivity
CHCHD7	8q12.1	7 (6.09%)	19 (7.76%)	-0.35	0.371	0.686	Mutual exclusivity

LYN	8q12.1	7 (6.09%)	19 (7.76%)	-0.35	0.371	0.686	Mutual exclusivity
MOS	8q12.1	7 (6.09%)	19 (7.76%)	-0.35	0.371	0.686	Mutual exclusivity
PLAG1	8q12.1	7 (6.09%)	19 (7.76%)	-0.35	0.371	0.686	Mutual exclusivity
RN7SL323P	8q12.1	7 (6.09%)	19 (7.76%)	-0.35	0.371	0.686	Mutual exclusivity
RN7SL798P	8q12.1	7 (6.09%)	19 (7.76%)	-0.35	0.371	0.686	Mutual exclusivity
RNA5SP265	8q12.1	7 (6.09%)	19 (7.76%)	-0.35	0.371	0.686	Mutual exclusivity
RPS20	8q12.1	7 (6.09%)	19 (7.76%)	-0.35	0.371	0.686	Mutual exclusivity
SNORD54	8q12.1	7 (6.09%)	19 (7.76%)	-0.35	0.371	0.686	Mutual exclusivity
TGS1	8q12.1	7 (6.09%)	19 (7.76%)	-0.35	0.371	0.686	Mutual exclusivity
TMEM68	8q12.1	7 (6.09%)	19 (7.76%)	-0.35	0.371	0.686	Mutual exclusivity
AADAC	3q25.1	1 (0.87%)	5 (2.04%)	-1.23	0.377	0.686	Mutual exclusivity
AADACL2	3q25.1	1 (0.87%)	5 (2.04%)	-1.23	0.377	0.686	Mutual exclusivity
ADAMTS16	5p15.32	1 (0.87%)	5 (2.04%)	-1.23	0.377	0.686	Mutual exclusivity
ALDH3A2	17p11.2	1 (0.87%)	5 (2.04%)	-1.23	0.377	0.686	Mutual exclusivity
ANKRD33B	5p15.2	1 (0.87%)	5 (2.04%)	-1.23	0.377	0.686	Mutual exclusivity
ARMC6	19p13.11	1 (0.87%)	5 (2.04%)	-1.23	0.377	0.686	Mutual exclusivity
ARRDC2	19p13.11	1 (0.87%)	5 (2.04%)	-1.23	0.377	0.686	Mutual exclusivity
ASB4	7q21.3	1 (0.87%)	5 (2.04%)	-1.23	0.377	0.686	Mutual exclusivity
ASNS	7q21.3	1 (0.87%)	5 (2.04%)	-1.23	0.377	0.686	Mutual exclusivity
BABAM2	2p23.2	1 (0.87%)	5 (2.04%)	-1.23	0.377	0.686	Mutual exclusivity
BET1	7q21.3	1 (0.87%)	5 (2.04%)	-1.23	0.377	0.686	Mutual exclusivity
BLACE	7q36.3	1 (0.87%)	5 (2.04%)	-1.23	0.377	0.686	Mutual exclusivity
C7ORF76	7q21.3	1 (0.87%)	5 (2.04%)	-1.23	0.377	0.686	Mutual exclusivity
CALCR	7q21.3	1 (0.87%)	5 (2.04%)	-1.23	0.377	0.686	Mutual exclusivity
CALD1	7q33	1 (0.87%)	5 (2.04%)	-1.23	0.377	0.686	Mutual exclusivity
CANX	5q35.3	1 (0.87%)	5 (2.04%)	-1.23	0.377	0.686	Mutual exclusivity
CASD1	7q21.3	1 (0.87%)	5 (2.04%)	-1.23	0.377	0.686	Mutual exclusivity
CCDC124	19p13.11	1 (0.87%)	5 (2.04%)	-1.23	0.377	0.686	Mutual exclusivity
CEP41	7q32.2	1 (0.87%)	5 (2.04%)	-1.23	0.377	0.686	Mutual exclusivity

CERS1	19p13.11	1 (0.87%)	5 (2.04%)	-1.23	0.377	0.686	Mutual exclusivity
CHCHD3	7q32.3-q33	1 (0.87%)	5 (2.04%)	-1.23	0.377	0.686	Mutual exclusivity
CLNS1A	11q14.1	1 (0.87%)	5 (2.04%)	-1.23	0.377	0.686	Mutual exclusivity
CLRN1	3q25.1	1 (0.87%)	5 (2.04%)	-1.23	0.377	0.686	Mutual exclusivity
CNPY1	7q36.3	1 (0.87%)	5 (2.04%)	-1.23	0.377	0.686	Mutual exclusivity
COL1A2	7q21.3	1 (0.87%)	5 (2.04%)	-1.23	0.377	0.686	Mutual exclusivity
COPE	19p13.11	1 (0.87%)	5 (2.04%)	-1.23	0.377	0.686	Mutual exclusivity
COPG2	7q32.2	1 (0.87%)	5 (2.04%)	-1.23	0.377	0.686	Mutual exclusivity
CP	3q24-q25.1	1 (0.87%)	5 (2.04%)	-1.23	0.377	0.686	Mutual exclusivity
CPA1	7q32.2	1 (0.87%)	5 (2.04%)	-1.23	0.377	0.686	Mutual exclusivity
CPA2	7q32.2	1 (0.87%)	5 (2.04%)	-1.23	0.377	0.686	Mutual exclusivity
CPA5	7q32.2	1 (0.87%)	5 (2.04%)	-1.23	0.377	0.686	Mutual exclusivity
DAP	5p15.2	1 (0.87%)	5 (2.04%)	-1.23	0.377	0.686	Mutual exclusivity
DDX49	19p13.11	1 (0.87%)	5 (2.04%)	-1.23	0.377	0.686	Mutual exclusivity
DIP2C	10p15.3	1 (0.87%)	5 (2.04%)	-1.23	0.377	0.686	Mutual exclusivity
EIF4E1B	5q35.2	1 (0.87%)	5 (2.04%)	-1.23	0.377	0.686	Mutual exclusivity
EN2	7q36.3	1 (0.87%)	5 (2.04%)	-1.23	0.377	0.686	Mutual exclusivity
ENPP7P8	11q13.4	1 (0.87%)	5 (2.04%)	-1.23	0.377	0.686	Mutual exclusivity
FKBP8	19p13.11	1 (0.87%)	5 (2.04%)	-1.23	0.377	0.686	Mutual exclusivity
GDF1	19p13.11	1 (0.87%)	5 (2.04%)	-1.23	0.377	0.686	Mutual exclusivity
GIMAP4	7q36.1	1 (0.87%)	5 (2.04%)	-1.23	0.377	0.686	Mutual exclusivity
GNG11	7q21.3	1 (0.87%)	5 (2.04%)	-1.23	0.377	0.686	Mutual exclusivity
GNGT1	7q21.3	1 (0.87%)	5 (2.04%)	-1.23	0.377	0.686	Mutual exclusivity
GPR171	3q25.1	1 (0.87%)	5 (2.04%)	-1.23	0.377	0.686	Mutual exclusivity
GPR37	7q31.33	1 (0.87%)	5 (2.04%)	-1.23	0.377	0.686	Mutual exclusivity
GPR87	3q25.1	1 (0.87%)	5 (2.04%)	-1.23	0.377	0.686	Mutual exclusivity
HBP1	7q22.3	1 (0.87%)	5 (2.04%)	-1.23	0.377	0.686	Mutual exclusivity
HEPACAM2	7q21.2	1 (0.87%)	5 (2.04%)	-1.23	0.377	0.686	Mutual exclusivity
HK3	5q35.2	1 (0.87%)	5 (2.04%)	-1.23	0.377	0.686	Mutual exclusivity

HMGB3P22	5q35.3	1 (0.87%)	5 (2.04%)	-1.23	0.377	0.686	Mutual exclusivity
HNRNPH1	5q35.3	1 (0.87%)	5 (2.04%)	-1.23	0.377	0.686	Mutual exclusivity
HOMER3	19p13.11	1 (0.87%)	5 (2.04%)	-1.23	0.377	0.686	Mutual exclusivity
HPS3	3q24	1 (0.87%)	5 (2.04%)	-1.23	0.377	0.686	Mutual exclusivity
HTR5A	7q36.2	1 (0.87%)	5 (2.04%)	-1.23	0.377	0.686	Mutual exclusivity
HYAL4	7q31.32	1 (0.87%)	5 (2.04%)	-1.23	0.377	0.686	Mutual exclusivity
IDO2	8p11.21	1 (0.87%)	5 (2.04%)	-1.23	0.377	0.686	Mutual exclusivity
IGSF10	3q25.1	1 (0.87%)	5 (2.04%)	-1.23	0.377	0.686	Mutual exclusivity
IQCN	19p13.11	1 (0.87%)	5 (2.04%)	-1.23	0.377	0.686	Mutual exclusivity
IRX1	5p15.33	1 (0.87%)	5 (2.04%)	-1.23	0.377	0.686	Mutual exclusivity
JAK3	19p13.11	1 (0.87%)	5 (2.04%)	-1.23	0.377	0.686	Mutual exclusivity
JUND	19p13.11	1 (0.87%)	5 (2.04%)	-1.23	0.377	0.686	Mutual exclusivity
KLF14	7q32.2	1 (0.87%)	5 (2.04%)	-1.23	0.377	0.686	Mutual exclusivity
KLHDC10	7q32.2	1 (0.87%)	5 (2.04%)	-1.23	0.377	0.686	Mutual exclusivity
KLHL26	19p13.11	1 (0.87%)	5 (2.04%)	-1.23	0.377	0.686	Mutual exclusivity
KXD1	19p13.11	1 (0.87%)	5 (2.04%)	-1.23	0.377	0.686	Mutual exclusivity
LINC00641	14q11.2	1 (0.87%)	5 (2.04%)	-1.23	0.377	0.686	Mutual exclusivity
LMAN2	5q35.3	1 (0.87%)	5 (2.04%)	-1.23	0.377	0.686	Mutual exclusivity
LSM4	19p13.11	1 (0.87%)	5 (2.04%)	-1.23	0.377	0.686	Mutual exclusivity
LTC4S	5q35.3	1 (0.87%)	5 (2.04%)	-1.23	0.377	0.686	Mutual exclusivity
MAML1	5q35.3	1 (0.87%)	5 (2.04%)	-1.23	0.377	0.686	Mutual exclusivity
MBNL1	3q25.1-q25.2	1 (0.87%)	5 (2.04%)	-1.23	0.377	0.686	Mutual exclusivity
MED12L	3q25.1	1 (0.87%)	5 (2.04%)	-1.23	0.377	0.686	Mutual exclusivity
MEST	7q32.2	1 (0.87%)	5 (2.04%)	-1.23	0.377	0.686	Mutual exclusivity
MIR-3188/3188		1 (0.87%)	5 (2.04%)	-1.23	0.377	0.686	Mutual exclusivity
MIR-4281/4281		1 (0.87%)	5 (2.04%)	-1.23	0.377	0.686	Mutual exclusivity
MIR-5186/5186		1 (0.87%)	5 (2.04%)	-1.23	0.377	0.686	Mutual exclusivity
MIR-591/591		1 (0.87%)	5 (2.04%)	-1.23	0.377	0.686	Mutual exclusivity
NEURL1B	5q35.1	1 (0.87%)	5 (2.04%)	-1.23	0.377	0.686	Mutual exclusivity

NSUN2	5p15.31	1 (0.87%)	5 (2.04%)	-1.23	0.377	0.686	Mutual exclusivity
OCM2	7q21.3	1 (0.87%)	5 (2.04%)	-1.23	0.377	0.686	Mutual exclusivity
OR5AU1	14q11.2	1 (0.87%)	5 (2.04%)	-1.23	0.377	0.686	Mutual exclusivity
OTOL1	3q26.1	1 (0.87%)	5 (2.04%)	-1.23	0.377	0.686	Mutual exclusivity
P2RY12	3q25.1	1 (0.87%)	5 (2.04%)	-1.23	0.377	0.686	Mutual exclusivity
P2RY13	3q25.1	1 (0.87%)	5 (2.04%)	-1.23	0.377	0.686	Mutual exclusivity
P2RY14	3q25.1	1 (0.87%)	5 (2.04%)	-1.23	0.377	0.686	Mutual exclusivity
PAK1	11q13.5-q14.1	1 (0.87%)	5 (2.04%)	-1.23	0.377	0.686	Mutual exclusivity
PAXIP1	7q36.2	1 (0.87%)	5 (2.04%)	-1.23	0.377	0.686	Mutual exclusivity
PEG10	7q21.3	1 (0.87%)	5 (2.04%)	-1.23	0.377	0.686	Mutual exclusivity
PIK3CG	7q22.3	1 (0.87%)	5 (2.04%)	-1.23	0.377	0.686	Mutual exclusivity
PLEKHM3	2q33.3	1 (0.87%)	5 (2.04%)	-1.23	0.377	0.686	Mutual exclusivity
PLXNA4	7q32.3	1 (0.87%)	5 (2.04%)	-1.23	0.377	0.686	Mutual exclusivity
PODXL	7q32.3	1 (0.87%)	5 (2.04%)	-1.23	0.377	0.686	Mutual exclusivity
PON1	7q21.3	1 (0.87%)	5 (2.04%)	-1.23	0.377	0.686	Mutual exclusivity
PON2	7q21.3	1 (0.87%)	5 (2.04%)	-1.23	0.377	0.686	Mutual exclusivity
PON3	7q21.3	1 (0.87%)	5 (2.04%)	-1.23	0.377	0.686	Mutual exclusivity
POT1	7q31.33	1 (0.87%)	5 (2.04%)	-1.23	0.377	0.686	Mutual exclusivity
PPP1R9A	7q21.3	1 (0.87%)	5 (2.04%)	-1.23	0.377	0.686	Mutual exclusivity
PRDM9	5p14.2	1 (0.87%)	5 (2.04%)	-1.23	0.377	0.686	Mutual exclusivity
PRKAR2B	7q22.3	1 (0.87%)	5 (2.04%)	-1.23	0.377	0.686	Mutual exclusivity
PRR26	10p15.3	1 (0.87%)	5 (2.04%)	-1.23	0.377	0.686	Mutual exclusivity
PXYLP1	3q23	1 (0.87%)	5 (2.04%)	-1.23	0.377	0.686	Mutual exclusivity
RGS14	5q35.3	1 (0.87%)	5 (2.04%)	-1.23	0.377	0.686	Mutual exclusivity
RN7SKP104	7q21.3	1 (0.87%)	5 (2.04%)	-1.23	0.377	0.686	Mutual exclusivity
RN7SKP129	7q21.3	1 (0.87%)	5 (2.04%)	-1.23	0.377	0.686	Mutual exclusivity
RN7SKP73	5p15.32	1 (0.87%)	5 (2.04%)	-1.23	0.377	0.686	Mutual exclusivity
RN7SKP79	5p15.31	1 (0.87%)	5 (2.04%)	-1.23	0.377	0.686	Mutual exclusivity
RN7SL155P	19p13.11	1 (0.87%)	5 (2.04%)	-1.23	0.377	0.686	Mutual exclusivity

RN7SL252P	7q21.3	1 (0.87%)	5 (2.04%)	-1.23	0.377	0.686	Mutual exclusivity
RN7SL513P	19p13.11	1 (0.87%)	5 (2.04%)	-1.23	0.377	0.686	Mutual exclusivity
RN7SL70P	19p13.11	1 (0.87%)	5 (2.04%)	-1.23	0.377	0.686	Mutual exclusivity
RN7SL7P	7q21.2	1 (0.87%)	5 (2.04%)	-1.23	0.377	0.686	Mutual exclusivity
RNA5SP145	3q25.1	1 (0.87%)	5 (2.04%)	-1.23	0.377	0.686	Mutual exclusivity
RNA5SP176	5p15.31	1 (0.87%)	5 (2.04%)	-1.23	0.377	0.686	Mutual exclusivity
RNA5SP177	5p15.31	1 (0.87%)	5 (2.04%)	-1.23	0.377	0.686	Mutual exclusivity
RNA5SP236	7q22.3	1 (0.87%)	5 (2.04%)	-1.23	0.377	0.686	Mutual exclusivity
RNA5SP245	7q32.2	1 (0.87%)	5 (2.04%)	-1.23	0.377	0.686	Mutual exclusivity
RNA5SP246	7q32.2	1 (0.87%)	5 (2.04%)	-1.23	0.377	0.686	Mutual exclusivity
RNA5SP468	19p13.11	1 (0.87%)	5 (2.04%)	-1.23	0.377	0.686	Mutual exclusivity
RNF130	5q35.3	1 (0.87%)	5 (2.04%)	-1.23	0.377	0.686	Mutual exclusivity
ROPN1L	5p15.2	1 (0.87%)	5 (2.04%)	-1.23	0.377	0.686	Mutual exclusivity
RPL18A	19p13.11	1 (0.87%)	5 (2.04%)	-1.23	0.377	0.686	Mutual exclusivity
SAMD9	7q21.2	1 (0.87%)	5 (2.04%)	-1.23	0.377	0.686	Mutual exclusivity
SAMD9L	7q21.2	1 (0.87%)	5 (2.04%)	-1.23	0.377	0.686	Mutual exclusivity
SCHIP1	3q25.32-q25.33	1 (0.87%)	5 (2.04%)	-1.23	0.377	0.686	Mutual exclusivity
SEM1	7q21.3	1 (0.87%)	5 (2.04%)	-1.23	0.377	0.686	Mutual exclusivity
SGCE	7q21.3	1 (0.87%)	5 (2.04%)	-1.23	0.377	0.686	Mutual exclusivity
SHH	7q36.3	1 (0.87%)	5 (2.04%)	-1.23	0.377	0.686	Mutual exclusivity
SLC25A13	7q21.3	1 (0.87%)	5 (2.04%)	-1.23	0.377	0.686	Mutual exclusivity
SLC25A42	19p13.11	1 (0.87%)	5 (2.04%)	-1.23	0.377	0.686	Mutual exclusivity
SLC5A5	19p13.11	1 (0.87%)	5 (2.04%)	-1.23	0.377	0.686	Mutual exclusivity
SNCB	5q35.2	1 (0.87%)	5 (2.04%)	-1.23	0.377	0.686	Mutual exclusivity
SNORD123	5p15.31	1 (0.87%)	5 (2.04%)	-1.23	0.377	0.686	Mutual exclusivity
SNORD29	11q12.3	1 (0.87%)	5 (2.04%)	-1.23	0.377	0.686	Mutual exclusivity
SPAM1	7q31.32	1 (0.87%)	5 (2.04%)	-1.23	0.377	0.686	Mutual exclusivity
SPTSSB	3q26.1	1 (0.87%)	5 (2.04%)	-1.23	0.377	0.686	Mutual exclusivity
SRD5A1	5p15.31	1 (0.87%)	5 (2.04%)	-1.23	0.377	0.686	Mutual exclusivity

SSMEM1	7q32.2	1 (0.87%)	5 (2.04%)	-1.23	0.377	0.686	Mutual exclusivity
SUCNR1	3q25.1	1 (0.87%)	5 (2.04%)	-1.23	0.377	0.686	Mutual exclusivity
SUGP2	19p13.11	1 (0.87%)	5 (2.04%)	-1.23	0.377	0.686	Mutual exclusivity
TAC1	7q21.3	1 (0.87%)	5 (2.04%)	-1.23	0.377	0.686	Mutual exclusivity
TAS2R1	5p15.31	1 (0.87%)	5 (2.04%)	-1.23	0.377	0.686	Mutual exclusivity
TCIM	8p11.21	1 (0.87%)	5 (2.04%)	-1.23	0.377	0.686	Mutual exclusivity
TENT4A	5p15.31	1 (0.87%)	5 (2.04%)	-1.23	0.377	0.686	Mutual exclusivity
TFPI2	7q21.3	1 (0.87%)	5 (2.04%)	-1.23	0.377	0.686	Mutual exclusivity
TMEM209	7q32.2	1 (0.87%)	5 (2.04%)	-1.23	0.377	0.686	Mutual exclusivity
TMEM229A	7q31.32	1 (0.87%)	5 (2.04%)	-1.23	0.377	0.686	Mutual exclusivity
TMEM59L	19p13.11	1 (0.87%)	5 (2.04%)	-1.23	0.377	0.686	Mutual exclusivity
TSGA13	7q32.2	1 (0.87%)	5 (2.04%)	-1.23	0.377	0.686	Mutual exclusivity
TSPAN17	5q35.2	1 (0.87%)	5 (2.04%)	-1.23	0.377	0.686	Mutual exclusivity
UBE2QL1	5p15.31	1 (0.87%)	5 (2.04%)	-1.23	0.377	0.686	Mutual exclusivity
UNC5A	5q35.2	1 (0.87%)	5 (2.04%)	-1.23	0.377	0.686	Mutual exclusivity
VPS50	7q21.2-q21.3	1 (0.87%)	5 (2.04%)	-1.23	0.377	0.686	Mutual exclusivity
ZBTB38	3q23	1 (0.87%)	5 (2.04%)	-1.23	0.377	0.686	Mutual exclusivity
ZC3HC1	7q32.2	1 (0.87%)	5 (2.04%)	-1.23	0.377	0.686	Mutual exclusivity
ZNF354C	5q35.3	1 (0.87%)	5 (2.04%)	-1.23	0.377	0.686	Mutual exclusivity
PEX2	8q21.13	12 (10.43%)	30 (12.24%)	-0.23	0.379	0.686	Mutual exclusivity
TRAM1	8q13.3	12 (10.43%)	30 (12.24%)	-0.23	0.379	0.686	Mutual exclusivity
ZFHX4	8q21.13	12 (10.43%)	30 (12.24%)	-0.23	0.379	0.686	Mutual exclusivity
CUL7	6p21.1	4 (3.48%)	12 (4.90%)	-0.49	0.38	0.686	Mutual exclusivity
GNMT	6p21.1	4 (3.48%)	12 (4.90%)	-0.49	0.38	0.686	Mutual exclusivity
KLC4	6p21.1	4 (3.48%)	12 (4.90%)	-0.49	0.38	0.686	Mutual exclusivity
KLHDC3	6p21.1	4 (3.48%)	12 (4.90%)	-0.49	0.38	0.686	Mutual exclusivity
MEA1	6p21.1	4 (3.48%)	12 (4.90%)	-0.49	0.38	0.686	Mutual exclusivity
MRPL2	6p21.1	4 (3.48%)	12 (4.90%)	-0.49	0.38	0.686	Mutual exclusivity
PEX6	6p21.1	4 (3.48%)	12 (4.90%)	-0.49	0.38	0.686	Mutual exclusivity

PPP2R5D	6p21.1	4 (3.48%)	12 (4.90%)	-0.49	0.38	0.686	Mutual exclusivity
PTK7	6p21.1	4 (3.48%)	12 (4.90%)	-0.49	0.38	0.686	Mutual exclusivity
RN7SL403P	6p21.1	4 (3.48%)	12 (4.90%)	-0.49	0.38	0.686	Mutual exclusivity
RRP36	6p21.1	4 (3.48%)	12 (4.90%)	-0.49	0.38	0.686	Mutual exclusivity
SRF	6p21.1	4 (3.48%)	12 (4.90%)	-0.49	0.38	0.686	Mutual exclusivity
SUPT3H	6p21.1	4 (3.48%)	12 (4.90%)	-0.49	0.38	0.686	Mutual exclusivity
TBCC	6p21.1	4 (3.48%)	12 (4.90%)	-0.49	0.38	0.686	Mutual exclusivity
ABAT	16p13.2	2 (1.74%)	2 (0.82%)	1.09	0.383	0.686	Co-occurrence
ABCA2	9q34.3	2 (1.74%)	2 (0.82%)	1.09	0.383	0.686	Co-occurrence
ABCB11	2q31.1	2 (1.74%)	2 (0.82%)	1.09	0.383	0.686	Co-occurrence
ABI2	2q33.2	2 (1.74%)	2 (0.82%)	1.09	0.383	0.686	Co-occurrence
ABLIM3	5q32	2 (1.74%)	2 (0.82%)	1.09	0.383	0.686	Co-occurrence
ACAN	15q26.1	2 (1.74%)	2 (0.82%)	1.09	0.383	0.686	Co-occurrence
ACBD7	10p13	2 (1.74%)	2 (0.82%)	1.09	0.383	0.686	Co-occurrence
ACSM2A	16p12.3	2 (1.74%)	2 (0.82%)	1.09	0.383	0.686	Co-occurrence
ADAM30	1p12	2 (1.74%)	2 (0.82%)	1.09	0.383	0.686	Co-occurrence
ADGRE2	19p13.12	2 (1.74%)	2 (0.82%)	1.09	0.383	0.686	Co-occurrence
ADGRE3	19p13.12	2 (1.74%)	2 (0.82%)	1.09	0.383	0.686	Co-occurrence
ADIG	20q11.23	2 (1.74%)	2 (0.82%)	1.09	0.383	0.686	Co-occurrence
ADPGK	15q24.1	2 (1.74%)	2 (0.82%)	1.09	0.383	0.686	Co-occurrence
ADRB2	5q32	2 (1.74%)	2 (0.82%)	1.09	0.383	0.686	Co-occurrence
AEBP1	7p13	2 (1.74%)	2 (0.82%)	1.09	0.383	0.686	Co-occurrence
AFAP1L1	5q32	2 (1.74%)	2 (0.82%)	1.09	0.383	0.686	Co-occurrence
AJM1	9q34.3	2 (1.74%)	2 (0.82%)	1.09	0.383	0.686	Co-occurrence
ALG1	16p13.3	2 (1.74%)	2 (0.82%)	1.09	0.383	0.686	Co-occurrence
ALPK3	15q25.3	2 (1.74%)	2 (0.82%)	1.09	0.383	0.686	Co-occurrence
ANAPC2	9q34.3	2 (1.74%)	2 (0.82%)	1.09	0.383	0.686	Co-occurrence
ANG	14q11.2	2 (1.74%)	2 (0.82%)	1.09	0.383	0.686	Co-occurrence
ANKDD1A	15q22.31	2 (1.74%)	2 (0.82%)	1.09	0.383	0.686	Co-occurrence

ANKHD1	5q31.3	2 (1.74%)	2 (0.82%)	1.09	0.383	0.686	Co-occurrence
ANKRD44	2q33.1	2 (1.74%)	2 (0.82%)	1.09	0.383	0.686	Co-occurrence
ANKS3	16p13.3	2 (1.74%)	2 (0.82%)	1.09	0.383	0.686	Co-occurrence
ANO8	19p13.11	2 (1.74%)	2 (0.82%)	1.09	0.383	0.686	Co-occurrence
ANP32BP1	15q24.2	2 (1.74%)	2 (0.82%)	1.09	0.383	0.686	Co-occurrence
AP2A2	11p15.5	2 (1.74%)	2 (0.82%)	1.09	0.383	0.686	Co-occurrence
AP3B2	15q25.2	2 (1.74%)	2 (0.82%)	1.09	0.383	0.686	Co-occurrence
APBB3	5q31.3	2 (1.74%)	2 (0.82%)	1.09	0.383	0.686	Co-occurrence
APEX1	14q11.2	2 (1.74%)	2 (0.82%)	1.09	0.383	0.686	Co-occurrence
ARHGAP12	10p11.22	2 (1.74%)	2 (0.82%)	1.09	0.383	0.686	Co-occurrence
ARHGAP27	17q21.31	2 (1.74%)	2 (0.82%)	1.09	0.383	0.686	Co-occurrence
ARHGEF37	5q32	2 (1.74%)	2 (0.82%)	1.09	0.383	0.686	Co-occurrence
ARID3C	9p13.3	2 (1.74%)	2 (0.82%)	1.09	0.383	0.686	Co-occurrence
ARIH1	15q24.1	2 (1.74%)	2 (0.82%)	1.09	0.383	0.686	Co-occurrence
ARL17A	17q21.31	2 (1.74%)	2 (0.82%)	1.09	0.383	0.686	Co-occurrence
ARL17B	17q21.31	2 (1.74%)	2 (0.82%)	1.09	0.383	0.686	Co-occurrence
ARL5B	10p12.31	2 (1.74%)	2 (0.82%)	1.09	0.383	0.686	Co-occurrence
ARL5C	17q12	2 (1.74%)	2 (0.82%)	1.09	0.383	0.686	Co-occurrence
ASF1B	19p13.12	2 (1.74%)	2 (0.82%)	1.09	0.383	0.686	Co-occurrence
ATP5F1C	10p14	2 (1.74%)	2 (0.82%)	1.09	0.383	0.686	Co-occurrence
B3GALT1	2q24.3	2 (1.74%)	2 (0.82%)	1.09	0.383	0.686	Co-occurrence
BAZ2B	2q24.2	2 (1.74%)	2 (0.82%)	1.09	0.383	0.686	Co-occurrence
BCL2L1	20q11.21	2 (1.74%)	2 (0.82%)	1.09	0.383	0.686	Co-occurrence
BICDL2	16p13.3	2 (1.74%)	2 (0.82%)	1.09	0.383	0.686	Co-occurrence
BLOC1S3	19q13.32	2 (1.74%)	2 (0.82%)	1.09	0.383	0.686	Co-occurrence
BLVRB	19q13.2	2 (1.74%)	2 (0.82%)	1.09	0.383	0.686	Co-occurrence
BNC1	15q25.2	2 (1.74%)	2 (0.82%)	1.09	0.383	0.686	Co-occurrence
BOLL	2q33.1	2 (1.74%)	2 (0.82%)	1.09	0.383	0.686	Co-occurrence
BRD3OS	9q34.2	2 (1.74%)	2 (0.82%)	1.09	0.383	0.686	Co-occurrence

BRD8	5q31.2	2 (1.74%)	2 (0.82%)	1.09	0.383	0.686	Co-occurrence
BRK1	3p25.3	2 (1.74%)	2 (0.82%)	1.09	0.383	0.686	Co-occurrence
BTBD1	15q25.2	2 (1.74%)	2 (0.82%)	1.09	0.383	0.686	Co-occurrence
C15ORF32	15q26.1	2 (1.74%)	2 (0.82%)	1.09	0.383	0.686	Co-occurrence
C15ORF40	15q25.2	2 (1.74%)	2 (0.82%)	1.09	0.383	0.686	Co-occurrence
C16ORF71	16p13.3	2 (1.74%)	2 (0.82%)	1.09	0.383	0.686	Co-occurrence
C16ORF72	16p13.2	2 (1.74%)	2 (0.82%)	1.09	0.383	0.686	Co-occurrence
C16ORF89	16p13.3	2 (1.74%)	2 (0.82%)	1.09	0.383	0.686	Co-occurrence
C17ORF98	17q12	2 (1.74%)	2 (0.82%)	1.09	0.383	0.686	Co-occurrence
C19ORF12	19q12	2 (1.74%)	2 (0.82%)	1.09	0.383	0.686	Co-occurrence
C19ORF47	19q13.2	2 (1.74%)	2 (0.82%)	1.09	0.383	0.686	Co-occurrence
C19ORF53	19p13.13	2 (1.74%)	2 (0.82%)	1.09	0.383	0.686	Co-occurrence
C19ORF54	19q13.2	2 (1.74%)	2 (0.82%)	1.09	0.383	0.686	Co-occurrence
C19ORF57	19p13.12	2 (1.74%)	2 (0.82%)	1.09	0.383	0.686	Co-occurrence
C19ORF67	19p13.12	2 (1.74%)	2 (0.82%)	1.09	0.383	0.686	Co-occurrence
C1QL3	10p13	2 (1.74%)	2 (0.82%)	1.09	0.383	0.686	Co-occurrence
C20ORF197	20q13.33	2 (1.74%)	2 (0.82%)	1.09	0.383	0.686	Co-occurrence
C8G	9q34.3	2 (1.74%)	2 (0.82%)	1.09	0.383	0.686	Co-occurrence
C9ORF139	9q34.3	2 (1.74%)	2 (0.82%)	1.09	0.383	0.686	Co-occurrence
C9ORF16	9q34.11	2 (1.74%)	2 (0.82%)	1.09	0.383	0.686	Co-occurrence
C9ORF62	9q34.3	2 (1.74%)	2 (0.82%)	1.09	0.383	0.686	Co-occurrence
CACNA1A	19p13.13	2 (1.74%)	2 (0.82%)	1.09	0.383	0.686	Co-occurrence
CACNB1	17q12	2 (1.74%)	2 (0.82%)	1.09	0.383	0.686	Co-occurrence
CACNB2	10p12.33-p12.31	2 (1.74%)	2 (0.82%)	1.09	0.383	0.686	Co-occurrence
CALCRL	2q32.1	2 (1.74%)	2 (0.82%)	1.09	0.383	0.686	Co-occurrence
CAMK1D	10p13	2 (1.74%)	2 (0.82%)	1.09	0.383	0.686	Co-occurrence
CARHSP1	16p13.2	2 (1.74%)	2 (0.82%)	1.09	0.383	0.686	Co-occurrence
CARMN	5q32	2 (1.74%)	2 (0.82%)	1.09	0.383	0.686	Co-occurrence
CASP16P	16p13.3	2 (1.74%)	2 (0.82%)	1.09	0.383	0.686	Co-occurrence

CASZ1	1p36.22	2 (1.74%)	2 (0.82%)	1.09	0.383	0.686	Co-occurrence
CC2D1A	19p13.12	2 (1.74%)	2 (0.82%)	1.09	0.383	0.686	Co-occurrence
CCDC13	3p22.1	2 (1.74%)	2 (0.82%)	1.09	0.383	0.686	Co-occurrence
CCDC130	19p13.13	2 (1.74%)	2 (0.82%)	1.09	0.383	0.686	Co-occurrence
CCDC148	2q24.1	2 (1.74%)	2 (0.82%)	1.09	0.383	0.686	Co-occurrence
CCDC150	2q33.1	2 (1.74%)	2 (0.82%)	1.09	0.383	0.686	Co-occurrence
CCDC170	6q25.1	2 (1.74%)	2 (0.82%)	1.09	0.383	0.686	Co-occurrence
CCDC173	2q31.1	2 (1.74%)	2 (0.82%)	1.09	0.383	0.686	Co-occurrence
CCDC183	9q34.3	2 (1.74%)	2 (0.82%)	1.09	0.383	0.686	Co-occurrence
CCDC78	16p13.3	2 (1.74%)	2 (0.82%)	1.09	0.383	0.686	Co-occurrence
CCL27	9p13.3	2 (1.74%)	2 (0.82%)	1.09	0.383	0.686	Co-occurrence
CCNB1IP1	14q11.2	2 (1.74%)	2 (0.82%)	1.09	0.383	0.686	Co-occurrence
CD14	5q31.3	2 (1.74%)	2 (0.82%)	1.09	0.383	0.686	Co-occurrence
CD248	11q13.2	2 (1.74%)	2 (0.82%)	1.09	0.383	0.686	Co-occurrence
CD3EAP	19q13.32	2 (1.74%)	2 (0.82%)	1.09	0.383	0.686	Co-occurrence
CDC123	10p14-p13	2 (1.74%)	2 (0.82%)	1.09	0.383	0.686	Co-occurrence
CDC23	5q31.2	2 (1.74%)	2 (0.82%)	1.09	0.383	0.686	Co-occurrence
CDC25C	5q31.2	2 (1.74%)	2 (0.82%)	1.09	0.383	0.686	Co-occurrence
CDC37L1	9p24.1	2 (1.74%)	2 (0.82%)	1.09	0.383	0.686	Co-occurrence
CDH26	20q13.33	2 (1.74%)	2 (0.82%)	1.09	0.383	0.686	Co-occurrence
CDK12	17q12	2 (1.74%)	2 (0.82%)	1.09	0.383	0.686	Co-occurrence
CDK9	9q34.11	2 (1.74%)	2 (0.82%)	1.09	0.383	0.686	Co-occurrence
CDNF	10p13	2 (1.74%)	2 (0.82%)	1.09	0.383	0.686	Co-occurrence
CELF2	10p14	2 (1.74%)	2 (0.82%)	1.09	0.383	0.686	Co-occurrence
CERK	22q13.31	2 (1.74%)	2 (0.82%)	1.09	0.383	0.686	Co-occurrence
CFLAR	2q33.1	2 (1.74%)	2 (0.82%)	1.09	0.383	0.686	Co-occurrence
CHD2	15q26.1	2 (1.74%)	2 (0.82%)	1.09	0.383	0.686	Co-occurrence
CHMP4B	20q11.22	2 (1.74%)	2 (0.82%)	1.09	0.383	0.686	Co-occurrence
CIAO3	16p13.3	2 (1.74%)	2 (0.82%)	1.09	0.383	0.686	Co-occurrence

CIITA	16p13.13	2 (1.74%)	2 (0.82%)	1.09	0.383	0.686	Co-occurrence
CILP	15q22.31	2 (1.74%)	2 (0.82%)	1.09	0.383	0.686	Co-occurrence
CIZ1	9q34.11	2 (1.74%)	2 (0.82%)	1.09	0.383	0.686	Co-occurrence
CKM	19q13.32	2 (1.74%)	2 (0.82%)	1.09	0.383	0.686	Co-occurrence
CLC	19q13.2	2 (1.74%)	2 (0.82%)	1.09	0.383	0.686	Co-occurrence
CLDN6	16p13.3	2 (1.74%)	2 (0.82%)	1.09	0.383	0.686	Co-occurrence
CLDN9	16p13.3	2 (1.74%)	2 (0.82%)	1.09	0.383	0.686	Co-occurrence
CLEC16A	16p13.13	2 (1.74%)	2 (0.82%)	1.09	0.383	0.686	Co-occurrence
CLEC17A	19p13.12	2 (1.74%)	2 (0.82%)	1.09	0.383	0.686	Co-occurrence
CLIC3	9q34.3	2 (1.74%)	2 (0.82%)	1.09	0.383	0.686	Co-occurrence
CLK1	2q33.1	2 (1.74%)	2 (0.82%)	1.09	0.383	0.686	Co-occurrence
CLPX	15q22.31	2 (1.74%)	2 (0.82%)	1.09	0.383	0.686	Co-occurrence
CNFN	19q13.2	2 (1.74%)	2 (0.82%)	1.09	0.383	0.686	Co-occurrence
CNIH2	11q13.2	2 (1.74%)	2 (0.82%)	1.09	0.383	0.686	Co-occurrence
COMMD4	15q24.2	2 (1.74%)	2 (0.82%)	1.09	0.383	0.686	Co-occurrence
COQ10B	2q33.1	2 (1.74%)	2 (0.82%)	1.09	0.383	0.686	Co-occurrence
COQ8B	19q13.2	2 (1.74%)	2 (0.82%)	1.09	0.383	0.686	Co-occurrence
COX4I2	20q11.21	2 (1.74%)	2 (0.82%)	1.09	0.383	0.686	Co-occurrence
CPAMD8	19p13.11	2 (1.74%)	2 (0.82%)	1.09	0.383	0.686	Co-occurrence
CPEB1	15q25.2	2 (1.74%)	2 (0.82%)	1.09	0.383	0.686	Co-occurrence
CRHR1	17q21.31	2 (1.74%)	2 (0.82%)	1.09	0.383	0.686	Co-occurrence
CSNK1A1	5q32	2 (1.74%)	2 (0.82%)	1.09	0.383	0.686	Co-occurrence
CTSH	15q25.1	2 (1.74%)	2 (0.82%)	1.09	0.383	0.686	Co-occurrence
CUBN	10p13	2 (1.74%)	2 (0.82%)	1.09	0.383	0.686	Co-occurrence
CWC25	17q12	2 (1.74%)	2 (0.82%)	1.09	0.383	0.686	Co-occurrence
CYP20A1	2q33.2	2 (1.74%)	2 (0.82%)	1.09	0.383	0.686	Co-occurrence
CYSRT1	9q34.3	2 (1.74%)	2 (0.82%)	1.09	0.383	0.686	Co-occurrence
DAG1	3p21.31	2 (1.74%)	2 (0.82%)	1.09	0.383	0.686	Co-occurrence
DAPL1	2q24.1	2 (1.74%)	2 (0.82%)	1.09	0.383	0.686	Co-occurrence

DBT	1p21.2	2 (1.74%)	2 (0.82%)	1.09	0.383	0.686	Co-occurrence
DCAF15	19p13.12	2 (1.74%)	2 (0.82%)	1.09	0.383	0.686	Co-occurrence
DCLRE1C	10p13	2 (1.74%)	2 (0.82%)	1.09	0.383	0.686	Co-occurrence
DCTN3	9p13.3	2 (1.74%)	2 (0.82%)	1.09	0.383	0.686	Co-occurrence
DDX3X	Xp11.4	2 (1.74%)	2 (0.82%)	1.09	0.383	0.686	Co-occurrence
DEXI	16p13.13	2 (1.74%)	2 (0.82%)	1.09	0.383	0.686	Co-occurrence
DHTKD1	10p14	2 (1.74%)	2 (0.82%)	1.09	0.383	0.686	Co-occurrence
DHX58	17q21.2	2 (1.74%)	2 (0.82%)	1.09	0.383	0.686	Co-occurrence
DMGDH	5q14.1	2 (1.74%)	2 (0.82%)	1.09	0.383	0.686	Co-occurrence
DMPK	19q13.32	2 (1.74%)	2 (0.82%)	1.09	0.383	0.686	Co-occurrence
DMWD	19q13.32	2 (1.74%)	2 (0.82%)	1.09	0.383	0.686	Co-occurrence
DNAJC18	5q31.2	2 (1.74%)	2 (0.82%)	1.09	0.383	0.686	Co-occurrence
DPH5	1p21.2	2 (1.74%)	2 (0.82%)	1.09	0.383	0.686	Co-occurrence
DPP7	9q34.3	2 (1.74%)	2 (0.82%)	1.09	0.383	0.686	Co-occurrence
DYRK1B	19q13.2	2 (1.74%)	2 (0.82%)	1.09	0.383	0.686	Co-occurrence
EBNA1BP2	1p34.2	2 (1.74%)	2 (0.82%)	1.09	0.383	0.686	Co-occurrence
ECHDC3	10p14	2 (1.74%)	2 (0.82%)	1.09	0.383	0.686	Co-occurrence
ECSCR	5q31.2	2 (1.74%)	2 (0.82%)	1.09	0.383	0.686	Co-occurrence
EDDM3A	14q11.2	2 (1.74%)	2 (0.82%)	1.09	0.383	0.686	Co-occurrence
EDDM3B	14q11.2	2 (1.74%)	2 (0.82%)	1.09	0.383	0.686	Co-occurrence
EDF1	9q34.3	2 (1.74%)	2 (0.82%)	1.09	0.383	0.686	Co-occurrence
EDN3	20q13.32	2 (1.74%)	2 (0.82%)	1.09	0.383	0.686	Co-occurrence
EEF2KMT	16p13.3	2 (1.74%)	2 (0.82%)	1.09	0.383	0.686	Co-occurrence
EEPD1	7p14.2	2 (1.74%)	2 (0.82%)	1.09	0.383	0.686	Co-occurrence
EFL1	15q25.2	2 (1.74%)	2 (0.82%)	1.09	0.383	0.686	Co-occurrence
EGR1	5q31.2	2 (1.74%)	2 (0.82%)	1.09	0.383	0.686	Co-occurrence
EID2	19q13.2	2 (1.74%)	2 (0.82%)	1.09	0.383	0.686	Co-occurrence
EID2B	19q13.2	2 (1.74%)	2 (0.82%)	1.09	0.383	0.686	Co-occurrence
EIF4EBP3	5q31.3	2 (1.74%)	2 (0.82%)	1.09	0.383	0.686	Co-occurrence

ELOB	16p13.3	2 (1.74%)	2 (0.82%)	1.09	0.383	0.686	Co-occurrence
EML2	19q13.32	2 (1.74%)	2 (0.82%)	1.09	0.383	0.686	Co-occurrence
ENG	9q34.11	2 (1.74%)	2 (0.82%)	1.09	0.383	0.686	Co-occurrence
ENTPD2	9q34.3	2 (1.74%)	2 (0.82%)	1.09	0.383	0.686	Co-occurrence
ENTR1	9q34.3	2 (1.74%)	2 (0.82%)	1.09	0.383	0.686	Co-occurrence
ERBB2	17q12	2 (1.74%)	2 (0.82%)	1.09	0.383	0.686	Co-occurrence
ERCC1	19q13.32	2 (1.74%)	2 (0.82%)	1.09	0.383	0.686	Co-occurrence
ERCC2	19q13.32	2 (1.74%)	2 (0.82%)	1.09	0.383	0.686	Co-occurrence
ERCC4	16p13.12	2 (1.74%)	2 (0.82%)	1.09	0.383	0.686	Co-occurrence
ESYT2	7q36.3	2 (1.74%)	2 (0.82%)	1.09	0.383	0.686	Co-occurrence
ETF1	5q31.2	2 (1.74%)	2 (0.82%)	1.09	0.383	0.686	Co-occurrence
EXOC3L2	19q13.32	2 (1.74%)	2 (0.82%)	1.09	0.383	0.686	Co-occurrence
EXTL2	1p21.2	2 (1.74%)	2 (0.82%)	1.09	0.383	0.686	Co-occurrence
F2RL3	19p13.11	2 (1.74%)	2 (0.82%)	1.09	0.383	0.686	Co-occurrence
FAH	15q25.1	2 (1.74%)	2 (0.82%)	1.09	0.383	0.686	Co-occurrence
FAM107B	10p13	2 (1.74%)	2 (0.82%)	1.09	0.383	0.686	Co-occurrence
FAM126B	2q33.1	2 (1.74%)	2 (0.82%)	1.09	0.383	0.686	Co-occurrence
FAM166A	9q34.3	2 (1.74%)	2 (0.82%)	1.09	0.383	0.686	Co-occurrence
FAM171A1	10p13	2 (1.74%)	2 (0.82%)	1.09	0.383	0.686	Co-occurrence
FAM171B	2q32.1	2 (1.74%)	2 (0.82%)	1.09	0.383	0.686	Co-occurrence
FAM173A	16p13.3	2 (1.74%)	2 (0.82%)	1.09	0.383	0.686	Co-occurrence
FAM174B	15q26.1	2 (1.74%)	2 (0.82%)	1.09	0.383	0.686	Co-occurrence
FAM215B	17q21.32	2 (1.74%)	2 (0.82%)	1.09	0.383	0.686	Co-occurrence
FAM217B	20q13.33	2 (1.74%)	2 (0.82%)	1.09	0.383	0.686	Co-occurrence
FAM219A	9p13.3	2 (1.74%)	2 (0.82%)	1.09	0.383	0.686	Co-occurrence
FAM53C	5q31.2	2 (1.74%)	2 (0.82%)	1.09	0.383	0.686	Co-occurrence
FBL	19q13.2	2 (1.74%)	2 (0.82%)	1.09	0.383	0.686	Co-occurrence
FBP1	9q22.32	2 (1.74%)	2 (0.82%)	1.09	0.383	0.686	Co-occurrence
FBXL16	16p13.3	2 (1.74%)	2 (0.82%)	1.09	0.383	0.686	Co-occurrence

FBXL20	17q12	2 (1.74%)	2 (0.82%)	1.09	0.383	0.686	Co-occurrence
FBXO38	5q32	2 (1.74%)	2 (0.82%)	1.09	0.383	0.686	Co-occurrence
FBXO46	19q13.32	2 (1.74%)	2 (0.82%)	1.09	0.383	0.686	Co-occurrence
FBXO47	17q12|17q12	2 (1.74%)	2 (0.82%)	1.09	0.383	0.686	Co-occurrence
FBXW5	9q34.3	2 (1.74%)	2 (0.82%)	1.09	0.383	0.686	Co-occurrence
FGG	4q32.1	2 (1.74%)	2 (0.82%)	1.09	0.383	0.686	Co-occurrence
FKBP6	7q11.23	2 (1.74%)	2 (0.82%)	1.09	0.383	0.686	Co-occurrence
FLYWCH1	16p13.3	2 (1.74%)	2 (0.82%)	1.09	0.383	0.686	Co-occurrence
FLYWCH2	16p13.3	2 (1.74%)	2 (0.82%)	1.09	0.383	0.686	Co-occurrence
FOSB	19q13.32	2 (1.74%)	2 (0.82%)	1.09	0.383	0.686	Co-occurrence
FOXA3	19q13.32	2 (1.74%)	2 (0.82%)	1.09	0.383	0.686	Co-occurrence
FPGS	9q34.11	2 (1.74%)	2 (0.82%)	1.09	0.383	0.686	Co-occurrence
FRRS1	1p21.2	2 (1.74%)	2 (0.82%)	1.09	0.383	0.686	Co-occurrence
FSD2	15q25.2	2 (1.74%)	2 (0.82%)	1.09	0.383	0.686	Co-occurrence
FSIP2	2q32.1	2 (1.74%)	2 (0.82%)	1.09	0.383	0.686	Co-occurrence
FSTL4	5q31.1	2 (1.74%)	2 (0.82%)	1.09	0.383	0.686	Co-occurrence
FUT7	9q34.3	2 (1.74%)	2 (0.82%)	1.09	0.383	0.686	Co-occurrence
GALT	9p13.3	2 (1.74%)	2 (0.82%)	1.09	0.383	0.686	Co-occurrence
GATA3	10p14	2 (1.74%)	2 (0.82%)	1.09	0.383	0.686	Co-occurrence
GCK	7p13	2 (1.74%)	2 (0.82%)	1.09	0.383	0.686	Co-occurrence
GCNT3	15q22.2	2 (1.74%)	2 (0.82%)	1.09	0.383	0.686	Co-occurrence
GDNF	5p13.2	2 (1.74%)	2 (0.82%)	1.09	0.383	0.686	Co-occurrence
GFRA3	5q31.2	2 (1.74%)	2 (0.82%)	1.09	0.383	0.686	Co-occurrence
GIPR	19q13.32	2 (1.74%)	2 (0.82%)	1.09	0.383	0.686	Co-occurrence
GMFG	19q13.2	2 (1.74%)	2 (0.82%)	1.09	0.383	0.686	Co-occurrence
GNAI2	3p21.31	2 (1.74%)	2 (0.82%)	1.09	0.383	0.686	Co-occurrence
GNAT1	3p21.31	2 (1.74%)	2 (0.82%)	1.09	0.383	0.686	Co-occurrence
GOLGA6L10	15q25.2	2 (1.74%)	2 (0.82%)	1.09	0.383	0.686	Co-occurrence
GOLGA6L9	15q25.2	2 (1.74%)	2 (0.82%)	1.09	0.383	0.686	Co-occurrence

GPR4	19q13.32	2 (1.74%)	2 (0.82%)	1.09	0.383	0.686	Co-occurrence
GRB7	17q12	2 (1.74%)	2 (0.82%)	1.09	0.383	0.686	Co-occurrence
GRIN1	9q34.3	2 (1.74%)	2 (0.82%)	1.09	0.383	0.686	Co-occurrence
GRIN2A	16p13.2	2 (1.74%)	2 (0.82%)	1.09	0.383	0.686	Co-occurrence
GRPEL2	5q32	2 (1.74%)	2 (0.82%)	1.09	0.383	0.686	Co-occurrence
GTF2A2	15q22.2	2 (1.74%)	2 (0.82%)	1.09	0.383	0.686	Co-occurrence
GTF2IRD2P1	7q11.23	2 (1.74%)	2 (0.82%)	1.09	0.383	0.686	Co-occurrence
GTPBP3	19p13.11	2 (1.74%)	2 (0.82%)	1.09	0.383	0.686	Co-occurrence
H2AFV	7p13	2 (1.74%)	2 (0.82%)	1.09	0.383	0.686	Co-occurrence
HACD1	10p12.33	2 (1.74%)	2 (0.82%)	1.09	0.383	0.686	Co-occurrence
HAGHL	16p13.3	2 (1.74%)	2 (0.82%)	1.09	0.383	0.686	Co-occurrence
HAPLN3	15q26.1	2 (1.74%)	2 (0.82%)	1.09	0.383	0.686	Co-occurrence
HAUS8	19p13.11	2 (1.74%)	2 (0.82%)	1.09	0.383	0.686	Co-occurrence
HCFC1R1	16p13.3	2 (1.74%)	2 (0.82%)	1.09	0.383	0.686	Co-occurrence
HDGFL3	15q25.2	2 (1.74%)	2 (0.82%)	1.09	0.383	0.686	Co-occurrence
HEYL	1p34.2	2 (1.74%)	2 (0.82%)	1.09	0.383	0.686	Co-occurrence
HHATL	3p22.1	2 (1.74%)	2 (0.82%)	1.09	0.383	0.686	Co-occurrence
HIBCH	2q32.2	2 (1.74%)	2 (0.82%)	1.09	0.383	0.686	Co-occurrence
HIGD1A	3p22.1	2 (1.74%)	2 (0.82%)	1.09	0.383	0.686	Co-occurrence
HIPK4	19q13.2	2 (1.74%)	2 (0.82%)	1.09	0.383	0.686	Co-occurrence
HM13	20q11.21	2 (1.74%)	2 (0.82%)	1.09	0.383	0.686	Co-occurrence
HNRNPA0	5q31.2	2 (1.74%)	2 (0.82%)	1.09	0.383	0.686	Co-occurrence
HNRNPL	19q13.2	2 (1.74%)	2 (0.82%)	1.09	0.383	0.686	Co-occurrence
HOMER2	15q25.2	2 (1.74%)	2 (0.82%)	1.09	0.383	0.686	Co-occurrence
HPCAL4	1p34.2	2 (1.74%)	2 (0.82%)	1.09	0.383	0.686	Co-occurrence
HSD17B7P2	10p11.1	2 (1.74%)	2 (0.82%)	1.09	0.383	0.686	Co-occurrence
HSD3B2	1p12	2 (1.74%)	2 (0.82%)	1.09	0.383	0.686	Co-occurrence
HSPA14	10p13	2 (1.74%)	2 (0.82%)	1.09	0.383	0.686	Co-occurrence
HSPA4	5q31.1	2 (1.74%)	2 (0.82%)	1.09	0.383	0.686	Co-occurrence

HSPA9	5q31.2	2 (1.74%)	2 (0.82%)	1.09	0.383	0.686	Co-occurrence
HSPB9	17q21.2	2 (1.74%)	2 (0.82%)	1.09	0.383	0.686	Co-occurrence
HSPD1	2q33.1	2 (1.74%)	2 (0.82%)	1.09	0.383	0.686	Co-occurrence
HSPE1	2q33.1	2 (1.74%)	2 (0.82%)	1.09	0.383	0.686	Co-occurrence
HVCN1	12q24.11	2 (1.74%)	2 (0.82%)	1.09	0.383	0.686	Co-occurrence
ID1	20q11.21	2 (1.74%)	2 (0.82%)	1.09	0.383	0.686	Co-occurrence
IGFBP4	17q21.2	2 (1.74%)	2 (0.82%)	1.09	0.383	0.686	Co-occurrence
IK	5q31.3	2 (1.74%)	2 (0.82%)	1.09	0.383	0.686	Co-occurrence
IL11RA	9p13.3	2 (1.74%)	2 (0.82%)	1.09	0.383	0.686	Co-occurrence
IL12RB2	1p31.3	2 (1.74%)	2 (0.82%)	1.09	0.383	0.686	Co-occurrence
IL17B	5q32	2 (1.74%)	2 (0.82%)	1.09	0.383	0.686	Co-occurrence
IL27RA	19p13.12	2 (1.74%)	2 (0.82%)	1.09	0.383	0.686	Co-occurrence
IL32	16p13.3	2 (1.74%)	2 (0.82%)	1.09	0.383	0.686	Co-occurrence
ILVBL	19p13.12	2 (1.74%)	2 (0.82%)	1.09	0.383	0.686	Co-occurrence
INPP1	2q32.2	2 (1.74%)	2 (0.82%)	1.09	0.383	0.686	Co-occurrence
INPP5E	9q34.3	2 (1.74%)	2 (0.82%)	1.09	0.383	0.686	Co-occurrence
ITFG1	16q12.1	2 (1.74%)	2 (0.82%)	1.09	0.383	0.686	Co-occurrence
ITGA8	10p13	2 (1.74%)	2 (0.82%)	1.09	0.383	0.686	Co-occurrence
ITGAV	2q32.1	2 (1.74%)	2 (0.82%)	1.09	0.383	0.686	Co-occurrence
ITGB6	2q24.2	2 (1.74%)	2 (0.82%)	1.09	0.383	0.686	Co-occurrence
ITIH2	10p14	2 (1.74%)	2 (0.82%)	1.09	0.383	0.686	Co-occurrence
ITIH5	10p14	2 (1.74%)	2 (0.82%)	1.09	0.383	0.686	Co-occurrence
ITPKC	19q13.2	2 (1.74%)	2 (0.82%)	1.09	0.383	0.686	Co-occurrence
ITPR2	12p11.23	2 (1.74%)	2 (0.82%)	1.09	0.383	0.686	Co-occurrence
JMJD8	16p13.3	2 (1.74%)	2 (0.82%)	1.09	0.383	0.686	Co-occurrence
KANSL1	17q21.31	2 (1.74%)	2 (0.82%)	1.09	0.383	0.686	Co-occurrence
KAT2A	17q21.2	2 (1.74%)	2 (0.82%)	1.09	0.383	0.686	Co-occurrence
KCNC1	11p15.1	2 (1.74%)	2 (0.82%)	1.09	0.383	0.686	Co-occurrence
KCNH4	17q21.2	2 (1.74%)	2 (0.82%)	1.09	0.383	0.686	Co-occurrence

KCTD18	2q33.1	2 (1.74%)	2 (0.82%)	1.09	0.383	0.686	Co-occurrence
KDM3B	5q31.2	2 (1.74%)	2 (0.82%)	1.09	0.383	0.686	Co-occurrence
KIAA0895	7p14.2	2 (1.74%)	2 (0.82%)	1.09	0.383	0.686	Co-occurrence
KIAA1217	10p12.2-p12.1	2 (1.74%)	2 (0.82%)	1.09	0.383	0.686	Co-occurrence
KIAA1755	20q11.23	2 (1.74%)	2 (0.82%)	1.09	0.383	0.686	Co-occurrence
KIF20A	5q31.2	2 (1.74%)	2 (0.82%)	1.09	0.383	0.686	Co-occurrence
KIF5B	10p11.22	2 (1.74%)	2 (0.82%)	1.09	0.383	0.686	Co-occurrence
KIN	10p14	2 (1.74%)	2 (0.82%)	1.09	0.383	0.686	Co-occurrence
KLC2	11q13.2	2 (1.74%)	2 (0.82%)	1.09	0.383	0.686	Co-occurrence
KLC3	19q13.32	2 (1.74%)	2 (0.82%)	1.09	0.383	0.686	Co-occurrence
KLHL10	17q21.2	2 (1.74%)	2 (0.82%)	1.09	0.383	0.686	Co-occurrence
KLHL11	17q21.2	2 (1.74%)	2 (0.82%)	1.09	0.383	0.686	Co-occurrence
KLHL3	5q31.2	2 (1.74%)	2 (0.82%)	1.09	0.383	0.686	Co-occurrence
KLHL33	14q11.2	2 (1.74%)	2 (0.82%)	1.09	0.383	0.686	Co-occurrence
KLHL40	3p22.1	2 (1.74%)	2 (0.82%)	1.09	0.383	0.686	Co-occurrence
KREMEN2	16p13.3	2 (1.74%)	2 (0.82%)	1.09	0.383	0.686	Co-occurrence
KRT10	17q21.2	2 (1.74%)	2 (0.82%)	1.09	0.383	0.686	Co-occurrence
KRT12	17q21.2	2 (1.74%)	2 (0.82%)	1.09	0.383	0.686	Co-occurrence
KRT16P3	17p11.2	2 (1.74%)	2 (0.82%)	1.09	0.383	0.686	Co-occurrence
KRT20	17q21.2	2 (1.74%)	2 (0.82%)	1.09	0.383	0.686	Co-occurrence
KRT222	17q21.2	2 (1.74%)	2 (0.82%)	1.09	0.383	0.686	Co-occurrence
KRT223P	17q21.2	2 (1.74%)	2 (0.82%)	1.09	0.383	0.686	Co-occurrence
KRT23	17q21.2	2 (1.74%)	2 (0.82%)	1.09	0.383	0.686	Co-occurrence
KRT24	17q21.2	2 (1.74%)	2 (0.82%)	1.09	0.383	0.686	Co-occurrence
KRT25	17q21.2	2 (1.74%)	2 (0.82%)	1.09	0.383	0.686	Co-occurrence
KRT26	17q21.2	2 (1.74%)	2 (0.82%)	1.09	0.383	0.686	Co-occurrence
KRT27	17q21.2	2 (1.74%)	2 (0.82%)	1.09	0.383	0.686	Co-occurrence
KRT28	17q21.2	2 (1.74%)	2 (0.82%)	1.09	0.383	0.686	Co-occurrence
KSR2	12q24.22-q24.23	2 (1.74%)	2 (0.82%)	1.09	0.383	0.686	Co-occurrence

LASP1	17q12	2 (1.74%)	2 (0.82%)	1.09	0.383	0.686	Co-occurrence
LCN12	9q34.3	2 (1.74%)	2 (0.82%)	1.09	0.383	0.686	Co-occurrence
LCN2	9q34.11	2 (1.74%)	2 (0.82%)	1.09	0.383	0.686	Co-occurrence
LCNL1	9q34.3	2 (1.74%)	2 (0.82%)	1.09	0.383	0.686	Co-occurrence
LDHA	11p15.1	2 (1.74%)	2 (0.82%)	1.09	0.383	0.686	Co-occurrence
LDHAL6A	11p15.1	2 (1.74%)	2 (0.82%)	1.09	0.383	0.686	Co-occurrence
LDHC	11p15.1	2 (1.74%)	2 (0.82%)	1.09	0.383	0.686	Co-occurrence
LEUTX	19q13.2	2 (1.74%)	2 (0.82%)	1.09	0.383	0.686	Co-occurrence
LGALS14	19q13.2	2 (1.74%)	2 (0.82%)	1.09	0.383	0.686	Co-occurrence
LGALS16	19q13.2	2 (1.74%)	2 (0.82%)	1.09	0.383	0.686	Co-occurrence
LGALS17A	19q13.2	2 (1.74%)	2 (0.82%)	1.09	0.383	0.686	Co-occurrence
LGALS9B	17p11.2	2 (1.74%)	2 (0.82%)	1.09	0.383	0.686	Co-occurrence
LINC00028	20q11.21	2 (1.74%)	2 (0.82%)	1.09	0.383	0.686	Co-occurrence
LINC00514	16p13.3	2 (1.74%)	2 (0.82%)	1.09	0.383	0.686	Co-occurrence
LINC00593	15q23	2 (1.74%)	2 (0.82%)	1.09	0.383	0.686	Co-occurrence
LINC00672	17q12	2 (1.74%)	2 (0.82%)	1.09	0.383	0.686	Co-occurrence
LINC00689	7q36.3	2 (1.74%)	2 (0.82%)	1.09	0.383	0.686	Co-occurrence
LINC00708	10p14	2 (1.74%)	2 (0.82%)	1.09	0.383	0.686	Co-occurrence
LINC00709	10p14	2 (1.74%)	2 (0.82%)	1.09	0.383	0.686	Co-occurrence
LINC00710	10p14	2 (1.74%)	2 (0.82%)	1.09	0.383	0.686	Co-occurrence
LINC00906	19q12	2 (1.74%)	2 (0.82%)	1.09	0.383	0.686	Co-occurrence
LINC00930	15q26.1	2 (1.74%)	2 (0.82%)	1.09	0.383	0.686	Co-occurrence
LINC02692	9q34.3	2 (1.74%)	2 (0.82%)	1.09	0.383	0.686	Co-occurrence
LIPC	15q21.3	2 (1.74%)	2 (0.82%)	1.09	0.383	0.686	Co-occurrence
LIPE	19q13.2	2 (1.74%)	2 (0.82%)	1.09	0.383	0.686	Co-occurrence
LOC105376839	17q21.31	2 (1.74%)	2 (0.82%)	1.09	0.383	0.686	Co-occurrence
LRP6	12p13.2	2 (1.74%)	2 (0.82%)	1.09	0.383	0.686	Co-occurrence
LRRC26	9q34.3	2 (1.74%)	2 (0.82%)	1.09	0.383	0.686	Co-occurrence
LRRC37A11P	17q12	2 (1.74%)	2 (0.82%)	1.09	0.383	0.686	Co-occurrence

LRRC37A2	17q21.31	2 (1.74%)	2 (0.82%)	1.09	0.383	0.686	Co-occurrence
LRRC37A4P	17q21.31	2 (1.74%)	2 (0.82%)	1.09	0.383	0.686	Co-occurrence
LRRC39	1p21.2	2 (1.74%)	2 (0.82%)	1.09	0.383	0.686	Co-occurrence
LTBP4	19q13.2	2 (1.74%)	2 (0.82%)	1.09	0.383	0.686	Co-occurrence
MALRD1	10p12.31	2 (1.74%)	2 (0.82%)	1.09	0.383	0.686	Co-occurrence
MAMDC4	9q34.3	2 (1.74%)	2 (0.82%)	1.09	0.383	0.686	Co-occurrence
MAN1B1	9q34.3	2 (1.74%)	2 (0.82%)	1.09	0.383	0.686	Co-occurrence
MAN2C1	15q24.2	2 (1.74%)	2 (0.82%)	1.09	0.383	0.686	Co-occurrence
MAPT	17q21.31	2 (1.74%)	2 (0.82%)	1.09	0.383	0.686	Co-occurrence
MARK4	19q13.32	2 (1.74%)	2 (0.82%)	1.09	0.383	0.686	Co-occurrence
MARS2	2q33.1	2 (1.74%)	2 (0.82%)	1.09	0.383	0.686	Co-occurrence
MCTP2	15q26.2	2 (1.74%)	2 (0.82%)	1.09	0.383	0.686	Co-occurrence
MED1	17q12	2 (1.74%)	2 (0.82%)	1.09	0.383	0.686	Co-occurrence
MED24	17q21.1	2 (1.74%)	2 (0.82%)	1.09	0.383	0.686	Co-occurrence
MED29	19q13.2	2 (1.74%)	2 (0.82%)	1.09	0.383	0.686	Co-occurrence
MEIG1	10p13	2 (1.74%)	2 (0.82%)	1.09	0.383	0.686	Co-occurrence
MESP1	15q26.1	2 (1.74%)	2 (0.82%)	1.09	0.383	0.686	Co-occurrence
MESP2	15q26.1	2 (1.74%)	2 (0.82%)	1.09	0.383	0.686	Co-occurrence
METRN	16p13.3	2 (1.74%)	2 (0.82%)	1.09	0.383	0.686	Co-occurrence
METTL22	16p13.2	2 (1.74%)	2 (0.82%)	1.09	0.383	0.686	Co-occurrence
MEX3B	15q25.2	2 (1.74%)	2 (0.82%)	1.09	0.383	0.686	Co-occurrence
MFGE8	15q26.1	2 (1.74%)	2 (0.82%)	1.09	0.383	0.686	Co-occurrence
MFSD14A	1p21.2	2 (1.74%)	2 (0.82%)	1.09	0.383	0.686	Co-occurrence
MFSD6	2q32.2	2 (1.74%)	2 (0.82%)	1.09	0.383	0.686	Co-occurrence
MIA	19q13.2	2 (1.74%)	2 (0.82%)	1.09	0.383	0.686	Co-occurrence
MIEN1	17q12	2 (1.74%)	2 (0.82%)	1.09	0.383	0.686	Co-occurrence
MINDY3	10p13	2 (1.74%)	2 (0.82%)	1.09	0.383	0.686	Co-occurrence
MIR-1272/1272		2 (1.74%)	2 (0.82%)	1.09	0.383	0.686	Co-occurrence
MIR-1827/1827		2 (1.74%)	2 (0.82%)	1.09	0.383	0.686	Co-occurrence

MIR-3175/3175		2 (1.74%)	2 (0.82%)	1.09	0.383	0.686	Co-occurrence
MIR-3193/3193		2 (1.74%)	2 (0.82%)	1.09	0.383	0.686	Co-occurrence
MIR-3621/3621		2 (1.74%)	2 (0.82%)	1.09	0.383	0.686	Co-occurrence
MIR-3646/3646		2 (1.74%)	2 (0.82%)	1.09	0.383	0.686	Co-occurrence
MIR-3655/3655		2 (1.74%)	2 (0.82%)	1.09	0.383	0.686	Co-occurrence
MIR-4292/4292		2 (1.74%)	2 (0.82%)	1.09	0.383	0.686	Co-occurrence
MIR-4479/4479		2 (1.74%)	2 (0.82%)	1.09	0.383	0.686	Co-occurrence
MIR-4515/4515		2 (1.74%)	2 (0.82%)	1.09	0.383	0.686	Co-occurrence
MIR-4533/4533		2 (1.74%)	2 (0.82%)	1.09	0.383	0.686	Co-occurrence
MIR-4657/4657		2 (1.74%)	2 (0.82%)	1.09	0.383	0.686	Co-occurrence
MIR-4785/4785		2 (1.74%)	2 (0.82%)	1.09	0.383	0.686	Co-occurrence
MIR-548AK/548AK		2 (1.74%)	2 (0.82%)	1.09	0.383	0.686	Co-occurrence
MIR-553/553		2 (1.74%)	2 (0.82%)	1.09	0.383	0.686	Co-occurrence
MIR-566/566		2 (1.74%)	2 (0.82%)	1.09	0.383	0.686	Co-occurrence
MIR-5707/5707		2 (1.74%)	2 (0.82%)	1.09	0.383	0.686	Co-occurrence
MIR-595/595		2 (1.74%)	2 (0.82%)	1.09	0.383	0.686	Co-occurrence
MIR-630/630		2 (1.74%)	2 (0.82%)	1.09	0.383	0.686	Co-occurrence
MIR-631/631		2 (1.74%)	2 (0.82%)	1.09	0.383	0.686	Co-occurrence
MIR-641/641		2 (1.74%)	2 (0.82%)	1.09	0.383	0.686	Co-occurrence
MIR-646/646		2 (1.74%)	2 (0.82%)	1.09	0.383	0.686	Co-occurrence
MMP17	12q24.33	2 (1.74%)	2 (0.82%)	1.09	0.383	0.686	Co-occurrence
MMP25	16p13.3	2 (1.74%)	2 (0.82%)	1.09	0.383	0.686	Co-occurrence
MOB4	2q33.1	2 (1.74%)	2 (0.82%)	1.09	0.383	0.686	Co-occurrence
MRI1	19p13.13	2 (1.74%)	2 (0.82%)	1.09	0.383	0.686	Co-occurrence
MRPL45P2	17q21.32	2 (1.74%)	2 (0.82%)	1.09	0.383	0.686	Co-occurrence
MRTFA	22q13.1-q13.2	2 (1.74%)	2 (0.82%)	1.09	0.383	0.686	Co-occurrence
MRTFB	16p13.12	2 (1.74%)	2 (0.82%)	1.09	0.383	0.686	Co-occurrence
MTFMT	15q22.31	2 (1.74%)	2 (0.82%)	1.09	0.383	0.686	Co-occurrence
MTRNR2L7	10p11.21	2 (1.74%)	2 (0.82%)	1.09	0.383	0.686	Co-occurrence

MYL7	7p13	2 (1.74%)	2 (0.82%)	1.09	0.383	0.686	Co-occurrence
MYO15A	17p11.2	2 (1.74%)	2 (0.82%)	1.09	0.383	0.686	Co-occurrence
MYOT	5q31.2	2 (1.74%)	2 (0.82%)	1.09	0.383	0.686	Co-occurrence
NAGPA	16p13.3	2 (1.74%)	2 (0.82%)	1.09	0.383	0.686	Co-occurrence
NAIF1	9q34.11	2 (1.74%)	2 (0.82%)	1.09	0.383	0.686	Co-occurrence
NANOS3	19p13.12	2 (1.74%)	2 (0.82%)	1.09	0.383	0.686	Co-occurrence
NCAPG2	7q36.3	2 (1.74%)	2 (0.82%)	1.09	0.383	0.686	Co-occurrence
NCF1B	7q11.23	2 (1.74%)	2 (0.82%)	1.09	0.383	0.686	Co-occurrence
NCOA6	20q11.22	2 (1.74%)	2 (0.82%)	1.09	0.383	0.686	Co-occurrence
NDOR1	9q34.3	2 (1.74%)	2 (0.82%)	1.09	0.383	0.686	Co-occurrence
NDUFA2	5q31.3	2 (1.74%)	2 (0.82%)	1.09	0.383	0.686	Co-occurrence
NDUFB3	2q33.1	2 (1.74%)	2 (0.82%)	1.09	0.383	0.686	Co-occurrence
NDUFB7	19p13.12	2 (1.74%)	2 (0.82%)	1.09	0.383	0.686	Co-occurrence
NEIL1	15q24.2	2 (1.74%)	2 (0.82%)	1.09	0.383	0.686	Co-occurrence
NEO1	15q24.1	2 (1.74%)	2 (0.82%)	1.09	0.383	0.686	Co-occurrence
NETO2	16q12.1	2 (1.74%)	2 (0.82%)	1.09	0.383	0.686	Co-occurrence
NEUROD2	17q12	2 (1.74%)	2 (0.82%)	1.09	0.383	0.686	Co-occurrence
NFKBIA	14q13.2	2 (1.74%)	2 (0.82%)	1.09	0.383	0.686	Co-occurrence
NFKBIB	19q13.2	2 (1.74%)	2 (0.82%)	1.09	0.383	0.686	Co-occurrence
NIF3L1	2q33.1	2 (1.74%)	2 (0.82%)	1.09	0.383	0.686	Co-occurrence
NKTR	3p22.1	2 (1.74%)	2 (0.82%)	1.09	0.383	0.686	Co-occurrence
NME5	5q31.2	2 (1.74%)	2 (0.82%)	1.09	0.383	0.686	Co-occurrence
NMT2	10p13	2 (1.74%)	2 (0.82%)	1.09	0.383	0.686	Co-occurrence
NOS2P3	17p11.2	2 (1.74%)	2 (0.82%)	1.09	0.383	0.686	Co-occurrence
NOTCH3	19p13.12	2 (1.74%)	2 (0.82%)	1.09	0.383	0.686	Co-occurrence
NPDC1	9q34.3	2 (1.74%)	2 (0.82%)	1.09	0.383	0.686	Co-occurrence
NPEPPS	17q21.32	2 (1.74%)	2 (0.82%)	1.09	0.383	0.686	Co-occurrence
NPY6R	5q31.2	2 (1.74%)	2 (0.82%)	1.09	0.383	0.686	Co-occurrence
NR3C1	5q31.3	2 (1.74%)	2 (0.82%)	1.09	0.383	0.686	Co-occurrence

NSF	17q21.31	2 (1.74%)	2 (0.82%)	1.09	0.383	0.686	Co-occurrence
NSUN5	7q11.23	2 (1.74%)	2 (0.82%)	1.09	0.383	0.686	Co-occurrence
NSUN5P2	7q11.23	2 (1.74%)	2 (0.82%)	1.09	0.383	0.686	Co-occurrence
NSUN6	10p12.31	2 (1.74%)	2 (0.82%)	1.09	0.383	0.686	Co-occurrence
NT5C1A	1p34.2	2 (1.74%)	2 (0.82%)	1.09	0.383	0.686	Co-occurrence
NT5C3B	17q21.2	2 (1.74%)	2 (0.82%)	1.09	0.383	0.686	Co-occurrence
NT5DC3	12q23.3	2 (1.74%)	2 (0.82%)	1.09	0.383	0.686	Co-occurrence
NUAK1	12q23.3	2 (1.74%)	2 (0.82%)	1.09	0.383	0.686	Co-occurrence
NUDT5	10p14	2 (1.74%)	2 (0.82%)	1.09	0.383	0.686	Co-occurrence
NUMBL	19q13.2	2 (1.74%)	2 (0.82%)	1.09	0.383	0.686	Co-occurrence
NWD1	19p13.11	2 (1.74%)	2 (0.82%)	1.09	0.383	0.686	Co-occurrence
OAZ2	15q22.31	2 (1.74%)	2 (0.82%)	1.09	0.383	0.686	Co-occurrence
OLAH	10p13	2 (1.74%)	2 (0.82%)	1.09	0.383	0.686	Co-occurrence
OLFM1	9q34.3	2 (1.74%)	2 (0.82%)	1.09	0.383	0.686	Co-occurrence
OPA3	19q13.32	2 (1.74%)	2 (0.82%)	1.09	0.383	0.686	Co-occurrence
OR11G2	14q11.2	2 (1.74%)	2 (0.82%)	1.09	0.383	0.686	Co-occurrence
OR11H4	14q11.2	2 (1.74%)	2 (0.82%)	1.09	0.383	0.686	Co-occurrence
OR11H6	14q11.2	2 (1.74%)	2 (0.82%)	1.09	0.383	0.686	Co-occurrence
OR11H7	14q11.2	2 (1.74%)	2 (0.82%)	1.09	0.383	0.686	Co-occurrence
OR1F1	16p13.3	2 (1.74%)	2 (0.82%)	1.09	0.383	0.686	Co-occurrence
OR6S1	14q11.2	2 (1.74%)	2 (0.82%)	1.09	0.383	0.686	Co-occurrence
ORC2	2q33.1	2 (1.74%)	2 (0.82%)	1.09	0.383	0.686	Co-occurrence
OSGEP	14q11.2	2 (1.74%)	2 (0.82%)	1.09	0.383	0.686	Co-occurrence
OXCT2	1p34.2	2 (1.74%)	2 (0.82%)	1.09	0.383	0.686	Co-occurrence
PAF1	19q13.2	2 (1.74%)	2 (0.82%)	1.09	0.383	0.686	Co-occurrence
PALM3	19p13.12	2 (1.74%)	2 (0.82%)	1.09	0.383	0.686	Co-occurrence
PALMD	1p21.2	2 (1.74%)	2 (0.82%)	1.09	0.383	0.686	Co-occurrence
PAQR4	16p13.3	2 (1.74%)	2 (0.82%)	1.09	0.383	0.686	Co-occurrence
PARN	16p13.12	2 (1.74%)	2 (0.82%)	1.09	0.383	0.686	Co-occurrence

PARP2	14q11.2	2 (1.74%)	2 (0.82%)	1.09	0.383	0.686	Co-occurrence
PAXX	9q34.3	2 (1.74%)	2 (0.82%)	1.09	0.383	0.686	Co-occurrence
PCLO	7q21.11	2 (1.74%)	2 (0.82%)	1.09	0.383	0.686	Co-occurrence
PCYOX1L	5q32	2 (1.74%)	2 (0.82%)	1.09	0.383	0.686	Co-occurrence
PDCD7	15q22.31	2 (1.74%)	2 (0.82%)	1.09	0.383	0.686	Co-occurrence
PGAP3	17q12	2 (1.74%)	2 (0.82%)	1.09	0.383	0.686	Co-occurrence
PHACTR3	20q13.32-q13.33	2 (1.74%)	2 (0.82%)	1.09	0.383	0.686	Co-occurrence
PHKB	16q12.1	2 (1.74%)	2 (0.82%)	1.09	0.383	0.686	Co-occurrence
PHOSPHO2	2q31.1	2 (1.74%)	2 (0.82%)	1.09	0.383	0.686	Co-occurrence
PHPT1	9q34.3	2 (1.74%)	2 (0.82%)	1.09	0.383	0.686	Co-occurrence
PIDD1	11p15.5	2 (1.74%)	2 (0.82%)	1.09	0.383	0.686	Co-occurrence
PIP4K2B	17q12	2 (1.74%)	2 (0.82%)	1.09	0.383	0.686	Co-occurrence
PIP4P1	14q11.2	2 (1.74%)	2 (0.82%)	1.09	0.383	0.686	Co-occurrence
PKD2L2	5q31.2	2 (1.74%)	2 (0.82%)	1.09	0.383	0.686	Co-occurrence
PKIG	20q13.12	2 (1.74%)	2 (0.82%)	1.09	0.383	0.686	Co-occurrence
PKMYT1	16p13.3	2 (1.74%)	2 (0.82%)	1.09	0.383	0.686	Co-occurrence
PKP4	2q24.1	2 (1.74%)	2 (0.82%)	1.09	0.383	0.686	Co-occurrence
PLA2R1	2q24.2	2 (1.74%)	2 (0.82%)	1.09	0.383	0.686	Co-occurrence
PLD3	19q13.2	2 (1.74%)	2 (0.82%)	1.09	0.383	0.686	Co-occurrence
PLEKHF1	19q12	2 (1.74%)	2 (0.82%)	1.09	0.383	0.686	Co-occurrence
PLEKHM1	17q21.31	2 (1.74%)	2 (0.82%)	1.09	0.383	0.686	Co-occurrence
PLEKHO2	15q22.31	2 (1.74%)	2 (0.82%)	1.09	0.383	0.686	Co-occurrence
PLPP6	9p24.1	2 (1.74%)	2 (0.82%)	1.09	0.383	0.686	Co-occurrence
PLXDC1	17q12	2 (1.74%)	2 (0.82%)	1.09	0.383	0.686	Co-occurrence
PLXDC2	10p12.31	2 (1.74%)	2 (0.82%)	1.09	0.383	0.686	Co-occurrence
PMM2	16p13.2	2 (1.74%)	2 (0.82%)	1.09	0.383	0.686	Co-occurrence
PMPCA	9q34.3	2 (1.74%)	2 (0.82%)	1.09	0.383	0.686	Co-occurrence
PNMT	17q12	2 (1.74%)	2 (0.82%)	1.09	0.383	0.686	Co-occurrence
PNP	14q11.2	2 (1.74%)	2 (0.82%)	1.09	0.383	0.686	Co-occurrence

PODNL1	19p13.12	2 (1.74%)	2 (0.82%)	1.09	0.383	0.686	Co-occurrence
POLD2	7p13	2 (1.74%)	2 (0.82%)	1.09	0.383	0.686	Co-occurrence
POM121	7q11.23	2 (1.74%)	2 (0.82%)	1.09	0.383	0.686	Co-occurrence
POP4	19q12	2 (1.74%)	2 (0.82%)	1.09	0.383	0.686	Co-occurrence
PP13004	7p14.2	2 (1.74%)	2 (0.82%)	1.09	0.383	0.686	Co-occurrence
PPIL3	2q33.1	2 (1.74%)	2 (0.82%)	1.09	0.383	0.686	Co-occurrence
PPM1N	19q13.32	2 (1.74%)	2 (0.82%)	1.09	0.383	0.686	Co-occurrence
PPP1R13L	19q13.32	2 (1.74%)	2 (0.82%)	1.09	0.383	0.686	Co-occurrence
PPP1R3D	20q13.33	2 (1.74%)	2 (0.82%)	1.09	0.383	0.686	Co-occurrence
PRKACA	19p13.12	2 (1.74%)	2 (0.82%)	1.09	0.383	0.686	Co-occurrence
PRM1	16p13.13	2 (1.74%)	2 (0.82%)	1.09	0.383	0.686	Co-occurrence
PRM2	16p13.13	2 (1.74%)	2 (0.82%)	1.09	0.383	0.686	Co-occurrence
PRM3	16p13.13	2 (1.74%)	2 (0.82%)	1.09	0.383	0.686	Co-occurrence
PRSS21	16p13.3	2 (1.74%)	2 (0.82%)	1.09	0.383	0.686	Co-occurrence
PRSS30P	16p13.3	2 (1.74%)	2 (0.82%)	1.09	0.383	0.686	Co-occurrence
PRSS33	16p13.3	2 (1.74%)	2 (0.82%)	1.09	0.383	0.686	Co-occurrence
PRSS41	16p13.3	2 (1.74%)	2 (0.82%)	1.09	0.383	0.686	Co-occurrence
PRX	19q13.2	2 (1.74%)	2 (0.82%)	1.09	0.383	0.686	Co-occurrence
PTER	10p13	2 (1.74%)	2 (0.82%)	1.09	0.383	0.686	Co-occurrence
PTGDS	9q34.3	2 (1.74%)	2 (0.82%)	1.09	0.383	0.686	Co-occurrence
PTGES2	9q34.11	2 (1.74%)	2 (0.82%)	1.09	0.383	0.686	Co-occurrence
PTPN2	18p11.21	2 (1.74%)	2 (0.82%)	1.09	0.383	0.686	Co-occurrence
PURB	7p13	2 (1.74%)	2 (0.82%)	1.09	0.383	0.686	Co-occurrence
QPCTL	19q13.32	2 (1.74%)	2 (0.82%)	1.09	0.383	0.686	Co-occurrence
RAB1B	11q13.2	2 (1.74%)	2 (0.82%)	1.09	0.383	0.686	Co-occurrence
RAB5C	17q21.2	2 (1.74%)	2 (0.82%)	1.09	0.383	0.686	Co-occurrence
RABL6	9q34.3	2 (1.74%)	2 (0.82%)	1.09	0.383	0.686	Co-occurrence
RAD17	5q13.2	2 (1.74%)	2 (0.82%)	1.09	0.383	0.686	Co-occurrence
RALGAPB	20q11.23	2 (1.74%)	2 (0.82%)	1.09	0.383	0.686	Co-occurrence

RAMAC	15q25.2	2 (1.74%)	2 (0.82%)	1.09	0.383	0.686	Co-occurrence
RASGRF1	15q25.1	2 (1.74%)	2 (0.82%)	1.09	0.383	0.686	Co-occurrence
RBFOX1	16p13.3	2 (1.74%)	2 (0.82%)	1.09	0.383	0.686	Co-occurrence
RBMS1	2q24.2	2 (1.74%)	2 (0.82%)	1.09	0.383	0.686	Co-occurrence
RBPMS2	15q22.31	2 (1.74%)	2 (0.82%)	1.09	0.383	0.686	Co-occurrence
REEP2	5q31.2	2 (1.74%)	2 (0.82%)	1.09	0.383	0.686	Co-occurrence
REM1	20q11.21	2 (1.74%)	2 (0.82%)	1.09	0.383	0.686	Co-occurrence
RFTN2	2q33.1	2 (1.74%)	2 (0.82%)	1.09	0.383	0.686	Co-occurrence
RFX1	19p13.12	2 (1.74%)	2 (0.82%)	1.09	0.383	0.686	Co-occurrence
RGMA	15q26.1	2 (1.74%)	2 (0.82%)	1.09	0.383	0.686	Co-occurrence
RHBDL1	16p13.3	2 (1.74%)	2 (0.82%)	1.09	0.383	0.686	Co-occurrence
RHOT2	16p13.3	2 (1.74%)	2 (0.82%)	1.09	0.383	0.686	Co-occurrence
RINL	19q13.2	2 (1.74%)	2 (0.82%)	1.09	0.383	0.686	Co-occurrence
RLN3	19p13.12	2 (1.74%)	2 (0.82%)	1.09	0.383	0.686	Co-occurrence
RMI2	16p13.13	2 (1.74%)	2 (0.82%)	1.09	0.383	0.686	Co-occurrence
RN7SKP145	5q32	2 (1.74%)	2 (0.82%)	1.09	0.383	0.686	Co-occurrence
RN7SKP24	9p13.3	2 (1.74%)	2 (0.82%)	1.09	0.383	0.686	Co-occurrence
RN7SKP42	2q32.1	2 (1.74%)	2 (0.82%)	1.09	0.383	0.686	Co-occurrence
RN7SL147P	3p25.2	2 (1.74%)	2 (0.82%)	1.09	0.383	0.686	Co-occurrence
RN7SL15P	Xp11.4	2 (1.74%)	2 (0.82%)	1.09	0.383	0.686	Co-occurrence
RN7SL199P	17q21.31	2 (1.74%)	2 (0.82%)	1.09	0.383	0.686	Co-occurrence
RN7SL270P	17q21.32	2 (1.74%)	2 (0.82%)	1.09	0.383	0.686	Co-occurrence
RN7SL274P	16p13.12	2 (1.74%)	2 (0.82%)	1.09	0.383	0.686	Co-occurrence
RN7SL337P	19p13.12	2 (1.74%)	2 (0.82%)	1.09	0.383	0.686	Co-occurrence
RN7SL387P	12q24.11	2 (1.74%)	2 (0.82%)	1.09	0.383	0.686	Co-occurrence
RN7SL393P	2q24.1	2 (1.74%)	2 (0.82%)	1.09	0.383	0.686	Co-occurrence
RN7SL493P	16p13.2	2 (1.74%)	2 (0.82%)	1.09	0.383	0.686	Co-occurrence
RN7SL566P	19q13.2	2 (1.74%)	2 (0.82%)	1.09	0.383	0.686	Co-occurrence
RN7SL567P	3p22.1	2 (1.74%)	2 (0.82%)	1.09	0.383	0.686	Co-occurrence

RN7SL619P	19p13.13	2 (1.74%)	2 (0.82%)	1.09	0.383	0.686	Co-occurrence
RN7SL63P	10p11.23	2 (1.74%)	2 (0.82%)	1.09	0.383	0.686	Co-occurrence
RN7SL656P	17q21.31	2 (1.74%)	2 (0.82%)	1.09	0.383	0.686	Co-occurrence
RN7SL670P	2q33.2	2 (1.74%)	2 (0.82%)	1.09	0.383	0.686	Co-occurrence
RN7SL743P	16p13.2	2 (1.74%)	2 (0.82%)	1.09	0.383	0.686	Co-occurrence
RN7SL823P	19p13.11	2 (1.74%)	2 (0.82%)	1.09	0.383	0.686	Co-occurrence
RN7SL825P	10p11.22	2 (1.74%)	2 (0.82%)	1.09	0.383	0.686	Co-occurrence
RN7SL835P	19p13.11	2 (1.74%)	2 (0.82%)	1.09	0.383	0.686	Co-occurrence
RN7SL836P	19q13.32	2 (1.74%)	2 (0.82%)	1.09	0.383	0.686	Co-occurrence
RN7SL842P	19p13.12	2 (1.74%)	2 (0.82%)	1.09	0.383	0.686	Co-occurrence
RNA5SP115	2q33.1	2 (1.74%)	2 (0.82%)	1.09	0.383	0.686	Co-occurrence
RNA5SP230	7p13	2 (1.74%)	2 (0.82%)	1.09	0.383	0.686	Co-occurrence
RNA5SP296	9q34.11	2 (1.74%)	2 (0.82%)	1.09	0.383	0.686	Co-occurrence
RNA5SP299	10p14	2 (1.74%)	2 (0.82%)	1.09	0.383	0.686	Co-occurrence
RNA5SP302	10p13	2 (1.74%)	2 (0.82%)	1.09	0.383	0.686	Co-occurrence
RNA5SP303	10p12.31	2 (1.74%)	2 (0.82%)	1.09	0.383	0.686	Co-occurrence
RNA5SP333	11p15.1	2 (1.74%)	2 (0.82%)	1.09	0.383	0.686	Co-occurrence
RNA5SP378	12q24.33	2 (1.74%)	2 (0.82%)	1.09	0.383	0.686	Co-occurrence
RNA5SP382	14q11.2	2 (1.74%)	2 (0.82%)	1.09	0.383	0.686	Co-occurrence
RNA5SP40	1p36.22	2 (1.74%)	2 (0.82%)	1.09	0.383	0.686	Co-occurrence
RNA5SP403	16p13.2	2 (1.74%)	2 (0.82%)	1.09	0.383	0.686	Co-occurrence
RNA5SP404	16p13.2	2 (1.74%)	2 (0.82%)	1.09	0.383	0.686	Co-occurrence
RNA5SP470	19q12	2 (1.74%)	2 (0.82%)	1.09	0.383	0.686	Co-occurrence
RNASE1	14q11.2	2 (1.74%)	2 (0.82%)	1.09	0.383	0.686	Co-occurrence
RNASE10	14q11.2	2 (1.74%)	2 (0.82%)	1.09	0.383	0.686	Co-occurrence
RNASE11	14q11.2	2 (1.74%)	2 (0.82%)	1.09	0.383	0.686	Co-occurrence
RNASE12	14q11.2	2 (1.74%)	2 (0.82%)	1.09	0.383	0.686	Co-occurrence
RNASE4	14q11.2	2 (1.74%)	2 (0.82%)	1.09	0.383	0.686	Co-occurrence
RNASE6	14q11.2	2 (1.74%)	2 (0.82%)	1.09	0.383	0.686	Co-occurrence

RNASE9	14q11.2	2 (1.74%)	2 (0.82%)	1.09	0.383	0.686	Co-occurrence
RNF208	9q34.3	2 (1.74%)	2 (0.82%)	1.09	0.383	0.686	Co-occurrence
RNF224	9q34.3	2 (1.74%)	2 (0.82%)	1.09	0.383	0.686	Co-occurrence
RNU6ATAC3P	17q21.32	2 (1.74%)	2 (0.82%)	1.09	0.383	0.686	Co-occurrence
RORA	15q22.2	2 (1.74%)	2 (0.82%)	1.09	0.383	0.686	Co-occurrence
RP2	Xp11.3	2 (1.74%)	2 (0.82%)	1.09	0.383	0.686	Co-occurrence
RPL19	17q12	2 (1.74%)	2 (0.82%)	1.09	0.383	0.686	Co-occurrence
RPL23	17q12	2 (1.74%)	2 (0.82%)	1.09	0.383	0.686	Co-occurrence
RPLP2	11p15.5	2 (1.74%)	2 (0.82%)	1.09	0.383	0.686	Co-occurrence
RPP38	10p13	2 (1.74%)	2 (0.82%)	1.09	0.383	0.686	Co-occurrence
RPP38-DT	10p13	2 (1.74%)	2 (0.82%)	1.09	0.383	0.686	Co-occurrence
RPPH1	14q11.2	2 (1.74%)	2 (0.82%)	1.09	0.383	0.686	Co-occurrence
RPS17	15q25.2	2 (1.74%)	2 (0.82%)	1.09	0.383	0.686	Co-occurrence
RSPH6A	19q13.32	2 (1.74%)	2 (0.82%)	1.09	0.383	0.686	Co-occurrence
RSU1	10p13	2 (1.74%)	2 (0.82%)	1.09	0.383	0.686	Co-occurrence
RTCA	1p21.2	2 (1.74%)	2 (0.82%)	1.09	0.383	0.686	Co-occurrence
RTN2	19q13.32	2 (1.74%)	2 (0.82%)	1.09	0.383	0.686	Co-occurrence
SAA2	11p15.1	2 (1.74%)	2 (0.82%)	1.09	0.383	0.686	Co-occurrence
SAA4	11p15.1	2 (1.74%)	2 (0.82%)	1.09	0.383	0.686	Co-occurrence
SAMD1	19p13.12	2 (1.74%)	2 (0.82%)	1.09	0.383	0.686	Co-occurrence
SAMD4B	19q13.2	2 (1.74%)	2 (0.82%)	1.09	0.383	0.686	Co-occurrence
SAPCD2	9q34.3	2 (1.74%)	2 (0.82%)	1.09	0.383	0.686	Co-occurrence
SARS2	19q13.2	2 (1.74%)	2 (0.82%)	1.09	0.383	0.686	Co-occurrence
SASS6	1p21.2	2 (1.74%)	2 (0.82%)	1.09	0.383	0.686	Co-occurrence
SATB2	2q33.1	2 (1.74%)	2 (0.82%)	1.09	0.383	0.686	Co-occurrence
SAXO2	15q25.2	2 (1.74%)	2 (0.82%)	1.09	0.383	0.686	Co-occurrence
SEC14L5	16p13.3	2 (1.74%)	2 (0.82%)	1.09	0.383	0.686	Co-occurrence
SEC61A2	10p14	2 (1.74%)	2 (0.82%)	1.09	0.383	0.686	Co-occurrence
SEC61G	7p11.2	2 (1.74%)	2 (0.82%)	1.09	0.383	0.686	Co-occurrence

SEH1L	18p11.21	2 (1.74%)	2 (0.82%)	1.09	0.383	0.686	Co-occurrence
SELENOV	19q13.2	2 (1.74%)	2 (0.82%)	1.09	0.383	0.686	Co-occurrence
SEMA3B	3p21.31	2 (1.74%)	2 (0.82%)	1.09	0.383	0.686	Co-occurrence
SEMA3F	3p21.31	2 (1.74%)	2 (0.82%)	1.09	0.383	0.686	Co-occurrence
SEPTIN12	16p13.3	2 (1.74%)	2 (0.82%)	1.09	0.383	0.686	Co-occurrence
SEPTIN7P9	10p11.1	2 (1.74%)	2 (0.82%)	1.09	0.383	0.686	Co-occurrence
SERTAD1	19q13.2	2 (1.74%)	2 (0.82%)	1.09	0.383	0.686	Co-occurrence
SERTAD3	19q13.2	2 (1.74%)	2 (0.82%)	1.09	0.383	0.686	Co-occurrence
SF3B1	2q33.1	2 (1.74%)	2 (0.82%)	1.09	0.383	0.686	Co-occurrence
SFSWAP	12q24.33	2 (1.74%)	2 (0.82%)	1.09	0.383	0.686	Co-occurrence
SFTA1P	10p14	2 (1.74%)	2 (0.82%)	1.09	0.383	0.686	Co-occurrence
SGO2	2q33.1	2 (1.74%)	2 (0.82%)	1.09	0.383	0.686	Co-occurrence
SH3GL3	15q25.2	2 (1.74%)	2 (0.82%)	1.09	0.383	0.686	Co-occurrence
SHKBP1	19q13.2	2 (1.74%)	2 (0.82%)	1.09	0.383	0.686	Co-occurrence
SIGMAR1	9p13.3	2 (1.74%)	2 (0.82%)	1.09	0.383	0.686	Co-occurrence
SIN3A	15q24.2	2 (1.74%)	2 (0.82%)	1.09	0.383	0.686	Co-occurrence
SIN3B	19p13.11	2 (1.74%)	2 (0.82%)	1.09	0.383	0.686	Co-occurrence
SIRT2	19q13.2	2 (1.74%)	2 (0.82%)	1.09	0.383	0.686	Co-occurrence
SIX5	19q13.32	2 (1.74%)	2 (0.82%)	1.09	0.383	0.686	Co-occurrence
SLC1A1	9p24.2	2 (1.74%)	2 (0.82%)	1.09	0.383	0.686	Co-occurrence
SLC25A22	11p15.5	2 (1.74%)	2 (0.82%)	1.09	0.383	0.686	Co-occurrence
SLC25A25	9q34.11	2 (1.74%)	2 (0.82%)	1.09	0.383	0.686	Co-occurrence
SLC25A33	1p36.22	2 (1.74%)	2 (0.82%)	1.09	0.383	0.686	Co-occurrence
SLC28A1	15q25.3	2 (1.74%)	2 (0.82%)	1.09	0.383	0.686	Co-occurrence
SLC30A7	1p21.2	2 (1.74%)	2 (0.82%)	1.09	0.383	0.686	Co-occurrence
SLC34A3	9q34.3	2 (1.74%)	2 (0.82%)	1.09	0.383	0.686	Co-occurrence
SLC35A3	1p21.2	2 (1.74%)	2 (0.82%)	1.09	0.383	0.686	Co-occurrence
SLC35A4	5q31.3	2 (1.74%)	2 (0.82%)	1.09	0.383	0.686	Co-occurrence
SLC38A3	3p21.31	2 (1.74%)	2 (0.82%)	1.09	0.383	0.686	Co-occurrence

SLCO3A1	15q26.1	2 (1.74%)	2 (0.82%)	1.09	0.383	0.686	Co-occurrence
SMARCE1	17q21.2	2 (1.74%)	2 (0.82%)	1.09	0.383	0.686	Co-occurrence
SNHG17	20q11.23	2 (1.74%)	2 (0.82%)	1.09	0.383	0.686	Co-occurrence
SNORA21	17q12	2 (1.74%)	2 (0.82%)	1.09	0.383	0.686	Co-occurrence
SNORA27	13q12.2	2 (1.74%)	2 (0.82%)	1.09	0.383	0.686	Co-occurrence
SNORA4	3q27.3	2 (1.74%)	2 (0.82%)	1.09	0.383	0.686	Co-occurrence
SNORA52	11p15.5	2 (1.74%)	2 (0.82%)	1.09	0.383	0.686	Co-occurrence
SNORA71A	20q11.23	2 (1.74%)	2 (0.82%)	1.09	0.383	0.686	Co-occurrence
SNORA71B	20q11.23	2 (1.74%)	2 (0.82%)	1.09	0.383	0.686	Co-occurrence
SNORA71C	20q11.23	2 (1.74%)	2 (0.82%)	1.09	0.383	0.686	Co-occurrence
SNORA71D	20q11.23	2 (1.74%)	2 (0.82%)	1.09	0.383	0.686	Co-occurrence
SNORA79	14q31.1	2 (1.74%)	2 (0.82%)	1.09	0.383	0.686	Co-occurrence
SNORD124	17q21.1	2 (1.74%)	2 (0.82%)	1.09	0.383	0.686	Co-occurrence
SNORD126	14q11.2	2 (1.74%)	2 (0.82%)	1.09	0.383	0.686	Co-occurrence
SNRPA	19q13.2	2 (1.74%)	2 (0.82%)	1.09	0.383	0.686	Co-occurrence
SNRPD2	19q13.32	2 (1.74%)	2 (0.82%)	1.09	0.383	0.686	Co-occurrence
SOCS1	16p13.13	2 (1.74%)	2 (0.82%)	1.09	0.383	0.686	Co-occurrence
SPATA6L	9p24.2-p24.1	2 (1.74%)	2 (0.82%)	1.09	0.383	0.686	Co-occurrence
SPATS2L	2q33.1	2 (1.74%)	2 (0.82%)	1.09	0.383	0.686	Co-occurrence
SPG21	15q22.31	2 (1.74%)	2 (0.82%)	1.09	0.383	0.686	Co-occurrence
SPINK13	5q32	2 (1.74%)	2 (0.82%)	1.09	0.383	0.686	Co-occurrence
SPPL2C	17q21.31	2 (1.74%)	2 (0.82%)	1.09	0.383	0.686	Co-occurrence
SRA1	5q31.3	2 (1.74%)	2 (0.82%)	1.09	0.383	0.686	Co-occurrence
SRRM2	16p13.3	2 (1.74%)	2 (0.82%)	1.09	0.383	0.686	Co-occurrence
SSNA1	9q34.3	2 (1.74%)	2 (0.82%)	1.09	0.383	0.686	Co-occurrence
ST8SIA2	15q26.1	2 (1.74%)	2 (0.82%)	1.09	0.383	0.686	Co-occurrence
ST8SIA6	10p12.33	2 (1.74%)	2 (0.82%)	1.09	0.383	0.686	Co-occurrence
STAB1	3p21.1	2 (1.74%)	2 (0.82%)	1.09	0.383	0.686	Co-occurrence
STAC2	17q12	2 (1.74%)	2 (0.82%)	1.09	0.383	0.686	Co-occurrence

STAG3L3	7q11.23	2 (1.74%)	2 (0.82%)	1.09	0.383	0.686	Co-occurrence
STAM	10p12.33	2 (1.74%)	2 (0.82%)	1.09	0.383	0.686	Co-occurrence
STARD5	15q25.1	2 (1.74%)	2 (0.82%)	1.09	0.383	0.686	Co-occurrence
STH	17q21.31	2 (1.74%)	2 (0.82%)	1.09	0.383	0.686	Co-occurrence
STK39	2q24.3	2 (1.74%)	2 (0.82%)	1.09	0.383	0.686	Co-occurrence
STUB1	16p13.3	2 (1.74%)	2 (0.82%)	1.09	0.383	0.686	Co-occurrence
SUGCT	7p14.1	2 (1.74%)	2 (0.82%)	1.09	0.383	0.686	Co-occurrence
SUV39H2	10p13	2 (1.74%)	2 (0.82%)	1.09	0.383	0.686	Co-occurrence
SVIL2P	10p11.23	2 (1.74%)	2 (0.82%)	1.09	0.383	0.686	Co-occurrence
SYCP2	20q13.33	2 (1.74%)	2 (0.82%)	1.09	0.383	0.686	Co-occurrence
SYDE1	19p13.12	2 (1.74%)	2 (0.82%)	1.09	0.383	0.686	Co-occurrence
SYMPK	19q13.32	2 (1.74%)	2 (0.82%)	1.09	0.383	0.686	Co-occurrence
SYN2	3p25.2	2 (1.74%)	2 (0.82%)	1.09	0.383	0.686	Co-occurrence
TAF3	10p14	2 (1.74%)	2 (0.82%)	1.09	0.383	0.686	Co-occurrence
TAMM41	3p25.2	2 (1.74%)	2 (0.82%)	1.09	0.383	0.686	Co-occurrence
TBC1D22A	22q13.31	2 (1.74%)	2 (0.82%)	1.09	0.383	0.686	Co-occurrence
TBC1D2B	15q24.3-q25.1	2 (1.74%)	2 (0.82%)	1.09	0.383	0.686	Co-occurrence
TCAP	17q12	2 (1.74%)	2 (0.82%)	1.09	0.383	0.686	Co-occurrence
TCTN1	12q24.11	2 (1.74%)	2 (0.82%)	1.09	0.383	0.686	Co-occurrence
TEP1	14q11.2	2 (1.74%)	2 (0.82%)	1.09	0.383	0.686	Co-occurrence
TFPI	2q32.1	2 (1.74%)	2 (0.82%)	1.09	0.383	0.686	Co-occurrence
THOC6	16p13.3	2 (1.74%)	2 (0.82%)	1.09	0.383	0.686	Co-occurrence
THRA	17q21.1	2 (1.74%)	2 (0.82%)	1.09	0.383	0.686	Co-occurrence
TLE3	15q23	2 (1.74%)	2 (0.82%)	1.09	0.383	0.686	Co-occurrence
TLR10	4p14	2 (1.74%)	2 (0.82%)	1.09	0.383	0.686	Co-occurrence
TM6SF1	15q25.2	2 (1.74%)	2 (0.82%)	1.09	0.383	0.686	Co-occurrence
TMCO6	5q31.3	2 (1.74%)	2 (0.82%)	1.09	0.383	0.686	Co-occurrence
TMEM114	16p13.2	2 (1.74%)	2 (0.82%)	1.09	0.383	0.686	Co-occurrence
TMEM151A	11q13.2	2 (1.74%)	2 (0.82%)	1.09	0.383	0.686	Co-occurrence

TMEM173	5q31.2	2 (1.74%)	2 (0.82%)	1.09	0.383	0.686	Co-occurrence
TMEM186	16p13.2	2 (1.74%)	2 (0.82%)	1.09	0.383	0.686	Co-occurrence
TMEM203	9q34.3	2 (1.74%)	2 (0.82%)	1.09	0.383	0.686	Co-occurrence
TMEM210	9q34.3	2 (1.74%)	2 (0.82%)	1.09	0.383	0.686	Co-occurrence
TMEM247	2p21	2 (1.74%)	2 (0.82%)	1.09	0.383	0.686	Co-occurrence
TMEM266	15q24.2	2 (1.74%)	2 (0.82%)	1.09	0.383	0.686	Co-occurrence
TMEM38A	19p13.11	2 (1.74%)	2 (0.82%)	1.09	0.383	0.686	Co-occurrence
TMEM99	17q21.2	2 (1.74%)	2 (0.82%)	1.09	0.383	0.686	Co-occurrence
TNFRSF12A	16p13.3	2 (1.74%)	2 (0.82%)	1.09	0.383	0.686	Co-occurrence
TNP2	16p13.13	2 (1.74%)	2 (0.82%)	1.09	0.383	0.686	Co-occurrence
TNS4	17q21.2	2 (1.74%)	2 (0.82%)	1.09	0.383	0.686	Co-occurrence
TPRN	9q34.3	2 (1.74%)	2 (0.82%)	1.09	0.383	0.686	Co-occurrence
TRAF2	9q34.3	2 (1.74%)	2 (0.82%)	1.09	0.383	0.686	Co-occurrence
TRAPPC6A	19q13.32	2 (1.74%)	2 (0.82%)	1.09	0.383	0.686	Co-occurrence
TRDMT1	10p13	2 (1.74%)	2 (0.82%)	1.09	0.383	0.686	Co-occurrence
TRIM50	7q11.23	2 (1.74%)	2 (0.82%)	1.09	0.383	0.686	Co-occurrence
TRIM74	7q11.23	2 (1.74%)	2 (0.82%)	1.09	0.383	0.686	Co-occurrence
TRMT13	1p21.2	2 (1.74%)	2 (0.82%)	1.09	0.383	0.686	Co-occurrence
TSG101	11p15.1	2 (1.74%)	2 (0.82%)	1.09	0.383	0.686	Co-occurrence
TTC5	14q11.2	2 (1.74%)	2 (0.82%)	1.09	0.383	0.686	Co-occurrence
TUBB4B	9q34.3	2 (1.74%)	2 (0.82%)	1.09	0.383	0.686	Co-occurrence
UAP1L1	9q34.3	2 (1.74%)	2 (0.82%)	1.09	0.383	0.686	Co-occurrence
UBAP1L	15q22.31	2 (1.74%)	2 (0.82%)	1.09	0.383	0.686	Co-occurrence
UBE2D2	5q31.2	2 (1.74%)	2 (0.82%)	1.09	0.383	0.686	Co-occurrence
UBE2Q2P2	15q25.2	2 (1.74%)	2 (0.82%)	1.09	0.383	0.686	Co-occurrence
UPF2	10p14	2 (1.74%)	2 (0.82%)	1.09	0.383	0.686	Co-occurrence
UPF3AP2	17p11.2	2 (1.74%)	2 (0.82%)	1.09	0.383	0.686	Co-occurrence
USP32P3	17p11.2	2 (1.74%)	2 (0.82%)	1.09	0.383	0.686	Co-occurrence
USP7	16p13.2	2 (1.74%)	2 (0.82%)	1.09	0.383	0.686	Co-occurrence

VASP	19q13.32	2 (1.74%)	2 (0.82%)	1.09	0.383	0.686	Co-occurrence
VAV3	1p13.3	2 (1.74%)	2 (0.82%)	1.09	0.383	0.686	Co-occurrence
VCAM1	1p21.2	2 (1.74%)	2 (0.82%)	1.09	0.383	0.686	Co-occurrence
VGLL4	3p25.3-p25.2	2 (1.74%)	2 (0.82%)	1.09	0.383	0.686	Co-occurrence
VHL	3p25.3	2 (1.74%)	2 (0.82%)	1.09	0.383	0.686	Co-occurrence
VIM	10p13	2 (1.74%)	2 (0.82%)	1.09	0.383	0.686	Co-occurrence
VIPR2	7q36.3	2 (1.74%)	2 (0.82%)	1.09	0.383	0.686	Co-occurrence
VPS13C	15q22.2	2 (1.74%)	2 (0.82%)	1.09	0.383	0.686	Co-occurrence
VSTM2B	19q12	2 (1.74%)	2 (0.82%)	1.09	0.383	0.686	Co-occurrence
WAC	10p12.1|10p12.1-p11.2	2 (1.74%)	2 (0.82%)	1.09	0.383	0.686	Co-occurrence
WDR24	16p13.3	2 (1.74%)	2 (0.82%)	1.09	0.383	0.686	Co-occurrence
WDR55	5q31.3	2 (1.74%)	2 (0.82%)	1.09	0.383	0.686	Co-occurrence
WDR60	7q36.3	2 (1.74%)	2 (0.82%)	1.09	0.383	0.686	Co-occurrence
WHAMM	15q25.2	2 (1.74%)	2 (0.82%)	1.09	0.383	0.686	Co-occurrence
WNT3	17q21.31-q21.32	2 (1.74%)	2 (0.82%)	1.09	0.383	0.686	Co-occurrence
WNT9B	17q21.32	2 (1.74%)	2 (0.82%)	1.09	0.383	0.686	Co-occurrence
YIF1A	11q13.2	2 (1.74%)	2 (0.82%)	1.09	0.383	0.686	Co-occurrence
YKT6	7p13	2 (1.74%)	2 (0.82%)	1.09	0.383	0.686	Co-occurrence
ZBTB47	3p22.1	2 (1.74%)	2 (0.82%)	1.09	0.383	0.686	Co-occurrence
ZC3H15	2q32.1	2 (1.74%)	2 (0.82%)	1.09	0.383	0.686	Co-occurrence
ZCCHC10	5q31.1	2 (1.74%)	2 (0.82%)	1.09	0.383	0.686	Co-occurrence
ZG16B	16p13.3	2 (1.74%)	2 (0.82%)	1.09	0.383	0.686	Co-occurrence
ZNF148	3q21.2	2 (1.74%)	2 (0.82%)	1.09	0.383	0.686	Co-occurrence
ZNF205	16p13.3	2 (1.74%)	2 (0.82%)	1.09	0.383	0.686	Co-occurrence
ZNF213	16p13.3	2 (1.74%)	2 (0.82%)	1.09	0.383	0.686	Co-occurrence
ZNF248	10p11.21	2 (1.74%)	2 (0.82%)	1.09	0.383	0.686	Co-occurrence
ZNF25	10p11.21	2 (1.74%)	2 (0.82%)	1.09	0.383	0.686	Co-occurrence
ZNF280D	15q21.3	2 (1.74%)	2 (0.82%)	1.09	0.383	0.686	Co-occurrence
ZNF333	19p13.12	2 (1.74%)	2 (0.82%)	1.09	0.383	0.686	Co-occurrence

ZNF33A	10p11.1	2 (1.74%)	2 (0.82%)	1.09	0.383	0.686	Co-occurrence
ZNF37A	10p11.1	2 (1.74%)	2 (0.82%)	1.09	0.383	0.686	Co-occurrence
ZNF500	16p13.3	2 (1.74%)	2 (0.82%)	1.09	0.383	0.686	Co-occurrence
ZNF609	15q22.31	2 (1.74%)	2 (0.82%)	1.09	0.383	0.686	Co-occurrence
ZSCAN10	16p13.3	2 (1.74%)	2 (0.82%)	1.09	0.383	0.686	Co-occurrence
ZSWIM2	2q32.1	2 (1.74%)	2 (0.82%)	1.09	0.383	0.686	Co-occurrence
ZSWIM4	19p13.13-p13.12	2 (1.74%)	2 (0.82%)	1.09	0.383	0.686	Co-occurrence
CA8	8q12.1	8 (6.96%)	21 (8.57%)	-0.3	0.383	0.686	Mutual exclusivity
TOX	8q12.1	8 (6.96%)	21 (8.57%)	-0.3	0.383	0.686	Mutual exclusivity
VEGFA	6p21.1	8 (6.96%)	21 (8.57%)	-0.3	0.383	0.686	Mutual exclusivity
C8ORF17	8q24.3	18 (15.65%)	43 (17.55%)	-0.17	0.388	0.686	Mutual exclusivity
COL22A1	8q24.23-q24.3	18 (15.65%)	43 (17.55%)	-0.17	0.388	0.686	Mutual exclusivity
FAM135B	8q24.23	18 (15.65%)	43 (17.55%)	-0.17	0.388	0.686	Mutual exclusivity
KCNK9	8q24.3	18 (15.65%)	43 (17.55%)	-0.17	0.388	0.686	Mutual exclusivity
OPLAH	8q24.3	18 (15.65%)	43 (17.55%)	-0.17	0.388	0.686	Mutual exclusivity
SPATC1	8q24.3	18 (15.65%)	43 (17.55%)	-0.17	0.388	0.686	Mutual exclusivity
TRAPPC9	8q24.3	18 (15.65%)	43 (17.55%)	-0.17	0.388	0.686	Mutual exclusivity
IL7	8q21.13	13 (11.30%)	32 (13.06%)	-0.21	0.388	0.686	Mutual exclusivity
PKIA	8q21.13	13 (11.30%)	32 (13.06%)	-0.21	0.388	0.686	Mutual exclusivity
RN7SKP29	8q13.3	13 (11.30%)	32 (13.06%)	-0.21	0.388	0.686	Mutual exclusivity
SULF1	8q13.2-q13.3	13 (11.30%)	32 (13.06%)	-0.21	0.388	0.686	Mutual exclusivity
ZC2HC1A	8q21.13	13 (11.30%)	32 (13.06%)	-0.21	0.388	0.686	Mutual exclusivity
NKAIN3	8q12.3	9 (7.83%)	23 (9.39%)	-0.26	0.394	0.686	Mutual exclusivity
REXO1L10P	8q21.2	14 (12.17%)	34 (13.88%)	-0.19	0.396	0.686	Mutual exclusivity
REXO1L11P	8q21.2	14 (12.17%)	34 (13.88%)	-0.19	0.396	0.686	Mutual exclusivity
REXO1L1P	8q21.2	14 (12.17%)	34 (13.88%)	-0.19	0.396	0.686	Mutual exclusivity
RN7SL107P	8q21.13	14 (12.17%)	34 (13.88%)	-0.19	0.396	0.686	Mutual exclusivity
ZNF704	8q21.13	14 (12.17%)	34 (13.88%)	-0.19	0.396	0.686	Mutual exclusivity
ABCB4	7q21.12	3 (2.61%)	4 (1.63%)	0.68	0.397	0.686	Co-occurrence

ABI3	17q21.32	3 (2.61%)	4 (1.63%)	0.68	0.397	0.686	Co-occurrence
ACTN3	11q13.2	3 (2.61%)	4 (1.63%)	0.68	0.397	0.686	Co-occurrence
AGPS	2q31.2	3 (2.61%)	4 (1.63%)	0.68	0.397	0.686	Co-occurrence
AKR1C4	10p15.1	3 (2.61%)	4 (1.63%)	0.68	0.397	0.686	Co-occurrence
AKR1C8P	10p15.1	3 (2.61%)	4 (1.63%)	0.68	0.397	0.686	Co-occurrence
ANKRD52	12q13.3	3 (2.61%)	4 (1.63%)	0.68	0.397	0.686	Co-occurrence
ATP13A3	3q29	3 (2.61%)	4 (1.63%)	0.68	0.397	0.686	Co-occurrence
ATP5MC1	17q21.32	3 (2.61%)	4 (1.63%)	0.68	0.397	0.686	Co-occurrence
AZGP1P1	7q22.1	3 (2.61%)	4 (1.63%)	0.68	0.397	0.686	Co-occurrence
B4GALNT2	17q21.32	3 (2.61%)	4 (1.63%)	0.68	0.397	0.686	Co-occurrence
BRCA1	17q21.31	3 (2.61%)	4 (1.63%)	0.68	0.397	0.686	Co-occurrence
C1QL1	17q21.31	3 (2.61%)	4 (1.63%)	0.68	0.397	0.686	Co-occurrence
CALCOCO2	17q21.32	3 (2.61%)	4 (1.63%)	0.68	0.397	0.686	Co-occurrence
CALR3	19p13.11	3 (2.61%)	4 (1.63%)	0.68	0.397	0.686	Co-occurrence
CBLN4	20q13.2	3 (2.61%)	4 (1.63%)	0.68	0.397	0.686	Co-occurrence
CCDC103	17q21.31	3 (2.61%)	4 (1.63%)	0.68	0.397	0.686	Co-occurrence
CD28	2q33.2	3 (2.61%)	4 (1.63%)	0.68	0.397	0.686	Co-occurrence
CDCA7	2q31.1	3 (2.61%)	4 (1.63%)	0.68	0.397	0.686	Co-occurrence
CDHR3	7q22.3	3 (2.61%)	4 (1.63%)	0.68	0.397	0.686	Co-occurrence
CEACAM19	19q13.31	3 (2.61%)	4 (1.63%)	0.68	0.397	0.686	Co-occurrence
CLDN12	7q21.13	3 (2.61%)	4 (1.63%)	0.68	0.397	0.686	Co-occurrence
COQ10A	12q13.3	3 (2.61%)	4 (1.63%)	0.68	0.397	0.686	Co-occurrence
CPN2	3q29	3 (2.61%)	4 (1.63%)	0.68	0.397	0.686	Co-occurrence
CROT	7q21.12	3 (2.61%)	4 (1.63%)	0.68	0.397	0.686	Co-occurrence
CS	12q13.3	3 (2.61%)	4 (1.63%)	0.68	0.397	0.686	Co-occurrence
DLX3	17q21.33	3 (2.61%)	4 (1.63%)	0.68	0.397	0.686	Co-occurrence
DLX4	17q21.33	3 (2.61%)	4 (1.63%)	0.68	0.397	0.686	Co-occurrence
DNAH5	5p15.2	3 (2.61%)	4 (1.63%)	0.68	0.397	0.686	Co-occurrence
EFTUD2	17q21.31	3 (2.61%)	4 (1.63%)	0.68	0.397	0.686	Co-occurrence

EME1	17q21.33	3 (2.61%)	4 (1.63%)	0.68	0.397	0.686	Co-occurrence
EPS15L1	19p13.11	3 (2.61%)	4 (1.63%)	0.68	0.397	0.686	Co-occurrence
FAM187A	17q21.31	3 (2.61%)	4 (1.63%)	0.68	0.397	0.686	Co-occurrence
FAM215A	17q21.31	3 (2.61%)	4 (1.63%)	0.68	0.397	0.686	Co-occurrence
FZD1	7q21.13	3 (2.61%)	4 (1.63%)	0.68	0.397	0.686	Co-occurrence
GFAP	17q21.31	3 (2.61%)	4 (1.63%)	0.68	0.397	0.686	Co-occurrence
GIP	17q21.32	3 (2.61%)	4 (1.63%)	0.68	0.397	0.686	Co-occurrence
GNGT2	17q21.32	3 (2.61%)	4 (1.63%)	0.68	0.397	0.686	Co-occurrence
GOLPH3	5p13.3	3 (2.61%)	4 (1.63%)	0.68	0.397	0.686	Co-occurrence
GP5	3q29	3 (2.61%)	4 (1.63%)	0.68	0.397	0.686	Co-occurrence
GPR179	17q12	3 (2.61%)	4 (1.63%)	0.68	0.397	0.686	Co-occurrence
HES1	3q29	3 (2.61%)	4 (1.63%)	0.68	0.397	0.686	Co-occurrence
HOXB1	17q21.32	3 (2.61%)	4 (1.63%)	0.68	0.397	0.686	Co-occurrence
HOXB13	17q21.32	3 (2.61%)	4 (1.63%)	0.68	0.397	0.686	Co-occurrence
HOXB2	17q21.32	3 (2.61%)	4 (1.63%)	0.68	0.397	0.686	Co-occurrence
HOXB3	17q21.32	3 (2.61%)	4 (1.63%)	0.68	0.397	0.686	Co-occurrence
HOXB4	17q21.32	3 (2.61%)	4 (1.63%)	0.68	0.397	0.686	Co-occurrence
HOXB5	17q21.32	3 (2.61%)	4 (1.63%)	0.68	0.397	0.686	Co-occurrence
HOXB6	17q21.32	3 (2.61%)	4 (1.63%)	0.68	0.397	0.686	Co-occurrence
HOXB7	17q21.32	3 (2.61%)	4 (1.63%)	0.68	0.397	0.686	Co-occurrence
HOXB8	17q21.32	3 (2.61%)	4 (1.63%)	0.68	0.397	0.686	Co-occurrence
HOXB9	17q21.32	3 (2.61%)	4 (1.63%)	0.68	0.397	0.686	Co-occurrence
IGF2BP1	17q21.32	3 (2.61%)	4 (1.63%)	0.68	0.397	0.686	Co-occurrence
IGSF23	19q13.31	3 (2.61%)	4 (1.63%)	0.68	0.397	0.686	Co-occurrence
KIF18B	17q21.31	3 (2.61%)	4 (1.63%)	0.68	0.397	0.686	Co-occurrence
KPNB1	17q21.32	3 (2.61%)	4 (1.63%)	0.68	0.397	0.686	Co-occurrence
LINC00884	3q29	3 (2.61%)	4 (1.63%)	0.68	0.397	0.686	Co-occurrence
LINC00887	3q29	3 (2.61%)	4 (1.63%)	0.68	0.397	0.686	Co-occurrence
LRRC59	17q21.33	3 (2.61%)	4 (1.63%)	0.68	0.397	0.686	Co-occurrence

MC3R	20q13.2	3 (2.61%)	4 (1.63%)	0.68	0.397	0.686	Co-occurrence
MFSD2A	1p34.2	3 (2.61%)	4 (1.63%)	0.68	0.397	0.686	Co-occurrence
MIR-1203/1203		3 (2.61%)	4 (1.63%)	0.68	0.397	0.686	Co-occurrence
MIR-3185/3185		3 (2.61%)	4 (1.63%)	0.68	0.397	0.686	Co-occurrence
MPP2	17q21.31	3 (2.61%)	4 (1.63%)	0.68	0.397	0.686	Co-occurrence
MRPL27	17q21.33	3 (2.61%)	4 (1.63%)	0.68	0.397	0.686	Co-occurrence
MRPL45	17q12	3 (2.61%)	4 (1.63%)	0.68	0.397	0.686	Co-occurrence
MYOM1	18p11.31	3 (2.61%)	4 (1.63%)	0.68	0.397	0.686	Co-occurrence
NGFR	17q21.33	3 (2.61%)	4 (1.63%)	0.68	0.397	0.686	Co-occurrence
NXPH3	17q21.33	3 (2.61%)	4 (1.63%)	0.68	0.397	0.686	Co-occurrence
OR2AE1	7q22.1	3 (2.61%)	4 (1.63%)	0.68	0.397	0.686	Co-occurrence
PFKFB3	10p15.1	3 (2.61%)	4 (1.63%)	0.68	0.397	0.686	Co-occurrence
PHB	17q21.33	3 (2.61%)	4 (1.63%)	0.68	0.397	0.686	Co-occurrence
PHOSPHO1	17q21.32	3 (2.61%)	4 (1.63%)	0.68	0.397	0.686	Co-occurrence
PRAC1	17q21.32	3 (2.61%)	4 (1.63%)	0.68	0.397	0.686	Co-occurrence
PVR	19q13.31	3 (2.61%)	4 (1.63%)	0.68	0.397	0.686	Co-occurrence
RAD50	5q31.1	3 (2.61%)	4 (1.63%)	0.68	0.397	0.686	Co-occurrence
RN7SL125P	17q21.32	3 (2.61%)	4 (1.63%)	0.68	0.397	0.686	Co-occurrence
RN7SL215P	3q29	3 (2.61%)	4 (1.63%)	0.68	0.397	0.686	Co-occurrence
RNA5SP401	15q26.2	3 (2.61%)	4 (1.63%)	0.68	0.397	0.686	Co-occurrence
RNA5SP487	20q13.2	3 (2.61%)	4 (1.63%)	0.68	0.397	0.686	Co-occurrence
RNF114	20q13.13	3 (2.61%)	4 (1.63%)	0.68	0.397	0.686	Co-occurrence
RNF41	12q13.3	3 (2.61%)	4 (1.63%)	0.68	0.397	0.686	Co-occurrence
RNU6ATAC	9q34.2	3 (2.61%)	4 (1.63%)	0.68	0.397	0.686	Co-occurrence
RPL9	4p14	3 (2.61%)	4 (1.63%)	0.68	0.397	0.686	Co-occurrence
SELENOP	5p12	3 (2.61%)	4 (1.63%)	0.68	0.397	0.686	Co-occurrence
SEPTIN8	5q31.1	3 (2.61%)	4 (1.63%)	0.68	0.397	0.686	Co-occurrence
SKAP1	17q21.32	3 (2.61%)	4 (1.63%)	0.68	0.397	0.686	Co-occurrence
SLC39A5	12q13.3	3 (2.61%)	4 (1.63%)	0.68	0.397	0.686	Co-occurrence

SMIM14	4p14	3 (2.61%)	4 (1.63%)	0.68	0.397	0.686	Co-occurrence
SNAI1	20q13.13	3 (2.61%)	4 (1.63%)	0.68	0.397	0.686	Co-occurrence
SNF8	17q21.32	3 (2.61%)	4 (1.63%)	0.68	0.397	0.686	Co-occurrence
SNX11	17q21.32	3 (2.61%)	4 (1.63%)	0.68	0.397	0.686	Co-occurrence
SOCS7	17q12	3 (2.61%)	4 (1.63%)	0.68	0.397	0.686	Co-occurrence
SOWAHA	5q31.1	3 (2.61%)	4 (1.63%)	0.68	0.397	0.686	Co-occurrence
SPOP	17q21.33	3 (2.61%)	4 (1.63%)	0.68	0.397	0.686	Co-occurrence
TBKBP1	17q21.32	3 (2.61%)	4 (1.63%)	0.68	0.397	0.686	Co-occurrence
TBX21	17q21.32	3 (2.61%)	4 (1.63%)	0.68	0.397	0.686	Co-occurrence
TMEM189	20q13.13	3 (2.61%)	4 (1.63%)	0.68	0.397	0.686	Co-occurrence
TRERNA1	20q13.13	3 (2.61%)	4 (1.63%)	0.68	0.397	0.686	Co-occurrence
TRIO	5p15.2	3 (2.61%)	4 (1.63%)	0.68	0.397	0.686	Co-occurrence
TTLL6	17q21.32	3 (2.61%)	4 (1.63%)	0.68	0.397	0.686	Co-occurrence
UBE2Z	17q21.32	3 (2.61%)	4 (1.63%)	0.68	0.397	0.686	Co-occurrence
USP22	17p11.2	3 (2.61%)	4 (1.63%)	0.68	0.397	0.686	Co-occurrence
UTP18	17q21.33	3 (2.61%)	4 (1.63%)	0.68	0.397	0.686	Co-occurrence
WDR5	9q34.2	3 (2.61%)	4 (1.63%)	0.68	0.397	0.686	Co-occurrence
XXYLT1	3q29	3 (2.61%)	4 (1.63%)	0.68	0.397	0.686	Co-occurrence
ZKSCAN1	7q22.1	3 (2.61%)	4 (1.63%)	0.68	0.397	0.686	Co-occurrence
ZNF652	17q21.32-q21.33	3 (2.61%)	4 (1.63%)	0.68	0.397	0.686	Co-occurrence
CRIP3	6p21.1	5 (4.35%)	14 (5.71%)	-0.39	0.397	0.686	Mutual exclusivity
SLC22A7	6p21.1	5 (4.35%)	14 (5.71%)	-0.39	0.397	0.686	Mutual exclusivity
ZNF318	6p21.1	5 (4.35%)	14 (5.71%)	-0.39	0.397	0.686	Mutual exclusivity
CYP3A4	7q22.1	4 (3.48%)	6 (2.45%)	0.51	0.402	0.686	Co-occurrence
DNAJB11	3q27.3	4 (3.48%)	6 (2.45%)	0.51	0.402	0.686	Co-occurrence
EHHADH	3q27.2	4 (3.48%)	6 (2.45%)	0.51	0.402	0.686	Co-occurrence
HRG	3q27.3	4 (3.48%)	6 (2.45%)	0.51	0.402	0.686	Co-occurrence
LINC00954	2p24.1	4 (3.48%)	6 (2.45%)	0.51	0.402	0.686	Co-occurrence
MAP3K13	3q27.2	4 (3.48%)	6 (2.45%)	0.51	0.402	0.686	Co-occurrence

RN7SL197P	20q13.13	4 (3.48%)	6 (2.45%)	0.51	0.402	0.686	Co-occurrence
SP2	17q21.32	4 (3.48%)	6 (2.45%)	0.51	0.402	0.686	Co-occurrence
SPAG9	17q21.33	4 (3.48%)	6 (2.45%)	0.51	0.402	0.686	Co-occurrence
TTC32	2p24.1	4 (3.48%)	6 (2.45%)	0.51	0.402	0.686	Co-occurrence
WWTR1	3q25.1	4 (3.48%)	6 (2.45%)	0.51	0.402	0.686	Co-occurrence
C6ORF223	6p21.1	8 (6.96%)	14 (5.71%)	0.28	0.403	0.686	Co-occurrence
BRD2	6p21.32	5 (4.35%)	8 (3.27%)	0.41	0.404	0.686	Co-occurrence
CYS1	2p25.1	5 (4.35%)	8 (3.27%)	0.41	0.404	0.686	Co-occurrence
GRHL1	2p25.1	5 (4.35%)	8 (3.27%)	0.41	0.404	0.686	Co-occurrence
KLF11	2p25.1	5 (4.35%)	8 (3.27%)	0.41	0.404	0.686	Co-occurrence
PLD1	3q26.31	5 (4.35%)	8 (3.27%)	0.41	0.404	0.686	Co-occurrence
TBC1D22B	6p21.2	5 (4.35%)	8 (3.27%)	0.41	0.404	0.686	Co-occurrence
AARS2	6p21.1	6 (5.22%)	10 (4.08%)	0.35	0.405	0.686	Co-occurrence
ABCF1	6p21.33	6 (5.22%)	10 (4.08%)	0.35	0.405	0.686	Co-occurrence
ABHD16A	6p21.33	6 (5.22%)	10 (4.08%)	0.35	0.405	0.686	Co-occurrence
AGER	6p21.32	6 (5.22%)	10 (4.08%)	0.35	0.405	0.686	Co-occurrence
AGPAT1	6p21.32	6 (5.22%)	10 (4.08%)	0.35	0.405	0.686	Co-occurrence
AIF1	6p21.33	6 (5.22%)	10 (4.08%)	0.35	0.405	0.686	Co-occurrence
APOM	6p21.33	6 (5.22%)	10 (4.08%)	0.35	0.405	0.686	Co-occurrence
ATAT1	6p21.33	6 (5.22%)	10 (4.08%)	0.35	0.405	0.686	Co-occurrence
ATF6B	6p21.32	6 (5.22%)	10 (4.08%)	0.35	0.405	0.686	Co-occurrence
ATP6V1G2	6p21.33	6 (5.22%)	10 (4.08%)	0.35	0.405	0.686	Co-occurrence
BAG6	6p21.33	6 (5.22%)	10 (4.08%)	0.35	0.405	0.686	Co-occurrence
BTNL2	6p21.32	6 (5.22%)	10 (4.08%)	0.35	0.405	0.686	Co-occurrence
C2	6p21.33	6 (5.22%)	10 (4.08%)	0.35	0.405	0.686	Co-occurrence
C4A	6p21.33	6 (5.22%)	10 (4.08%)	0.35	0.405	0.686	Co-occurrence
C4B	6p21.33	6 (5.22%)	10 (4.08%)	0.35	0.405	0.686	Co-occurrence
C6ORF136	6p21.33	6 (5.22%)	10 (4.08%)	0.35	0.405	0.686	Co-occurrence
C6ORF47	6p21.33	6 (5.22%)	10 (4.08%)	0.35	0.405	0.686	Co-occurrence

CFB	6p21.33	6 (5.22%)	10 (4.08%)	0.35	0.405	0.686	Co-occurrence
CLIC1	6p21.33	6 (5.22%)	10 (4.08%)	0.35	0.405	0.686	Co-occurrence
CSNK2B	6p21.33	6 (5.22%)	10 (4.08%)	0.35	0.405	0.686	Co-occurrence
CYP21A1P	6p21.33	6 (5.22%)	10 (4.08%)	0.35	0.405	0.686	Co-occurrence
CYP21A2	6p21.33	6 (5.22%)	10 (4.08%)	0.35	0.405	0.686	Co-occurrence
DDAH2	6p21.33	6 (5.22%)	10 (4.08%)	0.35	0.405	0.686	Co-occurrence
DDR1	6p21.33	6 (5.22%)	10 (4.08%)	0.35	0.405	0.686	Co-occurrence
DDX39B	6p21.33	6 (5.22%)	10 (4.08%)	0.35	0.405	0.686	Co-occurrence
DHX16	6p21.33	6 (5.22%)	10 (4.08%)	0.35	0.405	0.686	Co-occurrence
DXO	6p21.33	6 (5.22%)	10 (4.08%)	0.35	0.405	0.686	Co-occurrence
EGFL8	6p21.32	6 (5.22%)	10 (4.08%)	0.35	0.405	0.686	Co-occurrence
EHMT2	6p21.33	6 (5.22%)	10 (4.08%)	0.35	0.405	0.686	Co-occurrence
FKBPL	6p21.32	6 (5.22%)	10 (4.08%)	0.35	0.405	0.686	Co-occurrence
FLOT1	6p21.33	6 (5.22%)	10 (4.08%)	0.35	0.405	0.686	Co-occurrence
GFOD1	6p23	6 (5.22%)	10 (4.08%)	0.35	0.405	0.686	Co-occurrence
GPANK1	6p21.33	6 (5.22%)	10 (4.08%)	0.35	0.405	0.686	Co-occurrence
GPSM3	6p21.32	6 (5.22%)	10 (4.08%)	0.35	0.405	0.686	Co-occurrence
GTF2H4	6p21.33	6 (5.22%)	10 (4.08%)	0.35	0.405	0.686	Co-occurrence
HCG20	6p21.33	6 (5.22%)	10 (4.08%)	0.35	0.405	0.686	Co-occurrence
HCG23	6p21.32	6 (5.22%)	10 (4.08%)	0.35	0.405	0.686	Co-occurrence
HCG27	6p21.33	6 (5.22%)	10 (4.08%)	0.35	0.405	0.686	Co-occurrence
HCP5	6p21.33	6 (5.22%)	10 (4.08%)	0.35	0.405	0.686	Co-occurrence
HSP90AB1	6p21.1	6 (5.22%)	10 (4.08%)	0.35	0.405	0.686	Co-occurrence
HSPA1A	6p21.33	6 (5.22%)	10 (4.08%)	0.35	0.405	0.686	Co-occurrence
HSPA1B	6p21.33	6 (5.22%)	10 (4.08%)	0.35	0.405	0.686	Co-occurrence
HSPA1L	6p21.33	6 (5.22%)	10 (4.08%)	0.35	0.405	0.686	Co-occurrence
IER3	6p21.33	6 (5.22%)	10 (4.08%)	0.35	0.405	0.686	Co-occurrence
LINC00243	6p21.33	6 (5.22%)	10 (4.08%)	0.35	0.405	0.686	Co-occurrence
LSM2	6p21.33	6 (5.22%)	10 (4.08%)	0.35	0.405	0.686	Co-occurrence

LST1	6p21.33	6 (5.22%)	10 (4.08%)	0.35	0.405	0.686	Co-occurrence
LTA	6p21.33	6 (5.22%)	10 (4.08%)	0.35	0.405	0.686	Co-occurrence
LTB	6p21.33	6 (5.22%)	10 (4.08%)	0.35	0.405	0.686	Co-occurrence
LY6G5B	6p21.33	6 (5.22%)	10 (4.08%)	0.35	0.405	0.686	Co-occurrence
LY6G5C	6p21.33	6 (5.22%)	10 (4.08%)	0.35	0.405	0.686	Co-occurrence
LY6G6C	6p21.33	6 (5.22%)	10 (4.08%)	0.35	0.405	0.686	Co-occurrence
LY6G6D	6p21.33	6 (5.22%)	10 (4.08%)	0.35	0.405	0.686	Co-occurrence
LY6G6E	6p21.33	6 (5.22%)	10 (4.08%)	0.35	0.405	0.686	Co-occurrence
LY6G6F	6p21.33	6 (5.22%)	10 (4.08%)	0.35	0.405	0.686	Co-occurrence
MCCD1	6p21.33	6 (5.22%)	10 (4.08%)	0.35	0.405	0.686	Co-occurrence
MCUR1	6p23	6 (5.22%)	10 (4.08%)	0.35	0.405	0.686	Co-occurrence
MDC1	6p21.33	6 (5.22%)	10 (4.08%)	0.35	0.405	0.686	Co-occurrence
MICA	6p21.33	6 (5.22%)	10 (4.08%)	0.35	0.405	0.686	Co-occurrence
MICB	6p21.33	6 (5.22%)	10 (4.08%)	0.35	0.405	0.686	Co-occurrence
MIR-3135B/3135B		6 (5.22%)	10 (4.08%)	0.35	0.405	0.686	Co-occurrence
MIR-4642/4642		6 (5.22%)	10 (4.08%)	0.35	0.405	0.686	Co-occurrence
MIR-4647/4647		6 (5.22%)	10 (4.08%)	0.35	0.405	0.686	Co-occurrence
MPIG6B	6p21.33	6 (5.22%)	10 (4.08%)	0.35	0.405	0.686	Co-occurrence
MRPS18B	6p21.33	6 (5.22%)	10 (4.08%)	0.35	0.405	0.686	Co-occurrence
MSH5	6p21.33	6 (5.22%)	10 (4.08%)	0.35	0.405	0.686	Co-occurrence
NCR3	6p21.33	6 (5.22%)	10 (4.08%)	0.35	0.405	0.686	Co-occurrence
NELFE	6p21.33	6 (5.22%)	10 (4.08%)	0.35	0.405	0.686	Co-occurrence
NEU1	6p21.33	6 (5.22%)	10 (4.08%)	0.35	0.405	0.686	Co-occurrence
NFKBIE	6p21.1	6 (5.22%)	10 (4.08%)	0.35	0.405	0.686	Co-occurrence
NFKBIL1	6p21.33	6 (5.22%)	10 (4.08%)	0.35	0.405	0.686	Co-occurrence
NOL7	6p23	6 (5.22%)	10 (4.08%)	0.35	0.405	0.686	Co-occurrence
NOTCH4	6p21.32	6 (5.22%)	10 (4.08%)	0.35	0.405	0.686	Co-occurrence
PBX2	6p21.32	6 (5.22%)	10 (4.08%)	0.35	0.405	0.686	Co-occurrence
POU5F1	6p21.33	6 (5.22%)	10 (4.08%)	0.35	0.405	0.686	Co-occurrence

PPP1R10	6p21.33	6 (5.22%)	10 (4.08%)	0.35	0.405	0.686	Co-occurrence
PPP1R2P1	6p21.32	6 (5.22%)	10 (4.08%)	0.35	0.405	0.686	Co-occurrence
PPT2	6p21.32	6 (5.22%)	10 (4.08%)	0.35	0.405	0.686	Co-occurrence
PRRC2A	6p21.33	6 (5.22%)	10 (4.08%)	0.35	0.405	0.686	Co-occurrence
PRRT1	6p21.32	6 (5.22%)	10 (4.08%)	0.35	0.405	0.686	Co-occurrence
PSMB8	6p21.32	6 (5.22%)	10 (4.08%)	0.35	0.405	0.686	Co-occurrence
PSMB9	6p21.32	6 (5.22%)	10 (4.08%)	0.35	0.405	0.686	Co-occurrence
PSORS1C3	6p21.33	6 (5.22%)	10 (4.08%)	0.35	0.405	0.686	Co-occurrence
RN7SKP186	6p21.33	6 (5.22%)	10 (4.08%)	0.35	0.405	0.686	Co-occurrence
RN7SL353P	6p21.33	6 (5.22%)	10 (4.08%)	0.35	0.405	0.686	Co-occurrence
RNA5SP206	6p21.33	6 (5.22%)	10 (4.08%)	0.35	0.405	0.686	Co-occurrence
RNF144B	6p22.3	6 (5.22%)	10 (4.08%)	0.35	0.405	0.686	Co-occurrence
RNF39	6p22.1	6 (5.22%)	10 (4.08%)	0.35	0.405	0.686	Co-occurrence
RNF5	6p21.32	6 (5.22%)	10 (4.08%)	0.35	0.405	0.686	Co-occurrence
SAPCD1	6p21.33	6 (5.22%)	10 (4.08%)	0.35	0.405	0.686	Co-occurrence
SIRT5	6p23	6 (5.22%)	10 (4.08%)	0.35	0.405	0.686	Co-occurrence
SKIV2L	6p21.33	6 (5.22%)	10 (4.08%)	0.35	0.405	0.686	Co-occurrence
SLC35B2	6p21.1	6 (5.22%)	10 (4.08%)	0.35	0.405	0.686	Co-occurrence
SLC44A4	6p21.33	6 (5.22%)	10 (4.08%)	0.35	0.405	0.686	Co-occurrence
SNHG32	6p21.33	6 (5.22%)	10 (4.08%)	0.35	0.405	0.686	Co-occurrence
SNORA38	6p21.33	6 (5.22%)	10 (4.08%)	0.35	0.405	0.686	Co-occurrence
SNORD117	6p21.33	6 (5.22%)	10 (4.08%)	0.35	0.405	0.686	Co-occurrence
SNORD48	6p21.33	6 (5.22%)	10 (4.08%)	0.35	0.405	0.686	Co-occurrence
SNORD52	6p21.33	6 (5.22%)	10 (4.08%)	0.35	0.405	0.686	Co-occurrence
SNORD84	6p21.33	6 (5.22%)	10 (4.08%)	0.35	0.405	0.686	Co-occurrence
STK19	6p21.33	6 (5.22%)	10 (4.08%)	0.35	0.405	0.686	Co-occurrence
TAP1	6p21.32	6 (5.22%)	10 (4.08%)	0.35	0.405	0.686	Co-occurrence
TAP2	6p21.32	6 (5.22%)	10 (4.08%)	0.35	0.405	0.686	Co-occurrence
TCTE1	6p21.1	6 (5.22%)	10 (4.08%)	0.35	0.405	0.686	Co-occurrence

TNF	6p21.33	6 (5.22%)	10 (4.08%)	0.35	0.405	0.686	Co-occurrence
TNXB	6p21.33-p21.32	6 (5.22%)	10 (4.08%)	0.35	0.405	0.686	Co-occurrence
TRIM31	6p22.1	6 (5.22%)	10 (4.08%)	0.35	0.405	0.686	Co-occurrence
TSBP1	6p21.32	6 (5.22%)	10 (4.08%)	0.35	0.405	0.686	Co-occurrence
TUBB	6p21.33	6 (5.22%)	10 (4.08%)	0.35	0.405	0.686	Co-occurrence
VARS	6p21.33	6 (5.22%)	10 (4.08%)	0.35	0.405	0.686	Co-occurrence
VARS2	6p21.33	6 (5.22%)	10 (4.08%)	0.35	0.405	0.686	Co-occurrence
VWA7	6p21.33	6 (5.22%)	10 (4.08%)	0.35	0.405	0.686	Co-occurrence
ZBTB12	6p21.33	6 (5.22%)	10 (4.08%)	0.35	0.405	0.686	Co-occurrence
PREX2	8q13.2	10 (8.70%)	25 (10.20%)	-0.23	0.405	0.686	Mutual exclusivity
ABCC5	3q27.1	2 (1.74%)	7 (2.86%)	-0.72	0.41	0.686	Mutual exclusivity
ANK1	8p11.21	2 (1.74%)	7 (2.86%)	-0.72	0.41	0.686	Mutual exclusivity
AP2M1	3q27.1	2 (1.74%)	7 (2.86%)	-0.72	0.41	0.686	Mutual exclusivity
CPED1	7q31.31	2 (1.74%)	7 (2.86%)	-0.72	0.41	0.686	Mutual exclusivity
CPHL1P	3q25.1	2 (1.74%)	7 (2.86%)	-0.72	0.41	0.686	Mutual exclusivity
CTTN	11q13.3	2 (1.74%)	7 (2.86%)	-0.72	0.41	0.686	Mutual exclusivity
CYP2AB1P	3q27.1	2 (1.74%)	7 (2.86%)	-0.72	0.41	0.686	Mutual exclusivity
DNAJC5G	2p23.3	2 (1.74%)	7 (2.86%)	-0.72	0.41	0.686	Mutual exclusivity
DVL3	3q27.1	2 (1.74%)	7 (2.86%)	-0.72	0.41	0.686	Mutual exclusivity
EIF2B5	3q27.1	2 (1.74%)	7 (2.86%)	-0.72	0.41	0.686	Mutual exclusivity
GDF15	19p13.11	2 (1.74%)	7 (2.86%)	-0.72	0.41	0.686	Mutual exclusivity
HTR3C	3q27.1	2 (1.74%)	7 (2.86%)	-0.72	0.41	0.686	Mutual exclusivity
HTR3D	3q27.1	2 (1.74%)	7 (2.86%)	-0.72	0.41	0.686	Mutual exclusivity
HTR3E	3q27.1	2 (1.74%)	7 (2.86%)	-0.72	0.41	0.686	Mutual exclusivity
ING3	7q31.31	2 (1.74%)	7 (2.86%)	-0.72	0.41	0.686	Mutual exclusivity
KAT6A	8p11.21	2 (1.74%)	7 (2.86%)	-0.72	0.41	0.686	Mutual exclusivity
KLHL24	3q27.1	2 (1.74%)	7 (2.86%)	-0.72	0.41	0.686	Mutual exclusivity
KLHL29	2p24.1	2 (1.74%)	7 (2.86%)	-0.72	0.41	0.686	Mutual exclusivity
KLHL6	3q27.1	2 (1.74%)	7 (2.86%)	-0.72	0.41	0.686	Mutual exclusivity

LGSN	6q12	2 (1.74%)	7 (2.86%)	-0.72	0.41	0.686	Mutual exclusivity
LINC00888	3q27.1	2 (1.74%)	7 (2.86%)	-0.72	0.41	0.686	Mutual exclusivity
LRRC25	19p13.11	2 (1.74%)	7 (2.86%)	-0.72	0.41	0.686	Mutual exclusivity
MAP6D1	3q27.1	2 (1.74%)	7 (2.86%)	-0.72	0.41	0.686	Mutual exclusivity
MCF2L2	3q27.1	2 (1.74%)	7 (2.86%)	-0.72	0.41	0.686	Mutual exclusivity
MIR-4448/4448		2 (1.74%)	7 (2.86%)	-0.72	0.41	0.686	Mutual exclusivity
MPV17	2p23.3	2 (1.74%)	7 (2.86%)	-0.72	0.41	0.686	Mutual exclusivity
NRCAM	7q31.1	2 (1.74%)	7 (2.86%)	-0.72	0.41	0.686	Mutual exclusivity
OSR1	2p24.1	2 (1.74%)	7 (2.86%)	-0.72	0.41	0.686	Mutual exclusivity
PARL	3q27.1	2 (1.74%)	7 (2.86%)	-0.72	0.41	0.686	Mutual exclusivity
PC	11q13.2	2 (1.74%)	7 (2.86%)	-0.72	0.41	0.686	Mutual exclusivity
RNA5SP240	7q31.31	2 (1.74%)	7 (2.86%)	-0.72	0.41	0.686	Mutual exclusivity
RNA5SP241	7q31.31	2 (1.74%)	7 (2.86%)	-0.72	0.41	0.686	Mutual exclusivity
SNORA81	3q27.3	2 (1.74%)	7 (2.86%)	-0.72	0.41	0.686	Mutual exclusivity
ST6GAL1	3q27.3	2 (1.74%)	7 (2.86%)	-0.72	0.41	0.686	Mutual exclusivity
TRIM54	2p23.3	2 (1.74%)	7 (2.86%)	-0.72	0.41	0.686	Mutual exclusivity
TSPAN12	7q31.31	2 (1.74%)	7 (2.86%)	-0.72	0.41	0.686	Mutual exclusivity
UCN	2p23.3	2 (1.74%)	7 (2.86%)	-0.72	0.41	0.686	Mutual exclusivity
YEATS2	3q27.1	2 (1.74%)	7 (2.86%)	-0.72	0.41	0.686	Mutual exclusivity
ATP6V1H	8q11.23	7 (6.09%)	18 (7.35%)	-0.27	0.424	0.686	Mutual exclusivity
MRPS18A	6p21.1	7 (6.09%)	18 (7.35%)	-0.27	0.424	0.686	Mutual exclusivity
PCMTD1	8q11.23	7 (6.09%)	18 (7.35%)	-0.27	0.424	0.686	Mutual exclusivity
PXDNL	8q11.22-q11.23	7 (6.09%)	18 (7.35%)	-0.27	0.424	0.686	Mutual exclusivity
RN7SL250P	8q11.23	7 (6.09%)	18 (7.35%)	-0.27	0.424	0.686	Mutual exclusivity
RNU105C	8q11.23	7 (6.09%)	18 (7.35%)	-0.27	0.424	0.686	Mutual exclusivity
RP1	8q11.23-q12.1	7 (6.09%)	18 (7.35%)	-0.27	0.424	0.686	Mutual exclusivity
RSPH9	6p21.1	7 (6.09%)	18 (7.35%)	-0.27	0.424	0.686	Mutual exclusivity
SOX17	8q11.23	7 (6.09%)	18 (7.35%)	-0.27	0.424	0.686	Mutual exclusivity
ST18	8q11.23	7 (6.09%)	18 (7.35%)	-0.27	0.424	0.686	Mutual exclusivity

XKR4	8q12.1	7 (6.09%)	18 (7.35%)	-0.27	0.424	0.686	Mutual exclusivity
ADGRB1	8q24.3	18 (15.65%)	42 (17.14%)	-0.13	0.425	0.686	Mutual exclusivity
CYC1	8q24.3	18 (15.65%)	42 (17.14%)	-0.13	0.425	0.686	Mutual exclusivity
EXOSC4	8q24.3	18 (15.65%)	42 (17.14%)	-0.13	0.425	0.686	Mutual exclusivity
GPAA1	8q24.3	18 (15.65%)	42 (17.14%)	-0.13	0.425	0.686	Mutual exclusivity
HGH1	8q24.3	18 (15.65%)	42 (17.14%)	-0.13	0.425	0.686	Mutual exclusivity
LY6D	8q24.3	18 (15.65%)	42 (17.14%)	-0.13	0.425	0.686	Mutual exclusivity
LY6K	8q24.3	18 (15.65%)	42 (17.14%)	-0.13	0.425	0.686	Mutual exclusivity
LYNX1	8q24.3	18 (15.65%)	42 (17.14%)	-0.13	0.425	0.686	Mutual exclusivity
LYPD2	8q24.3	18 (15.65%)	42 (17.14%)	-0.13	0.425	0.686	Mutual exclusivity
MAF1	8q24.3	18 (15.65%)	42 (17.14%)	-0.13	0.425	0.686	Mutual exclusivity
MROH1	8q24.3	18 (15.65%)	42 (17.14%)	-0.13	0.425	0.686	Mutual exclusivity
PSCA	8q24.3	18 (15.65%)	42 (17.14%)	-0.13	0.425	0.686	Mutual exclusivity
SHARPIN	8q24.3	18 (15.65%)	42 (17.14%)	-0.13	0.425	0.686	Mutual exclusivity
SLURP1	8q24.3	18 (15.65%)	42 (17.14%)	-0.13	0.425	0.686	Mutual exclusivity
THEM6	8q24.3	18 (15.65%)	42 (17.14%)	-0.13	0.425	0.686	Mutual exclusivity
WDR97	8q24.3	18 (15.65%)	42 (17.14%)	-0.13	0.425	0.686	Mutual exclusivity
ANKRD66	6p12.3	3 (2.61%)	9 (3.67%)	-0.49	0.431	0.686	Mutual exclusivity
CAPZA2	7q31.2	3 (2.61%)	9 (3.67%)	-0.49	0.431	0.686	Mutual exclusivity
CAV1	7q31.2	3 (2.61%)	9 (3.67%)	-0.49	0.431	0.686	Mutual exclusivity
CRISP1	6p12.3	3 (2.61%)	9 (3.67%)	-0.49	0.431	0.686	Mutual exclusivity
CYP39A1	6p12.3	3 (2.61%)	9 (3.67%)	-0.49	0.431	0.686	Mutual exclusivity
DEFB114	6p12.3	3 (2.61%)	9 (3.67%)	-0.49	0.431	0.686	Mutual exclusivity
DEFB133	6p12.3	3 (2.61%)	9 (3.67%)	-0.49	0.431	0.686	Mutual exclusivity
EFHC1	6p12.2	3 (2.61%)	9 (3.67%)	-0.49	0.431	0.686	Mutual exclusivity
ENPP5	6p21.1	3 (2.61%)	9 (3.67%)	-0.49	0.431	0.686	Mutual exclusivity
GSTA1	6p12.2	3 (2.61%)	9 (3.67%)	-0.49	0.431	0.686	Mutual exclusivity
GSTA2	6p12.2	3 (2.61%)	9 (3.67%)	-0.49	0.431	0.686	Mutual exclusivity
GSTA3	6p12.2	3 (2.61%)	9 (3.67%)	-0.49	0.431	0.686	Mutual exclusivity

MED20	6p21.1	3 (2.61%)	9 (3.67%)	-0.49	0.431	0.686	Mutual exclusivity
MEP1A	6p12.3	3 (2.61%)	9 (3.67%)	-0.49	0.431	0.686	Mutual exclusivity
MET	7q31.2	3 (2.61%)	9 (3.67%)	-0.49	0.431	0.686	Mutual exclusivity
OARD1	6p21.1	3 (2.61%)	9 (3.67%)	-0.49	0.431	0.686	Mutual exclusivity
PGK2	6p12.3	3 (2.61%)	9 (3.67%)	-0.49	0.431	0.686	Mutual exclusivity
RN7SL285P	6p21.2	3 (2.61%)	9 (3.67%)	-0.49	0.431	0.686	Mutual exclusivity
RNA5SP239	7q31.2	3 (2.61%)	9 (3.67%)	-0.49	0.431	0.686	Mutual exclusivity
SLC25A27	6p12.3	3 (2.61%)	9 (3.67%)	-0.49	0.431	0.686	Mutual exclusivity
ST7	7q31.2	3 (2.61%)	9 (3.67%)	-0.49	0.431	0.686	Mutual exclusivity
TDRD6	6p12.3	3 (2.61%)	9 (3.67%)	-0.49	0.431	0.686	Mutual exclusivity
TMEM14A	6p12.2	3 (2.61%)	9 (3.67%)	-0.49	0.431	0.686	Mutual exclusivity
TRAM2	6p12.2	3 (2.61%)	9 (3.67%)	-0.49	0.431	0.686	Mutual exclusivity
TSPO2	6p21.1	3 (2.61%)	9 (3.67%)	-0.49	0.431	0.686	Mutual exclusivity
UNC5CL	6p21.1	3 (2.61%)	9 (3.67%)	-0.49	0.431	0.686	Mutual exclusivity
USP49	6p21.1	3 (2.61%)	9 (3.67%)	-0.49	0.431	0.686	Mutual exclusivity
SDCBP	8q12.1	9 (7.83%)	22 (8.98%)	-0.2	0.444	0.686	Mutual exclusivity
LINC01556	6p22.1	4 (3.48%)	11 (4.49%)	-0.37	0.447	0.686	Mutual exclusivity
MIR-586/586		4 (3.48%)	11 (4.49%)	-0.37	0.447	0.686	Mutual exclusivity
OR14J1	6p22.1	4 (3.48%)	11 (4.49%)	-0.37	0.447	0.686	Mutual exclusivity
OR2J1	6p22.1	4 (3.48%)	11 (4.49%)	-0.37	0.447	0.686	Mutual exclusivity
OR2J2	6p22.1	4 (3.48%)	11 (4.49%)	-0.37	0.447	0.686	Mutual exclusivity
OR2J3	6p22.1	4 (3.48%)	11 (4.49%)	-0.37	0.447	0.686	Mutual exclusivity
OR5V1	6p22.1	4 (3.48%)	11 (4.49%)	-0.37	0.447	0.686	Mutual exclusivity
PRPH2	6p21.1	4 (3.48%)	11 (4.49%)	-0.37	0.447	0.686	Mutual exclusivity
RN7SL471P	6p22.1	4 (3.48%)	11 (4.49%)	-0.37	0.447	0.686	Mutual exclusivity
SLC17A3	6p22.2	4 (3.48%)	11 (4.49%)	-0.37	0.447	0.686	Mutual exclusivity
UBR2	6p21.1	4 (3.48%)	11 (4.49%)	-0.37	0.447	0.686	Mutual exclusivity
ZSCAN12	6p22.1	4 (3.48%)	11 (4.49%)	-0.37	0.447	0.686	Mutual exclusivity
CPA6	8q13.2	10 (8.70%)	24 (9.80%)	-0.17	0.452	0.686	Mutual exclusivity

C8ORF34	8q13.2	11 (9.57%)	26 (10.61%)	-0.15	0.46	0.686	Mutual exclusivity
ABCC10	6p21.1	5 (4.35%)	13 (5.31%)	-0.29	0.46	0.686	Mutual exclusivity
DLK2	6p21.1	5 (4.35%)	13 (5.31%)	-0.29	0.46	0.686	Mutual exclusivity
DNPH1	6p21.1	5 (4.35%)	13 (5.31%)	-0.29	0.46	0.686	Mutual exclusivity
CEBPD	8q11.21	8 (6.96%)	15 (6.12%)	0.18	0.462	0.686	Co-occurrence
EFCAB1	8q11.21	8 (6.96%)	15 (6.12%)	0.18	0.462	0.686	Co-occurrence
MCM4	8q11.21	8 (6.96%)	15 (6.12%)	0.18	0.462	0.686	Co-occurrence
PPDPFL	8q11.21	8 (6.96%)	15 (6.12%)	0.18	0.462	0.686	Co-occurrence
PRKDC	8q11.21	8 (6.96%)	15 (6.12%)	0.18	0.462	0.686	Co-occurrence
RN7SKP294	8q11.21	8 (6.96%)	15 (6.12%)	0.18	0.462	0.686	Co-occurrence
SNAI2	8q11.21	8 (6.96%)	15 (6.12%)	0.18	0.462	0.686	Co-occurrence
UBE2V2	8q11.21	8 (6.96%)	15 (6.12%)	0.18	0.462	0.686	Co-occurrence
ABCA11P	4p16.3	0 (0.00%)	2 (0.82%)	<-10	0.463	0.686	Mutual exclusivity
ABCA12	2q35	0 (0.00%)	2 (0.82%)	<-10	0.463	0.686	Mutual exclusivity
ABCB5	7p21.1	0 (0.00%)	2 (0.82%)	<-10	0.463	0.686	Mutual exclusivity
ABCG2	4q22.1	0 (0.00%)	2 (0.82%)	<-10	0.463	0.686	Mutual exclusivity
ABHD12B	14q22.1	0 (0.00%)	2 (0.82%)	<-10	0.463	0.686	Mutual exclusivity
ABI3BP	3q12.2	0 (0.00%)	2 (0.82%)	<-10	0.463	0.686	Mutual exclusivity
ABLIM2	4p16.1	0 (0.00%)	2 (0.82%)	<-10	0.463	0.686	Mutual exclusivity
ACAD11	3q22.1	0 (0.00%)	2 (0.82%)	<-10	0.463	0.686	Mutual exclusivity
ACAD9	3q21.3	0 (0.00%)	2 (0.82%)	<-10	0.463	0.686	Mutual exclusivity
ACADL	2q34	0 (0.00%)	2 (0.82%)	<-10	0.463	0.686	Mutual exclusivity
ACKR4	3q22.1	0 (0.00%)	2 (0.82%)	<-10	0.463	0.686	Mutual exclusivity
ACOX3	4p16.1	0 (0.00%)	2 (0.82%)	<-10	0.463	0.686	Mutual exclusivity
ACP5	19p13.2	0 (0.00%)	2 (0.82%)	<-10	0.463	0.686	Mutual exclusivity
ACSL1	4q35.1	0 (0.00%)	2 (0.82%)	<-10	0.463	0.686	Mutual exclusivity
ACTRT1	Xq25	0 (0.00%)	2 (0.82%)	<-10	0.463	0.686	Mutual exclusivity
ADAM23	2q33.3	0 (0.00%)	2 (0.82%)	<-10	0.463	0.686	Mutual exclusivity
ADD1	4p16.3	0 (0.00%)	2 (0.82%)	<-10	0.463	0.686	Mutual exclusivity

ADGRG3	16q21	0 (0.00%)	2 (0.82%)	<-10	0.463	0.686	Mutual exclusivity
ADGRG4	Xq26.3	0 (0.00%)	2 (0.82%)	<-10	0.463	0.686	Mutual exclusivity
ADGRG5	16q21	0 (0.00%)	2 (0.82%)	<-10	0.463	0.686	Mutual exclusivity
ADGRG6	6q24.2	0 (0.00%)	2 (0.82%)	<-10	0.463	0.686	Mutual exclusivity
ADRA2C	4p16.3	0 (0.00%)	2 (0.82%)	<-10	0.463	0.686	Mutual exclusivity
AFAP1	4p16.1	0 (0.00%)	2 (0.82%)	<-10	0.463	0.686	Mutual exclusivity
AFDN	6q27	0 (0.00%)	2 (0.82%)	<-10	0.463	0.686	Mutual exclusivity
AFG1L	6q21	0 (0.00%)	2 (0.82%)	<-10	0.463	0.686	Mutual exclusivity
AFTPH	2p14	0 (0.00%)	2 (0.82%)	<-10	0.463	0.686	Mutual exclusivity
AGAP1	2q37.2	0 (0.00%)	2 (0.82%)	<-10	0.463	0.686	Mutual exclusivity
AGAP10P	10q11.22	0 (0.00%)	2 (0.82%)	<-10	0.463	0.686	Mutual exclusivity
AGAP4	10q11.22	0 (0.00%)	2 (0.82%)	<-10	0.463	0.686	Mutual exclusivity
AGAP9	10q11.22	0 (0.00%)	2 (0.82%)	<-10	0.463	0.686	Mutual exclusivity
AGFG1	2q36.3	0 (0.00%)	2 (0.82%)	<-10	0.463	0.686	Mutual exclusivity
AGTR2	Xq23	0 (0.00%)	2 (0.82%)	<-10	0.463	0.686	Mutual exclusivity
AHR	7p21.1	0 (0.00%)	2 (0.82%)	<-10	0.463	0.686	Mutual exclusivity
AHSP	16p11.2	0 (0.00%)	2 (0.82%)	<-10	0.463	0.686	Mutual exclusivity
AIG1	6q24.2	0 (0.00%)	2 (0.82%)	<-10	0.463	0.686	Mutual exclusivity
ALG1L2	3q22.1	0 (0.00%)	2 (0.82%)	<-10	0.463	0.686	Mutual exclusivity
ALOX5	10q11.21	0 (0.00%)	2 (0.82%)	<-10	0.463	0.686	Mutual exclusivity
ANAPC1	2q13	0 (0.00%)	2 (0.82%)	<-10	0.463	0.686	Mutual exclusivity
ANAPC4	4p15.2	0 (0.00%)	2 (0.82%)	<-10	0.463	0.686	Mutual exclusivity
ANGPTL5	11q22.1	0 (0.00%)	2 (0.82%)	<-10	0.463	0.686	Mutual exclusivity
ANKMY2	7p21.1	0 (0.00%)	2 (0.82%)	<-10	0.463	0.686	Mutual exclusivity
ANKRD20A8P	2q11.1	0 (0.00%)	2 (0.82%)	<-10	0.463	0.686	Mutual exclusivity
ANKS4B	16p12.2	0 (0.00%)	2 (0.82%)	<-10	0.463	0.686	Mutual exclusivity
ANTXRL	10q11.22	0 (0.00%)	2 (0.82%)	<-10	0.463	0.686	Mutual exclusivity
ANTXRLP1	10q11.22	0 (0.00%)	2 (0.82%)	<-10	0.463	0.686	Mutual exclusivity
ANXA4	2p13.3	0 (0.00%)	2 (0.82%)	<-10	0.463	0.686	Mutual exclusivity

ANXA8	10q11.22	0 (0.00%)	2 (0.82%)	<-10	0.463	0.686	Mutual exclusivity
ANXA8L1	10q11.22	0 (0.00%)	2 (0.82%)	<-10	0.463	0.686	Mutual exclusivity
AP2A1	19q13.33	0 (0.00%)	2 (0.82%)	<-10	0.463	0.686	Mutual exclusivity
AP2S1	19q13.32	0 (0.00%)	2 (0.82%)	<-10	0.463	0.686	Mutual exclusivity
AP4S1	14q12	0 (0.00%)	2 (0.82%)	<-10	0.463	0.686	Mutual exclusivity
ARAP2	4p14	0 (0.00%)	2 (0.82%)	<-10	0.463	0.686	Mutual exclusivity
ARAP3	5q31.3	0 (0.00%)	2 (0.82%)	<-10	0.463	0.686	Mutual exclusivity
ARHGAP35	19q13.32	0 (0.00%)	2 (0.82%)	<-10	0.463	0.686	Mutual exclusivity
ARHGAP42	11q22.1	0 (0.00%)	2 (0.82%)	<-10	0.463	0.686	Mutual exclusivity
ARHGEF6	Xq26.3	0 (0.00%)	2 (0.82%)	<-10	0.463	0.686	Mutual exclusivity
ARID1B	6q25.3	0 (0.00%)	2 (0.82%)	<-10	0.463	0.686	Mutual exclusivity
ARMC5	16p11.2	0 (0.00%)	2 (0.82%)	<-10	0.463	0.686	Mutual exclusivity
ARMC9	2q37.1	0 (0.00%)	2 (0.82%)	<-10	0.463	0.686	Mutual exclusivity
ARSK	5q15	0 (0.00%)	2 (0.82%)	<-10	0.463	0.686	Mutual exclusivity
ASAH2B	10q11.23	0 (0.00%)	2 (0.82%)	<-10	0.463	0.686	Mutual exclusivity
ASB1	2q37.3	0 (0.00%)	2 (0.82%)	<-10	0.463	0.686	Mutual exclusivity
ASB2	14q32.12	0 (0.00%)	2 (0.82%)	<-10	0.463	0.686	Mutual exclusivity
ASTE1	3q22.1	0 (0.00%)	2 (0.82%)	<-10	0.463	0.686	Mutual exclusivity
ATL1	14q22.1	0 (0.00%)	2 (0.82%)	<-10	0.463	0.686	Mutual exclusivity
ATP11C	Xq27.1	0 (0.00%)	2 (0.82%)	<-10	0.463	0.686	Mutual exclusivity
ATP1B4	Xq24	0 (0.00%)	2 (0.82%)	<-10	0.463	0.686	Mutual exclusivity
ATP2C1	3q22.1	0 (0.00%)	2 (0.82%)	<-10	0.463	0.686	Mutual exclusivity
ATP5ME	4p16.3	0 (0.00%)	2 (0.82%)	<-10	0.463	0.686	Mutual exclusivity
ATP8A1	4p13	0 (0.00%)	2 (0.82%)	<-10	0.463	0.686	Mutual exclusivity
AXL	19q13.2	0 (0.00%)	2 (0.82%)	<-10	0.463	0.686	Mutual exclusivity
B3GAT3	11q12.3	0 (0.00%)	2 (0.82%)	<-10	0.463	0.686	Mutual exclusivity
B3GNT2	2p15	0 (0.00%)	2 (0.82%)	<-10	0.463	0.686	Mutual exclusivity
B3GNT7	2q37.1|2q37.1	0 (0.00%)	2 (0.82%)	<-10	0.463	0.686	Mutual exclusivity
B3GNT8	19q13.2	0 (0.00%)	2 (0.82%)	<-10	0.463	0.686	Mutual exclusivity

B9D2	19q13.2	0 (0.00%)	2 (0.82%)	<-10	0.463	0.686	Mutual exclusivity
BANF2	20p12.1	0 (0.00%)	2 (0.82%)	<-10	0.463	0.686	Mutual exclusivity
BATF	14q24.3	0 (0.00%)	2 (0.82%)	<-10	0.463	0.686	Mutual exclusivity
BAX	19q13.33	0 (0.00%)	2 (0.82%)	<-10	0.463	0.686	Mutual exclusivity
BAZ1A	14q13.1-q13.2	0 (0.00%)	2 (0.82%)	<-10	0.463	0.686	Mutual exclusivity
BBC3	19q13.32	0 (0.00%)	2 (0.82%)	<-10	0.463	0.686	Mutual exclusivity
BCAT2	19q13.33	0 (0.00%)	2 (0.82%)	<-10	0.463	0.686	Mutual exclusivity
BCKDHA	19q13.2	0 (0.00%)	2 (0.82%)	<-10	0.463	0.686	Mutual exclusivity
BCKDHB	6q14.1	0 (0.00%)	2 (0.82%)	<-10	0.463	0.686	Mutual exclusivity
BCL9L	11q23.3	0 (0.00%)	2 (0.82%)	<-10	0.463	0.686	Mutual exclusivity
BCLAF1	6q23.3	0 (0.00%)	2 (0.82%)	<-10	0.463	0.686	Mutual exclusivity
BCYRN1	2p21	0 (0.00%)	2 (0.82%)	<-10	0.463	0.686	Mutual exclusivity
BFSP2	3q22.1	0 (0.00%)	2 (0.82%)	<-10	0.463	0.686	Mutual exclusivity
BIRC2	11q22.2	0 (0.00%)	2 (0.82%)	<-10	0.463	0.686	Mutual exclusivity
BIRC3	11q22.2	0 (0.00%)	2 (0.82%)	<-10	0.463	0.686	Mutual exclusivity
BLOC1S4	4p16.1	0 (0.00%)	2 (0.82%)	<-10	0.463	0.686	Mutual exclusivity
BMS1P1	10q11.22	0 (0.00%)	2 (0.82%)	<-10	0.463	0.686	Mutual exclusivity
BMS1P2	10q11.22	0 (0.00%)	2 (0.82%)	<-10	0.463	0.686	Mutual exclusivity
BOD1L1	4p15.33	0 (0.00%)	2 (0.82%)	<-10	0.463	0.686	Mutual exclusivity
BORCS5	12p13.2	0 (0.00%)	2 (0.82%)	<-10	0.463	0.686	Mutual exclusivity
BRMS1L	14q13.2	0 (0.00%)	2 (0.82%)	<-10	0.463	0.686	Mutual exclusivity
BRS3	Xq26.3	0 (0.00%)	2 (0.82%)	<-10	0.463	0.686	Mutual exclusivity
BST1	4p15.32	0 (0.00%)	2 (0.82%)	<-10	0.463	0.686	Mutual exclusivity
BVES	6q21	0 (0.00%)	2 (0.82%)	<-10	0.463	0.686	Mutual exclusivity
BZW2	7p21.1	0 (0.00%)	2 (0.82%)	<-10	0.463	0.686	Mutual exclusivity
C10ORF12	10q24.1	0 (0.00%)	2 (0.82%)	<-10	0.463	0.686	Mutual exclusivity
C10ORF25	10q11.21	0 (0.00%)	2 (0.82%)	<-10	0.463	0.686	Mutual exclusivity
C14ORF28	14q21.2	0 (0.00%)	2 (0.82%)	<-10	0.463	0.686	Mutual exclusivity
C16ORF58	16p11.2	0 (0.00%)	2 (0.82%)	<-10	0.463	0.686	Mutual exclusivity

C19ORF73	19q13.33	0 (0.00%)	2 (0.82%)	<-10	0.463	0.686	Mutual exclusivity
C1GALT1C1	Xq24	0 (0.00%)	2 (0.82%)	<-10	0.463	0.686	Mutual exclusivity
C1ORF210	1p34.2	0 (0.00%)	2 (0.82%)	<-10	0.463	0.686	Mutual exclusivity
C1QTNF7	4p15.32	0 (0.00%)	2 (0.82%)	<-10	0.463	0.686	Mutual exclusivity
C20ORF203	20q11.21	0 (0.00%)	2 (0.82%)	<-10	0.463	0.686	Mutual exclusivity
C22ORF15	22q11.23	0 (0.00%)	2 (0.82%)	<-10	0.463	0.686	Mutual exclusivity
C2CD2L	11q23.3	0 (0.00%)	2 (0.82%)	<-10	0.463	0.686	Mutual exclusivity
C2CD3	11q13.4	0 (0.00%)	2 (0.82%)	<-10	0.463	0.686	Mutual exclusivity
C3ORF67	3p14.2	0 (0.00%)	2 (0.82%)	<-10	0.463	0.686	Mutual exclusivity
C4ORF19	4p14	0 (0.00%)	2 (0.82%)	<-10	0.463	0.686	Mutual exclusivity
C4ORF48	4p16.3	0 (0.00%)	2 (0.82%)	<-10	0.463	0.686	Mutual exclusivity
C4ORF50	4p16.1	0 (0.00%)	2 (0.82%)	<-10	0.463	0.686	Mutual exclusivity
C5AR1	19q13.32	0 (0.00%)	2 (0.82%)	<-10	0.463	0.686	Mutual exclusivity
C5AR2	19q13.32	0 (0.00%)	2 (0.82%)	<-10	0.463	0.686	Mutual exclusivity
C5ORF24	5q31.1	0 (0.00%)	2 (0.82%)	<-10	0.463	0.686	Mutual exclusivity
C5ORF30	5q21.1	0 (0.00%)	2 (0.82%)	<-10	0.463	0.686	Mutual exclusivity
C6ORF118	6q27	0 (0.00%)	2 (0.82%)	<-10	0.463	0.686	Mutual exclusivity
C6ORF183	6q21	0 (0.00%)	2 (0.82%)	<-10	0.463	0.686	Mutual exclusivity
CALM1	14q32.11	0 (0.00%)	2 (0.82%)	<-10	0.463	0.686	Mutual exclusivity
CASP3	4q35.1	0 (0.00%)	2 (0.82%)	<-10	0.463	0.686	Mutual exclusivity
CAST	5q15	0 (0.00%)	2 (0.82%)	<-10	0.463	0.686	Mutual exclusivity
CAT	11p13	0 (0.00%)	2 (0.82%)	<-10	0.463	0.686	Mutual exclusivity
CC2D2A	4p15.32	0 (0.00%)	2 (0.82%)	<-10	0.463	0.686	Mutual exclusivity
CCDC149	4p15.2	0 (0.00%)	2 (0.82%)	<-10	0.463	0.686	Mutual exclusivity
CCDC151	19p13.2	0 (0.00%)	2 (0.82%)	<-10	0.463	0.686	Mutual exclusivity
CCDC159	19p13.2	0 (0.00%)	2 (0.82%)	<-10	0.463	0.686	Mutual exclusivity
CCDC160	Xq26.2	0 (0.00%)	2 (0.82%)	<-10	0.463	0.686	Mutual exclusivity
CCDC189	16p11.2	0 (0.00%)	2 (0.82%)	<-10	0.463	0.686	Mutual exclusivity
CCDC197	14q32.12	0 (0.00%)	2 (0.82%)	<-10	0.463	0.686	Mutual exclusivity

CCDC61	19q13.32	0 (0.00%)	2 (0.82%)	<-10	0.463	0.686	Mutual exclusivity
CCDC8	19q13.32	0 (0.00%)	2 (0.82%)	<-10	0.463	0.686	Mutual exclusivity
CCDC81	11q14.2	0 (0.00%)	2 (0.82%)	<-10	0.463	0.686	Mutual exclusivity
CCDC84	11q23.3	0 (0.00%)	2 (0.82%)	<-10	0.463	0.686	Mutual exclusivity
CCDC86	11q12.2	0 (0.00%)	2 (0.82%)	<-10	0.463	0.686	Mutual exclusivity
CCDC9	19q13.32	0 (0.00%)	2 (0.82%)	<-10	0.463	0.686	Mutual exclusivity
CCDC96	4p16.1	0 (0.00%)	2 (0.82%)	<-10	0.463	0.686	Mutual exclusivity
CCDC97	19q13.2	0 (0.00%)	2 (0.82%)	<-10	0.463	0.686	Mutual exclusivity
CCKAR	4p15.2	0 (0.00%)	2 (0.82%)	<-10	0.463	0.686	Mutual exclusivity
CCNO	5q11.2	0 (0.00%)	2 (0.82%)	<-10	0.463	0.686	Mutual exclusivity
CCR6	6q27	0 (0.00%)	2 (0.82%)	<-10	0.463	0.686	Mutual exclusivity
CCSER1	4q22.1	0 (0.00%)	2 (0.82%)	<-10	0.463	0.686	Mutual exclusivity
CCT4	2p15	0 (0.00%)	2 (0.82%)	<-10	0.463	0.686	Mutual exclusivity
CD109	6q13	0 (0.00%)	2 (0.82%)	<-10	0.463	0.686	Mutual exclusivity
CD27	12p13.31	0 (0.00%)	2 (0.82%)	<-10	0.463	0.686	Mutual exclusivity
CD33	19q13.41	0 (0.00%)	2 (0.82%)	<-10	0.463	0.686	Mutual exclusivity
CD40LG	Xq26.3	0 (0.00%)	2 (0.82%)	<-10	0.463	0.686	Mutual exclusivity
CDC20	1p34.2	0 (0.00%)	2 (0.82%)	<-10	0.463	0.686	Mutual exclusivity
CDC20B	5q11.2	0 (0.00%)	2 (0.82%)	<-10	0.463	0.686	Mutual exclusivity
CDCA3	12p13.31	0 (0.00%)	2 (0.82%)	<-10	0.463	0.686	Mutual exclusivity
CDCA7L	7p15.3	0 (0.00%)	2 (0.82%)	<-10	0.463	0.686	Mutual exclusivity
CDK1	10q21.2	0 (0.00%)	2 (0.82%)	<-10	0.463	0.686	Mutual exclusivity
CDR1	Xq27.1	0 (0.00%)	2 (0.82%)	<-10	0.463	0.686	Mutual exclusivity
CDS2	20p12.3	0 (0.00%)	2 (0.82%)	<-10	0.463	0.686	Mutual exclusivity
CEACAM18	19q13.41	0 (0.00%)	2 (0.82%)	<-10	0.463	0.686	Mutual exclusivity
CEACAM21	19q13.2	0 (0.00%)	2 (0.82%)	<-10	0.463	0.686	Mutual exclusivity
CEACAM4	19q13.2	0 (0.00%)	2 (0.82%)	<-10	0.463	0.686	Mutual exclusivity
CEACAMP3	19q13.2	0 (0.00%)	2 (0.82%)	<-10	0.463	0.686	Mutual exclusivity
CENPU	4q35.1	0 (0.00%)	2 (0.82%)	<-10	0.463	0.686	Mutual exclusivity

CENPV	17p11.2	0 (0.00%)	2 (0.82%)	<-10	0.463	0.686	Mutual exclusivity
CEP126	11q22.1	0 (0.00%)	2 (0.82%)	<-10	0.463	0.686	Mutual exclusivity
CEP135	4q12	0 (0.00%)	2 (0.82%)	<-10	0.463	0.686	Mutual exclusivity
CEP164P1	10q11.21	0 (0.00%)	2 (0.82%)	<-10	0.463	0.686	Mutual exclusivity
CEP57L1	6q21	0 (0.00%)	2 (0.82%)	<-10	0.463	0.686	Mutual exclusivity
CEP97	3q12.3	0 (0.00%)	2 (0.82%)	<-10	0.463	0.686	Mutual exclusivity
CFAP300	11q22.1	0 (0.00%)	2 (0.82%)	<-10	0.463	0.686	Mutual exclusivity
CFAP97	4q35.1	0 (0.00%)	2 (0.82%)	<-10	0.463	0.686	Mutual exclusivity
CGAS	6q13	0 (0.00%)	2 (0.82%)	<-10	0.463	0.686	Mutual exclusivity
CGB1	19q13.33	0 (0.00%)	2 (0.82%)	<-10	0.463	0.686	Mutual exclusivity
CGB2	19q13.33	0 (0.00%)	2 (0.82%)	<-10	0.463	0.686	Mutual exclusivity
CGB3	19q13.33	0 (0.00%)	2 (0.82%)	<-10	0.463	0.686	Mutual exclusivity
CGB5	19q13.33	0 (0.00%)	2 (0.82%)	<-10	0.463	0.686	Mutual exclusivity
CGB7	19q13.33	0 (0.00%)	2 (0.82%)	<-10	0.463	0.686	Mutual exclusivity
CGB8	19q13.33	0 (0.00%)	2 (0.82%)	<-10	0.463	0.686	Mutual exclusivity
CHCHD10	22q11.23	0 (0.00%)	2 (0.82%)	<-10	0.463	0.686	Mutual exclusivity
CHD1	5q15-q21.1	0 (0.00%)	2 (0.82%)	<-10	0.463	0.686	Mutual exclusivity
CHP2	16p12.2	0 (0.00%)	2 (0.82%)	<-10	0.463	0.686	Mutual exclusivity
CLDND2	19q13.41	0 (0.00%)	2 (0.82%)	<-10	0.463	0.686	Mutual exclusivity
CLEC14A	14q21.1	0 (0.00%)	2 (0.82%)	<-10	0.463	0.686	Mutual exclusivity
CLNK	4p16.1	0 (0.00%)	2 (0.82%)	<-10	0.463	0.686	Mutual exclusivity
CLOCK	4q12	0 (0.00%)	2 (0.82%)	<-10	0.463	0.686	Mutual exclusivity
CMYA5	5q14.1	0 (0.00%)	2 (0.82%)	<-10	0.463	0.686	Mutual exclusivity
CNBP	3q21.3	0 (0.00%)	2 (0.82%)	<-10	0.463	0.686	Mutual exclusivity
CNN1	19p13.2	0 (0.00%)	2 (0.82%)	<-10	0.463	0.686	Mutual exclusivity
CNOT11	2q11.2	0 (0.00%)	2 (0.82%)	<-10	0.463	0.686	Mutual exclusivity
CNTN5	11q22.1	0 (0.00%)	2 (0.82%)	<-10	0.463	0.686	Mutual exclusivity
COCH	14q12	0 (0.00%)	2 (0.82%)	<-10	0.463	0.686	Mutual exclusivity
COG7	16p12.2	0 (0.00%)	2 (0.82%)	<-10	0.463	0.686	Mutual exclusivity

COL19A1	6q13	0 (0.00%)	2 (0.82%)	<-10	0.463	0.686	Mutual exclusivity
COL4A3	2q36.3	0 (0.00%)	2 (0.82%)	<-10	0.463	0.686	Mutual exclusivity
COL4A6	Xq22.3	0 (0.00%)	2 (0.82%)	<-10	0.463	0.686	Mutual exclusivity
COL6A4P2	3q22.1	0 (0.00%)	2 (0.82%)	<-10	0.463	0.686	Mutual exclusivity
COL6A5	3q22.1	0 (0.00%)	2 (0.82%)	<-10	0.463	0.686	Mutual exclusivity
COL6A6	3q22.1	0 (0.00%)	2 (0.82%)	<-10	0.463	0.686	Mutual exclusivity
COL9A1	6q13	0 (0.00%)	2 (0.82%)	<-10	0.463	0.686	Mutual exclusivity
COMMD1	2p15	0 (0.00%)	2 (0.82%)	<-10	0.463	0.686	Mutual exclusivity
COMMD9	11p13	0 (0.00%)	2 (0.82%)	<-10	0.463	0.686	Mutual exclusivity
COPS7A	12p13.31	0 (0.00%)	2 (0.82%)	<-10	0.463	0.686	Mutual exclusivity
COPS7B	2q37.1	0 (0.00%)	2 (0.82%)	<-10	0.463	0.686	Mutual exclusivity
COX6A2	16p11.2	0 (0.00%)	2 (0.82%)	<-10	0.463	0.686	Mutual exclusivity
COX7B2	4p12	0 (0.00%)	2 (0.82%)	<-10	0.463	0.686	Mutual exclusivity
CPEB2	4p15.32	0 (0.00%)	2 (0.82%)	<-10	0.463	0.686	Mutual exclusivity
CPLX1	4p16.3	0 (0.00%)	2 (0.82%)	<-10	0.463	0.686	Mutual exclusivity
CPN1	10q24.2	0 (0.00%)	2 (0.82%)	<-10	0.463	0.686	Mutual exclusivity
CPNE4	3q22.1	0 (0.00%)	2 (0.82%)	<-10	0.463	0.686	Mutual exclusivity
CPO	2q33.3	0 (0.00%)	2 (0.82%)	<-10	0.463	0.686	Mutual exclusivity
CPZ	4p16.1	0 (0.00%)	2 (0.82%)	<-10	0.463	0.686	Mutual exclusivity
CREB1	2q33.3	0 (0.00%)	2 (0.82%)	<-10	0.463	0.686	Mutual exclusivity
CREG2	2q11.2	0 (0.00%)	2 (0.82%)	<-10	0.463	0.686	Mutual exclusivity
CRIPAK	4p16.3	0 (0.00%)	2 (0.82%)	<-10	0.463	0.686	Mutual exclusivity
CRMP1	4p16.2	0 (0.00%)	2 (0.82%)	<-10	0.463	0.686	Mutual exclusivity
CRYGB	2q33.3	0 (0.00%)	2 (0.82%)	<-10	0.463	0.686	Mutual exclusivity
CRYGC	2q33.3	0 (0.00%)	2 (0.82%)	<-10	0.463	0.686	Mutual exclusivity
CRYGD	2q33.3	0 (0.00%)	2 (0.82%)	<-10	0.463	0.686	Mutual exclusivity
CRYGEP	2q33.3	0 (0.00%)	2 (0.82%)	<-10	0.463	0.686	Mutual exclusivity
CRYM	16p12.2	0 (0.00%)	2 (0.82%)	<-10	0.463	0.686	Mutual exclusivity
CSF1R	5q32	0 (0.00%)	2 (0.82%)	<-10	0.463	0.686	Mutual exclusivity

CSGALNACT2	10q11.21	0 (0.00%)	2 (0.82%)	<-10	0.463	0.686	Mutual exclusivity
CSMD1	8p23.2	0 (0.00%)	2 (0.82%)	<-10	0.463	0.686	Mutual exclusivity
CT45A1	Xq26.3	0 (0.00%)	2 (0.82%)	<-10	0.463	0.686	Mutual exclusivity
CT45A2	Xq26.3	0 (0.00%)	2 (0.82%)	<-10	0.463	0.686	Mutual exclusivity
CT45A3	Xq26.3	0 (0.00%)	2 (0.82%)	<-10	0.463	0.686	Mutual exclusivity
CT45A4	Xq26.3	0 (0.00%)	2 (0.82%)	<-10	0.463	0.686	Mutual exclusivity
CT45A5	Xq26.3	0 (0.00%)	2 (0.82%)	<-10	0.463	0.686	Mutual exclusivity
CT45A6	Xq26.3	0 (0.00%)	2 (0.82%)	<-10	0.463	0.686	Mutual exclusivity
CT47A1	Xq24	0 (0.00%)	2 (0.82%)	<-10	0.463	0.686	Mutual exclusivity
CT47A10	Xq24	0 (0.00%)	2 (0.82%)	<-10	0.463	0.686	Mutual exclusivity
CT47A11	Xq24	0 (0.00%)	2 (0.82%)	<-10	0.463	0.686	Mutual exclusivity
CT47A12	Xq24	0 (0.00%)	2 (0.82%)	<-10	0.463	0.686	Mutual exclusivity
CT47A2	Xq24	0 (0.00%)	2 (0.82%)	<-10	0.463	0.686	Mutual exclusivity
CT47A3	Xq24	0 (0.00%)	2 (0.82%)	<-10	0.463	0.686	Mutual exclusivity
CT47A4	Xq24	0 (0.00%)	2 (0.82%)	<-10	0.463	0.686	Mutual exclusivity
CT47A5	Xq24	0 (0.00%)	2 (0.82%)	<-10	0.463	0.686	Mutual exclusivity
CT47A6	Xq24	0 (0.00%)	2 (0.82%)	<-10	0.463	0.686	Mutual exclusivity
CT47A7	Xq24	0 (0.00%)	2 (0.82%)	<-10	0.463	0.686	Mutual exclusivity
CT47A8	Xq24	0 (0.00%)	2 (0.82%)	<-10	0.463	0.686	Mutual exclusivity
CT47A9	Xq24	0 (0.00%)	2 (0.82%)	<-10	0.463	0.686	Mutual exclusivity
CT47B1	Xq24	0 (0.00%)	2 (0.82%)	<-10	0.463	0.686	Mutual exclusivity
CT55	Xq26.3	0 (0.00%)	2 (0.82%)	<-10	0.463	0.686	Mutual exclusivity
CT83	Xq23	0 (0.00%)	2 (0.82%)	<-10	0.463	0.686	Mutual exclusivity
CTBP1	4p16.3	0 (0.00%)	2 (0.82%)	<-10	0.463	0.686	Mutual exclusivity
CTBP2	10q26.13	0 (0.00%)	2 (0.82%)	<-10	0.463	0.686	Mutual exclusivity
CTNNA1	5q31.2	0 (0.00%)	2 (0.82%)	<-10	0.463	0.686	Mutual exclusivity
CTU1	19q13.41	0 (0.00%)	2 (0.82%)	<-10	0.463	0.686	Mutual exclusivity
CXCL14	5q31.1	0 (0.00%)	2 (0.82%)	<-10	0.463	0.686	Mutual exclusivity
CXCR2	2q35	0 (0.00%)	2 (0.82%)	<-10	0.463	0.686	Mutual exclusivity

CXCR2P1	2q35	0 (0.00%)	2 (0.82%)	<-10	0.463	0.686	Mutual exclusivity
CXCR5	11q23.3	0 (0.00%)	2 (0.82%)	<-10	0.463	0.686	Mutual exclusivity
CXORF66	Xq27.1	0 (0.00%)	2 (0.82%)	<-10	0.463	0.686	Mutual exclusivity
CYTL1	4p16.2	0 (0.00%)	2 (0.82%)	<-10	0.463	0.686	Mutual exclusivity
DACT2	6q27	0 (0.00%)	2 (0.82%)	<-10	0.463	0.686	Mutual exclusivity
DAZAP1	19p13.3	0 (0.00%)	2 (0.82%)	<-10	0.463	0.686	Mutual exclusivity
DCANP1	5q31.1	0 (0.00%)	2 (0.82%)	<-10	0.463	0.686	Mutual exclusivity
DCDC1	11p13	0 (0.00%)	2 (0.82%)	<-10	0.463	0.686	Mutual exclusivity
DCLK2	4q31.23-q31.3	0 (0.00%)	2 (0.82%)	<-10	0.463	0.686	Mutual exclusivity
DCTN5	16p12.2	0 (0.00%)	2 (0.82%)	<-10	0.463	0.686	Mutual exclusivity
DCUN1D5	11q22.3	0 (0.00%)	2 (0.82%)	<-10	0.463	0.686	Mutual exclusivity
DDHD1	14q22.1	0 (0.00%)	2 (0.82%)	<-10	0.463	0.686	Mutual exclusivity
DDX23	12q13.12	0 (0.00%)	2 (0.82%)	<-10	0.463	0.686	Mutual exclusivity
DDX24	14q32.12	0 (0.00%)	2 (0.82%)	<-10	0.463	0.686	Mutual exclusivity
DDX43	6q13	0 (0.00%)	2 (0.82%)	<-10	0.463	0.686	Mutual exclusivity
DEFB131A	4p16.1	0 (0.00%)	2 (0.82%)	<-10	0.463	0.686	Mutual exclusivity
DEPP1	10q11.21	0 (0.00%)	2 (0.82%)	<-10	0.463	0.686	Mutual exclusivity
DERL3	22q11.23	0 (0.00%)	2 (0.82%)	<-10	0.463	0.686	Mutual exclusivity
DGKD	2q37.1	0 (0.00%)	2 (0.82%)	<-10	0.463	0.686	Mutual exclusivity
DGKQ	4p16.3	0 (0.00%)	2 (0.82%)	<-10	0.463	0.686	Mutual exclusivity
DHDH	19q13.33	0 (0.00%)	2 (0.82%)	<-10	0.463	0.686	Mutual exclusivity
DHX29	5q11.2	0 (0.00%)	2 (0.82%)	<-10	0.463	0.686	Mutual exclusivity
DIAPH1	5q31.3	0 (0.00%)	2 (0.82%)	<-10	0.463	0.686	Mutual exclusivity
DIS3L2	2q37.1	0 (0.00%)	2 (0.82%)	<-10	0.463	0.686	Mutual exclusivity
DMAC2	19q13.2	0 (0.00%)	2 (0.82%)	<-10	0.463	0.686	Mutual exclusivity
DMD	Xp21.2-p21.1	0 (0.00%)	2 (0.82%)	<-10	0.463	0.686	Mutual exclusivity
DNAH11	7p15.3	0 (0.00%)	2 (0.82%)	<-10	0.463	0.686	Mutual exclusivity
DNAH3	16p12.3	0 (0.00%)	2 (0.82%)	<-10	0.463	0.686	Mutual exclusivity
DNAJB8	3q21.3	0 (0.00%)	2 (0.82%)	<-10	0.463	0.686	Mutual exclusivity

DNAJC13	3q22.1	0 (0.00%)	2 (0.82%)	<-10	0.463	0.686	Mutual exclusivity
DNAJC24	11p13	0 (0.00%)	2 (0.82%)	<-10	0.463	0.686	Mutual exclusivity
DOK7	4p16.3	0 (0.00%)	2 (0.82%)	<-10	0.463	0.686	Mutual exclusivity
DPAGT1	11q23.3	0 (0.00%)	2 (0.82%)	<-10	0.463	0.686	Mutual exclusivity
DPPA3P2	14q13.3	0 (0.00%)	2 (0.82%)	<-10	0.463	0.686	Mutual exclusivity
DPPA5	6q13	0 (0.00%)	2 (0.82%)	<-10	0.463	0.686	Mutual exclusivity
DRC7	16q21	0 (0.00%)	2 (0.82%)	<-10	0.463	0.686	Mutual exclusivity
DRD5	4p16.1	0 (0.00%)	2 (0.82%)	<-10	0.463	0.686	Mutual exclusivity
DSTNP2	12p13.31	0 (0.00%)	2 (0.82%)	<-10	0.463	0.686	Mutual exclusivity
DTHD1	4p14	0 (0.00%)	2 (0.82%)	<-10	0.463	0.686	Mutual exclusivity
DUSP16	12p13.2	0 (0.00%)	2 (0.82%)	<-10	0.463	0.686	Mutual exclusivity
DYNC2H1	11q22.3	0 (0.00%)	2 (0.82%)	<-10	0.463	0.686	Mutual exclusivity
DYTN	2q33.3	0 (0.00%)	2 (0.82%)	<-10	0.463	0.686	Mutual exclusivity
EARS2	16p12.2	0 (0.00%)	2 (0.82%)	<-10	0.463	0.686	Mutual exclusivity
ECE1	1p36.12	0 (0.00%)	2 (0.82%)	<-10	0.463	0.686	Mutual exclusivity
ECSIT	19p13.2	0 (0.00%)	2 (0.82%)	<-10	0.463	0.686	Mutual exclusivity
EEF1A1	6q13	0 (0.00%)	2 (0.82%)	<-10	0.463	0.686	Mutual exclusivity
EEF1B2	2q33.3	0 (0.00%)	2 (0.82%)	<-10	0.463	0.686	Mutual exclusivity
EFCAB12	3q21.3	0 (0.00%)	2 (0.82%)	<-10	0.463	0.686	Mutual exclusivity
EFNA2	19p13.3	0 (0.00%)	2 (0.82%)	<-10	0.463	0.686	Mutual exclusivity
EIF4G3	1p36.12	0 (0.00%)	2 (0.82%)	<-10	0.463	0.686	Mutual exclusivity
ELAVL3	19p13.2	0 (0.00%)	2 (0.82%)	<-10	0.463	0.686	Mutual exclusivity
ELF5	11p13	0 (0.00%)	2 (0.82%)	<-10	0.463	0.686	Mutual exclusivity
ELL2	5q15	0 (0.00%)	2 (0.82%)	<-10	0.463	0.686	Mutual exclusivity
ELOF1	19p13.2	0 (0.00%)	2 (0.82%)	<-10	0.463	0.686	Mutual exclusivity
ELOVL4	6q14.1	0 (0.00%)	2 (0.82%)	<-10	0.463	0.686	Mutual exclusivity
ELP2	18q12.2	0 (0.00%)	2 (0.82%)	<-10	0.463	0.686	Mutual exclusivity
EMB	5q11.1	0 (0.00%)	2 (0.82%)	<-10	0.463	0.686	Mutual exclusivity
EML3	11q12.3	0 (0.00%)	2 (0.82%)	<-10	0.463	0.686	Mutual exclusivity

ENO2	12p13.31	0 (0.00%)	2 (0.82%)	<-10	0.463	0.686	Mutual exclusivity
EPCAM	2p21	0 (0.00%)	2 (0.82%)	<-10	0.463	0.686	Mutual exclusivity
EPG5	18q12.3-q21.1	0 (0.00%)	2 (0.82%)	<-10	0.463	0.686	Mutual exclusivity
EPOR	19p13.2	0 (0.00%)	2 (0.82%)	<-10	0.463	0.686	Mutual exclusivity
ERFE	2q37.3	0 (0.00%)	2 (0.82%)	<-10	0.463	0.686	Mutual exclusivity
ERICH4	19q13.2	0 (0.00%)	2 (0.82%)	<-10	0.463	0.686	Mutual exclusivity
ERN2	16p12.2	0 (0.00%)	2 (0.82%)	<-10	0.463	0.686	Mutual exclusivity
ERO1A	14q22.1	0 (0.00%)	2 (0.82%)	<-10	0.463	0.686	Mutual exclusivity
ESM1	5q11.2	0 (0.00%)	2 (0.82%)	<-10	0.463	0.686	Mutual exclusivity
ESPNL	2q37.3	0 (0.00%)	2 (0.82%)	<-10	0.463	0.686	Mutual exclusivity
ESRP2	16q22.1	0 (0.00%)	2 (0.82%)	<-10	0.463	0.686	Mutual exclusivity
ESX1	Xq22.2	0 (0.00%)	2 (0.82%)	<-10	0.463	0.686	Mutual exclusivity
ETDB	Xq26.3	0 (0.00%)	2 (0.82%)	<-10	0.463	0.686	Mutual exclusivity
ETFB	19q13.41	0 (0.00%)	2 (0.82%)	<-10	0.463	0.686	Mutual exclusivity
ETS2	21q22.2	0 (0.00%)	2 (0.82%)	<-10	0.463	0.686	Mutual exclusivity
EVC	4p16.2	0 (0.00%)	2 (0.82%)	<-10	0.463	0.686	Mutual exclusivity
EVC2	4p16.2	0 (0.00%)	2 (0.82%)	<-10	0.463	0.686	Mutual exclusivity
EXOC1	4q12	0 (0.00%)	2 (0.82%)	<-10	0.463	0.686	Mutual exclusivity
EXOSC5	19q13.2	0 (0.00%)	2 (0.82%)	<-10	0.463	0.686	Mutual exclusivity
F9	Xq27.1	0 (0.00%)	2 (0.82%)	<-10	0.463	0.686	Mutual exclusivity
FAHD2A	2q11.1	0 (0.00%)	2 (0.82%)	<-10	0.463	0.686	Mutual exclusivity
FAM107A	3p14.3-p14.2	0 (0.00%)	2 (0.82%)	<-10	0.463	0.686	Mutual exclusivity
FAM120B	6q27	0 (0.00%)	2 (0.82%)	<-10	0.463	0.686	Mutual exclusivity
FAM122B	Xq26.3	0 (0.00%)	2 (0.82%)	<-10	0.463	0.686	Mutual exclusivity
FAM122C	Xq26.3	0 (0.00%)	2 (0.82%)	<-10	0.463	0.686	Mutual exclusivity
FAM172A	5q15	0 (0.00%)	2 (0.82%)	<-10	0.463	0.686	Mutual exclusivity
FAM174A	5q21.1	0 (0.00%)	2 (0.82%)	<-10	0.463	0.686	Mutual exclusivity
FAM178B	2q11.2	0 (0.00%)	2 (0.82%)	<-10	0.463	0.686	Mutual exclusivity
FAM181A	14q32.12	0 (0.00%)	2 (0.82%)	<-10	0.463	0.686	Mutual exclusivity

FAM199X	Xq22.2	0 (0.00%)	2 (0.82%)	<-10	0.463	0.686	Mutual exclusivity
FAM200B	4p15.32	0 (0.00%)	2 (0.82%)	<-10	0.463	0.686	Mutual exclusivity
FAM25BP	10q11.22	0 (0.00%)	2 (0.82%)	<-10	0.463	0.686	Mutual exclusivity
FAM25E	10q11.22	0 (0.00%)	2 (0.82%)	<-10	0.463	0.686	Mutual exclusivity
FAM25G	10q11.22	0 (0.00%)	2 (0.82%)	<-10	0.463	0.686	Mutual exclusivity
FAM3D	3p14.2	0 (0.00%)	2 (0.82%)	<-10	0.463	0.686	Mutual exclusivity
FAM53A	4p16.3	0 (0.00%)	2 (0.82%)	<-10	0.463	0.686	Mutual exclusivity
FAM86EP	4p16.3	0 (0.00%)	2 (0.82%)	<-10	0.463	0.686	Mutual exclusivity
FAM86HP	3q22.1	0 (0.00%)	2 (0.82%)	<-10	0.463	0.686	Mutual exclusivity
FAM90A26	4p16.1	0 (0.00%)	2 (0.82%)	<-10	0.463	0.686	Mutual exclusivity
FASTKD2	2q33.3	0 (0.00%)	2 (0.82%)	<-10	0.463	0.686	Mutual exclusivity
FBRS	16p11.2	0 (0.00%)	2 (0.82%)	<-10	0.463	0.686	Mutual exclusivity
FBXL5	4p15.32	0 (0.00%)	2 (0.82%)	<-10	0.463	0.686	Mutual exclusivity
FBXO11	2p16.3	0 (0.00%)	2 (0.82%)	<-10	0.463	0.686	Mutual exclusivity
FBXO33	14q21.1	0 (0.00%)	2 (0.82%)	<-10	0.463	0.686	Mutual exclusivity
FCHSD1	5q31.3	0 (0.00%)	2 (0.82%)	<-10	0.463	0.686	Mutual exclusivity
FCHSD2	11q13.4	0 (0.00%)	2 (0.82%)	<-10	0.463	0.686	Mutual exclusivity
FERD3L	7p21.1	0 (0.00%)	2 (0.82%)	<-10	0.463	0.686	Mutual exclusivity
FERMT2	14q22.1	0 (0.00%)	2 (0.82%)	<-10	0.463	0.686	Mutual exclusivity
FGF10	5p12	0 (0.00%)	2 (0.82%)	<-10	0.463	0.686	Mutual exclusivity
FGF13	Xq26.3-q27.1	0 (0.00%)	2 (0.82%)	<-10	0.463	0.686	Mutual exclusivity
FGF21	19q13.33	0 (0.00%)	2 (0.82%)	<-10	0.463	0.686	Mutual exclusivity
FGFR3	4p16.3	0 (0.00%)	2 (0.82%)	<-10	0.463	0.686	Mutual exclusivity
FGFRL1	4p16.3	0 (0.00%)	2 (0.82%)	<-10	0.463	0.686	Mutual exclusivity
FHL1	Xq26.3	0 (0.00%)	2 (0.82%)	<-10	0.463	0.686	Mutual exclusivity
FHOD3	18q12.2	0 (0.00%)	2 (0.82%)	<-10	0.463	0.686	Mutual exclusivity
FLACC1	2q33.1	0 (0.00%)	2 (0.82%)	<-10	0.463	0.686	Mutual exclusivity
FOLH1B	11q14.3	0 (0.00%)	2 (0.82%)	<-10	0.463	0.686	Mutual exclusivity
FOXN2	2p16.3	0 (0.00%)	2 (0.82%)	<-10	0.463	0.686	Mutual exclusivity

FOXR1	11q23.3	0 (0.00%)	2 (0.82%)	<-10	0.463	0.686	Mutual exclusivity
FPR1	19q13.41	0 (0.00%)	2 (0.82%)	<-10	0.463	0.686	Mutual exclusivity
FPR2	19q13.41	0 (0.00%)	2 (0.82%)	<-10	0.463	0.686	Mutual exclusivity
FPR3	19q13.41	0 (0.00%)	2 (0.82%)	<-10	0.463	0.686	Mutual exclusivity
FRMD1	6q27	0 (0.00%)	2 (0.82%)	<-10	0.463	0.686	Mutual exclusivity
FSHR	2p16.3	0 (0.00%)	2 (0.82%)	<-10	0.463	0.686	Mutual exclusivity
FTL	19q13.33	0 (0.00%)	2 (0.82%)	<-10	0.463	0.686	Mutual exclusivity
FUT1	19q13.33	0 (0.00%)	2 (0.82%)	<-10	0.463	0.686	Mutual exclusivity
FUT9	6q16.1	0 (0.00%)	2 (0.82%)	<-10	0.463	0.686	Mutual exclusivity
FUZ	19q13.33	0 (0.00%)	2 (0.82%)	<-10	0.463	0.686	Mutual exclusivity
FXN	9q21.11	0 (0.00%)	2 (0.82%)	<-10	0.463	0.686	Mutual exclusivity
FXYD4	10q11.21	0 (0.00%)	2 (0.82%)	<-10	0.463	0.686	Mutual exclusivity
G2E3	14q12	0 (0.00%)	2 (0.82%)	<-10	0.463	0.686	Mutual exclusivity
GABRA2	4p12	0 (0.00%)	2 (0.82%)	<-10	0.463	0.686	Mutual exclusivity
GABRG1	4p12	0 (0.00%)	2 (0.82%)	<-10	0.463	0.686	Mutual exclusivity
GAK	4p16.3	0 (0.00%)	2 (0.82%)	<-10	0.463	0.686	Mutual exclusivity
GAMT	19p13.3	0 (0.00%)	2 (0.82%)	<-10	0.463	0.686	Mutual exclusivity
GANAB	11q12.3	0 (0.00%)	2 (0.82%)	<-10	0.463	0.686	Mutual exclusivity
GAPDH	12p13.31	0 (0.00%)	2 (0.82%)	<-10	0.463	0.686	Mutual exclusivity
GAPT	5q11.2	0 (0.00%)	2 (0.82%)	<-10	0.463	0.686	Mutual exclusivity
GATA2	3q21.3	0 (0.00%)	2 (0.82%)	<-10	0.463	0.686	Mutual exclusivity
GEMIN2	14q21.1	0 (0.00%)	2 (0.82%)	<-10	0.463	0.686	Mutual exclusivity
GGA2	16p12.2	0 (0.00%)	2 (0.82%)	<-10	0.463	0.686	Mutual exclusivity
GIN1	5q21.1	0 (0.00%)	2 (0.82%)	<-10	0.463	0.686	Mutual exclusivity
GLUD2	Xq24	0 (0.00%)	2 (0.82%)	<-10	0.463	0.686	Mutual exclusivity
GNB3	12p13.31	0 (0.00%)	2 (0.82%)	<-10	0.463	0.686	Mutual exclusivity
GNG2	14q22.1	0 (0.00%)	2 (0.82%)	<-10	0.463	0.686	Mutual exclusivity
GNLY	2p11.2	0 (0.00%)	2 (0.82%)	<-10	0.463	0.686	Mutual exclusivity
GNPNAT1	14q22.1	0 (0.00%)	2 (0.82%)	<-10	0.463	0.686	Mutual exclusivity

GPR1	2q33.3	0 (0.00%)	2 (0.82%)	<-10	0.463	0.686	Mutual exclusivity
GPR101	Xq26.3	0 (0.00%)	2 (0.82%)	<-10	0.463	0.686	Mutual exclusivity
GPR137C	14q22.1	0 (0.00%)	2 (0.82%)	<-10	0.463	0.686	Mutual exclusivity
GPR150	5q15	0 (0.00%)	2 (0.82%)	<-10	0.463	0.686	Mutual exclusivity
GPR151	5q32	0 (0.00%)	2 (0.82%)	<-10	0.463	0.686	Mutual exclusivity
GPR162	12p13.31	0 (0.00%)	2 (0.82%)	<-10	0.463	0.686	Mutual exclusivity
GPR78	4p16.1	0 (0.00%)	2 (0.82%)	<-10	0.463	0.686	Mutual exclusivity
GPRIN2	10q11.22	0 (0.00%)	2 (0.82%)	<-10	0.463	0.686	Mutual exclusivity
GPX8	5q11.2	0 (0.00%)	2 (0.82%)	<-10	0.463	0.686	Mutual exclusivity
GRIA3	Xq25	0 (0.00%)	2 (0.82%)	<-10	0.463	0.686	Mutual exclusivity
GRID1	10q23.1-q23.2	0 (0.00%)	2 (0.82%)	<-10	0.463	0.686	Mutual exclusivity
GRK4	4p16.3	0 (0.00%)	2 (0.82%)	<-10	0.463	0.686	Mutual exclusivity
GRPEL1	4p16.1	0 (0.00%)	2 (0.82%)	<-10	0.463	0.686	Mutual exclusivity
GRXCR1	4p13	0 (0.00%)	2 (0.82%)	<-10	0.463	0.686	Mutual exclusivity
GRXCR2	5q32	0 (0.00%)	2 (0.82%)	<-10	0.463	0.686	Mutual exclusivity
GTF2A1L	2p16.3	0 (0.00%)	2 (0.82%)	<-10	0.463	0.686	Mutual exclusivity
GTF3C1	16p12.1	0 (0.00%)	2 (0.82%)	<-10	0.463	0.686	Mutual exclusivity
GUCY2EP	11q13.5	0 (0.00%)	2 (0.82%)	<-10	0.463	0.686	Mutual exclusivity
GUSBP11	22q11.23	0 (0.00%)	2 (0.82%)	<-10	0.463	0.686	Mutual exclusivity
GYS1	19q13.33	0 (0.00%)	2 (0.82%)	<-10	0.463	0.686	Mutual exclusivity
GZMA	5q11.2	0 (0.00%)	2 (0.82%)	<-10	0.463	0.686	Mutual exclusivity
GZMK	5q11.2	0 (0.00%)	2 (0.82%)	<-10	0.463	0.686	Mutual exclusivity
H1FX	3q21.3	0 (0.00%)	2 (0.82%)	<-10	0.463	0.686	Mutual exclusivity
H2AFX	11q23.3	0 (0.00%)	2 (0.82%)	<-10	0.463	0.686	Mutual exclusivity
H2AFY	5q31.1	0 (0.00%)	2 (0.82%)	<-10	0.463	0.686	Mutual exclusivity
HAS1	19q13.41	0 (0.00%)	2 (0.82%)	<-10	0.463	0.686	Mutual exclusivity
HCN1	5p12	0 (0.00%)	2 (0.82%)	<-10	0.463	0.686	Mutual exclusivity
HDAC3	5q31.3	0 (0.00%)	2 (0.82%)	<-10	0.463	0.686	Mutual exclusivity
HDAC9	7p21.1	0 (0.00%)	2 (0.82%)	<-10	0.463	0.686	Mutual exclusivity

HELT	4q35.1	0 (0.00%)	2 (0.82%)	<-10	0.463	0.686	Mutual exclusivity
HES6	2q37.3	0 (0.00%)	2 (0.82%)	<-10	0.463	0.686	Mutual exclusivity
HGFAC	4p16.3	0 (0.00%)	2 (0.82%)	<-10	0.463	0.686	Mutual exclusivity
HIF3A	19q13.32	0 (0.00%)	2 (0.82%)	<-10	0.463	0.686	Mutual exclusivity
HIVEP2	6q24.2	0 (0.00%)	2 (0.82%)	<-10	0.463	0.686	Mutual exclusivity
HMBS	11q23.3	0 (0.00%)	2 (0.82%)	<-10	0.463	0.686	Mutual exclusivity
HMCES	3q21.3	0 (0.00%)	2 (0.82%)	<-10	0.463	0.686	Mutual exclusivity
HMGXB3	5q32	0 (0.00%)	2 (0.82%)	<-10	0.463	0.686	Mutual exclusivity
HMHB1	5q31.3	0 (0.00%)	2 (0.82%)	<-10	0.463	0.686	Mutual exclusivity
HMX1	4p16.1	0 (0.00%)	2 (0.82%)	<-10	0.463	0.686	Mutual exclusivity
HNRNPF	10q11.21	0 (0.00%)	2 (0.82%)	<-10	0.463	0.686	Mutual exclusivity
HNRNPUL1	19q13.2	0 (0.00%)	2 (0.82%)	<-10	0.463	0.686	Mutual exclusivity
HOMER1	5q14.1	0 (0.00%)	2 (0.82%)	<-10	0.463	0.686	Mutual exclusivity
HPRT1	Xq26.2-q26.3	0 (0.00%)	2 (0.82%)	<-10	0.463	0.686	Mutual exclusivity
HRC	19q13.33	0 (0.00%)	2 (0.82%)	<-10	0.463	0.686	Mutual exclusivity
HS3ST1	4p15.33	0 (0.00%)	2 (0.82%)	<-10	0.463	0.686	Mutual exclusivity
HSD17B14	19q13.33	0 (0.00%)	2 (0.82%)	<-10	0.463	0.686	Mutual exclusivity
HSP90AB2P	4p15.33	0 (0.00%)	2 (0.82%)	<-10	0.463	0.686	Mutual exclusivity
HTATSF1	Xq26.3	0 (0.00%)	2 (0.82%)	<-10	0.463	0.686	Mutual exclusivity
HTR1E	6q14.3	0 (0.00%)	2 (0.82%)	<-10	0.463	0.686	Mutual exclusivity
HTR2C	Xq23	0 (0.00%)	2 (0.82%)	<-10	0.463	0.686	Mutual exclusivity
HTRA3	4p16.1	0 (0.00%)	2 (0.82%)	<-10	0.463	0.686	Mutual exclusivity
HTT	4p16.3	0 (0.00%)	2 (0.82%)	<-10	0.463	0.686	Mutual exclusivity
HYOU1	11q23.3	0 (0.00%)	2 (0.82%)	<-10	0.463	0.686	Mutual exclusivity
IDUA	4p16.3	0 (0.00%)	2 (0.82%)	<-10	0.463	0.686	Mutual exclusivity
IFFO1	12p13.31	0 (0.00%)	2 (0.82%)	<-10	0.463	0.686	Mutual exclusivity
IFFO2	1p36.13	0 (0.00%)	2 (0.82%)	<-10	0.463	0.686	Mutual exclusivity
IFI27	14q32.12	0 (0.00%)	2 (0.82%)	<-10	0.463	0.686	Mutual exclusivity
IFI27L1	14q32.12	0 (0.00%)	2 (0.82%)	<-10	0.463	0.686	Mutual exclusivity

IFI27L2	14q32.12	0 (0.00%)	2 (0.82%)	<-10	0.463	0.686	Mutual exclusivity
IFNAR2	21q22.11	0 (0.00%)	2 (0.82%)	<-10	0.463	0.686	Mutual exclusivity
IGFBP7	4q12	0 (0.00%)	2 (0.82%)	<-10	0.463	0.686	Mutual exclusivity
IGFL1	19q13.32	0 (0.00%)	2 (0.82%)	<-10	0.463	0.686	Mutual exclusivity
IGFL2	19q13.32	0 (0.00%)	2 (0.82%)	<-10	0.463	0.686	Mutual exclusivity
IGFL3	19q13.32	0 (0.00%)	2 (0.82%)	<-10	0.463	0.686	Mutual exclusivity
IGFL4	19q13.32	0 (0.00%)	2 (0.82%)	<-10	0.463	0.686	Mutual exclusivity
IGKC	2p11.2	0 (0.00%)	2 (0.82%)	<-10	0.463	0.686	Mutual exclusivity
IGKJ1	2p11.2	0 (0.00%)	2 (0.82%)	<-10	0.463	0.686	Mutual exclusivity
IGKJ2	2p11.2	0 (0.00%)	2 (0.82%)	<-10	0.463	0.686	Mutual exclusivity
IGKJ3	2p11.2	0 (0.00%)	2 (0.82%)	<-10	0.463	0.686	Mutual exclusivity
IGKJ4	2p11.2	0 (0.00%)	2 (0.82%)	<-10	0.463	0.686	Mutual exclusivity
IGKJ5	2p11.2	0 (0.00%)	2 (0.82%)	<-10	0.463	0.686	Mutual exclusivity
IGLON5	19q13.41	0 (0.00%)	2 (0.82%)	<-10	0.463	0.686	Mutual exclusivity
IL13RA2	Xq23	0 (0.00%)	2 (0.82%)	<-10	0.463	0.686	Mutual exclusivity
IL18R1	2q12.1	0 (0.00%)	2 (0.82%)	<-10	0.463	0.686	Mutual exclusivity
IL18RAP	2q12.1	0 (0.00%)	2 (0.82%)	<-10	0.463	0.686	Mutual exclusivity
IL1RL1	2q12.1	0 (0.00%)	2 (0.82%)	<-10	0.463	0.686	Mutual exclusivity
IL1RL2	2q12.1	0 (0.00%)	2 (0.82%)	<-10	0.463	0.686	Mutual exclusivity
IL22RA1	1p36.11	0 (0.00%)	2 (0.82%)	<-10	0.463	0.686	Mutual exclusivity
IL31RA	5q11.2	0 (0.00%)	2 (0.82%)	<-10	0.463	0.686	Mutual exclusivity
ILKAP	2q37.3	0 (0.00%)	2 (0.82%)	<-10	0.463	0.686	Mutual exclusivity
IMPG2	3q12.3	0 (0.00%)	2 (0.82%)	<-10	0.463	0.686	Mutual exclusivity
INAFM1	19q13.32	0 (0.00%)	2 (0.82%)	<-10	0.463	0.686	Mutual exclusivity
INO80D	2q33.3	0 (0.00%)	2 (0.82%)	<-10	0.463	0.686	Mutual exclusivity
INSM2	14q13.2	0 (0.00%)	2 (0.82%)	<-10	0.463	0.686	Mutual exclusivity
INTS6L	Xq26.3	0 (0.00%)	2 (0.82%)	<-10	0.463	0.686	Mutual exclusivity
IPCEF1	6q25.2	0 (0.00%)	2 (0.82%)	<-10	0.463	0.686	Mutual exclusivity
ISY1	3q21.3	0 (0.00%)	2 (0.82%)	<-10	0.463	0.686	Mutual exclusivity

ITGAD	16p11.2	0 (0.00%)	2 (0.82%)	<-10	0.463	0.686	Mutual exclusivity
ITGAX	16p11.2	0 (0.00%)	2 (0.82%)	<-10	0.463	0.686	Mutual exclusivity
ITGB1BP2	Xq13.1	0 (0.00%)	2 (0.82%)	<-10	0.463	0.686	Mutual exclusivity
ITGB8	7p21.1	0 (0.00%)	2 (0.82%)	<-10	0.463	0.686	Mutual exclusivity
IZUMO1	19q13.33	0 (0.00%)	2 (0.82%)	<-10	0.463	0.686	Mutual exclusivity
JAKMIP1	4p16.1	0 (0.00%)	2 (0.82%)	<-10	0.463	0.686	Mutual exclusivity
JMY	5q14.1	0 (0.00%)	2 (0.82%)	<-10	0.463	0.686	Mutual exclusivity
KANK2	19p13.2	0 (0.00%)	2 (0.82%)	<-10	0.463	0.686	Mutual exclusivity
KANSL1L	2q34	0 (0.00%)	2 (0.82%)	<-10	0.463	0.686	Mutual exclusivity
KCNA7	19q13.33	0 (0.00%)	2 (0.82%)	<-10	0.463	0.686	Mutual exclusivity
KCNE3	11q13.4	0 (0.00%)	2 (0.82%)	<-10	0.463	0.686	Mutual exclusivity
KCNIP3	2q11.1	0 (0.00%)	2 (0.82%)	<-10	0.463	0.686	Mutual exclusivity
KCNK12	2p16.3	0 (0.00%)	2 (0.82%)	<-10	0.463	0.686	Mutual exclusivity
KCTD8	4p13	0 (0.00%)	2 (0.82%)	<-10	0.463	0.686	Mutual exclusivity
KHDC3L	6q13	0 (0.00%)	2 (0.82%)	<-10	0.463	0.686	Mutual exclusivity
KIAA0232	4p16.1	0 (0.00%)	2 (0.82%)	<-10	0.463	0.686	Mutual exclusivity
KIAA1211	4q12	0 (0.00%)	2 (0.82%)	<-10	0.463	0.686	Mutual exclusivity
KIF25	6q27	0 (0.00%)	2 (0.82%)	<-10	0.463	0.686	Mutual exclusivity
KLF3	4p14	0 (0.00%)	2 (0.82%)	<-10	0.463	0.686	Mutual exclusivity
KLF7	2q33.3	0 (0.00%)	2 (0.82%)	<-10	0.463	0.686	Mutual exclusivity
KLHDC7A	1p36.13	0 (0.00%)	2 (0.82%)	<-10	0.463	0.686	Mutual exclusivity
KLHL13	Xq24	0 (0.00%)	2 (0.82%)	<-10	0.463	0.686	Mutual exclusivity
KLHL28	14q21.2	0 (0.00%)	2 (0.82%)	<-10	0.463	0.686	Mutual exclusivity
KLHL30	2q37.3	0 (0.00%)	2 (0.82%)	<-10	0.463	0.686	Mutual exclusivity
KLK13	19q13.41	0 (0.00%)	2 (0.82%)	<-10	0.463	0.686	Mutual exclusivity
KLK14	19q13.41	0 (0.00%)	2 (0.82%)	<-10	0.463	0.686	Mutual exclusivity
LAG3	12p13.31	0 (0.00%)	2 (0.82%)	<-10	0.463	0.686	Mutual exclusivity
LAMP2	Xq24	0 (0.00%)	2 (0.82%)	<-10	0.463	0.686	Mutual exclusivity
LARS	5q32	0 (0.00%)	2 (0.82%)	<-10	0.463	0.686	Mutual exclusivity

LDB2	4p15.32	0 (0.00%)	2 (0.82%)	<-10	0.463	0.686	Mutual exclusivity
LDLR	19p13.2	0 (0.00%)	2 (0.82%)	<-10	0.463	0.686	Mutual exclusivity
LDLRAD3	11p13	0 (0.00%)	2 (0.82%)	<-10	0.463	0.686	Mutual exclusivity
LDLRAP1	1p36.11	0 (0.00%)	2 (0.82%)	<-10	0.463	0.686	Mutual exclusivity
LDOC1	Xq27.1	0 (0.00%)	2 (0.82%)	<-10	0.463	0.686	Mutual exclusivity
LGI2	4p15.2	0 (0.00%)	2 (0.82%)	<-10	0.463	0.686	Mutual exclusivity
LHB	19q13.33	0 (0.00%)	2 (0.82%)	<-10	0.463	0.686	Mutual exclusivity
LHCGR	2p16.3	0 (0.00%)	2 (0.82%)	<-10	0.463	0.686	Mutual exclusivity
LIM2	19q13.41	0 (0.00%)	2 (0.82%)	<-10	0.463	0.686	Mutual exclusivity
LIMCH1	4p13	0 (0.00%)	2 (0.82%)	<-10	0.463	0.686	Mutual exclusivity
LIN28B	6q16.3-q21	0 (0.00%)	2 (0.82%)	<-10	0.463	0.686	Mutual exclusivity
LIN7B	19q13.33	0 (0.00%)	2 (0.82%)	<-10	0.463	0.686	Mutual exclusivity
LINC00222	6q21	0 (0.00%)	2 (0.82%)	<-10	0.463	0.686	Mutual exclusivity
LINC00261	20p11.21	0 (0.00%)	2 (0.82%)	<-10	0.463	0.686	Mutual exclusivity
LINC00342	2q11.1	0 (0.00%)	2 (0.82%)	<-10	0.463	0.686	Mutual exclusivity
LINC00471	2q37.1	0 (0.00%)	2 (0.82%)	<-10	0.463	0.686	Mutual exclusivity
LINC00472	6q13	0 (0.00%)	2 (0.82%)	<-10	0.463	0.686	Mutual exclusivity
LINC00473	6q27	0 (0.00%)	2 (0.82%)	<-10	0.463	0.686	Mutual exclusivity
LINC00491	5q21.1	0 (0.00%)	2 (0.82%)	<-10	0.463	0.686	Mutual exclusivity
LINC00492	5q21.1	0 (0.00%)	2 (0.82%)	<-10	0.463	0.686	Mutual exclusivity
LINC00517	14q21.1	0 (0.00%)	2 (0.82%)	<-10	0.463	0.686	Mutual exclusivity
LINC00519	14q21.3	0 (0.00%)	2 (0.82%)	<-10	0.463	0.686	Mutual exclusivity
LINC00609	14q13.2-q13.3	0 (0.00%)	2 (0.82%)	<-10	0.463	0.686	Mutual exclusivity
LINC00610	11p13	0 (0.00%)	2 (0.82%)	<-10	0.463	0.686	Mutual exclusivity
LINC00629	Xq26.3	0 (0.00%)	2 (0.82%)	<-10	0.463	0.686	Mutual exclusivity
LINC00632	Xq27.1	0 (0.00%)	2 (0.82%)	<-10	0.463	0.686	Mutual exclusivity
LINC00639	14q21.1	0 (0.00%)	2 (0.82%)	<-10	0.463	0.686	Mutual exclusivity
LINC00640	14q22.1	0 (0.00%)	2 (0.82%)	<-10	0.463	0.686	Mutual exclusivity
LINC00682	4p13	0 (0.00%)	2 (0.82%)	<-10	0.463	0.686	Mutual exclusivity

LINC00842	10q11.22	0 (0.00%)	2 (0.82%)	<-10	0.463	0.686	Mutual exclusivity
LINC00892	Xq26.3	0 (0.00%)	2 (0.82%)	<-10	0.463	0.686	Mutual exclusivity
LINC00907	18q12.3	0 (0.00%)	2 (0.82%)	<-10	0.463	0.686	Mutual exclusivity
LINC00955	4p16.3	0 (0.00%)	2 (0.82%)	<-10	0.463	0.686	Mutual exclusivity
LINC01565	3q21.3	0 (0.00%)	2 (0.82%)	<-10	0.463	0.686	Mutual exclusivity
LINC01587	4p16.2	0 (0.00%)	2 (0.82%)	<-10	0.463	0.686	Mutual exclusivity
LIPT2	11q13.4	0 (0.00%)	2 (0.82%)	<-10	0.463	0.686	Mutual exclusivity
LONRF3	Xq24	0 (0.00%)	2 (0.82%)	<-10	0.463	0.686	Mutual exclusivity
LRCH2	Xq23	0 (0.00%)	2 (0.82%)	<-10	0.463	0.686	Mutual exclusivity
LRP2BP	4q35.1	0 (0.00%)	2 (0.82%)	<-10	0.463	0.686	Mutual exclusivity
LRPAP1	4p16.3	0 (0.00%)	2 (0.82%)	<-10	0.463	0.686	Mutual exclusivity
LRRC23	12p13.31	0 (0.00%)	2 (0.82%)	<-10	0.463	0.686	Mutual exclusivity
LRRC32	11q13.5	0 (0.00%)	2 (0.82%)	<-10	0.463	0.686	Mutual exclusivity
LRRC72	7p21.1	0 (0.00%)	2 (0.82%)	<-10	0.463	0.686	Mutual exclusivity
LRRTM2	5q31.2	0 (0.00%)	2 (0.82%)	<-10	0.463	0.686	Mutual exclusivity
LUZP4	Xq23	0 (0.00%)	2 (0.82%)	<-10	0.463	0.686	Mutual exclusivity
LYAR	4p16.3	0 (0.00%)	2 (0.82%)	<-10	0.463	0.686	Mutual exclusivity
MAB21L4	2q37.3	0 (0.00%)	2 (0.82%)	<-10	0.463	0.686	Mutual exclusivity
MACC1	7p21.1	0 (0.00%)	2 (0.82%)	<-10	0.463	0.686	Mutual exclusivity
MAEA	4p16.3	0 (0.00%)	2 (0.82%)	<-10	0.463	0.686	Mutual exclusivity
MAGEC1	Xq27.2	0 (0.00%)	2 (0.82%)	<-10	0.463	0.686	Mutual exclusivity
MAGEC2	Xq27.2	0 (0.00%)	2 (0.82%)	<-10	0.463	0.686	Mutual exclusivity
MAGEC3	Xq27.2	0 (0.00%)	2 (0.82%)	<-10	0.463	0.686	Mutual exclusivity
MAL	2q11.1	0 (0.00%)	2 (0.82%)	<-10	0.463	0.686	Mutual exclusivity
MAMSTR	19q13.33	0 (0.00%)	2 (0.82%)	<-10	0.463	0.686	Mutual exclusivity
MAN1C1	1p36.11	0 (0.00%)	2 (0.82%)	<-10	0.463	0.686	Mutual exclusivity
MAN2B2	4p16.1	0 (0.00%)	2 (0.82%)	<-10	0.463	0.686	Mutual exclusivity
MANEA	6q16.1	0 (0.00%)	2 (0.82%)	<-10	0.463	0.686	Mutual exclusivity
MAP2	2q34	0 (0.00%)	2 (0.82%)	<-10	0.463	0.686	Mutual exclusivity

MAP3K4	6q26	0 (0.00%)	2 (0.82%)	<-10	0.463	0.686	Mutual exclusivity
MAP4K4	2q11.2	0 (0.00%)	2 (0.82%)	<-10	0.463	0.686	Mutual exclusivity
MAP4K5	14q22.1	0 (0.00%)	2 (0.82%)	<-10	0.463	0.686	Mutual exclusivity
MAP7D3	Xq26.3	0 (0.00%)	2 (0.82%)	<-10	0.463	0.686	Mutual exclusivity
8-Mar	10q11.21-q11.22	0 (0.00%)	2 (0.82%)	<-10	0.463	0.686	Mutual exclusivity
MBD4	3q21.3	0 (0.00%)	2 (0.82%)	<-10	0.463	0.686	Mutual exclusivity
MBIP	14q13.3	0 (0.00%)	2 (0.82%)	<-10	0.463	0.686	Mutual exclusivity
MCF2	Xq27.1	0 (0.00%)	2 (0.82%)	<-10	0.463	0.686	Mutual exclusivity
MCFD2	2p21	0 (0.00%)	2 (0.82%)	<-10	0.463	0.686	Mutual exclusivity
MCIDAS	5q11.2	0 (0.00%)	2 (0.82%)	<-10	0.463	0.686	Mutual exclusivity
MCTP1	5q15	0 (0.00%)	2 (0.82%)	<-10	0.463	0.686	Mutual exclusivity
MDH1	2p15	0 (0.00%)	2 (0.82%)	<-10	0.463	0.686	Mutual exclusivity
MDH1B	2q33.3	0 (0.00%)	2 (0.82%)	<-10	0.463	0.686	Mutual exclusivity
ME3	11q14.2	0 (0.00%)	2 (0.82%)	<-10	0.463	0.686	Mutual exclusivity
MED25	19q13.33	0 (0.00%)	2 (0.82%)	<-10	0.463	0.686	Mutual exclusivity
MERTK	2q13	0 (0.00%)	2 (0.82%)	<-10	0.463	0.686	Mutual exclusivity
METTL21A	2q33.3	0 (0.00%)	2 (0.82%)	<-10	0.463	0.686	Mutual exclusivity
MFF	2q36.3	0 (0.00%)	2 (0.82%)	<-10	0.463	0.686	Mutual exclusivity
MFSD10	4p16.3	0 (0.00%)	2 (0.82%)	<-10	0.463	0.686	Mutual exclusivity
MFSD9	2q12.1	0 (0.00%)	2 (0.82%)	<-10	0.463	0.686	Mutual exclusivity
MGST2	4q31.1	0 (0.00%)	2 (0.82%)	<-10	0.463	0.686	Mutual exclusivity
MIA2	14q21.1	0 (0.00%)	2 (0.82%)	<-10	0.463	0.686	Mutual exclusivity
MIA2	14q13.2	0 (0.00%)	2 (0.82%)	<-10	0.463	0.686	Mutual exclusivity
MIF	22q11.23	0 (0.00%)	2 (0.82%)	<-10	0.463	0.686	Mutual exclusivity
MIR-1183/1183		0 (0.00%)	2 (0.82%)	<-10	0.463	0.686	Mutual exclusivity
MIR-1256/1256		0 (0.00%)	2 (0.82%)	<-10	0.463	0.686	Mutual exclusivity
MIR-1288/1288		0 (0.00%)	2 (0.82%)	<-10	0.463	0.686	Mutual exclusivity
MIR-1471/1471		0 (0.00%)	2 (0.82%)	<-10	0.463	0.686	Mutual exclusivity
MIR-1913/1913		0 (0.00%)	2 (0.82%)	<-10	0.463	0.686	Mutual exclusivity

MIR-3146/3146		0 (0.00%)	2 (0.82%)	<-10	0.463	0.686	Mutual exclusivity
MIR-3191/3191		0 (0.00%)	2 (0.82%)	<-10	0.463	0.686	Mutual exclusivity
MIR-3656/3656		0 (0.00%)	2 (0.82%)	<-10	0.463	0.686	Mutual exclusivity
MIR-3661/3661		0 (0.00%)	2 (0.82%)	<-10	0.463	0.686	Mutual exclusivity
MIR-3672/3672		0 (0.00%)	2 (0.82%)	<-10	0.463	0.686	Mutual exclusivity
MIR-3920/3920		0 (0.00%)	2 (0.82%)	<-10	0.463	0.686	Mutual exclusivity
MIR-3945/3945		0 (0.00%)	2 (0.82%)	<-10	0.463	0.686	Mutual exclusivity
MIR-4274/4274		0 (0.00%)	2 (0.82%)	<-10	0.463	0.686	Mutual exclusivity
MIR-4282/4282		0 (0.00%)	2 (0.82%)	<-10	0.463	0.686	Mutual exclusivity
MIR-4308/4308		0 (0.00%)	2 (0.82%)	<-10	0.463	0.686	Mutual exclusivity
MIR-4455/4455		0 (0.00%)	2 (0.82%)	<-10	0.463	0.686	Mutual exclusivity
MIR-4461/4461		0 (0.00%)	2 (0.82%)	<-10	0.463	0.686	Mutual exclusivity
MIR-4492/4492		0 (0.00%)	2 (0.82%)	<-10	0.463	0.686	Mutual exclusivity
MIR-4506/4506		0 (0.00%)	2 (0.82%)	<-10	0.463	0.686	Mutual exclusivity
MIR-4644/4644		0 (0.00%)	2 (0.82%)	<-10	0.463	0.686	Mutual exclusivity
MIR-4780/4780		0 (0.00%)	2 (0.82%)	<-10	0.463	0.686	Mutual exclusivity
MIR-5091/5091		0 (0.00%)	2 (0.82%)	<-10	0.463	0.686	Mutual exclusivity
MIR-548P/548P		0 (0.00%)	2 (0.82%)	<-10	0.463	0.686	Mutual exclusivity
MIR-559/559		0 (0.00%)	2 (0.82%)	<-10	0.463	0.686	Mutual exclusivity
MIR-562/562		0 (0.00%)	2 (0.82%)	<-10	0.463	0.686	Mutual exclusivity
MIR-5696/5696		0 (0.00%)	2 (0.82%)	<-10	0.463	0.686	Mutual exclusivity
MIR-5704/5704		0 (0.00%)	2 (0.82%)	<-10	0.463	0.686	Mutual exclusivity
MIR-5705/5705		0 (0.00%)	2 (0.82%)	<-10	0.463	0.686	Mutual exclusivity
MIR-571/571		0 (0.00%)	2 (0.82%)	<-10	0.463	0.686	Mutual exclusivity
MIR-572/572		0 (0.00%)	2 (0.82%)	<-10	0.463	0.686	Mutual exclusivity
MIR-583/583		0 (0.00%)	2 (0.82%)	<-10	0.463	0.686	Mutual exclusivity
MIR-643/643		0 (0.00%)	2 (0.82%)	<-10	0.463	0.686	Mutual exclusivity
MIR-934/934		0 (0.00%)	2 (0.82%)	<-10	0.463	0.686	Mutual exclusivity
MIR-943/943		0 (0.00%)	2 (0.82%)	<-10	0.463	0.686	Mutual exclusivity

MIR503HG	Xq26.3	0 (0.00%)	2 (0.82%)	<-10	0.463	0.686	Mutual exclusivity
MIR924HG	18q12.2-q12.3	0 (0.00%)	2 (0.82%)	<-10	0.463	0.686	Mutual exclusivity
MLF2	12p13.31	0 (0.00%)	2 (0.82%)	<-10	0.463	0.686	Mutual exclusivity
MMGT1	Xq26.3	0 (0.00%)	2 (0.82%)	<-10	0.463	0.686	Mutual exclusivity
MMP1	11q22.2	0 (0.00%)	2 (0.82%)	<-10	0.463	0.686	Mutual exclusivity
MMP10	11q22.2	0 (0.00%)	2 (0.82%)	<-10	0.463	0.686	Mutual exclusivity
MMP11	22q11.23	0 (0.00%)	2 (0.82%)	<-10	0.463	0.686	Mutual exclusivity
MMP12	11q22.2	0 (0.00%)	2 (0.82%)	<-10	0.463	0.686	Mutual exclusivity
MMP13	11q22.2	0 (0.00%)	2 (0.82%)	<-10	0.463	0.686	Mutual exclusivity
MMP15	16q21	0 (0.00%)	2 (0.82%)	<-10	0.463	0.686	Mutual exclusivity
MMP20	11q22.2	0 (0.00%)	2 (0.82%)	<-10	0.463	0.686	Mutual exclusivity
MMP3	11q22.2	0 (0.00%)	2 (0.82%)	<-10	0.463	0.686	Mutual exclusivity
MMP7	11q22.2	0 (0.00%)	2 (0.82%)	<-10	0.463	0.686	Mutual exclusivity
MMP8	11q22.2	0 (0.00%)	2 (0.82%)	<-10	0.463	0.686	Mutual exclusivity
MOSPD1	Xq26.3	0 (0.00%)	2 (0.82%)	<-10	0.463	0.686	Mutual exclusivity
MPL	1p34.2	0 (0.00%)	2 (0.82%)	<-10	0.463	0.686	Mutual exclusivity
MPRIP	17p11.2	0 (0.00%)	2 (0.82%)	<-10	0.463	0.686	Mutual exclusivity
MREG	2q35	0 (0.00%)	2 (0.82%)	<-10	0.463	0.686	Mutual exclusivity
MRFAP1	4p16.1	0 (0.00%)	2 (0.82%)	<-10	0.463	0.686	Mutual exclusivity
MRFAP1L1	4p16.1	0 (0.00%)	2 (0.82%)	<-10	0.463	0.686	Mutual exclusivity
MROH2B	5p13.1	0 (0.00%)	2 (0.82%)	<-10	0.463	0.686	Mutual exclusivity
MRPL3	3q22.1	0 (0.00%)	2 (0.82%)	<-10	0.463	0.686	Mutual exclusivity
MRPL51	12p13.31	0 (0.00%)	2 (0.82%)	<-10	0.463	0.686	Mutual exclusivity
MRPS30	5p12	0 (0.00%)	2 (0.82%)	<-10	0.463	0.686	Mutual exclusivity
MRPS5	2q11.1	0 (0.00%)	2 (0.82%)	<-10	0.463	0.686	Mutual exclusivity
MS4A5	11q12.2	0 (0.00%)	2 (0.82%)	<-10	0.463	0.686	Mutual exclusivity
MSANTD1	4p16.3	0 (0.00%)	2 (0.82%)	<-10	0.463	0.686	Mutual exclusivity
MSH2	2p21-p16.3	0 (0.00%)	2 (0.82%)	<-10	0.463	0.686	Mutual exclusivity
MSH6	2p16.3	0 (0.00%)	2 (0.82%)	<-10	0.463	0.686	Mutual exclusivity

MSTN	2q32.2	0 (0.00%)	2 (0.82%)	<-10	0.463	0.686	Mutual exclusivity
MSX1	4p16.2	0 (0.00%)	2 (0.82%)	<-10	0.463	0.686	Mutual exclusivity
MTA2	11q12.3	0 (0.00%)	2 (0.82%)	<-10	0.463	0.686	Mutual exclusivity
MTFR2	6q23.3	0 (0.00%)	2 (0.82%)	<-10	0.463	0.686	Mutual exclusivity
MTMR7	8p22	0 (0.00%)	2 (0.82%)	<-10	0.463	0.686	Mutual exclusivity
MTO1	6q13	0 (0.00%)	2 (0.82%)	<-10	0.463	0.686	Mutual exclusivity
MTREX	5q11.2	0 (0.00%)	2 (0.82%)	<-10	0.463	0.686	Mutual exclusivity
MTX3	5q14.1	0 (0.00%)	2 (0.82%)	<-10	0.463	0.686	Mutual exclusivity
MYL5	4p16.3	0 (0.00%)	2 (0.82%)	<-10	0.463	0.686	Mutual exclusivity
MYO6	6q14.1	0 (0.00%)	2 (0.82%)	<-10	0.463	0.686	Mutual exclusivity
MYOM3	1p36.11	0 (0.00%)	2 (0.82%)	<-10	0.463	0.686	Mutual exclusivity
NAALAD2	11q14.3	0 (0.00%)	2 (0.82%)	<-10	0.463	0.686	Mutual exclusivity
NABP1	2q32.3	0 (0.00%)	2 (0.82%)	<-10	0.463	0.686	Mutual exclusivity
NAT8L	4p16.3	0 (0.00%)	2 (0.82%)	<-10	0.463	0.686	Mutual exclusivity
NBL1	1p36.13	0 (0.00%)	2 (0.82%)	<-10	0.463	0.686	Mutual exclusivity
NBPF3	1p36.12	0 (0.00%)	2 (0.82%)	<-10	0.463	0.686	Mutual exclusivity
NCAPD2	12p13.31	0 (0.00%)	2 (0.82%)	<-10	0.463	0.686	Mutual exclusivity
NCL	2q37.1	0 (0.00%)	2 (0.82%)	<-10	0.463	0.686	Mutual exclusivity
NCOR1	17p12-p11.2	0 (0.00%)	2 (0.82%)	<-10	0.463	0.686	Mutual exclusivity
NDFIP1	5q31.3	0 (0.00%)	2 (0.82%)	<-10	0.463	0.686	Mutual exclusivity
NDUFAB1	16p12.2	0 (0.00%)	2 (0.82%)	<-10	0.463	0.686	Mutual exclusivity
NDUFS1	2q33.3	0 (0.00%)	2 (0.82%)	<-10	0.463	0.686	Mutual exclusivity
NDUFS7	19p13.3	0 (0.00%)	2 (0.82%)	<-10	0.463	0.686	Mutual exclusivity
NEK11	3q22.1	0 (0.00%)	2 (0.82%)	<-10	0.463	0.686	Mutual exclusivity
NELFA	4p16.3	0 (0.00%)	2 (0.82%)	<-10	0.463	0.686	Mutual exclusivity
NEUROG1	5q31.1	0 (0.00%)	2 (0.82%)	<-10	0.463	0.686	Mutual exclusivity
NFATC3	16q22.1	0 (0.00%)	2 (0.82%)	<-10	0.463	0.686	Mutual exclusivity
NFIX	19p13.13	0 (0.00%)	2 (0.82%)	<-10	0.463	0.686	Mutual exclusivity
NFKBIZ	3q12.3	0 (0.00%)	2 (0.82%)	<-10	0.463	0.686	Mutual exclusivity

NID2	14q22.1	0 (0.00%)	2 (0.82%)	<-10	0.463	0.686	Mutual exclusivity
NIN	14q22.1	0 (0.00%)	2 (0.82%)	<-10	0.463	0.686	Mutual exclusivity
NKAPP1	Xq24	0 (0.00%)	2 (0.82%)	<-10	0.463	0.686	Mutual exclusivity
NKG7	19q13.41	0 (0.00%)	2 (0.82%)	<-10	0.463	0.686	Mutual exclusivity
NMU	4q12	0 (0.00%)	2 (0.82%)	<-10	0.463	0.686	Mutual exclusivity
NMUR1	2q37.1	0 (0.00%)	2 (0.82%)	<-10	0.463	0.686	Mutual exclusivity
NOA1	4q12	0 (0.00%)	2 (0.82%)	<-10	0.463	0.686	Mutual exclusivity
NONO	Xq13.1	0 (0.00%)	2 (0.82%)	<-10	0.463	0.686	Mutual exclusivity
NOP14	4p16.3	0 (0.00%)	2 (0.82%)	<-10	0.463	0.686	Mutual exclusivity
NPAS1	19q13.32	0 (0.00%)	2 (0.82%)	<-10	0.463	0.686	Mutual exclusivity
NPHP3	3q22.1	0 (0.00%)	2 (0.82%)	<-10	0.463	0.686	Mutual exclusivity
NPPC	2q37.1	0 (0.00%)	2 (0.82%)	<-10	0.463	0.686	Mutual exclusivity
NPY4R	10q11.22	0 (0.00%)	2 (0.82%)	<-10	0.463	0.686	Mutual exclusivity
NR2E1	6q21	0 (0.00%)	2 (0.82%)	<-10	0.463	0.686	Mutual exclusivity
NRP2	2q33.3	0 (0.00%)	2 (0.82%)	<-10	0.463	0.686	Mutual exclusivity
NRXN1	2p16.3	0 (0.00%)	2 (0.82%)	<-10	0.463	0.686	Mutual exclusivity
NSG1	4p16.3	0 (0.00%)	2 (0.82%)	<-10	0.463	0.686	Mutual exclusivity
NSUN7	4p14	0 (0.00%)	2 (0.82%)	<-10	0.463	0.686	Mutual exclusivity
NTF4	19q13.33	0 (0.00%)	2 (0.82%)	<-10	0.463	0.686	Mutual exclusivity
NUCB1	19q13.33	0 (0.00%)	2 (0.82%)	<-10	0.463	0.686	Mutual exclusivity
NUDT12	5q21.2	0 (0.00%)	2 (0.82%)	<-10	0.463	0.686	Mutual exclusivity
NUDT16	3q22.1	0 (0.00%)	2 (0.82%)	<-10	0.463	0.686	Mutual exclusivity
NUDT16P1	3q22.1	0 (0.00%)	2 (0.82%)	<-10	0.463	0.686	Mutual exclusivity
NXPE3	3q12.3	0 (0.00%)	2 (0.82%)	<-10	0.463	0.686	Mutual exclusivity
OGFRL1	6q13	0 (0.00%)	2 (0.82%)	<-10	0.463	0.686	Mutual exclusivity
OMP	11q13.5	0 (0.00%)	2 (0.82%)	<-10	0.463	0.686	Mutual exclusivity
ONECUT2	18q21.31	0 (0.00%)	2 (0.82%)	<-10	0.463	0.686	Mutual exclusivity
OOEP	6q13	0 (0.00%)	2 (0.82%)	<-10	0.463	0.686	Mutual exclusivity
OPCML	11q25	0 (0.00%)	2 (0.82%)	<-10	0.463	0.686	Mutual exclusivity

OR13A1	10q11.21	0 (0.00%)	2 (0.82%)	<-10	0.463	0.686	Mutual exclusivity
OR5AC1	3q11.2	0 (0.00%)	2 (0.82%)	<-10	0.463	0.686	Mutual exclusivity
OR5AC2	3q11.2	0 (0.00%)	2 (0.82%)	<-10	0.463	0.686	Mutual exclusivity
OR5G5P	11q12.1	0 (0.00%)	2 (0.82%)	<-10	0.463	0.686	Mutual exclusivity
OR5H1	3q11.2	0 (0.00%)	2 (0.82%)	<-10	0.463	0.686	Mutual exclusivity
OR5H14	3q11.2	0 (0.00%)	2 (0.82%)	<-10	0.463	0.686	Mutual exclusivity
OR5H15	3q11.2	0 (0.00%)	2 (0.82%)	<-10	0.463	0.686	Mutual exclusivity
OR5H6	3q11.2	0 (0.00%)	2 (0.82%)	<-10	0.463	0.686	Mutual exclusivity
OR8H1	11q12.1	0 (0.00%)	2 (0.82%)	<-10	0.463	0.686	Mutual exclusivity
OR8K1	11q12.1	0 (0.00%)	2 (0.82%)	<-10	0.463	0.686	Mutual exclusivity
OR8K3	11q12.1	0 (0.00%)	2 (0.82%)	<-10	0.463	0.686	Mutual exclusivity
ORC3	6q15	0 (0.00%)	2 (0.82%)	<-10	0.463	0.686	Mutual exclusivity
ORMDL1	2q32.2	0 (0.00%)	2 (0.82%)	<-10	0.463	0.686	Mutual exclusivity
OSGEPL1	2q32.2	0 (0.00%)	2 (0.82%)	<-10	0.463	0.686	Mutual exclusivity
OSTM1	6q21	0 (0.00%)	2 (0.82%)	<-10	0.463	0.686	Mutual exclusivity
OTOP1	4p16.3	0 (0.00%)	2 (0.82%)	<-10	0.463	0.686	Mutual exclusivity
OTUB2	14q32.12	0 (0.00%)	2 (0.82%)	<-10	0.463	0.686	Mutual exclusivity
OXCT1	5p13.1	0 (0.00%)	2 (0.82%)	<-10	0.463	0.686	Mutual exclusivity
P3H3	12p13.31	0 (0.00%)	2 (0.82%)	<-10	0.463	0.686	Mutual exclusivity
PACRG	6q26	0 (0.00%)	2 (0.82%)	<-10	0.463	0.686	Mutual exclusivity
PACRGL	4p15.31	0 (0.00%)	2 (0.82%)	<-10	0.463	0.686	Mutual exclusivity
PALB2	16p12.2	0 (0.00%)	2 (0.82%)	<-10	0.463	0.686	Mutual exclusivity
PAM	5q21.1	0 (0.00%)	2 (0.82%)	<-10	0.463	0.686	Mutual exclusivity
PARP8	5q11.1	0 (0.00%)	2 (0.82%)	<-10	0.463	0.686	Mutual exclusivity
PCDHA1	5q31.3	0 (0.00%)	2 (0.82%)	<-10	0.463	0.686	Mutual exclusivity
PCDHA10	5q31.3	0 (0.00%)	2 (0.82%)	<-10	0.463	0.686	Mutual exclusivity
PCDHA11	5q31.3	0 (0.00%)	2 (0.82%)	<-10	0.463	0.686	Mutual exclusivity
PCDHA12	5q31.3	0 (0.00%)	2 (0.82%)	<-10	0.463	0.686	Mutual exclusivity
PCDHA13	5q31.3	0 (0.00%)	2 (0.82%)	<-10	0.463	0.686	Mutual exclusivity

PCDHA14	5q31.3	0 (0.00%)	2 (0.82%)	<-10	0.463	0.686	Mutual exclusivity
PCDHA2	5q31.3	0 (0.00%)	2 (0.82%)	<-10	0.463	0.686	Mutual exclusivity
PCDHA3	5q31.3	0 (0.00%)	2 (0.82%)	<-10	0.463	0.686	Mutual exclusivity
PCDHA4	5q31.3	0 (0.00%)	2 (0.82%)	<-10	0.463	0.686	Mutual exclusivity
PCDHA5	5q31.3	0 (0.00%)	2 (0.82%)	<-10	0.463	0.686	Mutual exclusivity
PCDHA6	5q31.3	0 (0.00%)	2 (0.82%)	<-10	0.463	0.686	Mutual exclusivity
PCDHA7	5q31.3	0 (0.00%)	2 (0.82%)	<-10	0.463	0.686	Mutual exclusivity
PCDHA8	5q31.3	0 (0.00%)	2 (0.82%)	<-10	0.463	0.686	Mutual exclusivity
PCDHA9	5q31.3	0 (0.00%)	2 (0.82%)	<-10	0.463	0.686	Mutual exclusivity
PCDHAC1	5q31.3	0 (0.00%)	2 (0.82%)	<-10	0.463	0.686	Mutual exclusivity
PCDHAC2	5q31.3	0 (0.00%)	2 (0.82%)	<-10	0.463	0.686	Mutual exclusivity
PCDHB1	5q31.3	0 (0.00%)	2 (0.82%)	<-10	0.463	0.686	Mutual exclusivity
PCDHB10	5q31.3	0 (0.00%)	2 (0.82%)	<-10	0.463	0.686	Mutual exclusivity
PCDHB11	5q31.3	0 (0.00%)	2 (0.82%)	<-10	0.463	0.686	Mutual exclusivity
PCDHB12	5q31.3	0 (0.00%)	2 (0.82%)	<-10	0.463	0.686	Mutual exclusivity
PCDHB13	5q31.3	0 (0.00%)	2 (0.82%)	<-10	0.463	0.686	Mutual exclusivity
PCDHB14	5q31.3	0 (0.00%)	2 (0.82%)	<-10	0.463	0.686	Mutual exclusivity
PCDHB15	5q31.3	0 (0.00%)	2 (0.82%)	<-10	0.463	0.686	Mutual exclusivity
PCDHB16	5q31.3	0 (0.00%)	2 (0.82%)	<-10	0.463	0.686	Mutual exclusivity
PCDHB17P	5q31.3	0 (0.00%)	2 (0.82%)	<-10	0.463	0.686	Mutual exclusivity
PCDHB18P	5q31.3	0 (0.00%)	2 (0.82%)	<-10	0.463	0.686	Mutual exclusivity
PCDHB2	5q31.3	0 (0.00%)	2 (0.82%)	<-10	0.463	0.686	Mutual exclusivity
PCDHB3	5q31.3	0 (0.00%)	2 (0.82%)	<-10	0.463	0.686	Mutual exclusivity
PCDHB4	5q31.3	0 (0.00%)	2 (0.82%)	<-10	0.463	0.686	Mutual exclusivity
PCDHB5	5q31.3	0 (0.00%)	2 (0.82%)	<-10	0.463	0.686	Mutual exclusivity
PCDHB6	5q31.3	0 (0.00%)	2 (0.82%)	<-10	0.463	0.686	Mutual exclusivity
PCDHB7	5q31.3	0 (0.00%)	2 (0.82%)	<-10	0.463	0.686	Mutual exclusivity
PCDHB8	5q31.3	0 (0.00%)	2 (0.82%)	<-10	0.463	0.686	Mutual exclusivity
PCDHGA1	5q31.3	0 (0.00%)	2 (0.82%)	<-10	0.463	0.686	Mutual exclusivity

PCDHGA10	5q31.3	0 (0.00%)	2 (0.82%)	<-10	0.463	0.686	Mutual exclusivity
PCDHGA11	5q31.3	0 (0.00%)	2 (0.82%)	<-10	0.463	0.686	Mutual exclusivity
PCDHGA12	5q31.3	0 (0.00%)	2 (0.82%)	<-10	0.463	0.686	Mutual exclusivity
PCDHGA2	5q31.3	0 (0.00%)	2 (0.82%)	<-10	0.463	0.686	Mutual exclusivity
PCDHGA3	5q31.3	0 (0.00%)	2 (0.82%)	<-10	0.463	0.686	Mutual exclusivity
PCDHGA4	5q31.3	0 (0.00%)	2 (0.82%)	<-10	0.463	0.686	Mutual exclusivity
PCDHGA5	5q31.3	0 (0.00%)	2 (0.82%)	<-10	0.463	0.686	Mutual exclusivity
PCDHGA6	5q31.3	0 (0.00%)	2 (0.82%)	<-10	0.463	0.686	Mutual exclusivity
PCDHGA7	5q31.3	0 (0.00%)	2 (0.82%)	<-10	0.463	0.686	Mutual exclusivity
PCDHGA8	5q31.3	0 (0.00%)	2 (0.82%)	<-10	0.463	0.686	Mutual exclusivity
PCDHGA9	5q31.3	0 (0.00%)	2 (0.82%)	<-10	0.463	0.686	Mutual exclusivity
PCDHGB1	5q31.3	0 (0.00%)	2 (0.82%)	<-10	0.463	0.686	Mutual exclusivity
PCDHGB2	5q31.3	0 (0.00%)	2 (0.82%)	<-10	0.463	0.686	Mutual exclusivity
PCDHGB3	5q31.3	0 (0.00%)	2 (0.82%)	<-10	0.463	0.686	Mutual exclusivity
PCDHGB4	5q31.3	0 (0.00%)	2 (0.82%)	<-10	0.463	0.686	Mutual exclusivity
PCDHGB6	5q31.3	0 (0.00%)	2 (0.82%)	<-10	0.463	0.686	Mutual exclusivity
PCDHGB7	5q31.3	0 (0.00%)	2 (0.82%)	<-10	0.463	0.686	Mutual exclusivity
PCDHGB8P	5q31.3	0 (0.00%)	2 (0.82%)	<-10	0.463	0.686	Mutual exclusivity
PCDHGC3	5q31.3	0 (0.00%)	2 (0.82%)	<-10	0.463	0.686	Mutual exclusivity
PCDHGC4	5q31.3	0 (0.00%)	2 (0.82%)	<-10	0.463	0.686	Mutual exclusivity
PCDHGC5	5q31.3	0 (0.00%)	2 (0.82%)	<-10	0.463	0.686	Mutual exclusivity
PCGEM1	2q32.3	0 (0.00%)	2 (0.82%)	<-10	0.463	0.686	Mutual exclusivity
PCGF3	4p16.3	0 (0.00%)	2 (0.82%)	<-10	0.463	0.686	Mutual exclusivity
PCNP	3q12.3	0 (0.00%)	2 (0.82%)	<-10	0.463	0.686	Mutual exclusivity
PCSK1	5q15	0 (0.00%)	2 (0.82%)	<-10	0.463	0.686	Mutual exclusivity
PDCL2	4q12	0 (0.00%)	2 (0.82%)	<-10	0.463	0.686	Mutual exclusivity
PDE10A	6q27	0 (0.00%)	2 (0.82%)	<-10	0.463	0.686	Mutual exclusivity
PDE6B	4p16.3	0 (0.00%)	2 (0.82%)	<-10	0.463	0.686	Mutual exclusivity
PDE6D	2q37.1	0 (0.00%)	2 (0.82%)	<-10	0.463	0.686	Mutual exclusivity

PER2	2q37.3	0 (0.00%)	2 (0.82%)	<-10	0.463	0.686	Mutual exclusivity
PGLYRP1	19q13.32	0 (0.00%)	2 (0.82%)	<-10	0.463	0.686	Mutual exclusivity
PGR	11q22.1	0 (0.00%)	2 (0.82%)	<-10	0.463	0.686	Mutual exclusivity
PHACTR2	6q24.2	0 (0.00%)	2 (0.82%)	<-10	0.463	0.686	Mutual exclusivity
PHF6	Xq26.2	0 (0.00%)	2 (0.82%)	<-10	0.463	0.686	Mutual exclusivity
PHKG2	16p11.2	0 (0.00%)	2 (0.82%)	<-10	0.463	0.686	Mutual exclusivity
PHOX2B	4p13	0 (0.00%)	2 (0.82%)	<-10	0.463	0.686	Mutual exclusivity
PI4K2B	4p15.2	0 (0.00%)	2 (0.82%)	<-10	0.463	0.686	Mutual exclusivity
PIGG	4p16.3	0 (0.00%)	2 (0.82%)	<-10	0.463	0.686	Mutual exclusivity
PIGL	17p11.2	0 (0.00%)	2 (0.82%)	<-10	0.463	0.686	Mutual exclusivity
PIK3C3	18q12.3	0 (0.00%)	2 (0.82%)	<-10	0.463	0.686	Mutual exclusivity
PIK3R4	3q22.1	0 (0.00%)	2 (0.82%)	<-10	0.463	0.686	Mutual exclusivity
PLA2G15	16q22.1	0 (0.00%)	2 (0.82%)	<-10	0.463	0.686	Mutual exclusivity
PLA2G2C	1p36.12	0 (0.00%)	2 (0.82%)	<-10	0.463	0.686	Mutual exclusivity
PLA2G2D	1p36.12	0 (0.00%)	2 (0.82%)	<-10	0.463	0.686	Mutual exclusivity
PLA2G2F	1p36.12	0 (0.00%)	2 (0.82%)	<-10	0.463	0.686	Mutual exclusivity
PLA2G5	1p36.13	0 (0.00%)	2 (0.82%)	<-10	0.463	0.686	Mutual exclusivity
PLAC1	Xq26.3	0 (0.00%)	2 (0.82%)	<-10	0.463	0.686	Mutual exclusivity
PLAC8L1	5q32	0 (0.00%)	2 (0.82%)	<-10	0.463	0.686	Mutual exclusivity
PLEKHA4	19q13.33	0 (0.00%)	2 (0.82%)	<-10	0.463	0.686	Mutual exclusivity
PLEKHG1	6q25.1	0 (0.00%)	2 (0.82%)	<-10	0.463	0.686	Mutual exclusivity
PLK1	16p12.2	0 (0.00%)	2 (0.82%)	<-10	0.463	0.686	Mutual exclusivity
PLK2	5q11.2	0 (0.00%)	2 (0.82%)	<-10	0.463	0.686	Mutual exclusivity
PLS3	Xq23	0 (0.00%)	2 (0.82%)	<-10	0.463	0.686	Mutual exclusivity
PMS1	2q32.2	0 (0.00%)	2 (0.82%)	<-10	0.463	0.686	Mutual exclusivity
PNKP	19q13.33	0 (0.00%)	2 (0.82%)	<-10	0.463	0.686	Mutual exclusivity
PNMA8A	19q13.32	0 (0.00%)	2 (0.82%)	<-10	0.463	0.686	Mutual exclusivity
PNMA8B	19q13.32	0 (0.00%)	2 (0.82%)	<-10	0.463	0.686	Mutual exclusivity
PNN	14q21.1	0 (0.00%)	2 (0.82%)	<-10	0.463	0.686	Mutual exclusivity

POLD3	11q13.4	0 (0.00%)	2 (0.82%)	<-10	0.463	0.686	Mutual exclusivity
POLR2B	4q12	0 (0.00%)	2 (0.82%)	<-10	0.463	0.686	Mutual exclusivity
POPDC3	6q21	0 (0.00%)	2 (0.82%)	<-10	0.463	0.686	Mutual exclusivity
POU4F3	5q32	0 (0.00%)	2 (0.82%)	<-10	0.463	0.686	Mutual exclusivity
PPFIA3	19q13.33	0 (0.00%)	2 (0.82%)	<-10	0.463	0.686	Mutual exclusivity
PPIP5K2	5q21.1	0 (0.00%)	2 (0.82%)	<-10	0.463	0.686	Mutual exclusivity
PPM1K	4q22.1	0 (0.00%)	2 (0.82%)	<-10	0.463	0.686	Mutual exclusivity
PPP1R15A	19q13.33	0 (0.00%)	2 (0.82%)	<-10	0.463	0.686	Mutual exclusivity
PPP1R21	2p16.3	0 (0.00%)	2 (0.82%)	<-10	0.463	0.686	Mutual exclusivity
PPP2CA	5q31.1	0 (0.00%)	2 (0.82%)	<-10	0.463	0.686	Mutual exclusivity
PPP2R1A	19q13.41	0 (0.00%)	2 (0.82%)	<-10	0.463	0.686	Mutual exclusivity
PPP2R2C	4p16.1	0 (0.00%)	2 (0.82%)	<-10	0.463	0.686	Mutual exclusivity
PPP5C	19q13.32	0 (0.00%)	2 (0.82%)	<-10	0.463	0.686	Mutual exclusivity
PRIMPOL	4q35.1	0 (0.00%)	2 (0.82%)	<-10	0.463	0.686	Mutual exclusivity
PRKCSH	19p13.2	0 (0.00%)	2 (0.82%)	<-10	0.463	0.686	Mutual exclusivity
PRKD1	14q12	0 (0.00%)	2 (0.82%)	<-10	0.463	0.686	Mutual exclusivity
PRKG1	10q11.23-q21.1	0 (0.00%)	2 (0.82%)	<-10	0.463	0.686	Mutual exclusivity
PRKN	6q26	0 (0.00%)	2 (0.82%)	<-10	0.463	0.686	Mutual exclusivity
PRMT7	16q22.1	0 (0.00%)	2 (0.82%)	<-10	0.463	0.686	Mutual exclusivity
PROM2	2q11.1	0 (0.00%)	2 (0.82%)	<-10	0.463	0.686	Mutual exclusivity
PRPS1L1	7p21.1	0 (0.00%)	2 (0.82%)	<-10	0.463	0.686	Mutual exclusivity
PRR14	16p11.2	0 (0.00%)	2 (0.82%)	<-10	0.463	0.686	Mutual exclusivity
PRR5L	11p13-p12	0 (0.00%)	2 (0.82%)	<-10	0.463	0.686	Mutual exclusivity
PSAPL1	4p16.1	0 (0.00%)	2 (0.82%)	<-10	0.463	0.686	Mutual exclusivity
PSMC1	14q32.11	0 (0.00%)	2 (0.82%)	<-10	0.463	0.686	Mutual exclusivity
PSMC6	14q22.1	0 (0.00%)	2 (0.82%)	<-10	0.463	0.686	Mutual exclusivity
PTCSC3	14q13.3	0 (0.00%)	2 (0.82%)	<-10	0.463	0.686	Mutual exclusivity
PTGDR	14q22.1	0 (0.00%)	2 (0.82%)	<-10	0.463	0.686	Mutual exclusivity
PTGDR2	11q12.2	0 (0.00%)	2 (0.82%)	<-10	0.463	0.686	Mutual exclusivity

PTGER2	14q22.1	0 (0.00%)	2 (0.82%)	<-10	0.463	0.686	Mutual exclusivity
PTMA	2q37.1	0 (0.00%)	2 (0.82%)	<-10	0.463	0.686	Mutual exclusivity
PTMS	12p13.31	0 (0.00%)	2 (0.82%)	<-10	0.463	0.686	Mutual exclusivity
PTOV1	19q13.33	0 (0.00%)	2 (0.82%)	<-10	0.463	0.686	Mutual exclusivity
PTPN20	10q11.22	0 (0.00%)	2 (0.82%)	<-10	0.463	0.686	Mutual exclusivity
PTTG2	4p14	0 (0.00%)	2 (0.82%)	<-10	0.463	0.686	Mutual exclusivity
PWWP3A	19p13.3	0 (0.00%)	2 (0.82%)	<-10	0.463	0.686	Mutual exclusivity
PYGL	14q22.1	0 (0.00%)	2 (0.82%)	<-10	0.463	0.686	Mutual exclusivity
RAB28	4p15.33	0 (0.00%)	2 (0.82%)	<-10	0.463	0.686	Mutual exclusivity
RAB3C	5q11.2	0 (0.00%)	2 (0.82%)	<-10	0.463	0.686	Mutual exclusivity
RAB3D	19p13.2	0 (0.00%)	2 (0.82%)	<-10	0.463	0.686	Mutual exclusivity
RAD23B	9q31.2	0 (0.00%)	2 (0.82%)	<-10	0.463	0.686	Mutual exclusivity
RALGAPA1	14q13.2	0 (0.00%)	2 (0.82%)	<-10	0.463	0.686	Mutual exclusivity
RALY	20q11.22	0 (0.00%)	2 (0.82%)	<-10	0.463	0.686	Mutual exclusivity
RAP1A	1p13.2	0 (0.00%)	2 (0.82%)	<-10	0.463	0.686	Mutual exclusivity
RARS2	6q15	0 (0.00%)	2 (0.82%)	<-10	0.463	0.686	Mutual exclusivity
RASGEF1A	10q11.21	0 (0.00%)	2 (0.82%)	<-10	0.463	0.686	Mutual exclusivity
RASIP1	19q13.33	0 (0.00%)	2 (0.82%)	<-10	0.463	0.686	Mutual exclusivity
RASSF4	10q11.21	0 (0.00%)	2 (0.82%)	<-10	0.463	0.686	Mutual exclusivity
RBM27	5q32	0 (0.00%)	2 (0.82%)	<-10	0.463	0.686	Mutual exclusivity
RBMX	Xq26.3	0 (0.00%)	2 (0.82%)	<-10	0.463	0.686	Mutual exclusivity
RBMXL3	Xq23	0 (0.00%)	2 (0.82%)	<-10	0.463	0.686	Mutual exclusivity
RBP3	10q11.22	0 (0.00%)	2 (0.82%)	<-10	0.463	0.686	Mutual exclusivity
RBPJ	4p15.2	0 (0.00%)	2 (0.82%)	<-10	0.463	0.686	Mutual exclusivity
RELL2	5q31.3	0 (0.00%)	2 (0.82%)	<-10	0.463	0.686	Mutual exclusivity
REST	4q12	0 (0.00%)	2 (0.82%)	<-10	0.463	0.686	Mutual exclusivity
RET	10q11.21	0 (0.00%)	2 (0.82%)	<-10	0.463	0.686	Mutual exclusivity
RFESD	5q15	0 (0.00%)	2 (0.82%)	<-10	0.463	0.686	Mutual exclusivity
RFX8	2q11.2	0 (0.00%)	2 (0.82%)	<-10	0.463	0.686	Mutual exclusivity

RGL3	19p13.2	0 (0.00%)	2 (0.82%)	<-10	0.463	0.686	Mutual exclusivity
RGMB	5q15	0 (0.00%)	2 (0.82%)	<-10	0.463	0.686	Mutual exclusivity
RGS12	4p16.3	0 (0.00%)	2 (0.82%)	<-10	0.463	0.686	Mutual exclusivity
RGS6	14q24.2	0 (0.00%)	2 (0.82%)	<-10	0.463	0.686	Mutual exclusivity
RHOXF1	Xq24	0 (0.00%)	2 (0.82%)	<-10	0.463	0.686	Mutual exclusivity
RHOXF2	Xq24	0 (0.00%)	2 (0.82%)	<-10	0.463	0.686	Mutual exclusivity
RHOXF2B	Xq24	0 (0.00%)	2 (0.82%)	<-10	0.463	0.686	Mutual exclusivity
RIT2	18q12.3	0 (0.00%)	2 (0.82%)	<-10	0.463	0.686	Mutual exclusivity
RN7SKP113	4p16.2	0 (0.00%)	2 (0.82%)	<-10	0.463	0.686	Mutual exclusivity
RN7SKP115	11q22.1	0 (0.00%)	2 (0.82%)	<-10	0.463	0.686	Mutual exclusivity
RN7SKP119	2p21	0 (0.00%)	2 (0.82%)	<-10	0.463	0.686	Mutual exclusivity
RN7SKP170	4p15.32	0 (0.00%)	2 (0.82%)	<-10	0.463	0.686	Mutual exclusivity
RN7SKP178	2q33.3	0 (0.00%)	2 (0.82%)	<-10	0.463	0.686	Mutual exclusivity
RN7SKP199	4p12	0 (0.00%)	2 (0.82%)	<-10	0.463	0.686	Mutual exclusivity
RN7SKP200	2q33.3	0 (0.00%)	2 (0.82%)	<-10	0.463	0.686	Mutual exclusivity
RN7SKP21	14q13.3	0 (0.00%)	2 (0.82%)	<-10	0.463	0.686	Mutual exclusivity
RN7SKP211	6q21	0 (0.00%)	2 (0.82%)	<-10	0.463	0.686	Mutual exclusivity
RN7SKP212	3q22.1	0 (0.00%)	2 (0.82%)	<-10	0.463	0.686	Mutual exclusivity
RN7SKP23	16p12.2	0 (0.00%)	2 (0.82%)	<-10	0.463	0.686	Mutual exclusivity
RN7SKP237	4q31.1	0 (0.00%)	2 (0.82%)	<-10	0.463	0.686	Mutual exclusivity
RN7SKP260	2q33.3	0 (0.00%)	2 (0.82%)	<-10	0.463	0.686	Mutual exclusivity
RN7SKP266	7p21.1	0 (0.00%)	2 (0.82%)	<-10	0.463	0.686	Mutual exclusivity
RN7SKP275	4p16.2	0 (0.00%)	2 (0.82%)	<-10	0.463	0.686	Mutual exclusivity
RN7SKP292	4p16.1	0 (0.00%)	2 (0.82%)	<-10	0.463	0.686	Mutual exclusivity
RN7SKP297	11q13.4	0 (0.00%)	2 (0.82%)	<-10	0.463	0.686	Mutual exclusivity
RN7SKP30	4q12	0 (0.00%)	2 (0.82%)	<-10	0.463	0.686	Mutual exclusivity
RN7SKP31	Xq26.3	0 (0.00%)	2 (0.82%)	<-10	0.463	0.686	Mutual exclusivity
RN7SKP36	4p16.1	0 (0.00%)	2 (0.82%)	<-10	0.463	0.686	Mutual exclusivity
RN7SKP62	5q21.1	0 (0.00%)	2 (0.82%)	<-10	0.463	0.686	Mutual exclusivity

RN7SKP68	5q21.1	0 (0.00%)	2 (0.82%)	<-10	0.463	0.686	Mutual exclusivity
RN7SKP82	4p13	0 (0.00%)	2 (0.82%)	<-10	0.463	0.686	Mutual exclusivity
RN7SL101P	4p15.1	0 (0.00%)	2 (0.82%)	<-10	0.463	0.686	Mutual exclusivity
RN7SL193P	4p13	0 (0.00%)	2 (0.82%)	<-10	0.463	0.686	Mutual exclusivity
RN7SL211P	2p14	0 (0.00%)	2 (0.82%)	<-10	0.463	0.686	Mutual exclusivity
RN7SL222P	11q22.1	0 (0.00%)	2 (0.82%)	<-10	0.463	0.686	Mutual exclusivity
RN7SL248P	10q11.22	0 (0.00%)	2 (0.82%)	<-10	0.463	0.686	Mutual exclusivity
RN7SL255P	5q21.2	0 (0.00%)	2 (0.82%)	<-10	0.463	0.686	Mutual exclusivity
RN7SL268P	22q11.23	0 (0.00%)	2 (0.82%)	<-10	0.463	0.686	Mutual exclusivity
RN7SL297P	2q13	0 (0.00%)	2 (0.82%)	<-10	0.463	0.686	Mutual exclusivity
RN7SL325P	Xq26.3	0 (0.00%)	2 (0.82%)	<-10	0.463	0.686	Mutual exclusivity
RN7SL34P	19q13.2	0 (0.00%)	2 (0.82%)	<-10	0.463	0.686	Mutual exclusivity
RN7SL383P	5p12	0 (0.00%)	2 (0.82%)	<-10	0.463	0.686	Mutual exclusivity
RN7SL442P	17p11.2	0 (0.00%)	2 (0.82%)	<-10	0.463	0.686	Mutual exclusivity
RN7SL452P	14q22.1	0 (0.00%)	2 (0.82%)	<-10	0.463	0.686	Mutual exclusivity
RN7SL453P	10q11.22	0 (0.00%)	2 (0.82%)	<-10	0.463	0.686	Mutual exclusivity
RN7SL499P	2q37.1	0 (0.00%)	2 (0.82%)	<-10	0.463	0.686	Mutual exclusivity
RN7SL542P	7p15.3	0 (0.00%)	2 (0.82%)	<-10	0.463	0.686	Mutual exclusivity
RN7SL575P	2q11.1	0 (0.00%)	2 (0.82%)	<-10	0.463	0.686	Mutual exclusivity
RN7SL688P	11q23.3	0 (0.00%)	2 (0.82%)	<-10	0.463	0.686	Mutual exclusivity
RN7SL68P	5q31.3	0 (0.00%)	2 (0.82%)	<-10	0.463	0.686	Mutual exclusivity
RN7SL691P	4p13	0 (0.00%)	2 (0.82%)	<-10	0.463	0.686	Mutual exclusivity
RN7SL698P	3q21.3	0 (0.00%)	2 (0.82%)	<-10	0.463	0.686	Mutual exclusivity
RN7SL708P	19q13.33	0 (0.00%)	2 (0.82%)	<-10	0.463	0.686	Mutual exclusivity
RN7SL712P	Xq23	0 (0.00%)	2 (0.82%)	<-10	0.463	0.686	Mutual exclusivity
RN7SL727P	Xq27.1	0 (0.00%)	2 (0.82%)	<-10	0.463	0.686	Mutual exclusivity
RN7SL802P	5q21.1	0 (0.00%)	2 (0.82%)	<-10	0.463	0.686	Mutual exclusivity
RN7SL833P	19p13.2	0 (0.00%)	2 (0.82%)	<-10	0.463	0.686	Mutual exclusivity
RN7SL87P	5q31.3	0 (0.00%)	2 (0.82%)	<-10	0.463	0.686	Mutual exclusivity

RNA5SP100	2p11.2	0 (0.00%)	2 (0.82%)	<-10	0.463	0.686	Mutual exclusivity
RNA5SP101	2q11.2	0 (0.00%)	2 (0.82%)	<-10	0.463	0.686	Mutual exclusivity
RNA5SP116	2q33.3	0 (0.00%)	2 (0.82%)	<-10	0.463	0.686	Mutual exclusivity
RNA5SP118	2q34	0 (0.00%)	2 (0.82%)	<-10	0.463	0.686	Mutual exclusivity
RNA5SP136	3p13	0 (0.00%)	2 (0.82%)	<-10	0.463	0.686	Mutual exclusivity
RNA5SP152	4p16.1	0 (0.00%)	2 (0.82%)	<-10	0.463	0.686	Mutual exclusivity
RNA5SP153	4p16.1	0 (0.00%)	2 (0.82%)	<-10	0.463	0.686	Mutual exclusivity
RNA5SP154	4p16.1	0 (0.00%)	2 (0.82%)	<-10	0.463	0.686	Mutual exclusivity
RNA5SP156	4p15.33	0 (0.00%)	2 (0.82%)	<-10	0.463	0.686	Mutual exclusivity
RNA5SP157	4p15.31	0 (0.00%)	2 (0.82%)	<-10	0.463	0.686	Mutual exclusivity
RNA5SP160	4p14	0 (0.00%)	2 (0.82%)	<-10	0.463	0.686	Mutual exclusivity
RNA5SP183	5q11.2	0 (0.00%)	2 (0.82%)	<-10	0.463	0.686	Mutual exclusivity
RNA5SP184	5q11.2	0 (0.00%)	2 (0.82%)	<-10	0.463	0.686	Mutual exclusivity
RNA5SP185	5q11.2	0 (0.00%)	2 (0.82%)	<-10	0.463	0.686	Mutual exclusivity
RNA5SP188	5q21.1	0 (0.00%)	2 (0.82%)	<-10	0.463	0.686	Mutual exclusivity
RNA5SP193	5q31.2	0 (0.00%)	2 (0.82%)	<-10	0.463	0.686	Mutual exclusivity
RNA5SP196	5q32	0 (0.00%)	2 (0.82%)	<-10	0.463	0.686	Mutual exclusivity
RNA5SP226	6q27	0 (0.00%)	2 (0.82%)	<-10	0.463	0.686	Mutual exclusivity
RNA5SP310	10q11.22	0 (0.00%)	2 (0.82%)	<-10	0.463	0.686	Mutual exclusivity
RNA5SP311	10q11.22	0 (0.00%)	2 (0.82%)	<-10	0.463	0.686	Mutual exclusivity
RNA5SP312	10q11.22	0 (0.00%)	2 (0.82%)	<-10	0.463	0.686	Mutual exclusivity
RNA5SP318	10q21.1	0 (0.00%)	2 (0.82%)	<-10	0.463	0.686	Mutual exclusivity
RNA5SP344	11q13.5	0 (0.00%)	2 (0.82%)	<-10	0.463	0.686	Mutual exclusivity
RNA5SP385	14q22.1	0 (0.00%)	2 (0.82%)	<-10	0.463	0.686	Mutual exclusivity
RNA5SP454	18q12.3	0 (0.00%)	2 (0.82%)	<-10	0.463	0.686	Mutual exclusivity
RNA5SP455	18q12.3	0 (0.00%)	2 (0.82%)	<-10	0.463	0.686	Mutual exclusivity
RNA5SP515	Xq26.3	0 (0.00%)	2 (0.82%)	<-10	0.463	0.686	Mutual exclusivity
RND1	12q13.12	0 (0.00%)	2 (0.82%)	<-10	0.463	0.686	Mutual exclusivity
RNF149	2q11.2	0 (0.00%)	2 (0.82%)	<-10	0.463	0.686	Mutual exclusivity

RNF212	4p16.3	0 (0.00%)	2 (0.82%)	<-10	0.463	0.686	Mutual exclusivity
RNF40	16p11.2	0 (0.00%)	2 (0.82%)	<-10	0.463	0.686	Mutual exclusivity
RNU6ATAC11P	10q11.21	0 (0.00%)	2 (0.82%)	<-10	0.463	0.686	Mutual exclusivity
RNU6ATAC23P	Xq27.1	0 (0.00%)	2 (0.82%)	<-10	0.463	0.686	Mutual exclusivity
RNU6ATAC2P	5q11.2	0 (0.00%)	2 (0.82%)	<-10	0.463	0.686	Mutual exclusivity
RNU6ATAC31P	4q22.1	0 (0.00%)	2 (0.82%)	<-10	0.463	0.686	Mutual exclusivity
RNY3P1	5q14.1	0 (0.00%)	2 (0.82%)	<-10	0.463	0.686	Mutual exclusivity
RNY4P15	2p11.2	0 (0.00%)	2 (0.82%)	<-10	0.463	0.686	Mutual exclusivity
ROM1	11q12.3	0 (0.00%)	2 (0.82%)	<-10	0.463	0.686	Mutual exclusivity
RPE	2q34	0 (0.00%)	2 (0.82%)	<-10	0.463	0.686	Mutual exclusivity
RPL13P5	12p13.31	0 (0.00%)	2 (0.82%)	<-10	0.463	0.686	Mutual exclusivity
RPL24	3q12.3	0 (0.00%)	2 (0.82%)	<-10	0.463	0.686	Mutual exclusivity
RPL32P3	3q21.3	0 (0.00%)	2 (0.82%)	<-10	0.463	0.686	Mutual exclusivity
RPN1	3q21.3	0 (0.00%)	2 (0.82%)	<-10	0.463	0.686	Mutual exclusivity
RPS15	19p13.3	0 (0.00%)	2 (0.82%)	<-10	0.463	0.686	Mutual exclusivity
RPS25	11q23.3	0 (0.00%)	2 (0.82%)	<-10	0.463	0.686	Mutual exclusivity
RPS4XP5	2p15	0 (0.00%)	2 (0.82%)	<-10	0.463	0.686	Mutual exclusivity
RPS6KA1	1p36.11	0 (0.00%)	2 (0.82%)	<-10	0.463	0.686	Mutual exclusivity
RSU1P2	10q11.21	0 (0.00%)	2 (0.82%)	<-10	0.463	0.686	Mutual exclusivity
RTL8A	Xq26.3	0 (0.00%)	2 (0.82%)	<-10	0.463	0.686	Mutual exclusivity
RTL8B	Xq26.3	0 (0.00%)	2 (0.82%)	<-10	0.463	0.686	Mutual exclusivity
RTL8C	Xq26.3	0 (0.00%)	2 (0.82%)	<-10	0.463	0.686	Mutual exclusivity
RTRAF	14q22.1	0 (0.00%)	2 (0.82%)	<-10	0.463	0.686	Mutual exclusivity
RUFY4	2q35	0 (0.00%)	2 (0.82%)	<-10	0.463	0.686	Mutual exclusivity
RUVBL2	19q13.33	0 (0.00%)	2 (0.82%)	<-10	0.463	0.686	Mutual exclusivity
S100P	4p16.1	0 (0.00%)	2 (0.82%)	<-10	0.463	0.686	Mutual exclusivity
SAE1	19q13.32	0 (0.00%)	2 (0.82%)	<-10	0.463	0.686	Mutual exclusivity
SAGE1	Xq26.3	0 (0.00%)	2 (0.82%)	<-10	0.463	0.686	Mutual exclusivity
SAMD5	6q24.3	0 (0.00%)	2 (0.82%)	<-10	0.463	0.686	Mutual exclusivity

SAV1	14q22.1	0 (0.00%)	2 (0.82%)	<-10	0.463	0.686	Mutual exclusivity
SC5D	11q23.3-q24.1	0 (0.00%)	2 (0.82%)	<-10	0.463	0.686	Mutual exclusivity
SCARNA10	12p13.31	0 (0.00%)	2 (0.82%)	<-10	0.463	0.686	Mutual exclusivity
SCARNA22	4p16.3	0 (0.00%)	2 (0.82%)	<-10	0.463	0.686	Mutual exclusivity
SCFD1	14q12	0 (0.00%)	2 (0.82%)	<-10	0.463	0.686	Mutual exclusivity
SCIN	7p21.3	0 (0.00%)	2 (0.82%)	<-10	0.463	0.686	Mutual exclusivity
SCNN1B	16p12.2	0 (0.00%)	2 (0.82%)	<-10	0.463	0.686	Mutual exclusivity
SCNN1G	16p12.2	0 (0.00%)	2 (0.82%)	<-10	0.463	0.686	Mutual exclusivity
SDIM1	-	0 (0.00%)	2 (0.82%)	<-10	0.463	0.686	Mutual exclusivity
SEC23A	14q21.1	0 (0.00%)	2 (0.82%)	<-10	0.463	0.686	Mutual exclusivity
SEC61A1	3q21.3	0 (0.00%)	2 (0.82%)	<-10	0.463	0.686	Mutual exclusivity
SEL1L3	4p15.2	0 (0.00%)	2 (0.82%)	<-10	0.463	0.686	Mutual exclusivity
SENP7	3q12.3	0 (0.00%)	2 (0.82%)	<-10	0.463	0.686	Mutual exclusivity
SEPSECS	4p15.2	0 (0.00%)	2 (0.82%)	<-10	0.463	0.686	Mutual exclusivity
SERPINA11	14q32.13	0 (0.00%)	2 (0.82%)	<-10	0.463	0.686	Mutual exclusivity
SERPINA12	14q32.13	0 (0.00%)	2 (0.82%)	<-10	0.463	0.686	Mutual exclusivity
SERPINA4	14q32.13	0 (0.00%)	2 (0.82%)	<-10	0.463	0.686	Mutual exclusivity
SERPINA5	14q32.13	0 (0.00%)	2 (0.82%)	<-10	0.463	0.686	Mutual exclusivity
SERPINA9	14q32.13	0 (0.00%)	2 (0.82%)	<-10	0.463	0.686	Mutual exclusivity
SESN1	6q21	0 (0.00%)	2 (0.82%)	<-10	0.463	0.686	Mutual exclusivity
SETBP1	18q12.3	0 (0.00%)	2 (0.82%)	<-10	0.463	0.686	Mutual exclusivity
SFTA3	14q13.3	0 (0.00%)	2 (0.82%)	<-10	0.463	0.686	Mutual exclusivity
SH2D1A	Xq25	0 (0.00%)	2 (0.82%)	<-10	0.463	0.686	Mutual exclusivity
SH3BP2	4p16.3	0 (0.00%)	2 (0.82%)	<-10	0.463	0.686	Mutual exclusivity
SH3BP4	2q37.2	0 (0.00%)	2 (0.82%)	<-10	0.463	0.686	Mutual exclusivity
SH3RF2	5q32	0 (0.00%)	2 (0.82%)	<-10	0.463	0.686	Mutual exclusivity
SH3TC1	4p16.1	0 (0.00%)	2 (0.82%)	<-10	0.463	0.686	Mutual exclusivity
SHISA3	4p13	0 (0.00%)	2 (0.82%)	<-10	0.463	0.686	Mutual exclusivity
SHLD2P1	10q11.22	0 (0.00%)	2 (0.82%)	<-10	0.463	0.686	Mutual exclusivity

SHQ1	3p13	0 (0.00%)	2 (0.82%)	<-10	0.463	0.686	Mutual exclusivity
SIGLEC10	19q13.41	0 (0.00%)	2 (0.82%)	<-10	0.463	0.686	Mutual exclusivity
SIGLEC12	19q13.41	0 (0.00%)	2 (0.82%)	<-10	0.463	0.686	Mutual exclusivity
SIGLEC14	19q13.41	0 (0.00%)	2 (0.82%)	<-10	0.463	0.686	Mutual exclusivity
SIGLEC22P	19q13.41	0 (0.00%)	2 (0.82%)	<-10	0.463	0.686	Mutual exclusivity
SIGLEC5	19q13.41	0 (0.00%)	2 (0.82%)	<-10	0.463	0.686	Mutual exclusivity
SIGLEC6	19q13.41	0 (0.00%)	2 (0.82%)	<-10	0.463	0.686	Mutual exclusivity
SIGLEC7	19q13.41	0 (0.00%)	2 (0.82%)	<-10	0.463	0.686	Mutual exclusivity
SIGLEC8	19q13.41	0 (0.00%)	2 (0.82%)	<-10	0.463	0.686	Mutual exclusivity
SIGLEC9	19q13.41	0 (0.00%)	2 (0.82%)	<-10	0.463	0.686	Mutual exclusivity
SIGLECL1	19q13.41	0 (0.00%)	2 (0.82%)	<-10	0.463	0.686	Mutual exclusivity
SKP1	5q31.1	0 (0.00%)	2 (0.82%)	<-10	0.463	0.686	Mutual exclusivity
SLBP	4p16.3	0 (0.00%)	2 (0.82%)	<-10	0.463	0.686	Mutual exclusivity
SLC14A2	18q12.3	0 (0.00%)	2 (0.82%)	<-10	0.463	0.686	Mutual exclusivity
SLC17A5	6q13	0 (0.00%)	2 (0.82%)	<-10	0.463	0.686	Mutual exclusivity
SLC1A5	19q13.32	0 (0.00%)	2 (0.82%)	<-10	0.463	0.686	Mutual exclusivity
SLC25A2	5q31.3	0 (0.00%)	2 (0.82%)	<-10	0.463	0.686	Mutual exclusivity
SLC25A4	4q35.1	0 (0.00%)	2 (0.82%)	<-10	0.463	0.686	Mutual exclusivity
SLC25A53	Xq22.2	0 (0.00%)	2 (0.82%)	<-10	0.463	0.686	Mutual exclusivity
SLC26A1	4p16.3	0 (0.00%)	2 (0.82%)	<-10	0.463	0.686	Mutual exclusivity
SLC26A2	5q32	0 (0.00%)	2 (0.82%)	<-10	0.463	0.686	Mutual exclusivity
SLC2A11	22q11.23	0 (0.00%)	2 (0.82%)	<-10	0.463	0.686	Mutual exclusivity
SLC34A2	4p15.2	0 (0.00%)	2 (0.82%)	<-10	0.463	0.686	Mutual exclusivity
SLC37A4	11q23.3	0 (0.00%)	2 (0.82%)	<-10	0.463	0.686	Mutual exclusivity
SLC49A3	4p16.3	0 (0.00%)	2 (0.82%)	<-10	0.463	0.686	Mutual exclusivity
SLC5A2	16p11.2	0 (0.00%)	2 (0.82%)	<-10	0.463	0.686	Mutual exclusivity
SLC6A14	Xq23	0 (0.00%)	2 (0.82%)	<-10	0.463	0.686	Mutual exclusivity
SLC7A5	16q24.2	0 (0.00%)	2 (0.82%)	<-10	0.463	0.686	Mutual exclusivity
SLC7A6	16q22.1	0 (0.00%)	2 (0.82%)	<-10	0.463	0.686	Mutual exclusivity

SLC7A6OS	16q22.1	0 (0.00%)	2 (0.82%)	<-10	0.463	0.686	Mutual exclusivity
SLC9A2	2q12.1	0 (0.00%)	2 (0.82%)	<-10	0.463	0.686	Mutual exclusivity
SLC9A4	2q12.1	0 (0.00%)	2 (0.82%)	<-10	0.463	0.686	Mutual exclusivity
SLC9A6	Xq26.3	0 (0.00%)	2 (0.82%)	<-10	0.463	0.686	Mutual exclusivity
SLC9B1P2	2p11.1	0 (0.00%)	2 (0.82%)	<-10	0.463	0.686	Mutual exclusivity
SLCO4C1	5q21.1	0 (0.00%)	2 (0.82%)	<-10	0.463	0.686	Mutual exclusivity
SLCO6A1	5q21.1	0 (0.00%)	2 (0.82%)	<-10	0.463	0.686	Mutual exclusivity
SLF1	5q15	0 (0.00%)	2 (0.82%)	<-10	0.463	0.686	Mutual exclusivity
SLIT1	10q24.1	0 (0.00%)	2 (0.82%)	<-10	0.463	0.686	Mutual exclusivity
SLIT2	4p15.31	0 (0.00%)	2 (0.82%)	<-10	0.463	0.686	Mutual exclusivity
SMAD2	18q21.1	0 (0.00%)	2 (0.82%)	<-10	0.463	0.686	Mutual exclusivity
SMARCA4	19p13.2	0 (0.00%)	2 (0.82%)	<-10	0.463	0.686	Mutual exclusivity
SMARCB1	22q11.23|22q11	0 (0.00%)	2 (0.82%)	<-10	0.463	0.686	Mutual exclusivity
SMIM10	Xq26.3	0 (0.00%)	2 (0.82%)	<-10	0.463	0.686	Mutual exclusivity
SMIM10L2A	Xq26.3	0 (0.00%)	2 (0.82%)	<-10	0.463	0.686	Mutual exclusivity
SMIM10L2B	Xq26.3	0 (0.00%)	2 (0.82%)	<-10	0.463	0.686	Mutual exclusivity
SMIM20	4p15.2	0 (0.00%)	2 (0.82%)	<-10	0.463	0.686	Mutual exclusivity
SMOC2	6q27	0 (0.00%)	2 (0.82%)	<-10	0.463	0.686	Mutual exclusivity
SMPD3	16q22.1	0 (0.00%)	2 (0.82%)	<-10	0.463	0.686	Mutual exclusivity
SNHG1	11q12.3	0 (0.00%)	2 (0.82%)	<-10	0.463	0.686	Mutual exclusivity
SNORA41	2q33.3	0 (0.00%)	2 (0.82%)	<-10	0.463	0.686	Mutual exclusivity
SNORA75	2q37.1	0 (0.00%)	2 (0.82%)	<-10	0.463	0.686	Mutual exclusivity
SNORA7B	3q21.3	0 (0.00%)	2 (0.82%)	<-10	0.463	0.686	Mutual exclusivity
SNORD20	2q37.1	0 (0.00%)	2 (0.82%)	<-10	0.463	0.686	Mutual exclusivity
SNORD43	22q13.1	0 (0.00%)	2 (0.82%)	<-10	0.463	0.686	Mutual exclusivity
SNORD51	2q33.3	0 (0.00%)	2 (0.82%)	<-10	0.463	0.686	Mutual exclusivity
SNORD74	1q25.1	0 (0.00%)	2 (0.82%)	<-10	0.463	0.686	Mutual exclusivity
SNORD82	2q37.1	0 (0.00%)	2 (0.82%)	<-10	0.463	0.686	Mutual exclusivity
SNORD89	2q11.2	0 (0.00%)	2 (0.82%)	<-10	0.463	0.686	Mutual exclusivity

SNRNP70	19q13.33	0 (0.00%)	2 (0.82%)	<-10	0.463	0.686	Mutual exclusivity
SNX13	7p21.1	0 (0.00%)	2 (0.82%)	<-10	0.463	0.686	Mutual exclusivity
SNX25	4q35.1	0 (0.00%)	2 (0.82%)	<-10	0.463	0.686	Mutual exclusivity
SOD3	4p15.2	0 (0.00%)	2 (0.82%)	<-10	0.463	0.686	Mutual exclusivity
SOX3	Xq27.1	0 (0.00%)	2 (0.82%)	<-10	0.463	0.686	Mutual exclusivity
SP4	7p15.3	0 (0.00%)	2 (0.82%)	<-10	0.463	0.686	Mutual exclusivity
SP8	7p21.1	0 (0.00%)	2 (0.82%)	<-10	0.463	0.686	Mutual exclusivity
SPACA6	19q13.41	0 (0.00%)	2 (0.82%)	<-10	0.463	0.686	Mutual exclusivity
SPANXA1	Xq27.2	0 (0.00%)	2 (0.82%)	<-10	0.463	0.686	Mutual exclusivity
SPANXA2	Xq27.2	0 (0.00%)	2 (0.82%)	<-10	0.463	0.686	Mutual exclusivity
SPANXB1	Xq27.1	0 (0.00%)	2 (0.82%)	<-10	0.463	0.686	Mutual exclusivity
SPANXC	Xq27.2	0 (0.00%)	2 (0.82%)	<-10	0.463	0.686	Mutual exclusivity
SPANXD	Xq27.2	0 (0.00%)	2 (0.82%)	<-10	0.463	0.686	Mutual exclusivity
SPC24	19p13.2	0 (0.00%)	2 (0.82%)	<-10	0.463	0.686	Mutual exclusivity
SPON2	4p16.3	0 (0.00%)	2 (0.82%)	<-10	0.463	0.686	Mutual exclusivity
SPRY4	5q31.3	0 (0.00%)	2 (0.82%)	<-10	0.463	0.686	Mutual exclusivity
SPSB2	12p13.31	0 (0.00%)	2 (0.82%)	<-10	0.463	0.686	Mutual exclusivity
SRCAP	16p11.2	0 (0.00%)	2 (0.82%)	<-10	0.463	0.686	Mutual exclusivity
SSBP2	5q14.1	0 (0.00%)	2 (0.82%)	<-10	0.463	0.686	Mutual exclusivity
SSTR1	14q21.1	0 (0.00%)	2 (0.82%)	<-10	0.463	0.686	Mutual exclusivity
ST8SIA3	18q21.31	0 (0.00%)	2 (0.82%)	<-10	0.463	0.686	Mutual exclusivity
ST8SIA4	5q21.1	0 (0.00%)	2 (0.82%)	<-10	0.463	0.686	Mutual exclusivity
STAG2	Xq25	0 (0.00%)	2 (0.82%)	<-10	0.463	0.686	Mutual exclusivity
STIM2	4p15.2	0 (0.00%)	2 (0.82%)	<-10	0.463	0.686	Mutual exclusivity
STK32B	4p16.2	0 (0.00%)	2 (0.82%)	<-10	0.463	0.686	Mutual exclusivity
STON1	2p16.3	0 (0.00%)	2 (0.82%)	<-10	0.463	0.686	Mutual exclusivity
STX18	4p16.3-p16.2	0 (0.00%)	2 (0.82%)	<-10	0.463	0.686	Mutual exclusivity
STX5	11q12.3	0 (0.00%)	2 (0.82%)	<-10	0.463	0.686	Mutual exclusivity
STYX	14q22.1	0 (0.00%)	2 (0.82%)	<-10	0.463	0.686	Mutual exclusivity

SWSAP1	19p13.2	0 (0.00%)	2 (0.82%)	<-10	0.463	0.686	Mutual exclusivity
SYNE1	6q25.2	0 (0.00%)	2 (0.82%)	<-10	0.463	0.686	Mutual exclusivity
SYT15	10q11.22	0 (0.00%)	2 (0.82%)	<-10	0.463	0.686	Mutual exclusivity
SYT4	18q12.3	0 (0.00%)	2 (0.82%)	<-10	0.463	0.686	Mutual exclusivity
TACC3	4p16.3	0 (0.00%)	2 (0.82%)	<-10	0.463	0.686	Mutual exclusivity
TADA2B	4p16.1	0 (0.00%)	2 (0.82%)	<-10	0.463	0.686	Mutual exclusivity
TAF7	5q31.3	0 (0.00%)	2 (0.82%)	<-10	0.463	0.686	Mutual exclusivity
TAPBPL	12p13.31	0 (0.00%)	2 (0.82%)	<-10	0.463	0.686	Mutual exclusivity
TBC1D1	4p14	0 (0.00%)	2 (0.82%)	<-10	0.463	0.686	Mutual exclusivity
TBC1D14	4p16.1	0 (0.00%)	2 (0.82%)	<-10	0.463	0.686	Mutual exclusivity
TBC1D19	4p15.2	0 (0.00%)	2 (0.82%)	<-10	0.463	0.686	Mutual exclusivity
TBC1D8	2q11.2	0 (0.00%)	2 (0.82%)	<-10	0.463	0.686	Mutual exclusivity
TC2N	14q32.12	0 (0.00%)	2 (0.82%)	<-10	0.463	0.686	Mutual exclusivity
TCERG1	5q32	0 (0.00%)	2 (0.82%)	<-10	0.463	0.686	Mutual exclusivity
TCF7	5q31.1	0 (0.00%)	2 (0.82%)	<-10	0.463	0.686	Mutual exclusivity
TEKT4	2q11.1	0 (0.00%)	2 (0.82%)	<-10	0.463	0.686	Mutual exclusivity
TENT2	5q14.1	0 (0.00%)	2 (0.82%)	<-10	0.463	0.686	Mutual exclusivity
TEPP	16q21	0 (0.00%)	2 (0.82%)	<-10	0.463	0.686	Mutual exclusivity
TEX44	2q37.1	0 (0.00%)	2 (0.82%)	<-10	0.463	0.686	Mutual exclusivity
TGFB1	19q13.2	0 (0.00%)	2 (0.82%)	<-10	0.463	0.686	Mutual exclusivity
TGFB1I1	16p11.2	0 (0.00%)	2 (0.82%)	<-10	0.463	0.686	Mutual exclusivity
THBS4	5q14.1	0 (0.00%)	2 (0.82%)	<-10	0.463	0.686	Mutual exclusivity
THOC2	Xq25	0 (0.00%)	2 (0.82%)	<-10	0.463	0.686	Mutual exclusivity
THUMPD1	16p12.3	0 (0.00%)	2 (0.82%)	<-10	0.463	0.686	Mutual exclusivity
TIAM2	6q25.2-q25.3	0 (0.00%)	2 (0.82%)	<-10	0.463	0.686	Mutual exclusivity
TIFAB	5q31.1	0 (0.00%)	2 (0.82%)	<-10	0.463	0.686	Mutual exclusivity
TIGD6	5q32	0 (0.00%)	2 (0.82%)	<-10	0.463	0.686	Mutual exclusivity
TMED11P	4p16.3	0 (0.00%)	2 (0.82%)	<-10	0.463	0.686	Mutual exclusivity
TMEM108	3q22.1	0 (0.00%)	2 (0.82%)	<-10	0.463	0.686	Mutual exclusivity

TMEM125	1p34.2	0 (0.00%)	2 (0.82%)	<-10	0.463	0.686	Mutual exclusivity
TMEM128	4p16.3	0 (0.00%)	2 (0.82%)	<-10	0.463	0.686	Mutual exclusivity
TMEM129	4p16.3	0 (0.00%)	2 (0.82%)	<-10	0.463	0.686	Mutual exclusivity
TMEM133	11q22.1	0 (0.00%)	2 (0.82%)	<-10	0.463	0.686	Mutual exclusivity
TMEM154	4q31.3	0 (0.00%)	2 (0.82%)	<-10	0.463	0.686	Mutual exclusivity
TMEM159	16p12.3	0 (0.00%)	2 (0.82%)	<-10	0.463	0.686	Mutual exclusivity
TMEM160	19q13.32	0 (0.00%)	2 (0.82%)	<-10	0.463	0.686	Mutual exclusivity
TMEM165	4q12	0 (0.00%)	2 (0.82%)	<-10	0.463	0.686	Mutual exclusivity
TMEM175	4p16.3	0 (0.00%)	2 (0.82%)	<-10	0.463	0.686	Mutual exclusivity
TMEM182	2q12.1	0 (0.00%)	2 (0.82%)	<-10	0.463	0.686	Mutual exclusivity
TMEM196	7p21.1	0 (0.00%)	2 (0.82%)	<-10	0.463	0.686	Mutual exclusivity
TMEM205	19p13.2	0 (0.00%)	2 (0.82%)	<-10	0.463	0.686	Mutual exclusivity
TMEM242	6q25.3	0 (0.00%)	2 (0.82%)	<-10	0.463	0.686	Mutual exclusivity
TMEM33	4p13	0 (0.00%)	2 (0.82%)	<-10	0.463	0.686	Mutual exclusivity
TMEM72	10q11.21	0 (0.00%)	2 (0.82%)	<-10	0.463	0.686	Mutual exclusivity
TMEM91	19q13.2	0 (0.00%)	2 (0.82%)	<-10	0.463	0.686	Mutual exclusivity
TMX1	14q22.1	0 (0.00%)	2 (0.82%)	<-10	0.463	0.686	Mutual exclusivity
TNIP2	4p16.3	0 (0.00%)	2 (0.82%)	<-10	0.463	0.686	Mutual exclusivity
TNS1	2q35	0 (0.00%)	2 (0.82%)	<-10	0.463	0.686	Mutual exclusivity
TOGARAM1	14q21.2	0 (0.00%)	2 (0.82%)	<-10	0.463	0.686	Mutual exclusivity
TPGS2	18q12.2	0 (0.00%)	2 (0.82%)	<-10	0.463	0.686	Mutual exclusivity
TPI1	12p13.31	0 (0.00%)	2 (0.82%)	<-10	0.463	0.686	Mutual exclusivity
TRAF3IP1	2q37.3	0 (0.00%)	2 (0.82%)	<-10	0.463	0.686	Mutual exclusivity
TRAPPC4	11q23.3	0 (0.00%)	2 (0.82%)	<-10	0.463	0.686	Mutual exclusivity
TRAPPC6B	14q21.1	0 (0.00%)	2 (0.82%)	<-10	0.463	0.686	Mutual exclusivity
TRH	3q22.1	0 (0.00%)	2 (0.82%)	<-10	0.463	0.686	Mutual exclusivity
TRIM43	2q11.1	0 (0.00%)	2 (0.82%)	<-10	0.463	0.686	Mutual exclusivity
TRIM49	11q14.3	0 (0.00%)	2 (0.82%)	<-10	0.463	0.686	Mutual exclusivity
TRIM49C	11q14.3	0 (0.00%)	2 (0.82%)	<-10	0.463	0.686	Mutual exclusivity

TRIM49D1	11q14.3	0 (0.00%)	2 (0.82%)	<-10	0.463	0.686	Mutual exclusivity
TRIM64B	11q14.3	0 (0.00%)	2 (0.82%)	<-10	0.463	0.686	Mutual exclusivity
TRIM77	11q14.3	0 (0.00%)	2 (0.82%)	<-10	0.463	0.686	Mutual exclusivity
TRIM9	14q22.1	0 (0.00%)	2 (0.82%)	<-10	0.463	0.686	Mutual exclusivity
TRMT10C	3q12.3	0 (0.00%)	2 (0.82%)	<-10	0.463	0.686	Mutual exclusivity
TRMT44	4p16.1	0 (0.00%)	2 (0.82%)	<-10	0.463	0.686	Mutual exclusivity
TRPC6	11q22.1	0 (0.00%)	2 (0.82%)	<-10	0.463	0.686	Mutual exclusivity
TRPC7	5q31.1	0 (0.00%)	2 (0.82%)	<-10	0.463	0.686	Mutual exclusivity
TRPM4	19q13.33	0 (0.00%)	2 (0.82%)	<-10	0.463	0.686	Mutual exclusivity
TRPV2	17p11.2	0 (0.00%)	2 (0.82%)	<-10	0.463	0.686	Mutual exclusivity
TSKS	19q13.33	0 (0.00%)	2 (0.82%)	<-10	0.463	0.686	Mutual exclusivity
TSKU	11q13.5	0 (0.00%)	2 (0.82%)	<-10	0.463	0.686	Mutual exclusivity
TSPAN16	19p13.2	0 (0.00%)	2 (0.82%)	<-10	0.463	0.686	Mutual exclusivity
TTK	6q14.1	0 (0.00%)	2 (0.82%)	<-10	0.463	0.686	Mutual exclusivity
TTL	2q14.1	0 (0.00%)	2 (0.82%)	<-10	0.463	0.686	Mutual exclusivity
TULP2	19q13.33	0 (0.00%)	2 (0.82%)	<-10	0.463	0.686	Mutual exclusivity
TUT1	11q12.3	0 (0.00%)	2 (0.82%)	<-10	0.463	0.686	Mutual exclusivity
TWIST1	7p21.1	0 (0.00%)	2 (0.82%)	<-10	0.463	0.686	Mutual exclusivity
TWISTNB	7p21.1	0 (0.00%)	2 (0.82%)	<-10	0.463	0.686	Mutual exclusivity
TXNDC15	5q31.1	0 (0.00%)	2 (0.82%)	<-10	0.463	0.686	Mutual exclusivity
TXNDC16	14q22.1	0 (0.00%)	2 (0.82%)	<-10	0.463	0.686	Mutual exclusivity
UBA5	3q22.1	0 (0.00%)	2 (0.82%)	<-10	0.463	0.686	Mutual exclusivity
UBB	17p11.2	0 (0.00%)	2 (0.82%)	<-10	0.463	0.686	Mutual exclusivity
UBFD1	16p12.2	0 (0.00%)	2 (0.82%)	<-10	0.463	0.686	Mutual exclusivity
UBXN10	1p36.12	0 (0.00%)	2 (0.82%)	<-10	0.463	0.686	Mutual exclusivity
UCHL1	4p13	0 (0.00%)	2 (0.82%)	<-10	0.463	0.686	Mutual exclusivity
UGP2	2p15	0 (0.00%)	2 (0.82%)	<-10	0.463	0.686	Mutual exclusivity
UNC80	2q34	0 (0.00%)	2 (0.82%)	<-10	0.463	0.686	Mutual exclusivity
UPK2	11q23.3	0 (0.00%)	2 (0.82%)	<-10	0.463	0.686	Mutual exclusivity

USB1	16q21	0 (0.00%)	2 (0.82%)	<-10	0.463	0.686	Mutual exclusivity
USP17L10	4p16.1	0 (0.00%)	2 (0.82%)	<-10	0.463	0.686	Mutual exclusivity
USP17L11	4p16.1	0 (0.00%)	2 (0.82%)	<-10	0.463	0.686	Mutual exclusivity
USP17L12	4p16.1	0 (0.00%)	2 (0.82%)	<-10	0.463	0.686	Mutual exclusivity
USP17L13	4p16.1	0 (0.00%)	2 (0.82%)	<-10	0.463	0.686	Mutual exclusivity
USP17L15	4p16.1	0 (0.00%)	2 (0.82%)	<-10	0.463	0.686	Mutual exclusivity
USP17L17	4p16.1	0 (0.00%)	2 (0.82%)	<-10	0.463	0.686	Mutual exclusivity
USP17L18	4p16.1	0 (0.00%)	2 (0.82%)	<-10	0.463	0.686	Mutual exclusivity
USP17L19	4p16.1	0 (0.00%)	2 (0.82%)	<-10	0.463	0.686	Mutual exclusivity
USP17L20	4p16.1	0 (0.00%)	2 (0.82%)	<-10	0.463	0.686	Mutual exclusivity
USP17L21	4p16.1	0 (0.00%)	2 (0.82%)	<-10	0.463	0.686	Mutual exclusivity
USP17L22	4p16.1	0 (0.00%)	2 (0.82%)	<-10	0.463	0.686	Mutual exclusivity
USP17L23	4p16.1	0 (0.00%)	2 (0.82%)	<-10	0.463	0.686	Mutual exclusivity
USP17L24	4p16.1	0 (0.00%)	2 (0.82%)	<-10	0.463	0.686	Mutual exclusivity
USP17L25	4p16.1	0 (0.00%)	2 (0.82%)	<-10	0.463	0.686	Mutual exclusivity
USP17L26	4p16.1	0 (0.00%)	2 (0.82%)	<-10	0.463	0.686	Mutual exclusivity
USP17L27	4p16.1	0 (0.00%)	2 (0.82%)	<-10	0.463	0.686	Mutual exclusivity
USP17L28	4p16.1	0 (0.00%)	2 (0.82%)	<-10	0.463	0.686	Mutual exclusivity
USP17L29	4p16.1	0 (0.00%)	2 (0.82%)	<-10	0.463	0.686	Mutual exclusivity
USP17L30	4p16.1	0 (0.00%)	2 (0.82%)	<-10	0.463	0.686	Mutual exclusivity
USP17L5	4p16.1	0 (0.00%)	2 (0.82%)	<-10	0.463	0.686	Mutual exclusivity
USP31	16p12.2	0 (0.00%)	2 (0.82%)	<-10	0.463	0.686	Mutual exclusivity
USP37	2q35	0 (0.00%)	2 (0.82%)	<-10	0.463	0.686	Mutual exclusivity
USP40	2q37.1	0 (0.00%)	2 (0.82%)	<-10	0.463	0.686	Mutual exclusivity
USP48	1p36.12	0 (0.00%)	2 (0.82%)	<-10	0.463	0.686	Mutual exclusivity
USP5	12p13.31	0 (0.00%)	2 (0.82%)	<-10	0.463	0.686	Mutual exclusivity
UST	6q25.1	0 (0.00%)	2 (0.82%)	<-10	0.463	0.686	Mutual exclusivity
UVSSA	4p16.3	0 (0.00%)	2 (0.82%)	<-10	0.463	0.686	Mutual exclusivity
VAMP1	12p13.31	0 (0.00%)	2 (0.82%)	<-10	0.463	0.686	Mutual exclusivity

VGLL1	Xq26.3	0 (0.00%)	2 (0.82%)	<-10	0.463	0.686	Mutual exclusivity
VN1R2	19q13.42	0 (0.00%)	2 (0.82%)	<-10	0.463	0.686	Mutual exclusivity
VN1R4	19q13.42	0 (0.00%)	2 (0.82%)	<-10	0.463	0.686	Mutual exclusivity
VPREB3	22q11.23|22q11	0 (0.00%)	2 (0.82%)	<-10	0.463	0.686	Mutual exclusivity
VPS11	11q23.3	0 (0.00%)	2 (0.82%)	<-10	0.463	0.686	Mutual exclusivity
VPS54	2p15-p14	0 (0.00%)	2 (0.82%)	<-10	0.463	0.686	Mutual exclusivity
VSIG10L	19q13.41	0 (0.00%)	2 (0.82%)	<-10	0.463	0.686	Mutual exclusivity
VTA1	6q24.1-q24.2	0 (0.00%)	2 (0.82%)	<-10	0.463	0.686	Mutual exclusivity
VWA5B1	1p36.12	0 (0.00%)	2 (0.82%)	<-10	0.463	0.686	Mutual exclusivity
WASHC2A	10q11.23	0 (0.00%)	2 (0.82%)	<-10	0.463	0.686	Mutual exclusivity
WASHC2C	10q11.22	0 (0.00%)	2 (0.82%)	<-10	0.463	0.686	Mutual exclusivity
WDHD1	14q22.2-q22.3	0 (0.00%)	2 (0.82%)	<-10	0.463	0.686	Mutual exclusivity
WDPCP	2p15	0 (0.00%)	2 (0.82%)	<-10	0.463	0.686	Mutual exclusivity
WDR44	Xq24	0 (0.00%)	2 (0.82%)	<-10	0.463	0.686	Mutual exclusivity
WDR74	11q12.3	0 (0.00%)	2 (0.82%)	<-10	0.463	0.686	Mutual exclusivity
WFS1	4p16.1	0 (0.00%)	2 (0.82%)	<-10	0.463	0.686	Mutual exclusivity
WNT11	11q13.5	0 (0.00%)	2 (0.82%)	<-10	0.463	0.686	Mutual exclusivity
WTAPP1	11q22.2	0 (0.00%)	2 (0.82%)	<-10	0.463	0.686	Mutual exclusivity
WWP2	16q22.1	0 (0.00%)	2 (0.82%)	<-10	0.463	0.686	Mutual exclusivity
XPO6	16p12.1	0 (0.00%)	2 (0.82%)	<-10	0.463	0.686	Mutual exclusivity
YAP1	11q22.1	0 (0.00%)	2 (0.82%)	<-10	0.463	0.686	Mutual exclusivity
YIPF5	5q31.3	0 (0.00%)	2 (0.82%)	<-10	0.463	0.686	Mutual exclusivity
ZBTB11	3q12.3	0 (0.00%)	2 (0.82%)	<-10	0.463	0.686	Mutual exclusivity
ZBTB33	Xq24	0 (0.00%)	2 (0.82%)	<-10	0.463	0.686	Mutual exclusivity
ZC2HC1B	6q24.2	0 (0.00%)	2 (0.82%)	<-10	0.463	0.686	Mutual exclusivity
ZC3H4	19q13.32	0 (0.00%)	2 (0.82%)	<-10	0.463	0.686	Mutual exclusivity
ZC3H8	2q14.1	0 (0.00%)	2 (0.82%)	<-10	0.463	0.686	Mutual exclusivity
ZCCHC12	Xq24	0 (0.00%)	2 (0.82%)	<-10	0.463	0.686	Mutual exclusivity
ZCCHC18	Xq22.2	0 (0.00%)	2 (0.82%)	<-10	0.463	0.686	Mutual exclusivity

ZCCHC4	4p15.2	0 (0.00%)	2 (0.82%)	<-10	0.463	0.686	Mutual exclusivity
ZDBF2	2q33.3	0 (0.00%)	2 (0.82%)	<-10	0.463	0.686	Mutual exclusivity
ZEB2P1	4p15.32	0 (0.00%)	2 (0.82%)	<-10	0.463	0.686	Mutual exclusivity
ZFAND4	10q11.22	0 (0.00%)	2 (0.82%)	<-10	0.463	0.686	Mutual exclusivity
ZIC3	Xq26.3	0 (0.00%)	2 (0.82%)	<-10	0.463	0.686	Mutual exclusivity
ZNF137P	19q13.41	0 (0.00%)	2 (0.82%)	<-10	0.463	0.686	Mutual exclusivity
ZNF141	4p16.3	0 (0.00%)	2 (0.82%)	<-10	0.463	0.686	Mutual exclusivity
ZNF160	19q13.41-q13.42	0 (0.00%)	2 (0.82%)	<-10	0.463	0.686	Mutual exclusivity
ZNF175	19q13.41	0 (0.00%)	2 (0.82%)	<-10	0.463	0.686	Mutual exclusivity
ZNF2	2q11.1	0 (0.00%)	2 (0.82%)	<-10	0.463	0.686	Mutual exclusivity
ZNF22	10q11.21	0 (0.00%)	2 (0.82%)	<-10	0.463	0.686	Mutual exclusivity
ZNF28	19q13.41	0 (0.00%)	2 (0.82%)	<-10	0.463	0.686	Mutual exclusivity
ZNF292	6q14.3	0 (0.00%)	2 (0.82%)	<-10	0.463	0.686	Mutual exclusivity
ZNF319	16q21	0 (0.00%)	2 (0.82%)	<-10	0.463	0.686	Mutual exclusivity
ZNF320	19q13.41	0 (0.00%)	2 (0.82%)	<-10	0.463	0.686	Mutual exclusivity
ZNF321P	19q13.41	0 (0.00%)	2 (0.82%)	<-10	0.463	0.686	Mutual exclusivity
ZNF347	19q13.42	0 (0.00%)	2 (0.82%)	<-10	0.463	0.686	Mutual exclusivity
ZNF350	19q13.41	0 (0.00%)	2 (0.82%)	<-10	0.463	0.686	Mutual exclusivity
ZNF350-AS1	19q13.41	0 (0.00%)	2 (0.82%)	<-10	0.463	0.686	Mutual exclusivity
ZNF415	19q13.42	0 (0.00%)	2 (0.82%)	<-10	0.463	0.686	Mutual exclusivity
ZNF432	19q13.41	0 (0.00%)	2 (0.82%)	<-10	0.463	0.686	Mutual exclusivity
ZNF439	19p13.2	0 (0.00%)	2 (0.82%)	<-10	0.463	0.686	Mutual exclusivity
ZNF440	19p13.2	0 (0.00%)	2 (0.82%)	<-10	0.463	0.686	Mutual exclusivity
ZNF441	19p13.2	0 (0.00%)	2 (0.82%)	<-10	0.463	0.686	Mutual exclusivity
ZNF449	Xq26.3	0 (0.00%)	2 (0.82%)	<-10	0.463	0.686	Mutual exclusivity
ZNF468	19q13.41	0 (0.00%)	2 (0.82%)	<-10	0.463	0.686	Mutual exclusivity
ZNF480	19q13.41	0 (0.00%)	2 (0.82%)	<-10	0.463	0.686	Mutual exclusivity
ZNF487	10q11.21	0 (0.00%)	2 (0.82%)	<-10	0.463	0.686	Mutual exclusivity
ZNF488	10q11.22	0 (0.00%)	2 (0.82%)	<-10	0.463	0.686	Mutual exclusivity

ZNF491	19p13.2	0 (0.00%)	2 (0.82%)	<-10	0.463	0.686	Mutual exclusivity
ZNF514	2q11.1	0 (0.00%)	2 (0.82%)	<-10	0.463	0.686	Mutual exclusivity
ZNF518B	4p16.1	0 (0.00%)	2 (0.82%)	<-10	0.463	0.686	Mutual exclusivity
ZNF577	19q13.41	0 (0.00%)	2 (0.82%)	<-10	0.463	0.686	Mutual exclusivity
ZNF595	4p16.3	0 (0.00%)	2 (0.82%)	<-10	0.463	0.686	Mutual exclusivity
ZNF600	19q13.41	0 (0.00%)	2 (0.82%)	<-10	0.463	0.686	Mutual exclusivity
ZNF611	19q13.41	0 (0.00%)	2 (0.82%)	<-10	0.463	0.686	Mutual exclusivity
ZNF613	19q13.41	0 (0.00%)	2 (0.82%)	<-10	0.463	0.686	Mutual exclusivity
ZNF614	19q13.41	0 (0.00%)	2 (0.82%)	<-10	0.463	0.686	Mutual exclusivity
ZNF615	19q13.41	0 (0.00%)	2 (0.82%)	<-10	0.463	0.686	Mutual exclusivity
ZNF616	19q13.41	0 (0.00%)	2 (0.82%)	<-10	0.463	0.686	Mutual exclusivity
ZNF627	19p13.2	0 (0.00%)	2 (0.82%)	<-10	0.463	0.686	Mutual exclusivity
ZNF629	16p11.2	0 (0.00%)	2 (0.82%)	<-10	0.463	0.686	Mutual exclusivity
ZNF649	19q13.41	0 (0.00%)	2 (0.82%)	<-10	0.463	0.686	Mutual exclusivity
ZNF653	19p13.2	0 (0.00%)	2 (0.82%)	<-10	0.463	0.686	Mutual exclusivity
ZNF665	19q13.42	0 (0.00%)	2 (0.82%)	<-10	0.463	0.686	Mutual exclusivity
ZNF677	19q13.42	0 (0.00%)	2 (0.82%)	<-10	0.463	0.686	Mutual exclusivity
ZNF689	16p11.2	0 (0.00%)	2 (0.82%)	<-10	0.463	0.686	Mutual exclusivity
ZNF69	19p13.2	0 (0.00%)	2 (0.82%)	<-10	0.463	0.686	Mutual exclusivity
ZNF70	22q11.23	0 (0.00%)	2 (0.82%)	<-10	0.463	0.686	Mutual exclusivity
ZNF701	19q13.41	0 (0.00%)	2 (0.82%)	<-10	0.463	0.686	Mutual exclusivity
ZNF702P	19q13.41	0 (0.00%)	2 (0.82%)	<-10	0.463	0.686	Mutual exclusivity
ZNF718	4p16.3	0 (0.00%)	2 (0.82%)	<-10	0.463	0.686	Mutual exclusivity
ZNF721	4p16.3	0 (0.00%)	2 (0.82%)	<-10	0.463	0.686	Mutual exclusivity
ZNF732	4p16.3	0 (0.00%)	2 (0.82%)	<-10	0.463	0.686	Mutual exclusivity
ZNF75D	Xq26.3	0 (0.00%)	2 (0.82%)	<-10	0.463	0.686	Mutual exclusivity
ZNF766	19q13.41	0 (0.00%)	2 (0.82%)	<-10	0.463	0.686	Mutual exclusivity
ZNF816	19q13.41	0 (0.00%)	2 (0.82%)	<-10	0.463	0.686	Mutual exclusivity
ZNF823	19p13.2	0 (0.00%)	2 (0.82%)	<-10	0.463	0.686	Mutual exclusivity

ZNF83	19q13.41	0 (0.00%)	2 (0.82%)	<-10	0.463	0.686	Mutual exclusivity
ZNF833P	19p13.2	0 (0.00%)	2 (0.82%)	<-10	0.463	0.686	Mutual exclusivity
ZNF836	19q13.41	0 (0.00%)	2 (0.82%)	<-10	0.463	0.686	Mutual exclusivity
ZNF841	19q13.41	0 (0.00%)	2 (0.82%)	<-10	0.463	0.686	Mutual exclusivity
ZNF843	16p11.2	0 (0.00%)	2 (0.82%)	<-10	0.463	0.686	Mutual exclusivity
ZNF876P	4p16.3	0 (0.00%)	2 (0.82%)	<-10	0.463	0.686	Mutual exclusivity
ZNF888	19q13.41	0 (0.00%)	2 (0.82%)	<-10	0.463	0.686	Mutual exclusivity
ZP2	16p12.3-p12.2	0 (0.00%)	2 (0.82%)	<-10	0.463	0.686	Mutual exclusivity
ZPLD1	3q12.3	0 (0.00%)	2 (0.82%)	<-10	0.463	0.686	Mutual exclusivity
ZRANB3	2q21.3	0 (0.00%)	2 (0.82%)	<-10	0.463	0.686	Mutual exclusivity
CDC5L	6p21.1	6 (5.22%)	11 (4.49%)	0.22	0.473	0.686	Co-occurrence
PUM2	2p24.1	6 (5.22%)	11 (4.49%)	0.22	0.473	0.686	Co-occurrence
RANBP9	6p23	6 (5.22%)	11 (4.49%)	0.22	0.473	0.686	Co-occurrence
SLC29A1	6p21.1	6 (5.22%)	11 (4.49%)	0.22	0.473	0.686	Co-occurrence
SPATS1	6p21.1	6 (5.22%)	11 (4.49%)	0.22	0.473	0.686	Co-occurrence
TMEM151B	6p21.1	6 (5.22%)	11 (4.49%)	0.22	0.473	0.686	Co-occurrence
ACOT13	6p22.3	5 (4.35%)	9 (3.67%)	0.24	0.48	0.686	Co-occurrence
ALDH5A1	6p22.3	5 (4.35%)	9 (3.67%)	0.24	0.48	0.686	Co-occurrence
C6ORF62	6p22.3	5 (4.35%)	9 (3.67%)	0.24	0.48	0.686	Co-occurrence
CARMIL1	6p22.2	5 (4.35%)	9 (3.67%)	0.24	0.48	0.686	Co-occurrence
CMAHP	6p22.3	5 (4.35%)	9 (3.67%)	0.24	0.48	0.686	Co-occurrence
COX6A1P2	6p21.2	5 (4.35%)	9 (3.67%)	0.24	0.48	0.686	Co-occurrence
FGD2	6p21.2	5 (4.35%)	9 (3.67%)	0.24	0.48	0.686	Co-occurrence
GMNN	6p22.3	5 (4.35%)	9 (3.67%)	0.24	0.48	0.686	Co-occurrence
GPLD1	6p22.3	5 (4.35%)	9 (3.67%)	0.24	0.48	0.686	Co-occurrence
HS1BP3	2p24.1	5 (4.35%)	9 (3.67%)	0.24	0.48	0.686	Co-occurrence
IP6K3	6p21.31	5 (4.35%)	9 (3.67%)	0.24	0.48	0.686	Co-occurrence
KIAA0319	6p22.3	5 (4.35%)	9 (3.67%)	0.24	0.48	0.686	Co-occurrence
LAPTM4A	2p24.1	5 (4.35%)	9 (3.67%)	0.24	0.48	0.686	Co-occurrence

MRS2	6p22.3	5 (4.35%)	9 (3.67%)	0.24	0.48	0.686	Co-occurrence
NRSN1	6p22.3	5 (4.35%)	9 (3.67%)	0.24	0.48	0.686	Co-occurrence
RIPOR2	6p22.3	5 (4.35%)	9 (3.67%)	0.24	0.48	0.686	Co-occurrence
RN7SL334P	6p22.3	5 (4.35%)	9 (3.67%)	0.24	0.48	0.686	Co-occurrence
TDP2	6p22.3	5 (4.35%)	9 (3.67%)	0.24	0.48	0.686	Co-occurrence
AACSP1	5q35.3	1 (0.87%)	4 (1.63%)	-0.91	0.488	0.686	Mutual exclusivity
ABCB8	7q36.1	1 (0.87%)	4 (1.63%)	-0.91	0.488	0.686	Mutual exclusivity
ACTL6B	7q22.1	1 (0.87%)	4 (1.63%)	-0.91	0.488	0.686	Mutual exclusivity
ADAM2	8p11.22	1 (0.87%)	4 (1.63%)	-0.91	0.488	0.686	Mutual exclusivity
ADAM32	8p11.22	1 (0.87%)	4 (1.63%)	-0.91	0.488	0.686	Mutual exclusivity
ADAM9	8p11.22	1 (0.87%)	4 (1.63%)	-0.91	0.488	0.686	Mutual exclusivity
ADAMTS19	5q23.3	1 (0.87%)	4 (1.63%)	-0.91	0.488	0.686	Mutual exclusivity
ADARB2	10p15.3	1 (0.87%)	4 (1.63%)	-0.91	0.488	0.686	Mutual exclusivity
ADCY1	7p12.3	1 (0.87%)	4 (1.63%)	-0.91	0.488	0.686	Mutual exclusivity
AGAP3	7q36.1	1 (0.87%)	4 (1.63%)	-0.91	0.488	0.686	Mutual exclusivity
AGBL3	7q33	1 (0.87%)	4 (1.63%)	-0.91	0.488	0.686	Mutual exclusivity
AGFG2	7q22.1	1 (0.87%)	4 (1.63%)	-0.91	0.488	0.686	Mutual exclusivity
AGTR1	3q24	1 (0.87%)	4 (1.63%)	-0.91	0.488	0.686	Mutual exclusivity
AKR1B1	7q33	1 (0.87%)	4 (1.63%)	-0.91	0.488	0.686	Mutual exclusivity
ALDH3A1	17p11.2	1 (0.87%)	4 (1.63%)	-0.91	0.488	0.686	Mutual exclusivity
ANKH	5p15.2	1 (0.87%)	4 (1.63%)	-0.91	0.488	0.686	Mutual exclusivity
AOC1	7q36.1	1 (0.87%)	4 (1.63%)	-0.91	0.488	0.686	Mutual exclusivity
ARHGEF26	3q25.2	1 (0.87%)	4 (1.63%)	-0.91	0.488	0.686	Mutual exclusivity
ARHGEF40	14q11.2	1 (0.87%)	4 (1.63%)	-0.91	0.488	0.686	Mutual exclusivity
ARL14	3q25.33	1 (0.87%)	4 (1.63%)	-0.91	0.488	0.686	Mutual exclusivity
ARPC4	3p25.3	1 (0.87%)	4 (1.63%)	-0.91	0.488	0.686	Mutual exclusivity
ASIC3	7q36.1	1 (0.87%)	4 (1.63%)	-0.91	0.488	0.686	Mutual exclusivity
ATG9B	7q36.1	1 (0.87%)	4 (1.63%)	-0.91	0.488	0.686	Mutual exclusivity
ATP1B3	3q23	1 (0.87%)	4 (1.63%)	-0.91	0.488	0.686	Mutual exclusivity

AVL9	7p14.3	1 (0.87%)	4 (1.63%)	-0.91	0.488	0.686	Mutual exclusivity
B3GALNT1	3q26.1	1 (0.87%)	4 (1.63%)	-0.91	0.488	0.686	Mutual exclusivity
B3GNT3	19p13.11	1 (0.87%)	4 (1.63%)	-0.91	0.488	0.686	Mutual exclusivity
BOD1	5q35.2	1 (0.87%)	4 (1.63%)	-0.91	0.488	0.686	Mutual exclusivity
BRPF1	3p25.3	1 (0.87%)	4 (1.63%)	-0.91	0.488	0.686	Mutual exclusivity
C2ORF16	2p23.3	1 (0.87%)	4 (1.63%)	-0.91	0.488	0.686	Mutual exclusivity
C2ORF91	2p21	1 (0.87%)	4 (1.63%)	-0.91	0.488	0.686	Mutual exclusivity
C3ORF33	3q25.31	1 (0.87%)	4 (1.63%)	-0.91	0.488	0.686	Mutual exclusivity
C3ORF79	3q25.2	1 (0.87%)	4 (1.63%)	-0.91	0.488	0.686	Mutual exclusivity
C3ORF80	3q25.33	1 (0.87%)	4 (1.63%)	-0.91	0.488	0.686	Mutual exclusivity
C5ORF47	5q35.2	1 (0.87%)	4 (1.63%)	-0.91	0.488	0.686	Mutual exclusivity
C7ORF31	7p15.3	1 (0.87%)	4 (1.63%)	-0.91	0.488	0.686	Mutual exclusivity
C7ORF61	7q22.1	1 (0.87%)	4 (1.63%)	-0.91	0.488	0.686	Mutual exclusivity
CAMK1	3p25.3	1 (0.87%)	4 (1.63%)	-0.91	0.488	0.686	Mutual exclusivity
CAMK4	5q22.1	1 (0.87%)	4 (1.63%)	-0.91	0.488	0.686	Mutual exclusivity
CAMKMT	2p21	1 (0.87%)	4 (1.63%)	-0.91	0.488	0.686	Mutual exclusivity
CBWD1	9p24.3	1 (0.87%)	4 (1.63%)	-0.91	0.488	0.686	Mutual exclusivity
CCDC112	5q22.3	1 (0.87%)	4 (1.63%)	-0.91	0.488	0.686	Mutual exclusivity
CCDC121	2p23.3	1 (0.87%)	4 (1.63%)	-0.91	0.488	0.686	Mutual exclusivity
CCDC152	5p12	1 (0.87%)	4 (1.63%)	-0.91	0.488	0.686	Mutual exclusivity
CCNL1	3q25.31	1 (0.87%)	4 (1.63%)	-0.91	0.488	0.686	Mutual exclusivity
CCNYL2	10q11.21	1 (0.87%)	4 (1.63%)	-0.91	0.488	0.686	Mutual exclusivity
CDK5	7q36.1	1 (0.87%)	4 (1.63%)	-0.91	0.488	0.686	Mutual exclusivity
CEACAM22P	19q13.31	1 (0.87%)	4 (1.63%)	-0.91	0.488	0.686	Mutual exclusivity
CEP120	5q23.2	1 (0.87%)	4 (1.63%)	-0.91	0.488	0.686	Mutual exclusivity
CHST2	3q24	1 (0.87%)	4 (1.63%)	-0.91	0.488	0.686	Mutual exclusivity
CIDEC	3p25.3	1 (0.87%)	4 (1.63%)	-0.91	0.488	0.686	Mutual exclusivity
CLINT1	5q33.3	1 (0.87%)	4 (1.63%)	-0.91	0.488	0.686	Mutual exclusivity
CPA3	3q24	1 (0.87%)	4 (1.63%)	-0.91	0.488	0.686	Mutual exclusivity

CPB1	3q24	1 (0.87%)	4 (1.63%)	-0.91	0.488	0.686	Mutual exclusivity
CPEB4	5q35.2	1 (0.87%)	4 (1.63%)	-0.91	0.488	0.686	Mutual exclusivity
CPNE9	3p25.3	1 (0.87%)	4 (1.63%)	-0.91	0.488	0.686	Mutual exclusivity
CREB3L2	7q33	1 (0.87%)	4 (1.63%)	-0.91	0.488	0.686	Mutual exclusivity
CTNNBL1	20q11.23	1 (0.87%)	4 (1.63%)	-0.91	0.488	0.686	Mutual exclusivity
CYBRD1	2q31.1	1 (0.87%)	4 (1.63%)	-0.91	0.488	0.686	Mutual exclusivity
CYCS	7p15.3	1 (0.87%)	4 (1.63%)	-0.91	0.488	0.686	Mutual exclusivity
DEFB118	20q11.21	1 (0.87%)	4 (1.63%)	-0.91	0.488	0.686	Mutual exclusivity
DEFB119	20q11.21	1 (0.87%)	4 (1.63%)	-0.91	0.488	0.686	Mutual exclusivity
DHX36	3q25.2	1 (0.87%)	4 (1.63%)	-0.91	0.488	0.686	Mutual exclusivity
DIPK2A	3q24	1 (0.87%)	4 (1.63%)	-0.91	0.488	0.686	Mutual exclusivity
DLX1	2q31.1	1 (0.87%)	4 (1.63%)	-0.91	0.488	0.686	Mutual exclusivity
DLX2	2q31.1	1 (0.87%)	4 (1.63%)	-0.91	0.488	0.686	Mutual exclusivity
DMRT1	9p24.3	1 (0.87%)	4 (1.63%)	-0.91	0.488	0.686	Mutual exclusivity
DMRT2	9p24.3	1 (0.87%)	4 (1.63%)	-0.91	0.488	0.686	Mutual exclusivity
DMRT3	9p24.3	1 (0.87%)	4 (1.63%)	-0.91	0.488	0.686	Mutual exclusivity
DMXL1	5q23.1	1 (0.87%)	4 (1.63%)	-0.91	0.488	0.686	Mutual exclusivity
DOCK8	9p24.3	1 (0.87%)	4 (1.63%)	-0.91	0.488	0.686	Mutual exclusivity
DOCK8-AS1	9p24.3	1 (0.87%)	4 (1.63%)	-0.91	0.488	0.686	Mutual exclusivity
DYNC1I2	2q31.1	1 (0.87%)	4 (1.63%)	-0.91	0.488	0.686	Mutual exclusivity
FAM138C	9p24.3	1 (0.87%)	4 (1.63%)	-0.91	0.488	0.686	Mutual exclusivity
FAM170A	5q23.1	1 (0.87%)	4 (1.63%)	-0.91	0.488	0.686	Mutual exclusivity
FAM86C1	11q13.4	1 (0.87%)	4 (1.63%)	-0.91	0.488	0.686	Mutual exclusivity
FBXL7	5p15.1	1 (0.87%)	4 (1.63%)	-0.91	0.488	0.686	Mutual exclusivity
FBXO24	7q22.1	1 (0.87%)	4 (1.63%)	-0.91	0.488	0.686	Mutual exclusivity
FCHO1	19p13.11	1 (0.87%)	4 (1.63%)	-0.91	0.488	0.686	Mutual exclusivity
FEM1C	5q22.3	1 (0.87%)	4 (1.63%)	-0.91	0.488	0.686	Mutual exclusivity
FKBP9	7p14.3	1 (0.87%)	4 (1.63%)	-0.91	0.488	0.686	Mutual exclusivity
FOXD4	9p24.3	1 (0.87%)	4 (1.63%)	-0.91	0.488	0.686	Mutual exclusivity

GEMIN5	5q33.2	1 (0.87%)	4 (1.63%)	-0.91	0.488	0.686	Mutual exclusivity
GFM1	3q25.32	1 (0.87%)	4 (1.63%)	-0.91	0.488	0.686	Mutual exclusivity
GIGYF1	7q22.1	1 (0.87%)	4 (1.63%)	-0.91	0.488	0.686	Mutual exclusivity
GIMAP1	7q36.1	1 (0.87%)	4 (1.63%)	-0.91	0.488	0.686	Mutual exclusivity
GIMAP2	7q36.1	1 (0.87%)	4 (1.63%)	-0.91	0.488	0.686	Mutual exclusivity
GIMAP5	7q36.1	1 (0.87%)	4 (1.63%)	-0.91	0.488	0.686	Mutual exclusivity
GIMAP6	7q36.1	1 (0.87%)	4 (1.63%)	-0.91	0.488	0.686	Mutual exclusivity
GIMAP7	7q36.1	1 (0.87%)	4 (1.63%)	-0.91	0.488	0.686	Mutual exclusivity
GK5	3q23	1 (0.87%)	4 (1.63%)	-0.91	0.488	0.686	Mutual exclusivity
GLRA1	5q33.1	1 (0.87%)	4 (1.63%)	-0.91	0.488	0.686	Mutual exclusivity
GMPS	3q25.31	1 (0.87%)	4 (1.63%)	-0.91	0.488	0.686	Mutual exclusivity
GNB2	7q22.1	1 (0.87%)	4 (1.63%)	-0.91	0.488	0.686	Mutual exclusivity
GPN1	2p23.3	1 (0.87%)	4 (1.63%)	-0.91	0.488	0.686	Mutual exclusivity
GPR149	3q25.2	1 (0.87%)	4 (1.63%)	-0.91	0.488	0.686	Mutual exclusivity
GPR50	Xq28	1 (0.87%)	4 (1.63%)	-0.91	0.488	0.686	Mutual exclusivity
GRM6	5q35.3	1 (0.87%)	4 (1.63%)	-0.91	0.488	0.686	Mutual exclusivity
GTPBP4	10p15.3	1 (0.87%)	4 (1.63%)	-0.91	0.488	0.686	Mutual exclusivity
GYG1	3q24	1 (0.87%)	4 (1.63%)	-0.91	0.488	0.686	Mutual exclusivity
HAPLN4	19p13.11	1 (0.87%)	4 (1.63%)	-0.91	0.488	0.686	Mutual exclusivity
HAT1	2q31.1	1 (0.87%)	4 (1.63%)	-0.91	0.488	0.686	Mutual exclusivity
HLTF	3q24	1 (0.87%)	4 (1.63%)	-0.91	0.488	0.686	Mutual exclusivity
HNRNPC	14q11.2	1 (0.87%)	4 (1.63%)	-0.91	0.488	0.686	Mutual exclusivity
HS6ST2	Xq26.2	1 (0.87%)	4 (1.63%)	-0.91	0.488	0.686	Mutual exclusivity
HSD17B4	5q23.1	1 (0.87%)	4 (1.63%)	-0.91	0.488	0.686	Mutual exclusivity
HTRA4	8p11.22	1 (0.87%)	4 (1.63%)	-0.91	0.488	0.686	Mutual exclusivity
IDI1	10p15.3	1 (0.87%)	4 (1.63%)	-0.91	0.488	0.686	Mutual exclusivity
IDI2	10p15.3	1 (0.87%)	4 (1.63%)	-0.91	0.488	0.686	Mutual exclusivity
IDO1	8p11.21	1 (0.87%)	4 (1.63%)	-0.91	0.488	0.686	Mutual exclusivity
IFT80	3q25.33	1 (0.87%)	4 (1.63%)	-0.91	0.488	0.686	Mutual exclusivity

IL12A	3q25.33	1 (0.87%)	4 (1.63%)	-0.91	0.488	0.686	Mutual exclusivity
IL17RE	3p25.3	1 (0.87%)	4 (1.63%)	-0.91	0.488	0.686	Mutual exclusivity
IL9R	Xq28 and Yq12	1 (0.87%)	4 (1.63%)	-0.91	0.488	0.686	Mutual exclusivity
INSL3	19p13.11	1 (0.87%)	4 (1.63%)	-0.91	0.488	0.686	Mutual exclusivity
IQCJ	3q25.32	1 (0.87%)	4 (1.63%)	-0.91	0.488	0.686	Mutual exclusivity
ITCH	20q11.22	1 (0.87%)	4 (1.63%)	-0.91	0.488	0.686	Mutual exclusivity
ITGA6	2q31.1	1 (0.87%)	4 (1.63%)	-0.91	0.488	0.686	Mutual exclusivity
JAGN1	3p25.3	1 (0.87%)	4 (1.63%)	-0.91	0.488	0.686	Mutual exclusivity
KANK1	9p24.3	1 (0.87%)	4 (1.63%)	-0.91	0.488	0.686	Mutual exclusivity
KCNAB1	3q25.31	1 (0.87%)	4 (1.63%)	-0.91	0.488	0.686	Mutual exclusivity
KCNH2	7q36.1	1 (0.87%)	4 (1.63%)	-0.91	0.488	0.686	Mutual exclusivity
KCNN2	5q22.3	1 (0.87%)	4 (1.63%)	-0.91	0.488	0.686	Mutual exclusivity
KIAA1324L	7q21.12	1 (0.87%)	4 (1.63%)	-0.91	0.488	0.686	Mutual exclusivity
KPNA4	3q25.33	1 (0.87%)	4 (1.63%)	-0.91	0.488	0.686	Mutual exclusivity
KRT8P12	3q25.33	1 (0.87%)	4 (1.63%)	-0.91	0.488	0.686	Mutual exclusivity
LARP4B	10p15.3	1 (0.87%)	4 (1.63%)	-0.91	0.488	0.686	Mutual exclusivity
LEKR1	3q25.31	1 (0.87%)	4 (1.63%)	-0.91	0.488	0.686	Mutual exclusivity
LINC00200	10p15.3	1 (0.87%)	4 (1.63%)	-0.91	0.488	0.686	Mutual exclusivity
LINC00663	19p13.11	1 (0.87%)	4 (1.63%)	-0.91	0.488	0.686	Mutual exclusivity
LINC00664	19p12	1 (0.87%)	4 (1.63%)	-0.91	0.488	0.686	Mutual exclusivity
LINC00880	3q25.31	1 (0.87%)	4 (1.63%)	-0.91	0.488	0.686	Mutual exclusivity
LINC00881	3q25.31	1 (0.87%)	4 (1.63%)	-0.91	0.488	0.686	Mutual exclusivity
LINC00886	3q25.31	1 (0.87%)	4 (1.63%)	-0.91	0.488	0.686	Mutual exclusivity
LMBR1	7q36.3	1 (0.87%)	4 (1.63%)	-0.91	0.488	0.686	Mutual exclusivity
LRCH4	7q22.1	1 (0.87%)	4 (1.63%)	-0.91	0.488	0.686	Mutual exclusivity
LTBR	12p13.31	1 (0.87%)	4 (1.63%)	-0.91	0.488	0.686	Mutual exclusivity
LXN	3q25.32	1 (0.87%)	4 (1.63%)	-0.91	0.488	0.686	Mutual exclusivity
MAP6	11q13.5	1 (0.87%)	4 (1.63%)	-0.91	0.488	0.686	Mutual exclusivity
11-Mar	5p15.1	1 (0.87%)	4 (1.63%)	-0.91	0.488	0.686	Mutual exclusivity

MBNL3	Xq26.2	1 (0.87%)	4 (1.63%)	-0.91	0.488	0.686	Mutual exclusivity
MEPCE	7q22.1	1 (0.87%)	4 (1.63%)	-0.91	0.488	0.686	Mutual exclusivity
METAP1D	2q31.1	1 (0.87%)	4 (1.63%)	-0.91	0.488	0.686	Mutual exclusivity
MFSD1	3q25.32	1 (0.87%)	4 (1.63%)	-0.91	0.488	0.686	Mutual exclusivity
MIR-3919/3919		1 (0.87%)	4 (1.63%)	-0.91	0.488	0.686	Mutual exclusivity
MIR-4263/4263		1 (0.87%)	4 (1.63%)	-0.91	0.488	0.686	Mutual exclusivity
MIR-4330/4330		1 (0.87%)	4 (1.63%)	-0.91	0.488	0.686	Mutual exclusivity
MIR-4460/4460		1 (0.87%)	4 (1.63%)	-0.91	0.488	0.686	Mutual exclusivity
MIR-4637/4637		1 (0.87%)	4 (1.63%)	-0.91	0.488	0.686	Mutual exclusivity
MLF1	3q25.32	1 (0.87%)	4 (1.63%)	-0.91	0.488	0.686	Mutual exclusivity
MME	3q25.2	1 (0.87%)	4 (1.63%)	-0.91	0.488	0.686	Mutual exclusivity
MOSPD3	7q22.1	1 (0.87%)	4 (1.63%)	-0.91	0.488	0.686	Mutual exclusivity
MRPL33	2p23.2	1 (0.87%)	4 (1.63%)	-0.91	0.488	0.686	Mutual exclusivity
MTMR14	3p25.3	1 (0.87%)	4 (1.63%)	-0.91	0.488	0.686	Mutual exclusivity
NCAN	19p13.11	1 (0.87%)	4 (1.63%)	-0.91	0.488	0.686	Mutual exclusivity
NDRG2	14q11.2	1 (0.87%)	4 (1.63%)	-0.91	0.488	0.686	Mutual exclusivity
NMD3	3q26.1	1 (0.87%)	4 (1.63%)	-0.91	0.488	0.686	Mutual exclusivity
NOM1	7q36.3	1 (0.87%)	4 (1.63%)	-0.91	0.488	0.686	Mutual exclusivity
NOS3	7q36.1	1 (0.87%)	4 (1.63%)	-0.91	0.488	0.686	Mutual exclusivity
NPVF	7p15.3	1 (0.87%)	4 (1.63%)	-0.91	0.488	0.686	Mutual exclusivity
NREP	5q22.1	1 (0.87%)	4 (1.63%)	-0.91	0.488	0.686	Mutual exclusivity
NSG2	5q35.2	1 (0.87%)	4 (1.63%)	-0.91	0.488	0.686	Mutual exclusivity
NT5C3A	7p14.3	1 (0.87%)	4 (1.63%)	-0.91	0.488	0.686	Mutual exclusivity
NYAP1	7q22.1	1 (0.87%)	4 (1.63%)	-0.91	0.488	0.686	Mutual exclusivity
OCRL	Xq26.1	1 (0.87%)	4 (1.63%)	-0.91	0.488	0.686	Mutual exclusivity
OGG1	3p25.3	1 (0.87%)	4 (1.63%)	-0.91	0.488	0.686	Mutual exclusivity
OSMR	5p13.1	1 (0.87%)	4 (1.63%)	-0.91	0.488	0.686	Mutual exclusivity
P2RY1	3q25.2	1 (0.87%)	4 (1.63%)	-0.91	0.488	0.686	Mutual exclusivity
PAQR9	3q23	1 (0.87%)	4 (1.63%)	-0.91	0.488	0.686	Mutual exclusivity

PBX2P1	3q24	1 (0.87%)	4 (1.63%)	-0.91	0.488	0.686	Mutual exclusivity
PCOLCE	7q22.1	1 (0.87%)	4 (1.63%)	-0.91	0.488	0.686	Mutual exclusivity
PCOLCE2	3q23	1 (0.87%)	4 (1.63%)	-0.91	0.488	0.686	Mutual exclusivity
PDK1	2q31.1	1 (0.87%)	4 (1.63%)	-0.91	0.488	0.686	Mutual exclusivity
PGGT1B	5q22.3	1 (0.87%)	4 (1.63%)	-0.91	0.488	0.686	Mutual exclusivity
PKDCC	2p21	1 (0.87%)	4 (1.63%)	-0.91	0.488	0.686	Mutual exclusivity
PLCH1	3q25.31	1 (0.87%)	4 (1.63%)	-0.91	0.488	0.686	Mutual exclusivity
PLEKHA2	8p11.22	1 (0.87%)	4 (1.63%)	-0.91	0.488	0.686	Mutual exclusivity
PLEKHG6	12p13.31	1 (0.87%)	4 (1.63%)	-0.91	0.488	0.686	Mutual exclusivity
PLOD2	3q24	1 (0.87%)	4 (1.63%)	-0.91	0.488	0.686	Mutual exclusivity
PLPP3	1p32.2	1 (0.87%)	4 (1.63%)	-0.91	0.488	0.686	Mutual exclusivity
PLSCR1	3q24	1 (0.87%)	4 (1.63%)	-0.91	0.488	0.686	Mutual exclusivity
PLSCR2	3q24	1 (0.87%)	4 (1.63%)	-0.91	0.488	0.686	Mutual exclusivity
PLSCR4	3q24	1 (0.87%)	4 (1.63%)	-0.91	0.488	0.686	Mutual exclusivity
PLSCR5	3q24	1 (0.87%)	4 (1.63%)	-0.91	0.488	0.686	Mutual exclusivity
POP7	7q22.1	1 (0.87%)	4 (1.63%)	-0.91	0.488	0.686	Mutual exclusivity
PPM1L	3q25.33-q26.1	1 (0.87%)	4 (1.63%)	-0.91	0.488	0.686	Mutual exclusivity
PPP1CB	2p23.2	1 (0.87%)	4 (1.63%)	-0.91	0.488	0.686	Mutual exclusivity
PPP1R35	7q22.1	1 (0.87%)	4 (1.63%)	-0.91	0.488	0.686	Mutual exclusivity
PTN	7q33	1 (0.87%)	4 (1.63%)	-0.91	0.488	0.686	Mutual exclusivity
PTX3	3q25.32	1 (0.87%)	4 (1.63%)	-0.91	0.488	0.686	Mutual exclusivity
RAP2B	3q25.2	1 (0.87%)	4 (1.63%)	-0.91	0.488	0.686	Mutual exclusivity
RARRES1	3q25.32	1 (0.87%)	4 (1.63%)	-0.91	0.488	0.686	Mutual exclusivity
RASGEF1C	5q35.3	1 (0.87%)	4 (1.63%)	-0.91	0.488	0.686	Mutual exclusivity
RBKS	2p23.2	1 (0.87%)	4 (1.63%)	-0.91	0.488	0.686	Mutual exclusivity
RICTOR	5p13.1	1 (0.87%)	4 (1.63%)	-0.91	0.488	0.686	Mutual exclusivity
RN7SKP177	3q25.31	1 (0.87%)	4 (1.63%)	-0.91	0.488	0.686	Mutual exclusivity
RN7SKP207	5p13.3	1 (0.87%)	4 (1.63%)	-0.91	0.488	0.686	Mutual exclusivity
RN7SKP25	3q23	1 (0.87%)	4 (1.63%)	-0.91	0.488	0.686	Mutual exclusivity

RN7SKP46	3q25.32	1 (0.87%)	4 (1.63%)	-0.91	0.488	0.686	Mutual exclusivity
RN7SL174P	5q23.1	1 (0.87%)	4 (1.63%)	-0.91	0.488	0.686	Mutual exclusivity
RN7SL237P	20q11.23	1 (0.87%)	4 (1.63%)	-0.91	0.488	0.686	Mutual exclusivity
RN7SL300P	3q25.2	1 (0.87%)	4 (1.63%)	-0.91	0.488	0.686	Mutual exclusivity
RN7SL416P	7q22.1	1 (0.87%)	4 (1.63%)	-0.91	0.488	0.686	Mutual exclusivity
RN7SL505P	7p14.3	1 (0.87%)	4 (1.63%)	-0.91	0.488	0.686	Mutual exclusivity
RN7SL754P	10p15.3	1 (0.87%)	4 (1.63%)	-0.91	0.488	0.686	Mutual exclusivity
RNA5SP144	3q24	1 (0.87%)	4 (1.63%)	-0.91	0.488	0.686	Mutual exclusivity
RNA5SP146	3q25.31	1 (0.87%)	4 (1.63%)	-0.91	0.488	0.686	Mutual exclusivity
RNA5SP178	5p15.1	1 (0.87%)	4 (1.63%)	-0.91	0.488	0.686	Mutual exclusivity
RNA5SP189	5q21.3	1 (0.87%)	4 (1.63%)	-0.91	0.488	0.686	Mutual exclusivity
RNA5SP190	5q23.1	1 (0.87%)	4 (1.63%)	-0.91	0.488	0.686	Mutual exclusivity
RNA5SP200	5q35.1	1 (0.87%)	4 (1.63%)	-0.91	0.488	0.686	Mutual exclusivity
RNA5SP279	9p24.3	1 (0.87%)	4 (1.63%)	-0.91	0.488	0.686	Mutual exclusivity
RNA5SP297	10p15.3	1 (0.87%)	4 (1.63%)	-0.91	0.488	0.686	Mutual exclusivity
RNA5SP298	10p15.3	1 (0.87%)	4 (1.63%)	-0.91	0.488	0.686	Mutual exclusivity
RNASE13	14q11.2	1 (0.87%)	4 (1.63%)	-0.91	0.488	0.686	Mutual exclusivity
RNASE7	14q11.2	1 (0.87%)	4 (1.63%)	-0.91	0.488	0.686	Mutual exclusivity
RNASE8	14q11.2	1 (0.87%)	4 (1.63%)	-0.91	0.488	0.686	Mutual exclusivity
RP9P	7p14.3	1 (0.87%)	4 (1.63%)	-0.91	0.488	0.686	Mutual exclusivity
RPL26L1	5q35.1	1 (0.87%)	4 (1.63%)	-0.91	0.488	0.686	Mutual exclusivity
RPL37A	2q35	1 (0.87%)	4 (1.63%)	-0.91	0.488	0.686	Mutual exclusivity
RPRD1B	20q11.23	1 (0.87%)	4 (1.63%)	-0.91	0.488	0.686	Mutual exclusivity
RPUSD3	3p25.3	1 (0.87%)	4 (1.63%)	-0.91	0.488	0.686	Mutual exclusivity
RSRC1	3q25.32	1 (0.87%)	4 (1.63%)	-0.91	0.488	0.686	Mutual exclusivity
RUFY1	5q35.3	1 (0.87%)	4 (1.63%)	-0.91	0.488	0.686	Mutual exclusivity
SAMHD1	20q11.23	1 (0.87%)	4 (1.63%)	-0.91	0.488	0.686	Mutual exclusivity
SAP25	7q22.1	1 (0.87%)	4 (1.63%)	-0.91	0.488	0.686	Mutual exclusivity
SCARNA7	3q25.33	1 (0.87%)	4 (1.63%)	-0.91	0.488	0.686	Mutual exclusivity

SCNN1A	12p13.31	1 (0.87%)	4 (1.63%)	-0.91	0.488	0.686	Mutual exclusivity
SESTD1	2q31.2	1 (0.87%)	4 (1.63%)	-0.91	0.488	0.686	Mutual exclusivity
SFRP1	8p11.21	1 (0.87%)	4 (1.63%)	-0.91	0.488	0.686	Mutual exclusivity
SHOX2	3q25.32	1 (0.87%)	4 (1.63%)	-0.91	0.488	0.686	Mutual exclusivity
SKAP2	7p15.2	1 (0.87%)	4 (1.63%)	-0.91	0.488	0.686	Mutual exclusivity
SLC25A12	2q31.1	1 (0.87%)	4 (1.63%)	-0.91	0.488	0.686	Mutual exclusivity
SLC27A6	5q23.3	1 (0.87%)	4 (1.63%)	-0.91	0.488	0.686	Mutual exclusivity
SLC33A1	3q25.31	1 (0.87%)	4 (1.63%)	-0.91	0.488	0.686	Mutual exclusivity
SLC47A2	17p11.2	1 (0.87%)	4 (1.63%)	-0.91	0.488	0.686	Mutual exclusivity
SLC4A1AP	2p23.3	1 (0.87%)	4 (1.63%)	-0.91	0.488	0.686	Mutual exclusivity
SLC4A2	7q36.1	1 (0.87%)	4 (1.63%)	-0.91	0.488	0.686	Mutual exclusivity
SLC66A1L	3q25.32	1 (0.87%)	4 (1.63%)	-0.91	0.488	0.686	Mutual exclusivity
SLC9A9	3q24	1 (0.87%)	4 (1.63%)	-0.91	0.488	0.686	Mutual exclusivity
SMC4	3q25.33	1 (0.87%)	4 (1.63%)	-0.91	0.488	0.686	Mutual exclusivity
SMO	7q32.1	1 (0.87%)	4 (1.63%)	-0.91	0.488	0.686	Mutual exclusivity
SNORD53	2p23.2	1 (0.87%)	4 (1.63%)	-0.91	0.488	0.686	Mutual exclusivity
SNORD92	2p23.2	1 (0.87%)	4 (1.63%)	-0.91	0.488	0.686	Mutual exclusivity
SPDYA	2p23.2	1 (0.87%)	4 (1.63%)	-0.91	0.488	0.686	Mutual exclusivity
SPRY3	Xq28 and Yq12	1 (0.87%)	4 (1.63%)	-0.91	0.488	0.686	Mutual exclusivity
SRBD1	2p21	1 (0.87%)	4 (1.63%)	-0.91	0.488	0.686	Mutual exclusivity
SRRM3	7q11.23	1 (0.87%)	4 (1.63%)	-0.91	0.488	0.686	Mutual exclusivity
SSR3	3q25.31	1 (0.87%)	4 (1.63%)	-0.91	0.488	0.686	Mutual exclusivity
STARD4	5q22.1	1 (0.87%)	4 (1.63%)	-0.91	0.488	0.686	Mutual exclusivity
STC2	5q35.2	1 (0.87%)	4 (1.63%)	-0.91	0.488	0.686	Mutual exclusivity
SUGP1	19p13.11	1 (0.87%)	4 (1.63%)	-0.91	0.488	0.686	Mutual exclusivity
SUPT7L	2p23.3	1 (0.87%)	4 (1.63%)	-0.91	0.488	0.686	Mutual exclusivity
TACC1	8p11.22	1 (0.87%)	4 (1.63%)	-0.91	0.488	0.686	Mutual exclusivity
TADA3	3p25.3	1 (0.87%)	4 (1.63%)	-0.91	0.488	0.686	Mutual exclusivity
TFDP2	3q23	1 (0.87%)	4 (1.63%)	-0.91	0.488	0.686	Mutual exclusivity

TFR2	7q22.1	1 (0.87%)	4 (1.63%)	-0.91	0.488	0.686	Mutual exclusivity
TGM2	20q11.23	1 (0.87%)	4 (1.63%)	-0.91	0.488	0.686	Mutual exclusivity
TICAM2	5q22.3	1 (0.87%)	4 (1.63%)	-0.91	0.488	0.686	Mutual exclusivity
TIPARP	3q25.31	1 (0.87%)	4 (1.63%)	-0.91	0.488	0.686	Mutual exclusivity
TM2D2	8p11.22	1 (0.87%)	4 (1.63%)	-0.91	0.488	0.686	Mutual exclusivity
TM6SF2	19p13.11	1 (0.87%)	4 (1.63%)	-0.91	0.488	0.686	Mutual exclusivity
TMED7	5q22.3	1 (0.87%)	4 (1.63%)	-0.91	0.488	0.686	Mutual exclusivity
TMEM14EP	3q25.2	1 (0.87%)	4 (1.63%)	-0.91	0.488	0.686	Mutual exclusivity
TMEM176A	7q36.1	1 (0.87%)	4 (1.63%)	-0.91	0.488	0.686	Mutual exclusivity
TMEM176B	7q36.1	1 (0.87%)	4 (1.63%)	-0.91	0.488	0.686	Mutual exclusivity
TMEM232	5q22.1	1 (0.87%)	4 (1.63%)	-0.91	0.488	0.686	Mutual exclusivity
TMEM253	14q11.2	1 (0.87%)	4 (1.63%)	-0.91	0.488	0.686	Mutual exclusivity
TNFAIP8	5q23.1	1 (0.87%)	4 (1.63%)	-0.91	0.488	0.686	Mutual exclusivity
TNFRSF1A	12p13.31	1 (0.87%)	4 (1.63%)	-0.91	0.488	0.686	Mutual exclusivity
TOGARAM2	2p23.2	1 (0.87%)	4 (1.63%)	-0.91	0.488	0.686	Mutual exclusivity
TPPP2	14q11.2	1 (0.87%)	4 (1.63%)	-0.91	0.488	0.686	Mutual exclusivity
TRIM36	5q22.3	1 (0.87%)	4 (1.63%)	-0.91	0.488	0.686	Mutual exclusivity
TRIM59	3q25.33	1 (0.87%)	4 (1.63%)	-0.91	0.488	0.686	Mutual exclusivity
TRMT61B	2p23.2	1 (0.87%)	4 (1.63%)	-0.91	0.488	0.686	Mutual exclusivity
TRPC1	3q23	1 (0.87%)	4 (1.63%)	-0.91	0.488	0.686	Mutual exclusivity
TSC22D4	7q22.1	1 (0.87%)	4 (1.63%)	-0.91	0.488	0.686	Mutual exclusivity
TSPAN33	7q32.1	1 (0.87%)	4 (1.63%)	-0.91	0.488	0.686	Mutual exclusivity
TTLL3	3p25.3	1 (0.87%)	4 (1.63%)	-0.91	0.488	0.686	Mutual exclusivity
TUBB8	10p15.3	1 (0.87%)	4 (1.63%)	-0.91	0.488	0.686	Mutual exclusivity
U2SURP	3q23	1 (0.87%)	4 (1.63%)	-0.91	0.488	0.686	Mutual exclusivity
UNC93B6	11q13.4	1 (0.87%)	4 (1.63%)	-0.91	0.488	0.686	Mutual exclusivity
VAMP7	Xq28 and Yq12	1 (0.87%)	4 (1.63%)	-0.91	0.488	0.686	Mutual exclusivity
VEPH1	3q25.31-q25.32	1 (0.87%)	4 (1.63%)	-0.91	0.488	0.686	Mutual exclusivity
VSTM2L	20q11.23	1 (0.87%)	4 (1.63%)	-0.91	0.488	0.686	Mutual exclusivity

WASH6P	Xq28 and Yq12	1 (0.87%)	4 (1.63%)	-0.91	0.488	0.686	Mutual exclusivity
WASIR1	Xq28 and Yq12	1 (0.87%)	4 (1.63%)	-0.91	0.488	0.686	Mutual exclusivity
WDR37	10p15.3	1 (0.87%)	4 (1.63%)	-0.91	0.488	0.686	Mutual exclusivity
WDR43	2p23.2	1 (0.87%)	4 (1.63%)	-0.91	0.488	0.686	Mutual exclusivity
XPNPEP2	Xq26.1	1 (0.87%)	4 (1.63%)	-0.91	0.488	0.686	Mutual exclusivity
XRN1	3q23	1 (0.87%)	4 (1.63%)	-0.91	0.488	0.686	Mutual exclusivity
ZFP2	5q35.3	1 (0.87%)	4 (1.63%)	-0.91	0.488	0.686	Mutual exclusivity
ZMYND11	10p15.3	1 (0.87%)	4 (1.63%)	-0.91	0.488	0.686	Mutual exclusivity
ZNF100	19p12	1 (0.87%)	4 (1.63%)	-0.91	0.488	0.686	Mutual exclusivity
ZNF14	19p13.11	1 (0.87%)	4 (1.63%)	-0.91	0.488	0.686	Mutual exclusivity
ZNF219	14q11.2	1 (0.87%)	4 (1.63%)	-0.91	0.488	0.686	Mutual exclusivity
ZNF253	19p13.11	1 (0.87%)	4 (1.63%)	-0.91	0.488	0.686	Mutual exclusivity
ZNF354B	5q35.3	1 (0.87%)	4 (1.63%)	-0.91	0.488	0.686	Mutual exclusivity
ZNF429	19p12	1 (0.87%)	4 (1.63%)	-0.91	0.488	0.686	Mutual exclusivity
ZNF43	19p12	1 (0.87%)	4 (1.63%)	-0.91	0.488	0.686	Mutual exclusivity
ZNF430	19p12	1 (0.87%)	4 (1.63%)	-0.91	0.488	0.686	Mutual exclusivity
ZNF454	5q35.3	1 (0.87%)	4 (1.63%)	-0.91	0.488	0.686	Mutual exclusivity
ZNF486	19p12	1 (0.87%)	4 (1.63%)	-0.91	0.488	0.686	Mutual exclusivity
ZNF493	19p12	1 (0.87%)	4 (1.63%)	-0.91	0.488	0.686	Mutual exclusivity
ZNF506	19p13.11	1 (0.87%)	4 (1.63%)	-0.91	0.488	0.686	Mutual exclusivity
ZNF512	2p23.3	1 (0.87%)	4 (1.63%)	-0.91	0.488	0.686	Mutual exclusivity
ZNF626	19p12	1 (0.87%)	4 (1.63%)	-0.91	0.488	0.686	Mutual exclusivity
ZNF66	19p12	1 (0.87%)	4 (1.63%)	-0.91	0.488	0.686	Mutual exclusivity
ZNF682	19p12	1 (0.87%)	4 (1.63%)	-0.91	0.488	0.686	Mutual exclusivity
ZNF705E	11q13.4	1 (0.87%)	4 (1.63%)	-0.91	0.488	0.686	Mutual exclusivity
ZNF737	19p12	1 (0.87%)	4 (1.63%)	-0.91	0.488	0.686	Mutual exclusivity
ZNF826P	19p12	1 (0.87%)	4 (1.63%)	-0.91	0.488	0.686	Mutual exclusivity
ZNF85	19p12	1 (0.87%)	4 (1.63%)	-0.91	0.488	0.686	Mutual exclusivity
ZNF879	5q35.3	1 (0.87%)	4 (1.63%)	-0.91	0.488	0.686	Mutual exclusivity

ZNF90	19p12	1 (0.87%)	4 (1.63%)	-0.91	0.488	0.686	Mutual exclusivity
ZNF93	19p12	1 (0.87%)	4 (1.63%)	-0.91	0.488	0.686	Mutual exclusivity
ADI1	2p25.3	4 (3.48%)	7 (2.86%)	0.28	0.488	0.686	Co-occurrence
ALLC	2p25.3	4 (3.48%)	7 (2.86%)	0.28	0.488	0.686	Co-occurrence
ANKRD40CL	17q21.33	4 (3.48%)	7 (2.86%)	0.28	0.488	0.686	Co-occurrence
ATP6V1C2	2p25.1	4 (3.48%)	7 (2.86%)	0.28	0.488	0.686	Co-occurrence
B3GALT4	6p21.32	4 (3.48%)	7 (2.86%)	0.28	0.488	0.686	Co-occurrence
BAK1	6p21.31	4 (3.48%)	7 (2.86%)	0.28	0.488	0.686	Co-occurrence
C2ORF50	2p25.1	4 (3.48%)	7 (2.86%)	0.28	0.488	0.686	Co-occurrence
CLDN11	3q26.2	4 (3.48%)	7 (2.86%)	0.28	0.488	0.686	Co-occurrence
COLEC11	2p25.3	4 (3.48%)	7 (2.86%)	0.28	0.488	0.686	Co-occurrence
CUTA	6p21.32	4 (3.48%)	7 (2.86%)	0.28	0.488	0.686	Co-occurrence
DAXX	6p21.32	4 (3.48%)	7 (2.86%)	0.28	0.488	0.686	Co-occurrence
DCDC2C	2p25.3	4 (3.48%)	7 (2.86%)	0.28	0.488	0.686	Co-occurrence
E2F6	2p25.1	4 (3.48%)	7 (2.86%)	0.28	0.488	0.686	Co-occurrence
EIPR1	2p25.3	4 (3.48%)	7 (2.86%)	0.28	0.488	0.686	Co-occurrence
FAM83B	6p12.1	4 (3.48%)	7 (2.86%)	0.28	0.488	0.686	Co-occurrence
GGNBP1	6p21.31	4 (3.48%)	7 (2.86%)	0.28	0.488	0.686	Co-occurrence
GREB1	2p25.1	4 (3.48%)	7 (2.86%)	0.28	0.488	0.686	Co-occurrence
HCG25	6p21.32	4 (3.48%)	7 (2.86%)	0.28	0.488	0.686	Co-occurrence
HSD17B8	6p21.32	4 (3.48%)	7 (2.86%)	0.28	0.488	0.686	Co-occurrence
KCNF1	2p25.1	4 (3.48%)	7 (2.86%)	0.28	0.488	0.686	Co-occurrence
KCNS3	2p24.2	4 (3.48%)	7 (2.86%)	0.28	0.488	0.686	Co-occurrence
KIFC1	6p21.32	4 (3.48%)	7 (2.86%)	0.28	0.488	0.686	Co-occurrence
LINC00336	6p21.31	4 (3.48%)	7 (2.86%)	0.28	0.488	0.686	Co-occurrence
LINC00570	2p25.1	4 (3.48%)	7 (2.86%)	0.28	0.488	0.686	Co-occurrence
LUC7L3	17q21.33	4 (3.48%)	7 (2.86%)	0.28	0.488	0.686	Co-occurrence
MIR-1275/1275		4 (3.48%)	7 (2.86%)	0.28	0.488	0.686	Co-occurrence
MIR-4429/4429		4 (3.48%)	7 (2.86%)	0.28	0.488	0.686	Co-occurrence

MIR-548S/548S		4 (3.48%)	7 (2.86%)	0.28	0.488	0.686	Co-occurrence
MLN	6p21.31	4 (3.48%)	7 (2.86%)	0.28	0.488	0.686	Co-occurrence
NOL10	2p25.1	4 (3.48%)	7 (2.86%)	0.28	0.488	0.686	Co-occurrence
NT5C1B	2p24.2	4 (3.48%)	7 (2.86%)	0.28	0.488	0.686	Co-occurrence
PDIA6	2p25.1	4 (3.48%)	7 (2.86%)	0.28	0.488	0.686	Co-occurrence
PFDN6	6p21.32	4 (3.48%)	7 (2.86%)	0.28	0.488	0.686	Co-occurrence
PHF1	6p21.32	4 (3.48%)	7 (2.86%)	0.28	0.488	0.686	Co-occurrence
RDH14	2p24.2	4 (3.48%)	7 (2.86%)	0.28	0.488	0.686	Co-occurrence
RGL2	6p21.32	4 (3.48%)	7 (2.86%)	0.28	0.488	0.686	Co-occurrence
RING1	6p21.32	4 (3.48%)	7 (2.86%)	0.28	0.488	0.686	Co-occurrence
RN7SL26P	6p21.31	4 (3.48%)	7 (2.86%)	0.28	0.488	0.686	Co-occurrence
RN7SL674P	2p25.1	4 (3.48%)	7 (2.86%)	0.28	0.488	0.686	Co-occurrence
RN7SL832P	2p25.1	4 (3.48%)	7 (2.86%)	0.28	0.488	0.686	Co-occurrence
RNA5SP84	2p25.1	4 (3.48%)	7 (2.86%)	0.28	0.488	0.686	Co-occurrence
RNA5SP85	2p25.1	4 (3.48%)	7 (2.86%)	0.28	0.488	0.686	Co-occurrence
RNA5SP86	2p24.1	4 (3.48%)	7 (2.86%)	0.28	0.488	0.686	Co-occurrence
RNASEH1	2p25.3	4 (3.48%)	7 (2.86%)	0.28	0.488	0.686	Co-occurrence
RNY4P10	6p21.32	4 (3.48%)	7 (2.86%)	0.28	0.488	0.686	Co-occurrence
ROCK2	2p25.1	4 (3.48%)	7 (2.86%)	0.28	0.488	0.686	Co-occurrence
RPS18	6p21.32	4 (3.48%)	7 (2.86%)	0.28	0.488	0.686	Co-occurrence
RPS7	2p25.3	4 (3.48%)	7 (2.86%)	0.28	0.488	0.686	Co-occurrence
SLC39A7	6p21.32	4 (3.48%)	7 (2.86%)	0.28	0.488	0.686	Co-occurrence
SLC66A3	2p25.1	4 (3.48%)	7 (2.86%)	0.28	0.488	0.686	Co-occurrence
SYNGAP1	6p21.32	4 (3.48%)	7 (2.86%)	0.28	0.488	0.686	Co-occurrence
TAPBP	6p21.32	4 (3.48%)	7 (2.86%)	0.28	0.488	0.686	Co-occurrence
TRAPPC12	2p25.3	4 (3.48%)	7 (2.86%)	0.28	0.488	0.686	Co-occurrence
VPS52	6p21.32	4 (3.48%)	7 (2.86%)	0.28	0.488	0.686	Co-occurrence
WDR46	6p21.32	4 (3.48%)	7 (2.86%)	0.28	0.488	0.686	Co-occurrence
ZBTB22	6p21.32	4 (3.48%)	7 (2.86%)	0.28	0.488	0.686	Co-occurrence

ZBTB9	6p21.32	4 (3.48%)	7 (2.86%)	0.28	0.488	0.686	Co-occurrence
ASPH	8q12.3	9 (7.83%)	21 (8.57%)	-0.13	0.495	0.686	Mutual exclusivity
MIR-4470/4470		9 (7.83%)	21 (8.57%)	-0.13	0.495	0.686	Mutual exclusivity
RN7SKP97	8q12.3	9 (7.83%)	21 (8.57%)	-0.13	0.495	0.686	Mutual exclusivity
ACSF2	17q21.33	3 (2.61%)	5 (2.04%)	0.35	0.498	0.686	Co-occurrence
ACTL6A	3q26.33	3 (2.61%)	5 (2.04%)	0.35	0.498	0.686	Co-occurrence
ACTRT3	3q26.2	3 (2.61%)	5 (2.04%)	0.35	0.498	0.686	Co-occurrence
ANKUB1	3q25.1	3 (2.61%)	5 (2.04%)	0.35	0.498	0.686	Co-occurrence
ARRDC4	15q26.2	3 (2.61%)	5 (2.04%)	0.35	0.498	0.686	Co-occurrence
ATP13A4	3q29	3 (2.61%)	5 (2.04%)	0.35	0.498	0.686	Co-occurrence
BCL3	19q13.32	3 (2.61%)	5 (2.04%)	0.35	0.498	0.686	Co-occurrence
CALML5	10p15.1	3 (2.61%)	5 (2.04%)	0.35	0.498	0.686	Co-occurrence
CBLC	19q13.32	3 (2.61%)	5 (2.04%)	0.35	0.498	0.686	Co-occurrence
CCDC87	11q13.2	3 (2.61%)	5 (2.04%)	0.35	0.498	0.686	Co-occurrence
CCS	11q13.2	3 (2.61%)	5 (2.04%)	0.35	0.498	0.686	Co-occurrence
CDH9	5p14.1	3 (2.61%)	5 (2.04%)	0.35	0.498	0.686	Co-occurrence
CDK14	7q21.13	3 (2.61%)	5 (2.04%)	0.35	0.498	0.686	Co-occurrence
CEACAM16	19q13.31-q13.32	3 (2.61%)	5 (2.04%)	0.35	0.498	0.686	Co-occurrence
CENPA	2p23.3	3 (2.61%)	5 (2.04%)	0.35	0.498	0.686	Co-occurrence
CHAD	17q21.33	3 (2.61%)	5 (2.04%)	0.35	0.498	0.686	Co-occurrence
COMMD2	3q25.1	3 (2.61%)	5 (2.04%)	0.35	0.498	0.686	Co-occurrence
CTNND2	5p15.2	3 (2.61%)	5 (2.04%)	0.35	0.498	0.686	Co-occurrence
CTSF	11q13.2	3 (2.61%)	5 (2.04%)	0.35	0.498	0.686	Co-occurrence
CYP3A7	7q22.1	3 (2.61%)	5 (2.04%)	0.35	0.498	0.686	Co-occurrence
DUSP1	5q35.1	3 (2.61%)	5 (2.04%)	0.35	0.498	0.686	Co-occurrence
EGFEM1P	3q26.2	3 (2.61%)	5 (2.04%)	0.35	0.498	0.686	Co-occurrence
EIF2A	3q25.1	3 (2.61%)	5 (2.04%)	0.35	0.498	0.686	Co-occurrence
EIF5A2	3q26.2	3 (2.61%)	5 (2.04%)	0.35	0.498	0.686	Co-occurrence
ETV5	3q27.2	3 (2.61%)	5 (2.04%)	0.35	0.498	0.686	Co-occurrence

FAM117A	17q21.33	3 (2.61%)	5 (2.04%)	0.35	0.498	0.686	Co-occurrence
FAM169B	15q26.3	3 (2.61%)	5 (2.04%)	0.35	0.498	0.686	Co-occurrence
GNB4	3q26.33	3 (2.61%)	5 (2.04%)	0.35	0.498	0.686	Co-occurrence
GOLIM4	3q26.2	3 (2.61%)	5 (2.04%)	0.35	0.498	0.686	Co-occurrence
IGF1R	15q26.3	3 (2.61%)	5 (2.04%)	0.35	0.498	0.686	Co-occurrence
KAT7	17q21.33	3 (2.61%)	5 (2.04%)	0.35	0.498	0.686	Co-occurrence
KCNMB2	3q26.32	3 (2.61%)	5 (2.04%)	0.35	0.498	0.686	Co-occurrence
KCNMB3	3q26.32	3 (2.61%)	5 (2.04%)	0.35	0.498	0.686	Co-occurrence
KLB	4p14	3 (2.61%)	5 (2.04%)	0.35	0.498	0.686	Co-occurrence
LIAS	4p14	3 (2.61%)	5 (2.04%)	0.35	0.498	0.686	Co-occurrence
LINC00501	3q26.32	3 (2.61%)	5 (2.04%)	0.35	0.498	0.686	Co-occurrence
LINC00923	15q26.2	3 (2.61%)	5 (2.04%)	0.35	0.498	0.686	Co-occurrence
MB21D2	3q29	3 (2.61%)	5 (2.04%)	0.35	0.498	0.686	Co-occurrence
MECOM	3q26.2	3 (2.61%)	5 (2.04%)	0.35	0.498	0.686	Co-occurrence
MFN1	3q26.33	3 (2.61%)	5 (2.04%)	0.35	0.498	0.686	Co-occurrence
MRPL47	3q26.33	3 (2.61%)	5 (2.04%)	0.35	0.498	0.686	Co-occurrence
MTERF1	7q21.2	3 (2.61%)	5 (2.04%)	0.35	0.498	0.686	Co-occurrence
MTMR12	5p13.3	3 (2.61%)	5 (2.04%)	0.35	0.498	0.686	Co-occurrence
MYH16	7q22.1	3 (2.61%)	5 (2.04%)	0.35	0.498	0.686	Co-occurrence
NAALADL2	3q26.31	3 (2.61%)	5 (2.04%)	0.35	0.498	0.686	Co-occurrence
NDUFB5	3q26.33	3 (2.61%)	5 (2.04%)	0.35	0.498	0.686	Co-occurrence
OPA1	3q29	3 (2.61%)	5 (2.04%)	0.35	0.498	0.686	Co-occurrence
OSBPL7	17q21.32	3 (2.61%)	5 (2.04%)	0.35	0.498	0.686	Co-occurrence
PDCD10	3q26.1	3 (2.61%)	5 (2.04%)	0.35	0.498	0.686	Co-occurrence
PDZD2	5p13.3	3 (2.61%)	5 (2.04%)	0.35	0.498	0.686	Co-occurrence
PEX5L	3q26.33	3 (2.61%)	5 (2.04%)	0.35	0.498	0.686	Co-occurrence
PFDN1	5q31.3	3 (2.61%)	5 (2.04%)	0.35	0.498	0.686	Co-occurrence
PGPEP1L	15q26.3	3 (2.61%)	5 (2.04%)	0.35	0.498	0.686	Co-occurrence
PIK3CA	3q26.32	3 (2.61%)	5 (2.04%)	0.35	0.498	0.686	Co-occurrence

PLAAT1	3q29	3 (2.61%)	5 (2.04%)	0.35	0.498	0.686	Co-occurrence
RAB10	2p23.3	3 (2.61%)	5 (2.04%)	0.35	0.498	0.686	Co-occurrence
RN7SKP234	3q26.31	3 (2.61%)	5 (2.04%)	0.35	0.498	0.686	Co-occurrence
RN7SKP40	3q26.31	3 (2.61%)	5 (2.04%)	0.35	0.498	0.686	Co-occurrence
RN7SKP52	3q26.32	3 (2.61%)	5 (2.04%)	0.35	0.498	0.686	Co-occurrence
RN7SL447P	3q29	3 (2.61%)	5 (2.04%)	0.35	0.498	0.686	Co-occurrence
RNA5SP147	3q26.32	3 (2.61%)	5 (2.04%)	0.35	0.498	0.686	Co-occurrence
RNA5SP148	3q26.32	3 (2.61%)	5 (2.04%)	0.35	0.498	0.686	Co-occurrence
RNA5SP149	3q26.33	3 (2.61%)	5 (2.04%)	0.35	0.498	0.686	Co-occurrence
RNF13	3q25.1	3 (2.61%)	5 (2.04%)	0.35	0.498	0.686	Co-occurrence
RNY5P3	3q26.2	3 (2.61%)	5 (2.04%)	0.35	0.498	0.686	Co-occurrence
RPL22L1	3q26.2	3 (2.61%)	5 (2.04%)	0.35	0.498	0.686	Co-occurrence
RSAD1	17q21.33	3 (2.61%)	5 (2.04%)	0.35	0.498	0.686	Co-occurrence
SELENOT	3q25.1	3 (2.61%)	5 (2.04%)	0.35	0.498	0.686	Co-occurrence
SERP1	3q25.1	3 (2.61%)	5 (2.04%)	0.35	0.498	0.686	Co-occurrence
SERPINI1	3q26.1	3 (2.61%)	5 (2.04%)	0.35	0.498	0.686	Co-occurrence
SERPINI2	3q26.1	3 (2.61%)	5 (2.04%)	0.35	0.498	0.686	Co-occurrence
SLC35B1	17q21.33	3 (2.61%)	5 (2.04%)	0.35	0.498	0.686	Co-occurrence
SLC35F6	2p23.3	3 (2.61%)	5 (2.04%)	0.35	0.498	0.686	Co-occurrence
SLC7A14	3q26.2	3 (2.61%)	5 (2.04%)	0.35	0.498	0.686	Co-occurrence
SNORA18	11q21	3 (2.61%)	5 (2.04%)	0.35	0.498	0.686	Co-occurrence
TAC4	17q21.33	3 (2.61%)	5 (2.04%)	0.35	0.498	0.686	Co-occurrence
TBL1XR1	3q26.32	3 (2.61%)	5 (2.04%)	0.35	0.498	0.686	Co-occurrence
TERC	3q26.2	3 (2.61%)	5 (2.04%)	0.35	0.498	0.686	Co-occurrence
TSC22D2	3q25.1	3 (2.61%)	5 (2.04%)	0.35	0.498	0.686	Co-occurrence
UGDH	4p14	3 (2.61%)	5 (2.04%)	0.35	0.498	0.686	Co-occurrence
USP13	3q26.33	3 (2.61%)	5 (2.04%)	0.35	0.498	0.686	Co-occurrence
WDR49	3q26.1	3 (2.61%)	5 (2.04%)	0.35	0.498	0.686	Co-occurrence
ZBBX	3q26.1	3 (2.61%)	5 (2.04%)	0.35	0.498	0.686	Co-occurrence

ZMAT3	3q26.32	3 (2.61%)	5 (2.04%)	0.35	0.498	0.686	Co-occurrence
ZNF639	3q26.33	3 (2.61%)	5 (2.04%)	0.35	0.498	0.686	Co-occurrence
ABCF3	3q27.1	2 (1.74%)	6 (2.45%)	-0.49	0.502	0.686	Mutual exclusivity
ADCY3	2p23.3	2 (1.74%)	6 (2.45%)	-0.49	0.502	0.686	Mutual exclusivity
ADGRF3	2p23.3	2 (1.74%)	6 (2.45%)	-0.49	0.502	0.686	Mutual exclusivity
AHCY	20q11.22	2 (1.74%)	6 (2.45%)	-0.49	0.502	0.686	Mutual exclusivity
AKR1C2	10p15.1	2 (1.74%)	6 (2.45%)	-0.49	0.502	0.686	Mutual exclusivity
AKR1C3	10p15.1	2 (1.74%)	6 (2.45%)	-0.49	0.502	0.686	Mutual exclusivity
ALG3	3q27.1	2 (1.74%)	6 (2.45%)	-0.49	0.502	0.686	Mutual exclusivity
ANKIB1	7q21.2	2 (1.74%)	6 (2.45%)	-0.49	0.502	0.686	Mutual exclusivity
AP3M2	8p11.21	2 (1.74%)	6 (2.45%)	-0.49	0.502	0.686	Mutual exclusivity
ARHGAP4	Xq28	2 (1.74%)	6 (2.45%)	-0.49	0.502	0.686	Mutual exclusivity
ASB15	7q31.32	2 (1.74%)	6 (2.45%)	-0.49	0.502	0.686	Mutual exclusivity
ATAD2B	2p24.1-p23.3	2 (1.74%)	6 (2.45%)	-0.49	0.502	0.686	Mutual exclusivity
ATP11B	3q26.33	2 (1.74%)	6 (2.45%)	-0.49	0.502	0.686	Mutual exclusivity
AVPR2	Xq28	2 (1.74%)	6 (2.45%)	-0.49	0.502	0.686	Mutual exclusivity
B3GNT5	3q27.1	2 (1.74%)	6 (2.45%)	-0.49	0.502	0.686	Mutual exclusivity
C11ORF86	11q13.2	2 (1.74%)	6 (2.45%)	-0.49	0.502	0.686	Mutual exclusivity
CADPS2	7q31.32	2 (1.74%)	6 (2.45%)	-0.49	0.502	0.686	Mutual exclusivity
CAMK2N2	3q27.1	2 (1.74%)	6 (2.45%)	-0.49	0.502	0.686	Mutual exclusivity
CCDC50	3q28	2 (1.74%)	6 (2.45%)	-0.49	0.502	0.686	Mutual exclusivity
CENPO	2p23.3	2 (1.74%)	6 (2.45%)	-0.49	0.502	0.686	Mutual exclusivity
CHRD	3q27.1	2 (1.74%)	6 (2.45%)	-0.49	0.502	0.686	Mutual exclusivity
CLCN2	3q27.1	2 (1.74%)	6 (2.45%)	-0.49	0.502	0.686	Mutual exclusivity
CLDN1	3q28	2 (1.74%)	6 (2.45%)	-0.49	0.502	0.686	Mutual exclusivity
COG5	7q22.3	2 (1.74%)	6 (2.45%)	-0.49	0.502	0.686	Mutual exclusivity
CRYGS	3q27.3	2 (1.74%)	6 (2.45%)	-0.49	0.502	0.686	Mutual exclusivity
DBN1	5q35.3	2 (1.74%)	6 (2.45%)	-0.49	0.502	0.686	Mutual exclusivity
DCUN1D1	3q26.33	2 (1.74%)	6 (2.45%)	-0.49	0.502	0.686	Mutual exclusivity

DDX41	5q35.3	2 (1.74%)	6 (2.45%)	-0.49	0.502	0.686	Mutual exclusivity
DNAJC19	3q26.33	2 (1.74%)	6 (2.45%)	-0.49	0.502	0.686	Mutual exclusivity
DOK3	5q35.3	2 (1.74%)	6 (2.45%)	-0.49	0.502	0.686	Mutual exclusivity
ECE2	3q27.1	2 (1.74%)	6 (2.45%)	-0.49	0.502	0.686	Mutual exclusivity
EIF2B4	2p23.3	2 (1.74%)	6 (2.45%)	-0.49	0.502	0.686	Mutual exclusivity
EIF4G1	3q27.1	2 (1.74%)	6 (2.45%)	-0.49	0.502	0.686	Mutual exclusivity
EPAS1	2p21	2 (1.74%)	6 (2.45%)	-0.49	0.502	0.686	Mutual exclusivity
EPHB3	3q27.1	2 (1.74%)	6 (2.45%)	-0.49	0.502	0.686	Mutual exclusivity
F12	5q35.3	2 (1.74%)	6 (2.45%)	-0.49	0.502	0.686	Mutual exclusivity
FAM131A	3q27.1	2 (1.74%)	6 (2.45%)	-0.49	0.502	0.686	Mutual exclusivity
FAM193B	5q35.3	2 (1.74%)	6 (2.45%)	-0.49	0.502	0.686	Mutual exclusivity
FAM228A	2p23.3	2 (1.74%)	6 (2.45%)	-0.49	0.502	0.686	Mutual exclusivity
FAM228B	2p23.3	2 (1.74%)	6 (2.45%)	-0.49	0.502	0.686	Mutual exclusivity
FKBP1B	2p23.3	2 (1.74%)	6 (2.45%)	-0.49	0.502	0.686	Mutual exclusivity
FXR1	3q26.33	2 (1.74%)	6 (2.45%)	-0.49	0.502	0.686	Mutual exclusivity
GAREM2	2p23.3	2 (1.74%)	6 (2.45%)	-0.49	0.502	0.686	Mutual exclusivity
GATAD1	7q21.2	2 (1.74%)	6 (2.45%)	-0.49	0.502	0.686	Mutual exclusivity
GMNC	3q28	2 (1.74%)	6 (2.45%)	-0.49	0.502	0.686	Mutual exclusivity
GRK6	5q35.3	2 (1.74%)	6 (2.45%)	-0.49	0.502	0.686	Mutual exclusivity
GTF3C2	2p23.3	2 (1.74%)	6 (2.45%)	-0.49	0.502	0.686	Mutual exclusivity
HADHA	2p23.3	2 (1.74%)	6 (2.45%)	-0.49	0.502	0.686	Mutual exclusivity
HCFC1	Xq28	2 (1.74%)	6 (2.45%)	-0.49	0.502	0.686	Mutual exclusivity
ICE1	5p15.32	2 (1.74%)	6 (2.45%)	-0.49	0.502	0.686	Mutual exclusivity
IGF2BP2	3q27.2	2 (1.74%)	6 (2.45%)	-0.49	0.502	0.686	Mutual exclusivity
IGF2BP2-AS1	3q27.2	2 (1.74%)	6 (2.45%)	-0.49	0.502	0.686	Mutual exclusivity
IQUB	7q31.32	2 (1.74%)	6 (2.45%)	-0.49	0.502	0.686	Mutual exclusivity
IRAK1	Xq28	2 (1.74%)	6 (2.45%)	-0.49	0.502	0.686	Mutual exclusivity
ITSN2	2p23.3	2 (1.74%)	6 (2.45%)	-0.49	0.502	0.686	Mutual exclusivity
KRIT1	7q21.2	2 (1.74%)	6 (2.45%)	-0.49	0.502	0.686	Mutual exclusivity

L1CAM	Xq28	2 (1.74%)	6 (2.45%)	-0.49	0.502	0.686	Mutual exclusivity
LAMP3	3q27.1	2 (1.74%)	6 (2.45%)	-0.49	0.502	0.686	Mutual exclusivity
LCA10	Xq28	2 (1.74%)	6 (2.45%)	-0.49	0.502	0.686	Mutual exclusivity
LMOD2	7q31.32	2 (1.74%)	6 (2.45%)	-0.49	0.502	0.686	Mutual exclusivity
MAGEF1	3q27.1	2 (1.74%)	6 (2.45%)	-0.49	0.502	0.686	Mutual exclusivity
MASP1	3q27.3	2 (1.74%)	6 (2.45%)	-0.49	0.502	0.686	Mutual exclusivity
MCCC1	3q27.1	2 (1.74%)	6 (2.45%)	-0.49	0.502	0.686	Mutual exclusivity
MECP2	Xq28	2 (1.74%)	6 (2.45%)	-0.49	0.502	0.686	Mutual exclusivity
MFSD2B	2p23.3	2 (1.74%)	6 (2.45%)	-0.49	0.502	0.686	Mutual exclusivity
MINDY4B	3q25.1	2 (1.74%)	6 (2.45%)	-0.49	0.502	0.686	Mutual exclusivity
MIR-3163/3163		2 (1.74%)	6 (2.45%)	-0.49	0.502	0.686	Mutual exclusivity
MIR-718/718		2 (1.74%)	6 (2.45%)	-0.49	0.502	0.686	Mutual exclusivity
NAA10	Xq28	2 (1.74%)	6 (2.45%)	-0.49	0.502	0.686	Mutual exclusivity
NCOA1	2p23.3	2 (1.74%)	6 (2.45%)	-0.49	0.502	0.686	Mutual exclusivity
NDUFA5	7q31.32	2 (1.74%)	6 (2.45%)	-0.49	0.502	0.686	Mutual exclusivity
PDLIM7	5q35.3	2 (1.74%)	6 (2.45%)	-0.49	0.502	0.686	Mutual exclusivity
PFN4	2p23.3	2 (1.74%)	6 (2.45%)	-0.49	0.502	0.686	Mutual exclusivity
PGPEP1	19p13.11	2 (1.74%)	6 (2.45%)	-0.49	0.502	0.686	Mutual exclusivity
POLR2H	3q27.1	2 (1.74%)	6 (2.45%)	-0.49	0.502	0.686	Mutual exclusivity
PPM1G	2p23.3	2 (1.74%)	6 (2.45%)	-0.49	0.502	0.686	Mutual exclusivity
PRR7	5q35.3	2 (1.74%)	6 (2.45%)	-0.49	0.502	0.686	Mutual exclusivity
PSMD2	3q27.1	2 (1.74%)	6 (2.45%)	-0.49	0.502	0.686	Mutual exclusivity
PTRHD1	2p23.3	2 (1.74%)	6 (2.45%)	-0.49	0.502	0.686	Mutual exclusivity
PYDC2	3q28	2 (1.74%)	6 (2.45%)	-0.49	0.502	0.686	Mutual exclusivity
RBM14	11q13.2	2 (1.74%)	6 (2.45%)	-0.49	0.502	0.686	Mutual exclusivity
RBM4	11q13.2	2 (1.74%)	6 (2.45%)	-0.49	0.502	0.686	Mutual exclusivity
RBM4B	11q13.2	2 (1.74%)	6 (2.45%)	-0.49	0.502	0.686	Mutual exclusivity
RENBP	Xq28	2 (1.74%)	6 (2.45%)	-0.49	0.502	0.686	Mutual exclusivity
RN7SKP265	3q26.33	2 (1.74%)	6 (2.45%)	-0.49	0.502	0.686	Mutual exclusivity

RN7SKP27	2p24.1	2 (1.74%)	6 (2.45%)	-0.49	0.502	0.686	Mutual exclusivity
RN7SL117P	2p24.1	2 (1.74%)	6 (2.45%)	-0.49	0.502	0.686	Mutual exclusivity
RN7SL149P	8p11.21	2 (1.74%)	6 (2.45%)	-0.49	0.502	0.686	Mutual exclusivity
RN7SL229P	3q26.33	2 (1.74%)	6 (2.45%)	-0.49	0.502	0.686	Mutual exclusivity
RN7SL610P	2p23.3	2 (1.74%)	6 (2.45%)	-0.49	0.502	0.686	Mutual exclusivity
RN7SL703P	3q26.33	2 (1.74%)	6 (2.45%)	-0.49	0.502	0.686	Mutual exclusivity
RNA5SP150	3q26.33	2 (1.74%)	6 (2.45%)	-0.49	0.502	0.686	Mutual exclusivity
RNA5SP151	3q27.1	2 (1.74%)	6 (2.45%)	-0.49	0.502	0.686	Mutual exclusivity
RNA5SP87	2p24.1	2 (1.74%)	6 (2.45%)	-0.49	0.502	0.686	Mutual exclusivity
RNA5SP88	2p23.3	2 (1.74%)	6 (2.45%)	-0.49	0.502	0.686	Mutual exclusivity
RPL39L	3q27.3	2 (1.74%)	6 (2.45%)	-0.49	0.502	0.686	Mutual exclusivity
RTP1	3q27.3	2 (1.74%)	6 (2.45%)	-0.49	0.502	0.686	Mutual exclusivity
RTP2	3q27.3	2 (1.74%)	6 (2.45%)	-0.49	0.502	0.686	Mutual exclusivity
RTP4	3q27.3	2 (1.74%)	6 (2.45%)	-0.49	0.502	0.686	Mutual exclusivity
SELENOI	2p23.3	2 (1.74%)	6 (2.45%)	-0.49	0.502	0.686	Mutual exclusivity
SENP2	3q27.2	2 (1.74%)	6 (2.45%)	-0.49	0.502	0.686	Mutual exclusivity
SF3B6	2p23.3	2 (1.74%)	6 (2.45%)	-0.49	0.502	0.686	Mutual exclusivity
SLC13A1	7q31.32	2 (1.74%)	6 (2.45%)	-0.49	0.502	0.686	Mutual exclusivity
SNORD66	3q27.1	2 (1.74%)	6 (2.45%)	-0.49	0.502	0.686	Mutual exclusivity
SNX17	2p23.3	2 (1.74%)	6 (2.45%)	-0.49	0.502	0.686	Mutual exclusivity
SOX2	3q26.33	2 (1.74%)	6 (2.45%)	-0.49	0.502	0.686	Mutual exclusivity
SST	3q27.3	2 (1.74%)	6 (2.45%)	-0.49	0.502	0.686	Mutual exclusivity
TAS2R16	7q31.32|7q31	2 (1.74%)	6 (2.45%)	-0.49	0.502	0.686	Mutual exclusivity
TBCCD1	3q27.3	2 (1.74%)	6 (2.45%)	-0.49	0.502	0.686	Mutual exclusivity
THPO	3q27.1	2 (1.74%)	6 (2.45%)	-0.49	0.502	0.686	Mutual exclusivity
TM4SF1	3q25.1	2 (1.74%)	6 (2.45%)	-0.49	0.502	0.686	Mutual exclusivity
TM4SF18	3q25.1	2 (1.74%)	6 (2.45%)	-0.49	0.502	0.686	Mutual exclusivity
TM4SF4	3q25.1	2 (1.74%)	6 (2.45%)	-0.49	0.502	0.686	Mutual exclusivity
TMEM187	Xq28	2 (1.74%)	6 (2.45%)	-0.49	0.502	0.686	Mutual exclusivity

TP53I3	2p23.3	2 (1.74%)	6 (2.45%)	-0.49	0.502	0.686	Mutual exclusivity
UBXN2A	2p23.3	2 (1.74%)	6 (2.45%)	-0.49	0.502	0.686	Mutual exclusivity
UTS2B	3q28	2 (1.74%)	6 (2.45%)	-0.49	0.502	0.686	Mutual exclusivity
VPS8	3q27.2	2 (1.74%)	6 (2.45%)	-0.49	0.502	0.686	Mutual exclusivity
VWA5B2	3q27.1	2 (1.74%)	6 (2.45%)	-0.49	0.502	0.686	Mutual exclusivity
WASL	7q31.32	2 (1.74%)	6 (2.45%)	-0.49	0.502	0.686	Mutual exclusivity
WDCP	2p23.3	2 (1.74%)	6 (2.45%)	-0.49	0.502	0.686	Mutual exclusivity
ZNF513	2p23.3	2 (1.74%)	6 (2.45%)	-0.49	0.502	0.686	Mutual exclusivity
RNA5SP269	8q13.2	11 (9.57%)	25 (10.20%)	-0.09	0.507	0.686	Mutual exclusivity
ADCY10P1	6p21.1	3 (2.61%)	8 (3.27%)	-0.32	0.512	0.686	Mutual exclusivity
ANKS1A	6p21.31	3 (2.61%)	8 (3.27%)	-0.32	0.512	0.686	Mutual exclusivity
ANO1	11q13.3	3 (2.61%)	8 (3.27%)	-0.32	0.512	0.686	Mutual exclusivity
APOBEC2	6p21.1	3 (2.61%)	8 (3.27%)	-0.32	0.512	0.686	Mutual exclusivity
BAG2	6p12.1	3 (2.61%)	8 (3.27%)	-0.32	0.512	0.686	Mutual exclusivity
BEND6	6p12.1	3 (2.61%)	8 (3.27%)	-0.32	0.512	0.686	Mutual exclusivity
CDKN1A	6p21.2	3 (2.61%)	8 (3.27%)	-0.32	0.512	0.686	Mutual exclusivity
CLPS	6p21.31	3 (2.61%)	8 (3.27%)	-0.32	0.512	0.686	Mutual exclusivity
CLPSL1	6p21.31	3 (2.61%)	8 (3.27%)	-0.32	0.512	0.686	Mutual exclusivity
CLPSL2	6p21.31	3 (2.61%)	8 (3.27%)	-0.32	0.512	0.686	Mutual exclusivity
CRISP2	6p12.3	3 (2.61%)	8 (3.27%)	-0.32	0.512	0.686	Mutual exclusivity
CRISP3	6p12.3	3 (2.61%)	8 (3.27%)	-0.32	0.512	0.686	Mutual exclusivity
DEFB110	6p12.3	3 (2.61%)	8 (3.27%)	-0.32	0.512	0.686	Mutual exclusivity
DEFB112	6p12.3	3 (2.61%)	8 (3.27%)	-0.32	0.512	0.686	Mutual exclusivity
DEFB113	6p12.3	3 (2.61%)	8 (3.27%)	-0.32	0.512	0.686	Mutual exclusivity
DNAH8	6p21.2	3 (2.61%)	8 (3.27%)	-0.32	0.512	0.686	Mutual exclusivity
ELOVL5	6p12.1	3 (2.61%)	8 (3.27%)	-0.32	0.512	0.686	Mutual exclusivity
FBXO9	6p12.1	3 (2.61%)	8 (3.27%)	-0.32	0.512	0.686	Mutual exclusivity
FOXP4	6p21.1	3 (2.61%)	8 (3.27%)	-0.32	0.512	0.686	Mutual exclusivity
FRS3	6p21.1	3 (2.61%)	8 (3.27%)	-0.32	0.512	0.686	Mutual exclusivity

GCLC	6p12.1	3 (2.61%)	8 (3.27%)	-0.32	0.512	0.686	Mutual exclusivity
GCM1	6p12.1	3 (2.61%)	8 (3.27%)	-0.32	0.512	0.686	Mutual exclusivity
GLO1	6p21.2	3 (2.61%)	8 (3.27%)	-0.32	0.512	0.686	Mutual exclusivity
GLP1R	6p21.2	3 (2.61%)	8 (3.27%)	-0.32	0.512	0.686	Mutual exclusivity
GSTA4	6p12.2	3 (2.61%)	8 (3.27%)	-0.32	0.512	0.686	Mutual exclusivity
ICK	6p12.1	3 (2.61%)	8 (3.27%)	-0.32	0.512	0.686	Mutual exclusivity
IL17A	6p12.2	3 (2.61%)	8 (3.27%)	-0.32	0.512	0.686	Mutual exclusivity
IL17F	6p12.2	3 (2.61%)	8 (3.27%)	-0.32	0.512	0.686	Mutual exclusivity
KCNK16	6p21.2	3 (2.61%)	8 (3.27%)	-0.32	0.512	0.686	Mutual exclusivity
KCNK17	6p21.2	3 (2.61%)	8 (3.27%)	-0.32	0.512	0.686	Mutual exclusivity
KCTD20	6p21.31	3 (2.61%)	8 (3.27%)	-0.32	0.512	0.686	Mutual exclusivity
KIAA1586	6p12.1	3 (2.61%)	8 (3.27%)	-0.32	0.512	0.686	Mutual exclusivity
KLHL31	6p12.1	3 (2.61%)	8 (3.27%)	-0.32	0.512	0.686	Mutual exclusivity
LHFPL5	6p21.31	3 (2.61%)	8 (3.27%)	-0.32	0.512	0.686	Mutual exclusivity
LRRC1	6p12.1	3 (2.61%)	8 (3.27%)	-0.32	0.512	0.686	Mutual exclusivity
MAPK13	6p21.31	3 (2.61%)	8 (3.27%)	-0.32	0.512	0.686	Mutual exclusivity
MAPK14	6p21.31	3 (2.61%)	8 (3.27%)	-0.32	0.512	0.686	Mutual exclusivity
MCM3	6p12.2	3 (2.61%)	8 (3.27%)	-0.32	0.512	0.686	Mutual exclusivity
MDFI	6p21.1	3 (2.61%)	8 (3.27%)	-0.32	0.512	0.686	Mutual exclusivity
MDGA1	6p21.2	3 (2.61%)	8 (3.27%)	-0.32	0.512	0.686	Mutual exclusivity
MIR-133B/133B		3 (2.61%)	8 (3.27%)	-0.32	0.512	0.686	Mutual exclusivity
MIR-206/206		3 (2.61%)	8 (3.27%)	-0.32	0.512	0.686	Mutual exclusivity
MIR-5690/5690		3 (2.61%)	8 (3.27%)	-0.32	0.512	0.686	Mutual exclusivity
NCR2	6p21.1	3 (2.61%)	8 (3.27%)	-0.32	0.512	0.686	Mutual exclusivity
NFYA	6p21.1	3 (2.61%)	8 (3.27%)	-0.32	0.512	0.686	Mutual exclusivity
PAQR8	6p12.2	3 (2.61%)	8 (3.27%)	-0.32	0.512	0.686	Mutual exclusivity
PGC	6p21.1	3 (2.61%)	8 (3.27%)	-0.32	0.512	0.686	Mutual exclusivity
PPARD	6p21.31	3 (2.61%)	8 (3.27%)	-0.32	0.512	0.686	Mutual exclusivity
PRICKLE4	6p21.1	3 (2.61%)	8 (3.27%)	-0.32	0.512	0.686	Mutual exclusivity

PRIM2	6p11.2	3 (2.61%)	8 (3.27%)	-0.32	0.512	0.686	Mutual exclusivity
RAB23	6p12.1-p11.2	3 (2.61%)	8 (3.27%)	-0.32	0.512	0.686	Mutual exclusivity
RN7SKP256	6p12.1	3 (2.61%)	8 (3.27%)	-0.32	0.512	0.686	Mutual exclusivity
RN7SL200P	6p21.31	3 (2.61%)	8 (3.27%)	-0.32	0.512	0.686	Mutual exclusivity
RN7SL244P	6p12.1	3 (2.61%)	8 (3.27%)	-0.32	0.512	0.686	Mutual exclusivity
RN7SL465P	6p21.2	3 (2.61%)	8 (3.27%)	-0.32	0.512	0.686	Mutual exclusivity
RN7SL748P	6p21.31	3 (2.61%)	8 (3.27%)	-0.32	0.512	0.686	Mutual exclusivity
RNA5SP207	6p21.1	3 (2.61%)	8 (3.27%)	-0.32	0.512	0.686	Mutual exclusivity
SELENOS	15q26.3	3 (2.61%)	8 (3.27%)	-0.32	0.512	0.686	Mutual exclusivity
SLC26A8	6p21.31	3 (2.61%)	8 (3.27%)	-0.32	0.512	0.686	Mutual exclusivity
SNRPC	6p21.31	3 (2.61%)	8 (3.27%)	-0.32	0.512	0.686	Mutual exclusivity
SRPK1	6p21.31	3 (2.61%)	8 (3.27%)	-0.32	0.512	0.686	Mutual exclusivity
STK38	6p21.31	3 (2.61%)	8 (3.27%)	-0.32	0.512	0.686	Mutual exclusivity
TAF11	6p21.31	3 (2.61%)	8 (3.27%)	-0.32	0.512	0.686	Mutual exclusivity
TCP11	6p21.31	3 (2.61%)	8 (3.27%)	-0.32	0.512	0.686	Mutual exclusivity
TFAP2B	6p12.3	3 (2.61%)	8 (3.27%)	-0.32	0.512	0.686	Mutual exclusivity
TFAP2D	6p12.3	3 (2.61%)	8 (3.27%)	-0.32	0.512	0.686	Mutual exclusivity
TFEB	6p21.1	3 (2.61%)	8 (3.27%)	-0.32	0.512	0.686	Mutual exclusivity
TOMM6	6p21.1	3 (2.61%)	8 (3.27%)	-0.32	0.512	0.686	Mutual exclusivity
TREM1	6p21.1	3 (2.61%)	8 (3.27%)	-0.32	0.512	0.686	Mutual exclusivity
TREM2	6p21.1	3 (2.61%)	8 (3.27%)	-0.32	0.512	0.686	Mutual exclusivity
TREML1	6p21.1	3 (2.61%)	8 (3.27%)	-0.32	0.512	0.686	Mutual exclusivity
TREML2	6p21.1	3 (2.61%)	8 (3.27%)	-0.32	0.512	0.686	Mutual exclusivity
TREML3P	6p21.1	3 (2.61%)	8 (3.27%)	-0.32	0.512	0.686	Mutual exclusivity
TREML4	6p21.1	3 (2.61%)	8 (3.27%)	-0.32	0.512	0.686	Mutual exclusivity
UHRF1BP1	6p21.31	3 (2.61%)	8 (3.27%)	-0.32	0.512	0.686	Mutual exclusivity
ZNF451	6p12.1	3 (2.61%)	8 (3.27%)	-0.32	0.512	0.686	Mutual exclusivity
ABHD8	19p13.11	2 (1.74%)	3 (1.22%)	0.51	0.512	0.686	Co-occurrence
ABL1	9q34.12	2 (1.74%)	3 (1.22%)	0.51	0.512	0.686	Co-occurrence

ACSL6	5q31.1	2 (1.74%)	3 (1.22%)	0.51	0.512	0.686	Co-occurrence
ACSM2B	16p12.3	2 (1.74%)	3 (1.22%)	0.51	0.512	0.686	Co-occurrence
ACSM5	16p12.3	2 (1.74%)	3 (1.22%)	0.51	0.512	0.686	Co-occurrence
ADAM10	15q21.3	2 (1.74%)	3 (1.22%)	0.51	0.512	0.686	Co-occurrence
ADAMTS12	5p13.3-p13.2	2 (1.74%)	3 (1.22%)	0.51	0.512	0.686	Co-occurrence
ADCYAP1	18p11.32	2 (1.74%)	3 (1.22%)	0.51	0.512	0.686	Co-occurrence
AGL	1p21.2	2 (1.74%)	3 (1.22%)	0.51	0.512	0.686	Co-occurrence
AK3	9p24.1	2 (1.74%)	3 (1.22%)	0.51	0.512	0.686	Co-occurrence
AMACR	5p13.2	2 (1.74%)	3 (1.22%)	0.51	0.512	0.686	Co-occurrence
ANKLE1	19p13.11	2 (1.74%)	3 (1.22%)	0.51	0.512	0.686	Co-occurrence
ANKRD26P1	16q11.2	2 (1.74%)	3 (1.22%)	0.51	0.512	0.686	Co-occurrence
ANKRD30A	10p11.21	2 (1.74%)	3 (1.22%)	0.51	0.512	0.686	Co-occurrence
AP4M1	7q22.1	2 (1.74%)	3 (1.22%)	0.51	0.512	0.686	Co-occurrence
ARHGAP23P1	16p11.2	2 (1.74%)	3 (1.22%)	0.51	0.512	0.686	Co-occurrence
ATF7IP2	16p13.2-p13.13	2 (1.74%)	3 (1.22%)	0.51	0.512	0.686	Co-occurrence
ATG12	5q22.3	2 (1.74%)	3 (1.22%)	0.51	0.512	0.686	Co-occurrence
ATP2B2	3p25.3	2 (1.74%)	3 (1.22%)	0.51	0.512	0.686	Co-occurrence
ATXN7L1	7q22.3	2 (1.74%)	3 (1.22%)	0.51	0.512	0.686	Co-occurrence
B4GAT1	11q13.2	2 (1.74%)	3 (1.22%)	0.51	0.512	0.686	Co-occurrence
BABAM1	19p13.11	2 (1.74%)	3 (1.22%)	0.51	0.512	0.686	Co-occurrence
BAMBI	10p12.1	2 (1.74%)	3 (1.22%)	0.51	0.512	0.686	Co-occurrence
BEND7	10p13	2 (1.74%)	3 (1.22%)	0.51	0.512	0.686	Co-occurrence
BMP8B	1p34.2	2 (1.74%)	3 (1.22%)	0.51	0.512	0.686	Co-occurrence
BRD3	9q34.2	2 (1.74%)	3 (1.22%)	0.51	0.512	0.686	Co-occurrence
BRMS1	11q13.2	2 (1.74%)	3 (1.22%)	0.51	0.512	0.686	Co-occurrence
BZW1	2q33.1	2 (1.74%)	3 (1.22%)	0.51	0.512	0.686	Co-occurrence
C16ORF87	16q11.2	2 (1.74%)	3 (1.22%)	0.51	0.512	0.686	Co-occurrence
C1QTNF3	5p13.2	2 (1.74%)	3 (1.22%)	0.51	0.512	0.686	Co-occurrence
C7ORF13	7q36.3	2 (1.74%)	3 (1.22%)	0.51	0.512	0.686	Co-occurrence

C7ORF33	7q36.1	2 (1.74%)	3 (1.22%)	0.51	0.512	0.686	Co-occurrence
C8A	1p32.2	2 (1.74%)	3 (1.22%)	0.51	0.512	0.686	Co-occurrence
C8B	1p32.2	2 (1.74%)	3 (1.22%)	0.51	0.512	0.686	Co-occurrence
CACNA2D1	7q21.11	2 (1.74%)	3 (1.22%)	0.51	0.512	0.686	Co-occurrence
CASKIN1	16p13.3	2 (1.74%)	3 (1.22%)	0.51	0.512	0.686	Co-occurrence
CBX3P2	18p11.32	2 (1.74%)	3 (1.22%)	0.51	0.512	0.686	Co-occurrence
CCDC3	10p13	2 (1.74%)	3 (1.22%)	0.51	0.512	0.686	Co-occurrence
CCDC36	3p21.31	2 (1.74%)	3 (1.22%)	0.51	0.512	0.686	Co-occurrence
CD151	11p15.5	2 (1.74%)	3 (1.22%)	0.51	0.512	0.686	Co-occurrence
CD302	2q24.2	2 (1.74%)	3 (1.22%)	0.51	0.512	0.686	Co-occurrence
CDC14A	1p21.2	2 (1.74%)	3 (1.22%)	0.51	0.512	0.686	Co-occurrence
CDO1	5q22.3	2 (1.74%)	3 (1.22%)	0.51	0.512	0.686	Co-occurrence
CDRT15L2	17p11.2	2 (1.74%)	3 (1.22%)	0.51	0.512	0.686	Co-occurrence
CETN1	18p11.32	2 (1.74%)	3 (1.22%)	0.51	0.512	0.686	Co-occurrence
CGNL1	15q21.3	2 (1.74%)	3 (1.22%)	0.51	0.512	0.686	Co-occurrence
CHERP	19p13.11	2 (1.74%)	3 (1.22%)	0.51	0.512	0.686	Co-occurrence
CHID1	11p15.5	2 (1.74%)	3 (1.22%)	0.51	0.512	0.686	Co-occurrence
CHTF18	16p13.3	2 (1.74%)	3 (1.22%)	0.51	0.512	0.686	Co-occurrence
CLSTN2	3q23	2 (1.74%)	3 (1.22%)	0.51	0.512	0.686	Co-occurrence
CLUL1	18p11.32	2 (1.74%)	3 (1.22%)	0.51	0.512	0.686	Co-occurrence
CNTFR	9p13.3	2 (1.74%)	3 (1.22%)	0.51	0.512	0.686	Co-occurrence
COLEC12	18p11.32	2 (1.74%)	3 (1.22%)	0.51	0.512	0.686	Co-occurrence
COPS6	7q22.1	2 (1.74%)	3 (1.22%)	0.51	0.512	0.686	Co-occurrence
CPLANE1	5p13.2	2 (1.74%)	3 (1.22%)	0.51	0.512	0.686	Co-occurrence
CRACR2B	11p15.5	2 (1.74%)	3 (1.22%)	0.51	0.512	0.686	Co-occurrence
CSF2	5q31.1	2 (1.74%)	3 (1.22%)	0.51	0.512	0.686	Co-occurrence
CTLA4	2q33.2	2 (1.74%)	3 (1.22%)	0.51	0.512	0.686	Co-occurrence
CUL1	7q36.1	2 (1.74%)	3 (1.22%)	0.51	0.512	0.686	Co-occurrence
DBF4	7q21.12	2 (1.74%)	3 (1.22%)	0.51	0.512	0.686	Co-occurrence

DDA1	19p13.11	2 (1.74%)	3 (1.22%)	0.51	0.512	0.686	Co-occurrence
DLGAP1	18p11.31	2 (1.74%)	3 (1.22%)	0.51	0.512	0.686	Co-occurrence
DLL3	19q13.2	2 (1.74%)	3 (1.22%)	0.51	0.512	0.686	Co-occurrence
DNAJA2	16q11.2	2 (1.74%)	3 (1.22%)	0.51	0.512	0.686	Co-occurrence
EFCAB10	7q22.3	2 (1.74%)	3 (1.22%)	0.51	0.512	0.686	Co-occurrence
EFNA5	5q21.3	2 (1.74%)	3 (1.22%)	0.51	0.512	0.686	Co-occurrence
EME2	16p13.3	2 (1.74%)	3 (1.22%)	0.51	0.512	0.686	Co-occurrence
EMILIN2	18p11.32-p11.31	2 (1.74%)	3 (1.22%)	0.51	0.512	0.686	Co-occurrence
ENHO	9p13.3	2 (1.74%)	3 (1.22%)	0.51	0.512	0.686	Co-occurrence
ENOSF1	18p11.32	2 (1.74%)	3 (1.22%)	0.51	0.512	0.686	Co-occurrence
EP400	12q24.33	2 (1.74%)	3 (1.22%)	0.51	0.512	0.686	Co-occurrence
EPHX3	19p13.12	2 (1.74%)	3 (1.22%)	0.51	0.512	0.686	Co-occurrence
ESYT3	3q22.3	2 (1.74%)	3 (1.22%)	0.51	0.512	0.686	Co-occurrence
EXOSC2	9q34.12	2 (1.74%)	3 (1.22%)	0.51	0.512	0.686	Co-occurrence
FAHD1	16p13.3	2 (1.74%)	3 (1.22%)	0.51	0.512	0.686	Co-occurrence
FAM13B	5q31.2	2 (1.74%)	3 (1.22%)	0.51	0.512	0.686	Co-occurrence
FBXL17	5q21.3	2 (1.74%)	3 (1.22%)	0.51	0.512	0.686	Co-occurrence
FER	5q21.3	2 (1.74%)	3 (1.22%)	0.51	0.512	0.686	Co-occurrence
FGA	4q31.3	2 (1.74%)	3 (1.22%)	0.51	0.512	0.686	Co-occurrence
FRMD4A	10p13	2 (1.74%)	3 (1.22%)	0.51	0.512	0.686	Co-occurrence
FYB2	1p32.2	2 (1.74%)	3 (1.22%)	0.51	0.512	0.686	Co-occurrence
GALNT11	7q36.1|7q36.1	2 (1.74%)	3 (1.22%)	0.51	0.512	0.686	Co-occurrence
GNG13	16p13.3	2 (1.74%)	3 (1.22%)	0.51	0.512	0.686	Co-occurrence
GOSR2	17q21.32	2 (1.74%)	3 (1.22%)	0.51	0.512	0.686	Co-occurrence
GPNMB	7p15.3	2 (1.74%)	3 (1.22%)	0.51	0.512	0.686	Co-occurrence
GPR88	1p21.2	2 (1.74%)	3 (1.22%)	0.51	0.512	0.686	Co-occurrence
GPT2	16q11.2	2 (1.74%)	3 (1.22%)	0.51	0.512	0.686	Co-occurrence
HAGH	16p13.3	2 (1.74%)	3 (1.22%)	0.51	0.512	0.686	Co-occurrence
HERC2P4	16p11.2	2 (1.74%)	3 (1.22%)	0.51	0.512	0.686	Co-occurrence

HERC2P5	16p11.2	2 (1.74%)	3 (1.22%)	0.51	0.512	0.686	Co-occurrence
HERC2P8	16p11.2	2 (1.74%)	3 (1.22%)	0.51	0.512	0.686	Co-occurrence
HIBADH	7p15.2	2 (1.74%)	3 (1.22%)	0.51	0.512	0.686	Co-occurrence
HINT1	5q23.3	2 (1.74%)	3 (1.22%)	0.51	0.512	0.686	Co-occurrence
HNF4A	20q13.12	2 (1.74%)	3 (1.22%)	0.51	0.512	0.686	Co-occurrence
HS3ST6	16p13.3	2 (1.74%)	3 (1.22%)	0.51	0.512	0.686	Co-occurrence
HTR4	5q32	2 (1.74%)	3 (1.22%)	0.51	0.512	0.686	Co-occurrence
ICOS	2q33.2	2 (1.74%)	3 (1.22%)	0.51	0.512	0.686	Co-occurrence
IGF2BP3	7p15.3	2 (1.74%)	3 (1.22%)	0.51	0.512	0.686	Co-occurrence
IGFALS	16p13.3	2 (1.74%)	3 (1.22%)	0.51	0.512	0.686	Co-occurrence
IL20RB	3q22.3	2 (1.74%)	3 (1.22%)	0.51	0.512	0.686	Co-occurrence
IL3	5q31.1	2 (1.74%)	3 (1.22%)	0.51	0.512	0.686	Co-occurrence
IRF2BP1	19q13.32	2 (1.74%)	3 (1.22%)	0.51	0.512	0.686	Co-occurrence
KBTBD13	15q22.31	2 (1.74%)	3 (1.22%)	0.51	0.512	0.686	Co-occurrence
KLF6	10p15.2	2 (1.74%)	3 (1.22%)	0.51	0.512	0.686	Co-occurrence
KLHL23	2q31.1	2 (1.74%)	3 (1.22%)	0.51	0.512	0.686	Co-occurrence
KLHL5	4p14	2 (1.74%)	3 (1.22%)	0.51	0.512	0.686	Co-occurrence
LGALS13	19q13.2	2 (1.74%)	3 (1.22%)	0.51	0.512	0.686	Co-occurrence
LIFR	5p13.1	2 (1.74%)	3 (1.22%)	0.51	0.512	0.686	Co-occurrence
LINC00273	16p11.2	2 (1.74%)	3 (1.22%)	0.51	0.512	0.686	Co-occurrence
LINC00700	10p15.3	2 (1.74%)	3 (1.22%)	0.51	0.512	0.686	Co-occurrence
LINC00701	10p15.3	2 (1.74%)	3 (1.22%)	0.51	0.512	0.686	Co-occurrence
LINC00707	10p14	2 (1.74%)	3 (1.22%)	0.51	0.512	0.686	Co-occurrence
LINC00926	15q21.3	2 (1.74%)	3 (1.22%)	0.51	0.512	0.686	Co-occurrence
LOC105371954	18p11.32	2 (1.74%)	3 (1.22%)	0.51	0.512	0.686	Co-occurrence
LRRC37A17P	17q21.32	2 (1.74%)	3 (1.22%)	0.51	0.512	0.686	Co-occurrence
LY75	2q24.2	2 (1.74%)	3 (1.22%)	0.51	0.512	0.686	Co-occurrence
LYRM7	5q23.3-q31.1	2 (1.74%)	3 (1.22%)	0.51	0.512	0.686	Co-occurrence
MALSU1	7p15.3	2 (1.74%)	3 (1.22%)	0.51	0.512	0.686	Co-occurrence

MAPK8IP3	16p13.3	2 (1.74%)	3 (1.22%)	0.51	0.512	0.686	Co-occurrence
7-Mar	2q24.2	2 (1.74%)	3 (1.22%)	0.51	0.512	0.686	Co-occurrence
MCM10	10p13	2 (1.74%)	3 (1.22%)	0.51	0.512	0.686	Co-occurrence
MCM7	7q22.1	2 (1.74%)	3 (1.22%)	0.51	0.512	0.686	Co-occurrence
MED26	19p13.11	2 (1.74%)	3 (1.22%)	0.51	0.512	0.686	Co-occurrence
MEIOB	16p13.3	2 (1.74%)	3 (1.22%)	0.51	0.512	0.686	Co-occurrence
METTL4	18p11.32	2 (1.74%)	3 (1.22%)	0.51	0.512	0.686	Co-occurrence
MIR-1265/1265		2 (1.74%)	3 (1.22%)	0.51	0.512	0.686	Co-occurrence
MIR-3936/3936		2 (1.74%)	3 (1.22%)	0.51	0.512	0.686	Co-occurrence
MIR-4293/4293		2 (1.74%)	3 (1.22%)	0.51	0.512	0.686	Co-occurrence
MIR-4516/4516		2 (1.74%)	3 (1.22%)	0.51	0.512	0.686	Co-occurrence
MIR-4530/4530		2 (1.74%)	3 (1.22%)	0.51	0.512	0.686	Co-occurrence
MIR-4669/4669		2 (1.74%)	3 (1.22%)	0.51	0.512	0.686	Co-occurrence
MIR-662/662		2 (1.74%)	3 (1.22%)	0.51	0.512	0.686	Co-occurrence
MPP6	7p15.3	2 (1.74%)	3 (1.22%)	0.51	0.512	0.686	Co-occurrence
MRAS	3q22.3	2 (1.74%)	3 (1.22%)	0.51	0.512	0.686	Co-occurrence
MRC1	10p12.33	2 (1.74%)	3 (1.22%)	0.51	0.512	0.686	Co-occurrence
MRPL34	19p13.11	2 (1.74%)	3 (1.22%)	0.51	0.512	0.686	Co-occurrence
MRPS22	3q23	2 (1.74%)	3 (1.22%)	0.51	0.512	0.686	Co-occurrence
MRPS34	16p13.3	2 (1.74%)	3 (1.22%)	0.51	0.512	0.686	Co-occurrence
MSLN	16p13.3	2 (1.74%)	3 (1.22%)	0.51	0.512	0.686	Co-occurrence
MSLNL	16p13.3	2 (1.74%)	3 (1.22%)	0.51	0.512	0.686	Co-occurrence
MSRB1	16p13.3	2 (1.74%)	3 (1.22%)	0.51	0.512	0.686	Co-occurrence
MYLK3	16q11.2	2 (1.74%)	3 (1.22%)	0.51	0.512	0.686	Co-occurrence
MYO9B	19p13.11	2 (1.74%)	3 (1.22%)	0.51	0.512	0.686	Co-occurrence
MYPOP	19q13.32	2 (1.74%)	3 (1.22%)	0.51	0.512	0.686	Co-occurrence
N4BP2	4p14	2 (1.74%)	3 (1.22%)	0.51	0.512	0.686	Co-occurrence
NCK1	3q22.3	2 (1.74%)	3 (1.22%)	0.51	0.512	0.686	Co-occurrence
NDC80	18p11.32	2 (1.74%)	3 (1.22%)	0.51	0.512	0.686	Co-occurrence

NME3	16p13.3	2 (1.74%)	3 (1.22%)	0.51	0.512	0.686	Co-occurrence
NME9	3q22.3	2 (1.74%)	3 (1.22%)	0.51	0.512	0.686	Co-occurrence
NPAS4	11q13.2	2 (1.74%)	3 (1.22%)	0.51	0.512	0.686	Co-occurrence
NPR3	5p13.3	2 (1.74%)	3 (1.22%)	0.51	0.512	0.686	Co-occurrence
NR2F6	19p13.11	2 (1.74%)	3 (1.22%)	0.51	0.512	0.686	Co-occurrence
NT5DC2	3p21.1	2 (1.74%)	3 (1.22%)	0.51	0.512	0.686	Co-occurrence
NUB1	7q36.1	2 (1.74%)	3 (1.22%)	0.51	0.512	0.686	Co-occurrence
NUBP2	16p13.3	2 (1.74%)	3 (1.22%)	0.51	0.512	0.686	Co-occurrence
NUP155	5p13.2	2 (1.74%)	3 (1.22%)	0.51	0.512	0.686	Co-occurrence
NUP42	7p15.3	2 (1.74%)	3 (1.22%)	0.51	0.512	0.686	Co-occurrence
OCEL1	19p13.11	2 (1.74%)	3 (1.22%)	0.51	0.512	0.686	Co-occurrence
OPTN	10p13	2 (1.74%)	3 (1.22%)	0.51	0.512	0.686	Co-occurrence
ORC6	16q11.2	2 (1.74%)	3 (1.22%)	0.51	0.512	0.686	Co-occurrence
P4HA2	5q31.1	2 (1.74%)	3 (1.22%)	0.51	0.512	0.686	Co-occurrence
PDIA4	7q36.1	2 (1.74%)	3 (1.22%)	0.51	0.512	0.686	Co-occurrence
PDLIM4	5q31.1	2 (1.74%)	3 (1.22%)	0.51	0.512	0.686	Co-occurrence
PDS5A	4p14	2 (1.74%)	3 (1.22%)	0.51	0.512	0.686	Co-occurrence
PIF1	15q22.31	2 (1.74%)	3 (1.22%)	0.51	0.512	0.686	Co-occurrence
PIGU	20q11.22	2 (1.74%)	3 (1.22%)	0.51	0.512	0.686	Co-occurrence
PILRB	7q22.1	2 (1.74%)	3 (1.22%)	0.51	0.512	0.686	Co-occurrence
PITRM1	10p15.2	2 (1.74%)	3 (1.22%)	0.51	0.512	0.686	Co-occurrence
PLCL2	3p24.3	2 (1.74%)	3 (1.22%)	0.51	0.512	0.686	Co-occurrence
PLEKHG2	19q13.2	2 (1.74%)	3 (1.22%)	0.51	0.512	0.686	Co-occurrence
PNPLA2	11p15.5	2 (1.74%)	3 (1.22%)	0.51	0.512	0.686	Co-occurrence
POLR2L	11p15.5	2 (1.74%)	3 (1.22%)	0.51	0.512	0.686	Co-occurrence
PPARGC1B	5q32	2 (1.74%)	3 (1.22%)	0.51	0.512	0.686	Co-occurrence
PPIA	7p13	2 (1.74%)	3 (1.22%)	0.51	0.512	0.686	Co-occurrence
PPIE	1p34.2	2 (1.74%)	3 (1.22%)	0.51	0.512	0.686	Co-occurrence
PPP1R1B	17q12	2 (1.74%)	3 (1.22%)	0.51	0.512	0.686	Co-occurrence

PRDM12	9q34.12	2 (1.74%)	3 (1.22%)	0.51	0.512	0.686	Co-occurrence
PRKCQ	10p15.1	2 (1.74%)	3 (1.22%)	0.51	0.512	0.686	Co-occurrence
PROM1	4p15.32	2 (1.74%)	3 (1.22%)	0.51	0.512	0.686	Co-occurrence
PRPF18	10p13	2 (1.74%)	3 (1.22%)	0.51	0.512	0.686	Co-occurrence
PRR23B	3q23	2 (1.74%)	3 (1.22%)	0.51	0.512	0.686	Co-occurrence
PRR23C	3q23	2 (1.74%)	3 (1.22%)	0.51	0.512	0.686	Co-occurrence
PRR25	16p13.3	2 (1.74%)	3 (1.22%)	0.51	0.512	0.686	Co-occurrence
PRSS22	16p13.3	2 (1.74%)	3 (1.22%)	0.51	0.512	0.686	Co-occurrence
PTPRN2	7q36.3	2 (1.74%)	3 (1.22%)	0.51	0.512	0.686	Co-occurrence
PUS1	12q24.33	2 (1.74%)	3 (1.22%)	0.51	0.512	0.686	Co-occurrence
PXMP4	20q11.22	2 (1.74%)	3 (1.22%)	0.51	0.512	0.686	Co-occurrence
RAB26	16p13.3	2 (1.74%)	3 (1.22%)	0.51	0.512	0.686	Co-occurrence
RAB6B	3q22.1	2 (1.74%)	3 (1.22%)	0.51	0.512	0.686	Co-occurrence
RAI14	5p13.2	2 (1.74%)	3 (1.22%)	0.51	0.512	0.686	Co-occurrence
RAPH1	2q33.2	2 (1.74%)	3 (1.22%)	0.51	0.512	0.686	Co-occurrence
RBM47	4p14	2 (1.74%)	3 (1.22%)	0.51	0.512	0.686	Co-occurrence
RHOH	4p14	2 (1.74%)	3 (1.22%)	0.51	0.512	0.686	Co-occurrence
RIN1	11q13.2	2 (1.74%)	3 (1.22%)	0.51	0.512	0.686	Co-occurrence
RINT1	7q22.3	2 (1.74%)	3 (1.22%)	0.51	0.512	0.686	Co-occurrence
RN7SKP122	5q21.3	2 (1.74%)	3 (1.22%)	0.51	0.512	0.686	Co-occurrence
RN7SKP146	18p11.32	2 (1.74%)	3 (1.22%)	0.51	0.512	0.686	Co-occurrence
RN7SKP72	18p11.32	2 (1.74%)	3 (1.22%)	0.51	0.512	0.686	Co-occurrence
RN7SL146P	19p13.11	2 (1.74%)	3 (1.22%)	0.51	0.512	0.686	Co-occurrence
RN7SL189P	14q11.2	2 (1.74%)	3 (1.22%)	0.51	0.512	0.686	Co-occurrence
RN7SL198P	10p13	2 (1.74%)	3 (1.22%)	0.51	0.512	0.686	Co-occurrence
RN7SL37P	5p13.2	2 (1.74%)	3 (1.22%)	0.51	0.512	0.686	Co-occurrence
RN7SL39P	18p11.31	2 (1.74%)	3 (1.22%)	0.51	0.512	0.686	Co-occurrence
RN7SL521P	7q36.1	2 (1.74%)	3 (1.22%)	0.51	0.512	0.686	Co-occurrence
RN7SL541P	5q31.1	2 (1.74%)	3 (1.22%)	0.51	0.512	0.686	Co-occurrence

RN7SL72P	7q36.1	2 (1.74%)	3 (1.22%)	0.51	0.512	0.686	Co-occurrence
RN7SL782P	5q21.3	2 (1.74%)	3 (1.22%)	0.51	0.512	0.686	Co-occurrence
RN7SL868P	5q32	2 (1.74%)	3 (1.22%)	0.51	0.512	0.686	Co-occurrence
RNA5SP140	3q22.1	2 (1.74%)	3 (1.22%)	0.51	0.512	0.686	Co-occurrence
RNA5SP159	4p14	2 (1.74%)	3 (1.22%)	0.51	0.512	0.686	Co-occurrence
RNA5SP301	10p13	2 (1.74%)	3 (1.22%)	0.51	0.512	0.686	Co-occurrence
RNA5SP406	16p11.1	2 (1.74%)	3 (1.22%)	0.51	0.512	0.686	Co-occurrence
RNA5SP407	16p11.1	2 (1.74%)	3 (1.22%)	0.51	0.512	0.686	Co-occurrence
RNA5SP408	16p11.1	2 (1.74%)	3 (1.22%)	0.51	0.512	0.686	Co-occurrence
RNA5SP409	16p11.1	2 (1.74%)	3 (1.22%)	0.51	0.512	0.686	Co-occurrence
RNA5SP410	16p11.1	2 (1.74%)	3 (1.22%)	0.51	0.512	0.686	Co-occurrence
RNA5SP413	16p11.1	2 (1.74%)	3 (1.22%)	0.51	0.512	0.686	Co-occurrence
RNA5SP415	16p11.1	2 (1.74%)	3 (1.22%)	0.51	0.512	0.686	Co-occurrence
RNA5SP416	16p11.1	2 (1.74%)	3 (1.22%)	0.51	0.512	0.686	Co-occurrence
RNA5SP417	16p11.1	2 (1.74%)	3 (1.22%)	0.51	0.512	0.686	Co-occurrence
RNA5SP418	16p11.1	2 (1.74%)	3 (1.22%)	0.51	0.512	0.686	Co-occurrence
RNA5SP419	16p11.1	2 (1.74%)	3 (1.22%)	0.51	0.512	0.686	Co-occurrence
RNA5SP420	16p11.1	2 (1.74%)	3 (1.22%)	0.51	0.512	0.686	Co-occurrence
RNA5SP421	16p11.1	2 (1.74%)	3 (1.22%)	0.51	0.512	0.686	Co-occurrence
RNA5SP422	16p11.1	2 (1.74%)	3 (1.22%)	0.51	0.512	0.686	Co-occurrence
RNA5SP423	16p11.1	2 (1.74%)	3 (1.22%)	0.51	0.512	0.686	Co-occurrence
RNA5SP469	19p12	2 (1.74%)	3 (1.22%)	0.51	0.512	0.686	Co-occurrence
RNASE2	14q11.2	2 (1.74%)	3 (1.22%)	0.51	0.512	0.686	Co-occurrence
RNASE3	14q11.2	2 (1.74%)	3 (1.22%)	0.51	0.512	0.686	Co-occurrence
RNF32	7q36.3	2 (1.74%)	3 (1.22%)	0.51	0.512	0.686	Co-occurrence
RNU4ATAC6P	10p12.1	2 (1.74%)	3 (1.22%)	0.51	0.512	0.686	Co-occurrence
RNY1	7q36.1	2 (1.74%)	3 (1.22%)	0.51	0.512	0.686	Co-occurrence
RNY3	7q36.1	2 (1.74%)	3 (1.22%)	0.51	0.512	0.686	Co-occurrence
RNY4	7q36.1	2 (1.74%)	3 (1.22%)	0.51	0.512	0.686	Co-occurrence

RNY5	7q36.1	2 (1.74%)	3 (1.22%)	0.51	0.512	0.686	Co-occurrence
ROCK1P1	18p11.32	2 (1.74%)	3 (1.22%)	0.51	0.512	0.686	Co-occurrence
RPL3L	16p13.3	2 (1.74%)	3 (1.22%)	0.51	0.512	0.686	Co-occurrence
RPRML	17q21.32	2 (1.74%)	3 (1.22%)	0.51	0.512	0.686	Co-occurrence
RPS16	19q13.2	2 (1.74%)	3 (1.22%)	0.51	0.512	0.686	Co-occurrence
RPUSD1	16p13.3	2 (1.74%)	3 (1.22%)	0.51	0.512	0.686	Co-occurrence
RXFP3	5p13.2	2 (1.74%)	3 (1.22%)	0.51	0.512	0.686	Co-occurrence
SERBP1	1p31.3	2 (1.74%)	3 (1.22%)	0.51	0.512	0.686	Co-occurrence
SFMBT2	10p14	2 (1.74%)	3 (1.22%)	0.51	0.512	0.686	Co-occurrence
SH3TC2	5q32	2 (1.74%)	3 (1.22%)	0.51	0.512	0.686	Co-occurrence
SHCBP1	16q11.2	2 (1.74%)	3 (1.22%)	0.51	0.512	0.686	Co-occurrence
SLC22A4	5q31.1	2 (1.74%)	3 (1.22%)	0.51	0.512	0.686	Co-occurrence
SLC25A40	7q21.12	2 (1.74%)	3 (1.22%)	0.51	0.512	0.686	Co-occurrence
SLC29A2	11q13.2	2 (1.74%)	3 (1.22%)	0.51	0.512	0.686	Co-occurrence
SLC35E1	19p13.11	2 (1.74%)	3 (1.22%)	0.51	0.512	0.686	Co-occurrence
SLC39A12	10p12.33	2 (1.74%)	3 (1.22%)	0.51	0.512	0.686	Co-occurrence
SLC45A2	5p13.2	2 (1.74%)	3 (1.22%)	0.51	0.512	0.686	Co-occurrence
SLC6A10P	16p11.2	2 (1.74%)	3 (1.22%)	0.51	0.512	0.686	Co-occurrence
SMCHD1	18p11.32	2 (1.74%)	3 (1.22%)	0.51	0.512	0.686	Co-occurrence
SMG1	16p12.3	2 (1.74%)	3 (1.22%)	0.51	0.512	0.686	Co-occurrence
SMIM7	19p13.11	2 (1.74%)	3 (1.22%)	0.51	0.512	0.686	Co-occurrence
SP3	2q31.1	2 (1.74%)	3 (1.22%)	0.51	0.512	0.686	Co-occurrence
SPOCK1	5q31.2	2 (1.74%)	3 (1.22%)	0.51	0.512	0.686	Co-occurrence
SPSB3	16p13.3	2 (1.74%)	3 (1.22%)	0.51	0.512	0.686	Co-occurrence
SPTBN4	19q13.2	2 (1.74%)	3 (1.22%)	0.51	0.512	0.686	Co-occurrence
STAG3L5P	7q22.1	2 (1.74%)	3 (1.22%)	0.51	0.512	0.686	Co-occurrence
STARD3	17q12	2 (1.74%)	3 (1.22%)	0.51	0.512	0.686	Co-occurrence
SUB1	5p13.3	2 (1.74%)	3 (1.22%)	0.51	0.512	0.686	Co-occurrence
SUPT5H	19q13.2	2 (1.74%)	3 (1.22%)	0.51	0.512	0.686	Co-occurrence

TAF6	7q22.1	2 (1.74%)	3 (1.22%)	0.51	0.512	0.686	Co-occurrence
TARS	5p13.3	2 (1.74%)	3 (1.22%)	0.51	0.512	0.686	Co-occurrence
TAX1BP1	7p15.2	2 (1.74%)	3 (1.22%)	0.51	0.512	0.686	Co-occurrence
TCF12	15q21.3	2 (1.74%)	3 (1.22%)	0.51	0.512	0.686	Co-occurrence
TECR	19p13.12	2 (1.74%)	3 (1.22%)	0.51	0.512	0.686	Co-occurrence
TGIF1	18p11.31	2 (1.74%)	3 (1.22%)	0.51	0.512	0.686	Co-occurrence
THOC1	18p11.32	2 (1.74%)	3 (1.22%)	0.51	0.512	0.686	Co-occurrence
TIMM50	19q13.2	2 (1.74%)	3 (1.22%)	0.51	0.512	0.686	Co-occurrence
TMEM156	4p14	2 (1.74%)	3 (1.22%)	0.51	0.512	0.686	Co-occurrence
TMEM236	10p12.33	2 (1.74%)	3 (1.22%)	0.51	0.512	0.686	Co-occurrence
TMEM243	7q21.12	2 (1.74%)	3 (1.22%)	0.51	0.512	0.686	Co-occurrence
TOPBP1	3q22.1	2 (1.74%)	3 (1.22%)	0.51	0.512	0.686	Co-occurrence
TP53TG1	7q21.12	2 (1.74%)	3 (1.22%)	0.51	0.512	0.686	Co-occurrence
TP53TG3	16p11.2	2 (1.74%)	3 (1.22%)	0.51	0.512	0.686	Co-occurrence
TP53TG3B	16p11.2	2 (1.74%)	3 (1.22%)	0.51	0.512	0.686	Co-occurrence
TP53TG3C	16p11.2	2 (1.74%)	3 (1.22%)	0.51	0.512	0.686	Co-occurrence
TP53TG3D	16p11.2	2 (1.74%)	3 (1.22%)	0.51	0.512	0.686	Co-occurrence
TRAF7	16p13.3	2 (1.74%)	3 (1.22%)	0.51	0.512	0.686	Co-occurrence
TSPAN4	11p15.5	2 (1.74%)	3 (1.22%)	0.51	0.512	0.686	Co-occurrence
TTC7A	2p21	2 (1.74%)	3 (1.22%)	0.51	0.512	0.686	Co-occurrence
TWSG1	18p11.22	2 (1.74%)	3 (1.22%)	0.51	0.512	0.686	Co-occurrence
TYMS	18p11.32	2 (1.74%)	3 (1.22%)	0.51	0.512	0.686	Co-occurrence
TYMSOS	18p11.32	2 (1.74%)	3 (1.22%)	0.51	0.512	0.686	Co-occurrence
USE1	19p13.11	2 (1.74%)	3 (1.22%)	0.51	0.512	0.686	Co-occurrence
USHBP1	19p13.11	2 (1.74%)	3 (1.22%)	0.51	0.512	0.686	Co-occurrence
USP14	18p11.32	2 (1.74%)	3 (1.22%)	0.51	0.512	0.686	Co-occurrence
USP6NL	10p14	2 (1.74%)	3 (1.22%)	0.51	0.512	0.686	Co-occurrence
VPS35	16q11.2	2 (1.74%)	3 (1.22%)	0.51	0.512	0.686	Co-occurrence
WDR86	7q36.1	2 (1.74%)	3 (1.22%)	0.51	0.512	0.686	Co-occurrence

WDR93	15q26.1	2 (1.74%)	3 (1.22%)	0.51	0.512	0.686	Co-occurrence
WNT8A	5q31.2	2 (1.74%)	3 (1.22%)	0.51	0.512	0.686	Co-occurrence
YES1	18p11.32	2 (1.74%)	3 (1.22%)	0.51	0.512	0.686	Co-occurrence
ZFP36	19q13.2	2 (1.74%)	3 (1.22%)	0.51	0.512	0.686	Co-occurrence
ZMIZ2	7p13	2 (1.74%)	3 (1.22%)	0.51	0.512	0.686	Co-occurrence
ZNF212	7q36.1	2 (1.74%)	3 (1.22%)	0.51	0.512	0.686	Co-occurrence
ZNF267	16p11.2	2 (1.74%)	3 (1.22%)	0.51	0.512	0.686	Co-occurrence
ZNF282	7q36.1	2 (1.74%)	3 (1.22%)	0.51	0.512	0.686	Co-occurrence
ZNF341	20q11.22	2 (1.74%)	3 (1.22%)	0.51	0.512	0.686	Co-occurrence
ZNF398	7q36.1	2 (1.74%)	3 (1.22%)	0.51	0.512	0.686	Co-occurrence
ZNF425	7q36.1	2 (1.74%)	3 (1.22%)	0.51	0.512	0.686	Co-occurrence
ZNF431	19p12	2 (1.74%)	3 (1.22%)	0.51	0.512	0.686	Co-occurrence
ZNF592	15q25.3	2 (1.74%)	3 (1.22%)	0.51	0.512	0.686	Co-occurrence
ZNF714	19p12	2 (1.74%)	3 (1.22%)	0.51	0.512	0.686	Co-occurrence
ZNF746	7q36.1	2 (1.74%)	3 (1.22%)	0.51	0.512	0.686	Co-occurrence
ZNF777	7q36.1	2 (1.74%)	3 (1.22%)	0.51	0.512	0.686	Co-occurrence
ZNF783	7q36.1	2 (1.74%)	3 (1.22%)	0.51	0.512	0.686	Co-occurrence
ZNF786	7q36.1	2 (1.74%)	3 (1.22%)	0.51	0.512	0.686	Co-occurrence
BTN2A1	6p22.2	4 (3.48%)	10 (4.08%)	-0.23	0.52	0.686	Mutual exclusivity
BTN2A2	6p22.2	4 (3.48%)	10 (4.08%)	-0.23	0.52	0.686	Mutual exclusivity
BTN2A3P	6p22.2	4 (3.48%)	10 (4.08%)	-0.23	0.52	0.686	Mutual exclusivity
BTN3A1	6p22.2	4 (3.48%)	10 (4.08%)	-0.23	0.52	0.686	Mutual exclusivity
BTN3A2	6p22.2	4 (3.48%)	10 (4.08%)	-0.23	0.52	0.686	Mutual exclusivity
BTN3A3	6p22.2	4 (3.48%)	10 (4.08%)	-0.23	0.52	0.686	Mutual exclusivity
GABBR1	6p22.1	4 (3.48%)	10 (4.08%)	-0.23	0.52	0.686	Mutual exclusivity
GPX5	6p22.1	4 (3.48%)	10 (4.08%)	-0.23	0.52	0.686	Mutual exclusivity
GPX6	6p22.1	4 (3.48%)	10 (4.08%)	-0.23	0.52	0.686	Mutual exclusivity
HCG14	6p22.1	4 (3.48%)	10 (4.08%)	-0.23	0.52	0.686	Mutual exclusivity
HCG15	6p21	4 (3.48%)	10 (4.08%)	-0.23	0.52	0.686	Mutual exclusivity

HCG16	6p21.3	4 (3.48%)	10 (4.08%)	-0.23	0.52	0.686	Mutual exclusivity
HFE	6p22.2	4 (3.48%)	10 (4.08%)	-0.23	0.52	0.686	Mutual exclusivity
HIST1H1A	6p22.2	4 (3.48%)	10 (4.08%)	-0.23	0.52	0.686	Mutual exclusivity
HIST1H1C	6p22.2	4 (3.48%)	10 (4.08%)	-0.23	0.52	0.686	Mutual exclusivity
HIST1H1D	6p22.2	4 (3.48%)	10 (4.08%)	-0.23	0.52	0.686	Mutual exclusivity
HIST1H1E	6p22.2	4 (3.48%)	10 (4.08%)	-0.23	0.52	0.686	Mutual exclusivity
HIST1H1T	6p22.2	4 (3.48%)	10 (4.08%)	-0.23	0.52	0.686	Mutual exclusivity
HIST1H2AB	6p22.2	4 (3.48%)	10 (4.08%)	-0.23	0.52	0.686	Mutual exclusivity
HIST1H2AC	6p22.2	4 (3.48%)	10 (4.08%)	-0.23	0.52	0.686	Mutual exclusivity
HIST1H2AD	6p22.2	4 (3.48%)	10 (4.08%)	-0.23	0.52	0.686	Mutual exclusivity
HIST1H2AE	6p22.2	4 (3.48%)	10 (4.08%)	-0.23	0.52	0.686	Mutual exclusivity
HIST1H2BB	6p22.2	4 (3.48%)	10 (4.08%)	-0.23	0.52	0.686	Mutual exclusivity
HIST1H2BC	6p22.2	4 (3.48%)	10 (4.08%)	-0.23	0.52	0.686	Mutual exclusivity
HIST1H2BD	6p22.2	4 (3.48%)	10 (4.08%)	-0.23	0.52	0.686	Mutual exclusivity
HIST1H2BE	6p22.2	4 (3.48%)	10 (4.08%)	-0.23	0.52	0.686	Mutual exclusivity
HIST1H2BF	6p22.2	4 (3.48%)	10 (4.08%)	-0.23	0.52	0.686	Mutual exclusivity
HIST1H2BG	6p22.2	4 (3.48%)	10 (4.08%)	-0.23	0.52	0.686	Mutual exclusivity
HIST1H2BH	6p22.2	4 (3.48%)	10 (4.08%)	-0.23	0.52	0.686	Mutual exclusivity
HIST1H2BI	6p22.2	4 (3.48%)	10 (4.08%)	-0.23	0.52	0.686	Mutual exclusivity
HIST1H3A	6p22.2	4 (3.48%)	10 (4.08%)	-0.23	0.52	0.686	Mutual exclusivity
HIST1H3B	6p22.2	4 (3.48%)	10 (4.08%)	-0.23	0.52	0.686	Mutual exclusivity
HIST1H3C	6p22.2	4 (3.48%)	10 (4.08%)	-0.23	0.52	0.686	Mutual exclusivity
HIST1H3D	6p22.2	4 (3.48%)	10 (4.08%)	-0.23	0.52	0.686	Mutual exclusivity
HIST1H3E	6p22.2	4 (3.48%)	10 (4.08%)	-0.23	0.52	0.686	Mutual exclusivity
HIST1H3F	6p22.2	4 (3.48%)	10 (4.08%)	-0.23	0.52	0.686	Mutual exclusivity
HIST1H3G	6p22.2	4 (3.48%)	10 (4.08%)	-0.23	0.52	0.686	Mutual exclusivity
HIST1H4A	6p22.2	4 (3.48%)	10 (4.08%)	-0.23	0.52	0.686	Mutual exclusivity
HIST1H4B	6p22.2	4 (3.48%)	10 (4.08%)	-0.23	0.52	0.686	Mutual exclusivity
HIST1H4C	6p22.2	4 (3.48%)	10 (4.08%)	-0.23	0.52	0.686	Mutual exclusivity

HIST1H4D	6p22.2	4 (3.48%)	10 (4.08%)	-0.23	0.52	0.686	Mutual exclusivity
HIST1H4E	6p22.2	4 (3.48%)	10 (4.08%)	-0.23	0.52	0.686	Mutual exclusivity
HIST1H4F	6p22.2	4 (3.48%)	10 (4.08%)	-0.23	0.52	0.686	Mutual exclusivity
HIST1H4G	6p22.2	4 (3.48%)	10 (4.08%)	-0.23	0.52	0.686	Mutual exclusivity
HIST1H4H	6p22.2	4 (3.48%)	10 (4.08%)	-0.23	0.52	0.686	Mutual exclusivity
HMGCLL1	6p12.1	4 (3.48%)	10 (4.08%)	-0.23	0.52	0.686	Mutual exclusivity
LINC00533	6p22.1	4 (3.48%)	10 (4.08%)	-0.23	0.52	0.686	Mutual exclusivity
MAS1L	6p22.1	4 (3.48%)	10 (4.08%)	-0.23	0.52	0.686	Mutual exclusivity
NKAPL	6p22.1	4 (3.48%)	10 (4.08%)	-0.23	0.52	0.686	Mutual exclusivity
OPN5	6p12.3	4 (3.48%)	10 (4.08%)	-0.23	0.52	0.686	Mutual exclusivity
OR10C1	6p22.1	4 (3.48%)	10 (4.08%)	-0.23	0.52	0.686	Mutual exclusivity
OR11A1	6p22.1	4 (3.48%)	10 (4.08%)	-0.23	0.52	0.686	Mutual exclusivity
OR12D1	6p22.1	4 (3.48%)	10 (4.08%)	-0.23	0.52	0.686	Mutual exclusivity
OR12D2	6p22.1	4 (3.48%)	10 (4.08%)	-0.23	0.52	0.686	Mutual exclusivity
OR12D3	6p22.1	4 (3.48%)	10 (4.08%)	-0.23	0.52	0.686	Mutual exclusivity
OR2B3	6p22.1	4 (3.48%)	10 (4.08%)	-0.23	0.52	0.686	Mutual exclusivity
OR2H1	6p22.1	4 (3.48%)	10 (4.08%)	-0.23	0.52	0.686	Mutual exclusivity
OR2W1	6p22.1	4 (3.48%)	10 (4.08%)	-0.23	0.52	0.686	Mutual exclusivity
PGBD1	6p22.1	4 (3.48%)	10 (4.08%)	-0.23	0.52	0.686	Mutual exclusivity
PRSS16	6p22.1	4 (3.48%)	10 (4.08%)	-0.23	0.52	0.686	Mutual exclusivity
RUNX2	6p21.1	4 (3.48%)	10 (4.08%)	-0.23	0.52	0.686	Mutual exclusivity
SLC17A1	6p22.2	4 (3.48%)	10 (4.08%)	-0.23	0.52	0.686	Mutual exclusivity
SLC17A2	6p22.2	4 (3.48%)	10 (4.08%)	-0.23	0.52	0.686	Mutual exclusivity
SLC17A4	6p22.2	4 (3.48%)	10 (4.08%)	-0.23	0.52	0.686	Mutual exclusivity
TRIM27	6p22.1	4 (3.48%)	10 (4.08%)	-0.23	0.52	0.686	Mutual exclusivity
TRIM38	6p22.2	4 (3.48%)	10 (4.08%)	-0.23	0.52	0.686	Mutual exclusivity
UBD	6p22.1	4 (3.48%)	10 (4.08%)	-0.23	0.52	0.686	Mutual exclusivity
ZBED9	6p22.1	4 (3.48%)	10 (4.08%)	-0.23	0.52	0.686	Mutual exclusivity
ZFAND3	6p21.2	4 (3.48%)	10 (4.08%)	-0.23	0.52	0.686	Mutual exclusivity

ZKSCAN3	6p22.1	4 (3.48%)	10 (4.08%)	-0.23	0.52	0.686	Mutual exclusivity
ZKSCAN4	6p22.1	4 (3.48%)	10 (4.08%)	-0.23	0.52	0.686	Mutual exclusivity
ZKSCAN8	6p22.1	4 (3.48%)	10 (4.08%)	-0.23	0.52	0.686	Mutual exclusivity
ZNF192P1	6p22.1	4 (3.48%)	10 (4.08%)	-0.23	0.52	0.686	Mutual exclusivity
ZNF311	6p22.1	4 (3.48%)	10 (4.08%)	-0.23	0.52	0.686	Mutual exclusivity
ZSCAN23	6p22.1	4 (3.48%)	10 (4.08%)	-0.23	0.52	0.686	Mutual exclusivity
ZSCAN31	6p22.1|6p22.3-p22.1	4 (3.48%)	10 (4.08%)	-0.23	0.52	0.686	Mutual exclusivity
ZSCAN9	6p22.1	4 (3.48%)	10 (4.08%)	-0.23	0.52	0.686	Mutual exclusivity
SPIDR	8q11.21	8 (6.96%)	16 (6.53%)	0.09	0.52	0.686	Co-occurrence
SCARNA15	15q25.2	7 (6.09%)	14 (5.71%)	0.09	0.529	0.686	Co-occurrence
LRRC73	6p21.1	6 (5.22%)	14 (5.71%)	-0.13	0.533	0.686	Mutual exclusivity
POLR1C	6p21.1	6 (5.22%)	14 (5.71%)	-0.13	0.533	0.686	Mutual exclusivity
TJAP1	6p21.1	6 (5.22%)	14 (5.71%)	-0.13	0.533	0.686	Mutual exclusivity
TTBK1	6p21.1	6 (5.22%)	14 (5.71%)	-0.13	0.533	0.686	Mutual exclusivity
YIPF3	6p21.1	6 (5.22%)	14 (5.71%)	-0.13	0.533	0.686	Mutual exclusivity
AADACL3	1p36.21	1 (0.87%)	1 (0.41%)	1.09	0.537	0.686	Co-occurrence
AADACL4	1p36.21	1 (0.87%)	1 (0.41%)	1.09	0.537	0.686	Co-occurrence
AAMP	2q35	1 (0.87%)	1 (0.41%)	1.09	0.537	0.686	Co-occurrence
ABCB9	12q24.31	1 (0.87%)	1 (0.41%)	1.09	0.537	0.686	Co-occurrence
ABCC2	10q24.2	1 (0.87%)	1 (0.41%)	1.09	0.537	0.686	Co-occurrence
ABCC8	11p15.1	1 (0.87%)	1 (0.41%)	1.09	0.537	0.686	Co-occurrence
ABHD14A	3p21.2	1 (0.87%)	1 (0.41%)	1.09	0.537	0.686	Co-occurrence
ABHD14B	3p21.2	1 (0.87%)	1 (0.41%)	1.09	0.537	0.686	Co-occurrence
ABHD3	18q11.2	1 (0.87%)	1 (0.41%)	1.09	0.537	0.686	Co-occurrence
ABHD4	14q11.2	1 (0.87%)	1 (0.41%)	1.09	0.537	0.686	Co-occurrence
ABHD5	3p21.33	1 (0.87%)	1 (0.41%)	1.09	0.537	0.686	Co-occurrence
ABTB1	3q21.3	1 (0.87%)	1 (0.41%)	1.09	0.537	0.686	Co-occurrence
ACAA2	18q21.1	1 (0.87%)	1 (0.41%)	1.09	0.537	0.686	Co-occurrence
ACAT1	11q22.3	1 (0.87%)	1 (0.41%)	1.09	0.537	0.686	Co-occurrence

ACIN1	14q11.2	1 (0.87%)	1 (0.41%)	1.09	0.537	0.686	Co-occurrence
ACOT11	1p32.3	1 (0.87%)	1 (0.41%)	1.09	0.537	0.686	Co-occurrence
ACP7	19q13.2	1 (0.87%)	1 (0.41%)	1.09	0.537	0.686	Co-occurrence
ACTN1	14q24.1|14q22-q24	1 (0.87%)	1 (0.41%)	1.09	0.537	0.686	Co-occurrence
ACTN4	19q13.2	1 (0.87%)	1 (0.41%)	1.09	0.537	0.686	Co-occurrence
ACTR1A	10q24.32	1 (0.87%)	1 (0.41%)	1.09	0.537	0.686	Co-occurrence
ACTR5	20q11.23	1 (0.87%)	1 (0.41%)	1.09	0.537	0.686	Co-occurrence
ACY1	3p21.2	1 (0.87%)	1 (0.41%)	1.09	0.537	0.686	Co-occurrence
ADAM33	20p13	1 (0.87%)	1 (0.41%)	1.09	0.537	0.686	Co-occurrence
ADAMTS6	5q12.3	1 (0.87%)	1 (0.41%)	1.09	0.537	0.686	Co-occurrence
ADGRG7	3q12.2	1 (0.87%)	1 (0.41%)	1.09	0.537	0.686	Co-occurrence
ADORA2A	22q11.23	1 (0.87%)	1 (0.41%)	1.09	0.537	0.686	Co-occurrence
AFG3L2	18p11.21	1 (0.87%)	1 (0.41%)	1.09	0.537	0.686	Co-occurrence
AGAP11	10q23.2	1 (0.87%)	1 (0.41%)	1.09	0.537	0.686	Co-occurrence
AGGF1	5q13.3	1 (0.87%)	1 (0.41%)	1.09	0.537	0.686	Co-occurrence
AGMAT	1p36.21	1 (0.87%)	1 (0.41%)	1.09	0.537	0.686	Co-occurrence
AGMO	7p21.2	1 (0.87%)	1 (0.41%)	1.09	0.537	0.686	Co-occurrence
AGPAT3	21q22.3	1 (0.87%)	1 (0.41%)	1.09	0.537	0.686	Co-occurrence
AGTRAP	1p36.22	1 (0.87%)	1 (0.41%)	1.09	0.537	0.686	Co-occurrence
AIFM3	22q11.21	1 (0.87%)	1 (0.41%)	1.09	0.537	0.686	Co-occurrence
AIRN	6q25.3	1 (0.87%)	1 (0.41%)	1.09	0.537	0.686	Co-occurrence
AJUBA	14q11.2	1 (0.87%)	1 (0.41%)	1.09	0.537	0.686	Co-occurrence
AK2	1p35.1	1 (0.87%)	1 (0.41%)	1.09	0.537	0.686	Co-occurrence
AKIRIN1	1p34.3	1 (0.87%)	1 (0.41%)	1.09	0.537	0.686	Co-occurrence
ALDH18A1	10q24.1	1 (0.87%)	1 (0.41%)	1.09	0.537	0.686	Co-occurrence
ALDH1A1	9q21.13	1 (0.87%)	1 (0.41%)	1.09	0.537	0.686	Co-occurrence
ALDOA	16p11.2	1 (0.87%)	1 (0.41%)	1.09	0.537	0.686	Co-occurrence
ALPK1	4q25	1 (0.87%)	1 (0.41%)	1.09	0.537	0.686	Co-occurrence
ALX3	1p13.3	1 (0.87%)	1 (0.41%)	1.09	0.537	0.686	Co-occurrence

AMIGO1	1p13.3	1 (0.87%)	1 (0.41%)	1.09	0.537	0.686	Co-occurrence
AMIGO3	3p21.31	1 (0.87%)	1 (0.41%)	1.09	0.537	0.686	Co-occurrence
AMPD2	1p13.3	1 (0.87%)	1 (0.41%)	1.09	0.537	0.686	Co-occurrence
AMPH	7p14.1	1 (0.87%)	1 (0.41%)	1.09	0.537	0.686	Co-occurrence
AMY1A	1p21.1	1 (0.87%)	1 (0.41%)	1.09	0.537	0.686	Co-occurrence
AMY1B	1p21.1	1 (0.87%)	1 (0.41%)	1.09	0.537	0.686	Co-occurrence
AMY1C	1p21.1	1 (0.87%)	1 (0.41%)	1.09	0.537	0.686	Co-occurrence
AMY2A	1p21.1	1 (0.87%)	1 (0.41%)	1.09	0.537	0.686	Co-occurrence
AMY2B	1p21.1	1 (0.87%)	1 (0.41%)	1.09	0.537	0.686	Co-occurrence
ANGPTL2	9q33.3	1 (0.87%)	1 (0.41%)	1.09	0.537	0.686	Co-occurrence
ANKDD1B	5q13.3	1 (0.87%)	1 (0.41%)	1.09	0.537	0.686	Co-occurrence
ANKRD13A	12q24.11	1 (0.87%)	1 (0.41%)	1.09	0.537	0.686	Co-occurrence
ANKRD28	3p25.1	1 (0.87%)	1 (0.41%)	1.09	0.537	0.686	Co-occurrence
ANKRD31	5q13.3	1 (0.87%)	1 (0.41%)	1.09	0.537	0.686	Co-occurrence
ANKRD33	12q13.13	1 (0.87%)	1 (0.41%)	1.09	0.537	0.686	Co-occurrence
ANKRD42	11q14.1	1 (0.87%)	1 (0.41%)	1.09	0.537	0.686	Co-occurrence
ANKRD62	18p11.21	1 (0.87%)	1 (0.41%)	1.09	0.537	0.686	Co-occurrence
ANO5	11p14.3	1 (0.87%)	1 (0.41%)	1.09	0.537	0.686	Co-occurrence
ANO7L1	1p36.13	1 (0.87%)	1 (0.41%)	1.09	0.537	0.686	Co-occurrence
ANXA10	4q32.3	1 (0.87%)	1 (0.41%)	1.09	0.537	0.686	Co-occurrence
ANXA11	10q22.3	1 (0.87%)	1 (0.41%)	1.09	0.537	0.686	Co-occurrence
ANXA3	4q21.21	1 (0.87%)	1 (0.41%)	1.09	0.537	0.686	Co-occurrence
ANXA7	10q22.2	1 (0.87%)	1 (0.41%)	1.09	0.537	0.686	Co-occurrence
AP1AR	4q25	1 (0.87%)	1 (0.41%)	1.09	0.537	0.686	Co-occurrence
AP1G2	14q11.2	1 (0.87%)	1 (0.41%)	1.09	0.537	0.686	Co-occurrence
AP5S1	20p13	1 (0.87%)	1 (0.41%)	1.09	0.537	0.686	Co-occurrence
APBB1	11p15.4	1 (0.87%)	1 (0.41%)	1.09	0.537	0.686	Co-occurrence
APEH	3p21.31	1 (0.87%)	1 (0.41%)	1.09	0.537	0.686	Co-occurrence
APLP2	11q24.3	1 (0.87%)	1 (0.41%)	1.09	0.537	0.686	Co-occurrence

APOO	Xp22.11	1 (0.87%)	1 (0.41%)	1.09	0.537	0.686	Co-occurrence
AQP3	9p13.3	1 (0.87%)	1 (0.41%)	1.09	0.537	0.686	Co-occurrence
ARFGEF2	20q13.13	1 (0.87%)	1 (0.41%)	1.09	0.537	0.686	Co-occurrence
ARHGAP19	10q24.1	1 (0.87%)	1 (0.41%)	1.09	0.537	0.686	Co-occurrence
ARHGAP32	11q24.3	1 (0.87%)	1 (0.41%)	1.09	0.537	0.686	Co-occurrence
ARHGAP40	20q11.23	1 (0.87%)	1 (0.41%)	1.09	0.537	0.686	Co-occurrence
ARHGEF19	1p36.13	1 (0.87%)	1 (0.41%)	1.09	0.537	0.686	Co-occurrence
ARID5B	10q21.2	1 (0.87%)	1 (0.41%)	1.09	0.537	0.686	Co-occurrence
ARIH2	3p21.31	1 (0.87%)	1 (0.41%)	1.09	0.537	0.686	Co-occurrence
ARIH2OS	3p21.31	1 (0.87%)	1 (0.41%)	1.09	0.537	0.686	Co-occurrence
ARMH3	10q24.32	1 (0.87%)	1 (0.41%)	1.09	0.537	0.686	Co-occurrence
ARPC2	2q35	1 (0.87%)	1 (0.41%)	1.09	0.537	0.686	Co-occurrence
ARRDC5	19p13.3	1 (0.87%)	1 (0.41%)	1.09	0.537	0.686	Co-occurrence
ARSB	5q14.1	1 (0.87%)	1 (0.41%)	1.09	0.537	0.686	Co-occurrence
ART1	11p15.4	1 (0.87%)	1 (0.41%)	1.09	0.537	0.686	Co-occurrence
ART5	11p15.4	1 (0.87%)	1 (0.41%)	1.09	0.537	0.686	Co-occurrence
ARVCF	22q11.21	1 (0.87%)	1 (0.41%)	1.09	0.537	0.686	Co-occurrence
ASCC2	22q12.2	1 (0.87%)	1 (0.41%)	1.09	0.537	0.686	Co-occurrence
ASCL2	11p15.5	1 (0.87%)	1 (0.41%)	1.09	0.537	0.686	Co-occurrence
ASPHD1	16p11.2	1 (0.87%)	1 (0.41%)	1.09	0.537	0.686	Co-occurrence
ATP5PB	1p13.2	1 (0.87%)	1 (0.41%)	1.09	0.537	0.686	Co-occurrence
ATPAF1	1p33	1 (0.87%)	1 (0.41%)	1.09	0.537	0.686	Co-occurrence
ATPAF2	17p11.2	1 (0.87%)	1 (0.41%)	1.09	0.537	0.686	Co-occurrence
ATXN3L	Xp22.2	1 (0.87%)	1 (0.41%)	1.09	0.537	0.686	Co-occurrence
ATXN7L2	1p13.3	1 (0.87%)	1 (0.41%)	1.09	0.537	0.686	Co-occurrence
B3GAT2	6q13	1 (0.87%)	1 (0.41%)	1.09	0.537	0.686	Co-occurrence
B4GALNT3	12p13.33	1 (0.87%)	1 (0.41%)	1.09	0.537	0.686	Co-occurrence
BANF1	11q13.1	1 (0.87%)	1 (0.41%)	1.09	0.537	0.686	Co-occurrence
BARX2	11q24.3	1 (0.87%)	1 (0.41%)	1.09	0.537	0.686	Co-occurrence

BCAT1	12p12.1	1 (0.87%)	1 (0.41%)	1.09	0.537	0.686	Co-occurrence
BCL7A	12q24.31	1 (0.87%)	1 (0.41%)	1.09	0.537	0.686	Co-occurrence
BCOR	Xp11.4	1 (0.87%)	1 (0.41%)	1.09	0.537	0.686	Co-occurrence
BCRP2	22q11.21	1 (0.87%)	1 (0.41%)	1.09	0.537	0.686	Co-occurrence
BDP1	5q13.2	1 (0.87%)	1 (0.41%)	1.09	0.537	0.686	Co-occurrence
BHLHE41	12p12.1	1 (0.87%)	1 (0.41%)	1.09	0.537	0.686	Co-occurrence
BICC1	10q21.1	1 (0.87%)	1 (0.41%)	1.09	0.537	0.686	Co-occurrence
BICRA	19q13.33	1 (0.87%)	1 (0.41%)	1.09	0.537	0.686	Co-occurrence
BLNK	10q24.1	1 (0.87%)	1 (0.41%)	1.09	0.537	0.686	Co-occurrence
BMP2K	4q21.21	1 (0.87%)	1 (0.41%)	1.09	0.537	0.686	Co-occurrence
BOLA2	16p11.2	1 (0.87%)	1 (0.41%)	1.09	0.537	0.686	Co-occurrence
BOLA2B	16p11.2	1 (0.87%)	1 (0.41%)	1.09	0.537	0.686	Co-occurrence
BPIFA1	20q11.21	1 (0.87%)	1 (0.41%)	1.09	0.537	0.686	Co-occurrence
BPIFA2	20q11.21	1 (0.87%)	1 (0.41%)	1.09	0.537	0.686	Co-occurrence
BPIFA3	20q11.21	1 (0.87%)	1 (0.41%)	1.09	0.537	0.686	Co-occurrence
BPIFA4P	20q11.21	1 (0.87%)	1 (0.41%)	1.09	0.537	0.686	Co-occurrence
BPIFB1	20q11.21	1 (0.87%)	1 (0.41%)	1.09	0.537	0.686	Co-occurrence
BPIFB3	20q11.21	1 (0.87%)	1 (0.41%)	1.09	0.537	0.686	Co-occurrence
BPIFB4	20q11.21	1 (0.87%)	1 (0.41%)	1.09	0.537	0.686	Co-occurrence
BPIFB6	20q11.21	1 (0.87%)	1 (0.41%)	1.09	0.537	0.686	Co-occurrence
BRCA2	13q13.1	1 (0.87%)	1 (0.41%)	1.09	0.537	0.686	Co-occurrence
BSPH1	19q13.33	1 (0.87%)	1 (0.41%)	1.09	0.537	0.686	Co-occurrence
BTD	3p25.1	1 (0.87%)	1 (0.41%)	1.09	0.537	0.686	Co-occurrence
C10ORF131	10q24.1	1 (0.87%)	1 (0.41%)	1.09	0.537	0.686	Co-occurrence
C10ORF95	10q24.32	1 (0.87%)	1 (0.41%)	1.09	0.537	0.686	Co-occurrence
C11ORF21	11p15.5	1 (0.87%)	1 (0.41%)	1.09	0.537	0.686	Co-occurrence
C11ORF42	11p15.4	1 (0.87%)	1 (0.41%)	1.09	0.537	0.686	Co-occurrence
C11ORF68	11q13.1	1 (0.87%)	1 (0.41%)	1.09	0.537	0.686	Co-occurrence
C12ORF65	12q24.31	1 (0.87%)	1 (0.41%)	1.09	0.537	0.686	Co-occurrence

C12ORF76	12q24.11	1 (0.87%)	1 (0.41%)	1.09	0.537	0.686	Co-occurrence
C12ORF77	12p12.1	1 (0.87%)	1 (0.41%)	1.09	0.537	0.686	Co-occurrence
C14ORF119	14q11.2	1 (0.87%)	1 (0.41%)	1.09	0.537	0.686	Co-occurrence
C14ORF93	14q11.2	1 (0.87%)	1 (0.41%)	1.09	0.537	0.686	Co-occurrence
C16ORF54	16p11.2	1 (0.87%)	1 (0.41%)	1.09	0.537	0.686	Co-occurrence
C16ORF92	16p11.2	1 (0.87%)	1 (0.41%)	1.09	0.537	0.686	Co-occurrence
C1ORF127	1p36.22	1 (0.87%)	1 (0.41%)	1.09	0.537	0.686	Co-occurrence
C1ORF134	1p36.13	1 (0.87%)	1 (0.41%)	1.09	0.537	0.686	Co-occurrence
C1ORF158	1p36.21	1 (0.87%)	1 (0.41%)	1.09	0.537	0.686	Co-occurrence
C1ORF167	1p36.22	1 (0.87%)	1 (0.41%)	1.09	0.537	0.686	Co-occurrence
C1ORF194	1p13.3	1 (0.87%)	1 (0.41%)	1.09	0.537	0.686	Co-occurrence
C1ORF195	1p36.21	1 (0.87%)	1 (0.41%)	1.09	0.537	0.686	Co-occurrence
C1QTNF5	11q23.3	1 (0.87%)	1 (0.41%)	1.09	0.537	0.686	Co-occurrence
C20ORF194	20p13	1 (0.87%)	1 (0.41%)	1.09	0.537	0.686	Co-occurrence
C20ORF27	20p13	1 (0.87%)	1 (0.41%)	1.09	0.537	0.686	Co-occurrence
C22ORF39	22q11.21	1 (0.87%)	1 (0.41%)	1.09	0.537	0.686	Co-occurrence
C3ORF20	3p25.1	1 (0.87%)	1 (0.41%)	1.09	0.537	0.686	Co-occurrence
C3ORF56	3q21.3	1 (0.87%)	1 (0.41%)	1.09	0.537	0.686	Co-occurrence
C5ORF64	5q12.1	1 (0.87%)	1 (0.41%)	1.09	0.537	0.686	Co-occurrence
C9ORF106	9q34.11	1 (0.87%)	1 (0.41%)	1.09	0.537	0.686	Co-occurrence
CAB39	2q37.1	1 (0.87%)	1 (0.41%)	1.09	0.537	0.686	Co-occurrence
CABCOCO1	10q21.2	1 (0.87%)	1 (0.41%)	1.09	0.537	0.686	Co-occurrence
CABP5	19q13.33	1 (0.87%)	1 (0.41%)	1.09	0.537	0.686	Co-occurrence
CADM4	19q13.31	1 (0.87%)	1 (0.41%)	1.09	0.537	0.686	Co-occurrence
CALN1	7q11.22	1 (0.87%)	1 (0.41%)	1.09	0.537	0.686	Co-occurrence
CAMK2N1	1p36.12	1 (0.87%)	1 (0.41%)	1.09	0.537	0.686	Co-occurrence
CAPG	2p11.2	1 (0.87%)	1 (0.41%)	1.09	0.537	0.686	Co-occurrence
CAPN7	3p25.1	1 (0.87%)	1 (0.41%)	1.09	0.537	0.686	Co-occurrence
CARD8	19q13.33	1 (0.87%)	1 (0.41%)	1.09	0.537	0.686	Co-occurrence

CARS	11p15.4	1 (0.87%)	1 (0.41%)	1.09	0.537	0.686	Co-occurrence
CARTPT	5q13.2	1 (0.87%)	1 (0.41%)	1.09	0.537	0.686	Co-occurrence
CASP9	1p36.21	1 (0.87%)	1 (0.41%)	1.09	0.537	0.686	Co-occurrence
CATSPER1	11q13.1	1 (0.87%)	1 (0.41%)	1.09	0.537	0.686	Co-occurrence
CAV3	3p25.3	1 (0.87%)	1 (0.41%)	1.09	0.537	0.686	Co-occurrence
CAVIN3	11p15.4	1 (0.87%)	1 (0.41%)	1.09	0.537	0.686	Co-occurrence
CC2D2B	10q24.1	1 (0.87%)	1 (0.41%)	1.09	0.537	0.686	Co-occurrence
CCDC116	22q11.21	1 (0.87%)	1 (0.41%)	1.09	0.537	0.686	Co-occurrence
CCDC125	5q13.2	1 (0.87%)	1 (0.41%)	1.09	0.537	0.686	Co-occurrence
CCDC174	3p25.1	1 (0.87%)	1 (0.41%)	1.09	0.537	0.686	Co-occurrence
CCDC71	3p21.31	1 (0.87%)	1 (0.41%)	1.09	0.537	0.686	Co-occurrence
CCDC73	11p13	1 (0.87%)	1 (0.41%)	1.09	0.537	0.686	Co-occurrence
CCDC77	12p13.33	1 (0.87%)	1 (0.41%)	1.09	0.537	0.686	Co-occurrence
CCDC88A	2p16.1	1 (0.87%)	1 (0.41%)	1.09	0.537	0.686	Co-occurrence
CCDC90B	11q14.1	1 (0.87%)	1 (0.41%)	1.09	0.537	0.686	Co-occurrence
CCKBR	11p15.4	1 (0.87%)	1 (0.41%)	1.09	0.537	0.686	Co-occurrence
CCL19	9p13.3	1 (0.87%)	1 (0.41%)	1.09	0.537	0.686	Co-occurrence
CCNB1	5q13.2	1 (0.87%)	1 (0.41%)	1.09	0.537	0.686	Co-occurrence
CCNG2	4q21.1	1 (0.87%)	1 (0.41%)	1.09	0.537	0.686	Co-occurrence
CCNJ	10q24.1	1 (0.87%)	1 (0.41%)	1.09	0.537	0.686	Co-occurrence
CCR9	3p21.31	1 (0.87%)	1 (0.41%)	1.09	0.537	0.686	Co-occurrence
CCT6P1	7q11.21	1 (0.87%)	1 (0.41%)	1.09	0.537	0.686	Co-occurrence
CCT6P3	7q11.21	1 (0.87%)	1 (0.41%)	1.09	0.537	0.686	Co-occurrence
CD177	19q13.31	1 (0.87%)	1 (0.41%)	1.09	0.537	0.686	Co-occurrence
CD2BP2	16p11.2	1 (0.87%)	1 (0.41%)	1.09	0.537	0.686	Co-occurrence
CD53	1p13.3	1 (0.87%)	1 (0.41%)	1.09	0.537	0.686	Co-occurrence
CD81	11p15.5	1 (0.87%)	1 (0.41%)	1.09	0.537	0.686	Co-occurrence
CDC25B	20p13	1 (0.87%)	1 (0.41%)	1.09	0.537	0.686	Co-occurrence
CDC45	22q11.21	1 (0.87%)	1 (0.41%)	1.09	0.537	0.686	Co-occurrence

CDC7	1p22.1	1 (0.87%)	1 (0.41%)	1.09	0.537	0.686	Co-occurrence
CDH24	14q11.2	1 (0.87%)	1 (0.41%)	1.09	0.537	0.686	Co-occurrence
CDHR5	11p15.5	1 (0.87%)	1 (0.41%)	1.09	0.537	0.686	Co-occurrence
CDIPT	16p11.2	1 (0.87%)	1 (0.41%)	1.09	0.537	0.686	Co-occurrence
CDK13	7p14.1	1 (0.87%)	1 (0.41%)	1.09	0.537	0.686	Co-occurrence
CDK2AP1	12q24.31	1 (0.87%)	1 (0.41%)	1.09	0.537	0.686	Co-occurrence
CDK5RAP1	20q11.21	1 (0.87%)	1 (0.41%)	1.09	0.537	0.686	Co-occurrence
CDK7	5q13.2	1 (0.87%)	1 (0.41%)	1.09	0.537	0.686	Co-occurrence
CDKL5	Xp22.13	1 (0.87%)	1 (0.41%)	1.09	0.537	0.686	Co-occurrence
CDKN1C	11p15.4	1 (0.87%)	1 (0.41%)	1.09	0.537	0.686	Co-occurrence
CEACAMP10	19q13.31	1 (0.87%)	1 (0.41%)	1.09	0.537	0.686	Co-occurrence
CEBPE	14q11.2	1 (0.87%)	1 (0.41%)	1.09	0.537	0.686	Co-occurrence
CECR3	22q11.1	1 (0.87%)	1 (0.41%)	1.09	0.537	0.686	Co-occurrence
CECR9	-	1 (0.87%)	1 (0.41%)	1.09	0.537	0.686	Co-occurrence
CELA2A	1p36.21	1 (0.87%)	1 (0.41%)	1.09	0.537	0.686	Co-occurrence
CELA2B	1p36.21	1 (0.87%)	1 (0.41%)	1.09	0.537	0.686	Co-occurrence
CELF5	19p13.3	1 (0.87%)	1 (0.41%)	1.09	0.537	0.686	Co-occurrence
CENPB	20p13	1 (0.87%)	1 (0.41%)	1.09	0.537	0.686	Co-occurrence
CENPH	5q13.2	1 (0.87%)	1 (0.41%)	1.09	0.537	0.686	Co-occurrence
CENPS	1p36.22	1 (0.87%)	1 (0.41%)	1.09	0.537	0.686	Co-occurrence
CEP250	20q11.22	1 (0.87%)	1 (0.41%)	1.09	0.537	0.686	Co-occurrence
CEPT1	1p13.3	1 (0.87%)	1 (0.41%)	1.09	0.537	0.686	Co-occurrence
CERCAM	9q34.11	1 (0.87%)	1 (0.41%)	1.09	0.537	0.686	Co-occurrence
CERS4	19p13.2	1 (0.87%)	1 (0.41%)	1.09	0.537	0.686	Co-occurrence
CFAP36	2p16.1	1 (0.87%)	1 (0.41%)	1.09	0.537	0.686	Co-occurrence
CFAP70	10q22.2	1 (0.87%)	1 (0.41%)	1.09	0.537	0.686	Co-occurrence
CHCHD4	3p25.1	1 (0.87%)	1 (0.41%)	1.09	0.537	0.686	Co-occurrence
CHCHD6	3q21.3	1 (0.87%)	1 (0.41%)	1.09	0.537	0.686	Co-occurrence
CHI3L2	1p13.2	1 (0.87%)	1 (0.41%)	1.09	0.537	0.686	Co-occurrence

CHIA	1p13.2	1 (0.87%)	1 (0.41%)	1.09	0.537	0.686	Co-occurrence
CHIAP2	1p13.2	1 (0.87%)	1 (0.41%)	1.09	0.537	0.686	Co-occurrence
CHMP1B	18p11.21	1 (0.87%)	1 (0.41%)	1.09	0.537	0.686	Co-occurrence
CHRNA10	11p15.4	1 (0.87%)	1 (0.41%)	1.09	0.537	0.686	Co-occurrence
CIDEA	18p11.21|18	1 (0.87%)	1 (0.41%)	1.09	0.537	0.686	Co-occurrence
CISD1	10q21.1	1 (0.87%)	1 (0.41%)	1.09	0.537	0.686	Co-occurrence
CKMT2	5q14.1	1 (0.87%)	1 (0.41%)	1.09	0.537	0.686	Co-occurrence
CLCC1	1p13.3	1 (0.87%)	1 (0.41%)	1.09	0.537	0.686	Co-occurrence
CLCN6	1p36.22	1 (0.87%)	1 (0.41%)	1.09	0.537	0.686	Co-occurrence
CLDN5	22q11.21	1 (0.87%)	1 (0.41%)	1.09	0.537	0.686	Co-occurrence
CLSTN1	1p36.22	1 (0.87%)	1 (0.41%)	1.09	0.537	0.686	Co-occurrence
CLTCL1	22q11.21	1 (0.87%)	1 (0.41%)	1.09	0.537	0.686	Co-occurrence
CMPK1	1p33	1 (0.87%)	1 (0.41%)	1.09	0.537	0.686	Co-occurrence
CMTM8	3p22.3	1 (0.87%)	1 (0.41%)	1.09	0.537	0.686	Co-occurrence
CNGA4	11p15.4	1 (0.87%)	1 (0.41%)	1.09	0.537	0.686	Co-occurrence
CNOT6L	4q21.1	1 (0.87%)	1 (0.41%)	1.09	0.537	0.686	Co-occurrence
CNR2	1p36.11	1 (0.87%)	1 (0.41%)	1.09	0.537	0.686	Co-occurrence
CNTLN	9p22.2	1 (0.87%)	1 (0.41%)	1.09	0.537	0.686	Co-occurrence
COA8	14q32.33	1 (0.87%)	1 (0.41%)	1.09	0.537	0.686	Co-occurrence
COL11A1	1p21.1	1 (0.87%)	1 (0.41%)	1.09	0.537	0.686	Co-occurrence
COL13A1	10q22.1	1 (0.87%)	1 (0.41%)	1.09	0.537	0.686	Co-occurrence
COL4A3BP	5q13.3	1 (0.87%)	1 (0.41%)	1.09	0.537	0.686	Co-occurrence
COL4A4	2q36.3	1 (0.87%)	1 (0.41%)	1.09	0.537	0.686	Co-occurrence
COL6A3	2q37.3	1 (0.87%)	1 (0.41%)	1.09	0.537	0.686	Co-occurrence
COL6A4P1	3p25.1	1 (0.87%)	1 (0.41%)	1.09	0.537	0.686	Co-occurrence
COLQ	3p25.1	1 (0.87%)	1 (0.41%)	1.09	0.537	0.686	Co-occurrence
COMMD7	20q11.21	1 (0.87%)	1 (0.41%)	1.09	0.537	0.686	Co-occurrence
COMT	22q11.21	1 (0.87%)	1 (0.41%)	1.09	0.537	0.686	Co-occurrence
COQ4	9q34.11	1 (0.87%)	1 (0.41%)	1.09	0.537	0.686	Co-occurrence

CORO1A	16p11.2	1 (0.87%)	1 (0.41%)	1.09	0.537	0.686	Co-occurrence
CORT	1p36.22	1 (0.87%)	1 (0.41%)	1.09	0.537	0.686	Co-occurrence
CPLANE2	1p36.13	1 (0.87%)	1 (0.41%)	1.09	0.537	0.686	Co-occurrence
CRCP	7q11.21	1 (0.87%)	1 (0.41%)	1.09	0.537	0.686	Co-occurrence
CRK	17p13.3	1 (0.87%)	1 (0.41%)	1.09	0.537	0.686	Co-occurrence
CRKL	22q11.21	1 (0.87%)	1 (0.41%)	1.09	0.537	0.686	Co-occurrence
CRX	19q13.33	1 (0.87%)	1 (0.41%)	1.09	0.537	0.686	Co-occurrence
CSDC2	22q13.2	1 (0.87%)	1 (0.41%)	1.09	0.537	0.686	Co-occurrence
CSE1L	20q13.13	1 (0.87%)	1 (0.41%)	1.09	0.537	0.686	Co-occurrence
CSF1	1p13.3	1 (0.87%)	1 (0.41%)	1.09	0.537	0.686	Co-occurrence
CST6	11q13.1	1 (0.87%)	1 (0.41%)	1.09	0.537	0.686	Co-occurrence
CT62	15q23	1 (0.87%)	1 (0.41%)	1.09	0.537	0.686	Co-occurrence
CTIF	18q21.1	1 (0.87%)	1 (0.41%)	1.09	0.537	0.686	Co-occurrence
CTNNBIP1	1p36.22	1 (0.87%)	1 (0.41%)	1.09	0.537	0.686	Co-occurrence
CTPS1	1p34.2	1 (0.87%)	1 (0.41%)	1.09	0.537	0.686	Co-occurrence
CTRC	1p36.21	1 (0.87%)	1 (0.41%)	1.09	0.537	0.686	Co-occurrence
CTSG	14q12	1 (0.87%)	1 (0.41%)	1.09	0.537	0.686	Co-occurrence
CUEDC2	10q24.32	1 (0.87%)	1 (0.41%)	1.09	0.537	0.686	Co-occurrence
CUL5	11q22.3	1 (0.87%)	1 (0.41%)	1.09	0.537	0.686	Co-occurrence
CWC27	5q12.3	1 (0.87%)	1 (0.41%)	1.09	0.537	0.686	Co-occurrence
CXADRP3	18p11.21	1 (0.87%)	1 (0.41%)	1.09	0.537	0.686	Co-occurrence
CXCL13	4q21.1	1 (0.87%)	1 (0.41%)	1.09	0.537	0.686	Co-occurrence
CXCR6	3p21.31	1 (0.87%)	1 (0.41%)	1.09	0.537	0.686	Co-occurrence
CYB561D1	1p13.3	1 (0.87%)	1 (0.41%)	1.09	0.537	0.686	Co-occurrence
CYB5RL	1p32.3	1 (0.87%)	1 (0.41%)	1.09	0.537	0.686	Co-occurrence
CYLD	16q12.1	1 (0.87%)	1 (0.41%)	1.09	0.537	0.686	Co-occurrence
CYMP	1p13.3	1 (0.87%)	1 (0.41%)	1.09	0.537	0.686	Co-occurrence
CYP27C1	2q14.3	1 (0.87%)	1 (0.41%)	1.09	0.537	0.686	Co-occurrence
CYP4B1	1p33	1 (0.87%)	1 (0.41%)	1.09	0.537	0.686	Co-occurrence

CYP4Z2P	1p33	1 (0.87%)	1 (0.41%)	1.09	0.537	0.686	Co-occurrence
DAD1	14q11.2	1 (0.87%)	1 (0.41%)	1.09	0.537	0.686	Co-occurrence
DALRD3	3p21.31	1 (0.87%)	1 (0.41%)	1.09	0.537	0.686	Co-occurrence
DCTPP1	16p11.2	1 (0.87%)	1 (0.41%)	1.09	0.537	0.686	Co-occurrence
DDAH1	1p22.3	1 (0.87%)	1 (0.41%)	1.09	0.537	0.686	Co-occurrence
DDI2	1p36.21	1 (0.87%)	1 (0.41%)	1.09	0.537	0.686	Co-occurrence
DDIT4	10q22.1	1 (0.87%)	1 (0.41%)	1.09	0.537	0.686	Co-occurrence
DDX10	11q22.3	1 (0.87%)	1 (0.41%)	1.09	0.537	0.686	Co-occurrence
DDX11	12p11.21	1 (0.87%)	1 (0.41%)	1.09	0.537	0.686	Co-occurrence
DEAF1	11p15.5	1 (0.87%)	1 (0.41%)	1.09	0.537	0.686	Co-occurrence
DENND2D	1p13.3-p13.2	1 (0.87%)	1 (0.41%)	1.09	0.537	0.686	Co-occurrence
DEPDC1B	5q12.1	1 (0.87%)	1 (0.41%)	1.09	0.537	0.686	Co-occurrence
DEPDC5	22q12.2-q12.3	1 (0.87%)	1 (0.41%)	1.09	0.537	0.686	Co-occurrence
DESI1	22q13.2	1 (0.87%)	1 (0.41%)	1.09	0.537	0.686	Co-occurrence
DFFA	1p36.22	1 (0.87%)	1 (0.41%)	1.09	0.537	0.686	Co-occurrence
DGCR2	22q11.21	1 (0.87%)	1 (0.41%)	1.09	0.537	0.686	Co-occurrence
DGCR5	22q11.21	1 (0.87%)	1 (0.41%)	1.09	0.537	0.686	Co-occurrence
DGCR6	22q11.21|22q11	1 (0.87%)	1 (0.41%)	1.09	0.537	0.686	Co-occurrence
DGCR6L	22q11.21	1 (0.87%)	1 (0.41%)	1.09	0.537	0.686	Co-occurrence
DGCR8	22q11.21	1 (0.87%)	1 (0.41%)	1.09	0.537	0.686	Co-occurrence
DHRS2	14q11.2	1 (0.87%)	1 (0.41%)	1.09	0.537	0.686	Co-occurrence
DHRS4L2	14q11.2	1 (0.87%)	1 (0.41%)	1.09	0.537	0.686	Co-occurrence
DHX35	20q11.23-q12	1 (0.87%)	1 (0.41%)	1.09	0.537	0.686	Co-occurrence
DIMT1	5q12.1	1 (0.87%)	1 (0.41%)	1.09	0.537	0.686	Co-occurrence
DKK3	11p15.3	1 (0.87%)	1 (0.41%)	1.09	0.537	0.686	Co-occurrence
DLGAP4	20q11.23	1 (0.87%)	1 (0.41%)	1.09	0.537	0.686	Co-occurrence
DMAC1	9p24.1	1 (0.87%)	1 (0.41%)	1.09	0.537	0.686	Co-occurrence
DNAAF2	14q21.3	1 (0.87%)	1 (0.41%)	1.09	0.537	0.686	Co-occurrence
DNAJB12	10q22.1	1 (0.87%)	1 (0.41%)	1.09	0.537	0.686	Co-occurrence

DNAJC16	1p36.21	1 (0.87%)	1 (0.41%)	1.09	0.537	0.686	Co-occurrence
DNAJC25	9q31.3	1 (0.87%)	1 (0.41%)	1.09	0.537	0.686	Co-occurrence
DNAJC9	10q22.2	1 (0.87%)	1 (0.41%)	1.09	0.537	0.686	Co-occurrence
DNMT3B	20q11.21	1 (0.87%)	1 (0.41%)	1.09	0.537	0.686	Co-occurrence
DNTT	10q24.1	1 (0.87%)	1 (0.41%)	1.09	0.537	0.686	Co-occurrence
DOC2A	16p11.2	1 (0.87%)	1 (0.41%)	1.09	0.537	0.686	Co-occurrence
DPH3	3p25.1	1 (0.87%)	1 (0.41%)	1.09	0.537	0.686	Co-occurrence
DPY19L1	7p14.2	1 (0.87%)	1 (0.41%)	1.09	0.537	0.686	Co-occurrence
DPY19L2P1	7p14.2	1 (0.87%)	1 (0.41%)	1.09	0.537	0.686	Co-occurrence
DPYD	1p21.3	1 (0.87%)	1 (0.41%)	1.09	0.537	0.686	Co-occurrence
DRAM2	1p13.3	1 (0.87%)	1 (0.41%)	1.09	0.537	0.686	Co-occurrence
DRAP1	11q13.1	1 (0.87%)	1 (0.41%)	1.09	0.537	0.686	Co-occurrence
DRAXIN	1p36.22	1 (0.87%)	1 (0.41%)	1.09	0.537	0.686	Co-occurrence
DRC3	17p11.2	1 (0.87%)	1 (0.41%)	1.09	0.537	0.686	Co-occurrence
DRD4	11p15.5	1 (0.87%)	1 (0.41%)	1.09	0.537	0.686	Co-occurrence
DUPD1	10q22.2	1 (0.87%)	1 (0.41%)	1.09	0.537	0.686	Co-occurrence
DUSP26	8p12	1 (0.87%)	1 (0.41%)	1.09	0.537	0.686	Co-occurrence
E2F2	1p36.12	1 (0.87%)	1 (0.41%)	1.09	0.537	0.686	Co-occurrence
EAF1	3p25.1	1 (0.87%)	1 (0.41%)	1.09	0.537	0.686	Co-occurrence
ECD	10q22.2	1 (0.87%)	1 (0.41%)	1.09	0.537	0.686	Co-occurrence
ECPAS	9q31.3	1 (0.87%)	1 (0.41%)	1.09	0.537	0.686	Co-occurrence
EDEM2	20q11.22	1 (0.87%)	1 (0.41%)	1.09	0.537	0.686	Co-occurrence
EFCAB14	1p33	1 (0.87%)	1 (0.41%)	1.09	0.537	0.686	Co-occurrence
EFHD1	2q37.1	1 (0.87%)	1 (0.41%)	1.09	0.537	0.686	Co-occurrence
EFHD2	1p36.21	1 (0.87%)	1 (0.41%)	1.09	0.537	0.686	Co-occurrence
EHD2	19q13.33	1 (0.87%)	1 (0.41%)	1.09	0.537	0.686	Co-occurrence
EI24	11q24.2	1 (0.87%)	1 (0.41%)	1.09	0.537	0.686	Co-occurrence
EIF1AD	11q13.1	1 (0.87%)	1 (0.41%)	1.09	0.537	0.686	Co-occurrence
EIF2S2	20q11.22	1 (0.87%)	1 (0.41%)	1.09	0.537	0.686	Co-occurrence

EIF3K	19q13.2	1 (0.87%)	1 (0.41%)	1.09	0.537	0.686	Co-occurrence
EIF4E2	2q37.1	1 (0.87%)	1 (0.41%)	1.09	0.537	0.686	Co-occurrence
EIF5AL1	10q22.3	1 (0.87%)	1 (0.41%)	1.09	0.537	0.686	Co-occurrence
ELMO1	7p14.2-p14.1	1 (0.87%)	1 (0.41%)	1.09	0.537	0.686	Co-occurrence
ELMO2	20q13.12	1 (0.87%)	1 (0.41%)	1.09	0.537	0.686	Co-occurrence
ELMOD3	2p11.2	1 (0.87%)	1 (0.41%)	1.09	0.537	0.686	Co-occurrence
ELOA	1p36.11	1 (0.87%)	1 (0.41%)	1.09	0.537	0.686	Co-occurrence
ELOVL3	10q24.32	1 (0.87%)	1 (0.41%)	1.09	0.537	0.686	Co-occurrence
ELOVL7	5q12.1	1 (0.87%)	1 (0.41%)	1.09	0.537	0.686	Co-occurrence
ELSPBP1	19q13.33	1 (0.87%)	1 (0.41%)	1.09	0.537	0.686	Co-occurrence
ENTPD1	10q24.1	1 (0.87%)	1 (0.41%)	1.09	0.537	0.686	Co-occurrence
EPB41L2	6q23.1-q23.2	1 (0.87%)	1 (0.41%)	1.09	0.537	0.686	Co-occurrence
EPDR1	7p14.1	1 (0.87%)	1 (0.41%)	1.09	0.537	0.686	Co-occurrence
EPHA2	1p36.13	1 (0.87%)	1 (0.41%)	1.09	0.537	0.686	Co-occurrence
EPS8L2	11p15.5	1 (0.87%)	1 (0.41%)	1.09	0.537	0.686	Co-occurrence
EPS8L3	1p13.3	1 (0.87%)	1 (0.41%)	1.09	0.537	0.686	Co-occurrence
ERCC8	5q12.1	1 (0.87%)	1 (0.41%)	1.09	0.537	0.686	Co-occurrence
ESCO1	18q11.2	1 (0.87%)	1 (0.41%)	1.09	0.537	0.686	Co-occurrence
ESS2	22q11.21|22q11.2	1 (0.87%)	1 (0.41%)	1.09	0.537	0.686	Co-occurrence
ETHE1	19q13.31	1 (0.87%)	1 (0.41%)	1.09	0.537	0.686	Co-occurrence
EVPLL	17p11.2	1 (0.87%)	1 (0.41%)	1.09	0.537	0.686	Co-occurrence
EXOSC10	1p36.22	1 (0.87%)	1 (0.41%)	1.09	0.537	0.686	Co-occurrence
FAM138D	12p13.33	1 (0.87%)	1 (0.41%)	1.09	0.537	0.686	Co-occurrence
FAM149B1	10q22.2	1 (0.87%)	1 (0.41%)	1.09	0.537	0.686	Co-occurrence
FAM151A	-	1 (0.87%)	1 (0.41%)	1.09	0.537	0.686	Co-occurrence
FAM160A2	11p15.4	1 (0.87%)	1 (0.41%)	1.09	0.537	0.686	Co-occurrence
FAM169A	5q13.3	1 (0.87%)	1 (0.41%)	1.09	0.537	0.686	Co-occurrence
FAM183BP	7p14.1	1 (0.87%)	1 (0.41%)	1.09	0.537	0.686	Co-occurrence
FAM216A	12q24.11	1 (0.87%)	1 (0.41%)	1.09	0.537	0.686	Co-occurrence

FAM225A	9q32	1 (0.87%)	1 (0.41%)	1.09	0.537	0.686	Co-occurrence
FAM225B	9q32	1 (0.87%)	1 (0.41%)	1.09	0.537	0.686	Co-occurrence
FAM230A	22q11.21	1 (0.87%)	1 (0.41%)	1.09	0.537	0.686	Co-occurrence
FAM230B	22q11.21	1 (0.87%)	1 (0.41%)	1.09	0.537	0.686	Co-occurrence
FAM230C	13q11	1 (0.87%)	1 (0.41%)	1.09	0.537	0.686	Co-occurrence
FAM241A	4q25	1 (0.87%)	1 (0.41%)	1.09	0.537	0.686	Co-occurrence
FAM241B	10q22.1	1 (0.87%)	1 (0.41%)	1.09	0.537	0.686	Co-occurrence
FAM25A	10q23.2	1 (0.87%)	1 (0.41%)	1.09	0.537	0.686	Co-occurrence
FAM83D	20q11.23	1 (0.87%)	1 (0.41%)	1.09	0.537	0.686	Co-occurrence
FAM9C	Xp22.2	1 (0.87%)	1 (0.41%)	1.09	0.537	0.686	Co-occurrence
FBLIM1	1p36.21	1 (0.87%)	1 (0.41%)	1.09	0.537	0.686	Co-occurrence
FBLN2	3p25.1	1 (0.87%)	1 (0.41%)	1.09	0.537	0.686	Co-occurrence
FBP2	9q22.32	1 (0.87%)	1 (0.41%)	1.09	0.537	0.686	Co-occurrence
FBXL15	10q24.32	1 (0.87%)	1 (0.41%)	1.09	0.537	0.686	Co-occurrence
FBXO2	1p36.22	1 (0.87%)	1 (0.41%)	1.09	0.537	0.686	Co-occurrence
FBXO27	19q13.2	1 (0.87%)	1 (0.41%)	1.09	0.537	0.686	Co-occurrence
FBXO42	1p36.13	1 (0.87%)	1 (0.41%)	1.09	0.537	0.686	Co-occurrence
FBXO44	1p36.22	1 (0.87%)	1 (0.41%)	1.09	0.537	0.686	Co-occurrence
FBXO6	1p36.22	1 (0.87%)	1 (0.41%)	1.09	0.537	0.686	Co-occurrence
FCHO2	5q13.2	1 (0.87%)	1 (0.41%)	1.09	0.537	0.686	Co-occurrence
FEM1A	19p13.3	1 (0.87%)	1 (0.41%)	1.09	0.537	0.686	Co-occurrence
FER1L4	20q11.22	1 (0.87%)	1 (0.41%)	1.09	0.537	0.686	Co-occurrence
FGD5	3p25.1	1 (0.87%)	1 (0.41%)	1.09	0.537	0.686	Co-occurrence
FGD5P1	3p25.1	1 (0.87%)	1 (0.41%)	1.09	0.537	0.686	Co-occurrence
FITM2	20q13.12	1 (0.87%)	1 (0.41%)	1.09	0.537	0.686	Co-occurrence
FKBP15	9q32	1 (0.87%)	1 (0.41%)	1.09	0.537	0.686	Co-occurrence
FKSG49	5q13.3	1 (0.87%)	1 (0.41%)	1.09	0.537	0.686	Co-occurrence
FLVCR2	14q24.3	1 (0.87%)	1 (0.41%)	1.09	0.537	0.686	Co-occurrence
FNDC5	1p35.1	1 (0.87%)	1 (0.41%)	1.09	0.537	0.686	Co-occurrence

FOXD1	5q13.2	1 (0.87%)	1 (0.41%)	1.09	0.537	0.686	Co-occurrence
FOXD2	1p33	1 (0.87%)	1 (0.41%)	1.09	0.537	0.686	Co-occurrence
FOXE3	1p33	1 (0.87%)	1 (0.41%)	1.09	0.537	0.686	Co-occurrence
FRAS1	4q21.21	1 (0.87%)	1 (0.41%)	1.09	0.537	0.686	Co-occurrence
FRAT1	10q24.1	1 (0.87%)	1 (0.41%)	1.09	0.537	0.686	Co-occurrence
FRAT2	10q24.1	1 (0.87%)	1 (0.41%)	1.09	0.537	0.686	Co-occurrence
FUCA1	1p36.11	1 (0.87%)	1 (0.41%)	1.09	0.537	0.686	Co-occurrence
FUT10	8p12	1 (0.87%)	1 (0.41%)	1.09	0.537	0.686	Co-occurrence
FYCO1	3p21.31	1 (0.87%)	1 (0.41%)	1.09	0.537	0.686	Co-occurrence
GABBR2	9q22.33	1 (0.87%)	1 (0.41%)	1.09	0.537	0.686	Co-occurrence
GADD45B	19p13.3	1 (0.87%)	1 (0.41%)	1.09	0.537	0.686	Co-occurrence
GADD45G	9q22.2	1 (0.87%)	1 (0.41%)	1.09	0.537	0.686	Co-occurrence
GADL1	3p24.1-p23	1 (0.87%)	1 (0.41%)	1.09	0.537	0.686	Co-occurrence
GAL3ST1	22q12.2	1 (0.87%)	1 (0.41%)	1.09	0.537	0.686	Co-occurrence
GAL3ST3	11q13.1	1 (0.87%)	1 (0.41%)	1.09	0.537	0.686	Co-occurrence
GBF1	10q24.32	1 (0.87%)	1 (0.41%)	1.09	0.537	0.686	Co-occurrence
GDAP1L1	20q13.12	1 (0.87%)	1 (0.41%)	1.09	0.537	0.686	Co-occurrence
GDF5	20q11.22	1 (0.87%)	1 (0.41%)	1.09	0.537	0.686	Co-occurrence
GDF5OS	20q11.2	1 (0.87%)	1 (0.41%)	1.09	0.537	0.686	Co-occurrence
GDPD3	16p11.2	1 (0.87%)	1 (0.41%)	1.09	0.537	0.686	Co-occurrence
GFM2	5q13.3	1 (0.87%)	1 (0.41%)	1.09	0.537	0.686	Co-occurrence
GFRA4	20p13	1 (0.87%)	1 (0.41%)	1.09	0.537	0.686	Co-occurrence
GGT2	22q11.21	1 (0.87%)	1 (0.41%)	1.09	0.537	0.686	Co-occurrence
GGT3P	22q11.21	1 (0.87%)	1 (0.41%)	1.09	0.537	0.686	Co-occurrence
GGTLC3	22q11.21	1 (0.87%)	1 (0.41%)	1.09	0.537	0.686	Co-occurrence
GID4	17p11.2|17p11.2	1 (0.87%)	1 (0.41%)	1.09	0.537	0.686	Co-occurrence
GIT2	12q24.11	1 (0.87%)	1 (0.41%)	1.09	0.537	0.686	Co-occurrence
GJA9	1p34.3	1 (0.87%)	1 (0.41%)	1.09	0.537	0.686	Co-occurrence
GLRA3	4q34.1	1 (0.87%)	1 (0.41%)	1.09	0.537	0.686	Co-occurrence

GLT8D1	3p21.1	1 (0.87%)	1 (0.41%)	1.09	0.537	0.686	Co-occurrence
GLTP	12q24.11	1 (0.87%)	1 (0.41%)	1.09	0.537	0.686	Co-occurrence
GLUD1	10q23.2	1 (0.87%)	1 (0.41%)	1.09	0.537	0.686	Co-occurrence
GMPPB	3p21.31	1 (0.87%)	1 (0.41%)	1.09	0.537	0.686	Co-occurrence
GNAI3	1p13.3	1 (0.87%)	1 (0.41%)	1.09	0.537	0.686	Co-occurrence
GNAL	18p11.21	1 (0.87%)	1 (0.41%)	1.09	0.537	0.686	Co-occurrence
GNAT2	1p13.3	1 (0.87%)	1 (0.41%)	1.09	0.537	0.686	Co-occurrence
GNB1L	22q11.21	1 (0.87%)	1 (0.41%)	1.09	0.537	0.686	Co-occurrence
GNG10	9q31.3	1 (0.87%)	1 (0.41%)	1.09	0.537	0.686	Co-occurrence
GNG7	19p13.3	1 (0.87%)	1 (0.41%)	1.09	0.537	0.686	Co-occurrence
GNL3	3p21.1	1 (0.87%)	1 (0.41%)	1.09	0.537	0.686	Co-occurrence
GOLT1B	12p12.1	1 (0.87%)	1 (0.41%)	1.09	0.537	0.686	Co-occurrence
GP1BB	22q11.21	1 (0.87%)	1 (0.41%)	1.09	0.537	0.686	Co-occurrence
GPBAR1	2q35	1 (0.87%)	1 (0.41%)	1.09	0.537	0.686	Co-occurrence
GPR141	7p14.1	1 (0.87%)	1 (0.41%)	1.09	0.537	0.686	Co-occurrence
GPR34	Xp11.4	1 (0.87%)	1 (0.41%)	1.09	0.537	0.686	Co-occurrence
GPR55	2q37.1	1 (0.87%)	1 (0.41%)	1.09	0.537	0.686	Co-occurrence
GPR61	1p13.3	1 (0.87%)	1 (0.41%)	1.09	0.537	0.686	Co-occurrence
GPR62	3p21.2	1 (0.87%)	1 (0.41%)	1.09	0.537	0.686	Co-occurrence
GPR82	Xp11.4	1 (0.87%)	1 (0.41%)	1.09	0.537	0.686	Co-occurrence
GPSM2	1p13.3	1 (0.87%)	1 (0.41%)	1.09	0.537	0.686	Co-occurrence
GREB1L	18q11.1-q11.2	1 (0.87%)	1 (0.41%)	1.09	0.537	0.686	Co-occurrence
GRIP2	3p25.1	1 (0.87%)	1 (0.41%)	1.09	0.537	0.686	Co-occurrence
GRM5	11q14.2-q14.3	1 (0.87%)	1 (0.41%)	1.09	0.537	0.686	Co-occurrence
GSC2	22q11.21	1 (0.87%)	1 (0.41%)	1.09	0.537	0.686	Co-occurrence
GSTM1	1p13.3	1 (0.87%)	1 (0.41%)	1.09	0.537	0.686	Co-occurrence
GSTM2	1p13.3	1 (0.87%)	1 (0.41%)	1.09	0.537	0.686	Co-occurrence
GSTM3	1p13.3	1 (0.87%)	1 (0.41%)	1.09	0.537	0.686	Co-occurrence
GSTM4	1p13.3	1 (0.87%)	1 (0.41%)	1.09	0.537	0.686	Co-occurrence

GSTM5	1p13.3	1 (0.87%)	1 (0.41%)	1.09	0.537	0.686	Co-occurrence
GTSF1L	20q13.12	1 (0.87%)	1 (0.41%)	1.09	0.537	0.686	Co-occurrence
GUSB	7q11.21	1 (0.87%)	1 (0.41%)	1.09	0.537	0.686	Co-occurrence
GYS2	12p12.1	1 (0.87%)	1 (0.41%)	1.09	0.537	0.686	Co-occurrence
GZMB	14q12	1 (0.87%)	1 (0.41%)	1.09	0.537	0.686	Co-occurrence
GZMH	14q12	1 (0.87%)	1 (0.41%)	1.09	0.537	0.686	Co-occurrence
H6PD	1p36.22	1 (0.87%)	1 (0.41%)	1.09	0.537	0.686	Co-occurrence
HACD2	3q21.1	1 (0.87%)	1 (0.41%)	1.09	0.537	0.686	Co-occurrence
HACL1	3p25.1	1 (0.87%)	1 (0.41%)	1.09	0.537	0.686	Co-occurrence
HDAC11	3p25.1	1 (0.87%)	1 (0.41%)	1.09	0.537	0.686	Co-occurrence
HECTD4	12q24.13	1 (0.87%)	1 (0.41%)	1.09	0.537	0.686	Co-occurrence
HFM1	1p22.2	1 (0.87%)	1 (0.41%)	1.09	0.537	0.686	Co-occurrence
HIC2	22q11.21	1 (0.87%)	1 (0.41%)	1.09	0.537	0.686	Co-occurrence
HIRA	22q11.21	1 (0.87%)	1 (0.41%)	1.09	0.537	0.686	Co-occurrence
HIRIP3	16p11.2	1 (0.87%)	1 (0.41%)	1.09	0.537	0.686	Co-occurrence
HJURP	2q37.1	1 (0.87%)	1 (0.41%)	1.09	0.537	0.686	Co-occurrence
HMGCL	1p36.11	1 (0.87%)	1 (0.41%)	1.09	0.537	0.686	Co-occurrence
HMGCR	5q13.3	1 (0.87%)	1 (0.41%)	1.09	0.537	0.686	Co-occurrence
HNRNPCL1	1p36.21	1 (0.87%)	1 (0.41%)	1.09	0.537	0.686	Co-occurrence
HNRNPR	1p36.12	1 (0.87%)	1 (0.41%)	1.09	0.537	0.686	Co-occurrence
HPD	12q24.31	1 (0.87%)	1 (0.41%)	1.09	0.537	0.686	Co-occurrence
HPGD	4q34.1	1 (0.87%)	1 (0.41%)	1.09	0.537	0.686	Co-occurrence
HPGDS	4q22.3	1 (0.87%)	1 (0.41%)	1.09	0.537	0.686	Co-occurrence
HPS6	10q24.32	1 (0.87%)	1 (0.41%)	1.09	0.537	0.686	Co-occurrence
HS3ST5	6q21-q22.1	1 (0.87%)	1 (0.41%)	1.09	0.537	0.686	Co-occurrence
HSCB	22q12.1	1 (0.87%)	1 (0.41%)	1.09	0.537	0.686	Co-occurrence
HSPA12B	20p13	1 (0.87%)	1 (0.41%)	1.09	0.537	0.686	Co-occurrence
HTR1A	5q12.3	1 (0.87%)	1 (0.41%)	1.09	0.537	0.686	Co-occurrence
HUWE1	Xp11.22	1 (0.87%)	1 (0.41%)	1.09	0.537	0.686	Co-occurrence

HYAL2	3p21.31	1 (0.87%)	1 (0.41%)	1.09	0.537	0.686	Co-occurrence
ID3	1p36.12	1 (0.87%)	1 (0.41%)	1.09	0.537	0.686	Co-occurrence
IER2	19p13.13	1 (0.87%)	1 (0.41%)	1.09	0.537	0.686	Co-occurrence
IFIT2	10q23.31	1 (0.87%)	1 (0.41%)	1.09	0.537	0.686	Co-occurrence
IFIT3	10q23.31	1 (0.87%)	1 (0.41%)	1.09	0.537	0.686	Co-occurrence
IFT52	20q13.12	1 (0.87%)	1 (0.41%)	1.09	0.537	0.686	Co-occurrence
IFT81	12q24.11	1 (0.87%)	1 (0.41%)	1.09	0.537	0.686	Co-occurrence
IGF2	11p15.5	1 (0.87%)	1 (0.41%)	1.09	0.537	0.686	Co-occurrence
IGF2R	6q25.3	1 (0.87%)	1 (0.41%)	1.09	0.537	0.686	Co-occurrence
IGSF21	1p36.13	1 (0.87%)	1 (0.41%)	1.09	0.537	0.686	Co-occurrence
IKZF1	7p12.2	1 (0.87%)	1 (0.41%)	1.09	0.537	0.686	Co-occurrence
IL31	12q24.31	1 (0.87%)	1 (0.41%)	1.09	0.537	0.686	Co-occurrence
IMPA2	18p11.21	1 (0.87%)	1 (0.41%)	1.09	0.537	0.686	Co-occurrence
IMPDH2	3p21.31	1 (0.87%)	1 (0.41%)	1.09	0.537	0.686	Co-occurrence
INO80E	16p11.2	1 (0.87%)	1 (0.41%)	1.09	0.537	0.686	Co-occurrence
INPP5K	17p13.3	1 (0.87%)	1 (0.41%)	1.09	0.537	0.686	Co-occurrence
INS	11p15.5	1 (0.87%)	1 (0.41%)	1.09	0.537	0.686	Co-occurrence
INTS4P1	7q11.21	1 (0.87%)	1 (0.41%)	1.09	0.537	0.686	Co-occurrence
INTS4P2	7q11.21	1 (0.87%)	1 (0.41%)	1.09	0.537	0.686	Co-occurrence
IPMK	10q21.1	1 (0.87%)	1 (0.41%)	1.09	0.537	0.686	Co-occurrence
IPO11	5q12.1	1 (0.87%)	1 (0.41%)	1.09	0.537	0.686	Co-occurrence
IQCF1	3p21.2	1 (0.87%)	1 (0.41%)	1.09	0.537	0.686	Co-occurrence
IQCF5	3p21.2	1 (0.87%)	1 (0.41%)	1.09	0.537	0.686	Co-occurrence
IQGAP2	5q13.3	1 (0.87%)	1 (0.41%)	1.09	0.537	0.686	Co-occurrence
IQSEC2	Xp11.22	1 (0.87%)	1 (0.41%)	1.09	0.537	0.686	Co-occurrence
IQSEC3	12p13.33	1 (0.87%)	1 (0.41%)	1.09	0.537	0.686	Co-occurrence
IRF7	11p15.5	1 (0.87%)	1 (0.41%)	1.09	0.537	0.686	Co-occurrence
IRGC	19q13.31	1 (0.87%)	1 (0.41%)	1.09	0.537	0.686	Co-occurrence
IRGQ	19q13.31	1 (0.87%)	1 (0.41%)	1.09	0.537	0.686	Co-occurrence

ITGAL	16p11.2	1 (0.87%)	1 (0.41%)	1.09	0.537	0.686	Co-occurrence
ITGB2	21q22.3	1 (0.87%)	1 (0.41%)	1.09	0.537	0.686	Co-occurrence
ITIH1	3p21.1	1 (0.87%)	1 (0.41%)	1.09	0.537	0.686	Co-occurrence
ITIH3	3p21.1	1 (0.87%)	1 (0.41%)	1.09	0.537	0.686	Co-occurrence
ITIH4	3p21.1	1 (0.87%)	1 (0.41%)	1.09	0.537	0.686	Co-occurrence
ITM2C	2q37.1	1 (0.87%)	1 (0.41%)	1.09	0.537	0.686	Co-occurrence
JPH4	14q11.2	1 (0.87%)	1 (0.41%)	1.09	0.537	0.686	Co-occurrence
KAT2B	3p24.3	1 (0.87%)	1 (0.41%)	1.09	0.537	0.686	Co-occurrence
KAT6B	10q22.2	1 (0.87%)	1 (0.41%)	1.09	0.537	0.686	Co-occurrence
KAZN	1p36.21	1 (0.87%)	1 (0.41%)	1.09	0.537	0.686	Co-occurrence
KCNA10	1p13.3	1 (0.87%)	1 (0.41%)	1.09	0.537	0.686	Co-occurrence
KCNC4	1p13.3	1 (0.87%)	1 (0.41%)	1.09	0.537	0.686	Co-occurrence
KCNH8	3p24.3	1 (0.87%)	1 (0.41%)	1.09	0.537	0.686	Co-occurrence
KCNJ11	11p15.1	1 (0.87%)	1 (0.41%)	1.09	0.537	0.686	Co-occurrence
KCNN4	19q13.31	1 (0.87%)	1 (0.41%)	1.09	0.537	0.686	Co-occurrence
KCNQ1	11p15.5-p15.4	1 (0.87%)	1 (0.41%)	1.09	0.537	0.686	Co-occurrence
KCNQ1DN	11p15.4|11p15.5	1 (0.87%)	1 (0.41%)	1.09	0.537	0.686	Co-occurrence
KCNQ1OT1	11p15.5	1 (0.87%)	1 (0.41%)	1.09	0.537	0.686	Co-occurrence
KCTD10	12q24.11	1 (0.87%)	1 (0.41%)	1.09	0.537	0.686	Co-occurrence
KCTD13	16p11.2	1 (0.87%)	1 (0.41%)	1.09	0.537	0.686	Co-occurrence
KDM5A	12p13.33	1 (0.87%)	1 (0.41%)	1.09	0.537	0.686	Co-occurrence
KDM5C	Xp11.22	1 (0.87%)	1 (0.41%)	1.09	0.537	0.686	Co-occurrence
KIAA0754	1p34.3	1 (0.87%)	1 (0.41%)	1.09	0.537	0.686	Co-occurrence
KIAA2013	1p36.22	1 (0.87%)	1 (0.41%)	1.09	0.537	0.686	Co-occurrence
KIF1B	1p36.22	1 (0.87%)	1 (0.41%)	1.09	0.537	0.686	Co-occurrence
KIF22	16p11.2	1 (0.87%)	1 (0.41%)	1.09	0.537	0.686	Co-occurrence
KIF2A	5q12.1	1 (0.87%)	1 (0.41%)	1.09	0.537	0.686	Co-occurrence
KIF3B	20q11.21	1 (0.87%)	1 (0.41%)	1.09	0.537	0.686	Co-occurrence
KIRREL3	11q24.2	1 (0.87%)	1 (0.41%)	1.09	0.537	0.686	Co-occurrence

KLC1	14q32.33	1 (0.87%)	1 (0.41%)	1.09	0.537	0.686	Co-occurrence
KLF15	3q21.3	1 (0.87%)	1 (0.41%)	1.09	0.537	0.686	Co-occurrence
KLHL22	22q11.21	1 (0.87%)	1 (0.41%)	1.09	0.537	0.686	Co-occurrence
KLHL42	12p11.22	1 (0.87%)	1 (0.41%)	1.09	0.537	0.686	Co-occurrence
KPTN	19q13.32	1 (0.87%)	1 (0.41%)	1.09	0.537	0.686	Co-occurrence
KRT18	12q13.13	1 (0.87%)	1 (0.41%)	1.09	0.537	0.686	Co-occurrence
KRT8	12q13.13	1 (0.87%)	1 (0.41%)	1.09	0.537	0.686	Co-occurrence
L3MBTL1	20q13.12	1 (0.87%)	1 (0.41%)	1.09	0.537	0.686	Co-occurrence
LAMA3	18q11.2	1 (0.87%)	1 (0.41%)	1.09	0.537	0.686	Co-occurrence
LAMTOR5	1p13.3	1 (0.87%)	1 (0.41%)	1.09	0.537	0.686	Co-occurrence
LARP6	15q23	1 (0.87%)	1 (0.41%)	1.09	0.537	0.686	Co-occurrence
LARP7	4q25	1 (0.87%)	1 (0.41%)	1.09	0.537	0.686	Co-occurrence
LDB1	10q24.32	1 (0.87%)	1 (0.41%)	1.09	0.537	0.686	Co-occurrence
LGI1	10q23.33	1 (0.87%)	1 (0.41%)	1.09	0.537	0.686	Co-occurrence
LHX8	1p31.1	1 (0.87%)	1 (0.41%)	1.09	0.537	0.686	Co-occurrence
LIF	22q12.2	1 (0.87%)	1 (0.41%)	1.09	0.537	0.686	Co-occurrence
LIG1	19q13.33	1 (0.87%)	1 (0.41%)	1.09	0.537	0.686	Co-occurrence
LINC00167	11q24.3	1 (0.87%)	1 (0.41%)	1.09	0.537	0.686	Co-occurrence
LINC00174	7q11.21	1 (0.87%)	1 (0.41%)	1.09	0.537	0.686	Co-occurrence
LINC00265	7p14.1	1 (0.87%)	1 (0.41%)	1.09	0.537	0.686	Co-occurrence
LINC00312	3p25.3	1 (0.87%)	1 (0.41%)	1.09	0.537	0.686	Co-occurrence
LINC00595	10q22.3	1 (0.87%)	1 (0.41%)	1.09	0.537	0.686	Co-occurrence
LINC00607	2q35	1 (0.87%)	1 (0.41%)	1.09	0.537	0.686	Co-occurrence
LINC00620	3p25.1	1 (0.87%)	1 (0.41%)	1.09	0.537	0.686	Co-occurrence
LINC00645	14q12	1 (0.87%)	1 (0.41%)	1.09	0.537	0.686	Co-occurrence
LINC00648	14q21.3	1 (0.87%)	1 (0.41%)	1.09	0.537	0.686	Co-occurrence
LINC00844	10q21.1	1 (0.87%)	1 (0.41%)	1.09	0.537	0.686	Co-occurrence
LINC00857	10q22.3	1 (0.87%)	1 (0.41%)	1.09	0.537	0.686	Co-occurrence
LINC00871	14q21.2	1 (0.87%)	1 (0.41%)	1.09	0.537	0.686	Co-occurrence

LINC00879	3q11.2	1 (0.87%)	1 (0.41%)	1.09	0.537	0.686	Co-occurrence
LINC00958	11p15.3	1 (0.87%)	1 (0.41%)	1.09	0.537	0.686	Co-occurrence
LINGO3	19p13.3	1 (0.87%)	1 (0.41%)	1.09	0.537	0.686	Co-occurrence
LIPA	10q23.31	1 (0.87%)	1 (0.41%)	1.09	0.537	0.686	Co-occurrence
LMCD1	3p25.3	1 (0.87%)	1 (0.41%)	1.09	0.537	0.686	Co-occurrence
LMNB2	19p13.3	1 (0.87%)	1 (0.41%)	1.09	0.537	0.686	Co-occurrence
LMNTD1	12p12.1	1 (0.87%)	1 (0.41%)	1.09	0.537	0.686	Co-occurrence
LOC654780	16q24.1	1 (0.87%)	1 (0.41%)	1.09	0.537	0.686	Co-occurrence
LOC91370	22q12.2	1 (0.87%)	1 (0.41%)	1.09	0.537	0.686	Co-occurrence
LRAT	4q32.1	1 (0.87%)	1 (0.41%)	1.09	0.537	0.686	Co-occurrence
LRIF1	1p13.3	1 (0.87%)	1 (0.41%)	1.09	0.537	0.686	Co-occurrence
LRMDA	10q22.2-q22.3	1 (0.87%)	1 (0.41%)	1.09	0.537	0.686	Co-occurrence
LRMP	12p12.1	1 (0.87%)	1 (0.41%)	1.09	0.537	0.686	Co-occurrence
LRP10	14q11.2	1 (0.87%)	1 (0.41%)	1.09	0.537	0.686	Co-occurrence
LRR1	14q21.3	1 (0.87%)	1 (0.41%)	1.09	0.537	0.686	Co-occurrence
LRRC36	16q22.1	1 (0.87%)	1 (0.41%)	1.09	0.537	0.686	Co-occurrence
LRRC37A5P	9q31.3	1 (0.87%)	1 (0.41%)	1.09	0.537	0.686	Co-occurrence
LRRC38	1p36.21	1 (0.87%)	1 (0.41%)	1.09	0.537	0.686	Co-occurrence
LRRC3B	3p24.1	1 (0.87%)	1 (0.41%)	1.09	0.537	0.686	Co-occurrence
LRRC43	12q24.31	1 (0.87%)	1 (0.41%)	1.09	0.537	0.686	Co-occurrence
LRRC49	15q23	1 (0.87%)	1 (0.41%)	1.09	0.537	0.686	Co-occurrence
LRRC70	5q12.1	1 (0.87%)	1 (0.41%)	1.09	0.537	0.686	Co-occurrence
LRRC8C	1p22.2	1 (0.87%)	1 (0.41%)	1.09	0.537	0.686	Co-occurrence
LRRC8D	1p22.2	1 (0.87%)	1 (0.41%)	1.09	0.537	0.686	Co-occurrence
LRRFIP1	2q37.3	1 (0.87%)	1 (0.41%)	1.09	0.537	0.686	Co-occurrence
LRSAM1	9q33.3-q34.11	1 (0.87%)	1 (0.41%)	1.09	0.537	0.686	Co-occurrence
LSM3	3p25.1	1 (0.87%)	1 (0.41%)	1.09	0.537	0.686	Co-occurrence
LSM7	19p13.3	1 (0.87%)	1 (0.41%)	1.09	0.537	0.686	Co-occurrence
LYPD3	19q13.31	1 (0.87%)	1 (0.41%)	1.09	0.537	0.686	Co-occurrence

LYPD5	19q13.31	1 (0.87%)	1 (0.41%)	1.09	0.537	0.686	Co-occurrence
LYSMD3	5q14.3	1 (0.87%)	1 (0.41%)	1.09	0.537	0.686	Co-occurrence
LZIC	1p36.22	1 (0.87%)	1 (0.41%)	1.09	0.537	0.686	Co-occurrence
LZTR1	22q11.21|22q11.1-q11.2	1 (0.87%)	1 (0.41%)	1.09	0.537	0.686	Co-occurrence
MAD2L2	1p36.22	1 (0.87%)	1 (0.41%)	1.09	0.537	0.686	Co-occurrence
MAK16	8p12	1 (0.87%)	1 (0.41%)	1.09	0.537	0.686	Co-occurrence
MAN1A1	6q22.31	1 (0.87%)	1 (0.41%)	1.09	0.537	0.686	Co-occurrence
MANSC1	12p13.2	1 (0.87%)	1 (0.41%)	1.09	0.537	0.686	Co-occurrence
MANSC4	12p11.22	1 (0.87%)	1 (0.41%)	1.09	0.537	0.686	Co-occurrence
MAP1B	5q13.2	1 (0.87%)	1 (0.41%)	1.09	0.537	0.686	Co-occurrence
MAP4K1	19q13.2	1 (0.87%)	1 (0.41%)	1.09	0.537	0.686	Co-occurrence
MAPK3	16p11.2	1 (0.87%)	1 (0.41%)	1.09	0.537	0.686	Co-occurrence
MASP2	1p36.22	1 (0.87%)	1 (0.41%)	1.09	0.537	0.686	Co-occurrence
MAT2A	2p11.2	1 (0.87%)	1 (0.41%)	1.09	0.537	0.686	Co-occurrence
MATN4	20q13.12	1 (0.87%)	1 (0.41%)	1.09	0.537	0.686	Co-occurrence
MAZ	16p11.2	1 (0.87%)	1 (0.41%)	1.09	0.537	0.686	Co-occurrence
MBL1P	10q22.3	1 (0.87%)	1 (0.41%)	1.09	0.537	0.686	Co-occurrence
MBLAC2	5q14.3	1 (0.87%)	1 (0.41%)	1.09	0.537	0.686	Co-occurrence
MCCC2	5q13.2	1 (0.87%)	1 (0.41%)	1.09	0.537	0.686	Co-occurrence
MCHR1	22q13.2	1 (0.87%)	1 (0.41%)	1.09	0.537	0.686	Co-occurrence
MCM2	3q21.3	1 (0.87%)	1 (0.41%)	1.09	0.537	0.686	Co-occurrence
MDS2	1p36.11	1 (0.87%)	1 (0.41%)	1.09	0.537	0.686	Co-occurrence
MED15	22q11.21	1 (0.87%)	1 (0.41%)	1.09	0.537	0.686	Co-occurrence
MEIS3	19q13.32	1 (0.87%)	1 (0.41%)	1.09	0.537	0.686	Co-occurrence
MEOX2	7p21.2	1 (0.87%)	1 (0.41%)	1.09	0.537	0.686	Co-occurrence
METTL6	3p25.1	1 (0.87%)	1 (0.41%)	1.09	0.537	0.686	Co-occurrence
MFN2	1p36.22	1 (0.87%)	1 (0.41%)	1.09	0.537	0.686	Co-occurrence
MFRP	11q23.3	1 (0.87%)	1 (0.41%)	1.09	0.537	0.686	Co-occurrence
MFSD13A	10q24.32	1 (0.87%)	1 (0.41%)	1.09	0.537	0.686	Co-occurrence

MGAT2	14q21.3	1 (0.87%)	1 (0.41%)	1.09	0.537	0.686	Co-occurrence
MGLL	3q21.3	1 (0.87%)	1 (0.41%)	1.09	0.537	0.686	Co-occurrence
MIB1	18q11.2	1 (0.87%)	1 (0.41%)	1.09	0.537	0.686	Co-occurrence
MID2	Xq22.3	1 (0.87%)	1 (0.41%)	1.09	0.537	0.686	Co-occurrence
MIIP	1p36.22	1 (0.87%)	1 (0.41%)	1.09	0.537	0.686	Co-occurrence
MIR-1200/1200		1 (0.87%)	1 (0.41%)	1.09	0.537	0.686	Co-occurrence
MIR-1273D/1273D		1 (0.87%)	1 (0.41%)	1.09	0.537	0.686	Co-occurrence
MIR-1286/1286		1 (0.87%)	1 (0.41%)	1.09	0.537	0.686	Co-occurrence
MIR-1587/1587		1 (0.87%)	1 (0.41%)	1.09	0.537	0.686	Co-occurrence
MIR-197/197		1 (0.87%)	1 (0.41%)	1.09	0.537	0.686	Co-occurrence
MIR-3134/3134		1 (0.87%)	1 (0.41%)	1.09	0.537	0.686	Co-occurrence
MIR-3618/3618		1 (0.87%)	1 (0.41%)	1.09	0.537	0.686	Co-occurrence
MIR-3911/3911		1 (0.87%)	1 (0.41%)	1.09	0.537	0.686	Co-occurrence
MIR-3937/3937		1 (0.87%)	1 (0.41%)	1.09	0.537	0.686	Co-occurrence
MIR-4270/4270		1 (0.87%)	1 (0.41%)	1.09	0.537	0.686	Co-occurrence
MIR-4276/4276		1 (0.87%)	1 (0.41%)	1.09	0.537	0.686	Co-occurrence
MIR-4307/4307		1 (0.87%)	1 (0.41%)	1.09	0.537	0.686	Co-occurrence
MIR-4442/4442		1 (0.87%)	1 (0.41%)	1.09	0.537	0.686	Co-occurrence
MIR-4450/4450		1 (0.87%)	1 (0.41%)	1.09	0.537	0.686	Co-occurrence
MIR-4497/4497		1 (0.87%)	1 (0.41%)	1.09	0.537	0.686	Co-occurrence
MIR-4518/4518		1 (0.87%)	1 (0.41%)	1.09	0.537	0.686	Co-occurrence
MIR-4686/4686		1 (0.87%)	1 (0.41%)	1.09	0.537	0.686	Co-occurrence
MIR-4744/4744		1 (0.87%)	1 (0.41%)	1.09	0.537	0.686	Co-occurrence
MIR-4767/4767		1 (0.87%)	1 (0.41%)	1.09	0.537	0.686	Co-occurrence
MIR-4803/4803		1 (0.87%)	1 (0.41%)	1.09	0.537	0.686	Co-occurrence
MIR-5096/5096		1 (0.87%)	1 (0.41%)	1.09	0.537	0.686	Co-occurrence
MIR-544B/544B		1 (0.87%)	1 (0.41%)	1.09	0.537	0.686	Co-occurrence
MIR-548N/548N		1 (0.87%)	1 (0.41%)	1.09	0.537	0.686	Co-occurrence
MIR-548Y/548Y		1 (0.87%)	1 (0.41%)	1.09	0.537	0.686	Co-occurrence

MIR-563/563		1 (0.87%)	1 (0.41%)	1.09	0.537	0.686	Co-occurrence
MIR-5687/5687		1 (0.87%)	1 (0.41%)	1.09	0.537	0.686	Co-occurrence
MIR-5697/5697		1 (0.87%)	1 (0.41%)	1.09	0.537	0.686	Co-occurrence
MIR-606/606		1 (0.87%)	1 (0.41%)	1.09	0.537	0.686	Co-occurrence
MIR-648/648		1 (0.87%)	1 (0.41%)	1.09	0.537	0.686	Co-occurrence
MIR-649/649		1 (0.87%)	1 (0.41%)	1.09	0.537	0.686	Co-occurrence
MIR137HG	1p21.3	1 (0.87%)	1 (0.41%)	1.09	0.537	0.686	Co-occurrence
MIR22HG	17p13.3	1 (0.87%)	1 (0.41%)	1.09	0.537	0.686	Co-occurrence
MLPH	2q37.3	1 (0.87%)	1 (0.41%)	1.09	0.537	0.686	Co-occurrence
MLXIP	12q24.31	1 (0.87%)	1 (0.41%)	1.09	0.537	0.686	Co-occurrence
MMP14	14q11.2	1 (0.87%)	1 (0.41%)	1.09	0.537	0.686	Co-occurrence
MND1	4q31.3	1 (0.87%)	1 (0.41%)	1.09	0.537	0.686	Co-occurrence
MPLKIP	7p14.1	1 (0.87%)	1 (0.41%)	1.09	0.537	0.686	Co-occurrence
MPPE1	18p11.21	1 (0.87%)	1 (0.41%)	1.09	0.537	0.686	Co-occurrence
MRGPRE	11p15.4	1 (0.87%)	1 (0.41%)	1.09	0.537	0.686	Co-occurrence
MRGPRG	11p15.4	1 (0.87%)	1 (0.41%)	1.09	0.537	0.686	Co-occurrence
MROH2A	2q37.1	1 (0.87%)	1 (0.41%)	1.09	0.537	0.686	Co-occurrence
MROH7	1p32.3	1 (0.87%)	1 (0.41%)	1.09	0.537	0.686	Co-occurrence
MRPL1	4q21.1	1 (0.87%)	1 (0.41%)	1.09	0.537	0.686	Co-occurrence
MRPL37	1p32.3	1 (0.87%)	1 (0.41%)	1.09	0.537	0.686	Co-occurrence
MRPL40	22q11.21	1 (0.87%)	1 (0.41%)	1.09	0.537	0.686	Co-occurrence
MRPL52	14q11.2	1 (0.87%)	1 (0.41%)	1.09	0.537	0.686	Co-occurrence
MRPS16	10q22.2	1 (0.87%)	1 (0.41%)	1.09	0.537	0.686	Co-occurrence
MRPS25	3p25.1	1 (0.87%)	1 (0.41%)	1.09	0.537	0.686	Co-occurrence
MRPS27	5q13.2	1 (0.87%)	1 (0.41%)	1.09	0.537	0.686	Co-occurrence
MRPS35	12p11.22	1 (0.87%)	1 (0.41%)	1.09	0.537	0.686	Co-occurrence
MRPS36	5q13.2	1 (0.87%)	1 (0.41%)	1.09	0.537	0.686	Co-occurrence
MSS51	10q22.2	1 (0.87%)	1 (0.41%)	1.09	0.537	0.686	Co-occurrence
MST1	3p21.31	1 (0.87%)	1 (0.41%)	1.09	0.537	0.686	Co-occurrence

MTHFD2P1	3q11.2	1 (0.87%)	1 (0.41%)	1.09	0.537	0.686	Co-occurrence
MTHFR	1p36.22	1 (0.87%)	1 (0.41%)	1.09	0.537	0.686	Co-occurrence
MTMR3	22q12.2	1 (0.87%)	1 (0.41%)	1.09	0.537	0.686	Co-occurrence
MTRNR2L12	3q11.2	1 (0.87%)	1 (0.41%)	1.09	0.537	0.686	Co-occurrence
MUC2	11p15.5	1 (0.87%)	1 (0.41%)	1.09	0.537	0.686	Co-occurrence
MUC5AC	11p15.5	1 (0.87%)	1 (0.41%)	1.09	0.537	0.686	Co-occurrence
MUC5B	11p15.5	1 (0.87%)	1 (0.41%)	1.09	0.537	0.686	Co-occurrence
MUC6	11p15.5	1 (0.87%)	1 (0.41%)	1.09	0.537	0.686	Co-occurrence
MUSTN1	3p21.1	1 (0.87%)	1 (0.41%)	1.09	0.537	0.686	Co-occurrence
MVB12B	9q33.3	1 (0.87%)	1 (0.41%)	1.09	0.537	0.686	Co-occurrence
MVK	12q24.11	1 (0.87%)	1 (0.41%)	1.09	0.537	0.686	Co-occurrence
MVP	16p11.2	1 (0.87%)	1 (0.41%)	1.09	0.537	0.686	Co-occurrence
MYCBP	1p34.3	1 (0.87%)	1 (0.41%)	1.09	0.537	0.686	Co-occurrence
MYL9	20q11.23	1 (0.87%)	1 (0.41%)	1.09	0.537	0.686	Co-occurrence
MYLPF	16p11.2	1 (0.87%)	1 (0.41%)	1.09	0.537	0.686	Co-occurrence
MYO1C	17p13.3	1 (0.87%)	1 (0.41%)	1.09	0.537	0.686	Co-occurrence
MYO1H	12q24.11	1 (0.87%)	1 (0.41%)	1.09	0.537	0.686	Co-occurrence
MYOD1	11p15.1	1 (0.87%)	1 (0.41%)	1.09	0.537	0.686	Co-occurrence
MYSM1	1p32.1	1 (0.87%)	1 (0.41%)	1.09	0.537	0.686	Co-occurrence
NAP1L4	11p15.4	1 (0.87%)	1 (0.41%)	1.09	0.537	0.686	Co-occurrence
NAPA	19q13.32-q13.33	1 (0.87%)	1 (0.41%)	1.09	0.537	0.686	Co-occurrence
NCCRP1	19q13.2	1 (0.87%)	1 (0.41%)	1.09	0.537	0.686	Co-occurrence
NCR3LG1	11p15.1	1 (0.87%)	1 (0.41%)	1.09	0.537	0.686	Co-occurrence
NDUFA4	7p21.3	1 (0.87%)	1 (0.41%)	1.09	0.537	0.686	Co-occurrence
NDUFAF2	5q12.1	1 (0.87%)	1 (0.41%)	1.09	0.537	0.686	Co-occurrence
NDUFAF3	3p21.31	1 (0.87%)	1 (0.41%)	1.09	0.537	0.686	Co-occurrence
NDUFS4	5q11.2	1 (0.87%)	1 (0.41%)	1.09	0.537	0.686	Co-occurrence
NDUFS5	1p34.3	1 (0.87%)	1 (0.41%)	1.09	0.537	0.686	Co-occurrence
NECAP2	1p36.13	1 (0.87%)	1 (0.41%)	1.09	0.537	0.686	Co-occurrence

NEK3	13q14.3	1 (0.87%)	1 (0.41%)	1.09	0.537	0.686	Co-occurrence
NEK4	3p21.1	1 (0.87%)	1 (0.41%)	1.09	0.537	0.686	Co-occurrence
NEK5	13q14.3	1 (0.87%)	1 (0.41%)	1.09	0.537	0.686	Co-occurrence
NEK9	14q24.3	1 (0.87%)	1 (0.41%)	1.09	0.537	0.686	Co-occurrence
NEUROD4	12q13.2	1 (0.87%)	1 (0.41%)	1.09	0.537	0.686	Co-occurrence
NEUROG2	4q25	1 (0.87%)	1 (0.41%)	1.09	0.537	0.686	Co-occurrence
NEUROG3	10q22.1	1 (0.87%)	1 (0.41%)	1.09	0.537	0.686	Co-occurrence
NFIC	19p13.3	1 (0.87%)	1 (0.41%)	1.09	0.537	0.686	Co-occurrence
NFKB2	10q24.32	1 (0.87%)	1 (0.41%)	1.09	0.537	0.686	Co-occurrence
NFX1	9p13.3	1 (0.87%)	1 (0.41%)	1.09	0.537	0.686	Co-occurrence
NGDN	14q11.2	1 (0.87%)	1 (0.41%)	1.09	0.537	0.686	Co-occurrence
NGLY1	3p24.2	1 (0.87%)	1 (0.41%)	1.09	0.537	0.686	Co-occurrence
NIBAN2	9q34.11	1 (0.87%)	1 (0.41%)	1.09	0.537	0.686	Co-occurrence
NINJ2	12p13.33	1 (0.87%)	1 (0.41%)	1.09	0.537	0.686	Co-occurrence
NLGN4X	Xp22.32-p22.31	1 (0.87%)	1 (0.41%)	1.09	0.537	0.686	Co-occurrence
NME8	7p14.1	1 (0.87%)	1 (0.41%)	1.09	0.537	0.686	Co-occurrence
NMNAT1	1p36.22	1 (0.87%)	1 (0.41%)	1.09	0.537	0.686	Co-occurrence
NOL4	18q12.1	1 (0.87%)	1 (0.41%)	1.09	0.537	0.686	Co-occurrence
NOLC1	10q24.32	1 (0.87%)	1 (0.41%)	1.09	0.537	0.686	Co-occurrence
NOVA1	14q12	1 (0.87%)	1 (0.41%)	1.09	0.537	0.686	Co-occurrence
NPAT	11q22.3	1 (0.87%)	1 (0.41%)	1.09	0.537	0.686	Co-occurrence
NPIPB1P	18p11.21	1 (0.87%)	1 (0.41%)	1.09	0.537	0.686	Co-occurrence
NPPA	1p36.22	1 (0.87%)	1 (0.41%)	1.09	0.537	0.686	Co-occurrence
NPSR1	7p14.3	1 (0.87%)	1 (0.41%)	1.09	0.537	0.686	Co-occurrence
NR2C2	3p25.1	1 (0.87%)	1 (0.41%)	1.09	0.537	0.686	Co-occurrence
NRAP	10q25.3	1 (0.87%)	1 (0.41%)	1.09	0.537	0.686	Co-occurrence
NSA2	5q13.3	1 (0.87%)	1 (0.41%)	1.09	0.537	0.686	Co-occurrence
NUBPL	14q12	1 (0.87%)	1 (0.41%)	1.09	0.537	0.686	Co-occurrence
NUCB2	11p15.1	1 (0.87%)	1 (0.41%)	1.09	0.537	0.686	Co-occurrence

NUP98	11p15.4	1 (0.87%)	1 (0.41%)	1.09	0.537	0.686	Co-occurrence
NUTM2B	10q22.3	1 (0.87%)	1 (0.41%)	1.09	0.537	0.686	Co-occurrence
NUTM2E	10q22.3	1 (0.87%)	1 (0.41%)	1.09	0.537	0.686	Co-occurrence
OCSTAMP	20q13.12	1 (0.87%)	1 (0.41%)	1.09	0.537	0.686	Co-occurrence
ODF2	9q34.11	1 (0.87%)	1 (0.41%)	1.09	0.537	0.686	Co-occurrence
OPALIN	10q24.1	1 (0.87%)	1 (0.41%)	1.09	0.537	0.686	Co-occurrence
OR10AG1	11q12.1	1 (0.87%)	1 (0.41%)	1.09	0.537	0.686	Co-occurrence
OR10G2	14q11.2	1 (0.87%)	1 (0.41%)	1.09	0.537	0.686	Co-occurrence
OR10G3	14q11.2	1 (0.87%)	1 (0.41%)	1.09	0.537	0.686	Co-occurrence
OR2K2	9q31.3	1 (0.87%)	1 (0.41%)	1.09	0.537	0.686	Co-occurrence
OR4A15	11q11	1 (0.87%)	1 (0.41%)	1.09	0.537	0.686	Co-occurrence
OR4A16	11q11	1 (0.87%)	1 (0.41%)	1.09	0.537	0.686	Co-occurrence
OR4A5	11q11	1 (0.87%)	1 (0.41%)	1.09	0.537	0.686	Co-occurrence
OR4C11	11q11	1 (0.87%)	1 (0.41%)	1.09	0.537	0.686	Co-occurrence
OR4C15	11q11	1 (0.87%)	1 (0.41%)	1.09	0.537	0.686	Co-occurrence
OR4C16	11q11	1 (0.87%)	1 (0.41%)	1.09	0.537	0.686	Co-occurrence
OR4C46	11q11	1 (0.87%)	1 (0.41%)	1.09	0.537	0.686	Co-occurrence
OR4C6	11q11	1 (0.87%)	1 (0.41%)	1.09	0.537	0.686	Co-occurrence
OR4E2	14q11.2	1 (0.87%)	1 (0.41%)	1.09	0.537	0.686	Co-occurrence
OR4P4	11q11	1 (0.87%)	1 (0.41%)	1.09	0.537	0.686	Co-occurrence
OR4S2	11q11	1 (0.87%)	1 (0.41%)	1.09	0.537	0.686	Co-occurrence
OR52B4	11p15.4	1 (0.87%)	1 (0.41%)	1.09	0.537	0.686	Co-occurrence
OR52K2	11p15.4	1 (0.87%)	1 (0.41%)	1.09	0.537	0.686	Co-occurrence
OR5AS1	11q12.1	1 (0.87%)	1 (0.41%)	1.09	0.537	0.686	Co-occurrence
OR5D13	11q11	1 (0.87%)	1 (0.41%)	1.09	0.537	0.686	Co-occurrence
OR5D14	11q11	1 (0.87%)	1 (0.41%)	1.09	0.537	0.686	Co-occurrence
OR5D16	11q12.1	1 (0.87%)	1 (0.41%)	1.09	0.537	0.686	Co-occurrence
OR5D18	11q12.1	1 (0.87%)	1 (0.41%)	1.09	0.537	0.686	Co-occurrence
OR5F1	11q12.1	1 (0.87%)	1 (0.41%)	1.09	0.537	0.686	Co-occurrence

OR5I1	11q12.1	1 (0.87%)	1 (0.41%)	1.09	0.537	0.686	Co-occurrence
OR5J2	11q12.1	1 (0.87%)	1 (0.41%)	1.09	0.537	0.686	Co-occurrence
OR5L1	11q12.1	1 (0.87%)	1 (0.41%)	1.09	0.537	0.686	Co-occurrence
OR5L2	11q12.1	1 (0.87%)	1 (0.41%)	1.09	0.537	0.686	Co-occurrence
OR5M10	11q12.1	1 (0.87%)	1 (0.41%)	1.09	0.537	0.686	Co-occurrence
OR5M11	11q12.1	1 (0.87%)	1 (0.41%)	1.09	0.537	0.686	Co-occurrence
OR5T2	11q12.1	1 (0.87%)	1 (0.41%)	1.09	0.537	0.686	Co-occurrence
OR5W2	11q12.1	1 (0.87%)	1 (0.41%)	1.09	0.537	0.686	Co-occurrence
OR6J1	14q11.2	1 (0.87%)	1 (0.41%)	1.09	0.537	0.686	Co-occurrence
OR8H2	11q12.1	1 (0.87%)	1 (0.41%)	1.09	0.537	0.686	Co-occurrence
OR8H3	11q12.1	1 (0.87%)	1 (0.41%)	1.09	0.537	0.686	Co-occurrence
OR8I2	11q12.1	1 (0.87%)	1 (0.41%)	1.09	0.537	0.686	Co-occurrence
OR8J3	11q12.1	1 (0.87%)	1 (0.41%)	1.09	0.537	0.686	Co-occurrence
OR8K5	11q12.1	1 (0.87%)	1 (0.41%)	1.09	0.537	0.686	Co-occurrence
OSBPL5	11p15.4	1 (0.87%)	1 (0.41%)	1.09	0.537	0.686	Co-occurrence
OTOG	11p15.1	1 (0.87%)	1 (0.41%)	1.09	0.537	0.686	Co-occurrence
OVGP1	1p13.2	1 (0.87%)	1 (0.41%)	1.09	0.537	0.686	Co-occurrence
OXNAD1	3p25.1-p24.3	1 (0.87%)	1 (0.41%)	1.09	0.537	0.686	Co-occurrence
OXSM	3p24.2	1 (0.87%)	1 (0.41%)	1.09	0.537	0.686	Co-occurrence
OXTR	3p25.3	1 (0.87%)	1 (0.41%)	1.09	0.537	0.686	Co-occurrence
P2RX6	22q11.21	1 (0.87%)	1 (0.41%)	1.09	0.537	0.686	Co-occurrence
P2RX6P	22q11.21	1 (0.87%)	1 (0.41%)	1.09	0.537	0.686	Co-occurrence
P2RY2	11q13.4	1 (0.87%)	1 (0.41%)	1.09	0.537	0.686	Co-occurrence
P4HTM	3p21.31|3p21.3	1 (0.87%)	1 (0.41%)	1.09	0.537	0.686	Co-occurrence
PAGR1	16p11.2	1 (0.87%)	1 (0.41%)	1.09	0.537	0.686	Co-occurrence
PAK4	19q13.2	1 (0.87%)	1 (0.41%)	1.09	0.537	0.686	Co-occurrence
PAQR3	4q21.21	1 (0.87%)	1 (0.41%)	1.09	0.537	0.686	Co-occurrence
PARP3	3p21.2	1 (0.87%)	1 (0.41%)	1.09	0.537	0.686	Co-occurrence
PART1	5q12.1	1 (0.87%)	1 (0.41%)	1.09	0.537	0.686	Co-occurrence

PAX8	2q14.1	1 (0.87%)	1 (0.41%)	1.09	0.537	0.686	Co-occurrence
PCBP4	3p21.2	1 (0.87%)	1 (0.41%)	1.09	0.537	0.686	Co-occurrence
PDPN	1p36.21	1 (0.87%)	1 (0.41%)	1.09	0.537	0.686	Co-occurrence
PDZRN3	3p13	1 (0.87%)	1 (0.41%)	1.09	0.537	0.686	Co-occurrence
PEX26	22q11.21	1 (0.87%)	1 (0.41%)	1.09	0.537	0.686	Co-occurrence
PGAP2	11p15.4	1 (0.87%)	1 (0.41%)	1.09	0.537	0.686	Co-occurrence
PGCP1	1p13.2	1 (0.87%)	1 (0.41%)	1.09	0.537	0.686	Co-occurrence
PGD	1p36.22	1 (0.87%)	1 (0.41%)	1.09	0.537	0.686	Co-occurrence
PHF14	7p21.3	1 (0.87%)	1 (0.41%)	1.09	0.537	0.686	Co-occurrence
PHF5A	22q13.2	1 (0.87%)	1 (0.41%)	1.09	0.537	0.686	Co-occurrence
PHLDA2	11p15.4	1 (0.87%)	1 (0.41%)	1.09	0.537	0.686	Co-occurrence
PHLDB3	19q13.31	1 (0.87%)	1 (0.41%)	1.09	0.537	0.686	Co-occurrence
PHYHIP	8p21.3	1 (0.87%)	1 (0.41%)	1.09	0.537	0.686	Co-occurrence
PI3	20q13.12	1 (0.87%)	1 (0.41%)	1.09	0.537	0.686	Co-occurrence
PI4KA	22q11.21	1 (0.87%)	1 (0.41%)	1.09	0.537	0.686	Co-occurrence
PI4KAP1	22q11.21	1 (0.87%)	1 (0.41%)	1.09	0.537	0.686	Co-occurrence
PI4KAP2	22q11.21	1 (0.87%)	1 (0.41%)	1.09	0.537	0.686	Co-occurrence
PICALM	11q14.2	1 (0.87%)	1 (0.41%)	1.09	0.537	0.686	Co-occurrence
PID1	2q36.3	1 (0.87%)	1 (0.41%)	1.09	0.537	0.686	Co-occurrence
PIFO	1p13.2	1 (0.87%)	1 (0.41%)	1.09	0.537	0.686	Co-occurrence
PIGB	15q21.3	1 (0.87%)	1 (0.41%)	1.09	0.537	0.686	Co-occurrence
PIK3AP1	10q24.1	1 (0.87%)	1 (0.41%)	1.09	0.537	0.686	Co-occurrence
PIK3C2A	11p15.1	1 (0.87%)	1 (0.41%)	1.09	0.537	0.686	Co-occurrence
PIK3CD	1p36.22	1 (0.87%)	1 (0.41%)	1.09	0.537	0.686	Co-occurrence
PIK3CD-AS1	1p36.22	1 (0.87%)	1 (0.41%)	1.09	0.537	0.686	Co-occurrence
PINLYP	19q13.31	1 (0.87%)	1 (0.41%)	1.09	0.537	0.686	Co-occurrence
PITPNA	17p13.3	1 (0.87%)	1 (0.41%)	1.09	0.537	0.686	Co-occurrence
PITX3	10q24.32	1 (0.87%)	1 (0.41%)	1.09	0.537	0.686	Co-occurrence
PLA2G4C	19q13.33	1 (0.87%)	1 (0.41%)	1.09	0.537	0.686	Co-occurrence

PLAC9	10q22.3	1 (0.87%)	1 (0.41%)	1.09	0.537	0.686	Co-occurrence
PLAUR	19q13.31	1 (0.87%)	1 (0.41%)	1.09	0.537	0.686	Co-occurrence
PLCG1	20q12	1 (0.87%)	1 (0.41%)	1.09	0.537	0.686	Co-occurrence
PLEKHM2	1p36.21	1 (0.87%)	1 (0.41%)	1.09	0.537	0.686	Co-occurrence
PLIN3	19p13.3	1 (0.87%)	1 (0.41%)	1.09	0.537	0.686	Co-occurrence
PLOD1	1p36.22	1 (0.87%)	1 (0.41%)	1.09	0.537	0.686	Co-occurrence
PLPPR1	9q31.1	1 (0.87%)	1 (0.41%)	1.09	0.537	0.686	Co-occurrence
PLXNA1	3q21.3	1 (0.87%)	1 (0.41%)	1.09	0.537	0.686	Co-occurrence
PMCHL2	5q13.2	1 (0.87%)	1 (0.41%)	1.09	0.537	0.686	Co-occurrence
PMM1	22q13.2	1 (0.87%)	1 (0.41%)	1.09	0.537	0.686	Co-occurrence
PNPLA4	Xp22.31	1 (0.87%)	1 (0.41%)	1.09	0.537	0.686	Co-occurrence
POC5	5q13.3	1 (0.87%)	1 (0.41%)	1.09	0.537	0.686	Co-occurrence
PODXL2	3q21.3	1 (0.87%)	1 (0.41%)	1.09	0.537	0.686	Co-occurrence
POLE2	14q21.3	1 (0.87%)	1 (0.41%)	1.09	0.537	0.686	Co-occurrence
POLK	5q13.3	1 (0.87%)	1 (0.41%)	1.09	0.537	0.686	Co-occurrence
POLR3A	10q22.3	1 (0.87%)	1 (0.41%)	1.09	0.537	0.686	Co-occurrence
POLR3G	5q14.3	1 (0.87%)	1 (0.41%)	1.09	0.537	0.686	Co-occurrence
POM121L4P	22q11.21	1 (0.87%)	1 (0.41%)	1.09	0.537	0.686	Co-occurrence
POM121L7P	22q11.21	1 (0.87%)	1 (0.41%)	1.09	0.537	0.686	Co-occurrence
POMGNT2	3p22.1	1 (0.87%)	1 (0.41%)	1.09	0.537	0.686	Co-occurrence
POTEC	18p11.21	1 (0.87%)	1 (0.41%)	1.09	0.537	0.686	Co-occurrence
PPIL2	22q11.21	1 (0.87%)	1 (0.41%)	1.09	0.537	0.686	Co-occurrence
PPP1CC	12q24.11	1 (0.87%)	1 (0.41%)	1.09	0.537	0.686	Co-occurrence
PPP1R14C	6q25.1	1 (0.87%)	1 (0.41%)	1.09	0.537	0.686	Co-occurrence
PPP1R16B	20q11.23	1 (0.87%)	1 (0.41%)	1.09	0.537	0.686	Co-occurrence
PPP4C	16p11.2	1 (0.87%)	1 (0.41%)	1.09	0.537	0.686	Co-occurrence
PPRC1	10q24.32	1 (0.87%)	1 (0.41%)	1.09	0.537	0.686	Co-occurrence
PRAMEF1	1p36.21	1 (0.87%)	1 (0.41%)	1.09	0.537	0.686	Co-occurrence
PRAMEF10	1p36.21	1 (0.87%)	1 (0.41%)	1.09	0.537	0.686	Co-occurrence

PRAMEF11	1p36.21	1 (0.87%)	1 (0.41%)	1.09	0.537	0.686	Co-occurrence
PRAMEF12	1p36.21	1 (0.87%)	1 (0.41%)	1.09	0.537	0.686	Co-occurrence
PRAMEF13	1p36.21	1 (0.87%)	1 (0.41%)	1.09	0.537	0.686	Co-occurrence
PRAMEF14	1p36.21	1 (0.87%)	1 (0.41%)	1.09	0.537	0.686	Co-occurrence
PRAMEF15	1p36.21	1 (0.87%)	1 (0.41%)	1.09	0.537	0.686	Co-occurrence
PRAMEF16	1p36.21	1 (0.87%)	1 (0.41%)	1.09	0.537	0.686	Co-occurrence
PRAMEF17	1p36.21	1 (0.87%)	1 (0.41%)	1.09	0.537	0.686	Co-occurrence
PRAMEF18	1p36.21	1 (0.87%)	1 (0.41%)	1.09	0.537	0.686	Co-occurrence
PRAMEF19	1p36.21	1 (0.87%)	1 (0.41%)	1.09	0.537	0.686	Co-occurrence
PRAMEF2	1p36.21	1 (0.87%)	1 (0.41%)	1.09	0.537	0.686	Co-occurrence
PRAMEF20	1p36.21	1 (0.87%)	1 (0.41%)	1.09	0.537	0.686	Co-occurrence
PRAMEF22	1p36.21	1 (0.87%)	1 (0.41%)	1.09	0.537	0.686	Co-occurrence
PRAMEF4	1p36.21	1 (0.87%)	1 (0.41%)	1.09	0.537	0.686	Co-occurrence
PRAMEF5	1p36.21	1 (0.87%)	1 (0.41%)	1.09	0.537	0.686	Co-occurrence
PRAMEF6	1p36.21	1 (0.87%)	1 (0.41%)	1.09	0.537	0.686	Co-occurrence
PRAMEF7	1p36.21	1 (0.87%)	1 (0.41%)	1.09	0.537	0.686	Co-occurrence
PRAMEF8	1p36.21	1 (0.87%)	1 (0.41%)	1.09	0.537	0.686	Co-occurrence
PRAMEF9	1p36.21	1 (0.87%)	1 (0.41%)	1.09	0.537	0.686	Co-occurrence
PRDM2	1p36.21	1 (0.87%)	1 (0.41%)	1.09	0.537	0.686	Co-occurrence
PRELID3A	18p11.21	1 (0.87%)	1 (0.41%)	1.09	0.537	0.686	Co-occurrence
PRKAR2A	3p21.31	1 (0.87%)	1 (0.41%)	1.09	0.537	0.686	Co-occurrence
PRKG2	4q21.21	1 (0.87%)	1 (0.41%)	1.09	0.537	0.686	Co-occurrence
PRLH	2q37.3	1 (0.87%)	1 (0.41%)	1.09	0.537	0.686	Co-occurrence
PRMT3	11p15.1	1 (0.87%)	1 (0.41%)	1.09	0.537	0.686	Co-occurrence
PROCR	20q11.22	1 (0.87%)	1 (0.41%)	1.09	0.537	0.686	Co-occurrence
PRODH	22q11.21	1 (0.87%)	1 (0.41%)	1.09	0.537	0.686	Co-occurrence
PROK1	1p13.3	1 (0.87%)	1 (0.41%)	1.09	0.537	0.686	Co-occurrence
PRPF8	17p13.3	1 (0.87%)	1 (0.41%)	1.09	0.537	0.686	Co-occurrence
PRPSAP2	17p11.2	1 (0.87%)	1 (0.41%)	1.09	0.537	0.686	Co-occurrence

PRR14L	22q12.2	1 (0.87%)	1 (0.41%)	1.09	0.537	0.686	Co-occurrence
PRR33	11p15.5	1 (0.87%)	1 (0.41%)	1.09	0.537	0.686	Co-occurrence
PRRG4	11p13	1 (0.87%)	1 (0.41%)	1.09	0.537	0.686	Co-occurrence
PRRT2	16p11.2	1 (0.87%)	1 (0.41%)	1.09	0.537	0.686	Co-occurrence
PRSS3	9p13.3	1 (0.87%)	1 (0.41%)	1.09	0.537	0.686	Co-occurrence
PRXL2A	10q23.1	1 (0.87%)	1 (0.41%)	1.09	0.537	0.686	Co-occurrence
PSD	10q24.32	1 (0.87%)	1 (0.41%)	1.09	0.537	0.686	Co-occurrence
PSG1	19q13.2	1 (0.87%)	1 (0.41%)	1.09	0.537	0.686	Co-occurrence
PSG10P	19q13.2	1 (0.87%)	1 (0.41%)	1.09	0.537	0.686	Co-occurrence
PSG11	19q13.31	1 (0.87%)	1 (0.41%)	1.09	0.537	0.686	Co-occurrence
PSG2	19q13.31	1 (0.87%)	1 (0.41%)	1.09	0.537	0.686	Co-occurrence
PSG3	19q13.2	1 (0.87%)	1 (0.41%)	1.09	0.537	0.686	Co-occurrence
PSG4	19q13.31	1 (0.87%)	1 (0.41%)	1.09	0.537	0.686	Co-occurrence
PSG5	19q13.31	1 (0.87%)	1 (0.41%)	1.09	0.537	0.686	Co-occurrence
PSG6	19q13.31	1 (0.87%)	1 (0.41%)	1.09	0.537	0.686	Co-occurrence
PSG7	19q13.31	1 (0.87%)	1 (0.41%)	1.09	0.537	0.686	Co-occurrence
PSG8	19q13.2	1 (0.87%)	1 (0.41%)	1.09	0.537	0.686	Co-occurrence
PSG9	19q13.31	1 (0.87%)	1 (0.41%)	1.09	0.537	0.686	Co-occurrence
PSMA5	1p13.3	1 (0.87%)	1 (0.41%)	1.09	0.537	0.686	Co-occurrence
PSMB11	14q11.2	1 (0.87%)	1 (0.41%)	1.09	0.537	0.686	Co-occurrence
PSMB5	14q11.2	1 (0.87%)	1 (0.41%)	1.09	0.537	0.686	Co-occurrence
PSMD9	12q24.31	1 (0.87%)	1 (0.41%)	1.09	0.537	0.686	Co-occurrence
PTCD2	5q13.2	1 (0.87%)	1 (0.41%)	1.09	0.537	0.686	Co-occurrence
PTGR1	9q31.3	1 (0.87%)	1 (0.41%)	1.09	0.537	0.686	Co-occurrence
PTHLH	12p11.22	1 (0.87%)	1 (0.41%)	1.09	0.537	0.686	Co-occurrence
PTPN11	12q24.13	1 (0.87%)	1 (0.41%)	1.09	0.537	0.686	Co-occurrence
PTPRD	9p24.1-p23	1 (0.87%)	1 (0.41%)	1.09	0.537	0.686	Co-occurrence
PTPRT	20q12-q13.11	1 (0.87%)	1 (0.41%)	1.09	0.537	0.686	Co-occurrence
PTTG1IP	21q22.3	1 (0.87%)	1 (0.41%)	1.09	0.537	0.686	Co-occurrence

PUDP	Xp22.31	1 (0.87%)	1 (0.41%)	1.09	0.537	0.686	Co-occurrence
QPRT	16p11.2	1 (0.87%)	1 (0.41%)	1.09	0.537	0.686	Co-occurrence
QRICH1	3p21.31	1 (0.87%)	1 (0.41%)	1.09	0.537	0.686	Co-occurrence
QSER1	11p13	1 (0.87%)	1 (0.41%)	1.09	0.537	0.686	Co-occurrence
R3HDML	20q13.12	1 (0.87%)	1 (0.41%)	1.09	0.537	0.686	Co-occurrence
RAB17	2q37.3	1 (0.87%)	1 (0.41%)	1.09	0.537	0.686	Co-occurrence
RAB27A	15q21.3	1 (0.87%)	1 (0.41%)	1.09	0.537	0.686	Co-occurrence
RAB5IF	20q11.23	1 (0.87%)	1 (0.41%)	1.09	0.537	0.686	Co-occurrence
RAD18	3p25.3	1 (0.87%)	1 (0.41%)	1.09	0.537	0.686	Co-occurrence
RAD9B	12q24.11	1 (0.87%)	1 (0.41%)	1.09	0.537	0.686	Co-occurrence
RADX	Xq22.3	1 (0.87%)	1 (0.41%)	1.09	0.537	0.686	Co-occurrence
RAI1	17p11.2	1 (0.87%)	1 (0.41%)	1.09	0.537	0.686	Co-occurrence
RALA	7p14.1	1 (0.87%)	1 (0.41%)	1.09	0.537	0.686	Co-occurrence
RAMP1	2q37.3	1 (0.87%)	1 (0.41%)	1.09	0.537	0.686	Co-occurrence
RANBP1	22q11.21	1 (0.87%)	1 (0.41%)	1.09	0.537	0.686	Co-occurrence
RANGAP1	22q13.2	1 (0.87%)	1 (0.41%)	1.09	0.537	0.686	Co-occurrence
RARB	3p24.2	1 (0.87%)	1 (0.41%)	1.09	0.537	0.686	Co-occurrence
RASSF8	12p12.1	1 (0.87%)	1 (0.41%)	1.09	0.537	0.686	Co-occurrence
RBBP4	1p35.1	1 (0.87%)	1 (0.41%)	1.09	0.537	0.686	Co-occurrence
RBBP7	Xp22.2	1 (0.87%)	1 (0.41%)	1.09	0.537	0.686	Co-occurrence
RBM15	1p13.3	1 (0.87%)	1 (0.41%)	1.09	0.537	0.686	Co-occurrence
RBM44	2q37.3	1 (0.87%)	1 (0.41%)	1.09	0.537	0.686	Co-occurrence
RBP7	1p36.22	1 (0.87%)	1 (0.41%)	1.09	0.537	0.686	Co-occurrence
RBPJL	20q13.12	1 (0.87%)	1 (0.41%)	1.09	0.537	0.686	Co-occurrence
RBSN	3p25.1	1 (0.87%)	1 (0.41%)	1.09	0.537	0.686	Co-occurrence
REM2	14q11.2	1 (0.87%)	1 (0.41%)	1.09	0.537	0.686	Co-occurrence
REP15	12p11.22	1 (0.87%)	1 (0.41%)	1.09	0.537	0.686	Co-occurrence
REPS2	Xp22.2	1 (0.87%)	1 (0.41%)	1.09	0.537	0.686	Co-occurrence
RETSAT	2p11.2	1 (0.87%)	1 (0.41%)	1.09	0.537	0.686	Co-occurrence

RGS7BP	5q12.3	1 (0.87%)	1 (0.41%)	1.09	0.537	0.686	Co-occurrence
RHBDD1	2q36.3	1 (0.87%)	1 (0.41%)	1.09	0.537	0.686	Co-occurrence
RHBDL2	1p34.3	1 (0.87%)	1 (0.41%)	1.09	0.537	0.686	Co-occurrence
RHOF	12q24.31	1 (0.87%)	1 (0.41%)	1.09	0.537	0.686	Co-occurrence
RILP	17p13.3	1 (0.87%)	1 (0.41%)	1.09	0.537	0.686	Co-occurrence
RIMBP3	22q11.21	1 (0.87%)	1 (0.41%)	1.09	0.537	0.686	Co-occurrence
RIMBP3B	22q11.21	1 (0.87%)	1 (0.41%)	1.09	0.537	0.686	Co-occurrence
RIMBP3C	22q11.21	1 (0.87%)	1 (0.41%)	1.09	0.537	0.686	Co-occurrence
RN7SK	6p12.2	1 (0.87%)	1 (0.41%)	1.09	0.537	0.686	Co-occurrence
RN7SKP100	20q12	1 (0.87%)	1 (0.41%)	1.09	0.537	0.686	Co-occurrence
RN7SKP127	16p11.2	1 (0.87%)	1 (0.41%)	1.09	0.537	0.686	Co-occurrence
RN7SKP131	22q11.21	1 (0.87%)	1 (0.41%)	1.09	0.537	0.686	Co-occurrence
RN7SKP15	12p11.22	1 (0.87%)	1 (0.41%)	1.09	0.537	0.686	Co-occurrence
RN7SKP157	5q12.1	1 (0.87%)	1 (0.41%)	1.09	0.537	0.686	Co-occurrence
RN7SKP173	20q11.23	1 (0.87%)	1 (0.41%)	1.09	0.537	0.686	Co-occurrence
RN7SKP221	22q11.21	1 (0.87%)	1 (0.41%)	1.09	0.537	0.686	Co-occurrence
RN7SKP262	12p12.1	1 (0.87%)	1 (0.41%)	1.09	0.537	0.686	Co-occurrence
RN7SKP269	1p36.22	1 (0.87%)	1 (0.41%)	1.09	0.537	0.686	Co-occurrence
RN7SKP272	1p22.2	1 (0.87%)	1 (0.41%)	1.09	0.537	0.686	Co-occurrence
RN7SKP285	1p21.1	1 (0.87%)	1 (0.41%)	1.09	0.537	0.686	Co-occurrence
RN7SKP33	20q13.12	1 (0.87%)	1 (0.41%)	1.09	0.537	0.686	Co-occurrence
RN7SKP58	3p22.1	1 (0.87%)	1 (0.41%)	1.09	0.537	0.686	Co-occurrence
RN7SKP63	22q11.21	1 (0.87%)	1 (0.41%)	1.09	0.537	0.686	Co-occurrence
RN7SKP75	7q11.22	1 (0.87%)	1 (0.41%)	1.09	0.537	0.686	Co-occurrence
RN7SL1	14q21.3	1 (0.87%)	1 (0.41%)	1.09	0.537	0.686	Co-occurrence
RN7SL105P	17p13.3	1 (0.87%)	1 (0.41%)	1.09	0.537	0.686	Co-occurrence
RN7SL110P	3p25.1	1 (0.87%)	1 (0.41%)	1.09	0.537	0.686	Co-occurrence
RN7SL113P	2p11.2	1 (0.87%)	1 (0.41%)	1.09	0.537	0.686	Co-occurrence
RN7SL116P	20q11.23	1 (0.87%)	1 (0.41%)	1.09	0.537	0.686	Co-occurrence

RN7SL127P	4q21.21	1 (0.87%)	1 (0.41%)	1.09	0.537	0.686	Co-occurrence
RN7SL132P	7p14.3	1 (0.87%)	1 (0.41%)	1.09	0.537	0.686	Co-occurrence
RN7SL144P	Xp11.4	1 (0.87%)	1 (0.41%)	1.09	0.537	0.686	Co-occurrence
RN7SL153P	5q13.2	1 (0.87%)	1 (0.41%)	1.09	0.537	0.686	Co-occurrence
RN7SL168P	22q11.21	1 (0.87%)	1 (0.41%)	1.09	0.537	0.686	Co-occurrence
RN7SL169P	5q12.3	1 (0.87%)	1 (0.41%)	1.09	0.537	0.686	Co-occurrence
RN7SL182P	3p21.31	1 (0.87%)	1 (0.41%)	1.09	0.537	0.686	Co-occurrence
RN7SL20P	22q12.2	1 (0.87%)	1 (0.41%)	1.09	0.537	0.686	Co-occurrence
RN7SL233P	18q11.2	1 (0.87%)	1 (0.41%)	1.09	0.537	0.686	Co-occurrence
RN7SL280P	22q11.22	1 (0.87%)	1 (0.41%)	1.09	0.537	0.686	Co-occurrence
RN7SL292P	7p12.1	1 (0.87%)	1 (0.41%)	1.09	0.537	0.686	Co-occurrence
RN7SL322P	19q13.33	1 (0.87%)	1 (0.41%)	1.09	0.537	0.686	Co-occurrence
RN7SL368P	19q13.31	1 (0.87%)	1 (0.41%)	1.09	0.537	0.686	Co-occurrence
RN7SL377P	7q11.23	1 (0.87%)	1 (0.41%)	1.09	0.537	0.686	Co-occurrence
RN7SL397P	3q13.33	1 (0.87%)	1 (0.41%)	1.09	0.537	0.686	Co-occurrence
RN7SL419P	4q31.3	1 (0.87%)	1 (0.41%)	1.09	0.537	0.686	Co-occurrence
RN7SL441P	12q24.11	1 (0.87%)	1 (0.41%)	1.09	0.537	0.686	Co-occurrence
RN7SL443P	20q13.12	1 (0.87%)	1 (0.41%)	1.09	0.537	0.686	Co-occurrence
RN7SL457P	8p12	1 (0.87%)	1 (0.41%)	1.09	0.537	0.686	Co-occurrence
RN7SL496P	7p14.1	1 (0.87%)	1 (0.41%)	1.09	0.537	0.686	Co-occurrence
RN7SL4P	3p25.1	1 (0.87%)	1 (0.41%)	1.09	0.537	0.686	Co-occurrence
RN7SL517P	3p22.1	1 (0.87%)	1 (0.41%)	1.09	0.537	0.686	Co-occurrence
RN7SL518P	10q22.3	1 (0.87%)	1 (0.41%)	1.09	0.537	0.686	Co-occurrence
RN7SL621P	8p12	1 (0.87%)	1 (0.41%)	1.09	0.537	0.686	Co-occurrence
RN7SL625P	7q11.23	1 (0.87%)	1 (0.41%)	1.09	0.537	0.686	Co-occurrence
RN7SL649P	1p36.22	1 (0.87%)	1 (0.41%)	1.09	0.537	0.686	Co-occurrence
RN7SL666P	20q13.11	1 (0.87%)	1 (0.41%)	1.09	0.537	0.686	Co-occurrence
RN7SL680P	20q12	1 (0.87%)	1 (0.41%)	1.09	0.537	0.686	Co-occurrence
RN7SL731P	1p36.22	1 (0.87%)	1 (0.41%)	1.09	0.537	0.686	Co-occurrence

RN7SL732P	Xp11.4	1 (0.87%)	1 (0.41%)	1.09	0.537	0.686	Co-occurrence
RN7SL812P	22q11.21	1 (0.87%)	1 (0.41%)	1.09	0.537	0.686	Co-occurrence
RN7SL814P	5q13.3	1 (0.87%)	1 (0.41%)	1.09	0.537	0.686	Co-occurrence
RN7SL83P	7p14.1	1 (0.87%)	1 (0.41%)	1.09	0.537	0.686	Co-occurrence
RNA5SP124	3p25.1	1 (0.87%)	1 (0.41%)	1.09	0.537	0.686	Co-occurrence
RNA5SP186	5q13.3	1 (0.87%)	1 (0.41%)	1.09	0.537	0.686	Co-occurrence
RNA5SP231	7q11.22	1 (0.87%)	1 (0.41%)	1.09	0.537	0.686	Co-occurrence
RNA5SP232	7q11.22	1 (0.87%)	1 (0.41%)	1.09	0.537	0.686	Co-occurrence
RNA5SP233	7q11.23	1 (0.87%)	1 (0.41%)	1.09	0.537	0.686	Co-occurrence
RNA5SP262	8p12	1 (0.87%)	1 (0.41%)	1.09	0.537	0.686	Co-occurrence
RNA5SP263	8p12	1 (0.87%)	1 (0.41%)	1.09	0.537	0.686	Co-occurrence
RNA5SP294	9q31.3	1 (0.87%)	1 (0.41%)	1.09	0.537	0.686	Co-occurrence
RNA5SP321	10q22.3	1 (0.87%)	1 (0.41%)	1.09	0.537	0.686	Co-occurrence
RNA5SP324	10q24.1	1 (0.87%)	1 (0.41%)	1.09	0.537	0.686	Co-occurrence
RNA5SP354	12p11.23	1 (0.87%)	1 (0.41%)	1.09	0.537	0.686	Co-occurrence
RNA5SP384	14q21.3	1 (0.87%)	1 (0.41%)	1.09	0.537	0.686	Co-occurrence
RNA5SP41	1p36.21	1 (0.87%)	1 (0.41%)	1.09	0.537	0.686	Co-occurrence
RNA5SP426	16q12.1	1 (0.87%)	1 (0.41%)	1.09	0.537	0.686	Co-occurrence
RNA5SP44	1p34.3	1 (0.87%)	1 (0.41%)	1.09	0.537	0.686	Co-occurrence
RNA5SP451	18q11.2	1 (0.87%)	1 (0.41%)	1.09	0.537	0.686	Co-occurrence
RNA5SP502	Xp11.4	1 (0.87%)	1 (0.41%)	1.09	0.537	0.686	Co-occurrence
RNA5SP54	1p13.3	1 (0.87%)	1 (0.41%)	1.09	0.537	0.686	Co-occurrence
RNF122	8p12	1 (0.87%)	1 (0.41%)	1.09	0.537	0.686	Co-occurrence
RNF123	3p21.31	1 (0.87%)	1 (0.41%)	1.09	0.537	0.686	Co-occurrence
RNF180	5q12.3	1 (0.87%)	1 (0.41%)	1.09	0.537	0.686	Co-occurrence
RNF26	11q23.3	1 (0.87%)	1 (0.41%)	1.09	0.537	0.686	Co-occurrence
RNPC3	1p21.1	1 (0.87%)	1 (0.41%)	1.09	0.537	0.686	Co-occurrence
RNU4ATAC16P	12p13.33	1 (0.87%)	1 (0.41%)	1.09	0.537	0.686	Co-occurrence
RNU4ATAC17P	3p25.3	1 (0.87%)	1 (0.41%)	1.09	0.537	0.686	Co-occurrence

RNU6ATAC16P	3p21.1	1 (0.87%)	1 (0.41%)	1.09	0.537	0.686	Co-occurrence
RNU6ATAC30P	14q21.3	1 (0.87%)	1 (0.41%)	1.09	0.537	0.686	Co-occurrence
RNU6V	1p13.3	1 (0.87%)	1 (0.41%)	1.09	0.537	0.686	Co-occurrence
ROBO2	3p12.3	1 (0.87%)	1 (0.41%)	1.09	0.537	0.686	Co-occurrence
RPL11	1p36.11	1 (0.87%)	1 (0.41%)	1.09	0.537	0.686	Co-occurrence
RPL12	9q33.3	1 (0.87%)	1 (0.41%)	1.09	0.537	0.686	Co-occurrence
RPL26P30	11p15.5	1 (0.87%)	1 (0.41%)	1.09	0.537	0.686	Co-occurrence
RPL29	3p21.2	1 (0.87%)	1 (0.41%)	1.09	0.537	0.686	Co-occurrence
RPL29P30	15q23	1 (0.87%)	1 (0.41%)	1.09	0.537	0.686	Co-occurrence
RPL36AL	14q21.3	1 (0.87%)	1 (0.41%)	1.09	0.537	0.686	Co-occurrence
RPL6	12q24.13	1 (0.87%)	1 (0.41%)	1.09	0.537	0.686	Co-occurrence
RPRD1A	18q12.2	1 (0.87%)	1 (0.41%)	1.09	0.537	0.686	Co-occurrence
RPS24	10q22.3	1 (0.87%)	1 (0.41%)	1.09	0.537	0.686	Co-occurrence
RPS29	14q21.3	1 (0.87%)	1 (0.41%)	1.09	0.537	0.686	Co-occurrence
RRAGC	1p34.3	1 (0.87%)	1 (0.41%)	1.09	0.537	0.686	Co-occurrence
RRM1	11p15.4	1 (0.87%)	1 (0.41%)	1.09	0.537	0.686	Co-occurrence
RRP12	10q24.1	1 (0.87%)	1 (0.41%)	1.09	0.537	0.686	Co-occurrence
RRP9	3p21.2	1 (0.87%)	1 (0.41%)	1.09	0.537	0.686	Co-occurrence
RSC1A1	1p36.21	1 (0.87%)	1 (0.41%)	1.09	0.537	0.686	Co-occurrence
RSL24D1	15q21.3	1 (0.87%)	1 (0.41%)	1.09	0.537	0.686	Co-occurrence
RTL10	22q11.21	1 (0.87%)	1 (0.41%)	1.09	0.537	0.686	Co-occurrence
RTN3	11q13.1	1 (0.87%)	1 (0.41%)	1.09	0.537	0.686	Co-occurrence
RTN4R	22q11.21	1 (0.87%)	1 (0.41%)	1.09	0.537	0.686	Co-occurrence
RYR1	19q13.2	1 (0.87%)	1 (0.41%)	1.09	0.537	0.686	Co-occurrence
S100PBP	1p35.1	1 (0.87%)	1 (0.41%)	1.09	0.537	0.686	Co-occurrence
SALL2	14q11.2	1 (0.87%)	1 (0.41%)	1.09	0.537	0.686	Co-occurrence
SART1	11q13.1	1 (0.87%)	1 (0.41%)	1.09	0.537	0.686	Co-occurrence
SCARB2	4q21.1	1 (0.87%)	1 (0.41%)	1.09	0.537	0.686	Co-occurrence
SCARF1	17p13.3	1 (0.87%)	1 (0.41%)	1.09	0.537	0.686	Co-occurrence

SCARF2	22q11.21	1 (0.87%)	1 (0.41%)	1.09	0.537	0.686	Co-occurrence
SCARNA11	12p13.31	1 (0.87%)	1 (0.41%)	1.09	0.537	0.686	Co-occurrence
SCARNA2	1p13.3	1 (0.87%)	1 (0.41%)	1.09	0.537	0.686	Co-occurrence
SCARNA21	17p13.1	1 (0.87%)	1 (0.41%)	1.09	0.537	0.686	Co-occurrence
SCML2	Xp22.13	1 (0.87%)	1 (0.41%)	1.09	0.537	0.686	Co-occurrence
SCP2	1p32.3	1 (0.87%)	1 (0.41%)	1.09	0.537	0.686	Co-occurrence
SCT	11p15.5	1 (0.87%)	1 (0.41%)	1.09	0.537	0.686	Co-occurrence
SDC4	20q13.12	1 (0.87%)	1 (0.41%)	1.09	0.537	0.686	Co-occurrence
SDF2L1	22q11.21	1 (0.87%)	1 (0.41%)	1.09	0.537	0.686	Co-occurrence
SDSL	12q24.13	1 (0.87%)	1 (0.41%)	1.09	0.537	0.686	Co-occurrence
SEC14L6	22q12.2	1 (0.87%)	1 (0.41%)	1.09	0.537	0.686	Co-occurrence
SEMG1	20q13.12	1 (0.87%)	1 (0.41%)	1.09	0.537	0.686	Co-occurrence
SEMG2	20q13.12	1 (0.87%)	1 (0.41%)	1.09	0.537	0.686	Co-occurrence
SEPHS2	16p11.2	1 (0.87%)	1 (0.41%)	1.09	0.537	0.686	Co-occurrence
SEPTIN1	16p11.2	1 (0.87%)	1 (0.41%)	1.09	0.537	0.686	Co-occurrence
SEPTIN5	22q11.21	1 (0.87%)	1 (0.41%)	1.09	0.537	0.686	Co-occurrence
SERPINA13P	14q32.13	1 (0.87%)	1 (0.41%)	1.09	0.537	0.686	Co-occurrence
SERPIND1	22q11.21	1 (0.87%)	1 (0.41%)	1.09	0.537	0.686	Co-occurrence
SERPINF1	17p13.3	1 (0.87%)	1 (0.41%)	1.09	0.537	0.686	Co-occurrence
SERPINF2	17p13.3	1 (0.87%)	1 (0.41%)	1.09	0.537	0.686	Co-occurrence
SETD1B	12q24.31	1 (0.87%)	1 (0.41%)	1.09	0.537	0.686	Co-occurrence
SEZ6L2	16p11.2	1 (0.87%)	1 (0.41%)	1.09	0.537	0.686	Co-occurrence
SF3B2	11q13.1	1 (0.87%)	1 (0.41%)	1.09	0.537	0.686	Co-occurrence
SFRP4	7p14.1	1 (0.87%)	1 (0.41%)	1.09	0.537	0.686	Co-occurrence
SFTPA1	10q22.3	1 (0.87%)	1 (0.41%)	1.09	0.537	0.686	Co-occurrence
SFTPA2	10q22.3	1 (0.87%)	1 (0.41%)	1.09	0.537	0.686	Co-occurrence
SFTPD	10q22.3	1 (0.87%)	1 (0.41%)	1.09	0.537	0.686	Co-occurrence
SGK2	20q13.12	1 (0.87%)	1 (0.41%)	1.09	0.537	0.686	Co-occurrence
SH2B3	12q24.12	1 (0.87%)	1 (0.41%)	1.09	0.537	0.686	Co-occurrence

SH2D4B	10q23.1	1 (0.87%)	1 (0.41%)	1.09	0.537	0.686	Co-occurrence
SH3BP5	3p25.1	1 (0.87%)	1 (0.41%)	1.09	0.537	0.686	Co-occurrence
SHISAL2B	5q12.3	1 (0.87%)	1 (0.41%)	1.09	0.537	0.686	Co-occurrence
SHMT1	17p11.2	1 (0.87%)	1 (0.41%)	1.09	0.537	0.686	Co-occurrence
SIAH1	16q12.1	1 (0.87%)	1 (0.41%)	1.09	0.537	0.686	Co-occurrence
SIGLEC1	20p13	1 (0.87%)	1 (0.41%)	1.09	0.537	0.686	Co-occurrence
SINHCAF	12p11.21	1 (0.87%)	1 (0.41%)	1.09	0.537	0.686	Co-occurrence
SIPA1L3	19q13.13-q13.2	1 (0.87%)	1 (0.41%)	1.09	0.537	0.686	Co-occurrence
SKINT1L	1p33	1 (0.87%)	1 (0.41%)	1.09	0.537	0.686	Co-occurrence
SLC12A8	3q21.2	1 (0.87%)	1 (0.41%)	1.09	0.537	0.686	Co-occurrence
SLC13A3	20q13.12	1 (0.87%)	1 (0.41%)	1.09	0.537	0.686	Co-occurrence
SLC16A12	10q23.31	1 (0.87%)	1 (0.41%)	1.09	0.537	0.686	Co-occurrence
SLC16A4	1p13.3	1 (0.87%)	1 (0.41%)	1.09	0.537	0.686	Co-occurrence
SLC22A1	6q25.3	1 (0.87%)	1 (0.41%)	1.09	0.537	0.686	Co-occurrence
SLC22A18	11p15.4	1 (0.87%)	1 (0.41%)	1.09	0.537	0.686	Co-occurrence
SLC22A18AS	11p15.4	1 (0.87%)	1 (0.41%)	1.09	0.537	0.686	Co-occurrence
SLC22A2	6q25.3	1 (0.87%)	1 (0.41%)	1.09	0.537	0.686	Co-occurrence
SLC22A3	6q25.3	1 (0.87%)	1 (0.41%)	1.09	0.537	0.686	Co-occurrence
SLC25A1	22q11.21	1 (0.87%)	1 (0.41%)	1.09	0.537	0.686	Co-occurrence
SLC25A17	22q13.2	1 (0.87%)	1 (0.41%)	1.09	0.537	0.686	Co-occurrence
SLC25A20	3p21.31	1 (0.87%)	1 (0.41%)	1.09	0.537	0.686	Co-occurrence
SLC25A34	1p36.21	1 (0.87%)	1 (0.41%)	1.09	0.537	0.686	Co-occurrence
SLC27A4	9q34.11	1 (0.87%)	1 (0.41%)	1.09	0.537	0.686	Co-occurrence
SLC28A2	15q21.1	1 (0.87%)	1 (0.41%)	1.09	0.537	0.686	Co-occurrence
SLC29A3	10q22.1	1 (0.87%)	1 (0.41%)	1.09	0.537	0.686	Co-occurrence
SLC2A10	20q13.12	1 (0.87%)	1 (0.41%)	1.09	0.537	0.686	Co-occurrence
SLC2A8	9q33.3	1 (0.87%)	1 (0.41%)	1.09	0.537	0.686	Co-occurrence
SLC30A5	5q13.1-q13.2	1 (0.87%)	1 (0.41%)	1.09	0.537	0.686	Co-occurrence
SLC31A2	9q32	1 (0.87%)	1 (0.41%)	1.09	0.537	0.686	Co-occurrence

SLC32A1	20q11.23	1 (0.87%)	1 (0.41%)	1.09	0.537	0.686	Co-occurrence
SLC38A4	12q13.11	1 (0.87%)	1 (0.41%)	1.09	0.537	0.686	Co-occurrence
SLC43A2	17p13.3	1 (0.87%)	1 (0.41%)	1.09	0.537	0.686	Co-occurrence
SLC43A3	11q12.1	1 (0.87%)	1 (0.41%)	1.09	0.537	0.686	Co-occurrence
SLC44A5	1p31.1	1 (0.87%)	1 (0.41%)	1.09	0.537	0.686	Co-occurrence
SLC4A11	20p13	1 (0.87%)	1 (0.41%)	1.09	0.537	0.686	Co-occurrence
SLC6A12	12p13.33	1 (0.87%)	1 (0.41%)	1.09	0.537	0.686	Co-occurrence
SLC6A13	12p13.33	1 (0.87%)	1 (0.41%)	1.09	0.537	0.686	Co-occurrence
SLC6A17	1p13.3	1 (0.87%)	1 (0.41%)	1.09	0.537	0.686	Co-occurrence
SLC6A6	3p25.1	1 (0.87%)	1 (0.41%)	1.09	0.537	0.686	Co-occurrence
SLC6A9	1p34.1	1 (0.87%)	1 (0.41%)	1.09	0.537	0.686	Co-occurrence
SLC7A4	22q11.21	1 (0.87%)	1 (0.41%)	1.09	0.537	0.686	Co-occurrence
SLC7A8	14q11.2	1 (0.87%)	1 (0.41%)	1.09	0.537	0.686	Co-occurrence
SLC8A2	19q13.32	1 (0.87%)	1 (0.41%)	1.09	0.537	0.686	Co-occurrence
SLC9A7	Xp11.3|Xp11.3	1 (0.87%)	1 (0.41%)	1.09	0.537	0.686	Co-occurrence
SLPI	20q13.12	1 (0.87%)	1 (0.41%)	1.09	0.537	0.686	Co-occurrence
SLX1A	16p11.2	1 (0.87%)	1 (0.41%)	1.09	0.537	0.686	Co-occurrence
SLX1B	16p11.2	1 (0.87%)	1 (0.41%)	1.09	0.537	0.686	Co-occurrence
SMAP1	6q13	1 (0.87%)	1 (0.41%)	1.09	0.537	0.686	Co-occurrence
SMARCAD1	4q22.3	1 (0.87%)	1 (0.41%)	1.09	0.537	0.686	Co-occurrence
SMCR2	17p11.2	1 (0.87%)	1 (0.41%)	1.09	0.537	0.686	Co-occurrence
SMCR5	-	1 (0.87%)	1 (0.41%)	1.09	0.537	0.686	Co-occurrence
SMCR8	17p11.2	1 (0.87%)	1 (0.41%)	1.09	0.537	0.686	Co-occurrence
SMG9	19q13.31	1 (0.87%)	1 (0.41%)	1.09	0.537	0.686	Co-occurrence
SMIM15	5q12.1	1 (0.87%)	1 (0.41%)	1.09	0.537	0.686	Co-occurrence
SMIM24	19p13.3	1 (0.87%)	1 (0.41%)	1.09	0.537	0.686	Co-occurrence
SMPD1	11p15.4	1 (0.87%)	1 (0.41%)	1.09	0.537	0.686	Co-occurrence
SMPD4P1	22q11.21	1 (0.87%)	1 (0.41%)	1.09	0.537	0.686	Co-occurrence
SMYD4	17p13.3	1 (0.87%)	1 (0.41%)	1.09	0.537	0.686	Co-occurrence

SNAP29	22q11.21	1 (0.87%)	1 (0.41%)	1.09	0.537	0.686	Co-occurrence
SNORA25	11q21	1 (0.87%)	1 (0.41%)	1.09	0.537	0.686	Co-occurrence
SNORA50A	16q21	1 (0.87%)	1 (0.41%)	1.09	0.537	0.686	Co-occurrence
SNORA54	11p15.4	1 (0.87%)	1 (0.41%)	1.09	0.537	0.686	Co-occurrence
SNORA65	9q33.3	1 (0.87%)	1 (0.41%)	1.09	0.537	0.686	Co-occurrence
SNORA70	Xq28	1 (0.87%)	1 (0.41%)	1.09	0.537	0.686	Co-occurrence
SNORD121A	9p13.3	1 (0.87%)	1 (0.41%)	1.09	0.537	0.686	Co-occurrence
SNORD121B	9p13.3	1 (0.87%)	1 (0.41%)	1.09	0.537	0.686	Co-occurrence
SNORD13	8p12	1 (0.87%)	1 (0.41%)	1.09	0.537	0.686	Co-occurrence
SNORD19	3p21.1	1 (0.87%)	1 (0.41%)	1.09	0.537	0.686	Co-occurrence
SNORD19B	3p21.1	1 (0.87%)	1 (0.41%)	1.09	0.537	0.686	Co-occurrence
SNORD23	19q13.33	1 (0.87%)	1 (0.41%)	1.09	0.537	0.686	Co-occurrence
SNORD5	11q21	1 (0.87%)	1 (0.41%)	1.09	0.537	0.686	Co-occurrence
SNORD69	3p21.1	1 (0.87%)	1 (0.41%)	1.09	0.537	0.686	Co-occurrence
SNRPD1	18q11.2	1 (0.87%)	1 (0.41%)	1.09	0.537	0.686	Co-occurrence
SNX7	1p21.3	1 (0.87%)	1 (0.41%)	1.09	0.537	0.686	Co-occurrence
SOWAHB	4q21.1	1 (0.87%)	1 (0.41%)	1.09	0.537	0.686	Co-occurrence
SPACA5	Xp11.23	1 (0.87%)	1 (0.41%)	1.09	0.537	0.686	Co-occurrence
SPACA5B	Xp11.23	1 (0.87%)	1 (0.41%)	1.09	0.537	0.686	Co-occurrence
SPATA21	1p36.13	1 (0.87%)	1 (0.41%)	1.09	0.537	0.686	Co-occurrence
SPATA3	2q37.1	1 (0.87%)	1 (0.41%)	1.09	0.537	0.686	Co-occurrence
SPATA42	1p13.3	1 (0.87%)	1 (0.41%)	1.09	0.537	0.686	Co-occurrence
SPATA48	7p12.2	1 (0.87%)	1 (0.41%)	1.09	0.537	0.686	Co-occurrence
SPCS1	3p21.1	1 (0.87%)	1 (0.41%)	1.09	0.537	0.686	Co-occurrence
SPEF1	20p13	1 (0.87%)	1 (0.41%)	1.09	0.537	0.686	Co-occurrence
SPHKAP	2q36.3	1 (0.87%)	1 (0.41%)	1.09	0.537	0.686	Co-occurrence
SPN	16p11.2	1 (0.87%)	1 (0.41%)	1.09	0.537	0.686	Co-occurrence
SPOCK2	10q22.1	1 (0.87%)	1 (0.41%)	1.09	0.537	0.686	Co-occurrence
SPPL2B	19p13.3	1 (0.87%)	1 (0.41%)	1.09	0.537	0.686	Co-occurrence

SPSB1	1p36.22	1 (0.87%)	1 (0.41%)	1.09	0.537	0.686	Co-occurrence
SPX	12p12.1	1 (0.87%)	1 (0.41%)	1.09	0.537	0.686	Co-occurrence
SREBF1	17p11.2	1 (0.87%)	1 (0.41%)	1.09	0.537	0.686	Co-occurrence
SREK1IP1	5q12.3	1 (0.87%)	1 (0.41%)	1.09	0.537	0.686	Co-occurrence
SRM	1p36.22	1 (0.87%)	1 (0.41%)	1.09	0.537	0.686	Co-occurrence
SRRM5	19q13.31	1 (0.87%)	1 (0.41%)	1.09	0.537	0.686	Co-occurrence
SRSF6	20q13.11	1 (0.87%)	1 (0.41%)	1.09	0.537	0.686	Co-occurrence
SSBP3-AS1	1p32.3	1 (0.87%)	1 (0.41%)	1.09	0.537	0.686	Co-occurrence
SSPN	12p12.1	1 (0.87%)	1 (0.41%)	1.09	0.537	0.686	Co-occurrence
SSUH2	3p25.3	1 (0.87%)	1 (0.41%)	1.09	0.537	0.686	Co-occurrence
SSX6P	Xp11.23	1 (0.87%)	1 (0.41%)	1.09	0.537	0.686	Co-occurrence
ST14	11q24.3	1 (0.87%)	1 (0.41%)	1.09	0.537	0.686	Co-occurrence
ST3GAL3	1p34.1	1 (0.87%)	1 (0.41%)	1.09	0.537	0.686	Co-occurrence
STARD3NL	7p14.1	1 (0.87%)	1 (0.41%)	1.09	0.537	0.686	Co-occurrence
STRIP1	1p13.3	1 (0.87%)	1 (0.41%)	1.09	0.537	0.686	Co-occurrence
STS	Xp22.31	1 (0.87%)	1 (0.41%)	1.09	0.537	0.686	Co-occurrence
STXBP1	9q34.11	1 (0.87%)	1 (0.41%)	1.09	0.537	0.686	Co-occurrence
SULT1A3	16p11.2	1 (0.87%)	1 (0.41%)	1.09	0.537	0.686	Co-occurrence
SULT1A4	16p11.2	1 (0.87%)	1 (0.41%)	1.09	0.537	0.686	Co-occurrence
SULT1B1	4q13.3	1 (0.87%)	1 (0.41%)	1.09	0.537	0.686	Co-occurrence
SULT2A1	19q13.33	1 (0.87%)	1 (0.41%)	1.09	0.537	0.686	Co-occurrence
SVEP1	9q31.3	1 (0.87%)	1 (0.41%)	1.09	0.537	0.686	Co-occurrence
SWI5	9q34.11	1 (0.87%)	1 (0.41%)	1.09	0.537	0.686	Co-occurrence
SYCN	19q13.2	1 (0.87%)	1 (0.41%)	1.09	0.537	0.686	Co-occurrence
SYNC	1p35.1	1 (0.87%)	1 (0.41%)	1.09	0.537	0.686	Co-occurrence
SYPL2	1p13.3	1 (0.87%)	1 (0.41%)	1.09	0.537	0.686	Co-occurrence
SYS1	20q13.12	1 (0.87%)	1 (0.41%)	1.09	0.537	0.686	Co-occurrence
SYT9	11p15.4	1 (0.87%)	1 (0.41%)	1.09	0.537	0.686	Co-occurrence
SZRD1	1p36.13	1 (0.87%)	1 (0.41%)	1.09	0.537	0.686	Co-occurrence

TAF13	1p13.3	1 (0.87%)	1 (0.41%)	1.09	0.537	0.686	Co-occurrence
TANGO2	22q11.21	1 (0.87%)	1 (0.41%)	1.09	0.537	0.686	Co-occurrence
TAOK2	16p11.2	1 (0.87%)	1 (0.41%)	1.09	0.537	0.686	Co-occurrence
TARDBP	1p36.22	1 (0.87%)	1 (0.41%)	1.09	0.537	0.686	Co-occurrence
TBC1D10B	16p11.2	1 (0.87%)	1 (0.41%)	1.09	0.537	0.686	Co-occurrence
TBC1D13	9q34.11	1 (0.87%)	1 (0.41%)	1.09	0.537	0.686	Co-occurrence
TBX1	22q11.21	1 (0.87%)	1 (0.41%)	1.09	0.537	0.686	Co-occurrence
TBX20	7p14.2	1 (0.87%)	1 (0.41%)	1.09	0.537	0.686	Co-occurrence
TBX6	16p11.2	1 (0.87%)	1 (0.41%)	1.09	0.537	0.686	Co-occurrence
TCAIM	3p21.31	1 (0.87%)	1 (0.41%)	1.09	0.537	0.686	Co-occurrence
TCEANC2	1p32.3	1 (0.87%)	1 (0.41%)	1.09	0.537	0.686	Co-occurrence
TCF7L2	10q25.2-q25.3	1 (0.87%)	1 (0.41%)	1.09	0.537	0.686	Co-occurrence
TCHP	12q24.11	1 (0.87%)	1 (0.41%)	1.09	0.537	0.686	Co-occurrence
TCTN3	10q24.1	1 (0.87%)	1 (0.41%)	1.09	0.537	0.686	Co-occurrence
TECTB	10q25.2	1 (0.87%)	1 (0.41%)	1.09	0.537	0.686	Co-occurrence
TEF	22q13.2	1 (0.87%)	1 (0.41%)	1.09	0.537	0.686	Co-occurrence
TET1	10q21.3	1 (0.87%)	1 (0.41%)	1.09	0.537	0.686	Co-occurrence
TEX101	19q13.31	1 (0.87%)	1 (0.41%)	1.09	0.537	0.686	Co-occurrence
TEX38	1p33	1 (0.87%)	1 (0.41%)	1.09	0.537	0.686	Co-occurrence
TFAP2E	1p34.3	1 (0.87%)	1 (0.41%)	1.09	0.537	0.686	Co-occurrence
TFG	3q12.2	1 (0.87%)	1 (0.41%)	1.09	0.537	0.686	Co-occurrence
TGIF2	20q11.23	1 (0.87%)	1 (0.41%)	1.09	0.537	0.686	Co-occurrence
TH	11p15.5	1 (0.87%)	1 (0.41%)	1.09	0.537	0.686	Co-occurrence
THAP10	15q23	1 (0.87%)	1 (0.41%)	1.09	0.537	0.686	Co-occurrence
THAP7	22q11.21	1 (0.87%)	1 (0.41%)	1.09	0.537	0.686	Co-occurrence
THTPA	14q11.2	1 (0.87%)	1 (0.41%)	1.09	0.537	0.686	Co-occurrence
TIFA	4q25	1 (0.87%)	1 (0.41%)	1.09	0.537	0.686	Co-occurrence
TIMM13	19p13.3	1 (0.87%)	1 (0.41%)	1.09	0.537	0.686	Co-occurrence
TLCD2	17p13.3	1 (0.87%)	1 (0.41%)	1.09	0.537	0.686	Co-occurrence

TLCD3B	16p11.2	1 (0.87%)	1 (0.41%)	1.09	0.537	0.686	Co-occurrence
TLR7	Xp22.2	1 (0.87%)	1 (0.41%)	1.09	0.537	0.686	Co-occurrence
TLR8	Xp22.2	1 (0.87%)	1 (0.41%)	1.09	0.537	0.686	Co-occurrence
TMEM131L	4q31.3	1 (0.87%)	1 (0.41%)	1.09	0.537	0.686	Co-occurrence
TMEM167B	1p13.3	1 (0.87%)	1 (0.41%)	1.09	0.537	0.686	Co-occurrence
TMEM171	5q13.2	1 (0.87%)	1 (0.41%)	1.09	0.537	0.686	Co-occurrence
TMEM174	5q13.2	1 (0.87%)	1 (0.41%)	1.09	0.537	0.686	Co-occurrence
TMEM191A	22q11.21	1 (0.87%)	1 (0.41%)	1.09	0.537	0.686	Co-occurrence
TMEM191C	22q11.21	1 (0.87%)	1 (0.41%)	1.09	0.537	0.686	Co-occurrence
TMEM219	16p11.2	1 (0.87%)	1 (0.41%)	1.09	0.537	0.686	Co-occurrence
TMEM254	10q22.3	1 (0.87%)	1 (0.41%)	1.09	0.537	0.686	Co-occurrence
TMEM26	10q21.2	1 (0.87%)	1 (0.41%)	1.09	0.537	0.686	Co-occurrence
TMEM43	3p25.1	1 (0.87%)	1 (0.41%)	1.09	0.537	0.686	Co-occurrence
TMEM45A	3q12.2	1 (0.87%)	1 (0.41%)	1.09	0.537	0.686	Co-occurrence
TMEM51	1p36.21	1 (0.87%)	1 (0.41%)	1.09	0.537	0.686	Co-occurrence
TMEM61	1p32.3	1 (0.87%)	1 (0.41%)	1.09	0.537	0.686	Co-occurrence
TMEM80	11p15.5	1 (0.87%)	1 (0.41%)	1.09	0.537	0.686	Co-occurrence
TMEM82	1p36.21	1 (0.87%)	1 (0.41%)	1.09	0.537	0.686	Co-occurrence
TMPRSS9	19p13.3	1 (0.87%)	1 (0.41%)	1.09	0.537	0.686	Co-occurrence
TMSB4X	Xp22.2	1 (0.87%)	1 (0.41%)	1.09	0.537	0.686	Co-occurrence
TNC	9q33.1	1 (0.87%)	1 (0.41%)	1.09	0.537	0.686	Co-occurrence
TNFRSF1B	1p36.22	1 (0.87%)	1 (0.41%)	1.09	0.537	0.686	Co-occurrence
TNFRSF8	1p36.22	1 (0.87%)	1 (0.41%)	1.09	0.537	0.686	Co-occurrence
TNFSF15	9q32	1 (0.87%)	1 (0.41%)	1.09	0.537	0.686	Co-occurrence
TNNT3	11p15.5	1 (0.87%)	1 (0.41%)	1.09	0.537	0.686	Co-occurrence
TNPO1	5q13.2	1 (0.87%)	1 (0.41%)	1.09	0.537	0.686	Co-occurrence
TOB2	22q13.2	1 (0.87%)	1 (0.41%)	1.09	0.537	0.686	Co-occurrence
TOP1	20q12	1 (0.87%)	1 (0.41%)	1.09	0.537	0.686	Co-occurrence
TOP2B	3p24.2	1 (0.87%)	1 (0.41%)	1.09	0.537	0.686	Co-occurrence

TOP3A	17p11.2	1 (0.87%)	1 (0.41%)	1.09	0.537	0.686	Co-occurrence
TOPAZ1	3p21.31	1 (0.87%)	1 (0.41%)	1.09	0.537	0.686	Co-occurrence
TOX2	20q13.12	1 (0.87%)	1 (0.41%)	1.09	0.537	0.686	Co-occurrence
TP53RK	20q13.12	1 (0.87%)	1 (0.41%)	1.09	0.537	0.686	Co-occurrence
TPPP3	16q22.1	1 (0.87%)	1 (0.41%)	1.09	0.537	0.686	Co-occurrence
TPRA1	3q21.3	1 (0.87%)	1 (0.41%)	1.09	0.537	0.686	Co-occurrence
TPRX1	19q13.33	1 (0.87%)	1 (0.41%)	1.09	0.537	0.686	Co-occurrence
TPRX2P	19q13.33	1 (0.87%)	1 (0.41%)	1.09	0.537	0.686	Co-occurrence
TPRXL	3p25.1	1 (0.87%)	1 (0.41%)	1.09	0.537	0.686	Co-occurrence
TPST1	7q11.21	1 (0.87%)	1 (0.41%)	1.09	0.537	0.686	Co-occurrence
TPST2	22q12.1	1 (0.87%)	1 (0.41%)	1.09	0.537	0.686	Co-occurrence
TRAC	14q11.2	1 (0.87%)	1 (0.41%)	1.09	0.537	0.686	Co-occurrence
TRAF1	9q33.2	1 (0.87%)	1 (0.41%)	1.09	0.537	0.686	Co-occurrence
TRAJ1	14q11.2	1 (0.87%)	1 (0.41%)	1.09	0.537	0.686	Co-occurrence
TRAJ10	14q11.2	1 (0.87%)	1 (0.41%)	1.09	0.537	0.686	Co-occurrence
TRAJ11	14q11.2	1 (0.87%)	1 (0.41%)	1.09	0.537	0.686	Co-occurrence
TRAJ12	14q11.2	1 (0.87%)	1 (0.41%)	1.09	0.537	0.686	Co-occurrence
TRAJ13	14q11.2	1 (0.87%)	1 (0.41%)	1.09	0.537	0.686	Co-occurrence
TRAJ14	14q11.2	1 (0.87%)	1 (0.41%)	1.09	0.537	0.686	Co-occurrence
TRAJ16	14q11.2	1 (0.87%)	1 (0.41%)	1.09	0.537	0.686	Co-occurrence
TRAJ17	14q11.2	1 (0.87%)	1 (0.41%)	1.09	0.537	0.686	Co-occurrence
TRAJ18	14q11.2	1 (0.87%)	1 (0.41%)	1.09	0.537	0.686	Co-occurrence
TRAJ19	14q11.2	1 (0.87%)	1 (0.41%)	1.09	0.537	0.686	Co-occurrence
TRAJ2	14q11.2	1 (0.87%)	1 (0.41%)	1.09	0.537	0.686	Co-occurrence
TRAJ20	14q11.2	1 (0.87%)	1 (0.41%)	1.09	0.537	0.686	Co-occurrence
TRAJ21	14q11.2	1 (0.87%)	1 (0.41%)	1.09	0.537	0.686	Co-occurrence
TRAJ22	14q11.2	1 (0.87%)	1 (0.41%)	1.09	0.537	0.686	Co-occurrence
TRAJ23	14q11.2	1 (0.87%)	1 (0.41%)	1.09	0.537	0.686	Co-occurrence
TRAJ24	14q11.2	1 (0.87%)	1 (0.41%)	1.09	0.537	0.686	Co-occurrence

TRAJ25	14q11.2	1 (0.87%)	1 (0.41%)	1.09	0.537	0.686	Co-occurrence
TRAJ26	14q11.2	1 (0.87%)	1 (0.41%)	1.09	0.537	0.686	Co-occurrence
TRAJ27	14q11.2	1 (0.87%)	1 (0.41%)	1.09	0.537	0.686	Co-occurrence
TRAJ28	14q11.2	1 (0.87%)	1 (0.41%)	1.09	0.537	0.686	Co-occurrence
TRAJ29	14q11.2	1 (0.87%)	1 (0.41%)	1.09	0.537	0.686	Co-occurrence
TRAJ3	14q11.2	1 (0.87%)	1 (0.41%)	1.09	0.537	0.686	Co-occurrence
TRAJ30	14q11.2	1 (0.87%)	1 (0.41%)	1.09	0.537	0.686	Co-occurrence
TRAJ31	14q11.2	1 (0.87%)	1 (0.41%)	1.09	0.537	0.686	Co-occurrence
TRAJ32	14q11.2	1 (0.87%)	1 (0.41%)	1.09	0.537	0.686	Co-occurrence
TRAJ33	14q11.2	1 (0.87%)	1 (0.41%)	1.09	0.537	0.686	Co-occurrence
TRAJ34	14q11.2	1 (0.87%)	1 (0.41%)	1.09	0.537	0.686	Co-occurrence
TRAJ35	14q11.2	1 (0.87%)	1 (0.41%)	1.09	0.537	0.686	Co-occurrence
TRAJ36	14q11.2	1 (0.87%)	1 (0.41%)	1.09	0.537	0.686	Co-occurrence
TRAJ37	14q11.2	1 (0.87%)	1 (0.41%)	1.09	0.537	0.686	Co-occurrence
TRAJ38	14q11.2	1 (0.87%)	1 (0.41%)	1.09	0.537	0.686	Co-occurrence
TRAJ39	14q11.2	1 (0.87%)	1 (0.41%)	1.09	0.537	0.686	Co-occurrence
TRAJ4	14q11.2	1 (0.87%)	1 (0.41%)	1.09	0.537	0.686	Co-occurrence
TRAJ40	14q11.2	1 (0.87%)	1 (0.41%)	1.09	0.537	0.686	Co-occurrence
TRAJ41	14q11.2	1 (0.87%)	1 (0.41%)	1.09	0.537	0.686	Co-occurrence
TRAJ42	14q11.2	1 (0.87%)	1 (0.41%)	1.09	0.537	0.686	Co-occurrence
TRAJ43	14q11.2	1 (0.87%)	1 (0.41%)	1.09	0.537	0.686	Co-occurrence
TRAJ44	14q11.2	1 (0.87%)	1 (0.41%)	1.09	0.537	0.686	Co-occurrence
TRAJ45	14q11.2	1 (0.87%)	1 (0.41%)	1.09	0.537	0.686	Co-occurrence
TRAJ46	14q11.2	1 (0.87%)	1 (0.41%)	1.09	0.537	0.686	Co-occurrence
TRAJ47	14q11.2	1 (0.87%)	1 (0.41%)	1.09	0.537	0.686	Co-occurrence
TRAJ48	14q11.2	1 (0.87%)	1 (0.41%)	1.09	0.537	0.686	Co-occurrence
TRAJ49	14q11.2	1 (0.87%)	1 (0.41%)	1.09	0.537	0.686	Co-occurrence
TRAJ5	14q11.2	1 (0.87%)	1 (0.41%)	1.09	0.537	0.686	Co-occurrence
TRAJ50	14q11.2	1 (0.87%)	1 (0.41%)	1.09	0.537	0.686	Co-occurrence

TRAJ52	14q11.2	1 (0.87%)	1 (0.41%)	1.09	0.537	0.686	Co-occurrence
TRAJ53	14q11.2	1 (0.87%)	1 (0.41%)	1.09	0.537	0.686	Co-occurrence
TRAJ54	14q11.2	1 (0.87%)	1 (0.41%)	1.09	0.537	0.686	Co-occurrence
TRAJ56	14q11.2	1 (0.87%)	1 (0.41%)	1.09	0.537	0.686	Co-occurrence
TRAJ57	14q11.2	1 (0.87%)	1 (0.41%)	1.09	0.537	0.686	Co-occurrence
TRAJ58	14q11.2	1 (0.87%)	1 (0.41%)	1.09	0.537	0.686	Co-occurrence
TRAJ59	14q11.2	1 (0.87%)	1 (0.41%)	1.09	0.537	0.686	Co-occurrence
TRAJ6	14q11.2	1 (0.87%)	1 (0.41%)	1.09	0.537	0.686	Co-occurrence
TRAJ61	14q11.2	1 (0.87%)	1 (0.41%)	1.09	0.537	0.686	Co-occurrence
TRAJ7	14q11.2	1 (0.87%)	1 (0.41%)	1.09	0.537	0.686	Co-occurrence
TRAJ8	14q11.2	1 (0.87%)	1 (0.41%)	1.09	0.537	0.686	Co-occurrence
TRAJ9	14q11.2	1 (0.87%)	1 (0.41%)	1.09	0.537	0.686	Co-occurrence
TRAPPC10	21q22.3	1 (0.87%)	1 (0.41%)	1.09	0.537	0.686	Co-occurrence
TRAV10	14q11.2	1 (0.87%)	1 (0.41%)	1.09	0.537	0.686	Co-occurrence
TRAV14DV4	14q11.2	1 (0.87%)	1 (0.41%)	1.09	0.537	0.686	Co-occurrence
TRAV16	14q11.2	1 (0.87%)	1 (0.41%)	1.09	0.537	0.686	Co-occurrence
TRAV17	14q11.2	1 (0.87%)	1 (0.41%)	1.09	0.537	0.686	Co-occurrence
TRAV18	14q11.2	1 (0.87%)	1 (0.41%)	1.09	0.537	0.686	Co-occurrence
TRAV19	14q11.2	1 (0.87%)	1 (0.41%)	1.09	0.537	0.686	Co-occurrence
TRAV2	14q11.2	1 (0.87%)	1 (0.41%)	1.09	0.537	0.686	Co-occurrence
TRAV20	14q11.2	1 (0.87%)	1 (0.41%)	1.09	0.537	0.686	Co-occurrence
TRAV21	14q11.2	1 (0.87%)	1 (0.41%)	1.09	0.537	0.686	Co-occurrence
TRAV22	14q11.2	1 (0.87%)	1 (0.41%)	1.09	0.537	0.686	Co-occurrence
TRAV23DV6	14q11.2	1 (0.87%)	1 (0.41%)	1.09	0.537	0.686	Co-occurrence
TRAV24	14q11.2	1 (0.87%)	1 (0.41%)	1.09	0.537	0.686	Co-occurrence
TRAV25	14q11.2	1 (0.87%)	1 (0.41%)	1.09	0.537	0.686	Co-occurrence
TRAV27	14q11.2	1 (0.87%)	1 (0.41%)	1.09	0.537	0.686	Co-occurrence
TRAV29DV5	14q11.2	1 (0.87%)	1 (0.41%)	1.09	0.537	0.686	Co-occurrence
TRAV3	14q11.2	1 (0.87%)	1 (0.41%)	1.09	0.537	0.686	Co-occurrence

TRAV30	14q11.2	1 (0.87%)	1 (0.41%)	1.09	0.537	0.686	Co-occurrence
TRAV34	14q11.2	1 (0.87%)	1 (0.41%)	1.09	0.537	0.686	Co-occurrence
TRAV35	14q11.2	1 (0.87%)	1 (0.41%)	1.09	0.537	0.686	Co-occurrence
TRAV36DV7	14q11.2	1 (0.87%)	1 (0.41%)	1.09	0.537	0.686	Co-occurrence
TRAV39	14q11.2	1 (0.87%)	1 (0.41%)	1.09	0.537	0.686	Co-occurrence
TRAV4	14q11.2	1 (0.87%)	1 (0.41%)	1.09	0.537	0.686	Co-occurrence
TRAV40	14q11.2	1 (0.87%)	1 (0.41%)	1.09	0.537	0.686	Co-occurrence
TRAV41	14q11.2	1 (0.87%)	1 (0.41%)	1.09	0.537	0.686	Co-occurrence
TRAV5	14q11.2	1 (0.87%)	1 (0.41%)	1.09	0.537	0.686	Co-occurrence
TRAV6	14q11.2	1 (0.87%)	1 (0.41%)	1.09	0.537	0.686	Co-occurrence
TRAV7	14q11.2	1 (0.87%)	1 (0.41%)	1.09	0.537	0.686	Co-occurrence
TRDC	14q11.2	1 (0.87%)	1 (0.41%)	1.09	0.537	0.686	Co-occurrence
TRDD1	14q11.2	1 (0.87%)	1 (0.41%)	1.09	0.537	0.686	Co-occurrence
TRDD2	14q11.2	1 (0.87%)	1 (0.41%)	1.09	0.537	0.686	Co-occurrence
TRDD3	14q11.2	1 (0.87%)	1 (0.41%)	1.09	0.537	0.686	Co-occurrence
TRDJ1	14q11.2	1 (0.87%)	1 (0.41%)	1.09	0.537	0.686	Co-occurrence
TRDJ2	14q11.2	1 (0.87%)	1 (0.41%)	1.09	0.537	0.686	Co-occurrence
TRDJ3	14q11.2	1 (0.87%)	1 (0.41%)	1.09	0.537	0.686	Co-occurrence
TRDJ4	14q11.2	1 (0.87%)	1 (0.41%)	1.09	0.537	0.686	Co-occurrence
TRDV1	14q11.2	1 (0.87%)	1 (0.41%)	1.09	0.537	0.686	Co-occurrence
TRDV2	14q11.2	1 (0.87%)	1 (0.41%)	1.09	0.537	0.686	Co-occurrence
TRDV3	14q11.2	1 (0.87%)	1 (0.41%)	1.09	0.537	0.686	Co-occurrence
TRGC1	7p14.1	1 (0.87%)	1 (0.41%)	1.09	0.537	0.686	Co-occurrence
TRGC2	7p14.1	1 (0.87%)	1 (0.41%)	1.09	0.537	0.686	Co-occurrence
TRGJ1	7p14.1	1 (0.87%)	1 (0.41%)	1.09	0.537	0.686	Co-occurrence
TRGJ2	7p14.1	1 (0.87%)	1 (0.41%)	1.09	0.537	0.686	Co-occurrence
TRGJP	7p14.1	1 (0.87%)	1 (0.41%)	1.09	0.537	0.686	Co-occurrence
TRGJP1	7p14.1	1 (0.87%)	1 (0.41%)	1.09	0.537	0.686	Co-occurrence
TRGJP2	7p14.1	1 (0.87%)	1 (0.41%)	1.09	0.537	0.686	Co-occurrence

TRGV1	7p14.1	1 (0.87%)	1 (0.41%)	1.09	0.537	0.686	Co-occurrence
TRGV10	7p14.1	1 (0.87%)	1 (0.41%)	1.09	0.537	0.686	Co-occurrence
TRGV11	7p14.1	1 (0.87%)	1 (0.41%)	1.09	0.537	0.686	Co-occurrence
TRGV2	7p14.1	1 (0.87%)	1 (0.41%)	1.09	0.537	0.686	Co-occurrence
TRGV3	7p14.1	1 (0.87%)	1 (0.41%)	1.09	0.537	0.686	Co-occurrence
TRGV4	7p14.1	1 (0.87%)	1 (0.41%)	1.09	0.537	0.686	Co-occurrence
TRGV5	7p14.1	1 (0.87%)	1 (0.41%)	1.09	0.537	0.686	Co-occurrence
TRGV8	7p14.1	1 (0.87%)	1 (0.41%)	1.09	0.537	0.686	Co-occurrence
TRGV9	7p14.1	1 (0.87%)	1 (0.41%)	1.09	0.537	0.686	Co-occurrence
TRIM2	4q31.3	1 (0.87%)	1 (0.41%)	1.09	0.537	0.686	Co-occurrence
TRIM21	11p15.4	1 (0.87%)	1 (0.41%)	1.09	0.537	0.686	Co-occurrence
TRIM48	11q11	1 (0.87%)	1 (0.41%)	1.09	0.537	0.686	Co-occurrence
TRIM51	11q12.1	1 (0.87%)	1 (0.41%)	1.09	0.537	0.686	Co-occurrence
TRIM51HP	11q11	1 (0.87%)	1 (0.41%)	1.09	0.537	0.686	Co-occurrence
TRIM62	1p35.1	1 (0.87%)	1 (0.41%)	1.09	0.537	0.686	Co-occurrence
TRMT2A	22q11.21	1 (0.87%)	1 (0.41%)	1.09	0.537	0.686	Co-occurrence
TRPC2	11p15.4	1 (0.87%)	1 (0.41%)	1.09	0.537	0.686	Co-occurrence
TRPM1	15q13.3	1 (0.87%)	1 (0.41%)	1.09	0.537	0.686	Co-occurrence
TRPM3	9q21.12-q21.13	1 (0.87%)	1 (0.41%)	1.09	0.537	0.686	Co-occurrence
TRPM5	11p15.5	1 (0.87%)	1 (0.41%)	1.09	0.537	0.686	Co-occurrence
TRPV4	12q24.11	1 (0.87%)	1 (0.41%)	1.09	0.537	0.686	Co-occurrence
TRUB2	9q34.11	1 (0.87%)	1 (0.41%)	1.09	0.537	0.686	Co-occurrence
TSGA10IP	11q13.1	1 (0.87%)	1 (0.41%)	1.09	0.537	0.686	Co-occurrence
TSPAN14	10q23.1	1 (0.87%)	1 (0.41%)	1.09	0.537	0.686	Co-occurrence
TSPAN15	10q22.1	1 (0.87%)	1 (0.41%)	1.09	0.537	0.686	Co-occurrence
TSPAN32	11p15.5	1 (0.87%)	1 (0.41%)	1.09	0.537	0.686	Co-occurrence
TSSC2	11p15.4	1 (0.87%)	1 (0.41%)	1.09	0.537	0.686	Co-occurrence
TSSC4	11p15.5	1 (0.87%)	1 (0.41%)	1.09	0.537	0.686	Co-occurrence
TSSK2	22q11.21	1 (0.87%)	1 (0.41%)	1.09	0.537	0.686	Co-occurrence

TTI2	8p12	1 (0.87%)	1 (0.41%)	1.09	0.537	0.686	Co-occurrence
TUBA3C	13q12.11	1 (0.87%)	1 (0.41%)	1.09	0.537	0.686	Co-occurrence
TUBA3FP	22q11.21	1 (0.87%)	1 (0.41%)	1.09	0.537	0.686	Co-occurrence
TUBA8	22q11.21	1 (0.87%)	1 (0.41%)	1.09	0.537	0.686	Co-occurrence
TUBB6	18p11.21	1 (0.87%)	1 (0.41%)	1.09	0.537	0.686	Co-occurrence
TXLNG	Xp22.2	1 (0.87%)	1 (0.41%)	1.09	0.537	0.686	Co-occurrence
TXNRD2	22q11.21	1 (0.87%)	1 (0.41%)	1.09	0.537	0.686	Co-occurrence
TYW1B	7q11.22-q11.23	1 (0.87%)	1 (0.41%)	1.09	0.537	0.686	Co-occurrence
UACA	15q23	1 (0.87%)	1 (0.41%)	1.09	0.537	0.686	Co-occurrence
UBE2F	2q37.3	1 (0.87%)	1 (0.41%)	1.09	0.537	0.686	Co-occurrence
UBE2L3	22q11.21	1 (0.87%)	1 (0.41%)	1.09	0.537	0.686	Co-occurrence
UBE2R2	9p13.3	1 (0.87%)	1 (0.41%)	1.09	0.537	0.686	Co-occurrence
UBE4B	1p36.22	1 (0.87%)	1 (0.41%)	1.09	0.537	0.686	Co-occurrence
UBL4B	1p13.3	1 (0.87%)	1 (0.41%)	1.09	0.537	0.686	Co-occurrence
UFD1	22q11.21	1 (0.87%)	1 (0.41%)	1.09	0.537	0.686	Co-occurrence
UHRF1	19p13.3	1 (0.87%)	1 (0.41%)	1.09	0.537	0.686	Co-occurrence
ULK4	3p22.1	1 (0.87%)	1 (0.41%)	1.09	0.537	0.686	Co-occurrence
UMPS	3q21.2	1 (0.87%)	1 (0.41%)	1.09	0.537	0.686	Co-occurrence
UNC13C	15q21.3	1 (0.87%)	1 (0.41%)	1.09	0.537	0.686	Co-occurrence
UNC5B	10q22.1	1 (0.87%)	1 (0.41%)	1.09	0.537	0.686	Co-occurrence
UPB1	22q11.23	1 (0.87%)	1 (0.41%)	1.09	0.537	0.686	Co-occurrence
UQCC1	20q11.22	1 (0.87%)	1 (0.41%)	1.09	0.537	0.686	Co-occurrence
URM1	9q34.11	1 (0.87%)	1 (0.41%)	1.09	0.537	0.686	Co-occurrence
USH1C	11p15.1	1 (0.87%)	1 (0.41%)	1.09	0.537	0.686	Co-occurrence
USP18	22q11.21	1 (0.87%)	1 (0.41%)	1.09	0.537	0.686	Co-occurrence
USP41	22q11.21	1 (0.87%)	1 (0.41%)	1.09	0.537	0.686	Co-occurrence
USP47	11p15.3	1 (0.87%)	1 (0.41%)	1.09	0.537	0.686	Co-occurrence
USP9X	Xp11.4	1 (0.87%)	1 (0.41%)	1.09	0.537	0.686	Co-occurrence
UTRN	6q24.2	1 (0.87%)	1 (0.41%)	1.09	0.537	0.686	Co-occurrence

VCX	Xp22.31	1 (0.87%)	1 (0.41%)	1.09	0.537	0.686	Co-occurrence
VCX2	Xp22.31	1 (0.87%)	1 (0.41%)	1.09	0.537	0.686	Co-occurrence
VCX3A	Xp22.31	1 (0.87%)	1 (0.41%)	1.09	0.537	0.686	Co-occurrence
VIPR1	3p22.1	1 (0.87%)	1 (0.41%)	1.09	0.537	0.686	Co-occurrence
VKORC1L1	7q11.21	1 (0.87%)	1 (0.41%)	1.09	0.537	0.686	Co-occurrence
VPS29	12q24.11	1 (0.87%)	1 (0.41%)	1.09	0.537	0.686	Co-occurrence
VPS41	7p14.1	1 (0.87%)	1 (0.41%)	1.09	0.537	0.686	Co-occurrence
VWC2	7p12.2	1 (0.87%)	1 (0.41%)	1.09	0.537	0.686	Co-occurrence
WDR47	1p13.3	1 (0.87%)	1 (0.41%)	1.09	0.537	0.686	Co-occurrence
WDR6	3p21.31	1 (0.87%)	1 (0.41%)	1.09	0.537	0.686	Co-occurrence
WDR66	12q24.31	1 (0.87%)	1 (0.41%)	1.09	0.537	0.686	Co-occurrence
WDR77	1p13.2	1 (0.87%)	1 (0.41%)	1.09	0.537	0.686	Co-occurrence
WDR81	17p13.3	1 (0.87%)	1 (0.41%)	1.09	0.537	0.686	Co-occurrence
WNK1	12p13.33	1 (0.87%)	1 (0.41%)	1.09	0.537	0.686	Co-occurrence
WNT7A	3p25.1	1 (0.87%)	1 (0.41%)	1.09	0.537	0.686	Co-occurrence
XPC	3p25.1	1 (0.87%)	1 (0.41%)	1.09	0.537	0.686	Co-occurrence
XRCC1	19q13.31	1 (0.87%)	1 (0.41%)	1.09	0.537	0.686	Co-occurrence
XRCC6	22q13.2	1 (0.87%)	1 (0.41%)	1.09	0.537	0.686	Co-occurrence
YAE1	7p14.1	1 (0.87%)	1 (0.41%)	1.09	0.537	0.686	Co-occurrence
YARS	1p35.1	1 (0.87%)	1 (0.41%)	1.09	0.537	0.686	Co-occurrence
YDJC	22q11.21	1 (0.87%)	1 (0.41%)	1.09	0.537	0.686	Co-occurrence
YPEL1	22q11.21-q11.22	1 (0.87%)	1 (0.41%)	1.09	0.537	0.686	Co-occurrence
YPEL3	16p11.2	1 (0.87%)	1 (0.41%)	1.09	0.537	0.686	Co-occurrence
ZAR1L	13q13.1	1 (0.87%)	1 (0.41%)	1.09	0.537	0.686	Co-occurrence
ZBTB7C	18q21.1	1 (0.87%)	1 (0.41%)	1.09	0.537	0.686	Co-occurrence
ZBTB8A	1p35.1	1 (0.87%)	1 (0.41%)	1.09	0.537	0.686	Co-occurrence
ZBTB8OS	1p35.1	1 (0.87%)	1 (0.41%)	1.09	0.537	0.686	Co-occurrence
ZC3H7B	22q13.2	1 (0.87%)	1 (0.41%)	1.09	0.537	0.686	Co-occurrence
ZDHHC1	16q22.1	1 (0.87%)	1 (0.41%)	1.09	0.537	0.686	Co-occurrence

ZDHHC8	22q11.21	1 (0.87%)	1 (0.41%)	1.09	0.537	0.686	Co-occurrence
ZFHX2	14q11.2	1 (0.87%)	1 (0.41%)	1.09	0.537	0.686	Co-occurrence
ZFP37	9q32	1 (0.87%)	1 (0.41%)	1.09	0.537	0.686	Co-occurrence
ZG16	16p11.2	1 (0.87%)	1 (0.41%)	1.09	0.537	0.686	Co-occurrence
ZGRF1	4q25	1 (0.87%)	1 (0.41%)	1.09	0.537	0.686	Co-occurrence
ZHX3	20q12	1 (0.87%)	1 (0.41%)	1.09	0.537	0.686	Co-occurrence
ZMIZ1	10q22.3	1 (0.87%)	1 (0.41%)	1.09	0.537	0.686	Co-occurrence
ZNF107	7q11.21	1 (0.87%)	1 (0.41%)	1.09	0.537	0.686	Co-occurrence
ZNF112	19q13.31	1 (0.87%)	1 (0.41%)	1.09	0.537	0.686	Co-occurrence
ZNF114	19q13.33	1 (0.87%)	1 (0.41%)	1.09	0.537	0.686	Co-occurrence
ZNF117	7q11.21	1 (0.87%)	1 (0.41%)	1.09	0.537	0.686	Co-occurrence
ZNF138	7q11.21	1 (0.87%)	1 (0.41%)	1.09	0.537	0.686	Co-occurrence
ZNF155	19q13.31	1 (0.87%)	1 (0.41%)	1.09	0.537	0.686	Co-occurrence
ZNF182	Xp11.23	1 (0.87%)	1 (0.41%)	1.09	0.537	0.686	Co-occurrence
ZNF195	11p15.4	1 (0.87%)	1 (0.41%)	1.09	0.537	0.686	Co-occurrence
ZNF221	19q13.31	1 (0.87%)	1 (0.41%)	1.09	0.537	0.686	Co-occurrence
ZNF222	19q13.31	1 (0.87%)	1 (0.41%)	1.09	0.537	0.686	Co-occurrence
ZNF223	19q13.31	1 (0.87%)	1 (0.41%)	1.09	0.537	0.686	Co-occurrence
ZNF224	19q13.31	1 (0.87%)	1 (0.41%)	1.09	0.537	0.686	Co-occurrence
ZNF225	19q13.31	1 (0.87%)	1 (0.41%)	1.09	0.537	0.686	Co-occurrence
ZNF226	19q13.31	1 (0.87%)	1 (0.41%)	1.09	0.537	0.686	Co-occurrence
ZNF227	19q13.31	1 (0.87%)	1 (0.41%)	1.09	0.537	0.686	Co-occurrence
ZNF230	19q13.31	1 (0.87%)	1 (0.41%)	1.09	0.537	0.686	Co-occurrence
ZNF233	19q13.31	1 (0.87%)	1 (0.41%)	1.09	0.537	0.686	Co-occurrence
ZNF234	19q13.31	1 (0.87%)	1 (0.41%)	1.09	0.537	0.686	Co-occurrence
ZNF235	19q13.31	1 (0.87%)	1 (0.41%)	1.09	0.537	0.686	Co-occurrence
ZNF273	7q11.21	1 (0.87%)	1 (0.41%)	1.09	0.537	0.686	Co-occurrence
ZNF283	19q13.31	1 (0.87%)	1 (0.41%)	1.09	0.537	0.686	Co-occurrence
ZNF284	19q13.31	1 (0.87%)	1 (0.41%)	1.09	0.537	0.686	Co-occurrence

ZNF285	19q13.31	1 (0.87%)	1 (0.41%)	1.09	0.537	0.686	Co-occurrence
ZNF326	1p22.2	1 (0.87%)	1 (0.41%)	1.09	0.537	0.686	Co-occurrence
ZNF334	20q13.12	1 (0.87%)	1 (0.41%)	1.09	0.537	0.686	Co-occurrence
ZNF365	10q21.2	1 (0.87%)	1 (0.41%)	1.09	0.537	0.686	Co-occurrence
ZNF366	5q13.2|5q13.2	1 (0.87%)	1 (0.41%)	1.09	0.537	0.686	Co-occurrence
ZNF404	19q13.31	1 (0.87%)	1 (0.41%)	1.09	0.537	0.686	Co-occurrence
ZNF428	19q13.31	1 (0.87%)	1 (0.41%)	1.09	0.537	0.686	Co-occurrence
ZNF445	3p21.31	1 (0.87%)	1 (0.41%)	1.09	0.537	0.686	Co-occurrence
ZNF45	19q13.31	1 (0.87%)	1 (0.41%)	1.09	0.537	0.686	Co-occurrence
ZNF48	16p11.2	1 (0.87%)	1 (0.41%)	1.09	0.537	0.686	Co-occurrence
ZNF483	9q31.3	1 (0.87%)	1 (0.41%)	1.09	0.537	0.686	Co-occurrence
ZNF503	10q22.2	1 (0.87%)	1 (0.41%)	1.09	0.537	0.686	Co-occurrence
ZNF518A	10q24.1	1 (0.87%)	1 (0.41%)	1.09	0.537	0.686	Co-occurrence
ZNF536	19q12	1 (0.87%)	1 (0.41%)	1.09	0.537	0.686	Co-occurrence
ZNF541	19q13.33	1 (0.87%)	1 (0.41%)	1.09	0.537	0.686	Co-occurrence
ZNF575	19q13.31	1 (0.87%)	1 (0.41%)	1.09	0.537	0.686	Co-occurrence
ZNF576	19q13.31	1 (0.87%)	1 (0.41%)	1.09	0.537	0.686	Co-occurrence
ZNF618	9q32	1 (0.87%)	1 (0.41%)	1.09	0.537	0.686	Co-occurrence
ZNF630	Xp11.23	1 (0.87%)	1 (0.41%)	1.09	0.537	0.686	Co-occurrence
ZNF679	7q11.21	1 (0.87%)	1 (0.41%)	1.09	0.537	0.686	Co-occurrence
ZNF680	7q11.21	1 (0.87%)	1 (0.41%)	1.09	0.537	0.686	Co-occurrence
ZNF727	7q11.21	1 (0.87%)	1 (0.41%)	1.09	0.537	0.686	Co-occurrence
ZNF736	7q11.21	1 (0.87%)	1 (0.41%)	1.09	0.537	0.686	Co-occurrence
ZNF74	22q11.21	1 (0.87%)	1 (0.41%)	1.09	0.537	0.686	Co-occurrence
ZNF747	16p11.2	1 (0.87%)	1 (0.41%)	1.09	0.537	0.686	Co-occurrence
ZNF768	16p11.2	1 (0.87%)	1 (0.41%)	1.09	0.537	0.686	Co-occurrence
ZNF771	16p11.2	1 (0.87%)	1 (0.41%)	1.09	0.537	0.686	Co-occurrence
ZNF79	9q33.3	1 (0.87%)	1 (0.41%)	1.09	0.537	0.686	Co-occurrence
ZNF81	Xp11.23	1 (0.87%)	1 (0.41%)	1.09	0.537	0.686	Co-occurrence

ZNF883	9q32	1 (0.87%)	1 (0.41%)	1.09	0.537	0.686	Co-occurrence
ZNF92	7q11.21	1 (0.87%)	1 (0.41%)	1.09	0.537	0.686	Co-occurrence
ZPBP	7p12.2	1 (0.87%)	1 (0.41%)	1.09	0.537	0.686	Co-occurrence
ZSWIM6	5q12.1	1 (0.87%)	1 (0.41%)	1.09	0.537	0.686	Co-occurrence
ZSWIM9	19q13.33	1 (0.87%)	1 (0.41%)	1.09	0.537	0.686	Co-occurrence
ZWINT	10q21.1	1 (0.87%)	1 (0.41%)	1.09	0.537	0.686	Co-occurrence
LINC00293	8q11.1	7 (6.09%)	16 (6.53%)	-0.1	0.538	0.686	Mutual exclusivity
POLH	6p21.1	7 (6.09%)	16 (6.53%)	-0.1	0.538	0.686	Mutual exclusivity
RN7SKP32	8q11.1	7 (6.09%)	16 (6.53%)	-0.1	0.538	0.686	Mutual exclusivity
XPO5	6p21.1	7 (6.09%)	16 (6.53%)	-0.1	0.538	0.686	Mutual exclusivity
MRPL14	6p21.1	6 (5.22%)	12 (4.90%)	0.09	0.54	0.686	Co-occurrence
NSMAF	8q12.1	10 (8.70%)	22 (8.98%)	-0.05	0.552	0.686	Mutual exclusivity
C6ORF15	6p21.33	5 (4.35%)	10 (4.08%)	0.09	0.553	0.686	Co-occurrence
CCHCR1	6p21.33	5 (4.35%)	10 (4.08%)	0.09	0.553	0.686	Co-occurrence
CDSN	6p21.33	5 (4.35%)	10 (4.08%)	0.09	0.553	0.686	Co-occurrence
HCG21	6p21.33	5 (4.35%)	10 (4.08%)	0.09	0.553	0.686	Co-occurrence
HCG22	6p21.33	5 (4.35%)	10 (4.08%)	0.09	0.553	0.686	Co-occurrence
KAAG1	6p22.3	5 (4.35%)	10 (4.08%)	0.09	0.553	0.686	Co-occurrence
MUC21	6p21.33	5 (4.35%)	10 (4.08%)	0.09	0.553	0.686	Co-occurrence
MUC22	6p21.33	5 (4.35%)	10 (4.08%)	0.09	0.553	0.686	Co-occurrence
MUCL3	6p21.33	5 (4.35%)	10 (4.08%)	0.09	0.553	0.686	Co-occurrence
PSORS1C1	6p21.33	5 (4.35%)	10 (4.08%)	0.09	0.553	0.686	Co-occurrence
PSORS1C2	6p21.33	5 (4.35%)	10 (4.08%)	0.09	0.553	0.686	Co-occurrence
SFTA2	6p21.33	5 (4.35%)	10 (4.08%)	0.09	0.553	0.686	Co-occurrence
TCF19	6p21.33	5 (4.35%)	10 (4.08%)	0.09	0.553	0.686	Co-occurrence
CLVS1	8q12.2-q12.3	10 (8.70%)	21 (8.57%)	0.02	0.556	0.686	Co-occurrence
ARMC12	6p21.31	4 (3.48%)	8 (3.27%)	0.09	0.569	0.686	Co-occurrence
BMP5	6p12.1	4 (3.48%)	8 (3.27%)	0.09	0.569	0.686	Co-occurrence
BTBD9	6p21.2	4 (3.48%)	8 (3.27%)	0.09	0.569	0.686	Co-occurrence

C2ORF48	2p25.1	4 (3.48%)	8 (3.27%)	0.09	0.569	0.686	Co-occurrence
C6ORF141	6p12.3	4 (3.48%)	8 (3.27%)	0.09	0.569	0.686	Co-occurrence
CENPQ	6p12.3	4 (3.48%)	8 (3.27%)	0.09	0.569	0.686	Co-occurrence
COL11A2	6p21.32	4 (3.48%)	8 (3.27%)	0.09	0.569	0.686	Co-occurrence
DAAM2	6p21.2	4 (3.48%)	8 (3.27%)	0.09	0.569	0.686	Co-occurrence
GFRAL	6p12.1	4 (3.48%)	8 (3.27%)	0.09	0.569	0.686	Co-occurrence
GLYATL3	6p12.3	4 (3.48%)	8 (3.27%)	0.09	0.569	0.686	Co-occurrence
GRM4	6p21.31	4 (3.48%)	8 (3.27%)	0.09	0.569	0.686	Co-occurrence
HCRTR2	6p12.1	4 (3.48%)	8 (3.27%)	0.09	0.569	0.686	Co-occurrence
HPCAL1	2p25.1	4 (3.48%)	8 (3.27%)	0.09	0.569	0.686	Co-occurrence
ILRUN	6p21.31	4 (3.48%)	8 (3.27%)	0.09	0.569	0.686	Co-occurrence
KCNK5	6p21.2	4 (3.48%)	8 (3.27%)	0.09	0.569	0.686	Co-occurrence
KNG1	3q27.3	4 (3.48%)	8 (3.27%)	0.09	0.569	0.686	Co-occurrence
LINC00299	2p25.1	4 (3.48%)	8 (3.27%)	0.09	0.569	0.686	Co-occurrence
LINC00951	6p21.2	4 (3.48%)	8 (3.27%)	0.09	0.569	0.686	Co-occurrence
LPIN1	2p25.1	4 (3.48%)	8 (3.27%)	0.09	0.569	0.686	Co-occurrence
LPP	3q27.3-q28	4 (3.48%)	8 (3.27%)	0.09	0.569	0.686	Co-occurrence
MATN3	2p24.1	4 (3.48%)	8 (3.27%)	0.09	0.569	0.686	Co-occurrence
MIR-4261/4261		4 (3.48%)	8 (3.27%)	0.09	0.569	0.686	Co-occurrence
MMUT	6p12.3	4 (3.48%)	8 (3.27%)	0.09	0.569	0.686	Co-occurrence
MOCS1	6p21.2	4 (3.48%)	8 (3.27%)	0.09	0.569	0.686	Co-occurrence
MTCH1	6p21.2	4 (3.48%)	8 (3.27%)	0.09	0.569	0.686	Co-occurrence
NTSR2	2p25.1	4 (3.48%)	8 (3.27%)	0.09	0.569	0.686	Co-occurrence
ODC1	2p25.1	4 (3.48%)	8 (3.27%)	0.09	0.569	0.686	Co-occurrence
PKHD1	6p12.3-p12.2	4 (3.48%)	8 (3.27%)	0.09	0.569	0.686	Co-occurrence
RHAG	6p12.3	4 (3.48%)	8 (3.27%)	0.09	0.569	0.686	Co-occurrence
RN7SL580P	6p12.2	4 (3.48%)	8 (3.27%)	0.09	0.569	0.686	Co-occurrence
RN7SL66P	2p25.1	4 (3.48%)	8 (3.27%)	0.09	0.569	0.686	Co-occurrence
RPS10	6p21.31	4 (3.48%)	8 (3.27%)	0.09	0.569	0.686	Co-occurrence

RRM2	2p25.1	4 (3.48%)	8 (3.27%)	0.09	0.569	0.686	Co-occurrence
RXRB	6p21.32	4 (3.48%)	8 (3.27%)	0.09	0.569	0.686	Co-occurrence
SNORA80B	2p25.1	4 (3.48%)	8 (3.27%)	0.09	0.569	0.686	Co-occurrence
SPDEF	6p21.31	4 (3.48%)	8 (3.27%)	0.09	0.569	0.686	Co-occurrence
TAF1B	2p25.1	4 (3.48%)	8 (3.27%)	0.09	0.569	0.686	Co-occurrence
TDRG1	6p21.2	4 (3.48%)	8 (3.27%)	0.09	0.569	0.686	Co-occurrence
WDR35	2p24.1	4 (3.48%)	8 (3.27%)	0.09	0.569	0.686	Co-occurrence
SNTG1	8q11.21	8 (6.96%)	17 (6.94%)	0	0.576	0.686	Co-occurrence
ACP1	2p25.3	3 (2.61%)	6 (2.45%)	0.09	0.59	0.686	Co-occurrence
ADAMTS17	15q26.3	3 (2.61%)	6 (2.45%)	0.09	0.59	0.686	Co-occurrence
AGBL5	2p23.3	3 (2.61%)	6 (2.45%)	0.09	0.59	0.686	Co-occurrence
AKAP9	7q21.2	3 (2.61%)	6 (2.45%)	0.09	0.59	0.686	Co-occurrence
ALDH1A3	15q26.3	3 (2.61%)	6 (2.45%)	0.09	0.59	0.686	Co-occurrence
ALKAL2	2p25.3	3 (2.61%)	6 (2.45%)	0.09	0.59	0.686	Co-occurrence
APOB	2p24.1	3 (2.61%)	6 (2.45%)	0.09	0.59	0.686	Co-occurrence
ASB7	15q26.3	3 (2.61%)	6 (2.45%)	0.09	0.59	0.686	Co-occurrence
ATP13A5	3q29	3 (2.61%)	6 (2.45%)	0.09	0.59	0.686	Co-occurrence
BCL6	3q27.3	3 (2.61%)	6 (2.45%)	0.09	0.59	0.686	Co-occurrence
C3ORF70	3q27.2	3 (2.61%)	6 (2.45%)	0.09	0.59	0.686	Co-occurrence
CCDC39	3q26.33	3 (2.61%)	6 (2.45%)	0.09	0.59	0.686	Co-occurrence
CERS3	15q26.3	3 (2.61%)	6 (2.45%)	0.09	0.59	0.686	Co-occurrence
CMPK2	2p25.2	3 (2.61%)	6 (2.45%)	0.09	0.59	0.686	Co-occurrence
CYP51A1	7q21.2	3 (2.61%)	6 (2.45%)	0.09	0.59	0.686	Co-occurrence
DGKG	3q27.2-q27.3	3 (2.61%)	6 (2.45%)	0.09	0.59	0.686	Co-occurrence
DNM1P46	15q26.3	3 (2.61%)	6 (2.45%)	0.09	0.59	0.686	Co-occurrence
DPYSL5	2p23.3	3 (2.61%)	6 (2.45%)	0.09	0.59	0.686	Co-occurrence
EMILIN1	2p23.3	3 (2.61%)	6 (2.45%)	0.09	0.59	0.686	Co-occurrence
FAM110C	2p25.3	3 (2.61%)	6 (2.45%)	0.09	0.59	0.686	Co-occurrence
FETUB	3q27.3	3 (2.61%)	6 (2.45%)	0.09	0.59	0.686	Co-occurrence

FGF12	3q28-q29	3 (2.61%)	6 (2.45%)	0.09	0.59	0.686	Co-occurrence
GPR160	3q26.2	3 (2.61%)	6 (2.45%)	0.09	0.59	0.686	Co-occurrence
IRX4	5p15.33	3 (2.61%)	6 (2.45%)	0.09	0.59	0.686	Co-occurrence
KHK	2p23.3	3 (2.61%)	6 (2.45%)	0.09	0.59	0.686	Co-occurrence
LINC00487	2p25.2	3 (2.61%)	6 (2.45%)	0.09	0.59	0.686	Co-occurrence
LINS1	15q26.3	3 (2.61%)	6 (2.45%)	0.09	0.59	0.686	Co-occurrence
LIPH	3q27.2	3 (2.61%)	6 (2.45%)	0.09	0.59	0.686	Co-occurrence
LPCAT1	5p15.33	3 (2.61%)	6 (2.45%)	0.09	0.59	0.686	Co-occurrence
LRATD1	2p24.3	3 (2.61%)	6 (2.45%)	0.09	0.59	0.686	Co-occurrence
LRRC31	3q26.2	3 (2.61%)	6 (2.45%)	0.09	0.59	0.686	Co-occurrence
LRRC34	3q26.2	3 (2.61%)	6 (2.45%)	0.09	0.59	0.686	Co-occurrence
LRRC46	17q21.32	3 (2.61%)	6 (2.45%)	0.09	0.59	0.686	Co-occurrence
LRRD1	7q21.2	3 (2.61%)	6 (2.45%)	0.09	0.59	0.686	Co-occurrence
LRRIQ4	3q26.2	3 (2.61%)	6 (2.45%)	0.09	0.59	0.686	Co-occurrence
LRRK1	15q26.3	3 (2.61%)	6 (2.45%)	0.09	0.59	0.686	Co-occurrence
LYSMD4	15q26.3	3 (2.61%)	6 (2.45%)	0.09	0.59	0.686	Co-occurrence
MAP3K20	2q31.1	3 (2.61%)	6 (2.45%)	0.09	0.59	0.686	Co-occurrence
MAPRE3	2p23.3	3 (2.61%)	6 (2.45%)	0.09	0.59	0.686	Co-occurrence
MEF2A	15q26.3	3 (2.61%)	6 (2.45%)	0.09	0.59	0.686	Co-occurrence
MIR-4277/4277		3 (2.61%)	6 (2.45%)	0.09	0.59	0.686	Co-occurrence
MIR-4457/4457		3 (2.61%)	6 (2.45%)	0.09	0.59	0.686	Co-occurrence
MRPL10	17q21.32	3 (2.61%)	6 (2.45%)	0.09	0.59	0.686	Co-occurrence
MRPL36	5p15.33	3 (2.61%)	6 (2.45%)	0.09	0.59	0.686	Co-occurrence
MYNN	3q26.2	3 (2.61%)	6 (2.45%)	0.09	0.59	0.686	Co-occurrence
NDUFS6	5p15.33	3 (2.61%)	6 (2.45%)	0.09	0.59	0.686	Co-occurrence
OST4	2p23.3	3 (2.61%)	6 (2.45%)	0.09	0.59	0.686	Co-occurrence
PHC3	3q26.2	3 (2.61%)	6 (2.45%)	0.09	0.59	0.686	Co-occurrence
PRKCI	3q26.2	3 (2.61%)	6 (2.45%)	0.09	0.59	0.686	Co-occurrence
PXDN	2p25.3	3 (2.61%)	6 (2.45%)	0.09	0.59	0.686	Co-occurrence

RAPGEF4	2q31.1	3 (2.61%)	6 (2.45%)	0.09	0.59	0.686	Co-occurrence
RHOD	11q13.2	3 (2.61%)	6 (2.45%)	0.09	0.59	0.686	Co-occurrence
RN7SKP222	3q28	3 (2.61%)	6 (2.45%)	0.09	0.59	0.686	Co-occurrence
RN7SL484P	15q26.3	3 (2.61%)	6 (2.45%)	0.09	0.59	0.686	Co-occurrence
RNA5SP402	15q26.3	3 (2.61%)	6 (2.45%)	0.09	0.59	0.686	Co-occurrence
RSAD2	2p25.2	3 (2.61%)	6 (2.45%)	0.09	0.59	0.686	Co-occurrence
SAMD7	3q26.2	3 (2.61%)	6 (2.45%)	0.09	0.59	0.686	Co-occurrence
SCRN2	17q21.32	3 (2.61%)	6 (2.45%)	0.09	0.59	0.686	Co-occurrence
SDHAP3	5p15.33	3 (2.61%)	6 (2.45%)	0.09	0.59	0.686	Co-occurrence
SEC62	3q26.2	3 (2.61%)	6 (2.45%)	0.09	0.59	0.686	Co-occurrence
SH3YL1	2p25.3	3 (2.61%)	6 (2.45%)	0.09	0.59	0.686	Co-occurrence
SKIL	3q26.2	3 (2.61%)	6 (2.45%)	0.09	0.59	0.686	Co-occurrence
SLC2A2	3q26.2	3 (2.61%)	6 (2.45%)	0.09	0.59	0.686	Co-occurrence
SLC6A3	5p15.33	3 (2.61%)	6 (2.45%)	0.09	0.59	0.686	Co-occurrence
SNTG2	2p25.3	3 (2.61%)	6 (2.45%)	0.09	0.59	0.686	Co-occurrence
SP6	17q21.32	3 (2.61%)	6 (2.45%)	0.09	0.59	0.686	Co-occurrence
SPATA41	15q26.3	3 (2.61%)	6 (2.45%)	0.09	0.59	0.686	Co-occurrence
SYNM	15q26.3	3 (2.61%)	6 (2.45%)	0.09	0.59	0.686	Co-occurrence
SYT12	11q13.2	3 (2.61%)	6 (2.45%)	0.09	0.59	0.686	Co-occurrence
TDRD15	2p24.1	3 (2.61%)	6 (2.45%)	0.09	0.59	0.686	Co-occurrence
TMEM18	2p25.3	3 (2.61%)	6 (2.45%)	0.09	0.59	0.686	Co-occurrence
TMEM214	2p23.3	3 (2.61%)	6 (2.45%)	0.09	0.59	0.686	Co-occurrence
TMEM41A	3q27.2	3 (2.61%)	6 (2.45%)	0.09	0.59	0.686	Co-occurrence
TPO	2p25.3	3 (2.61%)	6 (2.45%)	0.09	0.59	0.686	Co-occurrence
TTC14	3q26.33	3 (2.61%)	6 (2.45%)	0.09	0.59	0.686	Co-occurrence
TTC23	15q26.3	3 (2.61%)	6 (2.45%)	0.09	0.59	0.686	Co-occurrence
DCDC2	6p22.3	5 (4.35%)	11 (4.49%)	-0.05	0.595	0.686	Mutual exclusivity
ABT1	6p22.2	4 (3.48%)	9 (3.67%)	-0.08	0.596	0.686	Mutual exclusivity
BTN1A1	6p22.2	4 (3.48%)	9 (3.67%)	-0.08	0.596	0.686	Mutual exclusivity

DST	6p12.1	4 (3.48%)	9 (3.67%)	-0.08	0.596	0.686	Mutual exclusivity
FKBP5	6p21.31	4 (3.48%)	9 (3.67%)	-0.08	0.596	0.686	Mutual exclusivity
GUSBP2	6p22.2	4 (3.48%)	9 (3.67%)	-0.08	0.596	0.686	Mutual exclusivity
HCG11	6p22.2	4 (3.48%)	9 (3.67%)	-0.08	0.596	0.686	Mutual exclusivity
HCG24	6p21.32	4 (3.48%)	9 (3.67%)	-0.08	0.596	0.686	Mutual exclusivity
HIST1H1B	6p22.1	4 (3.48%)	9 (3.67%)	-0.08	0.596	0.686	Mutual exclusivity
HIST1H2AA	6p22.2	4 (3.48%)	9 (3.67%)	-0.08	0.596	0.686	Mutual exclusivity
HIST1H2AG	6p22.1	4 (3.48%)	9 (3.67%)	-0.08	0.596	0.686	Mutual exclusivity
HIST1H2AH	6p22.1	4 (3.48%)	9 (3.67%)	-0.08	0.596	0.686	Mutual exclusivity
HIST1H2AI	6p22.1	4 (3.48%)	9 (3.67%)	-0.08	0.596	0.686	Mutual exclusivity
HIST1H2AJ	6p22.1	4 (3.48%)	9 (3.67%)	-0.08	0.596	0.686	Mutual exclusivity
HIST1H2AK	6p22.1	4 (3.48%)	9 (3.67%)	-0.08	0.596	0.686	Mutual exclusivity
HIST1H2AL	6p22.1	4 (3.48%)	9 (3.67%)	-0.08	0.596	0.686	Mutual exclusivity
HIST1H2AM	6p22.1	4 (3.48%)	9 (3.67%)	-0.08	0.596	0.686	Mutual exclusivity
HIST1H2BA	6p22.2	4 (3.48%)	9 (3.67%)	-0.08	0.596	0.686	Mutual exclusivity
HIST1H2BJ	6p22.1	4 (3.48%)	9 (3.67%)	-0.08	0.596	0.686	Mutual exclusivity
HIST1H2BK	6p22.1	4 (3.48%)	9 (3.67%)	-0.08	0.596	0.686	Mutual exclusivity
HIST1H2BL	6p22.1	4 (3.48%)	9 (3.67%)	-0.08	0.596	0.686	Mutual exclusivity
HIST1H2BM	6p22.1	4 (3.48%)	9 (3.67%)	-0.08	0.596	0.686	Mutual exclusivity
HIST1H2BN	6p22.1	4 (3.48%)	9 (3.67%)	-0.08	0.596	0.686	Mutual exclusivity
HIST1H2BO	6p22.1	4 (3.48%)	9 (3.67%)	-0.08	0.596	0.686	Mutual exclusivity
HIST1H3H	6p22.1	4 (3.48%)	9 (3.67%)	-0.08	0.596	0.686	Mutual exclusivity
HIST1H3I	6p22.1	4 (3.48%)	9 (3.67%)	-0.08	0.596	0.686	Mutual exclusivity
HIST1H3J	6p22.1	4 (3.48%)	9 (3.67%)	-0.08	0.596	0.686	Mutual exclusivity
HIST1H4I	6p22.1	4 (3.48%)	9 (3.67%)	-0.08	0.596	0.686	Mutual exclusivity
HIST1H4J	6p22.1	4 (3.48%)	9 (3.67%)	-0.08	0.596	0.686	Mutual exclusivity
HIST1H4K	6p22.1	4 (3.48%)	9 (3.67%)	-0.08	0.596	0.686	Mutual exclusivity
HIST1H4L	6p22.1	4 (3.48%)	9 (3.67%)	-0.08	0.596	0.686	Mutual exclusivity
HMGA1	6p21.31	4 (3.48%)	9 (3.67%)	-0.08	0.596	0.686	Mutual exclusivity

HMGN4	6p22.2	4 (3.48%)	9 (3.67%)	-0.08	0.596	0.686	Mutual exclusivity
KIF6	6p21.2	4 (3.48%)	9 (3.67%)	-0.08	0.596	0.686	Mutual exclusivity
LINC00240	6p22.2	4 (3.48%)	9 (3.67%)	-0.08	0.596	0.686	Mutual exclusivity
LRFN2	6p21.2-p21.1	4 (3.48%)	9 (3.67%)	-0.08	0.596	0.686	Mutual exclusivity
MIR-3143/3143		4 (3.48%)	9 (3.67%)	-0.08	0.596	0.686	Mutual exclusivity
MLIP	6p12.1	4 (3.48%)	9 (3.67%)	-0.08	0.596	0.686	Mutual exclusivity
MOG	6p22.1	4 (3.48%)	9 (3.67%)	-0.08	0.596	0.686	Mutual exclusivity
NUDT3	6p21.31	4 (3.48%)	9 (3.67%)	-0.08	0.596	0.686	Mutual exclusivity
OR2B2	6p22.1	4 (3.48%)	9 (3.67%)	-0.08	0.596	0.686	Mutual exclusivity
OR2B6	6p22.1	4 (3.48%)	9 (3.67%)	-0.08	0.596	0.686	Mutual exclusivity
OR2H2	6p22.1	4 (3.48%)	9 (3.67%)	-0.08	0.596	0.686	Mutual exclusivity
PACSIN1	6p21.31	4 (3.48%)	9 (3.67%)	-0.08	0.596	0.686	Mutual exclusivity
POM121L2	6p22.1	4 (3.48%)	9 (3.67%)	-0.08	0.596	0.686	Mutual exclusivity
PTCHD4	6p12.3	4 (3.48%)	9 (3.67%)	-0.08	0.596	0.686	Mutual exclusivity
RNY5P5	6p22.3	4 (3.48%)	9 (3.67%)	-0.08	0.596	0.686	Mutual exclusivity
SCGN	6p22.2	4 (3.48%)	9 (3.67%)	-0.08	0.596	0.686	Mutual exclusivity
SMIM29	6p21.31	4 (3.48%)	9 (3.67%)	-0.08	0.596	0.686	Mutual exclusivity
SNORD32B	6p22.1	4 (3.48%)	9 (3.67%)	-0.08	0.596	0.686	Mutual exclusivity
TRI-TAT2-2	6p22.2	4 (3.48%)	9 (3.67%)	-0.08	0.596	0.686	Mutual exclusivity
TRI-TAT2-3	6p22.1	4 (3.48%)	9 (3.67%)	-0.08	0.596	0.686	Mutual exclusivity
ZFP57	6p22.1	4 (3.48%)	9 (3.67%)	-0.08	0.596	0.686	Mutual exclusivity
ZNF165	6p22.1	4 (3.48%)	9 (3.67%)	-0.08	0.596	0.686	Mutual exclusivity
ZNF184	6p22.1	4 (3.48%)	9 (3.67%)	-0.08	0.596	0.686	Mutual exclusivity
ZNF322	6p22.2	4 (3.48%)	9 (3.67%)	-0.08	0.596	0.686	Mutual exclusivity
ZNF391	6p22.1	4 (3.48%)	9 (3.67%)	-0.08	0.596	0.686	Mutual exclusivity
ZSCAN12P1	6p22.1	4 (3.48%)	9 (3.67%)	-0.08	0.596	0.686	Mutual exclusivity
ZSCAN16	6p22.1	4 (3.48%)	9 (3.67%)	-0.08	0.596	0.686	Mutual exclusivity
CAPN11	6p21.1	6 (5.22%)	13 (5.31%)	-0.02	0.596	0.686	Mutual exclusivity
TMEM63B	6p21.1	6 (5.22%)	13 (5.31%)	-0.02	0.596	0.686	Mutual exclusivity

ABHD1	2p23.3	3 (2.61%)	7 (2.86%)	-0.13	0.598	0.686	Mutual exclusivity
ADAM17	2p25.1	3 (2.61%)	7 (2.86%)	-0.13	0.598	0.686	Mutual exclusivity
ADIPOQ	3q27.3	3 (2.61%)	7 (2.86%)	-0.13	0.598	0.686	Mutual exclusivity
AHSG	3q27.3	3 (2.61%)	7 (2.86%)	-0.13	0.598	0.686	Mutual exclusivity
ASAP2	2p25.1|2p24	3 (2.61%)	7 (2.86%)	-0.13	0.598	0.686	Mutual exclusivity
ATRAID	2p23.3	3 (2.61%)	7 (2.86%)	-0.13	0.598	0.686	Mutual exclusivity
AUTS2	7q11.22	3 (2.61%)	7 (2.86%)	-0.13	0.598	0.686	Mutual exclusivity
BNIP5	6p21.31	3 (2.61%)	7 (2.86%)	-0.13	0.598	0.686	Mutual exclusivity
BRPF3	6p21.31	3 (2.61%)	7 (2.86%)	-0.13	0.598	0.686	Mutual exclusivity
C6ORF89	6p21.2	3 (2.61%)	7 (2.86%)	-0.13	0.598	0.686	Mutual exclusivity
CAD	2p23.3	3 (2.61%)	7 (2.86%)	-0.13	0.598	0.686	Mutual exclusivity
CCDC167	6p21.2	3 (2.61%)	7 (2.86%)	-0.13	0.598	0.686	Mutual exclusivity
CGREF1	2p23.3	3 (2.61%)	7 (2.86%)	-0.13	0.598	0.686	Mutual exclusivity
CHSY1	15q26.3	3 (2.61%)	7 (2.86%)	-0.13	0.598	0.686	Mutual exclusivity
CMTR1	6p21.2	3 (2.61%)	7 (2.86%)	-0.13	0.598	0.686	Mutual exclusivity
CPNE5	6p21.2	3 (2.61%)	7 (2.86%)	-0.13	0.598	0.686	Mutual exclusivity
CPSF3	2p25.1	3 (2.61%)	7 (2.86%)	-0.13	0.598	0.686	Mutual exclusivity
DDX1	2p24.3	3 (2.61%)	7 (2.86%)	-0.13	0.598	0.686	Mutual exclusivity
DDX11L9	15q26.3	3 (2.61%)	7 (2.86%)	-0.13	0.598	0.686	Mutual exclusivity
DEF6	6p21.31	3 (2.61%)	7 (2.86%)	-0.13	0.598	0.686	Mutual exclusivity
DNM1P47	15q26.3	3 (2.61%)	7 (2.86%)	-0.13	0.598	0.686	Mutual exclusivity
EIF4A2	3q27.3	3 (2.61%)	7 (2.86%)	-0.13	0.598	0.686	Mutual exclusivity
ETV7	6p21.31	3 (2.61%)	7 (2.86%)	-0.13	0.598	0.686	Mutual exclusivity
FADD	11q13.3	3 (2.61%)	7 (2.86%)	-0.13	0.598	0.686	Mutual exclusivity
FAM138E	15q26.3	3 (2.61%)	7 (2.86%)	-0.13	0.598	0.686	Mutual exclusivity
FAM49A	2p24.2	3 (2.61%)	7 (2.86%)	-0.13	0.598	0.686	Mutual exclusivity
FANCE	6p21.31	3 (2.61%)	7 (2.86%)	-0.13	0.598	0.686	Mutual exclusivity
GDF7	2p24.1	3 (2.61%)	7 (2.86%)	-0.13	0.598	0.686	Mutual exclusivity
GEN1	2p24.2	3 (2.61%)	7 (2.86%)	-0.13	0.598	0.686	Mutual exclusivity

IAH1	2p25.1	3 (2.61%)	7 (2.86%)	-0.13	0.598	0.686	Mutual exclusivity
ID2	2p25.1	3 (2.61%)	7 (2.86%)	-0.13	0.598	0.686	Mutual exclusivity
ITGB1BP1	2p25.1	3 (2.61%)	7 (2.86%)	-0.13	0.598	0.686	Mutual exclusivity
KIDINS220	2p25.1	3 (2.61%)	7 (2.86%)	-0.13	0.598	0.686	Mutual exclusivity
LDAH	2p24.1	3 (2.61%)	7 (2.86%)	-0.13	0.598	0.686	Mutual exclusivity
LINC00276	2p24.3	3 (2.61%)	7 (2.86%)	-0.13	0.598	0.686	Mutual exclusivity
LINC00680	6p11.2	3 (2.61%)	7 (2.86%)	-0.13	0.598	0.686	Mutual exclusivity
LRRC28	15q26.3	3 (2.61%)	7 (2.86%)	-0.13	0.598	0.686	Mutual exclusivity
MBOAT2	2p25.1	3 (2.61%)	7 (2.86%)	-0.13	0.598	0.686	Mutual exclusivity
MIR-3125/3125		3 (2.61%)	7 (2.86%)	-0.13	0.598	0.686	Mutual exclusivity
MIR-4262/4262		3 (2.61%)	7 (2.86%)	-0.13	0.598	0.686	Mutual exclusivity
MIR-4462/4462		3 (2.61%)	7 (2.86%)	-0.13	0.598	0.686	Mutual exclusivity
MIR-548K/548K		3 (2.61%)	7 (2.86%)	-0.13	0.598	0.686	Mutual exclusivity
MIR-548U/548U		3 (2.61%)	7 (2.86%)	-0.13	0.598	0.686	Mutual exclusivity
MIR-569/569		3 (2.61%)	7 (2.86%)	-0.13	0.598	0.686	Mutual exclusivity
MSGN1	2p24.2	3 (2.61%)	7 (2.86%)	-0.13	0.598	0.686	Mutual exclusivity
MYCN	2p24.3	3 (2.61%)	7 (2.86%)	-0.13	0.598	0.686	Mutual exclusivity
MYCNOS	2p24.3	3 (2.61%)	7 (2.86%)	-0.13	0.598	0.686	Mutual exclusivity
MYT1L	2p25.3	3 (2.61%)	7 (2.86%)	-0.13	0.598	0.686	Mutual exclusivity
NBAS	2p24.3	3 (2.61%)	7 (2.86%)	-0.13	0.598	0.686	Mutual exclusivity
OR4F13P	15q26.3	3 (2.61%)	7 (2.86%)	-0.13	0.598	0.686	Mutual exclusivity
OR4F15	15q26.3	3 (2.61%)	7 (2.86%)	-0.13	0.598	0.686	Mutual exclusivity
OR4F4	15q26.3	3 (2.61%)	7 (2.86%)	-0.13	0.598	0.686	Mutual exclusivity
OR4F6	15q26.3	3 (2.61%)	7 (2.86%)	-0.13	0.598	0.686	Mutual exclusivity
PCSK6	15q26.3	3 (2.61%)	7 (2.86%)	-0.13	0.598	0.686	Mutual exclusivity
PI16	6p21.2	3 (2.61%)	7 (2.86%)	-0.13	0.598	0.686	Mutual exclusivity
PNPLA1	6p21.31	3 (2.61%)	7 (2.86%)	-0.13	0.598	0.686	Mutual exclusivity
PPFIA1	11q13.3	3 (2.61%)	7 (2.86%)	-0.13	0.598	0.686	Mutual exclusivity
PPIL1	6p21.2	3 (2.61%)	7 (2.86%)	-0.13	0.598	0.686	Mutual exclusivity

PREB	2p23.3	3 (2.61%)	7 (2.86%)	-0.13	0.598	0.686	Mutual exclusivity
PRR30	2p23.3	3 (2.61%)	7 (2.86%)	-0.13	0.598	0.686	Mutual exclusivity
PXT1	6p21.31	3 (2.61%)	7 (2.86%)	-0.13	0.598	0.686	Mutual exclusivity
RAB44	6p21.2	3 (2.61%)	7 (2.86%)	-0.13	0.598	0.686	Mutual exclusivity
RAD51AP2	2p24.2	3 (2.61%)	7 (2.86%)	-0.13	0.598	0.686	Mutual exclusivity
RFC4	3q27.3	3 (2.61%)	7 (2.86%)	-0.13	0.598	0.686	Mutual exclusivity
RN7SKP112	2p25.1	3 (2.61%)	7 (2.86%)	-0.13	0.598	0.686	Mutual exclusivity
RN7SKP168	2p24.2	3 (2.61%)	7 (2.86%)	-0.13	0.598	0.686	Mutual exclusivity
RN7SL104P	2p24.3	3 (2.61%)	7 (2.86%)	-0.13	0.598	0.686	Mutual exclusivity
RN7SL209P	15q26.3	3 (2.61%)	7 (2.86%)	-0.13	0.598	0.686	Mutual exclusivity
RN7SL273P	6p21.2	3 (2.61%)	7 (2.86%)	-0.13	0.598	0.686	Mutual exclusivity
RN7SL502P	6p21.31	3 (2.61%)	7 (2.86%)	-0.13	0.598	0.686	Mutual exclusivity
RN7SL531P	2p21	3 (2.61%)	7 (2.86%)	-0.13	0.598	0.686	Mutual exclusivity
RNF144A	2p25.1	3 (2.61%)	7 (2.86%)	-0.13	0.598	0.686	Mutual exclusivity
RNF8	6p21.2	3 (2.61%)	7 (2.86%)	-0.13	0.598	0.686	Mutual exclusivity
RNU6ATAC37P	2p25.1	3 (2.61%)	7 (2.86%)	-0.13	0.598	0.686	Mutual exclusivity
RPL10A	6p21.31	3 (2.61%)	7 (2.86%)	-0.13	0.598	0.686	Mutual exclusivity
SAYSD1	6p21.2	3 (2.61%)	7 (2.86%)	-0.13	0.598	0.686	Mutual exclusivity
SCUBE3	6p21.31	3 (2.61%)	7 (2.86%)	-0.13	0.598	0.686	Mutual exclusivity
SLC30A3	2p23.3	3 (2.61%)	7 (2.86%)	-0.13	0.598	0.686	Mutual exclusivity
SLC5A6	2p23.3	3 (2.61%)	7 (2.86%)	-0.13	0.598	0.686	Mutual exclusivity
SMC6	2p24.2	3 (2.61%)	7 (2.86%)	-0.13	0.598	0.686	Mutual exclusivity
SNORA8	11q21	3 (2.61%)	7 (2.86%)	-0.13	0.598	0.686	Mutual exclusivity
SNORD2	3q27.3	3 (2.61%)	7 (2.86%)	-0.13	0.598	0.686	Mutual exclusivity
SNRPA1	15q26.3	3 (2.61%)	7 (2.86%)	-0.13	0.598	0.686	Mutual exclusivity
SOX11	2p25.2	3 (2.61%)	7 (2.86%)	-0.13	0.598	0.686	Mutual exclusivity
SRSF3	6p21.31-p21.2	3 (2.61%)	7 (2.86%)	-0.13	0.598	0.686	Mutual exclusivity
TARSL2	15q26.3	3 (2.61%)	7 (2.86%)	-0.13	0.598	0.686	Mutual exclusivity
TCF23	2p23.3	3 (2.61%)	7 (2.86%)	-0.13	0.598	0.686	Mutual exclusivity

TEAD3	6p21.31	3 (2.61%)	7 (2.86%)	-0.13	0.598	0.686	Mutual exclusivity
TINAG	6p12.1	3 (2.61%)	7 (2.86%)	-0.13	0.598	0.686	Mutual exclusivity
TM2D3	15q26.3	3 (2.61%)	7 (2.86%)	-0.13	0.598	0.686	Mutual exclusivity
TRIB2	2p24.3	3 (2.61%)	7 (2.86%)	-0.13	0.598	0.686	Mutual exclusivity
TULP1	6p21.31	3 (2.61%)	7 (2.86%)	-0.13	0.598	0.686	Mutual exclusivity
VSNL1	2p24.2	3 (2.61%)	7 (2.86%)	-0.13	0.598	0.686	Mutual exclusivity
WASH3P	15q26.3	3 (2.61%)	7 (2.86%)	-0.13	0.598	0.686	Mutual exclusivity
YWHAQ	2p25.1	3 (2.61%)	7 (2.86%)	-0.13	0.598	0.686	Mutual exclusivity
ZNF76	6p21.31	3 (2.61%)	7 (2.86%)	-0.13	0.598	0.686	Mutual exclusivity
ABCD1	Xq28	2 (1.74%)	5 (2.04%)	-0.23	0.603	0.686	Mutual exclusivity
ADAM18	8p11.22	2 (1.74%)	5 (2.04%)	-0.23	0.603	0.686	Mutual exclusivity
ADCY2	5p15.31	2 (1.74%)	5 (2.04%)	-0.23	0.603	0.686	Mutual exclusivity
AGXT2	5p13.2	2 (1.74%)	5 (2.04%)	-0.23	0.603	0.686	Mutual exclusivity
AKR1C1	10p15.1	2 (1.74%)	5 (2.04%)	-0.23	0.603	0.686	Mutual exclusivity
AKR1D1	7q33	2 (1.74%)	5 (2.04%)	-0.23	0.603	0.686	Mutual exclusivity
ASXL2	2p23.3	2 (1.74%)	5 (2.04%)	-0.23	0.603	0.686	Mutual exclusivity
ATP2B3	Xq28	2 (1.74%)	5 (2.04%)	-0.23	0.603	0.686	Mutual exclusivity
ATP6AP1	Xq28	2 (1.74%)	5 (2.04%)	-0.23	0.603	0.686	Mutual exclusivity
BAIAP2L1	7q21.3-q22.1	2 (1.74%)	5 (2.04%)	-0.23	0.603	0.686	Mutual exclusivity
BBS1	11q13.2	2 (1.74%)	5 (2.04%)	-0.23	0.603	0.686	Mutual exclusivity
BCAP31	Xq28	2 (1.74%)	5 (2.04%)	-0.23	0.603	0.686	Mutual exclusivity
BGN	Xq28	2 (1.74%)	5 (2.04%)	-0.23	0.603	0.686	Mutual exclusivity
BHLHA15	7q21.3	2 (1.74%)	5 (2.04%)	-0.23	0.603	0.686	Mutual exclusivity
BRCC3	Xq28	2 (1.74%)	5 (2.04%)	-0.23	0.603	0.686	Mutual exclusivity
BRI3	7q21.3	2 (1.74%)	5 (2.04%)	-0.23	0.603	0.686	Mutual exclusivity
BRIX1	5p13.2	2 (1.74%)	5 (2.04%)	-0.23	0.603	0.686	Mutual exclusivity
C2ORF70	2p23.3	2 (1.74%)	5 (2.04%)	-0.23	0.603	0.686	Mutual exclusivity
C5ORF17	5p14.2	2 (1.74%)	5 (2.04%)	-0.23	0.603	0.686	Mutual exclusivity
C5ORF22	5p13.3	2 (1.74%)	5 (2.04%)	-0.23	0.603	0.686	Mutual exclusivity

CCDC71L	7q22.3	2 (1.74%)	5 (2.04%)	-0.23	0.603	0.686	Mutual exclusivity
CCNQ	Xq28	2 (1.74%)	5 (2.04%)	-0.23	0.603	0.686	Mutual exclusivity
CDH10	5p14.2-p14.1	2 (1.74%)	5 (2.04%)	-0.23	0.603	0.686	Mutual exclusivity
CDH6	5p13.3	2 (1.74%)	5 (2.04%)	-0.23	0.603	0.686	Mutual exclusivity
CDK6	7q21.2	2 (1.74%)	5 (2.04%)	-0.23	0.603	0.686	Mutual exclusivity
CETN2	Xq28	2 (1.74%)	5 (2.04%)	-0.23	0.603	0.686	Mutual exclusivity
CIB4	2p23.3	2 (1.74%)	5 (2.04%)	-0.23	0.603	0.686	Mutual exclusivity
CLDN16	3q28	2 (1.74%)	5 (2.04%)	-0.23	0.603	0.686	Mutual exclusivity
CLIC2	Xq28	2 (1.74%)	5 (2.04%)	-0.23	0.603	0.686	Mutual exclusivity
CLPTM1L	5p15.33	2 (1.74%)	5 (2.04%)	-0.23	0.603	0.686	Mutual exclusivity
CMC4	Xq28	2 (1.74%)	5 (2.04%)	-0.23	0.603	0.686	Mutual exclusivity
CPA4	7q32.2	2 (1.74%)	5 (2.04%)	-0.23	0.603	0.686	Mutual exclusivity
CSAG1	Xq28	2 (1.74%)	5 (2.04%)	-0.23	0.603	0.686	Mutual exclusivity
CSAG2	Xq28	2 (1.74%)	5 (2.04%)	-0.23	0.603	0.686	Mutual exclusivity
CSAG3	Xq28	2 (1.74%)	5 (2.04%)	-0.23	0.603	0.686	Mutual exclusivity
CSAG4	Xq28	2 (1.74%)	5 (2.04%)	-0.23	0.603	0.686	Mutual exclusivity
CTAG1A	Xq28	2 (1.74%)	5 (2.04%)	-0.23	0.603	0.686	Mutual exclusivity
CTAG1B	Xq28	2 (1.74%)	5 (2.04%)	-0.23	0.603	0.686	Mutual exclusivity
CTAG2	Xq28	2 (1.74%)	5 (2.04%)	-0.23	0.603	0.686	Mutual exclusivity
CYP3A5	7q22.1	2 (1.74%)	5 (2.04%)	-0.23	0.603	0.686	Mutual exclusivity
DKC1	Xq28	2 (1.74%)	5 (2.04%)	-0.23	0.603	0.686	Mutual exclusivity
DLG2	11q14.1	2 (1.74%)	5 (2.04%)	-0.23	0.603	0.686	Mutual exclusivity
DLX5	7q21.3	2 (1.74%)	5 (2.04%)	-0.23	0.603	0.686	Mutual exclusivity
DLX6	7q21.3	2 (1.74%)	5 (2.04%)	-0.23	0.603	0.686	Mutual exclusivity
DNAJC21	5p13.2	2 (1.74%)	5 (2.04%)	-0.23	0.603	0.686	Mutual exclusivity
DNAJC27	2p23.3	2 (1.74%)	5 (2.04%)	-0.23	0.603	0.686	Mutual exclusivity
DNASE1L1	Xq28	2 (1.74%)	5 (2.04%)	-0.23	0.603	0.686	Mutual exclusivity
DNMT3A	2p23.3	2 (1.74%)	5 (2.04%)	-0.23	0.603	0.686	Mutual exclusivity
DPP3	11q13.2	2 (1.74%)	5 (2.04%)	-0.23	0.603	0.686	Mutual exclusivity

DPP6	7q36.2	2 (1.74%)	5 (2.04%)	-0.23	0.603	0.686	Mutual exclusivity
DRC1	2p23.3	2 (1.74%)	5 (2.04%)	-0.23	0.603	0.686	Mutual exclusivity
DROSHA	5p13.3	2 (1.74%)	5 (2.04%)	-0.23	0.603	0.686	Mutual exclusivity
DTNB	2p23.3	2 (1.74%)	5 (2.04%)	-0.23	0.603	0.686	Mutual exclusivity
DUSP9	Xq28	2 (1.74%)	5 (2.04%)	-0.23	0.603	0.686	Mutual exclusivity
EFR3B	2p23.3	2 (1.74%)	5 (2.04%)	-0.23	0.603	0.686	Mutual exclusivity
EGFR	7p11.2	2 (1.74%)	5 (2.04%)	-0.23	0.603	0.686	Mutual exclusivity
ELL	19p13.11	2 (1.74%)	5 (2.04%)	-0.23	0.603	0.686	Mutual exclusivity
EMD	Xq28	2 (1.74%)	5 (2.04%)	-0.23	0.603	0.686	Mutual exclusivity
ERGIC1	5q35.1	2 (1.74%)	5 (2.04%)	-0.23	0.603	0.686	Mutual exclusivity
ERICH6	3q25.1	2 (1.74%)	5 (2.04%)	-0.23	0.603	0.686	Mutual exclusivity
F8	Xq28	2 (1.74%)	5 (2.04%)	-0.23	0.603	0.686	Mutual exclusivity
F8A1	Xq28	2 (1.74%)	5 (2.04%)	-0.23	0.603	0.686	Mutual exclusivity
F8A2	Xq28	2 (1.74%)	5 (2.04%)	-0.23	0.603	0.686	Mutual exclusivity
F8A3	Xq28	2 (1.74%)	5 (2.04%)	-0.23	0.603	0.686	Mutual exclusivity
FABP5P3	7q36.1	2 (1.74%)	5 (2.04%)	-0.23	0.603	0.686	Mutual exclusivity
FAM133B	7q21.2	2 (1.74%)	5 (2.04%)	-0.23	0.603	0.686	Mutual exclusivity
FAM3A	Xq28	2 (1.74%)	5 (2.04%)	-0.23	0.603	0.686	Mutual exclusivity
FAM50A	Xq28	2 (1.74%)	5 (2.04%)	-0.23	0.603	0.686	Mutual exclusivity
FLNA	Xq28	2 (1.74%)	5 (2.04%)	-0.23	0.603	0.686	Mutual exclusivity
FUNDC2	Xq28	2 (1.74%)	5 (2.04%)	-0.23	0.603	0.686	Mutual exclusivity
G6PD	Xq28	2 (1.74%)	5 (2.04%)	-0.23	0.603	0.686	Mutual exclusivity
GAB3	Xq28	2 (1.74%)	5 (2.04%)	-0.23	0.603	0.686	Mutual exclusivity
GDI1	Xq28	2 (1.74%)	5 (2.04%)	-0.23	0.603	0.686	Mutual exclusivity
GPAT4	8p11.21	2 (1.74%)	5 (2.04%)	-0.23	0.603	0.686	Mutual exclusivity
H2AFB1	Xq28	2 (1.74%)	5 (2.04%)	-0.23	0.603	0.686	Mutual exclusivity
H2AFB2	Xq28	2 (1.74%)	5 (2.04%)	-0.23	0.603	0.686	Mutual exclusivity
H2AFB3	Xq28	2 (1.74%)	5 (2.04%)	-0.23	0.603	0.686	Mutual exclusivity
HADHB	2p23.3	2 (1.74%)	5 (2.04%)	-0.23	0.603	0.686	Mutual exclusivity

HAUS7	Xq28	2 (1.74%)	5 (2.04%)	-0.23	0.603	0.686	Mutual exclusivity
HBEGF	5q31.3	2 (1.74%)	5 (2.04%)	-0.23	0.603	0.686	Mutual exclusivity
IDH3G	Xq28	2 (1.74%)	5 (2.04%)	-0.23	0.603	0.686	Mutual exclusivity
IFT172	2p23.3	2 (1.74%)	5 (2.04%)	-0.23	0.603	0.686	Mutual exclusivity
IKBKG	Xq28	2 (1.74%)	5 (2.04%)	-0.23	0.603	0.686	Mutual exclusivity
IL1RAP	3q28	2 (1.74%)	5 (2.04%)	-0.23	0.603	0.686	Mutual exclusivity
INSIG1	7q36.3	2 (1.74%)	5 (2.04%)	-0.23	0.603	0.686	Mutual exclusivity
ISYNA1	19p13.11	2 (1.74%)	5 (2.04%)	-0.23	0.603	0.686	Mutual exclusivity
KCNK3	2p23.3	2 (1.74%)	5 (2.04%)	-0.23	0.603	0.686	Mutual exclusivity
KHDRBS2	6q11.1	2 (1.74%)	5 (2.04%)	-0.23	0.603	0.686	Mutual exclusivity
KIF3C	2p23.3	2 (1.74%)	5 (2.04%)	-0.23	0.603	0.686	Mutual exclusivity
KMT2C	7q36.1	2 (1.74%)	5 (2.04%)	-0.23	0.603	0.686	Mutual exclusivity
KRTCAP3	2p23.3	2 (1.74%)	5 (2.04%)	-0.23	0.603	0.686	Mutual exclusivity
LAGE3	Xq28	2 (1.74%)	5 (2.04%)	-0.23	0.603	0.686	Mutual exclusivity
LMTK2	7q21.3	2 (1.74%)	5 (2.04%)	-0.23	0.603	0.686	Mutual exclusivity
MAGEA1	Xq28	2 (1.74%)	5 (2.04%)	-0.23	0.603	0.686	Mutual exclusivity
MAGEA12	Xq28	2 (1.74%)	5 (2.04%)	-0.23	0.603	0.686	Mutual exclusivity
MAGEA2	Xq28	2 (1.74%)	5 (2.04%)	-0.23	0.603	0.686	Mutual exclusivity
MAGEA2B	Xq28	2 (1.74%)	5 (2.04%)	-0.23	0.603	0.686	Mutual exclusivity
MAGEA3	Xq28	2 (1.74%)	5 (2.04%)	-0.23	0.603	0.686	Mutual exclusivity
MAGEA6	Xq28	2 (1.74%)	5 (2.04%)	-0.23	0.603	0.686	Mutual exclusivity
MED10	5p15.31	2 (1.74%)	5 (2.04%)	-0.23	0.603	0.686	Mutual exclusivity
MGAT4B	5q35.3	2 (1.74%)	5 (2.04%)	-0.23	0.603	0.686	Mutual exclusivity
MIR-4636/4636		2 (1.74%)	5 (2.04%)	-0.23	0.603	0.686	Mutual exclusivity
MIR-944/944		2 (1.74%)	5 (2.04%)	-0.23	0.603	0.686	Mutual exclusivity
MPP1	Xq28	2 (1.74%)	5 (2.04%)	-0.23	0.603	0.686	Mutual exclusivity
MRPL11	11q13.2	2 (1.74%)	5 (2.04%)	-0.23	0.603	0.686	Mutual exclusivity
MTCP1	Xq28	2 (1.74%)	5 (2.04%)	-0.23	0.603	0.686	Mutual exclusivity
NET1	10p15.1	2 (1.74%)	5 (2.04%)	-0.23	0.603	0.686	Mutual exclusivity

NPTX2	7q22.1	2 (1.74%)	5 (2.04%)	-0.23	0.603	0.686	Mutual exclusivity
NRBP1	2p23.3	2 (1.74%)	5 (2.04%)	-0.23	0.603	0.686	Mutual exclusivity
NSDHL	Xq28	2 (1.74%)	5 (2.04%)	-0.23	0.603	0.686	Mutual exclusivity
OPN1LW	Xq28	2 (1.74%)	5 (2.04%)	-0.23	0.603	0.686	Mutual exclusivity
OPN1MW	Xq28	2 (1.74%)	5 (2.04%)	-0.23	0.603	0.686	Mutual exclusivity
OPN1MW2	Xq28	2 (1.74%)	5 (2.04%)	-0.23	0.603	0.686	Mutual exclusivity
OSTN	3q28	2 (1.74%)	5 (2.04%)	-0.23	0.603	0.686	Mutual exclusivity
OTOF	2p23.3	2 (1.74%)	5 (2.04%)	-0.23	0.603	0.686	Mutual exclusivity
P3H2	3q28	2 (1.74%)	5 (2.04%)	-0.23	0.603	0.686	Mutual exclusivity
PDK4	7q21.3	2 (1.74%)	5 (2.04%)	-0.23	0.603	0.686	Mutual exclusivity
PDZD4	Xq28	2 (1.74%)	5 (2.04%)	-0.23	0.603	0.686	Mutual exclusivity
PELI3	11q13.2	2 (1.74%)	5 (2.04%)	-0.23	0.603	0.686	Mutual exclusivity
PEX1	7q21.2	2 (1.74%)	5 (2.04%)	-0.23	0.603	0.686	Mutual exclusivity
PFN2	3q25.1	2 (1.74%)	5 (2.04%)	-0.23	0.603	0.686	Mutual exclusivity
PLAT	8p11.21	2 (1.74%)	5 (2.04%)	-0.23	0.603	0.686	Mutual exclusivity
PLXNA3	Xq28	2 (1.74%)	5 (2.04%)	-0.23	0.603	0.686	Mutual exclusivity
PLXNB3	Xq28	2 (1.74%)	5 (2.04%)	-0.23	0.603	0.686	Mutual exclusivity
PNCK	Xq28	2 (1.74%)	5 (2.04%)	-0.23	0.603	0.686	Mutual exclusivity
PNMA3	Xq28	2 (1.74%)	5 (2.04%)	-0.23	0.603	0.686	Mutual exclusivity
PNMA5	Xq28	2 (1.74%)	5 (2.04%)	-0.23	0.603	0.686	Mutual exclusivity
PNMA6A	Xq28	2 (1.74%)	5 (2.04%)	-0.23	0.603	0.686	Mutual exclusivity
PNMA6B	Xq28	2 (1.74%)	5 (2.04%)	-0.23	0.603	0.686	Mutual exclusivity
POMC	2p23.3	2 (1.74%)	5 (2.04%)	-0.23	0.603	0.686	Mutual exclusivity
PRLR	5p13.2	2 (1.74%)	5 (2.04%)	-0.23	0.603	0.686	Mutual exclusivity
RAB39B	Xq28	2 (1.74%)	5 (2.04%)	-0.23	0.603	0.686	Mutual exclusivity
RAD1	5p13.2	2 (1.74%)	5 (2.04%)	-0.23	0.603	0.686	Mutual exclusivity
RBM48	7q21.2	2 (1.74%)	5 (2.04%)	-0.23	0.603	0.686	Mutual exclusivity
RN7SKP223	7q33	2 (1.74%)	5 (2.04%)	-0.23	0.603	0.686	Mutual exclusivity
RN7SKP280	7q36.2	2 (1.74%)	5 (2.04%)	-0.23	0.603	0.686	Mutual exclusivity

RN7SKP296	3q28	2 (1.74%)	5 (2.04%)	-0.23	0.603	0.686	Mutual exclusivity
RN7SKP298	3q26.1	2 (1.74%)	5 (2.04%)	-0.23	0.603	0.686	Mutual exclusivity
RN7SL190P	Xq28	2 (1.74%)	5 (2.04%)	-0.23	0.603	0.686	Mutual exclusivity
RN7SL478P	7q21.3	2 (1.74%)	5 (2.04%)	-0.23	0.603	0.686	Mutual exclusivity
RN7SL667P	Xq28	2 (1.74%)	5 (2.04%)	-0.23	0.603	0.686	Mutual exclusivity
RN7SL687P	Xq28	2 (1.74%)	5 (2.04%)	-0.23	0.603	0.686	Mutual exclusivity
RN7SL697P	Xq28	2 (1.74%)	5 (2.04%)	-0.23	0.603	0.686	Mutual exclusivity
RN7SL856P	2p23.3	2 (1.74%)	5 (2.04%)	-0.23	0.603	0.686	Mutual exclusivity
RNA5SP250	7q36.1	2 (1.74%)	5 (2.04%)	-0.23	0.603	0.686	Mutual exclusivity
RPL10	Xq28	2 (1.74%)	5 (2.04%)	-0.23	0.603	0.686	Mutual exclusivity
SDHAF3	7q21.3	2 (1.74%)	5 (2.04%)	-0.23	0.603	0.686	Mutual exclusivity
SEMA5A	5p15.31	2 (1.74%)	5 (2.04%)	-0.23	0.603	0.686	Mutual exclusivity
SIAH2	3q25.1	2 (1.74%)	5 (2.04%)	-0.23	0.603	0.686	Mutual exclusivity
SLC10A3	Xq28	2 (1.74%)	5 (2.04%)	-0.23	0.603	0.686	Mutual exclusivity
SLC47A1	17p11.2	2 (1.74%)	5 (2.04%)	-0.23	0.603	0.686	Mutual exclusivity
SLC6A8	Xq28	2 (1.74%)	5 (2.04%)	-0.23	0.603	0.686	Mutual exclusivity
SMIM9	Xq28	2 (1.74%)	5 (2.04%)	-0.23	0.603	0.686	Mutual exclusivity
SNORA36A	Xq28	2 (1.74%)	5 (2.04%)	-0.23	0.603	0.686	Mutual exclusivity
SNORA56	Xq28	2 (1.74%)	5 (2.04%)	-0.23	0.603	0.686	Mutual exclusivity
SNORA59B	17p11.2	2 (1.74%)	5 (2.04%)	-0.23	0.603	0.686	Mutual exclusivity
SRPK3	Xq28	2 (1.74%)	5 (2.04%)	-0.23	0.603	0.686	Mutual exclusivity
SSBP4	19p13.11	2 (1.74%)	5 (2.04%)	-0.23	0.603	0.686	Mutual exclusivity
SSR4	Xq28	2 (1.74%)	5 (2.04%)	-0.23	0.603	0.686	Mutual exclusivity
TAZ	Xq28	2 (1.74%)	5 (2.04%)	-0.23	0.603	0.686	Mutual exclusivity
TBC1D9B	5q35.3	2 (1.74%)	5 (2.04%)	-0.23	0.603	0.686	Mutual exclusivity
TECPR1	7q21.3	2 (1.74%)	5 (2.04%)	-0.23	0.603	0.686	Mutual exclusivity
TEX28	Xq28	2 (1.74%)	5 (2.04%)	-0.23	0.603	0.686	Mutual exclusivity
TEX28P1	Xq28	2 (1.74%)	5 (2.04%)	-0.23	0.603	0.686	Mutual exclusivity
TEX28P2	Xq28	2 (1.74%)	5 (2.04%)	-0.23	0.603	0.686	Mutual exclusivity

TKTL1	Xq28	2 (1.74%)	5 (2.04%)	-0.23	0.603	0.686	Mutual exclusivity
TMEM207	3q28	2 (1.74%)	5 (2.04%)	-0.23	0.603	0.686	Mutual exclusivity
TMLHE	Xq28	2 (1.74%)	5 (2.04%)	-0.23	0.603	0.686	Mutual exclusivity
TP63	3q28	2 (1.74%)	5 (2.04%)	-0.23	0.603	0.686	Mutual exclusivity
TRA2B	3q27.2	2 (1.74%)	5 (2.04%)	-0.23	0.603	0.686	Mutual exclusivity
TREX2	Xq28	2 (1.74%)	5 (2.04%)	-0.23	0.603	0.686	Mutual exclusivity
UBL4A	Xq28	2 (1.74%)	5 (2.04%)	-0.23	0.603	0.686	Mutual exclusivity
VBP1	Xq28	2 (1.74%)	5 (2.04%)	-0.23	0.603	0.686	Mutual exclusivity
XRCC2	7q36.1	2 (1.74%)	5 (2.04%)	-0.23	0.603	0.686	Mutual exclusivity
ZFP92	Xq28	2 (1.74%)	5 (2.04%)	-0.23	0.603	0.686	Mutual exclusivity
ZNF185	Xq28	2 (1.74%)	5 (2.04%)	-0.23	0.603	0.686	Mutual exclusivity
ZNF275	Xq28	2 (1.74%)	5 (2.04%)	-0.23	0.603	0.686	Mutual exclusivity
ZNF655	7q22.1	2 (1.74%)	5 (2.04%)	-0.23	0.603	0.686	Mutual exclusivity
ZSCAN25	7q22.1	2 (1.74%)	5 (2.04%)	-0.23	0.603	0.686	Mutual exclusivity
A4GNT	3q22.3	1 (0.87%)	3 (1.22%)	-0.49	0.617	0.686	Mutual exclusivity
ABCA13	7p12.3	1 (0.87%)	3 (1.22%)	-0.49	0.617	0.686	Mutual exclusivity
ABCF2	7q36.1	1 (0.87%)	3 (1.22%)	-0.49	0.617	0.686	Mutual exclusivity
ABHD6	3p14.3	1 (0.87%)	3 (1.22%)	-0.49	0.617	0.686	Mutual exclusivity
ACHE	7q22.1	1 (0.87%)	3 (1.22%)	-0.49	0.617	0.686	Mutual exclusivity
ACSM1	16p12.3	1 (0.87%)	3 (1.22%)	-0.49	0.617	0.686	Mutual exclusivity
ACSM3	16p12.3	1 (0.87%)	3 (1.22%)	-0.49	0.617	0.686	Mutual exclusivity
ACTL10	20q11.22	1 (0.87%)	3 (1.22%)	-0.49	0.617	0.686	Mutual exclusivity
ADGRA2	8p11.23	1 (0.87%)	3 (1.22%)	-0.49	0.617	0.686	Mutual exclusivity
ADGRE5	19p13.12	1 (0.87%)	3 (1.22%)	-0.49	0.617	0.686	Mutual exclusivity
ADRB3	8p11.23	1 (0.87%)	3 (1.22%)	-0.49	0.617	0.686	Mutual exclusivity
ALDH16A1	19q13.33	1 (0.87%)	3 (1.22%)	-0.49	0.617	0.686	Mutual exclusivity
ALS2	2q33.1	1 (0.87%)	3 (1.22%)	-0.49	0.617	0.686	Mutual exclusivity
ANKRD12	18p11.22	1 (0.87%)	3 (1.22%)	-0.49	0.617	0.686	Mutual exclusivity
ANKRD24	19p13.3	1 (0.87%)	3 (1.22%)	-0.49	0.617	0.686	Mutual exclusivity

ANLN	7p14.2	1 (0.87%)	3 (1.22%)	-0.49	0.617	0.686	Mutual exclusivity
ANO10	3p22.1-p21.33	1 (0.87%)	3 (1.22%)	-0.49	0.617	0.686	Mutual exclusivity
AP3S1	5q22.3-q23.1	1 (0.87%)	3 (1.22%)	-0.49	0.617	0.686	Mutual exclusivity
APC	5q22.2	1 (0.87%)	3 (1.22%)	-0.49	0.617	0.686	Mutual exclusivity
ARL14EPL	5q23.1	1 (0.87%)	3 (1.22%)	-0.49	0.617	0.686	Mutual exclusivity
ARMC8	3q22.3	1 (0.87%)	3 (1.22%)	-0.49	0.617	0.686	Mutual exclusivity
ASB10	7q36.1	1 (0.87%)	3 (1.22%)	-0.49	0.617	0.686	Mutual exclusivity
ASH2L	8p11.23	1 (0.87%)	3 (1.22%)	-0.49	0.617	0.686	Mutual exclusivity
ASIP	20q11.22	1 (0.87%)	3 (1.22%)	-0.49	0.617	0.686	Mutual exclusivity
ATF2	2q31.1	1 (0.87%)	3 (1.22%)	-0.49	0.617	0.686	Mutual exclusivity
ATL2	2p22.2-p22.1	1 (0.87%)	3 (1.22%)	-0.49	0.617	0.686	Mutual exclusivity
ATOX1	5q33.1	1 (0.87%)	3 (1.22%)	-0.49	0.617	0.686	Mutual exclusivity
ATP10B	5q34	1 (0.87%)	3 (1.22%)	-0.49	0.617	0.686	Mutual exclusivity
ATP5MC3	2q31.1	1 (0.87%)	3 (1.22%)	-0.49	0.617	0.686	Mutual exclusivity
ATP6V0A4	7q34	1 (0.87%)	3 (1.22%)	-0.49	0.617	0.686	Mutual exclusivity
BAG4	8p11.23	1 (0.87%)	3 (1.22%)	-0.49	0.617	0.686	Mutual exclusivity
BAIAP3	16p13.3	1 (0.87%)	3 (1.22%)	-0.49	0.617	0.686	Mutual exclusivity
BBS9	7p14.3	1 (0.87%)	3 (1.22%)	-0.49	0.617	0.686	Mutual exclusivity
BLCAP	20q11.23	1 (0.87%)	3 (1.22%)	-0.49	0.617	0.686	Mutual exclusivity
BMPR1A	10q23.2	1 (0.87%)	3 (1.22%)	-0.49	0.617	0.686	Mutual exclusivity
BMPR2	2q33.1-q33.2	1 (0.87%)	3 (1.22%)	-0.49	0.617	0.686	Mutual exclusivity
BPESC1	3q23	1 (0.87%)	3 (1.22%)	-0.49	0.617	0.686	Mutual exclusivity
BRF2	8p11.23	1 (0.87%)	3 (1.22%)	-0.49	0.617	0.686	Mutual exclusivity
C16ORF90	16p13.3	1 (0.87%)	3 (1.22%)	-0.49	0.617	0.686	Mutual exclusivity
C1QTNF2	5q33.3	1 (0.87%)	3 (1.22%)	-0.49	0.617	0.686	Mutual exclusivity
C1R	12p13.31	1 (0.87%)	3 (1.22%)	-0.49	0.617	0.686	Mutual exclusivity
C1RL	12p13.31	1 (0.87%)	3 (1.22%)	-0.49	0.617	0.686	Mutual exclusivity
C20ORF144	20q11.22	1 (0.87%)	3 (1.22%)	-0.49	0.617	0.686	Mutual exclusivity
C3ORF84	3p21.31	1 (0.87%)	3 (1.22%)	-0.49	0.617	0.686	Mutual exclusivity

C5ORF15	5q31.1	1 (0.87%)	3 (1.22%)	-0.49	0.617	0.686	Mutual exclusivity
C7ORF71	7p15.2	1 (0.87%)	3 (1.22%)	-0.49	0.617	0.686	Mutual exclusivity
C8ORF86	8p11.22	1 (0.87%)	3 (1.22%)	-0.49	0.617	0.686	Mutual exclusivity
CABIN1	22q11.23	1 (0.87%)	3 (1.22%)	-0.49	0.617	0.686	Mutual exclusivity
CAND2	3p25.2	1 (0.87%)	3 (1.22%)	-0.49	0.617	0.686	Mutual exclusivity
CARM1P1	9p24.2	1 (0.87%)	3 (1.22%)	-0.49	0.617	0.686	Mutual exclusivity
CASTOR3	7q22.1	1 (0.87%)	3 (1.22%)	-0.49	0.617	0.686	Mutual exclusivity
CBX3	7p15.2	1 (0.87%)	3 (1.22%)	-0.49	0.617	0.686	Mutual exclusivity
CCDC126	7p15.3	1 (0.87%)	3 (1.22%)	-0.49	0.617	0.686	Mutual exclusivity
CCDC146	7q11.23	1 (0.87%)	3 (1.22%)	-0.49	0.617	0.686	Mutual exclusivity
CCDC155	19q13.33	1 (0.87%)	3 (1.22%)	-0.49	0.617	0.686	Mutual exclusivity
CCM2	7p13	1 (0.87%)	3 (1.22%)	-0.49	0.617	0.686	Mutual exclusivity
CCNJL	5q33.3	1 (0.87%)	3 (1.22%)	-0.49	0.617	0.686	Mutual exclusivity
CD4	12p13.31	1 (0.87%)	3 (1.22%)	-0.49	0.617	0.686	Mutual exclusivity
CDC42BPB	14q32.32	1 (0.87%)	3 (1.22%)	-0.49	0.617	0.686	Mutual exclusivity
CDK15	2q33.1	1 (0.87%)	3 (1.22%)	-0.49	0.617	0.686	Mutual exclusivity
CDV3	3q22.1	1 (0.87%)	3 (1.22%)	-0.49	0.617	0.686	Mutual exclusivity
CEACAM20	19q13.31	1 (0.87%)	3 (1.22%)	-0.49	0.617	0.686	Mutual exclusivity
CEP70	3q22.3	1 (0.87%)	3 (1.22%)	-0.49	0.617	0.686	Mutual exclusivity
CHD8	14q11.2	1 (0.87%)	3 (1.22%)	-0.49	0.617	0.686	Mutual exclusivity
CHN1	2q31.1	1 (0.87%)	3 (1.22%)	-0.49	0.617	0.686	Mutual exclusivity
CHN2	7p14.3	1 (0.87%)	3 (1.22%)	-0.49	0.617	0.686	Mutual exclusivity
CHPF2	7q36.1	1 (0.87%)	3 (1.22%)	-0.49	0.617	0.686	Mutual exclusivity
CHRM2	7q33	1 (0.87%)	3 (1.22%)	-0.49	0.617	0.686	Mutual exclusivity
CHRNA1	2q31.1	1 (0.87%)	3 (1.22%)	-0.49	0.617	0.686	Mutual exclusivity
CHSY3	5q23.3	1 (0.87%)	3 (1.22%)	-0.49	0.617	0.686	Mutual exclusivity
CIR1	2q31.1	1 (0.87%)	3 (1.22%)	-0.49	0.617	0.686	Mutual exclusivity
CLDN18	3q22.3	1 (0.87%)	3 (1.22%)	-0.49	0.617	0.686	Mutual exclusivity
CLEC2L	7q34	1 (0.87%)	3 (1.22%)	-0.49	0.617	0.686	Mutual exclusivity

CLUAP1	16p13.3	1 (0.87%)	3 (1.22%)	-0.49	0.617	0.686	Mutual exclusivity
CNGB1	16q21	1 (0.87%)	3 (1.22%)	-0.49	0.617	0.686	Mutual exclusivity
CNOT3	19q13.42	1 (0.87%)	3 (1.22%)	-0.49	0.617	0.686	Mutual exclusivity
CNOT8	5q33.2	1 (0.87%)	3 (1.22%)	-0.49	0.617	0.686	Mutual exclusivity
CNPY4	7q22.1	1 (0.87%)	3 (1.22%)	-0.49	0.617	0.686	Mutual exclusivity
COLGALT1	19p13.11	1 (0.87%)	3 (1.22%)	-0.49	0.617	0.686	Mutual exclusivity
COMMD10	5q23.1	1 (0.87%)	3 (1.22%)	-0.49	0.617	0.686	Mutual exclusivity
COPB2	3q23	1 (0.87%)	3 (1.22%)	-0.49	0.617	0.686	Mutual exclusivity
COX7A2L	2p21	1 (0.87%)	3 (1.22%)	-0.49	0.617	0.686	Mutual exclusivity
CPS1	2q34	1 (0.87%)	3 (1.22%)	-0.49	0.617	0.686	Mutual exclusivity
CPVL	7p14.3	1 (0.87%)	3 (1.22%)	-0.49	0.617	0.686	Mutual exclusivity
CRAMP1	16p13.3	1 (0.87%)	3 (1.22%)	-0.49	0.617	0.686	Mutual exclusivity
CRELD1	3p25.3	1 (0.87%)	3 (1.22%)	-0.49	0.617	0.686	Mutual exclusivity
CRYGN	7q36.1	1 (0.87%)	3 (1.22%)	-0.49	0.617	0.686	Mutual exclusivity
CSNK1G3	5q23.2	1 (0.87%)	3 (1.22%)	-0.49	0.617	0.686	Mutual exclusivity
CTAGE15	7q35	1 (0.87%)	3 (1.22%)	-0.49	0.617	0.686	Mutual exclusivity
CTAGE6	7q35	1 (0.87%)	3 (1.22%)	-0.49	0.617	0.686	Mutual exclusivity
CTXN3	5q23.2	1 (0.87%)	3 (1.22%)	-0.49	0.617	0.686	Mutual exclusivity
CXORF40A	Xq28	1 (0.87%)	3 (1.22%)	-0.49	0.617	0.686	Mutual exclusivity
CXORF56	Xq24	1 (0.87%)	3 (1.22%)	-0.49	0.617	0.686	Mutual exclusivity
DBR1	3q22.3	1 (0.87%)	3 (1.22%)	-0.49	0.617	0.686	Mutual exclusivity
DCAF17	2q31.1	1 (0.87%)	3 (1.22%)	-0.49	0.617	0.686	Mutual exclusivity
DCP2	5q22.2	1 (0.87%)	3 (1.22%)	-0.49	0.617	0.686	Mutual exclusivity
DDHD2	8p11.23	1 (0.87%)	3 (1.22%)	-0.49	0.617	0.686	Mutual exclusivity
DDX39A	19p13.12	1 (0.87%)	3 (1.22%)	-0.49	0.617	0.686	Mutual exclusivity
DDX51	12q24.33	1 (0.87%)	3 (1.22%)	-0.49	0.617	0.686	Mutual exclusivity
DEFB108B	11q13.4	1 (0.87%)	3 (1.22%)	-0.49	0.617	0.686	Mutual exclusivity
DEFB115	20q11.21	1 (0.87%)	3 (1.22%)	-0.49	0.617	0.686	Mutual exclusivity
DEFB116	20q11.21	1 (0.87%)	3 (1.22%)	-0.49	0.617	0.686	Mutual exclusivity

DHCR7	11q13.4	1 (0.87%)	3 (1.22%)	-0.49	0.617	0.686	Mutual exclusivity
DIAPH2	Xq21.33	1 (0.87%)	3 (1.22%)	-0.49	0.617	0.686	Mutual exclusivity
DISP3	1p36.22	1 (0.87%)	3 (1.22%)	-0.49	0.617	0.686	Mutual exclusivity
DMTF1	7q21.12	1 (0.87%)	3 (1.22%)	-0.49	0.617	0.686	Mutual exclusivity
DNAJB1	19p13.12	1 (0.87%)	3 (1.22%)	-0.49	0.617	0.686	Mutual exclusivity
DNAJB6	7q36.3	1 (0.87%)	3 (1.22%)	-0.49	0.617	0.686	Mutual exclusivity
DNASE1	16p13.3	1 (0.87%)	3 (1.22%)	-0.49	0.617	0.686	Mutual exclusivity
DPYSL3	5q32	1 (0.87%)	3 (1.22%)	-0.49	0.617	0.686	Mutual exclusivity
DSN1	20q11.23	1 (0.87%)	3 (1.22%)	-0.49	0.617	0.686	Mutual exclusivity
DTWD2	5q23.1	1 (0.87%)	3 (1.22%)	-0.49	0.617	0.686	Mutual exclusivity
DTX2	7q11.23	1 (0.87%)	3 (1.22%)	-0.49	0.617	0.686	Mutual exclusivity
DZIP1L	3q22.3	1 (0.87%)	3 (1.22%)	-0.49	0.617	0.686	Mutual exclusivity
E2F1	20q11.22	1 (0.87%)	3 (1.22%)	-0.49	0.617	0.686	Mutual exclusivity
EIF4EBP1	8p11.23	1 (0.87%)	3 (1.22%)	-0.49	0.617	0.686	Mutual exclusivity
EML4	2p21	1 (0.87%)	3 (1.22%)	-0.49	0.617	0.686	Mutual exclusivity
EP400P1	12q24.33	1 (0.87%)	3 (1.22%)	-0.49	0.617	0.686	Mutual exclusivity
EPB41L4A	5q22.1-q22.2	1 (0.87%)	3 (1.22%)	-0.49	0.617	0.686	Mutual exclusivity
EPHB4	7q22.1	1 (0.87%)	3 (1.22%)	-0.49	0.617	0.686	Mutual exclusivity
ERLIN2	8p11.23	1 (0.87%)	3 (1.22%)	-0.49	0.617	0.686	Mutual exclusivity
EVX1	7p15.2	1 (0.87%)	3 (1.22%)	-0.49	0.617	0.686	Mutual exclusivity
FABP6	5q33.3	1 (0.87%)	3 (1.22%)	-0.49	0.617	0.686	Mutual exclusivity
FAIM	3q22.3	1 (0.87%)	3 (1.22%)	-0.49	0.617	0.686	Mutual exclusivity
FAM221A	7p15.3	1 (0.87%)	3 (1.22%)	-0.49	0.617	0.686	Mutual exclusivity
FASTK	7q36.1	1 (0.87%)	3 (1.22%)	-0.49	0.617	0.686	Mutual exclusivity
FAT2	5q33.1	1 (0.87%)	3 (1.22%)	-0.49	0.617	0.686	Mutual exclusivity
FAXDC2	5q33.2	1 (0.87%)	3 (1.22%)	-0.49	0.617	0.686	Mutual exclusivity
FBN2	5q23.3	1 (0.87%)	3 (1.22%)	-0.49	0.617	0.686	Mutual exclusivity
FCGRT	19q13.33	1 (0.87%)	3 (1.22%)	-0.49	0.617	0.686	Mutual exclusivity
FGFBP2	4p15.32	1 (0.87%)	3 (1.22%)	-0.49	0.617	0.686	Mutual exclusivity

FGFR1	8p11.23	1 (0.87%)	3 (1.22%)	-0.49	0.617	0.686	Mutual exclusivity
FGL2	7q11.23	1 (0.87%)	3 (1.22%)	-0.49	0.617	0.686	Mutual exclusivity
FLT3LG	19q13.33	1 (0.87%)	3 (1.22%)	-0.49	0.617	0.686	Mutual exclusivity
FMC1	7q34	1 (0.87%)	3 (1.22%)	-0.49	0.617	0.686	Mutual exclusivity
FOXL2	3q22.3	1 (0.87%)	3 (1.22%)	-0.49	0.617	0.686	Mutual exclusivity
FOXL2NB	3q22.3	1 (0.87%)	3 (1.22%)	-0.49	0.617	0.686	Mutual exclusivity
FRG1BP	20q11.1	1 (0.87%)	3 (1.22%)	-0.49	0.617	0.686	Mutual exclusivity
FZD7	2q33.1	1 (0.87%)	3 (1.22%)	-0.49	0.617	0.686	Mutual exclusivity
G3BP1	5q33.1	1 (0.87%)	3 (1.22%)	-0.49	0.617	0.686	Mutual exclusivity
GABRA1	5q34	1 (0.87%)	3 (1.22%)	-0.49	0.617	0.686	Mutual exclusivity
GABRA6	5q34	1 (0.87%)	3 (1.22%)	-0.49	0.617	0.686	Mutual exclusivity
GABRB2	5q34	1 (0.87%)	3 (1.22%)	-0.49	0.617	0.686	Mutual exclusivity
GABRG2	5q34	1 (0.87%)	3 (1.22%)	-0.49	0.617	0.686	Mutual exclusivity
GAL3ST4	7q22.1	1 (0.87%)	3 (1.22%)	-0.49	0.617	0.686	Mutual exclusivity
GALNT17	7q11.22	1 (0.87%)	3 (1.22%)	-0.49	0.617	0.686	Mutual exclusivity
GALNT9	12q24.33	1 (0.87%)	3 (1.22%)	-0.49	0.617	0.686	Mutual exclusivity
GBX1	7q36.1	1 (0.87%)	3 (1.22%)	-0.49	0.617	0.686	Mutual exclusivity
GDPD5	11q13.4-q13.5	1 (0.87%)	3 (1.22%)	-0.49	0.617	0.686	Mutual exclusivity
GGT5	22q11.23	1 (0.87%)	3 (1.22%)	-0.49	0.617	0.686	Mutual exclusivity
GHRH	20q11.23	1 (0.87%)	3 (1.22%)	-0.49	0.617	0.686	Mutual exclusivity
GIPC1	19p13.12	1 (0.87%)	3 (1.22%)	-0.49	0.617	0.686	Mutual exclusivity
GNPTG	16p13.3	1 (0.87%)	3 (1.22%)	-0.49	0.617	0.686	Mutual exclusivity
GOT1L1	8p11.23	1 (0.87%)	3 (1.22%)	-0.49	0.617	0.686	Mutual exclusivity
GPC2	7q22.1	1 (0.87%)	3 (1.22%)	-0.49	0.617	0.686	Mutual exclusivity
GPR155	2q31.1	1 (0.87%)	3 (1.22%)	-0.49	0.617	0.686	Mutual exclusivity
GRK7	3q23	1 (0.87%)	3 (1.22%)	-0.49	0.617	0.686	Mutual exclusivity
GRM3	7q21.11-q21.12	1 (0.87%)	3 (1.22%)	-0.49	0.617	0.686	Mutual exclusivity
GSAP	7q11.23	1 (0.87%)	3 (1.22%)	-0.49	0.617	0.686	Mutual exclusivity
GSDME	7p15.3	1 (0.87%)	3 (1.22%)	-0.49	0.617	0.686	Mutual exclusivity

GSS	20q11.22	1 (0.87%)	3 (1.22%)	-0.49	0.617	0.686	Mutual exclusivity
GTF2I	7q11.23	1 (0.87%)	3 (1.22%)	-0.49	0.617	0.686	Mutual exclusivity
GTF2IRD1P1	7q11.21	1 (0.87%)	3 (1.22%)	-0.49	0.617	0.686	Mutual exclusivity
HGF	7q21.11	1 (0.87%)	3 (1.22%)	-0.49	0.617	0.686	Mutual exclusivity
HIPK2	7q34	1 (0.87%)	3 (1.22%)	-0.49	0.617	0.686	Mutual exclusivity
HNRNPA1P57	2p22.1	1 (0.87%)	3 (1.22%)	-0.49	0.617	0.686	Mutual exclusivity
HNRNPA2B1	7p15.2	1 (0.87%)	3 (1.22%)	-0.49	0.617	0.686	Mutual exclusivity
HOTAIRM1	7p15.2	1 (0.87%)	3 (1.22%)	-0.49	0.617	0.686	Mutual exclusivity
HOTTIP	7p15.2	1 (0.87%)	3 (1.22%)	-0.49	0.617	0.686	Mutual exclusivity
HOXA1	7p15.2	1 (0.87%)	3 (1.22%)	-0.49	0.617	0.686	Mutual exclusivity
HOXA10	7p15.2	1 (0.87%)	3 (1.22%)	-0.49	0.617	0.686	Mutual exclusivity
HOXA11	7p15.2	1 (0.87%)	3 (1.22%)	-0.49	0.617	0.686	Mutual exclusivity
HOXA13	7p15.2	1 (0.87%)	3 (1.22%)	-0.49	0.617	0.686	Mutual exclusivity
HOXA2	7p15.2	1 (0.87%)	3 (1.22%)	-0.49	0.617	0.686	Mutual exclusivity
HOXA3	7p15.2	1 (0.87%)	3 (1.22%)	-0.49	0.617	0.686	Mutual exclusivity
HOXA4	7p15.2	1 (0.87%)	3 (1.22%)	-0.49	0.617	0.686	Mutual exclusivity
HOXA5	7p15.2	1 (0.87%)	3 (1.22%)	-0.49	0.617	0.686	Mutual exclusivity
HOXA6	7p15.2	1 (0.87%)	3 (1.22%)	-0.49	0.617	0.686	Mutual exclusivity
HOXA7	7p15.2	1 (0.87%)	3 (1.22%)	-0.49	0.617	0.686	Mutual exclusivity
HOXA9	7p15.2	1 (0.87%)	3 (1.22%)	-0.49	0.617	0.686	Mutual exclusivity
HSPB1	7q11.23	1 (0.87%)	3 (1.22%)	-0.49	0.617	0.686	Mutual exclusivity
HUS1	7p12.3	1 (0.87%)	3 (1.22%)	-0.49	0.617	0.686	Mutual exclusivity
IDS	Xq28	1 (0.87%)	3 (1.22%)	-0.49	0.617	0.686	Mutual exclusivity
IFT140	16p13.3	1 (0.87%)	3 (1.22%)	-0.49	0.617	0.686	Mutual exclusivity
IL17RC	3p25.3|3p25.3-p24.1	1 (0.87%)	3 (1.22%)	-0.49	0.617	0.686	Mutual exclusivity
IL1R1	2q11.2-q12.1	1 (0.87%)	3 (1.22%)	-0.49	0.617	0.686	Mutual exclusivity
IL1R2	2q11.2	1 (0.87%)	3 (1.22%)	-0.49	0.617	0.686	Mutual exclusivity
IQCA1L	7q36.1	1 (0.87%)	3 (1.22%)	-0.49	0.617	0.686	Mutual exclusivity
ISOC1	5q23.3	1 (0.87%)	3 (1.22%)	-0.49	0.617	0.686	Mutual exclusivity

JAKMIP2	5q32	1 (0.87%)	3 (1.22%)	-0.49	0.617	0.686	Mutual exclusivity
JPT2	16p13.3	1 (0.87%)	3 (1.22%)	-0.49	0.617	0.686	Mutual exclusivity
KBTBD12	3q21.3	1 (0.87%)	3 (1.22%)	-0.49	0.617	0.686	Mutual exclusivity
KBTBD2	7p14.3	1 (0.87%)	3 (1.22%)	-0.49	0.617	0.686	Mutual exclusivity
KCNG3	2p21	1 (0.87%)	3 (1.22%)	-0.49	0.617	0.686	Mutual exclusivity
KCNV2	9p24.2	1 (0.87%)	3 (1.22%)	-0.49	0.617	0.686	Mutual exclusivity
KCTD21	11q14.1	1 (0.87%)	3 (1.22%)	-0.49	0.617	0.686	Mutual exclusivity
KDM7A	7q34	1 (0.87%)	3 (1.22%)	-0.49	0.617	0.686	Mutual exclusivity
KIAA0087	7p15.2	1 (0.87%)	3 (1.22%)	-0.49	0.617	0.686	Mutual exclusivity
KIAA1549	7q34	1 (0.87%)	3 (1.22%)	-0.49	0.617	0.686	Mutual exclusivity
KIAA2026	9p24.1	1 (0.87%)	3 (1.22%)	-0.49	0.617	0.686	Mutual exclusivity
KIF24	9p13.3	1 (0.87%)	3 (1.22%)	-0.49	0.617	0.686	Mutual exclusivity
KLHDC8B	3p21.31	1 (0.87%)	3 (1.22%)	-0.49	0.617	0.686	Mutual exclusivity
KLHL7	7p15.3	1 (0.87%)	3 (1.22%)	-0.49	0.617	0.686	Mutual exclusivity
KLRG2	7q34	1 (0.87%)	3 (1.22%)	-0.49	0.617	0.686	Mutual exclusivity
KMT2E	7q22.3	1 (0.87%)	3 (1.22%)	-0.49	0.617	0.686	Mutual exclusivity
LAMA1	18p11.31	1 (0.87%)	3 (1.22%)	-0.49	0.617	0.686	Mutual exclusivity
LAMTOR4	7q22.1	1 (0.87%)	3 (1.22%)	-0.49	0.617	0.686	Mutual exclusivity
LANCL2	7p11.2	1 (0.87%)	3 (1.22%)	-0.49	0.617	0.686	Mutual exclusivity
LARP1	5q33.2	1 (0.87%)	3 (1.22%)	-0.49	0.617	0.686	Mutual exclusivity
LDLRAD4	18p11.21	1 (0.87%)	3 (1.22%)	-0.49	0.617	0.686	Mutual exclusivity
LENG1	19q13.42	1 (0.87%)	3 (1.22%)	-0.49	0.617	0.686	Mutual exclusivity
LETM2	8p11.23	1 (0.87%)	3 (1.22%)	-0.49	0.617	0.686	Mutual exclusivity
LHFPL3	7q22.2-q22.3	1 (0.87%)	3 (1.22%)	-0.49	0.617	0.686	Mutual exclusivity
LHFPL4	3p25.3	1 (0.87%)	3 (1.22%)	-0.49	0.617	0.686	Mutual exclusivity
LILRA3	19q13.4	1 (0.87%)	3 (1.22%)	-0.49	0.617	0.686	Mutual exclusivity
LILRA4	19q13.42	1 (0.87%)	3 (1.22%)	-0.49	0.617	0.686	Mutual exclusivity
LILRA5	19q13.42	1 (0.87%)	3 (1.22%)	-0.49	0.617	0.686	Mutual exclusivity
LILRA6	19q13.42	1 (0.87%)	3 (1.22%)	-0.49	0.617	0.686	Mutual exclusivity

LILRB2	19q13.42	1 (0.87%)	3 (1.22%)	-0.49	0.617	0.686	Mutual exclusivity
LILRB3	19q13.42	1 (0.87%)	3 (1.22%)	-0.49	0.617	0.686	Mutual exclusivity
LILRB5	19q13.42	1 (0.87%)	3 (1.22%)	-0.49	0.617	0.686	Mutual exclusivity
LINC00349	13q11	1 (0.87%)	3 (1.22%)	-0.49	0.617	0.686	Mutual exclusivity
LINC00387	13q11	1 (0.87%)	3 (1.22%)	-0.49	0.617	0.686	Mutual exclusivity
LINC00388	13q11	1 (0.87%)	3 (1.22%)	-0.49	0.617	0.686	Mutual exclusivity
LINC00489	20q11.23	1 (0.87%)	3 (1.22%)	-0.49	0.617	0.686	Mutual exclusivity
LINC00504	4p15.33	1 (0.87%)	3 (1.22%)	-0.49	0.617	0.686	Mutual exclusivity
LINC00893	Xq28	1 (0.87%)	3 (1.22%)	-0.49	0.617	0.686	Mutual exclusivity
LINC01588	14q21.3	1 (0.87%)	3 (1.22%)	-0.49	0.617	0.686	Mutual exclusivity
LMF1	16p13.3	1 (0.87%)	3 (1.22%)	-0.49	0.617	0.686	Mutual exclusivity
LRRC30	18p11.23	1 (0.87%)	3 (1.22%)	-0.49	0.617	0.686	Mutual exclusivity
LRTOMT	11q13.4	1 (0.87%)	3 (1.22%)	-0.49	0.617	0.686	Mutual exclusivity
LSM1	8p11.23	1 (0.87%)	3 (1.22%)	-0.49	0.617	0.686	Mutual exclusivity
LUC7L2	7q34	1 (0.87%)	3 (1.22%)	-0.49	0.617	0.686	Mutual exclusivity
LVRN	5q23.1	1 (0.87%)	3 (1.22%)	-0.49	0.617	0.686	Mutual exclusivity
MAGI2	7q21.11	1 (0.87%)	3 (1.22%)	-0.49	0.617	0.686	Mutual exclusivity
MAN2A1	5q21.3	1 (0.87%)	3 (1.22%)	-0.49	0.617	0.686	Mutual exclusivity
MAP11	7q22.1	1 (0.87%)	3 (1.22%)	-0.49	0.617	0.686	Mutual exclusivity
MAP1LC3A	20q11.22	1 (0.87%)	3 (1.22%)	-0.49	0.617	0.686	Mutual exclusivity
MAP1S	19p13.11	1 (0.87%)	3 (1.22%)	-0.49	0.617	0.686	Mutual exclusivity
MAP2K2	19p13.3	1 (0.87%)	3 (1.22%)	-0.49	0.617	0.686	Mutual exclusivity
MAP4K3	2p22.1	1 (0.87%)	3 (1.22%)	-0.49	0.617	0.686	Mutual exclusivity
MBLAC1	7q22.1	1 (0.87%)	3 (1.22%)	-0.49	0.617	0.686	Mutual exclusivity
MBOAT7	19q13.42	1 (0.87%)	3 (1.22%)	-0.49	0.617	0.686	Mutual exclusivity
MCC	5q22.2	1 (0.87%)	3 (1.22%)	-0.49	0.617	0.686	Mutual exclusivity
MDH2	7q11.23	1 (0.87%)	3 (1.22%)	-0.49	0.617	0.686	Mutual exclusivity
MEFV	16p13.3	1 (0.87%)	3 (1.22%)	-0.49	0.617	0.686	Mutual exclusivity
METTL17	14q11.2	1 (0.87%)	3 (1.22%)	-0.49	0.617	0.686	Mutual exclusivity

METTL8	2q31.1	1 (0.87%)	3 (1.22%)	-0.49	0.617	0.686	Mutual exclusivity
MFAP3	5q33.2	1 (0.87%)	3 (1.22%)	-0.49	0.617	0.686	Mutual exclusivity
MINAR2	5q23.3	1 (0.87%)	3 (1.22%)	-0.49	0.617	0.686	Mutual exclusivity
MIR-3142/3142		1 (0.87%)	3 (1.22%)	-0.49	0.617	0.686	Mutual exclusivity
MIR-3147/3147		1 (0.87%)	3 (1.22%)	-0.49	0.617	0.686	Mutual exclusivity
MIR-3165/3165		1 (0.87%)	3 (1.22%)	-0.49	0.617	0.686	Mutual exclusivity
MIR-3650/3650		1 (0.87%)	3 (1.22%)	-0.49	0.617	0.686	Mutual exclusivity
MIR-378H/378H		1 (0.87%)	3 (1.22%)	-0.49	0.617	0.686	Mutual exclusivity
MIR-3907/3907		1 (0.87%)	3 (1.22%)	-0.49	0.617	0.686	Mutual exclusivity
MIR-4658/4658		1 (0.87%)	3 (1.22%)	-0.49	0.617	0.686	Mutual exclusivity
MIR-4752/4752		1 (0.87%)	3 (1.22%)	-0.49	0.617	0.686	Mutual exclusivity
MIR-639/639		1 (0.87%)	3 (1.22%)	-0.49	0.617	0.686	Mutual exclusivity
MIR-933/933		1 (0.87%)	3 (1.22%)	-0.49	0.617	0.686	Mutual exclusivity
MKRN2	3p25.2	1 (0.87%)	3 (1.22%)	-0.49	0.617	0.686	Mutual exclusivity
MKRN2OS	3p25.2	1 (0.87%)	3 (1.22%)	-0.49	0.617	0.686	Mutual exclusivity
MLLT10	10p12.31	1 (0.87%)	3 (1.22%)	-0.49	0.617	0.686	Mutual exclusivity
MNX1	7q36.3	1 (0.87%)	3 (1.22%)	-0.49	0.617	0.686	Mutual exclusivity
MOXD2P	7q34	1 (0.87%)	3 (1.22%)	-0.49	0.617	0.686	Mutual exclusivity
MSL2	3q22.3	1 (0.87%)	3 (1.22%)	-0.49	0.617	0.686	Mutual exclusivity
MTCL1	18p11.22	1 (0.87%)	3 (1.22%)	-0.49	0.617	0.686	Mutual exclusivity
MTRNR2L4	16p13.3	1 (0.87%)	3 (1.22%)	-0.49	0.617	0.686	Mutual exclusivity
MUC13	3q21.2	1 (0.87%)	3 (1.22%)	-0.49	0.617	0.686	Mutual exclusivity
MYH7B	20q11.22	1 (0.87%)	3 (1.22%)	-0.49	0.617	0.686	Mutual exclusivity
MYO1G	7p13	1 (0.87%)	3 (1.22%)	-0.49	0.617	0.686	Mutual exclusivity
NAA60	16p13.3	1 (0.87%)	3 (1.22%)	-0.49	0.617	0.686	Mutual exclusivity
NACAD	7p13	1 (0.87%)	3 (1.22%)	-0.49	0.617	0.686	Mutual exclusivity
NADSYN1	11q13.4	1 (0.87%)	3 (1.22%)	-0.49	0.617	0.686	Mutual exclusivity
NDRG3	20q11.23	1 (0.87%)	3 (1.22%)	-0.49	0.617	0.686	Mutual exclusivity
NDUFV2	18p11.22	1 (0.87%)	3 (1.22%)	-0.49	0.617	0.686	Mutual exclusivity

NECAB3	20q11.22	1 (0.87%)	3 (1.22%)	-0.49	0.617	0.686	Mutual exclusivity
NFE2L3	7p15.2	1 (0.87%)	3 (1.22%)	-0.49	0.617	0.686	Mutual exclusivity
NIBAN3	19p13.11	1 (0.87%)	3 (1.22%)	-0.49	0.617	0.686	Mutual exclusivity
NKRF	Xq24	1 (0.87%)	3 (1.22%)	-0.49	0.617	0.686	Mutual exclusivity
NLN	5q12.3	1 (0.87%)	3 (1.22%)	-0.49	0.617	0.686	Mutual exclusivity
NLRC3	16p13.3	1 (0.87%)	3 (1.22%)	-0.49	0.617	0.686	Mutual exclusivity
NMNAT3	3q23	1 (0.87%)	3 (1.22%)	-0.49	0.617	0.686	Mutual exclusivity
NNAT	20q11.23	1 (0.87%)	3 (1.22%)	-0.49	0.617	0.686	Mutual exclusivity
NOC4L	12q24.33	1 (0.87%)	3 (1.22%)	-0.49	0.617	0.686	Mutual exclusivity
NOP58	2q33.1	1 (0.87%)	3 (1.22%)	-0.49	0.617	0.686	Mutual exclusivity
NPC1L1	7p13	1 (0.87%)	3 (1.22%)	-0.49	0.617	0.686	Mutual exclusivity
NPY	7p15.3	1 (0.87%)	3 (1.22%)	-0.49	0.617	0.686	Mutual exclusivity
NRG1	8p12	1 (0.87%)	3 (1.22%)	-0.49	0.617	0.686	Mutual exclusivity
NSD3	8p11.23	1 (0.87%)	3 (1.22%)	-0.49	0.617	0.686	Mutual exclusivity
NUDCD3	7p13	1 (0.87%)	3 (1.22%)	-0.49	0.617	0.686	Mutual exclusivity
NUMA1	11q13.4	1 (0.87%)	3 (1.22%)	-0.49	0.617	0.686	Mutual exclusivity
NUPR1	16p11.2	1 (0.87%)	3 (1.22%)	-0.49	0.617	0.686	Mutual exclusivity
NWD2	4p14	1 (0.87%)	3 (1.22%)	-0.49	0.617	0.686	Mutual exclusivity
OGDH	7p13	1 (0.87%)	3 (1.22%)	-0.49	0.617	0.686	Mutual exclusivity
OLA1	2q31.1	1 (0.87%)	3 (1.22%)	-0.49	0.617	0.686	Mutual exclusivity
OR2A12	7q35	1 (0.87%)	3 (1.22%)	-0.49	0.617	0.686	Mutual exclusivity
OR2A2	7q35	1 (0.87%)	3 (1.22%)	-0.49	0.617	0.686	Mutual exclusivity
OR2A25	7q35	1 (0.87%)	3 (1.22%)	-0.49	0.617	0.686	Mutual exclusivity
OR2A5	7q35	1 (0.87%)	3 (1.22%)	-0.49	0.617	0.686	Mutual exclusivity
OR2C1	16p13.3	1 (0.87%)	3 (1.22%)	-0.49	0.617	0.686	Mutual exclusivity
OR2F1	7q35	1 (0.87%)	3 (1.22%)	-0.49	0.617	0.686	Mutual exclusivity
OR2F2	7q35	1 (0.87%)	3 (1.22%)	-0.49	0.617	0.686	Mutual exclusivity
OR6B1	7q35	1 (0.87%)	3 (1.22%)	-0.49	0.617	0.686	Mutual exclusivity
OSBPL3	7p15.3	1 (0.87%)	3 (1.22%)	-0.49	0.617	0.686	Mutual exclusivity

PARD3B	2q33.3	1 (0.87%)	3 (1.22%)	-0.49	0.617	0.686	Mutual exclusivity
PARP12	7q34	1 (0.87%)	3 (1.22%)	-0.49	0.617	0.686	Mutual exclusivity
PDILT	16p12.3	1 (0.87%)	3 (1.22%)	-0.49	0.617	0.686	Mutual exclusivity
PHF2P2	13q12.11	1 (0.87%)	3 (1.22%)	-0.49	0.617	0.686	Mutual exclusivity
PHTF2	7q11.23-q21.11	1 (0.87%)	3 (1.22%)	-0.49	0.617	0.686	Mutual exclusivity
PIK3CB	3q22.3	1 (0.87%)	3 (1.22%)	-0.49	0.617	0.686	Mutual exclusivity
PKN1	19p13.12	1 (0.87%)	3 (1.22%)	-0.49	0.617	0.686	Mutual exclusivity
PLPBP	8p11.23	1 (0.87%)	3 (1.22%)	-0.49	0.617	0.686	Mutual exclusivity
PLPP5	8p11.23	1 (0.87%)	3 (1.22%)	-0.49	0.617	0.686	Mutual exclusivity
POMZP3	7q11.23	1 (0.87%)	3 (1.22%)	-0.49	0.617	0.686	Mutual exclusivity
PPFIBP1	12p11.23-p11.22	1 (0.87%)	3 (1.22%)	-0.49	0.617	0.686	Mutual exclusivity
PRKAA2	1p32.2	1 (0.87%)	3 (1.22%)	-0.49	0.617	0.686	Mutual exclusivity
PRORP	14q13.2	1 (0.87%)	3 (1.22%)	-0.49	0.617	0.686	Mutual exclusivity
PRR16	5q23.1	1 (0.87%)	3 (1.22%)	-0.49	0.617	0.686	Mutual exclusivity
PRR23A	3q23	1 (0.87%)	3 (1.22%)	-0.49	0.617	0.686	Mutual exclusivity
PRRC1	5q23.2	1 (0.87%)	3 (1.22%)	-0.49	0.617	0.686	Mutual exclusivity
PTGER1	19p13.12	1 (0.87%)	3 (1.22%)	-0.49	0.617	0.686	Mutual exclusivity
PTH2	19q13.33	1 (0.87%)	3 (1.22%)	-0.49	0.617	0.686	Mutual exclusivity
PTPN12	7q11.23	1 (0.87%)	3 (1.22%)	-0.49	0.617	0.686	Mutual exclusivity
PTPRM	18p11.23	1 (0.87%)	3 (1.22%)	-0.49	0.617	0.686	Mutual exclusivity
PTTG1	5q33.3	1 (0.87%)	3 (1.22%)	-0.49	0.617	0.686	Mutual exclusivity
PUM3	9p24.2	1 (0.87%)	3 (1.22%)	-0.49	0.617	0.686	Mutual exclusivity
PUS7	7q22.3	1 (0.87%)	3 (1.22%)	-0.49	0.617	0.686	Mutual exclusivity
PVRIG	7q22.1	1 (0.87%)	3 (1.22%)	-0.49	0.617	0.686	Mutual exclusivity
RAB11FIP1	8p11.23	1 (0.87%)	3 (1.22%)	-0.49	0.617	0.686	Mutual exclusivity
RAB12	18p11.22	1 (0.87%)	3 (1.22%)	-0.49	0.617	0.686	Mutual exclusivity
RAF1	3p25.2	1 (0.87%)	3 (1.22%)	-0.49	0.617	0.686	Mutual exclusivity
RALBP1	18p11.22	1 (0.87%)	3 (1.22%)	-0.49	0.617	0.686	Mutual exclusivity
RAMP3	7p13	1 (0.87%)	3 (1.22%)	-0.49	0.617	0.686	Mutual exclusivity

RANBP6	9p24.1	1 (0.87%)	3 (1.22%)	-0.49	0.617	0.686	Mutual exclusivity
RASA2	3q23	1 (0.87%)	3 (1.22%)	-0.49	0.617	0.686	Mutual exclusivity
RBL1	20q11.23	1 (0.87%)	3 (1.22%)	-0.49	0.617	0.686	Mutual exclusivity
RBP1	3q23	1 (0.87%)	3 (1.22%)	-0.49	0.617	0.686	Mutual exclusivity
RBP2	3q23	1 (0.87%)	3 (1.22%)	-0.49	0.617	0.686	Mutual exclusivity
RCN3	19q13.33	1 (0.87%)	3 (1.22%)	-0.49	0.617	0.686	Mutual exclusivity
REEP5	5q22.2	1 (0.87%)	3 (1.22%)	-0.49	0.617	0.686	Mutual exclusivity
RETREG1	5p15.1	1 (0.87%)	3 (1.22%)	-0.49	0.617	0.686	Mutual exclusivity
RFX3	9p24.2	1 (0.87%)	3 (1.22%)	-0.49	0.617	0.686	Mutual exclusivity
RHEB	7q36.1	1 (0.87%)	3 (1.22%)	-0.49	0.617	0.686	Mutual exclusivity
RN7SKP124	3q23	1 (0.87%)	3 (1.22%)	-0.49	0.617	0.686	Mutual exclusivity
RN7SKP185	20q11.23	1 (0.87%)	3 (1.22%)	-0.49	0.617	0.686	Mutual exclusivity
RN7SKP193	14q21.3	1 (0.87%)	3 (1.22%)	-0.49	0.617	0.686	Mutual exclusivity
RN7SKP218	7p12.1	1 (0.87%)	3 (1.22%)	-0.49	0.617	0.686	Mutual exclusivity
RN7SKP230	5q21.3	1 (0.87%)	3 (1.22%)	-0.49	0.617	0.686	Mutual exclusivity
RN7SKP232	5q33.1	1 (0.87%)	3 (1.22%)	-0.49	0.617	0.686	Mutual exclusivity
RN7SKP57	5q22.1	1 (0.87%)	3 (1.22%)	-0.49	0.617	0.686	Mutual exclusivity
RN7SKP60	5q34	1 (0.87%)	3 (1.22%)	-0.49	0.617	0.686	Mutual exclusivity
RN7SKP89	5q22.3	1 (0.87%)	3 (1.22%)	-0.49	0.617	0.686	Mutual exclusivity
RN7SL40P	2q33.1	1 (0.87%)	3 (1.22%)	-0.49	0.617	0.686	Mutual exclusivity
RN7SL456P	7q35	1 (0.87%)	3 (1.22%)	-0.49	0.617	0.686	Mutual exclusivity
RN7SL50P	18p11.23	1 (0.87%)	3 (1.22%)	-0.49	0.617	0.686	Mutual exclusivity
RN7SL549P	7q22.1	1 (0.87%)	3 (1.22%)	-0.49	0.617	0.686	Mutual exclusivity
RN7SL558P	4p14	1 (0.87%)	3 (1.22%)	-0.49	0.617	0.686	Mutual exclusivity
RN7SL592P	9p24.2	1 (0.87%)	3 (1.22%)	-0.49	0.617	0.686	Mutual exclusivity
RN7SL65P	2q31.1	1 (0.87%)	3 (1.22%)	-0.49	0.617	0.686	Mutual exclusivity
RN7SL709P	8p11.23	1 (0.87%)	3 (1.22%)	-0.49	0.617	0.686	Mutual exclusivity
RN7SL724P	3q23	1 (0.87%)	3 (1.22%)	-0.49	0.617	0.686	Mutual exclusivity
RN7SL750P	7q22.1	1 (0.87%)	3 (1.22%)	-0.49	0.617	0.686	Mutual exclusivity

RN7SL753P	2q33.1	1 (0.87%)	3 (1.22%)	-0.49	0.617	0.686	Mutual exclusivity
RN7SL76P	7q36.1	1 (0.87%)	3 (1.22%)	-0.49	0.617	0.686	Mutual exclusivity
RN7SL8P	7q22.3	1 (0.87%)	3 (1.22%)	-0.49	0.617	0.686	Mutual exclusivity
RNA5SP142	3q22.3	1 (0.87%)	3 (1.22%)	-0.49	0.617	0.686	Mutual exclusivity
RNA5SP191	5q23.3	1 (0.87%)	3 (1.22%)	-0.49	0.617	0.686	Mutual exclusivity
RNA5SP197	5q33.1	1 (0.87%)	3 (1.22%)	-0.49	0.617	0.686	Mutual exclusivity
RNA5SP198	5q33.1	1 (0.87%)	3 (1.22%)	-0.49	0.617	0.686	Mutual exclusivity
RNA5SP199	5q33.2	1 (0.87%)	3 (1.22%)	-0.49	0.617	0.686	Mutual exclusivity
RNA5SP228	7p15.3	1 (0.87%)	3 (1.22%)	-0.49	0.617	0.686	Mutual exclusivity
RNA5SP229	7p14.3	1 (0.87%)	3 (1.22%)	-0.49	0.617	0.686	Mutual exclusivity
RNA5SP249	7q35	1 (0.87%)	3 (1.22%)	-0.49	0.617	0.686	Mutual exclusivity
RNA5SP342	11q13.4	1 (0.87%)	3 (1.22%)	-0.49	0.617	0.686	Mutual exclusivity
RNA5SP480	20q11.21	1 (0.87%)	3 (1.22%)	-0.49	0.617	0.686	Mutual exclusivity
RNF121	11q13.4	1 (0.87%)	3 (1.22%)	-0.49	0.617	0.686	Mutual exclusivity
RNF7	3q23	1 (0.87%)	3 (1.22%)	-0.49	0.617	0.686	Mutual exclusivity
RNU6ATAC10P	5q23.3	1 (0.87%)	3 (1.22%)	-0.49	0.617	0.686	Mutual exclusivity
RNY4P4	3q22.3	1 (0.87%)	3 (1.22%)	-0.49	0.617	0.686	Mutual exclusivity
RP9	7p14.3	1 (0.87%)	3 (1.22%)	-0.49	0.617	0.686	Mutual exclusivity
RPGRIP1	14q11.2	1 (0.87%)	3 (1.22%)	-0.49	0.617	0.686	Mutual exclusivity
RPL13A	19q13.33	1 (0.87%)	3 (1.22%)	-0.49	0.617	0.686	Mutual exclusivity
RPL32	3p25.2	1 (0.87%)	3 (1.22%)	-0.49	0.617	0.686	Mutual exclusivity
RPN2	20q11.23	1 (0.87%)	3 (1.22%)	-0.49	0.617	0.686	Mutual exclusivity
RPS11	19q13.33	1 (0.87%)	3 (1.22%)	-0.49	0.617	0.686	Mutual exclusivity
RPS20P22	8p11.23	1 (0.87%)	3 (1.22%)	-0.49	0.617	0.686	Mutual exclusivity
RPS9	19q13.42	1 (0.87%)	3 (1.22%)	-0.49	0.617	0.686	Mutual exclusivity
RSBN1L	7q11.23	1 (0.87%)	3 (1.22%)	-0.49	0.617	0.686	Mutual exclusivity
RYK	3q22.2	1 (0.87%)	3 (1.22%)	-0.49	0.617	0.686	Mutual exclusivity
SCRN3	2q31.1	1 (0.87%)	3 (1.22%)	-0.49	0.617	0.686	Mutual exclusivity
SEMA3E	7q21.11	1 (0.87%)	3 (1.22%)	-0.49	0.617	0.686	Mutual exclusivity

SEMA6A	5q23.1	1 (0.87%)	3 (1.22%)	-0.49	0.617	0.686	Mutual exclusivity
SEPTIN7P2	7p12.3	1 (0.87%)	3 (1.22%)	-0.49	0.617	0.686	Mutual exclusivity
SERPINH1	11q13.5	1 (0.87%)	3 (1.22%)	-0.49	0.617	0.686	Mutual exclusivity
SGCD	5q33.2-q33.3	1 (0.87%)	3 (1.22%)	-0.49	0.617	0.686	Mutual exclusivity
SGF29	16p11.2	1 (0.87%)	3 (1.22%)	-0.49	0.617	0.686	Mutual exclusivity
SLC12A2	5q23.3	1 (0.87%)	3 (1.22%)	-0.49	0.617	0.686	Mutual exclusivity
SLC12A9	7q22.1	1 (0.87%)	3 (1.22%)	-0.49	0.617	0.686	Mutual exclusivity
SLC17A7	19q13.33	1 (0.87%)	3 (1.22%)	-0.49	0.617	0.686	Mutual exclusivity
SLC25A36	3q23	1 (0.87%)	3 (1.22%)	-0.49	0.617	0.686	Mutual exclusivity
SLC25A46	5q22.1	1 (0.87%)	3 (1.22%)	-0.49	0.617	0.686	Mutual exclusivity
SLC35G2	3q22.3	1 (0.87%)	3 (1.22%)	-0.49	0.617	0.686	Mutual exclusivity
SLC36A1	5q33.1	1 (0.87%)	3 (1.22%)	-0.49	0.617	0.686	Mutual exclusivity
SLC36A2	5q33.1	1 (0.87%)	3 (1.22%)	-0.49	0.617	0.686	Mutual exclusivity
SLC36A3	5q33.1	1 (0.87%)	3 (1.22%)	-0.49	0.617	0.686	Mutual exclusivity
SLC39A2	14q11.2	1 (0.87%)	3 (1.22%)	-0.49	0.617	0.686	Mutual exclusivity
SLC40A1	2q32.2	1 (0.87%)	3 (1.22%)	-0.49	0.617	0.686	Mutual exclusivity
SLC4A9	5q31.3	1 (0.87%)	3 (1.22%)	-0.49	0.617	0.686	Mutual exclusivity
SLC6A11	3p25.3	1 (0.87%)	3 (1.22%)	-0.49	0.617	0.686	Mutual exclusivity
SLC8A1	2p22.1	1 (0.87%)	3 (1.22%)	-0.49	0.617	0.686	Mutual exclusivity
SLCO2A1	3q22.1-q22.2	1 (0.87%)	3 (1.22%)	-0.49	0.617	0.686	Mutual exclusivity
SLU7	5q33.3	1 (0.87%)	3 (1.22%)	-0.49	0.617	0.686	Mutual exclusivity
SLX4	16p13.3	1 (0.87%)	3 (1.22%)	-0.49	0.617	0.686	Mutual exclusivity
SMARCA2	9p24.3	1 (0.87%)	3 (1.22%)	-0.49	0.617	0.686	Mutual exclusivity
SMARCD3	7q36.1	1 (0.87%)	3 (1.22%)	-0.49	0.617	0.686	Mutual exclusivity
SMCO2	12p11.23	1 (0.87%)	3 (1.22%)	-0.49	0.617	0.686	Mutual exclusivity
SNHG15	7p13	1 (0.87%)	3 (1.22%)	-0.49	0.617	0.686	Mutual exclusivity
SNORA13	5q22.1	1 (0.87%)	3 (1.22%)	-0.49	0.617	0.686	Mutual exclusivity
SNORA49	12q24.33	1 (0.87%)	3 (1.22%)	-0.49	0.617	0.686	Mutual exclusivity
SNORA5A	7p13	1 (0.87%)	3 (1.22%)	-0.49	0.617	0.686	Mutual exclusivity

SNORA5B	7p13	1 (0.87%)	3 (1.22%)	-0.49	0.617	0.686	Mutual exclusivity
SNORA5C	7p13	1 (0.87%)	3 (1.22%)	-0.49	0.617	0.686	Mutual exclusivity
SNORA7A	3p25.2	1 (0.87%)	3 (1.22%)	-0.49	0.617	0.686	Mutual exclusivity
SNORD11	2q33.1	1 (0.87%)	3 (1.22%)	-0.49	0.617	0.686	Mutual exclusivity
SNORD11B	2q33.1	1 (0.87%)	3 (1.22%)	-0.49	0.617	0.686	Mutual exclusivity
SNORD32A	19q13.33	1 (0.87%)	3 (1.22%)	-0.49	0.617	0.686	Mutual exclusivity
SNORD34	19q13.33	1 (0.87%)	3 (1.22%)	-0.49	0.617	0.686	Mutual exclusivity
SNORD35A	19q13.33	1 (0.87%)	3 (1.22%)	-0.49	0.617	0.686	Mutual exclusivity
SNORD35B	19q13.33	1 (0.87%)	3 (1.22%)	-0.49	0.617	0.686	Mutual exclusivity
SNORD70	2q33.1	1 (0.87%)	3 (1.22%)	-0.49	0.617	0.686	Mutual exclusivity
SNORD75	1q25.1	1 (0.87%)	3 (1.22%)	-0.49	0.617	0.686	Mutual exclusivity
SNORD8	14q11.2	1 (0.87%)	3 (1.22%)	-0.49	0.617	0.686	Mutual exclusivity
SNORD9	14q11.2	1 (0.87%)	3 (1.22%)	-0.49	0.617	0.686	Mutual exclusivity
SNX10	7p15.2	1 (0.87%)	3 (1.22%)	-0.49	0.617	0.686	Mutual exclusivity
SNX29P2	16p11.2	1 (0.87%)	3 (1.22%)	-0.49	0.617	0.686	Mutual exclusivity
SOGA1	20q11.23	1 (0.87%)	3 (1.22%)	-0.49	0.617	0.686	Mutual exclusivity
SOX14	3q22.3	1 (0.87%)	3 (1.22%)	-0.49	0.617	0.686	Mutual exclusivity
SP9	2q31.1	1 (0.87%)	3 (1.22%)	-0.49	0.617	0.686	Mutual exclusivity
SPANXN3	Xq27.3	1 (0.87%)	3 (1.22%)	-0.49	0.617	0.686	Mutual exclusivity
SPARC	5q33.1	1 (0.87%)	3 (1.22%)	-0.49	0.617	0.686	Mutual exclusivity
SPDYE3	7q22.1	1 (0.87%)	3 (1.22%)	-0.49	0.617	0.686	Mutual exclusivity
SPSB4	3q23	1 (0.87%)	3 (1.22%)	-0.49	0.617	0.686	Mutual exclusivity
SRL	16p13.3	1 (0.87%)	3 (1.22%)	-0.49	0.617	0.686	Mutual exclusivity
SRP19	5q22.2	1 (0.87%)	3 (1.22%)	-0.49	0.617	0.686	Mutual exclusivity
SRPK2	7q22.3	1 (0.87%)	3 (1.22%)	-0.49	0.617	0.686	Mutual exclusivity
SRRT	7q22.1	1 (0.87%)	3 (1.22%)	-0.49	0.617	0.686	Mutual exclusivity
SSC4D	7q11.23	1 (0.87%)	3 (1.22%)	-0.49	0.617	0.686	Mutual exclusivity
STAG1	3q22.3	1 (0.87%)	3 (1.22%)	-0.49	0.617	0.686	Mutual exclusivity
STAG3	7q22.1	1 (0.87%)	3 (1.22%)	-0.49	0.617	0.686	Mutual exclusivity

STAR	8p11.23	1 (0.87%)	3 (1.22%)	-0.49	0.617	0.686	Mutual exclusivity
STIM1	11p15.4	1 (0.87%)	3 (1.22%)	-0.49	0.617	0.686	Mutual exclusivity
STK31	7p15.3	1 (0.87%)	3 (1.22%)	-0.49	0.617	0.686	Mutual exclusivity
STK32A	5q32	1 (0.87%)	3 (1.22%)	-0.49	0.617	0.686	Mutual exclusivity
STYXL1	7q11.23	1 (0.87%)	3 (1.22%)	-0.49	0.617	0.686	Mutual exclusivity
SUMO1	2q33.1	1 (0.87%)	3 (1.22%)	-0.49	0.617	0.686	Mutual exclusivity
SUPT16H	14q11.2	1 (0.87%)	3 (1.22%)	-0.49	0.617	0.686	Mutual exclusivity
SVOPL	7q34	1 (0.87%)	3 (1.22%)	-0.49	0.617	0.686	Mutual exclusivity
TAPT1	4p15.32	1 (0.87%)	3 (1.22%)	-0.49	0.617	0.686	Mutual exclusivity
TBRG4	7p13	1 (0.87%)	3 (1.22%)	-0.49	0.617	0.686	Mutual exclusivity
TBXAS1	7q34	1 (0.87%)	3 (1.22%)	-0.49	0.617	0.686	Mutual exclusivity
TCAF1	7q35	1 (0.87%)	3 (1.22%)	-0.49	0.617	0.686	Mutual exclusivity
TCAF2	7q35	1 (0.87%)	3 (1.22%)	-0.49	0.617	0.686	Mutual exclusivity
THUMPD2	2p22.1|2p22-p21	1 (0.87%)	3 (1.22%)	-0.49	0.617	0.686	Mutual exclusivity
TIGD7	16p13.3	1 (0.87%)	3 (1.22%)	-0.49	0.617	0.686	Mutual exclusivity
TIMD4	5q33.3	1 (0.87%)	3 (1.22%)	-0.49	0.617	0.686	Mutual exclusivity
TLDC2	20q11.23	1 (0.87%)	3 (1.22%)	-0.49	0.617	0.686	Mutual exclusivity
TLK1	2q31.1	1 (0.87%)	3 (1.22%)	-0.49	0.617	0.686	Mutual exclusivity
TMC4	19q13.42	1 (0.87%)	3 (1.22%)	-0.49	0.617	0.686	Mutual exclusivity
TMEM178A	2p22.1	1 (0.87%)	3 (1.22%)	-0.49	0.617	0.686	Mutual exclusivity
TMEM204	16p13.3	1 (0.87%)	3 (1.22%)	-0.49	0.617	0.686	Mutual exclusivity
TMEM213	7q34	1 (0.87%)	3 (1.22%)	-0.49	0.617	0.686	Mutual exclusivity
TMEM40	3p25.2	1 (0.87%)	3 (1.22%)	-0.49	0.617	0.686	Mutual exclusivity
TMEM60	7q11.23	1 (0.87%)	3 (1.22%)	-0.49	0.617	0.686	Mutual exclusivity
TMUB1	7q36.1	1 (0.87%)	3 (1.22%)	-0.49	0.617	0.686	Mutual exclusivity
TNFAIP2	14q32.32	1 (0.87%)	3 (1.22%)	-0.49	0.617	0.686	Mutual exclusivity
TRA2A	7p15.3	1 (0.87%)	3 (1.22%)	-0.49	0.617	0.686	Mutual exclusivity
TRAP1	16p13.3	1 (0.87%)	3 (1.22%)	-0.49	0.617	0.686	Mutual exclusivity
TRIM24	7q33-q34	1 (0.87%)	3 (1.22%)	-0.49	0.617	0.686	Mutual exclusivity

TRIM42	3q23	1 (0.87%)	3 (1.22%)	-0.49	0.617	0.686	Mutual exclusivity
TRIP6	7q22.1	1 (0.87%)	3 (1.22%)	-0.49	0.617	0.686	Mutual exclusivity
TSEN2	3p25.2	1 (0.87%)	3 (1.22%)	-0.49	0.617	0.686	Mutual exclusivity
TSEN34	19q13.42	1 (0.87%)	3 (1.22%)	-0.49	0.617	0.686	Mutual exclusivity
TSLP	5q22.1	1 (0.87%)	3 (1.22%)	-0.49	0.617	0.686	Mutual exclusivity
TSR3	16p13.3	1 (0.87%)	3 (1.22%)	-0.49	0.617	0.686	Mutual exclusivity
TSSK1B	5q22.2	1 (0.87%)	3 (1.22%)	-0.49	0.617	0.686	Mutual exclusivity
TTC26	7q34	1 (0.87%)	3 (1.22%)	-0.49	0.617	0.686	Mutual exclusivity
TTI1	20q11.23	1 (0.87%)	3 (1.22%)	-0.49	0.617	0.686	Mutual exclusivity
UBE2A	Xq24	1 (0.87%)	3 (1.22%)	-0.49	0.617	0.686	Mutual exclusivity
UBE2I	16p13.3	1 (0.87%)	3 (1.22%)	-0.49	0.617	0.686	Mutual exclusivity
UBE3C	7q36.3	1 (0.87%)	3 (1.22%)	-0.49	0.617	0.686	Mutual exclusivity
UBN2	7q34	1 (0.87%)	3 (1.22%)	-0.49	0.617	0.686	Mutual exclusivity
UBR4	1p36.13	1 (0.87%)	3 (1.22%)	-0.49	0.617	0.686	Mutual exclusivity
UFSP1	7q22.1	1 (0.87%)	3 (1.22%)	-0.49	0.617	0.686	Mutual exclusivity
UNC13A	19p13.11	1 (0.87%)	3 (1.22%)	-0.49	0.617	0.686	Mutual exclusivity
UNKL	16p13.3	1 (0.87%)	3 (1.22%)	-0.49	0.617	0.686	Mutual exclusivity
UPK3B	7q11.2	1 (0.87%)	3 (1.22%)	-0.49	0.617	0.686	Mutual exclusivity
VDAC1	5q31.1	1 (0.87%)	3 (1.22%)	-0.49	0.617	0.686	Mutual exclusivity
VIL1	2q35	1 (0.87%)	3 (1.22%)	-0.49	0.617	0.686	Mutual exclusivity
VLDLR	9p24.2	1 (0.87%)	3 (1.22%)	-0.49	0.617	0.686	Mutual exclusivity
VOPP1	7p11.2	1 (0.87%)	3 (1.22%)	-0.49	0.617	0.686	Mutual exclusivity
WDR19	4p14	1 (0.87%)	3 (1.22%)	-0.49	0.617	0.686	Mutual exclusivity
WDR36	5q22.1	1 (0.87%)	3 (1.22%)	-0.49	0.617	0.686	Mutual exclusivity
WIPF1	2q31.1	1 (0.87%)	3 (1.22%)	-0.49	0.617	0.686	Mutual exclusivity
YWHAG	7q11.23	1 (0.87%)	3 (1.22%)	-0.49	0.617	0.686	Mutual exclusivity
ZAN	7q22.1	1 (0.87%)	3 (1.22%)	-0.49	0.617	0.686	Mutual exclusivity
ZBED8	5q33.3	1 (0.87%)	3 (1.22%)	-0.49	0.617	0.686	Mutual exclusivity
ZBTB7A	19p13.3	1 (0.87%)	3 (1.22%)	-0.49	0.617	0.686	Mutual exclusivity

ZC3HAV1	7q34	1 (0.87%)	3 (1.22%)	-0.49	0.617	0.686	Mutual exclusivity
ZC3HAV1L	7q34	1 (0.87%)	3 (1.22%)	-0.49	0.617	0.686	Mutual exclusivity
ZNF174	16p13.3	1 (0.87%)	3 (1.22%)	-0.49	0.617	0.686	Mutual exclusivity
ZNF180	19q13.31	1 (0.87%)	3 (1.22%)	-0.49	0.617	0.686	Mutual exclusivity
ZNF200	16p13.3	1 (0.87%)	3 (1.22%)	-0.49	0.617	0.686	Mutual exclusivity
ZNF263	16p13.3	1 (0.87%)	3 (1.22%)	-0.49	0.617	0.686	Mutual exclusivity
ZNF287	17p11.2	1 (0.87%)	3 (1.22%)	-0.49	0.617	0.686	Mutual exclusivity
ZNF385B	2q31.2-q31.3	1 (0.87%)	3 (1.22%)	-0.49	0.617	0.686	Mutual exclusivity
ZNF479	7p11.2	1 (0.87%)	3 (1.22%)	-0.49	0.617	0.686	Mutual exclusivity
ZNF597	16p13.3	1 (0.87%)	3 (1.22%)	-0.49	0.617	0.686	Mutual exclusivity
ZNF622	5p15.1	1 (0.87%)	3 (1.22%)	-0.49	0.617	0.686	Mutual exclusivity
ZNF624	17p11.2	1 (0.87%)	3 (1.22%)	-0.49	0.617	0.686	Mutual exclusivity
ZNF703	8p11.23	1 (0.87%)	3 (1.22%)	-0.49	0.617	0.686	Mutual exclusivity
ZNF716	7p11.2	1 (0.87%)	3 (1.22%)	-0.49	0.617	0.686	Mutual exclusivity
ZNF75A	16p13.3	1 (0.87%)	3 (1.22%)	-0.49	0.617	0.686	Mutual exclusivity
ZP3	7q11.23	1 (0.87%)	3 (1.22%)	-0.49	0.617	0.686	Mutual exclusivity
ZRSR2P1	5q22.2	1 (0.87%)	3 (1.22%)	-0.49	0.617	0.686	Mutual exclusivity
ZSCAN32	16p13.3	1 (0.87%)	3 (1.22%)	-0.49	0.617	0.686	Mutual exclusivity
ABCC1	16p13.11	2 (1.74%)	4 (1.63%)	0.09	0.623	0.686	Co-occurrence
ABCC6	16p13.11	2 (1.74%)	4 (1.63%)	0.09	0.623	0.686	Co-occurrence
ABCC6P1	16p12.3	2 (1.74%)	4 (1.63%)	0.09	0.623	0.686	Co-occurrence
ACTR3B	7q36.1-q36.2	2 (1.74%)	4 (1.63%)	0.09	0.623	0.686	Co-occurrence
ACTR3C	7q36.1	2 (1.74%)	4 (1.63%)	0.09	0.623	0.686	Co-occurrence
ADAM3A	8p11.22	2 (1.74%)	4 (1.63%)	0.09	0.623	0.686	Co-occurrence
ADAM5	8p11.22	2 (1.74%)	4 (1.63%)	0.09	0.623	0.686	Co-occurrence
AKAP10	17p11.2	2 (1.74%)	4 (1.63%)	0.09	0.623	0.686	Co-occurrence
AKR1C7P	10p15.1	2 (1.74%)	4 (1.63%)	0.09	0.623	0.686	Co-occurrence
AKR1E2	10p15.1	2 (1.74%)	4 (1.63%)	0.09	0.623	0.686	Co-occurrence
ANKRD16	10p15.1	2 (1.74%)	4 (1.63%)	0.09	0.623	0.686	Co-occurrence

ARL6IP1	16p12.3	2 (1.74%)	4 (1.63%)	0.09	0.623	0.686	Co-occurrence
ARPC1A	7q22.1	2 (1.74%)	4 (1.63%)	0.09	0.623	0.686	Co-occurrence
ARPC1B	7q22.1	2 (1.74%)	4 (1.63%)	0.09	0.623	0.686	Co-occurrence
ASB13	10p15.1	2 (1.74%)	4 (1.63%)	0.09	0.623	0.686	Co-occurrence
ATP5MF	7q22.1	2 (1.74%)	4 (1.63%)	0.09	0.623	0.686	Co-occurrence
ATP6V0E2	7q36.1	2 (1.74%)	4 (1.63%)	0.09	0.623	0.686	Co-occurrence
ATR	3q23	2 (1.74%)	4 (1.63%)	0.09	0.623	0.686	Co-occurrence
B9D1	17p11.2	2 (1.74%)	4 (1.63%)	0.09	0.623	0.686	Co-occurrence
BCHE	3q26.1	2 (1.74%)	4 (1.63%)	0.09	0.623	0.686	Co-occurrence
BFAR	16p13.12	2 (1.74%)	4 (1.63%)	0.09	0.623	0.686	Co-occurrence
BMERB1	16p13.11	2 (1.74%)	4 (1.63%)	0.09	0.623	0.686	Co-occurrence
BUD31	7q22.1	2 (1.74%)	4 (1.63%)	0.09	0.623	0.686	Co-occurrence
C5ORF49	5p15.31	2 (1.74%)	4 (1.63%)	0.09	0.623	0.686	Co-occurrence
CALM2	2p21	2 (1.74%)	4 (1.63%)	0.09	0.623	0.686	Co-occurrence
CAPSL	5p13.2	2 (1.74%)	4 (1.63%)	0.09	0.623	0.686	Co-occurrence
CCDC144CP	17p11.2	2 (1.74%)	4 (1.63%)	0.09	0.623	0.686	Co-occurrence
CDC42SE2	5q31.1	2 (1.74%)	4 (1.63%)	0.09	0.623	0.686	Co-occurrence
CHRNA9	4p14	2 (1.74%)	4 (1.63%)	0.09	0.623	0.686	Co-occurrence
CNTNAP2	7q35-q36.1	2 (1.74%)	4 (1.63%)	0.09	0.623	0.686	Co-occurrence
CPSF4	7q22.1	2 (1.74%)	4 (1.63%)	0.09	0.623	0.686	Co-occurrence
DEFB121	20q11.21	2 (1.74%)	4 (1.63%)	0.09	0.623	0.686	Co-occurrence
DEFB122	20q11.21	2 (1.74%)	4 (1.63%)	0.09	0.623	0.686	Co-occurrence
DEFB123	20q11.21	2 (1.74%)	4 (1.63%)	0.09	0.623	0.686	Co-occurrence
DEFB124	20q11.21	2 (1.74%)	4 (1.63%)	0.09	0.623	0.686	Co-occurrence
DGKI	7q33	2 (1.74%)	4 (1.63%)	0.09	0.623	0.686	Co-occurrence
EZH2	7q36.1	2 (1.74%)	4 (1.63%)	0.09	0.623	0.686	Co-occurrence
FAM200A	7q22.1	2 (1.74%)	4 (1.63%)	0.09	0.623	0.686	Co-occurrence
FASTKD3	5p15.31	2 (1.74%)	4 (1.63%)	0.09	0.623	0.686	Co-occurrence
FBH1	10p15.1	2 (1.74%)	4 (1.63%)	0.09	0.623	0.686	Co-occurrence

FNDC4	2p23.3	2 (1.74%)	4 (1.63%)	0.09	0.623	0.686	Co-occurrence
FNIP1	5q31.1	2 (1.74%)	4 (1.63%)	0.09	0.623	0.686	Co-occurrence
FOPNL	16p13.11	2 (1.74%)	4 (1.63%)	0.09	0.623	0.686	Co-occurrence
GABRQ	Xq28	2 (1.74%)	4 (1.63%)	0.09	0.623	0.686	Co-occurrence
GCKR	2p23.3	2 (1.74%)	4 (1.63%)	0.09	0.623	0.686	Co-occurrence
GCOM1	15q21.3	2 (1.74%)	4 (1.63%)	0.09	0.623	0.686	Co-occurrence
GDI2	10p15.1	2 (1.74%)	4 (1.63%)	0.09	0.623	0.686	Co-occurrence
GIMAP8	7q36.1	2 (1.74%)	4 (1.63%)	0.09	0.623	0.686	Co-occurrence
GINS4	8p11.21	2 (1.74%)	4 (1.63%)	0.09	0.623	0.686	Co-occurrence
GOLGA7	8p11.21	2 (1.74%)	4 (1.63%)	0.09	0.623	0.686	Co-occurrence
IKBKB	8p11.21	2 (1.74%)	4 (1.63%)	0.09	0.623	0.686	Co-occurrence
IL15RA	10p15.1	2 (1.74%)	4 (1.63%)	0.09	0.623	0.686	Co-occurrence
IL2RA	10p15.1	2 (1.74%)	4 (1.63%)	0.09	0.623	0.686	Co-occurrence
IL7R	5p13.2	2 (1.74%)	4 (1.63%)	0.09	0.623	0.686	Co-occurrence
JAZF1	7p15.2-p15.1	2 (1.74%)	4 (1.63%)	0.09	0.623	0.686	Co-occurrence
KRBA1	7q36.1	2 (1.74%)	4 (1.63%)	0.09	0.623	0.686	Co-occurrence
LINC00702	10p15.1	2 (1.74%)	4 (1.63%)	0.09	0.623	0.686	Co-occurrence
LINC00703	10p15.1	2 (1.74%)	4 (1.63%)	0.09	0.623	0.686	Co-occurrence
LINC00705	10p15.1	2 (1.74%)	4 (1.63%)	0.09	0.623	0.686	Co-occurrence
LINC01192	3q26.1	2 (1.74%)	4 (1.63%)	0.09	0.623	0.686	Co-occurrence
LINC01194	5p15.2	2 (1.74%)	4 (1.63%)	0.09	0.623	0.686	Co-occurrence
LINC02656	10p15.1	2 (1.74%)	4 (1.63%)	0.09	0.623	0.686	Co-occurrence
LMBRD2	5p13.2	2 (1.74%)	4 (1.63%)	0.09	0.623	0.686	Co-occurrence
LPIN2	18p11.31	2 (1.74%)	4 (1.63%)	0.09	0.623	0.686	Co-occurrence
LRRC61	7q36.1	2 (1.74%)	4 (1.63%)	0.09	0.623	0.686	Co-occurrence
MANCR	10p15.1	2 (1.74%)	4 (1.63%)	0.09	0.623	0.686	Co-occurrence
MAPK7	17p11.2	2 (1.74%)	4 (1.63%)	0.09	0.623	0.686	Co-occurrence
MARF1	16p13.11	2 (1.74%)	4 (1.63%)	0.09	0.623	0.686	Co-occurrence
MFAP4	17p11.2	2 (1.74%)	4 (1.63%)	0.09	0.623	0.686	Co-occurrence

MIR-1263/1263		2 (1.74%)	4 (1.63%)	0.09	0.623	0.686	Co-occurrence
MIR-4279/4279		2 (1.74%)	4 (1.63%)	0.09	0.623	0.686	Co-occurrence
MIR-4458/4458		2 (1.74%)	4 (1.63%)	0.09	0.623	0.686	Co-occurrence
MIR-4468/4468		2 (1.74%)	4 (1.63%)	0.09	0.623	0.686	Co-occurrence
MIR-484/484		2 (1.74%)	4 (1.63%)	0.09	0.623	0.686	Co-occurrence
MIR-720/720		2 (1.74%)	4 (1.63%)	0.09	0.623	0.686	Co-occurrence
MPV17L	16p13.11	2 (1.74%)	4 (1.63%)	0.09	0.623	0.686	Co-occurrence
MRNIP	5q35.3	2 (1.74%)	4 (1.63%)	0.09	0.623	0.686	Co-occurrence
MTRNR2L9	6q11.1	2 (1.74%)	4 (1.63%)	0.09	0.623	0.686	Co-occurrence
MTRR	5p15.31	2 (1.74%)	4 (1.63%)	0.09	0.623	0.686	Co-occurrence
MYH11	16p13.11	2 (1.74%)	4 (1.63%)	0.09	0.623	0.686	Co-occurrence
MYL12A	18p11.31	2 (1.74%)	4 (1.63%)	0.09	0.623	0.686	Co-occurrence
MYL12B	18p11.31	2 (1.74%)	4 (1.63%)	0.09	0.623	0.686	Co-occurrence
MYZAP	15q21.3	2 (1.74%)	4 (1.63%)	0.09	0.623	0.686	Co-occurrence
NABP2	12q13.3	2 (1.74%)	4 (1.63%)	0.09	0.623	0.686	Co-occurrence
NADK2	5p13.2	2 (1.74%)	4 (1.63%)	0.09	0.623	0.686	Co-occurrence
NAMPT	7q22.3	2 (1.74%)	4 (1.63%)	0.09	0.623	0.686	Co-occurrence
NDE1	16p13.11	2 (1.74%)	4 (1.63%)	0.09	0.623	0.686	Co-occurrence
NIPBL	5p13.2	2 (1.74%)	4 (1.63%)	0.09	0.623	0.686	Co-occurrence
NOMO1	16p13.11	2 (1.74%)	4 (1.63%)	0.09	0.623	0.686	Co-occurrence
NOMO2	16p12.3	2 (1.74%)	4 (1.63%)	0.09	0.623	0.686	Co-occurrence
NOMO3	16p13.11	2 (1.74%)	4 (1.63%)	0.09	0.623	0.686	Co-occurrence
NPIPA1	16p13.11	2 (1.74%)	4 (1.63%)	0.09	0.623	0.686	Co-occurrence
NPIPA2	16p13.11	2 (1.74%)	4 (1.63%)	0.09	0.623	0.686	Co-occurrence
NPIPA3	16p13.11	2 (1.74%)	4 (1.63%)	0.09	0.623	0.686	Co-occurrence
NPIPA5	16p13.11	2 (1.74%)	4 (1.63%)	0.09	0.623	0.686	Co-occurrence
NPIPA7	16p13.11	2 (1.74%)	4 (1.63%)	0.09	0.623	0.686	Co-occurrence
NPIPA8	16p12.3	2 (1.74%)	4 (1.63%)	0.09	0.623	0.686	Co-occurrence
NPIPP1	16p13.11	2 (1.74%)	4 (1.63%)	0.09	0.623	0.686	Co-occurrence

NTAN1	16p13.11	2 (1.74%)	4 (1.63%)	0.09	0.623	0.686	Co-occurrence
OTULIN	5p15.2	2 (1.74%)	4 (1.63%)	0.09	0.623	0.686	Co-occurrence
OTULINL	5p15.2	2 (1.74%)	4 (1.63%)	0.09	0.623	0.686	Co-occurrence
PDAP1	7q22.1	2 (1.74%)	4 (1.63%)	0.09	0.623	0.686	Co-occurrence
PDXDC1	16p13.11	2 (1.74%)	4 (1.63%)	0.09	0.623	0.686	Co-occurrence
PHYH	10p13	2 (1.74%)	4 (1.63%)	0.09	0.623	0.686	Co-occurrence
PILRA	7q22.1	2 (1.74%)	4 (1.63%)	0.09	0.623	0.686	Co-occurrence
PJA2	5q21.3	2 (1.74%)	4 (1.63%)	0.09	0.623	0.686	Co-occurrence
PKD1P5	16p12.3	2 (1.74%)	4 (1.63%)	0.09	0.623	0.686	Co-occurrence
PKD1P6	16p13.11	2 (1.74%)	4 (1.63%)	0.09	0.623	0.686	Co-occurrence
PLA2G10	16p13.12	2 (1.74%)	4 (1.63%)	0.09	0.623	0.686	Co-occurrence
PLS1	3q23	2 (1.74%)	4 (1.63%)	0.09	0.623	0.686	Co-occurrence
POLR2M	15q21.3	2 (1.74%)	4 (1.63%)	0.09	0.623	0.686	Co-occurrence
PTCD1	7q22.1	2 (1.74%)	4 (1.63%)	0.09	0.623	0.686	Co-occurrence
RANBP3L	5p13.2	2 (1.74%)	4 (1.63%)	0.09	0.623	0.686	Co-occurrence
RAPGEF6	5q31.1	2 (1.74%)	4 (1.63%)	0.09	0.623	0.686	Co-occurrence
RARRES2	7q36.1	2 (1.74%)	4 (1.63%)	0.09	0.623	0.686	Co-occurrence
RASGRF2	5q14.1	2 (1.74%)	4 (1.63%)	0.09	0.623	0.686	Co-occurrence
RBM17	10p15.1	2 (1.74%)	4 (1.63%)	0.09	0.623	0.686	Co-occurrence
REPIN1	7q36.1	2 (1.74%)	4 (1.63%)	0.09	0.623	0.686	Co-occurrence
RFC1	4p14	2 (1.74%)	4 (1.63%)	0.09	0.623	0.686	Co-occurrence
RN7SKP150	5q35.3	2 (1.74%)	4 (1.63%)	0.09	0.623	0.686	Co-occurrence
RN7SKP78	10p15.1	2 (1.74%)	4 (1.63%)	0.09	0.623	0.686	Co-occurrence
RN7SL118P	Xq24	2 (1.74%)	4 (1.63%)	0.09	0.623	0.686	Co-occurrence
RN7SL445P	10p15.1	2 (1.74%)	4 (1.63%)	0.09	0.623	0.686	Co-occurrence
RN7SL569P	7q36.1	2 (1.74%)	4 (1.63%)	0.09	0.623	0.686	Co-occurrence
RN7SL811P	7q36.1	2 (1.74%)	4 (1.63%)	0.09	0.623	0.686	Co-occurrence
RN7SL845P	7q36.1	2 (1.74%)	4 (1.63%)	0.09	0.623	0.686	Co-occurrence
RNA5SP112	2q31.2	2 (1.74%)	4 (1.63%)	0.09	0.623	0.686	Co-occurrence

RNA5SP143	3q23	2 (1.74%)	4 (1.63%)	0.09	0.623	0.686	Co-occurrence
RNA5SP181	5p13.2	2 (1.74%)	4 (1.63%)	0.09	0.623	0.686	Co-occurrence
RNF112	17p11.2	2 (1.74%)	4 (1.63%)	0.09	0.623	0.686	Co-occurrence
RPS15A	16p12.3	2 (1.74%)	4 (1.63%)	0.09	0.623	0.686	Co-occurrence
RRN3	16p13.11	2 (1.74%)	4 (1.63%)	0.09	0.623	0.686	Co-occurrence
SEPHS1	10p13	2 (1.74%)	4 (1.63%)	0.09	0.623	0.686	Co-occurrence
SI	3q26.1	2 (1.74%)	4 (1.63%)	0.09	0.623	0.686	Co-occurrence
SKP2	5p13.2	2 (1.74%)	4 (1.63%)	0.09	0.623	0.686	Co-occurrence
SLC1A3	5p13.2	2 (1.74%)	4 (1.63%)	0.09	0.623	0.686	Co-occurrence
SLC25A43	Xq24	2 (1.74%)	4 (1.63%)	0.09	0.623	0.686	Co-occurrence
SLC25A5	Xq24	2 (1.74%)	4 (1.63%)	0.09	0.623	0.686	Co-occurrence
SLITRK3	3q26.1	2 (1.74%)	4 (1.63%)	0.09	0.623	0.686	Co-occurrence
SPECC1	17p11.2	2 (1.74%)	4 (1.63%)	0.09	0.623	0.686	Co-occurrence
SPEF2	5p13.2	2 (1.74%)	4 (1.63%)	0.09	0.623	0.686	Co-occurrence
SQSTM1	5q35.3	2 (1.74%)	4 (1.63%)	0.09	0.623	0.686	Co-occurrence
SSPO	7q36.1	2 (1.74%)	4 (1.63%)	0.09	0.623	0.686	Co-occurrence
STPG4	2p21	2 (1.74%)	4 (1.63%)	0.09	0.623	0.686	Co-occurrence
SYPL1	7q22.3	2 (1.74%)	4 (1.63%)	0.09	0.623	0.686	Co-occurrence
TASOR2	10p15.1	2 (1.74%)	4 (1.63%)	0.09	0.623	0.686	Co-occurrence
THADA	2p21	2 (1.74%)	4 (1.63%)	0.09	0.623	0.686	Co-occurrence
TTC23L	5p13.2	2 (1.74%)	4 (1.63%)	0.09	0.623	0.686	Co-occurrence
TUBAL3	10p15.1	2 (1.74%)	4 (1.63%)	0.09	0.623	0.686	Co-occurrence
UCMA	10p13	2 (1.74%)	4 (1.63%)	0.09	0.623	0.686	Co-occurrence
UCN3	10p15.1	2 (1.74%)	4 (1.63%)	0.09	0.623	0.686	Co-occurrence
UGT3A1	5p13.2	2 (1.74%)	4 (1.63%)	0.09	0.623	0.686	Co-occurrence
UGT3A2	5p13.2	2 (1.74%)	4 (1.63%)	0.09	0.623	0.686	Co-occurrence
ULK2	17p11.2	2 (1.74%)	4 (1.63%)	0.09	0.623	0.686	Co-occurrence
VAV2	9q34.2	2 (1.74%)	4 (1.63%)	0.09	0.623	0.686	Co-occurrence
XYLT1	16p12.3	2 (1.74%)	4 (1.63%)	0.09	0.623	0.686	Co-occurrence

ZBED6CL	7q36.1	2 (1.74%)	4 (1.63%)	0.09	0.623	0.686	Co-occurrence
ZCWPW1	7q22.1	2 (1.74%)	4 (1.63%)	0.09	0.623	0.686	Co-occurrence
ZDHHC24	11q13.2	2 (1.74%)	4 (1.63%)	0.09	0.623	0.686	Co-occurrence
ZFR	5p13.3	2 (1.74%)	4 (1.63%)	0.09	0.623	0.686	Co-occurrence
ZIC1	3q24	2 (1.74%)	4 (1.63%)	0.09	0.623	0.686	Co-occurrence
ZIC4	3q24	2 (1.74%)	4 (1.63%)	0.09	0.623	0.686	Co-occurrence
ZKSCAN5	7q22.1	2 (1.74%)	4 (1.63%)	0.09	0.623	0.686	Co-occurrence
ZNF394	7q22.1	2 (1.74%)	4 (1.63%)	0.09	0.623	0.686	Co-occurrence
ZNF467	7q36.1	2 (1.74%)	4 (1.63%)	0.09	0.623	0.686	Co-occurrence
ZNF708	19p12	2 (1.74%)	4 (1.63%)	0.09	0.623	0.686	Co-occurrence
ZNF738	19p12	2 (1.74%)	4 (1.63%)	0.09	0.623	0.686	Co-occurrence
ZNF767P	7q36.1	2 (1.74%)	4 (1.63%)	0.09	0.623	0.686	Co-occurrence
ZNF775	7q36.1	2 (1.74%)	4 (1.63%)	0.09	0.623	0.686	Co-occurrence
ZNF789	7q22.1	2 (1.74%)	4 (1.63%)	0.09	0.623	0.686	Co-occurrence
ZNF862	7q36.1	2 (1.74%)	4 (1.63%)	0.09	0.623	0.686	Co-occurrence
A1CF	10q11.23	0 (0.00%)	1 (0.41%)	<-10	0.681	0.686	Mutual exclusivity
AAAS	12q13.13	0 (0.00%)	1 (0.41%)	<-10	0.681	0.686	Mutual exclusivity
AARS	16q22.1	0 (0.00%)	1 (0.41%)	<-10	0.681	0.686	Mutual exclusivity
AASDHPPT	11q22.3	0 (0.00%)	1 (0.41%)	<-10	0.681	0.686	Mutual exclusivity
ABCA1	9q31.1	0 (0.00%)	1 (0.41%)	<-10	0.681	0.686	Mutual exclusivity
ABCB6	2q35	0 (0.00%)	1 (0.41%)	<-10	0.681	0.686	Mutual exclusivity
ABCG4	11q23.3	0 (0.00%)	1 (0.41%)	<-10	0.681	0.686	Mutual exclusivity
ABHD12	20p11.21	0 (0.00%)	1 (0.41%)	<-10	0.681	0.686	Mutual exclusivity
ABRAXAS2	10q26.13	0 (0.00%)	1 (0.41%)	<-10	0.681	0.686	Mutual exclusivity
ABTB2	11p13	0 (0.00%)	1 (0.41%)	<-10	0.681	0.686	Mutual exclusivity
ACAT2	6q25.3	0 (0.00%)	1 (0.41%)	<-10	0.681	0.686	Mutual exclusivity
ACKR3	2q37.3	0 (0.00%)	1 (0.41%)	<-10	0.681	0.686	Mutual exclusivity
ACOT12	5q14.1	0 (0.00%)	1 (0.41%)	<-10	0.681	0.686	Mutual exclusivity
ACOT8	20q13.12	0 (0.00%)	1 (0.41%)	<-10	0.681	0.686	Mutual exclusivity

ACOXL	2q13	0 (0.00%)	1 (0.41%)	<-10	0.681	0.686	Mutual exclusivity
ACSL3	2q36.1	0 (0.00%)	1 (0.41%)	<-10	0.681	0.686	Mutual exclusivity
ACSL5	10q25.2	0 (0.00%)	1 (0.41%)	<-10	0.681	0.686	Mutual exclusivity
ACTA2	10q23.31	0 (0.00%)	1 (0.41%)	<-10	0.681	0.686	Mutual exclusivity
ACTBL2	5q11.2	0 (0.00%)	1 (0.41%)	<-10	0.681	0.686	Mutual exclusivity
ACTR10	14q23.1	0 (0.00%)	1 (0.41%)	<-10	0.681	0.686	Mutual exclusivity
ACTR1B	2q11.2	0 (0.00%)	1 (0.41%)	<-10	0.681	0.686	Mutual exclusivity
ACTR2	2p14	0 (0.00%)	1 (0.41%)	<-10	0.681	0.686	Mutual exclusivity
ACTR3	2q14.1	0 (0.00%)	1 (0.41%)	<-10	0.681	0.686	Mutual exclusivity
ACYP1	14q24.3	0 (0.00%)	1 (0.41%)	<-10	0.681	0.686	Mutual exclusivity
ACYP2	2p16.2	0 (0.00%)	1 (0.41%)	<-10	0.681	0.686	Mutual exclusivity
ADA	20q13.12	0 (0.00%)	1 (0.41%)	<-10	0.681	0.686	Mutual exclusivity
ADAM12	10q26.2	0 (0.00%)	1 (0.41%)	<-10	0.681	0.686	Mutual exclusivity
ADAM29	4q34.1	0 (0.00%)	1 (0.41%)	<-10	0.681	0.686	Mutual exclusivity
ADAMTS15	11q24.3	0 (0.00%)	1 (0.41%)	<-10	0.681	0.686	Mutual exclusivity
ADAMTS8	11q24.3	0 (0.00%)	1 (0.41%)	<-10	0.681	0.686	Mutual exclusivity
ADARB1	21q22.3	0 (0.00%)	1 (0.41%)	<-10	0.681	0.686	Mutual exclusivity
ADAT2	6q24.2	0 (0.00%)	1 (0.41%)	<-10	0.681	0.686	Mutual exclusivity
ADCY6	12q13.12	0 (0.00%)	1 (0.41%)	<-10	0.681	0.686	Mutual exclusivity
ADGB	6q24.3	0 (0.00%)	1 (0.41%)	<-10	0.681	0.686	Mutual exclusivity
ADGRE1	19p13.3-p13.2	0 (0.00%)	1 (0.41%)	<-10	0.681	0.686	Mutual exclusivity
ADGRE4P	19p13.2	0 (0.00%)	1 (0.41%)	<-10	0.681	0.686	Mutual exclusivity
ADH7	4q23	0 (0.00%)	1 (0.41%)	<-10	0.681	0.686	Mutual exclusivity
ADIPOR2	12p13.33	0 (0.00%)	1 (0.41%)	<-10	0.681	0.686	Mutual exclusivity
ADORA2B	17p12	0 (0.00%)	1 (0.41%)	<-10	0.681	0.686	Mutual exclusivity
ADRA1D	20p13	0 (0.00%)	1 (0.41%)	<-10	0.681	0.686	Mutual exclusivity
ADRA2B	2q11.2	0 (0.00%)	1 (0.41%)	<-10	0.681	0.686	Mutual exclusivity
AFF3	2q11.2	0 (0.00%)	1 (0.41%)	<-10	0.681	0.686	Mutual exclusivity
AGPAT4	6q26	0 (0.00%)	1 (0.41%)	<-10	0.681	0.686	Mutual exclusivity

AGXT	2q37.3	0 (0.00%)	1 (0.41%)	<-10	0.681	0.686	Mutual exclusivity
AHSA2P	2p15	0 (0.00%)	1 (0.41%)	<-10	0.681	0.686	Mutual exclusivity
AIRE	21q22.3	0 (0.00%)	1 (0.41%)	<-10	0.681	0.686	Mutual exclusivity
AKAP12	6q25.1	0 (0.00%)	1 (0.41%)	<-10	0.681	0.686	Mutual exclusivity
AKAP3	12p13.32	0 (0.00%)	1 (0.41%)	<-10	0.681	0.686	Mutual exclusivity
AKAP6	14q12	0 (0.00%)	1 (0.41%)	<-10	0.681	0.686	Mutual exclusivity
AKIRIN2	6q15	0 (0.00%)	1 (0.41%)	<-10	0.681	0.686	Mutual exclusivity
AKNA	9q32	0 (0.00%)	1 (0.41%)	<-10	0.681	0.686	Mutual exclusivity
AKR7A2	1p36.13	0 (0.00%)	1 (0.41%)	<-10	0.681	0.686	Mutual exclusivity
AKR7A3	1p36.13	0 (0.00%)	1 (0.41%)	<-10	0.681	0.686	Mutual exclusivity
AKR7L	1p36.13|1p35-p36.1	0 (0.00%)	1 (0.41%)	<-10	0.681	0.686	Mutual exclusivity
ALCAM	3q13.11	0 (0.00%)	1 (0.41%)	<-10	0.681	0.686	Mutual exclusivity
ALDH1L1	3q21.3	0 (0.00%)	1 (0.41%)	<-10	0.681	0.686	Mutual exclusivity
ALDH4A1	1p36.13	0 (0.00%)	1 (0.41%)	<-10	0.681	0.686	Mutual exclusivity
ALDOB	9q31.1	0 (0.00%)	1 (0.41%)	<-10	0.681	0.686	Mutual exclusivity
ALG1L	3q21.2	0 (0.00%)	1 (0.41%)	<-10	0.681	0.686	Mutual exclusivity
ALG9	11q23.1	0 (0.00%)	1 (0.41%)	<-10	0.681	0.686	Mutual exclusivity
ALKBH8	11q22.3	0 (0.00%)	1 (0.41%)	<-10	0.681	0.686	Mutual exclusivity
ALPG	2q37.1	0 (0.00%)	1 (0.41%)	<-10	0.681	0.686	Mutual exclusivity
ALPI	2q37.1	0 (0.00%)	1 (0.41%)	<-10	0.681	0.686	Mutual exclusivity
ALPL	1p36.12	0 (0.00%)	1 (0.41%)	<-10	0.681	0.686	Mutual exclusivity
ALPP	2q37.1	0 (0.00%)	1 (0.41%)	<-10	0.681	0.686	Mutual exclusivity
AMN	14q32.32	0 (0.00%)	1 (0.41%)	<-10	0.681	0.686	Mutual exclusivity
AMOTL1	11q21	0 (0.00%)	1 (0.41%)	<-10	0.681	0.686	Mutual exclusivity
ANGPTL6	19p13.2	0 (0.00%)	1 (0.41%)	<-10	0.681	0.686	Mutual exclusivity
ANGPTL8	19p13.2	0 (0.00%)	1 (0.41%)	<-10	0.681	0.686	Mutual exclusivity
ANKK1	11q23.2	0 (0.00%)	1 (0.41%)	<-10	0.681	0.686	Mutual exclusivity
ANKMY1	2q37.3	0 (0.00%)	1 (0.41%)	<-10	0.681	0.686	Mutual exclusivity
ANKRD2	10q24.2	0 (0.00%)	1 (0.41%)	<-10	0.681	0.686	Mutual exclusivity

ANKRD22	10q23.31	0 (0.00%)	1 (0.41%)	<-10	0.681	0.686	Mutual exclusivity
ANKRD23	2q11.2	0 (0.00%)	1 (0.41%)	<-10	0.681	0.686	Mutual exclusivity
ANKRD34B	5q14.1	0 (0.00%)	1 (0.41%)	<-10	0.681	0.686	Mutual exclusivity
ANKRD36	2q11.2	0 (0.00%)	1 (0.41%)	<-10	0.681	0.686	Mutual exclusivity
ANKRD36B	2q11.2	0 (0.00%)	1 (0.41%)	<-10	0.681	0.686	Mutual exclusivity
ANKRD36C	2q11.1	0 (0.00%)	1 (0.41%)	<-10	0.681	0.686	Mutual exclusivity
ANKRD39	2q11.2	0 (0.00%)	1 (0.41%)	<-10	0.681	0.686	Mutual exclusivity
ANKRD49	11q21	0 (0.00%)	1 (0.41%)	<-10	0.681	0.686	Mutual exclusivity
ANKZF1	2q35	0 (0.00%)	1 (0.41%)	<-10	0.681	0.686	Mutual exclusivity
ANO2	12p13.31	0 (0.00%)	1 (0.41%)	<-10	0.681	0.686	Mutual exclusivity
ANO7	2q37.3	0 (0.00%)	1 (0.41%)	<-10	0.681	0.686	Mutual exclusivity
AP1M2	19p13.2	0 (0.00%)	1 (0.41%)	<-10	0.681	0.686	Mutual exclusivity
AP1S3	2q36.1	0 (0.00%)	1 (0.41%)	<-10	0.681	0.686	Mutual exclusivity
AP3B1	5q14.1	0 (0.00%)	1 (0.41%)	<-10	0.681	0.686	Mutual exclusivity
AP4E1	15q21.2	0 (0.00%)	1 (0.41%)	<-10	0.681	0.686	Mutual exclusivity
AP5M1	14q22.3	0 (0.00%)	1 (0.41%)	<-10	0.681	0.686	Mutual exclusivity
APC2	19p13.3	0 (0.00%)	1 (0.41%)	<-10	0.681	0.686	Mutual exclusivity
APLNR	11q12.1	0 (0.00%)	1 (0.41%)	<-10	0.681	0.686	Mutual exclusivity
APOLD1	12p13.1	0 (0.00%)	1 (0.41%)	<-10	0.681	0.686	Mutual exclusivity
AQP12A	2q37.3	0 (0.00%)	1 (0.41%)	<-10	0.681	0.686	Mutual exclusivity
AQP12B	2q37.3	0 (0.00%)	1 (0.41%)	<-10	0.681	0.686	Mutual exclusivity
ARF3	12q13.12	0 (0.00%)	1 (0.41%)	<-10	0.681	0.686	Mutual exclusivity
ARF4	3p14.3	0 (0.00%)	1 (0.41%)	<-10	0.681	0.686	Mutual exclusivity
ARFIP1	4q31.3	0 (0.00%)	1 (0.41%)	<-10	0.681	0.686	Mutual exclusivity
ARHGAP10	4q31.23	0 (0.00%)	1 (0.41%)	<-10	0.681	0.686	Mutual exclusivity
ARHGEF1	19q13.2	0 (0.00%)	1 (0.41%)	<-10	0.681	0.686	Mutual exclusivity
ARHGEF17	11q13.4	0 (0.00%)	1 (0.41%)	<-10	0.681	0.686	Mutual exclusivity
ARHGEF3	3p14.3	0 (0.00%)	1 (0.41%)	<-10	0.681	0.686	Mutual exclusivity
ARID1A	1p36.11	0 (0.00%)	1 (0.41%)	<-10	0.681	0.686	Mutual exclusivity

ARID4A	14q23.1	0 (0.00%)	1 (0.41%)	<-10	0.681	0.686	Mutual exclusivity
ARID5A	2q11.2	0 (0.00%)	1 (0.41%)	<-10	0.681	0.686	Mutual exclusivity
ARL15	5q11.2	0 (0.00%)	1 (0.41%)	<-10	0.681	0.686	Mutual exclusivity
ARL3	10q24.32	0 (0.00%)	1 (0.41%)	<-10	0.681	0.686	Mutual exclusivity
ARL4A	7p21.3	0 (0.00%)	1 (0.41%)	<-10	0.681	0.686	Mutual exclusivity
ARL4C	2q37.1	0 (0.00%)	1 (0.41%)	<-10	0.681	0.686	Mutual exclusivity
ARMC2	6q21	0 (0.00%)	1 (0.41%)	<-10	0.681	0.686	Mutual exclusivity
ARMH4	14q23.1	0 (0.00%)	1 (0.41%)	<-10	0.681	0.686	Mutual exclusivity
ARMS2	10q26.13	0 (0.00%)	1 (0.41%)	<-10	0.681	0.686	Mutual exclusivity
ARMT1	6q25.1	0 (0.00%)	1 (0.41%)	<-10	0.681	0.686	Mutual exclusivity
ARRDC3	5q14.3	0 (0.00%)	1 (0.41%)	<-10	0.681	0.686	Mutual exclusivity
ASAP3	1p36.12	0 (0.00%)	1 (0.41%)	<-10	0.681	0.686	Mutual exclusivity
ASB18	2q37.2	0 (0.00%)	1 (0.41%)	<-10	0.681	0.686	Mutual exclusivity
ASB3	2p16.2	0 (0.00%)	1 (0.41%)	<-10	0.681	0.686	Mutual exclusivity
ASIC4	2q35	0 (0.00%)	1 (0.41%)	<-10	0.681	0.686	Mutual exclusivity
ASIC5	4q32.1	0 (0.00%)	1 (0.41%)	<-10	0.681	0.686	Mutual exclusivity
ASTL	2q11.2	0 (0.00%)	1 (0.41%)	<-10	0.681	0.686	Mutual exclusivity
ASXL1	20q11.21	0 (0.00%)	1 (0.41%)	<-10	0.681	0.686	Mutual exclusivity
ATG10	5q14.1-q14.2	0 (0.00%)	1 (0.41%)	<-10	0.681	0.686	Mutual exclusivity
ATG14	14q22.3	0 (0.00%)	1 (0.41%)	<-10	0.681	0.686	Mutual exclusivity
ATG16L1	2q37.1	0 (0.00%)	1 (0.41%)	<-10	0.681	0.686	Mutual exclusivity
ATG16L2	11q13.4	0 (0.00%)	1 (0.41%)	<-10	0.681	0.686	Mutual exclusivity
ATG2B	14q32.2	0 (0.00%)	1 (0.41%)	<-10	0.681	0.686	Mutual exclusivity
ATG4A	Xq22.3	0 (0.00%)	1 (0.41%)	<-10	0.681	0.686	Mutual exclusivity
ATG4B	2q37.3	0 (0.00%)	1 (0.41%)	<-10	0.681	0.686	Mutual exclusivity
ATG4D	19p13.2	0 (0.00%)	1 (0.41%)	<-10	0.681	0.686	Mutual exclusivity
ATG5	6q21	0 (0.00%)	1 (0.41%)	<-10	0.681	0.686	Mutual exclusivity
ATG9A	2q35	0 (0.00%)	1 (0.41%)	<-10	0.681	0.686	Mutual exclusivity
ATIC	2q35	0 (0.00%)	1 (0.41%)	<-10	0.681	0.686	Mutual exclusivity

ATP1A1	1p13.1	0 (0.00%)	1 (0.41%)	<-10	0.681	0.686	Mutual exclusivity
ATP1A1-AS1	1p13.1	0 (0.00%)	1 (0.41%)	<-10	0.681	0.686	Mutual exclusivity
ATP1A3	19q13.2	0 (0.00%)	1 (0.41%)	<-10	0.681	0.686	Mutual exclusivity
ATP5F1A	18q21.1	0 (0.00%)	1 (0.41%)	<-10	0.681	0.686	Mutual exclusivity
ATP5MG	11q23.3	0 (0.00%)	1 (0.41%)	<-10	0.681	0.686	Mutual exclusivity
ATP6AP1L	5q14.2	0 (0.00%)	1 (0.41%)	<-10	0.681	0.686	Mutual exclusivity
ATRN	20p13	0 (0.00%)	1 (0.41%)	<-10	0.681	0.686	Mutual exclusivity
ATXN3	14q32.12	0 (0.00%)	1 (0.41%)	<-10	0.681	0.686	Mutual exclusivity
AUH	9q22.31	0 (0.00%)	1 (0.41%)	<-10	0.681	0.686	Mutual exclusivity
AUNIP	1p36.11	0 (0.00%)	1 (0.41%)	<-10	0.681	0.686	Mutual exclusivity
AVP	20p13	0 (0.00%)	1 (0.41%)	<-10	0.681	0.686	Mutual exclusivity
AVPI1	10q24.2	0 (0.00%)	1 (0.41%)	<-10	0.681	0.686	Mutual exclusivity
B4GALT1	9p21.1	0 (0.00%)	1 (0.41%)	<-10	0.681	0.686	Mutual exclusivity
BAAT	9q31.1	0 (0.00%)	1 (0.41%)	<-10	0.681	0.686	Mutual exclusivity
BACH2	6q15	0 (0.00%)	1 (0.41%)	<-10	0.681	0.686	Mutual exclusivity
BAG1	9p13.3	0 (0.00%)	1 (0.41%)	<-10	0.681	0.686	Mutual exclusivity
BARD1	2q35	0 (0.00%)	1 (0.41%)	<-10	0.681	0.686	Mutual exclusivity
BBS12	4q27	0 (0.00%)	1 (0.41%)	<-10	0.681	0.686	Mutual exclusivity
BBX	3q13.12	0 (0.00%)	1 (0.41%)	<-10	0.681	0.686	Mutual exclusivity
BCCIP	10q26.2	0 (0.00%)	1 (0.41%)	<-10	0.681	0.686	Mutual exclusivity
BCKDK	16p11.2	0 (0.00%)	1 (0.41%)	<-10	0.681	0.686	Mutual exclusivity
BCL11A	2p16.1	0 (0.00%)	1 (0.41%)	<-10	0.681	0.686	Mutual exclusivity
BCL2L11	2q13	0 (0.00%)	1 (0.41%)	<-10	0.681	0.686	Mutual exclusivity
BCL2L14	12p13.2	0 (0.00%)	1 (0.41%)	<-10	0.681	0.686	Mutual exclusivity
BCL7C	16p11.2	0 (0.00%)	1 (0.41%)	<-10	0.681	0.686	Mutual exclusivity
BCR	22q11.23	0 (0.00%)	1 (0.41%)	<-10	0.681	0.686	Mutual exclusivity
BCS1L	2q35	0 (0.00%)	1 (0.41%)	<-10	0.681	0.686	Mutual exclusivity
BDKRB1	14q32.2	0 (0.00%)	1 (0.41%)	<-10	0.681	0.686	Mutual exclusivity
BDKRB2	14q32.2	0 (0.00%)	1 (0.41%)	<-10	0.681	0.686	Mutual exclusivity

BFSP1	20p12.1	0 (0.00%)	1 (0.41%)	<-10	0.681	0.686	Mutual exclusivity
BHMT	5q14.1	0 (0.00%)	1 (0.41%)	<-10	0.681	0.686	Mutual exclusivity
BHMT2	5q14.1	0 (0.00%)	1 (0.41%)	<-10	0.681	0.686	Mutual exclusivity
BLOC1S2	10q24.31	0 (0.00%)	1 (0.41%)	<-10	0.681	0.686	Mutual exclusivity
BMS1	10q11.21	0 (0.00%)	1 (0.41%)	<-10	0.681	0.686	Mutual exclusivity
BNIP3P1	14q12	0 (0.00%)	1 (0.41%)	<-10	0.681	0.686	Mutual exclusivity
BOK	2q37.3	0 (0.00%)	1 (0.41%)	<-10	0.681	0.686	Mutual exclusivity
BORCS7	10q24.32	0 (0.00%)	1 (0.41%)	<-10	0.681	0.686	Mutual exclusivity
BPIFB2	20q11.21	0 (0.00%)	1 (0.41%)	<-10	0.681	0.686	Mutual exclusivity
BRF1	14q32.33	0 (0.00%)	1 (0.41%)	<-10	0.681	0.686	Mutual exclusivity
BSCL2	11q12.3	0 (0.00%)	1 (0.41%)	<-10	0.681	0.686	Mutual exclusivity
BTBD16	10q26.13	0 (0.00%)	1 (0.41%)	<-10	0.681	0.686	Mutual exclusivity
BTBD18	11q12.1	0 (0.00%)	1 (0.41%)	<-10	0.681	0.686	Mutual exclusivity
BUB1	2q13	0 (0.00%)	1 (0.41%)	<-10	0.681	0.686	Mutual exclusivity
C10ORF62	10q24.2	0 (0.00%)	1 (0.41%)	<-10	0.681	0.686	Mutual exclusivity
C10ORF90	10q26.2	0 (0.00%)	1 (0.41%)	<-10	0.681	0.686	Mutual exclusivity
C11ORF44	11q24.3	0 (0.00%)	1 (0.41%)	<-10	0.681	0.686	Mutual exclusivity
C11ORF45	11q24.3	0 (0.00%)	1 (0.41%)	<-10	0.681	0.686	Mutual exclusivity
C11ORF54	11q21	0 (0.00%)	1 (0.41%)	<-10	0.681	0.686	Mutual exclusivity
C11ORF74	11p12	0 (0.00%)	1 (0.41%)	<-10	0.681	0.686	Mutual exclusivity
C11ORF87	11q22.3	0 (0.00%)	1 (0.41%)	<-10	0.681	0.686	Mutual exclusivity
C11ORF95	11q13.1	0 (0.00%)	1 (0.41%)	<-10	0.681	0.686	Mutual exclusivity
C11ORF98	11q12.3	0 (0.00%)	1 (0.41%)	<-10	0.681	0.686	Mutual exclusivity
C12ORF10	12q13.13	0 (0.00%)	1 (0.41%)	<-10	0.681	0.686	Mutual exclusivity
C12ORF4	12p13.32	0 (0.00%)	1 (0.41%)	<-10	0.681	0.686	Mutual exclusivity
C14ORF132	14q32.2	0 (0.00%)	1 (0.41%)	<-10	0.681	0.686	Mutual exclusivity
C16ORF78	16q12.1	0 (0.00%)	1 (0.41%)	<-10	0.681	0.686	Mutual exclusivity
C18ORF21	18q12.2	0 (0.00%)	1 (0.41%)	<-10	0.681	0.686	Mutual exclusivity
C18ORF25	18q21.1	0 (0.00%)	1 (0.41%)	<-10	0.681	0.686	Mutual exclusivity

C18ORF32	18q21.1	0 (0.00%)	1 (0.41%)	<-10	0.681	0.686	Mutual exclusivity
C19ORF24	19p13.3	0 (0.00%)	1 (0.41%)	<-10	0.681	0.686	Mutual exclusivity
C1ORF50	1p34.2	0 (0.00%)	1 (0.41%)	<-10	0.681	0.686	Mutual exclusivity
C1QA	1p36.12	0 (0.00%)	1 (0.41%)	<-10	0.681	0.686	Mutual exclusivity
C1QB	1p36.12	0 (0.00%)	1 (0.41%)	<-10	0.681	0.686	Mutual exclusivity
C1QC	1p36.12	0 (0.00%)	1 (0.41%)	<-10	0.681	0.686	Mutual exclusivity
C1QL2	2q14.2	0 (0.00%)	1 (0.41%)	<-10	0.681	0.686	Mutual exclusivity
C20ORF141	20p13	0 (0.00%)	1 (0.41%)	<-10	0.681	0.686	Mutual exclusivity
C21ORF58	21q22.3	0 (0.00%)	1 (0.41%)	<-10	0.681	0.686	Mutual exclusivity
C2ORF15	2q11.2	0 (0.00%)	1 (0.41%)	<-10	0.681	0.686	Mutual exclusivity
C2ORF42	2p13.3	0 (0.00%)	1 (0.41%)	<-10	0.681	0.686	Mutual exclusivity
C2ORF49	2q12.2	0 (0.00%)	1 (0.41%)	<-10	0.681	0.686	Mutual exclusivity
C2ORF72	2q37.1	0 (0.00%)	1 (0.41%)	<-10	0.681	0.686	Mutual exclusivity
C2ORF73	2p16.2	0 (0.00%)	1 (0.41%)	<-10	0.681	0.686	Mutual exclusivity
C2ORF74	2p15	0 (0.00%)	1 (0.41%)	<-10	0.681	0.686	Mutual exclusivity
C2ORF76	2q14.2	0 (0.00%)	1 (0.41%)	<-10	0.681	0.686	Mutual exclusivity
C2ORF80	2q33.3	0 (0.00%)	1 (0.41%)	<-10	0.681	0.686	Mutual exclusivity
C2ORF83	2q36.3	0 (0.00%)	1 (0.41%)	<-10	0.681	0.686	Mutual exclusivity
C3	19p13.3	0 (0.00%)	1 (0.41%)	<-10	0.681	0.686	Mutual exclusivity
C3ORF22	3q21.3	0 (0.00%)	1 (0.41%)	<-10	0.681	0.686	Mutual exclusivity
C3ORF38	3p11.1	0 (0.00%)	1 (0.41%)	<-10	0.681	0.686	Mutual exclusivity
C4ORF33	4q28.2	0 (0.00%)	1 (0.41%)	<-10	0.681	0.686	Mutual exclusivity
C6ORF120	6q27	0 (0.00%)	1 (0.41%)	<-10	0.681	0.686	Mutual exclusivity
C6ORF163	6q15	0 (0.00%)	1 (0.41%)	<-10	0.681	0.686	Mutual exclusivity
C6ORF99	6q25.3	0 (0.00%)	1 (0.41%)	<-10	0.681	0.686	Mutual exclusivity
C9ORF57	9q21.13	0 (0.00%)	1 (0.41%)	<-10	0.681	0.686	Mutual exclusivity
CA11	19q13.33	0 (0.00%)	1 (0.41%)	<-10	0.681	0.686	Mutual exclusivity
CACNA1C	12p13.33	0 (0.00%)	1 (0.41%)	<-10	0.681	0.686	Mutual exclusivity
CACNA2D4	12p13.33	0 (0.00%)	1 (0.41%)	<-10	0.681	0.686	Mutual exclusivity

CACNB3	12q13.12	0 (0.00%)	1 (0.41%)	<-10	0.681	0.686	Mutual exclusivity
CADM2	3p12.1	0 (0.00%)	1 (0.41%)	<-10	0.681	0.686	Mutual exclusivity
CAHM	6q26	0 (0.00%)	1 (0.41%)	<-10	0.681	0.686	Mutual exclusivity
CALR	19p13.13	0 (0.00%)	1 (0.41%)	<-10	0.681	0.686	Mutual exclusivity
CAPN10	2q37.3	0 (0.00%)	1 (0.41%)	<-10	0.681	0.686	Mutual exclusivity
CAPS	19p13.3	0 (0.00%)	1 (0.41%)	<-10	0.681	0.686	Mutual exclusivity
CAPZB	1p36.13	0 (0.00%)	1 (0.41%)	<-10	0.681	0.686	Mutual exclusivity
CARD16	11q22.3	0 (0.00%)	1 (0.41%)	<-10	0.681	0.686	Mutual exclusivity
CARD17	11q22.3	0 (0.00%)	1 (0.41%)	<-10	0.681	0.686	Mutual exclusivity
CARD18	11q22.3	0 (0.00%)	1 (0.41%)	<-10	0.681	0.686	Mutual exclusivity
CASC16	16q12.1-q12.2	0 (0.00%)	1 (0.41%)	<-10	0.681	0.686	Mutual exclusivity
CASP1	11q22.3	0 (0.00%)	1 (0.41%)	<-10	0.681	0.686	Mutual exclusivity
CASP12	11q22.3	0 (0.00%)	1 (0.41%)	<-10	0.681	0.686	Mutual exclusivity
CASP4	11q22.3	0 (0.00%)	1 (0.41%)	<-10	0.681	0.686	Mutual exclusivity
CASP5	11q22.3	0 (0.00%)	1 (0.41%)	<-10	0.681	0.686	Mutual exclusivity
CATSPERB	14q32.12	0 (0.00%)	1 (0.41%)	<-10	0.681	0.686	Mutual exclusivity
CATSPERD	19p13.3	0 (0.00%)	1 (0.41%)	<-10	0.681	0.686	Mutual exclusivity
CBL	11q23.3	0 (0.00%)	1 (0.41%)	<-10	0.681	0.686	Mutual exclusivity
CBLB	3q13.11	0 (0.00%)	1 (0.41%)	<-10	0.681	0.686	Mutual exclusivity
CBLIF	11q12.1	0 (0.00%)	1 (0.41%)	<-10	0.681	0.686	Mutual exclusivity
CBLN1	16q12.1	0 (0.00%)	1 (0.41%)	<-10	0.681	0.686	Mutual exclusivity
CBLN2	18q22.3	0 (0.00%)	1 (0.41%)	<-10	0.681	0.686	Mutual exclusivity
CBR4	4q32.3	0 (0.00%)	1 (0.41%)	<-10	0.681	0.686	Mutual exclusivity
CBWD2	2q14.1	0 (0.00%)	1 (0.41%)	<-10	0.681	0.686	Mutual exclusivity
CBX3P4	12p13.33	0 (0.00%)	1 (0.41%)	<-10	0.681	0.686	Mutual exclusivity
CCAR1	10q21.3	0 (0.00%)	1 (0.41%)	<-10	0.681	0.686	Mutual exclusivity
CCDC102A	16q21	0 (0.00%)	1 (0.41%)	<-10	0.681	0.686	Mutual exclusivity
CCDC114	19q13.33	0 (0.00%)	1 (0.41%)	<-10	0.681	0.686	Mutual exclusivity
CCDC138	2q13	0 (0.00%)	1 (0.41%)	<-10	0.681	0.686	Mutual exclusivity

CCDC14	3q21.1	0 (0.00%)	1 (0.41%)	<-10	0.681	0.686	Mutual exclusivity
CCDC140	2q36.1	0 (0.00%)	1 (0.41%)	<-10	0.681	0.686	Mutual exclusivity
CCDC144B	17p11.2	0 (0.00%)	1 (0.41%)	<-10	0.681	0.686	Mutual exclusivity
CCDC153	11q23.3	0 (0.00%)	1 (0.41%)	<-10	0.681	0.686	Mutual exclusivity
CCDC162P	6q21	0 (0.00%)	1 (0.41%)	<-10	0.681	0.686	Mutual exclusivity
CCDC171	9p22.3	0 (0.00%)	1 (0.41%)	<-10	0.681	0.686	Mutual exclusivity
CCDC198	14q22.3	0 (0.00%)	1 (0.41%)	<-10	0.681	0.686	Mutual exclusivity
CCDC54	3q13.12	0 (0.00%)	1 (0.41%)	<-10	0.681	0.686	Mutual exclusivity
CCDC65	12q13.12	0 (0.00%)	1 (0.41%)	<-10	0.681	0.686	Mutual exclusivity
CCDC82	11q21	0 (0.00%)	1 (0.41%)	<-10	0.681	0.686	Mutual exclusivity
CCDC83	11q14.1-q14.2	0 (0.00%)	1 (0.41%)	<-10	0.681	0.686	Mutual exclusivity
CCDC85A	2p16.1	0 (0.00%)	1 (0.41%)	<-10	0.681	0.686	Mutual exclusivity
CCDC88C	14q32.11-q32.12	0 (0.00%)	1 (0.41%)	<-10	0.681	0.686	Mutual exclusivity
CCDC89	11q14.1	0 (0.00%)	1 (0.41%)	<-10	0.681	0.686	Mutual exclusivity
CCDC93	2q14.1	0 (0.00%)	1 (0.41%)	<-10	0.681	0.686	Mutual exclusivity
CCDC9B	15q15.1	0 (0.00%)	1 (0.41%)	<-10	0.681	0.686	Mutual exclusivity
CCL20	2q36.3	0 (0.00%)	1 (0.41%)	<-10	0.681	0.686	Mutual exclusivity
CCM2L	20q11.21	0 (0.00%)	1 (0.41%)	<-10	0.681	0.686	Mutual exclusivity
CCN5	20q13.12	0 (0.00%)	1 (0.41%)	<-10	0.681	0.686	Mutual exclusivity
CCND2	12p13.32	0 (0.00%)	1 (0.41%)	<-10	0.681	0.686	Mutual exclusivity
CCNH	5q14.3	0 (0.00%)	1 (0.41%)	<-10	0.681	0.686	Mutual exclusivity
CCNT1	12q13.11-q13.12	0 (0.00%)	1 (0.41%)	<-10	0.681	0.686	Mutual exclusivity
CCNT2	2q21.3	0 (0.00%)	1 (0.41%)	<-10	0.681	0.686	Mutual exclusivity
CD164	6q21	0 (0.00%)	1 (0.41%)	<-10	0.681	0.686	Mutual exclusivity
CD3D	11q23.3	0 (0.00%)	1 (0.41%)	<-10	0.681	0.686	Mutual exclusivity
CD3E	11q23.3	0 (0.00%)	1 (0.41%)	<-10	0.681	0.686	Mutual exclusivity
CD3G	11q23.3	0 (0.00%)	1 (0.41%)	<-10	0.681	0.686	Mutual exclusivity
CD40	20q13.12	0 (0.00%)	1 (0.41%)	<-10	0.681	0.686	Mutual exclusivity
CD47	3q13.12	0 (0.00%)	1 (0.41%)	<-10	0.681	0.686	Mutual exclusivity

CD58	1p13.1	0 (0.00%)	1 (0.41%)	<-10	0.681	0.686	Mutual exclusivity
CD70	19p13.3	0 (0.00%)	1 (0.41%)	<-10	0.681	0.686	Mutual exclusivity
CD79A	19q13.2	0 (0.00%)	1 (0.41%)	<-10	0.681	0.686	Mutual exclusivity
CD8A	2p11.2	0 (0.00%)	1 (0.41%)	<-10	0.681	0.686	Mutual exclusivity
CD8B	2p11.2	0 (0.00%)	1 (0.41%)	<-10	0.681	0.686	Mutual exclusivity
CD8B2	2q12.2	0 (0.00%)	1 (0.41%)	<-10	0.681	0.686	Mutual exclusivity
CD93	20p11.21	0 (0.00%)	1 (0.41%)	<-10	0.681	0.686	Mutual exclusivity
CDA	1p36.12	0 (0.00%)	1 (0.41%)	<-10	0.681	0.686	Mutual exclusivity
CDC37	19p13.2	0 (0.00%)	1 (0.41%)	<-10	0.681	0.686	Mutual exclusivity
CDC42	1p36.12	0 (0.00%)	1 (0.41%)	<-10	0.681	0.686	Mutual exclusivity
CDH22	20q13.12	0 (0.00%)	1 (0.41%)	<-10	0.681	0.686	Mutual exclusivity
CDK5R2	2q35	0 (0.00%)	1 (0.41%)	<-10	0.681	0.686	Mutual exclusivity
CDKN1B	12p13.1	0 (0.00%)	1 (0.41%)	<-10	0.681	0.686	Mutual exclusivity
CDKN2D	19p13.2	0 (0.00%)	1 (0.41%)	<-10	0.681	0.686	Mutual exclusivity
CEACAM3	19q13.2	0 (0.00%)	1 (0.41%)	<-10	0.681	0.686	Mutual exclusivity
CEACAM5	19q13.2	0 (0.00%)	1 (0.41%)	<-10	0.681	0.686	Mutual exclusivity
CEACAM6	19q13.2	0 (0.00%)	1 (0.41%)	<-10	0.681	0.686	Mutual exclusivity
CEACAM7	19q13.2	0 (0.00%)	1 (0.41%)	<-10	0.681	0.686	Mutual exclusivity
CELA3A	1p36.12	0 (0.00%)	1 (0.41%)	<-10	0.681	0.686	Mutual exclusivity
CELA3B	1p36.12	0 (0.00%)	1 (0.41%)	<-10	0.681	0.686	Mutual exclusivity
CELF4	18q12.2	0 (0.00%)	1 (0.41%)	<-10	0.681	0.686	Mutual exclusivity
CENPT	16q22.1	0 (0.00%)	1 (0.41%)	<-10	0.681	0.686	Mutual exclusivity
CENPVL1	Xp11.22	0 (0.00%)	1 (0.41%)	<-10	0.681	0.686	Mutual exclusivity
CEP128	14q31.1	0 (0.00%)	1 (0.41%)	<-10	0.681	0.686	Mutual exclusivity
CEP295	11q21	0 (0.00%)	1 (0.41%)	<-10	0.681	0.686	Mutual exclusivity
CEP57	11q21	0 (0.00%)	1 (0.41%)	<-10	0.681	0.686	Mutual exclusivity
CEP68	2p14	0 (0.00%)	1 (0.41%)	<-10	0.681	0.686	Mutual exclusivity
CER1	9p22.3	0 (0.00%)	1 (0.41%)	<-10	0.681	0.686	Mutual exclusivity
CES5AP1	22q11.23	0 (0.00%)	1 (0.41%)	<-10	0.681	0.686	Mutual exclusivity

CETN3	5q14.3	0 (0.00%)	1 (0.41%)	<-10	0.681	0.686	Mutual exclusivity
CFAP206	6q15	0 (0.00%)	1 (0.41%)	<-10	0.681	0.686	Mutual exclusivity
CFAP410	21q22.3	0 (0.00%)	1 (0.41%)	<-10	0.681	0.686	Mutual exclusivity
CFAP65	2q35	0 (0.00%)	1 (0.41%)	<-10	0.681	0.686	Mutual exclusivity
CFL2	14q13.1	0 (0.00%)	1 (0.41%)	<-10	0.681	0.686	Mutual exclusivity
CGA	6q14.3	0 (0.00%)	1 (0.41%)	<-10	0.681	0.686	Mutual exclusivity
CGGBP1	3p11.1	0 (0.00%)	1 (0.41%)	<-10	0.681	0.686	Mutual exclusivity
CH25H	10q23.31	0 (0.00%)	1 (0.41%)	<-10	0.681	0.686	Mutual exclusivity
CHAC2	2p16.2	0 (0.00%)	1 (0.41%)	<-10	0.681	0.686	Mutual exclusivity
CHAT	10q11.23	0 (0.00%)	1 (0.41%)	<-10	0.681	0.686	Mutual exclusivity
CHCHD5	2q14.1	0 (0.00%)	1 (0.41%)	<-10	0.681	0.686	Mutual exclusivity
CHD9	16q12.2	0 (0.00%)	1 (0.41%)	<-10	0.681	0.686	Mutual exclusivity
CHMP2B	3p11.2	0 (0.00%)	1 (0.41%)	<-10	0.681	0.686	Mutual exclusivity
CHMP3	2p11.2	0 (0.00%)	1 (0.41%)	<-10	0.681	0.686	Mutual exclusivity
CHMP5	9p13.3	0 (0.00%)	1 (0.41%)	<-10	0.681	0.686	Mutual exclusivity
CHORDC1	11q14.3	0 (0.00%)	1 (0.41%)	<-10	0.681	0.686	Mutual exclusivity
CHPF	2q35	0 (0.00%)	1 (0.41%)	<-10	0.681	0.686	Mutual exclusivity
CHRM1	11q12.3	0 (0.00%)	1 (0.41%)	<-10	0.681	0.686	Mutual exclusivity
CHRND	2q37.1	0 (0.00%)	1 (0.41%)	<-10	0.681	0.686	Mutual exclusivity
CHRNG	2q37.1	0 (0.00%)	1 (0.41%)	<-10	0.681	0.686	Mutual exclusivity
CHST10	2q11.2	0 (0.00%)	1 (0.41%)	<-10	0.681	0.686	Mutual exclusivity
CHST13	3q21.3	0 (0.00%)	1 (0.41%)	<-10	0.681	0.686	Mutual exclusivity
CHTF8	16q22.1	0 (0.00%)	1 (0.41%)	<-10	0.681	0.686	Mutual exclusivity
CHUK	10q24.31	0 (0.00%)	1 (0.41%)	<-10	0.681	0.686	Mutual exclusivity
CHURC1	14q23.3	0 (0.00%)	1 (0.41%)	<-10	0.681	0.686	Mutual exclusivity
CIAO1	2q11.2	0 (0.00%)	1 (0.41%)	<-10	0.681	0.686	Mutual exclusivity
CIP2A	3q13.13	0 (0.00%)	1 (0.41%)	<-10	0.681	0.686	Mutual exclusivity
CIRBP	19p13.3	0 (0.00%)	1 (0.41%)	<-10	0.681	0.686	Mutual exclusivity
CKAP2L	2q14.1	0 (0.00%)	1 (0.41%)	<-10	0.681	0.686	Mutual exclusivity

CLDN19	1p34.2	0 (0.00%)	1 (0.41%)	<-10	0.681	0.686	Mutual exclusivity
CLDN20	6q25.3	0 (0.00%)	1 (0.41%)	<-10	0.681	0.686	Mutual exclusivity
CLDND1	3q11.2	0 (0.00%)	1 (0.41%)	<-10	0.681	0.686	Mutual exclusivity
CLEC18A	16q22.1	0 (0.00%)	1 (0.41%)	<-10	0.681	0.686	Mutual exclusivity
CLEC18C	16q22.1	0 (0.00%)	1 (0.41%)	<-10	0.681	0.686	Mutual exclusivity
CLEC4C	12p13.31	0 (0.00%)	1 (0.41%)	<-10	0.681	0.686	Mutual exclusivity
CLHC1	2p16.1	0 (0.00%)	1 (0.41%)	<-10	0.681	0.686	Mutual exclusivity
CLIC4	1p36.11	0 (0.00%)	1 (0.41%)	<-10	0.681	0.686	Mutual exclusivity
CLMN	14q32.13	0 (0.00%)	1 (0.41%)	<-10	0.681	0.686	Mutual exclusivity
CLP1	11q12.1	0 (0.00%)	1 (0.41%)	<-10	0.681	0.686	Mutual exclusivity
CLRN3	10q26.2	0 (0.00%)	1 (0.41%)	<-10	0.681	0.686	Mutual exclusivity
CLU	8p21.1	0 (0.00%)	1 (0.41%)	<-10	0.681	0.686	Mutual exclusivity
CMSS1	3q12.1	0 (0.00%)	1 (0.41%)	<-10	0.681	0.686	Mutual exclusivity
CMTM4	16q21-q22.1	0 (0.00%)	1 (0.41%)	<-10	0.681	0.686	Mutual exclusivity
CMTR2	16q22.2	0 (0.00%)	1 (0.41%)	<-10	0.681	0.686	Mutual exclusivity
CNDP1	18q22.3	0 (0.00%)	1 (0.41%)	<-10	0.681	0.686	Mutual exclusivity
CNDP2	18q22.3	0 (0.00%)	1 (0.41%)	<-10	0.681	0.686	Mutual exclusivity
CNEP1R1	16q12.1	0 (0.00%)	1 (0.41%)	<-10	0.681	0.686	Mutual exclusivity
CNGA3	2q11.2	0 (0.00%)	1 (0.41%)	<-10	0.681	0.686	Mutual exclusivity
CNKSR3	6q25.2	0 (0.00%)	1 (0.41%)	<-10	0.681	0.686	Mutual exclusivity
CNNM1	10q24.2	0 (0.00%)	1 (0.41%)	<-10	0.681	0.686	Mutual exclusivity
CNNM3	2q11.2	0 (0.00%)	1 (0.41%)	<-10	0.681	0.686	Mutual exclusivity
CNNM4	2q11.2	0 (0.00%)	1 (0.41%)	<-10	0.681	0.686	Mutual exclusivity
CNOT9	2q35	0 (0.00%)	1 (0.41%)	<-10	0.681	0.686	Mutual exclusivity
CNPPD1	2q35	0 (0.00%)	1 (0.41%)	<-10	0.681	0.686	Mutual exclusivity
CNTN3	3p12.3	0 (0.00%)	1 (0.41%)	<-10	0.681	0.686	Mutual exclusivity
COA4	11q13.4	0 (0.00%)	1 (0.41%)	<-10	0.681	0.686	Mutual exclusivity
COA5	2q11.2	0 (0.00%)	1 (0.41%)	<-10	0.681	0.686	Mutual exclusivity
COL12A1	6q13-q14.1	0 (0.00%)	1 (0.41%)	<-10	0.681	0.686	Mutual exclusivity

COL17A1	10q25.1	0 (0.00%)	1 (0.41%)	<-10	0.681	0.686	Mutual exclusivity
COL18A1	21q22.3	0 (0.00%)	1 (0.41%)	<-10	0.681	0.686	Mutual exclusivity
COL25A1	4q25	0 (0.00%)	1 (0.41%)	<-10	0.681	0.686	Mutual exclusivity
COL27A1	9q32	0 (0.00%)	1 (0.41%)	<-10	0.681	0.686	Mutual exclusivity
COL4A5	Xq22.3	0 (0.00%)	1 (0.41%)	<-10	0.681	0.686	Mutual exclusivity
COL6A1	21q22.3	0 (0.00%)	1 (0.41%)	<-10	0.681	0.686	Mutual exclusivity
COL6A2	21q22.3	0 (0.00%)	1 (0.41%)	<-10	0.681	0.686	Mutual exclusivity
COL8A1	3q12.1	0 (0.00%)	1 (0.41%)	<-10	0.681	0.686	Mutual exclusivity
COPS3	17p11.2	0 (0.00%)	1 (0.41%)	<-10	0.681	0.686	Mutual exclusivity
COPS8	2q37.3	0 (0.00%)	1 (0.41%)	<-10	0.681	0.686	Mutual exclusivity
COPS9	2q37.3	0 (0.00%)	1 (0.41%)	<-10	0.681	0.686	Mutual exclusivity
COPZ1	12q13.13	0 (0.00%)	1 (0.41%)	<-10	0.681	0.686	Mutual exclusivity
CORO2A	9q22.33	0 (0.00%)	1 (0.41%)	<-10	0.681	0.686	Mutual exclusivity
COX5B	2q11.2	0 (0.00%)	1 (0.41%)	<-10	0.681	0.686	Mutual exclusivity
COX5BP2	6q23.3	0 (0.00%)	1 (0.41%)	<-10	0.681	0.686	Mutual exclusivity
COX7A2	6q14.1	0 (0.00%)	1 (0.41%)	<-10	0.681	0.686	Mutual exclusivity
COX7C	5q14.3	0 (0.00%)	1 (0.41%)	<-10	0.681	0.686	Mutual exclusivity
COX8A	11q13.1	0 (0.00%)	1 (0.41%)	<-10	0.681	0.686	Mutual exclusivity
CPEB3	10q23.32	0 (0.00%)	1 (0.41%)	<-10	0.681	0.686	Mutual exclusivity
CPOX	3q11.2	0 (0.00%)	1 (0.41%)	<-10	0.681	0.686	Mutual exclusivity
CPSF2	14q32.12	0 (0.00%)	1 (0.41%)	<-10	0.681	0.686	Mutual exclusivity
CPXM1	20p13	0 (0.00%)	1 (0.41%)	<-10	0.681	0.686	Mutual exclusivity
CRACR2A	12p13.32	0 (0.00%)	1 (0.41%)	<-10	0.681	0.686	Mutual exclusivity
CRB3	19p13.3	0 (0.00%)	1 (0.41%)	<-10	0.681	0.686	Mutual exclusivity
CREBL2	12p13.1	0 (0.00%)	1 (0.41%)	<-10	0.681	0.686	Mutual exclusivity
CREBZF	11q14.1	0 (0.00%)	1 (0.41%)	<-10	0.681	0.686	Mutual exclusivity
CRHBP	5q13.3	0 (0.00%)	1 (0.41%)	<-10	0.681	0.686	Mutual exclusivity
CRPPA	7p21.2	0 (0.00%)	1 (0.41%)	<-10	0.681	0.686	Mutual exclusivity
CRTAC1	10q24.2	0 (0.00%)	1 (0.41%)	<-10	0.681	0.686	Mutual exclusivity

CRYBA2	2q35	0 (0.00%)	1 (0.41%)	<-10	0.681	0.686	Mutual exclusivity
CRYBB1	22q12.1	0 (0.00%)	1 (0.41%)	<-10	0.681	0.686	Mutual exclusivity
CRYBB2	22q11.23	0 (0.00%)	1 (0.41%)	<-10	0.681	0.686	Mutual exclusivity
CRYBG1	6q21	0 (0.00%)	1 (0.41%)	<-10	0.681	0.686	Mutual exclusivity
CRYGA	2q33.3	0 (0.00%)	1 (0.41%)	<-10	0.681	0.686	Mutual exclusivity
CSKMT	11q12.3	0 (0.00%)	1 (0.41%)	<-10	0.681	0.686	Mutual exclusivity
CSTF3	11p13	0 (0.00%)	1 (0.41%)	<-10	0.681	0.686	Mutual exclusivity
CTF1	16p11.2	0 (0.00%)	1 (0.41%)	<-10	0.681	0.686	Mutual exclusivity
CTNND1	11q12.1	0 (0.00%)	1 (0.41%)	<-10	0.681	0.686	Mutual exclusivity
CTRL	16q22.1	0 (0.00%)	1 (0.41%)	<-10	0.681	0.686	Mutual exclusivity
CTSA	20q13.12	0 (0.00%)	1 (0.41%)	<-10	0.681	0.686	Mutual exclusivity
CTSC	11q14.2	0 (0.00%)	1 (0.41%)	<-10	0.681	0.686	Mutual exclusivity
CTSL	9q21.33	0 (0.00%)	1 (0.41%)	<-10	0.681	0.686	Mutual exclusivity
CTSL3P	9q21.33	0 (0.00%)	1 (0.41%)	<-10	0.681	0.686	Mutual exclusivity
CUL3	2q36.2	0 (0.00%)	1 (0.41%)	<-10	0.681	0.686	Mutual exclusivity
CWC15	11q21	0 (0.00%)	1 (0.41%)	<-10	0.681	0.686	Mutual exclusivity
CWF19L1	10q24.31	0 (0.00%)	1 (0.41%)	<-10	0.681	0.686	Mutual exclusivity
CWF19L2	11q22.3	0 (0.00%)	1 (0.41%)	<-10	0.681	0.686	Mutual exclusivity
CXADR	21q21.1	0 (0.00%)	1 (0.41%)	<-10	0.681	0.686	Mutual exclusivity
CXCL12	10q11.21	0 (0.00%)	1 (0.41%)	<-10	0.681	0.686	Mutual exclusivity
CXCR3	Xq13.1	0 (0.00%)	1 (0.41%)	<-10	0.681	0.686	Mutual exclusivity
CXORF49	Xq13.1	0 (0.00%)	1 (0.41%)	<-10	0.681	0.686	Mutual exclusivity
CXORF49B	Xq13.1	0 (0.00%)	1 (0.41%)	<-10	0.681	0.686	Mutual exclusivity
CYP17A1	10q24.32	0 (0.00%)	1 (0.41%)	<-10	0.681	0.686	Mutual exclusivity
CYP19A1	15q21.2	0 (0.00%)	1 (0.41%)	<-10	0.681	0.686	Mutual exclusivity
CYP27A1	2q35	0 (0.00%)	1 (0.41%)	<-10	0.681	0.686	Mutual exclusivity
CYP2C18	10q23.33	0 (0.00%)	1 (0.41%)	<-10	0.681	0.686	Mutual exclusivity
CYP2C19	10q23.33	0 (0.00%)	1 (0.41%)	<-10	0.681	0.686	Mutual exclusivity
CYP2C8	10q23.33	0 (0.00%)	1 (0.41%)	<-10	0.681	0.686	Mutual exclusivity

CYP2C9	10q23.33	0 (0.00%)	1 (0.41%)	<-10	0.681	0.686	Mutual exclusivity
CYTH2	19q13.33	0 (0.00%)	1 (0.41%)	<-10	0.681	0.686	Mutual exclusivity
CYTOR	2p11.2	0 (0.00%)	1 (0.41%)	<-10	0.681	0.686	Mutual exclusivity
D2HGDH	2q37.3	0 (0.00%)	1 (0.41%)	<-10	0.681	0.686	Mutual exclusivity
DAND5	19p13.13	0 (0.00%)	1 (0.41%)	<-10	0.681	0.686	Mutual exclusivity
DAPK1	9q21.33	0 (0.00%)	1 (0.41%)	<-10	0.681	0.686	Mutual exclusivity
DAW1	2q36.3	0 (0.00%)	1 (0.41%)	<-10	0.681	0.686	Mutual exclusivity
DBI	2q14.2	0 (0.00%)	1 (0.41%)	<-10	0.681	0.686	Mutual exclusivity
DBIL5P2	2p15	0 (0.00%)	1 (0.41%)	<-10	0.681	0.686	Mutual exclusivity
DBNDD2	20q13.12	0 (0.00%)	1 (0.41%)	<-10	0.681	0.686	Mutual exclusivity
DBP	19q13.33	0 (0.00%)	1 (0.41%)	<-10	0.681	0.686	Mutual exclusivity
DCAF12L1	Xq25	0 (0.00%)	1 (0.41%)	<-10	0.681	0.686	Mutual exclusivity
DCAF12L2	Xq25	0 (0.00%)	1 (0.41%)	<-10	0.681	0.686	Mutual exclusivity
DCBLD2	3q12.1|3	0 (0.00%)	1 (0.41%)	<-10	0.681	0.686	Mutual exclusivity
DCP1A	3p21.1	0 (0.00%)	1 (0.41%)	<-10	0.681	0.686	Mutual exclusivity
DCP1B	12p13.33	0 (0.00%)	1 (0.41%)	<-10	0.681	0.686	Mutual exclusivity
DCUN1D3	16p12.3	0 (0.00%)	1 (0.41%)	<-10	0.681	0.686	Mutual exclusivity
DDI1	11q22.3	0 (0.00%)	1 (0.41%)	<-10	0.681	0.686	Mutual exclusivity
DDIAS	11q14.1	0 (0.00%)	1 (0.41%)	<-10	0.681	0.686	Mutual exclusivity
DDN	12q13.12	0 (0.00%)	1 (0.41%)	<-10	0.681	0.686	Mutual exclusivity
DDOST	1p36.12	0 (0.00%)	1 (0.41%)	<-10	0.681	0.686	Mutual exclusivity
DDRGK1	20p13	0 (0.00%)	1 (0.41%)	<-10	0.681	0.686	Mutual exclusivity
DDX19B	16q22.1	0 (0.00%)	1 (0.41%)	<-10	0.681	0.686	Mutual exclusivity
DDX20	1p13.2	0 (0.00%)	1 (0.41%)	<-10	0.681	0.686	Mutual exclusivity
DDX21	10q22.1	0 (0.00%)	1 (0.41%)	<-10	0.681	0.686	Mutual exclusivity
DDX28	16q22.1	0 (0.00%)	1 (0.41%)	<-10	0.681	0.686	Mutual exclusivity
DDX47	12p13.1	0 (0.00%)	1 (0.41%)	<-10	0.681	0.686	Mutual exclusivity
DDX50	10q22.1	0 (0.00%)	1 (0.41%)	<-10	0.681	0.686	Mutual exclusivity
DEDD2	19q13.2	0 (0.00%)	1 (0.41%)	<-10	0.681	0.686	Mutual exclusivity

DEFA4	8p23.1	0 (0.00%)	1 (0.41%)	<-10	0.681	0.686	Mutual exclusivity
DEFA6	8p23.1	0 (0.00%)	1 (0.41%)	<-10	0.681	0.686	Mutual exclusivity
DEFB1	8p23.1	0 (0.00%)	1 (0.41%)	<-10	0.681	0.686	Mutual exclusivity
DENND1C	19p13.3	0 (0.00%)	1 (0.41%)	<-10	0.681	0.686	Mutual exclusivity
DENND6A	3p14.3	0 (0.00%)	1 (0.41%)	<-10	0.681	0.686	Mutual exclusivity
DEPDC7	11p13	0 (0.00%)	1 (0.41%)	<-10	0.681	0.686	Mutual exclusivity
DES	2q35	0 (0.00%)	1 (0.41%)	<-10	0.681	0.686	Mutual exclusivity
DEUP1	11q21	0 (0.00%)	1 (0.41%)	<-10	0.681	0.686	Mutual exclusivity
DGKB	7p21.2	0 (0.00%)	1 (0.41%)	<-10	0.681	0.686	Mutual exclusivity
DGLUCY	14q32.11	0 (0.00%)	1 (0.41%)	<-10	0.681	0.686	Mutual exclusivity
DHDDS	1p36.11	0 (0.00%)	1 (0.41%)	<-10	0.681	0.686	Mutual exclusivity
DHFR	5q14.1	0 (0.00%)	1 (0.41%)	<-10	0.681	0.686	Mutual exclusivity
DHH	12q13.12	0 (0.00%)	1 (0.41%)	<-10	0.681	0.686	Mutual exclusivity
DHX32	10q26.2	0 (0.00%)	1 (0.41%)	<-10	0.681	0.686	Mutual exclusivity
DICER1	14q32.13	0 (0.00%)	1 (0.41%)	<-10	0.681	0.686	Mutual exclusivity
DIO2	14q31.1	0 (0.00%)	1 (0.41%)	<-10	0.681	0.686	Mutual exclusivity
DIP2A	21q22.3	0 (0.00%)	1 (0.41%)	<-10	0.681	0.686	Mutual exclusivity
DIPK1C	18q22.3	0 (0.00%)	1 (0.41%)	<-10	0.681	0.686	Mutual exclusivity
DIRAS2	9q22.2	0 (0.00%)	1 (0.41%)	<-10	0.681	0.686	Mutual exclusivity
DIRC3	2q35	0 (0.00%)	1 (0.41%)	<-10	0.681	0.686	Mutual exclusivity
DISC1FP1	11q14.3	0 (0.00%)	1 (0.41%)	<-10	0.681	0.686	Mutual exclusivity
DISP2	15q15.1	0 (0.00%)	1 (0.41%)	<-10	0.681	0.686	Mutual exclusivity
DKK1	10q21.1	0 (0.00%)	1 (0.41%)	<-10	0.681	0.686	Mutual exclusivity
DLGAP5	14q22.3	0 (0.00%)	1 (0.41%)	<-10	0.681	0.686	Mutual exclusivity
DLL1	6q27	0 (0.00%)	1 (0.41%)	<-10	0.681	0.686	Mutual exclusivity
DLST	14q24.3	0 (0.00%)	1 (0.41%)	<-10	0.681	0.686	Mutual exclusivity
DMP1	4q22.1	0 (0.00%)	1 (0.41%)	<-10	0.681	0.686	Mutual exclusivity
DMRTC2	19q13.2	0 (0.00%)	1 (0.41%)	<-10	0.681	0.686	Mutual exclusivity
DNA2	10q21.3	0 (0.00%)	1 (0.41%)	<-10	0.681	0.686	Mutual exclusivity

DNAH12	3p14.3	0 (0.00%)	1 (0.41%)	<-10	0.681	0.686	Mutual exclusivity
DNAJB13	11q13.4	0 (0.00%)	1 (0.41%)	<-10	0.681	0.686	Mutual exclusivity
DNAJB2	2q35	0 (0.00%)	1 (0.41%)	<-10	0.681	0.686	Mutual exclusivity
DNAJB3	2q37.1	0 (0.00%)	1 (0.41%)	<-10	0.681	0.686	Mutual exclusivity
DNER	2q36.3	0 (0.00%)	1 (0.41%)	<-10	0.681	0.686	Mutual exclusivity
DNM2	19p13.2	0 (0.00%)	1 (0.41%)	<-10	0.681	0.686	Mutual exclusivity
DNMT1	19p13.2	0 (0.00%)	1 (0.41%)	<-10	0.681	0.686	Mutual exclusivity
DNMT3L	21q22.3	0 (0.00%)	1 (0.41%)	<-10	0.681	0.686	Mutual exclusivity
DNPEP	2q35	0 (0.00%)	1 (0.41%)	<-10	0.681	0.686	Mutual exclusivity
DNTTIP1	20q13.12	0 (0.00%)	1 (0.41%)	<-10	0.681	0.686	Mutual exclusivity
DOCK1	10q26.2	0 (0.00%)	1 (0.41%)	<-10	0.681	0.686	Mutual exclusivity
DOCK10	2q36.2	0 (0.00%)	1 (0.41%)	<-10	0.681	0.686	Mutual exclusivity
DOCK6	19p13.2	0 (0.00%)	1 (0.41%)	<-10	0.681	0.686	Mutual exclusivity
DPEP2	16q22.1	0 (0.00%)	1 (0.41%)	<-10	0.681	0.686	Mutual exclusivity
DPEP3	16q22.1	0 (0.00%)	1 (0.41%)	<-10	0.681	0.686	Mutual exclusivity
DPH1	17p13.3	0 (0.00%)	1 (0.41%)	<-10	0.681	0.686	Mutual exclusivity
DPP10	2q14.1	0 (0.00%)	1 (0.41%)	<-10	0.681	0.686	Mutual exclusivity
DRD2	11q23.2	0 (0.00%)	1 (0.41%)	<-10	0.681	0.686	Mutual exclusivity
DRICH1	22q11.23	0 (0.00%)	1 (0.41%)	<-10	0.681	0.686	Mutual exclusivity
DSPP	4q22.1	0 (0.00%)	1 (0.41%)	<-10	0.681	0.686	Mutual exclusivity
DSTN	20p12.1	0 (0.00%)	1 (0.41%)	<-10	0.681	0.686	Mutual exclusivity
DTD1	20p11.23	0 (0.00%)	1 (0.41%)	<-10	0.681	0.686	Mutual exclusivity
DTD2	14q12	0 (0.00%)	1 (0.41%)	<-10	0.681	0.686	Mutual exclusivity
DTNA	18q12.1	0 (0.00%)	1 (0.41%)	<-10	0.681	0.686	Mutual exclusivity
DTYMK	2q37.3	0 (0.00%)	1 (0.41%)	<-10	0.681	0.686	Mutual exclusivity
DUBR	3q13.12	0 (0.00%)	1 (0.41%)	<-10	0.681	0.686	Mutual exclusivity
DUS2	16q22.1	0 (0.00%)	1 (0.41%)	<-10	0.681	0.686	Mutual exclusivity
DUS3L	19p13.3	0 (0.00%)	1 (0.41%)	<-10	0.681	0.686	Mutual exclusivity
DUSP2	2q11.2	0 (0.00%)	1 (0.41%)	<-10	0.681	0.686	Mutual exclusivity

DUSP28	2q37.3	0 (0.00%)	1 (0.41%)	<-10	0.681	0.686	Mutual exclusivity
DYNLT1	6q25.3	0 (0.00%)	1 (0.41%)	<-10	0.681	0.686	Mutual exclusivity
DYRK1A	21q22.13	0 (0.00%)	1 (0.41%)	<-10	0.681	0.686	Mutual exclusivity
DYRK4	12p13.32	0 (0.00%)	1 (0.41%)	<-10	0.681	0.686	Mutual exclusivity
DZANK1	20p11.23	0 (0.00%)	1 (0.41%)	<-10	0.681	0.686	Mutual exclusivity
DZIP3	3q13.13	0 (0.00%)	1 (0.41%)	<-10	0.681	0.686	Mutual exclusivity
EAPP	14q13.1	0 (0.00%)	1 (0.41%)	<-10	0.681	0.686	Mutual exclusivity
EBF2	8p21.2	0 (0.00%)	1 (0.41%)	<-10	0.681	0.686	Mutual exclusivity
EBF4	20p13	0 (0.00%)	1 (0.41%)	<-10	0.681	0.686	Mutual exclusivity
EBLN2	3p13	0 (0.00%)	1 (0.41%)	<-10	0.681	0.686	Mutual exclusivity
ECEL1	2q37.1	0 (0.00%)	1 (0.41%)	<-10	0.681	0.686	Mutual exclusivity
ECEL1P2	2q37.1	0 (0.00%)	1 (0.41%)	<-10	0.681	0.686	Mutual exclusivity
ECRG4	2q12.2	0 (0.00%)	1 (0.41%)	<-10	0.681	0.686	Mutual exclusivity
EDA2R	Xq12	0 (0.00%)	1 (0.41%)	<-10	0.681	0.686	Mutual exclusivity
EDAR	2q13	0 (0.00%)	1 (0.41%)	<-10	0.681	0.686	Mutual exclusivity
EDC4	16q22.1	0 (0.00%)	1 (0.41%)	<-10	0.681	0.686	Mutual exclusivity
EDIL3	5q14.3	0 (0.00%)	1 (0.41%)	<-10	0.681	0.686	Mutual exclusivity
EDRF1	10q26.2	0 (0.00%)	1 (0.41%)	<-10	0.681	0.686	Mutual exclusivity
EED	11q14.2	0 (0.00%)	1 (0.41%)	<-10	0.681	0.686	Mutual exclusivity
EEFSEC	3q21.3	0 (0.00%)	1 (0.41%)	<-10	0.681	0.686	Mutual exclusivity
EFCAB11	14q32.11	0 (0.00%)	1 (0.41%)	<-10	0.681	0.686	Mutual exclusivity
EFCAB8	20q11.21	0 (0.00%)	1 (0.41%)	<-10	0.681	0.686	Mutual exclusivity
EFEMP1	2p16.1	0 (0.00%)	1 (0.41%)	<-10	0.681	0.686	Mutual exclusivity
EGF	4q25	0 (0.00%)	1 (0.41%)	<-10	0.681	0.686	Mutual exclusivity
EGLN3	14q13.1	0 (0.00%)	1 (0.41%)	<-10	0.681	0.686	Mutual exclusivity
EIF2AK3	2p11.2	0 (0.00%)	1 (0.41%)	<-10	0.681	0.686	Mutual exclusivity
EIF2B2	14q24.3	0 (0.00%)	1 (0.41%)	<-10	0.681	0.686	Mutual exclusivity
EIF3G	19p13.2	0 (0.00%)	1 (0.41%)	<-10	0.681	0.686	Mutual exclusivity
EIF3M	11p13	0 (0.00%)	1 (0.41%)	<-10	0.681	0.686	Mutual exclusivity

EIF5B	2q11.2	0 (0.00%)	1 (0.41%)	<-10	0.681	0.686	Mutual exclusivity
ELMOD1	11q22.3	0 (0.00%)	1 (0.41%)	<-10	0.681	0.686	Mutual exclusivity
ELOVL1	1p34.2	0 (0.00%)	1 (0.41%)	<-10	0.681	0.686	Mutual exclusivity
ELP4	11p13	0 (0.00%)	1 (0.41%)	<-10	0.681	0.686	Mutual exclusivity
EMC1	1p36.13	0 (0.00%)	1 (0.41%)	<-10	0.681	0.686	Mutual exclusivity
EML6	2p16.1	0 (0.00%)	1 (0.41%)	<-10	0.681	0.686	Mutual exclusivity
EMP3	19q13.33	0 (0.00%)	1 (0.41%)	<-10	0.681	0.686	Mutual exclusivity
EN1	2q14.2	0 (0.00%)	1 (0.41%)	<-10	0.681	0.686	Mutual exclusivity
ENDOD1	11q21	0 (0.00%)	1 (0.41%)	<-10	0.681	0.686	Mutual exclusivity
ENTPD4	8p21.3	0 (0.00%)	1 (0.41%)	<-10	0.681	0.686	Mutual exclusivity
ENTPD6	20p11.21	0 (0.00%)	1 (0.41%)	<-10	0.681	0.686	Mutual exclusivity
ENTPD7	10q24.2	0 (0.00%)	1 (0.41%)	<-10	0.681	0.686	Mutual exclusivity
EPB41L4B	9q31.3	0 (0.00%)	1 (0.41%)	<-10	0.681	0.686	Mutual exclusivity
EPHA4	2q36.1	0 (0.00%)	1 (0.41%)	<-10	0.681	0.686	Mutual exclusivity
EPHA7	6q16.1	0 (0.00%)	1 (0.41%)	<-10	0.681	0.686	Mutual exclusivity
EPHA8	1p36.12	0 (0.00%)	1 (0.41%)	<-10	0.681	0.686	Mutual exclusivity
EPHB2	1p36.12	0 (0.00%)	1 (0.41%)	<-10	0.681	0.686	Mutual exclusivity
EPM2A	6q24.3	0 (0.00%)	1 (0.41%)	<-10	0.681	0.686	Mutual exclusivity
EPPIN	20q13.12	0 (0.00%)	1 (0.41%)	<-10	0.681	0.686	Mutual exclusivity
EPS8	12p12.3	0 (0.00%)	1 (0.41%)	<-10	0.681	0.686	Mutual exclusivity
ERAP1	5q15	0 (0.00%)	1 (0.41%)	<-10	0.681	0.686	Mutual exclusivity
ERAP2	5q15	0 (0.00%)	1 (0.41%)	<-10	0.681	0.686	Mutual exclusivity
ERBB4	2q34	0 (0.00%)	1 (0.41%)	<-10	0.681	0.686	Mutual exclusivity
ERC1	12p13.33	0 (0.00%)	1 (0.41%)	<-10	0.681	0.686	Mutual exclusivity
ERF	19q13.2	0 (0.00%)	1 (0.41%)	<-10	0.681	0.686	Mutual exclusivity
ERG	21q22.2	0 (0.00%)	1 (0.41%)	<-10	0.681	0.686	Mutual exclusivity
ERI2	16p12.3	0 (0.00%)	1 (0.41%)	<-10	0.681	0.686	Mutual exclusivity
ERLEC1	2p16.2	0 (0.00%)	1 (0.41%)	<-10	0.681	0.686	Mutual exclusivity
ERLIN1	10q24.31	0 (0.00%)	1 (0.41%)	<-10	0.681	0.686	Mutual exclusivity

ERMAP	1p34.2	0 (0.00%)	1 (0.41%)	<-10	0.681	0.686	Mutual exclusivity
ERMARD	6q27	0 (0.00%)	1 (0.41%)	<-10	0.681	0.686	Mutual exclusivity
ESR1	6q25.1-q25.2	0 (0.00%)	1 (0.41%)	<-10	0.681	0.686	Mutual exclusivity
ETNPPL	4q25	0 (0.00%)	1 (0.41%)	<-10	0.681	0.686	Mutual exclusivity
ETV1	7p21.2	0 (0.00%)	1 (0.41%)	<-10	0.681	0.686	Mutual exclusivity
EXOC5	14q22.3	0 (0.00%)	1 (0.41%)	<-10	0.681	0.686	Mutual exclusivity
EXOSC1	10q24.1	0 (0.00%)	1 (0.41%)	<-10	0.681	0.686	Mutual exclusivity
EXOSC6	16q22.1	0 (0.00%)	1 (0.41%)	<-10	0.681	0.686	Mutual exclusivity
EXTL3	8p21.1	0 (0.00%)	1 (0.41%)	<-10	0.681	0.686	Mutual exclusivity
EYA4	6q23.2	0 (0.00%)	1 (0.41%)	<-10	0.681	0.686	Mutual exclusivity
EZHIP	Xp11.22	0 (0.00%)	1 (0.41%)	<-10	0.681	0.686	Mutual exclusivity
EZR	6q25.3	0 (0.00%)	1 (0.41%)	<-10	0.681	0.686	Mutual exclusivity
F2R	5q13.3	0 (0.00%)	1 (0.41%)	<-10	0.681	0.686	Mutual exclusivity
F2RL1	5q13.3	0 (0.00%)	1 (0.41%)	<-10	0.681	0.686	Mutual exclusivity
F2RL2	5q13.3	0 (0.00%)	1 (0.41%)	<-10	0.681	0.686	Mutual exclusivity
FAHD2B	2q11.2	0 (0.00%)	1 (0.41%)	<-10	0.681	0.686	Mutual exclusivity
FAHD2CP	2q11.2	0 (0.00%)	1 (0.41%)	<-10	0.681	0.686	Mutual exclusivity
FAM106A	17p11.2	0 (0.00%)	1 (0.41%)	<-10	0.681	0.686	Mutual exclusivity
FAM110A	20p13	0 (0.00%)	1 (0.41%)	<-10	0.681	0.686	Mutual exclusivity
FAM124B	2q36.2	0 (0.00%)	1 (0.41%)	<-10	0.681	0.686	Mutual exclusivity
FAM135A	6q13	0 (0.00%)	1 (0.41%)	<-10	0.681	0.686	Mutual exclusivity
FAM138B	2q14.1	0 (0.00%)	1 (0.41%)	<-10	0.681	0.686	Mutual exclusivity
FAM13A	4q22.1	0 (0.00%)	1 (0.41%)	<-10	0.681	0.686	Mutual exclusivity
FAM149A	4q35.1	0 (0.00%)	1 (0.41%)	<-10	0.681	0.686	Mutual exclusivity
FAM151B	5q14.1	0 (0.00%)	1 (0.41%)	<-10	0.681	0.686	Mutual exclusivity
FAM156A	Xp11.22	0 (0.00%)	1 (0.41%)	<-10	0.681	0.686	Mutual exclusivity
FAM156B	Xp11.22	0 (0.00%)	1 (0.41%)	<-10	0.681	0.686	Mutual exclusivity
FAM160A1	4q31.3	0 (0.00%)	1 (0.41%)	<-10	0.681	0.686	Mutual exclusivity
FAM161A	2p15	0 (0.00%)	1 (0.41%)	<-10	0.681	0.686	Mutual exclusivity

FAM168A	11q13.4	0 (0.00%)	1 (0.41%)	<-10	0.681	0.686	Mutual exclusivity
FAM181B	11q14.1	0 (0.00%)	1 (0.41%)	<-10	0.681	0.686	Mutual exclusivity
FAM183A	1p34.2	0 (0.00%)	1 (0.41%)	<-10	0.681	0.686	Mutual exclusivity
FAM184A	6q22.31	0 (0.00%)	1 (0.41%)	<-10	0.681	0.686	Mutual exclusivity
FAM186A	12q13.12	0 (0.00%)	1 (0.41%)	<-10	0.681	0.686	Mutual exclusivity
FAM189A2	9q21.12	0 (0.00%)	1 (0.41%)	<-10	0.681	0.686	Mutual exclusivity
FAM207A	21q22.3	0 (0.00%)	1 (0.41%)	<-10	0.681	0.686	Mutual exclusivity
FAM234B	12p13.1	0 (0.00%)	1 (0.41%)	<-10	0.681	0.686	Mutual exclusivity
FAM245A	10q23.2	0 (0.00%)	1 (0.41%)	<-10	0.681	0.686	Mutual exclusivity
FAM43B	1p36.12	0 (0.00%)	1 (0.41%)	<-10	0.681	0.686	Mutual exclusivity
FAM71D	14q23.3	0 (0.00%)	1 (0.41%)	<-10	0.681	0.686	Mutual exclusivity
FAM76B	11q21	0 (0.00%)	1 (0.41%)	<-10	0.681	0.686	Mutual exclusivity
FAM81B	5q15	0 (0.00%)	1 (0.41%)	<-10	0.681	0.686	Mutual exclusivity
FAM83E	19q13.33	0 (0.00%)	1 (0.41%)	<-10	0.681	0.686	Mutual exclusivity
FAM86JP	3q21.2	0 (0.00%)	1 (0.41%)	<-10	0.681	0.686	Mutual exclusivity
FANCL	2p16.1	0 (0.00%)	1 (0.41%)	<-10	0.681	0.686	Mutual exclusivity
FANCM	14q21.2	0 (0.00%)	1 (0.41%)	<-10	0.681	0.686	Mutual exclusivity
FANK1	10q26.2	0 (0.00%)	1 (0.41%)	<-10	0.681	0.686	Mutual exclusivity
FARP2	2q37.3	0 (0.00%)	1 (0.41%)	<-10	0.681	0.686	Mutual exclusivity
FARSB	2q36.1	0 (0.00%)	1 (0.41%)	<-10	0.681	0.686	Mutual exclusivity
FAS	10q23.31	0 (0.00%)	1 (0.41%)	<-10	0.681	0.686	Mutual exclusivity
FASTKD5	20p13	0 (0.00%)	1 (0.41%)	<-10	0.681	0.686	Mutual exclusivity
FAT3	11q14.3	0 (0.00%)	1 (0.41%)	<-10	0.681	0.686	Mutual exclusivity
FBLN5	14q32.12	0 (0.00%)	1 (0.41%)	<-10	0.681	0.686	Mutual exclusivity
FBXL14	12p13.33	0 (0.00%)	1 (0.41%)	<-10	0.681	0.686	Mutual exclusivity
FBXL19	16p11.2	0 (0.00%)	1 (0.41%)	<-10	0.681	0.686	Mutual exclusivity
FBXO16	8p21.1	0 (0.00%)	1 (0.41%)	<-10	0.681	0.686	Mutual exclusivity
FBXO30	6q24.3	0 (0.00%)	1 (0.41%)	<-10	0.681	0.686	Mutual exclusivity
FBXO34	14q22.3	0 (0.00%)	1 (0.41%)	<-10	0.681	0.686	Mutual exclusivity

FBXO36	2q36.3	0 (0.00%)	1 (0.41%)	<-10	0.681	0.686	Mutual exclusivity
FBXO5	6q25.2	0 (0.00%)	1 (0.41%)	<-10	0.681	0.686	Mutual exclusivity
FBXW10	17p11.2	0 (0.00%)	1 (0.41%)	<-10	0.681	0.686	Mutual exclusivity
FBXW7	4q31.3	0 (0.00%)	1 (0.41%)	<-10	0.681	0.686	Mutual exclusivity
FDX2	19p13.2	0 (0.00%)	1 (0.41%)	<-10	0.681	0.686	Mutual exclusivity
FECH	18q21.31	0 (0.00%)	1 (0.41%)	<-10	0.681	0.686	Mutual exclusivity
FER1L5	2q11.2	0 (0.00%)	1 (0.41%)	<-10	0.681	0.686	Mutual exclusivity
FEV	2q35	0 (0.00%)	1 (0.41%)	<-10	0.681	0.686	Mutual exclusivity
FFAR4	10q23.33	0 (0.00%)	1 (0.41%)	<-10	0.681	0.686	Mutual exclusivity
FGF2	4q28.1	0 (0.00%)	1 (0.41%)	<-10	0.681	0.686	Mutual exclusivity
FGF23	12p13.32	0 (0.00%)	1 (0.41%)	<-10	0.681	0.686	Mutual exclusivity
FGF6	12p13.32	0 (0.00%)	1 (0.41%)	<-10	0.681	0.686	Mutual exclusivity
FGFR1OP	6q27	0 (0.00%)	1 (0.41%)	<-10	0.681	0.686	Mutual exclusivity
FHDC1	4q31.3	0 (0.00%)	1 (0.41%)	<-10	0.681	0.686	Mutual exclusivity
FHL2	2q12.2	0 (0.00%)	1 (0.41%)	<-10	0.681	0.686	Mutual exclusivity
FILIP1	6q14.1	0 (0.00%)	1 (0.41%)	<-10	0.681	0.686	Mutual exclusivity
FILIP1L	3q12.1	0 (0.00%)	1 (0.41%)	<-10	0.681	0.686	Mutual exclusivity
FIP1L1	4q12	0 (0.00%)	1 (0.41%)	<-10	0.681	0.686	Mutual exclusivity
FKBP11	12q13.12	0 (0.00%)	1 (0.41%)	<-10	0.681	0.686	Mutual exclusivity
FKBP3	14q21.2	0 (0.00%)	1 (0.41%)	<-10	0.681	0.686	Mutual exclusivity
FKBP4	12p13.33	0 (0.00%)	1 (0.41%)	<-10	0.681	0.686	Mutual exclusivity
FLCN	17p11.2	0 (0.00%)	1 (0.41%)	<-10	0.681	0.686	Mutual exclusivity
FN1	2q35	0 (0.00%)	1 (0.41%)	<-10	0.681	0.686	Mutual exclusivity
FNDC1	6q25.3	0 (0.00%)	1 (0.41%)	<-10	0.681	0.686	Mutual exclusivity
FNIP2	4q32.1	0 (0.00%)	1 (0.41%)	<-10	0.681	0.686	Mutual exclusivity
FOXA2	20p11.21	0 (0.00%)	1 (0.41%)	<-10	0.681	0.686	Mutual exclusivity
FOXD4L1	2q14.1	0 (0.00%)	1 (0.41%)	<-10	0.681	0.686	Mutual exclusivity
FOXG1	14q12	0 (0.00%)	1 (0.41%)	<-10	0.681	0.686	Mutual exclusivity
FOXI2	10q26.2	0 (0.00%)	1 (0.41%)	<-10	0.681	0.686	Mutual exclusivity

FOXI3	2p11.2	0 (0.00%)	1 (0.41%)	<-10	0.681	0.686	Mutual exclusivity
FOXM1	12p13.33	0 (0.00%)	1 (0.41%)	<-10	0.681	0.686	Mutual exclusivity
FOXO3	6q21	0 (0.00%)	1 (0.41%)	<-10	0.681	0.686	Mutual exclusivity
FOXO6	1p34.2	0 (0.00%)	1 (0.41%)	<-10	0.681	0.686	Mutual exclusivity
FREM1	9p22.3	0 (0.00%)	1 (0.41%)	<-10	0.681	0.686	Mutual exclusivity
FSCB	14q21.2	0 (0.00%)	1 (0.41%)	<-10	0.681	0.686	Mutual exclusivity
FSD1L	9q31.2	0 (0.00%)	1 (0.41%)	<-10	0.681	0.686	Mutual exclusivity
FSIP1	15q14	0 (0.00%)	1 (0.41%)	<-10	0.681	0.686	Mutual exclusivity
FST	5q11.2	0 (0.00%)	1 (0.41%)	<-10	0.681	0.686	Mutual exclusivity
FTCD	21q22.3	0 (0.00%)	1 (0.41%)	<-10	0.681	0.686	Mutual exclusivity
FUCA2	6q24.2	0 (0.00%)	1 (0.41%)	<-10	0.681	0.686	Mutual exclusivity
FUS	16p11.2	0 (0.00%)	1 (0.41%)	<-10	0.681	0.686	Mutual exclusivity
FUT2	19q13.33	0 (0.00%)	1 (0.41%)	<-10	0.681	0.686	Mutual exclusivity
FUT3	19p13.3	0 (0.00%)	1 (0.41%)	<-10	0.681	0.686	Mutual exclusivity
FUT4	11q21	0 (0.00%)	1 (0.41%)	<-10	0.681	0.686	Mutual exclusivity
FUT5	19p13.3	0 (0.00%)	1 (0.41%)	<-10	0.681	0.686	Mutual exclusivity
FUT6	19p13.3	0 (0.00%)	1 (0.41%)	<-10	0.681	0.686	Mutual exclusivity
FZD3	8p21.1	0 (0.00%)	1 (0.41%)	<-10	0.681	0.686	Mutual exclusivity
FZD4	11q14.2	0 (0.00%)	1 (0.41%)	<-10	0.681	0.686	Mutual exclusivity
GACAT1	2q12.3	0 (0.00%)	1 (0.41%)	<-10	0.681	0.686	Mutual exclusivity
GADD45GIP1	19p13.13	0 (0.00%)	1 (0.41%)	<-10	0.681	0.686	Mutual exclusivity
GAL3ST2	2q37.3	0 (0.00%)	1 (0.41%)	<-10	0.681	0.686	Mutual exclusivity
GALE	1p36.11	0 (0.00%)	1 (0.41%)	<-10	0.681	0.686	Mutual exclusivity
GALNT1	18q12.2	0 (0.00%)	1 (0.41%)	<-10	0.681	0.686	Mutual exclusivity
GALNT8	12p13.32	0 (0.00%)	1 (0.41%)	<-10	0.681	0.686	Mutual exclusivity
GATB	4q31.3	0 (0.00%)	1 (0.41%)	<-10	0.681	0.686	Mutual exclusivity
GATD3A	21q22.3	0 (0.00%)	1 (0.41%)	<-10	0.681	0.686	Mutual exclusivity
GBX2	2q37.2	0 (0.00%)	1 (0.41%)	<-10	0.681	0.686	Mutual exclusivity
GCC2	2q12.3	0 (0.00%)	1 (0.41%)	<-10	0.681	0.686	Mutual exclusivity

GCNA	Xq13.1	0 (0.00%)	1 (0.41%)	<-10	0.681	0.686	Mutual exclusivity
GDA	9q21.13	0 (0.00%)	1 (0.41%)	<-10	0.681	0.686	Mutual exclusivity
GGTLC2	22q11.22	0 (0.00%)	1 (0.41%)	<-10	0.681	0.686	Mutual exclusivity
GIGYF2	2q37.1	0 (0.00%)	1 (0.41%)	<-10	0.681	0.686	Mutual exclusivity
GINM1	6q25.1	0 (0.00%)	1 (0.41%)	<-10	0.681	0.686	Mutual exclusivity
GINS1	20p11.21	0 (0.00%)	1 (0.41%)	<-10	0.681	0.686	Mutual exclusivity
GJB7	6q14.3-q15	0 (0.00%)	1 (0.41%)	<-10	0.681	0.686	Mutual exclusivity
GJE1	6q24.1	0 (0.00%)	1 (0.41%)	<-10	0.681	0.686	Mutual exclusivity
GLB1L	2q35	0 (0.00%)	1 (0.41%)	<-10	0.681	0.686	Mutual exclusivity
GLRA4	Xq22.2	0 (0.00%)	1 (0.41%)	<-10	0.681	0.686	Mutual exclusivity
GLRX5	14q32.13	0 (0.00%)	1 (0.41%)	<-10	0.681	0.686	Mutual exclusivity
GMPPA	2q35	0 (0.00%)	1 (0.41%)	<-10	0.681	0.686	Mutual exclusivity
GNAZ	22q11.22-q11.23	0 (0.00%)	1 (0.41%)	<-10	0.681	0.686	Mutual exclusivity
GNG3	11q12.3	0 (0.00%)	1 (0.41%)	<-10	0.681	0.686	Mutual exclusivity
GNPDA2	4p12	0 (0.00%)	1 (0.41%)	<-10	0.681	0.686	Mutual exclusivity
GNRH2	20p13	0 (0.00%)	1 (0.41%)	<-10	0.681	0.686	Mutual exclusivity
GOLGA7B	10q24.2	0 (0.00%)	1 (0.41%)	<-10	0.681	0.686	Mutual exclusivity
GOPC	6q22.1	0 (0.00%)	1 (0.41%)	<-10	0.681	0.686	Mutual exclusivity
GOT1	10q24.2	0 (0.00%)	1 (0.41%)	<-10	0.681	0.686	Mutual exclusivity
GPAT2	2q11.2	0 (0.00%)	1 (0.41%)	<-10	0.681	0.686	Mutual exclusivity
GPATCH3	1p36.11	0 (0.00%)	1 (0.41%)	<-10	0.681	0.686	Mutual exclusivity
GPBP1	5q11.2	0 (0.00%)	1 (0.41%)	<-10	0.681	0.686	Mutual exclusivity
GPC1	2q37.3	0 (0.00%)	1 (0.41%)	<-10	0.681	0.686	Mutual exclusivity
GPM6A	4q34.2	0 (0.00%)	1 (0.41%)	<-10	0.681	0.686	Mutual exclusivity
GPN2	1p36.11	0 (0.00%)	1 (0.41%)	<-10	0.681	0.686	Mutual exclusivity
GPR108	19p13.3	0 (0.00%)	1 (0.41%)	<-10	0.681	0.686	Mutual exclusivity
GPR15	3q11.2	0 (0.00%)	1 (0.41%)	<-10	0.681	0.686	Mutual exclusivity
GPR156	3q13.33	0 (0.00%)	1 (0.41%)	<-10	0.681	0.686	Mutual exclusivity
GPR173	Xp11.22	0 (0.00%)	1 (0.41%)	<-10	0.681	0.686	Mutual exclusivity

GPR176	15q14-q15.1	0 (0.00%)	1 (0.41%)	<-10	0.681	0.686	Mutual exclusivity
GPR19	12p13.1	0 (0.00%)	1 (0.41%)	<-10	0.681	0.686	Mutual exclusivity
GPR31	6q27	0 (0.00%)	1 (0.41%)	<-10	0.681	0.686	Mutual exclusivity
GPR33	14q12	0 (0.00%)	1 (0.41%)	<-10	0.681	0.686	Mutual exclusivity
GPR35	2q37.3	0 (0.00%)	1 (0.41%)	<-10	0.681	0.686	Mutual exclusivity
GPR45	2q12.1	0 (0.00%)	1 (0.41%)	<-10	0.681	0.686	Mutual exclusivity
GPR68	14q32.11	0 (0.00%)	1 (0.41%)	<-10	0.681	0.686	Mutual exclusivity
GPR75	2p16.2	0 (0.00%)	1 (0.41%)	<-10	0.681	0.686	Mutual exclusivity
GPR83	11q21	0 (0.00%)	1 (0.41%)	<-10	0.681	0.686	Mutual exclusivity
GPR84	12q13.13	0 (0.00%)	1 (0.41%)	<-10	0.681	0.686	Mutual exclusivity
GPRC5A	12p13.1	0 (0.00%)	1 (0.41%)	<-10	0.681	0.686	Mutual exclusivity
GPRC5D	12p13.1	0 (0.00%)	1 (0.41%)	<-10	0.681	0.686	Mutual exclusivity
GPRIN3	4q22.1	0 (0.00%)	1 (0.41%)	<-10	0.681	0.686	Mutual exclusivity
GPX2	14q23.3	0 (0.00%)	1 (0.41%)	<-10	0.681	0.686	Mutual exclusivity
GRHL3	1p36.11	0 (0.00%)	1 (0.41%)	<-10	0.681	0.686	Mutual exclusivity
GRIK2	6q16.3	0 (0.00%)	1 (0.41%)	<-10	0.681	0.686	Mutual exclusivity
GRIK4	11q23.3	0 (0.00%)	1 (0.41%)	<-10	0.681	0.686	Mutual exclusivity
GRIK5	19q13.2	0 (0.00%)	1 (0.41%)	<-10	0.681	0.686	Mutual exclusivity
GRIN2D	19q13.33	0 (0.00%)	1 (0.41%)	<-10	0.681	0.686	Mutual exclusivity
GRIN3A	9q31.1	0 (0.00%)	1 (0.41%)	<-10	0.681	0.686	Mutual exclusivity
GRK5	10q26.11	0 (0.00%)	1 (0.41%)	<-10	0.681	0.686	Mutual exclusivity
GRM1	6q24.3	0 (0.00%)	1 (0.41%)	<-10	0.681	0.686	Mutual exclusivity
GRWD1	19q13.33	0 (0.00%)	1 (0.41%)	<-10	0.681	0.686	Mutual exclusivity
GSC	14q32.13	0 (0.00%)	1 (0.41%)	<-10	0.681	0.686	Mutual exclusivity
GSG1	12p13.1	0 (0.00%)	1 (0.41%)	<-10	0.681	0.686	Mutual exclusivity
GSK3A	19q13.2	0 (0.00%)	1 (0.41%)	<-10	0.681	0.686	Mutual exclusivity
GSPT2	Xp11.22	0 (0.00%)	1 (0.41%)	<-10	0.681	0.686	Mutual exclusivity
GTF2H5	6q25.3	0 (0.00%)	1 (0.41%)	<-10	0.681	0.686	Mutual exclusivity
GTSCR1	18q22.2	0 (0.00%)	1 (0.41%)	<-10	0.681	0.686	Mutual exclusivity

GUCY1A2	11q22.3	0 (0.00%)	1 (0.41%)	<-10	0.681	0.686	Mutual exclusivity
GUF1	4p12	0 (0.00%)	1 (0.41%)	<-10	0.681	0.686	Mutual exclusivity
H2BFM	Xq22.2	0 (0.00%)	1 (0.41%)	<-10	0.681	0.686	Mutual exclusivity
H2BFWT	Xq22.2	0 (0.00%)	1 (0.41%)	<-10	0.681	0.686	Mutual exclusivity
HAPLN1	5q14.3	0 (0.00%)	1 (0.41%)	<-10	0.681	0.686	Mutual exclusivity
HAS3	16q22.1	0 (0.00%)	1 (0.41%)	<-10	0.681	0.686	Mutual exclusivity
HAUS1	18q21.1	0 (0.00%)	1 (0.41%)	<-10	0.681	0.686	Mutual exclusivity
HCK	20q11.21	0 (0.00%)	1 (0.41%)	<-10	0.681	0.686	Mutual exclusivity
HDAC4	2q37.3	0 (0.00%)	1 (0.41%)	<-10	0.681	0.686	Mutual exclusivity
HDLBP	2q37.3	0 (0.00%)	1 (0.41%)	<-10	0.681	0.686	Mutual exclusivity
HEATR3	16q12.1	0 (0.00%)	1 (0.41%)	<-10	0.681	0.686	Mutual exclusivity
HEBP1	12p13.1	0 (0.00%)	1 (0.41%)	<-10	0.681	0.686	Mutual exclusivity
HELLS	10q23.33	0 (0.00%)	1 (0.41%)	<-10	0.681	0.686	Mutual exclusivity
HEPHL1	11q21	0 (0.00%)	1 (0.41%)	<-10	0.681	0.686	Mutual exclusivity
HERC3	4q22.1	0 (0.00%)	1 (0.41%)	<-10	0.681	0.686	Mutual exclusivity
HERC5	4q22.1	0 (0.00%)	1 (0.41%)	<-10	0.681	0.686	Mutual exclusivity
HERC6	4q22.1	0 (0.00%)	1 (0.41%)	<-10	0.681	0.686	Mutual exclusivity
HGD	3q13.33	0 (0.00%)	1 (0.41%)	<-10	0.681	0.686	Mutual exclusivity
HHLA2	3q13.13	0 (0.00%)	1 (0.41%)	<-10	0.681	0.686	Mutual exclusivity
HIKESHI	11q14.2	0 (0.00%)	1 (0.41%)	<-10	0.681	0.686	Mutual exclusivity
HINFP	11q23.3	0 (0.00%)	1 (0.41%)	<-10	0.681	0.686	Mutual exclusivity
HK1	10q22.1	0 (0.00%)	1 (0.41%)	<-10	0.681	0.686	Mutual exclusivity
HKDC1	10q22.1	0 (0.00%)	1 (0.41%)	<-10	0.681	0.686	Mutual exclusivity
HMBOX1	8p21.1-p12	0 (0.00%)	1 (0.41%)	<-10	0.681	0.686	Mutual exclusivity
HMGN2	1p36.11	0 (0.00%)	1 (0.41%)	<-10	0.681	0.686	Mutual exclusivity
HMGN3	6q14.1	0 (0.00%)	1 (0.41%)	<-10	0.681	0.686	Mutual exclusivity
HNRNPA1	12q13.13	0 (0.00%)	1 (0.41%)	<-10	0.681	0.686	Mutual exclusivity
HNRNPUL2	11q12.3	0 (0.00%)	1 (0.41%)	<-10	0.681	0.686	Mutual exclusivity
HOGA1	10q24.2	0 (0.00%)	1 (0.41%)	<-10	0.681	0.686	Mutual exclusivity

HOPX	4q12	0 (0.00%)	1 (0.41%)	<-10	0.681	0.686	Mutual exclusivity
HP1BP3	1p36.12	0 (0.00%)	1 (0.41%)	<-10	0.681	0.686	Mutual exclusivity
HPS1	10q24.2	0 (0.00%)	1 (0.41%)	<-10	0.681	0.686	Mutual exclusivity
HPSE2	10q24.2	0 (0.00%)	1 (0.41%)	<-10	0.681	0.686	Mutual exclusivity
HSD11B1L	19p13.3	0 (0.00%)	1 (0.41%)	<-10	0.681	0.686	Mutual exclusivity
HSD17B11	4q22.1	0 (0.00%)	1 (0.41%)	<-10	0.681	0.686	Mutual exclusivity
HSD17B13	4q22.1	0 (0.00%)	1 (0.41%)	<-10	0.681	0.686	Mutual exclusivity
HSD3B7	16p11.2	0 (0.00%)	1 (0.41%)	<-10	0.681	0.686	Mutual exclusivity
HSPB3	5q11.2	0 (0.00%)	1 (0.41%)	<-10	0.681	0.686	Mutual exclusivity
HSPG2	1p36.12	0 (0.00%)	1 (0.41%)	<-10	0.681	0.686	Mutual exclusivity
HTR1B	6q14.1	0 (0.00%)	1 (0.41%)	<-10	0.681	0.686	Mutual exclusivity
HTR1D	1p36.12	0 (0.00%)	1 (0.41%)	<-10	0.681	0.686	Mutual exclusivity
HTR1F	3p11.2-p11.1	0 (0.00%)	1 (0.41%)	<-10	0.681	0.686	Mutual exclusivity
HTR2B	2q37.1	0 (0.00%)	1 (0.41%)	<-10	0.681	0.686	Mutual exclusivity
HTR5BP	2q14.1	0 (0.00%)	1 (0.41%)	<-10	0.681	0.686	Mutual exclusivity
HTR6	1p36.13	0 (0.00%)	1 (0.41%)	<-10	0.681	0.686	Mutual exclusivity
HTR7P1	12p13.1	0 (0.00%)	1 (0.41%)	<-10	0.681	0.686	Mutual exclusivity
HTRA1	10q26.13	0 (0.00%)	1 (0.41%)	<-10	0.681	0.686	Mutual exclusivity
HYPM	Xp11.4	0 (0.00%)	1 (0.41%)	<-10	0.681	0.686	Mutual exclusivity
IBSP	4q22.1	0 (0.00%)	1 (0.41%)	<-10	0.681	0.686	Mutual exclusivity
ICAM1	19p13.2	0 (0.00%)	1 (0.41%)	<-10	0.681	0.686	Mutual exclusivity
ICAM3	19p13.2	0 (0.00%)	1 (0.41%)	<-10	0.681	0.686	Mutual exclusivity
ICAM4	19p13.2	0 (0.00%)	1 (0.41%)	<-10	0.681	0.686	Mutual exclusivity
ICAM5	19p13.2	0 (0.00%)	1 (0.41%)	<-10	0.681	0.686	Mutual exclusivity
ICOSLG	21q22.3	0 (0.00%)	1 (0.41%)	<-10	0.681	0.686	Mutual exclusivity
IDH1	2q34	0 (0.00%)	1 (0.41%)	<-10	0.681	0.686	Mutual exclusivity
IDH3B	20p13	0 (0.00%)	1 (0.41%)	<-10	0.681	0.686	Mutual exclusivity
IFIT1	10q23.31	0 (0.00%)	1 (0.41%)	<-10	0.681	0.686	Mutual exclusivity
IFIT1B	10q23.31	0 (0.00%)	1 (0.41%)	<-10	0.681	0.686	Mutual exclusivity

IFIT5	10q23.31	0 (0.00%)	1 (0.41%)	<-10	0.681	0.686	Mutual exclusivity
IFNAR1	21q22.11	0 (0.00%)	1 (0.41%)	<-10	0.681	0.686	Mutual exclusivity
IFT57	3q13.12-q13.13	0 (0.00%)	1 (0.41%)	<-10	0.681	0.686	Mutual exclusivity
IGFBP2	2q35	0 (0.00%)	1 (0.41%)	<-10	0.681	0.686	Mutual exclusivity
IGFBP5	2q35	0 (0.00%)	1 (0.41%)	<-10	0.681	0.686	Mutual exclusivity
IGLC1	22q11.22	0 (0.00%)	1 (0.41%)	<-10	0.681	0.686	Mutual exclusivity
IGLC2	22q11.22	0 (0.00%)	1 (0.41%)	<-10	0.681	0.686	Mutual exclusivity
IGLC3	22q11.22	0 (0.00%)	1 (0.41%)	<-10	0.681	0.686	Mutual exclusivity
IGLC7	22q11.22	0 (0.00%)	1 (0.41%)	<-10	0.681	0.686	Mutual exclusivity
IGLJ1	22q11.22	0 (0.00%)	1 (0.41%)	<-10	0.681	0.686	Mutual exclusivity
IGLJ2	22q11.22	0 (0.00%)	1 (0.41%)	<-10	0.681	0.686	Mutual exclusivity
IGLJ3	22q11.22	0 (0.00%)	1 (0.41%)	<-10	0.681	0.686	Mutual exclusivity
IGLJ4	22q11.22	0 (0.00%)	1 (0.41%)	<-10	0.681	0.686	Mutual exclusivity
IGLJ5	22q11.22	0 (0.00%)	1 (0.41%)	<-10	0.681	0.686	Mutual exclusivity
IGLJ6	22q11.22	0 (0.00%)	1 (0.41%)	<-10	0.681	0.686	Mutual exclusivity
IGLJ7	22q11.22	0 (0.00%)	1 (0.41%)	<-10	0.681	0.686	Mutual exclusivity
IGLL1	22q11.23	0 (0.00%)	1 (0.41%)	<-10	0.681	0.686	Mutual exclusivity
IGLL5	22q11.22	0 (0.00%)	1 (0.41%)	<-10	0.681	0.686	Mutual exclusivity
IGSF3	1p13.1	0 (0.00%)	1 (0.41%)	<-10	0.681	0.686	Mutual exclusivity
IGSF9B	11q25	0 (0.00%)	1 (0.41%)	<-10	0.681	0.686	Mutual exclusivity
IHH	2q35	0 (0.00%)	1 (0.41%)	<-10	0.681	0.686	Mutual exclusivity
IKZF2	2q34	0 (0.00%)	1 (0.41%)	<-10	0.681	0.686	Mutual exclusivity
IL10RA	11q23.3	0 (0.00%)	1 (0.41%)	<-10	0.681	0.686	Mutual exclusivity
IL15	4q31.21	0 (0.00%)	1 (0.41%)	<-10	0.681	0.686	Mutual exclusivity
IL18	11q23.1	0 (0.00%)	1 (0.41%)	<-10	0.681	0.686	Mutual exclusivity
IL1A	2q14.1	0 (0.00%)	1 (0.41%)	<-10	0.681	0.686	Mutual exclusivity
IL1B	2q14.1	0 (0.00%)	1 (0.41%)	<-10	0.681	0.686	Mutual exclusivity
IL1F10	2q14.1	0 (0.00%)	1 (0.41%)	<-10	0.681	0.686	Mutual exclusivity
IL1RN	2q14.1	0 (0.00%)	1 (0.41%)	<-10	0.681	0.686	Mutual exclusivity

IL20RA	6q23.3	0 (0.00%)	1 (0.41%)	<-10	0.681	0.686	Mutual exclusivity
IL36A	2q14.1	0 (0.00%)	1 (0.41%)	<-10	0.681	0.686	Mutual exclusivity
IL36B	2q14.1	0 (0.00%)	1 (0.41%)	<-10	0.681	0.686	Mutual exclusivity
IL36G	2q14.1	0 (0.00%)	1 (0.41%)	<-10	0.681	0.686	Mutual exclusivity
IL36RN	2q14.1	0 (0.00%)	1 (0.41%)	<-10	0.681	0.686	Mutual exclusivity
IL37	2q14.1	0 (0.00%)	1 (0.41%)	<-10	0.681	0.686	Mutual exclusivity
ILF3	19p13.2	0 (0.00%)	1 (0.41%)	<-10	0.681	0.686	Mutual exclusivity
IMMP1L	11p13	0 (0.00%)	1 (0.41%)	<-10	0.681	0.686	Mutual exclusivity
IMPG1	6q14.1	0 (0.00%)	1 (0.41%)	<-10	0.681	0.686	Mutual exclusivity
INAFM2	15q15.1	0 (0.00%)	1 (0.41%)	<-10	0.681	0.686	Mutual exclusivity
ING5	2q37.3	0 (0.00%)	1 (0.41%)	<-10	0.681	0.686	Mutual exclusivity
INGX	Xq13.1	0 (0.00%)	1 (0.41%)	<-10	0.681	0.686	Mutual exclusivity
INHA	2q35	0 (0.00%)	1 (0.41%)	<-10	0.681	0.686	Mutual exclusivity
INHBB	2q14.2	0 (0.00%)	1 (0.41%)	<-10	0.681	0.686	Mutual exclusivity
INKA2	1p13.2	0 (0.00%)	1 (0.41%)	<-10	0.681	0.686	Mutual exclusivity
INO80C	18q12.2	0 (0.00%)	1 (0.41%)	<-10	0.681	0.686	Mutual exclusivity
INPP4A	2q11.2	0 (0.00%)	1 (0.41%)	<-10	0.681	0.686	Mutual exclusivity
INPP5D	2q37.1	0 (0.00%)	1 (0.41%)	<-10	0.681	0.686	Mutual exclusivity
INPP5F	10q26.11	0 (0.00%)	1 (0.41%)	<-10	0.681	0.686	Mutual exclusivity
INSIG2	2q14.1-q14.2	0 (0.00%)	1 (0.41%)	<-10	0.681	0.686	Mutual exclusivity
INSYN2A	10q26.2	0 (0.00%)	1 (0.41%)	<-10	0.681	0.686	Mutual exclusivity
INTS5	11q12.3	0 (0.00%)	1 (0.41%)	<-10	0.681	0.686	Mutual exclusivity
INTS9	8p21.1	0 (0.00%)	1 (0.41%)	<-10	0.681	0.686	Mutual exclusivity
IQCA1	2q37.2-q37.3	0 (0.00%)	1 (0.41%)	<-10	0.681	0.686	Mutual exclusivity
IRAK1BP1	6q14.1	0 (0.00%)	1 (0.41%)	<-10	0.681	0.686	Mutual exclusivity
IRS1	2q36.3	0 (0.00%)	1 (0.41%)	<-10	0.681	0.686	Mutual exclusivity
ISL1	5q11.1	0 (0.00%)	1 (0.41%)	<-10	0.681	0.686	Mutual exclusivity
ITFG2	12p13.33	0 (0.00%)	1 (0.41%)	<-10	0.681	0.686	Mutual exclusivity
ITGA1	5q11.2	0 (0.00%)	1 (0.41%)	<-10	0.681	0.686	Mutual exclusivity

ITGA2	5q11.2	0 (0.00%)	1 (0.41%)	<-10	0.681	0.686	Mutual exclusivity
ITGA5	12q13.13	0 (0.00%)	1 (0.41%)	<-10	0.681	0.686	Mutual exclusivity
ITGAM	16p11.2	0 (0.00%)	1 (0.41%)	<-10	0.681	0.686	Mutual exclusivity
ITPA	20p13	0 (0.00%)	1 (0.41%)	<-10	0.681	0.686	Mutual exclusivity
ITPRIPL1	2q11.2	0 (0.00%)	1 (0.41%)	<-10	0.681	0.686	Mutual exclusivity
IYD	6q25.1	0 (0.00%)	1 (0.41%)	<-10	0.681	0.686	Mutual exclusivity
IZUMO1R	11q21	0 (0.00%)	1 (0.41%)	<-10	0.681	0.686	Mutual exclusivity
JAG2	14q32.33	0 (0.00%)	1 (0.41%)	<-10	0.681	0.686	Mutual exclusivity
JAM3	11q25	0 (0.00%)	1 (0.41%)	<-10	0.681	0.686	Mutual exclusivity
JAML	11q23.3	0 (0.00%)	1 (0.41%)	<-10	0.681	0.686	Mutual exclusivity
JRKL	11q21	0 (0.00%)	1 (0.41%)	<-10	0.681	0.686	Mutual exclusivity
KANSL2	12q13.11	0 (0.00%)	1 (0.41%)	<-10	0.681	0.686	Mutual exclusivity
KANSL3	2q11.2	0 (0.00%)	1 (0.41%)	<-10	0.681	0.686	Mutual exclusivity
KAT8	16p11.2	0 (0.00%)	1 (0.41%)	<-10	0.681	0.686	Mutual exclusivity
KATNA1	6q25.1	0 (0.00%)	1 (0.41%)	<-10	0.681	0.686	Mutual exclusivity
KATNB1	16q21	0 (0.00%)	1 (0.41%)	<-10	0.681	0.686	Mutual exclusivity
KBTBD3	11q22.3	0 (0.00%)	1 (0.41%)	<-10	0.681	0.686	Mutual exclusivity
KCNA1	12p13.32	0 (0.00%)	1 (0.41%)	<-10	0.681	0.686	Mutual exclusivity
KCNA5	12p13.32	0 (0.00%)	1 (0.41%)	<-10	0.681	0.686	Mutual exclusivity
KCNA6	12p13.32	0 (0.00%)	1 (0.41%)	<-10	0.681	0.686	Mutual exclusivity
KCND3	1p13.2	0 (0.00%)	1 (0.41%)	<-10	0.681	0.686	Mutual exclusivity
KCNE4	2q36.1	0 (0.00%)	1 (0.41%)	<-10	0.681	0.686	Mutual exclusivity
KCNIP2	10q24.32	0 (0.00%)	1 (0.41%)	<-10	0.681	0.686	Mutual exclusivity
KCNJ13	2q37.1	0 (0.00%)	1 (0.41%)	<-10	0.681	0.686	Mutual exclusivity
KCNJ14	19q13.33	0 (0.00%)	1 (0.41%)	<-10	0.681	0.686	Mutual exclusivity
KCNJ5	11q24.3	0 (0.00%)	1 (0.41%)	<-10	0.681	0.686	Mutual exclusivity
KCNJ6	21q22.13	0 (0.00%)	1 (0.41%)	<-10	0.681	0.686	Mutual exclusivity
KCNK13	14q32.11	0 (0.00%)	1 (0.41%)	<-10	0.681	0.686	Mutual exclusivity
KCNK15	20q13.12	0 (0.00%)	1 (0.41%)	<-10	0.681	0.686	Mutual exclusivity

KCNS1	20q13.12	0 (0.00%)	1 (0.41%)	<-10	0.681	0.686	Mutual exclusivity
KDELR1	19q13.33	0 (0.00%)	1 (0.41%)	<-10	0.681	0.686	Mutual exclusivity
KDF1	1p36.11	0 (0.00%)	1 (0.41%)	<-10	0.681	0.686	Mutual exclusivity
KDM1A	1p36.12	0 (0.00%)	1 (0.41%)	<-10	0.681	0.686	Mutual exclusivity
KDM3A	2p11.2	0 (0.00%)	1 (0.41%)	<-10	0.681	0.686	Mutual exclusivity
KDM4B	19p13.3	0 (0.00%)	1 (0.41%)	<-10	0.681	0.686	Mutual exclusivity
KDM4D	11q21	0 (0.00%)	1 (0.41%)	<-10	0.681	0.686	Mutual exclusivity
KDM4E	11q21	0 (0.00%)	1 (0.41%)	<-10	0.681	0.686	Mutual exclusivity
KDR	4q12	0 (0.00%)	1 (0.41%)	<-10	0.681	0.686	Mutual exclusivity
KEAP1	19p13.2	0 (0.00%)	1 (0.41%)	<-10	0.681	0.686	Mutual exclusivity
KHSRP	19p13.3	0 (0.00%)	1 (0.41%)	<-10	0.681	0.686	Mutual exclusivity
KIAA1109	4q27	0 (0.00%)	1 (0.41%)	<-10	0.681	0.686	Mutual exclusivity
KIAA1211L	2q11.2	0 (0.00%)	1 (0.41%)	<-10	0.681	0.686	Mutual exclusivity
KIAA1328	18q12.2	0 (0.00%)	1 (0.41%)	<-10	0.681	0.686	Mutual exclusivity
KIAA1841	2p15	0 (0.00%)	1 (0.41%)	<-10	0.681	0.686	Mutual exclusivity
KIF17	1p36.12	0 (0.00%)	1 (0.41%)	<-10	0.681	0.686	Mutual exclusivity
KIF18A	11p14.1	0 (0.00%)	1 (0.41%)	<-10	0.681	0.686	Mutual exclusivity
KIF1A	2q37.3	0 (0.00%)	1 (0.41%)	<-10	0.681	0.686	Mutual exclusivity
KIF1BP	10q22.1	0 (0.00%)	1 (0.41%)	<-10	0.681	0.686	Mutual exclusivity
KIF20B	10q23.31	0 (0.00%)	1 (0.41%)	<-10	0.681	0.686	Mutual exclusivity
KL	13q13.1	0 (0.00%)	1 (0.41%)	<-10	0.681	0.686	Mutual exclusivity
KLF9	9q21.12	0 (0.00%)	1 (0.41%)	<-10	0.681	0.686	Mutual exclusivity
KLHDC4	16q24.2	0 (0.00%)	1 (0.41%)	<-10	0.681	0.686	Mutual exclusivity
KLHL8	4q22.1	0 (0.00%)	1 (0.41%)	<-10	0.681	0.686	Mutual exclusivity
KMT2D	12q13.12	0 (0.00%)	1 (0.41%)	<-10	0.681	0.686	Mutual exclusivity
KRCC1	2p11.2	0 (0.00%)	1 (0.41%)	<-10	0.681	0.686	Mutual exclusivity
KRI1	19p13.2	0 (0.00%)	1 (0.41%)	<-10	0.681	0.686	Mutual exclusivity
KRT16P1	17p11.2	0 (0.00%)	1 (0.41%)	<-10	0.681	0.686	Mutual exclusivity
KRT17P2	17p11.2	0 (0.00%)	1 (0.41%)	<-10	0.681	0.686	Mutual exclusivity

KTN1	14q22.3	0 (0.00%)	1 (0.41%)	<-10	0.681	0.686	Mutual exclusivity
LACTBL1	1p36.12	0 (0.00%)	1 (0.41%)	<-10	0.681	0.686	Mutual exclusivity
LAMA2	6q22.33	0 (0.00%)	1 (0.41%)	<-10	0.681	0.686	Mutual exclusivity
LANCL3	Xp21.1	0 (0.00%)	1 (0.41%)	<-10	0.681	0.686	Mutual exclusivity
LARP4	12q13.12	0 (0.00%)	1 (0.41%)	<-10	0.681	0.686	Mutual exclusivity
LATS1	6q25.1	0 (0.00%)	1 (0.41%)	<-10	0.681	0.686	Mutual exclusivity
LCA5	6q14.1	0 (0.00%)	1 (0.41%)	<-10	0.681	0.686	Mutual exclusivity
LCAT	16q22.1	0 (0.00%)	1 (0.41%)	<-10	0.681	0.686	Mutual exclusivity
LCOR	10q24.1	0 (0.00%)	1 (0.41%)	<-10	0.681	0.686	Mutual exclusivity
LCT	2q21.3	0 (0.00%)	1 (0.41%)	<-10	0.681	0.686	Mutual exclusivity
LDLRAD2	1p36.12	0 (0.00%)	1 (0.41%)	<-10	0.681	0.686	Mutual exclusivity
LGALS3	14q22.3	0 (0.00%)	1 (0.41%)	<-10	0.681	0.686	Mutual exclusivity
LGALS9C	17p11.2	0 (0.00%)	1 (0.41%)	<-10	0.681	0.686	Mutual exclusivity
LGALSL	2p14	0 (0.00%)	1 (0.41%)	<-10	0.681	0.686	Mutual exclusivity
LHFPL2	5q14.1	0 (0.00%)	1 (0.41%)	<-10	0.681	0.686	Mutual exclusivity
LIMS1	2q12.3	0 (0.00%)	1 (0.41%)	<-10	0.681	0.686	Mutual exclusivity
LIMS3	2q13	0 (0.00%)	1 (0.41%)	<-10	0.681	0.686	Mutual exclusivity
LIMS4	2q13	0 (0.00%)	1 (0.41%)	<-10	0.681	0.686	Mutual exclusivity
LIN28A	1p36.11	0 (0.00%)	1 (0.41%)	<-10	0.681	0.686	Mutual exclusivity
LINC00114	21q22.2	0 (0.00%)	1 (0.41%)	<-10	0.681	0.686	Mutual exclusivity
LINC00163	21q22.3	0 (0.00%)	1 (0.41%)	<-10	0.681	0.686	Mutual exclusivity
LINC00205	21q22.3	0 (0.00%)	1 (0.41%)	<-10	0.681	0.686	Mutual exclusivity
LINC00242	6q27	0 (0.00%)	1 (0.41%)	<-10	0.681	0.686	Mutual exclusivity
LINC00271	6q23.3	0 (0.00%)	1 (0.41%)	<-10	0.681	0.686	Mutual exclusivity
LINC00290	4q34.3	0 (0.00%)	1 (0.41%)	<-10	0.681	0.686	Mutual exclusivity
LINC00305	18q22.1	0 (0.00%)	1 (0.41%)	<-10	0.681	0.686	Mutual exclusivity
LINC00309	2p14	0 (0.00%)	1 (0.41%)	<-10	0.681	0.686	Mutual exclusivity
LINC00315	21q22.3	0 (0.00%)	1 (0.41%)	<-10	0.681	0.686	Mutual exclusivity
LINC00316	21q22.3	0 (0.00%)	1 (0.41%)	<-10	0.681	0.686	Mutual exclusivity

LINC00334	21q22.3	0 (0.00%)	1 (0.41%)	<-10	0.681	0.686	Mutual exclusivity
LINC00339	1p36.12	0 (0.00%)	1 (0.41%)	<-10	0.681	0.686	Mutual exclusivity
LINC00341	14q32.13	0 (0.00%)	1 (0.41%)	<-10	0.681	0.686	Mutual exclusivity
LINC00423	13q13.1	0 (0.00%)	1 (0.41%)	<-10	0.681	0.686	Mutual exclusivity
LINC00457	13q13.2	0 (0.00%)	1 (0.41%)	<-10	0.681	0.686	Mutual exclusivity
LINC00461	5q14.3	0 (0.00%)	1 (0.41%)	<-10	0.681	0.686	Mutual exclusivity
LINC00484	9q22.2	0 (0.00%)	1 (0.41%)	<-10	0.681	0.686	Mutual exclusivity
LINC00494	20q13.13	0 (0.00%)	1 (0.41%)	<-10	0.681	0.686	Mutual exclusivity
LINC00520	14q22.3	0 (0.00%)	1 (0.41%)	<-10	0.681	0.686	Mutual exclusivity
LINC00574	6q27	0 (0.00%)	1 (0.41%)	<-10	0.681	0.686	Mutual exclusivity
LINC00583	9p23	0 (0.00%)	1 (0.41%)	<-10	0.681	0.686	Mutual exclusivity
LINC00601	10q26.2	0 (0.00%)	1 (0.41%)	<-10	0.681	0.686	Mutual exclusivity
LINC00608	2q35	0 (0.00%)	1 (0.41%)	<-10	0.681	0.686	Mutual exclusivity
LINC00635	3q13.12	0 (0.00%)	1 (0.41%)	<-10	0.681	0.686	Mutual exclusivity
LINC00636	3q13.12	0 (0.00%)	1 (0.41%)	<-10	0.681	0.686	Mutual exclusivity
LINC00642	14q32.11	0 (0.00%)	1 (0.41%)	<-10	0.681	0.686	Mutual exclusivity
LINC00656	20p11.21	0 (0.00%)	1 (0.41%)	<-10	0.681	0.686	Mutual exclusivity
LINC00849	10q21.3	0 (0.00%)	1 (0.41%)	<-10	0.681	0.686	Mutual exclusivity
LINC00851	20p11.23	0 (0.00%)	1 (0.41%)	<-10	0.681	0.686	Mutual exclusivity
LINC00863	10q23.2	0 (0.00%)	1 (0.41%)	<-10	0.681	0.686	Mutual exclusivity
LINC00865	10q23.31	0 (0.00%)	1 (0.41%)	<-10	0.681	0.686	Mutual exclusivity
LINC00866	10q24.2	0 (0.00%)	1 (0.41%)	<-10	0.681	0.686	Mutual exclusivity
LINC00882	3q13.12	0 (0.00%)	1 (0.41%)	<-10	0.681	0.686	Mutual exclusivity
LINC00900	11q23.3	0 (0.00%)	1 (0.41%)	<-10	0.681	0.686	Mutual exclusivity
LINC00909	18q22.3	0 (0.00%)	1 (0.41%)	<-10	0.681	0.686	Mutual exclusivity
LINC00940	12p13.33	0 (0.00%)	1 (0.41%)	<-10	0.681	0.686	Mutual exclusivity
LINC00942	12p13.33	0 (0.00%)	1 (0.41%)	<-10	0.681	0.686	Mutual exclusivity
LINC00973	-	0 (0.00%)	1 (0.41%)	<-10	0.681	0.686	Mutual exclusivity
LINC01547	21q22.3	0 (0.00%)	1 (0.41%)	<-10	0.681	0.686	Mutual exclusivity

LINC01548	21q22.11	0 (0.00%)	1 (0.41%)	<-10	0.681	0.686	Mutual exclusivity
LINC01549	21q21.1	0 (0.00%)	1 (0.41%)	<-10	0.681	0.686	Mutual exclusivity
LINC01551	14q12	0 (0.00%)	1 (0.41%)	<-10	0.681	0.686	Mutual exclusivity
LINC01558	6q27	0 (0.00%)	1 (0.41%)	<-10	0.681	0.686	Mutual exclusivity
LINC01590	6q15	0 (0.00%)	1 (0.41%)	<-10	0.681	0.686	Mutual exclusivity
LIPF	10q23.31	0 (0.00%)	1 (0.41%)	<-10	0.681	0.686	Mutual exclusivity
LIPK	10q23.31	0 (0.00%)	1 (0.41%)	<-10	0.681	0.686	Mutual exclusivity
LIPM	10q23.31	0 (0.00%)	1 (0.41%)	<-10	0.681	0.686	Mutual exclusivity
LIPN	10q23.31	0 (0.00%)	1 (0.41%)	<-10	0.681	0.686	Mutual exclusivity
LIPT1	2q11.2	0 (0.00%)	1 (0.41%)	<-10	0.681	0.686	Mutual exclusivity
LIX1	5q15	0 (0.00%)	1 (0.41%)	<-10	0.681	0.686	Mutual exclusivity
LMAN2L	2q11.2	0 (0.00%)	1 (0.41%)	<-10	0.681	0.686	Mutual exclusivity
LMBR1L	12q13.12	0 (0.00%)	1 (0.41%)	<-10	0.681	0.686	Mutual exclusivity
LMBRD1	6q13	0 (0.00%)	1 (0.41%)	<-10	0.681	0.686	Mutual exclusivity
LMTK3	19q13.33	0 (0.00%)	1 (0.41%)	<-10	0.681	0.686	Mutual exclusivity
LNP1	3q12.2	0 (0.00%)	1 (0.41%)	<-10	0.681	0.686	Mutual exclusivity
LNPEP	5q15	0 (0.00%)	1 (0.41%)	<-10	0.681	0.686	Mutual exclusivity
LNX1	4q12	0 (0.00%)	1 (0.41%)	<-10	0.681	0.686	Mutual exclusivity
LOC105379831		0 (0.00%)	1 (0.41%)	<-10	0.681	0.686	Mutual exclusivity
LOH12CR2	12p13.2	0 (0.00%)	1 (0.41%)	<-10	0.681	0.686	Mutual exclusivity
LONP1	19p13.3	0 (0.00%)	1 (0.41%)	<-10	0.681	0.686	Mutual exclusivity
LONRF2	2q11.2	0 (0.00%)	1 (0.41%)	<-10	0.681	0.686	Mutual exclusivity
LOXHD1	18q21.1	0 (0.00%)	1 (0.41%)	<-10	0.681	0.686	Mutual exclusivity
LOXL2	8p21.3	0 (0.00%)	1 (0.41%)	<-10	0.681	0.686	Mutual exclusivity
LOXL4	10q24.2	0 (0.00%)	1 (0.41%)	<-10	0.681	0.686	Mutual exclusivity
LPA	6q25.3-q26	0 (0.00%)	1 (0.41%)	<-10	0.681	0.686	Mutual exclusivity
LPAL2	6q25.3	0 (0.00%)	1 (0.41%)	<-10	0.681	0.686	Mutual exclusivity
LRBA	4q31.3	0 (0.00%)	1 (0.41%)	<-10	0.681	0.686	Mutual exclusivity
LRFN5	14q21.1	0 (0.00%)	1 (0.41%)	<-10	0.681	0.686	Mutual exclusivity

LRP11	6q25.1	0 (0.00%)	1 (0.41%)	<-10	0.681	0.686	Mutual exclusivity
LRP1B	2q22.1-q22.2	0 (0.00%)	1 (0.41%)	<-10	0.681	0.686	Mutual exclusivity
LRRC3	21q22.3	0 (0.00%)	1 (0.41%)	<-10	0.681	0.686	Mutual exclusivity
LRRC3DN	21q22.3	0 (0.00%)	1 (0.41%)	<-10	0.681	0.686	Mutual exclusivity
LRRC55	11q12.1	0 (0.00%)	1 (0.41%)	<-10	0.681	0.686	Mutual exclusivity
LRRC9	14q23.1	0 (0.00%)	1 (0.41%)	<-10	0.681	0.686	Mutual exclusivity
LRRN4CL	11q12.3	0 (0.00%)	1 (0.41%)	<-10	0.681	0.686	Mutual exclusivity
LRTM2	12p13.33	0 (0.00%)	1 (0.41%)	<-10	0.681	0.686	Mutual exclusivity
LSS	21q22.3	0 (0.00%)	1 (0.41%)	<-10	0.681	0.686	Mutual exclusivity
LTV1	6q24.2	0 (0.00%)	1 (0.41%)	<-10	0.681	0.686	Mutual exclusivity
LUCAT1	5q14.3	0 (0.00%)	1 (0.41%)	<-10	0.681	0.686	Mutual exclusivity
LURAP1L	9p23	0 (0.00%)	1 (0.41%)	<-10	0.681	0.686	Mutual exclusivity
LUZP1	1p36.12	0 (0.00%)	1 (0.41%)	<-10	0.681	0.686	Mutual exclusivity
LYG1	2q11.2	0 (0.00%)	1 (0.41%)	<-10	0.681	0.686	Mutual exclusivity
LYG2	2q11.2	0 (0.00%)	1 (0.41%)	<-10	0.681	0.686	Mutual exclusivity
LYL1	19p13.13	0 (0.00%)	1 (0.41%)	<-10	0.681	0.686	Mutual exclusivity
LYPD4	19q13.2	0 (0.00%)	1 (0.41%)	<-10	0.681	0.686	Mutual exclusivity
LYPLA2	1p36.11	0 (0.00%)	1 (0.41%)	<-10	0.681	0.686	Mutual exclusivity
LYRM1	16p12.3	0 (0.00%)	1 (0.41%)	<-10	0.681	0.686	Mutual exclusivity
LZTS3	20p13	0 (0.00%)	1 (0.41%)	<-10	0.681	0.686	Mutual exclusivity
MACO1	1p36.11|1p36.11	0 (0.00%)	1 (0.41%)	<-10	0.681	0.686	Mutual exclusivity
MAD2L1	4q27	0 (0.00%)	1 (0.41%)	<-10	0.681	0.686	Mutual exclusivity
MAGED1	Xp11.22	0 (0.00%)	1 (0.41%)	<-10	0.681	0.686	Mutual exclusivity
MAGED4	Xp11.22	0 (0.00%)	1 (0.41%)	<-10	0.681	0.686	Mutual exclusivity
MAGED4B	Xp11.22	0 (0.00%)	1 (0.41%)	<-10	0.681	0.686	Mutual exclusivity
MALL	2q13	0 (0.00%)	1 (0.41%)	<-10	0.681	0.686	Mutual exclusivity
MALT1	18q21.32	0 (0.00%)	1 (0.41%)	<-10	0.681	0.686	Mutual exclusivity
MAML2	11q21	0 (0.00%)	1 (0.41%)	<-10	0.681	0.686	Mutual exclusivity
MAOA	Xp11.3	0 (0.00%)	1 (0.41%)	<-10	0.681	0.686	Mutual exclusivity

MAOB	Xp11.3	0 (0.00%)	1 (0.41%)	<-10	0.681	0.686	Mutual exclusivity
MAP3K1	5q11.2	0 (0.00%)	1 (0.41%)	<-10	0.681	0.686	Mutual exclusivity
MAP3K19	2q21.3	0 (0.00%)	1 (0.41%)	<-10	0.681	0.686	Mutual exclusivity
MAP3K5	6q23.3	0 (0.00%)	1 (0.41%)	<-10	0.681	0.686	Mutual exclusivity
MAP7	6q23.3	0 (0.00%)	1 (0.41%)	<-10	0.681	0.686	Mutual exclusivity
MAPK1	22q11.22	0 (0.00%)	1 (0.41%)	<-10	0.681	0.686	Mutual exclusivity
MAPK1IP1L	14q22.3	0 (0.00%)	1 (0.41%)	<-10	0.681	0.686	Mutual exclusivity
MAPRE1	20q11.21	0 (0.00%)	1 (0.41%)	<-10	0.681	0.686	Mutual exclusivity
MAPRE2	18q12.1-q12.2	0 (0.00%)	1 (0.41%)	<-10	0.681	0.686	Mutual exclusivity
4-Mar	2q35	0 (0.00%)	1 (0.41%)	<-10	0.681	0.686	Mutual exclusivity
5-Mar	10q23.32-q23.33	0 (0.00%)	1 (0.41%)	<-10	0.681	0.686	Mutual exclusivity
MARCO	2q14.2	0 (0.00%)	1 (0.41%)	<-10	0.681	0.686	Mutual exclusivity
MARK2	11q13.1	0 (0.00%)	1 (0.41%)	<-10	0.681	0.686	Mutual exclusivity
MARVELD1	10q24.2	0 (0.00%)	1 (0.41%)	<-10	0.681	0.686	Mutual exclusivity
MAS1	6q25.3	0 (0.00%)	1 (0.41%)	<-10	0.681	0.686	Mutual exclusivity
MAVS	20p13	0 (0.00%)	1 (0.41%)	<-10	0.681	0.686	Mutual exclusivity
MBD3L2	19p13.2	0 (0.00%)	1 (0.41%)	<-10	0.681	0.686	Mutual exclusivity
MBD3L3	19p13.2	0 (0.00%)	1 (0.41%)	<-10	0.681	0.686	Mutual exclusivity
MBD3L4	19p13.2	0 (0.00%)	1 (0.41%)	<-10	0.681	0.686	Mutual exclusivity
MBD3L5	19p13.2	0 (0.00%)	1 (0.41%)	<-10	0.681	0.686	Mutual exclusivity
MCM3AP	21q22.3	0 (0.00%)	1 (0.41%)	<-10	0.681	0.686	Mutual exclusivity
MED17	11q21	0 (0.00%)	1 (0.41%)	<-10	0.681	0.686	Mutual exclusivity
MED18	1p35.3	0 (0.00%)	1 (0.41%)	<-10	0.681	0.686	Mutual exclusivity
MED19	11q12.1	0 (0.00%)	1 (0.41%)	<-10	0.681	0.686	Mutual exclusivity
MED8	1p34.2	0 (0.00%)	1 (0.41%)	<-10	0.681	0.686	Mutual exclusivity
MED9	17p11.2	0 (0.00%)	1 (0.41%)	<-10	0.681	0.686	Mutual exclusivity
MEF2C	5q14.3	0 (0.00%)	1 (0.41%)	<-10	0.681	0.686	Mutual exclusivity
MEIS1	2p14	0 (0.00%)	1 (0.41%)	<-10	0.681	0.686	Mutual exclusivity
MEPE	4q22.1	0 (0.00%)	1 (0.41%)	<-10	0.681	0.686	Mutual exclusivity

MGAT4A	2q11.2	0 (0.00%)	1 (0.41%)	<-10	0.681	0.686	Mutual exclusivity
MICAL1	6q21	0 (0.00%)	1 (0.41%)	<-10	0.681	0.686	Mutual exclusivity
MICOS10	1p36.13	0 (0.00%)	1 (0.41%)	<-10	0.681	0.686	Mutual exclusivity
MICOS13	19p13.3	0 (0.00%)	1 (0.41%)	<-10	0.681	0.686	Mutual exclusivity
MIER3	5q11.2	0 (0.00%)	1 (0.41%)	<-10	0.681	0.686	Mutual exclusivity
MIR-1181/1181		0 (0.00%)	1 (0.41%)	<-10	0.681	0.686	Mutual exclusivity
MIR-1202/1202		0 (0.00%)	1 (0.41%)	<-10	0.681	0.686	Mutual exclusivity
MIR-1260B/1260B		0 (0.00%)	1 (0.41%)	<-10	0.681	0.686	Mutual exclusivity
MIR-1261/1261		0 (0.00%)	1 (0.41%)	<-10	0.681	0.686	Mutual exclusivity
MIR-1273C/1273C		0 (0.00%)	1 (0.41%)	<-10	0.681	0.686	Mutual exclusivity
MIR-1280/1280		0 (0.00%)	1 (0.41%)	<-10	0.681	0.686	Mutual exclusivity
MIR-1290/1290		0 (0.00%)	1 (0.41%)	<-10	0.681	0.686	Mutual exclusivity
MIR-1539/1539		0 (0.00%)	1 (0.41%)	<-10	0.681	0.686	Mutual exclusivity
MIR-1825/1825		0 (0.00%)	1 (0.41%)	<-10	0.681	0.686	Mutual exclusivity
MIR-1976/1976		0 (0.00%)	1 (0.41%)	<-10	0.681	0.686	Mutual exclusivity
MIR-3115/3115		0 (0.00%)	1 (0.41%)	<-10	0.681	0.686	Mutual exclusivity
MIR-3131/3131		0 (0.00%)	1 (0.41%)	<-10	0.681	0.686	Mutual exclusivity
MIR-3132/3132		0 (0.00%)	1 (0.41%)	<-10	0.681	0.686	Mutual exclusivity
MIR-3133/3133		0 (0.00%)	1 (0.41%)	<-10	0.681	0.686	Mutual exclusivity
MIR-3166/3166		0 (0.00%)	1 (0.41%)	<-10	0.681	0.686	Mutual exclusivity
MIR-346/346		0 (0.00%)	1 (0.41%)	<-10	0.681	0.686	Mutual exclusivity
MIR-3649/3649		0 (0.00%)	1 (0.41%)	<-10	0.681	0.686	Mutual exclusivity
MIR-3660/3660		0 (0.00%)	1 (0.41%)	<-10	0.681	0.686	Mutual exclusivity
MIR-378F/378F		0 (0.00%)	1 (0.41%)	<-10	0.681	0.686	Mutual exclusivity
MIR-3918/3918		0 (0.00%)	1 (0.41%)	<-10	0.681	0.686	Mutual exclusivity
MIR-3921/3921		0 (0.00%)	1 (0.41%)	<-10	0.681	0.686	Mutual exclusivity
MIR-3929/3929		0 (0.00%)	1 (0.41%)	<-10	0.681	0.686	Mutual exclusivity
MIR-3939/3939		0 (0.00%)	1 (0.41%)	<-10	0.681	0.686	Mutual exclusivity
MIR-3941/3941		0 (0.00%)	1 (0.41%)	<-10	0.681	0.686	Mutual exclusivity

MIR-4253/4253		0 (0.00%)	1 (0.41%)	<-10	0.681	0.686	Mutual exclusivity
MIR-4265/4265		0 (0.00%)	1 (0.41%)	<-10	0.681	0.686	Mutual exclusivity
MIR-4266/4266		0 (0.00%)	1 (0.41%)	<-10	0.681	0.686	Mutual exclusivity
MIR-4267/4267		0 (0.00%)	1 (0.41%)	<-10	0.681	0.686	Mutual exclusivity
MIR-4268/4268		0 (0.00%)	1 (0.41%)	<-10	0.681	0.686	Mutual exclusivity
MIR-4269/4269		0 (0.00%)	1 (0.41%)	<-10	0.681	0.686	Mutual exclusivity
MIR-4280/4280		0 (0.00%)	1 (0.41%)	<-10	0.681	0.686	Mutual exclusivity
MIR-4288/4288		0 (0.00%)	1 (0.41%)	<-10	0.681	0.686	Mutual exclusivity
MIR-4290/4290		0 (0.00%)	1 (0.41%)	<-10	0.681	0.686	Mutual exclusivity
MIR-4296/4296		0 (0.00%)	1 (0.41%)	<-10	0.681	0.686	Mutual exclusivity
MIR-4300/4300		0 (0.00%)	1 (0.41%)	<-10	0.681	0.686	Mutual exclusivity
MIR-4318/4318		0 (0.00%)	1 (0.41%)	<-10	0.681	0.686	Mutual exclusivity
MIR-4319/4319		0 (0.00%)	1 (0.41%)	<-10	0.681	0.686	Mutual exclusivity
MIR-4322/4322		0 (0.00%)	1 (0.41%)	<-10	0.681	0.686	Mutual exclusivity
MIR-4323/4323		0 (0.00%)	1 (0.41%)	<-10	0.681	0.686	Mutual exclusivity
MIR-4418/4418		0 (0.00%)	1 (0.41%)	<-10	0.681	0.686	Mutual exclusivity
MIR-4419A/4419A		0 (0.00%)	1 (0.41%)	<-10	0.681	0.686	Mutual exclusivity
MIR-4431/4431		0 (0.00%)	1 (0.41%)	<-10	0.681	0.686	Mutual exclusivity
MIR-4432/4432		0 (0.00%)	1 (0.41%)	<-10	0.681	0.686	Mutual exclusivity
MIR-4438/4438		0 (0.00%)	1 (0.41%)	<-10	0.681	0.686	Mutual exclusivity
MIR-4439/4439		0 (0.00%)	1 (0.41%)	<-10	0.681	0.686	Mutual exclusivity
MIR-4440/4440		0 (0.00%)	1 (0.41%)	<-10	0.681	0.686	Mutual exclusivity
MIR-4441/4441		0 (0.00%)	1 (0.41%)	<-10	0.681	0.686	Mutual exclusivity
MIR-4453/4453		0 (0.00%)	1 (0.41%)	<-10	0.681	0.686	Mutual exclusivity
MIR-4459/4459		0 (0.00%)	1 (0.41%)	<-10	0.681	0.686	Mutual exclusivity
MIR-4463/4463		0 (0.00%)	1 (0.41%)	<-10	0.681	0.686	Mutual exclusivity
MIR-4466/4466		0 (0.00%)	1 (0.41%)	<-10	0.681	0.686	Mutual exclusivity
MIR-448/448		0 (0.00%)	1 (0.41%)	<-10	0.681	0.686	Mutual exclusivity
MIR-4484/4484		0 (0.00%)	1 (0.41%)	<-10	0.681	0.686	Mutual exclusivity

MIR-4490/4490		0 (0.00%)	1 (0.41%)	<-10	0.681	0.686	Mutual exclusivity
MIR-4519/4519		0 (0.00%)	1 (0.41%)	<-10	0.681	0.686	Mutual exclusivity
MIR-4692/4692		0 (0.00%)	1 (0.41%)	<-10	0.681	0.686	Mutual exclusivity
MIR-4698/4698		0 (0.00%)	1 (0.41%)	<-10	0.681	0.686	Mutual exclusivity
MIR-5092/5092		0 (0.00%)	1 (0.41%)	<-10	0.681	0.686	Mutual exclusivity
MIR-5100/5100		0 (0.00%)	1 (0.41%)	<-10	0.681	0.686	Mutual exclusivity
MIR-548AB/548AB		0 (0.00%)	1 (0.41%)	<-10	0.681	0.686	Mutual exclusivity
MIR-548AC/548AC		0 (0.00%)	1 (0.41%)	<-10	0.681	0.686	Mutual exclusivity
MIR-548AI/548AI		0 (0.00%)	1 (0.41%)	<-10	0.681	0.686	Mutual exclusivity
MIR-548J/548J		0 (0.00%)	1 (0.41%)	<-10	0.681	0.686	Mutual exclusivity
MIR-548L/548L		0 (0.00%)	1 (0.41%)	<-10	0.681	0.686	Mutual exclusivity
MIR-5694/5694		0 (0.00%)	1 (0.41%)	<-10	0.681	0.686	Mutual exclusivity
MIR-5702/5702		0 (0.00%)	1 (0.41%)	<-10	0.681	0.686	Mutual exclusivity
MIR-581/581		0 (0.00%)	1 (0.41%)	<-10	0.681	0.686	Mutual exclusivity
MIR-607/607		0 (0.00%)	1 (0.41%)	<-10	0.681	0.686	Mutual exclusivity
MIR-610/610		0 (0.00%)	1 (0.41%)	<-10	0.681	0.686	Mutual exclusivity
MIR-613/613		0 (0.00%)	1 (0.41%)	<-10	0.681	0.686	Mutual exclusivity
MIR-614/614		0 (0.00%)	1 (0.41%)	<-10	0.681	0.686	Mutual exclusivity
MIR-638/638		0 (0.00%)	1 (0.41%)	<-10	0.681	0.686	Mutual exclusivity
MIR-650/650		0 (0.00%)	1 (0.41%)	<-10	0.681	0.686	Mutual exclusivity
MIR-762/762		0 (0.00%)	1 (0.41%)	<-10	0.681	0.686	Mutual exclusivity
MIR-764/764		0 (0.00%)	1 (0.41%)	<-10	0.681	0.686	Mutual exclusivity
MIS18BP1	14q21.2	0 (0.00%)	1 (0.41%)	<-10	0.681	0.686	Mutual exclusivity
MITD1	2q11.2	0 (0.00%)	1 (0.41%)	<-10	0.681	0.686	Mutual exclusivity
MKKS	20p12.2	0 (0.00%)	1 (0.41%)	<-10	0.681	0.686	Mutual exclusivity
MLH3	14q24.3	0 (0.00%)	1 (0.41%)	<-10	0.681	0.686	Mutual exclusivity
MMP21	10q26.2	0 (0.00%)	1 (0.41%)	<-10	0.681	0.686	Mutual exclusivity
MMP9	20q13.12	0 (0.00%)	1 (0.41%)	<-10	0.681	0.686	Mutual exclusivity
MMRN1	4q22.1	0 (0.00%)	1 (0.41%)	<-10	0.681	0.686	Mutual exclusivity

MMS19	10q24.1	0 (0.00%)	1 (0.41%)	<-10	0.681	0.686	Mutual exclusivity
MOCOS	18q12.2	0 (0.00%)	1 (0.41%)	<-10	0.681	0.686	Mutual exclusivity
MOCS2	5q11.2	0 (0.00%)	1 (0.41%)	<-10	0.681	0.686	Mutual exclusivity
MOGAT1	2q36.1	0 (0.00%)	1 (0.41%)	<-10	0.681	0.686	Mutual exclusivity
MORC3	21q22.12	0 (0.00%)	1 (0.41%)	<-10	0.681	0.686	Mutual exclusivity
MORF4L2	Xq22.2	0 (0.00%)	1 (0.41%)	<-10	0.681	0.686	Mutual exclusivity
MORN4	10q24.2	0 (0.00%)	1 (0.41%)	<-10	0.681	0.686	Mutual exclusivity
MPDZ	9p23	0 (0.00%)	1 (0.41%)	<-10	0.681	0.686	Mutual exclusivity
MPP5	14q23.3	0 (0.00%)	1 (0.41%)	<-10	0.681	0.686	Mutual exclusivity
MPZL2	11q23.3	0 (0.00%)	1 (0.41%)	<-10	0.681	0.686	Mutual exclusivity
MPZL3	11q23.3	0 (0.00%)	1 (0.41%)	<-10	0.681	0.686	Mutual exclusivity
MRE11	11q21	0 (0.00%)	1 (0.41%)	<-10	0.681	0.686	Mutual exclusivity
MRPL16	11q12.1	0 (0.00%)	1 (0.41%)	<-10	0.681	0.686	Mutual exclusivity
MRPL18	6q25.3	0 (0.00%)	1 (0.41%)	<-10	0.681	0.686	Mutual exclusivity
MRPL30	2q11.2	0 (0.00%)	1 (0.41%)	<-10	0.681	0.686	Mutual exclusivity
MRPL4	19p13.2	0 (0.00%)	1 (0.41%)	<-10	0.681	0.686	Mutual exclusivity
MRPL44	2q36.1	0 (0.00%)	1 (0.41%)	<-10	0.681	0.686	Mutual exclusivity
MRPL48	11q13.4	0 (0.00%)	1 (0.41%)	<-10	0.681	0.686	Mutual exclusivity
MRPL50	9q31.1	0 (0.00%)	1 (0.41%)	<-10	0.681	0.686	Mutual exclusivity
MRPS26	20p13	0 (0.00%)	1 (0.41%)	<-10	0.681	0.686	Mutual exclusivity
MRPS9	2q12.1	0 (0.00%)	1 (0.41%)	<-10	0.681	0.686	Mutual exclusivity
MRTO4	1p36.13	0 (0.00%)	1 (0.41%)	<-10	0.681	0.686	Mutual exclusivity
MS4A14	11q12.2	0 (0.00%)	1 (0.41%)	<-10	0.681	0.686	Mutual exclusivity
MS4A2	11q12.1	0 (0.00%)	1 (0.41%)	<-10	0.681	0.686	Mutual exclusivity
MS4A3	11q12.1	0 (0.00%)	1 (0.41%)	<-10	0.681	0.686	Mutual exclusivity
MS4A4A	11q12.2	0 (0.00%)	1 (0.41%)	<-10	0.681	0.686	Mutual exclusivity
MS4A4E	11q12.2	0 (0.00%)	1 (0.41%)	<-10	0.681	0.686	Mutual exclusivity
MS4A6A	11q12.2	0 (0.00%)	1 (0.41%)	<-10	0.681	0.686	Mutual exclusivity
MS4A6E	11q12.2	0 (0.00%)	1 (0.41%)	<-10	0.681	0.686	Mutual exclusivity

MS4A7	11q12.2	0 (0.00%)	1 (0.41%)	<-10	0.681	0.686	Mutual exclusivity
MSH3	5q14.1	0 (0.00%)	1 (0.41%)	<-10	0.681	0.686	Mutual exclusivity
MSL3P1	2q37.1	0 (0.00%)	1 (0.41%)	<-10	0.681	0.686	Mutual exclusivity
MSR1	8p22	0 (0.00%)	1 (0.41%)	<-10	0.681	0.686	Mutual exclusivity
MTERF4	2q37.3	0 (0.00%)	1 (0.41%)	<-10	0.681	0.686	Mutual exclusivity
MTFR1L	1p36.11	0 (0.00%)	1 (0.41%)	<-10	0.681	0.686	Mutual exclusivity
MTHFD1L	6q25.1	0 (0.00%)	1 (0.41%)	<-10	0.681	0.686	Mutual exclusivity
MTIF2	2p16.1	0 (0.00%)	1 (0.41%)	<-10	0.681	0.686	Mutual exclusivity
MTLN	2q13	0 (0.00%)	1 (0.41%)	<-10	0.681	0.686	Mutual exclusivity
MTMR2	11q21	0 (0.00%)	1 (0.41%)	<-10	0.681	0.686	Mutual exclusivity
MTNR1B	11q14.3	0 (0.00%)	1 (0.41%)	<-10	0.681	0.686	Mutual exclusivity
MTRF1L	6q25.2	0 (0.00%)	1 (0.41%)	<-10	0.681	0.686	Mutual exclusivity
MTRNR2L2	5q14.1	0 (0.00%)	1 (0.41%)	<-10	0.681	0.686	Mutual exclusivity
MUL1	1p36.12	0 (0.00%)	1 (0.41%)	<-10	0.681	0.686	Mutual exclusivity
MYCT1	6q25.2	0 (0.00%)	1 (0.41%)	<-10	0.681	0.686	Mutual exclusivity
MYH15	3q13.13	0 (0.00%)	1 (0.41%)	<-10	0.681	0.686	Mutual exclusivity
MYL1	2q34	0 (0.00%)	1 (0.41%)	<-10	0.681	0.686	Mutual exclusivity
MYLK	3q21.1	0 (0.00%)	1 (0.41%)	<-10	0.681	0.686	Mutual exclusivity
N4BP1	16q12.1	0 (0.00%)	1 (0.41%)	<-10	0.681	0.686	Mutual exclusivity
N4BP2L1	13q13.1	0 (0.00%)	1 (0.41%)	<-10	0.681	0.686	Mutual exclusivity
N4BP2L2	13q13.1	0 (0.00%)	1 (0.41%)	<-10	0.681	0.686	Mutual exclusivity
NAA15	4q31.1	0 (0.00%)	1 (0.41%)	<-10	0.681	0.686	Mutual exclusivity
NAA30	14q22.3	0 (0.00%)	1 (0.41%)	<-10	0.681	0.686	Mutual exclusivity
NAA40	11q13.1	0 (0.00%)	1 (0.41%)	<-10	0.681	0.686	Mutual exclusivity
NACC1	19p13.13	0 (0.00%)	1 (0.41%)	<-10	0.681	0.686	Mutual exclusivity
NANOG	12p13.31	0 (0.00%)	1 (0.41%)	<-10	0.681	0.686	Mutual exclusivity
NANOGNB	12p13.31	0 (0.00%)	1 (0.41%)	<-10	0.681	0.686	Mutual exclusivity
NAP1L5	4q22.1|4q21-q22	0 (0.00%)	1 (0.41%)	<-10	0.681	0.686	Mutual exclusivity
NARS	18q21.31	0 (0.00%)	1 (0.41%)	<-10	0.681	0.686	Mutual exclusivity

NBPF22P	5q14.3	0 (0.00%)	1 (0.41%)	<-10	0.681	0.686	Mutual exclusivity
NCAPH	2q11.2	0 (0.00%)	1 (0.41%)	<-10	0.681	0.686	Mutual exclusivity
NCK2	2q12.2	0 (0.00%)	1 (0.41%)	<-10	0.681	0.686	Mutual exclusivity
NCKAP5	2q21.2	0 (0.00%)	1 (0.41%)	<-10	0.681	0.686	Mutual exclusivity
NCMAP	1p36.11	0 (0.00%)	1 (0.41%)	<-10	0.681	0.686	Mutual exclusivity
NCOA3	20q13.12	0 (0.00%)	1 (0.41%)	<-10	0.681	0.686	Mutual exclusivity
NCOA5	20q13.12	0 (0.00%)	1 (0.41%)	<-10	0.681	0.686	Mutual exclusivity
NDNF	4q27	0 (0.00%)	1 (0.41%)	<-10	0.681	0.686	Mutual exclusivity
NDUFA10	2q37.3	0 (0.00%)	1 (0.41%)	<-10	0.681	0.686	Mutual exclusivity
NDUFA11	19p13.3	0 (0.00%)	1 (0.41%)	<-10	0.681	0.686	Mutual exclusivity
NDUFA9	12p13.32	0 (0.00%)	1 (0.41%)	<-10	0.681	0.686	Mutual exclusivity
NDUFB1	14q32.12	0 (0.00%)	1 (0.41%)	<-10	0.681	0.686	Mutual exclusivity
NETO1	18q22.3	0 (0.00%)	1 (0.41%)	<-10	0.681	0.686	Mutual exclusivity
NEU2	2q37.1	0 (0.00%)	1 (0.41%)	<-10	0.681	0.686	Mutual exclusivity
NEU4	2q37.3	0 (0.00%)	1 (0.41%)	<-10	0.681	0.686	Mutual exclusivity
NEURL2	20q13.12	0 (0.00%)	1 (0.41%)	<-10	0.681	0.686	Mutual exclusivity
NEURL3	2q11.2	0 (0.00%)	1 (0.41%)	<-10	0.681	0.686	Mutual exclusivity
NFE2	12q13.13	0 (0.00%)	1 (0.41%)	<-10	0.681	0.686	Mutual exclusivity
NFIB	9p23-p22.3	0 (0.00%)	1 (0.41%)	<-10	0.681	0.686	Mutual exclusivity
NFRKB	11q24.3	0 (0.00%)	1 (0.41%)	<-10	0.681	0.686	Mutual exclusivity
NGEF	2q37.1	0 (0.00%)	1 (0.41%)	<-10	0.681	0.686	Mutual exclusivity
NGF	1p13.2	0 (0.00%)	1 (0.41%)	<-10	0.681	0.686	Mutual exclusivity
NHEJ1	2q35	0 (0.00%)	1 (0.41%)	<-10	0.681	0.686	Mutual exclusivity
NHSL1	6q24.1	0 (0.00%)	1 (0.41%)	<-10	0.681	0.686	Mutual exclusivity
NINL	20p11.21	0 (0.00%)	1 (0.41%)	<-10	0.681	0.686	Mutual exclusivity
NIPAL3	1p36.11	0 (0.00%)	1 (0.41%)	<-10	0.681	0.686	Mutual exclusivity
NIPSNAP3A	9q31.1	0 (0.00%)	1 (0.41%)	<-10	0.681	0.686	Mutual exclusivity
NIPSNAP3B	9q31.1	0 (0.00%)	1 (0.41%)	<-10	0.681	0.686	Mutual exclusivity
NIT2	3q12.2	0 (0.00%)	1 (0.41%)	<-10	0.681	0.686	Mutual exclusivity

NKAIN2	6q22.31	0 (0.00%)	1 (0.41%)	<-10	0.681	0.686	Mutual exclusivity
NLRX1	11q23.3	0 (0.00%)	1 (0.41%)	<-10	0.681	0.686	Mutual exclusivity
NMBR	6q24.1	0 (0.00%)	1 (0.41%)	<-10	0.681	0.686	Mutual exclusivity
NMS	2q11.2	0 (0.00%)	1 (0.41%)	<-10	0.681	0.686	Mutual exclusivity
NOB1	16q22.1	0 (0.00%)	1 (0.41%)	<-10	0.681	0.686	Mutual exclusivity
NOP56	20p13	0 (0.00%)	1 (0.41%)	<-10	0.681	0.686	Mutual exclusivity
NOX3	6q25.3	0 (0.00%)	1 (0.41%)	<-10	0.681	0.686	Mutual exclusivity
NOX4	11q14.3	0 (0.00%)	1 (0.41%)	<-10	0.681	0.686	Mutual exclusivity
NPAS2	2q11.2	0 (0.00%)	1 (0.41%)	<-10	0.681	0.686	Mutual exclusivity
NPAS3	14q13.1	0 (0.00%)	1 (0.41%)	<-10	0.681	0.686	Mutual exclusivity
NPHP1	2q13	0 (0.00%)	1 (0.41%)	<-10	0.681	0.686	Mutual exclusivity
NPS	10q26.2	0 (0.00%)	1 (0.41%)	<-10	0.681	0.686	Mutual exclusivity
NR0B2	1p36.11	0 (0.00%)	1 (0.41%)	<-10	0.681	0.686	Mutual exclusivity
NR2F1	5q15	0 (0.00%)	1 (0.41%)	<-10	0.681	0.686	Mutual exclusivity
NR3C2	4q31.23	0 (0.00%)	1 (0.41%)	<-10	0.681	0.686	Mutual exclusivity
NRG3	10q23.1	0 (0.00%)	1 (0.41%)	<-10	0.681	0.686	Mutual exclusivity
NRIP2	12p13.33	0 (0.00%)	1 (0.41%)	<-10	0.681	0.686	Mutual exclusivity
NRK	Xq22.3	0 (0.00%)	1 (0.41%)	<-10	0.681	0.686	Mutual exclusivity
NRN1L	16q22.1	0 (0.00%)	1 (0.41%)	<-10	0.681	0.686	Mutual exclusivity
NRTN	19p13.3	0 (0.00%)	1 (0.41%)	<-10	0.681	0.686	Mutual exclusivity
NRXN3	14q24.3-q31.1	0 (0.00%)	1 (0.41%)	<-10	0.681	0.686	Mutual exclusivity
NT5DC4	2q14.1	0 (0.00%)	1 (0.41%)	<-10	0.681	0.686	Mutual exclusivity
NT5E	6q14.3	0 (0.00%)	1 (0.41%)	<-10	0.681	0.686	Mutual exclusivity
NT5M	17p11.2	0 (0.00%)	1 (0.41%)	<-10	0.681	0.686	Mutual exclusivity
NTF3	12p13.31	0 (0.00%)	1 (0.41%)	<-10	0.681	0.686	Mutual exclusivity
NTM	11q25	0 (0.00%)	1 (0.41%)	<-10	0.681	0.686	Mutual exclusivity
NTN5	19q13.33	0 (0.00%)	1 (0.41%)	<-10	0.681	0.686	Mutual exclusivity
NUDC	1p36.11	0 (0.00%)	1 (0.41%)	<-10	0.681	0.686	Mutual exclusivity
NUDT11	Xp11.22	0 (0.00%)	1 (0.41%)	<-10	0.681	0.686	Mutual exclusivity

NUDT14	14q32.33	0 (0.00%)	1 (0.41%)	<-10	0.681	0.686	Mutual exclusivity
NUDT9	4q22.1	0 (0.00%)	1 (0.41%)	<-10	0.681	0.686	Mutual exclusivity
NUP210P1	3q21.3	0 (0.00%)	1 (0.41%)	<-10	0.681	0.686	Mutual exclusivity
NUP43	6q25.1	0 (0.00%)	1 (0.41%)	<-10	0.681	0.686	Mutual exclusivity
NUP93	16q13	0 (0.00%)	1 (0.41%)	<-10	0.681	0.686	Mutual exclusivity
NUTF2	16q22.1	0 (0.00%)	1 (0.41%)	<-10	0.681	0.686	Mutual exclusivity
NUTM2A	10q23.2	0 (0.00%)	1 (0.41%)	<-10	0.681	0.686	Mutual exclusivity
NUTM2D	10q23.2	0 (0.00%)	1 (0.41%)	<-10	0.681	0.686	Mutual exclusivity
NXF1	11q12.3	0 (0.00%)	1 (0.41%)	<-10	0.681	0.686	Mutual exclusivity
NYAP2	2q36.3	0 (0.00%)	1 (0.41%)	<-10	0.681	0.686	Mutual exclusivity
OBSL1	2q35	0 (0.00%)	1 (0.41%)	<-10	0.681	0.686	Mutual exclusivity
OGT	Xq13.1	0 (0.00%)	1 (0.41%)	<-10	0.681	0.686	Mutual exclusivity
OLMALINC	10q24.31	0 (0.00%)	1 (0.41%)	<-10	0.681	0.686	Mutual exclusivity
OOSP1	11q12.1	0 (0.00%)	1 (0.41%)	<-10	0.681	0.686	Mutual exclusivity
OOSP2	11q12.1	0 (0.00%)	1 (0.41%)	<-10	0.681	0.686	Mutual exclusivity
OPRM1	6q25.2	0 (0.00%)	1 (0.41%)	<-10	0.681	0.686	Mutual exclusivity
OR10V1	11q12.1	0 (0.00%)	1 (0.41%)	<-10	0.681	0.686	Mutual exclusivity
OR13D1	9q31.1	0 (0.00%)	1 (0.41%)	<-10	0.681	0.686	Mutual exclusivity
OR5AK2	11q12.1	0 (0.00%)	1 (0.41%)	<-10	0.681	0.686	Mutual exclusivity
OR5AP2	11q12.1	0 (0.00%)	1 (0.41%)	<-10	0.681	0.686	Mutual exclusivity
OR5AR1	11q12.1	0 (0.00%)	1 (0.41%)	<-10	0.681	0.686	Mutual exclusivity
OR5H2	3q11.2	0 (0.00%)	1 (0.41%)	<-10	0.681	0.686	Mutual exclusivity
OR5H8	3q11.2	0 (0.00%)	1 (0.41%)	<-10	0.681	0.686	Mutual exclusivity
OR5K1	3q11.2	0 (0.00%)	1 (0.41%)	<-10	0.681	0.686	Mutual exclusivity
OR5K2	3q11.2	0 (0.00%)	1 (0.41%)	<-10	0.681	0.686	Mutual exclusivity
OR5K3	3q11.2	0 (0.00%)	1 (0.41%)	<-10	0.681	0.686	Mutual exclusivity
OR5K4	3q11.2	0 (0.00%)	1 (0.41%)	<-10	0.681	0.686	Mutual exclusivity
OR5M1	11q12.1	0 (0.00%)	1 (0.41%)	<-10	0.681	0.686	Mutual exclusivity
OR5M3	11q12.1	0 (0.00%)	1 (0.41%)	<-10	0.681	0.686	Mutual exclusivity

OR5M8	11q12.1	0 (0.00%)	1 (0.41%)	<-10	0.681	0.686	Mutual exclusivity
OR5M9	11q12.1	0 (0.00%)	1 (0.41%)	<-10	0.681	0.686	Mutual exclusivity
OR5R1	11q12.1	0 (0.00%)	1 (0.41%)	<-10	0.681	0.686	Mutual exclusivity
OR5T1	11q12.1	0 (0.00%)	1 (0.41%)	<-10	0.681	0.686	Mutual exclusivity
OR5T3	11q12.1	0 (0.00%)	1 (0.41%)	<-10	0.681	0.686	Mutual exclusivity
OR6B2	2q37.3	0 (0.00%)	1 (0.41%)	<-10	0.681	0.686	Mutual exclusivity
OR6B3	2q37.3	0 (0.00%)	1 (0.41%)	<-10	0.681	0.686	Mutual exclusivity
OR8J1	11q12.1	0 (0.00%)	1 (0.41%)	<-10	0.681	0.686	Mutual exclusivity
OR8U1	11q12.1	0 (0.00%)	1 (0.41%)	<-10	0.681	0.686	Mutual exclusivity
OR9G1	11q12.1	0 (0.00%)	1 (0.41%)	<-10	0.681	0.686	Mutual exclusivity
OR9G4	11q12.1	0 (0.00%)	1 (0.41%)	<-10	0.681	0.686	Mutual exclusivity
ORAI3	16p11.2	0 (0.00%)	1 (0.41%)	<-10	0.681	0.686	Mutual exclusivity
OSBP	11q12.1	0 (0.00%)	1 (0.41%)	<-10	0.681	0.686	Mutual exclusivity
OSBPL11	3q21.2	0 (0.00%)	1 (0.41%)	<-10	0.681	0.686	Mutual exclusivity
OSTC	4q25	0 (0.00%)	1 (0.41%)	<-10	0.681	0.686	Mutual exclusivity
OSTCP1	6q25.3	0 (0.00%)	1 (0.41%)	<-10	0.681	0.686	Mutual exclusivity
OTOS	2q37.3	0 (0.00%)	1 (0.41%)	<-10	0.681	0.686	Mutual exclusivity
OTP	5q14.1	0 (0.00%)	1 (0.41%)	<-10	0.681	0.686	Mutual exclusivity
OTUB1	11q13.1	0 (0.00%)	1 (0.41%)	<-10	0.681	0.686	Mutual exclusivity
OTUD3	1p36.13	0 (0.00%)	1 (0.41%)	<-10	0.681	0.686	Mutual exclusivity
OTX1	2p15	0 (0.00%)	1 (0.41%)	<-10	0.681	0.686	Mutual exclusivity
OTX2	14q22.3	0 (0.00%)	1 (0.41%)	<-10	0.681	0.686	Mutual exclusivity
OXT	20p13	0 (0.00%)	1 (0.41%)	<-10	0.681	0.686	Mutual exclusivity
P2RX3	11q12.1	0 (0.00%)	1 (0.41%)	<-10	0.681	0.686	Mutual exclusivity
P2RY11	19p13.2	0 (0.00%)	1 (0.41%)	<-10	0.681	0.686	Mutual exclusivity
P2RY6	11q13.4	0 (0.00%)	1 (0.41%)	<-10	0.681	0.686	Mutual exclusivity
P3H1	1p34.2	0 (0.00%)	1 (0.41%)	<-10	0.681	0.686	Mutual exclusivity
PAAF1	11q13.4	0 (0.00%)	1 (0.41%)	<-10	0.681	0.686	Mutual exclusivity
PABPC1L	20q13.12	0 (0.00%)	1 (0.41%)	<-10	0.681	0.686	Mutual exclusivity

PAFAH1B2	11q23.3	0 (0.00%)	1 (0.41%)	<-10	0.681	0.686	Mutual exclusivity
PANK1	10q23.31	0 (0.00%)	1 (0.41%)	<-10	0.681	0.686	Mutual exclusivity
PANK2	20p13	0 (0.00%)	1 (0.41%)	<-10	0.681	0.686	Mutual exclusivity
PANX1	11q21	0 (0.00%)	1 (0.41%)	<-10	0.681	0.686	Mutual exclusivity
PAPOLG	2p16.1	0 (0.00%)	1 (0.41%)	<-10	0.681	0.686	Mutual exclusivity
PAQR7	1p36.11	0 (0.00%)	1 (0.41%)	<-10	0.681	0.686	Mutual exclusivity
PARP11	12p13.32	0 (0.00%)	1 (0.41%)	<-10	0.681	0.686	Mutual exclusivity
PASK	2q37.3	0 (0.00%)	1 (0.41%)	<-10	0.681	0.686	Mutual exclusivity
PATL1	11q12.1	0 (0.00%)	1 (0.41%)	<-10	0.681	0.686	Mutual exclusivity
PAX3	2q36.1	0 (0.00%)	1 (0.41%)	<-10	0.681	0.686	Mutual exclusivity
PAX6	11p13	0 (0.00%)	1 (0.41%)	<-10	0.681	0.686	Mutual exclusivity
PAX7	1p36.13	0 (0.00%)	1 (0.41%)	<-10	0.681	0.686	Mutual exclusivity
PCBP3	21q22.3	0 (0.00%)	1 (0.41%)	<-10	0.681	0.686	Mutual exclusivity
PCDH7	4p15.1	0 (0.00%)	1 (0.41%)	<-10	0.681	0.686	Mutual exclusivity
PCED1A	20p13	0 (0.00%)	1 (0.41%)	<-10	0.681	0.686	Mutual exclusivity
PCED1B	12q13.11	0 (0.00%)	1 (0.41%)	<-10	0.681	0.686	Mutual exclusivity
PCF11	11q14.1	0 (0.00%)	1 (0.41%)	<-10	0.681	0.686	Mutual exclusivity
PCGF6	10q24.33	0 (0.00%)	1 (0.41%)	<-10	0.681	0.686	Mutual exclusivity
PCIF1	20q13.12	0 (0.00%)	1 (0.41%)	<-10	0.681	0.686	Mutual exclusivity
PCMT1	6q25.1	0 (0.00%)	1 (0.41%)	<-10	0.681	0.686	Mutual exclusivity
PCNT	21q22.3	0 (0.00%)	1 (0.41%)	<-10	0.681	0.686	Mutual exclusivity
PCSK7	11q23.3	0 (0.00%)	1 (0.41%)	<-10	0.681	0.686	Mutual exclusivity
PDCD1	2q37.3	0 (0.00%)	1 (0.41%)	<-10	0.681	0.686	Mutual exclusivity
PDCD2	6q27	0 (0.00%)	1 (0.41%)	<-10	0.681	0.686	Mutual exclusivity
PDCD4	10q25.2	0 (0.00%)	1 (0.41%)	<-10	0.681	0.686	Mutual exclusivity
PDCL3	2q11.2	0 (0.00%)	1 (0.41%)	<-10	0.681	0.686	Mutual exclusivity
PDE12	3p14.3	0 (0.00%)	1 (0.41%)	<-10	0.681	0.686	Mutual exclusivity
PDE4A	19p13.2	0 (0.00%)	1 (0.41%)	<-10	0.681	0.686	Mutual exclusivity
PDE8B	5q13.3	0 (0.00%)	1 (0.41%)	<-10	0.681	0.686	Mutual exclusivity

PDGFC	4q32.1	0 (0.00%)	1 (0.41%)	<-10	0.681	0.686	Mutual exclusivity
PDGFD	11q22.3	0 (0.00%)	1 (0.41%)	<-10	0.681	0.686	Mutual exclusivity
PDHX	11p13	0 (0.00%)	1 (0.41%)	<-10	0.681	0.686	Mutual exclusivity
PDIA5	3q21.1	0 (0.00%)	1 (0.41%)	<-10	0.681	0.686	Mutual exclusivity
PDPR	16q22.1	0 (0.00%)	1 (0.41%)	<-10	0.681	0.686	Mutual exclusivity
PDS5B	13q13.1	0 (0.00%)	1 (0.41%)	<-10	0.681	0.686	Mutual exclusivity
PDSS2	6q21	0 (0.00%)	1 (0.41%)	<-10	0.681	0.686	Mutual exclusivity
PDXDC2P-NPIP	16q22.1	0 (0.00%)	1 (0.41%)	<-10	0.681	0.686	Mutual exclusivity
PDZD3	11q23.3	0 (0.00%)	1 (0.41%)	<-10	0.681	0.686	Mutual exclusivity
PELI1	2p14	0 (0.00%)	1 (0.41%)	<-10	0.681	0.686	Mutual exclusivity
PELI2	14q22.3	0 (0.00%)	1 (0.41%)	<-10	0.681	0.686	Mutual exclusivity
PELO	5q11.2	0 (0.00%)	1 (0.41%)	<-10	0.681	0.686	Mutual exclusivity
PEMT	17p11.2	0 (0.00%)	1 (0.41%)	<-10	0.681	0.686	Mutual exclusivity
PEX13	2p15	0 (0.00%)	1 (0.41%)	<-10	0.681	0.686	Mutual exclusivity
PEX3	6q24.2	0 (0.00%)	1 (0.41%)	<-10	0.681	0.686	Mutual exclusivity
PEX5	12p13.31	0 (0.00%)	1 (0.41%)	<-10	0.681	0.686	Mutual exclusivity
PEX7	6q23.3	0 (0.00%)	1 (0.41%)	<-10	0.681	0.686	Mutual exclusivity
PFKL	21q22.3	0 (0.00%)	1 (0.41%)	<-10	0.681	0.686	Mutual exclusivity
PGAM1	10q24.1	0 (0.00%)	1 (0.41%)	<-10	0.681	0.686	Mutual exclusivity
PGF	14q24.3	0 (0.00%)	1 (0.41%)	<-10	0.681	0.686	Mutual exclusivity
PHACTR4	1p35.3	0 (0.00%)	1 (0.41%)	<-10	0.681	0.686	Mutual exclusivity
PHF10	6q27	0 (0.00%)	1 (0.41%)	<-10	0.681	0.686	Mutual exclusivity
PHGR1	15q15.1	0 (0.00%)	1 (0.41%)	<-10	0.681	0.686	Mutual exclusivity
PHIP	6q14.1	0 (0.00%)	1 (0.41%)	<-10	0.681	0.686	Mutual exclusivity
PI4K2A	10q24.2	0 (0.00%)	1 (0.41%)	<-10	0.681	0.686	Mutual exclusivity
PICSAR	21q22.3	0 (0.00%)	1 (0.41%)	<-10	0.681	0.686	Mutual exclusivity
PIGT	20q13.12	0 (0.00%)	1 (0.41%)	<-10	0.681	0.686	Mutual exclusivity
PIGY	4q22.1	0 (0.00%)	1 (0.41%)	<-10	0.681	0.686	Mutual exclusivity
PIKFYVE	2q34	0 (0.00%)	1 (0.41%)	<-10	0.681	0.686	Mutual exclusivity

PINK1	1p36.12	0 (0.00%)	1 (0.41%)	<-10	0.681	0.686	Mutual exclusivity
PITHD1	1p36.11	0 (0.00%)	1 (0.41%)	<-10	0.681	0.686	Mutual exclusivity
PIWIL4	11q21	0 (0.00%)	1 (0.41%)	<-10	0.681	0.686	Mutual exclusivity
PKD2	4q22.1	0 (0.00%)	1 (0.41%)	<-10	0.681	0.686	Mutual exclusivity
PKD2L1	10q24.31	0 (0.00%)	1 (0.41%)	<-10	0.681	0.686	Mutual exclusivity
PKIB	6q22.31	0 (0.00%)	1 (0.41%)	<-10	0.681	0.686	Mutual exclusivity
PLA2G2A	1p36.13	0 (0.00%)	1 (0.41%)	<-10	0.681	0.686	Mutual exclusivity
PLA2G2E	1p36.13	0 (0.00%)	1 (0.41%)	<-10	0.681	0.686	Mutual exclusivity
PLAGL1	6q24.2	0 (0.00%)	1 (0.41%)	<-10	0.681	0.686	Mutual exclusivity
PLAGL2	20q11.21	0 (0.00%)	1 (0.41%)	<-10	0.681	0.686	Mutual exclusivity
PLCB2	15q15.1	0 (0.00%)	1 (0.41%)	<-10	0.681	0.686	Mutual exclusivity
PLCD4	2q35	0 (0.00%)	1 (0.41%)	<-10	0.681	0.686	Mutual exclusivity
PLCG2	16q23.3	0 (0.00%)	1 (0.41%)	<-10	0.681	0.686	Mutual exclusivity
PLD6	17p11.2	0 (0.00%)	1 (0.41%)	<-10	0.681	0.686	Mutual exclusivity
PLEKHA1	10q26.13	0 (0.00%)	1 (0.41%)	<-10	0.681	0.686	Mutual exclusivity
PLEKHB1	11q13.4	0 (0.00%)	1 (0.41%)	<-10	0.681	0.686	Mutual exclusivity
PLG	6q26	0 (0.00%)	1 (0.41%)	<-10	0.681	0.686	Mutual exclusivity
PLGLA	2q12.2	0 (0.00%)	1 (0.41%)	<-10	0.681	0.686	Mutual exclusivity
PLGLB1	2p11.2	0 (0.00%)	1 (0.41%)	<-10	0.681	0.686	Mutual exclusivity
PLGLB2	2p11.2	0 (0.00%)	1 (0.41%)	<-10	0.681	0.686	Mutual exclusivity
PLP1	Xq22.2	0 (0.00%)	1 (0.41%)	<-10	0.681	0.686	Mutual exclusivity
PLTP	20q13.12	0 (0.00%)	1 (0.41%)	<-10	0.681	0.686	Mutual exclusivity
PNLDC1	6q25.3	0 (0.00%)	1 (0.41%)	<-10	0.681	0.686	Mutual exclusivity
PNOC	8p21.1	0 (0.00%)	1 (0.41%)	<-10	0.681	0.686	Mutual exclusivity
PNPT1	2p16.1	0 (0.00%)	1 (0.41%)	<-10	0.681	0.686	Mutual exclusivity
PNRC2	1p36.11	0 (0.00%)	1 (0.41%)	<-10	0.681	0.686	Mutual exclusivity
POFUT1	20q11.21	0 (0.00%)	1 (0.41%)	<-10	0.681	0.686	Mutual exclusivity
POFUT2	21q22.3	0 (0.00%)	1 (0.41%)	<-10	0.681	0.686	Mutual exclusivity
POLR1B	2q14.1	0 (0.00%)	1 (0.41%)	<-10	0.681	0.686	Mutual exclusivity

POLR2G	11q12.3	0 (0.00%)	1 (0.41%)	<-10	0.681	0.686	Mutual exclusivity
POLR3F	20p11.23	0 (0.00%)	1 (0.41%)	<-10	0.681	0.686	Mutual exclusivity
POM121L1P	22q11.22	0 (0.00%)	1 (0.41%)	<-10	0.681	0.686	Mutual exclusivity
POU1F1	3p11.2	0 (0.00%)	1 (0.41%)	<-10	0.681	0.686	Mutual exclusivity
POU2F2	19q13.2	0 (0.00%)	1 (0.41%)	<-10	0.681	0.686	Mutual exclusivity
POU3F3	2q12.1	0 (0.00%)	1 (0.41%)	<-10	0.681	0.686	Mutual exclusivity
POU5F2	5q15	0 (0.00%)	1 (0.41%)	<-10	0.681	0.686	Mutual exclusivity
PPAN	19p13.2	0 (0.00%)	1 (0.41%)	<-10	0.681	0.686	Mutual exclusivity
PPIL4	6q25.1	0 (0.00%)	1 (0.41%)	<-10	0.681	0.686	Mutual exclusivity
PPIL6	6q21	0 (0.00%)	1 (0.41%)	<-10	0.681	0.686	Mutual exclusivity
PPM1F	22q11.22	0 (0.00%)	1 (0.41%)	<-10	0.681	0.686	Mutual exclusivity
PPP1R7	2q37.3	0 (0.00%)	1 (0.41%)	<-10	0.681	0.686	Mutual exclusivity
PPP2CB	8p12	0 (0.00%)	1 (0.41%)	<-10	0.681	0.686	Mutual exclusivity
PPP3R2	9q31.1	0 (0.00%)	1 (0.41%)	<-10	0.681	0.686	Mutual exclusivity
PPP4R2	3p13	0 (0.00%)	1 (0.41%)	<-10	0.681	0.686	Mutual exclusivity
PPP4R3B	2p16.1	0 (0.00%)	1 (0.41%)	<-10	0.681	0.686	Mutual exclusivity
PRAME	22q11.22	0 (0.00%)	1 (0.41%)	<-10	0.681	0.686	Mutual exclusivity
PRAMENP	22q11.22	0 (0.00%)	1 (0.41%)	<-10	0.681	0.686	Mutual exclusivity
PRCP	11q14.1	0 (0.00%)	1 (0.41%)	<-10	0.681	0.686	Mutual exclusivity
PRDM1	6q21	0 (0.00%)	1 (0.41%)	<-10	0.681	0.686	Mutual exclusivity
PRDM5	4q27	0 (0.00%)	1 (0.41%)	<-10	0.681	0.686	Mutual exclusivity
PRDX3	10q26.11	0 (0.00%)	1 (0.41%)	<-10	0.681	0.686	Mutual exclusivity
PREP	6q21	0 (0.00%)	1 (0.41%)	<-10	0.681	0.686	Mutual exclusivity
PRG2	11q12.1	0 (0.00%)	1 (0.41%)	<-10	0.681	0.686	Mutual exclusivity
PRG3	11q12.1	0 (0.00%)	1 (0.41%)	<-10	0.681	0.686	Mutual exclusivity
PRICKLE2	3p14.1	0 (0.00%)	1 (0.41%)	<-10	0.681	0.686	Mutual exclusivity
PRKAG1	12q13.12	0 (0.00%)	1 (0.41%)	<-10	0.681	0.686	Mutual exclusivity
PRKAG3	2q35	0 (0.00%)	1 (0.41%)	<-10	0.681	0.686	Mutual exclusivity
PRMT2	21q22.3	0 (0.00%)	1 (0.41%)	<-10	0.681	0.686	Mutual exclusivity

PRMT8	12p13.32	0 (0.00%)	1 (0.41%)	<-10	0.681	0.686	Mutual exclusivity
PRND	20p13	0 (0.00%)	1 (0.41%)	<-10	0.681	0.686	Mutual exclusivity
PRNP	20p13	0 (0.00%)	1 (0.41%)	<-10	0.681	0.686	Mutual exclusivity
PRNT	20p13	0 (0.00%)	1 (0.41%)	<-10	0.681	0.686	Mutual exclusivity
PROKR2	20p12.3	0 (0.00%)	1 (0.41%)	<-10	0.681	0.686	Mutual exclusivity
PRORSD1P	2p16.1	0 (0.00%)	1 (0.41%)	<-10	0.681	0.686	Mutual exclusivity
PROX2	14q24.3	0 (0.00%)	1 (0.41%)	<-10	0.681	0.686	Mutual exclusivity
PRPF39	14q21.2	0 (0.00%)	1 (0.41%)	<-10	0.681	0.686	Mutual exclusivity
PRPH	12q13.12	0 (0.00%)	1 (0.41%)	<-10	0.681	0.686	Mutual exclusivity
PRR21	2q37.3	0 (0.00%)	1 (0.41%)	<-10	0.681	0.686	Mutual exclusivity
PRR22	19p13.3	0 (0.00%)	1 (0.41%)	<-10	0.681	0.686	Mutual exclusivity
PRR32	Xq25	0 (0.00%)	1 (0.41%)	<-10	0.681	0.686	Mutual exclusivity
PRSS23	11q14.2	0 (0.00%)	1 (0.41%)	<-10	0.681	0.686	Mutual exclusivity
PRSS36	16p11.2	0 (0.00%)	1 (0.41%)	<-10	0.681	0.686	Mutual exclusivity
PRSS53	16p11.2	0 (0.00%)	1 (0.41%)	<-10	0.681	0.686	Mutual exclusivity
PRSS56	2q37.1	0 (0.00%)	1 (0.41%)	<-10	0.681	0.686	Mutual exclusivity
PRSS8	16p11.2	0 (0.00%)	1 (0.41%)	<-10	0.681	0.686	Mutual exclusivity
PSD4	2q14.1	0 (0.00%)	1 (0.41%)	<-10	0.681	0.686	Mutual exclusivity
PSKH1	16q22.1	0 (0.00%)	1 (0.41%)	<-10	0.681	0.686	Mutual exclusivity
PSMA3	14q23.1	0 (0.00%)	1 (0.41%)	<-10	0.681	0.686	Mutual exclusivity
PSMB1	6q27	0 (0.00%)	1 (0.41%)	<-10	0.681	0.686	Mutual exclusivity
PSMB10	16q22.1	0 (0.00%)	1 (0.41%)	<-10	0.681	0.686	Mutual exclusivity
PSMD1	2q37.1	0 (0.00%)	1 (0.41%)	<-10	0.681	0.686	Mutual exclusivity
PSMD10	Xq22.3	0 (0.00%)	1 (0.41%)	<-10	0.681	0.686	Mutual exclusivity
PSME4	2p16.2	0 (0.00%)	1 (0.41%)	<-10	0.681	0.686	Mutual exclusivity
PSTPIP2	18q21.1|tdb7990	0 (0.00%)	1 (0.41%)	<-10	0.681	0.686	Mutual exclusivity
PTENP1	9p13.3	0 (0.00%)	1 (0.41%)	<-10	0.681	0.686	Mutual exclusivity
PTH2R	2q34	0 (0.00%)	1 (0.41%)	<-10	0.681	0.686	Mutual exclusivity
PTK2B	8p21.2	0 (0.00%)	1 (0.41%)	<-10	0.681	0.686	Mutual exclusivity

PTPRA	20p13	0 (0.00%)	1 (0.41%)	<-10	0.681	0.686	Mutual exclusivity
PTPRE	10q26.2	0 (0.00%)	1 (0.41%)	<-10	0.681	0.686	Mutual exclusivity
PTPRN	2q35	0 (0.00%)	1 (0.41%)	<-10	0.681	0.686	Mutual exclusivity
PTPRS	19p13.3	0 (0.00%)	1 (0.41%)	<-10	0.681	0.686	Mutual exclusivity
PUM1	1p35.2	0 (0.00%)	1 (0.41%)	<-10	0.681	0.686	Mutual exclusivity
PURG	8p12	0 (0.00%)	1 (0.41%)	<-10	0.681	0.686	Mutual exclusivity
PUS10	2p16.1-p15	0 (0.00%)	1 (0.41%)	<-10	0.681	0.686	Mutual exclusivity
PWP2	21q22.3	0 (0.00%)	1 (0.41%)	<-10	0.681	0.686	Mutual exclusivity
PYCARD	16p11.2	0 (0.00%)	1 (0.41%)	<-10	0.681	0.686	Mutual exclusivity
PYCARD-AS1	16p11.2	0 (0.00%)	1 (0.41%)	<-10	0.681	0.686	Mutual exclusivity
PYDC1	16p11.2	0 (0.00%)	1 (0.41%)	<-10	0.681	0.686	Mutual exclusivity
PYGB	20p11.21	0 (0.00%)	1 (0.41%)	<-10	0.681	0.686	Mutual exclusivity
PYROXD2	10q24.2	0 (0.00%)	1 (0.41%)	<-10	0.681	0.686	Mutual exclusivity
PYURF	4q22.1	0 (0.00%)	1 (0.41%)	<-10	0.681	0.686	Mutual exclusivity
QKI	6q26	0 (0.00%)	1 (0.41%)	<-10	0.681	0.686	Mutual exclusivity
QRSL1	6q21	0 (0.00%)	1 (0.41%)	<-10	0.681	0.686	Mutual exclusivity
QTRT1	19p13.2	0 (0.00%)	1 (0.41%)	<-10	0.681	0.686	Mutual exclusivity
R3HCC1L	10q24.2	0 (0.00%)	1 (0.41%)	<-10	0.681	0.686	Mutual exclusivity
R3HDM1	2q21.3	0 (0.00%)	1 (0.41%)	<-10	0.681	0.686	Mutual exclusivity
RAB15	14q23.3	0 (0.00%)	1 (0.41%)	<-10	0.681	0.686	Mutual exclusivity
RAB1A	2p14	0 (0.00%)	1 (0.41%)	<-10	0.681	0.686	Mutual exclusivity
RAB30	11q14.1	0 (0.00%)	1 (0.41%)	<-10	0.681	0.686	Mutual exclusivity
RAB32	6q24.3	0 (0.00%)	1 (0.41%)	<-10	0.681	0.686	Mutual exclusivity
RAB33B	4q31.1	0 (0.00%)	1 (0.41%)	<-10	0.681	0.686	Mutual exclusivity
RAB36	22q11.23	0 (0.00%)	1 (0.41%)	<-10	0.681	0.686	Mutual exclusivity
RAB38	11q14.2	0 (0.00%)	1 (0.41%)	<-10	0.681	0.686	Mutual exclusivity
RAB39A	11q22.3	0 (0.00%)	1 (0.41%)	<-10	0.681	0.686	Mutual exclusivity
RAB3GAP1	2q21.3	0 (0.00%)	1 (0.41%)	<-10	0.681	0.686	Mutual exclusivity
RAB6A	11q13.4	0 (0.00%)	1 (0.41%)	<-10	0.681	0.686	Mutual exclusivity

RAB9B	Xq22.2	0 (0.00%)	1 (0.41%)	<-10	0.681	0.686	Mutual exclusivity
RABAC1	19q13.2	0 (0.00%)	1 (0.41%)	<-10	0.681	0.686	Mutual exclusivity
RABL2A	2q14.1	0 (0.00%)	1 (0.41%)	<-10	0.681	0.686	Mutual exclusivity
RABL3	3q13.33	0 (0.00%)	1 (0.41%)	<-10	0.681	0.686	Mutual exclusivity
RAD23A	19p13.13	0 (0.00%)	1 (0.41%)	<-10	0.681	0.686	Mutual exclusivity
RAD51AP1	12p13.32	0 (0.00%)	1 (0.41%)	<-10	0.681	0.686	Mutual exclusivity
RAD52	12p13.33	0 (0.00%)	1 (0.41%)	<-10	0.681	0.686	Mutual exclusivity
RAET1E	6q25.1	0 (0.00%)	1 (0.41%)	<-10	0.681	0.686	Mutual exclusivity
RAET1G	6q25.1	0 (0.00%)	1 (0.41%)	<-10	0.681	0.686	Mutual exclusivity
RAET1K	6q25.1	0 (0.00%)	1 (0.41%)	<-10	0.681	0.686	Mutual exclusivity
RAET1L	6q25.1	0 (0.00%)	1 (0.41%)	<-10	0.681	0.686	Mutual exclusivity
RAG1	11p12	0 (0.00%)	1 (0.41%)	<-10	0.681	0.686	Mutual exclusivity
RAG2	11p12	0 (0.00%)	1 (0.41%)	<-10	0.681	0.686	Mutual exclusivity
RANBP10	16q22.1	0 (0.00%)	1 (0.41%)	<-10	0.681	0.686	Mutual exclusivity
RANBP2	2q13	0 (0.00%)	1 (0.41%)	<-10	0.681	0.686	Mutual exclusivity
RANBP3	19p13.3	0 (0.00%)	1 (0.41%)	<-10	0.681	0.686	Mutual exclusivity
RAP1GAP	1p36.12	0 (0.00%)	1 (0.41%)	<-10	0.681	0.686	Mutual exclusivity
RAP1GDS1	4q23	0 (0.00%)	1 (0.41%)	<-10	0.681	0.686	Mutual exclusivity
RAPGEF2	4q32.1	0 (0.00%)	1 (0.41%)	<-10	0.681	0.686	Mutual exclusivity
RASA1	5q14.3	0 (0.00%)	1 (0.41%)	<-10	0.681	0.686	Mutual exclusivity
RASD1	17p11.2	0 (0.00%)	1 (0.41%)	<-10	0.681	0.686	Mutual exclusivity
RASSF2	20p13	0 (0.00%)	1 (0.41%)	<-10	0.681	0.686	Mutual exclusivity
RAVER1	19p13.2	0 (0.00%)	1 (0.41%)	<-10	0.681	0.686	Mutual exclusivity
RBBP9	20p11.23	0 (0.00%)	1 (0.41%)	<-10	0.681	0.686	Mutual exclusivity
RBM20	10q25.2	0 (0.00%)	1 (0.41%)	<-10	0.681	0.686	Mutual exclusivity
RCAN3	1p36.11	0 (0.00%)	1 (0.41%)	<-10	0.681	0.686	Mutual exclusivity
RCN1	11p13	0 (0.00%)	1 (0.41%)	<-10	0.681	0.686	Mutual exclusivity
RCOR2	11q13.1	0 (0.00%)	1 (0.41%)	<-10	0.681	0.686	Mutual exclusivity
RDH12	14q24.1	0 (0.00%)	1 (0.41%)	<-10	0.681	0.686	Mutual exclusivity

RDX	11q22.3	0 (0.00%)	1 (0.41%)	<-10	0.681	0.686	Mutual exclusivity
REL	2p16.1	0 (0.00%)	1 (0.41%)	<-10	0.681	0.686	Mutual exclusivity
RELT	11q13.4	0 (0.00%)	1 (0.41%)	<-10	0.681	0.686	Mutual exclusivity
REPS1	6q24.1	0 (0.00%)	1 (0.41%)	<-10	0.681	0.686	Mutual exclusivity
RESP18	2q35	0 (0.00%)	1 (0.41%)	<-10	0.681	0.686	Mutual exclusivity
RETNLB	3q13.13	0 (0.00%)	1 (0.41%)	<-10	0.681	0.686	Mutual exclusivity
RETREG2	2q35	0 (0.00%)	1 (0.41%)	<-10	0.681	0.686	Mutual exclusivity
REV1	2q11.2	0 (0.00%)	1 (0.41%)	<-10	0.681	0.686	Mutual exclusivity
RFC3	13q13.2	0 (0.00%)	1 (0.41%)	<-10	0.681	0.686	Mutual exclusivity
RFX6	6q22.1	0 (0.00%)	1 (0.41%)	<-10	0.681	0.686	Mutual exclusivity
RGL4	22q11.23	0 (0.00%)	1 (0.41%)	<-10	0.681	0.686	Mutual exclusivity
RGPD1	2p11.2	0 (0.00%)	1 (0.41%)	<-10	0.681	0.686	Mutual exclusivity
RGPD2	2p11.2	0 (0.00%)	1 (0.41%)	<-10	0.681	0.686	Mutual exclusivity
RGPD3	2q12.2	0 (0.00%)	1 (0.41%)	<-10	0.681	0.686	Mutual exclusivity
RGPD4	2q12.3	0 (0.00%)	1 (0.41%)	<-10	0.681	0.686	Mutual exclusivity
RGPD5	2q13	0 (0.00%)	1 (0.41%)	<-10	0.681	0.686	Mutual exclusivity
RGPD6	2q13	0 (0.00%)	1 (0.41%)	<-10	0.681	0.686	Mutual exclusivity
RGS17	6q25.2	0 (0.00%)	1 (0.41%)	<-10	0.681	0.686	Mutual exclusivity
RHCE	1p36.11	0 (0.00%)	1 (0.41%)	<-10	0.681	0.686	Mutual exclusivity
RHD	1p36.11	0 (0.00%)	1 (0.41%)	<-10	0.681	0.686	Mutual exclusivity
RHEBL1	12q13.12	0 (0.00%)	1 (0.41%)	<-10	0.681	0.686	Mutual exclusivity
RHNO1	12p13.33	0 (0.00%)	1 (0.41%)	<-10	0.681	0.686	Mutual exclusivity
RIMS4	20q13.12	0 (0.00%)	1 (0.41%)	<-10	0.681	0.686	Mutual exclusivity
RIOK2	5q15	0 (0.00%)	1 (0.41%)	<-10	0.681	0.686	Mutual exclusivity
RMND1	6q25.1	0 (0.00%)	1 (0.41%)	<-10	0.681	0.686	Mutual exclusivity
RMND5A	2p11.2	0 (0.00%)	1 (0.41%)	<-10	0.681	0.686	Mutual exclusivity
RN7SKP137	4q27	0 (0.00%)	1 (0.41%)	<-10	0.681	0.686	Mutual exclusivity
RN7SKP154	2q21.2	0 (0.00%)	1 (0.41%)	<-10	0.681	0.686	Mutual exclusivity
RN7SKP163	6q14.1	0 (0.00%)	1 (0.41%)	<-10	0.681	0.686	Mutual exclusivity

RN7SKP182	18q12.2	0 (0.00%)	1 (0.41%)	<-10	0.681	0.686	Mutual exclusivity
RN7SKP191	9q31.1	0 (0.00%)	1 (0.41%)	<-10	0.681	0.686	Mutual exclusivity
RN7SKP192	11q12.1	0 (0.00%)	1 (0.41%)	<-10	0.681	0.686	Mutual exclusivity
RN7SKP208	2p16.1	0 (0.00%)	1 (0.41%)	<-10	0.681	0.686	Mutual exclusivity
RN7SKP209	6q14.3	0 (0.00%)	1 (0.41%)	<-10	0.681	0.686	Mutual exclusivity
RN7SKP213	2q35	0 (0.00%)	1 (0.41%)	<-10	0.681	0.686	Mutual exclusivity
RN7SKP228	7p21.3	0 (0.00%)	1 (0.41%)	<-10	0.681	0.686	Mutual exclusivity
RN7SKP243	11q13.4	0 (0.00%)	1 (0.41%)	<-10	0.681	0.686	Mutual exclusivity
RN7SKP244	4q22.1	0 (0.00%)	1 (0.41%)	<-10	0.681	0.686	Mutual exclusivity
RN7SKP248	4q22.1	0 (0.00%)	1 (0.41%)	<-10	0.681	0.686	Mutual exclusivity
RN7SKP253	4q31.1	0 (0.00%)	1 (0.41%)	<-10	0.681	0.686	Mutual exclusivity
RN7SKP259	11q12.1	0 (0.00%)	1 (0.41%)	<-10	0.681	0.686	Mutual exclusivity
RN7SKP26	18q21.1	0 (0.00%)	1 (0.41%)	<-10	0.681	0.686	Mutual exclusivity
RN7SKP268	6q25.1	0 (0.00%)	1 (0.41%)	<-10	0.681	0.686	Mutual exclusivity
RN7SKP283	2q36.3	0 (0.00%)	1 (0.41%)	<-10	0.681	0.686	Mutual exclusivity
RN7SKP284	3p12.1	0 (0.00%)	1 (0.41%)	<-10	0.681	0.686	Mutual exclusivity
RN7SKP295	5q14.3	0 (0.00%)	1 (0.41%)	<-10	0.681	0.686	Mutual exclusivity
RN7SKP34	5q14.3	0 (0.00%)	1 (0.41%)	<-10	0.681	0.686	Mutual exclusivity
RN7SKP35	4q31.3	0 (0.00%)	1 (0.41%)	<-10	0.681	0.686	Mutual exclusivity
RN7SKP38	2q35	0 (0.00%)	1 (0.41%)	<-10	0.681	0.686	Mutual exclusivity
RN7SKP43	2q35	0 (0.00%)	1 (0.41%)	<-10	0.681	0.686	Mutual exclusivity
RN7SKP53	11q22.1	0 (0.00%)	1 (0.41%)	<-10	0.681	0.686	Mutual exclusivity
RN7SKP69	20p12.1	0 (0.00%)	1 (0.41%)	<-10	0.681	0.686	Mutual exclusivity
RN7SKP99	14q23.1	0 (0.00%)	1 (0.41%)	<-10	0.681	0.686	Mutual exclusivity
RN7SL111P	2q14.1	0 (0.00%)	1 (0.41%)	<-10	0.681	0.686	Mutual exclusivity
RN7SL163P	21q21.1	0 (0.00%)	1 (0.41%)	<-10	0.681	0.686	Mutual exclusivity
RN7SL167P	11q25	0 (0.00%)	1 (0.41%)	<-10	0.681	0.686	Mutual exclusivity
RN7SL173P	6q25.3	0 (0.00%)	1 (0.41%)	<-10	0.681	0.686	Mutual exclusivity
RN7SL183P	6q15	0 (0.00%)	1 (0.41%)	<-10	0.681	0.686	Mutual exclusivity

RN7SL186P	1p36.12	0 (0.00%)	1 (0.41%)	<-10	0.681	0.686	Mutual exclusivity
RN7SL204P	2q37.2	0 (0.00%)	1 (0.41%)	<-10	0.681	0.686	Mutual exclusivity
RN7SL208P	5q13.3	0 (0.00%)	1 (0.41%)	<-10	0.681	0.686	Mutual exclusivity
RN7SL210P	2q11.2	0 (0.00%)	1 (0.41%)	<-10	0.681	0.686	Mutual exclusivity
RN7SL213P	14q24.1	0 (0.00%)	1 (0.41%)	<-10	0.681	0.686	Mutual exclusivity
RN7SL223P	11q21	0 (0.00%)	1 (0.41%)	<-10	0.681	0.686	Mutual exclusivity
RN7SL225P	11q14.2	0 (0.00%)	1 (0.41%)	<-10	0.681	0.686	Mutual exclusivity
RN7SL234P	6q25.1	0 (0.00%)	1 (0.41%)	<-10	0.681	0.686	Mutual exclusivity
RN7SL259P	11q12.3	0 (0.00%)	1 (0.41%)	<-10	0.681	0.686	Mutual exclusivity
RN7SL263P	22q11.23	0 (0.00%)	1 (0.41%)	<-10	0.681	0.686	Mutual exclusivity
RN7SL277P	1p36.13	0 (0.00%)	1 (0.41%)	<-10	0.681	0.686	Mutual exclusivity
RN7SL279P	16q22.1	0 (0.00%)	1 (0.41%)	<-10	0.681	0.686	Mutual exclusivity
RN7SL28P	4q35.1	0 (0.00%)	1 (0.41%)	<-10	0.681	0.686	Mutual exclusivity
RN7SL304P	1p36.13	0 (0.00%)	1 (0.41%)	<-10	0.681	0.686	Mutual exclusivity
RN7SL313P	2q11.2	0 (0.00%)	1 (0.41%)	<-10	0.681	0.686	Mutual exclusivity
RN7SL32P	2q37.1	0 (0.00%)	1 (0.41%)	<-10	0.681	0.686	Mutual exclusivity
RN7SL335P	4q27	0 (0.00%)	1 (0.41%)	<-10	0.681	0.686	Mutual exclusivity
RN7SL341P	2p14	0 (0.00%)	1 (0.41%)	<-10	0.681	0.686	Mutual exclusivity
RN7SL357P	4q12	0 (0.00%)	1 (0.41%)	<-10	0.681	0.686	Mutual exclusivity
RN7SL359P	2q37.1	0 (0.00%)	1 (0.41%)	<-10	0.681	0.686	Mutual exclusivity
RN7SL361P	2p16.1	0 (0.00%)	1 (0.41%)	<-10	0.681	0.686	Mutual exclusivity
RN7SL366P	6q26	0 (0.00%)	1 (0.41%)	<-10	0.681	0.686	Mutual exclusivity
RN7SL371P	7q11.22	0 (0.00%)	1 (0.41%)	<-10	0.681	0.686	Mutual exclusivity
RN7SL378P	5q14.1	0 (0.00%)	1 (0.41%)	<-10	0.681	0.686	Mutual exclusivity
RN7SL386P	1p36.12	0 (0.00%)	1 (0.41%)	<-10	0.681	0.686	Mutual exclusivity
RN7SL401P	18q22.3	0 (0.00%)	1 (0.41%)	<-10	0.681	0.686	Mutual exclusivity
RN7SL420P	1p13.1	0 (0.00%)	1 (0.41%)	<-10	0.681	0.686	Mutual exclusivity
RN7SL424P	4q12	0 (0.00%)	1 (0.41%)	<-10	0.681	0.686	Mutual exclusivity
RN7SL461P	14q22.3	0 (0.00%)	1 (0.41%)	<-10	0.681	0.686	Mutual exclusivity

RN7SL468P	2q14.2	0 (0.00%)	1 (0.41%)	<-10	0.681	0.686	Mutual exclusivity
RN7SL47P	6q21	0 (0.00%)	1 (0.41%)	<-10	0.681	0.686	Mutual exclusivity
RN7SL492P	4q12	0 (0.00%)	1 (0.41%)	<-10	0.681	0.686	Mutual exclusivity
RN7SL51P	2p15	0 (0.00%)	1 (0.41%)	<-10	0.681	0.686	Mutual exclusivity
RN7SL555P	20p13	0 (0.00%)	1 (0.41%)	<-10	0.681	0.686	Mutual exclusivity
RN7SL596P	11q13.1	0 (0.00%)	1 (0.41%)	<-10	0.681	0.686	Mutual exclusivity
RN7SL598P	14q23.1	0 (0.00%)	1 (0.41%)	<-10	0.681	0.686	Mutual exclusivity
RN7SL5P	9p23	0 (0.00%)	1 (0.41%)	<-10	0.681	0.686	Mutual exclusivity
RN7SL627P	17p11.2	0 (0.00%)	1 (0.41%)	<-10	0.681	0.686	Mutual exclusivity
RN7SL629P	5q14.3	0 (0.00%)	1 (0.41%)	<-10	0.681	0.686	Mutual exclusivity
RN7SL632P	2p16.1	0 (0.00%)	1 (0.41%)	<-10	0.681	0.686	Mutual exclusivity
RN7SL635P	2p14	0 (0.00%)	1 (0.41%)	<-10	0.681	0.686	Mutual exclusivity
RN7SL638P	20p11.23	0 (0.00%)	1 (0.41%)	<-10	0.681	0.686	Mutual exclusivity
RN7SL643P	6q14.3	0 (0.00%)	1 (0.41%)	<-10	0.681	0.686	Mutual exclusivity
RN7SL660P	14q12	0 (0.00%)	1 (0.41%)	<-10	0.681	0.686	Mutual exclusivity
RN7SL679P	1p36.11	0 (0.00%)	1 (0.41%)	<-10	0.681	0.686	Mutual exclusivity
RN7SL681P	4q22.1	0 (0.00%)	1 (0.41%)	<-10	0.681	0.686	Mutual exclusivity
RN7SL705P	18q21.33	0 (0.00%)	1 (0.41%)	<-10	0.681	0.686	Mutual exclusivity
RN7SL733P	10q23.2	0 (0.00%)	1 (0.41%)	<-10	0.681	0.686	Mutual exclusivity
RN7SL744P	12q13.13	0 (0.00%)	1 (0.41%)	<-10	0.681	0.686	Mutual exclusivity
RN7SL749P	10q26.11	0 (0.00%)	1 (0.41%)	<-10	0.681	0.686	Mutual exclusivity
RN7SL764P	2q35	0 (0.00%)	1 (0.41%)	<-10	0.681	0.686	Mutual exclusivity
RN7SL775P	17p11.2	0 (0.00%)	1 (0.41%)	<-10	0.681	0.686	Mutual exclusivity
RN7SL795P	18q22.2	0 (0.00%)	1 (0.41%)	<-10	0.681	0.686	Mutual exclusivity
RN7SL797P	6q16.1	0 (0.00%)	1 (0.41%)	<-10	0.681	0.686	Mutual exclusivity
RN7SL801P	5q11.2	0 (0.00%)	1 (0.41%)	<-10	0.681	0.686	Mutual exclusivity
RN7SL807P	2q36.1	0 (0.00%)	1 (0.41%)	<-10	0.681	0.686	Mutual exclusivity
RN7SL822P	4q12	0 (0.00%)	1 (0.41%)	<-10	0.681	0.686	Mutual exclusivity
RN7SL834P	2q37.1	0 (0.00%)	1 (0.41%)	<-10	0.681	0.686	Mutual exclusivity

RN7SL846P	10q26.11	0 (0.00%)	1 (0.41%)	<-10	0.681	0.686	Mutual exclusivity
RN7SL849P	9p23	0 (0.00%)	1 (0.41%)	<-10	0.681	0.686	Mutual exclusivity
RN7SL93P	Xq23	0 (0.00%)	1 (0.41%)	<-10	0.681	0.686	Mutual exclusivity
RNA5SP117	2q34	0 (0.00%)	1 (0.41%)	<-10	0.681	0.686	Mutual exclusivity
RNA5SP119	2q34	0 (0.00%)	1 (0.41%)	<-10	0.681	0.686	Mutual exclusivity
RNA5SP120	2q35	0 (0.00%)	1 (0.41%)	<-10	0.681	0.686	Mutual exclusivity
RNA5SP121	2q36.3	0 (0.00%)	1 (0.41%)	<-10	0.681	0.686	Mutual exclusivity
RNA5SP122	2q37.3	0 (0.00%)	1 (0.41%)	<-10	0.681	0.686	Mutual exclusivity
RNA5SP137	3q21.2	0 (0.00%)	1 (0.41%)	<-10	0.681	0.686	Mutual exclusivity
RNA5SP138	3q21.3	0 (0.00%)	1 (0.41%)	<-10	0.681	0.686	Mutual exclusivity
RNA5SP139	3q21.3	0 (0.00%)	1 (0.41%)	<-10	0.681	0.686	Mutual exclusivity
RNA5SP161	4q12	0 (0.00%)	1 (0.41%)	<-10	0.681	0.686	Mutual exclusivity
RNA5SP166	4q31.23	0 (0.00%)	1 (0.41%)	<-10	0.681	0.686	Mutual exclusivity
RNA5SP167	4q31.23	0 (0.00%)	1 (0.41%)	<-10	0.681	0.686	Mutual exclusivity
RNA5SP182	5q11.2	0 (0.00%)	1 (0.41%)	<-10	0.681	0.686	Mutual exclusivity
RNA5SP187	5q14.3	0 (0.00%)	1 (0.41%)	<-10	0.681	0.686	Mutual exclusivity
RNA5SP209	6q14.1	0 (0.00%)	1 (0.41%)	<-10	0.681	0.686	Mutual exclusivity
RNA5SP210	6q14.1	0 (0.00%)	1 (0.41%)	<-10	0.681	0.686	Mutual exclusivity
RNA5SP211	6q21	0 (0.00%)	1 (0.41%)	<-10	0.681	0.686	Mutual exclusivity
RNA5SP212	6q21	0 (0.00%)	1 (0.41%)	<-10	0.681	0.686	Mutual exclusivity
RNA5SP219	6q23.3	0 (0.00%)	1 (0.41%)	<-10	0.681	0.686	Mutual exclusivity
RNA5SP221	6q24.2	0 (0.00%)	1 (0.41%)	<-10	0.681	0.686	Mutual exclusivity
RNA5SP222	6q24.3	0 (0.00%)	1 (0.41%)	<-10	0.681	0.686	Mutual exclusivity
RNA5SP223	6q25.2	0 (0.00%)	1 (0.41%)	<-10	0.681	0.686	Mutual exclusivity
RNA5SP224	6q25.2	0 (0.00%)	1 (0.41%)	<-10	0.681	0.686	Mutual exclusivity
RNA5SP225	6q25.2	0 (0.00%)	1 (0.41%)	<-10	0.681	0.686	Mutual exclusivity
RNA5SP259	8p21.1	0 (0.00%)	1 (0.41%)	<-10	0.681	0.686	Mutual exclusivity
RNA5SP261	8p12	0 (0.00%)	1 (0.41%)	<-10	0.681	0.686	Mutual exclusivity
RNA5SP319	10q21.3	0 (0.00%)	1 (0.41%)	<-10	0.681	0.686	Mutual exclusivity

RNA5SP322	10q23.1	0 (0.00%)	1 (0.41%)	<-10	0.681	0.686	Mutual exclusivity
RNA5SP328	10q26.2	0 (0.00%)	1 (0.41%)	<-10	0.681	0.686	Mutual exclusivity
RNA5SP341	11q12.1	0 (0.00%)	1 (0.41%)	<-10	0.681	0.686	Mutual exclusivity
RNA5SP345	11q21	0 (0.00%)	1 (0.41%)	<-10	0.681	0.686	Mutual exclusivity
RNA5SP346	11q21	0 (0.00%)	1 (0.41%)	<-10	0.681	0.686	Mutual exclusivity
RNA5SP347	11q22.1	0 (0.00%)	1 (0.41%)	<-10	0.681	0.686	Mutual exclusivity
RNA5SP348	11q22.3	0 (0.00%)	1 (0.41%)	<-10	0.681	0.686	Mutual exclusivity
RNA5SP349	11q22.3	0 (0.00%)	1 (0.41%)	<-10	0.681	0.686	Mutual exclusivity
RNA5SP387	14q24.3	0 (0.00%)	1 (0.41%)	<-10	0.681	0.686	Mutual exclusivity
RNA5SP392	15q15.1	0 (0.00%)	1 (0.41%)	<-10	0.681	0.686	Mutual exclusivity
RNA5SP456	18q21.1	0 (0.00%)	1 (0.41%)	<-10	0.681	0.686	Mutual exclusivity
RNA5SP46	1p34.2	0 (0.00%)	1 (0.41%)	<-10	0.681	0.686	Mutual exclusivity
RNA5SP460	18q22.3	0 (0.00%)	1 (0.41%)	<-10	0.681	0.686	Mutual exclusivity
RNA5SP476	20p11.23	0 (0.00%)	1 (0.41%)	<-10	0.681	0.686	Mutual exclusivity
RNA5SP478	20p11.21	0 (0.00%)	1 (0.41%)	<-10	0.681	0.686	Mutual exclusivity
RNA5SP482	20q11.21	0 (0.00%)	1 (0.41%)	<-10	0.681	0.686	Mutual exclusivity
RNA5SP485	20q13.12	0 (0.00%)	1 (0.41%)	<-10	0.681	0.686	Mutual exclusivity
RNA5SP486	20q13.13	0 (0.00%)	1 (0.41%)	<-10	0.681	0.686	Mutual exclusivity
RNA5SP493	22q11.22	0 (0.00%)	1 (0.41%)	<-10	0.681	0.686	Mutual exclusivity
RNA5SP504	Xp11.22	0 (0.00%)	1 (0.41%)	<-10	0.681	0.686	Mutual exclusivity
RNA5SP511	Xq22.2	0 (0.00%)	1 (0.41%)	<-10	0.681	0.686	Mutual exclusivity
RNA5SP93	2p16.1	0 (0.00%)	1 (0.41%)	<-10	0.681	0.686	Mutual exclusivity
RNA5SP94	2p16.1	0 (0.00%)	1 (0.41%)	<-10	0.681	0.686	Mutual exclusivity
RNA5SP95	2p16.1	0 (0.00%)	1 (0.41%)	<-10	0.681	0.686	Mutual exclusivity
RNASET2	6q27	0 (0.00%)	1 (0.41%)	<-10	0.681	0.686	Mutual exclusivity
RND3	2q23.3	0 (0.00%)	1 (0.41%)	<-10	0.681	0.686	Mutual exclusivity
RNF103	2p11.2	0 (0.00%)	1 (0.41%)	<-10	0.681	0.686	Mutual exclusivity
RNF165	18q21.1	0 (0.00%)	1 (0.41%)	<-10	0.681	0.686	Mutual exclusivity
RNF186	1p36.13	0 (0.00%)	1 (0.41%)	<-10	0.681	0.686	Mutual exclusivity

RNF20	9q31.1	0 (0.00%)	1 (0.41%)	<-10	0.681	0.686	Mutual exclusivity
RNF217	6q22.31	0 (0.00%)	1 (0.41%)	<-10	0.681	0.686	Mutual exclusivity
RNF24	20p13	0 (0.00%)	1 (0.41%)	<-10	0.681	0.686	Mutual exclusivity
RNF25	2q35	0 (0.00%)	1 (0.41%)	<-10	0.681	0.686	Mutual exclusivity
RNPEPL1	2q37.3	0 (0.00%)	1 (0.41%)	<-10	0.681	0.686	Mutual exclusivity
RNU4ATAC15P	9p21.1	0 (0.00%)	1 (0.41%)	<-10	0.681	0.686	Mutual exclusivity
RNU4ATAC18P	6q25.3	0 (0.00%)	1 (0.41%)	<-10	0.681	0.686	Mutual exclusivity
RNU6ATAC12P	11q25	0 (0.00%)	1 (0.41%)	<-10	0.681	0.686	Mutual exclusivity
RNU6ATAC26P	3p14.3	0 (0.00%)	1 (0.41%)	<-10	0.681	0.686	Mutual exclusivity
RNU6ATAC34P	20p11.23	0 (0.00%)	1 (0.41%)	<-10	0.681	0.686	Mutual exclusivity
RNU6ATAC36P	5q13.3	0 (0.00%)	1 (0.41%)	<-10	0.681	0.686	Mutual exclusivity
RNU6ATAC38P	20q13.12	0 (0.00%)	1 (0.41%)	<-10	0.681	0.686	Mutual exclusivity
RNU6ATAC6P	3p11.1	0 (0.00%)	1 (0.41%)	<-10	0.681	0.686	Mutual exclusivity
RNY1P4	13q13.1	0 (0.00%)	1 (0.41%)	<-10	0.681	0.686	Mutual exclusivity
RNY4P17	4q32.3	0 (0.00%)	1 (0.41%)	<-10	0.681	0.686	Mutual exclusivity
RNY4P19	2q36.3	0 (0.00%)	1 (0.41%)	<-10	0.681	0.686	Mutual exclusivity
RNY4P20	6q25.1	0 (0.00%)	1 (0.41%)	<-10	0.681	0.686	Mutual exclusivity
RNY4P3	16q12.1	0 (0.00%)	1 (0.41%)	<-10	0.681	0.686	Mutual exclusivity
RNY4P6	11q23.3	0 (0.00%)	1 (0.41%)	<-10	0.681	0.686	Mutual exclusivity
RNY5P4	4q28.2	0 (0.00%)	1 (0.41%)	<-10	0.681	0.686	Mutual exclusivity
ROPN1	3q21.1	0 (0.00%)	1 (0.41%)	<-10	0.681	0.686	Mutual exclusivity
ROPN1B	3q21.2	0 (0.00%)	1 (0.41%)	<-10	0.681	0.686	Mutual exclusivity
ROS1	6q22.1	0 (0.00%)	1 (0.41%)	<-10	0.681	0.686	Mutual exclusivity
RPA1	17p13.3	0 (0.00%)	1 (0.41%)	<-10	0.681	0.686	Mutual exclusivity
RPA4	Xq21.33	0 (0.00%)	1 (0.41%)	<-10	0.681	0.686	Mutual exclusivity
RPL10L	14q21.2	0 (0.00%)	1 (0.41%)	<-10	0.681	0.686	Mutual exclusivity
RPL13AP3	14q22.3	0 (0.00%)	1 (0.41%)	<-10	0.681	0.686	Mutual exclusivity
RPL17	18q21.1	0 (0.00%)	1 (0.41%)	<-10	0.681	0.686	Mutual exclusivity
RPL18	19q13.33	0 (0.00%)	1 (0.41%)	<-10	0.681	0.686	Mutual exclusivity

RPL23AP7	2q14.1	0 (0.00%)	1 (0.41%)	<-10	0.681	0.686	Mutual exclusivity
RPL31	2q11.2	0 (0.00%)	1 (0.41%)	<-10	0.681	0.686	Mutual exclusivity
RPL36	19p13.3	0 (0.00%)	1 (0.41%)	<-10	0.681	0.686	Mutual exclusivity
RPS19	19q13.2	0 (0.00%)	1 (0.41%)	<-10	0.681	0.686	Mutual exclusivity
RPS23	5q14.2	0 (0.00%)	1 (0.41%)	<-10	0.681	0.686	Mutual exclusivity
RPS27A	2p16.1	0 (0.00%)	1 (0.41%)	<-10	0.681	0.686	Mutual exclusivity
RPS6KA5	14q32.11	0 (0.00%)	1 (0.41%)	<-10	0.681	0.686	Mutual exclusivity
RPS6KL1	14q24.3	0 (0.00%)	1 (0.41%)	<-10	0.681	0.686	Mutual exclusivity
RPSAP58	19p12	0 (0.00%)	1 (0.41%)	<-10	0.681	0.686	Mutual exclusivity
RRBP1	20p12.1	0 (0.00%)	1 (0.41%)	<-10	0.681	0.686	Mutual exclusivity
RSPH14	22q11.22-q11.23	0 (0.00%)	1 (0.41%)	<-10	0.681	0.686	Mutual exclusivity
RSPH3	6q25.3	0 (0.00%)	1 (0.41%)	<-10	0.681	0.686	Mutual exclusivity
RSRP1	1p36.11	0 (0.00%)	1 (0.41%)	<-10	0.681	0.686	Mutual exclusivity
RTN4	2p16.1	0 (0.00%)	1 (0.41%)	<-10	0.681	0.686	Mutual exclusivity
RTN4IP1	6q21	0 (0.00%)	1 (0.41%)	<-10	0.681	0.686	Mutual exclusivity
RTN4RL1	17p13.3	0 (0.00%)	1 (0.41%)	<-10	0.681	0.686	Mutual exclusivity
RTN4RL2	11q12.1	0 (0.00%)	1 (0.41%)	<-10	0.681	0.686	Mutual exclusivity
RTP5	2q37.3	0 (0.00%)	1 (0.41%)	<-10	0.681	0.686	Mutual exclusivity
RTTN	18q22.2	0 (0.00%)	1 (0.41%)	<-10	0.681	0.686	Mutual exclusivity
RUVBL1	3q21.3	0 (0.00%)	1 (0.41%)	<-10	0.681	0.686	Mutual exclusivity
RWDD1	6q22.1	0 (0.00%)	1 (0.41%)	<-10	0.681	0.686	Mutual exclusivity
S100B	21q22.3	0 (0.00%)	1 (0.41%)	<-10	0.681	0.686	Mutual exclusivity
S100Z	5q13.3	0 (0.00%)	1 (0.41%)	<-10	0.681	0.686	Mutual exclusivity
S1PR2	19p13.2	0 (0.00%)	1 (0.41%)	<-10	0.681	0.686	Mutual exclusivity
S1PR5	19p13.2	0 (0.00%)	1 (0.41%)	<-10	0.681	0.686	Mutual exclusivity
SAFB	19p13.3	0 (0.00%)	1 (0.41%)	<-10	0.681	0.686	Mutual exclusivity
SAFB2	19p13.3	0 (0.00%)	1 (0.41%)	<-10	0.681	0.686	Mutual exclusivity
SAG	2q37.1	0 (0.00%)	1 (0.41%)	<-10	0.681	0.686	Mutual exclusivity
SASH1	6q24.3-q25.1	0 (0.00%)	1 (0.41%)	<-10	0.681	0.686	Mutual exclusivity

SCAF8	6q25.2	0 (0.00%)	1 (0.41%)	<-10	0.681	0.686	Mutual exclusivity
SCAMP1	5q14.1	0 (0.00%)	1 (0.41%)	<-10	0.681	0.686	Mutual exclusivity
SCARA3	8p21.1	0 (0.00%)	1 (0.41%)	<-10	0.681	0.686	Mutual exclusivity
SCARNA13	14q32.13	0 (0.00%)	1 (0.41%)	<-10	0.681	0.686	Mutual exclusivity
SCARNA5	2q37.1	0 (0.00%)	1 (0.41%)	<-10	0.681	0.686	Mutual exclusivity
SCARNA6	2q37.1	0 (0.00%)	1 (0.41%)	<-10	0.681	0.686	Mutual exclusivity
SCARNA9	11q21	0 (0.00%)	1 (0.41%)	<-10	0.681	0.686	Mutual exclusivity
SCD	10q24.31	0 (0.00%)	1 (0.41%)	<-10	0.681	0.686	Mutual exclusivity
SCFD2	4q12	0 (0.00%)	1 (0.41%)	<-10	0.681	0.686	Mutual exclusivity
SCG2	2q36.1	0 (0.00%)	1 (0.41%)	<-10	0.681	0.686	Mutual exclusivity
SCLT1	4q28.2	0 (0.00%)	1 (0.41%)	<-10	0.681	0.686	Mutual exclusivity
SCLY	2q37.3	0 (0.00%)	1 (0.41%)	<-10	0.681	0.686	Mutual exclusivity
SCMH1	1p34.2	0 (0.00%)	1 (0.41%)	<-10	0.681	0.686	Mutual exclusivity
SCN2B	11q23.3	0 (0.00%)	1 (0.41%)	<-10	0.681	0.686	Mutual exclusivity
SCN4B	11q23.3	0 (0.00%)	1 (0.41%)	<-10	0.681	0.686	Mutual exclusivity
SCRT2	20p13	0 (0.00%)	1 (0.41%)	<-10	0.681	0.686	Mutual exclusivity
SDHAF4	6q13	0 (0.00%)	1 (0.41%)	<-10	0.681	0.686	Mutual exclusivity
SDHD	11q23.1	0 (0.00%)	1 (0.41%)	<-10	0.681	0.686	Mutual exclusivity
SEC1P	19q13.33	0 (0.00%)	1 (0.41%)	<-10	0.681	0.686	Mutual exclusivity
SEC22A	3q21.1	0 (0.00%)	1 (0.41%)	<-10	0.681	0.686	Mutual exclusivity
SEC23B	20p11.23	0 (0.00%)	1 (0.41%)	<-10	0.681	0.686	Mutual exclusivity
SEC63	6q21	0 (0.00%)	1 (0.41%)	<-10	0.681	0.686	Mutual exclusivity
SELENOH	11q12.1	0 (0.00%)	1 (0.41%)	<-10	0.681	0.686	Mutual exclusivity
SELENON	1p36.11	0 (0.00%)	1 (0.41%)	<-10	0.681	0.686	Mutual exclusivity
SEMA4C	2q11.2	0 (0.00%)	1 (0.41%)	<-10	0.681	0.686	Mutual exclusivity
SENP6	6q14.1	0 (0.00%)	1 (0.41%)	<-10	0.681	0.686	Mutual exclusivity
SEPTIN10	2q13	0 (0.00%)	1 (0.41%)	<-10	0.681	0.686	Mutual exclusivity
SEPTIN2	2q37.3	0 (0.00%)	1 (0.41%)	<-10	0.681	0.686	Mutual exclusivity
SERAC1	6q25.3	0 (0.00%)	1 (0.41%)	<-10	0.681	0.686	Mutual exclusivity

SERINC1	6q22.31	0 (0.00%)	1 (0.41%)	<-10	0.681	0.686	Mutual exclusivity
SERINC5	5q14.1	0 (0.00%)	1 (0.41%)	<-10	0.681	0.686	Mutual exclusivity
SERPINA3	14q32.13	0 (0.00%)	1 (0.41%)	<-10	0.681	0.686	Mutual exclusivity
SERPINA7	Xq22.3	0 (0.00%)	1 (0.41%)	<-10	0.681	0.686	Mutual exclusivity
SERPINE2	2q36.1	0 (0.00%)	1 (0.41%)	<-10	0.681	0.686	Mutual exclusivity
SERPING1	11q12.1	0 (0.00%)	1 (0.41%)	<-10	0.681	0.686	Mutual exclusivity
SERTAD2	2p14	0 (0.00%)	1 (0.41%)	<-10	0.681	0.686	Mutual exclusivity
SESN2	1p35.3	0 (0.00%)	1 (0.41%)	<-10	0.681	0.686	Mutual exclusivity
SESN3	11q21	0 (0.00%)	1 (0.41%)	<-10	0.681	0.686	Mutual exclusivity
SETD1A	16p11.2	0 (0.00%)	1 (0.41%)	<-10	0.681	0.686	Mutual exclusivity
SETD7	4q31.1	0 (0.00%)	1 (0.41%)	<-10	0.681	0.686	Mutual exclusivity
SETD9	5q11.2	0 (0.00%)	1 (0.41%)	<-10	0.681	0.686	Mutual exclusivity
SF3B5	6q24.2	0 (0.00%)	1 (0.41%)	<-10	0.681	0.686	Mutual exclusivity
SFR1	10q25.1	0 (0.00%)	1 (0.41%)	<-10	0.681	0.686	Mutual exclusivity
SFRP5	10q24.2	0 (0.00%)	1 (0.41%)	<-10	0.681	0.686	Mutual exclusivity
SFXN4	10q26.11	0 (0.00%)	1 (0.41%)	<-10	0.681	0.686	Mutual exclusivity
SGPP2	2q36.1	0 (0.00%)	1 (0.41%)	<-10	0.681	0.686	Mutual exclusivity
SH2D3A	19p13.3	0 (0.00%)	1 (0.41%)	<-10	0.681	0.686	Mutual exclusivity
SH2D4A	8p21.3	0 (0.00%)	1 (0.41%)	<-10	0.681	0.686	Mutual exclusivity
SH2D5	1p36.12	0 (0.00%)	1 (0.41%)	<-10	0.681	0.686	Mutual exclusivity
SH3BGRL2	6q14.1	0 (0.00%)	1 (0.41%)	<-10	0.681	0.686	Mutual exclusivity
SH3D19	4q31.3	0 (0.00%)	1 (0.41%)	<-10	0.681	0.686	Mutual exclusivity
SH3RF1	4q32.3-q33	0 (0.00%)	1 (0.41%)	<-10	0.681	0.686	Mutual exclusivity
SH3RF3	2q13	0 (0.00%)	1 (0.41%)	<-10	0.681	0.686	Mutual exclusivity
SHFL	19p13.2	0 (0.00%)	1 (0.41%)	<-10	0.681	0.686	Mutual exclusivity
SHLD2	10q23.2	0 (0.00%)	1 (0.41%)	<-10	0.681	0.686	Mutual exclusivity
SHPRH	6q24.3	0 (0.00%)	1 (0.41%)	<-10	0.681	0.686	Mutual exclusivity
SIDT2	11q23.3	0 (0.00%)	1 (0.41%)	<-10	0.681	0.686	Mutual exclusivity
SIPA1L1	14q24.2	0 (0.00%)	1 (0.41%)	<-10	0.681	0.686	Mutual exclusivity

SLC12A3	16q13	0 (0.00%)	1 (0.41%)	<-10	0.681	0.686	Mutual exclusivity
SLC12A4	16q22.1	0 (0.00%)	1 (0.41%)	<-10	0.681	0.686	Mutual exclusivity
SLC12A5	20q13.12	0 (0.00%)	1 (0.41%)	<-10	0.681	0.686	Mutual exclusivity
SLC16A14	2q36.3	0 (0.00%)	1 (0.41%)	<-10	0.681	0.686	Mutual exclusivity
SLC18A3	10q11.23	0 (0.00%)	1 (0.41%)	<-10	0.681	0.686	Mutual exclusivity
SLC19A1	21q22.3	0 (0.00%)	1 (0.41%)	<-10	0.681	0.686	Mutual exclusivity
SLC19A3	2q36.3	0 (0.00%)	1 (0.41%)	<-10	0.681	0.686	Mutual exclusivity
SLC1A2	11p13	0 (0.00%)	1 (0.41%)	<-10	0.681	0.686	Mutual exclusivity
SLC1A4	2p14	0 (0.00%)	1 (0.41%)	<-10	0.681	0.686	Mutual exclusivity
SLC20A1	2q14.1	0 (0.00%)	1 (0.41%)	<-10	0.681	0.686	Mutual exclusivity
SLC22A10	11q12.3	0 (0.00%)	1 (0.41%)	<-10	0.681	0.686	Mutual exclusivity
SLC22A24	11q12.3	0 (0.00%)	1 (0.41%)	<-10	0.681	0.686	Mutual exclusivity
SLC22A25	11q12.3	0 (0.00%)	1 (0.41%)	<-10	0.681	0.686	Mutual exclusivity
SLC22A6	11q12.3	0 (0.00%)	1 (0.41%)	<-10	0.681	0.686	Mutual exclusivity
SLC22A8	11q12.3	0 (0.00%)	1 (0.41%)	<-10	0.681	0.686	Mutual exclusivity
SLC22A9	11q12.3	0 (0.00%)	1 (0.41%)	<-10	0.681	0.686	Mutual exclusivity
SLC23A2	20p13	0 (0.00%)	1 (0.41%)	<-10	0.681	0.686	Mutual exclusivity
SLC23A3	2q35	0 (0.00%)	1 (0.41%)	<-10	0.681	0.686	Mutual exclusivity
SLC24A2	9p22.1-p21.3	0 (0.00%)	1 (0.41%)	<-10	0.681	0.686	Mutual exclusivity
SLC25A16	10q21.3	0 (0.00%)	1 (0.41%)	<-10	0.681	0.686	Mutual exclusivity
SLC25A23	19p13.3	0 (0.00%)	1 (0.41%)	<-10	0.681	0.686	Mutual exclusivity
SLC25A28	10q24.2	0 (0.00%)	1 (0.41%)	<-10	0.681	0.686	Mutual exclusivity
SLC25A41	19p13.3	0 (0.00%)	1 (0.41%)	<-10	0.681	0.686	Mutual exclusivity
SLC2A1	1p34.2	0 (0.00%)	1 (0.41%)	<-10	0.681	0.686	Mutual exclusivity
SLC35A1	6q15	0 (0.00%)	1 (0.41%)	<-10	0.681	0.686	Mutual exclusivity
SLC35C2	20q13.12	0 (0.00%)	1 (0.41%)	<-10	0.681	0.686	Mutual exclusivity
SLC35D3	6q23.3	0 (0.00%)	1 (0.41%)	<-10	0.681	0.686	Mutual exclusivity
SLC35F1	6q22.2-q22.31	0 (0.00%)	1 (0.41%)	<-10	0.681	0.686	Mutual exclusivity
SLC35F2	11q22.3	0 (0.00%)	1 (0.41%)	<-10	0.681	0.686	Mutual exclusivity

SLC35F4	14q22.3-q23.1	0 (0.00%)	1 (0.41%)	<-10	0.681	0.686	Mutual exclusivity
SLC35F5	2q14.1	0 (0.00%)	1 (0.41%)	<-10	0.681	0.686	Mutual exclusivity
SLC36A4	11q21	0 (0.00%)	1 (0.41%)	<-10	0.681	0.686	Mutual exclusivity
SLC3A2	11q12.3	0 (0.00%)	1 (0.41%)	<-10	0.681	0.686	Mutual exclusivity
SLC41A3	3q21.2-q21.3	0 (0.00%)	1 (0.41%)	<-10	0.681	0.686	Mutual exclusivity
SLC43A1	11q12.1	0 (0.00%)	1 (0.41%)	<-10	0.681	0.686	Mutual exclusivity
SLC44A1	9q31.1-q31.2	0 (0.00%)	1 (0.41%)	<-10	0.681	0.686	Mutual exclusivity
SLC44A2	19p13.2	0 (0.00%)	1 (0.41%)	<-10	0.681	0.686	Mutual exclusivity
SLC4A3	2q35	0 (0.00%)	1 (0.41%)	<-10	0.681	0.686	Mutual exclusivity
SLC52A3	20p13	0 (0.00%)	1 (0.41%)	<-10	0.681	0.686	Mutual exclusivity
SLC5A7	2q12.3	0 (0.00%)	1 (0.41%)	<-10	0.681	0.686	Mutual exclusivity
SLC66A1	1p36.13	0 (0.00%)	1 (0.41%)	<-10	0.681	0.686	Mutual exclusivity
SLC7A2	8p22	0 (0.00%)	1 (0.41%)	<-10	0.681	0.686	Mutual exclusivity
SLFNL1	1p34.2	0 (0.00%)	1 (0.41%)	<-10	0.681	0.686	Mutual exclusivity
SLMAP	3p14.3	0 (0.00%)	1 (0.41%)	<-10	0.681	0.686	Mutual exclusivity
SLN	11q22.3	0 (0.00%)	1 (0.41%)	<-10	0.681	0.686	Mutual exclusivity
SMARCAL1	2q35	0 (0.00%)	1 (0.41%)	<-10	0.681	0.686	Mutual exclusivity
SMC5	9q21.12	0 (0.00%)	1 (0.41%)	<-10	0.681	0.686	Mutual exclusivity
SMCO4	11q21	0 (0.00%)	1 (0.41%)	<-10	0.681	0.686	Mutual exclusivity
SMIM26	20p11.23	0 (0.00%)	1 (0.41%)	<-10	0.681	0.686	Mutual exclusivity
SMIM8	6q15	0 (0.00%)	1 (0.41%)	<-10	0.681	0.686	Mutual exclusivity
SMOX	20p13	0 (0.00%)	1 (0.41%)	<-10	0.681	0.686	Mutual exclusivity
SMPD2	6q21	0 (0.00%)	1 (0.41%)	<-10	0.681	0.686	Mutual exclusivity
SMTNL1	11q12.1	0 (0.00%)	1 (0.41%)	<-10	0.681	0.686	Mutual exclusivity
SMU1	9p21.1	0 (0.00%)	1 (0.41%)	<-10	0.681	0.686	Mutual exclusivity
SMUG1	12q13.13	0 (0.00%)	1 (0.41%)	<-10	0.681	0.686	Mutual exclusivity
SNAP25	20p12.2	0 (0.00%)	1 (0.41%)	<-10	0.681	0.686	Mutual exclusivity
SNCA	4q22.1	0 (0.00%)	1 (0.41%)	<-10	0.681	0.686	Mutual exclusivity
SNED1	2q37.3	0 (0.00%)	1 (0.41%)	<-10	0.681	0.686	Mutual exclusivity

SNHG10	14q32.13	0 (0.00%)	1 (0.41%)	<-10	0.681	0.686	Mutual exclusivity
SNHG5	6q14.3	0 (0.00%)	1 (0.41%)	<-10	0.681	0.686	Mutual exclusivity
SNORA11B	14q32.11	0 (0.00%)	1 (0.41%)	<-10	0.681	0.686	Mutual exclusivity
SNORA11D	Xp11.22	0 (0.00%)	1 (0.41%)	<-10	0.681	0.686	Mutual exclusivity
SNORA11E	Xp11.22	0 (0.00%)	1 (0.41%)	<-10	0.681	0.686	Mutual exclusivity
SNORA12	10q24.31	0 (0.00%)	1 (0.41%)	<-10	0.681	0.686	Mutual exclusivity
SNORA20	6q25.3	0 (0.00%)	1 (0.41%)	<-10	0.681	0.686	Mutual exclusivity
SNORA29	6q25.3	0 (0.00%)	1 (0.41%)	<-10	0.681	0.686	Mutual exclusivity
SNORA35	Xq23	0 (0.00%)	1 (0.41%)	<-10	0.681	0.686	Mutual exclusivity
SNORA47	5q13.3	0 (0.00%)	1 (0.41%)	<-10	0.681	0.686	Mutual exclusivity
SNORA70B	2p15	0 (0.00%)	1 (0.41%)	<-10	0.681	0.686	Mutual exclusivity
SNORA70E	11q14.1	0 (0.00%)	1 (0.41%)	<-10	0.681	0.686	Mutual exclusivity
SNORA80E	1q22	0 (0.00%)	1 (0.41%)	<-10	0.681	0.686	Mutual exclusivity
SNORC	2q37.1	0 (0.00%)	1 (0.41%)	<-10	0.681	0.686	Mutual exclusivity
SNORD103C	1p35.2	0 (0.00%)	1 (0.41%)	<-10	0.681	0.686	Mutual exclusivity
SNORD105	19p13.2	0 (0.00%)	1 (0.41%)	<-10	0.681	0.686	Mutual exclusivity
SNORD105B	19p13.2	0 (0.00%)	1 (0.41%)	<-10	0.681	0.686	Mutual exclusivity
SNORD110	20p13	0 (0.00%)	1 (0.41%)	<-10	0.681	0.686	Mutual exclusivity
SNORD119	20p13	0 (0.00%)	1 (0.41%)	<-10	0.681	0.686	Mutual exclusivity
SNORD127	14q21.2	0 (0.00%)	1 (0.41%)	<-10	0.681	0.686	Mutual exclusivity
SNORD27	11q12.3	0 (0.00%)	1 (0.41%)	<-10	0.681	0.686	Mutual exclusivity
SNORD28	11q12.3	0 (0.00%)	1 (0.41%)	<-10	0.681	0.686	Mutual exclusivity
SNORD30	11q12.3	0 (0.00%)	1 (0.41%)	<-10	0.681	0.686	Mutual exclusivity
SNORD57	20p13	0 (0.00%)	1 (0.41%)	<-10	0.681	0.686	Mutual exclusivity
SNORD58A	18q21.1	0 (0.00%)	1 (0.41%)	<-10	0.681	0.686	Mutual exclusivity
SNORD58B	18q21.1	0 (0.00%)	1 (0.41%)	<-10	0.681	0.686	Mutual exclusivity
SNORD58C	18q21.1	0 (0.00%)	1 (0.41%)	<-10	0.681	0.686	Mutual exclusivity
SNORD6	11q21	0 (0.00%)	1 (0.41%)	<-10	0.681	0.686	Mutual exclusivity
SNORD61	Xq26.3	0 (0.00%)	1 (0.41%)	<-10	0.681	0.686	Mutual exclusivity

SNORD86	20p13	0 (0.00%)	1 (0.41%)	<-10	0.681	0.686	Mutual exclusivity
SNORD98	10q21.3	0 (0.00%)	1 (0.41%)	<-10	0.681	0.686	Mutual exclusivity
SNRNP200	2q11.2	0 (0.00%)	1 (0.41%)	<-10	0.681	0.686	Mutual exclusivity
SNRPB	20p13	0 (0.00%)	1 (0.41%)	<-10	0.681	0.686	Mutual exclusivity
SNTB2	16q22.1	0 (0.00%)	1 (0.41%)	<-10	0.681	0.686	Mutual exclusivity
SNX14	6q14.3	0 (0.00%)	1 (0.41%)	<-10	0.681	0.686	Mutual exclusivity
SNX18	5q11.2	0 (0.00%)	1 (0.41%)	<-10	0.681	0.686	Mutual exclusivity
SNX19	11q24.3-q25	0 (0.00%)	1 (0.41%)	<-10	0.681	0.686	Mutual exclusivity
SNX21	20q13.12	0 (0.00%)	1 (0.41%)	<-10	0.681	0.686	Mutual exclusivity
SNX3	6q21	0 (0.00%)	1 (0.41%)	<-10	0.681	0.686	Mutual exclusivity
SNX4	3q21.2	0 (0.00%)	1 (0.41%)	<-10	0.681	0.686	Mutual exclusivity
SNX6	14q13.1	0 (0.00%)	1 (0.41%)	<-10	0.681	0.686	Mutual exclusivity
SNX9	6q25.3	0 (0.00%)	1 (0.41%)	<-10	0.681	0.686	Mutual exclusivity
SOBP	6q21	0 (0.00%)	1 (0.41%)	<-10	0.681	0.686	Mutual exclusivity
SOCS4	14q22.3	0 (0.00%)	1 (0.41%)	<-10	0.681	0.686	Mutual exclusivity
SOCS6	18q22.2	0 (0.00%)	1 (0.41%)	<-10	0.681	0.686	Mutual exclusivity
SOD2	6q25.3	0 (0.00%)	1 (0.41%)	<-10	0.681	0.686	Mutual exclusivity
SORBS2	4q35.1	0 (0.00%)	1 (0.41%)	<-10	0.681	0.686	Mutual exclusivity
SORL1	11q24.1	0 (0.00%)	1 (0.41%)	<-10	0.681	0.686	Mutual exclusivity
SOSTDC1	7p21.2	0 (0.00%)	1 (0.41%)	<-10	0.681	0.686	Mutual exclusivity
SOWAHC	2q13	0 (0.00%)	1 (0.41%)	<-10	0.681	0.686	Mutual exclusivity
SP100	2q37.1	0 (0.00%)	1 (0.41%)	<-10	0.681	0.686	Mutual exclusivity
SP110	2q37.1	0 (0.00%)	1 (0.41%)	<-10	0.681	0.686	Mutual exclusivity
SP140	2q37.1	0 (0.00%)	1 (0.41%)	<-10	0.681	0.686	Mutual exclusivity
SP140L	2q37.1	0 (0.00%)	1 (0.41%)	<-10	0.681	0.686	Mutual exclusivity
SP7	12q13.13	0 (0.00%)	1 (0.41%)	<-10	0.681	0.686	Mutual exclusivity
SPACA1	6q15	0 (0.00%)	1 (0.41%)	<-10	0.681	0.686	Mutual exclusivity
SPACA4	19q13.33	0 (0.00%)	1 (0.41%)	<-10	0.681	0.686	Mutual exclusivity
SPAG16	2q34	0 (0.00%)	1 (0.41%)	<-10	0.681	0.686	Mutual exclusivity

SPANXN5	Xp11.22	0 (0.00%)	1 (0.41%)	<-10	0.681	0.686	Mutual exclusivity
SPARCL1	4q22.1	0 (0.00%)	1 (0.41%)	<-10	0.681	0.686	Mutual exclusivity
SPATA19	11q25	0 (0.00%)	1 (0.41%)	<-10	0.681	0.686	Mutual exclusivity
SPATA25	20q13.12	0 (0.00%)	1 (0.41%)	<-10	0.681	0.686	Mutual exclusivity
SPATA31D1	9q21.32	0 (0.00%)	1 (0.41%)	<-10	0.681	0.686	Mutual exclusivity
SPATC1L	21q22.3	0 (0.00%)	1 (0.41%)	<-10	0.681	0.686	Mutual exclusivity
SPEG	2q35	0 (0.00%)	1 (0.41%)	<-10	0.681	0.686	Mutual exclusivity
SPHK2	19q13.33	0 (0.00%)	1 (0.41%)	<-10	0.681	0.686	Mutual exclusivity
SPINDOC	11q13.1	0 (0.00%)	1 (0.41%)	<-10	0.681	0.686	Mutual exclusivity
SPINK2	4q12	0 (0.00%)	1 (0.41%)	<-10	0.681	0.686	Mutual exclusivity
SPINK4	9p13.3	0 (0.00%)	1 (0.41%)	<-10	0.681	0.686	Mutual exclusivity
SPINT3	20q13.12	0 (0.00%)	1 (0.41%)	<-10	0.681	0.686	Mutual exclusivity
SPINT4	20q13.12	0 (0.00%)	1 (0.41%)	<-10	0.681	0.686	Mutual exclusivity
SPP1	4q22.1	0 (0.00%)	1 (0.41%)	<-10	0.681	0.686	Mutual exclusivity
SPP2	2q37.1	0 (0.00%)	1 (0.41%)	<-10	0.681	0.686	Mutual exclusivity
SPRED2	2p14	0 (0.00%)	1 (0.41%)	<-10	0.681	0.686	Mutual exclusivity
SPTB	14q23.3	0 (0.00%)	1 (0.41%)	<-10	0.681	0.686	Mutual exclusivity
SPTBN1	2p16.2	0 (0.00%)	1 (0.41%)	<-10	0.681	0.686	Mutual exclusivity
SPTLC1	9q22.31	0 (0.00%)	1 (0.41%)	<-10	0.681	0.686	Mutual exclusivity
SPTSSA	14q13.1	0 (0.00%)	1 (0.41%)	<-10	0.681	0.686	Mutual exclusivity
SPZ1	5q14.1	0 (0.00%)	1 (0.41%)	<-10	0.681	0.686	Mutual exclusivity
SRD5A3	4q12	0 (0.00%)	1 (0.41%)	<-10	0.681	0.686	Mutual exclusivity
SRGN	10q22.1	0 (0.00%)	1 (0.41%)	<-10	0.681	0.686	Mutual exclusivity
SRPX	Xp11.4	0 (0.00%)	1 (0.41%)	<-10	0.681	0.686	Mutual exclusivity
SRRM1	1p36.11	0 (0.00%)	1 (0.41%)	<-10	0.681	0.686	Mutual exclusivity
SRSF10	1p36.11	0 (0.00%)	1 (0.41%)	<-10	0.681	0.686	Mutual exclusivity
SRSF8	11q21	0 (0.00%)	1 (0.41%)	<-10	0.681	0.686	Mutual exclusivity
SSR4P1	21q22.3	0 (0.00%)	1 (0.41%)	<-10	0.681	0.686	Mutual exclusivity
SSRP1	11q12.1	0 (0.00%)	1 (0.41%)	<-10	0.681	0.686	Mutual exclusivity

SSTR4	20p11.21	0 (0.00%)	1 (0.41%)	<-10	0.681	0.686	Mutual exclusivity
SSX2	Xp11.22	0 (0.00%)	1 (0.41%)	<-10	0.681	0.686	Mutual exclusivity
SSX2B	Xp11.22	0 (0.00%)	1 (0.41%)	<-10	0.681	0.686	Mutual exclusivity
SSX7	Xp11.22	0 (0.00%)	1 (0.41%)	<-10	0.681	0.686	Mutual exclusivity
ST3GAL6	3q12.1	0 (0.00%)	1 (0.41%)	<-10	0.681	0.686	Mutual exclusivity
ST6GAL2	2q12.3	0 (0.00%)	1 (0.41%)	<-10	0.681	0.686	Mutual exclusivity
ST8SIA5	18q21.1	0 (0.00%)	1 (0.41%)	<-10	0.681	0.686	Mutual exclusivity
STAMBPL1	10q23.31	0 (0.00%)	1 (0.41%)	<-10	0.681	0.686	Mutual exclusivity
STARD13	13q13.1-q13.2	0 (0.00%)	1 (0.41%)	<-10	0.681	0.686	Mutual exclusivity
STARD7	2q11.2	0 (0.00%)	1 (0.41%)	<-10	0.681	0.686	Mutual exclusivity
STEAP3	2q14.2	0 (0.00%)	1 (0.41%)	<-10	0.681	0.686	Mutual exclusivity
STK11IP	2q35	0 (0.00%)	1 (0.41%)	<-10	0.681	0.686	Mutual exclusivity
STK16	2q35	0 (0.00%)	1 (0.41%)	<-10	0.681	0.686	Mutual exclusivity
STK25	2q37.3	0 (0.00%)	1 (0.41%)	<-10	0.681	0.686	Mutual exclusivity
STK36	2q35	0 (0.00%)	1 (0.41%)	<-10	0.681	0.686	Mutual exclusivity
STK4	20q13.12	0 (0.00%)	1 (0.41%)	<-10	0.681	0.686	Mutual exclusivity
STMN1	1p36.11	0 (0.00%)	1 (0.41%)	<-10	0.681	0.686	Mutual exclusivity
STMN4	8p21.2	0 (0.00%)	1 (0.41%)	<-10	0.681	0.686	Mutual exclusivity
STOX1	10q22.1	0 (0.00%)	1 (0.41%)	<-10	0.681	0.686	Mutual exclusivity
STPG1	1p36.11	0 (0.00%)	1 (0.41%)	<-10	0.681	0.686	Mutual exclusivity
STPG2	4q22.3-q23	0 (0.00%)	1 (0.41%)	<-10	0.681	0.686	Mutual exclusivity
STX10	19p13.13	0 (0.00%)	1 (0.41%)	<-10	0.681	0.686	Mutual exclusivity
STX11	6q24.2	0 (0.00%)	1 (0.41%)	<-10	0.681	0.686	Mutual exclusivity
STX1B	16p11.2	0 (0.00%)	1 (0.41%)	<-10	0.681	0.686	Mutual exclusivity
STX3	11q12.1	0 (0.00%)	1 (0.41%)	<-10	0.681	0.686	Mutual exclusivity
STX4	16p11.2	0 (0.00%)	1 (0.41%)	<-10	0.681	0.686	Mutual exclusivity
STXBP5	6q24.3	0 (0.00%)	1 (0.41%)	<-10	0.681	0.686	Mutual exclusivity
SUFU	10q24.32	0 (0.00%)	1 (0.41%)	<-10	0.681	0.686	Mutual exclusivity
SULF2	20q13.12	0 (0.00%)	1 (0.41%)	<-10	0.681	0.686	Mutual exclusivity

SULT1C2	2q12.3	0 (0.00%)	1 (0.41%)	<-10	0.681	0.686	Mutual exclusivity
SULT1C2P1	2q12.3	0 (0.00%)	1 (0.41%)	<-10	0.681	0.686	Mutual exclusivity
SULT1C3	2q12.3	0 (0.00%)	1 (0.41%)	<-10	0.681	0.686	Mutual exclusivity
SULT1C4	2q12.3	0 (0.00%)	1 (0.41%)	<-10	0.681	0.686	Mutual exclusivity
SULT2B1	19q13.33	0 (0.00%)	1 (0.41%)	<-10	0.681	0.686	Mutual exclusivity
SUMO3	21q22.3	0 (0.00%)	1 (0.41%)	<-10	0.681	0.686	Mutual exclusivity
SUMO4	6q25.1	0 (0.00%)	1 (0.41%)	<-10	0.681	0.686	Mutual exclusivity
SUN5	20q11.21	0 (0.00%)	1 (0.41%)	<-10	0.681	0.686	Mutual exclusivity
SUPV3L1	10q22.1	0 (0.00%)	1 (0.41%)	<-10	0.681	0.686	Mutual exclusivity
SVBP	1p34.2	0 (0.00%)	1 (0.41%)	<-10	0.681	0.686	Mutual exclusivity
SYF2	1p36.11	0 (0.00%)	1 (0.41%)	<-10	0.681	0.686	Mutual exclusivity
SYK	9q22.2	0 (0.00%)	1 (0.41%)	<-10	0.681	0.686	Mutual exclusivity
SYNCRIP	6q14.3	0 (0.00%)	1 (0.41%)	<-10	0.681	0.686	Mutual exclusivity
SYNE3	14q32.13	0 (0.00%)	1 (0.41%)	<-10	0.681	0.686	Mutual exclusivity
SYNGR4	19q13.33	0 (0.00%)	1 (0.41%)	<-10	0.681	0.686	Mutual exclusivity
SYNJ2	6q25.3	0 (0.00%)	1 (0.41%)	<-10	0.681	0.686	Mutual exclusivity
SYTL2	11q14.1	0 (0.00%)	1 (0.41%)	<-10	0.681	0.686	Mutual exclusivity
SYTL3	6q25.3	0 (0.00%)	1 (0.41%)	<-10	0.681	0.686	Mutual exclusivity
SYTL5	Xp11.4	0 (0.00%)	1 (0.41%)	<-10	0.681	0.686	Mutual exclusivity
TAB2	6q25.1	0 (0.00%)	1 (0.41%)	<-10	0.681	0.686	Mutual exclusivity
TACC2	10q26.13	0 (0.00%)	1 (0.41%)	<-10	0.681	0.686	Mutual exclusivity
TACR2	10q22.1	0 (0.00%)	1 (0.41%)	<-10	0.681	0.686	Mutual exclusivity
TAF1	Xq13.1	0 (0.00%)	1 (0.41%)	<-10	0.681	0.686	Mutual exclusivity
TAF1D	11q21	0 (0.00%)	1 (0.41%)	<-10	0.681	0.686	Mutual exclusivity
TAF6L	11q12.3	0 (0.00%)	1 (0.41%)	<-10	0.681	0.686	Mutual exclusivity
TAGAP	6q25.3	0 (0.00%)	1 (0.41%)	<-10	0.681	0.686	Mutual exclusivity
TAGLN	11q23.3	0 (0.00%)	1 (0.41%)	<-10	0.681	0.686	Mutual exclusivity
TAS1R2	1p36.13	0 (0.00%)	1 (0.41%)	<-10	0.681	0.686	Mutual exclusivity
TBC1D23	3q12.1-q12.2	0 (0.00%)	1 (0.41%)	<-10	0.681	0.686	Mutual exclusivity

TBC1D27P	17p11.2	0 (0.00%)	1 (0.41%)	<-10	0.681	0.686	Mutual exclusivity
TBC1D28	17p11.2	0 (0.00%)	1 (0.41%)	<-10	0.681	0.686	Mutual exclusivity
TBCA	5q14.1	0 (0.00%)	1 (0.41%)	<-10	0.681	0.686	Mutual exclusivity
TBP	6q27	0 (0.00%)	1 (0.41%)	<-10	0.681	0.686	Mutual exclusivity
TBPL2	14q22.3	0 (0.00%)	1 (0.41%)	<-10	0.681	0.686	Mutual exclusivity
TCEA3	1p36.12	0 (0.00%)	1 (0.41%)	<-10	0.681	0.686	Mutual exclusivity
TCEAL1	Xq22.2	0 (0.00%)	1 (0.41%)	<-10	0.681	0.686	Mutual exclusivity
TCEAL3	Xq22.2	0 (0.00%)	1 (0.41%)	<-10	0.681	0.686	Mutual exclusivity
TCEAL4	Xq22.2	0 (0.00%)	1 (0.41%)	<-10	0.681	0.686	Mutual exclusivity
TCL1A	14q32.13	0 (0.00%)	1 (0.41%)	<-10	0.681	0.686	Mutual exclusivity
TCL1B	14q32.13	0 (0.00%)	1 (0.41%)	<-10	0.681	0.686	Mutual exclusivity
TCL6	14q32.13	0 (0.00%)	1 (0.41%)	<-10	0.681	0.686	Mutual exclusivity
TCN1	11q12.1	0 (0.00%)	1 (0.41%)	<-10	0.681	0.686	Mutual exclusivity
TCP1	6q25.3	0 (0.00%)	1 (0.41%)	<-10	0.681	0.686	Mutual exclusivity
TCP10	6q27	0 (0.00%)	1 (0.41%)	<-10	0.681	0.686	Mutual exclusivity
TCP10L2	6q27	0 (0.00%)	1 (0.41%)	<-10	0.681	0.686	Mutual exclusivity
TCP11L1	11p13	0 (0.00%)	1 (0.41%)	<-10	0.681	0.686	Mutual exclusivity
TCTE3	6q27	0 (0.00%)	1 (0.41%)	<-10	0.681	0.686	Mutual exclusivity
TDO2	4q32.1	0 (0.00%)	1 (0.41%)	<-10	0.681	0.686	Mutual exclusivity
TDP1	14q32.11	0 (0.00%)	1 (0.41%)	<-10	0.681	0.686	Mutual exclusivity
TEAD4	12p13.33	0 (0.00%)	1 (0.41%)	<-10	0.681	0.686	Mutual exclusivity
TECTA	11q23.3	0 (0.00%)	1 (0.41%)	<-10	0.681	0.686	Mutual exclusivity
TENT5A	6q14.1	0 (0.00%)	1 (0.41%)	<-10	0.681	0.686	Mutual exclusivity
TEX12	11q23.1	0 (0.00%)	1 (0.41%)	<-10	0.681	0.686	Mutual exclusivity
TEX13A	Xq22.3	0 (0.00%)	1 (0.41%)	<-10	0.681	0.686	Mutual exclusivity
TEX13B	Xq22.3	0 (0.00%)	1 (0.41%)	<-10	0.681	0.686	Mutual exclusivity
TEX36	10q26.13	0 (0.00%)	1 (0.41%)	<-10	0.681	0.686	Mutual exclusivity
TEX37	2p11.2	0 (0.00%)	1 (0.41%)	<-10	0.681	0.686	Mutual exclusivity
TEX46	1p36.12	0 (0.00%)	1 (0.41%)	<-10	0.681	0.686	Mutual exclusivity

TEX49	12q13.12	0 (0.00%)	1 (0.41%)	<-10	0.681	0.686	Mutual exclusivity
TFB1M	6q25.3	0 (0.00%)	1 (0.41%)	<-10	0.681	0.686	Mutual exclusivity
TGFBRAP1	2q12.1-q12.2	0 (0.00%)	1 (0.41%)	<-10	0.681	0.686	Mutual exclusivity
TGM3	20p13	0 (0.00%)	1 (0.41%)	<-10	0.681	0.686	Mutual exclusivity
TGM6	20p13	0 (0.00%)	1 (0.41%)	<-10	0.681	0.686	Mutual exclusivity
THAP11	16q22.1	0 (0.00%)	1 (0.41%)	<-10	0.681	0.686	Mutual exclusivity
THAP4	2q37.3	0 (0.00%)	1 (0.41%)	<-10	0.681	0.686	Mutual exclusivity
THBD	20p11.21	0 (0.00%)	1 (0.41%)	<-10	0.681	0.686	Mutual exclusivity
THBS2	6q27	0 (0.00%)	1 (0.41%)	<-10	0.681	0.686	Mutual exclusivity
THEGL	4q12	0 (0.00%)	1 (0.41%)	<-10	0.681	0.686	Mutual exclusivity
THEMIS	6q22.33	0 (0.00%)	1 (0.41%)	<-10	0.681	0.686	Mutual exclusivity
TIGAR	12p13.32	0 (0.00%)	1 (0.41%)	<-10	0.681	0.686	Mutual exclusivity
TIGD1	2q37.1	0 (0.00%)	1 (0.41%)	<-10	0.681	0.686	Mutual exclusivity
TIGD2	4q22.1	0 (0.00%)	1 (0.41%)	<-10	0.681	0.686	Mutual exclusivity
TIGD4	4q31.3	0 (0.00%)	1 (0.41%)	<-10	0.681	0.686	Mutual exclusivity
TIMM10	11q12.1	0 (0.00%)	1 (0.41%)	<-10	0.681	0.686	Mutual exclusivity
TINCR	19p13.3	0 (0.00%)	1 (0.41%)	<-10	0.681	0.686	Mutual exclusivity
TJP2	9q21.11	0 (0.00%)	1 (0.41%)	<-10	0.681	0.686	Mutual exclusivity
TKT	3p21.1	0 (0.00%)	1 (0.41%)	<-10	0.681	0.686	Mutual exclusivity
TLE1	9q21.32	0 (0.00%)	1 (0.41%)	<-10	0.681	0.686	Mutual exclusivity
TLR3	4q35.1	0 (0.00%)	1 (0.41%)	<-10	0.681	0.686	Mutual exclusivity
TM9SF4	20q11.21	0 (0.00%)	1 (0.41%)	<-10	0.681	0.686	Mutual exclusivity
TMC1	9q21.13	0 (0.00%)	1 (0.41%)	<-10	0.681	0.686	Mutual exclusivity
TMC2	20p13	0 (0.00%)	1 (0.41%)	<-10	0.681	0.686	Mutual exclusivity
TMCO4	1p36.13	0 (0.00%)	1 (0.41%)	<-10	0.681	0.686	Mutual exclusivity
TMEM106B	7p21.3	0 (0.00%)	1 (0.41%)	<-10	0.681	0.686	Mutual exclusivity
TMEM126A	11q14.1	0 (0.00%)	1 (0.41%)	<-10	0.681	0.686	Mutual exclusivity
TMEM126B	11q14.1	0 (0.00%)	1 (0.41%)	<-10	0.681	0.686	Mutual exclusivity
TMEM127	2q11.2	0 (0.00%)	1 (0.41%)	<-10	0.681	0.686	Mutual exclusivity

TMEM131	2q11.2	0 (0.00%)	1 (0.41%)	<-10	0.681	0.686	Mutual exclusivity
TMEM135	11q14.2	0 (0.00%)	1 (0.41%)	<-10	0.681	0.686	Mutual exclusivity
TMEM143	19q13.33	0 (0.00%)	1 (0.41%)	<-10	0.681	0.686	Mutual exclusivity
TMEM161B	5q14.3	0 (0.00%)	1 (0.41%)	<-10	0.681	0.686	Mutual exclusivity
TMEM167A	5q14.2	0 (0.00%)	1 (0.41%)	<-10	0.681	0.686	Mutual exclusivity
TMEM179B	11q12.3	0 (0.00%)	1 (0.41%)	<-10	0.681	0.686	Mutual exclusivity
TMEM181	6q25.3	0 (0.00%)	1 (0.41%)	<-10	0.681	0.686	Mutual exclusivity
TMEM192	4q32.3	0 (0.00%)	1 (0.41%)	<-10	0.681	0.686	Mutual exclusivity
TMEM198	2q35	0 (0.00%)	1 (0.41%)	<-10	0.681	0.686	Mutual exclusivity
TMEM223	11q12.3	0 (0.00%)	1 (0.41%)	<-10	0.681	0.686	Mutual exclusivity
TMEM229B	14q24.1	0 (0.00%)	1 (0.41%)	<-10	0.681	0.686	Mutual exclusivity
TMEM239	20p13	0 (0.00%)	1 (0.41%)	<-10	0.681	0.686	Mutual exclusivity
TMEM244	6q22.33	0 (0.00%)	1 (0.41%)	<-10	0.681	0.686	Mutual exclusivity
TMEM246	9q31.1	0 (0.00%)	1 (0.41%)	<-10	0.681	0.686	Mutual exclusivity
TMEM260	14q22.3	0 (0.00%)	1 (0.41%)	<-10	0.681	0.686	Mutual exclusivity
TMEM30A	6q14.1	0 (0.00%)	1 (0.41%)	<-10	0.681	0.686	Mutual exclusivity
TMEM30CP	3q12.1	0 (0.00%)	1 (0.41%)	<-10	0.681	0.686	Mutual exclusivity
TMEM31	Xq22.2	0 (0.00%)	1 (0.41%)	<-10	0.681	0.686	Mutual exclusivity
TMEM45B	11q24.3	0 (0.00%)	1 (0.41%)	<-10	0.681	0.686	Mutual exclusivity
TMEM50A	1p36.11	0 (0.00%)	1 (0.41%)	<-10	0.681	0.686	Mutual exclusivity
TMOD1	9q22.33	0 (0.00%)	1 (0.41%)	<-10	0.681	0.686	Mutual exclusivity
TMPRSS4	11q23.3	0 (0.00%)	1 (0.41%)	<-10	0.681	0.686	Mutual exclusivity
TMSB15B	Xq22.2	0 (0.00%)	1 (0.41%)	<-10	0.681	0.686	Mutual exclusivity
TMX2	11q12.1	0 (0.00%)	1 (0.41%)	<-10	0.681	0.686	Mutual exclusivity
TNFAIP8L3	15q21.2	0 (0.00%)	1 (0.41%)	<-10	0.681	0.686	Mutual exclusivity
TNFRSF13B	17p11.2	0 (0.00%)	1 (0.41%)	<-10	0.681	0.686	Mutual exclusivity
TNFSF14	19p13.3	0 (0.00%)	1 (0.41%)	<-10	0.681	0.686	Mutual exclusivity
TNFSF9	19p13.3	0 (0.00%)	1 (0.41%)	<-10	0.681	0.686	Mutual exclusivity
TNIP3	4q27	0 (0.00%)	1 (0.41%)	<-10	0.681	0.686	Mutual exclusivity

TNKS1BP1	11q12.1	0 (0.00%)	1 (0.41%)	<-10	0.681	0.686	Mutual exclusivity
TNKS2	10q23.32	0 (0.00%)	1 (0.41%)	<-10	0.681	0.686	Mutual exclusivity
TNNC2	20q13.12	0 (0.00%)	1 (0.41%)	<-10	0.681	0.686	Mutual exclusivity
TNP1	2q35	0 (0.00%)	1 (0.41%)	<-10	0.681	0.686	Mutual exclusivity
TOMM34	20q13.12	0 (0.00%)	1 (0.41%)	<-10	0.681	0.686	Mutual exclusivity
TOMM70	3q12.2	0 (0.00%)	1 (0.41%)	<-10	0.681	0.686	Mutual exclusivity
TOP3B	22q11.22	0 (0.00%)	1 (0.41%)	<-10	0.681	0.686	Mutual exclusivity
TP53AIP1	11q24.3	0 (0.00%)	1 (0.41%)	<-10	0.681	0.686	Mutual exclusivity
TP53TG5	20q13.12	0 (0.00%)	1 (0.41%)	<-10	0.681	0.686	Mutual exclusivity
TRAF3	14q32.32	0 (0.00%)	1 (0.41%)	<-10	0.681	0.686	Mutual exclusivity
TRAF6	11p12	0 (0.00%)	1 (0.41%)	<-10	0.681	0.686	Mutual exclusivity
TRDN	6q22.31	0 (0.00%)	1 (0.41%)	<-10	0.681	0.686	Mutual exclusivity
TRIM16L	17p11.2	0 (0.00%)	1 (0.41%)	<-10	0.681	0.686	Mutual exclusivity
TRIM35	8p21.2	0 (0.00%)	1 (0.41%)	<-10	0.681	0.686	Mutual exclusivity
TRIM72	16p11.2	0 (0.00%)	1 (0.41%)	<-10	0.681	0.686	Mutual exclusivity
TRIM8	10q24.32	0 (0.00%)	1 (0.41%)	<-10	0.681	0.686	Mutual exclusivity
TRIP10	19p13.3	0 (0.00%)	1 (0.41%)	<-10	0.681	0.686	Mutual exclusivity
TRIP11	14q32.12	0 (0.00%)	1 (0.41%)	<-10	0.681	0.686	Mutual exclusivity
TRIP12	2q36.3	0 (0.00%)	1 (0.41%)	<-10	0.681	0.686	Mutual exclusivity
TRMT1	19p13.13	0 (0.00%)	1 (0.41%)	<-10	0.681	0.686	Mutual exclusivity
TRPM2	21q22.3	0 (0.00%)	1 (0.41%)	<-10	0.681	0.686	Mutual exclusivity
TRPM8	2q37.1	0 (0.00%)	1 (0.41%)	<-10	0.681	0.686	Mutual exclusivity
TSGA10	2q11.2	0 (0.00%)	1 (0.41%)	<-10	0.681	0.686	Mutual exclusivity
TSNAXIP1	16q22.1	0 (0.00%)	1 (0.41%)	<-10	0.681	0.686	Mutual exclusivity
TSPAN9	12p13.33-p13.32	0 (0.00%)	1 (0.41%)	<-10	0.681	0.686	Mutual exclusivity
TSPEAR	21q22.3	0 (0.00%)	1 (0.41%)	<-10	0.681	0.686	Mutual exclusivity
TSPEAR-AS2	21q22.3	0 (0.00%)	1 (0.41%)	<-10	0.681	0.686	Mutual exclusivity
TSPY26P	20q11.21	0 (0.00%)	1 (0.41%)	<-10	0.681	0.686	Mutual exclusivity
TSPYL2	Xp11.22	0 (0.00%)	1 (0.41%)	<-10	0.681	0.686	Mutual exclusivity

TSPYL6	2p16.2	0 (0.00%)	1 (0.41%)	<-10	0.681	0.686	Mutual exclusivity
TSTD2	9q22.33	0 (0.00%)	1 (0.41%)	<-10	0.681	0.686	Mutual exclusivity
TTC19	17p12	0 (0.00%)	1 (0.41%)	<-10	0.681	0.686	Mutual exclusivity
TTC37	5q15	0 (0.00%)	1 (0.41%)	<-10	0.681	0.686	Mutual exclusivity
TTC39B	9p22.3	0 (0.00%)	1 (0.41%)	<-10	0.681	0.686	Mutual exclusivity
TTC7B	14q32.11	0 (0.00%)	1 (0.41%)	<-10	0.681	0.686	Mutual exclusivity
TTC9	14q24.2	0 (0.00%)	1 (0.41%)	<-10	0.681	0.686	Mutual exclusivity
TTC9C	11q12.3	0 (0.00%)	1 (0.41%)	<-10	0.681	0.686	Mutual exclusivity
TTLL2	6q27	0 (0.00%)	1 (0.41%)	<-10	0.681	0.686	Mutual exclusivity
TTLL4	2q35	0 (0.00%)	1 (0.41%)	<-10	0.681	0.686	Mutual exclusivity
TTLL5	14q24.3	0 (0.00%)	1 (0.41%)	<-10	0.681	0.686	Mutual exclusivity
TUBA1A	12q13.12	0 (0.00%)	1 (0.41%)	<-10	0.681	0.686	Mutual exclusivity
TUBA1B	12q13.12	0 (0.00%)	1 (0.41%)	<-10	0.681	0.686	Mutual exclusivity
TUBA1C	12q13.12	0 (0.00%)	1 (0.41%)	<-10	0.681	0.686	Mutual exclusivity
TUBA4A	2q35	0 (0.00%)	1 (0.41%)	<-10	0.681	0.686	Mutual exclusivity
TUBA4B	2q35	0 (0.00%)	1 (0.41%)	<-10	0.681	0.686	Mutual exclusivity
TUBB4A	19p13.3	0 (0.00%)	1 (0.41%)	<-10	0.681	0.686	Mutual exclusivity
TULP3	12p13.33	0 (0.00%)	1 (0.41%)	<-10	0.681	0.686	Mutual exclusivity
TULP4	6q25.3	0 (0.00%)	1 (0.41%)	<-10	0.681	0.686	Mutual exclusivity
TUNAR	14q32.2	0 (0.00%)	1 (0.41%)	<-10	0.681	0.686	Mutual exclusivity
TVP23B	17p11.2	0 (0.00%)	1 (0.41%)	<-10	0.681	0.686	Mutual exclusivity
TWIST2	2q37.3	0 (0.00%)	1 (0.41%)	<-10	0.681	0.686	Mutual exclusivity
TXNDC9	2q11.2	0 (0.00%)	1 (0.41%)	<-10	0.681	0.686	Mutual exclusivity
TXNRD3	3q21.3	0 (0.00%)	1 (0.41%)	<-10	0.681	0.686	Mutual exclusivity
TXNRD3NB	3q21.3	0 (0.00%)	1 (0.41%)	<-10	0.681	0.686	Mutual exclusivity
TYK2	19p13.2	0 (0.00%)	1 (0.41%)	<-10	0.681	0.686	Mutual exclusivity
TYR	11q14.3	0 (0.00%)	1 (0.41%)	<-10	0.681	0.686	Mutual exclusivity
TYRP1	9p23	0 (0.00%)	1 (0.41%)	<-10	0.681	0.686	Mutual exclusivity
UBE2C	20q13.12	0 (0.00%)	1 (0.41%)	<-10	0.681	0.686	Mutual exclusivity

UBE2G2	21q22.3	0 (0.00%)	1 (0.41%)	<-10	0.681	0.686	Mutual exclusivity
UBE2L6	11q12.1	0 (0.00%)	1 (0.41%)	<-10	0.681	0.686	Mutual exclusivity
UBE3D	6q14.1	0 (0.00%)	1 (0.41%)	<-10	0.681	0.686	Mutual exclusivity
UBE4A	11q23.3	0 (0.00%)	1 (0.41%)	<-10	0.681	0.686	Mutual exclusivity
UBOX5	20p13	0 (0.00%)	1 (0.41%)	<-10	0.681	0.686	Mutual exclusivity
UBTD1	10q24.1-q24.2	0 (0.00%)	1 (0.41%)	<-10	0.681	0.686	Mutual exclusivity
UBXN1	11q12.3	0 (0.00%)	1 (0.41%)	<-10	0.681	0.686	Mutual exclusivity
UBXN4	2q21.3	0 (0.00%)	1 (0.41%)	<-10	0.681	0.686	Mutual exclusivity
UBXN8	8p12	0 (0.00%)	1 (0.41%)	<-10	0.681	0.686	Mutual exclusivity
UCP2	11q13.4	0 (0.00%)	1 (0.41%)	<-10	0.681	0.686	Mutual exclusivity
UCP3	11q13.4	0 (0.00%)	1 (0.41%)	<-10	0.681	0.686	Mutual exclusivity
UGT1A1	2q37.1	0 (0.00%)	1 (0.41%)	<-10	0.681	0.686	Mutual exclusivity
UGT1A10	2q37.1	0 (0.00%)	1 (0.41%)	<-10	0.681	0.686	Mutual exclusivity
UGT1A3	2q37.1	0 (0.00%)	1 (0.41%)	<-10	0.681	0.686	Mutual exclusivity
UGT1A4	2q37.1	0 (0.00%)	1 (0.41%)	<-10	0.681	0.686	Mutual exclusivity
UGT1A5	2q37.1	0 (0.00%)	1 (0.41%)	<-10	0.681	0.686	Mutual exclusivity
UGT1A6	2q37.1	0 (0.00%)	1 (0.41%)	<-10	0.681	0.686	Mutual exclusivity
UGT1A7	2q37.1	0 (0.00%)	1 (0.41%)	<-10	0.681	0.686	Mutual exclusivity
UGT1A8	2q37.1	0 (0.00%)	1 (0.41%)	<-10	0.681	0.686	Mutual exclusivity
UGT1A9	2q37.1	0 (0.00%)	1 (0.41%)	<-10	0.681	0.686	Mutual exclusivity
ULBP1	6q25.1	0 (0.00%)	1 (0.41%)	<-10	0.681	0.686	Mutual exclusivity
ULBP2	6q25.1	0 (0.00%)	1 (0.41%)	<-10	0.681	0.686	Mutual exclusivity
ULBP3	6q25.1	0 (0.00%)	1 (0.41%)	<-10	0.681	0.686	Mutual exclusivity
UNC50	2q11.2	0 (0.00%)	1 (0.41%)	<-10	0.681	0.686	Mutual exclusivity
UNC93A	6q27	0 (0.00%)	1 (0.41%)	<-10	0.681	0.686	Mutual exclusivity
UQCC3	11q12.3	0 (0.00%)	1 (0.41%)	<-10	0.681	0.686	Mutual exclusivity
UROC1	3q21.3	0 (0.00%)	1 (0.41%)	<-10	0.681	0.686	Mutual exclusivity
UROS	10q26.2	0 (0.00%)	1 (0.41%)	<-10	0.681	0.686	Mutual exclusivity
USP32P2	17p11.2	0 (0.00%)	1 (0.41%)	<-10	0.681	0.686	Mutual exclusivity

USP34	2p15	0 (0.00%)	1 (0.41%)	<-10	0.681	0.686	Mutual exclusivity
UTP4	16q22.1	0 (0.00%)	1 (0.41%)	<-10	0.681	0.686	Mutual exclusivity
UXS1	2q12.2	0 (0.00%)	1 (0.41%)	<-10	0.681	0.686	Mutual exclusivity
VAV1	19p13.3	0 (0.00%)	1 (0.41%)	<-10	0.681	0.686	Mutual exclusivity
VCAN	5q14.2-q14.3	0 (0.00%)	1 (0.41%)	<-10	0.681	0.686	Mutual exclusivity
VGLL3	3p12.1	0 (0.00%)	1 (0.41%)	<-10	0.681	0.686	Mutual exclusivity
VIP	6q25.2	0 (0.00%)	1 (0.41%)	<-10	0.681	0.686	Mutual exclusivity
VKORC1	16p11.2	0 (0.00%)	1 (0.41%)	<-10	0.681	0.686	Mutual exclusivity
VMAC	19p13.3	0 (0.00%)	1 (0.41%)	<-10	0.681	0.686	Mutual exclusivity
VPREB1	22q11.22	0 (0.00%)	1 (0.41%)	<-10	0.681	0.686	Mutual exclusivity
VPS16	20p13	0 (0.00%)	1 (0.41%)	<-10	0.681	0.686	Mutual exclusivity
VPS26A	10q22.1	0 (0.00%)	1 (0.41%)	<-10	0.681	0.686	Mutual exclusivity
VPS37A	8p22	0 (0.00%)	1 (0.41%)	<-10	0.681	0.686	Mutual exclusivity
VRK2	2p16.1	0 (0.00%)	1 (0.41%)	<-10	0.681	0.686	Mutual exclusivity
VSIG1	Xq22.3	0 (0.00%)	1 (0.41%)	<-10	0.681	0.686	Mutual exclusivity
VSTM5	11q21	0 (0.00%)	1 (0.41%)	<-10	0.681	0.686	Mutual exclusivity
VWA3B	2q11.2	0 (0.00%)	1 (0.41%)	<-10	0.681	0.686	Mutual exclusivity
VWC2L	2q34-q35	0 (0.00%)	1 (0.41%)	<-10	0.681	0.686	Mutual exclusivity
VWDE	7p21.3	0 (0.00%)	1 (0.41%)	<-10	0.681	0.686	Mutual exclusivity
WASH2P	2q14.1	0 (0.00%)	1 (0.41%)	<-10	0.681	0.686	Mutual exclusivity
WBP1L	10q24.32	0 (0.00%)	1 (0.41%)	<-10	0.681	0.686	Mutual exclusivity
WDFY1	2q36.1	0 (0.00%)	1 (0.41%)	<-10	0.681	0.686	Mutual exclusivity
WDR17	4q34.2	0 (0.00%)	1 (0.41%)	<-10	0.681	0.686	Mutual exclusivity
WDR27	6q27	0 (0.00%)	1 (0.41%)	<-10	0.681	0.686	Mutual exclusivity
WDR41	5q13.3-q14.1	0 (0.00%)	1 (0.41%)	<-10	0.681	0.686	Mutual exclusivity
WDR7	18q21.31	0 (0.00%)	1 (0.41%)	<-10	0.681	0.686	Mutual exclusivity
WFDC10A	20q13.12	0 (0.00%)	1 (0.41%)	<-10	0.681	0.686	Mutual exclusivity
WFDC10B	20q13.12	0 (0.00%)	1 (0.41%)	<-10	0.681	0.686	Mutual exclusivity
WFDC11	20q13.12	0 (0.00%)	1 (0.41%)	<-10	0.681	0.686	Mutual exclusivity

WFDC12	20q13.12	0 (0.00%)	1 (0.41%)	<-10	0.681	0.686	Mutual exclusivity
WFDC13	20q13.12	0 (0.00%)	1 (0.41%)	<-10	0.681	0.686	Mutual exclusivity
WFDC2	20q13.12	0 (0.00%)	1 (0.41%)	<-10	0.681	0.686	Mutual exclusivity
WFDC3	20q13.12	0 (0.00%)	1 (0.41%)	<-10	0.681	0.686	Mutual exclusivity
WFDC5	20q13.12	0 (0.00%)	1 (0.41%)	<-10	0.681	0.686	Mutual exclusivity
WFDC6	20q13.12	0 (0.00%)	1 (0.41%)	<-10	0.681	0.686	Mutual exclusivity
WFDC8	20q13.12	0 (0.00%)	1 (0.41%)	<-10	0.681	0.686	Mutual exclusivity
WFDC9	20q13.12	0 (0.00%)	1 (0.41%)	<-10	0.681	0.686	Mutual exclusivity
WHRN	9q32	0 (0.00%)	1 (0.41%)	<-10	0.681	0.686	Mutual exclusivity
WNT1	12q13.12	0 (0.00%)	1 (0.41%)	<-10	0.681	0.686	Mutual exclusivity
WNT10A	2q35	0 (0.00%)	1 (0.41%)	<-10	0.681	0.686	Mutual exclusivity
WNT10B	12q13.12	0 (0.00%)	1 (0.41%)	<-10	0.681	0.686	Mutual exclusivity
WNT4	1p36.12	0 (0.00%)	1 (0.41%)	<-10	0.681	0.686	Mutual exclusivity
WNT5B	12p13.33	0 (0.00%)	1 (0.41%)	<-10	0.681	0.686	Mutual exclusivity
WNT6	2q35	0 (0.00%)	1 (0.41%)	<-10	0.681	0.686	Mutual exclusivity
WRN	8p12	0 (0.00%)	1 (0.41%)	<-10	0.681	0.686	Mutual exclusivity
WT1	11p13	0 (0.00%)	1 (0.41%)	<-10	0.681	0.686	Mutual exclusivity
WTAP	6q25.3	0 (0.00%)	1 (0.41%)	<-10	0.681	0.686	Mutual exclusivity
XAGE1A	Xp11.22	0 (0.00%)	1 (0.41%)	<-10	0.681	0.686	Mutual exclusivity
XAGE1B	Xp11.22	0 (0.00%)	1 (0.41%)	<-10	0.681	0.686	Mutual exclusivity
XAGE2	Xp11.22	0 (0.00%)	1 (0.41%)	<-10	0.681	0.686	Mutual exclusivity
XAGE3	Xp11.22	0 (0.00%)	1 (0.41%)	<-10	0.681	0.686	Mutual exclusivity
XAGE5	Xp11.22	0 (0.00%)	1 (0.41%)	<-10	0.681	0.686	Mutual exclusivity
XK	Xp21.1	0 (0.00%)	1 (0.41%)	<-10	0.681	0.686	Mutual exclusivity
XPO1	2p15	0 (0.00%)	1 (0.41%)	<-10	0.681	0.686	Mutual exclusivity
XRCC4	5q14.2	0 (0.00%)	1 (0.41%)	<-10	0.681	0.686	Mutual exclusivity
YBEY	21q22.3	0 (0.00%)	1 (0.41%)	<-10	0.681	0.686	Mutual exclusivity
YBX1	1p34.2	0 (0.00%)	1 (0.41%)	<-10	0.681	0.686	Mutual exclusivity
YIPF7	4p12	0 (0.00%)	1 (0.41%)	<-10	0.681	0.686	Mutual exclusivity

YLPM1	14q24.3	0 (0.00%)	1 (0.41%)	<-10	0.681	0.686	Mutual exclusivity
YPEL4	11q12.1	0 (0.00%)	1 (0.41%)	<-10	0.681	0.686	Mutual exclusivity
YWHAB	20q13.12	0 (0.00%)	1 (0.41%)	<-10	0.681	0.686	Mutual exclusivity
ZAP70	2q11.2	0 (0.00%)	1 (0.41%)	<-10	0.681	0.686	Mutual exclusivity
ZBED3	5q13.3	0 (0.00%)	1 (0.41%)	<-10	0.681	0.686	Mutual exclusivity
ZBTB16	11q23.2	0 (0.00%)	1 (0.41%)	<-10	0.681	0.686	Mutual exclusivity
ZBTB2	6q25.1	0 (0.00%)	1 (0.41%)	<-10	0.681	0.686	Mutual exclusivity
ZBTB3	11q12.3	0 (0.00%)	1 (0.41%)	<-10	0.681	0.686	Mutual exclusivity
ZBTB40	1p36.12	0 (0.00%)	1 (0.41%)	<-10	0.681	0.686	Mutual exclusivity
ZBTB44	11q24.3	0 (0.00%)	1 (0.41%)	<-10	0.681	0.686	Mutual exclusivity
ZC2HC1C	14q24.3	0 (0.00%)	1 (0.41%)	<-10	0.681	0.686	Mutual exclusivity
ZC3H12D	6q25.1	0 (0.00%)	1 (0.41%)	<-10	0.681	0.686	Mutual exclusivity
ZCCHC7	9p13.2	0 (0.00%)	1 (0.41%)	<-10	0.681	0.686	Mutual exclusivity
ZCCHC9	5q14.1	0 (0.00%)	1 (0.41%)	<-10	0.681	0.686	Mutual exclusivity
ZDHHC16	10q24.1	0 (0.00%)	1 (0.41%)	<-10	0.681	0.686	Mutual exclusivity
ZDHHC2	8p22	0 (0.00%)	1 (0.41%)	<-10	0.681	0.686	Mutual exclusivity
ZDHHC21	9p22.3	0 (0.00%)	1 (0.41%)	<-10	0.681	0.686	Mutual exclusivity
ZDHHC5	11q12.1	0 (0.00%)	1 (0.41%)	<-10	0.681	0.686	Mutual exclusivity
ZDHHC6	10q25.2	0 (0.00%)	1 (0.41%)	<-10	0.681	0.686	Mutual exclusivity
ZDHHC8P1	22q11.23	0 (0.00%)	1 (0.41%)	<-10	0.681	0.686	Mutual exclusivity
ZFAND2B	2q35	0 (0.00%)	1 (0.41%)	<-10	0.681	0.686	Mutual exclusivity
ZFP36L1	14q24.1	0 (0.00%)	1 (0.41%)	<-10	0.681	0.686	Mutual exclusivity
ZFYVE16	5q14.1	0 (0.00%)	1 (0.41%)	<-10	0.681	0.686	Mutual exclusivity
ZFYVE26	14q24.1	0 (0.00%)	1 (0.41%)	<-10	0.681	0.686	Mutual exclusivity
ZFYVE27	10q24.2	0 (0.00%)	1 (0.41%)	<-10	0.681	0.686	Mutual exclusivity
ZGLP1	19p13.2	0 (0.00%)	1 (0.41%)	<-10	0.681	0.686	Mutual exclusivity
ZMYM3	Xq13.1	0 (0.00%)	1 (0.41%)	<-10	0.681	0.686	Mutual exclusivity
ZMYND8	20q13.12	0 (0.00%)	1 (0.41%)	<-10	0.681	0.686	Mutual exclusivity
ZNF142	2q35	0 (0.00%)	1 (0.41%)	<-10	0.681	0.686	Mutual exclusivity

ZNF189	9q31.1	0 (0.00%)	1 (0.41%)	<-10	0.681	0.686	Mutual exclusivity
ZNF24	18q12.2	0 (0.00%)	1 (0.41%)	<-10	0.681	0.686	Mutual exclusivity
ZNF259P1	6q21	0 (0.00%)	1 (0.41%)	<-10	0.681	0.686	Mutual exclusivity
ZNF271P	18q12.2	0 (0.00%)	1 (0.41%)	<-10	0.681	0.686	Mutual exclusivity
ZNF280A	22q11.22	0 (0.00%)	1 (0.41%)	<-10	0.681	0.686	Mutual exclusivity
ZNF280B	22q11.22	0 (0.00%)	1 (0.41%)	<-10	0.681	0.686	Mutual exclusivity
ZNF286B	17p11.2	0 (0.00%)	1 (0.41%)	<-10	0.681	0.686	Mutual exclusivity
ZNF335	20q13.12	0 (0.00%)	1 (0.41%)	<-10	0.681	0.686	Mutual exclusivity
ZNF33B	10q11.21	0 (0.00%)	1 (0.41%)	<-10	0.681	0.686	Mutual exclusivity
ZNF343	20p13	0 (0.00%)	1 (0.41%)	<-10	0.681	0.686	Mutual exclusivity
ZNF385A	12q13.13	0 (0.00%)	1 (0.41%)	<-10	0.681	0.686	Mutual exclusivity
ZNF395	8p21.1	0 (0.00%)	1 (0.41%)	<-10	0.681	0.686	Mutual exclusivity
ZNF396	18q12.2	0 (0.00%)	1 (0.41%)	<-10	0.681	0.686	Mutual exclusivity
ZNF397	18q12.2	0 (0.00%)	1 (0.41%)	<-10	0.681	0.686	Mutual exclusivity
ZNF407	18q22.3	0 (0.00%)	1 (0.41%)	<-10	0.681	0.686	Mutual exclusivity
ZNF423	16q12.1	0 (0.00%)	1 (0.41%)	<-10	0.681	0.686	Mutual exclusivity
ZNF436	1p36.12	0 (0.00%)	1 (0.41%)	<-10	0.681	0.686	Mutual exclusivity
ZNF436-AS1	1p36.12	0 (0.00%)	1 (0.41%)	<-10	0.681	0.686	Mutual exclusivity
ZNF526	19q13.2	0 (0.00%)	1 (0.41%)	<-10	0.681	0.686	Mutual exclusivity
ZNF557	19p13.2	0 (0.00%)	1 (0.41%)	<-10	0.681	0.686	Mutual exclusivity
ZNF574	19q13.2	0 (0.00%)	1 (0.41%)	<-10	0.681	0.686	Mutual exclusivity
ZNF646	16p11.2	0 (0.00%)	1 (0.41%)	<-10	0.681	0.686	Mutual exclusivity
ZNF654	3p11.1	0 (0.00%)	1 (0.41%)	<-10	0.681	0.686	Mutual exclusivity
ZNF668	16p11.2	0 (0.00%)	1 (0.41%)	<-10	0.681	0.686	Mutual exclusivity
ZNF675	19p12	0 (0.00%)	1 (0.41%)	<-10	0.681	0.686	Mutual exclusivity
ZNF681	19p12	0 (0.00%)	1 (0.41%)	<-10	0.681	0.686	Mutual exclusivity
ZNF691	1p34.2	0 (0.00%)	1 (0.41%)	<-10	0.681	0.686	Mutual exclusivity
ZNRF4	19p13.3	0 (0.00%)	1 (0.41%)	<-10	0.681	0.686	Mutual exclusivity
ZRANB1	10q26.13	0 (0.00%)	1 (0.41%)	<-10	0.681	0.686	Mutual exclusivity

ZSCAN30	18q12.2	0 (0.00%)	1 (0.41%)	<-10	0.681	0.686	Mutual exclusivity
ZSWIM1	20q13.12	0 (0.00%)	1 (0.41%)	<-10	0.681	0.686	Mutual exclusivity
ZSWIM3	20q13.12	0 (0.00%)	1 (0.41%)	<-10	0.681	0.686	Mutual exclusivity
ZSWIM7	17p12	0 (0.00%)	1 (0.41%)	<-10	0.681	0.686	Mutual exclusivity
ZXDC	3q21.3	0 (0.00%)	1 (0.41%)	<-10	0.681	0.686	Mutual exclusivity
ABCA17P	16p13.3	1 (0.87%)	2 (0.82%)	0.09	0.686	0.686	Co-occurrence
ABCA3	16p13.3	1 (0.87%)	2 (0.82%)	0.09	0.686	0.686	Co-occurrence
ABCC11	16q12.1	1 (0.87%)	2 (0.82%)	0.09	0.686	0.686	Co-occurrence
ABCC12	16q12.1	1 (0.87%)	2 (0.82%)	0.09	0.686	0.686	Co-occurrence
ABI1	10p12.1	1 (0.87%)	2 (0.82%)	0.09	0.686	0.686	Co-occurrence
ACBD5	10p12.1	1 (0.87%)	2 (0.82%)	0.09	0.686	0.686	Co-occurrence
ACKR2	3p22.1	1 (0.87%)	2 (0.82%)	0.09	0.686	0.686	Co-occurrence
ACO2	22q13.2	1 (0.87%)	2 (0.82%)	0.09	0.686	0.686	Co-occurrence
ACPP	3q22.1	1 (0.87%)	2 (0.82%)	0.09	0.686	0.686	Co-occurrence
ACRBP	12p13.31	1 (0.87%)	2 (0.82%)	0.09	0.686	0.686	Co-occurrence
ACSS2	20q11.22	1 (0.87%)	2 (0.82%)	0.09	0.686	0.686	Co-occurrence
ACVRL1	12q13.13	1 (0.87%)	2 (0.82%)	0.09	0.686	0.686	Co-occurrence
ADCY5	3q21.1	1 (0.87%)	2 (0.82%)	0.09	0.686	0.686	Co-occurrence
ADCY9	16p13.3	1 (0.87%)	2 (0.82%)	0.09	0.686	0.686	Co-occurrence
ADCYAP1R1	7p14.3	1 (0.87%)	2 (0.82%)	0.09	0.686	0.686	Co-occurrence
ADGRL1	19p13.12	1 (0.87%)	2 (0.82%)	0.09	0.686	0.686	Co-occurrence
ADIRF	10q23.2	1 (0.87%)	2 (0.82%)	0.09	0.686	0.686	Co-occurrence
ADORA3	1p13.2	1 (0.87%)	2 (0.82%)	0.09	0.686	0.686	Co-occurrence
AHCYL1	1p13.3	1 (0.87%)	2 (0.82%)	0.09	0.686	0.686	Co-occurrence
AKAIN1	18p11.31	1 (0.87%)	2 (0.82%)	0.09	0.686	0.686	Co-occurrence
AKAP14	Xq24	1 (0.87%)	2 (0.82%)	0.09	0.686	0.686	Co-occurrence
ALKBH5	17p11.2|17p11.2	1 (0.87%)	2 (0.82%)	0.09	0.686	0.686	Co-occurrence
ALPK2	18q21.31-q21.32	1 (0.87%)	2 (0.82%)	0.09	0.686	0.686	Co-occurrence
AMDHD2	16p13.3	1 (0.87%)	2 (0.82%)	0.09	0.686	0.686	Co-occurrence

AMOTL2	3q22.2	1 (0.87%)	2 (0.82%)	0.09	0.686	0.686	Co-occurrence
ANAPC13	3q22.2	1 (0.87%)	2 (0.82%)	0.09	0.686	0.686	Co-occurrence
ANAPC15	11q13.4	1 (0.87%)	2 (0.82%)	0.09	0.686	0.686	Co-occurrence
ANGPTL7	1p36.22	1 (0.87%)	2 (0.82%)	0.09	0.686	0.686	Co-occurrence
ANHX	12q24.33	1 (0.87%)	2 (0.82%)	0.09	0.686	0.686	Co-occurrence
ANKAR	2q32.2	1 (0.87%)	2 (0.82%)	0.09	0.686	0.686	Co-occurrence
ANKLE2	12q24.33	1 (0.87%)	2 (0.82%)	0.09	0.686	0.686	Co-occurrence
ANKRA2	5q13.2	1 (0.87%)	2 (0.82%)	0.09	0.686	0.686	Co-occurrence
ANKRD20A5P	18p11.21	1 (0.87%)	2 (0.82%)	0.09	0.686	0.686	Co-occurrence
ANKRD26	10p12.1	1 (0.87%)	2 (0.82%)	0.09	0.686	0.686	Co-occurrence
ANKRD30B	18p11.21	1 (0.87%)	2 (0.82%)	0.09	0.686	0.686	Co-occurrence
ANO9	11p15.5	1 (0.87%)	2 (0.82%)	0.09	0.686	0.686	Co-occurrence
AOAH	7p14.2	1 (0.87%)	2 (0.82%)	0.09	0.686	0.686	Co-occurrence
AOPEP	9q22.32	1 (0.87%)	2 (0.82%)	0.09	0.686	0.686	Co-occurrence
APBB1IP	10p12.1	1 (0.87%)	2 (0.82%)	0.09	0.686	0.686	Co-occurrence
APCDD1	18p11.22	1 (0.87%)	2 (0.82%)	0.09	0.686	0.686	Co-occurrence
AQP1	7p14.3	1 (0.87%)	2 (0.82%)	0.09	0.686	0.686	Co-occurrence
ARF6	14q21.3	1 (0.87%)	2 (0.82%)	0.09	0.686	0.686	Co-occurrence
ARHGAP21	10p12.1|10p12.3	1 (0.87%)	2 (0.82%)	0.09	0.686	0.686	Co-occurrence
ARHGAP26	5q31.3	1 (0.87%)	2 (0.82%)	0.09	0.686	0.686	Co-occurrence
ARHGAP28	18p11.31	1 (0.87%)	2 (0.82%)	0.09	0.686	0.686	Co-occurrence
ARHGDIG	16p13.3	1 (0.87%)	2 (0.82%)	0.09	0.686	0.686	Co-occurrence
ARHGEF28	5q13.2	1 (0.87%)	2 (0.82%)	0.09	0.686	0.686	Co-occurrence
ARL6	3q11.2	1 (0.87%)	2 (0.82%)	0.09	0.686	0.686	Co-occurrence
ARMC3	10p12.2	1 (0.87%)	2 (0.82%)	0.09	0.686	0.686	Co-occurrence
ARMC4	10p12.1	1 (0.87%)	2 (0.82%)	0.09	0.686	0.686	Co-occurrence
ARMC4P1	10p12.1	1 (0.87%)	2 (0.82%)	0.09	0.686	0.686	Co-occurrence
ARNTL2	12p11.23	1 (0.87%)	2 (0.82%)	0.09	0.686	0.686	Co-occurrence
ASL	7q11.21	1 (0.87%)	2 (0.82%)	0.09	0.686	0.686	Co-occurrence

ASNSD1	2q32.2	1 (0.87%)	2 (0.82%)	0.09	0.686	0.686	Co-occurrence
ATG7	3p25.3	1 (0.87%)	2 (0.82%)	0.09	0.686	0.686	Co-occurrence
ATM	11q22.3	1 (0.87%)	2 (0.82%)	0.09	0.686	0.686	Co-occurrence
ATN1	12p13.31	1 (0.87%)	2 (0.82%)	0.09	0.686	0.686	Co-occurrence
ATP6V0C	16p13.3	1 (0.87%)	2 (0.82%)	0.09	0.686	0.686	Co-occurrence
ATP6V1E2	2p21|2p16-p12	1 (0.87%)	2 (0.82%)	0.09	0.686	0.686	Co-occurrence
AXIN1	16p13.3	1 (0.87%)	2 (0.82%)	0.09	0.686	0.686	Co-occurrence
B4GALNT4	11p15.5	1 (0.87%)	2 (0.82%)	0.09	0.686	0.686	Co-occurrence
BBS5	2q31.1	1 (0.87%)	2 (0.82%)	0.09	0.686	0.686	Co-occurrence
BET1L	11p15.5	1 (0.87%)	2 (0.82%)	0.09	0.686	0.686	Co-occurrence
BLVRA	7p13	1 (0.87%)	2 (0.82%)	0.09	0.686	0.686	Co-occurrence
BMI1	10p12.2	1 (0.87%)	2 (0.82%)	0.09	0.686	0.686	Co-occurrence
BMPER	7p14.3	1 (0.87%)	2 (0.82%)	0.09	0.686	0.686	Co-occurrence
BMS1P18	14q11.2	1 (0.87%)	2 (0.82%)	0.09	0.686	0.686	Co-occurrence
BRICD5	16p13.3	1 (0.87%)	2 (0.82%)	0.09	0.686	0.686	Co-occurrence
BSN	3p21.31	1 (0.87%)	2 (0.82%)	0.09	0.686	0.686	Co-occurrence
BST2	19p13.11	1 (0.87%)	2 (0.82%)	0.09	0.686	0.686	Co-occurrence
BTF3	5q13.2	1 (0.87%)	2 (0.82%)	0.09	0.686	0.686	Co-occurrence
C10ORF113	10p12.31	1 (0.87%)	2 (0.82%)	0.09	0.686	0.686	Co-occurrence
C10ORF126	10p12.1	1 (0.87%)	2 (0.82%)	0.09	0.686	0.686	Co-occurrence
C10ORF67	10p12.2	1 (0.87%)	2 (0.82%)	0.09	0.686	0.686	Co-occurrence
C11ORF65	11q22.3	1 (0.87%)	2 (0.82%)	0.09	0.686	0.686	Co-occurrence
C12ORF57	12p13.31	1 (0.87%)	2 (0.82%)	0.09	0.686	0.686	Co-occurrence
C16ORF91	16p13.3	1 (0.87%)	2 (0.82%)	0.09	0.686	0.686	Co-occurrence
C16ORF96	16p13.3	1 (0.87%)	2 (0.82%)	0.09	0.686	0.686	Co-occurrence
C1ORF162	1p13.2	1 (0.87%)	2 (0.82%)	0.09	0.686	0.686	Co-occurrence
C1QTNF8	16p13.3	1 (0.87%)	2 (0.82%)	0.09	0.686	0.686	Co-occurrence
C1S	12p13.31	1 (0.87%)	2 (0.82%)	0.09	0.686	0.686	Co-occurrence
C20ORF173	20q11.22	1 (0.87%)	2 (0.82%)	0.09	0.686	0.686	Co-occurrence

C2ORF66	2q33.1	1 (0.87%)	2 (0.82%)	0.09	0.686	0.686	Co-occurrence
C2ORF69	2q33.1	1 (0.87%)	2 (0.82%)	0.09	0.686	0.686	Co-occurrence
C2ORF88	2q32.2	1 (0.87%)	2 (0.82%)	0.09	0.686	0.686	Co-occurrence
C3ORF36	3q22.1	1 (0.87%)	2 (0.82%)	0.09	0.686	0.686	Co-occurrence
C4ORF45	4q32.1	1 (0.87%)	2 (0.82%)	0.09	0.686	0.686	Co-occurrence
C5ORF46	5q32	1 (0.87%)	2 (0.82%)	0.09	0.686	0.686	Co-occurrence
C5ORF51	5p13.1	1 (0.87%)	2 (0.82%)	0.09	0.686	0.686	Co-occurrence
C6	5p13.1	1 (0.87%)	2 (0.82%)	0.09	0.686	0.686	Co-occurrence
C7	5p13.1	1 (0.87%)	2 (0.82%)	0.09	0.686	0.686	Co-occurrence
C7ORF25	7p14.1	1 (0.87%)	2 (0.82%)	0.09	0.686	0.686	Co-occurrence
C7ORF57	7p12.3	1 (0.87%)	2 (0.82%)	0.09	0.686	0.686	Co-occurrence
C7ORF65	7p12.3	1 (0.87%)	2 (0.82%)	0.09	0.686	0.686	Co-occurrence
C7ORF69	7p12.3	1 (0.87%)	2 (0.82%)	0.09	0.686	0.686	Co-occurrence
C9ORF24	9p13.3	1 (0.87%)	2 (0.82%)	0.09	0.686	0.686	Co-occurrence
CABP1	12q24.31	1 (0.87%)	2 (0.82%)	0.09	0.686	0.686	Co-occurrence
CACNA1H	16p13.3	1 (0.87%)	2 (0.82%)	0.09	0.686	0.686	Co-occurrence
CALM3	19q13.32	1 (0.87%)	2 (0.82%)	0.09	0.686	0.686	Co-occurrence
CAMLG	5q31.1	1 (0.87%)	2 (0.82%)	0.09	0.686	0.686	Co-occurrence
CAPN15	16p13.3	1 (0.87%)	2 (0.82%)	0.09	0.686	0.686	Co-occurrence
CARD6	5p13.1	1 (0.87%)	2 (0.82%)	0.09	0.686	0.686	Co-occurrence
CARF	2q33.2	1 (0.87%)	2 (0.82%)	0.09	0.686	0.686	Co-occurrence
CASC1	12p12.1	1 (0.87%)	2 (0.82%)	0.09	0.686	0.686	Co-occurrence
CASC10	10p12.31	1 (0.87%)	2 (0.82%)	0.09	0.686	0.686	Co-occurrence
CASP10	2q33.1	1 (0.87%)	2 (0.82%)	0.09	0.686	0.686	Co-occurrence
CASP14	19p13.12	1 (0.87%)	2 (0.82%)	0.09	0.686	0.686	Co-occurrence
CASP8	2q33.1	1 (0.87%)	2 (0.82%)	0.09	0.686	0.686	Co-occurrence
CASTOR2	7q11.23	1 (0.87%)	2 (0.82%)	0.09	0.686	0.686	Co-occurrence
CATIP	2q35	1 (0.87%)	2 (0.82%)	0.09	0.686	0.686	Co-occurrence
CAVIN2	2q32.3	1 (0.87%)	2 (0.82%)	0.09	0.686	0.686	Co-occurrence

CCDC105	19p13.12	1 (0.87%)	2 (0.82%)	0.09	0.686	0.686	Co-occurrence
CCDC144A	17p11.2	1 (0.87%)	2 (0.82%)	0.09	0.686	0.686	Co-occurrence
CCDC154	16p13.3	1 (0.87%)	2 (0.82%)	0.09	0.686	0.686	Co-occurrence
CCDC91	12p11.22	1 (0.87%)	2 (0.82%)	0.09	0.686	0.686	Co-occurrence
CCL24	7q11.23	1 (0.87%)	2 (0.82%)	0.09	0.686	0.686	Co-occurrence
CCL26	7q11.23	1 (0.87%)	2 (0.82%)	0.09	0.686	0.686	Co-occurrence
CCNF	16p13.3	1 (0.87%)	2 (0.82%)	0.09	0.686	0.686	Co-occurrence
CCNYL1	2q33.3	1 (0.87%)	2 (0.82%)	0.09	0.686	0.686	Co-occurrence
CCT6A	7p11.2	1 (0.87%)	2 (0.82%)	0.09	0.686	0.686	Co-occurrence
CD274	9p24.1	1 (0.87%)	2 (0.82%)	0.09	0.686	0.686	Co-occurrence
CD36	7q21.11	1 (0.87%)	2 (0.82%)	0.09	0.686	0.686	Co-occurrence
CD37	19q13.33	1 (0.87%)	2 (0.82%)	0.09	0.686	0.686	Co-occurrence
CDIP1	16p13.3	1 (0.87%)	2 (0.82%)	0.09	0.686	0.686	Co-occurrence
CDKL1	14q21.3	1 (0.87%)	2 (0.82%)	0.09	0.686	0.686	Co-occurrence
CELF6	15q23	1 (0.87%)	2 (0.82%)	0.09	0.686	0.686	Co-occurrence
CEMP1	16p13.3	1 (0.87%)	2 (0.82%)	0.09	0.686	0.686	Co-occurrence
CEP192	18p11.21	1 (0.87%)	2 (0.82%)	0.09	0.686	0.686	Co-occurrence
CEP63	3q22.2	1 (0.87%)	2 (0.82%)	0.09	0.686	0.686	Co-occurrence
CEP76	18p11.21	1 (0.87%)	2 (0.82%)	0.09	0.686	0.686	Co-occurrence
CERKL	2q31.3	1 (0.87%)	2 (0.82%)	0.09	0.686	0.686	Co-occurrence
CERS6	2q24.3	1 (0.87%)	2 (0.82%)	0.09	0.686	0.686	Co-occurrence
CFAP157	9q34.11	1 (0.87%)	2 (0.82%)	0.09	0.686	0.686	Co-occurrence
CHADL	22q13.2	1 (0.87%)	2 (0.82%)	0.09	0.686	0.686	Co-occurrence
CHCHD2	7p11.2	1 (0.87%)	2 (0.82%)	0.09	0.686	0.686	Co-occurrence
CHD4	12p13.31	1 (0.87%)	2 (0.82%)	0.09	0.686	0.686	Co-occurrence
CHFR	12q24.33	1 (0.87%)	2 (0.82%)	0.09	0.686	0.686	Co-occurrence
CIDECP1	3p25.3	1 (0.87%)	2 (0.82%)	0.09	0.686	0.686	Co-occurrence
CLCN7	16p13.3	1 (0.87%)	2 (0.82%)	0.09	0.686	0.686	Co-occurrence
CLCNKA	1p36.13	1 (0.87%)	2 (0.82%)	0.09	0.686	0.686	Co-occurrence

CLCNKB	1p36.13	1 (0.87%)	2 (0.82%)	0.09	0.686	0.686	Co-occurrence
CLPB	11q13.4	1 (0.87%)	2 (0.82%)	0.09	0.686	0.686	Co-occurrence
COA1	7p13	1 (0.87%)	2 (0.82%)	0.09	0.686	0.686	Co-occurrence
COBL	7p12.1	1 (0.87%)	2 (0.82%)	0.09	0.686	0.686	Co-occurrence
COL3A1	2q32.2	1 (0.87%)	2 (0.82%)	0.09	0.686	0.686	Co-occurrence
COL5A2	2q32.2	1 (0.87%)	2 (0.82%)	0.09	0.686	0.686	Co-occurrence
COMMD3	10p12.2	1 (0.87%)	2 (0.82%)	0.09	0.686	0.686	Co-occurrence
COMTD1	10q22.2	1 (0.87%)	2 (0.82%)	0.09	0.686	0.686	Co-occurrence
COPG1	3q21.3	1 (0.87%)	2 (0.82%)	0.09	0.686	0.686	Co-occurrence
CORO7	16p13.3	1 (0.87%)	2 (0.82%)	0.09	0.686	0.686	Co-occurrence
CREB3L3	19p13.3	1 (0.87%)	2 (0.82%)	0.09	0.686	0.686	Co-occurrence
CREB5	7p15.1	1 (0.87%)	2 (0.82%)	0.09	0.686	0.686	Co-occurrence
CREBBP	16p13.3	1 (0.87%)	2 (0.82%)	0.09	0.686	0.686	Co-occurrence
CRHR2	7p14.3	1 (0.87%)	2 (0.82%)	0.09	0.686	0.686	Co-occurrence
CRIPT	2p21	1 (0.87%)	2 (0.82%)	0.09	0.686	0.686	Co-occurrence
CRYBG3	3q11.2	1 (0.87%)	2 (0.82%)	0.09	0.686	0.686	Co-occurrence
CTDSP1	2q35	1 (0.87%)	2 (0.82%)	0.09	0.686	0.686	Co-occurrence
CTSD	11p15.5	1 (0.87%)	2 (0.82%)	0.09	0.686	0.686	Co-occurrence
CWC22	2q31.3	1 (0.87%)	2 (0.82%)	0.09	0.686	0.686	Co-occurrence
CXCR1	2q35	1 (0.87%)	2 (0.82%)	0.09	0.686	0.686	Co-occurrence
CXXC5	5q31.2	1 (0.87%)	2 (0.82%)	0.09	0.686	0.686	Co-occurrence
CYP2A13	19q13.2	1 (0.87%)	2 (0.82%)	0.09	0.686	0.686	Co-occurrence
CYP2F1	19q13.2	1 (0.87%)	2 (0.82%)	0.09	0.686	0.686	Co-occurrence
CYP2S1	19q13.2	1 (0.87%)	2 (0.82%)	0.09	0.686	0.686	Co-occurrence
CYP4A11	1p33	1 (0.87%)	2 (0.82%)	0.09	0.686	0.686	Co-occurrence
CYP4A22	1p33	1 (0.87%)	2 (0.82%)	0.09	0.686	0.686	Co-occurrence
CYP4F35P	18p11.21	1 (0.87%)	2 (0.82%)	0.09	0.686	0.686	Co-occurrence
CYP4X1	1p33|1	1 (0.87%)	2 (0.82%)	0.09	0.686	0.686	Co-occurrence
CYP4Z1	1p33	1 (0.87%)	2 (0.82%)	0.09	0.686	0.686	Co-occurrence

CYP8B1	3p22.1	1 (0.87%)	2 (0.82%)	0.09	0.686	0.686	Co-occurrence
DACT3	19q13.32	1 (0.87%)	2 (0.82%)	0.09	0.686	0.686	Co-occurrence
DAZL	3p24.3	1 (0.87%)	2 (0.82%)	0.09	0.686	0.686	Co-occurrence
DBNL	7p13	1 (0.87%)	2 (0.82%)	0.09	0.686	0.686	Co-occurrence
DCAF12	9p13.3	1 (0.87%)	2 (0.82%)	0.09	0.686	0.686	Co-occurrence
DDT	22q11.23	1 (0.87%)	2 (0.82%)	0.09	0.686	0.686	Co-occurrence
DDTL	22q11.23	1 (0.87%)	2 (0.82%)	0.09	0.686	0.686	Co-occurrence
DDX11L10	16p13.3	1 (0.87%)	2 (0.82%)	0.09	0.686	0.686	Co-occurrence
DDX4	5q11.2	1 (0.87%)	2 (0.82%)	0.09	0.686	0.686	Co-occurrence
DDX46	5q31.1	1 (0.87%)	2 (0.82%)	0.09	0.686	0.686	Co-occurrence
DDX56	7p13	1 (0.87%)	2 (0.82%)	0.09	0.686	0.686	Co-occurrence
DECR2	16p13.3	1 (0.87%)	2 (0.82%)	0.09	0.686	0.686	Co-occurrence
DHRS3	1p36.21	1 (0.87%)	2 (0.82%)	0.09	0.686	0.686	Co-occurrence
DHRS9	2q31.1	1 (0.87%)	2 (0.82%)	0.09	0.686	0.686	Co-occurrence
DHX34	19q13.32	1 (0.87%)	2 (0.82%)	0.09	0.686	0.686	Co-occurrence
DIRC1	2q32.2	1 (0.87%)	2 (0.82%)	0.09	0.686	0.686	Co-occurrence
DKKL1	19q13.33	1 (0.87%)	2 (0.82%)	0.09	0.686	0.686	Co-occurrence
DLG5	10q22.3	1 (0.87%)	2 (0.82%)	0.09	0.686	0.686	Co-occurrence
DMAC2L	14q21.3	1 (0.87%)	2 (0.82%)	0.09	0.686	0.686	Co-occurrence
DNAH7	2q32.3	1 (0.87%)	2 (0.82%)	0.09	0.686	0.686	Co-occurrence
DNAJA3	16p13.3	1 (0.87%)	2 (0.82%)	0.09	0.686	0.686	Co-occurrence
DNAJC1	10p12.31	1 (0.87%)	2 (0.82%)	0.09	0.686	0.686	Co-occurrence
DNAJC10	2q32.1	1 (0.87%)	2 (0.82%)	0.09	0.686	0.686	Co-occurrence
DNASE1L2	16p13.3	1 (0.87%)	2 (0.82%)	0.09	0.686	0.686	Co-occurrence
DNASE1L3	3p14.3	1 (0.87%)	2 (0.82%)	0.09	0.686	0.686	Co-occurrence
DND1	5q31.3	1 (0.87%)	2 (0.82%)	0.09	0.686	0.686	Co-occurrence
DNM1	9q34.11	1 (0.87%)	2 (0.82%)	0.09	0.686	0.686	Co-occurrence
DNMBP	10q24.2	1 (0.87%)	2 (0.82%)	0.09	0.686	0.686	Co-occurrence
DPP9	19p13.3	1 (0.87%)	2 (0.82%)	0.09	0.686	0.686	Co-occurrence

DRG2	17p11.2	1 (0.87%)	2 (0.82%)	0.09	0.686	0.686	Co-occurrence
DUSP13	10q22.2	1 (0.87%)	2 (0.82%)	0.09	0.686	0.686	Co-occurrence
DUSP19	2q32.1	1 (0.87%)	2 (0.82%)	0.09	0.686	0.686	Co-occurrence
DYM	18q21.1	1 (0.87%)	2 (0.82%)	0.09	0.686	0.686	Co-occurrence
DYNLRB1	20q11.22	1 (0.87%)	2 (0.82%)	0.09	0.686	0.686	Co-occurrence
E4F1	16p13.3	1 (0.87%)	2 (0.82%)	0.09	0.686	0.686	Co-occurrence
EBLN1	10p12.31	1 (0.87%)	2 (0.82%)	0.09	0.686	0.686	Co-occurrence
ECI1	16p13.3	1 (0.87%)	2 (0.82%)	0.09	0.686	0.686	Co-occurrence
EEF1DP3	13q13.1	1 (0.87%)	2 (0.82%)	0.09	0.686	0.686	Co-occurrence
EFCC1	3q21.3	1 (0.87%)	2 (0.82%)	0.09	0.686	0.686	Co-occurrence
EGFLAM	5p13.2-p13.1	1 (0.87%)	2 (0.82%)	0.09	0.686	0.686	Co-occurrence
EMC3	3p25.3	1 (0.87%)	2 (0.82%)	0.09	0.686	0.686	Co-occurrence
EMG1	12p13.31	1 (0.87%)	2 (0.82%)	0.09	0.686	0.686	Co-occurrence
ENC1	5q13.3	1 (0.87%)	2 (0.82%)	0.09	0.686	0.686	Co-occurrence
ENKUR	10p12.1	1 (0.87%)	2 (0.82%)	0.09	0.686	0.686	Co-occurrence
EPB41L3	18p11.31	1 (0.87%)	2 (0.82%)	0.09	0.686	0.686	Co-occurrence
EPHA6	3q11.2	1 (0.87%)	2 (0.82%)	0.09	0.686	0.686	Co-occurrence
EPHB1	3q22.2	1 (0.87%)	2 (0.82%)	0.09	0.686	0.686	Co-occurrence
EPS15P1	7p12.3	1 (0.87%)	2 (0.82%)	0.09	0.686	0.686	Co-occurrence
ERGIC3	20q11.22	1 (0.87%)	2 (0.82%)	0.09	0.686	0.686	Co-occurrence
ERICH2	2q31.1	1 (0.87%)	2 (0.82%)	0.09	0.686	0.686	Co-occurrence
ERMP1	9p24.1	1 (0.87%)	2 (0.82%)	0.09	0.686	0.686	Co-occurrence
ETFRF1	12p12.1	1 (0.87%)	2 (0.82%)	0.09	0.686	0.686	Co-occurrence
EXOC3L4	14q32.32	1 (0.87%)	2 (0.82%)	0.09	0.686	0.686	Co-occurrence
EXPH5	11q22.3	1 (0.87%)	2 (0.82%)	0.09	0.686	0.686	Co-occurrence
EYA2	20q13.12	1 (0.87%)	2 (0.82%)	0.09	0.686	0.686	Co-occurrence
FABP1	2p11.2	1 (0.87%)	2 (0.82%)	0.09	0.686	0.686	Co-occurrence
FAM114A1	4p14	1 (0.87%)	2 (0.82%)	0.09	0.686	0.686	Co-occurrence
FAM131C	1p36.13	1 (0.87%)	2 (0.82%)	0.09	0.686	0.686	Co-occurrence

FAM210A	18p11.21	1 (0.87%)	2 (0.82%)	0.09	0.686	0.686	Co-occurrence
FAM234A	16p13.3	1 (0.87%)	2 (0.82%)	0.09	0.686	0.686	Co-occurrence
FAM238A	10p12.1	1 (0.87%)	2 (0.82%)	0.09	0.686	0.686	Co-occurrence
FAM99A	11p15.5	1 (0.87%)	2 (0.82%)	0.09	0.686	0.686	Co-occurrence
FAM99B	11p15.5	1 (0.87%)	2 (0.82%)	0.09	0.686	0.686	Co-occurrence
FANCD2	3p25.3	1 (0.87%)	2 (0.82%)	0.09	0.686	0.686	Co-occurrence
FANCD2OS	3p25.3	1 (0.87%)	2 (0.82%)	0.09	0.686	0.686	Co-occurrence
FASTKD1	2q31.1	1 (0.87%)	2 (0.82%)	0.09	0.686	0.686	Co-occurrence
FBRSL1	12q24.33	1 (0.87%)	2 (0.82%)	0.09	0.686	0.686	Co-occurrence
FBXO17	19q13.2	1 (0.87%)	2 (0.82%)	0.09	0.686	0.686	Co-occurrence
FBXO4	5p13.1	1 (0.87%)	2 (0.82%)	0.09	0.686	0.686	Co-occurrence
FKBP14	7p14.3	1 (0.87%)	2 (0.82%)	0.09	0.686	0.686	Co-occurrence
FKBP9P1	7p11.2	1 (0.87%)	2 (0.82%)	0.09	0.686	0.686	Co-occurrence
FKRP	19q13.32	1 (0.87%)	2 (0.82%)	0.09	0.686	0.686	Co-occurrence
FLII	17p11.2	1 (0.87%)	2 (0.82%)	0.09	0.686	0.686	Co-occurrence
FLNB	3p14.3	1 (0.87%)	2 (0.82%)	0.09	0.686	0.686	Co-occurrence
FOLR1	11q13.4	1 (0.87%)	2 (0.82%)	0.09	0.686	0.686	Co-occurrence
FOLR2	11q13.4	1 (0.87%)	2 (0.82%)	0.09	0.686	0.686	Co-occurrence
FOLR3	11q13.4	1 (0.87%)	2 (0.82%)	0.09	0.686	0.686	Co-occurrence
FOS	14q24.3	1 (0.87%)	2 (0.82%)	0.09	0.686	0.686	Co-occurrence
FRY	13q13.1	1 (0.87%)	2 (0.82%)	0.09	0.686	0.686	Co-occurrence
FRZB	2q32.1	1 (0.87%)	2 (0.82%)	0.09	0.686	0.686	Co-occurrence
FTCDNL1	2q33.1	1 (0.87%)	2 (0.82%)	0.09	0.686	0.686	Co-occurrence
FZD5	2q33.3	1 (0.87%)	2 (0.82%)	0.09	0.686	0.686	Co-occurrence
GABRR3	3q11.2	1 (0.87%)	2 (0.82%)	0.09	0.686	0.686	Co-occurrence
GAD1	2q31.1	1 (0.87%)	2 (0.82%)	0.09	0.686	0.686	Co-occurrence
GAD2	10p12.1	1 (0.87%)	2 (0.82%)	0.09	0.686	0.686	Co-occurrence
GALNT15	3p25.1	1 (0.87%)	2 (0.82%)	0.09	0.686	0.686	Co-occurrence
GARS	7p14.3	1 (0.87%)	2 (0.82%)	0.09	0.686	0.686	Co-occurrence

GASK1A	3p22.1	1 (0.87%)	2 (0.82%)	0.09	0.686	0.686	Co-occurrence
GCH1	14q22.2	1 (0.87%)	2 (0.82%)	0.09	0.686	0.686	Co-occurrence
GCNT4	5q13.3	1 (0.87%)	2 (0.82%)	0.09	0.686	0.686	Co-occurrence
GGCT	7p14.3	1 (0.87%)	2 (0.82%)	0.09	0.686	0.686	Co-occurrence
GGT7	20q11.22	1 (0.87%)	2 (0.82%)	0.09	0.686	0.686	Co-occurrence
GHRHR	7p14.3	1 (0.87%)	2 (0.82%)	0.09	0.686	0.686	Co-occurrence
GHRL	3p25.3	1 (0.87%)	2 (0.82%)	0.09	0.686	0.686	Co-occurrence
GHRLOS	3p25.3	1 (0.87%)	2 (0.82%)	0.09	0.686	0.686	Co-occurrence
GLDC	9p24.1	1 (0.87%)	2 (0.82%)	0.09	0.686	0.686	Co-occurrence
GLE1	9q34.11	1 (0.87%)	2 (0.82%)	0.09	0.686	0.686	Co-occurrence
GLI3	7p14.1	1 (0.87%)	2 (0.82%)	0.09	0.686	0.686	Co-occurrence
GLIS2	16p13.3	1 (0.87%)	2 (0.82%)	0.09	0.686	0.686	Co-occurrence
GLS	2q32.2	1 (0.87%)	2 (0.82%)	0.09	0.686	0.686	Co-occurrence
GLYR1	16p13.3	1 (0.87%)	2 (0.82%)	0.09	0.686	0.686	Co-occurrence
GNAI1	7q21.11	1 (0.87%)	2 (0.82%)	0.09	0.686	0.686	Co-occurrence
GNAT3	7q21.11	1 (0.87%)	2 (0.82%)	0.09	0.686	0.686	Co-occurrence
GNG8	19q13.32	1 (0.87%)	2 (0.82%)	0.09	0.686	0.686	Co-occurrence
GOLGA2	9q34.11	1 (0.87%)	2 (0.82%)	0.09	0.686	0.686	Co-occurrence
GOLGA3	12q24.33	1 (0.87%)	2 (0.82%)	0.09	0.686	0.686	Co-occurrence
GORASP2	2q31.1	1 (0.87%)	2 (0.82%)	0.09	0.686	0.686	Co-occurrence
GP2	16p12.3	1 (0.87%)	2 (0.82%)	0.09	0.686	0.686	Co-occurrence
GP9	3q21.3	1 (0.87%)	2 (0.82%)	0.09	0.686	0.686	Co-occurrence
GPAM	10q25.2	1 (0.87%)	2 (0.82%)	0.09	0.686	0.686	Co-occurrence
GPR158	10p12.1	1 (0.87%)	2 (0.82%)	0.09	0.686	0.686	Co-occurrence
GRAMD2A	15q23	1 (0.87%)	2 (0.82%)	0.09	0.686	0.686	Co-occurrence
GSTT1	22q11.23	1 (0.87%)	2 (0.82%)	0.09	0.686	0.686	Co-occurrence
GSTT2	22q11.23	1 (0.87%)	2 (0.82%)	0.09	0.686	0.686	Co-occurrence
GSTT2B	22q11.23	1 (0.87%)	2 (0.82%)	0.09	0.686	0.686	Co-occurrence
GSTTP2	22q11.23	1 (0.87%)	2 (0.82%)	0.09	0.686	0.686	Co-occurrence

GTF2IRD1	7q11.23	1 (0.87%)	2 (0.82%)	0.09	0.686	0.686	Co-occurrence
GTF2IRD2	7q11.23	1 (0.87%)	2 (0.82%)	0.09	0.686	0.686	Co-occurrence
GTF2IRD2B	7q11.23	1 (0.87%)	2 (0.82%)	0.09	0.686	0.686	Co-occurrence
GTF3C3	2q33.1	1 (0.87%)	2 (0.82%)	0.09	0.686	0.686	Co-occurrence
GULP1	2q32.1-q32.2	1 (0.87%)	2 (0.82%)	0.09	0.686	0.686	Co-occurrence
H19	11p15.5	1 (0.87%)	2 (0.82%)	0.09	0.686	0.686	Co-occurrence
HAAO	2p21	1 (0.87%)	2 (0.82%)	0.09	0.686	0.686	Co-occurrence
HARS	5q31.3	1 (0.87%)	2 (0.82%)	0.09	0.686	0.686	Co-occurrence
HARS2	5q31.3	1 (0.87%)	2 (0.82%)	0.09	0.686	0.686	Co-occurrence
HAUS3	4p16.3	1 (0.87%)	2 (0.82%)	0.09	0.686	0.686	Co-occurrence
HAUS4	14q11.2	1 (0.87%)	2 (0.82%)	0.09	0.686	0.686	Co-occurrence
HBA1	16p13.3	1 (0.87%)	2 (0.82%)	0.09	0.686	0.686	Co-occurrence
HBA2	16p13.3	1 (0.87%)	2 (0.82%)	0.09	0.686	0.686	Co-occurrence
HBM	16p13.3	1 (0.87%)	2 (0.82%)	0.09	0.686	0.686	Co-occurrence
HBQ1	16p13.3	1 (0.87%)	2 (0.82%)	0.09	0.686	0.686	Co-occurrence
HBZ	16p13.3	1 (0.87%)	2 (0.82%)	0.09	0.686	0.686	Co-occurrence
HEATR5A	14q12	1 (0.87%)	2 (0.82%)	0.09	0.686	0.686	Co-occurrence
HECTD1	14q12	1 (0.87%)	2 (0.82%)	0.09	0.686	0.686	Co-occurrence
HECW1	7p14.1-p13	1 (0.87%)	2 (0.82%)	0.09	0.686	0.686	Co-occurrence
HECW2	2q32.3	1 (0.87%)	2 (0.82%)	0.09	0.686	0.686	Co-occurrence
HEG1	3q21.2	1 (0.87%)	2 (0.82%)	0.09	0.686	0.686	Co-occurrence
HERPUD2	7p14.2	1 (0.87%)	2 (0.82%)	0.09	0.686	0.686	Co-occurrence
HEXA	15q23	1 (0.87%)	2 (0.82%)	0.09	0.686	0.686	Co-occurrence
HEXB	5q13.3	1 (0.87%)	2 (0.82%)	0.09	0.686	0.686	Co-occurrence
HIP1	7q11.23	1 (0.87%)	2 (0.82%)	0.09	0.686	0.686	Co-occurrence
HMGA1P4	9q34.11	1 (0.87%)	2 (0.82%)	0.09	0.686	0.686	Co-occurrence
HMOX2	16p13.3	1 (0.87%)	2 (0.82%)	0.09	0.686	0.686	Co-occurrence
HRAS	11p15.5	1 (0.87%)	2 (0.82%)	0.09	0.686	0.686	Co-occurrence
HRH1	3p25.3	1 (0.87%)	2 (0.82%)	0.09	0.686	0.686	Co-occurrence

HSD17B10	Xp11.22	1 (0.87%)	2 (0.82%)	0.09	0.686	0.686	Co-occurrence
HSPB7	1p36.13	1 (0.87%)	2 (0.82%)	0.09	0.686	0.686	Co-occurrence
ICA1L	2q33.2	1 (0.87%)	2 (0.82%)	0.09	0.686	0.686	Co-occurrence
IFITM1	11p15.5	1 (0.87%)	2 (0.82%)	0.09	0.686	0.686	Co-occurrence
IFITM10	11p15.5	1 (0.87%)	2 (0.82%)	0.09	0.686	0.686	Co-occurrence
IFITM2	11p15.5	1 (0.87%)	2 (0.82%)	0.09	0.686	0.686	Co-occurrence
IFITM3	11p15.5	1 (0.87%)	2 (0.82%)	0.09	0.686	0.686	Co-occurrence
IFITM5	11p15.5	1 (0.87%)	2 (0.82%)	0.09	0.686	0.686	Co-occurrence
IGFBP1	7p12.3	1 (0.87%)	2 (0.82%)	0.09	0.686	0.686	Co-occurrence
IGFBP3	7p12.3	1 (0.87%)	2 (0.82%)	0.09	0.686	0.686	Co-occurrence
IL18BP	11q13.4	1 (0.87%)	2 (0.82%)	0.09	0.686	0.686	Co-occurrence
IL1RAPL2	Xq22.3	1 (0.87%)	2 (0.82%)	0.09	0.686	0.686	Co-occurrence
IL33	9p24.1	1 (0.87%)	2 (0.82%)	0.09	0.686	0.686	Co-occurrence
ING4	12p13.31	1 (0.87%)	2 (0.82%)	0.09	0.686	0.686	Co-occurrence
INHBA	7p14.1	1 (0.87%)	2 (0.82%)	0.09	0.686	0.686	Co-occurrence
INMT	7p14.3	1 (0.87%)	2 (0.82%)	0.09	0.686	0.686	Co-occurrence
INPPL1	11q13.4	1 (0.87%)	2 (0.82%)	0.09	0.686	0.686	Co-occurrence
INSL4	9p24.1	1 (0.87%)	2 (0.82%)	0.09	0.686	0.686	Co-occurrence
INSL6	9p24.1	1 (0.87%)	2 (0.82%)	0.09	0.686	0.686	Co-occurrence
IQSEC1	3p25.2-p25.1	1 (0.87%)	2 (0.82%)	0.09	0.686	0.686	Co-occurrence
IRAK2	3p25.3	1 (0.87%)	2 (0.82%)	0.09	0.686	0.686	Co-occurrence
ITGA4	2q31.3	1 (0.87%)	2 (0.82%)	0.09	0.686	0.686	Co-occurrence
ITGB5	3q21.2	1 (0.87%)	2 (0.82%)	0.09	0.686	0.686	Co-occurrence
ITPR1	3p26.1	1 (0.87%)	2 (0.82%)	0.09	0.686	0.686	Co-occurrence
ITPRID1	7p14.3	1 (0.87%)	2 (0.82%)	0.09	0.686	0.686	Co-occurrence
ITPRID2	2q31.3	1 (0.87%)	2 (0.82%)	0.09	0.686	0.686	Co-occurrence
JADE3	Xp11.3	1 (0.87%)	2 (0.82%)	0.09	0.686	0.686	Co-occurrence
JAK2	9p24.1	1 (0.87%)	2 (0.82%)	0.09	0.686	0.686	Co-occurrence
JCAD	10p11.23	1 (0.87%)	2 (0.82%)	0.09	0.686	0.686	Co-occurrence

KALRN	3q21.1-q21.2	1 (0.87%)	2 (0.82%)	0.09	0.686	0.686	Co-occurrence
KCNA2	1p13.3	1 (0.87%)	2 (0.82%)	0.09	0.686	0.686	Co-occurrence
KCNA3	1p13.3	1 (0.87%)	2 (0.82%)	0.09	0.686	0.686	Co-occurrence
KCNU1	8p11.23	1 (0.87%)	2 (0.82%)	0.09	0.686	0.686	Co-occurrence
KCTD16	5q31.3	1 (0.87%)	2 (0.82%)	0.09	0.686	0.686	Co-occurrence
KCTD5	16p13.3	1 (0.87%)	2 (0.82%)	0.09	0.686	0.686	Co-occurrence
KCTD7	7q11.21	1 (0.87%)	2 (0.82%)	0.09	0.686	0.686	Co-occurrence
KDM4C	9p24.1	1 (0.87%)	2 (0.82%)	0.09	0.686	0.686	Co-occurrence
KIAA1257	3q21.3	1 (0.87%)	2 (0.82%)	0.09	0.686	0.686	Co-occurrence
KIAA1522	1p35.1	1 (0.87%)	2 (0.82%)	0.09	0.686	0.686	Co-occurrence
KIF12	9q32	1 (0.87%)	2 (0.82%)	0.09	0.686	0.686	Co-occurrence
KIFC3	16q21	1 (0.87%)	2 (0.82%)	0.09	0.686	0.686	Co-occurrence
KLHDC1	14q21.3	1 (0.87%)	2 (0.82%)	0.09	0.686	0.686	Co-occurrence
KLHDC2	14q21.3	1 (0.87%)	2 (0.82%)	0.09	0.686	0.686	Co-occurrence
KLHL41	2q31.1	1 (0.87%)	2 (0.82%)	0.09	0.686	0.686	Co-occurrence
KRAS	12p12.1	1 (0.87%)	2 (0.82%)	0.09	0.686	0.686	Co-occurrence
KRBOX1	3p22.1	1 (0.87%)	2 (0.82%)	0.09	0.686	0.686	Co-occurrence
KRT16P2	17p11.2	1 (0.87%)	2 (0.82%)	0.09	0.686	0.686	Co-occurrence
KRT17P1	17p11.2	1 (0.87%)	2 (0.82%)	0.09	0.686	0.686	Co-occurrence
KY	3q22.2	1 (0.87%)	2 (0.82%)	0.09	0.686	0.686	Co-occurrence
L2HGDH	14q21.3	1 (0.87%)	2 (0.82%)	0.09	0.686	0.686	Co-occurrence
L3MBTL2	22q13.2	1 (0.87%)	2 (0.82%)	0.09	0.686	0.686	Co-occurrence
L3MBTL4	18p11.31	1 (0.87%)	2 (0.82%)	0.09	0.686	0.686	Co-occurrence
LAMB2	3p21.31	1 (0.87%)	2 (0.82%)	0.09	0.686	0.686	Co-occurrence
LAMTOR1	11q13.4	1 (0.87%)	2 (0.82%)	0.09	0.686	0.686	Co-occurrence
LANCL1	2q34	1 (0.87%)	2 (0.82%)	0.09	0.686	0.686	Co-occurrence
LETM1	4p16.3	1 (0.87%)	2 (0.82%)	0.09	0.686	0.686	Co-occurrence
LINC00442	13q12.11	1 (0.87%)	2 (0.82%)	0.09	0.686	0.686	Co-occurrence
LINC00525	7p12.3	1 (0.87%)	2 (0.82%)	0.09	0.686	0.686	Co-occurrence

LINC00526	18p11.31	1 (0.87%)	2 (0.82%)	0.09	0.686	0.686	Co-occurrence
LINC00605	14q32.32	1 (0.87%)	2 (0.82%)	0.09	0.686	0.686	Co-occurrence
LINC00606	3p25.3	1 (0.87%)	2 (0.82%)	0.09	0.686	0.686	Co-occurrence
LINC00614	10p12.1	1 (0.87%)	2 (0.82%)	0.09	0.686	0.686	Co-occurrence
LINC00667	18p11.31	1 (0.87%)	2 (0.82%)	0.09	0.686	0.686	Co-occurrence
LINC00668	18p11.31	1 (0.87%)	2 (0.82%)	0.09	0.686	0.686	Co-occurrence
LINC00677	14q32.33	1 (0.87%)	2 (0.82%)	0.09	0.686	0.686	Co-occurrence
LINC00690	3p24.3	1 (0.87%)	2 (0.82%)	0.09	0.686	0.686	Co-occurrence
LINC00837	10p12.1	1 (0.87%)	2 (0.82%)	0.09	0.686	0.686	Co-occurrence
LINC00845	10q21.2	1 (0.87%)	2 (0.82%)	0.09	0.686	0.686	Co-occurrence
LINC00852	3p25.3	1 (0.87%)	2 (0.82%)	0.09	0.686	0.686	Co-occurrence
LINC00853	1p33	1 (0.87%)	2 (0.82%)	0.09	0.686	0.686	Co-occurrence
LINC00957	7p13	1 (0.87%)	2 (0.82%)	0.09	0.686	0.686	Co-occurrence
LINC00972	7q21.11	1 (0.87%)	2 (0.82%)	0.09	0.686	0.686	Co-occurrence
LINC01545	Xp11.3	1 (0.87%)	2 (0.82%)	0.09	0.686	0.686	Co-occurrence
LINC01599	14q21.3	1 (0.87%)	2 (0.82%)	0.09	0.686	0.686	Co-occurrence
LLGL1	17p11.2	1 (0.87%)	2 (0.82%)	0.09	0.686	0.686	Co-occurrence
LMNTD2	11p15.5	1 (0.87%)	2 (0.82%)	0.09	0.686	0.686	Co-occurrence
LONP2	16q12.1	1 (0.87%)	2 (0.82%)	0.09	0.686	0.686	Co-occurrence
LPAR5	12p13.31	1 (0.87%)	2 (0.82%)	0.09	0.686	0.686	Co-occurrence
LPCAT3	12p13.31	1 (0.87%)	2 (0.82%)	0.09	0.686	0.686	Co-occurrence
LRCOL1	12q24.33	1 (0.87%)	2 (0.82%)	0.09	0.686	0.686	Co-occurrence
LRP2	2q31.1	1 (0.87%)	2 (0.82%)	0.09	0.686	0.686	Co-occurrence
LRRC37A6P	10p12.1	1 (0.87%)	2 (0.82%)	0.09	0.686	0.686	Co-occurrence
LRRC56	11p15.5	1 (0.87%)	2 (0.82%)	0.09	0.686	0.686	Co-occurrence
LSM5	7p14.3	1 (0.87%)	2 (0.82%)	0.09	0.686	0.686	Co-occurrence
LSP1	11p15.5	1 (0.87%)	2 (0.82%)	0.09	0.686	0.686	Co-occurrence
LUC7L	16p13.3	1 (0.87%)	2 (0.82%)	0.09	0.686	0.686	Co-occurrence
LYZL1	10p12.1-p11.23	1 (0.87%)	2 (0.82%)	0.09	0.686	0.686	Co-occurrence

MAML3	4q31.1	1 (0.87%)	2 (0.82%)	0.09	0.686	0.686	Co-occurrence
MANBAL	20q11.23	1 (0.87%)	2 (0.82%)	0.09	0.686	0.686	Co-occurrence
MAP3K8	10p11.23	1 (0.87%)	2 (0.82%)	0.09	0.686	0.686	Co-occurrence
MASTL	10p12.1	1 (0.87%)	2 (0.82%)	0.09	0.686	0.686	Co-occurrence
MATR3	5q31.2	1 (0.87%)	2 (0.82%)	0.09	0.686	0.686	Co-occurrence
MC2R	18p11.21	1 (0.87%)	2 (0.82%)	0.09	0.686	0.686	Co-occurrence
MC5R	18p11.21	1 (0.87%)	2 (0.82%)	0.09	0.686	0.686	Co-occurrence
MCRIP2	16p13.3	1 (0.87%)	2 (0.82%)	0.09	0.686	0.686	Co-occurrence
MED15P1	14q11.2	1 (0.87%)	2 (0.82%)	0.09	0.686	0.686	Co-occurrence
MED15P6	14q11.2	1 (0.87%)	2 (0.82%)	0.09	0.686	0.686	Co-occurrence
METTL26	16p13.3	1 (0.87%)	2 (0.82%)	0.09	0.686	0.686	Co-occurrence
METTL3	14q11.2	1 (0.87%)	2 (0.82%)	0.09	0.686	0.686	Co-occurrence
METTL5	2q31.1	1 (0.87%)	2 (0.82%)	0.09	0.686	0.686	Co-occurrence
MGRN1	16p13.3	1 (0.87%)	2 (0.82%)	0.09	0.686	0.686	Co-occurrence
MICAL3	22q11.21	1 (0.87%)	2 (0.82%)	0.09	0.686	0.686	Co-occurrence
MIEF2	17p11.2	1 (0.87%)	2 (0.82%)	0.09	0.686	0.686	Co-occurrence
MINDY4	7p14.3	1 (0.87%)	2 (0.82%)	0.09	0.686	0.686	Co-occurrence
MIR-1258/1258		1 (0.87%)	2 (0.82%)	0.09	0.686	0.686	Co-occurrence
MIR-3154/3154		1 (0.87%)	2 (0.82%)	0.09	0.686	0.686	Co-occurrence
MIR-3176/3176		1 (0.87%)	2 (0.82%)	0.09	0.686	0.686	Co-occurrence
MIR-3178/3178		1 (0.87%)	2 (0.82%)	0.09	0.686	0.686	Co-occurrence
MIR-320E/320E		1 (0.87%)	2 (0.82%)	0.09	0.686	0.686	Co-occurrence
MIR-3714/3714		1 (0.87%)	2 (0.82%)	0.09	0.686	0.686	Co-occurrence
MIR-378B/378B		1 (0.87%)	2 (0.82%)	0.09	0.686	0.686	Co-occurrence
MIR-3943/3943		1 (0.87%)	2 (0.82%)	0.09	0.686	0.686	Co-occurrence
MIR-3960/3960		1 (0.87%)	2 (0.82%)	0.09	0.686	0.686	Co-occurrence
MIR-3976/3976		1 (0.87%)	2 (0.82%)	0.09	0.686	0.686	Co-occurrence
MIR-4298/4298		1 (0.87%)	2 (0.82%)	0.09	0.686	0.686	Co-occurrence
MIR-4324/4324		1 (0.87%)	2 (0.82%)	0.09	0.686	0.686	Co-occurrence

MIR-4437/4437		1 (0.87%)	2 (0.82%)	0.09	0.686	0.686	Co-occurrence
MIR-4480/4480		1 (0.87%)	2 (0.82%)	0.09	0.686	0.686	Co-occurrence
MIR-4504/4504		1 (0.87%)	2 (0.82%)	0.09	0.686	0.686	Co-occurrence
MIR-4526/4526		1 (0.87%)	2 (0.82%)	0.09	0.686	0.686	Co-occurrence
MIR-4651/4651		1 (0.87%)	2 (0.82%)	0.09	0.686	0.686	Co-occurrence
MIR-4675/4675		1 (0.87%)	2 (0.82%)	0.09	0.686	0.686	Co-occurrence
MIR-4775/4775		1 (0.87%)	2 (0.82%)	0.09	0.686	0.686	Co-occurrence
MIR-4788/4788		1 (0.87%)	2 (0.82%)	0.09	0.686	0.686	Co-occurrence
MIR-4801/4801		1 (0.87%)	2 (0.82%)	0.09	0.686	0.686	Co-occurrence
MIR-548Q/548Q		1 (0.87%)	2 (0.82%)	0.09	0.686	0.686	Co-occurrence
MIR-603/603		1 (0.87%)	2 (0.82%)	0.09	0.686	0.686	Co-occurrence
MIR-604/604		1 (0.87%)	2 (0.82%)	0.09	0.686	0.686	Co-occurrence
MIR-938/938		1 (0.87%)	2 (0.82%)	0.09	0.686	0.686	Co-occurrence
MIR-940/940		1 (0.87%)	2 (0.82%)	0.09	0.686	0.686	Co-occurrence
MIR210HG	11p15.5	1 (0.87%)	2 (0.82%)	0.09	0.686	0.686	Co-occurrence
MKX	10p12.1	1 (0.87%)	2 (0.82%)	0.09	0.686	0.686	Co-occurrence
MLANA	9p24.1	1 (0.87%)	2 (0.82%)	0.09	0.686	0.686	Co-occurrence
MLST8	16p13.3	1 (0.87%)	2 (0.82%)	0.09	0.686	0.686	Co-occurrence
MMAB	12q24.11	1 (0.87%)	2 (0.82%)	0.09	0.686	0.686	Co-occurrence
MMRN2	10q23.2	1 (0.87%)	2 (0.82%)	0.09	0.686	0.686	Co-occurrence
MPG	16p13.3	1 (0.87%)	2 (0.82%)	0.09	0.686	0.686	Co-occurrence
MPP7	10p12.1	1 (0.87%)	2 (0.82%)	0.09	0.686	0.686	Co-occurrence
MROH8	20q11.23	1 (0.87%)	2 (0.82%)	0.09	0.686	0.686	Co-occurrence
MRPL23	11p15.5	1 (0.87%)	2 (0.82%)	0.09	0.686	0.686	Co-occurrence
MRPL28	16p13.3	1 (0.87%)	2 (0.82%)	0.09	0.686	0.686	Co-occurrence
MRPL32	7p14.1	1 (0.87%)	2 (0.82%)	0.09	0.686	0.686	Co-occurrence
MRPS12	19q13.2	1 (0.87%)	2 (0.82%)	0.09	0.686	0.686	Co-occurrence
MRPS17	7p11.2	1 (0.87%)	2 (0.82%)	0.09	0.686	0.686	Co-occurrence
MRPS24	7p13	1 (0.87%)	2 (0.82%)	0.09	0.686	0.686	Co-occurrence

MSRB2	10p12.2	1 (0.87%)	2 (0.82%)	0.09	0.686	0.686	Co-occurrence
MTA3	2p21	1 (0.87%)	2 (0.82%)	0.09	0.686	0.686	Co-occurrence
MTOR	1p36.22	1 (0.87%)	2 (0.82%)	0.09	0.686	0.686	Co-occurrence
MTPAP	10p11.23	1 (0.87%)	2 (0.82%)	0.09	0.686	0.686	Co-occurrence
MTURN	7p14.3	1 (0.87%)	2 (0.82%)	0.09	0.686	0.686	Co-occurrence
MUC8	12q24.33	1 (0.87%)	2 (0.82%)	0.09	0.686	0.686	Co-occurrence
MVB12A	19p13.11	1 (0.87%)	2 (0.82%)	0.09	0.686	0.686	Co-occurrence
MXD4	4p16.3	1 (0.87%)	2 (0.82%)	0.09	0.686	0.686	Co-occurrence
MYO1B	2q32.3	1 (0.87%)	2 (0.82%)	0.09	0.686	0.686	Co-occurrence
MYO3A	10p12.1	1 (0.87%)	2 (0.82%)	0.09	0.686	0.686	Co-occurrence
MYO3B	2q31.1	1 (0.87%)	2 (0.82%)	0.09	0.686	0.686	Co-occurrence
MYO9A	15q23	1 (0.87%)	2 (0.82%)	0.09	0.686	0.686	Co-occurrence
MYORG	9p13.3	1 (0.87%)	2 (0.82%)	0.09	0.686	0.686	Co-occurrence
MZB1	5q31.2	1 (0.87%)	2 (0.82%)	0.09	0.686	0.686	Co-occurrence
NAB1	2q32.2	1 (0.87%)	2 (0.82%)	0.09	0.686	0.686	Co-occurrence
NAMPTP1	10p11.21	1 (0.87%)	2 (0.82%)	0.09	0.686	0.686	Co-occurrence
NANOS2	19q13.32	1 (0.87%)	2 (0.82%)	0.09	0.686	0.686	Co-occurrence
NAPG	18p11.22	1 (0.87%)	2 (0.82%)	0.09	0.686	0.686	Co-occurrence
NBEAL1	2q33.2	1 (0.87%)	2 (0.82%)	0.09	0.686	0.686	Co-occurrence
NCF1	7q11.23	1 (0.87%)	2 (0.82%)	0.09	0.686	0.686	Co-occurrence
NCKAP1	2q32.1	1 (0.87%)	2 (0.82%)	0.09	0.686	0.686	Co-occurrence
NDUFA1	Xq24	1 (0.87%)	2 (0.82%)	0.09	0.686	0.686	Co-occurrence
NEBL	10p12.31	1 (0.87%)	2 (0.82%)	0.09	0.686	0.686	Co-occurrence
NEMF	14q21.3	1 (0.87%)	2 (0.82%)	0.09	0.686	0.686	Co-occurrence
NEMP2	2q32.2	1 (0.87%)	2 (0.82%)	0.09	0.686	0.686	Co-occurrence
NEUROD1	2q31.3	1 (0.87%)	2 (0.82%)	0.09	0.686	0.686	Co-occurrence
NEUROD6	7p14.3	1 (0.87%)	2 (0.82%)	0.09	0.686	0.686	Co-occurrence
NHLRC4	16p13.3	1 (0.87%)	2 (0.82%)	0.09	0.686	0.686	Co-occurrence
NIPSNAP2	7p11.2	1 (0.87%)	2 (0.82%)	0.09	0.686	0.686	Co-occurrence

NKAP	Xq24	1 (0.87%)	2 (0.82%)	0.09	0.686	0.686	Co-occurrence
NLRP6	11p15.5	1 (0.87%)	2 (0.82%)	0.09	0.686	0.686	Co-occurrence
NME4	16p13.3	1 (0.87%)	2 (0.82%)	0.09	0.686	0.686	Co-occurrence
NMRAL1	16p13.3	1 (0.87%)	2 (0.82%)	0.09	0.686	0.686	Co-occurrence
NOD1	7p14.3	1 (0.87%)	2 (0.82%)	0.09	0.686	0.686	Co-occurrence
NOP2	12p13.31	1 (0.87%)	2 (0.82%)	0.09	0.686	0.686	Co-occurrence
NOSTRIN	2q24.3	1 (0.87%)	2 (0.82%)	0.09	0.686	0.686	Co-occurrence
NOVA2	19q13.32	1 (0.87%)	2 (0.82%)	0.09	0.686	0.686	Co-occurrence
NPRL3	16p13.3	1 (0.87%)	2 (0.82%)	0.09	0.686	0.686	Co-occurrence
NR2E3	15q23	1 (0.87%)	2 (0.82%)	0.09	0.686	0.686	Co-occurrence
NSD2	4p16.3	1 (0.87%)	2 (0.82%)	0.09	0.686	0.686	Co-occurrence
NSUN5P1	7q11.23	1 (0.87%)	2 (0.82%)	0.09	0.686	0.686	Co-occurrence
NTN3	16p13.3	1 (0.87%)	2 (0.82%)	0.09	0.686	0.686	Co-occurrence
NUDT16L1	16p13.3	1 (0.87%)	2 (0.82%)	0.09	0.686	0.686	Co-occurrence
NUDT2	9p13.3	1 (0.87%)	2 (0.82%)	0.09	0.686	0.686	Co-occurrence
NUP210	3p25.1	1 (0.87%)	2 (0.82%)	0.09	0.686	0.686	Co-occurrence
NUP35	2q32.1	1 (0.87%)	2 (0.82%)	0.09	0.686	0.686	Co-occurrence
NUPR2	7p11.2	1 (0.87%)	2 (0.82%)	0.09	0.686	0.686	Co-occurrence
NXNL1	19p13.11	1 (0.87%)	2 (0.82%)	0.09	0.686	0.686	Co-occurrence
ODF3	11p15.5	1 (0.87%)	2 (0.82%)	0.09	0.686	0.686	Co-occurrence
OR11H12	14q11.2	1 (0.87%)	2 (0.82%)	0.09	0.686	0.686	Co-occurrence
OR1I1	19p13.12	1 (0.87%)	2 (0.82%)	0.09	0.686	0.686	Co-occurrence
OR4K1	14q11.2	1 (0.87%)	2 (0.82%)	0.09	0.686	0.686	Co-occurrence
OR4K13	14q11.2	1 (0.87%)	2 (0.82%)	0.09	0.686	0.686	Co-occurrence
OR4K14	14q11.2	1 (0.87%)	2 (0.82%)	0.09	0.686	0.686	Co-occurrence
OR4K15	14q11.2	1 (0.87%)	2 (0.82%)	0.09	0.686	0.686	Co-occurrence
OR4K17	14q11.2	1 (0.87%)	2 (0.82%)	0.09	0.686	0.686	Co-occurrence
OR4K2	14q11.2	1 (0.87%)	2 (0.82%)	0.09	0.686	0.686	Co-occurrence
OR4K5	14q11.2	1 (0.87%)	2 (0.82%)	0.09	0.686	0.686	Co-occurrence

OR4L1	14q11.2	1 (0.87%)	2 (0.82%)	0.09	0.686	0.686	Co-occurrence
OR4M1	14q11.2	1 (0.87%)	2 (0.82%)	0.09	0.686	0.686	Co-occurrence
OR4N2	14q11.2	1 (0.87%)	2 (0.82%)	0.09	0.686	0.686	Co-occurrence
OR4N5	14q11.2	1 (0.87%)	2 (0.82%)	0.09	0.686	0.686	Co-occurrence
OR4Q2	14q11.2	1 (0.87%)	2 (0.82%)	0.09	0.686	0.686	Co-occurrence
OR4Q3	14q11.2	1 (0.87%)	2 (0.82%)	0.09	0.686	0.686	Co-occurrence
OR7A10	19p13.12	1 (0.87%)	2 (0.82%)	0.09	0.686	0.686	Co-occurrence
OR7A17	19p13.12	1 (0.87%)	2 (0.82%)	0.09	0.686	0.686	Co-occurrence
OR7A5	19p13.12	1 (0.87%)	2 (0.82%)	0.09	0.686	0.686	Co-occurrence
OR7C1	19p13.12	1 (0.87%)	2 (0.82%)	0.09	0.686	0.686	Co-occurrence
OR7C2	19p13.12	1 (0.87%)	2 (0.82%)	0.09	0.686	0.686	Co-occurrence
OTUD1	10p12.2	1 (0.87%)	2 (0.82%)	0.09	0.686	0.686	Co-occurrence
OXA1L	14q11.2	1 (0.87%)	2 (0.82%)	0.09	0.686	0.686	Co-occurrence
OXER1	2p21	1 (0.87%)	2 (0.82%)	0.09	0.686	0.686	Co-occurrence
P2RX2	12q24.33	1 (0.87%)	2 (0.82%)	0.09	0.686	0.686	Co-occurrence
PACS1	11q13.1-q13.2	1 (0.87%)	2 (0.82%)	0.09	0.686	0.686	Co-occurrence
PAIP2	5q31.2	1 (0.87%)	2 (0.82%)	0.09	0.686	0.686	Co-occurrence
PAM16	16p13.3	1 (0.87%)	2 (0.82%)	0.09	0.686	0.686	Co-occurrence
PARP6	15q23	1 (0.87%)	2 (0.82%)	0.09	0.686	0.686	Co-occurrence
PBRM1	3p21.1	1 (0.87%)	2 (0.82%)	0.09	0.686	0.686	Co-occurrence
PCCB	3q22.3	1 (0.87%)	2 (0.82%)	0.09	0.686	0.686	Co-occurrence
PDCD1LG2	9p24.1	1 (0.87%)	2 (0.82%)	0.09	0.686	0.686	Co-occurrence
PDE1A	2q32.1	1 (0.87%)	2 (0.82%)	0.09	0.686	0.686	Co-occurrence
PDE1C	7p14.3	1 (0.87%)	2 (0.82%)	0.09	0.686	0.686	Co-occurrence
PDE4D	5q11.2-q12.1	1 (0.87%)	2 (0.82%)	0.09	0.686	0.686	Co-occurrence
PDE6A	5q32	1 (0.87%)	2 (0.82%)	0.09	0.686	0.686	Co-occurrence
PDIA2	16p13.3	1 (0.87%)	2 (0.82%)	0.09	0.686	0.686	Co-occurrence
PDPK1	16p13.3	1 (0.87%)	2 (0.82%)	0.09	0.686	0.686	Co-occurrence
PDSS1	10p12.1	1 (0.87%)	2 (0.82%)	0.09	0.686	0.686	Co-occurrence

PDZK1IP1	1p33	1 (0.87%)	2 (0.82%)	0.09	0.686	0.686	Co-occurrence
PECR	2q35	1 (0.87%)	2 (0.82%)	0.09	0.686	0.686	Co-occurrence
PGAM2	7p13	1 (0.87%)	2 (0.82%)	0.09	0.686	0.686	Co-occurrence
PGAM5	12q24.33	1 (0.87%)	2 (0.82%)	0.09	0.686	0.686	Co-occurrence
PGAP1	2q33.1	1 (0.87%)	2 (0.82%)	0.09	0.686	0.686	Co-occurrence
PGGHG	11p15.5	1 (0.87%)	2 (0.82%)	0.09	0.686	0.686	Co-occurrence
PGLS	19p13.11	1 (0.87%)	2 (0.82%)	0.09	0.686	0.686	Co-occurrence
PGP	16p13.3	1 (0.87%)	2 (0.82%)	0.09	0.686	0.686	Co-occurrence
PHB2	12p13.31	1 (0.87%)	2 (0.82%)	0.09	0.686	0.686	Co-occurrence
PHKG1	7p11.2	1 (0.87%)	2 (0.82%)	0.09	0.686	0.686	Co-occurrence
PHOX2A	11q13.4	1 (0.87%)	2 (0.82%)	0.09	0.686	0.686	Co-occurrence
PHRF1	11p15.5	1 (0.87%)	2 (0.82%)	0.09	0.686	0.686	Co-occurrence
PIANP	12p13.31	1 (0.87%)	2 (0.82%)	0.09	0.686	0.686	Co-occurrence
PIAS4	19p13.3	1 (0.87%)	2 (0.82%)	0.09	0.686	0.686	Co-occurrence
PIGF	2p21	1 (0.87%)	2 (0.82%)	0.09	0.686	0.686	Co-occurrence
PIGQ	16p13.3	1 (0.87%)	2 (0.82%)	0.09	0.686	0.686	Co-occurrence
PIP4K2A	10p12.2	1 (0.87%)	2 (0.82%)	0.09	0.686	0.686	Co-occurrence
PKD1L1	7p12.3	1 (0.87%)	2 (0.82%)	0.09	0.686	0.686	Co-occurrence
PKM	15q23	1 (0.87%)	2 (0.82%)	0.09	0.686	0.686	Co-occurrence
PKN3	9q34.11	1 (0.87%)	2 (0.82%)	0.09	0.686	0.686	Co-occurrence
PKP3	11p15.5	1 (0.87%)	2 (0.82%)	0.09	0.686	0.686	Co-occurrence
PLBD2	12q24.13	1 (0.87%)	2 (0.82%)	0.09	0.686	0.686	Co-occurrence
PLCXD3	5p13.1	1 (0.87%)	2 (0.82%)	0.09	0.686	0.686	Co-occurrence
PLEKHA8	7p14.3	1 (0.87%)	2 (0.82%)	0.09	0.686	0.686	Co-occurrence
PLGRKT	9p24.1	1 (0.87%)	2 (0.82%)	0.09	0.686	0.686	Co-occurrence
PLVAP	19p13.11	1 (0.87%)	2 (0.82%)	0.09	0.686	0.686	Co-occurrence
PMS2P3	7q11.23	1 (0.87%)	2 (0.82%)	0.09	0.686	0.686	Co-occurrence
PMS2P4	7q11.21	1 (0.87%)	2 (0.82%)	0.09	0.686	0.686	Co-occurrence
PNKD	2q35	1 (0.87%)	2 (0.82%)	0.09	0.686	0.686	Co-occurrence

POGLUT3	11q22.3	1 (0.87%)	2 (0.82%)	0.09	0.686	0.686	Co-occurrence
POLE	12q24.33	1 (0.87%)	2 (0.82%)	0.09	0.686	0.686	Co-occurrence
POLM	7p13	1 (0.87%)	2 (0.82%)	0.09	0.686	0.686	Co-occurrence
POLN	4p16.3	1 (0.87%)	2 (0.82%)	0.09	0.686	0.686	Co-occurrence
POLR2J4	7p13	1 (0.87%)	2 (0.82%)	0.09	0.686	0.686	Co-occurrence
POLR3H	22q13.2	1 (0.87%)	2 (0.82%)	0.09	0.686	0.686	Co-occurrence
POLR3K	16p13.3	1 (0.87%)	2 (0.82%)	0.09	0.686	0.686	Co-occurrence
POM121C	7q11.23	1 (0.87%)	2 (0.82%)	0.09	0.686	0.686	Co-occurrence
POM121L12	7p12.1	1 (0.87%)	2 (0.82%)	0.09	0.686	0.686	Co-occurrence
POM121L9P	22q11.23	1 (0.87%)	2 (0.82%)	0.09	0.686	0.686	Co-occurrence
POR	7q11.23	1 (0.87%)	2 (0.82%)	0.09	0.686	0.686	Co-occurrence
POTEG	14q11.2	1 (0.87%)	2 (0.82%)	0.09	0.686	0.686	Co-occurrence
POTEM	14q11.2	1 (0.87%)	2 (0.82%)	0.09	0.686	0.686	Co-occurrence
PPARG	3p25.2	1 (0.87%)	2 (0.82%)	0.09	0.686	0.686	Co-occurrence
PPIG	2q31.1	1 (0.87%)	2 (0.82%)	0.09	0.686	0.686	Co-occurrence
PPL	16p13.3	1 (0.87%)	2 (0.82%)	0.09	0.686	0.686	Co-occurrence
PPP1R17	7p14.3	1 (0.87%)	2 (0.82%)	0.09	0.686	0.686	Co-occurrence
PPP1R1C	2q31.3-q32.1	1 (0.87%)	2 (0.82%)	0.09	0.686	0.686	Co-occurrence
PPP2R2B	5q32	1 (0.87%)	2 (0.82%)	0.09	0.686	0.686	Co-occurrence
PPP2R3A	3q22.2-q22.3	1 (0.87%)	2 (0.82%)	0.09	0.686	0.686	Co-occurrence
PPP4R1	18p11.22	1 (0.87%)	2 (0.82%)	0.09	0.686	0.686	Co-occurrence
PPP4R4	14q32.12-q32.13	1 (0.87%)	2 (0.82%)	0.09	0.686	0.686	Co-occurrence
PPP5D1	19q13.32	1 (0.87%)	2 (0.82%)	0.09	0.686	0.686	Co-occurrence
PPTC7	12q24.11	1 (0.87%)	2 (0.82%)	0.09	0.686	0.686	Co-occurrence
PRELID2	5q32	1 (0.87%)	2 (0.82%)	0.09	0.686	0.686	Co-occurrence
PRKAA1	5p13.1	1 (0.87%)	2 (0.82%)	0.09	0.686	0.686	Co-occurrence
PRKD2	19q13.32	1 (0.87%)	2 (0.82%)	0.09	0.686	0.686	Co-occurrence
PRMT5	14q11.2	1 (0.87%)	2 (0.82%)	0.09	0.686	0.686	Co-occurrence
PROB1	5q31.2	1 (0.87%)	2 (0.82%)	0.09	0.686	0.686	Co-occurrence

PRR15	7p14.3	1 (0.87%)	2 (0.82%)	0.09	0.686	0.686	Co-occurrence
PRR35	16p13.3	1 (0.87%)	2 (0.82%)	0.09	0.686	0.686	Co-occurrence
PRRT3	3p25.3	1 (0.87%)	2 (0.82%)	0.09	0.686	0.686	Co-occurrence
PRSS27	16p13.3	1 (0.87%)	2 (0.82%)	0.09	0.686	0.686	Co-occurrence
PRSS29P	16p13.3	1 (0.87%)	2 (0.82%)	0.09	0.686	0.686	Co-occurrence
PRTFDC1	10p12.1	1 (0.87%)	2 (0.82%)	0.09	0.686	0.686	Co-occurrence
PSD2	5q31.2	1 (0.87%)	2 (0.82%)	0.09	0.686	0.686	Co-occurrence
PSMA2	7p14.1	1 (0.87%)	2 (0.82%)	0.09	0.686	0.686	Co-occurrence
PSMA6	14q13.2	1 (0.87%)	2 (0.82%)	0.09	0.686	0.686	Co-occurrence
PSMB2	1p34.3	1 (0.87%)	2 (0.82%)	0.09	0.686	0.686	Co-occurrence
PSMD13	11p15.5	1 (0.87%)	2 (0.82%)	0.09	0.686	0.686	Co-occurrence
PSMG2	18p11.21	1 (0.87%)	2 (0.82%)	0.09	0.686	0.686	Co-occurrence
PSPH	7p11.2	1 (0.87%)	2 (0.82%)	0.09	0.686	0.686	Co-occurrence
PTCHD3	10p12.1	1 (0.87%)	2 (0.82%)	0.09	0.686	0.686	Co-occurrence
PTCHD3P1	10p11.23	1 (0.87%)	2 (0.82%)	0.09	0.686	0.686	Co-occurrence
PTDSS2	11p15.5	1 (0.87%)	2 (0.82%)	0.09	0.686	0.686	Co-occurrence
PTF1A	10p12.2	1 (0.87%)	2 (0.82%)	0.09	0.686	0.686	Co-occurrence
PTGER4	5p13.1	1 (0.87%)	2 (0.82%)	0.09	0.686	0.686	Co-occurrence
PTGIR	19q13.32	1 (0.87%)	2 (0.82%)	0.09	0.686	0.686	Co-occurrence
PTPN6	12p13.31	1 (0.87%)	2 (0.82%)	0.09	0.686	0.686	Co-occurrence
PTPRF	1p34.2	1 (0.87%)	2 (0.82%)	0.09	0.686	0.686	Co-occurrence
PTRH1	9q34.11	1 (0.87%)	2 (0.82%)	0.09	0.686	0.686	Co-occurrence
PTX4	16p13.3	1 (0.87%)	2 (0.82%)	0.09	0.686	0.686	Co-occurrence
PXK	3p14.3	1 (0.87%)	2 (0.82%)	0.09	0.686	0.686	Co-occurrence
PXMP2	12q24.33	1 (0.87%)	2 (0.82%)	0.09	0.686	0.686	Co-occurrence
QARS	3p21.31	1 (0.87%)	2 (0.82%)	0.09	0.686	0.686	Co-occurrence
RAB11FIP3	16p13.3	1 (0.87%)	2 (0.82%)	0.09	0.686	0.686	Co-occurrence
RAB18	10p12.1	1 (0.87%)	2 (0.82%)	0.09	0.686	0.686	Co-occurrence
RAB2B	14q11.2	1 (0.87%)	2 (0.82%)	0.09	0.686	0.686	Co-occurrence

RAB31	18p11.22	1 (0.87%)	2 (0.82%)	0.09	0.686	0.686	Co-occurrence
RAB40C	16p13.3	1 (0.87%)	2 (0.82%)	0.09	0.686	0.686	Co-occurrence
RAB43	3q21.3	1 (0.87%)	2 (0.82%)	0.09	0.686	0.686	Co-occurrence
RABGEF1	7q11.21	1 (0.87%)	2 (0.82%)	0.09	0.686	0.686	Co-occurrence
RALGAPA2	20p11.23	1 (0.87%)	2 (0.82%)	0.09	0.686	0.686	Co-occurrence
RASA4CP	7p13	1 (0.87%)	2 (0.82%)	0.09	0.686	0.686	Co-occurrence
RASSF7	11p15.5	1 (0.87%)	2 (0.82%)	0.09	0.686	0.686	Co-occurrence
RBM23	14q11.2	1 (0.87%)	2 (0.82%)	0.09	0.686	0.686	Co-occurrence
RCC1L	7q11.23	1 (0.87%)	2 (0.82%)	0.09	0.686	0.686	Co-occurrence
RCL1	9p24.1	1 (0.87%)	2 (0.82%)	0.09	0.686	0.686	Co-occurrence
RFTN1	3p24.3	1 (0.87%)	2 (0.82%)	0.09	0.686	0.686	Co-occurrence
RGS11	16p13.3	1 (0.87%)	2 (0.82%)	0.09	0.686	0.686	Co-occurrence
RHBDD2	7q11.23	1 (0.87%)	2 (0.82%)	0.09	0.686	0.686	Co-occurrence
RHBDF1	16p13.3	1 (0.87%)	2 (0.82%)	0.09	0.686	0.686	Co-occurrence
RHOBTB1	10q21.2	1 (0.87%)	2 (0.82%)	0.09	0.686	0.686	Co-occurrence
RHOG	11p15.4	1 (0.87%)	2 (0.82%)	0.09	0.686	0.686	Co-occurrence
RHOQ	2p21	1 (0.87%)	2 (0.82%)	0.09	0.686	0.686	Co-occurrence
RIBC1	Xp11.22	1 (0.87%)	2 (0.82%)	0.09	0.686	0.686	Co-occurrence
RIC1	9p24.1	1 (0.87%)	2 (0.82%)	0.09	0.686	0.686	Co-occurrence
RIC8A	11p15.5	1 (0.87%)	2 (0.82%)	0.09	0.686	0.686	Co-occurrence
RIOX2	3q11.2	1 (0.87%)	2 (0.82%)	0.09	0.686	0.686	Co-occurrence
RLN1	9p24.1	1 (0.87%)	2 (0.82%)	0.09	0.686	0.686	Co-occurrence
RLN2	9p24.1	1 (0.87%)	2 (0.82%)	0.09	0.686	0.686	Co-occurrence
RN7SKP114	9p13.3	1 (0.87%)	2 (0.82%)	0.09	0.686	0.686	Co-occurrence
RN7SKP132	10p12.1	1 (0.87%)	2 (0.82%)	0.09	0.686	0.686	Co-occurrence
RN7SKP179	2q32.2	1 (0.87%)	2 (0.82%)	0.09	0.686	0.686	Co-occurrence
RN7SKP219	10p12.31	1 (0.87%)	2 (0.82%)	0.09	0.686	0.686	Co-occurrence
RN7SKP220	10p12.1	1 (0.87%)	2 (0.82%)	0.09	0.686	0.686	Co-occurrence
RN7SKP224	2p16.3	1 (0.87%)	2 (0.82%)	0.09	0.686	0.686	Co-occurrence

RN7SKP241	10p12.1	1 (0.87%)	2 (0.82%)	0.09	0.686	0.686	Co-occurrence
RN7SKP246	5q31.3	1 (0.87%)	2 (0.82%)	0.09	0.686	0.686	Co-occurrence
RN7SKP37	10p12.31	1 (0.87%)	2 (0.82%)	0.09	0.686	0.686	Co-occurrence
RN7SKP39	10p12.1	1 (0.87%)	2 (0.82%)	0.09	0.686	0.686	Co-occurrence
RN7SKP64	5q31.2	1 (0.87%)	2 (0.82%)	0.09	0.686	0.686	Co-occurrence
RN7SL123P	9p24.1	1 (0.87%)	2 (0.82%)	0.09	0.686	0.686	Co-occurrence
RN7SL156P	20q11.23	1 (0.87%)	2 (0.82%)	0.09	0.686	0.686	Co-occurrence
RN7SL2	14q21.3	1 (0.87%)	2 (0.82%)	0.09	0.686	0.686	Co-occurrence
RN7SL231P	19p13.12	1 (0.87%)	2 (0.82%)	0.09	0.686	0.686	Co-occurrence
RN7SL241P	10p11.23	1 (0.87%)	2 (0.82%)	0.09	0.686	0.686	Co-occurrence
RN7SL25P	9p24.1	1 (0.87%)	2 (0.82%)	0.09	0.686	0.686	Co-occurrence
RN7SL267P	2q32.1	1 (0.87%)	2 (0.82%)	0.09	0.686	0.686	Co-occurrence
RN7SL282P	18p11.31	1 (0.87%)	2 (0.82%)	0.09	0.686	0.686	Co-occurrence
RN7SL284P	10q22.3	1 (0.87%)	2 (0.82%)	0.09	0.686	0.686	Co-occurrence
RN7SL3	14q21.3	1 (0.87%)	2 (0.82%)	0.09	0.686	0.686	Co-occurrence
RN7SL314P	10p11.21	1 (0.87%)	2 (0.82%)	0.09	0.686	0.686	Co-occurrence
RN7SL35P	7q21.11	1 (0.87%)	2 (0.82%)	0.09	0.686	0.686	Co-occurrence
RN7SL362P	18p11.21	1 (0.87%)	2 (0.82%)	0.09	0.686	0.686	Co-occurrence
RN7SL364P	19q13.32	1 (0.87%)	2 (0.82%)	0.09	0.686	0.686	Co-occurrence
RN7SL43P	7q11.21	1 (0.87%)	2 (0.82%)	0.09	0.686	0.686	Co-occurrence
RN7SL560P	9q34.11	1 (0.87%)	2 (0.82%)	0.09	0.686	0.686	Co-occurrence
RN7SL589P	4p16.3	1 (0.87%)	2 (0.82%)	0.09	0.686	0.686	Co-occurrence
RN7SL607P	20p11.23	1 (0.87%)	2 (0.82%)	0.09	0.686	0.686	Co-occurrence
RN7SL620P	17p11.2	1 (0.87%)	2 (0.82%)	0.09	0.686	0.686	Co-occurrence
RN7SL650P	14q11.2	1 (0.87%)	2 (0.82%)	0.09	0.686	0.686	Co-occurrence
RN7SL671P	4p16.3	1 (0.87%)	2 (0.82%)	0.09	0.686	0.686	Co-occurrence
RN7SL717P	2q33.1	1 (0.87%)	2 (0.82%)	0.09	0.686	0.686	Co-occurrence
RN7SL718P	19q13.2	1 (0.87%)	2 (0.82%)	0.09	0.686	0.686	Co-occurrence
RN7SL791P	5q32	1 (0.87%)	2 (0.82%)	0.09	0.686	0.686	Co-occurrence

RN7SL813P	2q24.3	1 (0.87%)	2 (0.82%)	0.09	0.686	0.686	Co-occurrence
RN7SL817P	2p21	1 (0.87%)	2 (0.82%)	0.09	0.686	0.686	Co-occurrence
RN7SL820P	2q32.3	1 (0.87%)	2 (0.82%)	0.09	0.686	0.686	Co-occurrence
RN7SL838P	11p15.5	1 (0.87%)	2 (0.82%)	0.09	0.686	0.686	Co-occurrence
RN7SL850P	16p13.3	1 (0.87%)	2 (0.82%)	0.09	0.686	0.686	Co-occurrence
RN7SL862P	18p11.22	1 (0.87%)	2 (0.82%)	0.09	0.686	0.686	Co-occurrence
RN7SL869P	7q21.11	1 (0.87%)	2 (0.82%)	0.09	0.686	0.686	Co-occurrence
RNA5SP113	2q31.3	1 (0.87%)	2 (0.82%)	0.09	0.686	0.686	Co-occurrence
RNA5SP114	2q32.1	1 (0.87%)	2 (0.82%)	0.09	0.686	0.686	Co-occurrence
RNA5SP123	3p25.2	1 (0.87%)	2 (0.82%)	0.09	0.686	0.686	Co-occurrence
RNA5SP141	3q22.2	1 (0.87%)	2 (0.82%)	0.09	0.686	0.686	Co-occurrence
RNA5SP158	4p14	1 (0.87%)	2 (0.82%)	0.09	0.686	0.686	Co-occurrence
RNA5SP194	5q31.2	1 (0.87%)	2 (0.82%)	0.09	0.686	0.686	Co-occurrence
RNA5SP195	5q31.2	1 (0.87%)	2 (0.82%)	0.09	0.686	0.686	Co-occurrence
RNA5SP234	7q21.11	1 (0.87%)	2 (0.82%)	0.09	0.686	0.686	Co-occurrence
RNA5SP235	7q21.11	1 (0.87%)	2 (0.82%)	0.09	0.686	0.686	Co-occurrence
RNA5SP24	13q12.11	1 (0.87%)	2 (0.82%)	0.09	0.686	0.686	Co-occurrence
RNA5SP264	8p11.23	1 (0.87%)	2 (0.82%)	0.09	0.686	0.686	Co-occurrence
RNA5SP282	9p13.3	1 (0.87%)	2 (0.82%)	0.09	0.686	0.686	Co-occurrence
RNA5SP300	10p13	1 (0.87%)	2 (0.82%)	0.09	0.686	0.686	Co-occurrence
RNA5SP304	10p12.2	1 (0.87%)	2 (0.82%)	0.09	0.686	0.686	Co-occurrence
RNA5SP305	10p12.1	1 (0.87%)	2 (0.82%)	0.09	0.686	0.686	Co-occurrence
RNA5SP306	10p12.1	1 (0.87%)	2 (0.82%)	0.09	0.686	0.686	Co-occurrence
RNA5SP307	10p12.1	1 (0.87%)	2 (0.82%)	0.09	0.686	0.686	Co-occurrence
RNA5SP308	10p12.1	1 (0.87%)	2 (0.82%)	0.09	0.686	0.686	Co-occurrence
RNA5SP309	10p11.22	1 (0.87%)	2 (0.82%)	0.09	0.686	0.686	Co-occurrence
RNA5SP355	12p11.22	1 (0.87%)	2 (0.82%)	0.09	0.686	0.686	Co-occurrence
RNA5SP377	12q24.33	1 (0.87%)	2 (0.82%)	0.09	0.686	0.686	Co-occurrence
RNA5SP379	12q24.33	1 (0.87%)	2 (0.82%)	0.09	0.686	0.686	Co-occurrence

RNA5SP380	14q11.2	1 (0.87%)	2 (0.82%)	0.09	0.686	0.686	Co-occurrence
RNA5SP399	15q23	1 (0.87%)	2 (0.82%)	0.09	0.686	0.686	Co-occurrence
RNA5SP449	18p11.22	1 (0.87%)	2 (0.82%)	0.09	0.686	0.686	Co-occurrence
RNA5SP450	18p11.22	1 (0.87%)	2 (0.82%)	0.09	0.686	0.686	Co-occurrence
RNF113A	Xq24	1 (0.87%)	2 (0.82%)	0.09	0.686	0.686	Co-occurrence
RNF4	4p16.3	1 (0.87%)	2 (0.82%)	0.09	0.686	0.686	Co-occurrence
RNH1	11p15.5	1 (0.87%)	2 (0.82%)	0.09	0.686	0.686	Co-occurrence
RNMT	18p11.21	1 (0.87%)	2 (0.82%)	0.09	0.686	0.686	Co-occurrence
RNPS1	16p13.3	1 (0.87%)	2 (0.82%)	0.09	0.686	0.686	Co-occurrence
RNU4ATAC12P	12q24.33	1 (0.87%)	2 (0.82%)	0.09	0.686	0.686	Co-occurrence
RNU4ATAC13P	5q22.2	1 (0.87%)	2 (0.82%)	0.09	0.686	0.686	Co-occurrence
RNU4ATAC14P	14q24.3	1 (0.87%)	2 (0.82%)	0.09	0.686	0.686	Co-occurrence
RNU6ATAC18P	1p36.21	1 (0.87%)	2 (0.82%)	0.09	0.686	0.686	Co-occurrence
RNU6ATAC19P	2q31.3	1 (0.87%)	2 (0.82%)	0.09	0.686	0.686	Co-occurrence
RNU6ATAC39P	10p13	1 (0.87%)	2 (0.82%)	0.09	0.686	0.686	Co-occurrence
RNU6ATAC9P	14q22.2	1 (0.87%)	2 (0.82%)	0.09	0.686	0.686	Co-occurrence
ROCK1	18q11.1	1 (0.87%)	2 (0.82%)	0.09	0.686	0.686	Co-occurrence
ROGDI	16p13.3	1 (0.87%)	2 (0.82%)	0.09	0.686	0.686	Co-occurrence
RPL13AP17	7q21.11	1 (0.87%)	2 (0.82%)	0.09	0.686	0.686	Co-occurrence
RPL37	5p13.1	1 (0.87%)	2 (0.82%)	0.09	0.686	0.686	Co-occurrence
RPL39	Xq24	1 (0.87%)	2 (0.82%)	0.09	0.686	0.686	Co-occurrence
RPL39P5	3q22.2	1 (0.87%)	2 (0.82%)	0.09	0.686	0.686	Co-occurrence
RPL6P27	18p11.31	1 (0.87%)	2 (0.82%)	0.09	0.686	0.686	Co-occurrence
RPP14	3p14.3	1 (0.87%)	2 (0.82%)	0.09	0.686	0.686	Co-occurrence
RPP25L	9p13.3	1 (0.87%)	2 (0.82%)	0.09	0.686	0.686	Co-occurrence
RXFP2	13q13.1	1 (0.87%)	2 (0.82%)	0.09	0.686	0.686	Co-occurrence
SAMD4A	14q22.2	1 (0.87%)	2 (0.82%)	0.09	0.686	0.686	Co-occurrence
SAMD8	10q22.2	1 (0.87%)	2 (0.82%)	0.09	0.686	0.686	Co-occurrence
SAR1B	5q31.1	1 (0.87%)	2 (0.82%)	0.09	0.686	0.686	Co-occurrence

SBDS	7q11.21	1 (0.87%)	2 (0.82%)	0.09	0.686	0.686	Co-occurrence
SCARNA12	12p13.31	1 (0.87%)	2 (0.82%)	0.09	0.686	0.686	Co-occurrence
SCGB1C1	11p15.5	1 (0.87%)	2 (0.82%)	0.09	0.686	0.686	Co-occurrence
SCGB3A2	5q32	1 (0.87%)	2 (0.82%)	0.09	0.686	0.686	Co-occurrence
SCRN1	7p14.3	1 (0.87%)	2 (0.82%)	0.09	0.686	0.686	Co-occurrence
SDS	12q24.13	1 (0.87%)	2 (0.82%)	0.09	0.686	0.686	Co-occurrence
SEC13	3p25.3	1 (0.87%)	2 (0.82%)	0.09	0.686	0.686	Co-occurrence
SEC24A	5q31.1	1 (0.87%)	2 (0.82%)	0.09	0.686	0.686	Co-occurrence
SEMA3A	7q21.11	1 (0.87%)	2 (0.82%)	0.09	0.686	0.686	Co-occurrence
SEMA3C	7q21.11	1 (0.87%)	2 (0.82%)	0.09	0.686	0.686	Co-occurrence
SEMA3D	7q21.11	1 (0.87%)	2 (0.82%)	0.09	0.686	0.686	Co-occurrence
SEMA5B	3q21.1	1 (0.87%)	2 (0.82%)	0.09	0.686	0.686	Co-occurrence
SENP8	15q23	1 (0.87%)	2 (0.82%)	0.09	0.686	0.686	Co-occurrence
SEPTIN14	7p11.2	1 (0.87%)	2 (0.82%)	0.09	0.686	0.686	Co-occurrence
SEPTIN6	Xq24	1 (0.87%)	2 (0.82%)	0.09	0.686	0.686	Co-occurrence
SEPTIN7	7p14.2	1 (0.87%)	2 (0.82%)	0.09	0.686	0.686	Co-occurrence
SET	9q34.11	1 (0.87%)	2 (0.82%)	0.09	0.686	0.686	Co-occurrence
SETD5	3p25.3	1 (0.87%)	2 (0.82%)	0.09	0.686	0.686	Co-occurrence
SH2D3C	9q34.11	1 (0.87%)	2 (0.82%)	0.09	0.686	0.686	Co-occurrence
SIGIRR	11p15.5	1 (0.87%)	2 (0.82%)	0.09	0.686	0.686	Co-occurrence
SIK3	11q23.3	1 (0.87%)	2 (0.82%)	0.09	0.686	0.686	Co-occurrence
SIL1	5q31.2	1 (0.87%)	2 (0.82%)	0.09	0.686	0.686	Co-occurrence
SIRT3	11p15.5	1 (0.87%)	2 (0.82%)	0.09	0.686	0.686	Co-occurrence
SIRT6	19p13.3	1 (0.87%)	2 (0.82%)	0.09	0.686	0.686	Co-occurrence
SKIDA1	10p12.31	1 (0.87%)	2 (0.82%)	0.09	0.686	0.686	Co-occurrence
SLA2	20q11.23	1 (0.87%)	2 (0.82%)	0.09	0.686	0.686	Co-occurrence
SLC11A1	2q35	1 (0.87%)	2 (0.82%)	0.09	0.686	0.686	Co-occurrence
SLC14A1	18q12.3	1 (0.87%)	2 (0.82%)	0.09	0.686	0.686	Co-occurrence
SLC1A6	19p13.12	1 (0.87%)	2 (0.82%)	0.09	0.686	0.686	Co-occurrence

SLC23A1	5q31.2	1 (0.87%)	2 (0.82%)	0.09	0.686	0.686	Co-occurrence
SLC27A1	19p13.11	1 (0.87%)	2 (0.82%)	0.09	0.686	0.686	Co-occurrence
SLC39A10	2q32.3	1 (0.87%)	2 (0.82%)	0.09	0.686	0.686	Co-occurrence
SLC39A6	18q12.2	1 (0.87%)	2 (0.82%)	0.09	0.686	0.686	Co-occurrence
SLC6A1	3p25.3	1 (0.87%)	2 (0.82%)	0.09	0.686	0.686	Co-occurrence
SLC6A16	19q13.33	1 (0.87%)	2 (0.82%)	0.09	0.686	0.686	Co-occurrence
SLC7A7	14q11.2	1 (0.87%)	2 (0.82%)	0.09	0.686	0.686	Co-occurrence
SLC8B1	12q24.13	1 (0.87%)	2 (0.82%)	0.09	0.686	0.686	Co-occurrence
SMC1A	Xp11.22	1 (0.87%)	2 (0.82%)	0.09	0.686	0.686	Co-occurrence
SMIM22	16p13.3	1 (0.87%)	2 (0.82%)	0.09	0.686	0.686	Co-occurrence
SMIM4	3p21.1	1 (0.87%)	2 (0.82%)	0.09	0.686	0.686	Co-occurrence
SMYD1	2p11.2	1 (0.87%)	2 (0.82%)	0.09	0.686	0.686	Co-occurrence
SNCG	10q23.2	1 (0.87%)	2 (0.82%)	0.09	0.686	0.686	Co-occurrence
SNORA1	11q21	1 (0.87%)	2 (0.82%)	0.09	0.686	0.686	Co-occurrence
SNORA14A	7q11.23	1 (0.87%)	2 (0.82%)	0.09	0.686	0.686	Co-occurrence
SNORA15	7p11.2	1 (0.87%)	2 (0.82%)	0.09	0.686	0.686	Co-occurrence
SNORA17B	9q34.3	1 (0.87%)	2 (0.82%)	0.09	0.686	0.686	Co-occurrence
SNORA22	7q11.21	1 (0.87%)	2 (0.82%)	0.09	0.686	0.686	Co-occurrence
SNORA57	11q12.3	1 (0.87%)	2 (0.82%)	0.09	0.686	0.686	Co-occurrence
SNORA59A	1p36.21	1 (0.87%)	2 (0.82%)	0.09	0.686	0.686	Co-occurrence
SNORA74A	5q31.2	1 (0.87%)	2 (0.82%)	0.09	0.686	0.686	Co-occurrence
SNORD41	19p13.13	1 (0.87%)	2 (0.82%)	0.09	0.686	0.686	Co-occurrence
SNORD72	5p13.1	1 (0.87%)	2 (0.82%)	0.09	0.686	0.686	Co-occurrence
SNRK	3p22.1	1 (0.87%)	2 (0.82%)	0.09	0.686	0.686	Co-occurrence
SNRNP25	16p13.3	1 (0.87%)	2 (0.82%)	0.09	0.686	0.686	Co-occurrence
SOCS5	2p21	1 (0.87%)	2 (0.82%)	0.09	0.686	0.686	Co-occurrence
SOS2	14q21.3	1 (0.87%)	2 (0.82%)	0.09	0.686	0.686	Co-occurrence
SOWAHD	Xq24	1 (0.87%)	2 (0.82%)	0.09	0.686	0.686	Co-occurrence
SOX8	16p13.3	1 (0.87%)	2 (0.82%)	0.09	0.686	0.686	Co-occurrence

SP5	2q31.1	1 (0.87%)	2 (0.82%)	0.09	0.686	0.686	Co-occurrence
SPAG6	10p12.2	1 (0.87%)	2 (0.82%)	0.09	0.686	0.686	Co-occurrence
SPATA24	5q31.2	1 (0.87%)	2 (0.82%)	0.09	0.686	0.686	Co-occurrence
SPDYE1	7p13	1 (0.87%)	2 (0.82%)	0.09	0.686	0.686	Co-occurrence
SPECC1L	22q11.23	1 (0.87%)	2 (0.82%)	0.09	0.686	0.686	Co-occurrence
SPEN	1p36.21-p36.13	1 (0.87%)	2 (0.82%)	0.09	0.686	0.686	Co-occurrence
SPINK1	5q32	1 (0.87%)	2 (0.82%)	0.09	0.686	0.686	Co-occurrence
SPINK14	5q32	1 (0.87%)	2 (0.82%)	0.09	0.686	0.686	Co-occurrence
SPINK5	5q32	1 (0.87%)	2 (0.82%)	0.09	0.686	0.686	Co-occurrence
SPINK6	5q32	1 (0.87%)	2 (0.82%)	0.09	0.686	0.686	Co-occurrence
SPINK7	5q32	1 (0.87%)	2 (0.82%)	0.09	0.686	0.686	Co-occurrence
SPINK9	5q32	1 (0.87%)	2 (0.82%)	0.09	0.686	0.686	Co-occurrence
SPIRE1	18p11.21	1 (0.87%)	2 (0.82%)	0.09	0.686	0.686	Co-occurrence
SPTAN1	9q34.11	1 (0.87%)	2 (0.82%)	0.09	0.686	0.686	Co-occurrence
SRARP	1p36.13	1 (0.87%)	2 (0.82%)	0.09	0.686	0.686	Co-occurrence
SRC	20q11.23	1 (0.87%)	2 (0.82%)	0.09	0.686	0.686	Co-occurrence
SRGAP3	3p25.3	1 (0.87%)	2 (0.82%)	0.09	0.686	0.686	Co-occurrence
SSB	2q31.1	1 (0.87%)	2 (0.82%)	0.09	0.686	0.686	Co-occurrence
SSTR5	16p13.3	1 (0.87%)	2 (0.82%)	0.09	0.686	0.686	Co-occurrence
STAG3L1	7q11.23	1 (0.87%)	2 (0.82%)	0.09	0.686	0.686	Co-occurrence
STAG3L2	7q11.23	1 (0.87%)	2 (0.82%)	0.09	0.686	0.686	Co-occurrence
STAG3L4	7q11.21	1 (0.87%)	2 (0.82%)	0.09	0.686	0.686	Co-occurrence
STAT1	2q32.2	1 (0.87%)	2 (0.82%)	0.09	0.686	0.686	Co-occurrence
STAT4	2q32.2-q32.3	1 (0.87%)	2 (0.82%)	0.09	0.686	0.686	Co-occurrence
STIL	1p33	1 (0.87%)	2 (0.82%)	0.09	0.686	0.686	Co-occurrence
STK17A	7p13	1 (0.87%)	2 (0.82%)	0.09	0.686	0.686	Co-occurrence
STK17B	2q32.3	1 (0.87%)	2 (0.82%)	0.09	0.686	0.686	Co-occurrence
STRN4	19q13.32	1 (0.87%)	2 (0.82%)	0.09	0.686	0.686	Co-occurrence
STXBP6	14q12	1 (0.87%)	2 (0.82%)	0.09	0.686	0.686	Co-occurrence

SUMF2	7p11.2	1 (0.87%)	2 (0.82%)	0.09	0.686	0.686	Co-occurrence
SUN3	7p12.3	1 (0.87%)	2 (0.82%)	0.09	0.686	0.686	Co-occurrence
SVIL	10p11.23	1 (0.87%)	2 (0.82%)	0.09	0.686	0.686	Co-occurrence
SYT8	11p15.5	1 (0.87%)	2 (0.82%)	0.09	0.686	0.686	Co-occurrence
TAF9	5q13.2	1 (0.87%)	2 (0.82%)	0.09	0.686	0.686	Co-occurrence
TAL1	1p33	1 (0.87%)	2 (0.82%)	0.09	0.686	0.686	Co-occurrence
TATDN2	3p25.3	1 (0.87%)	2 (0.82%)	0.09	0.686	0.686	Co-occurrence
TBC1D24	16p13.3	1 (0.87%)	2 (0.82%)	0.09	0.686	0.686	Co-occurrence
TBC1D5	3p24.3	1 (0.87%)	2 (0.82%)	0.09	0.686	0.686	Co-occurrence
TEAD1	11p15.3	1 (0.87%)	2 (0.82%)	0.09	0.686	0.686	Co-occurrence
TEAD2	19q13.33	1 (0.87%)	2 (0.82%)	0.09	0.686	0.686	Co-occurrence
TEDC2	16p13.3	1 (0.87%)	2 (0.82%)	0.09	0.686	0.686	Co-occurrence
TELO2	16p13.3	1 (0.87%)	2 (0.82%)	0.09	0.686	0.686	Co-occurrence
TFAP4	16p13.3	1 (0.87%)	2 (0.82%)	0.09	0.686	0.686	Co-occurrence
TGFBR3	1p22.1	1 (0.87%)	2 (0.82%)	0.09	0.686	0.686	Co-occurrence
THNSL1	10p12.1	1 (0.87%)	2 (0.82%)	0.09	0.686	0.686	Co-occurrence
THNSL2	2p11.2	1 (0.87%)	2 (0.82%)	0.09	0.686	0.686	Co-occurrence
THSD4	15q23	1 (0.87%)	2 (0.82%)	0.09	0.686	0.686	Co-occurrence
THSD7A	7p21.3	1 (0.87%)	2 (0.82%)	0.09	0.686	0.686	Co-occurrence
THUMPD3	3p25.3	1 (0.87%)	2 (0.82%)	0.09	0.686	0.686	Co-occurrence
TIMP4	3p25.2	1 (0.87%)	2 (0.82%)	0.09	0.686	0.686	Co-occurrence
TLR1	4p14	1 (0.87%)	2 (0.82%)	0.09	0.686	0.686	Co-occurrence
TLR6	4p14	1 (0.87%)	2 (0.82%)	0.09	0.686	0.686	Co-occurrence
TMBIM1	2q35	1 (0.87%)	2 (0.82%)	0.09	0.686	0.686	Co-occurrence
TMED10	14q24.3	1 (0.87%)	2 (0.82%)	0.09	0.686	0.686	Co-occurrence
TMED4	7p13	1 (0.87%)	2 (0.82%)	0.09	0.686	0.686	Co-occurrence
TMEFF2	2q32.3	1 (0.87%)	2 (0.82%)	0.09	0.686	0.686	Co-occurrence
TMEM120A	7q11.23	1 (0.87%)	2 (0.82%)	0.09	0.686	0.686	Co-occurrence
TMEM123	11q22.2	1 (0.87%)	2 (0.82%)	0.09	0.686	0.686	Co-occurrence

TMEM164	Xq23	1 (0.87%)	2 (0.82%)	0.09	0.686	0.686	Co-occurrence
TMEM169	2q35	1 (0.87%)	2 (0.82%)	0.09	0.686	0.686	Co-occurrence
TMEM200C	18p11.31	1 (0.87%)	2 (0.82%)	0.09	0.686	0.686	Co-occurrence
TMEM202	15q23-q24.1	1 (0.87%)	2 (0.82%)	0.09	0.686	0.686	Co-occurrence
TMEM221	19p13.11	1 (0.87%)	2 (0.82%)	0.09	0.686	0.686	Co-occurrence
TMEM248	7q11.21	1 (0.87%)	2 (0.82%)	0.09	0.686	0.686	Co-occurrence
TMEM255A	Xq24	1 (0.87%)	2 (0.82%)	0.09	0.686	0.686	Co-occurrence
TMEM8A	16p13.3	1 (0.87%)	2 (0.82%)	0.09	0.686	0.686	Co-occurrence
TNNI2	11p15.5	1 (0.87%)	2 (0.82%)	0.09	0.686	0.686	Co-occurrence
TNS3	7p12.3	1 (0.87%)	2 (0.82%)	0.09	0.686	0.686	Co-occurrence
TOR2A	9q34.11	1 (0.87%)	2 (0.82%)	0.09	0.686	0.686	Co-occurrence
TOX4	14q11.2	1 (0.87%)	2 (0.82%)	0.09	0.686	0.686	Co-occurrence
TPD52L3	9p24.1	1 (0.87%)	2 (0.82%)	0.09	0.686	0.686	Co-occurrence
TPSAB1	16p13.3	1 (0.87%)	2 (0.82%)	0.09	0.686	0.686	Co-occurrence
TPSB2	16p13.3	1 (0.87%)	2 (0.82%)	0.09	0.686	0.686	Co-occurrence
TPSD1	16p13.3	1 (0.87%)	2 (0.82%)	0.09	0.686	0.686	Co-occurrence
TPSG1	16p13.3	1 (0.87%)	2 (0.82%)	0.09	0.686	0.686	Co-occurrence
TRIL	7p14.3	1 (0.87%)	2 (0.82%)	0.09	0.686	0.686	Co-occurrence
TRIM73	7q11.23	1 (0.87%)	2 (0.82%)	0.09	0.686	0.686	Co-occurrence
TRPC4AP	20q11.22	1 (0.87%)	2 (0.82%)	0.09	0.686	0.686	Co-occurrence
TSPAN7	Xp11.4	1 (0.87%)	2 (0.82%)	0.09	0.686	0.686	Co-occurrence
TTC16	9q34.11	1 (0.87%)	2 (0.82%)	0.09	0.686	0.686	Co-occurrence
TTC33	5p13.1	1 (0.87%)	2 (0.82%)	0.09	0.686	0.686	Co-occurrence
TXNDC2	18p11.22	1 (0.87%)	2 (0.82%)	0.09	0.686	0.686	Co-occurrence
TYW1	7q11.21	1 (0.87%)	2 (0.82%)	0.09	0.686	0.686	Co-occurrence
TYW5	2q33.1	1 (0.87%)	2 (0.82%)	0.09	0.686	0.686	Co-occurrence
UBALD1	16p13.3	1 (0.87%)	2 (0.82%)	0.09	0.686	0.686	Co-occurrence
UBAP1	9p13.3	1 (0.87%)	2 (0.82%)	0.09	0.686	0.686	Co-occurrence
UBAP2	9p13.3	1 (0.87%)	2 (0.82%)	0.09	0.686	0.686	Co-occurrence

UBE2D4	7p13	1 (0.87%)	2 (0.82%)	0.09	0.686	0.686	Co-occurrence
UBE2E3	2q31.3	1 (0.87%)	2 (0.82%)	0.09	0.686	0.686	Co-occurrence
UBE3B	12q24.11	1 (0.87%)	2 (0.82%)	0.09	0.686	0.686	Co-occurrence
UBIAD1	1p36.22	1 (0.87%)	2 (0.82%)	0.09	0.686	0.686	Co-occurrence
UBN1	16p13.3	1 (0.87%)	2 (0.82%)	0.09	0.686	0.686	Co-occurrence
UBR3	2q31.1	1 (0.87%)	2 (0.82%)	0.09	0.686	0.686	Co-occurrence
UHRF2	9p24.1	1 (0.87%)	2 (0.82%)	0.09	0.686	0.686	Co-occurrence
UMOD	16p12.3	1 (0.87%)	2 (0.82%)	0.09	0.686	0.686	Co-occurrence
UPF3B	Xq24	1 (0.87%)	2 (0.82%)	0.09	0.686	0.686	Co-occurrence
UPP1	7p12.3	1 (0.87%)	2 (0.82%)	0.09	0.686	0.686	Co-occurrence
URGCP	7p13	1 (0.87%)	2 (0.82%)	0.09	0.686	0.686	Co-occurrence
USP19	3p21.31	1 (0.87%)	2 (0.82%)	0.09	0.686	0.686	Co-occurrence
USP32P1	17p11.2	1 (0.87%)	2 (0.82%)	0.09	0.686	0.686	Co-occurrence
UTP15	5q13.2	1 (0.87%)	2 (0.82%)	0.09	0.686	0.686	Co-occurrence
VAPA	18p11.22	1 (0.87%)	2 (0.82%)	0.09	0.686	0.686	Co-occurrence
VASN	16p13.3	1 (0.87%)	2 (0.82%)	0.09	0.686	0.686	Co-occurrence
VCPKMT	14q21.3	1 (0.87%)	2 (0.82%)	0.09	0.686	0.686	Co-occurrence
VDAC2	10q22.2	1 (0.87%)	2 (0.82%)	0.09	0.686	0.686	Co-occurrence
VPS13D	1p36.22-p36.21	1 (0.87%)	2 (0.82%)	0.09	0.686	0.686	Co-occurrence
VSTM2A	7p11.2	1 (0.87%)	2 (0.82%)	0.09	0.686	0.686	Co-occurrence
VTI1A	10q25.2	1 (0.87%)	2 (0.82%)	0.09	0.686	0.686	Co-occurrence
WASH4P	16p13.3	1 (0.87%)	2 (0.82%)	0.09	0.686	0.686	Co-occurrence
WASIR2	16p13.3	1 (0.87%)	2 (0.82%)	0.09	0.686	0.686	Co-occurrence
WDR12	2q33.2	1 (0.87%)	2 (0.82%)	0.09	0.686	0.686	Co-occurrence
WDR34	9q34.11	1 (0.87%)	2 (0.82%)	0.09	0.686	0.686	Co-occurrence
WDR75	2q32.2	1 (0.87%)	2 (0.82%)	0.09	0.686	0.686	Co-occurrence
WDR90	16p13.3	1 (0.87%)	2 (0.82%)	0.09	0.686	0.686	Co-occurrence
WFIKKN1	16p13.3	1 (0.87%)	2 (0.82%)	0.09	0.686	0.686	Co-occurrence
WIPF3	7p14.3	1 (0.87%)	2 (0.82%)	0.09	0.686	0.686	Co-occurrence

XIAP	Xq25	1 (0.87%)	2 (0.82%)	0.09	0.686	0.686	Co-occurrence
XRCC5	2q35	1 (0.87%)	2 (0.82%)	0.09	0.686	0.686	Co-occurrence
YME1L1	10p12.1	1 (0.87%)	2 (0.82%)	0.09	0.686	0.686	Co-occurrence
YTHDC2	5q22.2	1 (0.87%)	2 (0.82%)	0.09	0.686	0.686	Co-occurrence
ZBTB14	18p11.31	1 (0.87%)	2 (0.82%)	0.09	0.686	0.686	Co-occurrence
ZBTB17	1p36.13	1 (0.87%)	2 (0.82%)	0.09	0.686	0.686	Co-occurrence
ZBTB49	4p16.3	1 (0.87%)	2 (0.82%)	0.09	0.686	0.686	Co-occurrence
ZDHHC12	9q34.11	1 (0.87%)	2 (0.82%)	0.09	0.686	0.686	Co-occurrence
ZER1	9q34.11	1 (0.87%)	2 (0.82%)	0.09	0.686	0.686	Co-occurrence
ZFP36L2	2p21	1 (0.87%)	2 (0.82%)	0.09	0.686	0.686	Co-occurrence
ZFYVE28	4p16.3	1 (0.87%)	2 (0.82%)	0.09	0.686	0.686	Co-occurrence
ZMAT2	5q31.3	1 (0.87%)	2 (0.82%)	0.09	0.686	0.686	Co-occurrence
ZNF10	12q24.33	1 (0.87%)	2 (0.82%)	0.09	0.686	0.686	Co-occurrence
ZNF140	12q24.33	1 (0.87%)	2 (0.82%)	0.09	0.686	0.686	Co-occurrence
ZNF229	19q13.31	1 (0.87%)	2 (0.82%)	0.09	0.686	0.686	Co-occurrence
ZNF26	12q24.33	1 (0.87%)	2 (0.82%)	0.09	0.686	0.686	Co-occurrence
ZNF268	12q24.33	1 (0.87%)	2 (0.82%)	0.09	0.686	0.686	Co-occurrence
ZNF384	12p13.31	1 (0.87%)	2 (0.82%)	0.09	0.686	0.686	Co-occurrence
ZNF438	10p11.23	1 (0.87%)	2 (0.82%)	0.09	0.686	0.686	Co-occurrence
ZNF519	18p11.21	1 (0.87%)	2 (0.82%)	0.09	0.686	0.686	Co-occurrence
ZNF605	12q24.33	1 (0.87%)	2 (0.82%)	0.09	0.686	0.686	Co-occurrence
ZNF662	3p22.1	1 (0.87%)	2 (0.82%)	0.09	0.686	0.686	Co-occurrence
ZNF688	16p11.2	1 (0.87%)	2 (0.82%)	0.09	0.686	0.686	Co-occurrence
ZNF713	7p11.2	1 (0.87%)	2 (0.82%)	0.09	0.686	0.686	Co-occurrence
ZNF764	16p11.2	1 (0.87%)	2 (0.82%)	0.09	0.686	0.686	Co-occurrence
ZNF785	16p11.2	1 (0.87%)	2 (0.82%)	0.09	0.686	0.686	Co-occurrence
ZNF804A	2q32.1	1 (0.87%)	2 (0.82%)	0.09	0.686	0.686	Co-occurrence
ZNF84	12q24.33	1 (0.87%)	2 (0.82%)	0.09	0.686	0.686	Co-occurrence
ZNF891	12q24.33	1 (0.87%)	2 (0.82%)	0.09	0.686	0.686	Co-occurrence

ZNRF2	7p14.3	1 (0.87%)	2 (0.82%)	0.09	0.686	0.686	Co-occurrence
