# Supplementary material for: Prognostic potential of PRPF3 in hepatocellular carcinoma
Source: Aging (Albany NY). 2020 Jan 11;12(1):912–30. doi: 10.18632/aging.102665 (PMC6977647; doi:10.18632/aging.102665)
Supplement: Supplementary Table 8 [file aging-12-102665-s001..pdf]

Supplementary Table 8. Functional annotation of PRPF3 AMP co-occurrence genes.

GO term	Total	Expected	Hits	P-Value	FDR
Acute inflammatory response	118	5.33	18	5.47E-06	4.49E-03
Immune effector process	576	26	47	6.15E-05	2.25E-02
Adaptive immune response	241	10.9	25	9.36E-05	2.25E-02
Regulation of response to external stimulus	461	20.8	39	1.23E-04	2.25E-02
Adaptive immune response based on somatic recombination of immune receptors built fromimmunoglobulin superfamily domains	218	9.84	23	1.37E-04	2.25E-02
Leukocyte migration	309	13.9	27	8.11E-04	1.08E-01
Regulation of immune effector process	249	11.2	23	9.19E-04	1.08E-01
Inflammatory response	569	25.7	42	1.18E-03	1.21E-01
Protein maturation	153	6.91	16	1.51E-03	1.35E-01
Protein processing	140	6.32	15	1.64E-03	1.35E-01
Immune response	1430	64.4	87	2.14E-03	1.57E-01
Regulation of defense response	519	23.4	38	2.30E-03	1.57E-01
Protein secretion	196	8.85	18	3.38E-03	1.93E-01
Striated muscle contraction	108	4.88	12	3.43E-03	1.93E-01
Positive regulation of hydrolase activity	497	22.4	36	3.54E-03	1.93E-01
Cytokine secretion	110	4.97	12	3.99E-03	1.93E-01
Protein autoprocessing	8	0.361	3	4.32E-03	1.93E-01
Positive regulation of immune system process	739	33.4	49	4.41E-03	1.93E-01
Regulation of immune system process	1190	53.9	73	4.47E-03	1.93E-01
Regulation of muscle contraction	127	5.73	13	4.84E-03	1.98E-01
Response to carbohydrate stimulus	143	6.46	14	5.26E-03	2.05E-01
Production of molecular mediator of immune response	130	5.87	13	5.89E-03	2.13E-01
Negative regulation of immune system process	207	9.34	18	5.98E-03	2.13E-01
Keratinocyte differentiation	117	5.28	12	6.55E-03	2.24E-01
Response to wounding	1310	59.3	78	6.95E-03	2.28E-01
Leukocyte chemotaxis	150	6.77	14	7.96E-03	2.51E-01
Response to bacterium	385	17.4	28	8.96E-03	2.72E-01
Regulation of angiogenesis	169	7.63	15	9.61E-03	2.81E-01
Epidermis development	319	14.4	24	1.01E-02	2.85E-01
Response to abiotic stimulus	876	39.5	54	1.20E-02	3.23E-01
Negative regulation of MAP kinase activity	69	3.11	8	1.22E-02	3.23E-01
Regulation of immune response	727	32.8	46	1.28E-02	3.28E-01
Positive regulation of immune response	487	22	33	1.32E-02	3.28E-01
Regulation of G_protein coupled receptor protein signaling pathway	129	5.82	12	1.38E-02	3.32E-01
B cell activation	194	8.76	16	1.48E-02	3.46E-01
Response to hypoxia	245	11.1	19	1.54E-02	3.51E-01
Defense response to bacterium	152	6.86	13	2.02E-02	4.46E-01
Response to biotic stimulus	749	33.8	46	2.07E-02	4.46E-01
Amyloid precursor protein metabolic process	24	1.08	4	2.13E-02	4.48E-01
Cellular monovalent inorganic cation homeostasis	36	1.63	5	2.18E-02	4.48E-01
Response to other organism	716	32.3	44	2.31E-02	4.61E-01
Cell migration	1050	47.6	61	2.62E-02	5.12E-01

Monovalent inorganic cation homeostasis	80	3.61	8	2.76E-02	5.26E-01
Positive regulation of catalytic activity	1070	48.3	61	3.35E-02	6.25E-01
Positive regulation of NF_kappaB transcription factor activity	115	5.19	10	3.51E-02	6.35E-01
Inactivation of MAPK activity	28	1.26	4	3.56E-02	6.35E-01
Regulation of lymphocyte activation	360	16.3	24	3.68E-02	6.42E-01
Negative regulation of angiogenesis	56	2.53	6	3.97E-02	6.67E-01
Regulation of cell migration	456	20.6	29	3.99E-02	6.67E-01
Positive regulation of defense response	273	12.3	19	4.10E-02	6.72E-01
Regulation of cellular pH	30	1.35	4	4.45E-02	7.15E-01
Establishment of protein localization	1460	65.7	79	4.65E-02	7.33E-01
Interleukin_1 secretion	31	1.4	4	4.93E-02	7.53E-01
**Pathway**					
Complement and coagulation cascades	79	3.42	12	1.33E-04	3.00E-02
Systemic lupus erythematosus	133	5.76	16	1.89E-04	3.00E-02
Leishmaniasis	74	3.2	10	1.23E-03	1.30E-01
Staphylococcus aureus infection	68	2.94	9	2.48E-03	1.60E-01
ECM-receptor interaction	82	3.55	10	2.70E-03	1.60E-01
Hematopoietic cell lineage	97	4.2	11	3.02E-03	1.60E-01
Fc gamma R-mediated phagocytosis	91	3.94	10	5.76E-03	2.62E-01
Small cell lung cancer	93	4.03	10	6.71E-03	2.67E-01
Aldosterone synthesis and secretion	98	4.24	10	9.62E-03	3.11E-01
Adrenergic signaling in cardiomyocytes	145	6.28	13	9.94E-03	3.11E-01
Hypertrophic cardiomyopathy (HCM)	85	3.68	9	1.09E-02	3.11E-01
Insulin secretion	86	3.72	9	1.17E-02	3.11E-01
Terpenoid backbone biosynthesis	22	0.953	4	1.36E-02	3.33E-01
Malaria	49	2.12	6	1.83E-02	4.16E-01
Amoebiasis	96	4.16	9	2.28E-02	4.28E-01
Tuberculosis	179	7.75	14	2.31E-02	4.28E-01
Alcoholism	180	7.79	14	2.41E-02	4.28E-01
Cell adhesion molecules (CAMs)	146	6.32	12	2.42E-02	4.28E-01
PI3K-Akt signaling pathway	354	15.3	23	3.31E-02	5.54E-01
VEGF signaling pathway	59	2.55	6	4.13E-02	6.04E-01
Dilated cardiomyopathy	91	3.94	8	4.28E-02	6.04E-01
Gastric acid secretion	75	3.25	7	4.29E-02	6.04E-01
Ras signaling pathway	232	10	16	4.39E-02	6.04E-01
Necroptosis	162	7.01	12	4.82E-02	6.04E-01
Focal adhesion	199	8.62	14	4.98E-02	6.04E-01
