# Supplementary material for: Prognostic potential of PRPF3 in hepatocellular carcinoma
Source: Aging (Albany NY). 2020 Jan 11;12(1):912–30. doi: 10.18632/aging.102665 (PMC6977647; doi:10.18632/aging.102665)
Supplement: Supplementary Table 9 [file aging-12-102665-s008..pdf]

Supplementary Table 9. Correlation of PRPF3 with various immune signatures.

| | | None adjusted | | Tumor purity adjusted | |
|---|---|---|---|---|---|
| marker gene | Class | Correlation coefficient | P-value | Correlation coefficient | P-value |
| CCL2 | chemokine | -0.1523 | 4.23E-03 | -0.0963 | 7.27E-02 |
| CCL3 | chemokine | -0.0882 | 9.88E-02 | -0.0191 | 7.23E-01 |
| CCL4 | chemokine | -0.1020 | 5.61E-02 | -0.0193 | 7.19E-01 |
| CCL5 | chemokine | -0.1204 | 2.41E-02 | -0.0480 | 3.72E-01 |
| CCL7 | chemokine | -0.0840 | 1.16E-01 | -0.0431 | 4.23E-01 |
| CCL8 | chemokine | -0.0725 | 1.75E-01 | -0.0247 | 6.46E-01 |
| CCL11 | chemokine | -0.0550 | 3.04E-01 | -0.0004 | 9.93E-01 |
| CCL13 | chemokine | -0.0874 | 1.02E-01 | -0.0202 | 7.07E-01 |
| CCL14 | chemokine | -0.3149 | 1.62E-09 | -0.3120 | 2.70E-09 |
| CCL15 | chemokine | 0.0382 | 4.76E-01 | 0.0090 | 8.68E-01 |
| CCL16 | chemokine | -0.3167 | 1.28E-09 | -0.3306 | 2.55E-10 |
| CCL17 | chemokine | -0.0368 | 4.92E-01 | 0.0327 | 5.43E-01 |
| CCL18 | chemokine | -0.1415 | 7.94E-03 | -0.0889 | 9.77E-02 |
| CCL19 | chemokine | -0.0462 | 3.89E-01 | 0.0090 | 8.67E-01 |
| CCL20 | chemokine | 0.1877 | 4.06E-04 | 0.2188 | 3.84E-05 |
| CCL21 | chemokine | -0.0931 | 8.15E-02 | -0.0494 | 3.58E-01 |
| CCL22 | chemokine | -0.1249 | 1.92E-02 | -0.0621 | 2.48E-01 |
| CCL23 | chemokine | -0.1505 | 4.72E-03 | -0.1071 | 4.59E-02 |
| CCL24 | chemokine | -0.0808 | 1.31E-01 | -0.0472 | 3.80E-01 |
| CCL25 | chemokine | 0.1248 | 1.93E-02 | 0.1203 | 2.49E-02 |
| CCL26 | chemokine | 0.1730 | 1.14E-03 | 0.2174 | 4.33E-05 |
| CCL27 | chemokine | 0.1678 | 1.61E-03 | 0.1777 | 8.68E-04 |
| CCL28 | chemokine | 0.0723 | 1.76E-01 | 0.0757 | 1.59E-01 |
| CX3CL1 | chemokine | -0.2139 | 5.35E-05 | -0.2003 | 1.69E-04 |
| CXCL1 | chemokine | 0.0470 | 3.80E-01 | 0.0979 | 6.81E-02 |
| CXCL2 | chemokine | -0.1421 | 7.65E-03 | -0.1147 | 3.25E-02 |

| | | | | | |
|---|---|---|---|---|---|
| CXCL3 | chemokine | 0.0707 | 1.86E-01 | 0.1257 | 1.89E-02 |
| CXCL5 | chemokine | 0.0640 | 2.32E-01 | 0.1099 | 4.04E-02 |
| CXCL6 | chemokine | -0.0638 | 2.33E-01 | -0.0230 | 6.69E-01 |
| CXCL9 | chemokine | -0.0535 | 3.18E-01 | 0.0093 | 8.63E-01 |
| CXCL10 | chemokine | -0.0535 | 3.18E-01 | -0.0027 | 9.61E-01 |
| CXCL11 | chemokine | -0.0706 | 1.87E-01 | -0.0185 | 7.31E-01 |
| CXCL12 | chemokine | -0.1784 | 7.84E-04 | -0.1356 | 1.13E-02 |
| CXCL13 | chemokine | 0.0582 | 2.77E-01 | 0.1186 | 2.69E-02 |
| CXCL14 | chemokine | -0.0705 | 1.88E-01 | -0.0287 | 5.93E-01 |
| CXCL16 | chemokine | 0.0088 | 8.69E-01 | 0.0330 | 5.39E-01 |
| CXCL17 | chemokine | 0.0627 | 2.41E-01 | 0.0810 | 1.31E-01 |
| XCL1 | chemokine | 0.0367 | 4.93E-01 | 0.0992 | 6.46E-02 |
| XCL2 | chemokine | -0.0189 | 7.24E-01 | 0.0503 | 3.50E-01 |
| CCR1 | receptor | -0.1671 | 1.68E-03 | -0.0996 | 6.36E-02 |
| CCR2 | receptor | -0.1156 | 3.03E-02 | -0.0454 | 3.99E-01 |
| CCR3 | receptor | 0.0780 | 1.45E-01 | 0.1181 | 2.76E-02 |
| CCR4 | receptor | -0.1389 | 9.19E-03 | -0.0907 | 9.13E-02 |
| CCR5 | receptor | -0.0790 | 1.40E-01 | 0.0154 | 7.75E-01 |
| CCR6 | receptor | 0.1354 | 1.11E-02 | 0.1725 | 1.23E-03 |
| CCR7 | receptor | -0.0941 | 7.82E-02 | -0.0267 | 6.19E-01 |
| CCR8 | receptor | -0.0200 | 7.08E-01 | 0.0438 | 4.15E-01 |
| CCR9 | receptor | -0.0387 | 4.69E-01 | -0.0066 | 9.02E-01 |
| CCR10 | receptor | 0.0607 | 2.57E-01 | 0.0900 | 9.38E-02 |
| CXCR1 | receptor | -0.1047 | 5.00E-02 | -0.0748 | 1.64E-01 |
| CXCR2 | receptor | -0.0767 | 1.51E-01 | -0.0334 | 5.34E-01 |
| CXCR3 | receptor | -0.0428 | 4.24E-01 | 0.0471 | 3.81E-01 |
| CXCR4 | receptor | -0.0025 | 9.62E-01 | 0.0912 | 8.95E-02 |
| CXCR5 | receptor | 0.0022 | 9.67E-01 | 0.0718 | 1.82E-01 |
| CXCR6 | receptor | -0.0798 | 1.36E-01 | 0.0047 | 9.30E-01 |

| | | | | | |
|---|---|---|---|---|---|
| XCR1 | receptor | -0.0976 | 6.78E-02 | -0.0446 | 4.05E-01 |
| CX3CR1 | receptor | -0.1435 | 7.08E-03 | -0.1080 | 4.34E-02 |
| B2M | MHC | -0.2467 | 2.91E-06 | -0.2118 | 6.53E-05 |
| HLA-A | MHC | -0.0877 | 1.01E-01 | -0.0417 | 4.37E-01 |
| HLA-B | MHC | -0.1148 | 3.15E-02 | -0.0678 | 2.06E-01 |
| HLA-C | MHC | -0.1371 | 1.01E-02 | -0.1001 | 6.14E-02 |
| HLA-DMA | MHC | -0.0695 | 1.94E-01 | -0.0043 | 9.35E-01 |
| HLA-DMB | MHC | -0.0692 | 1.96E-01 | 0.0169 | 7.53E-01 |
| HLA-DOA | MHC | -0.1057 | 4.78E-02 | -0.0259 | 6.29E-01 |
| HLA-DOB | MHC | 0.0488 | 3.62E-01 | 0.1353 | 1.13E-02 |
| HLA-DPA1 | MHC | -0.1399 | 8.66E-03 | -0.0697 | 1.93E-01 |
| HLA-DPB1 | MHC | -0.1093 | 4.07E-02 | -0.0323 | 5.47E-01 |
| HLA-DQA1 | MHC | -0.0808 | 1.31E-01 | -0.0047 | 9.30E-01 |
| HLA-DQA2 | MHC | -0.0006 | 9.91E-01 | 0.0772 | 1.49E-01 |
| HLA-DQB1 | MHC | -0.0946 | 7.67E-02 | -0.0230 | 6.67E-01 |
| HLA-DRA | MHC | -0.1168 | 2.87E-02 | -0.0461 | 3.90E-01 |
| HLA-DRB1 | MHC | -0.1109 | 3.79E-02 | -0.0458 | 3.93E-01 |
| HLA-E | MHC | -0.2333 | 1.00E-05 | -0.1968 | 2.12E-04 |
| HLA-F | MHC | -0.0670 | 2.11E-01 | -0.0194 | 7.17E-01 |
| HLA-G | MHC | -0.0998 | 6.19E-02 | -0.0682 | 2.03E-01 |
| TAP1 | MHC | 0.0890 | 9.58E-02 | 0.1562 | 3.38E-03 |
| TAP2 | MHC | -0.0098 | 8.54E-01 | 0.0486 | 3.65E-01 |
| TAPBP | MHC | -0.0772 | 1.49E-01 | -0.0418 | 4.35E-01 |
| ADORA2A | Immunoinhibitor | 0.1235 | 2.07E-02 | 0.1719 | 1.24E-03 |
| BTLA | Immunoinhibitor | -0.0500 | 3.51E-01 | 0.0276 | 6.07E-01 |
| CD160 | Immunoinhibitor | 0.0574 | 2.84E-01 | 0.1050 | 4.96E-02 |
| CD244 | Immunoinhibitor | -0.1133 | 3.38E-02 | -0.0554 | 3.01E-01 |
| CD274 | Immunoinhibitor | -0.1401 | 8.57E-03 | -0.0897 | 9.40E-02 |
| CD96 | Immunoinhibitor | -0.1295 | 1.52E-02 | -0.0568 | 2.90E-01 |

| | | | | | |
|---|---|---|---|---|---|
| CSF1R | Immunoinhibitor | -0.1458 | 6.20E-03 | -0.0746 | 1.64E-01 |
| CTLA4 | Immunoinhibitor | 0.0755 | 1.58E-01 | 0.1751 | 1.01E-03 |
| HAVCR2 | Immunoinhibitor | -0.0969 | 6.99E-02 | -0.0108 | 8.40E-01 |
| IDO1 | Immunoinhibitor | -0.0184 | 7.31E-01 | 0.0344 | 5.21E-01 |
| IL10 | Immunoinhibitor | -0.1173 | 2.79E-02 | -0.0503 | 3.48E-01 |
| IL10RB | Immunoinhibitor | 0.0618 | 2.48E-01 | 0.0772 | 1.49E-01 |
| KDR | Immunoinhibitor | -0.2891 | 3.48E-08 | -0.2781 | 1.23E-07 |
| KIR2DL1 | Immunoinhibitor | -0.0627 | 2.42E-01 | -0.0562 | 2.94E-01 |
| KIR2DL3 | Immunoinhibitor | -0.0680 | 2.04E-01 | -0.0388 | 4.70E-01 |
| LAG3 | Immunoinhibitor | 0.0527 | 3.24E-01 | 0.1163 | 2.96E-02 |
| LGALS9 | Immunoinhibitor | -0.0109 | 8.39E-01 | 0.0772 | 1.49E-01 |
| PDCD1 | Immunoinhibitor | 0.0514 | 3.37E-01 | 0.1319 | 1.35E-02 |
| PDCD1LG2 | Immunoinhibitor | -0.1893 | 3.61E-04 | -0.1356 | 1.11E-02 |
| PVRL2 | Immunoinhibitor | 0.0715 | 1.81E-01 | 0.0649 | 2.26E-01 |
| TGFB1 | Immunoinhibitor | -0.0548 | 3.06E-01 | -0.0022 | 9.67E-01 |
| TGFBR1 | Immunoinhibitor | 0.0739 | 1.67E-01 | 0.1164 | 2.95E-02 |
| TIGIT | Immunoinhibitor | -0.0336 | 5.30E-01 | 0.0568 | 2.89E-01 |
| VTCN1 | Immunoinhibitor | 0.0275 | 6.08E-01 | 0.0659 | 2.18E-01 |
| C10orf54 | Immunostimulator | -0.1008 | 5.93E-02 | -0.0466 | 3.84E-01 |
| CD27 | Immunostimulator | -0.0050 | 9.26E-01 | 0.0874 | 1.03E-01 |
| CD276 | Immunostimulator | 0.1045 | 5.04E-02 | 0.1336 | 1.24E-02 |
| CD28 | Immunostimulator | -0.1067 | 4.59E-02 | -0.0379 | 4.80E-01 |
| CD40 | Immunostimulator | -0.1267 | 1.76E-02 | -0.0933 | 8.14E-02 |
| CD40LG | Immunostimulator | -0.1460 | 6.13E-03 | -0.0879 | 1.01E-01 |
| CD48 | Immunostimulator | -0.1062 | 4.69E-02 | -0.0248 | 6.44E-01 |
| CD70 | Immunostimulator | -0.0018 | 9.74E-01 | 0.0865 | 1.06E-01 |
| CD80 | Immunostimulator | -0.0329 | 5.39E-01 | 0.0491 | 3.60E-01 |
| CD86 | Immunostimulator | -0.1016 | 5.73E-02 | -0.0127 | 8.12E-01 |
| CXCL12 | Immunostimulator | -0.1784 | 7.84E-04 | -0.1372 | 1.02E-02 |

| | | | | | |
|---|---|---|---|---|---|
| CXCR4 | Immunostimulator | -0.0025 | 9.62E-01 | 0.0847 | 1.14E-01 |
| ENTPD1 | Immunostimulator | -0.0342 | 5.23E-01 | 0.0334 | 5.33E-01 |
| HHLA2 | Immunostimulator | 0.0811 | 1.29E-01 | 0.1092 | 4.12E-02 |
| ICOS | Immunostimulator | 0.0203 | 7.05E-01 | 0.1211 | 2.35E-02 |
| ICOSLG | Immunostimulator | -0.2541 | 1.41E-06 | -0.2452 | 3.46E-06 |
| IL2RA | Immunostimulator | -0.0240 | 6.54E-01 | 0.0742 | 1.66E-01 |
| IL6 | Immunostimulator | -0.1381 | 9.58E-03 | -0.0852 | 1.12E-01 |
| IL6R | Immunostimulator | 0.0118 | 8.26E-01 | -0.0076 | 8.88E-01 |
| KLRC1 | Immunostimulator | -0.0973 | 6.86E-02 | -0.0478 | 3.72E-01 |
| KLRK1 | Immunostimulator | -0.1338 | 1.21E-02 | -0.0723 | 1.77E-01 |
| LTA | Immunostimulator | 0.0212 | 6.93E-01 | 0.1270 | 1.75E-02 |
| MICB | Immunostimulator | 0.1305 | 1.44E-02 | 0.1769 | 8.90E-04 |
| NT5E | Immunostimulator | -0.0227 | 6.72E-01 | -0.0163 | 7.61E-01 |
| PVR | Immunostimulator | -0.0438 | 4.14E-01 | -0.0653 | 2.23E-01 |
| RAET1E | Immunostimulator | 0.0041 | 9.39E-01 | 0.0232 | 6.65E-01 |
| TMEM173 | Immunostimulator | -0.1723 | 1.19E-03 | -0.1165 | 2.93E-02 |
| TMIGD2 | Immunostimulator | -0.1258 | 1.84E-02 | -0.0773 | 1.49E-01 |
| TNFRSF13B | Immunostimulator | 0.0333 | 5.34E-01 | 0.1043 | 5.12E-02 |
| TNFRSF13C | Immunostimulator | 0.1302 | 1.46E-02 | 0.1955 | 2.34E-04 |
| TNFRSF14 | Immunostimulator | -0.0725 | 1.76E-01 | -0.0385 | 4.73E-01 |
| TNFRSF17 | Immunostimulator | -0.0347 | 5.17E-01 | 0.0390 | 4.67E-01 |
| TNFRSF18 | Immunostimulator | 0.0829 | 1.21E-01 | 0.1717 | 1.26E-03 |
| TNFRSF25 | Immunostimulator | 0.1293 | 1.53E-02 | 0.1654 | 1.90E-03 |
| TNFRSF4 | Immunostimulator | 0.1376 | 9.87E-03 | 0.1928 | 2.84E-04 |
| TNFRSF8 | Immunostimulator | -0.0503 | 3.48E-01 | 0.0490 | 3.61E-01 |
| TNFRSF9 | Immunostimulator | 0.0102 | 8.49E-01 | 0.0685 | 2.01E-01 |
| TNFSF13 | Immunostimulator | -0.1781 | 8.01E-04 | -0.1350 | 1.15E-02 |
| TNFSF13B | Immunostimulator | -0.0846 | 1.14E-01 | -0.0028 | 9.58E-01 |
| TNFSF14 | Immunostimulator | 0.0940 | 7.87E-02 | 0.1128 | 3.49E-02 |

| | | | | | |
|---|---|---|---|---|---|
| TNFSF15 | Immunostimulator | 0.0261 | 6.25E-01 | 0.0663 | 2.16E-01 |
| TNFSF18 | Immunostimulator | -0.0601 | 2.61E-01 | -0.0331 | 5.37E-01 |
| TNFSF4 | Immunostimulator | 0.2993 | 1.07E-08 | 0.3064 | 4.84E-09 |
| TNFSF9 | Immunostimulator | 0.1600 | 2.65E-03 | 0.2116 | 6.59E-05 |
| ULBP1 | Immunostimulator | 0.1350 | 1.13E-02 | 0.1625 | 2.30E-03 |
| ADRM1 | Activated CD8 T cell | 0.0434 | 4.17E-01 | 0.0510 | 3.42E-01 |
| AHSA1 | Activated CD8 T cell | 0.0464 | 3.86E-01 | 0.0498 | 3.53E-01 |
| C1GALT1C1 | Activated CD8 T cell | -0.0042 | 9.38E-01 | 0.0134 | 8.03E-01 |
| CCT6B | Activated CD8 T cell | -0.2883 | 3.82E-08 | -0.3117 | 2.53E-09 |
| CD37 | Activated CD8 T cell | -0.0824 | 1.23E-01 | 0.0095 | 8.60E-01 |
| CD3D | Activated CD8 T cell | 0.0226 | 6.73E-01 | 0.1133 | 3.41E-02 |
| CD3E | Activated CD8 T cell | -0.0931 | 8.14E-02 | -0.0112 | 8.35E-01 |
| CD3G | Activated CD8 T cell | -0.0688 | 1.99E-01 | 0.0126 | 8.14E-01 |
| CD69 | Activated CD8 T cell | -0.1511 | 4.56E-03 | -0.0892 | 9.56E-02 |
| CD8A | Activated CD8 T cell | -0.0993 | 6.32E-02 | -0.0288 | 5.91E-01 |
| CETN3 | Activated CD8 T cell | 0.0194 | 7.17E-01 | 0.0206 | 7.01E-01 |
| CSE1L | Activated CD8 T cell | 0.2510 | 1.91E-06 | 0.2442 | 3.78E-06 |
| GEMIN6 | Activated CD8 T cell | 0.0557 | 2.98E-01 | 0.0379 | 4.80E-01 |
| GNLY | Activated CD8 T cell | -0.0378 | 4.80E-01 | -0.0003 | 9.95E-01 |
| GPT2 | Activated CD8 T cell | -0.2661 | 4.22E-07 | -0.2872 | 4.52E-08 |
| GZMA | Activated CD8 T cell | -0.1467 | 5.90E-03 | -0.0849 | 1.13E-01 |
| GZMH | Activated CD8 T cell | -0.1656 | 1.85E-03 | -0.1192 | 2.57E-02 |
| GZMK | Activated CD8 T cell | -0.1558 | 3.44E-03 | -0.0950 | 7.60E-02 |
| IL2RB | Activated CD8 T cell | -0.1291 | 1.55E-02 | -0.0558 | 2.98E-01 |
| LCK | Activated CD8 T cell | -0.0842 | 1.15E-01 | 0.0036 | 9.47E-01 |
| MPZL1 | Activated CD8 T cell | 0.3774 | 2.51E-13 | 0.4119 | 9.18E-16 |
| NKG7 | Activated CD8 T cell | -0.1198 | 2.47E-02 | -0.0651 | 2.25E-01 |
| PIK3IP1 | Activated CD8 T cell | -0.1298 | 1.49E-02 | -0.0972 | 6.93E-02 |
| PTRH2 | Activated CD8 T cell | 0.1665 | 1.74E-03 | 0.1594 | 2.78E-03 |

| | | | | | |
|---|---|---|---|---|---|
| TIMM13 | Activated CD8 T cell | -0.0588 | 2.72E-01 | -0.0703 | 1.89E-01 |
| ZAP70 | Activated CD8 T cell | -0.0428 | 4.24E-01 | 0.0398 | 4.58E-01 |
| ACTN4 | Central memory CD8 T | 0.0200 | 7.08E-01 | 0.0167 | 7.56E-01 |
| ADAM12 | Central memory CD8 T | 0.0836 | 1.18E-01 | 0.1524 | 4.27E-03 |
| ADCY9 | Central memory CD8 T | -0.1317 | 1.35E-02 | -0.1302 | 1.48E-02 |
| F13A1 | Central memory CD8 T | -0.1228 | 2.14E-02 | -0.0585 | 2.75E-01 |
| FCER1G | Central memory CD8 T | -0.0687 | 1.99E-01 | 0.0184 | 7.31E-01 |
| FCGR3B | Central memory CD8 T | -0.0510 | 3.41E-01 | -0.0127 | 8.12E-01 |
| FGF7 | Central memory CD8 T | -0.1948 | 2.41E-04 | -0.1586 | 2.93E-03 |
| FKBP4 | Central memory CD8 T | 0.0591 | 2.70E-01 | 0.0496 | 3.55E-01 |
| GLUD1 | Central memory CD8 T | -0.2082 | 8.53E-05 | -0.2291 | 1.51E-05 |
| GM2A | Central memory CD8 T | 0.0466 | 3.84E-01 | 0.0829 | 1.22E-01 |
| GUSB | Central memory CD8 T | -0.1621 | 2.31E-03 | -0.1644 | 2.03E-03 |
| IL1RN | Central memory CD8 T | -0.0734 | 1.70E-01 | -0.0650 | 2.25E-01 |
| NOL11 | Central memory CD8 T | 0.4122 | 7.86E-16 | 0.3962 | 1.34E-14 |
| NTRK1 | Central memory CD8 T | 0.0389 | 4.68E-01 | 0.0925 | 8.39E-02 |
| RARA | Central memory CD8 T | 0.1511 | 4.55E-03 | 0.1363 | 1.07E-02 |
| RNF128 | Central memory CD8 T | -0.1689 | 1.50E-03 | -0.1819 | 6.27E-04 |
| SIGLEC1 | Central memory CD8 T | -0.1521 | 4.30E-03 | -0.0874 | 1.02E-01 |
| TNFRSF11A | Central memory CD8 T | 0.0905 | 9.04E-02 | 0.1342 | 1.20E-02 |
| TOX4 | Central memory CD8 T | 0.1910 | 3.19E-04 | 0.1813 | 6.54E-04 |
| UBA52 | Central memory CD8 T | 0.0840 | 1.16E-01 | 0.0788 | 1.41E-01 |
| ULBP1 | Central memory CD8 T | 0.1350 | 1.13E-02 | 0.1625 | 2.30E-03 |
| ACAP1 | Effector memeory CD8 T | -0.0730 | 1.72E-01 | 0.0161 | 7.65E-01 |
| APOL3 | Effector memeory CD8 T | -0.0748 | 1.62E-01 | -0.0354 | 5.09E-01 |
| ARHGAP10 | Effector memeory CD8 T | -0.1793 | 7.40E-04 | -0.1390 | 9.23E-03 |
| ATP10D | Effector memeory CD8 T | -0.0792 | 1.39E-01 | -0.0353 | 5.10E-01 |
| C3AR1 | Effector memeory CD8 T | -0.1005 | 6.00E-02 | -0.0140 | 7.94E-01 |
| CCR5 | Effector memeory CD8 T | -0.0790 | 1.40E-01 | 0.0165 | 7.58E-01 |

| | | | | | |
|---|---|---|---|---|---|
| CD160 | Effector memeory CD8 T | 0.0574 | 2.84E-01 | 0.1050 | 4.96E-02 |
| CD55 | Effector memeory CD8 T | -0.0283 | 5.98E-01 | -0.0077 | 8.86E-01 |
| CFLAR | Effector memeory CD8 T | -0.0568 | 2.88E-01 | -0.0246 | 6.47E-01 |
| CMKLR1 | Effector memeory CD8 T | -0.1315 | 1.37E-02 | -0.0594 | 2.68E-01 |
| DAPP1 | Effector memeory CD8 T | -0.0708 | 1.86E-01 | 0.0195 | 7.17E-01 |
| FCRL6 | Effector memeory CD8 T | -0.1781 | 8.03E-04 | -0.1349 | 1.15E-02 |
| FLT3LG | Effector memeory CD8 T | -0.1174 | 2.78E-02 | -0.0720 | 1.79E-01 |
| GZMM | Effector memeory CD8 T | -0.1083 | 4.27E-02 | -0.0452 | 3.99E-01 |
| HAPLN3 | Effector memeory CD8 T | 0.0078 | 8.84E-01 | 0.1115 | 3.71E-02 |
| HLA-DMB | Effector memeory CD8 T | -0.0692 | 1.96E-01 | 0.0169 | 7.53E-01 |
| HLA-DPA1 | Effector memeory CD8 T | -0.1399 | 8.66E-03 | -0.0697 | 1.93E-01 |
| HLA-DPB1 | Effector memeory CD8 T | -0.1093 | 4.07E-02 | -0.0323 | 5.47E-01 |
| IFI16 | Effector memeory CD8 T | -0.0708 | 1.86E-01 | 0.0104 | 8.47E-01 |
| LIME1 | Effector memeory CD8 T | -0.1964 | 2.13E-04 | -0.2246 | 2.23E-05 |
| LTK | Effector memeory CD8 T | -0.0224 | 6.76E-01 | 0.0132 | 8.06E-01 |
| NFKBIA | Effector memeory CD8 T | -0.0905 | 9.06E-02 | -0.0776 | 1.47E-01 |
| SETD7 | Effector memeory CD8 T | -0.3218 | 6.73E-10 | -0.3210 | 7.84E-10 |
| SIK1 | Effector memeory CD8 T | -0.1384 | 9.44E-03 | -0.1501 | 4.89E-03 |
| TRIB2 | Effector memeory CD8 T | -0.0148 | 7.82E-01 | -0.0120 | 8.23E-01 |
| AIM2 | Activated CD4 T cell | 0.0519 | 3.32E-01 | 0.1657 | 1.86E-03 |
| BIRC3 | Activated CD4 T cell | 0.1174 | 2.78E-02 | 0.1768 | 8.95E-04 |
| BRIP1 | Activated CD4 T cell | 0.1941 | 2.54E-04 | 0.1915 | 3.13E-04 |
| CCL20 | Activated CD4 T cell | 0.1877 | 4.06E-04 | 0.2190 | 3.59E-05 |
| CCL4 | Activated CD4 T cell | -0.1020 | 5.61E-02 | -0.0259 | 6.29E-01 |
| CCL5 | Activated CD4 T cell | -0.1204 | 2.41E-02 | -0.0516 | 3.35E-01 |
| CCNB1 | Activated CD4 T cell | 0.3650 | 1.67E-12 | 0.3762 | 3.31E-13 |
| CCR7 | Activated CD4 T cell | -0.0941 | 7.82E-02 | -0.0267 | 6.19E-01 |
| DUSP2 | Activated CD4 T cell | -0.0274 | 6.08E-01 | 0.0430 | 4.23E-01 |
| ESCO2 | Activated CD4 T cell | 0.3049 | 5.53E-09 | 0.3218 | 7.13E-10 |

| | | | | | |
|---|---|---|---|---|---|
| ETS1 | Activated CD4 T cell | -0.2332 | 1.02E-05 | -0.1945 | 2.52E-04 |
| EXO1 | Activated CD4 T cell | 0.4795 | 1.41E-21 | 0.4902 | 1.48E-22 |
| EXOC6 | Activated CD4 T cell | 0.2337 | 9.66E-06 | 0.2355 | 8.47E-06 |
| IARS | Activated CD4 T cell | 0.2431 | 4.09E-06 | 0.2496 | 2.26E-06 |
| ITK | Activated CD4 T cell | -0.1523 | 4.23E-03 | -0.0884 | 9.87E-02 |
| KIF11 | Activated CD4 T cell | 0.4089 | 1.39E-15 | 0.4189 | 2.64E-16 |
| KNTC1 | Activated CD4 T cell | 0.4255 | 7.19E-17 | 0.4308 | 3.02E-17 |
| NUF2 | Activated CD4 T cell | 0.5126 | 6.60E-25 | 0.5176 | 2.25E-25 |
| PRC1 | Activated CD4 T cell | 0.3915 | 2.66E-14 | 0.3994 | 7.75E-15 |
| PSAT1 | Activated CD4 T cell | -0.0417 | 4.37E-01 | -0.0360 | 5.02E-01 |
| RGS1 | Activated CD4 T cell | 0.0037 | 9.45E-01 | 0.0929 | 8.27E-02 |
| RTKN2 | Activated CD4 T cell | 0.3646 | 1.79E-12 | 0.3849 | 8.36E-14 |
| SAMSN1 | Activated CD4 T cell | -0.1064 | 4.64E-02 | -0.0189 | 7.25E-01 |
| SELL | Activated CD4 T cell | -0.0732 | 1.71E-01 | -0.0034 | 9.50E-01 |
| TRAT1 | Activated CD4 T cell | -0.1266 | 1.77E-02 | -0.0603 | 2.60E-01 |
| ABHD3 | Central memory CD4 T | 0.1600 | 2.64E-03 | 0.1562 | 3.39E-03 |
| AHNAK | Central memory CD4 T | -0.0653 | 2.22E-01 | -0.0558 | 2.98E-01 |
| ANXA2P2 | Central memory CD4 T | 0.0712 | 1.83E-01 | 0.1127 | 3.51E-02 |
| AQP3 | Central memory CD4 T | 0.0006 | 9.91E-01 | -0.0011 | 9.83E-01 |
| ATHL1 | Central memory CD4 T | 0.0010 | 9.85E-01 | 0.0034 | 9.49E-01 |
| BMI1 | Central memory CD4 T | -0.0071 | 8.94E-01 | -0.0159 | 7.68E-01 |
| BZW2 | Central memory CD4 T | 0.1441 | 6.85E-03 | 0.1497 | 5.00E-03 |
| CD63 | Central memory CD4 T | -0.0214 | 6.90E-01 | -0.0053 | 9.22E-01 |
| COL4A1 | Central memory CD4 T | -0.1194 | 2.53E-02 | -0.0864 | 1.06E-01 |
| CYLD | Central memory CD4 T | -0.2229 | 2.50E-05 | -0.1922 | 2.98E-04 |
| ELMO2 | Central memory CD4 T | 0.0439 | 4.12E-01 | 0.0752 | 1.60E-01 |
| FYN | Central memory CD4 T | -0.3358 | 1.06E-10 | -0.3103 | 3.00E-09 |
| GLIPR1 | Central memory CD4 T | -0.1144 | 3.21E-02 | -0.0411 | 4.44E-01 |
| GSS | Central memory CD4 T | -0.1226 | 2.16E-02 | -0.1199 | 2.49E-02 |

| | | | | | |
|---|---|---|---|---|---|
| IFITM2 | Central memory CD4 T ( | -0.2071 | 9.25E-05 | -0.1881 | 4.02E-04 |
| ITGB1 | Central memory CD4 T ( | -0.0724 | 1.76E-01 | -0.0623 | 2.45E-01 |
| ITGB2 | Central memory CD4 T ( | -0.1039 | 5.17E-02 | -0.0115 | 8.31E-01 |
| KLF5 | Central memory CD4 T ( | 0.2133 | 5.63E-05 | 0.2116 | 6.59E-05 |
| LSP1 | Central memory CD4 T ( | -0.0248 | 6.43E-01 | 0.0770 | 1.50E-01 |
| NDUFB9 | Central memory CD4 T ( | -0.0172 | 7.48E-01 | -0.0287 | 5.92E-01 |
| PKM2 | Central memory CD4 T ( | 0.2230 | 2.47E-05 | 0.2894 | 3.51E-08 |
| SFXN3 | Central memory CD4 T ( | 0.0038 | 9.43E-01 | 0.0601 | 2.62E-01 |
| SIRPG | Central memory CD4 T ( | 0.0218 | 6.84E-01 | 0.1109 | 3.82E-02 |
| SMAD4 | Central memory CD4 T ( | 0.0663 | 2.15E-01 | 0.0696 | 1.94E-01 |
| STX4 | Central memory CD4 T ( | 0.1309 | 1.41E-02 | 0.1517 | 4.45E-03 |
| TRADD | Central memory CD4 T ( | -0.0821 | 1.25E-01 | -0.0665 | 2.14E-01 |
| VIM | Central memory CD4 T ( | -0.1284 | 1.61E-02 | -0.0702 | 1.90E-01 |
| XRCC6 | Central memory CD4 T ( | 0.2489 | 2.36E-06 | 0.2521 | 1.79E-06 |
| ATM | Effector memeory CD4 T | 0.0529 | 3.23E-01 | 0.0795 | 1.38E-01 |
| CASP3 | Effector memeory CD4 T | 0.0527 | 3.25E-01 | 0.0808 | 1.32E-01 |
| CASQ1 | Effector memeory CD4 T | 0.1262 | 1.80E-02 | 0.1484 | 5.40E-03 |
| CD300E | Effector memeory CD4 T | -0.1406 | 8.34E-03 | -0.0961 | 7.25E-02 |
| DARS | Effector memeory CD4 T | 0.1210 | 2.34E-02 | 0.1221 | 2.24E-02 |
| DOCK9 | Effector memeory CD4 T | -0.0892 | 9.53E-02 | -0.0650 | 2.25E-01 |
| EXOSC9 | Effector memeory CD4 T | 0.1669 | 1.70E-03 | 0.1917 | 3.10E-04 |
| EZH2 | Effector memeory CD4 T | 0.4368 | 8.79E-18 | 0.4417 | 3.81E-18 |
| GDE1 | Effector memeory CD4 T | -0.2230 | 2.48E-05 | -0.1983 | 1.89E-04 |
| IL34 | cytokine and cytokine re | -0.0274 | 6.09E-01 | 0.0312 | 5.61E-01 |
| NCOA4 | Effector memeory CD4 T | -0.1985 | 1.82E-04 | -0.2168 | 4.30E-05 |
| NEFL | Effector memeory CD4 T | -0.0344 | 5.21E-01 | 0.0019 | 9.72E-01 |
| PDGFRL | Effector memeory CD4 T | 0.1669 | 1.71E-03 | 0.1819 | 6.30E-04 |
| PTGS1 | Effector memeory CD4 T | -0.0081 | 8.80E-01 | 0.0799 | 1.36E-01 |
| REPS1 | Effector memeory CD4 T | 0.0577 | 2.81E-01 | 0.0762 | 1.55E-01 |

| | | | | | |
|---|---|---|---|---|---|
| SCG2 | Effector memeory CD4 T | 0.0347 | 5.17E-01 | 0.0879 | 1.01E-01 |
| SDPR | Effector memeory CD4 T | -0.2143 | 5.18E-05 | -0.2094 | 7.92E-05 |
| SIGLEC14 | Effector memeory CD4 T | -0.1073 | 4.46E-02 | -0.0483 | 3.68E-01 |
| SIGLEC6 | Effector memeory CD4 T | -0.0304 | 5.70E-01 | 0.0198 | 7.12E-01 |
| TAL1 | Effector memeory CD4 T | -0.2188 | 3.56E-05 | -0.1936 | 2.69E-04 |
| TFEC | Effector memeory CD4 T | -0.1046 | 5.02E-02 | -0.0173 | 7.48E-01 |
| TIPIN | Effector memeory CD4 T | 0.3645 | 1.81E-12 | 0.3781 | 2.46E-13 |
| TPK1 | Effector memeory CD4 T | -0.2495 | 2.21E-06 | -0.2176 | 4.02E-05 |
| UQCRB | Effector memeory CD4 T | -0.0336 | 5.31E-01 | -0.0461 | 3.90E-01 |
| USP9Y | Effector memeory CD4 T | -0.0283 | 5.97E-01 | -0.0186 | 7.28E-01 |
| WIPF1 | Effector memeory CD4 T | -0.1186 | 2.62E-02 | -0.0413 | 4.41E-01 |
| ZCRB1 | Effector memeory CD4 T | 0.0872 | 1.03E-01 | 0.0883 | 9.90E-02 |
| B3GAT1 | T follicular helper cell | 0.0912 | 8.79E-02 | 0.0998 | 6.21E-02 |
| CDK5R1 | T follicular helper cell | 0.2532 | 1.55E-06 | 0.2533 | 1.58E-06 |
| PDCD1 | T follicular helper cell | 0.0514 | 3.37E-01 | 0.1319 | 1.35E-02 |
| BCL6 | T follicular helper cell | 0.0004 | 9.93E-01 | -0.0008 | 9.89E-01 |
| CD200 | T follicular helper cell | -0.0938 | 7.93E-02 | -0.0525 | 3.27E-01 |
| CD83 | T follicular helper cell | 0.0691 | 1.97E-01 | 0.1407 | 8.38E-03 |
| CD84 | T follicular helper cell | -0.1467 | 5.90E-03 | -0.0815 | 1.28E-01 |
| FGF2 | T follicular helper cell | -0.1023 | 5.55E-02 | -0.0863 | 1.07E-01 |
| GPR18 | T follicular helper cell | -0.0863 | 1.06E-01 | -0.0183 | 7.33E-01 |
| CEBPA | T follicular helper cell | 0.1961 | 2.19E-04 | 0.1803 | 7.01E-04 |
| CECR1 | T follicular helper cell | -0.2006 | 1.55E-04 | -0.1500 | 4.92E-03 |
| CLEC10A | T follicular helper cell | -0.0965 | 7.09E-02 | -0.0246 | 6.46E-01 |
| CLEC4A | T follicular helper cell | -0.0447 | 4.03E-01 | 0.0452 | 3.99E-01 |
| CSF1R | T follicular helper cell | -0.1458 | 6.20E-03 | -0.0746 | 1.64E-01 |
| CTSS | T follicular helper cell | -0.0043 | 9.35E-01 | 0.0804 | 1.33E-01 |
| DPP4 | T follicular helper cell | -0.0804 | 1.33E-01 | -0.0955 | 7.45E-02 |
| LRRC32 | T follicular helper cell | -0.1808 | 6.65E-04 | -0.1479 | 5.56E-03 |

| | | | | | |
|---|---|---|---|---|---|
| MICA | T follicular helper cell | 0.1629 | 2.21E-03 | 0.1522 | 4.33E-03 |
| NCAM1 | T follicular helper cell | -0.0309 | 5.64E-01 | 0.0147 | 7.85E-01 |
| NRP1 | T follicular helper cell | -0.1440 | 6.90E-03 | -0.1279 | 1.67E-02 |
| PDCD1LG2 | T follicular helper cell | -0.1893 | 3.61E-04 | -0.1356 | 1.11E-02 |
| PDCD6 | T follicular helper cell | 0.1226 | 2.16E-02 | 0.1209 | 2.37E-02 |
| PRDX1 | T follicular helper cell | 0.0685 | 2.00E-01 | 0.0967 | 7.07E-02 |
| RAE1 | T follicular helper cell | 0.3348 | 1.23E-10 | 0.3335 | 1.54E-10 |
| RAET1E | T follicular helper cell | 0.0041 | 9.39E-01 | 0.0232 | 6.65E-01 |
| SIGLEC7 | T follicular helper cell | -0.1441 | 6.83E-03 | -0.0751 | 1.61E-01 |
| SIGLEC9 | T follicular helper cell | -0.1018 | 5.67E-02 | -0.0191 | 7.22E-01 |
| TYRO3 | T follicular helper cell | 0.2739 | 1.85E-07 | 0.2885 | 3.91E-08 |
| CHST12 | T follicular helper cell | 0.0716 | 1.81E-01 | 0.1111 | 3.77E-02 |
| CLIC3 | T follicular helper cell | 0.0285 | 5.94E-01 | 0.0903 | 9.17E-02 |
| IVNS1ABP | T follicular helper cell | 0.1678 | 1.60E-03 | 0.1798 | 7.25E-04 |
| LGMN | T follicular helper cell | -0.1517 | 4.40E-03 | -0.0955 | 7.42E-02 |
| ACP5 | Gamma delta T cell | -0.0262 | 6.25E-01 | 0.0196 | 7.15E-01 |
| AQP9 | Gamma delta T cell | -0.2571 | 1.05E-06 | -0.2913 | 2.83E-08 |
| BTN3A2 | Gamma delta T cell | -0.0810 | 1.30E-01 | -0.0303 | 5.72E-01 |
| C1orf54 | Gamma delta T cell | -0.0024 | 9.64E-01 | 0.0715 | 1.82E-01 |
| CARD8 | Gamma delta T cell | 0.0706 | 1.87E-01 | 0.0850 | 1.12E-01 |
| CCL18 | Gamma delta T cell | -0.1415 | 7.94E-03 | -0.0926 | 8.37E-02 |
| CD209 | Gamma delta T cell | -0.0979 | 6.69E-02 | -0.0369 | 4.91E-01 |
| CD33 | Gamma delta T cell | -0.0918 | 8.61E-02 | -0.0054 | 9.20E-01 |
| CD36 | Gamma delta T cell | -0.1361 | 1.07E-02 | -0.1349 | 1.15E-02 |
| CDK5 | Gamma delta T cell | -0.1028 | 5.42E-02 | -0.1064 | 4.67E-02 |
| IL10RB | Gamma delta T cell | 0.0618 | 2.48E-01 | 0.0772 | 1.49E-01 |
| KLRF1 | Gamma delta T cell | -0.1361 | 1.07E-02 | -0.1021 | 5.64E-02 |
| LGALS1 | Gamma delta T cell | -0.0155 | 7.72E-01 | 0.0236 | 6.60E-01 |
| MAPK7 | Gamma delta T cell | 0.2240 | 2.28E-05 | 0.2309 | 1.28E-05 |

| | | | | | |
|---|---|---|---|---|---|
| KLHL7 | Gamma delta T cell | 0.0690 | 1.97E-01 | 0.0545 | 3.10E-01 |
| KRT80 | Gamma delta T cell | 0.1435 | 7.08E-03 | 0.1829 | 5.84E-04 |
| LAMC1 | Gamma delta T cell | 0.2689 | 3.15E-07 | 0.2678 | 3.69E-07 |
| LCORL | Gamma delta T cell | -0.0095 | 8.59E-01 | -0.0009 | 9.86E-01 |
| LMNB1 | Gamma delta T cell | 0.3572 | 5.28E-12 | 0.3751 | 3.91E-13 |
| MEIS3P1 | Gamma delta T cell | -0.2197 | 3.29E-05 | -0.1872 | 4.32E-04 |
| MPL | Gamma delta T cell | -0.0983 | 6.59E-02 | -0.0926 | 8.35E-02 |
| FABP1 | Gamma delta T cell | -0.2080 | 8.66E-05 | -0.1994 | 1.73E-04 |
| FABP5 | Gamma delta T cell | 0.0812 | 1.29E-01 | 0.1460 | 6.22E-03 |
| FADD | Gamma delta T cell | 0.0364 | 4.96E-01 | 0.0500 | 3.51E-01 |
| MFAP3L | Gamma delta T cell | -0.2741 | 1.82E-07 | -0.2645 | 5.14E-07 |
| MINPP1 | Gamma delta T cell | -0.1670 | 1.69E-03 | -0.1743 | 1.06E-03 |
| RPS24 | Gamma delta T cell | 0.1326 | 1.29E-02 | 0.1239 | 2.04E-02 |
| RPS7 | Gamma delta T cell | 0.1639 | 2.07E-03 | 0.1503 | 4.85E-03 |
| RPS9 | Gamma delta T cell | 0.0715 | 1.82E-01 | 0.0711 | 1.84E-01 |
| DBNL | Gamma delta T cell | -0.0277 | 6.05E-01 | -0.0034 | 9.50E-01 |
| CCL13 | Gamma delta T cell | -0.0874 | 1.02E-01 | -0.0245 | 6.47E-01 |
| CD70 | Type 1 T helper cell | -0.0018 | 9.74E-01 | 0.0865 | 1.06E-01 |
| TBX21 | Type 1 T helper cell | -0.1340 | 1.20E-02 | -0.0763 | 1.54E-01 |
| ADAM8 | Type 1 T helper cell | 0.0767 | 1.52E-01 | 0.1570 | 3.22E-03 |
| AHCYL2 | Type 1 T helper cell | -0.1757 | 9.46E-04 | -0.1862 | 4.64E-04 |
| ALCAM | Type 1 T helper cell | -0.0376 | 4.83E-01 | -0.0501 | 3.50E-01 |
| B3GALNT1 | Type 1 T helper cell | 0.1609 | 2.50E-03 | 0.2106 | 7.20E-05 |
| BBS12 | Type 1 T helper cell | -0.0091 | 8.65E-01 | 0.0408 | 4.46E-01 |
| BST1 | Type 1 T helper cell | -0.0576 | 2.82E-01 | -0.0143 | 7.90E-01 |
| CD151 | Type 1 T helper cell | -0.0274 | 6.09E-01 | -0.0138 | 7.97E-01 |
| CD47 | Type 1 T helper cell | 0.0072 | 8.93E-01 | 0.0349 | 5.16E-01 |
| CD48 | Type 1 T helper cell | -0.1062 | 4.69E-02 | -0.0248 | 6.44E-01 |
| CD52 | Type 1 T helper cell | -0.0714 | 1.82E-01 | 0.0150 | 7.80E-01 |

| | | | | | |
|---|---|---|---|---|---|
| CD53 | Type 1 T helper cell | -0.1088 | 4.16E-02 | -0.0165 | 7.58E-01 |
| CD59 | Type 1 T helper cell | -0.1626 | 2.24E-03 | -0.1370 | 1.03E-02 |
| CD6 | Type 1 T helper cell | -0.0676 | 2.06E-01 | 0.0202 | 7.07E-01 |
| CD68 | Type 1 T helper cell | -0.2191 | 3.47E-05 | -0.1764 | 9.18E-04 |
| CD7 | Type 1 T helper cell | 0.0490 | 3.60E-01 | 0.1352 | 1.13E-02 |
| CD96 | Type 1 T helper cell | -0.1295 | 1.52E-02 | -0.0568 | 2.90E-01 |
| CFHR3 | Type 1 T helper cell | -0.2526 | 1.64E-06 | -0.2429 | 4.29E-06 |
| CHRM3 | Type 1 T helper cell | 0.0240 | 6.54E-01 | 0.0402 | 4.54E-01 |
| CLEC7A | Type 1 T helper cell | -0.0897 | 9.33E-02 | -0.0022 | 9.67E-01 |
| COL23A1 | Type 1 T helper cell | -0.0937 | 7.95E-02 | -0.0743 | 1.66E-01 |
| COL4A4 | Type 1 T helper cell | -0.1814 | 6.37E-04 | -0.1480 | 5.52E-03 |
| COL5A3 | Type 1 T helper cell | -0.1061 | 4.69E-02 | -0.1036 | 5.27E-02 |
| DAB1 | Type 1 T helper cell | -0.1010 | 5.87E-02 | -0.1142 | 3.27E-02 |
| DLEU7 | Type 1 T helper cell | 0.0748 | 1.62E-01 | 0.1073 | 4.48E-02 |
| DOC2B | Type 1 T helper cell | 0.0097 | 8.56E-01 | 0.0330 | 5.38E-01 |
| EMP1 | Type 1 T helper cell | -0.2527 | 1.63E-06 | -0.2226 | 2.65E-05 |
| F12 | Type 1 T helper cell | -0.2317 | 1.16E-05 | -0.2325 | 1.11E-05 |
| FURIN | Type 1 T helper cell | -0.0925 | 8.35E-02 | -0.0754 | 1.59E-01 |
| GAB3 | Type 1 T helper cell | -0.1285 | 1.60E-02 | -0.0538 | 3.15E-01 |
| GATM | Type 1 T helper cell | -0.2524 | 1.68E-06 | -0.2653 | 4.77E-07 |
| GFPT2 | Type 1 T helper cell | -0.0245 | 6.48E-01 | 0.0301 | 5.74E-01 |
| GPR25 | Type 1 T helper cell | -0.0560 | 2.95E-01 | -0.0268 | 6.17E-01 |
| GREM2 | Type 1 T helper cell | -0.1656 | 1.85E-03 | -0.1798 | 7.28E-04 |
| HAVCR1 | Type 1 T helper cell | 0.0884 | 9.83E-02 | 0.1130 | 3.47E-02 |
| HSD11B1 | Type 1 T helper cell | -0.2476 | 2.66E-06 | -0.2541 | 1.46E-06 |
| HUNK | Type 1 T helper cell | 0.2149 | 4.92E-05 | 0.2180 | 3.90E-05 |
| IGF2 | Type 1 T helper cell | 0.0164 | 7.60E-01 | 0.0021 | 9.68E-01 |
| RCSD1 | Type 1 T helper cell | -0.1344 | 1.17E-02 | -0.0660 | 2.18E-01 |
| RYR1 | Type 1 T helper cell | -0.0158 | 7.68E-01 | 0.0531 | 3.22E-01 |

| | | | | | |
|---|---|---|---|---|---|
| SAV1 | Type 1 T helper cell | -0.2056 | 1.05E-04 | -0.1967 | 2.13E-04 |
| SELE | Type 1 T helper cell | -0.2871 | 4.39E-08 | -0.2617 | 6.88E-07 |
| SELP | Type 1 T helper cell | -0.2391 | 5.89E-06 | -0.2076 | 9.11E-05 |
| SH3KBP1 | Type 1 T helper cell | -0.1487 | 5.25E-03 | -0.1173 | 2.82E-02 |
| SIT1 | Type 1 T helper cell | -0.0112 | 8.34E-01 | 0.0910 | 8.92E-02 |
| SLC35B3 | Type 1 T helper cell | 0.0014 | 9.79E-01 | -0.0135 | 8.02E-01 |
| SIGLEC10 | Type 1 T helper cell | -0.0099 | 8.53E-01 | 0.0873 | 1.03E-01 |
| SKAP1 | Type 1 T helper cell | -0.1401 | 8.57E-03 | -0.1035 | 5.32E-02 |
| THUMPD2 | Type 1 T helper cell | 0.3504 | 1.41E-11 | 0.3418 | 4.99E-11 |
| TIGIT | Type 1 T helper cell | -0.0336 | 5.30E-01 | 0.0568 | 2.89E-01 |
| ZEB2 | Type 1 T helper cell | -0.1821 | 6.08E-04 | -0.1297 | 1.52E-02 |
| ENC1 | Type 1 T helper cell | 0.0719 | 1.79E-01 | 0.0796 | 1.37E-01 |
| FAM134B | Type 1 T helper cell | -0.2021 | 1.38E-04 | -0.1955 | 2.32E-04 |
| FBXO30 | Type 1 T helper cell | 0.1887 | 3.80E-04 | 0.2161 | 4.58E-05 |
| FCGR2C | Type 1 T helper cell | -0.0734 | 1.70E-01 | -0.0081 | 8.79E-01 |
| STAC | Type 1 T helper cell | -0.0319 | 5.51E-01 | 0.0184 | 7.32E-01 |
| LTC4S | Type 1 T helper cell | -0.1724 | 1.18E-03 | -0.1375 | 9.99E-03 |
| MAN1B1 | Type 1 T helper cell | 0.1187 | 2.62E-02 | 0.1315 | 1.38E-02 |
| MDH1 | Type 1 T helper cell | -0.1539 | 3.86E-03 | -0.1575 | 3.13E-03 |
| MMD | Type 1 T helper cell | 0.1977 | 1.94E-04 | 0.2294 | 1.46E-05 |
| RGS16 | Type 1 T helper cell | -0.0303 | 5.72E-01 | 0.0021 | 9.68E-01 |
| IL12A | Type 1 T helper cell | 0.2515 | 1.82E-06 | 0.3056 | 5.29E-09 |
| P2RX5 | Type 1 T helper cell | 0.1311 | 1.40E-02 | 0.1770 | 8.79E-04 |
| CD97 | Type 1 T helper cell | 0.0326 | 5.43E-01 | 0.1045 | 5.08E-02 |
| ITGB4 | Type 1 T helper cell | 0.0743 | 1.65E-01 | 0.0980 | 6.72E-02 |
| ICAM3 | Type 1 T helper cell | -0.2445 | 3.57E-06 | -0.2264 | 1.90E-05 |
| METRNL | Type 1 T helper cell | -0.0196 | 7.15E-01 | 0.0344 | 5.22E-01 |
| TNFRSF1A | Type 1 T helper cell | -0.1330 | 1.26E-02 | -0.1167 | 2.90E-02 |
| IRF1 | Type 1 T helper cell | 0.0257 | 6.31E-01 | 0.1074 | 4.47E-02 |

| | | | | | |
|---|---|---|---|---|---|
| HTR2B | Type 1 T helper cell | -0.2965 | 1.50E-08 | -0.2732 | 2.09E-07 |
| CALD1 | Type 1 T helper cell | -0.3581 | 4.62E-12 | -0.3488 | 1.90E-11 |
| MOCOS | Type 1 T helper cell | -0.2132 | 5.68E-05 | -0.2089 | 8.25E-05 |
| TRAF3IP2 | Type 1 T helper cell | -0.0064 | 9.04E-01 | 0.0082 | 8.78E-01 |
| TLR8 | Type 1 T helper cell | -0.1118 | 3.63E-02 | -0.0328 | 5.41E-01 |
| TRAF1 | Type 1 T helper cell | 0.0998 | 6.17E-02 | 0.1577 | 3.09E-03 |
| DUSP14 | Type 1 T helper cell | -0.0250 | 6.41E-01 | -0.0002 | 9.98E-01 |
| IL17RA | Type 17 T helper cell | -0.0121 | 8.21E-01 | 0.0106 | 8.43E-01 |
| C2CD4A | Type 17 T helper cell | -0.0536 | 3.17E-01 | -0.0265 | 6.21E-01 |
| C2CD4B | Type 17 T helper cell | -0.1332 | 1.25E-02 | -0.1054 | 4.87E-02 |
| CA2 | Type 17 T helper cell | -0.2169 | 4.15E-05 | -0.2174 | 4.11E-05 |
| CCDC65 | Type 17 T helper cell | 0.0720 | 1.78E-01 | 0.0993 | 6.34E-02 |
| CEACAM3 | Type 17 T helper cell | 0.0065 | 9.03E-01 | 0.0590 | 2.71E-01 |
| IL17C | Type 17 T helper cell | 0.0363 | 4.98E-01 | 0.0498 | 3.53E-01 |
| IL17RC | Type 17 T helper cell | -0.1943 | 2.51E-04 | -0.1941 | 2.59E-04 |
| IL17RE | Type 17 T helper cell | 0.1561 | 3.37E-03 | 0.1390 | 9.20E-03 |
| IL23A | Type 17 T helper cell | -0.0577 | 2.81E-01 | -0.0275 | 6.08E-01 |
| ILDR1 | Type 17 T helper cell | 0.1737 | 1.09E-03 | 0.1919 | 3.06E-04 |
| LONRF3 | Type 17 T helper cell | -0.0556 | 2.99E-01 | -0.0575 | 2.84E-01 |
| SH2D6 | Type 17 T helper cell | 0.0443 | 4.08E-01 | 0.0555 | 3.01E-01 |
| TNIP2 | Type 17 T helper cell | -0.0016 | 9.76E-01 | 0.0137 | 7.98E-01 |
| ABCA1 | Type 17 T helper cell | -0.0178 | 7.39E-01 | -0.0040 | 9.40E-01 |
| ABCB1 | Type 17 T helper cell | -0.3005 | 9.27E-09 | -0.3009 | 9.30E-09 |
| ADAMTS12 | Type 17 T helper cell | -0.1375 | 9.92E-03 | -0.0971 | 6.95E-02 |
| ANK1 | Type 17 T helper cell | -0.0200 | 7.09E-01 | 0.0436 | 4.16E-01 |
| ANKRD22 | Type 17 T helper cell | 0.0330 | 5.38E-01 | 0.1030 | 5.43E-02 |
| B3GALT2 | Type 17 T helper cell | -0.1122 | 3.56E-02 | -0.0660 | 2.18E-01 |
| CAMTA1 | Type 17 T helper cell | -0.0925 | 8.34E-02 | -0.1017 | 5.73E-02 |
| CCR9 | Type 17 T helper cell | -0.0387 | 4.69E-01 | -0.0049 | 9.27E-01 |

| | | | | | |
|---|---|---|---|---|---|
| CD40 | Type 17 T helper cell | -0.1267 | 1.76E-02 | -0.0933 | 8.14E-02 |
| GPR44 | Type 17 T helper cell | 0.0385 | 4.72E-01 | 0.0259 | 6.29E-01 |
| IFT80 | Type 17 T helper cell | 0.1933 | 2.70E-04 | 0.2149 | 5.05E-05 |
| ASB2 | Type 2 T helper cell | -0.0257 | 6.32E-01 | 0.0636 | 2.35E-01 |
| CSRP2 | Type 2 T helper cell | -0.0217 | 6.85E-01 | -0.0112 | 8.35E-01 |
| DAPK1 | Type 2 T helper cell | 0.0326 | 5.43E-01 | 0.0324 | 5.46E-01 |
| DLC1 | Type 2 T helper cell | -0.2257 | 1.97E-05 | -0.2253 | 2.10E-05 |
| DNAJC12 | Type 2 T helper cell | -0.0795 | 1.37E-01 | -0.0818 | 1.27E-01 |
| DUSP6 | Type 2 T helper cell | -0.1679 | 1.60E-03 | -0.1473 | 5.75E-03 |
| GNAI1 | Type 2 T helper cell | -0.0268 | 6.17E-01 | -0.0504 | 3.48E-01 |
| LAMP3 | Type 2 T helper cell | 0.1309 | 1.42E-02 | 0.1855 | 4.85E-04 |
| NRP2 | Type 2 T helper cell | -0.2349 | 8.66E-06 | -0.1981 | 1.92E-04 |
| OSBPL1A | Type 2 T helper cell | -0.1650 | 1.93E-03 | -0.1464 | 6.06E-03 |
| PDE4B | Type 2 T helper cell | -0.1400 | 8.61E-03 | -0.0886 | 9.79E-02 |
| PHLDA1 | Type 2 T helper cell | -0.2380 | 6.55E-06 | -0.2253 | 2.09E-05 |
| PLA2G4A | Type 2 T helper cell | -0.1100 | 3.94E-02 | -0.0368 | 4.93E-01 |
| RAB27B | Type 2 T helper cell | -0.0193 | 7.19E-01 | 0.0098 | 8.55E-01 |
| RBMS3 | Type 2 T helper cell | -0.1895 | 3.58E-04 | -0.1568 | 3.28E-03 |
| RNF125 | Type 2 T helper cell | -0.3492 | 1.66E-11 | -0.3395 | 6.86E-11 |
| TMPRSS3 | Type 2 T helper cell | 0.2176 | 3.92E-05 | 0.2582 | 9.75E-07 |
| GATA3 | Type 2 T helper cell | -0.1091 | 4.11E-02 | -0.0367 | 4.94E-01 |
| BIRC5 | Type 2 T helper cell | 0.4282 | 4.43E-17 | 0.4360 | 1.13E-17 |
| CDC25C | Type 2 T helper cell | 0.3943 | 1.66E-14 | 0.4036 | 3.80E-15 |
| CDC7 | Type 2 T helper cell | 0.3435 | 3.70E-11 | 0.3840 | 9.60E-14 |
| CENPF | Type 2 T helper cell | 0.4502 | 6.38E-19 | 0.4536 | 3.64E-19 |
| CXCR6 | Type 2 T helper cell | -0.0798 | 1.36E-01 | 0.0037 | 9.45E-01 |
| DHFR | Type 2 T helper cell | 0.0688 | 1.98E-01 | 0.0597 | 2.65E-01 |
| EVI5 | Type 2 T helper cell | -0.1170 | 2.84E-02 | -0.0982 | 6.65E-02 |
| GSTA4 | Type 2 T helper cell | 0.1699 | 1.40E-03 | 0.1581 | 3.02E-03 |

| | | | | | |
|---|---|---|---|---|---|
| HELLS | Type 2 T helper cell | 0.3926 | 2.21E-14 | 0.4088 | 1.58E-15 |
| IL26 | Type 2 T helper cell | 0.0385 | 4.72E-01 | 0.0829 | 1.21E-01 |
| LAIR2 | Type 2 T helper cell | 0.1020 | 5.63E-02 | 0.1566 | 3.32E-03 |
| CCL3L1 | Regulatory T cell | 0.0686 | 2.00E-01 | 0.1156 | 3.06E-02 |
| CD72 | Regulatory T cell | -0.0927 | 8.28E-02 | -0.0216 | 6.87E-01 |
| CLEC5A | Regulatory T cell | 0.0246 | 6.46E-01 | 0.0956 | 7.41E-02 |
| FOXP3 | Regulatory T cell | -0.1162 | 2.96E-02 | -0.0696 | 1.94E-01 |
| ITGA4 | Regulatory T cell | -0.0999 | 6.15E-02 | -0.0279 | 6.04E-01 |
| L1CAM | Regulatory T cell | -0.0303 | 5.71E-01 | 0.0264 | 6.22E-01 |
| LIPA | Regulatory T cell | -0.2191 | 3.46E-05 | -0.1901 | 3.48E-04 |
| LRP1 | Regulatory T cell | -0.2941 | 1.96E-08 | -0.2817 | 8.34E-08 |
| LRRC42 | Regulatory T cell | 0.2462 | 3.05E-06 | 0.2652 | 4.80E-07 |
| MARCO | Regulatory T cell | -0.1679 | 1.59E-03 | -0.1209 | 2.37E-02 |
| MMP12 | Regulatory T cell | 0.0677 | 2.06E-01 | 0.1349 | 1.15E-02 |
| MNDA | Regulatory T cell | -0.0487 | 3.63E-01 | 0.0504 | 3.47E-01 |
| MRC1 | Regulatory T cell | -0.2220 | 2.70E-05 | -0.1825 | 6.00E-04 |
| MS4A6A | Regulatory T cell | -0.1529 | 4.08E-03 | -0.0856 | 1.10E-01 |
| PELO | Regulatory T cell | 0.0672 | 2.09E-01 | 0.0980 | 6.71E-02 |
| PLEK | Regulatory T cell | -0.0666 | 2.13E-01 | 0.0275 | 6.08E-01 |
| PRSS23 | Regulatory T cell | -0.0529 | 3.23E-01 | 0.0028 | 9.59E-01 |
| PTGIR | Regulatory T cell | -0.1101 | 3.92E-02 | -0.0619 | 2.48E-01 |
| ST8SIA4 | Regulatory T cell | -0.0614 | 2.51E-01 | 0.0279 | 6.03E-01 |
| STAB1 | Regulatory T cell | -0.0976 | 6.79E-02 | -0.0282 | 5.99E-01 |
| ADAM28 | Activated B cell | -0.0065 | 9.04E-01 | 0.0888 | 9.72E-02 |
| CD180 | Activated B cell | -0.0938 | 7.93E-02 | -0.0130 | 8.08E-01 |
| CD79B | Activated B cell | -0.0228 | 6.70E-01 | 0.0400 | 4.56E-01 |
| BLK | Activated B cell | -0.0573 | 2.84E-01 | -0.0158 | 7.69E-01 |
| CD19 | Activated B cell | 0.0837 | 1.18E-01 | 0.1519 | 4.40E-03 |
| MS4A1 | Activated B cell | -0.0295 | 5.81E-01 | 0.0330 | 5.38E-01 |

| | | | | | |
|---|---|---|---|---|---|
| TNFRSF17 | Activated B cell | -0.0347 | 5.17E-01 | 0.0390 | 4.67E-01 |
| GNG7 | Activated B cell | -0.1215 | 2.28E-02 | -0.1390 | 9.24E-03 |
| MICAL3 | Activated B cell | -0.0785 | 1.42E-01 | -0.0960 | 7.27E-02 |
| SPIB | Activated B cell | 0.1598 | 2.67E-03 | 0.2453 | 3.41E-06 |
| HLA-DOB | Activated B cell | 0.0488 | 3.62E-01 | 0.1353 | 1.13E-02 |
| PNOC | Activated B cell | 0.0237 | 6.58E-01 | 0.1046 | 5.05E-02 |
| FCRL2 | Activated B cell | -0.0006 | 9.90E-01 | 0.0569 | 2.89E-01 |
| BACH2 | Activated B cell | -0.0896 | 9.37E-02 | -0.0616 | 2.51E-01 |
| CR2 | Activated B cell | 0.0959 | 7.29E-02 | 0.1540 | 3.88E-03 |
| TCL1A | Activated B cell | -0.0173 | 7.47E-01 | 0.0380 | 4.79E-01 |
| AKNA | Activated B cell | -0.0061 | 9.10E-01 | 0.0748 | 1.62E-01 |
| ARHGAP25 | Activated B cell | -0.1409 | 8.19E-03 | -0.0712 | 1.84E-01 |
| CCL21 | Activated B cell | -0.0931 | 8.15E-02 | -0.0508 | 3.44E-01 |
| CD27 | Activated B cell | -0.0050 | 9.26E-01 | 0.0874 | 1.03E-01 |
| CD38 | Activated B cell | -0.0243 | 6.50E-01 | 0.0585 | 2.75E-01 |
| CLEC17A | Activated B cell | 0.0298 | 5.78E-01 | 0.0805 | 1.33E-01 |
| CLEC9A | Activated B cell | -0.1617 | 2.37E-03 | -0.1168 | 2.89E-02 |
| CLECL1 | Activated B cell | 0.0804 | 1.33E-01 | 0.1805 | 6.90E-04 |
| CD22 | Immature B cell | 0.0049 | 9.27E-01 | 0.0603 | 2.61E-01 |
| CYBB | Immature B cell | -0.0917 | 8.61E-02 | -0.0033 | 9.51E-01 |
| FAM129C | Immature B cell | -0.0009 | 9.86E-01 | 0.0412 | 4.42E-01 |
| FCRL1 | Immature B cell | 0.0122 | 8.20E-01 | 0.0592 | 2.69E-01 |
| FCRL3 | Immature B cell | 0.0050 | 9.26E-01 | 0.0697 | 1.93E-01 |
| FCRL5 | Immature B cell | -0.0174 | 7.45E-01 | 0.0582 | 2.78E-01 |
| FCRLA | Immature B cell | 0.0283 | 5.98E-01 | 0.0911 | 8.86E-02 |
| HDAC9 | Immature B cell | -0.0778 | 1.46E-01 | -0.0053 | 9.21E-01 |
| HLA-DQA1 | Immature B cell | -0.0808 | 1.31E-01 | -0.0047 | 9.30E-01 |
| HVCN1 | Immature B cell | -0.1183 | 2.67E-02 | -0.0467 | 3.84E-01 |
| KIAA0226 | Immature B cell | 0.2169 | 4.18E-05 | 0.2564 | 1.17E-06 |

| | | | | | |
|---|---|---|---|---|---|
| NCF1 | Immature  B cell | -0.0440 | 4.11E-01 | 0.0565 | 2.92E-01 |
| NCF1B | Immature  B cell | -0.0289 | 5.90E-01 | 0.0655 | 2.22E-01 |
| P2RY10 | Immature  B cell | -0.0860 | 1.08E-01 | -0.0125 | 8.16E-01 |
| SP100 | Immature  B cell | -0.2514 | 1.84E-06 | -0.2255 | 2.05E-05 |
| TXNIP | Immature  B cell | -0.0964 | 7.12E-02 | -0.0695 | 1.95E-01 |
| STAP1 | Immature  B cell | -0.0158 | 7.68E-01 | 0.0451 | 4.00E-01 |
| TAGAP | Immature  B cell | -0.1191 | 2.56E-02 | -0.0443 | 4.09E-01 |
| ZCCHC2 | Immature  B cell | 0.0341 | 5.24E-01 | 0.0439 | 4.13E-01 |
| AICDA | Memory B cell | 0.0325 | 5.44E-01 | 0.0795 | 1.38E-01 |
| CCNA2 | Memory B cell | 0.3181 | 1.08E-09 | 0.3340 | 1.45E-10 |
| CDKN3 | Memory B cell | 0.3838 | 9.23E-14 | 0.3901 | 3.60E-14 |
| CLCN5 | Memory B cell | -0.0732 | 1.71E-01 | -0.0911 | 8.88E-02 |
| ENPP1 | Memory B cell | -0.0555 | 2.99E-01 | -0.0778 | 1.46E-01 |
| FCER1A | Memory B cell | -0.0852 | 1.11E-01 | -0.0473 | 3.78E-01 |
| MYC | Memory B cell | -0.0345 | 5.20E-01 | -0.0229 | 6.70E-01 |
| RUNX2 | Memory B cell | -0.0722 | 1.77E-01 | -0.0008 | 9.88E-01 |
| SORL1 | Memory B cell | -0.2278 | 1.63E-05 | -0.2207 | 3.10E-05 |
| SOX5 | Memory B cell | -0.1269 | 1.74E-02 | -0.1291 | 1.57E-02 |
| STAT5A | Memory B cell | -0.0783 | 1.43E-01 | -0.0251 | 6.40E-01 |
| STAT5B | Memory B cell | -0.0517 | 3.35E-01 | -0.0673 | 2.09E-01 |
| TLR9 | Memory B cell | 0.1557 | 3.46E-03 | 0.2191 | 3.57E-05 |
| AKT3 | Natural killer cell | -0.0904 | 9.08E-02 | -0.0522 | 3.30E-01 |
| AXL | Natural killer cell | -0.1324 | 1.31E-02 | -0.0706 | 1.88E-01 |
| BST2 | Natural killer cell | -0.1506 | 4.69E-03 | -0.1296 | 1.52E-02 |
| CDH2 | Natural killer cell | -0.0429 | 4.23E-01 | -0.0498 | 3.53E-01 |
| CRTAM | Natural killer cell | -0.0648 | 2.26E-01 | 0.0138 | 7.97E-01 |
| CSF2RA | Natural killer cell | -0.0144 | 7.88E-01 | 0.0631 | 2.39E-01 |
| CTSZ | Natural killer cell | -0.1659 | 1.81E-03 | -0.1415 | 8.00E-03 |
| CXCL1 | Natural killer cell | 0.0470 | 3.80E-01 | 0.0949 | 7.62E-02 |

| | | | | | |
|---|---|---|---|---|---|
| CYTH1 | Natural killer cell | 0.1029 | 5.41E-02 | 0.1040 | 5.19E-02 |
| DAXX | Natural killer cell | 0.2419 | 4.56E-06 | 0.2459 | 3.24E-06 |
| DGKH | Natural killer cell | 0.0362 | 4.99E-01 | 0.0234 | 6.62E-01 |
| DLL4 | Natural killer cell | -0.0373 | 4.86E-01 | -0.0246 | 6.46E-01 |
| DPYD | Natural killer cell | -0.1223 | 2.19E-02 | -0.1065 | 4.64E-02 |
| ERBB3 | Natural killer cell | 0.2169 | 4.18E-05 | 0.1920 | 3.03E-04 |
| F11R | Natural killer cell | 0.1307 | 1.43E-02 | 0.1171 | 2.85E-02 |
| FAM27A | Natural killer cell | 0.1241 | 2.00E-02 | 0.1655 | 1.89E-03 |
| FAM49A | Natural killer cell | -0.0824 | 1.24E-01 | -0.0372 | 4.88E-01 |
| FASLG | Natural killer cell | -0.0697 | 1.93E-01 | 0.0003 | 9.95E-01 |
| FCGR1A | Natural killer cell | -0.0432 | 4.20E-01 | 0.0529 | 3.23E-01 |
| FN1 | Natural killer cell | -0.0944 | 7.73E-02 | -0.0796 | 1.37E-01 |
| FSTL1 | Natural killer cell | -0.1634 | 2.14E-03 | -0.1238 | 2.05E-02 |
| FUCA1 | Natural killer cell | -0.3162 | 1.37E-09 | -0.3009 | 9.35E-09 |
| GBP3 | Natural killer cell | -0.1478 | 5.53E-03 | -0.1112 | 3.76E-02 |
| GLS2 | Natural killer cell | -0.1984 | 1.83E-04 | -0.1956 | 2.32E-04 |
| GRB2 | Natural killer cell | 0.1967 | 2.09E-04 | 0.2178 | 3.97E-05 |
| LST1 | Natural killer cell | -0.0969 | 6.99E-02 | -0.0245 | 6.48E-01 |
| BCL2 | Natural killer cell | -0.1633 | 2.14E-03 | -0.1152 | 3.12E-02 |
| CDC5L | Natural killer cell | 0.0868 | 1.05E-01 | 0.0730 | 1.73E-01 |
| FGF18 | Natural killer cell | -0.1455 | 6.31E-03 | -0.1213 | 2.33E-02 |
| FUT5 | Natural killer cell | -0.1268 | 1.75E-02 | -0.1157 | 3.05E-02 |
| FZR1 | Natural killer cell | -0.0074 | 8.91E-01 | -0.0138 | 7.97E-01 |
| IGFBP5 | Natural killer cell | -0.1836 | 5.45E-04 | -0.1570 | 3.24E-03 |
| KANK2 | Natural killer cell | -0.0323 | 5.46E-01 | -0.0443 | 4.09E-01 |
| LDB3 | Natural killer cell | -0.0797 | 1.36E-01 | -0.0584 | 2.76E-01 |
| ABAT | CD56bright natural killer | -0.3198 | 8.73E-10 | -0.3252 | 4.58E-10 |
| C11orf75 | CD56bright natural killer | 0.0859 | 1.08E-01 | 0.0860 | 1.08E-01 |
| C5orf15 | CD56bright natural killer | -0.0525 | 3.27E-01 | -0.0371 | 4.89E-01 |

| | | | | | |
|---|---|---|---|---|---|
| CDHR1 | CD56bright natural killer | -0.0976 | 6.78E-02 | -0.0481 | 3.70E-01 |
| DCAF12 | CD56bright natural killer | 0.1115 | 3.68E-02 | 0.1126 | 3.53E-02 |
| DYNLL1 | CD56bright natural killer | 0.1226 | 2.16E-02 | 0.1283 | 1.63E-02 |
| GPR137B | CD56bright natural killer | 0.0015 | 9.78E-01 | 0.0251 | 6.40E-01 |
| HCP5 | CD56bright natural killer | -0.0137 | 7.97E-01 | 0.0309 | 5.65E-01 |
| HDGFRP2 | CD56bright natural killer | 0.1523 | 4.24E-03 | 0.1439 | 7.02E-03 |
| KRT86 | CD56bright natural killer | -0.0064 | 9.04E-01 | 0.0197 | 7.13E-01 |
| MLST8 | CD56bright natural killer | 0.0241 | 6.52E-01 | 0.0283 | 5.98E-01 |
| ELMOD3 | CD56bright natural killer | 0.0548 | 3.06E-01 | 0.0377 | 4.83E-01 |
| ENTPD5 | CD56bright natural killer | -0.2093 | 7.79E-05 | -0.2077 | 9.06E-05 |
| FAM119A | CD56bright natural killer | 0.2664 | 4.09E-07 | 0.2701 | 2.89E-07 |
| FAM179A | CD56bright natural killer | -0.0324 | 5.45E-01 | 0.0083 | 8.77E-01 |
| CLIC2 | CD56bright natural killer | -0.1201 | 2.45E-02 | -0.0547 | 3.07E-01 |
| COX7A2L | CD56bright natural killer | -0.0184 | 7.31E-01 | -0.0250 | 6.41E-01 |
| CREB3L4 | CD56bright natural killer | 0.3609 | 3.09E-12 | 0.3422 | 4.73E-11 |
| CSF1 | CD56bright natural killer | -0.1049 | 4.96E-02 | -0.0473 | 3.78E-01 |
| CSNK2A2 | CD56bright natural killer | -0.1690 | 1.49E-03 | -0.1622 | 2.33E-03 |
| CSTA | CD56bright natural killer | 0.0239 | 6.56E-01 | 0.0317 | 5.55E-01 |
| CSTB | CD56bright natural killer | 0.0486 | 3.64E-01 | 0.0603 | 2.61E-01 |
| CTPS | CD56bright natural killer | -0.0309 | 5.64E-01 | -0.0157 | 7.69E-01 |
| CTSD | CD56bright natural killer | -0.1943 | 2.51E-04 | -0.1782 | 8.10E-04 |
| FST | CD56bright natural killer | -0.1577 | 3.05E-03 | -0.1592 | 2.82E-03 |
| GATA2 | CD56bright natural killer | -0.1516 | 4.41E-03 | -0.1352 | 1.14E-02 |
| GMPR | CD56bright natural killer | -0.1142 | 3.24E-02 | -0.1131 | 3.45E-02 |
| HDC | CD56bright natural killer | -0.1686 | 1.53E-03 | -0.1527 | 4.20E-03 |
| HEY1 | CD56bright natural killer | 0.1261 | 1.81E-02 | 0.1317 | 1.37E-02 |
| HOXA1 | CD56bright natural killer | -0.0282 | 5.99E-01 | 0.0346 | 5.19E-01 |
| HS2ST1 | CD56bright natural killer | 0.0466 | 3.85E-01 | 0.0576 | 2.82E-01 |
| HS3ST1 | CD56bright natural killer | -0.1074 | 4.44E-02 | -0.0619 | 2.48E-01 |

| | | | | | |
|---|---|---|---|---|---|
| BCL11B | CD56bright natural killer | -0.0529 | 3.23E-01 | 0.0239 | 6.56E-01 |
| CDH3 | CD56bright natural killer | -0.1345 | 1.16E-02 | -0.0854 | 1.11E-01 |
| MYL6B | CD56bright natural killer | 0.1273 | 1.70E-02 | 0.1289 | 1.58E-02 |
| NAA16 | CD56bright natural killer | 0.1563 | 3.32E-03 | 0.1565 | 3.32E-03 |
| CYP27B1 | CD56bright natural killer | 0.1604 | 2.57E-03 | 0.1814 | 6.51E-04 |
| EIF3M | CD56bright natural killer | 0.2033 | 1.26E-04 | 0.2077 | 9.06E-05 |
| CYP27A1 | CD56dim natural killer c | -0.1891 | 3.67E-04 | -0.2030 | 1.31E-04 |
| DDX55 | CD56dim natural killer c | 0.4036 | 3.51E-15 | 0.4005 | 6.43E-15 |
| DYRK2 | CD56dim natural killer c | 0.1970 | 2.04E-04 | 0.2320 | 1.16E-05 |
| RPL37A | CD56dim natural killer c | 0.1308 | 1.42E-02 | 0.1199 | 2.49E-02 |
| NOTCH3 | CD56dim natural killer c | -0.1150 | 3.12E-02 | -0.0860 | 1.08E-01 |
| AKR7A3 | CD56dim natural killer c | -0.3630 | 2.26E-12 | -0.3691 | 9.75E-13 |
| GPRC5C | CD56dim natural killer c | -0.0593 | 2.68E-01 | -0.0775 | 1.48E-01 |
| GRIN1 | CD56dim natural killer c | 0.2177 | 3.91E-05 | 0.2286 | 1.56E-05 |
| HLA-E | CD56dim natural killer c | -0.2333 | 1.00E-05 | -0.1968 | 2.12E-04 |
| PORCN | CD56dim natural killer c | 0.1514 | 4.48E-03 | 0.1633 | 2.18E-03 |
| PSMC4 | CD56dim natural killer c | 0.0558 | 2.97E-01 | 0.0571 | 2.87E-01 |
| UPP1 | CD56dim natural killer c | 0.0765 | 1.52E-01 | 0.1179 | 2.74E-02 |
| IL21R | CD56dim natural killer c | 0.0268 | 6.16E-01 | 0.1426 | 7.53E-03 |
| CCR2 | Myeloid derived suppres | -0.1156 | 3.03E-02 | -0.0455 | 3.96E-01 |
| CD14 | Myeloid derived suppres | -0.2750 | 1.65E-07 | -0.2616 | 6.90E-07 |
| CD2 | Myeloid derived suppres | -0.0622 | 2.45E-01 | 0.0271 | 6.13E-01 |
| CD86 | Myeloid derived suppres | -0.1016 | 5.73E-02 | -0.0127 | 8.12E-01 |
| CXCR4 | Myeloid derived suppres | -0.0025 | 9.62E-01 | 0.0847 | 1.14E-01 |
| FCGR2A | Myeloid derived suppres | -0.0186 | 7.29E-01 | 0.0725 | 1.76E-01 |
| FCGR2B | Myeloid derived suppres | -0.0921 | 8.48E-02 | -0.0288 | 5.91E-01 |
| FCGR3A | Myeloid derived suppres | -0.0822 | 1.24E-01 | -0.0041 | 9.38E-01 |
| FERMT3 | Myeloid derived suppres | -0.0529 | 3.23E-01 | 0.0562 | 2.95E-01 |
| GPSM3 | Myeloid derived suppres | -0.0862 | 1.07E-01 | 0.0082 | 8.78E-01 |

| | | | | | |
|---|---|---|---|---|---|
| IL18BP | Myeloid derived suppres | -0.0831 | 1.20E-01 | -0.0117 | 8.28E-01 |
| IL4R | Myeloid derived suppres | 0.0556 | 2.99E-01 | 0.0930 | 8.24E-02 |
| ITGAL | Myeloid derived suppres | 0.0231 | 6.67E-01 | 0.0946 | 7.70E-02 |
| ITGAM | Myeloid derived suppres | -0.0052 | 9.22E-01 | 0.0806 | 1.32E-01 |
| PARVG | Myeloid derived suppres | -0.0948 | 7.60E-02 | -0.0008 | 9.88E-01 |
| PSAP | Myeloid derived suppres | -0.1645 | 1.99E-03 | -0.1298 | 1.51E-02 |
| PTGER2 | Myeloid derived suppres | -0.1714 | 1.27E-03 | -0.1166 | 2.92E-02 |
| PTGES2 | Myeloid derived suppres | 0.1119 | 3.62E-02 | 0.1128 | 3.48E-02 |
| S100A8 | Myeloid derived suppres | -0.0363 | 4.98E-01 | 0.0078 | 8.84E-01 |
| S100A9 | Myeloid derived suppres | 0.0595 | 2.67E-01 | 0.1301 | 1.48E-02 |
| BTN2A2 | Natural killer T cell | 0.0583 | 2.76E-01 | 0.1024 | 5.56E-02 |
| CD101 | Natural killer T cell | 0.0334 | 5.33E-01 | 0.1120 | 3.63E-02 |
| CD109 | Natural killer T cell | 0.0797 | 1.36E-01 | 0.1248 | 1.95E-02 |
| CNPY3 | Natural killer T cell | 0.2505 | 2.01E-06 | 0.2564 | 1.17E-06 |
| CNPY4 | Natural killer T cell | 0.0812 | 1.29E-01 | 0.1242 | 2.01E-02 |
| CREB1 | Natural killer T cell | 0.1921 | 2.94E-04 | 0.2092 | 8.05E-05 |
| CRTC2 | Natural killer T cell | 0.5377 | 1.10E-27 | 0.5287 | 1.35E-26 |
| CRTC3 | Natural killer T cell | 0.0942 | 7.79E-02 | 0.1320 | 1.34E-02 |
| CSF2 | Natural killer T cell | 0.0846 | 1.14E-01 | 0.1266 | 1.78E-02 |
| KLRC1 | Natural killer T cell | -0.0973 | 6.86E-02 | -0.0478 | 3.72E-01 |
| FUT4 | Natural killer T cell | 0.2197 | 3.30E-05 | 0.2486 | 2.50E-06 |
| ICAM2 | Natural killer T cell | -0.0254 | 6.36E-01 | 0.0145 | 7.87E-01 |
| IL32 | Natural killer T cell | -0.1477 | 5.56E-03 | -0.1228 | 2.16E-02 |
| LAMP2 | Natural killer T cell | -0.1342 | 1.18E-02 | -0.1217 | 2.27E-02 |
| LILRB5 | Natural killer T cell | -0.2697 | 2.89E-07 | -0.2362 | 7.95E-06 |
| KLRG1 | Natural killer T cell | -0.0066 | 9.02E-01 | 0.0549 | 3.06E-01 |
| HSPA4 | Natural killer T cell | 0.0046 | 9.32E-01 | 0.0193 | 7.20E-01 |
| HSPB6 | Natural killer T cell | -0.0794 | 1.38E-01 | -0.0499 | 3.52E-01 |
| ISM2 | Natural killer T cell | 0.0966 | 7.08E-02 | 0.1057 | 4.81E-02 |

| | | | | | |
|---|---|---|---|---|---|
| ITIH2 | Natural killer T cell | -0.1148 | 3.16E-02 | -0.1312 | 1.41E-02 |
| KDM4C | Natural killer T cell | 0.0544 | 3.10E-01 | 0.0517 | 3.35E-01 |
| KIR2DS4 | Natural killer T cell | -0.0241 | 6.52E-01 | -0.0154 | 7.74E-01 |
| KIRREL3 | Natural killer T cell | 0.1328 | 1.28E-02 | 0.1643 | 2.05E-03 |
| SDCBP | Natural killer T cell | -0.2351 | 8.54E-06 | -0.2129 | 5.96E-05 |
| NFATC2IP | Natural killer T cell | 0.1647 | 1.96E-03 | 0.1710 | 1.32E-03 |
| MICB | Natural killer T cell | 0.1305 | 1.44E-02 | 0.1769 | 8.90E-04 |
| KIR2DL1 | Natural killer T cell | -0.0627 | 2.42E-01 | -0.0562 | 2.94E-01 |
| KIR2DL3 | Natural killer T cell | -0.0680 | 2.04E-01 | -0.0388 | 4.70E-01 |
| KIR3DL1 | Natural killer T cell | -0.0961 | 7.20E-02 | -0.0751 | 1.61E-01 |
| KIR3DL2 | Natural killer T cell | 0.0168 | 7.53E-01 | 0.0577 | 2.82E-01 |
| NCR1 | Natural killer T cell | -0.1054 | 4.84E-02 | -0.0681 | 2.04E-01 |
| FOSL1 | Natural killer T cell | -0.1283 | 1.62E-02 | -0.0897 | 9.37E-02 |
| TSLP | Natural killer T cell | -0.2759 | 1.49E-07 | -0.2653 | 4.76E-07 |
| SLC7A7 | Natural killer T cell | 0.0228 | 6.71E-01 | 0.1122 | 3.59E-02 |
| SPP1 | Natural killer T cell | 0.1227 | 2.15E-02 | 0.1651 | 1.94E-03 |
| TREM2 | Natural killer T cell | 0.0568 | 2.89E-01 | 0.1314 | 1.39E-02 |
| UBASH3A | Natural killer T cell | -0.0738 | 1.68E-01 | 0.0040 | 9.41E-01 |
| YBX2 | Natural killer T cell | 0.0552 | 3.02E-01 | 0.0569 | 2.88E-01 |
| CCDC88A | Natural killer T cell | 0.0698 | 1.92E-01 | 0.0981 | 6.67E-02 |
| CLEC1A | Natural killer T cell | -0.2488 | 2.36E-06 | -0.2196 | 3.41E-05 |
| THBD | Natural killer T cell | -0.2245 | 2.18E-05 | -0.1895 | 3.64E-04 |
| PDPN | Natural killer T cell | -0.0352 | 5.11E-01 | 0.0326 | 5.43E-01 |
| VCAM1 | Natural killer T cell | -0.0579 | 2.79E-01 | -0.0062 | 9.08E-01 |
| EMR1 | Natural killer T cell | -0.1261 | 1.81E-02 | -0.0687 | 2.00E-01 |
| ABCD1 | Activated dendritic cell | 0.2790 | 1.07E-07 | 0.2918 | 2.69E-08 |
| C1QC | Activated dendritic cell | -0.1353 | 1.12E-02 | -0.0626 | 2.43E-01 |
| CAPG | Activated dendritic cell | 0.0707 | 1.86E-01 | 0.1237 | 2.06E-02 |
| CCL3L3 | Activated dendritic cell | 0.0157 | 7.70E-01 | 0.0394 | 4.63E-01 |

| | | | | | |
|---|---|---|---|---|---|
| CD207 | Activated dendritic cell | -0.0001 | 9.98E-01 | 0.0436 | 4.16E-01 |
| CD302 | Activated dendritic cell | -0.3144 | 1.71E-09 | -0.3349 | 1.28E-10 |
| ATP5B | Activated dendritic cell | -0.2612 | 6.95E-07 | -0.2563 | 1.18E-06 |
| ATP5L | Activated dendritic cell | 0.0165 | 7.58E-01 | 0.0164 | 7.60E-01 |
| ATP6V1A | Activated dendritic cell | -0.1582 | 2.96E-03 | -0.1580 | 3.04E-03 |
| BCL2L1 | Activated dendritic cell | -0.0737 | 1.68E-01 | -0.0823 | 1.25E-01 |
| C1QB | Activated dendritic cell | -0.1325 | 1.30E-02 | -0.0616 | 2.51E-01 |
| SNURF | Activated dendritic cell | 0.0822 | 1.24E-01 | 0.0899 | 9.32E-02 |
| SPCS3 | Activated dendritic cell | -0.3019 | 7.89E-09 | -0.2782 | 1.22E-07 |
| CCNA1 | Activated dendritic cell | -0.0115 | 8.30E-01 | 0.0158 | 7.69E-01 |
| NOS2 | Activated dendritic cell | -0.0869 | 1.04E-01 | -0.0770 | 1.51E-01 |
| SRA1 | Activated dendritic cell | -0.0363 | 4.98E-01 | -0.0352 | 5.11E-01 |
| TNFRSF6B | Activated dendritic cell | 0.0769 | 1.50E-01 | 0.1105 | 3.89E-02 |
| TREM1 | Activated dendritic cell | 0.1459 | 6.17E-03 | 0.2086 | 8.42E-05 |
| TREML1 | Activated dendritic cell | 0.0978 | 6.72E-02 | 0.1397 | 8.87E-03 |
| RHOA | Activated dendritic cell | 0.0520 | 3.31E-01 | 0.0812 | 1.30E-01 |
| SLC25A37 | Activated dendritic cell | 0.0351 | 5.12E-01 | 0.0448 | 4.03E-01 |
| TNFSF14 | Activated dendritic cell | 0.0940 | 7.87E-02 | 0.1128 | 3.49E-02 |
| TREML4 | Activated dendritic cell | 0.0905 | 9.03E-02 | 0.1273 | 1.72E-02 |
| VNN2 | Activated dendritic cell | 0.0603 | 2.60E-01 | 0.1195 | 2.54E-02 |
| XPO6 | Activated dendritic cell | 0.1665 | 1.75E-03 | 0.2163 | 4.47E-05 |
| CLEC4C | Activated dendritic cell | 0.0340 | 5.25E-01 | 0.0710 | 1.85E-01 |
| TNFAIP2 | Activated dendritic cell | 0.0834 | 1.19E-01 | 0.1205 | 2.42E-02 |
| UBD | Activated dendritic cell | 0.1675 | 1.64E-03 | 0.2117 | 6.54E-05 |
| ACTR3 | Activated dendritic cell | 0.0734 | 1.70E-01 | 0.1177 | 2.77E-02 |
| RAB1A | Activated dendritic cell | -0.0354 | 5.09E-01 | -0.0218 | 6.84E-01 |
| SLA | Activated dendritic cell | -0.1431 | 7.25E-03 | -0.0683 | 2.02E-01 |
| HLA-DQA2 | Activated dendritic cell | -0.0006 | 9.91E-01 | 0.0772 | 1.49E-01 |
| SIGLEC5 | Activated dendritic cell | -0.1104 | 3.87E-02 | -0.0425 | 4.28E-01 |

| | | | | | |
|---|---|---|---|---|---|
| SLAMF9 | Activated dendritic cell | -0.0597 | 2.65E-01 | -0.0415 | 4.39E-01 |
| CBX6 | Plasmacytoid dendritic c | 0.0522 | 3.30E-01 | 0.0757 | 1.58E-01 |
| DAB2 | Plasmacytoid dendritic c | -0.0764 | 1.53E-01 | -0.0218 | 6.85E-01 |
| DDX17 | Plasmacytoid dendritic c | 0.3297 | 2.42E-10 | 0.3314 | 2.04E-10 |
| HIGD1A | Plasmacytoid dendritic c | -0.3652 | 1.64E-12 | -0.3719 | 6.37E-13 |
| IDH3A | Plasmacytoid dendritic c | -0.1865 | 4.45E-04 | -0.1736 | 1.11E-03 |
| IL3RA | Plasmacytoid dendritic c | -0.0822 | 1.24E-01 | -0.0468 | 3.83E-01 |
| MAGED1 | Plasmacytoid dendritic c | 0.0973 | 6.87E-02 | 0.1161 | 2.98E-02 |
| NUCB2 | Plasmacytoid dendritic c | 0.0843 | 1.15E-01 | 0.0966 | 7.12E-02 |
| OFD1 | Plasmacytoid dendritic c | 0.3656 | 1.52E-12 | 0.3809 | 1.58E-13 |
| OGT | Plasmacytoid dendritic c | 0.2757 | 1.54E-07 | 0.2823 | 7.77E-08 |
| PDIA4 | Plasmacytoid dendritic c | -0.1374 | 9.96E-03 | -0.1259 | 1.85E-02 |
| SERTAD2 | Plasmacytoid dendritic c | 0.1385 | 9.40E-03 | 0.1257 | 1.86E-02 |
| SIRPA | Plasmacytoid dendritic c | -0.0755 | 1.58E-01 | -0.0526 | 3.26E-01 |
| TMED2 | Plasmacytoid dendritic c | -0.0156 | 7.71E-01 | -0.0108 | 8.40E-01 |
| ENG | Plasmacytoid dendritic c | -0.3153 | 1.53E-09 | -0.2884 | 3.94E-08 |
| FCAR | Plasmacytoid dendritic c | -0.1192 | 2.55E-02 | -0.0675 | 2.08E-01 |
| IGF1 | Plasmacytoid dendritic c | -0.2493 | 2.26E-06 | -0.2426 | 4.41E-06 |
| ITGA2B | Plasmacytoid dendritic c | 0.0448 | 4.03E-01 | 0.0572 | 2.86E-01 |
| GABARAP | Plasmacytoid dendritic c | -0.1211 | 2.33E-02 | -0.1088 | 4.18E-02 |
| GPX1 | Plasmacytoid dendritic c | -0.1517 | 4.39E-03 | -0.1328 | 1.29E-02 |
| KRT23 | Plasmacytoid dendritic c | 0.0987 | 6.46E-02 | 0.1216 | 2.29E-02 |
| PROK2 | Plasmacytoid dendritic c | 0.0018 | 9.73E-01 | 0.0393 | 4.64E-01 |
| RALB | Plasmacytoid dendritic c | -0.0712 | 1.83E-01 | -0.0410 | 4.45E-01 |
| RNF141 | Plasmacytoid dendritic c | -0.1081 | 4.29E-02 | -0.0979 | 6.72E-02 |
| SEC14L1 | Plasmacytoid dendritic c | -0.1361 | 1.07E-02 | -0.1007 | 5.99E-02 |
| SEPX1 | Plasmacytoid dendritic c | -0.1818 | 6.22E-04 | -0.1853 | 4.93E-04 |
| EMP3 | Plasmacytoid dendritic c | -0.0397 | 4.58E-01 | 0.0502 | 3.49E-01 |
| CD300LF | Plasmacytoid dendritic c | -0.0441 | 4.10E-01 | 0.0370 | 4.91E-01 |

| | | | | | |
|---|---|---|---|---|---|
| ABTB1 | Plasmacytoid dendritic c | -0.0098 | 8.55E-01 | 0.0035 | 9.48E-01 |
| KLHL21 | Plasmacytoid dendritic c | -0.0516 | 3.35E-01 | -0.0511 | 3.41E-01 |
| PHRF1 | Plasmacytoid dendritic c | 0.0651 | 2.24E-01 | 0.0685 | 2.01E-01 |
| ACADM | Immature dendritic cell | -0.3155 | 1.50E-09 | -0.3152 | 1.63E-09 |
| AHCYL1 | Immature dendritic cell | -0.2087 | 8.15E-05 | -0.1976 | 1.99E-04 |
| ALDH1A2 | Immature dendritic cell | -0.0777 | 1.46E-01 | -0.0490 | 3.61E-01 |
| ALDH3A2 | Immature dendritic cell | -0.1092 | 4.08E-02 | -0.1514 | 4.54E-03 |
| ALDH9A1 | Immature dendritic cell | -0.1650 | 1.92E-03 | -0.1757 | 9.65E-04 |
| ALOX15 | Immature dendritic cell | -0.0227 | 6.71E-01 | -0.0004 | 9.94E-01 |
| AMT | Immature dendritic cell | -0.1775 | 8.35E-04 | -0.1972 | 2.05E-04 |
| ARL1 | Immature dendritic cell | -0.0266 | 6.20E-01 | -0.0074 | 8.90E-01 |
| ATIC | Immature dendritic cell | 0.1906 | 3.30E-04 | 0.1969 | 2.10E-04 |
| ATP5A1 | Immature dendritic cell | -0.3175 | 1.17E-09 | -0.3099 | 3.14E-09 |
| CAPZA1 | Immature dendritic cell | 0.0863 | 1.06E-01 | 0.1226 | 2.18E-02 |
| LILRA5 | Immature dendritic cell | -0.1596 | 2.71E-03 | -0.1046 | 5.07E-02 |
| RDX | Immature dendritic cell | -0.0545 | 3.09E-01 | -0.0536 | 3.18E-01 |
| RRAGD | Immature dendritic cell | 0.1492 | 5.10E-03 | 0.1548 | 3.69E-03 |
| TACSTD2 | Immature dendritic cell | -0.0276 | 6.06E-01 | 0.0059 | 9.12E-01 |
| INPP5F | Immature dendritic cell | 0.1385 | 9.39E-03 | 0.1619 | 2.38E-03 |
| RAB38 | Immature dendritic cell | 0.0012 | 9.83E-01 | 0.0670 | 2.11E-01 |
| PLAU | Immature dendritic cell | -0.0828 | 1.22E-01 | -0.0096 | 8.57E-01 |
| CSF3R | Immature dendritic cell | -0.0577 | 2.81E-01 | 0.0256 | 6.33E-01 |
| SLC18A2 | Immature dendritic cell | -0.0883 | 9.87E-02 | -0.0762 | 1.55E-01 |
| AMPD2 | Immature dendritic cell | -0.0479 | 3.71E-01 | -0.0459 | 3.92E-01 |
| CLTB | Immature dendritic cell | -0.0843 | 1.15E-01 | -0.0715 | 1.82E-01 |
| C1orf162 | Immature dendritic cell | -0.1404 | 8.44E-03 | -0.0714 | 1.83E-01 |
| AIF1 | Macrophage | -0.0976 | 6.77E-02 | -0.0195 | 7.16E-01 |
| CCL14 | Macrophage | -0.3149 | 1.62E-09 | -0.3118 | 2.51E-09 |
| CCL23 | Macrophage | -0.1505 | 4.72E-03 | -0.1116 | 3.69E-02 |

| | | | | | |
|---|---|---|---|---|---|
| CCL26 | Macrophage | 0.1730 | 1.14E-03 | 0.2150 | 5.01E-05 |
| CD300LB | Macrophage | 0.0175 | 7.44E-01 | 0.0888 | 9.73E-02 |
| CNR1 | Macrophage | -0.0588 | 2.72E-01 | -0.0315 | 5.57E-01 |
| CNR2 | Macrophage | 0.0249 | 6.42E-01 | 0.0781 | 1.45E-01 |
| EIF1 | Macrophage | 0.0690 | 1.97E-01 | 0.0612 | 2.54E-01 |
| EIF4A1 | Macrophage | -0.0296 | 5.80E-01 | -0.0126 | 8.14E-01 |
| FPR1 | Macrophage | -0.0968 | 7.01E-02 | -0.0184 | 7.31E-01 |
| FPR2 | Macrophage | -0.1563 | 3.32E-03 | -0.0996 | 6.26E-02 |
| FRAT2 | Macrophage | 0.1170 | 2.84E-02 | 0.1257 | 1.86E-02 |
| GPR27 | Macrophage | 0.0164 | 7.59E-01 | 0.0284 | 5.96E-01 |
| GPR77 | Macrophage | 0.0377 | 4.82E-01 | 0.0580 | 2.79E-01 |
| RNASE2 | Macrophage | 0.1210 | 2.34E-02 | 0.1733 | 1.13E-03 |
| MS4A2 | Macrophage | -0.1085 | 4.22E-02 | -0.0671 | 2.10E-01 |
| BASP1 | Macrophage | -0.1179 | 2.72E-02 | -0.0444 | 4.08E-01 |
| IGSF6 | Macrophage | -0.1161 | 2.96E-02 | -0.0441 | 4.10E-01 |
| HK3 | Macrophage | -0.0863 | 1.07E-01 | -0.0028 | 9.58E-01 |
| VNN1 | Macrophage | -0.1206 | 2.38E-02 | -0.0983 | 6.62E-02 |
| FES | Macrophage | 0.0972 | 6.89E-02 | 0.1205 | 2.41E-02 |
| NPL | Macrophage | 0.0486 | 3.64E-01 | 0.0970 | 6.98E-02 |
| FZD2 | Macrophage | 0.0372 | 4.87E-01 | 0.1164 | 2.94E-02 |
| FAM198B | Macrophage | -0.2512 | 1.87E-06 | -0.2219 | 2.81E-05 |
| HNMT | Macrophage | -0.2426 | 4.28E-06 | -0.2644 | 5.20E-07 |
| SLC15A3 | Macrophage | -0.1142 | 3.25E-02 | -0.0489 | 3.62E-01 |
| CD4 | Macrophage | -0.2115 | 6.49E-05 | -0.1649 | 1.96E-03 |
| TXNDC3 | Macrophage | -0.0410 | 4.44E-01 | 0.0205 | 7.03E-01 |
| FRMD4A | Macrophage | 0.0817 | 1.27E-01 | 0.1086 | 4.23E-02 |
| CRYBB1 | Macrophage | -0.0579 | 2.79E-01 | 0.0151 | 7.78E-01 |
| HRH1 | Macrophage | -0.0759 | 1.56E-01 | -0.0165 | 7.58E-01 |
| WNT5B | Macrophage | -0.0934 | 8.07E-02 | -0.0949 | 7.62E-02 |

| | | | | | |
|---|---|---|---|---|---|
| GIPR | Eosinophil | 0.1137 | 3.32E-02 | 0.1416 | 7.99E-03 |
| LRMP | Eosinophil | -0.1068 | 4.55E-02 | -0.0527 | 3.26E-01 |
| FOSB | Eosinophil | -0.1961 | 2.18E-04 | -0.1735 | 1.12E-03 |
| RRP12 | Eosinophil | 0.1292 | 1.54E-02 | 0.1375 | 9.99E-03 |
| GPR183 | Eosinophil | -0.1183 | 2.66E-02 | -0.0507 | 3.44E-01 |
| NR4A3 | Eosinophil | -0.1533 | 3.99E-03 | -0.1257 | 1.87E-02 |
| ST3GAL6 | Eosinophil | -0.3021 | 7.66E-09 | -0.3151 | 1.67E-09 |
| DEPDC5 | Eosinophil | -0.0057 | 9.16E-01 | -0.0271 | 6.14E-01 |
| PDE6C | Eosinophil | -0.0259 | 6.29E-01 | -0.0317 | 5.54E-01 |
| PKD2L2 | Eosinophil | 0.0662 | 2.16E-01 | 0.0867 | 1.05E-01 |
| GPR65 | Eosinophil | -0.1336 | 1.23E-02 | -0.0552 | 3.04E-01 |
| IL5RA | Eosinophil | -0.1301 | 1.47E-02 | -0.0837 | 1.18E-01 |
| P2RY14 | Eosinophil | -0.1761 | 9.22E-04 | -0.1460 | 6.20E-03 |
| DACH1 | Eosinophil | -0.0441 | 4.10E-01 | -0.0167 | 7.55E-01 |
| DAPK2 | Eosinophil | -0.0566 | 2.90E-01 | -0.0530 | 3.23E-01 |
| EMR3 | Eosinophil | 0.0276 | 6.06E-01 | 0.0744 | 1.65E-01 |
| ADAMTS3 | Mast cell | -0.0441 | 4.10E-01 | 0.0175 | 7.45E-01 |
| CPA3 | Mast cell | -0.0982 | 6.62E-02 | -0.0556 | 2.99E-01 |
| CMA1 | Mast cell | -0.1377 | 9.81E-03 | -0.1128 | 3.49E-02 |
| CTSG | Mast cell | -0.1636 | 2.10E-03 | -0.1401 | 8.68E-03 |
| ARHGAP15 | Mast cell | -0.0827 | 1.22E-01 | -0.0075 | 8.88E-01 |
| CPM | Mast cell | -0.0626 | 2.42E-01 | -0.0358 | 5.04E-01 |
| FCN1 | Mast cell | -0.1561 | 3.38E-03 | -0.0989 | 6.45E-02 |
| FTL | Mast cell | -0.0072 | 8.93E-01 | 0.0068 | 8.99E-01 |
| HSPA6 | Mast cell | 0.1476 | 5.61E-03 | 0.2072 | 9.39E-05 |
| ITGA9 | Mast cell | -0.2025 | 1.33E-04 | -0.1661 | 1.82E-03 |
| RNASE3 | Mast cell | -0.0042 | 9.37E-01 | 0.0261 | 6.26E-01 |
| S100A4 | Mast cell | 0.0065 | 9.04E-01 | 0.1078 | 4.38E-02 |
| SIGLEC8 | Mast cell | -0.1251 | 1.90E-02 | -0.0731 | 1.73E-01 |

| | | | | | |
|---|---|---|---|---|---|
| SLC6A4 | Mast cell | 0.0066 | 9.02E-01 | 0.0012 | 9.83E-01 |
| PTGS2 | Mast cell | -0.1324 | 1.30E-02 | -0.0786 | 1.42E-01 |
| EGR3 | Mast cell | -0.1751 | 9.90E-04 | -0.1266 | 1.78E-02 |
| PILRA | Mast cell | -0.0575 | 2.83E-01 | 0.0429 | 4.24E-01 |
| ASGR2 | Monocyte | -0.1628 | 2.21E-03 | -0.1834 | 5.64E-04 |
| CFP | Monocyte | -0.2118 | 6.36E-05 | -0.1726 | 1.19E-03 |
| ASGR1 | Monocyte | -0.1443 | 6.76E-03 | -0.1686 | 1.55E-03 |
| CD1D | Monocyte | -0.1235 | 2.06E-02 | -0.0840 | 1.17E-01 |
| UPK3A | Monocyte | 0.1514 | 4.47E-03 | 0.1558 | 3.47E-03 |
| ACTG1 | Monocyte | 0.0918 | 8.58E-02 | 0.1117 | 3.67E-02 |
| ANXA5 | Monocyte | 0.0120 | 8.23E-01 | 0.0692 | 1.97E-01 |
| ATP6V1B2 | Monocyte | -0.1244 | 1.97E-02 | -0.0870 | 1.04E-01 |
| CFL1 | Monocyte | 0.0483 | 3.67E-01 | 0.0912 | 8.83E-02 |
| DAZAP2 | Monocyte | -0.0972 | 6.89E-02 | -0.0643 | 2.30E-01 |
| CTBS | Monocyte | -0.3146 | 1.66E-09 | -0.3047 | 5.91E-09 |
| EMR4P | Monocyte | -0.0597 | 2.65E-01 | -0.0017 | 9.75E-01 |
| HIVEP2 | Monocyte | 0.0275 | 6.08E-01 | 0.0512 | 3.40E-01 |
| MARCKSL1 | Monocyte | 0.1779 | 8.16E-04 | 0.2016 | 1.47E-04 |
| MBP | Monocyte | -0.0804 | 1.33E-01 | -0.0588 | 2.73E-01 |
| MMP15 | Monocyte | -0.1081 | 4.30E-02 | -0.1060 | 4.74E-02 |
| PNPLA6 | Monocyte | -0.0060 | 9.11E-01 | 0.0111 | 8.36E-01 |
| TMBIM6 | Monocyte | -0.3895 | 3.69E-14 | -0.3983 | 9.35E-15 |
| PQBP1 | Monocyte | 0.2340 | 9.45E-06 | 0.2297 | 1.43E-05 |
| TEX264 | Monocyte | -0.2255 | 2.00E-05 | -0.2473 | 2.82E-06 |
| IKZF1 | Monocyte | -0.1434 | 7.14E-03 | -0.0704 | 1.89E-01 |
| CREB5 | Neutrophil | 0.0197 | 7.13E-01 | 0.0402 | 4.53E-01 |
| CDA | Neutrophil | -0.1103 | 3.90E-02 | -0.0936 | 8.03E-02 |
| CHST15 | Neutrophil | -0.1642 | 2.02E-03 | -0.1515 | 4.50E-03 |
| S100A12 | Neutrophil | -0.0851 | 1.12E-01 | -0.0613 | 2.52E-01 |

| | | | | | |
|---|---|---|---|---|---|
| APOBEC3A | Neutrophil | 0.0425 | 4.27E-01 | 0.0881 | 9.99E-02 |
| CASP5 | Neutrophil | 0.0306 | 5.68E-01 | 0.0932 | 8.16E-02 |
| MMP25 | Neutrophil | -0.0669 | 2.11E-01 | 0.0089 | 8.68E-01 |
| HAL | Neutrophil | -0.0553 | 3.02E-01 | -0.0486 | 3.65E-01 |
| C1orf183 | Neutrophil | 0.0036 | 9.46E-01 | 0.0186 | 7.29E-01 |
| FFAR2 | Neutrophil | 0.0418 | 4.35E-01 | 0.0898 | 9.35E-02 |
| MAK | Neutrophil | 0.0255 | 6.34E-01 | 0.0430 | 4.23E-01 |
| CXCR1 | Neutrophil | -0.1047 | 5.00E-02 | -0.0779 | 1.46E-01 |
| STEAP4 | Neutrophil | -0.3596 | 3.73E-12 | -0.3469 | 2.46E-11 |
| MGAM | Neutrophil | 0.0519 | 3.32E-01 | 0.0909 | 8.94E-02 |
| BTNL8 | Neutrophil | 0.1728 | 1.15E-03 | 0.1840 | 5.41E-04 |
| CXCR2 | Neutrophil | -0.0767 | 1.51E-01 | -0.0375 | 4.84E-01 |
| TNFRSF10C | Neutrophil | -0.0303 | 5.72E-01 | -0.0079 | 8.83E-01 |
| VNN3 | Neutrophil | -0.0447 | 4.03E-01 | -0.0256 | 6.33E-01 |
| CCL2 | TAM | -0.1523 | 4.23E-03 | -0.0973 | 6.91E-02 |
| CD68 | TAM | -0.2191 | 3.47E-05 | -0.1764 | 9.18E-04 |
| IL10 | TAM | -0.1173 | 2.79E-02 | -0.0503 | 3.48E-01 |
| NOS2 | M1 Macrophage | -0.0869 | 1.04E-01 | -0.0770 | 1.51E-01 |
| IRF5 | M1 Macrophage | 0.1479 | 5.51E-03 | 0.1661 | 1.82E-03 |
| PTGS2 | M1 Macrophage | -0.1324 | 1.30E-02 | -0.0786 | 1.42E-01 |
| CD163 | M2 Macrophage | -0.2106 | 7.02E-05 | -0.1624 | 2.31E-03 |
| VSIG4 | M2 Macrophage | -0.1559 | 3.41E-03 | -0.0925 | 8.42E-02 |
| MS4A4A | M2 Macrophage | -0.1698 | 1.41E-03 | -0.1094 | 4.08E-02 |
| ADPRH | regulatory T cell marker | -0.0293 | 5.85E-01 | 0.0378 | 4.81E-01 |
| IL1R1 | regulatory T cell marker | -0.2291 | 1.47E-05 | -0.2244 | 2.26E-05 |
| KSR1 | regulatory T cell marker | 0.0331 | 5.37E-01 | 0.0698 | 1.93E-01 |
| SOCS2 | regulatory T cell marker | -0.2577 | 9.88E-07 | -0.2579 | 1.01E-06 |
| SSH1 | regulatory T cell marker | -0.0199 | 7.11E-01 | 0.0060 | 9.11E-01 |
| NFAT5 | regulatory T cell marker | -0.0353 | 5.10E-01 | -0.0211 | 6.94E-01 |

| | | | | | |
|---|---|---|---|---|---|
| JAK1 | regulatory T cell marker | -0.2072 | 9.23E-05 | -0.1832 | 5.74E-04 |
| CHST7 | regulatory T cell marker | -0.1566 | 3.27E-03 | -0.1353 | 1.13E-02 |
| METTL7A | regulatory T cell marker | -0.3642 | 1.90E-12 | -0.3925 | 2.44E-14 |
| ENTPD1 | regulatory T cell marker | -0.0342 | 5.23E-01 | 0.0334 | 5.33E-01 |
| LY75 | regulatory T cell marker | -0.1373 | 1.00E-02 | -0.0786 | 1.42E-01 |
| ANKRD10 | regulatory T cell marker | 0.2411 | 4.90E-06 | 0.2450 | 3.52E-06 |
| IKZF2 | regulatory T cell marker | -0.1789 | 7.62E-04 | -0.1376 | 9.94E-03 |
| LAX1 | regulatory T cell marker | -0.0740 | 1.67E-01 | -0.0129 | 8.10E-01 |
| LAYN | regulatory T cell marker | -0.0250 | 6.40E-01 | 0.0239 | 6.56E-01 |
| CSF1 | regulatory T cell marker | -0.1049 | 4.96E-02 | -0.0473 | 3.78E-01 |
| ACSL4 | regulatory T cell marker | 0.1088 | 4.17E-02 | 0.1301 | 1.49E-02 |
| CHRNA6 | regulatory T cell marker | 0.0589 | 2.71E-01 | 0.1107 | 3.84E-02 |
| TMPRSS6 | regulatory T cell marker | -0.0907 | 8.99E-02 | -0.0980 | 6.70E-02 |
| CADM1 | regulatory T cell marker | -0.1215 | 2.29E-02 | -0.1504 | 4.81E-03 |
| CHST2 | regulatory T cell marker | -0.0581 | 2.78E-01 | 0.0087 | 8.71E-01 |
| ZBTB38 | regulatory T cell marker | -0.1331 | 1.26E-02 | -0.1339 | 1.22E-02 |
| MAGEH1 | regulatory T cell marker | 0.0959 | 7.27E-02 | 0.1152 | 3.12E-02 |
| NPTN | regulatory T cell marker | -0.0004 | 9.94E-01 | 0.0039 | 9.42E-01 |
| RRAGB | regulatory T cell marker | -0.0372 | 4.88E-01 | -0.0421 | 4.32E-01 |
| ADAT2 | regulatory T cell marker | 0.3058 | 4.95E-09 | 0.3265 | 3.90E-10 |
| LIMA1 | regulatory T cell marker | 0.0162 | 7.62E-01 | 0.0636 | 2.35E-01 |
| ICOS | regulatory T cell marker | 0.0203 | 7.05E-01 | 0.1211 | 2.35E-02 |
| IL21R | regulatory T cell marker | 0.0268 | 6.16E-01 | 0.1426 | 7.53E-03 |
| THADA | regulatory T cell marker | 0.0912 | 8.78E-02 | 0.0873 | 1.03E-01 |
| CSF2RB | regulatory T cell marker | -0.1148 | 3.16E-02 | -0.0330 | 5.38E-01 |
| SSTR3 | regulatory T cell marker | 0.1566 | 3.26E-03 | 0.1961 | 2.23E-04 |
| LAPTM4B | regulatory T cell marker | 0.2726 | 2.14E-07 | 0.2798 | 1.03E-07 |
| NDFIP2 | regulatory T cell marker | -0.1748 | 1.01E-03 | -0.1806 | 6.87E-04 |
| GCNT1 | regulatory T cell marker | -0.0271 | 6.13E-01 | 0.0503 | 3.49E-01 |

| | | | | | |
|---|---|---|---|---|---|
| HS3ST3B1 | regulatory T cell marker | -0.1521 | 4.30E-03 | -0.1680 | 1.61E-03 |
| IL1R2 | regulatory T cell marker | -0.0042 | 9.38E-01 | 0.0050 | 9.26E-01 |
| VDR | regulatory T cell marker | 0.1057 | 4.78E-02 | 0.1404 | 8.51E-03 |
| CTLA4 | regulatory T cell marker | 0.0755 | 1.58E-01 | 0.1751 | 1.01E-03 |
| CASP1 | regulatory T cell marker | -0.0669 | 2.12E-01 | 0.0272 | 6.12E-01 |
| TMEM184C | regulatory T cell marker | -0.1970 | 2.04E-04 | -0.1883 | 3.98E-04 |
| CTSC | regulatory T cell marker | 0.0083 | 8.76E-01 | 0.0905 | 9.09E-02 |
| HSDL2 | regulatory T cell marker | -0.2154 | 4.71E-05 | -0.2458 | 3.26E-06 |
| LEPROT | regulatory T cell marker | -0.1936 | 2.64E-04 | -0.1909 | 3.29E-04 |
| PTPRJ | regulatory T cell marker | -0.0274 | 6.08E-01 | -0.0200 | 7.10E-01 |
| CD274 | regulatory T cell marker | -0.1401 | 8.57E-03 | -0.0897 | 9.40E-02 |
| TIGIT | regulatory T cell marker | -0.0336 | 5.30E-01 | 0.0568 | 2.89E-01 |
| TRAF3 | regulatory T cell marker | 0.0115 | 8.30E-01 | 0.0634 | 2.37E-01 |
| NETO2 | regulatory T cell marker | 0.0461 | 3.89E-01 | 0.0663 | 2.16E-01 |
| SLC35F2 | regulatory T cell marker | 0.0777 | 1.46E-01 | 0.1487 | 5.31E-03 |
| ACP5 | regulatory T cell marker | -0.0262 | 6.25E-01 | 0.0196 | 7.15E-01 |
| AHCYL1 | regulatory T cell marker | -0.2087 | 8.15E-05 | -0.1976 | 1.99E-04 |
| RNF145 | regulatory T cell marker | 0.0398 | 4.58E-01 | 0.0787 | 1.42E-01 |
| BCL2L1 | regulatory T cell marker | -0.0737 | 1.68E-01 | -0.0823 | 1.25E-01 |
| TNFRSF4 | regulatory T cell marker | 0.1376 | 9.87E-03 | 0.1928 | 2.84E-04 |
| NFE2L3 | regulatory T cell marker | 0.2003 | 1.59E-04 | 0.2749 | 1.74E-07 |
| CCR8 | regulatory T cell marker | -0.0200 | 7.08E-01 | 0.0463 | 3.88E-01 |
| HTATIP2 | regulatory T cell marker | -0.0315 | 5.56E-01 | -0.0212 | 6.93E-01 |
| TNFRSF18 | regulatory T cell marker | 0.0829 | 1.21E-01 | 0.1717 | 1.26E-03 |
| FKBP1A | regulatory T cell marker | 0.1230 | 2.11E-02 | 0.1586 | 2.92E-03 |
| LTA | regulatory T cell marker | 0.0212 | 6.93E-01 | 0.1270 | 1.75E-02 |
| BATF | regulatory T cell marker | -0.0389 | 4.68E-01 | 0.0324 | 5.46E-01 |
| CD177 | regulatory T cell marker | 0.0416 | 4.38E-01 | 0.0908 | 9.00E-02 |
| TFRC | regulatory T cell marker | 0.1399 | 8.67E-03 | 0.1429 | 7.40E-03 |

| | | | | | |
|---|---|---|---|---|---|
| ETV7 | regulatory T cell marker | 0.1022 | 5.58E-02 | 0.1655 | 1.89E-03 |
| IL12RB2 | regulatory T cell marker | -0.1550 | 3.61E-03 | -0.1187 | 2.64E-02 |
| ZNF282 | regulatory T cell marker | 0.1325 | 1.30E-02 | 0.1101 | 3.96E-02 |
| ERI1 | regulatory T cell marker | 0.0332 | 5.35E-01 | 0.0653 | 2.23E-01 |
| GRSF1 | regulatory T cell marker | -0.1915 | 3.07E-04 | -0.1794 | 7.47E-04 |
| NAB1 | regulatory T cell marker | 0.1206 | 2.38E-02 | 0.1197 | 2.51E-02 |
| C10orf54 | immune checkpoint ger | -0.1008 | 5.93E-02 | -0.0466 | 3.84E-01 |
| CD200 | immune checkpoint ger | -0.0938 | 7.93E-02 | -0.0525 | 3.27E-01 |
| CD40LG | immune checkpoint ger | -0.1460 | 6.13E-03 | -0.0879 | 1.01E-01 |
| ADORA2A | immune checkpoint ger | 0.1235 | 2.07E-02 | 0.1719 | 1.24E-03 |
| TNFSF14 | immune checkpoint ger | 0.0940 | 7.87E-02 | 0.1128 | 3.49E-02 |
| BTLA | immune checkpoint ger | -0.0500 | 3.51E-01 | 0.0276 | 6.07E-01 |
| CD160 | immune checkpoint ger | 0.0574 | 2.84E-01 | 0.1050 | 4.96E-02 |
| CD28 | immune checkpoint ger | -0.1067 | 4.59E-02 | -0.0379 | 4.80E-01 |
| CD44 | immune checkpoint ger | -0.1328 | 1.28E-02 | -0.0810 | 1.30E-01 |
| CD48 | immune checkpoint ger | -0.1062 | 4.69E-02 | -0.0248 | 6.44E-01 |
| CD200R1 | immune checkpoint ger | -0.1457 | 6.26E-03 | -0.0806 | 1.33E-01 |
| NRP1 | immune checkpoint ger | -0.1440 | 6.90E-03 | -0.1279 | 1.67E-02 |
| TMIGD2 | immune checkpoint ger | -0.1258 | 1.84E-02 | -0.0773 | 1.49E-01 |
| VTCN1 | immune checkpoint ger | 0.0275 | 6.08E-01 | 0.0659 | 2.18E-01 |
| ICOS | immune checkpoint ger | 0.0203 | 7.05E-01 | 0.1211 | 2.35E-02 |
| TNFSF15 | immune checkpoint ger | 0.0261 | 6.25E-01 | 0.0663 | 2.16E-01 |
| CD40 | immune checkpoint ger | -0.1267 | 1.76E-02 | -0.0933 | 8.14E-02 |
| HHLA2 | immune checkpoint ger | 0.0811 | 1.29E-01 | 0.1092 | 4.12E-02 |
| IDO2 | immune checkpoint ger | -0.1843 | 5.21E-04 | -0.1619 | 2.39E-03 |
| PDCD1LG2 | immune checkpoint ger | -0.1893 | 3.61E-04 | -0.1356 | 1.11E-02 |
| TNFRSF14 | immune checkpoint ger | -0.0725 | 1.76E-01 | -0.0385 | 4.73E-01 |
| TNFRSF25 | immune checkpoint ger | 0.1293 | 1.53E-02 | 0.1654 | 1.90E-03 |
| CD27 | immune checkpoint ger | -0.0050 | 9.26E-01 | 0.0874 | 1.03E-01 |

| | | | | | |
|---|---|---|---|---|---|
| CD86 | immune checkpoint ger | -0.1016 | 5.73E-02 | -0.0127 | 8.12E-01 |
| CTLA4 | immune checkpoint ger | 0.0755 | 1.58E-01 | 0.1751 | 1.01E-03 |
| LAIR1 | immune checkpoint ger | -0.0166 | 7.57E-01 | 0.0947 | 7.68E-02 |
| LGALS9 | immune checkpoint ger | -0.0109 | 8.39E-01 | 0.0772 | 1.49E-01 |
| CD244 | immune checkpoint ger | -0.1133 | 3.38E-02 | -0.0554 | 3.01E-01 |
| CD274 | immune checkpoint ger | -0.1401 | 8.57E-03 | -0.0897 | 9.40E-02 |
| CD276 | immune checkpoint ger | 0.1045 | 5.04E-02 | 0.1336 | 1.24E-02 |
| CD80 | immune checkpoint ger | -0.0329 | 5.39E-01 | 0.0491 | 3.60E-01 |
| HAVCR2 | immune checkpoint ger | -0.0969 | 6.99E-02 | -0.0108 | 8.40E-01 |
| ICOSLG | immune checkpoint ger | -0.2541 | 1.41E-06 | -0.2452 | 3.46E-06 |
| PDCD1 | immune checkpoint ger | 0.0514 | 3.37E-01 | 0.1319 | 1.35E-02 |
| TIGIT | immune checkpoint ger | -0.0336 | 5.30E-01 | 0.0568 | 2.89E-01 |
| TNFRSF4 | immune checkpoint ger | 0.1376 | 9.87E-03 | 0.1928 | 2.84E-04 |
| TNFSF18 | immune checkpoint ger | -0.0601 | 2.61E-01 | -0.0331 | 5.37E-01 |
| KIR3DL1 | immune checkpoint ger | -0.0961 | 7.20E-02 | -0.0751 | 1.61E-01 |
| LAG3 | immune checkpoint ger | 0.0527 | 3.24E-01 | 0.1163 | 2.96E-02 |
| TNFRSF18 | immune checkpoint ger | 0.0829 | 1.21E-01 | 0.1717 | 1.26E-03 |
| TNFRSF8 | immune checkpoint ger | -0.0503 | 3.48E-01 | 0.0490 | 3.61E-01 |
| TNFSF4 | immune checkpoint ger | 0.2993 | 1.07E-08 | 0.3064 | 4.84E-09 |
| CD70 | immune checkpoint ger | -0.0018 | 9.74E-01 | 0.0865 | 1.06E-01 |
| TNFRSF9 | immune checkpoint ger | 0.0102 | 8.49E-01 | 0.0685 | 2.01E-01 |
| TNFSF9 | immune checkpoint ger | 0.1600 | 2.65E-03 | 0.2116 | 6.59E-05 |
| IDO1 | immune checkpoint ger | -0.0184 | 7.31E-01 | 0.0344 | 5.21E-01 |
| ENG | immune cell infiltrate ge | -0.3153 | 1.53E-09 | -0.2884 | 3.94E-08 |
| PTPRC | immune cell infiltrate ge | -0.1326 | 1.29E-02 | -0.0602 | 2.61E-01 |
| MS4A1 | immune cell infiltrate ge | -0.0295 | 5.81E-01 | 0.0330 | 5.38E-01 |
| CD1A | immune cell infiltrate ge | 0.0465 | 3.85E-01 | 0.1167 | 2.90E-02 |
| TPSB2 | immune cell infiltrate ge | -0.1039 | 5.17E-02 | -0.0678 | 2.05E-01 |
| PDPN | immune cell infiltrate ge | -0.0352 | 5.11E-01 | 0.0326 | 5.43E-01 |

| | | | | | |
|---|---|---|---|---|---|
| CXCR5 | immune cell infiltrate ge | 0.0022 | 9.67E-01 | 0.0718 | 1.80E-01 |
| IL3RA | immune cell infiltrate ge | -0.0822 | 1.24E-01 | -0.0468 | 3.83E-01 |
| CSF3 | immune cell infiltrate ge | -0.1143 | 3.23E-02 | -0.0948 | 7.64E-02 |
| B3GAT1 | immune cell infiltrate ge | 0.0912 | 8.79E-02 | 0.0998 | 6.21E-02 |
| CD247 | immune cell infiltrate ge | -0.1030 | 5.38E-02 | -0.0277 | 6.05E-01 |
| CD8A | immune cell infiltrate ge | -0.0993 | 6.32E-02 | -0.0288 | 5.91E-01 |
| FOXP3 | immune cell infiltrate ge | -0.1162 | 2.96E-02 | -0.0696 | 1.94E-01 |
| CSF2 | immune cell infiltrate ge | 0.0846 | 1.14E-01 | 0.1266 | 1.78E-02 |
| CD68 | immune cell infiltrate ge | -0.2191 | 3.47E-05 | -0.1764 | 9.18E-04 |
| GIMAP6 | tumor-infiltrating lympho | -0.2432 | 4.04E-06 | -0.2040 | 1.22E-04 |
| CFH | tumor-infiltrating lympho | -0.1616 | 2.39E-03 | -0.1543 | 3.81E-03 |
| ITGA4 | tumor-infiltrating lympho | -0.0999 | 6.15E-02 | -0.0279 | 6.04E-01 |
| FAM65B | tumor-infiltrating lympho | -0.1584 | 2.93E-03 | -0.1160 | 3.00E-02 |
| GVIN1 | tumor-infiltrating lympho | -0.1749 | 1.00E-03 | -0.1215 | 2.30E-02 |
| ARHGAP15 | tumor-infiltrating lympho | -0.0827 | 1.22E-01 | -0.0075 | 8.88E-01 |
| ARHGAP25 | tumor-infiltrating lympho | -0.1409 | 8.19E-03 | -0.0712 | 1.84E-01 |
| GIMAP4 | tumor-infiltrating lympho | -0.1817 | 6.27E-04 | -0.1266 | 1.78E-02 |
| GIMAP7 | tumor-infiltrating lympho | -0.2464 | 2.99E-06 | -0.2090 | 8.15E-05 |
| GPSM3 | tumor-infiltrating lympho | -0.0862 | 1.07E-01 | 0.0082 | 8.78E-01 |
| IL16 | tumor-infiltrating lympho | -0.1227 | 2.15E-02 | -0.0535 | 3.19E-01 |
| PIK3CD | tumor-infiltrating lympho | -0.0714 | 1.82E-01 | 0.0312 | 5.61E-01 |
| PRKCB | tumor-infiltrating lympho | -0.1239 | 2.03E-02 | -0.0483 | 3.68E-01 |
| SELL | tumor-infiltrating lympho | -0.0732 | 1.71E-01 | -0.0034 | 9.50E-01 |
| GIMAP5 | tumor-infiltrating lympho | -0.1013 | 5.79E-02 | -0.0487 | 3.64E-01 |
| INPP5D | tumor-infiltrating lympho | -0.1520 | 4.31E-03 | -0.0869 | 1.05E-01 |
| NLRC3 | tumor-infiltrating lympho | -0.1434 | 7.13E-03 | -0.0794 | 1.38E-01 |
| PRKCQ | tumor-infiltrating lympho | -0.1132 | 3.41E-02 | -0.0444 | 4.07E-01 |
| TRAT1 | tumor-infiltrating lympho | -0.1266 | 1.77E-02 | -0.0603 | 2.60E-01 |
| ARHGAP30 | tumor-infiltrating lympho | -0.0961 | 7.21E-02 | -0.0034 | 9.50E-01 |

| | | | | | |
|---|---|---|---|---|---|
| DOCK11 | tumor-infiltrating lympho | -0.0965 | 7.08E-02 | -0.0309 | 5.64E-01 |
| FYB | tumor-infiltrating lympho | -0.1196 | 2.50E-02 | -0.0418 | 4.35E-01 |
| IKZF1 | tumor-infiltrating lympho | -0.1434 | 7.14E-03 | -0.0704 | 1.89E-01 |
| IL7R | tumor-infiltrating lympho | -0.1851 | 4.91E-04 | -0.1372 | 1.02E-02 |
| P2RY8 | tumor-infiltrating lympho | -0.1740 | 1.06E-03 | -0.1397 | 8.89E-03 |
| PARVG | tumor-infiltrating lympho | -0.0948 | 7.60E-02 | -0.0008 | 9.88E-01 |
| PVRIG | tumor-infiltrating lympho | 0.0283 | 5.97E-01 | 0.0902 | 9.19E-02 |
| TRAF3IP3 | tumor-infiltrating lympho | -0.0918 | 8.60E-02 | -0.0132 | 8.06E-01 |
| CCR7 | tumor-infiltrating lympho | -0.0941 | 7.82E-02 | -0.0267 | 6.19E-01 |
| EVI2B | tumor-infiltrating lympho | -0.1371 | 1.02E-02 | -0.0612 | 2.54E-01 |
| GPR18 | tumor-infiltrating lympho | -0.0863 | 1.06E-01 | -0.0183 | 7.33E-01 |
| ITK | tumor-infiltrating lympho | -0.1523 | 4.23E-03 | -0.0884 | 9.87E-02 |
| ITM2C | tumor-infiltrating lympho | 0.1535 | 3.94E-03 | 0.1711 | 1.32E-03 |
| LAX1 | tumor-infiltrating lympho | -0.0740 | 1.67E-01 | -0.0129 | 8.10E-01 |
| MPEG1 | tumor-infiltrating lympho | -0.1786 | 7.74E-04 | -0.1224 | 2.20E-02 |
| MS4A1 | tumor-infiltrating lympho | -0.0295 | 5.81E-01 | 0.0330 | 5.38E-01 |
| PTPRC | tumor-infiltrating lympho | -0.1326 | 1.29E-02 | -0.0602 | 2.61E-01 |
| SELPLG | tumor-infiltrating lympho | -0.1259 | 1.82E-02 | -0.0461 | 3.90E-01 |
| SLAMF6 | tumor-infiltrating lympho | -0.1649 | 1.94E-03 | -0.1049 | 4.98E-02 |
| STAT4 | tumor-infiltrating lympho | -0.0100 | 8.52E-01 | 0.0470 | 3.81E-01 |
| CD28 | tumor-infiltrating lympho | -0.1067 | 4.59E-02 | -0.0379 | 4.80E-01 |
| CD48 | tumor-infiltrating lympho | -0.1062 | 4.69E-02 | -0.0248 | 6.44E-01 |
| CORO1A | tumor-infiltrating lympho | -0.0339 | 5.27E-01 | 0.0770 | 1.51E-01 |
| DOCK2 | tumor-infiltrating lympho | -0.1236 | 2.05E-02 | -0.0431 | 4.22E-01 |
| KLRD1 | tumor-infiltrating lympho | -0.1774 | 8.43E-04 | -0.1309 | 1.43E-02 |
| LCP2 | tumor-infiltrating lympho | -0.1347 | 1.15E-02 | -0.0590 | 2.71E-01 |
| MAL | tumor-infiltrating lympho | -0.0654 | 2.21E-01 | 0.0006 | 9.92E-01 |
| PLAC8 | tumor-infiltrating lympho | -0.0429 | 4.23E-01 | 0.0296 | 5.82E-01 |
| SASH3 | tumor-infiltrating lympho | -0.0895 | 9.41E-02 | 0.0071 | 8.94E-01 |

| | | | | | |
|---|---|---|---|---|---|
| SH2D1A | tumor-infiltrating lympho | -0.1275 | 1.68E-02 | -0.0591 | 2.70E-01 |
| STK10 | tumor-infiltrating lympho | -0.1245 | 1.97E-02 | -0.0555 | 3.01E-01 |
| ACAP1 | tumor-infiltrating lympho | -0.0730 | 1.72E-01 | 0.0161 | 7.65E-01 |
| ARHGAP9 | tumor-infiltrating lympho | -0.0374 | 4.85E-01 | 0.0715 | 1.82E-01 |
| CCR2 | tumor-infiltrating lympho | -0.1156 | 3.03E-02 | -0.0455 | 3.96E-01 |
| CD2 | tumor-infiltrating lympho | -0.0622 | 2.45E-01 | 0.0271 | 6.13E-01 |
| CD3E | tumor-infiltrating lympho | -0.0931 | 8.14E-02 | -0.0112 | 8.35E-01 |
| CD52 | tumor-infiltrating lympho | -0.0714 | 1.82E-01 | 0.0150 | 7.80E-01 |
| CD6 | tumor-infiltrating lympho | -0.0676 | 2.06E-01 | 0.0202 | 7.07E-01 |
| GPR171 | tumor-infiltrating lympho | -0.1050 | 4.93E-02 | -0.0332 | 5.36E-01 |
| GZMK | tumor-infiltrating lympho | -0.1558 | 3.44E-03 | -0.0950 | 7.60E-02 |
| HCLS1 | tumor-infiltrating lympho | -0.1452 | 6.42E-03 | -0.0716 | 1.82E-01 |
| IL2RG | tumor-infiltrating lympho | -0.0033 | 9.51E-01 | 0.0984 | 6.60E-02 |
| IRF4 | tumor-infiltrating lympho | -0.0078 | 8.84E-01 | 0.0752 | 1.61E-01 |
| LAT | tumor-infiltrating lympho | 0.0675 | 2.07E-01 | 0.1582 | 3.00E-03 |
| LPXN | tumor-infiltrating lympho | -0.2089 | 8.06E-05 | -0.1624 | 2.31E-03 |
| MFNG | tumor-infiltrating lympho | -0.0604 | 2.59E-01 | 0.0085 | 8.74E-01 |
| MS4A6A | tumor-infiltrating lympho | -0.1529 | 4.08E-03 | -0.0856 | 1.10E-01 |
| NCKAP1L | tumor-infiltrating lympho | -0.1033 | 5.32E-02 | -0.0136 | 8.00E-01 |
| PAX5 | tumor-infiltrating lympho | -0.0388 | 4.69E-01 | -0.0157 | 7.69E-01 |
| PTPRCAP | tumor-infiltrating lympho | -0.0611 | 2.54E-01 | 0.0216 | 6.88E-01 |
| TARP | tumor-infiltrating lympho | -0.1938 | 2.60E-04 | -0.1473 | 5.75E-03 |
| TCL1A | tumor-infiltrating lympho | -0.0173 | 7.47E-01 | 0.0380 | 4.79E-01 |
| C1orf38 | tumor-infiltrating lympho | -0.1210 | 2.34E-02 | -0.0396 | 4.60E-01 |
| CD247 | tumor-infiltrating lympho | -0.1030 | 5.38E-02 | -0.0277 | 6.05E-01 |
| CD38 | tumor-infiltrating lympho | -0.0243 | 6.50E-01 | 0.0585 | 2.75E-01 |
| CD53 | tumor-infiltrating lympho | -0.1088 | 4.16E-02 | -0.0165 | 7.58E-01 |
| CFHR1 | tumor-infiltrating lympho | -0.0986 | 6.49E-02 | -0.1080 | 4.35E-02 |
| CSF2RB | tumor-infiltrating lympho | -0.1148 | 3.16E-02 | -0.0330 | 5.38E-01 |

| | | | | | |
|---|---|---|---|---|---|
| FCRL3 | tumor-infiltrating lympho | 0.0050 | 9.26E-01 | 0.0697 | 1.93E-01 |
| GLYR1 | tumor-infiltrating lympho | -0.1165 | 2.91E-02 | -0.1210 | 2.36E-02 |
| ICOS | tumor-infiltrating lympho | 0.0203 | 7.05E-01 | 0.1211 | 2.35E-02 |
| IFFO1 | tumor-infiltrating lympho | 0.0321 | 5.49E-01 | 0.0419 | 4.35E-01 |
| IL10RA | tumor-infiltrating lympho | -0.1408 | 8.25E-03 | -0.0724 | 1.76E-01 |
| IL2RB | tumor-infiltrating lympho | -0.1291 | 1.55E-02 | -0.0558 | 2.98E-01 |
| KLHL6 | tumor-infiltrating lympho | -0.1347 | 1.15E-02 | -0.0599 | 2.64E-01 |
| LCK | tumor-infiltrating lympho | -0.0842 | 1.15E-01 | 0.0036 | 9.47E-01 |
| LY9 | tumor-infiltrating lympho | -0.0723 | 1.77E-01 | 0.0149 | 7.82E-01 |
| PLEK | tumor-infiltrating lympho | -0.0666 | 2.13E-01 | 0.0275 | 6.08E-01 |
| TBC1D10C | tumor-infiltrating lympho | -0.0428 | 4.24E-01 | 0.0312 | 5.61E-01 |
| CD3G | tumor-infiltrating lympho | -0.0688 | 1.99E-01 | 0.0126 | 8.14E-01 |
| CD40 | tumor-infiltrating lympho | -0.1267 | 1.76E-02 | -0.0933 | 8.14E-02 |
| CD79A | tumor-infiltrating lympho | -0.0195 | 7.15E-01 | 0.0482 | 3.69E-01 |
| CYBB | tumor-infiltrating lympho | -0.0917 | 8.61E-02 | -0.0033 | 9.51E-01 |
| FCRL5 | tumor-infiltrating lympho | -0.0174 | 7.45E-01 | 0.0582 | 2.78E-01 |
| LILRB1 | tumor-infiltrating lympho | -0.1398 | 8.73E-03 | -0.0703 | 1.90E-01 |
| PAG1 | tumor-infiltrating lympho | 0.0380 | 4.78E-01 | 0.0809 | 1.31E-01 |
| SIRPG | tumor-infiltrating lympho | 0.0218 | 6.84E-01 | 0.1109 | 3.82E-02 |
| SLAMF1 | tumor-infiltrating lympho | -0.1092 | 4.10E-02 | -0.0322 | 5.49E-01 |
| TBX21 | tumor-infiltrating lympho | -0.1340 | 1.20E-02 | -0.0763 | 1.54E-01 |
| VAMP5 | tumor-infiltrating lympho | -0.2166 | 4.26E-05 | -0.2004 | 1.61E-04 |
| CCL5 | tumor-infiltrating lympho | -0.1204 | 2.41E-02 | -0.0516 | 3.35E-01 |
| CD27 | tumor-infiltrating lympho | -0.0050 | 9.26E-01 | 0.0874 | 1.03E-01 |
| CD3D | tumor-infiltrating lympho | 0.0226 | 6.73E-01 | 0.1133 | 3.41E-02 |
| CD86 | tumor-infiltrating lympho | -0.1016 | 5.73E-02 | -0.0127 | 8.12E-01 |
| CD8A | tumor-infiltrating lympho | -0.0993 | 6.32E-02 | -0.0288 | 5.91E-01 |
| CLEC2D | tumor-infiltrating lympho | 0.1773 | 8.48E-04 | 0.2265 | 1.88E-05 |
| CST7 | tumor-infiltrating lympho | -0.1461 | 6.11E-03 | -0.0847 | 1.14E-01 |

| | | | | | |
|---|---|---|---|---|---|
| HCST | tumor-infiltrating lympho | -0.0941 | 7.83E-02 | -0.0161 | 7.64E-01 |
| KLRK1 | tumor-infiltrating lympho | -0.1338 | 1.21E-02 | -0.0723 | 1.77E-01 |
| NCF4 | tumor-infiltrating lympho | -0.0373 | 4.86E-01 | 0.0654 | 2.22E-01 |
| SIT1 | tumor-infiltrating lympho | -0.0112 | 8.34E-01 | 0.0910 | 8.92E-02 |
| XCL1 | tumor-infiltrating lympho | 0.0367 | 4.93E-01 | 0.0999 | 6.19E-02 |
| XCL2 | tumor-infiltrating lympho | -0.0189 | 7.24E-01 | 0.0475 | 3.75E-01 |
| DOK2 | tumor-infiltrating lympho | -0.0807 | 1.31E-01 | 0.0150 | 7.79E-01 |
| F5 | tumor-infiltrating lympho | -0.0327 | 5.42E-01 | -0.0632 | 2.38E-01 |
| MGC29506 | tumor-infiltrating lympho | -0.0263 | 6.23E-01 | 0.0498 | 3.53E-01 |
| TIGIT | tumor-infiltrating lympho | -0.0336 | 5.30E-01 | 0.0568 | 2.89E-01 |
| TNFRSF4 | tumor-infiltrating lympho | 0.1376 | 9.87E-03 | 0.1928 | 2.84E-04 |
| SPNS1 | tumor-infiltrating lympho | 0.1269 | 1.74E-02 | 0.1473 | 5.76E-03 |
| CEP55 | cancer-testis antigen ge | 0.3634 | 2.14E-12 | 0.3928 | 2.33E-14 |
| KIF2C | cancer-testis antigen ge | 0.4323 | 2.04E-17 | 0.4499 | 7.61E-19 |
| OIP5 | cancer-testis antigen ge | 0.3761 | 3.07E-13 | 0.3830 | 1.12E-13 |
| TTK | cancer-testis antigen ge | 0.4439 | 2.24E-18 | 0.4540 | 3.38E-19 |
| CASC5 | cancer-testis antigen ge | 0.1124 | 3.52E-02 | 0.1198 | 2.50E-02 |
| NUF2 | cancer-testis antigen ge | 0.5126 | 6.60E-25 | 0.5176 | 2.25E-25 |
| PBK | cancer-testis antigen ge | 0.3455 | 2.81E-11 | 0.3510 | 1.38E-11 |
| FANCA | cancer-testis antigen ge | 0.2039 | 1.20E-04 | 0.2270 | 1.80E-05 |
| KIF20B | cancer-testis antigen ge | 0.3000 | 9.84E-09 | 0.3051 | 5.66E-09 |
| ATAD2 | cancer-testis antigen ge | 0.3250 | 4.43E-10 | 0.3219 | 6.98E-10 |
| MAGEA6 | cancer-testis antigen ge | -0.0395 | 4.61E-01 | -0.0012 | 9.82E-01 |
| MAGEA3 | cancer-testis antigen ge | -0.0409 | 4.45E-01 | -0.0065 | 9.04E-01 |
| ZNF165 | cancer-testis antigen ge | 0.2083 | 8.42E-05 | 0.2085 | 8.52E-05 |
| ACTL8 | cancer-testis antigen ge | 0.0686 | 2.00E-01 | 0.0722 | 1.78E-01 |
| GPATCH2 | cancer-testis antigen ge | 0.3214 | 7.08E-10 | 0.3158 | 1.52E-09 |
| MAGEA1 | cancer-testis antigen ge | -0.0410 | 4.44E-01 | -0.0157 | 7.69E-01 |
| MAGEA12 | cancer-testis antigen ge | -0.0002 | 9.96E-01 | 0.0302 | 5.74E-01 |

| | | | | | |
|---|---|---|---|---|---|
| MAGEA2 | cancer-testis antigen ge | 0.0052 | 9.22E-01 | 0.0322 | 5.48E-01 |
| MAGEA4 | cancer-testis antigen ge | 0.1820 | 6.12E-04 | 0.1977 | 1.97E-04 |
| PRAME | cancer-testis antigen ge | 0.1907 | 3.26E-04 | 0.2034 | 1.27E-04 |
| CSAG1 | cancer-testis antigen ge | 0.0125 | 8.16E-01 | 0.0397 | 4.59E-01 |
| MAGEC1 | cancer-testis antigen ge | 0.0737 | 1.68E-01 | 0.0944 | 7.78E-02 |
| MAGEC2 | cancer-testis antigen ge | 0.0597 | 2.65E-01 | 0.0869 | 1.05E-01 |
| XAGE1D | cancer-testis antigen ge | 0.0442 | 4.09E-01 | 0.0752 | 1.60E-01 |
| CAGE1 | cancer-testis antigen ge | 0.1816 | 6.28E-04 | 0.1817 | 6.35E-04 |
| CSAG2 | cancer-testis antigen ge | 0.0554 | 3.01E-01 | 0.0887 | 9.77E-02 |
| CTAG1B | cancer-testis antigen ge | 0.0662 | 2.16E-01 | 0.1013 | 5.82E-02 |
| GAGE12D | cancer-testis antigen ge | 0.1601 | 2.63E-03 | 0.1874 | 4.23E-04 |
| GAGE4 | cancer-testis antigen ge | 0.1111 | 3.75E-02 | 0.1452 | 6.49E-03 |
| IMP3 | cancer-testis antigen ge | -0.0984 | 6.55E-02 | -0.1104 | 3.90E-02 |
| MAGEA9B | cancer-testis antigen ge | 0.1581 | 2.97E-03 | 0.1781 | 8.17E-04 |
| MAGEB6 | cancer-testis antigen ge | 0.0403 | 4.52E-01 | 0.0536 | 3.17E-01 |
| ODF2 | cancer-testis antigen ge | 0.1962 | 2.16E-04 | 0.2331 | 1.05E-05 |
| PIWIL2 | cancer-testis antigen ge | -0.1147 | 3.17E-02 | -0.1071 | 4.52E-02 |
| POTEE | cancer-testis antigen ge | -0.0434 | 4.18E-01 | -0.0169 | 7.53E-01 |
| SLCO6A1 | cancer-testis antigen ge | 0.0644 | 2.28E-01 | 0.0923 | 8.47E-02 |
| SSX1 | cancer-testis antigen ge | 0.0185 | 7.30E-01 | 0.0482 | 3.68E-01 |
| SSX2 | cancer-testis antigen ge | 0.0485 | 3.65E-01 | 0.0759 | 1.56E-01 |
| SSX6 | cancer-testis antigen ge | 0.0567 | 2.89E-01 | 0.0801 | 1.35E-01 |
| ARX | cancer-testis antigen ge | 0.0275 | 6.07E-01 | 0.0492 | 3.59E-01 |
| BAGE | cancer-testis antigen ge | 0.0165 | 7.58E-01 | 0.0376 | 4.84E-01 |
| CTAG2 | cancer-testis antigen ge | 0.0686 | 2.00E-01 | 0.1038 | 5.23E-02 |
| DDX53 | cancer-testis antigen ge | -0.0090 | 8.66E-01 | 0.0060 | 9.11E-01 |
| DSCR8 | cancer-testis antigen ge | 0.0026 | 9.61E-01 | 0.0187 | 7.27E-01 |
| GAGE12J | cancer-testis antigen ge | 0.1331 | 1.26E-02 | 0.1620 | 2.37E-03 |
| GAGE2A | cancer-testis antigen ge | 0.1633 | 2.15E-03 | 0.1854 | 4.88E-04 |

| | | | | | |
|---|---|---|---|---|---|
| HORMAD1 | cancer-testis antigen ge | 0.0808 | 1.31E-01 | 0.1149 | 3.17E-02 |
| HORMAD2 | cancer-testis antigen ge | -0.1827 | 5.84E-04 | -0.1854 | 4.90E-04 |
| IGSF11 | cancer-testis antigen ge | 0.0191 | 7.22E-01 | 0.0575 | 2.83E-01 |
| KDM5B | cancer-testis antigen ge | 0.2376 | 6.82E-06 | 0.2419 | 4.71E-06 |
| MAGEA11 | cancer-testis antigen ge | 0.0635 | 2.35E-01 | 0.0991 | 6.40E-02 |
| MAGEA8 | cancer-testis antigen ge | 0.1081 | 4.30E-02 | 0.1282 | 1.64E-02 |
| PAGE1 | cancer-testis antigen ge | 0.1173 | 2.80E-02 | 0.1315 | 1.38E-02 |
| PAGE5 | cancer-testis antigen ge | -0.0686 | 2.00E-01 | -0.0428 | 4.24E-01 |
| SSX4 | cancer-testis antigen ge | 0.0667 | 2.12E-01 | 0.0938 | 7.97E-02 |
| TPTE | cancer-testis antigen ge | 0.0709 | 1.85E-01 | 0.0847 | 1.14E-01 |
| BAGE2 | cancer-testis antigen ge | 0.0375 | 4.84E-01 | 0.0439 | 4.13E-01 |
| CALR3 | cancer-testis antigen ge | 0.0988 | 6.45E-02 | 0.0851 | 1.12E-01 |
| DCAF12 | cancer-testis antigen ge | 0.1115 | 3.68E-02 | 0.1126 | 3.53E-02 |
| FAM133A | cancer-testis antigen ge | 0.1069 | 4.53E-02 | 0.1529 | 4.14E-03 |
| MAGEA5 | cancer-testis antigen ge | 0.2001 | 1.60E-04 | 0.1979 | 1.95E-04 |
| MAGEB2 | cancer-testis antigen ge | 0.0110 | 8.37E-01 | 0.0346 | 5.19E-01 |
| RQCD1 | cancer-testis antigen ge | 0.1801 | 7.00E-04 | 0.2066 | 9.91E-05 |
| SPA17 | cancer-testis antigen ge | 0.1985 | 1.82E-04 | 0.2116 | 6.64E-05 |
| SSX5 | cancer-testis antigen ge | 0.0303 | 5.71E-01 | 0.0598 | 2.65E-01 |
| TEX15 | cancer-testis antigen ge | 0.0034 | 9.50E-01 | 0.0194 | 7.18E-01 |
| ACRBP | cancer-testis antigen ge | 0.0130 | 8.08E-01 | 0.0433 | 4.19E-01 |
| ANKRD45 | cancer-testis antigen ge | 0.0743 | 1.65E-01 | 0.0975 | 6.84E-02 |
| CABYR | cancer-testis antigen ge | 0.2820 | 7.70E-08 | 0.2930 | 2.34E-08 |
| COX6B2 | cancer-testis antigen ge | 0.0723 | 1.77E-01 | 0.0955 | 7.43E-02 |
| CXorf48 | cancer-testis antigen ge | 0.1454 | 6.34E-03 | 0.1719 | 1.25E-03 |
| FATE1 | cancer-testis antigen ge | 0.1276 | 1.67E-02 | 0.1223 | 2.21E-02 |
| KIAA0100 | cancer-testis antigen ge | 0.2157 | 4.61E-05 | 0.2348 | 9.07E-06 |
| LY6K | cancer-testis antigen ge | 0.0061 | 9.10E-01 | 0.0034 | 9.50E-01 |
| NR6A1 | cancer-testis antigen ge | 0.1811 | 6.54E-04 | 0.1820 | 6.22E-04 |

| | | | | | |
|---|---|---|---|---|---|
| NXF2 | cancer-testis antigen ge | 0.0253 | 6.36E-01 | 0.0441 | 4.11E-01 |
| OTOA | cancer-testis antigen ge | -0.1843 | 5.20E-04 | -0.1471 | 5.81E-03 |
| PAGE2 | cancer-testis antigen ge | 0.0937 | 7.95E-02 | 0.1178 | 2.76E-02 |
| PAGE2B | cancer-testis antigen ge | 0.0378 | 4.81E-01 | 0.0563 | 2.94E-01 |
| PLAC1 | cancer-testis antigen ge | 0.1768 | 8.78E-04 | 0.2250 | 2.14E-05 |
| SPAG4 | cancer-testis antigen ge | -0.0326 | 5.42E-01 | -0.0175 | 7.44E-01 |
| SSX3 | cancer-testis antigen ge | 0.0492 | 3.58E-01 | 0.0809 | 1.31E-01 |
| TAF7L | cancer-testis antigen ge | -0.0821 | 1.25E-01 | -0.0921 | 8.55E-02 |
| TMEFF1 | cancer-testis antigen ge | 0.1395 | 8.89E-03 | 0.1676 | 1.65E-03 |
| TULP2 | cancer-testis antigen ge | 0.0675 | 2.07E-01 | 0.0732 | 1.72E-01 |
| XAGE5 | cancer-testis antigen ge | 0.0318 | 5.53E-01 | 0.0606 | 2.58E-01 |
| CCDC110 | cancer-testis antigen ge | 0.0343 | 5.22E-01 | 0.0407 | 4.48E-01 |
| CCDC62 | cancer-testis antigen ge | 0.1209 | 2.35E-02 | 0.1455 | 6.38E-03 |
| CEP290 | cancer-testis antigen ge | 0.2194 | 3.37E-05 | 0.2262 | 1.93E-05 |
| CTCFL | cancer-testis antigen ge | 0.0701 | 1.90E-01 | 0.0593 | 2.68E-01 |
| CTNNA2 | cancer-testis antigen ge | -0.0179 | 7.39E-01 | -0.0402 | 4.53E-01 |
| DKKL1 | cancer-testis antigen ge | 0.1356 | 1.10E-02 | 0.1472 | 5.80E-03 |
| FBXO39 | cancer-testis antigen ge | -0.0880 | 9.99E-02 | -0.0680 | 2.04E-01 |
| LYPD6B | cancer-testis antigen ge | 0.1481 | 5.43E-03 | 0.1805 | 6.94E-04 |
| MAEL | cancer-testis antigen ge | 0.1675 | 1.64E-03 | 0.1750 | 1.01E-03 |
| MAGEB1 | cancer-testis antigen ge | 0.0339 | 5.27E-01 | 0.0554 | 3.02E-01 |
| NOL4 | cancer-testis antigen ge | -0.1038 | 5.20E-02 | -0.0922 | 8.50E-02 |
| PAGE4 | cancer-testis antigen ge | -0.1301 | 1.47E-02 | -0.1431 | 7.33E-03 |
| PIWIL1 | cancer-testis antigen ge | 0.0686 | 2.00E-01 | 0.0809 | 1.31E-01 |
| RBM46 | cancer-testis antigen ge | 0.0252 | 6.37E-01 | 0.0237 | 6.58E-01 |
| SPAG1 | cancer-testis antigen ge | 0.0598 | 2.64E-01 | 0.0819 | 1.26E-01 |
| TEKT5 | cancer-testis antigen ge | -0.0109 | 8.39E-01 | -0.0208 | 6.98E-01 |
| TEX14 | cancer-testis antigen ge | 0.1750 | 9.92E-04 | 0.1681 | 1.59E-03 |
| TMEFF2 | cancer-testis antigen ge | -0.0655 | 2.21E-01 | -0.0663 | 2.16E-01 |

| | | | | | |
|---|---|---|---|---|---|
| TSSK6 | cancer-testis antigen ge | 0.0131 | 8.07E-01 | -0.0111 | 8.36E-01 |
| AKAP3 | cancer-testis antigen ge | -0.1961 | 2.19E-04 | -0.2005 | 1.59E-04 |
| ARMC3 | cancer-testis antigen ge | 0.1995 | 1.68E-04 | 0.2081 | 8.80E-05 |
| CCNA1 | cancer-testis antigen ge | -0.0115 | 8.30E-01 | 0.0158 | 7.69E-01 |
| CTAGE1 | cancer-testis antigen ge | -0.1644 | 2.00E-03 | -0.1790 | 7.66E-04 |
| CTAGE5 | cancer-testis antigen ge | -0.2618 | 6.56E-07 | -0.2975 | 1.38E-08 |
| ELOVL4 | cancer-testis antigen ge | 0.0130 | 8.08E-01 | 0.0592 | 2.70E-01 |
| HSPB9 | cancer-testis antigen ge | 0.0331 | 5.36E-01 | 0.0092 | 8.63E-01 |
| LDHC | cancer-testis antigen ge | 0.0125 | 8.16E-01 | 0.0043 | 9.35E-01 |
| PRSS50 | cancer-testis antigen ge | 0.1578 | 3.02E-03 | 0.1386 | 9.42E-03 |
| ROPN1 | cancer-testis antigen ge | 0.0279 | 6.02E-01 | 0.0134 | 8.03E-01 |
| SPAG6 | cancer-testis antigen ge | 0.2224 | 2.61E-05 | 0.2262 | 1.94E-05 |
| SPAG9 | cancer-testis antigen ge | 0.0347 | 5.17E-01 | 0.0434 | 4.18E-01 |
| SYCE1 | cancer-testis antigen ge | 0.0104 | 8.47E-01 | 0.0308 | 5.66E-01 |
| TEX101 | cancer-testis antigen ge | -0.0264 | 6.22E-01 | -0.0029 | 9.58E-01 |
| TFDP3 | cancer-testis antigen ge | -0.0587 | 2.73E-01 | -0.0473 | 3.78E-01 |
| TPPP2 | cancer-testis antigen ge | -0.3604 | 3.33E-12 | -0.3769 | 2.93E-13 |
| TSGA10 | cancer-testis antigen ge | 0.0403 | 4.52E-01 | 0.0486 | 3.65E-01 |
| C21orf99 | cancer-testis antigen ge | 0.0724 | 1.76E-01 | 0.1055 | 4.85E-02 |
| CCDC36 | cancer-testis antigen ge | 0.0523 | 3.28E-01 | 0.0837 | 1.18E-01 |
| CRISP2 | cancer-testis antigen ge | 0.0599 | 2.63E-01 | 0.0903 | 9.16E-02 |
| CT47B1 | cancer-testis antigen ge | 0.0312 | 5.60E-01 | 0.0530 | 3.23E-01 |
| FMR1NB | cancer-testis antigen ge | 0.0617 | 2.49E-01 | 0.0669 | 2.12E-01 |
| IL13RA2 | cancer-testis antigen ge | -0.0023 | 9.65E-01 | 0.0410 | 4.44E-01 |
| MAGEC3 | cancer-testis antigen ge | 0.1431 | 7.27E-03 | 0.1447 | 6.68E-03 |
| MORC1 | cancer-testis antigen ge | -0.0006 | 9.92E-01 | 0.0063 | 9.06E-01 |
| RGS22 | cancer-testis antigen ge | -0.0546 | 3.07E-01 | -0.0426 | 4.26E-01 |
| SPAG17 | cancer-testis antigen ge | 0.0832 | 1.20E-01 | 0.1151 | 3.13E-02 |
| SPAG8 | cancer-testis antigen ge | 0.1661 | 1.79E-03 | 0.1722 | 1.22E-03 |

| | | | | | |
|---|---|---|---|---|---|
| SPEF2 | cancer-testis antigen ge | 0.3994 | 7.15E-15 | 0.3918 | 2.75E-14 |
| TDRD6 | cancer-testis antigen ge | -0.0666 | 2.13E-01 | -0.0548 | 3.07E-01 |
| TMEM108 | cancer-testis antigen ge | -0.0186 | 7.28E-01 | 0.0205 | 7.03E-01 |
| XAGE3 | cancer-testis antigen ge | -0.0255 | 6.34E-01 | -0.0210 | 6.96E-01 |
| DDX43 | cancer-testis antigen ge | -0.0059 | 9.12E-01 | 0.0188 | 7.27E-01 |
| GPAT2 | cancer-testis antigen ge | -0.0720 | 1.79E-01 | -0.0485 | 3.66E-01 |
| HEMGN | cancer-testis antigen ge | -0.1278 | 1.66E-02 | -0.1072 | 4.51E-02 |
| NLRP4 | cancer-testis antigen ge | 0.0237 | 6.59E-01 | 0.0610 | 2.55E-01 |
| PRSS55 | cancer-testis antigen ge | 0.1575 | 3.09E-03 | 0.1570 | 3.24E-03 |
| RNF17 | cancer-testis antigen ge | 0.1465 | 5.95E-03 | 0.1802 | 7.06E-04 |
| TDRD1 | cancer-testis antigen ge | 0.0407 | 4.47E-01 | 0.0618 | 2.49E-01 |
| HLA-DOA | HLA genes | -0.1057 | 4.78E-02 | -0.0259 | 6.29E-01 |
| HLA-DOB | HLA genes | 0.0488 | 3.62E-01 | 0.1353 | 1.13E-02 |
| HLA-DPB1 | HLA genes | -0.1093 | 4.07E-02 | -0.0323 | 5.47E-01 |
| HLA-DQA1 | HLA genes | -0.0808 | 1.31E-01 | -0.0047 | 9.30E-01 |
| HLA-DQB2 | HLA genes | 0.0010 | 9.85E-01 | 0.0884 | 9.86E-02 |
| HLA-J | HLA genes | 0.0583 | 2.76E-01 | 0.0975 | 6.86E-02 |
| HLA-A | HLA genes | -0.0877 | 1.01E-01 | -0.0417 | 4.37E-01 |
| HLA-DPA1 | HLA genes | -0.1399 | 8.66E-03 | -0.0697 | 1.93E-01 |
| HLA-DPB2 | HLA genes | -0.0360 | 5.02E-01 | 0.0350 | 5.13E-01 |
| HLA-DQA2 | HLA genes | -0.0006 | 9.91E-01 | 0.0772 | 1.49E-01 |
| HLA-DRA | HLA genes | -0.1168 | 2.87E-02 | -0.0461 | 3.90E-01 |
| HLA-E | HLA genes | -0.2333 | 1.00E-05 | -0.1968 | 2.12E-04 |
| HLA-H | HLA genes | -0.0638 | 2.33E-01 | -0.0172 | 7.48E-01 |
| HLA-B | HLA genes | -0.1148 | 3.15E-02 | -0.0678 | 2.06E-01 |
| HLA-DMB | HLA genes | -0.0692 | 1.96E-01 | 0.0169 | 7.53E-01 |
| HLA-DQB1 | HLA genes | -0.0946 | 7.67E-02 | -0.0230 | 6.67E-01 |
| HLA-DRB1 | HLA genes | -0.1109 | 3.79E-02 | -0.0458 | 3.93E-01 |
| HLA-F | HLA genes | -0.0670 | 2.11E-01 | -0.0194 | 7.17E-01 |

| | | | | | |
|---|---|---|---|---|---|
| HLA-C | HLA genes | -0.1371 | 1.01E-02 | -0.1001 | 6.14E-02 |
| HLA-DMA | HLA genes | -0.0695 | 1.94E-01 | -0.0043 | 9.35E-01 |
| HLA-DRB5 | HLA genes | -0.0813 | 1.29E-01 | -0.0249 | 6.42E-01 |
| HLA-G | HLA genes | -0.0998 | 6.19E-02 | -0.0682 | 2.03E-01 |
| HLA-L | HLA genes | -0.0499 | 3.51E-01 | -0.0081 | 8.79E-01 |
| HLA-DRB6 | HLA genes | -0.0602 | 2.61E-01 | 0.0117 | 8.28E-01 |
| TNFAIP8L3 | cytokine and cytokine re | 0.0670 | 2.10E-01 | 0.1001 | 6.14E-02 |
| CCL14 | cytokine and cytokine re | -0.3149 | 1.62E-09 | -0.3118 | 2.51E-09 |
| CX3CR1 | cytokine and cytokine re | -0.1435 | 7.08E-03 | -0.1080 | 4.34E-02 |
| CCL21 | cytokine and cytokine re | -0.0931 | 8.15E-02 | -0.0508 | 3.44E-01 |
| IL1R1 | cytokine and cytokine re | -0.2291 | 1.47E-05 | -0.2244 | 2.26E-05 |
| IL33 | cytokine and cytokine re | -0.2170 | 4.13E-05 | -0.1888 | 3.83E-04 |
| CCL19 | cytokine and cytokine re | -0.0462 | 3.89E-01 | 0.0081 | 8.80E-01 |
| CCR6 | cytokine and cytokine re | 0.1354 | 1.11E-02 | 0.1727 | 1.18E-03 |
| IL16 | cytokine and cytokine re | -0.1227 | 2.15E-02 | -0.0535 | 3.19E-01 |
| IL17D | cytokine and cytokine re | 0.0691 | 1.97E-01 | 0.0889 | 9.69E-02 |
| TGFB2 | cytokine and cytokine re | -0.0374 | 4.85E-01 | 0.0004 | 9.94E-01 |
| TGFBR2 | cytokine and cytokine re | -0.2787 | 1.11E-07 | -0.2633 | 5.83E-07 |
| BMP3 | cytokine and cytokine re | -0.0447 | 4.04E-01 | -0.0101 | 8.51E-01 |
| CXCL12 | cytokine and cytokine re | -0.1784 | 7.84E-04 | -0.1372 | 1.02E-02 |
| TNFSF8 | cytokine and cytokine re | -0.1765 | 8.96E-04 | -0.1221 | 2.23E-02 |
| BMPR2 | cytokine and cytokine re | -0.1177 | 2.75E-02 | -0.1026 | 5.52E-02 |
| IL7R | cytokine and cytokine re | -0.1851 | 4.91E-04 | -0.1372 | 1.02E-02 |
| ILK | cytokine and cytokine re | -0.0012 | 9.82E-01 | 0.0176 | 7.43E-01 |
| TGFB1I1 | cytokine and cytokine re | -0.0918 | 8.58E-02 | -0.0549 | 3.06E-01 |
| BMP5 | cytokine and cytokine re | -0.1467 | 5.91E-03 | -0.1139 | 3.31E-02 |
| BMP6 | cytokine and cytokine re | -0.1225 | 2.17E-02 | -0.0942 | 7.86E-02 |
| BMPER | cytokine and cytokine re | -0.1948 | 2.40E-04 | -0.1732 | 1.14E-03 |
| CCR4 | cytokine and cytokine re | -0.1389 | 9.19E-03 | -0.0863 | 1.07E-01 |

| | | | | | |
|---|---|---|---|---|---|
| CCR7 | cytokine and cytokine re | -0.0941 | 7.82E-02 | -0.0267 | 6.19E-01 |
| CX3CL1 | cytokine and cytokine re | -0.2139 | 5.35E-05 | -0.2003 | 1.62E-04 |
| IL11RA | cytokine and cytokine re | 0.2033 | 1.25E-04 | 0.1860 | 4.69E-04 |
| IL34 | cytokine and cytokine re | -0.0274 | 6.09E-01 | 0.0312 | 5.61E-01 |
| TGFB1 | cytokine and cytokine re | -0.0548 | 3.06E-01 | -0.0022 | 9.67E-01 |
| TGFB3 | cytokine and cytokine re | -0.1000 | 6.13E-02 | -0.0633 | 2.38E-01 |
| TGFBR3 | cytokine and cytokine re | -0.1884 | 3.86E-04 | -0.1860 | 4.70E-04 |
| TNFRSF19 | cytokine and cytokine re | -0.0027 | 9.60E-01 | -0.0098 | 8.55E-01 |
| TNFSF10 | cytokine and cytokine re | -0.0518 | 3.33E-01 | -0.0399 | 4.57E-01 |
| TNFSF14 | cytokine and cytokine re | 0.0940 | 7.87E-02 | 0.1128 | 3.49E-02 |
| BMP4 | cytokine and cytokine re | 0.0253 | 6.36E-01 | 0.0157 | 7.70E-01 |
| BMPR1B | cytokine and cytokine re | -0.0493 | 3.58E-01 | 0.0026 | 9.61E-01 |
| CCL16 | cytokine and cytokine re | -0.3167 | 1.28E-09 | -0.3310 | 2.16E-10 |
| CSF1 | cytokine and cytokine re | -0.1049 | 4.96E-02 | -0.0473 | 3.78E-01 |
| CSF1R | cytokine and cytokine re | -0.1458 | 6.20E-03 | -0.0746 | 1.64E-01 |
| CXCL14 | cytokine and cytokine re | -0.0705 | 1.88E-01 | -0.0252 | 6.39E-01 |
| IFNGR1 | cytokine and cytokine re | -0.1416 | 7.90E-03 | -0.1256 | 1.88E-02 |
| IL12B | cytokine and cytokine re | -0.0531 | 3.22E-01 | -0.0011 | 9.83E-01 |
| IL17RE | cytokine and cytokine re | 0.1561 | 3.37E-03 | 0.1390 | 9.20E-03 |
| IL28RA | cytokine and cytokine re | -0.0047 | 9.30E-01 | -0.0130 | 8.09E-01 |
| IL4R | cytokine and cytokine re | 0.0556 | 2.99E-01 | 0.0930 | 8.24E-02 |
| IL5RA | cytokine and cytokine re | -0.1301 | 1.47E-02 | -0.0837 | 1.18E-01 |
| IL6ST | cytokine and cytokine re | -0.1679 | 1.59E-03 | -0.1635 | 2.15E-03 |
| TNFSF12 | cytokine and cytokine re | -0.0232 | 6.65E-01 | 0.0208 | 6.98E-01 |
| BMP7 | cytokine and cytokine re | 0.0957 | 7.33E-02 | 0.1077 | 4.42E-02 |
| BMP8A | cytokine and cytokine re | 0.2117 | 6.42E-05 | 0.2402 | 5.51E-06 |
| CCL2 | cytokine and cytokine re | -0.1523 | 4.23E-03 | -0.0973 | 6.91E-02 |
| CCR2 | cytokine and cytokine re | -0.1156 | 3.03E-02 | -0.0455 | 3.96E-01 |
| CCR9 | cytokine and cytokine re | -0.0387 | 4.69E-01 | -0.0049 | 9.27E-01 |

| | | | | | |
|---|---|---|---|---|---|
| CSF3 | cytokine and cytokine re | -0.1143 | 3.23E-02 | -0.0948 | 7.64E-02 |
| CXCR5 | cytokine and cytokine re | 0.0022 | 9.67E-01 | 0.0718 | 1.80E-01 |
| IFNAR2 | cytokine and cytokine re | 0.0583 | 2.76E-01 | 0.0953 | 7.51E-02 |
| IL17B | cytokine and cytokine re | 0.0076 | 8.87E-01 | 0.0324 | 5.45E-01 |
| IL2RG | cytokine and cytokine re | -0.0033 | 9.51E-01 | 0.0984 | 6.60E-02 |
| IL31RA | cytokine and cytokine re | -0.0130 | 8.08E-01 | -0.0017 | 9.74E-01 |
| IL3RA | cytokine and cytokine re | -0.0822 | 1.24E-01 | -0.0468 | 3.83E-01 |
| IL6R | cytokine and cytokine re | 0.0118 | 8.26E-01 | -0.0076 | 8.88E-01 |
| TNFAIP3 | cytokine and cytokine re | 0.0293 | 5.84E-01 | 0.0688 | 1.99E-01 |
| TNFAIP8L2 | cytokine and cytokine re | -0.0450 | 4.01E-01 | 0.0465 | 3.86E-01 |
| TNFRSF11B | cytokine and cytokine re | 0.0436 | 4.15E-01 | 0.0936 | 8.03E-02 |
| CCL17 | cytokine and cytokine re | -0.0368 | 4.92E-01 | 0.0287 | 5.93E-01 |
| CCL22 | cytokine and cytokine re | -0.1249 | 1.92E-02 | -0.0660 | 2.18E-01 |
| CCL23 | cytokine and cytokine re | -0.1505 | 4.72E-03 | -0.1116 | 3.69E-02 |
| CCR5 | cytokine and cytokine re | -0.0790 | 1.40E-01 | 0.0165 | 7.58E-01 |
| CCRL2 | cytokine and cytokine re | -0.0480 | 3.70E-01 | 0.0284 | 5.96E-01 |
| CSF2RA | cytokine and cytokine re | -0.0144 | 7.88E-01 | 0.0631 | 2.39E-01 |
| CSF2RB | cytokine and cytokine re | -0.1148 | 3.16E-02 | -0.0330 | 5.38E-01 |
| CXCL2 | cytokine and cytokine re | -0.1421 | 7.65E-03 | -0.1198 | 2.50E-02 |
| CXCR2 | cytokine and cytokine re | -0.0767 | 1.51E-01 | -0.0375 | 4.84E-01 |
| CXCR4 | cytokine and cytokine re | -0.0025 | 9.62E-01 | 0.0847 | 1.14E-01 |
| IFNAR1 | cytokine and cytokine re | -0.2075 | 8.97E-05 | -0.2017 | 1.45E-04 |
| IL10RA | cytokine and cytokine re | -0.1408 | 8.25E-03 | -0.0724 | 1.76E-01 |
| IL10RB | cytokine and cytokine re | 0.0618 | 2.48E-01 | 0.0772 | 1.49E-01 |
| IL12A | cytokine and cytokine re | 0.2515 | 1.82E-06 | 0.3056 | 5.29E-09 |
| IL13RA1 | cytokine and cytokine re | -0.1626 | 2.25E-03 | -0.1514 | 4.53E-03 |
| IL17RC | cytokine and cytokine re | -0.1943 | 2.51E-04 | -0.1941 | 2.59E-04 |
| IL21R | cytokine and cytokine re | 0.0268 | 6.16E-01 | 0.1426 | 7.53E-03 |
| IL24 | cytokine and cytokine re | -0.0667 | 2.13E-01 | -0.0245 | 6.48E-01 |

| | | | | | |
|---|---|---|---|---|---|
| IL2RB | cytokine and cytokine re | -0.1291 | 1.55E-02 | -0.0558 | 2.98E-01 |
| IL6 | cytokine and cytokine re | -0.1381 | 9.58E-03 | -0.0852 | 1.12E-01 |
| ILDR1 | cytokine and cytokine re | 0.1737 | 1.09E-03 | 0.1919 | 3.06E-04 |
| TGFA | cytokine and cytokine re | 0.1087 | 4.18E-02 | 0.1389 | 9.27E-03 |
| TGFBR1 | cytokine and cytokine re | 0.0739 | 1.67E-01 | 0.1164 | 2.95E-02 |
| TGFBRAP1 | cytokine and cytokine re | -0.0115 | 8.30E-01 | -0.0079 | 8.82E-01 |
| TNFRSF10D | cytokine and cytokine re | -0.0493 | 3.58E-01 | -0.0566 | 2.91E-01 |
| TNFRSF13B | cytokine and cytokine re | 0.0333 | 5.34E-01 | 0.1043 | 5.12E-02 |
| TNFRSF21 | cytokine and cytokine re | 0.0215 | 6.88E-01 | 0.0535 | 3.18E-01 |
| TNFSF13 | cytokine and cytokine re | -0.1781 | 8.01E-04 | -0.1350 | 1.15E-02 |
| TNFSF15 | cytokine and cytokine re | 0.0261 | 6.25E-01 | 0.0663 | 2.16E-01 |
| BMPR1A | cytokine and cytokine re | 0.1024 | 5.52E-02 | 0.0933 | 8.12E-02 |
| CCL3L3 | cytokine and cytokine re | 0.0157 | 7.70E-01 | 0.0394 | 4.63E-01 |
| CCR1 | cytokine and cytokine re | -0.1671 | 1.68E-03 | -0.1049 | 4.99E-02 |
| CCR10 | cytokine and cytokine re | 0.0607 | 2.57E-01 | 0.0883 | 9.92E-02 |
| CCRN4L | cytokine and cytokine re | -0.0787 | 1.41E-01 | -0.0544 | 3.10E-01 |
| CSF3R | cytokine and cytokine re | -0.0577 | 2.81E-01 | 0.0256 | 6.33E-01 |
| CXCL6 | cytokine and cytokine re | -0.0638 | 2.33E-01 | -0.0195 | 7.17E-01 |
| CXCR2P1 | cytokine and cytokine re | -0.0799 | 1.35E-01 | -0.0191 | 7.22E-01 |
| CXCR6 | cytokine and cytokine re | -0.0798 | 1.36E-01 | 0.0037 | 9.45E-01 |
| EPOR | cytokine and cytokine re | 0.1250 | 1.91E-02 | 0.1240 | 2.03E-02 |
| IL10 | cytokine and cytokine re | -0.1173 | 2.79E-02 | -0.0503 | 3.48E-01 |
| IL13RA2 | cytokine and cytokine re | -0.0023 | 9.65E-01 | 0.0410 | 4.44E-01 |
| IL17RA | cytokine and cytokine re | -0.0121 | 8.21E-01 | 0.0106 | 8.43E-01 |
| IL17RD | cytokine and cytokine re | 0.0197 | 7.13E-01 | 0.0630 | 2.40E-01 |
| IL17REL | cytokine and cytokine re | 0.0873 | 1.02E-01 | 0.1281 | 1.65E-02 |
| IL18 | cytokine and cytokine re | -0.1213 | 2.30E-02 | -0.0458 | 3.93E-01 |
| IL18BP | cytokine and cytokine re | -0.0831 | 1.20E-01 | -0.0117 | 8.28E-01 |
| IL1R2 | cytokine and cytokine re | -0.0042 | 9.38E-01 | 0.0050 | 9.26E-01 |

| | | | | | |
|---|---|---|---|---|---|
| IL1RL1 | cytokine and cytokine re | -0.2771 | 1.32E-07 | -0.2502 | 2.14E-06 |
| IL20 | cytokine and cytokine re | -0.0402 | 4.53E-01 | -0.0190 | 7.23E-01 |
| IL20RA | cytokine and cytokine re | 0.1167 | 2.88E-02 | 0.1418 | 7.88E-03 |
| IL23R | cytokine and cytokine re | -0.0093 | 8.62E-01 | 0.0154 | 7.74E-01 |
| IL7 | cytokine and cytokine re | -0.1180 | 2.70E-02 | -0.0811 | 1.30E-01 |
| TGFBI | cytokine and cytokine re | -0.0714 | 1.82E-01 | -0.0848 | 1.13E-01 |
| TNF | cytokine and cytokine re | -0.0444 | 4.07E-01 | 0.0298 | 5.78E-01 |
| TNFAIP8 | cytokine and cytokine re | -0.0053 | 9.22E-01 | 0.0848 | 1.13E-01 |
| TNFRSF10B | cytokine and cytokine re | -0.0753 | 1.59E-01 | -0.0703 | 1.90E-01 |
| TNFRSF11A | cytokine and cytokine re | 0.0905 | 9.04E-02 | 0.1342 | 1.20E-02 |
| TNFRSF14 | cytokine and cytokine re | -0.0725 | 1.76E-01 | -0.0385 | 4.73E-01 |
| TNFRSF1B | cytokine and cytokine re | -0.1330 | 1.26E-02 | -0.0795 | 1.38E-01 |
| TNFRSF25 | cytokine and cytokine re | 0.1293 | 1.53E-02 | 0.1654 | 1.90E-03 |
| TNFSF11 | cytokine and cytokine re | -0.0568 | 2.89E-01 | -0.0487 | 3.64E-01 |
| BMP2K | cytokine and cytokine re | -0.1504 | 4.76E-03 | -0.1142 | 3.27E-02 |
| CCL11 | cytokine and cytokine re | -0.0550 | 3.04E-01 | -0.0017 | 9.75E-01 |
| CCL13 | cytokine and cytokine re | -0.0874 | 1.02E-01 | -0.0245 | 6.47E-01 |
| CCL14-CCL15 | cytokine and cytokine re | -0.1260 | 1.82E-02 | -0.1376 | 9.96E-03 |
| CCL15 | cytokine and cytokine re | 0.0382 | 4.76E-01 | 0.0113 | 8.34E-01 |
| CCL5 | cytokine and cytokine re | -0.1204 | 2.41E-02 | -0.0516 | 3.35E-01 |
| CXCL1 | cytokine and cytokine re | 0.0470 | 3.80E-01 | 0.0949 | 7.62E-02 |
| CXCL3 | cytokine and cytokine re | 0.0707 | 1.86E-01 | 0.1197 | 2.51E-02 |
| CXCL5 | cytokine and cytokine re | 0.0640 | 2.32E-01 | 0.1056 | 4.85E-02 |
| CXCL9 | cytokine and cytokine re | -0.0535 | 3.18E-01 | 0.0078 | 8.84E-01 |
| CXCR3 | cytokine and cytokine re | -0.0428 | 4.24E-01 | 0.0464 | 3.86E-01 |
| IL18RAP | cytokine and cytokine re | -0.1015 | 5.75E-02 | -0.0352 | 5.11E-01 |
| IL1B | cytokine and cytokine re | -0.0218 | 6.84E-01 | 0.0624 | 2.44E-01 |
| IL1RAP | cytokine and cytokine re | -0.0066 | 9.02E-01 | 0.0018 | 9.74E-01 |
| IL1RAPL1 | cytokine and cytokine re | 0.0324 | 5.45E-01 | 0.0453 | 3.98E-01 |

| | | | | | |
|---|---|---|---|---|---|
| IL1RAPL2 | cytokine and cytokine re | -0.1245 | 1.96E-02 | -0.1179 | 2.75E-02 |
| IL1RL2 | cytokine and cytokine re | -0.0603 | 2.60E-01 | -0.0405 | 4.51E-01 |
| IL20RB | cytokine and cytokine re | 0.1131 | 3.41E-02 | 0.1357 | 1.10E-02 |
| IL22RA1 | cytokine and cytokine re | 0.1442 | 6.80E-03 | 0.1438 | 7.04E-03 |
| IL22RA2 | cytokine and cytokine re | 0.0271 | 6.13E-01 | 0.0700 | 1.91E-01 |
| IL26 | cytokine and cytokine re | 0.0385 | 4.72E-01 | 0.0829 | 1.21E-01 |
| IL27RA | cytokine and cytokine re | 0.1167 | 2.89E-02 | 0.1818 | 6.32E-04 |
| IL9R | cytokine and cytokine re | 0.0227 | 6.72E-01 | 0.0681 | 2.04E-01 |
| TNFRSF10A | cytokine and cytokine re | -0.1087 | 4.19E-02 | -0.0917 | 8.65E-02 |
| TNFRSF10C | cytokine and cytokine re | -0.0303 | 5.72E-01 | -0.0079 | 8.83E-01 |
| TNFRSF13C | cytokine and cytokine re | 0.1302 | 1.46E-02 | 0.1955 | 2.34E-04 |
| TNFRSF1A | cytokine and cytokine re | -0.1330 | 1.26E-02 | -0.1167 | 2.90E-02 |
| XCL1 | cytokine and cytokine re | 0.0367 | 4.93E-01 | 0.0999 | 6.19E-02 |
| XCL2 | cytokine and cytokine re | -0.0189 | 7.24E-01 | 0.0475 | 3.75E-01 |
| BMP1 | cytokine and cytokine re | -0.2029 | 1.30E-04 | -0.2065 | 9.93E-05 |
| BMP10 | cytokine and cytokine re | -0.1357 | 1.09E-02 | -0.1158 | 3.03E-02 |
| BMP2 | cytokine and cytokine re | -0.0325 | 5.44E-01 | -0.0180 | 7.37E-01 |
| CCL24 | cytokine and cytokine re | -0.0808 | 1.31E-01 | -0.0496 | 3.55E-01 |
| CCL26 | cytokine and cytokine re | 0.1730 | 1.14E-03 | 0.2150 | 5.01E-05 |
| CCL28 | cytokine and cytokine re | 0.0723 | 1.76E-01 | 0.0801 | 1.35E-01 |
| CCL4 | cytokine and cytokine re | -0.1020 | 5.61E-02 | -0.0259 | 6.29E-01 |
| CCL8 | cytokine and cytokine re | -0.0725 | 1.75E-01 | -0.0248 | 6.44E-01 |
| CSF2 | cytokine and cytokine re | 0.0846 | 1.14E-01 | 0.1266 | 1.78E-02 |
| CXCL10 | cytokine and cytokine re | -0.0535 | 3.18E-01 | -0.0068 | 9.00E-01 |
| CXCL11 | cytokine and cytokine re | -0.0706 | 1.87E-01 | -0.0210 | 6.96E-01 |
| CXCL13 | cytokine and cytokine re | 0.0582 | 2.77E-01 | 0.1117 | 3.68E-02 |
| CXCL16 | cytokine and cytokine re | 0.0088 | 8.69E-01 | 0.0309 | 5.64E-01 |
| CXCL17 | cytokine and cytokine re | 0.0627 | 2.41E-01 | 0.0752 | 1.61E-01 |
| IL12RB1 | cytokine and cytokine re | -0.0708 | 1.86E-01 | 0.0233 | 6.63E-01 |

| | | | | | |
|---|---|---|---|---|---|
| IL17RB | cytokine and cytokine re | 0.1917 | 3.05E-04 | 0.1845 | 5.21E-04 |
| IL18R1 | cytokine and cytokine re | -0.1460 | 6.15E-03 | -0.1029 | 5.44E-02 |
| IL1F9 | cytokine and cytokine re | 0.1106 | 3.84E-02 | 0.1037 | 5.26E-02 |
| IL2 | cytokine and cytokine re | 0.0258 | 6.30E-01 | 0.0567 | 2.90E-01 |
| IL23A | cytokine and cytokine re | -0.0577 | 2.81E-01 | -0.0275 | 6.08E-01 |
| IL2RA | cytokine and cytokine re | -0.0240 | 6.54E-01 | 0.0742 | 1.66E-01 |
| ILKAP | cytokine and cytokine re | 0.2683 | 3.36E-07 | 0.2747 | 1.78E-07 |
| TNFAIP2 | cytokine and cytokine re | 0.0834 | 1.19E-01 | 0.1205 | 2.42E-02 |
| TNFAIP6 | cytokine and cytokine re | 0.0525 | 3.27E-01 | 0.1128 | 3.50E-02 |
| TNFRSF4 | cytokine and cytokine re | 0.1376 | 9.87E-03 | 0.1928 | 2.84E-04 |
| TNFRSF6B | cytokine and cytokine re | 0.0769 | 1.50E-01 | 0.1105 | 3.89E-02 |
| TNFSF13B | cytokine and cytokine re | -0.0846 | 1.14E-01 | -0.0028 | 9.58E-01 |
| TNFSF18 | cytokine and cytokine re | -0.0601 | 2.61E-01 | -0.0331 | 5.37E-01 |
| BMP8B | cytokine and cytokine re | 0.2278 | 1.63E-05 | 0.2261 | 1.95E-05 |
| CCL18 | cytokine and cytokine re | -0.1415 | 7.94E-03 | -0.0926 | 8.37E-02 |
| CCL20 | cytokine and cytokine re | 0.1877 | 4.06E-04 | 0.2190 | 3.59E-05 |
| CCL27 | cytokine and cytokine re | 0.1678 | 1.61E-03 | 0.1795 | 7.40E-04 |
| CCL4L2 | cytokine and cytokine re | -0.0088 | 8.70E-01 | 0.0633 | 2.37E-01 |
| CCR3 | cytokine and cytokine re | 0.0780 | 1.45E-01 | 0.1210 | 2.36E-02 |
| CCR8 | cytokine and cytokine re | -0.0200 | 7.08E-01 | 0.0463 | 3.88E-01 |
| IFNG | cytokine and cytokine re | -0.0017 | 9.74E-01 | 0.0659 | 2.19E-01 |
| IFNGR2 | cytokine and cytokine re | 0.1868 | 4.35E-04 | 0.2263 | 1.93E-05 |
| IL15RA | cytokine and cytokine re | 0.0421 | 4.32E-01 | 0.1020 | 5.65E-02 |
| IL1F7 | cytokine and cytokine re | 0.2478 | 2.61E-06 | 0.2567 | 1.13E-06 |
| IL1RN | cytokine and cytokine re | -0.0734 | 1.70E-01 | -0.0650 | 2.25E-01 |
| IL27 | cytokine and cytokine re | -0.1580 | 2.99E-03 | -0.1665 | 1.77E-03 |
| IL32 | cytokine and cytokine re | -0.1477 | 5.56E-03 | -0.1228 | 2.16E-02 |
| IL4I1 | cytokine and cytokine re | 0.1036 | 5.26E-02 | 0.2026 | 1.36E-04 |
| IL5 | cytokine and cytokine re | 0.0633 | 2.37E-01 | 0.0594 | 2.68E-01 |

| | | | | | |
|---|---|---|---|---|---|
| IL8 | cytokine and cytokine re | 0.0344 | 5.20E-01 | 0.0804 | 1.33E-01 |
| ILDR2 | cytokine and cytokine re | 0.0806 | 1.32E-01 | 0.0922 | 8.49E-02 |
| ILF3 | cytokine and cytokine re | 0.3800 | 1.68E-13 | 0.3776 | 2.64E-13 |
| ILVBL | cytokine and cytokine re | -0.1956 | 2.27E-04 | -0.2094 | 7.90E-05 |
| TNFRSF17 | cytokine and cytokine re | -0.0347 | 5.17E-01 | 0.0390 | 4.67E-01 |
| TNFRSF18 | cytokine and cytokine re | 0.0829 | 1.21E-01 | 0.1717 | 1.26E-03 |
| TNFRSF8 | cytokine and cytokine re | -0.0503 | 3.48E-01 | 0.0490 | 3.61E-01 |
| TNFSF12-TNFSF13 | cytokine and cytokine re | -0.0883 | 9.85E-02 | -0.0308 | 5.66E-01 |
| TNFSF4 | cytokine and cytokine re | 0.2993 | 1.07E-08 | 0.3064 | 4.84E-09 |
| CCL25 | cytokine and cytokine re | 0.1248 | 1.93E-02 | 0.1203 | 2.45E-02 |
| CCL3L1 | cytokine and cytokine re | 0.0686 | 2.00E-01 | 0.1156 | 3.06E-02 |
| CXCR1 | cytokine and cytokine re | -0.1047 | 5.00E-02 | -0.0779 | 1.46E-01 |
| EPO | cytokine and cytokine re | 0.1397 | 8.75E-03 | 0.1616 | 2.43E-03 |
| IL12RB2 | cytokine and cytokine re | -0.1550 | 3.61E-03 | -0.1187 | 2.64E-02 |
| IL13 | cytokine and cytokine re | 0.0453 | 3.98E-01 | 0.0609 | 2.56E-01 |
| IL15 | cytokine and cytokine re | -0.0542 | 3.12E-01 | 0.0285 | 5.95E-01 |
| IL29 | cytokine and cytokine re | -0.0077 | 8.86E-01 | 0.0251 | 6.40E-01 |
| ILF2 | cytokine and cytokine re | 0.5512 | 2.77E-29 | 0.5525 | 2.33E-29 |
| TNFAIP1 | cytokine and cytokine re | -0.0866 | 1.05E-01 | -0.0961 | 7.25E-02 |
| TNFAIP8L1 | cytokine and cytokine re | -0.2070 | 9.35E-05 | -0.2046 | 1.16E-04 |
| TNFRSF12A | cytokine and cytokine re | -0.0546 | 3.08E-01 | -0.0411 | 4.43E-01 |
| TNFRSF9 | cytokine and cytokine re | 0.0102 | 8.49E-01 | 0.0685 | 2.01E-01 |
| TNFSF9 | cytokine and cytokine re | 0.1600 | 2.65E-03 | 0.2116 | 6.59E-05 |
| CCL3 | cytokine and cytokine re | -0.0882 | 9.88E-02 | -0.0266 | 6.20E-01 |
| CCL7 | cytokine and cytokine re | -0.0840 | 1.16E-01 | -0.0422 | 4.31E-01 |
| IL11 | cytokine and cytokine re | 0.2062 | 9.98E-05 | 0.2296 | 1.44E-05 |
| IL17C | cytokine and cytokine re | 0.0363 | 4.98E-01 | 0.0498 | 3.53E-01 |
| IL1A | cytokine and cytokine re | 0.0793 | 1.38E-01 | 0.1077 | 4.40E-02 |
| CXCL10 | pro-inflammatory genes | -0.0535 | 3.18E-01 | -0.0068 | 9.00E-01 |

| | | | | | |
|---|---|---|---|---|---|
| CXCL9 | pro-inflammatory genes | -0.0535 | 3.18E-01 | 0.0078 | 8.84E-01 |
| IFNG | pro-inflammatory genes | -0.0017 | 9.74E-01 | 0.0659 | 2.19E-01 |
| GZMB | pro-inflammatory genes | -0.1180 | 2.70E-02 | -0.0699 | 1.92E-01 |
| CXCL13 | pro-inflammatory genes | 0.0582 | 2.77E-01 | 0.1117 | 3.68E-02 |
| STAT1 | pro-inflammatory genes | 0.0456 | 3.94E-01 | 0.0954 | 7.47E-02 |
| IRF1 | pro-inflammatory genes | 0.0257 | 6.31E-01 | 0.1074 | 4.47E-02 |
| CCL5 | pro-inflammatory genes | -0.1204 | 2.41E-02 | -0.0516 | 3.35E-01 |
| GNLY | pro-inflammatory genes | -0.0378 | 4.80E-01 | -0.0003 | 9.95E-01 |
| TBX21 | pro-inflammatory genes | -0.1340 | 1.20E-02 | -0.0763 | 1.54E-01 |
| CD8B | pro-inflammatory genes | -0.0556 | 2.99E-01 | 0.0240 | 6.55E-01 |
| PRF1 | pro-inflammatory genes | -0.1626 | 2.24E-03 | -0.1094 | 4.07E-02 |
| IL12A | pro-inflammatory genes | 0.2515 | 1.82E-06 | 0.3056 | 5.29E-09 |
| IL12B | pro-inflammatory genes | -0.0531 | 3.22E-01 | -0.0011 | 9.83E-01 |
| CD19 | pro-inflammatory genes | 0.0837 | 1.18E-01 | 0.1519 | 4.40E-03 |
